# Transcriptomics as an Early Warning of Domoic Acid Exposure in Pacific Razor Clams (*Siliqua patula*)

**DOI:** 10.3390/toxins17040194

**Published:** 2025-04-11

**Authors:** Lizabeth Bowen, Shannon Waters, Brenda Ballachey, Heather Coletti, Zachary Forster, Jie Li, Bradley Jenner

**Affiliations:** 1 U.S. Geological Survey, Western Ecological Research Center, Davis Field Station, Davis, CA 95616, USA; swaters@usgs.gov; 2 U.S. Geological Survey, Alaska Science Center, Anchorage, AK 99508, USA; beballachey@gmail.com; 3 National Park Service, Anchorage, AK 99501, USA; heather_coletti@nps.gov; 4 Washington Department of Fish and Wildlife, Long Beach, WA 98640, USA; zachary.forster@dfw.wa.gov; 5 University of California Davis, Bioinformatics Core, Davis, CA 95616, USA; jjsli@ucdavis.edu (J.L.); bnjenner@ucdavis.edu (B.J.)

**Keywords:** harmful algal bloom (HAB), domoic acid, transcriptome, sublethal effects, razor clam (*Siliqua patula*)

## Abstract

As oceans warm, harmful algal blooms (HABs) are expected to increase, including blooms of *Pseudo-nitzschia*, a diatom that produces domoic acid (DA), which is a potent neurotoxin. Regulatory limits for human consumption (0.075–0.1 mg/kg/day; acute exposure) exist for the Pacific razor clam; however, fisheries currently do not have regulatory limits for chronic low-level exposure to DA even though razor clams can retain DA for over a year after an algal bloom. For bivalves, exposure to marine toxins may disrupt important cellular processes, leading to concerns about effects on their overall health and potential population- and ecosystem-level impacts. Transcriptomics was used to identify differentially expressed genes in razor clams (N = 30) from Long Beach, WA, collected prior to, during, and after a DA-producing bloom. Differentially expressed genes were identified that may indicate exposure of razor clams to DA, including clams with tissue DA concentrations that fall below regulatory limits for human consumption. Targeting these genes in real-time PCR assays may provide an early warning system for routine monitoring of DA in clams. Our results suggest DA exposure is associated with physiological responses ranging from decreased immune function to the potential disruption of cell communication, including retinoic acid catabolic processes, cell adhesion, collagen fibril organization, and immune effector processes. This work may also allow us to examine potential drivers of population-level change and whether chronic lower-level exposure to DA negatively impacts razor clam function, consequently affecting individual and population health.

## 1. Introduction

As climate change progresses and temperatures rise in the nearshore, the frequency and severity of harmful algal blooms (HABs) in marine environments are increasing [1,2,3,4,5]. Further, warming waters will expand the geographic range and duration of conditions favorable to algal blooms [6], and thus algal toxins are a growing concern for humans and other species that rely on nearshore resources, and for nearshore communities overall. Methods for assessing HABs, the toxins they produce, and the effects on species that may be exposed to these toxins could help to monitor and mitigate potentially dangerous situations.

Marine toxins associated with harmful algal blooms (HABs) are generally classified according to their chemical properties and the symptoms they cause in humans. Numerous types of algae can produce toxic HABs. The most common marine algae associated with toxic HABs include the dinoflagellates (e.g., Florida red tide *Karenia brevis*) and diatoms (e.g., *Pseudo-nitzschia*) [4]. Additional types of algae can produce non-toxic excessive biomass HAB conditions that cause hypoxia and odor/aesthetic issues, including cyanobacteria, green algae (e.g., *Cladophora*), and brown algae (e.g., *Sargassum*) (EPA; https://www.epa.gov/habs/learn-about-harmful-algae-cyanobacteria-and-cyanotoxins, accessed on 20 November 2024). Domoic acid (DA), an amnesic shellfish poisoning neurotoxin, is produced by some diatom species of the genus *Pseudo-nitzschia. Pseudo-nitzschia* is widespread in the world’s oceans and blooms producing DA are not uncommon. DA is a potent neurotoxin; it is excitotoxic in the central nervous system, and in vertebrates, it can lead to severe illness or death [7].

Benthic invertebrates are often used as sentinel species of ecosystem health as they are relatively sedentary, accessible, and may bioaccumulate contaminants, pathogens, and toxins. Mussels (*Mytilus* spp.) have been widely used [8], but there are other bivalve candidates, including the Pacific razor clam (*Siliqua patula*; [9,10]). The use of razor clams as sentinels has been evaluated in China, using the Chinese razor clam (*Sinonovacula constricta*), an important aquaculture species [9]. The Pacific razor clam has merit as a bioindicator species in the northeast Pacific as it is valuable ecologically and economically [9]. Pacific razor clams are harvested by humans for commercial and personal use (subsistence and recreation), and also are eaten by sea otters and other animals inhabiting coastal areas. They can be found in intertidal and subtidal areas (to about 55 m depth), from the eastern Aleutian Islands, Alaska, to central California, USA.

The half-life of DA in marine waters can range from days to several weeks, but in tissues, persistence is more variable, ranging from a few hours to perhaps several years [11]. After algal blooms, marine “snow” (typically comprising senescent algae, plant debris, and fecal particles) drifts to the sea floor, where it will release algal organic matter and DA into sediment [7,12], potentially concentrating DA in razor clam habitats. The accumulation of DA in filter-feeding marine organisms and its subsequent transfer to higher-trophic-level predators can result in significant wildlife mortality [13] and, in people, amnesic shellfish poisoning (ASP), a severe neurological illness [14]. For example, in 2015, the West Coast of the U.S. experienced a massive *Pseudo-nitzschia* bloom that resulted in widespread DA production and mortality of marine mammals and seabirds as well as contamination of shellfish, which triggered closures of the economically and culturally important razor clam fisheries [3,14,15].

The standard approach for evaluating the risk of DA exposure to humans is to quantify the abundance of *Pseudo-nitzschia* in water and DA levels in shellfish tissues [16]. Federal regulations require that at tissue concentrations of 20 ppm or more, a shellfishery is closed. However, there are no regulatory limits that address the risk of chronic low-level exposure despite the ability of razor clams to retain DA for over a year after an algal bloom [17] and despite recent studies that have shown a risk to human health from consumption of razor clams with tissue DA levels below 20 ppm [18].

To date, potential direct effects of DA and other algal toxins on the health of exposed bivalve populations have been addressed in several bivalve species [19,20,21]. However, methods are lacking for measuring the influence of the toxins on the physiology of individual bivalves, populations, and communities, including the Pacific razor clam. If razor clams and other bivalves are affected by these toxins, this could cause changes in their populations, with ramifications for many higher-trophic-level consumers and potentially the nearshore ecosystem. Linkages between the health of marine ecosystems and the health of humans and organisms that depend on these ecosystems are widely recognized in the “One Health” research model, a key component of which is the use of marine organisms as sentinels for potential emerging threats ([22]; see graphical abstract).

Gene-based health diagnostics provides an opportunity for an alternate, holistic assessment of health not only in individuals or populations but potentially in ecosystems [23]. Gene transcription is driven by many stimuli, including toxins, infectious agents, contaminants, trauma, or nutritional stress. The earliest observable signs of impaired health are altered levels of gene transcripts in individuals across populations. Increasingly, gene expression-based diagnostics is being used to monitor ecosystem and wildlife population health [24,25,26,27], including studies looking at the effects of algal toxins in HABs [6]. For Pacific razor clams, gene transcription has been used to compare clams from declining versus stable populations to identify potential drivers of abundance [9,10], but no work has been conducted using gene transcription to examine the effects of DA or other toxins on razor clams.

A method for assessing the effects of DA exposure on the welfare of the Pacific razor clam could be of value in monitoring, for the health of the clams directly as well as perhaps extending to the nearshore community and ecosystem, and potentially contributing to safety standards for human consumption of razor clams. In this study, our objectives are to (1) use transcriptomics to assess the potential effects of DA on the molecular physiology of razor clams exposed to varying levels of DA and (2) identify candidate genes that merit further evaluation for a gene transcription panel to monitor DA in razor clams.

## 2. Results

### 2.1. Pseudo-Nitzschia and Domoic Acid Levels

**Figure 1:**
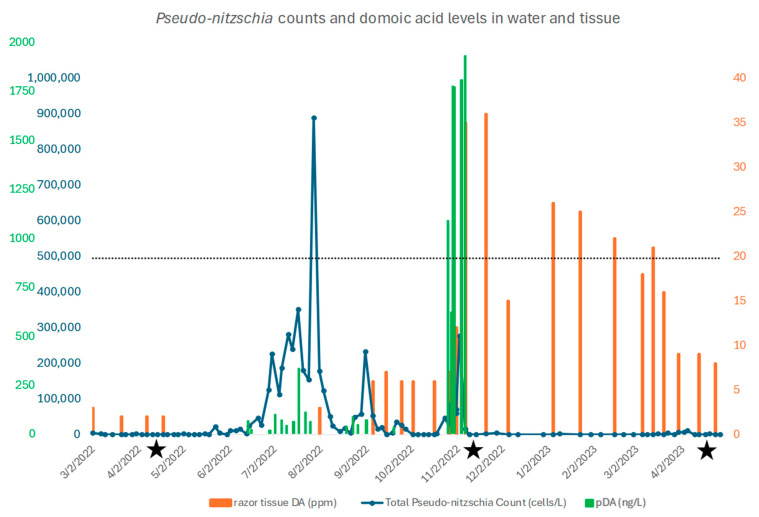
Particulate DA in water (pDA) levels (ng/dL) over time (green bars) and tissue DA levels (ppm; orange bars), superimposed on *Pseudo-nitzschia* count (cells/L) from water samples (blue line). Sampling events are indicated by black stars. Although a large spike in *Pseudo-nitzschia* occurred in August 2022, there was little DA in the *Pseudo-nitzschia* cells or in razor clam tissue at that time. However, by the second sampling time (8 November 2022), DA was detected in both water and tissue samples. The fishery is closed when razor clam tissue DA levels reach 20 ppm, represented by the dashed black line.

The pre-bloom (6 April 2022) water sample levels of *Pseudo-nitzschia* were negligible (~0 cells/L) and increased to over 800,000 cells/L prior to the “bloom” sampling when the levels were at ~300,000 cells/L (8 November 2022) (Figure 1). *Pseudo-nitzschia* levels were again negligible (~0 cells/L) during the post-bloom water sampling (13 April 2023) (Figure 1). Particulate DA levels reached a high three months after the peak *Pseudo-nitzschia* cell count (2 November 2022) of approximately 2000 ng/L (Figure 1). Razor clam tissue DA levels (ppm) followed pDA levels, with a peak of approximately 36 ppm just prior to our second sampling and decreasing to approximately 9 ppm during our post-bloom sampling (Figure 1).

### 2.2. Differential Expression and Functional Annotation

Sample RIN scores ranged from 9.20 to 9.80. We annotated a total of 147,888 genes, of which 87,769 were BLAST-characterized, 71,753 were KEGG-annotated, and 84,976 were GO-annotated. A total of 60,119 genes were BLAST Uncharacterized.

Table 1 displays the top 20 significantly down-regulated genes and the top 20 significantly up-regulated genes in bloom vs. pre-bloom samples. Among the top down-regulated genes (Table 1, Supplementary File S1) were genes involved in neurotransmitter uptake, cytoskeletal organization, glycosylation, ATP binding, fatty acid transport, response to L-glutamate, and immune function processes. The top up-regulated genes were involved in GTPase activity and microtubule-based movement. Many of the top up-regulated genes did not result in a BlastX identification (indicated as “Uncharacterized” in the Description column).

Table 2 displays the top 20 significantly down-regulated genes and the top 20 significantly up-regulated genes between the bloom and post-bloom samples. Among the top down-regulated genes (Table 2, Supplementary File S2) were genes involved in hormone biosynthetic processes, carboxylic acid transport, response to hypoxia, and glycosylation. The top up-regulated genes were involved in immune response and xenobiotic metabolic response. As in Table 1, many of the top down-regulated and up-regulated genes did not result in a BlastX identification.

Table 3 displays the top 20 significantly down-regulated genes and the top 20 significantly up-regulated genes between the pre-bloom and post-bloom samples. Among the top down-regulated genes (Table 3, Supplementary File S3) were genes involved in hormone biosynthetic processes, carboxylic acid transport, response to hypoxia, and glycosylation. The top up-regulated genes were involved in protein folding and calcium ion processes. Again, many of the top down-regulated and up-regulated genes did not result in a BlastX identification.

**Figure 2:**
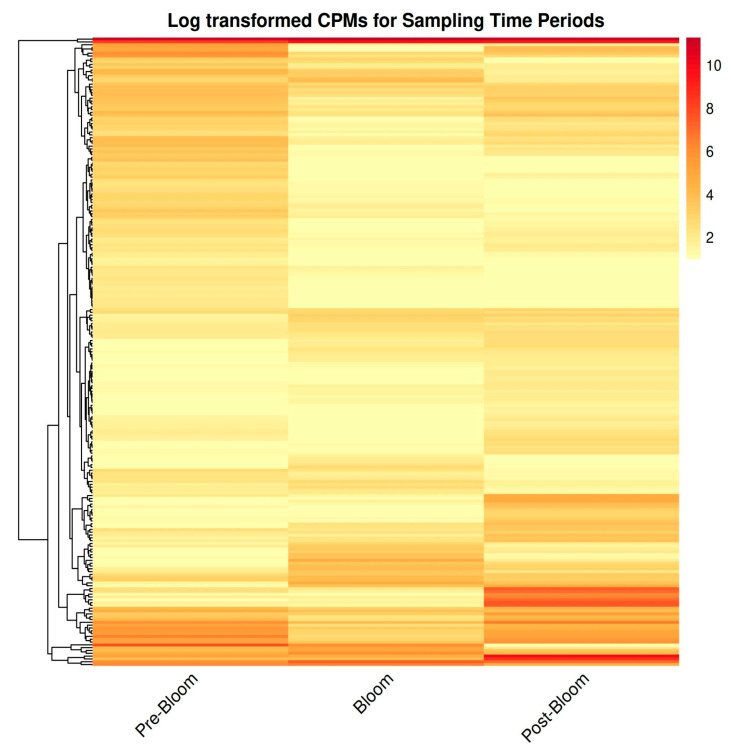
Heatmap showing the log-transformed counts per million (CPM) of 223 significantly differentially expressed genes of razor clams sampled prior to a DA-producing bloom (6 April 2022; “Pre-Bloom”), during a DA-producing bloom (8 November 2022; “Bloom”), and after a DA-producing bloom (13 April 2023; “Post-Bloom”). Transcription signal intensity is denoted by color; differences in color across the three sampling periods indicate differences in gene expression. The tree structures to the left demonstrate similarities between genes and samples (i.e., the smaller the distance between tree branches, the greater the similarity), according to the hierarchical cluster analysis results.

The number of differentially expressed genes (DEGs) between the pre-bloom and post-bloom samples with absolute fold change > 1.5 and adjusted *p*-value < 0.05 was 127, of which 52 were up-regulated and 75 were down-regulated (Figure 2, Supplementary File S2).

### 2.3. Enriched GO Terms

Significantly enriched biological processes were distributed uniquely according to DA exposure status (pre-bloom, bloom, post-bloom) (Table 4). There was a considerable overlap of superclusters identified in each comparison. Superclusters of enriched biological processes identified in both the pre-bloom/bloom and bloom/post-bloom comparisons included cerebellar cortex morphogenesis, protein galactosylation, cilium movement involved in cell motility, and homophilic cell adhesion via plasma membrane adhesion molecules. Superclusters of enriched biological processes identified in both the bloom/post-bloom and pre-bloom/post-bloom comparisons included the nucleoside triphosphate biosynthetic process. Superclusters of enriched biological processes identified in both the pre-bloom/bloom and pre-bloom/post-bloom comparisons included defense response to Gram-positive bacterium, cilium movement involved in cell motility, and homophilic cell adhesion via plasma membrane adhesion molecules. The only supercluster identified in all three comparisons was locomotion. Superclusters unique to the pre-bloom/bloom comparison included retinoic acid catabolic process, cell adhesion, collagen fibril organization, and immune effector process. Superclusters unique to the bloom/post-bloom comparison included complement activation, classical pathway, cilium assembly, mitochondrial transmembrane transport, binding of sperm to zona pellucida, and cellular organofluorine metabolic process. Superclusters unique to the pre-bloom/post-bloom comparison included negative regulation of necroptotic process, gonad morphogenesis, positive regulation of anion channel activity, viral genome replication, and cilium-dependent cell motility.

## 3. Discussion

Although there are many studies about the effects of DA in mammals, there is less knowledge about the effects of DA on bivalve mollusks; most studies of invertebrates simply report body burdens of DA [19,28,29] or depuration rates [30,31]. Mafra et al. [30,32] reported no physiological effects in oysters from DA exposure, but several publications have shown that DA can exert physiological and sublethal effects on marine bivalves (Dizer et al., 2001; Jones et al., 1995a, 1995b; Liu et al., 2007; Liu et al., 2008; Ventoso et al., 2019) [20,29,33,34,35,36], including oxidative stress (Hégaret et al., 2011) [37], DNA damage [20], stress response [33,34], and impaired growth and survival [35,36]. The molecular mechanisms of DA absorption and excretion in bivalve mollusks are poorly understood, and very few studies have examined the transcriptional effects of DA exposure on mollusks [21,29,38]. The molecular mechanisms of DA uptake and elimination in bivalves and how the toxin (and the toxin-producing species) affects gene expression remain knowledge gaps in this field.

Gene expression levels were assessed in Pacific razor clams prior to a *Pseudo-nitzschia* bloom, during a DA spike in water and tissues, and post-bloom, when *Pseudo-nitzschia* cells and DA in both water and tissue were low. We coupled GE sampling with ongoing *Pseudo-nitzschia* monitoring that occurs along the coast of Washington state and is in place to manage human harvests of razor clams and potential impacts on human health. This monitoring provided us with information on DA levels in the water (pDA) and in the razor clam tissues. Monitored levels of *Pseudo-nitzschia*, pDA, and tissue DA in the pre-bloom (6 April 2022) samples were negligible. Monitored levels of *Pseudo-nitzschia*, pDA, and tissue DA in the bloom (8 November 2022) samples increased dramatically, causing closure of the fishery (closure triggered by levels of 20 ppm or greater in tissues). By the third sampling event (13 April 2023; post-bloom), monitored *Pseudo-nitzschia* and pDA levels were again negligible, razor clam tissue DA levels (ppm) had decreased to approximately 9 ppm, and the fishery was reopened (Figure 1).

Razor clam spawning generally occurs in May and June, potentially overlapping with one or more of our sampling periods. Levels of expression of genes involved in hormone biosynthesis were lower in razor clams sampled during a bloom than in razor clams sampled post-bloom, and lower in clams sampled pre-bloom in comparison with clams sampled post-bloom. This suggests that razor clams sampled post-bloom (13 April 2023) were perhaps becoming gravid. Given this consideration, and the results of both the DEG and GO analyses, we have identified pre-bloom and during the bloom as the focal timepoints for addressing the effects of DA on razor clam physiology.

The metabolism of xenobiotics (such as toxins) has three phases: phase I (functionalization) and phase II (conjugation) are catalyzed by metabolizing enzymes, while phase III consists of the export from the cell by transmembrane transporter proteins. Ventoso et al. [29] found that the Pfam domains of some phase I (cytochrome P450s and aldo-keto reductases) and phase II (glutathione S-transferases and sulfotransferases) drug-metabolizing enzymes were functionally enriched, suggesting that glutathione S-transferases might play a role in DA detoxification. While we identified cytochrome P450s and glutathione S-transferases in the DEGs, neither reached a level of statistical significance between sampling periods (Supplementary File S4).

One of the top down-regulated genes in razor clams sampled during the DA bloom was the sodium- and chloride-dependent glycine transporter 2 (GlyT2). GlyT2 is a membrane protein which recaptures glycine, which, in addition to acting as an inhibitory neurotransmitter, is also a co-agonist at N-methyl-D-aspartate (NMDA) glutamate receptors [21,39]. In humans, GlyT2 is encoded by the *SLC6A5* gene. Inactivation of GlyT2 in knockout mice is lethal during the second post-natal week as the absence of GlyT2 disrupts inhibitory transmission by reducing glycine release. GlyT2’s main physiological role is to recapture glycine released in the synaptic cleft and to maintain high glycine concentrations in presynaptic neurons. Therefore, chronic inhibition of GlyT2 will deplete intracellular storage of glycine and limit its accumulation in synaptic vesicles [40]. It is of interest that genes of the SLC6 family were up-regulated in *M. galloprovincialis* [21,38] and down-regulated in *A. opercularis* [29] after exposure to DA-producing *Pseudo-nitzschia,* indicating that GlyT2 plays an important role in DA processing across various species.

Also down-regulated in DA-exposed razor clams was Beta-1,4-galactosyltransferase (galt-1). Studies have shown that galt-1 plays a role in the activation of lymphocytes (Sun et al., 2014) [41]. Down-regulation of galt-1 suggests a decreased immune function potential in razor clams exposed to DA. This is consistent with findings from Ventoso et al. [21], in which genes involved in immunologic processes were down-regulated in king scallops (*P. maximus*) exposed to DA.

Fatty acid-binding protein homolog 6 (part of the fatty acid transport pathway) was also down-regulated in razor clams exposed to DA in our study. Interestingly, Du et al. [7] found similar genes, related to the metabolic pathways of glycine fermentation, fatty acid synthesis, and ß-oxidation, involved in the biotransformation of DA by anaerobic microbial communities. Du et al. [7] found that the addition of glycine, glucose, or glycine–glucose in experimental, anaerobic conditions had a significantly positive effect on DA biotransformation, with glycine identified as the most effective co-substrate for DA anaerobic biotransformation.

There were many genes up-regulated in the bloom samples in comparison with both the pre-bloom and post-bloom samples, including lysosomal-associated transmembrane protein 4A, uncharacterized genes, and Dynein-associated genes. We do know that continued transcription of genes can be physiologically costly [9]. Perhaps the largest cost is the reallocation of nutrients and energy from one portion of an individual’s resource budget to other metabolic functions. Mitigation of stressors imposes demands on organisms above those normally required to sustain life and may result in a reduction in fitness, evidenced by decreased reproductive capability, increased susceptibility to disease, or disadvantageous behavioral changes [42,43].

Based on the REVIGO table of the superclusters, we identified enriched biological processes in the comparison between the pre-bloom and bloom samples. For example, the retinoic acid catabolic process was enriched, consistent with the findings of Hidayat et al. [44], who identified that DA exposure affected genes associated with retinoic acid metabolism in zebrafish. Retinoic acid plays a part in neuron differentiation, regeneration, and synaptic plasticity. Abnormal retinoic acid levels may contribute to nervous system dysfunction [44]. Consistent with our findings of enhanced biological processes associated with cell adhesion, Neely et al. [45] identified increased cell adhesion proteins in the cerebrospinal fluid from sea lions with DA toxicity. Some cell adhesion molecules are involved in synapse formation, axon growth, myelin formation, and neuron differentiation [45]. Collagen fibril organization was also enriched. In human and animal studies, exposure to DA has been shown to induce cardiac lesions; collagen fiber accumulation is a marker of cardiac fibrosis [46]. Ventoso et al. [29] also identified enriched collagen processes in queen scallops *(Aequipecten opercularis*) after exposure to DA; collagens are components of the extracellular matrix and primarily have a structural function. Also of note was the enriched immune effector process in the pre-bloom/bloom clams. Although the exact mechanism of DA-induced immune dysfunction is not completely understood, it is known that DA-induced neurotoxicity is mediated through glutamate cell surface receptors. Glutamate receptors act as mediators of excitatory neurotransmission and have been identified outside the central nervous system, including in cells of the immune system [47].

This study was not in a controlled laboratory setting, and therefore the possibility exists that other algal toxins or environmental stressors may have contributed to the molecular differences among sampling timepoints. However, as seen in ancillary data collected by ORHAB (Supplementary File S5), significant exposure to toxins other than DA was not identified until the very end of the study when there were minor detections of paralytic shellfish toxins during the post-bloom sampling.

In our previous studies of the Pacific razor clam, we were only able to amplify and identify five target genes and two reference genes in which we had complete confidence [9,10]. Similar difficulties were found in transcriptomic studies of the Chinese razor clam, in which only 9.9% of the 147,669 transcribed sequences had significant matches in GenBank [48]. The limited number of genetic studies on razor clams is incongruent with their value as a commercial species and popularity in sport, recreational, and subsistence fishing. Studies to date have focused on the Chinese razor clam, identifying genes responding to heavy metals [49], anthropogenic sound [50], and bacterial challenge (Niu et al., 2014, 2016; Peng et al., 2016) [50,51,52], and have provided a foundation for the development of protocols for using razor clams as indicators of ecosystem health.

## 4. Conclusions

This study elucidates genes with potential to identify razor clams exposed to DA, even when tissue levels of DA in the clams may fall below established regulatory limits for human consumption. Our results suggest that DA exposure is associated with physiological responses ranging from decreased immune function to the potential disruption of cell communication. The utility of targeting these genes in real-time PCR assays for routine monitoring lies in the potential of an early warning system prior to the detection of DA in the tissues. This work may also allow us to examine potential drivers of population-level change and whether chronic lower-level exposure to DA negatively impacts razor clam function and, consequently, affects individual and population health. Our work has identified unique expression patterns in exposed razor clams relative to unexposed individuals that may relate to DA exposure. These transcripts present potentially useful targets for future hypothesis-driven studies using qPCR. Overall, the results provide a foundation for continued transcriptomic studies of chronic low-level exposure of razor clams to DA.

## 5. Materials and Methods

### 5.1. Harmful Algal Bloom Monitoring

*Pseudo-nitzschia* cell counts and particulate domoic acid (pDA) data were collected as part of the Olympic Region Harmful Algal Bloom (ORHAB) Monitoring Program to inform the potential status of DA levels both in water and tissues during this study. As part of ORHAB, WDFW routinely collects water samples from the surf zone on Long Beach (46°29.798, 124°03.633). The sampling frequency varies seasonally: twice weekly during the “bloom” season (March–November) and once weekly during the winter months.

### 5.2. Cell Counts and Particulate Domoic Acid

Seawater collected from the surf zone on Long Beach was brought back to the Willapa Bay Field Station in Ocean Park, WA, where it was processed using standardized methods. Cell counts were performed on 10× concentrated water using a Palmer-Maloney counting chamber and a light microscope at 20× magnification. Seawater samples were screened for pDA using an Enzyme-Linked Immunosorbent Assay (ELISA) when cell counts of *Pseudo-nitzschia* reached a predetermined threshold (30,000 cells/L large-type *Pseudo-nitzschia*). Seawater for pDA was filtered through a 0.45-micron cellulose nitrate membrane filter to concentrate cells, and then the DA was extracted in deionized water for analysis. Samples in this study were run with the DAK-36 DA kit (Mercury Science, Durham, NC, USA) and the results quantified in ng/L of DA.

### 5.3. Domoic Acid Tissue Testing

Domoic acid in razor clam tissue is monitored by the Washington State Department of Health (DOH). The DOH Public Health Lab (PHL) located in Shoreline, WA, follows a National Shellfish Sanitation Program-validated High-Pressure Liquid Chromatography (HPLC) testing method to screen for DA in razor clam tissue [53,54]. The DA test results used in this study were collected for routine testing prior to the opening of the recreational fishery on Long Beach. Samples of 12 clams (separate from the clams used for transcriptomics) were collected by WDFW and delivered to the PHL where they were shucked, cleaned (gills and guts removed), and homogenized in a laboratory blender. A sub-sample of the 12-clam matrix was then prepared using standardized methods for HPLC analysis and results reported in parts per million (ppm). In summary, when *Pseudo-nitzschia* cell counts reach a defined action level (30,000 cells/L large-type *Pseudo-nitzschia*), this triggers additional screening for DA in clams (ppm) and in water (ng/L). If the results indicate elevated DA levels, managers are notified, and further water and clam tissue samples are collected to determine response actions for event response. Fisheries are closed when tissue DA levels meet or exceed 20 ppm.

### 5.4. Sample Collection

In collaboration with the Washington Department of Fish and Wildlife (WDFW), we collected gill tissue from Pacific razor clams from Long Beach, WA, for gene transcription analyses. This beach is also regularly monitored for DA and other algal toxins through routine water and razor clam tissue toxin testing. Ten razor clams were sampled from Long Beach at each of three timepoints: pre-bloom (6 April 2022), during the bloom (8 November 2022), and post-bloom (13 April 2023). Gill tissues were excised and placed immediately into individual tubes pre-filled with RNAlater. Samples were then frozen for transport to the USGS Davis Field Station genetics lab, where they were stored at −80 °C.

### 5.5. RNA Extraction

We extracted total RNA from homogenized gills using the RNeasy Lipid Tissue Mini Kit (Qiagen; www.qiagen.com). To remove contaminating genomic (g)DNA, we treated the spin columns with 10 U μL^−1^ of RNase-free DNase I (DNase, Amersham Pharmacia Biotech Inc.; www.apbiotech.com) at 20 °C for 15 min. We then stored RNA at −80 °C pending further analyses.

The samples were then sent to the UC Davis Genome Center DNA Technologies Core Facility for further DNase treatment followed by a microbead clean-up to further purify the RNA.

Specifically, the total RNA samples were DNAse-digested in a volume of 50 μL with two units of RNAse-free DNAse I (NEB, Ipswich, MA, USA) in the accompanying DNAse buffer at 37 °C for 10 min. The digestion reaction was stopped and cleaned up by the addition of 90 μL RNAClean XP beads (Beckman Coulter, Brea, CA, USA) according to the protocol of the manufacturer. The RNA was eluted from the beads in 12 μL molecular biology-grade water. Quality assurance of the total RNA showed Bioanalyzer RIN scores ≥ 7 and enough material for library preparation.

### 5.6. RNAseq

GE profiling was carried out using a 3_-Tag-RNA-Seq protocol. The fragment size distribution of the libraries was verified via micro-capillary gel electrophoresis on a Bioanalyzer 2100 (Agilent, Santa Clara, CA, USA). The libraries were quantified by fluorometry on a Qubit fluorometer (Life Technologies, Carlsbad, CA, USA) and pooled in equimolar ratios. Up to 48 libraries were sequenced per lane on a HiSeq 4000 sequencer (Illumina, San Diego, CA, USA) with single-end 100 bp reads. Prior to RNAseq analysis, we trimmed low-quality ends off reads (Q = 30) using Trimgalore (v0.6.7; https://www.bioinformatics.babraham.ac.uk/projects/trim_galore/; accessed on 4 November 2024), removed adapter sequences and N bases from read ends, and removed reads less than 30 base pairs. We created a transcript reference index using isoseq and used Salmon (v1.9.0; [55]) to align reads to isoseq transcript references. We quantified expression at the transcript level.

### 5.7. Differential Expression Analysis

In order to characterize the molecular response to DA exposure, we conducted differential expression (DE) analyses. We used the limma-voom pipeline as a framework for differential expression analysis, which consisted of normalization and moderated paired *t*-tests implemented in the limma framework (limma version 3.34.9 [56,57], edgeR version 3.20.9, and R v4.3.3 [58]). We used the Benjamini–Hochberg procedure for multiple comparisons [59].

### 5.8. Functional Annotation

We carried out functional annotation using a variety of tools: We used TransDecoder (v5.5.0; https://github.com/TransDecoder/TransDecoder; accessed on 4 November 2024) to find coding regions within transcripts. We used https://gensoft.pasteur.fr/docs/STAR/2.7.9a/STARsolo.html and BLASTX (v2.10.1; [60]; accessed on 8 November 2024) to align transcripts to protein databases, hmmer (v3.1b2; hmmer.org) to identify homologous sequences in the Pfam database, Pfam [61] and SignalP (v4.1c; [62]) to detect the presence and position of signal peptide cleavage sites, RNAMMER (v1.2; [63]) to annotate ribosomal RNA genes, TMHMM (v2.0c; [64]) to predict transmembrane protein topology, and Trinotate (v3.2.2; [65]) as a wrapper for other tools. The GO annotation was not performed using any particular species, but instead through the multispecies UniProt and Swissprot protein database that is utilized by Trinotate.

Differentially expressed (DE) genes were annotated based on Gene Ontology (GO) pathway analysis. GO annotations explain the function of a particular gene and are created by associating a gene or gene product with a GO term. Together, these statements comprise a “snapshot” of current biological knowledge describing gene functions at the molecular level, the location in the cell of these functions, and what biological processes (pathways, programs) they help to carry out. GO terms were assigned using trinotate, which uses Pfam and blast (https://github.com/Trinotate/Trinotate; accessed on 5 December 2024).

### 5.9. GO Enrichment

GO annotations explain the function of a particular gene and are created by associating a gene or gene product with a GO term. Together, these statements comprise a “snapshot” of current biological knowledge describing gene functions at the molecular level, the location in the cell of these functions, and what biological processes (pathways, programs) they help to carry out.

Therefore, multiple functions of individual genes can be accommodated by association with the following three classes of GO terms: cellular component, molecular function, and biological process. A particular gene can have any number of associated annotations in any of those categories.

GO enrichment analyses were conducted using Kolmogorov–Smirnov tests to compare DE *p*-values annotated and not annotated with a given GO term, implemented using the Bioconductor package topGO, version 2.30.1. A GO term is a group of genes associated with a cellular biological process that are predefined by the GO bioinformatics initiative using a controlled vocabulary. GO terms describe three main aspects of the biological domain: molecular function, cellular component, and biological process. Biological process is the largest of the three ontology aspects in GO, and also the most diverse. Because biological process concepts span the entire range of how biologists characterize biological systems, we have chosen to focus on biological processes throughout this paper. Through the enrichment analysis of the differentially expressed genes, we can find out which biological functions or pathways are significantly associated with differentially expressed genes. Analysis and interpretation of transcriptome data often results in long lists of genes that require an impractically large amount of manual literature searching to interpret [66]. A common approach to mitigating this is to perform pathway enrichment analysis, which summarizes the large gene list as a smaller list of more easily interpretable pathways which are then statistically tested for over-representation relative to what is expected by chance [66]. GO provides a framework and set of concepts for describing the functions of gene products from all organisms and is specifically designed for supporting the computational representation of biological systems. A GO annotation is an association between a specific gene product and a GO concept; a biological process represents a specific objective that the organism is genetically “programmed” to achieve [67].

### 5.10. Superclusters

Significant GO terms were input into the Revigo Web server. Revigo summarizes lists of GO terms by finding a representative subset of the terms using a simple clustering algorithm that relies on semantic similarity measures. Representative terms are joined into “superclusters” of loosely related terms [68].

## Figures and Tables

**Table 1:** Top 20 significantly differentially expressed genes between razor clams sampled during a *Pseudo-nitzschia* bloom and razor clams sampled pre-bloom (a—down-regulated; b—up-regulated). Fold change (base 2) is noted. Due to the capabilities of these genes to differentiate between bloom and pre-bloom razor clams, they are potential candidates for use in DA-specific assay panels for use with razor clams. A positive fold change indicates greater expression in the bloom razors compared with the pre-bloom razors. A negative fold change indicates lower expression in the bloom razors compared with the pre-bloom razors. Genes with no Blastx results are identified as “Uncharacterized”.

(**a**) Top 20 down-regulated genes in bloom compared with pre-bloom razor clams				
**Transcript_ID**	**Description**	**logFC**	**adj.P.Val**	**Representative GO ID and Function**
a_HQ_transcript/79367	Sodium- and chloride-dependent glycine transporter 2	−4.83	1.85 × 10^−07^	GO:0001504 neurotransmitter uptake
a_HQ_transcript/131580	Spectrin alpha chain, non-erythrocytic 1;	−4.73	5.88 × 10^−07^	GO:0030036 actin cytoskeleton organization
a_HQ_transcript/122962	Uncharacterized	−4.51	4.56 × 10^−06^	Uncharacterized
a_HQ_transcript/79902	Beta-1,4-galactosyltransferase galt-1	−4.34	4.56 × 10^−06^	GO:0070085 glycosylation
a_HQ_transcript/75321	Myosin heavy chain, striated muscle	−3.52	4.56 × 10^−06^	GO:0005524 ATP binding
a_HQ_transcript/141107	Fatty acid-binding protein homolog 6	−4.25	0.00	GO:0015908 fatty acid transport
a_HQ_transcript/133860	Tubulin alpha-1A chain	−3.89	0.00	GO:1902065 response to L-glutamate
a_HQ_transcript/145194	Full = Septin-7	−5.52	0.00	GO:0030154 cell differentiation55
a_HQ_transcript/34261	Nuclear receptor ROR-alpha	−2.56	0.00	GO:0071356 cellular response to tumor necrosis factor
a_HQ_transcript/48432	Complement C3	−6.27	0.00	GO:0006954 inflammatory response
a_HQ_transcript/123862	Uncharacterized	−3.88	0.00	Uncharacterized
a_HQ_transcript/60848	Metalloprotease mig-17	−3.65	0.00	GO:0016540 protein autoprocessing
a_HQ_transcript/108352	Ankyrin repeat and EF-hand domain-containing protein 1	−3.03	0.00	Uncharacterized
a_HQ_transcript/98226	Nuclear receptor ROR-alpha	−2.44	0.00	GO:0071356 cellular response to tumor necrosis factor
a_HQ_transcript/49343	Polyubiquitin-A	−3.91	0.00	GO:0019941 modification-dependent protein catabolic process
a_HQ_transcript/33295	Collagen alpha-2(I) chain	−4.12	0.00	GO:0007179 transforming growth factor beta receptor signaling pathway
a_HQ_transcript/76594	Glutamine synthetase	−5.21	0.00	GO:0006536 glutamate metabolic process
a_HQ_transcript/66242	Glutamine synthetase	−4.47	0.00	GO:0006536 glutamate metabolic process
a_HQ_transcript/102242	Uncharacterized	−2.17	0.00	Uncharacterized
a_HQ_transcript/7159	Uncharacterized	−1.70	0.00	Uncharacterized
(**b**) Top 20 up-regulated genes in bloom compared with pre-bloom razor clams				
**Transcript_ID**	**Description**	**logFC**	**adj.P.Val**	**Representative GO ID and Function**
a_HQ_transcript/118139	Uncharacterized	6.80	0.00	Uncharacterized
a_HQ_transcript/53710	Uncharacterized	3.02	0.00	Uncharacterized
a_HQ_transcript/138234	Adapter molecule	5.01	0.00	GO:0043087 regulation of GTPase activity
a_HQ_transcript/114723	Uncharacterized	3.02	0.00	Uncharacterized
a_HQ_transcript/135178	Uncharacterized	5.39	0.00	Uncharacterized
a_HQ_transcript/41636	Uncharacterized	3.52	0.00	Uncharacterized
a_HQ_transcript/152158	Uncharacterized	1.42	0.01	Uncharacterized
a_HQ_transcript/70915	Dynein beta chain, ciliary	3.24	0.01	GO:0007018 microtubule-based movement
a_HQ_transcript/51541	Opioid growth factor receptor-like protein 1	3.85	0.01	GO:0140625 opioid growth factor receptor activity
a_HQ_transcript/105284	Isoamyl acetate-hydrolyzing esterase 1 homolog	3.92	0.01	GO:0016042 lipid catabolic process
a_HQ_transcript/101470	Lysosomal-associated transmembrane protein 4A	4.15	0.01	GO:0005765 lysosomal membrane
a_HQ_transcript/79577	Uncharacterized	4.41	0.01	Uncharacterized
a_HQ_transcript/149006	Uncharacterized	4.76	0.01	Uncharacterized
a_HQ_transcript/130597	Uncharacterized	2.93	0.01	Uncharacterized
a_HQ_transcript/95361	Uncharacterized	3.71	0.01	Uncharacterized
a_HQ_transcript/110997	Uncharacterized	5.25	0.02	Uncharacterized
a_HQ_transcript/62554	Heat shock 70 kDa protein 12A	1.76	0.02	GO:0005524 ATP binding
a_HQ_transcript/84948	Coiled-coil domain-containing protein 81	4.20	0.02	GO:0005737 cytoplasm
a_HQ_transcript/91398	Transcription elongation regulator 1	5.34	0.02	GO:0006397 mRNA processing
a_HQ_transcript/135047	Uncharacterized	4.42	0.02	Uncharacterized

**Table 2:** Top 20 significantly differentially expressed genes between razor clams sampled during a *Pseudo-nitzschia* bloom (bloom) and razor clams sampled post-bloom (a—down-regulated; b—up-regulated). Fold change (base 2) is noted. A positive fold change indicates greater expression in the bloom razors compared with the post-bloom razors. A negative fold change indicates lower expression in the bloom razors compared with the post-bloom razors. Genes with no Blastx results are identified as “Uncharacterized”.

(**a**) Top 20 down-regulated genes in bloom compared with post-bloom razor clams				
**Transcript_ID**	**Description**	**logFC**	**adj.P.Val**	**Representative GO ID and Function**
a_HQ_transcript/72650	a_HQ_transcript/72650	−5.47	7.62 × 10^−07^	GO:0098609 cell-cell adhesion
a_HQ_transcript/45368	Cilia- and flagella- associated protein 210	−7.24	3.35 × 10^−06^	GO:0005879 axonemal microtubule
a_HQ_transcript/23807	Steroid 17-alpha-hydroxylase	−4.50	3.35 × 10^−06^	GO:0042446 hormone biosynthetic process
a_HQ_transcript/27296	Uncharacterized	−7.17	8.17 × 10^−06^	Uncharacterized
a_HQ_transcript/1070	Uncharacterized	−6.16	8.17 × 10^−06^	Uncharacterized
a_HQ_transcript/55144	ATP-dependent translocase ABCB1	−4.07	8.17 × 10^−06^	GO:1905039 carboxylic acid transmembrane transport
a_HQ_transcript/95382	Uncharacterized	−6.62	9.54 × 10^−06^	Uncharacterized
a_HQ_transcript/66970	ATP-dependent translocase ABCB1	−2.94	9.54 × 10^−06^	GO:1905039 carboxylic acid transmembrane transport
a_HQ_transcript/102242	Uncharacterized	−3.02	9.54 × 10^−06^	Uncharacterized
a_HQ_transcript/34261	Nuclear receptor ROR-alpha	−4.89	9.54 × 10^−06^	GO:0071456 cellular response to hypoxia
a_HQ_transcript/125280	Uncharacterized	−4.91	2.74 × 10^−05^	Uncharacterized
a_HQ_transcript/146424	Uncharacterized	−2.57	2.74 × 10^−05^	Uncharacterized
a_HQ_transcript/58336	Nose resistant to fluoxetine protein 6	−5.68	2.74 × 10^−05^	GO:0006869 lipid transport
a_HQ_transcript/129123	Uncharacterized	−9.53	2.74 × 10^−05^	Uncharacterized
a_HQ_transcript/73314	Uncharacterized	−3.72	5.25 × 10^−05^	Uncharacterized
a_HQ_transcript/22218	Steroid 17-alpha-hydroxylase	−4.21	5.58 × 10^−05^	GO:0042446 hormone biosynthetic process
a_HQ_transcript/92691	Adenylate kinase isoenzyme 1	−3.66	5.62 × 10^−05^	GO:0006172 ADP biosynthetic process
a_HQ_transcript/79902	Beta-1,4-galactosyltransferase galt-1	−7.11	6.74 × 10^−05^	GO:0070085 glycosylation
a_HQ_transcript/35228	Steroid 17-alpha-hydroxylase	−5.73	9.66 × 10^−05^	GO:0006694 steroid biosynthetic process
a_HQ_transcript/95521	S-crystallin SL11	−5.60	0.0001407	GO:0005212 structural constituent of eye lens
(**b**) Top 20 up-regulated genes in bloom compared with post-bloom razor clams				
**Transcript_ID**	**Description**	**logFC**	**adj.P.Val**	**Representative GO ID and Function**
a_HQ_transcript/75035	Uncharacterized	5.34	0.00	Uncharacterized
a_HQ_transcript/135178	Uncharacterized	7.34	0.00	Uncharacterized
a_HQ_transcript/36072	Macrophage-expressed gene 1 protein	3.18	0.01	GO:0002250 adaptive immune response
a_HQ_transcript/53566	ATP-dependent translocase ABCB1	4.10	0.01	GO:1905039 carboxylic acid transmembrane transport
a_HQ_transcript/101470	Lysosomal-associated transmembrane protein 4A	4.16	0.01	GO:0005765 lysosomal membrane
a_HQ_transcript/52617	Dynein regulatory complex protein 10	2.03	0.01	GO:0031514 motile cilium
a_HQ_transcript/146734	Uncharacterized	4.00	0.01	Uncharacterized
a_HQ_transcript/130548	Uncharacterized	4.54	0.01	Uncharacterized
a_HQ_transcript/47331	Uncharacterized	3.63	0.01	Uncharacterized
a_HQ_transcript/131746	Uncharacterized	5.99	0.01	Uncharacterized
a_HQ_transcript/15084	Sodium-dependent phosphate transport protein 2B	3.73	0.01	GO:0006412 translation
a_HQ_transcript/17220	NADPH--cytochrome P450 reductase	4.47	0.01	GO:0006805 xenobiotic metabolic process
a_HQ_transcript/49759	Phospholipase A and acyltransferase 3	1.68	0.02	GO:0046485 ether lipid metabolic process
a_HQ_transcript/8232	Uncharacterized	2.92	0.02	Uncharacterized
a_HQ_transcript/57283	Fibropellin-3	2.44	0.02	GO:0005509 calcium ion binding
a_HQ_transcript/76697	Skeletal aspartic acid-rich protein 1	5.11	0.02	GO:0005576 extracellular region
a_HQ_transcript/22375	Steroid 17-alpha-hydroxylase	1.53	0.02	GO:0042446 hormone biosynthetic process
a_HQ_transcript/52827	Tektin-4	4.93	0.02	GO:0030030 cell projection organization
a_HQ_transcript/107872	Fascin	3.86	0.02	GO:0030036 actin cytoskeleton organization
a_HQ_transcript/109347	Malate dehydrogenase	3.24	0.03	GO:0006108 malate metabolic process

**Table 3:** Top 20 significantly differentially expressed genes between razor clams sampled prior to a *Pseudo-nitzschia* bloom (pre-bloom) and razor clams sampled post-bloom (a—down-regulated; b—up-regulated). Fold change (base 2) is noted. A positive fold change indicates greater expression in the pre-bloom razors compared with the post-bloom razors. A negative fold change indicates lower expression in the pre-bloom razors compared with the post-bloom razors. Genes with no Blastx results are identified as “Uncharacterized”.

(**a**) Top 20 down-regulated genes in pre-bloom compared with post-bloom razor clams				
**Transcript_ID**	**Description**	**logFC**	**adj.P.Val**	**Representative GO ID and Function**
a_HQ_transcript/72650	protocadherin	−7.28	2.02 × 10^−15^	GO:0098609 cell-cell adhesion
a_HQ_transcript/45368	Cilia and flagella associate protein	−5.47	7.62 × 10^−07^	GO:0005879 axonemal microtubule
a_HQ_transcript/23807	Steroid 17-alpha-hydroxylase	−7.24	3.35 × 10^−06^	GO:0042446 hormone biosynthetic process
a_HQ_transcript/27296	Uncharacterized	−4.50	3.35 × 10^−06^	Uncharacterized
a_HQ_transcript/1070	Uncharacterized	−7.17	8.17 × 10^−06^	Uncharacterized
a_HQ_transcript/55144	ATP-dependent translocase ABCB1	−6.16	8.17 × 10^−06^	GO:1905039 carboxylic acid transmembrane transport
a_HQ_transcript/95382	Uncharacterized	−4.07	8.17 × 10^−06^	Uncharacterized
a_HQ_transcript/66970	ATP-dependent translocase ABCB1	−6.62	9.54 × 10^−06^	GO:1905039 carboxylic acid transmembrane transport
a_HQ_transcript/125280	Uncharacterized	−4.89	9.54 × 10^−06^	Uncharacterized
a_HQ_transcript/34261	Nuclear receptor ROR-alpha	−3.02	9.54 × 10^−06^	GO:0071456 cellular response to hypoxia
a_HQ_transcript/102242	Uncharacterized	−2.94	9.54 × 10^−06^	Uncharacterized
a_HQ_transcript/73314	Uncharacterized	−9.53	2.74 × 10^−05^	Uncharacterized
a_HQ_transcript/129123	Uncharacterized	−5.68	2.74 × 10^−05^	Uncharacterized
a_HQ_transcript/146424	Uncharacterized	−4.91	2.74 × 10^−05^	Uncharacterized
a_HQ_transcript/58336	Nose resistant to fluoxetine protein 6	−2.57	2.74 × 10^−05^	GO:0006869 lipid transport
a_HQ_transcript/22218	Steroid 17-alpha-hydroxylase	−3.72	5.25 × 10^−05^	GO:0042446 hormone biosynthetic process
a_HQ_transcript/92691	Adenylate kinase isoenzyme 1	−4.21	5.58 × 10^−05^	GO:0046034 ATP metabolic process
a_HQ_transcript/79902	Beta-1,4-galactosyltransferase galt-1	−3.66	5.62 × 10^−05^	GO:0070085 glycosylation
a_HQ_transcript/35228	Steroid 17-alpha-hydroxylase	−7.11	6.74 × 10^−05^	GO:0006694 steroid biosynthetic process
a_HQ_transcript/95521	S-crystallin SL11	−5.73	9.66 × 10^−05^	GO:0005212 structural constituent of eye lens
(**b**) Top 20 up-regulated genes in pre-bloom compared with post-bloom razor clams				
**Transcript_ID**	**Description**	**logFC**	**adj.P.Val**	**Representative GO ID and Function**
a_HQ_transcript/135178	Uncharacterized	5.34	0.00	Uncharacterized
a_HQ_transcript/107478	Tubulin alpha-1A chain	7.34	0.00	GO:0071277 cellular response to calcium ion
a_HQ_transcript/70915	Dynein beta chain	3.18	0.01	GO:0030030 cell projection organization
a_HQ_transcript/101470	Lysosomal-associated transmembrane protein 4A	4.10	0.01	GO:0005765 lysosomal membrane
a_HQ_transcript/136081	NADH dehydrogenase [ubiquinone] 1 beta subcomplex subunit 3	4.16	0.01	GO:0032981 mitochondrial respiratory chain complex I assembly
a_HQ_transcript/90430	Uncharacterized	2.03	0.01	Uncharacterized
a_HQ_transcript/62043	U2 small nuclear ribonucleoprotein A	4.00	0.01	GO:0000398 mRNA splicing, via spliceosome
a_HQ_transcript/140232	Peptidyl-prolyl cis-trans isomerase	4.54	0.01	GO:0006457 protein folding
a_HQ_transcript/131746	Uncharacterized	3.63	0.01	Uncharacterized
a_HQ_transcript/119340	Syntaxin-12	5.99	0.01	GO:0033344 cholesterol efflux
a_HQ_transcript/114019	Uncharacterized	3.73	0.01	Uncharacterized
a_HQ_transcript/80242	Soluble calcium-activated nucleotidase 1	4.47	0.01	GO:0030166 proteoglycan biosynthetic process
a_HQ_transcript/60560	Uncharacterized	1.68	0.02	Uncharacterized
a_HQ_transcript/57283	Fibropellin-3	2.92	0.02	GO:0005509 calcium ion binding
a_HQ_transcript/112742	Poly [ADP-ribose] polymerase 2	2.44	0.02	GO:0006284 base-excision repair
a_HQ_transcript/115926	Uncharacterized	5.11	0.02	Uncharacterized
a_HQ_transcript/135559	Uncharacterized	1.53	0.02	Uncharacterized
a_HQ_transcript/107872	Fascin	4.93	0.02	GO:0030036 actin cytoskeleton organization
a_HQ_transcript/73908	Uncharacterized	3.86	0.02	Uncharacterized
a_HQ_transcript/101113	N-alpha-acetyltransferase 40	3.24	0.03	GO:0006629 lipid metabolic process

**Table 4:** REVIGO table of the superclusters representing the top 50 significantly enriched biological processes associated with DA exposure in Pacific razor clam (*Siliqua patula*) gill tissue for pre-bloom (6 April 2022) vs. bloom (8 November 2022), bloom vs. post-bloom (13 April 2023), and pre-bloom vs. post-bloom comparisons.

Supercluster	Bloom vs. Pre-Bloom	Bloom vs. Post-Bloom	Pre-Bloom vs. Post-Bloom
Basement membrane assembly			X
Binding of sperm to zona pellucida		X	
Cell adhesion	X		
Cellular organofluorine metabolic process		X	
Cerebellar cortex morphogenesis	X	X	
Cilium assembly		X	
Cilium movement involved in cell motility	X	X	
Cilium-dependent cell motility			X
Collagen fibril organization	X		
Complement activation, classical pathway		X	
Defense response to Gram-positive bacterium	X		X
Epithelial cilium movement involved in extracellular fluid movement			X
Gonad morphogenesis			X
Homophilic cell adhesion via plasma membrane adhesion molecules	X	X	X
Immune effector process	X		
Locomotion		X	
Maintenance of location in cell	X		
Mitochondrial transmembrane transport		X	
Negative regulation of necroptotic process			X
Nucleoside triphosphate biosynthetic process		X	X
Positive regulation of anion channel activity			X
Positive regulation of epithelial cell differentiation	X		
Protein galactosylation	X	X	
Retinoic acid catabolic process	X		
Viral genome replication			X

## Data Availability

The original contributions presented in this study are included in the article/Supplementary Materials. Further inquiries can be directed to the corresponding author(s).

## Supplementary Materials

The following supporting information can be downloaded at https://www.mdpi.com/article/10.3390/toxins17040194/s1: Supplementary File S1. Differential Gene Expression values (Transcript ID, log FC, Average Expression, t value, P value, adjusted P value, and B value) for razor clam bloom vs. pre-bloom samples. Supplementary File S2. Differential Gene Expression values (Transcript ID, log FC, Average Expression, t value, P value, adjusted P value, and B value) for razor clam pre-bloom vs. post-bloom samples. Supplementary File S3. Differential Gene Expression values (Transcript ID, log FC, Average Expression, t value, P value, adjusted P value, and B value) for razor clam bloom vs. post-bloom samples. Supplementary File S4. Functional categories assigned to DEGs. Supplementary File S5. Environmental data collected at the time of sampling.
