# Supplementary material for: Transcriptomics as an Early Warning of Domoic Acid Exposure in Pacific Razor Clams (Siliqua patula)
Source: Toxins (Basel). 2025 Apr 11;17(4):194. doi: 10.3390/toxins17040194 (PMC12031274; doi:10.3390/toxins17040194)
Supplement: Supplementary file 1 [file Supplementary File S1. bloom vs pre-bloom.pdf › Supplementary File S1. bloom vs pre-bloom.pdf]

Supplementary File S1. Differential Gene Expression values (Transcript ID, log FC, Average Expression, t value, P value, adjusted P value, and B value) for razor clam bloom vs. pre-bloom samples.

Transcript_ID	logFC	AveExpr	t	P.Value	adj.P.Val	B
a_HQ_transcript/79367	-4.82636415195852	-0.635081666507411	-10.7242093402647	3.18935511898983e-12	1.85390834356641e-07	15.513310372443
a_HQ_transcript/131580	-4.7335059059033	-0.663421011542513	-9.96576627586738	2.02310806283148e-11	5.87996127381341e-07	14.0484211653683
a_HQ_transcript/79902	-4.33806346583056	-0.433956745393014	-8.85874041955901	3.43251354995642e-10	4.56099945329989e-06	11.9667089487186
a_HQ_transcript/75321	-3.5222005522686	-0.718949747060596	-8.8227735003764	3.773344114401e-10	4.56099945329989e-06	11.7753552705064
a_HQ_transcript/122962	-4.50582754760238	-0.568058149532234	-8.80799174428493	3.92323790023731e-10	4.56099945329989e-06	11.621139199102
a_HQ_transcript/133860	-3.89419488251191	-0.702438422772455	-7.49551841895427	1.39028469246497e-08	0.000125198132079533	8.87317564081969
a_HQ_transcript/141107	-4.24707900451526	0.967925269374051	-7.46654545189305	1.50768463486914e-08	0.000125198132079533	7.88731439210387
a_HQ_transcript/145194	-5.52052807344657	-0.355350496250831	-7.13091723496524	3.88155314755571e-08	0.000282033651701398	8.12950737828569
a_HQ_transcript/118139	6.79788981385947	0.211863343642185	7.01991505908363	5.32036719183397e-08	0.000336995893042733	7.90444560425847
a_HQ_transcript/34261	-2.56004266487178	4.96248036288928	-6.98975444125236	5.79747958028373e-08	0.000336995893042733	8.26333326775231
a_HQ_transcript/48432	-6.27428145921098	-0.357750321668499	-6.80809753175624	9.74244444583374e-08	0.000514826191588567	7.21934538989907
a_HQ_transcript/123862	-3.87600781880918	-0.609327696419562	-6.71467981982322	1.27379244687451e-07	0.000617025061266013	7.02030298765683
a_HQ_transcript/60848	-3.64920422564462	-0.531145402215251	-6.67107946344986	1.44394688408097e-07	0.00064564418829122	6.89206644787745
a_HQ_transcript/108352	-3.02793086292033	-0.721329883259728	-6.59609556116464	1.7920856004423e-07	0.000744073941303645	6.53918037560753
a_HQ_transcript/98226	-2.44021371209504	5.14605637708665	-6.4616673005311	2.64250936471795e-07	0.0010240252290155	6.86539450420323
a_HQ_transcript/49343	-3.91353002917631	-0.33365300045546	-6.36918071089915	3.45454856806668e-07	0.00125503749477862	5.96565204169056
a_HQ_transcript/33295	-4.11631734552856	-0.180984139802306	-6.2422463258988	4.99486450659818e-07	0.00170789108258552	5.99963135732621
a_HQ_transcript/53710	3.01689592337017	2.16555175186338	6.14392312280069	6.65061855887154e-07	0.00211452366159117	5.73136819731963
a_HQ_transcript/138234	5.0057114559935	1.53452389582318	6.13071704428316	6.91163459438346e-07	0.00211452366159117	5.60992067622593
a_HQ_transcript/102242	-2.16609656330572	5.80058899474801	-6.06406726439397	8.39500982672064e-07	0.00234948612159169	5.78065992816262
a_HQ_transcript/76594	-5.20553750680739	1.83731124271816	-6.05102440092323	8.72082102864625e-07	0.00234948612159169	5.31074221656552
a_HQ_transcript/66242	-4.46654681565156	3.57604687805596	-6.04435840138128	8.89221970049154e-07	0.00234948612159169	5.53368438412512
a_HQ_transcript/135178	5.39339992602946	0.484900470760079	5.95046606822854	1.16995377384948e-06	0.00283605056477038	5.18475277047509
a_HQ_transcript/114723	3.01881628085941	4.13856618085362	5.95017384923413	1.17095399040891e-06	0.00283605056477038	5.38804785186627
a_HQ_transcript/41636	3.52289351071742	-0.0392623948241094	5.79032528542335	1.86971678444541e-06	0.00434731588984971	4.69392463176767
a_HQ_transcript/7159	-1.70411722965451	3.28779169983435	-5.75349574617333	2.082848416048e-06	0.00465660818184763	4.83345295433335
a_HQ_transcript/22985	-4.65396410474909	-0.206997450037532	-5.70765495671623	2.38250912103274e-06	0.00512927741434782	4.18048994452302
a_HQ_transcript/91960	-5.39491415905828	-0.213604041511221	-5.68957428293624	2.51227135914867e-06	0.00521547534159264	4.54867194286726
a_HQ_transcript/113447	-4.81868291837634	-0.5146548048098	-5.63244983098901	2.97068410773664e-06	0.00571047276418769	4.3260289904389
a_HQ_transcript/147165	-4.46871898114141	-0.117843247084957	-5.60888342439136	3.18342306323175e-06	0.00571047276418769	4.1026246515583
a_HQ_transcript/152513	-3.61516824974847	-0.417952952153582	-5.60484735798916	3.22135963155061e-06	0.00571047276418769	4.30294433811274
a_HQ_transcript/86802	-3.99453426596597	-0.275290269662923	-5.60414069183141	3.22804831731737e-06	0.00571047276418769	4.18919179311882
a_HQ_transcript/51541	3.8499898651123	1.87901076571752	5.58919374328572	3.37282940613352e-06	0.00571047276418769	4.2395139306591
a_HQ_transcript/152158	1.42421208571993	6.78005724023949	5.58747082087515	3.38993102697854e-06	0.00571047276418769	4.42305816374335
a_HQ_transcript/70915	3.23648757853506	-0.448088062605346	5.58263597786117	3.43838677997814e-06	0.00571047276418769	4.30834466942068
a_HQ_transcript/114092	-4.51463125112426	-0.522692033621738	-5.56649047089961	3.60528466149789e-06	0.00582133296676526	4.14317115122172
a_HQ_transcript/105284	3.91657671204859	-0.0869311327277208	5.54674280139901	3.82049191321269e-06	0.00600209605219534	4.09801515869281
a_HQ_transcript/84715	-3.94099609908466	0.419343577774182	-5.52214358767453	4.10665486505067e-06	0.00616512276979613	3.81837664618175
a_HQ_transcript/14699	-3.95734318432449	-0.0116256591485831	-5.51968704810467	4.13638501276578e-06	0.00616512276979613	4.04914318146224
a_HQ_transcript/101470	4.15378445126313	-0.628658497946083	5.47792066505204	4.67614901347127e-06	0.00679537974637645	3.9242379614443
a_HQ_transcript/72041	-3.60024377173333	-0.535814678882066	-5.39375950877634	5.98740749053515e-06	0.00848868347828847	3.72106549723893
a_HQ_transcript/133941	-3.36073571837051	3.72042937018381	-5.37867331798762	6.2586756019723e-06	0.00866200703312967	3.85588103755446
a_HQ_transcript/108929	-3.29968933188488	0.590367307439213	-5.36296550541139	6.55418728876787e-06	0.00867065783710106	3.61276385503954
a_HQ_transcript/64014	-5.59894016365938	0.0649184940469495	-5.36018556495337	6.60792077353441e-06	0.00867065783710106	3.74052305791488
a_HQ_transcript/21477	-2.91512267969367	4.6205154870355	-5.35484329502647	6.71242091022481e-06	0.00867065783710106	3.8199507354699

a_HQ_transcript/7957	-6.46933580431793	0.484731296961551	-5.34030646012605	7.0052148379143e-06	0.00868202404456815	3.63182461274884
a_HQ_transcript/87882	-3.84306238639854	-0.225267978392436	-5.33881270143934	7.03601538703208e-06	0.00868202404456815	3.56769641224617
a_HQ_transcript/60314	-3.84311384721265	0.137906351513572	-5.33242306693891	7.16930144060128e-06	0.00868202404456815	3.33237173071819
a_HQ_transcript/79577	4.40769316294134	2.85389516685434	5.30086090976444	7.86562252762579e-06	0.00933087563848637	3.60978126018157
a_HQ_transcript/149006	4.75653847778261	0.19716484757054	5.28139948780599	8.32824671045639e-06	0.00968208649570818	3.43426193456561
a_HQ_transcript/16534	-3.25281049947133	1.3445274787515	-5.24696016554344	9.21450444515246e-06	0.0105023669487808	2.99793382633783
a_HQ_transcript/141915	-3.99078905997741	-0.297641686820032	-5.21010024046201	1.02676086794795e-05	0.0113217099018119	3.26090318282467
a_HQ_transcript/38496	-4.89510110729879	0.81822932127722	-5.20827011660615	1.03229188135844e-05	0.0113217099018119	3.24249762664684
a_HQ_transcript/58336	-1.72927814972074	3.11861714951177	-5.19246340212627	1.08131745055372e-05	0.0116285277678491	3.34016750726655
a_HQ_transcript/125181	-6.18815150689849	-0.160414790893547	-5.18654149614416	1.10027702179965e-05	0.0116285277678491	3.201852041555
a_HQ_transcript/130597	2.92888951097439	-0.198006455453038	5.15947409756485	1.19124666324444e-05	0.0122167530104784	3.10331945141534
a_HQ_transcript/138347	-4.7979825930836	-0.311340340399027	-5.15699912756826	1.19992999147579e-05	0.0122167530104784	3.13087237140207
a_HQ_transcript/10309	-4.1956452867853	-0.200356676938866	-5.15162995778197	1.21898512697452e-05	0.0122167530104784	3.08819259732785
a_HQ_transcript/134402	-5.71395130537636	1.0064524672045	-5.13972334984238	1.26232504821259e-05	0.0124366831190681	3.08325137297879
a_HQ_transcript/61128	-2.3039648211658	2.87377873468814	-5.12599676911359	1.31419961922459e-05	0.0125890040371773	3.0704479560012
a_HQ_transcript/119977	-3.75302689304245	1.20816070268976	-5.12421158691301	1.32110040990197e-05	0.0125890040371773	2.91031218408873
a_HQ_transcript/95361	3.70961693598818	0.294295765842848	5.11378755376937	1.36212290966559e-05	0.0127705613698454	2.70891109826559
a_HQ_transcript/68297	-3.30519798001588	0.597602534038179	-5.09584834126665	1.43571505281761e-05	0.0130952309181399	2.6516868849946
a_HQ_transcript/150765	-2.91465909380978	2.95595375052447	-5.09440416285862	1.4418090743892e-05	0.0130952309181399	3.07894388330421
a_HQ_transcript/10793	-4.7437035817738	-0.0803394432176864	-5.07687243066265	1.51787632124537e-05	0.0135740176617463	2.81569458981014
a_HQ_transcript/144370	-3.0839422488388	6.04793452237314	-5.06257275860072	1.58287116132877e-05	0.0139407780099573	2.99730815593117
a_HQ_transcript/113201	-3.61443274091275	-0.415547323160588	-5.04302900168592	1.67620777643617e-05	0.0145424784520421	2.85416661160235
a_HQ_transcript/107942	-4.33581842977419	0.155423523123545	-4.9966898990359	1.91999994821293e-05	0.0164126113220178	2.70039405078931
a_HQ_transcript/110997	5.24510883564346	0.538608703896695	4.98221951937952	2.00313193364308e-05	0.0168750801505515	2.67397079249587
a_HQ_transcript/3769	-1.41761741830633	3.12274499544304	-4.97116395873535	2.06905123458187e-05	0.0171814014519679	2.7547959393962
a_HQ_transcript/139351	-2.9513356195698	2.54282895694462	-4.92792524813317	2.34825235735708e-05	0.0191653146456137	2.57974474235737
a_HQ_transcript/45335	-3.54056869358869	-0.307597445910407	-4.91858943425593	2.41328922044801e-05	0.0191653146456137	2.39368479597137
a_HQ_transcript/15918	-3.95054677891273	0.0525427977209765	-4.91655951873837	2.42766574706333e-05	0.0191653146456137	2.3945262597352
a_HQ_transcript/104320	-4.93727122674128	-0.173232446788346	-4.91484913705903	2.43984531340389e-05	0.0191653146456137	2.51712339672263
a_HQ_transcript/65118	-2.5231436029391	1.40741501649825	-4.90223589710687	2.53155958564843e-05	0.0194208584030001	2.21848369591069
a_HQ_transcript/62554	1.75970222914683	3.63245379741918	4.89950883373271	2.55183502893509e-05	0.0194208584030001	2.56864038159475
a_HQ_transcript/97980	-1.72921506543341	2.80902843530591	-4.88885750287632	2.63258493110591e-05	0.0194208584030001	2.46450648009815
a_HQ_transcript/128228	-3.91941150160451	-0.392981869522164	-4.88495912319349	2.66276998193678e-05	0.0194208584030001	2.39333177780423
a_HQ_transcript/93493	-2.5540268220037	4.56167307165769	-4.88423128190036	2.66844362635913e-05	0.0194208584030001	2.5319284535704
a_HQ_transcript/84948	4.2025713550831	1.21233368766258	4.87951370872127	2.70551033141685e-05	0.0194208584030001	2.3858344661005
a_HQ_transcript/96323	-1.68638129150527	2.72398882307808	-4.87942010895612	2.70625091288709e-05	0.0194208584030001	2.43672222307024
a_HQ_transcript/93341	-3.90478321320263	0.285831909147395	-4.86357612901021	2.83455960585316e-05	0.0200935708254918	2.19157537642659
a_HQ_transcript/139434	-5.62923516381778	-0.0666270334310415	-4.84212517224009	3.01795608181289e-05	0.0211358736293518	2.27310560483209
a_HQ_transcript/91398	5.34113390281011	0.911710689357241	4.81782373719059	3.23999586310744e-05	0.0224207713727035	2.31008978606629
a_HQ_transcript/151861	-4.04305495044831	-0.0229913321702823	-4.81223521641696	3.29330937356682e-05	0.0225215867372579	2.23851570542058
a_HQ_transcript/36309	-7.80881111264416	1.37323091670793	-4.80375486972675	3.37587851898227e-05	0.0225640413782968	2.30221356836381
a_HQ_transcript/137269	-3.81289288317976	-0.356251571476699	-4.80064164836968	3.40670347118876e-05	0.0225640413782968	2.21704948538456
a_HQ_transcript/135047	4.41652677208457	0.462520160341094	4.79971112055817	3.41597103167169e-05	0.0225640413782968	2.19689047158544
a_HQ_transcript/117566	-5.66891596663281	-0.327324054662147	-4.78371014601624	3.57930436970449e-05	0.0232519226458134	2.18174833683118
a_HQ_transcript/40062	-4.46391861567029	-0.279792072277588	-4.78172398270022	3.60011188777045e-05	0.0232519226458134	2.12635490974614
a_HQ_transcript/150310	-8.15341102969839	0.607206036726857	-4.77289436631911	3.69407558108759e-05	0.0235966181733472	2.19171843371018
a_HQ_transcript/7408	2.53006693675762	-0.810811751688042	4.75261626375502	3.91918512232427e-05	0.0247624339989636	2.07056697245482
a_HQ_transcript/86660	-3.77021193846042	5.45706332630578	-4.74419821112759	4.01658474326251e-05	0.0251049503178885	2.1563847505359
a_HQ_transcript/45953	-3.82019418566126	-0.285465669061069	-4.69150264303923	4.68321877787462e-05	0.0289602277787549	1.92972262219892
a_HQ_transcript/152550	0.648421391482667	11.1182790651046	4.68079114681653	4.83158056686541e-05	0.0293979954592815	1.52179719570015

a_HQ_transcript/104293	-6.21550749160569	-0.0257753145920844	-4.67384625470204	4.93025134186189e-05	0.0293979954592815	1.89483746708376
a_HQ_transcript/90430	1.81957729877903	5.01372702198626	4.67322347642484	4.93919651953424e-05	0.0293979954592815	1.9324372174943
a_HQ_transcript/16078	-3.9654262308724	-0.539909101766448	-4.67203515354254	4.95630944641066e-05	0.0293979954592815	1.89238693285111
a_HQ_transcript/61459	-3.20488217970128	1.34202999446122	-4.66166150304009	5.10821603961325e-05	0.0299929678738019	1.61211383848551
a_HQ_transcript/132228	-5.79258826366346	0.197734663073961	-4.65135117479679	5.26376386658021e-05	0.0305972066036574	1.86746820083298
a_HQ_transcript/134114	-4.8349754422705	0.14912714193675	-4.62841318445306	5.6268483675757e-05	0.032218579486354	1.81237484351467
a_HQ_transcript/111966	-5.24854781555384	1.22661426339283	-4.62678466665919	5.65354924925699e-05	0.032218579486354	1.76772015340963
a_HQ_transcript/145496	-3.76998561286788	1.80951738329361	-4.62181879260865	5.73574572352991e-05	0.0323696531473152	1.82656588778733
a_HQ_transcript/94439	5.6381208170644	0.689423716049023	4.61468048399496	5.85597758540903e-05	0.0326337677513451	1.79287659335355
a_HQ_transcript/9025	4.49555163352127	0.490140431580227	4.61240487573185	5.8948279897661e-05	0.0326337677513451	1.68669289413642
a_HQ_transcript/141208	4.36512687203729	-0.132645711253313	4.59044613041726	6.28307514182547e-05	0.0344549614947199	1.69180679157454
a_HQ_transcript/103117	-4.55087848580485	1.12904764021098	-4.57820082684495	6.5104617144015e-05	0.0353682353770776	1.56828529229714
a_HQ_transcript/17025	4.65354145767303	0.791000148918188	4.56526288676265	6.75955749941942e-05	0.0363814405857641	1.62814901067321
a_HQ_transcript/6210	-3.34579721743303	-0.502949709856226	-4.55120086962928	7.04099312728905e-05	0.0375485182112897	1.56304154192473
a_HQ_transcript/66096	2.383910469004	-0.822597907086957	4.54328654636131	7.20445350803018e-05	0.0380709521377071	1.5443503645728
a_HQ_transcript/77421	-1.80205979107148	3.52413368284294	-4.53675729876799	7.34212705194965e-05	0.0384489334482639	1.60276176111816
a_HQ_transcript/146850	1.3798553168615	3.16598710466506	4.5275594784235	7.54049217470319e-05	0.038992219400695	1.58169974677698
a_HQ_transcript/133467	-0.996568691419844	3.76623892788093	-4.52575472575044	7.58003164099666e-05	0.038992219400695	1.5707014593385
a_HQ_transcript/19170	-2.80014576709287	-0.481267269716107	-4.50149671762732	8.13180313567525e-05	0.0414636361991694	1.42908705885994
a_HQ_transcript/119826	-5.00447428450114	0.371406008741976	-4.49595378924028	8.26334958369656e-05	0.0417679986609664	1.47358191599867
a_HQ_transcript/120643	2.65349647964244	3.62658286932305	4.48160639817757	8.61368916079528e-05	0.0428400715038541	1.46090802984074
a_HQ_transcript/128603	-1.86797562013477	3.24776159174336	-4.48123914770908	8.62284676223323e-05	0.0428400715038541	1.45927130845369
a_HQ_transcript/115362	-3.36359328850472	0.00907514567694103	-4.46231951986794	9.10785515050256e-05	0.0443600927294051	1.37143976749911
a_HQ_transcript/82126	2.02654779729852	-0.756109324733384	4.46035339446689	9.15977662202287e-05	0.0443600927294051	1.35555833206239
a_HQ_transcript/9687	-2.77610055686374	4.35034479092834	-4.45786078132864	9.22602187077616e-05	0.0443600927294051	1.37093778007889
a_HQ_transcript/49067	2.34709699446384	1.23465829233036	4.45755973787542	9.23405453526359e-05	0.0443600927294051	1.17428946188315
a_HQ_transcript/62111	-1.97528711342318	3.76777027217547	-4.4513759858583	9.40058998733791e-05	0.044423673077094	1.38085006131551
a_HQ_transcript/150782	-5.18532952385089	0.35988805736438	-4.44951451804134	9.4512995472391e-05	0.044423673077094	1.33679033436377
a_HQ_transcript/84204	-2.08684644276382	-0.758590781835724	-4.44859088065167	9.47656114361351e-05	0.044423673077094	1.33141800925886
a_HQ_transcript/73739	3.61652195487594	-0.401085480706151	4.42508526709749	0.000101423530511807	0.0471643758527226	1.25819525947129
a_HQ_transcript/146751	0.981157752965258	8.97542690122055	4.41390625565908	0.000104749894670551	0.0483246180746808	0.944663309920283
a_HQ_transcript/96011	3.22409925593187	0.539180317833389	4.40405780560486	0.00010776936498023	0.0493261232092191	1.052365522019
a_HQ_transcript/68413	-5.68865621314156	0.556677424698185	-4.39823122228788	0.000109596009337494	0.0497702877403893	1.18084073144031
a_HQ_transcript/123601	4.24635780559809	6.13080723064274	4.38806842584271	0.000112855420500227	0.050840031773096	1.20182512228369
a_HQ_transcript/118932	-2.21325795865697	-0.788995815682508	-4.38547960257065	0.000113700869297111	0.050840031773096	1.16665769053783
a_HQ_transcript/110068	-2.72459392505866	-0.740420798614906	-4.3819258800491	0.000114871609728512	0.0509714269488471	1.14875029774527
a_HQ_transcript/132199	-3.51225428355393	-0.34278539044225	-4.37637988042705	0.00011672247526116	0.0514003336513692	1.16763527285216
a_HQ_transcript/8680	-4.6627072155368	0.763810850700416	-4.36151936326696	0.000121828033464153	0.053245262625596	1.06351459558164
a_HQ_transcript/128312	4.03171636318426	-0.286184926203821	4.35249944949809	0.000125033652461802	0.0535127482813019	1.06565449246112
a_HQ_transcript/37063	-4.10970504393452	-0.237459334225681	-4.35178448201962	0.000125291273849965	0.0535127482813019	1.08137669575378
a_HQ_transcript/141218	-1.37972003370838	3.60940193035952	-4.35053082894607	0.000125744260910456	0.0535127482813019	1.11728747157508
a_HQ_transcript/76138	3.93349122126073	0.0613702787056159	4.34556114080938	0.000127555909181296	0.0535127482813019	0.939610385142172
a_HQ_transcript/150751	0.6389869524342	11.3713790172458	4.34536224026959	0.000127628949058899	0.0535127482813019	0.538222969897886
a_HQ_transcript/44579	-5.46729135448149	-0.251261063420382	-4.3404173506842	0.000129458107036715	0.0535127482813019	1.04446429974673
a_HQ_transcript/25345	-3.23572587111184	0.435909734431057	-4.33975541520277	0.000129704914593127	0.0535127482813019	0.975452883346779
a_HQ_transcript/127299	-2.37218505864582	3.30019777251091	-4.33653038441039	0.000130914037812201	0.0535127482813019	1.07961503949694
a_HQ_transcript/38749	-4.61953249723303	0.30483119501361	-4.33364752693908	0.000132004268715514	0.0535127482813019	0.976872783912567
a_HQ_transcript/111815	1.98220794775745	4.66931044676028	4.33359632144916	0.000132023714080141	0.0535127482813019	1.02130928563989
a_HQ_transcript/104414	3.60268018789595	0.879895918565103	4.3321695307974	0.000132566676171681	0.0535127482813019	0.9582135300721
a_HQ_transcript/121392	4.19097716677653	4.35399889487758	4.32635964607472	0.000134800419875117	0.054039164182764	1.01430537928707

a_HQ_transcript/59762	3.83183132038776	0.128022035228718	4.32378079625809	0.00013580375864801	0.0540684991965171	0.913621923475115
a_HQ_transcript/89147	1.16511396395373	3.84029266842845	4.31755680610034	0.000138255687161096	0.0542942257308593	1.01027161662429
a_HQ_transcript/112044	-4.13192488544582	0.725525924865289	-4.31709594261306	0.000138438967221541	0.0542942257308593	1.02811687561672
a_HQ_transcript/108656	-6.43140178585557	0.0708451691603729	-4.31429648035436	0.000139557432657489	0.0542942257308593	0.994211547057635
a_HQ_transcript/135029	-3.51600047071874	0.177199938540398	-4.31292927507437	0.000140106899594496	0.0542942257308593	0.98056316641306
a_HQ_transcript/152307	0.661852501351248	11.3015658412455	4.30729830896211	0.000142392472734479	0.0548145010272171	0.444231107592175
a_HQ_transcript/3864	-1.55558473750868	4.0799346235528	-4.30192937579582	0.000144605857619803	0.0553003242876573	0.951188113066226
a_HQ_transcript/139533	6.05178397174585	0.769540411713427	4.28507438143043	0.000151776834384791	0.0576632930007787	0.923425576685356
a_HQ_transcript/7777	-2.82618523880297	-0.510399116877064	-4.28139441320627	0.000153388424515881	0.0578971580536308	0.899466483676439
a_HQ_transcript/46521	3.39194141541633	-0.454764068876786	4.26543029819473	0.000160576852213428	0.060205637309463	0.860329787287487
a_HQ_transcript/24702	2.60341019014532	-0.721815363212724	4.26326762208041	0.000161575822671969	0.060205637309463	0.847239966908482
a_HQ_transcript/118088	-3.99062449396589	-0.316337857585814	-4.26099604435792	0.000162631679264721	0.0602130844095524	0.846186624672173
a_HQ_transcript/61619	4.32941662048643	0.408944576751481	4.24393970154793	0.000170779426575059	0.062829534860475	0.805007955256978
a_HQ_transcript/79095	-2.441313211043	-0.784961027564212	-4.23272977694736	0.000176351594978851	0.0644714812134004	0.772816008228263
a_HQ_transcript/27739	5.66504584403821	0.0804643526006884	4.22794146537916	0.000178785870919974	0.0649529069052264	0.777276106808764
a_HQ_transcript/57741	-5.72583025934915	1.11130072342566	-4.22334762586561	0.000181152314130202	0.0652839173352336	0.786395138267314
a_HQ_transcript/111564	-3.46228014622431	-0.435313314734951	-4.2218254581079	0.000181943204794726	0.0652839173352336	0.660620999508877
a_HQ_transcript/25486	-1.05189782622149	4.12741469041592	-4.21803869748052	0.000183925489641216	0.0655903120359791	0.758522341289753
a_HQ_transcript/133278	-2.71139289880046	-0.752533068621888	-4.21459890521218	0.000185744532625977	0.0658351109297732	0.749086861481143
a_HQ_transcript/103916	-3.76548644846484	-0.332011754401766	-4.20618681236441	0.000190267859269936	0.0670296371129869	0.715911052193571
a_HQ_transcript/28622	-4.25958044238802	0.157744552283701	-4.20146853457705	0.000192852156761251	0.0675307841458915	0.727148952407712
a_HQ_transcript/80504	3.37185121276746	1.49244930389979	4.19650319550241	0.000195609016041725	0.0676988907093885	0.715526309163442
a_HQ_transcript/124735	3.02264802078409	1.78671798316128	4.19640927524675	0.000195661533842163	0.0676988907093885	0.693273690543784
a_HQ_transcript/142002	-3.95435126921898	0.609717661685521	-4.1939873222274	0.000197020622218724	0.0677657676232544	0.510543617933617
a_HQ_transcript/147389	3.67822669816109	1.02998612496236	4.18558870262734	0.00020180576082249	0.0690033250887628	0.651728707837128
a_HQ_transcript/141706	-2.44409927941932	-0.87608748586883	-4.18034082888463	0.000204853505253694	0.06956923485644	0.643352912314282
a_HQ_transcript/113538	6.42649896198392	0.66054716329608	4.17848410577117	0.000205942600950567	0.06956923485644	0.666900780574768
a_HQ_transcript/18636	-3.46783330592265	-0.553810800190859	-4.17651999746103	0.000207100874172733	0.06956923485644	0.640382084413624
a_HQ_transcript/104817	2.34635273739559	-0.164028662976738	4.17458518879727	0.000208248122505859	0.06956923485644	0.629115683683962
a_HQ_transcript/33824	-3.59748338130854	-0.505250522603711	-4.17166629865919	0.000209990701660998	0.069750511463717	0.624386203235898
a_HQ_transcript/151643	-2.4843341678993	-0.874308408868962	-4.16616653115376	0.000213313054310507	0.0699249546817901	0.608968733620043
a_HQ_transcript/146023	-2.25274133585189	-0.883790641179142	-4.16580388811418	0.00021353392776885	0.0699249546817901	0.604899248112058
a_HQ_transcript/137388	-2.67985270377423	-0.668039416878115	-4.16483569165834	0.00021412472359893	0.0699249546817901	0.627331315793715
a_HQ_transcript/149075	-2.41501413224126	-0.801961837119902	-4.15956424662907	0.00021736963452727	0.0699744946908674	0.594076306486749
a_HQ_transcript/74281	-3.55471659654863	2.21928107210851	-4.1558112861592	0.000219709188261066	0.0699744946908674	0.539850307451515
a_HQ_transcript/118352	-4.11219017798592	2.67912558548757	-4.15546371292455	0.000219927107916676	0.0699744946908674	0.60915610934231
a_HQ_transcript/150084	0.907162002055349	9.44685449199647	4.15545154309491	0.000219934741935965	0.0699744946908674	0.208445979926742
a_HQ_transcript/121500	-2.3334715060203	-0.880155257123607	-4.15487702775402	0.000220295426101513	0.0699744946908674	0.579216967737649
a_HQ_transcript/71185	-4.13877065572285	-0.101749658524449	-4.15224559883232	0.000221954886010767	0.0701184435545318	0.599632710973357
a_HQ_transcript/104522	-5.5792814477185	0.215060120018818	-4.14904600579797	0.000223989182125952	0.0703786117763099	0.574782470757057
a_HQ_transcript/69200	-2.41793002229334	-0.876361181855213	-4.13371418639315	0.000233993825033489	0.0731268444169174	0.528321202748465
a_HQ_transcript/39600	-3.17051788181863	0.183337286467848	-4.12708911598113	0.000238451417477182	0.0741214117385756	0.501672297107606
a_HQ_transcript/74225	-2.5499506555481	-0.863793117187421	-4.12365257423937	0.000240796353473945	0.074452183163476	0.505011189961182
a_HQ_transcript/91820	-2.39542502649255	-0.863764969796631	-4.11670873534498	0.000245603737938415	0.0755367940681704	0.486165371256361
a_HQ_transcript/130323	-8.0884426751712	0.277758515482276	-4.11166848574683	0.000249152054815037	0.076202379245842	0.491331247444583
a_HQ_transcript/78367	-2.30652745711577	-0.856930620891761	-4.10992695434893	0.000250389733621591	0.076202379245842	0.468273472203128
a_HQ_transcript/146668	0.606310039952653	10.1690936090227	4.10750465819438	0.000252121250681312	0.0763297086437672	0.0135337975313146
a_HQ_transcript/142864	4.11931875649581	-0.139649813451738	4.09943927247524	0.000257971637037218	0.0773472171290898	0.464201564778233
a_HQ_transcript/12544	-3.09885571277838	0.205876109105444	-4.09920518453867	0.000258143409768845	0.0773472171290898	0.465360189240384
a_HQ_transcript/79537	2.31736287173214	-0.750286793997967	4.08978961573294	0.000265146245003961	0.0787890634477312	0.415453198812604

a_HQ_transcript/22664	3.58161802506569	1.50610831401627	4.08910000632492	0.000265666398908535	0.0787890634477312	0.420778838882335
a_HQ_transcript/126548	2.81060558486041	-0.715021304056201	4.07879409504145	0.000273560064983613	0.0807182713571952	0.394028256619617
a_HQ_transcript/131090	-4.34455558335548	1.41721874597731	-4.07644843825246	0.000275388545013011	0.0808474007298802	0.344690511983123
a_HQ_transcript/44113	4.18120583171865	1.5554428809958	4.0743098052669	0.000277066079494464	0.0809311410495187	0.298378928982612
a_HQ_transcript/138268	5.11079767125807	1.66347098067858	4.06979703535734	0.000280638796815128	0.0813008426901608	0.374871759906283
a_HQ_transcript/137581	-1.6080102776505	-0.797475100847019	-4.06685436492008	0.000282992751354318	0.0813008426901608	0.370187593255269
a_HQ_transcript/122717	-2.58711876549467	-0.868785322522082	-4.06206085014613	0.000286868745918074	0.0813008426901608	0.354281218050415
a_HQ_transcript/140433	-2.7361079315062	1.74830557242684	-4.06176719689583	0.000287107874431589	0.0813008426901608	0.366311645114332
a_HQ_transcript/1104	-1.30882801248939	4.75192415476932	-4.05919348514954	0.000289212078148157	0.0813008426901608	0.294230529939335
a_HQ_transcript/175	2.66048538071846	3.44212541272994	4.05624207835991	0.000291643672061615	0.0813008426901608	0.352531130163678
a_HQ_transcript/150401	-3.35194354383995	4.54339001997534	-4.05540668514195	0.000292335560907966	0.0813008426901608	0.275762989711101
a_HQ_transcript/4580	3.13675406714653	-0.137905505164463	4.05208535036182	0.000295102290397759	0.0813008426901608	0.305708938352146
a_HQ_transcript/10911	-1.80247418291131	2.02697939210488	-4.05038852790366	0.000296525653289859	0.0813008426901608	0.267056144308666
a_HQ_transcript/36469	3.1387449214249	-0.435034440019614	4.04979198581861	0.000297027652267656	0.0813008426901608	0.335620463353003
a_HQ_transcript/99370	2.86679909309068	1.79484417953802	4.04944507110936	0.0002973199687304	0.0813008426901608	0.30901262304759
a_HQ_transcript/18767	-3.96925362929306	0.0200001211249429	-4.04922887680425	0.000297502280333665	0.0813008426901608	0.328145578629336
a_HQ_transcript/135061	6.55214177376289	2.18967698527701	4.04791766410304	0.000298610342944063	0.0813008426901608	0.334261199184898
a_HQ_transcript/100332	-5.06228309273288	0.464155404467543	-4.04721325654041	0.000299207282168921	0.0813008426901608	0.310031923757141
a_HQ_transcript/99016	-3.81044698798385	0.0727762463134926	-4.0470903875721	0.00029931152518054	0.0813008426901608	0.312487179649203
a_HQ_transcript/63058	-4.29069214026158	0.483199468118627	-4.04090883160085	0.00030460215733697	0.0820693342587054	0.265077945171213
a_HQ_transcript/22643	-2.08440941824143	3.48314055182867	-4.0394494275808	0.00030586452112066	0.0820693342587054	0.312109437771802
a_HQ_transcript/151611	0.64276057843967	10.5795535332608	4.03808830362817	0.000307046489309689	0.0820693342587054	-0.219893703723353
a_HQ_transcript/125971	3.51776867272711	4.8411034197085	4.03723673883796	0.000307788240923441	0.0820693342587054	0.250762221715571
a_HQ_transcript/122014	0.725687673370902	6.46884583457073	4.03120017658342	0.000313096885995927	0.0827256682277599	0.107912439897773
a_HQ_transcript/150400	0.597625049727821	10.6730497226356	4.03048577681928	0.000313731040504613	0.0827256682277599	-0.252483069178692
a_HQ_transcript/124861	-2.57618420570179	-0.220681268593106	-4.02849901151245	0.000315501258758797	0.0827256682277599	0.227710877406156
a_HQ_transcript/67896	-3.67857554220331	-0.18402334014433	-4.02800563321527	0.000315942374527985	0.0827256682277599	0.24806798246859
a_HQ_transcript/152746	3.47897313323285	5.25306092881334	4.02272445414121	0.000320702103899335	0.0835953896657425	0.260012006780542
a_HQ_transcript/43654	4.27091036702253	0.963659035774034	4.02035268190501	0.000322862461091924	0.0837828086533541	0.253151500523018
a_HQ_transcript/71767	-2.1636281167692	-0.847325487626962	-4.01586651081932	0.000326987756293056	0.0844475289985452	0.229063079071408
a_HQ_transcript/144359	5.46798252576073	1.04357052616772	4.01441924102726	0.000328329575310886	0.0844475289985452	0.234638809513487
a_HQ_transcript/92596	2.38140833355902	-0.736992740101095	4.01280069576075	0.000329836572481895	0.0844614109481392	0.227991650991127
a_HQ_transcript/2272	-2.29139187668958	1.85559474029028	-4.00723901095307	0.000335066713011958	0.0848317596534569	0.179079295996718
a_HQ_transcript/18762	-1.37161245092875	3.96692844143605	-4.00716830879414	0.000335133719964989	0.0848317596534569	0.197622384862029
a_HQ_transcript/113730	6.68942491572575	2.17339719122656	4.00661239486887	0.000335661036338685	0.0848317596534569	0.211415829420251
a_HQ_transcript/96656	-3.50600900607277	0.555842927022215	-4.00410260321616	0.000338051839921187	0.0850661357183497	0.179881004425764
a_HQ_transcript/149772	4.72295532692528	0.293051140878907	3.99860519324282	0.000343346971394505	0.0859625101491573	0.20132021635588
a_HQ_transcript/26454	3.31817265193088	0.571011436923702	3.99590458173628	0.000345977843605282	0.0859625101491573	0.139196721370315
a_HQ_transcript/15379	2.19564021812528	3.32489770332158	3.99583021590256	0.000346050567280877	0.0859625101491573	0.20486265880344
a_HQ_transcript/152866	-3.36880882834126	-0.333295194286172	-3.99348033847247	0.00034835627010485	0.0861605227741358	0.200510185882493
a_HQ_transcript/12110	3.5896425109387	0.486421716078627	3.99149877286983	0.000350312255852716	0.0861605227741358	0.184005543669803
a_HQ_transcript/1242	-1.42140344963791	3.57140981932085	-3.98907467064666	0.000352719675451598	0.0861605227741358	0.165167779578526
a_HQ_transcript/78823	-1.73102212820283	2.76233598355347	-3.9863722087255	0.000355422612353531	0.0861605227741358	0.18466013496561
a_HQ_transcript/58481	3.11983419358387	0.342933942793215	3.98623841682924	0.000355556952633886	0.0861605227741358	0.153944019098229
a_HQ_transcript/145676	4.24728565266548	-0.133950962721917	3.98605498494396	0.000355741217069099	0.0861605227741358	0.173323807973216
a_HQ_transcript/141222	2.67634088290261	4.39347191229227	3.97725321828182	0.000364693513625723	0.0877295438377231	0.079227970947076
a_HQ_transcript/8273	4.20292650915324	2.8098858542665	3.97672480480519	0.000365237916472767	0.0877295438377231	0.159492660584656
a_HQ_transcript/55891	1.03347830851617	2.78088471824576	3.96774523161253	0.000374611579754402	0.0890812580859204	0.137526178471691
a_HQ_transcript/60560	1.34101127437836	4.39688825615139	3.96726344238326	0.000375121105549039	0.0890812580859204	0.0687524981811167
a_HQ_transcript/62537	-4.95713482234335	-0.0890696367580293	-3.96694059949852	0.00037546291341609	0.0890812580859204	0.120584790846234

a_HQ_transcript/59392	-2.0109751425426	1.93036126468611	-3.96440745571649	0.000378155448475402	0.0893553654836511	0.0931493151539655
a_HQ_transcript/124985	-2.96550219288594	-0.333708127300771	-3.95108070819782	0.00039263448583667	0.0912091544995049	0.0857495675385405
a_HQ_transcript/105029	-2.39363185434579	-0.722773300856166	-3.95080031455578	0.000392944868795261	0.0912091544995049	0.0881606760386573
a_HQ_transcript/72446	-2.09417774224501	4.38406251876546	-3.95046070942846	0.000393321117163143	0.0912091544995049	0.0572578686858289
a_HQ_transcript/18283	5.75747861068173	1.19117604938879	3.95016456461849	0.000393649503129559	0.0912091544995049	0.0777357129078915
a_HQ_transcript/131462	1.41939045297502	3.31315568323478	3.94998720421559	0.000393846300911363	0.0912091544995049	0.0862679570421943
a_HQ_transcript/1462	1.63621282494897	4.65725224599906	3.94590358962484	0.000398404125048019	0.091845398952784	0.00290994789051791
a_HQ_transcript/119311	-3.57585398005928	0.117533386564774	-3.94470313961301	0.000399753749226781	0.091845398952784	0.0533756314490565
a_HQ_transcript/8048	-2.43607865320685	-0.342240039111848	-3.9427815342517	0.000401923444721407	0.0919803385620706	0.00385954649662867
a_HQ_transcript/81764	6.01795178555361	3.93287216330815	3.94123084538585	0.000403682719073318	0.0920206631148778	0.0451148535420423
a_HQ_transcript/44509	4.37337806093827	1.55754151490414	3.9351223171958	0.00041068637633811	0.093251475327272	0.0141496761897963
a_HQ_transcript/29459	-3.34185564494465	-0.10497610809266	-3.92936854072031	0.000417391747950682	0.0940567740387987	0.038035210384245
a_HQ_transcript/139051	2.79741964858818	2.75069587792049	3.9289621762183	0.000417869340505719	0.0940567740387987	0.0318970915953072
a_HQ_transcript/122843	4.37456449793958	1.28451579277317	3.92792794298041	0.000419087263901199	0.0940567740387987	0.0381025344879742
a_HQ_transcript/135559	1.24152375265839	3.96075426711963	3.9219244427334	0.000426225799091252	0.0952909740368318	-0.0259440714774728
a_HQ_transcript/147574	4.9183739345526	0.57958623422573	3.92017516353587	0.000428328041705339	0.0953940705296857	-0.00124690870449751
a_HQ_transcript/105405	-4.15276121414988	0.257196868134334	-3.91772918823925	0.00043128453824193	0.095685907018805	0.000321135964577834
a_HQ_transcript/75138	-4.72679213980568	0.256703529367295	-3.90961456669039	0.000441236199335233	0.0975215885739865	-0.00761649668575082
a_HQ_transcript/36045	-2.93101233569728	-0.854567457313395	-3.90763846195614	0.000443693398305306	0.0976932191541319	-0.0208670660098669
a_HQ_transcript/145193	-8.99846764164841	1.1848572621725	-3.90225405847997	0.000450456630038619	0.098808086758056	-0.0277489131597282
a_HQ_transcript/28460	-3.00238721329415	3.69708598323494	-3.90056383215539	0.000452600356966764	0.0989050885329476	-0.0623029124542454
a_HQ_transcript/111594	3.01718067075941	1.89525173296024	3.88824139104548	0.000468532647229886	0.102003242390183	-0.117365763720776
a_HQ_transcript/23487	2.38324883704132	-0.103762205004484	3.88492622709981	0.000472911553742626	0.102572398492356	-0.111647401783743
a_HQ_transcript/128310	3.17578499999531	1.34758796619019	3.87267203419671	0.000489446921049008	0.105758258387557	-0.184034906431431
a_HQ_transcript/24735	-1.78598411642438	3.42115179030048	-3.87061792932348	0.000492273282490554	0.105758258387557	-0.131416799047201
a_HQ_transcript/52370	-1.44506949631203	-0.780932968893372	-3.87004950545865	0.000493058216746285	0.105758258387557	-0.110984871852891
a_HQ_transcript/39793	-2.27957372955703	-0.833006015604848	-3.86795354097476	0.00049596308522118	0.105767096897044	-0.12736240600788
a_HQ_transcript/131005	-4.63446128027597	1.4941941449399	-3.86648007990976	0.000498015186090219	0.105767096897044	-0.115979856811692
a_HQ_transcript/51635	-5.10878816258638	0.226554793858516	-3.86517488792754	0.000499839849350358	0.105767096897044	-0.119154953000652
a_HQ_transcript/50793	-4.2225361621877	1.66706734126596	-3.86431187150995	0.000501049927263249	0.105767096897044	-0.119806450433708
a_HQ_transcript/134030	3.04521016585162	-0.572066308817819	3.86334282873932	0.000502412073790675	0.105767096897044	-0.12561755567722
a_HQ_transcript/61033	-2.39369819765314	-0.456581982394408	-3.8615267622396	0.000504974577950517	0.105767096897044	-0.152254656197317
a_HQ_transcript/37259	-3.30057496355057	0.434339966970085	-3.86091806512436	0.000505836308446499	0.105767096897044	-0.231845920454372
a_HQ_transcript/125531	-4.52019934220686	-0.0597066764795738	-3.85832665392041	0.000509521029619413	0.106155693224793	-0.135134591045651
a_HQ_transcript/19952	-3.32953330782374	1.70011758063452	-3.8545651758576	0.000514916053554466	0.106896572717907	-0.168476560920396
a_HQ_transcript/37021	-2.20287926075393	2.77210606744151	-3.85025395140151	0.000521168059080045	0.107809455296103	-0.154632243593229
a_HQ_transcript/29553	3.67823917329153	0.575733965990473	3.84700994490681	0.000525921130856356	0.108406891824178	-0.174750991086171
a_HQ_transcript/139656	-5.3322676933426	0.383084528807095	-3.84128206112419	0.00053441688003853	0.10870772843682	-0.182502065157796
a_HQ_transcript/57908	4.78184870132881	0.21404782806651	3.83993760055253	0.000536430313023767	0.10870772843682	-0.185828272378924
a_HQ_transcript/7649	2.91084053749445	2.64518665178	3.83763141397868	0.000539901228249146	0.10870772843682	-0.188008984016624
a_HQ_transcript/73927	4.23073085886152	1.51525980334215	3.83759959791325	0.000539949265537986	0.10870772843682	-0.200883611613631
a_HQ_transcript/149255	5.71842846820869	1.03241956281322	3.83726056249138	0.000540461414721168	0.10870772843682	-0.186235098351033
a_HQ_transcript/8617	3.28750417168623	0.897140938059447	3.8367848443189	0.0005411808357357	0.10870772843682	-0.308122791115176
a_HQ_transcript/78892	2.98980665561294	-0.626200737953787	3.83572201169583	0.000542791512606488	0.10870772843682	-0.197540219461411
a_HQ_transcript/133232	7.83711547043378	1.84041998132949	3.83566523179977	0.000542877691358312	0.10870772843682	-0.190225288842933
a_HQ_transcript/141853	3.88733393242998	-0.688145721857639	3.83478729495767	0.000544211894011743	0.10870772843682	-0.197740688077451
a_HQ_transcript/90922	6.74845489817342	1.39589634938861	3.83230342631104	0.00054800396023203	0.109090322603998	-0.199843648342922
a_HQ_transcript/146160	0.905839812419851	6.43192468000786	3.82712500142787	0.000555992688244581	0.110302877072631	-0.44037119437503
a_HQ_transcript/98995	3.61905722521287	0.115050662994042	3.82427443950503	0.000560438519412474	0.110806701552409	-0.259025295598695
a_HQ_transcript/61604	-3.87281543621088	0.0276477821066253	-3.8210834532209	0.000565456359980632	0.111107448628181	-0.251278907278516

a_HQ_transcript/81914	3.25616459441535	1.27361481309715	3.82087701102776	0.000565782493702547	0.111107448628181	-0.250364270370403
a_HQ_transcript/152143	0.55596327871866	11.4804402593886	3.81782262602214	0.000570629212772154	0.111681935622962	-0.923984932127101
a_HQ_transcript/28118	1.78309247929863	2.56538280058768	3.81538134096804	0.000574532107121639	0.112068464170358	-0.251233088211261
a_HQ_transcript/16467	-1.70703451005366	2.91490914604922	-3.81011010111475	0.000583048111850254	0.113349232928534	-0.252955136340213
a_HQ_transcript/108706	4.67843021314726	0.348945288664174	3.80084479786986	0.000598315509379674	0.115492879012567	-0.28073902840458
a_HQ_transcript/96222	-2.70938481990059	-0.0208134362081223	-3.79935028967002	0.000600814334558902	0.115492879012567	-0.307372116238966
a_HQ_transcript/110640	2.56377380156749	3.37409714938222	3.79866225313103	0.000601968146532962	0.115492879012567	-0.284292060612653
a_HQ_transcript/50167	-0.981539334734272	3.06830696523158	-3.7982364823052	0.000602683228331057	0.115492879012567	-0.294252117676421
a_HQ_transcript/77900	5.62846049963818	1.30830507409233	3.79618434286921	0.000606141405238192	0.115492879012567	-0.288210938444123
a_HQ_transcript/56817	2.70970759485733	1.62187484020721	3.79497595138856	0.000608186759245784	0.115492879012567	-0.316105795791431
a_HQ_transcript/23195	3.05091236553516	0.309653868986922	3.79450577058169	0.000608984412838249	0.115492879012567	-0.311248952419124
a_HQ_transcript/109187	6.57105214066633	2.54169827406811	3.79392586407244	0.000609969616309833	0.115492879012567	-0.359859490125849
a_HQ_transcript/139521	3.08031436645715	1.03011588051256	3.79031068359495	0.000616146510466773	0.116283230313872	-0.331848498555099
a_HQ_transcript/149178	3.53954024215286	0.223597358567106	3.78914870896836	0.000618144752391042	0.116283230313872	-0.320393328196588
a_HQ_transcript/118243	-6.52567613054109	0.410821550500586	-3.78790993133535	0.000620282015956389	0.11630888072101	-0.31320912095233
a_HQ_transcript/140809	-3.42611438182713	0.236601576032631	-3.78347365687822	0.000627995055671312	0.117376516386052	-0.328205854218247
a_HQ_transcript/38696	-5.88835534712991	0.59449512282276	-3.7818401509007	0.000630858554905261	0.117533801536965	-0.325450123810858
a_HQ_transcript/137077	3.12454192106746	-0.630723316132006	3.77903238802646	0.000635810204425206	0.117825322629986	-0.333882850704029
a_HQ_transcript/151713	0.563572450436271	11.0267166558663	3.77865575925064	0.000636477279552294	0.117825322629986	-0.967974073783304
a_HQ_transcript/37167	4.50856103155122	-0.0764416365610908	3.77604213375918	0.000641125254436975	0.118277891721137	-0.340233327431688
a_HQ_transcript/30049	3.86140761057063	2.5608819730413	3.77499786068927	0.000642991566609541	0.118277891721137	-0.351681512071348
a_HQ_transcript/150843	0.743054961099317	10.5923776373038	3.76905783038301	0.000653708443964501	0.119869919339964	-0.951655136275463
a_HQ_transcript/90114	-2.33209382785431	2.2686343186492	-3.76618714828134	0.000658949727262006	0.120451036938006	-0.360463563924938
a_HQ_transcript/67722	3.46790040748794	3.77363754073229	3.76191579736965	0.00066682405208444	0.121216554342075	-0.372185111038585
a_HQ_transcript/93588	-3.49888435707109	2.48901874217388	-3.76165472001473	0.000667308309067302	0.121216554342075	-0.37673316037844
a_HQ_transcript/66184	2.23860356219258	-0.657415499671533	3.76029277783628	0.00066984004428791	0.121230444934635	-0.388152990525193
a_HQ_transcript/114019	2.7598269428707	1.66711044969896	3.7586469034351	0.000672912048173317	0.121230444934635	-0.403061210449301
a_HQ_transcript/145761	0.909091024809857	9.82268818421468	3.75765952030599	0.000674761554558967	0.121230444934635	-0.897698905243131
a_HQ_transcript/12317	-4.1927024092183	-0.273929345102581	-3.75714510252647	0.00067572708778595	0.121230444934635	-0.385036961650886
a_HQ_transcript/76273	4.18908911660179	1.63269811318871	3.7556141146463	0.000678608619073132	0.121286190578762	-0.38825480819825
a_HQ_transcript/5152	5.24122854966803	2.19703334416937	3.75476554722912	0.000680210881652157	0.121286190578762	-0.40766094200867
a_HQ_transcript/42438	-2.74361323713764	-0.527335423789143	-3.75112157695456	0.00068713331542069	0.122145826785241	-0.399591627788656
a_HQ_transcript/81336	2.59671299928509	1.39658398729016	3.74853491259377	0.000692088869250656	0.122538098472118	-0.406006272579609
a_HQ_transcript/128116	3.13641961730556	0.0177255591145662	3.74777234376213	0.000693556193182748	0.122538098472118	-0.424771523606818
a_HQ_transcript/125268	-5.7158990389545	1.08112696396047	-3.74450481740173	0.000699878589542345	0.123280432281568	-0.418626010356141
a_HQ_transcript/150636	0.615277537748263	11.1537946600316	3.74016811438897	0.000708356305645868	0.124051635680128	-1.09488999438322
a_HQ_transcript/113995	-1.31783221898575	4.19716829686885	-3.74008231680925	0.000708525031754103	0.124051635680128	-0.524455885414429
a_HQ_transcript/133357	3.6854154632569	1.17515322169947	3.73779417125951	0.000713039242447996	0.124259192430031	-0.444889476850241
a_HQ_transcript/26517	-1.44828303913811	2.95116316853579	-3.7373161371929	0.000713985863467352	0.124259192430031	-0.430735879587598
a_HQ_transcript/133757	-1.02473278838932	4.19245953946784	-3.7350183221854	0.000718553149044552	0.124680768500483	-0.533024358923155
a_HQ_transcript/142844	1.08348755646018	7.40346684706942	3.73306809906963	0.000722451795402725	0.124984160604671	-0.747485919000415
a_HQ_transcript/91809	-1.05638581336156	3.11197366733153	-3.72793358963481	0.000732814597124947	0.12619526388057	-0.474153217294926
a_HQ_transcript/28934	-1.59512429507274	3.05968492031727	-3.72745179113377	0.000733794370899269	0.12619526388057	-0.454726275930768
a_HQ_transcript/42985	6.46541834837856	3.1989223280056	3.72357807356208	0.000741718241710411	0.127008647582309	-0.4886944771096
a_HQ_transcript/103666	2.93521181809534	-0.490423080293469	3.72300673019931	0.000742893961223247	0.127008647582309	-0.469976409365217
a_HQ_transcript/17401	-5.83020448313982	1.42398384638313	-3.72167664881808	0.000745638039379988	0.127103952941583	-0.470105245273966
a_HQ_transcript/152319	0.563670439219617	11.2502111139063	3.7119689324929	0.000765966159192369	0.130187371057117	-1.17644785574914
a_HQ_transcript/121462	4.04607944933616	1.88191245781842	3.71087291670315	0.000768294759602346	0.130202442525263	-0.504668946531032
a_HQ_transcript/115559	-7.08412255266614	1.00267545391119	-3.7082332242788	0.000773931306337633	0.130776392368587	-0.51162232052494
a_HQ_transcript/47799	3.29173809962272	1.26427451111994	3.70695696637252	0.000776670881452015	0.130858913034907	-0.540610097843075

a_HQ_transcript/93267	-5.74651352485275	0.895913169009124	-3.70492727227945	0.00078104715936106	0.131215922772658	-0.542125498580945
a_HQ_transcript/145641	-2.16580219191166	2.56878910183902	-3.70367937954716	0.000783749646076509	0.131290488262638	-0.512363827338319
a_HQ_transcript/140415	3.74146421781478	1.68769228586744	3.70239279252219	0.00078654543263964	0.131380209507118	-0.557959224654017
a_HQ_transcript/25727	-3.76809970251638	0.222511014289561	-3.69712571734854	0.000798092184507435	0.132926941263748	-0.530379504375082
a_HQ_transcript/55367	-0.828999027047857	4.94534130083143	-3.69432141823792	0.000804306848095177	0.133579281331647	-0.660419174518573
a_HQ_transcript/1384	-2.90408523617085	0.0628052698247346	-3.69134299304879	0.000810958814391171	0.133996841916189	-0.544017172901804
a_HQ_transcript/24750	3.68335404240002	-0.0133194430120313	3.69113226496425	0.000811431467700567	0.133996841916189	-0.556124845496658
a_HQ_transcript/83146	4.93147703372927	2.05972612560551	3.68972887468342	0.000814586031164806	0.13413670487124	-0.55049878050086
a_HQ_transcript/140744	4.57518797504728	1.25145410122607	3.6876792809388	0.000819214529064823	0.134517802670847	-0.550788493043225
a_HQ_transcript/133449	5.95258054799008	1.20253125783492	3.68626817538729	0.000822415979745208	0.1346630875229	-0.558193974946082
a_HQ_transcript/21797	-2.07498477330305	-0.470318427587378	-3.68334923559134	0.000829076900723614	0.135121722382364	-0.56132640958096
a_HQ_transcript/22613	3.65937120000265	0.147946901974443	3.68221033535519	0.000831689999978213	0.135121722382364	-0.565179347504775
a_HQ_transcript/129089	-3.35978167095586	-0.63883822963645	-3.68199253812776	0.000832190624361521	0.135121722382364	-0.565479447025923
a_HQ_transcript/136253	2.30491804083458	-0.637015448373451	3.6777525934503	0.000841994911262616	0.136332813932795	-0.574696789551425
a_HQ_transcript/151422	3.6372315649754	-0.0859254712241455	3.66740548165704	0.000866393776194582	0.139403212761347	-0.607333303890856
a_HQ_transcript/118635	-5.1621682285824	0.411441758876412	-3.66672566966032	0.000868020577503103	0.139403212761347	-0.60383249322033
a_HQ_transcript/150630	-4.39176276625328	0.924828395441957	-3.66667063520358	0.000868152405374477	0.139403212761347	-0.602708873433796
a_HQ_transcript/113126	5.42772941761319	3.13648009931417	3.66566667684669	0.000870560666591595	0.1394048221147	-0.680691445237257
a_HQ_transcript/132595	3.4258814240797	-0.531118096860999	3.66391166253007	0.000874786101730292	0.139696611322468	-0.610342540851925
a_HQ_transcript/89181	3.40109200527645	-0.15921076446934	3.66094253505852	0.000881979993443967	0.140459542627153	-0.621148607267024
a_HQ_transcript/82032	1.81428955890767	2.24834355098723	3.65811701698226	0.000888879170628306	0.140623268066707	-0.626354836194962
a_HQ_transcript/72030	4.49358970948831	0.210780030500611	3.65724348565592	0.00089102267223954	0.140623268066707	-0.624078369327162
a_HQ_transcript/66998	6.22746116662225	0.0416359269929084	3.65703895696391	0.000891525275299103	0.140623268066707	-0.628080534532329
a_HQ_transcript/139239	-4.36896884474378	-0.11610175092083	-3.65641353618388	0.000893063872249774	0.140623268066707	-0.628009573257193
a_HQ_transcript/150778	0.551580359564879	11.0631424002664	3.65513015133566	0.000896229189474301	0.140623268066707	-1.30476657334321
a_HQ_transcript/117184	3.82301461277546	-0.244925247550908	3.65460672960882	0.000897523266803408	0.140623268066707	-0.63074755023922
a_HQ_transcript/26542	5.25360640994054	1.68735891206339	3.65168055990698	0.00090479118763168	0.141380919770576	-0.637406697672776
a_HQ_transcript/99504	3.09288942749532	3.9118048458859	3.64955640261826	0.000910102794490102	0.141513188945617	-0.677044541488423
a_HQ_transcript/83389	2.5593698828233	1.40864473581528	3.64939537463429	0.000910506686375941	0.141513188945617	-0.64536563512796
a_HQ_transcript/138056	-4.37026430373931	3.34143418395562	-3.64612654555106	0.000918743244993314	0.142412552919924	-0.701499905968861
a_HQ_transcript/119318	-3.71639682056499	-0.109638241464251	-3.64131132649077	0.000931007930559553	0.143298819190957	-0.668455531677294
a_HQ_transcript/101094	4.87672752267516	-0.104853626551314	3.64115607218676	0.000931406001798445	0.143298819190957	-0.665076722893565
a_HQ_transcript/53631	4.65475199730254	1.94070277559647	3.64049723305685	0.000933097097370295	0.143298819190957	-0.695771585995393
a_HQ_transcript/7587	7.38721896625548	3.46632845631937	3.63979747699941	0.000934896475178851	0.143298819190957	-0.716447753159412
a_HQ_transcript/147669	1.06500288186566	3.41502198644025	3.63899387184089	0.000936967041407763	0.143298819190957	-0.700519967952956
a_HQ_transcript/146707	1.04292973321729	8.51780477214996	3.63810898791783	0.000939252169552617	0.143298819190957	-1.08156424066599
a_HQ_transcript/150312	-4.45556273185056	0.83002446420306	-3.63697910125714	0.000942177843018008	0.143368883923955	-0.680676054949105
a_HQ_transcript/76282	2.71038918332194	1.13117050408028	3.63468702555773	0.000948139975158324	0.143828155912539	-0.684318753104985
a_HQ_transcript/151376	0.634317286431485	10.7586027512166	3.63391946187037	0.000950144712882175	0.143828155912539	-1.33051687079134
a_HQ_transcript/134227	3.97640997665897	1.04597844074065	3.63279709270406	0.000953088353150983	0.143898284466502	-0.684064539608716
a_HQ_transcript/123183	-2.23981928478304	-0.619278992475098	-3.6303030652467	0.000959645461827305	0.14451365648989	-0.692401449500529
a_HQ_transcript/21471	4.65054230243836	0.716220666338817	3.62677833711659	0.000968993892086131	0.145544384907449	-0.709962558386465
a_HQ_transcript/3685	1.34331643672465	3.38412587007607	3.62538355241306	0.000972717500862142	0.145727120850811	-0.739547620870111
a_HQ_transcript/76645	1.77516427797308	3.20221669592716	3.62258741581118	0.000980224027851161	0.146474196120648	-0.750957983548773
a_HQ_transcript/145301	1.04535114696975	4.17514827043528	3.62109723020319	0.000984247471864441	0.146698300114195	-0.807569605784349
a_HQ_transcript/102738	2.53409064641283	-0.231823745664619	3.61670074719633	0.000996211205571792	0.148101700658509	-0.747396139055149
a_HQ_transcript/22098	-3.33665455098505	-0.836500119813226	-3.61274221127786	0.00100710355211032	0.149339069584359	-0.730359577964133
a_HQ_transcript/80308	-0.945884023477468	3.39537095685775	-3.61110229755595	0.00101164964890445	0.149631477841012	-0.778793902369576
a_HQ_transcript/73786	3.94195714459223	2.38512710490362	3.60982322281757	0.00101520922595281	0.149776857579149	-0.748383133077723
a_HQ_transcript/110460	1.90305695756458	2.11560433682352	3.60533412363953	0.00102779826665868	0.151154675446562	-0.75433982559327

a_HQ_transcript/106473	3.68142362490721	0.52076936125152	3.60464327208259	0.0010297490276087	0.151154675446562	-0.757198601180072
a_HQ_transcript/145580	3.94815476377115	-0.161840114123825	3.60234835520105	0.0010362549504221	0.15126311577326	-0.755172281518051
a_HQ_transcript/123148	-7.16568772064905	0.404767546001654	-3.60177236471218	0.00103789407578345	0.15126311577326	-0.757047953022563
a_HQ_transcript/6968	-2.46308831478337	-0.781665089189109	-3.6016317859771	0.00103829450855923	0.15126311577326	-0.757488637302711
a_HQ_transcript/138045	1.34580224713581	5.05391494424227	3.60050894641948	0.00104149824125242	0.151350524418802	-0.920726943528584
a_HQ_transcript/135539	-6.35473361171707	0.613288804841466	-3.59653666127017	0.00105290908654747	0.152627180505813	-0.768915520995834
a_HQ_transcript/119756	3.50393731871871	1.12048140427902	3.59557289299656	0.00105569580268455	0.152650461737431	-0.78049583609733
a_HQ_transcript/87328	4.09489115363383	1.39301253778397	3.5937092594066	0.00106110471475252	0.153051848285694	-0.775784732263475
a_HQ_transcript/13257	-2.82721400450545	-0.3109342299502	-3.58430835028666	0.00108880069494004	0.156657937612561	-0.798436284908407
a_HQ_transcript/17604	1.60039523520505	3.96283722942871	3.58195959542828	0.00109582876986828	0.157279838851613	-0.894638357227864
a_HQ_transcript/146811	6.65231210155047	1.1546941461468	3.57989359627008	0.00110204701877588	0.157782731791636	-0.813446895077766
a_HQ_transcript/29487	-4.07177722892822	1.17442256756307	-3.57633815844868	0.00111282815660082	0.158639838218757	-0.820271607099954
a_HQ_transcript/120640	2.86724048052376	0.458293738239472	3.57612037472286	0.00111349184546609	0.158639838218757	-0.827621585815069
a_HQ_transcript/11309	-0.770569367153187	3.48786398871885	-3.57292806720208	0.00112326433005961	0.159449811569877	-0.887519708381254
a_HQ_transcript/62282	2.96160286765478	0.289510889051061	3.57247330544179	0.00112466320437052	0.159449811569877	-0.847268014133268
a_HQ_transcript/39479	-3.8658964308964	0.198675604979196	-3.57125379284524	0.0011284228171836	0.159593580333938	-0.839519258131312
a_HQ_transcript/54951	-2.61797241619153	1.91032895318548	-3.56510529557624	0.00114756370876639	0.161906755493137	-0.843370154537098
a_HQ_transcript/76922	3.36935239175033	-0.0516269031121331	3.55780676310163	0.00117069230181799	0.164769980920281	-0.866549477164636
a_HQ_transcript/8249	1.51223867234114	1.37706320643648	3.55589249211615	0.00117683268672698	0.165234131434941	-0.865185229319531
a_HQ_transcript/12760	3.06690996139523	1.16144885455498	3.55477881964606	0.00118041930823224	0.165338345901021	-0.873030770818374
a_HQ_transcript/74226	-3.41576551460219	0.60198115203413	-3.55234703557436	0.00118828770283239	0.166040354784234	-0.874018397530737
a_HQ_transcript/76865	4.53783496245896	1.16318975140537	3.54939327283822	0.00119791320888437	0.166535507907889	-0.88158378615339
a_HQ_transcript/24483	2.17762578516512	1.28331627015184	3.54811730884547	0.00120209448550326	0.166535507907889	-0.905827226663889
a_HQ_transcript/61704	3.91622635923892	1.05688608542473	3.54779174939028	0.00120316358473511	0.166535507907889	-0.884827450914528
a_HQ_transcript/149699	4.18054291404175	0.872795902799154	3.54660622038329	0.00120706449143112	0.166535507907889	-0.928515774270028
a_HQ_transcript/112744	3.1413770366461	-0.765340094775649	3.54639774359818	0.00120775173280788	0.166535507907889	-0.887517906755857
a_HQ_transcript/151047	3.01553113649249	-0.632760287316694	3.54601294786465	0.00120902120040478	0.166535507907889	-0.888444414390468
a_HQ_transcript/143840	-5.01249653980685	1.03213512981729	-3.54512964419051	0.00121194015722506	0.16654292543541	-0.905322764408332
a_HQ_transcript/2807	-1.27123824516438	2.2372717988717	-3.53687548010429	0.00123954782537101	0.169637898273631	-0.91855171805916
a_HQ_transcript/21389	-0.944092044916954	2.89031487727696	-3.53665335007538	0.00124029911172401	0.169637898273631	-0.917723289717255
a_HQ_transcript/126172	1.74576624532159	1.56057941702021	3.53455775452665	0.00124740849388375	0.170209767447123	-0.93439039836055
a_HQ_transcript/9578	2.29088084656399	-0.745170907631604	3.53304477732586	0.00125256576662734	0.170363652286175	-0.919033307876155
a_HQ_transcript/53847	-1.63412157229307	1.57815876304481	-3.53250873594121	0.00125439793522025	0.170363652286175	-0.941878747691227
a_HQ_transcript/15450	2.82804002660532	0.545838658668681	3.53066258245751	0.00126072768684688	0.170676110381787	-0.938248099978988
a_HQ_transcript/116250	2.09595782354734	-0.637885894301649	3.53012663990979	0.00126257100647138	0.170676110381787	-0.927161164708528
a_HQ_transcript/32810	-1.32891231355181	3.05735796254023	-3.52469388823187	0.00128140387464675	0.172820056671616	-0.962101008304733
a_HQ_transcript/124897	3.3673215309059	-0.157388788960999	3.52162122856655	0.0012921751401265	0.173805065915459	-0.955071008700547
a_HQ_transcript/87734	-5.01740796713201	0.541375272803474	-3.51962431363448	0.00129922216063024	0.173805065915459	-0.950819097089581
a_HQ_transcript/63481	5.28075432684537	2.37221371809165	3.51954807224313	0.00129949194678531	0.173805065915459	-0.958233260540172
a_HQ_transcript/72471	-2.90631424811981	1.17197265627676	-3.51921602417888	0.0013006675556225	0.173805065915459	-0.964782007339341
a_HQ_transcript/137781	-5.18657808133771	0.639985715983226	-3.51593950413522	0.0013123231737249	0.174960370280461	-0.966512003892482
a_HQ_transcript/136797	1.84022642513516	-0.838967395247612	3.51425637421235	0.00131834972375498	0.17525591834465	-0.963040192203453
a_HQ_transcript/17156	-2.51687391886995	-0.0211199201034729	-3.51363815702267	0.00132056998752678	0.17525591834465	-0.968478550480071
a_HQ_transcript/60388	-3.1291651189693	2.04650051354734	-3.51203233836027	0.00132635399223864	0.175442514221059	-0.975863509818693
a_HQ_transcript/30722	4.20907223517311	0.0677963240828536	3.51157315243671	0.00132801242529015	0.175442514221059	-0.973134497024673
a_HQ_transcript/139578	-1.95432258205696	-0.828893916546048	-3.5105965735306	0.00133154617207246	0.175510466871265	-0.971898218978785
a_HQ_transcript/43715	-3.27044111425239	0.108470564374149	-3.50743319379953	0.00134305524964073	0.176626958260445	-0.981686177944769
a_HQ_transcript/69121	2.12843349323333	0.9237838550546	3.50418704327659	0.00135496521474796	0.177437982339255	-0.996994444346859
a_HQ_transcript/12366	-3.0674013184203	-0.250368650219673	-3.50341897662131	0.00135779808296342	0.177437982339255	-0.988362704989589
a_HQ_transcript/1516	-3.3474832943319	2.45863296268301	-3.5032316534905	0.00135848985496092	0.177437982339255	-0.998984552026024

a_HQ_transcript/104840	1.87042679795573	2.27342471435832	3.5020689379206	0.00136279129742788	0.177437982339255	-0.996535819543328
a_HQ_transcript/81947	2.83876580527061	0.131622079057636	3.50154305483917	0.00136474110312946	0.177437982339255	-0.993071289072443
a_HQ_transcript/20832	3.38583161731975	-0.149320404693588	3.50079011804985	0.00136753743614069	0.177437982339255	-1.00231360304334
a_HQ_transcript/28374	3.80509798964188	2.09226758989569	3.49934867003886	0.00137290625739977	0.177737850623906	-1.01580061805317
a_HQ_transcript/93615	-4.3632630241327	0.155032642877145	-3.49793981801982	0.00137817331336982	0.178023240799024	-1.00170380038124
a_HQ_transcript/47944	4.68560409794391	1.75961603383632	3.49529370704166	0.00138811864048547	0.178546378782233	-1.00755066362997
a_HQ_transcript/35950	-2.73485701136856	2.17551452180639	-3.49522801533312	0.00138836641910214	0.178546378782233	-1.01524906683262
a_HQ_transcript/54847	4.32724191566257	0.93977761474199	3.4920572680931	0.00140037681503614	0.179693385219472	-1.01501094111322
a_HQ_transcript/5796	-2.99256437034826	0.021670670063453	-3.49113304335576	0.00140389648001384	0.179748225969701	-1.0215297647728
a_HQ_transcript/140930	-4.53330713532173	0.176712885739091	-3.48834822684326	0.00141455336342586	0.180714632767514	-1.02372483201077
a_HQ_transcript/29244	-5.64304082769487	1.36581677164864	-3.48708627962848	0.0014194082001261	0.180755004905623	-1.05106194643847
a_HQ_transcript/110048	2.8422479222963	-0.62182913577816	3.48665045638152	0.00142108858453533	0.180755004905623	-1.0275096547943
a_HQ_transcript/81274	4.46770396718658	2.06151164892272	3.48583564232593	0.00142423537328434	0.180759724406708	-1.02970002275558
a_HQ_transcript/3740	-1.69768815007348	3.09943870432845	-3.48439019557421	0.00142983418365955	0.181021191484146	-1.04738175210483
a_HQ_transcript/101901	4.54035954660544	0.844929220539708	3.48369773503649	0.0014325238797603	0.181021191484146	-1.03792336821089
a_HQ_transcript/122419	2.4127793227039	-0.683537671192475	3.48041454855183	0.00144534315201624	0.181851545422129	-1.04201292976135
a_HQ_transcript/48996	2.91637750907892	0.268341850892705	3.4804123231951	0.00144535187835507	0.181851545422129	-1.04254916587773
a_HQ_transcript/150752	-3.78486055273639	0.0975356589527258	-3.47736993342693	0.0014573296805727	0.182575821007022	-1.04909261361152
a_HQ_transcript/123687	3.69269099169299	1.79286045125112	3.47645093141823	0.00146096654037763	0.182575821007022	-1.05920654851956
a_HQ_transcript/93097	-2.39985459254309	-0.789028286237927	-3.47644682293635	0.00146098281891281	0.182575821007022	-1.05588216459833
a_HQ_transcript/85485	2.78216091969689	-0.418783856430265	3.47510378337264	0.00146631355931015	0.182575821007022	-1.05442987583279
a_HQ_transcript/19457	-3.1947051480695	2.38368649668913	-3.47456744346485	0.00146844761221687	0.182575821007022	-1.06207794668796
a_HQ_transcript/101542	-5.82657830622338	0.285614888714687	-3.47418931140263	0.00146995396764531	0.182575821007022	-1.05779853534434
a_HQ_transcript/41344	3.67633990873942	0.991263204919926	3.47314789924745	0.00147411031792	0.182701672835935	-1.0609360228827
a_HQ_transcript/7314	3.99179776185609	0.326776693304067	3.47180771518918	0.00147947575555179	0.182701792471181	-1.07989449599701
a_HQ_transcript/139753	-3.34661451563271	0.252150316270012	-3.47157795947965	0.00148039747202598	0.182701792471181	-1.06361635473015
a_HQ_transcript/85246	-2.07887941759443	-0.442149394999655	-3.46821497078979	0.00149395234260504	0.183645490185374	-1.07039962427358
a_HQ_transcript/134148	3.9582429467321	-0.355089412784535	3.46750426208283	0.00149683219437777	0.183645490185374	-1.07231374423535
a_HQ_transcript/144563	1.50480865464211	3.44729377340236	3.46733420793616	0.00149752206076017	0.183645490185374	-1.14537779114277
a_HQ_transcript/53674	3.14921111215649	-0.60139242171414	3.46598160470723	0.0015030201467196	0.183931694923194	-1.07584804497816
a_HQ_transcript/22072	4.11858814719346	0.491348421570578	3.46518500509386	0.00150626727326134	0.183941815252385	-1.08767187028833
a_HQ_transcript/35753	-3.17021311739049	-0.745722149456915	-3.46208651795076	0.00151896181823877	0.185103171007512	-1.08574566063591
a_HQ_transcript/4797	-3.49240112368333	3.0849331886457	-3.46064226276673	0.00152491410788171	0.185439764148427	-1.08958393774306
a_HQ_transcript/29614	3.50786776857713	0.0192269427452337	3.45887186836369	0.00153224118844263	0.185941786642574	-1.092513158519
a_HQ_transcript/77868	2.41166442192946	1.73390188736211	3.45600928004638	0.00154416029596921	0.186507758432011	-1.09937146383879
a_HQ_transcript/88101	3.21658616358595	1.02926255670406	3.4558765363831	0.0015447151685809	0.186507758432011	-1.10541193944854
a_HQ_transcript/85782	-4.1855538490496	1.30322926982843	-3.45544251018197	0.00154653075220598	0.186507758432011	-1.105369182934
a_HQ_transcript/147086	0.848155953255038	4.78122170784228	3.45443278357481	0.00155076251519044	0.186630897480311	-1.29569638711749
a_HQ_transcript/112680	4.0074784860239	0.74196678909842	3.45135345535711	0.00156373694787805	0.187760741619561	-1.11907909466973
a_HQ_transcript/39679	-3.66976086031022	0.12535427985625	-3.45067468088449	0.00156661092219735	0.187760741619561	-1.11730861425844
a_HQ_transcript/23099	-1.24873473417563	2.24311658084314	-3.44942183805764	0.0015719289015802	0.188010459240852	-1.11597112287187
a_HQ_transcript/88795	3.74932870494053	-0.125413138962578	3.44864382736623	0.00157524009106777	0.188019622204492	-1.12238669853962
a_HQ_transcript/68245	-2.47846814013364	-0.694773313358569	-3.44763321172347	0.00157955126891042	0.188147860982018	-1.11856455438025
a_HQ_transcript/6106	-1.37831011694065	2.37419550773845	-3.44626217279581	0.00158541813748168	0.188328672026366	-1.122458953185
a_HQ_transcript/65027	2.89116153045208	-0.199255447387923	3.44576541264243	0.0015875490175633	0.188328672026366	-1.13405160141455
a_HQ_transcript/20776	2.68842993367278	-0.396992528146668	3.44426993249936	0.00159398062032974	0.188416930678809	-1.12620652363945
a_HQ_transcript/140018	-5.0296918452697	0.598543786647138	-3.44408543176664	0.00159477583770255	0.188416930678809	-1.13706639330842
a_HQ_transcript/149561	-0.825232888723005	4.68727051004167	-3.44175933725247	0.00160483436576293	0.18922071402245	-1.27482188419584
a_HQ_transcript/52393	2.91739344960845	0.272427466950051	3.44016192038817	0.00161177727712169	0.189654634746011	-1.1361723114614
a_HQ_transcript/72167	1.50654785995683	2.06753636714097	3.43917579891491	0.00161607771672308	0.189776495995311	-1.13884801891187

a_HQ_transcript/22387	2.28098180021308	1.5534409541615	3.43717352913882	0.0016248435458233	0.190421180708905	-1.14634543682385
a_HQ_transcript/2516	1.9773834571654	2.73609043273441	3.43204588157964	0.00164750126610103	0.192345935351324	-1.15957835129059
a_HQ_transcript/76949	-1.86851530016255	2.84708914887523	-3.43195956095284	0.0016478852842857	0.192345935351324	-1.16997147185172
a_HQ_transcript/11903	-3.91674890036135	1.08774767311427	-3.42957781790302	0.0016585150847607	0.192591448142393	-1.16473832289324
a_HQ_transcript/57283	2.04129113342105	0.651609081932315	3.42941606032706	0.00165923940203067	0.192591448142393	-1.18200010182355
a_HQ_transcript/127365	-1.45301282694254	4.54824810568363	-3.42926226310929	0.0016599283567186	0.192591448142393	-1.33841476501262
a_HQ_transcript/126638	-2.50431782777468	0.74215525025657	-3.42808996799852	0.00166518885829768	0.192816928197466	-1.16624443373814
a_HQ_transcript/116290	-2.87100299499045	0.601640809685978	-3.42680563553444	0.00167097052373766	0.192930342445625	-1.16616919467613
a_HQ_transcript/98628	3.00308225072507	2.18530008396637	3.42565279354012	0.00167617670532122	0.192930342445625	-1.16882954169267
a_HQ_transcript/113674	-3.83254990282974	0.578514764979022	-3.42488112171464	0.0016796702552076	0.192930342445625	-1.1731299321147
a_HQ_transcript/107245	-3.58872680299366	2.14680587881222	-3.42434493542599	0.00168210182726274	0.192930342445625	-1.18322676647894
a_HQ_transcript/43265	-1.67762440698849	0.75081015387653	-3.42384782275765	0.001684359227854	0.192930342445625	-1.19012933045136
a_HQ_transcript/81625	-0.993005841032659	3.57336221223891	-3.42346872486234	0.0016860826789564	0.192930342445625	-1.2321411441554
a_HQ_transcript/147702	-2.92852572474822	-0.216569611951782	-3.42232288257929	0.00169130221502562	0.193147377514753	-1.17664500580038
a_HQ_transcript/132233	-1.09121554136477	2.90605645120205	-3.41890232906058	0.00170697612516638	0.194555114124845	-1.22323430124104
a_HQ_transcript/67063	2.51324817435315	-0.607134042101055	3.41564875152347	0.00172201449429732	0.195885046036233	-1.19203717857714
a_HQ_transcript/15440	3.07545215250391	1.42721144284445	3.41439042037025	0.00172786473227902	0.196166642886552	-1.19578126105101
a_HQ_transcript/141110	-3.57230990578509	-0.825002274992967	-3.41271850563406	0.0017356673808511	0.196591061206664	-1.20150438679408
a_HQ_transcript/10306	-1.56505744906607	3.76311048000667	-3.41154938628939	0.00174114365613378	0.196591061206664	-1.27828829781894
a_HQ_transcript/131358	-7.29006028337828	0.435842246217098	-3.4108072710071	0.00174462841709118	0.196591061206664	-1.20465629027817
a_HQ_transcript/137239	4.24613782512455	1.88337206762696	3.41020518688006	0.00174746055862378	0.196591061206664	-1.20448275981471
a_HQ_transcript/111908	4.94442544484465	0.437368509385275	3.40998163306947	0.00174851325770447	0.196591061206664	-1.20511804832551
a_HQ_transcript/106960	3.57817758394134	0.96100396871538	3.40713706442096	0.00176196148738743	0.197720651233314	-1.21152059646081
a_HQ_transcript/65866	-4.90708053326272	0.192397405266128	-3.40416429705939	0.00177612205130021	0.198288898567602	-1.23577659047959
a_HQ_transcript/56168	-3.12368792425725	0.271553830687574	-3.40343459143225	0.00177961463979751	0.198288898567602	-1.22079072735447
a_HQ_transcript/150140	0.561200794461262	10.3833025852604	3.40329241743852	0.00178029589496315	0.198288898567602	-1.88462849690929
a_HQ_transcript/404	-2.76618911979619	-0.746964000510233	-3.40321429689871	0.0017806703318932	0.198288898567602	-1.22200346866397
a_HQ_transcript/146794	-6.0749509887417	0.640412469479781	-3.40174654252167	0.00178771946541089	0.19851180810153	-1.22627023937664
a_HQ_transcript/49780	4.25119713019875	0.892638913405245	3.40090199647895	0.00179178768777549	0.19851180810153	-1.2309851365133
a_HQ_transcript/77962	4.5789880397998	2.88837176608068	3.40042797068122	0.00179407499245283	0.19851180810153	-1.259440884199
a_HQ_transcript/9377	-3.49644383547518	1.57826163962603	-3.39982960630567	0.00179696627263634	0.19851180810153	-1.22924423903311
a_HQ_transcript/124728	-2.66010269891453	-0.776738743017324	-3.39868976763191	0.00180248633841006	0.19851180810153	-1.23129478865991
a_HQ_transcript/146268	-4.80340414686811	0.0914783564368897	-3.39815380838149	0.0018050875379104	0.19851180810153	-1.23300670579617
a_HQ_transcript/35066	2.57354348488857	2.1542426828925	3.39723690775346	0.00180954594862021	0.19851180810153	-1.24403295675573
a_HQ_transcript/32571	-4.46538566483763	0.0879201926078694	-3.39670701869985	0.00181212734706797	0.19851180810153	-1.23743203310206
a_HQ_transcript/108725	-4.3488578395757	0.460386400027293	-3.39437188307524	0.00182354537854423	0.19851180810153	-1.24754278083483
a_HQ_transcript/65760	4.04714687734004	2.35914392265274	3.39430832012433	0.00182385714468443	0.19851180810153	-1.2842496962278
a_HQ_transcript/25906	2.69022212393694	-0.287753128455338	3.39428895749009	0.00182395212549651	0.19851180810153	-1.241262952394
a_HQ_transcript/135805	2.52164758242994	-0.668870259307493	3.3928947690684	0.00183080363788138	0.19851180810153	-1.24423458370251
a_HQ_transcript/130610	-1.47559369661641	6.23511907957121	-3.39277649983465	0.00183138598948486	0.19851180810153	-1.53597373339022
a_HQ_transcript/55373	-6.76872015451662	1.19849670702052	-3.39157616515241	0.00183730645546921	0.19851180810153	-1.35144861028211
a_HQ_transcript/60873	3.33280563566056	0.128706169808097	3.3909158054205	0.00184057141601884	0.19851180810153	-1.25226823204178
a_HQ_transcript/126605	3.06332845366328	-0.138383955604883	3.39085544755293	0.00184087011621842	0.19851180810153	-1.25364026276879
a_HQ_transcript/129305	-3.11925915820771	-0.0855682679723796	-3.38983149097706	0.00184594459615306	0.19851180810153	-1.25120260775811
a_HQ_transcript/152939	-3.10280584002059	-0.566191838733094	-3.38953082435998	0.00184743717780348	0.19851180810153	-1.25293886809067
a_HQ_transcript/112040	4.53130559510443	5.61818207381944	3.38912173492636	0.00184946985885924	0.19851180810153	-1.48556325165697
a_HQ_transcript/102792	-3.95731287764006	-0.196932567396272	-3.38871191976784	0.00185150830044595	0.19851180810153	-1.25606962674509
a_HQ_transcript/88888	-3.98207722661896	6.26972418304058	-3.38813355422459	0.00185438879368172	0.19851180810153	-1.54501568553117
a_HQ_transcript/134743	1.08052222310058	5.4479304548287	3.38654395336994	0.00186232784220924	0.198829150007941	-1.46993094146475
a_HQ_transcript/18934	2.6797923898134	1.37057443091926	3.38617118436868	0.00186419430832521	0.198829150007941	-1.26430142681468

a_HQ_transcript/149557	-2.58483131195851	-0.779194839352485	-3.3845103166471	0.001872532189123	0.199069391888687	-1.26478702425406
a_HQ_transcript/116659	-3.62082267021881	-0.822837088852058	-3.3843584988774	0.00187329612859743	0.199069391888687	-1.26749658681991
a_HQ_transcript/24444	-6.47662642903282	1.31573898059689	-3.3815192501105	0.00188763836668145	0.200227450690619	-1.29395372751087
a_HQ_transcript/74216	1.98293880280847	0.836112995578303	3.3756817253343	0.00191745823401151	0.202673474275715	-1.30216581821328
a_HQ_transcript/119615	-3.55051922666684	0.101590718869024	-3.37538064820643	0.00191900843411942	0.202673474275715	-1.28649130550785
a_HQ_transcript/26848	-2.873351462658	1.54337998475625	-3.374586141107	0.00192310501073529	0.202673474275715	-1.30120234743559
a_HQ_transcript/14021	3.93648116282219	0.284610427046796	3.37428791197737	0.00192464488370828	0.202673474275715	-1.28750237178767
a_HQ_transcript/131155	-4.31888435553534	-0.0610866038466513	-3.37292525986081	0.0019316958643905	0.203033982807624	-1.29218161897242
a_HQ_transcript/109917	4.49382769537622	0.48650879851166	3.37227792950365	0.0019350541301167	0.203033982807624	-1.32013013632017
a_HQ_transcript/144384	-2.77123528784422	0.54085168985131	-3.37153469018964	0.00193891687291875	0.203036327243088	-1.30346679306599
a_HQ_transcript/103648	-0.907201670687047	5.13925821941938	-3.37031569033269	0.00194526826048319	0.203036327243088	-1.50005237546753
a_HQ_transcript/150442	0.711912937692973	8.02072652064514	3.36962502301632	0.00194887572383223	0.203036327243088	-1.7285288288552
a_HQ_transcript/138431	-1.4544447825219	3.65763653605831	-3.36931550954609	0.00195049444456513	0.203036327243088	-1.37640606397988
a_HQ_transcript/5695	-0.862419701627577	3.14457087709527	-3.36892453110379	0.0019525410633238	0.203036327243088	-1.35737615489587
a_HQ_transcript/74801	-6.73545004167851	2.87829083759658	-3.36821753765238	0.0019562471330098	0.203058452406417	-1.36297193705703
a_HQ_transcript/25037	2.56978439209291	0.0286120303137619	3.36726934982296	0.00196122815131382	0.203212602459126	-1.30537940946663
a_HQ_transcript/139204	2.58340446176836	2.55871937016849	3.36416210227465	0.00197763661100034	0.204548151110725	-1.31360265404328
a_HQ_transcript/4575	-1.29363979127236	3.16881014033055	-3.3616863050733	0.00199080482182484	0.20554440973896	-1.37342749813488
a_HQ_transcript/43109	-3.1911585901869	-0.76427473671085	-3.35978786400623	0.0020009591746446	0.206046930972069	-1.32354058938834
a_HQ_transcript/101808	2.99259952021953	3.3334838902275	3.3594518851613	0.0020027614230529	0.206046930972069	-1.36569572578388
a_HQ_transcript/30556	1.95596484429296	2.75640322887719	3.35619733983301	0.00202030019606511	0.207484116248891	-1.3499752076119
a_HQ_transcript/148118	-6.15517065040853	1.96234765666265	-3.35442822354833	0.00202989572247104	0.20774283419243	-1.38035305424927
a_HQ_transcript/56161	4.96243220661121	1.39083732817625	3.35441508678466	0.00202996713840662	0.20774283419243	-1.34478716607936
a_HQ_transcript/75499	3.49100570437716	0.211715273586239	3.35227711745894	0.00204162205268689	0.208568377291008	-1.34591691956967
a_HQ_transcript/94939	2.23992188352568	1.44371394235738	3.35006240791238	0.002053763004295	0.209083332046131	-1.34333475255842
a_HQ_transcript/152542	-2.71493324569006	-0.135957256306587	-3.35004536410219	0.00205385670586191	0.209083332046131	-1.34556713703003
a_HQ_transcript/73675	-3.12550832934858	0.471893376690002	-3.34925030607966	0.00205823224891461	0.209162454833756	-1.35193823582692
a_HQ_transcript/35282	-2.74161435269669	-0.753309118222675	-3.34833830713019	0.00206326238136207	0.209307706289379	-1.34852608696313
a_HQ_transcript/42415	1.87549064037771	1.91844245942307	3.34650438458466	0.00207341311293433	0.209850330198167	-1.34978383543954
a_HQ_transcript/60359	3.85715778544808	1.19556386562382	3.34606870771571	0.00207583161065142	0.209850330198167	-1.35305585936845
a_HQ_transcript/152170	0.636335268583084	11.026655230374	3.34487605601661	0.00208246602401215	0.210108869278753	-2.1128682915975
a_HQ_transcript/124438	2.73675237890354	1.06851711632149	3.34431066530359	0.00208561824893064	0.210108869278753	-1.35605362784747
a_HQ_transcript/127644	1.36768863957363	3.18417121126658	3.34339560412705	0.00209072969369596	0.210241226044221	-1.43617556237283
a_HQ_transcript/108544	3.17604927041938	0.816974212346731	3.34264330633089	0.00209494095608965	0.210241226044221	-1.36283662165846
a_HQ_transcript/125858	3.76979341289772	-0.419704356014774	3.34149617049138	0.00210137813548788	0.210241226044221	-1.36477387787101
a_HQ_transcript/7634	-1.49949456669058	3.34023902457538	-3.34149236212764	0.0021013995377734	0.210241226044221	-1.42716122901816
a_HQ_transcript/30504	-4.59884651134355	0.376238627288023	-3.34018442083223	0.00210876230039581	0.210615352229223	-1.37705979396206
a_HQ_transcript/113651	-3.05953491888443	0.288451348748444	-3.33946290079182	0.00211283451868832	0.210660111324725	-1.37170585871609
a_HQ_transcript/135245	2.4726171656324	-0.505295647191211	3.336830291968	0.00212775687142008	0.211784677092305	-1.37261008655016
a_HQ_transcript/12525	6.01791668870031	0.256062963365437	3.33555007492381	0.00213504996997752	0.212018426151486	-1.38952861032233
a_HQ_transcript/50205	2.70545460487333	2.25103345041289	3.3351384165474	0.00213740018106198	0.212018426151486	-1.39140503678939
a_HQ_transcript/147040	2.12668006368303	2.71865064684595	3.33428448839772	0.00214228328065756	0.212140787969442	-1.39432834204881
a_HQ_transcript/70934	-3.713205772121	-0.818757598656869	-3.33277043126655	0.00215096756939214	0.21263850828848	-1.3869759780158
a_HQ_transcript/137233	-2.15312161735915	-0.549165156623605	-3.32902711560022	0.00217258351169613	0.213974270484269	-1.40794735369492
a_HQ_transcript/126092	-3.9049851983385	0.572516309926773	-3.32872947047769	0.00217431118630338	0.213974270484269	-1.4020709387904
a_HQ_transcript/75378	3.83273546601415	0.997876276646052	3.32852085452466	0.0021755228780657	0.213974270484269	-1.39049996519754
a_HQ_transcript/12483	-0.762540657691898	5.06795720846974	-3.32517650305448	0.00219503627283	0.21530205230685	-1.61632484827932
a_HQ_transcript/39827	-3.8017777708846	-0.815790878479751	-3.3249386277123	0.00219643058453691	0.21530205230685	-1.40513730829525
a_HQ_transcript/107915	0.581412042980769	5.07076569333031	3.32340811260735	0.00220542205840505	0.215720794941164	-1.6369035257452
a_HQ_transcript/78093	-2.56599344424556	0.440642458688717	-3.32294924076221	0.00220812470736981	0.215720794941164	-1.42545362908716

a_HQ_transcript/149230	-2.25501302326555	-0.594709988670314	-3.32145163023581	0.0022169673912792	0.216221276040733	-1.41305167075776
a_HQ_transcript/90742	5.76468630417589	1.91829889194847	3.31878067549188	0.00223282242844053	0.217402851122934	-1.47850224025697
a_HQ_transcript/66516	3.44034083172789	-0.180821528459195	3.31814604362137	0.00223660560907621	0.217407041545789	-1.42143732514133
a_HQ_transcript/56208	-1.8773610205406	3.24887425233202	-3.31706780363018	0.00224304732404764	0.21753590295169	-1.51623386233615
a_HQ_transcript/13620	-3.9614405249918	0.0725581244498658	-3.31667206972179	0.00224541600899763	0.21753590295169	-1.43163109159025
a_HQ_transcript/143058	-3.16309019618897	0.222310148714732	-3.31525064730174	0.00225394378594022	0.217998742743982	-1.42942045518112
a_HQ_transcript/91594	3.50810212557187	-0.0144787346276181	3.30924356450348	0.00229032700180515	0.221149714220813	-1.44307027563103
a_HQ_transcript/92279	5.14694165197222	1.3633451952527	3.30811899287725	0.00229720045358349	0.221174779749738	-1.43789200558953
a_HQ_transcript/8000	1.740239435834	2.51175953898311	3.30795629399589	0.00229819651405247	0.221174779749738	-1.44994878572442
a_HQ_transcript/150813	0.592995635126288	10.8167481202588	3.30701540568262	0.00230396485532659	0.22136342001723	-2.18497789775581
a_HQ_transcript/119245	2.8414320682671	2.23979962827697	3.30499719831063	0.00231638477478939	0.221724936727597	-1.46374402215099
a_HQ_transcript/104007	4.04328170834899	1.37066288238958	3.30295869147121	0.00232899470212527	0.221724936727597	-1.50198651774522
a_HQ_transcript/102599	-3.7045727059243	0.025661574298661	-3.30254272380606	0.00233157588623147	0.221724936727597	-1.45244608147628
a_HQ_transcript/135634	3.63902996226514	-0.0508273279567906	3.30173202991301	0.00233661432136443	0.221724936727597	-1.45937174188477
a_HQ_transcript/38259	3.15608680351394	0.460096601628088	3.30162412832777	0.0023372857118571	0.221724936727597	-1.4546128362505
a_HQ_transcript/142734	0.598960183098523	9.12866256521016	3.3013319589441	0.00233910458997744	0.221724936727597	-2.00708260886592
a_HQ_transcript/92870	-0.675594098318737	4.46224153140557	-3.30123202381027	0.00233972703957592	0.221724936727597	-1.63191599417842
a_HQ_transcript/140343	-7.15404026128584	1.75386161090484	-3.30078571052213	0.00234250885477632	0.221724936727597	-1.47313236962803
a_HQ_transcript/13599	3.65540817030788	-0.0860143341882213	3.30037713131819	0.00234505825501202	0.221724936727597	-1.45410255916167
a_HQ_transcript/87579	-2.76033346192205	-0.173501913389187	-3.30024683792671	0.00234587180166997	0.221724936727597	-1.46157843777789
a_HQ_transcript/145809	0.551025187730552	10.5476474406513	3.29919026290747	0.00235247900337838	0.221769291129641	-2.16635414881704
a_HQ_transcript/75999	-2.09316231721701	-0.145930526787466	-3.29895199482422	0.0023539714531205	0.221769291129641	-1.45721606221405
a_HQ_transcript/174	-3.12904124817365	4.28593451715157	-3.29739113113322	0.00236377074834561	0.222059835130975	-1.55419902706814
a_HQ_transcript/44871	2.57415912035161	-0.511377712982897	3.29681784727643	0.00236737969401121	0.222059835130975	-1.46523522100606
a_HQ_transcript/80062	-2.16203057371766	-0.816947819637428	-3.29663751811949	0.00236851599541021	0.222059835130975	-1.46475939252061
a_HQ_transcript/152426	0.543045496402969	10.849199700992	3.29566065165729	0.00237468057048694	0.222279278907028	-2.21112016373211
a_HQ_transcript/150381	3.35040876112131	4.62273275000762	3.29396192124588	0.00238543711468425	0.222726550531694	-1.52613504225895
a_HQ_transcript/584	-0.975895585407921	2.84225656648271	-3.2936964636156	0.00238712222992783	0.222726550531694	-1.49496634716404
a_HQ_transcript/18083	-4.0078841254538	-0.123484844773696	-3.29236432709853	0.00239559580794102	0.222804596758371	-1.47886599937113
a_HQ_transcript/149643	-0.782330372295265	4.41739210441918	-3.29150798227027	0.00240105812346921	0.222804596758371	-1.63976188282494
a_HQ_transcript/136887	1.05363457154309	4.57611265093374	3.29046907074236	0.00240770097470128	0.222804596758371	-1.69240964433774
a_HQ_transcript/88021	4.77162619315071	3.77448482305959	3.28979369315483	0.00241202880452408	0.222804596758371	-1.63409773662492
a_HQ_transcript/84985	0.809501935079979	3.82600337786398	3.28968691220172	0.00241271373990636	0.222804596758371	-1.62717120373962
a_HQ_transcript/146571	3.29930767099124	0.412952069073622	3.28872047768822	0.00241892130816343	0.222804596758371	-1.4811529794283
a_HQ_transcript/114476	6.03228367958469	1.58053310905582	3.28866801551849	0.00241925871844608	0.222804596758371	-1.48944355852574
a_HQ_transcript/80712	-1.46403248024451	5.64295105592227	-3.28851097002951	0.00242026902533294	0.222804596758371	-1.76613543364656
a_HQ_transcript/144029	1.1247683581658	3.09079876237071	3.28817128017808	0.00242245570381383	0.222804596758371	-1.55291362569893
a_HQ_transcript/143124	-2.83015257375296	-0.371094221103905	-3.28725422634784	0.00242836848370922	0.222995581707819	-1.50166245015107
a_HQ_transcript/119736	2.56522919453643	-0.361433480827093	3.28590599196174	0.00243708642545645	0.223255465337135	-1.48636530602243
a_HQ_transcript/32328	-2.51290335501017	0.669735171232379	-3.28562917711057	0.00243888006621733	0.223255465337135	-1.48828402585178
a_HQ_transcript/85316	-2.25342569086479	3.75257367323179	-3.28345371855296	0.0024530201464724	0.223644796217123	-1.5714717187604
a_HQ_transcript/550	-3.90573933518671	0.90820946034979	-3.2833351640128	0.0024537929792947	0.223644796217123	-1.50228781213991
a_HQ_transcript/112543	-2.65058426231563	-0.744493232091076	-3.28319982011091	0.00245467554339603	0.223644796217123	-1.4964500409649
a_HQ_transcript/99976	2.48371513495829	2.10009626197243	3.28098341773401	0.00246917177649038	0.223945667924784	-1.50736152651606
a_HQ_transcript/40412	3.88856057539965	0.975349272384559	3.28064119567944	0.00247141734563893	0.223945667924784	-1.49840743099137
a_HQ_transcript/66562	-1.93424233345012	2.01767661625053	-3.28035167347154	0.00247331863846373	0.223945667924784	-1.51131197643417
a_HQ_transcript/99280	-4.08536457511868	0.467524283558182	-3.28025239356216	0.00247397093201145	0.223945667924784	-1.51963645910238
a_HQ_transcript/75764	2.52945202952548	-0.140703119525503	3.27934011438103	0.0024799725430362	0.223945667924784	-1.50471409180015
a_HQ_transcript/124151	-4.06888804459959	0.928142531153043	-3.27916993795007	0.00248109362344414	0.223945667924784	-1.52919057866861
a_HQ_transcript/144724	3.05791964110207	1.0745057789303	3.27749740248049	0.00249213770968513	0.224593768664461	-1.50689009087964

a_HQ_transcript/56469	3.76875749980319	0.731834626052807	3.27652075439182	0.00249860844902412	0.224828346632932	-1.51266062190423
a_HQ_transcript/77040	3.00923145751083	0.272628737784213	3.27537554237606	0.00250621647783541	0.225164376234338	-1.52787065607565
a_HQ_transcript/125031	-2.59917633271805	1.39654384779449	-3.27458885849426	0.00251145551996602	0.225286861828063	-1.51729382814748
a_HQ_transcript/107374	4.13235548731023	0.704993160284647	3.27159741094406	0.00253147335783387	0.226504003397468	-1.54562594967801
a_HQ_transcript/104260	4.43155542300886	3.35184224475695	3.27139738954053	0.00253281726892984	0.226504003397468	-1.6193381411289
a_HQ_transcript/4012	-1.05363500574899	3.76264733997459	-3.2704931106068	0.00253890149304824	0.226699333314759	-1.64683484389464
a_HQ_transcript/56214	-0.831307592340715	3.47261383391939	-3.26460493842965	0.0025788618638081	0.229914236839628	-1.63635587457316
a_HQ_transcript/71238	7.18248247855968	3.16472832749053	3.26389206254461	0.00258374045433318	0.229996424394302	-1.62243305491924
a_HQ_transcript/44797	4.33337951974837	1.14320381064376	3.26314150819666	0.00258888643616215	0.230102126546229	-1.54311957153825
a_HQ_transcript/87146	-2.79912411677335	-0.181742534385022	-3.26081586816707	0.0026048938931937	0.231163524708796	-1.54240586361279
a_HQ_transcript/119741	-2.15435824806171	-0.773156312739671	-3.26025306798363	0.00260878186431616	0.231163524708796	-1.54723712416996
a_HQ_transcript/149628	-2.8130841209423	-0.738520425868969	-3.25966451498589	0.00261285367772448	0.231171930865706	-1.55170209406872
a_HQ_transcript/151582	-6.35795164419951	1.75094302404589	-3.25854684903075	0.00262060281029258	0.231299104213268	-1.60601250678243
a_HQ_transcript/139610	-6.0711129545507	9.91518478646703	-3.2583097752115	0.00262224934070575	0.231299104213268	-2.25840772953483
a_HQ_transcript/143594	6.10937010252665	2.53177575897612	3.25716476522763	0.00263021562413174	0.231650263332621	-1.68255684820411
a_HQ_transcript/59598	4.41743937233279	1.30022483804235	3.25578789030923	0.00263982572453024	0.232144916362321	-1.55361062449072
a_HQ_transcript/96791	4.91980540048761	1.3717907742256	3.25461111664939	0.00264806578504582	0.232517776364265	-1.55663979777285
a_HQ_transcript/70185	3.24585556045231	0.0477023206483836	3.25247740592449	0.0026630693044841	0.232770190594809	-1.581795013149
a_HQ_transcript/105431	3.05950823634202	0.14691434026342	3.25188669990456	0.00266723728637482	0.232770190594809	-1.58134252688383
a_HQ_transcript/127493	-1.22296673835595	2.85508250717096	-3.25145218294693	0.00267030718826374	0.232770190594809	-1.61121494079893
a_HQ_transcript/141985	4.62020986729642	0.248731607518871	3.25116237697987	0.00267235657095451	0.232770190594809	-1.57951123479048
a_HQ_transcript/96569	2.92074859701828	1.42955139137318	3.25092027028822	0.00267406979744463	0.232770190594809	-1.5865740239568
a_HQ_transcript/144641	-6.15138006984947	1.19647549172545	-3.25079352669654	0.00267496709532983	0.232770190594809	-1.60681112770965
a_HQ_transcript/36126	3.44353265114962	1.6512808882487	3.25004486098164	0.00268027324597707	0.232883293336555	-1.56803506693505
a_HQ_transcript/108176	0.963797496188461	3.73853517966631	3.24753454841396	0.00269813854133597	0.234085667359369	-1.72357755561511
a_HQ_transcript/123944	4.49699154109173	0.43594842240801	3.24655075333853	0.00270517096874655	0.234346017990014	-1.58822114950218
a_HQ_transcript/114608	2.21427558782459	0.2640813502785	3.24553365101416	0.00271245989951039	0.234603173426771	-1.5763852937291
a_HQ_transcript/25529	2.65357439767772	-0.311092424358277	3.24501118303998	0.00271621139065884	0.234603173426771	-1.57916534869629
a_HQ_transcript/107967	3.56948171134181	0.358903426387338	3.24384127686975	0.00272462968917512	0.234758768143308	-1.59162215171267
a_HQ_transcript/35758	-2.24870623556346	0.853972297568522	-3.24363866503079	0.00272609015443045	0.234758768143308	-1.58173513307648
a_HQ_transcript/135376	3.91491848368066	0.318010677378739	3.24281074350218	0.00273206574977146	0.234795912350618	-1.58354193983923
a_HQ_transcript/43607	3.93786339643803	1.59468179300015	3.24246013515427	0.00273460006642871	0.234795912350618	-1.58320798499465
a_HQ_transcript/919	1.19205835440536	3.94245418057285	3.24129481522329	0.00274303954597646	0.235006698951249	-1.71412536393319
a_HQ_transcript/150693	-3.69959234818598	0.371141564229502	-3.24087316737796	0.00274609933053916	0.235006698951249	-1.59085293864741
a_HQ_transcript/82917	-3.07556821188922	-0.726245162972704	-3.24044857346583	0.00274918378899755	0.235006698951249	-1.59642982615296
a_HQ_transcript/83683	-7.36591057767189	1.02196765157303	-3.23910248398595	0.00275898433929015	0.235286693735187	-1.61462208040473
a_HQ_transcript/149629	1.27950542890716	6.03065606061855	3.23888721866374	0.00276055472624892	0.235286693735187	-1.90141795041611
a_HQ_transcript/77836	-3.237228304146	3.92041676771514	-3.23781752920991	0.00276837091511262	0.235389676813491	-1.7563662411668
a_HQ_transcript/49002	-2.05053490688033	2.42177039829324	-3.23756023292256	0.00277025412320439	0.235389676813491	-1.60382904796108
a_HQ_transcript/147780	4.77025642902777	0.402277807184098	3.23706101281968	0.00277391151626138	0.235389676813491	-1.60202579119968
a_HQ_transcript/4889	-4.27655124369193	1.29845798374218	-3.23584637209559	0.00278282950809887	0.235802206482174	-1.62110987317554
a_HQ_transcript/58862	3.89205764986902	0.0631027072241708	3.23439232683256	0.00279354124587642	0.236365306463325	-1.61431189379746
a_HQ_transcript/142765	7.36829506759116	4.81157083637022	3.23261694096588	0.00280667365825096	0.237131288381994	-1.7085379824883
a_HQ_transcript/150215	-8.60412569524477	2.32244581415272	-3.22903715647562	0.00283333261523905	0.239036223887685	-1.68267889869859
a_HQ_transcript/56047	-1.11211922369088	2.98246290258034	-3.22655591425218	0.00285195234356834	0.240226163001466	-1.68750360648209
a_HQ_transcript/1101	2.00812629406663	4.36529489149479	3.22594633914744	0.00285654456148348	0.240226163001466	-1.74233798244203
a_HQ_transcript/1802	-3.33841318145929	-0.351358574875878	-3.2255101022878	0.00285983527382697	0.240226163001466	-1.62074307208455
a_HQ_transcript/134045	-3.06854404918762	-0.183673745849482	-3.22130410560662	0.00289174905246234	0.242556405370174	-1.65135476570907
a_HQ_transcript/59292	-2.26561111970057	0.960648891957569	-3.21923941256254	0.00290753943579252	0.243529470207129	-1.66459832789194
a_HQ_transcript/20759	-1.8114955409437	2.41785860988972	-3.21752131285539	0.00292074184846747	0.24395031349431	-1.64296039384335

a_HQ_transcript/116327	-3.8103005675056	0.912773064743037	-3.2174933091494	0.00292095751087324	0.24395031349431	-1.70226644214326
a_HQ_transcript/125454	-5.61285463712888	0.785331537291449	-3.21434806208318	0.00294527667949351	0.245628468903298	-1.66711546785691
a_HQ_transcript/82825	-2.69403333637879	-0.739917451590094	-3.21366734444403	0.00295056539232381	0.245716998746416	-1.65515956996138
a_HQ_transcript/120303	0.656987207282204	4.90964150008061	3.21308110366001	0.00295512734698517	0.24574483894929	-1.88209124203852
a_HQ_transcript/26007	1.42199004381717	4.28755175981544	3.2118508932664	0.00296472237861676	0.246190546320335	-1.82997379143769
a_HQ_transcript/22025	-3.0529112556309	0.205846402053418	-3.20822125338306	0.00299320533306859	0.248040453427389	-1.6753586598594
a_HQ_transcript/115459	3.45247651713201	1.01812071455433	3.20792597023115	0.00299553396480228	0.248040453427389	-1.65952186996707
a_HQ_transcript/25181	-0.879363022191214	3.32615235944944	-3.20561917560143	0.00301378516194478	0.248044760562634	-1.74600326823889
a_HQ_transcript/30111	4.011431631201	0.333263257237459	3.20513608375665	0.00301762076314861	0.248044760562634	-1.68076464480256
a_HQ_transcript/59214	2.80534861902057	-0.274459572144813	3.20484141161283	0.0030199626559381	0.248044760562634	-1.67194317952652
a_HQ_transcript/86305	5.60583846033795	3.47461570511698	3.20447549289106	0.00302287319099977	0.248044760562634	-1.7725614741521
a_HQ_transcript/78843	2.00895852835335	2.20212480121043	3.20419720909695	0.00302508846490604	0.248044760562634	-1.66834503502832
a_HQ_transcript/37904	-4.02315890434339	1.24774391050617	-3.20399798694941	0.00302667532093503	0.248044760562634	-1.67977453956774
a_HQ_transcript/150684	0.552352877737362	10.9785849140332	3.20387841377597	0.0030276281335614	0.248044760562634	-2.4592755649587
a_HQ_transcript/31572	1.72999743112646	-0.487004151315449	3.20287770331246	0.00303561346891558	0.248044760562634	-1.67285842833326
a_HQ_transcript/83984	3.225914994969	0.599886581688963	3.20266612523768	0.00303730436239142	0.248044760562634	-1.67183354172869
a_HQ_transcript/41759	2.47500599895576	1.60941573786818	3.20247777562909	0.00303881037408846	0.248044760562634	-1.6739525702196
a_HQ_transcript/113067	2.31414589788317	-0.445366114668533	3.20199152010674	0.00304270168527694	0.248044760562634	-1.67260026143662
a_HQ_transcript/55903	-1.73216421262013	2.33257479644826	-3.20148096408227	0.0030467925791653	0.248044760562634	-1.67369371078526
a_HQ_transcript/143204	3.62807171946555	2.65081583786864	3.19937085399023	0.00306375581739706	0.249076920494624	-1.72334236076605
a_HQ_transcript/12890	-1.88876438361202	2.6707969677158	-3.19877070207775	0.00306859688679765	0.24912206680974	-1.70488451962585
a_HQ_transcript/47077	4.08563656537253	1.65927248996608	3.19663645274317	0.00308587178283868	0.249794638877485	-1.6978641008303
a_HQ_transcript/105334	-1.86673986656937	0.709553808988061	-3.19658145670507	0.0030863181514924	0.249794638877485	-1.68770577241783
a_HQ_transcript/5290	-2.77041079905815	4.55309536325384	-3.19615600607798	0.00308977335110294	0.249794638877485	-1.82329143486735
a_HQ_transcript/140995	-2.27854594246741	-0.769265294877837	-3.19385751571021	0.00310850390318082	0.250959881783465	-1.69731135089194
a_HQ_transcript/50233	4.22682786359744	0.570203659721079	3.19250188767011	0.00311960171782301	0.251394597539107	-1.69404022529406
a_HQ_transcript/152515	-8.18528528212279	0.522870009136609	-3.19214396004925	0.00312253818165488	0.251394597539107	-1.69569248451459
a_HQ_transcript/125817	-2.05158821212149	-0.803144596641732	-3.19148569205165	0.00312794554157023	0.251481629931389	-1.70299347543227
a_HQ_transcript/152129	0.526861256282312	11.2125170496601	3.1899647960348	0.00314047314807698	0.251796009346458	-2.52582045960982
a_HQ_transcript/144715	2.85058011893987	3.87964226607826	3.1899592018408	0.00314051931558254	0.251796009346458	-1.79118321451734
a_HQ_transcript/126945	3.04218428229354	1.49299409624862	3.18783930151964	0.00315806099200213	0.252853676781129	-1.70474221505302
a_HQ_transcript/11038	2.74483647402369	1.91572087210986	3.18670958575895	0.00316744723807585	0.253256359085108	-1.73352494050961
a_HQ_transcript/24311	3.65442962037001	4.33189734978172	3.18524892165842	0.00317962259602739	0.25388063497511	-1.79714003122431
a_HQ_transcript/89489	4.75113348412727	1.70448688992633	3.18375656386051	0.00319210820469726	0.254295396143101	-1.75072212084097
a_HQ_transcript/130093	1.89972082536777	3.4798064035318	3.18312876520436	0.0031973745495982	0.254295396143101	-1.82880290612864
a_HQ_transcript/149969	2.13786623277676	-0.643921770659923	3.18306125637048	0.00319794134634955	0.254295396143101	-1.73714854520764
a_HQ_transcript/141952	4.2864751184938	2.05671243288502	3.18036655456098	0.00322064413261896	0.255455120183308	-1.75159108340536
a_HQ_transcript/147395	-2.26348745646121	-0.798032775120454	-3.18028719209821	0.00322131508213537	0.255455120183308	-1.72771749826041
a_HQ_transcript/126094	2.57956863849196	1.6791615053488	3.17926396068468	0.00322997766850961	0.255661722876097	-1.72278832477035
a_HQ_transcript/115345	3.20957335532298	0.453872535710096	3.17867844968813	0.00323494453420441	0.255661722876097	-1.72784190665465
a_HQ_transcript/143908	3.26850616473558	0.0208320200542809	3.17829254664158	0.00323822211249479	0.255661722876097	-1.73628414813082
a_HQ_transcript/114766	-5.6987670266176	1.30444481133064	-3.17790540618374	0.00324151338012117	0.255661722876097	-1.73815204843854
a_HQ_transcript/139334	2.11172746887912	1.42015863059112	3.17653659141168	0.00325317591371583	0.256233888228284	-1.73235879681474
a_HQ_transcript/101080	2.91235078268511	-0.329782383218423	3.175185453111	0.00326472704608772	0.256694554137001	-1.73139652511206
a_HQ_transcript/52451	-3.71299148076103	0.332780888030809	-3.1748201695521	0.00326785662781071	0.256694554137001	-1.73372769959863
a_HQ_transcript/48829	2.78529399128444	0.307740795521341	3.17383448657716	0.00327631577659832	0.257011718572344	-1.74193004644355
a_HQ_transcript/71709	4.01626007150918	2.07542927189809	3.17218714565249	0.00329049987529931	0.257561271124271	-1.83770315305254
a_HQ_transcript/140778	-5.40201088037965	3.3416648885653	-3.17179730877508	0.00329386503790108	0.257561271124271	-1.85988968321317
a_HQ_transcript/9649	1.63450760699809	2.20208682569083	3.17147912287153	0.00329661412256499	0.257561271124271	-1.75488924584712
a_HQ_transcript/116145	-6.99674382575795	4.25397913461628	-3.16889745211485	0.00331900037783012	0.258646429365278	-1.9727424903541

a_HQ_transcript/9169	-5.10143552155414	2.05658483720519	-3.16843992394837	0.00332298278481143	0.258646429365278	-1.78138578577507
a_HQ_transcript/107437	3.56886194335278	-0.493010656615711	3.16834010455256	0.00332385223534034	0.258646429365278	-1.75780869812085
a_HQ_transcript/70149	-3.16502004789257	-0.170809174444635	-3.16778326524994	0.00332870641479969	0.258677869624969	-1.75137669801876
a_HQ_transcript/147146	1.40403245615348	4.33677018276846	3.16555481694392	0.00334820031310694	0.259845377570468	-1.91023278289987
a_HQ_transcript/126368	-1.49915507708213	2.09165370930781	-3.16444720666773	0.00335792981214139	0.260252992160207	-1.76155249905662
a_HQ_transcript/10131	-4.12469547175869	0.0321415385946196	-3.16360485679137	0.00336534722388989	0.260480563821933	-1.7685832321905
a_HQ_transcript/36169	2.39218405406629	-0.444511929073763	3.16084576357196	0.00338975215899327	0.262020629651543	-1.76499520022895
a_HQ_transcript/120600	-6.67156401589281	0.914448981306943	-3.16028545704205	0.00339472876890576	0.26205683118055	-1.79695911560048
a_HQ_transcript/130371	1.1320842464462	5.22607692757961	3.15924322610087	0.00340400431584465	0.262424353940872	-2.04393222624059
a_HQ_transcript/89652	-3.15457938645451	0.983524905479177	-3.1578024189286	0.00341686682052908	0.262764307311959	-1.78989902025728
a_HQ_transcript/85168	-6.59867955087013	1.33291944140327	-3.15706954579874	0.00342342713500003	0.262764307311959	-1.80916689885233
a_HQ_transcript/43141	5.68334277214874	1.41187989669233	3.15683618621041	0.00342551856959669	0.262764307311959	-1.80532330518778
a_HQ_transcript/4721	2.22697984939679	1.2307348961038	3.15562092716577	0.00343642974200802	0.262764307311959	-1.77862606988478
a_HQ_transcript/72747	-3.00938271089835	2.43858244399204	-3.15474475396125	0.00344431695995127	0.262764307311959	-1.84564956094323
a_HQ_transcript/61164	-2.81109817780776	-0.583478406853472	-3.15412597675649	0.00344989750649264	0.262764307311959	-1.78372195646545
a_HQ_transcript/114456	-2.11219224788491	4.71873562844405	-3.1536701239433	0.00345401419717004	0.262764307311959	-1.92340061457851
a_HQ_transcript/55468	2.26577959421134	-0.620896800382023	3.15361837985071	0.00345448178032101	0.262764307311959	-1.79245321852968
a_HQ_transcript/43082	-2.94361666379773	-0.216920690320758	-3.15346652871921	0.00345585432411723	0.262764307311959	-1.7910898813179
a_HQ_transcript/92367	-2.83176402167647	-0.211866673887687	-3.15324283197472	0.00345787720787065	0.262764307311959	-1.78832838114277
a_HQ_transcript/7874	2.3161360248444	0.944856474131387	3.15239455261872	0.00346555842207692	0.262764307311959	-1.78397434955084
a_HQ_transcript/8230	-1.11752910277901	4.99476016186289	-3.1520135884335	0.00346901335553588	0.262764307311959	-2.04778598432333
a_HQ_transcript/12224	-1.94147461173474	1.84298640276824	-3.15195754742347	0.00346952186347532	0.262764307311959	-1.78451546002644
a_HQ_transcript/5728	2.48495782792918	0.0996769439171692	3.15124810478811	0.0034759653800182	0.262764307311959	-1.78579626634769
a_HQ_transcript/120826	5.30625578241432	3.7441499821414	3.15084303832547	0.0034796495052131	0.262764307311959	-1.88670446357045
a_HQ_transcript/17948	-1.11091402690821	3.54393019131592	-3.15072310063051	0.00348074106506689	0.262764307311959	-1.88641557091059
a_HQ_transcript/18266	2.10607421615461	2.2699767417836	3.14812285419901	0.00350448634007401	0.264092561456805	-1.8487897663376
a_HQ_transcript/150815	-2.82141978259909	0.906697661713194	-3.147802488769	0.00350742254068012	0.264092561456805	-1.79756148996197
a_HQ_transcript/34275	2.25083301389445	1.76461211492882	3.14467514339813	0.00353620840760428	0.26591555280365	-1.79833660702012
a_HQ_transcript/140967	4.63486942023371	0.138599421447653	3.14231468526629	0.00355808413995398	0.267214877115304	-1.83358255791098
a_HQ_transcript/104220	-3.63904255279888	0.683033745340974	-3.14117671006149	0.0035686763600112	0.267664541231911	-1.80530586679212
a_HQ_transcript/131225	0.797073980764745	4.15483966700982	3.13930226398001	0.00358618905307126	0.268631439789853	-2.01623565276963
a_HQ_transcript/4895	-2.60875584627339	-0.320980955271136	-3.13841542333465	0.00359450314769586	0.268907694941139	-1.82275944274626
a_HQ_transcript/82692	1.80693317024581	3.94211560171588	3.13767984509137	0.00360141307265652	0.269078327875807	-1.95401810613711
a_HQ_transcript/4511	-3.29096892784336	0.0337146413695399	-3.13660553724434	0.00361152768747989	0.269487652654469	-1.8173081777188
a_HQ_transcript/25462	4.7195037495178	1.63067430793308	3.13603083438702	0.00361694960692566	0.269546213783814	-1.93364570367669
a_HQ_transcript/13199	3.40937691639855	1.42533272052827	3.13511342551719	0.00362562076631923	0.269846458264538	-1.84184768170088
a_HQ_transcript/99797	3.07944972450646	0.0126551946477211	3.13201878003692	0.00365501679405688	0.271686465735215	-1.8331634474014
a_HQ_transcript/40262	1.63226570861184	-0.763648093304977	3.13062739306465	0.00366830731776354	0.272326140187687	-1.85073535288099
a_HQ_transcript/151281	3.20917670624522	-0.315892417255363	3.13000851890309	0.00367423355781817	0.272418173786804	-1.83471638011961
a_HQ_transcript/27449	2.96470307518592	0.379842330909118	3.12746362720401	0.00369869886674321	0.273584400975989	-1.83595472643495
a_HQ_transcript/57417	-3.28101330821555	0.0475932557141504	-3.12739339971314	0.00369937618991067	0.273584400975989	-1.85060957903081
a_HQ_transcript/71319	2.61294050720468	1.07001707899922	3.12657527641525	0.00370727543970427	0.27382021189216	-1.84506069127359
a_HQ_transcript/133333	1.66881250411658	-0.76277114861345	3.12393157998161	0.00373291098926966	0.275063897965391	-1.86543200079669
a_HQ_transcript/19691	-2.54574816426067	-0.103838174791805	-3.12384831137455	0.00373372116458676	0.275063897965391	-1.86383732427669
a_HQ_transcript/128290	3.93497922967587	1.92487116932537	3.12301000928419	0.00374188687829418	0.275063897965391	-1.91503709218674
a_HQ_transcript/138038	2.55341391714296	-0.423003902488576	3.12289156720442	0.00374304196412441	0.275063897965391	-1.85578676585252
a_HQ_transcript/6008	-2.00031508093104	1.58346698287136	-3.12218082898542	0.00374998043858553	0.275076041159001	-1.85332427816964
a_HQ_transcript/110355	-3.58421913578082	0.665867321541563	-3.12190548767574	0.0037526717010578	0.275076041159001	-1.85070033009285
a_HQ_transcript/142470	-3.36340033713752	0.341390527536779	-3.12103572361381	0.00376118508253277	0.275352854505623	-1.8814202112712
a_HQ_transcript/124594	-1.45445570550463	2.22616667338742	-3.1201211683442	0.00377015668777793	0.275645904394182	-1.85327385969384

a_HQ_transcript/63697	-2.80167454144934	0.0998772354785428	-3.11920383072914	0.00377917602008163	0.275645904394182	-1.85281771517923
a_HQ_transcript/105804	1.59839920720958	3.03425239828393	3.11917963907695	0.00377941415156488	0.275645904394182	-1.97541613871389
a_HQ_transcript/44662	3.13383622529234	1.17836720927396	3.11708188909575	0.00380011776356846	0.276562161485067	-1.86250784639611
a_HQ_transcript/39443	2.63505173817041	1.65403493655624	3.11694296259328	0.00380149268900648	0.276562161485067	-1.85825175733982
a_HQ_transcript/123763	0.624531411084709	5.51096420936184	3.11515240786508	0.00381925579002286	0.277507125703061	-2.17350975900849
a_HQ_transcript/77158	-1.93123679475002	2.47939099955213	-3.11424860591349	0.00382825183762687	0.277508216105511	-1.88038373943255
a_HQ_transcript/152430	-1.01220210478232	4.49682559224954	-3.11338676627634	0.00383684896429113	0.277508216105511	-2.09737671735712
a_HQ_transcript/127638	-1.26066028266327	2.71084027360202	-3.11288582401381	0.00384185445189945	0.277508216105511	-1.89363719061314
a_HQ_transcript/5789	-2.11745267410919	2.55294562468485	-3.11278274394595	0.00384288521238661	0.277508216105511	-1.87884922948279
a_HQ_transcript/131115	2.10854904995335	-0.66747634651296	3.11265124436038	0.00384420053843553	0.277508216105511	-1.87856073178665
a_HQ_transcript/118161	-4.17802534250151	1.17063089540699	-3.11228008942539	0.00384791532791498	0.277508216105511	-1.89480474259731
a_HQ_transcript/150646	0.465881745484086	10.7041683169053	3.11161371917912	0.00385459340910832	0.277645360204025	-2.64882817847544
a_HQ_transcript/93898	-1.92082446789159	3.05228316628448	-3.11065378011299	0.00386423287470427	0.277725110811653	-1.90520903225594
a_HQ_transcript/5731	3.75631759757665	1.33950772237088	3.11055200279415	0.00386525623875976	0.277725110811653	-1.8940528270428
a_HQ_transcript/150443	4.67786010257339	0.276198620085716	3.1082982045026	0.00388798412574512	0.278149148704418	-1.90183840481048
a_HQ_transcript/108769	-3.62537411503673	1.11082735409154	-3.10822346233619	0.00388874001601021	0.278149148704418	-1.90577352310361
a_HQ_transcript/141659	2.64867028544005	-0.0542672513177952	3.10789378202107	0.00389207583977996	0.278149148704418	-1.90202221645232
a_HQ_transcript/118545	3.6579697103672	1.28117480704031	3.10762445040416	0.00389480305125275	0.278149148704418	-1.88795672142151
a_HQ_transcript/141558	1.09765186671121	2.51596542988431	3.10732866803524	0.00389780019074512	0.278149148704418	-1.93718717335542
a_HQ_transcript/50068	-2.16213145206774	2.59820549187951	-3.10684689667313	0.00390268663352589	0.278149148704418	-1.88333727045157
a_HQ_transcript/125931	-3.27138658497728	1.66425034753675	-3.10665312687189	0.00390465361517349	0.278149148704418	-1.90923566824359
a_HQ_transcript/70094	-2.10681055372539	2.39254885347564	-3.10456737177625	0.00392588609271101	0.278872557321215	-1.90315557729274
a_HQ_transcript/94573	-3.36434163430703	1.00844153449072	-3.10442521851283	0.00392733716327366	0.278872557321215	-1.88463277608393
a_HQ_transcript/30821	-2.54940483059737	1.88850711723041	-3.10414969954404	0.00393015105362775	0.278872557321215	-1.89951511841017
a_HQ_transcript/113367	-1.71513617209345	2.869178206175	-3.10343849662285	0.00393742346282275	0.278872557321215	-2.02953633026214
a_HQ_transcript/54942	2.57801428211588	1.39270748940955	3.10330434982702	0.0039387966136925	0.278872557321215	-1.88889543107623
a_HQ_transcript/9969	-0.950439718538139	3.73810211752688	-3.10168938549275	0.00395536342088588	0.279704823514908	-2.04048078094001
a_HQ_transcript/117764	-3.35310377651673	0.413812663220845	-3.10074935314941	0.00396503701648064	0.280048203759401	-1.9240772117098
a_HQ_transcript/26362	-1.50599174312137	1.44600227319971	-3.09925105068089	0.00398050206819323	0.280750358867466	-1.90192856234079
a_HQ_transcript/80894	2.44353299035086	-0.381476044123838	3.09885127169485	0.00398463814453722	0.280750358867466	-1.92182376553699
a_HQ_transcript/82258	2.64291719834218	-0.0896019965993885	3.09804640979085	0.00399297755434839	0.28099733568906	-1.96116042791502
a_HQ_transcript/98825	-3.39680263283953	-0.729808049629882	-3.09531564423005	0.00402139549822194	0.282108600640724	-1.92421579703167
a_HQ_transcript/113946	-4.21565567571946	0.469546608787631	-3.09526476705728	0.00402192677374287	0.282108600640724	-1.95236909658637
a_HQ_transcript/1637	-4.42653143942023	0.236235894477849	-3.09513057750752	0.00402332834315924	0.282108600640724	-1.91797593014598
a_HQ_transcript/37014	1.91114366805907	-0.751397935277389	3.09403637574012	0.00403477427182683	0.282401994954891	-1.93378994312872
a_HQ_transcript/8352	2.79521285352778	2.47089371016473	3.09336909994553	0.00404176949786759	0.282401994954891	-1.91104777883825
a_HQ_transcript/4095	2.30102087772264	1.21442586723739	3.09102347066012	0.00406645083008416	0.282401994954891	-1.92323979367876
a_HQ_transcript/115672	3.69897267502898	0.0265459935906671	3.09088080666095	0.00406795658510065	0.282401994954891	-1.93487802783471
a_HQ_transcript/58980	-2.37402275014093	2.38419453710309	-3.08920112849458	0.00408572468847024	0.282401994954891	-1.95620524042507
a_HQ_transcript/150824	-3.96295640593934	1.62737343351282	-3.08883724544634	0.00408958364134672	0.282401994954891	-1.96626873503507
a_HQ_transcript/5655	-0.836982396797997	3.50766943384138	-3.08878759241583	0.00409011047625029	0.282401994954891	-2.06098476138963
a_HQ_transcript/110928	-3.36582763393421	0.320656902043874	-3.08856887676822	0.00409243188783425	0.282401994954891	-1.93941614253525
a_HQ_transcript/106592	1.81405892415791	4.30880527053964	3.08834976078229	0.00409475880259893	0.282401994954891	-2.1423340240219
a_HQ_transcript/38870	2.0338763226613	-0.745003828408128	3.0881894598201	0.00409646192325155	0.282401994954891	-1.9437314573173
a_HQ_transcript/142637	7.23473082016721	1.89495225830159	3.08761572643077	0.00410256307624224	0.282401994954891	-1.95234704790244
a_HQ_transcript/132074	4.59960312491283	1.75104777612156	3.08744093279349	0.00410442356725056	0.282401994954891	-1.95024309572328
a_HQ_transcript/115977	-2.47349102974314	-0.281213686873194	-3.08723142665953	0.0041066545907943	0.282401994954891	-1.92731878572032
a_HQ_transcript/121864	3.33530156643488	0.541494369129625	3.08720974831585	0.00410688550842624	0.282401994954891	-1.94921269694786
a_HQ_transcript/121437	-3.02766425003516	4.2599014756826	-3.08701767836	0.00410893197524069	0.282401994954891	-2.12235742048972
a_HQ_transcript/120275	4.01209357364485	-0.237137217558273	3.08666256122437	0.00411271822640865	0.282401994954891	-1.96112542753882

a_HQ_transcript/32596	3.41496485305034	0.177105670387754	3.08666220408378	0.00411272203590017	0.282401994954891	-1.92806483046619
a_HQ_transcript/127617	3.02712316225874	1.43303874519912	3.08645229586462	0.00411496163168856	0.282401994954891	-1.92523899570777
a_HQ_transcript/148643	-2.42238371780668	-0.761956891745828	-3.08593507740749	0.00412048498592202	0.282447584034995	-1.93846255092568
a_HQ_transcript/148283	2.40491197245585	-0.756287941503513	3.085362137349	0.00412661160301992	0.282534368975668	-1.94008899334923
a_HQ_transcript/133884	1.99121739345781	-0.7611246339105	3.08404507745165	0.00414072810985225	0.283167345375872	-1.94956202657463
a_HQ_transcript/109860	-3.28621887720684	0.00616637740217527	-3.08249195077135	0.0041574337137328	0.283758214450796	-1.95124990609782
a_HQ_transcript/8604	1.28935420811639	2.53883420095243	3.08233443398629	0.00415913154954718	0.283758214450796	-1.96828653013771
a_HQ_transcript/70148	2.74534112648826	1.1211720284485	3.08127106494132	0.00417061057627911	0.284207797863953	-1.94983011164919
a_HQ_transcript/24658	-3.63975370284567	-0.714196890302528	-3.08046167884737	0.0041793680101676	0.284228827787227	-1.95814624477709
a_HQ_transcript/147327	3.92212491206285	0.663523048953136	3.08033884366971	0.00418069859203962	0.284228827787227	-1.94292943092244
a_HQ_transcript/130230	2.46821112020706	1.22160017022118	3.07931531116737	0.0041918013956652	0.284406486173486	-1.93837472715219
a_HQ_transcript/77398	3.22942115132428	0.299406421087548	3.07800570341322	0.00420604821275948	0.284406486173486	-1.94371663141373
a_HQ_transcript/88670	2.0245660883073	2.91789975722518	3.07744971040559	0.00421211057025922	0.284406486173486	-1.97252264076603
a_HQ_transcript/143355	-1.94097137796266	-0.791997407767235	-3.07715279816029	0.00421535139446952	0.284406486173486	-1.9543773975672
a_HQ_transcript/150639	5.71826139426221	1.89183999388877	3.07677367828487	0.00421949296136661	0.284406486173486	-2.02067222140828
a_HQ_transcript/76767	2.3832466262007	0.816254396994093	3.07664148125943	0.00422093801059403	0.284406486173486	-1.94539672914942
a_HQ_transcript/145114	-4.3776315772473	1.21655531588386	-3.07608878676538	0.00422698461521796	0.284406486173486	-1.95382639021086
a_HQ_transcript/111909	1.8994461910616	-0.764095423545154	3.07542889667504	0.00423421472699512	0.284406486173486	-1.96002469058038
a_HQ_transcript/107895	-1.76316638302183	2.14955326631116	-3.07504693599855	0.00423840505325778	0.284406486173486	-1.95756923143499
a_HQ_transcript/43910	2.77513406458948	1.87152884960909	3.07494691899873	0.00423950294647866	0.284406486173486	-1.98885290322979
a_HQ_transcript/110250	-1.35278778605921	2.45094470368161	-3.07487135496902	0.00424033259673264	0.284406486173486	-1.95772933191351
a_HQ_transcript/129334	3.59817824970064	0.872875552882875	3.07455916000289	0.00424376195361514	0.284406486173486	-1.95027033255967
a_HQ_transcript/45664	-3.73246385691431	0.844679359160174	-3.07427208804653	0.0042469176644403	0.284406486173486	-1.95389359378412
a_HQ_transcript/149596	1.32933984377132	-0.78001158982172	3.07339122692314	0.00425661465253887	0.284554404321082	-1.98132863286596
a_HQ_transcript/130406	4.204154902843	0.161448357937312	3.07318235763133	0.00425891707540843	0.284554404321082	-1.9539685008981
a_HQ_transcript/37274	-4.43376085080349	0.815459939247835	-3.0719760228888	0.00427223796150804	0.284900378056128	-1.95614548244983
a_HQ_transcript/103161	4.70959952818955	-0.421683997630085	3.07182596111946	0.00427389777155491	0.284900378056128	-1.97613818247319
a_HQ_transcript/44280	-1.23140647020021	2.56204577851133	-3.0710410063203	0.00428259000753763	0.285152797202918	-1.97510561327269
a_HQ_transcript/54252	3.56739151906151	0.971484266324871	3.0703196067445	0.00429059324507538	0.285358814816638	-1.9696193184849
a_HQ_transcript/142954	3.77935966737796	1.1158506756194	3.06616469480439	0.00433696483134779	0.287508478444904	-1.98659804049475
a_HQ_transcript/145625	-5.48507929473773	2.56640321911931	-3.0660558133658	0.00433818638769258	0.287508478444904	-2.12015519738521
a_HQ_transcript/101030	1.92705368278358	-0.771425697156111	3.06597477126979	0.00433909582228008	0.287508478444904	-1.9941234681371
a_HQ_transcript/98390	-5.00202910852277	2.60102040738343	-3.0656537848527	0.00434269962969009	0.287508478444904	-2.0334715534911
a_HQ_transcript/16214	3.29723912900782	0.754207926979024	3.06476227075566	0.00435272381052701	0.28775916256937	-2.02968340430163
a_HQ_transcript/145717	1.99739717388367	1.17551549956374	3.06405897651356	0.00436064712477187	0.28775916256937	-1.98172221584695
a_HQ_transcript/14301	-2.8032175553927	-0.355046255815741	-3.0634726982352	0.0043672625865365	0.28775916256937	-1.97723587786947
a_HQ_transcript/110935	1.24608323958695	3.04322715720587	3.06334532271805	0.0043687011273743	0.28775916256937	-2.07675726581032
a_HQ_transcript/130354	-2.99533896958335	2.7784745816421	-3.06277482376155	0.0043751496852962	0.28775916256937	-2.08168638231976
a_HQ_transcript/112415	-5.1803892901972	6.28841633987902	-3.06268297301513	0.00437618875088294	0.28775916256937	-2.38239711049241
a_HQ_transcript/64557	-2.14045805943322	2.17500853076425	-3.06111626241492	0.00439394830342012	0.288182791320105	-1.99637765635424
a_HQ_transcript/16849	1.14901928876699	2.35989993160063	3.06082367257697	0.00439727253780302	0.288182791320105	-1.98455610176592
a_HQ_transcript/147164	-4.09954587707348	0.0109127327344784	-3.06080327232847	0.00439750440236948	0.288182791320105	-2.00000233496347
a_HQ_transcript/70788	-1.47302384926711	2.7490922180653	-3.05947108395415	0.00441267086783109	0.288805501868148	-2.08939231090416
a_HQ_transcript/56400	1.62358132678333	1.54419184751328	3.05909657004321	0.00441694348955381	0.288805501868148	-1.98146644278935
a_HQ_transcript/126246	2.62976918918567	3.01496241127309	3.05810539181104	0.00442827022446533	0.288946966315809	-2.0097483763659
a_HQ_transcript/150039	-6.7838132351821	1.76240402581215	-3.05803735113538	0.0044290487714593	0.288946966315809	-2.07772885282759
a_HQ_transcript/94704	-1.61941215319082	2.64549191598335	-3.05683689042977	0.00444280625092056	0.289519553535325	-2.00285534024114
a_HQ_transcript/108058	-3.44810732567748	0.338842201706913	-3.05623851588379	0.00444967884003836	0.289626142553421	-1.99124126308555
a_HQ_transcript/55391	-4.89123879855351	1.10956001273927	-3.05568267270602	0.00445607196462204	0.289626142553421	-2.03049624426933
a_HQ_transcript/8520	1.88985341929533	1.92285130547645	3.05489854875147	0.00446510550384449	0.289626142553421	-2.00033854033678

a_HQ_transcript/119948	3.30358957800686	1.49818416067174	3.05442041988205	0.00447062232678162	0.289626142553421	-2.00590229782967
a_HQ_transcript/85941	1.35717794908664	1.91986924425247	3.05429016669375	0.00447212635420623	0.289626142553421	-1.99409979244657
a_HQ_transcript/99377	2.68354865857238	-0.297133392658959	3.05409877146552	0.0044743372559347	0.289626142553421	-2.00037802694532
a_HQ_transcript/36935	3.26525335729964	0.764290046586996	3.05339745924754	0.00448244731810172	0.289723174761669	-1.99702082412385
a_HQ_transcript/42913	1.75918187914011	1.61280959513926	3.05310748127313	0.00448580472896887	0.289723174761669	-2.04424742381212
a_HQ_transcript/43269	-4.58317453539862	1.6158924558669	-3.05256919167782	0.00449204345000633	0.289804108392861	-2.08739875068856
a_HQ_transcript/146691	-5.25687056122979	3.09286752858893	-3.05182659779468	0.0045006635336047	0.290038325810836	-2.13413543628293
a_HQ_transcript/25864	1.48487726999477	3.86686601618741	3.04992089582103	0.0045228568460181	0.291145761622747	-2.21192071197036
a_HQ_transcript/138853	5.98955537010873	2.11998331345421	3.04897644867262	0.00453389402522155	0.291533398117343	-2.06142916092089
a_HQ_transcript/123466	1.65849361004488	2.29567593614603	3.04671956886252	0.00456037225064874	0.292607794653147	-2.01665130033523
a_HQ_transcript/151416	-2.04908474446917	5.13117127363224	-3.04669420783474	0.00456067062269047	0.292607794653147	-2.16277622188968
a_HQ_transcript/76999	-1.9862577139345	-0.586262797810553	-3.04470452945385	0.00458413688186394	0.292835232645101	-2.02348482350744
a_HQ_transcript/4932	-3.75709743318679	0.05718276491831	-3.04461676959831	0.00458517455053077	0.292835232645101	-2.03319155781133
a_HQ_transcript/42249	2.20487367779955	0.177372487906686	3.04446213160015	0.00458700352489507	0.292835232645101	-2.01367224179284
a_HQ_transcript/49701	2.40230445784654	1.79599919345939	3.04438397807027	0.00458792814533739	0.292835232645101	-2.04816151180229
a_HQ_transcript/131007	-1.01126412942332	3.30542770646802	-3.04353741077488	0.00459795505233223	0.292835232645101	-2.13324198314278
a_HQ_transcript/20124	-0.921994681117189	3.36613631109945	-3.0434522407589	0.00459896497093928	0.292835232645101	-2.13398964395167
a_HQ_transcript/61868	-2.61161281103944	0.0881895119364266	-3.04319886139547	0.00460197070432945	0.292835232645101	-2.02463473087378
a_HQ_transcript/95770	2.1104777699531	-0.652149674440607	3.04288024909642	0.00460575290984169	0.292835232645101	-2.02912983884343
a_HQ_transcript/8193	2.5608858416968	0.398134356931899	3.0425601754685	0.00460955542716535	0.292835232645101	-2.01906678592304
a_HQ_transcript/114484	-2.41226547721703	-0.764841418875178	-3.04146357596474	0.00462260573373809	0.293343696605598	-2.03636964086918
a_HQ_transcript/142505	1.42896015815981	-0.803922904146225	3.04008643708314	0.00463904422851968	0.293906290710289	-2.04894525616738
a_HQ_transcript/73426	-2.29200562989538	1.38941478634058	-3.03969183613055	0.00464376465733325	0.293906290710289	-2.0235747493271
a_HQ_transcript/150826	-3.25415852816278	-0.464157797257034	-3.03945167258201	0.00464663984934551	0.293906290710289	-2.06420772875768
a_HQ_transcript/147194	-1.91671407160709	0.537885817767293	-3.03853583351292	0.00465761957977828	0.293955826593025	-2.02581657031293
a_HQ_transcript/105584	-1.26084987310689	2.37549778468382	-3.03851134748711	0.0046579134726467	0.293955826593025	-2.05437116164999
a_HQ_transcript/20800	-2.93436746131668	-0.0684608408515279	-3.03812156940501	0.00466259413912003	0.293955826593025	-2.02597382177123
a_HQ_transcript/97847	2.1165994994869	0.834885194690524	3.03625831321652	0.00468503073363987	0.295050342887344	-2.03746724726546
a_HQ_transcript/94851	-2.82351865980255	-0.168195789217506	-3.03498383401002	0.00470043634835987	0.295612415938289	-2.04237865076801
a_HQ_transcript/22783	-2.50532616295758	1.57000482469007	-3.03437095820741	0.0047078617192594	0.295612415938289	-2.05923831590047
a_HQ_transcript/26167	2.0288518494028	-0.19992517526354	3.03425957575937	0.00470921237886829	0.295612415938289	-2.03782669056239
a_HQ_transcript/21407	-1.26128356849325	2.22887922855942	-3.03314612844596	0.00472273458237811	0.295948274368051	-2.04619901535889
a_HQ_transcript/36902	3.49577288240588	0.146222382114003	3.03298081408348	0.00472474536563362	0.295948274368051	-2.06250515191016
a_HQ_transcript/86920	2.56747683488226	2.86576473758325	3.03108429692729	0.00474787150847755	0.296279319026351	-2.11102282003731
a_HQ_transcript/61341	-2.43773149789961	0.755339495979422	-3.03096858162952	0.00474928600470801	0.296279319026351	-2.04273378480401
a_HQ_transcript/145176	7.27729217726873	3.91424539970164	3.03093171856891	0.00474973670013148	0.296279319026351	-2.24889475786696
a_HQ_transcript/103029	3.33093504415746	0.400072124415375	3.03087596115737	0.00475041847874621	0.296279319026351	-2.05373203356572
a_HQ_transcript/139182	1.19102530843892	2.73792469724412	3.02996708883839	0.00476154486579582	0.296475364798794	-2.09489787227518
a_HQ_transcript/835	4.32749319636779	1.17285419959405	3.02978617325245	0.00476376257091374	0.296475364798794	-2.07013388233975
a_HQ_transcript/98783	4.77009254553104	3.06002452691006	3.02851478391679	0.00477937515758089	0.297039029667876	-2.10200872864428
a_HQ_transcript/48284	-2.99514373529859	0.173390724305537	-3.02819565525301	0.0047833016366629	0.297039029667876	-2.05461057017094
a_HQ_transcript/10072	5.23302016859432	2.23849281565863	3.02780195875696	0.00478814978665703	0.297039029667876	-2.16584992169483
a_HQ_transcript/120311	-5.13589686230704	2.33194967766962	-3.02669495358074	0.00480180686834427	0.29727796305077	-2.16845352958199
a_HQ_transcript/23081	-2.10204322367627	1.98978259500987	-3.0266607285714	0.00480222968801048	0.29727796305077	-2.05107302561338
a_HQ_transcript/74477	-3.27453720075439	0.474124892719801	-3.02550118210348	0.00481657569633695	0.297407161958219	-2.05265012278895
a_HQ_transcript/85109	-2.81604155781334	-0.109492178255256	-3.0253109600341	0.00481893300946252	0.297407161958219	-2.05297112367572
a_HQ_transcript/148135	0.478901873192047	10.8921069463397	3.02525182789319	0.00481966602265074	0.297407161958219	-2.88286447758995
a_HQ_transcript/13881	-2.85894383676844	0.325598068773211	-3.02357006940822	0.00484055765113713	0.2983795706737	-2.08017487429061
a_HQ_transcript/2633	-3.74227547885514	2.22166069519852	-3.0219253324488	0.00486107224930837	0.299326703080293	-2.09524663737514
a_HQ_transcript/119688	3.13842269902324	-0.579400368946345	3.02005097274234	0.00488455119982672	0.299929322014721	-2.08583924174009

a_HQ_transcript/58526	4.48776612901914	0.171481954584229	3.01972490592329	0.00488864657941657	0.299929322014721	-2.06495082210407
a_HQ_transcript/114529	-1.94283096687081	-0.76417512809254	-3.01953605058339	0.00489102007669231	0.299929322014721	-2.06811490092836
a_HQ_transcript/77462	-3.5715771373866	0.87763879349684	-3.01917303270686	0.00489558547748	0.299929322014721	-2.13942755335568
a_HQ_transcript/106508	3.25029176092003	1.74385298422196	3.0190878107019	0.00489665783429622	0.299929322014721	-2.07965106186901
a_HQ_transcript/42870	2.86598011024737	2.34523288291204	3.0185874191469	0.00490295879500834	0.299999146143416	-2.07302533263578
a_HQ_transcript/67736	1.61436550866901	1.18298035457009	3.01765177769601	0.00491476103594952	0.300405078336145	-2.06958326747338
a_HQ_transcript/139895	-3.29471451270521	-0.0714859597313601	-3.01590614786891	0.00493685240901985	0.301111394817916	-2.09691724291042
a_HQ_transcript/136469	1.8261961328632	2.79669434985221	3.01540326475554	0.00494323392623684	0.301111394817916	-2.11598441822985
a_HQ_transcript/31529	4.52286448607869	-0.0597793620006147	3.01535688261883	0.00494382290203158	0.301111394817916	-2.09448073766438
a_HQ_transcript/48059	4.61966975610117	0.491228142797462	3.01496518274699	0.00494879948545573	0.301111394817916	-2.07710432885501
a_HQ_transcript/152837	0.816964040799668	5.7776709924514	3.01469637871757	0.00495221740720355	0.301111394817916	-2.43964041358778
a_HQ_transcript/87587	-4.82826177202427	1.05225208094889	-3.01412088121184	0.00495954253564582	0.30124168078581	-2.15106390733864
a_HQ_transcript/53414	2.52516916481128	1.65668296355981	3.01306706209748	0.00497298247885756	0.301742719760994	-2.09963266912756
a_HQ_transcript/90868	3.44068223221107	0.972750894810144	3.01182098950456	0.0049889187881754	0.302394026401522	-2.11619640097353
a_HQ_transcript/104286	0.729292228090048	5.57644027094245	3.00980514416727	0.00501480221678093	0.303343541719019	-2.42405199171296
a_HQ_transcript/129099	-2.59989893901746	0.518181973656472	-3.00961984111515	0.00501718786878995	0.303343541719019	-2.08908754908315
a_HQ_transcript/8927	2.92635512410424	-0.0872267372792493	3.00938292293334	0.00502023959423507	0.303343541719019	-2.09474567948215
a_HQ_transcript/125749	3.58497437056151	1.16477926726462	3.00776599020213	0.00504111410443048	0.30409382255124	-2.11017105996275
a_HQ_transcript/124870	-4.18199784378738	2.24874223444668	-3.00721036734371	0.00504830609301505	0.30409382255124	-2.18545985537045
a_HQ_transcript/60970	3.68459640381961	1.41468837077935	3.00720691121632	0.00504835085951601	0.30409382255124	-2.13844369742262
a_HQ_transcript/34663	-2.86614880873199	0.532055989067777	-3.00676816499955	0.00505403689778164	0.304121176805643	-2.09740999417455
a_HQ_transcript/103042	2.31632649287319	-0.508370409571849	3.00550190443406	0.00507048128799219	0.304795177154509	-2.10719048787173
a_HQ_transcript/118708	2.89527970439909	-0.389740440764623	3.00402777771238	0.00508968887632776	0.304925076845084	-2.13303087280833
a_HQ_transcript/117826	-5.30921023763971	0.132942237446205	-3.00385248206141	0.00509197751624967	0.304925076845084	-2.1225205350972
a_HQ_transcript/87571	2.9898317096968	0.595525130990341	3.00374364723452	0.00509339894080769	0.304925076845084	-2.12086486147763
a_HQ_transcript/129640	1.09575777124085	2.82753694088765	3.00356312083474	0.00509575751202581	0.304925076845084	-2.1710574102361
a_HQ_transcript/119063	2.17425339456403	1.03471805969663	3.00315447033593	0.00510110033157798	0.304925076845084	-2.09931497339287
a_HQ_transcript/80637	-1.77852657966628	2.19206716135474	-3.00269623276187	0.00510709776641293	0.304925076845084	-2.10443211546264
a_HQ_transcript/50050	3.65511072295536	0.514370353043316	3.00252332486195	0.00510936252489526	0.304925076845084	-2.14260149126828
a_HQ_transcript/141870	4.00670820069895	1.01874512524987	3.00187233788445	0.005117897712433	0.305070433258027	-2.12234965660477
a_HQ_transcript/69386	1.88472735545041	1.59003963566578	3.00153738039232	0.00512229464044582	0.305070433258027	-2.11246919304812
a_HQ_transcript/120	3.15233082016394	1.26793292274613	2.99750812281184	0.0051754665997721	0.30792172212032	-2.1111231843495
a_HQ_transcript/126013	-0.728051904348601	4.56717944716269	-2.99666442754194	0.00518666625600736	0.308149226263879	-2.33645724845817
a_HQ_transcript/27425	-2.62222103388473	3.37897956461185	-2.99642167924467	0.00518989286595681	0.308149226263879	-2.20767168675135
a_HQ_transcript/69450	-3.4563530047739	-0.454531227205592	-2.99252052845193	0.00524200787948114	0.310865999090372	-2.17217211445334
a_HQ_transcript/103976	2.08742032998962	-0.145583717635996	2.99214250963245	0.00524708398493216	0.310865999090372	-2.12267162225113
a_HQ_transcript/150082	-6.24755211793248	9.90310617504109	-2.99175275661218	0.00525232252231649	0.310865999090372	-2.46682790829084
a_HQ_transcript/149338	-2.43695610677029	-0.528797599602068	-2.99087330635218	0.00526416107476734	0.310865999090372	-2.15924802238181
a_HQ_transcript/54819	-3.23196958175934	1.74166190012684	-2.99043262709261	0.00527010267247571	0.310865999090372	-2.19545200439989
a_HQ_transcript/68679	2.36784791182318	-0.630323749397343	2.98948500863387	0.00528290070738299	0.310865999090372	-2.1455197839453
a_HQ_transcript/142455	-2.70412280653182	-0.691742109521582	-2.98947992062769	0.00528296950249604	0.310865999090372	-2.14875277513897
a_HQ_transcript/57843	2.10688378081675	3.46519195366236	2.98942468804339	0.00528371635870411	0.310865999090372	-2.18584806120238
a_HQ_transcript/68491	3.8750345301865	0.576276095757148	2.98941992685688	0.0052837807442418	0.310865999090372	-2.15699991061077
a_HQ_transcript/148504	0.998219176841483	9.93605626803255	2.98850714673804	0.00529613797448864	0.311044498648206	-2.84972599629512
a_HQ_transcript/86859	-3.13660715180638	0.676194102275048	-2.98802283498939	0.00530270567547863	0.311044498648206	-2.15139904645708
a_HQ_transcript/130871	-2.95213472047308	-0.11194380161293	-2.98783244833398	0.00530528959526816	0.311044498648206	-2.14469641246315
a_HQ_transcript/44694	3.72159125666651	0.427737676811361	2.98744603127476	0.00531053768913221	0.311044498648206	-2.19668395943251
a_HQ_transcript/91640	-2.69381481158225	0.750000624802697	-2.98722293908883	0.00531356983136645	0.311044498648206	-2.13402234092992
a_HQ_transcript/30381	-2.70644630572851	-0.332471334197819	-2.98480857961651	0.00534648922520841	0.312656665676976	-2.1524353106772
a_HQ_transcript/74466	3.36872995858989	0.277019970777107	2.98415150352574	0.00535548164296755	0.312867775821525	-2.14819633850114

a_HQ_transcript/91592	-3.32418830628155	1.068940088098	-2.98290307996257	0.00537260631902655	0.313553072401983	-2.1909875612706
a_HQ_transcript/106139	2.64637449008769	0.25344191885569	2.9813697289072	0.00539371012926598	0.314468989362059	-2.15413077592212
a_HQ_transcript/105936	-2.40111430920156	0.199938039768482	-2.97918378539836	0.00542393109070124	0.315652343840474	-2.17366607305682
a_HQ_transcript/113931	3.19478240552541	2.94124115850824	2.97911624500539	0.00542486738743176	0.315652343840474	-2.26659628013675
a_HQ_transcript/38666	2.62205202162405	1.37387798157028	2.97852939245276	0.00543300921447273	0.315809959618871	-2.15059328468583
a_HQ_transcript/141414	1.78936651054744	-0.868988482003771	2.97808193174564	0.00543922490990698	0.31585541015292	-2.17345991418249
a_HQ_transcript/116607	1.93219874930339	-0.857712885920764	2.97592192401163	0.00546932422317307	0.316893370470036	-2.17802370376066
a_HQ_transcript/56281	1.94341609709159	-0.856835735953374	2.97589766483011	0.00546966316176816	0.316893370470036	-2.17807357279419
a_HQ_transcript/67191	1.98478583387253	-0.853661660096046	2.97562642403941	0.00547345416928015	0.316893370470036	-2.17865932004708
a_HQ_transcript/5052	-1.1876482846423	2.6048941624431	-2.97507113371485	0.00548122294545122	0.317027390421083	-2.19245609296933
a_HQ_transcript/135951	1.50009228916317	3.67290861229949	2.97464971236469	0.00548712577799277	0.317053327259606	-2.34577974412075
a_HQ_transcript/35957	1.00960882448376	3.62423337171974	2.97377729958122	0.00549936470729775	0.317198414433765	-2.32108725788653
a_HQ_transcript/22509	2.50409952311479	0.112574421802472	2.97369286877952	0.00550055053931384	0.317198414433765	-2.17259773123031
a_HQ_transcript/18364	1.81892336368755	-0.286965282877011	2.97216350736311	0.00552207227829947	0.318123902272539	-2.16572863626726
a_HQ_transcript/13402	3.59137320197541	1.78087178769425	2.97118427804223	0.00553589407108604	0.318316405749854	-2.1709855890049
a_HQ_transcript/125101	-5.88671292124608	0.614050417544298	-2.97102888104136	0.00553809049525482	0.318316405749854	-2.18464186811334
a_HQ_transcript/92211	2.56273333123572	-0.401169092275462	2.97076358339767	0.00554184218653406	0.318316405749854	-2.18286731632469
a_HQ_transcript/27217	-3.39272530589719	0.17269200902319	-2.97000761236022	0.00555254586899683	0.318616373418606	-2.1933665125734
a_HQ_transcript/143375	1.79058259083278	-0.8687971682936	2.96886822138199	0.00556871521248027	0.318728982418987	-2.19339740527148
a_HQ_transcript/86016	1.69253610399138	-0.87679167091489	2.96875832341798	0.00557027714600951	0.318728982418987	-2.19378443125576
a_HQ_transcript/133781	1.29154367548309	2.62626082348301	2.96871042808185	0.00557095799163381	0.318728982418987	-2.27336214635783
a_HQ_transcript/99560	3.12611898027258	2.78498519042116	2.9669685326689	0.00559577296163643	0.319259158628203	-2.27250594080044
a_HQ_transcript/38691	2.19831574631399	-0.837377033406671	2.96684532374119	0.00559753213540029	0.319259158628203	-2.19780531551112
a_HQ_transcript/148478	4.68946088133475	1.5729994507375	2.96678948058544	0.0055983296343531	0.319259158628203	-2.27181420121499
a_HQ_transcript/45285	-2.67319425882342	2.92703379097624	-2.96651898214242	0.0056021941542934	0.319259158628203	-2.2799933195381
a_HQ_transcript/39175	-2.82007655659516	0.60441385389127	-2.96567833018413	0.00561422034061575	0.319631145895506	-2.17825987986327
a_HQ_transcript/112100	4.04940762508635	0.255350152347765	2.96511791884599	0.00562225099421034	0.319775152437826	-2.20108585526584
a_HQ_transcript/8712	1.91967965762646	-0.858551598246018	2.96447225003671	0.00563151681977508	0.319929397895678	-2.20279013094066
a_HQ_transcript/54863	-2.18916976074889	-0.443125857152645	-2.96410949618411	0.00563672892632919	0.319929397895678	-2.19451318053259
a_HQ_transcript/125680	1.1389256908992	4.66651624732539	2.96377948369505	0.0056414745534522	0.319929397895678	-2.44313743246021
a_HQ_transcript/102201	2.14075060707957	-0.841499583145002	2.96331647081178	0.0056481391009091	0.319940097577535	-2.20536640274496
a_HQ_transcript/112944	3.37239513939698	0.089381059110321	2.96190502120835	0.00566850119254279	0.319940097577535	-2.20653735127595
a_HQ_transcript/32701	2.01630585247365	-0.850929924303028	2.96184347477276	0.00566939065806657	0.319940097577535	-2.20846197126096
a_HQ_transcript/87007	-1.46735881655904	2.58964417737117	-2.96180732313149	0.00566991318088245	0.319940097577535	-2.20584482613282
a_HQ_transcript/45	-0.968360681495545	3.44154005668649	-2.96173026428908	0.00567102711331607	0.319940097577535	-2.31094157184022
a_HQ_transcript/99693	1.85363925969197	-0.863699492414651	2.96100034590007	0.00568158877322662	0.319940097577535	-2.21034010083954
a_HQ_transcript/57974	-3.51414383425338	0.199180183405051	-2.96011076101635	0.005694485841556	0.319940097577535	-2.22893830611102
a_HQ_transcript/108676	-1.99825000012002	-0.601546981187379	-2.95968891527821	0.00570061134723498	0.319940097577535	-2.20942595420178
a_HQ_transcript/143348	1.68289141672058	-0.877572790187168	2.95931125113378	0.00570610057732588	0.319940097577535	-2.2142134176489
a_HQ_transcript/104828	-1.40631339176128	4.01238821582006	-2.9587815802974	0.00571380757826116	0.319940097577535	-2.367737740828
a_HQ_transcript/45580	1.78892978501679	-0.868921009901111	2.95862682205354	0.00571606124857232	0.319940097577535	-2.21552544538281
a_HQ_transcript/53341	1.7884098024043	-0.868964119289551	2.9586026304318	0.00571641361536859	0.319940097577535	-2.21557835079383
a_HQ_transcript/119272	-1.32244263437329	6.42720454116305	-2.95857153726469	0.0057168665377813	0.319940097577535	-2.60587920123596
a_HQ_transcript/36607	2.67529323107921	1.0200371108461	2.95844431494642	0.00571872008985445	0.319940097577535	-2.19228316347672
a_HQ_transcript/122760	-0.790259235180733	4.21994917811763	-2.95683439127411	0.00574222473240273	0.320395679888381	-2.44959260990983
a_HQ_transcript/125986	2.89587817552234	-0.099068243213256	2.95670856192063	0.00574406565628987	0.320395679888381	-2.20405943971347
a_HQ_transcript/40596	2.54458597858184	0.605742467200801	2.95657921650433	0.00574595860154108	0.320395679888381	-2.2100250313052
a_HQ_transcript/96858	-0.93191112375158	3.53475232385408	-2.95637756386563	0.00574891092285269	0.320395679888381	-2.36070327455433
a_HQ_transcript/4694	1.18677529594284	1.83488991084549	2.95566798610349	0.00575931096651826	0.320572603423244	-2.20621358717685
a_HQ_transcript/50722	1.52952948545436	-0.573123525699976	2.95540871942049	0.00576311537601999	0.320572603423244	-2.21045198970793

a_HQ_transcript/117686	1.63986839100907	-0.881134007372932	2.9545853875225	0.00577521244770273	0.320938383518226	-2.22449366248873
a_HQ_transcript/50054	-2.48057167933298	-0.209424592366275	-2.95412740963261	0.005781951798495	0.32100601159782	-2.25493202677705
a_HQ_transcript/51590	1.64482949039241	0.791859666763752	2.95349964720571	0.00579120165728661	0.321212757571332	-2.22536221698467
a_HQ_transcript/16947	-2.16748460765514	-0.684979810829969	-2.95291697766834	0.00579979957460102	0.32137105161271	-2.22777082767988
a_HQ_transcript/102016	1.71912159161655	-0.874597921445683	2.95225163349848	0.00580963216633643	0.32137105161271	-2.22938627019751
a_HQ_transcript/146230	7.81652691651899	3.70731856472836	2.95207421866387	0.00581225669243637	0.32137105161271	-2.43894513627058
a_HQ_transcript/109174	3.4268973958195	-0.264876917393089	2.95122452830076	0.00582484180583942	0.32137105161271	-2.21682482031471
a_HQ_transcript/131980	-1.80484999039791	2.29539505798904	-2.95100102548825	0.0058281564639909	0.32137105161271	-2.26483018238855
a_HQ_transcript/56169	1.61102141697381	-0.883563897658248	2.95065220991301	0.00583333312720687	0.32137105161271	-2.23304374574143
a_HQ_transcript/45010	1.60768292828845	-0.88384596703697	2.95019943853146	0.00584005903277586	0.32137105161271	-2.23402775934823
a_HQ_transcript/78789	-1.68456401128205	1.76753305336128	-2.94979610882216	0.0058460566414571	0.32137105161271	-2.21350508743547
a_HQ_transcript/151981	3.54831031729267	2.64459259784663	2.94941819562533	0.00585168157048759	0.32137105161271	-2.3105314130138
a_HQ_transcript/131611	-5.59598023306164	0.313787900043514	-2.94931646331221	0.00585319664435412	0.32137105161271	-2.26592788125011
a_HQ_transcript/136846	-4.63069146568187	-0.778860236040724	-2.94908465456435	0.0058566502956962	0.32137105161271	-2.24936728340456
a_HQ_transcript/143453	1.5975775897692	-0.884700929961349	2.94883313968456	0.00586039971630664	0.32137105161271	-2.23699687057315
a_HQ_transcript/13001	1.66047358421604	-0.879475596625017	2.94721312042023	0.00588460420109365	0.321900659105366	-2.24034637838321
a_HQ_transcript/47499	2.03267440331829	-0.836768805048501	2.94694194938979	0.00588866492730494	0.321900659105366	-2.24059971342035
a_HQ_transcript/119950	-2.82680191984581	0.351434841996755	-2.94681515121968	0.00589056460818113	0.321900659105366	-2.28901391878269
a_HQ_transcript/150698	0.491180151052141	10.4118500031519	2.9465016586819	0.00589526381071721	0.321900659105366	-3.02193830546134
a_HQ_transcript/120931	0.83297709314289	4.06813386100051	2.94512341346219	0.0059159654392235	0.321900659105366	-2.45545666022658
a_HQ_transcript/9186	1.42189470291438	-0.82433061207414	2.94498887876427	0.0059179898550541	0.321900659105366	-2.24812141824225
a_HQ_transcript/117232	-1.92850133366275	-0.0856038032264867	-2.94473924237201	0.00592174799809755	0.321900659105366	-2.23049690009576
a_HQ_transcript/26275	1.63222476441005	-0.881895462324548	2.94436540259501	0.00592738016442358	0.321900659105366	-2.24654249721052
a_HQ_transcript/7911	2.17553426345277	1.85041702022246	2.9442207839782	0.00592956030259992	0.321900659105366	-2.23464895781712
a_HQ_transcript/9777	-2.71531248643411	1.43026494270847	-2.94414277518271	0.00593073660575989	0.321900659105366	-2.24307636849171
a_HQ_transcript/48031	0.920747219967931	3.30835689899763	2.94412709161296	0.00593097312658009	0.321900659105366	-2.36063320236258
a_HQ_transcript/42181	-2.81386070055493	-0.0859438030715466	-2.94271186217788	0.00595235256122735	0.32223122237575	-2.25212716210761
a_HQ_transcript/49788	1.61407505682322	2.8641936457846	2.94251011913066	0.00595540614034615	0.32223122237575	-2.3030652380483
a_HQ_transcript/19456	2.65230231978945	1.13257725395931	2.94208551676783	0.00596183773871026	0.32223122237575	-2.2427937552935
a_HQ_transcript/112122	-1.68415340759131	-0.672820666855959	-2.9416791830046	0.00596799874615878	0.32223122237575	-2.24722493609771
a_HQ_transcript/137152	1.54354510124178	-0.889302766075328	2.94162986955526	0.00596874686610879	0.32223122237575	-2.25264337040108
a_HQ_transcript/76448	1.95708479077495	-0.789151516059185	2.94152589205035	0.00597032456817167	0.32223122237575	-2.25806628366161
a_HQ_transcript/133342	2.2793809755088	-0.450656278284544	2.94015446013695	0.00599117079776677	0.323056378601657	-2.23836414753256
a_HQ_transcript/52543	3.58219967871253	1.09620446859304	2.93940392783718	0.00600260816707281	0.323299819407786	-2.23369131202196
a_HQ_transcript/81900	2.11298361225723	4.43442588005627	2.93912859023613	0.00600680919626358	0.323299819407786	-2.50468941698872
a_HQ_transcript/121818	-3.83393478239036	4.49916091563539	-2.93843790752203	0.00601735964890714	0.323544194305953	-2.52413957645932
a_HQ_transcript/1052	1.07980516330883	4.00163046838214	2.93786192136748	0.00602617141744219	0.323544194305953	-2.47073712472611
a_HQ_transcript/79498	-2.98765789622686	1.01572218027843	-2.93773937317578	0.00602804779853679	0.323544194305953	-2.23519503028847
a_HQ_transcript/129652	2.28316237829722	-0.830263469476569	2.93650226276998	0.00604702051407718	0.324263107419076	-2.26338370903822
a_HQ_transcript/139744	2.96995570039318	0.0727132034481835	2.93602479687359	0.00605435811975309	0.324357353718901	-2.26890650466494
a_HQ_transcript/61885	-1.24299889623974	3.12738398035482	-2.93380037340537	0.00608865335995363	0.325894330117297	-2.34737617766062
a_HQ_transcript/62164	3.13350643071173	0.235255158956885	2.93261567086503	0.0061069932290795	0.326574303641038	-2.24593508999777
a_HQ_transcript/140146	2.11999408687694	-0.667295686141479	2.9309590877923	0.00613272535722699	0.326574303641038	-2.27503359291979
a_HQ_transcript/24055	-4.61237369157078	-0.778873639096903	-2.93082668850542	0.00613478635089555	0.326574303641038	-2.28900208542071
a_HQ_transcript/101149	-2.29415643939651	-0.710271524478767	-2.93067675663571	0.00613712105330491	0.326574303641038	-2.27329536576846
a_HQ_transcript/85238	-3.67768892168668	1.22600069789421	-2.93017909327657	0.00614487654654801	0.326574303641038	-2.33921371296908
a_HQ_transcript/64642	4.39014170641237	4.23671679403691	2.92998743717688	0.00614786574107932	0.326574303641038	-2.36830658213993
a_HQ_transcript/25690	-2.33610635139905	1.20082173460952	-2.92983475309874	0.00615024808252528	0.326574303641038	-2.25166573039113
a_HQ_transcript/26398	2.44546481611974	0.680434841828755	2.92969890118313	0.00615236852148645	0.326574303641038	-2.26185534204192
a_HQ_transcript/140499	0.9387837548445	4.74055011144543	2.92965914912413	0.00615298912006187	0.326574303641038	-2.55595007575392

a_HQ_transcript/63882	-2.45715240893532	-0.66046449911364	-2.92936781386944	0.00615753916856899	0.326574303641038	-2.2799633509695
a_HQ_transcript/143510	-2.70748524180998	2.5304793600296	-2.92853912750436	0.00617049885434184	0.326963315775007	-2.37391192022593
a_HQ_transcript/142965	-1.29559635287327	2.6015994059614	-2.92714129518023	0.0061924175516899	0.327363060907019	-2.33359377209314
a_HQ_transcript/36871	1.89915813761463	-0.885880580069148	2.92697330779045	0.00619505660677982	0.327363060907019	-2.28405068273011
a_HQ_transcript/43555	2.55168733246697	-0.607704926427642	2.92673112730843	0.00619886308787182	0.327363060907019	-2.28232158860137
a_HQ_transcript/52649	2.14050394409406	0.459688186679673	2.92623161884718	0.00620672108939415	0.327363060907019	-2.26351425195843
a_HQ_transcript/82146	-3.60052533914543	0.442506496824395	-2.9255078700983	0.00621812336503914	0.327363060907019	-2.27352214730111
a_HQ_transcript/23954	1.9591622429854	-0.783041332543945	2.92547989638228	0.00621856447173807	0.327363060907019	-2.28175449736475
a_HQ_transcript/25503	4.07188747717788	2.16597476287565	2.92527403293724	0.00622181155922481	0.327363060907019	-2.31556485254395
a_HQ_transcript/69237	2.2917383779774	-0.606796167688325	2.92500966913326	0.00622598371562827	0.327363060907019	-2.2880776098781
a_HQ_transcript/17603	3.32013738540722	0.0215896413151688	2.92437531190142	0.00623600581042558	0.327363060907019	-2.28531995648503
a_HQ_transcript/97579	0.750821602954635	4.80036887168172	2.92413707319039	0.00623977362320171	0.327363060907019	-2.54012683044864
a_HQ_transcript/11386	-2.13073399057296	-0.621299295694767	-2.92344188818902	0.00625078042930707	0.327363060907019	-2.30554255357253
a_HQ_transcript/70641	1.37209352907457	3.20038600006201	2.92322940685595	0.00625414827485034	0.327363060907019	-2.38857878281848
a_HQ_transcript/38556	-1.57424015779316	1.72528745064609	-2.92299645205786	0.00625784258987946	0.327363060907019	-2.2713701891859
a_HQ_transcript/84916	-5.69462073811893	1.4773522501974	-2.92284216453951	0.00626029049042102	0.327363060907019	-2.35752070655426
a_HQ_transcript/131771	1.80807557406132	-0.836192980829042	2.92224713167909	0.00626973963105551	0.327363060907019	-2.29361240622493
a_HQ_transcript/109232	-1.96028144201396	-0.542941485577148	-2.92192622277455	0.00627484124722496	0.327363060907019	-2.27407874207438
a_HQ_transcript/52156	3.02300832775204	1.78805417306076	2.92192537851549	0.00627485467389962	0.327363060907019	-2.34632935847209
a_HQ_transcript/55411	4.71286537587771	0.614891981265066	2.92163875104946	0.006279414617935	0.327363060907019	-2.30725485463959
a_HQ_transcript/129080	-2.21237700311625	-0.504087414325286	-2.92017572184382	0.00630273853326717	0.327869447672096	-2.31044053450826
a_HQ_transcript/114004	2.06612598708835	-0.717656833048814	2.91951018991649	0.00631337555048194	0.327869447672096	-2.2912375460999
a_HQ_transcript/118748	1.61905596635381	6.93060164441443	2.9193647441639	0.00631570241996122	0.327869447672096	-2.73590269583745
a_HQ_transcript/3255	-1.22589557138845	2.8534311363413	-2.91926805402394	0.00631724973439695	0.327869447672096	-2.31531048915776
a_HQ_transcript/23937	-2.46286467843367	1.46948357523975	-2.91909857489412	0.00631996273861769	0.327869447672096	-2.31104672860542
a_HQ_transcript/81502	3.36451656524382	0.397495179423371	2.91891073935179	0.0063229708718762	0.327869447672096	-2.30906995930104
a_HQ_transcript/104896	-3.05601148129543	0.423360613927291	-2.91847900678025	0.00632989005757494	0.327935694533615	-2.35641885602614
a_HQ_transcript/142088	2.10791204149435	0.868142227335969	2.91809403844885	0.00633606578460763	0.327963340986351	-2.27875076359542
a_HQ_transcript/25283	2.4556338307397	0.723422363128544	2.91649211347634	0.0063618250949662	0.328736549734215	-2.27839565810989
a_HQ_transcript/68875	-0.965502742850713	3.71259850892839	-2.91646173612811	0.00636231452055794	0.328736549734215	-2.43096952612515
a_HQ_transcript/37696	-2.40870437095212	-0.0926499259257768	-2.91565546619325	0.006375317725166	0.328876909241115	-2.31640025061116
a_HQ_transcript/102803	4.99164458451864	2.18504642833	2.91520512429083	0.00638259154617284	0.328876909241115	-2.353954174184
a_HQ_transcript/21591	2.6906902728605	-0.377388219441981	2.91502066319542	0.00638557317468004	0.328876909241115	-2.30474762424285
a_HQ_transcript/88736	-3.03087839396642	0.532985712734508	-2.91470821749575	0.00639062653705857	0.328876909241115	-2.39253268244828
a_HQ_transcript/115274	3.40950543890897	3.48003330174103	2.91432247446064	0.00639687057110139	0.328876909241115	-2.39559024085984
a_HQ_transcript/66920	2.88839701223896	1.03540165694218	2.91384656791082	0.00640458199679016	0.328876909241115	-2.28374513595375
a_HQ_transcript/90361	-2.17339543540475	-0.106465390522652	-2.91335065944985	0.0064126268345287	0.328876909241115	-2.3183731774569
a_HQ_transcript/49430	-0.782541411957234	3.39368203855085	-2.91302617686855	0.0064178958750779	0.328876909241115	-2.4339322569105
a_HQ_transcript/152540	-3.41795390407493	0.414514433407318	-2.91281148440809	0.00642138435189172	0.328876909241115	-2.28592559562644
a_HQ_transcript/40327	-4.77077741168618	1.69884065017746	-2.91252611989655	0.00642602392118752	0.328876909241115	-2.33768896191078
a_HQ_transcript/55503	-1.1224456479003	3.15478381079899	-2.91213510559679	0.00643238630967213	0.328876909241115	-2.42312418697013
a_HQ_transcript/148106	-2.15242297762038	-0.772773870793106	-2.91210203558354	0.00643292468013948	0.328876909241115	-2.30885390117742
a_HQ_transcript/27898	-2.88382893696052	1.00419192488302	-2.91128164874968	0.00644629392791645	0.329023680780127	-2.33405818847238
a_HQ_transcript/112664	3.53226823364758	3.72185050258964	2.91123124131395	0.006447116233288	0.329023680780127	-2.40391712265108
a_HQ_transcript/67287	3.03602116967561	0.0967532290258041	2.90951696313271	0.00647514038705866	0.330164000367496	-2.32142371090003
a_HQ_transcript/109062	1.42322288702124	3.50978533937334	2.90845919794786	0.00649248933863336	0.330592349680632	-2.46313068373173
a_HQ_transcript/104826	-2.69184374565318	-0.646315812206742	-2.9083114710914	0.00649491576065375	0.330592349680632	-2.30662197361924
a_HQ_transcript/143465	8.81998556497203	2.08594905669036	2.90793430311034	0.00650111463683315	0.330618365362937	-2.36587915966019
a_HQ_transcript/135324	3.07572720714574	4.71153270609897	2.9075415561759	0.00650757547397489	0.3306576461112	-2.54347458709521
a_HQ_transcript/14400	-1.11536248808623	2.65481407477658	-2.90677414023378	0.00652021719754556	0.330756877876049	-2.32782609812021

a_HQ_transcript/106099	1.55929024334905	3.30711836761193	2.90673220178133	0.00652090871948033	0.330756877876049	-2.43075307484131
a_HQ_transcript/115631	2.31397296054275	-0.680103006522589	2.90532377068409	0.00654417240822226	0.331647474930378	-2.32856996278653
a_HQ_transcript/138357	-0.940276296744961	4.15463951148769	-2.90489147575638	0.00655132846311558	0.331686950691082	-2.54268240240094
a_HQ_transcript/32043	-6.29581118940916	4.19351835123073	-2.90395967579826	0.00656677818630083	0.331686950691082	-2.56845245451644
a_HQ_transcript/132694	3.16024502728479	0.723416853356966	2.9039180789526	0.00656746868194508	0.331686950691082	-2.31568183514368
a_HQ_transcript/12643	-2.69500729368395	-0.080270026604455	-2.90389957001251	0.00656777594696937	0.331686950691082	-2.344489511998
a_HQ_transcript/110995	3.10491465563376	3.29047768429239	2.90330623374186	0.0065776330297836	0.331896399961164	-2.42160921006389
a_HQ_transcript/46431	-3.00609928556313	-0.180371638952356	-2.90222560699343	0.00659562121981098	0.332367642114511	-2.34761907389355
a_HQ_transcript/142912	2.97961465860325	0.316338126226614	2.90205844262182	0.00659840797894552	0.332367642114511	-2.32918039511409
a_HQ_transcript/90724	1.95538728991354	3.15180918088798	2.90043794012985	0.00662548047184593	0.333131949174936	-2.46712710359765
a_HQ_transcript/150904	-3.63378572831221	1.38427834785896	-2.90040545459376	0.00662602424939424	0.333131949174936	-2.36896920366305
a_HQ_transcript/148986	5.19169826401068	2.83539306331607	2.90012177368228	0.00663077458703896	0.333131949174936	-2.42447935283477
a_HQ_transcript/150829	0.4930360953687	10.3033934007669	2.89866067918236	0.00665529190590404	0.334074963649732	-3.11990919243874
a_HQ_transcript/98517	-2.08400346981349	-0.589765886551079	-2.89740467288576	0.00667643593951157	0.334605563504628	-2.34162225768843
a_HQ_transcript/52254	4.31180382225425	1.51316812305583	2.89734897757555	0.00667737499424319	0.334605563504628	-2.43085257648979
a_HQ_transcript/132368	2.03900981177019	2.87442636825146	2.89572300294624	0.00670484465768867	0.335692687564278	-2.38983527438751
a_HQ_transcript/3882	0.991980318119907	-0.828582608888635	2.89492488441541	0.00671836713786949	0.336080245258243	-2.37401526082052
a_HQ_transcript/15113	-2.79369723797092	2.53369907693457	-2.89347221391747	0.0067430454909781	0.336654640624392	-2.38366460975873
a_HQ_transcript/111917	-1.84827819186942	-0.0586630950864065	-2.89340322929911	0.00674421953713913	0.336654640624392	-2.35836625145296
a_HQ_transcript/110117	2.80630198736635	-0.790032072976621	2.89322672457768	0.00674722433814024	0.336654640624392	-2.35779222852125
a_HQ_transcript/99913	-2.81257265232338	1.74017077388772	-2.89113231793108	0.00678297554037407	0.337961029842394	-2.40899005541309
a_HQ_transcript/139705	2.18652462188105	-0.724440183411743	2.89101197792198	0.00678503512637754	0.337961029842394	-2.36064226065956
a_HQ_transcript/78344	2.33671608170039	0.20308047505256	2.88993702250156	0.00680345878824437	0.338523191315949	-2.34944421232997
a_HQ_transcript/76318	4.30844509768454	2.29485905802874	2.88967429596424	0.00680796880416228	0.338523191315949	-2.36608856284919
a_HQ_transcript/24506	-3.43423673164105	-0.0438025298072756	-2.88922117033852	0.00681575385228835	0.338596306454917	-2.35359692048123
a_HQ_transcript/101655	-4.7920452115716	0.583369508604704	-2.88887725825741	0.00682166811209448	0.338596306454917	-2.39219617234393
a_HQ_transcript/120548	2.50971944956038	0.66934430791517	2.88857243205404	0.00682691424382677	0.338596306454917	-2.34276317129759
a_HQ_transcript/19460	-2.1414665717479	1.94364235995916	-2.88726975191163	0.00684937643848388	0.339208661312226	-2.34545799367056
a_HQ_transcript/135593	3.75896169017123	1.26661798752454	2.88697601363282	0.00685445097401754	0.339208661312226	-2.39638032433503
a_HQ_transcript/41365	-3.24211194675687	2.05229344612201	-2.88655105471604	0.00686179869237155	0.339208661312226	-2.40311509299365
a_HQ_transcript/77832	-4.22206369633794	3.11205107205735	-2.88650456463188	0.0068626029745248	0.339208661312226	-2.49075803834411
a_HQ_transcript/9024	-2.29853247970123	1.45714054022461	-2.88604202672024	0.0068706097442269	0.339315890579797	-2.34684551131647
a_HQ_transcript/113852	-2.97905511320776	0.563545169280385	-2.88416739335631	0.00690315059422695	0.340633563447559	-2.36233414305409
a_HQ_transcript/8241	3.18929332406877	-0.14134553082255	2.88359622525318	0.00691309397371873	0.340676075684452	-2.37312863104761
a_HQ_transcript/129263	2.97731962711916	1.03607612086997	2.88311608261693	0.00692146311495929	0.340676075684452	-2.3946511939903
a_HQ_transcript/139070	1.96251171899609	-0.875701159300155	2.8827691852166	0.00692751564379988	0.340676075684452	-2.37826971197483
a_HQ_transcript/137937	0.916148932344875	4.80667955193312	2.88257581817764	0.00693089159382758	0.340676075684452	-2.65290598559167
a_HQ_transcript/24745	-2.57357410460888	2.07725586572385	-2.88243700307137	0.00693331608750872	0.340676075684452	-2.40484873953922
a_HQ_transcript/66229	1.29438308981524	3.09934017995	2.88206986637157	0.00693973220372802	0.34070333913708	-2.45614045511696
a_HQ_transcript/6822	2.84404986729505	-0.35091477126042	2.88166783141333	0.00694676460445532	0.340760787280826	-2.35894091194827
a_HQ_transcript/111610	-2.4125556578099	-0.450797280017876	-2.87787048758753	0.00701351887150521	0.343539599850605	-2.40796750350525
a_HQ_transcript/67277	-3.45302284262912	0.390597484736971	-2.87756571197087	0.00701890262443454	0.343539599850605	-2.44119647113185
a_HQ_transcript/147218	1.71562788008805	3.07026579084875	2.87743890531013	0.00702114376242979	0.343539599850605	-2.49563505475748
a_HQ_transcript/1636	-2.60451178574796	1.48285343547296	-2.87655325615813	0.0070368151776431	0.343579610208259	-2.36142558056179
a_HQ_transcript/45240	2.27510260785344	-0.174952146282692	2.87623227500754	0.00704250299332967	0.343579610208259	-2.37514860732492
a_HQ_transcript/14779	1.64156928810513	-0.830372008786135	2.87606617130919	0.00704544806149522	0.343579610208259	-2.39240305385652
a_HQ_transcript/100665	-2.20173906595781	-0.185107856719741	-2.87519423025852	0.00706092682035117	0.343579610208259	-2.39924999748073
a_HQ_transcript/54126	-1.76805596905795	1.57580793005986	-2.87514196947777	0.00706185557122591	0.343579610208259	-2.36873789397197
a_HQ_transcript/98187	-3.69895475429385	1.90490269533344	-2.87461061180193	0.00707130509410251	0.343579610208259	-2.4676956526495
a_HQ_transcript/7066	-0.796203628817346	3.7808922988854	-2.87430141497877	0.0070768092322775	0.343579610208259	-2.57093732647834

a_HQ_transcript/138170	4.27244054163597	3.68367330422776	2.8741512686973	0.00707948349833873	0.343579610208259	-2.51460898421184
a_HQ_transcript/142263	-3.56772631612982	1.82634915729	-2.87397183266789	0.0070826806910729	0.343579610208259	-2.49577214852023
a_HQ_transcript/118394	-2.41268746915836	0.00349530673583749	-2.87358083553285	0.00708965217888769	0.343579610208259	-2.45372022612607
a_HQ_transcript/123379	3.10111841173067	0.795655256978204	2.87325791612054	0.00709541469788826	0.343579610208259	-2.37758564514953
a_HQ_transcript/16425	2.291026576136	0.332671364763217	2.87315165134848	0.00709731196174259	0.343579610208259	-2.38820839998764
a_HQ_transcript/117629	1.15791162280015	3.62016552498455	2.87306826270277	0.00709880112613747	0.343579610208259	-2.56039829498202
a_HQ_transcript/141007	3.11645880471323	-0.389329836361584	2.87268925725322	0.00710557314912863	0.343621261241721	-2.39623886882143
a_HQ_transcript/116128	-2.24888366730767	-0.392123356191579	-2.87227978930007	0.00711289629007202	0.343630183584388	-2.37690814417191
a_HQ_transcript/20499	-3.48877975675578	0.781886210549576	-2.87201806187454	0.00711758087385774	0.343630183584388	-2.37987830775749
a_HQ_transcript/130713	-4.85674561204426	-0.768780242325043	-2.87091292176131	0.00713739342185047	0.344009425818358	-2.41853329831329
a_HQ_transcript/42395	-1.85418937529181	3.62774093567343	-2.87086440973665	0.00713826431312691	0.344009425818358	-2.5100500344515
a_HQ_transcript/102093	1.06600330192695	4.63258836910597	2.87031693593189	0.00714809952096096	0.344009425818358	-2.6438703192017
a_HQ_transcript/15094	1.06498682163717	2.27905649781879	2.87009444277113	0.00715210018113189	0.344009425818358	-2.42614302127612
a_HQ_transcript/33315	-1.22638395095993	2.28015247747973	-2.8697108967326	0.00715900167726233	0.344009425818358	-2.39258671397641
a_HQ_transcript/138833	-4.86101721730079	-0.768590797750592	-2.86960296597636	0.00716094490160014	0.344009425818358	-2.42134726777866
a_HQ_transcript/60257	-3.70032898506703	0.424044624911647	-2.86838885585159	0.00718283832616784	0.344776239656056	-2.42848332925479
a_HQ_transcript/8038	-3.22393110198549	0.830660467034009	-2.86713266083914	0.00720555678567652	0.345525190860911	-2.38579031579469
a_HQ_transcript/41965	2.22567581411367	1.83649305989851	2.86675318820955	0.00721243285652948	0.345525190860911	-2.394552490975
a_HQ_transcript/55399	-2.76954050860709	-0.0493211218590425	-2.86630131039482	0.00722062894967555	0.345525190860911	-2.4145641204617
a_HQ_transcript/21454	-2.52053120618566	0.575451472458327	-2.86621373850881	0.00722221832672735	0.345525190860911	-2.38052509366897
a_HQ_transcript/133136	3.92361375988848	0.602942397883645	2.86494039419345	0.00724536589825639	0.345684760431274	-2.39851124633419
a_HQ_transcript/116742	2.19653514225744	-0.427624327060777	2.86465464503766	0.00725056996668042	0.345684760431274	-2.41609486611141
a_HQ_transcript/84783	-1.24640492075885	2.72831206294589	-2.86461035853613	0.00725137682715951	0.345684760431274	-2.43676922852844
a_HQ_transcript/75633	-1.59035689603346	2.19105626865575	-2.86452697578439	0.00725289621511931	0.345684760431274	-2.40666171233845
a_HQ_transcript/152715	3.47119081563495	0.0644755307231715	2.86439572447771	0.00725528846212074	0.345684760431274	-2.42481763645346
a_HQ_transcript/94486	2.33729047436554	2.54177130285713	2.86353681977375	0.00727096154779154	0.346016267917981	-2.4751902829354
a_HQ_transcript/83059	2.84112155653635	0.981810032769953	2.86281510802145	0.00728415573487443	0.346016267917981	-2.42712585689071
a_HQ_transcript/44510	3.36765322067002	0.859065043955452	2.86267611454152	0.00728669936470929	0.346016267917981	-2.40775837323711
a_HQ_transcript/92797	4.0899807039143	1.77554395402741	2.8623387664826	0.00729287642113615	0.346016267917981	-2.39986593672111
a_HQ_transcript/36208	2.55039236004947	0.158840720473849	2.86221025091602	0.00729523091501537	0.346016267917981	-2.44240485783681
a_HQ_transcript/51450	4.02160139392917	2.29162345949878	2.8620612198413	0.00729796216053269	0.346016267917981	-2.48589925329321
a_HQ_transcript/5012	3.60778815983698	0.121275095849628	2.86040567761974	0.0073283673759858	0.347174685274085	-2.40774209074018
a_HQ_transcript/15461	-3.59693574434716	0.0814126112958593	-2.85982393304692	0.00733907972842635	0.347397711972726	-2.43170072801181
a_HQ_transcript/81219	-4.93226559410953	1.40829975234695	-2.85923056011333	0.0073500213450238	0.347397711972726	-2.49570434370574
a_HQ_transcript/51126	2.29357303722666	1.43526087387864	2.85917728698818	0.00735100443377465	0.347397711972726	-2.3989665321299
a_HQ_transcript/115368	3.23143508717857	-0.23927366406866	2.85779491062125	0.00737655764145823	0.348322130448971	-2.42738236968187
a_HQ_transcript/33980	3.36921000262258	0.267640529258305	2.85626639917081	0.00740490917575862	0.348337130928781	-2.41935373023959
a_HQ_transcript/114275	5.15252589485259	-0.0623724401926017	2.85624424885307	0.00740532078060746	0.348337130928781	-2.43260492873809
a_HQ_transcript/139125	3.33775229346697	-0.186773090139543	2.85610137523058	0.00740797622340245	0.348337130928781	-2.40989891687622
a_HQ_transcript/105386	2.3359718510905	1.74321469009021	2.85598456834854	0.00741014785467103	0.348337130928781	-2.41347391204162
a_HQ_transcript/95338	2.73158267694592	0.0808400733324763	2.85584391897522	0.00741276354952489	0.348337130928781	-2.43651753532599
a_HQ_transcript/11534	1.74967387093092	3.44642122969538	2.85584030139219	0.00741283083813141	0.348337130928781	-2.53991019386758
a_HQ_transcript/142781	-2.51933275289217	-0.731095132534237	-2.85516669266563	0.0074253702444226	0.348363830657411	-2.43294438410883
a_HQ_transcript/130445	3.33717401436792	1.62566685022421	2.85516589425871	0.00742538511878152	0.348363830657411	-2.41076645859685
a_HQ_transcript/117871	-1.00063963844965	2.41876777437169	-2.85466584818655	0.00743470649432405	0.348519854114571	-2.42433068353913
a_HQ_transcript/20752	2.42091403385932	-0.522491581627566	2.85371190831144	0.0074525193748824	0.349073365208029	-2.43882900189125
a_HQ_transcript/151843	0.472982187587882	11.6823770235735	2.85327660438268	0.00746066108409103	0.349173355471855	-3.40121842619385
a_HQ_transcript/15608	2.95467316788701	-0.157865066472164	2.85218807227957	0.00748105698549894	0.349774017712992	-2.46746329105927
a_HQ_transcript/147604	-2.68631803867654	-0.722207984417764	-2.85146797402518	0.00749457824905746	0.349774017712992	-2.44402006537722
a_HQ_transcript/43883	-4.8654384427548	1.88355853527445	-2.85134521443977	0.00749688558797713	0.349774017712992	-2.48303618683924

a_HQ_transcript/114556	-2.69491049154641	0.211298328309799	-2.85130910328955	0.0074975644451966	0.349774017712992	-2.42630877286804
a_HQ_transcript/99065	3.17263566078151	4.32873514967222	2.85037723133239	0.0075151027575236	0.34999155850923	-2.69474163112587
a_HQ_transcript/19779	3.56990603072152	0.399777064976197	2.8502962558322	0.0075166285741243	0.34999155850923	-2.43634164382252
a_HQ_transcript/47928	2.69063428911766	0.268842467192991	2.85010196983469	0.00752029067881277	0.34999155850923	-2.42409848440407
a_HQ_transcript/39199	-1.06056050011932	3.04521529286248	-2.84935583054413	0.00753437024852362	0.350115767392507	-2.51467562936302
a_HQ_transcript/112506	4.18018674667148	-0.231524091309711	2.84914943383306	0.00753826929583302	0.350115767392507	-2.4527534682243
a_HQ_transcript/123685	-3.8467468055292	1.07461897637536	-2.84879161882084	0.00754503327601462	0.350115767392507	-2.45186986069931
a_HQ_transcript/133344	-2.24714705739998	1.8598964743425	-2.84863769171326	0.0075479447981519	0.350115767392507	-2.44352099738121
a_HQ_transcript/141115	2.35300315371843	2.61794198098973	2.8482788531874	0.00755473629960658	0.350115767392507	-2.46273440657348
a_HQ_transcript/99805	3.14102182245429	-0.523731176519898	2.84804852022316	0.0075590986801128	0.350115767392507	-2.44632511831684
a_HQ_transcript/27820	-3.55250950653105	2.05665425033071	-2.84737369000595	0.0075718931994258	0.350414478608376	-2.5221532053832
a_HQ_transcript/122371	5.05532537279204	1.04966295428599	2.84687393219354	0.00758138150005842	0.350414478608376	-2.43342213589627
a_HQ_transcript/104273	-3.7602213612926	2.25034716694742	-2.84675543412662	0.00758363291510696	0.350414478608376	-2.4779518550165
a_HQ_transcript/135162	-1.87868698302813	-0.766781582216687	-2.84604801023482	0.00759708673082852	0.350622433839347	-2.45123052421511
a_HQ_transcript/66549	2.47696557428274	1.12877776122509	2.84575124235158	0.0076027373355816	0.350622433839347	-2.42164548442236
a_HQ_transcript/117682	-3.58637093515738	0.360963540366927	-2.84457232114709	0.00762522351622625	0.350622433839347	-2.45674689733563
a_HQ_transcript/68147	-2.16655630563481	-0.721307025838766	-2.84437641043615	0.00762896626077297	0.350622433839347	-2.44069000062258
a_HQ_transcript/66167	2.24483483374337	2.39360933802142	2.84413239363593	0.00763363044925679	0.350622433839347	-2.48285721155563
a_HQ_transcript/16041	3.47990052799449	0.370699108227822	2.84399560570917	0.00763624621184754	0.350622433839347	-2.46171205374707
a_HQ_transcript/33211	-3.03085954888039	0.245336596110922	-2.84399081710331	0.00763633779842828	0.350622433839347	-2.4427702295768
a_HQ_transcript/45853	-2.82941088990143	1.75885869695841	-2.84372622536833	0.00764139996463512	0.350622433839347	-2.47453155479011
a_HQ_transcript/127686	3.20122565243463	-0.175628743354277	2.84335863477421	0.00764843792604311	0.350622433839347	-2.46329680493126
a_HQ_transcript/99695	1.77137435891593	-0.76000884610994	2.84299196079419	0.00765546439235333	0.350622433839347	-2.44142637271523
a_HQ_transcript/61942	3.41459334464458	1.54596713068303	2.84244614864347	0.00766593484134775	0.350622433839347	-2.54631572697817
a_HQ_transcript/5834	2.72854280076013	2.18515171773294	2.84242917674612	0.00766626063256978	0.350622433839347	-2.45443715557335
a_HQ_transcript/120092	-2.42697386470452	1.04113672637803	-2.84241419697844	0.00766654819381039	0.350622433839347	-2.42803755098705
a_HQ_transcript/110702	-3.91697906401521	-0.144681201846631	-2.84161000530381	0.00768200082636656	0.350979606529331	-2.47155933222042
a_HQ_transcript/125892	-0.825583637162594	3.12707925942875	-2.84115923751387	0.0076906751453877	0.350979606529331	-2.55020179932557
a_HQ_transcript/117929	4.28719335627058	0.504707169922867	2.8407558943019	0.00769844463612898	0.350979606529331	-2.50657561398104
a_HQ_transcript/102404	-3.29581782606218	1.59267971641354	-2.8406212850461	0.00770103921485897	0.350979606529331	-2.45399542145377
a_HQ_transcript/149615	0.685949312177502	7.49034356046908	2.84043930297075	0.00770454820278397	0.350979606529331	-2.96408300059081
a_HQ_transcript/31368	-5.50019632465384	2.28273594165859	-2.83995143645414	0.00771396266712839	0.351133298288832	-2.51857431708212
a_HQ_transcript/60906	1.98666138580535	4.39101111762445	2.83812491669937	0.00774930525718092	0.352377047674684	-2.70342083664085
a_HQ_transcript/88097	3.44699965508214	2.14807720799444	2.83716746805024	0.00776789216917818	0.352377047674684	-2.54707977926661
a_HQ_transcript/10604	1.92973260579251	0.593449459124728	2.83705619169982	0.00777005508078285	0.352377047674684	-2.43934024372618
a_HQ_transcript/57671	4.0957438909951	1.41657671200124	2.83654753753274	0.00777994913271057	0.352377047674684	-2.45635013158169
a_HQ_transcript/57110	2.48560818316446	2.72314751337131	2.83649449453886	0.00778098157449737	0.352377047674684	-2.51674551627152
a_HQ_transcript/29811	2.53234735062562	1.94063201862318	2.8364597351316	0.00778165820973341	0.352377047674684	-2.45935807903985
a_HQ_transcript/145082	0.587929171004917	5.2626179953192	2.8359863498112	0.00779087873709071	0.352377047674684	-2.81652708423785
a_HQ_transcript/119438	-0.650594643163713	4.43970809822411	-2.83589438077658	0.0077926712834598	0.352377047674684	-2.7223245148975
a_HQ_transcript/137589	4.34150469202921	-0.0376410459824209	2.83497004656593	0.00781070873678726	0.352377047674684	-2.4823318515578
a_HQ_transcript/98546	3.67754280634619	-0.188568130884793	2.83488793941207	0.00781231286613868	0.352377047674684	-2.47707101424496
a_HQ_transcript/62001	-3.15903634884806	0.258551123649382	-2.83413050494537	0.00782712544856179	0.352377047674684	-2.44809620920533
a_HQ_transcript/57527	-3.55958044850601	0.119991273788033	-2.83409116019203	0.00782789560326263	0.352377047674684	-2.45537651588735
a_HQ_transcript/95028	-7.17347148139863	3.28071474372262	-2.83373897616311	0.00783479260042721	0.352377047674684	-2.71561137518751
a_HQ_transcript/56013	-2.70743898428933	0.27464976114522	-2.83244084261936	0.00786026381186834	0.352377047674684	-2.48534747054956
a_HQ_transcript/56015	-4.3626190030297	-0.374416338369184	-2.8320808404273	0.00786734129365417	0.352377047674684	-2.49356477354562
a_HQ_transcript/28553	-0.615110957576243	3.98396948247315	-2.83200543088623	0.00786882456763873	0.352377047674684	-2.66339787943303
a_HQ_transcript/141672	-0.713857132794273	3.75101835340982	-2.83114040619816	0.00788585799255594	0.352377047674684	-2.68393728213629
a_HQ_transcript/127633	-1.53310003252874	3.29827951970259	-2.83091305896722	0.00789034047536036	0.352377047674684	-2.61651605212668

a_HQ_transcript/54116	-1.12578947767492	2.09235789910299	-2.83054620647354	0.00789757854257114	0.352377047674684	-2.49248086243079
a_HQ_transcript/57745	-2.28921258205133	1.47332719922873	-2.83053008851482	0.0078978966954833	0.352377047674684	-2.45163798190951
a_HQ_transcript/146414	-4.26682708411607	1.05887788995866	-2.830388933614	0.00790068346978672	0.352377047674684	-2.48139272689055
a_HQ_transcript/75652	-2.93304981343456	0.739255875400429	-2.83027189796517	0.00790299476428424	0.352377047674684	-2.47824865290044
a_HQ_transcript/1221	-2.94290273406443	2.79261181787914	-2.83020284675021	0.00790435872876064	0.352377047674684	-2.60296910786656
a_HQ_transcript/7580	1.62316195120124	3.46640596188252	2.82991801943408	0.00790998723701791	0.352377047674684	-2.57984822208909
a_HQ_transcript/125945	-4.65019793779955	2.84631749879296	-2.82982301135304	0.00791186553861519	0.352377047674684	-2.71747152392643
a_HQ_transcript/131778	-2.23713178488987	1.41413934590402	-2.82977569516026	0.00791280113154792	0.352377047674684	-2.49109298875317
a_HQ_transcript/36767	-4.99596052379647	3.93434299400628	-2.8297629906486	0.00791305235815386	0.352377047674684	-2.77600022740084
a_HQ_transcript/45163	2.88823994031858	0.16741692693703	2.8295902210964	0.00791646954841417	0.352377047674684	-2.49745524039353
a_HQ_transcript/60507	2.72055572203552	1.94232282470609	2.82955901856089	0.00791708684735648	0.352377047674684	-2.4791212857724
a_HQ_transcript/62556	4.39300799983865	1.80598935711516	2.82922274980097	0.00792374232136306	0.352403438145518	-2.45461912355218
a_HQ_transcript/144437	-2.82664405296722	1.39458828540901	-2.827901179509	0.00794994982127494	0.35302358463626	-2.49939855336103
a_HQ_transcript/129727	6.01580980151885	2.80951136418131	2.82740824168333	0.00795974583783478	0.35302358463626	-2.59948471418055
a_HQ_transcript/137931	3.28828409804696	0.160158846572208	2.82714986551069	0.00796488499137583	0.35302358463626	-2.50204471060745
a_HQ_transcript/83770	3.078636132167	0.549325022627976	2.82704329410024	0.00796700562322372	0.35302358463626	-2.48345330811104
a_HQ_transcript/76614	-1.05190080326864	1.19786766524299	-2.82640888341428	0.00797964051651392	0.35302358463626	-2.48854249336693
a_HQ_transcript/93983	2.36116796582439	-0.199100364926456	2.82640314971351	0.00797975479416525	0.35302358463626	-2.47519395739768
a_HQ_transcript/87744	-1.24945524631686	2.74454417430346	-2.82638087801455	0.00798019870306988	0.35302358463626	-2.50366195337284
a_HQ_transcript/129492	-1.98297253279212	-0.713046890841299	-2.82586238790045	0.00799053954101689	0.35321222999257	-2.49468151872614
a_HQ_transcript/9677	-2.79988101990607	0.539537039389047	-2.82305406799947	0.00804676749169148	0.355427432186202	-2.49847435237082
a_HQ_transcript/41968	-2.96263207042541	-0.162541885242806	-2.82271817046818	0.00805351756104805	0.355455481236599	-2.56819896336843
a_HQ_transcript/138314	-2.21700085871529	-0.708985630750343	-2.82203146203305	0.00806733389872365	0.355560708889932	-2.50268957146198
a_HQ_transcript/130319	3.0475744913576	1.86737185602378	2.8218777031161	0.00807043051525813	0.355560708889932	-2.51283100846284
a_HQ_transcript/73253	4.66471195299329	1.92236191270791	2.82166875716897	0.00807464035107472	0.355560708889932	-2.58105520400304
a_HQ_transcript/47677	-2.32155593578942	1.92980649444044	-2.82138430756905	0.00808037473736426	0.355560708889932	-2.47664113929205
a_HQ_transcript/106515	1.23567833710009	3.17373785129028	2.8207361389532	0.00809345579720445	0.355560708889932	-2.59760215632542
a_HQ_transcript/67021	2.85385485543189	1.50437758935717	2.82072930310146	0.00809359386103046	0.355560708889932	-2.48069730360787
a_HQ_transcript/58418	-2.71853888691949	1.15899343951445	-2.8203838629742	0.00810057359408451	0.355560708889932	-2.48297845347674
a_HQ_transcript/50375	-2.10455474530144	-0.642217403243245	-2.8199226544352	0.00810990125287762	0.355560708889932	-2.50528616639632
a_HQ_transcript/85227	-3.03506405027494	0.714490586365199	-2.81987066128204	0.00811095341295158	0.355560708889932	-2.48543870076135
a_HQ_transcript/26061	-2.89990599293848	0.571164262747944	-2.81904415253309	0.00812769624206256	0.356026169674915	-2.48594513604064
a_HQ_transcript/126761	-0.637950086004631	4.72984854794593	-2.81745621229689	0.00815995445825272	0.35699313058375	-2.77924245920716
a_HQ_transcript/82290	-2.5559826848878	-0.485871356324725	-2.81735306794385	0.00816205392488653	0.35699313058375	-2.5439834391175
a_HQ_transcript/97484	3.36594398224547	0.972132186421288	2.81560614745872	0.00819768877374269	0.358282145142944	-2.51283566023477
a_HQ_transcript/58574	-2.26986131420801	0.544710978861163	-2.81317133918216	0.00824759862469027	0.360192646773851	-2.50262086716035
a_HQ_transcript/152348	-3.82565204530335	2.6840528781738	-2.81187204796202	0.00827434835482922	0.360867717572527	-2.63516014107431
a_HQ_transcript/104722	2.51085523201237	-0.391616036157807	2.81154724592771	0.0082810480348839	0.360867717572527	-2.51155375965492
a_HQ_transcript/119641	2.36467702739684	5.37654286329173	2.81151658729364	0.00828168069160732	0.360867717572527	-2.82771196268604
a_HQ_transcript/119961	-3.26233570249753	0.569057905362259	-2.81104506337341	0.00829141653050333	0.360920512239108	-2.5700602543103
a_HQ_transcript/135883	-3.79231311635376	0.144518335323342	-2.81085662024746	0.00829531042443311	0.360920512239108	-2.60030742871449
a_HQ_transcript/37168	10.1165855094548	1.93742346561916	2.80967130011088	0.00831984254842837	0.361559227357628	-2.54323694886122
a_HQ_transcript/30536	-3.33052122208088	0.159223135273645	-2.80954644361743	0.00832243060494954	0.361559227357628	-2.53751037836259
a_HQ_transcript/128576	-1.09373124994012	3.00633115967974	-2.80891931687499	0.0083354412436097	0.361854016884649	-2.5911969534132
a_HQ_transcript/82237	4.95986515206243	0.700244667983968	2.80826062595185	0.00834912721914214	0.361877863172208	-2.61397968067325
a_HQ_transcript/90891	4.29494785159764	-0.173154902717644	2.80814854224051	0.00835145813506724	0.361877863172208	-2.56060063425807
a_HQ_transcript/75746	3.68842849139445	0.0837647154774381	2.80781444687532	0.00835840966419929	0.361877863172208	-2.53764349138873
a_HQ_transcript/31424	2.65857114864482	0.58546482365684	2.8076951739684	0.00836089268924227	0.361877863172208	-2.49702854399032
a_HQ_transcript/6264	-2.32234243415924	-0.60531631817894	-2.80693324603655	0.00837677081011239	0.362295337537361	-2.53818663254082
a_HQ_transcript/40829	2.73861186463972	0.678849997898723	2.8064385061994	0.00838709599471197	0.36247220519005	-2.52570968302579

a_HQ_transcript/17356	-2.25472659215272	-0.570307471132133	-2.80570813728499	0.00840236050809979	0.362862118584565	-2.54608967632159
a_HQ_transcript/3687	-3.56260225754919	0.270324753231844	-2.80517642272159	0.00841348954412689	0.363072991997778	-2.53119787749273
a_HQ_transcript/26058	2.71090460480781	-0.400719698488461	2.80419689638401	0.00843402760349075	0.36368928526388	-2.51714011560421
a_HQ_transcript/19321	2.15601977844562	2.64302462887497	2.80359438649536	0.00844668391960358	0.363709159446777	-2.61050830183851
a_HQ_transcript/83780	-3.4765973143533	0.579285150063156	-2.80307874227931	0.00845752964359757	0.363709159446777	-2.53841203123648
a_HQ_transcript/71347	-3.78686738755346	2.45235127185772	-2.80303208202724	0.00845851170738263	0.363709159446777	-2.62747869396157
a_HQ_transcript/119236	2.90744844819778	-0.15777014251827	2.80298434020362	0.0084595166455416	0.363709159446777	-2.52222922737876
a_HQ_transcript/146162	3.70526244695099	1.88977242270798	2.80131158525369	0.00849479777572085	0.364956101335626	-2.67651204383528
a_HQ_transcript/132750	-1.11914561921242	3.91419371202011	-2.80101382134928	0.00850109251468306	0.364956798887369	-2.7128996796353
a_HQ_transcript/138678	3.3387793949398	1.20825488892041	2.80039755768285	0.00851413421063165	0.365039749658222	-2.55435964954796
a_HQ_transcript/79479	1.9476631666313	1.33955958066004	2.80032907739548	0.00851558458120955	0.365039749658222	-2.54421732859335
a_HQ_transcript/105100	-4.16337162834284	1.13554372239948	-2.79931633206221	0.00853706090439544	0.365623241281976	-2.61380618552907
a_HQ_transcript/124362	2.51870164828322	3.61096539215592	2.79854994365425	0.00855334663174954	0.365623241281976	-2.64592272089809
a_HQ_transcript/9749	-2.30539444723719	4.34655412216043	-2.79853344622475	0.0085536975208032	0.365623241281976	-2.70506722520455
a_HQ_transcript/100929	-1.9317634669742	1.04590010966358	-2.79850248673629	0.00855435604430717	0.365623241281976	-2.55439614046705
a_HQ_transcript/23114	-2.70494037883496	1.1595119368012	-2.79760530130627	0.00857346022670224	0.366170533473731	-2.55358531009032
a_HQ_transcript/136920	3.36024917213536	-0.15087329392727	2.79663711144847	0.00859412109478384	0.366783458882228	-2.53231442342781
a_HQ_transcript/137759	3.56509890495609	1.26045182289491	2.79578257802312	0.00861239524258472	0.367034835325679	-2.5753222062927
a_HQ_transcript/20373	-2.23392214950197	0.446270954903448	-2.79495662949038	0.00863009263000419	0.367034835325679	-2.55638941431711
a_HQ_transcript/130317	2.72072748869621	-0.796474507062807	2.79468166616439	0.0086359917388529	0.367034835325679	-2.56582179693475
a_HQ_transcript/17438	-3.26388435196138	1.99632486867837	-2.79450169137343	0.00863985498920483	0.367034835325679	-2.56254309455902
a_HQ_transcript/114651	1.16169015820644	2.56152860492642	2.79391491875442	0.00865246158659637	0.367034835325679	-2.58929914661942
a_HQ_transcript/72268	1.69861692598177	-0.816951844123457	2.79356053342392	0.00866008375518922	0.367034835325679	-2.56650867663823
a_HQ_transcript/150123	-3.27260299128729	-0.637144969495944	-2.79332917223889	0.00866506329005329	0.367034835325679	-2.57742417291994
a_HQ_transcript/27213	2.30311339302527	0.663443009961167	2.79306098710492	0.00867083872839359	0.367034835325679	-2.55454186837981
a_HQ_transcript/92964	-2.00967687750657	-0.767407270870732	-2.79305154221984	0.00867104219210103	0.367034835325679	-2.56361830545075
a_HQ_transcript/123343	-2.31005421738378	-0.59230838741585	-2.7928060249787	0.00867633274268244	0.367034835325679	-2.56265928047228
a_HQ_transcript/100922	-2.15525409054287	0.0561281292904957	-2.79275699170574	0.00867738970209481	0.367034835325679	-2.55846546593
a_HQ_transcript/10245	-2.24304910076376	3.97149335441607	-2.79265072285758	0.00867968084261481	0.367034835325679	-2.69114154208161
a_HQ_transcript/81439	-5.16749287452745	1.11106702163153	-2.79231281233705	0.00868696990193219	0.367034835325679	-2.65241600436024
a_HQ_transcript/148697	2.37784625392627	-0.284172888585488	2.79127736748373	0.00870934110670557	0.367034835325679	-2.57162051399102
a_HQ_transcript/117078	2.57850212608687	1.14585059901852	2.79123811302802	0.0087101902737509 8	0.367034835325679	-2.54829842995973
a_HQ_transcript/97244	3.97187894254618	-0.233535849549143	2.79122223494768	0.00871053377629919	0.367034835325679	-2.57196266904425
a_HQ_transcript/16525	0.997655374976809	3.48272232238253	2.79119771913471	0.00871106417033599	0.367034835325679	-2.72063576782186
a_HQ_transcript/95126	2.95823999332553	1.14480404914649	2.79053985416221	0.00872530820941903	0.367034835325679	-2.5365812069726
a_HQ_transcript/134690	2.10525533049595	1.48714486476901	2.79038088420442	0.00872875347883608	0.367034835325679	-2.54913616123649
a_HQ_transcript/74003	3.65733693751008	4.11762013979938	2.79012576456451	0.00873428520608708	0.367034835325679	-2.82296510920644
a_HQ_transcript/145402	0.668808703311222	4.2347447063323	2.78951344523919	0.00874757542336581	0.367034835325679	-2.8141706957372
a_HQ_transcript/141607	2.76012528519855	1.54349459198913	2.78946190718499	0.00874869490424722	0.367034835325679	-2.55112376031823
a_HQ_transcript/117621	2.09533086836832	-0.0825397140568681	2.78917387913719	0.008754953754796	0.367034835325679	-2.56878051539743
a_HQ_transcript/44690	2.54370774691929	-0.601069754171882	2.78912447434415	0.00875602774155435	0.367034835325679	-2.57142607554142
a_HQ_transcript/81332	-1.11354412463792	3.39871376652504	-2.78851283281695	0.00876933412255542	0.367034835325679	-2.66056158391424
a_HQ_transcript/9292	1.13934546350818	2.18770281074178	2.78837350240286	0.00877236792493885	0.367034835325679	-2.58625023083531
a_HQ_transcript/12738	-3.24198037354022	3.18694490934425	-2.78820158459539	0.00877611264233515	0.367034835325679	-2.59797413357585
a_HQ_transcript/127450	2.67536827707451	2.30166945943985	2.78816956944187	0.00877681016210249	0.367034835325679	-2.58980061770393
a_HQ_transcript/100847	2.66144486841896	1.69582186800167	2.78690881817117	0.00880431960383272	0.367826077801362	-2.53784148590153
a_HQ_transcript/138147	3.38518688229201	0.294259101733174	2.78672273799256	0.00880838666906647	0.367826077801362	-2.57441666515131
a_HQ_transcript/35341	2.57814431877049	-0.045950570887242	2.78623564814802	0.00881904107321781	0.368006618452265	-2.56340861715798
a_HQ_transcript/148338	-2.23785787795279	-0.745085973132692	-2.78519055369422	0.0088419416973373	0.368412426378376	-2.58295289956086
a_HQ_transcript/125814	3.92768944333046	0.333246139283335	2.78464063323458	0.00885401412731191	0.368412426378376	-2.60931770543638

a_HQ_transcript/102855	-3.79131358913835	-0.19243221402358	-2.78458015401909	0.00885534277013795	0.368412426378376	-2.65470078452233
a_HQ_transcript/148648	2.69446326575957	7.52609342417568	2.78442111491941	0.00885883752340938	0.368412426378376	-2.98319895735768
a_HQ_transcript/11493	-2.7567935755109	-0.0134207465722158	-2.78434749239405	0.00886045575414551	0.368412426378376	-2.54695310025392
a_HQ_transcript/77822	-2.74665226722275	-0.017571516146904	-2.78389527253206	0.00887040165424383	0.368562335495272	-2.57622981052777
a_HQ_transcript/28712	1.58123837015098	3.8552641319891	2.7832584616638	0.00888442504733168	0.368881327965211	-2.80505283126101
a_HQ_transcript/104407	3.53992114292143	-0.247343513586262	2.78240939711449	0.0089031547630801	0.369395132097302	-2.57064230625036
a_HQ_transcript/145751	4.31881895331261	2.0508016064191	2.78190650362076	0.00891426559962124	0.369592318669603	-2.58202540156547
a_HQ_transcript/127787	3.12501163585222	1.2469000909732	2.78114949770656	0.00893101518000018	0.370022844178938	-2.56350484017238
a_HQ_transcript/134525	2.67545104790995	-0.181353311551246	2.77988720133377	0.00895901026968558	0.370918339712452	-2.58271040002053
a_HQ_transcript/60196	-2.69198860506601	1.49344705256849	-2.77907335837662	0.00897710304505062	0.371165355740279	-2.62753199977195
a_HQ_transcript/52946	-2.1640929641949	1.34643419617003	-2.77884473421337	0.00898219179188527	0.371165355740279	-2.604752080177
a_HQ_transcript/151987	0.549574095369498	11.1700226035346	2.77849347990052	0.00899001530733196	0.371165355740279	-3.50487467052623
a_HQ_transcript/60903	4.45702198001116	1.95866094136533	2.77847092742122	0.00899051783791482	0.371165355740279	-2.66908178797949
a_HQ_transcript/151506	-6.03577063826359	0.320209851081572	-2.77776058747385	0.0090063595798845	0.371265593890358	-2.61028007998676
a_HQ_transcript/140073	0.616670789063276	3.83455004048985	2.77753212527121	0.00901146019951437	0.371265593890358	-2.79005880774069
a_HQ_transcript/26698	-1.33365585671992	2.10895273756903	-2.77750316510918	0.0090121069532634	0.371265593890358	-2.58121741615324
a_HQ_transcript/131754	-2.30822227976606	0.623697667602495	-2.77686416967488	0.00902638837650232	0.371546945653411	-2.60690280466205
a_HQ_transcript/147062	2.57787953686487	-0.380925902269248	2.77634378584343	0.00903803446783486	0.371546945653411	-2.60735565506103
a_HQ_transcript/146357	2.66889307829556	0.279767919890269	2.77592638825684	0.00904738588804352	0.371546945653411	-2.6173051732765
a_HQ_transcript/101608	3.06667302124984	1.3801695612504	2.77587924356371	0.00904844269060885	0.371546945653411	-2.56047742403171
a_HQ_transcript/106613	2.91465765232069	0.440799606535641	2.77570165864156	0.00905242449622178	0.371546945653411	-2.56265222026856
a_HQ_transcript/132985	2.1161525084446	1.02556072816034	2.77540753271271	0.00905902298203371	0.371546945653411	-2.57205032229634
a_HQ_transcript/87009	-4.19420908940602	0.994590469481474	-2.77520008460247	0.00906367961974499	0.371546945653411	-2.70348276822743
a_HQ_transcript/38730	-2.4077356471337	1.63990892766345	-2.77432152890308	0.00908342556388557	0.372093982507076	-2.58290692172917
a_HQ_transcript/133051	-3.14995708925633	1.71901375605485	-2.77374820739283	0.00909633288374262	0.372360308356473	-2.73408014679366
a_HQ_transcript/131484	9.23611766097915	5.71428487628262	2.77298544164024	0.00911353175914452	0.372801811467666	-2.82958228651539
a_HQ_transcript/38717	3.13813541453889	-0.00828570757639579	2.77179289760307	0.00914048215659007	0.373641312797656	-2.61832841451926
a_HQ_transcript/69175	-2.95685504413848	0.0908807915530408	-2.77079501229207	0.00916309061537462	0.374302270759308	-2.57748689879852
a_HQ_transcript/103486	2.97699131365917	1.99792708674113	2.77011357104341	0.00917855959101309	0.374670865102815	-2.64846501697493
a_HQ_transcript/25035	3.94103017480981	1.61957698650174	2.76954613990475	0.00919145909458372	0.374934129298219	-2.70352338007312
a_HQ_transcript/7698	-2.69433840358313	-0.0135009974995303	-2.76908256419435	0.00920201019626658	0.375101296415557	-2.5917409273369
a_HQ_transcript/150406	6.76285340917528	4.711927238303	2.76865849423581	0.0092116720392093	0.375232005813005	-2.80194868265903
a_HQ_transcript/14052	-2.53076794661775	2.04992831500673	-2.76675967600377	0.00925505022098084	0.376692711963047	-2.59845805728698
a_HQ_transcript/30374	2.05976280606485	-0.602412310148363	2.76637492999604	0.00926386287942252	0.376692711963047	-2.61593899304264
a_HQ_transcript/108017	1.60475560129233	-0.696576843382374	2.76623925020677	0.00926697251078925	0.376692711963047	-2.61386236974503
a_HQ_transcript/139004	2.46097706864095	0.136530231951554	2.7654980026303	0.00928397829799702	0.377120258914026	-2.61561259234845
a_HQ_transcript/82997	2.9797407965803	1.19128614018504	2.76467313181241	0.00930293682069578	0.37723996605577	-2.63157549068621
a_HQ_transcript/135313	2.6733339222643	0.720559592254162	2.76465834630908	0.00930327697460526	0.37723996605577	-2.61268356685499
a_HQ_transcript/91264	-1.96036049767482	-0.567672929516957	-2.7645228511841	0.00930639470348152	0.37723996605577	-2.618672259512
a_HQ_transcript/108901	-3.27973184154917	0.115004009557374	-2.76421074830679	0.00931357986910608	0.37726813284418	-2.61142027425651
a_HQ_transcript/49426	3.02151558803305	2.59813716689579	2.76387375111563	0.00932134395965148	0.377319694767842	-2.74590323303825
a_HQ_transcript/145126	-2.15461063082807	-0.684650925603416	-2.76346043871597	0.00933087452667689	0.37744264056136	-2.62690149471389
a_HQ_transcript/123472	-3.8047441706501	0.257331419978486	-2.76267166335534	0.00934908813513935	0.37791640828886	-2.64735279236355
a_HQ_transcript/131822	2.59454444745186	0.270055758896437	2.76080212984814	0.00939239012381081	0.379402955605889	-2.59856191273565
a_HQ_transcript/6065	-0.993173541427603	3.87445044736122	-2.75995441572863	0.00941208641958003	0.379934555137047	-2.83324849074258
a_HQ_transcript/69279	-3.23274951400068	0.916379358599418	-2.75927623394874	0.00942787144259579	0.38030764137072	-2.65460613077417
a_HQ_transcript/1738	-2.41762351964646	0.323478390254349	-2.7586734909995	0.00944192131766899	0.380388695930702	-2.62754962998
a_HQ_transcript/136346	3.18457099277354	-0.31419349287662	2.75857576757372	0.00944420108008934	0.380388695930702	-2.63299896753424
a_HQ_transcript/76160	-2.50656141494854	0.134208428810266	-2.7583481650653	0.00944951274642056	0.380388695930702	-2.61806271529081
a_HQ_transcript/118019	-3.00039449388472	-0.166192552017838	-2.75796646508005	0.0094584269149766	0.380484041324401	-2.64728895221513

a_HQ_transcript/133626	-4.71455157771587	0.805037102747631	-2.75695283727182	0.00948213713360593	0.381174043777486	-2.66992888185677
a_HQ_transcript/54922	3.23449858085136	0.936507777736159	2.75650040715905	0.00949273801603041	0.381269186116274	-2.63450223861876
a_HQ_transcript/108054	-0.721479530694469	3.79559736911045	-2.75629211722102	0.00949762216997599	0.381269186116274	-2.81098425697429
a_HQ_transcript/32350	-2.087248473557	0.462663268940183	-2.75593346929353	0.00950603753581796	0.381343650712234	-2.60458144299475
a_HQ_transcript/88608	-1.79136412751047	-0.601142155600073	-2.75467005067513	0.00953573801283962	0.381824489535967	-2.64104434190532
a_HQ_transcript/119726	5.16942764049334	0.0192904007761825	2.7543055780581	0.00954432211829329	0.381824489535967	-2.64836374642775
a_HQ_transcript/50010	-2.3495947066504	-0.613163619729629	-2.75412741247684	0.00954852091918946	0.381824489535967	-2.6488273206497
a_HQ_transcript/18995	2.87332405198763	1.0884400938637	2.75340806691237	0.00956549115358787	0.381824489535967	-2.61116242902431
a_HQ_transcript/107029	1.87403548388328	2.54901899009035	2.75339952261861	0.00956569289279515	0.381824489535967	-2.68804386464035
a_HQ_transcript/143310	0.553727363711655	10.8656151971974	2.75294096258421	0.00957652577133065	0.381824489535967	-3.52893235889548
a_HQ_transcript/39367	2.99604714293317	0.49018067288121	2.75276542768874	0.00958067558025016	0.381824489535967	-2.62805249036037
a_HQ_transcript/152136	0.545982735674282	10.7735831134425	2.75275237862407	0.00958098413929579	0.381824489535967	-3.5131030969187
a_HQ_transcript/148311	5.68254024405866	2.26402431480133	2.75265870383267	0.00958319945171078	0.381824489535967	-2.70563911180855
a_HQ_transcript/47850	-1.05940156178845	4.55116718284259	-2.75263709237804	0.00958371060819184	0.381824489535967	-2.88709697413926
a_HQ_transcript/103149	-2.17011353303689	-0.685195230823262	-2.75229112430941	0.00959189694540334	0.381869902050046	-2.65024474321076
a_HQ_transcript/50984	2.10482511437893	3.0412734621769	2.75200892147199	0.00959857929130105	0.381869902050046	-2.72148052970094
a_HQ_transcript/65355	-2.62485055499142	-0.443949220978259	-2.75175655343623	0.009604558849387	0.381869902050046	-2.64621524467993
a_HQ_transcript/37567	3.0404479226335	0.257991333410494	2.75105812846903	0.00962112530799131	0.382267103146219	-2.65108579972301
a_HQ_transcript/46923	5.91042152033962	2.82720968346209	2.75052785673268	0.00963372100872592	0.3823617915169	-2.81249404647547
a_HQ_transcript/66635	2.43408087400903	-0.435683419467123	2.75040403751623	0.00963666433684728	0.3823617915169	-2.64739111527702
a_HQ_transcript/53434	-4.68538077327998	2.01964964248412	-2.74909826869922	0.00966775508946855	0.38333374341107	-2.72623217043464
a_HQ_transcript/1504	-2.49629452641189	0.269416018367837	-2.74839195220019	0.00968461165738116	0.383705845099762	-2.6510600637494
a_HQ_transcript/137224	2.20883416959847	2.19467004310386	2.74790654148865	0.0096962120918162	0.383705845099762	-2.64536370689103
a_HQ_transcript/140567	1.61419515397484	-0.833255624428039	2.74787598706136	0.00969694272040239	0.383705845099762	-2.6474810324252
a_HQ_transcript/145672	4.34409286888367	1.21639670682043	2.74739397344204	0.00970847560018914	0.383738378857036	-2.64410869314845
a_HQ_transcript/6416	-1.14634454343856	3.37150829275057	-2.74713461478569	0.00971468642568579	0.383738378857036	-2.82005583251315
a_HQ_transcript/27836	3.06541763179826	1.05559948634594	2.74697945847063	0.00971840370307488	0.383738378857036	-2.63264845113229
a_HQ_transcript/138936	-6.22789915374403	5.81225672245773	-2.74673883092328	0.00972417134696556	0.383738378857036	-3.02618867658434
a_HQ_transcript/11168	2.45101201429192	1.25214293956032	2.74586597276057	0.00974511983301997	0.384074761786185	-2.63451339770065
a_HQ_transcript/149350	2.5472232073459	0.965139873735623	2.74583307092841	0.00974591029511806	0.384074761786185	-2.6464840485046
a_HQ_transcript/100417	-2.14591309586024	0.449722779267622	-2.74467395208135	0.00977379614748345	0.384538045529182	-2.64946600555516
a_HQ_transcript/21473	2.68960014472295	1.75307944291581	2.74460456356261	0.00977546784021562	0.384538045529182	-2.66321744420216
a_HQ_transcript/84811	-2.58946419190079	2.99859075466337	-2.74447567279292	0.00977857375534817	0.384538045529182	-2.75555078251789
a_HQ_transcript/52403	2.79960346346706	3.88238056746065	2.74424529162026	0.00978412760352431	0.384538045529182	-2.77209775284199
a_HQ_transcript/135627	2.0076298371691	2.46811502562535	2.74394288393656	0.00979142227352124	0.384564725618407	-2.71732396834221
a_HQ_transcript/137601	-0.648573796838933	3.84539691869063	-2.7429197950881	0.00981613880213899	0.385062663399526	-2.86691414904491
a_HQ_transcript/32998	-2.47557751359661	-0.558917697613199	-2.74286975998548	0.0098173490771762	0.385062663399526	-2.65412693513082
a_HQ_transcript/116627	-2.73404942930316	0.219453825855275	-2.74253359168088	0.00982548409795642	0.385121874339859	-2.62894500830241
a_HQ_transcript/21416	-2.29041793473074	3.02394013635331	-2.74210061905047	0.00983597096891143	0.385273127008682	-2.75600761044465
a_HQ_transcript/151327	0.548753155699346	10.5062821237869	2.74178876080814	0.00984353083519721	0.385309602955113	-3.50267564514309
a_HQ_transcript/97275	-5.36051139014851	1.23584031531763	-2.74079985201896	0.0098675391546265	0.385732326869866	-2.72913638560811
a_HQ_transcript/99611	2.84955847800832	-0.0954942375369324	2.7407579817116	0.00986855686796363	0.385732326869866	-2.66373448379566
a_HQ_transcript/59900	-1.28521926906618	4.10359392134851	-2.74052432791299	0.00987423792978188	0.385732326869866	-2.85579590757223
a_HQ_transcript/119415	1.99065866362735	-0.665536283228923	2.73925327214157	0.0099051958249197	0.386532023961982	-2.66126011346631
a_HQ_transcript/152154	-4.14034299891727	0.72759034167581	-2.7391379757688	0.00990800845897592	0.386532023961982	-2.69092405740286
a_HQ_transcript/139522	3.04937749990331	0.99740581774405	2.73848729278583	0.0099238957100567	0.38672992600178	-2.67338904383374
a_HQ_transcript/24695	1.60366456566601	-0.516373536563721	2.73838532848865	0.00992638744829783	0.38672992600178	-2.6981452976465
a_HQ_transcript/75084	-2.21424249231321	-0.511101459840549	-2.7378315101899	0.00993993149169816	0.386760040725617	-2.67949680314548
a_HQ_transcript/2596	2.68167876792324	-0.438006059334472	2.73780960296473	0.00994046760329052	0.386760040725617	-2.68186879672938
a_HQ_transcript/86594	2.55474309525878	0.267212692117154	2.73647481570988	0.00997318327467644	0.387773376180864	-2.66959002022484

a_HQ_transcript/16336	1.69913508604571	-0.690883707000009	2.73619151248763	0.00998013993876501	0.387784474839928	-2.68590735341116
a_HQ_transcript/30698	-0.698409582170994	3.91203353672289	-2.73562614139692	0.00999403644908931	0.388065030536181	-2.86284183946178
a_HQ_transcript/6797	-2.27844237547474	-0.0144285293754142	-2.73488755917272	0.010012217554319	0.388309472644635	-2.6748779662133
a_HQ_transcript/66934	-2.36434698884609	2.21264381524972	-2.7348277090265	0.0100136921878322	0.388309472644635	-2.66819325297981
a_HQ_transcript/54233	-2.50902314890858	-0.175603719944248	-2.73455399582075	0.0100204387215532	0.38831204133763	-2.69041537683221
a_HQ_transcript/96264	-0.963317801682908	4.88788558710405	-2.73372259828267	0.0100409571375837	0.388703539222398	-3.00310698547745
a_HQ_transcript/60343	2.90370452135026	4.98139121378699	2.73342186836817	0.0100483886148367	0.388703539222398	-3.02225455373804
a_HQ_transcript/138402	-2.25024414559685	-0.684173955435153	-2.73333232089437	0.0100506024540886	0.388703539222398	-2.69050202502165
a_HQ_transcript/129610	4.5462239388132	0.941750001488154	2.73219104213982	0.0100788575689939	0.38953712285271	-2.72877606467614
a_HQ_transcript/8930	-2.45652467752243	-0.578372956993074	-2.73112760442767	0.0101052521208744	0.390138973727968	-2.71399512866441
a_HQ_transcript/42675	-1.40245561369928	3.20910438395566	-2.73102294441378	0.0101078532623576	0.390138973727968	-2.7751073177919
a_HQ_transcript/109936	-2.36125432203292	0.336959925815619	-2.7294349767214	0.0101473960142733	0.39122810213171	-2.678191220984
a_HQ_transcript/952	0.999037693715505	4.58923099232078	2.72932379993518	0.0101501698710404	0.39122810213171	-3.02253647003609
a_HQ_transcript/91872	2.67090658573381	0.451153463438364	2.72907971944892	0.0101562621476182	0.39122810213171	-2.66707446390144
a_HQ_transcript/77004	2.80953831837029	-0.218949235915165	2.72833055870855	0.0101749825594526	0.391638369707653	-2.71609590797661
a_HQ_transcript/126565	2.68339855559128	0.186051356216734	2.72810069154327	0.0101807330376085	0.391638369707653	-2.71992388997372
a_HQ_transcript/134058	3.71317543767598	0.475888271749724	2.72784129615402	0.0101872258441401	0.391638369707653	-2.71173276684948
a_HQ_transcript/99666	-2.47887138096007	2.03883755475562	-2.72757630329131	0.0101938627402918	0.391638369707653	-2.66764019485314
a_HQ_transcript/38164	1.89969269037462	-0.612281017110095	2.72694821567259	0.0102096096361126	0.391984272739731	-2.72136900171321
a_HQ_transcript/152713	2.29909927204367	-0.337865482704764	2.72632433799406	0.0102252734062653	0.392163324775111	-2.70623342638303
a_HQ_transcript/6162	-2.82286765461348	1.68849988051018	-2.72622512917484	0.0102277663150129	0.392163324775111	-2.69176397628808
a_HQ_transcript/64277	1.15127613828686	4.96444768813339	2.72589707222596	0.0102360137258934	0.392198216071521	-2.99319934784545
a_HQ_transcript/12907	1.66090730618759	4.78869461724495	2.72565233133279	0.0102421705889858	0.392198216071521	-2.88268115753379
a_HQ_transcript/96151	2.03362825155333	-0.259530641304851	2.7249631727646	0.0102595260469712	0.392370592540134	-2.71236879022599
a_HQ_transcript/29480	4.14050563242205	0.984006106966524	2.72493752846461	0.0102601723895714	0.392370592540134	-2.76387720539205
a_HQ_transcript/151978	0.816035450291653	5.78890044074433	2.72426952780366	0.0102770221363858	0.392756569851304	-3.07000043752404
a_HQ_transcript/22295	-1.86745937636397	-0.756223183863271	-2.72341534210319	0.0102986057791948	0.39312801878268	-2.70618569543423
a_HQ_transcript/80863	2.57881540911079	1.02577446126878	2.7233496317279	0.0103002679019753	0.39312801878268	-2.70000212732376
a_HQ_transcript/82419	2.71462267359851	0.498307700220609	2.72283338208781	0.0103133349618563	0.393368461064818	-2.6691106272914
a_HQ_transcript/32068	-1.29440423181541	1.22330755524084	-2.72147587759749	0.0103477691393877	0.394154536572797	-2.67928656532597
a_HQ_transcript/5698	-3.27705226795982	1.33579883631299	-2.72119035982021	0.0103550251335055	0.394154536572797	-2.69590604496437
a_HQ_transcript/93343	-9.3869447983883	1.29484886356033	-2.72100523887165	0.010359732227748	0.394154536572797	-2.75399356270869
a_HQ_transcript/104071	3.31092850107193	1.19598715606934	2.72070439504927	0.010367386071366	0.394154536572797	-2.68092182485415
a_HQ_transcript/55938	-0.941004867417979	3.5682922094892	-2.72068623290419	0.0103678483075249	0.394154536572797	-2.90493766987892
a_HQ_transcript/112244	2.02166860870952	2.11979483840904	2.71910612888641	0.0104081363033163	0.394832532606451	-2.69659725323385
a_HQ_transcript/61016	-2.40656682613361	-0.410075619299822	-2.71904804219567	0.0104096201159497	0.394832532606451	-2.72865110201374
a_HQ_transcript/104843	4.27441971050908	1.34790571548318	2.71830152633518	0.0104287072444565	0.394832532606451	-2.6932953285961
a_HQ_transcript/65186	2.24000785723171	-0.525241227198211	2.7180058492626	0.0104362762014768	0.394832532606451	-2.72322016409437
a_HQ_transcript/152378	-2.79399960434932	0.77434551320572	-2.71778004179079	0.0104420600285419	0.394832532606451	-2.7177989220177
a_HQ_transcript/45291	-3.42396949980081	-0.311038726047612	-2.71768770646437	0.0104444259625974	0.394832532606451	-2.7531385636275
a_HQ_transcript/104287	2.44361473413123	1.00831957727315	2.7176561368659	0.0104452349937489	0.394832532606451	-2.69005677418865
a_HQ_transcript/11963	-1.02721127603545	3.32108495997154	-2.71763600327572	0.0104457509858434	0.394832532606451	-2.87291360079752
a_HQ_transcript/119262	3.48921367103726	-0.324033170759877	2.7171824738531	0.0104573805160429	0.394832532606451	-2.73103830457621
a_HQ_transcript/56410	2.72458067156868	-0.193281979807942	2.71714903528585	0.0104582384340702	0.394832532606451	-2.67925557356259
a_HQ_transcript/150134	0.432095699407686	10.5355650427678	2.71706481728965	0.0104603994669339	0.394832532606451	-3.56447850601963
a_HQ_transcript/4130	-3.16399328956024	0.718221877590833	-2.71641483280103	0.0104770920479919	0.394998020738347	-2.77596522782575
a_HQ_transcript/151403	0.591131815980821	4.6710686093679	2.71616924703962	0.0104834055105995	0.394998020738347	-3.03246850761659
a_HQ_transcript/120571	-3.01001630071882	1.07425216185584	-2.71610064577071	0.0104851697288616	0.394998020738347	-2.68607875535456
a_HQ_transcript/27444	2.50502184087369	3.01535295855122	2.71515148395053	0.0105096076894015	0.395562715488155	-2.79276165757215
a_HQ_transcript/51212	3.83616790281928	0.773256687868663	2.71499004409824	0.0105137695332576	0.395562715488155	-2.68231012383215

a_HQ_transcript/148830	0.55186612666618	10.1047952056659	2.71456514265154	0.01052473062497	0.395718979151524	-3.51131316702923
a_HQ_transcript/70783	-2.31032275604883	1.31855075470162	-2.71428123232933	0.0105320605240775	0.395738599963527	-2.69083201934475
a_HQ_transcript/138087	-1.79287307899654	1.78172741802147	-2.71321711592584	0.0105595758028638	0.396500772328467	-2.69757425103467
a_HQ_transcript/104717	-4.81627803537767	4.31561951407472	-2.71296953947644	0.0105659870688273	0.396500772328467	-2.99561371852664
a_HQ_transcript/49502	2.86906045893268	1.31325806518117	2.71208433555238	0.0105889400405242	0.396940164395532	-2.71514311885097
a_HQ_transcript/40868	1.46428070945617	-0.758063873438954	2.71199136270734	0.0105913534781425	0.396940164395532	-2.74817299846131
a_HQ_transcript/97240	4.76013459916553	1.37967231006771	2.71166591235596	0.0105998057199415	0.396974044436857	-2.8547087256161
a_HQ_transcript/130832	2.32231936896383	0.762188719192445	2.71143078486118	0.0106059160991336	0.396974044436857	-2.73115905867451
a_HQ_transcript/109303	3.9215333634363	-0.168153875696984	2.7108266975722	0.0106216298545856	0.397178965344498	-2.74316815247714
a_HQ_transcript/96529	-2.05804944028947	-0.125007768619524	-2.71050561782666	0.0106299907196019	0.397178965344498	-2.7137769386738
a_HQ_transcript/8211	-2.73494022881638	1.93740868205173	-2.71043273411538	0.0106318894521752	0.397178965344498	-2.73845442449478
a_HQ_transcript/70898	2.62603444245553	-0.0158473040017255	2.7101229187346	0.010639964140117	0.397225327897701	-2.69882138940741
a_HQ_transcript/48120	-0.963308174009243	3.88811703708866	-2.70895843028355	0.0106703651348802	0.398104611399431	-2.94728203866893
a_HQ_transcript/12410	-3.68966875109615	0.828509198270581	-2.70795185812568	0.0106967085372339	0.39883147777571	-2.70648171989921
a_HQ_transcript/64328	1.86949963013658	0.46648660196084	2.70686277757198	0.0107252794270716	0.398865353368661	-2.73633582388406
a_HQ_transcript/36981	-6.57872159317178	1.26959253608986	-2.70676684585754	0.0107277994937222	0.398865353368661	-2.75007446984653
a_HQ_transcript/31168	4.59001353849832	1.2299339256535	2.70670203050185	0.0107295024646556	0.398865353368661	-2.72331075652796
a_HQ_transcript/1890	2.79030082574458	1.5244700734865	2.70658480184786	0.0107325831911517	0.398865353368661	-2.73758204012515
a_HQ_transcript/62242	0.764900231645634	5.43081131637773	2.70636432784596	0.0107383793966416	0.398865353368661	-3.10213271898355
a_HQ_transcript/81551	1.30598405626997	3.43552862383613	2.70634878372227	0.0107387881575481	0.398865353368661	-2.90741725050475
a_HQ_transcript/93833	-4.51565560663894	1.51706048909455	-2.70522765387442	0.0107683084898124	0.399400122585303	-2.78839837088372
a_HQ_transcript/76653	-0.93923419671217	4.68442088322553	-2.7051187311118	0.0107711805403225	0.399400122585303	-3.01986344123677
a_HQ_transcript/11721	3.59641926664914	1.16677286780246	2.70501944676751	0.0107737990678117	0.399400122585303	-2.72591289823663
a_HQ_transcript/103405	2.73285087286175	-0.0184264271335771	2.70424046126538	0.0107943646017905	0.399698482489924	-2.79352122895312
a_HQ_transcript/96071	3.63247248991544	0.850367959887618	2.70419372311293	0.0107955996681321	0.399698482489924	-2.70416312823232
a_HQ_transcript/145738	0.724894104662317	3.50210700706238	2.70350776784249	0.0108137413163951	0.400115312055642	-2.93429660930121
a_HQ_transcript/12349	2.59449889396339	0.561038854462856	2.70253438794913	0.0108395332218251	0.400537789683405	-2.84099367346334
a_HQ_transcript/108296	4.66651039773013	6.79311603890329	2.70224260147042	0.0108472759017567	0.400537789683405	-3.23358874096297
a_HQ_transcript/115658	-4.58631538330769	3.44931071469098	-2.70219520403988	0.0108485340983977	0.400537789683405	-2.99240662965307
a_HQ_transcript/105908	-2.55575193730272	1.23202078649702	-2.70137869570238	0.0108702301755049	0.400537789683405	-2.81939915836534
a_HQ_transcript/97255	6.03356074714188	3.55356714836876	2.70075836695184	0.0108867403724185	0.400537789683405	-2.90688330543484
a_HQ_transcript/57904	-0.780312645560594	3.46092752305227	-2.70070973858246	0.010888035612965	0.400537789683405	-2.87473648757159
a_HQ_transcript/85006	-2.69186196872502	-0.447226957694568	-2.70024687284067	0.010900371443706	0.400537789683405	-2.7867898917862
a_HQ_transcript/119095	-2.3037507382739	-0.473872687188901	-2.70021421503607	0.010901242297104	0.400537789683405	-2.76709820737801
a_HQ_transcript/127629	2.79927418182585	1.35903453753951	2.70009330911105	0.0109044669389529	0.400537789683405	-2.7193110209686
a_HQ_transcript/97927	-2.33560608629851	1.27537728386044	-2.69994841236571	0.0109083326002026	0.400537789683405	-2.79456759233737
a_HQ_transcript/5408	3.34832897248151	0.730482760735382	2.69991691264692	0.0109091731412864	0.400537789683405	-2.71389062433138
a_HQ_transcript/134551	3.11758527531259	0.312140989830657	2.69988956401299	0.0109099029633138	0.400537789683405	-2.71380523704694
a_HQ_transcript/133826	-3.20673035106928	0.083481601107979	-2.69970844390903	0.0109147374562778	0.400537789683405	-2.74476915297725
a_HQ_transcript/105390	3.00460482784014	-0.0843694544056848	2.69895086204165	0.0109349805735695	0.400810624428811	-2.76352181362253
a_HQ_transcript/108200	6.60987574089728	3.06517359707166	2.69891413317974	0.010935962880954	0.400810624428811	-2.89845080644606
a_HQ_transcript/58820	2.74306372261114	0.553982219821627	2.69855627049592	0.0109455381539758	0.400892672422337	-2.75934041736624
a_HQ_transcript/116601	-1.70812417551008	-0.579751141832203	-2.69831511998766	0.0109519949732774	0.400892672422337	-2.75532308302242
a_HQ_transcript/134625	-1.86487071379737	-0.717505884689717	-2.69779662261643	0.010965889763714	0.40114867223736	-2.75428224616751
a_HQ_transcript/130774	2.39978092772528	-0.187970577702	2.69674006394106	0.0109942543529082	0.401339806885057	-2.75679273996331
a_HQ_transcript/106990	1.44034669820308	3.07998650195695	2.69622437959838	0.0110081232710332	0.401339806885057	-2.90747425815422
a_HQ_transcript/16014	1.28601081130304	2.27685320133065	2.696199096261	0.0110088036641743	0.401339806885057	-2.74373457755621
a_HQ_transcript/100440	-1.94803095900283	2.21973261629878	-2.69614477106731	0.0110102657271774	0.401339806885057	-2.81217790323111
a_HQ_transcript/3049	2.70525668717658	0.0491057997777523	2.69591145192188	0.011016547136568	0.401339806885057	-2.72986230201641
a_HQ_transcript/42017	3.56435237241117	1.54031709835705	2.69562066989721	0.0110243802216208	0.401339806885057	-2.72891471306623

a_HQ_transcript/17576	2.37528488549547	0.588507925175651	2.69560684432656	0.0110247527836697	0.401339806885057	-2.73676183950683
a_HQ_transcript/33614	2.18914914646941	1.77213141487263	2.69554757820562	0.0110263499792774	0.401339806885057	-2.77489970197792
a_HQ_transcript/63838	-3.66274510912758	0.922878523566924	-2.69522851986792	0.0110349521555139	0.401401563764524	-2.79183790138491
a_HQ_transcript/47577	-1.55191664530081	2.90925006122325	-2.69282220676824	0.0111000302775343	0.403351867239707	-2.90395810054893
a_HQ_transcript/15884	-3.38388168719509	0.110399530723596	-2.69273313206612	0.0111024461117453	0.403351867239707	-2.79890035665428
a_HQ_transcript/151258	0.40408316719825	11.3877448166531	2.69162716439064	0.0111324822692647	0.404006597791889	-3.71992712955052
a_HQ_transcript/138144	7.66849434238535	1.67491606390046	2.69155780524003	0.0111343684568987	0.404006597791889	-2.77691510797738
a_HQ_transcript/44455	2.17108227279548	1.1392675259475	2.69082292783776	0.0111543713312454	0.404272657023408	-2.73347390103081
a_HQ_transcript/149503	3.53392170920618	0.836656310818502	2.69077743351629	0.0111556107532608	0.404272657023408	-2.84410788933985
a_HQ_transcript/132069	3.05991730295627	0.277062900131637	2.69040557725305	0.011165746197066	0.404387847316543	-2.7735055150521
a_HQ_transcript/43832	5.19100564915426	2.73140438994761	2.69010354451702	0.0111739848026256	0.404434239481332	-2.87853206612505
a_HQ_transcript/83269	2.33529908775791	-0.12086933747366	2.688828864805	0.0112088167411903	0.404974043631791	-2.79537024972274
a_HQ_transcript/28557	2.82484272688193	0.067214033811376	2.68856757212378	0.0112159692800925	0.404974043631791	-2.78006035903971
a_HQ_transcript/130535	1.93766681965152	-0.555391781273905	2.6883559298322	0.011221765813621	0.404974043631791	-2.75795293844418
a_HQ_transcript/137955	-3.37523195541716	0.24625442848388	-2.68773277108197	0.0112388492691918	0.404974043631791	-2.78490254170696
a_HQ_transcript/116376	2.88358639020437	0.889301311610181	2.68759339019792	0.0112426736020718	0.404974043631791	-2.74450479940359
a_HQ_transcript/30874	2.32316567481737	-0.330191079462661	2.68736744802732	0.0112488755718676	0.404974043631791	-2.7592819879168
a_HQ_transcript/74367	1.00979022558883	2.95189817607514	2.68736231805148	0.0112490164233542	0.404974043631791	-2.88493529254685
a_HQ_transcript/122120	3.74453657780797	2.45460154498485	2.68726941875529	0.0112515674018887	0.404974043631791	-2.82193169388905
a_HQ_transcript/16017	1.72202002403035	0.620820672374702	2.68695099193675	0.0112603153567656	0.404974043631791	-2.73804357198619
a_HQ_transcript/62262	2.40961629053661	-0.47965970712981	2.68679270129526	0.0112646663340847	0.404974043631791	-2.76466083153886
a_HQ_transcript/83791	2.32591124777314	-0.558658300587977	2.68663060917477	0.0112691234220201	0.404974043631791	-2.78780539247514
a_HQ_transcript/107729	-3.95296440125825	1.32953642423599	-2.68650777522665	0.0112725021090737	0.404974043631791	-2.85038885056608
a_HQ_transcript/143288	3.70719642262786	1.10445449740734	2.6852441516376	0.0113073141790365	0.405524217066664	-2.78622104026108
a_HQ_transcript/54009	1.93302733792518	1.41946558868645	2.68518930440959	0.0113088274459624	0.405524217066664	-2.78775442044414
a_HQ_transcript/74987	2.61616558609118	0.976571187075951	2.6850116751179	0.0113137296326119	0.405524217066664	-2.74530594463758
a_HQ_transcript/91821	-2.98671481242995	1.01607011870786	-2.6849395078946	0.011315721856629	0.405524217066664	-2.81196842499961
a_HQ_transcript/65351	-1.93931436756125	2.82875551038343	-2.68441736715404	0.0113301456073779	0.405542434773226	-2.8239575021416
a_HQ_transcript/136654	-5.00570298501186	0.597571407407183	-2.68419085027734	0.0113364082773117	0.405542434773226	-2.79523188917296
a_HQ_transcript/148013	-3.06185745446149	-0.153688676800702	-2.68404154146313	0.0113405380799853	0.405542434773226	-2.83701246927216
a_HQ_transcript/25790	-1.2726735530337	2.16554395251482	-2.68391146522654	0.0113441370585822	0.405542434773226	-2.78850107312968
a_HQ_transcript/150825	5.53357394880804	2.63105870414508	2.68362814970289	0.0113519795678635	0.405573367130158	-2.79574872641603
a_HQ_transcript/118511	5.34571558284567	0.236432473913873	2.68281242858815	0.0113745878224124	0.405859052798345	-2.8054067274822
a_HQ_transcript/97525	2.71806939158847	0.075992980858504	2.68276518765119	0.0113758984165344	0.405859052798345	-2.78664009258626
a_HQ_transcript/116730	2.29463324852607	-0.525205050649882	2.68258414353285	0.0113809223792544	0.405859052798345	-2.79635016328391
a_HQ_transcript/90855	-2.9957281011425	4.60530027582086	-2.68206989851413	0.011395203886011	0.405868663009443	-3.10830718730246
a_HQ_transcript/14841	2.9109592951094	2.70001282980164	2.68188181038853	0.011400431586048	0.405868663009443	-2.81278318413421
a_HQ_transcript/58138	-3.95618852756734	0.451555933081312	-2.68147727087531	0.0114116828583421	0.405868663009443	-2.80966845296927
a_HQ_transcript/128153	3.92015278078554	1.8514564486389	2.68146422741608	0.0114120458016332	0.405868663009443	-2.78778083150072
a_HQ_transcript/127956	-2.03317955439421	3.14277091632795	-2.68131842817748	0.0114161034960852	0.405868663009443	-2.86131390148579
a_HQ_transcript/94290	1.69777871840002	2.08840001385764	2.68085005447406	0.0114291477292275	0.406065302958402	-2.80035652857405
a_HQ_transcript/34519	2.53983579767586	1.42537608641246	2.68061834664479	0.0114356059204326	0.406065302958402	-2.76571766820383
a_HQ_transcript/42472	-2.41967275028084	0.399355102192216	-2.68001619846003	0.0114524049222844	0.406413549036964	-2.83652413238152
a_HQ_transcript/144950	-1.8119606329213	-0.525486406515749	-2.67968412979888	0.0114616789231308	0.406494492034013	-2.79708112574501
a_HQ_transcript/136166	-0.76055735783971	3.99261321051105	-2.67884655791237	0.011485101581444	0.407076819954984	-3.00642021996788
a_HQ_transcript/19671	1.08198953135737	3.32704872001467	2.67795159040805	0.0115101784122358	0.40765317736654	-3.00262184801613
a_HQ_transcript/75648	3.11175378575783	0.879078468084921	2.67738685546202	0.0115260283329479	0.40765317736654	-2.76983548309601
a_HQ_transcript/68166	5.24044124075199	1.08875554252346	2.67690212046083	0.0115396491225891	0.40765317736654	-2.83247871363947
a_HQ_transcript/68972	1.86869594809561	1.66867915639907	2.67685218725715	0.0115410530670013	0.40765317736654	-2.76711210535078
a_HQ_transcript/80874	-3.54196619338595	0.792823472846054	-2.67653573041859	0.01154995439722	0.40765317736654	-2.83297630283503

a_HQ_transcript/85284	3.00511367024768	1.75153827009762	2.67611752131716	0.0115617276018314	0.40765317736654	-2.78541181058113
a_HQ_transcript/50075	-2.6560898019254	0.890036108878107	-2.67601836942809	0.0115645205073005	0.40765317736654	-2.78313972781781
a_HQ_transcript/66503	2.81444146076789	3.70490624485292	2.67581697653842	0.0115701952601563	0.40765317736654	-2.9548393510943
a_HQ_transcript/147090	2.61318276668478	0.864983696155596	2.67580221232327	0.0115706113808501	0.40765317736654	-2.75640227829891
a_HQ_transcript/149622	-2.86959384035767	1.04167501771981	-2.67577093506304	0.0115714929578653	0.40765317736654	-2.76655441480892
a_HQ_transcript/124535	1.98668791676332	-0.656702065498605	2.67527050026602	0.01158560663357	0.40773155107681	-2.79773969444634
a_HQ_transcript/28511	2.35152206138146	0.556549655527175	2.67519467925205	0.0115877463938014	0.40773155107681	-2.76207876187383
a_HQ_transcript/35466	1.04058421540269	2.74537344543548	2.67451580914754	0.011606921277006	0.408159177247309	-2.86157240530693
a_HQ_transcript/63790	-3.02722531700158	0.512904263851015	-2.67355675191549	0.0116340603218342	0.40864795144924	-2.78674930539577
a_HQ_transcript/87333	2.91379430472932	0.803406231875577	2.67277678202311	0.011656175031916	0.40864795144924	-2.77812725556921
a_HQ_transcript/53365	-2.24637761492697	0.103221595360788	-2.67273853765749	0.0116572603881087	0.40864795144924	-2.78854495858986
a_HQ_transcript/90941	0.875396600109303	3.60013147568893	2.67248871867663	0.0116643524349698	0.40864795144924	-3.00385968064209
a_HQ_transcript/135829	-4.24828728938304	2.7018663863306	-2.67210483439353	0.0116752582316978	0.40864795144924	-2.97483018294681
a_HQ_transcript/106751	1.23985340173554	3.61028859005415	2.67179306226443	0.0116841223514453	0.40864795144924	-2.89299388967298
a_HQ_transcript/148386	-4.24087322269078	-0.273173319829959	-2.67166520200045	0.0116877594039289	0.40864795144924	-2.87557750681235
a_HQ_transcript/99131	2.1188414400138	-0.490763758980347	2.67165287097633	0.0116881102219407	0.40864795144924	-2.80506462679936
a_HQ_transcript/65780	-1.84617115058195	-0.726898710329288	-2.67157310418051	0.0116903798257804	0.40864795144924	-2.81188027375189
a_HQ_transcript/112457	1.13359065212973	3.729969682685	2.67140612257405	0.0116951322759952	0.40864795144924	-2.98903026058761
a_HQ_transcript/144451	-1.72909340278152	-0.806579878445917	-2.67130004746982	0.0116981522022353	0.40864795144924	-2.80538529171911
a_HQ_transcript/67538	-4.69181523547054	0.820624101104741	-2.67033292113521	0.0117257193919542	0.409364935024333	-2.84969584044016
a_HQ_transcript/39087	2.44632688971235	0.692830761187934	2.66935156362073	0.0117537538382005	0.410097360808473	-2.82105061750512
a_HQ_transcript/138777	4.39032587047684	4.99737975487056	2.66900894411766	0.0117635560893861	0.410193154387422	-3.11513816561318
a_HQ_transcript/83357	-1.84324374693227	1.69205794626349	-2.66848798612692	0.0117784750916726	0.410270839385854	-2.77419082219192
a_HQ_transcript/76759	3.92608612912712	0.272772321458112	2.66843825941613	0.0117799000642546	0.410270839385854	-2.82584651038228
a_HQ_transcript/84180	-1.16680047036884	2.23706275230795	-2.66735195767979	0.0118110691533764	0.410940933696216	-2.82098201634547
a_HQ_transcript/6078	2.97235254288146	0.10887645928321	2.66727503027206	0.0118132793181664	0.410940933696216	-2.78426307892749
a_HQ_transcript/79552	2.86291407478457	-0.271858683556855	2.66700947532543	0.0118209117998457	0.410960503051094	-2.83605773704883
a_HQ_transcript/90231	-2.79313532059525	0.627213153489634	-2.66600987561203	0.0118496829631666	0.41138836546073	-2.78663607481405
a_HQ_transcript/133673	2.31707963878032	-0.107537300930402	2.66597323420035	0.0118507388345969	0.41138836546073	-2.78475053350469
a_HQ_transcript/111722	-2.09321477589875	-0.521840439892193	-2.66566354648247	0.0118596663883431	0.41138836546073	-2.83142635745859
a_HQ_transcript/85681	2.27000306955838	1.32297008442479	2.66557462607617	0.0118622309028754	0.41138836546073	-2.84975206128374
a_HQ_transcript/26690	2.92895701462376	1.1700354384191	2.66535368136085	0.011868605299987	0.41138836546073	-2.78715709353087
a_HQ_transcript/134331	2.70578955519998	1.37642886305719	2.66463960857326	0.0118892284947 6	0.411721166919242	-2.79674625176888
a_HQ_transcript/115993	2.07867066555676	-0.463502715976688	2.66449923392971	0.0118932865738551	0.411721166919242	-2.79041921744789
a_HQ_transcript/145375	0.593716576682348	9.29997164898242	2.66384447930452	0.0119122317995714	0.411721166919242	-3.52854765998996
a_HQ_transcript/55908	2.90768098371423	0.159618003288695	2.66372469657266	0.0119157007221266	0.411721166919242	-2.80374154020853
a_HQ_transcript/75458	3.02171316552094	0.253798467743441	2.66357999881419	0.0119198924364503	0.411721166919242	-2.79788716770819
a_HQ_transcript/35132	3.29311123912332	0.705438944220983	2.66355196431099	0.0119207047193278	0.411721166919242	-2.85519936412424
a_HQ_transcript/129103	2.69744347077008	-0.348371074163964	2.66265204792692	0.0119468065468793	0.412228066659666	-2.83129867508754
a_HQ_transcript/77831	3.07904170645217	0.847499853781728	2.66255706394878	0.0119495646215514	0.412228066659666	-2.8356081168641
a_HQ_transcript/144161	3.69803308365781	2.49710345468933	2.66226294154293	0.0119581088769465	0.412278145195223	-2.9086231541514
a_HQ_transcript/8129	-3.54785953843636	0.191409109266186	-2.66198556819028	0.0119661717632747	0.412311578100551	-2.84020177160168
a_HQ_transcript/63886	3.66825052888021	0.903313644667535	2.66163251086648	0.0119764419784018	0.412420983009799	-2.85299631995395
a_HQ_transcript/132994	-3.2943996291904	1.25355986498581	-2.66093171525591	0.0119968519022941	0.412759457708254	-2.83502497151852
a_HQ_transcript/152917	3.54771937363787	3.98826695740773	2.66080750315856	0.0120004728104691	0.412759457708254	-2.96390918737046
a_HQ_transcript/147102	1.30335285420243	3.39412417536799	2.66012028083925	0.012020524356922	0.4128746691506	-3.02669382816306
a_HQ_transcript/44539	-1.98567530419678	-0.740948433511741	-2.66000426661605	0.0120239124452961	0.4128746691506	-2.83477309578383
a_HQ_transcript/94300	1.77460794406878	-0.362235391711147	2.6599625484678	0.0120251310017886	0.4128746691506	-2.82197126366324
a_HQ_transcript/144235	4.83852935452833	1.61339922634816	2.65959706935613	0.0120358112755313	0.41299742492567	-2.84438716034632
a_HQ_transcript/37937	-0.651636975115991	3.25866719936478	-2.65767805752589	0.0120920343992772	0.414510020430988	-2.98501112347055

a_HQ_transcript/95155	-4.43638412179395	1.17843430560758	-2.65760586668943	0.0120941541881874	0.414510020430988	-2.86255471970839
a_HQ_transcript/148134	-2.82671470051103	2.0816513470346	-2.6569767706757	0.0121126413532612	0.414717027051606	-2.84461790133787
a_HQ_transcript/31027	-2.89702242132935	0.204735609539924	-2.6567805520791	0.0121184129625716	0.414717027051606	-2.81861661951666
a_HQ_transcript/121913	0.662819230867845	3.81585082540507	2.6566723182459	0.0121215976631	0.414717027051606	-3.06261851863558
a_HQ_transcript/51555	3.69839938471834	2.99729850567463	2.65552505840616	0.0121554026699183	0.415143990288448	-3.05640665398728
a_HQ_transcript/84851	-2.89223678118704	1.04475691148897	-2.65524910887387	0.0121635467909335	0.415143990288448	-2.89331134917182
a_HQ_transcript/113168	2.0021931775336	0.701107884183317	2.65471849739974	0.0121792209866508	0.415143990288448	-2.80769731717415
a_HQ_transcript/11982	-1.95160385065262	0.821744858578161	-2.65457726587201	0.0121833961019341	0.415143990288448	-2.80165449060378
a_HQ_transcript/92809	-0.754613244360831	5.14503872875393	-2.65449954980327	0.0121856941261005	0.415143990288448	-3.20303607538459
a_HQ_transcript/103133	-3.3613334376626	1.09737623605412	-2.65373666811437	0.0122082734848969	0.415143990288448	-2.8365482476364
a_HQ_transcript/130198	4.04857444174546	1.77213316476269	2.65352154039542	0.0122146477248639	0.415143990288448	-2.83443771184064
a_HQ_transcript/67995	2.9461204939967	1.9863706976354	2.65316103071456	0.0122253365529897	0.415143990288448	-2.91185514690419
a_HQ_transcript/59014	-2.58858863140988	0.0865051655211927	-2.65303579710205	0.0122290516594566	0.415143990288448	-2.83279942385874
a_HQ_transcript/9949	-4.15099937450604	1.46782236353287	-2.65296391998565	0.0122311843962009	0.415143990288448	-2.96480644810147
a_HQ_transcript/16936	-3.13976327378528	0.903308961830179	-2.65269960056784	0.0122390302433431	0.415143990288448	-2.82010243759039
a_HQ_transcript/147639	3.23638731592724	-0.201051054121989	2.65261703440719	0.0122414820274956	0.415143990288448	-2.89688904236504
a_HQ_transcript/146567	-3.05314969927766	-0.036936194463213	-2.6526029636765	0.0122418999001747	0.415143990288448	-2.86917845870415
a_HQ_transcript/59351	3.03413790328092	0.385615895088874	2.652495409294	0.01224509448793	0.415143990288448	-2.82437567920557
a_HQ_transcript/83190	3.71213679761836	1.12641808969861	2.65234462617112	0.0122495743596415	0.415143990288448	-2.84794249021787
a_HQ_transcript/78828	-3.08594455119317	1.23877974187594	-2.65207374657239	0.0122576261973026	0.415143990288448	-2.8470716396042
a_HQ_transcript/70581	-3.30254876778748	0.620762646487716	-2.65194108584866	0.012261571297863	0.415143990288448	-2.86187469430126
a_HQ_transcript/106189	2.29782549082378	2.36621209514592	2.65190544872401	0.0122626312848415	0.415143990288448	-2.8401410727876
a_HQ_transcript/123037	-3.76171122190734	0.868704295640679	-2.6516166749195	0.0122712236760402	0.415193067427744	-2.90919029842466
a_HQ_transcript/80436	-1.93460958532676	1.95341030370166	-2.65097022551999	0.0122904788443731	0.41540071886531	-2.85383319172369
a_HQ_transcript/131410	1.93780602102675	2.9244264386331	2.65069589574538	0.0122986585016961	0.41540071886531	-2.90624718596436
a_HQ_transcript/148544	-2.97513130300811	0.103628965654171	-2.65069115698117	0.0122987998411643	0.41540071886531	-2.88669297274249
a_HQ_transcript/12329	1.852715167607	1.09165928591513	2.65018116626201	0.0123140197426808	0.415673251801714	-2.86469464527603
a_HQ_transcript/82663	2.36431378723736	0.0860466320091622	2.64985692497211	0.0123237053076534	0.41575875921258	-2.9146175850496
a_HQ_transcript/150914	7.71167586758042	5.80897851324879	2.64909753195116	0.0123464171311244	0.415826946996838	-3.10650672320926
a_HQ_transcript/50179	3.79835188165653	1.09711887143508	2.64908123806517	0.0123469048712173	0.415826946996838	-2.85232551187849
a_HQ_transcript/148964	0.586512072149272	6.71831945073112	2.64907179916237	0.0123471874228692	0.415826946996838	-3.32547566636421
a_HQ_transcript/49854	-1.04189119094811	5.0344836786967	-2.64857238713022	0.0123621457693089	0.416089640578107	-3.18667243965056
a_HQ_transcript/150561	2.03596902704523	-0.670219011993511	2.64765145102537	0.0123897736091692	0.416778217797331	-2.8657686784106
a_HQ_transcript/136804	2.20672399761545	0.930346664274878	2.64726599874948	0.0124013540500035	0.416842430617988	-2.84757646026873
a_HQ_transcript/109033	-3.25977287736581	1.09999387392943	-2.64710787481945	0.0124061075897639	0.416842430617988	-2.80789815887848
a_HQ_transcript/123670	1.02600898672501	2.76211850269151	2.64687219245935	0.0124131958333288	0.416842430617988	-2.92389874063017
a_HQ_transcript/2960	-3.09891496149424	1.38313281346119	-2.64655855023328	0.0124226345710981	0.416918534843412	-2.82314093573361
a_HQ_transcript/125721	2.52516395952434	1.62170067294747	2.64570454729711	0.0124483686163494	0.417541125753698	-2.97567996916849
a_HQ_transcript/143324	4.31185256495302	0.723089607721181	2.64387302801938	0.0125037252084381	0.419028934224585	-2.86359727556681
a_HQ_transcript/58603	1.28887331801889	3.82312019417367	2.64376019808338	0.0125071428722759	0.419028934224585	-3.06241549418497
a_HQ_transcript/63198	2.97469351354197	1.96547625823202	2.64333395766259	0.0125200616753854	0.419220129646777	-2.88170311191832
a_HQ_transcript/3638	-3.31679090313169	1.51360578017456	-2.6430039579762	0.0125300720326141	0.419293842936489	-2.95309605742411
a_HQ_transcript/96466	-2.16904691063132	1.83368795991826	-2.64275941594517	0.0125374948611489	0.419293842936489	-2.85180867933058
a_HQ_transcript/128396	-2.59212619156329	0.0131284043112554	-2.642548410689	0.0125439029876575	0.419293842936489	-2.87613412649499
a_HQ_transcript/79759	0.945383837146658	2.30845209925511	2.64121406810123	0.0125844966202416	0.420221681095498	-2.90469672004961
a_HQ_transcript/95221	-0.964723748949834	2.6189016130591	-2.6409742450909	0.0125918054491091	0.420221681095498	-2.92614697369327
a_HQ_transcript/15829	-1.54603315202269	2.2401997418409	-2.64092362590954	0.0125933486180216	0.420221681095498	-2.84035286389871
a_HQ_transcript/148768	2.58384906790006	0.312092155327815	2.64066983792288	0.0126010882020124	0.420238092609502	-2.83903124894981
a_HQ_transcript/83845	-2.82288953469063	0.898763519474452	-2.6399491072904	0.0126230918146988	0.420238092609502	-2.86359669015238
a_HQ_transcript/5683	-3.14703034541838	1.26140635601165	-2.63986003401435	0.012625813654637	0.420238092609502	-3.00550911423527

a_HQ_transcript/108738	2.77381100489304	0.251710472371583	2.63984587119051	0.0126262464825479	0.420238092609502	-2.84502090874719
a_HQ_transcript/53369	1.31666380312943	0.768503228333336	2.63972345847679	0.0126299880916047	0.420238092609502	-2.88933954993961
a_HQ_transcript/98861	1.58812043599498	2.22987861778765	2.63898229975485	0.0126526639352261	0.420594121385155	-2.90888378823703
a_HQ_transcript/150849	-2.6910809808144	-0.142395888063735	-2.63886538260685	0.0126562444707281	0.420594121385155	-2.86027187611933
a_HQ_transcript/143879	1.07742125737696	2.7813430027348	2.63866460864334	0.0126623952729153	0.420594121385155	-2.98701633568482
a_HQ_transcript/11661	1.55840700748643	2.50720070666002	2.63809621659931	0.0126798232365738	0.42093247578273	-2.90075262709215
a_HQ_transcript/38768	-1.94734677400327	-0.720775257972862	-2.63760272638764	0.0126949725806657	0.42119484370373	-2.87822255412538
a_HQ_transcript/111789	3.66312005942248	0.916401461505288	2.63655045496199	0.0127273315793281	0.422027569904839	-2.86283508571468
a_HQ_transcript/19265	-3.24252594700944	0.131108448575498	-2.63602891997817	0.0127433978881413	0.422319402760478	-2.89777494930387
a_HQ_transcript/151697	-1.6657260745201	-0.489196100510387	-2.63561487514287	0.0127561662442373	0.422464621701136	-2.87568565740995
a_HQ_transcript/130436	1.54107867206782	-0.78736154593196	2.63541560734119	0.0127623155055601	0.422464621701136	-2.89217734445215
a_HQ_transcript/62873	-3.3082619413329	0.255212160739168	-2.63485226970721	0.0127797145494632	0.422589576228432	-2.88474562598956
a_HQ_transcript/3725	-2.69445960281982	-0.325651741114005	-2.63482264137017	0.0127806302472059	0.422589576228432	-2.89868671462132
a_HQ_transcript/13313	2.15470389783376	-0.468389459814054	2.63441642754156	0.0127931908782342	0.422726604690232	-2.90286447876707
a_HQ_transcript/66809	-1.03470627265525	2.46879632873061	-2.63405752401947	0.012804298124152	0.422726604690232	-2.90313838050273
a_HQ_transcript/148429	1.52450107282232	6.42519768143398	2.63390628450169	0.0128089813154514	0.422726604690232	-3.30818210731763
a_HQ_transcript/127945	-2.32909496020713	-0.243278221674152	-2.63352937490217	0.0128206593703827	0.422726604690232	-2.86266987060668
a_HQ_transcript/10504	3.39228998752382	1.52592518622471	2.63304713130202	0.0128356154347468	0.422726604690232	-2.88114040605426
a_HQ_transcript/25698	3.24767499787414	2.79772782378135	2.63298882450982	0.0128374248267082	0.422726604690232	-2.95677611323437
a_HQ_transcript/150540	0.529691736987609	9.84884710407886	2.63287613395721	0.0128409225391925	0.422726604690232	-3.67309150118257
a_HQ_transcript/98601	-1.73651122215067	1.13213381798004	-2.63237225414853	0.012856572856975	0.422726604690232	-2.84053081225978
a_HQ_transcript/110430	-1.14338080924697	4.06480795310766	-2.63232727269718	0.0128579708220334	0.422726604690232	-3.14212680623614
a_HQ_transcript/91253	-1.83339129175803	1.27069231942894	-2.63211738045313	0.012864495860446	0.422726604690232	-2.92300513681767
a_HQ_transcript/7713	2.04929355022807	-0.626188353866006	2.63151191438991	0.0128833355011224	0.422726604690232	-2.89438387316971
a_HQ_transcript/132635	4.16725552561864	0.156997299731403	2.63129399864231	0.0128901223996921	0.422726604690232	-2.86541297165877
a_HQ_transcript/8562	1.75841721335007	0.33556045729174	2.63126081127607	0.0128911562972111	0.422726604690232	-2.85086491934715
a_HQ_transcript/123152	-1.9786386100423	1.96731429612485	-2.63102293420147	0.0128985692111069	0.422726604690232	-2.88893392045996
a_HQ_transcript/112481	-5.5677850783436	2.05303223908276	-2.6309171387932	0.0129018673587921	0.422726604690232	-2.90595140531472
a_HQ_transcript/59877	-0.770291400767583	4.59162797399868	-2.630673643104	0.0129094612469923	0.422726604690232	-3.19736023825408
a_HQ_transcript/42141	2.12866472167706	-0.640570222704122	2.63058351060173	0.0129122732538192	0.422726604690232	-2.90111293799042
a_HQ_transcript/142218	-1.90846043394342	-0.593839856970367	-2.63047444806326	0.0129156766090327	0.422726604690232	-2.90775777405034
a_HQ_transcript/70905	-4.66794590257886	3.95488914365575	-2.62966754014624	0.0129408824096285	0.423306234348809	-3.18980082466417
a_HQ_transcript/148241	0.922468052776197	4.80549466754517	2.62938212432693	0.0129498089849779	0.423306234348809	-3.20789588612214
a_HQ_transcript/1375	3.16144236761745	0.349412451443475	2.62920878341068	0.0129552331218437	0.423306234348809	-2.91688570130529
a_HQ_transcript/48359	3.55320867815811	1.09323168555089	2.62890921409891	0.0129646121201436	0.423374704112194	-2.85667408235458
a_HQ_transcript/29096	1.66321354624898	-0.650738163673073	2.62720646582354	0.013018041693597	0.424692319782509	-2.91944254278711
a_HQ_transcript/139107	3.47990566158464	0.282473973595071	2.62694359866357	0.0130263081848655	0.424692319782509	-2.90982178871175
a_HQ_transcript/150040	2.54587948794946	-0.344066140998727	2.62682865552225	0.0130299243749245	0.424692319782509	-2.89469251984396
a_HQ_transcript/42672	-2.07019100174415	0.0205799293202235	-2.62623235780381	0.0130486992229125	0.424692319782509	-2.88423857843905
a_HQ_transcript/42295	-2.56209429920435	0.462740029853352	-2.62621547836021	0.0130492310480327	0.424692319782509	-2.88507817916937
a_HQ_transcript/119643	1.45981184025812	2.2181408541143	2.62610905323941	0.0130525846744965	0.424692319782509	-2.92676773579531
a_HQ_transcript/151405	2.9437169285342	0.347837737093437	2.62599741763262	0.0130561033486675	0.424692319782509	-2.90730781164196
a_HQ_transcript/41618	4.21801126795689	1.2884985515124	2.62549230379576	0.0130720351457095	0.424796005434822	-2.89151957946643
a_HQ_transcript/64417	-1.95349306070537	1.11897319176841	-2.62540249606199	0.0130748696542641	0.424796005434822	-2.865448914286
a_HQ_transcript/134738	3.99409326723704	0.783113888575242	2.62482838096773	0.0130930032956596	0.424796005434822	-2.87557545263143
a_HQ_transcript/84929	1.25158638146112	2.48939520808427	2.6245745597169	0.0131010277502179	0.424796005434822	-2.93113820289237
a_HQ_transcript/147227	-4.21871042109457	5.64531015312621	-2.62418527047503	0.013113343810256	0.424796005434822	-3.16775274146283
a_HQ_transcript/20675	-4.40583938568888	1.15464768884449	-2.62400589566644	0.013119022348599	0.424796005434822	-2.95188419438555
a_HQ_transcript/77170	-3.65838848920487	-0.38083476546602	-2.62374289316757	0.0131273524343161	0.424796005434822	-2.92370253588363
a_HQ_transcript/151690	-3.64746783227697	1.30190485463116	-2.62362952701263	0.0131309445926879	0.424796005434822	-2.98596619403968

a_HQ_transcript/137273	4.83269766358191	1.61591802492476	2.62343887625875	0.0131369876675222	0.424796005434822	-2.96081045511414
a_HQ_transcript/100955	0.83630084922448	4.68090034555448	2.62337115559247	0.013139134834983	0.424796005434822	-3.22363700133376
a_HQ_transcript/6115	-4.24955765251091	0.862435039264105	-2.62315037765284	0.013146137127129	0.424796005434822	-2.97243320637835
a_HQ_transcript/80219	3.58551776525614	1.93960247462586	2.62311068252316	0.0131473964817312	0.424796005434822	-2.94666553813875
a_HQ_transcript/10061	1.69659631885183	-0.642546543643175	2.62289332920329	0.0131542941402195	0.424796005434822	-2.91608102150339
a_HQ_transcript/87336	1.16137451864504	2.58747213474798	2.62246527438905	0.0131678881517882	0.424998890886808	-2.8999143662986
a_HQ_transcript/14517	4.5338918831524	2.14901887466382	2.62131941901148	0.0132043417929236	0.425592603228532	-2.98894434867151
a_HQ_transcript/86237	4.22735877990674	0.525092242709218	2.62109465878346	0.0132115031378468	0.425592603228532	-2.94458348191563
a_HQ_transcript/134563	4.50097078871309	0.66236311167769	2.62097867238973	0.0132152001191923	0.425592603228532	-2.91409796793023
a_HQ_transcript/6211	1.17133495201199	2.51965174113409	2.62096707190091	0.0132155699289069	0.425592603228532	-2.91512531154314
a_HQ_transcript/95045	2.36871917916569	3.3678467213227	2.62024195832706	0.013238704689247	0.425735663916856	-3.04207239466236
a_HQ_transcript/65652	2.10987621542075	-0.320298016171718	2.62017721268378	0.0132407722205343	0.425735663916856	-2.92812324652074
a_HQ_transcript/39704	1.52625984885323	-0.82957199904792	2.62007150269772	0.0132441485134774	0.425735663916856	-2.92186395876184
a_HQ_transcript/127441	1.28926591534284	2.71517192837155	2.61990998811776	0.013249308698486	0.425735663916856	-2.96198627149011
a_HQ_transcript/82384	-3.28479197703156	0.0190298512698235	-2.61921608847758	0.0132714990683703	0.426213092732723	-2.92875254311644
a_HQ_transcript/106352	1.98567872564495	-0.517634662030842	2.61888155026369	0.0132822096147899	0.426321524289622	-2.92337526748846
a_HQ_transcript/135131	3.26357050871265	1.04455659155575	2.61836253408594	0.0132988422133705	0.42654103207144	-2.9627957099068
a_HQ_transcript/9554	2.54655313519993	0.962255260832805	2.61807902052899	0.013307935933504	0.42654103207144	-2.89844832036424
a_HQ_transcript/33509	2.68335565159003	0.190027383529391	2.61798159014541	0.0133110623482245	0.42654103207144	-2.86979611298955
a_HQ_transcript/118911	9.15979995792862	1.5690620564676	2.61749825408384	0.0133265820203207	0.426622395321973	-3.12290662265039
a_HQ_transcript/81857	-1.94261822598582	-0.772289353007589	-2.61744539934929	0.0133282801731473	0.426622395321973	-2.92211499810367
a_HQ_transcript/11883	3.16705884876146	0.71403075124622	2.61608592121905	0.013372027229015	0.427786590970687	-2.88636580085179
a_HQ_transcript/88936	1.72641672427932	-0.817070176822501	2.61583692179414	0.0133800542274329	0.427786590970687	-2.93103831339982
a_HQ_transcript/76072	4.81430767874567	2.36877393044239	2.61481582759254	0.0134130178726982	0.427786590970687	-2.92828256640662
a_HQ_transcript/3965	-1.14460592843187	3.12225380934197	-2.61473763884348	0.0134155451063296	0.427786590970687	-3.0110075191193
a_HQ_transcript/141411	-3.73187023653698	0.0643904678890125	-2.61445052633974	0.0134248289953791	0.427786590970687	-3.04443342002638
a_HQ_transcript/107473	4.68469458081459	3.22219703659754	2.61404627440541	0.0134379107003266	0.427786590970687	-3.04019372060184
a_HQ_transcript/56266	3.56107233008826	0.8531003003129	2.61390518420935	0.0134424791918221	0.427786590970687	-2.88227645303705
a_HQ_transcript/138844	2.51299590136835	1.81611532871927	2.61372180621882	0.0134484191052232	0.427786590970687	-2.93995170229407
a_HQ_transcript/6185	1.2541723443556	2.08078306216163	2.61361417912037	0.0134519064530402	0.427786590970687	-2.89953097635243
a_HQ_transcript/9198	0.83468188591589	3.67514698907487	2.61351094985252	0.0134552520871766	0.427786590970687	-3.12110435080555
a_HQ_transcript/142397	-1.94339147479096	-0.716856504500977	-2.61341329317675	0.0134584178230632	0.427786590970687	-2.93020779575672
a_HQ_transcript/87705	-2.04772687119054	1.5347297164701	-2.61314540917093	0.0134671053525458	0.427786590970687	-2.87845566028774
a_HQ_transcript/94729	-4.26387682219466	0.212282668069997	-2.61307192756865	0.0134694892808023	0.427786590970687	-2.9889761795698
a_HQ_transcript/81660	-1.36434369396263	4.28244208146239	-2.61279353690223	0.0134785245141882	0.427786590970687	-3.06254297214664
a_HQ_transcript/56186	2.51107249532822	2.08740475770228	2.61255997096852	0.0134861092721633	0.427786590970687	-2.99477743647836
a_HQ_transcript/95523	1.52421076000745	1.75567673843433	2.61251626188115	0.0134875291076099	0.427786590970687	-2.90051512204084
a_HQ_transcript/81433	-1.12859744046499	2.4343150375337	-2.61236723728772	0.0134923710252719	0.427786590970687	-2.9668115869403
a_HQ_transcript/21481	2.46802330979608	1.79278398800686	2.61222111258328	0.0134971202835164	0.427786590970687	-2.92794938963134
a_HQ_transcript/129290	-3.81070669368003	3.44015865805062	-2.61196801189576	0.0135053500679349	0.427814162805951	-3.10394608678011
a_HQ_transcript/1565	-1.13618759622812	4.34798107390386	-2.61139015309911	0.0135241570257618	0.427965923881851	-3.19149224496475
a_HQ_transcript/24742	-2.33771698901805	3.19635975346136	-2.6112093186729	0.0135300474233324	0.427965923881851	-3.07094031428801
a_HQ_transcript/134117	1.20595894087876	4.08773010066833	2.61101960004337	0.0135362297587138	0.427965923881851	-3.15338320468527
a_HQ_transcript/132084	3.91697914343687	2.00847837236729	2.61091649266524	0.0135395907999368	0.427965923881851	-2.97380046330945
a_HQ_transcript/90739	1.70484989964155	-0.730764964771927	2.61061310974732	0.0135494847944176	0.427969494591008	-2.94158906661574
a_HQ_transcript/9286	-1.43552677694648	4.0121746896797	-2.61039371774031	0.0135566438206741	0.427969494591008	-3.16151216710374
a_HQ_transcript/73290	-1.57631501281017	2.00703549726116	-2.61014666792853	0.013564709539251	0.427969494591008	-2.90456559705285
a_HQ_transcript/147183	1.82786005821258	-0.488533786242636	2.60982122683728	0.0135753413572936	0.427969494591008	-2.932212101305
a_HQ_transcript/151706	0.551468595459065	10.7682292690346	2.60957366267217	0.0135834341750481	0.427969494591008	-3.83677892901566
a_HQ_transcript/16313	-2.9344665945446	2.77006935569572	-2.6095600596834	0.0135838789829413	0.427969494591008	-3.00577561943032

a_HQ_transcript/10205	0.686021657560479	3.69973050272349	2.6091123275148	0.0135985270163798	0.428198904879808	-3.14783087758379
a_HQ_transcript/80581	5.94418666798321	1.13224867819443	2.60843904498939	0.0136205816513932	0.428461522498636	-3.05266234449717
a_HQ_transcript/21102	2.85828627821605	0.449450123358231	2.608407703767	0.0136216090967774	0.428461522498636	-2.91595292159974
a_HQ_transcript/147654	-1.29973625495305	3.75808088449831	-2.60643619636568	0.0136863842879001	0.430150259385805	-3.14420026084357
a_HQ_transcript/114226	1.08604312421153	3.01852329827301	2.60632344593077	0.0136900973689743	0.430150259385805	-3.03959929280267
a_HQ_transcript/93296	-2.87247018913106	0.106086560586247	-2.60514295348237	0.0137290291021999	0.431002742812171	-2.91476766147264
a_HQ_transcript/66368	-3.12150564369909	0.224129828302898	-2.60498711198617	0.0137341762712009	0.431002742812171	-2.97924492168109
a_HQ_transcript/28001	1.87262757807889	0.414857552994847	2.60467819786673	0.0137443844282904	0.431002742812171	-2.91569496282416
a_HQ_transcript/33246	2.1099106580787	0.677585357042339	2.60427610079961	0.0137576823434938	0.431002742812171	-2.96621614553208
a_HQ_transcript/94369	2.74905500490537	0.792822470973217	2.6040038025501	0.013766694378371	0.431002742812171	-2.97764185782235
a_HQ_transcript/146228	2.68906322108675	1.00182011147277	2.60395415371318	0.013768338153496	0.431002742812171	-2.92205551612375
a_HQ_transcript/113206	3.1253251144974	0.653366475768906	2.60384713212295	0.0137718820440844	0.431002742812171	-2.92425716737222
a_HQ_transcript/7572	-2.84631208428703	-0.238591195329866	-2.60370630884894	0.0137765465205239	0.431002742812171	-2.94576006326181
a_HQ_transcript/14263	3.20664011804796	1.49845427788419	2.60321127856297	0.0137929549484503	0.431283961938419	-2.93625319269731
a_HQ_transcript/11533	0.911429576544747	3.49988145974809	2.60290883049188	0.0138029888690249	0.431365665042301	-3.14338592228034
a_HQ_transcript/18973	-6.26951557042447	4.53434490381731	-2.60210002831237	0.0138298545666759	0.431973017867673	-3.29399721708842
a_HQ_transcript/43241	-2.41215900196201	-0.675041634001185	-2.6011666143662	0.0138609194827429	0.432651774307371	-2.95524776229911
a_HQ_transcript/144280	1.79860756645246	1.95430906246477	2.60099999522363	0.0138664715031419	0.432651774307371	-2.9268570106602
a_HQ_transcript/113253	-2.60817870101101	0.144846793395641	-2.60074023598828	0.0138751312001171	0.432689713734123	-2.98156899545612
a_HQ_transcript/6688	1.61250617381163	2.12726848822015	2.60043973245942	0.0138851554373429	0.432742040467836	-2.96671412104832
a_HQ_transcript/141671	4.31414838000622	0.578156850337974	2.60017976283791	0.0138938329349274	0.432742040467836	-3.00860194816906
a_HQ_transcript/133844	2.0667695004454	-0.31814686311746	2.60002074955631	0.0138991430903084	0.432742040467836	-3.00506076869144
a_HQ_transcript/49952	-3.18106329392558	0.563451263576945	-2.59975764189172	0.0139079335333929	0.432783918859243	-2.9559858012979
a_HQ_transcript/140616	1.48695752027929	1.21255212116124	2.59901025567998	0.0139329317676632	0.433284076133882	-2.93996734421574
a_HQ_transcript/89093	2.56527086761218	1.16668873657753	2.59883157008405	0.0139389145054081	0.433284076133882	-2.97178478386924
a_HQ_transcript/40611	-3.31291574566657	0.454776337353309	-2.59851461375191	0.013949532650443	0.433382380494361	-2.93634622724
a_HQ_transcript/44357	-3.22438545095713	1.80155000499274	-2.59807329272359	0.0139643294962923	0.433587163064782	-3.04421486363383
a_HQ_transcript/54467	-0.674317121235439	2.96561286691803	-2.59771734565266	0.0139762744368272	0.433587163064782	-3.0844376221813
a_HQ_transcript/67446	3.00605510822465	0.0947335025673843	2.59756704506655	0.0139813210855366	0.433587163064782	-2.99157026715173
a_HQ_transcript/40840	-0.923540592081171	3.42894953842879	-2.59727299195052	0.0139911993854513	0.433587163064782	-3.12952063355342
a_HQ_transcript/143028	-0.917167364064621	3.60764057100168	-2.59675847976164	0.0140084992022865	0.433587163064782	-3.14061007565679
a_HQ_transcript/139941	6.2431613563509	2.89643286718707	2.59670426774095	0.0140103231621748	0.433587163064782	-3.12597933114806
a_HQ_transcript/112116	7.71586512297027	2.6722614564543	2.59655137773961	0.0140154683176145	0.433587163064782	-3.0771155722388
a_HQ_transcript/138752	-3.73146894564125	0.506118715819469	-2.59632543265033	0.0140230751656086	0.433587163064782	-2.98884759563779
a_HQ_transcript/27555	-1.72168816420593	-0.436792145593458	-2.59600149351528	0.0140339878102735	0.433587163064782	-2.97963780794492
a_HQ_transcript/139244	-4.71418025367383	2.4817146512767	-2.59574049296901	0.0140427859251448	0.433587163064782	-3.15501487101509
a_HQ_transcript/127466	-1.88630110360657	-0.788795502624355	-2.59560210964962	0.0140474527792332	0.433587163064782	-2.96509781606983
a_HQ_transcript/145016	5.29008871930217	2.39130147114509	2.59549209462281	0.0140511639596354	0.433587163064782	-3.1098431422453
a_HQ_transcript/92832	3.37196706924219	4.589862566824	2.59543490742891	0.0140530934354192	0.433587163064782	-3.30125740873028
a_HQ_transcript/26841	2.91582282035762	3.81517758920621	2.59442069538307	0.0140873532578472	0.43429746370366	-3.11917724799784
a_HQ_transcript/130975	1.05075587852663	3.92186108898315	2.59421074999743	0.0140944547851473	0.43429746370366	-3.19087878203549
a_HQ_transcript/77943	-4.61611678421773	1.90981355031382	-2.59409033606423	0.0140985293491743	0.43429746370366	-3.00680468492194
a_HQ_transcript/122521	3.16484117753455	1.9631659497466	2.59360838302138	0.0141148485545825	0.434569871176256	-2.97354028707932
a_HQ_transcript/28593	-2.90654194878422	-0.455819752503447	-2.59301104443697	0.0141350989805854	0.43476047474281	-2.97797624963624
a_HQ_transcript/15500	4.41981738385952	0.544103458002948	2.59273041192212	0.0141446219841471	0.43476047474281	-3.01191687298423
a_HQ_transcript/41054	3.16182739591792	0.127879149986194	2.59265510914682	0.0141471783214628	0.43476047474281	-2.9309689261298
a_HQ_transcript/53864	1.77370702924925	-0.790229652367296	2.59232984789056	0.0141582250124115	0.43476047474281	-2.97804837381922
a_HQ_transcript/137369	-2.23124163993504	2.37263012108941	-2.59232363229549	0.0141584361871755	0.43476047474281	-3.00602175379761
a_HQ_transcript/129127	1.98704426375931	-0.740057562094781	2.58990544732896	0.0142408149442694	0.437059182196669	-2.98266522348038
a_HQ_transcript/90352	2.36858218714597	3.33294336446017	2.58911899402369	0.0142677017301875	0.437581455219011	-3.11418395123852

a_HQ_transcript/45229	-2.87870478022315	-0.580608843175	-2.58896744742167	0.014272888093436	0.437581455219011	-2.99371671152494
a_HQ_transcript/12199	-1.76044125021121	1.99990986125003	-2.58782486784309	0.0143120465160558	0.438488527184477	-2.93353170311528
a_HQ_transcript/119076	-2.87726583061774	-0.540475090097882	-2.58761941216998	0.0143190983888675	0.438488527184477	-2.99186897500752
a_HQ_transcript/133064	4.27522227795734	0.175930412379207	2.58744447887325	0.0143251051665862	0.438488527184477	-3.03463189520503
a_HQ_transcript/128879	1.07581640907758	3.29813315515279	2.58714779693982	0.0143352978080721	0.438569574204007	-3.08532207230681
a_HQ_transcript/78068	-1.51710195895022	2.97840194720487	-2.58683953172587	0.0143458954871555	0.438662921029655	-3.03681770802704
a_HQ_transcript/88979	-2.17688610392819	-0.474879982808844	-2.58592201727145	0.0143774810028069	0.439397589764015	-2.98875494955818
a_HQ_transcript/94513	-3.60562232796612	3.06128801813286	-2.58552996263479	0.0143909970747265	0.439579547009829	-3.17517424250434
a_HQ_transcript/63798	-3.54218019962484	0.677415840957072	-2.58454749587634	0.0144249191024072	0.440384294949961	-2.94053844664662
a_HQ_transcript/121296	-2.1779214751505	2.82043059135432	-2.58398692301734	0.0144443072674173	0.440475591837691	-3.11144496391664
a_HQ_transcript/51191	-0.822745053384337	3.03860474952123	-2.58389115438763	0.0144476219575028	0.440475591837691	-3.05863783876333
a_HQ_transcript/28735	-2.38361619149906	0.027903257354879	-2.58360829992361	0.0144574160550005	0.440475591837691	-2.976158932814
a_HQ_transcript/100461	1.38262025418623	2.41314506498858	2.58358508129119	0.0144582202935988	0.440475591837691	-2.99164265279167
a_HQ_transcript/41287	-3.54473493001331	1.56117288606057	-2.58294875442388	0.0144802772249528	0.440869180661165	-3.01438604557005
a_HQ_transcript/104646	3.12888944312855	-0.0257648554733919	2.58238285437838	0.014499919032259	0.440869180661165	-2.98007264898028
a_HQ_transcript/36480	4.56355755767692	1.17906827202245	2.58220071791419	0.0145062460273791	0.440869180661165	-3.06656076464856
a_HQ_transcript/130126	2.71372715868408	0.216872968044628	2.58216361725306	0.0145075351298075	0.440869180661165	-3.0065950554
a_HQ_transcript/47009	1.3982012183863	-0.73224991593166	2.58211968411502	0.014509061770658	0.440869180661165	-3.02251615412546
a_HQ_transcript/149637	-2.8140731773822	-0.701903737438539	-2.58178010514232	0.014520866864386	0.440997361072639	-3.00723207181272
a_HQ_transcript/124298	1.82514502222985	-0.736870063344472	2.5810800139763	0.0145452327388853	0.441108102333789	-2.99649528404476
a_HQ_transcript/148930	0.477785581145154	10.397755469937	2.58091792771281	0.0145508793400523	0.441108102333789	-3.84900044148009
a_HQ_transcript/28399	-1.99205173075055	3.59857223344096	-2.58089807103595	0.0145515712263507	0.441108102333789	-3.21739514698579
a_HQ_transcript/57496	2.80446179992477	0.571501204438162	2.58041884225564	0.0145682786861925	0.441108102333789	-2.94492446680134
a_HQ_transcript/49824	-3.37489229909082	0.963662071168108	-2.58036912597151	0.0145700129686669	0.441108102333789	-3.01792860227554
a_HQ_transcript/104493	-2.34270840986562	0.479347694908165	-2.58036821729513	0.0145700446683332	0.441108102333789	-3.00388195816768
a_HQ_transcript/10323	-3.33907989489518	2.12080040002593	-2.57994712515258	0.0145847415419852	0.441155582095851	-3.05297156091113
a_HQ_transcript/82351	-0.697675416938941	3.1531658376406	-2.57988841508726	0.0145867917146337	0.441155582095851	-3.11354622178215
a_HQ_transcript/18142	2.02576919304601	0.716927144914125	2.57946134603535	0.0146017130915356	0.441186793671286	-2.94383208096069
a_HQ_transcript/60773	1.67075699988195	2.56601145851024	2.5794244300327	0.0146030035615117	0.441186793671286	-3.04653813015844
a_HQ_transcript/121464	2.29018560910904	2.6051151062584	2.57873124487079	0.0146272547228566	0.44136471393344	-3.09152066424228
a_HQ_transcript/59554	2.10469042296622	-0.457989605278142	2.57868375685067	0.0146289174552341	0.44136471393344	-3.00512130823436
a_HQ_transcript/125621	3.26653887432969	0.489557606256586	2.57840513473565	0.0146386765647619	0.44136471393344	-2.97601474082583
a_HQ_transcript/53307	-4.04802866324584	1.52339109018395	-2.57818846785546	0.0146462697592689	0.44136471393344	-2.98563391121523
a_HQ_transcript/27526	-1.10268781173259	2.65334849679766	-2.57805162054174	0.0146510675101968	0.44136471393344	-3.02835182105466
a_HQ_transcript/118955	-0.644604779084305	4.42710913954761	-2.57795515199298	0.0146544504867111	0.44136471393344	-3.28418130046276
a_HQ_transcript/86810	-3.38635724164854	1.78827010499376	-2.57711478545802	0.0146839510961111	0.441841470696902	-3.04725397946261
a_HQ_transcript/11935	2.68869563502867	0.918243334968316	2.57687140613916	0.0146925050152244	0.441841470696902	-2.99548694192253
a_HQ_transcript/122341	3.04700861280782	0.0134321332391704	2.57626965951716	0.0147136740008142	0.441841470696902	-2.9871834559758
a_HQ_transcript/52957	-2.27383023792586	1.56068402347532	-2.57626369491118	0.0147138839717778	0.441841470696902	-3.00160765472839
a_HQ_transcript/8976	-3.31374292530586	2.44481259364475	-2.57625263138306	0.0147142734465059	0.441841470696902	-3.00866883623526
a_HQ_transcript/86152	2.10462571291586	-0.679566524591489	2.57603634889163	0.0147218892531636	0.441841470696902	-3.0082499709189
a_HQ_transcript/125561	-1.22796579830373	3.50668203247506	-2.57578903112888	0.0147306023375837	0.441841470696902	-3.18282211419901
a_HQ_transcript/2033	-2.1033578577492	-0.649320766496729	-2.57577520730084	0.0147310894957782	0.441841470696902	-3.01148592227987
a_HQ_transcript/41499	-0.924147447369329	2.58466265954313	-2.57542975103883	0.0147432683616872	0.441978702077438	-3.0071839909775
a_HQ_transcript/135103	-2.77733777504184	0.920170496677374	-2.57497784113834	0.014759214206024	0.442228661529774	-2.99719842296199
a_HQ_transcript/142913	-3.93141319334231	1.79684243306771	-2.57436521857539	0.0147808562579663	0.442501575501583	-3.13266746993869
a_HQ_transcript/140437	2.1970339666072	0.00980434968363509	2.57376507530846	0.0148020858208834	0.442501575501583	-3.00937023562689
a_HQ_transcript/32498	1.82640697154029	1.95086635104178	2.57368170165524	0.0148050373165159	0.442501575501583	-2.98523982646833
a_HQ_transcript/7987	2.15987575858038	0.774655390458525	2.57368100598301	0.0148050619461652	0.442501575501583	-2.95642671695195
a_HQ_transcript/141855	2.89434716741976	0.855549122043187	2.5735562491012	0.0148094794621117	0.442501575501583	-2.96875064547833

a_HQ_transcript/135199	-2.51105030644918	1.42842990399676	-2.57342867947246	0.0148139978310983	0.442501575501583	-3.00700235529338
a_HQ_transcript/20623	-2.85289279211957	-0.125838880917933	-2.57299333451746	0.0148294268170405	0.442734936836636	-3.01620231888854
a_HQ_transcript/15108	2.21772497815588	0.553756780603605	2.57214384215816	0.0148595761930453	0.443407312602331	-2.95627829567712
a_HQ_transcript/140311	2.44815124966467	-0.430622477777253	2.5715706564462	0.014879951068982	0.443596988414886	-3.03446181292955
a_HQ_transcript/16209	-1.62955002762317	1.88315054491686	-2.57153567380966	0.0148811954206067	0.443596988414886	-2.96386650908767
a_HQ_transcript/135602	-3.27928260140703	3.80680351221498	-2.57054257934932	0.0149165603722184	0.444294611201053	-3.12276083020777
a_HQ_transcript/70663	-1.72842423320585	-0.353155425650744	-2.57044932825468	0.0149198850995124	0.444294611201053	-2.99720376438576
a_HQ_transcript/127527	-2.46595303479732	2.33278213702916	-2.56950250461315	0.0149536813645655	0.444857594395796	-3.12217091041965
a_HQ_transcript/144722	-2.83109531562325	0.39298786092591	-2.56935527913854	0.0149589428177158	0.444857594395796	-3.08336307570246
a_HQ_transcript/120558	-2.16578505804805	4.54039773254993	-2.56927675170467	0.0149617498803293	0.444857594395796	-3.37636701375697
a_HQ_transcript/136490	4.3594435219454	1.20715705353058	2.56833498649957	0.0149954523129304	0.445452609288686	-3.0328173219397
a_HQ_transcript/101613	3.02961385354907	0.919567734507747	2.568193470046	0.0150005227263416	0.445452609288686	-3.07348357702153
a_HQ_transcript/103731	2.04106550507625	-0.672210639515029	2.5680754710474	0.0150047517373253	0.445452609288686	-2.99766190778257
a_HQ_transcript/98348	2.9050413608438	0.0865644545180643	2.56716198087461	0.0150375278529412	0.446090291655385	-3.0333084771202
a_HQ_transcript/88661	1.52250520319071	-0.810965828763441	2.56704916804458	0.0150415801617904	0.446090291655385	-3.02926154675794
a_HQ_transcript/42745	-3.463437471393	3.92110826936199	-2.56591214128362	0.0150824791044946	0.446953534577991	-3.26255737209979
a_HQ_transcript/54403	2.15225101325133	0.771510391994146	2.56567972309876	0.0150908518077649	0.446953534577991	-2.99059723683266
a_HQ_transcript/24432	-3.82294733840557	0.298704299574108	-2.56520635781937	0.0151079177068525	0.446953534577991	-3.02986533569388
a_HQ_transcript/149545	2.36442899361345	-0.299001022193618	2.56511062798827	0.0151113711466842	0.446953534577991	-3.01660078930451
a_HQ_transcript/8839	-1.59462048776625	-0.801903134029476	-2.56500775356111	0.0151150831375209	0.446953534577991	-3.02285113321437
a_HQ_transcript/108457	2.15782437459629	2.16279043021906	2.56495956116406	0.0151168223400139	0.446953534577991	-3.00336899763593
a_HQ_transcript/149966	5.64076419192379	4.95683361570384	2.56467630514035	0.0151270484177713	0.447028505555776	-3.11820959345532
a_HQ_transcript/127584	3.86305436726752	1.68202350070716	2.56439419979982	0.0151372392877688	0.447036985209478	-3.02206893027376
a_HQ_transcript/72283	-0.663297728500667	2.9992802347832	-2.56421837551456	0.0151435940236325	0.447036985209478	-3.13283470278687
a_HQ_transcript/104275	5.77230772065498	3.9395492799531	2.56398332631002	0.0151520931373546	0.447036985209478	-3.23647524371403
a_HQ_transcript/108348	-2.91932269360861	0.115449158335491	-2.56376463026444	0.0151600048855172	0.447036985209478	-3.01273583071055
a_HQ_transcript/98929	2.9172290660807	-0.135026409446204	2.56341227475025	0.0151727600267272	0.447036985209478	-3.05448782012855
a_HQ_transcript/133762	0.687845510443805	3.76613756752053	2.56327406439176	0.0151777658902528	0.447036985209478	-3.25092225061941
a_HQ_transcript/152914	2.58320502602109	0.157070934269514	2.56297833290498	0.0151884821481684	0.447036985209478	-3.0516328378769
a_HQ_transcript/109336	1.56602373645908	2.76826242249826	2.56285864927073	0.0151928210384803	0.447036985209478	-3.12396884265211
a_HQ_transcript/64052	2.85405105610753	1.93315614172111	2.56227600681044	0.0152139598868031	0.447036985209478	-3.07442782382549
a_HQ_transcript/44064	-3.26723439771201	0.691155060532953	-2.56226964656336	0.0152141907923133	0.447036985209478	-3.0002371563032
a_HQ_transcript/138445	1.14222751640008	3.30375952218101	2.5620585350858	0.0152218569183821	0.447036985209478	-3.2144009337998
a_HQ_transcript/131102	1.16226803001932	3.11838211335706	2.56155984580226	0.015239980036807	0.447036985209478	-3.13316382115678
a_HQ_transcript/139267	1.45657438250669	1.55244648790259	2.56136370095946	0.0152471136796573	0.447036985209478	-2.98630086159194
a_HQ_transcript/66361	2.30462241400864	-0.533614524374919	2.56135734971359	0.0152473447211712	0.447036985209478	-3.01778013178785
a_HQ_transcript/133176	3.48927116727476	1.61356388474399	2.56121822241385	0.0152524066131633	0.447036985209478	-3.07119496630399
a_HQ_transcript/44260	-2.7103559997689	1.60094976554653	-2.5610163560565	0.0152597539031019	0.447036985209478	-2.99518354751815
a_HQ_transcript/8099	-2.02639028795814	1.71762048875691	-2.56098353597223	0.0152609487572682	0.447036985209478	-3.01735431755545
a_HQ_transcript/68600	2.82238460482738	0.660564064316254	2.56067726706477	0.0152721029955597	0.447036985209478	-3.10175482021118
a_HQ_transcript/110496	1.4535161759296	2.1935443261415	2.56052611963737	0.0152776105145266	0.447036985209478	-3.01567242888655
a_HQ_transcript/142942	-2.91387024843447	3.05979756016816	-2.5604291023305	0.0152811466007988	0.447036985209478	-3.19093532815791
a_HQ_transcript/135291	-3.75820407706557	0.62800284307466	-2.55988510706463	0.0153009881044929	0.447392271900385	-3.07264065696136
a_HQ_transcript/48234	-1.18135298386364	2.77869795372324	-2.55894436816986	0.0153353563070482	0.447785973127743	-3.07994189819737
a_HQ_transcript/92341	2.99120286979126	1.48413316748047	2.55876602324165	0.0153418798289456	0.447785973127743	-3.11701902988035
a_HQ_transcript/97789	-2.51147919457581	1.10227493780963	-2.55875291694867	0.0153423593332711	0.447785973127743	-3.03708083115847
a_HQ_transcript/121538	2.76695446807907	-0.442305859054625	2.55867345996896	0.0153452666265907	0.447785973127743	-3.04480064720303
a_HQ_transcript/103547	3.46332886094108	1.48016745018617	2.55840428685019	0.0153551193146743	0.447848658064922	-2.97664588384244
a_HQ_transcript/141282	1.16369412907627	2.0139903236724	2.55815402331811	0.0153642850707816	0.447891255062384	-3.01990085781373
a_HQ_transcript/26145	2.13155380972257	4.91100154627316	2.55741247062643	0.0153914735963546	0.448359988679287	-3.29817145675079

a_HQ_transcript/97652	-3.06531016657251	-0.247749608817616	-2.55729482757886	0.0153957909682744	0.448359988679287	-3.04940946602611
a_HQ_transcript/116680	-2.39342016364348	-0.462686974531439	-2.55678553845969	0.0154144941914127	0.448507225061442	-3.01580684820363
a_HQ_transcript/1798	-3.51509078665113	-0.044506677021253	-2.55659192996176	0.0154216097906598	0.448507225061442	-3.05480732915742
a_HQ_transcript/78435	2.60275218456908	0.781236751144724	2.55639871490263	0.0154287139451461	0.448507225061442	-3.02341007647991
a_HQ_transcript/30292	3.20339032827535	0.88957404938953	2.55631724907636	0.015431710193416	0.448507225061442	-3.05923872318258
a_HQ_transcript/80007	1.82586800876532	-0.778649615226634	2.55495848421821	0.0154817635358601	0.449737106852811	-3.05232394988425
a_HQ_transcript/16202	-1.48729470785659	3.35332470296159	-2.55438472596403	0.0155029441379957	0.450127440985722	-3.0929759428011
a_HQ_transcript/124274	-2.48401284234773	0.585931350155499	-2.55397257205117	0.0155181754877504	0.450344735273068	-3.02983114187285
a_HQ_transcript/79054	-1.98493906601094	-0.742363185883263	-2.55363272597914	0.0155307450274491	0.450474843636087	-3.04972342213266
a_HQ_transcript/17955	-1.66823259059441	3.79719758341298	-2.5534324119321	0.0155381582282266	0.450474843636087	-3.31314254256142
a_HQ_transcript/98321	2.3659689755892	-0.237732960841936	2.55309050300046	0.0155508190827058	0.450617154356691	-3.02494633461596
a_HQ_transcript/40053	-0.962453807779906	2.61976433514298	-2.55245830199984	0.0155742544410993	0.451071381241764	-3.09708855785752
a_HQ_transcript/120087	2.78913939290133	2.23414730796159	2.55211098193142	0.0155871432451749	0.451219851870281	-3.15740802384716
a_HQ_transcript/91128	3.34019192726374	-0.116363091281767	2.551724672798	0.0156014904388305	0.451351765838612	-3.06110173920748
a_HQ_transcript/134153	1.77299528590943	-0.622552840180427	2.55157022975459	0.0156072297229495	0.451351765838612	-3.08301417723756
a_HQ_transcript/120343	-1.75357470775604	2.24738512051227	-2.55134295428354	0.0156156790793627	0.451371553219888	-3.13317585189236
a_HQ_transcript/9373	1.81118328087629	2.43799212300987	2.5502744468246	0.0156554591358396	0.452296485411572	-3.12401509013245
a_HQ_transcript/60548	-2.90258959378244	0.553224573943673	-2.54987592250176	0.0156703198834902	0.452486508443578	-3.02331056081803
a_HQ_transcript/50773	2.3593364242312	0.600558110481188	2.54968068067186	0.0156776050785399	0.452486508443578	-3.03679873189809
a_HQ_transcript/67871	2.63365825974046	0.994705962070114	2.54911488786024	0.0156987345098047	0.452871483665474	-3.0407792192081
a_HQ_transcript/98845	2.76388437316836	0.320352232638106	2.54889906273691	0.0157068013631081	0.45287943930295	-3.05253929382479
a_HQ_transcript/135144	-3.34908593268733	0.272249214915805	-2.54841883945779	0.0157247642629891	0.45309437293149	-3.11486910924649
a_HQ_transcript/121489	2.19281449550226	-0.824282340811155	2.54810252170917	0.015736606550935	0.45309437293149	-3.06652868638449
a_HQ_transcript/713	-1.01737043972257	3.14427675675474	-2.54807492731162	0.0157376400176968	0.45309437293149	-3.23441911047937
a_HQ_transcript/29072	-2.16499975455802	2.63673587648911	-2.54756780723275	0.0157566438282667	0.453416926955192	-3.15536405383537
a_HQ_transcript/84075	3.05868050071988	1.01275566080647	2.54707714203605	0.0157750511059448	0.453552414106144	-3.08876271801785
a_HQ_transcript/59831	1.63640889868918	-0.804014415854375	2.54624992363056	0.0158061289627424	0.453552414106144	-3.07004799802653
a_HQ_transcript/44674	-1.86082357476821	-0.662205238309182	-2.54587072850273	0.0158203938065309	0.453552414106144	-3.06342404574972
a_HQ_transcript/135811	2.78751088761877	2.63377286553622	2.54582607590944	0.0158220743602208	0.453552414106144	-3.06025795144345
a_HQ_transcript/71523	4.97731531507361	1.78164799407977	2.54556067922344	0.0158320662712411	0.453552414106144	-3.15010845856847
a_HQ_transcript/54398	-1.43177787657987	3.58243109277881	-2.54547860675343	0.0158351573899952	0.453552414106144	-3.20627590845266
a_HQ_transcript/148499	2.55607492959962	1.75297415941788	2.54543954111678	0.0158366289252084	0.453552414106144	-3.05205366411343
a_HQ_transcript/142405	1.25921519859497	3.88131424735265	2.54533481358338	0.0158405744516682	0.453552414106144	-3.29366688521339
a_HQ_transcript/145624	0.893037746335973	4.46879432151069	2.54532739761326	0.0158408538767241	0.453552414106144	-3.36596927192346
a_HQ_transcript/1464	-1.48396880974391	1.36962267935379	-2.54506622251919	0.0158506975405134	0.453552414106144	-3.01767377262204
a_HQ_transcript/16318	3.27831098866568	1.67127559443058	2.54506570983277	0.0158507168691474	0.453552414106144	-3.08912973134265
a_HQ_transcript/152689	-3.57169606603475	-0.351315428012166	-2.54495254154883	0.0158549839227857	0.453552414106144	-3.10589322923692
a_HQ_transcript/145876	1.17391670772137	8.6481686917514	2.544683168592	0.0158651449855182	0.453619846393608	-3.71919765937139
a_HQ_transcript/151226	1.52623511077162	-0.8348761696644	2.54445914119855	0.0158736001232611	0.453638460159746	-3.07033276267302
a_HQ_transcript/72608	2.07225389823814	1.81797034050188	2.54422194251693	0.0158825568836438	0.453671384045428	-3.06743751586418
a_HQ_transcript/148671	0.918930708905665	8.9913145421429	2.54298084834714	0.0159294971732183	0.454460333751664	-3.75533569445689
a_HQ_transcript/127027	3.6584674707498	1.32921874263349	2.54294064420291	0.0159310198940061	0.454460333751664	-3.03683341064122
a_HQ_transcript/37428	-1.00874605105009	3.77602635177662	-2.54287168575645	0.0159336319877837	0.454460333751664	-3.31501296592015
a_HQ_transcript/32529	-3.8402998861103	3.17675322103965	-2.5419847833625	0.0159672623145968	0.45516493816651	-3.14261842110672
a_HQ_transcript/143050	2.87922769936547	0.551237663903008	2.54180739283473	0.0159739965913102	0.45516493816651	-3.05435105802817
a_HQ_transcript/42287	2.88749306110495	1.15854607170766	2.54132228248724	0.0159924261784802	0.455466804949876	-3.06736400408145
a_HQ_transcript/126046	1.34353988985707	2.82523570105232	2.54035335818146	0.0160292946537298	0.456293261328113	-3.10409358154143
a_HQ_transcript/148328	-3.45007652940123	1.13764566616209	-2.53988127517835	0.0160472861600945	0.456516567598937	-3.11669865681303
a_HQ_transcript/16655	-2.62774541754685	3.44651112457091	-2.53957423309005	0.0160589977747957	0.456516567598937	-3.2808107516445
a_HQ_transcript/36472	3.92352662873071	3.63123810851312	2.53952961816232	0.016060700191643	0.456516567598937	-3.29610218127925

a_HQ_transcript/82703	2.97639178833676	0.715689705155463	2.53902870069968	0.0160798256009478	0.456836804756547	-3.09683614343266
a_HQ_transcript/150854	0.551397602841052	10.5355300754324	2.53855105117043	0.0160980821278665	0.457132055656386	-3.95376445321528
a_HQ_transcript/89053	2.70649951341242	-0.271563405299404	2.53800353123876	0.0161190326588544	0.457330880471594	-3.07080029930534
a_HQ_transcript/59328	-2.28510740962576	-0.651452270826099	-2.53778597364278	0.0161273643316057	0.457330880471594	-3.08132417048362
a_HQ_transcript/1157	-1.42889153655264	2.56959877616174	-2.537751451304	0.0161286867768849	0.457330880471594	-3.04783409932248
a_HQ_transcript/79359	-1.03266287114545	3.16751272141306	-2.5375298088796	0.0161371795961282	0.457334994708069	-3.20322468888668
a_HQ_transcript/37111	2.13485881799704	1.74705240826609	2.53733709405143	0.0161445673193806	0.457334994708069	-3.1288327312035
a_HQ_transcript/95607	-2.7245498961874	0.386244995274354	-2.53683233094896	0.0161639321561256	0.457660520395163	-3.0462104795134
a_HQ_transcript/118615	-1.93712790803831	2.71008521218416	-2.53640534868555	0.0161803296638616	0.457901753992672	-3.17798108836161
a_HQ_transcript/42983	1.75744188585755	-0.474862948171139	2.53579782048849	0.0162036870854806	0.458339621851492	-3.07429420566867
a_HQ_transcript/145914	1.56765687891296	-0.708265416496787	2.53506694837988	0.0162318277446669	0.458912297248052	-3.0668411624975
a_HQ_transcript/46680	-2.24278182396228	1.06637512138029	-2.53474867361199	0.0162440962674669	0.459035891023489	-3.06331382454119
a_HQ_transcript/142234	-2.19772826839426	3.76450408359746	-2.53380081540597	0.0162806838286791	0.459846253446772	-3.36883445916525
a_HQ_transcript/140916	-3.13501303203501	1.14991905290724	-2.53342540804479	0.0162951955771705	0.460032602481675	-3.11382241515923
a_HQ_transcript/136519	2.12290632769561	3.84721545558762	2.53294174005249	0.0163139097769806	0.460127592568006	-3.30931990549943
a_HQ_transcript/54927	1.67840613244827	1.80264920706811	2.53292928814922	0.0163143918298008	0.460127592568006	-3.06338327053641
a_HQ_transcript/82820	-1.03153836074615	4.21668165101441	-2.53262232864841	0.0163262793498372	0.460137864314343	-3.346671980872
a_HQ_transcript/93300	1.74004907764113	-0.769629868474312	2.53244247369758	0.0163332482322306	0.460137864314343	-3.09698779476213
a_HQ_transcript/126788	2.04563481550788	-0.446023934423143	2.53230688176221	0.016338503852615	0.460137864314343	-3.05836964545844
a_HQ_transcript/70009	3.86939027675832	1.69257923623279	2.53200327674613	0.0163502773926332	0.460246452435342	-3.14050557614248
a_HQ_transcript/69575	-2.50362492925323	0.513940713723866	-2.5315406905018	0.0163682310798984	0.460359472971217	-3.06056578083589
a_HQ_transcript/102588	-1.79134065921242	-0.717461647316218	-2.53138181879199	0.0163744013154794	0.460359472971217	-3.09096012872993
a_HQ_transcript/125669	0.684702908393487	5.85971605410927	2.53128785391539	0.0163780517152573	0.460359472971217	-3.52269502482988
a_HQ_transcript/98006	1.61138831234955	3.6018534890693	2.53087373593889	0.0163941485141759	0.460589204848729	-3.33167979073238
a_HQ_transcript/108857	1.04755210884794	4.43749559898376	2.53055413668717	0.0164065813019252	0.460623264507819	-3.380459559526
a_HQ_transcript/22995	-1.42701218828629	2.46611639637039	-2.53043522254654	0.0164112094136336	0.460623264507819	-3.16486846195925
a_HQ_transcript/94576	3.00692709991039	2.83666673768181	2.52973138077743	0.0164386273539291	0.461170140361579	-3.21904346039913
a_HQ_transcript/48895	2.45332125670087	2.00450901101108	2.52927729675561	0.0164563383905081	0.461227373282236	-3.12342778067569
a_HQ_transcript/61272	2.26121657721659	3.66048066855307	2.52912270737413	0.0164623719754048	0.461227373282236	-3.33688638020904
a_HQ_transcript/122772	-4.05159370971958	-0.607618682397647	-2.52906892656308	0.0164644715035893	0.461227373282236	-3.12247196896453
a_HQ_transcript/142923	3.023637030958	-0.307313637864045	2.5287212650267	0.0164780496647462	0.461385390612892	-3.10124673270344
a_HQ_transcript/29924	-1.57545440868957	3.04661661825094	-2.52788386743349	0.0165107970568783	0.462079735831595	-3.22640641788866
a_HQ_transcript/122307	-3.95133743049447	0.85408426234795	-2.52762717314456	0.0165208473495637	0.462138505647468	-3.17364612034209
a_HQ_transcript/28612	-1.98755268686865	3.11918270904335	-2.52731515636323	0.0165330712399275	0.46225799183959	-3.27823000890079
a_HQ_transcript/12790	2.50018072036735	0.0182557856007093	2.5269551066339	0.0165471872458384	0.462327133969174	-3.11713954315559
a_HQ_transcript/152122	-2.69782876299535	-0.687543345576771	-2.52684639906022	0.0165514513795391	0.462327133969174	-3.11464690487919
a_HQ_transcript/74830	1.35449191170077	1.49449118433344	2.52554086487398	0.0166027408367615	0.46353704099869	-3.05285233845634
a_HQ_transcript/135390	2.11369106214063	1.05911351985422	2.52509341501281	0.0166203530053007	0.463805991114796	-3.09420298559939
a_HQ_transcript/45288	2.73227906130291	0.495200566884286	2.52441037536152	0.0166472713729977	0.464334256415361	-3.09115163230876
a_HQ_transcript/20355	-2.46850734780297	0.867819665239528	-2.5238861878669	0.0166679565841587	0.464688239963539	-3.06537283158584
a_HQ_transcript/95013	1.27974294860087	2.24374346822697	2.52273497826187	0.0167134679001598	0.465234668779531	-3.08820700179728
a_HQ_transcript/22485	2.36834613346549	0.813150880549206	2.52268650872567	0.0167153865710257	0.465234668779531	-3.05083245216491
a_HQ_transcript/88346	-2.82165797933977	1.93296391285966	-2.52255737370285	0.0167204993798508	0.465234668779531	-3.0970374802914
a_HQ_transcript/32324	-4.45094037623623	2.16418459026176	-2.5222273015342	0.0167335743686028	0.465234668779531	-3.16933612640605
a_HQ_transcript/135617	1.90600333783501	1.71759767873664	2.52201460446068	0.0167420048093262	0.465234668779531	-3.07970663999408
a_HQ_transcript/14995	-0.960029674378451	2.99402497635736	-2.52184303956012	0.0167488077813257	0.465234668779531	-3.1507552135927
a_HQ_transcript/137339	3.31887484991083	-0.0393132924501294	2.52156917080238	0.0167596726152245	0.465234668779531	-3.14181633397978
a_HQ_transcript/110066	0.906488802477886	2.8761022162478	2.52099762865061	0.0167823675058161	0.465234668779531	-3.15303423644333
a_HQ_transcript/93061	2.27400033363754	1.21547501588839	2.52028441478945	0.0168107275042184	0.465234668779531	-3.09175060279585
a_HQ_transcript/115295	-3.95387731461694	2.12453020260629	-2.51991864818078	0.0168252887926527	0.465234668779531	-3.1430658872329

a_HQ_transcript/24271	-0.948207381795779	4.3426626134843	-2.51988239130543	0.0168267328215207	0.465234668779531	-3.39476966116763
a_HQ_transcript/131570	3.17940277940701	3.66497033409771	2.51987144061762	0.016827168984883	0.465234668779531	-3.30725154802752
a_HQ_transcript/92217	-3.79171299400707	0.618089762674387	-2.51950819124434	0.0168416430088346	0.465234668779531	-3.16042635954721
a_HQ_transcript/42906	2.18850729501129	2.09052625331184	2.51946743092407	0.0168432678565749	0.465234668779531	-3.11797341444201
a_HQ_transcript/59314	3.01444644241885	1.72097662279734	2.51946026792644	0.0168435534133758	0.465234668779531	-3.07150418412441
a_HQ_transcript/132845	-0.855175764133738	2.87444418237566	-2.51943053194701	0.0168447389019225	0.465234668779531	-3.17007149363137
a_HQ_transcript/139915	2.42670426818663	0.217167929183003	2.51914686933501	0.0168560515720526	0.465234668779531	-3.08699576084223
a_HQ_transcript/135094	2.39281768153617	-0.0377921642223226	2.51897739598541	0.0168628136204856	0.465234668779531	-3.12042596173603
a_HQ_transcript/123920	1.50539749815706	4.18407755597806	2.51883473052178	0.0168685079554522	0.465234668779531	-3.41784269900969
a_HQ_transcript/29748	-1.88525512299448	2.03653543378805	-2.51843699880479	0.0168843923032635	0.465234668779531	-3.10353349145109
a_HQ_transcript/139176	1.89459765149909	-0.795078653884797	2.51834720796026	0.0168879802107528	0.465234668779531	-3.12525556299624
a_HQ_transcript/36780	2.28312441557642	0.432513722207022	2.51833159994233	0.0168886039551584	0.465234668779531	-3.06898357742065
a_HQ_transcript/118185	-1.68597016026298	-0.662701549025665	-2.51829482186714	0.0168900738038186	0.465234668779531	-3.10461731462027
a_HQ_transcript/93921	-3.41506778796417	0.777789267645461	-2.51828022152863	0.016890657344032	0.465234668779531	-3.10579852216047
a_HQ_transcript/81768	-2.59865074343806	-0.214572703021379	-2.51819380806261	0.0168941114602729	0.465234668779531	-3.12388451700381
a_HQ_transcript/127371	2.67065895420871	-0.182555651237492	2.5181553044883	0.0168956507327551	0.465234668779531	-3.09980729841029
a_HQ_transcript/130346	-2.78606894700599	1.00027666616124	-2.5179125854803	0.0169053569705332	0.465281529348086	-3.07725180034974
a_HQ_transcript/142310	2.60013888330961	1.58378247800509	2.51756657868371	0.0169192024979772	0.465442216186663	-3.19663030892445
a_HQ_transcript/107170	4.39343945789519	1.40470988392381	2.5170162254158	0.016941246439807	0.46562575206933	-3.17230502898978
a_HQ_transcript/8269	2.14105109659189	0.887922980966595	2.51686862344789	0.0169471629964633	0.46562575206933	-3.07596922086758
a_HQ_transcript/70986	-1.11394428349944	3.70862683849851	-2.51680022816496	0.0169499052329119	0.46562575206933	-3.29817800670566
a_HQ_transcript/113258	-3.00593270081017	-0.182465943052866	-2.51554047026657	0.0170004868004211	0.466794660715576	-3.15056164989426
a_HQ_transcript/25338	1.60603762538102	2.68031757727679	2.51470748337013	0.0170340088035005	0.467494269938565	-3.12539612702253
a_HQ_transcript/3632	1.30949974282603	3.34765402495452	2.51390969530965	0.0170661711693998	0.467522996113147	-3.31114919748604
a_HQ_transcript/96043	-0.851947812934881	3.96745718664858	-2.51385710239061	0.0170682933798375	0.467522996113147	-3.36243792574124
a_HQ_transcript/136755	1.61749458680339	1.56985253724765	2.51378503747146	0.017071201710742	0.467522996113147	-3.08766330545933
a_HQ_transcript/5122	3.39692489573309	-0.101883920960073	2.5137625833091	0.0170721079884832	0.467522996113147	-3.09796140375982
a_HQ_transcript/137540	0.9682061982219	3.90962350653617	2.51365205726261	0.017076569600029	0.467522996113147	-3.37692630806901
a_HQ_transcript/111201	2.69073941830752	1.86608312494304	2.51348255102438	0.0170834141478527	0.467522996113147	-3.08175476522843
a_HQ_transcript/5006	-1.97397156757899	1.9545372120337	-2.51326264194933	0.0170922976808498	0.467522996113147	-3.18002993920983
a_HQ_transcript/4571	-1.48055171779835	4.90036424039036	-2.51279982298436	0.0171110077503693	0.467522996113147	-3.4824807056214
a_HQ_transcript/92738	1.81698661804075	-0.767638093763712	2.51266330292391	0.0171165303437992	0.467522996113147	-3.13567721179671
a_HQ_transcript/133936	3.26835973365467	3.48432015321296	2.51253511430637	0.0171217173977584	0.467522996113147	-3.30519511213101
a_HQ_transcript/131291	-3.4699887155209	3.01745000790377	-2.51197175150077	0.0171445305478801	0.467522996113147	-3.1797775240065
a_HQ_transcript/133645	-2.95646182020202	0.304624606805914	-2.51177255504349	0.0171526036018273	0.467522996113147	-3.11493372194242
a_HQ_transcript/24960	5.83047899654569	1.33096343711503	2.51168204167152	0.0171562730895131	0.467522996113147	-3.20011812163324
a_HQ_transcript/72748	-1.09563398649258	2.83397302538561	-2.51135036761445	0.0171697255905537	0.467522996113147	-3.18143705240703
a_HQ_transcript/74968	-2.9165249390323	2.54422401653615	-2.51135011035262	0.0171697360286954	0.467522996113147	-3.15979124704617
a_HQ_transcript/152123	1.80970761564584	1.58108971441515	2.51118403539224	0.0171764755698569	0.467522996113147	-3.0765207724416
a_HQ_transcript/138660	4.45222643756982	3.64802304718523	2.51111252737043	0.0171793782074416	0.467522996113147	-3.27436812717445
a_HQ_transcript/93815	3.11469569159689	1.30228110859018	2.51110141455068	0.0171798293369406	0.467522996113147	-3.13755249354513
a_HQ_transcript/152745	-6.77834057562494	1.71527581036851	-2.50995613997501	0.0172263805676973	0.468570449059012	-3.19021813200871
a_HQ_transcript/62977	-0.794108151313171	4.30493649987136	-2.50937250319041	0.0172501477690349	0.468997469372526	-3.41855223227673
a_HQ_transcript/141451	-2.6202217842044	-0.554993351203678	-2.50846886279384	0.0172870057100397	0.46966802643114	-3.11737645992859
a_HQ_transcript/87751	2.37521061172446	3.25251521813348	2.50837174573296	0.0172909712455725	0.46966802643114	-3.19720861155376
a_HQ_transcript/2009	2.96477022570343	2.49509941989426	2.50791231891654	0.0173097421217644	0.469690450327359	-3.14976303841972
a_HQ_transcript/91919	1.47550888520415	-0.836918722303201	2.50734193687849	0.0173330723272455	0.469690450327359	-3.14731883243492
a_HQ_transcript/131045	1.49764730001001	-0.776601527365985	2.50726465263186	0.0173362356853649	0.469690450327359	-3.13852525165754
a_HQ_transcript/85730	-2.29079088533401	-0.469604461996592	-2.50721878878405	0.0173381132106746	0.469690450327359	-3.16958850163536
a_HQ_transcript/150451	3.86252060379251	5.93530341810208	2.50716368258114	0.0173403693363915	0.469690450327359	-3.56622432633461

a_HQ_transcript/61573	-3.3321434926005	1.79300952258823	-2.5069050668937	0.0173509610261174	0.469690450327359	-3.20318660760529
a_HQ_transcript/106746	-2.45784060683246	0.824465693397302	-2.50683308615872	0.0173539100762725	0.469690450327359	-3.15094885158651
a_HQ_transcript/143298	5.78352128627109	2.27051379923657	2.50628052251814	0.0173765639228551	0.469690450327359	-3.27482746020239
a_HQ_transcript/89347	2.98890718007513	0.224687943400289	2.50625377293587	0.0173776612827467	0.469690450327359	-3.17755683595191
a_HQ_transcript/4602	2.52389415345608	2.49262941267055	2.50614539906793	0.0173821078022193	0.469690450327359	-3.23043215755575
a_HQ_transcript/77834	-1.41744380886368	3.18037982837398	-2.50600254162567	0.0173879707582945	0.469690450327359	-3.29317838480996
a_HQ_transcript/3423	3.30627806028849	0.248240924551183	2.50589561106113	0.0173923604406151	0.469690450327359	-3.14070988330253
a_HQ_transcript/108970	4.51864026604053	2.32887664373873	2.50578650658593	0.0173968404134807	0.469690450327359	-3.1566782562284
a_HQ_transcript/76673	4.40471005032295	0.652598511590003	2.50537966264709	0.0174135552943153	0.46972413801222	-3.08857070644967
a_HQ_transcript/105193	2.96002026324566	2.02678920745393	2.50522864981472	0.0174197632923096	0.46972413801222	-3.1861453653769
a_HQ_transcript/33959	-0.725596988640708	3.58230532132369	-2.5051662095972	0.0174223307451546	0.46972413801222	-3.304887107155
a_HQ_transcript/72439	-3.17563194523115	0.0499798878155138	-2.50455903262552	0.0174473151029191	0.470179662634439	-3.14035653538216
a_HQ_transcript/55050	3.61501420959978	1.6772922790004	2.50393405590766	0.01747306621142 7	0.470655418321514	-3.19308444144622
a_HQ_transcript/90355	3.82441610192749	1.54176557548142	2.50360774149333	0.0174865252930818	0.470799787974183	-3.17041030088658
a_HQ_transcript/89153	-2.33652905066721	0.108337104079301	-2.50315394015307	0.0175052584753059	0.470828406863898	-3.09203059572056
a_HQ_transcript/1181	-2.79327712877162	-0.064032413772081	-2.50300506012815	0.0175114083377151	0.470828406863898	-3.14072845257237
a_HQ_transcript/5930	-2.09945381507674	-0.567820891073395	-2.50282205675388	0.017518970465386	0.470828406863898	-3.17379243816705
a_HQ_transcript/150524	0.564433830848411	9.96042091396823	2.5026367490836	0.0175266308637029	0.470828406863898	-3.97564109973788
a_HQ_transcript/10476	-2.26773170837399	-0.266176560242797	-2.50260152001844	0.0175280875387675	0.470828406863898	-3.12766883343125
a_HQ_transcript/150207	0.522801960512655	10.661021378805	2.50234594282964	0.0175386586412636	0.470894757274537	-4.05823759816241
a_HQ_transcript/142502	2.4007599165152	1.63049141908581	2.50105108060465	0.0175923062283142	0.471998101350163	-3.11781014223679
a_HQ_transcript/152199	3.30688606935794	3.46604370049432	2.50093336745778	0.0175971906696902	0.471998101350163	-3.30202030448627
a_HQ_transcript/115944	1.0616720505302	4.80607776856552	2.50076659193458	0.0176041130561374	0.471998101350163	-3.47371476213192
a_HQ_transcript/129720	-1.34751441695946	3.23374701292839	-2.49994910715389	0.0176380806998738	0.472690804482372	-3.22769590633096
a_HQ_transcript/70859	-1.03776948824179	3.49092124066996	-2.49956041689906	0.0176542523988151	0.472906167483099	-3.26368500861986
a_HQ_transcript/24457	1.5264831648946	3.37449243728278	2.4990549446058	0.0176753032227953	0.473251969477036	-3.31088940206524
a_HQ_transcript/118472	-3.15831009060418	1.06218132615815	-2.49851298412187	0.0176978991930518	0.473638804923441	-3.18679603887324
a_HQ_transcript/19603	2.29647730462349	0.676991606432592	2.49777632538716	0.0177286551875247	0.474100979066121	-3.09255604242249
a_HQ_transcript/131024	-2.04041008906455	-0.580123156285671	-2.49769934272117	0.0177318720916433	0.474100979066121	-3.15025633231118
a_HQ_transcript/13184	2.47968706888063	-0.483281337864571	2.49744631916357	0.0177424490541689	0.474100979066121	-3.1752320037347
a_HQ_transcript/53424	1.99223092478004	1.15830198286288	2.49721438151956	0.0177521496580839	0.474100979066121	-3.10485739535086
a_HQ_transcript/143472	1.95747938843638	-0.534124925960965	2.49712356091169	0.0177559494809205	0.474100979066121	-3.16590889148261
a_HQ_transcript/144644	-1.51596932073327	3.94441139600877	-2.49677879999121	0.0177703806434889	0.474268450892893	-3.29473173385245
a_HQ_transcript/100189	0.965103820936675	3.1920288857081	2.49644009157585	0.0177845689274381	0.474429289864215	-3.30781139976493
a_HQ_transcript/137776	3.73193205742841	2.044696943138	2.49601196795582	0.0178025176168859	0.474480223363093	-3.13427658013073
a_HQ_transcript/41658	2.04448734763972	-0.507213044614683	2.49591370355703	0.0178066396038257	0.474480223363093	-3.15755588999116
a_HQ_transcript/142425	-1.21250808794279	4.23604888536963	-2.49581058220523	0.0178109662706143	0.474480223363093	-3.43942093694652
a_HQ_transcript/23400	0.677588946232227	3.87812686751268	2.49495853414982	0.0178467525788522	0.475048916727097	-3.40790760938327
a_HQ_transcript/88249	-1.78424229800267	3.29362751385905	-2.49491319412383	0.0178486587209603	0.475048916727097	-3.28813267115789
a_HQ_transcript/113782	-0.84020732522126	3.74549340960429	-2.49443757625935	0.0178686654422392	0.47536374591601	-3.3243841856219
a_HQ_transcript/45843	2.65110144970224	1.65700438920129	2.49387086613401	0.0178925307674045	0.475780891330142	-3.19502429809496
a_HQ_transcript/127881	0.827913439929233	4.584964767493	2.49344129986736	0.0179106401441639	0.47604466863281	-3.47816203057102
a_HQ_transcript/43879	1.97366077664186	2.19012475101822	2.49295911313401	0.0179309878703319	0.476085987110801	-3.17608225942825
a_HQ_transcript/123979	3.29763152963782	2.88267459687034	2.49289107432561	0.0179338607348425	0.476085987110801	-3.2856564519848
a_HQ_transcript/40571	2.79523732404077	0.86778775546886	2.49282228739537	0.017936765616788	0.476085987110801	-3.20304731787717
a_HQ_transcript/104010	-3.61027103837517	1.50458209658351	-2.49241338483093	0.0179540425331099	0.476229771447694	-3.14225222241478
a_HQ_transcript/95669	-1.93719312367589	-0.311165502435681	-2.49227063612196	0.0179600775297953	0.476229771447694	-3.17209018185042
a_HQ_transcript/100192	-0.646241404460529	4.90507862793733	-2.49211259785378	0.0179667610924992	0.476229771447694	-3.50244315598243
a_HQ_transcript/54028	-2.91487682884047	1.44508989179865	-2.49191728973688	0.0179750239753017	0.476231628822397	-3.15820575843701
a_HQ_transcript/78324	-0.673195020229719	2.83558202146635	-2.49147582152378	0.0179937139642601	0.476420375141937	-3.26555173205607

a_HQ_transcript/64473	3.50068797417474	0.674871072837607	2.49136189172223	0.0179985401839336	0.476420375141937	-3.22031028637641
a_HQ_transcript/104960	-3.29218411916581	0.990565014566847	-2.49113193357928	0.0180082851338166	0.476461355602408	-3.16575766453871
a_HQ_transcript/19756	2.72363177170511	-0.173757452990773	2.49070909146886	0.0180262165584168	0.476478764278485	-3.17440917623786
a_HQ_transcript/9487	2.70553995702182	0.784161054589518	2.49063595547001	0.0180293196887606	0.476478764278485	-3.2059263112617
a_HQ_transcript/1453	-0.857725312273452	4.91108927372037	-2.49053664220174	0.0180335342935017	0.476478764278485	-3.50632956493136
a_HQ_transcript/123294	2.04476070976406	1.13433438953692	2.49030126827433	0.018043526574969	0.476526175715493	-3.1230671449867
a_HQ_transcript/93779	-2.57683846696428	0.379332319482912	-2.48938198600289	0.0180826012959311	0.47734125709804	-3.16358930891492
a_HQ_transcript/49751	-2.11434051761535	0.380221483040163	-2.48907298129788	0.0180957531523035	0.477471602014115	-3.11790476818323
a_HQ_transcript/19528	-5.39508938771448	-0.709882890545854	-2.48868414905138	0.0181123150802757	0.477691765420265	-3.18315852818995
a_HQ_transcript/50588	-1.15568501508483	2.95539421610021	-2.48810339667783	0.018137077519434	0.478127910226602	-3.26204621424474
a_HQ_transcript/143403	1.50195030745304	4.62380015103236	2.48610844102605	0.0182223759742706	0.479651903198431	-3.52800660531634
a_HQ_transcript/80482	1.57102917264843	-0.653368547941772	2.48598809067958	0.0182275335361487	0.479651903198431	-3.17770765191747
a_HQ_transcript/91657	-0.970825424013931	4.06417663501191	-2.48592085793552	0.0182304153483138	0.479651903198431	-3.43101008900553
a_HQ_transcript/103345	2.28224764902002	2.74371297034214	2.48575389756539	0.0182375736131432	0.479651903198431	-3.17921257536354
a_HQ_transcript/83849	-3.1283610574025	0.131867154195574	-2.48563049506368	0.0182428660309084	0.479651903198431	-3.32822979415691
a_HQ_transcript/36543	3.8701828396756	1.58761098373215	2.48554500807745	0.0182465331739246	0.479651903198431	-3.24725691787411
a_HQ_transcript/123572	-3.58392690724697	1.00506365043961	-2.48536576636655	0.0182542243139703	0.479651903198431	-3.23765038303467
a_HQ_transcript/2898	1.89847410068758	1.33831087172779	2.48507415368232	0.0182667435615868	0.479651903198431	-3.14285928618912
a_HQ_transcript/79644	-1.38147896418599	3.53969705777479	-2.4850180560511	0.0182691527952334	0.479651903198431	-3.38345550101459
a_HQ_transcript/115233	-2.11577498002227	0.497784274100161	-2.48452808265522	0.018290208133943	0.479987908988641	-3.14834519510499
a_HQ_transcript/22099	2.4244572536769	2.47586513712794	2.4838816927644	0.0183180190665391	0.480400899555324	-3.18462357236587
a_HQ_transcript/145156	-2.18387442283188	-0.446039030406076	-2.48366172129252	0.0183274921704267	0.480400899555324	-3.19021878441178
a_HQ_transcript/31200	1.41453812682964	2.51562641850537	2.48344087595295	0.0183370074206597	0.480400899555324	-3.21684556422167
a_HQ_transcript/118958	3.79336715063105	0.711249191335802	2.48339456076245	0.0183390035114448	0.480400899555324	-3.23593116034958
a_HQ_transcript/107647	-2.44901081190249	-0.0819326611295031	-2.48292876354495	0.0183590894937213	0.480710429770734	-3.17568714678968
a_HQ_transcript/134687	-2.55611133634776	0.311427288614216	-2.48223056209294	0.0183892348819982	0.481282955975142	-3.13630707632397
a_HQ_transcript/15123	2.53675824846843	3.33471255980739	2.48135603038262	0.0184270574059373	0.482055802381783	-3.31565760435464
a_HQ_transcript/149524	0.916319547496262	3.65760567639503	2.48055746346796	0.018461656741276	0.482581628413346	-3.40300293144192
a_HQ_transcript/87918	-3.59371022051829	1.72912693078601	-2.48050892379199	0.0184637617256964	0.482581628413346	-3.35183777708351
a_HQ_transcript/31538	2.21016724460627	0.221275506634731	2.48007008653972	0.018482802434371	0.482862175238254	-3.15313670149826
a_HQ_transcript/115065	2.43381345619265	5.42156014611335	2.4795001935532	0.018507556352459	0.483291660222703	-3.60218903788546
a_HQ_transcript/28989	2.59757107224354	0.133870627517671	2.47887177097234	0.0185348877518662	0.483706442845234	-3.1645965141329
a_HQ_transcript/149691	-1.61359948277637	3.60096589306861	-2.47875241020426	0.0185400831726394	0.483706442845234	-3.43206045691869
a_HQ_transcript/140053	1.82183358005809	-0.724314534873195	2.47818291158857	0.0185648901209884	0.48413635394922	-3.20014123035548
a_HQ_transcript/136512	1.20340322917089	3.91709509960608	2.47777507469882	0.0185826738684932	0.484287294570755	-3.48746480340994
a_HQ_transcript/75807	1.52021252632349	-0.688529150573	2.47766810334371	0.0185873409404651	0.484287294570755	-3.20025641199217
a_HQ_transcript/117416	0.921315471638034	4.17949633942946	2.4771059438984	0.0186118851335412	0.48470952466061	-3.39076226776166
a_HQ_transcript/143504	-2.0905738151403	-0.492349980492204	-2.4768112499176	0.0186247634849161	0.484827698992926	-3.19309595262848
a_HQ_transcript/130762	-2.03282136011184	-0.673028673706079	-2.47624203403552	0.0186496617479518	0.4851888588326	-3.18621940820183
a_HQ_transcript/92895	2.87849652455361	1.30028982458091	2.47611251517404	0.0186553313289785	0.4851888588326	-3.26661290368487
a_HQ_transcript/121646	1.58923346128689	-0.00097170602467236	2.47582578184551	0.0186678884557188	0.4852983095501	-3.13519993083763
a_HQ_transcript/52066	2.37422918330777	0.022575661122852	2.47542929210046	0.018685264971967	0.485532893290344	-3.23486606867273
a_HQ_transcript/61248	1.68359958519957	0.795484310588961	2.47466485346156	0.0187188089668969	0.486187188394898	-3.20195366295154
a_HQ_transcript/95489	3.83042538955809	0.988485018285014	2.47396086016404	0.0187497493234372	0.486536963493313	-3.24791068764543
a_HQ_transcript/42619	2.2066791899536	1.05315484425885	2.47386492606019	0.0187539692284713	0.486536963493313	-3.15620672780459
a_HQ_transcript/72003	2.59526347577631	2.09816431235766	2.47378726393708	0.0187573860306309	0.486536963493313	-3.19795632469573
a_HQ_transcript/76214	0.887296622713722	3.08167012781092	2.47341078503599	0.0187739575721146	0.48674063601796	-3.33140251135314
a_HQ_transcript/46058	1.98301628351523	-0.248078787719178	2.47322851525176	0.0187819853872193	0.48674063601796	-3.14736525056962
a_HQ_transcript/61011	-2.33536485222061	0.082231068146705	-2.47262020998269	0.0188088000737441	0.487218329182975	-3.26305957112185
a_HQ_transcript/147209	3.43408561821443	-0.280272243987501	2.47232272097636	0.0188219264283803	0.487341175692157	-3.21624926904605

a_HQ_transcript/139055	2.72167350484416	-0.0115611040284528	2.47205444943173	0.0188337707837816	0.487430733802163	-3.16098113707053
a_HQ_transcript/122415	2.5895073180261	-0.0287005140072506	2.47142208869876	0.018861716891884	0.487839201339478	-3.19443275105957
a_HQ_transcript/58907	-2.81290783355947	1.15948218574463	-2.47114252039677	0.0188740840283145	0.487839201339478	-3.19799900266999
a_HQ_transcript/24990	-3.88363063830193	1.49626816283011	-2.47112789964011	0.018874731004206	0.487839201339478	-3.30939949844724
a_HQ_transcript/73692	1.5738015757553	1.33038408547651	2.47071672849562	0.0188929338466889	0.487887924054572	-3.15420845003733
a_HQ_transcript/85100	-3.27944303636013	1.52921734727883	-2.47070614078452	0.0188934027843181	0.487887924054572	-3.26440957633642
a_HQ_transcript/76460	1.30568949662854	0.935149039648768	2.47046369415871	0.0189041438406202	0.487948522720948	-3.25671452564622
a_HQ_transcript/136375	-3.43172212989304	1.06684449379245	-2.4702090177549	0.0189154327260789	0.488023201731699	-3.22692715387464
a_HQ_transcript/5201	0.981566970870299	2.87688644718371	2.46988756523217	0.0189296903606868	0.488167664908559	-3.29360253844103
a_HQ_transcript/95840	0.549198072910918	5.30563991863783	2.46935068432123	0.0189535249828605	0.488167664908559	-3.58845000236228
a_HQ_transcript/129786	-5.22807672522645	2.73316809139033	-2.4687760453368	0.0189790662752533	0.488167664908559	-3.25945120588772
a_HQ_transcript/75870	-4.33179044629384	1.19718288797536	-2.46871536563635	0.0189817651760766	0.488167664908559	-3.27953629573813
a_HQ_transcript/74621	-1.3046958188004	2.25666528506373	-2.4687034826549	0.0189822937462982	0.488167664908559	-3.20704192523943
a_HQ_transcript/104307	3.55469458481774	0.0475370555814256	2.4686562762901	0.0189843936788026	0.488167664908559	-3.22879236979035
a_HQ_transcript/70520	-2.04823873836664	-0.391152302624007	-2.46860713151746	0.0189865800653974	0.488167664908559	-3.22448711752944
a_HQ_transcript/119143	-2.60257051158614	0.0745587347787094	-2.46857033564318	0.0189882172164577	0.488167664908559	-3.25736439308134
a_HQ_transcript/151702	0.880852617250526	2.93163435105767	2.46790576979047	0.0190178078400843	0.488241507471318	-3.30038805887342
a_HQ_transcript/37639	2.28460586844097	0.497152489519489	2.46775946207742	0.0190243280361389	0.488241507471318	-3.17940944884664
a_HQ_transcript/56475	3.54259581253236	0.035681169315658	2.46758373197055	0.019032162142545	0.488241507471318	-3.25230828022247
a_HQ_transcript/116883	-4.51780618988963	2.9635387020222	-2.46753766502602	0.0190342163102988	0.488241507471318	-3.33005230559233
a_HQ_transcript/150864	-3.25601950393581	-0.603556328978074	-2.46742704414707	0.0190391498259996	0.488241507471318	-3.22864569410203
a_HQ_transcript/60101	2.06060719435243	1.76004723917794	2.46737467114476	0.0190414859867444	0.488241507471318	-3.15075528499663
a_HQ_transcript/8663	1.85457011208457	-0.417476823243301	2.46670754501625	0.01907126689929	0.488565006189758	-3.18977568253693
a_HQ_transcript/50443	-2.43191981154428	0.358375348349827	-2.46638127253355	0.0190858474062987	0.488565006189758	-3.18215269627382
a_HQ_transcript/131015	-1.45605371909971	4.09510150459333	-2.46607769313202	0.0190994229573248	0.488565006189758	-3.54331320128005
a_HQ_transcript/143217	3.35554365026082	4.55140205081586	2.46601364949137	0.0191022880070723	0.488565006189758	-3.37556479740363
a_HQ_transcript/23743	-0.799573682912804	4.00441032979254	-2.46546511144498	0.019126843454284	0.488565006189758	-3.48046248907346
a_HQ_transcript/29112	2.45757251294771	-0.482284915200681	2.46544714662332	0.0191276481421338	0.488565006189758	-3.22943118137799
a_HQ_transcript/10395	-1.55951400804359	3.268143057006	-2.46533174411109	0.0191328180382889	0.488565006189758	-3.27794529763959
a_HQ_transcript/49709	-0.890735247658363	2.50473754727364	-2.46522606872313	0.019137553291597	0.488565006189758	-3.3014487982275
a_HQ_transcript/148098	6.52372865834694	1.77607189716101	2.46467865164051	0.0191620998960294	0.488565006189758	-3.2846368574583
a_HQ_transcript/145910	3.2649293054819	1.51756958829892	2.46458523596668	0.0191662916016261	0.488565006189758	-3.30483614901107
a_HQ_transcript/152356	5.05857488994054	1.36813398281218	2.46399888659898	0.019192621158173	0.488565006189758	-3.27543736521625
a_HQ_transcript/4562	2.68554532038228	-0.105637262035856	2.46392698871968	0.0191958519512573	0.488565006189758	-3.22671891667305
a_HQ_transcript/74703	3.51277657455368	0.0272272357631742	2.46389995025396	0.0191970670766566	0.488565006189758	-3.22195888245054
a_HQ_transcript/118741	-2.11281029391464	-0.597210403833437	-2.4635086832898	0.0192146587312927	0.488565006189758	-3.18659193910527
a_HQ_transcript/113428	-4.65588874567782	6.90624975540092	-2.46341325451115	0.0192189515157687	0.488565006189758	-3.80715956400561
a_HQ_transcript/17534	2.48768454440376	1.69784692444324	2.46319340884983	0.0192288444279795	0.488565006189758	-3.28382506598215
a_HQ_transcript/41933	-0.450624302455637	4.95667045431558	-2.46301663440452	0.0192368025404142	0.488565006189758	-3.5854905726575
a_HQ_transcript/95102	-4.25451390162099	0.738364266379904	-2.46296719656103	0.0192390286945626	0.488565006189758	-3.32085564040608
a_HQ_transcript/102387	2.69362734230433	-0.00466661434291933	2.46280489667399	0.0192463386096825	0.488565006189758	-3.22350719305037
a_HQ_transcript/102542	1.17174565250269	3.11712215116553	2.46267118470591	0.019252362845394	0.488565006189758	-3.27751514370559
a_HQ_transcript/102484	2.98183076677392	0.0739562795824906	2.46253771643803	0.0192583778207931	0.488565006189758	-3.22656244778579
a_HQ_transcript/97039	1.47396678409686	4.34466860196544	2.46253330346416	0.0192585767283377	0.488565006189758	-3.50227568809188
a_HQ_transcript/110687	-2.75452349501956	0.4215115470501	-2.46228854267918	0.0192696118601193	0.488565006189758	-3.22041078407442
a_HQ_transcript/103114	3.67121735701836	4.36788413068123	2.46198270994724	0.0192834085683513	0.488565006189758	-3.53246041898524
a_HQ_transcript/116434	2.08223701069889	-0.0170221085049335	2.46185381414021	0.019289226013245	0.488565006189758	-3.19331254075524
a_HQ_transcript/143812	-1.96112114263683	-0.160976892016935	-2.4616607647964	0.0192979418961951	0.488565006189758	-3.19771731533302
a_HQ_transcript/17253	-3.53535385453204	1.11091015162209	-2.46160832378163	0.0193003101497067	0.488565006189758	-3.16980987239662
a_HQ_transcript/43331	-1.64746367176089	1.6600072876158	-2.46130200867587	0.0193141487505189	0.488565006189758	-3.16956293273578

a_HQ_transcript/102815	-2.40593088883421	-0.0742093958959827	-2.4612696458597	0.0193156113566526	0.488565006189758	-3.2880416134333
a_HQ_transcript/138122	2.1170512429629	1.75338657108221	2.46123277252701	0.0193172779346227	0.488565006189758	-3.21309614426303
a_HQ_transcript/91488	1.82249367053806	0.339205168382602	2.46097120272589	0.0193291039782714	0.488565006189758	-3.17714737833945
a_HQ_transcript/13395	1.5253102080214	3.53990477930979	2.46094525197287	0.019330277619566	0.488565006189758	-3.40239160785873
a_HQ_transcript/112682	-2.54086019727497	0.78554546911448	-2.46091895450112	0.0193314670079212	0.488565006189758	-3.25380186713988
a_HQ_transcript/33473	-0.938820232795022	2.89181441653776	-2.46032384516212	0.0193584006500364	0.488836099442579	-3.36795828149113
a_HQ_transcript/133368	1.32509217902598	-0.73980571874932	2.4603103264157	0.0193590128839254	0.488836099442579	-3.25793594649697
a_HQ_transcript/117291	0.994374204811004	3.94099351627545	2.45983869310106	0.0193803831891005	0.488879323788379	-3.53395315594845
a_HQ_transcript/26137	-1.68942995295085	2.81857718545178	-2.4595297224707	0.0193943947298989	0.488879323788379	-3.32413241311919
a_HQ_transcript/20684	2.38887923632772	0.234461246884105	2.45943321300776	0.0193987732449783	0.488879323788379	-3.27584752179548
a_HQ_transcript/72504	-2.39690116301197	1.33250621845276	-2.45921183636634	0.019408820243351	0.488879323788379	-3.2467833516222
a_HQ_transcript/30355	-2.64108386955968	-0.33122829634063	-2.45920341006997	0.0194092027578927	0.488879323788379	-3.26528559798893
a_HQ_transcript/30109	1.35698400280906	0.930068183851426	2.45884579049354	0.0194254433718294	0.488879323788379	-3.23867312314128
a_HQ_transcript/44117	3.49278171743294	2.30366338769401	2.4587881330349	0.0194280629382735	0.488879323788379	-3.33191627279038
a_HQ_transcript/77318	2.11728503621109	-0.440158974482778	2.45858876422127	0.0194371234026401	0.488879323788379	-3.23571529583734
a_HQ_transcript/67792	-3.31257555812619	2.74036060709732	-2.45851295684674	0.0194405695386396	0.488879323788379	-3.46733964510967
a_HQ_transcript/129210	1.51644370759822	2.15741698438093	2.45821376272911	0.0194541760900915	0.488879323788379	-3.22125330790467
a_HQ_transcript/32220	-2.41103004591599	1.31458150419907	-2.45808885604345	0.0194598590884381	0.488879323788379	-3.23867261453871
a_HQ_transcript/95861	2.34857668247051	1.52066695982036	2.45799668682339	0.0194640535720435	0.488879323788379	-3.21470702747356
a_HQ_transcript/77556	4.70349734504861	1.05844871323824	2.45786473865493	0.0194700597744649	0.488879323788379	-3.23007740743519
a_HQ_transcript/122141	3.92744297206137	0.316601390119509	2.45738913820237	0.019491722884674	0.489094614014374	-3.34439122190216
a_HQ_transcript/17188	-4.90934602913458	0.630301166419818	-2.45730709499508	0.0194954620952262	0.489094614014374	-3.23034773901789
a_HQ_transcript/6103	-3.91248560880575	1.50238206837472	-2.45695773383849	0.0195113919597177	0.489231341466216	-3.21806855094173
a_HQ_transcript/80279	-0.810508258562085	2.88356359043891	-2.45669079711513	0.0195235715230757	0.489231341466216	-3.36688328674016
a_HQ_transcript/16544	-2.54487279650081	-0.0313892337400529	-2.45657542426106	0.0195288378084183	0.489231341466216	-3.19721713511656
a_HQ_transcript/3678	2.0998596711885	-0.134393886820298	2.45639624292138	0.0195370192564131	0.489231341466216	-3.17211059045046
a_HQ_transcript/70184	1.89214445808765	0.493112950404814	2.45563061852145	0.0195720130846109	0.489231341466216	-3.277310968794
a_HQ_transcript/131643	-2.29694946393507	-0.175300932720315	-2.45548415570321	0.0195787138754071	0.489231341466216	-3.23665000500631
a_HQ_transcript/94969	2.45632909912334	-0.401967017898982	2.45547661129562	0.0195790590948415	0.489231341466216	-3.24364405263535
a_HQ_transcript/90199	2.51157939426213	0.0332788057139667	2.45513079521087	0.0195948890309274	0.489231341466216	-3.21268329182495
a_HQ_transcript/333	0.710909142705441	2.81816180664298	2.45466804825672	0.019616089842437	0.489231341466216	-3.32182844643387
a_HQ_transcript/133905	1.69068569578098	1.83728134113293	2.45452866468891	0.0196224798248715	0.489231341466216	-3.21436058141379
a_HQ_transcript/111117	-1.83509880082858	2.06050804463266	-2.45448295226625	0.0196245759062613	0.489231341466216	-3.27781293232854
a_HQ_transcript/4671	1.71497501282323	-0.660811951341069	2.45445916071894	0.0196256669164719	0.489231341466216	-3.24284132464089
a_HQ_transcript/130320	7.10584703709275	4.2975837197053	2.45434263514224	0.0196310112363367	0.489231341466216	-3.45936054146692
a_HQ_transcript/42713	1.47443082839255	3.07020359610922	2.45433765135522	0.0196312398420024	0.489231341466216	-3.3009371638096
a_HQ_transcript/113667	4.05232103000364	2.47212323452815	2.45420228793461	0.0196374498749173	0.489231341466216	-3.44027686788955
a_HQ_transcript/54429	2.90655424084929	1.6098684853261	2.45418541864273	0.0196382239088633	0.489231341466216	-3.20926957540877
a_HQ_transcript/3983	1.39012398712592	1.95828399306043	2.45394745674039	0.0196491455707149	0.489231341466216	-3.21918966901407
a_HQ_transcript/36155	1.00776823635993	2.07203015982514	2.45387639436952	0.0196524081737478	0.489231341466216	-3.23019983780909
a_HQ_transcript/118402	-3.10393634921712	1.03793263709327	-2.45302203100953	0.0196916723206877	0.489409447358635	-3.17578811477796
a_HQ_transcript/148956	-1.96108343838362	-0.697768816311487	-2.45288141550459	0.0196981414799828	0.489409447358635	-3.25290004991939
a_HQ_transcript/23983	-2.76396271336168	2.85545591346125	-2.45253817910151	0.0197139405748557	0.489409447358635	-3.30435305145805
a_HQ_transcript/44581	0.681016796767282	4.74646357542234	2.45248909071439	0.0197162010483384	0.489409447358635	-3.5961479511006
a_HQ_transcript/40942	0.749635463064931	3.03354489087741	2.4524302385076	0.0197189114485494	0.489409447358635	-3.38664183774993
a_HQ_transcript/78546	-2.00450141110881	1.31302479575074	-2.4523259258256	0.0197237163378509	0.489409447358635	-3.24926364461696
a_HQ_transcript/28720	-1.12235472760649	1.88397695981455	-2.45203804139116	0.0197369825269893	0.489409447358635	-3.20695639270936
a_HQ_transcript/62856	-2.0417579536514	1.42734683939377	-2.45203149239152	0.0197372844104907	0.489409447358635	-3.22777602123909
a_HQ_transcript/37767	1.96626300830507	-0.230464311865806	2.45194487226333	0.0197412776595713	0.489409447358635	-3.27466578883533
a_HQ_transcript/119034	3.253079514216	0.580286262547463	2.45189108175817	0.019743757811313	0.489409447358635	-3.20891816316864

a_HQ_transcript/122915	1.12742973804517	2.53343606321176	2.45170637239421	0.0197522764861025	0.489411904341076	-3.3092430775017
a_HQ_transcript/145771	4.03934484548887	1.36966705092731	2.45141070168144	0.019765919614945	0.489414202816344	-3.29741933837604
a_HQ_transcript/71104	2.36061443543636	0.343283259163886	2.45133945491393	0.0197692084402143	0.489414202816344	-3.20000532885624
a_HQ_transcript/55827	2.75575747757343	0.786327790452804	2.45110652177059	0.0197799643667403	0.489472017330727	-3.22816589102189
a_HQ_transcript/148286	-2.63929583824727	-0.237252530447676	-2.4507304913598	0.0197973392372722	0.489693504333684	-3.27241599771468
a_HQ_transcript/15937	3.29607381007088	1.91440067693248	2.4504107310059	0.0198121250591782	0.489850789213064	-3.30729615315913
a_HQ_transcript/151840	-0.911076808775731	2.82241466420774	-2.44818337912476	0.0199153986885876	0.492087371710457	-3.34416666066698
a_HQ_transcript/108202	-4.29373383756599	1.01263282569813	-2.44809481289957	0.0199195153047534	0.492087371710457	-3.25508201555285
a_HQ_transcript/99937	2.7337988734623	1.01527928379681	2.44742425148996	0.0199507086631859	0.492459922959451	-3.24782134855274
a_HQ_transcript/62368	-1.7967726359225	2.44586532119443	-2.44740639286818	0.0199515400249365	0.492459922959451	-3.30881738014678
a_HQ_transcript/36798	2.75275849395118	0.208509838998226	2.44713196852041	0.0199643191165896	0.492566189137997	-3.25301962087834
a_HQ_transcript/28299	-1.99949501128128	0.383392652481279	-2.44664433839039	0.0199870449837314	0.492587049130385	-3.20457506508584
a_HQ_transcript/123195	2.22429686421931	0.393794069345531	2.44646799041546	0.0199952694469237	0.492587049130385	-3.21538524411496
a_HQ_transcript/117393	1.55499140312472	0.961110224585175	2.44635826989567	0.0200003881185661	0.492587049130385	-3.17877493019552
a_HQ_transcript/318	2.24432030708603	0.149855571175508	2.4459026284776	0.0200216574645821	0.492587049130385	-3.22461987477705
a_HQ_transcript/125159	1.67374829530033	2.69169043687068	2.4458409021463	0.0200245404383284	0.492587049130385	-3.26992540106625
a_HQ_transcript/63417	-2.86273773073564	0.0834072361751882	-2.44558901090112	0.0200363091343811	0.492587049130385	-3.29041808992523
a_HQ_transcript/27975	-2.82644743854435	3.43987050296386	-2.44546462068268	0.0200421231409862	0.492587049130385	-3.42596485710744
a_HQ_transcript/36330	2.44308440464687	2.01542600958707	2.44539260001197	0.0200454900957565	0.492587049130385	-3.30109100167777
a_HQ_transcript/115035	2.91823115393393	-0.675120919411784	2.4453847803829	0.0200458556932374	0.492587049130385	-3.27038497463306
a_HQ_transcript/145808	2.84309568519928	2.50430310604722	2.4452981503891	0.0200499063831973	0.492587049130385	-3.36505119065698
a_HQ_transcript/16667	-1.89948305961813	2.401170833406	-2.44481533231306	0.0200724959332738	0.492879052239098	-3.2414983188242
a_HQ_transcript/68201	-1.32584442419259	1.83008281505011	-2.44461946813043	0.0200816664303874	0.492879052239098	-3.1990817371287
a_HQ_transcript/14568	2.91719393281988	1.14873995261342	2.44450069204942	0.0200872294721033	0.492879052239098	-3.27165881996737
a_HQ_transcript/105116	2.27412903430868	0.908843691879919	2.44364942334781	0.0201271410131695	0.493481586246194	-3.20661780962828
a_HQ_transcript/44006	-1.59139408563524	3.24720115498598	-2.44361482216483	0.020128764811962	0.493481586246194	-3.30355704455826
a_HQ_transcript/58642	2.43290294635588	0.57584648305023	2.44330012286091	0.0201435388258899	0.493635592272904	-3.25249602536925
a_HQ_transcript/38699	-2.24644031214124	0.557003733946034	-2.44298224821906	0.020158471959924	0.493793366239554	-3.24531417989877
a_HQ_transcript/58492	3.14124798835016	0.526963973355411	2.44246620292142	0.0201827362891375	0.494110093053997	-3.30366660209576
a_HQ_transcript/150710	0.476664896997067	10.680696450313	2.44220502281462	0.0201950270757529	0.494110093053997	-4.18748144423351
a_HQ_transcript/103269	2.51849121828386	0.116564871880682	2.44180633318244	0.0202138020524922	0.494110093053997	-3.2347486598
a_HQ_transcript/111420	-2.39837838462628	1.40422472745218	-2.44157293288279	0.0202248006644809	0.494110093053997	-3.23040657161661
a_HQ_transcript/59586	1.04605006101607	3.55033326843369	2.44129871972025	0.020237729495524	0.494110093053997	-3.48122659738513
a_HQ_transcript/100575	-2.1852754598397	1.24782706153412	-2.44101009021848	0.0202513461891173	0.494110093053997	-3.26371749149579
a_HQ_transcript/65168	-2.1236144422002	-0.517632249459021	-2.4409573815846	0.0202538337307435	0.494110093053997	-3.27408975403596
a_HQ_transcript/9999	2.22254837060876	1.28355790255735	2.44089826775224	0.0202566238923553	0.494110093053997	-3.25554675346974
a_HQ_transcript/98134	3.57605548446161	0.199166671784206	2.44038846215881	0.0202807011826583	0.494110093053997	-3.27166963659783
a_HQ_transcript/55464	-2.26891207228474	2.26654453407428	-2.43984359381255	0.0203064633046051	0.494110093053997	-3.25197181752737
a_HQ_transcript/152819	-3.1173826424427	2.01565964982939	-2.43975395265185	0.0203107045214598	0.494110093053997	-3.34050591572946
a_HQ_transcript/121563	3.83217933346126	0.302563178735408	2.43971089691289	0.0203127419168615	0.494110093053997	-3.31285048237588
a_HQ_transcript/145005	5.19633999106027	3.14295466276577	2.43970332449694	0.0203131002624906	0.494110093053997	-3.53890182024256
a_HQ_transcript/92356	1.64524448484003	1.38883626638233	2.43942282768481	0.0203263781377435	0.494110093053997	-3.20161381544347
a_HQ_transcript/635	3.27603926619497	0.264258329896411	2.4394220384322	0.0203264155097603	0.494110093053997	-3.27145192908594
a_HQ_transcript/14178	2.5695643174152	1.80736105943482	2.43934797883443	0.0203299225957571	0.494110093053997	-3.28427643533166
a_HQ_transcript/4159	2.28165238531236	-0.466486528121899	2.43914087263636	0.0203397330239977	0.494110093053997	-3.22067748602833
a_HQ_transcript/147835	0.898851136597628	5.08896738985364	2.43900550438429	0.0203461476270273	0.494110093053997	-3.6493457938803
a_HQ_transcript/78650	-3.17722853332237	0.519833239093352	-2.43881818226652	0.0203550271775091	0.494110093053997	-3.28360351825752
a_HQ_transcript/144596	0.833507968259437	4.45400231580315	2.43875400897193	0.0203580699700506	0.494110093053997	-3.57873841970762
a_HQ_transcript/28331	2.11229322143389	1.9217127621914	2.43871562119349	0.0203598903344198	0.494110093053997	-3.27096071552111
a_HQ_transcript/21265	2.10857824772501	1.51181311883106	2.43854785321779	0.0203678477047038	0.494110093053997	-3.23021756344651

a_HQ_transcript/25968	2.37316852202865	1.06363528391486	2.43825990993655	0.0203815117026583	0.494110093053997	-3.26232090657675
a_HQ_transcript/142036	4.0191721334756	0.514511457892196	2.43792089313394	0.0203976100544465	0.494110093053997	-3.2606458983904
a_HQ_transcript/1549	-0.911808882593539	3.671401795823	-2.43775818712375	0.0204053403359833	0.494110093053997	-3.45660509517215
a_HQ_transcript/23543	-2.53447716146809	0.197715354132512	-2.43774044791706	0.0204061833002676	0.494110093053997	-3.28798465903115
a_HQ_transcript/123551	-2.99202822128513	1.32686526375944	-2.43751685120809	0.0204168113053256	0.494110093053997	-3.2396937067272
a_HQ_transcript/60381	2.96062428600263	0.957264369780682	2.43739520202745	0.0204225956589267	0.494110093053997	-3.29524235335427
a_HQ_transcript/66953	-0.848763232994792	2.70070479532472	-2.43737249419597	0.0204236755698001	0.494110093053997	-3.36030782632269
a_HQ_transcript/125646	-2.95176820339752	0.371866944625638	-2.43725100172754	0.0204294542430887	0.494110093053997	-3.24579509448175
a_HQ_transcript/145616	-3.88322899125973	0.997512170589359	-2.43681501232794	0.020450203948403	0.494110093053997	-3.28294684725431
a_HQ_transcript/137885	0.950285351607012	3.20894059718123	2.43649634835438	0.0204653820377837	0.494110093053997	-3.44742324061402
a_HQ_transcript/120671	-2.09247881119654	-0.47363898365041	-2.43641044466782	0.0204694754224337	0.494110093053997	-3.27960703211754
a_HQ_transcript/57250	-2.44679594613863	-0.273973587429086	-2.43641019750122	0.020469487201213	0.494110093053997	-3.25306108724246
a_HQ_transcript/127609	2.19131997568015	-0.225362285336843	2.43628706142019	0.0204753560474912	0.494110093053997	-3.26365663576152
a_HQ_transcript/48556	1.35357303397852	2.388577105334	2.43624385688323	0.0204774156029294	0.494110093053997	-3.29054542079836
a_HQ_transcript/23143	-2.75824909313315	0.594452122215151	-2.43595638672546	0.02049112408679	0.494235709924038	-3.23804228232971
a_HQ_transcript/114655	-3.07785820844908	-0.039172403234931	-2.43569352087845	0.0205036666021595	0.494333111675788	-3.2491970046785
a_HQ_transcript/48314	3.10523211816354	0.169683875500521	2.43532928458632	0.0205210575359356	0.494505304675963	-3.29368035044231
a_HQ_transcript/76229	-3.0588215636506	-0.41864004581994	-2.43505062699984	0.0205343714886715	0.494505304675963	-3.29354001074591
a_HQ_transcript/83330	-3.03842962309542	0.148526215655769	-2.43500964428435	0.0205363302623138	0.494505304675963	-3.25828076289408
a_HQ_transcript/105081	-2.97421973087364	-0.491794226999283	-2.43469020256278	0.0205516038600648	0.494532504101557	-3.29045159132763
a_HQ_transcript/62818	0.923285922059058	2.42262909894626	2.43463017534698	0.0205544751223053	0.494532504101557	-3.32811924518494
a_HQ_transcript/142384	-3.83245654125608	-0.0901315232971941	-2.43419984264638	0.0205750698042493	0.494823193041541	-3.32852345151162
a_HQ_transcript/92976	2.05992464377384	-0.503788168800383	2.43346223947063	0.0206104134973417	0.495468203380265	-3.26016173764673
a_HQ_transcript/46251	-2.21619248551702	0.827958399604647	-2.43289992468271	0.0206373951008878	0.495757034829947	-3.2734274164597
a_HQ_transcript/118963	-6.50474642954917	2.06868349112142	-2.43285638336003	0.020639485691723	0.495757034829947	-3.40668706244475
a_HQ_transcript/50091	-1.99492332112725	0.792275924635634	-2.43227812803005	0.0206672683379027	0.496110834012153	-3.25896327327427
a_HQ_transcript/51887	-4.02734676836886	1.33292673339376	-2.43218570177694	0.0206717121767454	0.496110834012153	-3.33611417214022
a_HQ_transcript/95281	-0.59260354560167	2.9382225512462	-2.43187588783548	0.0206866143370929	0.496110834012153	-3.3750481175593
a_HQ_transcript/3133	2.22977442826776	2.37337934414054	2.43181786492808	0.0206894063486504	0.496110834012153	-3.30999447618804
a_HQ_transcript/116821	-3.91154555400027	2.5927299166766	-2.43166239438749	0.0206968891494541	0.496110834012153	-3.44558123174806
a_HQ_transcript/139963	3.09223838187587	-0.167281787442789	2.43147886360031	0.0207057256673924	0.4961180633117	-3.31462874843413
a_HQ_transcript/118098	1.08087387672411	4.96338606839472	2.4311611588791	0.020721030436712	0.496280204872352	-3.63041992257411
a_HQ_transcript/11362	0.8233557411914	2.64752875861772	2.43020238481958	0.0207672799693457	0.496916729421335	-3.36315798965511
a_HQ_transcript/97108	-1.54086497424096	-0.810236414071117	-2.4301324747644	0.0207706559841882	0.496916729421335	-3.27716675412019
a_HQ_transcript/20135	2.72193045271657	0.949701101618371	2.42994907646992	0.0207795148195605	0.496916729421335	-3.23675249596196
a_HQ_transcript/139988	2.15614578055945	-0.729096602503011	2.4299017443553	0.0207818017000975	0.496916729421335	-3.28707446243137
a_HQ_transcript/80768	2.7920593509857	1.09581543884856	2.42940710519541	0.0208057142345211	0.497283946144836	-3.35546578104544
a_HQ_transcript/92929	4.73728677178227	2.44374495156454	2.428846776889	0.0208328327138791	0.497341901585093	-3.31827410235426
a_HQ_transcript/93516	3.05823149581225	1.38135224822874	2.42839544108952	0.0208546996630682	0.497341901585093	-3.27050899444214
a_HQ_transcript/94186	3.24678021181208	-0.230790547278423	2.42836657456441	0.0208560989407622	0.497341901585093	-3.30549042129099
a_HQ_transcript/88654	2.09182220642117	0.409360990789653	2.42802382089569	0.0208727201472556	0.497341901585093	-3.21038787317126
a_HQ_transcript/123276	1.14518227116221	3.8425451187156	2.42800424810776	0.0208736696579285	0.497341901585093	-3.53007922962753
a_HQ_transcript/98088	1.78548089347794	3.94214008057241	2.42795650848672	0.0208759857568401	0.497341901585093	-3.52881974095424
a_HQ_transcript/13348	-0.797872100358268	3.33508109151075	-2.42773024595318	0.0208869661241428	0.497341901585093	-3.42140154233522
a_HQ_transcript/91619	2.94198093691478	1.55692684685957	2.42758969608383	0.0208937895652725	0.497341901585093	-3.2744079212844
a_HQ_transcript/119681	-2.19326690168363	1.69949650006394	-2.42750169289405	0.0208980629950944	0.497341901585093	-3.33251230019085
a_HQ_transcript/99741	-2.63802523191297	0.32743726509201	-2.4274806002553	0.0208990873708039	0.497341901585093	-3.22543298141313
a_HQ_transcript/52578	4.78728828427045	2.75946990175497	2.4274153870649	0.0209022547751924	0.497341901585093	-3.35707234657147
a_HQ_transcript/8010	-2.92837153050379	1.79244398508142	-2.42715546924278	0.0209148833318684	0.497438763631279	-3.32452932911961
a_HQ_transcript/14345	1.50750668198761	2.63239712649101	2.42672423585142	0.0209358508975682	0.497733799989303	-3.33147921505408

a_HQ_transcript/102799	1.75089898459109	4.43159502631022	2.4263566391316	0.0209537394301769	0.497810464747449	-3.49307586743456
a_HQ_transcript/148772	-3.53486294535051	0.390639013071874	-2.42630602305702	0.0209562036752857	0.497810464747449	-3.30624157300198
a_HQ_transcript/117242	2.27739848266109	0.348847349015765	2.42530969811943	0.0210047636009619	0.498332316760784	-3.30779354840948
a_HQ_transcript/43658	-1.9449282281535	0.148678986464039	-2.42512535066319	0.0210137597577175	0.498332316760784	-3.2578609664593
a_HQ_transcript/7734	0.820761136708802	2.86739932899008	2.42510511585599	0.0210147474303022	0.498332316760784	-3.43546557709335
a_HQ_transcript/118830	4.10776092417063	1.41189778316441	2.42458569402302	0.021040115198772	0.498332316760784	-3.33485546739265
a_HQ_transcript/118823	2.25814041055714	0.610227097132458	2.42415656617631	0.0210610942007425	0.498332316760784	-3.26851234095637
a_HQ_transcript/137349	2.54280309628496	0.617782306169766	2.4241381985588	0.0210619925736423	0.498332316760784	-3.27750055970946
a_HQ_transcript/38643	2.3141821919713	1.02809322457161	2.42408798723506	0.0210644486225436	0.498332316760784	-3.23065280950395
a_HQ_transcript/42545	-1.15617038747886	2.50872496744071	-2.42371639566498	0.021082632859381	0.498332316760784	-3.34096304499591
a_HQ_transcript/42704	4.32425384125039	0.274010226876503	2.42363680543809	0.021086529553611	0.498332316760784	-3.34226160064393
a_HQ_transcript/99637	-6.02675086391998	1.70600606662858	-2.42342804668778	0.0210967533878224	0.498332316760784	-3.44909385076585
a_HQ_transcript/38305	-2.82052929871059	-0.490128921082445	-2.42341268384597	0.0210975059522144	0.498332316760784	-3.35611994666468
a_HQ_transcript/512	-2.36811024014806	0.839333299274555	-2.42306125147703	0.0211147279066909	0.498332316760784	-3.26926793924215
a_HQ_transcript/37688	3.26753865061915	0.0447425000942337	2.42299699202917	0.021117878328312	0.498332316760784	-3.29571000297334
a_HQ_transcript/116047	2.12744067849944	-0.281124291169653	2.42294935190895	0.0211202142375124	0.498332316760784	-3.29510484010395
a_HQ_transcript/143525	3.52882215159192	0.162189321416026	2.42293372535221	0.0211209804963081	0.498332316760784	-3.34791350464838
a_HQ_transcript/116859	1.81899497401853	-0.733247681973748	2.42292747768645	0.0211212868619214	0.498332316760784	-3.31085626647251
a_HQ_transcript/152336	4.73864041368537	7.31814874649568	2.42287392187116	0.0211239132345612	0.498332316760784	-3.85011721118299
a_HQ_transcript/32585	0.775584112080012	4.06648019079743	2.42242419487844	0.0211459795553285	0.498650506933928	-3.57189593739051
a_HQ_transcript/76914	5.79758068164193	0.909604598363383	2.4210828580862	0.0212119184619859	0.500002593819269	-3.40845592643221
a_HQ_transcript/30343	-0.775381869662479	3.65693494152745	-2.42004354578697	0.0212631388980583	0.501006784704633	-3.49446927441324
a_HQ_transcript/141524	2.66561791415412	-0.0905367146458994	2.41926008050483	0.0213018248969644	0.501473200538036	-3.3110936664645
a_HQ_transcript/51696	2.73877238019648	0.102808367905956	2.41880783997427	0.0213241848421108	0.501473200538036	-3.31704222024522
a_HQ_transcript/129666	2.18010538261035	3.89479423927264	2.41875783250864	0.0213266586535826	0.501473200538036	-3.51786215947882
a_HQ_transcript/10100	-2.19944067316619	0.595896378299769	-2.41864731849852	0.0213321265812841	0.501473200538036	-3.27353091005293
a_HQ_transcript/56786	0.865679859112166	3.34977586117881	2.41842896948684	0.0213429336418143	0.501473200538036	-3.50105239289273
a_HQ_transcript/126367	-1.64214349958844	2.10086922030142	-2.41837399721695	0.0213456552490476	0.501473200538036	-3.24454876625461
a_HQ_transcript/148015	0.775029391677464	4.37109701151011	2.41828795621156	0.0213499156646642	0.501473200538036	-3.60205444388939
a_HQ_transcript/89062	1.83283860858157	-0.239868813523857	2.41800034546761	0.0213641626528325	0.501473200538036	-3.3161423134779
a_HQ_transcript/138068	-7.67241663210635	3.37321503787847	-2.4178905927432	0.021369601609453	0.501473200538036	-3.44318479742436
a_HQ_transcript/97960	-2.38574587450037	0.0442629828539688	-2.41777306219579	0.0213754274062161	0.501473200538036	-3.25265697933689
a_HQ_transcript/62204	2.94159097646571	-0.338545804352328	2.41772456961188	0.0213778315258267	0.501473200538036	-3.32363057534146
a_HQ_transcript/144816	1.60955502715147	3.42273883946457	2.41739657722552	0.0213940988921904	0.501652351918211	-3.45811563579157
a_HQ_transcript/147933	0.747404925645002	3.54152299508388	2.41720620524062	0.0214035459012151	0.501671498445901	-3.51378494925665
a_HQ_transcript/46436	2.45516864262864	0.884090563725615	2.41672602993733	0.0214273909781708	0.502015019462868	-3.266032705542
a_HQ_transcript/71756	-1.07292615522104	3.2943179665892	-2.41656335443135	0.021435474785075	0.502015019462868	-3.41234823589612
a_HQ_transcript/68592	-2.22478619456514	1.04457347273767	-2.41627158278449	0.0214499807054808	0.502082146993658	-3.2983693461266
a_HQ_transcript/13606	2.72121466561255	1.70261903832328	2.41615828094147	0.0214556161081105	0.502082146993658	-3.28327757817874
a_HQ_transcript/67212	2.88951663267572	0.860316038858445	2.41583776437012	0.0214715652535804	0.502253177086568	-3.29889934296126
a_HQ_transcript/89761	-1.20854536507266	2.29832053005019	-2.41509101439489	0.0215087659931413	0.502646983238671	-3.34299250912586
a_HQ_transcript/113897	5.31163657879108	2.84858307314586	2.41483504700483	0.0215215309893389	0.502646983238671	-3.49111221928002
a_HQ_transcript/41381	-1.11756553143667	3.59988013394482	-2.41472326781223	0.0215271075393627	0.502646983238671	-3.54495441492188
a_HQ_transcript/98743	-1.53598104564879	-0.731960347920069	-2.41465588002503	0.0215304700832588	0.502646983238671	-3.30993193828821
a_HQ_transcript/74532	-2.11850866312879	0.650336493168585	-2.41458696055084	0.0215339095507298	0.502646983238671	-3.31619988595116
a_HQ_transcript/41882	-0.963759081252386	3.06433536201208	-2.41445925453176	0.0215402841186267	0.502646983238671	-3.42753432638337
a_HQ_transcript/116788	0.92886602813729	3.54989833770263	2.41408476744508	0.0215589869100802	0.502881537363219	-3.54592795150059
a_HQ_transcript/145088	0.529986354408236	10.3232301432275	2.41354448912755	0.0215859957788622	0.503309571854675	-4.20598970774245
a_HQ_transcript/23508	2.59063740406856	0.744584782363273	2.41329616799201	0.0215984198496475	0.503370482284116	-3.28826717461472
a_HQ_transcript/4573	4.05472107505942	0.533831837752114	2.41314617503372	0.0216059274927551	0.503370482284116	-3.27549599809835

a_HQ_transcript/114454	0.903587207780214	-0.658599811028516	2.41272424964334	0.0216270589998724	0.503524036266784	-3.36442447732281
a_HQ_transcript/33448	-1.9245763617018	-0.181428223707839	-2.41266868888373	0.0216298430800674	0.503524036266784	-3.29241961289176
a_HQ_transcript/74486	-1.14696056573081	2.58014426557693	-2.41226782560135	0.0216499395120893	0.50357791857121	-3.40257744446573
a_HQ_transcript/120084	2.38858601806282	-0.386484946175061	2.41217467127092	0.0216546120413353	0.50357791857121	-3.32659669076802
a_HQ_transcript/93015	-3.49066545088073	0.407810327794263	-2.4121041989196	0.0216581474750211	0.50357791857121	-3.355306892535
a_HQ_transcript/107861	3.03674346787143	0.824051128825894	2.41163754657644	0.0216815716030284	0.503920989260629	-3.33124103250174
a_HQ_transcript/143547	0.821329194552664	2.89506644096248	2.41087775274407	0.0217197596528766	0.504475650181858	-3.4253310492797
a_HQ_transcript/103317	1.52277070288489	1.38359125806235	2.41059962156674	0.021733754129028	0.504475650181858	-3.2927712513923
a_HQ_transcript/130798	-2.6551196791771	1.65442505411626	-2.41010960110223	0.0217584300238078	0.504475650181858	-3.28949320978749
a_HQ_transcript/37150	-0.721891093375149	2.68135257390769	-2.4099927121802	0.0217643199504237	0.504475650181858	-3.39277603289577
a_HQ_transcript/33258	-1.15029089219169	1.71294739941266	-2.40990371091599	0.021768805616703	0.504475650181858	-3.30696055328719
a_HQ_transcript/150779	3.33759526522562	-0.0205883520072698	2.40987304313825	0.0217703514685647	0.504475650181858	-3.37024181095301
a_HQ_transcript/9674	-2.06284192083978	0.357044812286746	-2.40972105091067	0.0217780143227944	0.504475650181858	-3.33060008077065
a_HQ_transcript/116130	1.69611914572567	-0.279166916431934	2.40949321031589	0.0217895057550415	0.504475650181858	-3.30586210349241
a_HQ_transcript/140661	1.99574331971214	-0.545939859641167	2.40936795451193	0.0217958255433815	0.504475650181858	-3.32840860965158
a_HQ_transcript/91726	2.77978755743919	0.433331449143595	2.4093227952661	0.0217981044648235	0.504475650181858	-3.34427224060063
a_HQ_transcript/14075	-3.93696132085326	3.69317882595967	-2.40926736440171	0.0218009020309804	0.504475650181858	-3.5015927687791
a_HQ_transcript/39898	2.38633910545935	-0.506119650198758	2.4090785375238	0.0218104344763148	0.504495398025955	-3.33764800802724
a_HQ_transcript/26937	3.77348485956299	1.78671506184555	2.40817043512446	0.0218563307464494	0.505355924275898	-3.37793149487274
a_HQ_transcript/49406	-1.80545349689587	3.18101221601767	-2.40792328696062	0.0218688370369883	0.505444039477558	-3.54000716199887
a_HQ_transcript/75296	-3.631921801363	0.0397218295723804	-2.40750368328087	0.0218900849102497	0.505734044381159	-3.34294785803328
a_HQ_transcript/44427	-3.5469177265263	1.28264148315902	-2.40695360323505	0.0219179683062395	0.505985491263103	-3.36680434592658
a_HQ_transcript/11397	-1.86457852134132	-0.0157148262469473	-2.40694552878156	0.0219183778385716	0.505985491263103	-3.27929111643214
a_HQ_transcript/84511	-3.35475337305837	0.773019297406557	-2.40626989630331	0.0219526702724481	0.506257445083052	-3.32899698365477
a_HQ_transcript/93410	3.76287345144623	0.482663543511584	2.40622081133046	0.0219551635334324	0.506257445083052	-3.3433499689328
a_HQ_transcript/151286	0.576030525700017	7.24235793022897	2.40619870596647	0.0219562864549679	0.506257445083052	-3.86065635546158
a_HQ_transcript/47836	-2.9354684455407	1.17158956514481	-2.40592635278048	0.0219701259057405	0.506375685427788	-3.36755968400983
a_HQ_transcript/152462	2.21785289438702	-0.722099618302625	2.40535483759705	0.0219991928940919	0.506844662920243	-3.34322067780025
a_HQ_transcript/42818	1.48627933284856	1.73827308508658	2.40465756702137	0.0220347031388162	0.507314565668855	-3.30500287277292
a_HQ_transcript/17144	1.76641404961017	-0.792080479788042	2.40448894816824	0.0220432983039338	0.507314565668855	-3.34648711090645
a_HQ_transcript/144857	-2.86273296321547	1.09727689406321	-2.40425724383937	0.0220551141605795	0.507314565668855	-3.31455340934209
a_HQ_transcript/71580	2.16301730485904	-0.271509718541725	2.40419739174488	0.0220581672791202	0.507314565668855	-3.35688222338961
a_HQ_transcript/125525	1.83608612936639	-0.564342374991027	2.40409823212153	0.0220632263626973	0.507314565668855	-3.3122262645717
a_HQ_transcript/148088	-3.88496268683138	-0.603292306337257	-2.40386720791488	0.0220750172196744	0.507384974671899	-3.36593590734766
a_HQ_transcript/32223	-2.06853694046631	0.369332467438836	-2.4036600462705	0.0220855950713804	0.507427458620237	-3.31282597024602
a_HQ_transcript/116465	-2.62944470671612	1.59842057240823	-2.40338202820559	0.0220997981615697	0.507474274647903	-3.33412963087648
a_HQ_transcript/65622	1.81046367309247	-0.514952560608747	2.40315975197734	0.0221111595507666	0.507474274647903	-3.33149129832805
a_HQ_transcript/111050	3.66807209738987	-0.279865535998191	2.4031076472699	0.0221138235907504	0.507474274647903	-3.35944714922016
a_HQ_transcript/20107	2.12475228626437	0.0926983853603617	2.40263791578148	0.022137853485776	0.50766372349991	-3.29101920908804
a_HQ_transcript/22153	-0.995518107243112	2.27970749555781	-2.40258885906681	0.0221403644333295	0.50766372349991	-3.40837367733063
a_HQ_transcript/53538	-5.17045443277361	2.69721343950039	-2.40243425044636	0.0221482797067811	0.50766372349991	-3.5468581740525
a_HQ_transcript/147199	-3.88542297478812	1.09597842105624	-2.40218401904936	0.0221610958970321	0.507757265393253	-3.43148963133298
a_HQ_transcript/128261	-1.94621565934341	0.743346926241197	-2.40124909291015	0.0222090400666958	0.508048771955148	-3.31182818904653
a_HQ_transcript/127769	-3.7036670921574	0.392705525347372	-2.40113191533894	0.0222150557280786	0.508048771955148	-3.39380894858464
a_HQ_transcript/141759	-1.96646086089618	-0.65482526323979	-2.40106961166513	0.0222182548773129	0.508048771955148	-3.34510210811532
a_HQ_transcript/48275	-2.47412837717024	1.70498513586386	-2.40106894360412	0.0222182891829684	0.508048771955148	-3.39507362278777
a_HQ_transcript/256	-1.05670742798034	5.55555938049792	-2.40102933613906	0.0222203231552697	0.508048771955148	-3.78721710367745
a_HQ_transcript/40762	3.56159299682643	0.853754833452016	2.40091375180509	0.0222262597557449	0.508048771955148	-3.28310417467076
a_HQ_transcript/9688	-1.39886911833319	2.26985346994553	-2.40018996977303	0.022263467205622	0.508699222377514	-3.32179509086083
a_HQ_transcript/72846	2.71015329807157	1.22379794886517	2.39950356338277	0.0222988056281327	0.509306472908486	-3.4068438237198

a_HQ_transcript/105337	-6.02264740632603	0.925512458141765	-2.39928899494831	0.022309862769463	0.509320786851812	-3.36015353183019
a_HQ_transcript/8220	2.21709005805328	-0.374931143675493	2.39890932862159	0.0223294399570546	0.509320786851812	-3.35561952922391
a_HQ_transcript/8775	3.14948006880822	0.0300667473396573	2.39878010141385	0.0223361070175389	0.509320786851812	-3.3832019700929
a_HQ_transcript/118535	7.06042369647527	2.10542040088084	2.39865688624073	0.0223424655927977	0.509320786851812	-3.45275528453623
a_HQ_transcript/119997	-2.08370102256144	2.12075722026377	-2.3986418315345	0.0223432426106544	0.509320786851812	-3.31724168864564
a_HQ_transcript/137788	1.55105863273607	2.0047957913755	2.39801997875781	0.0223753597544043	0.509553337637179	-3.44567215918386
a_HQ_transcript/12957	-2.12913448471669	1.04031797890753	-2.39793387211529	0.0223798102558927	0.509553337637179	-3.3552559784156
a_HQ_transcript/49330	2.03684106940098	2.25109000438016	2.39785834746504	0.02238371448012	0.509553337637179	-3.31241580144003
a_HQ_transcript/3122	-2.18846482072243	-0.18468746666035	-2.39776562594604	0.0223885085384901	0.509553337637179	-3.31392446550424
a_HQ_transcript/112581	-1.13735512048191	3.74428436817936	-2.39758241720924	0.0223979838791079	0.509569474334554	-3.55144057979885
a_HQ_transcript/61654	2.848831951673	1.69604617639462	2.39733168902549	0.0224109571576564	0.509665147754401	-3.34488512084283
a_HQ_transcript/42794	-3.74653490453435	0.0890347243303459	-2.39653611856944	0.0224521671449508	0.510099366466395	-3.35822971879509
a_HQ_transcript/113182	2.06305180339239	0.425437861782542	2.39651014193543	0.0224535138773677	0.510099366466395	-3.3173627256154
a_HQ_transcript/107433	-2.89027928214397	1.00155186148242	-2.39645492205845	0.0224563769403301	0.510099366466395	-3.40603524803536
a_HQ_transcript/116341	-2.91474477932975	0.038573486853004	-2.39610677198491	0.0224744356157665	0.510310153700498	-3.31186753565275
a_HQ_transcript/130496	2.81305992383331	2.44917656032309	2.39522782780008	0.0225200855555345	0.510929302453222	-3.34176719606556
a_HQ_transcript/4607	-1.00230449477196	2.82360024909609	-2.39511114071698	0.0225261522972371	0.510929302453222	-3.40944152318181
a_HQ_transcript/37595	2.55604311398298	0.448063870033069	2.39494886823772	0.0225345915646078	0.510929302453222	-3.35270309553672
a_HQ_transcript/95314	-1.51232570520413	2.38947472843058	-2.39484347910699	0.0225400740526647	0.510929302453222	-3.38706518718459
a_HQ_transcript/126303	-3.08238664053585	2.29628648972237	-2.3947362784531	0.0225456520229926	0.510929302453222	-3.40772206525589
a_HQ_transcript/76812	-6.59207156508084	0.955327797743086	-2.39452474019488	0.0225566626730421	0.51097961335097	-3.44853567298912
a_HQ_transcript/27765	1.24742158283466	3.11698326532718	2.39430391483411	0.0225681619365797	0.511004652299856	-3.4981873170839
a_HQ_transcript/13607	2.94542498264791	5.86712876239904	2.39416593052338	0.0225753500396716	0.511004652299856	-3.85532309440448
a_HQ_transcript/114046	-0.774304629555986	3.42632024005564	-2.39382644301142	0.0225930440376251	0.511206097243702	-3.51490535764975
a_HQ_transcript/31601	-2.90778302898587	1.65850027848808	-2.39341674068496	0.0226144144037471	0.511490537144362	-3.47680058816684
a_HQ_transcript/128522	-2.37233817374386	0.752962498452215	-2.39290902320643	0.0226409228097755	0.511835009743961	-3.31234458631535
a_HQ_transcript/137285	-3.95905646582565	1.24451618585278	-2.39278782015502	0.0226472551104712	0.511835009743961	-3.49499584526309
a_HQ_transcript/10013	1.01269486398055	3.28872846000577	2.39239442673535	0.0226678191993246	0.512100658538026	-3.54938662601668
a_HQ_transcript/150592	-2.01440266257906	-0.328097966938443	-2.39180723107223	0.0226985456103317	0.512556780071639	-3.38673634972841
a_HQ_transcript/130672	3.092955764152	2.19474229117596	2.39167166913101	0.0227056445892594	0.512556780071639	-3.41131253880875
a_HQ_transcript/115596	3.23051581325855	3.2055355149892	2.39134176551623	0.0227229291018139	0.512733132231982	-3.43590242630421
a_HQ_transcript/146630	3.42513168770572	1.56981332132044	2.39091081759264	0.0227455256072315	0.512733132231982	-3.33989294470494
a_HQ_transcript/144865	3.3928941985265	-0.0070576176323995	2.39090029011822	0.022746077864777	0.512733132231982	-3.36174215888511
a_HQ_transcript/31015	2.39779375044315	-0.181891570275085	2.3908495496182	0.0227487398160315	0.512733132231982	-3.33832433633209
a_HQ_transcript/35449	-1.20444793855718	2.63559291005558	-2.39032729951414	0.0227761545951171	0.513152059808127	-3.39914381224584
a_HQ_transcript/83378	-1.8219370767817	-0.434129657416057	-2.3899761325959	0.0227946054869533	0.513327454313635	-3.38041599728252
a_HQ_transcript/97973	-1.42865509703643	2.30759607708561	-2.3898430500311	0.0228016014147709	0.513327454313635	-3.33945676263436
a_HQ_transcript/142325	-3.8010808131148	0.0149026860478106	-2.38956059723644	0.0228164559527974	0.513463008758889	-3.3553659698264
a_HQ_transcript/149301	-0.891319776033814	3.60081656290013	-2.3890666074459	0.0228424566258008	0.513654480882383	-3.56440368446331
a_HQ_transcript/15392	2.18696737901828	1.66363492369513	2.38906317262933	0.0228426375082741	0.513654480882383	-3.31637478734851
a_HQ_transcript/74143	4.74686442910262	0.770786801771874	2.3882585773576	0.0228850445614168	0.514278013205446	-3.43846805771795
a_HQ_transcript/18372	1.6916654383486	2.31965987652431	2.38817594005919	0.0228894040977982	0.514278013205446	-3.42011455189156
a_HQ_transcript/1228	2.22625568331661	1.25633073122538	2.38803372338868	0.0228969085152714	0.514278013205446	-3.34189598876097
a_HQ_transcript/120375	0.777593578487239	2.85845303986524	2.38698065131008	0.0229525461055841	0.515328543849129	-3.46542329243147
a_HQ_transcript/122526	-2.56460538301143	1.63239861575301	-2.3865730872451	0.0229741121381237	0.515421731012995	-3.33728248751254
a_HQ_transcript/137308	2.50495719829542	0.117501098364903	2.38655714646374	0.0229749560100245	0.515421731012995	-3.37243255601185
a_HQ_transcript/134256	3.24052906892848	0.770678732192022	2.38639960077445	0.0229832976669709	0.515421731012995	-3.39236826173067
a_HQ_transcript/72998	-2.89876204484115	0.791016934109521	-2.38610009424858	0.0229991633908085	0.51557862305473	-3.42256866649194
a_HQ_transcript/101422	2.36403199397888	-0.651528010392673	2.3852530185966	0.0230440893199002	0.516386593672767	-3.38195344283433
a_HQ_transcript/85891	3.41322922379591	0.949741927691241	2.38505745984156	0.0230544723946565	0.516420181640305	-3.48092084547614

a_HQ_transcript/148644	1.37746891940089	5.52656936876611	2.38471873811357	0.0230724666794502	0.516511840972417	-3.79458348306491
a_HQ_transcript/94849	-2.01897872582448	0.849246790775635	-2.38464593654751	0.0230763358623274	0.516511840972417	-3.37112178737871
a_HQ_transcript/131131	1.92771414895702	-0.796124597747565	2.38440918060574	0.0230889228002857	0.516594651476139	-3.37904090331774
a_HQ_transcript/59588	2.78688657711512	0.437764445838993	2.38411822292027	0.0231043998729724	0.516742037636067	-3.41431000329948
a_HQ_transcript/68535	3.0297396031141	2.70130927689376	2.38361706242682	0.023131080480607	0.517139786991048	-3.40812798651989
a_HQ_transcript/87127	-1.73413333244024	1.37822058817186	-2.38297480916054	0.0231653134554023	0.517674725917347	-3.34767977542999
a_HQ_transcript/62805	2.23557061446053	-0.145738722039779	2.38251613206339	0.0231897897345884	0.517674725917347	-3.37488015690017
a_HQ_transcript/87714	0.839177597499475	2.53187299946865	2.38235398450589	0.0231984479957563	0.517674725917347	-3.38581739061048
a_HQ_transcript/52652	-3.58814963946706	0.66734951007453	-2.38216529578774	0.0232085271832051	0.517674725917347	-3.41975834162341
a_HQ_transcript/47433	-2.52563003118486	-0.0674644903193485	-2.38205459074646	0.0232144425670985	0.517674725917347	-3.42205413564211
a_HQ_transcript/18849	0.738279269737889	3.20469053185957	2.38195430753776	0.0232198022557288	0.517674725917347	-3.55452919571651
a_HQ_transcript/100472	2.33131569323558	0.238085460580431	2.38194964704464	0.0232200513655559	0.517674725917347	-3.3662256690327
a_HQ_transcript/72896	1.94425460305443	0.169494191890684	2.38183362150875	0.0232262538740786	0.517674725917347	-3.32064693928146
a_HQ_transcript/123935	2.42971957098053	-0.155568609390557	2.38148866225481	0.0232447036414364	0.517887364227449	-3.34928212731428
a_HQ_transcript/139508	2.57722573911483	-0.318436012594818	2.38116812808399	0.0232618589903813	0.518071011261641	-3.3949303627809
a_HQ_transcript/152597	0.742256165531473	10.2430094008101	2.38086720376317	0.0232779752635349	0.518231384955478	-4.24560058347086
a_HQ_transcript/131211	4.45806531636602	1.94193335660029	2.38025123581765	0.0233109956108417	0.518767822690279	-3.49101339727189
a_HQ_transcript/101456	-1.40561450380805	2.04905134544456	-2.37958115292448	0.0233469652183506	0.519278855254198	-3.33763407649227
a_HQ_transcript/123108	-2.56341736771336	2.56640463159248	-2.3792190443139	0.0233664239419952	0.519278855254198	-3.52042155815213
a_HQ_transcript/61046	-3.55260377188466	0.565457973524256	-2.37919058392737	0.0233679539492263	0.519278855254198	-3.41944517068228
a_HQ_transcript/123324	-3.11557111647694	0.567712083983244	-2.37915824647229	0.0233696924949247	0.519278855254198	-3.45704508903843
a_HQ_transcript/126187	-3.28862477445177	1.87774973545226	-2.37839226758246	0.0234109078714243	0.519907749453632	-3.31910052339041
a_HQ_transcript/137878	0.802349071900622	3.86059786354117	2.37803703420135	0.0234300444713797	0.519907749453632	-3.65286427136058
a_HQ_transcript/126452	0.51967782977232	4.94354633820793	2.37790261727863	0.0234372892817649	0.519907749453632	-3.74834273554987
a_HQ_transcript/78834	-1.73709777599118	-0.791036598708112	-2.37785002140679	0.0234401246504905	0.519907749453632	-3.3855258477838
a_HQ_transcript/51344	3.19894560000167	1.47794272141203	2.37740567528136	0.0234640911526537	0.519907749453632	-3.33681232449222
a_HQ_transcript/97289	2.69494681556529	0.282214341117641	2.37739794938816	0.0234645080574956	0.519907749453632	-3.33505627082457
a_HQ_transcript/119943	0.608248577831341	4.62578340821037	2.37693309249493	0.023489605057476	0.519907749453632	-3.72345419410631
a_HQ_transcript/138727	4.06608270320481	-0.108818797650087	2.37663826806643	0.0235055348610037	0.519907749453632	-3.39242740236628
a_HQ_transcript/97806	-4.22900136677396	4.49721464793364	-2.3765879802971	0.0235082529631635	0.519907749453632	-3.62254664828614
a_HQ_transcript/31341	3.12889563563957	3.32297193605578	2.3765603701907	0.0235097454372456	0.519907749453632	-3.53371179098953
a_HQ_transcript/91914	2.61714371752066	-0.134621830226495	2.37600023493028	0.0235400423295068	0.519907749453632	-3.33373655721596
a_HQ_transcript/91582	1.86265835037171	-0.650809156212179	2.37591763066502	0.023544513270688	0.519907749453632	-3.34981455333817
a_HQ_transcript/79327	3.55214653080196	1.1465958084715	2.37583162317335	0.0235491692301222	0.519907749453632	-3.3578426380194
a_HQ_transcript/55689	2.48173684360695	2.2905682087708	2.37578977856974	0.0235514347626932	0.519907749453632	-3.49530791234063
a_HQ_transcript/112776	-3.48521834472077	0.482381170696598	-2.37572780523098	0.0235547904601523	0.519907749453632	-3.42191302661738
a_HQ_transcript/22706	-1.54612542527772	2.30270183313332	-2.37565103942106	0.0235589477338768	0.519907749453632	-3.41062780550753
a_HQ_transcript/1463	1.58615100398636	2.0240144585244	2.37546621351588	0.0235689597647916	0.519907749453632	-3.37675508993496
a_HQ_transcript/141834	2.31220770630492	0.1883931805465	2.37542219820702	0.0235713446462629	0.519907749453632	-3.43446808248717
a_HQ_transcript/35045	2.33629044785951	-0.609095542343625	2.3753590846019	0.0235747647130121	0.519907749453632	-3.39263814083171
a_HQ_transcript/149836	-2.01394632871474	-0.639111026629591	-2.37532007029471	0.0235768790868389	0.519907749453632	-3.39015575541983
a_HQ_transcript/140955	-3.32435574202829	3.76986699946979	-2.37458301543955	0.0236168560122431	0.519995260346498	-3.64697920079758
a_HQ_transcript/44589	4.16320326893733	0.574504234728703	2.37443997719187	0.0236246213523074	0.519995260346498	-3.49481927120906
a_HQ_transcript/98256	-0.91199076447672	2.77813543029702	-2.37437347055721	0.0236282326908517	0.519995260346498	-3.43246639958281
a_HQ_transcript/48201	-2.05115046266022	0.392705067265923	-2.3742206504459	0.023636532786664	0.519995260346498	-3.36213970995261
a_HQ_transcript/20520	2.85061762342183	1.05341636414443	2.37416878916911	0.0236393501218647	0.519995260346498	-3.44400506697178
a_HQ_transcript/55583	2.40477864020584	3.08529937260257	2.37395215450448	0.02365112197676	0.519995260346498	-3.56808267629938
a_HQ_transcript/84085	-2.10650739576813	0.587973695144415	-2.37394296583304	0.023651621403697	0.519995260346498	-3.36351982004548
a_HQ_transcript/142920	4.35238003246097	5.68631686794343	2.37392840032658	0.0236524130944835	0.519995260346498	-3.83770407230384
a_HQ_transcript/65491	0.669065873571543	2.99876275799862	2.37314631000171	0.0236949580231347	0.520361825127043	-3.53835861681223

a_HQ_transcript/23761	4.1340406412872	0.504080899224988	2.37314532228504	0.0236950117978205	0.520361825127043	-3.36837950816631
a_HQ_transcript/97781	-2.55172725255839	0.549464575845843	-2.373128226031	0.0236959425941247	0.520361825127043	-3.44906216757994
a_HQ_transcript/879	2.00391970612054	1.98688224120463	2.37251520789096	0.0237293399730841	0.520628692684496	-3.40921159125059
a_HQ_transcript/115546	-3.73480625619858	2.63101754458815	-2.37238587786375	0.0237363913598974	0.520628692684496	-3.453556567439
a_HQ_transcript/24418	-1.873843213631	1.22062897296816	-2.37232920298725	0.0237394820109141	0.520628692684496	-3.33390629593267
a_HQ_transcript/143544	-0.843758192437588	4.46978445891338	-2.37224780694755	0.0237439214200833	0.520628692684496	-3.70770328752839
a_HQ_transcript/1142	-1.93469728085132	1.41360363286384	-2.37103368917779	0.0238102299358569	0.521813763867327	-3.35666404552773
a_HQ_transcript/45641	3.74509882012127	0.17589692603652	2.37073241552148	0.0238267098690437	0.521813763867327	-3.38511711585587
a_HQ_transcript/15294	1.11982286595943	-0.730322750566291	2.37066834927509	0.0238302156832607	0.521813763867327	-3.43410130561842
a_HQ_transcript/124502	2.17031617384237	1.5295967135313	2.37049141934006	0.0238399000225516	0.521813763867327	-3.3600116326375
a_HQ_transcript/37399	1.1424181173626	2.28077484784582	2.37025730308027	0.0238527199704442	0.521813763867327	-3.40530280673006
a_HQ_transcript/150281	2.97811435194337	2.92932942102104	2.3701405231728	0.0238591170474971	0.521813763867327	-3.50970031528271
a_HQ_transcript/122052	-3.50923970809678	0.969033302563539	-2.37010967904999	0.0238608069150728	0.521813763867327	-3.46032102032684
a_HQ_transcript/50902	1.19382108659131	2.24155793409575	2.3693711449868	0.0239013016827686	0.522365318113262	-3.3913443946977
a_HQ_transcript/139329	1.42985484552944	-0.626063921415617	2.3691650522519	0.0239126131348225	0.522365318113262	-3.43529580428389
a_HQ_transcript/52124	-3.49331911751239	0.973929647416815	-2.3690740418065	0.0239176098121511	0.522365318113262	-3.46914806975742
a_HQ_transcript/135436	0.743210104365069	3.86608375591895	2.36897121443217	0.0239232564051901	0.522365318113262	-3.6506814067532
a_HQ_transcript/511	-0.608063588367863	5.02870677803857	-2.36849950935428	0.0239491748038604	0.522365318113262	-3.77399308427774
a_HQ_transcript/148571	-5.58035596909469	2.08441787229556	-2.36830164403046	0.0239600543331898	0.522365318113262	-3.51866251339991
a_HQ_transcript/46862	1.83478418910243	-0.23288950779908	2.36829124888498	0.0239606260292069	0.522365318113262	-3.36278748025835
a_HQ_transcript/136657	-6.900321576671	2.43523495549708	-2.36805791866768	0.0239734616180904	0.522365318113262	-3.50243461671889
a_HQ_transcript/736	1.86533669180767	3.89145009281112	2.36804066747323	0.0239744108608446	0.522365318113262	-3.49558674322773
a_HQ_transcript/139677	-3.49974364920325	0.848593050013697	-2.36801374522453	0.0239758923191265	0.522365318113262	-3.39610103346278
a_HQ_transcript/58211	2.55548542590978	1.47602209877969	2.36767801273816	0.0239943737439291	0.522459137022658	-3.45282759353242
a_HQ_transcript/31536	-2.2922510776179	2.63984088472601	-2.36760899485183	0.0239981746464784	0.522459137022658	-3.52582777624832
a_HQ_transcript/26395	1.66457048946608	1.39073466467913	2.36706529405848	0.0240281360449864	0.522915571704594	-3.35837160016031
a_HQ_transcript/132873	2.41816160149855	-0.408077741107008	2.36682971193981	0.0240411286640241	0.52300251758323	-3.41637579627515
a_HQ_transcript/23780	2.21089050075094	-0.0189414434385591	2.36607016373333	0.0240830620139693	0.523547364053478	-3.43437497597257
a_HQ_transcript/141720	-4.53709732523978	2.68776564764141	-2.3658579751092	0.0240947884337819	0.523547364053478	-3.59056648132069
a_HQ_transcript/118673	-4.60550719047944	1.44287623938378	-2.36583507658126	0.0240960542105976	0.523547364053478	-3.42630480460429
a_HQ_transcript/122489	2.32339758900321	-0.534990891461593	2.36572389162087	0.0241022011114627	0.523547364053478	-3.43930858442836
a_HQ_transcript/134081	-0.98427297057009	2.62357465329206	-2.36521862975403	0.0241301526197064	0.523895082183592	-3.46083369546765
a_HQ_transcript/149757	2.34735204413698	2.52063158846627	2.36510876452431	0.0241362343464021	0.523895082183592	-3.46769120317839
a_HQ_transcript/481	4.93860052131401	3.48176911618515	2.36489121058076	0.0241482814186865	0.523935750781137	-3.69278888855281
a_HQ_transcript/131848	0.414888062829556	10.5618012800022	2.36468191886245	0.0241598761163841	0.523935750781137	-4.33115191375704
a_HQ_transcript/99477	3.06450117344038	2.60570011206745	2.36454931944008	0.0241672246969457	0.523935750781137	-3.42884270342095
a_HQ_transcript/64288	2.4285545702763	-0.0705688421860635	2.36442417634906	0.0241741619115574	0.523935750781137	-3.42629884389516
a_HQ_transcript/68781	1.25109323605364	1.71194727611718	2.36404618771515	0.0241951263994785	0.524194672884414	-3.35543897939335
a_HQ_transcript/134700	3.5959245905154	0.399478396271799	2.36265812240525	0.024272254582922	0.525624174418148	-3.44306819246664
a_HQ_transcript/120766	2.58987520039259	2.05740429299484	2.36241354916848	0.0242858674352741	0.525624174418148	-3.34631519039136
a_HQ_transcript/101593	-1.93084627502771	-0.613385548341171	-2.36237102277174	0.0242882351446316	0.525624174418148	-3.40542169844687
a_HQ_transcript/146460	2.70759450379142	-0.39439287521266	2.36199591828282	0.0243091286156655	0.525655273899276	-3.41502371459421
a_HQ_transcript/50025	3.16219041347817	1.53601017689799	2.36190601677946	0.0243141385861962	0.525655273899276	-3.4327357057626
a_HQ_transcript/149952	-0.89252546549657	3.80327171643728	-2.36172995407246	0.0243239528027506	0.525655273899276	-3.65770772343609
a_HQ_transcript/140157	2.18244775265461	-0.311155037888674	2.3616960259767	0.0243258444603127	0.525655273899276	-3.40460574087251
a_HQ_transcript/120620	-2.71997038548077	1.89642879765382	-2.3614400131655	0.0243401227304344	0.525691102952509	-3.46501044519541
a_HQ_transcript/98454	-2.88015767454201	1.45501064297756	-2.36129262443148	0.0243483462974066	0.525691102952509	-3.44082613901962
a_HQ_transcript/82616	-0.974157494319597	2.69306941290779	-2.36117996978541	0.0243546335716197	0.525691102952509	-3.49719719147383
a_HQ_transcript/55390	-3.32557120082767	0.32768332643504	-2.36080059032053	0.0243758176293563	0.525941975766088	-3.51988783649147
a_HQ_transcript/135457	4.49132080317417	1.54974309331816	2.36064783151493	0.0243843522001378	0.525941975766088	-3.51015148859702

a_HQ_transcript/78671	-1.92031756159432	1.09383516995214	-2.36020788129349	0.0244089471757947	0.525975029696705	-3.38638126460892
a_HQ_transcript/6912	-3.13321460734969	1.10171246585553	-2.36005994631488	0.0244172223840638	0.525975029696705	-3.42076454525002
a_HQ_transcript/12026	1.61833184208325	-0.518580047400236	2.35981888506986	0.0244307123525014	0.525975029696705	-3.38087831557201
a_HQ_transcript/65467	1.84881040108646	-0.379584536222084	2.35973615172747	0.0244353437297823	0.525975029696705	-3.44827666551932
a_HQ_transcript/137235	2.77716947142401	3.2732124072776	2.35949183095619	0.0244490253491626	0.525975029696705	-3.58579986175702
a_HQ_transcript/145087	-2.85345482325512	3.02843369082459	-2.35945678783839	0.0244509882838551	0.525975029696705	-3.52049672778481
a_HQ_transcript/74916	1.88042438597182	-0.654186382361202	2.35903352428858	0.024474708612085	0.525975029696705	-3.43080502510699
a_HQ_transcript/151513	-3.44020866818218	-0.374873167589601	-2.35894269733066	0.0244798014097694	0.525975029696705	-3.44290437080077
a_HQ_transcript/116740	4.84231678534602	1.95931419633396	2.35888511252854	0.0244830307697608	0.525975029696705	-3.47029141765635
a_HQ_transcript/14243	2.81432767594538	1.52199871516486	2.35877088896189	0.0244894375787323	0.525975029696705	-3.39898831114294
a_HQ_transcript/71772	-3.19113658185279	1.11412859734153	-2.358308144182	0.0245154085244615	0.525975029696705	-3.54733281313009
a_HQ_transcript/17193	-3.94854307265715	2.64561871221809	-2.35829559447465	0.0245161132079934	0.525975029696705	-3.53059637592313
a_HQ_transcript/16606	2.26904029269969	-0.206838608764943	2.35759933941212	0.0245552376791728	0.525975029696705	-3.41357528827403
a_HQ_transcript/73100	-5.20401103773019	5.35856207687871	-2.35753690727476	0.0245587486748569	0.525975029696705	-3.66165405772869
a_HQ_transcript/137845	-1.93242987450691	0.258434653120023	-2.35700116903512	0.0245888956942514	0.525975029696705	-3.50219179435749
a_HQ_transcript/129646	-2.9995793128672	-0.0345923069607002	-2.35692487373594	0.0245931917048301	0.525975029696705	-3.42947303760174
a_HQ_transcript/123590	3.59929554090066	2.96099167325413	2.35691085928659	0.0245939808997853	0.525975029696705	-3.6006587495242
a_HQ_transcript/115000	-3.42813350530884	-0.313020116912041	-2.3568543225675	0.0245971648821985	0.525975029696705	-3.3968695126533
a_HQ_transcript/43920	-1.2978073125771	2.0627101959258	-2.35678887834217	0.0246008509758288	0.525975029696705	-3.392289444675
a_HQ_transcript/135316	-1.44930624082705	3.66464374902575	-2.35665927996546	0.0246081519782325	0.525975029696705	-3.64500955057621
a_HQ_transcript/125201	3.31925886020374	0.774191601919058	2.35658469398189	0.0246123547147604	0.525975029696705	-3.50659124573355
a_HQ_transcript/57876	2.09523880643624	-0.211803234875671	2.35647104019876	0.0246187600765854	0.525975029696705	-3.42517239769699
a_HQ_transcript/80044	1.6765984399496	-0.767928620826765	2.35606112445768	0.024641874885162	0.525975029696705	-3.43666614580547
a_HQ_transcript/107407	2.32277737644942	0.395154585876855	2.35586308162295	0.0246530494020994	0.525975029696705	-3.42688012648637
a_HQ_transcript/73535	-2.1275505212874	-0.51211409579031	-2.35583130466112	0.0246548428366694	0.525975029696705	-3.43034304391852
a_HQ_transcript/148780	1.91304774429792	3.99122721998074	2.3556809687729	0.0246633291269014	0.525975029696705	-3.69506033110847
a_HQ_transcript/149114	1.72989760464887	-0.813280778903796	2.35556286572383	0.0246699977666534	0.525975029696705	-3.40309476841491
a_HQ_transcript/106864	-1.22508231186157	5.19370735365934	-2.35552871397453	0.02467192643561	0.525975029696705	-3.82141440171389
a_HQ_transcript/16948	-2.91886993263667	-0.465030687500786	-2.35540459191897	0.0246789371939431	0.525975029696705	-3.43769223938992
a_HQ_transcript/97634	-2.0423087249656	0.811690148392656	-2.35537044597752	0.0246808661681649	0.525975029696705	-3.4072087514863
a_HQ_transcript/149924	3.20554837896539	0.37789347551465	2.35531643087571	0.0246839178710297	0.525975029696705	-3.44139827830807
a_HQ_transcript/83012	3.07896290158251	-0.0166935523157701	2.3553009346893	0.0246847934255422	0.525975029696705	-3.4073910388785
a_HQ_transcript/364	2.3930156680062	3.09627074170538	2.35511199063683	0.0246954712721401	0.525975029696705	-3.52709398784093
a_HQ_transcript/61204	2.93671968209977	2.33069852023104	2.35501692313575	0.0247008454304693	0.525975029696705	-3.47299446896188
a_HQ_transcript/101524	2.20677684319479	1.08727135579143	2.35498616367651	0.0247025844872007	0.525975029696705	-3.49558610203684
a_HQ_transcript/105363	-3.38862257193037	-0.290573530380092	-2.3546958177642	0.0247190053284836	0.526131944977698	-3.39309610608353
a_HQ_transcript/54472	5.84147919145025	2.16954416354801	2.35424575959507	0.0247444784285103	0.526164429319392	-3.62297356915975
a_HQ_transcript/122186	0.742787825407483	4.42850256041152	2.35420560055141	0.0247467525688387	0.526164429319392	-3.76213213746323
a_HQ_transcript/6794	-1.12041043049665	2.98231987503905	-2.35418910045479	0.0247476869969587	0.526164429319392	-3.57327590556004
a_HQ_transcript/63088	-3.14345709775546	0.605224769116128	-2.35375981507578	0.0247720093873977	0.526488980501152	-3.40699457584245
a_HQ_transcript/111613	1.11654575354044	2.08443532129624	2.35327107893864	0.0247997264977711	0.526742244839555	-3.45144015882305
a_HQ_transcript/95118	-0.93869766591833	2.64050995461486	-2.35323014268808	0.0248020493415542	0.526742244839555	-3.47259654913883
a_HQ_transcript/94626	-2.20323671352351	0.94642413602658	-2.35302093778154	0.0248139233243838	0.526801948502477	-3.46361028031179
a_HQ_transcript/122549	1.91142300564246	-0.615811062495567	2.35263305140098	0.0248359524807352	0.527044940788018	-3.45869899995749
a_HQ_transcript/112916	3.44302348687245	0.0331121650348788	2.35249596677345	0.0248437421384402	0.527044940788018	-3.46512938038893
a_HQ_transcript/140390	-3.58428316959778	1.89831955154939	-2.35223552785475	0.0248585473432502	0.527044940788018	-3.5439134337849
a_HQ_transcript/6340	-2.26862042047965	0.780157505547146	-2.35178961049432	0.0248839150268031	0.527044940788018	-3.41743161067738
a_HQ_transcript/82375	2.22793277857476	-0.400727692312885	2.35150279379863	0.0249002440637816	0.527044940788018	-3.44204799637159
a_HQ_transcript/95018	1.89208458462609	1.17138041066297	2.3514329177463	0.0249042237148456	0.527044940788018	-3.47003901046244
a_HQ_transcript/17013	1.98304674456554	3.57316203403736	2.35137007347768	0.0249078033768742	0.527044940788018	-3.59487129916615

a_HQ_transcript/124206	-1.92911179666175	2.42662492258851	-2.35127011922291	0.0249134978150419	0.527044940788018	-3.52956521144922
a_HQ_transcript/22215	3.37879116903033	2.970770110382	2.35123915783451	0.024915261938292	0.527044940788018	-3.54771067029478
a_HQ_transcript/98145	-2.50596057933325	0.425438706531371	-2.35120948300161	0.0249169528621781	0.527044940788018	-3.43990602157457
a_HQ_transcript/128240	-3.30776025585721	0.566821741470021	-2.35106643112112	0.0249251056672561	0.527044940788018	-3.48143244844314
a_HQ_transcript/128544	-3.39324505149421	-0.493462618384114	-2.35065863730465	0.0249483598962799	0.527344823291257	-3.45157127481257
a_HQ_transcript/59577	1.61038462770652	1.14555924848026	2.35021859166171	0.0249734753146966	0.527683814283056	-3.36905019730769
a_HQ_transcript/68330	-0.765132404964973	2.43632876978065	-2.34967411179273	0.0250045829738657	0.52814912758171	-3.42378527997249
a_HQ_transcript/14592	-2.32555798860584	1.13766340021048	-2.34951227945842	0.0250138356725485	0.528152648010861	-3.3792635697834
a_HQ_transcript/2019	0.980262376163345	1.98308317154558	2.34922693007793	0.0250301579645169	0.528234068718825	-3.41912961731971
a_HQ_transcript/135646	2.99408542934865	3.5208497629061	2.34912716873856	0.0250358666962628	0.528234068718825	-3.54482752787074
a_HQ_transcript/107580	3.07167601753833	1.13742231412643	2.34893811943408	0.0250466880677094	0.528270640057986	-3.44310555292596
a_HQ_transcript/109407	-1.802171629974	3.42580472426079	-2.34869126129974	0.0250608248524666	0.528377086334486	-3.6089466489437
a_HQ_transcript/134852	2.4197977496251	-0.335373315670098	2.34846063889735	0.0250740383975703	0.528381818575449	-3.4412797746115
a_HQ_transcript/73187	-2.21345117337322	2.45754526727385	-2.34837007066907	0.0250792292432161	0.528381818575449	-3.5323536176377
a_HQ_transcript/76395	2.7410588374748	1.05770067921754	2.34805303402942	0.0250974076127478	0.528412312412788	-3.40915363903343
a_HQ_transcript/84922	-0.757002878770887	5.3443511505529	-2.34802775474082	0.0250988575999812	0.528412312412788	-3.84559768534879
a_HQ_transcript/67360	2.42474759710643	-0.410290791281405	2.34770605417494	0.0251173165551866	0.528609477451082	-3.45435782209684
a_HQ_transcript/72441	-3.10628157068807	-0.376839574521693	-2.3473486836647	0.0251378366421533	0.528849861865757	-3.46650761246767
a_HQ_transcript/10465	-2.74175631414386	2.40285205387993	-2.34638268408504	0.0251933800014002	0.529826625441892	-3.51081951709403
a_HQ_transcript/77954	2.23163428110925	2.71783191208592	2.34620814938315	0.0252034273008464	0.529846228623362	-3.55991992912369
a_HQ_transcript/144172	-2.19669632665656	-0.187699049070833	-2.34571512052398	0.0252318287036059	0.530229379807965	-3.42634602512808
a_HQ_transcript/10272	-1.48966589070337	1.69164530713532	-2.34557516369934	0.0252398963310047	0.530229379807965	-3.40060437830611
a_HQ_transcript/25646	2.98961747036783	1.6796663130408	2.34540762006116	0.0252495572358292	0.530240701952414	-3.46540405331566
a_HQ_transcript/95531	-2.47446607540592	0.868865045056293	-2.34487422083016	0.0252803364167687	0.530695339557216	-3.45036115491775
a_HQ_transcript/143441	-2.4751642437609	1.96023877503393	-2.34435210014318	0.0253104976813414	0.531136682029247	-3.50965126931125
a_HQ_transcript/126738	1.86223032325076	-0.642060690236644	2.34406943035343	0.0253268402140339	0.531199601212594	-3.47850771775643
a_HQ_transcript/72322	2.51310779150043	0.340138019620181	2.34387282218695	0.0253382127333083	0.531199601212594	-3.4410435459415
a_HQ_transcript/101716	-2.50684666372251	-0.321253666345212	-2.34382618045262	0.0253409113364045	0.531199601212594	-3.44754323343317
a_HQ_transcript/56592	1.95944575447628	1.28767985586707	2.3433094702471	0.0253708246317745	0.53140362609075	-3.37975969830265
a_HQ_transcript/1595	-3.80266108532324	2.39918542470554	-2.34304044060445	0.0253864119000757	0.53140362609075	-3.58787380129456
a_HQ_transcript/116568	1.0147183167048	2.80323560124244	2.34297380147026	0.0253902742344136	0.53140362609075	-3.53246367330506
a_HQ_transcript/115162	2.07091704321445	2.67744757396401	2.34276653879016	0.0254022903626	0.53140362609075	-3.50022126710077
a_HQ_transcript/81741	-2.40351901811897	0.320254508648731	-2.34271173442288	0.0254054685259395	0.53140362609075	-3.48005438548457
a_HQ_transcript/93443	3.7016330574103	0.698489912876028	2.34271125907896	0.0254054960932114	0.53140362609075	-3.51473511153274
a_HQ_transcript/89501	-2.62424699356959	2.13504727003536	-2.34236234832953	0.0254257382596486	0.531538423115749	-3.45134667820684
a_HQ_transcript/84752	2.22434907569004	2.51259947408061	2.34208594419952	0.0254417842950738	0.531538423115749	-3.50634466797068
a_HQ_transcript/69417	-1.80467104312918	1.46450386601607	-2.34180777162911	0.0254579422480404	0.531538423115749	-3.48802512896012
a_HQ_transcript/122977	-2.94779928352197	0.40236438219044	-2.3416576713876	0.0254666648400386	0.531538423115749	-3.37358726912377
a_HQ_transcript/140160	-1.5529044374319	3.39591868965944	-2.34159359984139	0.0254703889753315	0.531538423115749	-3.60382319342744
a_HQ_transcript/135828	3.59367758199025	0.608096727556274	2.34156393009181	0.0254721136857145	0.531538423115749	-3.44649971387571
a_HQ_transcript/74950	-3.27603466574244	-0.16131238850675	-2.34149793402657	0.0254759504335342	0.531538423115749	-3.43475324355523
a_HQ_transcript/130474	1.80874306237827	2.710167627462	2.34130250776311	0.0254873148047266	0.531584727294276	-3.59154617299756
a_HQ_transcript/30001	-2.58834697872118	0.695017344427105	-2.34052040311617	0.0255328414702554	0.532269960878928	-3.41995282646821
a_HQ_transcript/99033	1.70957318392579	2.55265903066717	2.34000846331065	0.0255626815635307	0.532269960878928	-3.59041295942677
a_HQ_transcript/129233	2.64790491097217	1.58376361245055	2.33984192087796	0.0255723958384606	0.532269960878928	-3.44450094696678
a_HQ_transcript/15857	2.10241633049783	1.43431828024898	2.33972359702502	0.0255792995956802	0.532269960878928	-3.4256891914423
a_HQ_transcript/44563	1.91626970328555	3.03152948955362	2.33955366658208	0.0255892173565588	0.532269960878928	-3.58280074562946
a_HQ_transcript/28583	2.73247671076618	1.52056580611611	2.33952918613386	0.02559064641163	0.532269960878928	-3.37947584675495
a_HQ_transcript/125365	-3.09911100560438	0.192600535677557	-2.33948728663826	0.0255930924775342	0.532269960878928	-3.49127723833009
a_HQ_transcript/3304	2.22414777576697	0.535168580083523	2.33948161098932	0.0255934238345824	0.532269960878928	-3.46515350442084

a_HQ_transcript/43892	3.68811260528123	1.31016084780004	2.33788963105171	0.0256865206010781	0.534015046315976	-3.58183300786701
a_HQ_transcript/23347	-2.87700085382245	1.43936854723822	-2.33745675932573	0.0257118872714762	0.534182458571439	-3.41023736158731
a_HQ_transcript/130036	3.36150897211968	2.5904844036949	2.33743858535543	0.0257129527780568	0.534182458571439	-3.5222353666722
a_HQ_transcript/3216	1.26350541742435	2.5003767791053	2.33692545530298	0.0257430531470703	0.534485369160155	-3.54616257194828
a_HQ_transcript/96307	1.77611064014605	-0.518455040328095	2.3367977628043	0.0257505485814735	0.534485369160155	-3.48845998740901
a_HQ_transcript/73747	-1.93097164480454	0.501405753711913	-2.33671992868574	0.0257551183425818	0.534485369160155	-3.48333560864644
a_HQ_transcript/105136	1.82313935588851	1.91336297270955	2.33636512166111	0.0257759589077171	0.534618657215324	-3.40177206126995
a_HQ_transcript/27654	-2.38182406502773	-0.257914988069408	-2.33626900983698	0.0257816069255077	0.534618657215324	-3.47102130595533
a_HQ_transcript/127681	-2.61397349535181	2.7830986046218	-2.33605294068003	0.0257943083324432	0.534618657215324	-3.6748989566138
a_HQ_transcript/146945	1.07940783659944	3.90187858829789	2.33598454428873	0.0257983301247073	0.534618657215324	-3.66200842209802
a_HQ_transcript/143849	-2.13699962590589	-0.544543668870126	-2.33569245514302	0.0258155117127904	0.534683874751988	-3.46606990024965
a_HQ_transcript/84268	-3.73997139501502	1.25082141468617	-2.33561832354909	0.0258198740095794	0.534683874751988	-3.53322297749839
a_HQ_transcript/100326	1.84313020590654	1.59462495520607	2.33530219901429	0.0258384839432797	0.534878701800201	-3.42777631639657
a_HQ_transcript/85567	-0.907815164301752	4.21149009577714	-2.33498818943076	0.0258569813846207	0.535071062273133	-3.77138392655395
a_HQ_transcript/106076	-2.1458617543395	1.14293089224874	-2.33457678864018	0.0258812340034241	0.535382338132042	-3.39602300392944
a_HQ_transcript/104778	-3.72050597273082	-0.373914742086823	-2.33426209929634	0.0258997992438379	0.535575784576951	-3.49709472613593
a_HQ_transcript/92871	-4.80388817051086	1.90814533619866	-2.3336640657941	0.0259351137055873	0.536012323877626	-3.57868194679101
a_HQ_transcript/145315	-1.98425570145537	0.947357073228893	-2.33359233774328	0.0259393522410501	0.536012323877626	-3.44541577762313
a_HQ_transcript/114897	0.935982742941309	2.89895045831779	2.3329148083571	0.0259794195556672	0.536596802896387	-3.62801777986065
a_HQ_transcript/136097	2.39952353602427	2.94651326320047	2.33258696936	0.0259988271860202	0.536596802896387	-3.64890340124174
a_HQ_transcript/68301	3.5863385035825	5.07193748300905	2.33249441957753	0.0260043083824387	0.536596802896387	-3.80985861305005
a_HQ_transcript/44292	1.61132113493004	-0.731188538978144	2.33249013366734	0.0260045622378049	0.536596802896387	-3.48106123795074
a_HQ_transcript/132295	1.31881391273845	2.8156257561832	2.33229978747212	0.0260158387437276	0.536638990239673	-3.53102745613604
a_HQ_transcript/115708	1.47599778007242	-0.668408447522179	2.33188564860639	0.0260403884752473	0.536954842599921	-3.47638228100573
a_HQ_transcript/151080	3.08304143675177	3.02590358189079	2.33162540947798	0.026055825904393	0.537082641195233	-3.56635398384569
a_HQ_transcript/139501	1.17195979959207	2.37190496303458	2.33117527115249	0.0260825477272318	0.53714245933594	-3.46834889766827
a_HQ_transcript/49584	-2.76974472452006	-0.246798880002253	-2.33112995008959	0.0260852395206355	0.53714245933594	-3.48362849465316
a_HQ_transcript/146138	1.87470082385482	0.499826115852726	2.33110957176351	0.0260864499502023	0.53714245933594	-3.44472781322749
a_HQ_transcript/87567	3.75789122799019	0.14433760425997	2.33052132846592	0.0261214122640177	0.537339536544298	-3.52261621189084
a_HQ_transcript/18246	0.971320906330253	3.74418287905434	2.3302770281252	0.0261359447347562	0.537339536544298	-3.66340665293652
a_HQ_transcript/64386	1.92640430736631	0.355670735829018	2.33015026934178	0.0261434879990866	0.537339536544298	-3.45959699372079
a_HQ_transcript/69036	0.834838684627716	-0.63110017896714	2.3301090022326	0.0261459441804022	0.537339536544298	-3.56940302611372
a_HQ_transcript/4952	-0.592683741466685	2.99877214593504	-2.32983166168572	0.0261624566553454	0.537339536544298	-3.61294844686448
a_HQ_transcript/106349	1.75534389348911	0.600145398073569	2.32973027089381	0.0261684956772487	0.537339536544298	-3.45798277852188
a_HQ_transcript/88267	1.42041809532361	-0.621211879354121	2.32972707100007	0.0261686862893176	0.537339536544298	-3.52835480727478
a_HQ_transcript/100597	1.1220187765726	4.37862975549881	2.32970546013441	0.0261699736436297	0.537339536544298	-3.63875901894028
a_HQ_transcript/1290	1.98812080254088	1.23509607782075	2.32876919062481	0.0262258020175568	0.538295699038328	-3.46154874269934
a_HQ_transcript/6245	-1.20803603135426	2.72944275671105	-2.32798968255622	0.0262723650636883	0.539061078864128	-3.5005794848734
a_HQ_transcript/137495	-2.03281197996833	-0.646790444213152	-2.32749436345033	0.0263019912224932	0.539478527092831	-3.48363766902503
a_HQ_transcript/15107	-1.73381415013311	2.46842781139002	-2.32719475338925	0.0263199262365811	0.539655969058196	-3.47990478608634
a_HQ_transcript/836	-2.36130300340996	0.933918619680509	-2.32650853813346	0.0263610455641203	0.53979771173289	-3.44686943591731
a_HQ_transcript/64356	2.46511149250982	-0.00913584232241956	2.32647475354103	0.0263630715005144	0.53979771173289	-3.51736753799267
a_HQ_transcript/82858	-2.94905754978689	-0.208674211160044	-2.32644965848692	0.0263645764487073	0.53979771173289	-3.46587380768391
a_HQ_transcript/29104	-2.61336012322532	1.39397004764044	-2.32638543812733	0.0263684280917183	0.53979771173289	-3.44167252719328
a_HQ_transcript/98561	1.44578929314704	-0.805276381262406	2.32630470061355	0.0263732710797104	0.53979771173289	-3.49352804437746
a_HQ_transcript/108285	3.02871538201299	0.409930222141377	2.32581252243201	0.0264028114675874	0.540150122501292	-3.46345283378367
a_HQ_transcript/57651	-2.12119121222924	0.201451152801725	-2.32491421490608	0.0264568047403937	0.540150122501292	-3.47197154716225
a_HQ_transcript/145062	0.916262384045726	4.00204630703483	2.32474242170047	0.0264671418274303	0.540150122501292	-3.75870522013234
a_HQ_transcript/115202	-3.65426128921317	2.08909422480667	-2.32450679232789	0.0264813259816602	0.540150122501292	-3.59092557055575
a_HQ_transcript/62833	-1.39571152463341	-0.819317632208447	-2.32440283793485	0.0264875858962088	0.540150122501292	-3.48587741180229

a_HQ_transcript/69985	2.03964273128536	1.90051554929719	2.32437617194077	0.0264891918817243	0.540150122501292	-3.52341505210961
a_HQ_transcript/131764	-3.14124238461469	0.871579699293071	-2.32433332790685	0.0264917723897749	0.540150122501292	-3.44448604687792
a_HQ_transcript/114967	-2.50726444996575	-0.21856779235816	-2.32403589395769	0.0265096931837382	0.540150122501292	-3.46778388321875
a_HQ_transcript/12532	2.76163936199756	0.539527213086485	2.32403387613467	0.0265098147976872	0.540150122501292	-3.40743220080806
a_HQ_transcript/106133	-2.67429152838643	1.34688590392745	-2.32402151389336	0.0265105598794828	0.540150122501292	-3.4031996834887
a_HQ_transcript/870	1.45927792748906	-0.633744633682827	2.323999675738	0.0265118761281566	0.540150122501292	-3.48947908503438
a_HQ_transcript/57852	2.0700836823828	-0.104532639227977	2.32388057641411	0.0265190556277649	0.540150122501292	-3.49327941246934
a_HQ_transcript/75025	2.76431606160019	4.36187553185684	2.3235117188581	0.026541302106547	0.540150122501292	-3.80081965771512
a_HQ_transcript/51319	-1.07011219978522	3.8086639605869	-2.32326868392066	0.0265559692074532	0.540150122501292	-3.74254595268376
a_HQ_transcript/102351	-2.06451334206231	-0.123914610367516	-2.32326667102601	0.026556090715748	0.540150122501292	-3.43152654159859
a_HQ_transcript/130101	-1.35296464886664	3.05731657102906	-2.32324340986782	0.0265574949109653	0.540150122501292	-3.69258421816408
a_HQ_transcript/25423	4.7705989929716	0.985107635141741	2.3232236593398	0.0265586872343237	0.540150122501292	-3.53338707722938
a_HQ_transcript/11230	0.982696674994209	3.62351455649568	2.32315234299463	0.0265629929469734	0.540150122501292	-3.70763468965286
a_HQ_transcript/151121	3.05775711332963	-0.413858033533393	2.32306261041404	0.0265684114330466	0.540150122501292	-3.5326245061323
a_HQ_transcript/99992	-0.767437964412631	3.31723511097139	-2.32281940659083	0.026583102275856	0.540150122501292	-3.62765412553742
a_HQ_transcript/72238	1.02686497380792	2.76791407159769	2.32270918044373	0.0265897629559872	0.540150122501292	-3.61092051706157
a_HQ_transcript/142440	-4.96425804216183	2.52056140975403	-2.32262381469655	0.0265949224229063	0.540150122501292	-3.60033096324446
a_HQ_transcript/4692	1.96506249849807	0.844226341375092	2.3223420130772	0.0266119608079469	0.540307390095822	-3.42156397953303
a_HQ_transcript/148409	-3.58038064890802	-0.257478587461242	-2.32204877972501	0.0266297008543247	0.540389158168501	-3.55156913822044
a_HQ_transcript/117343	-2.80662925971979	1.29415797285373	-2.32175926902021	0.0266472261707565	0.540389158168501	-3.55811488347052
a_HQ_transcript/20152	2.49125447163231	-0.381202866957938	2.3216810298048	0.0266519641122928	0.540389158168501	-3.49826257440636
a_HQ_transcript/93634	2.73994818477081	3.89570305633306	2.32166104736945	0.0266531743130521	0.540389158168501	-3.77188540847886
a_HQ_transcript/58653	2.44498351540517	0.124787922079055	2.32131377953821	0.0266742139047164	0.540470998879995	-3.46868242322394
a_HQ_transcript/108244	0.659436382902845	3.54543590252942	2.32124332172668	0.0266784844971584	0.540470998879995	-3.71880908915949
a_HQ_transcript/117415	2.24982486418064	-0.282960931155902	2.32096888722898	0.0266951244249906	0.540470998879995	-3.48087847025798
a_HQ_transcript/143369	-1.95283358681155	-0.674983002640402	-2.32058501136742	0.026718415900187	0.540470998879995	-3.48788428376033
a_HQ_transcript/88626	-2.79374052484137	1.88506828276499	-2.32045159771874	0.026726515004901	0.540470998879995	-3.48643859504202
a_HQ_transcript/125715	0.513124913736967	5.38502311219705	2.32043099472447	0.0267277659427659	0.540470998879995	-3.89973017901408
a_HQ_transcript/136299	1.59626351444541	-0.727698171997005	2.32041171185502	0.0267289367754429	0.540470998879995	-3.48318860958932
a_HQ_transcript/16056	-1.95762307585168	0.277272806741332	-2.32026887457495	0.0267376111266481	0.540470998879995	-3.50338977717767
a_HQ_transcript/142710	2.89287379036039	2.02722648705685	2.32019488223674	0.0267421056000665	0.540470998879995	-3.52199970488589
a_HQ_transcript/69654	1.53498350892078	-0.782515920424788	2.32006181184774	0.0267501903347396	0.540470998879995	-3.50170230258109
a_HQ_transcript/97611	-1.69457962771683	-0.756496863771032	-2.31896717937195	0.0268167777082498	0.541487520942553	-3.49016094022897
a_HQ_transcript/102871	2.87203392192698	0.624364834038615	2.31892852356599	0.0268191331680706	0.541487520942553	-3.50742518064458
a_HQ_transcript/3064	-0.764690131747902	2.8399346069405	-2.31818294995231	0.0268645771363769	0.54221671520254	-3.64961107195889
a_HQ_transcript/140401	-2.40727204107154	-0.529928421230428	-2.317612582064	0.0268993889684653	0.542730885789293	-3.50795170480303
a_HQ_transcript/137272	2.98897034653373	2.5686355299407	2.31716884045721	0.0269265004687685	0.542975492248385	-3.48833995234027
a_HQ_transcript/47709	2.28842403672794	1.28818968635243	2.31710841093484	0.0269301944699989	0.542975492248385	-3.49293407900945
a_HQ_transcript/116647	-2.12217247182918	1.76673049441246	-2.31669264607731	0.0269556222050055	0.542996694073849	-3.52902045371998
a_HQ_transcript/133640	2.94119452283032	1.2781560204706	2.31651598263348	0.0269664333159568	0.542996694073849	-3.5986671121558
a_HQ_transcript/87851	4.16551305266789	0.697324529501294	2.31650038677962	0.0269673879092392	0.542996694073849	-3.52766819218528
a_HQ_transcript/10214	1.81025642644041	-0.648794121745595	2.31648039502395	0.0269686116121525	0.542996694073849	-3.5109943050188
a_HQ_transcript/70217	2.28015126059551	-0.0828118176811575	2.3160336209112	0.0269959719116517	0.543359368171914	-3.46848603786651
a_HQ_transcript/76741	-3.98124136838241	0.959376320950637	-2.31548763278799	0.0270294420812395	0.543714235851501	-3.52338781966371
a_HQ_transcript/108206	-2.09599614502577	-0.140659273494319	-2.31544087048382	0.0270323104460989	0.543714235851501	-3.50773851134299
a_HQ_transcript/147131	1.14067518378075	8.94492047340245	2.31479352007179	0.0270720466904578	0.544200655009019	-4.21627890919373
a_HQ_transcript/35879	2.99604311207869	0.265778821898991	2.31467260989543	0.0270794743509558	0.544200655009019	-3.53170297576269
a_HQ_transcript/88242	1.77184746611132	-0.622643401617032	2.31450491006285	0.0270897794037924	0.544200655009019	-3.48262715138568
a_HQ_transcript/82655	3.70977445760434	4.01588937994127	2.31443717636223	0.0270939426024653	0.544200655009019	-3.73630913606419
a_HQ_transcript/120577	1.81769065814992	-0.376502778528321	2.31409087912899	0.0271152365190006	0.544440230872701	-3.46723153283297

a_HQ_transcript/86246	4.8394563449488	1.41997403499951	2.31384261580421	0.0271305116015051	0.544489908010976	-3.48453618856515
a_HQ_transcript/146727	2.83082703798737	0.345087854299635	2.3137462185198	0.027136444802983	0.544489908010976	-3.52496117157685
a_HQ_transcript/67556	-1.87100779631236	0.821027736702904	-2.31325018244104	0.0271669940892684	0.544741122030475	-3.44856599027257
a_HQ_transcript/37990	4.23557270628196	1.96569088313547	2.31310326615003	0.0271760481556934	0.544741122030475	-3.63129014550932
a_HQ_transcript/149109	-1.84273150019202	1.26863325598089	-2.31299821979099	0.0271825235581926	0.544741122030475	-3.50157902332015
a_HQ_transcript/91030	2.48469003635935	1.30900850170719	2.31281240807238	0.0271939810142817	0.544741122030475	-3.49301680525209
a_HQ_transcript/63962	2.85870538076913	0.910318610142244	2.31270201291223	0.0272007902260555	0.544741122030475	-3.46416938300509
a_HQ_transcript/59915	2.56723737009632	-0.443228798388009	2.31250268370105	0.0272130888201806	0.544741122030475	-3.51080387516994
a_HQ_transcript/115719	-1.67066334376381	3.14601813873125	-2.31247876845866	0.0272145647257174	0.544741122030475	-3.58162535008874
a_HQ_transcript/108462	2.5585348185428	3.568161430761	2.31211838630574	0.0272368141056759	0.544997405625332	-3.73018159622551
a_HQ_transcript/149696	2.4957791765984	0.550473433689368	2.31196773454743	0.0272461199550512	0.544997405625332	-3.49839124938905
a_HQ_transcript/116096	2.63634656365295	0.575621281762112	2.31145978517508	0.0272775174512131	0.545437748333029	-3.55960952921898
a_HQ_transcript/147688	1.71027764285445	-0.662731157121188	2.31124633082162	0.0272907212905884	0.545514115261115	-3.53999071253405
a_HQ_transcript/146624	2.29485089318317	1.60617774876464	2.31103355845238	0.0273038886858575	0.545589701454631	-3.52961939973319
a_HQ_transcript/139848	-0.839827747719635	2.76964334431212	-2.31085105474687	0.0273151874772425	0.545627909854691	-3.59669678743578
a_HQ_transcript/29575	-1.09267565573241	1.81464914299607	-2.30965846936919	0.0273891243162283	0.546899668473368	-3.4704756003833
a_HQ_transcript/139771	-2.54063845877185	2.91851987190936	-2.30945224743883	0.0274019277827344	0.546899668473368	-3.64811795421758
a_HQ_transcript/46408	2.73858999549122	2.17063924658173	2.3092919436993	0.0274118841103505	0.546899668473368	-3.53272292568301
a_HQ_transcript/151593	6.7421565947156	0.534712132310577	2.30915847861896	0.0274201760027393	0.546899668473368	-3.55966695188819
a_HQ_transcript/82614	5.59756195528156	1.55278325434616	2.30868535172865	0.0274495885782538	0.546899668473368	-3.56096719442142
a_HQ_transcript/90232	1.85886771499931	-0.765566995938051	2.30844045955873	0.0274648238137672	0.546899668473368	-3.51333300507408
a_HQ_transcript/138427	1.77896621396149	7.72052838693783	2.30831739457948	0.0274724828150525	0.546899668473368	-4.15219427424205
a_HQ_transcript/117883	2.68721877768981	-0.228153724418118	2.30820491996365	0.0274794844064635	0.546899668473368	-3.53301073213418
a_HQ_transcript/110790	-2.50003243438183	1.614006809763	-2.30801854560731	0.027491089832647	0.546899668473368	-3.47100116305359
a_HQ_transcript/19935	-2.21562588494223	1.82044616175746	-2.30793145555512	0.0274965143974345	0.546899668473368	-3.48298389147173
a_HQ_transcript/135708	-3.35150427942195	1.21152241592237	-2.30792711318132	0.0274967848954911	0.546899668473368	-3.61606981352823
a_HQ_transcript/137278	2.79820582193153	1.51477883145937	2.30789888269231	0.0274985435065718	0.546899668473368	-3.58978497614948
a_HQ_transcript/94405	0.784098299079083	5.58206649762996	2.30785680035429	0.027501165203476	0.546899668473368	-3.94527316182211
a_HQ_transcript/147262	-2.43579207938315	3.21193129387615	-2.30697851336647	0.0275559333066214	0.54780139919538	-3.75658991284777
a_HQ_transcript/11680	2.28980957517156	2.72655009599195	2.30639261062564	0.0275925236624347	0.548226536222244	-3.57032478525595
a_HQ_transcript/132312	4.33700053104585	5.25555373187262	2.30633407622923	0.0275961816161968	0.548226536222244	-3.72967344210327
a_HQ_transcript/10019	-1.6258861862345	1.63561743823803	-2.30575132228076	0.027632623168925	0.548762938012735	-3.45571563050987
a_HQ_transcript/60628	-2.49790049158884	0.469241510994596	-2.30535164312331	0.0276576415394295	0.548921798469643	-3.5937877210362
a_HQ_transcript/45287	-2.45020799504135	1.21239579467628	-2.30521420604494	0.027666249290541	0.548921798469643	-3.57447744953169
a_HQ_transcript/138749	1.80844948140504	1.20676072626497	2.30517105315128	0.0276689524758473	0.548921798469643	-3.48389401696545
a_HQ_transcript/106785	-3.97288191419083	0.892372263613771	-2.30472685210074	0.0276967919909614	0.549202218756653	-3.48962817429868
a_HQ_transcript/16404	2.35295602937299	1.63297182935701	2.30464405978302	0.027701983646341	0.549202218756653	-3.53101136770519
a_HQ_transcript/52339	2.38590059596205	1.71317071678482	2.30408407782347	0.0277371214766681	0.549711352606807	-3.46772780367992
a_HQ_transcript/108957	2.97385303730361	1.21080254722372	2.30350507036645	0.0277734953989571	0.550079700693042	-3.54257631094341
a_HQ_transcript/151224	5.499204002342	5.39686450172441	2.30348695791932	0.0277746339377594	0.550079700693042	-3.8681350351454
a_HQ_transcript/10507	-0.713789659863507	3.99475411901705	-2.30300955157579	0.0278046586316788	0.550486783699667	-3.81429991871412
a_HQ_transcript/55608	-5.71114842563974	1.1366025997457	-2.3023459616962	0.0278464412791871	0.5511262984939	-3.54115550011333
a_HQ_transcript/144257	-2.90060775336134	1.02711281006204	-2.30166770168428	0.0278892061455508	0.551428898747079	-3.6518723005529
a_HQ_transcript/110938	-3.56469372839929	-0.304564597669084	-2.30152267957795	0.0278983575953342	0.551428898747079	-3.54925180350299
a_HQ_transcript/29288	-0.588987465342276	3.0015926538614	-2.30142113446472	0.02790476709265	0.551428898747079	-3.68634696154882
a_HQ_transcript/67738	-2.61302083400636	0.822262979691473	-2.30138724784212	0.0279069063017292	0.551428898747079	-3.60085029899571
a_HQ_transcript/78521	3.88647313998916	1.55887786856624	2.3013515044842	0.0279091628838754	0.551428898747079	-3.50458710120853
a_HQ_transcript/131189	-0.95128313902066	2.72201271437976	-2.30106760955324	0.0279270918421703	0.551555704442454	-3.51328582440503
a_HQ_transcript/44569	-2.15823087403071	-0.0857923735133284	-2.30094943388055	0.0279345581110409	0.551555704442454	-3.50291275408679
a_HQ_transcript/2362	-3.20342283391938	1.07819002973255	-2.30061201490753	0.0279558859510685	0.551709432896885	-3.55422031644967

a_HQ_transcript/21826	-2.70012033264383	0.559160968555227	-2.30052597870373	0.0279613265433908	0.551709432896885	-3.54957491514288
a_HQ_transcript/141738	0.851532701436493	3.092996887413	2.29984985614896	0.0280041151334296	0.552366204437054	-3.67295514072823
a_HQ_transcript/140919	-2.25771908482826	-0.419354704436647	-2.29930755101559	0.0280384777198397	0.552670528963158	-3.53811934760752
a_HQ_transcript/149423	3.82523230294127	3.15393228437386	2.29917775474614	0.0280467077593882	0.552670528963158	-3.63230251479664
a_HQ_transcript/124603	1.41852698331153	2.7681740009139	2.29915631548369	0.0280480673761581	0.552670528963158	-3.62068295454912
a_HQ_transcript/149320	1.89741620788569	1.62961320304258	2.29890492538812	0.0280640142527553	0.552797363769623	-3.4812086462589
a_HQ_transcript/152130	1.06803739377146	2.95222633067192	2.29861540637232	0.0280823899588516	0.552971938864542	-3.69589098167892
a_HQ_transcript/128737	2.67891462490517	-0.00706809685225789	2.29834186373484	0.0280997616151492	0.553126631617132	-3.52850273865454
a_HQ_transcript/98558	-3.40609410774725	0.407894864247736	-2.29800187518438	0.0281213665054608	0.553364520050585	-3.54769195993645
a_HQ_transcript/98745	-3.29938505256874	0.864401807082216	-2.2976620064774	0.0281429787490751	0.553602392123938	-3.52627027914213
a_HQ_transcript/110966	2.08199987844051	-0.394331397727207	2.29744664264941	0.0281566814806942	0.553684567357845	-3.54510840569297
a_HQ_transcript/90554	5.62671274386781	1.65125830338042	2.2972480520395	0.0281693223307923	0.553745812798206	-3.61035179041348
a_HQ_transcript/119069	8.49807141534305	3.69576255054094	2.29702741059904	0.0281833727784696	0.553834716993536	-3.76137342342495
a_HQ_transcript/82056	-2.35195402935856	-0.551735509044621	-2.29676965728929	0.0281997945119446	0.553970143761513	-3.53721334758201
a_HQ_transcript/42273	1.59433955821666	1.81693213316696	2.29652840717735	0.0282151726247624	0.554084984571686	-3.49153677070816
a_HQ_transcript/29649	4.787359688877	1.78189578198596	2.29615243013005	0.0282391537694576	0.554368635701125	-3.64225634356616
a_HQ_transcript/141689	3.54774572607493	-0.00251369786488605	2.29588249227836	0.0282563826902493	0.554485822871701	-3.53980931367764
a_HQ_transcript/139330	2.11174018238593	0.30617735255778	2.29576004145771	0.0282642013000422	0.554485822871701	-3.51000250096694
a_HQ_transcript/141941	-2.79872007647778	-0.608265394411931	-2.29536818768732	0.0282892346713545	0.554789687239033	-3.55327904096817
a_HQ_transcript/26948	2.02107273188128	2.18504859095734	2.2952040511037	0.028299726388466	0.554808261554386	-3.54593847396914
a_HQ_transcript/108732	1.64685920603861	-0.753033654863558	2.29493757876188	0.0283167669436561	0.554955168206623	-3.53438548668399
a_HQ_transcript/70790	-2.11868522064645	1.19602594715671	-2.29466707322987	0.0283340748759493	0.555107214152067	-3.49648972048239
a_HQ_transcript/5282	-1.88165132963969	1.6776032126959	-2.29401710507554	0.0283757012032978	0.555730955253606	-3.5441957585315
a_HQ_transcript/101011	2.32079664282495	0.0497654641768518	2.29382156488203	0.0283882350908869	0.555730955253606	-3.52476955941777
a_HQ_transcript/65734	-2.30830118666733	2.38845202717231	-2.29372239594514	0.0283945936055465	0.555730955253606	-3.54691574254858
a_HQ_transcript/135174	-2.53624968258549	0.37110545715957	-2.29339706566771	0.0284154621488185	0.555952199187654	-3.49957556579802
a_HQ_transcript/119315	0.933766292287166	3.74893756098523	2.29286477912205	0.0284496358621673	0.556353705669772	-3.80078147387399
a_HQ_transcript/144730	2.15040463552843	2.23318155628156	2.29272357108883	0.0284587078755876	0.556353705669772	-3.56209223292659
a_HQ_transcript/115734	-1.60086196326696	-0.772152411138274	-2.29258062935703	0.0284678939270481	0.556353705669772	-3.52645838321086
a_HQ_transcript/14918	3.36910406544332	0.263125303040007	2.29241632588395	0.0284784560770738	0.556353705669772	-3.5411035180382
a_HQ_transcript/10835	2.32665724237998	0.747018475757346	2.29233260166116	0.0284838395966357	0.556353705669772	-3.49527456376575
a_HQ_transcript/76167	3.99254823110272	4.27552752409825	2.29185802960118	0.0285143722116509	0.55676299224684	-3.81867805318033
a_HQ_transcript/98719	1.64483736293824	-0.60800649043256	2.29164088134639	0.0285283527448385	0.556810187810507	-3.54733932370207
a_HQ_transcript/1630	1.86348267544242	0.831888825671776	2.29150190554463	0.0285373035866611	0.556810187810507	-3.49906374153356
a_HQ_transcript/120681	2.10494003946583	1.09880265425992	2.29123437708593	0.0285545410848031	0.556810187810507	-3.55494281976707
a_HQ_transcript/100633	-2.97409816706938	-0.481903368511459	-2.29122562044636	0.0285551054545678	0.556810187810507	-3.56937270000693
a_HQ_transcript/137856	-2.09700899296	-0.425850001435116	-2.29087262408406	0.0285778646165917	0.556993516911963	-3.54638870150578
a_HQ_transcript/70088	2.78354473941776	0.562154615525067	2.29046798474343	0.0286039735251573	0.556993516911963	-3.54489970390024
a_HQ_transcript/110978	-1.07642673555105	2.39980565408252	-2.29042584998425	0.0286066934593466	0.556993516911963	-3.56901652936711
a_HQ_transcript/70011	-0.780588140864889	4.32282070113276	-2.29036455212539	0.0286106508493709	0.556993516911963	-3.84159931097405
a_HQ_transcript/123933	0.690393372724188	4.53282516643799	2.29033718024266	0.0286124181375434	0.556993516911963	-3.89136038796049
a_HQ_transcript/131167	-2.57322220909203	0.0938292755168527	-2.29008222167317	0.0286288844735339	0.557075511346897	-3.56221322324927
a_HQ_transcript/28242	-3.45557500126865	-0.110164302806877	-2.28997522478562	0.0286357973421506	0.557075511346897	-3.54460619829643
a_HQ_transcript/15060	2.85538273950409	1.00397018112401	2.28973373962909	0.0286514047740183	0.557192658649761	-3.50470742230862
a_HQ_transcript/138400	0.774699282742276	4.13032987362556	2.28921221119975	0.0286851378409797	0.557366677807594	-3.8418295128448
a_HQ_transcript/61706	-3.03604061422399	0.611555043988983	-2.28915313761882	0.02868896104428	0.557366677807594	-3.52237417640902
a_HQ_transcript/150219	7.05314119764422	1.8054577312085	2.28900853159736	0.0286983217883051	0.557366677807594	-3.62983887379296
a_HQ_transcript/83374	2.61308519703011	1.34410949305648	2.28900257474907	0.028698707450422	0.557366677807594	-3.56620279772582
a_HQ_transcript/37584	-2.04948003733771	-0.389409733075264	-2.28875006202201	0.0287150600871384	0.557467552531272	-3.56868151525579
a_HQ_transcript/63159	2.49856781478952	0.439548527744002	2.28850848970622	0.0287307120829261	0.557467552531272	-3.5477452280797

a_HQ_transcript/33569	2.66041890964328	0.733478769570379	2.28840103919332	0.0287376765047192	0.557467552531272	-3.544407830858
a_HQ_transcript/116814	1.9383592147853	-0.198934454916627	2.28833029162307	0.0287422628498524	0.557467552531272	-3.57777371108623
a_HQ_transcript/71953	-1.82039572305424	3.9583067007821	-2.28796412028723	0.0287660111397739	0.557734147196822	-3.82730081375387
a_HQ_transcript/40229	0.654740463746823	3.40312699867641	2.2878225430116	0.0287751979673009	0.557734147196822	-3.77364477663131
a_HQ_transcript/102893	1.12898351468517	3.04679422291776	2.28755368304383	0.0287926513347332	0.557757369974196	-3.74997934751687
a_HQ_transcript/80473	2.53232362949928	0.921132347442323	2.28738772065505	0.028803429741839	0.557757369974196	-3.56800860287208
a_HQ_transcript/10668	-2.65874999504621	1.50548204884336	-2.28719332629497	0.0288160592816568	0.557757369974196	-3.55436334007382
a_HQ_transcript/137435	-0.714063110236049	2.81706504869959	-2.28705784859165	0.0288248640350019	0.557757369974196	-3.67457715064321
a_HQ_transcript/84868	2.1738958822098	-0.321296885705544	2.28693350140739	0.0288329475439843	0.557757369974196	-3.55638676549455
a_HQ_transcript/55537	2.53946991952256	1.29525695647977	2.28691043728299	0.0288344471112705	0.557757369974196	-3.49161533830578
a_HQ_transcript/131862	1.19614011457885	3.14551297302813	2.28677024704922	0.0288435634142312	0.557757369974196	-3.62546487537707
a_HQ_transcript/95511	-1.09989089901255	3.50933375695765	-2.28626230339019	0.0288766157675628	0.558210815210139	-3.82443552802137
a_HQ_transcript/8670	-2.4151372374254	-0.381637009202384	-2.2860758783818	0.0288887551668847	0.558259827240916	-3.55438279216213
a_HQ_transcript/82819	2.05718781410486	-0.488049700140614	2.28583930790814	0.0289041664954487	0.558365076312354	-3.55614365563413
a_HQ_transcript/17005	2.60122432733694	0.838301869992225	2.28568131481518	0.0289144630423948	0.558365076312354	-3.49489578361072
a_HQ_transcript/145030	1.68926698263765	3.88467405851438	2.28548505921801	0.0289272577903667	0.558365076312354	-3.71693415486711
a_HQ_transcript/101002	1.95304254790247	1.12106448315585	2.28540276040037	0.0289326247222132	0.558365076312354	-3.48795949651288
a_HQ_transcript/9682	-0.649094805594014	3.02222126598611	-2.28518973577504	0.0289465208079854	0.558447846507326	-3.68850513257827
a_HQ_transcript/133298	3.65100501428716	0.733671729253914	2.28405844677728	0.0290204181574739	0.559447806246717	-3.59960528508797
a_HQ_transcript/127923	-2.16071625353248	2.22839328005095	-2.28401809898792	0.0290230568652206	0.559447806246717	-3.64456844133805
a_HQ_transcript/77047	-2.29095559299187	0.842997389260516	-2.28395435901463	0.0290272258402164	0.559447806246717	-3.5687714990711
a_HQ_transcript/51796	3.00316006814577	1.81824585250421	2.28359570424278	0.029050694060351	0.559664175490189	-3.53154409671246
a_HQ_transcript/120219	-0.781700658501947	5.76607998767452	-2.28348855569559	0.0290577085333985	0.559664175490189	-3.99565012777287
a_HQ_transcript/60677	3.19868331357686	3.64163781641624	2.28331876560424	0.0290688269544875	0.559692869562918	-3.71587335317344
a_HQ_transcript/66172	1.4995611681467	-0.711332136089949	2.28312954700599	0.0290812221322118	0.55974611923881	-3.57277234310431
a_HQ_transcript/73733	-1.81393761827629	2.50927750088722	-2.28275593056408	0.0291057106704444	0.56003202577014	-3.5776958658674
a_HQ_transcript/92393	1.48675203967484	2.02760383444868	2.28255259832317	0.0291190458004815	0.56010320790549	-3.56529347340131
a_HQ_transcript/143978	3.23583859835248	2.22115303116636	2.28220535546532	0.0291418317341415	0.560356068488977	-3.64486853354636
a_HQ_transcript/71779	-1.05410526043909	2.15093297355699	-2.28188338311262	0.029162973770019	0.560476631245123	-3.52128184624912
a_HQ_transcript/149009	-4.67219286320929	-0.0305395313654294	-2.28173346712882	0.0291728225844947	0.560476631245123	-3.61969436260575
a_HQ_transcript/63285	-1.79557297184245	1.17385913408245	-2.28153102572812	0.0291861268377785	0.560476631245123	-3.58958793828078
a_HQ_transcript/149708	-3.28952287084625	0.503833768730975	-2.28152276021817	0.0291866701551574	0.560476631245123	-3.62056078316658
a_HQ_transcript/63764	-1.82345884093079	-0.647444736487184	-2.28117650708701	0.0292094386200525	0.560728615622989	-3.56311446649917
a_HQ_transcript/56578	-0.841020398565032	2.50014911268077	-2.2808218918528	0.0292327735223693	0.560991303832381	-3.59590215494482
a_HQ_transcript/136135	3.70229597252845	5.86238143275761	2.28066903958704	0.0292428369021668	0.560999215659787	-4.00065918895693
a_HQ_transcript/137283	1.92221675412076	3.24735206085579	2.28029768191754	0.0292672990806018	0.561071957072402	-3.78541505383022
a_HQ_transcript/40503	3.04417276328018	1.3879418161349	2.28019437797439	0.0292741072215591	0.561071957072402	-3.64825458743297
a_HQ_transcript/106780	-2.77232531623708	0.465065713924073	-2.28017194694594	0.0292755857039739	0.561071957072402	-3.57899154702215
a_HQ_transcript/152882	-1.61907903827801	-0.776886493688434	-2.27968061209426	0.0293079876181844	0.561480009191906	-3.56628170494321
a_HQ_transcript/65515	-1.05386772177371	2.4207010520436	-2.27955622322809	0.0293161957730772	0.561480009191906	-3.56734884181332
a_HQ_transcript/133093	4.22293037713376	0.886250995582271	2.279323513206	0.0293315577360722	0.561569541210678	-3.57332194179379
a_HQ_transcript/100176	-2.53023531780091	-0.238089185790301	-2.27919275265118	0.0293401922766452	0.561569541210678	-3.57254549364134
a_HQ_transcript/116335	2.7101602244355	0.292437849037435	2.27889882710792	0.0293596103112688	0.561709585617515	-3.54203762519133
a_HQ_transcript/117027	-4.76870186830163	1.26300371536889	-2.2787895016836	0.0293668357881164	0.561709585617515	-3.5775156616312
a_HQ_transcript/107834	1.83504828087327	2.51787334587246	2.27860819446026	0.0293788221753376	0.561754005068429	-3.66317327354816
a_HQ_transcript/140339	-1.81166344269896	-0.419677953494129	-2.27824084244164	0.0294031216553444	0.562033757179172	-3.54272370357424
a_HQ_transcript/100941	2.62499145046453	0.799840296824119	2.2777425745027	0.0294361098257527	0.562479353041208	-3.5669496022935
a_HQ_transcript/117698	-1.73792161823111	1.67056794883204	-2.27697304028839	0.0294871228146074	0.562677411511568	-3.49954010303825
a_HQ_transcript/138588	3.24390075573669	2.8801419475661	2.27657812578	0.0295133527848689	0.562677411511568	-3.68670757379097
a_HQ_transcript/126700	-0.673366583489677	2.73458792509964	-2.27651438670119	0.0295175651247878	0.562677411511568	-3.62098039563643

a_HQ_transcript/78200	2.87291922906129	-0.0886258929997212	2.27641896558314	0.0295239020834956	0.562677411511568	-3.54320001402132
a_HQ_transcript/110716	3.80526589453846	1.91769376237758	2.27623130510315	0.0295363682705159	0.562677411511568	-3.57233514499608
a_HQ_transcript/67947	2.48565255868214	1.63804932022967	2.27622421306619	0.0295368394836744	0.562677411511568	-3.58754086113034
a_HQ_transcript/59698	1.61140562974808	-0.358693766018262	2.27621686856048	0.0295373274786352	0.562677411511568	-3.54856781724037
a_HQ_transcript/2118	1.67291675421925	-0.29903459639157	2.27605499362508	0.0295480848646993	0.562677411511568	-3.58499206294622
a_HQ_transcript/64685	2.11626065293698	-0.380890401270036	2.27602469398857	0.0295500988163109	0.562677411511568	-3.55542827030053
a_HQ_transcript/62015	2.47540214029879	0.666057501589139	2.27595266740483	0.0295548867640905	0.562677411511568	-3.56296803103264
a_HQ_transcript/78564	-3.1169192573598	0.893736575997255	-2.27587413999482	0.0295601076479676	0.562677411511568	-3.60195994957903
a_HQ_transcript/128050	-2.31618039933906	0.373111858409186	-2.27583613894394	0.0295626344404819	0.562677411511568	-3.58070835019687
a_HQ_transcript/118512	2.64277283969986	0.161382076313598	2.27531222878183	0.0295974904580652	0.563156440375258	-3.65239784173076
a_HQ_transcript/87813	1.49542674425999	0.364879292045229	2.27484389324265	0.0296286803649651	0.563341706988714	-3.5318153818583
a_HQ_transcript/110888	-0.77643449843541	5.62113501284667	-2.27475717941275	0.0296344585200489	0.563341706988714	-4.01578288534078
a_HQ_transcript/97444	2.04385487008493	-0.520456670939349	2.27472952302867	0.0296363016097489	0.563341706988714	-3.58700178265805
a_HQ_transcript/129038	3.27278525276887	0.60524864758335	2.27432259443411	0.0296634322611287	0.563524561704254	-3.60778468152584
a_HQ_transcript/60971	3.20485595621668	0.419618678396553	2.27429443796514	0.0296653103291876	0.563524561704254	-3.55496297153735
a_HQ_transcript/89498	2.68020253941664	0.393433536919549	2.27366676317497	0.0297072047249204	0.564136032750791	-3.59440575756569
a_HQ_transcript/28365	1.92572626325518	-0.171940647580763	2.27328965761995	0.029732400369026	0.564358998344788	-3.53722800422514
a_HQ_transcript/1094	-0.494762856482649	4.106767729213	-2.27320044422001	0.0297383638165787	0.564358998344788	-3.88248125449162
a_HQ_transcript/14751	1.10207300958254	2.47613363213737	2.27301230806191	0.0297509432586182	0.564413456180469	-3.63149085026068
a_HQ_transcript/135235	-1.91269468782134	-0.155765040308593	-2.27267839542054	0.0297732816144835	0.564541264483837	-3.61073504205586
a_HQ_transcript/64861	-0.687595350798543	3.00528050157135	-2.27249780121866	0.0297853694361445	0.564541264483837	-3.73031248435408
a_HQ_transcript/23064	2.51250123208291	0.103164563671048	2.27247618916057	0.029786816304912	0.564541264483837	-3.5774694572884
a_HQ_transcript/97482	0.97380630553826	4.388064509608	2.27199024176073	0.0298193658646775	0.564619859747541	-3.90351809330198
a_HQ_transcript/10923	2.39042690778746	1.00251794215287	2.27175224082278	0.0298353192298563	0.564619859747541	-3.50584452332796
a_HQ_transcript/46196	-2.09138562419856	0.950784109022629	-2.27170468636271	0.029838507757782	0.564619859747541	-3.58115425317458
a_HQ_transcript/110519	2.30436691481698	-0.425934749693353	2.27168085164416	0.0298401059917168	0.564619859747541	-3.58799319241559
a_HQ_transcript/103982	1.84490268163137	-0.695694469413254	2.2715698191363	0.029847552276721	0.564619859747541	-3.59292476172372
a_HQ_transcript/132401	3.51610466728394	2.69395968803047	2.2715446038344	0.0298492435487922	0.564619859747541	-3.7048503452698
a_HQ_transcript/133267	3.45679055384236	3.6607443612888	2.27135862208974	0.0298617206103458	0.564672119596025	-3.70312265888148
a_HQ_transcript/31347	-1.24012402107125	2.0422680300582	-2.27105117429879	0.0298823568200356	0.564833767805959	-3.52657419934135
a_HQ_transcript/93104	2.34882193647124	0.839208717158304	2.27094177007112	0.0298897032371857	0.564833767805959	-3.55870829879222
a_HQ_transcript/130153	-2.92822350493824	3.97739960015997	-2.27071962117848	0.0299046253776921	0.564932097482772	-3.94910297769344
a_HQ_transcript/1182	-0.672568243257595	4.51213358548113	-2.27022430326035	0.029937920899814	0.565227831431585	-3.9090535941151
a_HQ_transcript/93626	-1.22722652511943	2.53961898064885	-2.27008880301932	0.0299470351008052	0.565227831431585	-3.65090036740001
a_HQ_transcript/71014	-2.67790010442355	0.126622935572411	-2.26991660841513	0.0299586210883447	0.565227831431585	-3.55874383814297
a_HQ_transcript/124272	-4.01107587834565	1.60246377160297	-2.26967003608514	0.0299752185456314	0.565227831431585	-3.68082777509894
a_HQ_transcript/20797	3.02860522922016	0.575865383504627	2.2696538675023	0.0299763071859702	0.565227831431585	-3.51606962747698
a_HQ_transcript/105631	2.38838405431901	-0.406954761589802	2.26954479102794	0.0299836522992141	0.565227831431585	-3.6126942699623
a_HQ_transcript/61780	-4.56502256128617	0.791310459964849	-2.26947508690492	0.0299883469607591	0.565227831431585	-3.64629714205875
a_HQ_transcript/147773	-1.47370271475056	4.9100992700293	-2.26885136052458	0.0300303851585102	0.565800979746098	-3.99174273945473
a_HQ_transcript/147792	2.57878437418601	0.0894647298318924	2.26872138147567	0.0300391522156496	0.565800979746098	-3.58326917299178
a_HQ_transcript/109106	2.80945760989164	3.62931139784078	2.26859088252112	0.0300479566555912	0.565800979746098	-3.71026833166527
a_HQ_transcript/19695	-3.16084528327819	3.27747606886463	-2.26844564113819	0.0300577584589703	0.565802261561861	-3.64869269273885
a_HQ_transcript/144520	-3.16641107824776	0.600595868468274	-2.26797270281473	0.0300896952424573	0.566220072856444	-3.61330272810635
a_HQ_transcript/31803	-1.06659065163057	2.8670693181996	-2.26776095696258	0.0301040039938444	0.566306001344397	-3.68970061129886
a_HQ_transcript/130744	-2.11417861725732	-0.257631463730159	-2.26759158173456	0.0301154539491161	0.566338112958338	-3.59870448719098
a_HQ_transcript/72445	-2.64002917433667	0.0739193969753997	-2.26741615436295	0.030127317163977	0.566363393641851	-3.5657743740574
a_HQ_transcript/92856	1.63466387273349	1.45311957295245	2.26697168458326	0.0301573930891337	0.566363393641851	-3.52093265017749
a_HQ_transcript/69992	2.91368673109246	0.511252564290081	2.26688602329255	0.030163192631166	0.566363393641851	-3.54231414731452
a_HQ_transcript/5931	2.47165697675542	0.738198727297892	2.26681377892641	0.0301680845841318	0.566363393641851	-3.54333798260722

a_HQ_transcript/126004	2.07602974437141	1.55998723997273	2.26680267970886	0.0301688362194448	0.566363393641851	-3.64847283774008
a_HQ_transcript/60223	-1.05688779960252	-0.707275136896085	-2.26670785255439	0.0301752585691717	0.566363393641851	-3.61551341761569
a_HQ_transcript/16374	2.38813666048639	1.94076648298809	2.26650012498747	0.0301893316101898	0.566444631322502	-3.54600381112273
a_HQ_transcript/122252	0.907346990030329	3.27808426836721	2.26611937183428	0.0302151420376754	0.566449090535551	-3.81579906252658
a_HQ_transcript/60730	-1.84398471488733	-0.366920023560177	-2.26608305837482	0.0302176046839876	0.566449090535551	-3.56311301100226
a_HQ_transcript/143308	2.53001349870703	2.57852163282187	2.2660653769002	0.0302188038423951	0.566449090535551	-3.66507665318463
a_HQ_transcript/44884	1.92892939971632	-0.427619121836105	2.26572557894487	0.0302418572676477	0.566496726863477	-3.57523335442755
a_HQ_transcript/89154	-4.08706888631273	2.34600322595342	-2.26562348863917	0.030248786622229	0.566496726863477	-3.58247325683839
a_HQ_transcript/51365	2.89187433735321	0.250680146672831	2.2655970382803	0.0302505821666707	0.566496726863477	-3.57910692483238
a_HQ_transcript/146366	2.84793377201929	2.99073211077185	2.26525051625408	0.03027411416584	0.566754817466006	-3.7631429991067
a_HQ_transcript/99770	2.29543837538298	-0.645917976276991	2.26483598953899	0.0303022859178363	0.567099573674175	-3.60580356598865
a_HQ_transcript/98639	-2.29135021750414	2.3092797440378	-2.26389560646906	0.0303662829073417	0.567815714322795	-3.56976377938056
a_HQ_transcript/76657	-1.68521003394835	4.93767340035128	-2.26388193043403	0.0303672145141088	0.567815714322795	-3.89116764275332
a_HQ_transcript/8893	1.75016976882345	3.4062465547851	2.26371425123536	0.0303786388515412	0.567815714322795	-3.74679731991118
a_HQ_transcript/57348	2.6715602235572	0.946802802182736	2.26369168738461	0.0303801764691297	0.567815714322795	-3.56367772459164
a_HQ_transcript/126890	-2.85551624667305	-0.0214045547713802	-2.26350638115797	0.0303928068410148	0.567815714322795	-3.57842310675241
a_HQ_transcript/103631	1.04096007114858	2.87197656209622	2.26334863227885	0.030403562637751	0.567815714322795	-3.64394264851752
a_HQ_transcript/14379	1.81123450640362	1.88673736123953	2.263269922134	0.0304089306132477	0.567815714322795	-3.58825038359331
a_HQ_transcript/15589	-1.11253661492504	3.94079906224815	-2.26304596092262	0.0304242092678281	0.567863380355961	-3.90178629616712
a_HQ_transcript/5851	2.50640898798667	0.925001744816603	2.26278084339426	0.0304423045274944	0.567863380355961	-3.52834640032157
a_HQ_transcript/14936	1.66149235693448	-0.435547813429978	2.26273028253502	0.0304457565926451	0.567863380355961	-3.60924622428091
a_HQ_transcript/19429	-0.770131014553861	3.50386143914028	-2.26254326241155	0.0304585285322972	0.567863380355961	-3.83098363516666
a_HQ_transcript/3058	-2.23995087840474	0.20044655836019	-2.26237737491978	0.030469861316089	0.567863380355961	-3.55997163508796
a_HQ_transcript/36447	-4.04072666459272	1.48437686292107	-2.26213934960098	0.0304861288932565	0.567863380355961	-3.56971417671151
a_HQ_transcript/141928	2.78046272640355	0.333436338131405	2.26202145954274	0.0304941888487122	0.567863380355961	-3.68963162603184
a_HQ_transcript/128736	-3.08233377206921	0.709569950572079	-2.26195688446174	0.0304986045558123	0.567863380355961	-3.60946001234442
a_HQ_transcript/62960	-6.70281115991138	3.46232838980306	-2.26171257983002	0.030515315544749	0.567863380355961	-3.74324113111869
a_HQ_transcript/49150	-2.65172198641099	0.404751003228548	-2.26171122998501	0.0305154079000515	0.567863380355961	-3.55795504051755
a_HQ_transcript/25627	-1.20902280957469	2.74620709012943	-2.26157481637185	0.0305247425043779	0.567863380355961	-3.62367682280509
a_HQ_transcript/145110	-2.38942126418201	0.753363834766854	-2.26151679525497	0.0305287135909093	0.567863380355961	-3.63437500505022
a_HQ_transcript/83385	-2.68182882925005	-0.164373784926324	-2.26117104771831	0.0305523869021688	0.568121927654917	-3.65028093672937
a_HQ_transcript/114799	0.429669336032283	6.84531366996146	2.26046092002299	0.0306010610388612	0.568752761980249	-4.15981972191346
a_HQ_transcript/96755	-2.20686433646731	0.852001816941737	-2.26033342551389	0.030609807242828	0.568752761980249	-3.55490207359703
a_HQ_transcript/30830	-2.65041513874225	-0.39823426843053	-2.26024805026508	0.0306156652944571	0.568752761980249	-3.6122036906756
a_HQ_transcript/100838	1.69240196151033	0.967848863014784	2.25971199951716	0.0306524696216083	0.568873148293308	-3.50853640967997
a_HQ_transcript/2579	1.88697325703099	-0.307808791692925	2.25951545321928	0.0306659741105815	0.568873148293308	-3.60077836831289
a_HQ_transcript/58238	1.90165119634483	0.708848157024624	2.25950258207671	0.0306668586596822	0.568873148293308	-3.53044349265245
a_HQ_transcript/143237	1.52916901087434	3.21727325441572	2.25948109146165	0.0306683356196117	0.568873148293308	-3.6649583332875
a_HQ_transcript/108246	-2.52289064184557	3.12472756550396	-2.25944118451924	0.0306710784260808	0.568873148293308	-3.81853219023045
a_HQ_transcript/120617	-1.99785346113236	-0.554162249218701	-2.25923451015983	0.030685286693308	0.56895513394214	-3.60399333306011
a_HQ_transcript/26059	-3.16548124021667	0.81431539840608	-2.25892443210618	0.030706614758259	0.568964407108428	-3.56256449984675
a_HQ_transcript/106829	2.70997984500936	6.30538178863086	2.25881353277499	0.0307142459676351	0.568964407108428	-3.78324858251358
a_HQ_transcript/24712	-2.64800095463354	1.64180186385433	-2.25872713584859	0.0307201922971878	0.568964407108428	-3.62141350568445
a_HQ_transcript/149123	2.57316793119002	0.184550134122758	2.25865817469882	0.0307249393392746	0.568964407108428	-3.60443274921607
a_HQ_transcript/133119	0.662931519172313	3.26005346893551	2.25848120946763	0.0307371240164664	0.569008772238585	-3.81998554341386
a_HQ_transcript/133389	2.49788275763127	0.671838200834238	2.25825037707853	0.0307530241605493	0.569121868323594	-3.59334393493432
a_HQ_transcript/123569	2.05198927077627	3.16507356939311	2.25790970017025	0.0307765040894883	0.569265562323873	-3.75678122541945
a_HQ_transcript/150715	2.64486036787002	0.122271206762234	2.25741322012893	0.0308107510237502	0.569265562323873	-3.55910489118319
a_HQ_transcript/40008	-2.325869194441	2.23489249099429	-2.25725424133016	0.030821724531836	0.569265562323873	-3.58669679432721
a_HQ_transcript/41597	1.68505437248482	3.47750638137227	2.25725217674201	0.0308218670630767	0.569265562323873	-3.7154500541644

a_HQ_transcript/39822	2.18761102193883	-0.51362146928483	2.25711846992029	0.0308310989296054	0.569265562323873	-3.6161240248688
a_HQ_transcript/134341	-2.52745362218703	-0.398396126102439	-2.25685740077077	0.0308491317630941	0.569265562323873	-3.61642356274767
a_HQ_transcript/106726	-3.20785683460995	-0.353190226240157	-2.25658767174356	0.0308677727101944	0.569265562323873	-3.62436829941246
a_HQ_transcript/150719	1.58047521966667	-0.778320700228933	2.25642729548729	0.0308788610933759	0.569265562323873	-3.62151212235236
a_HQ_transcript/63606	-1.60235162479284	1.04738524529702	-2.25623862068779	0.030891910612464	0.569265562323873	-3.57106114765693
a_HQ_transcript/69330	1.99713069844987	0.749649098455955	2.2561670560896	0.0308968616076548	0.569265562323873	-3.552981750108
a_HQ_transcript/1900	2.9013811379185	2.00980535294337	2.25596827573262	0.0309106174060854	0.569265562323873	-3.65538073678498
a_HQ_transcript/22173	-1.08385192838896	2.84064134667525	-2.25574069392999	0.030926373046685	0.569265562323873	-3.71965018258532
a_HQ_transcript/75862	-2.02932908513143	-0.546348455402347	-2.25568510800772	0.0309302223931239	0.569265562323873	-3.6161715783996
a_HQ_transcript/118131	1.94235376235007	-0.153275780747526	2.25549662466379	0.0309432781405336	0.569265562323873	-3.57815536582624
a_HQ_transcript/117975	-2.24797695795048	-0.151570975886364	-2.25527607340247	0.0309585614324379	0.569265562323873	-3.59149012660448
a_HQ_transcript/49308	-1.20861374764596	4.62152455392508	-2.25515736292133	0.0309667903848562	0.569265562323873	-3.90390364509809
a_HQ_transcript/117150	1.91880472625254	2.60012403022111	2.25511605361588	0.0309696543863618	0.569265562323873	-3.65223094518621
a_HQ_transcript/114614	-3.45758283377793	1.23739052372041	-2.25506138031229	0.0309734452884732	0.569265562323873	-3.58418204456494
a_HQ_transcript/36843	-3.42204627915496	0.587534564609421	-2.25492661250045	0.0309827915114277	0.569265562323873	-3.67958865663766
a_HQ_transcript/103862	2.81618150140954	2.13949163059977	2.25486324415946	0.0309871870165958	0.569265562323873	-3.60442667294202
a_HQ_transcript/4158	-2.66482562343986	0.610968097992939	-2.25475413922487	0.0309947563240231	0.569265562323873	-3.62862732321091
a_HQ_transcript/118732	2.39803824707115	4.84275217841343	2.25451233109209	0.0310115380197184	0.569265562323873	-4.01587964012547
a_HQ_transcript/57004	-1.87269191367977	0.742478898862105	-2.25426677526813	0.0310285881582485	0.569265562323873	-3.61443212687488
a_HQ_transcript/40799	-2.55125031743775	1.17501999309375	-2.25424140922227	0.0310303499261493	0.569265562323873	-3.63568031362173
a_HQ_transcript/95813	2.50980333473355	-0.0549124652926457	2.25396334007536	0.031049668771422	0.569265562323873	-3.62087990408512
a_HQ_transcript/79313	1.18974779201368	1.18138117865304	2.25392765242895	0.0310521489513918	0.569265562323873	-3.54094751462674
a_HQ_transcript/45225	1.92421522908477	1.34762424010829	2.2539198996785	0.0310526877668556	0.569265562323873	-3.52877790797097
a_HQ_transcript/107863	2.30122774947937	0.17514248957239	2.25377633719712	0.0310626668637571	0.569265562323873	-3.59164311882325
a_HQ_transcript/120494	1.92754354607943	1.66362468972816	2.25361574079748	0.0310738334088087	0.569265562323873	-3.5247350387727
a_HQ_transcript/640	2.54270723920382	-0.0110551532653355	2.25343598050357	0.0310863367277673	0.569265562323873	-3.57193988501667
a_HQ_transcript/75745	-1.56529322790524	-0.714903084580798	-2.25323235428317	0.0311005055101879	0.569265562323873	-3.61420957112532
a_HQ_transcript/63607	-2.0126366119491	1.72796811743961	-2.25317142927248	0.0311047459399402	0.569265562323873	-3.62371805631904
a_HQ_transcript/132052	-4.32603532037712	1.7350679391696	-2.25282534704456	0.0311288433929276	0.569265562323873	-3.73114237152306
a_HQ_transcript/1884	-1.25576856934267	2.50955676204298	-2.25267476263676	0.0311393337077875	0.569265562323873	-3.65143765355133
a_HQ_transcript/79915	-0.594896523311965	4.2918209366785	-2.2521414599019	0.0311765112499826	0.569265562323873	-3.92744288725411
a_HQ_transcript/139467	2.61700447311281	-0.109901774919006	2.25212732036478	0.0311774974859225	0.569265562323873	-3.61997734255703
a_HQ_transcript/75991	2.17496390484308	1.33713104686511	2.25199174616214	0.0311869552394385	0.569265562323873	-3.63175512153517
a_HQ_transcript/16348	-1.34439434433812	2.14375711433573	-2.25192530871808	0.0311915909034868	0.569265562323873	-3.58434379625836
a_HQ_transcript/17131	-1.63695638546167	1.25029413437615	-2.2518165091117	0.0311991837179859	0.569265562323873	-3.60531787305698
a_HQ_transcript/136885	3.02452322048661	1.57259773393659	2.25176921612665	0.0312024846780273	0.569265562323873	-3.6305859455711
a_HQ_transcript/143229	2.97444853097902	0.110124546713616	2.25169372230184	0.0312077546525816	0.569265562323873	-3.63435623998045
a_HQ_transcript/146539	3.76178638498437	0.451222512600605	2.25166319279506	0.0312098860435165	0.569265562323873	-3.65096148331203
a_HQ_transcript/67981	-2.29757918873193	0.222930413943468	-2.25152768221889	0.0312193481739007	0.569265562323873	-3.60604215162898
a_HQ_transcript/64906	-3.11150129352248	0.370419964790798	-2.25149722903922	0.0312214749450194	0.569265562323873	-3.63649827576551
a_HQ_transcript/11633	1.28783706979419	2.01825885990579	2.2514469873853	0.0312249839761874	0.569265562323873	-3.59502331681145
a_HQ_transcript/126931	0.714867188633517	3.70218622937176	2.25132235112718	0.0312336904832952	0.569265562323873	-3.89738791894391
a_HQ_transcript/134213	6.21381908767732	2.42473617933333	2.25113351366338	0.0312468859394045	0.569265562323873	-3.66203634597454
a_HQ_transcript/68630	3.2468595895526	2.15424724975807	2.25107388455718	0.0312510537011428	0.569265562323873	-3.63112572579137
a_HQ_transcript/23292	-1.42705439854855	-0.493739043040551	-2.25094744314202	0.0312598929439014	0.569265562323873	-3.61707367863015
a_HQ_transcript/3221	1.73932574997129	-0.580073888460852	2.2509026494139	0.0312630249138098	0.569265562323873	-3.62473320836547
a_HQ_transcript/141178	-2.21214396640628	0.321459417479086	-2.25085849470175	0.0312661124795234	0.569265562323873	-3.57659391282996
a_HQ_transcript/908	-2.5202684575542	2.60282610885585	-2.25070538123892	0.0312768212235248	0.569265562323873	-3.78910165746428
a_HQ_transcript/94232	2.5098588957493	0.391432427994449	2.25059207372346	0.0312847480622771	0.569265562323873	-3.63636164864147
a_HQ_transcript/150942	2.72948593440528	0.626072038071416	2.25044173456102	0.0312952683682137	0.569265562323873	-3.61104138484405

a_HQ_transcript/72980	-0.880165867070822	3.76242022854571	-2.25036070658791	0.0313009397916918	0.569265562323873	-3.82960734484605
a_HQ_transcript/148517	0.964221546115587	3.33711053801769	2.25023563466924	0.031309695811907	0.569265562323873	-3.76193181570658
a_HQ_transcript/17658	-0.882876895706104	1.85997518492567	-2.25010265989009	0.0313190075060512	0.569265562323873	-3.5922677414164
a_HQ_transcript/49371	-1.71455405804084	-0.555718804696506	-2.24973203673247	0.0313449738907126	0.569540556256169	-3.61718517868339
a_HQ_transcript/139896	1.49071784798811	5.07486905759985	2.24955497092417	0.031357386192729	0.569540556256169	-4.00969053525205
a_HQ_transcript/113934	-3.1533096581542	1.16542862343765	-2.24919557912646	0.0313825931009109	0.569540556256169	-3.71739639076793
a_HQ_transcript/117613	2.64961505649905	0.921076264732279	2.24912573228019	0.0313874941071653	0.569540556256169	-3.61262681893426
a_HQ_transcript/39343	2.51141824140625	1.95521892494558	2.24904560991212	0.0313931169711631	0.569540556256169	-3.57263629646638
a_HQ_transcript/107809	1.69423831353268	1.24488369516838	2.24895423681339	0.0313995304974806	0.569540556256169	-3.61423441864226
a_HQ_transcript/24370	3.05772619266218	2.2593259394145	2.24890875961577	0.0314027230044216	0.569540556256169	-3.67580104925088
a_HQ_transcript/144170	-3.2200294331938	2.82988963876109	-2.24825928814777	0.0314483478110991	0.570190131492067	-3.68012137408236
a_HQ_transcript/48954	1.5709089486113	0.883678470381754	2.24786744453047	0.0314759032506927	0.570511788012555	-3.61118287326216
a_HQ_transcript/109214	3.21619931760736	0.927260206494384	2.24764962765444	0.031491230055723	0.570544588637089	-3.58976557672829
a_HQ_transcript/61821	2.27866550399124	2.45951015948952	2.24751610181788	0.0315006289861462	0.570544588637089	-3.62673275310284
a_HQ_transcript/137533	3.88260430181966	2.1634529547944	2.24742335642766	0.0315071588481464	0.570544588637089	-3.72758697074508
a_HQ_transcript/47724	2.79773588207177	0.360209680415683	2.24691378985107	0.0315430572335628	0.570696159175581	-3.5906712298512
a_HQ_transcript/146721	-0.851545643157875	2.45667397160291	-2.24691371529963	0.0315430624883112	0.570696159175581	-3.65421999653134
a_HQ_transcript/30601	3.3387988386833	1.33025072702739	2.24688647192142	0.0315449827868005	0.570696159175581	-3.68535189908153
a_HQ_transcript/97667	2.04689970718389	-0.492623856517986	2.24644842552963	0.0315758736816983	0.571077282317909	-3.63209797368255
a_HQ_transcript/43353	2.05788914563498	0.23497565279675	2.24629121128969	0.0315869669983717	0.571100223229036	-3.60730872226839
a_HQ_transcript/136447	-0.862912772130921	4.26348169577361	-2.24534543062126	0.0316537767964706	0.572126211266029	-3.96744649903439
a_HQ_transcript/145939	4.58947303053927	6.25459029273808	2.24520937186239	0.0316633983904971	0.572126211266029	-4.02645229033251
a_HQ_transcript/65826	1.67260891222756	2.63943036075383	2.24445551997758	0.03171675568942	0.572759724022952	-3.67429353703136
a_HQ_transcript/63932	1.04933753091269	2.27963002240967	2.24443561041358	0.0317181659721629	0.572759724022952	-3.6492391223536
a_HQ_transcript/13104	4.09882483023231	0.825490435791986	2.24420909996969	0.0317342146731458	0.572871562273484	-3.70101436287047
a_HQ_transcript/31849	2.70490499036489	0.569152847172796	2.24361959585012	0.0317760163309825	0.57327851190681	-3.70478049401592
a_HQ_transcript/2201	-0.640921838503838	4.72007957385377	-2.24352031191087	0.0317830613999152	0.57327851190681	-3.99157202788074
a_HQ_transcript/754	-2.22002105202356	1.07342297739953	-2.24347404834527	0.0317863446854468	0.57327851190681	-3.57899348518783
a_HQ_transcript/107822	-1.27347509386665	3.06412169363838	-2.24322983310426	0.0318036814741736	0.573413274420212	-3.79554024721293
a_HQ_transcript/50772	4.54161397575763	1.52702400326985	2.24306318938504	0.0318155163450853	0.573448785769648	-3.77509878339482
a_HQ_transcript/82048	-2.49977965171719	2.35890418743772	-2.24220499058455	0.0318765273500175	0.57422922041871	-3.67804590474778
a_HQ_transcript/8954	2.32021329388817	0.0731293210133558	2.24213663926459	0.0318813910859359	0.57422922041871	-3.66117355752896
a_HQ_transcript/96526	4.05075020322129	2.28780983576353	2.24175084865014	0.0319088556164137	0.57422922041871	-3.63957221478727
a_HQ_transcript/103379	1.25323982573754	2.99557237085411	2.24173181040637	0.0319102115030374	0.57422922041871	-3.78018079709805
a_HQ_transcript/51786	3.0297830768876	0.960854378057146	2.24169315471125	0.031912964685912	0.57422922041871	-3.61207531527901
a_HQ_transcript/17096	1.66524195967727	1.69479196647305	2.24162123142166	0.0319180878608046	0.57422922041871	-3.57200933852098
a_HQ_transcript/35142	1.96091894828742	0.239176682418321	2.24147993224217	0.0319281549046856	0.574232607765954	-3.6530256724818
a_HQ_transcript/47613	1.83273611918569	1.70026646919492	2.24129289812442	0.0319414847732975	0.57425494978382	-3.5571739083592
a_HQ_transcript/52442	4.09744064505095	0.55315344634513	2.24118530111389	0.031949155443175	0.57425494978382	-3.72391258727498
a_HQ_transcript/64058	2.11303608370738	1.63700327865481	2.24014897217384	0.0320231207228723	0.57512454791357	-3.59781164937683
a_HQ_transcript/112974	2.9198652771636	2.08913164493901	2.24013691927149	0.0320239818700311	0.57512454791357	-3.74595532028736
a_HQ_transcript/140265	2.11211057502954	2.24662357520462	2.24005679612472	0.0320297069793	0.57512454791357	-3.61541392906127
a_HQ_transcript/113249	2.26724764653165	-0.692762175537421	2.23995317201129	0.0320371126848359	0.57512454791357	-3.64264986036407
a_HQ_transcript/50854	1.45851624391635	-0.432330391813001	2.23963984078393	0.0320595148726815	0.575136289189629	-3.64865994088313
a_HQ_transcript/92295	1.42326692017081	-0.779641029846662	2.23962298806673	0.0320607201864412	0.575136289189629	-3.65153196525795
a_HQ_transcript/142605	-2.34779815481584	1.84822459191581	-2.23940423947636	0.0320763688692786	0.575136289189629	-3.6975733171236
a_HQ_transcript/131968	2.68215379922166	0.81351287666267	2.23930174042535	0.0320837037305733	0.575136289189629	-3.71017530174951
a_HQ_transcript/146216	2.62396212415662	-0.574595494167599	2.23925235553083	0.0320872382645535	0.575136289189629	-3.64340915342705
a_HQ_transcript/101683	3.00408835444009	-0.221230874822995	2.23907377985115	0.0321000220465209	0.575188064586982	-3.64748580462114
a_HQ_transcript/52449	1.98362379306033	-0.755772698233894	2.23885347557296	0.032115799365745	0.575269671941844	-3.64583622320948

a_HQ_transcript/89654	1.65555443222735	-0.77534067784514	2.23873384727415	0.0321243695830448	0.575269671941844	-3.65331162888294
a_HQ_transcript/28213	3.18752045456933	2.47278566469272	2.23843690922836	0.0321456512015676	0.575470018896906	-3.68054165822617
a_HQ_transcript/60503	-2.52274751424465	1.08267940927835	-2.23830153761934	0.0321553575106171	0.575470018896906	-3.63359407280928
a_HQ_transcript/143570	2.40082758569111	0.865283214031922	2.23799983806082	0.0321769991909447	0.575680088941592	-3.60229939322223
a_HQ_transcript/97881	2.38230391674117	0.349523275042297	2.23771243508369	0.0321976274857477	0.575871904766629	-3.63930734285121
a_HQ_transcript/59709	-1.9591984650053	-0.644064502100094	-2.23740356008535	0.0322198101485764	0.575924381392291	-3.64530884350421
a_HQ_transcript/9006	1.64014484738845	-0.773408896612649	2.23739566629249	0.0322203772413936	0.575924381392291	-3.64693242288417
a_HQ_transcript/31735	-2.38155627349581	2.21261940476414	-2.23717876292893	0.0322359631558007	0.576025842705313	-3.67906217604264
a_HQ_transcript/4579	-2.47467195593245	0.365936580201981	-2.23685644430788	0.0322591363255427	0.576262776991747	-3.71233341616823
a_HQ_transcript/39943	2.2651786412808	1.18990217611991	2.23652662436617	0.0322828642626545	0.576509472767921	-3.56998866533658
a_HQ_transcript/59498	2.69491266247371	0.404908096482376	2.23638148025905	0.0322933111903311	0.576518916729596	-3.72364691077394
a_HQ_transcript/146078	-2.81719315889543	0.051601007283617	-2.23596722281856	0.0323231445553236	0.576874346549539	-3.67121357237675
a_HQ_transcript/149548	-2.64254875349536	2.86450177748427	-2.23514455242184	0.0323824636441247	0.577755631278601	-3.76976608284259
a_HQ_transcript/49729	2.64188540420974	0.757677857297572	2.2345786476467	0.0324233251387942	0.577986953098367	-3.63563909130513
a_HQ_transcript/125673	2.81145766308235	0.218846956687099	2.23435726748828	0.0324393225975917	0.577986953098367	-3.6560964980055
a_HQ_transcript/13215	1.96436942204327	1.11472342707225	2.23403001030191	0.0324629839416727	0.577986953098367	-3.59003937288837
a_HQ_transcript/92140	2.04740316959994	0.229239363976126	2.23398874331829	0.0324659687244598	0.577986953098367	-3.64542779830642
a_HQ_transcript/21433	1.38050082390833	2.33491413754309	2.23398757860278	0.0324660529702609	0.577986953098367	-3.72152829835407
a_HQ_transcript/82390	-1.07241768591753	3.41432690499784	-2.23393397206814	0.0324699306316769	0.577986953098367	-3.86380781315085
a_HQ_transcript/60083	-2.75892930976832	2.67733864521929	-2.23368203006389	0.0324881605705723	0.577986953098367	-3.84305921156912
a_HQ_transcript/120542	-5.42115804951882	2.69286791552641	-2.23356430618807	0.0324966819411054	0.577986953098367	-3.72963086803629
a_HQ_transcript/140068	-4.49455193339812	1.68592863473488	-2.23355899669215	0.0324970663129138	0.577986953098367	-3.74818686132103
a_HQ_transcript/54243	2.29611653302792	2.00932907655678	2.2334903327403	0.0325020374884714	0.577986953098367	-3.70183616169042
a_HQ_transcript/36061	2.90643768405091	1.77494727206138	2.23341744076037	0.0325073155131844	0.577986953098367	-3.69017169511405
a_HQ_transcript/92313	-2.54500638130875	0.957086425799921	-2.2330241425017	0.0325358070447825	0.577986953098367	-3.70136691303433
a_HQ_transcript/35939	2.10597328803951	0.79000048269135	2.2326378780779	0.0325638108071477	0.577986953098367	-3.57054645517068
a_HQ_transcript/90003	-1.1819562770148	4.08204561847904	-2.23248676556159	0.0325747721813566	0.577986953098367	-3.93420827370663
a_HQ_transcript/78785	-2.32671060313631	0.226644048980057	-2.23245593434717	0.0325770090168606	0.577986953098367	-3.64225474058437
a_HQ_transcript/83218	-1.51779792565763	1.59700461899384	-2.23243185078005	0.0325787563995416	0.577986953098367	-3.61407043532849
a_HQ_transcript/115770	5.86958246315896	3.56297289445278	2.23238487340849	0.032582165083395	0.577986953098367	-3.78746318143157
a_HQ_transcript/122008	2.95480115081923	2.90869564835642	2.23236773255755	0.032583408905223	0.577986953098367	-3.60810968326536
a_HQ_transcript/60319	-1.74121924691699	0.182532622269093	-2.23233323627199	0.0325859122490758	0.577986953098367	-3.66881650808061
a_HQ_transcript/131086	1.9467009882656	-0.268599860730385	2.23221772611619	0.0325942959031181	0.577986953098367	-3.65326894125497
a_HQ_transcript/95209	-2.14720525249452	0.141884868831878	-2.23200601923926	0.0326096664719366	0.578083163367103	-3.60268207501364
a_HQ_transcript/55123	2.64890503765088	1.27449323810477	2.23182390727875	0.0326228935551947	0.5781413282245	-3.66367947636348
a_HQ_transcript/30236	-2.06588954314017	-0.290137157633144	-2.23164907308339	0.0326355965655963	0.57819017286345	-3.63309495295201
a_HQ_transcript/19798	0.971269396683185	2.20675114992614	2.23087776537361	0.0326916907554958	0.578493457099715	-3.67248143335696
a_HQ_transcript/119108	3.84822874979132	1.36132777673259	2.23087129494233	0.0326921616897844	0.578493457099715	-3.75664288194056
a_HQ_transcript/91321	1.65659001247233	-0.171952004420277	2.23058424466461	0.0327130600491463	0.578493457099715	-3.62131602448391
a_HQ_transcript/27259	1.30522459754081	3.7307270658075	2.23055736658869	0.0327150174886955	0.578493457099715	-3.91015311771321
a_HQ_transcript/138401	2.5904645279167	2.34368827051553	2.23053592097071	0.0327165793759353	0.578493457099715	-3.71103181204942
a_HQ_transcript/49720	-0.625023910296218	3.20957392167276	-2.23043295786581	0.0327240791231057	0.578493457099715	-3.81811102743925
a_HQ_transcript/112336	0.974821596155705	2.79600094975759	2.230370781427	0.0327286087492098	0.578493457099715	-3.7422985365867
a_HQ_transcript/128519	-2.82146449594677	1.71797968990767	-2.23018925092524	0.032741836672331	0.578493457099715	-3.69006609723134
a_HQ_transcript/66994	2.17055053974408	0.899317234343275	2.23013136129717	0.0327460560314673	0.578493457099715	-3.58773137503833
a_HQ_transcript/83483	2.44306535013831	-0.159494737036455	2.22987132264134	0.0327650152888313	0.578493457099715	-3.68646040069843
a_HQ_transcript/142104	0.881501359813856	2.59762569237869	2.22970810760177	0.0327769202189936	0.578493457099715	-3.7275997256844
a_HQ_transcript/119675	3.66921343971628	0.719694334194331	2.22969734298755	0.0327777055278158	0.578493457099715	-3.67088056082508
a_HQ_transcript/3283	0.863340106034083	3.60566046126883	2.2295890074926	0.0327856098447475	0.578493457099715	-3.86573243269493
a_HQ_transcript/118502	-1.52210573101978	1.00595336579352	-2.22950083733729	0.0327920441292245	0.578493457099715	-3.61431796970064

a_HQ_transcript/129644	4.90606723361304	-0.37106142076696	2.22920174905458	0.0328138787531485	0.578544311286199	-3.6878274142062
a_HQ_transcript/51408	-1.53355385394579	1.59802283587412	-2.22913956590889	0.0328184200030248	0.578544311286199	-3.62924320398733
a_HQ_transcript/4453	-1.97760539973219	2.5763783349412	-2.22898615337784	0.0328296261645199	0.578544311286199	-3.73190548419834
a_HQ_transcript/37361	-2.54855196634021	1.53725459921451	-2.22891618020248	0.0328347385585805	0.578544311286199	-3.68056265350947
a_HQ_transcript/134926	1.84662202564721	-0.455079669619594	2.22853195572063	0.0328628235555835	0.578863699284533	-3.66137759646971
a_HQ_transcript/59097	2.58976118003847	2.16961115359919	2.22815338942661	0.0328905160090288	0.579175981391345	-3.64078176477664
a_HQ_transcript/78323	-2.7573290756458	-0.0836876662430947	-2.22788286158657	0.0329103181593312	0.579349174429317	-3.70423961228614
a_HQ_transcript/61465	2.41673561135008	1.06329768101138	2.22753157164576	0.0329360478860368	0.579626579327746	-3.61843541505935
a_HQ_transcript/71476	-2.61178371932611	0.748044234904434	-2.22733125008907	0.0329507282156641	0.579709421828125	-3.63910384143394
a_HQ_transcript/147136	6.71494210654387	3.86051698849019	2.22706609621131	0.0329701687181624	0.579781775350462	-3.76399009441844
a_HQ_transcript/93742	-4.05106298343623	0.791699198198789	-2.22700309626813	0.0329747892462948	0.579781775350462	-3.64699680867559
a_HQ_transcript/149094	-3.10571804767429	-0.522376814176943	-2.22586945550424	0.0330580316478599	0.580963025486065	-3.64611354184548
a_HQ_transcript/6436	-1.14055730258763	1.80837974155509	-2.22581600212111	0.0330619613320242	0.580963025486065	-3.63685412601526
a_HQ_transcript/59266	-1.77610570546604	0.645980358579258	-2.22549967901804	0.0330852247360307	0.581035504269439	-3.58509398923568
a_HQ_transcript/22968	2.53850885825886	1.21836381352704	2.22533961388361	0.0330970020179491	0.581035504269439	-3.64863403081175
a_HQ_transcript/126850	-1.9294996144903	-0.404836428906828	-2.22533799230334	0.033097121349884	0.581035504269439	-3.62854479838219
a_HQ_transcript/118487	1.54384742119738	2.34381890182987	2.22516311891728	0.0331099925268215	0.581035504269439	-3.63123021046486
a_HQ_transcript/99985	3.22868369721431	1.92897681117961	2.22508063695736	0.0331160649883817	0.581035504269439	-3.79381357207323
a_HQ_transcript/149372	-2.93618979072091	-0.482527847372243	-2.22420067701187	0.0331809112289939	0.581652246016541	-3.68419902291194
a_HQ_transcript/89590	-1.09258229301382	2.02854350524053	-2.22410516215501	0.0331879567708631	0.581652246016541	-3.63740747348639
a_HQ_transcript/1650	-2.6687123828229	0.842748951674011	-2.22406774977597	0.0331907168163817	0.581652246016541	-3.63223630654355
a_HQ_transcript/143457	-6.32780813397509	3.64758345832929	-2.2240606347182	0.0331912417429959	0.581652246016541	-4.00925666748232
a_HQ_transcript/55671	1.25816742418362	1.16170227179701	2.22360131624669	0.0332251445499287	0.582070886798751	-3.6323627895651
a_HQ_transcript/123928	-1.03723712729566	2.24570608013888	-2.22339252537237	0.0332405658840587	0.582165596176127	-3.68168825536275
a_HQ_transcript/29656	3.39988934078811	1.17916871323564	2.22314855530858	0.0332585936786222	0.582305883539444	-3.68606300499757
a_HQ_transcript/140759	2.35079219662133	0.138266906383264	2.22298621746389	0.0332705942348572	0.58234059069069	-3.63370566309579
a_HQ_transcript/134366	1.38126770138739	3.06918286790976	2.22269282961595	0.0332922922797955	0.582544962564706	-3.78892125050328
a_HQ_transcript/40636	1.65384985220882	2.13902202859538	2.22226042700885	0.033324294514901	0.582755868130625	-3.68020927432704
a_HQ_transcript/119020	3.41546821325108	0.182842749153785	2.22212113318155	0.0333346095520765	0.582755868130625	-3.7141883165202
a_HQ_transcript/109185	-2.10888931963893	2.55324759299188	-2.22187513648361	0.0333528331702958	0.582755868130625	-3.73076227791943
a_HQ_transcript/150258	-4.85199630711463	0.911777518379109	-2.22140675182909	0.0333875561113511	0.582755868130625	-3.71169913761632
a_HQ_transcript/109828	3.33007651044131	1.29200700896145	2.22131852459153	0.0333941003158284	0.582755868130625	-3.69703207033169
a_HQ_transcript/150572	-1.63404542764299	-0.706562099611611	-2.22121220572643	0.0334019879818216	0.582755868130625	-3.65197271492739
a_HQ_transcript/104899	-3.06820735828339	1.21307650221039	-2.22117937083264	0.0334044242992759	0.582755868130625	-3.67068800296512
a_HQ_transcript/113878	3.03079272714173	2.23300532374605	2.22081064297633	0.0334317944752754	0.582755868130625	-3.77682322812017
a_HQ_transcript/118421	2.9516570001937	1.91882324928064	2.22076497220381	0.0334351859524127	0.582755868130625	-3.77591732725439
a_HQ_transcript/23698	-1.21250571322358	3.3776792411478	-2.22054911928829	0.0334512191946906	0.582755868130625	-3.85636613503634
a_HQ_transcript/95695	2.40138773341929	1.32195283554144	2.22051883747904	0.0334534690340111	0.582755868130625	-3.65254203832983
a_HQ_transcript/71884	1.94203221549355	0.318979648048633	2.22034411839483	0.0334664527354355	0.582755868130625	-3.6362674306767
a_HQ_transcript/12520	-2.33082714088119	0.0211726726475276	-2.22028255016364	0.0334710290602484	0.582755868130625	-3.60875809848639
a_HQ_transcript/142953	2.63717814133607	0.227940324699926	2.22015576757502	0.0334804544881928	0.582755868130625	-3.63998134002094
a_HQ_transcript/81288	3.41304992546735	1.72284656117464	2.22012881529132	0.0334824585143477	0.582755868130625	-3.7100036826754
a_HQ_transcript/140558	1.23963717136376	2.96589835647059	2.22011292633424	0.0334836399816272	0.582755868130625	-3.88310468740516
a_HQ_transcript/144182	-3.83904491746184	1.51692774022362	-2.22006910973315	0.0334868982793242	0.582755868130625	-3.78543901348424
a_HQ_transcript/41974	-2.76156830261046	1.22245016617759	-2.21996750345772	0.0334944550348445	0.582755868130625	-3.70438941640737
a_HQ_transcript/47086	-2.01771586211057	-0.29431642124993	-2.21996249070846	0.0334948278871528	0.582755868130625	-3.62912141078647
a_HQ_transcript/35612	4.30553181899112	2.02558795365268	2.21972031537244	0.0335128455082375	0.582788901154291	-3.7600058123375
a_HQ_transcript/120824	-0.639055616211123	3.27523328307016	-2.21955105473432	0.033525443492626	0.582788901154291	-3.86637296736494
a_HQ_transcript/97514	-2.62840173381205	-0.288790228644965	-2.21953277378745	0.0335268043879017	0.582788901154291	-3.68266182673707
a_HQ_transcript/89856	4.06650134456235	2.10716248964099	2.21913722651394	0.0335562623700379	0.583055325283841	-3.81959621778893

a_HQ_transcript/30169	-1.52840122621273	2.16609395742188	-2.21905763889905	0.0335621923754427	0.583055325283841	-3.67569761320836
a_HQ_transcript/148001	8.12783438531613	1.24629024828703	2.21880963824978	0.0335806767073183	0.583184880760606	-3.78993662910146
a_HQ_transcript/52418	1.54885735179671	-0.76366179722619	2.21865435465005	0.0335922551596679	0.583184880760606	-3.64472373397677
a_HQ_transcript/13937	-2.51526690130864	-0.302298607195435	-2.21855388726252	0.0335997482395278	0.583184880760606	-3.68966265859468
a_HQ_transcript/147798	0.535636669171829	4.07554926236654	2.21818005938287	0.0336276422875466	0.583494803250898	-3.95220090622996
a_HQ_transcript/149007	3.18618494850607	0.374989139880222	2.21801824830081	0.0336397226273832	0.583530228852441	-3.65520656924594
a_HQ_transcript/138828	-4.54352386341098	0.765919238029248	-2.21655829467334	0.0337488941767861	0.585249320020352	-3.66194136086622
a_HQ_transcript/104211	-2.41399622439485	2.62924701633141	-2.21618944623061	0.0337765258195847	0.585413601792676	-3.74545472008518
a_HQ_transcript/38991	2.01584636430746	1.68289505496122	2.21604140998988	0.0337876213890268	0.585413601792676	-3.65533789090637
a_HQ_transcript/26746	-2.2149109697399	2.53254763650553	-2.2158868764817	0.0337992074184076	0.585413601792676	-3.75346244217794
a_HQ_transcript/138157	3.3062370299296	1.67811226478688	2.21585052389217	0.0338019334418437	0.585413601792676	-3.71429495539932
a_HQ_transcript/58731	4.32789611195842	1.51852257722783	2.21575999142848	0.0338087231836294	0.585413601792676	-3.85050001508492
a_HQ_transcript/62878	2.30114456417052	1.12579201267168	2.21548636138019	0.0338292522715438	0.585474782062816	-3.62106780764533
a_HQ_transcript/128390	2.83289983021717	3.69458725911195	2.21541786967543	0.0338343926047987	0.585474782062816	-3.81607262287097
a_HQ_transcript/114062	4.01858943975444	0.353302761826207	2.21522450139143	0.0338489087527975	0.585474782062816	-3.75382694076156
a_HQ_transcript/884	-0.938653071471098	3.29950858255971	-2.21516303521192	0.0338535241828365	0.585474782062816	-3.874345981158
a_HQ_transcript/17028	0.916150690523843	2.58256870704884	2.21504195943665	0.0338626172807457	0.585474782062816	-3.80826707027896
a_HQ_transcript/149459	1.00366259748855	5.01041935710776	2.21483918857909	0.0338778507803366	0.585563993505622	-4.07449737470577
a_HQ_transcript/61545	1.90832761423322	1.06265268844535	2.21427771316384	0.0339200645535242	0.586119355638304	-3.6428602447281
a_HQ_transcript/125046	2.74255251420785	-0.0547398797857036	2.21412698250664	0.0339314050432126	0.586129020724908	-3.72947405933303
a_HQ_transcript/69676	-1.05267292817937	2.39265636730816	-2.21400226788895	0.0339407907335542	0.586129020724908	-3.72989867315005
a_HQ_transcript/87832	1.8996870375153	-0.0683549304214584	2.21341447838487	0.0339850574788095	0.586719162794249	-3.75620715855536
a_HQ_transcript/139029	-2.17672897896997	1.34353117773185	-2.2130712661643	0.0340109288490792	0.586746903289428	-3.75026782842576
a_HQ_transcript/139761	-4.24814342805583	2.04554756203656	-2.21303765338885	0.0340134635301316	0.586746903289428	-3.89086833408964
a_HQ_transcript/105642	2.75538964173375	0.344975666355261	2.21296522020183	0.0340189261653554	0.586746903289428	-3.74316528699015
a_HQ_transcript/103915	2.74835122277316	2.1490154687184	2.21271886240171	0.0340375114060335	0.586746903289428	-3.74384825170276
a_HQ_transcript/61593	1.78623547602261	1.33548010133994	2.21259611792673	0.0340467746422335	0.586746903289428	-3.66912001432725
a_HQ_transcript/114034	-2.65577856086752	0.131166822340349	-2.21250659780576	0.0340535319364841	0.586746903289428	-3.69962705921983
a_HQ_transcript/125783	2.39265968709872	1.35828473816364	2.2124563855173	0.0340573226620309	0.586746903289428	-3.68701690032113
a_HQ_transcript/49563	4.10334143413194	1.37956560551261	2.21190755766546	0.0340987804729574	0.587211026338827	-3.80950946153591
a_HQ_transcript/132713	1.83870672049273	1.57676470723937	2.21183233216041	0.0341044664347626	0.587211026338827	-3.70289751034529
a_HQ_transcript/87058	1.58655199314834	2.15647375102104	2.21169349346582	0.0341149628616329	0.58721781498993	-3.69312826507821
a_HQ_transcript/69886	-2.23149706346471	-0.533112375489588	-2.21118576718617	0.0341533723825021	0.587704922986999	-3.69048913702679
a_HQ_transcript/150642	-1.60366295335938	-0.630061059315265	-2.21060177223993	0.0341975994485291	0.588184442861822	-3.68783751971196
a_HQ_transcript/44069	-5.02217498119275	1.94485455004806	-2.21055061210372	0.0342014763431214	0.588184442861822	-3.66408259846506
a_HQ_transcript/121111	1.85890667720016	-0.508301644409726	2.21007209455654	0.0342377572436482	0.588316029909033	-3.70443551866269
a_HQ_transcript/110971	-1.6074290950059	-0.39060104116296	-2.21006534988287	0.0342382688664227	0.588316029909033	-3.68118174529647
a_HQ_transcript/134682	2.23885005896285	0.0774193187218544	2.20984218365897	0.0342552011795513	0.588316029909033	-3.72174416102074
a_HQ_transcript/134108	-2.9889256765055	2.259407265813	-2.20975380628366	0.0342619087142615	0.588316029909033	-3.87045561374676
a_HQ_transcript/138758	-3.79607803412667	1.71820612196106	-2.20926695857979	0.0342988798144378	0.588316029909033	-3.87089834848852
a_HQ_transcript/41257	-2.82357661720783	-0.174039315351961	-2.2092273003409	0.0343018930222821	0.588316029909033	-3.7487457440944
a_HQ_transcript/131063	-0.540510444122836	3.6273578791668	-2.20918685247271	0.034304966469441	0.588316029909033	-3.93730264915208
a_HQ_transcript/133777	2.01279044469045	-0.473823567629626	2.20908906015589	0.0343123982742931	0.588316029909033	-3.70607928355475
a_HQ_transcript/150718	-2.5695258052704	1.74675949758752	-2.20902820386833	0.0343170238227974	0.588316029909033	-3.69240652255883
a_HQ_transcript/115748	2.68756529538579	-0.225779107747864	2.20859091569726	0.034350277503773	0.588316029909033	-3.71920530706606
a_HQ_transcript/107965	2.34713965920592	0.869297165929791	2.20838825398479	0.0343656987278206	0.588316029909033	-3.68605091100672
a_HQ_transcript/149823	0.791445672994332	4.18876381761363	2.20837343541497	0.0343668265663521	0.588316029909033	-3.96488609244731
a_HQ_transcript/43028	1.17860055874541	3.07187965272963	2.20836813660447	0.0343672298658433	0.588316029909033	-3.8134203641326
a_HQ_transcript/7402	3.81375720987668	2.29638861400905	2.20834811983225	0.0343687534070904	0.588316029909033	-3.86444137798417
a_HQ_transcript/93313	2.73151272369145	1.23543933159503	2.20830325540323	0.0343721684031564	0.588316029909033	-3.64830606959789

a_HQ_transcript/128665	1.99979095530617	0.230171950906254	2.20827519002176	0.0343743048415076	0.588316029909033	-3.66707323116917
a_HQ_transcript/130763	-4.44851889414724	1.9015876155058	-2.20761531110657	0.034424571431832	0.588316029909033	-3.78290569262106
a_HQ_transcript/43166	-2.6059961437997	2.51722723373995	-2.20757983519903	0.0344272756862588	0.588316029909033	-3.74269689070675
a_HQ_transcript/52365	2.78549723335428	0.318309393791696	2.20756644827499	0.0344282961928996	0.588316029909033	-3.6982152840219
a_HQ_transcript/24040	-1.83850437078348	1.28456193635542	-2.20752842155076	0.0344311951784409	0.588316029909033	-3.63491334556266
a_HQ_transcript/4923	1.5972941736918	1.57224240378742	2.20744075415615	0.0344378793756831	0.588316029909033	-3.67399196117145
a_HQ_transcript/89369	1.99161914773207	2.34879655213873	2.20738084107437	0.0344424481135795	0.588316029909033	-3.75766427822444
a_HQ_transcript/107013	2.5441962135245	0.168231946089836	2.20734654280553	0.0344450638096156	0.588316029909033	-3.74040309894566
a_HQ_transcript/41434	2.52360515758546	1.81904834857962	2.20708211059999	0.0344652362206298	0.588316029909033	-3.73290058395103
a_HQ_transcript/76879	-2.22265395356336	1.26851362501486	-2.20706289484651	0.0344667025204139	0.588316029909033	-3.67986477033903
a_HQ_transcript/89360	2.91012802591042	0.385479826408661	2.20698987531648	0.0344722749427155	0.588316029909033	-3.6872295666012
a_HQ_transcript/136390	3.04949851528448	0.47415655008958	2.20681291401772	0.0344857829319935	0.588373815753132	-3.80319422042447
a_HQ_transcript/71315	1.64406646545614	-0.758258312135396	2.20643782802243	0.0345144300193621	0.588530962036812	-3.71160994744299
a_HQ_transcript/57918	2.26456538581213	-0.102510417200745	2.20625979138079	0.0345280349601975	0.588530962036812	-3.67320428005545
a_HQ_transcript/61865	-0.919208815893965	4.1670692606364	-2.2061778466556	0.0345342985016799	0.588530962036812	-4.05687360141439
a_HQ_transcript/1389	2.23335087517417	1.41447651720351	2.2061544896336	0.0345360840091125	0.588530962036812	-3.61188783479985
a_HQ_transcript/32009	-2.18643808349057	0.141289207531396	-2.20580916526041	0.0345624916625714	0.588530962036812	-3.74430740565418
a_HQ_transcript/35112	2.10873989322685	1.11939377793594	2.20580547769939	0.0345627737551819	0.588530962036812	-3.6203275175031
a_HQ_transcript/20451	-3.15790883724662	1.20776660858362	-2.20563804620544	0.0345755841687787	0.588530962036812	-3.76428184965428
a_HQ_transcript/117912	1.80511777320857	0.140971048672163	2.20554003262798	0.0345830852912249	0.588530962036812	-3.67669317142809
a_HQ_transcript/121014	3.0717594998752	0.0892451741955036	2.20550043371891	0.0345861162661325	0.588530962036812	-3.68823189207821
a_HQ_transcript/134564	-6.94829373240689	1.12705658671138	-2.2053004665405	0.0346014257564865	0.588562457550411	-3.75878141039191
a_HQ_transcript/139856	1.40774156663005	2.07057066749106	2.20521177755919	0.0346082177248022	0.588562457550411	-3.68536825031602
a_HQ_transcript/30104	1.90850885184138	-0.502385174890553	2.20493694294388	0.0346292726419564	0.58874827731256	-3.71204836537119
a_HQ_transcript/117679	-4.84601728198045	1.23697998503373	-2.20416616601505	0.0346883824887224	0.589580788685514	-3.75062611354158
a_HQ_transcript/83405	2.14705531508044	1.85057502715787	2.20358456454135	0.0347330443903553	0.590167320760763	-3.68958407370611
a_HQ_transcript/40680	2.00573385513161	-0.22746915596958	2.20342557154011	0.0347452626006638	0.590202403404847	-3.70503989503185
a_HQ_transcript/15283	4.67826054407049	1.29060303136288	2.20287193070383	0.0347878384751429	0.590363326015253	-3.71831544445288
a_HQ_transcript/131699	-0.765847498024565	3.58411231851509	-2.20274862745064	0.0347973270389571	0.590363326015253	-3.87818994210474
a_HQ_transcript/27890	-1.56414147889696	-0.514445534321511	-2.20269240531046	0.0348016542727164	0.590363326015253	-3.63053138294115
a_HQ_transcript/145048	1.89618705837152	0.270821540287345	2.20259781566001	0.0348089356128177	0.590363326015253	-3.66427932951043
a_HQ_transcript/12157	-1.83839565128714	0.101678304006131	-2.20259183231152	0.034809396245925	0.590363326015253	-3.63460501384595
a_HQ_transcript/151250	-4.19833965938907	6.85945998786983	-2.20251029882195	0.0348156737128456	0.590363326015253	-4.43300419425515
a_HQ_transcript/83297	-2.55180173820631	2.22786814293662	-2.20219080401382	0.0348402821639972	0.590608317768687	-3.6972058545848
a_HQ_transcript/54902	2.09628746189263	0.692359269428562	2.20195886060497	0.0348581568775631	0.590739050431193	-3.63058247248927
a_HQ_transcript/63288	1.95407618313231	2.25656917244131	2.20141791696593	0.0348998765262041	0.591273687763098	-3.76005123276159
a_HQ_transcript/112753	-1.01565503473814	3.4848068298214	-2.20114202789763	0.0349211713211847	0.591432024497	-3.81722029362875
a_HQ_transcript/114225	1.88458298633913	2.80440027781913	2.2006515681261	0.0349590566476186	0.591432024497	-3.8070784286846
a_HQ_transcript/150458	9.2692761523449	3.23815651224765	2.20060799956965	0.034962423854506	0.591432024497	-3.72392910583358
a_HQ_transcript/25414	-2.64857736474354	0.544425926119604	-2.200465218145	0.0349734607834917	0.591432024497	-3.70356018412674
a_HQ_transcript/127767	-2.8351839686433	0.633383925515145	-2.20039941327648	0.0349785485128344	0.591432024497	-3.79070353939322
a_HQ_transcript/61340	-1.81575205737664	0.605426099159833	-2.20038235878836	0.034979867195174	0.591432024497	-3.65942110630467
a_HQ_transcript/81052	-2.26592507009731	-0.217681541021621	-2.20037488773229	0.0349804448840608	0.591432024497	-3.73936195815094
a_HQ_transcript/99249	-2.22005791135105	0.342581007579486	-2.20011359553081	0.0350006542972119	0.591601637972764	-3.70295039741839
a_HQ_transcript/131379	2.43989117772745	3.6735510842647	2.1997982898293	0.0350250552217525	0.591768386024627	-3.98438387657256
a_HQ_transcript/41114	-3.25154325257863	0.68284707131425	-2.19962731243342	0.0350382931985254	0.591768386024627	-3.64886281803091
a_HQ_transcript/76176	-1.83635258037086	-0.665495967260262	-2.19942144569812	0.0350542384227548	0.591768386024627	-3.71920897604843
a_HQ_transcript/50553	0.981790034693137	3.85691764587503	2.19940334888402	0.0350556404051957	0.591768386024627	-3.99232065913067
a_HQ_transcript/130570	0.694666570669237	4.13228932423885	2.19932873045897	0.0350614217153319	0.591768386024627	-4.04970517629019
a_HQ_transcript/140764	2.40249723275384	0.0202074660993775	2.19914802934635	0.0350754256620528	0.59183290069196	-3.71066689873516

a_HQ_transcript/136721	0.904241636600681	2.48490721100857	2.19894179114867	0.0350914147821732	0.59193086432332	-3.77046365167676
a_HQ_transcript/144876	-2.70734359297839	-0.342057105398419	-2.19842918746322	0.0351311838356322	0.592127374156278	-3.76565136686451
a_HQ_transcript/112489	-2.70858048410137	0.71982601038005	-2.19838313919176	0.0351347583432724	0.592127374156278	-3.66539973128468
a_HQ_transcript/77408	2.31769049841645	0.667706138547425	2.19837029633244	0.0351357553311001	0.592127374156278	-3.72783193847798
a_HQ_transcript/6009	-0.799741480666212	2.19152199007066	-2.1982665389736	0.035143810914519	0.592127374156278	-3.68937028329625
a_HQ_transcript/138567	2.48320526393816	2.42247489606467	2.19784426997096	0.0351766123370287	0.592485947091642	-3.70219513909258
a_HQ_transcript/110546	5.0101759619142	0.377834168677597	2.19751112020074	0.0352025103600505	0.592485947091642	-3.74163274736061
a_HQ_transcript/31349	-2.90233994598095	2.17501463816149	-2.19746913059613	0.0352057757087716	0.592485947091642	-4.00445334766105
a_HQ_transcript/52009	-1.87302302838112	-0.330810709796633	-2.19710896322301	0.0352337954690857	0.592485947091642	-3.72296228024364
a_HQ_transcript/89389	-1.46760200416447	2.78601821127637	-2.19697062051539	0.0352445633395999	0.592485947091642	-3.82831696607128
a_HQ_transcript/133349	2.26225490470994	0.0229023822580873	2.19679515453623	0.0352582249178207	0.592485947091642	-3.70957792985494
a_HQ_transcript/103313	5.55295521254375	1.43691710476751	2.1967801750787	0.0352593914201006	0.592485947091642	-3.75289493245718
a_HQ_transcript/129832	-1.02947590441894	2.86761872744001	-2.19663729747944	0.0352705195260501	0.592485947091642	-3.74724080751321
a_HQ_transcript/9935	-0.87438584602059	2.2426161995881	-2.19649309386243	0.0352817540893733	0.592485947091642	-3.7752010451118
a_HQ_transcript/134530	3.07568237792239	0.192658999526258	2.19649112735217	0.0352819073176263	0.592485947091642	-3.75251129070577
a_HQ_transcript/35711	-0.897853553524795	2.64459136433824	-2.19648851730228	0.0352821106906758	0.592485947091642	-3.72997354253576
a_HQ_transcript/135307	-2.32099926848176	0.300287317855744	-2.19639946133914	0.0352890504889899	0.592485947091642	-3.71587854253712
a_HQ_transcript/87850	2.24183178515927	-0.442984262306738	2.19628978280737	0.0352975990018297	0.592485947091642	-3.77609355870134
a_HQ_transcript/110575	6.74803679749563	2.89526278175813	2.19598684144946	0.0353212203228999	0.592537769641049	-4.01190635452734
a_HQ_transcript/22650	1.54534179962342	-0.80763724497809	2.19587643267626	0.0353298327620209	0.592537769641049	-3.73427520888839
a_HQ_transcript/124598	2.70866982174542	3.03226177576112	2.19585804380968	0.0353312673681509	0.592537769641049	-3.9269638415878
a_HQ_transcript/31316	-2.76258106763903	-0.0256365247256823	-2.19559091700252	0.0353521131134824	0.592562619991128	-3.74271661868663
a_HQ_transcript/82388	-2.3865829190767	1.88075310031518	-2.1955677938344	0.0353539180899239	0.592562619991128	-3.76808333713464
a_HQ_transcript/127666	2.76427358053121	8.37105782433989	2.19544721875752	0.0353633314194402	0.592562619991128	-4.44073650957437
a_HQ_transcript/60633	1.03516730323324	3.45691050072338	2.19502250034503	0.0353965071290181	0.592841519013654	-3.88074819890199
a_HQ_transcript/6229	2.31109276886106	0.966152737243912	2.19494400918618	0.035402641290965	0.592841519013654	-3.6467298638664
a_HQ_transcript/137404	-1.5217853893392	0.721745533659628	-2.19478484138761	0.0354150833259874	0.592841519013654	-3.66065911472751
a_HQ_transcript/14169	0.881698351449568	3.33923021482645	2.19471209289588	0.0354207713242228	0.592841519013654	-3.94925022432084
a_HQ_transcript/116126	-2.64439619117052	1.01403326624572	-2.19445462945134	0.0354409082119455	0.593007804416802	-3.75087672722609
a_HQ_transcript/16975	2.36901071627036	0.268908299806469	2.19398809394223	0.0354774232103563	0.593447958668084	-3.76901844267164
a_HQ_transcript/17621	-2.40190815693169	1.1649175166954	-2.19379213254152	0.0354927708133212	0.593533884302857	-3.70327668657227
a_HQ_transcript/11873	-0.61263398715156	3.65816938024294	-2.19350095402761	0.0355155867210136	0.593577826316665	-3.97611843130147
a_HQ_transcript/134943	3.06290814398932	3.20839781786532	2.19334669467199	0.0355276793431466	0.593577826316665	-3.9673488930831
a_HQ_transcript/145838	5.24263562440356	7.76841745821765	2.19326577626054	0.0355340241282057	0.593577826316665	-4.12432345650681
a_HQ_transcript/137840	0.770559507529755	3.89681086650182	2.19309613201729	0.0355473291584992	0.593577826316665	-4.0066703461144
a_HQ_transcript/24452	2.51085224390846	0.3626246054326	2.19308874682937	0.035547908472479	0.593577826316665	-3.72862133361519
a_HQ_transcript/110632	2.50652402203673	0.343869273624207	2.1928684697112	0.0355651914759997	0.593577826316665	-3.6808786847893
a_HQ_transcript/92811	-1.88524512865073	1.61782422736723	-2.19284696100698	0.0355668794567324	0.593577826316665	-3.74066522623408
a_HQ_transcript/122738	2.19036512519566	0.23746596759579	2.19268962925045	0.0355792288640577	0.593613494664164	-3.71420863129488
a_HQ_transcript/12679	-4.07413848577299	0.74803627364528	-2.1924614458777	0.035597146412451	0.593742016259096	-3.68523897866148
a_HQ_transcript/149160	-2.20664196941736	0.972603891465497	-2.1920426747598	0.0356300503570488	0.594074660260216	-3.6391040749482
a_HQ_transcript/6164	1.6952774511185	1.53182566679865	2.19188775979617	0.0356422293083661	0.594074660260216	-3.70235466029069
a_HQ_transcript/47827	1.69941918370426	0.16912721956176	2.19177183197098	0.0356513456377516	0.594074660260216	-3.70547961293357
a_HQ_transcript/92178	0.631248787705979	4.75477398396194	2.19163708460235	0.0356619445130258	0.594074660260216	-4.12008965400426
a_HQ_transcript/35016	-5.13281937491643	0.394667751315809	-2.19153466974217	0.035670002078656	0.594074660260216	-3.75226038340102
a_HQ_transcript/146652	-1.75234267951861	-0.406322598983237	-2.19112030287625	0.0357026192891005	0.594074660260216	-3.72866902614807
a_HQ_transcript/128466	2.11034473984313	0.178493706220096	2.19107254891129	0.0357063799896174	0.594074660260216	-3.65569711730671
a_HQ_transcript/68351	1.72701590501202	2.09814133746078	2.19096581735866	0.0357147865467841	0.594074660260216	-3.75329434272413
a_HQ_transcript/106580	1.71528205711806	-0.692063389178478	2.19088945095654	0.0357208025204962	0.594074660260216	-3.73916688560549
a_HQ_transcript/48061	-2.42272279986026	1.49481999694248	-2.1908125515921	0.0357268613935715	0.594074660260216	-3.75356516535428

a_HQ_transcript/150954	4.66710264515444	2.88153440616728	2.19077892706544	0.0357295109460108	0.594074660260216	-3.98341832955712
a_HQ_transcript/122839	-2.94180760033438	0.114397903495959	-2.19050554733494	0.0357510592879584	0.594262960906619	-3.79512088326131
a_HQ_transcript/144210	2.72678368333394	3.22788119461188	2.18980771447011	0.0358061164738066	0.594795728657034	-4.00221553777313
a_HQ_transcript/96864	2.14151535060302	1.46864724776545	2.18958024164543	0.0358240798276271	0.594795728657034	-3.73337392465429
a_HQ_transcript/46615	-2.37838234595506	0.281182070311122	-2.18957092350096	0.0358248158457352	0.594795728657034	-3.69667158923428
a_HQ_transcript/98119	2.55378834054604	2.09992099101965	2.18925741095828	0.0358495873223685	0.594795728657034	-3.76471883308001
a_HQ_transcript/5398	1.33623118548191	2.6218745577163	2.18906658254907	0.0358646726739262	0.594795728657034	-3.75029146763114
a_HQ_transcript/37706	2.07743885765969	0.476544970302987	2.18899993280954	0.0358699427979704	0.594795728657034	-3.65244745202312
a_HQ_transcript/6450	2.44492420688918	2.32797219603194	2.1888993812136	0.0358778949150149	0.594795728657034	-3.71580429244408
a_HQ_transcript/151950	1.96658414697201	-0.026222945560434	2.18868334791202	0.0358949852160904	0.594795728657034	-3.66782931075929
a_HQ_transcript/22852	1.99564251830451	-0.387802974362868	2.1885980742932	0.0359017331742838	0.594795728657034	-3.70098039663327
a_HQ_transcript/114108	1.5940602846586	1.99172098930074	2.18850705803773	0.0359089368132692	0.594795728657034	-3.70890346475962
a_HQ_transcript/116623	3.14067622753491	1.05045297707385	2.18849944672295	0.0359095392823695	0.594795728657034	-3.7022642218561
a_HQ_transcript/22132	1.77975564857322	1.71763167316759	2.18827569036806	0.0359272546136426	0.594795728657034	-3.68152975412073
a_HQ_transcript/131162	2.09464324172555	1.24885009155821	2.18823673504245	0.035930339597659	0.594795728657034	-3.67394001188732
a_HQ_transcript/74787	1.34337183808667	-0.714412106973712	2.18803328840465	0.0359464549611093	0.594795728657034	-3.74665863196132
a_HQ_transcript/11973	-2.12149409168691	-0.635519696361625	-2.18793659991936	0.035954116085157	0.594795728657034	-3.73261623752899
a_HQ_transcript/80273	-2.44226297372616	-0.496453109625841	-2.18792285441625	0.0359552053300415	0.594795728657034	-3.73848015467389
a_HQ_transcript/89595	4.14244329882607	0.751224267865865	2.18772460003701	0.0359709190205267	0.594795728657034	-3.84189213967093
a_HQ_transcript/98959	-2.08108415971131	2.20783798256356	-2.18771580266024	0.0359716164446963	0.594795728657034	-3.86695696272724
a_HQ_transcript/86164	1.63755917442563	2.19787872028053	2.18764123237494	0.0359775285913524	0.594795728657034	-3.75918985596035
a_HQ_transcript/9728	-1.68630190010826	0.0814958939403977	-2.1870231895609	0.0360265621771151	0.595378392653118	-3.70956602538277
a_HQ_transcript/133061	-2.08585135926124	1.84484951484263	-2.18661729914231	0.0360587966767565	0.595378392653118	-3.77050667516139
a_HQ_transcript/16647	1.51867874069471	-0.771209876828777	2.18655260531893	0.0360639368266146	0.595378392653118	-3.74784711731023
a_HQ_transcript/106011	2.11584239231466	2.50562198646943	2.18635695055589	0.0360794862573732	0.595378392653118	-3.78688619098312
a_HQ_transcript/23624	4.41067238336131	2.10500795871484	2.18634464907217	0.0360804641030209	0.595378392653118	-3.75903310405159
a_HQ_transcript/11858	2.22809258445259	-0.0986033434166181	2.18625401497411	0.0360876693414122	0.595378392653118	-3.69691387155738
a_HQ_transcript/148078	-2.82758434671731	0.529147938047113	-2.18622909259253	0.0360896508483079	0.595378392653118	-3.75802876299004
a_HQ_transcript/67432	1.43383660114022	1.33388031619442	2.186165433639	0.0360947126291906	0.595378392653118	-3.66731101390184
a_HQ_transcript/151911	-3.8902793534766	1.33466298233831	-2.18547205927212	0.0361498866544353	0.595822132869979	-3.80654951630797
a_HQ_transcript/80558	3.20580487280957	0.133240613836717	2.18540476127267	0.0361552457751262	0.595822132869979	-3.76359193769607
a_HQ_transcript/49677	-2.71374835899	2.30741404246765	-2.18531913657172	0.0361620653246459	0.595822132869979	-3.81654149040615
a_HQ_transcript/405	1.19486863014033	2.77869169544669	2.18531223559137	0.0361626150007791	0.595822132869979	-3.93795154637584
a_HQ_transcript/84628	3.64518719851172	0.758927130526044	2.18484558732735	0.0361998017107086	0.59604171989445	-3.76592017305551
a_HQ_transcript/50383	3.04649545448894	0.778761923036341	2.18483544517845	0.0362006103064259	0.59604171989445	-3.72889710738511
a_HQ_transcript/17083	2.18271422533681	2.9377679997492	2.1847590140575	0.0362067043928452	0.59604171989445	-3.84394858671379
a_HQ_transcript/58619	-2.37491574960751	4.34382109905472	-2.18430230308273	0.0362431384233474	0.596464909632434	-4.10497427459554
a_HQ_transcript/3696	-1.80612185560629	1.23164964907997	-2.18409617464529	0.0362595929856854	0.596464909632434	-3.69110070171157
a_HQ_transcript/89491	-2.43758434942763	3.92472626402929	-2.18370716257053	0.036290664704596	0.596464909632434	-4.09818150070679
a_HQ_transcript/134176	-4.88066156472705	-0.390954216998913	-2.18368841951218	0.03629216237586l	0.596464909632434	-3.77907473645477
a_HQ_transcript/110454	-1.24309632361029	-0.827111306995701	-2.18363710791297	0.0362962627308563	0.596464909632434	-3.73665835936669
a_HQ_transcript/18506	-1.6319647854743	2.02407146885021	-2.18360474799306	0.0362988488529119	0.596464909632434	-3.68960174428686
a_HQ_transcript/91227	1.83402642643077	-0.281696088010509	2.18353729832642	0.0363042397859818	0.596464909632434	-3.78267978982133
a_HQ_transcript/76388	-2.21515216704977	-0.24711097130456	-2.18315922524667	0.0363344705990185	0.596744584035709	-3.74157150401946
a_HQ_transcript/136353	2.32696947542476	-0.245686538218871	2.18306767385845	0.0363417944447841	0.596744584035709	-3.74259630511301
a_HQ_transcript/133897	0.750432893923299	5.08035718521087	2.1822376154912	0.0364082568024708	0.59758221570468	-4.1586748520215
a_HQ_transcript/22349	1.35391215013034	3.27008453558206	2.18207650062126	0.036421169741373	0.59758221570468	-3.85575482302475
a_HQ_transcript/10981	-1.71554075150082	3.45738904475287	-2.18174651296175	0.036447630143269	0.59758221570468	-3.93773836066846
a_HQ_transcript/61057	-1.7428645289277	1.97976901947184	-2.18151703856117	0.0364660408875786	0.59758221570468	-3.72637435743937
a_HQ_transcript/136394	-2.84872495295535	-0.458202486547369	-2.18138559723885	0.0364765901666695	0.59758221570468	-3.75676072190652

a_HQ_transcript/149333	0.667945273629679	5.46001507272614	2.18138095281599	0.0364769629706928	0.59758221570468	-4.17874780107957
a_HQ_transcript/62285	4.99809660821571	3.20284587002936	2.18122129546491	0.0364897806012739	0.59758221570468	-3.9099253903109
a_HQ_transcript/138787	0.583411896348135	5.83727141750922	2.18118407590784	0.036492769243292	0.59758221570468	-4.2067239981229
a_HQ_transcript/113770	2.48682978744066	1.69490201676837	2.18087925883438	0.0365172535431666	0.59758221570468	-3.7682268431616
a_HQ_transcript/112923	1.80591352394683	-0.515191241269518	2.1808496141332	0.0365196355222059	0.59758221570468	-3.7503742730264
a_HQ_transcript/144612	-2.21508831700332	-0.288994854032686	-2.18069851208735	0.0365317788631745	0.59758221570468	-3.79277172619803
a_HQ_transcript/41145	4.43885700886143	0.952430841428155	2.18067624941505	0.0365335683107358	0.59758221570468	-3.79349991923097
a_HQ_transcript/145166	-0.839398395051718	3.67979861319474	-2.18063751165546	0.0365366821928192	0.59758221570468	-3.95752169851146
a_HQ_transcript/72129	1.28587507528581	-0.679881153162186	2.18063688416015	0.0365367326351231	0.59758221570468	-3.78878708651834
a_HQ_transcript/73636	-3.29673438281247	2.70294002400121	-2.18018556346102	0.0365730289196699	0.597603430159102	-3.86448318190181
a_HQ_transcript/127411	2.35434010955073	0.758915427791417	2.18015741933741	0.0365752934013812	0.597603430159102	-3.72067781380353
a_HQ_transcript/137274	1.35372314625056	-0.526489569185173	2.17977352079615	0.0366061944355286	0.597603430159102	-3.80543242316724
a_HQ_transcript/138113	-2.80020222707813	0.0383156592768042	-2.17969832646031	0.0366122497562094	0.597603430159102	-3.74555000030146
a_HQ_transcript/134219	-2.28810443154683	-0.291802476831869	-2.17966242443706	0.0366151412232397	0.597603430159102	-3.7641082443924
a_HQ_transcript/30265	-1.65941485957761	1.98130476877423	-2.17960185935917	0.0366200194577336	0.597603430159102	-3.85202957902392
a_HQ_transcript/138850	1.52571569485436	2.10998110370147	2.17958011105179	0.036621771323799	0.597603430159102	-3.79810144066802
a_HQ_transcript/81652	-2.70372793783022	0.627263469307177	-2.17933299505212	0.0366416822183529	0.597603430159102	-3.79336445676735
a_HQ_transcript/106256	2.41776099155318	1.01235867662256	2.17926044812058	0.0366475293791723	0.597603430159102	-3.75992402797417
a_HQ_transcript/4279	1.36660686144305	1.45663176636653	2.17910662861921	0.0366599297237891	0.597603430159102	-3.6850310799487
a_HQ_transcript/112716	1.68029355538196	-0.191787552292668	2.1790363795549	0.0366655941807386	0.597603430159102	-3.73953864828199
a_HQ_transcript/113675	-2.87030875478836	1.08315328182199	-2.1786095087226	0.0367000312218881	0.597603430159102	-3.88171641531405
a_HQ_transcript/150707	-1.48933616667837	-0.754831056511801	-2.17857576790445	0.0367027544310126	0.597603430159102	-3.74688386015152
a_HQ_transcript/48606	1.75126436907503	2.42978219811497	2.17834843858096	0.0367211067925782	0.597603430159102	-3.75034262375912
a_HQ_transcript/90493	2.16154170304833	0.180827455863445	2.17823152497098	0.0367305484462504	0.597603430159102	-3.68738402541899
a_HQ_transcript/112595	3.69454712684457	0.771050821314317	2.17823081584293	0.0367306057202767	0.597603430159102	-3.84731977827004
a_HQ_transcript/64389	-2.46251542722471	0.647439867430769	-2.17817604582491	0.0367350295616181	0.597603430159102	-3.74503601784898
a_HQ_transcript/37463	1.68208463935803	-0.137408052482935	2.17809565643851	0.0367415235706624	0.597603430159102	-3.69020399918674
a_HQ_transcript/116447	-2.36053890543689	2.20694044312533	-2.17798786918857	0.0367502324373433	0.597603430159102	-3.71232084050676
a_HQ_transcript/59234	2.46286320955441	0.362024299158931	2.17795329830671	0.0367530260441749	0.597603430159102	-3.72162870447512
a_HQ_transcript/81069	-3.4377773357951	1.12403699903764	-2.17790883596818	0.0367566192389263	0.597603430159102	-3.84324647331025
a_HQ_transcript/72890	2.07265549692392	1.96506350673824	2.17781494854203	0.0367642077182932	0.597603430159102	-3.72526370711911
a_HQ_transcript/105749	1.96032989938257	0.376362972205109	2.17764182142935	0.0367782044301835	0.597663815241182	-3.66661896097761
a_HQ_transcript/132122	3.99283222863381	1.26360288162898	2.1773055211562	0.0368054066848815	0.597938703124312	-3.77275885414798
a_HQ_transcript/41531	2.50792577915544	1.61392170077451	2.17702951057449	0.0368277456937978	0.598134451435898	-3.71737358069651
a_HQ_transcript/58524	1.75841581450524	0.295802277776246	2.1768343251789	0.036843550381665	0.598223993459616	-3.69828824136751
a_HQ_transcript/104332	3.64547292302024	0.707685667052023	2.17649286146998	0.0368712141552529	0.598455192473175	-3.80041550641374
a_HQ_transcript/77219	1.95977879364552	-0.501265284080557	2.17640444246143	0.0368783804610328	0.598455192473175	-3.77185539643448
a_HQ_transcript/53167	3.3511700938744	1.6162264492744	2.17613974613729	0.0368998413536097	0.598593800481929	-3.80498743417218
a_HQ_transcript/17813	-1.70675707261561	0.443372049353885	-2.17595431217451	0.0369148824870966	0.598593800481929	-3.65954247106526
a_HQ_transcript/133723	1.02151693055057	6.44017608282652	2.17591816148336	0.036917815419896	0.598593800481929	-4.29698959793454
a_HQ_transcript/48727	-2.30084307143932	1.15598690231175	-2.17543753003921	0.0369568291464475	0.599059276247825	-3.81688351136207
a_HQ_transcript/137713	-1.47292715565954	0.200099652703703	-2.17525702440573	0.0369714906020874	0.599078196781203	-3.78601755581659
a_HQ_transcript/151283	4.30126076246507	6.28151362175281	2.17516941164632	0.0369786087608546	0.599078196781203	-4.07339189876449
a_HQ_transcript/67108	1.19350285826544	2.72419445416944	2.17449830019128	0.0370331741818291	0.599452108384343	-3.9249897580859
a_HQ_transcript/12944	2.25173986410882	2.82893496715228	2.17447786799518	0.0370348365675821	0.599452108384343	-3.81258139061707
a_HQ_transcript/64958	1.47688161714738	-0.800170256099016	2.17438061503573	0.037042750085737	0.599452108384343	-3.7697632241781
a_HQ_transcript/14816	-1.14096331343502	2.03757461053813	-2.17428318400476	0.0370506796041872	0.599452108384343	-3.68739846566873
a_HQ_transcript/7509	1.78183562791206	-0.0305919126334047	2.17425158207513	0.0370532518824825	0.599452108384343	-3.71366179664176
a_HQ_transcript/115999	1.54300505965662	-0.771839982662627	2.17369726065482	0.0370983974398948	0.599921450023873	-3.77055880966437
a_HQ_transcript/95866	2.30930006004919	2.45288815887821	2.17353016788405	0.0371120155717667	0.599921450023873	-3.7533434154518

a_HQ_transcript/22471	0.653405858684762	2.8934526580736	2.173515332789	0.0371132248535275	0.599921450023873	-3.87858771707539
a_HQ_transcript/132783	0.951780538452551	2.59533278743518	2.1729315249169	0.0371608417915188	0.600065918964998	-3.82668174051686
a_HQ_transcript/88566	-2.83952659477198	3.82036389215588	-2.1727226849379	0.0371778885517427	0.600065918964998	-3.90391168854617
a_HQ_transcript/60538	1.68218010050333	2.8270695044551	2.17263822820257	0.0371847843901817	0.600065918964998	-3.85709572375312
a_HQ_transcript/47134	-2.09277041482247	0.68515008908533	-2.17261673708939	0.0371865393076586	0.600065918964998	-3.7986965405107
a_HQ_transcript/142190	-5.45054562555768	4.7221635537572	-2.17248232515033	0.0371975167673657	0.600065918964998	-4.01842928774247
a_HQ_transcript/150408	2.47094471239729	-0.0274488289200584	2.17236265566032	0.0372072926360034	0.600065918964998	-3.77595256793702
a_HQ_transcript/149583	3.17585252487456	1.48332049015387	2.17230265046574	0.0372121953557917	0.600065918964998	-3.81289365149196
a_HQ_transcript/119505	-3.08337083096488	2.2081380886855	-2.17228646416656	0.0372135179546537	0.600065918964998	-3.73834704093476
a_HQ_transcript/7623	-2.54741270617631	-0.300049407750817	-2.17224889392771	0.0372165880182134	0.600065918964998	-3.75589248141626
a_HQ_transcript/126955	2.81656570638152	0.333795043623237	2.17190570356384	0.0372446423674724	0.600065918964998	-3.77469757136025
a_HQ_transcript/62622	-2.93149278533238	-0.0699216155537796	-2.17189192417856	0.0372457691666848	0.600065918964998	-3.77844714218395
a_HQ_transcript/12449	-0.764067409759379	3.0566013536645	-2.17188860753695	0.0372460403871751	0.600065918964998	-3.88096856732352
a_HQ_transcript/82507	-3.7193187616598	-0.270795546260376	-2.17145601841182	0.0372814307201873	0.600208088853157	-3.77696773959358
a_HQ_transcript/152145	3.27789100469719	1.93280863622713	2.17131545260636	0.037292936924311	0.600208088853157	-3.8997625393674
a_HQ_transcript/135077	2.32327860606511	1.15225310893463	2.17103518331917	0.037315888185819	0.600208088853157	-3.76109445707834
a_HQ_transcript/102662	-3.53310030166629	2.11671210261185	-2.17100370490819	0.0373184667395725	0.600208088853157	-3.80741377841834
a_HQ_transcript/40606	1.45104342312944	-0.580080981840969	2.17094473981909	0.0373232972913367	0.600208088853157	-3.79790686238208
a_HQ_transcript/88984	-2.64027066441621	1.59599631652382	-2.17086999072497	0.037329421704416	0.600208088853157	-3.80003215139975
a_HQ_transcript/89106	2.26991057894226	1.17932822938357	2.17086976782548	0.0373294399685647	0.600208088853157	-3.74007594384656
a_HQ_transcript/52853	1.6699295393261	-0.707980056497221	2.17071527513765	0.0373421008507641	0.600208088853157	-3.77541826249617
a_HQ_transcript/9327	1.7341508556714	-0.742030300425397	2.17058676931444	0.0373526349878816	0.600208088853157	-3.77521837592624
a_HQ_transcript/47767	1.65438709290747	2.43715114870296	2.17048089488851	0.037361315926928	0.600208088853157	-3.74262758363209
a_HQ_transcript/9538	0.848678315097391	2.78205900683531	2.17039394218283	0.0373684467650629	0.600208088853157	-3.88579094180053
a_HQ_transcript/126129	1.68055056688527	1.84769641493122	2.17003492522491	0.03739790194745	0.600515260884358	-3.72265195101041
a_HQ_transcript/138804	2.36202342889549	0.0332028519951948	2.16982453460703	0.0374151728388022	0.600626668537391	-3.71881394950326
a_HQ_transcript/144593	1.16510986369896	4.30202449387303	2.1693345146324	0.0374554259424976	0.600685944301858	-4.09329586442761
a_HQ_transcript/126701	-2.06004990310601	1.23807356792577	-2.16928939834972	0.0374591339954388	0.600685944301858	-3.77167790426585
a_HQ_transcript/148991	2.88115252448605	-0.32722562351729	2.16905597295504	0.0374783241684461	0.600685944301858	-3.73593181407587
a_HQ_transcript/146361	-5.31603175134376	2.85449288375217	-2.16893379044575	0.0374883724242093	0.600685944301858	-3.84971742812665
a_HQ_transcript/148736	2.41596903532866	2.47499763948427	2.16886462544598	0.037494061597847	0.600685944301858	-3.79473148737136
a_HQ_transcript/130547	-1.90465304708041	-0.37698030115995	-2.16881013025305	0.0374985446465634	0.600685944301858	-3.79125726448024
a_HQ_transcript/39283	-1.61403545132914	1.9056631141026	-2.16880176794747	0.037499232614123	0.600685944301858	-3.82312086403393
a_HQ_transcript/135261	1.78727786532261	1.09191418820378	2.16877376956711	0.037501536124956	0.600685944301858	-3.68982744203226
a_HQ_transcript/119203	1.53430588725793	1.26309231057046	2.16855214301356	0.0375197744531849	0.600812520499926	-3.7048414352877
a_HQ_transcript/145638	4.77644379476364	0.675276647940323	2.16820261241936	0.0375485544459858	0.600866713504373	-3.78061518902384
a_HQ_transcript/16681	-1.66749604609015	-0.383596527697311	-2.16813419617133	0.0375541900696357	0.600866713504373	-3.72722043196441
a_HQ_transcript/125115	1.45054996142123	2.81115276675385	2.16804026207441	0.0375619288913761	0.600866713504373	-3.7642161855665
a_HQ_transcript/20448	1.98680154023638	-0.582458313664225	2.16800897815797	0.0375645065523481	0.600866713504373	-3.74253669444702
a_HQ_transcript/22712	1.11248458759369	1.21516709933396	2.16774146097883	0.0375865552562858	0.600911928507512	-3.73135285555821
a_HQ_transcript/121135	2.31892988799256	1.24265900432851	2.16771789033986	0.037588498495076	0.600911928507512	-3.68591459274207
a_HQ_transcript/111193	1.91373958971599	1.21072900283494	2.16759845529824	0.0375983464764282	0.600911928507512	-3.6858260332348
a_HQ_transcript/105373	-2.9896102384279	0.71828720734364	-2.16689110695042	0.0376567177758074	0.601545035857813	-3.82015634695442
a_HQ_transcript/113922	-2.54511091883263	-0.561814975706351	-2.16658360318303	0.0376821185317515	0.601545035857813	-3.76679346055045
a_HQ_transcript/107544	-3.08857420126468	-0.414622210851763	-2.16651073819784	0.0376881396381196	0.601545035857813	-3.77554937428981
a_HQ_transcript/14820	0.640930307423031	3.08987875486225	2.16628455400757	0.037706835539735	0.601545035857813	-3.98354221781348
a_HQ_transcript/125527	2.48548529352861	0.0570201899465	2.16623223833782	0.0377111610165372	0.601545035857813	-3.77081064599898
a_HQ_transcript/150246	2.42994585913082	-0.654161111192919	2.16620416214969	0.0377134825471313	0.601545035857813	-3.77274172193095
a_HQ_transcript/9056	-0.617159357663588	3.00567645115085	-2.16609272180753	0.0377226984487746	0.601545035857813	-3.89532786043842
a_HQ_transcript/136713	1.7152470641756	0.78497377002371	2.16600516675948	0.0377299404887029	0.601545035857813	-3.73871206237437

a_HQ_transcript/133539	-3.375895806201	-0.377526139142721	-2.16599118729795	0.0377310969023119	0.601545035857813	-3.79220926948648
a_HQ_transcript/124450	-2.42749194397817	-0.269641091465026	-2.16555998446993	0.0377667825026527	0.601948871213106	-3.77712363437645
a_HQ_transcript/131732	0.853751894122266	7.63408651488206	2.1654232702097	0.03777810300886	0.60196424662802	-4.39913238058817
a_HQ_transcript/71944	2.33246079033673	-0.30724182452014	2.16474664795422	0.0378341746041991	0.602657264982713	-3.77082527505988
a_HQ_transcript/152028	-1.85261212137174	-0.675996445669783	-2.16464829800201	0.0378423310140879	0.602657264982713	-3.77622013811173
a_HQ_transcript/125918	1.44755369352745	-0.711151153402208	2.16416793287005	0.0378821913995732	0.603126820507913	-3.77317773639581
a_HQ_transcript/107819	-1.27943651353438	2.79635942697601	-2.16394170306716	0.0379009767401305	0.603260672494607	-3.91483937312415
a_HQ_transcript/89040	2.30050384198083	1.17481278811358	2.1635292940102	0.0379352430937773	0.603640791282531	-3.81338143119712
a_HQ_transcript/122357	-2.83708515904576	1.0702897342106	-2.16331717680699	0.0379528782785625	0.603756132615294	-3.78735030389955
a_HQ_transcript/92094	-3.31458631717269	2.93459473140427	-2.16309590869611	0.0379712820331684	0.603810575136141	-3.95185495375779
a_HQ_transcript/144600	3.16116283606082	3.46713743427319	2.16297826044359	0.0379810705425791	0.603810575136141	-4.00938612911696
a_HQ_transcript/144082	-1.70781842468406	0.731578647291187	-2.16287667760344	0.0379895241920981	0.603810575136141	-3.69274740436608
a_HQ_transcript/150742	0.924063286425576	4.44179479124496	2.1626916893633	0.0380049230785394	0.603810575136141	-4.12232552356423
a_HQ_transcript/111217	-0.635345100596124	3.37398874666604	-2.16265186820119	0.0380082386186199	0.603810575136141	-3.96160045101724
a_HQ_transcript/93851	1.89987108583867	-0.449354000192066	2.16217387152059	0.0380480570747025	0.603913476321431	-3.78971289091842
a_HQ_transcript/146593	2.67250180210955	2.27008187335007	2.16216580356161	0.0380487294765806	0.603913476321431	-3.87573928980956
a_HQ_transcript/86109	2.70266017991906	1.01474094255629	2.16210988876633	0.0380533898321503	0.603913476321431	-3.71728449220272
a_HQ_transcript/86444	2.25821525868519	0.7395677226531	2.16199216918699	0.0380632031190737	0.603913476321431	-3.73651562773829
a_HQ_transcript/91069	-2.23062205576728	-0.505531069947037	-2.16195067309463	0.038066662834464	0.603913476321431	-3.78233449895545
a_HQ_transcript/149620	3.90396915922766	0.870979626501447	2.16181175878543	0.0380782467798258	0.603932422596921	-3.71085609315905
a_HQ_transcript/152014	4.25014680913363	0.500556726237393	2.16109409693805	0.0381381419560296	0.604717380147323	-3.79566660799583
a_HQ_transcript/38348	2.46371528545496	0.510597516398661	2.1609089123279	0.0381536108340952	0.604797679455764	-3.7739577311988
a_HQ_transcript/26106	-2.73363030896763	1.33394708998716	-2.16046360048803	0.038190831563706	0.605026961167167	-3.70733681113397
a_HQ_transcript/113103	-3.11492829230331	1.82456558272929	-2.16040698055377	0.0381955663708116	0.605026961167167	-3.8043413430687
a_HQ_transcript/71886	-2.21825541813458	-0.0512176348617432	-2.16032478400671	0.0382024409357028	0.605026961167167	-3.76302719558313
a_HQ_transcript/62784	2.83617504926059	0.538583663805872	2.16023789485235	0.038209709166747	0.605026961167167	-3.7230467774175
a_HQ_transcript/141427	-2.22880788899534	0.141076500756023	-2.16006982889224	0.0382237712848415	0.605041515528645	-3.71816297188593
a_HQ_transcript/148434	2.26211777269715	0.557339780705214	2.15995521309717	0.0382333638552613	0.605041515528645	-3.71283022783649
a_HQ_transcript/64534	-1.87186751105673	-0.462261669940228	-2.15985378289333	0.0382418546664644	0.605041515528645	-3.78439329129689
a_HQ_transcript/132651	-2.77065220095548	0.32076135746216	-2.15954881912231	0.0382673935546329	0.605125227923648	-3.79779430924392
a_HQ_transcript/33694	2.15203669124521	0.169241416653147	2.1595419834447	0.0382679661754633	0.605125227923648	-3.74811547053814
a_HQ_transcript/152705	0.821058155541691	5.74744815060885	2.15933123393666	0.0382856242822822	0.605143415732731	-4.25758348681109
a_HQ_transcript/130704	0.885612212126905	3.75551825930941	2.1591916695726	0.0382973219752747	0.605143415732731	-4.05535781764131
a_HQ_transcript/45565	0.677893954542567	2.85057089276291	2.15915557287788	0.0383003479645045	0.605143415732731	-3.89992088340633
a_HQ_transcript/135861	3.6210491811612	1.27896436680093	2.1590246020652	0.038311329045473	0.605152427922623	-3.86938963583177
a_HQ_transcript/124144	-0.99266779575216	3.14847362065932	-2.15832206654192	0.0383702800525232	0.605654475032945	-3.95788490507229
a_HQ_transcript/147554	2.55087196952348	0.58909100501383	2.15826322601568	0.0383752211254706	0.605654475032945	-3.74017776438442
a_HQ_transcript/123689	-2.64787631711936	0.395853682526477	-2.15818264937351	0.0383819883845711	0.605654475032945	-3.77701585983996
a_HQ_transcript/83366	2.87757284756113	3.33228722152816	2.1580861538868	0.0383900939902916	0.605654475032945	-3.95314253039525
a_HQ_transcript/122711	2.23656106666155	1.67691662163242	2.15802526362483	0.0383952095461121	0.605654475032945	-3.82381640499902
a_HQ_transcript/105803	-2.2039513025762	0.789026448124008	-2.15757418791488	0.0384331245221164	0.60576480331011	-3.70880090046756
a_HQ_transcript/139542	-2.46704789832873	1.97621246068924	-2.15736295883965	0.0384508907358332	0.60576480331011	-3.79776134322081
a_HQ_transcript/106634	-2.23007306397361	-0.326647609011828	-2.15730201691831	0.0384560178420859	0.60576480331011	-3.78080226333908
a_HQ_transcript/10554	-3.77747837671296	1.76816328903359	-2.1569478797981	0.0384858237939568	0.60576480331011	-3.84876471576614
a_HQ_transcript/89966	-1.15645360027523	2.87757321605664	-2.15689298439658	0.0384904459041897	0.60576480331011	-3.91809173842435
a_HQ_transcript/127045	1.78609811088822	-0.652528991928406	2.15685118076073	0.0384939660385248	0.60576480331011	-3.79817822112492
a_HQ_transcript/90002	-3.26997785697883	1.12266914520313	-2.15683910374961	0.0384949830536411	0.60576480331011	-3.88590180371909
a_HQ_transcript/48756	2.60751867817511	1.85225781957017	2.15677568257518	0.0385003241955128	0.60576480331011	-3.74059850667432
a_HQ_transcript/150518	1.49896537448235	-0.771909796476643	2.15670804035415	0.0385060215475596	0.60576480331011	-3.80442381959072
a_HQ_transcript/111077	1.45197365795809	-0.790830818028537	2.15670335772641	0.0385064159825016	0.60576480331011	-3.8012646551641

a_HQ_transcript/27207	2.25888782624054	0.161992795264626	2.15643876453613	0.0385287094772127	0.605904735014966	-3.71646798161406
a_HQ_transcript/99578	3.25171870340843	1.03582942441181	2.15635038845363	0.0385361582258176	0.605904735014966	-3.84054724011784
a_HQ_transcript/121781	2.76844852472027	1.00068129967013	2.1557307695271	0.0385884185710746	0.606261180392836	-3.79866574591581
a_HQ_transcript/85398	-3.6086321181699	3.09308466694632	-2.15533763819895	0.0386216090020232	0.606261180392836	-3.99916330018929
a_HQ_transcript/152558	2.35184154677355	-0.149017378297626	2.15527642879891	0.0386267789381869	0.606261180392836	-3.79236395467928
a_HQ_transcript/55881	-1.72610320894581	0.281357436885037	-2.15511526193879	0.0386403945351995	0.606261180392836	-3.77676795814532
a_HQ_transcript/21295	-0.886975177728781	2.0824552088165	-2.15496889460258	0.0386527635452954	0.606261180392836	-3.76330022857016
a_HQ_transcript/114913	2.16233517591348	-0.365975213883444	2.15476546739082	0.0386699603392042	0.606261180392836	-3.74917376366208
a_HQ_transcript/81324	1.73576460139806	-0.742355070078244	2.15475995255578	0.038670426632492	0.606261180392836	-3.76052547494399
a_HQ_transcript/117070	-1.86337200423507	0.366879186061257	-2.15466072591457	0.038678817350801	0.606261180392836	-3.80496446986212
a_HQ_transcript/27273	-2.48089286414232	1.27205473963219	-2.15453182697097	0.0386897196099433	0.606261180392836	-3.78169035418909
a_HQ_transcript/102840	-2.56824173422482	0.442107188400197	-2.1544924778264	0.0386930483007183	0.606261180392836	-3.72214143831432
a_HQ_transcript/151315	3.0303788493986	0.343210265549405	2.1544813361667	0.0386939908614831	0.606261180392836	-3.84938324157636
a_HQ_transcript/47729	1.48857194187285	-0.393194632044416	2.15439423868147	0.0387013598274061	0.606261180392836	-3.87096792423964
a_HQ_transcript/2719	1.28446106022334	1.89321025259393	2.15432318656616	0.0387073721834821	0.606261180392836	-3.76853758996495
a_HQ_transcript/4752	1.54501697766097	-0.758004240396097	2.1543104396039	0.0387084509060104	0.606261180392836	-3.80486125802469
a_HQ_transcript/137803	4.17412156004897	2.65318775021481	2.15422980924007	0.038715274938381	0.606261180392836	-3.92255984676444
a_HQ_transcript/8258	-0.853963148539395	3.16559229357578	-2.15392646828613	0.0387409573356288	0.606493730249175	-3.96931592353986
a_HQ_transcript/44556	1.10285306896028	5.47181680254108	2.15371346714597	0.0387590001520884	0.606493730249175	-4.24161211328255
a_HQ_transcript/37510	1.86288125207461	-0.365185321179931	2.15361600531829	0.0387672584007833	0.606493730249175	-3.81307010209627
a_HQ_transcript/115795	2.81618672285902	0.0488582475029622	2.15338665777871	0.0387866979141454	0.606493730249175	-3.83170880909895
a_HQ_transcript/142000	0.915619978767973	3.82098859414591	2.15324737076432	0.0387985081199113	0.606493730249175	-4.08339163191149
a_HQ_transcript/28045	-1.37074201615763	-0.665497000221312	-2.15322831057412	0.0388001244902428	0.606493730249175	-3.81620732312123
a_HQ_transcript/66251	4.75274406924349	0.697373985065519	2.15288449173284	0.0388292918024353	0.606493730249175	-3.81223978700682
a_HQ_transcript/45606	-1.8244233418853	0.794475610865787	-2.15275556802812	0.0388402338584683	0.606493730249175	-3.76651824240796
a_HQ_transcript/23423	2.60518381768329	0.708060564610035	2.15247155443881	0.0388643484252933	0.606493730249175	-3.68077515899358
a_HQ_transcript/35908	1.69408047602115	1.04161151333636	2.15232380617362	0.0388768984575475	0.606493730249175	-3.72238962698346
a_HQ_transcript/4855	-1.37636187498677	2.29303248148948	-2.15209346074265	0.0388964716433381	0.606493730249175	-3.8551647880958
a_HQ_transcript/26350	-2.24292329901247	1.38753608580095	-2.15205938650863	0.0388993677831862	0.606493730249175	-3.81488726185769
a_HQ_transcript/63314	-2.55327317400195	1.31450556622016	-2.15203707810703	0.0389012639898086	0.606493730249175	-3.75200285744956
a_HQ_transcript/151211	2.40265479060643	2.13415953216544	2.15174310074946	0.0389262596418952	0.606493730249175	-3.83598656839532
a_HQ_transcript/114228	2.01051700011844	1.02192466662778	2.15155747795115	0.0389420497148925	0.606493730249175	-3.73399146675023
a_HQ_transcript/108510	1.86493428545807	0.111253135449206	2.15151234135715	0.0389458901364985	0.606493730249175	-3.7654090484582
a_HQ_transcript/40612	1.84787583329317	0.42736965152369	2.15146718299445	0.038949732747124	0.606493730249175	-3.73946635558742
a_HQ_transcript/106399	2.89476885073028	0.532888425836725	2.1514117843178	0.0389544471856876	0.606493730249175	-3.82306617469682
a_HQ_transcript/136935	-0.536246924506635	6.10121848959297	-2.15125049353	0.0389681759520744	0.606493730249175	-4.2921714241103
a_HQ_transcript/32853	2.69621431801296	3.36552234703404	2.15124198427113	0.0389689003634722	0.606493730249175	-3.97016596730715
a_HQ_transcript/18988	-5.53061828230984	3.23946130923866	-2.1511850716628	0.0389737457632024	0.606493730249175	-3.88307710519085
a_HQ_transcript/85959	1.15508195276569	1.57620209130337	2.15100112240102	0.0389894104139185	0.606493730249175	-3.74034484883367
a_HQ_transcript/138329	2.38774162847301	1.72086168821843	2.15082747231418	0.0390042031470274	0.606493730249175	-3.80435714163279
a_HQ_transcript/138709	3.80492617091117	0.595943318110993	2.15064016034241	0.0390201652868812	0.606493730249175	-3.83721833250714
a_HQ_transcript/60603	-2.09003519505324	0.736253798397588	-2.15046618218635	0.0390349963603698	0.606493730249175	-3.77940379713767
a_HQ_transcript/115455	0.979010187206789	2.59393974161571	2.15031411902016	0.0390479633544732	0.606493730249175	-3.91841269169928
a_HQ_transcript/111396	3.41332595099898	1.5068720396868	2.15022338283448	0.0390557025912439	0.606493730249175	-3.96810541213357
a_HQ_transcript/69936	-1.55099987732704	0.41620956011069	-2.15018918726832	0.0390586196156683	0.606493730249175	-3.70524416506784
a_HQ_transcript/99831	2.027398781249	1.86403568826557	2.15018144246562	0.0390592803065309	0.606493730249175	-3.79211115108198
a_HQ_transcript/126644	2.00417373328036	0.184771466965305	2.15006925558106	0.0390688518191163	0.606493730249175	-3.75425625629943
a_HQ_transcript/3719	2.94938056457242	2.1056949499095	2.15005931312234	0.0390697001860871	0.606493730249175	-3.77146559825084
a_HQ_transcript/79420	3.07932216678631	0.493078114952787	2.14982462899377	0.039089729993221	0.606493730249175	-3.83985346633507
a_HQ_transcript/151126	-3.03013421978088	1.09343272518341	-2.14968699321695	0.0391014811677704	0.606493730249175	-3.87472612035205

a_HQ_transcript/72808	-0.78988006886718	3.01888547359354	-2.14962846534374	0.0391064791572648	0.606493730249175	-3.95298197843228
a_HQ_transcript/7985	-2.51364096686665	1.47041120032914	-2.14955877916316	0.0391124307510594	0.606493730249175	-3.87450734835885
a_HQ_transcript/115667	1.99717200408251	1.41580856318174	2.14954198484017	0.0391138652015402	0.606493730249175	-3.71834509633419
a_HQ_transcript/138447	2.51105156911939	6.99676799795951	2.14951494840451	0.0391161745579438	0.606493730249175	-4.43129663785964
a_HQ_transcript/123040	-1.39525630734651	2.06678212538579	-2.14922958832801	0.0391405563918011	0.606611724620932	-3.78221403734448
a_HQ_transcript/101544	3.01716802542348	0.220468434973906	2.14918161981469	0.0391446562595155	0.606611724620932	-3.79831206247526
a_HQ_transcript/38354	1.72176801092228	-0.340210189671687	2.14869509490735	0.0391862610987105	0.607094612245694	-3.80464700743594
a_HQ_transcript/100527	-1.63000071929491	2.0696010572988	-2.14811102908197	0.0392362589711975	0.607524542826774	-3.81193730760008
a_HQ_transcript/103271	-0.995619904035149	2.8417801815589	-2.14808849703001	0.0392381889206188	0.607524542826774	-3.96716626897515
a_HQ_transcript/132232	2.5880844689409	1.30968911122242	2.14800470895376	0.0392453664037045	0.607524542826774	-3.87506821402323
a_HQ_transcript/50991	2.40962756452877	2.03039134149801	2.1477534196606	0.0392668994290979	0.607530514933112	-3.75405922677769
a_HQ_transcript/140500	1.37358750959762	3.8922019195014	2.14769729184777	0.039271710465405	0.607530514933112	-4.07939730226266
a_HQ_transcript/32130	-0.944457911983538	1.99049170436144	-2.14752929833659	0.0392861132808284	0.607530514933112	-3.74768766334415
a_HQ_transcript/6544	-1.73756979798762	2.00887479736276	-2.14751244338163	0.0392875585885214	0.607530514933112	-3.75494508350359
a_HQ_transcript/63324	1.03031405534608	3.29475023235021	2.14697404720147	0.0393337507709154	0.608083049152066	-3.91070971345278
a_HQ_transcript/59524	-2.0219841893912	0.863627233100816	-2.14667218422009	0.0393596704779364	0.608107837806851	-3.72796854270686
a_HQ_transcript/1292	2.63823413362978	1.84636489210023	2.14656057578508	0.0393692576669259	0.608107837806851	-3.8643225403492
a_HQ_transcript/142832	2.33772716279292	0.0506896104133269	2.14649574685533	0.0393748274385206	0.608107837806851	-3.80772904974341
a_HQ_transcript/10380	4.35131854245348	3.6998279098717	2.14646813009644	0.0393772003424337	0.608107837806851	-3.99633683246906
a_HQ_transcript/5323	2.78110667875296	-0.0966837397841608	2.14633198311043	0.0393889003031447	0.608126957987037	-3.86234409428443
a_HQ_transcript/36093	1.90298485691287	1.45179155048705	2.14616160212051	0.039403546555716	0.608191543863691	-3.75631433356642
a_HQ_transcript/144725	-1.08333797310417	3.1749493135497	-2.14557407862653	0.03945408822594	0.6086249615157	-3.98445884686645
a_HQ_transcript/85082	2.2446316895202	0.318609450373891	2.14526567140462	0.0394806419758653	0.6086249615157	-3.78654812224253
a_HQ_transcript/89830	-2.52822792534255	0.907091247336465	-2.14510228768227	0.039494715685851	0.6086249615157	-3.81236770574717
a_HQ_transcript/18620	-1.07306899667213	1.85952054632219	-2.14505108632902	0.0394991270359337	0.6086249615157	-3.73208774922142
a_HQ_transcript/137948	-3.15147887934739	0.491483786150586	-2.14463589708506	0.0395349146204119	0.6086249615157	-3.86749102101681
a_HQ_transcript/123904	-2.88781666920287	1.84764233187798	-2.14416902633195	0.0395751913294591	0.6086249615157	-3.82085609513829
a_HQ_transcript/5244	2.21696933580166	1.88376440471885	2.14408846647405	0.0395821448736935	0.6086249615157	-3.83767618261173
a_HQ_transcript/2005	1.58159672260405	2.73759972010444	2.1438815920607	0.0396000062610536	0.6086249615157	-3.86197895158235
a_HQ_transcript/49955	1.20642579449695	-0.736868132852571	2.14374259617384	0.0396120110851556	0.6086249615157	-3.81514267907549
a_HQ_transcript/140542	-2.37450728300376	-0.526737740144954	-2.14357209458371	0.039626741413496	0.6086249615157	-3.77544401189951
a_HQ_transcript/35122	1.4022996497565	-0.292326461398267	2.14355996482204	0.0396277895384269	0.6086249615157	-3.74124165717874
a_HQ_transcript/56749	-0.912648764679024	1.88091104857775	-2.14339648603337	0.0396419180376694	0.6086249615157	-3.76101012993308
a_HQ_transcript/135884	-3.51315454744902	-0.246663647919154	-2.14329009945817	0.0396511147989194	0.6086249615157	-3.80178072963873
a_HQ_transcript/92358	-2.40786882896838	0.523952476576765	-2.1431976275288	0.0396591102249203	0.6086249615157	-3.78770350490153
a_HQ_transcript/12195	1.7841667447558	0.140304525378754	2.14310573162885	0.0396670572637475	0.6086249615157	-3.82816759017753
a_HQ_transcript/104943	2.09435909311474	1.9049030584254	2.14309942666527	0.0396676025606931	0.6086249615157	-3.79045476268572
a_HQ_transcript/137331	3.22459056440113	-0.129399320200325	2.14309823375934	0.0396677057322144	0.6086249615157	-3.8210046400929
a_HQ_transcript/42566	1.62086688907675	0.330505658411838	2.14291471145944	0.0396835809661113	0.6086249615157	-3.80015226794486
a_HQ_transcript/67322	-2.15418563909331	-0.0916279981609078	-2.14289201529913	0.0396855446445312	0.6086249615157	-3.77915738630781
a_HQ_transcript/152486	0.664235891311938	5.8027447558607	2.14281456283549	0.0396922465039381	0.6086249615157	-4.2780632954032
a_HQ_transcript/104063	1.8551760185296	-0.409440278156475	2.14279814681357	0.0396936670896999	0.6086249615157	-3.80590621980462
a_HQ_transcript/117426	3.44285765882515	1.01152019375696	2.14266761377204	0.0397049645744226	0.6086249615157	-3.90369031229832
a_HQ_transcript/108096	2.74921149275334	0.163523165299755	2.14266497807002	0.0397051927208073	0.6086249615157	-3.84692458420158
a_HQ_transcript/34750	1.16368587953113	2.26657974380154	2.14263434688294	0.0397078442419298	0.6086249615157	-3.83830748295496
a_HQ_transcript/148493	2.08846222639335	-0.449836208054297	2.14261929253345	0.0397091474460976	0.6086249615157	-3.81592506369059
a_HQ_transcript/81534	3.97613186571811	2.24793026405743	2.14255475253226	0.0397147348863363	0.6086249615157	-3.93059906619685
a_HQ_transcript/62968	1.41961831451933	-0.682531827266601	2.14251450252013	0.0397182198157235	0.6086249615157	-3.82504786665179
a_HQ_transcript/134972	-1.38139006159887	-0.772739677508907	-2.14243852566989	0.0397247987887174	0.6086249615157	-3.80960978195331
a_HQ_transcript/129408	-0.689203541633552	4.31462823885376	-2.14212628249576	0.0397518466507398	0.608641593772595	-4.12042526948438

a_HQ_transcript/63536	-1.25442168032309	3.245282097087	-2.14211969345146	0.0397524175985419	0.608641593772595	-3.93829709199599
a_HQ_transcript/137010	3.97661733294222	1.47940658097755	2.14204730989588	0.0397586901911056	0.608641593772595	-3.77766953437856
a_HQ_transcript/129006	2.89069778451149	0.915410772772274	2.14163471796888	0.0397944612422933	0.608641593772595	-3.84135127104609
a_HQ_transcript/55058	-2.10262065757391	-0.538949095849856	-2.14141213878731	0.0398137703587831	0.608641593772595	-3.81323207090687
a_HQ_transcript/37123	2.00978631869601	3.14021692174165	2.1413633761205	0.0398180017123252	0.608641593772595	-4.01728342203844
a_HQ_transcript/82303	4.76416671159937	1.95520894845675	2.14126592624686	0.0398264590676004	0.608641593772595	-3.93856166363872
a_HQ_transcript/149833	-3.96584800266211	8.2884183037794	-2.14124521042174	0.0398282571317207	0.608641593772595	-4.68507425301653
a_HQ_transcript/100546	2.43875412439698	1.99911922851414	2.14123675540303	0.0398289910196881	0.608641593772595	-3.82627943469812
a_HQ_transcript/14093	-0.552179596449298	3.56159695812889	-2.1412183169773	0.039830591499982	0.608641593772595	-4.03739114984307
a_HQ_transcript/2284	2.94784624258402	-0.010241474783165	2.14100462066459	0.0398491447948356	0.608652406926163	-3.80931957725883
a_HQ_transcript/143696	2.92905850336072	1.11378742490661	2.14050295441718	0.0398927300325906	0.608652406926163	-3.83222730381182
a_HQ_transcript/143240	0.463359135239329	10.4576411735256	2.14036939786486	0.0399043406826226	0.608652406926163	-4.76122202872416
a_HQ_transcript/130	2.81751408327088	-0.0738834240202523	2.14034723216779	0.0399062679329623	0.608652406926163	-3.77556616822979
a_HQ_transcript/16896	-1.3397591312994	3.51403316925606	-2.14021893100521	0.039917425012371	0.608652406926163	-3.97161120833025
a_HQ_transcript/80379	2.39285219692863	0.142365989508395	2.14020546228209	0.0399185964141974	0.608652406926163	-3.74368187458422
a_HQ_transcript/127867	-3.35582442530859	3.4918796412463	-2.14020358582321	0.0399187596160057	0.608652406926163	-4.05652430050164
a_HQ_transcript/142664	-4.27916205621611	1.55445846310575	-2.13991997226887	0.0399434332317701	0.608652406926163	-3.88588068876167
a_HQ_transcript/80294	3.19512410622933	1.46551956368661	2.13977984673459	0.0399556287717671	0.608652406926163	-3.83589445203625
a_HQ_transcript/65150	3.22867273179818	2.00349253089693	2.13967006116757	0.039965186043673	0.608652406926163	-3.84804093590192
a_HQ_transcript/136489	-3.00212538181287	0.253070283463984	-2.13964758067671	0.0399671433104277	0.608652406926163	-3.84478075615427
a_HQ_transcript/141623	1.42469419472782	2.8924582226361	2.13963465399423	0.0399682688121868	0.608652406926163	-3.89044892623822
a_HQ_transcript/112616	1.00408198779739	2.86906838156268	2.13959388793632	0.0399718184198942	0.608652406926163	-4.00149001126896
a_HQ_transcript/128703	0.414262382955297	6.4255644243446	2.13952414561716	0.0399778917155947	0.608652406926163	-4.33718884450158
a_HQ_transcript/59978	0.541793129328652	6.04078456857897	2.13900226346376	0.0400233642224842	0.609185157246547	-4.31749021130638
a_HQ_transcript/78124	2.06025436475109	0.834681624739248	2.13868188506744	0.0400513020895174	0.609383018499456	-3.86563625656126
a_HQ_transcript/6914	1.88038884692424	-0.679557894854858	2.13861277883427	0.0400573306097994	0.609383018499456	-3.83242916278381
a_HQ_transcript/128945	2.39265924006202	0.624465133797752	2.13825049551035	0.0400889477744048	0.609581683718559	-3.74889103876089
a_HQ_transcript/133082	-1.77308259910303	-0.646877898782514	-2.13810348258392	0.0401017841920581	0.609581683718559	-3.82145535092531
a_HQ_transcript/64797	-1.53035106095948	1.23907658355783	-2.13810272472361	0.0401018503739982	0.609581683718559	-3.81087610726431
a_HQ_transcript/31446	1.6668282579154	-0.437891283647144	2.13778762559417	0.0401293755488647	0.609596018694843	-3.81586094969159
a_HQ_transcript/48497	-0.919680500576296	4.37340358984208	-2.13774327113255	0.0401332514351328	0.609596018694843	-4.17506234136874
a_HQ_transcript/1297	-4.18170475011479	1.83481837109396	-2.13734787363438	0.0401678176762553	0.609596018694843	-3.90226791945191
a_HQ_transcript/151266	1.9917900223963	1.79794000850786	2.13724267202602	0.0401770190069595	0.609596018694843	-3.75234038636934
a_HQ_transcript/9250	2.28998411824232	1.20839002257393	2.1372169545747	0.0401792686370447	0.609596018694843	-3.73222826170104
a_HQ_transcript/133618	3.11092664389507	0.570262145656468	2.13718266889209	0.0401822679456827	0.609596018694843	-3.88515858742838
a_HQ_transcript/40147	1.33368503902442	0.916146115431721	2.13714622291243	0.0401854564547763	0.609596018694843	-3.84644624885908
a_HQ_transcript/26760	-3.0072667621562	-0.579097339447317	-2.13710068398585	0.0401894407839834	0.609596018694843	-3.8397596455313
a_HQ_transcript/147204	-2.70672380637775	-0.0647763182087915	-2.13701226739444	0.0401971776021424	0.609596018694843	-3.84718342534334
a_HQ_transcript/51751	-1.44122549617953	3.33477483802697	-2.13679818161421	0.0402159164697619	0.609647061713837	-4.00289136934242
a_HQ_transcript/95711	1.49778667213795	-0.739552893139189	2.13672268189475	0.040222526787413	0.609647061713837	-3.82801995155102
a_HQ_transcript/14722	2.85963585486444	-0.190323011064037	2.13661441782053	0.0402320074445066	0.609647061713837	-3.82514279909618
a_HQ_transcript/65756	0.922540643671688	2.37501534365	2.13646098924511	0.0402454465415257	0.609684566286716	-3.92560618204598
a_HQ_transcript/125216	0.684578425745122	5.15234435827715	2.13634670195668	0.0402554597682428	0.609684566286716	-4.21503400752309
a_HQ_transcript/150185	-2.66874370256727	1.14244837085918	-2.13620583875128	0.0402678044727098	0.609712669546672	-3.78662423572739
a_HQ_transcript/137751	3.688483197913	4.35353351013668	2.13600932654187	0.0402850316513557	0.609717158561306	-4.1579735734259
a_HQ_transcript/60795	-1.9301700939136	-0.560622885384447	-2.13593869845333	0.0402912248369661	0.609717158561306	-3.82780840774867
a_HQ_transcript/70128	2.81048574492217	1.59306208629897	2.13584356013771	0.0402995685933206	0.609717158561306	-3.76070980666222
a_HQ_transcript/14837	2.05264490132937	1.32859760643186	2.13525430623389	0.0403512811026875	0.610239624517459	-3.77667906713468
a_HQ_transcript/69284	1.71640096722721	0.51331425348136	2.13521084389164	0.0403550976576712	0.610239624517459	-3.89315980277762
a_HQ_transcript/141767	-1.70381812743374	-0.294724377818591	-2.13473808690445	0.0403966325196312	0.610708830975584	-3.78420330604264

a_HQ_transcript/64913	2.24515665608178	1.53596929064485	2.13460386874774	0.0404084313919571	0.610728367122122	-3.87184231079819
a_HQ_transcript/79919	2.1926202085113	0.0333318945591351	2.13387482209633	0.0404725739668455	0.611538804144735	-3.76185295090869
a_HQ_transcript/1646	1.13426739856061	3.52801342393589	2.13362339974465	0.040494715403401	0.611624008842252	-4.04142900057527
a_HQ_transcript/123371	-1.77013743241742	4.21084674279766	-2.13345619313097	0.0405094463537315	0.611624008842252	-4.13904432053348
a_HQ_transcript/94036	-1.38114578516041	3.15322071562494	-2.1334524179208	0.0405097790056887	0.611624008842252	-4.10041091613795
a_HQ_transcript/39090	-1.39329033153062	2.44934018517624	-2.13331931650165	0.0405215087618235	0.611642238719106	-3.81511867131044
a_HQ_transcript/72427	-2.08405954735331	-0.339112891259619	-2.13287729596354	0.0405604840824739	0.611696739328078	-3.84418562871863
a_HQ_transcript/137924	-2.91717985350127	2.04451100772627	-2.13286673997115	0.0405614152672997	0.611696739328078	-3.83000686601919
a_HQ_transcript/116303	1.9354862267946	-0.630212900400287	2.13281722714007	0.0405657832382677	0.611696739328078	-3.84446082240873
a_HQ_transcript/22338	-2.00547472369752	0.680033065419391	-2.13275633179387	0.0405711559350977	0.611696739328078	-3.72947031628147
a_HQ_transcript/4913	1.25896021203163	3.27896859156905	2.13260488006815	0.0405845210101801	0.611696739328078	-3.98094795576423
a_HQ_transcript/111774	1.72290101199696	-0.78246359792219	2.13256252858423	0.0405882590763212	0.611696739328078	-3.83265611436482
a_HQ_transcript/136041	1.26248602759067	2.92013948493613	2.13233589366711	0.0406082677284554	0.611817659191275	-3.86052022272322
a_HQ_transcript/71427	-2.71096023299394	-0.242360135880415	-2.13223324256923	0.0406173332442047	0.611817659191275	-3.83324448919246
a_HQ_transcript/19218	-1.29582411863021	2.35839203805088	-2.13185525012114	0.0406507306960288	0.612162091683618	-3.82209876041659
a_HQ_transcript/86979	-1.80068581465758	-0.12388922946703	-2.13153008479377	0.0406794801018984	0.61228151869831	-3.8735680793061
a_HQ_transcript/78842	2.92646912994237	0.74915512498503	2.13152728264272	0.040679727931683	0.61228151869831	-3.88793680643364
a_HQ_transcript/106894	2.06194467390258	-0.503743769451073	2.13130628075593	0.0406992781514034	0.612407770724734	-3.84358085066918
a_HQ_transcript/133975	2.33126810965985	1.72818247306424	2.13119430313033	0.0407091870712974	0.612407770724734	-3.84914604306448
a_HQ_transcript/32882	-1.03174915605784	3.00720807890917	-2.12937807744912	0.0408702042258385	0.614609877638992	-3.96444876447033
a_HQ_transcript/114001	-3.54128757838452	0.916867412214576	-2.12922034415244	0.0408842146568001	0.614609877638992	-3.818041494438
a_HQ_transcript/68323	-1.1341584105021	2.97386329407398	-2.1289974090074	0.0409040238096598	0.614609877638992	-3.88464878084679
a_HQ_transcript/91896	2.81368111594675	0.79638924571459	2.12898587676812	0.0409050487511834	0.614609877638992	-3.9421544926231
a_HQ_transcript/135027	-0.886093495705526	2.59537792091668	-2.12873431980084	0.0409274118429969	0.614609877638992	-3.85605788817823
a_HQ_transcript/50874	-1.69492643861196	2.64760038669671	-2.12867089277266	0.0409330521352453	0.614609877638992	-3.89852762290279
a_HQ_transcript/106163	-3.11295139181568	3.95770543697506	-2.12861937421604	0.0409376339656308	0.614609877638992	-4.15767131906657
a_HQ_transcript/124231	2.30578516348689	1.61271962593732	2.12851618404432	0.0409468126062066	0.614609877638992	-3.79010887703905
a_HQ_transcript/44974	-0.773078745185142	2.78634810473161	-2.12839441512891	0.0409576461502398	0.614609877638992	-3.94174631395315
a_HQ_transcript/147536	-3.65541383712665	1.67413082133193	-2.1283533098875	0.0409613037774129	0.614609877638992	-3.96274968240139
a_HQ_transcript/118592	1.80445453333065	5.37164368589543	2.12809464841422	0.0409843266413321	0.614772502570606	-4.30097185521562
a_HQ_transcript/1410	-1.13330666048238	1.83681229018877	-2.12790036187074	0.0410016271842658	0.614772502570606	-3.81618220538031
a_HQ_transcript/29960	1.90375023677545	1.97421989115601	2.12750124590053	0.0410371873886018	0.614772502570606	-3.85489660072123
a_HQ_transcript/39083	-3.95515736777009	4.18171255945978	-2.12747476281726	0.0410395479300071	0.614772502570606	-4.26039031405277
a_HQ_transcript/48699	-1.59411273773182	1.96634835811171	-2.12734856316081	0.0410507982553935	0.614772502570606	-3.83225536307562
a_HQ_transcript/31372	1.71926940598304	-0.686081401638949	2.12733335652641	0.0410521540663281	0.614772502570606	-3.81159367148943
a_HQ_transcript/56359	2.55206736095261	0.52141443402353	2.12718099812634	0.0410657404064911	0.614772502570606	-3.8297012469901
a_HQ_transcript/126422	3.25881287272226	-0.252439872404547	2.12713944228252	0.0410694467805643	0.614772502570606	-3.84020605075135
a_HQ_transcript/2708	-1.63587503308683	0.0524444964068988	-2.1271234047812	0.0410708772478334	0.614772502570606	-3.76337049638415
a_HQ_transcript/23739	2.32651017766198	0.194135112190149	2.12698915065656	0.0410828537965071	0.614772502570606	-3.80751648902213
a_HQ_transcript/103040	1.02222112655533	3.78548154518546	2.12692609293581	0.0410884801212291	0.614772502570606	-4.10564030527562
a_HQ_transcript/25219	-2.46639475136864	-0.513089429378068	-2.12655935895665	0.0411212154970625	0.614948923355748	-3.85825010116596
a_HQ_transcript/36514	2.58207723605431	-0.623009517857108	2.12655696019606	0.041121429691092	0.614948923355748	-3.8546950099908
a_HQ_transcript/123866	8.49558497858366	5.75036555498643	2.12602995819073	0.0411685115921309	0.61500857662314	-4.13162331149661
a_HQ_transcript/108292	2.01320513754799	-0.042342693037054	2.12600668321098	0.0411705920598072	0.61500857662314	-3.84373921657785
a_HQ_transcript/135975	-3.43536009277101	1.45545865254423	-2.12600106857385	0.0411710939462057	0.61500857662314	-3.75652704346897
a_HQ_transcript/132280	3.1657023987928	1.37200289541979	2.12586931751454	0.0411828725889363	0.61500857662314	-3.95397205346577
a_HQ_transcript/152280	-5.37990563463554	3.57732372695418	-2.12583584973642	0.0411858651088553	0.61500857662314	-4.00170400032712
a_HQ_transcript/151486	-1.91459122384179	3.17433149732469	-2.12565960963571	0.0412016267895719	0.61500857662314	-4.00995010880886
a_HQ_transcript/6462	-1.60286927497242	0.885583013912473	-2.1255727416447	0.0412093976212305	0.61500857662314	-3.73155990308721
a_HQ_transcript/121757	2.69833985306283	4.13571850860241	2.12552920183235	0.0412132929916358	0.61500857662314	-3.98210338434113

a_HQ_transcript/8875	1.44728547376999	1.80321291133336	2.12544708176109	0.0412206409049641	0.61500857662314	-3.79745932510236
a_HQ_transcript/91857	0.63217282994655	4.1625399710423	2.12505143165013	0.0412560590331581	0.615240442308625	-4.16452839188736
a_HQ_transcript/56408	-4.13354122867913	1.73236730101862	-2.1250370155201	0.0412573500570985	0.615240442308625	-3.90415252958096
a_HQ_transcript/54978	1.56361460761932	-0.702507590983224	2.12459289291055	0.0412971406153664	0.615480904956029	-3.85691312446633
a_HQ_transcript/7935	-3.39764054250576	3.55017216268004	-2.12409343092201	0.0413419298544852	0.615480904956029	-3.84243110383275
a_HQ_transcript/85999	1.37064345230374	-0.444251398893082	2.12405750883299	0.0413451528254214	0.615480904956029	-3.80903901081798
a_HQ_transcript/87960	0.499721895441332	5.00673689061273	2.12404198382108	0.0413465458161608	0.615480904956029	-4.25556092951897
a_HQ_transcript/128402	0.845425289570749	4.54816846261352	2.12388175521659	0.0413609248507323	0.615480904956029	-4.21309651476527
a_HQ_transcript/147402	-2.21225183515269	-0.336603140553103	-2.12386140667836	0.0413627512608034	0.615480904956029	-3.8517993142651
a_HQ_transcript/118461	3.20939901555965	0.509069042722821	2.1237725184571	0.0413707303791046	0.615480904956029	-3.87423604692314
a_HQ_transcript/151245	-5.35492853261535	2.48218346573544	-2.1237043427009	0.0413768511515172	0.615480904956029	-3.93923774771382
a_HQ_transcript/11276	-2.18602039048883	1.14237534292423	-2.12357083441551	0.0413888397576944	0.615480904956029	-3.82682017239455
a_HQ_transcript/84461	-2.83173987816714	0.571948507116084	-2.12355229265932	0.0413905049899811	0.615480904956029	-3.86526338608258
a_HQ_transcript/151585	-5.76987629115086	5.58204170375838	-2.12340216389873	0.0414039902174348	0.615480904956029	-4.18467491318211
a_HQ_transcript/142978	3.13276039181904	0.157848987723306	2.12323850396064	0.0414186953098811	0.615480904956029	-3.84915723794936
a_HQ_transcript/143128	4.48668394374411	2.9078605958528	2.12318523288094	0.0414234827949649	0.615480904956029	-3.91360233773144
a_HQ_transcript/13120	1.03343299645408	2.75644350082365	2.12311556283564	0.0414297447990822	0.615480904956029	-3.97990760642989
a_HQ_transcript/108796	2.88525721292508	1.0937711827	2.12298893315869	0.041441128531986	0.615480904956029	-3.87980682342618
a_HQ_transcript/47069	-0.802607723132573	4.21208559877313	-2.12295174839191	0.0414444718881112	0.615480904956029	-4.18592492849753
a_HQ_transcript/44115	1.89736629613765	1.06736914562732	2.1228516007716	0.0414534775478746	0.615480904956029	-3.73655780901136
a_HQ_transcript/89717	-2.87692340574062	1.75565273716847	-2.12254764674922	0.0414808208818898	0.615729610884191	-3.93473936350235
a_HQ_transcript/55461	2.86341770468764	-0.0375335275704244	2.12236447934109	0.0414973061142134	0.615817056371457	-3.87923560742284
a_HQ_transcript/133930	1.52245646473823	3.04090299388453	2.12209573033817	0.0415215042733957	0.615927117460523	-3.96849194548528
a_HQ_transcript/745	-0.818470233404238	4.7129660241521	-2.12204676162052	0.0415259147627269	0.615927117460523	-4.2020600123145
a_HQ_transcript/107721	7.50271009638738	1.92779821884845	2.12175837943278	0.0415518970420013	0.616155273279962	-3.99806466316776
a_HQ_transcript/96528	-2.15265441960663	1.68425607216066	-2.12137839207928	0.0415861546333131	0.616505992482842	-3.82676123020064
a_HQ_transcript/117014	5.37072607516157	4.56408557207134	2.12124665664755	0.0415980370220529	0.616523193211062	-4.27038621778149
a_HQ_transcript/57730	1.01602980918461	2.73690017232612	2.12110256872417	0.0416110370354918	0.616523193211062	-4.01191078810769
a_HQ_transcript/38322	1.94983548823836	-0.196350522315628	2.12101284684852	0.0416191338109036	0.616523193211062	-3.78986908426384
a_HQ_transcript/106404	0.991162508493566	3.92237554810683	2.12060105293499	0.0416563132529663	0.616569738971045	-4.097234525921
a_HQ_transcript/3002	3.5191086153679	1.03172290713985	2.12031208559002	0.0416824206738657	0.616569738971045	-3.82466740005756
a_HQ_transcript/141625	-2.69160197952152	2.95637438990255	-2.1202636604782	0.0416867971698318	0.616569738971045	-4.03586922314494
a_HQ_transcript/128905	1.3073769120217	1.82584734286413	2.12017816401211	0.0416945250416701	0.616569738971045	-3.83001623032657
a_HQ_transcript/135855	-2.10538741859687	1.25709281507988	-2.12014706170525	0.0416973366380157	0.616569738971045	-3.80559229431733
a_HQ_transcript/78910	2.33513302705389	0.528521957389963	2.12008089971946	0.0417033181290836	0.616569738971045	-3.79067340835438
a_HQ_transcript/151454	1.85259271155547	2.4738039077447	2.1198522378516	0.0417239965614098	0.616569738971045	-3.88553165276429
a_HQ_transcript/19273	2.53435960179877	2.00033047799763	2.11973553220331	0.041734554028577	0.616569738971045	-3.9323853380154
a_HQ_transcript/10211	-2.85933625427662	-0.0659921011212734	-2.11949567674172	0.0417562593458421	0.616569738971045	-3.88041549667409
a_HQ_transcript/68722	0.550401599482342	4.57090539578653	2.11947374148639	0.0417582448388353	0.616569738971045	-4.21667514391238
a_HQ_transcript/107184	-2.27946198343083	0.549014497306021	-2.11946602407929	0.0417589434080406	0.616569738971045	-3.87900695535398
a_HQ_transcript/134681	0.629803834673974	4.55769870649627	2.11933975581466	0.0417703745099412	0.616569738971045	-4.22750709883606
a_HQ_transcript/18244	2.16705116708616	-0.0139033149275461	2.11926091276418	0.0417775135991453	0.616569738971045	-3.84194277096988
a_HQ_transcript/147961	-3.16808760116557	3.53510704870855	-2.11912811565065	0.0417895405697508	0.616569738971045	-4.27257603869941
a_HQ_transcript/127682	1.56834289343674	1.5356342019864	2.11904229227001	0.0417973149246382	0.616569738971045	-3.79026663978813
a_HQ_transcript/150465	-3.00564330429609	1.28504850123964	-2.11902490744224	0.0417988898941696	0.616569738971045	-3.97136272280962
a_HQ_transcript/123440	-5.22620047139512	0.075901730012406	-2.11891201349647	0.0418091187420513	0.616569738971045	-3.8980589981921
a_HQ_transcript/98375	-1.95494170916707	3.0629619153542	-2.11886693418309	0.0418132038092461	0.616569738971045	-3.87639767819567
a_HQ_transcript/152373	3.04941605510603	0.150768834618688	2.118686628159	0.0418295465951201	0.616654294821492	-3.90694774472729
a_HQ_transcript/81010	3.04281149335621	0.468461860649507	2.11856591190595	0.0418404913756856	0.616659250173898	-3.84626801991389
a_HQ_transcript/66882	5.06194608829375	1.49198528959342	2.11820111467109	0.0418735812557344	0.616800816128979	-3.8847183238036

a_HQ_transcript/24322	2.49533938031399	0.769622195527376	2.11813011706433	0.0418800239713123	0.616800816128979	-3.87985623112973
a_HQ_transcript/145089	1.65495209973221	2.95443367540165	2.11810911590862	0.0418819299005828	0.616800816128979	-3.94998240359173
a_HQ_transcript/140504	-0.792334629779998	4.04246398747441	-2.11787847035897	0.0419028668557664	0.616828739712731	-4.14891824623914
a_HQ_transcript/148861	-1.83033594686403	-0.413264610516915	-2.11780474105201	0.0419095616219557	0.616828739712731	-3.86310442833846
a_HQ_transcript/110631	1.04619200132259	1.82651990904405	2.11762606203469	0.041925789957643	0.616828739712731	-3.79785302040113
a_HQ_transcript/147929	4.01537986890575	0.178850557240678	2.11749463658968	0.0419377300882687	0.616828739712731	-3.87192458941797
a_HQ_transcript/90687	-2.15752980032774	6.01056370669424	-2.11738784505343	0.0419474344213942	0.616828739712731	-4.28634440659483
a_HQ_transcript/11137	-1.72969739446204	1.35217937205525	-2.1173490473472	0.0419509605290058	0.616828739712731	-3.78739280990354
a_HQ_transcript/20701	2.56565303735113	1.83245252103779	2.11727042499645	0.0419581068817805	0.616828739712731	-3.89142114145829
a_HQ_transcript/34336	3.61522847191736	0.308804074342576	2.11699717655013	0.041982952093693	0.616898013608418	-3.87510331094861
a_HQ_transcript/151984	2.33783275982044	1.93822170762174	2.11698516492289	0.0419840445539998	0.616898013608418	-3.81825054962634
a_HQ_transcript/89503	2.0821059790535	3.15645892422822	2.11661519730897	0.0420177055245346	0.617236590025309	-4.0987985668317
a_HQ_transcript/16358	3.42184093046858	1.51357065793279	2.11624678478917	0.0420512487495676	0.617412238714659	-3.98117298406399
a_HQ_transcript/134180	-2.23592702476356	0.283452749081214	-2.11617957759915	0.042057370386488	0.617412238714659	-3.87879034130105
a_HQ_transcript/132534	0.860313313630937	2.70654430747397	2.11613394410723	0.0420615274103711	0.617412238714659	-4.00877350908567
a_HQ_transcript/147653	0.761724998728308	4.68794224391891	2.1156478061817	0.0421058351659123	0.617502435943631	-4.23421696592356
a_HQ_transcript/77578	2.32415351422382	1.32628465906402	2.11536821068958	0.0421313368969607	0.617502435943631	-3.83873939640802
a_HQ_transcript/89380	2.14046527357715	0.754357566691815	2.11523097392708	0.04214385913866	0.617502435943631	-3.87119345043877
a_HQ_transcript/115389	2.98160747677984	2.38873772955959	2.11518888121068	0.0421477005795498	0.617502435943631	-3.87827118911134
a_HQ_transcript/76927	-1.84690789551466	-0.668652838997862	-2.11513543462956	0.0421525786368742	0.617502435943631	-3.86271587872855
a_HQ_transcript/1178	1.41131606194449	2.1946536984117	2.11499604895527	0.0421653026871461	0.617502435943631	-3.82328956270373
a_HQ_transcript/136185	-1.61552232485335	-0.648220031216103	-2.11497404105838	0.0421673120249453	0.617502435943631	-3.85840418384366
a_HQ_transcript/96232	1.14274878490126	2.89981976313427	2.11484917466949	0.0421787140265093	0.617502435943631	-3.92782441813374
a_HQ_transcript/133565	-3.01976659803123	3.14607242528312	-2.1145171654048	0.042209044270308	0.617502435943631	-3.93705089816532
a_HQ_transcript/120390	1.23660505512372	4.44931174133831	2.11438245419709	0.0422213561298901	0.617502435943631	-4.18711140122308
a_HQ_transcript/140940	1.80188121313423	-0.304509633818473	2.11428098276691	0.0422306321565629	0.617502435943631	-3.93949147658784
a_HQ_transcript/140	1.74110892098878	2.6931580483649	2.11426271760528	0.0422323020607405	0.617502435943631	-3.96758448206619
a_HQ_transcript/74859	2.91003187022586	1.53649046179411	2.1142620561671	0.0422323625342488	0.617502435943631	-3.85306660211894
a_HQ_transcript/124417	-0.936840196579455	3.07566065586028	-2.11420310850917	0.0422377522674325	0.617502435943631	-4.0217010041518
a_HQ_transcript/730	2.17504860326562	0.514567641981721	2.1140261129286	0.0422539390790314	0.617502435943631	-3.78909075167448
a_HQ_transcript/32559	1.89133294643928	0.875096569838251	2.11378347870717	0.042276137676879	0.617502435943631	-3.92651597922332
a_HQ_transcript/121519	-4.26750612045192	1.13599840643931	-2.11376282059136	0.0422780281640607	0.617502435943631	-3.8812575041752
a_HQ_transcript/30116	-0.456104713668881	4.2505947579804	-2.11359409473044	0.0422934715837497	0.617502435943631	-4.19837524948389
a_HQ_transcript/114084	2.5342705497112	3.17690189567947	2.11354961690437	0.0422975434552946	0.617502435943631	-4.14640929955728
a_HQ_transcript/48533	-0.815566226240467	4.93112120982809	-2.11352031589925	0.0423002261035205	0.617502435943631	-4.25877928503841
a_HQ_transcript/59981	-2.18402149345835	2.8818019724502	-2.11351746666297	0.0423004869728667	0.617502435943631	-3.93770414532707
a_HQ_transcript/20406	2.47464045810582	1.07739528341365	2.113486528384	0.0423033197003151	0.617502435943631	-3.75104574077668
a_HQ_transcript/78255	2.18911183517143	2.35746652961006	2.1133695645383	0.0423140304977564	0.617502435943631	-3.83163069064565
a_HQ_transcript/145147	2.23305884377264	0.71701950250565	2.11327570014296	0.0423226277318921	0.617502435943631	-3.89333625674145
a_HQ_transcript/57832	-2.47068106643025	-0.323734252085203	-2.11253954486693	0.0423901073423262	0.617997529320481	-3.85096442221154
a_HQ_transcript/112388	0.524674453602302	3.9880154559337	2.11249905575472	0.0423938215343791	0.617997529320481	-4.16127887709984
a_HQ_transcript/23710	1.97978704982597	1.32853568104832	2.11246352782459	0.0423970808590203	0.617997529320481	-3.91912446964163
a_HQ_transcript/77262	-2.33710078935179	1.246675433673	-2.11244165795814	0.0423990873061189	0.617997529320481	-3.92064337460497
a_HQ_transcript/123307	-2.52063208687488	-0.183886344764625	-2.11217978766053	0.0424231190793281	0.618034356527771	-3.86425267647913
a_HQ_transcript/141655	1.24374548314313	3.00853812080913	2.11210582757431	0.0424299085610319	0.618034356527771	-3.9338315307299
a_HQ_transcript/49164	-1.69211485130546	1.92360635146837	-2.11206659122765	0.0424335108192667	0.618034356527771	-3.81209638974576
a_HQ_transcript/113786	-2.68492953431312	1.42958378725807	-2.11188762161709	0.042449945311879	0.618082566859618	-3.93250891264898
a_HQ_transcript/122820	3.59878474346726	0.790702002267205	2.11179898078149	0.0424580871433811	0.618082566859618	-3.90033851531907
a_HQ_transcript/86294	2.09860038414236	0.610903871702727	2.1115820746664	0.0424780162162723	0.618098175948094	-3.86763726877642
a_HQ_transcript/131620	1.79777237425319	1.98684630414386	2.11155585097101	0.0424804261786512	0.618098175948094	-3.94433578809546

a_HQ_transcript/77198	-2.64407470956484	0.383088496325852	-2.11121044958213	0.0425121799228196	0.618248566979537	-3.84496283233198
a_HQ_transcript/144327	2.31227425544521	-0.695845960609535	2.11104099945493	0.0425277656389578	0.618248566979537	-3.88258519881396
a_HQ_transcript/132975	1.0105298042052	2.71474957276969	2.11092556351223	0.0425383861233471	0.618248566979537	-3.93140700828527
a_HQ_transcript/94779	1.62136720119978	-0.0948749866139183	2.11086612089929	0.0425438559535484	0.618248566979537	-3.83375508572134
a_HQ_transcript/6801	3.63312735395437	1.94754886964396	2.11086518448299	0.04254394212631	0.618248566979537	-3.83241398130942
a_HQ_transcript/19558	2.30517616817904	-0.0734924930638658	2.11065934453027	0.0425628880881855	0.618300767535321	-3.87292988233651
a_HQ_transcript/111918	2.23958793704747	-0.200723333263471	2.11058379439788	0.0425698437611379	0.618300767535321	-3.81466406311914
a_HQ_transcript/70799	1.9025405836811	2.18838676951629	2.11046183019558	0.042581074760019	0.618300767535321	-3.85798922813556
a_HQ_transcript/100062	1.20930310701943	-0.618972850727016	2.11036403818142	0.0425900817714599	0.618300767535321	-3.88079513486451
a_HQ_transcript/49361	2.45273222091048	0.0297383917254891	2.11024825507467	0.0426007480094425	0.618301193581241	-3.84349868148693
a_HQ_transcript/88264	1.47242065810995	-0.749999294953304	2.11008988077358	0.0426153416853981	0.618358607461013	-3.88274207790017
a_HQ_transcript/34472	2.72282802451132	0.337381630487029	2.10966579980886	0.0426544411467342	0.618397622587304	-3.82816918871732
a_HQ_transcript/93760	2.88143948691589	0.73099494743621	2.1096428710073	0.042656556042892	0.618397622587304	-3.85164576886512
a_HQ_transcript/133643	-3.16526783747665	0.610848364044119	-2.1096203773993	0.0426586308880158	0.618397622587304	-3.90599767258034
a_HQ_transcript/86986	-2.06413653620796	1.10485404253718	-2.10959919697039	0.0426605846850931	0.618397622587304	-3.94898744900483
a_HQ_transcript/99055	1.56755763450083	-0.722145281441115	2.10914342844169	0.0427026464193406	0.618853011982905	-3.83391097770057
a_HQ_transcript/8086	-2.03478370991704	1.36159619522001	-2.10876133195044	0.0427379374138771	0.619060643199962	-3.92578026242074
a_HQ_transcript/46926	2.25749474599593	0.106601347017606	2.10864497432375	0.0427486894992584	0.619060643199962	-3.81247323430601
a_HQ_transcript/47648	2.86160740933199	0.41610282933015	2.10854650688751	0.0427577903018036	0.619060643199962	-3.81124658881135
a_HQ_transcript/21853	2.34236851769712	0.393845408435853	2.10852721660083	0.0427595733974651	0.619060643199962	-3.80960293537274
a_HQ_transcript/5947	1.9092841484282	-0.469015438021356	2.10838582208241	0.0427726451934258	0.619095697162214	-3.86825039518019
a_HQ_transcript/79898	0.4936406823473	4.00178586448399	2.1081326952666	0.0427960553686054	0.619240884825349	-4.15843385080634
a_HQ_transcript/125750	1.03515016472098	6.65829859820027	2.10804701309441	0.0428039821639873	0.619240884825349	-4.47173935540728
a_HQ_transcript/93084	-2.00774527343897	-0.00563518738633298	-2.1076755284231	0.0428383646986042	0.619297655408589	-3.80691744978246
a_HQ_transcript/95962	-2.90049567103426	0.722169291579933	-2.10738450412669	0.0428653173357984	0.619297655408589	-3.88369439641062
a_HQ_transcript/122253	-0.691906858540203	4.99359149158137	-2.1072402557563	0.0428786821709926	0.619297655408589	-4.26154687626304
a_HQ_transcript/37685	1.98757777162733	-0.667035740987816	2.10689202336147	0.0429109616320331	0.619297655408589	-3.88682056310069
a_HQ_transcript/46507	3.29299020542642	0.546463743457361	2.10688206750657	0.042911884807627	0.619297655408589	-3.90516959279852
a_HQ_transcript/122016	-2.2449851668589	0.180563095795652	-2.10686214650502	0.0429137320731498	0.619297655408589	-3.89086447430474
a_HQ_transcript/95537	2.96620564666687	1.63312755964677	2.1067123761359	0.0429276224644129	0.619297655408589	-3.84887522213165
a_HQ_transcript/15653	-4.94820213482369	2.30992341047602	-2.10668606261459	0.0429300633117697	0.619297655408589	-4.0022065802808
a_HQ_transcript/113898	-1.4123949559064	2.45462886526022	-2.10668104575073	0.0429305286909696	0.619297655408589	-3.95131118714408
a_HQ_transcript/38374	2.22094759935515	0.771434138360567	2.10653793543158	0.0429438059085718	0.619297655408589	-3.85213673998736
a_HQ_transcript/152012	2.62320810419256	-0.127344772634056	2.10647631870339	0.042949523586783	0.619297655408589	-3.87129738640039
a_HQ_transcript/109270	-2.49409903793159	0.589663427688607	-2.10619907529759	0.0429752585121241	0.619297655408589	-3.86091591253622
a_HQ_transcript/28568	-2.63749887966787	0.978831382852616	-2.10603110896626	0.0429908565023793	0.619297655408589	-3.86864928837668
a_HQ_transcript/79635	-1.34819620204917	4.24175054815334	-2.10598640944622	0.0429950083126527	0.619297655408589	-4.08001024316227
a_HQ_transcript/14171	2.41230515013215	-0.196413597321292	2.10592208740901	0.0430009833367459	0.619297655408589	-3.8962679918786
a_HQ_transcript/11947	2.65079080874165	0.642359619106926	2.10588984920741	0.0430039782947453	0.619297655408589	-3.81763677448088
a_HQ_transcript/109597	-0.695976185403182	3.83626028715716	-2.10580082430293	0.0430122497467394	0.619297655408589	-4.14293360456712
a_HQ_transcript/620	-2.57383159660241	1.01689353086475	-2.10579366713384	0.0430129147923264	0.619297655408589	-3.93269116254583
a_HQ_transcript/149254	0.358020846590303	10.4369574592955	2.10565314760999	0.0430259737366827	0.619297655408589	-4.82399449970861
a_HQ_transcript/100634	1.70823081254558	0.505541043038119	2.10554769269398	0.0430357763138258	0.619297655408589	-3.81736043460932
a_HQ_transcript/144479	1.09690720045544	5.90073901666059	2.10537958947466	0.0430514064565797	0.619297655408589	-4.38122282865967
a_HQ_transcript/152035	-1.73858750311089	2.04997274635871	-2.10536745512917	0.0430525348954286	0.619297655408589	-3.8888184752253
a_HQ_transcript/136878	-2.2088336269859	-0.50366731263493	-2.10511254867295	0.0430762460812622	0.619297655408589	-3.87184773597824
a_HQ_transcript/150927	2.12449520909027	-0.64719840966433	2.10511079243338	0.0430764094852479	0.619297655408589	-3.88969898316391
a_HQ_transcript/67842	2.31073436684079	2.20818370246505	2.10508422729728	0.0430788812246406	0.619297655408589	-3.863084470765
a_HQ_transcript/138033	1.93637503063851	-0.426773227646861	2.10496170831324	0.0430902825634727	0.619297655408589	-3.88122101280108
a_HQ_transcript/37857	1.06528261180728	2.84610204739625	2.10479993851522	0.0431053405814157	0.619297655408589	-4.01244941450006

a_HQ_transcript/135482	-2.32490812061859	0.298789855601824	-2.10479050020823	0.0431062192709736	0.619297655408589	-3.89862295988886
a_HQ_transcript/105108	-0.484842910716428	4.75535529927611	-2.10434366093885	0.0431478373394212	0.619581392320388	-4.24950387163406
a_HQ_transcript/125890	0.866565055330885	3.76551457730386	2.1042288979267	0.0431585319599741	0.619581392320388	-4.13839040448621
a_HQ_transcript/117936	-1.96753049498625	-0.447025853179761	-2.10410801685479	0.0431697992492769	0.619581392320388	-3.81008818841812
a_HQ_transcript/105177	1.32100253166572	1.57139356800462	2.10395271573482	0.0431842786382433	0.619581392320388	-3.80246003896095
a_HQ_transcript/106319	-5.56118508838247	2.20869837646688	-2.10391112136789	0.043188157388282	0.619581392320388	-4.00880353548259
a_HQ_transcript/122789	-0.620902338927519	3.43097094308035	-2.10380234766217	0.0431983021893756	0.619581392320388	-4.13419043407881
a_HQ_transcript/150352	1.87946985241294	2.39538644655544	2.10367521581573	0.0432101618363408	0.619581392320388	-3.92565956794605
a_HQ_transcript/133735	-2.20624240176159	-0.561845534666325	-2.10366365869596	0.0432112400988655	0.619581392320388	-3.88282173113484
a_HQ_transcript/38774	1.9906753902821	0.149357367532575	2.10329874663557	0.0432452981014274	0.619916815792792	-3.82865992943888
a_HQ_transcript/98914	-0.777204059391668	3.2962949989103	-2.10273922083289	0.0432975659030705	0.620088424280229	-4.08588339964258
a_HQ_transcript/6568	2.78732735242755	-0.0431801035425254	2.10272465503966	0.0432989273043329	0.620088424280229	-3.86260640260781
a_HQ_transcript/118657	0.470834676480637	4.58972702190848	2.10263964950605	0.0433068731562607	0.620088424280229	-4.24435478978065
a_HQ_transcript/81075	-2.7157243168114	1.34393904092919	-2.10261581980541	0.0433091008575427	0.620088424280229	-3.90813776815313
a_HQ_transcript/136997	2.51476806518032	1.27324887205253	2.10259970207218	0.0433106076688985	0.620088424280229	-3.8163922762797
a_HQ_transcript/113268	1.50277127123671	-0.823623806044075	2.10219876025688	0.0433481057542269	0.62047246768818	-3.89337140785402
a_HQ_transcript/91680	2.48686219695914	0.153051232568586	2.10206307472281	0.0433608022425192	0.620501406389256	-3.87429320995853
a_HQ_transcript/79209	-2.42999789406418	-0.460581596264331	-2.1017265323995	0.0433923076714675	0.620799424151381	-3.89158966035756
a_HQ_transcript/72961	2.78454616047161	0.454681390232523	2.10092981186353	0.0434669733763242	0.621593642560667	-3.8668901582738
a_HQ_transcript/57142	2.97815193367425	-0.058261261702949	2.10081463960634	0.0434777762838464	0.621593642560667	-3.9223569934089
a_HQ_transcript/83932	1.47680305506653	-0.776934463661478	2.10079197852101	0.0434799021237901	0.621593642560667	-3.89887881330339
a_HQ_transcript/90210	1.35949294587947	-0.479767685715695	2.10057437622629	0.0435003201060002	0.621601476285774	-3.85740369064277
a_HQ_transcript/123185	0.660402714154255	3.38562030458256	2.10055820923423	0.0435018374196692	0.621601476285774	-4.10249246125264
a_HQ_transcript/123759	-2.30501587861189	3.18372001568503	-2.1002209353768	0.0435335021047836	0.62168348354064	-4.19173954230216
a_HQ_transcript/133919	-2.67648954958648	1.64509547573746	-2.10015037163174	0.0435401295014382	0.62168348354064	-4.05922744425938
a_HQ_transcript/65546	2.35856759249371	1.07802927662048	2.10011864876619	0.0435431092258532	0.62168348354064	-3.85026808514233
a_HQ_transcript/148076	4.32436625985705	1.8039534790453	2.09994231641127	0.0435596753850736	0.62168348354064	-4.02969400237469
a_HQ_transcript/139403	1.05495889497496	1.93682823783651	2.0999276666119	0.043561051962239	0.62168348354064	-3.83494679553312
a_HQ_transcript/80907	3.9127186821015	1.71225744792562	2.09974478702161	0.0435782395882679	0.621776119486214	-3.96107378800349
a_HQ_transcript/86869	1.94123314447583	0.0531943786340672	2.0993567986913	0.0436147238553577	0.621909728738403	-3.84111936357303
a_HQ_transcript/44364	-2.67705434846724	0.739655262409535	-2.09926817961427	0.0436230608868685	0.621909728738403	-3.8061063347306
a_HQ_transcript/43843	0.730300093506205	2.77483721440192	2.09924320822058	0.0436254103791014	0.621909728738403	-4.00203031086488
a_HQ_transcript/124746	-2.51508522470618	2.85321355631859	-2.0991901835504	0.0436303997005781	0.621909728738403	-3.96710554072615
a_HQ_transcript/54366	-2.1421337747037	0.0436316671373895	-2.09874301894049	0.0436724954210499	0.622357149751113	-3.83272459858387
a_HQ_transcript/147524	-2.21242579567835	1.08743130624998	-2.0985710374404	0.0436886951761465	0.622435410097805	-3.82503782486099
a_HQ_transcript/139479	1.75687269842832	0.286689001471076	2.09815532176766	0.0437278753433416	0.622825770036102	-3.90799560837266
a_HQ_transcript/86462	1.42290531919621	-0.668637672549297	2.09805299688115	0.0437375239692982	0.622825770036102	-3.85906988158246
a_HQ_transcript/132211	4.07485597540159	5.0415786072604	2.09748361574303	0.0437912475753811	0.623287788973477	-3.9930686702564
a_HQ_transcript/49931	1.75861584736682	1.24089323006795	2.09737914309249	0.0438011113557849	0.623287788973477	-3.88893346854043
a_HQ_transcript/124775	-1.13778284791602	1.92323302393697	-2.0973682814189	0.0438021369728299	0.623287788973477	-3.83289076578412
a_HQ_transcript/32485	2.17236237460334	-0.252994495111627	2.09630020651689	0.0439030939738051	0.624335789475665	-3.89309633614117
a_HQ_transcript/139666	-0.699558220304167	4.15530140700492	-2.09606148917872	0.0439256861952344	0.624335789475665	-4.24014893813623
a_HQ_transcript/89439	-3.48027764732114	2.21932275700546	-2.09593282922845	0.043937866838398	0.624335789475665	-3.93132566022522
a_HQ_transcript/101720	-3.1075805607253	1.97450399077551	-2.09579951739236	0.0439504910376975	0.624335789475665	-3.90522633535037
a_HQ_transcript/125785	1.92260509155223	-0.59606655680236	2.09567434629449	0.0439623472519022	0.624335789475665	-3.90590737536504
a_HQ_transcript/123332	-0.795147994909112	2.90997944452315	-2.0956665194351	0.0439630887063572	0.624335789475665	-3.96384847444112
a_HQ_transcript/83143	-2.22935294310699	-0.0953408812418874	-2.09548708633272	0.0439800898021255	0.624335789475665	-3.90689917234628
a_HQ_transcript/124192	2.18536425879241	1.47261614463267	2.09529566670373	0.0439982330106388	0.624335789475665	-3.91507484443867
a_HQ_transcript/144599	4.50209015473224	3.25380102544542	2.09490547848848	0.0440352364535725	0.624335789475665	-4.13793247712505
a_HQ_transcript/146580	2.60530027766065	0.459743307954424	2.0948792935132	0.0440377206858654	0.624335789475665	-3.97806301154872

a_HQ_transcript/141104	-2.76442189347871	0.672984011839098	-2.09487777747308	0.0440378645200705	0.624335789475665	-3.98213272868611
a_HQ_transcript/1790	-2.08468030765708	0.2673347771482	-2.09467989309198	0.0440566423496271	0.624335789475665	-3.92724410664105
a_HQ_transcript/67172	2.24577954256104	-0.117729855002972	2.09467094136062	0.0440574919728536	0.624335789475665	-3.89406533127955
a_HQ_transcript/40702	-3.21715395858766	1.22730257510605	-2.09456572005216	0.0440674797813741	0.624335789475665	-4.04241689946918
a_HQ_transcript/13770	3.58565289440021	1.24863799580987	2.09448727084918	0.0440749276315409	0.624335789475665	-3.88415429183471
a_HQ_transcript/64366	2.38061340199313	1.18147489562673	2.09447462476514	0.0440761283359214	0.624335789475665	-3.84674030144758
a_HQ_transcript/132732	-2.29047401818794	2.64493619034838	-2.09429611053518	0.0440930807616542	0.624335789475665	-3.89277082773926
a_HQ_transcript/113007	1.74321529945175	-0.580644865383865	2.09421751141984	0.0441005466739929	0.624335789475665	-3.90285354070544
a_HQ_transcript/57680	-2.35086424885711	-0.0822909165779144	-2.09419576270391	0.0441026127215009	0.624335789475665	-3.90961619041343
a_HQ_transcript/60156	2.80827610502366	0.295044310793663	2.09416385505683	0.044105643984572	0.624335789475665	-3.84395849602313
a_HQ_transcript/12497	-0.628481313390667	3.18659211359676	-2.09404108204441	0.0441173092777993	0.624335789475665	-4.05600054100523
a_HQ_transcript/138700	1.83529143175896	2.01895644839367	2.09399052699536	0.0441221135641287	0.624335789475665	-3.96282536383549
a_HQ_transcript/67940	1.92449778970006	1.96774472270081	2.09385688209078	0.0441348161705147	0.624335789475665	-3.86074134014316
a_HQ_transcript/119446	-2.2849382358083	-0.235676445748675	-2.09379764925217	0.0441404471332133	0.624335789475665	-3.90182761336808
a_HQ_transcript/83987	1.97392330536248	-0.0514809906709088	2.09367636304538	0.0441519791716568	0.624335789475665	-3.9129619367449
a_HQ_transcript/144192	1.10407734444645	2.25017927741021	2.09357610463731	0.044161513870417	0.624335789475665	-3.94841348500525
a_HQ_transcript/137901	2.39280667104685	0.383627246107016	2.09331717973442	0.0441861463619678	0.624335789475665	-3.83530473951419
a_HQ_transcript/141313	3.13662414590833	1.31882339334331	2.09325576753264	0.0441919905136446	0.624335789475665	-3.90503330532563
a_HQ_transcript/74437	3.12181308733367	3.67046782180757	2.09319280990377	0.0441979824409273	0.624335789475665	-4.10622512284653
a_HQ_transcript/60099	-2.88116897141583	0.427148111282185	-2.0931925477549	0.0441980073921753	0.624335789475665	-3.91540370373015
a_HQ_transcript/71436	-1.65621768376012	-0.704187940970099	-2.09284023437347	0.0442315517140584	0.624657832369968	-3.85903552727319
a_HQ_transcript/149928	2.39959334927316	1.2678241063111	2.09243549366267	0.0442701154729261	0.624826529076924	-3.82996158708143
a_HQ_transcript/148508	6.25488134000283	3.22029615741395	2.09243196198621	0.0442704521022372	0.624826529076924	-4.18197380004852
a_HQ_transcript/8953	-3.03921730308771	0.302924118079694	-2.09228754718905	0.0442842192442213	0.624826529076924	-3.99341894082484
a_HQ_transcript/93147	2.40901474817213	1.31530601871315	2.0922064087141	0.0442919558781866	0.624826529076924	-3.84124268484621
a_HQ_transcript/10548	2.01610885276202	0.795731273357881	2.09215096952834	0.044297242745768	0.624826529076924	-3.8655429451282
a_HQ_transcript/85978	1.79416752141413	-0.56898198584928	2.09184705491263	0.044326234968901	0.625083790944269	-3.94148478715875
a_HQ_transcript/56063	2.65417735954665	-0.283667675192474	2.09159133061956	0.0443506430091724	0.625223672652847	-3.89549730504381
a_HQ_transcript/117750	3.0415685817054	-0.0291433796352406	2.09151777038257	0.0443576662885415	0.625223672652847	-3.90994199779147
a_HQ_transcript/542	1.66644571242136	1.00773840493636	2.09049629901548	0.0444552944163559	0.626447843353682	-3.80008721107381
a_HQ_transcript/151373	-2.85090861317287	0.955395646554693	-2.09028827828174	0.0444751994354864	0.626576440326213	-3.97315852972252
a_HQ_transcript/39714	-0.864998709249253	2.54260850529545	-2.08981313378855	0.0445206944174091	0.627065404675347	-3.91344338888368
a_HQ_transcript/147935	-1.86211658784751	1.90498072439576	-2.08947598787187	0.0445530009780683	0.627368420749311	-3.9550390958746
a_HQ_transcript/71054	-1.88782798513063	4.24049034289088	-2.08912260257249	0.0445868858675382	0.627447491899938	-4.22948461675456
a_HQ_transcript/108728	2.88621572000291	1.25774674693178	2.08906314121066	0.044592589646386	0.627447491899938	-3.95385989406286
a_HQ_transcript/13123	1.5083588905287	1.83383132913088	2.08903581279801	0.0445952113163586	0.627447491899938	-3.9449706124849
a_HQ_transcript/146306	1.7181085794345	-0.727891933178983	2.08865179825632	0.0446320649992484	0.627447491899938	-3.90591109590341
a_HQ_transcript/82702	-1.80996026933059	0.0327036828866656	-2.08863644862946	0.0446335386538053	0.627447491899938	-3.89177803474126
a_HQ_transcript/135230	2.10094530162028	3.65736027168642	2.08860025888937	0.0446370132519298	0.627447491899938	-4.02691367929074
a_HQ_transcript/46815	-1.35339676434388	2.8966334113821	-2.08855699219864	0.0446411676255618	0.627447491899938	-3.98303025639912
a_HQ_transcript/21522	2.10240889331569	1.05373390586419	2.08833268623469	0.0446627104679676	0.627447491899938	-3.86827288954665
a_HQ_transcript/116991	-2.44212647598833	-0.495719350427132	-2.08809766774983	0.0446852920076453	0.627447491899938	-3.90121975272465
a_HQ_transcript/94373	1.66313944319486	-0.744086883908628	2.08801383710616	0.0446933492375126	0.627447491899938	-3.91984488497245
a_HQ_transcript/113949	1.61888223371488	-0.670951918510168	2.0879973819679	0.0446949309437387	0.627447491899938	-3.87689902261705
a_HQ_transcript/20143	1.1044049488367	1.93785728822468	2.08792080556643	0.0447022922957807	0.627447491899938	-3.86431530594715
a_HQ_transcript/30293	1.83112128867682	2.90838210228072	2.0879042509308	0.0447038838470935	0.627447491899938	-3.99017084607567
a_HQ_transcript/85447	-1.12781358522751	3.35588290070023	-2.08784338788508	0.0447097356084768	0.627447491899938	-4.06940259238989
a_HQ_transcript/98125	0.728697600311022	3.81382091027913	2.08745211416743	0.0447473713046828	0.627599162672869	-4.1931220529208
a_HQ_transcript/27929	0.908631312151351	1.93133506976232	2.08735997118987	0.0447562383867749	0.627599162672869	-3.87295136632843
a_HQ_transcript/1596	0.708209941835587	2.82715731064376	2.08715970851619	0.0447755153665276	0.627599162672869	-4.01461617883384

a_HQ_transcript/65936	1.70062479345191	0.521685055896909	2.08711731933391	0.0447795966240896	0.627599162672869	-3.80655208316471
a_HQ_transcript/103819	1.06606082622235	2.38361548442576	2.08696735845482	0.0447940375885281	0.627599162672869	-3.92308771675388
a_HQ_transcript/72521	-2.28813623412693	0.44522854740456	-2.08691520078888	0.0447990612416639	0.627599162672869	-3.87662647866511
a_HQ_transcript/70785	3.89309581463763	0.941266525698555	2.08661313368632	0.0448281651204076	0.627599162672869	-3.98584785051724
a_HQ_transcript/52398	1.07543613296502	3.18579134681974	2.08659617480847	0.0448297995867451	0.627599162672869	-4.06028300299327
a_HQ_transcript/138065	2.33491294582361	0.385957779962725	2.08655328073739	0.0448339338753726	0.627599162672869	-3.8789310445206
a_HQ_transcript/32546	1.58443688438988	0.980084306325518	2.0865390035577	0.0448353100371869	0.627599162672869	-3.86370470667519
a_HQ_transcript/115098	2.01217867474991	-0.695399636158922	2.08649752349064	0.0448393084671832	0.627599162672869	-3.92171932595763
a_HQ_transcript/5592	1.79828450826618	1.9415141838608	2.08638330091438	0.044850320465587	0.627602173332605	-3.92763788247637
a_HQ_transcript/32971	-3.07852392607057	1.20705409506832	-2.08607548776721	0.0448800080733878	0.62778499091321	-3.91878575303118
a_HQ_transcript/95812	-2.02840965279738	-0.356230955498091	-2.08588574439233	0.0448983168613041	0.62778499091321	-3.91435972792823
a_HQ_transcript/121732	-1.73173508491665	0.506726453327629	-2.0856932148315	0.0449169012309109	0.62778499091321	-3.91065359179949
a_HQ_transcript/132403	3.05568117054163	0.666047414484463	2.08567004824047	0.0449191378981391	0.62778499091321	-3.90622802165962
a_HQ_transcript/5482	1.76484443327755	-0.188456161106188	2.08551592866312	0.0449340201984064	0.62778499091321	-3.85847317236802
a_HQ_transcript/50871	-1.42454718238836	3.30166056567641	-2.08550982116486	0.0449346100484098	0.62778499091321	-3.96147956395185
a_HQ_transcript/117707	2.90810647897766	0.56079685516195	2.08546451887635	0.0449389854663822	0.62778499091321	-3.84145346633121
a_HQ_transcript/137413	1.9330910047232	-0.642773396874071	2.08486885242721	0.0449965515248375	0.628066624738481	-3.92535343355653
a_HQ_transcript/83461	3.31749823073566	0.242478897660542	2.0848534620559	0.0449980397341599	0.628066624738481	-3.95399775543618
a_HQ_transcript/123120	-2.1270069402819	0.598468941268999	-2.08484971283794	0.0449984022804713	0.628066624738481	-3.90516130481166
a_HQ_transcript/129427	-2.16342482217912	1.06549604959377	-2.08480873127346	0.0450023653322972	0.628066624738481	-3.84415540155531
a_HQ_transcript/144409	2.92750429394324	1.09280802896855	2.08460082262951	0.045022475524929	0.628196461188928	-3.99862813103191
a_HQ_transcript/95416	-2.34930721269183	-0.067061471566361	-2.08432716320131	0.045048957620168	0.628351321103176	-3.91596966682834
a_HQ_transcript/38931	1.06579463981044	2.6033896305044	2.08420080600787	0.0450611898759203	0.628351321103176	-3.9914167458398
a_HQ_transcript/33154	1.61008720680972	1.56126889791562	2.08415108925123	0.0450660036071969	0.628351321103176	-3.87786059727942
a_HQ_transcript/11184	-1.23475267974149	2.61395229123617	-2.08389526386352	0.0450907805919952	0.628451499039306	-4.05706059129448
a_HQ_transcript/50455	1.77062442159771	-0.394654271887974	2.08383913658682	0.0450962181908898	0.628451499039306	-3.86231369294558
a_HQ_transcript/50059	1.40435147778656	1.40415938182719	2.08373218763511	0.045106580984374	0.628451499039306	-3.82278542025688
a_HQ_transcript/3493	1.53341602310859	0.523884286490679	2.08363051461883	0.0451164345150534	0.628451499039306	-3.8097624213844
a_HQ_transcript/680	-2.68136219343373	1.71299398388672	-2.08337286809767	0.0451414125525999	0.628648785064093	-3.90242085146592
a_HQ_transcript/132488	-3.28354086494375	0.783044204782299	-2.08320712388816	0.0451574873907973	0.628652080769765	-3.94888609849823
a_HQ_transcript/68853	4.53571482265411	2.09994903859367	2.08308600232631	0.0451692376578916	0.628652080769765	-4.07087407176161
a_HQ_transcript/74963	2.17445958089556	0.0265567290511605	2.08295207400955	0.0451822334759481	0.628652080769765	-3.90552532592121
a_HQ_transcript/89855	-3.52801372465275	0.570459439409332	-2.08292450508188	0.0451849090534007	0.628652080769765	-3.95632197837112
a_HQ_transcript/144870	3.08364767057886	1.50614367164937	2.08254624815922	0.0452216331820749	0.628946377102961	-3.93285548268696
a_HQ_transcript/81390	-1.08609269120139	1.6871191133278	-2.08236278663625	0.0452394545350179	0.628946377102961	-3.84602126224816
a_HQ_transcript/124355	-2.34562530176859	-0.470349323964793	-2.08235976939299	0.0452397476801623	0.628946377102961	-3.91508001136415
a_HQ_transcript/79916	4.05787084141011	2.38033352449936	2.08222803709188	0.0452525479782434	0.628946377102961	-3.96264030313296
a_HQ_transcript/79538	-0.887754889799116	2.35834273155422	-2.08211819386888	0.0452632237783359	0.628946377102961	-3.94033242575889
a_HQ_transcript/131746	1.32194011842934	2.75660515046323	2.08203838390471	0.0452709820017683	0.628946377102961	-4.08885604507438
a_HQ_transcript/125653	1.49557128867323	-0.379434266351144	2.08182810695178	0.0452914283685767	0.62906774101318	-3.93144840047644
a_HQ_transcript/73425	1.09088549892998	3.365884927259	2.08166888210674	0.0453069160804945	0.62906774101318	-4.14419811731439
a_HQ_transcript/120502	3.83163222544672	1.69494693419131	2.0816147350243	0.0453121840012074	0.62906774101318	-3.87567886456336
a_HQ_transcript/33599	-3.0335085794414	1.98121333943784	-2.08144419779318	0.045328778944361	0.629116969665796	-4.06940540708143
a_HQ_transcript/140398	-2.50068180936225	-0.380283703893148	-2.08123323518553	0.0453493150889486	0.629116969665796	-3.93160492280863
a_HQ_transcript/52247	-2.91053152867042	-0.153429781766781	-2.08120294339568	0.0453522645152937	0.629116969665796	-3.85378589785757
a_HQ_transcript/93251	2.0588088507851	-0.513441583927593	2.08113354980456	0.0453590218116803	0.629116969665796	-3.88218953747469
a_HQ_transcript/29624	-0.748987287369782	3.34932996056024	-2.08080359172392	0.0453911640830689	0.629225886134904	-4.11666099529994
a_HQ_transcript/60826	-2.70393004750147	0.87998956166281	-2.08062676186234	0.0454083979102964	0.629225886134904	-3.95986426915232
a_HQ_transcript/32539	2.55531123661444	3.17770333165576	2.08058206815751	0.0454127546693597	0.629225886134904	-4.1228803726356
a_HQ_transcript/124384	1.7259914758447	0.621624934900413	2.08035399195455	0.0454349933636486	0.629225886134904	-3.86246349158051

a_HQ_transcript/144904	2.00146810164533	0.0959452762190691	2.08031411215396	0.0454388828515325	0.629225886134904	-3.87344256928525
a_HQ_transcript/9591	2.55526656235218	0.359410812112315	2.08024208326732	0.0454459085926437	0.629225886134904	-3.93198324919611
a_HQ_transcript/99330	2.42186146745216	3.52535706169609	2.08009049074971	0.0454606981479414	0.629225886134904	-4.00157168649699
a_HQ_transcript/100659	3.17911956086507	0.153795442254749	2.07997942793757	0.0454715362680919	0.629225886134904	-3.93035261093363
a_HQ_transcript/29562	1.84014414648521	-0.674101306324079	2.07996625237771	0.0454728221628645	0.629225886134904	-3.92345589278361
a_HQ_transcript/152212	-1.07667089029695	1.74054448487808	-2.07994267868423	0.045475122964023	0.629225886134904	-3.88240651776851
a_HQ_transcript/36840	3.77470903533842	0.509711545499256	2.07938486230854	0.0455295960080984	0.629694928727916	-3.88892570344972
a_HQ_transcript/137088	3.67883889862162	0.933174244077058	2.07937369428903	0.0455306871979671	0.629694928727916	-4.01525133026395
a_HQ_transcript/103813	3.95529723705916	2.53493494757026	2.07899600149667	0.0455676038775188	0.630005420516297	-3.98647120130842
a_HQ_transcript/94129	-2.50590481498883	0.855670155471819	-2.07892226567575	0.0455748140873767	0.630005420516297	-3.96932000345775
a_HQ_transcript/124319	1.87824736866728	-0.536491732436401	2.07877878737394	0.0455888469038596	0.630049570334652	-3.92459659807338
a_HQ_transcript/72517	-1.84181400240412	0.311077956466138	-2.07860069940226	0.0456062699986243	0.630121837635363	-3.90200350873452
a_HQ_transcript/44411	-0.897666063086752	4.0740448977806	-2.07850375972761	0.0456157564817232	0.630121837635363	-4.18712400776831
a_HQ_transcript/103139	1.56502971136867	0.562476242299517	2.0779771755378	0.0456673182264622	0.630650313397728	-3.83032286189624
a_HQ_transcript/94609	2.32110481507791	-0.59193581451595	2.0778036440039	0.0456843212321437	0.630650313397728	-3.90063975911818
a_HQ_transcript/2833	2.24200330901711	0.632427496604013	2.07778078021551	0.0456865618930263	0.630650313397728	-3.8916747754468
a_HQ_transcript/71651	-2.89369530160415	1.04657196772793	-2.07754310820681	0.0457098595883461	0.63069936967004	-3.94999586255063
a_HQ_transcript/48268	-2.58320876170185	-0.220843739959942	-2.07752315398913	0.0457118160683299	0.63069936967004	-3.98481983596475
a_HQ_transcript/18176	-2.23571699499852	0.900518487069887	-2.07673963917559	0.045788696855425	0.631481632886333	-3.96774806362076
a_HQ_transcript/13323	1.38963222628852	3.28528679003224	2.07672392232424	0.0457902402046499	0.631481632886333	-4.17049911561412
a_HQ_transcript/136188	2.28531581365333	4.35192905319126	2.07653850000271	0.0458084516006756	0.631582939906088	-4.29590532524935
a_HQ_transcript/114425	1.53706926033444	-0.642599955783326	2.0764219779673	0.0458198991682771	0.631590964869246	-3.94028642398954
a_HQ_transcript/8157	3.31235192589188	1.26644425548877	2.07624816223662	0.0458369801722905	0.631622263739082	-3.98409118315274
a_HQ_transcript/90649	2.97860485468863	1.34481265255896	2.0761777430315	0.0458439019184418	0.631622263739082	-3.88278629253625
a_HQ_transcript/65573	2.2878875597357	0.423063720173664	2.07604775469053	0.0458566813399553	0.631648619177469	-3.82733577329042
a_HQ_transcript/20875	2.34621474268424	-0.0991808254505686	2.07576051156885	0.0458849319383244	0.631802290312766	-3.97492128772295
a_HQ_transcript/79026	-2.39050515463006	0.537083896249105	-2.07571330784555	0.0458895759307132	0.631802290312766	-3.95630140780886
a_HQ_transcript/108172	-2.58252969125241	1.97172048280626	-2.07534347474627	0.0459259751559138	0.632153702075055	-4.00008645112412
a_HQ_transcript/112041	-3.11088124754303	-0.13208231055892	-2.07518848928922	0.0459412364942768	0.632214061301922	-3.99020694774524
a_HQ_transcript/115199	3.13154173670509	0.558070152877055	2.07504465380423	0.0459554038990538	0.632259341501585	-3.93083412366142
a_HQ_transcript/6698	1.86990789610582	2.52780677911372	2.07483417649202	0.0459761422822146	0.632394983099993	-3.93485300951429
a_HQ_transcript/89624	-0.967658401689845	3.15111429603456	-2.07471208623399	0.0459881756455457	0.632410852596235	-4.09584351833935
a_HQ_transcript/53487	2.18264729962402	0.541795183550948	2.07430618878834	0.04602820134228	0.632765048515238	-3.93725074411678
a_HQ_transcript/83951	2.20074368876336	3.17314003779149	2.07419532501355	0.0460391389959373	0.632765048515238	-4.06068137003285
a_HQ_transcript/48946	1.38036498832418	1.41985171956917	2.07401853947777	0.0460565851296166	0.632765048515238	-3.82650784005182
a_HQ_transcript/22766	1.46332677949849	0.18279247686905	2.07400952145851	0.0460574752316951	0.632765048515238	-3.8697628946049
a_HQ_transcript/100302	3.24598589050864	3.15310863652475	2.07340231346467	0.0461174431303493	0.633207944776732	-4.10726117682677
a_HQ_transcript/124799	0.563582203776653	3.14077271894622	2.07332643833495	0.0461249413961776	0.633207944776732	-4.11609663700591
a_HQ_transcript/65353	1.42762598367877	1.26418156031229	2.07306034374735	0.0461512463546421	0.633207944776732	-3.84400292263276
a_HQ_transcript/90382	0.571449127406011	3.53379765021695	2.07276829542098	0.0461801322006769	0.633207944776732	-4.16531338209891
a_HQ_transcript/108601	-1.56268255469059	3.8492738689149	-2.07263760886956	0.0461930632727851	0.633207944776732	-4.11861209133749
a_HQ_transcript/100862	2.44290578079678	0.494483050316629	2.07250610613471	0.0462060783239236	0.633207944776732	-3.90251552283993
a_HQ_transcript/17614	2.94602701132394	0.266595101304378	2.07241514453448	0.0462150828395993	0.633207944776732	-3.98384001444241
a_HQ_transcript/52592	3.08704416482197	0.70150852556652	2.07234427131013	0.0462220998283485	0.633207944776732	-3.94334309811857
a_HQ_transcript/112437	-1.23842112285431	-0.649740804654916	-2.07230985494923	0.0462255076486381	0.633207944776732	-3.95380805845304
a_HQ_transcript/120741	-4.96319979795735	1.62355263924459	-2.07223617976634	0.046232803521474	0.633207944776732	-4.00171163880079
a_HQ_transcript/40946	1.31033066645456	1.1784274569775	2.07219115334035	0.0462372628775484	0.633207944776732	-3.83740528016188
a_HQ_transcript/103037	-2.92423464517915	0.222489750391095	-2.07197618324957	0.0462585584470227	0.633207944776732	-3.89058919703193
a_HQ_transcript/125278	2.24411557032689	-0.666825193159555	2.07192793981487	0.0462633387705405	0.633207944776732	-3.93740966870587
a_HQ_transcript/104420	4.36096632981108	3.95059770590013	2.07190105062344	0.0462660033433843	0.633207944776732	-4.41224904965083

a_HQ_transcript/106173	2.23233499668819	-0.169112218059155	2.07182219866362	0.0462738179239451	0.633207944776732	-3.86419075345699
a_HQ_transcript/2729	2.49739952265485	1.35434319366162	2.07173497072381	0.0462824639560394	0.633207944776732	-3.90834750955806
a_HQ_transcript/109728	3.70343821502418	1.00963033398034	2.07157475695229	0.0462983480501872	0.633207944776732	-4.11695383513286
a_HQ_transcript/74393	-1.99303291503942	0.204572252607067	-2.07132460637988	0.0463231583625268	0.633207944776732	-3.97564129534251
a_HQ_transcript/144853	-1.68227984854944	4.26270716293757	-2.07127664611252	0.046327916472183	0.633207944776732	-4.27778906041407
a_HQ_transcript/115086	-2.01219304064067	0.660040355515746	-2.07120656385129	0.046334870065908	0.633207944776732	-3.97073791812817
a_HQ_transcript/77611	-2.30749114754078	0.294729505835048	-2.07119933272178	0.0463355875941771	0.633207944776732	-3.96709599139007
a_HQ_transcript/71353	-3.27421919554479	3.47485030097898	-2.07119423862055	0.0463360930759462	0.633207944776732	-4.09559862251368
a_HQ_transcript/62085	2.72174230893956	0.00179546786409989	2.07115225368769	0.0463402593772402	0.633207944776732	-3.94461972342936
a_HQ_transcript/7808	-2.39168015920547	-0.465021633301609	-2.0705513623338	0.046399923946361	0.633874213667232	-3.9100743319021
a_HQ_transcript/146263	2.39531118690338	1.47201569912751	2.07011132159281	0.0464436600142658	0.634322619668524	-4.03776920322634
a_HQ_transcript/24358	-4.47228982567644	4.45917274038593	-2.06975476147294	0.0464791254904681	0.634417124162498	-4.11461264731385
a_HQ_transcript/108314	1.20674384103331	3.90166534472184	2.0697132170719	0.0464832592814915	0.634417124162498	-4.23931959181291
a_HQ_transcript/143650	1.78131305891538	-0.645129531171799	2.06968692355495	0.046485875731667	0.634417124162498	-3.95088621823986
a_HQ_transcript/115085	-2.80597900359412	1.57761866504064	-2.06960291758885	0.0464942359780526	0.634417124162498	-3.98318676949083
a_HQ_transcript/148878	6.38695344890209	1.2096186394046	2.06904083124068	0.0465502087082408	0.634641402026851	-3.99833473452428
a_HQ_transcript/6677	-1.81060396575838	1.12337334092593	-2.0689206928535	0.0465621798199749	0.634641402026851	-3.86518677384377
a_HQ_transcript/138913	-3.05174937804428	-0.0466444892199533	-2.06890600703687	0.0465636433646014	0.634641402026851	-3.95383513974553
a_HQ_transcript/100942	2.51994404582758	0.582690050665024	2.0687898129763	0.0465752243460168	0.634641402026851	-3.98693016931178
a_HQ_transcript/46501	-1.21963534960263	3.30775822768091	-2.06877812622282	0.0465763892970101	0.634641402026851	-4.04362366626826
a_HQ_transcript/102521	3.13086176407443	1.97152320333159	2.06873271233556	0.0465809164567569	0.634641402026851	-3.90090929772548
a_HQ_transcript/7864	-1.04445238217681	2.79727960171783	-2.06854584000275	0.0465995492178038	0.634641402026851	-4.01830414462379
a_HQ_transcript/17239	2.77289285773613	0.393651559044518	2.06844104774856	0.0466100007688769	0.634641402026851	-3.9529001248288
a_HQ_transcript/94072	1.51949479228294	0.678574457015984	2.06842717904252	0.0466113841315885	0.634641402026851	-3.9056442853439
a_HQ_transcript/13910	0.972101394853111	2.60972116045723	2.06834228842757	0.0466198525091979	0.634641402026851	-4.04065055694899
a_HQ_transcript/102498	2.0351327654048	-0.159584005631003	2.06809731726517	0.0466442975337641	0.634672130354065	-3.85732824439735
a_HQ_transcript/93955	3.15013221046442	1.7938847686065	2.06781652140363	0.0466723312931438	0.634672130354065	-3.94667210399555
a_HQ_transcript/86427	-0.688950467163378	2.86897015828122	-2.06779747666943	0.0466742331955305	0.634672130354065	-4.04661498947672
a_HQ_transcript/135729	2.58955253798781	0.626893258066488	2.06769683105691	0.0466842853029843	0.634672130354065	-4.00037577330439
a_HQ_transcript/310	-1.12617301227049	2.67228989842859	-2.06767028838453	0.0466869366034869	0.634672130354065	-3.97057623210944
a_HQ_transcript/48593	1.78788334959448	3.4801744564258	2.06766343768178	0.0466876209295689	0.634672130354065	-4.05792141086571
a_HQ_transcript/5259	2.55705340775983	0.858195934211011	2.06748223187327	0.0467057250327849	0.634769788334281	-3.91014160511229
a_HQ_transcript/135271	4.09421445190364	2.15584027921643	2.06725507056306	0.0467284292387054	0.634901242913181	-4.05700503632098
a_HQ_transcript/35521	1.87890725920239	-0.54610641195706	2.06707944859334	0.0467459888762495	0.634901242913181	-3.9507306077036
a_HQ_transcript/61315	3.50277915340993	0.208785258076132	2.06705769088741	0.04674816473418	0.634901242913181	-3.93012146059941
a_HQ_transcript/128702	-3.85979717475416	4.08661807971754	-2.06670498463153	0.0467834492168061	0.635151862372694	-4.16187238155629
a_HQ_transcript/90925	-1.73424329984604	2.10521635433205	-2.06658222237746	0.0467957357649325	0.635151862372694	-3.98009873666507
a_HQ_transcript/15544	-2.62401593256504	0.275009145500034	-2.06654336479967	0.0467996253820095	0.635151862372694	-3.97362659305455
a_HQ_transcript/124980	2.29249305787785	0.317979539041	2.06643648827921	0.0468103251170627	0.635151862372694	-3.94464384484738
a_HQ_transcript/110748	-0.9816181003868	1.88142955475713	-2.06621273961301	0.0468327322540192	0.635181510972957	-3.88867216456242
a_HQ_transcript/70969	2.31445621504244	1.92070495834553	2.0661292263442	0.046841098045343	0.635181510972957	-4.01064004266032
a_HQ_transcript/19033	-0.728635078501863	3.23014345217143	-2.06602420089224	0.0468516206484511	0.635181510972957	-4.08608276809923
a_HQ_transcript/68832	1.08744028941354	1.99584605828898	2.06597830794075	0.0468562193616164	0.635181510972957	-3.90115354388676
a_HQ_transcript/109756	1.82166279698347	-0.157427283666383	2.06567555119185	0.0468865671263629	0.635317087901368	-3.86244120426832
a_HQ_transcript/112967	2.31835927871099	0.493102496048899	2.06566046413349	0.0468880798771138	0.635317087901368	-3.85259350078134
a_HQ_transcript/150073	1.89178108209966	0.714671592643198	2.06535440056536	0.0469187775693985	0.635479192539897	-3.97731991432055
a_HQ_transcript/142653	2.15255804724701	3.40542751823504	2.06532319482017	0.0469219084499938	0.635479192539897	-4.07315668489993
a_HQ_transcript/128666	1.87178489662987	0.0942836465689907	2.06519214556492	0.0469350586641128	0.635509221995702	-3.97043890535254
a_HQ_transcript/107984	1.14107771986269	1.77817759449469	2.06503167431036	0.0469511656643538	0.635528995897046	-3.9452940760736
a_HQ_transcript/106797	-4.6083891689191	0.466427660888843	-2.06494640773621	0.0469597261156942	0.635528995897046	-3.97575420873003

a_HQ_transcript/139821	1.515625088989	2.15632496137276	2.06485087529386	0.0469693188544885	0.635528995897046	-4.01068340484669
a_HQ_transcript/56692	1.0292351385579	2.05734461562227	2.06473809736997	0.0469806454909904	0.635534317221385	-3.94506498055736
a_HQ_transcript/83963	3.35220636376136	0.633235425962697	2.06459077858312	0.0469954447949555	0.635586601917443	-3.93186736017693
a_HQ_transcript/67966	3.08383298138255	0.014809909912079	2.06436713973575	0.0470179188782253	0.635742635160148	-3.95646338360068
a_HQ_transcript/12783	-1.53453293124449	3.6771122812083	-2.06417179109843	0.0470375577403883	0.635746037921438	-4.19677266941126
a_HQ_transcript/13504	2.30164656882884	1.38286604437988	2.064030542228	0.0470517623218167	0.635746037921438	-3.89087688894627
a_HQ_transcript/144844	0.892005932360415	3.53185110559037	2.06398409492105	0.0470564340834665	0.635746037921438	-4.19089532383182
a_HQ_transcript/147088	1.39322485462396	1.19422347992937	2.06392957281846	0.0470619185448656	0.635746037921438	-3.87661746590756
a_HQ_transcript/105480	1.122763641882	2.217135464688	2.06354935787469	0.0471001805895383	0.635789882558765	-3.90793491459008
a_HQ_transcript/119792	2.42975500362703	1.0847656932193	2.06353736012404	0.0471013884006732	0.635789882558765	-3.87382690064709
a_HQ_transcript/28195	-2.31834536478692	2.00019276415331	-2.06353651949484	0.0471014730276667	0.635789882558765	-3.97656805309119
a_HQ_transcript/148086	1.12291712444599	4.43836375135613	2.06340651624119	0.0471145621976782	0.635789882558765	-4.27317250748937
a_HQ_transcript/151555	-2.22650213627215	2.05050074949606	-2.06326728246454	0.0471285842782896	0.635789882558765	-3.99962339256983
a_HQ_transcript/38823	0.829122411415492	4.83864071778728	2.06319319321541	0.0471360472221748	0.635789882558765	-4.34595926360467
a_HQ_transcript/8443	-0.918790829766205	2.89301330986003	-2.06305086198438	0.047150387038148	0.635789882558765	-3.93648556303152
a_HQ_transcript/104626	0.537637755679623	4.41350508789465	2.06301482333778	0.0471540185267899	0.635789882558765	-4.32140952996019
a_HQ_transcript/25769	2.97164443499326	1.51169012903723	2.06291970943579	0.0471636040048409	0.635789882558765	-4.07397594310377
a_HQ_transcript/142073	3.79103385442689	0.627455508199034	2.06274482859732	0.0471812327850923	0.635880060127949	-3.96336643811444
a_HQ_transcript/145589	-1.7521014064231	0.605964241021311	-2.0625969588524	0.0471961432489761	0.635933568562004	-3.87909534261677
a_HQ_transcript/44345	1.49380297818241	-0.198932346456058	2.06204018217046	0.0472523231019129	0.63654299820811	-3.957983727621
a_HQ_transcript/13529	-2.08593386195587	1.05586833894269	-2.06173471462112	0.0472831703651476	0.636557874750877	-3.87433460754474
a_HQ_transcript/106775	2.48479017330842	1.61371830438827	2.06171835022341	0.0472848234035881	0.636557874750877	-3.91978978073317
a_HQ_transcript/14386	2.06779475255844	0.0607668023177811	2.06170392764521	0.0472862803326157	0.636557874750877	-3.89584393921766
a_HQ_transcript/88152	1.6575428266491	2.20459348835865	2.0613196520072	0.0473251133459829	0.636933130024379	-3.97552127466978
a_HQ_transcript/19064	2.49047786531128	1.05434908579747	2.06111941397342	0.0473453595387776	0.636943277644684	-3.84734680342649
a_HQ_transcript/21578	2.07777560352675	1.49395290275683	2.06096097554772	0.0473613847473203	0.636943277644684	-3.86948656732533
a_HQ_transcript/101889	-1.46502180686809	3.18872029676735	-2.06092259530455	0.0473652674238448	0.636943277644684	-4.0758340095023
a_HQ_transcript/57975	-1.78202107692873	0.66273674324051	-2.06087880519217	0.0473696977232654	0.636943277644684	-3.91649443414581
a_HQ_transcript/115715	2.20189757881683	1.561199555305	2.06047043154579	0.0474110309433356	0.637351620414942	-3.91206841627693
a_HQ_transcript/96495	2.79763191194155	-0.19229552969673	2.05956941751783	0.0475023386267155	0.637365430516675	-3.92892226763062
a_HQ_transcript/63817	2.38479353583828	1.52918181674194	2.05941569507205	0.0475179321134452	0.637365430516675	-3.91177139222332
a_HQ_transcript/94921	0.753392409837964	3.52295978949203	2.05939270991216	0.0475202640973392	0.637365430516675	-4.21908676799724
a_HQ_transcript/135292	-2.38481737962483	0.142754091367529	-2.05928760725267	0.0475309286823853	0.637365430516675	-4.04664829182884
a_HQ_transcript/11524	0.781629242904252	2.71426328044936	2.05928132123581	0.0475315665803613	0.637365430516675	-4.10656501028592
a_HQ_transcript/129272	1.59560727262729	-0.604229071791191	2.05927612945951	0.047532093441706	0.637365430516675	-3.98646190216405
a_HQ_transcript/145767	1.67600510619177	-0.623730726536934	2.05925415848525	0.0475343231125579	0.637365430516675	-4.00668193061234
a_HQ_transcript/42175	1.03946693403627	1.25409698004235	2.05905065912303	0.0475549791229965	0.637365430516675	-3.88971205983058
a_HQ_transcript/102061	-2.65599550504621	0.955825665324959	-2.05895240594849	0.0475649550447176	0.637365430516675	-3.98480184042816
a_HQ_transcript/99202	2.66401307703748	-0.123119637903561	2.05885205209592	0.047575146154426	0.637365430516675	-3.95391059073414
a_HQ_transcript/100451	-2.16718625642362	2.36825931142983	-2.05883061819214	0.0475773230538438	0.637365430516675	-4.05161869228848
a_HQ_transcript/97055	3.922607068086	1.97417788078896	2.05872755490919	0.0475877917313828	0.637365430516675	-4.01962859728883
a_HQ_transcript/116500	1.452435053903	-0.652326604546874	2.05872110908456	0.0475884465348746	0.637365430516675	-3.97052735853774
a_HQ_transcript/49377	2.79129250554048	0.157672128192129	2.05855832018538	0.047604986183934	0.637365430516675	-3.9839694318752
a_HQ_transcript/118164	1.92924442420122	0.94263293778706	2.05841918293596	0.0476191267897342	0.637365430516675	-3.89088322477539
a_HQ_transcript/77308	2.07876964022175	0.571442391522014	2.05835081931545	0.0476260759807905	0.637365430516675	-3.9556649225739
a_HQ_transcript/101108	0.938876057381885	-0.186222203261479	2.05825573868213	0.0476357424505293	0.637365430516675	-3.99379732853488
a_HQ_transcript/146438	1.15726149456384	3.23612851929688	2.05824999402452	0.0476363265422929	0.637365430516675	-4.14394532140614
a_HQ_transcript/144297	4.9372342391498	0.702538584993647	2.0581141571072	0.0476501396840029	0.637365430516675	-3.98010732241535
a_HQ_transcript/94794	-0.624289149718097	2.7484479653512	-2.05807932722164	0.0476536820731472	0.637365430516675	-4.02024715838603
a_HQ_transcript/120422	-0.756616461951515	4.24218842788882	-2.05805680163585	0.0476559731712971	0.637365430516675	-4.32450266820769

a_HQ_transcript/136946	3.47928685672027	4.17542110290432	2.05785161286223	0.047676847572246	0.637365430516675	-4.1457588226723
a_HQ_transcript/120727	0.471316204492446	7.46912280723881	2.05783950251956	0.0476780798409763	0.637365430516675	-4.58664103041333
a_HQ_transcript/113207	1.81681769939288	2.59556477311472	2.05781312989162	0.0476807634428694	0.637365430516675	-4.02110176883805
a_HQ_transcript/113281	1.82420038390253	1.12383681413056	2.0577599059206	0.0476861797639179	0.637365430516675	-3.90462326670612
a_HQ_transcript/81945	1.12725763665619	1.67525692114029	2.05756459207026	0.0477060604545796	0.637484570598575	-3.9436777856955
a_HQ_transcript/30634	-1.83184975137398	0.71042500244662	-2.05719765336769	0.0477434302825604	0.637800614358572	-3.92409357505071
a_HQ_transcript/111327	-2.16601642914013	-0.107973092992403	-2.05702773948236	0.0477607433976226	0.637800614358572	-3.96250352841426
a_HQ_transcript/151434	0.702405918378073	4.37955022162174	2.05700924121446	0.0477626285835203	0.637800614358572	-4.31395830834971
a_HQ_transcript/115537	-1.3734293666805	1.77519963986125	-2.05665048684308	0.0477992027166123	0.638037445968235	-3.91670289225605
a_HQ_transcript/146717	0.848160422599845	5.28535916722543	2.0566199510102	0.047802316907371	0.638037445968235	-4.39790783950429
a_HQ_transcript/19758	2.31302169747815	1.36670270468419	2.05650244784602	0.0478143021082205	0.63805090747168	-3.89828391087987
a_HQ_transcript/145067	1.77477663002529	-0.759274483868411	2.05592573998815	0.0478731640312001	0.638689758734359	-3.9764504462159
a_HQ_transcript/103919	-2.37281388701717	2.81507594588428	-2.05535333686754	0.0479316495856357	0.639323296721853	-4.21484545340886
a_HQ_transcript/36031	0.868616128617047	2.57840250550408	2.05507994463541	0.047959605725911	0.639410037971779	-4.05649479022527
a_HQ_transcript/97542	1.05740569237056	1.9338217097439	2.05507459541181	0.0479601528619075	0.639410037971779	-3.90772020484888
a_HQ_transcript/109179	-0.651844851975283	5.70888789158556	-2.05487650336349	0.0479804182262702	0.639533536036834	-4.40262274067168
a_HQ_transcript/64563	4.24692827130095	3.52417043529185	2.05470211244704	0.0479982651303335	0.639585599581024	-4.10090961110667
a_HQ_transcript/64826	2.06960177792818	0.936725046730775	2.05462332183848	0.0480063303566613	0.639585599581024	-3.96953667907437
a_HQ_transcript/37498	-0.91046356458454	4.26632714611838	-2.05429974932279	0.048039464636548	0.63974313823775	-4.30774586313851
a_HQ_transcript/6664	-0.517545177133718	3.42544236040213	-2.05396817615679	0.0480734390502024	0.63974313823775	-4.17785543874807
a_HQ_transcript/148759	1.09874369247557	3.00873225500057	2.05363620559063	0.0481074753422268	0.63974313823775	-4.07410036650845
a_HQ_transcript/126631	-3.49119174409702	2.5688185957909	-2.05340010937818	0.0481316947132644	0.63974313823775	-4.12841733447583
a_HQ_transcript/135232	2.63592822680693	2.14778956910835	2.05336547474122	0.0481352485268717	0.63974313823775	-3.99822170583527
a_HQ_transcript/53718	3.07682779927623	2.65452011307611	2.0532574108907	0.0481463382961516	0.63974313823775	-4.03098728926165
a_HQ_transcript/64422	3.12658062967719	0.173704598885763	2.05325084309073	0.0481470123717931	0.63974313823775	-3.89916283126882
a_HQ_transcript/87365	-0.772462778039396	2.77737125706461	-2.05313462342905	0.0481589417631197	0.63974313823775	-4.08673423874966
a_HQ_transcript/32844	-0.970019381396642	3.61385767307604	-2.05309976687421	0.0481625201278807	0.63974313823775	-4.21958319942228
a_HQ_transcript/102979	2.27350891540352	1.21455707546316	2.05295348842319	0.0481775395838081	0.63974313823775	-3.92290507038619
a_HQ_transcript/133609	4.03821318014206	2.35704930072597	2.0529139873274	0.0481815961500878	0.63974313823775	-4.1754925247212
a_HQ_transcript/124168	-3.34219336436364	2.33569595694673	-2.0528221723592	0.048191026251141	0.63974313823775	-4.08428267876063
a_HQ_transcript/119016	3.68745724517336	2.65328803721689	2.0527971526438	0.0481935962489667	0.63974313823775	-4.08844152032817
a_HQ_transcript/132556	-0.985983966577135	3.00028181330199	-2.05278577053644	0.048194765446465	0.63974313823775	-4.07957844984666
a_HQ_transcript/90825	0.707905185098842	3.69337300521953	2.05262493596778	0.0482112894264997	0.63974313823775	-4.24660013140738
a_HQ_transcript/1669	1.8445080159849	1.18074189683312	2.05254160081648	0.0482198531536141	0.63974313823775	-3.85756362005396
a_HQ_transcript/5032	-1.95922656935239	1.19384621970805	-2.05252745193516	0.0482213072605676	0.63974313823775	-3.96020447536746
a_HQ_transcript/117083	2.0473517459669	3.86937978608034	2.05247553058585	0.0482266436448218	0.63974313823775	-4.15888534071135
a_HQ_transcript/122438	-1.9807725006913	2.81796339103382	-2.05246949003619	0.0482272645155144	0.63974313823775	-4.0179614609295
a_HQ_transcript/2604	0.722859858393639	3.72215168748462	2.05229382225642	0.0482453233920809	0.639836677648843	-4.21913628888322
a_HQ_transcript/70963	-0.68788264155783	2.71003781643252	-2.05201953683047	0.0482735321763548	0.640064753272616	-4.04185525334374
a_HQ_transcript/123600	0.861049569044165	3.89885891216714	2.05186852800253	0.048289068810722	0.640124741580307	-4.28080282873041
a_HQ_transcript/109183	-0.884895742285163	3.51466719147254	-2.05140487753333	0.0483367992343521	0.640611369332972	-4.21817648346896
a_HQ_transcript/3726	-1.1175881452588	4.49197055807217	-2.05128240681465	0.0483494138834995	0.640626853968363	-4.36014953167264
a_HQ_transcript/14696	2.00202948295751	-0.220667804739949	2.05114752375169	0.0483633103686814	0.640626853968363	-3.96980933691273
a_HQ_transcript/130288	3.54084403431456	1.13789139319776	2.05107097486165	0.0483711984687299	0.640626853968363	-3.95571694609023
a_HQ_transcript/136075	3.08167107518763	1.32834327497863	2.05096567127952	0.0483820514884584	0.640626853968363	-4.03568817873296
a_HQ_transcript/36230	2.65820687285465	1.13388534839408	2.05038724617294	0.0484417045251281	0.640627530827613	-3.95353685871958
a_HQ_transcript/22707	1.56510165676133	2.22283136366242	2.05031600411583	0.0484490561939199	0.640627530827613	-3.99865400472768
a_HQ_transcript/96470	1.49875579563711	1.41420746860752	2.05029654181504	0.0484510647341209	0.640627530827613	-3.89042997481987
a_HQ_transcript/24609	1.89929036355378	-0.0510616392911008	2.05026124999365	0.0484547070925099	0.640627530827613	-3.89744559344569
a_HQ_transcript/151124	0.44212159736732	10.2629299833837	2.05024531254365	0.048456352025687	0.640627530827613	-4.90161113194325

a_HQ_transcript/121963	1.40267587779352	-0.540317457798132	2.05023603747229	0.0484573093452451	0.640627530827613	-4.0313550176276
a_HQ_transcript/134414	3.7367729016234	2.49840410326149	2.05021724092749	0.0484592494675374	0.640627530827613	-4.13335341692664
a_HQ_transcript/6768	1.35487528477278	1.04764923962603	2.04999120447401	0.0484825856104388	0.640790299309593	-3.91094478551286
a_HQ_transcript/150330	-0.733420097525391	2.89247255110739	-2.04938822227297	0.0485448861777436	0.641374461498619	-4.1109994894937
a_HQ_transcript/144085	2.17612262350637	2.11739090238732	2.04934986809358	0.048548851338321	0.641374461498619	-4.00442457929579
a_HQ_transcript/4877	-2.44440756527617	1.55584111369322	-2.04814433487322	0.0486736278038845	0.642876763686481	-3.96376583968986
a_HQ_transcript/145554	-1.9187173481846	2.10587622760047	-2.04784138303255	0.0487050285305756	0.642951297170673	-3.91758215783987
a_HQ_transcript/84029	-2.22591056664881	-0.570153445637739	-2.04777740235159	0.0487116623548211	0.642951297170673	-3.99103276690692
a_HQ_transcript/131353	1.1329038135583	2.64351264591608	2.0477316618971	0.048716405431353	0.642951297170673	-4.11059063968155
a_HQ_transcript/65264	3.7411886194308	2.75390777839022	2.04758522539705	0.0487315929572585	0.642951297170673	-4.06379558127417
a_HQ_transcript/70777	1.07053784108685	1.95510082402677	2.0475564709792	0.0487345756835602	0.642951297170673	-3.97384126109962
a_HQ_transcript/14710	1.71620430943539	-0.383558157309739	2.04738924076534	0.0487519258300144	0.643029173858714	-3.96277790656545
a_HQ_transcript/139960	1.89835650471348	-0.650058947670878	2.04712256931065	0.0487796042372986	0.643029173858714	-3.98910859756652
a_HQ_transcript/104084	-2.42184101636191	0.263857497973649	-2.04712127080857	0.0487797390454022	0.643029173858714	-3.94682363456587
a_HQ_transcript/139975	1.17731627013302	2.07452204796807	2.04707322035442	0.0487847277855238	0.643029173858714	-4.01614552304777
a_HQ_transcript/32545	-2.31513391620807	2.8172597955	-2.04685151959243	0.0488077512182982	0.643186797283437	-4.14984224687701
a_HQ_transcript/43103	-1.75951844311433	3.12321922417523	-2.04649965485058	0.0488443116626174	0.643522699076297	-4.08531222228941
a_HQ_transcript/114574	-0.792848283207114	2.87079055315171	-2.04622239033441	0.0488731377267581	0.643744114961069	-4.05976735830229
a_HQ_transcript/17228	-1.01659578921594	3.61000547030917	-2.04605357800651	0.0488906957751866	0.643744114961069	-4.24617144792705
a_HQ_transcript/130276	0.757101879264251	3.82344341642325	2.04601853527027	0.0488943412392156	0.643744114961069	-4.27281125232082
a_HQ_transcript/107917	-3.79802799882508	2.3823699870474	-2.04584543520373	0.0489123521844385	0.643835418427772	-4.08724191087549
a_HQ_transcript/27629	2.11910557371857	1.02730474533069	2.04498896534317	0.0490015530622558	0.644863544578403	-3.94772638642462
a_HQ_transcript/151473	2.02356379337123	-0.561780344740474	2.0448760570673	0.0490133230503675	0.644872440532314	-4.01312666495589
a_HQ_transcript/1248	-1.45342058510309	2.25000996905717	-2.04465121828964	0.0490367684943139	0.644889536906029	-4.00371578210689
a_HQ_transcript/9351	-2.32435484799083	0.655237881260596	-2.04447702563274	0.0490549394985065	0.644889536906029	-4.05738788013222
a_HQ_transcript/82877	1.92780180186193	-0.129191425092112	2.04443927426482	0.049058878333005	0.644889536906029	-3.98347227646346
a_HQ_transcript/102342	-0.787279140165167	2.68827004118651	-2.04443811146681	0.0490589996593459	0.644889536906029	-4.01996002348829
a_HQ_transcript/5703	1.45363325260287	0.53077994033045	2.04430929544198	0.0490724419545287	0.644920394739509	-3.90178518510748
a_HQ_transcript/101297	-2.23875538938139	0.0809751939826598	-2.04404466532197	0.0491000669594354	0.645062308043198	-3.97712216312772
a_HQ_transcript/9043	-2.71405634815729	0.59511638164853	-2.04399326012487	0.0491054347834288	0.645062308043198	-3.89948542625817
a_HQ_transcript/119919	5.33071592814674	1.66874667997725	2.0438077152906	0.0491248139986352	0.645147871030226	-3.93799876288328
a_HQ_transcript/28429	-0.842850906290462	2.82426281593925	-2.04371839191821	0.0491341457653938	0.645147871030226	-4.00534902358786
a_HQ_transcript/76367	1.44385235958531	-0.784240380213963	2.0430191626151	0.049207249253706	0.645638496093063	-3.98608907600423
a_HQ_transcript/16437	-2.08532756191993	0.0310460918161647	-2.04287043566391	0.0492228107679572	0.645638496093063	-3.97925166272752
a_HQ_transcript/64945	-2.89255447670329	1.64470121183349	-2.0428578998264	0.0492241226080355	0.645638496093063	-4.06395269490974
a_HQ_transcript/1689	2.09361625575327	1.40974924682704	2.04284468581525	0.0492255054503056	0.645638496093063	-3.97783752295771
a_HQ_transcript/8167	0.9627388465203	1.37315566724357	2.04282995124554	0.0492270474587885	0.645638496093063	-3.88458856254484
a_HQ_transcript/128790	1.03579009868065	2.33179493965385	2.04264623616127	0.0492462772493513	0.64566684394137	-4.01423756379551
a_HQ_transcript/1076	-2.94951396260848	2.0283332957484	-2.04259707455229	0.0492514242023815	0.64566684394137	-4.10503249318158
a_HQ_transcript/128808	1.8855384983028	1.50822557864057	2.04226232884703	0.0492864828143245	0.645980760548152	-3.96933358528496
a_HQ_transcript/148586	0.639918629001167	8.75711854410776	2.04203138486958	0.0493106828257496	0.646114598865156	-4.73735960665138
a_HQ_transcript/6397	1.69427309847384	0.303368239372727	2.04186117823514	0.0493285250072359	0.646114598865156	-3.92738485045601
a_HQ_transcript/81730	1.8094477386512	-0.374786040602268	2.04165162368208	0.0493504996807866	0.646114598865156	-3.975845762343
a_HQ_transcript/131718	-1.4427274797532	3.44703392155803	-2.04164853416561	0.0493508237232998	0.646114598865156	-4.16781531759198
a_HQ_transcript/25789	-2.51797020779259	-0.382175842570364	-2.04152361884855	0.0493639269733788	0.646114598865156	-4.00352723624416
a_HQ_transcript/30250	1.89647701911646	0.854671131151609	2.04144862266982	0.049371795319488	0.646114598865156	-3.99117854104545
a_HQ_transcript/81132	-0.647340129408204	3.62507107477577	-2.04139015875816	0.0493779299195237	0.646114598865156	-4.22576596167648
a_HQ_transcript/64029	1.31473354441674	2.11065847675797	2.04131690549588	0.0493856173059092	0.646114598865156	-3.94987256985029
a_HQ_transcript/87562	2.03591728605493	0.597118721761867	2.04104325271974	0.049414344428042	0.646344962401716	-3.94618573223125
a_HQ_transcript/88061	-2.01666799477296	-0.170542523162896	-2.0407895973119	0.0494409853937566	0.646408684529874	-3.93792112343832

a_HQ_transcript/57637	4.45274766473106	2.93943581392476	2.04078510828598	0.049441456981486	0.646408684529874	-4.18565338306364
a_HQ_transcript/138721	4.83145108648656	0.882986494818969	2.04052900689321	0.0494683678590844	0.646615085881012	-4.04921831157683
a_HQ_transcript/16075	1.83495976606469	1.74896936423603	2.04021311456394	0.0495015792168116	0.646673406661914	-3.95822256983787
a_HQ_transcript/85252	2.1391086286932	0.04541362525661	2.04016967695162	0.049506147562794	0.646673406661914	-3.9852238815065
a_HQ_transcript/84195	-3.13606910069201	3.86118421508516	-2.04016913486161	0.049506204576891	0.646673406661914	-4.27273103561532
a_HQ_transcript/152127	-4.65179127647216	6.01762385203351	-2.03908962711244	0.0496198557532428	0.648012351207481	-4.69982327814202
a_HQ_transcript/16135	3.18959959048023	2.53246747184438	2.03832947190814	0.0497000226373419	0.648756877530623	-4.06237594620444
a_HQ_transcript/144476	-1.9311697238589	1.59184464878043	-2.03830003951995	0.0497031288966277	0.648756877530623	-3.91597293243465
a_HQ_transcript/63802	-2.70629398236043	1.41769409095938	-2.03823163949034	0.0497103484124931	0.648756877530623	-3.95410018218712
a_HQ_transcript/50680	-0.983469952446859	1.93790567105778	-2.03812055868561	0.0497220747778261	0.648764256495056	-3.94328911236947
a_HQ_transcript/117543	2.01818185298887	0.828873917933488	2.0379455362184	0.0497405561391067	0.648859750281417	-3.97852860805479
a_HQ_transcript/147470	-0.447093798159092	6.39395028697279	-2.03773169264449	0.0497631449659793	0.649008770604094	-4.52670574027197
a_HQ_transcript/143260	-3.94902636451561	2.24741410116324	-2.03752550084442	0.0497849340400925	0.649102760279231	-4.17525476388936
a_HQ_transcript/125600	2.5298275701107	0.955296561972675	2.0374521696218	0.0497926852478168	0.649102760279231	-3.9557080481836
a_HQ_transcript/20585	-1.64138217849194	-0.42508908268694	-2.03718263760656	0.0498211842391818	0.649328654137929	-3.96820294929602
a_HQ_transcript/137244	-1.02587605205853	3.26219394790416	-2.03663165583574	0.0498794868778489	0.649699860974796	-4.19601607115094
a_HQ_transcript/30129	1.99136171529773	0.0135991302543586	2.03662204689134	0.0498805041878944	0.649699860974796	-3.92101273357191
a_HQ_transcript/37678	-1.08342559401863	3.15911833216796	-2.03659661221596	0.0498831970742244	0.649699860974796	-4.17004317304227
a_HQ_transcript/15960	1.79054126365453	1.53095742338698	2.03632658301469	0.0499117941762716	0.649706424740602	-3.89033642231233
a_HQ_transcript/145360	-5.13438755931376	4.24036564406851	-2.03623499581228	0.049921496869255	0.649706424740602	-4.17313434650836
a_HQ_transcript/85726	-0.544585475025024	4.16151914653626	-2.03609941231855	0.0499358635407019	0.649706424740602	-4.32617942400747
a_HQ_transcript/13280	-0.924174740628562	1.87854514745969	-2.0360907108483	0.0499367856880234	0.649706424740602	-3.96550663615673
a_HQ_transcript/152090	-3.39418196791554	3.88449813798645	-2.03606427946564	0.0499395868727809	0.649706424740602	-4.42527871952596
a_HQ_transcript/140806	-1.32704858596127	-0.723943832181478	-2.03583320812631	0.0499640815687836	0.649727865190332	-3.99130974707104
a_HQ_transcript/91517	0.627519443991775	4.52208823466959	2.03576876640343	0.0499709145868624	0.649727865190332	-4.35725200994327
a_HQ_transcript/81677	-1.69258875573014	0.729070151949158	-2.03549758978497	0.0499996775306145	0.649727865190332	-4.00937791279365
a_HQ_transcript/125691	4.6545115016952	2.71913990439814	2.03547717363422	0.0500018436022209	0.649727865190332	-4.15502391218818
a_HQ_transcript/34622	1.33992055421983	2.53564813757591	2.03545826505769	0.0500038497996773	0.649727865190332	-4.06052694227021
a_HQ_transcript/139113	1.13998008004224	2.63590415196707	2.03541632288008	0.0500083001111606	0.649727865190332	-4.12637537849006
a_HQ_transcript/130359	-3.1018224017218	2.42410505751085	-2.0351697809641	0.0500344666811005	0.649836838942863	-4.13668196783414
a_HQ_transcript/142519	3.14716974152546	4.7230161722151	2.03512664207698	0.0500390464338744	0.649836838942863	-4.38522326284651
a_HQ_transcript/4975	-1.36136806312154	1.19232004603915	-2.0345798488953	0.0500971275020418	0.650199169213331	-3.94026822431381
a_HQ_transcript/101265	-2.86047268630051	-0.258521490856796	-2.03448481084267	0.0501072285946637	0.650199169213331	-4.00163556884534
a_HQ_transcript/29012	-1.58400737414014	1.23079000646922	-2.03433303444417	0.0501233638107689	0.650199169213331	-3.99685858142716
a_HQ_transcript/125336	0.498098368503074	3.59293348485479	2.03427132059522	0.0501299258591193	0.650199169213331	-4.25637746969898
a_HQ_transcript/105294	1.42447613280944	-0.495191255383507	2.03425546200919	0.0501316122280556	0.650199169213331	-4.01313002211476
a_HQ_transcript/37884	-0.647323391675202	3.70674648022258	-2.03420962222133	0.0501364870151834	0.650199169213331	-4.23682752910914
a_HQ_transcript/73550	2.36298206028009	0.786574949504494	2.03412726481579	0.0501452462768952	0.650199169213331	-4.01832901197062
a_HQ_transcript/144247	3.57022178954662	1.11544181174403	2.03377193100109	0.0501830537975602	0.650335136389756	-4.11174161226697
a_HQ_transcript/84371	1.42087789460953	-0.594566742871663	2.03361733811484	0.0501995102845222	0.650335136389756	-4.03242991831552
a_HQ_transcript/52907	2.63951305913612	1.83782789473671	2.03359222696488	0.0502021838252624	0.650335136389756	-3.98695322727924
a_HQ_transcript/143381	1.61424076986164	-0.367706227623942	2.03347446476396	0.0502147234293	0.650335136389756	-4.00175305600336
a_HQ_transcript/92759	1.91736530405678	0.487905200037062	2.03325077912135	0.0502385495818777	0.650335136389756	-3.94088923981627
a_HQ_transcript/94303	1.65125542841462	-0.597267032878308	2.0331417947113	0.0502501617824637	0.650335136389756	-3.99827638692786
a_HQ_transcript/7430	-1.04782154038068	1.68867090411726	-2.03311201332793	0.0502533353741836	0.650335136389756	-3.95851696147798
a_HQ_transcript/124528	1.96976238864333	1.80944660808959	2.03300457453187	0.0502647858288422	0.650335136389756	-3.9402872502556
a_HQ_transcript/112092	1.95634854564276	-0.356960614027328	2.03281962748665	0.0502845022033803	0.650335136389756	-3.97295337629976
a_HQ_transcript/119232	3.19541875344983	3.56890838242954	2.03281627239422	0.0502848599373496	0.650335136389756	-4.10481683964081
a_HQ_transcript/46092	-0.52790074270619	3.12464307101978	-2.03280412742755	0.0502861549030868	0.650335136389756	-4.1736066324417
a_HQ_transcript/22719	2.05796895490454	-0.316645016987076	2.03276817743109	0.05028998826851	0.650335136389756	-3.95974404766197

a_HQ_transcript/127992	2.34934450705004	1.48216852026126	2.03265658499369	0.0503018890561164	0.650344352102744	-3.98672760032598
a_HQ_transcript/23105	-2.18241752487566	0.73218737080711	-2.03207717490099	0.0503637200156624	0.650998958654753	-4.02933063899876
a_HQ_transcript/51479	-1.86774871096976	3.04452872444192	-2.03193376308799	0.0503790342970018	0.651052135530485	-4.16549278416217
a_HQ_transcript/110062	3.0812388646638	1.37534531347275	2.03148090674033	0.0504274195290557	0.651532572212702	-3.95944097040073
a_HQ_transcript/148232	-2.91946606772569	1.43525930757951	-2.03136980130212	0.0504392967620327	0.651541209374097	-4.03048803325528
a_HQ_transcript/98789	0.816747906434386	1.91649418176754	2.03115103174395	0.0504626905186646	0.651657623876335	-4.07465537797349
a_HQ_transcript/134573	-1.07827903114206	-0.740896550773956	-2.03107586538192	0.0504707305032215	0.651657623876335	-4.02751722871055
a_HQ_transcript/17728	1.83451576406163	0.386952259158405	2.03020722119677	0.0505637243971786	0.652625267460175	-3.99531007462285
a_HQ_transcript/117637	1.97648745291561	-0.259171586010226	2.03016611276646	0.0505681290366197	0.652625267460175	-4.02908183989793
a_HQ_transcript/127742	1.08436291839547	2.34588521148509	2.02994495233239	0.0505918314567412	0.652767012852163	-4.05035873173305
a_HQ_transcript/36453	2.73751584766359	0.858071063656352	2.02985409524618	0.0506015717023095	0.652767012852163	-3.92725004926094
a_HQ_transcript/147143	1.9217155282894	0.503327303523323	2.02973000510677	0.0506148773207194	0.652783337380796	-3.90556770175738
a_HQ_transcript/27617	2.10919053789339	1.0502156728195	2.02959693569814	0.0506291491528425	0.652783337380796	-3.94895027380665
a_HQ_transcript/146198	4.5464679324339	3.6177087533595	2.02952815384308	0.0506365274609484	0.652783337380796	-4.27112870068807
a_HQ_transcript/125164	1.3758648213188	1.9318702499417	2.0291794675601	0.0506739459385571	0.653120871289678	-4.00575445753689
a_HQ_transcript/7197	-0.947187589994127	4.06560513434765	-2.02889809734988	0.0507041582120731	0.653234079832742	-4.28352116196835
a_HQ_transcript/12937	-1.34444079214758	4.25615607971139	-2.0286990291196	0.050725542802775	0.653234079832742	-4.31218345015161
a_HQ_transcript/60011	-2.10366840507958	1.68617099645045	-2.02868572772415	0.0507269719659074	0.653234079832742	-3.93735681765063
a_HQ_transcript/86954	2.09270746536587	0.495117181351238	2.02867912954835	0.050727680917372	0.653234079832742	-4.00311448512418
a_HQ_transcript/105496	-3.53017385134603	2.00785678589973	-2.02845319003084	0.0507519625878611	0.653402011363718	-4.0569785562619
a_HQ_transcript/80371	1.92785397422089	-0.120683194095061	2.02813963158245	0.050785677523857	0.653454687079473	-4.04302674104509
a_HQ_transcript/77790	-1.6732546160307	-0.340162408323341	-2.02812243952652	0.050787526643255	0.653454687079473	-4.01996316955104
a_HQ_transcript/150570	0.369587181503212	10.5286636010698	2.0280067855746	0.0507999675249164	0.653454687079473	-4.98122876612309
a_HQ_transcript/123331	-3.61260221451011	0.0907010414005655	-2.02796648529667	0.0508043032483965	0.653454687079473	-4.01666764277916
a_HQ_transcript/122666	1.88804021600419	0.779278889574818	2.02789251383578	0.0508122623451558	0.653454687079473	-3.96436774433697
a_HQ_transcript/108853	3.63346200845632	0.874011174205189	2.02772700889506	0.0508300741145026	0.653539161275781	-4.05665807880301
a_HQ_transcript/6539	3.22326435248507	1.05241142837153	2.02715485949427	0.0508916914353741	0.65418669609806	-4.028637879308
a_HQ_transcript/7049	-1.03230511876201	2.82400073306975	-2.02676640667499	0.0509335629489068	0.654580178442197	-4.20695460782444
a_HQ_transcript/80832	-1.73895972199391	3.08668451297814	-2.02654091501899	0.050957882646708	0.654650031600803	-4.14533435784386
a_HQ_transcript/70703	-2.08800541985043	2.17453574902256	-2.02650717240843	0.0509615227255992	0.654650031600803	-4.03443094424803
a_HQ_transcript/137636	2.16014643411045	-0.463352469695242	2.02632746920156	0.0509809125519108	0.654754415558433	-4.05020168951192
a_HQ_transcript/69996	1.72004992440351	-0.639276018184256	2.02567380864281	0.0510514965129896	0.655516101017685	-3.96666643210121
a_HQ_transcript/51642	-2.01169913501793	-0.484174329869428	-2.02545724100257	0.0510749009240032	0.655539223843124	-4.00573050117355
a_HQ_transcript/21918	-2.52654756930073	2.09861926404866	-2.02537642363785	0.0510836372447998	0.655539223843124	-4.23603683735851
a_HQ_transcript/82593	1.35104640914795	-0.80542190692518	2.02532373733284	0.051089333315626	0.655539223843124	-4.02893905521027
a_HQ_transcript/118389	-0.509863083636131	4.57893564589166	-2.02523981676107	0.0510984073636319	0.655539223843124	-4.40654688193916
a_HQ_transcript/11773	-1.39486343307345	2.8804160040814	-2.02465340291967	0.0511618537263464	0.65620834805937	-4.04493283530801
a_HQ_transcript/18269	-2.42259538512389	0.773386427104649	-2.02425031753304	0.0512055050933951	0.65662333996666	-3.95893885315656
a_HQ_transcript/150986	-3.27793657945194	1.10901520667484	-2.02394909080895	0.0512381471670532	0.656897004527232	-4.16585376313917
a_HQ_transcript/44284	1.98939802423213	0.816419112162716	2.02376142746139	0.0512584922951543	0.657012930569511	-4.04157549169786
a_HQ_transcript/138849	2.09148395426292	-0.683533846565047	2.02348504200705	0.051288468929629	0.657252231468579	-4.01899624575718
a_HQ_transcript/139736	-1.12251047865338	3.08854460833823	-2.02327086904878	0.0513117085852963	0.657405112771898	-4.13135964246327
a_HQ_transcript/31456	3.10883620244143	-0.215141383765861	2.02303160986555	0.0513376812244864	0.657592933939389	-4.02946595844472
a_HQ_transcript/8558	5.71791145796024	1.97601239184044	2.02257753711233	0.0513870044884454	0.658079708505035	-4.00296834934882
a_HQ_transcript/89164	-2.27636082311834	-0.139451533376786	-2.0224604875359	0.0513997256247972	0.658097632404892	-4.02668318082075
a_HQ_transcript/13847	-0.670331305494887	3.53474391471828	-2.02195890354207	0.0514542698187005	0.658543490696531	-4.24812349111073
a_HQ_transcript/87717	0.6308543525024	3.06820570362407	2.02178381444315	0.0514733216202065	0.658543490696531	-4.22265557580177
a_HQ_transcript/3392	-0.623813216567977	3.35816538237806	-2.02178325142119	0.0514733828937367	0.658543490696531	-4.23308841606109
a_HQ_transcript/138469	0.666576720049585	4.16055089314891	2.02172368824636	0.0514798654989857	0.658543490696531	-4.39337417323915
a_HQ_transcript/39749	1.63804039999674	2.62621158444832	2.02146350233946	0.0515081914216068	0.658760869297065	-4.18011310387852

a_HQ_transcript/136242	2.8936000069097	0.825894286341278	2.02126405757678	0.0515299138167573	0.658795308326067	-4.09493548857876
a_HQ_transcript/82461	-0.767259491130776	4.03231144641882	-2.02117859148671	0.0515392247548371	0.658795308326067	-4.33439771939833
a_HQ_transcript/1643	2.67770458457591	1.28659352657651	2.02112664415717	0.0515448847761312	0.658795308326067	-3.98324722164434
a_HQ_transcript/6218	-0.822234953353149	2.96027265653227	-2.02086844985534	0.0515730249099139	0.659010066380187	-4.13097645612157
a_HQ_transcript/54133	4.72196446188296	3.6385356847838	2.02063122011791	0.0515988920106536	0.659129988554885	-4.29834179035536
a_HQ_transcript/121013	1.03177467367502	4.12072794293159	2.02057440797434	0.0516050883896449	0.659129988554885	-4.33790185310799
a_HQ_transcript/79744	3.19592507189858	0.304934849003804	2.0203390925012	0.0516307606838978	0.659310509624766	-4.0389773602705
a_HQ_transcript/35156	-2.2400114026105	2.08209220490128	-2.01999654395861	0.0516681518087864	0.659310509624766	-3.99288654717288
a_HQ_transcript/63998	2.76617816786653	1.14566168328468	2.0198003145044	0.051689582050826	0.659310509624766	-4.11530491917313
a_HQ_transcript/54161	1.31364589128806	1.94774588175276	2.0196891863951	0.0517017218160703	0.659310509624766	-3.97812862815082
a_HQ_transcript/84422	5.35101252810521	1.16910696858968	2.01965780582518	0.0517051503183386	0.659310509624766	-4.06209436734386
a_HQ_transcript/85480	-1.36384468787868	0.985124516438812	-2.01956567628389	0.0517152171345932	0.659310509624766	-4.03306205994546
a_HQ_transcript/147821	-3.10468804158266	3.96155235279757	-2.01955719691658	0.0517161437448391	0.659310509624766	-4.24075346191198
a_HQ_transcript/33028	1.50169721860795	1.96573232261213	2.01951054670008	0.0517212418578669	0.659310509624766	-4.0020931467101
a_HQ_transcript/103303	1.00251539250398	3.61533164463041	2.01950998360721	0.0517213033974837	0.659310509624766	-4.28980641307381
a_HQ_transcript/152382	0.721031212357686	4.26496052954001	2.01846880060354	0.0518352025062486	0.660315316293405	-4.35747589344098
a_HQ_transcript/8858	1.53722626583011	0.0323306118632134	2.01832147006777	0.0518513372942011	0.660315316293405	-3.93511606036623
a_HQ_transcript/3541	-2.8645737345909	1.34760474197475	-2.01826632672864	0.0518573774042788	0.660315316293405	-4.15346530148649
a_HQ_transcript/11955	1.76234830522305	2.51245309349605	2.01826015708866	0.0518580532323602	0.660315316293405	-4.00012577348087
a_HQ_transcript/36020	2.25538210248303	0.475640804641326	2.01821970749089	0.0518624843097688	0.660315316293405	-3.93484187344476
a_HQ_transcript/35618	1.76559195878343	2.02879829384951	2.0181667497929	0.0518682860961273	0.660315316293405	-4.02193399362628
a_HQ_transcript/150386	-1.6078139481315	3.45384297213637	-2.01765212590649	0.0519246953640426	0.660888699829444	-4.13082921378221
a_HQ_transcript/122288	1.52308621221575	-0.753584511725249	2.01750803858245	0.051940498770845	0.660945120961401	-4.04061551937646
a_HQ_transcript/148607	1.58819667542921	-0.687521577211529	2.01723100875322	0.0519708950564297	0.661051787301232	-4.04068239147398
a_HQ_transcript/28923	-1.83474005391336	1.42828405658953	-2.01690191736227	0.052007023876331	0.661051787301232	-3.99100125736053
a_HQ_transcript/144551	1.23136046751713	4.72291902652378	2.01688075309611	0.052009348116641	0.661051787301232	-4.27535315649619
a_HQ_transcript/150495	1.40727682978937	3.17639370515853	2.01683065799244	0.0520148498772678	0.661051787301232	-4.16288168258212
a_HQ_transcript/83191	1.45792520599979	-0.15968346714237	2.01673753333707	0.0520250787685718	0.661051787301232	-4.00423127458688
a_HQ_transcript/89092	1.72819079631841	1.10281726773852	2.01663255529718	0.052036611756567	0.661051787301232	-3.94411775344406
a_HQ_transcript/144365	1.36235427939015	-0.480079117962015	2.01662652928583	0.0520372738478463	0.661051787301232	-4.09214239885315
a_HQ_transcript/69225	-3.66701419588293	1.44125755718889	-2.01660299264419	0.0520398599416879	0.661051787301232	-4.11663059702436
a_HQ_transcript/140941	3.35725743963139	4.41902747819788	2.01641406149476	0.052060622870148	0.661119334342958	-4.3803975331415
a_HQ_transcript/79523	-2.14483396554007	0.15318237505385	-2.01624188168957	0.0520795511933352	0.661119334342958	-4.01167533978489
a_HQ_transcript/50138	2.3184864691895	0.288094591202809	2.01611206050574	0.0520938268685979	0.661119334342958	-4.03096889122689
a_HQ_transcript/148850	-6.05865811224729	1.70888269098595	-2.01601033468797	0.0521050154539292	0.661119334342958	-4.05628683690547
a_HQ_transcript/130421	-2.26401922444565	-0.353722333122149	-2.01601013627141	0.052105037279359	0.661119334342958	-4.03451130546265
a_HQ_transcript/141346	3.52207500648515	1.13803511340995	2.01593394745805	0.0521134184895306	0.661119334342958	-3.96591326821826
a_HQ_transcript/77595	2.33731830699557	0.439705603067987	2.0156863126857	0.0521406678964624	0.661320694629187	-4.03666957159969
a_HQ_transcript/94645	1.96221842494772	-0.0502785962860882	2.01501381584664	0.0522147314869634	0.66197411801214	-4.04152912041463
a_HQ_transcript/27373	-2.60443428056606	1.78401142135207	-2.01491307352511	0.0522258343899878	0.66197411801214	-4.02497905326848
a_HQ_transcript/112433	3.34532496164562	0.912223680123995	2.0149083905294	0.0522263505574538	0.66197411801214	-3.96150728387913
a_HQ_transcript/107716	4.41821649285415	1.74884787158509	2.01455243937887	0.0522655971434562	0.662219919694967	-4.06079993359963
a_HQ_transcript/48924	-1.71646154631877	-0.675493909511763	-2.01445298954638	0.0522765669287797	0.662219919694967	-4.02402319129115
a_HQ_transcript/66196	-1.86535767564132	2.53210186560537	-2.01442259154431	0.0522799203736616	0.662219919694967	-4.0296892820705
a_HQ_transcript/129318	-2.3449603778981	-0.58475529689682	-2.01423265395562	0.0523008781591551	0.662341055693979	-4.04585888317681
a_HQ_transcript/137036	0.818806267170229	2.45283126139332	2.01404716622646	0.0523213520395265	0.662395799214299	-4.09157328604894
a_HQ_transcript/10519	-4.01979044042107	0.750376884657192	-2.0137985433122	0.0523488056931843	0.662395799214299	-4.07923100999576
a_HQ_transcript/104353	2.45756542177434	0.180548837852313	2.01356779410536	0.052374296956574	0.662395799214299	-3.97731781526936
a_HQ_transcript/145345	-1.785699189788	0.243558084739105	-2.01352556801087	0.0523789629214426	0.662395799214299	-3.94211078531675
a_HQ_transcript/113846	-3.42124531603222	4.66999653635109	-2.01351119590378	0.0523805511159056	0.662395799214299	-4.53147648139156

a_HQ_transcript/135139	-2.0968286947371	-0.307814585696645	-2.01336539714247	0.0523966650380676	0.662395799214299	-4.03971268714122
a_HQ_transcript/62470	1.91948607881974	1.89167473827315	2.01336106561277	0.0523971438322778	0.662395799214299	-3.97842030626455
a_HQ_transcript/67243	3.12099033549619	1.59931425824466	2.01327627503673	0.0524065170972817	0.662395799214299	-4.10569602541712
a_HQ_transcript/119628	2.26966513075075	3.09718108689849	2.01326503159531	0.0524077601256978	0.662395799214299	-4.30810060418755
a_HQ_transcript/80266	2.20568503108017	2.02713032386558	2.01300355361156	0.0524366753266776	0.662397064322994	-4.08056734912564
a_HQ_transcript/118215	0.467394846478194	3.93848247161733	2.01257753018983	0.0524838164633768	0.662397064322994	-4.35020460721522
a_HQ_transcript/25801	-2.1406865772238	0.29674191315499	-2.01251766136209	0.0524904441504374	0.662397064322994	-4.0997506973964
a_HQ_transcript/130991	4.38713101988928	0.255282265365244	2.01238278033947	0.0525053786306662	0.662397064322994	-4.06548384894931
a_HQ_transcript/10492	-1.81136029997051	2.24199347603064	-2.0123731092966	0.0525064495838338	0.662397064322994	-4.03231662441704
a_HQ_transcript/114140	0.798659019107196	4.57206135996378	2.01233956698408	0.0525101641447386	0.662397064322994	-4.39704109157691
a_HQ_transcript/120260	-2.31999476883297	0.602240498487232	-2.01226190876582	0.0525187650953106	0.662397064322994	-4.1362818671329
a_HQ_transcript/77502	-2.20728928522503	0.984060734223466	-2.01220758792288	0.0525247820729814	0.662397064322994	-3.97461317917591
a_HQ_transcript/103667	-0.858075675882747	2.24353983866993	-2.01219398729781	0.0525262886730255	0.662397064322994	-4.032069668292
a_HQ_transcript/27930	3.60435960748515	0.713921270411004	2.01218831986854	0.0525269164898272	0.662397064322994	-3.95404205060839
a_HQ_transcript/101266	2.01313996770075	0.945027980665597	2.01213150481733	0.0525332106132845	0.662397064322994	-3.99423062411341
a_HQ_transcript/133867	-1.99695383098361	-0.279514830980527	-2.01197109564904	0.0525509847311375	0.662402475181435	-3.97395035273627
a_HQ_transcript/74872	2.16170876394128	-0.00601607021280902	2.01183983707888	0.0525655327335165	0.662402475181435	-4.01784077514808
a_HQ_transcript/37574	1.51185897855875	-0.622329323809638	2.01181914471497	0.0525678264865806	0.662402475181435	-4.05022188822024
a_HQ_transcript/47393	-2.51170867428199	-0.163511739970701	-2.01155674971127	0.0525969206248663	0.662625444751241	-4.09599668553355
a_HQ_transcript/84216	1.57408632254621	3.30772810716161	2.0111216239335	0.0526451980495117	0.662768013648069	-4.22643006406258
a_HQ_transcript/100285	1.20540416772044	-0.648304786656668	2.01111877595964	0.0526455141612693	0.662768013648069	-4.08485171831994
a_HQ_transcript/137299	2.13692292843492	0.385417555016511	2.01105495814935	0.0526525980746655	0.662768013648069	-3.99493924338566
a_HQ_transcript/11732	2.40157576952777	2.48954730559904	2.01094510844467	0.0526647935792892	0.662768013648069	-4.08713340720994
a_HQ_transcript/148449	0.841363288602946	3.00916334477039	2.01094102823291	0.0526652466116232	0.662768013648069	-4.17961097527345
a_HQ_transcript/32377	-1.41840089288897	2.11333675372075	-2.01057864170928	0.0527054965542055	0.663130974827459	-4.03626233070606
a_HQ_transcript/5286	-3.46350423761829	2.17109150307286	-2.01043034742064	0.0527219752216963	0.663194757776836	-4.06838705153
a_HQ_transcript/138677	2.22213795382622	1.32910739577777	2.01018042167	0.0527497575142883	0.663324170166903	-4.06845784507841
a_HQ_transcript/132920	0.468152115434206	4.69810968423242	2.01013250053954	0.0527550859943847	0.663324170166903	-4.43483302106899
a_HQ_transcript/146217	2.16608846014479	0.141450178192561	2.00979144623832	0.0527930223420405	0.663657613040253	-4.01140771134503
a_HQ_transcript/10382	0.751916188790115	3.84849438543985	2.00937701955385	0.0528391521924184	0.663797257130595	-4.31032511177879
a_HQ_transcript/71509	-1.64325860465134	2.11832478278998	-2.00937295554007	0.0528396047324061	0.663797257130595	-4.08905973154313
a_HQ_transcript/117265	1.24788405105586	1.70813479230338	2.00931487607751	0.0528460724231237	0.663797257130595	-3.97003094272428
a_HQ_transcript/87115	-2.12108988532918	-0.234820754088946	-2.00928132337593	0.0528498091453412	0.663797257130595	-3.99812413613576
a_HQ_transcript/49109	-0.571973438478442	3.00377360252896	-2.0087614858634	0.0529077323211171	0.664168290070582	-4.15286528272033
a_HQ_transcript/14796	-0.928028922189058	2.09412128672166	-2.008736877886	0.0529104756537494	0.664168290070582	-3.97942234347314
a_HQ_transcript/5743	1.1613541855805	-0.710576824978093	2.00865188828414	0.0529199513730857	0.664168290070582	-4.05647234644978
a_HQ_transcript/79955	-0.673968523207084	3.72765185108864	-2.0085923841477	0.0529265865323641	0.664168290070582	-4.28787594071635
a_HQ_transcript/43189	2.0618931059862	1.23790442874233	2.00850050672947	0.0529368329849442	0.664168290070582	-3.95011093826039
a_HQ_transcript/69262	-2.37740199254417	1.16028300218761	-2.00837517276114	0.0529508134116832	0.664168290070582	-3.93946935702535
a_HQ_transcript/72236	2.01209199163632	1.50663420270583	2.00828315232187	0.0529610799227223	0.664168290070582	-3.98806490273894
a_HQ_transcript/68254	1.80621907505078	-0.485001592911891	2.0081964247474	0.0529707575138869	0.664168290070582	-4.04849109242125
a_HQ_transcript/81071	1.89918410277368	-0.247338891132898	2.00807919816238	0.0529838408276733	0.664189066127021	-3.95284071259111
a_HQ_transcript/49355	-1.1730027531177	2.59135662878125	-2.00782161125435	0.0530125992683118	0.664304609637135	-4.06601948366759
a_HQ_transcript/95467	4.31969976151841	1.49244773632017	2.0076815036343	0.0530282473945671	0.664304609637135	-4.10868678803443
a_HQ_transcript/101074	2.15981494230154	0.26981376261343	2.00746710864945	0.0530522002319496	0.664304609637135	-4.00578546449625
a_HQ_transcript/22335	-0.926037407209924	1.96125599942356	-2.00744892114084	0.0530542326289848	0.664304609637135	-3.97092104920055
a_HQ_transcript/119674	-2.17908282654261	1.0130970984351	-2.00744388501065	0.053054795412768	0.664304609637135	-4.06318566383883
a_HQ_transcript/38357	-1.40060799443631	4.30164718240174	-2.00718114187257	0.053084164001606	0.664304609637135	-4.34466520883343
a_HQ_transcript/139868	0.739478547934655	4.37596672356086	2.00701875936903	0.0531023217040456	0.664304609637135	-4.41906770022762
a_HQ_transcript/62990	2.63737312988533	0.0548269985380808	2.00685688860754	0.0531204275866592	0.664304609637135	-4.05907244966676

a_HQ_transcript/152305	-3.09912546015003	0.204483964775227	-2.0068545625137	0.0531206878087492	0.664304609637135	-4.01345083716956
a_HQ_transcript/40063	-1.50096674290218	2.26949685944383	-2.00679982073576	0.0531268121395356	0.664304609637135	-4.06322111010887
a_HQ_transcript/129782	1.08759132137872	1.30948238251446	2.00678241551746	0.0531287595076553	0.664304609637135	-3.97055321931545
a_HQ_transcript/133107	0.68994426732373	4.12356383199234	2.00667669669003	0.0531405891097426	0.664304609637135	-4.3475672092358
a_HQ_transcript/124778	2.61055531846328	1.07266805616749	2.00661090835072	0.0531479517787652	0.664304609637135	-4.00751790413873
a_HQ_transcript/112551	1.67731606614685	1.52160423156509	2.00656532013559	0.0531530542840338	0.664304609637135	-3.99252656490948
a_HQ_transcript/113065	-2.46321166724703	5.49366064639289	-2.00636550881433	0.0531754234119156	0.6644075318761	-4.43450360397429
a_HQ_transcript/121335	-6.4862874097846	1.53215400098661	-2.00628758274304	0.0531841495633686	0.6644075318761	-4.05010008832085
a_HQ_transcript/104606	2.62461029615216	0.0777438866852245	2.00598302086637	0.0532182663698246	0.664526999838745	-4.08803760145128
a_HQ_transcript/127319	-0.632936591237642	3.64731902775472	-2.0058792305835	0.0532298972534502	0.664526999838745	-4.30837161902961
a_HQ_transcript/13523	2.57168143574379	1.11643768843192	2.00584880341154	0.0532333073858178	0.664526999838745	-4.05455700167074
a_HQ_transcript/143993	2.46074199656833	-0.359143973977998	2.00575416989348	0.0532439146798363	0.664526999838745	-4.01999019186048
a_HQ_transcript/96590	1.55236887770006	-0.765412772038833	2.00551002864306	0.0532712885469078	0.664526999838745	-4.06027421758763
a_HQ_transcript/11823	1.69195557796351	2.03373965531739	2.00509026899157	0.0533183820435068	0.664526999838745	-3.98382948232673
a_HQ_transcript/79953	-1.4627128237103	2.30051626827319	-2.00495142639611	0.05333396701876	0.664526999838745	-4.10713713114664
a_HQ_transcript/70589	-1.65120303734325	-0.571857093761789	-2.00494177940194	0.053335050034389	0.664526999838745	-4.04683645297072
a_HQ_transcript/79823	1.99134638431211	2.95580685789775	2.00482644755273	0.0533479992030324	0.664526999838745	-4.09788136155608
a_HQ_transcript/22307	2.39903048200231	2.38302809950501	2.00478769270652	0.0533523511159532	0.664526999838745	-4.03904984316218
a_HQ_transcript/151355	0.652008061605059	7.88413013677182	2.00452807739029	0.0533815122002205	0.664526999838745	-4.69292800744791
a_HQ_transcript/26939	2.16933048380506	-0.244858106248522	2.00442529946919	0.0533930604966105	0.664526999838745	-4.01052900719397
a_HQ_transcript/2888	-0.875195547912716	3.82445043066171	-2.00431518732093	0.0534054353012362	0.664526999838745	-4.27468597247593
a_HQ_transcript/62615	-2.73105639639058	3.46022297555725	-2.00421846696998	0.0534163071544133	0.664526999838745	-4.36358561117039
a_HQ_transcript/3994	2.02651334891692	-0.260264574510963	2.00419170576551	0.0534193155897585	0.664526999838745	-4.0279483732155
a_HQ_transcript/117386	1.19067209650704	2.35622325909956	2.00417630983904	0.0534210464328112	0.664526999838745	-4.06354603306116
a_HQ_transcript/107746	2.06567292355542	1.63983162437805	2.00403240054165	0.0534372273945181	0.664526999838745	-4.06847957236618
a_HQ_transcript/80465	-3.26347601333713	1.77461692416527	-2.00393039731202	0.0534486990896171	0.664526999838745	-4.10019109275199
a_HQ_transcript/34136	-2.36420878743585	-0.278234504021221	-2.00371473980053	0.0534729598888231	0.664526999838745	-4.07444317614719
a_HQ_transcript/38693	3.00402267721162	1.35422543821997	2.00371130995679	0.053473345813298	0.664526999838745	-4.04829361114171
a_HQ_transcript/127225	2.08675594423751	1.00185290630989	2.00368058638762	0.0534768029240237	0.664526999838745	-3.94820603119283
a_HQ_transcript/83589	-2.3994093382394	0.524681741205464	-2.00367273394053	0.053477686536915	0.664526999838745	-4.05674553052939
a_HQ_transcript/78326	1.9743301587119	0.14025271930773	2.00344724404914	0.0535030656982148	0.664526999838745	-4.07192097874045
a_HQ_transcript/152242	1.64651985293227	-0.751875135967566	2.00341169387125	0.0535070678741747	0.664526999838745	-4.06350817087852
a_HQ_transcript/140032	3.59672446319429	2.7077921129609	2.00327193054526	0.0535228047173112	0.664526999838745	-4.10168706156913
a_HQ_transcript/131501	-1.19673244068456	2.70845322420088	-2.00323948829022	0.053526458175706	0.664526999838745	-4.1364160269762
a_HQ_transcript/57773	-2.00364827860484	1.11609398999341	-2.00304520923972	0.0535483413142062	0.664526999838745	-4.00610989398199
a_HQ_transcript/121665	-1.00757866186501	3.52889959883843	-2.00304510504052	0.0535483530530572	0.664526999838745	-4.26118950546592
a_HQ_transcript/69457	1.92108380922103	-0.0841218559658393	2.00299116506069	0.05355443011381	0.664526999838745	-4.05148913838372
a_HQ_transcript/122146	-2.37655769306932	0.492373305042377	-2.0028591409959	0.0535693069344059	0.664526999838745	-4.08734071493558
a_HQ_transcript/144928	3.70101855260835	0.596963832581774	2.00282315076669	0.0535733630348175	0.664526999838745	-4.00294398017372
a_HQ_transcript/13783	-1.76149670205587	2.87015087505542	-2.00279618291588	0.0535764024886446	0.664526999838745	-4.19125794071065
a_HQ_transcript/50644	0.515663798227419	3.01886489100558	2.00275343529506	0.0535812207348338	0.664526999838745	-4.25102032910257
a_HQ_transcript/43951	1.41901706820511	-0.670531763208945	2.00265657602033	0.0535921395111699	0.664526999838745	-4.04534968075394
a_HQ_transcript/22094	2.89288806639021	-0.112859581995744	2.00264151656741	0.0535938373115201	0.664526999838745	-4.04830314084501
a_HQ_transcript/75599	2.66570372881223	0.461101626126922	2.00250748600462	0.0536089499664465	0.664572626071572	-4.11273737966163
a_HQ_transcript/132334	-1.04830811323947	4.5456102460942	-2.00222942727913	0.053640314433151	0.664590569091535	-4.27453856511038
a_HQ_transcript/82954	1.36622055801189	3.10362866530065	2.00188998704746	0.0536786243400629	0.664590569091535	-4.11873406644531
a_HQ_transcript/48772	1.08722401341579	-0.684737475738369	2.00179244495076	0.053689637557502	0.664590569091535	-4.05480657505347
a_HQ_transcript/136027	-1.29973577933322	-0.340263447288554	-2.0017581951799	0.0536935050758034	0.664590569091535	-4.02489819456355
a_HQ_transcript/136026	-4.95607807617258	2.57036370263698	-2.00175725587333	0.053693611146668	0.664590569091535	-4.11967846190439
a_HQ_transcript/59630	-1.22643980816574	0.587451187511501	-2.00166416652576	0.0537041241365426	0.664590569091535	-3.94348086734224

a_HQ_transcript/106127	0.499253816918508	2.9724423477037	2.0015612194446	0.0537157524975133	0.664590569091535	-4.23298143434809
a_HQ_transcript/141593	-4.10220562512341	3.50663755196513	-2.00154404782679	0.0537176923271914	0.664590569091535	-4.1663756798479
a_HQ_transcript/4738	1.62786300786268	3.32636980020563	2.00145311069207	0.053727966261373	0.664590569091535	-4.25671152508457
a_HQ_transcript/1653	0.459276738823933	6.23636046779252	2.00143799308354	0.0537296743913496	0.664590569091535	-4.56815977323281
a_HQ_transcript/124075	1.70667844112573	3.49573756980616	2.00138057146105	0.0537361628600712	0.664590569091535	-4.17279616633478
a_HQ_transcript/12731	-2.69546180347269	1.31029381701017	-2.00123209168279	0.0537529437968077	0.664656693686628	-3.98432852883694
a_HQ_transcript/106920	0.975060297108731	2.47856887011472	2.00098943481556	0.0537803783247413	0.664850717757248	-4.15250670782201
a_HQ_transcript/27754	2.30685958829821	2.33284992290508	2.00066061393215	0.0538175739809257	0.664850717757248	-4.08910701814806
a_HQ_transcript/83127	1.3266875255111	-0.196465422451726	2.00053813929048	0.0538314338452958	0.664850717757248	-3.97295424214114
a_HQ_transcript/78856	2.63845360351273	0.0748393361774717	2.00052653515949	0.0538327471904979	0.664850717757248	-3.92998508064428
a_HQ_transcript/110481	-3.79526691341234	2.08039116819313	-2.00050860224933	0.05383477687633	0.664850717757248	-4.15905217600453
a_HQ_transcript/96794	2.31804994059972	0.0508621910405105	2.00048665183895	0.0538372613625682	0.664850717757248	-4.0254844589051
a_HQ_transcript/129043	1.98448858845876	-0.407388573691172	2.00031894680579	0.0538562465843813	0.664943904302659	-4.08900238624508
a_HQ_transcript/103449	2.43651876774551	2.66374252376617	1.99996302352284	0.0538965586234818	0.665043791486881	-4.10183335917839
a_HQ_transcript/45449	2.11803374481484	0.581465348785548	1.99993098445109	0.0539001886764656	0.665043791486881	-3.96824224290043
a_HQ_transcript/73268	1.94942664035201	0.417629967515413	1.99985064119734	0.053909292571858	0.665043791486881	-3.97122644447933
a_HQ_transcript/128661	1.34845991664496	2.75037682812523	1.99984350809571	0.0539101009063822	0.665043791486881	-4.19628143546172
a_HQ_transcript/148056	-1.70683817732659	-0.608203756171949	-1.9995087640316	0.0539480465474743	0.665263469792639	-4.04736536605784
a_HQ_transcript/121934	2.5064277996519	0.244427366408921	1.99948449802514	0.0539507981799219	0.665263469792639	-4.05359817514596
a_HQ_transcript/128468	-2.26382930586072	1.02455200789511	-1.99926663337788	0.05397550833046	0.665273097768304	-4.07247299430757
a_HQ_transcript/30297	1.50272099707038	1.20969881490876	1.99919817802434	0.0539832745589502	0.665273097768304	-3.94742130538535
a_HQ_transcript/143644	2.34513731917368	-0.524617897851781	1.99917491568625	0.0539859138826915	0.665273097768304	-4.06266383783362
a_HQ_transcript/146889	2.20995110828352	0.548023007194594	1.99859587980626	0.0540516470908705	0.665679556044027	-3.99326100547078
a_HQ_transcript/123943	3.28424659910041	0.605230793492662	1.99850363425422	0.0540621254255827	0.665679556044027	-4.12645112212584
a_HQ_transcript/29155	2.02191659266233	-0.293203676429176	1.99846034584771	0.0540670432433964	0.665679556044027	-4.0019349778337
a_HQ_transcript/748	2.46781639538636	-0.206639956159784	1.99836048419141	0.0540783896054874	0.665679556044027	-4.07178511422687
a_HQ_transcript/142446	0.507125226219633	5.22803019006398	1.99832143244778	0.054082827261661	0.665679556044027	-4.49693866159053
a_HQ_transcript/87291	-1.43283459984238	-0.781677531931105	-1.99827935478769	0.0540876091246205	0.665679556044027	-4.05553632834546
a_HQ_transcript/133882	2.87968669984928	1.02976633518631	1.99808254527255	0.0541099801975171	0.665689107629655	-4.1198786969027
a_HQ_transcript/144113	1.3815953158874	1.75519892521365	1.99801741615286	0.0541173851159987	0.665689107629655	-4.01549777987576
a_HQ_transcript/125160	0.524770018568985	4.42274847948691	1.99797030881763	0.0541227415816431	0.665689107629655	-4.43521858776102
a_HQ_transcript/26224	-1.91230769083771	1.24557357661751	-1.99782005907264	0.0541398292241891	0.665758407688526	-4.05620085943078
a_HQ_transcript/121081	2.07024117076131	2.10676150226	1.99765954953681	0.0541580888988411	0.665842087883214	-4.0895066341984
a_HQ_transcript/119630	-2.25385012230847	0.135469986865228	-1.99737232702148	0.054190776949198	0.66608550418518	-4.09265369655676
a_HQ_transcript/90475	3.36050489150265	0.212024813621886	1.99728423681656	0.054200805718344	0.66608550418518	-4.10851990618234
a_HQ_transcript/144045	2.70958897719556	0.788605696650673	1.99717279433828	0.0542134953858145	0.666100625615436	-4.04418511212903
a_HQ_transcript/130884	-2.62694716099291	-0.156508939145355	-1.99704056385903	0.0542285554940621	0.666144859205165	-4.07200142630259
a_HQ_transcript/134164	3.05914994783612	1.96516169230983	1.99686721776879	0.0542483039097383	0.666246653214719	-4.0755443058859
a_HQ_transcript/142327	0.813363333090557	2.9666210775651	1.99614894349169	0.054330200075044	0.666692773669011	-4.16522567174966
a_HQ_transcript/69404	2.10005387592474	-0.0929934007130288	1.99613388595006	0.0543319180595741	0.666692773669011	-4.02751022575944
a_HQ_transcript/7686	2.22755312075369	1.75749524428698	1.9961333681081	0.0543319771434006	0.666692773669011	-3.95014439899593
a_HQ_transcript/146070	0.428258645224124	10.1070032650202	1.99603960341483	0.0543426762680195	0.666692773669011	-4.9767312634284
a_HQ_transcript/151668	2.02980977140489	-0.323517551011899	1.9959009422112	0.054358501729953	0.666692773669011	-4.0751848628196
a_HQ_transcript/63538	-1.42279591625518	0.587424808885737	-1.99571284934968	0.0543799752779147	0.666692773669011	-4.07473695992224
a_HQ_transcript/120014	-1.69194610784004	1.82741839641218	-1.9956544114513	0.0543866483252381	0.666692773669011	-4.09716093637172
a_HQ_transcript/17654	1.39354758203918	4.22958595710184	1.99551440824145	0.0544026382562414	0.666692773669011	-4.30267921637536
a_HQ_transcript/39471	-2.42311686645292	3.83232306089946	-1.99548806776903	0.0544056470911491	0.666692773669011	-4.30289937995656
a_HQ_transcript/113153	-3.82786997120905	4.1879043671999	-1.99534789580224	0.0544216611800483	0.666692773669011	-4.50199452295129
a_HQ_transcript/44820	1.69989883983318	0.225042726359463	1.99533977119709	0.0544225895099677	0.666692773669011	-3.96476945183174
a_HQ_transcript/44835	0.765834773810959	4.15876340379775	1.99532035078948	0.0544248085717125	0.666692773669011	-4.39875667836771

a_HQ_transcript/44818	2.94536211091637	0.586884417479297	1.99524227373975	0.0544337307981202	0.666692773669011	-4.09493898154346
a_HQ_transcript/93645	2.55514436721588	1.15292180836238	1.99512319763883	0.0544473406345724	0.666718983864847	-4.02057976792454
a_HQ_transcript/111167	1.76966162679077	2.40749798736419	1.99486614146811	0.0544767310691051	0.666938379019575	-4.07087539752843
a_HQ_transcript/77516	1.96897897644029	1.96678172268073	1.99456544985723	0.0545111281196153	0.667157949612596	-4.13540178954052
a_HQ_transcript/146664	1.4457998551164	2.31597813697148	1.99447904751155	0.0545210154586208	0.667157949612596	-4.06885147158692
a_HQ_transcript/101605	3.2008949691329	0.676029504994761	1.99440842537034	0.05452909817316	0.667157949612596	-4.0082856765626
a_HQ_transcript/78925	1.72944338419327	-0.424466829822596	1.99424160083895	0.0545481954187441	0.667233283114945	-4.11244930098722
a_HQ_transcript/107692	-0.721543307313466	4.37427687572363	-1.99411202689482	0.0545630324291464	0.667233283114945	-4.38920536170152
a_HQ_transcript/23727	-2.31771340469911	4.38704195096927	-1.99405388211739	0.0545696915071644	0.667233283114945	-4.38712440411445
a_HQ_transcript/131977	1.7628575323083	-0.205017464617057	1.99364968673408	0.0546160019186467	0.667345162684285	-4.00674146711243
a_HQ_transcript/2157	-0.784041106731466	2.60780991866699	-1.99323930460655	0.0546630562582315	0.667345162684285	-4.1793335089854
a_HQ_transcript/43408	1.74114146763749	2.60752639216309	1.99312017102547	0.0546767227168391	0.667345162684285	-4.09597115129606
a_HQ_transcript/58573	1.72353177255492	0.900011079784173	1.9928330690041	0.0547096699969656	0.667345162684285	-3.96362086707113
a_HQ_transcript/122730	2.21138111567436	0.221137540229004	1.992788007155	0.0547148427814385	0.667345162684285	-4.08109316704257
a_HQ_transcript/115146	-3.28113649329508	2.0124337842809	-1.99263214371375	0.0547327381033576	0.667345162684285	-4.22741420830976
a_HQ_transcript/3548	1.07429396502902	1.71196689021174	1.99246635204916	0.0547517789307399	0.667345162684285	-4.06927318232484
a_HQ_transcript/48046	1.24027913666096	2.7713106869393	1.99244828933147	0.0547538537453711	0.667345162684285	-4.17038564128273
a_HQ_transcript/9802	1.43908590838073	1.69555763370969	1.99223136331247	0.0547787767996685	0.667345162684285	-4.00166015218729
a_HQ_transcript/88434	-2.08550163677141	-0.267674839680281	-1.99214599120889	0.0547885880815857	0.667345162684285	-4.09714734333321
a_HQ_transcript/95995	1.92365414029909	0.0409129913172557	1.99212556415151	0.0547909358628461	0.667345162684285	-4.14607026161703
a_HQ_transcript/140696	0.502100913165478	5.60644926768583	1.99195273858268	0.0548108030611971	0.667345162684285	-4.53599563273127
a_HQ_transcript/96775	1.70633442946933	0.600033006444069	1.99175920733504	0.0548330579449465	0.667345162684285	-3.98729390251432
a_HQ_transcript/139272	2.14469951551126	-0.350237177476424	1.99175430086486	0.0548336222608064	0.667345162684285	-4.07227585358246
a_HQ_transcript/12816	1.56863859793855	0.351082724077416	1.99171215522657	0.0548384698345692	0.667345162684285	-4.03387521335033
a_HQ_transcript/124840	-4.16202101411579	-0.153990668333672	-1.99165390309895	0.0548451705855735	0.667345162684285	-4.09769135481973
a_HQ_transcript/75729	-0.913196010526303	3.34164789813717	-1.991627697313	0.0548481852740395	0.667345162684285	-4.30343126155709
a_HQ_transcript/63478	0.902528963274063	3.86450140377275	1.99159118080617	0.0548523863390359	0.667345162684285	-4.382065927863
a_HQ_transcript/48364	1.58539434359486	-0.0673497322504485	1.99158762700294	0.0548527952036019	0.667345162684285	-4.05333588800862
a_HQ_transcript/93208	2.79323885498788	2.25913179852763	1.99147774592762	0.0548654383181898	0.667345162684285	-4.15539194346811
a_HQ_transcript/80975	-1.6559942910528	2.43241218895783	-1.99141558844929	0.0548725913955293	0.667345162684285	-4.07312340230667
a_HQ_transcript/142096	1.51478988803197	1.18129268343786	1.991390759122	0.0548754489798249	0.667345162684285	-4.06618970928232
a_HQ_transcript/125313	3.09117381106506	0.275149547595889	1.9913581406146	0.0548792032109662	0.667345162684285	-4.12580648599987
a_HQ_transcript/141429	1.75891906215404	2.69884958079547	1.99131689226606	0.0548839510155895	0.667345162684285	-4.15793045206819
a_HQ_transcript/26584	1.31040228504414	2.54815888470345	1.99127110774723	0.0548892213662128	0.667345162684285	-4.08155928464722
a_HQ_transcript/63753	-2.22267230948002	2.30594285930095	-1.99115222315009	0.0549029084798075	0.667345162684285	-4.14751271574274
a_HQ_transcript/45632	2.26949256039515	0.223152540204655	1.99110664899794	0.0549081561945455	0.667345162684285	-4.07960686640018
a_HQ_transcript/89288	-4.20372243077211	1.46084807474811	-1.9910829932561	0.0549108802484545	0.667345162684285	-4.08519322804564
a_HQ_transcript/142101	-1.64188008659565	1.64784207373599	-1.99100315091036	0.0549200752865536	0.667345162684285	-4.02240777372841
a_HQ_transcript/4664	0.988449302697342	5.22995236364373	1.99097549997918	0.0549232600172313	0.667345162684285	-4.52316318790183
a_HQ_transcript/82084	2.46313215674898	1.33938256892012	1.99074669673586	0.0549496189006978	0.667524265846223	-4.01758108557953
a_HQ_transcript/15310	2.03300243819743	-0.212684579020603	1.99063959531598	0.0549619611267614	0.667524265846223	-4.02818860102812
a_HQ_transcript/54044	-1.7612360326121	0.306300499249557	-1.99054858013379	0.054972451496798	0.667524265846223	-4.02132717494609
a_HQ_transcript/98176	2.01462396709315	0.259840749673893	1.99019966398638	0.0550126836039605	0.667739248333404	-4.07335415138783
a_HQ_transcript/152177	-1.98648304286069	1.43660049644963	-1.99010262597652	0.0550238772276844	0.667739248333404	-4.06942148902387
a_HQ_transcript/132466	-3.41733781282115	2.27377593618341	-1.99009620413862	0.0550246180759188	0.667739248333404	-4.11697612211474
a_HQ_transcript/116629	2.96604903092632	0.757789696157894	1.98968421560004	0.0550721648671854	0.668094583772302	-4.07597492373971
a_HQ_transcript/149318	2.67666203787153	2.40193995612128	1.98946901511216	0.0550970149803421	0.668094583772302	-4.22335586346679
a_HQ_transcript/59363	-2.54541695724116	2.61115075199114	-1.98942502037142	0.0551020964436326	0.668094583772302	-4.262031449152
a_HQ_transcript/151596	2.13432691562927	1.69964705411811	1.98925601436253	0.0551216207109186	0.668094583772302	-4.03203552259811
a_HQ_transcript/103814	1.89045968624543	0.395587307126841	1.98925559062919	0.0551216696699141	0.668094583772302	-4.01121155302095

a_HQ_transcript/43635	-2.70865568678703	0.646923738873395	-1.98924528595366	0.0551228603043621	0.668094583772302	-4.03222904206563
a_HQ_transcript/110872	-0.606233155430635	3.70847320343463	-1.98876961679073	0.0551778450292423	0.668474442682372	-4.3568509961263
a_HQ_transcript/66730	1.62278933681178	2.24041355373368	1.98868442150235	0.0551876981829808	0.668474442682372	-4.14525914849187
a_HQ_transcript/89076	-0.971144223626912	3.19581585539997	-1.9886757456326	0.055188701665853	0.668474442682372	-4.25400883717665
a_HQ_transcript/33283	2.72000058114049	0.00945360231717933	1.98855981579077	0.0552021120630881	0.668497577083996	-4.02723740856205
a_HQ_transcript/23474	1.56788348435556	-0.328728842855428	1.98839735142166	0.0552209102092451	0.66858593389773	-4.08845169820322
a_HQ_transcript/111042	3.27384905473938	1.96815135140799	1.98819771610975	0.0552440169112758	0.668588614397059	-4.00022358652292
a_HQ_transcript/16955	-2.24812991534537	-0.0103245886415416	-1.9881966907433	0.0552441356136298	0.668588614397059	-4.00605435941806
a_HQ_transcript/45097	2.04324452410282	-0.700584563655418	1.98781773358532	0.0552880211226977	0.668980452085797	-4.07846632558357
a_HQ_transcript/149707	4.79197992204388	3.36702860251179	1.98750789533785	0.0553239248349762	0.66916043585298	-4.20422436701272
a_HQ_transcript/125619	-2.14757519130767	0.0847215050063924	-1.98749068622753	0.0553259196034513	0.66916043585298	-4.12489983758099
a_HQ_transcript/74189	-0.852629876010334	2.25427022135463	-1.98734597578582	0.055342695980774	0.669170716344742	-4.06737163393856
a_HQ_transcript/11992	-2.32839260874021	0.970922236935925	-1.98723464931939	0.0553556051505589	0.669170716344742	-4.03828725337934
a_HQ_transcript/102385	-3.00257775680592	-0.0229737352895876	-1.98711784714683	0.0553691520936337	0.669170716344742	-4.04103116644849
a_HQ_transcript/51120	1.24336651330299	0.769518997383505	1.98708624670054	0.0553728176716593	0.669170716344742	-4.10441195633774
a_HQ_transcript/15956	-3.16765084262695	1.52253308933198	-1.98684277974551	0.0554010663665149	0.669372934057946	-4.09680085030458
a_HQ_transcript/104155	2.78569664645721	4.15315632189296	1.98665406662812	0.0554229707920319	0.669401208091545	-4.42647195977988
a_HQ_transcript/130269	1.41783628721724	2.76036756852007	1.98662419764192	0.0554264384555568	0.669401208091545	-4.12945780875005
a_HQ_transcript/4205	-1.65744995266197	3.51549789525026	-1.98645582705978	0.055445989110142	0.669498224967664	-4.19575720143272
a_HQ_transcript/135606	0.627562145294752	2.84158960167285	1.98632597395163	0.0554610713451988	0.669541257560481	-4.18107243358441
a_HQ_transcript/146355	0.601725554199169	4.90216481560812	1.98597673481207	0.0555016526727094	0.669892040398515	-4.49983088442846
a_HQ_transcript/140089	2.80682919221418	0.26174301474601	1.98579849595587	0.0555223738710681	0.670003020215371	-4.0133636166804
a_HQ_transcript/141626	1.63272291096066	-0.547998439116187	1.98559360254468	0.055546202133456	0.670124846829286	-4.09323183580496
a_HQ_transcript/72305	2.32049514885111	0.51657549614576	1.98545045180761	0.0555628552662784	0.670124846829286	-4.03392392453752
a_HQ_transcript/9679	1.47903116478168	-0.757012586327128	1.985273969311	0.0555833919683537	0.670124846829286	-4.09866492145884
a_HQ_transcript/142479	-2.49622557487432	0.461859112476813	-1.98521628749334	0.0555901056511822	0.670124846829286	-4.09663144937966
a_HQ_transcript/31198	1.29211419620394	2.02549519018928	1.98521623574631	0.0555901116744223	0.670124846829286	-4.05309361686356
a_HQ_transcript/111177	1.46408712531993	-0.780902987772408	1.98508596450028	0.0556052767643896	0.670130101259024	-4.0952144539928
a_HQ_transcript/44606	-2.63697274568969	0.77290546870394	-1.98495635033545	0.0556203689416257	0.670130101259024	-4.04608870175233
a_HQ_transcript/41707	-1.63991876284653	1.66060236944816	-1.98491330591554	0.0556253817917568	0.670130101259024	-4.0376829198259
a_HQ_transcript/46019	1.92275326388395	1.52805503910536	1.98456326516905	0.055666161325353	0.670130101259024	-4.03083451365333
a_HQ_transcript/148033	4.05774809535291	3.58512982744703	1.9843716771191	0.0556884922498091	0.670130101259024	-4.45244749986983
a_HQ_transcript/19752	3.32914908708337	3.26030058532699	1.98433191378732	0.0556931279220622	0.670130101259024	-4.16976976625336
a_HQ_transcript/7628	1.43615011141518	1.8366457545208	1.98427114699592	0.0557002128607405	0.670130101259024	-4.0385130539208
a_HQ_transcript/11360	-2.21857487783251	0.415643580552086	-1.98411198646881	0.0557187734730948	0.670130101259024	-4.04028486208621
a_HQ_transcript/133683	-3.42521165314327	2.07207859437248	-1.98410045061234	0.0557201189442929	0.670130101259024	-4.22684724798987
a_HQ_transcript/147775	2.79560024575495	0.0186560333399737	1.98405417204054	0.0557255168763731	0.670130101259024	-4.07772575325803
a_HQ_transcript/20173	1.00482852027519	1.74300254651332	1.98399788027115	0.0557320833621123	0.670130101259024	-4.02453481008688
a_HQ_transcript/45939	1.94423879567859	0.777381135377136	1.98390450413737	0.0557429772597428	0.670130101259024	-4.08281569787414
a_HQ_transcript/17856	-1.51195752011566	2.74835255163253	-1.98373308805381	0.0557629806622111	0.670130101259024	-4.22431476417679
a_HQ_transcript/91600	-1.51466499059375	3.49652815549307	-1.98352257900209	0.055787554578627	0.670130101259024	-4.27423387017814
a_HQ_transcript/45122	1.1541427985692	-0.279403725474585	1.98348391464609	0.0557920691142313	0.670130101259024	-4.02696708203637
a_HQ_transcript/7048	-1.06406387537763	2.80373813595464	-1.98343522639777	0.0557977545145886	0.670130101259024	-4.2216649137527
a_HQ_transcript/73206	-1.05446759212206	2.0064837897747	-1.98314958180962	0.0558311198374275	0.670130101259024	-4.05020726335933
a_HQ_transcript/26782	2.08485993155376	-0.695510736562858	1.98303181653138	0.0558448807223909	0.670130101259024	-4.09660672146023
a_HQ_transcript/136760	4.39514233326232	2.17847644582343	1.98286323578413	0.0558645845445477	0.670130101259024	-4.16699581192114
a_HQ_transcript/62970	-1.61831443466876	1.54890789989143	-1.98285612382888	0.0558654159274623	0.670130101259024	-4.03040289618202
a_HQ_transcript/48635	1.43125763936921	3.62876168627387	1.98276838850914	0.0558756730159689	0.670130101259024	-4.33443728004847
a_HQ_transcript/111761	-4.52907574940613	5.7510091200295	-1.98264764245456	0.0558897920552287	0.670130101259024	-4.65727034290593
a_HQ_transcript/18654	-0.692746427222592	1.86430508364078	-1.98263898539622	0.0558908044590624	0.670130101259024	-4.06682362035637

a_HQ_transcript/82618	-5.85530158419944	3.57343169307903	-1.98260799930531	0.0558944282732853	0.670130101259024	-4.32813487407965
a_HQ_transcript/57834	-2.7076204292286	0.167134166581598	-1.9825446193846	0.055901841174145	0.670130101259024	-4.09221420207554
a_HQ_transcript/74634	1.77639081926728	-0.314928370070414	1.98252195565045	0.0559044921272001	0.670130101259024	-4.07647994423066
a_HQ_transcript/11496	2.16328295666839	0.704096389186724	1.98246686579088	0.055910936386724	0.670130101259024	-4.05763354708473
a_HQ_transcript/79933	1.84175876371974	2.25170887071939	1.98244626604014	0.0559133462549248	0.670130101259024	-4.14112869452916
a_HQ_transcript/142084	-2.75950091294596	1.43079677656051	-1.98192985693054	0.0559737881185412	0.670523635309899	-4.09306320318276
a_HQ_transcript/78705	2.04760975724734	0.976992978774714	1.9818608218041	0.055981872481967	0.670523635309899	-4.02639934642299
a_HQ_transcript/86786	-0.953461463824514	2.10655699011261	-1.98173241824142	0.0559969118966403	0.670523635309899	-4.04116081175724
a_HQ_transcript/97565	-0.864365769376259	2.10761621678989	-1.98168792194114	0.0560021243982398	0.670523635309899	-4.10798605261558
a_HQ_transcript/126475	-9.77376456603049	2.6591553766609	-1.98167312515519	0.056003857855587	0.670523635309899	-4.2064121948429
a_HQ_transcript/134716	-3.78318290365077	1.77950380458426	-1.98146858404722	0.0560278248288496	0.670672446798058	-4.21480177438444
a_HQ_transcript/90464	-4.91916309074014	3.30782526699415	-1.9813606873349	0.0560404711573234	0.670685712874798	-4.09167749910956
a_HQ_transcript/142423	4.62529266884197	1.96961385403646	1.98105071030947	0.0560768166981662	0.670932049954679	-4.15907209600096
a_HQ_transcript/50160	-2.30978208544637	-0.57924121627074	-1.98089596733175	0.0560949683636279	0.670932049954679	-4.10009468756111
a_HQ_transcript/34579	-0.711233679163606	2.90757680538966	-1.98088989041309	0.0560956813029821	0.670932049954679	-4.21472303664111
a_HQ_transcript/79534	1.53024388568404	-0.23199707250814	1.98054793172974	0.0561358123526311	0.671273914921568	-4.0547138178948
a_HQ_transcript/103062	1.7536609387233	-0.0252440126263736	1.9803277438828	0.0561616660485787	0.671444945304769	-4.08045600562916
a_HQ_transcript/143042	-2.29013746462111	-0.17424586400574	-1.98010918845177	0.056187338330075	0.671589439529895	-4.09353593196031
a_HQ_transcript/137579	1.46607207025539	2.6619316511101	1.97997473233585	0.0562031370924469	0.671589439529895	-4.09133073881953
a_HQ_transcript/146616	1.66252529497899	2.65791973296935	1.9799298399139	0.056208412870096	0.671589439529895	-4.13355899468577
a_HQ_transcript/5039	2.51763741448674	2.56379701735119	1.97974524872748	0.0562301106440429	0.671599591205523	-4.14401966756151
a_HQ_transcript/61365	-3.07557111115591	1.75769768530346	-1.97972603006765	0.0562323701210653	0.671599591205523	-4.16918800522051
a_HQ_transcript/10827	2.92039456030858	-0.00705798622371632	1.97957290806537	0.056250375016037	0.671676622623706	-4.10509258312062
a_HQ_transcript/88405	-1.07085866612383	3.60411523930512	-1.97928258478276	0.0562845265517464	0.671908487827704	-4.26970655862175
a_HQ_transcript/6510	-1.06980773723591	2.35208340812857	-1.9792113310387	0.0562929110879596	0.671908487827704	-4.16059140542708
a_HQ_transcript/115215	1.88855082553654	-0.569515754844278	1.97879333630533	0.0563421190786665	0.672310703802789	-4.10335914156162
a_HQ_transcript/133304	-1.50695749665994	1.74548802028991	-1.97872862013773	0.056349741070176	0.672310703802789	-4.00649945959205
a_HQ_transcript/111570	-4.21894830171132	0.757811611003011	-1.97858003152665	0.0563672445954024	0.672381529620675	-4.11858045016182
a_HQ_transcript/29629	2.20133126816404	0.0877940974394883	1.97847755521396	0.0563793189172089	0.672387576942864	-4.11812480312437
a_HQ_transcript/132171	2.21948694197289	0.898427952410692	1.97802399243948	0.0564327872292488	0.672790392649761	-4.05065917528622
a_HQ_transcript/61538	2.63425539957092	1.29630829514273	1.97799468746222	0.0564362433691205	0.672790392649761	-4.00362162257343
a_HQ_transcript/121308	1.41483361611533	-0.704509909952916	1.9776806854366	0.0564732873850091	0.67279599691879	-4.06125757157895
a_HQ_transcript/54348	1.3407586875421	-0.610824899980468	1.97764315380801	0.0564777165508859	0.67279599691879	-4.10265315950552
a_HQ_transcript/120764	-1.9736515993518	-0.506957107586893	-1.97762980896642	0.0564792914693114	0.67279599691879	-4.08512161926875
a_HQ_transcript/122949	-0.945724606778549	3.17489847963337	-1.97759829312535	0.0564830110267633	0.67279599691879	-4.26997470402344
a_HQ_transcript/55258	-1.33675635356841	2.32225830208704	-1.97732115772191	0.0565157282436214	0.673047787614265	-4.1067694015881
a_HQ_transcript/20230	1.81817675731079	-0.660731833183598	1.97716368713415	0.0565343257844303	0.673068689684554	-4.05767688871756
a_HQ_transcript/86046	2.41806666666576	2.17392331572491	1.97703322612324	0.0565497374855004	0.673068689684554	-4.12593783285294
a_HQ_transcript/90806	2.28418026045333	-0.0952967225724233	1.9769841105485	0.0565555405849995	0.673068689684554	-4.05252140957287
a_HQ_transcript/55681	1.4213428655924	-0.508634700811385	1.97679097892431	0.0565783644866752	0.673068689684554	-4.06178958735601
a_HQ_transcript/43034	-2.28303157277267	1.1473880686608	-1.97668910711889	0.0565904067200661	0.673068689684554	-3.97603791258359
a_HQ_transcript/4621	2.1887513714083	1.28723280594266	1.97659885241418	0.0566010775643897	0.673068689684554	-4.08711288297108
a_HQ_transcript/4918	-2.17842505784845	0.9188283883405	-1.97649389886803	0.0566134884611617	0.673068689684554	-4.09466927768527
a_HQ_transcript/85798	-2.51986362119212	-0.381964005579539	-1.97646755288665	0.056616604280664	0.673068689684554	-4.0666952865857
a_HQ_transcript/46796	-1.95469844117106	0.209437143339249	-1.97638882598804	0.0566259158430613	0.673068689684554	-4.08669416026457
a_HQ_transcript/92626	1.64243134096596	3.09764016105777	1.97632662113098	0.0566332741750474	0.673068689684554	-4.25895036831075
a_HQ_transcript/102479	0.624995845285254	3.55322777944836	1.97587736151092	0.0566864426847667	0.673436102167344	-4.31863342947147
a_HQ_transcript/6894	-2.46176317674037	-0.58766196375786	-1.97586961572375	0.0566873597561384	0.673436102167344	-4.12056653602303
a_HQ_transcript/72581	-1.15364853625926	0.928406688220449	-1.97549176163053	0.0567321119145355	0.673816824684389	-4.07643036956139
a_HQ_transcript/4181	2.08839721288206	-0.233548044424121	1.97527860240019	0.0567573715663264	0.673816824684389	-4.09033511041133

a_HQ_transcript/124204	0.849748403006549	3.31476040853173	1.9752330615204	0.0567627694903946	0.673816824684389	-4.35354028225136
a_HQ_transcript/85762	-2.57686195085558	1.15143260813397	-1.97520770336442	0.0567657753660792	0.673816824684389	-4.05038537596286
a_HQ_transcript/66957	1.73775186535449	1.74663803167487	1.97454743470676	0.0568440903243929	0.674474077557564	-4.10052637459374
a_HQ_transcript/121128	1.90982676846731	1.72319389197534	1.97453935887891	0.0568450487863612	0.674474077557564	-4.0599866937214
a_HQ_transcript/88317	-1.45764189413007	2.73560209301264	-1.97427252292738	0.0568767255412173	0.674474077557564	-4.21043268386126
a_HQ_transcript/445	-2.27079395755165	1.43848435420578	-1.97417673892239	0.0568881000445822	0.674474077557564	-3.99977776483922
a_HQ_transcript/116674	-1.84862459583676	3.7724065166633	-1.97409502681661	0.0568978050526728	0.674474077557564	-4.40371081500748
a_HQ_transcript/139237	-2.32938349881553	1.04169780419536	-1.97388670194747	0.0569225544754527	0.674474077557564	-4.0341049858491
a_HQ_transcript/67206	2.63216431758376	0.426922166563166	1.97378802760605	0.0569342804604518	0.674474077557564	-4.11966870202904
a_HQ_transcript/129130	1.52290185810309	-0.0725914165421328	1.9737785157877	0.0569354109103891	0.674474077557564	-4.01416768951432
a_HQ_transcript/91061	-5.85547810181077	4.39436037446021	-1.97375223637128	0.0569385342386113	0.674474077557564	-4.40076580755854
a_HQ_transcript/35868	1.96999731300335	1.87310652691746	1.97359340036166	0.0569574151919512	0.674474077557564	-4.02670886409973
a_HQ_transcript/140663	-1.67456419052001	-0.355317718054626	-1.97357329783003	0.0569598051831724	0.674474077557564	-4.10533563815622
a_HQ_transcript/149286	1.80847263842024	1.22987915608715	1.97356842284075	0.0569603847840985	0.674474077557564	-4.10416644352001
a_HQ_transcript/20872	-2.3995491547154	1.87687514503774	-1.97338209780554	0.0569825413299573	0.674599014751071	-4.07603003312689
a_HQ_transcript/116213	1.96326992798318	1.20446000537703	1.97304865426946	0.0570222109650805	0.674906334847878	-4.07445325351946
a_HQ_transcript/107952	2.2982191556793	1.2595376969919	1.97296288763989	0.0570324184702881	0.674906334847878	-4.07244181188193
a_HQ_transcript/107113	2.08837507877933	-0.295074597376874	1.97287120057252	0.0570433323545903	0.674906334847878	-4.09770309649735
a_HQ_transcript/126737	-2.18975844830873	1.17563259076899	-1.97250971625495	0.057086379015915	0.675056371895643	-4.07691530475041
a_HQ_transcript/8111	-2.74744062750095	0.670955592150052	-1.97246366234552	0.0570918652867069	0.675056371895643	-4.12422941994142
a_HQ_transcript/148893	-2.24893794934256	1.0967310697741	-1.97230623026751	0.0571106231866462	0.675056371895643	-4.12209353702059
a_HQ_transcript/46235	1.72873370780721	-0.364243662831339	1.97228136955397	0.0571135858101443	0.675056371895643	-4.05525757528113
a_HQ_transcript/17140	-2.81323367202501	0.523028079389908	-1.97219201679157	0.057124234983503	0.675056371895643	-4.05208234286193
a_HQ_transcript/105578	0.573610636226542	3.02890243357996	1.9721797827757	0.0571256931832279	0.675056371895643	-4.2470754517476
a_HQ_transcript/34086	-1.69995780187388	1.44857863459608	-1.97207610417969	0.0571380521677264	0.67506518219626	-4.09800287435689
a_HQ_transcript/129713	2.14079740997445	0.559875701347952	1.97169912018255	0.0571830100791736	0.675226910701613	-3.9998649402775
a_HQ_transcript/43388	-1.09232210702564	2.71210759128647	-1.97147843291101	0.05720934282405	0.675226910701613	-4.17966783410422
a_HQ_transcript/300	-2.11354433045729	0.964276120304356	-1.97142934121734	0.0572152019551206	0.675226910701613	-4.03026572107838
a_HQ_transcript/145797	4.82674070669294	2.51559160749814	1.97137006465201	0.0572222773543377	0.675226910701613	-4.36666839876237
a_HQ_transcript/82027	-1.11195491255862	3.03701247692733	-1.9712595655436	0.0572354688372046	0.675226910701613	-4.19632623692742
a_HQ_transcript/84061	-2.20195261762388	0.421633362725639	-1.97125409312346	0.0572361222084087	0.675226910701613	-4.0479455102059
a_HQ_transcript/120296	-2.5853398855907	-0.0183251016377907	-1.97111419576157	0.0572528272432386	0.675226910701613	-4.05983677877036
a_HQ_transcript/24815	3.55864073045708	0.832420993887462	1.97092926228154	0.0572749165170588	0.675226910701613	-4.11779592665414
a_HQ_transcript/76596	1.60642665581196	-0.35082921831043	1.97088321300918	0.057280417999186	0.675226910701613	-4.0612155411501
a_HQ_transcript/148836	1.53266065945191	1.43271513321971	1.97088308109964	0.057280433759009	0.675226910701613	-4.04371524745045
a_HQ_transcript/18305	2.38085862208143	0.416213352208759	1.97087289674021	0.05728165054141	0.675226910701613	-4.0140146198377
a_HQ_transcript/125027	0.499916302880154	3.78200780784793	1.97079351505102	0.0572911354868627	0.675226910701613	-4.39670019097351
a_HQ_transcript/6013	-1.96071117498263	0.372243640911743	-1.97063212110913	0.0573104239054796	0.675305684837576	-4.04212356958974
a_HQ_transcript/117744	-2.21737208925223	-0.38798516100789	-1.97054319186527	0.0573210543797929	0.675305684837576	-4.09418403755332
a_HQ_transcript/76560	0.624468194355305	2.0403216729478	1.97039568888975	0.0573386904571639	0.675315762886381	-4.10235328689997
a_HQ_transcript/26229	3.16506947276692	1.36225550251841	1.97034171240309	0.0573451452932696	0.675315762886381	-3.98424793674432
a_HQ_transcript/63150	3.55012520582912	1.99929170708833	1.97003644625187	0.0573816627744933	0.675366462419441	-4.24666468622882
a_HQ_transcript/21613	1.81067436261702	3.7068026473845	1.97001318920477	0.0573844457294096	0.675366462419441	-4.41773146182718
a_HQ_transcript/56152	-2.06229461171191	1.59057844551805	-1.96982144856157	0.0574073940276379	0.675366462419441	-4.03008353287955
a_HQ_transcript/67838	-3.02575068598533	1.2771759572754	-1.96981465916293	0.0574082067567694	0.675366462419441	-4.09567242192306
a_HQ_transcript/109349	-2.02120730137287	1.96363245132116	-1.96966550362254	0.0574260640382059	0.675366462419441	-4.17998217202871
a_HQ_transcript/80666	-1.57351585176519	0.610407325505851	-1.96955851497065	0.0574388759686564	0.675366462419441	-4.0935433360004
a_HQ_transcript/5250	-2.30981688695547	3.83230921973846	-1.96955587536542	0.0574391920937869	0.675366462419441	-4.32013605775686
a_HQ_transcript/70944	-4.15789663566999	0.392477580474822	-1.96948227376195	0.0574480073989308	0.675366462419441	-4.12029785056763
a_HQ_transcript/102713	3.41612755025329	0.24378886845738	1.96943209533597	0.0574540179717887	0.675366462419441	-4.1157090510352

a_HQ_transcript/121843	-3.76645111158244	2.63006152514151	-1.96917432277133	0.0574849036274813	0.675398071215295	-4.60950696016592
a_HQ_transcript/105402	3.4149742645962	1.29566481486263	1.96912318729353	0.0574910322675539	0.675398071215295	-4.0771632318495
a_HQ_transcript/99848	-3.54508572333146	5.41023701507061	-1.9691187474352	0.0574915644160005	0.675398071215295	-4.60862185803748
a_HQ_transcript/11140	1.62187962000839	1.02151013548373	1.96898796772446	0.05750724121229	0.675445729882399	-4.02385967947178
a_HQ_transcript/144186	-0.925620499694243	2.19533006935734	-1.96875825896584	0.0575347858121472	0.675582445569161	-4.06058280397975
a_HQ_transcript/47437	2.59981411749522	1.31423934060117	1.96866264496691	0.05754625436193	0.675582445569161	-4.2601723323416
a_HQ_transcript/116665	-2.41350414004973	0.180970058872825	-1.96842124014973	0.0575752188371332	0.675582445569161	-4.10924709575104
a_HQ_transcript/116157	0.592386074277611	3.25926186498311	1.96838440217193	0.0575796398834848	0.675582445569161	-4.32408555922367
a_HQ_transcript/7562	1.89622158622702	0.382880207801086	1.96827762518351	0.0575924562081314	0.675582445569161	-4.06954033344361
a_HQ_transcript/148077	-2.69493989069298	1.22486673912913	-1.96819101186799	0.0576028541320747	0.675582445569161	-4.18410710455256
a_HQ_transcript/107865	-2.53048171913509	1.58224784190577	-1.96812625815199	0.0576106288790221	0.675582445569161	-4.15142680273918
a_HQ_transcript/16617	1.86946780942324	0.0507290479357669	1.96811600738866	0.0576118597351763	0.675582445569161	-4.11465237968507
a_HQ_transcript/49379	1.37927763591711	0.849402236358225	1.96792405421077	0.0576349126585788	0.675672299534974	-3.98671311913424
a_HQ_transcript/47731	1.82734878403435	0.253548921739405	1.96785864445885	0.0576427699799397	0.675672299534974	-4.21982957265405
a_HQ_transcript/132077	0.555303168258779	3.6639034405898	1.96727999593683	0.0577123205025152	0.676351162534315	-4.41407451081805
a_HQ_transcript/31152	-1.08255470689372	2.47600917048513	-1.96686538401485	0.0577621995601405	0.676561002655272	-4.14002996550247
a_HQ_transcript/141393	3.90999944418428	0.531887084794711	1.96678799857748	0.0577715134112609	0.676561002655272	-4.25377287875941
a_HQ_transcript/15174	-1.96342521436126	0.0672512210231763	-1.96677769226565	0.0577727539428669	0.676561002655272	-4.10665865345686
a_HQ_transcript/142643	1.31637308108749	1.0789010105416	1.96674422384609	0.0577767825691709	0.676561002655272	-4.03756647766201
a_HQ_transcript/120314	2.92255280627917	0.965014429492961	1.9665653999301	0.0577983119188771	0.676676792592244	-4.04592576934256
a_HQ_transcript/61480	-2.45884767771417	-0.273955826672597	-1.96628131378833	0.0578325285690673	0.676941043226489	-4.10690450393191
a_HQ_transcript/4508	-1.20965803851877	1.82249674600361	-1.96616892230117	0.0578460703827193	0.676963233180331	-4.11369348424548
a_HQ_transcript/137555	-1.76375463206437	1.68654042764864	-1.96581515112592	0.0578887135290768	0.67732591385229	-4.12636036006844
a_HQ_transcript/126877	1.17719724545605	8.82247935388001	1.9655073601643	0.0579258365123139	0.67762387296997	-4.88783330302419
a_HQ_transcript/101816	4.15615165481734	4.64927802582751	1.96532740745567	0.0579475503813189	0.677721354796264	-4.35774895801885
a_HQ_transcript/39452	2.85086648858033	0.0055748037993195	1.96524507055514	0.057957487866299	0.677721354796264	-4.13106031125553
a_HQ_transcript/8231	-1.39007259291384	1.89109719592557	-1.96510375677947	0.0579745468996739	0.677784485555962	-4.05394478184941
a_HQ_transcript/138800	0.585809022544051	4.71085416971305	1.96482886620858	0.0580077434855003	0.678036218243548	-4.50010207046747
a_HQ_transcript/138089	1.73245380642745	0.0820680001100358	1.96462385514535	0.0580325119770692	0.678038019652046	-4.14218855101042
a_HQ_transcript/134780	-0.594310348190425	2.73604392134594	-1.96448173753668	0.0580496873676248	0.678038019652046	-4.23261966262594
a_HQ_transcript/6784	-1.34665830560122	1.95003724415522	-1.96425843009732	0.0580766837503626	0.678038019652046	-4.17953433515419
a_HQ_transcript/152226	0.489185566441392	6.79940100706256	1.96409323132832	0.0580966622056688	0.678038019652046	-4.70873392393612
a_HQ_transcript/27024	-1.8538234829273	2.53144977476687	-1.96401000779578	0.0581067291783325	0.678038019652046	-4.12841344245163
a_HQ_transcript/11291	2.23277381523944	1.31941322345397	1.963957418745	0.0581130912924579	0.678038019652046	-4.01927105503429
a_HQ_transcript/117974	3.43348985302195	0.30385461722848	1.96392152712892	0.0581174337333223	0.678038019652046	-4.14112861136918
a_HQ_transcript/137247	1.12139817908442	1.39094600228725	1.96389385519549	0.0581207818870174	0.678038019652046	-4.22886620521389
a_HQ_transcript/86543	3.10334470882002	0.275160115577942	1.96383080980383	0.058128410663362	0.678038019652046	-4.12669188142922
a_HQ_transcript/120171	3.57783526656861	2.41892811265874	1.96367142195382	0.0581477011937083	0.678038019652046	-4.33431831650048
a_HQ_transcript/94061	1.85367263084206	-0.163929150208899	1.963639248241	0.0581515958166495	0.678038019652046	-4.12739974090094
a_HQ_transcript/78418	2.12779563344598	0.868706680167282	1.96355426362545	0.0581618842818309	0.678038019652046	-4.02578955933949
a_HQ_transcript/136643	-3.46391134196908	1.65266608560263	-1.96351995881172	0.0581660377627604	0.678038019652046	-4.20163975825245
a_HQ_transcript/150247	2.54903699335465	3.11681772068681	1.96328598735069	0.0581943729094868	0.678038019652046	-4.25117492084678
a_HQ_transcript/96937	-2.96140650467585	1.32012330422051	-1.96319431535368	0.0582054781309901	0.678038019652046	-4.18458659756291
a_HQ_transcript/24790	1.75456934940796	-0.343926344720828	1.96318418583031	0.058206705342848	0.678038019652046	-4.1498998337681
a_HQ_transcript/27481	1.29927003014947	1.06585982370532	1.9631816149062	0.0582070168189851	0.678038019652046	-4.03740593919025
a_HQ_transcript/13726	-2.23237236037157	0.825504647655969	-1.96309212416085	0.0582178598280237	0.678038019652046	-4.06490539777436
a_HQ_transcript/88981	1.97164268738725	-0.471692338535376	1.96280437218993	0.0582527367556227	0.678278076760319	-4.13001512546722
a_HQ_transcript/67550	4.2542318853838	2.9773411942352	1.96263502283872	0.0582732712031568	0.678278076760319	-4.31878861755474
a_HQ_transcript/89391	4.79567290610748	1.47092006889672	1.96263331959496	0.0582734777618538	0.678278076760319	-4.22240999253359
a_HQ_transcript/81946	2.70119522475696	2.72386047440306	1.96243754258912	0.0582972246079252	0.678406850349516	-4.26055884566519

a_HQ_transcript/122281	-2.64821726648917	0.491190114549381	-1.96225546855667	0.0583193168952748	0.678406850349516	-4.15699456931774
a_HQ_transcript/3365	-1.57888819141443	0.960165381386918	-1.96222846278995	0.0583225943092686	0.678406850349516	-4.07979141956815
a_HQ_transcript/33912	-2.01997409471902	-0.490242236559101	-1.96206647165387	0.0583422568789096	0.678406850349516	-4.11579037539072
a_HQ_transcript/52235	-2.03319043812167	0.586744437173036	-1.96199623254096	0.0583507843301649	0.678406850349516	-4.09224167018941
a_HQ_transcript/149758	2.17785079026157	1.22648557756043	1.96196508088058	0.0583545666760869	0.678406850349516	-4.12545423680421
a_HQ_transcript/139259	-1.70620326794715	-0.405636158600091	-1.96182876892792	0.0583711197918274	0.678463597532362	-4.12149357106635
a_HQ_transcript/81292	2.36422341287775	0.249161750684156	1.96156227066821	0.0584034939417179	0.678704177497837	-4.14600676603704
a_HQ_transcript/124354	1.88023786042321	0.327061457485172	1.96138978609345	0.0584244556478021	0.678812064340484	-4.05394691106433
a_HQ_transcript/4339	-1.85711854092669	1.7818253941431	-1.96101561046139	0.0584699509444482	0.679015228087642	-4.03410533988177
a_HQ_transcript/149291	2.33351421518585	0.740434251461135	1.96097860471656	0.0584744520745551	0.679015228087642	-4.11730528654494
a_HQ_transcript/16118	-1.25754618362882	2.01827474756272	-1.96095777477544	0.0584769858210627	0.679015228087642	-4.15381002057536
a_HQ_transcript/71596	-1.79552415674002	-0.583878343985227	-1.96082124168663	0.058493596019123	0.679072448452084	-4.12197763735028
a_HQ_transcript/93098	2.44981425896437	3.24029758891919	1.96061574880643	0.0585186033841586	0.679227112123477	-4.21872145597287
a_HQ_transcript/93121	2.89952788622454	2.29207774649158	1.95995746352967	0.0585987756831205	0.679948623725482	-4.23179532661827
a_HQ_transcript/37859	1.98256904029935	0.781101129973857	1.95988142136994	0.058608042973854	0.679948623725482	-4.00997070684117
a_HQ_transcript/146650	2.30878855340478	0.0379169289763571	1.95981730948704	0.0586158573060898	0.679948623725482	-4.13145015408647
a_HQ_transcript/134505	-2.89841984645156	0.13155704344505	-1.95959736443981	0.0586426723792112	0.680033141623228	-4.11691076933879
a_HQ_transcript/7684	0.988038014852824	1.93985547569602	1.95942968858884	0.0586631221007719	0.680033141623228	-4.17443237252307
a_HQ_transcript/29308	-3.34363410438155	0.390020322813127	-1.95941241853209	0.0586652287062163	0.680033141623228	-4.06995111994679
a_HQ_transcript/52465	1.75320759153655	1.8040194901553	1.95932587140528	0.0586757867345142	0.680033141623228	-4.17274403346008
a_HQ_transcript/20687	-1.68415528855559	2.05293585578294	-1.95927791491882	0.0586816377370994	0.680033141623228	-4.16907823694561
a_HQ_transcript/106382	3.9561742997213	-0.136168155552435	1.95887672991987	0.0587306047729869	0.680464938059434	-4.1442387649684
a_HQ_transcript/51279	-2.10368438430438	1.11065377786907	-1.9587801602951	0.0587423969749419	0.680465932913397	-4.09920498186825
a_HQ_transcript/120342	2.5701916826241	0.503537056747061	1.9586275283826	0.0587610391873313	0.680546261382984	-4.083854939794
a_HQ_transcript/122188	2.21451080271043	1.15113399012245	1.95834753849976	0.0587952500571037	0.680701102231776	-4.06237172264439
a_HQ_transcript/121237	-1.20356814378864	2.50931041331603	-1.95832643330619	0.0587978295194355	0.680701102231776	-4.17599856568669
a_HQ_transcript/64039	-1.30031514025067	0.0847835269673523	-1.95807929019114	0.0588280424991633	0.680819179536834	-4.07798762329402
a_HQ_transcript/116499	-2.15907187783027	-0.120653154256573	-1.95792073946648	0.0588474322553882	0.680819179536834	-4.09787629293268
a_HQ_transcript/130509	5.35718858159596	6.78584847684677	1.95781910516441	0.058859864411081	0.680819179536834	-4.53527208838073
a_HQ_transcript/133321	1.64695819942601	0.857407141052573	1.95772509610623	0.0588713658595461	0.680819179536834	-4.11998426173766
a_HQ_transcript/70586	1.45014353460023	-0.556578733101356	1.95769206919603	0.0588754069687442	0.680819179536834	-4.16694003210652
a_HQ_transcript/114562	1.45251065866645	2.24567419936	1.95766839927874	0.0588783033225238	0.680819179536834	-4.105549825352
a_HQ_transcript/88195	-0.734108249022851	2.61463478404729	-1.95749858581902	0.0588990860638379	0.680821458788133	-4.12748303143027
a_HQ_transcript/128668	1.92542616935049	-0.298355930527075	1.95741333625996	0.0589095217942441	0.680821458788133	-4.13368213515823
a_HQ_transcript/23160	-1.96967230390534	1.42601066025115	-1.95737971623278	0.0589136377942525	0.680821458788133	-4.03883969399829
a_HQ_transcript/102485	2.98362820217208	0.0393464926828456	1.95710652846618	0.0589470926238439	0.680966212873326	-4.13661576739801
a_HQ_transcript/54564	-0.889976456826468	2.46616957699966	-1.95700825948523	0.058959130770217	0.680966212873326	-4.19241382070344
a_HQ_transcript/152728	3.58279159717302	3.3586327554744	1.95670733056676	0.0589960084468875	0.680966212873326	-4.16399708979282
a_HQ_transcript/146476	1.93302940233924	3.59476302767228	1.95648071279107	0.0590237928032105	0.680966212873326	-4.31613302667462
a_HQ_transcript/79255	-1.76632639452762	-0.626530470848353	-1.95640140213076	0.0590335193305496	0.680966212873326	-4.12150300659582
a_HQ_transcript/92771	-1.77171915923723	0.129030005074074	-1.9559855143304	0.0590845459112325	0.680966212873326	-4.10861878867485
a_HQ_transcript/91752	2.03709168081239	0.334446455126552	1.9559125362279	0.0590935037734348	0.680966212873326	-4.08351429169211
a_HQ_transcript/148907	2.74689526249871	1.28749277877953	1.95588479785821	0.0590969088916926	0.680966212873326	-4.18172246896106
a_HQ_transcript/116833	-3.74072351257549	0.263182454588477	-1.955779307529	0.0591098602735581	0.680966212873326	-4.19971782411703
a_HQ_transcript/116865	0.80333090960555	2.55744455293844	1.95576636264188	0.0591114497281474	0.680966212873326	-4.15959596418185
a_HQ_transcript/77582	2.18918996411528	1.65596926208655	1.95574958177176	0.0591135102440836	0.680966212873326	-4.0477487573872
a_HQ_transcript/15820	1.62316426547794	-0.689604430270695	1.95557937436758	0.0591344134645221	0.680966212873326	-4.14281499091073
a_HQ_transcript/22106	-2.63582088976791	0.238917903615769	-1.95555866178783	0.0591369576204845	0.680966212873326	-4.04476073442282
a_HQ_transcript/136559	1.52077903539268	-0.550890010926551	1.95554773839757	0.0591382993945066	0.680966212873326	-4.04835170838867
a_HQ_transcript/54676	-2.40354194268673	1.37679106872257	-1.95509804136534	0.0591935608576731	0.680966212873326	-4.24234758510583

a_HQ_transcript/132022	-3.08543503037548	0.191008639107823	-1.95507834215189	0.0591959826398276	0.680966212873326	-4.19283617600207
a_HQ_transcript/104148	3.03927501156654	1.22750165429971	1.95506009507415	0.0591982259760662	0.680966212873326	-4.22642210348563
a_HQ_transcript/55096	-2.19920370594225	0.588529653642294	-1.95495900982715	0.0592106549580974	0.680966212873326	-4.10761593670488
a_HQ_transcript/5102	-2.07928251176135	0.177433683050915	-1.95495754735612	0.0592108347935098	0.680966212873326	-4.09049297050703
a_HQ_transcript/11072	-0.622416699822427	2.95675562369909	-1.95486819320634	0.0592218232889961	0.680966212873326	-4.26320920010089
a_HQ_transcript/60542	1.87643840136709	0.124495770035329	1.95464432796634	0.0592493613164619	0.680966212873326	-4.10500259424014
a_HQ_transcript/5801	-0.678514392335677	2.95174684668323	-1.95451675505461	0.0592650592411605	0.680966212873326	-4.22372249844831
a_HQ_transcript/7556	1.80207821929284	-0.137174913196794	1.95443346632299	0.0592753099189639	0.680966212873326	-4.09061256511941
a_HQ_transcript/86173	0.569532042348881	5.71758932258394	1.95439342837803	0.0592802380971149	0.680966212873326	-4.64383136846142
a_HQ_transcript/2730	-0.823893439691447	2.61556365979326	-1.95425922609845	0.059296759340635	0.680966212873326	-4.14290521108602
a_HQ_transcript/121953	2.82699625318471	0.482427769460833	1.95416594832931	0.0593082448444218	0.680966212873326	-4.11937714811132
a_HQ_transcript/6611	-0.711894493720393	2.87432428609697	-1.95403105088509	0.0593248584902443	0.680966212873326	-4.19646212003518
a_HQ_transcript/59117	2.56652413097644	0.574188424343755	1.9538683247944	0.0593449048226121	0.680966212873326	-4.13898756084537
a_HQ_transcript/2845	-3.25396325324597	2.5350171269611	-1.95386154233909	0.0593457404853322	0.680966212873326	-4.1723096978903
a_HQ_transcript/102001	-0.52600776137293	3.32897510357616	-1.9538453941778	0.0593477301328408	0.680966212873326	-4.35464260463039
a_HQ_transcript/64929	1.43328801065095	-0.647998315118224	1.95355266904986	0.0593838074282754	0.680966212873326	-4.14080672417487
a_HQ_transcript/26361	2.12456163813494	0.792296317556951	1.95345240684791	0.0593961687530614	0.680966212873326	-4.07543230668955
a_HQ_transcript/146130	2.29707125372261	1.89325834959041	1.95328698495205	0.0594165684988205	0.680966212873326	-4.10162494536323
a_HQ_transcript/85515	-2.75606363594773	2.26465623158227	-1.95328091322752	0.0594173173765815	0.680966212873326	-4.12727452265508
a_HQ_transcript/52223	2.34719138229937	0.149772363745804	1.953134731294	0.0594353497196142	0.680966212873326	-4.15552267708339
a_HQ_transcript/110288	0.921270669196611	7.35268426005776	1.95311139816633	0.059438228428518	0.680966212873326	-4.78301344451313
a_HQ_transcript/114774	3.08956832419209	-0.00615147301490745	1.95305665552472	0.0594449827401872	0.680966212873326	-4.15023820135085
a_HQ_transcript/52704	-3.75288000234677	1.35663020631247	-1.95298219216007	0.0594541713235982	0.680966212873326	-4.1736323687065
a_HQ_transcript/144016	-1.3812597432517	4.9304783396591	-1.9529659550132	0.0594561751085747	0.680966212873326	-4.55161975624732
a_HQ_transcript/21330	3.11658717913484	0.800817235194479	1.95294416116049	0.0594588647246383	0.680966212873326	-4.18037727797371
a_HQ_transcript/52288	-1.30095491068065	2.41639946490398	-1.95291552332035	0.0594623991293094	0.680966212873326	-4.18830157914092
a_HQ_transcript/127593	0.876158399309473	3.59387039873685	1.95281942685428	0.0594742604278279	0.680966212873326	-4.38277637243465
a_HQ_transcript/101498	1.51962302508805	-0.575864248891861	1.95274496566054	0.0594834526719235	0.680966212873326	-4.18092952729763
a_HQ_transcript/114940	3.37734098492019	1.45910368669841	1.95264047005382	0.0594963547481919	0.680966212873326	-4.24211713958713
a_HQ_transcript/47418	-1.37858969022954	5.89533640747235	-1.95259736723789	0.0595016773620918	0.680966212873326	-4.62871565340817
a_HQ_transcript/150449	0.966772618316091	2.65406401465271	1.95239242001861	0.0595269912234505	0.680966212873326	-4.26446949447373
a_HQ_transcript/2067	3.27466145246662	1.80356975173372	1.95236942020734	0.0595298326066824	0.680966212873326	-4.19700381215829
a_HQ_transcript/45473	-2.10987410136187	1.7802409445222	-1.952026830941	0.0595721698242939	0.680966212873326	-4.09820141592678
a_HQ_transcript/113719	-1.36904279101627	1.22048731552273	-1.95197921826862	0.0595780558715561	0.680966212873326	-4.01991023011223
a_HQ_transcript/71099	1.67062924825878	0.543165737958226	1.95188322877389	0.0595899239678476	0.680966212873326	-4.07934063566655
a_HQ_transcript/43062	1.43622268557845	1.53278679606695	1.95178057566471	0.0596026182210834	0.680966212873326	-4.14434371385804
a_HQ_transcript/5923	-2.80001758964807	1.55106896638837	-1.95160298418214	0.0596245850207292	0.680966212873326	-4.1187286843319
a_HQ_transcript/36331	2.19272073603888	2.27300950013975	1.95159193466837	0.0596259519989564	0.680966212873326	-4.15569276670459
a_HQ_transcript/126273	-2.03462546213819	1.86901505512242	-1.95155705788739	0.0596302669193205	0.680966212873326	-4.20623070684127
a_HQ_transcript/69490	1.91652962875481	-0.467544233884666	1.95148711740213	0.0596389207027663	0.680966212873326	-4.09081908485083
a_HQ_transcript/48419	2.64992918209593	0.0735428316415761	1.9514830782254	0.0596394205061228	0.680966212873326	-4.13600865409049
a_HQ_transcript/49691	-1.74619637380672	1.09923200853759	-1.95147915580533	0.0596399058656156	0.680966212873326	-4.04490042936817
a_HQ_transcript/151831	0.728261988465079	6.82555666048539	1.95142395573449	0.0596467366752401	0.680966212873326	-4.70856532027434
a_HQ_transcript/62956	-1.78807911135194	0.30274579171275	-1.95139203779002	0.0596506867155642	0.680966212873326	-4.03515338445015
a_HQ_transcript/35381	-0.560384256101581	3.11895533978701	-1.95131078290099	0.0596607435284959	0.680966212873326	-4.32937022920759
a_HQ_transcript/96897	2.10024075521815	0.616839379805019	1.95121326027896	0.0596728157208818	0.680966212873326	-4.01103003285958
a_HQ_transcript/150446	1.96451472442034	-0.707335285795767	1.951155301585	0.0596799913530003	0.680966212873326	-4.14966568117015
a_HQ_transcript/148560	0.486654481953638	8.94841698435387	1.95112246459375	0.0596840571007834	0.680966212873326	-4.93245009129124
a_HQ_transcript/118204	-2.05266741208012	1.47547216115683	-1.95107521535993	0.059689907737889	0.680966212873326	-4.09090672995726
a_HQ_transcript/131651	2.46422500571253	1.80705237432744	1.95055365683647	0.0597545228112491	0.680966212873326	-4.14449692624777

a_HQ_transcript/71528	1.83463041956711	-0.0406272598124384	1.95048658103569	0.0597628371318117	0.680966212873326	-4.14867188020907
a_HQ_transcript/123471	1.70688519049005	3.63175584678179	1.95048490273016	0.0597630451775392	0.680966212873326	-4.29060586022742
a_HQ_transcript/32729	1.52447166101627	0.858702658868283	1.95038842846017	0.0597750053555682	0.680966212873326	-4.10929392757384
a_HQ_transcript/143346	1.34083564331305	-0.488882631961047	1.9503061989402	0.0597852012129378	0.680966212873326	-4.1334347480492
a_HQ_transcript/134745	-0.9662745333445	3.48629781846978	-1.95006585633931	0.0598150105857685	0.680966212873326	-4.26508214504539
a_HQ_transcript/127038	-1.71791865524572	1.58130749460508	-1.95003936766309	0.0598182967300253	0.680966212873326	-4.07762098113584
a_HQ_transcript/23385	1.56343553445981	0.41397891739302	1.9499923171154	0.0598241341346017	0.680966212873326	-4.01282163948601
a_HQ_transcript/25943	-2.73753231648488	1.14288511441129	-1.94998558044565	0.0598249699712138	0.680966212873326	-4.11235208111457
a_HQ_transcript/35620	1.29282100117031	3.5996226471062	1.94997742054413	0.0598259824055636	0.680966212873326	-4.42079070397154
a_HQ_transcript/9190	1.42811688565397	1.42963589577846	1.94985174482107	0.0598415774154062	0.680966212873326	-4.17979028240878
a_HQ_transcript/134748	2.2482352877341	-0.327274953817512	1.94980415540859	0.0598474836720772	0.680966212873326	-4.13201212453013
a_HQ_transcript/141305	1.7081922299764	-0.492221911609919	1.94979255633299	0.0598489232941029	0.680966212873326	-4.13167449682391
a_HQ_transcript/122366	1.16798624455989	2.11087207014857	1.94975629439357	0.0598534241471726	0.680966212873326	-4.14216036885983
a_HQ_transcript/2623	-2.05574557255985	1.68941067034286	-1.94968685816737	0.0598620434318017	0.680966212873326	-4.08651548443381
a_HQ_transcript/62518	-2.40468285401755	0.104387864440377	-1.9495178060912	0.0598830327735069	0.680966212873326	-4.05579437824093
a_HQ_transcript/141861	2.57602926729341	1.53008546613489	1.94943700932875	0.0598930666764601	0.680966212873326	-4.02787279948935
a_HQ_transcript/133568	-3.72582779137489	1.68265503547315	-1.94940129381537	0.0598975025418673	0.680966212873326	-4.1756122928995
a_HQ_transcript/147794	2.77317358407074	2.26358242943357	1.94936890666217	0.0599015252715373	0.680966212873326	-4.28326512344735
a_HQ_transcript/10957	3.09197422557992	0.0490424489252743	1.94929891920186	0.0599102190447665	0.680966212873326	-4.14567111032857
a_HQ_transcript/149348	5.27506768895208	2.19538183443063	1.94899927386065	0.0599474530731253	0.681256217445675	-4.27483791060872
a_HQ_transcript/47166	2.24576630226132	0.468724326334772	1.94830353381797	0.0600339834036215	0.68193175269373	-4.2130905490932
a_HQ_transcript/80247	-0.638900606930738	2.80332175186626	-1.94827784876818	0.0600371799685497	0.68193175269373	-4.26967602427441
a_HQ_transcript/16395	-0.598523206870618	3.22371627564038	-1.94823838377271	0.0600420917679347	0.68193175269373	-4.30090170798749
a_HQ_transcript/152716	-0.913360069054016	3.34139962366093	-1.94757786837484	0.0601243510447959	0.682666007537376	-4.31514116845474
a_HQ_transcript/82837	-2.04978676405154	-0.473104105084505	-1.94753069930896	0.0601302291252299	0.682666007537376	-4.14873224509362
a_HQ_transcript/76349	2.0985882828624	-0.0954446209562645	1.94733201395797	0.0601549942252941	0.682696091734004	-4.13942402513979
a_HQ_transcript/87235	2.497868205589	-0.0515267328568894	1.94702629671598	0.060193117578527	0.682696091734004	-4.13676579366416
a_HQ_transcript/144413	-2.44590084094579	0.532897783097525	-1.9470101553142	0.0601951310155336	0.682696091734004	-4.14404218677424
a_HQ_transcript/18352	-1.30961390857779	1.61584162103181	-1.94678861310159	0.0602227715265689	0.682696091734004	-4.07444761071826
a_HQ_transcript/45508	1.99342225035956	1.40834315641376	1.94678798679655	0.0602228496825464	0.682696091734004	-4.16451346510383
a_HQ_transcript/62592	1.85113615518928	0.34701950075289	1.9467396476476	0.0602288821419448	0.682696091734004	-4.1080279552113
a_HQ_transcript/128680	1.80924579342807	-0.478277113602071	1.94669423013911	0.0602345504743145	0.682696091734004	-4.16929013001369
a_HQ_transcript/22363	2.22599095992611	0.271087011339393	1.94648468454449	0.0602607088064307	0.682696091734004	-4.10531930791741
a_HQ_transcript/28204	-2.31240712634159	1.31715356816783	-1.94647730848251	0.0602616297663494	0.682696091734004	-4.24079002792464
a_HQ_transcript/68310	-1.24189131211315	-0.322396723306934	-1.946267972417	0.0602877721272281	0.682696091734004	-4.09671268326106
a_HQ_transcript/33419	1.57384752087897	1.91372008704817	1.94626295383264	0.0602883989801081	0.682696091734004	-4.07341108278101
a_HQ_transcript/24339	2.63716178508637	0.288238097520053	1.94614960811814	0.0603025580814757	0.682696091734004	-4.18960718184169
a_HQ_transcript/113515	1.90802458073676	-0.164612980675344	1.94597445970981	0.0603244432228575	0.682696091734004	-4.08013676212723
a_HQ_transcript/77002	1.54145575329916	0.0527144192578866	1.94590675007183	0.0603329055227781	0.682696091734004	-4.10376473099653
a_HQ_transcript/13430	0.608221037992451	2.65812119125727	1.94589802806471	0.0603339956675602	0.682696091734004	-4.31193946452573
a_HQ_transcript/34877	-2.89652579198008	0.125833276987406	-1.94581030460022	0.0603449609835028	0.682696091734004	-4.16881497301972
a_HQ_transcript/107166	0.850570619958304	2.61605616925555	1.94580682775085	0.0603453956206411	0.682696091734004	-4.21239949520464
a_HQ_transcript/152469	1.34197084196417	3.36694482475944	1.94551260240327	0.0603821862480617	0.682696091734004	-4.34107453700479
a_HQ_transcript/113882	-2.01337713063462	0.25541042766934	-1.94538496648468	0.0603981521917046	0.682696091734004	-4.1534774735252
a_HQ_transcript/79889	-1.31569710753254	1.8558386546292	-1.94536737084879	0.0604003535123369	0.682696091734004	-4.10158514206447
a_HQ_transcript/142163	-1.57098321315647	-0.64464921090581	-1.94528656132781	0.0604104641665351	0.682696091734004	-4.14396846140019
a_HQ_transcript/91354	1.28705994729297	1.82909772423146	1.94528535499885	0.0604106151100553	0.682696091734004	-4.14276243906103
a_HQ_transcript/36959	2.28555737778563	0.682090936957191	1.94524400311183	0.0604157895178312	0.682696091734004	-4.14728661370855
a_HQ_transcript/149199	2.04464548417754	0.204377041004466	1.94510999398755	0.0604325608704883	0.682696091734004	-4.18289129324477
a_HQ_transcript/134381	-2.00578312634113	0.240161000895972	-1.94507551865544	0.0604368761397308	0.682696091734004	-4.06284274191101

a_HQ_transcript/23298	-2.53629138326208	0.35112970702295	-1.9450646129357	0.0604382412617531	0.682696091734004	-4.16788450467624
a_HQ_transcript/137567	1.06400683108385	3.9984318101332	1.94441140444788	0.0605200553312244	0.68348742496472	-4.38489632187927
a_HQ_transcript/126639	1.83258895533644	1.88731437635761	1.94412117996972	0.0605564366213157	0.683641412679873	-4.10099994919737
a_HQ_transcript/53405	1.33933457954056	-0.661056132277455	1.94411499416276	0.0605572122537962	0.683641412679873	-4.1532758427734
a_HQ_transcript/147397	-2.55106412436367	3.13063134398888	-1.94384579368193	0.0605909753936046	0.683889751005718	-4.44878429096505
a_HQ_transcript/17189	-1.50903368990732	1.35601574529029	-1.94357032366973	0.0606255417548188	0.68409416529523	-4.12515108490729
a_HQ_transcript/147368	1.74732849212139	-0.26155487309252	1.94351390375516	0.0606326235136427	0.68409416529523	-4.15021318211385
a_HQ_transcript/86	-1.03901912480529	1.76557906818888	-1.94307504341384	0.0606877332209698	0.684319655372302	-4.11225096754639
a_HQ_transcript/88705	-4.51543093504546	2.13360857449125	-1.9429739722531	0.060700431341547	0.684319655372302	-4.3019004847686
a_HQ_transcript/143692	1.90308015772814	-0.101784592285428	1.94293556639925	0.0607052570821526	0.684319655372302	-4.15769227972687
a_HQ_transcript/112423	1.80686040853546	0.408701190857199	1.94281298232422	0.0607206621403101	0.684319655372302	-4.09205611154661
a_HQ_transcript/78099	1.89921489596525	2.13444685308689	1.94278510739321	0.0607241656370737	0.684319655372302	-4.19184066374218
a_HQ_transcript/117828	1.02739123957548	5.17662573705748	1.94266038620585	0.0607398435273336	0.684319655372302	-4.54995478266875
a_HQ_transcript/149537	3.12612640760692	-0.448538019264006	1.94250900621153	0.0607588772376008	0.684319655372302	-4.17091077548079
a_HQ_transcript/141640	1.78743230877685	2.86577584138531	1.94250835094119	0.0607589596390258	0.684319655372302	-4.31522551045251
a_HQ_transcript/143319	2.81727942216062	0.219567889180786	1.94244421664784	0.0607670251106293	0.684319655372302	-4.14512179561281
a_HQ_transcript/150553	-1.05008201287903	2.22835770099009	-1.94231584365871	0.0607831719718455	0.684319655372302	-4.15617197736493
a_HQ_transcript/65136	1.63524728208076	1.59630523686723	1.94227922868049	0.0607877781159335	0.684319655372302	-4.07074254060471
a_HQ_transcript/69553	2.07284672297837	-0.242310908210799	1.94214118216567	0.0608051470127139	0.684319655372302	-4.1879385786484
a_HQ_transcript/8900	0.565492435352391	3.12109600010994	1.94201705452359	0.060820768321989	0.684319655372302	-4.37365555793661
a_HQ_transcript/45435	-2.02304396929325	1.30428974792594	-1.94199600200393	0.0608234181004328	0.684319655372302	-4.19529619209798
a_HQ_transcript/104128	0.681127817541711	4.9228188412514	1.94192596812676	0.0608322336447849	0.684319655372302	-4.58974515333955
a_HQ_transcript/77199	2.67046100534668	5.40257874885834	1.94182998903702	0.0608443168530492	0.684319655372302	-4.63642022757781
a_HQ_transcript/115810	5.51059701395074	2.18714597290546	1.94170887535813	0.060859567328886	0.684319655372302	-4.2664863716699
a_HQ_transcript/39440	2.05178565356493	0.426147083724693	1.94166957608089	0.060864516554411	0.684319655372302	-4.16927896037006
a_HQ_transcript/96805	1.39754920843332	-0.21561935870827	1.94151934497693	0.060883439396375	0.684400031953681	-4.11974769142103
a_HQ_transcript/82179	-1.45015332928933	-0.690342162629049	-1.9411864848472	0.0609253840330437	0.684627126435677	-4.149368777449
a_HQ_transcript/51962	-1.57075651847881	2.06597886386716	-1.94099820016249	0.0609491214031831	0.684627126435677	-4.13290021432099
a_HQ_transcript/126394	-0.952944262772611	2.9111481712814	-1.94094533032584	0.0609557882346982	0.684627126435677	-4.25234038701434
a_HQ_transcript/93749	-0.705053771660696	2.84887766172113	-1.94072706242028	0.0609833182828222	0.684627126435677	-4.28028722038733
a_HQ_transcript/95689	-2.10556934962247	0.0281145149735866	-1.94072128562619	0.0609840470540356	0.684627126435677	-4.09536715962254
a_HQ_transcript/15305	1.82912899520366	0.179724571411283	1.94053670644766	0.0610073366066931	0.684627126435677	-4.14584652622922
a_HQ_transcript/61620	-2.07186000870407	1.61184651571025	-1.94049837163205	0.0610121745257969	0.684627126435677	-4.02834749442778
a_HQ_transcript/106640	-4.80307589588774	0.951160275055079	-1.94032505622903	0.061034051375558	0.684627126435677	-4.31589860832211
a_HQ_transcript/79121	-2.29454825714511	0.659723000978268	-1.94027769274028	0.0610400310455345	0.684627126435677	-4.16441450623486
a_HQ_transcript/127784	-2.6392970636275	1.99581238852931	-1.94022913908655	0.0610461615016307	0.684627126435677	-4.28139942423598
a_HQ_transcript/152144	2.30198112550958	-0.0348314503961712	1.94015174410999	0.0610559346090346	0.684627126435677	-4.15824986086775
a_HQ_transcript/73662	-2.67547910259845	1.04886862873141	-1.93999797123293	0.0610753564155682	0.684627126435677	-4.05684302770613
a_HQ_transcript/71885	-1.09954668148695	2.49280940937427	-1.93992982635114	0.0610839649556309	0.684627126435677	-4.29235446419223
a_HQ_transcript/137457	-0.999100100617929	2.57731986152048	-1.93991592862381	0.0610857207432507	0.684627126435677	-4.21507843265802
a_HQ_transcript/133688	-2.42860246946751	0.741318193016965	-1.9398798772755	0.0610902755415414	0.684627126435677	-4.12393032377881
a_HQ_transcript/13562	1.53355283995648	3.36020919692328	1.93971366775201	0.0611112785866716	0.684627126435677	-4.36426769919686
a_HQ_transcript/1664	-1.2815852977743	4.11380550057317	-1.93967124077266	0.061116640868967	0.684627126435677	-4.4598829659194
a_HQ_transcript/30670	0.67942213295719	2.99869476429836	1.93959977749627	0.0611256739271793	0.684627126435677	-4.32149736331794
a_HQ_transcript/122742	-4.11597289068169	1.40228117043172	-1.93954793270542	0.0611322279055023	0.684627126435677	-4.21396644291934
a_HQ_transcript/125235	2.26570202045963	1.03956544918347	1.93949278630434	0.0611391999265002	0.684627126435677	-4.15451744826183
a_HQ_transcript/21296	-1.61159670104185	2.32205886766745	-1.93930273675314	0.0611632326937122	0.684764327815891	-4.21170036526629
a_HQ_transcript/135576	-1.45822037232825	2.73633523524523	-1.9390487961412	0.0611953575994658	0.684812225005574	-4.25149052665795
a_HQ_transcript/130451	-1.13044060683652	4.16717333417183	-1.93895965486352	0.0612066379356667	0.684812225005574	-4.38581775715273
a_HQ_transcript/53553	-0.484225083210207	2.92278999178772	-1.93892358257374	0.0612112031967263	0.684812225005574	-4.31422507094618

a_HQ_transcript/30890	1.49473047763078	-0.775328036735793	1.9388429495724	0.0612214090692142	0.684812225005574	-4.16254435865952
a_HQ_transcript/48318	-0.691236654961446	3.36908672502752	-1.9388033925599	0.0612264164147049	0.684812225005574	-4.31974348983165
a_HQ_transcript/141844	-2.58691851708758	2.25043903885724	-1.93842289648482	0.0612745998406212	0.685187159583898	-4.33544527704668
a_HQ_transcript/117272	0.977558598358659	2.34381848833691	1.93835253937764	0.0612835129830148	0.685187159583898	-4.16082562257044
a_HQ_transcript/134556	2.21782309545999	0.327159966700417	1.93797140671307	0.0613318160448695	0.685595346741572	-4.16071024805717
a_HQ_transcript/21222	2.45219618359473	1.30338645163531	1.93775689513239	0.0613590167954294	0.685767530914194	-4.04181155532299
a_HQ_transcript/67337	2.32651812552712	0.855446662419587	1.93743712162898	0.0613995844961587	0.685935839377011	-4.13283750435234
a_HQ_transcript/112295	1.3641731692261	1.7728919636834	1.93743341519815	0.0614000548444774	0.685935839377011	-4.07968232808077
a_HQ_transcript/144939	-3.04719724234965	1.85466577239964	-1.93721956870023	0.0614271973810007	0.685935839377011	-4.26596980126592
a_HQ_transcript/85889	1.87635831191928	-0.608120488617515	1.9371995172745	0.0614297429477229	0.685935839377011	-4.1659538026536
a_HQ_transcript/64308	2.48233333091311	1.48126781602572	1.93717324520574	0.0614330783752533	0.685935839377011	-4.13645904598155
a_HQ_transcript/81868	-1.98567241071095	0.493350810827302	-1.93692300482034	0.0614648560519755	0.685942883965116	-4.24138398951567
a_HQ_transcript/89003	-3.03581967175184	-0.376222185830141	-1.93682433152984	0.0614773903493182	0.685942883965116	-4.16986477206577
a_HQ_transcript/55795	2.21501448117688	1.77013703953306	1.93681219925948	0.061478931643487	0.685942883965116	-4.16148708907285
a_HQ_transcript/138718	3.53046371930756	1.85306254424795	1.93679661499797	0.0614809115307297	0.685942883965116	-4.22164596435651
a_HQ_transcript/83645	1.51906174040324	-0.32542408467109	1.9365179504475	0.0615163235211403	0.686206266289934	-4.17154946456199
a_HQ_transcript/136560	0.865975502400707	2.90279976579396	1.93610296722973	0.0615690912931645	0.686663111797595	-4.29231642057991
a_HQ_transcript/96708	-1.75817811881837	2.33693525752511	-1.93589954497283	0.061594972052553	0.686758826725972	-4.16913617284736
a_HQ_transcript/51147	-1.27076934601864	3.33564166640507	-1.93584979758703	0.0616013026862995	0.686758826725972	-4.343717605371
a_HQ_transcript/1072	1.25347919186246	2.28320195604016	1.93560772217687	0.061632116183898	0.686839336840334	-4.14933256579376
a_HQ_transcript/83554	-2.33178411113443	1.85995427218576	-1.93560740714759	0.0616321562923064	0.686839336840334	-4.18901219262918
a_HQ_transcript/143106	-2.9224249211713	2.53211755208895	-1.9353712465191	0.0616622297854	0.686903672369429	-4.22160588701223
a_HQ_transcript/65003	2.21947225239084	0.526319929542294	1.9351891220699	0.0616854308084165	0.686903672369429	-4.20960881351509
a_HQ_transcript/11739	2.27701993317026	1.07438117801021	1.93513077777302	0.0616928649479701	0.686903672369429	-4.11000525690267
a_HQ_transcript/39885	1.78446913372149	3.60029206837541	1.93512841256877	0.0616931663349816	0.686903672369429	-4.46161132836395
a_HQ_transcript/19974	1.52039067257879	-0.653362317204007	1.93509821211486	0.0616970147509082	0.686903672369429	-4.16408232393857
a_HQ_transcript/29199	1.86888523454872	-0.472235735129731	1.93480725844596	0.0617341013599647	0.687101834913175	-4.17334281114658
a_HQ_transcript/88754	-3.36179427005649	1.32126155693212	-1.93477311683045	0.0617384545099008	0.687101834913175	-4.07870962445879
a_HQ_transcript/23157	0.794037379666156	2.15130124983167	1.93446865438351	0.0617772860672678	0.687264896636972	-4.16221566272279
a_HQ_transcript/74915	1.94490215223201	1.07425656804714	1.9343934843856	0.0617868766069375	0.687264896636972	-4.10296987001677
a_HQ_transcript/48486	4.14280130468969	4.00823074495096	1.93431946748249	0.0617963212892487	0.687264896636972	-4.47643087753003
a_HQ_transcript/4654	-0.916216690666823	2.52124150616987	-1.93428751103571	0.0618003993724444	0.687264896636972	-4.17816857652278
a_HQ_transcript/104864	-1.72446193500633	-0.35847133068242	-1.93409684733014	0.061824735533812	0.68737476233462	-4.17439903036346
a_HQ_transcript/27296	1.00943562085119	-0.218201885112703	1.93402483627052	0.0618339291261996	0.68737476233462	-4.28209280007186
a_HQ_transcript/38763	1.58802804315397	-0.55829847679931	1.93385747163826	0.061855301003758	0.687418921512947	-4.19268996893216
a_HQ_transcript/44611	1.06102194466234	3.09809502236469	1.93380851784126	0.0618615534412715	0.687418921512947	-4.2293159052805
a_HQ_transcript/48022	2.1606474467088	-0.00338527106208021	1.93368663380542	0.061877122993836	0.687457442472968	-4.12371298781852
a_HQ_transcript/137571	0.654593443932927	3.95481924418907	1.93359623171748	0.0618886732118952	0.687457442472968	-4.50041448360769
a_HQ_transcript/44602	4.5571969609698	2.06617328320547	1.93327290614233	0.0619299981677589	0.687785046521875	-4.31419798543224
a_HQ_transcript/118485	-3.26707933169084	0.734048370700332	-1.93301239631083	0.0619633118840752	0.687816383488762	-4.13343957908818
a_HQ_transcript/76430	2.74847647686817	0.638167430774765	1.93300648837201	0.0619640675648225	0.687816383488762	-4.12870939166551
a_HQ_transcript/69351	0.841245612694799	2.52338384207421	1.932973257918	0.061968318200018	0.687816383488762	-4.22820761391219
a_HQ_transcript/61473	2.92403400131946	2.76435131006989	1.93276162374603	0.0619953950656167	0.687985552572388	-4.37910464472166
a_HQ_transcript/133535	-1.39846057885262	3.47716161217823	-1.93262459418503	0.0620129323430984	0.688048812987139	-4.2944933446044
a_HQ_transcript/35919	-3.05321541258584	1.08598666019145	-1.9324044713309	0.0620411130342669	0.688230118025929	-4.13801100045507
a_HQ_transcript/34582	-2.70551601604248	-0.540575476719659	-1.93195360224096	0.0620988690641819	0.688666025455289	-4.14082851108392
a_HQ_transcript/68224	1.5157405682915	-0.332109336338212	1.93185276411041	0.0621117927292248	0.688666025455289	-4.16801448432762
a_HQ_transcript/115768	1.87998913007802	2.28519747458822	1.93159975621558	0.0621442290997175	0.688666025455289	-4.24172597796691
a_HQ_transcript/152335	3.97737934504574	1.16520380587822	1.93159460933084	0.0621448890979307	0.688666025455289	-4.21396935134388
a_HQ_transcript/12839	-0.990165186057958	2.51344740485808	-1.93156034993004	0.0621492824232752	0.688666025455289	-4.23132234627934

a_HQ_transcript/92256	1.04957585223797	1.68356621398877	1.93154311460796	0.0621514927322194	0.688666025455289	-4.07221003087611
a_HQ_transcript/1105	-1.15126960496338	1.04614187725028	-1.93114478744267	0.0622025943556	0.689100896646144	-4.07604807128076
a_HQ_transcript/86965	-0.554273021388389	3.19706909620503	-1.93065692206012	0.0622652324395633	0.689202128137207	-4.3615961946084
a_HQ_transcript/106154	2.25988595172398	1.96703132788347	1.93064190481355	0.0622671614019734	0.689202128137207	-4.20260408517932
a_HQ_transcript/4001	-0.811282890451656	3.54117452281727	-1.93059474313496	0.0622732196467593	0.689202128137207	-4.44520557127357
a_HQ_transcript/104147	3.78528386830637	0.0658710476987819	1.93057866440869	0.0622752851873093	0.689202128137207	-4.14760414343954
a_HQ_transcript/13081	-2.4567005654907	0.531486940153619	-1.93053356199901	0.062281079548342	0.689202128137207	-4.12297604846433
a_HQ_transcript/68465	-0.846320807651664	3.48700595758053	-1.93051026636064	0.0622840725498923	0.689202128137207	-4.42885801153324
a_HQ_transcript/81373	-1.08019868227079	1.1173622137901	-1.93029881186519	0.062311245717497	0.689202128137207	-4.09441906415303
a_HQ_transcript/127316	1.28758034779757	3.02923440440016	1.93027186443264	0.0623147093608357	0.689202128137207	-4.29822030002559
a_HQ_transcript/132755	1.0827107820802	2.07881825521814	1.93024282712538	0.0623184418092686	0.689202128137207	-4.14833705239512
a_HQ_transcript/129249	2.17837772672384	0.0476401104025612	1.9298432620197	0.0623698214653559	0.689517913041306	-4.12749079682813
a_HQ_transcript/84593	3.35802445524422	2.11069418372723	1.92983628029946	0.0623707195632258	0.689517913041306	-4.2155943461074
a_HQ_transcript/111462	3.45764479234404	0.515844798852794	1.92911364817737	0.062463736343178	0.690171992774094	-4.07930421330588
a_HQ_transcript/102287	1.91642055431792	0.338606299869194	1.92909748453654	0.0624658182894297	0.690171992774094	-4.10957264483218
a_HQ_transcript/97765	-1.69132332419595	0.424808591523723	-1.92902967452602	0.0624745531640116	0.690171992774094	-4.1271530981216
a_HQ_transcript/4447	1.91616787993145	1.01652237237307	1.92881206315559	0.0625025916813047	0.690171992774094	-4.06026821905367
a_HQ_transcript/35870	-0.548337981615715	3.41703278661084	-1.92877651650548	0.0625071727861006	0.690171992774094	-4.40789833308596
a_HQ_transcript/137143	2.15454997683109	-0.65334737854649	1.92858163526211	0.062532293435853	0.690171992774094	-4.18611286322417
a_HQ_transcript/72365	2.64990908450375	3.16465300996498	1.92855171840882	0.0625361505618791	0.690171992774094	-4.31972766592241
a_HQ_transcript/113358	-2.31039315356887	0.912175570700357	-1.92849973476121	0.0625428532096383	0.690171992774094	-4.22288338737014
a_HQ_transcript/27894	-1.25959894862728	1.66339021511977	-1.92848458548442	0.0625448066382968	0.690171992774094	-4.14669313918004
a_HQ_transcript/145561	3.30390504043371	0.99339385920596	1.92839681265849	0.0625561255743878	0.690171992774094	-4.12864239959879
a_HQ_transcript/10810	-2.03198390786663	0.685801333598665	-1.92836296337438	0.0625604911561847	0.690171992774094	-4.22780011765794
a_HQ_transcript/129096	-1.82108160593789	1.03522429430439	-1.92823018571794	0.062577618191347	0.690229941219472	-4.13400982701032
a_HQ_transcript/29557	-2.29103342750123	0.022293748950214	-1.9281136271687	0.0625926564623554	0.690264833019122	-4.21371009877514
a_HQ_transcript/5103	-2.8107367432002	1.94100006061045	-1.9277566673091	0.0626387305316147	0.690517573641903	-4.27768636111373
a_HQ_transcript/23437	3.01994932611878	1.16934790054116	1.92766258793836	0.0626508785733025	0.690517573641903	-4.15984821978698
a_HQ_transcript/128647	0.992430426818221	4.08758364840788	1.92764838673173	0.0626527124876585	0.690517573641903	-4.49476682845402
a_HQ_transcript/7281	1.64433035701619	2.25258947711757	1.9275357093754	0.0626672650540325	0.690517573641903	-4.22428703101446
a_HQ_transcript/129974	0.982576923998817	3.18250030914066	1.92742081899391	0.0626821064494915	0.690517573641903	-4.32753215622236
a_HQ_transcript/135193	1.99963254521198	1.81190166894096	1.92735196120239	0.0626910028701231	0.690517573641903	-4.24554937225223
a_HQ_transcript/34395	2.57947002711293	2.90044756275914	1.92729216367831	0.0626987295912807	0.690517573641903	-4.31441591672654
a_HQ_transcript/67351	1.85250046174117	2.00116999331468	1.92716809434277	0.0627147638057839	0.690562375735566	-4.17331300284417
a_HQ_transcript/152066	-4.39778430063934	0.066468696045427	-1.92707685349808	0.0627265576638417	0.690562375735566	-4.21131800294296
a_HQ_transcript/54173	-2.78729572061495	1.32342067771007	-1.92673214956395	0.0627711316658396	0.690826334606403	-4.16775565374143
a_HQ_transcript/9381	0.981739506637873	1.86677278118867	1.92670763082697	0.0627743032512906	0.690826334606403	-4.24925659166238
a_HQ_transcript/112599	2.09942387929463	1.19933555102082	1.92660394891089	0.0627877164069842	0.690843153379742	-4.06176259828868
a_HQ_transcript/78956	2.57509238630659	-0.377097103491489	1.92650085159542	0.0628010563923398	0.690844657155189	-4.19398040853205
a_HQ_transcript/25115	2.1971611361189	-0.0447709007416408	1.92640020751897	0.0628140813128141	0.690844657155189	-4.09388948286026
a_HQ_transcript/93552	-4.08904562731275	3.16478706086401	-1.92609246922577	0.0628539219682885	0.690844657155189	-4.26899443628718
a_HQ_transcript/35809	2.89378383529004	1.84603847375214	1.92606045645357	0.0628580676858266	0.690844657155189	-4.07788792628577
a_HQ_transcript/28841	1.68169805335759	3.17438931088754	1.9260563162368	0.0628586038693905	0.690844657155189	-4.26341748221179
a_HQ_transcript/140773	2.11460137450061	1.40360453643606	1.92602612990849	0.0628625133043505	0.690844657155189	-4.07527555895134
a_HQ_transcript/95376	1.3545839016309	-0.388206485839146	1.92584597457395	0.0628858496168677	0.690844657155189	-4.17101474380516
a_HQ_transcript/149234	4.49457719330521	4.45603150227309	1.92576073352944	0.0628968938755888	0.690844657155189	-4.39952022758574
a_HQ_transcript/85089	-2.00029875683701	-0.234897647769858	-1.92574957241108	0.0628983400904745	0.690844657155189	-4.14640052694884
a_HQ_transcript/93217	1.22769966828931	-0.817634362555094	1.9256850444557	0.0629067019392103	0.690844657155189	-4.18392364516839
a_HQ_transcript/65178	2.77133106876296	1.05460030328743	1.92554801920577	0.0629244615317606	0.690909161299241	-4.08949097961371
a_HQ_transcript/87980	2.31874318784607	1.96008545058441	1.925216543256	0.0629674414611628	0.69110749838363	-4.41706512362802

a_HQ_transcript/92970	1.68881245686133	-0.306953356944928	1.92521231027675	0.0629679904830932	0.69110749838363	-4.13251024078168
a_HQ_transcript/102936	1.42561515425605	-0.757488963363874	1.92509107158796	0.0629837170282502	0.69110749838363	-4.19393975841258
a_HQ_transcript/112008	-0.974524896823385	2.26553226900977	-1.92497062870838	0.0629993437083924	0.69110749838363	-4.16861125573176
a_HQ_transcript/3166	-2.15235284548771	0.363205681362415	-1.92490918388725	0.063007317065923	0.69110749838363	-4.09988829250489
a_HQ_transcript/15370	2.11750320358351	0.707630686808349	1.9248345305213	0.0630170055983055	0.69110749838363	-4.17934357455447
a_HQ_transcript/115105	1.86774672590138	2.72467865519196	1.92460992311026	0.0630461629747116	0.69110749838363	-4.27482954037808
a_HQ_transcript/121769	1.62639167524813	-0.0853818103969239	1.92458617706722	0.0630492462455192	0.69110749838363	-4.17253665114819
a_HQ_transcript/52566	2.10156368949435	-0.587041290477572	1.92452531727237	0.0630571490935054	0.69110749838363	-4.18254872409362
a_HQ_transcript/144038	-2.87797480975311	2.50754317808262	-1.92449243645512	0.0630614191340967	0.69110749838363	-4.19984637604942
a_HQ_transcript/117040	2.51350915890671	0.228788009484187	1.92379738343037	0.0631517401635855	0.691775223769668	-4.22191612695468
a_HQ_transcript/43063	2.37485854966295	1.21234601895602	1.92371072230242	0.0631630094788733	0.691775223769668	-4.15697299465819
a_HQ_transcript/88187	-1.950093681687	0.530086256473123	-1.92364510559974	0.0631715433576918	0.691775223769668	-4.11600730160278
a_HQ_transcript/71778	-2.17811961057871	2.03993615157801	-1.92363860912606	0.0631723883205773	0.691775223769668	-4.255723157346
a_HQ_transcript/147730	-2.30919107046474	-0.165329386042347	-1.92356585699886	0.0631818514828167	0.691775223769668	-4.21853462407495
a_HQ_transcript/141503	2.62190640644372	-0.177064141080595	1.92344485082854	0.0631975939579584	0.691817277135256	-4.14722563276138
a_HQ_transcript/129551	2.40947523553325	3.10479465298978	1.92318966451554	0.0632308039148775	0.692050493308981	-4.48972114029125
a_HQ_transcript/118618	3.18506825326807	1.34426954733133	1.92298676988719	0.0632572193980159	0.692075296651998	-4.30454277514031
a_HQ_transcript/100075	-2.59949264993325	2.41677396393414	-1.92292545761479	0.0632652037108336	0.692075296651998	-4.19320276507549
a_HQ_transcript/131082	0.657216313341735	4.2667009217091	1.92275144002944	0.0632878696765663	0.692075296651998	-4.47943726742493
a_HQ_transcript/141278	1.53970398942478	2.03144021532007	1.92271742424221	0.0632923010874297	0.692075296651998	-4.18062215310004
a_HQ_transcript/51403	-0.802540984203538	5.69793549372312	-1.92259125125323	0.0633087406324175	0.692075296651998	-4.66815799718498
a_HQ_transcript/42073	2.72463058447291	1.54584495592594	1.92259031146843	0.0633088630943037	0.692075296651998	-4.22623024695777
a_HQ_transcript/12272	-2.3803715820554	3.76229653788649	-1.92253237936946	0.0633164125308857	0.692075296651998	-4.37844787739606
a_HQ_transcript/152318	-2.6811626584283	1.58822536384485	-1.92182904881379	0.0634081293439388	0.692905014728696	-4.30536395943977
a_HQ_transcript/64735	-1.68882136933536	-0.248254636573797	-1.92176749047858	0.0634161622343219	0.692905014728696	-4.17564701409783
a_HQ_transcript/30416	1.91884411920506	1.41245979720102	1.92159071964487	0.063439234362624	0.693026839885474	-4.11723856793708
a_HQ_transcript/92592	1.87173315688017	1.13100390238556	1.92148153557638	0.0634534886918623	0.693052309410103	-4.20624370080387
a_HQ_transcript/150436	-4.31654046854781	1.20719342244912	-1.92097094244367	0.0635201850149336	0.693589627971933	-4.17554347595925
a_HQ_transcript/108195	-1.66031258355039	0.150635929561083	-1.92088071635503	0.0635319771132044	0.693589627971933	-4.07705506948903
a_HQ_transcript/143759	-2.42773690561668	0.513117477812664	-1.92069287371869	0.0635565332769339	0.693589627971933	-4.19690296944023
a_HQ_transcript/121291	2.30355964662784	0.17225842065858	1.92059741582535	0.0635690153749774	0.693589627971933	-4.17852097630556
a_HQ_transcript/773	-1.89569749114957	1.15626629334052	-1.92044977132	0.0635883255808948	0.693589627971933	-4.06372258100224
a_HQ_transcript/91	-1.58130658114946	4.17785586444477	-1.92044080070369	0.0635894989978764	0.693589627971933	-4.60136971157896
a_HQ_transcript/6223	2.23159998909228	0.636430937694183	1.92038291917881	0.063597070740871	0.693589627971933	-4.08113346130793
a_HQ_transcript/74402	-2.06360044925661	-0.523101196913319	-1.92037474117627	0.063598140605051	0.693589627971933	-4.19321001125001
a_HQ_transcript/10	-1.89233929161746	3.43916731728337	-1.92018778418579	0.063622602970938	0.693726255016823	-4.4863388452692
a_HQ_transcript/764	3.37426276590839	1.07184534176958	1.91950674359071	0.0637117824361468	0.694462441248906	-4.25455802653355
a_HQ_transcript/142986	-2.09952014080607	-0.231410036912938	-1.91945892619684	0.0637180479844086	0.694462441248906	-4.19505324333347
a_HQ_transcript/117009	1.64921086131788	4.49182306686875	1.91939854139267	0.0637259610105571	0.694462441248906	-4.48813431872555
a_HQ_transcript/31959	1.80663799710006	1.82835020804911	1.91908811349683	0.063766653924974	0.694557211489947	-4.18613873559311
a_HQ_transcript/145817	-3.40051686070105	1.11229291017649	-1.91894003252269	0.0637860732555482	0.694557211489947	-4.19960530951053
a_HQ_transcript/7939	2.21420913808251	0.275102134114602	1.91878671344129	0.0638061848963399	0.694557211489947	-4.2246298231081
a_HQ_transcript/122848	2.50904063597469	0.433058394964001	1.9186962735932	0.0638180509190156	0.694557211489947	-4.12084330200501
a_HQ_transcript/2800	-1.41830969544138	1.94157541325575	-1.91867409341958	0.0638209613261335	0.694557211489947	-4.17048576661898
a_HQ_transcript/132168	-4.09692744312437	1.18064384111235	-1.91848427238124	0.0638458736903496	0.694557211489947	-4.20816616573911
a_HQ_transcript/108623	-0.940500810636817	3.05539664649406	-1.91845998688061	0.0638490615578581	0.694557211489947	-4.33332114075037
a_HQ_transcript/97590	2.03640106540246	0.280829272980828	1.91818612765819	0.063885019563616	0.694557211489947	-4.22150894743941
a_HQ_transcript/144692	0.762754933820225	4.38473607409065	1.918172288579	0.0638868371137628	0.694557211489947	-4.53612606878553
a_HQ_transcript/23388	1.92024985828835	-0.47620461190919	1.91814559563426	0.0638903429476166	0.694557211489947	-4.16054288071719
a_HQ_transcript/86985	-1.34477703361741	1.82222424123302	-1.91807811486793	0.0638992065685875	0.694557211489947	-4.16629747706581

a_HQ_transcript/144128	2.10059076784228	0.222478610077948	1.91806712588678	0.0639006500754997	0.694557211489947	-4.19903407024828
a_HQ_transcript/122577	2.35807009506131	2.18134613155427	1.9179857695743	0.0639113378754395	0.694557211489947	-4.28630097541612
a_HQ_transcript/10216	1.74794633354526	0.644194553601594	1.91798043713412	0.0639120384534312	0.694557211489947	-4.09792182833008
a_HQ_transcript/114423	2.78418367706159	1.72295389475819	1.91791242107141	0.0639209750114263	0.694557211489947	-4.1896452060496
a_HQ_transcript/114965	2.30387698436705	0.643752472193408	1.91783058671553	0.0639317285712996	0.694557211489947	-4.20073717154687
a_HQ_transcript/148807	3.11593899235182	-0.0226419490809533	1.91778449320985	0.0639377862421329	0.694557211489947	-4.24089818182717
a_HQ_transcript/24297	0.887498282688857	2.18618626752677	1.91761008461819	0.0639607117439913	0.694676429793484	-4.18693973361906
a_HQ_transcript/25534	-1.04858686490142	3.65391241993941	-1.91751490705023	0.0639732255537635	0.694682543431565	-4.4533139768383
a_HQ_transcript/105188	2.02618705320121	0.601294807225117	1.9173739789346	0.0639917584649328	0.694754003744792	-4.19055421503664
a_HQ_transcript/126628	0.380659580824984	7.41838005466559	1.91684538586128	0.0640613130320373	0.695153603126278	-4.83919753961719
a_HQ_transcript/64240	3.68102231468119	-0.103023109248922	1.91683789926837	0.0640622986193001	0.695153603126278	-4.16958023090646
a_HQ_transcript/18916	-1.85784835485244	1.15597029211524	-1.91682162259906	0.064064441438678	0.695153603126278	-4.1081048273767
a_HQ_transcript/29200	-4.97527593578522	1.24182648061781	-1.91635657785536	0.0641256906262806	0.695688343546928	-4.14961170888308
a_HQ_transcript/136182	-3.47386337208639	1.49857825260041	-1.91594517679268	0.0641799167846375	0.695910492341654	-4.17824259605878
a_HQ_transcript/16393	0.914765347612482	0.665900362096393	1.91574339584657	0.0642065277052969	0.695910492341654	-4.17538690841618
a_HQ_transcript/23267	-1.92642617810336	-0.0461809714807055	-1.91572516503978	0.0642089324579849	0.695910492341654	-4.15425232549282
a_HQ_transcript/148065	2.02770546619546	0.10006527305413	1.91563437980331	0.0642209087332691	0.695910492341654	-4.18660522638746
a_HQ_transcript/50339	1.86141774903423	-0.699560474188331	1.91538739853885	0.0642534999600149	0.695910492341654	-4.20733552084105
a_HQ_transcript/139066	0.750932534132825	-0.770364206695736	1.91527597214736	0.064268208272931	0.695910492341654	-4.25225329577315
a_HQ_transcript/62380	3.17346738505713	1.10029003272525	1.91521816760855	0.0642758396328681	0.695910492341654	-4.18496609272025
a_HQ_transcript/74777	1.46622927861326	-0.696288739738329	1.91520368874207	0.0642775125658835	0.695910492341654	-4.21365063254617
a_HQ_transcript/146782	1.56704759324932	-0.467949843853655	1.91518745721759	0.0642798943395759	0.695910492341654	-4.21713885188642
a_HQ_transcript/104560	-1.13768491464726	2.33813172543082	-1.9149505358839	0.0643111825973308	0.695910492341654	-4.26643200410589
a_HQ_transcript/38050	-2.26185816764685	1.31847969288902	-1.91483576083877	0.0643263447257326	0.695910492341654	-4.14672411352952
a_HQ_transcript/46499	1.9191204093239	0.513956781659877	1.91478072328554	0.0643336164471298	0.695910492341654	-4.24369577388499
a_HQ_transcript/5670	-1.7171621003177	2.69327924990206	-1.91465959378963	0.0643496229288798	0.695910492341654	-4.25595500648926
a_HQ_transcript/148410	-2.73821921351184	3.0705676396329	-1.9146209786174	0.0643547263978907	0.695910492341654	-4.2888898578478
a_HQ_transcript/4884	1.744261503931	3.78641371953244	1.91459121904831	0.0643586597287662	0.695910492341654	-4.48244127652776
a_HQ_transcript/142137	2.59447066297113	0.188966502836137	1.91437651073892	0.0643870439418771	0.695910492341654	-4.15780129767619
a_HQ_transcript/70299	1.45051884324696	-0.655384227244369	1.91437099130018	0.0643877737484037	0.695910492341654	-4.23327731237365
a_HQ_transcript/60864	0.760014667245692	2.12557419938105	1.91410829507732	0.0644225169595637	0.695910492341654	-4.1778302668518
a_HQ_transcript/42058	2.92488267011384	1.21472032090667	1.91398994266319	0.0644381750930271	0.695910492341654	-4.17172117452855
a_HQ_transcript/39533	2.22820895789272	2.10957608888052	1.91382880096457	0.0644594995723054	0.695910492341654	-4.2850949777864
a_HQ_transcript/142290	5.01661717128498	3.37236053380826	1.91381598895135	0.0644611952950979	0.695910492341654	-4.52550348027066
a_HQ_transcript/101224	1.9579285507587	0.0703028095085962	1.91377892441854	0.0644661011558278	0.695910492341654	-4.15093931987974
a_HQ_transcript/48980	-1.25120452248078	3.86677096830738	-1.91376977102652	0.064467312748236	0.695910492341654	-4.48045268249132
a_HQ_transcript/9428	-0.705958327706188	2.73948309694603	-1.91375640533594	0.0644690819385568	0.695910492341654	-4.23569678328878
a_HQ_transcript/86049	0.904938690188923	2.73233453240803	1.91373470032002	0.0644719550783958	0.695910492341654	-4.40581789206104
a_HQ_transcript/120010	2.17541377456269	0.0934687100237585	1.91371835141629	0.0644741192911117	0.695910492341654	-4.20266749126339
a_HQ_transcript/135347	2.1018997105796	0.140742759812704	1.91368902230698	0.0644780019368802	0.695910492341654	-4.22029768369023
a_HQ_transcript/9968	-0.69217618352525	3.15454411022161	-1.91359543272082	0.0644903982625103	0.695910492341654	-4.39607353014582
a_HQ_transcript/63003	1.40412287310177	1.92179313427874	1.91352568172768	0.0644996289812632	0.695910492341654	-4.19855109050781
a_HQ_transcript/108394	2.03676858105968	0.439229687694167	1.91347114856392	0.0645068508178489	0.695910492341654	-4.1940445027268
a_HQ_transcript/148776	-2.58814247019565	1.64050884301571	-1.91320595972425	0.064541979778348	0.695910492341654	-4.25103701972445
a_HQ_transcript/27166	2.08711711634369	0.172247485023454	1.91310733818716	0.0645550481619358	0.695910492341654	-4.12269994642143
a_HQ_transcript/29886	2.50755064633726	1.2711172244548	1.91308839382527	0.0645575587493282	0.695910492341654	-4.13803576371354
a_HQ_transcript/65590	1.38963921109893	-0.769701324703123	1.91304616768944	0.0645631550398179	0.695910492341654	-4.21372675035334
a_HQ_transcript/149879	2.43052815567915	0.50190116322108	1.91300315589443	0.0645688558855262	0.695910492341654	-4.1897220927225
a_HQ_transcript/17281	1.9358151880547	-0.382955367829592	1.91294050371079	0.064577160674561	0.695910492341654	-4.15132969270448
a_HQ_transcript/82680	1.62748668003447	-0.631340906926246	1.91269380121204	0.0646098710113479	0.696133935523194	-4.20795649270308

a_HQ_transcript/143823	-1.81980159381543	0.203253987855706	-1.91255791133935	0.0646278947884873	0.696199086038767	-4.19390768226411
a_HQ_transcript/119234	1.44356259115307	1.78089710106505	1.9124451168264	0.0646428586057838	0.69623125533389	-4.2119065993093
a_HQ_transcript/124798	1.45524759086855	-0.722420581704293	1.91230245386545	0.0646617891877752	0.696306128548906	-4.21432545458474
a_HQ_transcript/117806	3.29303956824855	0.528705026942139	1.91210673618919	0.0646877676234448	0.696407575248193	-4.19963855535807
a_HQ_transcript/9475	-1.64884491178932	-0.571225380370428	-1.91205097183967	0.0646951711110006	0.696407575248193	-4.19081827180735
a_HQ_transcript/868	-1.42066224806081	-0.773108731430832	-1.91163741118706	0.0647500998343228	0.696869802475378	-4.20578823952342
a_HQ_transcript/19724	2.04479308199139	1.22297376565646	1.91128138769535	0.0647974187746539	0.697249973812122	-4.20959330827153
a_HQ_transcript/53147	1.30817234116928	2.22813758487626	1.91116554377449	0.0648128219800081	0.697273654945875	-4.15525078102917
a_HQ_transcript/150580	1.62157460293152	-0.232934730996775	1.91107291854107	0.0648251401829768	0.697273654945875	-4.18129218180334
a_HQ_transcript/131904	1.07891546040962	3.58254136556136	1.91086399867878	0.0648529317957233	0.697273654945875	-4.4169559310286
a_HQ_transcript/126306	-0.566155688377168	3.97969492564475	-1.91050832980532	0.064900268389918	0.697273654945875	-4.50954573507579
a_HQ_transcript/148890	2.11253356865807	0.146357014864116	1.91031236462267	0.0649263624814009	0.697273654945875	-4.23225956988649
a_HQ_transcript/43373	0.880142225258994	3.56749943674602	1.91025718084949	0.0649337122086134	0.697273654945875	-4.49205577356883
a_HQ_transcript/51614	2.51035670035659	1.99145160890256	1.91021389232794	0.0649394781529144	0.697273654945875	-4.19023074796385
a_HQ_transcript/55408	1.94272170385875	0.152551052695155	1.91020391820176	0.064940806749174	0.697273654945875	-4.20886213152506
a_HQ_transcript/9537	-1.65963771289544	1.28314454720128	-1.91017543330526	0.0649446011883876	0.697273654945875	-4.12920667218198
a_HQ_transcript/54539	1.49762668603552	1.89876739122747	1.91016492900006	0.0649460005026984	0.697273654945875	-4.11242875485341
a_HQ_transcript/40888	-1.67842040592295	1.18249461070385	-1.90999040822265	0.0649692528188511	0.697273654945875	-4.10802411287887
a_HQ_transcript/144407	-6.08901145353414	1.68727403390583	-1.90991348602886	0.0649795038465397	0.697273654945875	-4.22602593850045
a_HQ_transcript/94395	1.80069550682018	0.308383791612569	1.90975948647604	0.0650000307754037	0.697273654945875	-4.19377134932109
a_HQ_transcript/123376	-1.68217139509441	-0.596999371889174	-1.90967148618523	0.0650117630346277	0.697273654945875	-4.19736368439141
a_HQ_transcript/3811	-2.49597968658725	2.76359101653922	-1.90953049209443	0.065030564273919	0.697273654945875	-4.24051950301902
a_HQ_transcript/111151	-1.68742950118876	1.71066255293593	-1.90952374351552	0.0650314642991464	0.697273654945875	-4.17643216998484
a_HQ_transcript/1745	1.25213214845395	1.02886299850768	1.90946496751881	0.0650393034232617	0.697273654945875	-4.08632503531522
a_HQ_transcript/25647	-2.41347279041239	2.64630771603396	-1.90937644347528	0.0650511116693814	0.697273654945875	-4.34753238968424
a_HQ_transcript/148257	-2.13381465896257	0.0373641157286538	-1.90915654509657	0.0650804519808831	0.697273654945875	-4.22147190685903
a_HQ_transcript/81944	-0.551950789386053	4.40086148948422	-1.90898915794508	0.0651027935487642	0.697273654945875	-4.55816255213588
a_HQ_transcript/146927	4.73922670255924	3.12181083868296	1.90895469351324	0.0651073944214885	0.697273654945875	-4.44911925714847
a_HQ_transcript/8638	1.87437808679823	-0.515271961331201	1.90891069063895	0.0651132690484568	0.697273654945875	-4.20700369159918
a_HQ_transcript/25335	1.4040630744443	1.21607171354967	1.90885223315897	0.0651210741527487	0.697273654945875	-4.10528287799841
a_HQ_transcript/92317	1.82840114683497	-0.658384548294019	1.90882305993936	0.065124969593963	0.697273654945875	-4.20647566411236
a_HQ_transcript/20003	-0.817840866800626	2.94382088565551	-1.90873361735121	0.0651369139359523	0.697273654945875	-4.36543017662486
a_HQ_transcript/150146	-2.38984209423337	3.93694986065015	-1.90871731603116	0.0651390910504848	0.697273654945875	-4.59139132623303
a_HQ_transcript/103511	4.67382613045429	1.5667035074861	1.90869655961232	0.0651418632543666	0.697273654945875	-4.22794993643883
a_HQ_transcript/149329	1.11854555879667	2.65847156253492	1.90869037635082	0.0651426891034784	0.697273654945875	-4.29593502004606
a_HQ_transcript/54197	0.672518350569359	4.04172583382755	1.90865444276458	0.0651474886459374	0.697273654945875	-4.55088137183185
a_HQ_transcript/103816	5.52146377483482	2.57185538488166	1.90831521348169	0.065192813535716	0.697508345809326	-4.31811265199973
a_HQ_transcript/117434	-2.0847907322394	5.01196365999034	-1.90830524680179	0.0651941456088916	0.697508345809326	-4.61100206030371
a_HQ_transcript/1610	-1.19500177139492	2.09639356616174	-1.90801581459514	0.0652328392365528	0.697508345809326	-4.15031566105046
a_HQ_transcript/117005	1.9537963698744	0.501918415593418	1.90792010617299	0.0652456386683281	0.697508345809326	-4.1552962037715
a_HQ_transcript/131158	2.16097251793204	2.75014362712105	1.90785095210718	0.0652548882390414	0.697508345809326	-4.22745822256119
a_HQ_transcript/25424	0.451928620221735	4.22533867863457	1.90771249408683	0.0652734108292594	0.697508345809326	-4.5531730138078
a_HQ_transcript/129875	-2.25078456637259	-0.44951493522275	-1.90768050491224	0.0652776909113563	0.697508345809326	-4.21342872803396
a_HQ_transcript/146060	3.77558858719675	-0.0246898457911692	1.90757050786481	0.0652924101255551	0.697508345809326	-4.20132918603862
a_HQ_transcript/11313	1.72164046592984	0.657296117598498	1.90749017966786	0.065303161022796	0.697508345809326	-4.27759066145462
a_HQ_transcript/24723	1.54252715810675	1.43187656360792	1.90745425385731	0.0653079697253372	0.697508345809326	-4.1358191246654
a_HQ_transcript/151084	3.43065589558104	2.34195676659473	1.90744622736573	0.0653090441203193	0.697508345809326	-4.30095694322633
a_HQ_transcript/65300	1.83090729681587	1.00535164909841	1.90728829025635	0.0653301880710006	0.697508345809326	-4.13444713597042
a_HQ_transcript/55968	-1.11254442826633	1.9558863181777	-1.9072504019686	0.0653352612726961	0.697508345809326	-4.13465739195619
a_HQ_transcript/85255	2.18068284099296	0.403110768335067	1.90718396973263	0.0653441572994224	0.697508345809326	-4.19804237660022

a_HQ_transcript/47035	1.69498673190683	0.660523225015372	1.9071447550063	0.0653494090847369	0.697508345809326	-4.25530425306629
a_HQ_transcript/46118	2.87898148520352	1.17971122146005	1.90699831051566	0.0653690247076276	0.697527785887974	-4.17952546858421
a_HQ_transcript/125970	2.40102646384183	0.467678321541965	1.90683468041122	0.0653909482803383	0.697527785887974	-4.11877251697679
a_HQ_transcript/5658	-2.31671525447994	-0.153383133669694	-1.90682492625856	0.065392255366562	0.697527785887974	-4.19042159117856
a_HQ_transcript/138711	-1.61973993233201	1.86378364944973	-1.90677288185609	0.0653992298563422	0.697527785887974	-4.17849992667864
a_HQ_transcript/44625	1.97678267484497	0.25215272730394	1.90657605958508	0.0654256118857183	0.697681153493493	-4.29433281812915
a_HQ_transcript/112922	-1.51068839671603	-0.408939910599333	-1.9062514508999	0.0654691424472116	0.698017298637476	-4.18455904101606
a_HQ_transcript/90732	4.14589958870124	2.80667473464366	1.9061064212878	0.0654885992323825	0.69802672439553	-4.36988458550887
a_HQ_transcript/152363	2.28863970475674	1.12289828520412	1.90590788579023	0.0655152423125983	0.69802672439553	-4.24843225736349
a_HQ_transcript/2220	-0.912642412966409	2.58048602164746	-1.90589301079444	0.0655172388847923	0.69802672439553	-4.22745713684976
a_HQ_transcript/140364	-1.2860125701613	3.71704479222531	-1.90580383210318	0.0655292098507346	0.69802672439553	-4.38420095590998
a_HQ_transcript/133795	-2.48251375163551	0.462785373230853	-1.90571236409623	0.0655414900847475	0.69802672439553	-4.2526529662509
a_HQ_transcript/115752	2.3499609490664	1.50920222628192	1.90559739833865	0.0655569278755488	0.69802672439553	-4.24591543885285
a_HQ_transcript/116439	1.62361985888753	-0.0117070369430119	1.90554225803635	0.0655643333188962	0.69802672439553	-4.20074574607953
a_HQ_transcript/81706	-2.19712327814165	0.54794314928907	-1.90549833530223	0.0655702327386833	0.69802672439553	-4.14510723651753
a_HQ_transcript/51413	3.78272597537958	1.92077004206955	1.90543974818814	0.065578102496628	0.69802672439553	-4.42363245438529
a_HQ_transcript/45445	-2.45314948073521	0.805765277922468	-1.90508402548352	0.0656259027092665	0.698301412479417	-4.18632624928887
a_HQ_transcript/124200	-2.30919854786632	3.21208389771515	-1.90502524949852	0.0656338036204724	0.698301412479417	-4.4392719455705
a_HQ_transcript/96464	2.20979140972124	1.44379345934332	1.90497954220118	0.0656399483516986	0.698301412479417	-4.31234954126772
a_HQ_transcript/15389	2.00918259399801	1.86778657142681	1.90483208665088	0.0656597751494766	0.698384521480106	-4.15459524655389
a_HQ_transcript/96597	-1.97115766221645	0.223073135847474	-1.90462581484669	0.0656875190177181	0.698551793900826	-4.09292983346722
a_HQ_transcript/37910	2.38273877899587	3.2796540832058	1.90442151239505	0.0657150079714843	0.698716294744181	-4.33635226155979
a_HQ_transcript/98982	-3.44729625314738	2.83624413956404	-1.90402111754083	0.0657689099923747	0.699161521586824	-4.28990963409802
a_HQ_transcript/116286	6.10350674024633	5.17816192751468	1.90365941178443	0.0658176363816546	0.699287860474723	-4.38096132020437
a_HQ_transcript/8616	-1.54482881266697	2.03026061903155	-1.9036217915328	0.0658227060974719	0.699287860474723	-4.36450807909858
a_HQ_transcript/26160	3.19818978561972	1.2711104262072	1.90348602646911	0.0658410046368842	0.699287860474723	-4.26549852491726
a_HQ_transcript/5689	0.926178511215539	1.89524373866333	1.90346463442216	0.0658438882765702	0.699287860474723	-4.20965397292167
a_HQ_transcript/147004	3.22322099364339	0.785352828026235	1.90318672782277	0.0658813598784337	0.699287860474723	-4.24937548729849
a_HQ_transcript/217	-2.03237452769925	0.934852147000705	-1.90318630265966	0.0658814172194879	0.699287860474723	-4.15860962434255
a_HQ_transcript/128255	-3.0045346482547	0.957731332198651	-1.90311906925921	0.0658904854198925	0.699287860474723	-4.27796661855296
a_HQ_transcript/129158	1.03570425765602	1.98436068409307	1.90310122999625	0.0658928916965093	0.699287860474723	-4.16899937003591
a_HQ_transcript/66176	-4.69895507902585	2.39582766570239	-1.90299292109217	0.0659075027375779	0.699287860474723	-4.24177367934327
a_HQ_transcript/110207	1.67939197240628	1.09518206918586	1.90295171402855	0.0659130623687294	0.699287860474723	-4.1192492437052
a_HQ_transcript/19412	-1.2395706721033	1.0303099394083	-1.90292813002253	0.0659162444899765	0.699287860474723	-4.10922254254092
a_HQ_transcript/76546	2.19432937995352	1.26786160911847	1.90286208744242	0.0659251561278813	0.699287860474723	-4.26654194817868
a_HQ_transcript/111155	-3.03921415566079	0.499578371146977	-1.90264333362367	0.0659546816987936	0.699386452586399	-4.16401575574587
a_HQ_transcript/27550	1.28103283185311	5.20321347563069	1.90261494245652	0.065958514538237	0.699386452586399	-4.67806691752005
a_HQ_transcript/140732	4.15187468832753	0.753003721233565	1.90244889568576	0.0659809348854253	0.699455697429411	-4.23220977405432
a_HQ_transcript/5477	2.6685641399471	2.9007948421299	1.90217747935048	0.0660175968327435	0.699455697429411	-4.29103112262843
a_HQ_transcript/147699	0.595194874026706	7.55573090461681	1.90217243892518	0.0660182778414475	0.699455697429411	-4.88863606289429
a_HQ_transcript/61552	3.49789678880061	1.68573257571584	1.90214720010697	0.0660216879334624	0.699455697429411	-4.23210040282175
a_HQ_transcript/18785	-1.26144628495056	4.45753574572338	-1.902088076984	0.0660296768295795	0.699455697429411	-4.59894480536932
a_HQ_transcript/136025	2.52368150084746	0.998755588605106	1.90203208718449	0.0660372431099058	0.699455697429411	-4.16682377035409
a_HQ_transcript/63947	1.514153658464	-0.255769000503118	1.90188053948705	0.0660577265255351	0.699545186277338	-4.15645185938904
a_HQ_transcript/13509	2.05065189944543	0.159524488143822	1.90163657820174	0.0660907122085291	0.699681983530153	-4.28361659841972
a_HQ_transcript/136391	-3.23016797206736	-0.222359561996215	-1.90153459747592	0.0661045050960672	0.699681983530153	-4.23344857057745
a_HQ_transcript/47207	-3.1462524413868	2.12436364160173	-1.90151796419922	0.066106754981207	0.699681983530153	-4.33872894139291
a_HQ_transcript/103642	1.91673851704269	1.60027959348263	1.9012954504058	0.066136859458032	0.699873177967683	-4.13667704830759
a_HQ_transcript/56082	-4.64855389872822	1.23627783810195	-1.90103412046208	0.0661722305650974	0.699944833433548	-4.14047389185044
a_HQ_transcript/54375	1.82560278411131	-0.174937639043488	1.900877375257	0.0661934539115664	0.699944833433548	-4.14963686641876

a_HQ_transcript/150260	5.66418828984676	0.279236202577854	1.90086310294693	0.0661953866779653	0.699944833433548	-4.25332377450862
a_HQ_transcript/128110	2.71112166868424	0.195591018649407	1.90068115469713	0.0662200305033823	0.699944833433548	-4.21344209382775
a_HQ_transcript/476	2.64447118757257	1.25994194582345	1.90065004339111	0.0662242451393872	0.699944833433548	-4.39426500640184
a_HQ_transcript/52852	4.27655339778101	1.38254163729234	1.90062496772901	0.0662276422974581	0.699944833433548	-4.35136639389968
a_HQ_transcript/44255	-1.22229156537641	1.37101563333858	-1.90062291158946	0.0662279208623127	0.699944833433548	-4.14724039942245
a_HQ_transcript/22821	0.476478920096018	5.15501534986901	1.900478921361	0.0662474311054828	0.699945695967709	-4.64804756357719
a_HQ_transcript/117882	1.8537025908659	2.62190136199732	1.90044457707893	0.0662520853842267	0.699945695967709	-4.27979275641173
a_HQ_transcript/95042	0.925756177241081	4.51623396746067	1.90028106347592	0.0662742483357169	0.700052608987561	-4.53864499830578
a_HQ_transcript/47056	2.97590171798336	1.29828416794345	1.90012967281718	0.0662947738172984	0.70014218976234	-4.19035459128844
a_HQ_transcript/120917	2.13936525787857	0.924370654293236	1.89985235830477	0.0663323862137543	0.700271019669083	-4.16464067786582
a_HQ_transcript/99974	-1.0682918500876	-0.785363418402318	-1.89983648439089	0.0663345397622782	0.700271019669083	-4.183433989876
a_HQ_transcript/94494	0.747217824393124	3.46022605324345	1.89977329086508	0.0663431135651948	0.700271019669083	-4.39552165117091
a_HQ_transcript/148822	-1.29365472439487	2.70020191173479	-1.89941719825015	0.0663914444089121	0.700454476404859	-4.43409884068024
a_HQ_transcript/126232	1.45398973935725	-0.7726916240976	1.89923394652816	0.0664163281630055	0.700454476404859	-4.23537161533995
a_HQ_transcript/124183	2.2372262492958	-0.106567432446189	1.89917965592827	0.0664237018277204	0.700454476404859	-4.14086411293938
a_HQ_transcript/19996	-2.34193459794765	1.29126939428993	-1.89914980911929	0.0664277558753891	0.700454476404859	-4.19918556243829
a_HQ_transcript/119860	0.702304934262738	3.80789545975785	1.89911505139811	0.0664324772337307	0.700454476404859	-4.5448656675674
a_HQ_transcript/7387	2.32707358956323	1.99148648633924	1.89911270952469	0.0664327953554223	0.700454476404859	-4.16546201086818
a_HQ_transcript/151442	0.434262302031956	10.1447130126896	1.89880953223612	0.0664739902550854	0.700691997096711	-5.1774095748464
a_HQ_transcript/51082	-1.43795670404821	-0.474441384740978	-1.89867166337624	0.0664927307790939	0.700691997096711	-4.1769018700848
a_HQ_transcript/26678	1.86375058823383	0.0401149927928168	1.8985326219341	0.0665116353036776	0.700691997096711	-4.25758800423077
a_HQ_transcript/129544	0.423780211870261	3.71724225668883	1.89848513441358	0.0665180929217476	0.700691997096711	-4.53133475211766
a_HQ_transcript/58200	-1.63956355190134	-0.500959694110783	-1.89842002000236	0.0665269484211717	0.700691997096711	-4.21525299485419
a_HQ_transcript/97483	2.18749457556622	-0.65227035226776	1.89832171484499	0.0665403197560108	0.700691997096711	-4.23574535132679
a_HQ_transcript/91343	1.85029828592213	0.508917026594067	1.89831556384833	0.0665411564832612	0.700691997096711	-4.25085064258663
a_HQ_transcript/102990	2.1055989163688	1.58406719719416	1.89823764438864	0.0665517567432381	0.700691997096711	-4.1250528248183
a_HQ_transcript/55643	-1.9448275130801	0.0571160620531449	-1.89810082634839	0.0665703731601666	0.700761074077176	-4.30291171849022
a_HQ_transcript/14019	-0.612079599969037	3.06764121351853	-1.89795067600006	0.0665908088286056	0.700849273146693	-4.38170585517593
a_HQ_transcript/96184	2.61265321650717	0.439221260588465	1.89778769649231	0.0666129966814943	0.700889058173045	-4.19923585884928
a_HQ_transcript/76948	-2.56609070778541	1.16689199157399	-1.89754556090261	0.0666459725201683	0.700889058173045	-4.24520801971435
a_HQ_transcript/72954	-1.08723996669542	2.29155765001173	-1.89748035426047	0.0666548552529339	0.700889058173045	-4.30310739180359
a_HQ_transcript/142897	-5.62002105170076	2.73783894609257	-1.8974718449275	0.0666560145065756	0.700889058173045	-4.30911946718649
a_HQ_transcript/98883	2.88290299869457	-0.0116395030572285	1.8973396591715	0.0666740248259917	0.700889058173045	-4.22388081632394
a_HQ_transcript/21129	3.18482440841793	0.574592856697563	1.89723064648114	0.0666888809682163	0.700889058173045	-4.14679650251319
a_HQ_transcript/15531	-2.35784807831087	0.403860406529977	-1.89713258491942	0.0667022471381387	0.700889058173045	-4.14534224443828
a_HQ_transcript/24086	-1.3594526984145	3.21160868491415	-1.89698922557702	0.0667217917245342	0.700889058173045	-4.50270836817903
a_HQ_transcript/88622	1.55978256442739	0.0219502891583466	1.89696598011322	0.0667249613095162	0.700889058173045	-4.2759762796539
a_HQ_transcript/147879	-3.388016112524	0.195432724725776	-1.89688276221685	0.0667363093695104	0.700889058173045	-4.26486047641115
a_HQ_transcript/38029	-2.40777228518068	0.089342904514038	-1.89684235346896	0.0667418203331967	0.700889058173045	-4.14545297812107
a_HQ_transcript/142205	2.01471541208916	0.750137318999234	1.89670431513149	0.0667606490229688	0.700889058173045	-4.15705002555419
a_HQ_transcript/41126	2.05094184224812	1.31998152739272	1.89666571447675	0.0667659150438873	0.700889058173045	-4.14881986575467
a_HQ_transcript/25745	-3.11505646135721	0.708571749186362	-1.8966036890983	0.0667743774877403	0.700889058173045	-4.21217856026594
a_HQ_transcript/152273	3.23060788075663	4.03527471129475	1.89648523733318	0.0667905410435624	0.700889058173045	-4.40439639320136
a_HQ_transcript/98107	1.51312640597102	-0.658417942474009	1.89642909028748	0.0667982038706045	0.700889058173045	-4.23963779472423
a_HQ_transcript/121659	-0.881869671964655	2.25221381408061	-1.8964174208718	0.0667997965822186	0.700889058173045	-4.23591637047372
a_HQ_transcript/125175	-2.28429733993966	-0.390992475009373	-1.89628692726513	0.0668176093584965	0.700889058173045	-4.23726192042673
a_HQ_transcript/107584	-2.12913729582184	-0.481505184295761	-1.89598789234973	0.0668584439727959	0.700889058173045	-4.25818424400926
a_HQ_transcript/123296	-2.22138689727405	0.830465906470887	-1.89598089227797	0.0668594001227571	0.700889058173045	-4.18287164474963
a_HQ_transcript/112353	1.612819005846	-0.633491762483669	1.89594444221076	0.0668643790807113	0.700889058173045	-4.23525612447719
a_HQ_transcript/17961	-1.45617414212862	2.33422365683605	-1.89590034503031	0.0668704030358685	0.700889058173045	-4.2791341005101

a_HQ_transcript/148063	1.55644203542348	-0.52011437740051	1.89588927230422	0.0668719157140742	0.700889058173045	-4.24256745997571
a_HQ_transcript/116436	-1.95515288194201	-0.156377404505594	-1.89555032045728	0.0669182352037241	0.701248093730318	-4.17889813412897
a_HQ_transcript/78265	0.87127796708819	4.0044881446532	1.89533102214991	0.0669482181533284	0.701311278496733	-4.56550447941056
a_HQ_transcript/19343	-2.05773458801968	0.662391729840892	-1.89520825225643	0.0669650085768361	0.701311278496733	-4.16452303227391
a_HQ_transcript/116333	-2.9343909217423	-0.0947700770813938	-1.89509914215845	0.0669799338850644	0.701311278496733	-4.27463207313712
a_HQ_transcript/125213	3.0926854897675	0.223095260621976	1.89491832480854	0.0670046744280313	0.701311278496733	-4.23366957337677
a_HQ_transcript/7020	-1.9820706546746	0.729863683854108	-1.89478411578091	0.0670230428226031	0.701311278496733	-4.1914340929897
a_HQ_transcript/142766	2.58622644359914	1.36047100243138	1.8947225015707	0.0670314770471309	0.701311278496733	-4.14360758129672
a_HQ_transcript/88036	1.7299786136433	0.237961864136053	1.89456290573787	0.0670533279968298	0.701311278496733	-4.28075829046162
a_HQ_transcript/68976	1.64118968344256	1.44796495599854	1.89434752296959	0.0670828267016879	0.701311278496733	-4.20633910505108
a_HQ_transcript/12905	1.49530267746539	2.321320577972	1.8943072346532	0.0670883458079836	0.701311278496733	-4.17978345634715
a_HQ_transcript/121948	1.94984336131858	0.130456657211493	1.89425171158283	0.0670959525678259	0.701311278496733	-4.14360002411846
a_HQ_transcript/135426	2.20205291214362	1.30888200021859	1.8942485285415	0.0670963886726881	0.701311278496733	-4.26067617345722
a_HQ_transcript/143945	0.907114691219477	5.02029432062945	1.89420331919969	0.067102583016637	0.701311278496733	-4.64817995621107
a_HQ_transcript/115931	-0.936650469609789	5.32460358478234	-1.89412568047151	0.0671132218121063	0.701311278496733	-4.69645813048246
a_HQ_transcript/48415	1.52453160955707	1.29736005661618	1.8940900100478	0.0671181101991682	0.701311278496733	-4.14036178630953
a_HQ_transcript/93333	-1.7478220881032	1.22421044612566	-1.89406568730882	0.0671214436396633	0.701311278496733	-4.14696393341203
a_HQ_transcript/41279	-2.90579484445313	0.244954040300527	-1.89406433298182	0.0671216292548782	0.701311278496733	-4.23307001715338
a_HQ_transcript/134116	0.914838615814064	2.54669441912012	1.89398257089927	0.0671328358509312	0.701311278496733	-4.33673600558841
a_HQ_transcript/96234	2.18721629765875	0.491394987571579	1.89391984907065	0.0671414338156193	0.701311278496733	-4.22672200633309
a_HQ_transcript/15784	3.49359678434684	-0.099735848614086	1.89381818505974	0.0671553720240926	0.701330841720527	-4.20328476615805
a_HQ_transcript/30157	-0.745379051943904	2.79065915900073	-1.89322972816512	0.0672360988511781	0.702008932364144	-4.30405211740767
a_HQ_transcript/86327	0.544690203431054	3.3669622496494	1.89316884399636	0.0672444559490014	0.702008932364144	-4.4698208107473
a_HQ_transcript/114622	3.21229755709957	-0.531994289429636	1.89294868275237	0.0672746832421607	0.702198381666424	-4.2443481355328
a_HQ_transcript/120366	2.19912065766471	0.565590128698971	1.89264983222741	0.0673157329918588	0.702500705089905	-4.2236294885402
a_HQ_transcript/87299	-4.25246269729521	3.61458473995517	-1.89228439975122	0.06736595766864	0.702778281886512	-4.43984148731994
a_HQ_transcript/40768	2.13962334672121	0.954581983160491	1.89224672107485	0.0673711380229993	0.702778281886512	-4.156907580312
a_HQ_transcript/57810	-2.72297789703454	0.991148589812909	-1.89216853365576	0.0673818889254232	0.702778281886512	-4.23033982693447
a_HQ_transcript/91237	-3.23357108715652	0.296501417963508	-1.89210452024885	0.0673906919769374	0.702778281886512	-4.28028665852671
a_HQ_transcript/130042	2.92341141326226	0.888620508953663	1.89146624012115	0.0674785217154104	0.703295619954865	-4.25153204700748
a_HQ_transcript/7667	-0.690128853752076	2.98775476777926	-1.8913643755075	0.0674925477833373	0.703295619954865	-4.41677114892933
a_HQ_transcript/3645	-0.498419801628511	3.06065980534414	-1.89121663280313	0.0675128954141399	0.703295619954865	-4.38094992432446
a_HQ_transcript/77740	2.09671330163421	-0.414166002776908	1.89119666369248	0.0675156460334988	0.703295619954865	-4.23570174304529
a_HQ_transcript/61393	1.65765921987256	-0.725287489235654	1.89109049819804	0.0675302712824516	0.703295619954865	-4.24704332027022
a_HQ_transcript/28224	2.80339692295691	-0.212598202732268	1.89108106255845	0.0675315712583905	0.703295619954865	-4.29193750536455
a_HQ_transcript/107814	-1.4960906327197	1.93384712606588	-1.89101521308455	0.067540644134749	0.703295619954865	-4.24719232365883
a_HQ_transcript/121069	2.92130841789075	3.04622911635918	1.89089841693464	0.0675567391305275	0.703295619954865	-4.39923566315636
a_HQ_transcript/38473	0.742796199421417	5.41706047949323	1.89086673048578	0.0675611062244834	0.703295619954865	-4.70820079758924
a_HQ_transcript/6170	0.702943062954986	2.4963944356301	1.89070785675948	0.067583006204502	0.703295619954865	-4.30472482129127
a_HQ_transcript/59514	-2.60417155958314	0.0920637497022046	-1.89050975266801	0.0676103224626446	0.703295619954865	-4.33078527290891
a_HQ_transcript/30007	-0.663622569253686	3.438431120566	-1.89043837095995	0.0676201675019198	0.703295619954865	-4.42904622172517
a_HQ_transcript/88111	2.5686319167696	0.110250319956989	1.89036881291345	0.0676297622070465	0.703295619954865	-4.23889040897143
a_HQ_transcript/71583	3.34571060817482	1.0864853524687	1.89035880806162	0.0676311423537487	0.703295619954865	-4.14950167056901
a_HQ_transcript/33516	-1.61438474763232	2.19629026005027	-1.89029152767375	0.0676404241609717	0.703295619954865	-4.36381858720221
a_HQ_transcript/89189	1.98206241753967	0.137479462570103	1.89027244649441	0.0676430567447295	0.703295619954865	-4.13061008052111
a_HQ_transcript/151645	5.53419172010845	6.17718256842215	1.89022582661447	0.0676494891468133	0.703295619954865	-4.4816071465888
a_HQ_transcript/65276	2.75530868181632	1.28931857892991	1.89016353996934	0.0676580840006125	0.703295619954865	-4.20499535434254
a_HQ_transcript/109646	2.28167805584947	-0.0489373445168054	1.88983414285116	0.0677035527150782	0.703443785200439	-4.23619176056703
a_HQ_transcript/4472	1.46323433336256	1.8404693794822	1.88980789413857	0.0677071771178664	0.703443785200439	-4.19504605266868
a_HQ_transcript/94110	-1.77718185931285	-0.291441489510181	-1.88979728099939	0.0677086426196748	0.703443785200439	-4.25536261730656

a_HQ_transcript/113461	3.09480681652603	0.012254503174523	1.88931804392904	0.067774845917905	0.703685830818172	-4.25241220331339
a_HQ_transcript/140185	1.90934078862993	2.39420250571877	1.88926257218832	0.0677825125530534	0.703685830818172	-4.18855576147943
a_HQ_transcript/76207	2.04954429377664	1.82501868348436	1.88923199008683	0.0677867395615554	0.703685830818172	-4.21900533286722
a_HQ_transcript/151632	-2.39013107518457	0.279509953202152	-1.88890705449213	0.0678316656460001	0.703685830818172	-4.25322740856129
a_HQ_transcript/108856	5.18210815218671	1.31464813205769	1.8887286112045	0.0678563483822932	0.703685830818172	-4.37044361781261
a_HQ_transcript/130077	1.98001948060059	2.22835991409693	1.88872769489969	0.0678564751478859	0.703685830818172	-4.28912850165536
a_HQ_transcript/32302	2.34508437464004	0.546771730778201	1.88872070569757	0.0678574420712103	0.703685830818172	-4.14332706705807
a_HQ_transcript/69422	-1.13346678364184	1.73789714350332	-1.88867738445693	0.0678634356261579	0.703685830818172	-4.18137347168674
a_HQ_transcript/135580	2.13599802912249	0.513988339757563	1.88861368881777	0.0678722488353561	0.703685830818172	-4.18817419921142
a_HQ_transcript/15196	-0.876340223515914	3.50946595552153	-1.88850523744039	0.0678872569119565	0.703685830818172	-4.34347047804722
a_HQ_transcript/95898	1.56989746560015	-0.403373774379444	1.88838820208383	0.0679034560890208	0.703685830818172	-4.15651611261455
a_HQ_transcript/111371	0.903503102746433	-0.40296609153634	1.88823819788648	0.0679242234356214	0.703685830818172	-4.28347254982577
a_HQ_transcript/143434	-1.23931267302327	0.216861323637011	-1.88814483227953	0.0679371522062701	0.703685830818172	-4.18351379795268
a_HQ_transcript/33987	1.72985855780082	1.65843707918171	1.88812965954934	0.0679392534449427	0.703685830818172	-4.24848760395889
a_HQ_transcript/49455	-2.58702292019828	1.55584315811561	-1.8880379904053	0.0679519496966507	0.703685830818172	-4.20212116155257
a_HQ_transcript/35954	-2.00346261166269	1.64802628861551	-1.88803535397069	0.0679523148752118	0.703685830818172	-4.34089194860617
a_HQ_transcript/34757	-1.96686414027875	1.88759330384458	-1.88795689324807	0.0679631834215605	0.703685830818172	-4.40146089436713
a_HQ_transcript/6617	-0.512131343904723	3.24203410409604	-1.88773156742365	0.067994404351481	0.703685830818172	-4.44300562818773
a_HQ_transcript/23946	-1.67295326125272	-0.645314337225476	-1.8876354123212	0.0680077312667286	0.703685830818172	-4.19646956062153
a_HQ_transcript/113478	1.40761126441972	-0.583175137130011	1.88762370864279	0.0680093535278381	0.703685830818172	-4.23467656528581
a_HQ_transcript/108221	0.982416240260241	2.76600004413368	1.88760182326553	0.0680123871759695	0.703685830818172	-4.38398847352611
a_HQ_transcript/120627	-2.13326021276922	0.177308895776029	-1.88758630890738	0.0680145377739049	0.703685830818172	-4.1622081818702
a_HQ_transcript/99629	2.17479345850859	0.62609907010091	1.8873577775675	0.0680462235330057	0.703685830818172	-4.22070313822577
a_HQ_transcript/17640	-1.91993423070071	1.77618286099601	-1.88715102634197	0.068074900432399	0.703685830818172	-4.23289915109827
a_HQ_transcript/105513	-1.5177403562026	2.64079667987167	-1.88708821782783	0.0680836141856019	0.703685830818172	-4.41166669218251
a_HQ_transcript/93081	1.43930456792311	-0.391937224414307	1.88679590874461	0.0681241803788926	0.703685830818172	-4.2845964853631
a_HQ_transcript/97087	1.89831945021497	0.994445461027506	1.88678481862197	0.0681257198579883	0.703685830818172	-4.11841462158986
a_HQ_transcript/135017	-2.84051269364032	-0.00117346866317596	-1.88677985514916	0.0681264088738296	0.703685830818172	-4.29953393657597
a_HQ_transcript/44833	1.8019553092271	-0.0864862449972668	1.88673060892879	0.0681332454255	0.703685830818172	-4.25164228148419
a_HQ_transcript/18684	3.95689594036364	0.854652781995605	1.88672728818567	0.0681337064452063	0.703685830818172	-4.19207505923214
a_HQ_transcript/35551	1.1950836896417	0.3831575830765	1.88662714690299	0.0681476103486822	0.703685830818172	-4.15507491799453
a_HQ_transcript/109993	1.77195578195038	-0.564639615845047	1.88654329000464	0.0681592551593468	0.703685830818172	-4.24102079382036
a_HQ_transcript/53417	1.93431158588075	1.16314055821752	1.88608993067906	0.0682222406743513	0.703685830818172	-4.14954789710455
a_HQ_transcript/67874	3.19954910401883	0.324446257243787	1.88607636328577	0.0682241263728233	0.703685830818172	-4.29861956402621
a_HQ_transcript/50260	-2.19882077399763	1.29454456327552	-1.88600265357439	0.0682343718870617	0.703685830818172	-4.2237364317084
a_HQ_transcript/109357	1.01543827661544	-0.611091536041889	1.88589343084497	0.0682495560793136	0.703685830818172	-4.25091021860895
a_HQ_transcript/60490	-1.9420339275816	0.286492943847691	-1.88583354015094	0.0682578833440279	0.703685830818172	-4.25200083644248
a_HQ_transcript/147500	2.28108701704502	0.269495575095761	1.88577872809802	0.0682655052351758	0.703685830818172	-4.23289566679128
a_HQ_transcript/130897	-2.0846304994017	0.440989651430131	-1.8856854808976	0.06827847340881	0.703685830818172	-4.15969930577244
a_HQ_transcript/7816	1.23425220327903	2.83466725467202	1.88566858782611	0.0682808230067087	0.703685830818172	-4.31705248396949
a_HQ_transcript/45612	1.81562359605719	-0.22744228626999	1.88565227038347	0.0682830926084545	0.703685830818172	-4.25668879237795
a_HQ_transcript/124218	4.31721053314896	2.67310680736507	1.88564188512923	0.068284537132768	0.703685830818172	-4.32192532612985
a_HQ_transcript/119773	3.39692368147202	3.03150785866098	1.88554422981354	0.068298121666032	0.703685830818172	-4.36394025528086
a_HQ_transcript/116369	1.31201170158845	2.77837739364397	1.88547941880613	0.0683071386110746	0.703685830818172	-4.36142870025384
a_HQ_transcript/95779	2.28223379365644	1.52071891007593	1.88547219491366	0.06830814371121	0.703685830818172	-4.13623259250317
a_HQ_transcript/91330	-1.82879086210627	-0.310725947152405	-1.88539602328716	0.0683187426654799	0.703685830818172	-4.185731205528
a_HQ_transcript/33266	2.09036606476674	0.752081329298419	1.88533917619569	0.0683266536156324	0.703685830818172	-4.17029854317237
a_HQ_transcript/147241	4.04829755555874	5.83342024543679	1.8852205683278	0.0683431618509649	0.703685830818172	-4.63558966459388
a_HQ_transcript/34796	1.73310377129186	-0.285606822394245	1.88517467975741	0.068349549694374	0.703685830818172	-4.25173267874901
a_HQ_transcript/10560	-1.27360246987986	2.10047323872895	-1.88505212607292	0.0683666120970452	0.703685830818172	-4.15957635073067

a_HQ_transcript/98834	-1.13064088109748	2.56085144850944	-1.88497065716292	0.0683779565441167	0.703685830818172	-4.22699706947577
a_HQ_transcript/2296	1.29274942605581	-0.61851432582497	1.88468735300031	0.0684174189121944	0.703685830818172	-4.18004642772859
a_HQ_transcript/109434	1.74571998242718	-0.603047753050747	1.88461086660488	0.068428076311295	0.703685830818172	-4.25105584418321
a_HQ_transcript/41008	2.87608078914963	0.18665740174294	1.88453983068147	0.0684379755358143	0.703685830818172	-4.25445634694475
a_HQ_transcript/106601	0.81129716552073	2.82643084234102	1.88447703876021	0.0684467269414527	0.703685830818172	-4.29245614441636
a_HQ_transcript/100686	1.41852768325825	-0.376175085617226	1.88444342355168	0.0684514123397815	0.703685830818172	-4.19079240919215
a_HQ_transcript/131742	3.06065567850785	-0.42436529910639	1.88443558941919	0.0684525043265696	0.703685830818172	-4.25255065394954
a_HQ_transcript/943	-2.21528018466408	0.697031627719132	-1.88442827494898	0.0684535238920172	0.703685830818172	-4.25680435399328
a_HQ_transcript/125744	3.47311470731176	4.43029332890046	1.88438896490257	0.0684590035508767	0.703685830818172	-4.64896700493078
a_HQ_transcript/129256	-0.552485051689466	3.3507313232318	-1.88434461746027	0.0684651858548981	0.703685830818172	-4.48317584610842
a_HQ_transcript/42219	0.939573331577463	2.86008710616523	1.88416974927037	0.0684895682274566	0.703685830818172	-4.39442678430605
a_HQ_transcript/126480	-2.84755988354173	0.317144968067302	-1.88411135504858	0.0684977119642009	0.703685830818172	-4.27629765465767
a_HQ_transcript/3045	-0.84625566522703	5.25502632641612	-1.88406342448938	0.0685043970468075	0.703685830818172	-4.67905398574631
a_HQ_transcript/118322	2.87784919549825	0.19081060952832	1.88404683247914	0.0685067113370499	0.703685830818172	-4.2435717879507
a_HQ_transcript/9456	1.32469974568062	-0.624426768003963	1.88385771593109	0.0685330944824005	0.703832458670137	-4.26149962160254
a_HQ_transcript/13249	-0.515504978896691	2.75974475433054	-1.88356312934453	0.0685742088788982	0.704007267978842	-4.35193949999925
a_HQ_transcript/99601	1.57509326402315	-0.532285734282634	1.88356220047768	0.0685743385510632	0.704007267978842	-4.2552432104695
a_HQ_transcript/112655	-2.56481606422855	3.15711010825498	-1.88312317802358	0.0686356508059807	0.704353130423874	-4.3908693887312
a_HQ_transcript/97205	-2.78551978673084	2.95454819299974	-1.88308640145816	0.0686407890245564	0.704353130423874	-4.47442298747512
a_HQ_transcript/125510	2.26883922905414	2.2229370778088	1.88306070514989	0.0686443793671078	0.704353130423874	-4.32865499894078
a_HQ_transcript/112366	-1.66434596390173	1.02327560971892	-1.88281094623407	0.0686792846249974	0.70446372561469	-4.2125295767213
a_HQ_transcript/43646	-1.69780230623162	3.77811424823057	-1.88281014919127	0.0686793960407798	0.70446372561469	-4.41443932567956
a_HQ_transcript/130420	-2.49881104961656	1.70661807318198	-1.88254062526774	0.0687170807466543	0.704677894258069	-4.34294036732835
a_HQ_transcript/23231	2.37558149842508	0.278057383821876	1.88248742384522	0.0687245214448974	0.704677894258069	-4.15338827907986
a_HQ_transcript/148139	1.48314976780843	-0.709043793460923	1.8823778149457	0.0687398534209525	0.704710793589617	-4.26607735978513
a_HQ_transcript/137991	1.40528780133066	-0.785144228928464	1.88227048091263	0.0687548700411654	0.704740448907223	-4.26295067311249
a_HQ_transcript/87464	2.08820800613719	0.38130247273692	1.88214720233604	0.068772120870721	0.704762456572946	-4.1284512266593
a_HQ_transcript/30827	2.56858552070473	2.11888290736939	1.88208186153599	0.0687812657607061	0.704762456572946	-4.1827830850418
a_HQ_transcript/137969	-1.78325730017812	-0.17180320395452	-1.88184485206684	0.0688144456251605	0.704925475926015	-4.26622879667171
a_HQ_transcript/85699	-2.34375694099854	0.302194719295593	-1.88179030600722	0.0688220836849334	0.704925475926015	-4.33959531931668
a_HQ_transcript/101726	2.18930167010295	0.064961050796563	1.88167265662625	0.0688385605555214	0.704925475926015	-4.27257294731789
a_HQ_transcript/1792	-0.489829887555178	4.2077087601503	-1.88162179987834	0.0688456841252406	0.704925475926015	-4.5979102311148
a_HQ_transcript/118226	-1.37909246383469	2.0551536314725	-1.88148464514125	0.0688648987241528	0.7049818245539	-4.29990155844744
a_HQ_transcript/39684	1.72783079490335	-0.260121117782151	1.88140938977259	0.0688754435322323	0.7049818245539	-4.24802790927114
a_HQ_transcript/15848	1.80213957802711	1.33825192454817	1.88109580662151	0.068919397855889	0.705234192720078	-4.20351105592685
a_HQ_transcript/148989	3.67679210321285	0.674704587450603	1.88103111245581	0.0689284689079055	0.705234192720078	-4.23530443072224
a_HQ_transcript/80242	1.7097368387748	0.601060938925951	1.88097386450752	0.0689364967491653	0.705234192720078	-4.22911824672673
a_HQ_transcript/130811	1.63380739141528	-0.228989812568345	1.88075419908693	0.0689673077428127	0.705358704709324	-4.22335314410133
a_HQ_transcript/100789	0.881509236486304	4.06911435811943	1.88071407523316	0.068972936924852	0.705358704709324	-4.53738817145747
a_HQ_transcript/63142	1.26136852759267	3.20270475297448	1.8806264063732	0.0689852378149256	0.70536040522533	-4.31904201093371
a_HQ_transcript/140141	-2.53456823858229	-0.19613584041014	-1.88033243417183	0.0690264990620553	0.705538302227358	-4.25248049037556
a_HQ_transcript/122279	2.32146525537332	-0.161915920949282	1.88032949485123	0.0690269117252784	0.705538302227358	-4.19184605763886
a_HQ_transcript/103705	1.73006106778355	1.06333586825685	1.88008964896794	0.0690605918157213	0.705758453070367	-4.12765270611259
a_HQ_transcript/100505	3.52044475688214	2.16175000072361	1.87994170099491	0.0690813742879883	0.705846743647773	-4.26886333865047
a_HQ_transcript/21655	2.14404287879584	1.36949934953786	1.87974636301744	0.0691088219389796	0.706003093439193	-4.21526009389559
a_HQ_transcript/80801	1.58745999074644	-0.570657808664246	1.87964484559031	0.0691230902238245	0.706024773946665	-4.30938828234757
a_HQ_transcript/121338	3.51133556840339	0.411956013262446	1.87945284543905	0.0691500827878219	0.706119999146955	-4.20573758067189
a_HQ_transcript/121190	4.26401247793266	1.38521296942136	1.87917684382992	0.0691889006659312	0.706119999146955	-4.4193256830849
a_HQ_transcript/140378	-0.539431794842156	2.95720072275887	-1.8789747940448	0.0692173295604544	0.706119999146955	-4.39512046046457
a_HQ_transcript/149145	2.82751406730038	2.76837587643156	1.87895479323643	0.0692201442686662	0.706119999146955	-4.28866975664781

a_HQ_transcript/78246	1.59180997172361	-0.447775352752182	1.87889943301629	0.069227935610046	0.706119999146955	-4.2629516363665
a_HQ_transcript/93948	-0.910298534172768	2.2371638549821	-1.87883614830539	0.0692368431594801	0.706119999146955	-4.22735616252187
a_HQ_transcript/128159	3.33327018163967	2.91249869976496	1.87883211040875	0.0692374115413042	0.706119999146955	-4.46291528024772
a_HQ_transcript/2256	-1.15021008101562	1.97622288134099	-1.87880441850089	0.0692413096136902	0.706119999146955	-4.18065104273247
a_HQ_transcript/53005	3.4169096277205	1.00560316662937	1.87880134493581	0.0692417422780354	0.706119999146955	-4.25774530874432
a_HQ_transcript/135652	1.35165191766777	3.86391865032643	1.87869498255807	0.0692567162926081	0.706148816814019	-4.49658174115654
a_HQ_transcript/125967	1.68090268554348	1.13538304399258	1.87847275020925	0.069288011811255	0.706344010972401	-4.16853236433613
a_HQ_transcript/100109	-1.52696794154268	1.72725278868646	-1.8781687158215	0.0693308466583659	0.706493593746978	-4.23524702796351
a_HQ_transcript/143582	1.54420315502921	0.909947546765524	1.87808993751813	0.0693419493003708	0.706493593746978	-4.18711874162603
a_HQ_transcript/135970	-2.1957284010624	0.118650106503223	-1.87799475278716	0.0693553662250395	0.706493593746978	-4.18731234201197
a_HQ_transcript/142020	3.22693020395903	0.968720534112633	1.87797207559635	0.0693585630558335	0.706493593746978	-4.24100672743829
a_HQ_transcript/103851	3.33087008335968	0.940526436434151	1.87781816992658	0.0693802626687411	0.706493593746978	-4.2707912601847
a_HQ_transcript/152276	-2.87124144500723	1.20279203933125	-1.87757990315823	0.0694138681074341	0.706493593746978	-4.37380742008603
a_HQ_transcript/112742	0.894218908084468	2.49628585847689	1.87748463355022	0.0694273089641121	0.706493593746978	-4.27748823878229
a_HQ_transcript/108050	1.89855032601327	2.76577279373866	1.87747041405938	0.069429315274558	0.706493593746978	-4.27167094240197
a_HQ_transcript/128598	1.53553600825883	-0.703093168843842	1.87731162501873	0.0694517231259292	0.706493593746978	-4.26971261773802
a_HQ_transcript/144059	-2.04800639268829	1.6973750004311	-1.87717815008741	0.0694705635298898	0.706493593746978	-4.3437601601727
a_HQ_transcript/132224	2.6584272352136	0.988541775576674	1.8770624920158	0.0694868925794197	0.706493593746978	-4.14872970826803
a_HQ_transcript/84719	1.74819019193449	-0.339626677947393	1.87702721997976	0.0694918730767206	0.706493593746978	-4.21950126745149
a_HQ_transcript/148543	-2.51794720633708	3.69549414499738	-1.87702276225057	0.0694925025407569	0.706493593746978	-4.67400064171642
a_HQ_transcript/19512	2.4467554306872	0.192410359512197	1.87700971725945	0.0694943446172681	0.706493593746978	-4.18122372688517
a_HQ_transcript/135623	2.14611892372234	0.622558057116102	1.87699860840877	0.069495913325455	0.706493593746978	-4.30538145384075
a_HQ_transcript/95031	2.4508597889734	2.9518113202391	1.87698984689229	0.0694971505822534	0.706493593746978	-4.35475477251958
a_HQ_transcript/13166	-1.03078400812472	2.6928299341246	-1.87685383731591	0.0695163595902868	0.706510771004797	-4.26120861315074
a_HQ_transcript/100034	1.06950012731729	3.18488876394653	1.87672050059001	0.0695351955321558	0.706510771004797	-4.35680905284026
a_HQ_transcript/119474	0.624496175336254	4.02757955693307	1.87671973650494	0.0695353034840084	0.706510771004797	-4.60595156902774
a_HQ_transcript/18722	1.45535785953585	0.972203502867843	1.87602306444956	0.0696337909920265	0.707387801954651	-4.11066812641161
a_HQ_transcript/78547	-3.8032279946555	3.34333568459461	-1.87570021214396	0.0696794227697447	0.707524679751406	-4.34975031270631
a_HQ_transcript/94030	-1.72051546722855	1.91723058309016	-1.87558154204549	0.0696962703876177	0.707524679751406	-4.20091954229457
a_HQ_transcript/45031	1.28424587930802	1.98482106450834	1.87554090908938	0.069702022735856	0.707524679751406	-4.23379468742244
a_HQ_transcript/126428	2.49580075967918	3.21217679657026	1.87551861323828	0.0697051793002097	0.707524679751406	-4.54067593199091
a_HQ_transcript/48659	3.52146239797818	2.22811006547957	1.87549781353674	0.0697081241559369	0.707524679751406	-4.24215308228981
a_HQ_transcript/26459	2.32875918913503	0.296390091459316	1.87526057376296	0.0697417205125507	0.70759829843146	-4.15236837497529
a_HQ_transcript/105783	1.88418849269762	2.00901534245651	1.87523859957338	0.0697448330583063	0.70759829843146	-4.2774252043636
a_HQ_transcript/147167	-2.1551846399004	-0.150405948011478	-1.87518873442388	0.0697518966765116	0.70759829843146	-4.26561647577618
a_HQ_transcript/107624	4.52983388219173	4.28799541638697	1.87505381028005	0.069771012357006	0.707668715108714	-4.47920905995203
a_HQ_transcript/128129	1.8246831625017	-0.62442886603827	1.87476248005464	0.0698123025675936	0.707892112290317	-4.26574588430812
a_HQ_transcript/14839	1.78671602447636	3.04301077979833	1.87472656666165	0.0698173940228528	0.707892112290317	-4.39216097379592
a_HQ_transcript/65238	-1.57937334623705	1.51072999102254	-1.87460827834417	0.0698341660574769	0.707938682348975	-4.13934018808783
a_HQ_transcript/147116	2.36149016444993	0.70837897992849	1.87445549713849	0.0698558339407598	0.708034858815778	-4.28393377802739
a_HQ_transcript/121568	1.85955513174325	-0.137431607145365	1.87412880738647	0.0699021854241984	0.708381142666982	-4.24738679886133
a_HQ_transcript/88749	1.45635569844662	0.299649246541361	1.87402791763331	0.069916505228556	0.708402756828569	-4.19308685718912
a_HQ_transcript/42840	-1.0494819707815	3.6816316705554	-1.87384038061243	0.0699431300217299	0.708544127012701	-4.53535121731159
a_HQ_transcript/93612	2.62007910116933	0.639986772402859	1.87370731801609	0.0699620263140997	0.708544127012701	-4.34854744169149
a_HQ_transcript/48585	1.03289299795271	1.50451078916573	1.87367211653049	0.0699670260296743	0.708544127012701	-4.21185096358281
a_HQ_transcript/95979	3.86264873567411	2.68626529868902	1.87336138071935	0.0700111735762343	0.708764411762972	-4.37470231343577
a_HQ_transcript/136224	-1.78676519117482	-0.243260954344166	-1.87323907096519	0.0700285571983816	0.708764411762972	-4.29870854110841
a_HQ_transcript/68091	-1.719682083821	0.321059816151912	-1.8731166057367	0.0700459666312359	0.708764411762972	-4.19907713998996
a_HQ_transcript/135689	3.2665687840922	0.443379879491521	1.87303798685173	0.0700571449068002	0.708764411762972	-4.23495980644491
a_HQ_transcript/43409	1.94249828056224	0.300596102255686	1.87297319463461	0.0700663584154611	0.708764411762972	-4.22095862456321

a_HQ_transcript/145083	3.65125108992485	2.76664147888609	1.87293014015872	0.0700724813741408	0.708764411762972	-4.36527305568125
a_HQ_transcript/3193	-0.508599981134663	2.87189100389822	-1.87291854262245	0.0700741307872591	0.708764411762972	-4.37068877900376
a_HQ_transcript/33861	1.13078882165725	1.40118841410782	1.8725409851756	0.0701278455940595	0.709184309097336	-4.17302262416645
a_HQ_transcript/13871	1.65894031306235	2.72487367735945	1.87232755211133	0.0701582261905489	0.709344330615063	-4.31664950834038
a_HQ_transcript/101789	-1.70676495633936	-0.581946943304431	-1.87214000369817	0.0701849316282625	0.709344330615063	-4.25551863030488
a_HQ_transcript/23106	1.05073930498054	4.81101894557035	1.87212161862948	0.0701875499889475	0.709344330615063	-4.67228333526683
a_HQ_transcript/61086	-2.14597161158228	1.17274400390894	-1.87197795185835	0.0702080135801789	0.709344330615063	-4.23768277550227
a_HQ_transcript/6967	0.831797189206117	3.88011908818361	1.87190918829909	0.0702178099287326	0.709344330615063	-4.54612763027666
a_HQ_transcript/129577	0.836855184326865	3.5898993975102	1.87170490836636	0.070246919437286	0.709344330615063	-4.56429163476037
a_HQ_transcript/143176	2.1960177456145	-0.428790886495598	1.87165592037952	0.0702539016738178	0.709344330615063	-4.27043046216255
a_HQ_transcript/134996	0.995014075876434	1.71586845708846	1.87165354676006	0.0702542399999041	0.709344330615063	-4.17900936945737
a_HQ_transcript/84082	1.81096246797403	1.05255560489433	1.87162187345413	0.0702587547182816	0.709344330615063	-4.20936699625015
a_HQ_transcript/12953	2.86090173259934	0.528192947683098	1.87149972592986	0.0702761679796212	0.709344330615063	-4.19130927594503
a_HQ_transcript/64764	-3.81467699161636	5.42854414484349	-1.87148755026616	0.0702779039363499	0.709344330615063	-4.83192727453778
a_HQ_transcript/27792	0.89620050929547	2.36477910858433	1.87135541425521	0.0702967457204614	0.709394204733201	-4.27712891333256
a_HQ_transcript/147764	5.29454783443065	0.988630080087967	1.87128173936437	0.0703072531906821	0.709394204733201	-4.37028306550165
a_HQ_transcript/5674	-1.2843034593636	-0.774033476179821	-1.87105948638992	0.0703389589502162	0.709590941662299	-4.26045482603964
a_HQ_transcript/84931	0.7944140003587	3.11688894449296	1.87079794154831	0.0703762856575566	0.709844305518385	-4.44141302601418
a_HQ_transcript/129598	-1.03079694394515	2.25528031332803	-1.87053669126893	0.0704135872998928	0.710097328689828	-4.23829281355258
a_HQ_transcript/19688	1.08060121955665	3.50879119807249	1.87004916783757	0.0704832418932214	0.710415059988691	-4.44145208903679
a_HQ_transcript/38692	1.84684175049212	0.368839244954188	1.8700221033002	0.0704871104535732	0.710415059988691	-4.23275571690397
a_HQ_transcript/104966	-1.6763383813161	-0.236410765526742	-1.86996393714706	0.0704954252443513	0.710415059988691	-4.19283842900458
a_HQ_transcript/59174	2.46901320340001	1.43545983385261	1.86989799043302	0.0705048532764806	0.710415059988691	-4.37058961220337
a_HQ_transcript/131443	-2.13274332254883	3.92263000708611	-1.86986907603377	0.0705089873473698	0.710415059988691	-4.37193646418209
a_HQ_transcript/136333	0.890604516613758	5.5528826080712	1.86980308643474	0.0705184230686545	0.710415059988691	-4.7379105978274
a_HQ_transcript/84346	0.879748955952067	2.6058001558587	1.86952294576404	0.07055849189239	0.710695549596404	-4.39337735434106
a_HQ_transcript/138582	1.23694731953048	2.50280227741473	1.86932136370309	0.0705873364809304	0.710862906265336	-4.35276406081936
a_HQ_transcript/10767	0.571462261745222	3.71419135705439	1.86906301773797	0.0706243182716891	0.710981463872188	-4.54479994297653
a_HQ_transcript/92706	0.593280716268556	2.92131310194769	1.8689844222765	0.070635572378263	0.710981463872188	-4.42516290537794
a_HQ_transcript/115481	-0.787842328113009	2.95182276435839	-1.86898281214367	0.0706358029497296	0.710981463872188	-4.44700439593075
a_HQ_transcript/64474	2.27046770497796	-0.245818731950163	1.86881554937932	0.0706597585428212	0.711099453701023	-4.22001224065028
a_HQ_transcript/102130	0.441916828823266	4.24119998597757	1.86863249757851	0.0706859834526077	0.711108414666635	-4.64162155969637
a_HQ_transcript/55565	2.41398561822325	0.883836514927543	1.86860595338503	0.0706897869988889	0.711108414666635	-4.26427356064618
a_HQ_transcript/72683	1.70989053542214	-0.258369932061173	1.86855318023932	0.0706973494418952	0.711108414666635	-4.21966509714773
a_HQ_transcript/85010	-3.00528014452125	0.876337717048836	-1.86817575632771	0.0707514548955058	0.711529510409336	-4.20578586199447
a_HQ_transcript/25794	-1.18056263864442	3.27642348244511	-1.86801950763767	0.070773864261415	0.71162034153308	-4.46817668024095
a_HQ_transcript/127675	2.05956131148903	0.421581805978913	1.86794207948801	0.0707849713519176	0.71162034153308	-4.13182263894294
a_HQ_transcript/959	-2.58297898753543	1.73349549144148	-1.86781595507788	0.0708030671376702	0.711621756051572	-4.22102993306519
a_HQ_transcript/36108	-2.54401614032155	1.59356526915112	-1.86771891614301	0.070816992566802	0.711621756051572	-4.21388612846409
a_HQ_transcript/138116	-2.54884194036371	1.12901771047696	-1.86768514759568	0.0708218390235058	0.711621756051572	-4.35877327027386
a_HQ_transcript/84475	5.05714571579949	1.66518197520462	1.8673226081627	0.070873888558253	0.711753349540733	-4.35350930109589
a_HQ_transcript/564	-0.89603954485556	5.12975902353961	-1.86731183531865	0.0708754357099949	0.711753349540733	-4.70353257975293
a_HQ_transcript/85043	1.7686912672627	0.264038317620218	1.86713052327977	0.0709014793507853	0.711753349540733	-4.25018425291589
a_HQ_transcript/13640	-1.52586557927239	3.41209779600843	-1.86710747404828	0.070904790727961	0.711753349540733	-4.49781304860634
a_HQ_transcript/147202	1.9191648280127	2.0981952471871	1.86698679806227	0.070922129858366	0.711753349540733	-4.22343432228947
a_HQ_transcript/8476	0.799626146773876	3.41876051161205	1.86695793816367	0.0709262770843786	0.711753349540733	-4.52177254626126
a_HQ_transcript/978	-2.15714360078359	2.78560239662367	-1.86680777845088	0.0709478586976879	0.711753349540733	-4.45037873589715
a_HQ_transcript/84571	2.23069956466336	1.41727205825195	1.86679816449311	0.0709492406497999	0.711753349540733	-4.24025247187423
a_HQ_transcript/145366	-0.728488776696541	4.05793968182302	-1.86678585729741	0.0709510097733527	0.711753349540733	-4.47766824017731
a_HQ_transcript/5861	2.72571772827772	1.6681180218688	1.86674153487182	0.0709573813065743	0.711753349540733	-4.38725830155891

a_HQ_transcript/125878	1.0639995819132	-0.829318340103552	1.86657693845543	0.0709810470232699	0.711867891885548	-4.29282989654922
a_HQ_transcript/145708	0.589522160122338	2.718776954135	1.86623820698804	0.0710297712314095	0.712233621277208	-4.37915048109483
a_HQ_transcript/28538	-1.87266792467654	0.561701306816976	-1.86615308499567	0.0710420199588022	0.712233621277208	-4.24233526328508
a_HQ_transcript/23892	0.781184528919837	2.53270192288883	1.86605644381524	0.0710559284494036	0.712250217090349	-4.35547099046152
a_HQ_transcript/148210	-2.51435997380408	-0.0726701528624422	-1.86578906405052	0.0710944216183692	0.712278676176338	-4.27146723063642
a_HQ_transcript/122040	4.46775969145622	0.144066223044812	1.86575685058886	0.0710990604173865	0.712278676176338	-4.29484773436486
a_HQ_transcript/3930	0.695322240473935	5.96884583713672	1.86566901520851	0.0711117101963698	0.712278676176338	-4.80207988204277
a_HQ_transcript/121730	0.657598833354069	2.90552000637959	1.86560592477384	0.0711207974763566	0.712278676176338	-4.40083365010387
a_HQ_transcript/123200	1.54176808957596	-0.386913149537856	1.86539075970719	0.0711517964363871	0.712278676176338	-4.34999040664179
a_HQ_transcript/76270	2.16331321715967	1.15770972881341	1.86537713129517	0.0711537602800924	0.712278676176338	-4.19468615051596
a_HQ_transcript/114644	-0.780313104109444	2.98994218979982	-1.86524696431183	0.0711725195852343	0.712278676176338	-4.39816635896523
a_HQ_transcript/98674	-2.56504361866413	1.43642650018963	-1.86519653654243	0.071179788235695	0.712278676176338	-4.39046122987046
a_HQ_transcript/56601	2.20893843388111	0.324740163016275	1.86519101380806	0.0711805843204107	0.712278676176338	-4.22111296889873
a_HQ_transcript/22559	2.12372270785315	0.659717785194048	1.86518602224446	0.0711813038451064	0.712278676176338	-4.17417651124336
a_HQ_transcript/51482	2.72336554081263	1.33300154962241	1.86499501099748	0.0712088424518674	0.71243159966302	-4.25127495082454
a_HQ_transcript/56618	-3.89931186600012	2.97724693756884	-1.86471686891048	0.0712489592880763	0.712612430119384	-4.3362214703069
a_HQ_transcript/107287	-1.18608402440076	1.2712172105889	-1.8646997048581	0.0712514355190934	0.712612430119384	-4.21899401408974
a_HQ_transcript/140998	-2.13432488418311	1.98637309511075	-1.86442700538256	0.0712907873495884	0.712732501185717	-4.21548826895837
a_HQ_transcript/87280	-0.720916145372976	3.76373537879897	-1.8643957455127	0.0712952994879869	0.712732501185717	-4.4936439626385
a_HQ_transcript/38846	-2.81298283377377	0.646216427047986	-1.86436157284355	0.0713002323483277	0.712732501185717	-4.24872748348786
a_HQ_transcript/44944	-2.01048834055204	0.870453102439969	-1.86427668890339	0.0713124866999747	0.712732501185717	-4.28648371647738
a_HQ_transcript/58144	-1.35426170493003	-0.42486379572096	-1.86418791140444	0.0713253050813914	0.712738066661702	-4.26473791818644
a_HQ_transcript/150200	-2.42130400714788	-0.2099606169301	-1.86390195103653	0.0713666076836087	0.712949317505974	-4.28243436176704
a_HQ_transcript/27442	-2.37627738374323	-0.228644089849897	-1.86373744268493	0.0713903776918599	0.712949317505974	-4.28147444104607
a_HQ_transcript/132017	3.01248194007816	0.264997292863454	1.86367973184414	0.071398718008672	0.712949317505974	-4.28538713106401
a_HQ_transcript/144734	3.41952071308359	3.66632484675582	1.86366894942567	0.0714002763663562	0.712949317505974	-4.4426939931831
a_HQ_transcript/97394	3.8983562554522	1.49765820146496	1.86356955700571	0.0714146426936822	0.712949317505974	-4.27629925089319
a_HQ_transcript/101744	-4.18751335010255	4.21127350421152	-1.86333167410935	0.0714490367054412	0.712949317505974	-4.48205353140118
a_HQ_transcript/44285	-2.12604530561592	2.48537727322205	-1.86329139450884	0.0714548618888786	0.712949317505974	-4.25208146164093
a_HQ_transcript/148744	-1.3440669480973	-0.727629427237804	-1.86327429730854	0.071457334586862	0.712949317505974	-4.28006431830333
a_HQ_transcript/31905	1.13156013745405	3.93115491966586	1.86320750622687	0.0714669950103916	0.712949317505974	-4.61372334715746
a_HQ_transcript/25759	-0.686244859479531	2.19790122744736	-1.86304598386239	0.0714903616572779	0.712949317505974	-4.27283270104115
a_HQ_transcript/46688	1.763623947613	-0.295417126525946	1.86300048223766	0.0714969453363064	0.712949317505974	-4.24049035023427
a_HQ_transcript/95163	3.61913473372967	0.160477563598519	1.86298182801897	0.0714996445855294	0.712949317505974	-4.17585728942115
a_HQ_transcript/57923	1.91726398913618	1.60510789626953	1.86293865145959	0.0715058925313073	0.712949317505974	-4.25284043701605
a_HQ_transcript/122894	0.629239743376653	2.84636529919504	1.86274274967211	0.0715342467514715	0.712990759195891	-4.47622907293489
a_HQ_transcript/135129	1.16050899058576	2.87946205442747	1.86265551507901	0.0715468759191442	0.712990759195891	-4.36084201723012
a_HQ_transcript/6150	2.12897340665974	0.927171667083451	1.86250438745987	0.0715687595629633	0.712990759195891	-4.18889494670796
a_HQ_transcript/28837	1.06160744238138	1.88747637334957	1.86248816915761	0.071571108353204	0.712990759195891	-4.33053168838072
a_HQ_transcript/113061	0.56043909893431	3.57189901318733	1.86248630496732	0.0715713783358799	0.712990759195891	-4.54734910775719
a_HQ_transcript/48716	2.57232523625642	2.61149222409729	1.86238461086005	0.0715861075799563	0.713015078160412	-4.39167233787753
a_HQ_transcript/130493	3.05887638811943	0.433912274596567	1.86228391230215	0.0716006951907674	0.713015078160412	-4.32989651321429
a_HQ_transcript/9061	1.64881621182817	-0.141267177512099	1.86208783106031	0.0716291076460451	0.713015078160412	-4.32979848971014
a_HQ_transcript/71076	2.92025826998366	1.02157048577238	1.86205102245128	0.0716344423441379	0.713015078160412	-4.27932654098919
a_HQ_transcript/68487	1.72148943323671	3.01008464713537	1.86201492474315	0.071639674341678	0.713015078160412	-4.47950170429402
a_HQ_transcript/129767	-2.55598840618875	2.03898974855275	-1.86196150718053	0.0716474172779894	0.713015078160412	-4.41820907415882
a_HQ_transcript/46705	2.12677482011831	1.95431988880385	1.86181894894863	0.0716680847679287	0.713098670213995	-4.26753616680117
a_HQ_transcript/152759	-2.46352072691585	-0.313518101115085	-1.86153407902536	0.0717093993169368	0.713330768055415	-4.28801212964095
a_HQ_transcript/117585	2.29354028729685	-0.631319652344633	1.86148889236984	0.0717159545918636	0.713330768055415	-4.2964585041801
a_HQ_transcript/5060	1.50888053333997	-0.471753690783468	1.86136880772941	0.0717333778917569	0.713382000015748	-4.27367016520421

a_HQ_transcript/65406	2.7061229407655	3.10060583264447	1.86073418429794	0.0718255168387594	0.713934658069201	-4.34507059091371
a_HQ_transcript/110208	1.87454533990078	0.567218945856792	1.86072076072617	0.0718274668593231	0.713934658069201	-4.18731482861883
a_HQ_transcript/145654	3.44188630168006	3.83077085666528	1.86065290003607	0.0718373255666409	0.713934658069201	-4.60474125184721
a_HQ_transcript/5046	-0.726898437992299	2.43029577494635	-1.86047639090133	0.0718629740021162	0.713934658069201	-4.34227172206455
a_HQ_transcript/103262	0.64103641048725	2.77197676737304	1.8604352419872	0.0718689544540083	0.713934658069201	-4.41269526270676
a_HQ_transcript/149730	-3.11603972892767	3.87230559665635	-1.86030556832235	0.0718878036011978	0.713934658069201	-4.51352230718444
a_HQ_transcript/60302	3.90545117008635	1.63975119811426	1.85998746620262	0.0719340603409528	0.713934658069201	-4.28416511089246
a_HQ_transcript/59209	-0.91545291399284	2.83564818625177	-1.85994586057358	0.0719401122991439	0.713934658069201	-4.30205113593076
a_HQ_transcript/47219	2.53561866353057	0.750996332625519	1.85981622729203	0.0719589715650162	0.713934658069201	-4.29807406944068
a_HQ_transcript/142790	0.540794548609496	2.90726238688884	1.8597657658697	0.0719663139225118	0.713934658069201	-4.4266153311674
a_HQ_transcript/721	-3.08402851750101	1.99669926756955	-1.85972871007544	0.0719717061114048	0.713934658069201	-4.3968890186784
a_HQ_transcript/144357	-1.66854956889172	-0.625519595756999	-1.85962437235888	0.0719868907165805	0.713934658069201	-4.28128776467569
a_HQ_transcript/91737	2.1010014876912	1.418254363172	1.85948692586038	0.0720068979405398	0.713934658069201	-4.22829953697308
a_HQ_transcript/138346	0.628021088067251	3.41881729883972	1.85947640934466	0.0720084289599596	0.713934658069201	-4.50973309106494
a_HQ_transcript/2399	-1.70569717725451	0.121989188201551	-1.85943283903295	0.0720147723270026	0.713934658069201	-4.25416277783814
a_HQ_transcript/118960	2.71343687046054	0.629030334771253	1.85940956344053	0.0720181611982443	0.713934658069201	-4.27346849918289
a_HQ_transcript/59958	-4.35661594506349	5.77705682361566	-1.85939652001645	0.0720200603497142	0.713934658069201	-5.0188426062349
a_HQ_transcript/50611	2.29599809881905	3.4650987953194	1.85939381065936	0.0720204548434984	0.713934658069201	-4.51928991411709
a_HQ_transcript/161	-3.23982782686421	1.6961025034457	-1.85938106982839	0.0720223099868875	0.713934658069201	-4.20265100093511
a_HQ_transcript/18396	-1.44158966392813	1.60383383516286	-1.85878759494191	0.072108768976061	0.714669824900337	-4.1748568648894
a_HQ_transcript/96996	-2.48239127438875	0.268241601590012	-1.85866231693771	0.0721270311713662	0.714728958051342	-4.23166572412253
a_HQ_transcript/86939	-2.03615132695775	-0.185695272831703	-1.85833111771329	0.0721753302845222	0.714978945296281	-4.29629200277423
a_HQ_transcript/127685	5.11923101797161	1.13873986712951	1.85831902337883	0.072177094537746	0.714978945296281	-4.36510935758553
a_HQ_transcript/31937	1.4382459925178	1.55787361345677	1.85809412300789	0.0722099084672713	0.714978945296281	-4.2276255407281
a_HQ_transcript/139212	3.68835273205172	5.97295520303772	1.85804000142141	0.0722178069469792	0.714978945296281	-4.98231143201993
a_HQ_transcript/89014	1.3014299204519	1.9672111304046	1.85803166095146	0.072219024217198	0.714978945296281	-4.25627393023839
a_HQ_transcript/92728	2.29710638356828	1.74580772534216	1.85798346142637	0.072226059158749	0.714978945296281	-4.26768126629002
a_HQ_transcript/61367	-3.83485792036175	2.14949795384423	-1.85781561557801	0.0722505616121582	0.715099718268608	-4.42257348698976
a_HQ_transcript/28993	-1.40487544675573	2.25087510277327	-1.85768087451038	0.0722702365205382	0.715172677641445	-4.28486307240619
a_HQ_transcript/75859	-2.29126370949104	-0.145649496971647	-1.85745629444332	0.0723030399334309	0.715375507276676	-4.26446855430977
a_HQ_transcript/24663	-2.51678246876023	1.44103980898159	-1.85707902118804	0.0723581752776812	0.715701625742083	-4.38295317305329
a_HQ_transcript/148843	2.74561264331773	0.203056280854515	1.8570622590141	0.0723606257653149	0.715701625742083	-4.33760123656634
a_HQ_transcript/147803	-2.13703742596033	0.4381578257683	-1.85688863000843	0.0723860130357736	0.71583092305945	-4.31314735927639
a_HQ_transcript/42053	-1.83000868010786	0.603856410200292	-1.85677017957824	0.0724033367154533	0.715842147639252	-4.25176528848464
a_HQ_transcript/137903	1.32156719128802	-0.719108222277628	1.85671247161201	0.0724117779403868	0.715842147639252	-4.30441762891929
a_HQ_transcript/121869	-4.10802117216758	1.45571066290665	-1.85650142094065	0.0724426565289411	0.715919213680001	-4.27642589519987
a_HQ_transcript/32538	-1.10199874502968	1.25016300323983	-1.85649083156256	0.0724442061461906	0.715919213680001	-4.2184538334925
a_HQ_transcript/139629	0.41804961363965	5.21643964974978	1.85640124841914	0.0724573166060504	0.715927060968298	-4.74837786605167
a_HQ_transcript/106653	-1.37127902418994	1.9492514417347	-1.85628996580358	0.0724736056061849	0.71596630636919	-4.26256125466306
a_HQ_transcript/127590	2.04877111097423	-0.492672733763485	1.85599843459322	0.0725162933799538	0.716160349442386	-4.32912613827509
a_HQ_transcript/61723	1.65583787628152	0.354225758948767	1.8559777061364	0.0725193293866954	0.716160349442386	-4.20902441051896
a_HQ_transcript/63347	1.06605936678315	1.83514642963171	1.85590182169215	0.0725304447794663	0.716160349442386	-4.26164988165432
a_HQ_transcript/145399	-1.47430218091464	-0.72853177409745	-1.85581933300528	0.0725425292030823	0.716160349442386	-4.26457752655351
a_HQ_transcript/103764	0.798104277360968	3.79794375960675	1.85564153382887	0.0725685822830811	0.71617430669791	-4.54048854616195
a_HQ_transcript/50300	1.31874587136954	2.22801542886611	1.85564152027075	0.0725685842700711	0.71617430669791	-4.30247026051135
a_HQ_transcript/149904	4.78066048295425	0.58227621132899	1.85509369761165	0.0726489076341444	0.716845306901638	-4.35336963927878
a_HQ_transcript/117972	-2.85391049592536	0.47984709754711	-1.85465391464718	0.0727134449914093	0.717230649211439	-4.30913798442316
a_HQ_transcript/48620	-1.8704743491293	1.95412182326401	-1.8546400491222	0.0727154805303718	0.717230649211439	-4.35273228061231
a_HQ_transcript/22798	-1.20190735895824	3.32627738874051	-1.85457536815173	0.0727249767143583	0.717230649211439	-4.38843476901604
a_HQ_transcript/15583	-0.755777094038526	2.88385450031764	-1.85434192923894	0.0727592580530696	0.717368569268481	-4.43211016781445

a_HQ_transcript/4722	1.28174794981426	1.78600451707577	1.85431207155508	0.0727636437587215	0.717368569268481	-4.19952812407243
a_HQ_transcript/121331	1.39141179088142	1.97140382354981	1.85402309151069	0.0728061028711623	0.717511757969057	-4.33742062003629
a_HQ_transcript/57087	-0.596056021240241	2.97659616252514	-1.85392962917173	0.0728198395977988	0.717511757969057	-4.43082766311789
a_HQ_transcript/102897	1.96470909598667	-0.0918326337540164	1.85390048443194	0.0728241236308433	0.717511757969057	-4.38824312949568
a_HQ_transcript/115209	5.89192381307597	4.45961077410244	1.8538069673795	0.0728378713128881	0.717511757969057	-4.60826842964323
a_HQ_transcript/85868	-3.06932243717413	1.20267146346865	-1.85375006011353	0.0728462381795487	0.717511757969057	-4.31350497482869
a_HQ_transcript/32793	2.37197985693067	1.26524392514353	1.85364643774009	0.072861475510869	0.717511757969057	-4.29845392631974
a_HQ_transcript/10325	-2.22742217012261	0.538556325401385	-1.85361264670102	0.0728664449627836	0.717511757969057	-4.27070255977012
a_HQ_transcript/102057	1.59433320979492	3.43196988916268	1.8535414473501	0.0728769167879389	0.717511757969057	-4.48167380389453
a_HQ_transcript/96615	1.65154874078848	1.23345486043854	1.85345483804412	0.0728896568084868	0.717515659773704	-4.22518566369759
a_HQ_transcript/135031	2.9673630315727	0.981415740285497	1.85322179240576	0.0729239467310193	0.717661043557119	-4.29310703300284
a_HQ_transcript/40175	1.78548928057931	1.55402535370714	1.8531866533549	0.0729291182268769	0.717661043557119	-4.26615540877938
a_HQ_transcript/87138	-3.49864813738614	2.18438684308883	-1.85263869535152	0.0730098031181987	0.718253946897266	-4.48161030995981
a_HQ_transcript/110039	-3.2109719636762	0.585295436193716	-1.85221561426889	0.073072152595452	0.718253946897266	-4.35664365818698
a_HQ_transcript/105657	-1.9383594102414	2.26671026253742	-1.85208580012315	0.0730912924484833	0.718253946897266	-4.32179635811604
a_HQ_transcript/32742	0.756596646052049	2.16338003562406	1.85203485326423	0.0730988052477405	0.718253946897266	-4.36481095886984
a_HQ_transcript/113506	1.40995154939647	-0.547087164824586	1.85203259714089	0.0730991379587418	0.718253946897266	-4.33841915021524
a_HQ_transcript/21179	2.00485739051264	-0.381533507429556	1.85191809299902	0.0731160256122267	0.718253946897266	-4.29603956838626
a_HQ_transcript/38946	1.6175446088898	1.04495395205297	1.85184208305637	0.0731272377896808	0.718253946897266	-4.18520734505764
a_HQ_transcript/20177	-1.72742745757071	-0.529978410036488	-1.85169763685453	0.073148549011791	0.718253946897266	-4.28011689503475
a_HQ_transcript/74125	-1.96477917382316	0.205759475839591	-1.85169140037656	0.0731494692454702	0.718253946897266	-4.27904683853024
a_HQ_transcript/144423	0.549269129375647	2.96839325283945	1.85166428023874	0.0731534711168946	0.718253946897266	-4.46461764570282
a_HQ_transcript/150722	0.599709892376915	10.2053006740432	1.85165913752779	0.0731542300009907	0.718253946897266	-5.23893235447949
a_HQ_transcript/105628	2.59235982241871	0.911449772680461	1.85160256945217	0.0731625779133505	0.718253946897266	-4.37823832251025
a_HQ_transcript/41324	1.21281832085388	3.25551108324599	1.85153535999108	0.0731724972653255	0.718253946897266	-4.53569405783229
a_HQ_transcript/105398	-2.07987647454496	0.579906098373225	-1.85150290085135	0.0731772882773837	0.718253946897266	-4.27824196995237
a_HQ_transcript/22386	1.39905023092037	-0.359426581349044	1.85138161665648	0.073195192361643	0.718253946897266	-4.31229725592837
a_HQ_transcript/36970	-0.796154273877001	2.41351705833387	-1.85133868366496	0.0732015310678504	0.718253946897266	-4.27695002814111
a_HQ_transcript/48011	1.66895007969287	-0.441631004926946	1.85119975915792	0.0732220453591577	0.718253946897266	-4.34152820699672
a_HQ_transcript/62548	-2.4083086594398	0.782132978150693	-1.85099736196916	0.0732519411529495	0.718253946897266	-4.3990963140647
a_HQ_transcript/21926	-1.8745604277914	3.60600587712128	-1.85099413937705	0.0732524172418217	0.718253946897266	-4.55771260214567
a_HQ_transcript/90075	1.55977263222622	3.50107190032886	1.85095022135758	0.0732589057239114	0.718253946897266	-4.54168532997771
a_HQ_transcript/85500	-0.441483634121655	3.6416647491625	-1.85081820014449	0.0732784136020324	0.718253946897266	-4.58041644450333
a_HQ_transcript/34620	2.44446788567898	1.51428383953253	1.85062208011288	0.0733074011357498	0.718253946897266	-4.23521437491743
a_HQ_transcript/110894	0.593800779556447	3.82505988794504	1.85056812913374	0.0733153770847729	0.718253946897266	-4.59963577561628
a_HQ_transcript/107145	2.66756674171763	0.377256809440872	1.85054410621191	0.0733189287996196	0.718253946897266	-4.20514739715921
a_HQ_transcript/118634	0.726812467838248	3.39614303969894	1.85048442512232	0.0733277531019083	0.718253946897266	-4.46686909947532
a_HQ_transcript/141639	-2.08444482964106	0.581495536810327	-1.85044806312422	0.0733331299450647	0.718253946897266	-4.26355331956344
a_HQ_transcript/68333	-2.00150226395193	0.465383693609404	-1.85039185658805	0.0733414418610006	0.718253946897266	-4.37147945723109
a_HQ_transcript/77037	2.13229010292155	0.994459806793381	1.85036872306176	0.0733448631195217	0.718253946897266	-4.32413733890036
a_HQ_transcript/131990	-4.05425204604544	1.50474407524593	-1.85010027050071	0.0733845750390278	0.718253946897266	-4.29544446032622
a_HQ_transcript/66734	1.31329750671339	1.63330992166273	1.85009842142652	0.0733848486345281	0.718253946897266	-4.2565605230069
a_HQ_transcript/40331	2.49150413522553	0.857989635017703	1.85007155102413	0.0733888245719973	0.718253946897266	-4.27360258499867
a_HQ_transcript/146180	1.56187463694601	2.23657459092437	1.84999571148701	0.073400047328374	0.718253946897266	-4.3652460506005
a_HQ_transcript/33585	2.24025476141064	-0.0657842708927099	1.84995381433585	0.0734062479105974	0.718253946897266	-4.25633542401022
a_HQ_transcript/83979	1.70398941975171	-0.261497470923843	1.84985910637005	0.073420265898161	0.718253946897266	-4.3480208446536
a_HQ_transcript/97929	-1.57643118203922	-0.22956092993361	-1.84982230082577	0.0734257142065372	0.718253946897266	-4.29157823181313
a_HQ_transcript/96100	1.81777228520588	-0.565261511842341	1.84976497713837	0.0734342004956381	0.718253946897266	-4.31380588847563
a_HQ_transcript/98630	0.575394395721097	3.03110529226747	1.84925158947616	0.0735102406435198	0.718876727477543	-4.49873055825649
a_HQ_transcript/30391	-0.695069516199102	3.34304212540394	-1.84890387502866	0.0735617804529306	0.719136640204578	-4.51367442930695

a_HQ_transcript/134932	-1.19332713724656	4.58990836978397	-1.84882391413094	0.0735736369911495	0.719136640204578	-4.68077714288924
a_HQ_transcript/22107	-1.64003391274846	1.09564104805572	-1.84878737210252	0.0735790559583965	0.719136640204578	-4.21426257174538
a_HQ_transcript/20702	1.24657178117717	2.91578415262376	1.8487384929587	0.0735863049810217	0.719136640204578	-4.42465711643363
a_HQ_transcript/46346	1.66735145290257	1.92812107250625	1.84838689245837	0.0736384671119804	0.719290347984667	-4.25779692271288
a_HQ_transcript/144604	3.31825457476488	0.595680605415644	1.84827813054685	0.0736546090266551	0.719290347984667	-4.39405312376822
a_HQ_transcript/92116	-0.556962309098222	4.98224695119248	-1.84815935312803	0.0736722408527153	0.719290347984667	-4.72931415160607
a_HQ_transcript/146993	-0.807157644599447	3.69343408766778	-1.84814149661683	0.0736748918615509	0.719290347984667	-4.58191978979251
a_HQ_transcript/32957	-2.44517677289213	1.65111231667342	-1.84808417815636	0.0736834020117791	0.719290347984667	-4.28721776789171
a_HQ_transcript/21687	0.67791322503415	3.44903170808204	1.84801892615057	0.073693091090042	0.719290347984667	-4.58472763519028
a_HQ_transcript/144269	1.40336620427199	-0.682809953415057	1.84799185821198	0.0736971106484361	0.719290347984667	-4.29264940522514
a_HQ_transcript/44880	-1.92159193714515	-0.00829268312730107	-1.84796548473511	0.0737010272604713	0.719290347984667	-4.3506451953658
a_HQ_transcript/119685	3.65962443451733	2.25480881806429	1.8478298946514	0.0737211659742097	0.719366113102041	-4.37858230469505
a_HQ_transcript/15034	-2.77702447212601	0.957685591812828	-1.84772848551199	0.0737362309908437	0.719386595512493	-4.35591323162175
a_HQ_transcript/48119	-1.42467280625334	1.55106430409477	-1.8476491622491	0.0737480168362742	0.719386595512493	-4.30511071478596
a_HQ_transcript/78750	2.22613959698715	0.689477855901712	1.8473959759631	0.0737856460211848	0.719632891261649	-4.31469277712274
a_HQ_transcript/141387	2.17816704279147	1.32262447464168	1.84722886945142	0.0738104908099944	0.719754438819553	-4.32956425149498
a_HQ_transcript/99956	2.61639292428268	0.665521691371788	1.84672155039718	0.0738859610113176	0.72029881815356	-4.26187285324846
a_HQ_transcript/145122	-2.62579483713391	0.393677627272771	-1.84668702261047	0.0738910998597866	0.72029881815356	-4.34766232264441
a_HQ_transcript/12132	-1.7043131710434	1.52602719388088	-1.84651100924698	0.0739173010782521	0.720433413326063	-4.31664456287756
a_HQ_transcript/589	-1.76690672957661	0.37771131946577	-1.84630981356013	0.0739472606523784	0.72047381791928	-4.1989812767671
a_HQ_transcript/63008	-0.744558436420251	2.14369006849829	-1.84622605200184	0.0739597364499037	0.72047381791928	-4.32455053348861
a_HQ_transcript/53054	1.70876812453976	-0.101379060047665	1.84621578900917	0.0739612651868463	0.72047381791928	-4.2904483874177
a_HQ_transcript/148923	4.24493420199495	0.890439685132065	1.84615027146883	0.073971025071261	0.72047381791928	-4.42492812906881
a_HQ_transcript/37814	-2.1794738036071	0.054095128696102	-1.84594549847971	0.0740015363820557	0.720554593276229	-4.27956676695498
a_HQ_transcript/53551	1.20065846780163	-0.480723763182648	1.84585398786034	0.0740151750045984	0.720554593276229	-4.32203516263171
a_HQ_transcript/64116	1.86628547525469	-0.562460172572017	1.84583544203958	0.0740179393113619	0.720554593276229	-4.30795001266263
a_HQ_transcript/99392	1.49636527003077	-0.583888324918495	1.8457618970514	0.074028902268195	0.720554593276229	-4.31981821508704
a_HQ_transcript/86202	1.90965355219681	1.5562793313874	1.84547775062393	0.0740712715008558	0.720846286589946	-4.33084071549166
a_HQ_transcript/77418	2.23951216864804	-0.271991566696504	1.84539333713108	0.0740838624391122	0.720848134559879	-4.30316571515876
a_HQ_transcript/128348	1.9328682717658	1.70051219116998	1.84530745386229	0.0740966744845449	0.720852132960272	-4.22181356736002
a_HQ_transcript/136281	0.649553407406596	2.74620952165587	1.84514983006396	0.0741201936954623	0.720932287215558	-4.43267888650673
a_HQ_transcript/31121	-1.90559600893446	-0.0612348181357098	-1.84508600735522	0.0741297185639862	0.720932287215558	-4.30483832602643
a_HQ_transcript/52342	1.68631693668064	0.454020680367408	1.84494000884497	0.0741515112434789	0.721023594105209	-4.22198153486576
a_HQ_transcript/80419	-4.59947421542351	2.28190808104558	-1.84478734419595	0.0741743048134812	0.721124601136985	-4.4631500548841
a_HQ_transcript/110396	0.980457016530647	4.65447855673837	1.84452430812968	0.074213591425194	0.721361425635673	-4.7287890883759
a_HQ_transcript/53152	3.7082994116382	1.61683394044111	1.84438014147538	0.0742351314552552	0.721361425635673	-4.3793917559456
a_HQ_transcript/65771	2.85008220627732	0.149093645737371	1.84437503824809	0.0742358940296001	0.721361425635673	-4.3213119830479
a_HQ_transcript/124281	0.818289229214768	2.65949528795615	1.84412451629446	0.0742733377172873	0.721604642291573	-4.49203247004433
a_HQ_transcript/55112	0.756069891151266	2.87237912613843	1.84398464917173	0.0742942496593218	0.721637565878321	-4.45267997814721
a_HQ_transcript/117269	0.591421442664963	4.82527161529801	1.8438540371121	0.0743137823954924	0.721637565878321	-4.75090045832501
a_HQ_transcript/13327	-0.69003923869376	2.92815010924844	-1.84380976348607	0.0743204044118803	0.721637565878321	-4.44497603421242
a_HQ_transcript/26018	-1.78225802115106	-0.428602179998364	-1.8437697812764	0.0743263849936951	0.721637565878321	-4.28973419820936
a_HQ_transcript/65086	-1.66921743996945	-0.430862311064791	-1.84351087910828	0.0743651218182196	0.721893086347606	-4.31775400626041
a_HQ_transcript/130938	-0.615460921440513	3.03126077389764	-1.84335623659266	0.0743882675943482	0.721997197983682	-4.45522590167787
a_HQ_transcript/105568	2.07117168075558	0.694957219826494	1.84323247103526	0.0744067963672915	0.722056470657416	-4.29003054322775
a_HQ_transcript/137786	-1.57616388596723	3.0230995802081	-1.84311240679893	0.0744247747910017	0.72211038375085	-4.53488675589235
a_HQ_transcript/115738	-0.413709452964354	4.66095141707458	-1.8429389398922	0.0744507562919193	0.722208529715933	-4.71264848657776
a_HQ_transcript/136117	1.17167689818708	-0.665237346386542	1.84285865624528	0.0744627836289123	0.722208529715933	-4.31866626753226
a_HQ_transcript/16260	-1.77485725068388	1.24390420851327	-1.84266614296399	0.0744916309162184	0.722208529715933	-4.27468611497644
a_HQ_transcript/133457	1.53862043454149	1.88423556447053	1.84259185719508	0.0745027648735867	0.722208529715933	-4.28585523047931

a_HQ_transcript/51438	3.69875163633013	2.28766283233763	1.84254637194555	0.0745095829088761	0.722208529715933	-4.38166732830402
a_HQ_transcript/108155	3.69744628135761	1.50858660807382	1.84253834483956	0.0745107861915015	0.722208529715933	-4.33771408108323
a_HQ_transcript/106871	1.54574455243472	2.98335143167313	1.84241899305605	0.0745286792727604	0.722208529715933	-4.36608625815181
a_HQ_transcript/148209	3.35003854038362	1.62041905022058	1.84238160046555	0.0745342858822923	0.722208529715933	-4.32327630206036
a_HQ_transcript/138699	2.57844624910777	0.258870208012418	1.84217445220603	0.0745653520275767	0.722389130443163	-4.23282198402011
a_HQ_transcript/140827	1.77464930531058	1.54834165091336	1.84203914489567	0.074585650119665	0.722398797650544	-4.24845642990127
a_HQ_transcript/29282	1.07300892937504	2.1602464456326	1.84195204659708	0.0745987186476634	0.722398797650544	-4.3249749518162
a_HQ_transcript/131802	-3.7731933416789	2.10242984237806	-1.84191929673199	0.0746036330562933	0.722398797650544	-4.46021558249152
a_HQ_transcript/139266	-1.88362451893392	-0.119501721708087	-1.84178065250591	0.0746244409217361	0.722479930362871	-4.3480659861352
a_HQ_transcript/38911	2.52114798243223	3.19092240212768	1.84162223485706	0.0746482224748297	0.722514505183589	-4.59403501653707
a_HQ_transcript/8684	2.2855595671891	0.94490103720069	1.84159127062587	0.0746528715615991	0.722514505183589	-4.26715988184419
a_HQ_transcript/85465	1.05384258810625	0.807507176090597	1.8414285104059	0.0746773130662913	0.722630739789809	-4.24504686721921
a_HQ_transcript/18321	3.09875573974986	2.40935754449594	1.84105580022494	0.0747333082678159	0.722749608236612	-4.40994156956608
a_HQ_transcript/69356	0.61409062045614	2.37311291917174	1.84105487805214	0.074733446857631	0.722749608236612	-4.3556617261369
a_HQ_transcript/106880	-0.583887189738763	3.76925136816079	-1.84094781501784	0.0747495384438587	0.722749608236612	-4.5630981297163
a_HQ_transcript/90585	1.82420449880653	-0.106552913184427	1.8407957859751	0.0747723935143859	0.722749608236612	-4.2895977386647
a_HQ_transcript/25316	1.29825109390972	1.77784312823036	1.8406974419931	0.0747871810985512	0.722749608236612	-4.25393648308817
a_HQ_transcript/117159	-2.09831307520613	1.85489516703671	-1.84068676343072	0.0747887869409224	0.722749608236612	-4.40570982563134
a_HQ_transcript/22071	-2.38710111910932	3.98085173496663	-1.84066469964942	0.0747921049860358	0.722749608236612	-4.49824900945263
a_HQ_transcript/11598	-1.62782079397249	-0.731927929568291	-1.84061046724374	0.0748002612201498	0.722749608236612	-4.30126507806738
a_HQ_transcript/38458	-2.28322857697267	0.137728962812619	-1.84043989509276	0.0748259192216619	0.722749608236612	-4.3984768065594
a_HQ_transcript/7655	0.906872417605311	2.5402357101061	1.84036401309312	0.0748373360488075	0.722749608236612	-4.39533350816464
a_HQ_transcript/49089	6.25571298830885	1.74915523057457	1.84025758192345	0.0748533516600201	0.722749608236612	-4.39046482761105
a_HQ_transcript/28699	-2.38016422944534	0.857035823384998	-1.8401040829383	0.0748764551277227	0.722749608236612	-4.27606995071736
a_HQ_transcript/136861	6.32522754509567	2.23727580173221	1.84006909351708	0.0748817223147511	0.722749608236612	-4.43550260635165
a_HQ_transcript/124460	-2.60363795185083	3.84963964703177	-1.84001540437582	0.0748898051089598	0.722749608236612	-4.60923300215074
a_HQ_transcript/77051	-1.97598852080251	-0.353248709313288	-1.83992210778791	0.0749038524995698	0.722749608236612	-4.36285681723607
a_HQ_transcript/120459	0.514028036319002	3.17380570539389	1.83990808848889	0.074905963538441	0.722749608236612	-4.49939134599785
a_HQ_transcript/102122	-1.74832011217218	1.80077347027081	-1.83965291687295	0.0749443963993155	0.722749608236612	-4.32544011304091
a_HQ_transcript/58891	-1.69711860515664	0.220121989189751	-1.83964067483825	0.0749462406659131	0.722749608236612	-4.18592619835838
a_HQ_transcript/103897	-1.85694945735451	1.64380604196151	-1.8394177603004	0.0749798295982427	0.722749608236612	-4.35953128131831
a_HQ_transcript/40689	1.4511174284635	0.221930302103259	1.83938659392664	0.0749845267959783	0.722749608236612	-4.22803770791092
a_HQ_transcript/53317	-2.37068668799986	0.318517429443706	-1.83935893881127	0.0749886950097145	0.722749608236612	-4.27534418780927
a_HQ_transcript/131181	-3.07864583230186	-0.242215215618569	-1.83918914536981	0.0750142908301583	0.722749608236612	-4.28033773892509
a_HQ_transcript/54659	1.2224718501369	-0.682696615120734	1.83918038013518	0.0750156123639663	0.722749608236612	-4.32701950229314
a_HQ_transcript/46331	-2.25833418616769	-0.419608688328112	-1.83915283294211	0.0750197657818332	0.722749608236612	-4.3269613061199
a_HQ_transcript/14656	1.43633744451934	-0.516670524434862	1.83913305718979	0.075022747584751	0.722749608236612	-4.27150267430577
a_HQ_transcript/138500	-2.36926219595507	0.647481953712932	-1.83911176804336	0.0750259576915687	0.722749608236612	-4.35719581128061
a_HQ_transcript/12626	-1.54538021423412	0.372512460058695	-1.83908304123102	0.0750302894808616	0.722749608236612	-4.20027739587183
a_HQ_transcript/137117	-1.9003922173953	-0.0172667612181004	-1.83893550879166	0.0750525396407954	0.722749608236612	-4.3083638059816
a_HQ_transcript/3679	-2.37653413177942	0.702827163399797	-1.83888949646278	0.0750594801606018	0.722749608236612	-4.29328471685788
a_HQ_transcript/149949	2.17573695473872	0.576385327014646	1.83886874935697	0.0750626098424929	0.722749608236612	-4.21090417443293
a_HQ_transcript/126157	1.64169015130847	4.13919910166533	1.83868665781191	0.0750900829691371	0.722846998868133	-4.62207986194179
a_HQ_transcript/59041	1.51413556472393	1.8810999886369	1.83863687533753	0.075097595412962	0.722846998868133	-4.31599396183245
a_HQ_transcript/134469	-0.94815250017551	4.81749870681815	-1.83845194411952	0.0751255081546737	0.722995950002462	-4.65307815093014
a_HQ_transcript/15011	1.7387085507773	0.506960292532472	1.83819513320219	0.0751642848174732	0.723249387166046	-4.32037862531817
a_HQ_transcript/28915	2.70101022832093	0.522528498529304	1.83795107325487	0.0752011520182597	0.72338801044585	-4.31021324347119
a_HQ_transcript/85745	1.94358003757556	1.82736610251856	1.83793499809842	0.0752035808409763	0.72338801044585	-4.32326379769138
a_HQ_transcript/68494	-1.07376981049863	1.41672094557662	-1.83780767815941	0.0752228201945075	0.723453357423285	-4.24146175746205
a_HQ_transcript/98574	0.513502654591447	3.35591762830601	1.83752079548081	0.0752661865325405	0.723668784441341	-4.55205398822493

a_HQ_transcript/70236	-0.788625790392077	1.74474196775053	-1.83737604458021	0.0752880757655613	0.723668784441341	-4.28258624589801
a_HQ_transcript/123986	3.8582606930137	4.83666291834072	1.83732663471101	0.0752955487714233	0.723668784441341	-4.73300659269955
a_HQ_transcript/80054	-3.18165010569418	0.504122682919878	-1.83728110741958	0.075302435116905	0.723668784441341	-4.39025210383029
a_HQ_transcript/99538	-3.40173260590127	0.37541239995209	-1.83720027267369	0.0753146633062353	0.723668784441341	-4.32837904295155
a_HQ_transcript/131079	-1.55779144811121	2.94293071368904	-1.83716554525178	0.0753199171805346	0.723668784441341	-4.53163707037436
a_HQ_transcript/83066	1.37642216201187	1.58582217219875	1.83666068342976	0.0753963325484761	0.724283262002615	-4.25723383268207
a_HQ_transcript/107132	-0.660251842940802	2.84245022960266	-1.83630532412663	0.0754501590686434	0.724344822016574	-4.41743911063316
a_HQ_transcript/64112	2.58709264944427	0.25723832732621	1.83627125945043	0.0754553205922625	0.724344822016574	-4.26463615259243
a_HQ_transcript/137906	2.27156497988268	0.875260001061074	1.83621135780056	0.0754643976988489	0.724344822016574	-4.29462572461825
a_HQ_transcript/119801	0.802483536338575	3.18307696721542	1.83611064038098	0.075479661864151	0.724344822016574	-4.47184226062607
a_HQ_transcript/56751	1.5360390506735	1.07504290424748	1.83608980850714	0.0754828193548031	0.724344822016574	-4.21114471876459
a_HQ_transcript/23323	-1.85377202675362	0.228352047537798	-1.83594322003861	0.0755050409838419	0.724344822016574	-4.29976404111634
a_HQ_transcript/121708	1.48563204180516	0.116699575043748	1.83594101827521	0.0755053747959923	0.724344822016574	-4.28664324855752
a_HQ_transcript/17037	-0.705061311561847	4.32433481700671	-1.83592280509511	0.0755081361677419	0.724344822016574	-4.69594304720626
a_HQ_transcript/61425	2.02916091978171	3.12255018115699	1.83574900607879	0.075534490857697	0.724344822016574	-4.47523748450614
a_HQ_transcript/50579	-1.37980928020767	0.252252022796872	-1.8355656928011	0.075562296791997	0.724344822016574	-4.25612476616444
a_HQ_transcript/108258	1.34627784329393	4.49571345401842	1.83547857968985	0.0755755136365017	0.724344822016574	-4.70124868999778
a_HQ_transcript/12688	-2.35882466245268	1.49302288135061	-1.83547772819179	0.0755756428359526	0.724344822016574	-4.43751826290894
a_HQ_transcript/45236	1.15476636620906	-0.434431147189672	1.83536234460423	0.0755931519559546	0.724344822016574	-4.25797301208309
a_HQ_transcript/148473	-0.626844276892739	3.87776261911603	-1.83533542873002	0.0755972368589515	0.724344822016574	-4.58327472606778
a_HQ_transcript/101491	-0.518508630658429	3.65255413343097	-1.83530440894065	0.0756019448287804	0.724344822016574	-4.62467664727783
a_HQ_transcript/25278	1.4266195757435	1.71600788202821	1.83530325437504	0.0756021200656233	0.724344822016574	-4.35657522932802
a_HQ_transcript/110047	-1.86905000751323	3.93898390360642	-1.83487809075813	0.0756666738350911	0.724843839269311	-4.64795700571308
a_HQ_transcript/141289	-2.95204654001876	1.6989559393321	-1.83457101513574	0.0757133272460751	0.725066380138801	-4.52811773253142
a_HQ_transcript/138939	1.74952488959474	0.712871385688222	1.83454461511789	0.0757173392965484	0.725066380138801	-4.20631070360005
a_HQ_transcript/124153	-2.20159974415513	0.78502195599118	-1.83447890733686	0.0757273257951876	0.725066380138801	-4.28149895647992
a_HQ_transcript/29771	-1.76394397808593	-0.325665676389079	-1.83428940506284	0.0757561333135115	0.72507745653903	-4.33732953928103
a_HQ_transcript/64738	2.0100587661038	0.0485702022861031	1.83419306737355	0.0757707818423761	0.72507745653903	-4.31848225370067
a_HQ_transcript/139447	-2.43845315426496	0.258773715056512	-1.83409066183572	0.0757863556634378	0.72507745653903	-4.24032080137769
a_HQ_transcript/28881	2.03691308557033	0.54767411409905	1.83407520894127	0.0757887059749558	0.72507745653903	-4.32171511278176
a_HQ_transcript/142796	1.51977280719976	1.652976928518	1.83406110171576	0.0757908516709872	0.72507745653903	-4.24239154662433
a_HQ_transcript/16965	1.86654587640734	0.946092120811303	1.83384155519561	0.0758242511801004	0.725277616027131	-4.32987604853973
a_HQ_transcript/42349	-2.77733902446382	1.88733653553602	-1.83362310584977	0.0758574962481526	0.72537791300186	-4.32607394579871
a_HQ_transcript/152040	2.48055582352762	0.309396212795853	1.83360099732031	0.0758608615639392	0.72537791300186	-4.36475825341962
a_HQ_transcript/12749	1.93606825736936	0.743697146834176	1.83352668828736	0.0758721736693385	0.72537791300186	-4.19382060006104
a_HQ_transcript/143926	1.19042348905469	-0.601174250183814	1.83307487397071	0.075940984616014	0.725916387725647	-4.30219552969394
a_HQ_transcript/105739	2.35911317015108	0.219365254352211	1.83289014502012	0.0759691340227689	0.725980450675684	-4.26367293613444
a_HQ_transcript/61318	-1.4389715689067	0.158559243749244	-1.83284708587774	0.0759756967502371	0.725980450675684	-4.30573810658338
a_HQ_transcript/15474	1.11424436435743	0.0367675429857593	1.83278503747094	0.0759851545195235	0.725980450675684	-4.21527263270313
a_HQ_transcript/122454	-1.23274120858379	3.94063954769705	-1.83263034850435	0.0760087374633237	0.726016464335969	-4.6596331215658
a_HQ_transcript/73708	-2.11802470674854	0.182786705818294	-1.83248409797735	0.0760310396793837	0.726016464335969	-4.25980818554177
a_HQ_transcript/46451	2.08297305287999	0.845321324778417	1.8323600153286	0.0760499658237684	0.726016464335969	-4.23761832219789
a_HQ_transcript/102993	0.646685045940789	3.19018044941743	1.83235051309433	0.0760514153513801	0.726016464335969	-4.53347649072784
a_HQ_transcript/116570	1.73322103971997	0.733964244077734	1.8322916412515	0.076060396539869	0.726016464335969	-4.26588430557053
a_HQ_transcript/101941	2.11171057923545	1.5689002319614	1.83221521444562	0.0760720571746127	0.726016464335969	-4.31370770533979
a_HQ_transcript/75137	1.63631114686974	1.7702614030128	1.83213859811823	0.0760837482566921	0.726016464335969	-4.34601548176652
a_HQ_transcript/83221	3.74117566621259	1.63598064724749	1.83202498922795	0.0761010869520049	0.726016464335969	-4.35226614446814
a_HQ_transcript/84036	1.83427427606409	0.6791017885778	1.83202337352719	0.0761013335604022	0.726016464335969	-4.27071080270976
a_HQ_transcript/142180	0.980980486509281	2.81945285210372	1.83189040134486	0.0761216317712229	0.726042818221881	-4.45850537061811
a_HQ_transcript/87957	0.810103188488931	3.2301953828726	1.83183560864609	0.076129997223956	0.726042818221881	-4.5444784824709

a_HQ_transcript/152547	0.951241197346524	4.1156350718436	1.83171012862903	0.0761491577900448	0.726042818221881	-4.66881127257049
a_HQ_transcript/6338	1.36205251849995	-0.44925187512949	1.83167804446294	0.0761540576434559	0.726042818221881	-4.30442891756963
a_HQ_transcript/120249	5.59441556282958	0.0150786026946238	1.8315085566064	0.0761799460809735	0.72613709936047	-4.3523881033537
a_HQ_transcript/10310	2.54025511320097	2.17469320602361	1.8314348884234	0.0761912008757587	0.72613709936047	-4.47432811079308
a_HQ_transcript/62910	-4.14417120232043	0.303807879405221	-1.83102786070319	0.0762534108836565	0.72613709936047	-4.34931686261047
a_HQ_transcript/71018	2.03941544852689	1.83892718104872	1.83097672692483	0.0762612292208237	0.72613709936047	-4.26458834079192
a_HQ_transcript/93782	1.97762310248367	-0.175832703684338	1.83087416048288	0.0762769136569791	0.72613709936047	-4.29259035121172
a_HQ_transcript/44831	0.962509380398418	2.0049372718255	1.83082375215387	0.0762846230956811	0.72613709936047	-4.26236111506676
a_HQ_transcript/47121	1.81475108717985	1.3711380355584	1.83081570436104	0.0762858539848939	0.72613709936047	-4.26679353433109
a_HQ_transcript/130948	-1.46927758876972	4.45922148205741	-1.83081196512996	0.0762864258964353	0.72613709936047	-4.71198730520117
a_HQ_transcript/64230	2.49786374908482	0.747184584081669	1.83075696749706	0.0762948381504428	0.72613709936047	-4.243847535234
a_HQ_transcript/69273	-3.48180594651001	1.58147652756878	-1.83075153683875	0.0762956688487176	0.72613709936047	-4.38502219196907
a_HQ_transcript/109223	-0.690004948394907	2.75120361490194	-1.83067504867458	0.0763073696472973	0.72613709936047	-4.43626730171378
a_HQ_transcript/27985	-1.28481817582201	3.41179596508746	-1.83061058135674	0.0763172327427281	0.72613709936047	-4.53581881666774
a_HQ_transcript/86044	1.44774117889272	0.127755811786541	1.83055103980981	0.0763263431924799	0.72613709936047	-4.28666219531783
a_HQ_transcript/53990	1.45061890321982	4.20848889969375	1.83029042267644	0.0763662311321832	0.726397689944615	-4.71091050449429
a_HQ_transcript/126912	1.87419018944503	0.115481108673288	1.8302030526576	0.0763796072575854	0.726406055410492	-4.32839356152982
a_HQ_transcript/108359	2.05696415288695	-0.210404983910394	1.8298693209066	0.076430719136093	0.726508652995677	-4.32872800247908
a_HQ_transcript/53846	-3.10859428684544	1.80945855697605	-1.82978194340408	0.0764441060458712	0.726508652995677	-4.36425824569216
a_HQ_transcript/140706	-2.05529862805263	-0.285466502419808	-1.82968777436948	0.0764585357108575	0.726508652995677	-4.37521021210062
a_HQ_transcript/149780	-1.89429932675645	0.687236759814965	-1.82967477009782	0.0764605285578822	0.726508652995677	-4.29723609311424
a_HQ_transcript/14124	-1.88576768765373	1.99196786968351	-1.82963023394469	0.0764673538618647	0.726508652995677	-4.42103127650565
a_HQ_transcript/5312	1.98346709149134	0.61271173214904	1.82960429288353	0.0764713296497488	0.726508652995677	-4.25713105213798
a_HQ_transcript/138549	1.04409907274365	2.08105180883646	1.82944612775231	0.0764955742250181	0.726508652995677	-4.37248368186172
a_HQ_transcript/103528	-2.42309169205668	4.10257564966309	-1.82940641532598	0.0765016626327832	0.726508652995677	-4.75796210873544
a_HQ_transcript/62433	-2.16890374508719	0.247129270434581	-1.82928648685239	0.0765200516657373	0.726508652995677	-4.22506629515488
a_HQ_transcript/1628	1.4646809611538	-0.638780969662816	1.82917851043104	0.0765366112768204	0.726508652995677	-4.33648653703556
a_HQ_transcript/120070	1.64122880886659	-0.506799808273531	1.82917153879674	0.0765376805741392	0.726508652995677	-4.33755215149469
a_HQ_transcript/95348	-0.967208621659731	2.89142575358312	-1.8291095105055	0.0765471949283297	0.726508652995677	-4.42185992738555
a_HQ_transcript/75047	1.25889918253412	-0.799231604599579	1.82907248480036	0.0765528746834318	0.726508652995677	-4.3486019319354
a_HQ_transcript/51711	1.99721885362666	0.655828961635203	1.8288816984594	0.0765821470696561	0.726667816660949	-4.1840273087425
a_HQ_transcript/61786	-1.72050324195071	4.35536030867173	-1.82855950840913	0.0766316024333223	0.726945668540118	-4.70235234239708
a_HQ_transcript/102257	-1.57473873613442	0.0617649786002532	-1.8285279942043	0.0766364412471416	0.726945668540118	-4.39284981588792
a_HQ_transcript/113593	-1.01092382625024	3.30861797548598	-1.82843817516407	0.0766502338427568	0.726957871237031	-4.45897699501693
a_HQ_transcript/60823	-0.738665651388288	3.13721295266384	-1.82823446849292	0.0766815228501876	0.727094130414561	-4.458447338704
a_HQ_transcript/62885	-2.07851516789366	1.5887509951477	-1.82814923761097	0.0766946174052375	0.727094130414561	-4.41385504593789
a_HQ_transcript/110164	2.67414403619643	1.17292946254659	1.82810036758449	0.0767021264743685	0.727094130414561	-4.37738025759613
a_HQ_transcript/97558	1.55939985236254	-0.617438718124486	1.82793443193597	0.0767276278106204	0.727182340273417	-4.38155653941182
a_HQ_transcript/58423	-0.793702531009421	1.53411603832956	-1.82787702516745	0.0767364518861331	0.727182340273417	-4.26023984562004
a_HQ_transcript/16668	-1.77302437843226	1.17033148772559	-1.82771021667495	0.0767620971675312	0.727306794483171	-4.2249689048578
a_HQ_transcript/123668	1.72370204990179	0.335518516254873	1.82754047883707	0.0767882003130837	0.727385829049238	-4.27505889842095
a_HQ_transcript/120442	-0.911628792012785	2.63549711267329	-1.82749324352243	0.0767954657458569	0.727385829049238	-4.47541134169576
a_HQ_transcript/27400	2.56754415551997	1.73176228435885	1.82717795454392	0.0768439764924704	0.727606473651722	-4.33548306793867
a_HQ_transcript/111576	1.74182333100892	-0.648992193930594	1.82716045423066	0.0768466698774694	0.727606473651722	-4.34999273733001
a_HQ_transcript/28180	-1.6793808832811	0.485165135962046	-1.82709780372001	0.076856312761863	0.727606473651722	-4.26452446866978
a_HQ_transcript/113970	1.78908733220145	-0.43230965186131	1.82697926780481	0.0768745600995376	0.727660711523518	-4.38719849224691
a_HQ_transcript/49956	-2.53568132695542	0.95378347630383	-1.8268332021813	0.0768970504234885	0.72774680983833	-4.48137074043136
a_HQ_transcript/123581	2.3669195802303	0.932407898340952	1.82675758631085	0.076908695513984	0.72774680983833	-4.27526504492335
a_HQ_transcript/50393	-1.31843280312536	2.29877373420521	-1.82659451707482	0.0769338138270787	0.727866004254627	-4.36818160430241
a_HQ_transcript/147774	1.52276843538955	4.41801428885192	1.82616707784514	0.076999687487544	0.728180574432804	-4.68361392717355

a_HQ_transcript/32891	-0.850313234705185	2.16945124998186	-1.82606640906095	0.0770152087860073	0.728180574432804	-4.32926150910591
a_HQ_transcript/95383	3.63300328298032	3.21726854770812	1.82595378638124	0.0770325763201868	0.728180574432804	-4.63103389692067
a_HQ_transcript/1355	2.24303475147522	0.390171359589281	1.82594265111402	0.0770342936702525	0.728180574432804	-4.46195655442865
a_HQ_transcript/87432	1.6599826019799	0.188111545199648	1.82582359540869	0.0770526572212718	0.728180574432804	-4.29978704839277
a_HQ_transcript/6200	-0.566217540865437	2.75138207001731	-1.8257776839863	0.0770597397502071	0.728180574432804	-4.42658672100965
a_HQ_transcript/8907	-2.4958283120738	3.04623529557113	-1.82574743696403	0.077064406112651	0.728180574432804	-4.51490466163221
a_HQ_transcript/41258	1.395438696019	-0.696177445858052	1.82572007496107	0.0770686275960267	0.728180574432804	-4.35199389123059
a_HQ_transcript/66120	-1.7482700307739	-0.0918643493793688	-1.82563269973974	0.0770821094003566	0.728180574432804	-4.31136370415596
a_HQ_transcript/73610	-2.14976221773148	0.41642887545505	-1.82556292067963	0.0770928776000475	0.728180574432804	-4.27743458479389
a_HQ_transcript/88858	1.70925095272898	1.15482683099232	1.82546275322049	0.0771083375341108	0.728180574432804	-4.29742666906893
a_HQ_transcript/2649	1.51340416394307	0.186629385024458	1.82540411206341	0.077117389488858	0.728180574432804	-4.35351808689264
a_HQ_transcript/86773	2.08973784088961	0.925372784382091	1.82515811758873	0.0771553715131694	0.728420892531673	-4.27373702769305
a_HQ_transcript/66347	1.61128538364447	0.874688445561403	1.8250278027842	0.0771754988342275	0.728492594387134	-4.24178029205288
a_HQ_transcript/44377	-1.85811479832804	0.730641544010526	-1.82492786553226	0.0771909373287527	0.728520020302929	-4.26512462878244
a_HQ_transcript/140632	-2.64966957060831	0.974652815687038	-1.82461824176669	0.0772387853081981	0.728666587278545	-4.41557092624004
a_HQ_transcript/60887	-3.06948612850245	0.666250028579049	-1.82454065409399	0.0772507793486176	0.728666587278545	-4.32769253090731
a_HQ_transcript/7988	0.816397302049782	1.79078080047154	1.82442435788549	0.0772687601967766	0.728666587278545	-4.30065622442559
a_HQ_transcript/137109	-1.78205424237574	-0.530574887692111	-1.82442369314492	0.077268862984238	0.728666587278545	-4.32275353885503
a_HQ_transcript/54866	1.61265582296512	-0.283049544439868	1.82442187115835	0.0772691447148526	0.728666587278545	-4.37807567779672
a_HQ_transcript/116367	0.775899088280621	3.58007865604299	1.8242751278154	0.0772918382586697	0.728762364038921	-4.57152069070304
a_HQ_transcript/150261	1.88781961671098	-0.556771428103618	1.82411320289781	0.0773168861947158	0.72888030501564	-4.33820039815082
a_HQ_transcript/37698	2.12358880835077	0.789107215769553	1.82371891348341	0.0773779072084415	0.729248073169894	-4.27373977888991
a_HQ_transcript/40287	-1.42369300908509	1.63605446543789	-1.82361575213821	0.0773938794377667	0.729248073169894	-4.23846450798295
a_HQ_transcript/121498	1.98402898207567	0.230109913985306	1.82350379330338	0.0774112169420066	0.729248073169894	-4.34223853239657
a_HQ_transcript/142454	4.52641973009117	1.45583513949109	1.82322623497179	0.0774542128107956	0.729248073169894	-4.4488139323125
a_HQ_transcript/95050	2.33220889506585	0.95779582786212	1.82314736695007	0.0774664337726277	0.729248073169894	-4.30540066444558
a_HQ_transcript/86459	1.72259107318859	-0.297278144854341	1.82308279103351	0.0774764413310201	0.729248073169894	-4.31924644755844
a_HQ_transcript/152592	-4.93337208465797	0.190747079640902	-1.82299324167975	0.0774903209350577	0.729248073169894	-4.38631958864413
a_HQ_transcript/90273	0.602264204839667	4.6323572975036	1.82299064948203	0.0774907227414665	0.729248073169894	-4.75352910253574
a_HQ_transcript/62929	-2.08411196475723	-0.0726548884424199	-1.82293129544609	0.0774999234638414	0.729248073169894	-4.33483583034593
a_HQ_transcript/84146	-2.34869987160547	1.06680398020602	-1.82292568399585	0.0775007933668636	0.729248073169894	-4.33112485062701
a_HQ_transcript/126879	-2.2179077554755	0.311398268901875	-1.82288275377121	0.0775074488081506	0.729248073169894	-4.34936803948691
a_HQ_transcript/80190	2.3261275590793	-0.0896631257572083	1.82276042681213	0.0775264157388881	0.729248073169894	-4.33573143431278
a_HQ_transcript/42246	-1.75415311254651	-0.287611970860425	-1.82267451015701	0.0775397395765614	0.729248073169894	-4.35058824972586
a_HQ_transcript/149293	5.01723356712142	2.96978768156816	1.82267379957388	0.0775398497809614	0.729248073169894	-4.51888228140214
a_HQ_transcript/122147	-1.37477298061217	-0.65338095662675	-1.82255837463775	0.0775577528166166	0.729248073169894	-4.33661649182342
a_HQ_transcript/81170	-2.04918637532472	0.046850958221677	-1.82245777622278	0.0775733590514722	0.729248073169894	-4.37716868501164
a_HQ_transcript/143807	-3.16824403225789	0.0401840201625209	-1.82234953625663	0.0775901537398996	0.729248073169894	-4.34947168669211
a_HQ_transcript/35152	3.23919253922532	1.51548246425549	1.82232521789412	0.0775939274427233	0.729248073169894	-4.41992289824773
a_HQ_transcript/130679	-1.70333607211778	2.36022190616575	-1.82231459023241	0.0775955766833548	0.729248073169894	-4.36963025713832
a_HQ_transcript/32781	-1.63243526871352	1.63571064314571	-1.82224221604236	0.0776068087776797	0.729248073169894	-4.28470916654264
a_HQ_transcript/121638	1.98555229946855	0.979565558137131	1.82192089479538	0.0776566928826646	0.729598875688303	-4.26288602418705
a_HQ_transcript/113137	2.11640239500315	0.655782841259221	1.82178590786782	0.0776776573252389	0.729677903199981	-4.3289794280488
a_HQ_transcript/15948	1.40986256634511	0.274244084758761	1.82157623864485	0.0777102300306566	0.729865931688803	-4.24926755880263
a_HQ_transcript/96847	1.57550008696132	0.647091918998343	1.82136792793428	0.0777426032137957	0.730052025785382	-4.34809466648668
a_HQ_transcript/144768	2.02185144409515	1.52794233826116	1.82118150830425	0.0777715840883559	0.730153049990169	-4.26041323600171
a_HQ_transcript/79450	1.82930760995758	-0.0717525966130861	1.82108081461257	0.0777872418012591	0.730153049990169	-4.24666318872072
a_HQ_transcript/81797	-1.68837675735508	1.61760878563577	-1.82104270097071	0.0777931691142414	0.730153049990169	-4.2891070925406
a_HQ_transcript/102314	1.38726403627162	-0.682712878212241	1.8209755979978	0.0778036056915618	0.730153049990169	-4.35740831950029
a_HQ_transcript/90864	-1.98005869759127	1.41233615365703	-1.82084635767526	0.0778237099008455	0.730223827137425	-4.31888152151318

a_HQ_transcript/20382	0.629080008330726	2.67821601825303	1.8206976940295	0.0778468410141977	0.73032297844953	-4.39527035994196
a_HQ_transcript/98104	-2.16215816602919	-0.131812646930635	-1.82054692390181	0.0778703058657542	0.730410784250377	-4.35561602533356
a_HQ_transcript/149064	2.90788043828564	0.608024842035506	1.82039746885496	0.0778935719946316	0.730410784250377	-4.38944734086525
a_HQ_transcript/146397	1.9811482740408	0.938618741283487	1.82030963183184	0.0779072486186474	0.730410784250377	-4.36912427197203
a_HQ_transcript/30987	-1.80916189266138	0.479632029580979	-1.82028785142721	0.0779106402432706	0.730410784250377	-4.27991306653511
a_HQ_transcript/120668	1.62469160423553	-0.678208624462007	1.82023398873102	0.0779190282331508	0.730410784250377	-4.35824447628399
a_HQ_transcript/121515	0.38996394830774	5.01514068778759	1.81988529352556	0.0779733488441629	0.730694213880195	-4.80195434460375
a_HQ_transcript/120666	1.61621922534802	-0.465399648182035	1.81985283758838	0.0779784065521217	0.730694213880195	-4.3591582199133
a_HQ_transcript/67024	-2.53913715976223	1.04022840110401	-1.81979785467183	0.0779869753460076	0.730694213880195	-4.34233295893153
a_HQ_transcript/148637	3.89586966785047	2.67123051319356	1.81968793013347	0.0780041089033717	0.730736960892054	-4.48214801510598
a_HQ_transcript/117802	-3.78536687943881	1.10101902274232	-1.81950656195127	0.0780323851449599	0.73077901055672	-4.48873991442512
a_HQ_transcript/107646	-0.571250799724882	3.46811995572196	-1.8194978643028	0.0780337413729281	0.73077901055672	-4.57848201675155
a_HQ_transcript/117446	2.79404014167711	0.878070704715772	1.81937867641034	0.0780523284140875	0.730835332805102	-4.27508891980225
a_HQ_transcript/143655	1.65327223316728	-0.53100548568016	1.81922954371436	0.0780755905813167	0.730935404946171	-4.35915693910323
a_HQ_transcript/119939	1.97904502584865	0.315161148728874	1.8189867117816	0.078113480875784	0.731172369782218	-4.31197124908625
a_HQ_transcript/129307	2.24913559366234	0.800331619921043	1.81869025038192	0.0781597604817379	0.731388545568111	-4.33164924041174
a_HQ_transcript/98286	-3.48387062760707	1.54499766391581	-1.81866238612769	0.0781641114786154	0.731388545568111	-4.40037962893558
a_HQ_transcript/30561	-0.636377288798804	3.34397690357441	-1.81855864984871	0.0781803116885931	0.731388545568111	-4.59191507760121
a_HQ_transcript/45261	-0.480503479714895	4.99709722520086	-1.81840750916611	0.0782039200332858	0.731388545568111	-4.78613127168527
a_HQ_transcript/63021	1.18994311423764	3.10645932004582	1.81821238215221	0.0782344080727643	0.731388545568111	-4.61480894143208
a_HQ_transcript/41118	-2.2929042663794	0.309129346568567	-1.81818214980541	0.0782391326964282	0.731388545568111	-4.25066632825544
a_HQ_transcript/107916	-2.57594098950681	0.678945342630294	-1.81814885403289	0.07824433634461	0.731388545568111	-4.33347809571216
a_HQ_transcript/43083	-1.75385612481347	-0.545867836311523	-1.81814144151821	0.078245494853675	0.731388545568111	-4.3481925995108
a_HQ_transcript/36748	1.71826693111108	-0.450455953238778	1.8180332128208	0.0782624116852075	0.731388545568111	-4.34139950415754
a_HQ_transcript/127907	-0.738923338660704	3.64328371576654	-1.8180064751234	0.0782665914366541	0.731388545568111	-4.6291825510477
a_HQ_transcript/50693	0.68714227448709	3.77253515325019	1.81791408982882	0.0782810349653098	0.731388545568111	-4.61579885106866
a_HQ_transcript/86193	1.80436264698323	-0.394024624085217	1.81784802887284	0.0782913643355567	0.731388545568111	-4.34990641388726
a_HQ_transcript/8072	1.67678509751144	1.73047332504286	1.81779132836063	0.0783002310213764	0.731388545568111	-4.30448509167054
a_HQ_transcript/118787	0.574885649097993	2.59352358424269	1.81771141621945	0.0783127289364148	0.731388545568111	-4.4657189816849
a_HQ_transcript/94139	1.36723534252363	-0.66868614979892	1.81752525472802	0.0783418503820587	0.731507883219821	-4.3651860418442
a_HQ_transcript/140194	-0.867688245539809	3.10205722291377	-1.81737225365943	0.078365791410996	0.731507883219821	-4.51317606572151
a_HQ_transcript/44742	1.0341005112439	-0.547189376778049	1.81737184792046	0.0783658549077789	0.731507883219821	-4.4011441333896
a_HQ_transcript/50400	1.15720524696983	1.97313514018443	1.81730801793934	0.0783758446306951	0.731507883219821	-4.42795234531008
a_HQ_transcript/117555	1.82840938116961	-0.364933071116031	1.81698009234495	0.0784271837859005	0.731869535897708	-4.31786593515857
a_HQ_transcript/101457	2.01698551360412	0.915621758806517	1.81679384202385	0.0784563553888583	0.732024241740539	-4.4336877827396
a_HQ_transcript/136729	-4.28981488317935	1.66161509726296	-1.81654401426246	0.0784954993677821	0.732271928623084	-4.51470820441425
a_HQ_transcript/143409	1.1877282711979	3.8192363442651	1.8163650149862	0.0785235558916086	0.732393005792367	-4.62971082001715
a_HQ_transcript/73373	0.94939805591537	0.486691448841674	1.81628801683694	0.0785356272841906	0.732393005792367	-4.31383172174668
a_HQ_transcript/107882	-1.23381448612649	-0.681151625532997	-1.81617259473083	0.0785537255562132	0.732393005792367	-4.35556565048441
a_HQ_transcript/81966	-1.82290702385788	1.76986868399205	-1.81613974665299	0.0785588768083437	0.732393005792367	-4.26063035533096
a_HQ_transcript/140337	3.68917464900216	2.52342822711371	1.8160488215464	0.0785731372294701	0.732408486349365	-4.48731432559051
a_HQ_transcript/120052	1.40717426415541	0.610483867019228	1.81571345552285	0.078625754090502	0.732781438796328	-4.22927469558583
a_HQ_transcript/28452	1.63951503733616	0.0305057373337017	1.81542865567489	0.0786704609840579	0.732911279066788	-4.31029081048265
a_HQ_transcript/78274	3.17738002455839	0.559328908656693	1.81537733672751	0.0786785191555698	0.732911279066788	-4.47944382372992
a_HQ_transcript/1081	2.25230230332298	0.860126686481533	1.81522541375281	0.0787023784290693	0.732911279066788	-4.29848080688105
a_HQ_transcript/105931	1.44287145275998	-0.307154282367532	1.81519994433785	0.078706378965044	0.732911279066788	-4.36436178134279
a_HQ_transcript/15912	1.36514744306903	0.198985991927828	1.81515955561595	0.0787127232637097	0.732911279066788	-4.30649662981538
a_HQ_transcript/22385	-1.97646681316124	1.56033298277066	-1.81501208111236	0.0787358923957419	0.732911279066788	-4.29233594249706
a_HQ_transcript/39731	-0.527766480237551	2.74170681343145	-1.81499651173817	0.0787383387769311	0.732911279066788	-4.46541401640322
a_HQ_transcript/148935	1.30282685191557	3.17852167429758	1.81498241223974	0.0787405542556443	0.732911279066788	-4.38429438166284

a_HQ_transcript/55934	1.98086476975636	0.739591902819552	1.81480773481848	0.0787680060244829	0.732941360204735	-4.26923043875918
a_HQ_transcript/11867	-0.931328114720443	3.22548229807459	-1.81462552821199	0.0787966497387074	0.732941360204735	-4.55572932915896
a_HQ_transcript/60754	-0.779144457448603	2.43719543365333	-1.81457761811543	0.0788041828978051	0.732941360204735	-4.36720857864726
a_HQ_transcript/115422	-3.6736928350376	3.88803437988808	-1.81456387659062	0.0788063436639168	0.732941360204735	-4.50255959496904
a_HQ_transcript/83399	2.41687610333848	-0.365719849861675	1.81447723242995	0.0788199690607621	0.732941360204735	-4.35902716768078
a_HQ_transcript/28056	-0.757292591075596	2.76975982954506	-1.81440809838896	0.0788308423079318	0.732941360204735	-4.41592671163995
a_HQ_transcript/80557	1.92856737444792	1.39709937955837	1.81427057153871	0.0788524760195298	0.732941360204735	-4.25302800938979
a_HQ_transcript/101229	1.24042258386105	-0.808490501098659	1.81426108147164	0.0788539690443955	0.732941360204735	-4.37189053000048
a_HQ_transcript/13438	2.40017673542123	3.37772013468216	1.81420454904226	0.0788628635078969	0.732941360204735	-4.50944438255655
a_HQ_transcript/16329	1.83324495815542	-0.232122877720751	1.8141099011554	0.0788777567340549	0.732941360204735	-4.3502997017398
a_HQ_transcript/48584	-2.80209458152471	2.07240929060602	-1.81407984904362	0.0788824860556156	0.732941360204735	-4.30118221830324
a_HQ_transcript/84647	-2.04638724515423	1.00810035166042	-1.81388061843071	0.0789138452192957	0.733083206290524	-4.30259039423358
a_HQ_transcript/113405	-0.760726649911599	3.05746394149887	-1.81380396824058	0.07892591288761	0.733083206290524	-4.4745136947579
a_HQ_transcript/133043	2.40353136681818	2.073693143638	1.81374252981589	0.0789355867769817	0.733083206290524	-4.34491630990403
a_HQ_transcript/49532	-1.31713871903019	0.373067606968261	-1.81346110205474	0.0789799123542205	0.733239805313912	-4.22643316296336
a_HQ_transcript/105788	1.73419729405745	-0.0840103181677689	1.81339871549245	0.0789897412622807	0.733239805313912	-4.24603110995915
a_HQ_transcript/103570	4.88429054339786	2.14028211082473	1.81297285632752	0.0790568625392333	0.733239805313912	-4.49364109957617
a_HQ_transcript/94347	2.72377540714843	0.228887465128678	1.81292188595487	0.0790648994246039	0.733239805313912	-4.3542493800039
a_HQ_transcript/62736	-3.28075359555823	0.1771173958998	-1.81279670661306	0.0790846403521144	0.733239805313912	-4.33886166026937
a_HQ_transcript/28942	0.893119207780336	1.93796545779234	1.81278376521481	0.0790866814648736	0.733239805313912	-4.36126565522299
a_HQ_transcript/92069	-1.66701252328657	0.0747371455511826	-1.81277774375707	0.0790876311823436	0.733239805313912	-4.3567129103818
a_HQ_transcript/66092	4.47976606863768	2.38780127295686	1.81272591788652	0.0790958056731573	0.733239805313912	-4.44514892137495
a_HQ_transcript/53862	-1.89779950117861	0.506198597341985	-1.81270182232498	0.0790996065092051	0.733239805313912	-4.24028848155873
a_HQ_transcript/138375	1.06830975248232	3.13585670688977	1.81267853849627	0.0791032794501158	0.733239805313912	-4.56634151878223
a_HQ_transcript/113380	-2.56090273574862	1.60618154518703	-1.81258262263693	0.079118411365268	0.733239805313912	-4.39012939752241
a_HQ_transcript/151409	1.99635084329096	-0.538964805405236	1.81251317425869	0.0791293692448647	0.733239805313912	-4.36997879164545
a_HQ_transcript/30731	1.60190558443388	1.96747501183545	1.81249617426799	0.0791320517771421	0.733239805313912	-4.27666899509522
a_HQ_transcript/17562	0.726053770568967	4.55143620865382	1.81239755713842	0.0791476147025457	0.733239805313912	-4.74512061035644
a_HQ_transcript/138214	-2.7854116965557	1.00651556976152	-1.81237895753363	0.0791505502275769	0.733239805313912	-4.30577026660025
a_HQ_transcript/41395	-1.35296900152019	1.43168391805684	-1.81230115627036	0.0791628303937277	0.733239805313912	-4.30130489696219
a_HQ_transcript/147467	3.15417512851326	0.528800375320494	1.8121498655754	0.079186714787708	0.733239805313912	-4.34675850698017
a_HQ_transcript/138811	3.10602807505548	0.82613981232681	1.8120160459934	0.0792078461125488	0.733239805313912	-4.45087165741158
a_HQ_transcript/76917	1.88278380822897	-0.528437599088847	1.81200355807401	0.0792098183129732	0.733239805313912	-4.36746449707799
a_HQ_transcript/149408	2.03130442542025	0.646102530975565	1.81197323628409	0.0792146071666515	0.733239805313912	-4.31727837065723
a_HQ_transcript/136165	-2.65331142814572	1.97202588485445	-1.81195588569375	0.079217347532538	0.733239805313912	-4.44390753987667
a_HQ_transcript/152498	-5.13138866925739	0.0913458887649162	-1.81176854618948	0.0792469412194272	0.733396943035005	-4.47606023021884
a_HQ_transcript/135488	-1.17190323147718	1.86377315977538	-1.81160718729046	0.0792724383369315	0.733410274042783	-4.43593640247336
a_HQ_transcript/126317	-1.36960937160813	1.66088671197882	-1.81146744689538	0.079294525047648	0.733410274042783	-4.25623651834219
a_HQ_transcript/57017	-3.05213927569162	1.30122302924998	-1.81134538290165	0.0793138221936805	0.733410274042783	-4.34084731904622
a_HQ_transcript/91694	1.60931544938935	-0.0136817735997739	1.8111764451559	0.0793405362220869	0.733410274042783	-4.32318911580005
a_HQ_transcript/94274	-1.54905007421529	1.30815485622036	-1.81116022727936	0.0793431011482599	0.733410274042783	-4.39510388885375
a_HQ_transcript/131615	-1.27759564119973	-0.729238481918009	-1.8111352500323	0.0793470515441478	0.733410274042783	-4.35196111353995
a_HQ_transcript/114704	1.22786890411602	1.77227629978347	1.81109091023102	0.0793540647295693	0.733410274042783	-4.299738752208
a_HQ_transcript/7823	-0.611494692546914	2.33885332703305	-1.81101248392346	0.0793664706376374	0.733410274042783	-4.32491399956458
a_HQ_transcript/129431	-2.2221476471095	2.37324943577606	-1.81089502096707	0.079385054665843	0.733410274042783	-4.49528287994629
a_HQ_transcript/139376	-1.51892800926097	3.50156998788667	-1.8108782338117	0.0793877108946062	0.733410274042783	-4.64343834387752
a_HQ_transcript/125119	0.88096328467796	2.1296991844914	1.81082939013459	0.0793954398514869	0.733410274042783	-4.34281140826458
a_HQ_transcript/151460	0.455527680340777	9.23318742311974	1.81080191591326	0.0793997876161443	0.733410274042783	-5.19167848794884
a_HQ_transcript/97417	-0.794031259301109	2.79186703769617	-1.81061364538128	0.0794295866667501	0.733568956746878	-4.4679001061197
a_HQ_transcript/42447	-3.68501001501896	2.10176325968354	-1.81027184626219	0.079483710205631	0.73363113537503	-4.32649140291007

a_HQ_transcript/17995	3.64261319096655	1.19264008761927	1.81026634946049	0.0794845808748055	0.73363113537503	-4.40766442442487
a_HQ_transcript/78974	0.959028034443025	1.44869801726385	1.81023245746331	0.0794899493971037	0.73363113537503	-4.27641505268396
a_HQ_transcript/139449	-2.72977479971817	-0.0805172720367076	-1.81016573272315	0.0795005195563674	0.73363113537503	-4.37304208623098
a_HQ_transcript/19626	2.04564342729996	-0.665199600590178	1.81006877088749	0.0795158818380767	0.73363113537503	-4.37344100231135
a_HQ_transcript/39907	1.96461625268528	1.25653815745028	1.81005701955315	0.0795177438484515	0.73363113537503	-4.23918588231506
a_HQ_transcript/28887	-1.32662675133407	2.25787584272671	-1.81001333534835	0.0795246659784968	0.73363113537503	-4.31500921567484
a_HQ_transcript/133601	-2.54472132819811	2.56825767451865	-1.80979762423893	0.0795588547356878	0.733830071100613	-4.55794589710883
a_HQ_transcript/6353	3.58269353493377	2.40560536333114	1.80962408017045	0.0795863693763133	0.73386369404586	-4.30459114014727
a_HQ_transcript/31346	-2.84492624688941	0.131878122227794	-1.8096153739722	0.0795877499185436	0.73386369404586	-4.29969813669135
a_HQ_transcript/95845	-0.969915409919006	3.05636313290432	-1.80946987800059	0.0796108242360053	0.733906949029248	-4.52765120608289
a_HQ_transcript/77393	-2.46473746292842	2.24566119669327	-1.80914481587991	0.0796623966458986	0.733906949029248	-4.45791205817361
a_HQ_transcript/112920	2.70464944067512	1.8526030679698	1.80913106174719	0.0796645794207418	0.733906949029248	-4.33138012245219
a_HQ_transcript/91528	-0.662307153652408	3.00262978269207	-1.80911539508989	0.0796670657743736	0.733906949029248	-4.52405532781993
a_HQ_transcript/68548	-1.41446287012925	-0.556486459050539	-1.80906045469858	0.0796757855293674	0.733906949029248	-4.33624616973828
a_HQ_transcript/20463	-1.73458697689913	0.0990110383111511	-1.80901584774542	0.0796828658303715	0.733906949029248	-4.32125083684354
a_HQ_transcript/40737	-2.49527216337821	1.68428669432192	-1.80896024799551	0.07969169172764	0.733906949029248	-4.36490038174286
a_HQ_transcript/105278	2.76207765946726	0.471236923373596	1.80894919378386	0.0796934465708886	0.733906949029248	-4.40048735219663
a_HQ_transcript/140479	-2.93363249790732	0.625842975769823	-1.80874556375624	0.0797257784807492	0.734049525264911	-4.38939391584176
a_HQ_transcript/28926	1.13690634842932	2.90716977670242	1.80857864491217	0.0797522897931051	0.734049525264911	-4.4526386561398
a_HQ_transcript/24868	-0.72194253797304	2.16788091078098	-1.8085448786744	0.0797576537138921	0.734049525264911	-4.37628239983932
a_HQ_transcript/119117	-2.38427450316744	0.988884116249416	-1.80848365178462	0.0797673806670211	0.734049525264911	-4.35094798519941
a_HQ_transcript/96837	-2.58267459252197	1.13588270901695	-1.80839109693626	0.0797820865249255	0.734049525264911	-4.4124165127987
a_HQ_transcript/30283	-3.98903735187167	0.0251281737603764	-1.8083746651841	0.0797846975747266	0.734049525264911	-4.38936860326053
a_HQ_transcript/106005	2.2044841736623	2.42941690598602	1.80821569992676	0.0798099613361731	0.734165759225996	-4.51345115112109
a_HQ_transcript/80384	-1.62961659604414	1.64422787647148	-1.80800785949041	0.0798430029118255	0.73419838198283	-4.30809305698468
a_HQ_transcript/17737	-1.48886016449786	0.165937687155784	-1.80793056256861	0.0798552942103204	0.73419838198283	-4.31698052118643
a_HQ_transcript/42967	-1.78549429159803	1.84336471481323	-1.80791999740714	0.079856974344691	0.73419838198283	-4.36327096950595
a_HQ_transcript/116547	1.32920873255892	-0.624214362160442	1.80787562789204	0.079864030575238	0.73419838198283	-4.38047957815331
a_HQ_transcript/139247	-2.27743478498902	1.02269206993533	-1.80768766424948	0.0798939289354521	0.734202358115859	-4.2956216954629
a_HQ_transcript/150411	1.65265040687935	-0.296732485452591	1.8075928598241	0.0799090125735438	0.734202358115859	-4.4515433302693
a_HQ_transcript/77823	-1.7927146801155	-0.460807091824741	-1.80743123874841	0.0799347325029245	0.734202358115859	-4.35968598198089
a_HQ_transcript/90500	-1.14375992658482	2.34105549149531	-1.80740488087517	0.0799389276892785	0.734202358115859	-4.35961765146012
a_HQ_transcript/53213	1.9217638118423	1.1440877584201	1.8073805034461	0.0799428078297378	0.734202358115859	-4.29410939253783
a_HQ_transcript/140946	6.19585455019081	2.83378363647276	1.80718581465565	0.0799738020727327	0.734202358115859	-4.49622225399164
a_HQ_transcript/59655	1.7814559742775	1.14342188445966	1.80714702100151	0.0799799792008675	0.734202358115859	-4.23082166187343
a_HQ_transcript/68159	1.49822520328065	2.6564613843405	1.80703914912744	0.0799971578124902	0.734202358115859	-4.37886259367378
a_HQ_transcript/151926	-2.37633837156758	2.1500360800096	-1.80701732272924	0.0800006340511787	0.734202358115859	-4.52510468871972
a_HQ_transcript/61574	1.63607817543477	-0.234973586794401	1.8069817294712	0.0800063031805104	0.734202358115859	-4.36122671809998
a_HQ_transcript/119402	-1.51639340528393	-0.377242378014149	-1.8069746261907	0.0800074345990242	0.734202358115859	-4.35263401430165
a_HQ_transcript/104233	-2.04662786007436	0.0153319629915913	-1.80692064941022	0.0800160325258733	0.734202358115859	-4.37178315864799
a_HQ_transcript/109747	2.28591064150472	0.2581403373121	1.80669944497384	0.0800512762467105	0.734367722820492	-4.342892763071
a_HQ_transcript/330	-0.577966736738441	3.66336723523695	-1.8065419759937	0.0800763732642122	0.734367722820492	-4.63359222872924
a_HQ_transcript/121043	1.31412851539547	2.60129559184055	1.80630781268751	0.0801137060047146	0.734367722820492	-4.35572043844795
a_HQ_transcript/49665	2.4655569516205	0.83723233819326	1.80628547452572	0.0801172681589614	0.734367722820492	-4.36969358473023
a_HQ_transcript/89514	1.96453124460869	0.529886220006425	1.80614929121692	0.0801389875389469	0.734367722820492	-4.32971897534611
a_HQ_transcript/139807	1.82296545793831	-0.609240651205264	1.80610033263552	0.0801467969880311	0.734367722820492	-4.38168969753083
a_HQ_transcript/77849	0.75113575653752	2.64052513726939	1.80607413319849	0.0801509763611785	0.734367722820492	-4.47170211315261
a_HQ_transcript/88939	-1.84668672477019	-0.0956514135076594	-1.80598287491572	0.0801655354648821	0.734367722820492	-4.37270621166278
a_HQ_transcript/21784	3.40163200266376	1.65476498116878	1.80596842836601	0.08016784043512	0.734367722820492	-4.47173759767644
a_HQ_transcript/126839	-1.26898701144112	3.02540660321491	-1.80586770835305	0.0801839120409851	0.734367722820492	-4.4467678465134

a_HQ_transcript/68610	-1.78049236289889	-0.653521508517036	-1.8058656762004	0.0801842363339889	0.734367722820492	-4.36753710643351
a_HQ_transcript/75401	1.39756520563369	2.53298290314843	1.80571980315997	0.0802075178163039	0.734367722820492	-4.45342454830497
a_HQ_transcript/29187	0.866458887846747	2.33472863807127	1.80567393433141	0.0802148397144663	0.734367722820492	-4.36773778771615
a_HQ_transcript/104126	1.95800655815109	0.617964821187678	1.80564542006434	0.0802193916444645	0.734367722820492	-4.30233363701138
a_HQ_transcript/109841	2.29103016174071	-0.0335508654455934	1.80561931580625	0.0802235590405678	0.734367722820492	-4.24711128463172
a_HQ_transcript/15509	-1.47698353615443	0.591905456880195	-1.80542232122286	0.0802550140391363	0.734417883869622	-4.35432926229563
a_HQ_transcript/103967	0.68456362411304	2.8021652691844	1.80536168580944	0.0802646980744261	0.734417883869622	-4.45175721577721
a_HQ_transcript/127723	2.51145784459001	2.07084697910006	1.80531936732192	0.0802714573161039	0.734417883869622	-4.31883173221592
a_HQ_transcript/87334	2.26575261802272	0.780157873240289	1.80526853724834	0.0802795766946666	0.734417883869622	-4.25319526158093
a_HQ_transcript/30518	2.55757339547769	0.817433740187083	1.80513457355828	0.0803009788262407	0.734498079813017	-4.29908384938943
a_HQ_transcript/105859	1.76002826401093	-0.556042500891386	1.80488563343974	0.0803407625521124	0.734746357084517	-4.37476213075123
a_HQ_transcript/107395	1.57563457884381	-0.61423895585785	1.80460260677315	0.0803860140832097	0.734984029364103	-4.37861895304776
a_HQ_transcript/142704	-3.30287762217032	1.90169553567252	-1.8045649323296	0.0803920392701791	0.734984029364103	-4.36245405782506
a_HQ_transcript/71563	1.50070030229213	4.19348746143934	1.80437677528613	0.0804221365388107	0.735143568600092	-4.75059774895273
a_HQ_transcript/17518	1.44817363667985	-0.63150653165782	1.80424957254624	0.0804424890900447	0.735147839928079	-4.37104674237116
a_HQ_transcript/122590	2.52409518478226	-0.244967806590978	1.80417998283579	0.0804536253579379	0.735147839928079	-4.35971169594545
a_HQ_transcript/14587	2.0466828559528	-0.233383372364301	1.80407627891379	0.0804702232693314	0.735147839928079	-4.36629830761267
a_HQ_transcript/79098	1.77909726326187	-0.403304538398683	1.80401660996846	0.080479774659747	0.735147839928079	-4.37524550915018
a_HQ_transcript/91447	-0.822768797717653	3.51046140390337	-1.8039252864035	0.0804943949677238	0.735147839928079	-4.5194804413567
a_HQ_transcript/127649	-0.553108767105497	3.19305129170125	-1.8038308601884	0.0805095143633748	0.735147839928079	-4.47938537851919
a_HQ_transcript/129632	-1.5347009783343	3.3261987987396	-1.80382075102761	0.0805111331713142	0.735147839928079	-4.55572705982554
a_HQ_transcript/50527	-1.54350342801868	1.98473268157343	-1.80369112221602	0.0805318934439923	0.735221910179423	-4.33832406006364
a_HQ_transcript/19308	1.32003803904224	2.41952814353644	1.80358708773799	0.0805485580316023	0.735258571177917	-4.3713700377594
a_HQ_transcript/86973	-2.45665801171605	0.759841478660926	-1.80336862107533	0.0805835622849627	0.735331157145643	-4.40469250583347
a_HQ_transcript/52217	1.30124544454706	-0.544975407420409	1.80330220137356	0.0805942070753943	0.735331157145643	-4.36522921748465
a_HQ_transcript/35818	1.41085409188037	-0.425787519646789	1.80330007511446	0.0805945478612297	0.735331157145643	-4.31034181285089
a_HQ_transcript/149393	-1.82353106128164	-0.564106338837548	-1.8032216970711	0.0806071107440826	0.735331157145643	-4.35946325019827
a_HQ_transcript/53123	-2.58899769489081	2.52984618269279	-1.80304944759658	0.0806347257264023	0.735440184543585	-4.55997108431342
a_HQ_transcript/54466	-2.03121139582545	2.56268870650709	-1.80298534558288	0.0806450045937722	0.735440184543585	-4.42417489146756
a_HQ_transcript/40743	-1.84846210737435	1.07438140287916	-1.80282927890602	0.0806700348074998	0.735440184543585	-4.3448283744531
a_HQ_transcript/112237	-2.08521737571227	1.56112257782606	-1.80269360308763	0.0806918000677723	0.735440184543585	-4.38483889759409
a_HQ_transcript/7356	1.46933055539453	1.08544973401939	1.80265722318184	0.0806976370195898	0.735440184543585	-4.26034100211206
a_HQ_transcript/111505	1.23907017470084	-0.770554502023197	1.80263328325437	0.0807014782423906	0.735440184543585	-4.3373735509199
a_HQ_transcript/9246	2.60384931753103	2.30592799102776	1.80255683473729	0.080713745643408	0.735440184543585	-4.34512155637722
a_HQ_transcript/13054	1.93109364694548	1.09118578829545	1.80251612394869	0.0807202789944273	0.735440184543585	-4.37398805526842
a_HQ_transcript/82649	0.777298839830564	3.63835730795465	1.80243295186891	0.08073362801753	0.735446533365144	-4.67042766328148
a_HQ_transcript/80817	3.01971049696292	1.87896228404415	1.80226708713469	0.0807602547169156	0.735451963981144	-4.46712146796277
a_HQ_transcript/24949	-2.80953029456114	0.658359811192282	-1.80224155949727	0.0807643533977843	0.735451963981144	-4.40286301455728
a_HQ_transcript/150266	-5.67923651142765	3.28445105947606	-1.80208813271444	0.0807889911056242	0.735451963981144	-4.54935253929471
a_HQ_transcript/72104	-2.25245822662815	0.339109528468881	-1.80202409305591	0.080799276664425	0.735451963981144	-4.34404206091059
a_HQ_transcript/77917	2.12442781288548	0.296949127438041	1.8019552749077	0.0808103309478042	0.735451963981144	-4.37122122932175
a_HQ_transcript/152190	-5.12099325958476	1.47826121616819	-1.80185832940012	0.0808259055188628	0.735451963981144	-4.56716525073403
a_HQ_transcript/103568	2.37713722296768	0.515249951728227	1.80184370335841	0.0808282554553837	0.735451963981144	-4.46708758982501
a_HQ_transcript/89635	-4.33224231158525	7.25178686623244	-1.80176203377827	0.0808413782085798	0.735451963981144	-4.96470717451585
a_HQ_transcript/22863	1.0373244145306	3.55329379192063	1.80169627100942	0.080851946354349	0.735451963981144	-4.56240815085059
a_HQ_transcript/118686	-1.85138771855667	-0.26998116475612	-1.80159308733049	0.0808685304451612	0.735451963981144	-4.36751222357461
a_HQ_transcript/17216	1.34048985213748	2.48273702964593	1.80156279755294	0.0808733992872191	0.735451963981144	-4.37521688886123
a_HQ_transcript/82277	-0.847463710698849	1.83055779541106	-1.80138090125292	0.0809026429167754	0.735583800632323	-4.31904536896925
a_HQ_transcript/62737	-1.52745927411748	2.18383741575804	-1.80128663952237	0.0809178009925229	0.735583800632323	-4.37009455809556
a_HQ_transcript/147953	-3.09997118036065	0.173438010161327	-1.80123652843419	0.0809258602574268	0.735583800632323	-4.38406929703032

a_HQ_transcript/8567	-1.06903313474889	1.68378309317158	-1.80115531289736	0.0809389234360534	0.735587514304395	-4.29838974880202
a_HQ_transcript/76522	2.15245847858212	2.07336063198121	1.80104769705113	0.0809562357532434	0.735629837715262	-4.4986980566718
a_HQ_transcript/59405	4.81016759257627	1.84328798645991	1.80088928677109	0.0809817251679269	0.735746439600071	-4.4128175607328
a_HQ_transcript/149638	-2.43191898367633	1.44643613879409	-1.80065401608081	0.0810195945868707	0.735965458610896	-4.32930663769964
a_HQ_transcript/12829	2.34847820153868	1.47113662083274	1.80048150396126	0.0810473718781691	0.735965458610896	-4.37444319039041
a_HQ_transcript/46239	2.45894897755878	1.21093921093785	1.80033533112939	0.0810709144412873	0.735965458610896	-4.25412588646984
a_HQ_transcript/121059	1.46309779921851	-0.704061233176376	1.80033148751691	0.0810715335710129	0.735965458610896	-4.38128710352643
a_HQ_transcript/1203	-2.61797640114992	0.213997096420145	-1.80020127988245	0.0810925098107313	0.735965458610896	-4.30965286695973
a_HQ_transcript/45116	-1.64093673366186	-0.340898544166373	-1.80004976300577	0.081116924738229	0.735965458610896	-4.34114359474894
a_HQ_transcript/86631	0.847406321645269	2.35071118036362	1.79990810575413	0.0811397565655218	0.735965458610896	-4.40991931041809
a_HQ_transcript/84517	-2.03693237227162	1.45408221748562	-1.79972819655565	0.0811687615777908	0.735965458610896	-4.35158174767965
a_HQ_transcript/125581	1.4686413356375	-0.44218301490114	1.7997211732846	0.0811698940501296	0.735965458610896	-4.28406248458685
a_HQ_transcript/143387	2.53596832377875	3.78909264569332	1.79958472932721	0.0811918977161415	0.735965458610896	-4.65439156232208
a_HQ_transcript/79244	-1.19416165575392	1.88158005077028	-1.79957005210366	0.0811942649437158	0.735965458610896	-4.35074284640023
a_HQ_transcript/138578	2.7532658134595	3.55193940880153	1.79956399569705	0.0811952417731162	0.735965458610896	-4.69385164029144
a_HQ_transcript/105241	4.23974966014441	2.36519192283269	1.79954350561621	0.0811985466637694	0.735965458610896	-4.45432542539329
a_HQ_transcript/135391	-0.811958617691604	3.59007627013724	-1.79946927516414	0.0812105204149594	0.735965458610896	-4.52003671887126
a_HQ_transcript/111233	1.19173690651949	2.54138772690801	1.79943198133122	0.0812165366667026	0.735965458610896	-4.41333092722475
a_HQ_transcript/29397	-1.57704220642409	1.69940315056492	-1.79939273112098	0.0812228689306257	0.735965458610896	-4.36938147891718
a_HQ_transcript/738	0.907658375214795	3.0396087836831	1.79935027620441	0.0812297186841222	0.735965458610896	-4.49308829099033
a_HQ_transcript/8841	2.12743149404339	1.52489080683508	1.79932540206751	0.0812337321505558	0.735965458610896	-4.45427078938864
a_HQ_transcript/58541	4.09958700039362	1.29337132444932	1.79920691822479	0.0812528519468081	0.736023964152105	-4.42353077656582
a_HQ_transcript/43526	2.1940466279961	-0.26412287210978	1.79902856882078	0.081281639484516	0.736079387454003	-4.38283194475068
a_HQ_transcript/66997	2.84618254556395	2.44694328117647	1.79897258379044	0.0812906778646761	0.736079387454003	-4.44418415678619
a_HQ_transcript/130196	2.1523084658144	-0.0315824265860388	1.79892052579891	0.0812990830184995	0.736079387454003	-4.45226303832828
a_HQ_transcript/62287	-1.96569785436857	0.992495135582673	-1.79885525405437	0.0813096226748237	0.736079387454003	-4.29956731826473
a_HQ_transcript/19010	1.59921200178162	-0.12359434143003	1.79869745034662	0.0813351085839756	0.73619545184823	-4.29436816885153
a_HQ_transcript/94817	-3.10787812717562	-0.0147721715269731	-1.79845674019463	0.0813739972704336	0.736295933390322	-4.36532637397998
a_HQ_transcript/111581	1.71428295292868	-0.0394653735417921	1.79838780075322	0.0813851379071322	0.736295933390322	-4.39384271759474
a_HQ_transcript/54085	-2.19438576141595	0.145818857356825	-1.79833359906979	0.0813938978266534	0.736295933390322	-4.42028597169007
a_HQ_transcript/88365	-2.92804416677089	0.853737190047065	-1.79824466659445	0.0814082725710141	0.736295933390322	-4.31483186701497
a_HQ_transcript/57063	1.63395851411466	0.114558727556972	1.79823680230077	0.0814095438325695	0.736295933390322	-4.32477468130514
a_HQ_transcript/139862	-1.97516976307202	1.30753902649287	-1.79813219369501	0.0814264553969732	0.736334318499573	-4.39693383564316
a_HQ_transcript/121116	-1.04435999113722	3.31602046049399	-1.79797127897429	0.081452475520746	0.736348801328769	-4.63270407364979
a_HQ_transcript/26518	4.86994022603171	2.54794774157995	1.79796560966198	0.0814533923848057	0.736348801328769	-4.46755920345201
a_HQ_transcript/61922	-1.22668619313724	3.20122758435705	-1.79783213930882	0.0814749802806402	0.736353801461814	-4.4741002016836
a_HQ_transcript/40217	0.621057762042588	4.36686146235522	1.79780555252808	0.0814792810866086	0.736353801461814	-4.75983381852229
a_HQ_transcript/99121	-1.28889468467889	4.15006019971674	-1.79772150532035	0.0814928782364676	0.736362198994153	-4.73139429663895
a_HQ_transcript/43038	1.34294976243273	-0.414698190083344	1.79749937591684	0.0815288235867284	0.736368284783456	-4.36434415632298
a_HQ_transcript/122782	0.429926257071791	4.05996235215232	1.7974807069842	0.0815318452376647	0.736368284783456	-4.74384921162992
a_HQ_transcript/121353	1.77280544983532	-0.481502798730645	1.79738485832889	0.0815473602720676	0.736368284783456	-4.37050643899433
a_HQ_transcript/100243	-1.9857259042236	1.02728033527871	-1.79733523420338	0.0815553939205849	0.736368284783456	-4.48887869135714
a_HQ_transcript/110231	0.553709579027072	3.51842701477869	1.79726597640912	0.0815666071865929	0.736368284783456	-4.67378889040913
a_HQ_transcript/17062	1.79144465930548	1.34193195999912	1.79721719112711	0.0815745066117583	0.736368284783456	-4.29460443405458
a_HQ_transcript/7574	-4.03992382065346	3.04398577037305	-1.79716950872979	0.0815822280829455	0.736368284783456	-4.69343750534147
a_HQ_transcript/70260	1.59853204510644	1.97294170000004	1.7970732911256	0.0815978110148438	0.736394590695674	-4.3606470620526
a_HQ_transcript/145363	0.468623903111667	3.67444784513811	1.79695163825288	0.0816175169355098	0.736407357101509	-4.66126698424032
a_HQ_transcript/95325	-2.47881791351557	2.18684543385785	-1.79684678984826	0.0816345040286826	0.736407357101509	-4.47506390196969
a_HQ_transcript/49643	-2.63773293466687	0.909402645785378	-1.79682995521327	0.0816372317843745	0.736407357101509	-4.28377198346609
a_HQ_transcript/24210	1.47887610225817	-0.600911742793392	1.79673985607563	0.0816518320758979	0.736424778108269	-4.38599008226923

a_HQ_transcript/88854	1.60108822582881	-0.310201787477441	1.79640999043746	0.0817053047162015	0.736686592336371	-4.3882865579889
a_HQ_transcript/112850	1.5133692838296	-0.5724367726203	1.79640441954347	0.081706208037307	0.736686592336371	-4.38601000800324
a_HQ_transcript/99424	1.59321803367743	1.25502502666306	1.79609033954303	0.0817571498603622	0.736884376334855	-4.36116109636465
a_HQ_transcript/603	2.22080065535391	2.14112855978006	1.79595767524176	0.0817786752827544	0.736884376334855	-4.33151656723098
a_HQ_transcript/106835	1.76805038368593	-0.250111308782474	1.79590065988006	0.0817879277779464	0.736884376334855	-4.37526070237528
a_HQ_transcript/77444	1.58249982708297	3.15698202713169	1.79582575497835	0.0818000847514952	0.736884376334855	-4.59394078207935
a_HQ_transcript/145220	1.94363837446543	3.9914814199678	1.79577310076152	0.0818086313842523	0.736884376334855	-4.65127148463005
a_HQ_transcript/68324	-1.49215620187486	1.77818626039965	-1.79576366661106	0.0818101627798393	0.736884376334855	-4.36110246252917
a_HQ_transcript/138812	0.683315330922526	3.01002710633842	1.79572226959631	0.0818168828252332	0.736884376334855	-4.53756817136992
a_HQ_transcript/149722	-2.17008007484297	2.21571638952948	-1.79563999300651	0.0818302403102115	0.736890504841515	-4.45649180983031
a_HQ_transcript/138955	0.450774964382818	4.03464161500822	1.79528022947878	0.0818886691413372	0.737286058063497	-4.71134005637476
a_HQ_transcript/18911	2.18077300934549	0.494804276799242	1.79520594967993	0.0819007372576743	0.737286058063497	-4.29248623204755
a_HQ_transcript/1180	-1.67453255250431	0.861323610679146	-1.79513529857313	0.0819122172270517	0.737286058063497	-4.36542382923572
a_HQ_transcript/21673	-1.73610733024646	0.0935383243095188	-1.79492900872873	0.0819457447031742	0.737427256674782	-4.37829774867985
a_HQ_transcript/114862	-1.7818974095787	-0.424567764682951	-1.79487878617414	0.081953908939909	0.737427256674782	-4.38448048191145
a_HQ_transcript/150604	0.759032598991885	2.76100750605464	1.79480464215485	0.0819659631395501	0.737427256674782	-4.52987477270605
a_HQ_transcript/53186	-1.89154471065045	0.403988776192424	-1.79464029091861	0.0819926884287981	0.73755354270956	-4.27739143158012
a_HQ_transcript/129881	4.55510498475998	2.95605302495069	1.79435821118096	0.0820385748846357	0.737779912434475	-4.49134444357669
a_HQ_transcript/107284	-1.43878379277098	1.96633691473115	-1.79432175149729	0.0820445074419462	0.737779912434475	-4.28621857911708
a_HQ_transcript/18429	2.48315699037136	1.14214168143197	1.79405608013514	0.0820877472951513	0.737779912434475	-4.35536741112761
a_HQ_transcript/133460	-1.81029842659171	2.80432460428195	-1.79397861143195	0.082100359503899	0.737779912434475	-4.37309718277165
a_HQ_transcript/121469	-1.20618806069751	1.86989843526558	-1.79395101055434	0.0821048534323143	0.737779912434475	-4.35999017998208
a_HQ_transcript/65869	-1.61718291179259	0.065538301923929	-1.79394732078535	0.0821054542101055	0.737779912434475	-4.40476409000693
a_HQ_transcript/75302	1.84086700803055	-0.368501632212684	1.79393221908817	0.0821079131466273	0.737779912434475	-4.39085252243452
a_HQ_transcript/145923	2.35861465739081	0.150483048894656	1.79376044645951	0.0821358864533969	0.737779912434475	-4.38965812913607
a_HQ_transcript/95495	-0.759977592463687	2.85669580457393	-1.79367904859356	0.0821491449832328	0.737779912434475	-4.45285809759515
a_HQ_transcript/13078	-1.47465723227642	3.3131423103773	-1.79363182409137	0.0821568380009905	0.737779912434475	-4.52351021992586
a_HQ_transcript/51480	-1.90314426382339	0.403807290552986	-1.79354100681744	0.0821716341345674	0.737779912434475	-4.34903109934904
a_HQ_transcript/20225	0.709825651591175	2.81348679570475	1.79347990938664	0.0821815895235107	0.737779912434475	-4.43547056608947
a_HQ_transcript/10914	1.35962831099307	-0.764637153812481	1.79342326559742	0.0821908201376588	0.737779912434475	-4.40234504812007
a_HQ_transcript/128085	1.21719975255691	10.3668466371729	1.79339426578073	0.0821955462586991	0.737779912434475	-5.38097247505536
a_HQ_transcript/76947	-1.81586252444659	1.98072878814225	-1.79275235056457	0.0823002187010506	0.738520787806615	-4.30522266454216
a_HQ_transcript/69449	-1.01403533853095	3.42159444170955	-1.79273225830264	0.0823034968244435	0.738520787806615	-4.67092840169443
a_HQ_transcript/41865	-1.87839475389731	1.01805773147739	-1.79260355514469	0.0823244978261918	0.738595216799024	-4.34644765873708
a_HQ_transcript/20155	-2.03141561779299	0.315963163822081	-1.7923038993729	0.0823734114555367	0.738920009427074	-4.32324706439632
a_HQ_transcript/106658	-2.24276571455231	0.0612286341481875	-1.7922005803086	0.0823902822233715	0.738957309841095	-4.43397619571557
a_HQ_transcript/147883	-0.573996048182833	5.22506222641486	-1.79208822961196	0.0824086310754949	0.739007853618693	-4.85323711538453
a_HQ_transcript/126423	3.93312066544715	2.39996326931631	1.79197927191457	0.0824264291019842	0.739015489369243	-4.62892718237681
a_HQ_transcript/36459	2.64709587998691	0.651317083391241	1.79192736136798	0.0824349097348984	0.739015489369243	-4.30477642290817
a_HQ_transcript/74296	1.49466498933581	-0.770887045255265	1.79163810324873	0.0824821794271257	0.739325231417111	-4.38189592751349
a_HQ_transcript/148962	1.35577864373036	-0.512750013421027	1.79142757450919	0.0825165978562294	0.739507190672409	-4.43012688628303
a_HQ_transcript/17838	2.38116711504032	0.172745582799388	1.79133459810912	0.0825318020417286	0.739507190672409	-4.42585001389866
a_HQ_transcript/40010	2.3905255144895	-0.0333392306149526	1.79126598219387	0.0825430241452694	0.739507190672409	-4.36993114290223
a_HQ_transcript/55144	-1.36034830045301	3.41384697766839	-1.79120274477035	0.0825533677448607	0.739507190672409	-4.5688107897777
a_HQ_transcript/130932	-3.06164379429987	0.630855056782185	-1.79103349351438	0.0825810571918299	0.739641246910122	-4.48375847989172
a_HQ_transcript/142884	1.47479199952706	-0.48303349558266	1.79071273013408	0.0826335555817193	0.739997430111567	-4.38038511764788
a_HQ_transcript/135585	2.10935167977844	0.5444847117821	1.79055229331796	0.0826598244169286	0.740118649677638	-4.34281700832293
a_HQ_transcript/86910	-3.98745110824821	1.69427356063325	-1.79012427530194	0.0827299398349498	0.74062741079708	-4.50707883031065
a_HQ_transcript/122220	2.59668142472436	1.09931220106482	1.78983305504845	0.0827776746689071	0.74062741079708	-4.33940830523306
a_HQ_transcript/136095	1.14004426972092	2.61578888804511	1.78966245658995	0.0828056488500223	0.74062741079708	-4.45152004260001

a_HQ_transcript/19274	-1.68108693208688	2.80478366882322	-1.78965652991447	0.0828066208310917	0.74062741079708	-4.31286553101323
a_HQ_transcript/127008	-1.92961637350993	1.04644348391115	-1.78959643014197	0.0828164778040818	0.74062741079708	-4.45289700501583
a_HQ_transcript/7204	-2.87173622977127	0.213224417394738	-1.78946873565577	0.0828374242994478	0.74062741079708	-4.43699994180404
a_HQ_transcript/134007	-3.49205451032407	3.33443884674762	-1.78930515910215	0.0828642633096939	0.74062741079708	-4.67961479231796
a_HQ_transcript/145947	1.4744733342284	1.15130162774891	1.7892712502266	0.0828698278698529	0.74062741079708	-4.27549726718921
a_HQ_transcript/86425	1.26283747205703	3.8152277502738	1.7892339343345	0.0828759518990613	0.74062741079708	-4.61936845399503
a_HQ_transcript/64488	-1.24904312404027	1.89778296038751	-1.78921631697085	0.0828788432737427	0.74062741079708	-4.33942420115776
a_HQ_transcript/141266	1.27443218046041	2.23220056291028	1.78910271053362	0.0828974905001042	0.74062741079708	-4.3540457887608
a_HQ_transcript/88433	0.373866021732006	3.89772099555293	1.78907552206963	0.0829019537099884	0.74062741079708	-4.72174113636207
a_HQ_transcript/124533	-1.80495342874482	1.55401774699593	-1.78904431741226	0.0829070764621594	0.74062741079708	-4.4124861647566
a_HQ_transcript/49490	2.44999478949991	0.804757973953536	1.7890120846701	0.0829123682729895	0.74062741079708	-4.37205036518813
a_HQ_transcript/54129	-1.59157569576641	-0.559590310874772	-1.78892083019284	0.0829273515310924	0.74062741079708	-4.37215096944953
a_HQ_transcript/38057	1.74593328530248	2.18168052516723	1.78874432992249	0.0829563379838245	0.74062741079708	-4.40691724804221
a_HQ_transcript/119594	1.8445298688394	1.36337078314256	1.78869615350904	0.0829642514375438	0.74062741079708	-4.46371231508495
a_HQ_transcript/118450	4.20311089548245	1.45393880774432	1.78869213333027	0.0829649118207117	0.74062741079708	-4.44088796227365
a_HQ_transcript/53537	-2.60562201872325	3.05404711787023	-1.78858001769432	0.0829833305281552	0.74062741079708	-4.64268539982633
a_HQ_transcript/59074	1.5782404571171	-0.300985334559071	1.78855550355152	0.0829873582500983	0.74062741079708	-4.36547247872463
a_HQ_transcript/110329	-1.3186307096617	-0.374176017708981	-1.78844253732989	0.0830059209646416	0.74062741079708	-4.39762887073311
a_HQ_transcript/118425	2.31281541624639	-0.30233991303476	1.7884046858231	0.0830121415467666	0.74062741079708	-4.44294913409776
a_HQ_transcript/105521	0.552538809079017	-0.807518985687392	1.78822581207981	0.0830415433146885	0.74062741079708	-4.45602699702214
a_HQ_transcript/149890	3.77833785428807	4.18404995784063	1.78818910451836	0.0830475780879177	0.74062741079708	-4.70597699717743
a_HQ_transcript/103144	1.01800734497809	3.11212676462168	1.78812196295825	0.0830586172140897	0.74062741079708	-4.57873084897559
a_HQ_transcript/9918	1.14981935456624	3.87026522413622	1.78808137848774	0.0830652905411954	0.74062741079708	-4.74452925130315
a_HQ_transcript/1881	0.657013942157237	4.01636186019015	1.787874979165	0.0830992359314806	0.74062741079708	-4.71168200453054
a_HQ_transcript/111629	1.88628142329594	-0.262744214942045	1.78785553060995	0.0831024351372811	0.74062741079708	-4.36970313144731
a_HQ_transcript/133859	3.76449116175818	4.7049666257111	1.78782505241566	0.0831074488825407	0.74062741079708	-4.73906526647523
a_HQ_transcript/128488	2.10366917656325	1.73904181057238	1.78774719537097	0.0831202577408751	0.74062741079708	-4.40484866454789
a_HQ_transcript/142694	2.59416057884239	0.34854200990991	1.78773149292039	0.0831228412739988	0.74062741079708	-4.33283944069778
a_HQ_transcript/90645	0.566405430709412	3.70956472606378	1.78771926498894	0.0831248531896614	0.74062741079708	-4.69424114910899
a_HQ_transcript/147335	1.21330185145548	-0.773834770951271	1.78764478431436	0.0831371087161256	0.74062741079708	-4.40088535249419
a_HQ_transcript/7732	1.79108817808652	1.10932950522229	1.78743029432956	0.0831724108273648	0.74077466299932	-4.31354924186558
a_HQ_transcript/38077	-2.12759118692698	0.29573058054926	-1.78731565368092	0.083191284316985	0.74077466299932	-4.40710715062717
a_HQ_transcript/80689	-3.51274913944185	0.0591564474795985	-1.78731209851627	0.0831918696679666	0.74077466299932	-4.39873590462064
a_HQ_transcript/45685	-1.57884082415741	-0.436567449623597	-1.78710875668977	0.0832253553173702	0.740959328210767	-4.42772636314944
a_HQ_transcript/44198	-2.29138712178	0.625721982235535	-1.78700492371948	0.0832424585884474	0.740998106099429	-4.40853778486781
a_HQ_transcript/70833	-2.68526067568013	0.972008206965717	-1.78668695753553	0.0832948522171913	0.741230198718877	-4.32248978114537
a_HQ_transcript/122278	1.76949446275055	-0.611558238548495	1.78651828542682	0.0833226569069324	0.741230198718877	-4.39889594895773
a_HQ_transcript/129002	-1.85178188278403	1.23037439057425	-1.7863726989868	0.0833466623734477	0.741230198718877	-4.31577078276206
a_HQ_transcript/128851	-2.99470114392158	-0.269972054698071	-1.7863074454952	0.0833574237977768	0.741230198718877	-4.42764555962322
a_HQ_transcript/81104	-2.14721776850699	0.726015135583625	-1.78624211859849	0.0833681985074954	0.741230198718877	-4.38059453263417
a_HQ_transcript/150064	0.870210699274865	4.71977568538049	1.78614520632142	0.0833841849350692	0.741230198718877	-4.79952162976622
a_HQ_transcript/42097	1.21257446883954	1.88005824497823	1.78589867615463	0.0834248636989349	0.741230198718877	-4.35492978833043
a_HQ_transcript/95039	0.894842957457619	3.27091395585408	1.78588331377828	0.083427399127774	0.741230198718877	-4.71035641496963
a_HQ_transcript/142732	3.52012027207257	3.91028635733935	1.78583930760273	0.0834346623321783	0.741230198718877	-4.65653584986769
a_HQ_transcript/8825	-1.30367273127943	1.99105162853143	-1.78573645819277	0.083451639682425	0.741230198718877	-4.33118907467582
a_HQ_transcript/76275	2.12092190587703	0.517402891494994	1.78573001872656	0.08345270274238	0.741230198718877	-4.37426547857361
a_HQ_transcript/102905	1.54723937186596	1.72978551304256	1.78571695982092	0.083454858608422	0.741230198718877	-4.32644756297881
a_HQ_transcript/30466	1.48798241879956	-0.67994042674167	1.78566708775727	0.0834630923121659	0.741230198718877	-4.40059964796091
a_HQ_transcript/51630	-1.82126282613359	0.556770307082903	-1.78564643420906	0.0834665023425105	0.741230198718877	-4.37873159839186
a_HQ_transcript/53034	1.56242393240711	-0.229292914103937	1.78564351395847	0.0834669845037259	0.741230198718877	-4.40949141927719

a_HQ_transcript/69338	-1.85647708329267	2.80314023887137	-1.78560975557779	0.0834725585056043	0.741230198718877	-4.46006790028397
a_HQ_transcript/34470	2.50473807540015	1.46037053238935	1.78548163612309	0.0834937157699899	0.74130482820803	-4.5248019906608
a_HQ_transcript/6138	1.80747461153632	1.77457115114707	1.78532498239138	0.0835195912749639	0.741421319735965	-4.41180670362662
a_HQ_transcript/144523	2.3939733042382	0.28384180118847	1.78520188451601	0.0835399289109036	0.741488622344328	-4.43640799209542
a_HQ_transcript/18531	2.30549224951407	0.213286526650712	1.78502373499532	0.0835693693450398	0.741636687219614	-4.36336474362206
a_HQ_transcript/95032	1.24564923727172	-0.43857465711974	1.7846940589175	0.083623873774252	0.741710160534655	-4.3645392848092
a_HQ_transcript/123526	-3.07517397262582	2.4451970303093	-1.78466938896002	0.0836279536007855	0.741710160534655	-4.52601540197847
a_HQ_transcript/129368	3.34038349416329	3.02928980412057	1.78463437751851	0.0836337439533135	0.741710160534655	-4.43266191237332
a_HQ_transcript/39064	1.94028905004193	2.14914273205873	1.7846113812917	0.0836375473594539	0.741710160534655	-4.48716758637844
a_HQ_transcript/52014	1.51768087960402	-0.692346530909253	1.78458779673667	0.0836414482229677	0.741710160534655	-4.41841411344671
a_HQ_transcript/132155	1.38531009580566	-0.446660281614639	1.7842937678625	0.0836900932683512	0.741983063520384	-4.41957780515561
a_HQ_transcript/19092	1.98149713939632	0.261525490521799	1.78424748681383	0.0836977523311168	0.741983063520384	-4.40669390328483
a_HQ_transcript/22982	-1.06843532330558	4.80568182169869	-1.7840617866405	0.0837284898734514	0.741995585319878	-4.82502942311254
a_HQ_transcript/47135	-0.677600441884151	2.46449754143107	-1.78392681225458	0.0837508371622046	0.741995585319878	-4.43197470848675
a_HQ_transcript/71126	-1.68745480487367	-0.218411114718043	-1.78375208509919	0.0837797736980239	0.741995585319878	-4.27644045625399
a_HQ_transcript/87183	2.43047329069689	1.60693296769132	1.78374009957295	0.0837817589294797	0.741995585319878	-4.35812886915693
a_HQ_transcript/40603	-3.31501768663668	2.54045037035366	-1.78373041981947	0.0837833622716582	0.741995585319878	-4.67692480528475
a_HQ_transcript/127432	4.42605723553454	2.50314265243979	1.78358097754361	0.0838081190009124	0.741995585319878	-4.5389318548738
a_HQ_transcript/41000	1.87229439908114	0.00519783705399295	1.78356560301764	0.0838106663089719	0.741995585319878	-4.30113580125457
a_HQ_transcript/129798	0.616013388576394	4.18921149666056	1.7835586648267	0.0838118158754676	0.741995585319878	-4.75783309708759
a_HQ_transcript/114872	-2.15111172288121	0.0150705399187037	-1.78354518987803	0.0838140485344467	0.741995585319878	-4.36927600414134
a_HQ_transcript/67955	-3.53053792267879	3.31340811056107	-1.7831623027299	0.0838775100164319	0.742377438075091	-4.69176974679659
a_HQ_transcript/149481	0.837051116803672	2.78160417278657	1.78313085232089	0.0838827245609207	0.742377438075091	-4.49086895990489
a_HQ_transcript/91389	1.47351777482945	-0.499315541158759	1.78298826265436	0.0839063696750605	0.742473657553953	-4.40526082130677
a_HQ_transcript/7166	-1.29743919155163	1.19459943232255	-1.78286939805891	0.0839260848637668	0.742535077771848	-4.34545166489047
a_HQ_transcript/119521	4.21837806656796	4.06461687486793	1.78248284203584	0.0839902271769178	0.742884882381987	-4.71057628748908
a_HQ_transcript/23527	-1.56744150547154	-0.345373535967475	-1.78242943831567	0.0839990918680851	0.742884882381987	-4.35336487700859
a_HQ_transcript/6515	-2.77858823393501	1.08058193047069	-1.78237091164496	0.0840088078478993	0.742884882381987	-4.33152466284979
a_HQ_transcript/113236	-1.76839845763202	1.14343690140482	-1.78232311860126	0.0840167426503437	0.742884882381987	-4.42622463900354
a_HQ_transcript/84116	-0.976744231964124	-0.696367500162709	-1.78219953376139	0.0840372636691789	0.74293710086518	-4.42847346081042
a_HQ_transcript/10500	2.56927126866432	1.79442765614388	1.78213361900735	0.0840482104199254	0.74293710086518	-4.43532614301102
a_HQ_transcript/7485	-2.46690107187699	1.03533731991228	-1.78191858828885	0.0840839299063608	0.743051230881946	-4.32640079065836
a_HQ_transcript/12453	0.628463396997357	3.81531293432004	1.78189684184805	0.0840875429976343	0.743051230881946	-4.71905752525714
a_HQ_transcript/109470	1.00385787726872	8.37588362174675	1.78182505555452	0.0840994709601624	0.743051230881946	-5.15816607269482
a_HQ_transcript/119175	0.396636151767635	4.1131097669535	1.78161905232709	0.0841337082756866	0.743240759065214	-4.76877803586164
a_HQ_transcript/143458	-3.05533651156509	1.56483717964473	-1.78146182502166	0.0841598470749378	0.743358697883602	-4.6300630587511
a_HQ_transcript/3741	-2.10674985393232	2.16689215750116	-1.78133558341114	0.0841808395274828	0.743388909060339	-4.53868478689926
a_HQ_transcript/115806	-2.84428074466301	2.0067009913538	-1.78121405354173	0.0842010526636539	0.743388909060339	-4.50794577342585
a_HQ_transcript/123349	-2.0465112076584	2.22827078467801	-1.78118856791119	0.0842052920154956	0.743388909060339	-4.30944273522553
a_HQ_transcript/10895	0.76869569558478	3.14427327581207	1.78104398576689	0.0842293456397725	0.743388909060339	-4.63254825984851
a_HQ_transcript/148301	1.7998967762724	0.741576694160512	1.78101301825118	0.0842344983532969	0.743388909060339	-4.42748762489467
a_HQ_transcript/42336	-1.65391381964474	-0.504896669949059	-1.78097995295847	0.0842400004125456	0.743388909060339	-4.36038267693373
a_HQ_transcript/88176	3.60432788289374	1.15454589797611	1.78061442153315	0.0843008450627846	0.743812920736118	-4.50254194864733
a_HQ_transcript/109573	-1.2093257902094	3.04469816306971	-1.78033258437869	0.0843477837608067	0.74399357127624	-4.68625139756892
a_HQ_transcript/37322	-0.774749445730216	3.12910455047163	-1.78024425685143	0.0843624988631553	0.74399357127624	-4.62123110180567
a_HQ_transcript/136087	0.631252360279421	2.63089269859537	1.78019315143037	0.0843710138663791	0.74399357127624	-4.53184463248375
a_HQ_transcript/80568	-1.55060379065034	1.90484129788517	-1.78018413514598	0.0843725162030859	0.74399357127624	-4.314320985582
a_HQ_transcript/54557	1.83356911123989	1.01913204899677	1.77991751909977	0.0844169512912146	0.744241375593678	-4.33581042356917
a_HQ_transcript/105375	2.52003106342724	1.71886616711597	1.77986188822101	0.0844262254105545	0.744241375593678	-4.32165422711862
a_HQ_transcript/8821	-0.714064412780689	2.79218421757	-1.77968232093464	0.0844561666221205	0.744392426597516	-4.48963223135187

a_HQ_transcript/33661	-1.69595576080643	0.64416344690561	-1.77947562358121	0.0844906426553959	0.744470900852115	-4.28684862015333
a_HQ_transcript/32001	-1.31791630008572	2.08518737689038	-1.77947537024332	0.0844906849181359	0.744470900852115	-4.36861226726964
a_HQ_transcript/141135	-1.98063045641855	1.2783121905218	-1.77917946411315	0.0845400612658905	0.744793070818988	-4.38298119988744
a_HQ_transcript/57724	1.89838587061655	1.47255145709243	1.77904940391122	0.0845617714760202	0.744838288617642	-4.35904347433008
a_HQ_transcript/320	-4.12969586134314	2.92180212933327	-1.77890757898134	0.0845854508847291	0.744838288617642	-4.55439011140083
a_HQ_transcript/2016	-1.71774739019051	1.57474027545625	-1.77885146756984	0.084594820921614	0.744838288617642	-4.36127595361899
a_HQ_transcript/124324	-1.28690942425883	1.73229275180395	-1.77884092165057	0.0845965820813111	0.744838288617642	-4.32762355465382
a_HQ_transcript/85041	-3.34761198378666	1.49185179450553	-1.77875718675199	0.0846105668410006	0.744838288617642	-4.44935335333814
a_HQ_transcript/146372	-2.06029377740385	3.64286395974031	-1.77868828100142	0.0846220764180929	0.744838288617642	-4.6270892797244
a_HQ_transcript/1349	-2.18311497519805	0.409189260919353	-1.77846438395129	0.084659483903636	0.744927612180604	-4.50125990927596
a_HQ_transcript/52513	2.38628504395944	0.780880050253032	1.77835923219026	0.0846770569106507	0.744927612180604	-4.46320817141102
a_HQ_transcript/144237	2.23271935238245	0.232128476800399	1.77830128358635	0.0846867426255926	0.744927612180604	-4.40222139517518
a_HQ_transcript/145748	0.918457790993346	3.99670066446308	1.77817498247195	0.0847078562471699	0.744927612180604	-4.69695124613458
a_HQ_transcript/12604	-0.444713862650097	4.83302289639497	-1.77812571260111	0.0847160938465592	0.744927612180604	-4.83843792901328
a_HQ_transcript/147892	1.84874307787414	1.50551263142685	1.77805370287739	0.0847281346199948	0.744927612180604	-4.33787607354087
a_HQ_transcript/73054	2.29067680917823	0.239653522077129	1.77804677656745	0.0847292928472909	0.744927612180604	-4.27691405667035
a_HQ_transcript/47961	-2.41071557367482	2.57398746617517	-1.77793537927432	0.084747922700569	0.744927612180604	-4.52187330774302
a_HQ_transcript/108402	1.39186142535812	2.57216513940914	1.77780897225788	0.0847690669482491	0.744927612180604	-4.59648129885599
a_HQ_transcript/68143	0.854091072812345	3.21873855697911	1.77779337250603	0.0847716766462586	0.744927612180604	-4.66103564985654
a_HQ_transcript/3330	-0.776086142515436	3.20233963817324	-1.77778430936035	0.0847731928601482	0.744927612180604	-4.67243952376877
a_HQ_transcript/51622	1.64240400569023	1.25083351049246	1.77770080113333	0.084787164402438	0.744937770916704	-4.28398256440288
a_HQ_transcript/12443	1.05468779640674	2.41256999353326	1.77753489386996	0.0848149276936119	0.745069082208595	-4.45700831788479
a_HQ_transcript/88028	1.77359623190042	-0.126345242881786	1.77745484923705	0.0848283252922845	0.745074175368678	-4.36455908079569
a_HQ_transcript/40615	-0.37845832849078	5.09115194505083	-1.77717744785163	0.0848747696543173	0.745369483376062	-4.8739335627652
a_HQ_transcript/131725	-1.77650816142621	2.01734420293576	-1.77693793129682	0.0849148884004002	0.745428083023544	-4.4507411676896
a_HQ_transcript/3387	1.6404191974785	1.2815716897319	1.77687943481664	0.0849246889312323	0.745428083023544	-4.33356445106087
a_HQ_transcript/93230	1.07232228671007	1.67308099706723	1.77687073967313	0.084926145801834	0.745428083023544	-4.31028723783807
a_HQ_transcript/76008	1.86487828817698	1.27855075009491	1.77682139236262	0.0849344143404126	0.745428083023544	-4.38559711161114
a_HQ_transcript/62370	-8.18010381682119	3.22988396745818	-1.77675486927363	0.0849455618969852	0.745428083023544	-4.38492749177804
a_HQ_transcript/142662	-0.759427315779372	2.51146922568697	-1.77650528378585	0.0849873970351609	0.745471296183377	-4.49183738471871
a_HQ_transcript/94833	-2.1271549796229	0.950155304644247	-1.77641942822616	0.0850017920427387	0.745471296183377	-4.41167079528467
a_HQ_transcript/18853	0.79276745213985	0.845515121217939	1.7763905110156	0.0850066409231618	0.745471296183377	-4.31400518238106
a_HQ_transcript/12670	-1.04816031326482	4.20614031187183	-1.77620331346465	0.0850380361464198	0.745471296183377	-4.76995012237578
a_HQ_transcript/146222	2.41115286389654	0.957668726861747	1.7761356667348	0.0850493837090123	0.745471296183377	-4.43333380093137
a_HQ_transcript/118954	2.88410365045463	0.875719589952646	1.77609961113969	0.0850554324645572	0.745471296183377	-4.37663482586035
a_HQ_transcript/96344	1.11163081903155	2.95468358889387	1.77607315316392	0.085059871336703	0.745471296183377	-4.51597447752414
a_HQ_transcript/9908	1.88900323761376	0.0200691408599067	1.7760668384825	0.0850609307839682	0.745471296183377	-4.37904787467066
a_HQ_transcript/17272	-2.14812837892308	0.977878300481055	-1.77602009518181	0.0850687735002564	0.745471296183377	-4.4262985777282
a_HQ_transcript/144111	-4.07277677731031	2.90993864748001	-1.7758447199291	0.0850982038848137	0.745471296183377	-4.5389431639551
a_HQ_transcript/47245	1.72731572098886	-0.667262427136083	1.77583923819707	0.0850991239338366	0.745471296183377	-4.42737366862197
a_HQ_transcript/151760	5.83742869039874	4.13349840372332	1.77579803071704	0.0851060404300412	0.745471296183377	-4.62066427627691
a_HQ_transcript/144736	1.2879009934448	1.9618465924682	1.77573150950649	0.0851172067294432	0.745471296183377	-4.31639005201721
a_HQ_transcript/137627	-2.658038009744	0.340997940077303	-1.775646143624	0.0851315381254712	0.745484490532899	-4.46326389153299
a_HQ_transcript/61591	1.97352123131916	0.489011172444346	1.77555740453618	0.0851464379862363	0.745502658120793	-4.36884194892484
a_HQ_transcript/110065	2.00842227448784	-0.575079074137024	1.77525091258434	0.0851979169185039	0.745552644280468	-4.41311337413154
a_HQ_transcript/101174	2.23380445381672	-0.617917836074971	1.77524915050574	0.0851982129564675	0.745552644280468	-4.4168342271588
a_HQ_transcript/139506	-3.69636819907693	1.16030709638778	-1.77522718724544	0.0852019029664041	0.745552644280468	-4.41841797314811
a_HQ_transcript/123137	2.31054900949378	3.90778992694562	1.77521797179283	0.0852034512791623	0.745552644280468	-4.70422612089941
a_HQ_transcript/13918	-1.46536593995901	1.33382754408442	-1.7750137397645	0.0852377709568165	0.745577471800123	-4.31366460207304
a_HQ_transcript/149555	0.816890243803139	3.74012040719048	1.77488286928209	0.0852597689185311	0.745577471800123	-4.70604418752888

a_HQ_transcript/15082	-2.00970204189598	0.279114414163112	-1.77484296074202	0.0852664780830168	0.745577471800123	-4.38584809524199
a_HQ_transcript/122601	1.84361467247504	0.645722960449492	1.77479440668067	0.0852746412786336	0.745577471800123	-4.33481614411472
a_HQ_transcript/116234	1.68672213978065	1.65375752193476	1.77475312817455	0.0852815817849026	0.745577471800123	-4.35166224084932
a_HQ_transcript/26581	-2.1343783640724	0.909670522095128	-1.77474322186375	0.0852832474882848	0.745577471800123	-4.38751401234946
a_HQ_transcript/105147	-0.676064266984586	3.00123340860059	-1.77459677979306	0.0853078743010457	0.745680619153562	-4.57104157302896
a_HQ_transcript/41951	-2.12973228640216	1.98300255441828	-1.77433937720019	0.0853511756600034	0.74579082731674	-4.61974395665121
a_HQ_transcript/59895	2.45437448855667	-0.0888566219738001	1.77425863293228	0.0853647626346169	0.74579082731674	-4.37411010505651
a_HQ_transcript/107932	1.36300551052172	0.418222182586119	1.7742005734106	0.0853745335393638	0.74579082731674	-4.38206431761969
a_HQ_transcript/137296	1.97278154725475	0.326203497192782	1.77414539168026	0.0853838210141135	0.74579082731674	-4.46021538606523
a_HQ_transcript/57971	-1.79547197673566	-0.25560537490175	-1.77414056659381	0.0853846331508551	0.74579082731674	-4.42603119103274
a_HQ_transcript/96989	1.2731759929929	1.86054418039436	1.77404607982815	0.0854005380530692	0.745817679679809	-4.33136913355333
a_HQ_transcript/77030	2.65918313266903	2.56918028248199	1.77395924502902	0.0854151571140412	0.745833296188221	-4.44809170082963
a_HQ_transcript/24323	-1.18874459578328	-0.68890582963637	-1.77383012217826	0.0854368994884578	0.745911098447743	-4.40970667223624
a_HQ_transcript/131882	0.899833433025635	2.07905250244445	1.77371777247784	0.0854558213215421	0.745957666920208	-4.32978934930266
a_HQ_transcript/26418	2.78575876465919	2.30638850967947	1.77364606850075	0.085467899492303	0.745957666920208	-4.55436668082619
a_HQ_transcript/92572	-0.61544165271841	3.47362855595859	-1.77355394940059	0.0854834186059665	0.745981107450476	-4.65409691363253
a_HQ_transcript/115466	1.81375751400825	0.766256553678681	1.77342500952106	0.0855051448428332	0.746058700003634	-4.36468161790316
a_HQ_transcript/85166	2.3862380784215	0.840617589948049	1.77331218215697	0.0855241599687758	0.746112617539396	-4.36909331847811
a_HQ_transcript/111535	3.20542751703443	1.2475107348603	1.77306450754761	0.085565913839	0.746271381255805	-4.51329307889988
a_HQ_transcript/46803	-0.86465682402076	1.98274653582502	-1.77305192622947	0.085568035302607	0.746271381255805	-4.42664751407397
a_HQ_transcript/15233	2.33335395572752	0.160136073601032	1.77277623540732	0.0856145334959956	0.746564896947979	-4.36485786313398
a_HQ_transcript/138963	2.11655901503517	-0.139563593598852	1.77253764931549	0.0856547908284808	0.746689862539763	-4.45557768798956
a_HQ_transcript/105258	1.40415737227721	0.894799751157245	1.77248552870018	0.0856635874233631	0.746689862539763	-4.37048024890667
a_HQ_transcript/142836	2.15429611140261	-0.0106869008916845	1.77240399139685	0.085677350317241	0.746689862539763	-4.43572040490136
a_HQ_transcript/20519	1.53531678536049	2.66387901635959	1.77238683308843	0.0856802467509671	0.746689862539763	-4.48768854140674
a_HQ_transcript/91749	2.66004669491894	-0.0109376323824606	1.77223827889412	0.0857053271244296	0.746766606593679	-4.44274101478127
a_HQ_transcript/1123	1.43500613432627	0.758279039550111	1.77209133871459	0.0857301411114152	0.746766606593679	-4.32851949981699
a_HQ_transcript/41134	2.2947461771667	-0.0946321884576493	1.77183458945346	0.0857735132884575	0.746766606593679	-4.3955198582556
a_HQ_transcript/71279	-1.58861029449664	1.21473438213037	-1.77181725092853	0.0857764429220523	0.746766606593679	-4.32998160783402
a_HQ_transcript/129199	-3.10510868446595	0.399570930756641	-1.7717676706386	0.0857848208076744	0.746766606593679	-4.4053485981215
a_HQ_transcript/152463	1.4119997158146	-0.657255764106463	1.77175523952276	0.0857869214780348	0.746766606593679	-4.43473800399254
a_HQ_transcript/74037	1.64390338241372	-0.166578989589136	1.771401993186	0.085846632942738	0.746766606593679	-4.43166909955755
a_HQ_transcript/30803	1.77638585070637	1.16240377524911	1.771385756671	0.0858493783482092	0.746766606593679	-4.37259631900241
a_HQ_transcript/150136	-0.590544527592513	2.9923692827851	-1.77130473385271	0.08586307947214	0.746766606593679	-4.60303963906042
a_HQ_transcript/122149	-3.36527963505554	0.154169986993424	-1.77125055624145	0.0858722420482018	0.746766606593679	-4.41407910695176
a_HQ_transcript/57883	-1.98793033861548	0.386953612839071	-1.7711580100489	0.0858878954723574	0.746766606593679	-4.46184067123667
a_HQ_transcript/79079	-1.18090911486742	2.45202136387537	-1.77107944827969	0.0859011854400217	0.746766606593679	-4.58945958094165
a_HQ_transcript/36519	-1.55479695173244	1.90769346168735	-1.77101209043707	0.0859125814704434	0.746766606593679	-4.39246329297797
a_HQ_transcript/66942	-1.66846259147608	-0.330164771000001	-1.77080282160716	0.0859479950638709	0.746766606593679	-4.40657681243283
a_HQ_transcript/95234	-1.76208674095145	2.17980990472377	-1.77072896112747	0.0859604970765901	0.746766606593679	-4.39616066585629
a_HQ_transcript/2463	2.849101049571	2.29739049860994	1.77068456559701	0.0859680124370301	0.746766606593679	-4.42219967464096
a_HQ_transcript/11339	-2.15304100745688	3.31094082428999	-1.770679711268	0.085968834220834	0.746766606593679	-4.54688391501682
a_HQ_transcript/150848	-3.17331677278078	1.18731362957944	-1.77066579616592	0.0859711899293478	0.746766606593679	-4.46369196095874
a_HQ_transcript/138049	2.20483952569426	0.51935481506138	1.770649783119	0.0859739008696271	0.746766606593679	-4.34330872609449
a_HQ_transcript/65741	1.94884905973878	0.241208700116424	1.77059860461445	0.0859825656559287	0.746766606593679	-4.40335159869115
a_HQ_transcript/117401	0.495585556238696	3.43779686943638	1.7705820933882	0.0859853612496937	0.746766606593679	-4.67097590659868
a_HQ_transcript/54986	1.03503864810229	-0.587096720344276	1.77051136366036	0.0859973377039229	0.746766606593679	-4.39860474112291
a_HQ_transcript/144587	-1.71798717127939	2.28816822069427	-1.77048831608203	0.0860012405863724	0.746766606593679	-4.44120254948324
a_HQ_transcript/102627	-2.38874202653102	0.577226487276973	-1.77043227714381	0.0860107308583258	0.746766606593679	-4.37803072056967
a_HQ_transcript/110533	1.70948098131401	1.19654684641862	1.77041839517135	0.0860130819264482	0.746766606593679	-4.39461894267334

a_HQ_transcript/13653	1.87983594771444	0.505768835151545	1.77035940501283	0.0860230731790406	0.746766606593679	-4.38803804452111
a_HQ_transcript/103616	-3.81184239984556	0.8596650680444	-1.77008805970911	0.0860690439818309	0.747054112076432	-4.49156376371542
a_HQ_transcript/53514	-0.789069016541694	2.47060005339972	-1.76997175324495	0.0860887547583988	0.747113636398359	-4.45337649957034
a_HQ_transcript/149211	1.71933191874795	-0.409305568873601	1.769838151867	0.0861114012606682	0.747198616581597	-4.43618741705031
a_HQ_transcript/37310	0.802450417213787	2.20156358063017	1.76971957960584	0.0861315044252639	0.747261505855483	-4.45195643739198
a_HQ_transcript/128051	-2.85539246524464	2.97813407114793	-1.76953885374176	0.0861621529651045	0.747330925740705	-4.65477745984642
a_HQ_transcript/96273	-1.29241008793332	2.02877267892261	-1.76950980908154	0.0861670793866866	0.747330925740705	-4.46227531563669
a_HQ_transcript/130482	1.84028913106767	0.0982676037182316	1.76944498216663	0.0861780758883833	0.747330925740705	-4.34939464247018
a_HQ_transcript/55451	2.43869837123651	0.604694479429572	1.76935936402054	0.0861926009963069	0.747345392409506	-4.37933959252414
a_HQ_transcript/14622	-1.85944965050098	2.05805756980969	-1.76900180302062	0.0862532835442936	0.747477168346774	-4.4152493539661
a_HQ_transcript/69670	1.87899868466319	-0.454841919929917	1.7689813259971	0.0862567598427404	0.747477168346774	-4.4323389632561
a_HQ_transcript/123383	0.93504479171594	2.46144278197816	1.7689774262874	0.0862574218935334	0.747477168346774	-4.45688586513857
a_HQ_transcript/42405	0.493657999367847	3.7997144082404	1.76882762434696	0.0862828569081222	0.747477168346774	-4.72934993407327
a_HQ_transcript/109239	2.42851402107941	4.07868922166362	1.76882542391428	0.0862832305689257	0.747477168346774	-4.74020147227731
a_HQ_transcript/152801	-2.04709426826218	-0.154826750121614	-1.76881527564923	0.0862849538880893	0.747477168346774	-4.39955819811269
a_HQ_transcript/113771	1.34583391753281	-0.729785010578226	1.76854622379878	0.0863306533142372	0.747537875618271	-4.42706406722783
a_HQ_transcript/109016	1.49702648188862	2.31486663785163	1.76827120077444	0.0863773881060682	0.747537875618271	-4.52216910643308
a_HQ_transcript/114390	2.56318164245974	0.395926899773843	1.7682295035082	0.086384475608367	0.747537875618271	-4.50205941383589
a_HQ_transcript/136153	1.50205580989573	-0.544956853886995	1.7682052859307	0.0863885922222129	0.747537875618271	-4.4015853457675
a_HQ_transcript/121579	1.99206961596649	0.0163070807919398	1.76815385438613	0.086397335339733	0.747537875618271	-4.37856952138121
a_HQ_transcript/136102	0.961197790855883	1.7553412196784	1.76815274873964	0.0863975233025784	0.747537875618271	-4.38958182391534
a_HQ_transcript/76676	1.3494084088399	0.582289201626283	1.76812098016952	0.0864029241916576	0.747537875618271	-4.36028692992952
a_HQ_transcript/82575	1.31197830009224	-0.762793383411477	1.76800483893788	0.0864226714780705	0.747537875618271	-4.44179585114643
a_HQ_transcript/13566	1.79813142770754	2.41487348749164	1.76793592616081	0.0864343903974346	0.747537875618271	-4.47757267175215
a_HQ_transcript/39030	-2.78813501527856	1.80155810233997	-1.76792890405683	0.0864355846123377	0.747537875618271	-4.35459627476512
a_HQ_transcript/28334	0.515232304304805	2.91551176725466	1.76789082449892	0.0864420608594662	0.747537875618271	-4.5402968820861
a_HQ_transcript/26553	4.80971193966891	0.455379849100586	1.76786178694611	0.0864469995948789	0.747537875618271	-4.39576631535812
a_HQ_transcript/74059	1.58806250833315	-0.378596598852712	1.76777227492295	0.0864622253878966	0.747537875618271	-4.43744332822536
a_HQ_transcript/107761	-2.50843968988088	1.32190334800668	-1.76768703101292	0.0864767272910924	0.747537875618271	-4.46373062074523
a_HQ_transcript/87724	1.66832076975884	-0.229056323339271	1.76757953977007	0.0864950168951425	0.747537875618271	-4.37864675507617
a_HQ_transcript/140989	2.48146588678642	1.41264603962786	1.76756362613789	0.0864977248728409	0.747537875618271	-4.4520057636752
a_HQ_transcript/97424	-1.71410062068444	-0.456409518718136	-1.76744773513685	0.0865174478767989	0.747597177074858	-4.43544173724453
a_HQ_transcript/105129	2.06687694766539	0.0321053315526079	1.76736971753992	0.0865307275048849	0.747598516288518	-4.41540436939229
a_HQ_transcript/126214	1.80660928726648	0.28394868109795	1.76729571474133	0.0865433253527635	0.747598516288518	-4.37613069297643
a_HQ_transcript/65683	-2.18874794897859	2.37439304771058	-1.76713152881261	0.0865712810367619	0.747728889168633	-4.4183167788345
a_HQ_transcript/8513	-1.23731400009153	3.15325170395346	-1.76690326262863	0.086610160244482	0.747842487235568	-4.53712485865296
a_HQ_transcript/33308	-2.7695375692785	3.33698239694673	-1.76689638967696	0.0866113311021477	0.747842487235568	-4.70132531776473
a_HQ_transcript/85899	1.59688590179781	-0.164324521845324	1.76642152857413	0.0866922595350942	0.747842487235568	-4.38747496712968
a_HQ_transcript/72643	-2.44462287916572	0.0130493088322419	-1.76636111752209	0.0867025596971926	0.747842487235568	-4.40150058467177
a_HQ_transcript/26423	1.31017121383555	1.25124144735293	1.76633994623916	0.0867061696728258	0.747842487235568	-4.31062854275082
a_HQ_transcript/145798	3.56555684079884	4.20130277874291	1.76625467994183	0.086720709957176	0.747842487235568	-4.82252330621199
a_HQ_transcript/81982	-0.882063890490809	1.81312735146949	-1.76609174655775	0.0867485003775351	0.747842487235568	-4.36737770863865
a_HQ_transcript/84211	2.70127399578383	2.74358457403015	1.76607582178279	0.0867512169598178	0.747842487235568	-4.49949170985259
a_HQ_transcript/140729	1.60743691127305	1.36544286892751	1.7660700591584	0.0867522000145453	0.747842487235568	-4.33614585618635
a_HQ_transcript/146789	1.36881016167131	-0.645187553323798	1.76602716811914	0.0867595171568996	0.747842487235568	-4.43509527139079
a_HQ_transcript/51901	-0.389196114970594	4.85462704294849	-1.76597202858472	0.0867689246388156	0.747842487235568	-4.84364106924856
a_HQ_transcript/82168	2.0617736917087	1.47587995426231	1.76593288463846	0.0867756035999183	0.747842487235568	-4.40361422263489
a_HQ_transcript/54877	1.95403745196395	3.82137476035654	1.7657557457606	0.0868058334652926	0.747842487235568	-4.6874489630142
a_HQ_transcript/88934	-1.78702121925719	1.00098255996975	-1.76574513506691	0.0868076445293853	0.747842487235568	-4.4288746700175
a_HQ_transcript/150823	-0.748928165902605	2.67899171406818	-1.76562636772471	0.0868279182634281	0.747842487235568	-4.50462888320245

a_HQ_transcript/115289	1.53278916508026	2.5028960043993	1.76562567767888	0.0868280360668027	0.747842487235568	-4.52738246870238
a_HQ_transcript/87794	-1.38053848381052	0.226410842981287	-1.76557420513651	0.0868368237466032	0.747842487235568	-4.46766399709009
a_HQ_transcript/100676	2.11972199847439	0.482873562500523	1.76549643688557	0.0868501022016328	0.747842487235568	-4.38402223863094
a_HQ_transcript/127757	-0.806263571601571	2.88986030999307	-1.76549361783267	0.0868505835699517	0.747842487235568	-4.61764510980449
a_HQ_transcript/132331	1.55390410995474	0.878613574664931	1.76545362358449	0.0868574130439558	0.747842487235568	-4.34498817923535
a_HQ_transcript/22877	-1.38860411504186	2.26948041012852	-1.76543463958744	0.0868606549367304	0.747842487235568	-4.52373934365791
a_HQ_transcript/87927	0.900292833166657	2.77755007936627	1.76535715608295	0.0868738878368011	0.747842487235568	-4.51432547175028
a_HQ_transcript/132972	-2.33919526985553	2.3557005656242	-1.76531938869082	0.0868803384995492	0.747842487235568	-4.56060383208114
a_HQ_transcript/139760	-3.55608406095194	6.10063754318197	-1.76522444383439	0.0868965568484508	0.747871343868337	-4.87702111808417
a_HQ_transcript/110565	2.08422710444179	1.48576933957646	1.76511375608746	0.0869154676046027	0.74792336060997	-4.44408318885718
a_HQ_transcript/117137	-1.51454581323246	-0.666466011080466	-1.76496253452954	0.0869413090911819	0.748002704867875	-4.42567045076943
a_HQ_transcript/70762	-2.4067451112446	0.396475680218804	-1.76490920106378	0.0869504245250522	0.748002704867875	-4.37489302557334
a_HQ_transcript/98930	0.353983486282241	-0.500487324602008	1.76476611682313	0.0869748836073528	0.74802660393089	-4.63140439621427
a_HQ_transcript/120326	-3.62293697180788	1.44182407120023	-1.76474239128283	0.0869789398563324	0.74802660393089	-4.49140655297383
a_HQ_transcript/136543	4.60119837086964	5.71637422547813	1.76455907296987	0.0870102863513411	0.748041394936066	-4.81281011256435
a_HQ_transcript/102592	0.554845121064724	3.28348467901436	1.76452902378899	0.0870154255178526	0.748041394936066	-4.66456578932827
a_HQ_transcript/103632	0.846008065918075	2.14190641805416	1.76450601014005	0.0870193616046177	0.748041394936066	-4.40401052074635
a_HQ_transcript/138271	-0.904664159557038	3.5526449533981	-1.76443133119008	0.0870321351836053	0.748041394936066	-4.68496602130211
a_HQ_transcript/136709	0.490839998041012	6.13611398352922	1.76419367632314	0.0870727957750791	0.748280229570342	-4.95770616691374
a_HQ_transcript/32519	2.09370427446474	2.86361603909581	1.76370746845832	0.087156031516776	0.748778864389916	-4.5507211132324
a_HQ_transcript/1158	-2.15160969912787	0.394862898188013	-1.76370425441113	0.0871565819650112	0.748778864389916	-4.38936902370704
a_HQ_transcript/61226	3.39620631446643	2.85453246595129	1.76357164242656	0.0871792960868901	0.748824995347046	-4.66839967015278
a_HQ_transcript/26923	-3.33604217784505	-0.379622403584119	-1.76332884496281	0.087220896015885	0.748824995347046	-4.45060639524006
a_HQ_transcript/127592	1.92376234346008	-0.583186555159612	1.76328297618026	0.0872287568685544	0.748824995347046	-4.4309309677498
a_HQ_transcript/72835	-1.25094901516126	-0.776630713969123	-1.76322159596122	0.0872392769600203	0.748824995347046	-4.41948633852289
a_HQ_transcript/144355	-2.33494682291683	0.299625718619977	-1.76321181683818	0.0872409531244819	0.748824995347046	-4.44120771587576
a_HQ_transcript/68303	1.79090110315239	1.36530191958264	1.76320097053996	0.0872428122370606	0.748824995347046	-4.31726879374
a_HQ_transcript/3786	-1.86902486319899	0.667557480197124	-1.76314662442916	0.0872521279501367	0.748824995347046	-4.47682917170185
a_HQ_transcript/149063	-2.5413091345185	1.00985531466352	-1.76303952810001	0.0872704882757041	0.748834275239881	-4.50640523170973
a_HQ_transcript/118568	3.21866655633606	0.851784594505884	1.76294360926248	0.0872869351294775	0.748834275239881	-4.3827297748075
a_HQ_transcript/56101	2.47289556801795	1.78096195940341	1.76291490911683	0.0872918567476162	0.748834275239881	-4.50338122262066
a_HQ_transcript/108749	6.89870308862781	2.02636733091278	1.76276096187983	0.0873182602477939	0.748903145415768	-4.58492718457887
a_HQ_transcript/86685	4.05770289361065	0.316274759692191	1.76271766949283	0.0873256865424721	0.748903145415768	-4.46322324600531
a_HQ_transcript/15341	-1.78247103523441	-0.0710068119565169	-1.76264276931714	0.0873385360372539	0.748903145415768	-4.36121084442926
a_HQ_transcript/147243	2.34742043601011	0.59000732582629	1.76254101938777	0.0873559942971614	0.748942365266283	-4.39700939424542
a_HQ_transcript/124514	-2.34221121050759	1.92309986571354	-1.76231188105426	0.087395320657601	0.749037382307289	-4.46301134628831
a_HQ_transcript/30673	-0.817075511686116	1.92407171928828	-1.76229986023393	0.0873973841693891	0.749037382307289	-4.4014185049688
a_HQ_transcript/25494	-1.36454656494865	3.21446463657527	-1.762176952536	0.0874184850470906	0.749037382307289	-4.70313834399662
a_HQ_transcript/39498	2.01658820041541	4.4414346286155	1.7621761607147	0.0874186210014562	0.749037382307289	-4.75625456457946
a_HQ_transcript/105983	-1.96844092183423	0.339101881386242	-1.76187755262554	0.0874699042295065	0.749366336485299	-4.38144436190137
a_HQ_transcript/20289	-0.606529582495182	2.55503209964002	-1.76161590103972	0.0875148613894818	0.749525846351935	-4.401102016458
a_HQ_transcript/99981	-2.02141934781627	-0.312677388448333	-1.76161070259479	0.0875157547875468	0.749525846351935	-4.4401998630126
a_HQ_transcript/151876	-3.96775041237802	0.429587781584227	-1.76154407354405	0.0875272062523558	0.749525846351935	-4.50676743038878
a_HQ_transcript/118801	0.721668945786584	2.63294886871926	1.76134018263966	0.0875622566257827	0.749715547671748	-4.49097647495618
a_HQ_transcript/69098	2.31390380458712	1.55357606627967	1.76095970484522	0.0876276952936398	0.750066146473425	-4.313417550528
a_HQ_transcript/146150	-1.57408598455038	3.23788937959481	-1.76095205251004	0.0876290118480083	0.750066146473425	-4.58918235978928
a_HQ_transcript/124695	-2.17836787769181	0.385561835966832	-1.76070209168583	0.0876720258123029	0.75032383928409	-4.44208537785333
a_HQ_transcript/135270	1.47780380207064	-0.721082205483202	1.76052943193507	0.0877017479953733	0.750467717867667	-4.44689265405906
a_HQ_transcript/67830	1.6160225518903	0.872184689299377	1.76002876527623	0.0877879823922359	0.750922401234486	-4.38693578801349
a_HQ_transcript/54442	2.36038637764978	0.25195545131165	1.76001057767071	0.0877911163564166	0.750922401234486	-4.44877787105177

a_HQ_transcript/51935	-1.30662012465787	1.41008548849914	-1.75995284072395	0.0878010658191018	0.750922401234486	-4.40310717934768
a_HQ_transcript/136377	-0.494503137886282	3.11111448889023	-1.7599209760607	0.0878065572734448	0.750922401234486	-4.5901837085342
a_HQ_transcript/103628	1.44564555223868	-0.681984390941804	1.75971376817907	0.0878422738898161	0.751117342846018	-4.45266404390032
a_HQ_transcript/112948	1.82083577862403	-0.557573416344152	1.75940904196189	0.0878948221066901	0.751456128756829	-4.44236645963188
a_HQ_transcript/10289	-0.729905865958582	2.88153049197842	-1.75928128374294	0.0879168611399406	0.75153401534448	-4.52885072957325
a_HQ_transcript/27161	2.78269300899729	3.78914325016031	1.75917350842305	0.0879354566334251	0.751582447167731	-4.79290273515024
a_HQ_transcript/79139	-3.29540492849026	0.389701994047183	-1.75891324142936	0.087980376641065	0.7517333146033	-4.47789591832097
a_HQ_transcript/146560	2.5905390089755	0.632709750444392	1.75891096505507	0.0879807696105158	0.7517333146033	-4.51729665000742
a_HQ_transcript/107986	3.09552513871692	1.32757319323828	1.75878149239029	0.0880031228552485	0.7517333146033	-4.403571843188
a_HQ_transcript/915	-0.687978440149703	2.59588034073892	-1.75877156094574	0.0880048377008577	0.7517333146033	-4.5531271606832
a_HQ_transcript/147732	1.66207030381995	-0.642882653028724	1.75867975106593	0.0880206916915479	0.751758267212209	-4.44475085475178
a_HQ_transcript/8748	-2.77589298599615	2.0690731552463	-1.75831948291249	0.0880829271038178	0.751845291582378	-4.57119028190397
a_HQ_transcript/98725	-0.56784438571756	2.97228863112533	-1.75825019315293	0.0880949009906316	0.751845291582378	-4.59243316068607
a_HQ_transcript/70059	-2.11468038533675	0.900827796883273	-1.75818415386221	0.0881063144457189	0.751845291582378	-4.41631515671314
a_HQ_transcript/129214	0.594873444660635	3.14509364829837	1.75817532988231	0.0881078395732141	0.751845291582378	-4.64459388226242
a_HQ_transcript/97938	0.739552422973714	1.30356863457076	1.75811325043892	0.0881185699491559	0.751845291582378	-4.38284763601665
a_HQ_transcript/90826	-3.86953870248683	0.599015585338006	-1.75810234153303	0.0881204556576203	0.751845291582378	-4.45271476531391
a_HQ_transcript/74358	-4.25920143047688	2.37993921080954	-1.75803338534594	0.0881323761802767	0.751845291582378	-4.6087922308041
a_HQ_transcript/86238	0.713933143905348	3.53455001933091	1.75787419984559	0.0881598999271368	0.751845291582378	-4.74100110023976
a_HQ_transcript/29740	-2.92305939452929	1.57832609147501	-1.75779225094868	0.0881740720142472	0.751845291582378	-4.58322500159015
a_HQ_transcript/127890	1.83168656387242	-0.158390696333444	1.7577797166395	0.088176239843417	0.751845291582378	-4.52602247753396
a_HQ_transcript/80074	-2.10306375043625	-0.026202219726076	-1.75761824329191	0.0882041709427532	0.751845291582378	-4.38844127089366
a_HQ_transcript/32731	2.06427926461433	0.911483640056343	1.75760855302442	0.0882058473689826	0.751845291582378	-4.39380427002084
a_HQ_transcript/146452	-4.00601526594424	2.50139449104098	-1.75759171832745	0.0882087598527645	0.751845291582378	-4.58176979315073
a_HQ_transcript/118360	-1.31654830896928	1.34028411231825	-1.75750934247617	0.0882230124422041	0.751845291582378	-4.39653021485539
a_HQ_transcript/40882	-0.786209409007199	2.19766855843345	-1.75749845896438	0.0882248956420899	0.751845291582378	-4.51138803222104
a_HQ_transcript/129904	-1.47902369936681	1.15260545922625	-1.75731713164702	0.0882562761279082	0.752002465371306	-4.34469753859067
a_HQ_transcript/129484	1.49964925018174	-0.481384617841928	1.75698583348983	0.0883136348691932	0.75238091274754	-4.44505487822975
a_HQ_transcript/45221	1.50941119909216	0.949842070379076	1.75678193413396	0.0883489522665803	0.75257149726726	-4.29257218229709
a_HQ_transcript/11916	1.67408039065695	0.614139246374474	1.75665366630603	0.0883711756348682	0.752577242763195	-4.30288239912745
a_HQ_transcript/129192	-1.80791418114275	0.939680506937446	-1.75662859164538	0.0883755205598261	0.752577242763195	-4.39961403267783
a_HQ_transcript/63621	-1.64440254296963	-0.620725562093172	-1.7565496408727	0.0883892022880953	0.752583499429091	-4.42361290704396
a_HQ_transcript/138158	-1.34211141644947	2.53729115729537	-1.75646583933852	0.0884037265808753	0.752596927166538	-4.60135175052741
a_HQ_transcript/119005	-0.786326155901038	3.85606377728256	-1.75618628809812	0.0884521923305279	0.752897445198559	-4.76350763926101
a_HQ_transcript/147488	-3.45686388139288	1.2843034679535	-1.75604534559399	0.0884766360044369	0.752897445198559	-4.39767846278604
a_HQ_transcript/147997	-1.99414515805662	0.0523915143191722	-1.75603814982428	0.0884778841204128	0.752897445198559	-4.49199385696421
a_HQ_transcript/145412	-1.85170329337189	0.00743542128710528	-1.75579223031807	0.0885205481177877	0.753075399619215	-4.44136064744606
a_HQ_transcript/122939	1.15561510230198	2.43173769147519	1.75576825977845	0.0885247076382827	0.753075399619215	-4.4663152219971
a_HQ_transcript/81605	1.54791590466408	3.2695868796738	1.75560648450279	0.0885527842236559	0.753146916468211	-4.5630969355496
a_HQ_transcript/63084	1.86123414941676	0.277890646250886	1.75547418206677	0.0885757512911312	0.753146916468211	-4.37724164388501
a_HQ_transcript/52146	1.56781334826452	0.0381121342276484	1.75545392285709	0.0885792686361193	0.753146916468211	-4.43141105709276
a_HQ_transcript/105158	-1.73217663630339	4.170049967397	-1.755367899322	0.088594205103782	0.753146916468211	-4.86243474222849
a_HQ_transcript/20589	-0.665866653772425	3.20559905689042	-1.75534663273957	0.0885978979976883	0.753146916468211	-4.64722797949315
a_HQ_transcript/137076	1.7292742378995	-0.208197022365545	1.755012066722	0.0886560117169934	0.753222407439708	-4.44769936045166
a_HQ_transcript/132128	-2.27167231648977	0.848023361386732	-1.75498607771263	0.088660527322186	0.753222407439708	-4.44424366188589
a_HQ_transcript/137401	-3.00885525676051	2.72851977102274	-1.7549828973609	0.0886610799234309	0.753222407439708	-4.56470822077037
a_HQ_transcript/152064	1.87392854559261	0.25694051828671	1.7549765931638	0.088662175316258	0.753222407439708	-4.4373715004264
a_HQ_transcript/20910	-1.55877843269498	-0.382417416363455	-1.75485772114552	0.0886828321934424	0.753222407439708	-4.45943296208197
a_HQ_transcript/7575	1.61559229940331	1.13089865260167	1.75484797211661	0.0886845265021566	0.753222407439708	-4.3851465976017
a_HQ_transcript/151779	-2.84865220712549	0.254014583709571	-1.75470912303645	0.0887086604025729	0.753317313642186	-4.45184546512008

a_HQ_transcript/100066	-1.36548775894839	2.20970248315595	-1.75432943112176	0.0887746844160114	0.753565805941129	-4.42195733493749
a_HQ_transcript/141784	2.62786253769811	1.25489818008857	1.75420616350059	0.0887961281310206	0.753565805941129	-4.45165936751499
a_HQ_transcript/145414	-2.28242591788844	0.351231213902075	-1.75403742979496	0.0888254882361962	0.753565805941129	-4.51334687469899
a_HQ_transcript/59806	2.22607583884186	1.80657440848804	1.75403451216741	0.0888259959829866	0.753565805941129	-4.35550102584563
a_HQ_transcript/63729	-1.90207856669314	-0.266376709051974	-1.75401575761677	0.0888292598448664	0.753565805941129	-4.37363320680124
a_HQ_transcript/5917	1.44511724524553	2.50755164019162	1.75400205608476	0.0888316443918337	0.753565805941129	-4.43880813910691
a_HQ_transcript/123640	1.24798246192273	-0.757420513738144	1.75397173069806	0.0888369222642935	0.753565805941129	-4.46404550838917
a_HQ_transcript/70394	-1.800824147873	1.00279569899215	-1.75394466274585	0.0888416334316433	0.753565805941129	-4.32895291900827
a_HQ_transcript/128836	2.54321025926255	3.19434133799637	1.75383998398078	0.0888598547156427	0.753610393188047	-4.63467804907675
a_HQ_transcript/118181	1.81379164071858	0.174237699817219	1.75347810761211	0.0889228702974119	0.75403480738847	-4.44517739480402
a_HQ_transcript/49100	2.23590662234464	0.0942203961982998	1.75331565931859	0.0889511705889585	0.754130040422187	-4.44011671412141
a_HQ_transcript/151014	0.606685234240955	3.25083017029233	1.75326470882629	0.0889600482929903	0.754130040422187	-4.6729558035775
a_HQ_transcript/110172	-2.5844708716693	2.3122667819098	-1.75315244742151	0.0889796115551041	0.754152817380254	-4.47198209341631
a_HQ_transcript/101784	1.21732337600154	0.440522618140674	1.75302061736749	0.0890025895829743	0.754152817380254	-4.36329298362933
a_HQ_transcript/118836	-1.31032177743831	2.11988559103617	-1.75298912508887	0.0890080794391105	0.754152817380254	-4.42927812169277
a_HQ_transcript/128389	-1.73874905539994	3.0199259483781	-1.75295154362276	0.089014631159612	0.754152817380254	-4.52047875951314
a_HQ_transcript/92755	1.41101301553004	-0.390943404831654	1.75278918545792	0.0890429403506998	0.754267386401672	-4.43990237526361
a_HQ_transcript/2136	-0.59080677245159	5.00304667068614	-1.75272516053923	0.0890541059880725	0.754267386401672	-4.88507091320393
a_HQ_transcript/149392	3.72921358238684	2.53128142672467	1.75244049157329	0.089103765175388	0.754493185102437	-4.48012815146962
a_HQ_transcript/119969	1.76025887006694	1.90532743330469	1.75242352825478	0.0891067250847824	0.754493185102437	-4.52626327296458
a_HQ_transcript/75553	-2.08132519185325	0.193121845321187	-1.75234774172686	0.089119949998764	0.754495259762329	-4.40825361812923
a_HQ_transcript/29653	-2.12001793887485	-0.202284743234197	-1.75225722813973	0.0891357469845407	0.754506507996116	-4.44495151415615
a_HQ_transcript/94989	-3.11757641806608	0.178089970324233	-1.75219139082276	0.0891472387991558	0.754506507996116	-4.41122393135854
a_HQ_transcript/149634	2.07661589998362	3.35163994119954	1.75209379983429	0.0891642754716927	0.754540836310752	-4.65816812942474
a_HQ_transcript/112125	-1.40804141376646	2.04058395893588	-1.75198573235634	0.0891831442498514	0.75459065632538	-4.41826412357076
a_HQ_transcript/78877	-1.06507370595904	1.47155478346335	-1.75158790165707	0.0892526352156307	0.755068720683188	-4.33276026096814
a_HQ_transcript/148528	1.9294319910714	1.0298226185375	1.75146249060047	0.089274550815133	0.755126264995953	-4.36386730542906
a_HQ_transcript/118603	2.26227516587038	1.07361055082113	1.75140030911913	0.0892854187193295	0.755126264995953	-4.4558783169469
a_HQ_transcript/67546	2.08073308187472	-0.0817389529088166	1.75129599444655	0.0893036530482872	0.755170605817696	-4.44927425685128
a_HQ_transcript/876	2.02330210337164	0.995563035906294	1.75110433117122	0.0893371641976352	0.755326971116788	-4.33971048838495
a_HQ_transcript/134417	-0.413367219061122	3.9645638211905	-1.75104161162016	0.0893481326279768	0.755326971116788	-4.8059262365565
a_HQ_transcript/108825	1.10225895252747	2.87172306086731	1.75075311919617	0.089398598961826	0.755597607723451	-4.51732356792057
a_HQ_transcript/140387	1.892032094088	1.37046190347634	1.75069486232967	0.0894087928215669	0.755597607723451	-4.47549183100104
a_HQ_transcript/98895	1.07065623779456	2.01112455553029	1.75060285200896	0.0894248948983646	0.755597607723451	-4.42866968182358
a_HQ_transcript/55899	2.02757406134941	-0.0699913847483947	1.75052080229016	0.0894392559016429	0.755597607723451	-4.39832154018918
a_HQ_transcript/86760	3.45930097191878	2.6020248635089	1.75044160343607	0.0894531197668464	0.755597607723451	-4.52954079598613
a_HQ_transcript/123043	-2.67373065991158	-0.0288539399716599	-1.75034541110638	0.0894699607980063	0.755597607723451	-4.48688550958401
a_HQ_transcript/80495	1.48160015250197	0.229102573488956	1.75033868536638	0.0894711384179829	0.755597607723451	-4.32072479468914
a_HQ_transcript/141368	-1.58489534401475	-0.411779280592482	-1.75021589206098	0.0894926407794082	0.755669410695154	-4.46125461609561
a_HQ_transcript/110408	2.5614824787218	1.53356964108776	1.74992379218848	0.0895438079486268	0.755991643927056	-4.38718169260243
a_HQ_transcript/141039	-3.13766957582795	0.870990562620012	-1.74976428842754	0.0895717586448287	0.755994508762399	-4.50264471966019
a_HQ_transcript/116633	1.99336942888831	-0.0145417186486874	1.74969057084519	0.0895846790542289	0.755994508762399	-4.47796064752706
a_HQ_transcript/140549	-0.779753261098647	5.24841934246434	-1.74962460420674	0.0895962422944484	0.755994508762399	-4.90136899003884
a_HQ_transcript/51024	-1.83020967745146	-0.270633143345289	-1.74956565315803	0.0896065768414568	0.755994508762399	-4.39816363091977
a_HQ_transcript/5795	1.18464033266907	2.30440152010587	1.7495485127613	0.0896095818659504	0.755994508762399	-4.42848124349918
a_HQ_transcript/84337	-2.92337624862916	1.6667314911473	-1.74947665144608	0.0896221813907531	0.755994508762399	-4.64490032802076
a_HQ_transcript/135365	2.2844394414348	0.609567656983618	1.74935305586994	0.0896438550325348	0.756008268583182	-4.37455806204646
a_HQ_transcript/56288	-7.47739822298592	4.5216822796013	-1.74930382162035	0.0896524899491081	0.756008268583182	-4.7380591573328
a_HQ_transcript/88229	-1.48760388163801	1.6300678729009	-1.74917264298275	0.0896755000492914	0.756008268583182	-4.3633100698092
a_HQ_transcript/142552	0.600108452716552	5.17823105420662	1.74917072631154	0.0896758362902739	0.756008268583182	-4.95236966354646

a_HQ_transcript/36383	-1.1390481082726	2.30110816889954	-1.7490256044067	0.0897012980619031	0.756113261853582	-4.49597298974068
a_HQ_transcript/130102	0.897763375967843	2.83990234419754	1.7487115831805	0.0897564142229776	0.756373372796623	-4.59060601880227
a_HQ_transcript/140035	-2.11438442828138	0.247284992596669	-1.74870152167743	0.0897581806625225	0.756373372796623	-4.40799324949333
a_HQ_transcript/50255	-2.30961107120115	-0.227472366529212	-1.74861076714072	0.0897741152318755	0.756397995390413	-4.408518790222
a_HQ_transcript/141996	1.8926247407441	-0.0753892214561332	1.74842422891755	0.0898068748653325	0.756479093791046	-4.47188337306606
a_HQ_transcript/149257	-2.49944182602916	4.96427310312785	-1.74834683624121	0.0898204694356838	0.756479093791046	-4.97036141848367
a_HQ_transcript/39877	1.01664685562723	3.46047146277237	1.74833366874474	0.08982278257201	0.756479093791046	-4.71989001826302
a_HQ_transcript/16837	-1.60026086288205	4.62526071824924	-1.74805755634347	0.0898712988435156	0.756738847097301	-4.86130809241318
a_HQ_transcript/117982	1.85545834399363	-0.0975094353144497	1.7479854382652	0.0898839744926892	0.756738847097301	-4.44629730733332
a_HQ_transcript/38234	-1.46712184621348	2.07262004935169	-1.74793590972279	0.0898926806221934	0.756738847097301	-4.51472702095168
a_HQ_transcript/3706	1.32411451031158	0.790484452072801	1.74770728590551	0.0899328773346105	0.756957553022091	-4.34187935452801
a_HQ_transcript/133753	1.93207683097041	-0.0274781386137399	1.74758894624935	0.089953689796337	0.756957553022091	-4.40717829080744
a_HQ_transcript/146088	1.92927961160301	-0.165507696850349	1.74753434959595	0.0899632931078399	0.756957553022091	-4.46874324845592
a_HQ_transcript/69125	-2.2926304585647	-0.239537130646954	-1.74749196129856	0.0899707496185939	0.756957553022091	-4.42804413907849
a_HQ_transcript/143286	1.05641920694452	2.1828415912566	1.74721392043241	0.0900196725974465	0.757259555534641	-4.53576423421191
a_HQ_transcript/118449	3.63194875657712	0.732546329211251	1.7467804085073	0.0900959963515505	0.757791936901017	-4.4568692562919
a_HQ_transcript/39015	-3.30161169052225	0.732796624431484	-1.746663293431	0.0901166248833265	0.757854830580057	-4.52148953333272
a_HQ_transcript/111568	2.61550957392883	0.809609663724335	1.74657381223116	0.0901323886922072	0.757854830580057	-4.48694778097265
a_HQ_transcript/123311	2.58524876771414	1.76858652345512	1.74646737174552	0.0901511432245838	0.757854830580057	-4.6681209426179
a_HQ_transcript/12350	2.9066141657462	0.697785253375342	1.74644194001043	0.0901556247154744	0.757854830580057	-4.42422480850652
a_HQ_transcript/65482	-1.93569743262122	-0.298752578869118	-1.74622443751864	0.0901939599030586	0.757942067090484	-4.40380685704098
a_HQ_transcript/147619	2.2202762761534	0.406613042021411	1.74614876110117	0.0902073012198777	0.757942067090484	-4.49049946472285
a_HQ_transcript/140385	-2.3800308617057	0.921279242757476	-1.74609736997962	0.0902163621261733	0.757942067090484	-4.54407111335941
a_HQ_transcript/142709	2.53194932096019	3.14225184266218	1.74608717754538	0.0902181592726234	0.757942067090484	-4.60591756482529
a_HQ_transcript/88031	2.96756920548131	2.71215631303026	1.7458531484899	0.090259431946436	0.757996111744057	-4.58761585716521
a_HQ_transcript/123662	1.26526920597379	-0.47867372794026	1.74577333442421	0.0902735113546873	0.757996111744057	-4.44620475186076
a_HQ_transcript/132824	-1.77388803426361	1.84446761812974	-1.74571376539095	0.0902840206917774	0.757996111744057	-4.54887693599234
a_HQ_transcript/53067	1.88324385015992	0.802985978320664	1.74568394859768	0.0902892814415099	0.757996111744057	-4.35200700185049
a_HQ_transcript/55241	5.71277886395676	0.802369213452086	1.74568105022705	0.0902897928316104	0.757996111744057	-4.48416967303826
a_HQ_transcript/35679	1.69353318096932	1.75269259390764	1.74554131754591	0.0903144502355085	0.758083625975606	-4.38653625953494
a_HQ_transcript/139958	2.57418402541184	-0.0428158650979177	1.74547417411288	0.0903263004663337	0.758083625975606	-4.43198701742163
a_HQ_transcript/8802	1.71546359950997	1.27985253902308	1.74522163234782	0.0903708836098787	0.75810511777783	-4.34422617985897
a_HQ_transcript/23659	2.04124591222318	0.119366220630226	1.7452120374488	0.0903725778365846	0.75810511777783	-4.40768289341035
a_HQ_transcript/114389	1.48246583474848	1.20592325805403	1.74515787439255	0.0903821422216331	0.75810511777783	-4.37725885009149
a_HQ_transcript/115882	5.78897161949673	3.4876399625146	1.74508786034229	0.0903945069177639	0.75810511777783	-4.58473187602846
a_HQ_transcript/49265	4.12571529753	0.911928962355158	1.74497790112003	0.0904139289284994	0.75810511777783	-4.50924260794347
a_HQ_transcript/118432	-1.20242301057901	1.73077896535001	-1.74486079419506	0.0904346172953722	0.75810511777783	-4.46031718779703
a_HQ_transcript/125287	2.45427851183905	1.86161058408329	1.74483942521488	0.0904383928163272	0.75810511777783	-4.38829695064496
a_HQ_transcript/120060	-0.478814313657051	4.5955050358179	-1.74483317871642	0.0904394964872191	0.75810511777783	-4.87482668473113
a_HQ_transcript/25892	2.56291351538401	0.482653021909273	1.74478188392158	0.0904485600051717	0.75810511777783	-4.36480136386894
a_HQ_transcript/118348	1.89993837169329	0.922430832150221	1.74457872619818	0.0904844644085037	0.75810511777783	-4.3440064955026
a_HQ_transcript/51765	-1.33122628116936	2.99883348079512	-1.74454528576035	0.0904903755427367	0.75810511777783	-4.62098163185422
a_HQ_transcript/125112	-2.63314569923993	0.776109228588691	-1.7444427030806	0.0905085107064332	0.75810511777783	-4.38867654006606
a_HQ_transcript/9394	-1.69424923419993	1.60813090538029	-1.74437387338473	0.0905206805360726	0.75810511777783	-4.37910948616414
a_HQ_transcript/76267	-2.00761454985768	-0.0860603273814094	-1.74426666722457	0.0905396384805058	0.75810511777783	-4.4375375230582
a_HQ_transcript/151853	2.86715193490597	0.80860969162204	1.74416085116733	0.0905583538816443	0.75810511777783	-4.48179877177446
a_HQ_transcript/32536	1.76216056715398	-0.0418564631260225	1.74414782858248	0.0905606573761978	0.75810511777783	-4.47890059843844
a_HQ_transcript/25392	-0.785670344150805	2.87055740895782	-1.74411987164253	0.0905656026945971	0.75810511777783	-4.50769270551296
a_HQ_transcript/38309	-1.52801445591299	-0.478847317482205	-1.74406574444767	0.0905751779298156	0.75810511777783	-4.46770673119393
a_HQ_transcript/104096	-1.50356492081185	1.29564330362886	-1.74402788678978	0.0905818755499667	0.75810511777783	-4.35585245352591

a_HQ_transcript/69085	0.622155752556418	2.63492505395168	1.74392028044411	0.0906009150958589	0.75810511777783	-4.57676679239045
a_HQ_transcript/74513	1.75783399437648	-0.214525562835842	1.7438879459785	0.0906066369179296	0.75810511777783	-4.448732744115
a_HQ_transcript/44481	1.35109082611296	3.20839555060969	1.74383625225711	0.090615785134881	0.75810511777783	-4.66734774054737
a_HQ_transcript/28744	1.6476270745209	-0.577021507888874	1.74363659377447	0.0906511259165149	0.758237791110885	-4.46235051917763
a_HQ_transcript/107812	2.21686904276442	0.360886972414502	1.74359927959099	0.0906577320434326	0.758237791110885	-4.42599240136027
a_HQ_transcript/142570	-2.35066378956837	2.64292813351753	-1.7433246317404	0.0907063683430488	0.758535430735828	-4.59673645630974
a_HQ_transcript/147447	2.31958529574891	0.804750331879004	1.74321026705766	0.0907266272068846	0.758561623090234	-4.37858785862465
a_HQ_transcript/43142	2.15691683355017	0.334145563824504	1.74315962017415	0.0907356001470273	0.758561623090234	-4.47697355189782
a_HQ_transcript/83815	-2.34789861076966	2.67999347887278	-1.74304354332133	0.0907561679150664	0.758624464850012	-4.51815742923779
a_HQ_transcript/85429	1.78262275011631	2.33252276360741	1.74295081886855	0.0907726006681413	0.758640384430522	-4.48268709830395
a_HQ_transcript/118731	-2.31889608051831	-0.246118029180168	-1.74273974296964	0.0908100171630807	0.758640384430522	-4.46170975164124
a_HQ_transcript/2119	1.05675229527711	2.60217580017302	1.74269335556358	0.0908182417937147	0.758640384430522	-4.54333958326318
a_HQ_transcript/52594	2.5794948012151	0.672290309865907	1.74247830190809	0.0908563796626298	0.758640384430522	-4.37678753132009
a_HQ_transcript/47942	2.07154763629067	0.278050982803833	1.74243012765533	0.0908649247889168	0.758640384430522	-4.45177444817102
a_HQ_transcript/145249	1.88037362435965	3.5226806787554	1.74227557769945	0.0908923433527738	0.758640384430522	-4.56025709191642
a_HQ_transcript/81657	-1.15731329652217	2.03806398844481	-1.74217147560799	0.0909108159319684	0.758640384430522	-4.42845876853166
a_HQ_transcript/125415	3.02032881713824	0.791858974401913	1.74215187722052	0.090914293955734	0.758640384430522	-4.4864758108935
a_HQ_transcript/143914	-2.92165624490642	0.211464091757464	-1.74201126771245	0.0909392504764833	0.758640384430522	-4.5090292100964
a_HQ_transcript/59896	2.87148222477687	1.05119226435477	1.7419606625322	0.090948233706635	0.758640384430522	-4.49267824149827
a_HQ_transcript/65613	0.869246902203938	2.22483232284428	1.74191857821374	0.09095570491601	0.758640384430522	-4.41194386249071
a_HQ_transcript/116999	-1.91298361985329	1.05071504721827	-1.74189184523479	0.0909604510774773	0.758640384430522	-4.4271175359513
a_HQ_transcript/65187	0.613253708653118	2.8108004204679	1.74180404396735	0.0909760407425933	0.758640384430522	-4.55851049294452
a_HQ_transcript/21536	1.31893595548741	2.32543531761116	1.74176393683549	0.090983162763155	0.758640384430522	-4.47056473372948
a_HQ_transcript/5652	0.842840600342829	2.81865393198348	1.74175886976489	0.090984062581168	0.758640384430522	-4.62880909167573
a_HQ_transcript/138519	2.17852827304474	0.8963315017277	1.74170971831016	0.0909927913589902	0.758640384430522	-4.36098477813169
a_HQ_transcript/136318	-1.90666016481017	0.375484508395243	-1.74170914296513	0.0909928935383459	0.758640384430522	-4.41725290576029
a_HQ_transcript/125587	1.90245002693512	0.24044113090578	1.74164976777129	0.0910034388936348	0.758640384430522	-4.36227732246576
a_HQ_transcript/79688	-2.0103348449211	0.996854963748445	-1.74155138597041	0.0910209142966826	0.758640384430522	-4.4390704128609
a_HQ_transcript/103145	-1.4621700281012	0.311484111781858	-1.7415488727869	0.0910213607464612	0.758640384430522	-4.43076115038561
a_HQ_transcript/143277	1.64067293952828	0.137368579616224	1.74141326991503	0.0910454523966662	0.758640384430522	-4.42915232829851
a_HQ_transcript/12246	-1.49648555772616	0.239282542823107	-1.74139141815018	0.0910493351546638	0.758640384430522	-4.50032082128619
a_HQ_transcript/150183	-3.70674211835992	0.184708695260183	-1.74123933432124	0.0910763622181165	0.758640384430522	-4.50525719449024
a_HQ_transcript/108294	-1.88997041488703	0.85197856204414	-1.74122519391264	0.0910788754759645	0.758640384430522	-4.36775309959814
a_HQ_transcript/98092	-1.67560604818056	-0.557423684914346	-1.74116818151144	0.0910890092167078	0.758640384430522	-4.45500326266489
a_HQ_transcript/4220	0.866470087706566	1.33566521412506	1.74112095810782	0.0910974037180884	0.758640384430522	-4.44221111051497
a_HQ_transcript/147148	4.0301649839371	0.826099930235484	1.74102538482848	0.0911143949558248	0.75867319151872	-4.53107823925394
a_HQ_transcript/13807	-0.636384475563601	3.83265983754902	-1.74095176589168	0.0911274849178496	0.758673509496529	-4.75358935601277
a_HQ_transcript/131416	-0.437106124229126	4.96526190075929	-1.74063806011177	0.0911832817370173	0.75883295473187	-4.90204418980494
a_HQ_transcript/136651	-1.64912503302967	1.70502823613452	-1.74049833895566	0.0912081422856751	0.75883295473187	-4.43142644981326
a_HQ_transcript/33279	-2.10125146601589	2.0331089829088	-1.74036137266833	0.0912325181956074	0.75883295473187	-4.43867685972911
a_HQ_transcript/81856	1.55815955836745	1.5762509439457	1.74031647995283	0.0912405089510849	0.75883295473187	-4.33841384724248
a_HQ_transcript/9845	1.6820138249807	1.46955026317938	1.74030363522964	0.0912427953778261	0.75883295473187	-4.41145576373557
a_HQ_transcript/30496	-4.02174311239087	2.13725430330258	-1.74029060067161	0.091245115645419	0.75883295473187	-4.59147352244089
a_HQ_transcript/124211	1.03792982375627	3.81130150952022	1.74000144501387	0.0912966006968561	0.75883295473187	-4.69155557172064
a_HQ_transcript/112809	3.5616341827165	1.39470613476111	1.73997736569888	0.0913008891947282	0.75883295473187	-4.57166817794844
a_HQ_transcript/110045	2.176555853864	2.36934608786512	1.73997095557136	0.0913020308595107	0.75883295473187	-4.44848878066422
a_HQ_transcript/2992	1.93226029014451	1.72523573222657	1.73993380348697	0.0913086480043638	0.75883295473187	-4.42631467804987
a_HQ_transcript/109088	-4.69089412240739	4.30672559587539	-1.73987909560885	0.0913183927399373	0.75883295473187	-4.81353957218388
a_HQ_transcript/144481	4.56569352214013	1.97037126040591	1.73984936832987	0.0913236882211509	0.75883295473187	-4.522471005153
a_HQ_transcript/37389	-1.08179908981221	2.3521044170612	-1.73981808389605	0.0913292613657458	0.75883295473187	-4.50568420069782

a_HQ_transcript/145657	0.634524306723143	4.46820075793039	1.73981730685217	0.091329399795351	0.75883295473187	-4.86944344900899
a_HQ_transcript/142080	1.6228249514978	-0.611234854172483	1.73954039559936	0.0913787425029883	0.75913442106813	-4.43007622557681
a_HQ_transcript/116276	1.57854124619034	0.070671472824453	1.73944468594316	0.0913958021827617	0.759167646367472	-4.426035354885
a_HQ_transcript/146192	1.74252263590935	2.27001836738817	1.73918687495635	0.091441768792589	0.759362827345326	-4.45988578688545
a_HQ_transcript/93194	1.87883435654802	-0.10361808908672	1.73916636096758	0.0914454271851308	0.759362827345326	-4.46838386700707
a_HQ_transcript/105085	3.01902595288608	0.776527417506888	1.73893084215751	0.0914874376011542	0.75940960680087	-4.41981421669393
a_HQ_transcript/78660	2.36542685184918	0.0823924836240681	1.73885519845659	0.0915009339088722	0.75940960680087	-4.35338947910846
a_HQ_transcript/132158	1.78964802601099	0.292474631439666	1.73880124733697	0.091510560861116	0.75940960680087	-4.46070694893869
a_HQ_transcript/96492	-0.591258158304348	3.79817051599992	-1.73879001228743	0.0915125657327065	0.75940960680087	-4.77683382456474
a_HQ_transcript/42850	1.92267008329709	1.71160882654669	1.73876862285444	0.0915163827353443	0.75940960680087	-4.42189046716162
a_HQ_transcript/94618	2.25703401490281	0.422171550485854	1.73864840723967	0.0915378380307037	0.759479224814266	-4.48219923699457
a_HQ_transcript/132629	-2.0469048447601	3.25827837347761	-1.73833856097831	0.09159315686484	0.759714365308518	-4.70187395963906
a_HQ_transcript/139356	-2.92696372350378	1.95227007459746	-1.73833304847141	0.091594141302425	0.759714365308518	-4.67996437289287
a_HQ_transcript/79211	-0.734920144968489	3.43106236106061	-1.73827007512118	0.0916053878758499	0.759714365308518	-4.70227714717475
a_HQ_transcript/142462	0.945132691283505	4.8329310766839	1.73803631266788	0.0916471462736887	0.75973516054418	-4.94274698112059
a_HQ_transcript/149040	-2.11460953193424	2.4073770307045	-1.73799240058047	0.0916549923405305	0.75973516054418	-4.61520051920874
a_HQ_transcript/9338	1.09943298381305	1.93138858730496	1.73796572410855	0.0916597590802827	0.75973516054418	-4.50961827941435
a_HQ_transcript/72277	-1.51705781982897	1.82349837279404	-1.73784814017874	0.0916807722889984	0.75973516054418	-4.46803430955868
a_HQ_transcript/106889	2.76222141949319	1.10225845211568	1.73775437589893	0.0916975316346119	0.75973516054418	-4.51941178695414
a_HQ_transcript/82522	-1.98776961178501	1.21974871673869	-1.73769500239337	0.0917081453352706	0.75973516054418	-4.38660441583592
a_HQ_transcript/4462	2.25014435939681	5.42090441755633	1.73763177823246	0.0917194485207504	0.75973516054418	-5.00674749441107
a_HQ_transcript/147323	-2.69808598187666	1.21530299777028	-1.73755258506438	0.0917336082903435	0.75973516054418	-4.55323038472097
a_HQ_transcript/66498	-2.6985141420133	2.10266520582304	-1.73754862902133	0.0917343156805977	0.75973516054418	-4.63560799101645
a_HQ_transcript/17946	2.07670989128113	-0.264543184001624	1.7375246936863	0.0917385957173754	0.75973516054418	-4.52468171347574
a_HQ_transcript/134555	1.65953345234492	-0.415023704505839	1.73729227357108	0.0917801650392431	0.759971144359134	-4.47944041001125
a_HQ_transcript/111994	1.22031070907516	-0.399786359636599	1.73687136458067	0.0918554867067131	0.760486502106227	-4.57993224315892
a_HQ_transcript/30655	2.73025406598528	0.0393253874047292	1.7367190426538	0.0918827575207365	0.760603948898515	-4.46572147899994
a_HQ_transcript/103349	1.84377103513379	1.05102542638692	1.73662918539051	0.0918988482239266	0.760628826649638	-4.446079013587
a_HQ_transcript/123732	1.37900709733139	3.06161684941662	1.73633086114581	0.0919522860130667	0.760832535912352	-4.63357353776511
a_HQ_transcript/34487	-1.22306629710798	4.16934613257389	-1.73627393793133	0.0919624854386086	0.760832535912352	-4.80337104924378
a_HQ_transcript/147469	1.23456504680795	1.98302092689902	1.73624842260727	0.091967057548984	0.760832535912352	-4.38572148265765
a_HQ_transcript/138071	2.00316286567751	0.264222877340352	1.73619954802966	0.0919758159554105	0.760832535912352	-4.53340035719728
a_HQ_transcript/112685	1.42467908486499	-0.466592066371191	1.73595893987482	0.0920189435645032	0.760972124856236	-4.50577029866625
a_HQ_transcript/110778	-0.950306927756279	3.69704284389502	-1.73593379974792	0.0920234507664382	0.760972124856236	-4.70781614715775
a_HQ_transcript/106713	-2.00106423050335	3.58517591051061	-1.73585522819421	0.0920375385209446	0.760972124856236	-4.62118250565405
a_HQ_transcript/2572	-2.71890777141403	2.37804656398718	-1.73581330564614	0.0920450559087565	0.760972124856236	-4.51666475083619
a_HQ_transcript/131219	3.2859345837414	-0.124410911733095	1.73569969318944	0.0920654310461158	0.761032334449462	-4.49658928166522
a_HQ_transcript/56954	-2.30648238067041	3.28533619410486	-1.73554396071285	0.0920933661031168	0.761155009930609	-4.71664948730117
a_HQ_transcript/89421	1.76487300904431	-0.355589741950151	1.73513200452222	0.092167296537132	0.761518103550448	-4.47039098896152
a_HQ_transcript/127152	1.78817173431812	0.84521642209611	1.73508672265194	0.0921754259489939	0.761518103550448	-4.50068418803634
a_HQ_transcript/24621	1.5484101061719	-0.452718413370188	1.73493221992437	0.0922031682156996	0.761518103550448	-4.44217437081581
a_HQ_transcript/8834	1.49631762077514	1.46493481565429	1.73492487060985	0.0922044880218815	0.761518103550448	-4.36828021886743
a_HQ_transcript/718	0.710315299155759	3.46417104165158	1.73483075691938	0.0922213905705211	0.761518103550448	-4.72231553410648
a_HQ_transcript/4742	1.05341098529524	1.7848743734482	1.73481832219574	0.0922236240059472	0.761518103550448	-4.38096103335982
a_HQ_transcript/105277	1.96138607705138	1.39384468282916	1.73474377792306	0.0922370140640276	0.761518103550448	-4.42466272864923
a_HQ_transcript/59497	-1.97252654311103	0.333009096773766	-1.73470353438992	0.092244243511066	0.761518103550448	-4.48908402137124
a_HQ_transcript/120460	1.82456615078872	-0.0852642562274168	1.73464252788249	0.0922552037779084	0.761518103550448	-4.46396609994523
a_HQ_transcript/104724	2.2082879175592	1.18249400962964	1.73446784990868	0.0922865920115066	0.761622580903019	-4.43951445129932
a_HQ_transcript/69876	-1.97295723208409	0.0811254732796222	-1.73442439068754	0.0922944026797585	0.761622580903019	-4.41763155646199
a_HQ_transcript/6072	-2.65478585169923	0.303323960132298	-1.73435336800968	0.0923071683605452	0.761622580903019	-4.4602591571054

a_HQ_transcript/29020	2.2423494770413	2.79060549612769	1.73427234632171	0.0923217330844203	0.761634643873288	-4.61520406650683
a_HQ_transcript/133575	1.05311456884081	-0.831690143033333	1.73398389239785	0.0923736021844478	0.761954412910116	-4.483843091051
a_HQ_transcript/144580	3.03691855989251	1.09756505991919	1.7336996846794	0.0924247316905632	0.762142955208593	-4.4691281789153
a_HQ_transcript/67772	1.82008425106873	0.61032292603965	1.73369702953751	0.0924252094677121	0.762142955208593	-4.36770709534829
a_HQ_transcript/53909	-0.519998465184644	3.13071378148457	-1.7335548605636	0.0924507949682236	0.762142955208593	-4.6421569064597
a_HQ_transcript/114152	2.40812825898516	2.8612552048955	1.73348956788424	0.0924625473874644	0.762142955208593	-4.68595215847313
a_HQ_transcript/133425	1.71811300022727	1.64575463650624	1.73345325186069	0.0924690846686748	0.762142955208593	-4.47357817760455
a_HQ_transcript/113318	2.85933600068541	0.426505596705612	1.73341967958602	0.0924751283905554	0.762142955208593	-4.52672589718028
a_HQ_transcript/2191	-0.617738905247252	2.58597582966954	-1.73319557931223	0.0925154796985212	0.762266588775531	-4.53972868626515
a_HQ_transcript/133561	2.81685448064581	0.536191667180035	1.73319070944624	0.0925163567267302	0.762266588775531	-4.52546692824949
a_HQ_transcript/3039	1.15660964450602	2.68760057990879	1.7328921892124	0.092570131437024	0.762452063189527	-4.58708098783837
a_HQ_transcript/50069	-1.95065144246875	0.166001918445635	-1.73271529724662	0.0926020087158691	0.762452063189527	-4.47066128298994
a_HQ_transcript/17680	1.33202651080867	-0.773743023859257	1.73265532601177	0.0926128180821057	0.762452063189527	-4.48425426119431
a_HQ_transcript/115122	1.82172838943338	0.0116306292811529	1.73262585150774	0.0926181310293818	0.762452063189527	-4.47257572617469
a_HQ_transcript/1196	-0.794131074002869	4.49828575912476	-1.73259647995345	0.092623425674109	0.762452063189527	-4.86836363374862
a_HQ_transcript/1092	0.821616529206048	2.57372509162918	1.73257942827365	0.0926264996010889	0.762452063189527	-4.50492745539834
a_HQ_transcript/43710	-3.31412029273216	2.75624024452811	-1.73250407380756	0.0926400848683005	0.762452063189527	-4.65946636793073
a_HQ_transcript/74666	-1.10007157880024	-0.746060138456965	-1.73248345735278	0.0926438019940069	0.762452063189527	-4.42448629805074
a_HQ_transcript/79217	1.94650113675788	-0.218634494290349	1.73216306853289	0.0927015838765554	0.762617733206545	-4.4822394198832
a_HQ_transcript/52234	2.77002602036374	1.32953092706224	1.73206221587502	0.0927197788413903	0.762617733206545	-4.46377918633547
a_HQ_transcript/14180	1.63397407169442	2.25333181507062	1.73204223139022	0.0927233846255221	0.762617733206545	-4.45644823222129
a_HQ_transcript/51086	-4.76823545393134	0.246490370248389	-1.73199770928354	0.0927314181361142	0.762617733206545	-4.51219009427835
a_HQ_transcript/74900	-1.31519781477739	1.22589478348748	-1.7319480528017	0.0927403787763528	0.762617733206545	-4.38522413844218
a_HQ_transcript/2521	0.91999805983922	2.54046511370287	1.73193546748036	0.0927426499455868	0.762617733206545	-4.5151511267843
a_HQ_transcript/149237	3.42065617019108	1.18582015806938	1.73176521697038	0.0927733782436711	0.762762507856876	-4.51514063686047
a_HQ_transcript/37002	1.41867622381145	1.27075714912654	1.73143822132537	0.0928324212602832	0.763138333041989	-4.45505009274314
a_HQ_transcript/146896	2.13359712501443	0.766099488852125	1.73136660125967	0.0928453573417394	0.763138333041989	-4.44756850013806
a_HQ_transcript/62450	-2.45043200911633	0.95106953757536	-1.73114573528281	0.0928852598905095	0.763138333041989	-4.46585078058479
a_HQ_transcript/10595	1.24593678760198	-0.583462064035262	1.73109413493767	0.0928945842948888	0.763138333041989	-4.53876287768341
a_HQ_transcript/21511	1.90684879449254	0.271433875531771	1.73096938249164	0.0929171308468275	0.763138333041989	-4.47970922452646
a_HQ_transcript/133607	-1.34193602890131	3.31898551312539	-1.73083475113605	0.0929414679739014	0.763138333041989	-4.72410956070175
a_HQ_transcript/44825	1.76554851785958	1.05533173166958	1.73074289268354	0.0929580761739242	0.763138333041989	-4.44589365560864
a_HQ_transcript/14869	1.69597040155609	-0.410003595362202	1.73066429222073	0.0929722892805949	0.763138333041989	-4.43769176035904
a_HQ_transcript/114005	-1.14786147930826	2.05038892037748	-1.73043786065512	0.0930132444839964	0.763138333041989	-4.50528275281472
a_HQ_transcript/9397	-1.50713563527477	0.280133460732611	-1.73038073795384	0.0930235787927092	0.763138333041989	-4.39624748248774
a_HQ_transcript/93402	0.612934640894285	4.64551165911486	1.73035767901992	0.0930277507548481	0.763138333041989	-4.88461409787883
a_HQ_transcript/49228	-0.691056450828768	3.30557878038422	-1.73030822925369	0.0930366980362034	0.763138333041989	-4.70892509965766
a_HQ_transcript/78287	2.45721492718489	-0.444394209578571	1.73030451287115	0.0930373704956803	0.763138333041989	-4.49596878200243
a_HQ_transcript/17822	-1.32184431341388	1.21518845588472	-1.73023964078259	0.0930491094095217	0.763138333041989	-4.40962931603784
a_HQ_transcript/139887	-1.59369723608361	1.42459629643331	-1.73009971767077	0.0930744333989326	0.763138333041989	-4.48725447716163
a_HQ_transcript/133163	2.29097326886878	1.11345838576111	1.73003319556774	0.0930864749339401	0.763138333041989	-4.43662721666241
a_HQ_transcript/151707	6.16996822636349	2.13424575386292	1.73001253654097	0.0930902148052425	0.763138333041989	-4.63085295128912
a_HQ_transcript/11422	-0.38278026623591	3.29231566000559	-1.72997966919896	0.0930961649888184	0.763138333041989	-4.7255740488499
a_HQ_transcript/103792	-1.57545032185257	1.96711325665511	-1.7298904766866	0.0931123136849612	0.763138333041989	-4.43994819860955
a_HQ_transcript/89749	-1.70100132901045	0.585714303516608	-1.72978047838106	0.0931322326007121	0.763138333041989	-4.42856770994455
a_HQ_transcript/91215	-1.33687821319178	2.3909528076141	-1.72976704920582	0.0931346646524315	0.763138333041989	-4.52604991166736
a_HQ_transcript/118780	-2.28909645777657	-0.260687793701415	-1.72969406473183	0.0931478832258769	0.763138333041989	-4.4707033061412
a_HQ_transcript/141995	1.41012696249968	1.86996952647373	1.72969211414001	0.093148236528672	0.763138333041989	-4.39209885587934
a_HQ_transcript/67535	1.56844164978055	-0.479285442883052	1.72968850784391	0.0931488897254428	0.763138333041989	-4.49668930305854
a_HQ_transcript/148970	0.812160091726364	3.6550660219483	1.72954153763924	0.0931755132368743	0.763138333041989	-4.71373264060373

a_HQ_transcript/125794	2.35632835130768	1.5889658086918	1.72940745162611	0.0931998083631611	0.763138333041989	-4.47374442656302
a_HQ_transcript/96325	-1.07864866203662	2.50939627355591	-1.7292589830593	0.0932267156786672	0.763138333041989	-4.53457669075213
a_HQ_transcript/13474	-1.61411510623746	-0.347797794826955	-1.72920798392164	0.0932359598800623	0.763138333041989	-4.51583375235399
a_HQ_transcript/63867	-1.21456235155497	2.24113623327103	-1.72915978985421	0.0932446963363748	0.763138333041989	-4.40323440656306
a_HQ_transcript/14960	-2.0146464198218	-0.26109956440206	-1.72912982248323	0.0932501290658465	0.763138333041989	-4.48339535549624
a_HQ_transcript/10133	2.09435234210173	1.42342189561519	1.72900195899556	0.093273312188261	0.763138333041989	-4.37855939917358
a_HQ_transcript/130165	0.427071864480231	5.06530830243729	1.72899425777564	0.0932747086625988	0.763138333041989	-4.94817772707048
a_HQ_transcript/111711	0.582140377434405	5.15806382739982	1.72898166310116	0.0932769925125897	0.763138333041989	-4.93115506603411
a_HQ_transcript/75734	1.43290733668295	-0.452618491950639	1.72895656366321	0.0932815440486153	0.763138333041989	-4.47545843673689
a_HQ_transcript/2936	-0.900028064803873	4.2325567466441	-1.72886396955706	0.0932983366913215	0.763138333041989	-4.85658610877664
a_HQ_transcript/12480	-2.03970053649791	-0.251639742367006	-1.72880858217989	0.0933083828250049	0.763138333041989	-4.48996014508626
a_HQ_transcript/30318	1.50191064055321	3.00964883299019	1.7287039982455	0.0933273546812745	0.763138333041989	-4.6463516474204
a_HQ_transcript/36626	1.26457531530761	-0.682419602571946	1.72864217319847	0.0933385714633223	0.763138333041989	-4.50170489959265
a_HQ_transcript/15661	-2.02092628235811	2.54738254730407	-1.72863416924397	0.0933400236858975	0.763138333041989	-4.50364679014822
a_HQ_transcript/63296	2.01543395594098	0.307451941183752	1.72860975644649	0.0933444532155088	0.763138333041989	-4.48850675318368
a_HQ_transcript/104562	3.26570614311359	3.77277624728762	1.72853862178188	0.0933573611041423	0.763138333041989	-4.70635082872687
a_HQ_transcript/121715	-0.786752933630936	1.98893609004277	-1.72833924796209	0.0933935468819313	0.763233910405304	-4.45478562194964
a_HQ_transcript/21352	-1.63829065359273	0.858868915148655	-1.72832951399066	0.0933953138713344	0.763233910405304	-4.37460221620947
a_HQ_transcript/38079	-0.962281416651747	2.3528179076074	-1.72820911323155	0.0934171723138919	0.763305228037941	-4.55494989660065
a_HQ_transcript/95107	1.58022885114022	0.896353294791331	1.72797293953473	0.0934600615051962	0.763473557268781	-4.39009220274654
a_HQ_transcript/36041	-0.656885074999564	1.7773906473075	-1.72791606427322	0.0934703925336468	0.763473557268781	-4.42610548160671
a_HQ_transcript/97493	1.8885985560133	3.99245164595474	1.72770622562141	0.0935085166798212	0.763473557268781	-4.67409022839786
a_HQ_transcript/62140	-1.79254211656273	1.81761058300677	-1.72766525846613	0.0935159612442098	0.763473557268781	-4.42802379168341
a_HQ_transcript/21055	-1.06483487353581	2.19015611729383	-1.72766334172903	0.0935163095664638	0.763473557268781	-4.47886881288848
a_HQ_transcript/8257	-1.72193312549648	0.581954547675837	-1.72759166365179	0.0935293361668034	0.763473557268781	-4.44218412691935
a_HQ_transcript/1402	-1.03556567070337	2.11167340213053	-1.72758958610547	0.0935297137577585	0.763473557268781	-4.42657376619025
a_HQ_transcript/110906	1.82966446253413	-0.596761558487795	1.72722275623276	0.0935964046046977	0.763528731782568	-4.49911670452974
a_HQ_transcript/69578	1.67045285259383	1.16587341878368	1.72720490925462	0.0935996502605147	0.763528731782568	-4.49093177525204
a_HQ_transcript/12955	-1.60351524238895	2.24641328277049	-1.72719008409109	0.0936023464402189	0.763528731782568	-4.55391018112522
a_HQ_transcript/146511	2.56282724589281	0.848203207206494	1.7271474447845	0.0936101014049466	0.763528731782568	-4.51164835897278
a_HQ_transcript/53488	1.31397695714898	-0.290918013916085	1.72708671743158	0.0936211470389818	0.763528731782568	-4.46485963147699
a_HQ_transcript/1208	-0.552054390127576	3.55986280146011	-1.72699541180867	0.0936377565811584	0.763528731782568	-4.76988745869897
a_HQ_transcript/78357	1.23821794794399	1.33796769416649	1.72688688431142	0.0936575021972504	0.763528731782568	-4.40634291062515
a_HQ_transcript/43866	-1.82294115654975	1.14855004156369	-1.72685048873995	0.0936641248313392	0.763528731782568	-4.40057439265144
a_HQ_transcript/42484	-0.488915574404452	4.2326440691144	-1.72670832791619	0.0936899965555327	0.763528731782568	-4.83326421086452
a_HQ_transcript/94792	1.76116363281453	-0.427906339869076	1.72662883544485	0.0937044659354348	0.763528731782568	-4.51678082644071
a_HQ_transcript/24349	1.54667555115737	-0.670677108255806	1.72662683088151	0.0937048308343368	0.763528731782568	-4.50543432474334
a_HQ_transcript/151705	-4.94039810350776	0.337660803774755	-1.72659856893908	0.0937099755988114	0.763528731782568	-4.60824400764952
a_HQ_transcript/151818	0.723630347371992	7.41556075948469	1.72654958637165	0.0937188928783893	0.763528731782568	-5.1577962058759
a_HQ_transcript/11087	-2.45865712354189	1.34742275698106	-1.72653240172678	0.093722021512855	0.763528731782568	-4.48338675154851
a_HQ_transcript/17569	2.26330426389463	0.512719231175627	1.72646934456795	0.0937335024428917	0.763528731782568	-4.42286967295513
a_HQ_transcript/12101	-1.7038005686838	0.310009419699638	-1.72624227624238	0.0937748549589673	0.763758549678415	-4.42390566391073
a_HQ_transcript/21805	-2.95566710114886	0.868862048942137	-1.72600046311479	0.0938189095558407	0.764010307461741	-4.53757341490246
a_HQ_transcript/150242	-0.728217809675688	6.22340695140081	-1.72570487175151	0.0938727853031067	0.76403759933641	-5.03896384667885
a_HQ_transcript/26269	-0.806968199945922	2.95741242181888	-1.72566711076334	0.0938796696522596	0.76403759933641	-4.58607663625007
a_HQ_transcript/18412	-3.03999332598291	2.05381531465982	-1.72564417646511	0.093883851098062	0.76403759933641	-4.49562338249113
a_HQ_transcript/145392	1.55974606161222	2.68863246312778	1.7256249737725	0.0938873523072368	0.76403759933641	-4.56037420152143
a_HQ_transcript/56064	0.693812141736394	3.49532160491822	1.72557906525207	0.0938957232093903	0.76403759933641	-4.75384736628637
a_HQ_transcript/119050	2.67235936182648	-0.159747587939787	1.7255494405695	0.0939011252693936	0.76403759933641	-4.46293779032938
a_HQ_transcript/99827	-1.33267678996672	-0.711990503392109	-1.72514578149943	0.0939747584796975	0.764271621166025	-4.47844923405965

a_HQ_transcript/75772	2.39323301465825	0.882372954210411	1.72508078986865	0.093986618413427	0.764271621166025	-4.44821775366915
a_HQ_transcript/119519	1.58118525581677	4.05163482584352	1.72506547578998	0.0939894131708122	0.764271621166025	-4.76219758421051
a_HQ_transcript/92819	1.29201329644091	1.74680171763632	1.72504162164782	0.0939937665941278	0.764271621166025	-4.44571691604964
a_HQ_transcript/53120	1.43068188253636	1.46268076268018	1.72466837087757	0.0940619075613286	0.764271621166025	-4.3734600756011
a_HQ_transcript/33255	2.97702445740159	1.50139107863886	1.72465432670077	0.0940644722866636	0.764271621166025	-4.5922841334743
a_HQ_transcript/127521	-2.59025692664809	2.7040420240995	-1.72456426763328	0.0940809201254997	0.764271621166025	-4.66025238357354
a_HQ_transcript/93978	0.722579693368579	2.60190307383714	1.72454851032828	0.0940837981916011	0.764271621166025	-4.53961218059439
a_HQ_transcript/40820	-0.509863221389059	2.82058504246307	-1.72452721304454	0.0940876882502218	0.764271621166025	-4.61135365835337
a_HQ_transcript/123941	-0.949859150482029	1.99949713954533	-1.72445660664233	0.0941005858387211	0.764271621166025	-4.4886088345153
a_HQ_transcript/148779	-0.409770476964495	6.05804455077159	-1.72441516552184	0.094108156530273	0.764271621166025	-5.02301306656806
a_HQ_transcript/140976	1.67001783422305	-0.495889190716008	1.72426687002026	0.0941352521534703	0.764271621166025	-4.50736621593325
a_HQ_transcript/148503	-0.97926243152857	2.1768231081588	-1.72424366955488	0.0941394917888939	0.764271621166025	-4.48515056530902
a_HQ_transcript/120618	3.74135831978766	2.0740561295719	1.72420805428536	0.0941460004079095	0.764271621166025	-4.55026958590596
a_HQ_transcript/151477	1.875293088913	-0.0680149610395041	1.72387244064138	0.0942073516701224	0.764271621166025	-4.49797868403634
a_HQ_transcript/83529	3.6014155760434	1.20549722411371	1.72377055307795	0.0942259836824072	0.764271621166025	-4.5266210589243
a_HQ_transcript/112767	2.53614333812074	1.02849851699236	1.72375210896515	0.0942293568576126	0.764271621166025	-4.45187266732438
a_HQ_transcript/70431	-3.24951654965408	1.27406603514481	-1.72372885056368	0.0942336106445866	0.764271621166025	-4.60280755256295
a_HQ_transcript/63358	-4.2900515810008	2.30047715736614	-1.72365457064405	0.0942471969628604	0.764271621166025	-4.48243518933337
a_HQ_transcript/80792	-1.67801188014573	2.84562469146979	-1.72361490327111	0.0942544530766278	0.764271621166025	-4.56628380102988
a_HQ_transcript/37177	-2.30927197992592	-0.126260578761233	-1.72350082187144	0.0942753239123736	0.764271621166025	-4.44657675971669
a_HQ_transcript/55392	1.76076725739396	0.0686469776233025	1.72349521459011	0.0942763498469281	0.764271621166025	-4.4095595658582
a_HQ_transcript/149695	-2.42321378223239	2.7439247427802	-1.72347511557331	0.0942800273344826	0.764271621166025	-4.47671852647896
a_HQ_transcript/19039	1.97180522919637	3.4254297585516	1.72344578084453	0.0942853948826569	0.764271621166025	-4.61009405984656
a_HQ_transcript/121530	-0.850390688193085	2.60234395918666	-1.7233764741167	0.0942980773612815	0.764271621166025	-4.63658868131257
a_HQ_transcript/116217	0.52465507757238	3.0719501764251	1.72330511390854	0.0943111371026673	0.764271621166025	-4.68763556962556
a_HQ_transcript/26741	1.81955128200823	-0.174827876523975	1.72327162558287	0.0943172663756635	0.764271621166025	-4.41200387770861
a_HQ_transcript/5583	-0.629550054104326	4.71064269593531	-1.72316222060795	0.0943372927860078	0.764271621166025	-4.93174449484813
a_HQ_transcript/117377	-1.50932194015785	-0.508145212703932	-1.72315587733848	0.0943384540209955	0.764271621166025	-4.45854305171093
a_HQ_transcript/23978	1.26685662332026	1.9274132287528	1.72314230756607	0.0943409382209758	0.764271621166025	-4.48906716894531
a_HQ_transcript/60817	1.31745215498317	1.42010241576299	1.72312113032524	0.0943448152196018	0.764271621166025	-4.53002217869268
a_HQ_transcript/70349	-1.81330934084572	4.57536447663973	-1.72308939485432	0.0943506254040634	0.764271621166025	-4.90903485334873
a_HQ_transcript/138742	0.485541675473023	4.81349910986527	1.72256082052266	0.094447441837145	0.764440788981576	-4.93123842181588
a_HQ_transcript/40633	1.742910130713	0.0595158834831839	1.72254504221327	0.0944503331549339	0.764440788981576	-4.45708290257023
a_HQ_transcript/45732	-0.870186064944115	2.59342376195695	-1.7223998246902	0.0944769472235941	0.764440788981576	-4.57026512588462
a_HQ_transcript/99483	2.55582724365228	1.42454814896667	1.72225298137292	0.0945038656474762	0.764440788981576	-4.56563925782346
a_HQ_transcript/136496	1.94882805132797	3.24711164122887	1.72224140078573	0.0945059888039046	0.764440788981576	-4.67375729885683
a_HQ_transcript/116540	-1.09246465590044	2.12896993742476	-1.72220778023049	0.0945121529406297	0.764440788981576	-4.49370832897438
a_HQ_transcript/22056	1.83497863611648	0.653265181168525	1.72210106775002	0.0945317202979284	0.764440788981576	-4.44096077793364
a_HQ_transcript/133322	2.13303909717245	0.487741442749459	1.72205016062737	0.0945410560905091	0.764440788981576	-4.5179419685304
a_HQ_transcript/10240	2.05001662138586	0.551453589765386	1.7220397447646	0.0945429663376404	0.764440788981576	-4.40219320637858
a_HQ_transcript/83828	-1.79995726832175	1.2060261712892	-1.72176403101919	0.0945935434235853	0.764440788981576	-4.46839837170109
a_HQ_transcript/21033	-1.76221544507944	1.40660166336346	-1.72172613419605	0.0946004970117214	0.764440788981576	-4.43120606379648
a_HQ_transcript/144945	0.930429219638368	4.47380675770406	1.7216215165686	0.0946196952493573	0.764440788981576	-4.91814956676048
a_HQ_transcript/58293	-1.00699705351348	3.46953284359753	-1.72158600833081	0.0946262120600805	0.764440788981576	-4.83857463273913
a_HQ_transcript/58254	2.18293730166001	0.902004978792529	1.72148660956982	0.0946444566709957	0.764440788981576	-4.46497431638308
a_HQ_transcript/136021	-5.80510078955477	3.08869292512107	-1.72146208291942	0.0946489589835709	0.764440788981576	-4.66576244719675
a_HQ_transcript/140781	5.48072425822107	1.36635708724827	1.72146131468947	0.0946491000090442	0.764440788981576	-4.58153813143364
a_HQ_transcript/119751	-2.55457353012125	1.24138519206622	-1.72142268265 93	0.0946561919936071	0.764440788981576	-4.44037021766735
a_HQ_transcript/101117	1.77921946730797	-0.101615217068314	1.72137505904162	0.0946649352476023	0.764440788981576	-4.4739148018438
a_HQ_transcript/149065	2.91248907245877	2.72182265953656	1.72130238907253	0.0946782780842498	0.764440788981576	-4.65512356701456

a_HQ_transcript/121618	2.96383335632704	1.37872521884886	1.72103633675496	0.0947271410536224	0.764440788981576	-4.52709973889488
a_HQ_transcript/43500	-0.8250063576533	1.96288083400511	-1.72099885938588	0.0947340258179632	0.764440788981576	-4.4163914542287
a_HQ_transcript/129424	-4.61269891985727	5.54151543894978	-1.720971607969	0.0947390322915113	0.764440788981576	-4.89052071272585
a_HQ_transcript/125433	2.0857518713263	-0.0397305706919331	1.72094120945179	0.0947446171939852	0.764440788981576	-4.48027861494263
a_HQ_transcript/133924	-2.0459319622099	2.24262265842504	-1.72091926281714	0.0947486494640289	0.764440788981576	-4.61570326753957
a_HQ_transcript/32116	-3.2368625390883	2.02954646247309	-1.72087936322987	0.0947559806108108	0.764440788981576	-4.68084300270555
a_HQ_transcript/62327	1.18583343812828	1.69752416642329	1.72084933293474	0.0947614986883892	0.764440788981576	-4.40804422312724
a_HQ_transcript/149651	5.67554117591271	2.21459556834163	1.72080075582016	0.0947704253211702	0.764440788981576	-4.64119914931112
a_HQ_transcript/83630	2.12167287871106	0.130049249637969	1.72067527049335	0.0947934880320102	0.764440788981576	-4.44686713155223
a_HQ_transcript/120896	-1.89354419711445	0.355571978673334	-1.72057487779841	0.0948119424036523	0.764440788981576	-4.44954611034044
a_HQ_transcript/148585	5.82235906626725	2.50469814270261	1.72054962335263	0.0948165851969331	0.764440788981576	-4.58327879930862
a_HQ_transcript/6631	-1.94076202197841	0.328342731346429	-1.72054063918573	0.0948182368978226	0.764440788981576	-4.45881235654143
a_HQ_transcript/63368	1.35043138275033	-0.418556735596797	1.7205072077683	0.0948243833336967	0.764440788981576	-4.46604293735727
a_HQ_transcript/40775	2.88618187335666	0.257062796310181	1.72048712215099	0.0948280762769986	0.764440788981576	-4.50291318118498
a_HQ_transcript/148278	1.78768019931106	0.403184635257333	1.72047109324469	0.0948310234395828	0.764440788981576	-4.42292629441981
a_HQ_transcript/66850	0.865681660248736	1.64865142127929	1.72035382171482	0.0948525879622716	0.764440788981576	-4.47730634185771
a_HQ_transcript/144447	1.70221777533756	-0.574957666978131	1.72030700183676	0.094861198602776	0.764440788981576	-4.45340784662916
a_HQ_transcript/81919	3.66009171160823	0.309071256039746	1.7201934265263	0.0948820889532655	0.764440788981576	-4.44879259082131
a_HQ_transcript/51923	-1.14295713149047	-0.750344633588228	-1.72018515193889	0.0948836110811744	0.764440788981576	-4.49050533136452
a_HQ_transcript/62632	1.51985982162129	3.36461290799178	1.72006169223473	0.0949063241871745	0.764440788981576	-4.78387195339074
a_HQ_transcript/126658	1.86112410950021	1.4088201746978	1.71967037314341	0.0949783459935633	0.764440788981576	-4.42221391441081
a_HQ_transcript/10276	1.78534281789094	1.25498679080755	1.7196484775908	0.09498237719678	0.764440788981576	-4.41424738288501
a_HQ_transcript/76097	1.47083802685559	0.537736710304807	1.71949508812814	0.095010621844183	0.764440788981576	-4.48505926436253
a_HQ_transcript/35765	1.92073942056737	0.909353818668498	1.7193903992001	0.0950299029723721	0.764440788981576	-4.47968169021753
a_HQ_transcript/10349	1.86120643346329	1.45355514062068	1.71938748601747	0.095030439555934	0.764440788981576	-4.44579344372323
a_HQ_transcript/14798	-0.680578626430381	2.58248657651943	-1.71938706524457	0.0950305170589478	0.764440788981576	-4.53368603937467
a_HQ_transcript/117810	1.95397850638748	-0.490883594808499	1.71938412165514	0.0950310592461356	0.764440788981576	-4.49960705070155
a_HQ_transcript/92214	0.650502145570499	3.36615733071853	1.71934930524499	0.0950374723655635	0.764440788981576	-4.74732189753846
a_HQ_transcript/126448	0.549783065086448	3.16698432632988	1.7193409803177	0.0950390058557726	0.764440788981576	-4.70148147841429
a_HQ_transcript/17693	2.02746322472003	0.0903991408704285	1.71929314439307	0.0950478178569174	0.764440788981576	-4.35848138151573
a_HQ_transcript/102995	-1.66884003669329	-0.344992393542821	-1.71926633557001	0.0950527566912059	0.764440788981576	-4.54275974266143
a_HQ_transcript/113695	1.61638607397682	-0.16723832121576	1.7192630300763	0.0950533656580731	0.764440788981576	-4.44700755631067
a_HQ_transcript/117818	1.99258055407683	0.119777372035845	1.71918120561354	0.0950684411164409	0.764440788981576	-4.47373401293635
a_HQ_transcript/120444	2.30426537033423	2.39535685336323	1.71917883908095	0.0950688771596771	0.764440788981576	-4.48331632141783
a_HQ_transcript/64294	2.57307192275616	0.939138796024041	1.71910945091273	0.0950816629565234	0.764440788981576	-4.46116618264399
a_HQ_transcript/89710	-0.580294901930411	3.49702062558873	-1.71878195147021	0.0951420290328404	0.764635275513911	-4.73249310031227
a_HQ_transcript/27818	-1.67127817963395	1.07851650066199	-1.71878107611038	0.0951421904258874	0.764635275513911	-4.43916544366755
a_HQ_transcript/126054	2.52882691842301	1.24698897696453	1.71876412213509	0.0951453163327848	0.764635275513911	-4.41859198304336
a_HQ_transcript/85595	1.98702358099894	0.0504596968056281	1.71851150723614	0.0951919026663217	0.764858080362057	-4.42404477111876
a_HQ_transcript/8836	-1.16976175773426	1.83645239309158	-1.71842940910167	0.0952070470254558	0.764858080362057	-4.42783774009216
a_HQ_transcript/139819	-2.06413990608402	1.08487111031622	-1.71839976992318	0.0952125149583651	0.764858080362057	-4.53255615393386
a_HQ_transcript/12571	-0.70373290718404	3.10809025430871	-1.71832171864653	0.0952269153731729	0.76486805814727	-4.70737496783325
a_HQ_transcript/23891	1.89696328727611	1.22055022373851	1.71812286896884	0.0952636112716369	0.765057087040303	-4.52736356384285
a_HQ_transcript/145134	3.00910132902114	1.1203612206176	1.71796406415406	0.0952929257659474	0.765186792225859	-4.51972160594658
a_HQ_transcript/7116	2.22736919875262	-0.16910553813874	1.71786854265699	0.095310562149241	0.765222701189375	-4.53194184786841
a_HQ_transcript/3186	-0.496443550987819	3.16750592555233	-1.71751932608975	0.0953750621974366	0.76563480395147	-4.68540712853786
a_HQ_transcript/53142	-1.35409906656709	0.614110415041601	-1.71731766202192	0.0954123260630288	0.765828181357599	-4.50373156524987
a_HQ_transcript/105658	-3.77562439065669	2.2495496135584	-1.71719152091395	0.0954356408558247	0.76583429577885	-4.59519500273792
a_HQ_transcript/32251	-1.63094500030985	-0.155089035655244	-1.71711468939571	0.0954498440449093	0.76583429577885	-4.46212480866457
a_HQ_transcript/92814	1.65461066794952	0.756460670002641	1.71709971341908	0.0954526127325518	0.76583429577885	-4.46660604876414

a_HQ_transcript/56094	1.71071075721924	2.10201268498715	1.71663792608772	0.0955380188228952	0.766413739737407	-4.44511136032349
a_HQ_transcript/1421	-1.60594704866198	2.09839669518698	-1.71637292888655	0.0955870581148432	0.766457067882387	-4.4300264175712
a_HQ_transcript/91850	-2.01996946466222	0.333842277346711	-1.71626000963879	0.0956079608933586	0.766457067882387	-4.49384699075821
a_HQ_transcript/56741	1.03020977858484	1.81203423598552	1.71602579760549	0.0956513287074344	0.766457067882387	-4.45190516439169
a_HQ_transcript/150562	3.09412234654488	8.85930605346144	1.71595203487644	0.0956649903772331	0.766457067882387	-5.30197629523775
a_HQ_transcript/75552	2.20982561431702	0.375030503723646	1.71571616550487	0.0957086869541792	0.766457067882387	-4.52187444089958
a_HQ_transcript/140518	-1.41867031017933	3.09841118688858	-1.71570872230849	0.0957100661344488	0.766457067882387	-4.57567702316887
a_HQ_transcript/45938	-1.77351410282661	1.8369431061807	-1.71565363637989	0.0957202737488134	0.766457067882387	-4.55666663396764
a_HQ_transcript/147797	0.613331632618164	4.70894742489166	1.71554726682761	0.0957399869779881	0.766457067882387	-4.93444332483744
a_HQ_transcript/81873	-1.44791964750664	3.6785443226758	-1.71546715291311	0.0957548365543148	0.766457067882387	-4.70309256222072
a_HQ_transcript/120412	-2.26534364822101	0.373235580605419	-1.71546347931852	0.095755517522531	0.766457067882387	-4.56253815299752
a_HQ_transcript/15823	1.30123767871375	-0.737521994122038	1.71542281157101	0.0957630563073651	0.766457067882387	-4.52508540257517
a_HQ_transcript/2751	-1.61051956508863	3.35886066666046	-1.71529540603659	0.0957866773306333	0.766457067882387	-4.80449407010581
a_HQ_transcript/120338	2.75833714104047	0.418658675824143	1.71521162875098	0.0958022123235051	0.766457067882387	-4.54518076878911
a_HQ_transcript/8818	1.43988105588734	-0.633631224745273	1.71520519119484	0.0958034061399792	0.766457067882387	-4.51249317102788
a_HQ_transcript/122367	0.581214895300715	3.97641486855085	1.71518796618616	0.0958066005033576	0.766457067882387	-4.80485078963184
a_HQ_transcript/109767	0.792519602481188	2.39169064559911	1.71512333759885	0.095818586615925	0.766457067882387	-4.52194503389203
a_HQ_transcript/135126	0.801649358062829	2.25103253492732	1.71509910724931	0.0958230807358121	0.766457067882387	-4.54916113431359
a_HQ_transcript/4870	1.28994132909656	2.85924058324688	1.71506737823655	0.095828965935943	0.766457067882387	-4.59174165428276
a_HQ_transcript/134209	1.17018688448381	-0.796632002398661	1.71506616780067	0.0958291904575095	0.766457067882387	-4.51295599617115
a_HQ_transcript/77025	0.714755146143031	2.73145057675845	1.71503799082702	0.0958344170779889	0.766457067882387	-4.66578358430134
a_HQ_transcript/68888	1.43139197620495	0.785778048137749	1.7149427589107	0.0958520836623539	0.766457067882387	-4.38120750339877
a_HQ_transcript/558	2.08360836918409	0.553037908555455	1.71490790505929	0.0958585501230218	0.766457067882387	-4.53895229550884
a_HQ_transcript/137197	2.54986236150573	0.386083760063098	1.71481490850715	0.0958758056298956	0.766457067882387	-4.52595902392033
a_HQ_transcript/126998	2.33733714079748	1.6797063083143	1.7147116314557	0.09589497173202	0.766457067882387	-4.43853268443186
a_HQ_transcript/5246	-1.36162732666872	1.68718735489138	-1.71459375030075	0.0959168519779828	0.766457067882387	-4.45888648737543
a_HQ_transcript/19908	-2.73043395202761	2.48218141863203	-1.71456797617606	0.0959216365413196	0.766457067882387	-4.71166208085389
a_HQ_transcript/121165	1.66455713613382	-0.356676834262674	1.71454380523369	0.0959261236805545	0.766457067882387	-4.53333163406378
a_HQ_transcript/2725	-2.34904167806422	0.861887794305561	-1.7145221470592	0.0959301444941722	0.766457067882387	-4.60065506350266
a_HQ_transcript/92888	2.0113121461994	0.792158114507045	1.7144851594009	0.0959370115341017	0.766457067882387	-4.47302575790917
a_HQ_transcript/119847	-1.46931620597443	1.38077402899344	-1.71444416896406	0.0959446222012515	0.766457067882387	-4.42351842679981
a_HQ_transcript/125011	-1.6316012288665	2.02559118095725	-1.71440348783728	0.0959521759389646	0.766457067882387	-4.43309878908547
a_HQ_transcript/15402	0.892230864692713	4.34791749318952	1.71400478343016	0.0960262343870695	0.766943247107938	-4.85167780789337
a_HQ_transcript/72315	1.68072819669399	-0.416206349070415	1.713919883426	0.096042010552769	0.766963867208594	-4.49240834764638
a_HQ_transcript/125095	0.475022438550937	3.31415408179729	1.71374548126542	0.0960744248679097	0.767063365106582	-4.71711549259786
a_HQ_transcript/119838	-1.14334504893864	2.09493105465689	-1.7136496950219	0.0960922315578082	0.767063365106582	-4.48689127856093
a_HQ_transcript/119720	2.03972371844848	1.30050194529255	1.71360165820618	0.0961011626560356	0.767063365106582	-4.50576453178332
a_HQ_transcript/141669	1.57376683003161	-0.659402370620651	1.71356889489073	0.0961072544741129	0.767063365106582	-4.51982964484487
a_HQ_transcript/24278	1.304247353002	1.086039796324	1.71330032758379	0.0961572024896545	0.767261193618772	-4.40635509547856
a_HQ_transcript/120991	-2.43994243503522	1.92086306468019	-1.71329367549701	0.096158439917299	0.767261193618772	-4.72320145110375
a_HQ_transcript/12956	1.36881588705832	1.70771279289488	1.71308383187715	0.0961974820769602	0.767330340342302	-4.42214532801929
a_HQ_transcript/39695	-2.35926826960745	0.586680336977718	-1.71307191118953	0.0961997003621169	0.767330340342302	-4.54036207198718
a_HQ_transcript/9852	1.44314085088253	2.24912841409349	1.71280576447136	0.0962492379647096	0.767330340342302	-4.55024575336249
a_HQ_transcript/68478	-1.64791516114047	2.28933926602771	-1.71276746971624	0.0962563674837624	0.767330340342302	-4.56827592045966
a_HQ_transcript/61812	-1.5020575519021	4.15421254260995	-1.71276425431506	0.0962569661306134	0.767330340342302	-4.8776701131192
a_HQ_transcript/3296	0.924038533471777	1.53606927617058	1.71276157390521	0.0962574651745858	0.767330340342302	-4.41440192915452
a_HQ_transcript/28124	2.29320390797154	0.296688827762339	1.71275058697496	0.0962595107654843	0.767330340342302	-4.4988272160247
a_HQ_transcript/143837	2.93895404915361	0.763234897113683	1.7126361244414	0.0962808240256908	0.767395000543721	-4.56963031793394
a_HQ_transcript/109776	1.87780106748055	-0.341867733456807	1.71246543649913	0.0963126139614071	0.767543134679006	-4.45701218830652
a_HQ_transcript/139058	0.857092692618488	4.15278821108482	1.71231295698377	0.0963410200831351	0.767664265303972	-4.86991654129158

a_HQ_transcript/70555	-1.94892770901441	0.734847611912057	-1.71204135591327	0.0963916352909343	0.767858687008434	-4.42398640424142
a_HQ_transcript/148373	-0.740704337408605	2.94933571144574	-1.71203631280054	0.0963925753290823	0.767858687008434	-4.57574467162027
a_HQ_transcript/24005	2.31713260264904	0.0766355865296054	1.71182592160821	0.0964317991676915	0.767858687008434	-4.50739674064199
a_HQ_transcript/32584	1.8144837764083	-0.194230506223153	1.7117670373159	0.0964427795295709	0.767858687008434	-4.53088776880332
a_HQ_transcript/147316	2.21104987245802	1.66346339001708	1.7117229360619	0.0964510039316084	0.767858687008434	-4.54307700587491
a_HQ_transcript/107530	-1.90356870642447	0.977381550667329	-1.71169921622845	0.0964554276642118	0.767858687008434	-4.52012836957923
a_HQ_transcript/150817	-2.07123822400623	0.511365057394211	-1.71144898098032	0.0965021067143151	0.767858687008434	-4.48416563886528
a_HQ_transcript/142509	3.55032159314623	1.76447012210221	1.71142604917978	0.0965063853734235	0.767858687008434	-4.47541083917963
a_HQ_transcript/40160	1.02252672000244	2.83732774537767	1.71136261741188	0.0965182214209076	0.767858687008434	-4.60420848264061
a_HQ_transcript/139452	2.31444540774582	0.580996144500628	1.71135242216357	0.0965201239160927	0.767858687008434	-4.58242061419299
a_HQ_transcript/18902	-1.75861495724906	0.0786666558257796	-1.71134580365224	0.0965213589872605	0.767858687008434	-4.40172340973962
a_HQ_transcript/95114	-4.07126036843728	0.734534136226097	-1.71130691826385	0.0965286156025577	0.767858687008434	-4.59725724968245
a_HQ_transcript/45333	-3.02108678816726	1.83686313433448	-1.71126120458747	0.0965371470660893	0.767858687008434	-4.7065704466703
a_HQ_transcript/15792	-2.11231031078592	2.3166151418975	-1.71090436314734	0.0966037654567358	0.768106863495159	-4.45515676758656
a_HQ_transcript/100605	2.07781106180181	1.02223738575014	1.71088623198045	0.0966071513741386	0.768106863495159	-4.39043752362007
a_HQ_transcript/125571	1.60814710482836	3.91275179417102	1.71088173798205	0.0966079906243654	0.768106863495159	-4.83720080551267
a_HQ_transcript/146258	0.805623178936671	2.72760114753387	1.71057269468516	0.0966657188280079	0.768198293538958	-4.6745525406599
a_HQ_transcript/84640	-2.00292324196382	0.0747821159834247	-1.71051425377582	0.0966766386303823	0.768198293538958	-4.51440975224723
a_HQ_transcript/149563	1.22457359227643	-0.736876584238887	1.71051392738331	0.0966766996203836	0.768198293538958	-4.52901614732443
a_HQ_transcript/42626	-0.970368595603134	1.96490826162316	-1.71048799147844	0.0966815461301079	0.768198293538958	-4.44666098512391
a_HQ_transcript/104280	1.55881330924865	-0.22155691368931	1.71046646769398	0.096685568323889	0.768198293538958	-4.5688255208718
a_HQ_transcript/36725	4.51155807222329	2.24720139732749	1.71031990211675	0.0967129610582023	0.768310919829327	-4.6224360615937
a_HQ_transcript/16295	2.34793311467098	0.670360893262775	1.71002047639135	0.096768943156174	0.768565090305622	-4.58134990673449
a_HQ_transcript/15314	-2.52845542084248	0.200475261531015	-1.71000734302185	0.0967713992558981	0.768565090305622	-4.49768106108738
a_HQ_transcript/54702	1.49117757452502	0.199513291175266	1.70986022732047	0.0967989152456886	0.768648607778147	-4.43099898474955
a_HQ_transcript/31731	2.85851379926545	1.61064899101644	1.70953621899881	0.0968595396836607	0.768648607778147	-4.56801441764564
a_HQ_transcript/4941	1.06966891021432	0.937781490717948	1.70946889630153	0.0968721402618851	0.768648607778147	-4.40796087411091
a_HQ_transcript/109446	-2.23028597124015	2.53916514665559	-1.70920654068083	0.0969212576306413	0.768648607778147	-4.71439802764628
a_HQ_transcript/142232	0.602387533849653	4.04699589965257	1.70912355156574	0.0969367989216904	0.768648607778147	-4.82888779941811
a_HQ_transcript/11625	-1.15176611088846	1.11898559740415	-1.7091218495765	0.0969371176733831	0.768648607778147	-4.36439577394093
a_HQ_transcript/9144	-0.505312949854911	3.02517828258784	-1.70903736013874	0.0969529421140652	0.768648607778147	-4.69210972047998
a_HQ_transcript/144506	-2.57653379426962	3.73046379738692	-1.70898329114105	0.0969630700957388	0.768648607778147	-4.89594225538616
a_HQ_transcript/143744	3.20759112889771	1.33937095637148	1.70892559000742	0.0969738794106131	0.768648607778147	-4.56872239087069
a_HQ_transcript/126981	3.00456564871468	1.72557334409289	1.70889442016861	0.0969797189632045	0.768648607778147	-4.46632961296308
a_HQ_transcript/152063	1.1738597484257	3.20004437510535	1.70886595925364	0.096985051266047	0.768648607778147	-4.71923133851106
a_HQ_transcript/21492	-1.85959975346996	0.78176797585637	-1.70884225657384	0.0969894922756093	0.768648607778147	-4.42011993601653
a_HQ_transcript/55366	-2.30359497066003	-0.155475563563733	-1.70883890438528	0.0969901203660653	0.768648607778147	-4.51509293245605
a_HQ_transcript/3906	-1.62340087305587	0.731840705883176	-1.70883424719427	0.0969909929769328	0.768648607778147	-4.44932782197752
a_HQ_transcript/4543	1.37658089465861	-0.355957744113977	1.70876346597289	0.0970042559564169	0.768648607778147	-4.51446350056846
a_HQ_transcript/148249	1.46737798743495	-0.621056317250128	1.70874495584475	0.0970077246330228	0.768648607778147	-4.52227484207254
a_HQ_transcript/124345	-1.59708851260016	2.78147751992592	-1.70867595220273	0.0970206563775256	0.768648607778147	-4.73937343931162
a_HQ_transcript/62283	1.29482826264851	4.29662625548719	1.70867461792994	0.0970209064433937	0.768648607778147	-4.91471841126923
a_HQ_transcript/121147	-1.5154713255581	2.05636270115044	-1.70856887527702	0.0970407261660152	0.768648607778147	-4.53233483287544
a_HQ_transcript/123119	1.86585330565719	0.622421055295255	1.70836213728144	0.0970794855921608	0.768648607778147	-4.43412769044948
a_HQ_transcript/127069	-1.32552853830969	1.69978853488748	-1.70836161639567	0.0970795832646518	0.768648607778147	-4.42375312352215
a_HQ_transcript/125965	0.87009094182369	5.37227999798184	1.70833825440635	0.0970839640096249	0.768648607778147	-5.04583825725977
a_HQ_transcript/95783	2.2931768684628	2.98665681465638	1.70832711534209	0.0970860528197625	0.768648607778147	-4.51478953178892
a_HQ_transcript/147820	0.402850485559439	10.3391999451983	1.70821093087689	0.0971078421058054	0.768716416440999	-5.51527676299368
a_HQ_transcript/27609	1.18579134911602	1.97188169931585	1.70798191784987	0.0971508032908007	0.76895178290954	-4.46197952253115
a_HQ_transcript/36265	-2.79387043588102	0.527082935915488	-1.70785906839381	0.0971738555108321	0.768975544112084	-4.49332824537753

a_HQ_transcript/128941	-1.92898891204068	2.52873860059687	-1.70776825980412	0.0971908983289065	0.768975544112084	-4.76523864818632
a_HQ_transcript/138318	3.59995923295523	1.65809368632331	1.70773959024222	0.0971962795080348	0.768975544112084	-4.55658834149264
a_HQ_transcript/1256	-1.96727105526595	1.8088929777337	-1.70759160478163	0.0972240598389092	0.768975544112084	-4.46718092342607
a_HQ_transcript/114475	0.669016708231018	4.81085679186866	1.70758343421969	0.097225593837886	0.768975544112084	-4.95652512778756
a_HQ_transcript/128688	1.48828404789388	1.74218567206504	1.70754303297996	0.0972331793494326	0.768975544112084	-4.45086517442933
a_HQ_transcript/109804	-1.9698602770388	0.335372567226255	-1.70726251896822	0.0972858607498751	0.769258363344979	-4.44427796483245
a_HQ_transcript/47413	2.20830539232563	0.21329583586844	1.70721169506517	0.0972954081907563	0.769258363344979	-4.50965959165872
a_HQ_transcript/151758	0.754888296408288	8.33430812595402	1.70708166206478	0.0973198388945542	0.769291659725707	-5.25620198600469
a_HQ_transcript/144845	1.51323062792349	2.71765371179409	1.70704840339137	0.0973260883846485	0.769291659725707	-4.58615150430218
a_HQ_transcript/140878	-2.21611065790308	0.128289069397668	-1.70694630075083	0.0973452761370589	0.769338709897343	-4.54200345847792
a_HQ_transcript/86091	1.86471316056836	3.81849879144345	1.70670982341906	0.0973897285541728	0.769585391707036	-4.66694818579528
a_HQ_transcript/142339	-4.58041006270047	2.3709768288893	-1.70658802051841	0.0974126313827355	0.769606537361825	-4.6375974999151
a_HQ_transcript/6578	-0.8259767433824	1.76264432861327	-1.70644963042541	0.0974386585989919	0.769606537361825	-4.4453012937303
a_HQ_transcript/122161	2.07961579565627	-0.11276650958508	1.70624194959802	0.0974777283330094	0.769606537361825	-4.4679324803372
a_HQ_transcript/116403	1.84445631398873	1.44271053746806	1.70617301074695	0.0974907002769141	0.769606537361825	-4.49631729252265
a_HQ_transcript/142213	2.12593935836314	0.5894069413449	1.70611459422168	0.0975016934074611	0.769606537361825	-4.51953348751259
a_HQ_transcript/83760	1.61238027123636	0.160231270578209	1.70610813731491	0.0975029085659367	0.769606537361825	-4.56636671587542
a_HQ_transcript/107144	2.32953386345831	-0.485552637536291	1.70609390753577	0.097505586585936	0.769606537361825	-4.51952948512746
a_HQ_transcript/91649	-1.44919357292789	0.0350396997620821	-1.7060413976962	0.0975154693799242	0.769606537361825	-4.38386075927028
a_HQ_transcript/10516	1.1428757430199	1.82300075551111	1.70603829320054	0.0975160536983776	0.769606537361825	-4.43404217939029
a_HQ_transcript/122336	1.02109758707392	4.52039882246986	1.70599180935035	0.0975248030932975	0.769606537361825	-4.88385203561807
a_HQ_transcript/121947	-0.826709528612608	3.70073556745129	-1.70574045518976	0.097572125453959	0.769776719665858	-4.76833745536634
a_HQ_transcript/81165	-1.58205061105057	-0.372235287233741	-1.70573658508755	0.097572854226845	0.769776719665858	-4.52285990972442
a_HQ_transcript/24480	1.426822205984	-0.321265189671818	1.70565870257967	0.0975875211275491	0.769787953331819	-4.44139757046643
a_HQ_transcript/126362	-0.519904917126762	6.5321567814806	-1.70548943816001	0.0976194035061579	0.769934964315596	-5.10450492470324
a_HQ_transcript/72908	-1.03664981451301	2.89584523153457	-1.70532394466087	0.0976505840212655	0.770076400486789	-4.65041865676726
a_HQ_transcript/76718	2.0924199531179	0.557434952808668	1.70523463504653	0.0976674142432443	0.770104646653731	-4.46621701449929
a_HQ_transcript/10802	0.894898134516928	3.49865029379607	1.70510693503544	0.0976914832739933	0.770189955208285	-4.75939035570134
a_HQ_transcript/119797	0.542586187081872	4.86845252190691	1.70470309700063	0.0977676317324854	0.770683069302687	-4.96454877823511
a_HQ_transcript/75135	1.60543427694826	1.19738618461994	1.70459605762124	0.0977878235976328	0.770683069302687	-4.47550275379165
a_HQ_transcript/148115	1.84729365315691	0.376226898973942	1.70456435105479	0.0977938053808254	0.770683069302687	-4.43188531362941
a_HQ_transcript/72373	-3.37818305265364	1.43359432331092	-1.70447457757384	0.0978107437685532	0.770712066392634	-4.63379568146954
a_HQ_transcript/131695	-0.57858058109301	2.46136668433819	-1.70438911007543	0.0978268719862204	0.77073467265045	-4.53024098240426
a_HQ_transcript/130670	-3.1447744293278	1.47266155833717	-1.70428058806677	0.0978473539290622	0.770745992648807	-4.64340148777475
a_HQ_transcript/100508	3.2970622846132	2.05401464184941	1.70424099348266	0.0978548277206887	0.770745992648807	-4.64261189940874
a_HQ_transcript/147932	-0.956835713203279	5.68046382332992	-1.70404077415975	0.0978926280143583	0.770934116636231	-5.06721643516012
a_HQ_transcript/33658	1.83398579636667	0.0379806959299176	1.70387827757355	0.0979233154389859	0.770934116636231	-4.54578731711437
a_HQ_transcript/33685	-1.89676162332281	1.21556114719505	-1.70385531085441	0.0979276533446299	0.770934116636231	-4.57169772527784
a_HQ_transcript/14854	1.56555952008761	4.97331935891311	1.70383355359454	0.0979317629583321	0.770934116636231	-5.01908277875635
a_HQ_transcript/115186	2.32122364697574	0.562908553547849	1.70367167337553	0.0979623441837671	0.771060844507647	-4.54097169271359
a_HQ_transcript/29033	-0.661543045068395	2.67968370132216	-1.70355239688511	0.0979848821327004	0.771060844507647	-4.54868291970588
a_HQ_transcript/136238	1.85738337646693	0.234789408220427	1.70339562749821	0.0980145111631764	0.771060844507647	-4.48330991896771
a_HQ_transcript/97728	-2.05945082286224	-0.013400361514818	-1.70334282534187	0.0980244923274644	0.771060844507647	-4.44431786653123
a_HQ_transcript/118470	-0.835973241807112	1.76367046851158	-1.70325414872148	0.0980412567347903	0.771060844507647	-4.49642431548597
a_HQ_transcript/41581	1.5138918765279	0.32814960651285	1.70313408041311	0.0980639595928104	0.771060844507647	-4.46969368103426
a_HQ_transcript/18387	3.69578242347778	1.19550200498537	1.7029739933552	0.0980942361455453	0.771060844507647	-4.57461785747784
a_HQ_transcript/82653	1.23868114459943	1.91837735912922	1.70296929426194	0.0980951249821684	0.771060844507647	-4.55728396190526
a_HQ_transcript/21209	1.78762685105935	1.31135407295006	1.70285452984376	0.0981168348384065	0.771060844507647	-4.54781090005172
a_HQ_transcript/41979	2.61741057705197	1.28062421781994	1.70267507450583	0.0981507902530956	0.771060844507647	-4.48422642617093
a_HQ_transcript/143039	1.75061714486427	1.86172372825556	1.7025250038983	0.0981791932138903	0.771060844507647	-4.54865673742663

a_HQ_transcript/86503	-1.2222597816791	2.91697363824497	-1.70251905706171	0.0981803188773601	0.771060844507647	-4.56777330748064
a_HQ_transcript/130204	-1.73363462432874	1.33801731745899	-1.70234289585781	0.0982136689327427	0.771060844507647	-4.45271099909364
a_HQ_transcript/119626	1.34271352466865	-0.153678855276981	1.70233302627739	0.0982155376778567	0.771060844507647	-4.54964176240792
a_HQ_transcript/108354	-3.00629921591519	0.89277463224337	-1.70222957038994	0.0982351282103723	0.771060844507647	-4.50523389420598
a_HQ_transcript/16217	2.01358576231316	1.06692618460278	1.7022022047544	0.0982403107465181	0.771060844507647	-4.47066103892914
a_HQ_transcript/141576	4.06870252794206	0.730938873953121	1.7021843290052	0.0982436962002463	0.771060844507647	-4.63668228025917
a_HQ_transcript/61402	-3.81364030359299	2.76697146031408	-1.70210622206502	0.0982584898671128	0.771060844507647	-4.71687573344707
a_HQ_transcript/110619	2.80399454320543	0.0950708980998267	1.70208798382179	0.0982619445080825	0.771060844507647	-4.52312714868947
a_HQ_transcript/18226	1.85700081004437	0.0489062594595178	1.70198848329521	0.0982807934286933	0.771060844507647	-4.44813253629463
a_HQ_transcript/598	2.21768535017402	1.2716160103525	1.70192867274507	0.0982921251173752	0.771060844507647	-4.57072640159258
a_HQ_transcript/141969	1.89988436625355	1.1108772080366	1.70191353370717	0.0982949935280636	0.771060844507647	-4.46894155254436
a_HQ_transcript/42276	-1.46683286307192	-0.652498094801829	-1.70186857142652	0.0983035129948146	0.771060844507647	-4.51875924238764
a_HQ_transcript/74778	-0.522118364617088	3.04138830374502	-1.70184711096748	0.0983075795467446	0.771060844507647	-4.70302544874171
a_HQ_transcript/58301	-1.3841898937156	2.42634459922059	-1.70184686510521	0.0983076261361097	0.771060844507647	-4.56417566811348
a_HQ_transcript/109095	-1.58682864344562	4.03639449619525	-1.70184151283025	0.098308640363378	0.771060844507647	-4.58886011663316
a_HQ_transcript/135658	1.73672138568535	2.59547196303217	1.70182836957779	0.0983111309760098	0.771060844507647	-4.5188955424932
a_HQ_transcript/137362	-1.37656407493234	-0.661250214073888	-1.70174236391669	0.0983274301297751	0.771060844507647	-4.51239952387547
a_HQ_transcript/123575	2.61119466448571	0.0625149948611586	1.70171538905538	0.098332542670231	0.771060844507647	-4.6287913527621
a_HQ_transcript/6280	1.34863016480929	-0.315724915533616	1.70160023094693	0.0983543710637706	0.771106816082373	-4.47023220618788
a_HQ_transcript/136317	2.49844147928795	0.637025262382807	1.70142494368845	0.0983876048013945	0.771106816082373	-4.57218511650108
a_HQ_transcript/96913	1.70224483304168	0.380236467594797	1.70140681750331	0.098391041986272	0.771106816082373	-4.47418848416362
a_HQ_transcript/22348	-0.807271326136371	2.31281772890432	-1.70140457066288	0.0983914680512483	0.771106816082373	-4.50984504653278
a_HQ_transcript/112533	1.26643864776344	2.07707029054853	1.70132736166825	0.0984061100137741	0.771111017165936	-4.5023326896347
a_HQ_transcript/79673	2.2061009250533	1.64540471972758	1.70126184753815	0.0984185355827497	0.771111017165936	-4.53604685792245
a_HQ_transcript/74234	1.455385731357	0.259200665953445	1.70111163179622	0.0984470308145226	0.771144952536988	-4.41279227162915
a_HQ_transcript/119158	3.85432080150268	2.83550075166159	1.70096979369766	0.098473943173761	0.771144952536988	-4.63218614488012
a_HQ_transcript/74312	1.13942369643517	-0.736694074524967	1.70096134550123	0.0984755463284211	0.771144952536988	-4.54465716119912
a_HQ_transcript/79690	1.54552068616575	-0.445127469840912	1.70090432599087	0.0984863670891288	0.771144952536988	-4.48320176238717
a_HQ_transcript/16691	4.03582241087461	4.36651971013427	1.70084838484692	0.0984969841705549	0.771144952536988	-4.8591285424456
a_HQ_transcript/94664	-0.756018325061671	4.5799024541563	-1.70081426148172	0.0985034609207758	0.771144952536988	-4.87815173692733
a_HQ_transcript/38431	2.29961117007901	1.07656044210931	1.70074962001856	0.0985157311027332	0.771144952536988	-4.39498176767392
a_HQ_transcript/15078	1.64079781320757	2.13414429392219	1.70055454028231	0.098552768698404	0.771247465395597	-4.61590131991702
a_HQ_transcript/50271	3.01432279198125	3.03879156104108	1.70046111288021	0.0985705108283554	0.771247465395597	-4.75074501757488
a_HQ_transcript/83314	-1.52898945895723	2.20289617742154	-1.70044775544451	0.0985730476615024	0.771247465395597	-4.56109727861545
a_HQ_transcript/56468	2.81967842655143	0.472297800914586	1.70040114799722	0.0985818997366034	0.771247465395597	-4.46486686162341
a_HQ_transcript/144391	-2.30160206268458	1.7981613750526	-1.70009707933436	0.0986396672955438	0.771292440925956	-4.63591588731266
a_HQ_transcript/80391	1.9778324293803	2.15440760873981	1.70007296014903	0.0986442507159016	0.771292440925956	-4.61241764218953
a_HQ_transcript/63433	3.594612681691	2.17536906215839	1.70002975823475	0.0986524609128297	0.771292440925956	-4.52138849651474
a_HQ_transcript/121368	0.441954825804605	4.5408282914516	1.69986414516732	0.0986839397124278	0.771292440925956	-4.94871814090218
a_HQ_transcript/36936	0.669477591152087	3.72033734025833	1.69986287642252	0.0986841809006833	0.771292440925956	-4.80965947366128
a_HQ_transcript/13014	-4.14918281380534	0.129486159142363	-1.69985095111243	0.0986864479249655	0.771292440925956	-4.56011977089892
a_HQ_transcript/33726	-2.15807201473254	3.28171800320214	-1.69982182479531	0.098691985076526	0.771292440925956	-4.80859117672263
a_HQ_transcript/138331	2.91791994237807	3.23164773337357	1.69981228108636	0.0986937994702597	0.771292440925956	-4.60184160972862
a_HQ_transcript/32378	-1.5185666373304	0.884962762933488	-1.69960284686501	0.0987336228870345	0.771499936977758	-4.48313232583449
a_HQ_transcript/54381	-2.23817765534651	2.96785241673001	-1.69927940302914	0.0987951513330969	0.771812595659338	-4.72493297279995
a_HQ_transcript/30682	1.81145049790636	1.64670041837658	1.69925291589886	0.0988001913759485	0.771812595659338	-4.52423293932447
a_HQ_transcript/138125	-3.2549604813571	3.06977988935239	-1.69881511831443	0.098883527803765	0.772251034509085	-4.66309293873937
a_HQ_transcript/32276	1.16671233355613	2.27769474424603	1.69869825680743	0.098905782759541	0.772251034509085	-4.50294269262146
a_HQ_transcript/66543	1.16830897994811	3.07512210516571	1.69862774038317	0.0989192138408718	0.772251034509085	-4.72703310357586
a_HQ_transcript/54994	-0.839693458615172	3.57292201237991	-1.6985599836286	0.0989321207301484	0.772251034509085	-4.8004365723977

a_HQ_transcript/102144	-3.14718193461858	1.64547726396929	-1.6985454632709	0.0989348868757594	0.772251034509085	-4.55104107153086
a_HQ_transcript/80012	-1.3836706879774	-0.680335572542431	-1.69849813957546	0.098943902545024	0.772251034509085	-4.52279815062816
a_HQ_transcript/126356	2.75122162185116	0.100036253467466	1.69846973804014	0.0989493136702392	0.772251034509085	-4.54346808869666
a_HQ_transcript/23441	0.729355055789364	4.67482139513239	1.69837849872598	0.0989666984646466	0.77228302434595	-4.94531640947559
a_HQ_transcript/132346	1.66343929066163	0.846476086265734	1.69827678456717	0.0989860821494488	0.772330601769552	-4.39766767532835
a_HQ_transcript/142996	-1.92040447589811	-0.0817906412230682	-1.69811609695428	0.0990167108769363	0.772465893149182	-4.5226202448817
a_HQ_transcript/148902	1.50837402226437	3.97422518193172	1.69772904578641	0.0990905193437664	0.772937964092116	-4.78210030683246
a_HQ_transcript/127224	2.17362933089185	1.62032940135039	1.6975024529384	0.0991337506362797	0.773161413266943	-4.43075448684365
a_HQ_transcript/142383	-2.54578024861201	0.361163196839169	-1.69742375142376	0.0991487696591101	0.773161413266943	-4.47920251807171
a_HQ_transcript/95976	-1.3303650446896	-0.570927468345521	-1.6973697898003	0.0991590685367647	0.773161413266943	-4.56339863552881
a_HQ_transcript/47929	2.83583620106107	0.815218767487174	1.69717731887695	0.0991958099649573	0.773254169811065	-4.52694664249727
a_HQ_transcript/71568	2.51695968480119	2.75849285517714	1.69716810080361	0.0991975699195072	0.773254169811065	-4.68721452207586
a_HQ_transcript/16054	0.986760169616403	2.53130682462069	1.69684617026459	0.0992590506528563	0.773629672345029	-4.57161649838024
a_HQ_transcript/141271	1.90430567550447	3.76746641708699	1.69672483461392	0.099282231001342	0.773686364777672	-4.94703851286831
a_HQ_transcript/140836	0.632039642624064	4.69566052365021	1.69666876336698	0.0992929445575528	0.773686364777672	-4.96108055948469
a_HQ_transcript/109338	1.97584746363673	1.169819651296	1.6964968562057	0.0993257969461307	0.773838617462094	-4.48864475939844
a_HQ_transcript/20457	-0.530356262869708	5.2418752026487	-1.69614182104198	0.0993936748137324	0.774145085498883	-5.004492475963
a_HQ_transcript/113933	2.20676994331928	1.88602241223367	1.69603217956722	0.0994146445891699	0.774145085498883	-4.51355380527673
a_HQ_transcript/130504	3.31533299475916	1.44952630715556	1.69591264297183	0.0994375110910318	0.774145085498883	-4.53706302190027
a_HQ_transcript/123478	0.449793836215934	4.70339493334057	1.69590887166933	0.099438232585865	0.774145085498883	-4.94604301378242
a_HQ_transcript/140789	1.13081176908161	6.11774383359658	1.69589428464761	0.0994410232970238	0.774145085498883	-5.11230152298518
a_HQ_transcript/9796	0.867403106312323	2.43596289555828	1.69584382535971	0.0994506774030306	0.774145085498883	-4.52761974978652
a_HQ_transcript/151617	0.395730191926215	5.25600235339034	1.6955730487582	0.0995024970100776	0.774145085498883	-4.99500893204398
a_HQ_transcript/50355	-0.700013566551215	3.60927371593982	-1.69551870186469	0.0995129003091911	0.774145085498883	-4.68281094788753
a_HQ_transcript/51588	1.76298497422466	1.38378222761996	1.69550472722136	0.0995155755379076	0.774145085498883	-4.57781690581987
a_HQ_transcript/116881	-1.21588321094189	0.0229468754169462	-1.69546700151619	0.0995227978388378	0.774145085498883	-4.50371198392423
a_HQ_transcript/111715	2.40732753086168	0.625606930787955	1.6954349755805	0.0995289293053925	0.774145085498883	-4.61718827790327
a_HQ_transcript/136057	3.40694090489563	4.26419917722415	1.69535385972497	0.0995444606030052	0.774145085498883	-4.91108618168933
a_HQ_transcript/94624	-0.975482003137265	2.30187198931323	-1.69516615632601	0.0995804080339751	0.774145085498883	-4.5545345556718
a_HQ_transcript/34679	1.59019190774808	-0.107774000539669	1.69515215294977	0.0995830902812009	0.774145085498883	-4.5491923134858
a_HQ_transcript/3959	1.74808171348194	4.68025032957485	1.69505124628171	0.0996024200195993	0.774145085498883	-4.94885942773116
a_HQ_transcript/45090	1.35363796538781	0.862606312548179	1.69497623348393	0.0996167915429731	0.774145085498883	-4.50485252705257
a_HQ_transcript/4198	1.83265909977859	0.903401542778868	1.69482885076265	0.099645033289054	0.774145085498883	-4.58941916618846
a_HQ_transcript/128030	-1.59193941991823	0.838804582773654	-1.69478061596219	0.0996542775809552	0.774145085498883	-4.44250480355641
a_HQ_transcript/101027	-1.85625401153211	-0.333237279975103	-1.69468380888094	0.0996728330035541	0.774145085498883	-4.52560287451698
a_HQ_transcript/114216	1.64612034340653	0.147673883289648	1.6946800208448	0.0996735591310832	0.774145085498883	-4.4669612220266
a_HQ_transcript/39873	1.99449536727107	0.994087210862973	1.69461640533109	0.0996857542298301	0.774145085498883	-4.6095118004616
a_HQ_transcript/29073	2.95065935515836	1.75998723304995	1.69451112834065	0.0997059385725907	0.774145085498883	-4.48892154849911
a_HQ_transcript/14409	-2.23566019707886	1.232213180033	-1.69448667330727	0.0997106277280169	0.774145085498883	-4.40362095591711
a_HQ_transcript/98451	4.36034129290733	2.58220853348651	1.69428668024652	0.099748982510372	0.774145085498883	-4.70327339583659
a_HQ_transcript/94213	-0.493338091384471	2.73557349339417	-1.69428331071839	0.0997496288258467	0.774145085498883	-4.64740023461028
a_HQ_transcript/141881	-1.96198428431508	1.42239512863235	-1.69427956245255	0.0997503477918153	0.774145085498883	-4.42659904303501
a_HQ_transcript/37441	1.76373676356439	-0.575201912404794	1.69427827341871	0.0997505950462111	0.774145085498883	-4.54496975829017
a_HQ_transcript/19901	1.83254679814236	2.32103077200039	1.69427543654187	0.0997511391999581	0.774145085498883	-4.66307698531489
a_HQ_transcript/14038	-2.50350120925541	0.525745713991007	-1.69425655000865	0.099754761971599	0.774145085498883	-4.6281387411878
a_HQ_transcript/40773	-1.5712315300713	0.48651842106112	-1.69420489081523	0.0997646716809821	0.774145085498883	-4.48425305393804
a_HQ_transcript/499	-2.0401824763302	1.78538180540161	-1.69412026669443	0.0997809067806747	0.774167718813009	-4.48162899947335
a_HQ_transcript/123138	3.40933689555045	0.953916674206821	1.69357971933625	0.0998846625824437	0.774751412911585	-4.5193391720354
a_HQ_transcript/139543	-1.74747473180461	0.505011570416178	-1.69352925865927	0.0998943528911325	0.774751412911585	-4.54092009115422
a_HQ_transcript/105174	1.96287079110168	-0.109478333060954	1.69350487701863	0.0998990353453309	0.774751412911585	-4.57592841229316

a_HQ_transcript/86179	2.0566346107088	1.03008922511011	1.69335300712246	0.099928205834216	0.774751412911585	-4.47715212948023
a_HQ_transcript/45873	1.82183350981007	-0.170912445938632	1.69325999520205	0.0999460746563201	0.774751412911585	-4.52321427080772
a_HQ_transcript/62014	-0.504735188146449	4.69499385967626	-1.69319304458067	0.0999589384079054	0.774751412911585	-4.96029389096598
a_HQ_transcript/139170	1.31723434092215	-0.616922399931043	1.69311268104862	0.0999743811123631	0.774751412911585	-4.54626445626145
a_HQ_transcript/54392	1.69625415069671	-0.459881880589733	1.6930943082991	0.0999779119104923	0.774751412911585	-4.52835892951377
a_HQ_transcript/137498	1.82816792343922	2.65405280165985	1.69298413044808	0.0999990876118902	0.774751412911585	-4.63821859940977
a_HQ_transcript/141589	-1.57169258554655	-0.507427408608671	-1.69295839131936	0.100004035099161	0.774751412911585	-4.52739401181069
a_HQ_transcript/74814	-1.62440057757537	-0.445295008577697	-1.69294780610045	0.100006069812996	0.774751412911585	-4.51947603991196
a_HQ_transcript/122606	0.74537386030909	3.32056711958787	1.69289574115653	0.100016078352748	0.774751412911585	-4.79073843889555
a_HQ_transcript/131591	2.30379077515431	0.345945934652922	1.69269528542931	0.100054620128929	0.774946696716106	-4.48483761280626
a_HQ_transcript/9667	-0.922916573163455	2.64536706095466	-1.69262178227077	0.10006875574229	0.774952922167312	-4.64901885913195
a_HQ_transcript/4192	2.14782864721287	0.951508952665395	1.69254360054564	0.100083792933559	0.774966127033695	-4.55830614778436
a_HQ_transcript/19027	1.38684168116662	1.70122347582379	1.6922546806282	0.100139379107317	0.775285562495167	-4.45258602166498
a_HQ_transcript/128995	1.52995979347988	-0.24165271016744	1.69197368409394	0.100193465582386	0.775285562495167	-4.60056258309563
a_HQ_transcript/126809	0.47670425336849	3.49592053522972	1.69195213378423	0.100197614612135	0.775285562495167	-4.82218140294655
a_HQ_transcript/104822	1.39399955962518	0.488089746508975	1.69190713286321	0.10020627899434	0.775285562495167	-4.45562314755342
a_HQ_transcript/33361	1.61438543379066	0.0576025977152872	1.691887663593	0.100210027760174	0.775285562495167	-4.49858076156765
a_HQ_transcript/60651	-1.53626759336921	1.28191615155089	-1.69188169404056	0.100211177208011	0.775285562495167	-4.44373179416976
a_HQ_transcript/126720	-2.62956984334767	1.31322437875274	-1.69184413428304	0.100218409657801	0.775285562495167	-4.58244954406832
a_HQ_transcript/33920	1.62942116399594	-0.353358540907079	1.69166794740576	0.100252341747479	0.775444859760142	-4.47899890313103
a_HQ_transcript/120956	-1.11275753558055	2.12566476088197	-1.69149317742449	0.100286010423424	0.775602077420542	-4.45458468981628
a_HQ_transcript/36672	2.88347969563015	0.961874200342959	1.69139235397051	0.100305437912845	0.775637718983082	-4.5781465866763
a_HQ_transcript/88438	1.19526010943192	1.47466652820133	1.69133076907237	0.100317306140153	0.775637718983082	-4.41980891532217
a_HQ_transcript/80670	0.565547048732032	3.05712607474585	1.69123899222508	0.10033499493037	0.775671310721178	-4.69146044529594
a_HQ_transcript/125424	1.98443180611132	0.666255704378657	1.6911411710916	0.10035385154227	0.775683085814765	-4.51354877729364
a_HQ_transcript/12040	-1.83566012810855	0.430977771746809	-1.69109264484612	0.100363206860942	0.775683085814765	-4.40735255493855
a_HQ_transcript/339	-2.64232692976945	1.19588152965769	-1.69102028615124	0.100377158161065	0.775687775802494	-4.52144493589253
a_HQ_transcript/7115	-0.607298499579845	3.53120653744925	-1.69089877895079	0.100400589301353	0.775696305590783	-4.78597250084355
a_HQ_transcript/152614	2.75062966425098	6.30554349137351	1.69080273417677	0.100419113560092	0.775696305590783	-5.1367539761617
a_HQ_transcript/1967	-1.08815081529776	0.428428309581109	-1.69078377429135	0.100422770710466	0.775696305590783	-4.4575914370923
a_HQ_transcript/33955	2.93823042694001	2.98093358622203	1.69073779301792	0.100431640446536	0.775696305590783	-4.60387384987521
a_HQ_transcript/146340	-1.73084744166506	-0.142422588291945	-1.69052093764781	0.100473480412335	0.775831866344767	-4.48968159193949
a_HQ_transcript/143971	6.3099171091213	2.39871596265563	1.69050847287521	0.10047588580105	0.775831866344767	-4.65236581937262
a_HQ_transcript/33298	-1.15512289201924	3.47186698361157	-1.69039340113964	0.100498094030506	0.775862937044754	-4.84750807187944
a_HQ_transcript/7130	-1.75168356686804	0.355211510367422	-1.69011224874733	0.100552372160053	0.775862937044754	-4.5197495094657
a_HQ_transcript/43430	-2.17609868514147	0.323628740033065	-1.69008545029858	0.100557547034834	0.775862937044754	-4.61064436766448
a_HQ_transcript/118492	-1.06606230828561	2.74807074566109	-1.69003350138552	0.100567579185401	0.775862937044754	-4.58895218448743
a_HQ_transcript/69211	-1.92189218253917	0.010027970678729	-1.68998534230227	0.100576880205906	0.775862937044754	-4.55308455728765
a_HQ_transcript/29498	-1.44682480379402	0.45427599726297	-1.68988131104261	0.100596974334315	0.775862937044754	-4.50759239459502
a_HQ_transcript/19239	1.55157810286963	-0.107924494015846	1.68977544031339	0.100617427202146	0.775862937044754	-4.54091538421305
a_HQ_transcript/23782	1.77277264963153	1.17033016513661	1.68975704343897	0.100620981596104	0.775862937044754	-4.48437273285052
a_HQ_transcript/117545	1.21178207851905	1.09390367428075	1.68970675807043	0.100630697585624	0.775862937044754	-4.43228503966349
a_HQ_transcript/36180	-1.17926349015496	2.02681670714616	-1.68963795781679	0.100643992233739	0.775862937044754	-4.48430966462885
a_HQ_transcript/14662	2.38048688078486	1.74548562247953	1.68962561437262	0.100646377579711	0.775862937044754	-4.40925103972654
a_HQ_transcript/96667	-1.86567676083221	0.032066597703648	-1.68962035027672	0.100647394870087	0.775862937044754	-4.51228999428157
a_HQ_transcript/43354	-1.3738050177978	2.96869181428215	-1.68958913685953	0.100653427061906	0.775862937044754	-4.59039708070071
a_HQ_transcript/130740	1.88210009737135	0.57395561328663	1.68949792829883	0.10067105542526	0.7758959307557	-4.40983384089897
a_HQ_transcript/24672	0.661487568584008	2.679627945465	1.68939648148291	0.100690665614871	0.775933844098975	-4.6063219964632
a_HQ_transcript/113682	-2.64531747310604	0.698475264374187	-1.68926899102791	0.100715314690362	0.775933844098975	-4.49864421778196
a_HQ_transcript/148042	2.39767778958192	-0.0869481250843262	1.68923605643898	0.100721683099848	0.775933844098975	-4.58653323148513

a_HQ_transcript/29521	2.82483416935814	3.95178395714693	1.68919149226015	0.100730300804038	0.775933844098975	-4.67941180597872
a_HQ_transcript/123237	1.51514974354547	2.6500573078903	1.68912728475842	0.100742718163621	0.775933844098975	-4.56110966350516
a_HQ_transcript/107450	-3.01069764500619	1.52523532730169	-1.68903283122388	0.100760987250957	0.775971736476366	-4.63378574424582
a_HQ_transcript/111292	1.34556781190418	-0.717677159737652	1.68890801650381	0.100785132998799	0.776054869645539	-4.53670998931681
a_HQ_transcript/10644	2.27293117383493	1.54505644939653	1.68872861359227	0.100819847431965	0.776217031242019	-4.52770050191188
a_HQ_transcript/18019	1.59252913230305	0.280625738750596	1.68866117246214	0.100832899857358	0.776217031242019	-4.55115191548665
a_HQ_transcript/151948	4.00465587059459	2.91242755408387	1.68849563047874	0.100864944505166	0.77636089700692	-4.65696049262511
a_HQ_transcript/87698	1.46721641367441	0.718824765287859	1.68841646790816	0.100880271332756	0.776376064084526	-4.41984257936767
a_HQ_transcript/37355	1.11398281751109	2.55419830096779	1.68828043623558	0.100906613238957	0.776475988132655	-4.62480853288496
a_HQ_transcript/7593	1.47312768988096	2.477710402508	1.68810842563268	0.10093993050928	0.776507318953932	-4.60818242745481
a_HQ_transcript/8447	0.474159579474627	4.53893200692112	1.68807492965699	0.100946419516053	0.776507318953932	-4.95761435471726
a_HQ_transcript/76218	1.83034700435482	0.944384754293781	1.68805252241986	0.100950760551453	0.776507318953932	-4.53407385287828
a_HQ_transcript/23647	1.38324497503748	0.42396245335559	1.68776656454119	0.101006173877271	0.776830758816888	-4.46074655644117
a_HQ_transcript/17749	-1.91310602114139	3.13858302933963	-1.68746901494627	0.101063860379843	0.777171593618138	-4.7153891105783
a_HQ_transcript/12016	-0.767078486442321	3.36254565637443	-1.6871713055452	0.101121605347518	0.777251611176141	-4.71246016217614
a_HQ_transcript/29720	1.52416466604232	0.0183018713504833	1.68714567692124	0.101126577668656	0.777251611176141	-4.50206213218757
a_HQ_transcript/2830	0.537203999685023	3.16494540557819	1.68700937240494	0.101153026126899	0.777251611176141	-4.80931501579279
a_HQ_transcript/19838	1.48368883959265	1.18197618767556	1.68690260331887	0.101173747572318	0.777251611176141	-4.48884014147022
a_HQ_transcript/128699	1.73826671070389	0.735455774490825	1.68686331654109	0.101181373130617	0.777251611176141	-4.49448743092383
a_HQ_transcript/117724	2.55944088889078	1.45150390507273	1.68682973528071	0.101187891628501	0.777251611176141	-4.5714764796836
a_HQ_transcript/5344	-1.84864540929035	-0.300772466671318	-1.68674828057495	0.101203704351303	0.777251611176141	-4.48648960343469
a_HQ_transcript/73588	2.28190385190134	-0.178784672679848	1.68672479067183	0.101208264805949	0.777251611176141	-4.49332789950143
a_HQ_transcript/150032	1.08372191707594	3.66607067995835	1.68668291663213	0.101216394879413	0.777251611176141	-4.84049239855404
a_HQ_transcript/32606	-0.993591707226712	0.933896184390461	-1.68664128694798	0.101224478049488	0.777251611176141	-4.50587585158803
a_HQ_transcript/56433	-1.84303879527775	1.66896664254161	-1.68660291610387	0.101231928932231	0.777251611176141	-4.51025796008381
a_HQ_transcript/64023	-3.18353531668044	2.9143341645941	-1.68658853039935	0.10123472247823	0.777251611176141	-4.72419368860156
a_HQ_transcript/67780	1.21596152284417	2.48046041565139	1.68641911762165	0.101267625415073	0.777324198585908	-4.64771974479001
a_HQ_transcript/114439	-0.352754013799822	4.54469569024699	-1.68622757095607	0.101304837876811	0.777324198585908	-4.94687231167214
a_HQ_transcript/96221	-2.870374321528	2.89099642850462	-1.6861173504555	0.101326255976241	0.777324198585908	-4.68849634411051
a_HQ_transcript/10607	2.74400603743995	2.41539751330774	1.68609658214997	0.101330292105633	0.777324198585908	-4.61860413329583
a_HQ_transcript/121669	2.06441337573944	1.47020736725047	1.68577555150848	0.101392698506396	0.777324198585908	-4.54182213636851
a_HQ_transcript/112926	0.40034669080881	3.58734827661392	1.68576920236656	0.101393933063823	0.777324198585908	-4.86480887067461
a_HQ_transcript/6895	1.74755772439546	2.91875029819331	1.68575957320602	0.101395805427612	0.777324198585908	-4.75807712920242
a_HQ_transcript/60091	-2.81248010044385	1.75041523422256	-1.68571690795474	0.101404101915084	0.777324198585908	-4.60457027696448
a_HQ_transcript/38500	1.4406571353312	-0.143306384009628	1.68570001292332	0.101407387401124	0.777324198585908	-4.47210159766832
a_HQ_transcript/136302	1.86513269945358	3.40651598352463	1.6856668685205	0.101413833072885	0.777324198585908	-4.55684842220091
a_HQ_transcript/81818	-1.54801216758552	-0.115599890417931	-1.68565074053461	0.101416969644589	0.777324198585908	-4.41313805011267
a_HQ_transcript/9260	1.49618278925759	-0.433851605536491	1.68564373668442	0.101418331778987	0.777324198585908	-4.55398119820262
a_HQ_transcript/138768	1.55456346181416	3.39010260307537	1.6856195419115	0.101423037384206	0.777324198585908	-4.78142570010312
a_HQ_transcript/145928	1.38455351571643	0.798582869550106	1.68551353384298	0.101443656879982	0.777324198585908	-4.46065324382227
a_HQ_transcript/34983	2.05570915731142	-0.451124762818234	1.68550783109276	0.101444766213747	0.777324198585908	-4.54564219906749
a_HQ_transcript/103893	-2.10292587520489	1.88876052975236	-1.68522500897621	0.101499795181014	0.777416493922167	-4.54708498189613
a_HQ_transcript/10387	-1.61252586575087	-0.0660569267299032	-1.68522434680489	0.101499924049495	0.777416493922167	-4.54208776038234
a_HQ_transcript/140626	2.11937161050735	4.35592297682059	1.68522204426984	0.101500372158453	0.777416493922167	-4.93429129265142
a_HQ_transcript/130149	2.07270836936829	0.194789606600886	1.68517099225742	0.101510308093677	0.777416493922167	-4.49664136070255
a_HQ_transcript/40253	1.5108305499128	1.0762049193282	1.68502122982599	0.10153946009905	0.777491318769491	-4.4612994420087
a_HQ_transcript/3562	0.929803157884954	2.44454654488039	1.68498337787318	0.101546829275013	0.777491318769491	-4.5823753957335
a_HQ_transcript/137868	-2.82028173168709	3.61205521457621	-1.68482920431116	0.101576849030848	0.777618738372863	-4.8696159693829
a_HQ_transcript/44418	1.40371053892015	0.400542448224898	1.68472273167675	0.101597585063059	0.777675062489529	-4.47482516902253
a_HQ_transcript/1772	0.671666505707352	3.10269561919171	1.68455755823233	0.101629760325981	0.777779151368409	-4.61601775604136

a_HQ_transcript/109763	3.17036899579581	0.701218562254566	1.68451555164724	0.101637944429439	0.777779151368409	-4.49521205875338
a_HQ_transcript/134487	0.695847461113411	3.37188382341851	1.6844342646366	0.101653783060624	0.777797959951025	-4.78459103464696
a_HQ_transcript/8013	-4.60082508198677	3.36698766031573	-1.6843468017755	0.101670827342024	0.777825987330503	-4.69564670635585
a_HQ_transcript/15447	-2.18949292255494	0.941942496500209	-1.68406708853052	0.101725352304767	0.777970682280682	-4.55853971884948
a_HQ_transcript/152182	-3.05306645647894	1.77115484432246	-1.68400323202518	0.101737803373256	0.777970682280682	-4.6724241621462
a_HQ_transcript/12990	2.12779700598477	-0.474332109955739	1.68397460992532	0.101743384668952	0.777970682280682	-4.54236560289971
a_HQ_transcript/78256	2.834376890285	2.81204794614674	1.68392441402803	0.101753173460699	0.777970682280682	-4.79385714129678
a_HQ_transcript/112431	0.324807755011548	4.12288107812267	1.68390653948802	0.101756659396161	0.777970682280682	-4.92150408068376
a_HQ_transcript/15272	1.4918298250246	0.457446422312225	1.68378281034196	0.101780792077513	0.777981906862141	-4.52673010264102
a_HQ_transcript/58788	-2.18956356381129	1.79228711944759	-1.68362201365224	0.101812161701132	0.777981906862141	-4.57007018160555
a_HQ_transcript/142685	-3.32299925242328	0.732576011431756	-1.68361041876124	0.101814424045098	0.777981906862141	-4.67388517267652
a_HQ_transcript/27566	-1.6709420480751	1.80932759691851	-1.68351567404656	0.101832911784934	0.777981906862141	-4.50941684751214
a_HQ_transcript/43568	-1.77962857058221	0.711666318785363	-1.68343406525063	0.1018488385264	0.777981906862141	-4.51085241774176
a_HQ_transcript/110339	2.31327470154433	0.572365351610103	1.68343046485885	0.101849541225274	0.777981906862141	-4.47166850549882
a_HQ_transcript/31881	-1.01720065152055	2.37366856128167	-1.68341881416209	0.101851815153126	0.777981906862141	-4.51011107244293
a_HQ_transcript/98643	1.93885585905347	1.57441640260113	1.6832294351485	0.101888783183616	0.778023454758448	-4.6035468103704
a_HQ_transcript/100869	-2.06327163477188	0.680798876324461	-1.68322296329267	0.101890046729968	0.778023454758448	-4.53495069503902
a_HQ_transcript/99823	1.639575709896	-0.368211990975299	1.68306629479325	0.101920638215742	0.778023454758448	-4.51578967734635
a_HQ_transcript/75829	0.908182698621196	3.95016207221909	1.68298576297475	0.101936366038189	0.778023454758448	-4.84431974395127
a_HQ_transcript/110943	7.21549789239837	3.82557072260139	1.6829430231239	0.101944713930033	0.778023454758448	-4.82084734189021
a_HQ_transcript/145289	1.4839937513571	2.83829396380131	1.68292192834041	0.101948834345465	0.778023454758448	-4.73624802627174
a_HQ_transcript/73542	1.58425065636562	-0.464014240443558	1.68280723186244	0.10197124028045	0.778023454758448	-4.55133041995179
a_HQ_transcript/79565	-1.36788173945853	3.55348937485079	-1.68273348996162	0.101985647912705	0.778023454758448	-4.88874147689318
a_HQ_transcript/2081	-0.813135438978751	3.13570158845192	-1.68272834587816	0.101986653022783	0.778023454758448	-4.70216216250681
a_HQ_transcript/72700	-2.03455730068454	2.96389967471315	-1.6826714985557	0.101997761054611	0.778023454758448	-4.66095322632313
a_HQ_transcript/65981	-1.55230735038932	-0.476065030324734	-1.68255252239176	0.102021012394537	0.778023454758448	-4.53510551970387
a_HQ_transcript/77224	-2.4967910905068	1.27665171451689	-1.68253557223847	0.102024325297754	0.778023454758448	-4.54153725477584
a_HQ_transcript/101931	2.99297354073329	3.16846982236326	1.68238153891357	0.102054435179432	0.778023454758448	-4.54642870434766
a_HQ_transcript/5341	-1.83598170228241	1.65034002547189	-1.68224404892941	0.102081317479065	0.778023454758448	-4.64488131976569
a_HQ_transcript/72780	-3.54098840029552	2.7007456954088	-1.68221151986335	0.102087678485695	0.778023454758448	-4.7586548422502
a_HQ_transcript/106694	2.43916655025913	0.71907452181941	1.68216787689297	0.102096213317016	0.778023454758448	-4.51803218428083
a_HQ_transcript/72888	-2.20840355258565	-0.0271816591154505	-1.68211306086101	0.102106933997247	0.778023454758448	-4.57073564792002
a_HQ_transcript/30015	-1.9176497356113	0.736479977390325	-1.68201504994286	0.102126104881466	0.778023454758448	-4.47097167160643
a_HQ_transcript/151883	0.8488619388339	6.72492548443099	1.68199765466898	0.102129507701118	0.778023454758448	-5.15521944821528
a_HQ_transcript/143198	-2.19121743248769	-0.00741557641387336	-1.68195311277751	0.102138221304142	0.778023454758448	-4.55434498652512
a_HQ_transcript/151131	-0.815581370906822	2.60494055514926	-1.68184679563161	0.102159022326334	0.778023454758448	-4.60645088598182
a_HQ_transcript/74186	3.62106621401636	1.54399122841447	1.68175564693461	0.10217685843975	0.778023454758448	-4.48686372034912
a_HQ_transcript/129982	-1.99275332683088	3.48730015493133	-1.68171893953266	0.10218404213151	0.778023454758448	-4.78359321428792
a_HQ_transcript/62161	1.51171630477807	2.0617646553725	1.68170870140756	0.102186045822443	0.778023454758448	-4.63160831290918
a_HQ_transcript/23606	-1.80732745395949	0.545851132371336	-1.68167893787391	0.102191870992994	0.778023454758448	-4.50429087524321
a_HQ_transcript/3587	1.77320037660939	1.14584599019673	1.68156859121046	0.102213469905409	0.778085984633528	-4.44327526923772
a_HQ_transcript/23373	-2.49598447358953	0.907415431852431	-1.68140881414525	0.10224475090783	0.778206037572368	-4.41736981531836
a_HQ_transcript/105730	3.0322510070661	0.23948154802249	1.681351282803	0.102256016291249	0.778206037572368	-4.48050091359703
a_HQ_transcript/49915	1.47914359407139	1.35064020979857	1.68125687101233	0.102274505584836	0.778244856739803	-4.52615747181983
a_HQ_transcript/112747	2.72527297884686	3.13505994509713	1.68115889066979	0.102293696675309	0.778289005280413	-4.77093231199924
a_HQ_transcript/40131	-2.01007131564933	-0.324992688547269	-1.68105754466781	0.102313550141177	0.778325896331987	-4.54512962906491
a_HQ_transcript/39463	2.88190001177938	2.51957073207367	1.68099744455541	0.102325325140535	0.778325896331987	-4.61711866707831
a_HQ_transcript/26496	-1.38569497996729	0.528133634060262	-1.68075347854599	0.102373135300733	0.778543492544061	-4.42897600568179
a_HQ_transcript/127281	-0.67211279222945	2.55207508669262	-1.68071478709525	0.102380719395245	0.778543492544061	-4.64508338063087
a_HQ_transcript/131557	-3.12465305063032	1.17796609321912	-1.68045662281391	0.102431335373498	0.778669489770603	-4.52590702752006

a_HQ_transcript/9193	0.940845457039364	1.71366239858302	1.68039971335094	0.102442495908629	0.778669489770603	-4.51614446301975
a_HQ_transcript/65079	-0.479280841691954	3.65303881438552	-1.6803922755856	0.102443954606247	0.778669489770603	-4.8533469678279
a_HQ_transcript/49491	0.593413230714149	3.92279509774193	1.68028437233377	0.102465118581965	0.778669489770603	-4.89498463163758
a_HQ_transcript/144130	4.54051225268908	3.05627650433596	1.68025507047986	0.102470866430359	0.778669489770603	-4.77221487566585
a_HQ_transcript/125555	-2.25774768233666	-0.3568697478138	-1.68014350637928	0.102492753288577	0.778669489770603	-4.54146983666763
a_HQ_transcript/17860	-1.3155089211593	0.723354797684967	-1.68011528563877	0.102498290304024	0.778669489770603	-4.54791260401991
a_HQ_transcript/55199	-1.44293369551777	0.374936255338636	-1.68008386941486	0.102504454578252	0.778669489770603	-4.49782997899865
a_HQ_transcript/130171	-1.68886240941435	-0.302790318628675	-1.67984944170804	0.102550462106374	0.778870032299801	-4.58275964374051
a_HQ_transcript/143523	2.77430563096531	1.33654535618899	1.67981281130268	0.102557652546495	0.778870032299801	-4.68362244689113
a_HQ_transcript/58780	0.953528255395986	0.865385227906271	1.67958549546087	0.102602283357348	0.778974230359246	-4.64463714118946
a_HQ_transcript/3760	-1.12918704252551	3.78480346798495	-1.67955698602787	0.102607881992418	0.778974230359246	-4.78633173639869
a_HQ_transcript/10642	-3.75649735563943	2.96777090012704	-1.67953817675876	0.102611575864656	0.778974230359246	-4.68881037384558
a_HQ_transcript/138665	3.47681915507137	2.34230000318142	1.67943470550007	0.102631898123821	0.779026766014818	-4.65801514710003
a_HQ_transcript/758	0.59165159697662	2.60755634112175	1.67918282142234	0.102681383388532	0.779175873979321	-4.68489206224114
a_HQ_transcript/28818	-0.975609546573701	4.0459163009382	-1.67913672786772	0.102690441099367	0.779175873979321	-4.85385825296678
a_HQ_transcript/35565	-2.54819337144972	1.79881689827759	-1.6791300387291	0.102691755617871	0.779175873979321	-4.50637238713886
a_HQ_transcript/86264	1.83097443263956	0.104670068846586	1.67898198142019	0.102720854739675	0.779221109781504	-4.74952650703658
a_HQ_transcript/89161	-1.49805488091329	2.30707110306495	-1.67896329417116	0.102724528011555	0.779221109781504	-4.58258726412325
a_HQ_transcript/119082	-3.36367876751507	2.9949615856096	-1.67883480429797	0.102749787671683	0.779311020065186	-4.7387852159228
a_HQ_transcript/134959	-2.32543058667924	0.333132569435454	-1.67873806188484	0.102768809550507	0.77934011270494	-4.5422781693276
a_HQ_transcript/110830	1.45638910244944	0.746165988282681	1.67860152240901	0.102795661473793	0.77934011270494	-4.41651459879292
a_HQ_transcript/133011	2.2257561826144	0.942659552254015	1.67852721145232	0.10281027795825	0.77934011270494	-4.61453441611388
a_HQ_transcript/141197	1.77856566836141	2.79084033666984	1.6784828478061	0.102819004825003	0.77934011270494	-4.66544574501271
a_HQ_transcript/27206	-1.18361863821418	2.31953987454121	-1.67847443397289	0.102820659997492	0.77934011270494	-4.52261037029666
a_HQ_transcript/94673	2.03923799624606	0.0883560707869452	1.67837555765255	0.102840112646634	0.779385928021322	-4.48452008137602
a_HQ_transcript/120072	-2.48545410930649	1.14979645319369	-1.67827407430903	0.102860081379152	0.77943564208152	-4.50405456385583
a_HQ_transcript/90970	2.13291453952481	0.347317444647803	1.678156025093	0.102883313814609	0.779510071091711	-4.45912340331145
a_HQ_transcript/140377	1.95350880511034	0.00636234145151747	1.67805272609564	0.102903646945725	0.779562516572541	-4.49390412044884
a_HQ_transcript/140994	-2.82551041573408	0.56866389913957	-1.67793508814121	0.102926806595937	0.779563255622924	-4.55041981034428
a_HQ_transcript/12503	1.5366260873685	0.123497940979531	1.67784306567803	0.102944926289969	0.779563255622924	-4.51845659178425
a_HQ_transcript/34123	1.62213057893372	0.187324944664651	1.67754245716697	0.103004136144466	0.779563255622924	-4.4717784482877
a_HQ_transcript/13897	2.32097382377568	3.61258335085837	1.67741205410394	0.103029830011675	0.779563255622924	-4.84777374226523
a_HQ_transcript/32847	2.87632617766017	1.03681894615519	1.67728579666729	0.103054712129835	0.779563255622924	-4.56073088006113
a_HQ_transcript/20238	0.700648379266269	2.67777128013829	1.67726714030443	0.103058389246739	0.779563255622924	-4.69053335256902
a_HQ_transcript/64965	-0.534736691067204	3.53524298468616	-1.67715471582041	0.103080550109094	0.779563255622924	-4.77077490522563
a_HQ_transcript/138479	1.18704603765479	3.24442826699331	1.67706575679226	0.103098088323253	0.779563255622924	-4.70733805905969
a_HQ_transcript/39903	1.85919753492222	-0.490802815861883	1.67699418549124	0.103112200360636	0.779563255622924	-4.56103162830308
a_HQ_transcript/44545	-0.855052384596515	3.26821342376957	-1.67692940352766	0.103124975099333	0.779563255622924	-4.7010919022699
a_HQ_transcript/43732	1.44072319507118	1.03362136772686	1.67684639917648	0.103141345142977	0.779563255622924	-4.48153991227569
a_HQ_transcript/148812	1.90437117734263	0.598185666769481	1.67683810177317	0.10314298166827	0.779563255622924	-4.47556264650215
a_HQ_transcript/10429	2.00117613655533	2.2542044511833	1.67683060153691	0.103144460984166	0.779563255622924	-4.52918897295481
a_HQ_transcript/9427	-2.05276777227984	0.071791195634767	-1.67679830632427	0.103150830961345	0.779563255622924	-4.53486120747602
a_HQ_transcript/144951	-0.927467542529943	1.90819520867463	-1.67675078950768	0.103160203875483	0.779563255622924	-4.54145576019477
a_HQ_transcript/148733	3.8116808925214	0.444545903682761	1.67647400999398	0.103214814013849	0.779563255622924	-4.61066446552389
a_HQ_transcript/81457	-2.25987550307533	0.145441177515624	-1.67642031156838	0.103225411797335	0.779563255622924	-4.59062170078321
a_HQ_transcript/142830	1.5140485626381	-0.39115186496209	1.67640388517592	0.103228653848816	0.779563255622924	-4.56798849808087
a_HQ_transcript/34843	-3.30259284214068	0.0103673806219472	-1.67636165279876	0.103236989576161	0.779563255622924	-4.58326023290854
a_HQ_transcript/77189	2.16112686916324	1.38321485811453	1.67628537159438	0.103252047200198	0.779563255622924	-4.51841810786124
a_HQ_transcript/86717	-1.72141068878025	-0.449282679451032	-1.67627040844225	0.103255001084727	0.779563255622924	-4.55957450520235
a_HQ_transcript/95984	2.22621076474956	1.7135038412829	1.67623084094565	0.103262812465899	0.779563255622924	-4.49727223543725

a_HQ_transcript/135957	1.43343336771241	-0.560834729814387	1.67622458310171	0.103264047929096	0.779563255622924	-4.56799218263999
a_HQ_transcript/69283	1.39446609002372	1.44234827013376	1.67622259105126	0.103264441214896	0.779563255622924	-4.53785520449622
a_HQ_transcript/6406	1.4672893740971	-0.457718395005402	1.67614150059574	0.10328045176921	0.779563255622924	-4.51804844665443
a_HQ_transcript/86084	1.06855862055338	1.93662682823827	1.67613639367441	0.103281460152325	0.779563255622924	-4.53899150428442
a_HQ_transcript/9070	-2.11541395127921	1.35244051928223	-1.67609087112597	0.103290449133067	0.779563255622924	-4.5569407627243
a_HQ_transcript/89144	-1.89797430476179	0.468393584682981	-1.67599397889983	0.103309583848192	0.779563255622924	-4.51952372844466
a_HQ_transcript/2063	1.351502860979	1.17555868274886	1.6759896765725	0.103310433559627	0.779563255622924	-4.43652964834125
a_HQ_transcript/34096	-1.11996695996258	2.76585143993145	-1.67595081729993	0.103318108545503	0.779563255622924	-4.58705713158664
a_HQ_transcript/80181	1.83010475265282	-0.336194024882632	1.67586805789885	0.103334455703351	0.779563255622924	-4.54999902707205
a_HQ_transcript/115941	0.657217279697716	5.2242907145861	1.67584418895817	0.103339170846507	0.779563255622924	-5.0432702893388
a_HQ_transcript/20729	-1.80131387892012	0.773474227613683	-1.67580803874161	0.103346312411063	0.779563255622924	-4.51423812818611
a_HQ_transcript/46703	1.07165814026242	1.69392873060365	1.67562036044901	0.103383395320091	0.779635058475558	-4.42518661595739
a_HQ_transcript/219	2.34284778789357	2.11752665577321	1.6756152412102	0.103384406973407	0.779635058475558	-4.55500314749054
a_HQ_transcript/36874	-2.19570750048359	0.260149496818693	-1.6755281463916	0.103401619732006	0.779635058475558	-4.47646734948876
a_HQ_transcript/130637	1.2386497228296	0.510411361655486	1.67541573403119	0.103423839581223	0.779635058475558	-4.44364445495528
a_HQ_transcript/109278	0.926183342703048	2.36290069268267	1.67526982711318	0.103452686013399	0.779635058475558	-4.631661858709
a_HQ_transcript/86178	1.6637790745428	-0.273712686425022	1.675257311468	0.10345516072249	0.779635058475558	-4.50456133120041
a_HQ_transcript/7886	0.998380769211978	1.87346195987196	1.675254701047	0.103455676885275	0.779635058475558	-4.51790431420578
a_HQ_transcript/150977	-1.8846923306573	0.809773224578597	-1.67521700739213	0.103463130351646	0.779635058475558	-4.55836925591859
a_HQ_transcript/71882	-2.9823491392686	0.933363190751042	-1.67503001741607	0.103500111971817	0.779812638846114	-4.5533892598082
a_HQ_transcript/22830	-0.614889112592787	3.46463196882178	-1.67494473767526	0.103516981675702	0.779818898026782	-4.77053882270157
a_HQ_transcript/147567	-1.75405913300253	2.2312073162184	-1.67489018732779	0.103527773810774	0.779818898026782	-4.56765943852458
a_HQ_transcript/94179	1.83074637108392	0.109422939838718	1.67481458631066	0.103542732116688	0.779830517294483	-4.53512180862573
a_HQ_transcript/27656	2.53350210729896	1.16556604791309	1.67467360198803	0.103570631868829	0.779918219351325	-4.55800908188402
a_HQ_transcript/109870	2.10078169753116	2.31536974360599	1.67462014951874	0.103581211350678	0.779918219351325	-4.59950961505963
a_HQ_transcript/81941	-0.584592985066643	3.99920399079243	-1.67433873857172	0.103636923918174	0.779983885995747	-4.93102167287594
a_HQ_transcript/5048	-0.623898165888282	2.71464610546207	-1.674262192028	0.103652082594716	0.779983885995747	-4.65422820709457
a_HQ_transcript/796	4.17469583337998	1.81913374065246	1.67414328119118	0.103675634420252	0.779983885995747	-4.63766558287037
a_HQ_transcript/150717	1.5253292569682	0.69241044290364	1.67413262415879	0.103677745400303	0.779983885995747	-4.56340678833491
a_HQ_transcript/1450	-1.13857867980896	3.81461295467759	-1.67412202770828	0.103679844415536	0.779983885995747	-4.80473449923421
a_HQ_transcript/9804	-1.04242690917859	1.79229800123736	-1.673959525165	0.10371203842444	0.779983885995747	-4.49920080918294
a_HQ_transcript/43060	1.50022177933619	-0.657919832991962	1.67393453337599	0.103716990382141	0.779983885995747	-4.57901666111728
a_HQ_transcript/145095	2.71637670212583	1.05011304990521	1.67393005341228	0.1037178780781	0.779983885995747	-4.60248085272459
a_HQ_transcript/146149	-0.818656699542853	3.45469749136793	-1.67388016966316	0.103727762871316	0.779983885995747	-4.7678156486317
a_HQ_transcript/149088	-2.02885178679312	-0.161894422243161	-1.67386822003584	0.103730130884925	0.779983885995747	-4.58294508433126
a_HQ_transcript/139885	2.6819100135662	1.6299162876749	1.67375122130798	0.103753318466544	0.779983885995747	-4.52027176233928
a_HQ_transcript/108159	0.601128686675087	2.83854509189135	1.67374585486309	0.103754382127455	0.779983885995747	-4.69682998091738
a_HQ_transcript/143357	1.98701581053627	-0.289320930057272	1.67364119368328	0.103775128401282	0.779983885995747	-4.52243482712943
a_HQ_transcript/84065	-1.68218929314845	-0.551840833077183	-1.67362776790637	0.103777789951333	0.779983885995747	-4.55436706984448
a_HQ_transcript/75006	2.17832338557508	-0.0508493445833776	1.67352184732169	0.103798789828125	0.780040860391632	-4.55397142596561
a_HQ_transcript/53678	1.5137479825498	-0.650583765366132	1.67323798311761	0.103855086334881	0.780363037548344	-4.57992856728917
a_HQ_transcript/144860	-1.37993721463217	-0.0623978402442207	-1.67304678064513	0.103893020292353	0.780484950519356	-4.58679265357609
a_HQ_transcript/45929	1.14554339429332	1.82980060895148	1.67302085266988	0.10389816520642	0.780484950519356	-4.57262453800801
a_HQ_transcript/119568	-2.0599683419279	0.339283699220823	-1.67273354454634	0.103955190225265	0.780732008787285	-4.52029616402774
a_HQ_transcript/148095	2.16259729385039	0.246142725138552	1.67267683768952	0.103966448496703	0.780732008787285	-4.57911865054075
a_HQ_transcript/12085	-1.3564863221693	1.92064855749288	-1.67262944723169	0.10397585791841	0.780732008787285	-4.58736501793353
a_HQ_transcript/121720	-2.70647102153724	0.803850161478925	-1.6725845214649	0.103984778627016	0.780732008787285	-4.46398134964265
a_HQ_transcript/151471	6.04669200742402	2.39903494059443	1.67240272575779	0.104020883484899	0.780864727750851	-4.73115821706271
a_HQ_transcript/3244	-0.801395713111831	3.19992163600208	-1.67234271146066	0.104032804688251	0.780864727750851	-4.74985459277263
a_HQ_transcript/42465	-1.13464102731494	1.45575793458519	-1.67220943698312	0.104059282310887	0.780864727750851	-4.51197948064145

a_HQ_transcript/146521	-1.88342222686879	0.159553980896696	-1.67195822535166	0.104109205723912	0.780864727750851	-4.61417630992376
a_HQ_transcript/152694	-2.05898995952607	2.75282651401678	-1.67194555573281	0.104111724090904	0.780864727750851	-4.77801983749247
a_HQ_transcript/40627	-1.32528390310545	2.02255115484	-1.67188618369979	0.104123526270444	0.780864727750851	-4.5870468630388
a_HQ_transcript/45803	1.76420129948561	3.19600161616934	1.67183793772392	0.104133117592634	0.780864727750851	-4.60197455849716
a_HQ_transcript/78584	0.97819937284717	-0.704964334564497	1.67178060627901	0.104144516065134	0.780864727750851	-4.58251243827627
a_HQ_transcript/87190	1.47541851800894	0.871547877577398	1.67172064388191	0.104156438725565	0.780864727750851	-4.55162321155522
a_HQ_transcript/4287	1.87971497195931	-0.270982975359735	1.6716781419673	0.104164890307226	0.780864727750851	-4.55636685862402
a_HQ_transcript/61494	1.87458686794337	2.51514476548257	1.67167239866251	0.104166032417366	0.780864727750851	-4.67742835990367
a_HQ_transcript/152935	1.23724286947986	-0.638543936019047	1.67164552936236	0.104171375768508	0.780864727750851	-4.59673951375621
a_HQ_transcript/482	2.69228985780618	0.20333755886742	1.67161678974961	0.104177091310691	0.780864727750851	-4.54100624139964
a_HQ_transcript/73494	2.59801962915408	1.34638038816119	1.67147487122152	0.104205318941299	0.78097560332901	-4.57692751665194
a_HQ_transcript/110713	0.798354541293778	4.26287158521976	1.67127181299415	0.104245718328808	0.781108445551089	-4.96299716437108
a_HQ_transcript/51605	1.8840065304633	2.96018285788889	1.67121113474354	0.104257793077599	0.781108445551089	-4.79549697480524
a_HQ_transcript/102530	-1.53984827806861	1.64094525074276	-1.67118317563107	0.104263357229406	0.781108445551089	-4.69002752131219
a_HQ_transcript/26743	1.62092846920264	0.625283378306207	1.67092479517233	0.104314789286926	0.781127201278259	-4.45089829346243
a_HQ_transcript/95780	1.44440697224511	-0.119414025248204	1.67088301417208	0.104323108006042	0.781127201278259	-4.49808004146299
a_HQ_transcript/138679	2.60331223948786	0.668982647796167	1.67087265767525	0.104325170100062	0.781127201278259	-4.73943463011681
a_HQ_transcript/25074	-2.03130429317764	0.441055235411106	-1.67084693280401	0.104330292355409	0.781127201278259	-4.58307733511476
a_HQ_transcript/140225	2.17764079676129	0.374618836653124	1.67083307864866	0.104333051037786	0.781127201278259	-4.54398856992998
a_HQ_transcript/92953	-1.70984031190454	1.54732214328773	-1.67073412070221	0.104352757614076	0.781174126798584	-4.48602037901797
a_HQ_transcript/32958	1.15779848447828	-0.557095660792671	1.67060731632116	0.104378014076552	0.781262580767681	-4.54026854869846
a_HQ_transcript/106075	0.68061164893665	3.60046029653141	1.67041808921441	0.104415713130704	0.781444131950765	-4.86928318217197
a_HQ_transcript/98353	1.47207916542851	1.39598730958654	1.67027932453245	0.104443365923575	0.78147839635442	-4.54975031701578
a_HQ_transcript/109694	1.32570049466198	0.160800153552211	1.67020499971037	0.104458179752027	0.78147839635442	-4.49415410067186
a_HQ_transcript/124565	3.02614622858158	2.24261327626163	1.66996568005764	0.104505890892127	0.78147839635442	-4.73307180014691
a_HQ_transcript/127269	2.22781832101534	2.20249208344668	1.66993892673716	0.104511225601076	0.78147839635442	-4.63289931818842
a_HQ_transcript/68043	1.96498320687957	1.41945931300729	1.66992316540839	0.104514368573016	0.78147839635442	-4.53002545685851
a_HQ_transcript/71776	0.507658494125806	3.72987917481157	1.66985001661384	0.1045289562272	0.78147839635442	-4.86172131889901
a_HQ_transcript/127719	1.95834433325966	1.54688437302189	1.66980052094453	0.104538827830859	0.78147839635442	-4.53919097297948
a_HQ_transcript/68627	-1.96693120149824	1.42196754797642	-1.66979705702528	0.104539518717029	0.78147839635442	-4.53476604045696
a_HQ_transcript/106471	-2.03489897268637	0.610087550992049	-1.66978818457094	0.104541288364505	0.78147839635442	-4.55907356221082
a_HQ_transcript/53292	-1.47246758809084	0.498783477718219	-1.66963532363592	0.104571781015138	0.781605823177051	-4.44361081410967
a_HQ_transcript/124975	1.39555595167127	-0.629175296561506	1.66922802364278	0.1046530651699	0.78189223010015	-4.51988204006691
a_HQ_transcript/94425	0.98053285292215	3.7839917355459	1.66921152439097	0.104656359003084	0.78189223010015	-4.73975769686157
a_HQ_transcript/116049	-2.08584992794677	1.33733895022393	-1.6691215877125	0.10467431505387	0.78189223010015	-4.5603474820776
a_HQ_transcript/107816	1.40651683810274	0.627536628444355	1.66906757194791	0.104685100647403	0.78189223010015	-4.54331731659543
a_HQ_transcript/135755	-1.43403183678076	4.18476297229773	-1.66902005909103	0.104694588536637	0.78189223010015	-4.96450469090441
a_HQ_transcript/1373	-1.64256543468102	0.194630082295701	-1.66893385355735	0.104711804828726	0.78189223010015	-4.53747830694155
a_HQ_transcript/115064	-1.94217432779808	0.268670963777692	-1.6688817163126	0.104722218411168	0.78189223010015	-4.50420702969938
a_HQ_transcript/7595	-0.718634209509954	2.35679008873931	-1.66882312275886	0.104733922565098	0.78189223010015	-4.64051097586842
a_HQ_transcript/135934	1.10137349922513	-0.707154310175073	1.66875311882313	0.104747907385485	0.78189223010015	-4.60128641132997
a_HQ_transcript/24487	1.29484759456638	-0.711367728974882	1.6687113364129	0.104756255076929	0.78189223010015	-4.57516715970456
a_HQ_transcript/43741	-1.59655229892525	0.740731115232313	-1.6687022874743	0.104758063033649	0.78189223010015	-4.60069231264709
a_HQ_transcript/140169	-1.97871059338984	0.613010208980485	-1.66855486041422	0.10478752226648	0.781985525379039	-4.44739547249512
a_HQ_transcript/147663	-1.13707266960959	1.85571228262219	-1.6685050933671	0.104797468392216	0.781985525379039	-4.54540006692825
a_HQ_transcript/121597	-1.09230619818881	2.97294481202925	-1.66835121189764	0.104828227126444	0.782114643358479	-4.70353180014152
a_HQ_transcript/68604	1.61735422315979	-0.290679557547355	1.66815684785098	0.104867088475028	0.782171671865084	-4.5453095410203
a_HQ_transcript/21191	-2.05033002246725	1.56149204767008	-1.66815448492863	0.104867560993787	0.782171671865084	-4.55891504377866
a_HQ_transcript/47214	1.9776490494709	-0.224193015535388	1.66811109130303	0.104876238826666	0.782171671865084	-4.55685321373965
a_HQ_transcript/87593	-1.28879538807187	1.22227954973011	-1.66791398045449	0.104915664452679	0.782246184761548	-4.47420954356718

a_HQ_transcript/8743	-1.82380351182502	0.620657661673575	-1.66784227763208	0.104930009327951	0.782246184761548	-4.57517442702378
a_HQ_transcript/59735	-0.484650354070218	3.60088206482857	-1.66782343228658	0.104933779800394	0.782246184761548	-4.84415607495891
a_HQ_transcript/136897	1.18759598573608	-0.713207397746442	1.66767934183972	0.10496261233965	0.782246184761548	-4.59219098477074
a_HQ_transcript/93009	-0.764164647287833	1.95912008608858	-1.66765948159163	0.104966586896885	0.782246184761548	-4.61068440992245
a_HQ_transcript/42809	1.41571382137821	1.40582814145444	1.66765754935341	0.104966973595171	0.782246184761548	-4.56358576490872
a_HQ_transcript/99303	3.06159199221092	3.36765607655391	1.6675529504399	0.104987908711986	0.782287108973324	-4.65216570840162
a_HQ_transcript/13985	2.79981441085698	0.541711591782797	1.66730497031591	0.105037554948249	0.782287108973324	-4.58632585169975
a_HQ_transcript/150931	0.745505994606271	8.38587891934274	1.66728484068537	0.105041585805753	0.782287108973324	-5.33008639876637
a_HQ_transcript/115995	-2.41346572350822	0.423782004498697	-1.66727766958745	0.105043021813165	0.782287108973324	-4.60241311606592
a_HQ_transcript/102423	2.30493163408794	2.50139157962257	1.66718479148242	0.105061622062841	0.782287108973324	-4.7223649657577
a_HQ_transcript/121440	-2.72554303946765	1.54563071747572	-1.66716012351225	0.105066562657857	0.782287108973324	-4.67163441791258
a_HQ_transcript/126359	1.87688514156108	-0.530494536738696	1.66711845186261	0.105074909252885	0.782287108973324	-4.57374657943537
a_HQ_transcript/113662	1.50075728737465	-0.585552235976285	1.6670923921584	0.105080129143678	0.782287108973324	-4.57575902992809
a_HQ_transcript/72435	-2.00255544261288	1.70346930948166	-1.6669571939334	0.105107213490358	0.782388539603985	-4.52440992030323
a_HQ_transcript/140015	-2.15978175678197	0.414891627496306	-1.66687007102806	0.105124669949299	0.782412728345069	-4.65354377332151
a_HQ_transcript/8842	-2.26208149797052	0.960709432322029	-1.6668066275191	0.10513738337984	0.782412728345069	-4.60090862344891
a_HQ_transcript/44190	-1.47884679254314	1.21101629167013	-1.66659782283649	0.105179234720664	0.782522892238625	-4.55336066909633
a_HQ_transcript/85308	1.13065251406458	1.65111908083047	1.66648626658871	0.105201599939475	0.782522892238625	-4.55681067647946
a_HQ_transcript/109796	-2.10720035201028	0.541300536989546	-1.66623928844256	0.105251129104077	0.782522892238625	-4.4937063950246
a_HQ_transcript/22451	-2.12224950378505	-0.191086723124763	-1.66615610998151	0.105267814127269	0.782522892238625	-4.57561852154529
a_HQ_transcript/121163	2.06540128720768	-0.0101670525917727	1.66614996387941	0.105269047079919	0.782522892238625	-4.54582747480575
a_HQ_transcript/48679	-2.11835989346835	6.88317662246573	-1.66608594300115	0.105281890845413	0.782522892238625	-4.92864280640753
a_HQ_transcript/91925	-1.51078697982223	-0.556721621894393	-1.66607970070436	0.105283143234565	0.782522892238625	-4.56711834652401
a_HQ_transcript/101892	1.17060964660363	-0.73967549421512	1.66600232786563	0.105298667538831	0.782522892238625	-4.59712845631632
a_HQ_transcript/96235	-2.83879508626805	0.505099886089735	-1.66598069696773	0.105303007963073	0.782522892238625	-4.61334252116691
a_HQ_transcript/7888	1.5945127093364	1.46538089320661	1.66593456667455	0.105312264896292	0.782522892238625	-4.41813056119707
a_HQ_transcript/10416	2.34328258403471	0.444856110100048	1.6658915641175	0.105320894797455	0.782522892238625	-4.53629581518779
a_HQ_transcript/152233	-1.70889268489146	-0.238155157746478	-1.66587539597664	0.105324139627507	0.782522892238625	-4.56022627703218
a_HQ_transcript/77782	-1.68624496404417	0.415030636655255	-1.66580371959379	0.105338525563825	0.782522892238625	-4.44781949443725
a_HQ_transcript/60840	1.73904177548486	1.86227202706297	1.66579310734668	0.105340655652478	0.782522892238625	-4.64255158080482
a_HQ_transcript/93745	-1.55257970263456	-0.151646885264961	-1.66567338095731	0.105364689593457	0.782594129114559	-4.522103339696
a_HQ_transcript/71595	1.68166268857053	2.45177711255168	1.66561120858005	0.105377171906476	0.782594129114559	-4.6357785068858
a_HQ_transcript/2581	1.91980515972564	-0.138584064152487	1.66548170763261	0.105403175680766	0.782627629445511	-4.55754234479716
a_HQ_transcript/143072	0.587022519412602	3.7403573639091	1.66542156147386	0.105415254833832	0.782627629445511	-4.87293122128222
a_HQ_transcript/85306	1.43544425508255	-0.648546560680786	1.66527717952459	0.105444255751803	0.782627629445511	-4.58516952976482
a_HQ_transcript/81685	1.84506932948614	0.0271755699747169	1.66525013304339	0.105449689111997	0.782627629445511	-4.56561930548367
a_HQ_transcript/96280	1.55302773754487	1.95573510311247	1.66516905354823	0.105465978542468	0.782627629445511	-4.52587257404082
a_HQ_transcript/44847	-2.5226519069147	1.19033307655266	-1.66511103023331	0.105477637109587	0.782627629445511	-4.62417368517756
a_HQ_transcript/87062	0.677456085637027	4.92154289970713	1.66502738415817	0.105494445915569	0.782627629445511	-5.03463602263365
a_HQ_transcript/1577	0.510723586877148	3.06445716117589	1.66502503573944	0.105494917865976	0.782627629445511	-4.75337931778065
a_HQ_transcript/130309	-1.58172647725476	-0.537968155877072	-1.66493174445553	0.105513667584222	0.782627629445511	-4.55849067524915
a_HQ_transcript/61764	-2.22040032225631	1.10373312165502	-1.66477368706436	0.105545440334	0.782627629445511	-4.49941107511995
a_HQ_transcript/25366	-1.95637419764268	3.23619555366269	-1.66468274623735	0.105563724889043	0.782627629445511	-4.73806367654242
a_HQ_transcript/41501	-0.929342955704271	5.86112081719108	-1.66466435165497	0.105567423621958	0.782627629445511	-5.11294365786711
a_HQ_transcript/105299	7.42708476620934	0.621099741873028	1.66465715086473	0.105568871566701	0.782627629445511	-4.52308357183184
a_HQ_transcript/66378	-1.56649991859139	1.18328256851458	-1.66465065939822	0.10557017689379	0.782627629445511	-4.44997114649157
a_HQ_transcript/74880	-0.771504913776637	2.37432871643842	-1.6644193753491	0.105616693053825	0.782711949873015	-4.55169546525568
a_HQ_transcript/129672	-1.77070839506914	1.50945842186046	-1.66440439459552	0.10561970658983	0.782711949873015	-4.54089005427073
a_HQ_transcript/141967	-4.50296019856231	1.37931481726817	-1.66439325743524	0.105621946992911	0.782711949873015	-4.54156555841385
a_HQ_transcript/126502	1.07131364165308	3.61686637155605	1.66425135779755	0.105650495639133	0.782755274227638	-4.80906908450612

a_HQ_transcript/44598	1.43158594289564	0.60338441228302	1.66423033638465	0.10565472546088	0.782755274227638	-4.4505882869827
a_HQ_transcript/113937	-2.17676509869466	-0.167610399420116	-1.66409433615315	0.10568209413228	0.782858260191304	-4.57848824723347
a_HQ_transcript/146847	1.27624676298759	-0.697578411482547	1.66401787486573	0.105697483777427	0.782872494522716	-4.59375835491247
a_HQ_transcript/32572	-0.927391414338429	1.89908898083867	-1.66371828518735	0.105757801221218	0.783145956197017	-4.53951576475357
a_HQ_transcript/126927	3.10265875734818	1.65404206006744	1.66370066295099	0.105761350057573	0.783145956197017	-4.58173654115595
a_HQ_transcript/87356	-1.89172516329811	0.872431865418179	-1.66358973643476	0.105783691151969	0.78321161626311	-4.49454680884119
a_HQ_transcript/17349	2.24757482186692	0.180192432722961	1.66348286249016	0.105805219745319	0.783261388234174	-4.51283909943818
a_HQ_transcript/84761	-1.12861962149882	1.9028780091035	-1.66342258749097	0.105817363091849	0.783261388234174	-4.44288387003467
a_HQ_transcript/116844	-1.56820705769562	1.72274623687941	-1.66313904351477	0.10587450300589	0.783498967087884	-4.50429476751038
a_HQ_transcript/126020	1.86442374913409	-0.332420829456212	1.66308110766329	0.105886181415835	0.783498967087884	-4.52333803651884
a_HQ_transcript/116175	2.1223946630434	0.735086567472764	1.6630626800406	0.105889896185013	0.783498967087884	-4.5365325857742
a_HQ_transcript/75990	1.91568501547027	-0.314684763212903	1.66291587391926	0.10591949425391	0.783618220948359	-4.63754825378309
a_HQ_transcript/92068	-2.70847097715201	0.550569996174034	-1.66278768823271	0.105945343809825	0.783620980678302	-4.5993814528358
a_HQ_transcript/111662	1.77036364647701	0.917530874921372	1.66278032315146	0.105946829189905	0.783620980678302	-4.47833103635959
a_HQ_transcript/96018	1.2668241244333	-0.476998466260182	1.66269205445503	0.105964632451453	0.783652945946316	-4.58709882705231
a_HQ_transcript/46719	2.88170605536907	0.466415386796401	1.66255035306439	0.105993217955702	0.783747628952307	-4.54897798555597
a_HQ_transcript/97957	-2.32126840997334	-0.281395896174854	-1.66242609136321	0.106018290607326	0.783747628952307	-4.58366831702679
a_HQ_transcript/114466	1.30126417656779	-0.692322939919181	1.66232125957703	0.106039446654749	0.783747628952307	-4.59481142293745
a_HQ_transcript/129283	1.83512552270802	0.680832736587744	1.66230410739568	0.106042908460878	0.783747628952307	-4.58105604078041
a_HQ_transcript/34324	4.27402038707068	3.41533974673005	1.66219606405581	0.106064716886783	0.783747628952307	-4.84046494027033
a_HQ_transcript/58989	1.0223978366326	2.83787542952023	1.66218201771035	0.106067552399075	0.783747628952307	-4.73369795475117
a_HQ_transcript/121626	-1.54496712140201	1.28863006793259	-1.66204773531686	0.106094662936707	0.783747628952307	-4.5907805740645
a_HQ_transcript/97011	-2.42362687854134	1.12432743862589	-1.66197085920349	0.106110186195035	0.783747628952307	-4.48364974043087
a_HQ_transcript/124793	1.91392376043204	0.348088042363042	1.66181771548402	0.106141115455155	0.783747628952307	-4.55207936008722
a_HQ_transcript/143667	-2.14564918248801	6.61794425297768	-1.66176334709481	0.106152097619983	0.783747628952307	-5.2404115043408
a_HQ_transcript/102906	-1.90583788557293	-0.0690988957754444	-1.66174019320369	0.106156774885859	0.783747628952307	-4.56451456996686
a_HQ_transcript/124217	0.653363447728445	4.15390723164688	1.66167271016545	0.106170407959304	0.783747628952307	-4.89793909166385
a_HQ_transcript/9119	2.00977320234971	1.12864425212357	1.66165011579869	0.10617497284812	0.783747628952307	-4.55799544421771
a_HQ_transcript/147118	1.46220820683287	-0.666107749030273	1.66162758208225	0.106179525645546	0.783747628952307	-4.58813499854687
a_HQ_transcript/59848	1.7681630047437	-0.453943963229035	1.66162091777415	0.106180872158637	0.783747628952307	-4.59003232826497
a_HQ_transcript/146917	0.670604917979526	5.18618508756701	1.66156007735977	0.106193165524848	0.783747628952307	-5.02924400303089
a_HQ_transcript/39115	-0.730393259241476	1.9889275598247	-1.66133778140602	0.106238092510492	0.783919797248114	-4.50402547768009
a_HQ_transcript/48385	-2.59380617814542	1.20483620452455	-1.66128562309442	0.106248636217497	0.783919797248114	-4.60751434363396
a_HQ_transcript/136964	-2.97995154461747	0.970318805408901	-1.66124449104296	0.106256951598505	0.783919797248114	-4.64457061543702
a_HQ_transcript/77770	-2.18197884911391	0.0707360619328018	-1.66095322017209	0.106315851256848	0.784141745738309	-4.63618577877795
a_HQ_transcript/145873	-1.60525857128018	0.370329415911763	-1.66093911644916	0.106318703944022	0.784141745738309	-4.55779369537099
a_HQ_transcript/98086	1.91235441777882	0.282343218064612	1.66077981876129	0.106350928673663	0.784141745738309	-4.64328412794219
a_HQ_transcript/123656	1.33648173325345	-0.2541191599728	1.66077518127791	0.106351866923195	0.784141745738309	-4.59477854444496
a_HQ_transcript/123987	0.930294069250671	-0.790021896625955	1.66076223945354	0.106354485332384	0.784141745738309	-4.60709856523668
a_HQ_transcript/14896	2.18537229660926	0.964221672185769	1.66068775868477	0.106369555432034	0.784153394819694	-4.55727967310343
a_HQ_transcript/67045	1.88969827294087	0.450135660931728	1.66045003640213	0.106417666926774	0.784383040542078	-4.5660319899916
a_HQ_transcript/117225	1.4617679267051	2.21537129280855	1.66040049942187	0.106427694755632	0.784383040542078	-4.55382729137713
a_HQ_transcript/107010	1.06375799677274	1.91174192721907	1.66003447469142	0.106501813865617	0.784829796701391	-4.54992979300037
a_HQ_transcript/3688	1.42781057614552	0.99738753501119	1.65995821969106	0.106517260704079	0.784844128559602	-4.53059883029444
a_HQ_transcript/18666	0.450411209961573	3.68287013210791	1.65959927390636	0.106589996670262	0.784910198345491	-4.88914369698096
a_HQ_transcript/3955	1.2445115799919	-0.475299098170859	1.65956511539258	0.106596920620799	0.784910198345491	-4.54162903487724
a_HQ_transcript/17990	-1.90026428096328	1.35222176664528	-1.65951365525081	0.106607352325664	0.784910198345491	-4.60079008625484
a_HQ_transcript/140535	1.39926138649686	4.54388911648751	1.65939215434349	0.106631985652481	0.784910198345491	-4.94775461818598
a_HQ_transcript/136916	-2.2310597155957	0.0570403294116725	-1.65938384097425	0.10663367129343	0.784910198345491	-4.55680087425503
a_HQ_transcript/145690	1.39528177395347	-0.623193699958573	1.65933231027462	0.106644120288776	0.784910198345491	-4.55133914454768

a_HQ_transcript/23249	-1.74621354806553	0.114806900131452	-1.65930136815391	0.1066503948999	0.784910198345491	-4.53754900611677
a_HQ_transcript/93708	-0.823476462571664	0.350063935987119	-1.65919755146858	0.106671449647713	0.784910198345491	-4.47975890319998
a_HQ_transcript/133175	1.29538397005258	5.86360475626666	1.65915569985302	0.106679938422127	0.784910198345491	-5.07849635851045
a_HQ_transcript/95145	-4.60882853117646	2.2241469616493	-1.65914976190344	0.106681142863414	0.784910198345491	-4.72647128299954
a_HQ_transcript/76386	0.822162283393497	3.37778299316896	1.65908994226204	0.106693277183593	0.784910198345491	-4.89119826318158
a_HQ_transcript/101123	1.67032291869021	0.934843695688683	1.65902635319196	0.10670617738153	0.784910198345491	-4.55570823969451
a_HQ_transcript/99402	-1.75816609857216	0.627690397325939	-1.6590240727979	0.106706640024912	0.784910198345491	-4.52616848472829
a_HQ_transcript/136369	1.69504235102443	0.792633352768934	1.65892593902305	0.106726550853735	0.784910198345491	-4.54636591728713
a_HQ_transcript/32221	0.648181638731194	2.74302173163555	1.65891498005733	0.106728774561705	0.784910198345491	-4.70419144186596
a_HQ_transcript/1003	-2.6412717418548	3.27456745556906	-1.65884503798506	0.106742967567424	0.784915271190288	-4.69198544672977
a_HQ_transcript/110798	-2.12766586002561	1.66201046024602	-1.65862806668363	0.106787006461332	0.785106973294366	-4.65298459046637
a_HQ_transcript/107868	1.84972914792451	0.836059773118845	1.65858351638167	0.106796050747291	0.785106973294366	-4.57924989046811
a_HQ_transcript/125790	-1.72212838970622	1.23675827357577	-1.65832206916262	0.106849140708228	0.785304327485579	-4.48196835794293
a_HQ_transcript/17361	-2.07723206765623	0.498828633815917	-1.65828232675985	0.106857212790646	0.785304327485579	-4.58810863969571
a_HQ_transcript/53079	1.93714927753953	1.22738861284311	1.65822966378356	0.106867909950696	0.785304327485579	-4.45400084387692
a_HQ_transcript/25346	2.2407042675543	-0.443464889824988	1.65803643847197	0.106907166422535	0.785304327485579	-4.58748242983322
a_HQ_transcript/7333	-2.12606933063715	0.4634982736397	-1.65802720148192	0.106909043347933	0.785304327485579	-4.5346149990169
a_HQ_transcript/62972	-1.05524850979926	3.50199795235679	-1.65800237723049	0.10691408768795	0.785304327485579	-4.86607455581923
a_HQ_transcript/115568	-2.41521401535534	1.28113685750319	-1.65793660020009	0.106927454672892	0.785304327485579	-4.60918805475819
a_HQ_transcript/19615	1.27793028076737	1.33946405555936	1.65791927515264	0.106930975640799	0.785304327485579	-4.52131457211338
a_HQ_transcript/121906	-1.62067592479795	0.209471793144847	-1.65774201609488	0.106967005507525	0.785469693802603	-4.53934616907225
a_HQ_transcript/71063	2.26216472489323	0.211623165108275	1.65763376795787	0.106989013090146	0.785532064279904	-4.58169642350502
a_HQ_transcript/124755	0.821537726098296	2.81936442318046	1.65747932908973	0.10702041806121	0.785558077334764	-4.74238486302692
a_HQ_transcript/124464	-0.849778832532725	4.66486362557367	-1.65739445408274	0.107037680552992	0.785558077334764	-5.02006794387219
a_HQ_transcript/7625	-4.28563656474882	3.27494510350915	-1.65736644651005	0.107043377441911	0.785558077334764	-4.77972614808445
a_HQ_transcript/45735	1.21128490795775	2.75530061927567	1.65731965729972	0.10705289517635	0.785558077334764	-4.76759668644505
a_HQ_transcript/122218	0.61416291841531	3.06722800668461	1.65728410570759	0.107060127453998	0.785558077334764	-4.76817986131798
a_HQ_transcript/147880	0.907275166874915	2.56246252417788	1.65718976280542	0.10707932164015	0.785599748617774	-4.6674766855289
a_HQ_transcript/151063	-1.51671270719688	-0.477378608868673	-1.65708682544049	0.107100267635401	0.785654260109866	-4.58398379953572
a_HQ_transcript/131933	0.586283518173934	3.79974146344773	1.65679792705185	0.107159071666765	0.785804658397323	-4.91757584033242
a_HQ_transcript/115218	1.29562046401225	-0.649038432098269	1.65677989763936	0.10716274236368	0.785804658397323	-4.5927343778009
a_HQ_transcript/3287	0.74217470297256	2.99284682379625	1.65673126099082	0.107172645058432	0.785804658397323	-4.81446267055028
a_HQ_transcript/106445	-1.99378562688015	1.11390675588977	-1.65672046151356	0.107174843995561	0.785804658397323	-4.56494336648656
a_HQ_transcript/15746	-0.493046000169043	2.40539237093559	-1.65651753418911	0.107216170026698	0.785862161650488	-4.66552614334968
a_HQ_transcript/139777	2.36768962743284	2.55994325230846	1.65642764102338	0.107234480938691	0.785862161650488	-4.66935615861035
a_HQ_transcript/101910	1.13143558058549	2.32943314705266	1.65637750017575	0.107244695573736	0.785862161650488	-4.6033991036685
a_HQ_transcript/36565	2.12453325679656	-0.504994337001232	1.65637586914187	0.1072450278596	0.785862161650488	-4.58824150854954
a_HQ_transcript/16182	-1.93499404414045	1.69806506366501	-1.65630910138423	0.107258630996339	0.785862161650488	-4.61373540095944
a_HQ_transcript/29119	2.10590717385342	0.678197771674935	1.65626693054196	0.107267223540952	0.785862161650488	-4.45989012276047
a_HQ_transcript/121923	-2.31255663105666	0.131465348145829	-1.65621736578023	0.107277323367338	0.785862161650488	-4.59902293906424
a_HQ_transcript/76493	1.56015642053474	0.380777754104753	1.65612257048309	0.107296642028911	0.78586771928921	-4.52857798098673
a_HQ_transcript/38154	1.74411652454554	1.36910305237528	1.6560809680179	0.107305121249629	0.78586771928921	-4.5616110669055
a_HQ_transcript/101973	1.30879710839422	-0.226609118573908	1.65597116031152	0.107327504412666	0.785880533492608	-4.50499870481808
a_HQ_transcript/73917	0.707652559297646	4.88605942490542	1.65592229070669	0.107337467219503	0.785880533492608	-4.99203200727497
a_HQ_transcript/130615	-1.17235742135557	-0.757092072938776	-1.65587342289025	0.107347430428215	0.785880533492608	-4.5885586586931
a_HQ_transcript/119297	-2.52154853522683	1.33562900693586	-1.65568532740969	0.107385786635588	0.786062335417886	-4.61974277246612
a_HQ_transcript/151928	0.343246360581452	4.56600254965902	1.65551546393655	0.107420434753598	0.786150591828054	-5.0214557629304
a_HQ_transcript/143792	0.935711300093332	3.47678190429232	1.65549361302305	0.107424892493983	0.786150591828054	-4.83444969858446
a_HQ_transcript/28690	2.90046525877684	2.20221054464498	1.65531607493579	0.107461117198886	0.786289033485888	-4.5458645132118
a_HQ_transcript/82159	2.26517065535312	3.21041861775326	1.65522363034721	0.107479983513765	0.786289033485888	-4.75295148422968

a_HQ_transcript/144800	1.4178329903424	-0.343675006808606	1.65510571792741	0.10750405134852	0.786289033485888	-4.58422204434398
a_HQ_transcript/49578	-1.94640895011686	0.25490673987871	-1.65509679285325	0.107505873282535	0.786289033485888	-4.56142893277117
a_HQ_transcript/48668	-5.48716989014997	3.42607801397602	-1.65506069218141	0.107513243010887	0.786289033485888	-4.63519893975363
a_HQ_transcript/22874	-1.48712210265927	-0.485833105759715	-1.65500324567717	0.107524971221775	0.786289033485888	-4.57754955091077
a_HQ_transcript/25922	2.64771540569558	2.35553044313932	1.65476335138051	0.107573959218561	0.786463016169997	-4.70441350353184
a_HQ_transcript/12606	0.87799952920527	1.74315686286527	1.65475422604038	0.107575823038254	0.786463016169997	-4.6054228700467
a_HQ_transcript/9083	1.97473967973505	2.04721191133078	1.65467071480598	0.107592881166959	0.786488807403545	-4.52677081627086
a_HQ_transcript/152232	-0.627509844670409	4.67642563384803	-1.65445678631499	0.107636588743347	0.786496194043575	-4.99887798484039
a_HQ_transcript/9799	1.62797209316255	0.851541295117191	1.65437819082112	0.107652650227344	0.786496194043575	-4.45461369613859
a_HQ_transcript/22369	1.66311778449328	0.0173815895260714	1.65431260944384	0.107666053714514	0.786496194043575	-4.5328830228539
a_HQ_transcript/47414	-1.71973012660771	1.86553521564454	-1.65426799570872	0.107675172636357	0.786496194043575	-4.62643246570692
a_HQ_transcript/135227	1.31381942681824	-0.760969036538939	1.65425748451902	0.107677321187084	0.786496194043575	-4.60968017924655
a_HQ_transcript/58910	-1.58414371374849	-0.337861961198664	-1.65407321241353	0.107714993290087	0.786496194043575	-4.51403315883779
a_HQ_transcript/111130	-2.20895472980316	0.461532840175856	-1.65405739642397	0.107718227178066	0.786496194043575	-4.6480391341863
a_HQ_transcript/112662	3.51259631607676	2.88430744369129	1.65405629493548	0.107718452401898	0.786496194043575	-4.77601853833471
a_HQ_transcript/34240	-2.20597921374166	2.37348766199731	-1.65399485808289	0.10773101515131	0.786496194043575	-4.62968018939372
a_HQ_transcript/116839	1.19184382173816	2.70063242986828	1.65388640597375	0.107753194651737	0.786496194043575	-4.81250695174412
a_HQ_transcript/151836	5.44518120582954	6.30484222973705	1.65382240547749	0.107766285144363	0.786496194043575	-5.34435351608231
a_HQ_transcript/129586	0.75085347232929	6.45359702394071	1.65380950894164	0.107768923127273	0.786496194043575	-5.19950177885464
a_HQ_transcript/84941	-1.36874139532989	1.16381544728216	-1.65380528513529	0.107769787117346	0.786496194043575	-4.46717721282207
a_HQ_transcript/143936	2.30941186658101	0.289451125376854	1.65353698897933	0.107824679522898	0.786704523168683	-4.54942189152491
a_HQ_transcript/137849	-1.95900654180157	-0.261514752116261	-1.65353346113897	0.10782540146031	0.786704523168683	-4.58625205660917
a_HQ_transcript/81975	-2.01200334880283	0.965937744483734	-1.65338705068889	0.10785536643343	0.786824390065562	-4.64099042596914
a_HQ_transcript/14826	-1.13836384364255	2.50567782223808	-1.65321861489783	0.107889847737922	0.786873078751203	-4.57514264649307
a_HQ_transcript/59020	-2.10671055972716	1.71545459986284	-1.6531588054276	0.107902093820413	0.786873078751203	-4.53563409486775
a_HQ_transcript/40173	-1.65617826659702	0.367527150529893	-1.65315608318865	0.107902651230489	0.786873078751203	-4.60726040575341
a_HQ_transcript/45108	1.86080995279235	0.377429526900573	1.65304635405717	0.107925121531993	0.786937107413769	-4.59482741098264
a_HQ_transcript/147320	0.923520003334107	6.12027731673526	1.65295456210813	0.107943921639733	0.786937107413769	-5.16656622515278
a_HQ_transcript/83544	1.94520969153587	0.176678663216373	1.65291490180499	0.107952045391505	0.786937107413769	-4.57250181193391
a_HQ_transcript/89263	1.58728668934595	3.11183735647415	1.65267246613207	0.108001715307399	0.787075422484059	-4.68044272431742
a_HQ_transcript/67749	-3.63312120296088	3.50842170060844	-1.65261765332644	0.108012947909606	0.787075422484059	-4.79074969423596
a_HQ_transcript/148469	-5.25164984738837	0.825104074000343	-1.65261436770133	0.108013621252349	0.787075422484059	-4.64831487244856
a_HQ_transcript/152084	3.01053230442983	0.477132362227127	1.65255796376317	0.108025180989847	0.787075422484059	-4.64504269304488
a_HQ_transcript/4955	-2.35426405743529	-0.318125463605675	-1.65211702375358	0.108115585016201	0.787635383609695	-4.61147261409841
a_HQ_transcript/73847	-1.78381755003982	1.25425404702085	-1.6520249178699	0.108134476997587	0.787674295603476	-4.60006007863918
a_HQ_transcript/110600	1.82843130077006	0.417889849574415	1.65157882874755	0.1082260137249	0.787856793100945	-4.51932474029407
a_HQ_transcript/120185	3.10446103222116	2.07819456711895	1.65152314414799	0.108237444613742	0.787856793100945	-4.65720611376185
a_HQ_transcript/54780	-1.67921834311989	0.108532062208204	-1.65152085026173	0.10823791552227	0.787856793100945	-4.64213731072132
a_HQ_transcript/126611	-1.38837540585867	-0.637080097042369	-1.65138205322531	0.108266412118722	0.787856793100945	-4.58009199244981
a_HQ_transcript/138199	3.74880737666728	1.76282699141122	1.65134655022154	0.108273702283487	0.787856793100945	-4.70659704649351
a_HQ_transcript/119325	-1.3712631382806	2.49746708420461	-1.65129799841211	0.108283672540004	0.787856793100945	-4.73093821026136
a_HQ_transcript/87091	1.33278630182607	0.211544826830577	1.65128475677841	0.108286391880287	0.787856793100945	-4.49106362261262
a_HQ_transcript/20670	2.37130100263562	0.676136860088826	1.65114219386767	0.108315672595146	0.787856793100945	-4.53367894180455
a_HQ_transcript/95891	1.59431170472704	1.49717758432143	1.65103050189954	0.108338617368378	0.787856793100945	-4.52831070550835
a_HQ_transcript/88484	-0.918390503744008	2.06322519118911	-1.65098401658608	0.108348167987543	0.787856793100945	-4.6639575766182
a_HQ_transcript/85828	0.74801697482031	3.2655233258784	1.65092056229266	0.108361206086238	0.787856793100945	-4.82744079424304
a_HQ_transcript/16213	-0.501799675543189	4.71509804019213	-1.65086317811083	0.10837299806392	0.787856793100945	-4.98744562332264
a_HQ_transcript/105202	-1.11591537223358	3.15797803767654	-1.65081783917726	0.108382315593507	0.787856793100945	-4.74961677308378
a_HQ_transcript/59997	2.14015553199938	1.1123037821928	1.65080186830184	0.108385597899802	0.787856793100945	-4.54837144374369
a_HQ_transcript/117894	1.81858620558592	-0.460713329626135	1.6507232015206	0.108401766559911	0.787856793100945	-4.60528516562164

a_HQ_transcript/72546	1.52517655394808	-0.574395543380385	1.65072061057982	0.108402299119052	0.787856793100945	-4.61227353550231
a_HQ_transcript/36060	-1.72937596037083	1.94541567105478	-1.65070958773617	0.1084045648516	0.787856793100945	-4.71784237651431
a_HQ_transcript/145619	1.54952098184248	-0.574150028522033	1.65066467041135	0.10841379796036	0.787856793100945	-4.58468093490666
a_HQ_transcript/3461	-0.672972848941606	2.48992456530428	-1.65061100069247	0.108424831046284	0.787856793100945	-4.64567848594168
a_HQ_transcript/104546	1.58103640032179	2.51723642387982	1.6505829039833	0.108430607363191	0.787856793100945	-4.6741010600414
a_HQ_transcript/3123	2.40654968578725	1.07620325810686	1.65036718448163	0.10847496496771	0.788070337675925	-4.62485947621373
a_HQ_transcript/35686	2.59695255374537	1.74449775118929	1.65029776598562	0.108489242431732	0.788070337675925	-4.59669247075671
a_HQ_transcript/59473	0.594614747370072	3.21844691150413	1.65024221221346	0.108500669426446	0.788070337675925	-4.8749914650615
a_HQ_transcript/111635	1.88300668047724	-0.464938893488656	1.6499349481375	0.108563889344433	0.788431004474416	-4.60185394340199
a_HQ_transcript/148167	0.839324149956089	3.65427103206655	1.64965063373301	0.108622414505302	0.78871877440007	-4.94807023996936
a_HQ_transcript/62398	1.55621618686409	-0.0997225112245534	1.6496106289231	0.108630651456217	0.78871877440007	-4.56410381032588
a_HQ_transcript/142642	2.18159302396336	0.687557876903721	1.64951003438258	0.108651366057897	0.788733157618655	-4.60473167201852
a_HQ_transcript/65514	-5.47459926509366	4.0318232877971	-1.64940109204127	0.10867380334139	0.788733157618655	-4.86151104330619
a_HQ_transcript/39557	-0.943502104928605	2.00509369491967	-1.64931859203386	0.108690797230679	0.788733157618655	-4.65736853640669
a_HQ_transcript/4822	-1.54294091920839	-0.5120606322092	-1.6492967523265	0.108695296284203	0.788733157618655	-4.57356108154111
a_HQ_transcript/114294	1.6539598997263	-0.201120141906001	1.64927160467489	0.108700476976381	0.788733157618655	-4.60251061927825
a_HQ_transcript/119900	2.35762203011236	2.81746082181614	1.64913928679173	0.108727739279612	0.788832504848391	-4.95339090487592
a_HQ_transcript/129345	2.588869094936	0.394907997903152	1.64902773476895	0.108750727456341	0.788900821862251	-4.5481665259933
a_HQ_transcript/6956	-1.74869662785112	1.24265945219111	-1.64877966811299	0.108801862422303	0.789152233470878	-4.56896478189176
a_HQ_transcript/11358	1.75431510950055	1.90730887090636	1.64872789558514	0.108812537009171	0.789152233470878	-4.48211572587922
a_HQ_transcript/44836	-2.11721041395233	2.16992675269354	-1.64851562307825	0.108856312939587	0.789274534597476	-4.68171902045535
a_HQ_transcript/180	1.62687250619047	-0.135788297081046	1.64848211179789	0.108863225140628	0.789274534597476	-4.60683509023751
a_HQ_transcript/125781	3.63558522001282	1.82450015854557	1.6484057070297	0.108878986121221	0.789274534597476	-4.48859931878446
a_HQ_transcript/143173	-4.03350561782115	1.26490709138617	-1.64838121044184	0.108884039742911	0.789274534597476	-4.67776343598917
a_HQ_transcript/68773	-0.900010634193638	2.39241464144729	-1.64831697863248	0.108897291623172	0.789274534597476	-4.55327504270219
a_HQ_transcript/68977	1.83067594037519	0.99963483476065	1.64820734940124	0.108919912681472	0.789340067865428	-4.49898249424591
a_HQ_transcript/39301	1.76261342424443	-0.320458194970877	1.64804946121199	0.10895249839868	0.789387662974828	-4.59330529163713
a_HQ_transcript/140786	-2.79107981337393	0.742961655955636	-1.64802754790481	0.10895702160903	0.789387662974828	-4.64558787169234
a_HQ_transcript/108444	1.49594205910391	-0.612135962010024	1.64797813954695	0.108967220749209	0.789387662974828	-4.61094906768203
a_HQ_transcript/28449	2.87723302789975	0.375242787836711	1.64772524781844	0.109019436398949	0.789667513893846	-4.55952236829249
a_HQ_transcript/55801	1.84155505275869	2.62032139251116	1.64759527463359	0.109046280579858	0.789742181202347	-4.68534772314527
a_HQ_transcript/117370	2.93101549405881	1.08460877196009	1.64745745813566	0.109074750664602	0.789742181202347	-4.60163910646801
a_HQ_transcript/75089	1.52557942401392	0.0217608161153516	1.64734446993835	0.109098096315999	0.789742181202347	-4.52341711331384
a_HQ_transcript/130747	2.34575791027585	2.09231014507276	1.64726485013649	0.109114549864031	0.789742181202347	-4.76622074932273
a_HQ_transcript/17318	1.31768710904938	-0.369320179766611	1.64725560093462	0.109116461358188	0.789742181202347	-4.64425411889234
a_HQ_transcript/72828	2.24475387491579	0.0209828037709706	1.6471806602776	0.109131950054524	0.789742181202347	-4.60133669479607
a_HQ_transcript/143609	2.43551502399941	0.650298126290177	1.6471777960201	0.109132542073882	0.789742181202347	-4.67692229337722
a_HQ_transcript/123850	2.67672884843424	2.19095959110349	1.64701196250004	0.109166823081468	0.789742181202347	-4.70705494247698
a_HQ_transcript/150402	1.714422196534	3.29247763366722	1.64699826947233	0.1091696540943	0.789742181202347	-4.68665577135701
a_HQ_transcript/1319	1.06605764659514	1.76799076150782	1.64693680720937	0.109182362072807	0.789742181202347	-4.5539467003665
a_HQ_transcript/78651	1.92427387488921	-0.567171255841636	1.64689502369635	0.109191001958742	0.789742181202347	-4.61704544984731
a_HQ_transcript/73574	1.81706609833684	-0.122533118243095	1.64687093189725	0.109195983855304	0.789742181202347	-4.60518123163238
a_HQ_transcript/132144	1.83804644832694	0.347402694127183	1.64682072732483	0.109206366166124	0.789742181202347	-4.59891262066998
a_HQ_transcript/29103	-1.85446357915087	1.20032792006417	-1.64670816043711	0.109229647979135	0.789812287315734	-4.56568939734654
a_HQ_transcript/135119	2.23966328842569	0.163831014532247	1.64650585785952	0.109271499831525	0.790012449221225	-4.65190269607877
a_HQ_transcript/57666	-1.36003327812945	1.7956997203078	-1.64644297376332	0.10928451183918	0.790012449221225	-4.57013522690393
a_HQ_transcript/138275	-0.7181455414906	2.42393912038802	-1.64627635753988	0.109318994347297	0.790102745966469	-4.63378618285192
a_HQ_transcript/2037	-1.69118166316185	3.15183173089833	-1.64622861668158	0.109328876343897	0.790102745966469	-4.80494412231294
a_HQ_transcript/9892	-1.08509405462946	2.60027559543497	-1.64597914078759	0.109380527989687	0.790102745966469	-4.62233470229649
a_HQ_transcript/16445	1.53725884603989	0.141204873889687	1.64580080719792	0.109417462660403	0.790102745966469	-4.48715064423393

a_HQ_transcript/3294	1.28820545311163	4.56254403109587	1.64579435519261	0.109418799128631	0.790102745966469	-4.89037872873523
a_HQ_transcript/32253	1.55536887688859	1.86560586799241	1.64577332780596	0.10942315483473	0.790102745966469	-4.55905994223616
a_HQ_transcript/8855	-1.80003169792195	1.23773069100572	-1.64575566601285	0.109426813487702	0.790102745966469	-4.62756564881912
a_HQ_transcript/124542	1.30497871898469	1.71547346489183	1.64570942931243	0.109436391932241	0.790102745966469	-4.56392684303461
a_HQ_transcript/35761	-0.691476856538616	3.47675206327251	-1.64569214153619	0.109439973464687	0.790102745966469	-4.83210637404429
a_HQ_transcript/20975	-0.869563727298125	2.55792993173383	-1.64562237590755	0.10945442789144	0.790102745966469	-4.72139722067304
a_HQ_transcript/83477	1.57299501405544	0.467299220094385	1.64561311935332	0.109456345833937	0.790102745966469	-4.70816983631575
a_HQ_transcript/3805	1.07544783828246	2.45019581730057	1.64552797227898	0.109473989468907	0.790102745966469	-4.628986132035
a_HQ_transcript/124461	1.6833822976248	-0.183090845675014	1.6454547224705	0.109489169710645	0.790102745966469	-4.65065020247812
a_HQ_transcript/98997	-3.71491465713746	2.76688991726845	-1.64538443602923	0.109503737463133	0.790102745966469	-4.80157834658862
a_HQ_transcript/30022	-2.41177374433681	3.36586892973451	-1.64529052436992	0.109523204342689	0.790102745966469	-4.81107685034535
a_HQ_transcript/70514	1.78417207163896	3.91043838472511	1.64526062850968	0.109529402034493	0.790102745966469	-4.93979893011293
a_HQ_transcript/151007	2.31850948146438	0.606614960093259	1.64493826437393	0.109596249570258	0.790102745966469	-4.63999743820094
a_HQ_transcript/94151	-2.13860485676619	2.22576273193097	-1.64486542965697	0.10961135772629	0.790102745966469	-4.72963510626998
a_HQ_transcript/140264	-2.9066484340458	2.6420469707415	-1.64481589971848	0.109621632739929	0.790102745966469	-4.91242206382291
a_HQ_transcript/66062	-1.05703206930341	3.07531549084011	-1.64469780916278	0.109646133906344	0.790102745966469	-4.78984990247689
a_HQ_transcript/30168	2.45177984930611	2.48709370061548	1.64462838328889	0.109660540346631	0.790102745966469	-4.67936848794555
a_HQ_transcript/54253	-1.40256038635831	3.30681388250507	-1.64452958854612	0.109681043768007	0.790102745966469	-4.90859533936266
a_HQ_transcript/54505	-0.916218072647337	2.07799959493844	-1.64451562648156	0.109683941648462	0.790102745966469	-4.56224423792165
a_HQ_transcript/7239	0.833940497482478	2.48875520659813	1.6444898426198	0.10968929335506	0.790102745966469	-4.6771694941135
a_HQ_transcript/35210	2.70637915898137	1.07103877291588	1.64440998575617	0.109705869841514	0.790102745966469	-4.53964828012778
a_HQ_transcript/143868	0.720715360279763	4.62642924707802	1.64419245833063	0.109751034144826	0.790102745966469	-5.03825939118654
a_HQ_transcript/135107	-0.570351906423795	5.1300635825026	-1.64412306195445	0.109765445857167	0.790102745966469	-5.07506858708565
a_HQ_transcript/2027	-0.680595779008416	2.8773460914162	-1.64406907914822	0.109776657678598	0.790102745966469	-4.70679602995161
a_HQ_transcript/65005	-0.642077839481071	2.56447588142834	-1.64399893237135	0.109791228049663	0.790102745966469	-4.70419734859945
a_HQ_transcript/23266	-1.8480767510179	2.94098428945108	-1.64392375965994	0.109806844146057	0.790102745966469	-4.87778758354792
a_HQ_transcript/72616	1.87753411788795	-0.0329123581109032	1.64380512028728	0.109831493580286	0.790102745966469	-4.56944009179471
a_HQ_transcript/71010	1.27276423049853	1.46505476053555	1.6438048423891	0.109831551323934	0.790102745966469	-4.58254604339865
a_HQ_transcript/1291	-1.86831779857913	4.76127890486055	-1.64372977246178	0.109847150803421	0.790102745966469	-4.90351144424797
a_HQ_transcript/47712	-2.6015374522879	1.74114254710246	-1.64368323900201	0.109856821344332	0.790102745966469	-4.62836566891687
a_HQ_transcript/69441	-2.16356599034793	1.47915366635903	-1.64359592827524	0.10987496807921	0.790102745966469	-4.58714806811479
a_HQ_transcript/89333	-2.51595695081169	0.835288888255824	-1.64357935842341	0.109878412250987	0.790102745966469	-4.78152457617217
a_HQ_transcript/115726	-3.56475171444678	0.611459423580348	-1.64357128845082	0.109880089668709	0.790102745966469	-4.56898390113417
a_HQ_transcript/68877	1.43816434249123	1.01840502513898	1.64354206086291	0.109886165163873	0.790102745966469	-4.50525109576091
a_HQ_transcript/22579	1.10283556658022	2.47543159789268	1.6435095453867	0.109892924410773	0.790102745966469	-4.61623775742682
a_HQ_transcript/4384	-1.77714337010916	1.21876985101814	-1.64348733473392	0.109897541710889	0.790102745966469	-4.59513317072829
a_HQ_transcript/130084	3.00442433388384	2.63393486285077	1.64322994012089	0.109951062339844	0.790102745966469	-4.78855732728369
a_HQ_transcript/134685	2.08565976099186	1.80908198747337	1.64316873169173	0.109963792714855	0.790102745966469	-4.63586202403805
a_HQ_transcript/11470	1.53949028585835	-0.661190761974227	1.64305682434111	0.109987070806345	0.790102745966469	-4.61122876131442
a_HQ_transcript/5663	1.54142292697	-0.347550847063679	1.64304537681216	0.109989452261453	0.790102745966469	-4.59777862611584
a_HQ_transcript/45968	2.89206098312099	0.260372111532548	1.64303659572704	0.109991279039166	0.790102745966469	-4.61146441638397
a_HQ_transcript/15433	1.66852475890083	-0.541068700051762	1.64297616924703	0.110003850574396	0.790102745966469	-4.61374244581512
a_HQ_transcript/96261	3.7260245331228	4.12312986460341	1.64288873696661	0.110022042682877	0.790102745966469	-4.72704083868207
a_HQ_transcript/148172	-1.87440035997907	1.30335440300363	-1.64285309968846	0.110029458475027	0.790102745966469	-4.65957049871155
a_HQ_transcript/83615	-2.23950918448516	1.26857057004334	-1.64281730866518	0.110036906676091	0.790102745966469	-4.54524296617364
a_HQ_transcript/144118	-2.39604348623452	1.98324598732863	-1.6426122930813	0.110079578963004	0.790102745966469	-4.61427811996801
a_HQ_transcript/64953	-2.09112517380798	4.08979660394926	-1.64260234358511	0.110081650215858	0.790102745966469	-4.94672441711436
a_HQ_transcript/112601	2.69648865047059	0.318059116966239	1.64254932933423	0.110092687088637	0.790102745966469	-4.66292848746248
a_HQ_transcript/23636	0.798801895366046	2.35394155965285	1.64254831993533	0.110092897241145	0.790102745966469	-4.58569202029928
a_HQ_transcript/113333	-1.96068875563779	2.50331902495896	-1.64245511854755	0.110112302798185	0.790102745966469	-4.53647501027254

a_HQ_transcript/81147	1.70535528417196	-0.597882963333274	1.64243788680144	0.110115890947552	0.790102745966469	-4.62299548243988
a_HQ_transcript/118038	1.46282690839721	3.18269092739126	1.64239839100109	0.11012411548351	0.790102745966469	-4.78383153875006
a_HQ_transcript/38418	-2.11489774649615	1.35630255757486	-1.64234573302723	0.110135081677248	0.790102745966469	-4.54016593652452
a_HQ_transcript/3977	0.383017551410331	4.46390583718856	1.64231420098647	0.110141648759101	0.790102745966469	-4.99865157086167
a_HQ_transcript/14764	-1.77411563472323	0.856525920349493	-1.64231248205556	0.110142006764899	0.790102745966469	-4.60655635605631
a_HQ_transcript/87045	-0.510283602516891	3.04571463272141	-1.64224951700664	0.110155121307288	0.790102745966469	-4.80015978531674
a_HQ_transcript/54915	1.98900443865296	0.585760766400809	1.64223518787016	0.110158106001569	0.790102745966469	-4.59911221197082
a_HQ_transcript/107872	1.7039454892894	0.0815440819830914	1.64211303796363	0.110183551988618	0.790102745966469	-4.63467592633904
a_HQ_transcript/110184	0.487298591700677	4.6581004292424	1.64211182239227	0.11018380523806	0.790102745966469	-5.03349097520874
a_HQ_transcript/131849	0.632262581510979	2.90666905730667	1.64209410508922	0.110187496476313	0.790102745966469	-4.7992447424136
a_HQ_transcript/146210	1.96722596201055	0.0537081531183762	1.64207527044356	0.110191420614794	0.790102745966469	-4.66276199614178
a_HQ_transcript/15898	1.55075275298073	-0.296645812858618	1.64197221087119	0.110212894794407	0.790102745966469	-4.57855802779175
a_HQ_transcript/81429	1.29065563580729	2.10087376317177	1.64161569563094	0.110287207372391	0.790102745966469	-4.58476173857185
a_HQ_transcript/151920	0.723606442866605	2.99892243892391	1.6416085098544	0.110288705612291	0.790102745966469	-4.79475761749047
a_HQ_transcript/100412	-1.93257904780027	1.74211569918782	-1.64154842662116	0.11030123367082	0.790102745966469	-4.64921218914781
a_HQ_transcript/72660	-0.863105071321749	1.89934787254567	-1.64154667072741	0.110301599812943	0.790102745966469	-4.68200598674573
a_HQ_transcript/22319	-4.12553520615205	2.85389087231615	-1.64143245342148	0.110325418769294	0.790102745966469	-4.85639308960648
a_HQ_transcript/133879	1.10868801549619	-0.610008788931831	1.6413899675146	0.110334279894668	0.790102745966469	-4.6535784317314
a_HQ_transcript/35342	1.58681973162589	-0.0880638268667408	1.64136870848602	0.110338714030917	0.790102745966469	-4.58002668344272
a_HQ_transcript/3031	-2.68123636806498	2.39118609991997	-1.64133121983362	0.110346533646081	0.790102745966469	-4.63629741490058
a_HQ_transcript/34743	-1.72607260133782	0.854585960832513	-1.64124490548065	0.110364539369568	0.790102745966469	-4.54540729881812
a_HQ_transcript/34509	1.99573355762437	1.08823165787886	1.64122120814183	0.110369483210175	0.790102745966469	-4.53341149138357
a_HQ_transcript/68367	-2.08914517306958	1.17845437920354	-1.64108241362481	0.110398442794377	0.790102745966469	-4.58712696772483
a_HQ_transcript/148205	1.86563513178718	-0.475693016683817	1.64105019120035	0.1104051669266	0.790102745966469	-4.61554464876227
a_HQ_transcript/33286	-2.437870188841	2.05639133905085	-1.64094232760189	0.110427678220564	0.790102745966469	-4.66075178326054
a_HQ_transcript/116476	3.1408232826454	2.40931024831935	1.64084731865892	0.11044750987112	0.790102745966469	-4.7111874552678
a_HQ_transcript/66522	-2.37270235139903	0.552461784099911	-1.64081769446398	0.110453694066603	0.790102745966469	-4.69354497138326
a_HQ_transcript/25687	-2.28307970803899	1.99953314613926	-1.64077594430439	0.110462410102353	0.790102745966469	-4.70351807265315
a_HQ_transcript/59037	2.35349454673605	2.05971854386804	1.64070088377415	0.110478081658151	0.790102745966469	-4.85181122187652
a_HQ_transcript/6580	1.54337042196474	-0.103583069226824	1.64069787275528	0.110478710353784	0.790102745966469	-4.66679265902529
a_HQ_transcript/96317	-5.15024880144257	1.98943966466175	-1.64066458401198	0.110485661183975	0.790102745966469	-4.58349484581522
a_HQ_transcript/142892	1.51799567608352	-0.605474538091333	1.64063297375322	0.11049226187406	0.790102745966469	-4.61489120830241
a_HQ_transcript/3258	1.57453321918494	3.07338793245578	1.64062009265144	0.110494951732305	0.790102745966469	-4.7721955038176
a_HQ_transcript/104621	-3.84585724368599	0.163397554361199	-1.64061049282617	0.110496956422816	0.790102745966469	-4.65401418159409
a_HQ_transcript/127512	-1.95741790503605	0.664970647518051	-1.64048963428163	0.110522197368023	0.790102745966469	-4.56637240369021
a_HQ_transcript/136011	-1.3230593840728	-0.508415838818459	-1.64042131070561	0.110536468650571	0.790102745966469	-4.59592217806676
a_HQ_transcript/85929	-1.57253681372635	-0.0629176221095496	-1.64033012471509	0.110555517758017	0.790102745966469	-4.62323434575613
a_HQ_transcript/18678	1.58226938663012	-0.391088393585822	1.64027412250111	0.110567218182635	0.790102745966469	-4.53366877401312
a_HQ_transcript/89688	1.71919327781913	-0.328741056021778	1.64025683584733	0.11057083005349	0.790102745966469	-4.61356733778208
a_HQ_transcript/115960	2.55942286196501	0.449726872684215	1.64018995796861	0.110584804425809	0.790102745966469	-4.64547280615894
a_HQ_transcript/120942	-2.01177547525225	2.24078289531698	-1.64017329277688	0.110588286904473	0.790102745966469	-4.79282852943911
a_HQ_transcript/152322	1.38394775365249	-0.47196191140737	1.63991945096309	0.110641342725424	0.790339223395571	-4.64484322841954
a_HQ_transcript/6069	-1.20779304902102	3.3342194453352	-1.63988483732597	0.110648578997955	0.790339223395571	-4.75961303768079
a_HQ_transcript/34224	1.29178427680747	2.08748343693862	1.63967388161757	0.11069268952214	0.790471761393764	-4.52420840693394
a_HQ_transcript/132601	0.745573422112635	3.38856506260451	1.63965676456086	0.11069626931045	0.790471761393764	-4.82029167501401
a_HQ_transcript/89379	1.53516192910976	0.612204527011376	1.63954362037553	0.110719934209421	0.790471761393764	-4.58153749766281
a_HQ_transcript/79147	2.62035169978781	1.50333438825932	1.6395359926631	0.110721529749312	0.790471761393764	-4.57244444310114
a_HQ_transcript/102734	1.52791485776029	1.6172826963313	1.63935317659143	0.110759977630094	0.790481976666884	-4.52542996568067
a_HQ_transcript/59592	2.11712264331056	0.183781816353541	1.63935110791326	0.11076020914704	0.790481976666884	-4.63758783127613
a_HQ_transcript/119316	3.62493217264082	1.76960851919301	1.63933414938383	0.110763757568672	0.790481976666884	-4.72814323098368

a_HQ_transcript/29011	2.21372325869859	0.677116955852764	1.63911844338102	0.110808900329948	0.790707065845714	-4.6488213586943
a_HQ_transcript/136620	-2.92603544885848	2.46734454107676	-1.63905221097031	0.110822764440232	0.79070892983697	-4.72311611215413
a_HQ_transcript/12603	-2.21100510652081	0.262510633091289	-1.63895742132317	0.110842608792068	0.790753456537229	-4.65146303156138
a_HQ_transcript/89133	1.17127851517319	-0.345104392386808	1.63838281757401	0.110962965779318	0.791353904502599	-4.62561148684255
a_HQ_transcript/125827	-2.07765527748623	-0.0278851597265993	-1.63837068276501	0.110965508710332	0.791353904502599	-4.49718420549502
a_HQ_transcript/147063	2.17928973866816	1.29476919828296	1.6383606193864	0.110967617595663	0.791353904502599	-4.57367008371114
a_HQ_transcript/29458	-1.70152357373347	-0.00411397232217531	-1.63815909470912	0.111009856152275	0.791558012563718	-4.59888556221958
a_HQ_transcript/144712	3.10594978129421	3.18841063230683	1.63791754019519	0.111060502260833	0.791822013420544	-4.72997143769399
a_HQ_transcript/138950	-1.53109713618008	4.71282213654656	-1.63770449231925	0.111105187296266	0.791948268006582	-4.98715693571041
a_HQ_transcript/1875	-2.31238319872163	0.958732063581219	-1.63770319671837	0.111105459083293	0.791948268006582	-4.63672988333698
a_HQ_transcript/136478	-0.714879478999717	2.34423102909878	-1.63734332051146	0.11118097403283	0.792321471611487	-4.63591382946666
a_HQ_transcript/49082	0.646508020192451	3.29961715467624	1.6373237658581	0.111185078515258	0.792321471611487	-4.83887447282708
a_HQ_transcript/126574	-2.01313397054689	0.680156492196583	-1.63715480043979	0.111220549229908	0.792372365252601	-4.61814764037912
a_HQ_transcript/130312	-1.4995893840656	0.646420004244482	-1.63709122638931	0.111233897676354	0.792372365252601	-4.52819987378713
a_HQ_transcript/134708	0.57920777780059	3.19145949550386	1.63700699973255	0.111251584524296	0.792372365252601	-4.78244905478443
a_HQ_transcript/116653	1.89146412207195	1.39428750384018	1.63697012735712	0.111259328125768	0.792372365252601	-4.50046973214751
a_HQ_transcript/131793	1.39865842474221	1.59125960221207	1.63695218363389	0.111263096664608	0.792372365252601	-4.51690091080726
a_HQ_transcript/95133	-1.96674019625265	0.331187832910887	-1.63690022604995	0.111274009385442	0.792372365252601	-4.5088236133175
a_HQ_transcript/56055	1.01485024361182	2.39753606605145	1.63682627851645	0.111289542209101	0.792385902686259	-4.61473706338845
a_HQ_transcript/117593	1.96894807797467	0.615697545649642	1.63669666027529	0.111316773093824	0.792482717256317	-4.66176588538009
a_HQ_transcript/149509	5.05701227779695	0.317048570763529	1.63659112260276	0.111338949069517	0.792489750917714	-4.65912762823533
a_HQ_transcript/145536	3.28216291298307	2.02824648067985	1.63645205232903	0.111368176613704	0.792489750917714	-4.75324403651779
a_HQ_transcript/131807	2.02656460636003	0.333684576531973	1.63639555028564	0.111380053110636	0.792489750917714	-4.5611345661464
a_HQ_transcript/114581	2.32308982521337	0.476536846447766	1.63638224821913	0.111382849302358	0.792489750917714	-4.64673309650421
a_HQ_transcript/43924	2.6596862420174	0.705186914635089	1.63636759907741	0.11138592872622	0.792489750917714	-4.61608729954224
a_HQ_transcript/70744	1.88209292462225	0.58842295011432	1.63608722981146	0.11144487923208	0.792812133154123	-4.60994930995363
a_HQ_transcript/40389	0.404805167949083	3.74382839922835	1.63579454042487	0.111506447653253	0.793029512207708	-4.938832420612
a_HQ_transcript/10980	-1.67014998422794	-0.168010087130682	-1.63564605159134	0.111537693629749	0.793029512207708	-4.61804456713939
a_HQ_transcript/257	-3.03815202962969	0.408653376151037	-1.63562944802617	0.111541187908189	0.793029512207708	-4.62965036755265
a_HQ_transcript/143935	2.71575055021609	1.78928508235506	1.63530025752697	0.111610485865003	0.793029512207708	-4.6780417833323
a_HQ_transcript/85102	2.84074400281287	2.42340307661393	1.63527920867147	0.111614918072571	0.793029512207708	-4.76533684634771
a_HQ_transcript/128740	1.31246763435051	-0.582010572896599	1.63521954712099	0.111627481653429	0.793029512207708	-4.63431606467411
a_HQ_transcript/58328	-1.59432699806184	0.760037500356818	-1.63517647888358	0.111636551725708	0.793029512207708	-4.61318988612316
a_HQ_transcript/19209	-1.68065545926082	1.09070265421831	-1.63517470296135	0.11163692574393	0.793029512207708	-4.58077536082392
a_HQ_transcript/32024	1.73699746598079	-0.439231192674776	1.63517326309636	0.111637228987552	0.793029512207708	-4.63318856231737
a_HQ_transcript/139616	-1.7821036517087	-0.218359278452125	-1.63510798357714	0.1116509779339	0.793029512207708	-4.56291734696377
a_HQ_transcript/69862	1.14125655991351	2.6102609776616	1.63507967515301	0.111656940590744	0.793029512207708	-4.7819119874032
a_HQ_transcript/57455	1.36332503039446	-0.638993275695766	1.63504911374988	0.111663378091924	0.793029512207708	-4.63628622646671
a_HQ_transcript/59303	1.36356097548458	1.76627793469588	1.6350397832264	0.111665343548864	0.793029512207708	-4.60900716042549
a_HQ_transcript/127266	2.42784307177902	0.980906910523581	1.63497273680015	0.111679467590446	0.793029512207708	-4.7510914092085
a_HQ_transcript/67584	1.25561257283447	1.00512937086592	1.6349698383037	0.111680078222755	0.793029512207708	-4.52318908021125
a_HQ_transcript/105529	1.24971972603914	1.27520558337577	1.63478418180396	0.111719196609117	0.793210389702545	-4.48886685652435
a_HQ_transcript/137201	2.09539066520198	0.754198098764929	1.63465099448307	0.111747266548444	0.793291884680397	-4.55278830207869
a_HQ_transcript/151983	1.74278111894015	0.054084118853561	1.63459877505261	0.111758273661137	0.793291884680397	-4.63003863258771
a_HQ_transcript/152349	2.46846808122225	1.21714145633479	1.63448921869728	0.111781369490232	0.793291884680397	-4.72765151650846
a_HQ_transcript/141062	3.74894473430644	0.741081362314393	1.63428830896781	0.111823733973917	0.793291884680397	-4.56807312839235
a_HQ_transcript/81317	-2.39859095510395	0.0940868251311863	-1.63424044120593	0.111833829481841	0.793291884680397	-4.65899264583483
a_HQ_transcript/34339	1.66095581797024	0.454629555043872	1.63409527888955	0.111864449412032	0.793291884680397	-4.51568442468352
a_HQ_transcript/26261	0.473584286105759	3.57362719627107	1.6340407209491	0.111875959424274	0.793291884680397	-4.9190918924638
a_HQ_transcript/113507	1.88766350384128	0.642129022926963	1.63399577865681	0.111885441570996	0.793291884680397	-4.49207273116301

a_HQ_transcript/11367	2.04570521537915	0.739468628834517	1.63397351744359	0.111890138597343	0.793291884680397	-4.68456751876807
a_HQ_transcript/124196	1.631244674286	-0.463508294528316	1.63397281145095	0.111890287561609	0.793291884680397	-4.62691001089484
a_HQ_transcript/19160	-1.51298707637393	3.7831364436416	-1.63394305280205	0.111896566777811	0.793291884680397	-4.97454720307242
a_HQ_transcript/12704	-1.13772963632546	1.14243957531651	-1.63386114517556	0.111913851177949	0.793291884680397	-4.58857054296081
a_HQ_transcript/140424	-1.6675316850469	-0.455259121296591	-1.63382688001428	0.111921082571805	0.793291884680397	-4.60866187610613
a_HQ_transcript/112675	1.10144359081529	4.33733524856471	1.63373406952751	0.111940671435098	0.793291884680397	-4.93983925781957
a_HQ_transcript/99966	-2.21742019964337	1.30720118353614	-1.63370465661266	0.111946880004755	0.793291884680397	-4.66019871158996
a_HQ_transcript/147577	2.93527431161117	1.33414532939171	1.63353287614732	0.111983145637757	0.793291884680397	-4.63471945839515
a_HQ_transcript/147454	1.76590886430949	2.13165159269804	1.63351792076772	0.11198630341894	0.793291884680397	-4.57261343730967
a_HQ_transcript/3961	-2.03294333303894	0.946233251446757	-1.63343764239759	0.112003255199437	0.793291884680397	-4.53944437896851
a_HQ_transcript/122082	1.89354368649495	2.81833318503709	1.6334376389802	0.112003255921108	0.793291884680397	-4.77829983041883
a_HQ_transcript/116099	-2.38809097088928	4.39141779389259	-1.63343590887994	0.1120036212767	0.793291884680397	-5.06579441064835
a_HQ_transcript/19031	-1.26870601559556	3.11616140992248	-1.63324467044607	0.112044012301523	0.793481280100261	-4.82385441645859
a_HQ_transcript/39177	1.0859226669406	2.37113896590039	1.63317234547958	0.112059291022101	0.793492808933203	-4.59331378061736
a_HQ_transcript/100139	-1.20658397441938	1.35992706428105	-1.63305538743203	0.112084002163711	0.793553390560153	-4.56500776942171
a_HQ_transcript/42976	1.442176556578	-0.582320802103123	1.63296538398909	0.112103021341762	0.793553390560153	-4.62643388011075
a_HQ_transcript/143562	1.73680771102725	1.19059010165034	1.63293803093448	0.112108802010734	0.793553390560153	-4.57377497624166
a_HQ_transcript/139935	3.1422610223572	2.73017311710595	1.63269399264324	0.112160386831456	0.793555815440363	-4.81009998159708
a_HQ_transcript/152159	2.48983010680001	0.547869049471275	1.63265035190917	0.112169613675315	0.793555815440363	-4.66877081696778
a_HQ_transcript/89223	1.79729258536712	-0.554267324575475	1.63262837462102	0.112174260511801	0.793555815440363	-4.61466441583964
a_HQ_transcript/91615	1.78206083685165	1.17965909716662	1.63256089854906	0.112188528518214	0.793555815440363	-4.51315294146151
a_HQ_transcript/1151	-3.62102035053597	0.419223092267782	-1.63255639140817	0.11218948161927	0.793555815440363	-4.70740690238809
a_HQ_transcript/19556	-2.17749018509581	2.34681561640162	-1.63254894731953	0.112191055795639	0.793555815440363	-4.73069390550031
a_HQ_transcript/81495	-1.77129908760907	0.33891360880515	-1.63237440420387	0.112227971065201	0.793720343360262	-4.56326967979953
a_HQ_transcript/132509	-1.99327227226876	2.06171677973947	-1.6322540895621	0.112253423034113	0.793747140322274	-4.69853639348858
a_HQ_transcript/141029	1.7088501918383	0.758940585769276	1.63217661510842	0.112269814893464	0.793747140322274	-4.48775524494852
a_HQ_transcript/40826	-2.22893176261885	-0.389488759925219	-1.63216285940982	0.11227272549769	0.793747140322274	-4.71126410265149
a_HQ_transcript/54112	1.92223198853679	1.55476290842142	1.63196842011892	0.112313874076272	0.793853605519226	-4.63236377936073
a_HQ_transcript/86927	-2.47958958714474	1.7436548472362	-1.63196263475022	0.112315098606353	0.793853605519226	-4.68440656548338
a_HQ_transcript/44442	-0.720807768600152	1.49920722724407	-1.63182335194862	0.11234458250389	0.79396545796792	-4.56297978192513
a_HQ_transcript/121679	1.66338740276969	-0.467205727515974	1.63165725766226	0.112379750303569	0.794035566503403	-4.66896581657497
a_HQ_transcript/24537	0.921320652719384	1.30178338710008	1.63161668843174	0.112388341567175	0.794035566503403	-4.53963879354515
a_HQ_transcript/4874	2.69680678678912	0.890071158294658	1.63149781236444	0.112413518831013	0.794035566503403	-4.57367480567203
a_HQ_transcript/76327	-1.83720792966052	1.12895008155325	-1.63135387727724	0.112444009678451	0.794035566503403	-4.68953547932565
a_HQ_transcript/34615	1.47267381166197	-0.20702415829134	1.63135132009935	0.112444551446275	0.794035566503403	-4.63718450229823
a_HQ_transcript/19479	-1.78198481847372	1.18746265629799	-1.63133231741678	0.112448577452663	0.794035566503403	-4.55365243850924
a_HQ_transcript/12809	0.639733507500389	3.67800303609686	1.63130396064466	0.112454585485873	0.794035566503403	-4.90356876973361
a_HQ_transcript/135702	3.01487993576873	-0.00659317950541791	1.63125747457631	0.112464435199649	0.794035566503403	-4.71199226008651
a_HQ_transcript/149154	1.42910339713199	-0.554445434120757	1.63119608506724	0.112477443823786	0.794035566503403	-4.62130700719566
a_HQ_transcript/85956	2.09191469661662	0.0589188090212698	1.63072960945703	0.112576331986674	0.794434770341111	-4.63901509227286
a_HQ_transcript/152254	0.488559791492231	10.8157297725339	1.63072380585108	0.112577562744634	0.794434770341111	-5.69322437478488
a_HQ_transcript/103033	-0.954306477806241	2.14309421295494	-1.63060332758563	0.112603114812595	0.794434770341111	-4.65207222707352
a_HQ_transcript/116848	-0.607857038434884	2.71086099317671	-1.63057450380239	0.112609228717901	0.794434770341111	-4.62708847577338
a_HQ_transcript/36347	-0.888477835473674	2.0068030884525	-1.63047645195773	0.112630028860022	0.794434770341111	-4.6067480372847
a_HQ_transcript/110918	-0.650354300268202	2.96985833465618	-1.63046119485186	0.112633265697722	0.794434770341111	-4.8478817724439
a_HQ_transcript/152135	-0.763299962517352	3.60795454219107	-1.63041189814848	0.112643724655776	0.794434770341111	-4.87596089888082
a_HQ_transcript/38816	2.56512486413009	-0.530575297235269	1.6303969892666	0.112646887933181	0.794434770341111	-4.63563376522694
a_HQ_transcript/5379	1.9093729796459	-0.276296111590135	1.6303493553073	0.112656995112885	0.794434770341111	-4.63398256416165
a_HQ_transcript/62344	1.40482738639109	3.45368589099806	1.63011658009748	0.112706397124588	0.794686736057506	-4.83373883237115
a_HQ_transcript/104238	-4.62519580138092	1.11478267534418	-1.62984954424927	0.112763092296416	0.794739827295327	-4.80437071780178

a_HQ_transcript/91717	0.715329147322079	2.57673617489194	1.62984476781897	0.112764106608575	0.794739827295327	-4.7360319306485
a_HQ_transcript/60578	-0.490627118201544	2.78005104500349	-1.62981234353851	0.112770992355902	0.794739827295327	-4.7085858437197
a_HQ_transcript/142118	1.46854249008294	2.46403732251377	1.6297613520646	0.112781821806748	0.794739827295327	-4.66532974633362
a_HQ_transcript/146124	-1.30927479188	1.02690470552368	-1.62975915716298	0.112782287974111	0.794739827295327	-4.53856786914396
a_HQ_transcript/29314	-0.518378090382881	3.89028335264418	-1.62963449335803	0.112808767485705	0.794807858271201	-4.89370645514411
a_HQ_transcript/125373	2.23911822131815	0.0587039213741545	1.62958496484257	0.112819289130809	0.794807858271201	-4.64035011947405
a_HQ_transcript/105017	1.77738362080878	-0.297869974529449	1.62926331551665	0.112887638759109	0.795034334067703	-4.68334749087463
a_HQ_transcript/41046	-1.26450235040543	1.25131219464718	-1.62919445704385	0.112902275438872	0.795034334067703	-4.56485405153941
a_HQ_transcript/18307	-0.620673702243048	4.26675065408931	-1.62904713926206	0.112933594825148	0.795034334067703	-5.03665703490787
a_HQ_transcript/76601	1.82336819305479	0.42887263419554	1.6289655540163	0.112950942723609	0.795034334067703	-4.63257700542887
a_HQ_transcript/6625	-0.957414437928049	1.35743684285933	-1.62870223734653	0.113006948119888	0.795034334067703	-4.51366285138046
a_HQ_transcript/68947	1.52016878697091	-0.689169625375597	1.62864690554754	0.11301871967182	0.795034334067703	-4.62934850617646
a_HQ_transcript/53223	0.480980228532354	3.08463572198285	1.62856004665601	0.113037200487839	0.795034334067703	-4.80501086576543
a_HQ_transcript/30106	1.83791341929527	0.379671073611413	1.62854346224924	0.113040729406662	0.795034334067703	-4.51480819231611
a_HQ_transcript/2641	1.65592018362348	-0.0572110164840745	1.62848171232443	0.11305386968496	0.795034334067703	-4.71915840703429
a_HQ_transcript/134087	-4.69059931607254	1.38665233936176	-1.62837936538796	0.11307565171162	0.795034334067703	-4.7000529346043
a_HQ_transcript/62822	1.12883009264535	-0.571730805508289	1.62837470175184	0.113076644334223	0.795034334067703	-4.68895179228595
a_HQ_transcript/106396	1.07206210928226	-0.820868339446342	1.62835947244639	0.113079885836305	0.795034334067703	-4.65038870685934
a_HQ_transcript/148347	1.75952160556776	2.68982488063509	1.6282083136758	0.113112063583449	0.795034334067703	-4.71887802989993
a_HQ_transcript/38748	2.19717873093085	1.14530875545583	1.6282079360923	0.113112143970548	0.795034334067703	-4.57300851244004
a_HQ_transcript/80002	1.11504289831938	2.24428558661475	1.62815467000447	0.113123484732368	0.795034334067703	-4.72508247765052
a_HQ_transcript/94998	2.14666243796346	4.22993876631477	1.62809125021386	0.113136988518584	0.795034334067703	-4.91858674859152
a_HQ_transcript/45137	-1.68399673985283	2.06385608662802	-1.62808718597847	0.113137853949376	0.795034334067703	-4.67325909393673
a_HQ_transcript/90095	-0.673840540864612	2.84435840670888	-1.62804195245046	0.113147486261891	0.795034334067703	-4.80549299504095
a_HQ_transcript/115624	3.10227135723731	1.91060122893203	1.62792259261304	0.113172906744025	0.795034334067703	-4.60953509052184
a_HQ_transcript/32944	-2.83210146080202	0.887907297128076	-1.62790523093278	0.113176604714048	0.795034334067703	-4.61223267951664
a_HQ_transcript/113037	0.9955014095956	4.23849664206024	1.62786631442905	0.113184894139137	0.795034334067703	-4.85897948818431
a_HQ_transcript/139281	1.49494816011014	-0.164746920609376	1.62781767563809	0.11319525516759	0.795034334067703	-4.6798595095911
a_HQ_transcript/144129	-2.05979051783351	-0.675996446488209	-1.62770822061168	0.113218574121892	0.795034334067703	-4.65615710246901
a_HQ_transcript/88334	1.7629970224011	-0.245480322735325	1.62770172700731	0.113219957682697	0.795034334067703	-4.58243910522785
a_HQ_transcript/77596	-0.484895075289337	3.66978952949244	-1.62767464200911	0.113225728702298	0.795034334067703	-4.9076182671198
a_HQ_transcript/151903	2.04804429377414	2.71147617346608	1.62767070717701	0.113226567119914	0.795034334067703	-4.70382146179161
a_HQ_transcript/84955	-2.46716811535898	0.781520157667187	-1.62765006929002	0.113230964638638	0.795034334067703	-4.65381977950367
a_HQ_transcript/73287	-0.393734488083312	3.98127512262606	-1.6275465542217	0.113253023742579	0.795034334067703	-4.95854607065043
a_HQ_transcript/146508	5.19406502463791	2.19114799882463	1.62751593602909	0.113259549171022	0.795034334067703	-4.67760533731285
a_HQ_transcript/45002	-0.435819967624584	3.62481012598788	-1.62746292451763	0.113270847854445	0.795034334067703	-4.93076768870852
a_HQ_transcript/54034	-0.680959389456473	2.53545141757543	-1.62737943255315	0.113288644916557	0.795034334067703	-4.70148971840154
a_HQ_transcript/129044	4.12360361286834	0.964006981075149	1.62737725059398	0.113289110051658	0.795034334067703	-4.80591265104439
a_HQ_transcript/60718	6.52263825109676	5.45437150562868	1.62712544084165	0.11334279970471	0.795282811041435	-4.92046484355114
a_HQ_transcript/96657	1.11348778698621	-0.756803555554833	1.62695502277526	0.113379147317584	0.795282811041435	-4.64640959517583
a_HQ_transcript/147482	1.5016244277409	1.31477752099151	1.62679401535228	0.113413496608422	0.795282811041435	-4.48001691823466
a_HQ_transcript/112803	-1.5334805144157	1.56660296024889	-1.62678736858684	0.113414914812359	0.795282811041435	-4.56661146488701
a_HQ_transcript/115509	1.64451070001892	0.901331872127735	1.62676116644342	0.11342050564143	0.795282811041435	-4.53493335768857
a_HQ_transcript/57715	1.51462040656664	0.964167074881314	1.62669963352676	0.113433635995593	0.795282811041435	-4.56829887387327
a_HQ_transcript/115887	-2.10515348366063	1.30900181887344	-1.6266771003753	0.113438444601467	0.795282811041435	-4.63236583309249
a_HQ_transcript/86223	-1.65295412886319	1.03719526984535	-1.62665323220159	0.1134435382862	0.795282811041435	-4.53826872227427
a_HQ_transcript/45180	1.24814778000614	1.46928591561255	1.62663396038842	0.113447651203475	0.795282811041435	-4.54963183050979
a_HQ_transcript/120939	-0.891710584032017	1.70514902904351	-1.62652397997334	0.113471125158782	0.795303260575761	-4.56923757066781
a_HQ_transcript/150844	1.51382738962615	-0.436165957264181	1.62641843903033	0.113493655328074	0.795303260575761	-4.65927490682387
a_HQ_transcript/102332	1.3459829426622	-0.571345187620159	1.62639507895864	0.113498642577107	0.795303260575761	-4.62836706801657

a_HQ_transcript/136690	2.09021452286448	0.888511716386979	1.62636391560345	0.113505296066208	0.795303260575761	-4.52551094334748
a_HQ_transcript/124308	-6.00985924817266	3.01942957774379	-1.62613971329824	0.113553173546607	0.795451009216479	-4.65880693473856
a_HQ_transcript/114761	-1.76109877626099	1.48118565253351	-1.62613700672738	0.11355375162535	0.795451009216479	-4.59689022919159
a_HQ_transcript/10053	-1.08343529662514	3.51258192341938	-1.62601044952011	0.113580784858845	0.795524271893137	-4.70818049723521
a_HQ_transcript/136151	0.798624055631288	4.15986960850542	1.62595991094647	0.113591581625259	0.795524271893137	-5.00607049575614
a_HQ_transcript/150296	1.59849582662379	0.240912270666615	1.62578777306568	0.113628362506421	0.795685996358661	-4.67978810110525
a_HQ_transcript/2071	3.52846496151966	2.87518855931106	1.62567915389593	0.113651576328463	0.795700105756444	-4.62157738408386
a_HQ_transcript/25100	0.823118526061907	1.13162108023428	1.62556034127698	0.113676973145086	0.795700105756444	-4.52009865947712
a_HQ_transcript/23489	0.491823959089745	3.40872889542317	1.6255593135702	0.113677192843168	0.795700105756444	-4.86619303630252
a_HQ_transcript/13861	2.40047288641815	1.30188286218462	1.62547164465319	0.113695935559606	0.795700105756444	-4.59494270561321
a_HQ_transcript/134110	-0.765976486014947	2.78424575559744	-1.62545162525128	0.113700215859813	0.795700105756444	-4.78973315896505
a_HQ_transcript/75650	1.88162595034365	-0.310685482342202	1.6253941279758	0.113712509952498	0.795700105756444	-4.59530134570869
a_HQ_transcript/123616	2.00523418652152	-0.534110072188795	1.62526779346755	0.113739526700398	0.79579335676947	-4.63172455145019
a_HQ_transcript/96097	1.8631234401639	-0.293156870171059	1.62498368639557	0.113800302575924	0.796063738412705	-4.68946693800268
a_HQ_transcript/5589	2.95437761029891	0.915696947899038	1.62485961094574	0.113826853055068	0.796063738412705	-4.75979429885798
a_HQ_transcript/21941	-1.39970588478837	3.24142999214112	-1.62482063139672	0.113835195208392	0.796063738412705	-4.87425702860514
a_HQ_transcript/41002	1.29547053214693	-0.717186123672446	1.62481931706822	0.113835476501335	0.796063738412705	-4.63259233840649
a_HQ_transcript/146736	0.386710386952035	5.36576064454869	1.62476712874987	0.113846646322337	0.796063738412705	-5.12659196795124
a_HQ_transcript/140471	-2.69543803858437	0.637478528826113	-1.62467875725392	0.113865562457134	0.796100242303138	-4.66817645697563
a_HQ_transcript/83617	-0.709455600583041	2.73216122154818	-1.62459226816299	0.113884078166036	0.796133938140152	-4.67469189837012
a_HQ_transcript/92984	-2.39940533172743	0.851881943116425	-1.62449507079824	0.113904889278054	0.796183670509226	-4.72209834253071
a_HQ_transcript/5576	-1.33888794569515	2.14089899768103	-1.62430375081949	0.113945862315495	0.796374303796452	-4.66310872653163
a_HQ_transcript/1371	-1.03456977499388	2.4504250299334	-1.6241899091208	0.113970248386431	0.796448977904116	-4.63822151481577
a_HQ_transcript/19941	-1.61558618711267	4.03407605733062	-1.62392556860898	0.114026889446032	0.796654063985873	-5.03831170607814
a_HQ_transcript/92455	0.520911699684292	2.73870896036857	1.6238954633936	0.114033341654446	0.796654063985873	-4.69289591483754
a_HQ_transcript/33221	0.630371406982863	3.6009621187256	1.62386107924713	0.114040711299657	0.796654063985873	-4.97775925061551
a_HQ_transcript/141702	1.53373087367123	2.06494197168586	1.62368820862111	0.11407776906457	0.796775830423875	-4.67751921542481
a_HQ_transcript/45709	1.68857907111406	1.23534907914939	1.62365188625297	0.114085556644266	0.796775830423875	-4.52447808086065
a_HQ_transcript/51839	3.66003616415906	2.58116640623257	1.62340641792742	0.114138196967053	0.797047707027974	-4.62138684033511
a_HQ_transcript/40254	2.19235912042535	0.166066293426783	1.62308093096253	0.114208028023162	0.797439549901548	-4.7275056034989
a_HQ_transcript/70098	-1.25668654239573	-0.310396284375499	-1.62280557913769	0.114267130394578	0.797756396297863	-4.64886248658488
a_HQ_transcript/64807	1.9016507464111	1.02847748733959	1.62255285288418	0.114321398513116	0.797982244557883	-4.58694443264727
a_HQ_transcript/148559	1.26989155023983	3.36021149685441	1.62247056805321	0.114339072183521	0.797982244557883	-4.87250346831371
a_HQ_transcript/40849	1.81631924997466	0.632132555866786	1.62237527750156	0.114359542119016	0.797982244557883	-4.58779098223109
a_HQ_transcript/123060	-1.15047468937431	1.64482836856466	-1.62231820524599	0.114371803598022	0.797982244557883	-4.50966590132639
a_HQ_transcript/48828	1.73752718350013	0.418845003552899	1.62231389285318	0.114372730122204	0.797982244557883	-4.59576070281533
a_HQ_transcript/132322	-1.64543045769923	1.51552284913549	-1.62225815580291	0.114384705868861	0.797982244557883	-4.54591023020114
a_HQ_transcript/24081	-1.70274281661458	-0.176827716024667	-1.62220756845187	0.11439557603738	0.797982244557883	-4.63158919083416
a_HQ_transcript/9842	1.83495478436327	0.28899852853211	1.62213335344686	0.114411524836409	0.797997734064172	-4.60523989191416
a_HQ_transcript/139340	1.94107476546001	0.0443476063957379	1.62201313166062	0.114437364375159	0.798082197528405	-4.55863648220185
a_HQ_transcript/94976	1.46304782808239	1.60054263702266	1.62184359698936	0.114473811010325	0.798240605375261	-4.54613248612927
a_HQ_transcript/152900	-6.69304452678863	4.2164820661476	-1.62156086384594	0.114534614364482	0.798325856074328	-4.86556102799682
a_HQ_transcript/150759	5.68748747808551	1.82639911941898	1.62153960940328	0.114539186326617	0.798325856074328	-4.6259427893809
a_HQ_transcript/76415	-2.00388915977144	1.1834630593256	-1.62144939770035	0.114558593094761	0.798325856074328	-4.62673239275167
a_HQ_transcript/72945	-2.14910668998612	0.915474100292532	-1.62144627029225	0.114559265926138	0.798325856074328	-4.6450047518725
a_HQ_transcript/20802	1.19288871677122	-0.641920014300467	1.62142123951472	0.11456465117126	0.798325856074328	-4.61964908244334
a_HQ_transcript/51841	1.65138201534613	0.163474343293735	1.62137682083882	0.114574208137795	0.798325856074328	-4.52433177006412
a_HQ_transcript/115613	1.59098825760573	-0.156693740393301	1.62123280820933	0.11460519790477	0.798325856074328	-4.55523701146644
a_HQ_transcript/13919	1.01700177872337	1.19980609695254	1.62114855937649	0.114623330428999	0.798325856074328	-4.57998001528883
a_HQ_transcript/121859	0.822096784404346	2.97326781144032	1.62110998744011	0.114631632893985	0.798325856074328	-4.92912810274088

a_HQ_transcript/26780	2.0249320737555	-0.298554181611	1.62100303701509	0.11465465616279	0.798325856074328	-4.67442408390575
a_HQ_transcript/14988	-1.91586623636084	0.903938441807077	-1.62084758927829	0.114688126259456	0.798325856074328	-4.59871274448781
a_HQ_transcript/66190	1.06630533735313	2.47832533745837	1.62084050270652	0.114689652290315	0.798325856074328	-4.65427007917828
a_HQ_transcript/62682	1.52832384263972	1.66484132051806	1.62079670068281	0.114699085041643	0.798325856074328	-4.55514670908929
a_HQ_transcript/107487	2.33322495843792	1.57335846645456	1.62066595736322	0.114727244371168	0.798325856074328	-4.67536446042665
a_HQ_transcript/112292	-1.90050421423011	0.562993772657303	-1.62066483481959	0.114727486167827	0.798325856074328	-4.70434559914642
a_HQ_transcript/51369	1.33123011636168	1.37108467994942	1.62063992077798	0.114732852777274	0.798325856074328	-4.5614872312366
a_HQ_transcript/83724	-2.44861384429887	2.49584725148345	-1.62053920625389	0.114754549297174	0.798325856074328	-4.66290190356733
a_HQ_transcript/37155	1.60553662451866	1.12814448027762	1.62051431393451	0.114759912268787	0.798325856074328	-4.61277598150253
a_HQ_transcript/81566	-0.724024223646481	4.09149539328459	-1.6204908143101	0.114764975378018	0.798325856074328	-4.95418154283874
a_HQ_transcript/58750	1.59364613291463	4.20687058593668	1.62045947679972	0.114771727484337	0.798325856074328	-4.96931687452928
a_HQ_transcript/145845	1.45179274263982	-0.224269999039433	1.62041397762362	0.114781531502396	0.798325856074328	-4.6443505923895
a_HQ_transcript/94983	1.94366283382534	0.271849656687357	1.62030480798984	0.114805057840481	0.798325856074328	-4.65427783180706
a_HQ_transcript/98148	-0.604733817650856	3.71486080901641	-1.62025587246507	0.114815604861003	0.798325856074328	-4.93119711287159
a_HQ_transcript/18525	2.13570443198603	0.873569210467296	1.62025565876275	0.114815650921783	0.798325856074328	-4.52506886779337
a_HQ_transcript/95641	1.91866067717388	0.156332055205644	1.61995094685481	0.114881343119612	0.798454534252795	-4.54318189236596
a_HQ_transcript/118631	-1.84746402862104	1.92396096038258	-1.61986469447469	0.114899943706178	0.798454534252795	-4.64261395191428
a_HQ_transcript/119109	2.97992173176221	0.662954945499775	1.61985549776755	0.114901927150138	0.798454534252795	-4.62940996203641
a_HQ_transcript/127477	0.81949674769103	2.35992871077377	1.61979737951753	0.114914462102626	0.798454534252795	-4.81144567101077
a_HQ_transcript/124896	2.35588306276373	1.40424190471839	1.61974948900027	0.1149247919822	0.798454534252795	-4.65401036122208
a_HQ_transcript/38967	-2.74222447272908	3.59202629177889	-1.61966440847507	0.114943145550205	0.798454534252795	-4.85119038665687
a_HQ_transcript/109018	-2.04325593827462	0.241553303070051	-1.61966332807432	0.114943378629732	0.798454534252795	-4.66913776934905
a_HQ_transcript/43086	-2.72171517241575	2.28093199928823	-1.61961593582061	0.114953603148429	0.798454534252795	-4.613323810046
a_HQ_transcript/102946	-1.33630259542507	0.519330422967351	-1.61952077840724	0.114974134898199	0.798454534252795	-4.54899897886548
a_HQ_transcript/11887	1.05588782054446	2.57532286844704	1.6194755565247	0.114983893308281	0.798454534252795	-4.72347414863702
a_HQ_transcript/130992	-0.823067157165867	3.45246200769132	-1.61946924621345	0.114985255061763	0.798454534252795	-4.91798481452094
a_HQ_transcript/72939	2.63180255589009	1.84473591522357	1.61934838184334	0.115011339931262	0.798540273235115	-4.66957534155351
a_HQ_transcript/148165	1.51534293288948	-0.643072866302428	1.619207453921	0.115041761054035	0.798656095371904	-4.65712216513666
a_HQ_transcript/125724	3.30586065365484	2.8802748005837	1.61907350099972	0.115070682671445	0.798751295617758	-4.71927850294254
a_HQ_transcript/56209	-2.18894651584197	1.55059604065341	-1.61897004256916	0.115093024355101	0.798751295617758	-4.57189369340281
a_HQ_transcript/123833	-1.03778360375663	1.76241290088732	-1.6189097352043	0.115106049281761	0.798751295617758	-4.54312321046221
a_HQ_transcript/125219	1.72011388406222	-0.0935230003288653	1.61888941100161	0.115110439089423	0.798751295617758	-4.64641409435563
a_HQ_transcript/99761	2.14879088856012	2.11952319858268	1.61871630801166	0.115147833045218	0.798915402154741	-4.76666155747048
a_HQ_transcript/105173	-0.507922289641868	5.25747921404759	-1.61843943016422	0.115207665378508	0.799186934312859	-5.11585328442887
a_HQ_transcript/102270	-1.6846123772742	0.496372516826892	-1.61840796497599	0.115214466514275	0.799186934312859	-4.66451985515478
a_HQ_transcript/98963	1.25417815013174	0.786051238806178	1.61829357226486	0.115239195047955	0.799263086713698	-4.47750775514362
a_HQ_transcript/2847	0.445992813551673	2.99696315499031	1.61795076563274	0.115313326499071	0.799593454659159	-4.86218968887115
a_HQ_transcript/94182	-1.95672811782952	1.78428958225689	-1.61784597765598	0.115335994580891	0.799593454659159	-4.53916325522514
a_HQ_transcript/26067	-2.0791815183113	0.0456557467500629	-1.61780945459977	0.115343896230938	0.799593454659159	-4.59312219733238
a_HQ_transcript/32434	1.02007835685023	1.61165202555666	1.61767653370146	0.115372657016153	0.799593454659159	-4.58948660987541
a_HQ_transcript/77651	1.0958094612553	-0.755797209099599	1.61763356187533	0.115381956315543	0.799593454659159	-4.64034299977764
a_HQ_transcript/69774	-1.98645190327424	1.46747994491688	-1.61759752988517	0.11538975427813	0.799593454659159	-4.59608871085298
a_HQ_transcript/21880	1.18273007324117	-0.499180330278675	1.6175183249755	0.115406897151565	0.799593454659159	-4.68471772679775
a_HQ_transcript/4535	2.19817684705841	0.874960307175204	1.61745709690719	0.115420150607385	0.799593454659159	-4.6416480272234
a_HQ_transcript/114186	2.54312957318399	0.056244371201838	1.61742241229971	0.115427659009118	0.799593454659159	-4.59633685740342
a_HQ_transcript/90260	-1.67410883601503	-0.465721081949983	-1.61739448758879	0.115433704344273	0.799593454659159	-4.6741183537738
a_HQ_transcript/143194	1.22489650850677	-0.722914782288096	1.61731186402813	0.115451592791658	0.799593454659159	-4.6636620392124
a_HQ_transcript/112494	-2.82256320074545	2.37922861413655	-1.61731045934176	0.115451896933566	0.799593454659159	-4.72622477009687
a_HQ_transcript/58076	-2.09830817954359	0.534421501765992	-1.617226786732	0.115470014869476	0.799604241521245	-4.60913607638678
a_HQ_transcript/18496	1.54433559058628	0.421245452512838	1.61706619957991	0.115504793945673	0.799604241521245	-4.5312555309816

a_HQ_transcript/53097	-0.946692035069883	1.98899632695819	-1.61691843326397	0.115536803969707	0.799604241521245	-4.57935854739828
a_HQ_transcript/112176	1.75491406905917	-0.310918881564672	1.61685647742895	0.115550227385034	0.799604241521245	-4.65653473620232
a_HQ_transcript/48076	2.6896712576176	2.43849386826082	1.61681684443722	0.115558814982943	0.799604241521245	-4.63949427720664
a_HQ_transcript/121799	0.81850188567687	2.11744173719818	1.616796076756	0.115563315092505	0.799604241521245	-4.56737928586144
a_HQ_transcript/66198	0.779024825986337	5.15555496142318	1.61678457125824	0.115565808259112	0.799604241521245	-5.08545690055419
a_HQ_transcript/29409	3.31217167626783	1.66202667442049	1.61677087093825	0.115568777087157	0.799604241521245	-4.77240729987031
a_HQ_transcript/105392	-1.90824464615716	1.57727841774214	-1.61673173671826	0.115577257728831	0.799604241521245	-4.5569567838343
a_HQ_transcript/148192	-2.11611406768539	0.631011620661714	-1.61665670221313	0.115593519629264	0.799621576699969	-4.59454635880589
a_HQ_transcript/149317	2.44624208310772	-0.400073331779888	1.61644768218852	0.115638829551423	0.79969019152933 5	-4.66450507153203
a_HQ_transcript/8826	-2.86378919812591	2.33173637786308	-1.61630334384162	0.115670126752277	0.799690191529335	-4.76116632733667
a_HQ_transcript/104492	-2.55624900963375	1.90044923537478	-1.61626463259708	0.115678521781401	0.799690191529335	-4.67013993038558
a_HQ_transcript/65098	-1.33160770638778	1.74643742622342	-1.61620467677683	0.115691524958122	0.799690191529335	-4.65852291960387
a_HQ_transcript/103051	-0.636818737897206	4.25511177137886	-1.61617905671608	0.115697081785798	0.799690191529335	-5.03304575127303
a_HQ_transcript/19699	0.814075193496219	2.87448512681658	1.61612366510667	0.115709096622883	0.799690191529335	-4.83477241077445
a_HQ_transcript/5816	1.87859233387985	0.637723393669247	1.61597647269453	0.115741028699556	0.799690191529335	-4.61152285351146
a_HQ_transcript/52433	1.28104790666144	1.62411243994448	1.61590150045698	0.115757296041792	0.799690191529335	-4.56697208801801
a_HQ_transcript/105148	-0.300612807894538	4.67365671077158	-1.6158645398335	0.115765316383978	0.799690191529335	-5.06917763495608
a_HQ_transcript/151077	-2.10689261736494	0.0170560184465977	-1.61581288756466	0.115776525533991	0.799690191529335	-4.68099876194695
a_HQ_transcript/67759	1.82149125090002	-0.177610725295385	1.61570562783253	0.115799805009539	0.799690191529335	-4.61895142994755
a_HQ_transcript/88665	-1.39689172906063	-0.500272561211111	-1.61569402996288	0.115802322422711	0.799690191529335	-4.63725499171631
a_HQ_transcript/102325	2.44480097715306	0.248207051527542	1.61559846416803	0.115823067479617	0.799690191529335	-4.65896667093069
a_HQ_transcript/70072	5.20820104167781	1.48757601767423	1.6155587318768	0.115831693312873	0.799690191529335	-4.7018336130427
a_HQ_transcript/108353	0.740589222528615	3.216194856897	1.61548223389676	0.115848302421524	0.799690191529335	-4.95416682051729
a_HQ_transcript/74100	1.7641248526713	0.402365511677515	1.61526346879669	0.115895811131468	0.799690191529335	-4.63062768673439
a_HQ_transcript/138283	0.600740608075054	3.74422676880516	1.61506018626137	0.115939971890803	0.799690191529335	-4.95816852944037
a_HQ_transcript/27420	3.24610721561388	2.21794468415715	1.61505637369869	0.115940800257761	0.799690191529335	-4.85921464535187
a_HQ_transcript/84764	0.945022821777948	2.68520525134596	1.61503028797293	0.115946468112423	0.799690191529335	-4.74723828473658
a_HQ_transcript/47470	1.68435078547165	-0.232224257710461	1.61501892260283	0.115948937628726	0.799690191529335	-4.66079692708145
a_HQ_transcript/134641	-1.85985340708492	1.88763914770666	-1.61499245642956	0.115954688479887	0.799690191529335	-4.75413523759113
a_HQ_transcript/11315	1.05974337279149	-0.783423295016214	1.61498256161353	0.115956838590826	0.799690191529335	-4.66024222053577
a_HQ_transcript/120621	-2.67086082971818	2.50710004788313	-1.61495878074154	0.115962006230102	0.799690191529335	-4.76791882698223
a_HQ_transcript/70531	-1.63331275083735	1.00558711050451	-1.61490113787797	0.115974532945536	0.799690191529335	-4.62634244668981
a_HQ_transcript/59819	6.33453143503438	4.85487900313877	1.61488693429552	0.115977619781878	0.799690191529335	-4.8895133184645
a_HQ_transcript/120847	2.1209179061839	1.33086684931196	1.61487062468394	0.115981164400685	0.799690191529335	-4.53965239781162
a_HQ_transcript/32884	-1.40339555646095	0.198837214531462	-1.61473425444443	0.116010805657601	0.799690191529335	-4.64649823768916
a_HQ_transcript/139410	2.65305156177381	0.459435776122367	1.61469967545086	0.116018322692471	0.799690191529335	-4.69444139486395
a_HQ_transcript/48131	3.7076122342097	1.06388972618171	1.61468759578383	0.116020948753362	0.799690191529335	-4.66625100194228
a_HQ_transcript/125246	2.58540469541382	2.63308930050861	1.61464736476252	0.116029695134333	0.799690191529335	-4.72958873763329
a_HQ_transcript/131040	3.60327060329561	2.49372230748741	1.61462734890972	0.116034046860805	0.799690191529335	-4.73155978414296
a_HQ_transcript/5954	1.72084851906889	0.170492004491551	1.61458306429512	0.116043675432665	0.799690191529335	-4.71256695862957
a_HQ_transcript/111730	-2.99951808741262	1.24066141636095	-1.61432547175436	0.116099695427208	0.79998140084597	-4.73412143967116
a_HQ_transcript/137391	1.84867424352554	0.379216459199589	1.61415112818542	0.116137623501411	0.800127043421629	-4.59204754505796
a_HQ_transcript/70001	-1.77552286040341	0.817354220253708	-1.61403165793779	0.116163619862858	0.800127043421629	-4.51204253229763
a_HQ_transcript/28455	0.554470783910829	2.90893623958333	1.61401543121651	0.116167151116962	0.800127043421629	-4.81771594151327
a_HQ_transcript/83922	-2.22859671852267	-0.243450746800601	-1.61397526738903	0.116175891936391	0.800127043421629	-4.65707312671002
a_HQ_transcript/149521	0.629391922141544	6.36904144397312	1.61390699868211	0.116190750437527	0.80013457427231	-5.20828366490656
a_HQ_transcript/19192	1.47760605283679	1.1120885985029	1.61381588247504	0.116210584070307	0.800176359966689	-4.50286863210603
a_HQ_transcript/97069	2.80905267775562	2.85193281977576	1.61374539409395	0.116225929470592	0.80018723537446	-4.84116326540313
a_HQ_transcript/96559	-1.82317865501794	1.65372130081577	-1.61365584399106	0.116245427032088	0.800213010538987	-4.60232515628052
a_HQ_transcript/77476	1.94208908486163	2.13402203329412	1.61353996817548	0.116270660428716	0.800213010538987	-4.65596457127665

a_HQ_transcript/142861	1.14982643862503	1.85324684561038	1.61346109719006	0.116287838140148	0.800213010538987	-4.58589525163111
a_HQ_transcript/122927	-0.996813924320245	5.104733137641	-1.61338759774248	0.116303847835317	0.800213010538987	-4.96785515451919
a_HQ_transcript/135249	1.09876720332554	2.19205497457335	1.613356287942	0.116310668305813	0.800213010538987	-4.7138073098246
a_HQ_transcript/149754	3.75224721755676	2.0051428396037	1.61330173009939	0.116322553873657	0.800213010538987	-4.78733636876902
a_HQ_transcript/42961	1.79841224870071	0.0430455332535702	1.61328573775049	0.116326038037683	0.800213010538987	-4.66440459175836
a_HQ_transcript/1789	0.465984056010858	3.839519694401	1.6129916721334	0.11639011977192	0.800318083287121	-4.97195518220825
a_HQ_transcript/46382	2.00685701363483	1.11997888269521	1.61297800291704	0.1163930992247	0.800318083287121	-4.69607808005181
a_HQ_transcript/131669	-2.5778180335624	4.00865944906187	-1.61288381538411	0.11641363080454	0.800318083287121	-4.97547097076546
a_HQ_transcript/38972	1.45481023411778	-0.483263498244288	1.61279049652058	0.116433975962699	0.800318083287121	-4.63239591820614
a_HQ_transcript/134238	-1.51525205282455	-0.166508152136872	-1.61274220714574	0.116444505045686	0.800318083287121	-4.65095521397088
a_HQ_transcript/130038	1.51871104732113	2.16299840508669	1.61266836676346	0.116460606819676	0.800318083287121	-4.64043463227284
a_HQ_transcript/66970	-1.35570494579178	2.79129939436016	-1.61263862051848	0.116467093859511	0.800318083287121	-4.70483650490206
a_HQ_transcript/23696	1.83221569421929	2.44697001140233	1.61261613199023	0.11647199833884	0.800318083287121	-4.61112874651837
a_HQ_transcript/139078	-0.459036588994682	3.65381728549882	-1.61260705044329	0.116473978963847	0.800318083287121	-4.93782524215197
a_HQ_transcript/46797	1.44176317492741	-0.484077954872542	1.61257531682411	0.116480900072613	0.800318083287121	-4.63466691675716
a_HQ_transcript/4063	1.57389658341677	2.43258297054226	1.61246674131522	0.116504582970308	0.800318083287121	-4.6263975775905
a_HQ_transcript/97522	1.69963153251752	1.58201292714409	1.61239823632593	0.116519527572265	0.800318083287121	-4.69760309714508
a_HQ_transcript/75916	2.20452970513618	-0.257446411665835	1.61228436264735	0.116544372998532	0.800318083287121	-4.65034521109686
a_HQ_transcript/41685	-0.429843424052543	4.30495659402203	-1.61227993704113	0.116545338683355	0.800318083287121	-5.00448971616096
a_HQ_transcript/97377	1.5234627316191	-0.347664594533844	1.61226849514479	0.116547835380978	0.800318083287121	-4.68424409355761
a_HQ_transcript/100149	1.51603555173228	-0.449097818829452	1.61207153811993	0.116590819601725	0.80049551461036	-4.62711462939801
a_HQ_transcript/135458	1.80880891153963	2.09472149098858	1.61202390694388	0.116601216663328	0.80049551461036	-4.72734560118549
a_HQ_transcript/18022	2.30376020429273	1.27346136038792	1.61188207907595	0.116632179746035	0.800574497532251	-4.59260382440341
a_HQ_transcript/111930	-1.77134797145211	0.16359868935858	-1.61180043712634	0.116650006433524	0.800574497532251	-4.65131883277658
a_HQ_transcript/27434	-1.98894085376021	-0.155285729270751	-1.61173200533579	0.116664950380839	0.800574497532251	-4.65282922757502
a_HQ_transcript/127572	-1.36161612175585	4.65342312243866	-1.61171890272001	0.116667811873722	0.800574497532251	-4.8996666750841
a_HQ_transcript/17825	-1.74917065783018	1.96505183683361	-1.61164388034563	0.116684197193089	0.800592423800741	-4.68138689229489
a_HQ_transcript/65568	2.50778812550044	1.07522496113893	1.61146977409216	0.116722230291152	0.800683338125173	-4.72799074206649
a_HQ_transcript/121303	0.723923340163073	2.73129392164177	1.61144888225585	0.116726794747794	0.800683338125173	-4.81612767248507
a_HQ_transcript/101348	-1.84616478261924	0.31160615853683	-1.61139406852498	0.116738771170707	0.800683338125173	-4.62463894535492
a_HQ_transcript/41174	1.7924183466746	0.431357160264091	1.61110479759454	0.116801991606704	0.800757965153708	-4.58201225602111
a_HQ_transcript/42025	1.47363541027084	2.13901404348271	1.61096512806646	0.116832526585204	0.800757965153708	-4.64949374422149
a_HQ_transcript/79961	1.30990426070346	3.09225096979694	1.61090856534307	0.116844894366524	0.800757965153708	-4.92082231264669
a_HQ_transcript/61507	1.1499107899973	0.173428005903043	1.61090579450629	0.116845500254373	0.800757965153708	-4.53355773455843
a_HQ_transcript/121522	1.65935721566298	-0.474155599087931	1.61087242672531	0.116852796856992	0.800757965153708	-4.67131098632476
a_HQ_transcript/54654	-0.727721665931927	2.42926305630934	-1.610681398101	0.116894576694903	0.800757965153708	-4.59602361481751
a_HQ_transcript/143082	-1.07601884368837	2.01759216655174	-1.61064972634126	0.116901504806241	0.800757965153708	-4.58007200790794
a_HQ_transcript/23362	2.00751267208837	0.0900354313753139	1.61059339196292	0.116913828632172	0.800757965153708	-4.68388535402327
a_HQ_transcript/10069	1.39402466848354	3.65630109042618	1.610588676799	0.116914860179667	0.800757965153708	-4.99984404349592
a_HQ_transcript/8992	-1.44402783932587	2.52865753614054	-1.61045877973385	0.11694328100708	0.800757965153708	-4.7640975562777
a_HQ_transcript/108579	-1.89016485140852	-0.119558692530757	-1.61042869207047	0.116949864846863	0.800757965153708	-4.65856293889341
a_HQ_transcript/109745	1.52663433141061	-0.505735013354522	1.61023911353198	0.116991355789375	0.800757965153708	-4.60252193879811
a_HQ_transcript/2748	0.890484166589028	1.95419589546056	1.61023477328318	0.116992305832876	0.800757965153708	-4.58795519326527
a_HQ_transcript/91617	2.32300262448342	0.708653487841122	1.61022897797676	0.11699357438598	0.800757965153708	-4.64744028066872
a_HQ_transcript/7785	1.77983729955587	-0.414604018324681	1.61018176995339	0.117003908321789	0.800757965153708	-4.60409482950839
a_HQ_transcript/150062	-4.17668882572988	1.95536833547979	-1.61006567859802	0.117029324154103	0.800757965153708	-4.69880731703278
a_HQ_transcript/35997	-1.97218812311529	-0.048325035550865	-1.61006538948956	0.11702938745416	0.800757965153708	-4.59507119669057
a_HQ_transcript/49374	5.6470858827452	3.4549892088425	1.61003863809956	0.117035244770519	0.800757965153708	-4.81215755541387
a_HQ_transcript/142027	-0.746380806487907	2.71693490218361	-1.60985184394321	0.117076150759157	0.800757965153708	-4.73692030958902
a_HQ_transcript/16558	1.33303231599722	0.209023548109252	1.60981564693093	0.11708407888908	0.800757965153708	-4.62050710499332

a_HQ_transcript/79068	-1.95646978617754	0.349008178171929	-1.6098119171345	0.117084895840981	0.800757965153708	-4.54840179903604
a_HQ_transcript/95794	1.23274794097757	2.07414520692193	1.60979601173265	0.117088379715935	0.800757965153708	-4.67386998153956
a_HQ_transcript/132842	-0.818586854346139	4.29044806253711	-1.60973431257865	0.117101894932902	0.800757965153708	-5.09672960807297
a_HQ_transcript/132725	1.60962475966895	-0.560890766536367	1.60970279110716	0.117108800214712	0.800757965153708	-4.66979019484351
a_HQ_transcript/101481	0.652934382501759	3.33787251375495	1.60958208952051	0.117135244918379	0.800757965153708	-4.89447041000636
a_HQ_transcript/127032	1.76721013589096	0.608385250138062	1.60956136140668	0.11713978676755	0.800757965153708	-4.61478246145275
a_HQ_transcript/98470	0.465940673855406	3.62474424053262	1.60947126371345	0.117159530241807	0.800757965153708	-4.89558104545443
a_HQ_transcript/152152	2.54546737872499	3.13016596462634	1.60946193436706	0.117161574775643	0.800757965153708	-4.78147303661039
a_HQ_transcript/124750	-2.65319278782596	0.172297304106027	-1.60941987115266	0.11717079332724	0.800757965153708	-4.59065434983435
a_HQ_transcript/46137	-1.75819905794972	2.24151530788943	-1.60940586786116	0.117173862413331	0.800757965153708	-4.79488610283352
a_HQ_transcript/113399	1.85795435467174	3.27346409011848	1.60937766598849	0.117180043587373	0.800757965153708	-4.94302689696341
a_HQ_transcript/93859	-1.70534593108472	1.12828546181033	-1.60933006880913	0.117190476354985	0.800757965153708	-4.62087158865447
a_HQ_transcript/34117	-0.314859456760633	4.25671182452102	-1.60910387469787	0.117240065996112	0.801002651177951	-4.9793692814841
a_HQ_transcript/141428	-0.646254360157417	3.542552321225	-1.60899799935583	0.117263283487079	0.80106712216911	-4.96114798121853
a_HQ_transcript/111387	1.99397693624333	0.250730205085645	1.60886704148297	0.117292006571884	0.801169184255053	-4.5169974150239
a_HQ_transcript/100932	2.75467974941603	0.689454403293059	1.60872620409502	0.117322902987373	0.801222879472812	-4.75434846318587
a_HQ_transcript/44330	1.53890150851445	-0.482602175766857	1.60870554739495	0.117327435144381	0.801222879472812	-4.61936644328117
a_HQ_transcript/19325	-0.383074969381607	3.64291885873815	-1.60850504192712	0.117371434264633	0.801299189398102	-4.91641631487094
a_HQ_transcript/49707	-1.1268756483066	3.79920133016001	-1.60824649004425	0.117428191153568	0.801299189398102	-4.97790129353873
a_HQ_transcript/135925	0.836289875003654	1.93563573724801	1.60811834036092	0.117456330717997	0.801299189398102	-4.59577871603829
a_HQ_transcript/4632	-1.31463795830068	2.35748616291592	-1.60796968582	0.117488979750357	0.801299189398102	-4.66293074887983
a_HQ_transcript/59199	-1.40789652773038	0.153097140197262	-1.60796291449546	0.117490467115459	0.801299189398102	-4.58039187707949
a_HQ_transcript/69256	1.34446750802711	-0.701931893528451	1.60795540282249	0.117492117120961	0.801299189398102	-4.66633388453189
a_HQ_transcript/136218	1.56041925443879	2.53249883177152	1.60793579171742	0.117496424963593	0.801299189398102	-4.73121102459794
a_HQ_transcript/37264	-0.701754286351478	3.1415559945072	-1.60783741531145	0.117518036620028	0.801299189398102	-4.83463755006033
a_HQ_transcript/142805	4.97194364068557	0.326871933851226	1.60783365049409	0.117518863752497	0.801299189398102	-4.67182465327327
a_HQ_transcript/99766	-1.31556730253088	1.72915748694157	-1.60755258764042	0.117580626921223	0.801299189398102	-4.70042733798825
a_HQ_transcript/98655	1.55704245173417	3.04691969693787	1.60755005564819	0.117581183444105	0.801299189398102	-4.89168809116397
a_HQ_transcript/776	-1.4852560862886	2.32912041949327	-1.60744611904206	0.117604030207227	0.801299189398102	-4.65403364412883
a_HQ_transcript/34680	1.99413928029583	0.452014699042237	1.607408227657	0.117612360186179	0.801299189398102	-4.58589231472835
a_HQ_transcript/114588	2.21758923219989	1.89745018071861	1.60739716891746	0.117614791412392	0.801299189398102	-4.70448696236607
a_HQ_transcript/10522	-1.38946944819528	1.31250517271883	-1.60732242738478	0.117631224165812	0.801299189398102	-4.61209226752329
a_HQ_transcript/35340	1.72115119363901	0.87705642093218	1.60731092011773	0.117633754333349	0.801299189398102	-4.52048805064139
a_HQ_transcript/5588	-1.05954735744703	2.24064000280528	-1.60726108058337	0.117644713348076	0.801299189398102	-4.78758322672465
a_HQ_transcript/123657	0.84584599558437	4.57325860893111	1.60725429368982	0.117646205755653	0.801299189398102	-4.98333742248462
a_HQ_transcript/17129	-0.866707668470278	1.71579548344687	-1.60723068724006	0.1176513968296	0.801299189398102	-4.56375644696096
a_HQ_transcript/146552	1.90926438342937	0.794887983088154	1.60722151707586	0.117653413405508	0.801299189398102	-4.71891006320108
a_HQ_transcript/98016	-1.28764254946787	-0.649146446485139	-1.60713797945889	0.117671785151979	0.801299189398102	-4.64506900296476
a_HQ_transcript/111991	1.71236120448191	0.109138971535117	1.60712041649397	0.117675647931229	0.801299189398102	-4.59412296198043
a_HQ_transcript/89082	-3.03525511176404	4.78872543905162	-1.60707212854627	0.117686268866213	0.801299189398102	-5.05550519920122
a_HQ_transcript/37076	-2.00409990848332	1.21381585628411	-1.60705028892945	0.117691072748844	0.801299189398102	-4.66669897944959
a_HQ_transcript/135040	-2.68600394834341	0.00689348809550776	-1.60704142181273	0.117693023222247	0.801299189398102	-4.6591590464441
a_HQ_transcript/44494	1.25647162630955	3.18193341189943	1.60702324443438	0.117697021729304	0.801299189398102	-4.77307207673029
a_HQ_transcript/99826	1.23801016378773	2.73070564674456	1.60693825016083	0.117715719536155	0.801325727325123	-4.7190811829889
a_HQ_transcript/93399	-1.4643373285498	0.158293364325304	-1.60688020258134	0.117728490767901	0.801325727325123	-4.6579572367143
a_HQ_transcript/68915	-0.70799116986368	2.74059497410087	-1.6066174942527	0.117786304285937	0.801509347158082	-4.85365655514162
a_HQ_transcript/19658	1.24601767064997	4.5706362853069	1.60657455710118	0.117795755565816	0.801509347158082	-5.04957516499456
a_HQ_transcript/54753	0.353983486282243	-0.735657115579295	1.60656965904856	0.117796833759488	0.801509347158082	-4.7946499863299
a_HQ_transcript/55797	-3.06944281407122	4.03454818363055	-1.60650016031374	0.117812133181281	0.801519625182761	-5.08800240327585
a_HQ_transcript/139648	2.19140604581199	1.08566159611132	1.60642465811999	0.117828756050926	0.801538903654559	-4.70338338369013

a_HQ_transcript/144188	4.5079940819726	2.78212089445396	1.60618481235882	0.117881574243351	0.801757749400747	-4.88721120342521
a_HQ_transcript/82803	-1.60919496429643	2.04627625355317	-1.60615331020268	0.117888513007986	0.801757749400747	-4.79193400590169
a_HQ_transcript/823	4.04330784629769	4.52152822094842	1.60607500880825	0.117905761373581	0.801781246738828	-5.31252398177753
a_HQ_transcript/107480	0.467966050022918	4.43747635513805	1.60562466627166	0.118005003830061	0.802362248524247	-5.04912071114792
a_HQ_transcript/32660	1.30829567959943	1.96785326332841	1.60554547105248	0.118022463251416	0.802387104547173	-4.66384980275917
a_HQ_transcript/33019	-0.480623015451291	2.26067147498262	-1.60518766117475	0.118101372648667	0.802820967473101	-4.70635273053757
a_HQ_transcript/84891	2.01098193932339	1.83536351820559	1.60513086421298	0.118113902316095	0.802820967473101	-4.56446618422066
a_HQ_transcript/95236	1.24435276689297	2.67524979521482	1.60497840907283	0.118147540006985	0.802955712092369	-4.71530511748084
a_HQ_transcript/48287	1.80125145075231	-0.365671208088102	1.60474133777101	0.118199863021808	0.803217399781583	-4.67060470945362
a_HQ_transcript/94404	-1.65778448741113	1.3125678983871	-1.60462318379067	0.118225947392958	0.803300744600567	-4.62212292179555
a_HQ_transcript/119835	1.2493189042115	3.66409745248989	1.60449870482308	0.118253433216156	0.803393591162776	-4.94055217761275
a_HQ_transcript/124973	1.5929570660992	-0.43865300968237	1.6042216197693	0.118314634356636	0.80364149138984	-4.67527786106551
a_HQ_transcript/83403	2.04863415446067	0.877018824420372	1.60417821797468	0.118324223079131	0.80364149138984	-4.65352511277125
a_HQ_transcript/123911	-4.60745009809875	2.09474582845381	-1.60406746657257	0.118348694184702	0.80364149138984	-4.99229991608148
a_HQ_transcript/30431	1.64842239761875	1.24704040304594	1.60401923523782	0.118359352450278	0.80364149138984	-4.56916928578227
a_HQ_transcript/94317	1.67487051434273	2.55284490419537	1.60398938706626	0.118365948758923	0.80364149138984	-4.73494651056419
a_HQ_transcript/56499	1.30480135579496	-0.679830878193701	1.60395804966142	0.118372874505915	0.80364149138984	-4.62397008915087
a_HQ_transcript/44792	1.36238615886731	1.88761221297807	1.60371698200041	0.118426162967255	0.803909377666772	-4.65061534453556
a_HQ_transcript/117008	0.648810038987039	4.34324469505386	1.60358444838773	0.118455468157874	0.803988965982341	-5.04680288936517
a_HQ_transcript/130848	2.33397663246541	0.145622647113172	1.6035359814565	0.118466186416863	0.803988965982341	-4.57462152405845
a_HQ_transcript/8528	-1.69633166045411	1.62390123508743	-1.60347631991383	0.118479381410073	0.803988965982341	-4.63042939194321
a_HQ_transcript/150657	-2.45301506760067	0.0883687805422809	-1.60330802829618	0.118516607978213	0.80405836818202	-4.73366311697828
a_HQ_transcript/87598	-2.11584443877195	-0.602427853241716	-1.60330501812918	0.118517273922783	0.80405836818202	-4.683757378849
a_HQ_transcript/76284	1.66973654669836	0.204006461899933	1.60312360853466	0.118557413156928	0.804235305127913	-4.61719411483693
a_HQ_transcript/57061	-0.674082835779367	2.91428364954136	-1.60306209929615	0.118571025408516	0.804235305127913	-4.76529125985369
a_HQ_transcript/45590	1.07932805986576	-0.141941917742849	1.60293146189708	0.11859994026504	0.804258510701736	-4.71607623534983
a_HQ_transcript/108284	0.432787964895068	4.3330304280547	1.60292162094068	0.11860211866459	0.804258510701736	-5.0611700954744
a_HQ_transcript/134271	-0.428114785060386	3.47096181295322	-1.6028225849248	0.118624043159469	0.804269348165478	-4.89764282077866
a_HQ_transcript/141136	-3.4727023093994	1.7677919125526	-1.60278940520838	0.118631389195754	0.804269348165478	-4.86205556915066
a_HQ_transcript/9879	-0.953542350208851	-0.65894024301331	-1.60260727879775	0.118671718889	0.804328669536149	-4.6948036170464
a_HQ_transcript/21674	-0.758902173391572	5.11166380622366	-1.60259014955487	0.118675512531535	0.804328669536149	-5.09992808189288
a_HQ_transcript/105709	0.938999475825273	4.21691244441227	1.60251245449147	0.118692721038444	0.804328669536149	-4.9791893315423
a_HQ_transcript/128080	0.464942181934477	3.71830387584495	1.60249996267535	0.118695488014057	0.804328669536149	-4.95668891936333
a_HQ_transcript/49719	-2.43599856059436	2.30505038379521	-1.60233333611448	0.118732401369812	0.804427338396892	-4.77803024200814
a_HQ_transcript/19361	0.719867001330576	2.27231783762684	1.60225863307474	0.118748953648762	0.804427338396892	-4.66107008853696
a_HQ_transcript/15539	-4.59363243344005	3.94840483065878	-1.60217627940151	0.118767203305727	0.804427338396892	-4.80214337124476
a_HQ_transcript/151096	4.85765876308112	1.79545757608225	1.60212828451846	0.118777840081311	0.804427338396892	-4.73240163947354
a_HQ_transcript/53900	1.24550672884963	-0.324126237164389	1.60212195393286	0.118779243143761	0.804427338396892	-4.68984778590037
a_HQ_transcript/147870	2.04485033124091	0.67324559517378	1.60201716074454	0.118802470677862	0.804490915140114	-4.66677000544804
a_HQ_transcript/81561	-1.23851431797787	2.45307917491919	-1.60191438370933	0.118825254948389	0.804507857580984	-4.65646589688785
a_HQ_transcript/28555	1.8216920081014	-0.37452155945541	1.6018274039293	0.118844539982642	0.804507857580984	-4.71843910420623
a_HQ_transcript/86518	1.71262369543701	0.851452125430425	1.60172064825848	0.118868213208913	0.804507857580984	-4.6596259765809
a_HQ_transcript/129734	3.75138230317225	1.36633484300347	1.60168219650226	0.118876740889617	0.804507857580984	-4.80260629389207
a_HQ_transcript/149437	-1.93804785573273	1.78978702742526	-1.60161264607072	0.118892166788144	0.804507857580984	-4.76053141477165
a_HQ_transcript/32992	-1.42770532786396	0.905344707614819	-1.6016057803546	0.118893689654797	0.804507857580984	-4.5075074025778
a_HQ_transcript/114196	0.423625895685561	3.87440183092125	1.60156897059412	0.118901854604979	0.804507857580984	-4.97602541041947
a_HQ_transcript/59275	-2.1296008394711	1.33620195233802	-1.60147456833875	0.118922796526058	0.804533983900565	-4.55979597706289
a_HQ_transcript/138063	-1.95491695399208	0.307679974716699	-1.60139626689044	0.118940168986397	0.804533983900565	-4.62737656690337
a_HQ_transcript/47500	-2.38339372433845	0.412230937281826	-1.60133812409195	0.118953070266375	0.804533983900565	-4.61343781010758
a_HQ_transcript/126561	0.680684703467276	4.07697019656204	1.60130203401953	0.118961078854001	0.804533983900565	-5.0052025289473

a_HQ_transcript/31382	-2.33330365231829	2.45331748741992	-1.60106617632413	0.119013427877078	0.804737541297335	-4.64083105875518
a_HQ_transcript/127722	-2.38738738308039	0.198211563464383	-1.60104167985078	0.119018865994584	0.804737541297335	-4.66427083004622
a_HQ_transcript/43736	1.46764057380065	-0.355578899337846	1.60074179988108	0.119085454648654	0.805037676020614	-4.70553207000328
a_HQ_transcript/52843	-1.99272709874337	0.333113423804893	-1.60070823201355	0.119092910329107	0.805037676020614	-4.7227284579986
a_HQ_transcript/12284	-1.82666841814089	0.686366490632999	-1.6006546890216	0.11910480342997	0.805037676020614	-4.60781109761556
a_HQ_transcript/79548	1.37060962588102	-0.361664510081347	1.6004980740921	0.119139596714232	0.805113457436079	-4.6620867593821
a_HQ_transcript/100934	1.34306939417904	-0.690072258157985	1.60047953156246	0.119143716640262	0.805113457436079	-4.69000314565468
a_HQ_transcript/53696	-3.71451461353326	1.25420042260304	-1.60032758665035	0.119177481365302	0.805163046817987	-4.6817313451607
a_HQ_transcript/5660	2.34242973473561	1.72313508248072	1.60017696443465	0.119210959909257	0.805163046817987	-4.54528606883788
a_HQ_transcript/144469	-2.58082002386562	3.7211736565838	-1.60015782761846	0.119215213968983	0.805163046817987	-4.7874081868465
a_HQ_transcript/113817	2.46867312907795	2.22591072849517	1.60014325776355	0.119218452889697	0.805163046817987	-4.66509490415627
a_HQ_transcript/90377	1.70469457575302	0.413195278760316	1.60007142829631	0.119234421841975	0.805163046817987	-4.60256508535705
a_HQ_transcript/32609	-1.49838752068137	1.81004001504435	-1.60003855836536	0.119241729989954	0.805163046817987	-4.6062122522033
a_HQ_transcript/111255	-1.96279639929811	1.26588679223136	-1.59999994708204	0.119250315112735	0.805163046817987	-4.61856607635853
a_HQ_transcript/143986	-2.96489843706047	1.21363924914628	-1.59992289130955	0.119267449787124	0.805163046817987	-4.71448390688262
a_HQ_transcript/139612	3.14022598634153	2.5004335458924	1.59988570719191	0.119275719036431	0.805163046817987	-4.8733802590176
a_HQ_transcript/104794	2.49854507909215	1.31848410277232	1.59970707841166	0.119315450236777	0.805260244473908	-4.66150621528161
a_HQ_transcript/46101	-1.77292335384785	0.305538440669788	-1.59963680941043	0.119331082672777	0.805260244473908	-4.62253489569459
a_HQ_transcript/144333	1.76855995807999	0.333360619324234	1.59939202884479	0.119385551044482	0.805260244473908	-4.62458877387838
a_HQ_transcript/90448	-1.71995069367475	0.413171940856123	-1.59938494219487	0.119387128263609	0.805260244473908	-4.61457051792876
a_HQ_transcript/94383	-1.9881170860714	1.1205382713681	-1.59933390467487	0.119398487781786	0.805260244473908	-4.80722875007306
a_HQ_transcript/148781	-1.75089475770348	2.13732238177466	-1.59928978162864	0.119408309046558	0.805260244473908	-4.62556229401211
a_HQ_transcript/141879	-2.99021606208807	1.42291846050767	-1.59927171636828	0.119412330349541	0.805260244473908	-4.76585599652365
a_HQ_transcript/150736	1.16215022705138	5.54015648834532	1.59926301202061	0.119414267965417	0.805260244473908	-5.20752591657433
a_HQ_transcript/114637	-1.9893645936946	1.24838514607398	-1.5992606364375	0.119414796782361	0.805260244473908	-4.61322679084479
a_HQ_transcript/63455	-1.73312855017852	1.43048940254632	-1.59908217913778	0.119454527779499	0.80543472807873	-4.68607483553879
a_HQ_transcript/140292	-2.78187905322773	0.383656662126916	-1.59886239836016	0.119503473757309	0.805671296980385	-4.68776222679361
a_HQ_transcript/152114	2.14115417604857	-0.285367509087399	1.59874285531227	0.1195301033314	0.805757375211369	-4.67480219440709
a_HQ_transcript/89413	-2.07397877400984	0.410734879750651	-1.59858046053949	0.119566286407566	0.805907826565283	-4.73410093505839
a_HQ_transcript/20065	2.49931226273108	0.620834117222489	1.59847560765963	0.119589653381107	0.805971869186897	-4.64027705274597
a_HQ_transcript/44778	1.87137344499391	0.0906652526524247	1.59832980867088	0.119622151615568	0.806097429759994	-4.67783840522308
a_HQ_transcript/34545	-1.02757131958473	2.12394981529069	-1.59821534902023	0.119647669461127	0.806163009165837	-4.69983762903779
a_HQ_transcript/96333	2.06449252638148	-0.18170314056184	1.59816174806985	0.119659620889809	0.806163009165837	-4.73020764601495
a_HQ_transcript/77898	1.10666291821396	3.50759280362782	1.59807204948938	0.119679623212765	0.806204327049673	-4.98521189539317
a_HQ_transcript/144022	2.79667074815341	1.66786952750211	1.59798489141918	0.119699061640276	0.806241837198834	-4.71354484002195
a_HQ_transcript/112285	5.87267489016827	3.19660996029999	1.59751214885774	0.119804540133953	0.806793445726865	-4.72398168007894
a_HQ_transcript/137207	1.36393802730481	-0.211254495322054	1.59744248762567	0.119820089411549	0.806793445726865	-4.66132791943162
a_HQ_transcript/119106	1.55324815753998	0.428000974767483	1.5974312620665	0.119822595254611	0.806793445726865	-4.64179840541894
a_HQ_transcript/131906	1.99234546937613	0.461134133188199	1.59724960065783	0.119863152879575	0.806801609056711	-4.68345333786225
a_HQ_transcript/55886	-1.77061191189887	0.124720556539241	-1.5972410021311	0.119865072860704	0.806801609056711	-4.6029462442614
a_HQ_transcript/122121	0.615280100575845	2.6186244795222	1.5972393271529	0.119865446872656	0.806801609056711	-4.82496364175449
a_HQ_transcript/123541	-1.05746829548477	1.55469559115554	-1.59707090825207	0.119903058622489	0.806947115691713	-4.52941244794348
a_HQ_transcript/92027	2.19804600556698	0.764903205097196	1.59698595941804	0.119922033287748	0.806947115691713	-4.67337841655459
a_HQ_transcript/140335	-2.36774541471325	1.62688615387438	-1.59695606468387	0.119928711334653	0.806947115691713	-4.87869482042892
a_HQ_transcript/67932	-1.90497494470594	1.8292271673615	-1.59688805733208	0.11994390431881	0.806955934055995	-4.74424060938799
a_HQ_transcript/85687	-1.92378493696846	2.47651443885017	-1.59663508941971	0.120000431723754	0.806988273408183	-4.74776457969299
a_HQ_transcript/111081	2.04095392731905	0.706099225874262	1.59662496531307	0.120002694478671	0.806988273408183	-4.60640215883732
a_HQ_transcript/107936	0.484351252096291	3.59524746886781	1.59652544745199	0.120024938749615	0.806988273408183	-5.00754007662086
a_HQ_transcript/143556	0.361116278494887	10.3214500766553	1.5964879958074	0.12003331083074	0.806988273408183	-5.6801868731829
a_HQ_transcript/108210	-1.56241323130827	3.42293702240084	-1.59644935082255	0.120041950177264	0.806988273408183	-4.79212664979414

a_HQ_transcript/6910	1.18221304734455	3.47024869851292	1.59644181892363	0.120043634043302	0.806988273408183	-4.92739873667107
a_HQ_transcript/102204	2.26752960484338	0.188275000280837	1.5964317200353	0.1200458918277	0.806988273408183	-4.72551388169513
a_HQ_transcript/7365	2.14440060462771	-0.181809422609483	1.59636157213575	0.120061575586731	0.807000377625522	-4.66903201487092
a_HQ_transcript/137736	0.981095545038402	4.8017057811822	1.59597428031516	0.120148197025644	0.807397339004839	-5.11513928844866
a_HQ_transcript/147951	4.21783450610256	0.941181529854237	1.5959491160766	0.120153827014198	0.807397339004839	-4.68353280522003
a_HQ_transcript/107782	1.46742244585304	2.34591884440474	1.59584418365349	0.120177305847931	0.807397339004839	-4.74970752872066
a_HQ_transcript/134952	-1.73566124011615	0.976110673286627	-1.59575547266035	0.12019715803732	0.807397339004839	-4.62813362822707
a_HQ_transcript/148456	2.01062201794172	1.07733205879485	1.59571296574179	0.120206671400115	0.807397339004839	-4.72262056731641
a_HQ_transcript/133131	2.23725410743174	1.18013651993393	1.59567173961115	0.120215898702394	0.807397339004839	-4.6481392392655
a_HQ_transcript/118848	-1.66557564693179	1.00602457785298	-1.59562080919725	0.120227298835055	0.807397339004839	-4.64322789118136
a_HQ_transcript/88985	1.51166335894746	-0.620506190489566	1.59560090900973	0.120231753482416	0.807397339004839	-4.6825323781581
a_HQ_transcript/81438	-0.679414346817473	3.03743465537729	-1.59536013738737	0.120285660810048	0.807666038069359	-4.87678693255417
a_HQ_transcript/120612	1.07372448285341	1.83333814859462	1.59527951997609	0.120303714991141	0.807693964542046	-4.65199038084149
a_HQ_transcript/46299	1.87928288931456	1.34868737388527	1.59497923956764	0.120370981995482	0.807708911246397	-4.50856520518501
a_HQ_transcript/100908	1.8097690814094	1.00054691814103	1.59494784931262	0.120378015629372	0.807708911246397	-4.58449569308609
a_HQ_transcript/149794	-2.91209357893636	2.85655294141077	-1.59490855111087	0.120386821676375	0.807708911246397	-4.82503796017748
a_HQ_transcript/80612	-1.37408390178946	1.00018022796907	-1.59489596969913	0.120389641064554	0.807708911246397	-4.63460197730971
a_HQ_transcript/147014	-1.8156428406205	-0.294305872260297	-1.59489444507962	0.120389982722592	0.807708911246397	-4.61477530262228
a_HQ_transcript/120644	-1.46652472471359	4.00752278685989	-1.59487014213702	0.120395428972716	0.807708911246397	-5.10081051992806
a_HQ_transcript/126667	-2.31769034754846	0.264501290341513	-1.59474953096565	0.120422460727457	0.807708911246397	-4.63368703236166
a_HQ_transcript/10676	-2.45331861725465	1.60370122646551	-1.59470923797767	0.12043149242635	0.807708911246397	-4.6418540208631
a_HQ_transcript/125330	1.00272964819632	2.9366508969025	1.59467848206024	0.120438386758216	0.807708911246397	-4.80638810293857
a_HQ_transcript/114239	3.38804388572544	3.7671912588306	1.59455992400772	0.120464966085732	0.807708911246397	-4.72833819403076
a_HQ_transcript/97757	1.98169095955013	-0.436559586698995	1.59452957157141	0.120471771519088	0.807708911246397	-4.67841435978241
a_HQ_transcript/134502	0.758244369752278	4.37335907716909	1.59428786489085	0.120525976718385	0.807708911246397	-5.02006595470677
a_HQ_transcript/47216	1.74231346417122	0.701304158541308	1.59421550062224	0.120542209032091	0.807708911246397	-4.63484873223442
a_HQ_transcript/76578	3.12583451608101	0.665901727354201	1.59419435447833	0.12054695274486	0.807708911246397	-4.6912205427825
a_HQ_transcript/134450	-2.0788621343885	2.10062872328792	-1.59418794379958	0.120548390882115	0.807708911246397	-4.81253926456944
a_HQ_transcript/81704	-1.50912465298837	-0.427796843787991	-1.59417908486476	0.120550378271116	0.807708911246397	-4.63215595967028
a_HQ_transcript/14702	1.13530096489442	1.97231312952964	1.59414087793592	0.120558949815605	0.807708911246397	-4.64092774249764
a_HQ_transcript/122447	2.4756164193751	0.372114437628978	1.59410254485035	0.120567550164413	0.807708911246397	-4.71454724242219
a_HQ_transcript/29346	1.70116508616854	2.93708346240284	1.59404448967285	0.120580576286537	0.807708911246397	-4.77102643337969
a_HQ_transcript/16712	1.60865850034683	-0.514214954657902	1.59398460075278	0.120594015062217	0.807708911246397	-4.68012324149507
a_HQ_transcript/2396	0.972415268714742	-0.669677733126735	1.59396641545546	0.120598095995241	0.807708911246397	-4.70065116846177
a_HQ_transcript/87806	-0.468985526873564	5.81153311698	-1.59378630449056	0.120638520512158	0.807708911246397	-5.19847230764779
a_HQ_transcript/139474	2.14119193950517	1.02068970266301	1.59373389401707	0.120650285722981	0.807708911246397	-4.63831019323589
a_HQ_transcript/103434	-2.318894490335	0.339023846099373	-1.59359446643963	0.12068158928738	0.807708911246397	-4.65321247653882
a_HQ_transcript/32938	-1.63353754439911	0.522692143997124	-1.59355498830987	0.120690453922751	0.807708911246397	-4.53856845619669
a_HQ_transcript/152302	0.658684475394077	3.9726271385297	1.59353236990041	0.120695533024511	0.807708911246397	-5.04880735232255
a_HQ_transcript/152358	3.52952608981175	5.10947637618577	1.59340393962505	0.120724376144906	0.807708911246397	-5.19651557476989
a_HQ_transcript/48884	1.52802970427153	-0.620221418513904	1.59336683989301	0.120732709125429	0.807708911246397	-4.69314724523671
a_HQ_transcript/107773	-1.16301890256063	-0.76425781461234	-1.5933564362962	0.120735045964595	0.807708911246397	-4.66519568412137
a_HQ_transcript/43977	-1.80770474460752	0.101969668088473	-1.59334529387093	0.120737548799298	0.807708911246397	-4.63616699348182
a_HQ_transcript/85820	-1.12101324225281	1.4990303830266	-1.59318542650324	0.120773463217677	0.807708911246397	-4.60067443981862
a_HQ_transcript/113131	1.64494214025451	-0.250890226625857	1.59315835994964	0.120779544621082	0.807708911246397	-4.70354081621658
a_HQ_transcript/139050	1.61620361093515	1.03020084358452	1.59309278542358	0.120794279163684	0.807708911246397	-4.5740307228538
a_HQ_transcript/2914	-1.40899625134431	2.95345411427055	-1.59306925342305	0.120799567143921	0.807708911246397	-4.68676724114099
a_HQ_transcript/95440	-1.75403439658733	-0.0243659169510676	-1.5929674968205	0.12082243550367	0.807708911246397	-4.61901531950617
a_HQ_transcript/106789	-3.12569159051026	3.76827039553838	-1.59295208520843	0.120825899354983	0.807708911246397	-5.07118436896404
a_HQ_transcript/26474	1.56252344191395	1.9769622997703	1.59292520220642	0.120831941663925	0.807708911246397	-4.72429858287585

a_HQ_transcript/76877	0.728334489939006	4.17761810549516	1.59291620231059	0.120833964564387	0.807708911246397	-5.05359691428982
a_HQ_transcript/94138	2.09401051136811	1.60374813652636	1.59284242891703	0.120850547605222	0.807726874921968	-4.69459102962657
a_HQ_transcript/102965	-2.09998260070719	4.95624146202181	-1.59261791280633	0.120901026585822	0.807934959169056	-4.91131572093107
a_HQ_transcript/53988	-1.1499763226805	3.03249634877714	-1.592562431928	0.120913503264504	0.807934959169056	-4.91461808495534
a_HQ_transcript/34071	0.649591406995865	2.3337585763715	1.59251852243545	0.12092337848835	0.807934959169056	-4.68753722056778
a_HQ_transcript/17309	-1.18340559867151	2.26168424693289	-1.59240535107378	0.120948833717819	0.808012160251627	-4.64712026388224
a_HQ_transcript/2178	-2.14086956720437	1.91985399812722	-1.59216156678878	0.12100368214951	0.808244168003401	-4.7290166598445
a_HQ_transcript/147370	-1.59107398633892	-0.423717123333157	-1.59204523022185	0.121029863606827	0.808244168003401	-4.61491760397145
a_HQ_transcript/64411	1.5857521865061	1.80482501940374	1.5920095449643	0.12103789547677	0.808244168003401	-4.54096544194178
a_HQ_transcript/1631	-0.498193690287364	2.94028371111653	-1.5918251942707	0.121079395214353	0.808244168003401	-4.82870620260409
a_HQ_transcript/107858	-0.411945954975565	5.71467933201407	-1.59174537765091	0.121097366586222	0.808244168003401	-5.2157385295981
a_HQ_transcript/135481	1.00375851275072	-0.813031171279037	1.59171197246975	0.121104888687148	0.808244168003401	-4.70298178777457
a_HQ_transcript/27641	1.20609107889425	-0.579209971109092	1.59170592042453	0.121106251513527	0.808244168003401	-4.62126862375655
a_HQ_transcript/98753	-2.74358715113486	1.88744644669972	-1.59164997186324	0.121118850853189	0.808244168003401	-4.8410402543887
a_HQ_transcript/48735	-1.48603685101772	0.555881890693316	-1.59164373886095	0.121120254560947	0.808244168003401	-4.53092194177123
a_HQ_transcript/82714	1.89639268665171	0.191460580200035	1.59156851055389	0.121137197456283	0.808244168003401	-4.62896721431321
a_HQ_transcript/142271	1.95539281576256	-0.235191603665719	1.59147327650806	0.121158648820422	0.808244168003401	-4.69476606851707
a_HQ_transcript/86216	2.74231159925394	1.18730937129595	1.59144767675355	0.121164415667258	0.808244168003401	-4.66166866693321
a_HQ_transcript/149710	8.50381604343049	4.75165495997957	1.59144766966305	0.121164417264562	0.808244168003401	-4.91801573927993
a_HQ_transcript/138599	-1.88898251341368	0.683109565076142	-1.591386374767	0.121178226055423	0.808244168003401	-4.57144056268256
a_HQ_transcript/79455	1.94033084726062	2.02822886797236	1.59120987217734	0.121217996551592	0.808416670898458	-4.638884541127
a_HQ_transcript/31969	1.7392355085194	1.03490080426153	1.59108739702775	0.12124559957825	0.808447420293905	-4.61555934798798
a_HQ_transcript/139534	0.702704732510322	2.38758837239161	1.59106599607974	0.121250423378101	0.808447420293905	-4.69971174301849
a_HQ_transcript/130987	2.00436999285028	0.399547653646328	1.59099431910376	0.121266580600309	0.808462415085993	-4.71696981687976
a_HQ_transcript/50344	-2.15535249490825	1.17372573804493	-1.59091631737789	0.121284165532246	0.808486923630547	-4.64806225064787
a_HQ_transcript/96117	1.34115372698024	-0.166337553812394	1.59079605439873	0.12131128205169	0.808574957355879	-4.67653933479437
a_HQ_transcript/83307	1.324489174975	-0.306848306149096	1.59056962035497	0.121362351157132	0.808629877637912	-4.60395306197979
a_HQ_transcript/148142	-2.86174880321787	1.9084375658841	-1.59055318427953	0.121366058774303	0.808629877637912	-4.80631033313951
a_HQ_transcript/54856	-1.48370310729944	1.04860696822414	-1.59039830895408	0.121400999794489	0.808629877637912	-4.63641269588427
a_HQ_transcript/135453	-2.67700994917671	1.38490666628918	-1.59028682653132	0.121426156150275	0.808629877637912	-4.73650167119156
a_HQ_transcript/65714	-0.724225238791274	2.01774673303981	-1.59026272568226	0.12143159514271	0.808629877637912	-4.61239351020109
a_HQ_transcript/119369	1.84486191899502	-0.0732507401847961	1.59015886318733	0.121455036736024	0.808629877637912	-4.65909184325632
a_HQ_transcript/129188	3.36822972045388	0.37096561963069	1.59015569223645	0.12145575247265	0.808629877637912	-4.66869707166093
a_HQ_transcript/105382	1.86786764537794	0.874808286862067	1.59009707518045	0.121468983943831	0.808629877637912	-4.64440642920213
a_HQ_transcript/13417	1.03967843499844	1.67300525076107	1.59006946274502	0.121475217233707	0.808629877637912	-4.64899532712425
a_HQ_transcript/5679	2.17627142192703	2.02836508517625	1.59001331561305	0.121487892813967	0.808629877637912	-4.69429973942985
a_HQ_transcript/131476	-0.62314689126315	2.57001958360573	-1.58997013548337	0.121497641745417	0.808629877637912	-4.72062542408613
a_HQ_transcript/136872	1.43307852986602	-0.598999122167818	1.58995419048614	0.121501241866004	0.808629877637912	-4.689981525848
a_HQ_transcript/93197	1.71202343134778	0.0113129305603249	1.5899529614592	0.121501519363887	0.808629877637912	-4.66064127029025
a_HQ_transcript/112086	1.53963421799441	-0.193394179799423	1.58974010458697	0.121549587429379	0.808629877637912	-4.7415419635399
a_HQ_transcript/113974	-1.81146618579978	1.06141676380056	-1.58972448457152	0.121553115407388	0.808629877637912	-4.62756955552258
a_HQ_transcript/7451	2.13612006441037	1.95250952410166	1.58960368115635	0.121580403216473	0.808629877637912	-4.67786490381465
a_HQ_transcript/81871	2.07764944753206	-0.350249903305897	1.58957964010168	0.121585834353938	0.808629877637912	-4.68168469980617
a_HQ_transcript/33406	-1.97072567358961	0.51067627825923	-1.5895769775657	0.121586435862177	0.808629877637912	-4.61730507992326
a_HQ_transcript/124023	2.6649974950235	0.932853942170138	1.58955371912844	0.121591690408015	0.808629877637912	-4.73128805869761
a_HQ_transcript/38388	1.61876998201508	1.14408400657839	1.58952691745489	0.121597745672189	0.808629877637912	-4.62437679769473
a_HQ_transcript/34056	0.599937655698583	3.31581585723849	1.58928561153848	0.121652274689113	0.808823863246908	-4.95156986645572
a_HQ_transcript/128397	-2.47794605337361	2.20934742445947	-1.58927287067066	0.121655154358125	0.808823863246908	-4.78776922122236
a_HQ_transcript/114733	-2.06199801746445	0.442864783697748	-1.58921311994652	0.121668659858088	0.808823863246908	-4.75079691302814
a_HQ_transcript/127047	1.94420343132901	-0.462185366221456	1.5891323613452	0.121686915733521	0.808826149083106	-4.68502606102008

a_HQ_transcript/110855	-3.33930821413806	0.389529180634787	-1.58908849510785	0.121696832849588	0.808826149083106	-4.78933400030554
a_HQ_transcript/31271	1.94848927642609	0.756665965847709	1.58882366560679	0.121756718568367	0.809130094916682	-4.65584622739663
a_HQ_transcript/95452	-6.79291245221071	3.45968253149863	-1.58873204191458	0.121777442985949	0.809130094916682	-4.64064922138876
a_HQ_transcript/137744	-1.724911593775	1.53726615079015	-1.58870162228933	0.121784324257261	0.809130094916682	-4.58121229171292
a_HQ_transcript/57633	-1.86495892039263	-0.0122965525649822	-1.58856014788379	0.121816331585651	0.809155926342988	-4.6270550307875
a_HQ_transcript/34660	-1.46344866222058	0.472349703974333	-1.58852688460682	0.121823858106011	0.809155926342988	-4.65013099048258
a_HQ_transcript/99359	1.84396825360737	0.74967486425489	1.58849951115825	0.121830052213418	0.809155926342988	-4.7516452006063
a_HQ_transcript/5504	-1.818643722047	1.57346785800489	-1.58843834823066	0.121843893188828	0.809155926342988	-4.82617842925435
a_HQ_transcript/91926	1.14506450647264	-0.64108242901932	1.58822260179389	0.121892726191867	0.809230633784347	-4.70673206999555
a_HQ_transcript/561	-1.28526070973878	2.09904093661363	-1.58813653695216	0.121912210955891	0.809230633784347	-4.68366244984917
a_HQ_transcript/59489	1.89880201896055	0.372194343330704	1.588114379809	0.12191722766412	0.809230633784347	-4.65326222895651
a_HQ_transcript/70512	-1.37488344330295	-0.291062901229694	-1.58804603838767	0.121932702246668	0.809230633784347	-4.6837474375853
a_HQ_transcript/100434	-0.913463779645635	3.4391609639446	-1.5880402940265	0.121934003018471	0.809230633784347	-4.83190422438983
a_HQ_transcript/137721	1.51691677765123	2.11672583840261	1.58799375218747	0.121944542522255	0.809230633784347	-4.69538856687676
a_HQ_transcript/86185	1.52207904413694	-0.353284830708323	1.5878913794616	0.121967727677911	0.809230633784347	-4.62191004622694
a_HQ_transcript/143025	-1.03021522707358	3.14010405929567	-1.58788456207782	0.121969271792441	0.809230633784347	-4.74937260514313
a_HQ_transcript/118727	-1.86988290029516	-0.142674265807218	-1.58783527111414	0.12198043650596	0.809230633784347	-4.68758509606738
a_HQ_transcript/59527	-1.30038927020285	1.87339911904105	-1.58765401880418	0.122021498484341	0.809410665742069	-4.63122377736632
a_HQ_transcript/119918	1.82444253046815	3.57009459338694	1.58750344429085	0.122055619129328	0.809528040025267	-4.89916417765648
a_HQ_transcript/149378	2.61971827518026	3.33464313820998	1.58745302358194	0.122067046360127	0.809528040025267	-4.92474618973218
a_HQ_transcript/6993	0.757431706557508	2.64778381321849	1.58709464656085	0.122148293301503	0.80997444593084	-4.86388688727472
a_HQ_transcript/131556	2.05757288346764	-0.0530827573095823	1.58703171146621	0.122162565756203	0.809976687838093	-4.71539821360683
a_HQ_transcript/39562	0.487034013692361	2.90351901494939	1.58688306453914	0.122196281401789	0.810107829074269	-4.83476657956434
a_HQ_transcript/19023	-1.59205541207408	1.42443946770675	-1.58679375214222	0.122216542628735	0.81014975366896	-4.66973679325853
a_HQ_transcript/39879	-0.732348327860268	3.57308107776404	-1.58657363296136	0.122266490144601	0.81036680087249	-4.95560764284207
a_HQ_transcript/27289	-2.4262796593493	2.18717880060002	-1.58650193301188	0.122282763272892	0.81036680087249	-4.67880131797987
a_HQ_transcript/135495	-1.52877572308132	1.17708881519888	-1.58639525908003	0.122306977426688	0.81036680087249	-4.69821742386886
a_HQ_transcript/625	1.75533029451384	2.30291851842664	1.58633042481595	0.122321696216657	0.81036680087249	-4.7082752802713
a_HQ_transcript/78606	-2.46467712905115	0.524755906156004	-1.58631174613446	0.122325936952918	0.81036680087249	-4.67583741447201
a_HQ_transcript/123705	0.432545094689606	4.13968325898724	1.58628093653324	0.122332932109415	0.81036680087249	-5.04105111155629
a_HQ_transcript/122060	0.519115956147636	3.03998583063521	1.58601986820882	0.122392219417928	0.810667152498324	-4.86262811055796
a_HQ_transcript/10509	-1.04852482563235	2.59651515376723	-1.58578441669514	0.12244570942546	0.810825492054051	-4.75913419723472
a_HQ_transcript/41859	1.40732468343202	4.81455400681183	1.58574060845602	0.122455663914743	0.810825492054051	-5.11720360165134
a_HQ_transcript/114701	-2.53184757133552	3.05250339254069	-1.5856431417121	0.122477813530239	0.810825492054051	-4.87540428358549
a_HQ_transcript/31009	-3.64570266302445	2.3958281844867	-1.5856137199649	0.122484500355873	0.810825492054051	-4.73808829198615
a_HQ_transcript/38725	-0.593255551471057	4.14838465822853	-1.58554666679386	0.12249974097622	0.810825492054051	-5.01019948416889
a_HQ_transcript/55189	-1.17483672146273	3.99524760514706	-1.58554632411145	0.122499818869025	0.810825492054051	-4.96080771719878
a_HQ_transcript/140912	-1.6013403845276	-0.0545610107224195	-1.58540794504585	0.122531276181902	0.810941366492268	-4.67099840461397
a_HQ_transcript/67809	1.66585091796504	2.93949238491987	1.5851357010214	0.122593183907904	0.811188764815334	-4.69082792460609
a_HQ_transcript/28796	1.54912748813648	0.367010614980498	1.58509453508932	0.122602547180758	0.811188764815334	-4.65609077228959
a_HQ_transcript/43783	-2.00651073168622	0.920810588451568	-1.5850592340335	0.122610576941528	0.811188764815334	-4.70505014937682
a_HQ_transcript/138428	-1.27525464231136	1.364771083306	-1.58497249510638	0.12263030885275	0.811188764815334	-4.6812824520321
a_HQ_transcript/77800	1.14310985567917	-0.388396697366952	1.58488637291521	0.122649903033435	0.811188764815334	-4.72829392270154
a_HQ_transcript/4999	-0.686247053115356	4.35598424250861	-1.58487544821022	0.122652388762076	0.811188764815334	-5.08702545555252
a_HQ_transcript/106179	-2.17119949145712	0.799099573267866	-1.58479635137517	0.122670387114477	0.811215501955671	-4.72150185700688
a_HQ_transcript/142956	3.64949140420056	1.25176175573055	1.58465946151073	0.122701541275859	0.811285786899412	-4.72019364115653
a_HQ_transcript/39384	-1.34828115387658	-0.562742287485647	-1.58461544216176	0.122711560819905	0.811285786899412	-4.67448355811176
a_HQ_transcript/143430	3.53831733908858	1.53806512125373	1.58456568972216	0.122722886116958	0.811285786899412	-4.60650451752665
a_HQ_transcript/61783	-1.85030708917076	1.03329382937044	-1.58436804282302	0.12276788551323	0.811433122259193	-4.6659725448904
a_HQ_transcript/122910	2.35998637441333	1.58418832593498	1.58422650248311	0.122800119096711	0.811433122259193	-4.89556460792284

a_HQ_transcript/142039	-2.68522362660656	1.28557203123939	-1.58420553229809	0.122804895314463	0.811433122259193	-4.70825891261701
a_HQ_transcript/4413	0.77700470297485	1.05690322169006	1.58414696408116	0.122818235751067	0.811433122259193	-4.59248168788184
a_HQ_transcript/120153	-2.16019990793004	0.700841667659994	-1.58411658363353	0.122825156155747	0.811433122259193	-4.75235994285531
a_HQ_transcript/77744	-1.62603353965041	-0.343815961183006	-1.5840477311491	0.12284084133929	0.811433122259193	-4.67675799897185
a_HQ_transcript/83592	1.11644108090246	1.40658832618357	1.58403874134416	0.122842889414411	0.811433122259193	-4.53647281191913
a_HQ_transcript/145269	0.555388966317221	5.49627656868691	1.58394018117115	0.122865345418241	0.811489239685437	-5.20460903923578
a_HQ_transcript/145834	0.557227893564022	4.07130684536927	1.58379930863497	0.122897447719184	0.811609048059615	-5.05408246707924
a_HQ_transcript/90520	2.8588945821847	1.34468002124758	1.58368865725368	0.122922667973042	0.811629943997967	-4.68734316987183
a_HQ_transcript/50081	2.16068358483029	0.305495171405906	1.58362332804649	0.122937560140808	0.811629943997967	-4.64615474314891
a_HQ_transcript/48742	-2.06507445331027	1.99313046620874	-1.58352396440964	0.122960213479187	0.811629943997967	-4.67611215500945
a_HQ_transcript/149828	-2.53067729197064	0.550742206967055	-1.58334036756992	0.123002079631861	0.811629943997967	-4.72644861601469
a_HQ_transcript/41853	-2.12275392023869	1.03214799844374	-1.58333301312245	0.123003756931816	0.811629943997967	-4.60264489603313
a_HQ_transcript/59157	1.449563350998	0.497725724480224	1.58332858805626	0.123004766148295	0.811629943997967	-4.67691187294942
a_HQ_transcript/142747	2.99644482140292	0.917045379552369	1.58328784398043	0.123014058890641	0.811629943997967	-4.78683209070089
a_HQ_transcript/112762	2.40533838969821	1.80876400603194	1.58309776388614	0.123057419164264	0.811629943997967	-4.72808450737144
a_HQ_transcript/47058	-2.44882990570837	0.184416305997191	-1.58309388020904	0.123058305222582	0.811629943997967	-4.67990453612316
a_HQ_transcript/71519	1.36334027060881	-0.571964651628616	1.58302937005574	0.123073023933255	0.811629943997967	-4.69161213543452
a_HQ_transcript/146156	1.27715897867998	0.768293027627474	1.58295797993804	0.123089314061503	0.811629943997967	-4.5726479362769
a_HQ_transcript/44695	-1.56918047387715	2.12327935004583	-1.58295245777094	0.123090574208629	0.811629943997967	-4.8352488088799
a_HQ_transcript/55570	1.87762611596695	0.545260338258817	1.58285005760727	0.123113943624873	0.811629943997967	-4.63526803879723
a_HQ_transcript/120187	1.91118406013089	0.684454931457831	1.58281442352733	0.123122076763451	0.811629943997967	-4.73023231362365
a_HQ_transcript/36712	-2.44903897475892	0.739017621467563	-1.58274047300861	0.123138956664474	0.811629943997967	-4.6681908573203
a_HQ_transcript/68028	-1.64295687349629	0.39296882374819	-1.58272495582077	0.123142498848488	0.811629943997967	-4.67067504993711
a_HQ_transcript/131145	1.73307530631462	1.46251033089085	1.58266469505386	0.123156255656694	0.811629943997967	-4.83714603949706
a_HQ_transcript/4156	-0.498617475435038	2.92752511024615	-1.5826078302887	0.123169238350205	0.811629943997967	-4.86927820872221
a_HQ_transcript/117830	1.76566651279215	1.23399927713086	1.58249896481679	0.12319409635266	0.811629943997967	-4.6373335789797
a_HQ_transcript/129567	-1.6518915520149	0.592247823944854	-1.582386115906	0.123219868248152	0.811629943997967	-4.67057965986147
a_HQ_transcript/87789	1.38677048017469	0.01832749071061	1.58217426680044	0.123268261233243	0.811629943997967	-4.65481692944485
a_HQ_transcript/151253	-4.25098299639126	4.99589383155874	-1.58212790523841	0.123278853740002	0.811629943997967	-5.22799914567948
a_HQ_transcript/5386	1.17784818139946	2.68434645114907	1.58212594796699	0.123279300946014	0.811629943997967	-4.75742276344587
a_HQ_transcript/148887	1.6570601713127	-0.386593818841552	1.58197566263584	0.123313642760222	0.811629943997967	-4.75172188706119
a_HQ_transcript/111374	4.1506040238404	2.27250886456179	1.5818670015729	0.123338477850063	0.811629943997967	-4.7620743628399
a_HQ_transcript/76504	-0.940104530451443	2.27820191726501	-1.5816982255827	0.123377060643665	0.811629943997967	-4.72593114418685
a_HQ_transcript/9566	-0.509889167872695	3.23387340171478	-1.58165117550687	0.123387818216337	0.811629943997967	-4.93630356338134
a_HQ_transcript/150658	0.548210819016767	3.4709595726438	1.58160868104583	0.123397534847653	0.811629943997967	-5.0062690057595
a_HQ_transcript/109367	0.448881085672843	3.08534701819059	1.58160120349602	0.123399244702098	0.811629943997967	-4.91231844845687
a_HQ_transcript/55673	0.528779069406461	4.02549882852288	1.58158854738424	0.123402138756879	0.811629943997967	-5.05713646398144
a_HQ_transcript/106202	-0.386260566197119	3.27439894024108	-1.58153442581093	0.123414515284391	0.811629943997967	-4.93012699279041
a_HQ_transcript/39044	-1.37468250000659	0.945095325308897	-1.58147392375721	0.123428352101346	0.811629943997967	-4.63255363075261
a_HQ_transcript/35834	1.41388447304967	2.03703609877118	1.58130297488059	0.123467454951408	0.811629943997967	-4.76880927140494
a_HQ_transcript/118291	3.03203768416656	0.805038500909521	1.58122749746419	0.123484722877685	0.811629943997967	-4.72531517635674
a_HQ_transcript/133169	1.48583992441685	-0.595992794234879	1.58121998045298	0.123486442747747	0.811629943997967	-4.69513759732845
a_HQ_transcript/9225	-1.12341468251202	1.910073566735	-1.58106239409823	0.123522502547722	0.811629943997967	-4.65983370416506
a_HQ_transcript/28784	1.5099195912642	0.483505702416466	1.58101928670729	0.123532368122662	0.811629943997967	-4.53029078993193
a_HQ_transcript/62674	1.69518365898086	0.21102098153105	1.58093265287486	0.123552197123299	0.811629943997967	-4.66556163180488
a_HQ_transcript/130892	-3.63976261346698	1.53140858352001	-1.58088572877144	0.123562938332466	0.811629943997967	-4.79823173404235
a_HQ_transcript/115066	-3.14698812462354	1.31608450610359	-1.58085568225275	0.123569816561681	0.811629943997967	-4.70245296952081
a_HQ_transcript/112807	1.25792262611967	-0.625582263720228	1.58079577093398	0.123583532354771	0.811629943997967	-4.68019292874916
a_HQ_transcript/79112	1.40493683669157	1.0014828953441	1.5806858666621	0.123608696513753	0.811629943997967	-4.57072385415185
a_HQ_transcript/135464	1.55586079223222	-0.350059497223776	1.58056965557353	0.123635309261257	0.811629943997967	-4.69811494693749

a_HQ_transcript/78848	1.6577241767726	1.37573542624903	1.58052182641393	0.12364626366621	0.811629943997967	-4.53500760865697
a_HQ_transcript/125272	1.8617917306892	0.449809384582024	1.58049120638921	0.12365327704593	0.811629943997967	-4.61773758792919
a_HQ_transcript/148683	3.048778416347	1.35330486209692	1.58036420510412	0.123682369591103	0.811629943997967	-4.67915162719774
a_HQ_transcript/4920	1.81940462319173	0.721442672769088	1.58033429028133	0.123689223078008	0.811629943997967	-4.59075970619864
a_HQ_transcript/37300	1.61478344190641	0.0321964705169322	1.58029784506452	0.123697573097542	0.811629943997967	-4.61036168354389
a_HQ_transcript/67309	-1.32808447877974	0.804767230483739	-1.58029081458692	0.123699183914023	0.811629943997967	-4.58308007736551
a_HQ_transcript/82391	-2.38014897551079	0.844634500733216	-1.5802733958662	0.123703174948903	0.811629943997967	-4.76976804195621
a_HQ_transcript/78455	2.05429429757361	0.817966713426467	1.58025841430955	0.123706607656822	0.811629943997967	-4.66526306407296
a_HQ_transcript/113213	-1.45662542212431	-0.488351165083381	-1.58024648306044	0.123709341506719	0.811629943997967	-4.68111466121805
a_HQ_transcript/145754	0.407092861963364	4.89132190379896	1.58024603862159	0.12370944334354	0.811629943997967	-5.16093091926849
a_HQ_transcript/147324	-2.05188443088926	0.448000548911422	-1.58019539962022	0.123721046996489	0.811629943997967	-4.67057798562004
a_HQ_transcript/139539	0.417989708753423	7.73803673678718	1.58006802203634	0.123750238810449	0.811629943997967	-5.40607773378397
a_HQ_transcript/106274	1.8147324110746	0.240054013351449	1.57997378611134	0.123771838990305	0.811629943997967	-4.68141128394749
a_HQ_transcript/114251	-1.75557741066902	0.312733584526554	-1.57987631180059	0.123794184692701	0.811629943997967	-4.72050302616508
a_HQ_transcript/85684	-0.618748125751787	2.96513583673545	-1.57986820951423	0.123796042266605	0.811629943997967	-4.85118077035968
a_HQ_transcript/116090	1.7304338033486	1.62226852228852	1.57971910920603	0.123830229872164	0.811629943997967	-4.71919266950348
a_HQ_transcript/27510	-1.73367020103517	2.24950691553604	-1.57966112777411	0.123843526670674	0.811629943997967	-4.86364854097385
a_HQ_transcript/26739	-0.392237714334131	3.11083318456395	-1.57963864733246	0.123848682391751	0.811629943997967	-4.85547608645066
a_HQ_transcript/142305	2.11127961239606	0.203052648117122	1.57963555479518	0.12384939165575	0.811629943997967	-4.70659024718742
a_HQ_transcript/17251	1.68228721454522	2.00300916272188	1.57953565204038	0.123872305832556	0.811629943997967	-4.82533588723147
a_HQ_transcript/35977	1.74386838094407	0.209001653054631	1.57944196503773	0.123893797480153	0.811629943997967	-4.70552371667689
a_HQ_transcript/149784	1.65930751692461	1.39200935168581	1.57940683291399	0.123901857517773	0.811629943997967	-4.62035839413493
a_HQ_transcript/123174	0.866440635378096	3.79999607837522	1.5793504055424	0.123914804022975	0.811629943997967	-5.04365789336212
a_HQ_transcript/58493	-1.83473820640869	0.647070610168674	-1.57928585915049	0.123929614683257	0.811629943997967	-4.60129348244956
a_HQ_transcript/23091	2.86706732153875	1.87044932104699	1.57928090278665	0.123930752018375	0.811629943997967	-4.86480221380033
a_HQ_transcript/86544	1.74757295959467	0.437053860016886	1.57928017832891	0.12393091826016	0.811629943997967	-4.54883952301293
a_HQ_transcript/57221	1.075735108948	4.95010964224422	1.57914614531421	0.123961678035963	0.811629943997967	-5.14104646725535
a_HQ_transcript/15066	2.56413137393379	1.60203575787482	1.57909921775903	0.123972449102771	0.811629943997967	-4.68771375104315
a_HQ_transcript/152258	5.33529412743454	5.31736898622923	1.57908618224194	0.12397544122093	0.811629943997967	-5.19808920169224
a_HQ_transcript/107103	-2.3009166157916	1.74981758138141	-1.5790423052025	0.123985513006635	0.811629943997967	-4.83560424048713
a_HQ_transcript/27405	0.760482294110401	2.67195968036383	1.5790024639488	0.123994658973853	0.811629943997967	-4.71999929688241
a_HQ_transcript/46315	1.61167450800758	1.02867246016605	1.57900013710547	0.123995193141561	0.811629943997967	-4.63325183062118
a_HQ_transcript/58102	-1.22165519160002	2.24697091192425	-1.57880079631626	0.124040962293302	0.811629943997967	-4.72745794816993
a_HQ_transcript/25641	1.34758144601713	1.32365726243488	1.5787083990372	0.124062181621717	0.811629943997967	-4.68619087842472
a_HQ_transcript/41157	-1.42924691190974	0.769204695517541	-1.5786030227976	0.124086385227111	0.811629943997967	-4.70026812196247
a_HQ_transcript/132586	-2.28876396354618	0.86340682148775	-1.5786003560288	0.124086997800595	0.811629943997967	-4.705261410172
a_HQ_transcript/1733	-1.75342299385453	-0.089296850421037	-1.57845721185359	0.124119882536987	0.811629943997967	-4.70857619105076
a_HQ_transcript/129084	1.83887726384517	-0.416402618863134	1.57844019136021	0.124123793154972	0.811629943997967	-4.70276268957985
a_HQ_transcript/2676	-1.25823619631883	1.91402450190921	-1.57841152811194	0.124130379032436	0.811629943997967	-4.69296248837457
a_HQ_transcript/37596	1.89960178287697	0.419999920901145	1.57838073242336	0.124137455192903	0.811629943997967	-4.64931621666437
a_HQ_transcript/85392	-0.881200466805011	2.44722648301099	-1.57833482553091	0.12414800418144	0.811629943997967	-4.7018404398605
a_HQ_transcript/4390	1.46572273333982	3.1627312988442	1.57828461432804	0.124159543099596	0.811629943997967	-4.96583994908927
a_HQ_transcript/3784	0.380081816655298	4.0034241135497	1.57821356854046	0.124175871460511	0.811629943997967	-5.05857010230552
a_HQ_transcript/64911	-2.07804126508163	0.162586226090179	-1.57820895181413	0.124176932577376	0.811629943997967	-4.60611545382338
a_HQ_transcript/8757	0.531582976757155	2.80928349099445	1.57819212968201	0.124180799069776	0.811629943997967	-4.86071764079584
a_HQ_transcript/126854	0.570875649221061	3.27979524578069	1.57797824134708	0.124229968924427	0.811629943997967	-4.97388493947336
a_HQ_transcript/144125	-2.56862616771581	1.99779134602828	-1.57796114518896	0.124233899771401	0.811629943997967	-4.86331429527968
a_HQ_transcript/58536	1.03306940037262	-0.5981280918441	1.57792943112918	0.124241191919264	0.811629943997967	-4.6987818567409
a_HQ_transcript/90734	1.43687045452301	-0.402295195088876	1.57788569490732	0.124251248946912	0.811629943997967	-4.75303467757945
a_HQ_transcript/112897	2.02849090076856	0.0143905462797453	1.57784679433992	0.124260194587138	0.811629943997967	-4.69985520286695

a_HQ_transcript/138476	0.808022883261516	2.48679902552323	1.57781349821002	0.124267851839051	0.811629943997967	-4.71178144065805
a_HQ_transcript/127294	2.57462856656528	1.34172464869771	1.5778126497824	0.124268046960574	0.811629943997967	-4.69622417438731
a_HQ_transcript/62880	1.47033715505916	2.53767242778872	1.57773792688748	0.124285232725262	0.811629943997967	-4.77374384780915
a_HQ_transcript/3313	-1.55177302016666	-0.330054592015814	-1.57770277181001	0.12429331882842	0.811629943997967	-4.57418079623882
a_HQ_transcript/61889	-1.35764119947283	1.44018938274582	-1.57758072684752	0.124321394014639	0.811629943997967	-4.57426594315551
a_HQ_transcript/77018	1.13483774903803	-0.206680847871355	1.57757039335872	0.124323771364968	0.811629943997967	-4.65632074119848
a_HQ_transcript/138991	2.41038697201537	1.18298939117292	1.57745493319664	0.124350336965474	0.811629943997967	-4.64650074239846
a_HQ_transcript/69377	1.16170294681407	2.07820173269661	1.57727093323902	0.124392682090712	0.811629943997967	-4.69459393074652
a_HQ_transcript/54741	0.983930321464904	0.520769189794354	1.57717730788815	0.124414233228858	0.811629943997967	-4.52574339076523
a_HQ_transcript/58343	-2.34193182665823	0.308516831095748	-1.57710325533759	0.124431281163228	0.811629943997967	-4.67835857193651
a_HQ_transcript/114807	1.67748370093359	-0.0987984341477976	1.57703412322689	0.124447198064278	0.811629943997967	-4.65972538717812
a_HQ_transcript/122642	2.07082457513712	0.909497028639377	1.57697404340835	0.124461032125222	0.811629943997967	-4.83091411480415
a_HQ_transcript/151407	-2.14405091701698	1.01464383144003	-1.57689282444493	0.124479735709855	0.811629943997967	-4.6797811594379
a_HQ_transcript/149213	1.83342046768912	0.439429841146371	1.57686362048083	0.124486461532652	0.811629943997967	-4.6735265955435
a_HQ_transcript/73141	-1.92589515386603	0.421767092836856	-1.57672598745968	0.124518163116718	0.811629943997967	-4.74066031222425
a_HQ_transcript/132347	-1.78194472075666	-0.377327630693752	-1.57671259581202	0.124521248022129	0.811629943997967	-4.69538111757445
a_HQ_transcript/112096	-4.4532862467378	1.66319174943408	-1.5766723126819	0.124530528036435	0.811629943997967	-4.70854309749462
a_HQ_transcript/76183	-2.54221489306278	0.306894332995929	-1.57653192735365	0.12456287297964	0.811629943997967	-4.74294369360449
a_HQ_transcript/23346	-0.488269322952325	4.69153581087554	-1.57652689278482	0.124564033077509	0.811629943997967	-5.13330937138075
a_HQ_transcript/106199	0.739341852966767	-0.735241072565648	1.57652186251615	0.124565192193314	0.811629943997967	-4.76056102294926
a_HQ_transcript/139699	-2.98722357117588	0.291174052116592	-1.57648139111752	0.124574518265571	0.811629943997967	-4.72065020483686
a_HQ_transcript/29029	3.50327300563336	0.291797113672515	1.57640522992916	0.124592070099875	0.811629943997967	-4.74192099032808
a_HQ_transcript/104087	1.32468213683384	-0.419148552501408	1.57640138470954	0.124592956308915	0.811629943997967	-4.69636110957585
a_HQ_transcript/44406	1.22011971932107	2.58859363094468	1.57638030415603	0.124597814842476	0.811629943997967	-4.77910334551986
a_HQ_transcript/150078	1.47541309434588	0.733725428151407	1.57619737589214	0.124639981665975	0.811629943997967	-4.75256475243592
a_HQ_transcript/7604	-2.53324010556943	0.544660254961578	-1.57615812665684	0.124649030530771	0.811629943997967	-4.73828436067595
a_HQ_transcript/146693	1.59594386259457	0.274177649664098	1.57606470261957	0.124670571488937	0.811629943997967	-4.71171840646153
a_HQ_transcript/71977	-1.64442015090607	0.230841671761445	-1.57605278717087	0.124673319075052	0.811629943997967	-4.68993083266986
a_HQ_transcript/145858	0.687463394355617	8.13791302301395	1.57604975502872	0.12467401826531	0.811629943997967	-5.47748618540217
a_HQ_transcript/139711	1.29385598827325	-0.330310242020376	1.57594194939407	0.124698879551854	0.811629943997967	-4.74374681235882
a_HQ_transcript/61100	0.942180085298606	-0.640757830552727	1.57590777727382	0.124706760901515	0.811629943997967	-4.6740864351015
a_HQ_transcript/48676	1.69117657999632	0.0471736281515415	1.57584819289125	0.124720504227247	0.811629943997967	-4.69977992154
a_HQ_transcript/111512	1.45716088101811	-0.514914645362345	1.57582463020718	0.124725939375635	0.811629943997967	-4.71155766111581
a_HQ_transcript/55147	-1.8233924585743	1.33545093679615	-1.57582390361824	0.124726106979253	0.811629943997967	-4.645905415587
a_HQ_transcript/122798	2.92473938010721	1.04914891811002	1.57581798501728	0.124727472240754	0.811629943997967	-4.70808935582566
a_HQ_transcript/141539	-5.04088446677454	3.23052140480719	-1.57581198839586	0.124728855511903	0.811629943997967	-5.08133491942592
a_HQ_transcript/5029	1.22192436024514	1.70788852241403	1.57577377061171	0.124737671696614	0.811629943997967	-4.60472669290709
a_HQ_transcript/17828	-1.52966707109789	0.337129772224444	-1.57575188112498	0.124742721453301	0.811629943997967	-4.73124449535645
a_HQ_transcript/85817	0.748945790549133	2.6142158750293	1.57568911424945	0.124757202272367	0.811629943997967	-4.86129342995504
a_HQ_transcript/14194	1.08172681240075	1.78436281009277	1.57566818379836	0.12476203139942	0.811629943997967	-4.63690560304052
a_HQ_transcript/144080	-1.46631950070512	0.300007440247399	-1.57562659303333	0.124771627779484	0.811629943997967	-4.56583779808552
a_HQ_transcript/113398	1.59042404522118	0.25767298685335	1.57555160659636	0.124788931179543	0.811651671881447	-4.61728261080351
a_HQ_transcript/40445	2.28003896566162	0.0126502580158503	1.57546065186069	0.124809921951952	0.811697375612334	-4.64664385534326
a_HQ_transcript/26388	1.43976992515722	0.104920371437984	1.57534687119521	0.124836184608522	0.811732490279602	-4.73554948269241
a_HQ_transcript/112458	-2.12445417813107	2.68758635560774	-1.57531626231833	0.124843250466206	0.811732490279602	-4.86928689079965
a_HQ_transcript/111526	1.76013807368651	0.456481400988914	1.57517601549479	0.124875629695037	0.811743971698324	-4.69151470752578
a_HQ_transcript/67875	1.99473097769543	0.547994052509658	1.57514783711182	0.124882136154238	0.811743971698324	-4.68549614775799
a_HQ_transcript/112034	2.86156729148554	4.30117076807174	1.57506441561212	0.124901400009024	0.811743971698324	-4.96374560968361
a_HQ_transcript/51235	0.635198715665996	2.97400513204559	1.57505450464033	0.124903688831011	0.811743971698324	-4.78811162699914
a_HQ_transcript/14939	-1.2458500352119	2.29526218714786	-1.57500621983746	0.124914840125955	0.811743971698324	-4.8800517509797

a_HQ_transcript/35031	1.71806193215093	0.161122361849335	1.57472757564241	0.124979208409764	0.811966427201527	-4.59364796257057
a_HQ_transcript/135724	-1.12101332156507	3.12476818188629	-1.57451497842766	0.125028337688892	0.811966427201527	-4.91453419202992
a_HQ_transcript/1079	-1.86615121191378	1.99202170063483	-1.57450821943126	0.125029899889749	0.811966427201527	-4.94819364379252
a_HQ_transcript/147833	0.763806922086786	-0.801193714903051	1.57448057282623	0.125036289990891	0.811966427201527	-4.72991593709049
a_HQ_transcript/142242	1.94792542526868	-0.434472502905495	1.57446559641704	0.125039751676155	0.811966427201527	-4.68506529986072
a_HQ_transcript/138559	0.695482690878483	2.71414997408863	1.57444236637582	0.125045121281583	0.811966427201527	-4.82925681165013
a_HQ_transcript/45298	1.31600396522722	-0.33271227720622	1.57442008677731	0.125050271369666	0.811966427201527	-4.66751762035638
a_HQ_transcript/63013	-1.51504730899268	0.304162005600156	-1.57436255289216	0.125063571537899	0.811966427201527	-4.60357254934491
a_HQ_transcript/127488	-0.473239959134953	5.84702349091583	-1.57431402822916	0.125074789931917	0.811966427201527	-5.25510127623444
a_HQ_transcript/28996	2.69621417302406	3.1388777103268	1.57421771341331	0.125097059340678	0.811993348550515	-4.78936275930608
a_HQ_transcript/41668	1.51093614245679	0.804796216289888	1.57417526532237	0.125106874993435	0.811993348550515	-4.61443135607789
a_HQ_transcript/62853	-2.13503086385378	0.652106897627906	-1.57403123978531	0.125140183992417	0.812118858447162	-4.71286265378457
a_HQ_transcript/39120	-0.865747687387886	2.69918813950892	-1.57386250231194	0.125179217359536	0.812209475329188	-4.94063414235016
a_HQ_transcript/60755	-2.84954151152655	1.44588252651297	-1.57367287363401	0.125223095256865	0.812209475329188	-4.75712306004764
a_HQ_transcript/111171	2.15204856061082	5.94458880209509	1.57354597560002	0.125252465009793	0.812209475329188	-5.29096684883827
a_HQ_transcript/106463	1.27306900177915	4.06261563378054	1.57343997373437	0.125277002782442	0.812209475329188	-4.93493735922508
a_HQ_transcript/139607	-1.83845515781125	0.112943666612831	-1.57335777355534	0.125296033536163	0.812209475329188	-4.63198040625019
a_HQ_transcript/119026	1.83711856479513	1.75303719001577	1.57332890030738	0.125302718748682	0.812209475329188	-4.80230289896607
a_HQ_transcript/37201	-2.65042631488352	0.836391038324541	-1.57331816819932	0.125305203698229	0.812209475329188	-4.61425653944185
a_HQ_transcript/67804	1.63972588661589	0.0962611259137335	1.57329850580349	0.125309756501878	0.812209475329188	-4.64846120946995
a_HQ_transcript/148019	1.61373723189567	1.24705890037428	1.57329702667471	0.12531009899779	0.812209475329188	-4.5928506770249
a_HQ_transcript/99604	2.03977187406503	1.82503400934695	1.57316162032013	0.125341455906958	0.812209475329188	-4.68781885034515
a_HQ_transcript/152818	1.66874607761826	0.357030487180482	1.57311267514379	0.125352792026751	0.812209475329188	-4.68346643947639
a_HQ_transcript/2605	-2.14518305292541	-0.21585441570388	-1.57310230115799	0.125355194837629	0.812209475329188	-4.66971084508348
a_HQ_transcript/92651	0.353983486282244	-0.777422126402508	1.57288504331621	0.125405524485013	0.812209475329188	-4.82578567973523
a_HQ_transcript/18032	1.3995229346541	1.1086694517448	1.57283183989755	0.125417852026106	0.812209475329188	-4.71160975852931
a_HQ_transcript/47617	-1.51341433803187	-0.481147389377458	-1.57278370325427	0.125429006417472	0.812209475329188	-4.69594878942665
a_HQ_transcript/113077	1.19308102091107	3.16315876490974	1.57264542258408	0.125461053798332	0.812209475329188	-4.73588481285738
a_HQ_transcript/116	2.42214483291931	3.1779134056478	1.57263785906948	0.12546280688113	0.812209475329188	-4.99304682520734
a_HQ_transcript/97956	2.49315058285415	0.809052596298763	1.57254807011079	0.125483619827338	0.812209475329188	-4.63068769342135
a_HQ_transcript/146900	-0.787630146374875	2.42667018186562	-1.57254622382721	0.125484047822652	0.812209475329188	-4.67695000236699
a_HQ_transcript/57708	1.02929452269352	3.86333882242668	1.57254442000157	0.125484465976746	0.812209475329188	-4.96159451217284
a_HQ_transcript/17599	1.16885377302483	2.70201705086665	1.57249890320573	0.125495017835269	0.812209475329188	-4.83809442865189
a_HQ_transcript/24683	2.24799649711831	0.857465402722845	1.57243897150974	0.125508912508564	0.812209475329188	-4.6302511917785
a_HQ_transcript/12996	0.737379725814879	2.3440542133422	1.57242573915834	0.125511980490017	0.812209475329188	-4.74984363191662
a_HQ_transcript/37262	0.560879837873965	4.46603208234263	1.57240719622139	0.125516279858021	0.812209475329188	-5.08665175772069
a_HQ_transcript/31344	2.71114116057105	0.798621035917436	1.57239512126299	0.125519079624159	0.812209475329188	-4.60276286029781
a_HQ_transcript/94708	0.479075560347919	3.94941803817688	1.57234694064231	0.125530251554363	0.812209475329188	-5.05040814566871
a_HQ_transcript/89371	1.91187686411758	2.36082638570602	1.57234193553352	0.125531412165521	0.812209475329188	-4.76527964353448
a_HQ_transcript/132529	-1.86921966952127	0.196026083057179	-1.57222257132483	0.125559093563752	0.812298162568035	-4.65811581251363
a_HQ_transcript/134467	1.37977463537872	3.07320504744464	1.57200632063247	0.125609256295107	0.812444496627482	-4.92269316580476
a_HQ_transcript/40528	-2.55382378085829	0.622219110445452	-1.57199828325792	0.125611121004793	0.812444496627482	-4.71158275917878
a_HQ_transcript/83517	1.71237366473077	-0.323143124705472	1.57190457210098	0.125632864111326	0.812444496627482	-4.65505817760957
a_HQ_transcript/114429	0.941086169041082	0.290260210920463	1.57188185836441	0.125638134673629	0.812444496627482	-4.76189514346844
a_HQ_transcript/139554	2.49638284852673	2.08881539847382	1.57182384578617	0.125651596901339	0.812444496627482	-4.6660787448446
a_HQ_transcript/132659	3.01786501077077	1.08910322676977	1.57169103214611	0.125682421671323	0.812553420855376	-4.73675864078783
a_HQ_transcript/94067	-1.81093254486732	0.501407321648826	-1.5713363685815	0.125764765893354	0.812951093473884	-4.8106660995551
a_HQ_transcript/33844	1.06547607648985	2.34353303118464	1.57130563692671	0.125771903103679	0.812951093473884	-4.7141865003094
a_HQ_transcript/100052	-1.77745548576717	0.0474981756615917	-1.57116671977372	0.125804169753958	0.812986836924807	-4.68261927781724
a_HQ_transcript/79126	-1.50979295020482	0.205048026070191	-1.57112699515987	0.1258133979346	0.812986836924807	-4.61017167223736

a_HQ_transcript/150399	0.484486416648463	5.15184958597463	1.57110119619558	0.125819391428839	0.812986836924807	-5.19023867902113
a_HQ_transcript/21395	1.12309831897757	2.50569622578105	1.57098816384073	0.125845653318732	0.813048798160007	-4.7262800299024
a_HQ_transcript/132810	1.46395776129668	-0.184009113116865	1.57093952639972	0.125856955096403	0.813048798160007	-4.7427826639979
a_HQ_transcript/33525	-0.913475887911376	1.49954732386387	-1.57062079682914	0.125931038080754	0.813436979837548	-4.76228244951589
a_HQ_transcript/136704	-3.39959431998404	1.23601733135384	-1.57025054568385	0.126017140959053	0.813561443492027	-4.71964824080124
a_HQ_transcript/837	-0.80523673008685	2.20320499906332	-1.5702275141268	0.126022498593197	0.813561443492027	-4.6755652061365
a_HQ_transcript/49925	1.53989500814709	-0.068858958283116	1.57021203125059	0.12602610034663	0.813561443492027	-4.7247386271735
a_HQ_transcript/18689	1.17789719843549	5.29796122140205	1.57019256174013	0.126030629622655	0.813561443492027	-5.20428435024106
a_HQ_transcript/72417	0.679131151619263	2.80619539708131	1.5701074088004	0.126050440676721	0.813561443492027	-4.84946131610947
a_HQ_transcript/87103	-4.48351235036962	1.44389071075704	-1.57008330303599	0.126056049406792	0.813561443492027	-4.7567103718938
a_HQ_transcript/79679	1.39331925114524	-0.0170441478597083	1.57005809914345	0.126061913857764	0.813561443492027	-4.68966548886896
a_HQ_transcript/87704	-1.6426310402269	-0.428510918622765	-1.5700559668418	0.126062410012699	0.813561443492027	-4.68482672116356
a_HQ_transcript/146634	2.15410424107379	0.126654866766497	1.56991720542426	0.126094701156297	0.813561443492027	-4.70344222126581
a_HQ_transcript/46449	-2.14500717079901	0.714875632609817	-1.56988025742313	0.126103300455176	0.813561443492027	-4.6251746560702
a_HQ_transcript/115662	-0.448626265417018	4.18096955056748	-1.56987612139425	0.126104263106647	0.813561443492027	-5.07745362167961
a_HQ_transcript/32947	-1.40778019558472	3.76663130378677	-1.56975386622061	0.126132720429959	0.813654730124587	-4.98824098753092
a_HQ_transcript/144465	2.32036979013076	2.19332649789105	1.56958256360302	0.126172603325848	0.813784864894922	-4.73506245702039
a_HQ_transcript/63382	1.14302598535469	-0.528935620528106	1.56953999919026	0.126182514821181	0.813784864894922	-4.7112568490233
a_HQ_transcript/54026	-2.50536954243618	1.24165586077777	-1.5694868432034	0.126194893548081	0.813784864894922	-4.78260428882367
a_HQ_transcript/110145	1.56503758038315	-0.0345427820034334	1.56933819426398	0.126229515497424	0.813843739776665	-4.75097974376582
a_HQ_transcript/15701	2.56693506585835	1.37659753861687	1.56932742039808	0.126232025148404	0.813843739776665	-4.64298172791061
a_HQ_transcript/68550	-1.86189143411952	2.24732644785628	-1.56918694346534	0.126264751392809	0.813905689821797	-4.70193147346253
a_HQ_transcript/53376	-2.04541049521232	1.24460157325091	-1.5690517072969	0.126296263252871	0.813905689821797	-4.99684882884261
a_HQ_transcript/94142	0.870616935296196	-0.812027653014932	1.56903325260628	0.126300563942794	0.813905689821797	-4.73618393840005
a_HQ_transcript/15193	-2.38442090871613	-0.168231070372998	-1.56901586992517	0.126304614920251	0.813905689821797	-4.72248002890099
a_HQ_transcript/144180	1.22626471900409	3.05658818976401	1.56898571040557	0.126311643749698	0.813905689821797	-4.81046818002117
a_HQ_transcript/85052	-0.674172186404648	2.46709824352904	-1.56890191498569	0.126331174371601	0.813916669964159	-4.7721970805808
a_HQ_transcript/83706	1.30781451834587	1.15445794772536	1.5688582521291	0.12634135206934	0.813916669964159	-4.62038794399254
a_HQ_transcript/78033	-2.69217035770664	0.296660030552627	-1.56860491658798	0.126400417107031	0.814206942109653	-4.76216681451726
a_HQ_transcript/73505	1.48336798349262	0.558808025714522	1.56850776161547	0.12642307470183	0.814262657758224	-4.75292475148566
a_HQ_transcript/32371	0.803024887322847	0.427525156946562	1.56833141710689	0.126464208610961	0.814437349671829	-4.60389237977148
a_HQ_transcript/6361	-2.64601069518895	5.26021612607153	-1.56812608001312	0.126512119036276	0.814607516865555	-5.19465307222754
a_HQ_transcript/99177	3.52243126038761	1.10986947445488	1.56809805177063	0.126518659893033	0.814607516865555	-4.83923419922636
a_HQ_transcript/7664	-1.56508825833844	3.01026395575414	-1.56802418097621	0.126535900192073	0.814628287336893	-5.02166456664029
a_HQ_transcript/4497	-2.42912964213127	0.76217041008235	-1.56794747155097	0.12655380500926	0.814653330850304	-4.69134703354087
a_HQ_transcript/32512	1.75958276700666	2.78150120809063	1.56786877975827	0.126572174677373	0.814681360828962	-4.69941469146235
a_HQ_transcript/119328	0.925167795150559	2.30404686464547	1.56772813220173	0.126605012596644	0.814694507121924	-4.68916071262915
a_HQ_transcript/139709	2.23606664264735	2.62256516438881	1.56771122208573	0.126608961181105	0.814694507121924	-4.85000440300256
a_HQ_transcript/97877	1.92164387054441	-0.0682045369537596	1.56763865543769	0.126625906943056	0.814694507121924	-4.73548909598815
a_HQ_transcript/133459	2.52451594115565	1.3046478550521	1.56761993326952	0.126630279243163	0.814694507121924	-4.71909628148467
a_HQ_transcript/15312	-0.555085382568874	2.07010344682136	-1.56755649482277	0.126645095318146	0.814699657000132	-4.67880821545045
a_HQ_transcript/104121	3.13318501834666	1.45733835004483	1.56740586474186	0.126680280692186	0.814835825614186	-4.74332791345219
a_HQ_transcript/129354	-2.74940513502173	1.03983628715035	-1.56725421885579	0.126715711389607	0.814844881304469	-4.74804012086001
a_HQ_transcript/16153	-1.57511345892809	2.37052348312156	-1.56722401655589	0.126722768849191	0.814844881304469	-4.8259303275496
a_HQ_transcript/31600	-1.76524276410401	2.76193177169828	-1.56718861010228	0.126731042786258	0.814844881304469	-4.86017899741248
a_HQ_transcript/97884	1.31542594425878	1.48031578415603	1.56713400806025	0.12674380329611	0.814844881304469	-4.65795570593424
a_HQ_transcript/132623	-0.957175466905333	0.516593215409346	-1.56700069907187	0.126774962034435	0.814844881304469	-4.64026647841816
a_HQ_transcript/74321	2.80339732539797	3.40247671914589	1.56695059894672	0.12678667371069	0.814844881304469	-4.78005218409343
a_HQ_transcript/9711	2.39065031412848	1.11323523133167	1.56693370611036	0.12679062287016	0.814844881304469	-4.61539775910845
a_HQ_transcript/48004	-3.60113323421956	3.2096931140786	-1.56691997287615	0.12679383346062	0.814844881304469	-4.91404589559611

a_HQ_transcript/91491	1.47536611701163	-0.683839906177028	1.56685953581796	0.126807963376923	0.814845599731793	-4.73484607336486
a_HQ_transcript/91840	1.442908826549	-0.674820721971635	1.56676188548013	0.126830796303329	0.81490223582623	-4.72468065445263
a_HQ_transcript/5187	-2.5474380106977	0.83458958602469	-1.56657932523055	0.126873492129549	0.814965698738201	-4.65327371381708
a_HQ_transcript/63099	1.83796050257196	1.16106135947482	1.56648726933817	0.126895025912731	0.814965698738201	-4.66420410851026
a_HQ_transcript/85976	-1.25568148199129	-0.251104359215314	-1.56646581925155	0.126900043960624	0.814965698738201	-4.65434257080404
a_HQ_transcript/26682	-1.6487714079969	0.324224766412636	-1.56639598161976	0.126916382943558	0.814965698738201	-4.65783282024056
a_HQ_transcript/91161	0.862290638838477	3.843472751192	1.5663302040243	0.126931773620535	0.814965698738201	-5.07746903106687
a_HQ_transcript/43718	-1.08174945712222	2.76157188013945	-1.56631659914045	0.126934957088239	0.814965698738201	-4.8123430436145
a_HQ_transcript/7206	1.352079802664	-0.360738066243869	1.56630011246439	0.126938814966551	0.814965698738201	-4.76613806349771
a_HQ_transcript/471	2.24167874882534	1.0738348161309	1.56618510261784	0.12696572990018	0.814969701310996	-4.67181609494965
a_HQ_transcript/16890	0.919716297631748	1.05245569039867	1.56614963514579	0.12697403103713	0.814969701310996	-4.63990808042176
a_HQ_transcript/4069	2.05769056191371	1.38299764992943	1.56611772830908	0.126981499187546	0.814969701310996	-4.73814642798023
a_HQ_transcript/39106	-2.24450616167761	3.34125899062887	-1.56586015689615	0.127041799745347	0.815172387110352	-4.99435411251755
a_HQ_transcript/51740	-2.10202781405938	3.02811736773854	-1.56585085303321	0.127043978327581	0.815172387110352	-5.0936012388806
a_HQ_transcript/21497	-1.41202915851781	1.94345367130265	-1.56580314028383	0.127055151170172	0.815172387110352	-4.8053918071647
a_HQ_transcript/144422	-1.52056037980629	-0.216990958509965	-1.56551701693534	0.127122169151077	0.815466592177486	-4.68877536127978
a_HQ_transcript/30915	2.3890363013591	3.21814482203445	1.56548758544965	0.12712906444936	0.815466592177486	-4.96086810477263
a_HQ_transcript/47804	2.60850358861809	1.87712064442375	1.56540121324273	0.127149301751941	0.815506412030986	-4.75188336617248
a_HQ_transcript/106407	3.12494217972324	0.48979854199346	1.56529274437357	0.127174720093433	0.815534868537376	-4.71605285813699
a_HQ_transcript/131261	3.27688162192715	0.991362307118693	1.56526254149683	0.127181798501433	0.815534868537376	-4.8416381230433
a_HQ_transcript/103497	2.66209854928796	3.90286572712162	1.56516509016272	0.127204639582924	0.815591362196801	-4.85470956931262
a_HQ_transcript/1294	-1.17117641993361	2.32414229486502	-1.56492599529001	0.12726069386387	0.815779432043187	-4.67117216710359
a_HQ_transcript/34695	-1.71224884722329	1.8325526328154	-1.56488512679489	0.127270277236443	0.815779432043187	-4.61080630657489
a_HQ_transcript/8682	-2.03790182494197	2.18948664822354	-1.56485021095907	0.127278465218016	0.815779432043187	-4.77339580804645
a_HQ_transcript/97166	4.34043007321759	1.8734770717251	1.56472938285515	0.127306803473267	0.815779432043187	-4.84131813242888
a_HQ_transcript/66814	-1.8571750396758	2.19601349910786	-1.56469040610055	0.127315945929201	0.815779432043187	-4.61983360493005
a_HQ_transcript/132298	2.21305202078565	0.833568751180073	1.56468089370175	0.127318177255295	0.815779432043187	-4.6806741332814
a_HQ_transcript/29952	-1.51526135855921	-0.516370426004091	-1.56459559026859	0.127338188328869	0.81580923809705	-4.66065647507551
a_HQ_transcript/150939	1.51861988692922	7.39049025060083	1.56454141498423	0.127350898473931	0.81580923809705	-5.37825114617738
a_HQ_transcript/122302	-1.21923732922516	0.440327983222764	-1.56447082963714	0.12736746015802	0.815825424139435	-4.58384205027204
a_HQ_transcript/124983	4.42085483038441	0.252507154044263	1.56430453551286	0.127406485255229	0.815878951144138	-4.75894246056884
a_HQ_transcript/1566	-0.798412912904109	2.5424791972665	-1.56426747770498	0.127415183127734	0.815878951144138	-4.71540079624301
a_HQ_transcript/148211	-1.39740996767922	0.369271619304699	-1.56420532068805	0.127429773144282	0.815878951144138	-4.75144117311701
a_HQ_transcript/87857	-0.445559595299291	3.24406352246471	-1.56419600270409	0.127431960456882	0.815878951144138	-4.92101469095306
a_HQ_transcript/120404	1.4943608867877	-0.269460976943481	1.56404395599617	0.127467656367281	0.815950290099493	-4.76921623735677
a_HQ_transcript/56925	3.04160327994627	1.90189489398231	1.56401815784417	0.127473713790901	0.815950290099493	-4.85416674417172
a_HQ_transcript/152520	1.61430657368566	-0.396020336808747	1.56389397823646	0.127502874525885	0.815950290099493	-4.76343875135172
a_HQ_transcript/6865	1.67589143139275	1.54178120193367	1.56385371311658	0.12751233103225	0.815950290099493	-4.66680349329247
a_HQ_transcript/118233	1.22682451392977	1.36934305486987	1.56384963632585	0.12751328852298	0.815950290099493	-4.69438322733788
a_HQ_transcript/54658	1.49921439043918	1.42765091904815	1.56375683854076	0.127535084950085	0.815999935936001	-4.68944549371762
a_HQ_transcript/82671	1.05316520104067	-0.451590332058733	1.56352341317092	0.127589925519756	0.816200149177946	-4.68691833860195
a_HQ_transcript/60278	1.97738078442249	0.494615144699756	1.56350411722219	0.127594459736788	0.816200149177946	-4.65761059616724
a_HQ_transcript/130869	3.10454997870124	1.37866611641927	1.56342424351091	0.127613230084255	0.816230395943833	-4.63861773573098
a_HQ_transcript/132638	2.57139685189997	-0.197781127505069	1.56312836285807	0.127682781715697	0.81658540384751	-4.71505207203163
a_HQ_transcript/129620	-0.323414444568357	6.02490191105701	-1.5628395566011	0.127750700145007	0.816659212166425	-5.27231849627029
a_HQ_transcript/111978	-2.01787026903893	1.86707324197169	-1.56279239523312	0.127761793853567	0.816659212166425	-4.77350782686095
a_HQ_transcript/19939	-2.73010733894217	1.80126001450058	-1.56273788827808	0.127774616431907	0.816659212166425	-4.74031837118011
a_HQ_transcript/69011	-1.75888720797914	0.368885204005859	-1.56272238915035	0.127778262740956	0.816659212166425	-4.7338175701695
a_HQ_transcript/12312	1.40065266167547	2.20644697197544	1.56267680648451	0.127788986963377	0.816659212166425	-4.72855179913128
a_HQ_transcript/132148	-0.384370542660463	3.28275841282999	-1.56260091713356	0.127806843056942	0.816659212166425	-4.9703939148502

a_HQ_transcript/138230	3.09285335854684	2.86660143823303	1.56257622146953	0.127812654166294	0.816659212166425	-4.70099002440559
a_HQ_transcript/77768	1.91612035000454	-0.0984335372177345	1.56254798684714	0.127819298287463	0.816659212166425	-4.73416456302985
a_HQ_transcript/138192	1.72414545686626	1.04618839813168	1.56254174795048	0.127820766451454	0.816659212166425	-4.64570473223948
a_HQ_transcript/109165	2.59976870945055	1.53583030094291	1.56240857435588	0.127852108707498	0.816735794188924	-4.73726493326386
a_HQ_transcript/88222	-1.72536125910656	2.01934321019725	-1.56237141982701	0.127860854099904	0.816735794188924	-4.79640820110969
a_HQ_transcript/131581	-3.39381726149623	2.27204096714361	-1.56220194076471	0.127900752071226	0.816812532710948	-4.74364309031546
a_HQ_transcript/82049	2.33947421123808	2.93563830091343	1.56220100869054	0.127900971523793	0.816812532710948	-4.89652227931366
a_HQ_transcript/138062	2.02336255185509	0.629116074676367	1.56207222656046	0.127931295628652	0.816843840667442	-4.6577174138891
a_HQ_transcript/73579	1.72499970620827	-0.50740957698497	1.56202501625972	0.127942413621625	0.816843840667442	-4.71601347929798
a_HQ_transcript/111649	1.53336872013237	1.03881663062616	1.56194618775881	0.127960979431099	0.816843840667442	-4.60446541539234
a_HQ_transcript/128090	1.71015218288301	1.75164984497749	1.56194149857869	0.127962083903071	0.816843840667442	-4.63064088148543
a_HQ_transcript/116211	2.18266983851767	-0.0215030462630966	1.56180467290244	0.127994314727345	0.816898845364307	-4.7258344096601
a_HQ_transcript/149034	-1.25401024852735	2.37191463118093	-1.56174675896206	0.128007958995765	0.816898845364307	-4.7160952912007
a_HQ_transcript/128427	1.8484261446717	2.35641625850375	1.56161623641644	0.128038713869883	0.816898845364307	-4.81133797769565
a_HQ_transcript/32339	-1.16157949895993	1.32342028361191	-1.56161614759498	0.128038734800827	0.816898845364307	-4.5925410292741
a_HQ_transcript/111074	1.55993668553473	-0.610200972316775	1.56157255452887	0.128049007924354	0.816898845364307	-4.73294448783497
a_HQ_transcript/115545	-0.869872258903706	1.89192366586216	-1.56146643632413	0.128074018498923	0.816898845364307	-4.70224447436453
a_HQ_transcript/139698	0.976287696470622	3.66051342363571	1.56139861100831	0.128090006057785	0.816898845364307	-4.89006109047766
a_HQ_transcript/34756	1.97858573864467	1.09299382727104	1.5613760100914	0.128095333831751	0.816898845364307	-4.64394133600589
a_HQ_transcript/50074	-2.27286998276014	1.16132670803012	-1.56136817161195	0.128097181659367	0.816898845364307	-4.73714883764566
a_HQ_transcript/32432	-2.51152286814118	2.12519482756646	-1.5612191742584	0.128132310124552	0.817033229806931	-4.63319844282398
a_HQ_transcript/135007	1.34721330911261	0.899372677781787	1.56094006515101	0.128198135569793	0.817306523653338	-4.66451160693951
a_HQ_transcript/41427	-2.38126687114574	0.272014144691746	-1.56078959948802	0.128233632993587	0.817306523653338	-4.72700706138519
a_HQ_transcript/45820	-0.377689921569366	4.43665946663192	-1.56078701396563	0.128234243032419	0.817306523653338	-5.13049341719308
a_HQ_transcript/89190	3.33066543842849	3.17360929763562	1.56075345985588	0.128242160141801	0.817306523653338	-4.92457430064867
a_HQ_transcript/134061	1.62709976781685	4.27522308693663	1.5606367367517	0.128269704115554	0.817306523653338	-5.14773570929439
a_HQ_transcript/41829	2.11262469825547	0.592548504683165	1.5605977861022	0.128278896645739	0.817306523653338	-4.70395905257338
a_HQ_transcript/148351	2.96488469200133	4.58685289400051	1.56052886131951	0.128295164520661	0.817306523653338	-5.13250222428047
a_HQ_transcript/57499	-1.95205287850539	0.307862147114966	-1.56052529675894	0.128296005886694	0.817306523653338	-4.66835312237205
a_HQ_transcript/9496	-1.4740123063155	1.40594548666699	-1.56050111429257	0.128301713947439	0.817306523653338	-4.63168850284563
a_HQ_transcript/120122	-2.14028346580336	0.440712041156907	-1.56031787563725	0.128344972550958	0.817442704977321	-4.73875044946022
a_HQ_transcript/61316	-2.79193297639719	1.65193094923394	-1.56026855564075	0.128356617940074	0.817442704977321	-4.77244266614975
a_HQ_transcript/130931	0.993287533587579	2.81547068626628	1.56023187175134	0.128365280261371	0.817442704977321	-4.82901113025228
a_HQ_transcript/55085	-0.463507531335722	3.21076804632375	-1.56014245951104	0.128386395542162	0.817455656854459	-4.93463587815395
a_HQ_transcript/31052	-1.59825940597925	0.452283796814242	-1.56008341233952	0.12840034146375	0.817455656854459	-4.58357139470297
a_HQ_transcript/91716	-4.37180502253219	1.99396209747072	-1.56004462424256	0.128409503212532	0.817455656854459	-4.84620060398746
a_HQ_transcript/105022	1.91042128406499	0.409427842529699	1.55986570297452	0.128451771300757	0.817544594330895	-4.76606362798606
a_HQ_transcript/141810	2.28782796966952	1.04521858326477	1.55978755991948	0.128470235247301	0.817544594330895	-4.76676487521561
a_HQ_transcript/56083	2.49014651007806	4.29799802451409	1.55975336175514	0.128478316402295	0.817544594330895	-5.18886574253916
a_HQ_transcript/145905	-0.830708116235241	4.2593396922016	-1.55965393252839	0.1285018142514	0.817544594330895	-5.0910109981723
a_HQ_transcript/40863	1.14264423665923	1.74619757648721	1.55956914281562	0.128521855143548	0.817544594330895	-4.66018041325482
a_HQ_transcript/131093	1.71919253086042	2.21907200777635	1.55956483038225	0.128522874498005	0.817544594330895	-4.65413844164543
a_HQ_transcript/146275	0.446050353103569	3.31507161644288	1.55951733760373	0.128534101071727	0.817544594330895	-4.94405951132421
a_HQ_transcript/117967	2.83997853507964	0.918896456554332	1.55946845074964	0.128545658016795	0.817544594330895	-4.73460279169442
a_HQ_transcript/44913	3.73462991013932	1.6500279881217	1.5593319702681	0.128577926734586	0.817544594330895	-4.8696269452842
a_HQ_transcript/48299	1.80539523838023	0.0797228372073374	1.55933080298947	0.128578202748135	0.817544594330895	-4.69152237682616
a_HQ_transcript/127329	1.37372246137073	0.931316937896556	1.55929179329699	0.128587427218515	0.817544594330895	-4.7133685015627
a_HQ_transcript/152347	-6.39782606470782	2.13895876855995	-1.55926536902054	0.128593675970221	0.817544594330895	-4.74383008983524
a_HQ_transcript/62625	1.98707671586572	0.0906253604836256	1.5592119330214	0.128606313146189	0.817544594330895	-4.68776429754488
a_HQ_transcript/97513	1.19299585755121	2.08431422352465	1.55873015867016	0.128720294437944	0.81796534837193	-4.68837877991117

a_HQ_transcript/72839	2.43180349138796	0.403561023781629	1.55871008692622	0.128725044922806	0.81796534837193	-4.76144012314257
a_HQ_transcript/102641	1.62167242915416	0.205367575085222	1.55869748421918	0.128728027744405	0.81796534837193	-4.72500145501315
a_HQ_transcript/16218	1.31079791926152	1.68077958476679	1.55869427079301	0.128728788310391	0.81796534837193	-4.62143663234263
a_HQ_transcript/65296	3.30190273202282	1.99288945826663	1.55850268569822	0.128774140006272	0.818164084630515	-4.79918150687704
a_HQ_transcript/88098	1.67490090503419	0.536745504768313	1.55830248114839	0.12882154596531	0.818375827745523	-4.70416619734194
a_HQ_transcript/48024	-1.11751805916345	1.40745301211801	-1.55821382776352	0.128842542521747	0.818419769610325	-4.72742870872148
a_HQ_transcript/90675	1.79393399398504	3.54825417044263	1.55808615248969	0.12887278586242	0.818512400241693	-5.01209360956196
a_HQ_transcript/78306	1.90560688495599	1.00344191410603	1.55803338214532	0.128885287630956	0.818512400241693	-4.70346831862083
a_HQ_transcript/75634	0.74402222259962	3.96339032995458	1.55775895202751	0.128950318488743	0.818749569686243	-5.09618429307046
a_HQ_transcript/92506	1.54474415492078	-0.217265401065001	1.5577569053675	0.128950803579644	0.818749569686243	-4.74305957585459
a_HQ_transcript/54316	1.6169566938612	2.24340490884209	1.55754963513009	0.12899993759833	0.81893288329492	-4.65240147921215
a_HQ_transcript/26110	-2.0692346403139	3.29196912206786	-1.55749381336025	0.129013172913965	0.81893288329492	-4.99031605881978
a_HQ_transcript/24894	-0.845850092722236	2.36418444305489	-1.5574568383027	0.12902194029065	0.81893288329492	-4.83023960413944
a_HQ_transcript/7952	2.11745740515727	0.228762162102394	1.55729510274128	0.129060296068477	0.819086897026799	-4.69133569874834
a_HQ_transcript/60836	-1.23243407004064	1.70672144515102	-1.55714197472315	0.129096619099087	0.819172414477396	-4.62252636031479
a_HQ_transcript/56160	-0.442447194948792	3.68059112865333	-1.55688973949857	0.129156469165617	0.819172414477396	-5.01494729128547
a_HQ_transcript/143561	1.65770593343227	-0.0131827047183621	1.55682256428642	0.129172412220551	0.819172414477396	-4.71045375530223
a_HQ_transcript/3766	-1.87472853457569	2.02280200982197	-1.55681746079226	0.129173623525687	0.819172414477396	-4.68937406302021
a_HQ_transcript/152785	3.2067924007463	2.30783270868884	1.55680242249857	0.129177192891264	0.819172414477396	-4.99441007858827
a_HQ_transcript/60119	-1.11171284504887	0.261324522370028	-1.55665743587334	0.129211609835435	0.819172414477396	-4.63421086050986
a_HQ_transcript/1649	2.32588535708257	1.24415853109078	1.55660454479509	0.12922416698178	0.819172414477396	-4.80917903516386
a_HQ_transcript/30270	-0.472042481862232	3.43539599995894	-1.55656362077424	0.12923388364732	0.819172414477396	-5.00512164427698
a_HQ_transcript/36978	1.40055022094878	-0.535224407380983	1.55656308827617	0.129234010083229	0.819172414477396	-4.68373154383756
a_HQ_transcript/14250	2.74441512986981	1.89689257378459	1.55644208141855	0.129262744460765	0.819172414477396	-4.81552511243212
a_HQ_transcript/4517	-2.20575089087642	1.55627766870713	-1.5564220144554	0.129267510078533	0.819172414477396	-4.62026976850093
a_HQ_transcript/134488	0.626532158585581	2.85504467941776	1.55641636502609	0.129268851763242	0.819172414477396	-4.88294747458109
a_HQ_transcript/118807	0.501585576479381	5.45898488078851	1.55641612983557	0.129268907618958	0.819172414477396	-5.25226586697579
a_HQ_transcript/113620	0.652581677199745	4.07440655946938	1.55634345505217	0.129286168191042	0.819172414477396	-5.08963214924423
a_HQ_transcript/148572	5.11200331721413	2.49655222531534	1.55631639545468	0.129292595439793	0.819172414477396	-4.81288039123128
a_HQ_transcript/125404	1.1530845555511	4.65834361035971	1.55628837283905	0.129299251700215	0.819172414477396	-5.20365487598558
a_HQ_transcript/38622	1.12348692211722	-0.571926436201109	1.55606771980361	0.129351673528734	0.819406817051104	-4.71792258913364
a_HQ_transcript/6118	-0.464507870139209	2.57538119342217	-1.55601398052049	0.129364443298892	0.819406817051104	-4.8162651996124
a_HQ_transcript/19777	1.81629951943739	0.34384120871853	1.5558593692796	0.129401188431388	0.81954446475744	-4.68955849026589
a_HQ_transcript/148728	1.46024213668285	-0.0734744236562399	1.55580390401437	0.129414372454042	0.81954446475744	-4.75871307032065
a_HQ_transcript/76631	-1.06130158728402	3.18081505960298	-1.55570521501782	0.129437833401428	0.819603745093487	-4.87334581332384
a_HQ_transcript/363	-1.45101637827895	1.4917404782795	-1.5556340320385	0.129454757596843	0.819621626139779	-4.6445106813748
a_HQ_transcript/1285	0.659441930023799	-0.522096645887377	1.55551660142558	0.129482681384519	0.819709137826109	-4.71610669236173
a_HQ_transcript/23558	-1.10608552660789	3.68371576452967	-1.55529539013439	0.129535296440399	0.819952925132037	-5.12001877906047
a_HQ_transcript/12258	2.24273337410848	2.58071140992559	1.55509322483635	0.129583396603187	0.820168083378707	-4.75103300787044
a_HQ_transcript/2756	0.840167342791816	1.72237472824716	1.55424696272881	0.129784900992509	0.821253980213037	-4.62226598266528
a_HQ_transcript/146674	-1.33332679359591	0.317521968374333	-1.554201061681	0.129795837814823	0.821253980213037	-4.71537257574702
a_HQ_transcript/75469	1.6651302058437	1.12665545834694	1.55408961264993	0.12982239584196	0.821253980213037	-4.64852534530221
a_HQ_transcript/106972	1.500982717641	0.0247285267678738	1.55395880716831	0.129853572094064	0.821253980213037	-4.65276231902462
a_HQ_transcript/47080	3.82782066203437	1.17687486591005	1.55394999690474	0.129855672155835	0.821253980213037	-4.65810732591803
a_HQ_transcript/11675	1.58053360460612	0.396568184685186	1.55392511916086	0.129861602297504	0.821253980213037	-4.65083373925713
a_HQ_transcript/138155	1.46299524336905	-0.122785156796286	1.55392494695209	0.129861643347911	0.821253980213037	-4.59133004940966
a_HQ_transcript/35021	-1.53555701451255	0.680912155803502	-1.5537187762219	0.129910797024513	0.821253980213037	-4.65836105141359
a_HQ_transcript/125425	1.64956278283727	-0.386680244581316	1.55364178170436	0.129929157354301	0.821253980213037	-4.74696793005213
a_HQ_transcript/32846	0.668332089659697	2.18704309832388	1.5536228310154	0.129933676712256	0.821253980213037	-4.75163065028119
a_HQ_transcript/18470	1.59859595903523	1.35937257776546	1.55361892706374	0.129934607742025	0.821253980213037	-4.76504923200375

a_HQ_transcript/35619	1.42376346423609	2.12116215851659	1.55358332479856	0.129943098560506	0.821253980213037	-4.6584181193258
a_HQ_transcript/34908	-0.942824863093199	1.93356816344416	-1.55357229979807	0.129945728014872	0.821253980213037	-4.6511609799224
a_HQ_transcript/35377	2.40795249648847	0.0611377268373934	1.55354281084782	0.129952761319837	0.821253980213037	-4.73695729115166
a_HQ_transcript/83202	1.83407558953661	-0.515648328250483	1.55327472286237	0.130016716219027	0.821528108147346	-4.74866485553253
a_HQ_transcript/104092	1.82396203690791	0.480563378250118	1.55324250138367	0.130024404675124	0.821528108147346	-4.75842886159762
a_HQ_transcript/19216	-0.985591721275185	1.66970311122098	-1.55301310506789	0.130079152237515	0.821734250081327	-4.66839761744142
a_HQ_transcript/131337	0.687095443869818	2.39250001588937	1.55293060198476	0.130098846941943	0.821734250081327	-4.82855757134723
a_HQ_transcript/50934	-1.33705494193144	1.43745566591366	-1.55286075942504	0.130115521285219	0.821734250081327	-4.69228216294198
a_HQ_transcript/138769	1.24732965121717	-0.607995016684632	1.55282410394366	0.130124273177838	0.821734250081327	-4.73170985570467
a_HQ_transcript/58247	1.64688026688075	0.140427796529567	1.55280969241594	0.130127714216877	0.821734250081327	-4.78333431784347
a_HQ_transcript/55205	0.648664530885394	2.95809362230201	1.55271515474713	0.130150288799119	0.821787528493936	-4.85091738767255
a_HQ_transcript/139152	1.52067062267222	-0.365084156246639	1.55239776631457	0.130226101019256	0.821983839789646	-4.73776095637365
a_HQ_transcript/16724	-1.13677693649667	1.6107769546753	-1.552352189806	0.130236990485694	0.821983839789646	-4.71725604687232
a_HQ_transcript/41508	1.55128292759645	0.82803937132844	1.55234272267172	0.130239252534092	0.821983839789646	-4.64481385399761
a_HQ_transcript/115401	-1.90158428371037	1.75153043099196	-1.55221979115449	0.130268628315569	0.821983839789646	-4.8325771380218
a_HQ_transcript/125746	1.42372235421748	-0.648280860724081	1.55219324294362	0.130274972996703	0.821983839789646	-4.74442295922291
a_HQ_transcript/110028	2.42347825262445	1.83049549018621	1.55213324303186	0.130289313128747	0.821983839789646	-4.81401804402745
a_HQ_transcript/3270	-3.41273617347581	2.91442018037655	-1.55208442956148	0.130300980618322	0.821983839789646	-4.88095018878568
a_HQ_transcript/16419	2.36115840392159	0.328368738858676	1.55206996887678	0.130304437201884	0.821983839789646	-4.60784697058913
a_HQ_transcript/137644	-3.03844372163749	1.78455218783669	-1.55205235386574	0.130308647874718	0.821983839789646	-4.9299488436847
a_HQ_transcript/631	-3.18032958734168	1.71099945811927	-1.55192963271183	0.130337986070408	0.822079693392003	-4.75329544423278
a_HQ_transcript/121731	1.43922049087105	0.527612814463908	1.55184116318871	0.130359139264955	0.822123906606629	-4.57303537637571
a_HQ_transcript/38616	1.53338842575044	-0.32746578607507	1.55156079584385	0.130426193929349	0.822457561371792	-4.73018131125665
a_HQ_transcript/91977	-2.02425651814308	1.37132050995905	-1.55131572489915	0.130484829789384	0.822700365665406	-4.69526030448183
a_HQ_transcript/122390	0.578386890564631	4.62403445783103	1.5512815650468	0.130493004600796	0.822700365665406	-5.15335375275486
a_HQ_transcript/8889	-1.6994027047358	0.213664310986209	-1.55101010886221	0.130557981698328	0.822704786816543	-4.73172022508659
a_HQ_transcript/77353	-2.59999174426648	2.57065504151086	-1.55099216455248	0.130562277862606	0.822704786816543	-4.75111814488165
a_HQ_transcript/102510	2.39682745963537	0.649874557855473	1.55094331712359	0.130573973325722	0.822704786816543	-4.6364839093764
a_HQ_transcript/6180	2.77884966895216	0.705512639504892	1.55093933654838	0.130574926426062	0.822704786816543	-4.64400944055843
a_HQ_transcript/144228	1.51552394822587	-0.541623188347429	1.55093236735598	0.130576595128059	0.822704786816543	-4.75607274314038
a_HQ_transcript/106812	1.04475359792365	2.47126792386326	1.5509238863327	0.130578625845882	0.822704786816543	-4.85902377847449
a_HQ_transcript/62168	-1.60562424333173	0.803772884511133	-1.55069018703363	0.130634593489139	0.822712542779818	-4.69829201654899
a_HQ_transcript/116169	2.26580627559599	0.864459583809033	1.55061923721873	0.130651588809035	0.822712542779818	-4.6685499079545
a_HQ_transcript/59307	1.07147076109241	1.29442229196371	1.55060919581271	0.13065399427259	0.822712542779818	-4.60612586527096
a_HQ_transcript/3542	1.42815559782857	2.21854769853103	1.55059848738373	0.130656559564076	0.822712542779818	-4.6740951082777
a_HQ_transcript/107893	2.76149522433274	3.63316634312086	1.55058728824223	0.13065924245349	0.822712542779818	-4.8868819553094
a_HQ_transcript/121121	1.41165666811067	-0.483113452358042	1.55054596206904	0.130669143023677	0.822712542779818	-4.73633195062468
a_HQ_transcript/66228	1.10883053243511	1.79087350108088	1.55046927946688	0.130687515597288	0.822712542779818	-4.66373089316193
a_HQ_transcript/105729	-2.68838797301158	0.609759922474314	-1.55040843834795	0.130702094162724	0.822712542779818	-4.69653740302366
a_HQ_transcript/116166	1.78598773206731	1.34683347324834	1.550386972597	0.130707238036258	0.822712542779818	-4.66474022248349
a_HQ_transcript/151284	-0.974022555148036	3.67940767599012	-1.55027518555726	0.13073402840636	0.822774406530616	-5.00947954799459
a_HQ_transcript/102036	3.80274780247178	1.47464398735957	1.55022784319218	0.130745375604241	0.822774406530616	-4.76550821527807
a_HQ_transcript/137307	0.560023687293355	3.66030130989477	1.55013347179379	0.130767997284539	0.822827684147616	-5.02463033642711
a_HQ_transcript/39167	-1.58705592837695	1.6207924323269	-1.54998023884594	0.130804735379289	0.822884968571772	-4.64611179520961
a_HQ_transcript/89862	0.747534790082813	2.55432665589084	1.54985757036355	0.130834151581693	0.822884968571772	-4.84366871695478
a_HQ_transcript/67771	0.812522349820276	2.60151872900255	1.54981241436398	0.130844981452876	0.822884968571772	-4.80948160744291
a_HQ_transcript/12991	2.13907646758361	0.422497655893245	1.54972382473853	0.130866230228131	0.822884968571772	-4.78536086760196
a_HQ_transcript/64593	-1.76212555823225	2.87376067612143	-1.54967554818989	0.130877810838893	0.822884968571772	-5.07090812847828
a_HQ_transcript/35226	1.46501206001458	1.18136384698052	1.54966692957334	0.130879878365885	0.822884968571772	-4.69949184440236
a_HQ_transcript/96608	3.01806111725146	1.35546110536442	1.54959749581302	0.130896535854621	0.822884968571772	-4.79662054038322

a_HQ_transcript/99648	0.489022880160721	2.4141083512573	1.54944056240836	0.130934191262716	0.822884968571772	-4.82380601945148
a_HQ_transcript/72051	0.489421706973196	4.11475035617702	1.54938569039896	0.130947359608949	0.822884968571772	-5.11914382181926
a_HQ_transcript/29118	2.2144276806863	2.77197035857353	1.54936715992767	0.130951806848719	0.822884968571772	-4.87733788613377
a_HQ_transcript/27610	1.82541574869071	1.17174178396609	1.54909846199083	0.13101630706596	0.822884968571772	-4.69433000920932
a_HQ_transcript/80127	1.99282236803137	0.663589861587873	1.54891310226518	0.131060817194747	0.822884968571772	-4.72055334472945
a_HQ_transcript/38423	1.84444211393419	0.128698087829724	1.54873586256007	0.131103388951507	0.822884968571772	-4.79060662054317
a_HQ_transcript/12227	-0.66483685506964	2.99087774955726	-1.54859696336041	0.131136759424732	0.822884968571772	-4.92881665814345
a_HQ_transcript/53410	-1.40407785247459	0.748246887289039	-1.5484543959136	0.131171018359993	0.822884968571772	-4.70865534704568
a_HQ_transcript/9579	1.35106910283617	0.609746861230298	1.54836894977836	0.1311915545332	0.822884968571772	-4.69175977530343
a_HQ_transcript/139644	1.54469071538695	-0.320925191063483	1.54833214841916	0.131200400196697	0.822884968571772	-4.7640084299864
a_HQ_transcript/116942	4.37008874660711	2.6321180088363	1.54829420227431	0.131209521530365	0.822884968571772	-4.85162703753577
a_HQ_transcript/133088	1.08765701555262	-0.692626813985528	1.54826513322611	0.131216509373159	0.822884968571772	-4.75353075540481
a_HQ_transcript/96609	1.64455719125069	1.19729222567563	1.54822067055618	0.131227198238015	0.822884968571772	-4.65249147918914
a_HQ_transcript/56373	0.583000902934968	4.35884139522829	1.54797081578584	0.131287276683731	0.822884968571772	-5.04992104726536
a_HQ_transcript/122981	2.7913072712859	1.42761912881025	1.54794233683541	0.13129412596259	0.822884968571772	-4.77628068934962
a_HQ_transcript/26991	2.66435824643912	2.61705022979841	1.54784801826082	0.131316811952057	0.822884968571772	-4.96032563385057
a_HQ_transcript/152966	0.353983486282244	-0.784339347933669	1.54783268843126	0.131320499462062	0.822884968571772	-4.84808666642828
a_HQ_transcript/82735	-2.42240053827369	1.26934854123462	-1.54778869895303	0.131331081366468	0.822884968571772	-4.62725200688385
a_HQ_transcript/83424	-2.08507126066324	1.92896947511625	-1.5477719184895	0.131335118179043	0.822884968571772	-4.8725907926149
a_HQ_transcript/54202	-2.0016057510635	0.372674477751269	-1.54767684396888	0.13135799179743	0.822884968571772	-4.73649259988261
a_HQ_transcript/139544	1.89549151187784	2.88857330565885	1.54751951235796	0.131395850712115	0.822884968571772	-4.74577756030424
a_HQ_transcript/66284	-0.747234356505177	1.9461077294586	-1.54746763059317	0.131408337030711	0.822884968571772	-4.68074526232702
a_HQ_transcript/102486	-0.881984141850546	3.78944367918769	-1.54745236842068	0.131412010341648	0.822884968571772	-5.00746257848578
a_HQ_transcript/111705	1.7218257726073	-0.478192844932715	1.5474310478857	0.131417141923196	0.822884968571772	-4.70502102353445
a_HQ_transcript/29154	0.746048655952647	2.5716985404287	1.54737774841702	0.131429971138421	0.822884968571772	-4.8057608947767
a_HQ_transcript/55960	1.93693968757452	0.623244114707772	1.54734706813061	0.131437356363129	0.822884968571772	-4.69467703620154
a_HQ_transcript/136379	1.54905417066976	0.722092237473709	1.54731603179725	0.131444827636421	0.822884968571772	-4.73033890485945
a_HQ_transcript/142264	-1.96947803087757	0.762184677884667	-1.54729158031297	0.131450714003515	0.822884968571772	-4.74217769462398
a_HQ_transcript/9166	-1.73469095746431	2.96261179838786	-1.5472769823547	0.131454228368236	0.822884968571772	-4.83460960318471
a_HQ_transcript/93371	-1.90060024100485	1.39698220069321	-1.54725588129511	0.131459308447553	0.822884968571772	-4.73136728952242
a_HQ_transcript/2046	-2.26201151612659	1.18652047776094	-1.54723855638625	0.131463479537774	0.822884968571772	-4.69914643802167
a_HQ_transcript/88022	1.41379479533846	0.860555003205861	1.54722895259101	0.131465791763368	0.822884968571772	-4.71919171654926
a_HQ_transcript/115438	-0.698152293121229	5.27161981457304	-1.5472160675903	0.131468894029228	0.822884968571772	-5.23432716547633
a_HQ_transcript/92203	1.87036221926123	0.0352363874985578	1.54718565408023	0.131476216793936	0.822884968571772	-4.78281813783075
a_HQ_transcript/100882	3.4705215153815	3.13348549448244	1.54717011499865	0.131479958319392	0.822884968571772	-4.86537142820101
a_HQ_transcript/80961	1.7285540205943	0.583234798770276	1.54713126435563	0.131489313218626	0.822884968571772	-4.63315185237359
a_HQ_transcript/60686	-1.97353642844052	0.196093112463644	-1.54707336983831	0.131503254720042	0.822884968571772	-4.72466209669885
a_HQ_transcript/443	-1.0518608432665	-0.553271063603864	-1.54707115105744	0.131503789045557	0.822884968571772	-4.71959537349807
a_HQ_transcript/137921	1.1178905802565	-0.740626481426761	1.54703977533788	0.131511345116569	0.822884968571772	-4.75748152516454
a_HQ_transcript/28921	-1.51390453545841	1.58482194472887	-1.54701439954438	0.131517456510422	0.822884968571772	-4.6604561811216
a_HQ_transcript/24192	0.674528332101852	-0.710871652994937	1.54700995291063	0.131518527441744	0.822884968571772	-4.8006863484829
a_HQ_transcript/112752	-2.04747723153552	1.14163436255611	-1.5469690477284	0.131528379414923	0.822884968571772	-4.87792209183967
a_HQ_transcript/25020	-1.63792791278183	0.714382984726843	-1.54694453195483	0.131534284301931	0.822884968571772	-4.64090840883285
a_HQ_transcript/64655	2.04603168292348	0.410040828273971	1.54693219756305	0.131537255253789	0.822884968571772	-4.69100677136861
a_HQ_transcript/95064	-1.80068947770896	0.290513650843648	-1.54685831831528	0.131555051489419	0.822884968571772	-4.73863991727495
a_HQ_transcript/52301	-2.42953414965269	3.864181815419	-1.54674515992245	0.131582313179523	0.822884968571772	-4.94539718940398
a_HQ_transcript/86653	-2.33154614783922	0.605647331900215	-1.54667274137836	0.13159976238451	0.822884968571772	-4.67645567777326
a_HQ_transcript/117985	0.822523895156355	1.28029945729915	1.54666202446549	0.131602344777684	0.822884968571772	-4.64720267862481
a_HQ_transcript/30392	0.686692150606735	2.57820811528048	1.54665956063025	0.131602938479776	0.822884968571772	-4.83518980173335
a_HQ_transcript/38784	1.95727992507987	0.60501415650542	1.54664069610446	0.131607484273102	0.822884968571772	-4.66690694904415

a_HQ_transcript/54842	0.869807052067616	2.73995231533797	1.54657556138534	0.131623180794882	0.822884968571772	-4.83645202980396
a_HQ_transcript/121535	0.847096968542771	5.36444971068332	1.5464083486184	0.131663483596852	0.822884968571772	-5.25217433713384
a_HQ_transcript/119165	-5.84073930353906	3.63170174906549	-1.54639924194035	0.131665678840068	0.822884968571772	-4.88786832104558
a_HQ_transcript/12346	-1.45455972946913	-0.0779973755968948	-1.54639338495477	0.131667090732597	0.822884968571772	-4.70324300108993
a_HQ_transcript/30241	-2.37701059819459	1.43806191601853	-1.54638065309969	0.131670159932428	0.822884968571772	-4.73833105607356
a_HQ_transcript/41354	-1.00595141299273	0.70468158987012	-1.54638052749088	0.131670190212557	0.822884968571772	-4.56147175082677
a_HQ_transcript/110185	-1.52570181653675	3.24863602713894	-1.54632692404735	0.131683112745228	0.822884968571772	-4.90736964508052
a_HQ_transcript/125509	2.54720791665042	0.303575208135964	1.54617318147688	0.131720182177962	0.823028136046499	-4.72871015394194
a_HQ_transcript/130789	0.494198213143528	4.1057754483735	1.54606286066171	0.131746787247499	0.823105895219544	-5.09054582155965
a_HQ_transcript/139646	-1.61064124074502	-0.0840176365189769	-1.54598197823543	0.131766295696682	0.823121261075395	-4.73313667392982
a_HQ_transcript/78397	1.46738511989973	-0.066884769131911	1.54593524868591	0.131777567705196	0.823121261075395	-4.65880168842872
a_HQ_transcript/62263	2.56198154048132	2.12309233066906	1.54585110036561	0.131797867766727	0.823159606483755	-4.68110480254561
a_HQ_transcript/50840	-0.770891465967604	2.89907615337804	-1.54554434050252	0.131871892430249	0.823317024880648	-4.79152417527669
a_HQ_transcript/116232	1.44648209242116	3.94856798502565	1.5454596801051	0.131892327887535	0.823317024880648	-5.08423080415765
a_HQ_transcript/144709	1.22499460972253	-0.573124281209846	1.54545811395301	0.13189270595193	0.823317024880648	-4.7465234947008
a_HQ_transcript/140555	-2.19716923982421	-0.0108633845833945	-1.5454361669707	0.131898003979783	0.823317024880648	-4.77155776391863
a_HQ_transcript/75720	-3.34578867768077	3.0750670978564	-1.54540891728971	0.131904582325601	0.823317024880648	-5.06865605712685
a_HQ_transcript/137144	2.55616515036371	1.22791177520327	1.54539453051281	0.131908055544892	0.823317024880648	-4.70400620224616
a_HQ_transcript/151660	-1.01901065690027	2.02303127792888	-1.54531521695121	0.131927204555678	0.823348136827618	-4.6955303794082
a_HQ_transcript/110897	0.958262645358496	-0.38864911672269	1.5452533137553	0.131942151673874	0.823353021202246	-4.68963882118178
a_HQ_transcript/72912	1.80672986276761	0.684809234737842	1.54498452532892	0.132007069134131	0.823560272808432	-4.58231614280862
a_HQ_transcript/133700	0.42646522254893	4.08579457158567	1.54496667536456	0.132011381153086	0.823560272808432	-5.14826082936505
a_HQ_transcript/144214	1.87271210322147	1.91360908365827	1.54493147314965	0.132019885292872	0.823560272808432	-4.7602042265008
a_HQ_transcript/144352	1.30867978941696	0.296942873443391	1.54487083703176	0.132034534788271	0.823560272808432	-4.74665804061439
a_HQ_transcript/114746	-4.89928203926966	0.525283070453247	-1.54482254084037	0.132046203939144	0.823560272808432	-4.78809330434081
a_HQ_transcript/31082	-1.50272266659271	-0.450859009649355	-1.54467555172823	0.132081724061213	0.823659470009608	-4.72537290647407
a_HQ_transcript/16516	2.50770524237686	1.14344550678801	1.5446394540028	0.132090448311134	0.823659470009608	-4.76615294739288
a_HQ_transcript/146368	2.78409247052956	1.88165036753745	1.54446025213682	0.132133765487635	0.823811542779918	-4.71352298447848
a_HQ_transcript/1166	1.40476199050943	1.63254720564193	1.54442130676517	0.132143180995045	0.823811542779918	-4.68680447229555
a_HQ_transcript/148243	-1.41356810986964	1.44079625542767	-1.5441616085988	0.132205980026667	0.824114660267032	-4.72257599264003
a_HQ_transcript/90042	-4.26286049091179	1.9902927850966	-1.544095653289	0.132221932892098	0.824118404613387	-4.80041687624816
a_HQ_transcript/65262	-1.73745603783252	1.09945944741777	-1.54404189818683	0.132234936000362	0.824118404613387	-4.78635884920457
a_HQ_transcript/36989	1.58743631331644	-0.286345205990675	1.54382636418618	0.132287083079925	0.824190698450446	-4.7394726638154
a_HQ_transcript/122462	-1.68184365730953	1.16521395716714	-1.54375457075238	0.132304456744802	0.824190698450446	-4.75137184873402
a_HQ_transcript/61779	1.81983256557067	1.26698440608734	1.54365363301097	0.132328886319717	0.824190698450446	-4.72439062360669
a_HQ_transcript/64599	-1.58477645981925	0.0561974732974745	-1.54358805103862	0.132344760832741	0.824190698450446	-4.67872029686128
a_HQ_transcript/126227	-2.10113199785085	0.868421339911417	-1.54357709673223	0.132347412539289	0.824190698450446	-4.74174967901228
a_HQ_transcript/72140	0.457783987929107	4.08350856128071	1.54354503526941	0.132355173898052	0.824190698450446	-5.08028539857826
a_HQ_transcript/37914	1.65288946684366	-0.235021224053214	1.54350004279953	0.132366066184792	0.824190698450446	-4.75329891688991
a_HQ_transcript/122433	1.76653616634798	0.170739520017643	1.54347717347708	0.132371602927917	0.824190698450446	-4.76073177791956
a_HQ_transcript/96028	-1.88759822039912	3.24811429593089	-1.54346383602419	0.132374832058671	0.824190698450446	-4.97921178104123
a_HQ_transcript/128391	-1.85058495557772	2.90725198222071	-1.54340810855007	0.132388324928293	0.824190698450446	-5.05595737649037
a_HQ_transcript/11938	2.2072056150264	2.08258375265681	1.5433109204279	0.132411859016646	0.824202528015094	-4.85425339840993
a_HQ_transcript/111038	1.34397759370031	0.81872255762102	1.54328315396474	0.13241858328401	0.824202528015094	-4.68794770671598
a_HQ_transcript/3771	-0.782717603893498	4.18884009130831	-1.54321049940584	0.132436179512927	0.824213092843155	-5.11596977642292
a_HQ_transcript/92005	-2.00557619419792	2.03502281615914	-1.5431590582216	0.132448639214284	0.824213092843155	-4.98266680280757
a_HQ_transcript/73806	-0.667069854004064	2.20466576876339	-1.54289271629582	0.132513165770806	0.824374764593452	-4.69498471840295
a_HQ_transcript/63467	-1.7249352655486	1.69641370321942	-1.54288472446607	0.132515102341974	0.824374764593452	-4.74571337675653
a_HQ_transcript/4485	1.35125298032594	1.10027697169414	1.54283926255883	0.132526119056387	0.824374764593452	-4.63150435479307
a_HQ_transcript/133045	0.773786372740829	2.10639299297855	1.54279619922562	0.132536555212555	0.824374764593452	-4.73605638912274

a_HQ_transcript/86276	1.00187937037758	2.09290425973842	1.54275916949707	0.132545529691206	0.824374764593452	-4.74724965372865
a_HQ_transcript/42363	2.31012031256804	0.390557043180614	1.54261211239133	0.132581175128773	0.824387452594807	-4.74233346706912
a_HQ_transcript/126807	3.02895385131956	0.440511742293385	1.54260500990528	0.132582896909838	0.824387452594807	-4.71943663615078
a_HQ_transcript/41275	0.872288682331691	3.0619777197727	1.54249366096189	0.132609892433163	0.824387452594807	-4.9148462638923
a_HQ_transcript/91878	-1.13320584754832	2.50940651859079	-1.54247832706061	0.132613610344872	0.824387452594807	-4.87715815366541
a_HQ_transcript/7727	1.32807810239691	2.31218517459333	1.54241887795211	0.132628025383673	0.824387452594807	-4.67165616003587
a_HQ_transcript/5138	-1.44377557712657	1.08096252388838	-1.54239975150998	0.13263266337508	0.824387452594807	-4.7418582209814
a_HQ_transcript/100618	1.72769378736113	0.564164728138254	1.54229555000737	0.132657933617452	0.824456363232682	-4.63429258928139
a_HQ_transcript/54154	-1.75846839440149	1.33013181371141	-1.54212420348047	0.132699495898538	0.824505557085034	-4.66451301880111
a_HQ_transcript/1263	-1.84027181239826	2.132789910374	-1.54210661443708	0.132703762941983	0.824505557085034	-4.82327212943481
a_HQ_transcript/11294	-0.763625445732051	2.35614902613485	-1.54208749239948	0.132708402010865	0.824505557085034	-4.7780686779672
a_HQ_transcript/3071	0.672927467311494	3.1261181273086	1.54173999150365	0.132792729759987	0.824825273767014	-5.00077569693488
a_HQ_transcript/126919	1.86955166234333	0.128601622510217	1.54171931619825	0.132797748387013	0.824825273767014	-4.7825752714997
a_HQ_transcript/38271	1.68409671288592	1.55635364987144	1.54164789440848	0.132815086160903	0.824825273767014	-4.61339355267178
a_HQ_transcript/58085	0.843636184761867	-0.713792505698418	1.54161838170088	0.132822250961691	0.824825273767014	-4.76895981246924
a_HQ_transcript/28776	1.12598048018148	1.07587412433616	1.54153393516648	0.132842753779034	0.824825273767014	-4.65482360073526
a_HQ_transcript/36454	-1.26942653311037	2.39293422312729	-1.54152468036923	0.132845000911898	0.824825273767014	-4.6742916955775
a_HQ_transcript/138781	1.48228630314392	4.16999031452609	1.54134342003244	0.132889018463964	0.824945394387991	-5.08242155752785
a_HQ_transcript/84442	3.68855296093284	0.836140302710584	1.54130893101977	0.132897395169248	0.824945394387991	-4.78370332347862
a_HQ_transcript/64904	-2.16982619824711	2.01776321605063	-1.54117514709369	0.132929892711286	0.824945394387991	-4.81621520748238
a_HQ_transcript/112712	-1.81363242329172	0.0848707567467185	-1.54113920886168	0.13293862358791	0.824945394387991	-4.79076992443805
a_HQ_transcript/128679	-1.39970453371317	-0.382267706571917	-1.54113655551603	0.132939268213258	0.824945394387991	-4.73306061082894
a_HQ_transcript/110374	1.55126207323984	0.232552986233476	1.54103683730532	0.132963496401462	0.824945394387991	-4.60652413961171
a_HQ_transcript/130677	4.19338890110492	1.59827330111731	1.54103603859182	0.132963690476553	0.824945394387991	-4.60048168910537
a_HQ_transcript/100463	1.50172196353676	1.13532489729665	1.54089300608185	0.132998448876205	0.824981760591579	-4.62761910056222
a_HQ_transcript/33119	1.52349867321566	0.693257360690018	1.54082408939454	0.133015198981329	0.824981760591579	-4.59634338571177
a_HQ_transcript/4194	1.32163675890275	-0.490030933319471	1.54073361960858	0.133037190131217	0.824981760591579	-4.69624528670341
a_HQ_transcript/7058	-2.8233644789837	2.69411230213735	-1.54072863051678	0.133038402951782	0.824981760591579	-4.92550270241656
a_HQ_transcript/133510	0.472016554966451	5.28839667487577	1.54066648777913	0.133053510257482	0.824981760591579	-5.25854622182078
a_HQ_transcript/102358	1.89878752466144	0.92394770942706	1.54066156552408	0.133054706949251	0.824981760591579	-4.66187033139807
a_HQ_transcript/112997	1.6516006217618	0.0302979779040302	1.54050282924581	0.133093303365779	0.825002349226075	-4.63547231145549
a_HQ_transcript/99299	1.6775737382549	-0.224218672815325	1.5404317688613	0.133110584498113	0.825002349226075	-4.74323465422538
a_HQ_transcript/25490	1.2383341002191	1.58196308219368	1.54039883315629	0.133118594728877	0.825002349226075	-4.62658016840133
a_HQ_transcript/110876	1.85382018448312	-0.0241442098271068	1.54029173109645	0.133144645518341	0.825002349226075	-4.75421574934462
a_HQ_transcript/44863	-1.13165852207376	-0.280806680350689	-1.5401826931108	0.133171171431263	0.825002349226075	-4.76170690562109
a_HQ_transcript/30042	1.39475007974243	-0.530671384820124	1.54016829267	0.133174674978436	0.825002349226075	-4.73360656799792
a_HQ_transcript/34607	0.870778725187731	2.49757842492966	1.54012848242076	0.13318436097919	0.825002349226075	-4.7914261724872
a_HQ_transcript/149869	0.925734601460277	2.31263336061119	1.5400868753328	0.133194484768147	0.825002349226075	-4.83106568132165
a_HQ_transcript/13163	1.82227207773387	2.07051129488041	1.54004191055643	0.133205426246757	0.825002349226075	-4.66528152819143
a_HQ_transcript/98869	2.87831119970173	1.80327594982469	1.53999871785615	0.133215937203723	0.825002349226075	-4.78866688284905
a_HQ_transcript/36511	-1.95901002324473	1.48985840215611	-1.53990892358266	0.133237790817835	0.825002349226075	-4.60976595462489
a_HQ_transcript/73333	2.0540902767731	0.194375700429466	1.53988893685269	0.13324265546833	0.825002349226075	-4.66057696929223
a_HQ_transcript/140084	-1.67987781788609	0.15919697067187	-1.53980336373559	0.133263485079511	0.825002349226075	-4.75784107287596
a_HQ_transcript/70176	1.17426004494279	1.15671120589267	1.53979311894426	0.133265978971902	0.825002349226075	-4.61627650135607
a_HQ_transcript/117670	-1.02206890982499	6.21484387942401	-1.53959809583225	0.13331346071092	0.825002349226075	-5.29115969410994
a_HQ_transcript/95691	-1.64360486592716	2.21558828687733	-1.53942824275954	0.133354825531577	0.825002349226075	-4.75861549604818
a_HQ_transcript/49622	-3.76278552049753	1.45548594260961	-1.53940159837856	0.13336131525943	0.825002349226075	-4.86890760660838
a_HQ_transcript/134583	4.17275704373011	0.850920326959126	1.53935304452073	0.133373142100056	0.825002349226075	-4.78062519060459
a_HQ_transcript/104133	-1.24871170649354	2.38070456174955	-1.5393428224437	0.133375632121165	0.825002349226075	-4.89216391886098
a_HQ_transcript/95487	1.79080530584934	0.314925286936744	1.53933281578317	0.133378069704897	0.825002349226075	-4.72255660333843

a_HQ_transcript/74352	-1.0119416568961	1.75699632848955	-1.53932028847729	0.133381121358923	0.825002349226075	-4.67201593983569
a_HQ_transcript/5558	-2.42511588406849	1.13980430439491	-1.53931272010038	0.133382965044336	0.825002349226075	-4.70339177374273
a_HQ_transcript/127416	0.475243668130018	5.48294704476433	1.53929685792269	0.133386829198127	0.825002349226075	-5.2531356003664
a_HQ_transcript/141540	-1.42577772374908	-0.0628571177140611	-1.5391856778938	0.133413916090081	0.825002349226075	-4.79407372787694
a_HQ_transcript/62250	-1.42728900221705	0.0165417327649797	-1.53918250837859	0.133414688347261	0.825002349226075	-4.73952619066883
a_HQ_transcript/54536	-0.676145514418922	1.65600588795156	-1.53913180912861	0.133427041788369	0.825002349226075	-4.65600710168484
a_HQ_transcript/120267	3.75362136252027	1.0415180230382	1.53899316255827	0.133460829311019	0.825057618224391	-4.75021036334905
a_HQ_transcript/135790	1.96435702063251	0.788092583502269	1.53894092938133	0.133473560089255	0.825057618224391	-4.72170175625723
a_HQ_transcript/21309	2.29227158159874	1.67725433994293	1.53888004390549	0.133488400931136	0.825057618224391	-4.72330246058215
a_HQ_transcript/146335	4.90151516620947	4.09480378422325	1.53886217951312	0.133492755632404	0.825057618224391	-5.10912480955048
a_HQ_transcript/135612	2.68103296135292	-0.315462758395627	1.53866871904746	0.133539921757588	0.825176796702889	-4.74891168936164
a_HQ_transcript/70277	1.93422898972201	-0.21675942998129	1.53835131253552	0.133617335455557	0.825176796702889	-4.807807738448
a_HQ_transcript/36229	1.42090775383831	0.83326095314644	1.53830753318799	0.133628015847849	0.825176796702889	-4.63692529666759
a_HQ_transcript/102689	-1.75204615318281	0.483484198376545	-1.53829412021424	0.133631288209936	0.825176796702889	-4.82727365608492
a_HQ_transcript/120373	-1.64358038880606	-0.300865919291982	-1.5382686436223	0.133637503910598	0.825176796702889	-4.75174952530734
a_HQ_transcript/55060	-1.66835074453324	0.450941358874813	-1.53819199603184	0.133656205565815	0.825176796702889	-4.79388162325275
a_HQ_transcript/113	1.95312428101277	3.14084173284727	1.53811738653379	0.13367441197106	0.825176796702889	-4.94340431925815
a_HQ_transcript/128221	-0.627134776820674	3.48081292947157	-1.53811053614219	0.133676083722208	0.825176796702889	-4.97598245432057
a_HQ_transcript/86861	2.07224023521111	0.328807845039788	1.53808339387042	0.133682707615626	0.825176796702889	-4.75468690851063
a_HQ_transcript/145004	-1.89985154864984	1.15383896984906	-1.53800267894608	0.133702407133659	0.825176796702889	-4.73077391664572
a_HQ_transcript/104639	1.69038213208893	-0.316663305682283	1.53790871401461	0.133725343447235	0.825176796702889	-4.75964033921738
a_HQ_transcript/51407	-1.80993421176797	-0.179536603281153	-1.5379083162771	0.133725440539503	0.825176796702889	-4.65777289244789
a_HQ_transcript/140769	6.02183996054157	3.81597606125333	1.5378560379028	0.133738202784463	0.825176796702889	-4.87574432943559
a_HQ_transcript/146252	2.65843223500122	1.05348299640817	1.53784920513577	0.133739870878708	0.825176796702889	-4.74317149310022
a_HQ_transcript/82465	3.07274229264687	0.133089203015777	1.53780182382914	0.133751438615583	0.825176796702889	-4.75299798922722
a_HQ_transcript/67438	1.48363465438853	4.57819306153933	1.5377827172102	0.133756103560776	0.825176796702889	-5.20007612665449
a_HQ_transcript/15359	-0.495003551936556	2.92876250674656	-1.53763408781751	0.13379239642389	0.825176796702889	-4.91126465360158
a_HQ_transcript/139	-0.777772097473384	2.93727558365722	-1.53752248293432	0.133819653748331	0.825176796702889	-4.93305115088784
a_HQ_transcript/149592	0.425903943843798	4.79842223873153	1.53749356465316	0.133826717209919	0.825176796702889	-5.1907821674293
a_HQ_transcript/13611	1.44573903078687	-0.479492621035706	1.537489295944	0.133827759892999	0.825176796702889	-4.77040685337268
a_HQ_transcript/45454	0.991427482772377	0.298683844506348	1.53748598551062	0.133828568510326	0.825176796702889	-4.72579722440624
a_HQ_transcript/50281	1.53518938426646	0.458364664017382	1.53746462810797	0.133833785434696	0.825176796702889	-4.6306257271703
a_HQ_transcript/1050	-0.406930808486412	3.46781540975992	-1.5373956606083	0.133850633093769	0.825176796702889	-5.03876651319296
a_HQ_transcript/36917	-1.46468436439045	-0.54329307379248	-1.53736119068707	0.133859054188632	0.825176796702889	-4.73346421666091
a_HQ_transcript/148358	-1.5508827378089	1.8189038554047	-1.53726069977313	0.133883606837977	0.825176796702889	-4.89178000666917
a_HQ_transcript/45617	2.38270920093366	1.2573166094183	1.53725491221586	0.133885021005828	0.825176796702889	-4.74891258130773
a_HQ_transcript/102566	-1.87672066232871	2.83229246234498	-1.53719042355652	0.133900779385184	0.825176796702889	-4.93390476095338
a_HQ_transcript/29527	-1.82280495532841	0.360660369167881	-1.53711923200537	0.133918177419449	0.825176796702889	-4.6865879191613
a_HQ_transcript/134080	1.50990007334424	1.5569836491451	1.53709656048396	0.133923718347355	0.825176796702889	-4.74362020865639
a_HQ_transcript/58466	2.88032715011942	2.31539853708027	1.5368996333255	0.133971855221706	0.825375500036414	-4.87417780330213
a_HQ_transcript/129625	-1.6421186858233	0.356615705603092	-1.5366494598872	0.134033027811466	0.825375500036414	-4.76781297491083
a_HQ_transcript/13294	-1.35047157019814	-0.00264673599133258	-1.53662853569533	0.134038145234276	0.825375500036414	-4.74675117293974
a_HQ_transcript/106604	-1.8665427719541	0.929995934743698	-1.53647450948619	0.13407582024078	0.825375500036414	-4.74508655371411
a_HQ_transcript/134243	1.36227437810742	3.69710588961706	1.53644859857982	0.134082158923467	0.825375500036414	-4.97461294362152
a_HQ_transcript/137506	2.31657171224222	0.448313712000682	1.5363973740159	0.134094690895286	0.825375500036414	-4.7087281462354
a_HQ_transcript/13829	1.09241980775596	2.59409571824282	1.53637941524658	0.134099084691338	0.825375500036414	-4.80554217124652
a_HQ_transcript/55236	1.43443576746264	0.380688959496202	1.53637331209969	0.134100577914989	0.825375500036414	-4.75849078891445
a_HQ_transcript/85551	1.82814365292477	0.308842779046536	1.53637261711383	0.134100747954242	0.825375500036414	-4.77092236392243
a_HQ_transcript/144760	0.622048606490741	2.84717018194384	1.53634585215041	0.13410729655744	0.825375500036414	-4.91314850227741
a_HQ_transcript/85257	-0.832312109660374	2.19861723758798	-1.53632597805101	0.134112159335328	0.825375500036414	-4.66844449482887

a_HQ_transcript/25862	-0.41618461450908	3.92548137298624	-1.53617651246624	0.134148735020869	0.825422624306365	-4.98560834332492
a_HQ_transcript/25659	0.824252616871747	-0.825357230263341	1.53615040387686	0.134155124875325	0.825422624306365	-4.78157299264365
a_HQ_transcript/87355	-1.41944454313375	1.27954254961913	-1.53608813130928	0.134170366549211	0.825422624306365	-4.77877206432402
a_HQ_transcript/118020	1.21346370468528	1.56388328436919	1.53605363827599	0.134178809578604	0.825422624306365	-4.65025659681323
a_HQ_transcript/105423	1.39395869587578	-0.386645146590375	1.53600458711557	0.134190816812812	0.825422624306365	-4.79502168630643
a_HQ_transcript/40939	1.68302178857155	-0.254889547117718	1.53583207367762	0.134233053286447	0.825595060991913	-4.74550335629491
a_HQ_transcript/25331	1.42538280262596	1.69674853983089	1.53558124651933	0.134294482502595	0.825791738488163	-4.75283136371533
a_HQ_transcript/27636	1.52044142832922	0.0296774267150294	1.53541422944613	0.134335398693843	0.825791738488163	-4.80839750528691
a_HQ_transcript/152827	1.67957355229021	-0.499717163297113	1.53527632096434	0.134369191404111	0.825791738488163	-4.77260428881951
a_HQ_transcript/86092	-2.07931323424346	1.54557294571152	-1.53513891531802	0.134402867741093	0.825791738488163	-4.67219390923423
a_HQ_transcript/40900	-2.13803866081544	1.57795724268149	-1.5351101385225	0.134409921424206	0.825791738488163	-4.73666530274898
a_HQ_transcript/54735	-0.657744278536704	2.36881659726209	-1.53510101190031	0.134412158577433	0.825791738488163	-4.74682737717
a_HQ_transcript/15906	-0.833389090150942	1.8191283048864	-1.53504248615701	0.134426505351947	0.825791738488163	-4.65485889399558
a_HQ_transcript/14859	2.06643501404977	0.560393220610214	1.53499905461751	0.134437152792518	0.825791738488163	-4.71049373380833
a_HQ_transcript/12666	-2.08609443672318	0.182868824140976	-1.53496077319408	0.13444653822432	0.825791738488163	-4.81040638697895
a_HQ_transcript/455	-0.758689340523081	3.61085667272332	-1.5349510187701	0.134448929794878	0.825791738488163	-5.07371958528376
a_HQ_transcript/148749	-2.64216653835783	-0.0135577494373698	-1.53477749529583	0.134491479692702	0.825791738488163	-4.7811382454206
a_HQ_transcript/109650	1.36746226341424	0.261290225885012	1.534765161065	0.134494504598804	0.825791738488163	-4.62430385684613
a_HQ_transcript/118326	2.40282197247043	1.00403993441683	1.53475087622732	0.134498007949897	0.825791738488163	-4.81925168698293
a_HQ_transcript/20133	0.842537747959305	2.74063272934959	1.53469399803928	0.134511958036451	0.825791738488163	-4.93040746085718
a_HQ_transcript/120969	4.0629806130928	4.8311507470068	1.53465516771899	0.13452148233018	0.825791738488163	-5.08552030810237
a_HQ_transcript/145721	0.470753905343152	4.03523695599335	1.53464214932054	0.134524675602644	0.825791738488163	-5.12845590536046
a_HQ_transcript/36402	1.03800222811132	0.425736783863283	1.53452876235619	0.134552490792858	0.825791738488163	-4.68310104113904
a_HQ_transcript/100136	-1.42960982333662	3.45339493915419	-1.53452151441632	0.134554268958143	0.825791738488163	-4.95060942025422
a_HQ_transcript/63710	0.934909750210295	2.24250924924292	1.53450982809971	0.134557136047695	0.825791738488163	-4.9081098907984
a_HQ_transcript/40472	1.21603023163578	2.05979788934912	1.53442698641457	0.134577461621338	0.825791738488163	-4.722312583628
a_HQ_transcript/103246	1.54927314916443	1.12866774089828	1.53440293232647	0.134583363863455	0.825791738488163	-4.72826105611193
a_HQ_transcript/70901	0.797522772144536	4.21271991933799	1.53437637902829	0.134589879589326	0.825791738488163	-5.06377701946342
a_HQ_transcript/89346	2.2228918540634	0.442101639775335	1.53435625684794	0.134594817398677	0.825791738488163	-4.79780205025905
a_HQ_transcript/34797	-0.512274566475576	2.75247237235919	-1.53425662026627	0.134619269515096	0.825791738488163	-4.8384009316781
a_HQ_transcript/62513	1.4502269553614	-0.350444214793393	1.5341449201518	0.134646686452098	0.825791738488163	-4.78393973850103
a_HQ_transcript/120750	1.54268028668163	-0.603494476787687	1.53413475206699	0.134649182445897	0.825791738488163	-4.77407661410909
a_HQ_transcript/21198	1.67531617449257	-0.164509774791029	1.53412163836482	0.134652401565506	0.825791738488163	-4.77384406182224
a_HQ_transcript/108113	2.21486614817959	-0.23188604397184	1.53406473553298	0.134666370654969	0.825791738488163	-4.75470234288971
a_HQ_transcript/116823	1.27278515938268	3.09374051874895	1.5339829002323	0.134686462477203	0.825791738488163	-4.96070426895902
a_HQ_transcript/50036	0.950068100135322	-0.788335833935537	1.53392950624999	0.134699572826371	0.825791738488163	-4.78302757259162
a_HQ_transcript/113482	-2.26304328555633	0.240405539924589	-1.5339056512842	0.134705430504142	0.825791738488163	-4.7879949619024
a_HQ_transcript/88007	2.9796977700798	1.52617993085616	1.53378571164606	0.134734885261848	0.825802647489108	-4.81272140883778
a_HQ_transcript/70880	2.02852576021226	0.791007089311304	1.53373552890311	0.134747210677776	0.825802647489108	-4.78353979077455
a_HQ_transcript/124547	1.37562967236129	3.34850842045206	1.53372486532766	0.134749829882917	0.825802647489108	-4.86118497800118
a_HQ_transcript/126684	-1.05737943360481	4.0043013497815	-1.53365575862737	0.134766804983786	0.82581961207016	-5.02735664022774
a_HQ_transcript/30500	-2.56570695469053	0.680660031179973	-1.53339208954558	0.134831587520169	0.826129495032403	-4.777002674401
a_HQ_transcript/110420	-2.65905365821328	0.371276104847786	-1.53317174313246	0.134885745150924	0.82626331437768	-4.8032653787187
a_HQ_transcript/125655	-1.35413911457322	0.472499934588382	-1.53312483997712	0.134897275468446	0.82626331437768	-4.6621097765856
a_HQ_transcript/143781	1.42990637832587	-0.648846468799546	1.53308400979006	0.13490731350228	0.82626331437768	-4.76650055630129
a_HQ_transcript/145704	0.592138388702423	3.27340914516076	1.53299530456504	0.13492912361668	0.82626331437768	-4.9993776546775
a_HQ_transcript/5432	-0.576994733908399	2.53300458815866	-1.53295473191302	0.134939100238498	0.82626331437768	-4.76143574037155
a_HQ_transcript/149169	1.85530608808527	3.31810333231416	1.53288536608385	0.134956158347195	0.82626331437768	-5.01059265733807
a_HQ_transcript/141677	1.86529804364968	-0.0609114849617621	1.53284393351305	0.13496634807616	0.82626331437768	-4.68156972324263
a_HQ_transcript/99650	0.504363841831802	2.2429992003314	1.53284069565507	0.134967144405727	0.82626331437768	-4.78506983413497

a_HQ_transcript/33508	-2.9287285997463	0.462748191854473	-1.53273207809659	0.134993860371285	0.826339839475785	-4.77894455045698
a_HQ_transcript/1013	2.33810494348552	2.54791495398075	1.53259878715754	0.135026650934066	0.826383026959689	-4.83744176490416
a_HQ_transcript/90757	0.877658820521731	-0.514622460568189	1.53258782150905	0.135029348851898	0.826383026959689	-4.80456164772603
a_HQ_transcript/24761	1.62304336125144	-0.504047370631669	1.53247581543159	0.135056908605518	0.826464678747401	-4.72704805339004
a_HQ_transcript/73167	1.03209725956269	-0.746994022109938	1.53237854556617	0.135080846117621	0.826502410104395	-4.77818171933655
a_HQ_transcript/29942	2.74545466946774	1.67602447713653	1.53230136522306	0.135099842163482	0.826502410104395	-4.81971686132498
a_HQ_transcript/130885	0.508604525509217	5.25933639671513	1.53227744299491	0.135105730470891	0.826502410104395	-5.27110095569991
a_HQ_transcript/109067	-0.618468131339091	3.39661784593359	-1.53208186513143	0.135153878527237	0.826644663890629	-4.95444222938198
a_HQ_transcript/15297	-0.730438351373762	1.79840446708659	-1.53206745559903	0.135157426466015	0.826644663890629	-4.73371636063638
a_HQ_transcript/72770	1.18365999191861	-0.252783670778946	1.5317033427813	0.135247103919241	0.827052974962488	-4.85231516039581
a_HQ_transcript/15776	-1.41752599555002	0.227919834136017	-1.53168086278666	0.135252642100079	0.827052974962488	-4.73626983609381
a_HQ_transcript/33108	2.6093701322938	1.07721001298572	1.53159728262841	0.135273234550772	0.827091887868653	-4.8141586564315
a_HQ_transcript/151825	0.972165545540329	3.83817649034749	1.53141492534224	0.135318172474644	0.827216418550249	-5.06943168000075
a_HQ_transcript/104117	-2.41131435842868	2.95498665389861	-1.53125569021707	0.135357422322452	0.827216418550249	-5.04032305207307
a_HQ_transcript/114903	0.668811267146178	4.5349212119364	1.53119893921102	0.135371413095807	0.827216418550249	-5.18845992485324
a_HQ_transcript/94678	1.5910418399522	-0.475226245824188	1.53117835199056	0.135376488733295	0.827216418550249	-4.79322503192097
a_HQ_transcript/700	-0.682538626448992	2.97461574355975	-1.53113620437804	0.135386880416915	0.827216418550249	-4.97681230933864
a_HQ_transcript/38724	1.39174587621264	0.202118513605677	1.53099309773546	0.135422168815719	0.827216418550249	-4.69529470521671
a_HQ_transcript/1110	-3.35355820849582	1.88500299141356	-1.53095089607722	0.135432576663028	0.827216418550249	-4.93587550835002
a_HQ_transcript/103484	1.60719096675754	2.07935424050108	1.53090748690833	0.135443282983347	0.827216418550249	-4.7623267016247
a_HQ_transcript/121065	1.75796076404553	-0.501095788244858	1.53080198126201	0.13546930746354	0.827216418550249	-4.76479422219296
a_HQ_transcript/94011	1.32290750756498	1.76902680404273	1.53076337495909	0.135478831271675	0.827216418550249	-4.68745450938959
a_HQ_transcript/10706	-1.08969552793066	1.60161331147608	-1.53069976397747	0.135494524676993	0.827216418550249	-4.62439709756218
a_HQ_transcript/128567	-1.69864801657444	-0.345936466655986	-1.53067757695024	0.135499998763197	0.827216418550249	-4.72506916078654
a_HQ_transcript/71804	0.923394072403563	-0.834697089030488	1.5305875572028	0.135522210686613	0.827216418550249	-4.77257659299268
a_HQ_transcript/74048	-2.39975505958429	0.64440335431537	-1.53057556234028	0.13552517058127	0.827216418550249	-4.66990882157315
a_HQ_transcript/81920	2.16944628904991	0.0640762684083277	1.53056736242269	0.135527194051977	0.827216418550249	-4.69662811647487
a_HQ_transcript/11012	-1.25594307140615	2.20554068636109	-1.53055581965589	0.135530042469606	0.827216418550249	-4.78121335747625
a_HQ_transcript/124348	4.17199485367069	0.505059272398645	1.53051258132628	0.135540712856293	0.827216418550249	-4.79784698920919
a_HQ_transcript/39431	0.967256089525937	4.45607633738552	1.53035227241553	0.135580279932673	0.827216418550249	-5.20952431910881
a_HQ_transcript/144664	1.50032005419094	0.31099853193086	1.53033600355099	0.135584295898107	0.827216418550249	-4.73384739655335
a_HQ_transcript/4587	-1.50005923867506	-0.227174065622876	-1.53028780792201	0.135596193542238	0.827216418550249	-4.77367748808691
a_HQ_transcript/124437	1.47640807940709	-0.462024769850914	1.53027396583811	0.13559961077548	0.827216418550249	-4.74428048711114
a_HQ_transcript/18572	1.49040971440915	-0.567751791208594	1.53021263361028	0.135614752868365	0.827216418550249	-4.77200331834809
a_HQ_transcript/141554	-1.80220603114224	0.12487150098652	-1.53018768127219	0.135620913652351	0.827216418550249	-4.75108412302596
a_HQ_transcript/100446	3.79088027115509	2.37317031756626	1.53012588077197	0.135636173297845	0.82722269242022	-4.87306705262772
a_HQ_transcript/55168	1.29374518511811	0.221208685260947	1.52997165058398	0.135674261534255	0.827312645375445	-4.76085203142193
a_HQ_transcript/145001	2.21899394166751	1.30787288836113	1.52995089593149	0.135679387702383	0.827312645375445	-4.83515759964731
a_HQ_transcript/141269	1.30901418316636	0.966288908217538	1.52982455988596	0.135710594675718	0.827343927997782	-4.78297547685056
a_HQ_transcript/83185	-1.36688383515417	-0.644954776272465	-1.52976685530301	0.135724850537286	0.827343927997782	-4.74201724637168
a_HQ_transcript/44571	-0.872756480199631	2.61824600631353	-1.52971284748274	0.135738194213583	0.827343927997782	-4.75100090028304
a_HQ_transcript/90084	1.39025717418671	-0.464631822604745	1.52966532260897	0.135749937029493	0.827343927997782	-4.76492325999778
a_HQ_transcript/140502	1.53888855191918	0.659304178436377	1.52960755433319	0.135764211970386	0.827343927997782	-4.6765861268963
a_HQ_transcript/58221	1.23264942984023	-0.645355624894351	1.52958446884634	0.13576991689325	0.827343927997782	-4.7237900528071
a_HQ_transcript/146297	1.81700616853813	3.30833379209911	1.52942266429651	0.135809907732907	0.827368559458792	-5.08532834901497
a_HQ_transcript/150013	2.09629045973028	-0.430764260131299	1.52938115643786	0.135820168150958	0.827368559458792	-4.75234423977031
a_HQ_transcript/7941	-1.32877598133314	-0.48937363784316	-1.52932242790741	0.135834686454528	0.827368559458792	-4.74927836950138
a_HQ_transcript/10383	-1.64416433429996	2.98791539553469	-1.52929237338794	0.135842116729425	0.827368559458792	-4.85179271041449
a_HQ_transcript/146632	0.538267909281309	3.61374611548472	1.52927308544904	0.135846885392865	0.827368559458792	-5.08592920271812
a_HQ_transcript/6855	-1.71468745145876	0.161052856501887	-1.52912532038349	0.135883422652322	0.827368559458792	-4.66157297331783

a_HQ_transcript/7891	1.55413487100406	0.41390251998667	1.52908995500344	0.135892168481811	0.827368559458792	-4.61377528404683
a_HQ_transcript/109820	0.97179173602295	-0.635755198785461	1.529063209005	0.135898783047361	0.827368559458792	-4.83276891743369
a_HQ_transcript/9621	-0.945477633133027	2.15127961777793	-1.52900349402852	0.135913552125328	0.827368559458792	-4.73658782083129
a_HQ_transcript/18065	0.583591358825688	3.26867209691265	1.52899240599057	0.135916294630333	0.827368559458792	-5.02128982603605
a_HQ_transcript/16349	1.31776406398977	1.37978090354198	1.52887573705849	0.13594515412618	0.82743325416391	-4.7173220148059
a_HQ_transcript/32280	0.764764669668876	2.05802524277652	1.52883435478306	0.135955391730655	0.82743325416391	-4.78150171055825
a_HQ_transcript/19317	0.723378572320224	2.57991114979388	1.52870893670083	0.135986422846283	0.827535467672604	-4.82449178310092
a_HQ_transcript/149087	-0.553658599739343	5.4289187885461	-1.52857817795628	0.136018781448981	0.827588111910372	-5.28372228669224
a_HQ_transcript/68285	0.390820806241998	-0.761668174051768	1.52855891719437	0.136023548396499	0.827588111910372	-4.85439542738712
a_HQ_transcript/44713	0.833683806778162	4.34078905231656	1.52844650487454	0.136051372607015	0.827650333821504	-5.1829617202162
a_HQ_transcript/58373	1.24590791613172	-0.487710211584345	1.52827126151836	0.136094757878696	0.827650333821504	-4.83253963755859
a_HQ_transcript/13875	0.841744987118569	2.49658299016269	1.52825951786008	0.1360976656741	0.827650333821504	-4.94551825070652
a_HQ_transcript/79280	1.22934929167976	2.15984396497799	1.5282277859434	0.136105522925004	0.827650333821504	-4.89808559768063
a_HQ_transcript/81201	-2.45110581225338	1.43520138656057	-1.52820286620782	0.13611169364481	0.827650333821504	-4.75607745942547
a_HQ_transcript/12331	1.50756631108544	1.41081883810211	1.52816768911024	0.13612040471636	0.827650333821504	-4.73011167542065
a_HQ_transcript/59774	1.13254926111305	0.757822206907196	1.52811503658719	0.136133444151999	0.827650333821504	-4.61235770921985
a_HQ_transcript/113486	3.40173280623039	1.34360537663021	1.52798369403707	0.136165975628289	0.827761538519263	-4.89988605312006
a_HQ_transcript/104770	-2.42407752971163	3.0070183909741	-1.52782841512606	0.136204443864132	0.827908806120911	-4.9964987938438
a_HQ_transcript/66175	-0.988433058021668	2.7622114237102	-1.52775621734333	0.136222332871333	0.827930966660901	-4.87040823324252
a_HQ_transcript/360	0.479589995120733	2.6175731107823	1.52757670849199	0.13626681940418	0.828114759887735	-4.81869393744794
a_HQ_transcript/133086	-2.07282261364806	0.395675630299121	-1.52735801590769	0.136321032447915	0.828273976196359	-4.82490073283998
a_HQ_transcript/80886	-1.57440115253391	1.00597001605888	-1.52735606225399	0.136321516829593	0.828273976196359	-4.63508937082542
a_HQ_transcript/46589	2.24047275847296	0.301360289829206	1.52726166567877	0.136344922823944	0.828325934349207	-4.75818489438407
a_HQ_transcript/88277	1.84919943438558	-0.00787132268658239	1.52709995366327	0.136385027484269	0.828325934349207	-4.77766376608121
a_HQ_transcript/109808	1.70208484626133	0.297166403478063	1.5270762265081	0.136390912632015	0.828325934349207	-4.65209771307357
a_HQ_transcript/48014	-0.540274627780439	3.08156990593714	-1.52704600813493	0.13639840812127	0.828325934349207	-4.97801581771618
a_HQ_transcript/108196	1.74482466693138	-0.482163592438916	1.52700440127182	0.136408729002689	0.828325934349207	-4.78246300159887
a_HQ_transcript/84223	0.485398973248794	3.11719458431945	1.52697683015177	0.136415568564632	0.828325934349207	-4.95530442525743
a_HQ_transcript/67143	0.96625170038986	1.53072094155377	1.52688528707645	0.136438279622577	0.828377304982362	-4.77541885840361
a_HQ_transcript/33796	-1.74769929478277	0.475877806115191	-1.52681105283258	0.136456698748874	0.828402609386374	-4.69098964946297
a_HQ_transcript/8165	-1.22654664927502	4.18509566269972	-1.5267087811429	0.136482077862845	0.828470156851654	-5.14911030539015
a_HQ_transcript/7689	1.7768425306605	1.74100870809097	1.52664682117687	0.136497455321462	0.828475764880697	-4.74143506487785
a_HQ_transcript/1211	-0.770922930637229	2.27144680224981	-1.52653318702863	0.136525661110236	0.828475764880697	-4.78550307899886
a_HQ_transcript/22463	-2.27781095334604	2.17671669496548	-1.52653279042594	0.136525759561523	0.828475764880697	-5.02792225351003
a_HQ_transcript/49495	1.08572261567031	-0.594621066258239	1.52632361369615	0.136577692863809	0.828494975057492	-4.77453894763683
a_HQ_transcript/20068	-1.04908039400924	-0.679623651024172	-1.52630318392219	0.136582765916047	0.828494975057492	-4.79262999151785
a_HQ_transcript/141408	0.491136391444265	10.4747707649473	1.52630087641826	0.136583338917168	0.828494975057492	-5.80007886035299
a_HQ_transcript/12121	1.52432238822088	0.220419582989076	1.52629041390516	0.136585937000687	0.828494975057492	-4.67481079677009
a_HQ_transcript/88350	0.591099528154197	2.75718993850618	1.52583092153826	0.136700078931824	0.828826368825153	-4.96664144361293
a_HQ_transcript/140282	-2.0788269252456	0.768271520321523	-1.52566965409349	0.136740157433388	0.828826368825153	-4.75813728988426
a_HQ_transcript/140289	-1.98619994280111	-0.366970378521874	-1.52564663654119	0.13674587857426	0.828826368825153	-4.73718150512192
a_HQ_transcript/45138	-1.28387407253566	-0.552381315543151	-1.52555617763208	0.136768364512471	0.828826368825153	-4.75578249069234
a_HQ_transcript/60632	-1.85868287060556	3.76438742764788	-1.52555445867192	0.136768791834077	0.828826368825153	-4.96309581175923
a_HQ_transcript/119820	0.443564398253486	3.47289795663371	1.52552298478979	0.136776616214069	0.828826368825153	-5.05462594186947
a_HQ_transcript/24955	-2.68077749115983	1.95767956258076	-1.52549795908024	0.136782837841838	0.828826368825153	-4.84665291674015
a_HQ_transcript/137227	1.70810982135436	-0.321308505288771	1.5253712834789	0.136814334098499	0.828826368825153	-4.78482202732603
a_HQ_transcript/83794	1.77483263274243	1.54600488558349	1.52534210746292	0.136821589168645	0.828826368825153	-4.80947818272174
a_HQ_transcript/86976	-1.4618709053157	1.8380816565908	-1.5253366256951	0.136822952330134	0.828826368825153	-4.84027391217674
a_HQ_transcript/77342	-3.55414968338546	-0.0482363379420865	-1.52529622884535	0.136832998228697	0.828826368825153	-4.8117911004204
a_HQ_transcript/85365	1.82109162194478	0.180398214554841	1.52524162785782	0.136846577362139	0.828826368825153	-4.67953034294044

a_HQ_transcript/81838	1.47786138973683	2.10680187293151	1.52518674535028	0.136860227605303	0.828826368825153	-4.746254422991
a_HQ_transcript/109085	1.32538736087882	0.813208703438172	1.52510950856232	0.136879439612524	0.828826368825153	-4.71754612889433
a_HQ_transcript/75509	0.996875361887529	1.40577820509167	1.5250868995552	0.136885063826533	0.828826368825153	-4.61936105636252
a_HQ_transcript/2635	2.03172162500271	0.201763709295546	1.52505918754054	0.136891957718592	0.828826368825153	-4.73547349732513
a_HQ_transcript/55375	-2.44251398022144	0.620987369515165	-1.52504204956861	0.136896221256168	0.828826368825153	-4.75169858073226
a_HQ_transcript/91773	1.90048758258505	0.862567775822207	1.52503301494498	0.136898468908167	0.828826368825153	-4.7452501191312
a_HQ_transcript/133297	1.93339299053859	0.982364342288629	1.5249806984967	0.136911484886098	0.828826368825153	-4.69735795635306
a_HQ_transcript/142463	-1.53435943085527	-0.386020019426743	-1.52462951377409	0.136998883130263	0.829269090762877	-4.78718783454618
a_HQ_transcript/135264	1.52473109242695	0.237930633644416	1.52425444640046	0.137092274704847	0.829680021744672	-4.73994623540591
a_HQ_transcript/84507	-2.32865476455788	1.43783249075323	-1.52424223025145	0.137095317383319	0.829680021744672	-4.6741219265769
a_HQ_transcript/9353	2.04619799268921	2.61543886885312	1.52397575538089	0.137161701891901	0.829995357856799	-4.93727055534331
a_HQ_transcript/6320	1.06253169543465	1.70396065607489	1.52390448116091	0.137179462199994	0.830016423312301	-4.7364484711976
a_HQ_transcript/73908	1.27606647459959	0.830560795652762	1.5237148058499	0.137226735063162	0.830161523117466	-4.83052339582849
a_HQ_transcript/38065	-2.4502815621285	0.660180839640277	-1.52369365813988	0.13723200653103	0.830161523117466	-4.75357944962989
a_HQ_transcript/83481	-1.56068216741825	-0.370596453645783	-1.52343845796367	0.137295632902677	0.830459994731197	-4.73514534789313
a_HQ_transcript/139642	1.51393061047957	-0.267642075120498	1.52312153448962	0.137374681232468	0.830590123231402	-4.789079359886
a_HQ_transcript/17649	-1.45067824425431	1.08601016997101	-1.52290764752708	0.137428050535014	0.830590123231402	-4.64208196425343
a_HQ_transcript/86658	-1.42711065275611	0.0947601318261153	-1.52286103803572	0.137439682804849	0.830590123231402	-4.68028846656058
a_HQ_transcript/64403	-0.70888293644116	2.47903056993978	-1.52283381734506	0.137446476604517	0.830590123231402	-4.74580366444948
a_HQ_transcript/99051	-1.83498410229862	0.521435585698073	-1.5228070924831	0.137453146917798	0.830590123231402	-4.79979053124043
a_HQ_transcript/93537	0.50661123456483	3.94097015865544	1.52278155524254	0.137459521054265	0.830590123231402	-5.1464660758335
a_HQ_transcript/48433	1.84800963968253	1.15916473915296	1.5227793052482	0.137460082667916	0.830590123231402	-4.68685669304345
a_HQ_transcript/146840	2.23777927915908	0.616982145254307	1.52276621943586	0.13746334901099	0.830590123231402	-4.79299516849817
a_HQ_transcript/94808	-2.6018756674561	0.720836697060493	-1.52274037815457	0.137469799424245	0.830590123231402	-4.85267358492171
a_HQ_transcript/141686	5.38338483440257	6.06743231586616	1.52271137742214	0.137477038779866	0.830590123231402	-5.0739513605175
a_HQ_transcript/124926	-1.60969147327638	0.998494361173447	-1.52252583762635	0.137523361737099	0.830590123231402	-4.73579090906235
a_HQ_transcript/60459	1.65893784572464	0.772327157963763	1.52250636578746	0.137528223921108	0.830590123231402	-4.66417632649567
a_HQ_transcript/99677	3.06335921181075	0.994505980795317	1.52248905253917	0.137532547214237	0.830590123231402	-4.77393801025529
a_HQ_transcript/43943	-1.42656252997123	2.70041654345597	-1.52234390018876	0.13756879753729	0.830590123231402	-5.00934534077209
a_HQ_transcript/79312	-0.664712616871751	3.04743061451989	-1.5223410063904	0.137569520312366	0.830590123231402	-4.88874623636575
a_HQ_transcript/46618	1.46496316643547	0.13781197731678	1.52230649873749	0.137578139418333	0.830590123231402	-4.74396183092453
a_HQ_transcript/76637	1.81514867505809	0.4638430496764	1.52228593704822	0.137583275395777	0.830590123231402	-4.70884862725699
a_HQ_transcript/128175	-1.72497777393452	-0.131198016620207	-1.52227240906846	0.137586654550707	0.830590123231402	-4.75735630018054
a_HQ_transcript/80559	1.93265524847774	3.44054023807348	1.52223484961476	0.137596036880101	0.830590123231402	-5.12553590306888
a_HQ_transcript/124015	-1.54781890375172	1.55172910445891	-1.52220727203431	0.137602926072089	0.830590123231402	-4.77192584239314
a_HQ_transcript/104545	1.96315769778571	1.89916650886968	1.52199951660322	0.137654834677962	0.830697034577265	-4.8179339964957
a_HQ_transcript/40469	1.2920911385812	1.09552485729061	1.52197998732016	0.137659714967225	0.830697034577265	-4.71462698940884
a_HQ_transcript/109676	2.52454260044959	1.76822193781258	1.52194578792413	0.137668261594958	0.830697034577265	-4.66850100935297
a_HQ_transcript/99090	1.77409380565169	0.341932084737567	1.52185448219837	0.137691081523362	0.830697034577265	-4.78082988442975
a_HQ_transcript/67933	-1.89364742797598	0.472781898145188	-1.52185043912107	0.137692092075281	0.830697034577265	-4.7255884795782
a_HQ_transcript/119350	0.688011017664027	2.25392623655015	1.52173999176151	0.137719700290534	0.830729753202388	-4.78922616260613
a_HQ_transcript/81992	1.45143803295198	0.616006193222686	1.52169464899448	0.137731035787189	0.830729753202388	-4.65652674508621
a_HQ_transcript/133738	2.16434671853554	0.491687541336556	1.52165609795538	0.137740673973089	0.830729753202388	-4.66482196738194
a_HQ_transcript/35301	2.21927366104529	0.939597700572542	1.52158890605498	0.1377574739915	0.830729753202388	-4.72622474938572
a_HQ_transcript/124499	-2.52675924052215	2.93216379319822	-1.52148157220852	0.137784314146415	0.830729753202388	-4.77882476991528
a_HQ_transcript/127358	-1.52830856526822	0.0206332458928981	-1.52147144464557	0.137786846886489	0.830729753202388	-4.86892418567193
a_HQ_transcript/111403	2.32776103018301	0.673006472836706	1.52142862798507	0.137797555057415	0.830729753202388	-4.74459905793767
a_HQ_transcript/112755	-1.50109830697924	0.179416458016655	-1.52111594572294	0.137875775230053	0.831004327925123	-4.71003987165993
a_HQ_transcript/124720	0.904581807144094	1.95512403161831	1.52109447242291	0.137881148275795	0.831004327925123	-4.87362022623078
a_HQ_transcript/8120	0.533516499185801	2.8508449323891	1.52103838777775	0.137895182563503	0.831004327925123	-4.89556663557935

a_HQ_transcript/127951	2.4462982066132	2.32730993533296	1.52101799959022	0.137900284667729	0.831004327925123	-4.93435016868939
a_HQ_transcript/101104	3.42644494463545	0.101919539144372	1.52092719683779	0.13792300972896	0.831055116567324	-4.76001301531419
a_HQ_transcript/26411	1.18937999670139	-0.574131126056745	1.52075954633137	0.137964975292977	0.831221816317388	-4.82379211535657
a_HQ_transcript/28569	1.36699105016096	-0.0939192442589915	1.52068579792215	0.137983438943467	0.831246900083518	-4.82865328233451
a_HQ_transcript/19131	-1.76628251342581	-0.0502913366864484	-1.52060773681277	0.138002984492624	0.831278495604895	-4.80021689587747
a_HQ_transcript/34268	0.799232775911088	1.77815708989716	1.52053736459914	0.138020606749984	0.831298500586788	-4.72739461167173
a_HQ_transcript/47716	-2.74142995094066	1.1734778112219	-1.52041923026894	0.138050193437943	0.831304632413738	-4.86501491194994
a_HQ_transcript/88874	-1.90838240028443	0.330915122340673	-1.52041909478737	0.138050227372175	0.831304632413738	-4.71088733652284
a_HQ_transcript/116150	2.38768548681862	-0.197228665080766	1.52033607828098	0.138071021881745	0.831333229737841	-4.84560523014873
a_HQ_transcript/129246	1.43025987968307	-0.596388984993434	1.52027952395229	0.138085189421073	0.831333229737841	-4.79365653072369
a_HQ_transcript/8699	1.73422764832861	3.00863772291015	1.52022886265404	0.138097881680921	0.831333229737841	-4.87524321589819
a_HQ_transcript/97817	-1.84442058790052	3.42981766912386	-1.52012725313765	0.138123340918997	0.831371020903463	-5.16982025365989
a_HQ_transcript/45030	1.48128392065345	-0.0840110041715034	1.52008964785537	0.138132764242459	0.831371020903463	-4.80025674672743
a_HQ_transcript/124989	-1.79858890730858	1.99573389545262	-1.51975729989117	0.138216068230927	0.831661070351556	-4.93035667555841
a_HQ_transcript/9831	1.45998051412774	1.75558677023352	1.51972062718514	0.138225262833086	0.831661070351556	-4.70039848650623
a_HQ_transcript/60600	1.57141328964888	-0.24895356993036	1.51971872900696	0.138225738758846	0.831661070351556	-4.78396451793265
a_HQ_transcript/65512	-1.66077937475579	0.300731813979888	-1.51966908737491	0.138238185757926	0.831661070351556	-4.68543533700416
a_HQ_transcript/80135	1.05419883502192	3.22093653577328	1.51955926899815	0.138265724511012	0.831740663807936	-4.96878146214654
a_HQ_transcript/110305	1.95913902642471	0.62491901628311	1.51926464717296	0.138339627603569	0.832099117688352	-4.63182491688507
a_HQ_transcript/72379	-2.60395166497391	2.56151225647095	-1.51913264765732	0.1383727487609	0.832109113211379	-4.87381482021497
a_HQ_transcript/62269	-1.4142046638021	1.20269137620464	-1.5191118063732	0.138377978814861	0.832109113211379	-4.85133546722437
a_HQ_transcript/65634	1.33653100377256	0.684656239997938	1.51904687801416	0.138394273401763	0.832109113211379	-4.71367791056845
a_HQ_transcript/128884	1.54374819009638	4.32914646049681	1.51902983879273	0.138398549864568	0.832109113211379	-5.13398766512769
a_HQ_transcript/45503	-0.964044925444131	0.600557395831578	-1.51885319032767	0.138442890949439	0.832289623033299	-4.68424683084029
a_HQ_transcript/150112	0.396721756807393	3.67108711459795	1.51874153926127	0.138470922740439	0.832372057606642	-5.09983044274606
a_HQ_transcript/66277	2.64672590164262	0.0760347040363902	1.5186056028837	0.138505057925224	0.83249115986738	-4.78482821720243
a_HQ_transcript/83600	2.04187522138177	0.0679655556752843	1.51844555966466	0.138545255309382	0.83252686234963	-4.82541713202331
a_HQ_transcript/124230	1.86316533426987	-0.20121733470394	1.51833927167525	0.138571956414519	0.83252686234963	-4.72438408169302
a_HQ_transcript/38529	-1.41216588957719	0.113970899453848	-1.51827267974492	0.138588687399755	0.83252686234963	-4.74148500491283
a_HQ_transcript/134761	1.92055772503607	0.36317861333738	1.51818585331522	0.138610504676638	0.83252686234963	-4.72347922203772
a_HQ_transcript/107520	-1.75203756597454	0.494926054461306	-1.51817653074111	0.138612847367357	0.83252686234963	-4.71046068936633
a_HQ_transcript/16161	1.01348523842391	2.82022986306648	1.51808199760633	0.138636604615244	0.83252686234963	-4.77898837087857
a_HQ_transcript/22614	-0.593430882429575	2.76776988023093	-1.51804084331946	0.138646948179268	0.83252686234963	-4.8756312411976
a_HQ_transcript/106921	-1.12273623274191	2.56440795451903	-1.51797209329454	0.138664228940136	0.83252686234963	-4.92931811852557
a_HQ_transcript/78696	1.35607015114972	0.612334480918938	1.51796140074546	0.138666916737108	0.83252686234963	-4.74650662132895
a_HQ_transcript/139438	2.32251043836289	2.51940821483906	1.51794439820264	0.138671190770128	0.83252686234963	-4.79668703825232
a_HQ_transcript/74514	1.55827191646299	0.0486325454439552	1.51790442451748	0.138681239617766	0.83252686234963	-4.73415327334414
a_HQ_transcript/101929	0.555042075428196	-0.720514175582602	1.51787319584152	0.138689090495921	0.83252686234963	-4.85630679610018
a_HQ_transcript/10573	1.38820043981429	1.4199965793055	1.51778036570585	0.138712430074638	0.83252686234963	-4.69093511430612
a_HQ_transcript/18779	1.90938544696135	0.146800514827028	1.51774136001028	0.138722237926141	0.83252686234963	-4.70486837229661
a_HQ_transcript/14058	-1.83249200374475	0.838623144665914	-1.51768389596627	0.138736688085843	0.83252686234963	-4.70712620137066
a_HQ_transcript/128939	3.62019362192229	2.82820953194771	1.51767011075922	0.138740154754694	0.83252686234963	-4.81607246156358
a_HQ_transcript/92887	2.41496978531152	2.48753609596525	1.51745156648315	0.138795123042058	0.832657882937414	-4.80514288472578
a_HQ_transcript/130542	0.493120829390737	3.86044212696273	1.51743392087159	0.138799562034511	0.832657882937414	-5.10798658884925
a_HQ_transcript/71661	-1.83908439990373	0.711791547832827	-1.51728327688203	0.138837463227698	0.832657882937414	-4.80761487327852
a_HQ_transcript/118249	-1.78675137635764	1.36488890549475	-1.51726501103268	0.138842059381148	0.832657882937414	-4.85411402608102
a_HQ_transcript/82039	1.22380704353129	3.15219153955614	1.51725088001125	0.138845615190591	0.832657882937414	-4.98723833977492
a_HQ_transcript/18243	0.411357757140526	2.55676888770548	1.5172416546915	0.138847936610796	0.832657882937414	-4.88301408630243
a_HQ_transcript/1015	2.428867241692	0.247253294869886	1.51710899747876	0.138881321366337	0.832772173342526	-4.78338230957299
a_HQ_transcript/91571	-2.27489844735645	2.23753223714663	-1.51691348296152	0.138930536698445	0.832944738821284	-5.0733588532207

a_HQ_transcript/36882	1.57632176733978	1.44767259672845	1.51688082390116	0.138938759076713	0.832944738821284	-4.7200730236873
a_HQ_transcript/9564	0.823911040205499	3.26040242262755	1.51671950485826	0.138979379169819	0.833102336019722	-4.95779251712614
a_HQ_transcript/131649	-1.4178150153165	1.00665001186839	-1.51640276689046	0.139059161548785	0.833377278310916	-4.65974007199217
a_HQ_transcript/16456	2.43745361809172	3.35261478916819	1.51634604620849	0.139073452682903	0.833377278310916	-5.10854877095567
a_HQ_transcript/116820	0.716515732339217	4.46362292108475	1.51633586436463	0.139076018188064	0.833377278310916	-5.15350592033857
a_HQ_transcript/103036	0.353983486282241	-0.809096849320308	1.51628639059161	0.139088484569276	0.833377278310916	-4.87511402300508
a_HQ_transcript/4269	2.59006507821521	0.420725806234878	1.51625287580676	0.139096930122703	0.833377278310916	-4.71414922861247
a_HQ_transcript/152613	1.38568687336045	-0.12046654695854	1.51619558822998	0.139111367254252	0.833377878569017	-4.73624206455618
a_HQ_transcript/113851	-1.69869952543047	0.274429680747377	-1.51582886811202	0.139203813567458	0.833795067826688	-4.79011182655576
a_HQ_transcript/112796	0.54652709026358	2.78996393630585	1.51580554523302	0.139209694695465	0.833795067826688	-4.98318813718078
a_HQ_transcript/87697	-1.20287718693865	1.8527512797346	-1.51566904967086	0.139244117611072	0.833808657085398	-4.71247158636942
a_HQ_transcript/97067	1.17463696693397	-0.423409576565664	1.51564352300453	0.13925055596082	0.833808657085398	-4.75261821279265
a_HQ_transcript/151551	1.81610469969489	0.287511020510894	1.51554698925727	0.139274905923873	0.833808657085398	-4.79794822876035
a_HQ_transcript/11714	0.784318843104951	3.3858108562596	1.51553325315474	0.1392783710385	0.833808657085398	-5.11511313136404
a_HQ_transcript/51845	1.23237445059315	2.6684175873788	1.51551218728125	0.139283685320314	0.833808657085398	-4.93507924904503
a_HQ_transcript/20356	0.896007900677342	1.57438021425824	1.51539044963931	0.139314399239869	0.833906641851002	-4.71989274610987
a_HQ_transcript/24	-0.677230823253272	2.00740638360863	-1.51507505061114	0.139393998532034	0.834211960875204	-4.69671114527941
a_HQ_transcript/78712	-0.963110008164105	0.943564621026005	-1.51502933037946	0.139405540282314	0.834211960875204	-4.75096159442997
a_HQ_transcript/223	-1.79503206781539	1.21960937374946	-1.51501776330346	0.139408460431147	0.834211960875204	-4.79620301150194
a_HQ_transcript/136640	0.490624569764437	2.75833293160747	1.51486371946368	0.139447354035586	0.83429472110893	-4.90184664578538
a_HQ_transcript/148049	-1.91060179681085	0.126914346788645	-1.51478923050044	0.13946616444172	0.83429472110893	-4.81338654288571
a_HQ_transcript/148470	-5.3433632547179	2.99796378432367	-1.5147716348086	0.139470608111275	0.83429472110893	-4.99018994759906
a_HQ_transcript/93150	1.5058068233367	0.382051235869295	1.51472081293323	0.139483443462716	0.83429472110893	-4.77791614665181
a_HQ_transcript/116840	1.42890359373278	0.0683799519302768	1.51467880150213	0.139494054405066	0.83429472110893	-4.66068500890458
a_HQ_transcript/105713	1.33812782477429	-0.487040500334307	1.51455602630741	0.139525067799244	0.834394356073505	-4.80246815042065
a_HQ_transcript/140257	1.63875089239046	1.10059701523546	1.51429072484459	0.139592102736593	0.83450094040627	-4.6754566181648
a_HQ_transcript/108506	0.581195965087982	4.29027558894803	1.51426085906226	0.139599650684953	0.83450094040627	-5.15678686559119
a_HQ_transcript/133286	-2.75385905261894	4.20499152622361	-1.51421244246358	0.139611887660371	0.83450094040627	-4.98040452687256
a_HQ_transcript/32142	-1.83074126465134	1.19344869831416	-1.51415865148782	0.139625483987161	0.83450094040627	-4.76813099685181
a_HQ_transcript/7787	0.985952033485029	2.43443943331434	1.51406040128302	0.13965032068044	0.83450094040627	-4.78438777659425
a_HQ_transcript/129688	4.87014969502043	5.71715244124781	1.51394496910898	0.139679505353661	0.83450094040627	-5.22261698997315
a_HQ_transcript/98965	-1.24999285404532	-0.64853105390986	-1.51387234951153	0.139697868256185	0.83450094040627	-4.7791769689204
a_HQ_transcript/50330	-1.20371431587031	1.45522610494477	-1.51382629432066	0.139709514976483	0.83450094040627	-4.69035555334623
a_HQ_transcript/62087	-1.21038709770271	0.199380641006867	-1.5138150404415	0.139712361045634	0.83450094040627	-4.74505477808723
a_HQ_transcript/87696	-1.11343475648345	1.89465716605059	-1.5138137363096	0.139712690859335	0.83450094040627	-4.74501712796969
a_HQ_transcript/63344	1.30134321538519	1.53282501809882	1.51377488081635	0.139722517663802	0.83450094040627	-4.76326443538521
a_HQ_transcript/19774	1.24699165657959	2.15094661844966	1.51372118446756	0.139736098732315	0.83450094040627	-4.87852210678212
a_HQ_transcript/68694	3.14783406153196	1.04958762958984	1.51371597492844	0.139737416403758	0.83450094040627	-4.79986050321266
a_HQ_transcript/3378	1.84689322964032	1.11057676578377	1.51369042804675	0.139743878232773	0.83450094040627	-4.81210476203439
a_HQ_transcript/82866	-1.57368376179825	0.762114721805017	-1.51361138709809	0.139763872376691	0.834534604366955	-4.70125305483952
a_HQ_transcript/73531	1.79686718589231	-0.431481856875313	1.5134140744512	0.139813794501356	0.834746943999057	-4.80038333506805
a_HQ_transcript/113760	1.59874341820072	2.02747622031332	1.51315830702558	0.139878527653894	0.835046903795175	-4.69864884887264
a_HQ_transcript/140691	-1.35395289941113	0.974707167365456	-1.51310205942418	0.139892766810443	0.835046903795175	-4.67812626466099
a_HQ_transcript/149940	3.05782027833051	3.41858518078094	1.5129078621141	0.139941937093911	0.835229025510301	-5.02088375305753
a_HQ_transcript/104294	0.999571729696787	1.3371813254433	1.51282289268888	0.139963455520192	0.835229025510301	-4.63278401860356
a_HQ_transcript/93584	0.804699430222391	2.6389516773219	1.51275630208059	0.139980321390524	0.835229025510301	-4.88044195530616
a_HQ_transcript/116523	1.49152842736994	0.890668800790111	1.51274715802591	0.139982637497148	0.835229025510301	-4.78890363971571
a_HQ_transcript/56933	1.81147635418343	0.765845413943574	1.51269787476008	0.139995121035419	0.835229025510301	-4.69756246194711
a_HQ_transcript/52425	-2.19954393676372	2.01632499515003	-1.51259698824628	0.140020678562987	0.835295772117132	-4.75502347744678
a_HQ_transcript/112319	2.18964741374925	1.03360868416439	1.51226026780144	0.140106006969188	0.835644602438471	-4.76760217945577

a_HQ_transcript/6469	-0.342551519191199	5.94135861141879	-1.51225278016481	0.140107904888614	0.835644602438471	-5.35156449290095
a_HQ_transcript/68010	-1.94294613079348	1.45142627529685	-1.51218001219021	0.140126350738913	0.835668874089621	-4.71267367224615
a_HQ_transcript/97362	1.65591206994427	0.511996393953507	1.51202213328834	0.140166377959205	0.835821831966834	-4.72254426317035
a_HQ_transcript/149624	0.987296283179707	2.73882269741118	1.51193850711348	0.140187583528498	0.835862535167148	-4.80362586533519
a_HQ_transcript/151693	-1.02606688373319	2.75484153005954	-1.51182430577686	0.140216546388512	0.835928943796781	-4.93069392856961
a_HQ_transcript/105097	-2.99865273098612	1.78464060952045	-1.51178118742073	0.140227482985178	0.835928943796781	-4.99359556292892
a_HQ_transcript/39353	-2.66194644299353	3.83321557002046	-1.51149065972592	0.140301190713886	0.836282569095239	-5.26243349432606
a_HQ_transcript/124641	1.48008651183121	0.333798983058187	1.51131373145187	0.140346093212411	0.836404408722104	-4.79353106583559
a_HQ_transcript/20881	1.9053226568885	2.04341939204589	1.51121790192904	0.140370418546254	0.836404408722104	-4.76541316078632
a_HQ_transcript/9779	0.382817119513707	3.48419674523054	1.51117951426381	0.140380163810181	0.836404408722104	-5.04091381939027
a_HQ_transcript/94714	1.83072139901448	-0.4481920239467	1.51115007686623	0.140387637288132	0.836404408722104	-4.78314276746693
a_HQ_transcript/102005	0.603692504717862	3.29968758828066	1.51105760130427	0.140411116788722	0.836404408722104	-5.05252833744481
a_HQ_transcript/39065	1.80412631592723	1.46927714428075	1.51102612870572	0.14041910838713	0.836404408722104	-4.68871167553001
a_HQ_transcript/110122	2.48282863169889	1.65050899786144	1.51101334508218	0.140422354540308	0.836404408722104	-4.83025756505028
a_HQ_transcript/84744	-4.75817014007141	2.71955762505698	-1.51087835636812	0.140456635991487	0.836483819643632	-4.91764511238586
a_HQ_transcript/48847	0.83128369709591	1.08098579883103	1.51084752249102	0.140464467443254	0.836483819643632	-4.6891013920863
a_HQ_transcript/150990	0.660612113474364	4.85848824257657	1.51073144459663	0.140493953051227	0.836573704462378	-5.24900338105398
a_HQ_transcript/140697	1.39698570992124	-0.543574729940826	1.51051221834384	0.140549653549106	0.836687210798005	-4.80074184980127
a_HQ_transcript/142469	-0.986595511487223	2.41089257014248	-1.51048198269221	0.140557337147679	0.836687210798005	-4.91246087159986
a_HQ_transcript/131290	-0.979846483635618	2.34207006174162	-1.5103615306313	0.140587950238351	0.836687210798005	-4.74897542916395
a_HQ_transcript/2837	-0.698006874660131	4.01717738046702	-1.51024045503707	0.140618727207936	0.836687210798005	-5.15854304296853
a_HQ_transcript/144637	3.03105691011001	1.60345076123123	1.51019741106277	0.140629670133749	0.836687210798005	-4.78068057763784
a_HQ_transcript/13885	-0.44455220902172	3.01947898590663	-1.51017662298299	0.140634955263214	0.836687210798005	-4.96294063722594
a_HQ_transcript/117962	-0.837653740077278	1.91017995833489	-1.51000017248153	0.140679822203804	0.836687210798005	-4.75077328377579
a_HQ_transcript/140361	0.978556777222576	5.43367530893259	1.50994878183056	0.140692891723995	0.836687210798005	-5.33533603235432
a_HQ_transcript/133382	1.20966332897116	-0.576279840008772	1.50991703789741	0.14070096523643	0.836687210798005	-4.79905758215446
a_HQ_transcript/65997	1.89096344889508	1.45597977770212	1.50988279591133	0.14070967450273	0.836687210798005	-4.6720959597279
a_HQ_transcript/132323	1.49788676068606	-0.619416767431302	1.50987532224847	0.140711575446635	0.836687210798005	-4.80691359192232
a_HQ_transcript/84564	1.53021477513234	-0.523604834917027	1.50984477729181	0.14071934484374	0.836687210798005	-4.81111982261447
a_HQ_transcript/148670	-1.5882970067315	1.66199337508645	-1.5098357986406	0.140721628713794	0.836687210798005	-4.9067600841334
a_HQ_transcript/71667	1.30839201405156	-0.386524399619667	1.50980414914009	0.14072967953127	0.836687210798005	-4.84116725778157
a_HQ_transcript/85421	0.353983486282242	-0.827289411987343	1.50977535882052	0.140737003368668	0.836687210798005	-4.88805444381117
a_HQ_transcript/128881	0.584952597873754	3.19424299644019	1.50975053950892	0.140743317285695	0.836687210798005	-5.03202510254973
a_HQ_transcript/82784	-1.00360539950471	4.83556935258992	-1.5096416718268	0.14077101540839	0.836766293451159	-5.24971694374084
a_HQ_transcript/10857	3.27978486117398	1.5523956464355	1.50952457138196	0.1408008130067	0.836857838287672	-4.87499212391255
a_HQ_transcript/147788	1.36788756429156	-0.368513165211755	1.50945141960631	0.140819429919502	0.836882918143419	-4.86560344366141
a_HQ_transcript/148150	0.582057526325692	3.6313113359068	1.50923800622748	0.140873754316863	0.837109418636679	-5.08888909042517
a_HQ_transcript/70376	1.92864301814248	0.400830362415023	1.50917743331294	0.140889176229872	0.837109418636679	-4.77903427495512
a_HQ_transcript/151474	0.353983486282245	-0.829249081316251	1.50906644226783	0.140917438164167	0.837109418636679	-4.88113262187734
a_HQ_transcript/86342	1.55539937454552	-0.0194345352368882	1.50898599841906	0.14093792463811	0.837109418636679	-4.76593614988139
a_HQ_transcript/2236	-0.495319226555683	3.22291609293164	-1.50894155655864	0.140949243585165	0.837109418636679	-5.00362772003191
a_HQ_transcript/135041	-2.43541955314239	1.43113488061766	-1.50891866924267	0.140955073066501	0.837109418636679	-4.9629871934063
a_HQ_transcript/146499	-4.38637263578958	2.46108964727995	-1.50890580249606	0.140958350358103	0.837109418636679	-4.97341036302118
a_HQ_transcript/5766	0.908754994239303	0.837383326948572	1.50877708390059	0.140991139671365	0.837218609338757	-4.69458864531536
a_HQ_transcript/69189	0.893995881299941	2.04950853876882	1.5084680985645	0.141069874529491	0.837442079385396	-4.73115338147025
a_HQ_transcript/54652	1.83861449748523	0.0984538883955576	1.50842277189903	0.141081427529274	0.837442079385396	-4.82673694893328
a_HQ_transcript/71788	-1.65914692006872	1.11599647782309	-1.50840598141799	0.141085707331846	0.837442079385396	-4.66969859976282
a_HQ_transcript/89866	1.77491232416667	0.203926335302444	1.50833990359835	0.141102551223964	0.837442079385396	-4.6789383044882
a_HQ_transcript/77773	-1.32949878432745	1.04497995665377	-1.50832911225074	0.141105302199228	0.837442079385396	-4.68827127052355
a_HQ_transcript/115908	1.47544963718321	-0.225490262667858	1.50829023173128	0.141115214140861	0.837442079385396	-4.77401031592661

a_HQ_transcript/37943	0.593216207004758	4.06906908251561	1.50819883413307	0.141138516643657	0.837494864787924	-5.15038507342878
a_HQ_transcript/152256	0.56493447616293	4.7804559831025	1.50813941746933	0.141153667027426	0.837499270896217	-5.23703754254988
a_HQ_transcript/144564	1.80773407810296	0.472541017780866	1.50808085458113	0.14116860099084	0.83750239216121	-4.68003562627539
a_HQ_transcript/5354	1.95952557692582	-0.113672444435947	1.50799933856815	0.141189390283896	0.837512961597207	-4.8291245342644
a_HQ_transcript/137624	3.26396592547769	0.76179966138228	1.50795412425122	0.141200922499361	0.837512961597207	-4.79084953970839
a_HQ_transcript/45677	1.71888629449576	3.04197693316191	1.50790439635106	0.141213606809356	0.837512961597207	-4.98758201936119
a_HQ_transcript/38178	3.49879655695576	2.98401007821552	1.50781171650227	0.141237249503754	0.837555301010931	-5.04414386404374
a_HQ_transcript/100373	0.66870933779664	6.16989022677731	1.50776345108829	0.141249563305294	0.837555301010931	-5.4040840625674
a_HQ_transcript/100392	-1.00595407731721	3.50083075273493	-1.5075298274309	0.141309179170724	0.837662356658944	-5.02111075360125
a_HQ_transcript/152757	1.22571052269771	-0.662801057363536	1.50750084505553	0.141316576276685	0.837662356658944	-4.80071325425937
a_HQ_transcript/3592	-1.43083396188661	1.13091716389655	-1.50743830290819	0.141332539828193	0.837662356658944	-4.61828455547934
a_HQ_transcript/6231	-1.0454160704773	0.0918549876923612	-1.50738833382431	0.141345295214075	0.837662356658944	-4.73721756030929
a_HQ_transcript/130281	-4.29053403901361	5.49923769105598	-1.50736469958216	0.141351328544465	0.837662356658944	-5.34538212501241
a_HQ_transcript/22577	1.69062799464105	2.09981461997061	1.50735391528097	0.141354081621036	0.837662356658944	-4.77156273604218
a_HQ_transcript/64669	-1.92642357084659	1.51235627244209	-1.50724087203171	0.141382942527404	0.837713270932941	-4.79250843162331
a_HQ_transcript/114271	-1.20190895820622	-0.560295828407614	-1.50708362615118	0.14142309662281	0.837713270932941	-4.77645532391101
a_HQ_transcript/41026	1.2279522617957	-0.472512041407851	1.50706437505945	0.141428013185571	0.837713270932941	-4.74919920931026
a_HQ_transcript/109998	-0.8676167821551	2.6822699513581	-1.50702138265445	0.141438993571154	0.837713270932941	-4.92115734423407
a_HQ_transcript/43290	-2.35417952452356	0.708521067002386	-1.50699918154191	0.141444664068418	0.837713270932941	-4.72843706838522
a_HQ_transcript/109744	-2.20126160276377	3.22540847163185	-1.50698164817648	0.141449142482226	0.837713270932941	-4.99243068137436
a_HQ_transcript/37555	0.543692635003782	4.51554789972369	1.50687598911953	0.141476132583159	0.837787758230835	-5.16250573382434
a_HQ_transcript/19999	-1.53891246740236	3.67791493099589	-1.50665521763115	0.141532540970295	0.837884259093488	-5.22866720086342
a_HQ_transcript/101205	-1.12661933390736	1.42409787779329	-1.50665169195595	0.1415334419473	0.837884259093488	-4.75063635152515
a_HQ_transcript/111666	0.449965296326597	2.85630345248764	1.50664296559378	0.141535671965988	0.837884259093488	-4.96044254418771
a_HQ_transcript/72108	-0.358122463728393	5.0127380217715	-1.50624025650672	0.14163861486567	0.837959109800878	-5.26879840231947
a_HQ_transcript/31297	-0.796134028878773	1.31358437173309	-1.50596689352705	0.141708527834911	0.837959109800878	-4.68717404225795
a_HQ_transcript/33470	-2.07904331166098	3.48775097297533	-1.505923648227	0.141719590422792	0.837959109800878	-4.98929527691104
a_HQ_transcript/124471	2.05482860086112	3.92009483427198	1.50591680671817	0.141721340613986	0.837959109800878	-4.9542601651298
a_HQ_transcript/80722	3.22362539243556	1.20680973877019	1.50591301420042	0.141722310821367	0.837959109800878	-4.86517301927246
a_HQ_transcript/27119	-1.95228239772554	-0.439250164268803	-1.50588081865453	0.141730547347607	0.837959109800878	-4.78748521280093
a_HQ_transcript/31650	2.89477778864576	2.36131096864923	1.50586549285432	0.141734468253574	0.837959109800878	-5.0796438875909
a_HQ_transcript/73863	-0.842946146930349	3.96511987545827	-1.50585607270949	0.14173687831796	0.837959109800878	-5.15779217065736
a_HQ_transcript/52574	1.49129477839543	-0.582986914460397	1.5058231752757	0.141745295106585	0.837959109800878	-4.748634594032
a_HQ_transcript/145836	1.09892370158817	3.30851305883523	1.50580187438647	0.141750745140575	0.837959109800878	-5.02028457209951
a_HQ_transcript/138004	1.37880392059902	-0.5951470883174	1.50579829045082	0.141751662140966	0.837959109800878	-4.77665064235475
a_HQ_transcript/131191	2.75078918562791	0.668763306609563	1.50576171355207	0.141761021128139	0.837959109800878	-4.85052783643011
a_HQ_transcript/70237	2.12677986004353	1.17327428460709	1.50575117715396	0.141763717184634	0.837959109800878	-4.67374562965684
a_HQ_transcript/143500	2.1766690476142	-0.275207257899909	1.50568766398545	0.141779969825067	0.837959109800878	-4.81068251754905
a_HQ_transcript/3648	1.07449881546181	3.01772978982656	1.50565247184394	0.141788975928753	0.837959109800878	-4.88765461153687
a_HQ_transcript/135293	0.560814996246729	4.44796636021773	1.50554435228673	0.141816647935684	0.837959109800878	-5.1955139836122
a_HQ_transcript/108763	-0.886259857778633	1.48997624322682	-1.50551390671124	0.141824440927878	0.837959109800878	-4.71255285170044
a_HQ_transcript/21720	1.41261911360489	0.0505199446118809	1.5054938457418	0.141829576015773	0.837959109800878	-4.6857911614352
a_HQ_transcript/109757	2.09101519584288	0.134444918523548	1.5054417797667	0.14184290425238	0.837959109800878	-4.73862895463943
a_HQ_transcript/19016	-1.59003261672078	2.7018979713631	-1.50541008962252	0.141851017024702	0.837959109800878	-4.79295305712869
a_HQ_transcript/10339	1.38178547353358	1.05831258355982	1.50540997385102	0.141851046663237	0.837959109800878	-4.67730414709875
a_HQ_transcript/19185	1.74769252308855	-0.383134557634939	1.50521448523061	0.141901100594448	0.838010859645954	-4.80154303577254
a_HQ_transcript/147599	-1.25651174937525	1.59941412657938	-1.50513673667538	0.141921011687597	0.838010859645954	-4.70523929324548
a_HQ_transcript/49900	0.514252482992457	2.66184112976564	1.50509117149804	0.141932681790397	0.838010859645954	-4.92198334822206
a_HQ_transcript/55703	-1.71403269637886	4.19664481806814	-1.50504693393027	0.141944012605223	0.838010859645954	-5.13045702511988
a_HQ_transcript/31608	-1.25511264710598	1.66588226037277	-1.50504565628841	0.141944339865557	0.838010859645954	-4.74085026893263

a_HQ_transcript/13133	1.40405695623596	0.0288278264337394	1.50499780816598	0.141956596313056	0.838010859645954	-4.74571318489493
a_HQ_transcript/89557	-1.78591379865898	1.27543663419178	-1.50498169677385	0.1419607234884	0.838010859645954	-4.78564502294361
a_HQ_transcript/29642	1.69584652214701	0.0273321848930142	1.50487087076201	0.141989115851145	0.838093351563295	-4.76443642389791
a_HQ_transcript/143891	1.51255827216632	1.5534977496834	1.50469358094123	0.142034544975327	0.838276376314935	-4.85383991370244
a_HQ_transcript/54522	-2.06782579612723	0.943114366407826	-1.50455845678639	0.142069177322767	0.838366836401607	-4.70784774733845
a_HQ_transcript/51618	0.907229125551462	3.13534016247167	1.50446416733433	0.142093347747689	0.838366836401607	-4.83428976022926
a_HQ_transcript/37913	1.50911220264229	1.7075937524044	1.50443217508667	0.142101549481806	0.838366836401607	-4.65649118019625
a_HQ_transcript/135396	-2.20817580530025	0.651448781602144	-1.50440871814648	0.142107563292476	0.838366836401607	-4.83557232884238
a_HQ_transcript/72865	1.39171607992668	2.20575422715454	1.50424434573938	0.142149710270517	0.838407646708422	-4.75408885426689
a_HQ_transcript/63614	1.2811020380985	1.24409322216358	1.50420140192852	0.142160723213032	0.838407646708422	-4.70644451631876
a_HQ_transcript/98356	5.10396047507485	2.20012748358969	1.50406293223115	0.142196238443926	0.838407646708422	-4.75090387803905
a_HQ_transcript/151545	2.39894075378309	0.0920365433374466	1.50389183918681	0.14224013088103	0.838407646708422	-4.85112178312039
a_HQ_transcript/21041	1.10755881481624	1.62209077925309	1.50385735735221	0.142248978214762	0.838407646708422	-4.69445287159391
a_HQ_transcript/142659	-3.16367681445332	4.3976838529499	-1.50384178615118	0.142252973611498	0.838407646708422	-5.07422373356571
a_HQ_transcript/98293	-0.551788235139017	2.70626853493531	-1.50384071778236	0.142253247746343	0.838407646708422	-4.85527155901116
a_HQ_transcript/113820	-0.851618751589652	1.3802360856184	-1.50370290201062	0.142288613724015	0.838407646708422	-4.68275950521305
a_HQ_transcript/132680	0.353983486282247	-0.844116892835941	1.50369425982533	0.142290831697819	0.838407646708422	-4.88557021301453
a_HQ_transcript/68289	1.27593674592792	-0.635050477344798	1.50365685011707	0.142300433033487	0.838407646708422	-4.80562105851156
a_HQ_transcript/90479	0.411327252661884	2.69861160798167	1.50364136225252	0.142304408201787	0.838407646708422	-4.92617696341256
a_HQ_transcript/5982	2.02975677106163	2.9270459092405	1.50361254864304	0.142311803838778	0.838407646708422	-4.91908118692453
a_HQ_transcript/90287	-0.493908407199912	2.84944070476825	-1.50359316803732	0.142316778464749	0.838407646708422	-4.89817344766368
a_HQ_transcript/96473	-2.1072470495368	0.104462503063399	-1.50356117478129	0.142324990819374	0.838407646708422	-4.80263642502687
a_HQ_transcript/16547	1.47042959227297	-0.536170579736221	1.5034927224684	0.142342563134727	0.838407646708422	-4.80498634260476
a_HQ_transcript/25391	1.30306088456651	-0.423635399169761	1.50348223146173	0.142345256423159	0.838407646708422	-4.86422639096171
a_HQ_transcript/36964	1.18518415035784	-0.515394086468999	1.50333791985397	0.142382308766442	0.838540916309598	-4.81897669293215
a_HQ_transcript/10905	2.3222025802675	2.51504501521746	1.50320933850199	0.142415328857413	0.838582841737209	-5.01765547405175
a_HQ_transcript/59211	-1.49823341961105	0.463681118734565	-1.50319784555558	0.142418280581299	0.838582841737209	-4.75431940024293
a_HQ_transcript/109	-1.47565905486716	3.48144866786496	-1.50273529837165	0.142537116991735	0.839167228002986	-5.00519666341872
a_HQ_transcript/141840	2.2881484306458	1.07193616438669	1.50269917445618	0.142546401205985	0.839167228002986	-4.84118676337997
a_HQ_transcript/108811	-0.979256495157269	2.46421920407172	-1.5025875530681	0.14257509210951	0.839167791474364	-4.7704639235046
a_HQ_transcript/43068	-1.28148523286414	1.56810540251129	-1.50258647194588	0.142575370021346	0.839167791474364	-4.774362347177
a_HQ_transcript/144854	1.90571682570758	0.285905730309913	1.50247998280015	0.14260274611174	0.839226591614819	-4.88590406455542
a_HQ_transcript/111271	0.940985851545683	1.21816750974463	1.5024352961999	0.142614235342196	0.839226591614819	-4.67685686854797
a_HQ_transcript/115496	1.24411543731479	-0.278884428488751	1.50226426012071	0.142658216757178	0.839312755616163	-4.79598588784742
a_HQ_transcript/88618	3.38315796701373	3.04472281437965	1.50222915464502	0.142667245372478	0.839312755616163	-5.05490767768443
a_HQ_transcript/104091	0.476287972395041	4.04848473812143	1.50205460115338	0.14271214479459	0.839312755616163	-5.12068953577468
a_HQ_transcript/77765	0.353983486282244	-0.848588974165972	1.50198298845004	0.142730568614359	0.839312755616163	-4.88697646617088
a_HQ_transcript/45492	2.26102660375099	2.03177767394687	1.50177083539483	0.142785160502099	0.839312755616163	-4.91716889314049
a_HQ_transcript/2328	1.06032973700267	2.93215731351527	1.50170609561335	0.142801822884405	0.839312755616163	-4.97698346682006
a_HQ_transcript/106527	-1.24974596693813	2.10153047121495	-1.50165259457332	0.14281559387744	0.839312755616163	-4.87740888378381
a_HQ_transcript/117350	0.480825448868246	4.39059760267423	1.50158296475055	0.142833517963813	0.839312755616163	-5.21347847651478
a_HQ_transcript/112988	-0.408523798656689	3.85758663216415	-1.50155932557776	0.14283960356279	0.839312755616163	-5.14384029174794
a_HQ_transcript/148547	0.436587184772899	5.02672741268386	1.50152714066489	0.142847889486667	0.839312755616163	-5.26805492991228
a_HQ_transcript/96338	-0.804522834056013	2.6483967490009	-1.50147333248699	0.142861743127243	0.839312755616163	-4.75461281619485
a_HQ_transcript/41465	1.79843826022672	0.946964743601899	1.50140167653295	0.14288019359353	0.839312755616163	-4.62514838314281
a_HQ_transcript/66532	2.74166916700847	1.09905507618588	1.50132753368036	0.142899286417908	0.839312755616163	-4.86565564229872
a_HQ_transcript/95972	-1.56428778358703	-0.424515539530379	-1.50130284733085	0.142905643953787	0.839312755616163	-4.78078715118627
a_HQ_transcript/70161	-1.99048411075235	1.07484254933895	-1.50124382644898	0.142920844666369	0.839312755616163	-4.70052342539156
a_HQ_transcript/27371	1.63532961171784	0.84249614486726	1.50124064712048	0.142921663533057	0.839312755616163	-4.74709803995738
a_HQ_transcript/145409	0.36357787853432	7.16899570562719	1.50118801890533	0.142935218984311	0.839312755616163	-5.4663078002084

a_HQ_transcript/89089	1.18688177223224	-0.570165583985438	1.50117693797261	0.142938073232125	0.839312755616163	-4.81995781288631
a_HQ_transcript/101777	-1.88387666910189	0.54617999020166	-1.50115497786946	0.142943729892671	0.839312755616163	-4.75499161252683
a_HQ_transcript/110082	2.25345619079701	0.763611599939453	1.501088426346	0.142960873868971	0.839312755616163	-4.84241694696215
a_HQ_transcript/131019	1.11805013375246	-0.747516903644405	1.50104305962992	0.14297256148857	0.839312755616163	-4.75147733717325
a_HQ_transcript/134981	1.77472080376297	-0.433798987763792	1.50099916534396	0.142983870503073	0.839312755616163	-4.80682264699596
a_HQ_transcript/18776	0.353983486282245	-0.851245731989613	1.50090771684978	0.143007433789246	0.839312755616163	-4.88785827096635
a_HQ_transcript/119454	-0.571863313475445	2.73002459475492	-1.50090101106076	0.143009161774473	0.839312755616163	-4.93845755936507
a_HQ_transcript/111306	1.43761918359315	-0.144242288358004	1.50078346510312	0.143039454397272	0.839312755616163	-4.86471323810425
a_HQ_transcript/70464	1.15723569374926	2.06521247239468	1.50071480129708	0.143057152045328	0.839312755616163	-4.77787975713841
a_HQ_transcript/43881	-1.89837586961517	0.903162396231667	-1.50068467623386	0.143064917138739	0.839312755616163	-4.63997032844679
a_HQ_transcript/59688	-2.10497770415705	4.41064138461667	-1.50056699838029	0.143095253248645	0.839312755616163	-5.13236011460486
a_HQ_transcript/14312	1.48655649587263	-0.494302717341666	1.50055339260626	0.143098761006823	0.839312755616163	-4.78399284350201
a_HQ_transcript/123250	2.3135672236475	0.721976058503031	1.50048550819734	0.143116263585228	0.839312755616163	-4.77526849395157
a_HQ_transcript/129617	1.88942266689799	0.794664483107428	1.50035406975295	0.143150157131617	0.839312755616163	-4.687206008972
a_HQ_transcript/143946	1.95060212798083	0.460643480082171	1.50032168591163	0.143158508822695	0.839312755616163	-4.69564929236361
a_HQ_transcript/71118	-1.50347245105996	-0.553643742207612	-1.50030644733478	0.143162438939214	0.839312755616163	-4.79630983142745
a_HQ_transcript/111748	-1.5381281538659	0.0603818282762866	-1.50029564982451	0.143165223731583	0.839312755616163	-4.70502789988687
a_HQ_transcript/146349	1.96309714231407	0.242185631535912	1.50018791663693	0.14319301164475	0.839312755616163	-4.79809778847261
a_HQ_transcript/71128	2.74032506809849	2.141606817822	1.49993315155245	0.143258741122082	0.839312755616163	-4.82376656178877
a_HQ_transcript/74901	-1.9498556332801	2.17670645440091	-1.49988677594271	0.143270708650568	0.839312755616163	-4.73904408085154
a_HQ_transcript/107398	-1.83350084036541	0.702498474568652	-1.49985150621931	0.143279810769978	0.839312755616163	-4.7343804415643
a_HQ_transcript/127431	1.16584882438308	0.630781786796705	1.49985111395692	0.143279912004431	0.839312755616163	-4.70294140255408
a_HQ_transcript/149026	2.2758598327129	-0.256987159676876	1.49981945378231	0.143288083001288	0.839312755616163	-4.81890402971362
a_HQ_transcript/30158	-1.72674906267704	-0.374853861777576	-1.49979232307412	0.143295085311203	0.839312755616163	-4.75576240715472
a_HQ_transcript/139158	-1.83853892688129	4.77638293808847	-1.49976312386288	0.14330262179909	0.839312755616163	-5.42903831008954
a_HQ_transcript/100441	0.353983486282242	-0.854043420834258	1.49973153956086	0.143310774251172	0.839312755616163	-4.88882122568423
a_HQ_transcript/6530	-1.3470225462011	2.5237890063118	-1.49964824066825	0.143332276915244	0.839312755616163	-5.0036047804986
a_HQ_transcript/140056	0.572181383058859	4.29204094147781	1.49956643842589	0.143353395756598	0.839312755616163	-5.20005767518475
a_HQ_transcript/132138	-0.539631501401606	4.91378570329491	-1.49951376767205	0.143366995059018	0.839312755616163	-5.23876091809071
a_HQ_transcript/140329	0.711418224380512	5.3975742984769	1.49946270580268	0.143380179944866	0.839312755616163	-5.29848388365464
a_HQ_transcript/138282	3.13968575039326	3.69790652564383	1.49935414420161	0.143408215296967	0.839312755616163	-4.95777004736762
a_HQ_transcript/1404	-0.876776720777561	4.58899877222191	-1.49929781953664	0.143422762521861	0.839312755616163	-5.20531046903263
a_HQ_transcript/93008	2.16709820379648	0.439949516003543	1.49921927847773	0.143443049652371	0.839312755616163	-4.77225272976813
a_HQ_transcript/101233	-0.823546435049708	2.33802211621954	-1.49921468477941	0.143444236274483	0.839312755616163	-4.84537368792738
a_HQ_transcript/94093	1.31984994133719	-0.275528889471857	1.49909153456617	0.143476050783967	0.839312755616163	-4.66893004758575
a_HQ_transcript/80915	-1.10021010371234	1.23218447018372	-1.49887794886843	0.143531241740528	0.839312755616163	-4.81731771650947
a_HQ_transcript/116645	0.422235112395439	5.07335621431316	1.49883790946282	0.143541589896622	0.839312755616163	-5.30064913634398
a_HQ_transcript/37846	0.353983486282246	-0.856132507434849	1.49881270679956	0.143548103814221	0.839312755616163	-4.88957232437839
a_HQ_transcript/52413	-0.879695319523034	1.51488452899665	-1.49868895861487	0.143580091395584	0.839312755616163	-4.7391954798749
a_HQ_transcript/9577	-0.781988060696092	2.43637481165971	-1.49866778936525	0.143585563993056	0.839312755616163	-4.83797915608405
a_HQ_transcript/44901	-1.40388413363146	1.36706843749855	-1.49859634425664	0.143604034958832	0.839312755616163	-4.92304507257676
a_HQ_transcript/149531	0.353983486282242	-0.856721658349321	1.49855068358528	0.143615840778382	0.839312755616163	-4.88978632774788
a_HQ_transcript/127789	0.641646751693042	3.83728115387433	1.49850858612508	0.143626726000084	0.839312755616163	-5.09827671873903
a_HQ_transcript/49758	1.77233130137391	1.34192665295909	1.49850110212192	0.143628661222577	0.839312755616163	-4.75736876517017
a_HQ_transcript/11946	-1.77271644089584	0.0421598287978192	-1.49848982598092	0.143631577060127	0.839312755616163	-4.78961750286331
a_HQ_transcript/44720	1.40305205046127	1.73285888274972	1.49839755843642	0.143655437820396	0.839312755616163	-4.7821106882309
a_HQ_transcript/34811	-0.503105572106327	3.42574527300062	-1.49838014529854	0.143659941285115	0.839312755616163	-5.0441233842031
a_HQ_transcript/132257	1.63638020693903	0.294025188481043	1.49833857288083	0.143670693390548	0.839312755616163	-4.79819432598704
a_HQ_transcript/18067	-1.5390975081711	0.149393823907545	-1.498275312763	0.143687055945479	0.839312755616163	-4.84871137045286
a_HQ_transcript/102343	4.78142847651892	3.79777025773454	1.49823640541477	0.14369712027401	0.839312755616163	-5.10657544181544

a_HQ_transcript/50996	2.14616532447019	2.73865714626332	1.49812918351245	0.143724858745344	0.839312755616163	-5.03747512537209
a_HQ_transcript/145862	0.544872646367222	3.36149465248427	1.49812894839401	0.14372491957557	0.839312755616163	-5.0678167526123
a_HQ_transcript/144684	0.661138645170571	3.6429441039939	1.49808209802847	0.143737041190346	0.839312755616163	-5.10312628928775
a_HQ_transcript/96572	0.905616611350432	1.82771639224016	1.497987122206	0.143761616842191	0.839312755616163	-4.76170271559832
a_HQ_transcript/141130	1.46950115986599	-0.697277904255367	1.49797355291007	0.143765128266763	0.839312755616163	-4.82290816926823
a_HQ_transcript/81723	0.353983486282246	-0.858125540744977	1.49790030227009	0.143784085051808	0.839312755616163	-4.89031715743212
a_HQ_transcript/107921	2.45619851456837	2.56737691008801	1.49789741587505	0.143784832072981	0.839312755616163	-4.86277611912239
a_HQ_transcript/59933	1.26309077007053	-0.607497737594396	1.49786872089218	0.143792258724023	0.839312755616163	-4.82586408291932
a_HQ_transcript/137229	1.89300706874316	1.83188182626662	1.49784709321203	0.143797856464629	0.839312755616163	-4.82129708923551
a_HQ_transcript/62337	-1.38815341310421	0.180014710023556	-1.49780058979292	0.143809893210331	0.839312755616163	-4.72872433369769
a_HQ_transcript/122605	0.353983486282242	-0.858477221998503	1.49773654780616	0.143826470887094	0.839312755616163	-4.89045073022637
a_HQ_transcript/446	0.589151408244478	3.83949594409522	1.49769633131257	0.143836881966791	0.839312755616163	-5.10164415324776
a_HQ_transcript/71718	-1.76301001390463	1.79884307318843	-1.49767199359838	0.143843182707394	0.839312755616163	-4.66734647681094
a_HQ_transcript/130957	2.71650153345847	2.29401209263809	1.49766403710807	0.143845242594788	0.839312755616163	-4.76366965031177
a_HQ_transcript/104448	1.01811587438763	-0.439695576248366	1.49765076030259	0.14384867993242	0.839312755616163	-4.84138596233065
a_HQ_transcript/93985	2.00816870850791	1.08553600068335	1.49758487411943	0.143865738704844	0.839312755616163	-4.71933010592999
a_HQ_transcript/39373	-1.35525276423929	-0.548059726877899	-1.4974964329273	0.143888639808374	0.839312755616163	-4.80110354292087
a_HQ_transcript/121214	0.8485592033935	2.09011019002441	1.49743505601624	0.143904534563056	0.839312755616163	-4.83379525067267
a_HQ_transcript/125838	1.93327197451083	2.10929180030394	1.49743476022636	0.143904611167056	0.839312755616163	-4.79393947317363
a_HQ_transcript/129311	-1.89184951246922	1.6156242869687	-1.49738734657888	0.143916890830577	0.839312755616163	-4.88920997748883
a_HQ_transcript/104947	-0.581326725473992	2.82866150623626	-1.49736918466411	0.143921594808722	0.839312755616163	-4.99018832862944
a_HQ_transcript/90769	1.82808558974231	-0.401715903540142	1.4973446103152	0.143927959818605	0.839312755616163	-4.82156699876359
a_HQ_transcript/42082	-2.26204779997149	1.50955400658673	-1.49734294323333	0.143928391618186	0.839312755616163	-4.69354389965217
a_HQ_transcript/108040	-2.03041421106262	2.90639199232152	-1.49717434401233	0.143972066751569	0.839483227619139	-5.03708063755372
a_HQ_transcript/33422	-0.989501388542256	2.17461778633093	-1.49709848933925	0.143991720149996	0.839513611723068	-4.83853363862066
a_HQ_transcript/120357	-4.29892942398205	2.57180253920386	-1.49701829565412	0.144012500095796	0.839550557172646	-4.93724776677818
a_HQ_transcript/75527	-1.8148641443812	0.725691491487662	-1.49692316357256	0.14403715402825	0.839599023111889	-4.81407697482006
a_HQ_transcript/69863	-2.51710547929226	2.09306122108951	-1.49687475110611	0.144049701649719	0.839599023111889	-4.95011423187114
a_HQ_transcript/107186	-1.40774335335927	2.67671138536871	-1.49674996777052	0.14408204723996	0.839689630235287	-4.88104580870751
a_HQ_transcript/105812	1.55022535679573	0.991068346220051	1.49666028553032	0.144105297732661	0.839689630235287	-4.74481149444192
a_HQ_transcript/58057	-1.74448056967638	1.43956932889089	-1.49658064499404	0.144125947395171	0.839689630235287	-4.79337515550859
a_HQ_transcript/98698	-1.19115902667265	3.73509460404739	-1.49652672299944	0.144139929951926	0.839689630235287	-4.99375236489428
a_HQ_transcript/3728	-1.68243139821894	0.995352523554839	-1.49647586335775	0.144153119404695	0.839689630235287	-4.67622742301428
a_HQ_transcript/119821	0.353983486282242	-0.861207938167279	1.49642600621067	0.14416604981983	0.839689630235287	-4.89151855535281
a_HQ_transcript/43885	4.28364825855502	1.87746309038145	1.49642478799574	0.144166365774638	0.839689630235287	-4.93678894263022
a_HQ_transcript/131950	-1.3443094872274	1.28054182264216	-1.49632326790189	0.144192697860181	0.839695494953171	-4.71103065988388
a_HQ_transcript/134076	0.353983486282241	-0.861573852549614	1.49624672322975	0.144212554420205	0.839695494953171	-4.89166447278439
a_HQ_transcript/63541	2.17248329363575	1.83627262544617	1.49623575315974	0.144215400361043	0.839695494953171	-4.82386123513429
a_HQ_transcript/59926	-1.3555871916739	1.81858965152541	-1.49618479257088	0.144228621543906	0.839695494953171	-4.79489012053745
a_HQ_transcript/65821	0.595406783772509	3.5110931239941	1.49614247613888	0.144239600831052	0.839695494953171	-5.00418537488561
a_HQ_transcript/117016	1.49279855876539	-0.456236417315108	1.49600065962374	0.144276400982391	0.839756701434894	-4.81684188746028
a_HQ_transcript/74795	1.40525851004768	1.35262353167248	1.4959419773455	0.144291630725546	0.839756701434894	-4.6920369094522
a_HQ_transcript/141352	1.2608155512603	3.97038591103966	1.49587416754392	0.144309230930113	0.839756701434894	-5.14707902390261
a_HQ_transcript/46964	-1.60398321485803	0.809245843384388	-1.49583400405359	0.144319656277025	0.839756701434894	-4.79001150016488
a_HQ_transcript/137075	0.471994340432611	3.48486479195235	1.49582363442484	0.144322348048008	0.839756701434894	-5.11275782839485
a_HQ_transcript/134035	-1.39161376371256	1.4149104505645	-1.49558246182055	0.144384963521882	0.840020937458945	-4.89199958643435
a_HQ_transcript/52863	1.35002289174194	1.14138975508553	1.49553741019061	0.144396662659816	0.840020937458945	-4.69506410580148
a_HQ_transcript/6444	-1.51236825604762	1.4252474317252	-1.49543598637138	0.144423003473861	0.840048642646528	-4.72476848527398
a_HQ_transcript/68069	-1.41537580293688	0.925661326249292	-1.49540778443684	0.144430328492455	0.840048642646528	-4.68930994399719
a_HQ_transcript/94102	-1.25806204438328	2.07029467732091	-1.49517846122553	0.144489902731064	0.840254433169758	-4.79823494696526

a_HQ_transcript/93162	-2.67190950738231	0.518735814780857	-1.49516030313725	0.14449462073295	0.840254433169758	-4.8434827108107
a_HQ_transcript/142460	0.547034330781714	3.76774719623692	1.49498487879183	0.144540207466147	0.840304909088948	-5.04120987313487
a_HQ_transcript/2278	-1.75895490108218	2.03210360231564	-1.49496488536808	0.144545403799058	0.840304909088948	-4.9369634868509
a_HQ_transcript/241	-3.09437554577761	2.61173591361184	-1.49496001678	0.144546669178028	0.840304909088948	-4.94903067482026
a_HQ_transcript/48720	-1.53222552753921	3.14704858901068	-1.49479690772067	0.144589067460071	0.840386801950828	-4.96668288977913
a_HQ_transcript/39549	1.47079817379278	-0.0554268016864907	1.49469545382194	0.144615444238329	0.840386801950828	-4.79306624320635
a_HQ_transcript/64916	-2.6147820532889	4.3055315755664	-1.49467462163717	0.144620860830156	0.840386801950828	-5.26446559632569
a_HQ_transcript/123926	2.18514011825507	0.253807575977376	1.49458562186165	0.144644003560044	0.840386801950828	-4.75740497832784
a_HQ_transcript/137711	3.51289036270133	2.65114000985389	1.49444358456819	0.144680943857511	0.840386801950828	-4.96202622254407
a_HQ_transcript/143981	2.29368489245272	1.0389687515216	1.49441652919783	0.14468798113074	0.840386801950828	-4.75406283996935
a_HQ_transcript/147051	-1.27736886074274	-0.380669499042465	-1.4943682245477	0.144700546158709	0.840386801950828	-4.78403305554296
a_HQ_transcript/1313	1.25876047515902	2.54493916694185	1.49433146481333	0.144710108704082	0.840386801950828	-4.93983516700908
a_HQ_transcript/41261	-1.39573492772199	-0.376098801677523	-1.49433134951986	0.144710138696909	0.840386801950828	-4.77485106258564
a_HQ_transcript/70080	-1.77258130945699	0.604924580630203	-1.49428465663863	0.144722285948051	0.840386801950828	-4.87084524262875
a_HQ_transcript/146640	2.11338820834532	-0.220128583521501	1.49411489253006	0.144766457327573	0.840386801950828	-4.80219779210312
a_HQ_transcript/128964	1.38048379636203	0.916886398256021	1.49409569672132	0.144771452616781	0.840386801950828	-4.88332365519429
a_HQ_transcript/57113	-1.78048096436935	4.02869395497622	-1.4940705698125	0.144777991553855	0.840386801950828	-4.94472211156864
a_HQ_transcript/3434	0.351887237599086	3.91942989934816	1.49396332945809	0.144805902063013	0.840386801950828	-5.17307240620817
a_HQ_transcript/35150	-1.27204339170351	1.18292179314482	-1.49394019403632	0.144811923882583	0.840386801950828	-4.68657335163064
a_HQ_transcript/132452	-1.37990597707706	-0.313650202064226	-1.49391990293462	0.144817205531564	0.840386801950828	-4.82241597855437
a_HQ_transcript/98651	2.280453647249	0.161898114127717	1.49391120026119	0.144819470831262	0.840386801950828	-4.82174152075462
a_HQ_transcript/6176	2.08348534030006	0.442480937649258	1.49390535824001	0.144820991521151	0.840386801950828	-4.77871341860337
a_HQ_transcript/140740	2.08198908348939	-0.124049172106443	1.49362890360358	0.144892967858675	0.840516102871857	-4.89756708714993
a_HQ_transcript/148717	2.80799535746726	1.00895976199223	1.49361422054466	0.144896791467875	0.840516102871857	-4.77307896842242
a_HQ_transcript/24829	0.780704213844994	2.02657249865904	1.49359731895607	0.144901192903604	0.840516102871857	-4.74875073831206
a_HQ_transcript/92637	0.353983486282243	-0.866741136243412	1.49355168225935	0.144913077942464	0.840516102871857	-4.89385325670031
a_HQ_transcript/18339	1.9386079369808	0.343810007779322	1.493391800235	0.144954721741332	0.840516102871857	-4.73335334603124
a_HQ_transcript/112254	1.37606256439791	1.77020422045527	1.49337478172436	0.144959155046144	0.840516102871857	-4.74408899290386
a_HQ_transcript/8628	1.07852416638819	4.10051076073967	1.49337151708533	0.144960005493734	0.840516102871857	-5.14243297278003
a_HQ_transcript/57294	-1.43963784013254	0.654608466527891	-1.49333448429382	0.144969652919445	0.840516102871857	-4.63535003005207
a_HQ_transcript/18978	-1.43351378177566	-0.522904521751839	-1.49325895018828	0.144989331931707	0.840516102871857	-4.79897076780326
a_HQ_transcript/72739	1.20846839700151	0.88386328251702	1.49315673691291	0.145015965117011	0.840516102871857	-4.70090830430212
a_HQ_transcript/15345	-1.25643988028597	1.88989773485598	-1.49309995703516	0.145030761650775	0.840516102871857	-4.7597769457902
a_HQ_transcript/68388	1.92751062988973	1.15465306898127	1.49307104225315	0.145038297154034	0.840516102871857	-4.64774579893067
a_HQ_transcript/145594	-2.58497736975772	0.510921814493547	-1.49303843564113	0.145046795164416	0.840516102871857	-4.83880295646024
a_HQ_transcript/55104	-1.03235637053684	1.3059780088601	-1.49297345773652	0.145063731045848	0.840516102871857	-4.64785474211016
a_HQ_transcript/75846	1.01805604332996	1.88418747440813	1.49296290429591	0.145066481850786	0.840516102871857	-4.72080226940652
a_HQ_transcript/82518	0.353983486282241	-0.867970931052665	1.4928777942044	0.145088667732895	0.840516102871857	-4.89439917961732
a_HQ_transcript/34406	-1.63938303679822	3.74755122496861	-1.49285914267399	0.145093530042021	0.840516102871857	-5.22266352575247
a_HQ_transcript/149657	1.5675042550818	2.44981396636186	1.4928207123839	0.145103548931996	0.840516102871857	-5.13621278136901
a_HQ_transcript/77972	0.419221629075707	5.88073175705342	1.49273848932206	0.14512498658913	0.840556518578415	-5.37226422706181
a_HQ_transcript/39828	1.63243843197675	0.0574441963197382	1.49252673990439	0.145180206770285	0.840623872587648	-4.77348179281153
a_HQ_transcript/78213	1.92152780679932	-0.180010565842822	1.49250087001929	0.14518695429369	0.840623872587648	-4.8096103865694
a_HQ_transcript/140568	1.61486821227869	0.986182790987519	1.49249469622884	0.145188564612267	0.840623872587648	-4.74938909708749
a_HQ_transcript/14147	-0.750988972194652	2.48551379704803	-1.49247208715816	0.145194461890655	0.840623872587648	-4.83232999710842
a_HQ_transcript/152525	-2.71984714909756	-0.150260686278866	-1.49232169673596	0.145233694137961	0.840767271472103	-4.82913950005625
a_HQ_transcript/60408	-2.96957967220065	3.1896524370115	-1.49224732584001	0.145253098362395	0.840771423919728	-5.27296015427802
a_HQ_transcript/9205	-1.36562735674797	1.48911004348828	-1.49220807688136	0.145263339705922	0.840771423919728	-4.74546998670965
a_HQ_transcript/121774	2.30822533999722	0.380219153344038	1.4921111892126	0.145288623361145	0.840834040097235	-4.81972575498835
a_HQ_transcript/81369	3.44352259248975	1.09390866909892	1.49182982945986	0.145362066556359	0.841111091930841	-4.88430056581361

a_HQ_transcript/30166	-1.61197827548665	1.81148276819452	-1.49181692616452	0.145365435410426	0.841111091930841	-4.80643349647253
a_HQ_transcript/111040	-1.01110608805886	1.50149526540283	-1.4917073126966	0.145394056342311	0.841114954859849	-4.69788279616397
a_HQ_transcript/130757	2.5068556823146	0.214453705134202	1.49170353381163	0.145395043119181	0.841114954859849	-4.82095268074968
a_HQ_transcript/84183	1.72444959806701	0.168069522132905	1.49161076405239	0.145419269687019	0.841171391020705	-4.72511627601659
a_HQ_transcript/150322	-1.27498396359915	1.42748837499933	-1.49155402626747	0.145434088197823	0.841173400871945	-4.67475844550026
a_HQ_transcript/134985	0.465479824651373	3.61984322330954	1.49144335976939	0.145462995040848	0.841256887447462	-5.11611770870658
a_HQ_transcript/143952	2.12546088387407	1.77948723806065	1.49127968172164	0.145505757301631	0.841264852677304	-4.72956669748757
a_HQ_transcript/68706	-2.67822574610333	2.41964025593683	-1.49127556964012	0.14550683174706	0.841264852677304	-4.85146774940947
a_HQ_transcript/12029	-0.904242409549524	2.37312245713799	-1.49121981464676	0.145521400591962	0.841264852677304	-4.92478414171188
a_HQ_transcript/146553	-1.48309563461428	-0.387187007820612	-1.49109246206741	0.145554682353539	0.841264852677304	-4.79753781762621
a_HQ_transcript/123531	0.353983486282241	-0.871131813941919	1.49107055477776	0.145560408122769	0.841264852677304	-4.89586051904164
a_HQ_transcript/147145	0.601324884193351	3.24949994006916	1.49104400351077	0.145567347900778	0.841264852677304	-4.98990484238529
a_HQ_transcript/97896	1.24872204617455	0.597377423987893	1.49097683706293	0.145584904564332	0.841264852677304	-4.7242621408751
a_HQ_transcript/24946	-2.12663642078789	0.395611643667369	-1.49089972236036	0.14560506369803	0.841264852677304	-4.80848690814593
a_HQ_transcript/60093	-0.942504167535491	2.10517353559093	-1.49083848056267	0.145621074969703	0.841264852677304	-4.70642751225764
a_HQ_transcript/142647	1.47791415862098	1.75514515154685	1.49081969451325	0.145625986743458	0.841264852677304	-4.78611900763182
a_HQ_transcript/112183	1.47741076341531	1.70063894358908	1.49073572151188	0.145647943827657	0.841264852677304	-4.80039151932483
a_HQ_transcript/39091	-0.399042945519542	3.71270169416928	-1.49063332097522	0.14567472289431	0.841264852677304	-5.12150489774837
a_HQ_transcript/11499	-2.13369676481908	0.623046466367752	-1.49057877633321	0.145688988634725	0.841264852677304	-4.72173091473031
a_HQ_transcript/76443	1.27729196434919	1.7755759059521	1.49047359128552	0.145716502152119	0.841264852677304	-4.75919784882818
a_HQ_transcript/141440	0.723609928213742	3.21297428992551	1.49045092598317	0.145722431317322	0.841264852677304	-5.05919068335241
a_HQ_transcript/2597	-1.12153623349038	2.69631939813288	-1.49039882025001	0.14573606273093	0.841264852677304	-4.86764423730598
a_HQ_transcript/128871	1.5824527633782	0.858418870593642	1.49038903123934	0.145738623754144	0.841264852677304	-4.74018210716583
a_HQ_transcript/3836	-0.7215753820551	2.63907030556091	-1.49015522294329	0.145799803926905	0.841264852677304	-4.8231986558901
a_HQ_transcript/141200	1.64157897842503	-0.220309746680218	1.49013406212223	0.145805342053882	0.841264852677304	-4.82537649819896
a_HQ_transcript/22491	0.783308966358334	1.27471269642669	1.49012041666746	0.145808913378075	0.841264852677304	-4.75045606379555
a_HQ_transcript/89046	3.03456767053867	0.292692153753587	1.49008521298485	0.145818127301756	0.841264852677304	-4.807938644996
a_HQ_transcript/41572	-0.659790120775287	2.55594147471298	-1.49007023158348	0.145822048552574	0.841264852677304	-4.85904895170783
a_HQ_transcript/107213	1.06567942368754	0.617986092634607	1.49003454142732	0.145831390479199	0.841264852677304	-4.72816208447621
a_HQ_transcript/45200	-2.47083795563519	0.483080859790943	-1.49001731338034	0.145835900105686	0.841264852677304	-4.70942984574337
a_HQ_transcript/131865	0.536253954462324	6.56014016437708	1.48997242956746	0.1458476494508	0.841264852677304	-5.45284038175312
a_HQ_transcript/83129	-1.82392639770964	0.111531418548069	-1.48992113053028	0.145861079053353	0.841264852677304	-4.80415467525782
a_HQ_transcript/23856	-1.03632497108127	1.91372217734272	-1.48988882336144	0.1458695372728	0.841264852677304	-4.8901073183541
a_HQ_transcript/15984	0.908880893635272	2.2256561786761	1.48986726382965	0.145875181912019	0.841264852677304	-4.83467055819607
a_HQ_transcript/22972	0.471225835211366	2.80571934075756	1.48978357035804	0.145897095894231	0.841264852677304	-5.02028775755512
a_HQ_transcript/52049	0.353983486282242	-0.873302988312266	1.48977622423921	0.145899019500088	0.841264852677304	-4.89690468028515
a_HQ_transcript/127734	3.04118452844468	0.690570118061136	1.48970771004522	0.145916961149903	0.841264852677304	-4.86512142045695
a_HQ_transcript/103384	-1.61568028608325	0.790677950117539	-1.48964987085949	0.145932108736181	0.841264852677304	-4.82739635171661
a_HQ_transcript/43212	2.79293033241579	2.76236192100316	1.48954025907797	0.145960818576016	0.841264852677304	-4.7779869176072
a_HQ_transcript/150668	0.353983486282245	-0.873877100716437	1.4894218616309	0.14599183466532	0.841264852677304	-4.89719019630123
a_HQ_transcript/147108	-1.72766410658763	0.783652059912462	-1.489311759785	0.146020682326974	0.841264852677304	-4.72694128465332
a_HQ_transcript/52617	-1.81800635256023	0.878139103882067	-1.48929810232777	0.146024261020341	0.841264852677304	-4.82178512348005
a_HQ_transcript/24414	-1.95944342415377	0.971639889203855	-1.48928121923772	0.146028685030409	0.841264852677304	-4.76830492766438
a_HQ_transcript/142409	1.70213908060847	0.448022898710313	1.48928061630611	0.14602884302335	0.841264852677304	-4.81781457960297
a_HQ_transcript/56490	0.986068931726392	-0.271087825797313	1.48922239811584	0.146044099234288	0.841264852677304	-4.77544825322763
a_HQ_transcript/146172	0.945412170684723	3.83839687207597	1.48921798052525	0.146045256926188	0.841264852677304	-5.14018022710403
a_HQ_transcript/90328	-0.454628492989259	4.62141836583013	-1.48917031040469	0.146057750020977	0.841264852677304	-5.23836343777611
a_HQ_transcript/1162	0.840412135744366	1.93364616082403	1.48911043620232	0.146073442699778	0.841271879248263	-4.72479274995599
a_HQ_transcript/18921	-1.78039499026387	0.748793399011386	-1.48902889042151	0.146094817544557	0.84131162613731	-4.72950825477209
a_HQ_transcript/142658	1.9080451555713	-0.338604521636257	1.48887907128936	0.146134094782864	0.841454448889382	-4.75677660738546

a_HQ_transcript/134364	2.20227571873732	2.75799385395842	1.48872153390109	0.146175404593488	0.841500531644252	-4.79408407552586
a_HQ_transcript/126167	1.6568270370326	0.143360043139759	1.48871992675694	0.146175826070647	0.841500531644252	-4.74228815485416
a_HQ_transcript/77810	-0.772611563017442	3.04923329666336	-1.48863886652392	0.146197085561053	0.841500531644252	-5.01228000852445
a_HQ_transcript/147529	0.472221677840475	4.67596874205528	1.48862773715742	0.146200004629014	0.841500531644252	-5.24533431647906
a_HQ_transcript/46906	-1.92064977146686	2.9771898666903	-1.48848421470259	0.146237652624705	0.841547926390698	-4.93671424133533
a_HQ_transcript/55221	-0.64139143795168	2.48030526622321	-1.48842747757461	0.146252537726937	0.841547926390698	-4.94682415484484
a_HQ_transcript/215	-0.51155295355655	2.14907503486642	-1.48836874938139	0.146267946467026	0.841547926390698	-4.81224531001834
a_HQ_transcript/144540	-5.30913044414883	2.04818746741319	-1.48835111603507	0.146272573249105	0.841547926390698	-4.91321192590659
a_HQ_transcript/13095	-0.965911031617145	5.5964881569434	-1.48825717289136	0.146297224797226	0.841547926390698	-5.32777210562715
a_HQ_transcript/111766	7.41227102078357	3.28929618327625	1.48823741606526	0.146302409594119	0.841547926390698	-4.93280121940937
a_HQ_transcript/104738	0.353983486282246	-0.875809954159611	1.48821008883547	0.146309581339533	0.841547926390698	-4.89816538834481
a_HQ_transcript/152385	0.820614582688724	8.28490852979101	1.48810236189491	0.146337855889578	0.841559623820148	-5.60480707134735
a_HQ_transcript/38547	-1.54712692209332	1.28849982432276	-1.48805215464794	0.146351035028569	0.841559623820148	-4.73866563253019
a_HQ_transcript/13337	-1.98773077430382	1.11006906855493	-1.48801756710116	0.146360114631012	0.841559623820148	-4.78481353375887
a_HQ_transcript/126220	1.49272216665739	-0.195450391327354	1.48795272305165	0.146377138105704	0.841559623820148	-4.72499756899461
a_HQ_transcript/6459	-0.957826746750157	1.87666857598868	-1.48790800789594	0.146388878075848	0.841559623820148	-4.81138930533584
a_HQ_transcript/73288	1.44289429321835	1.41774195789395	1.48787143362888	0.146398481215066	0.841559623820148	-4.7481907128274
a_HQ_transcript/129555	1.31929189404487	1.72667572381958	1.4877078559134	0.146441437245583	0.841723312984404	-4.7280893497757
a_HQ_transcript/27051	-2.04635003888472	0.0209895767570835	-1.4875882393097	0.146472855334761	0.841733139015211	-4.8197935995677
a_HQ_transcript/96435	2.29292793334837	0.785851319556454	1.48755879220102	0.146480590639602	0.841733139015211	-4.79729485167573
a_HQ_transcript/71477	0.353983486282241	-0.876845870988079	1.4875359589559	0.146486588808799	0.841733139015211	-4.89870713092575
a_HQ_transcript/143032	3.42895807187543	2.50219977773123	1.48744879416902	0.14650948833193	0.841747582003472	-4.88094388960574
a_HQ_transcript/134387	-1.79694670723056	1.28856420697703	-1.48741615384863	0.146518064180965	0.841747582003472	-4.78097218672419
a_HQ_transcript/43844	-1.56506703927774	1.16087516715351	-1.48735718426626	0.146533558743685	0.841753404748779	-4.81114478375558
a_HQ_transcript/121158	1.59814388508511	-0.355929186244957	1.48727064448775	0.146556299893035	0.841800849820389	-4.82217648255695
a_HQ_transcript/2182	0.656706563316372	4.22447425672441	1.48714341926256	0.146589737600313	0.841909719121726	-5.22663980332622
a_HQ_transcript/89812	-1.12688641684365	0.769016167406981	-1.48701407745578	0.146623737853795	0.842008462545892	-4.70997840108671
a_HQ_transcript/110412	1.5781559273996	-0.291113305133076	1.4869426061787	0.146642528311543	0.842008462545892	-4.82513512486626
a_HQ_transcript/106523	1.60019099844178	-0.0558133652614038	1.48691271844633	0.146650386643521	0.842008462545892	-4.82609041778551
a_HQ_transcript/145546	1.90096997139411	-0.29302539698243	1.48672319210305	0.146700226337571	0.842191040025683	-4.82104694203193
a_HQ_transcript/149118	0.353983486282247	-0.878187625423631	1.48663829378202	0.146722556423779	0.842191040025683	-4.89942765165483
a_HQ_transcript/72047	1.85161938403645	1.99487871540217	1.48662652773734	0.14672565136148	0.842191040025683	-4.86178136952624
a_HQ_transcript/13282	1.23748151438321	2.02206902177738	1.48656244070028	0.146742509718486	0.842204641085717	-4.77572657961407
a_HQ_transcript/143109	1.42728215871274	-0.273774965985246	1.4864859856357	0.146762623563653	0.842236921957551	-4.8093905069701
a_HQ_transcript/67791	1.69571043761791	0.300399143526933	1.4864162975385	0.146780959073172	0.842258992004475	-4.69686360022763
a_HQ_transcript/112555	1.43308550203683	0.421049123760312	1.48605911317427	0.146874965989314	0.842372784927832	-4.75250017116051
a_HQ_transcript/5402	1.80721619594311	1.04958590111022	1.48605225930964	0.146876770320625	0.842372784927832	-4.71598334988083
a_HQ_transcript/116085	0.885754235898053	4.32319516939188	1.48576894607636	0.14695137017927	0.842372784927832	-5.16225928442593
a_HQ_transcript/62306	1.32700403906279	0.310133848174582	1.48573116592257	0.146961320449303	0.842372784927832	-4.78227896753728
a_HQ_transcript/48293	-1.48933747944614	0.985027116317139	-1.48572048450941	0.146964133742213	0.842372784927832	-4.75145051360296
a_HQ_transcript/123899	0.60682699847488	2.86276668310024	1.48571812027972	0.146964756443791	0.842372784927832	-5.02703735389638
a_HQ_transcript/128864	-1.251388693795	1.68290467865895	-1.48566155763967	0.146979654798964	0.842372784927832	-4.89537432788589
a_HQ_transcript/108187	0.505212663319111	4.11837198968307	1.48558971327469	0.146998580036968	0.842372784927832	-5.21214606171429
a_HQ_transcript/72703	-0.980805594207527	1.59801400459069	-1.48557588328364	0.147002223356127	0.842372784927832	-4.70396597680193
a_HQ_transcript/141195	0.353983486282243	-0.879766974359443	1.48554369517249	0.147010703146982	0.842372784927832	-4.90030491529759
a_HQ_transcript/3995	2.04954265193476	0.337332934072017	1.48549121714369	0.147024529050647	0.842372784927832	-4.82427760641221
a_HQ_transcript/125627	-1.46065004605144	1.12706752941622	-1.48548740755718	0.14702553276788	0.842372784927832	-4.72167392481468
a_HQ_transcript/148566	1.47506177437124	-0.44762560479342	1.48547126388384	0.147029786225544	0.842372784927832	-4.84403647675718
a_HQ_transcript/22384	-1.45336131009492	0.431452509701821	-1.48543622374188	0.14703901877298	0.842372784927832	-4.7912719485511
a_HQ_transcript/138763	-3.19452285875778	1.13238999435176	-1.48535690869892	0.147059918800673	0.842372784927832	-4.95640692925153

a_HQ_transcript/143245	-2.17250010590454	0.907791788278549	-1.48531351531625	0.147071354244121	0.842372784927832	-4.8846332589338
a_HQ_transcript/24304	4.08269944331987	1.65275826222037	1.48521835058576	0.14709643546462	0.842372784927832	-4.73885362796878
a_HQ_transcript/92786	-1.68595325818629	1.94345945741862	-1.48518676744624	0.147104760141855	0.842372784927832	-4.78311231930829
a_HQ_transcript/145920	3.51208030608151	5.84868424948177	1.48505463512272	0.147139591639332	0.842372784927832	-5.21068113752249
a_HQ_transcript/146140	1.85762688530331	0.778242290550657	1.48498672963767	0.147157494820337	0.842372784927832	-4.68939904444974
a_HQ_transcript/145637	0.353983486282244	-0.880545655778445	1.48498290082539	0.147158504332954	0.842372784927832	-4.90075379717283
a_HQ_transcript/148660	2.16798951569702	0.204515174958971	1.48492059338938	0.14717493322004	0.842372784927832	-4.85148748883584
a_HQ_transcript/4927	1.41361250678514	1.46144495204969	1.48483975783875	0.147196249686879	0.842372784927832	-4.74538770214684
a_HQ_transcript/36258	-1.47167253700557	0.393369121994831	-1.48482929152095	0.147199009852604	0.842372784927832	-4.76123226615894
a_HQ_transcript/110104	0.473163019095494	3.52800064617151	1.48480115909049	0.147206429111213	0.842372784927832	-5.07474528198614
a_HQ_transcript/9857	2.64011671878918	2.79605598769456	1.48477158963979	0.147214227672193	0.842372784927832	-5.02226356394159
a_HQ_transcript/59233	1.44921880390692	0.0380623719150854	1.48467552990615	0.147239564468247	0.842372784927832	-4.80433423649737
a_HQ_transcript/66385	2.03152456660288	0.874654574965704	1.4846436035692	0.147247986158115	0.842372784927832	-4.75424611504568
a_HQ_transcript/129079	1.41636263011812	-0.554610817690906	1.48464239204419	0.147248305747869	0.842372784927832	-4.84091899654279
a_HQ_transcript/133851	0.888956896785068	3.04051130835996	1.48456835925437	0.147267836006672	0.842372784927832	-4.95329833102767
a_HQ_transcript/87884	1.62732330061471	4.58872241815586	1.48455766789222	0.147270656618295	0.842372784927832	-5.23968440812527
a_HQ_transcript/6458	1.59846950765952	1.11129860990858	1.48448057410127	0.147290996900397	0.842372784927832	-4.78371789300879
a_HQ_transcript/136081	1.09976439096337	0.802509157848773	1.48441922738145	0.147307184110312	0.842372784927832	-4.82937136580658
a_HQ_transcript/113085	2.5726475613286	0.827822007509005	1.48440885454445	0.147309921272454	0.842372784927832	-4.82159245953799
a_HQ_transcript/6913	-0.612557421380832	2.55411940891291	-1.48437532585655	0.147318769029537	0.842372784927832	-4.89093771175093
a_HQ_transcript/152523	2.66723755308973	0.876088664752254	1.4841347546999	0.14738226490001	0.842372784927832	-4.91063026090864
a_HQ_transcript/112106	-3.31270218315458	3.57662927310928	-1.48413242504536	0.147382879891387	0.842372784927832	-5.07767258385464
a_HQ_transcript/110379	-2.5598091457081	1.79666223830145	-1.48408107973487	0.14739643474981	0.842372784927832	-4.73804192411135
a_HQ_transcript/2155	-1.88362584301076	0.118347119128845	-1.4840607087695	0.147401812840544	0.842372784927832	-4.7656681492455
a_HQ_transcript/4087	-0.606892332405896	2.46090458324552	-1.48405025675644	0.1474045723129	0.842372784927832	-4.81392599031204
a_HQ_transcript/34650	-1.6106342052115	0.223035317545041	-1.48402639034295	0.147410873522727	0.842372784927832	-4.7593961138623
a_HQ_transcript/47491	-1.03530850167315	2.94248126632885	-1.48402316175574	0.147411725950777	0.842372784927832	-4.93459425418331
a_HQ_transcript/114194	-1.44015533867259	0.585923255406958	-1.48387718750335	0.147450270936499	0.842372784927832	-4.75935556213406
a_HQ_transcript/7401	-1.27979046897053	1.10010566283008	-1.48384047260657	0.147459966895577	0.842372784927832	-4.74300696468888
a_HQ_transcript/25768	-0.71576962287883	2.64707951094747	-1.4837890760305	0.147473540964823	0.842372784927832	-4.78585159114033
a_HQ_transcript/89059	-1.24154267563618	3.84583411456038	-1.48369854225412	0.14749745377733	0.842372784927832	-5.09158003197425
a_HQ_transcript/88765	-2.37397137563742	4.27503542558406	-1.48353952077356	0.147539463850709	0.842372784927832	-5.28560784056031
a_HQ_transcript/17076	-0.610934017696744	2.66789425798638	-1.48345436002154	0.147561965434264	0.842372784927832	-4.86484092141242
a_HQ_transcript/56010	-1.68070987957298	1.96914490667663	-1.48341399894826	0.147572630789865	0.842372784927832	-4.74176576570385
a_HQ_transcript/100662	-1.04252013035584	-0.729376683982948	-1.48338912470264	0.147579204080586	0.842372784927832	-4.81970223934389
a_HQ_transcript/117001	0.353983486282244	-0.831863271794232	1.48337034317102	0.14758416746034	0.842372784927832	-4.90204241564532
a_HQ_transcript/27780	-0.421455588397466	2.54841210293999	-1.48331583462342	0.14759857314494	0.842372784927832	-4.91070154684691
a_HQ_transcript/32547	1.78905869202255	0.116384854146536	1.48324364835203	0.14761765248294	0.842372784927832	-4.85554086391799
a_HQ_transcript/129628	1.7616637424063	0.959001002652491	1.48319742236266	0.147629871372233	0.842372784927832	-4.67292240116086
a_HQ_transcript/128523	0.353983486282245	-0.882924717822017	1.48319622035685	0.147630189108531	0.842372784927832	-4.90218137003885
a_HQ_transcript/28679	-1.28884218371268	2.69289333627166	-1.48318575028611	0.147632956773449	0.842372784927832	-5.02210871653613
a_HQ_transcript/86456	-1.77831833550166	0.0775811887141668	-1.483111028468	0.147652709989533	0.842372784927832	-4.84917819231845
a_HQ_transcript/139191	-3.0140454117207	0.0761142887872751	-1.4831065707685	0.147653888479241	0.842372784927832	-4.8590822220223
a_HQ_transcript/91498	-3.40671166005749	2.3681079885344	-1.48309371941869	0.147657286054865	0.842372784927832	-4.98595056318796
a_HQ_transcript/75906	0.470519308839619	3.27549215389703	1.48296208725369	0.147692089908804	0.842372784927832	-5.08605944708258
a_HQ_transcript/103294	-2.57976548080959	2.97182429155368	-1.48294565275106	0.14769643569059	0.842372784927832	-5.19761879040573
a_HQ_transcript/2442	1.12043707626406	2.72425304183378	1.48290238129163	0.147707878468187	0.842372784927832	-4.80342492926122
a_HQ_transcript/65272	1.11315242557205	-0.487522656360103	1.48282037856021	0.147729565353421	0.842372784927832	-4.78653466295697
a_HQ_transcript/42885	-2.39747567813471	2.07639259401487	-1.48278961631944	0.14773770155852	0.842372784927832	-4.9114703702132
a_HQ_transcript/83217	-2.16876262577278	2.15079448066799	-1.48260425308249	0.147786735264193	0.842372784927832	-4.8928541322576

a_HQ_transcript/7254	1.73469193858977	0.0853285677118825	1.482565227589	0.147797060245348	0.842372784927832	-4.83467733598833
a_HQ_transcript/5416	0.650425758976714	3.1033552030311	1.48249822578987	0.147814788267547	0.842372784927832	-4.99532522896013
a_HQ_transcript/145173	-1.51180951496041	-0.494872040419969	-1.48240277237245	0.147840047247059	0.842372784927832	-4.82245584012973
a_HQ_transcript/54502	1.34921450456974	0.901952126315316	1.48235099407061	0.147853750317315	0.842372784927832	-4.79171779187417
a_HQ_transcript/69317	-1.6554500905371	1.43035607510325	-1.48234756240865	0.147854658538738	0.842372784927832	-4.69853935517236
a_HQ_transcript/15761	-1.85677317667335	2.36739206528393	-1.48220611252625	0.147892098454402	0.842372784927832	-4.86619262377828
a_HQ_transcript/121442	0.353983486282244	-0.884206445695309	1.48218355038475	0.147898071056219	0.842372784927832	-4.90298877299593
a_HQ_transcript/75394	1.02474985658113	1.16600462144682	1.48216490161957	0.147903007863017	0.842372784927832	-4.70701109818709
a_HQ_transcript/31290	-1.14382071359819	0.237962860489915	-1.48215926865877	0.147904499078232	0.842372784927832	-4.76025183702031
a_HQ_transcript/143903	1.31800356591487	-0.457698075374977	1.48214630021894	0.147907932262464	0.842372784927832	-4.85699673935428
a_HQ_transcript/141863	1.65173934002517	-0.425627423521706	1.48208943215523	0.147922987912884	0.842372784927832	-4.8845089368579
a_HQ_transcript/77056	-1.7100507022912	0.7466508530188	-1.48208118234367	0.147925172127685	0.842372784927832	-4.85510080925722
a_HQ_transcript/147606	3.07929753605494	3.70677844935488	1.48198544075251	0.147950522496025	0.842372784927832	-5.05589066338681
a_HQ_transcript/103991	2.14753455265933	1.70167656011114	1.48194358953767	0.147961604912276	0.842372784927832	-4.82759551637209
a_HQ_transcript/124420	0.381823033263727	3.63454830138647	1.48186221534264	0.147983155116231	0.842372784927832	-5.15925972260191
a_HQ_transcript/118988	2.20452954571426	2.35024976860256	1.48175592358682	0.148011307979535	0.842372784927832	-4.89605827530977
a_HQ_transcript/72706	-2.00665808368728	0.745566732913572	-1.48174741949102	0.148013560593653	0.842372784927832	-4.81272722852171
a_HQ_transcript/151417	4.42040860634989	5.94916469651856	1.48166352103333	0.148035785571872	0.842372784927832	-5.30582601298699
a_HQ_transcript/29319	0.71789779942587	2.27150422974546	1.4816535601404	0.14803842442245	0.842372784927832	-4.77957934459403
a_HQ_transcript/96106	0.353983486282244	-0.884959459007243	1.48156793346036	0.148061110286759	0.842372784927832	-4.90347899336535
a_HQ_transcript/116480	0.686507943709687	3.1019974193039	1.48149469146074	0.148080517169069	0.842372784927832	-5.01444198741002
a_HQ_transcript/116636	1.51675252713674	-0.25815916468746	1.4814571495852	0.14809046540234	0.842372784927832	-4.85392673355498
a_HQ_transcript/35200	-1.51368311352013	-0.0355244392464686	-1.48144382752746	0.148093995747002	0.842372784927832	-4.74836754535759
a_HQ_transcript/148080	1.11111261958624	0.956896858409739	1.48143475682376	0.148096399521548	0.842372784927832	-4.74257003025991
a_HQ_transcript/126357	3.24215900344731	0.277614844211689	1.48138759878548	0.148108897102226	0.842372784927832	-4.87646948279308
a_HQ_transcript/60530	1.13541610342231	5.26559036179879	1.48129950895234	0.148132244475017	0.842372784927832	-5.31704249428583
a_HQ_transcript/102380	1.17145134503646	1.11075664135126	1.48126028994258	0.148142640047209	0.842372784927832	-4.71791072587708
a_HQ_transcript/128126	3.21253357862192	1.73116736957589	1.48122416028889	0.148152217256487	0.842372784927832	-4.94115645059162
a_HQ_transcript/15139	1.68294016256144	3.04026801015889	1.48114095345522	0.148174275515932	0.842372784927832	-5.03708153142388
a_HQ_transcript/142499	1.73009670724339	-0.0659162628647517	1.48112879069876	0.148177500101278	0.842372784927832	-4.8022587587644
a_HQ_transcript/131206	5.16067621264096	4.89240772262785	1.48106569809083	0.148194228089643	0.842385496811539	-5.33781371156954
a_HQ_transcript/21488	0.353983486282242	-0.885812438661354	1.48083219848896	0.148256149878635	0.842655077749615	-4.90406425764057
a_HQ_transcript/74157	0.353983486282243	-0.886091683288372	1.48059024712512	0.1483203348079	0.842937467903172	-4.90425658125134
a_HQ_transcript/78236	1.64102145716927	-0.514556450611839	1.4804826811878	0.148348877062756	0.84301725739602	-4.83381928034737
a_HQ_transcript/141297	-2.63378491063517	1.12214371451755	-1.48039567684006	0.148371966581276	0.843066048234251	-4.82469513676932
a_HQ_transcript/127534	1.35481764475332	-0.53322625885068	1.48024622227051	0.148411636063099	0.843097068763558	-4.83068221123374
a_HQ_transcript/139038	1.44588019770237	1.16790760554249	1.48018194144177	0.148428700625099	0.843097068763558	-4.73270861457565
a_HQ_transcript/44371	1.67383894519242	1.43636033744067	1.48016050775873	0.148434390950329	0.843097068763558	-4.66408025776816
a_HQ_transcript/39156	0.353983486282241	-0.886685623367346	1.48007383806509	0.148457402247577	0.843097068763558	-4.90466682806165
a_HQ_transcript/52267	1.58679240420372	2.27403459906082	1.47995848261682	0.148488034198591	0.843097068763558	-4.85207292165243
a_HQ_transcript/68637	0.353983486282242	-0.886834513159264	1.47994400183776	0.148491879839886	0.843097068763558	-4.90476992157881
a_HQ_transcript/48165	0.353983486282245	-0.886862968792255	1.47991917025698	0.148498474515139	0.843097068763558	-4.90478963618673
a_HQ_transcript/24982	-1.37687323284002	1.64638003521056	-1.47988586890572	0.148507318926727	0.843097068763558	-4.82835927644201
a_HQ_transcript/78637	1.33087347723638	-0.352446345360681	1.47983596755393	0.148520572873486	0.843097068763558	-4.75059269921193
a_HQ_transcript/139796	3.59237719101683	0.324324034631508	1.47970585937867	0.148555134435515	0.843097068763558	-4.86354851173256
a_HQ_transcript/131026	-2.74801935365156	2.79949113136656	-1.4796472082665	0.148570716450047	0.843097068763558	-4.95372741962568
a_HQ_transcript/88446	1.42758924256003	1.39813170059888	1.47961808194289	0.14857845501197	0.843097068763558	-4.79601396732351
a_HQ_transcript/70748	1.9658423351067	0.0727496097735416	1.47952573946867	0.148602991578525	0.843097068763558	-4.73889994382422
a_HQ_transcript/7276	1.71868905056245	0.258811373566788	1.47951378618543	0.148606167954165	0.843097068763558	-4.90032044154166
a_HQ_transcript/86539	1.61377911718409	0.360697275194367	1.47950383889613	0.148608811313416	0.843097068763558	-4.67167688305985

a_HQ_transcript/139600	-2.25710361158311	1.80303607048016	-1.47950127640251	0.14860949226795	0.843097068763558	-4.91230558242193
a_HQ_transcript/140618	1.73333705602106	3.96571561519192	1.47936514129008	0.148645672263855	0.843136394219456	-5.09336563207494
a_HQ_transcript/97593	0.701432977871729	2.6194751030772	1.47935896191646	0.148647314694232	0.843136394219456	-4.89902229464686
a_HQ_transcript/149239	-1.63750023872934	0.278819376029334	-1.47922213679108	0.148683685484458	0.843136394219456	-4.73110367647562
a_HQ_transcript/80444	1.29188848326289	-0.542444308635056	1.47921272130646	0.148686188565609	0.843136394219456	-4.83842102095633
a_HQ_transcript/139419	1.6671173931157	0.313733663804874	1.47920234117635	0.148688948134181	0.843136394219456	-4.80228319381714
a_HQ_transcript/55019	-1.7435445897164	0.660160901059382	-1.47904990702404	0.148729477628245	0.843163554521015	-4.76100485455496
a_HQ_transcript/31185	-1.12326257316501	2.55549904489175	-1.47902591154766	0.148735858397038	0.843163554521015	-4.85475827565711
a_HQ_transcript/62974	1.07150173251248	3.34097135453781	1.4790206641678	0.148737253785757	0.843163554521015	-4.91732475984525
a_HQ_transcript/23689	4.51145944029469	4.22903198198921	1.4788527490116	0.148781911481956	0.843334466174854	-5.12011223011647
a_HQ_transcript/57897	2.17387734027736	0.396051218181883	1.47877483050044	0.148802637855108	0.84336970877942	-4.7987301602986
a_HQ_transcript/135880	1.02746164794243	3.87297980322519	1.4787052133589	0.148821158009942	0.843392441532798	-5.18689827062064
a_HQ_transcript/116055	0.353983486282244	-0.888331825932569	1.47857470285684	0.148855882506144	0.843446855604911	-4.90585592788487
a_HQ_transcript/94980	1.41619249370456	1.08453915880128	1.47849946721958	0.148875903143662	0.843446855604911	-4.68458158078531
a_HQ_transcript/97655	-1.1167879985671	1.19219087274862	-1.47846118264405	0.148886091716109	0.843446855604911	-4.70046031556291
a_HQ_transcript/91765	0.69885843606145	0.860391967469187	1.47845100512406	0.148888800326211	0.843446855604911	-4.85873110246929
a_HQ_transcript/8692	-1.42652443318673	0.435253706244728	-1.47839525866854	0.148903637194157	0.843448706180272	-4.73246649802703
a_HQ_transcript/36518	0.990248990391781	1.78915094063195	1.47816080636605	0.148966049389458	0.843720015483818	-4.78064932613193
a_HQ_transcript/30014	0.73528673160884	2.07276948102904	1.47808668299012	0.148985785693171	0.843749586006687	-4.8147514930671
a_HQ_transcript/101873	1.60924295419957	0.691636867950768	1.47789228408773	0.14903755682808	0.843893006836933	-4.79806138569414
a_HQ_transcript/36799	1.05377325563705	-0.539039512693238	1.47784553825046	0.149050008038528	0.843893006836933	-4.88491331553123
a_HQ_transcript/59844	0.78240547435336	5.22347591223744	1.47778683284425	0.149065645973979	0.843893006836933	-5.25501566461454
a_HQ_transcript/48709	-2.32331715174355	2.69916675706446	-1.47777356025355	0.149069181705918	0.843893006836933	-4.90944193858466
a_HQ_transcript/46973	2.46072567982222	1.04475601282869	1.4775956247231	0.149116589044512	0.844079178885908	-4.88667168648594
a_HQ_transcript/29291	-1.57762087830786	0.449191148089888	-1.47745186919184	0.149154898604677	0.844208200100452	-4.77312178829164
a_HQ_transcript/1879	0.703027280008647	1.73042806798541	1.47740110495791	0.149168428695839	0.844208200100452	-4.7183426825184
a_HQ_transcript/62349	2.52038815931893	0.879463649515755	1.47734269566833	0.14918399762143	0.844214117381082	-4.77669728572771
a_HQ_transcript/29126	1.28090153205714	-0.346717818885535	1.47708683420863	0.149252212479542	0.844465013319239	-4.83112634780379
a_HQ_transcript/138948	2.441869412118	-0.364254879546316	1.47706741932055	0.149257389671791	0.844465013319239	-4.82241954979396
a_HQ_transcript/147011	-0.575999908671784	-0.657856863548859	-1.47677763632352	0.149334680524832	0.844710135006161	-4.89572213966445
a_HQ_transcript/136798	-0.523293275273673	2.6589725532281	-1.47668369971919	0.149359742132725	0.844710135006161	-4.88700986283922
a_HQ_transcript/14606	-0.442106067143647	2.89190676653843	-1.47665839430139	0.149366494009749	0.844710135006161	-4.98981074675827
a_HQ_transcript/34413	1.46332046988399	1.14995611441053	1.47665103759984	0.149368456937281	0.844710135006161	-4.82542909038595
a_HQ_transcript/50562	1.30997302506955	-0.274353151000216	1.47663261006264	0.149373373894308	0.844710135006161	-4.85168035841873
a_HQ_transcript/94496	0.353983486282241	-0.792480856331625	1.47649355012319	0.149410482952727	0.844837796991839	-4.92998977881056
a_HQ_transcript/114450	0.653768497366863	2.33444352598798	1.47625262987313	0.149474791529215	0.845119218170429	-4.81589578374077
a_HQ_transcript/141057	3.27743766715751	2.82581013537457	1.47614594898927	0.149503274799175	0.845120667812703	-5.00941719109954
a_HQ_transcript/13826	1.52763156234912	0.358584942774486	1.47609148268275	0.149517818705528	0.845120667812703	-4.79010951826241
a_HQ_transcript/31967	1.69451467021466	0.448924458495863	1.47608831420662	0.149518664805014	0.845120667812703	-4.75067441532231
a_HQ_transcript/105289	2.45359531849174	2.0167377282861	1.47598013060197	0.149547556094102	0.845133655424724	-4.78763570001954
a_HQ_transcript/26700	-1.88515383209963	0.643463563509442	-1.475970826865	0.14955004093894	0.845133655424724	-4.67872156880313
a_HQ_transcript/130186	-2.28065948682689	4.94882128812722	-1.47589558411436	0.149570138006542	0.84516506095502	-5.26277155516095
a_HQ_transcript/130066	-1.43055208016088	1.11346424376647	-1.47577711591943	0.149601784804804	0.845217395347071	-4.81538902592275
a_HQ_transcript/79612	2.05945445149642	0.626652225575474	1.47566214457942	0.149632502588229	0.845217395347071	-4.83693932780167
a_HQ_transcript/140700	-3.5178665948682	2.59033036629348	-1.47564680668858	0.149636600912001	0.845217395347071	-5.04381611706091
a_HQ_transcript/67861	1.59634919060949	2.13308215136444	1.47564320913983	0.149637562199228	0.845217395347071	-4.95579506944694
a_HQ_transcript/119775	0.435146175676824	3.66283116920209	1.47547547439992	0.149682387431057	0.845302660126155	-5.14901585132419
a_HQ_transcript/134657	1.91338665227244	0.728923616434907	1.47539163596982	0.149704796330954	0.845302660126155	-4.78285984712597
a_HQ_transcript/97206	0.617543697453895	3.64686669737376	1.47528227337115	0.149734031527798	0.845302660126155	-5.09672345744497
a_HQ_transcript/134863	-0.603069854298847	5.22290131832848	-1.47526683952272	0.149738157725742	0.845302660126155	-5.28156834888698

a_HQ_transcript/70713	-0.789871880444105	3.00818257777734	-1.4752299823258	0.149748011765604	0.845302660126155	-4.95603622474218
a_HQ_transcript/137303	3.13294384390057	1.85482287015179	1.47513885332855	0.14977237798864	0.845302660126155	-4.92577913421213
a_HQ_transcript/55756	1.32588144678201	-0.54566455926579	1.47509227075507	0.149784834537086	0.845302660126155	-4.79888420192233
a_HQ_transcript/122453	-0.592732849967794	2.67059060466562	-1.47507202477082	0.149790248731174	0.845302660126155	-4.92834438792476
a_HQ_transcript/131762	-3.63615616890337	0.142005713880941	-1.47502520298867	0.149802770440594	0.845302660126155	-4.86145723037972
a_HQ_transcript/148604	5.3495370128423	3.78631932610499	1.474972070998	0.149816980722748	0.845302660126155	-5.03765822246599
a_HQ_transcript/48918	-2.28577708262854	2.67741136080447	-1.47490645763961	0.149834530662395	0.845302660126155	-4.86535362227702
a_HQ_transcript/4302	2.07456039103107	0.492368838198739	1.47488163028879	0.149841171788505	0.845302660126155	-4.74397153691569
a_HQ_transcript/5891	0.608236401391453	2.9766446411046	1.47487963813271	0.149841704685176	0.845302660126155	-5.02618363792535
a_HQ_transcript/48154	1.80342141810167	0.907056854674522	1.47474253884558	0.149878382029427	0.845352190415785	-4.78733814919455
a_HQ_transcript/116281	-1.60197312972511	3.79576005012135	-1.47473809678503	0.1498795705069	0.845352190415785	-5.04304869595979
a_HQ_transcript/140527	-2.30788462363133	1.94664402082564	-1.47452865127894	0.149935616388507	0.84544863911363	-4.77772935840393
a_HQ_transcript/24013	0.769841615816717	2.38487769310868	1.474508675583	0.149940962592863	0.84544863911363	-4.89930792571945
a_HQ_transcript/13179	-2.19329403876296	0.736421284937319	-1.47445697248773	0.149954800880557	0.84544863911363	-4.78149919636922
a_HQ_transcript/124268	3.06455880350483	2.8746226947773	1.47441627280342	0.149965694832075	0.84544863911363	-4.79286968178128
a_HQ_transcript/70019	-0.529340919794331	3.57642933143897	-1.47437310738653	0.149977249468409	0.84544863911363	-5.1248223296299
a_HQ_transcript/151144	1.72801036194069	0.907937112621082	1.47434812062361	0.149983938317846	0.84544863911363	-4.83593800526347
a_HQ_transcript/109785	0.689492136203965	3.40885652381016	1.47423478140337	0.150014281731256	0.845537687236931	-5.05990046550219
a_HQ_transcript/111593	-1.55605278069854	2.18118066709486	-1.47405513934347	0.150062385916642	0.845572170651931	-4.79274641764823
a_HQ_transcript/132965	-0.572394136817222	2.13689282192713	-1.4740467684341	0.150064627761882	0.845572170651931	-4.76957569928419
a_HQ_transcript/70210	-2.06798226161679	0.721752929344609	-1.47396593100225	0.150086278518969	0.845572170651931	-4.83320258420971
a_HQ_transcript/102317	4.57288483383782	1.0866517951355	1.47392806814981	0.150096420215689	0.845572170651931	-5.01449408575044
a_HQ_transcript/10917	0.766203228506438	4.07513511855987	1.47390934061728	0.150101436652567	0.845572170651931	-5.16204802573332
a_HQ_transcript/128031	-3.62761795264303	1.28686727147479	-1.47381076940202	0.150127842564964	0.845572170651931	-4.80666186230302
a_HQ_transcript/6455	-0.710594098533292	2.86687743970977	-1.47375566267397	0.150142606537094	0.845572170651931	-5.03577613425626
a_HQ_transcript/88920	1.33271063175081	0.841761895747416	1.47374396255104	0.150145741335916	0.845572170651931	-4.71606209267158
a_HQ_transcript/107747	-1.10247788156515	-0.55714198931882	-1.4737231405236	0.150151320284015	0.845572170651931	-4.87219607524575
a_HQ_transcript/17767	2.27664901266212	1.89400089011208	1.47356038393104	0.1501949341578	0.845735845463972	-4.90592172115476
a_HQ_transcript/83409	-1.29198906082135	1.4958070173702	-1.47339150781937	0.150240198564666	0.845852388529894	-4.75275866088174
a_HQ_transcript/122473	1.42575131872455	2.26599599397658	1.4733310578436	0.15025640381127	0.845852388529894	-4.86441299929385
a_HQ_transcript/93774	1.25073746217479	-0.638119344520371	1.4732648772	0.1502741469173	0.845852388529894	-4.85845157990988
a_HQ_transcript/19314	1.89864789950394	0.784765567143944	1.47323464409276	0.150282253004136	0.845852388529894	-4.86467676612343
a_HQ_transcript/119471	3.22396789953224	2.36049709561188	1.47321176011168	0.150288388878626	0.845852388529894	-4.9084633443254
a_HQ_transcript/146458	1.87922326485509	0.59918408120985	1.4730887818742	0.15032136641761	0.845939051005423	-4.80004213416564
a_HQ_transcript/33425	-0.435445247943333	3.25431881277123	-1.47304580305559	0.150332892872385	0.845939051005423	-5.09014081280409
a_HQ_transcript/74430	1.05558711617157	1.56959108609917	1.4729811026339	0.150350246156297	0.845954806753772	-4.77769460345245
a_HQ_transcript/122449	0.616874946776688	3.04525467181492	1.47284781190893	0.150386001066178	0.845973513501994	-5.08196615745279
a_HQ_transcript/151823	1.95065603607294	2.86243014545373	1.47284472115447	0.15038683023395	0.845973513501994	-4.91622842107324
a_HQ_transcript/132147	1.73064122617945	0.115747322465976	1.47279257468836	0.150400820303157	0.845973513501994	-4.78826739527932
a_HQ_transcript/100594	0.418825420015962	4.17093191964542	1.47273849911196	0.150415329018716	0.845973513501994	-5.19832331238733
a_HQ_transcript/125748	-4.18045872529764	0.59943037960431	-1.47269746634084	0.150426339037239	0.845973513501994	-4.80039909280049
a_HQ_transcript/129122	-2.2511170139724	2.81317769032617	-1.47264006476888	0.150441742253315	0.845978290964562	-5.07731641020777
a_HQ_transcript/77953	-1.42049253711346	0.549605061034995	-1.47254361057235	0.150467627737628	0.846042006687255	-4.84796476091634
a_HQ_transcript/29723	-1.90163324145123	1.85272301155601	-1.47248585220254	0.150483130095118	0.846047333994487	-4.74567865345263
a_HQ_transcript/42448	1.53366035554887	1.63912121156725	1.47233614610016	0.150523317173132	0.846191429462266	-4.82711364711261
a_HQ_transcript/70074	-0.614367444663678	2.5190826378703	-1.47224802576295	0.150546976182634	0.846239257057075	-4.84752297789771
a_HQ_transcript/10343	4.46013075241529	2.94563380762891	1.472180111672	0.15056521213747	0.846239257057075	-5.1062387848093
a_HQ_transcript/87346	-2.4817672518647	0.386793268125725	-1.47214180246195	0.150575499513854	0.846239257057075	-4.84701466223919
a_HQ_transcript/89444	3.01450581278154	1.6315427655991	1.47198277485315	0.150618210040403	0.846317480678811	-4.78975667706385
a_HQ_transcript/118123	0.869817009294306	3.61451805678138	1.47198155638678	0.150618537324909	0.846317480678811	-5.08775307296947

a_HQ_transcript/83855	2.08586829971448	0.551220379065492	1.47184795397478	0.150654426860925	0.84643732114555	-4.78592225028648
a_HQ_transcript/84876	3.42339391041153	3.83832374242295	1.47169491357252	0.150695546394725	0.84646714529259	-4.9084752072706
a_HQ_transcript/125055	1.70060066981366	0.449275139173527	1.47167356621584	0.150701282802967	0.84646714529259	-4.78649658394948
a_HQ_transcript/130029	1.70401933337634	0.67347492040557	1.4716052669446	0.150719637182228	0.84646714529259	-4.74522221881586
a_HQ_transcript/12863	-1.09386770229392	2.18480456404249	-1.47156529757798	0.150730379162125	0.84646714529259	-4.87483830906523
a_HQ_transcript/113654	1.20713487494133	-0.225363665419827	1.47155723619022	0.150732545776968	0.84646714529259	-4.83617316905276
a_HQ_transcript/82902	1.23339962738012	-0.327541046846894	1.4714816590258	0.150752859443828	0.846499441050118	-4.85135613444637
a_HQ_transcript/126857	-1.96681461730357	1.04860860077643	-1.47139442222542	0.150776309708021	0.846549341322117	-4.8335655428056
a_HQ_transcript/129706	1.97338108728179	0.767527696139982	1.47111468611523	0.150851525643618	0.846889847654263	-4.79571791503518
a_HQ_transcript/11249	1.37334153486001	0.434004048826827	1.47096411433001	0.150892024036343	0.847018958775775	-4.79760447160191
a_HQ_transcript/22656	-2.42718192381128	2.24934982467585	-1.47092083328206	0.150903666685279	0.847018958775775	-5.05082323286492
a_HQ_transcript/148575	0.457657796368534	9.42971850783569	1.47068392054181	0.150967409164096	0.847294927091879	-5.76346790165906
a_HQ_transcript/42681	1.42453872826814	0.33750227313136	1.47058839375963	0.150993117164353	0.84735739665278	-4.79033084592753
a_HQ_transcript/71294	-3.11083883904835	2.48883529627494	-1.47041375007952	0.151040125990734	0.847423972831451	-5.01887573734593
a_HQ_transcript/7766	0.465107363856125	2.8636661970857	1.47037913396777	0.151049444992643	0.847423972831451	-5.00945328702352
a_HQ_transcript/9482	-0.864760940111546	2.42261077609393	-1.47024336696889	0.151085999245902	0.847423972831451	-4.96532973712121
a_HQ_transcript/1548	-1.20400705010413	1.89768890446791	-1.47020803724527	0.15109551266736	0.847423972831451	-4.8394435996449
a_HQ_transcript/137396	2.67883231814455	0.56987566485498	1.47010634081691	0.15112289966856	0.847423972831451	-4.88524440995891
a_HQ_transcript/71736	-2.12723595762164	2.01020902491316	-1.47008695737286	0.151128120108288	0.847423972831451	-4.93428811134956
a_HQ_transcript/136051	4.19703912968756	3.16678876730338	1.47008614525783	0.151128338834042	0.847423972831451	-5.06849437999448
a_HQ_transcript/135320	2.14668094710886	0.871639592119313	1.47005295250483	0.151137278805646	0.847423972831451	-4.78738858500653
a_HQ_transcript/98824	-0.650177951240329	3.97570141887367	-1.46994165190166	0.151167259036071	0.847423972831451	-5.10312303536756
a_HQ_transcript/19713	-0.653851835235607	2.7994912598751	-1.4699251019984	0.151171717367537	0.847423972831451	-5.01910560899206
a_HQ_transcript/39528	-1.63122389514281	0.703237883058478	-1.46986949789332	0.151186697167422	0.847423972831451	-4.78849046122114
a_HQ_transcript/145150	1.84957390724071	1.04762296283038	1.46984736376992	0.151192660451061	0.847423972831451	-4.76284562332591
a_HQ_transcript/14638	-1.35627686534379	1.23400855815505	-1.46978657654012	0.15120903845889	0.847423972831451	-4.84676445888414
a_HQ_transcript/84459	-3.81439592278277	0.180811194392388	-1.46976196405137	0.151215670246348	0.847423972831451	-4.87349732536929
a_HQ_transcript/123836	1.44735715497704	-0.452729123996902	1.4697323153439	0.15122365934112	0.847423972831451	-4.86009834345528
a_HQ_transcript/144433	1.26246573922363	2.14813052247689	1.46964491454258	0.15124721218379	0.847443463070912	-4.86542285108805
a_HQ_transcript/130780	2.16233757688975	1.32401449055461	1.46955734013138	0.151270814744234	0.847443463070912	-4.77393542336299
a_HQ_transcript/68767	1.24222951672784	-0.0573856004481497	1.46946287948965	0.151296276534403	0.847443463070912	-4.79306976289254
a_HQ_transcript/49946	-1.25814332758836	-0.497710696112356	-1.46933368690211	0.151331105824602	0.847443463070912	-4.79723440732349
a_HQ_transcript/32474	1.51078475457679	0.691291259419584	1.46933118131927	0.151331785372911	0.847443463070912	-4.78834927749199
a_HQ_transcript/143521	0.428926616608617	4.33894694039748	1.46930266226506	0.151339470770787	0.847443463070912	-5.212172530178
a_HQ_transcript/149709	2.00067122068135	-0.481933624815232	1.46917961130232	0.151372651733034	0.847443463070912	-4.83448740690757
a_HQ_transcript/13190	-1.17673404020087	0.688690257722628	-1.46915041559844	0.151380525269852	0.847443463070912	-4.7260283348782
a_HQ_transcript/72810	0.554243899824888	2.75011600398102	1.46913170075604	0.151385572485507	0.847443463070912	-4.91635776369131
a_HQ_transcript/39132	-0.423736597292246	2.78241007027961	-1.46879501893495	0.151476395298785	0.847443463070912	-4.93696527976989
a_HQ_transcript/133501	1.79508242708992	-0.384362260562289	1.4687392505947	0.151491443479753	0.847443463070912	-4.78527087522268
a_HQ_transcript/25974	1.03519369735808	2.85382850674359	1.46872302911721	0.151495820805726	0.847443463070912	-4.95370036640662
a_HQ_transcript/86716	1.41791712737604	0.0390785193766907	1.46869676751867	0.151502907647511	0.847443463070912	-4.79524816116189
a_HQ_transcript/82505	0.920596231843119	2.88246638218475	1.46867572884685	0.151508585243441	0.847443463070912	-4.96081484196519
a_HQ_transcript/8949	-0.562890055814685	3.08751014116509	-1.46864947665527	0.151515670022422	0.847443463070912	-4.99444932024469
a_HQ_transcript/144662	2.23321064037423	2.07387589975011	1.46852992277475	0.151547937823385	0.847443463070912	-4.92273714620729
a_HQ_transcript/49635	-1.45260991075244	2.24438922560525	-1.46848913124299	0.151558948782152	0.847443463070912	-4.89695529668853
a_HQ_transcript/51461	-1.93200753832384	1.12344674664599	-1.46839067459297	0.151585528055432	0.847443463070912	-4.8687880419406
a_HQ_transcript/38091	-1.47813287008834	-0.264242218539328	-1.46834601665232	0.151597585099674	0.847443463070912	-4.80964870389868
a_HQ_transcript/57317	1.54334857596391	1.58080082097111	1.46833896129755	0.151599490020629	0.847443463070912	-4.93287966324861
a_HQ_transcript/132448	0.735078612744551	2.47764592883189	1.46830183844914	0.151609513373308	0.847443463070912	-4.84688150134479
a_HQ_transcript/10454	1.46194090482869	-0.517221237601543	1.46822368686288	0.151630616414567	0.847443463070912	-4.85626045266513

a_HQ_transcript/138911	-2.96752305142204	1.52814976458239	-1.46816414404623	0.151646696156159	0.847443463070912	-5.0203377993988
a_HQ_transcript/41201	0.71093495072356	2.28381466855404	1.46808068522214	0.151669236784864	0.847443463070912	-4.87477339031492
a_HQ_transcript/129380	1.23681582511701	-0.375613682666249	1.46803767890386	0.151680853009556	0.847443463070912	-4.87949443715802
a_HQ_transcript/24637	2.22673835801984	0.201094769108983	1.46798677096957	0.151694604417209	0.847443463070912	-4.83829224294503
a_HQ_transcript/66267	1.37429176430709	1.10100463056259	1.46798391747923	0.151695375240195	0.847443463070912	-4.67774196762625
a_HQ_transcript/138099	-1.84715256542771	-0.284885554181567	-1.46795877785664	0.151702166426266	0.847443463070912	-4.83033042345963
a_HQ_transcript/71802	1.794936750981	0.289236561575118	1.46795601022529	0.151702914085528	0.847443463070912	-4.85221977968194
a_HQ_transcript/35949	1.26138608300357	1.88879284856708	1.46787097683518	0.151725886784805	0.847443463070912	-4.93563982013811
a_HQ_transcript/74336	-2.16856122986037	0.00183460163047852	-1.4676942758445	0.151773633353999	0.847443463070912	-4.87885089581588
a_HQ_transcript/60098	1.21979027759538	0.608536075259422	1.46766900224291	0.151780463540374	0.847443463070912	-4.73007993280357
a_HQ_transcript/39967	-2.02496734419337	1.54778673330751	-1.4676640848913	0.151781792482245	0.847443463070912	-4.74995868173754
a_HQ_transcript/39446	-0.477816773885209	3.45229162614807	-1.46763853332872	0.15178869808471	0.847443463070912	-5.13381407426923
a_HQ_transcript/16760	0.587520962150664	3.37137658353159	1.46754943574211	0.151812779686689	0.847443463070912	-5.12434175938244
a_HQ_transcript/137829	1.65429804219137	-0.195110103340646	1.46754507047881	0.151813959623293	0.847443463070912	-4.8338623873132
a_HQ_transcript/29058	0.357133732308739	4.03238576765889	1.4674774291786	0.151832244092628	0.847443463070912	-5.20256413965905
a_HQ_transcript/43297	-1.39731932195104	0.766418890593516	-1.46747161358078	0.151833816218841	0.847443463070912	-4.85661946700772
a_HQ_transcript/69522	1.86091119092865	2.61537950132636	1.46745843401058	0.151837379090216	0.847443463070912	-4.88287257071077
a_HQ_transcript/65722	0.839481528422855	1.00430163067088	1.46738104882284	0.151858300196082	0.847443463070912	-4.72197209279549
a_HQ_transcript/63195	2.92814031415173	0.848680790910304	1.46733489959756	0.151870777744607	0.847443463070912	-4.7902728040148
a_HQ_transcript/71214	-1.72799562047963	1.14448905437853	-1.46710458398369	0.151933061300354	0.847443463070912	-4.79121358272909
a_HQ_transcript/28651	0.778497606795337	1.97586043678249	1.46701205525639	0.151958089294574	0.847443463070912	-4.81740405721424
a_HQ_transcript/80753	-1.44096008472309	-0.297432159264518	-1.46694024602738	0.151977515158232	0.847443463070912	-4.86518748690792
a_HQ_transcript/117923	1.85129362449509	0.244954642403825	1.46693978743816	0.151977639222343	0.847443463070912	-4.89432054138386
a_HQ_transcript/18447	-1.16182257027084	1.91916855643727	-1.46692255076134	0.151982302393094	0.847443463070912	-4.79881583866118
a_HQ_transcript/3377	1.79415352848116	-0.181683706136391	1.46688356441545	0.151992850089624	0.847443463070912	-4.84622386853742
a_HQ_transcript/137900	1.89378651609786	0.0944296417674057	1.4668553937555	0.152000471981756	0.847443463070912	-4.94176870900702
a_HQ_transcript/113924	-1.81460432892621	1.39669790522292	-1.46684629784966	0.152002933047468	0.847443463070912	-4.73249898362433
a_HQ_transcript/92558	-1.44950933507187	2.20203392772379	-1.4667759241607	0.152021975024823	0.847443463070912	-4.86251049776309
a_HQ_transcript/126503	-0.470823967545346	4.94797070884017	-1.46673204613541	0.15203384866777	0.847443463070912	-5.29913365564452
a_HQ_transcript/114537	1.20619168249205	1.78788262351331	1.4667166589752	0.15203801269573	0.847443463070912	-4.74630816378585
a_HQ_transcript/117693	-2.45105370112729	0.968303330156231	-1.46671618240534	0.152038141665112	0.847443463070912	-4.97130235892778
a_HQ_transcript/91789	1.20349232478261	0.285344370110135	1.46659439227751	0.152071103378811	0.847443463070912	-4.76284221586146
a_HQ_transcript/78748	1.85076808876369	2.73954180278113	1.46657942694181	0.152075154043268	0.847443463070912	-5.02370076164172
a_HQ_transcript/61769	-1.68621863069668	0.29561830924877	-1.46657649071795	0.152075948800488	0.847443463070912	-4.85004213574635
a_HQ_transcript/136655	-0.528828764917065	4.07874394038114	-1.4665516816413	0.152082664085693	0.847443463070912	-5.22105688063228
a_HQ_transcript/65224	2.24758032461675	1.54724084717482	1.4665121752235	0.152093358113691	0.847443463070912	-4.81349964389913
a_HQ_transcript/149279	2.04966772939194	-0.154469983472568	1.46650967196747	0.152094035742516	0.847443463070912	-4.85325661859625
a_HQ_transcript/63036	-1.28152220275861	1.27664792366958	-1.46642460057184	0.152117065911314	0.847443463070912	-4.72838034164221
a_HQ_transcript/143303	0.345834514799702	4.2377004169767	1.46635474266307	0.152135979626488	0.847443463070912	-5.2311828527234
a_HQ_transcript/147216	1.36568019119121	0.127319851133412	1.46630190443205	0.152150286584678	0.847443463070912	-4.77736994317952
a_HQ_transcript/130492	-1.99587446479885	0.297279209779752	-1.46621635468381	0.152173453082187	0.847443463070912	-4.81304185089101
a_HQ_transcript/25830	1.15035979277625	1.72707245536127	1.46619672042094	0.152178770351102	0.847443463070912	-4.80353860023945
a_HQ_transcript/143893	-1.295796670715	1.32211440357014	-1.46604105151857	0.152220933190684	0.847443463070912	-4.77741022329018
a_HQ_transcript/145437	-2.3582208292326	0.0235329092119564	-1.46601673768808	0.152227519418901	0.847443463070912	-4.8449638439867
a_HQ_transcript/95076	1.48399770269708	-0.152010315215484	1.46592673885189	0.15225190064099	0.847443463070912	-4.75437338336774
a_HQ_transcript/113881	1.44784379411256	-0.635563244098821	1.46588824565175	0.152262329628417	0.847443463070912	-4.86992978593284
a_HQ_transcript/83453	1.94052271847807	3.73805268577764	1.46588374058863	0.152263550225256	0.847443463070912	-5.13130321338247
a_HQ_transcript/131635	-3.71965910161643	1.8605465620336	-1.46585260073924	0.152271987434876	0.847443463070912	-4.97984718279804
a_HQ_transcript/52098	1.80263092403366	-0.442771545451265	1.46581791397111	0.152281386105472	0.847443463070912	-4.84314282196253
a_HQ_transcript/16815	-1.4277523790547	2.66170963316283	-1.46575204601483	0.152299234852241	0.847443463070912	-5.01296626671316

a_HQ_transcript/32668	2.64425133069521	0.809888677254481	1.46568484865924	0.152317445553684	0.847443463070912	-4.84774358783928
a_HQ_transcript/136104	4.53666200616701	3.88601586802774	1.46566125365807	0.152323840286981	0.847443463070912	-4.98347958846718
a_HQ_transcript/142534	3.07803725468782	2.68105745572132	1.46561709063488	0.152335809954321	0.847443463070912	-4.91576071274376
a_HQ_transcript/145866	2.64888703477058	0.529525872250721	1.46559278094835	0.152342399018161	0.847443463070912	-4.931911112379
a_HQ_transcript/141478	0.423615824341277	3.15031776802638	1.46546819433809	0.152376171388749	0.847443463070912	-5.07504639251597
a_HQ_transcript/7231	-0.669981600397314	2.28483917308485	-1.46540498147502	0.152393309123838	0.847443463070912	-4.81266797554618
a_HQ_transcript/67697	1.85438140244211	4.24263551815038	1.46535505175716	0.152406846730455	0.847443463070912	-5.03465422519216
a_HQ_transcript/19259	-2.32058529347604	0.966673004163833	-1.4653127037318	0.152418329438828	0.847443463070912	-4.88073696434545
a_HQ_transcript/30358	1.31456566851851	-0.376131425931982	1.4652915191335	0.152424073921871	0.847443463070912	-4.84379865830245
a_HQ_transcript/44829	-0.810508557966629	3.00485272039581	-1.46527100626079	0.152429636421984	0.847443463070912	-4.92048923865103
a_HQ_transcript/54471	-0.528914216340321	4.30572877843063	-1.46524316010536	0.152437187755805	0.847443463070912	-5.21331791025792
a_HQ_transcript/21214	-1.28800529614316	3.35089581576658	-1.46518669998598	0.152452499552598	0.84744753696026	-5.0160733435061
a_HQ_transcript/126260	3.15762440364939	1.93224594078078	1.46501187861062	0.152499918297205	0.847568839722359	-5.0676614058867
a_HQ_transcript/99863	1.46418525175418	-0.487969183821111	1.46499873595938	0.152503483599232	0.847568839722359	-4.85220903163506
a_HQ_transcript/148921	-0.648074322002865	5.99165896557073	-1.46484289329255	0.152545765226849	0.847593099269133	-5.43400754436018
a_HQ_transcript/120424	-0.601770059830185	3.56469887684014	-1.46481889268079	0.152552277655176	0.847593099269133	-4.9762127833477
a_HQ_transcript/125796	1.39543966877966	-0.384587510516605	1.46475452601413	0.152569744274075	0.847593099269133	-4.88732075336611
a_HQ_transcript/4684	1.96790416201702	0.576107504396232	1.46465226840081	0.152597496302229	0.847593099269133	-4.79084711909604
a_HQ_transcript/150602	0.816275196290327	0.415609134534996	1.46460938610103	0.152609135466108	0.847593099269133	-4.81084190974534
a_HQ_transcript/53946	0.791323739202652	2.48046593428065	1.46458884671069	0.152614710541804	0.847593099269133	-4.84197138121163
a_HQ_transcript/68862	1.7220505679405	1.23535432773432	1.46458336387311	0.152616198794246	0.847593099269133	-4.77734138348902
a_HQ_transcript/83333	-2.57337525420474	2.50990511162163	-1.46455278020949	0.152624500585777	0.847593099269133	-4.99526790723936
a_HQ_transcript/17377	-1.66935087499801	2.66050928085167	-1.46448165726838	0.152643807963868	0.84759852284036	-5.02794327355376
a_HQ_transcript/48471	1.83817421686106	1.06948726585597	1.4644417568612	0.152654640373241	0.84759852284036	-4.82892311490068
a_HQ_transcript/78569	1.37512606328878	0.0322690211518823	1.46429542714511	0.152694372108735	0.847653528372046	-4.89825615452134
a_HQ_transcript/35691	1.32843853277652	-0.2325859751365	1.46424164016312	0.15270897852647	0.847653528372046	-4.88677264287147
a_HQ_transcript/115447	-1.44583802608364	2.06587243849543	-1.46417384159536	0.152727391521905	0.847653528372046	-4.84910915965302
a_HQ_transcript/148331	1.53853950317522	-0.0872417723179675	1.46415739875264	0.152731857398627	0.847653528372046	-4.8412873136671
a_HQ_transcript/83755	1.31187464991911	-0.191029082480745	1.46413677234745	0.152737459678104	0.847653528372046	-4.91396688239214
a_HQ_transcript/101599	-1.71266555417595	-0.0912726539339546	-1.46398759633441	0.152777981823332	0.84779747278536	-4.80910894768857
a_HQ_transcript/73737	-1.64142531580659	0.937770079963958	-1.46388789480011	0.152805069502	0.847866846125646	-4.8608587612012
a_HQ_transcript/74852	1.08975179139898	1.33524511607935	1.4637297225947	0.152848050786663	0.84787140827158	-4.68166113796449
a_HQ_transcript/13309	1.49334695628114	0.615704257742334	1.46371882551436	0.15285101228399	0.84787140827158	-4.83496547108529
a_HQ_transcript/3847	1.32488602956246	-0.275693799205111	1.46365395630673	0.152868642720903	0.84787140827158	-4.84091400376182
a_HQ_transcript/62105	1.62262657892499	1.25587457964838	1.46363833493362	0.152872888607869	0.84787140827158	-4.75642192459088
a_HQ_transcript/75510	-4.08099613154822	2.00631173410737	-1.46357574030515	0.152889902760723	0.84787140827158	-4.81259226360356
a_HQ_transcript/42881	1.85007323572948	0.9113140091676	1.46356284016553	0.152893409398271	0.84787140827158	-4.83996826855482
a_HQ_transcript/136613	-1.31078695632418	0.755624670129229	-1.46341967587293	0.152932329964251	0.847910554483866	-4.78602047292778
a_HQ_transcript/82862	1.06015920134715	2.53319557999729	1.46340204352526	0.152937124030092	0.847910554483866	-4.85518072471374
a_HQ_transcript/83573	-1.72615015784281	1.39634963561248	-1.46337591129692	0.152944229351833	0.847910554483866	-4.89678023382691
a_HQ_transcript/145629	-1.03470898105453	2.23713505349996	-1.46329385685234	0.152966541565606	0.847953378612012	-4.87614093015438
a_HQ_transcript/13237	-1.99187066440421	-0.376139411269499	-1.4631431565871	0.153007526674743	0.848050007440668	-4.87608245340815
a_HQ_transcript/33106	-1.35139529274331	2.26256063670057	-1.4631044369551	0.15301805844813	0.848050007440668	-4.93398947017564
a_HQ_transcript/120802	3.12997510383487	0.757879877328756	1.46306884137354	0.153027740986189	0.848050007440668	-4.94281130228303
a_HQ_transcript/116030	2.61994430104839	0.659865948669701	1.46290752393395	0.153071627890177	0.848212353288868	-4.83594098798374
a_HQ_transcript/39595	-0.708312492884399	3.34541684026499	-1.46284100240807	0.153089728192464	0.84823179109442	-5.11007804050763
a_HQ_transcript/34398	1.19400841421375	-0.0305854626627458	1.46252309094192	0.153176254450499	0.848630320119956	-4.701828027448
a_HQ_transcript/142811	-2.78736930282324	1.44958607270735	-1.46232039519758	0.153231442656372	0.848631935044253	-4.91534630083792
a_HQ_transcript/48872	1.870959187403	0.884559687974098	1.4622869009993	0.153240563684054	0.848631935044253	-4.88862699639803
a_HQ_transcript/9130	0.611796845107094	2.51601473127905	1.46228244517976	0.153241777110444	0.848631935044253	-4.93233183752062

a_HQ_transcript/45429	1.37139999892474	1.02072958428141	1.46220988981582	0.153261536750031	0.848631935044253	-4.83379535500726
a_HQ_transcript/74849	-2.13442476814882	4.45499540174348	-1.46219128537493	0.15326660378747	0.848631935044253	-5.12305475436351
a_HQ_transcript/9311	0.712059192874803	1.74863331476284	1.46216313591727	0.153274270724218	0.848631935044253	-4.82379605282701
a_HQ_transcript/150234	0.568457751258933	3.57162260359757	1.4620425060246	0.153307129583286	0.848631935044253	-5.09892129100087
a_HQ_transcript/150819	-1.35572625166857	3.27235322015137	-1.46200934006338	0.153316164774635	0.848631935044253	-4.94119165846792
a_HQ_transcript/34494	1.3450358422747	-0.334735936120642	1.46199454171155	0.153320196331991	0.848631935044253	-4.85103705034704
a_HQ_transcript/152688	0.887388411884106	6.6534416893525	1.46198594058822	0.153322539599414	0.848631935044253	-5.44757313579028
a_HQ_transcript/122844	1.40252026903965	0.818933586758098	1.46191727044563	0.153341248934631	0.848654681336023	-4.77717080454744
a_HQ_transcript/81312	-1.69392486861799	0.978481149202058	-1.46183126661141	0.15336468343582	0.848703571854277	-4.84240177995827
a_HQ_transcript/40081	-1.56400595569892	-0.244651842250642	-1.46171336875268	0.153396813117554	0.848766548733586	-4.79084252863599
a_HQ_transcript/151682	-1.69392847924376	-0.472711653012268	-1.46168235097333	0.153405267014091	0.848766548733586	-4.83114995629646
a_HQ_transcript/78151	1.67934283016883	0.172735269434768	1.46148471659762	0.153459140975433	0.848983815229843	-4.77187070926975
a_HQ_transcript/46858	1.50099509105659	3.08917512539274	1.46142220888296	0.153476183342029	0.848997295898883	-4.92560179699784
a_HQ_transcript/57218	1.41078533354403	3.96171491981199	1.46131297657268	0.153505968514492	0.849081257761004	-5.21145124946192
a_HQ_transcript/10656	3.62328519732358	2.77318125935908	1.46125335019686	0.153522229214715	0.849090403405608	-5.07397094304345
a_HQ_transcript/96284	-2.66829805626905	1.13182268742361	-1.46119829010882	0.153537245860366	0.849092667431389	-4.95539917939346
a_HQ_transcript/109497	2.38085564570841	2.59133351016296	1.46107303845259	0.15357141034313	0.849200812445347	-4.96082513538631
a_HQ_transcript/24707	1.13958056725507	0.745650309760339	1.46092364700984	0.153612167265472	0.849345387501889	-4.70085410940568
a_HQ_transcript/95623	2.33648136437107	1.01579325663305	1.46083540625816	0.153636245119789	0.849397722686235	-4.90277772957747
a_HQ_transcript/36013	-1.56202156982279	0.0709081704749045	-1.46076370253291	0.153655812801824	0.849425115220582	-4.85212926921385
a_HQ_transcript/39249	1.66148299516239	1.07304693492351	1.46068664607014	0.153676843436458	0.849460589128415	-4.74740779055022
a_HQ_transcript/28211	1.92928543024676	1.41640004088574	1.46057906162344	0.153706209751449	0.849468996982359	-4.81855877037091
a_HQ_transcript/94951	1.82723045297515	0.231458516409774	1.460529673468	0.153719692266983	0.849468996982359	-4.78537024823771
a_HQ_transcript/125502	-1.78980040225735	0.00867652400238915	-1.46050458939251	0.153726540350845	0.849468996982359	-4.96863974977363
a_HQ_transcript/80025	-0.799001586800764	2.25260138156346	-1.46041541146304	0.153750888357432	0.849468996982359	-4.81329595580504
a_HQ_transcript/57854	-0.63789122557217	1.81506114539381	-1.46041341551381	0.153751433341099	0.849468996982359	-4.77804828037194
a_HQ_transcript/122694	1.17048101707939	-0.683380372384107	1.46034350274273	0.153770523633147	0.849493727214175	-4.8763924106867
a_HQ_transcript/22535	0.812161284188453	3.67443770455114	1.46027209458356	0.153790024200454	0.849520719065285	-5.04796445540838
a_HQ_transcript/90689	-2.97308207838063	1.35257370406098	-1.46011341140706	0.15383336539475	0.849679386513306	-4.86649806417891
a_HQ_transcript/71155	-2.16731814035728	1.49901541699686	-1.46005912813862	0.15384819403401	0.849680553236002	-4.88524538195312
a_HQ_transcript/6672	1.78403940612064	3.38924451751856	1.45994656443912	0.1538789468467	0.849740402265238	-4.90617115484058
a_HQ_transcript/5708	-1.63064086298958	-0.191786653459339	-1.45991245174569	0.153888267524191	0.849740402265238	-4.8869846669155
a_HQ_transcript/124439	-2.01239814492459	2.75469378497755	-1.45982485370243	0.153912204164928	0.849791850655296	-4.90744283657768
a_HQ_transcript/32245	-0.960324001020273	4.08842987177229	-1.4597275826906	0.153938787466838	0.849857900833159	-5.20748745053167
a_HQ_transcript/146369	-2.08977886186232	0.230282496953846	-1.45947088924725	0.154008957035712	0.849953760899336	-4.90243548058355
a_HQ_transcript/95816	2.90745773971477	0.365834325384027	1.45938807515132	0.154031600475076	0.849953760899336	-4.85426276787962
a_HQ_transcript/135413	-4.18987339620742	3.03940137625536	-1.45937284250655	0.154035765748382	0.849953760899336	-4.93502837179673
a_HQ_transcript/89299	2.61665034391559	2.68680702435713	1.45937235359049	0.154035899440962	0.849953760899336	-5.00746575465331
a_HQ_transcript/124880	1.38688379165944	-0.496469149592789	1.45920914608972	0.154080533184063	0.849953760899336	-4.91561392345741
a_HQ_transcript/31478	1.48537212106609	-0.356721997382541	1.45908354794204	0.154114888580544	0.849953760899336	-4.9044620402498
a_HQ_transcript/92054	-2.30919499420093	1.88017648365779	-1.45892193019433	0.154159105534513	0.849953760899336	-5.00083276228312
a_HQ_transcript/120350	1.31753934211146	-0.418250972794932	1.45890826764804	0.154162843929127	0.849953760899336	-4.85960267331001
a_HQ_transcript/54368	2.47907411378372	2.28892349591462	1.45890213076742	0.154164523147739	0.849953760899336	-4.87018822501189
a_HQ_transcript/117154	-1.87418322156409	2.96797662454556	-1.45887205789924	0.154172752118388	0.849953760899336	-5.06303451425197
a_HQ_transcript/43429	-0.51058800277049	3.13409043750916	-1.45886527298677	0.154174608752002	0.849953760899336	-5.07906663854055
a_HQ_transcript/45666	-2.02170502088783	0.924379567453291	-1.45883147972379	0.154183856255926	0.849953760899336	-4.79897464193824
a_HQ_transcript/49238	1.00935947844171	-0.447983815073373	1.45881320735902	0.154188856660114	0.849953760899336	-4.831151734326
a_HQ_transcript/92473	-1.27976236617579	3.27309023551651	-1.45869049260727	0.15422244204554	0.849953760899336	-5.08307887225065
a_HQ_transcript/118563	1.43069959840271	0.507113958696127	1.45867270791877	0.154227309959395	0.849953760899336	-4.8347113927103
a_HQ_transcript/33490	-1.14654137676014	0.618028231657653	-1.45866298468645	0.154229971393477	0.849953760899336	-4.84459907825671

a_HQ_transcript/147440	0.805853731550748	5.98266346114565	1.45860307336399	0.154246371070584	0.849953760899336	-5.42135497205688
a_HQ_transcript/26860	1.58237910632157	-0.205452639660919	1.45860227110887	0.154246590683306	0.849953760899336	-4.90024759883883
a_HQ_transcript/126277	-1.25986743091955	0.145256510362652	-1.45859913429346	0.154247449370858	0.849953760899336	-4.89060992312932
a_HQ_transcript/38042	1.41187880188608	0.505682281386816	1.45858202812542	0.154252132165804	0.849953760899336	-4.89111412766176
a_HQ_transcript/42310	1.28622667665577	1.85644851541323	1.45850133273747	0.154274223965205	0.849953760899336	-4.85637302720161
a_HQ_transcript/104215	2.01676443109141	4.47244766432689	1.45843763079118	0.154291665284225	0.849953760899336	-5.34771911271168
a_HQ_transcript/116837	1.56984733851134	-0.111044765308543	1.45843473021756	0.154292459486131	0.849953760899336	-4.88700774908836
a_HQ_transcript/25436	1.34985212250845	2.46237416171617	1.45808881128856	0.154387198364757	0.850395059845219	-4.91615328706477
a_HQ_transcript/86498	2.11321584095076	0.572946251254053	1.4579336416273	0.154429710628851	0.850519479931764	-4.78141575643356
a_HQ_transcript/131821	1.15671286382082	3.16553263516468	1.45789955672432	0.154439050211254	0.850519479931764	-5.13173454444111
a_HQ_transcript/121369	0.647069090697561	4.34622205638872	1.4576744007572	0.15450075633494	0.850733158897178	-5.23596530577711
a_HQ_transcript/22628	1.00289171234592	2.21437436380996	1.45759560418725	0.154522355910175	0.850733158897178	-4.8648512939961
a_HQ_transcript/151232	1.39912441927385	1.99795527131784	1.45743297299408	0.154566943673262	0.850733158897178	-4.88307781012763
a_HQ_transcript/21730	1.92030891327207	1.1574201879396	1.45740155574824	0.154575558357646	0.850733158897178	-4.88119038053472
a_HQ_transcript/130197	-1.73724244063764	0.720983828798259	-1.45730918970807	0.154600887558674	0.850733158897178	-4.79578297094685
a_HQ_transcript/46433	1.89822394950032	0.519689367584483	1.4572177192909	0.154625974407747	0.850733158897178	-4.78964806559401
a_HQ_transcript/151792	6.05011543428727	4.44056318881526	1.4571752897562	0.154637612310539	0.850733158897178	-5.21380107040515
a_HQ_transcript/126090	0.933112233857588	3.06684060686672	1.45703097218266	0.154677202069022	0.850733158897178	-5.11727746322606
a_HQ_transcript/78039	0.764384001520726	2.0952335184097	1.45697494991173	0.154692572487046	0.850733158897178	-4.87695705015843
a_HQ_transcript/5775	-1.17847662451415	1.39880688537903	-1.45694963177848	0.154699519235171	0.850733158897178	-4.71517871883996
a_HQ_transcript/6117	1.26871338117305	2.22416168533032	1.45691685283609	0.154708513436281	0.850733158897178	-4.78186219570945
a_HQ_transcript/131531	-2.91691559789986	0.376505399567318	-1.45689978230456	0.154713197577308	0.850733158897178	-4.86892921965003
a_HQ_transcript/103989	-4.40310542946581	3.42569084243555	-1.45684287476985	0.154728813776379	0.850733158897178	-5.15649267665597
a_HQ_transcript/4043	-1.23221753713691	4.459267449466	-1.45683007041164	0.15473232763853	0.850733158897178	-5.27775191326757
a_HQ_transcript/139110	4.0242716212374	0.786313663773918	1.45675056669143	0.154754147028931	0.850733158897178	-4.83994064793266
a_HQ_transcript/128552	0.582261247154975	3.02745883592136	1.45674357802573	0.154756065149506	0.850733158897178	-5.06578529620885
a_HQ_transcript/101828	-1.97319010536609	-0.0702191184856751	-1.45672419121187	0.15476138618495	0.850733158897178	-4.87013839292998
a_HQ_transcript/100095	0.70515508812305	1.72799766690026	1.45663824357989	0.154784977702006	0.850733158897178	-4.81301476238722
a_HQ_transcript/60472	1.58388667229559	-0.122518978760994	1.45660963106457	0.154792832102766	0.850733158897178	-4.71183556703763
a_HQ_transcript/6659	-1.56598127913671	-0.260360711427326	-1.45655903325634	0.15480672244569	0.850733158897178	-4.83520223458857
a_HQ_transcript/113985	-1.8041662574797	1.12023949843177	-1.45650311077146	0.154822075695847	0.850733158897178	-4.86602518599395
a_HQ_transcript/14223	1.67630412043193	2.18941944417697	1.4564914338405	0.154825281693995	0.850733158897178	-4.92489728034918
a_HQ_transcript/134451	2.77301715194386	1.23960286079421	1.45644775034962	0.154837275826294	0.850733158897178	-4.94500832280445
a_HQ_transcript/108315	-2.06560213658491	1.2649956249152	-1.45641729967873	0.154845637073274	0.850733158897178	-4.89064779884448
a_HQ_transcript/145219	1.51070336017911	0.355234524546112	1.4563478005966	0.15486472170765	0.850733158897178	-4.82269587371139
a_HQ_transcript/2376	0.693111186211879	2.43055277588557	1.4562857271631	0.154881768819418	0.850733158897178	-4.87924147953778
a_HQ_transcript/103854	0.455244580465178	2.97071979384514	1.45624082005837	0.154894102503894	0.850733158897178	-5.04542071008946
a_HQ_transcript/34732	-1.06523321360012	-0.781021268462057	-1.45622967148803	0.154897164566811	0.850733158897178	-4.85455026486323
a_HQ_transcript/144922	2.38835469382155	4.02309387864949	1.45617083184676	0.154913326241647	0.850733158897178	-5.1605541571051
a_HQ_transcript/121619	2.49189693958033	2.37181341619548	1.45615776370894	0.154916915891251	0.850733158897178	-4.90359484379623
a_HQ_transcript/127378	-2.08889624549355	0.908080725557287	-1.45595579260147	0.154972403166433	0.850881589321219	-4.90880047579463
a_HQ_transcript/152202	1.10945944790389	11.4617719975372	1.4559319818588	0.154978945703225	0.850881589321219	-6.00410923239446
a_HQ_transcript/103494	1.50772668155104	-0.140908147405144	1.45587781290712	0.154993830658043	0.850881589321219	-4.86438912780088
a_HQ_transcript/141983	-1.15753832399575	3.46568461147392	-1.45583650516662	0.155005182275318	0.850881589321219	-5.12748978052239
a_HQ_transcript/139283	-3.14862328703294	0.0435138202691295	-1.45573973129611	0.155031778907981	0.850881589321219	-4.88634345709431
a_HQ_transcript/56566	1.58169470905312	1.17895611466643	1.45571542745246	0.155038458970797	0.850881589321219	-4.7513842852003
a_HQ_transcript/141092	1.21297197754239	3.4156225572682	1.45568649597294	0.155046411266349	0.850881589321219	-5.17410180835709
a_HQ_transcript/79959	1.60247102280791	-0.0167369470835119	1.45543201750421	0.155116372846071	0.85118517141475	-4.83480898545583
a_HQ_transcript/125933	2.03396529641294	2.90867312447976	1.45525773716993	0.155164300711757	0.8513677998653	-5.02036350539333
a_HQ_transcript/136852	2.18353212099002	1.39327453310257	1.45513581956312	0.155197835588939	0.851373244287292	-4.99738376492049

a_HQ_transcript/109839	1.40034256248958	0.795988982579104	1.4550326111849	0.155226228772463	0.851373244287292	-4.79018510475924
a_HQ_transcript/146841	-2.57556387082558	-0.171032106715774	-1.45501281996325	0.155231673916223	0.851373244287292	-4.91428171174041
a_HQ_transcript/95105	1.84857225776576	0.445576471605047	1.45498908416226	0.155238204529236	0.851373244287292	-4.77893163144543
a_HQ_transcript/49484	-0.987285914846644	2.24434856180544	-1.4549879172291	0.155238525602137	0.851373244287292	-4.81907752402853
a_HQ_transcript/14973	-2.34244520586994	0.57000025572646	-1.45489291366946	0.155264666888621	0.851373633188483	-4.88484553077561
a_HQ_transcript/48873	-2.28954261530997	3.01965651608501	-1.45488120253857	0.155267889578707	0.851373633188483	-4.86267789668732
a_HQ_transcript/46142	0.512492934281531	4.12589275892266	1.45478065580232	0.155295560397442	0.851385857670417	-5.23442869064486
a_HQ_transcript/115698	2.13316584431754	1.58598422411199	1.45476665970338	0.155299412484163	0.851385857670417	-4.84770709443489
a_HQ_transcript/139898	1.91944843019685	1.50304797328571	1.45452884306463	0.155364877348992	0.851561549032106	-4.87843115409613
a_HQ_transcript/140413	-1.4885308766084	4.99767059384956	-1.45449248429088	0.15537488792532	0.851561549032106	-5.33060839480112
a_HQ_transcript/86980	1.66817011110654	0.477892944966807	1.45449059086245	0.155375409252589	0.851561549032106	-4.7426925148105
a_HQ_transcript/80972	1.39292882718336	-0.532115513169355	1.45436771542156	0.155409244143128	0.851666686485505	-4.86887555990234
a_HQ_transcript/77250	-1.67321119812123	2.08419560628288	-1.45409574906736	0.155484153411116	0.851996876836478	-4.82675552119683
a_HQ_transcript/147679	2.96172325938884	0.185806621573895	1.4537736110514	0.155572918852821	0.852389432826872	-4.94554766153597
a_HQ_transcript/122311	0.960836979399978	2.88489938679047	1.45371509917037	0.155589046176796	0.852389432826872	-4.95602547995468
a_HQ_transcript/20137	3.28204233781509	-0.333287367725155	1.45362676718202	0.155613395176471	0.852389432826872	-4.88916259895262
a_HQ_transcript/91378	-1.20236521787288	1.02617680978334	-1.45360265787005	0.155620041512398	0.852389432826872	-4.75566645244434
a_HQ_transcript/58718	2.49559886366609	1.01625712914867	1.45356975460336	0.155629112486093	0.852389432826872	-4.93079478429054
a_HQ_transcript/101153	1.3942133403118	3.37025133659385	1.45350427943789	0.155647164329213	0.852407986445122	-5.2594961474165
a_HQ_transcript/120875	-2.97958760502545	1.22855774555215	-1.45330156596306	0.155703064031631	0.852533760514098	-5.07617343395582
a_HQ_transcript/23980	2.52558380697928	0.330062674799938	1.45328325354815	0.155708114597211	0.852533760514098	-4.82639703459938
a_HQ_transcript/137725	-0.635587790694989	3.59522092936891	-1.4532302010634	0.155722747211442	0.852533760514098	-5.13816109263561
a_HQ_transcript/63593	1.5727133040833	-0.4899709934294	1.45320406499467	0.155729956304738	0.852533760514098	-4.87592061539795
a_HQ_transcript/89365	2.13580693680799	2.65879833229916	1.45312445230097	0.155751917450865	0.852533760514098	-4.98515033500097
a_HQ_transcript/122158	2.36914785190585	0.129092946609237	1.45310193516944	0.155758129243389	0.852533760514098	-4.95425446960176
a_HQ_transcript/54075	2.90252367692698	2.56555479605918	1.45304307032871	0.155774369194101	0.85254237195318	-5.01265055647656
a_HQ_transcript/81541	-0.565779712303135	2.95536108053072	-1.45293298940305	0.155804742523865	0.852628325496823	-5.03540701508088
a_HQ_transcript/68980	1.66421843691516	0.411012101421713	1.45282454248249	0.155834669600442	0.852711821004847	-4.76808341372812
a_HQ_transcript/96824	-0.612017664167664	2.80841115433639	-1.45272995143457	0.155860776729181	0.85277440038722	-4.95468407405609
a_HQ_transcript/68585	-0.381423612759817	3.69443253606139	-1.45262598463598	0.155889475571416	0.852782376291989	-5.18687657525775
a_HQ_transcript/34078	0.952468194229518	-0.707148572918945	1.45261837599985	0.155891576012914	0.852782376291989	-4.8901337409253
a_HQ_transcript/17092	1.41623350994222	0.254907963158024	1.45250892217119	0.155921794344043	0.852836421370048	-4.8025107975596
a_HQ_transcript/59748	1.99678313234317	1.10802677857848	1.45239632145017	0.15595288633175	0.852836421370048	-4.87799199306155
a_HQ_transcript/59227	-1.27706327588563	1.63988271131272	-1.45237377878932	0.155959111538112	0.852836421370048	-4.811745197568
a_HQ_transcript/123465	3.18202025547825	3.5903534475966	1.45228616129026	0.155983309180778	0.852836421370048	-5.21130366089846
a_HQ_transcript/31614	2.16518350199054	1.28071931667823	1.4521186373493	0.156029583173764	0.852836421370048	-4.93524013583109
a_HQ_transcript/27147	-1.62468105250963	2.29293633394732	-1.45201384527744	0.156058534712215	0.852836421370048	-5.03657553791904
a_HQ_transcript/84669	-1.34016380014704	0.773911995894484	-1.4520072374459	0.156060360440673	0.852836421370048	-4.75821869912254
a_HQ_transcript/86074	-1.72335407721021	3.35220347935634	-1.45195935787412	0.156073589961394	0.852836421370048	-4.94762376622714
a_HQ_transcript/9423	-0.418471182647566	3.58222079036419	-1.45192821187678	0.156082196335409	0.852836421370048	-5.13781326221692
a_HQ_transcript/59358	-0.884202532750504	4.43412649699541	-1.45191130924271	0.156086867089767	0.852836421370048	-5.26313419842158
a_HQ_transcript/3490	0.963071519240431	-0.360376817662618	1.45190745535624	0.156087932061035	0.852836421370048	-4.88358003789104
a_HQ_transcript/96818	-2.07148503782478	0.740489290051435	-1.45173820517621	0.156134707831004	0.852836421370048	-4.8799916334195
a_HQ_transcript/1237	2.46732526711659	1.61685414573075	1.4516343515525	0.156163415425706	0.852836421370048	-4.82654569790795
a_HQ_transcript/121562	1.65000139509442	2.53165328558056	1.4515488066948	0.156187065192157	0.852836421370048	-4.97907799102591
a_HQ_transcript/52195	1.51518098933407	-0.34459654548525	1.45150970779913	0.156197875435581	0.852836421370048	-4.87992215217107
a_HQ_transcript/137613	-2.34513028421677	0.977880442136225	-1.45142241679159	0.156222012200161	0.852836421370048	-5.06167860329298
a_HQ_transcript/5842	1.58050989899313	-0.269009820545463	1.45140036026623	0.156228111500372	0.852836421370048	-4.79035865360994
a_HQ_transcript/9575	1.62317805565224	1.89710636311484	1.45133994185808	0.156244819996235	0.852836421370048	-4.8013760195142
a_HQ_transcript/64532	-1.71303749974411	1.37160873596138	-1.45130045346799	0.156255741136553	0.852836421370048	-4.87745333710774

a_HQ_transcript/17434	0.657949742254798	3.24644711551636	1.45119851147147	0.156283937613197	0.852836421370048	-5.05116740452106
a_HQ_transcript/54492	-1.29618124139049	3.33590553724436	-1.45119406813295	0.156285166702861	0.852836421370048	-4.9883890848221
a_HQ_transcript/1451	2.10448695343465	2.80257970679559	1.45118434905319	0.156287855163184	0.852836421370048	-4.96583140600512
a_HQ_transcript/83594	1.88386196505461	-0.327575295934594	1.45116160950849	0.156294145445716	0.852836421370048	-4.87022099404871
a_HQ_transcript/29086	-1.19835362477059	1.16431565990837	-1.45113981406067	0.15630017475789	0.852836421370048	-4.74804759544235
a_HQ_transcript/52694	-1.43148876119332	-0.297494802743985	-1.45109510611906	0.156312542970352	0.852836421370048	-4.8248415379346
a_HQ_transcript/92690	1.54560560605928	1.28170668024151	1.45093153309367	0.156357801192051	0.852836421370048	-4.72676947593739
a_HQ_transcript/70204	-0.900531281247132	3.33911010230122	-1.45090714041957	0.156364551173019	0.852836421370048	-5.06162943345658
a_HQ_transcript/72406	0.543625829168882	3.91053694502617	1.4509055010476	0.156365004831025	0.852836421370048	-5.21720605096799
a_HQ_transcript/148574	0.714803500814971	7.51474766646311	1.45082353394388	0.156387688649683	0.852836421370048	-5.57022498628949
a_HQ_transcript/37304	0.919401567622349	4.64579465583781	1.45081366229555	0.156390420735046	0.852836421370048	-5.32716879925877
a_HQ_transcript/52033	1.25382086234633	2.3432903631687	1.45080964974168	0.156391531263522	0.852836421370048	-4.94821140949407
a_HQ_transcript/146135	2.45044212519256	1.62827801782588	1.45063622059568	0.156439536103818	0.852836421370048	-4.92680247556624
a_HQ_transcript/25044	-2.41062474458955	1.00893995700947	-1.45061065753668	0.156446612896194	0.852836421370048	-4.88110665730213
a_HQ_transcript/150311	1.65305417475599	1.11600190800818	1.45052262214992	0.156470986264563	0.852836421370048	-4.80420431359367
a_HQ_transcript/101380	1.83140498760184	1.59192620613085	1.4505159650177	0.156472829472517	0.852836421370048	-4.73556400173873
a_HQ_transcript/824	0.981073614910694	1.7838602191776	1.45048092001777	0.156482532917176	0.852836421370048	-4.74956004497515
a_HQ_transcript/140866	0.611795313521627	3.89902684635146	1.4504652729816	0.156486865505397	0.852836421370048	-5.18686301520901
a_HQ_transcript/52603	-0.78904772590482	2.06754874831413	-1.45035732777794	0.156516757601268	0.852836421370048	-4.80824057161985
a_HQ_transcript/149180	-0.395373906563157	3.82249972844515	-1.45030575903075	0.156531039575227	0.852836421370048	-5.17964915628729
a_HQ_transcript/61854	1.91237324589077	1.18685074957755	1.4502761635376	0.15653923651955	0.852836421370048	-4.92274938418583
a_HQ_transcript/122616	1.14267415351144	1.70613448910792	1.45026311010568	0.156542851984174	0.852836421370048	-4.81613731865829
a_HQ_transcript/62618	1.40450066966783	-0.539030270048903	1.45023793684701	0.156549824515949	0.852836421370048	-4.8337299260787
a_HQ_transcript/117276	1.31019428472744	2.16179080251632	1.45019376553531	0.156562059756242	0.852836421370048	-4.7925311064246
a_HQ_transcript/12989	1.803592072059	0.767535467758569	1.45015396733632	0.156573084315282	0.852836421370048	-4.76795312602188
a_HQ_transcript/138416	-2.30097064941333	1.75773477101597	-1.45005378359487	0.156600839091718	0.852836421370048	-5.00117114449738
a_HQ_transcript/51252	-2.87321248317659	2.80109383915741	-1.45003076503274	0.156607216676474	0.852836421370048	-4.96679196271227
a_HQ_transcript/144131	-0.759973997257458	4.88729466351809	-1.45001545355116	0.156611459030901	0.852836421370048	-5.34709255401001
a_HQ_transcript/56318	-1.6327921023783	1.10283524362033	-1.44984491339816	0.156658716780712	0.852836421370048	-4.73140167770608
a_HQ_transcript/35787	1.67236270967803	-0.296411399445578	1.44983744261513	0.156660787240392	0.852836421370048	-4.86406882644064
a_HQ_transcript/70684	0.743622996751816	-0.628197451164677	1.44982495131014	0.156664249140649	0.852836421370048	-4.91422980924464
a_HQ_transcript/85972	-1.22437250540429	-0.187500632559314	-1.44982026621165	0.156665547607003	0.852836421370048	-4.88139661205821
a_HQ_transcript/53394	-1.37434861613024	2.99428349594652	-1.44979566674007	0.15667236544503	0.852836421370048	-5.04323584472991
a_HQ_transcript/130662	0.571901784324403	3.47755488834445	1.44976777511148	0.156680096001823	0.852836421370048	-5.14402965947801
a_HQ_transcript/139187	0.92315765708951	-0.758051014781651	1.44968965495152	0.15670174971297	0.852836421370048	-4.88111169181603
a_HQ_transcript/97082	-1.30001797223038	0.839706147531968	-1.44961377340073	0.156722785189406	0.852836421370048	-4.72341587540483
a_HQ_transcript/134950	2.45326361485447	2.42705213467539	1.44961274356587	0.156723070690112	0.852836421370048	-4.96267857582794
a_HQ_transcript/149485	3.51157948758395	3.03324162987507	1.44953253118436	0.156745309204348	0.852877593693751	-5.09272298525657
a_HQ_transcript/152577	-2.89514038990574	0.623437980306829	-1.44943919115969	0.156771190449272	0.852938577165413	-4.95111678454802
a_HQ_transcript/62455	-2.68279560153154	1.26647900777304	-1.44921204219244	0.156834188293284	0.852963687489054	-4.81754570450752
a_HQ_transcript/103448	-1.36416020796494	0.610891167759765	-1.44909318164947	0.156867161263013	0.852963687489054	-4.8276764033833
a_HQ_transcript/6655	3.04511088631702	1.91386070607562	1.4490663377684	0.156874608755178	0.852963687489054	-4.8807585093058
a_HQ_transcript/51644	-1.50096310952796	1.84477378082494	-1.44891916466148	0.156915445040244	0.852963687489054	-4.99130381432722
a_HQ_transcript/123391	2.02268338065243	-0.340159286235657	1.44888952054395	0.156923671446709	0.852963687489054	-4.87619181704269
a_HQ_transcript/152876	0.642302067177762	4.02637489280988	1.4488767720554	0.156927209327955	0.852963687489054	-5.2354653439326
a_HQ_transcript/102252	2.1125918320699	2.6259528109873	1.44881880778777	0.156943296008895	0.852963687489054	-5.05517791104283
a_HQ_transcript/82551	1.25510281898972	-0.324602841374999	1.44879772970357	0.156949146082221	0.852963687489054	-4.75866035859329
a_HQ_transcript/57137	-1.79542042331788	1.30497893806424	-1.44878873595832	0.156951642285317	0.852963687489054	-4.86518146307862
a_HQ_transcript/145931	-1.27598074491493	2.32511131769998	-1.4487240370906	0.156969600305893	0.852963687489054	-4.90301048902477
a_HQ_transcript/94937	1.22639764742249	-0.482808893233794	1.44871069330622	0.156973304251274	0.852963687489054	-4.88313294164693

a_HQ_transcript/92181	-1.05720253308673	0.603615742891752	-1.44870618175023	0.156974556577135	0.852963687489054	-4.78509997854699
a_HQ_transcript/38756	0.926647929030087	1.10254652827235	1.44869008227912	0.156979025562008	0.852963687489054	-4.78578574592466
a_HQ_transcript/32886	1.23157644517392	-0.579522628433189	1.44841774326348	0.157054638306135	0.852963687489054	-4.86811351213636
a_HQ_transcript/57135	1.33875766808378	0.490704939742965	1.44836066465715	0.157070489385029	0.852963687489054	-4.94689917627258
a_HQ_transcript/89473	-1.13740644378071	1.58100306992262	-1.44829508561845	0.157088702652648	0.852963687489054	-4.87423349984138
a_HQ_transcript/132169	-0.945591117323126	2.65892430902227	-1.44824444586143	0.157102767979523	0.852963687489054	-5.03639741607109
a_HQ_transcript/105916	3.18605944107986	3.63297583878933	1.44808087268984	0.157148207686788	0.852963687489054	-5.33826586149892
a_HQ_transcript/103280	1.06826302254288	0.0326107264293727	1.4480669461106	0.157152076893697	0.852963687489054	-4.77463624112777
a_HQ_transcript/77288	2.17326522897009	0.716379999311359	1.44805850728188	0.157154421481398	0.852963687489054	-4.930453557295
a_HQ_transcript/51999	-1.45641677468392	-0.393720947291738	-1.44799031826961	0.157173367678145	0.852963687489054	-4.89405113975324
a_HQ_transcript/47937	1.38266478774197	-0.351482045747507	1.44778341931096	0.157230865276827	0.852963687489054	-4.72571947430583
a_HQ_transcript/93818	1.67097361081197	2.77195015379996	1.44777432250246	0.157233393679348	0.852963687489054	-5.05902476966838
a_HQ_transcript/84671	1.25625129108067	1.39176402786314	1.44768191463136	0.157259079712001	0.852963687489054	-4.85978399845057
a_HQ_transcript/25726	1.45046666342638	-0.0644998234899911	1.44762414385701	0.157275139580051	0.852963687489054	-4.82249905040365
a_HQ_transcript/94544	-1.33559116006202	1.05080533435123	-1.44759376141167	0.157283586207604	0.852963687489054	-4.80429018973664
a_HQ_transcript/29481	0.337962409714451	3.98372436798012	1.4475540763785	0.157294619590665	0.852963687489054	-5.23768555042215
a_HQ_transcript/146856	2.08481504450893	0.088315191515009	1.44747754868754	0.157315897841986	0.852963687489054	-4.83139089679849
a_HQ_transcript/119510	1.4969737506934	0.465451068305749	1.44744920303966	0.157323779827542	0.852963687489054	-4.87815420659142
a_HQ_transcript/519	-1.68304010875592	1.10982159929043	-1.44732490981427	0.157358345353181	0.852963687489054	-4.77779285296751
a_HQ_transcript/145472	2.27359543558043	-0.157007311514454	1.44728867587619	0.157368423022512	0.852963687489054	-4.94557505793756
a_HQ_transcript/135486	2.43754571924261	5.2196709833448	1.4472461157005	0.157380260848722	0.852963687489054	-5.31934387146475
a_HQ_transcript/33496	-1.48604244249832	3.24200924135647	-1.44719181662224	0.157395364796799	0.852963687489054	-5.18911604295992
a_HQ_transcript/119793	1.42052985361318	1.73809117529804	1.44718209002547	0.157398070489213	0.852963687489054	-4.86967816962352
a_HQ_transcript/105440	-2.50055755188225	4.00148518036877	-1.44715726741816	0.157404975675882	0.852963687489054	-5.21212075564516
a_HQ_transcript/85524	1.99157723333732	1.57618785171169	1.44713020512405	0.157412504175392	0.852963687489054	-5.00204814288956
a_HQ_transcript/130720	1.67498036843284	-0.119243127119517	1.44712526614328	0.157413878188548	0.852963687489054	-4.87145393183892
a_HQ_transcript/129505	1.94737337404026	0.650700550732485	1.44705450945331	0.157433563582018	0.852963687489054	-4.90426871913026
a_HQ_transcript/136692	1.70173558341799	2.56369442034834	1.4470371323944	0.157438398395065	0.852963687489054	-4.950910859719
a_HQ_transcript/118427	1.38008477298933	1.95916881832646	1.44691722494507	0.157471763412276	0.852963687489054	-4.8658567197833
a_HQ_transcript/129761	3.95117464481509	2.41416844530383	1.44690741604908	0.157474493048603	0.852963687489054	-4.95436465566346
a_HQ_transcript/10392	-1.08953502960762	-0.43176919249608	-1.44689982385705	0.157476605842526	0.852963687489054	-4.76780375879555
a_HQ_transcript/111874	-1.94283579970141	0.579871151082998	-1.44689438794182	0.157478118590551	0.852963687489054	-4.84280672041583
a_HQ_transcript/149687	2.87100004946574	5.31861221895442	1.4468942737876	0.157478150358383	0.852963687489054	-5.07554865043172
a_HQ_transcript/134062	1.20730539836446	-0.640269993572188	1.44686736037728	0.1574856401988	0.852963687489054	-4.89147196790654
a_HQ_transcript/15219	-1.19373837341595	0.11718502219162	-1.44683902925206	0.15749352488593	0.852963687489054	-4.73389088876799
a_HQ_transcript/104336	-2.14069181441913	0.803717083345397	-1.44680830212483	0.157502076745684	0.852963687489054	-4.84077757996722
a_HQ_transcript/7479	1.1654323461704	0.0148092957522299	1.44680004387912	0.157504375212686	0.852963687489054	-4.76315910619857
a_HQ_transcript/73126	-1.46437052412244	-0.269585934254453	-1.4467995153425	0.157504522317955	0.852963687489054	-4.8313477891291
a_HQ_transcript/125205	1.97237255348732	1.95254466576598	1.44676614615034	0.157513810038798	0.852963687489054	-4.77139895088159
a_HQ_transcript/25446	-0.590161855866613	2.60593793583333	-1.446728912119	0.157524173981472	0.852963687489054	-4.96366306062001
a_HQ_transcript/89558	0.576827681246977	3.78844866792888	1.44653868785656	0.157577130576681	0.853161568951839	-5.19085632508838
a_HQ_transcript/2673	-0.916274554611992	-0.771087355176967	-1.44648588069694	0.157591834082219	0.853161568951839	-4.81799802536296
a_HQ_transcript/115521	0.359035629537744	5.03728196263121	1.44643949562435	0.15760475033383	0.853161568951839	-5.35567797113506
a_HQ_transcript/65261	-0.706396426518552	4.27960630513146	-1.44637102289245	0.157623818584439	0.853185336314019	-5.31140134245745
a_HQ_transcript/83580	2.34664075202004	1.06649049607976	1.44626460255587	0.157653458094703	0.853230643956992	-4.87339601371784
a_HQ_transcript/52874	-3.01503519064881	1.67940639474202	-1.44623556572271	0.157661546014693	0.853230643956992	-4.80103046596272
a_HQ_transcript/105261	0.62425143230308	3.5933035141666	1.44608617687789	0.157703161986022	0.853244598078439	-5.11659043572927
a_HQ_transcript/22758	1.10225447621799	-0.489126078802724	1.44604289279204	0.157715221469729	0.853244598078439	-4.84673457251688
a_HQ_transcript/141851	2.00722228834906	0.550824821201807	1.44603860982632	0.157716414796904	0.853244598078439	-4.91531569686538
a_HQ_transcript/84027	-0.541190571129931	2.86176174533076	-1.44587690315796	0.157761475020455	0.853244598078439	-5.01381797003294

a_HQ_transcript/93916	-0.47805860237303	2.61435259617954	-1.44586986939965	0.15776343523753	0.853244598078439	-4.8892297243396
a_HQ_transcript/83990	1.51900016539815	0.657881171081785	1.44586974282096	0.157763470513544	0.853244598078439	-4.80172736660213
a_HQ_transcript/79044	-1.03085714370072	2.36481846143406	-1.44584999153948	0.157768975043335	0.853244598078439	-4.81977886561629
a_HQ_transcript/122509	-1.68129660659441	1.77026937440066	-1.44580485708916	0.157781554237977	0.853244598078439	-4.90711978486703
a_HQ_transcript/146087	-3.78799732159128	2.26256859945329	-1.44557561278324	0.157845458035737	0.853510770669891	-5.04981583423966
a_HQ_transcript/128431	1.63213369579024	-0.0608284189160115	1.44544890123033	0.157880788760802	0.853549331679109	-4.90336678628329
a_HQ_transcript/61461	-0.41947258331219	3.44399826183278	-1.44538014716115	0.157899961941638	0.853549331679109	-5.10833285476838
a_HQ_transcript/71828	2.02468538876113	0.469358704642838	1.44535688492293	0.157906449409715	0.853549331679109	-4.87422769879952
a_HQ_transcript/138386	1.98382325582608	1.00944987500597	1.44533940210531	0.157911325228412	0.853549331679109	-4.90059479013071
a_HQ_transcript/111101	1.99493066752377	1.15171749894153	1.44524885710582	0.157936579417721	0.853606461031455	-4.93058693092121
a_HQ_transcript/83183	3.24436898210554	0.971726253158776	1.44517039311851	0.157958466643104	0.853645383881588	-4.78529585575289
a_HQ_transcript/126057	2.35347188046206	0.447365412351815	1.44502070711762	0.158000227641285	0.853647230624414	-4.80759901885408
a_HQ_transcript/149425	2.34606406579508	0.590229697969001	1.44498968121599	0.15800888467326	0.853647230624414	-4.98885556542826
a_HQ_transcript/131293	-1.1894244106634	-0.6408444156416	-1.44491432391719	0.158029912881356	0.853647230624414	-4.86862391264875
a_HQ_transcript/107056	1.99642583651758	0.300032415785839	1.4448791520221	0.158039728242966	0.853647230624414	-4.86942245032771
a_HQ_transcript/3571	-1.09272529103257	1.54187617630211	-1.44482064903851	0.15805605564246	0.853647230624414	-4.79530502558452
a_HQ_transcript/39536	1.42174807402209	2.25032005741866	1.44476285463289	0.158072186600005	0.853647230624414	-4.82178550308432
a_HQ_transcript/59075	-3.36801431892883	2.1763702529228	-1.44474853818008	0.158076182657115	0.853647230624414	-5.09354913684444
a_HQ_transcript/5696	-0.83511219338685	2.50101583094358	-1.44468489644173	0.15809394752509	0.853647230624414	-4.95779503980218
a_HQ_transcript/130209	1.63445773617756	0.948640280872086	1.44458993120624	0.158120458933275	0.853647230624414	-4.80406349525684
a_HQ_transcript/138084	-2.2762486011953	0.737303179133464	-1.44457743864552	0.158123946738855	0.853647230624414	-4.91892452806417
a_HQ_transcript/122917	1.48694588520735	0.381360657973509	1.44454507298719	0.158132983210169	0.853647230624414	-4.75613645193538
a_HQ_transcript/53498	1.39606708627709	1.57890105905277	1.44445055222556	0.158159375689836	0.853647230624414	-4.7909698560508
a_HQ_transcript/151626	0.536930104868863	6.09147724228339	1.44438899045487	0.158176567100265	0.853647230624414	-5.44094610384619
a_HQ_transcript/74134	3.00133355701073	1.54358401099273	1.44433930521157	0.158190443015104	0.853647230624414	-5.02141807643884
a_HQ_transcript/45996	0.391298842672547	5.01472993595717	1.44433535762822	0.158191545523253	0.853647230624414	-5.36127842840494
a_HQ_transcript/100105	-2.75070137182862	2.32403456200615	-1.4443273616977	0.158193778700217	0.853647230624414	-5.02070516681343
a_HQ_transcript/55103	3.07477805416346	1.1415628464027	1.44411558584797	0.158252934499671	0.853887178742865	-4.90968648027794
a_HQ_transcript/22648	-1.58491950055368	3.36625113852705	-1.44397139116611	0.158293222737431	0.85394315500359	-5.16221434645133
a_HQ_transcript/92874	1.99599816404733	-0.10176903407879	1.44394882969886	0.158299527184743	0.85394315500359	-4.9442387536985
a_HQ_transcript/105974	0.510370081528935	2.37584086872992	1.44392072484187	0.158307380923457	0.85394315500359	-4.93091246651058
a_HQ_transcript/83260	-0.551133094193349	4.00371039834446	-1.44375482219186	0.158353747744128	0.854096063301389	-5.23674900813357
a_HQ_transcript/134327	1.50518625677996	1.76246779706992	1.44371415761503	0.158365114407211	0.854096063301389	-4.94520077739569
a_HQ_transcript/140356	-1.90856586020207	1.89051863359437	-1.44362391779766	0.158390340773215	0.854152864687396	-4.77344368634768
a_HQ_transcript/57770	0.353983486282245	-0.877038658965845	1.44348545768827	0.158429053218627	0.854208297422681	-4.95063317609651
a_HQ_transcript/115637	-1.81011093022621	2.96046790321327	-1.44346540825901	0.158434659515377	0.854208297422681	-4.96706397118652
a_HQ_transcript/127709	1.21925011054176	2.28595586630761	1.44342948154977	0.15844470587	0.854208297422681	-4.95309825869527
a_HQ_transcript/26076	1.55088113997558	-0.00561565616262188	1.44320574610398	0.158507281423736	0.854466405879527	-4.8941960520268
a_HQ_transcript/141972	1.43044366331729	-0.228784147036532	1.44289821255131	0.158593326040631	0.854780407401456	-4.89489949427397
a_HQ_transcript/105932	1.27907101082891	-0.62460121236291	1.44285276856557	0.158606043921756	0.854780407401456	-4.87641027705904
a_HQ_transcript/72686	1.07741986606516	4.32776053133272	1.44278509908839	0.158624983290567	0.854780407401456	-5.2790761695465
a_HQ_transcript/113173	-1.54680385506251	0.417348353412177	-1.44276906150252	0.158629472161123	0.854780407401456	-4.82513361023163
a_HQ_transcript/145529	-2.12568052380686	2.95131837315824	-1.44262592901087	0.158669538958474	0.854780407401456	-5.00855281126188
a_HQ_transcript/47979	1.49213747751521	-0.292669784699284	1.44254999254838	0.15869079895777	0.854780407401456	-4.88804584261221
a_HQ_transcript/147073	2.06917618810289	-0.333392395698999	1.44253942356842	0.158693758143414	0.854780407401456	-4.83064712041094
a_HQ_transcript/111257	-0.74513175686277	2.07896227731044	-1.44250248236245	0.158704101574474	0.854780407401456	-4.92878656276451
a_HQ_transcript/19163	-1.38763817196793	-0.535128281472241	-1.44249572244597	0.158705994389381	0.854780407401456	-4.86779806620166
a_HQ_transcript/11814	0.353983486282244	-0.878997825219351	1.44247219804602	0.158712581494012	0.854780407401456	-4.95229279383162
a_HQ_transcript/91487	-2.32213126192386	-0.293003025594205	-1.44236676701779	0.158742106064106	0.854788697914491	-4.90827004347058
a_HQ_transcript/74581	1.40978565569893	-0.356694833163454	1.44236167754767	0.158743531413208	0.854788697914491	-4.906737326899

a_HQ_transcript/7264	-1.091070184801	1.77374005382071	-1.44228266610767	0.158765660534769	0.854828669466934	-4.84243685003953
a_HQ_transcript/43246	-2.41489898595357	1.15197444157383	-1.44221850462738	0.158783632352919	0.854846251867228	-4.80823764533294
a_HQ_transcript/49298	1.65014160797268	0.659201357235274	1.4421415559003	0.158805188041248	0.854847236859936	-4.82671707215741
a_HQ_transcript/145700	-2.36174265155154	2.18704772117941	-1.44199618564151	0.158845917015355	0.854847236859936	-5.04971652486031
a_HQ_transcript/98644	-2.64042060223397	1.10707526750846	-1.44191751212114	0.158867962744193	0.854847236859936	-4.89356151752921
a_HQ_transcript/40560	-1.20200381878016	1.6270852928916	-1.4419068160302	0.15887096016747	0.854847236859936	-4.78272092079587
a_HQ_transcript/35437	1.62411435113061	0.516485403564326	1.4417962650313	0.158901943094287	0.854847236859936	-4.83293909581942
a_HQ_transcript/17863	1.16460195576627	-0.401731332607594	1.44163044070239	0.158948425848552	0.854847236859936	-4.87663667478775
a_HQ_transcript/82848	3.0583366486608	0.747640295359243	1.44162813025228	0.158949073574355	0.854847236859936	-4.89560949720225
a_HQ_transcript/98893	1.6392156142287	-0.157228693410004	1.44158568221723	0.158960974089721	0.854847236859936	-4.89347869106877
a_HQ_transcript/94777	0.511480130609761	5.14676414646889	1.4415513141771	0.158970609855687	0.854847236859936	-5.34307685713961
a_HQ_transcript/132799	3.58346709219666	3.71076885893977	1.44150668802178	0.158983122379231	0.854847236859936	-4.93071837226405
a_HQ_transcript/120867	-0.545553498849989	4.2492516449248	-1.44139838541324	0.159013492095384	0.854847236859936	-5.26997266616094
a_HQ_transcript/78522	-2.12102682071249	1.32139782526111	-1.441379935967	0.15901866606163	0.854847236859936	-4.93818866752111
a_HQ_transcript/113726	1.51215031134979	1.61429463177119	1.4413461655296	0.159028136994923	0.854847236859936	-4.86544503473678
a_HQ_transcript/131221	2.40403082621348	2.00194152413984	1.44132866139232	0.159033046213307	0.854847236859936	-4.85062309688479
a_HQ_transcript/150600	4.50732312833633	7.02264806391144	1.44120746314502	0.159067040821666	0.854847236859936	-5.36523115858969
a_HQ_transcript/102282	1.04528274607293	2.99715192042923	1.44115092206257	0.159082901863756	0.854847236859936	-4.96307585989082
a_HQ_transcript/54786	0.711797757006804	3.6238843581694	1.44112742760635	0.159089492954109	0.854847236859936	-5.07550323073775
a_HQ_transcript/123694	-1.72763472043414	0.319914171538435	-1.44108016379722	0.159102752908867	0.854847236859936	-4.85340940391528
a_HQ_transcript/15970	3.42518354598398	2.45165484526929	1.44107833381795	0.159103266330751	0.854847236859936	-5.02180431592958
a_HQ_transcript/32870	-1.19713407057183	1.57757373215425	-1.44099303156056	0.159127200323281	0.854847236859936	-4.78958008288001
a_HQ_transcript/72137	-1.89506442443403	-0.0449382208439816	-1.44090574792317	0.159151693199615	0.854847236859936	-4.84693200334998
a_HQ_transcript/124482	-1.01054241205364	3.22164379336176	-1.44090100672377	0.159153023724721	0.854847236859936	-5.1605411841232
a_HQ_transcript/29170	-0.681123550469099	2.68036569170498	-1.44086634119012	0.159162752197999	0.854847236859936	-5.02515531122276
a_HQ_transcript/47748	0.895265416617221	-0.742977439011589	1.4408561305375	0.159165617787032	0.854847236859936	-4.90175791077024
a_HQ_transcript/148146	4.05522870693289	1.93906838972618	1.44072047955383	0.159203691705202	0.854847236859936	-5.12811065061568
a_HQ_transcript/72919	2.23066418653474	1.91778918654329	1.44063931099342	0.159226477185907	0.854847236859936	-4.97176490536861
a_HQ_transcript/94430	-1.75151020824289	0.0508937651766698	-1.44063062824102	0.159228914744168	0.854847236859936	-4.86762802881866
a_HQ_transcript/15658	-1.23429869012121	1.25092608693257	-1.44058044314263	0.159243004069983	0.854847236859936	-4.81281815823716
a_HQ_transcript/13559	1.34331309775759	1.2556508349951	1.44051361696162	0.159261766865378	0.854847236859936	-5.02029555236469
a_HQ_transcript/25927	1.41556395114266	1.91364861398889	1.44037629346143	0.159300328695959	0.854847236859936	-4.7947074031865
a_HQ_transcript/38196	-0.519494128836274	3.24901123973057	-1.44034556124417	0.159308959626205	0.854847236859936	-5.12876638808614
a_HQ_transcript/21824	1.84381298713686	0.874553235606097	1.44030485124187	0.159320393318252	0.854847236859936	-4.81247214779434
a_HQ_transcript/150704	3.51298260042069	4.70293573798099	1.44026566387414	0.159331399981271	0.854847236859936	-5.02017864555089
a_HQ_transcript/36669	1.13774337872224	-0.630365097411213	1.44026458092669	0.159331704160227	0.854847236859936	-4.90283852788663
a_HQ_transcript/133115	1.48891882010283	-0.490665979866338	1.44020938065438	0.159347209456145	0.854847236859936	-4.87469072734649
a_HQ_transcript/121965	1.58812920644256	-0.519393690004474	1.44016282505018	0.159360287465477	0.854847236859936	-4.88969224693985
a_HQ_transcript/103622	-2.03867797835847	0.340735567476047	-1.44003276723374	0.159396826717586	0.854847236859936	-4.95315694695915
a_HQ_transcript/7083	0.80477016391716	-0.504172062085649	1.43999906943976	0.159406295067226	0.854847236859936	-4.87101645730672
a_HQ_transcript/17370	-1.21561887280043	-0.0626602546703621	-1.43979912287539	0.159462484876364	0.854847236859936	-4.76753871029116
a_HQ_transcript/57166	1.17844367364524	3.14834118593396	1.43979095649475	0.159464780159587	0.854847236859936	-5.01779769411113
a_HQ_transcript/8471	-0.784091539038999	2.37087323086731	-1.43977922311049	0.159468078048225	0.854847236859936	-4.94615233856776
a_HQ_transcript/9382	1.77533577053685	0.0186098035596797	1.43973984493686	0.159479146420229	0.854847236859936	-4.8999055699539
a_HQ_transcript/49530	1.46955603450058	-0.153762363497079	1.43971910191096	0.159484977090758	0.854847236859936	-4.86565988332617
a_HQ_transcript/18504	1.51701476118011	4.15443255578662	1.43965333234781	0.159503465415397	0.854847236859936	-5.1553033350123
a_HQ_transcript/120183	1.54460044501279	-0.45121439525081	1.43964591135446	0.159505551619602	0.854847236859936	-4.84915640003079
a_HQ_transcript/65953	-1.49325068426284	0.828142003346869	-1.43962111985553	0.159512521211002	0.854847236859936	-4.76156194705321
a_HQ_transcript/27684	1.39835930762473	0.0175646099758769	1.43954556595487	0.159533763035741	0.854847236859936	-4.83430445162485
a_HQ_transcript/27113	1.96174981176471	0.948079711483728	1.43945461971814	0.159559335353957	0.854847236859936	-4.86416562192083

a_HQ_transcript/128167	0.859691014084781	3.59329787223115	1.43942802659395	0.159566813437837	0.854847236859936	-5.18347653898695
a_HQ_transcript/151035	4.05152375970239	4.46492178170337	1.43942241236259	0.159568392215712	0.854847236859936	-5.03682837783677
a_HQ_transcript/103783	2.83807986193029	2.96459396008756	1.43941678444789	0.159569974853891	0.854847236859936	-5.16907868543627
a_HQ_transcript/145014	2.54119285090341	1.80718996513434	1.43936754037029	0.15958382341842	0.854847236859936	-5.02462928514035
a_HQ_transcript/7193	1.39610587171249	2.73887583732864	1.43933015522317	0.159594337614817	0.854847236859936	-5.01622043856789
a_HQ_transcript/67355	1.24124617214175	1.26929050710659	1.43926634670296	0.159612284385509	0.854847236859936	-4.77491464383906
a_HQ_transcript/102274	-1.52449697596494	0.644407070385194	-1.43924478724313	0.159618348555568	0.854847236859936	-4.90945512650005
a_HQ_transcript/104374	0.549558147781653	4.76617174994371	1.43921112062476	0.159627818549887	0.854847236859936	-5.30745741876535
a_HQ_transcript/103326	-2.11320290219952	0.837928335216741	-1.43919386739715	0.15963267183713	0.854847236859936	-4.8319262382318
a_HQ_transcript/90791	-0.885869552804358	1.27144819832103	-1.43912166285393	0.159652984051055	0.854847236859936	-4.79969191304371
a_HQ_transcript/13034	2.12443548025976	0.648087184724454	1.43901511050983	0.1596829625498	0.854847236859936	-4.90123538965576
a_HQ_transcript/29644	1.30872195020692	-0.184416912280525	1.43900284023673	0.159686415076802	0.854847236859936	-4.83535653858058
a_HQ_transcript/112932	-0.732622919669565	3.27198120798767	-1.43900058539058	0.159687049536688	0.854847236859936	-5.1465372686524
a_HQ_transcript/130112	-2.03298050149897	-0.0403708638633359	-1.43897017921645	0.159695605303159	0.854847236859936	-4.87779064463862
a_HQ_transcript/46392	-0.553411153528679	2.960318157801	-1.43891165858886	0.15971207300697	0.854856664801946	-5.09382307910841
a_HQ_transcript/146738	-1.64898940145957	3.4028531101974	-1.43849047278792	0.159830634332681	0.855412495395457	-5.23352582877546
a_HQ_transcript/88944	2.37912758709659	0.310724340570109	1.43835469996197	0.159868868425751	0.855538352407667	-4.93238865792608
a_HQ_transcript/81817	1.38655301924031	1.07208774493222	1.4382757105023	0.159891115458181	0.855570485349799	-4.79651252530929
a_HQ_transcript/10630	4.36480040785737	1.35824878713997	1.43822886528454	0.159904310364028	0.855570485349799	-4.86709160762289
a_HQ_transcript/103166	-1.12909326898738	2.86393409610011	-1.43811186587613	0.159937269384124	0.855668071307905	-4.9546835458422
a_HQ_transcript/95938	2.13943609104982	3.78812627067057	1.43804966254988	0.159954794402066	0.855683074636784	-5.04249929150853
a_HQ_transcript/82339	2.74495364228774	0.861045261241506	1.43799252398191	0.159970893825855	0.855690449646573	-5.04275736857501
a_HQ_transcript/2796	-1.22457505439945	-0.606673892778942	-1.43789341340612	0.159998822367996	0.855761091885062	-4.87453845620754
a_HQ_transcript/46893	-1.54290312285595	3.45371437717388	-1.43780374329754	0.160024093988475	0.855817511763923	-5.30817675995752
a_HQ_transcript/20931	-1.43031682714643	0.173796408582674	-1.43771543002699	0.160048986299652	0.855824383206078	-4.87364272574482
a_HQ_transcript/143335	-1.79061185198465	-0.235676857557704	-1.43769471684591	0.16005482503842	0.855824383206078	-4.89050023589043
a_HQ_transcript/118435	-1.22456744474819	0.976478675296653	-1.43760368321049	0.160080488068995	0.855877004594214	-4.79140541884022
a_HQ_transcript/81994	4.39837818293618	3.36667667747116	1.43755535232615	0.16009411421265	0.855877004594214	-5.14748168778731
a_HQ_transcript/116101	1.76440569938071	0.342910116782209	1.43742418683421	0.160131098914388	0.855898484961803	-4.89703547369886
a_HQ_transcript/109116	1.54325621496649	1.07669451438704	1.4374169666817	0.160133134975298	0.855898484961803	-4.86288349633013
a_HQ_transcript/103259	-0.388629520639743	3.20758463136918	-1.43736829617887	0.160146860442488	0.855898484961803	-5.11040399648936
a_HQ_transcript/83363	-1.247036001529	2.81816790387263	-1.43721847611284	0.160189116729499	0.855898484961803	-5.04987389224777
a_HQ_transcript/2126	2.53017141990353	0.90986913687943	1.43720048524973	0.160194191590021	0.855898484961803	-4.9800520414572
a_HQ_transcript/123289	-1.74422853148764	1.19043435862797	-1.4371796500941	0.16020006892717	0.855898484961803	-4.89361035758205
a_HQ_transcript/126610	1.0425284331002	-0.568112198637941	1.4371756305838	0.160201202800448	0.855898484961803	-4.87282569615825
a_HQ_transcript/150508	1.15883146540162	3.71906733990959	1.43703266180789	0.160241537333515	0.855932116106817	-5.2204135384537
a_HQ_transcript/38573	1.30377692994671	2.17480538184111	1.43700294078337	0.160249923275289	0.855932116106817	-4.84133304781939
a_HQ_transcript/126299	2.74006090672617	4.49105651388584	1.43697969036064	0.160256483745098	0.855932116106817	-5.16371261328823
a_HQ_transcript/38814	0.714307896265836	4.86058791039183	1.43694455739638	0.16026639746261	0.855932116106817	-5.26601233534546
a_HQ_transcript/12108	0.717629209761873	4.16345573477088	1.43686242840828	0.160289574273777	0.855977250655593	-5.3155474130301
a_HQ_transcript/138057	1.54331784997667	2.33409663213129	1.4365568873357	0.160375821283802	0.856359153002464	-4.99599863247268
a_HQ_transcript/32370	-1.15656314662166	-0.665738584476301	-1.43638851177095	0.160423365403844	0.856503023359717	-4.88365186106277
a_HQ_transcript/36301	-2.39532248253693	0.74542477411487	-1.43625770805046	0.160460308075393	0.856503023359717	-4.81749548367558
a_HQ_transcript/67358	1.97434274603771	1.86598297903468	1.43618395245858	0.160481141707722	0.856503023359717	-4.79374832014432
a_HQ_transcript/62464	2.13406598803278	2.03157031558759	1.43617473415758	0.160483745737889	0.856503023359717	-5.1833945114778
a_HQ_transcript/149337	2.1620012909916	0.395509388211589	1.43606610825016	0.160514433425688	0.856503023359717	-4.89110496706517
a_HQ_transcript/67036	-1.51263341152042	1.15987672789928	-1.43601085710954	0.160530044095378	0.856503023359717	-4.86996219588766
a_HQ_transcript/32264	-0.540231818681709	3.0158533508855	-1.43600933524073	0.160530474101524	0.856503023359717	-4.99954082630837
a_HQ_transcript/89454	1.90491910239679	0.291749757087303	1.43599006139406	0.160535920032501	0.856503023359717	-4.8819285592894
a_HQ_transcript/1760	-0.426099482963328	4.11967234140833	-1.43596869977516	0.160541956045666	0.856503023359717	-5.22455152666429

a_HQ_transcript/68445	1.52505084843947	-0.0682187538896809	1.43587289725032	0.160569028547027	0.856503023359717	-4.89196916738112
a_HQ_transcript/145450	-1.37003149428951	7.49691275235708	-1.43585217998216	0.160574883442337	0.856503023359717	-5.63358530702885
a_HQ_transcript/130591	-1.25321062336289	0.401893553467053	-1.43561296136196	0.160642501110326	0.856503023359717	-4.77022412840171
a_HQ_transcript/130575	-1.63593573433751	4.66366604949601	-1.43559565371203	0.160647394173602	0.856503023359717	-5.25839229522599
a_HQ_transcript/73357	-1.54518970030009	0.0423298957421033	-1.43554831398381	0.160660778238252	0.856503023359717	-4.91325159552272
a_HQ_transcript/50578	0.997481918620823	4.01373918386726	1.43552595324077	0.160667100457585	0.856503023359717	-5.0209682358114
a_HQ_transcript/137390	0.896354061071448	4.62412777058898	1.43550555464285	0.160672868076617	0.856503023359717	-5.30811006025778
a_HQ_transcript/152760	2.97272359832364	3.7283844634585	1.43547877384704	0.160680440484225	0.856503023359717	-5.13354570773212
a_HQ_transcript/111841	2.88196739800244	2.4365858525848	1.43544306485679	0.160690537823688	0.856503023359717	-4.97391447674678
a_HQ_transcript/42977	0.657552750240985	4.00649893307053	1.43540245200089	0.160702022426475	0.856503023359717	-5.22830709225849
a_HQ_transcript/148823	-2.09052395170115	1.29649498828088	-1.43537452690807	0.160709919529129	0.856503023359717	-4.96833381235599
a_HQ_transcript/109693	-2.12497418968877	1.45347460451569	-1.43523835271301	0.160748433434301	0.856503023359717	-4.93152703004885
a_HQ_transcript/1002	1.62240591771862	2.35753777067404	1.43518946960664	0.160762260736253	0.856503023359717	-4.84135949412136
a_HQ_transcript/124335	1.45177118950614	0.839040517907246	1.43514959273133	0.160773541190457	0.856503023359717	-4.72602870140002
a_HQ_transcript/2476	-1.54208984600071	2.05927256953231	-1.43513019209876	0.160779029508432	0.856503023359717	-4.91514643521293
a_HQ_transcript/76412	2.31037197561122	0.158732465123049	1.43508987780037	0.160790434645807	0.856503023359717	-4.80273284362033
a_HQ_transcript/147921	-1.88799564499256	0.48215169245115	-1.435074126384	0.160794890982168	0.856503023359717	-4.85000594913084
a_HQ_transcript/74742	-1.58949882509889	2.29202736852551	-1.43505393452775	0.160800603735284	0.856503023359717	-4.92572353346599
a_HQ_transcript/138103	0.754648520069155	4.54144873361657	1.43496708136661	0.160825178375993	0.856555430514907	-5.3077770151852
a_HQ_transcript/33476	-1.30189515741749	1.29549634681603	-1.43478833832454	0.160875762114205	0.856677574894155	-4.85954705084128
a_HQ_transcript/126501	-2.72525142954623	1.52599207984137	-1.4347359616636	0.16089058693297	0.856677574894155	-5.0037718828622
a_HQ_transcript/82276	1.66831247283455	1.23677697972812	1.43472982019033	0.160892325301395	0.856677574894155	-4.76317687670277
a_HQ_transcript/26159	-0.650233097711814	1.76246459653521	-1.43455409628224	0.160942070943394	0.856754772421178	-4.8394597769136
a_HQ_transcript/26683	-1.44501261170166	2.35399662388987	-1.43453303074422	0.160948035197977	0.856754772421178	-4.85822914357733
a_HQ_transcript/37915	3.34159196426391	3.62484548442739	1.43452241437257	0.160951041061782	0.856754772421178	-5.1560530209197
a_HQ_transcript/64972	1.00512426953789	-0.580736478873717	1.43435907229803	0.160997294474878	0.856778973254063	-4.86455521516134
a_HQ_transcript/38715	-0.583373096485563	3.29713453711963	-1.43426730377807	0.161023285085306	0.856778973254063	-5.13695287242442
a_HQ_transcript/59780	2.57759667778384	0.191775717581312	1.43423460338236	0.161032547268001	0.856778973254063	-4.8679237434577
a_HQ_transcript/48033	1.1183591468231	1.03770276164552	1.43420492544827	0.161040953721894	0.856778973254063	-4.74505156568363
a_HQ_transcript/118812	4.67942144980193	3.77051373316807	1.43416928568607	0.161051049358009	0.856778973254063	-5.12148501738661
a_HQ_transcript/43558	0.69444537535704	1.01313326837555	1.43414718480121	0.161057310102592	0.856778973254063	-4.93231473049467
a_HQ_transcript/21226	1.35056319869993	1.0796063390522	1.43414205211215	0.161058764119652	0.856778973254063	-4.83679082674956
a_HQ_transcript/25076	2.92472816673046	0.529833910153995	1.43405393909805	0.161083726888589	0.856787563103136	-4.87265204068921
a_HQ_transcript/36857	1.63609075554087	0.306613313410533	1.43403229860357	0.161089858194918	0.856787563103136	-4.90832395346056
a_HQ_transcript/14283	-1.32444114675668	3.49957168163083	-1.43389394088421	0.161129062840512	0.856911603791482	-5.06765650647936
a_HQ_transcript/13415	1.50785024168577	-0.0344727378339913	1.43384594897897	0.161142663450397	0.856911603791482	-4.89400597114066
a_HQ_transcript/27095	-1.84015887160131	0.745499339513963	-1.43358156254895	0.161217605203343	0.856991401458777	-4.86541447068676
a_HQ_transcript/72956	1.80420465057455	3.31145463643348	1.43357574965392	0.161219253209083	0.856991401458777	-5.12378420131904
a_HQ_transcript/17465	1.63232077033829	0.191253276981692	1.43341818317273	0.161263929734042	0.856991401458777	-4.92377460011869
a_HQ_transcript/2640	0.445968668878466	4.23231273981468	1.43339118058541	0.161271587051089	0.856991401458777	-5.28251324536448
a_HQ_transcript/29190	-0.797882351368565	1.81025142117983	-1.43333840454674	0.161286553959982	0.856991401458777	-4.82246481355512
a_HQ_transcript/145337	0.449013875427567	3.80250282894864	1.43330804128341	0.161295165262789	0.856991401458777	-5.22848866666834
a_HQ_transcript/82061	-1.59381393655869	1.42911385990638	-1.43328604695814	0.161301403284156	0.856991401458777	-4.82687489754269
a_HQ_transcript/125922	0.329063849889824	4.76941717042504	1.43323084380941	0.161317060818027	0.856991401458777	-5.34722282082749
a_HQ_transcript/125888	-3.75742180362054	3.01283270066498	-1.43323018583508	0.161317247449687	0.856991401458777	-5.11101755735097
a_HQ_transcript/31404	-1.18930265616036	0.892918111290278	-1.43322719984894	0.161318094414275	0.856991401458777	-4.80298214535849
a_HQ_transcript/85077	-1.3024111252075	1.7822077583257	-1.43304314756898	0.161370306988458	0.856991401458777	-4.80973695149816
a_HQ_transcript/107651	-1.43765164410762	-0.223078978172413	-1.43296895000973	0.161391359380162	0.856991401458777	-4.82849574222154
a_HQ_transcript/79544	1.27740142153065	-0.366464972963018	1.43290299480585	0.161410074955156	0.856991401458777	-4.88116390203551
a_HQ_transcript/139792	-0.578334255569216	4.10197951198365	-1.43283056160472	0.161430630715072	0.856991401458777	-5.23090489045939

a_HQ_transcript/124388	-0.247266051225656	5.91143431112999	-1.43281157692884	0.161436018703118	0.856991401458777	-5.45450455628084
a_HQ_transcript/65548	2.00189744928289	-0.185793261084914	1.43279256010717	0.161441415956838	0.856991401458777	-4.88633580646013
a_HQ_transcript/140409	1.7928471716174	0.272616926307625	1.43278039743752	0.161444867976157	0.856991401458777	-4.87770816010617
a_HQ_transcript/26574	2.05247379748873	0.599717344422703	1.43276303166123	0.161449796846753	0.856991401458777	-4.78628987046434
a_HQ_transcript/5830	-0.729852573933614	1.63220015885182	-1.43275893338522	0.161450960064162	0.856991401458777	-4.83912256306926
a_HQ_transcript/21489	1.01518482506595	1.71378101858308	1.43268106387528	0.161473063096412	0.856991401458777	-4.76972831425108
a_HQ_transcript/132649	2.71506074569026	-0.0210351713625405	1.43267795943503	0.161473944332241	0.856991401458777	-4.87740049967382
a_HQ_transcript/86771	1.57569837254458	-0.128795071411749	1.43262680371909	0.161488466096302	0.856991401458777	-4.94262614933287
a_HQ_transcript/144120	2.69815042732911	2.30214540140877	1.43259757960844	0.16149676251685	0.856991401458777	-4.83147460199102
a_HQ_transcript/88603	2.01681849771669	0.514127363213266	1.43248743646727	0.16152803403391	0.857037925448161	-4.82694650248163
a_HQ_transcript/93559	-1.67702362656319	1.03548596257035	-1.43240281891778	0.161552061649723	0.857037925448161	-4.75801736408098
a_HQ_transcript/9736	0.980906641548182	-0.285641420344799	1.43239462182192	0.161554389409941	0.857037925448161	-4.77506428197599
a_HQ_transcript/134610	3.85875145323736	5.0308633261887	1.4323512800374	0.16156669778044	0.857037925448161	-5.2363942876058
a_HQ_transcript/101289	-0.515214864025228	3.69476203399297	-1.43227946556116	0.161587093568903	0.857037925448161	-5.19827812010409
a_HQ_transcript/92985	1.32095655321354	2.9183974428757	1.43225517168932	0.161593993650424	0.857037925448161	-4.99512597261284
a_HQ_transcript/118361	0.72160842495097	-0.728115560277924	1.43215055685007	0.161623709602715	0.857055785709362	-4.91905018726502
a_HQ_transcript/55653	0.723916192031265	2.3807549754649	1.43213950282638	0.161626849761664	0.857055785709362	-4.99819950078237
a_HQ_transcript/130728	0.594981478760927	3.01793485566859	1.4320239134336	0.161659688571734	0.857151726470653	-5.0178247832258
a_HQ_transcript/107899	2.17410473525798	0.669000749733951	1.43194086077269	0.161683286989056	0.857198659804804	-4.78716587012981
a_HQ_transcript/72253	1.41801741551437	-0.521662784492202	1.43163526782558	0.161770140990501	0.857538821264266	-4.79447240621933
a_HQ_transcript/145534	-2.04776594027671	0.783367555344136	-1.43151548670741	0.161804194596052	0.857538821264266	-4.84691199086672
a_HQ_transcript/45451	-1.57664719407477	0.937313766016739	-1.43146989522506	0.161817157679012	0.857538821264266	-4.83129118907531
a_HQ_transcript/5972	-2.07651969479011	0.299663578398423	-1.43145978670144	0.161820031959237	0.857538821264266	-4.87121925598198
a_HQ_transcript/91049	1.69848100774205	2.60437344918384	1.4314330824693	0.161827625294364	0.857538821264266	-4.98158530798973
a_HQ_transcript/70968	1.51596582802503	0.214825040814053	1.43133331164709	0.161855997568395	0.857538821264266	-4.89764190956907
a_HQ_transcript/122608	-1.50510825259323	0.0887358820915003	-1.43128428251573	0.161869941641211	0.857538821264266	-4.79076822089447
a_HQ_transcript/124523	0.650950947613308	4.02308938875462	1.43125416767541	0.161878506887609	0.857538821264266	-5.25880932385481
a_HQ_transcript/104269	1.80288915868338	1.04473720451604	1.43124814115425	0.161880220990449	0.857538821264266	-4.81292904803442
a_HQ_transcript/36605	1.85177519867841	0.934501368098388	1.43106034972669	0.161933641049611	0.85756970743126	-4.94511444487972
a_HQ_transcript/109670	1.12456144826385	0.952657501959194	1.43093613812614	0.161968982533596	0.85756970743126	-4.8630464317935
a_HQ_transcript/35242	-1.10463424020767	0.546649519953217	-1.43087486502532	0.16198641859437	0.85756970743126	-4.78708620998882
a_HQ_transcript/97	-1.19308576510548	3.79603803342918	-1.43083719931669	0.161997137599266	0.85756970743126	-5.18361009057256
a_HQ_transcript/115390	-1.58860593163443	2.22013669106949	-1.4308105458696	0.162004723044744	0.85756970743126	-4.95467621395927
a_HQ_transcript/18349	1.86859228185817	0.355984573410093	1.43073165430189	0.162027176857348	0.85756970743126	-4.92541919048537
a_HQ_transcript/141818	-1.08080316294431	2.93926571429653	-1.43066300744462	0.16204671686001	0.85756970743126	-5.08910547501479
a_HQ_transcript/111144	2.4395502694732	3.53745671896295	1.43061926464231	0.162059169012236	0.85756970743126	-5.15793535474686
a_HQ_transcript/138670	-1.75114859534079	1.83273064539402	-1.43053173415418	0.162084088362687	0.85756970743126	-4.83740639795234
a_HQ_transcript/80813	1.26694119556963	1.95058266940181	1.43044351530557	0.162109206746792	0.85756970743126	-4.98301566707936
a_HQ_transcript/117418	1.74310806837074	9.13004978721585	1.43034816496639	0.16213635912357	0.85756970743126	-5.79783891487189
a_HQ_transcript/95969	-1.23048612606555	3.737348081488	-1.43029286916858	0.162152107040488	0.85756970743126	-5.28803000029394
a_HQ_transcript/124872	-3.42938407449495	1.12791789126043	-1.43028651946019	0.162153915477285	0.85756970743126	-4.98316418921373
a_HQ_transcript/26062	1.11441137578984	0.0362425854162211	1.43027743567988	0.162156502622617	0.85756970743126	-4.81215941297637
a_HQ_transcript/111768	4.30118071236633	1.27161852331916	1.43014882232443	0.162193136403753	0.85756970743126	-4.79325609743375
a_HQ_transcript/123728	1.98254854620179	2.79705235003657	1.43004500234154	0.162222712893903	0.85756970743126	-4.99792762927607
a_HQ_transcript/72275	0.648581047464455	3.06384851154465	1.42999253580736	0.162237661308204	0.85756970743126	-4.95799304912122
a_HQ_transcript/87623	-1.02219426229272	3.02526566632251	-1.42991898606304	0.162258618441053	0.85756970743126	-5.00214036616397
a_HQ_transcript/37623	1.33327172719677	3.14081383149733	1.42985767789605	0.162276089114312	0.85756970743126	-5.11106541566522
a_HQ_transcript/35362	1.26042528087518	1.41407519510702	1.42984827684473	0.162278768214939	0.85756970743126	-4.81277029534114
a_HQ_transcript/37484	0.426293166116521	0.176600139320739	1.42980871925456	0.162290041673466	0.85756970743126	-4.93338780524695
a_HQ_transcript/22468	2.71497189462609	0.267219040252975	1.42978125466043	0.16229786913085	0.85756970743126	-4.83737570752904

a_HQ_transcript/110110	1.6525328629532	0.0422064986075188	1.42976463897235	0.162302604775756	0.85756970743126	-4.84671108723172
a_HQ_transcript/127667	-1.22808881320747	0.863576254048055	-1.42975524577134	0.162305281984576	0.85756970743126	-4.78335127381017
a_HQ_transcript/41884	1.56131962835002	-0.5327416124138	1.42967852150841	0.162327150902074	0.85756970743126	-4.84624004289063
a_HQ_transcript/1641	1.18658032312114	-0.226999035079305	1.42967751952965	0.162327436514095	0.85756970743126	-4.87789971392704
a_HQ_transcript/51045	1.12572504118764	0.914627019912327	1.42964293626456	0.162337294647073	0.85756970743126	-4.75902171051041
a_HQ_transcript/93366	1.65912726694914	-0.0162606145136589	1.42962420247718	0.162342635004419	0.85756970743126	-4.79368931500252
a_HQ_transcript/77290	-2.43921416605069	1.21294671540068	-1.42960930206992	0.162346882696207	0.85756970743126	-4.88267377802151
a_HQ_transcript/151194	2.61209108235136	1.37115398687735	1.42958239472266	0.162354553455049	0.85756970743126	-4.95184059739499
a_HQ_transcript/123480	-2.22297629356769	1.4010076740184	-1.42955765734583	0.162361605850219	0.85756970743126	-4.91149272985827
a_HQ_transcript/115926	1.49488228892553	1.15136973431188	1.42954840541126	0.162364243552534	0.85756970743126	-4.98092136601576
a_HQ_transcript/354	-2.03053683636305	2.15395894176156	-1.42951802690139	0.162372904624079	0.85756970743126	-4.90143435442188
a_HQ_transcript/20260	1.87198489180925	0.755567360864122	1.42935921583298	0.162418188426182	0.85768333431569	-4.8136552512814
a_HQ_transcript/34629	0.944210117626095	0.686235613522386	1.42933908608552	0.162423928986841	0.85768333431569	-4.83576193450776
a_HQ_transcript/110483	-3.46831233035731	2.09531501421882	-1.42919826082803	0.162464093729582	0.857749388300686	-5.03003945773589
a_HQ_transcript/20804	-2.16556812575779	1.93813262225655	-1.42919175163127	0.162465950405838	0.857749388300686	-4.92178715211852
a_HQ_transcript/13844	-1.33507191769951	0.507694805163296	-1.42913179436308	0.16248305334015	0.857754689070844	-4.78878421265206
a_HQ_transcript/135842	-0.377251919314762	3.59230349495471	-1.42904493768563	0.162507831907477	0.857754689070844	-5.2025504185526
a_HQ_transcript/51884	1.71262338406516	-0.00521533673081662	1.42903305043478	0.162511223347392	0.857754689070844	-4.88082311889341
a_HQ_transcript/124318	0.483385923990994	3.03234023226432	1.42882198285264	0.162571450362097	0.857960566655956	-5.15270383343498
a_HQ_transcript/58861	1.22248907369308	1.49200377730731	1.42876622374677	0.162587363869143	0.857960566655956	-4.81131342302067
a_HQ_transcript/140604	-1.66142175931105	2.77855174512829	-1.42874119044502	0.162594508709779	0.857960566655956	-4.97862708570515
a_HQ_transcript/70598	2.07043078445493	2.00408841313082	1.42857053137577	0.162643223705796	0.858007256760454	-4.93077031776079
a_HQ_transcript/8550	2.16292888911408	-0.399590725822935	1.42855791311287	0.162646826073303	0.858007256760454	-4.90990913732611
a_HQ_transcript/145712	1.4915555186891	-0.331931162191807	1.42855178099574	0.162648576744267	0.858007256760454	-4.96187034221093
a_HQ_transcript/65792	-1.36329796269816	0.350633039980098	-1.42847681592781	0.162669979881012	0.858007256760454	-4.84933759249294
a_HQ_transcript/12005	1.8429111213886	0.302214358361863	1.42845166788842	0.162677160348833	0.858007256760454	-4.75915063261737
a_HQ_transcript/118047	1.95895552578578	0.482470231060095	1.42823555387468	0.162738877251658	0.858120527654116	-4.87764004419997
a_HQ_transcript/52027	-0.925903659111972	0.996411655230638	-1.42818386397062	0.162753641368835	0.858120527654116	-4.7282247552837
a_HQ_transcript/15562	1.93973399918001	1.11567446777386	1.42817010541687	0.162757571384035	0.858120527654116	-4.97300704262229
a_HQ_transcript/46944	1.23832522812552	-0.426663207735474	1.42816970141779	0.162757686784108	0.858120527654116	-4.9038940023296
a_HQ_transcript/118442	1.96467075335822	0.911479655552691	1.42806148505878	0.162788600506113	0.858141902655032	-4.94193822446643
a_HQ_transcript/124531	1.31766103477496	2.14773830052002	1.42805215196645	0.162791266869272	0.858141902655032	-4.8209995933536
a_HQ_transcript/41897	-0.556583185036885	2.53017654346434	-1.42798801150003	0.162809592036701	0.858160678809334	-4.97197354164646
a_HQ_transcript/89571	1.49095774368219	1.27301495656927	1.42762307255652	0.162913887424229	0.858406949652302	-4.77005133721565
a_HQ_transcript/106104	-3.22409773660442	2.63359177797245	-1.4275370894167	0.162938468098513	0.858406949652302	-5.01645593458602
a_HQ_transcript/66785	0.774381346453609	3.2826821077964	1.42745210899601	0.162962764993488	0.858406949652302	-5.06494056759605
a_HQ_transcript/116419	0.537809726702532	3.41659390968166	1.42738392934718	0.162982260418688	0.858406949652302	-5.14804102601055
a_HQ_transcript/131287	1.11310934586119	-0.664563356446184	1.42738074681385	0.162983170483596	0.858406949652302	-4.91976694880989
a_HQ_transcript/58723	1.33626240898509	-0.363477441512452	1.4273094023218	0.163003572930947	0.858406949652302	-4.90071505682809
a_HQ_transcript/139009	1.32493610585138	-0.614967565804295	1.42727250901438	0.163014124133205	0.858406949652302	-4.91603795141593
a_HQ_transcript/115007	3.88926545178769	2.35330796235423	1.42726012514192	0.163017665946331	0.858406949652302	-5.06107064439066
a_HQ_transcript/66586	1.62621958888449	1.27411714755365	1.42724722747641	0.163021354769891	0.858406949652302	-4.83273943672848
a_HQ_transcript/122905	1.3495716268584	-0.147426039949143	1.42722420514559	0.163027939483067	0.858406949652302	-4.93754412211852
a_HQ_transcript/27854	0.633491440332645	3.10838868119125	1.42721118108051	0.163031664643375	0.858406949652302	-4.97108465481664
a_HQ_transcript/3908	-1.43311656129217	0.761503481260489	-1.42718771222006	0.163038377407431	0.858406949652302	-4.75511313215041
a_HQ_transcript/134120	1.71442089589338	0.012970661174221	1.42706314504074	0.163074010828029	0.858406949652302	-4.94343186365373
a_HQ_transcript/76720	2.18618450670242	2.20544514590566	1.42702514221967	0.163084883057006	0.858406949652302	-4.88019027164805
a_HQ_transcript/77660	1.25616032515329	1.37174578668136	1.42696012474504	0.163103485235964	0.858406949652302	-4.79957171660159
a_HQ_transcript/111005	1.36438223780628	0.253423592684549	1.42691059336964	0.163117657803689	0.858406949652302	-4.88084565521917
a_HQ_transcript/10024	-2.24959306199234	1.23153725421106	-1.426890117498	0.163123516912914	0.858406949652302	-4.79826343160359

a_HQ_transcript/143292	0.470111912998213	3.12376919310646	1.42686446517883	0.16313085748141	0.858406949652302	-5.12433457426544
a_HQ_transcript/117683	-1.25886023191408	2.26518723005924	-1.42682506337213	0.163142133057761	0.858406949652302	-4.95404032340031
a_HQ_transcript/31873	1.61967036812457	0.49493203548289	1.42679175646549	0.163151664942173	0.858406949652302	-4.91824805016131
a_HQ_transcript/148355	-2.34615626317203	1.80192811981278	-1.42669056085834	0.163180628142835	0.858481632065049	-4.85736285010466
a_HQ_transcript/80073	0.70274629074554	2.90040231082441	1.42647829434703	0.163241394133253	0.85871923139987	-5.07449443567797
a_HQ_transcript/39453	0.875220728314136	-0.669426232828627	1.42625891902458	0.163304213937389	0.85871923139987	-4.89514083886888
a_HQ_transcript/95847	-2.10736406437107	2.17930751242693	-1.42617860234607	0.163327218002668	0.85871923139987	-5.01624325414552
a_HQ_transcript/143995	0.522692792779582	3.98053073922471	1.42614875963537	0.163335766114927	0.85871923139987	-5.26835644216205
a_HQ_transcript/10010	2.31587108705224	1.19583247188309	1.42609843681529	0.16335018132592	0.85871923139987	-4.81149086805991
a_HQ_transcript/79309	1.01509448627455	1.876358462506	1.4260747496565	0.163356966972536	0.85871923139987	-4.94451276467509
a_HQ_transcript/151532	-2.20175366075995	2.54408956364955	-1.42603702298742	0.163367774968652	0.85871923139987	-5.14788466850721
a_HQ_transcript/28105	1.31285660099104	-0.23239608423522	1.42598554358905	0.163382523778582	0.85871923139987	-4.84495282542857
a_HQ_transcript/129287	1.63778494643858	0.145989812629783	1.42593234414309	0.163397766484532	0.85871923139987	-4.91460573675797
a_HQ_transcript/126043	2.10280034502522	0.0267762990524093	1.42589484347686	0.163408511850012	0.85871923139987	-4.88961300603741
a_HQ_transcript/135004	-3.28655272976461	1.3345869100224	-1.42588269493331	0.163411992988791	0.85871923139987	-5.09588272679515
a_HQ_transcript/131632	1.36995653062524	2.08075461070677	1.42581638287184	0.163430995597247	0.85871923139987	-4.92999568178742
a_HQ_transcript/136587	-0.939465525967265	-0.761493868770773	-1.4257229529133	0.163457772160141	0.85871923139987	-4.89467339180699
a_HQ_transcript/102280	-2.40654140298963	1.6966594141526	-1.42571658126091	0.163459598370174	0.85871923139987	-4.91567415871066
a_HQ_transcript/156	1.6945369123812	2.33571109045583	1.42557592732714	0.16349991597202	0.85871923139987	-4.89062216680032
a_HQ_transcript/146902	1.73984559649283	0.096357352841547	1.42557111354421	0.163501295952679	0.85871923139987	-4.85585444872779
a_HQ_transcript/117690	-5.69047582804998	2.75455709166306	-1.42549198325371	0.163523981771974	0.85871923139987	-4.96544928923319
a_HQ_transcript/69531	-1.26290025849241	2.14572972596297	-1.42543240910901	0.163541062688846	0.85871923139987	-4.825102747491
a_HQ_transcript/104991	-1.31660609799966	3.54155779599553	-1.42542721727632	0.163542551341929	0.85871923139987	-5.07943401206809
a_HQ_transcript/89113	0.415661680935368	4.14879524664587	1.42540247842214	0.163549644855138	0.85871923139987	-5.27962911589479
a_HQ_transcript/128014	1.3231811373132	-0.072804351217475	1.42539802461486	0.163550921946561	0.85871923139987	-4.89164936987596
a_HQ_transcript/16781	0.687720610713114	2.03706545775006	1.42531787039082	0.163573906834403	0.85871923139987	-4.85912105691782
a_HQ_transcript/16040	-1.21690553403405	1.02881999836225	-1.42523507961683	0.163597650449689	0.85871923139987	-4.76467682298594
a_HQ_transcript/23887	1.98969304496987	-0.266950574107345	1.42519339401582	0.163609606516897	0.85871923139987	-4.9004122107676
a_HQ_transcript/117115	1.06082163107641	-0.597453763126918	1.42514864003876	0.163622443408367	0.85871923139987	-4.89746991213563
a_HQ_transcript/6925	-1.94513064301875	0.545727282323292	-1.42514011465248	0.163624888855608	0.85871923139987	-4.78493485931002
a_HQ_transcript/75644	-1.92214441833613	0.878613277984616	-1.42510321070151	0.163635474827496	0.85871923139987	-4.83314832098827
a_HQ_transcript/3318	1.88419169995937	2.20368268821476	1.42508941463875	0.163639432394308	0.85871923139987	-4.83965251070785
a_HQ_transcript/139867	2.34707850518904	1.87953321138098	1.42495057478416	0.163679264482146	0.85874742054681	-4.96091075622862
a_HQ_transcript/1258	-1.19094338594138	1.84128947555669	-1.42494932180839	0.163679623986085	0.85874742054681	-4.83015381333484
a_HQ_transcript/112782	-1.62572104046991	2.05768410452433	-1.4249018553248	0.163693243533147	0.85874742054681	-4.94256512950163
a_HQ_transcript/3597	-1.21546882459377	-0.513169065155766	-1.42486472578247	0.163703897727071	0.85874742054681	-4.88558925301627
a_HQ_transcript/58585	1.23632423353711	-0.314191590568903	1.42478787368628	0.163725951908139	0.858785610225257	-4.89492137808496
a_HQ_transcript/144750	-2.04149757967295	1.34399000345612	-1.42466987690686	0.163759817904877	0.858885743496771	-4.88924053187257
a_HQ_transcript/129070	1.98698344505534	0.86254187422527	1.42449693578771	0.163809463337684	0.859068611051327	-4.93017541182624
a_HQ_transcript/22197	1.33519866541515	0.487503976185695	1.42436110251485	0.163848464708659	0.859162786299313	-4.77749894646586
a_HQ_transcript/41082	-1.90935901355438	1.95150603427528	-1.42433144208486	0.16385698198655	0.859162786299313	-5.10490471455737
a_HQ_transcript/25937	2.28329517397567	1.38374764723688	1.42401774912394	0.163947083313054	0.859557026758463	-4.85977057331175
a_HQ_transcript/126083	0.296783610563845	4.64697671356669	1.42392260260895	0.163974419753775	0.859557026758463	-5.35453352701471
a_HQ_transcript/116610	2.69015513147363	1.68952959359271	1.42386388071073	0.163991292871321	0.859557026758463	-4.78256410402565
a_HQ_transcript/118952	-2.50739522728786	1.33305982789009	-1.42386378763082	0.163991319617936	0.859557026758463	-4.98404463289
a_HQ_transcript/84938	1.4714133172002	-0.118371935030405	1.4237055598752	0.164036791513259	0.859698257338051	-4.89189172873093
a_HQ_transcript/118522	-1.53765916749061	1.79709904419478	-1.423627417725	0.164059251870161	0.859698257338051	-4.95888872093176
a_HQ_transcript/9603	1.74446309498483	0.708385467644544	1.42352760643126	0.164087944103942	0.859698257338051	-4.92810038718978
a_HQ_transcript/107418	1.74444637099665	1.44540867286061	1.42348596871079	0.164099914652395	0.859698257338051	-4.80857122297324
a_HQ_transcript/10968	1.47734752610805	0.750004881377424	1.42344856277744	0.164110669179231	0.859698257338051	-4.72912195560257

a_HQ_transcript/79045	0.496572645358795	2.79460184143974	1.42344555564654	0.164111533779301	0.859698257338051	-5.08026136203334
a_HQ_transcript/110111	1.8706934465695	1.51277061845689	1.42338822143203	0.164128019005041	0.859698257338051	-4.85659374603357
a_HQ_transcript/82068	1.44870109026882	0.0719464930994318	1.42335403480866	0.16413784925788	0.859698257338051	-4.83332777366026
a_HQ_transcript/97438	-1.73524271248318	4.11266925126776	-1.42328578946869	0.164157474372564	0.859698257338051	-5.07785077772817
a_HQ_transcript/5319	-1.43051198161605	2.75497976747221	-1.42325558123476	0.164166161857493	0.859698257338051	-5.02964550899624
a_HQ_transcript/112208	0.907150350762427	-0.578765403863154	1.42316202950277	0.164193068386456	0.859761704276001	-4.88578191750447
a_HQ_transcript/43064	-1.29417892005218	-0.367186478563228	-1.42299114564337	0.164242225478771	0.859941639581156	-4.88800787261401
a_HQ_transcript/62696	-2.16519699205427	-0.0101198665925457	-1.42284660030732	0.16428381498905	0.859991318980074	-4.90275349420322
a_HQ_transcript/21133	1.75053505411419	-0.418086113374812	1.42281646618944	0.164292486416194	0.859991318980074	-4.89556987620999
a_HQ_transcript/148253	-2.78476274525743	0.527931154880322	-1.42276443723844	0.16430745917402	0.859991318980074	-4.90326612093687
a_HQ_transcript/149865	-3.18055435351715	0.175061236752468	-1.4227525055198	0.164310893004967	0.859991318980074	-4.9409676270792
a_HQ_transcript/63816	-2.23276521555198	1.63663870155198	-1.42263180678443	0.164345632091059	0.860086001391411	-4.85428258408206
a_HQ_transcript/86635	-1.01848320237536	2.34760886845865	-1.42258683927812	0.16435857596091	0.860086001391411	-4.80545625098855
a_HQ_transcript/76830	-1.32033204952808	-0.215798557592847	-1.42246072130756	0.164394883230789	0.860116640035982	-4.80091880872719
a_HQ_transcript/125215	1.87035646937151	0.550083802232568	1.42240712488473	0.164410314665092	0.860116640035982	-4.91460042334491
a_HQ_transcript/105222	-1.84099619285381	2.06417337078409	-1.42228996756359	0.164444050472121	0.860116640035982	-5.04808594275166
a_HQ_transcript/150740	1.40544374325466	-0.302188129946341	1.42222020724478	0.164464140759568	0.860116640035982	-4.92804149095927
a_HQ_transcript/130134	1.1450689231047	-0.223203700454703	1.42219353397191	0.164471822911702	0.860116640035982	-4.92384401947818
a_HQ_transcript/125114	0.451235237519712	3.17463382713773	1.42217069711681	0.164478400364351	0.860116640035982	-5.06953813889521
a_HQ_transcript/91805	0.486019417840597	4.70496481266476	1.42208817036383	0.164502171381694	0.860116640035982	-5.34883429517321
a_HQ_transcript/145458	2.09892112832457	-0.143278905336313	1.42197307251186	0.164535328704205	0.860116640035982	-4.95756525750317
a_HQ_transcript/132916	-2.75255838438602	0.924409572822535	-1.42195185442286	0.164541441773943	0.860116640035982	-5.13380496050399
a_HQ_transcript/111380	1.02801067900404	-0.665586909837699	1.42192016410615	0.164550572294056	0.860116640035982	-4.92814028869131
a_HQ_transcript/149369	-1.10557750337106	2.26688700025423	-1.42191850215717	0.164551051140889	0.860116640035982	-5.01298934543149
a_HQ_transcript/144689	0.713100164769933	5.20936213282375	1.42183136523347	0.164576158889037	0.860116640035982	-5.38771380208372
a_HQ_transcript/35627	-0.331579873317849	3.5306852145372	-1.42175755588445	0.164597428773951	0.860116640035982	-5.16204196679705
a_HQ_transcript/67204	1.43192210302243	0.409511501917259	1.4217551730462	0.164598115480451	0.860116640035982	-4.8715475429762
a_HQ_transcript/44806	1.27984824311898	2.46280654482793	1.42167725603086	0.164620571509494	0.860116640035982	-4.94762505948973
a_HQ_transcript/41017	1.29199861013308	-0.642724721742563	1.42163571165387	0.164632545769799	0.860116640035982	-4.91313805944341
a_HQ_transcript/26297	-1.13886173637394	1.485092288771	-1.42163077264884	0.164633969375896	0.860116640035982	-4.75077954888293
a_HQ_transcript/139981	2.38934776920824	2.22583900748364	1.42155176137601	0.164656744700725	0.860116640035982	-5.03338842975649
a_HQ_transcript/36793	0.737952538872458	3.82098400266729	1.42148659256184	0.164675531748242	0.860116640035982	-5.25770366935006
a_HQ_transcript/1254	-0.846656642992104	4.34998572551421	-1.42147281689791	0.164679503251323	0.860116640035982	-5.34103799931342
a_HQ_transcript/131035	0.457737964400424	4.12091352904977	1.42144093004548	0.164688696472219	0.860116640035982	-5.29535307783904
a_HQ_transcript/146938	2.37238919491967	2.00897221716557	1.42143653513216	0.164689963590705	0.860116640035982	-4.88764375610942
a_HQ_transcript/69218	1.45337616978995	-0.240545718114992	1.42135507858103	0.164713450109391	0.860127987188652	-4.90197889245893
a_HQ_transcript/51348	2.02721142615454	0.152588624066192	1.42132636232668	0.164721730549547	0.860127987188652	-4.93140710111041
a_HQ_transcript/15909	-0.854279778459063	1.86949355226226	-1.4210121299345	0.16481236207856	0.860314250893911	-4.83397951570725
a_HQ_transcript/99885	1.8761621494991	0.0993769632691723	1.42098037675307	0.164821522579398	0.860314250893911	-4.78255193342762
a_HQ_transcript/121587	-1.92324916980298	1.13970405549812	-1.42095970003088	0.16482748783927	0.860314250893911	-4.98023377516144
a_HQ_transcript/126529	2.55508404952663	0.404236091043041	1.42084163285553	0.164861553624023	0.860314250893911	-4.91193337153698
a_HQ_transcript/61893	1.48811639077801	0.364274806903749	1.42080847764302	0.164871120856439	0.860314250893911	-4.75460290299892
a_HQ_transcript/10197	0.769226388929127	2.66370474313725	1.4207254522797	0.164895080485077	0.860314250893911	-4.9042972444313
a_HQ_transcript/147099	0.645842299584289	3.72117506969053	1.42071406030521	0.164898368218317	0.860314250893911	-5.20432171127042
a_HQ_transcript/18241	-0.806065318176368	2.09764494454233	-1.42065454796906	0.164915544367293	0.860314250893911	-4.87281067323822
a_HQ_transcript/147674	1.45449161400416	0.471543757881627	1.42063736305834	0.164920504451437	0.860314250893911	-4.97126614851729
a_HQ_transcript/117993	3.18930900589133	1.51661439906385	1.42061188389478	0.16492785872371	0.860314250893911	-5.03787221961695
a_HQ_transcript/21587	1.47150658984293	1.28288141174934	1.42059843231828	0.164931741473275	0.860314250893911	-4.87385094504492
a_HQ_transcript/25127	1.03691998489806	1.96554817062092	1.42057846188496	0.164937506000424	0.860314250893911	-4.89337810053302
a_HQ_transcript/151657	3.86305133693036	1.47703538293117	1.4204211726782	0.164982913563365	0.860314250893911	-4.96622316024925

a_HQ_transcript/37523	2.03024597912078	0.954404319740248	1.42040646559104	0.164987159831638	0.860314250893911	-5.03762158695995
a_HQ_transcript/72530	-2.46718110588961	1.76116261177023	-1.42039179703392	0.164991395061179	0.860314250893911	-4.90932513259261
a_HQ_transcript/43484	1.52493316013939	-0.260456107059235	1.42034052538769	0.165006199316241	0.860314250893911	-4.85028919190431
a_HQ_transcript/29231	2.24007360978951	0.715623426996526	1.42026429824308	0.16502821119521	0.860314250893911	-4.86958226700455
a_HQ_transcript/132866	-1.58780959047943	2.23593831852443	-1.42023758423294	0.165035925865421	0.860314250893911	-4.8819552937859
a_HQ_transcript/88138	0.441961025613344	4.42999357900027	1.42019771308945	0.165047440678743	0.860314250893911	-5.33863967437979
a_HQ_transcript/150128	1.72178110085924	-0.328936739185138	1.42013162745876	0.165066527647614	0.860314250893911	-4.92120745964146
a_HQ_transcript/97622	-0.80377294516052	4.78304699042636	-1.42008607510725	0.165079685170755	0.860314250893911	-5.3276943255292
a_HQ_transcript/135321	0.997057966023432	4.36820937047271	1.42005075452092	0.165089887878619	0.860314250893911	-5.30982637272261
a_HQ_transcript/85970	-3.62659949386381	2.05917416947545	-1.42002333208893	0.165097809467409	0.860314250893911	-4.95334513478855
a_HQ_transcript/18867	-0.550372465218322	5.01456881296375	-1.41989435895545	0.165135070285504	0.860431280526691	-5.39754990824825
a_HQ_transcript/8479	-1.50522335604632	-0.15412897304376	-1.41976246974047	0.165173180420462	0.860482791396982	-4.88416224090314
a_HQ_transcript/142169	0.822458169794161	2.83361092288008	1.4197576861386	0.165174562799469	0.860482791396982	-5.09503899562317
a_HQ_transcript/58345	1.69387761914768	1.81434177520681	1.41967753566311	0.165197726269624	0.860526340406907	-4.78683957536006
a_HQ_transcript/75915	3.21878814647037	1.5309484066748	1.41961412497219	0.16521605375841	0.860544692909397	-4.99409684411681
a_HQ_transcript/141421	1.43000151284312	-0.629764724341763	1.41952481405924	0.165241869862493	0.860546591754449	-4.92441888072887
a_HQ_transcript/74817	-0.759917632400913	2.48428380728393	-1.41951043347057	0.165246026994962	0.860546591754449	-4.89536613167205
a_HQ_transcript/51272	3.17003924273748	1.03656474332133	1.41942273498933	0.165271380605449	0.860574896146922	-4.82633692955481
a_HQ_transcript/5959	-0.596206228096902	1.70298262671081	-1.41935637821689	0.165290566368945	0.860574896146922	-4.81287735183772
a_HQ_transcript/150578	2.78295160654935	0.308947717980709	1.41932160661167	0.165300620599219	0.860574896146922	-4.86501022594771
a_HQ_transcript/27965	-1.25622363315911	2.19659701015651	-1.41928607801607	0.165310894211211	0.860574896146922	-5.00036504318859
a_HQ_transcript/119761	0.519646960144719	2.38617723037951	1.41923561822999	0.165325486259164	0.860574896146922	-4.93048277901921
a_HQ_transcript/151520	1.88104151946618	0.568641536385532	1.41909360892136	0.165366558200661	0.860688448667551	-4.91297245858721
a_HQ_transcript/98476	1.3696973635406	0.986628954915038	1.41900134148448	0.165393248101116	0.860688448667551	-4.77142584141531
a_HQ_transcript/100947	2.68298358839389	0.76452974941044	1.41898073049946	0.165399210637033	0.860688448667551	-4.96530500427657
a_HQ_transcript/3446	-1.16787220260645	3.31908983829065	-1.41893045899402	0.16541375435193	0.860688448667551	-5.01968357357705
a_HQ_transcript/149582	-0.581211118928199	3.2758633632335	-1.41890425799727	0.165421334787261	0.860688448667551	-5.01338603754684
a_HQ_transcript/137920	1.29743976988625	0.127278084944076	1.41880911425754	0.165448863939932	0.860729089476525	-4.87910227337138
a_HQ_transcript/58578	-0.667597001062882	4.21697412542118	-1.41874164044261	0.165468389186471	0.860729089476525	-5.27166880347195
a_HQ_transcript/49839	0.64747033956866	3.31010492058469	1.41870166215553	0.165479958766047	0.860729089476525	-5.13155051679308
a_HQ_transcript/34013	1.94796531812419	0.560208679325498	1.41863274038253	0.1654999059872	0.860729089476525	-4.94252648291261
a_HQ_transcript/84308	-0.717156939029629	2.51783000615323	-1.41860763210191	0.165507173253156	0.860729089476525	-5.01235881124499
a_HQ_transcript/89880	-0.912190380010751	1.31183321644705	-1.41857025968813	0.165517990678651	0.860729089476525	-4.85659634058455
a_HQ_transcript/135913	-1.722274513654	0.0996957767947406	-1.41842488397243	0.165560074898014	0.860733918602269	-4.91962080350959
a_HQ_transcript/60185	1.51556328512386	4.93192988917329	1.4184141257923	0.165563189574224	0.860733918602269	-5.27918644972238
a_HQ_transcript/31445	1.45903146499228	1.08617793704973	1.41841325768541	0.165563440907954	0.860733918602269	-4.7379997544587
a_HQ_transcript/65171	1.89901554460156	0.678797234851465	1.41833196071221	0.165586979284928	0.860733918602269	-4.97274932419379
a_HQ_transcript/76697	1.1188511466551	0.0438519363553065	1.41831131586682	0.165592957124435	0.860733918602269	-4.99402991113021
a_HQ_transcript/31513	1.54881511890811	-0.621953822364669	1.41825668857053	0.165608775607253	0.860739172791347	-4.91718887379395
a_HQ_transcript/82666	-1.74578283299161	2.20638438253066	-1.41819858983344	0.165625600624358	0.86074965695956	-5.04756421969808
a_HQ_transcript/132035	-1.2299072642309	-0.559591713332741	-1.41813579450462	0.165643787257369	0.860759686782655	-4.90570949564507
a_HQ_transcript/59102	1.20125581978491	-0.494452150865865	1.41808967053164	0.165657146573726	0.860759686782655	-4.85197353295984
a_HQ_transcript/149003	1.31601040539651	-0.269739882452618	1.41803057180599	0.165674265128484	0.860771691400473	-4.85119724961143
a_HQ_transcript/109248	-1.44538072780954	-0.315962350883414	-1.41787497457446	0.165719342130381	0.860928940866455	-4.88363111801428
a_HQ_transcript/109563	-2.11459391699985	5.09177426331969	-1.41774551355583	0.165756854748891	0.861046867993165	-5.31767461439854
a_HQ_transcript/59786	1.0635656517432	-0.41088890054246	1.41763881674646	0.16578777623615	0.861071317041541	-4.96684161743545
a_HQ_transcript/81252	-1.62461322196562	1.53197590987732	-1.41748877704879	0.16583126648054	0.861071317041541	-4.92916323681209
a_HQ_transcript/55751	0.841302237595954	-0.318779536727831	1.41747162925543	0.165836237481756	0.861071317041541	-4.88304529142721
a_HQ_transcript/102854	1.40914551967636	-0.251198289315361	1.41743601322571	0.165846562644367	0.861071317041541	-4.83747903165794
a_HQ_transcript/72401	-1.3697039267282	2.97174685065574	-1.41736900393516	0.165865990151665	0.861071317041541	-4.9788574152463

a_HQ_transcript/122459	0.574898986339935	2.73722467956177	1.417327275886	0.165878088961014	0.861071317041541	-5.01764646101588
a_HQ_transcript/71314	-1.69182022064639	-0.189321936125263	-1.41731825556852	0.165880704441825	0.861071317041541	-4.90819515801126
a_HQ_transcript/4387	1.90310660736886	1.06311088813889	1.4173066261479	0.165884076491503	0.861071317041541	-4.96166211669696
a_HQ_transcript/138134	1.92122792403091	0.101526157002165	1.41722445170039	0.165907905211033	0.861071317041541	-4.95429254243256
a_HQ_transcript/136969	-2.07640577660338	-0.188321177875287	-1.41720444907046	0.165913705926201	0.861071317041541	-4.8936587668431
a_HQ_transcript/121688	-1.43210581347491	-0.0630056969207112	-1.41716720044697	0.165924508363995	0.861071317041541	-4.81557100485863
a_HQ_transcript/15118	0.892271979445277	2.44014501086791	1.41693836497264	0.165990884870489	0.861327853089961	-4.90334776061031
a_HQ_transcript/56941	-1.39187725234359	-0.330791219247185	-1.416894615739	0.166003577246195	0.861327853089961	-4.8879382160834
a_HQ_transcript/1329	1.50914104814857	0.133303162645283	1.4167467756576	0.166046473749483	0.861396399858451	-4.77297635122986
a_HQ_transcript/37159	1.32422690790293	0.361585502008945	1.416729135879	0.166051592597747	0.861396399858451	-4.84910394104148
a_HQ_transcript/81123	-3.56607768955355	3.4331283094322	-1.41669587399442	0.166061245128231	0.861396399858451	-5.07338319245291
a_HQ_transcript/134440	-1.84391438206119	-0.337797439876503	-1.41663925786076	0.166077676029341	0.861404760616895	-4.89222285179723
a_HQ_transcript/117376	2.49570706689127	1.75368045672081	1.4164045970883	0.166145791955035	0.861656964754473	-4.88593292099021
a_HQ_transcript/103983	-1.4328716544317	1.68106823932595	-1.4163696174141	0.166155947528435	0.861656964754473	-4.87307140719462
a_HQ_transcript/109622	1.10712541096922	2.63287125221456	1.41625406131293	0.166189500154879	0.861728793443704	-4.89378165260894
a_HQ_transcript/151835	-2.93713109430093	4.16733328861356	-1.41621980480458	0.166199447827164	0.861728793443704	-5.21301847429626
a_HQ_transcript/93280	2.85170397759449	0.629177298322626	1.41615454707636	0.16621839917223	0.861750187931092	-4.99338080012106
a_HQ_transcript/123017	1.42585649004627	1.83920305238257	1.41590856865273	0.166289848480468	0.861832595960545	-4.83997847790844
a_HQ_transcript/142052	1.24988433787421	-0.375660999706842	1.41590460894443	0.166290998853849	0.861832595960545	-4.87797429805198
a_HQ_transcript/8572	-2.40576459764551	1.60431948490472	-1.41587176947041	0.166300539610269	0.861832595960545	-4.98856723469385
a_HQ_transcript/131197	-1.08494892252872	2.82082065435879	-1.41586554373151	0.166302348404568	0.861832595960545	-5.03888233244906
a_HQ_transcript/124703	0.322379030233487	4.26992007842198	1.41575167144648	0.166335435006362	0.861832595960545	-5.30902551056373
a_HQ_transcript/10231	1.10543474093472	-0.66361924514438	1.41572092686437	0.166344369005207	0.861832595960545	-4.9297442653421
a_HQ_transcript/88411	2.4878966691463	1.23790706217645	1.41567319368888	0.166358240429307	0.861832595960545	-4.95139422430817
a_HQ_transcript/136461	1.48482978317706	-0.0121902484987342	1.41566197428835	0.166361500957591	0.861832595960545	-4.84976289707184
a_HQ_transcript/59356	0.384739562040131	-0.824522702862087	1.41559902017143	0.166379797313309	0.861832595960545	-4.99279131250645
a_HQ_transcript/11882	-0.94178888257031	4.60066391887971	-1.4155387905698	0.166397303325093	0.861832595960545	-5.25680233555943
a_HQ_transcript/52211	1.48158044466799	0.376863613976231	1.41553850785082	0.166397385502085	0.861832595960545	-4.97745513338734
a_HQ_transcript/132856	-4.72943887766763	4.31682566816248	-1.41543202112741	0.166428339918217	0.861860860926148	-5.17230624682953
a_HQ_transcript/35710	1.31363804313628	1.26145882402733	1.41541544815417	0.16643315789062	0.861860860926148	-4.82756340406325
a_HQ_transcript/12546	0.672426502284407	2.2777691206137	1.41532050695181	0.166460760611017	0.861860860926148	-4.93267940118469
a_HQ_transcript/137480	-1.8255593717499	0.227519936091176	-1.41531572659326	0.166462150523291	0.861860860926148	-4.93836876816502
a_HQ_transcript/62722	0.685730460785591	2.98203501322877	1.41523858570029	0.166484580872066	0.861900224165607	-5.12380189834409
a_HQ_transcript/24766	-1.27135762611502	2.0971506312082	-1.41516466777174	0.166506076306622	0.861934740720574	-4.80781936299812
a_HQ_transcript/139180	1.43033562655109	-0.63679802790732	1.41504726681749	0.166540221152438	0.862022529305442	-4.93007368789403
a_HQ_transcript/128314	1.35236400240505	-0.058043332566033	1.41497451257537	0.166561383731166	0.862022529305442	-4.95390900930526
a_HQ_transcript/114935	-0.368219447691729	3.9218369114057	-1.41493892147857	0.16657173715429	0.862022529305442	-5.23439031678621
a_HQ_transcript/10233	1.99291236780045	0.260006831085393	1.41490242673931	0.166582353972062	0.862022529305442	-4.8300828726271
a_HQ_transcript/37470	-0.936243684558018	1.61214529855169	-1.41478184636362	0.166617436234457	0.862076696130078	-4.80527060414911
a_HQ_transcript/11408	-1.87229599430954	3.39466154393657	-1.41475916118398	0.166624037024543	0.862076696130078	-5.22265889978108
a_HQ_transcript/61000	-0.521749870752137	2.63813913552565	-1.41468810461771	0.166644713946119	0.862076696130078	-4.99633142967834
a_HQ_transcript/100414	-1.30904360343284	-0.624236268506902	-1.41466257234275	0.166652144137312	0.862076696130078	-4.91267918868399
a_HQ_transcript/144230	-1.53790454166313	0.568280695923022	-1.41432464498883	0.166750509509619	0.862227432243292	-4.84441319664077
a_HQ_transcript/106986	2.39217310056773	1.34866638181154	1.41422826887408	0.166778571458815	0.862227432243292	-5.0155521255396
a_HQ_transcript/25435	1.18322713379926	1.83174815707598	1.41417214132259	0.166794915896957	0.862227432243292	-4.82066762160371
a_HQ_transcript/136874	0.330172594804774	4.56695382744637	1.41415717581886	0.166799274089758	0.862227432243292	-5.35204455271295
a_HQ_transcript/144123	0.921034856854235	2.61652274204245	1.4141393296824	0.166804471285733	0.862227432243292	-5.06672517354586
a_HQ_transcript/134340	2.18002652704472	-0.347891518494111	1.41410381447595	0.166814814490911	0.862227432243292	-4.91671338895308
a_HQ_transcript/130046	-1.95650381176437	1.86439461377727	-1.41409141877895	0.166818424647459	0.862227432243292	-5.04848735770593
a_HQ_transcript/125208	1.2562427041033	-0.00512254918519434	1.41408585464219	0.166820045181782	0.862227432243292	-4.94066547493936

a_HQ_transcript/11638	0.783113641971062	3.51668611154004	1.41406442100734	0.166826287764656	0.862227432243292	-5.13728124147486
a_HQ_transcript/71455	1.87452182399138	0.898999697199527	1.41400882081281	0.1668424822731	0.862227432243292	-4.79383853907084
a_HQ_transcript/6903	2.06741573190688	2.49344803302119	1.41393879903805	0.166862879072783	0.862227432243292	-5.01055432564259
a_HQ_transcript/64419	2.20923798989587	0.880007208427863	1.41389118704551	0.166876749196906	0.862227432243292	-4.88942951745957
a_HQ_transcript/140557	1.22846219301143	-0.698537369900491	1.41387850322041	0.1668804443481	0.862227432243292	-4.92429215173908
a_HQ_transcript/143416	-1.04864060073003	4.73047903206131	-1.41384931058904	0.166888949218437	0.862227432243292	-5.35987444738474
a_HQ_transcript/2966	0.747791725247822	4.76985681920341	1.41377633399214	0.166910211434251	0.8622409660839	-5.31087338316894
a_HQ_transcript/119606	-1.60588416480024	0.768380517574551	-1.41373849901589	0.166921235744256	0.8622409660839	-4.91374338666891
a_HQ_transcript/27724	1.70665163108518	0.961670703110956	1.41365032122859	0.166946931104776	0.86229706870965	-4.96874085694328
a_HQ_transcript/39801	1.77974227030719	1.84856539672849	1.4135878149553	0.16696514756734	0.862301851674836	-4.8855218347915
a_HQ_transcript/87878	-1.37654857207004	1.62751565849899	-1.41351875491769	0.16698527583826	0.862301851674836	-4.87793508437117
a_HQ_transcript/126915	1.33478408171009	-0.408276816214977	1.41349444824532	0.166992360726391	0.862301851674836	-4.88113144605497
a_HQ_transcript/109968	-1.45755475624918	3.50315737760746	-1.4132668042049	0.167058725692229	0.86242349643745	-4.93955313398944
a_HQ_transcript/105	1.7750476359251	-0.166286362654496	1.41320819521521	0.167075815301778	0.86242349643745	-4.91491652708263
a_HQ_transcript/52441	0.54057870281903	3.07617427176031	1.41319710968549	0.167079047850851	0.86242349643745	-5.08460708454115
a_HQ_transcript/120843	0.531874573403062	3.42023049745789	1.41317471367397	0.167085578695253	0.86242349643745	-5.156794877764
a_HQ_transcript/85963	1.34448785090742	1.48818447170862	1.41310210706437	0.167106752705785	0.86242349643745	-4.77102920435937
a_HQ_transcript/85469	1.76557970424184	1.37915994550259	1.41306118881466	0.167118686480822	0.86242349643745	-4.75332826240326
a_HQ_transcript/95814	-1.41229937957966	3.28069118377701	-1.41305745766194	0.16711977470189	0.86242349643745	-5.22347374304228
a_HQ_transcript/42524	-2.54222135631079	0.994513551127208	-1.41293493123951	0.167155513626548	0.862531353402929	-4.89549082825895
a_HQ_transcript/51625	0.678994597864229	2.42885455718526	1.41284602262397	0.167181450557041	0.862588616898606	-4.94209557989223
a_HQ_transcript/112245	-2.42830666878699	1.79526063764794	-1.41275756782637	0.167207258237376	0.862645203410152	-5.22197425824567
a_HQ_transcript/113175	0.6379783403362	3.65619148932865	1.41264846808599	0.167239093625898	0.862646355963047	-5.26272130515249
a_HQ_transcript/43714	2.00419904974305	2.62229549327159	1.41261553049793	0.167248705776073	0.862646355963047	-4.95137837321241
a_HQ_transcript/143295	1.9240217514896	0.86927291464498	1.41260423229163	0.167252003022701	0.862646355963047	-5.05839152378114
a_HQ_transcript/92164	1.14072776079374	-0.470608178854129	1.411985314084	0.167432705010767	0.863241849907751	-4.9201867112422
a_HQ_transcript/70196	1.20478974555324	-0.592423660780937	1.41191954292778	0.167451916855051	0.863241849907751	-4.89949144320791
a_HQ_transcript/65411	-1.38877637787949	1.81359241297331	-1.41191855795243	0.167452204580863	0.863241849907751	-4.9833941971811
a_HQ_transcript/128061	1.63460503918756	3.2406512026885	1.41187414339932	0.167465179130041	0.863241849907751	-5.12868104283975
a_HQ_transcript/124443	1.24026679379435	-0.512289354911117	1.41178597383315	0.167490937902579	0.863241849907751	-4.94567666775697
a_HQ_transcript/148415	3.61675692146481	2.14574829432077	1.41178213912682	0.167492058284174	0.863241849907751	-4.99515860030613
a_HQ_transcript/98728	0.596225011953194	2.6194207440462	1.41173731495734	0.167505154945469	0.863241849907751	-4.99036680562075
a_HQ_transcript/90691	1.28590951789988	0.00255018644275031	1.41171384908298	0.167512011491862	0.863241849907751	-4.92117623395524
a_HQ_transcript/68514	1.47512306434789	0.697458239530535	1.41163694060205	0.167534485099655	0.863241849907751	-4.92010559059835
a_HQ_transcript/86807	1.64818566078554	1.27357365742178	1.41163153186348	0.167536065689047	0.863241849907751	-4.89135066384413
a_HQ_transcript/106757	1.05364175184619	4.23723516218215	1.41162381826222	0.167538319846065	0.863241849907751	-5.27234117280709
a_HQ_transcript/148419	1.55457425351401	0.0579270965328834	1.41150068192161	0.167574307387691	0.863241849907751	-4.95257420669451
a_HQ_transcript/3433	-1.44154498191624	0.983034842845861	-1.41138474125966	0.167608197490628	0.863241849907751	-4.85330421553572
a_HQ_transcript/148242	1.94042418011338	-0.0640609865398165	1.41136298831802	0.167614556597105	0.863241849907751	-4.89765576661744
a_HQ_transcript/119121	1.35284705639173	2.46757394393899	1.41130186913192	0.167632424777727	0.863241849907751	-4.97396968660068
a_HQ_transcript/43802	1.99004443853089	0.538063116775328	1.41129383653969	0.167634773215607	0.863241849907751	-4.99943179091691
a_HQ_transcript/45882	1.50490999228375	1.42012486580941	1.41129321355016	0.167634955356171	0.863241849907751	-4.94349421809624
a_HQ_transcript/53335	-1.10454975421546	0.51943127518309	-1.41117412993593	0.167669774137968	0.863241849907751	-4.8248191614922
a_HQ_transcript/115989	1.52610931281279	0.783255611100398	1.41107169276593	0.167699730224205	0.863241849907751	-4.79377229079127
a_HQ_transcript/77300	0.419503933002354	4.14168729466371	1.41105805629232	0.167703718306611	0.863241849907751	-5.28208840916376
a_HQ_transcript/99079	2.71013984930726	1.13008657955642	1.41103408866	0.167710727989526	0.863241849907751	-4.90652377517086
a_HQ_transcript/82393	1.60515082425954	1.47972104910949	1.41102390628905	0.167713706041804	0.863241849907751	-4.92776066936889
a_HQ_transcript/121193	0.682893541986637	6.09643538851897	1.4109864002454	0.167724675845366	0.863241849907751	-5.46595224317131
a_HQ_transcript/68370	-1.64043987561697	0.206339050810335	-1.41095905713177	0.167732673540831	0.863241849907751	-4.76477001463349
a_HQ_transcript/113799	-3.23087276624673	2.68023478862049	-1.41092847965343	0.167741617623416	0.863241849907751	-5.29831667523739

a_HQ_transcript/132336	-1.37164274232517	0.62335256245231	-1.41082131945454	0.167772965536569	0.863241849907751	-4.81326690822998
a_HQ_transcript/121644	1.83044852391385	0.815744003011103	1.41075573235209	0.167792154211035	0.863241849907751	-4.96601081894066
a_HQ_transcript/125409	2.61411179892716	3.42899725085593	1.41073037893136	0.167799572267847	0.863241849907751	-5.14538892014038
a_HQ_transcript/12361	-1.44597798098986	2.47730621264116	-1.41064530556945	0.167824465428729	0.863241849907751	-4.94979815946158
a_HQ_transcript/122911	0.592213848172182	4.05696643870799	1.41064307434479	0.16782511834235	0.863241849907751	-5.2588835104641
a_HQ_transcript/79014	-1.42379671407401	1.24391496308861	-1.41055491577945	0.167850917399646	0.863241849907751	-4.95844240451154
a_HQ_transcript/3835	-0.937110366691403	3.81691646139848	-1.41055347134458	0.167851340130498	0.863241849907751	-5.17339851547787
a_HQ_transcript/41111	1.5332820048914	1.88333840431332	1.41048941380339	0.167870088164659	0.863241849907751	-4.87642520726211
a_HQ_transcript/81667	-3.27145860556946	2.01234785585903	-1.41046047846565	0.167878557351124	0.863241849907751	-5.12560077029868
a_HQ_transcript/61788	0.631562384591951	-0.62622845546623	1.41043083696761	0.167887233574304	0.863241849907751	-4.91058526553646
a_HQ_transcript/141168	1.37979937899479	-0.0785138436648508	1.41031432050926	0.16792134197249	0.863250202550659	-4.86255477364108
a_HQ_transcript/82011	1.12667359108183	1.51440243986526	1.41024152626016	0.16794265413098	0.863250202550659	-4.80374618652556
a_HQ_transcript/134355	1.59527486803643	0.711990094590989	1.41023691386917	0.167944004584086	0.863250202550659	-4.75645859924114
a_HQ_transcript/1773	1.11451456660563	-0.553130846185065	1.41022237504564	0.167948261434169	0.863250202550659	-4.87965794574213
a_HQ_transcript/115161	-2.22129106959276	3.56463705128085	-1.41008462632016	0.167988597355351	0.863381183649144	-5.01938896396859
a_HQ_transcript/140042	-1.10329985395105	1.54978193923686	-1.40996785556714	0.168022796415936	0.863480603842765	-4.9011436294142
a_HQ_transcript/108271	1.18348253420737	-0.363105901153892	1.40983043369292	0.168063050649798	0.863547638365717	-4.89065627199817
a_HQ_transcript/118737	1.21273181436383	1.33134920285777	1.40978017308894	0.168077775104411	0.863547638365717	-4.80877654126035
a_HQ_transcript/46120	1.83476025203273	-0.0810208219307	1.40976942122897	0.168080925124027	0.863547638365717	-4.9068143327156
a_HQ_transcript/32278	0.745065008343983	1.80328343649874	1.40967415945528	0.168108836410152	0.863547638365717	-4.86946344056192
a_HQ_transcript/79032	0.537291312139598	4.16423122576535	1.40966977763961	0.168110120350717	0.863547638365717	-5.26380952427964
a_HQ_transcript/108344	2.89115473601461	2.34819462342831	1.40959531694269	0.168131939678069	0.863583404577787	-5.04702913028039
a_HQ_transcript/146667	1.7407219323754	0.0151471690480264	1.40952297515293	0.168153140229758	0.863585099340368	-4.83537583649728
a_HQ_transcript/10969	-0.828460315181541	3.11364818947959	-1.40940381351135	0.168188066429375	0.863585099340368	-5.21246359735616
a_HQ_transcript/99399	1.76420475246006	2.39967186501445	1.40938151970416	0.168194601362575	0.863585099340368	-4.92766875587247
a_HQ_transcript/5667	2.90487821071416	2.05049265826149	1.40934817414925	0.168204376241869	0.863585099340368	-4.9563886017999
a_HQ_transcript/50151	1.46700446662417	1.17959220994859	1.40927825143685	0.168224874757952	0.863585099340368	-4.84295232570476
a_HQ_transcript/108697	3.82285309006644	1.93299424316881	1.409275476711	0.168225688236021	0.863585099340368	-5.05194812349511
a_HQ_transcript/16592	2.73573306607747	0.849287472980021	1.40923939784008	0.168236265911959	0.863585099340368	-5.05064549260016
a_HQ_transcript/126180	-1.68398299854564	0.79990722483078	-1.40911023774898	0.168274137610622	0.863631681102558	-4.91430513693564
a_HQ_transcript/118076	0.817378390089989	4.24484062099105	1.40910710795138	0.168275055397873	0.863631681102558	-5.33688281510326
a_HQ_transcript/136633	-0.564996436013924	2.74512699412907	-1.40888848579173	0.168339174268545	0.86378824457819	-4.99767164832705
a_HQ_transcript/148873	2.02299038816631	1.06342369243905	1.40886089393911	0.168347267942904	0.86378824457819	-4.81461305295589
a_HQ_transcript/142971	1.56853379472518	0.361806556890346	1.40885109818421	0.168350141460678	0.86378824457819	-4.92607830104029
a_HQ_transcript/95357	5.20554702830967	3.13130343556642	1.408756341434	0.16837793969487	0.863854622999415	-5.02595819955887
a_HQ_transcript/137246	-2.01192053182644	4.08929900175823	-1.40868428413256	0.168399081137643	0.86388684038204	-5.23853081243465
a_HQ_transcript/34882	-1.54891478258789	1.39920745052259	-1.40859208629351	0.168426134807374	0.863949379110752	-4.87863358425134
a_HQ_transcript/105718	0.360012920746825	4.08410180254148	1.40848354935511	0.16845798724397	0.86403652011978	-5.29080669095496
a_HQ_transcript/144382	2.02855014817288	0.0552001801523609	1.40842173512364	0.168476130038668	0.864053333941036	-4.89528261146594
a_HQ_transcript/52391	-1.79586645586157	2.01023191036644	-1.40826944038743	0.168520835874957	0.864100224205379	-5.03093614546848
a_HQ_transcript/59600	1.56286197453024	0.1210231669628	1.40824095708626	0.168529198129112	0.864100224205379	-4.96594733743939
a_HQ_transcript/35002	-2.70398721913747	5.03118912558024	-1.40823867105891	0.168529869285308	0.864100224205379	-5.50454997832692
a_HQ_transcript/14590	2.17514626476824	-0.018058522062467	1.40811332248432	0.168566673664956	0.864140908590579	-4.96935633185691
a_HQ_transcript/8635	1.98758805259651	1.58896307268261	1.40811038404447	0.168567536514392	0.864140908590579	-5.04436311870552
a_HQ_transcript/72448	-0.922809919289389	3.0268860530609	-1.40788176397274	0.168634679609783	0.864344601328097	-5.00909118013494
a_HQ_transcript/140046	1.26633267714694	-0.433466820631606	1.40787382997923	0.168637010109791	0.864344601328097	-4.94074947205923
a_HQ_transcript/91907	1.36836218760814	3.16292112137158	1.40762368112409	0.16871050081997	0.864527835878765	-5.17387428898609
a_HQ_transcript/61524	-2.3382754488937	0.801318497458247	-1.40760788321106	0.168715142900038	0.864527835878765	-4.96211671993048
a_HQ_transcript/138505	2.0891912297236	-0.0907710573187695	1.40760027556954	0.16871737837546	0.864527835878765	-4.93662159937754
a_HQ_transcript/143896	2.20243825570693	1.23404334899618	1.40737004296856	0.168785042319917	0.86459798557109	-4.84242232124191

a_HQ_transcript/58659	-0.636428505518126	2.57582757242897	-1.40736779950379	0.16878570176522	0.86459798557109	-4.9882805097021
a_HQ_transcript/16889	0.598522202023305	3.03019845379445	1.40726621753913	0.168815562944915	0.86459798557109	-5.08716588399798
a_HQ_transcript/121366	1.95207938134006	1.73264446842155	1.40726410922479	0.168816182751953	0.86459798557109	-5.02210072885218
a_HQ_transcript/18932	1.33235598487495	-0.353126620985478	1.40725490781738	0.168818887823514	0.86459798557109	-4.83749375614893
a_HQ_transcript/148094	1.23506982856907	-0.47630822362883	1.40703939769012	0.168882254204017	0.86459798557109	-4.9736440444055
a_HQ_transcript/7901	2.98188108672914	1.63960294831458	1.40701909438087	0.168888224942582	0.86459798557109	-4.97833644777361
a_HQ_transcript/150157	0.440380279936778	5.09835073042736	1.40699317013477	0.168895848910938	0.86459798557109	-5.38731422994872
a_HQ_transcript/135796	2.57847449402121	3.97363273859307	1.40696506308666	0.168904115116986	0.86459798557109	-5.21978093028124
a_HQ_transcript/101258	1.35570173397235	-0.178527820108782	1.4069581854787	0.168906137851065	0.86459798557109	-5.03397371321266
a_HQ_transcript/70681	-1.70956574878339	0.283525310128704	-1.40691918033314	0.168917609791907	0.86459798557109	-4.90959850327758
a_HQ_transcript/15600	1.78935710311386	0.367573019572641	1.40685456782455	0.168936614550518	0.86459798557109	-4.86247886411083
a_HQ_transcript/149424	-1.84784050253545	1.71606844775039	-1.40684153724472	0.168940447495133	0.86459798557109	-4.95047292171912
a_HQ_transcript/151923	1.53919149249127	5.54025319580861	1.4068072693596	0.168950527718249	0.86459798557109	-5.32337050008861
a_HQ_transcript/52371	1.23649223859706	2.11611506161827	1.40675981925566	0.168964486395542	0.86459798557109	-4.87604025207393
a_HQ_transcript/142828	-1.82317920910608	-0.660209991775647	-1.40674429635027	0.168969053056833	0.86459798557109	-4.95156157667376
a_HQ_transcript/35639	-1.57125738120712	1.09576826634728	-1.40651243345694	0.169037276002842	0.864757581164086	-4.82861313323387
a_HQ_transcript/116734	2.81006405793535	1.03857047275183	1.40649551780573	0.16904225408171	0.864757581164086	-4.85303130606861
a_HQ_transcript/67694	0.910607280846199	3.30476661850616	1.40648661781	0.169044873292862	0.864757581164086	-5.18058240652901
a_HQ_transcript/103217	-0.683465276394063	3.39653355075538	-1.40630993821111	0.169096875549612	0.864884153126956	-5.10727593771127
a_HQ_transcript/124032	1.26927268165594	-0.14966092728593	1.40628686831974	0.169103666657194	0.864884153126956	-5.01966082417653
a_HQ_transcript/151100	2.42286287688723	2.27837208080057	1.40625090800074	0.169114252760133	0.864884153126956	-5.09114853751037
a_HQ_transcript/14184	3.50926355102875	1.51228000223755	1.40615915295418	0.169141266233778	0.864946206003082	-4.97026017056705
a_HQ_transcript/122974	0.83730556037737	2.95843372931104	1.40603371664685	0.169178201240016	0.865058979739587	-5.03982028776831
a_HQ_transcript/55917	1.40326705567188	2.1825167715986	1.40587262349292	0.169225644771637	0.865162192493894	-4.93821032493515
a_HQ_transcript/52140	-0.47652784412489	3.00852023139116	-1.40582959216445	0.169238319691873	0.865162192493894	-5.14304513496266
a_HQ_transcript/46712	1.5927714705127	3.25704129975869	1.40581357544981	0.169243037621251	0.865162192493894	-5.07635662829801
a_HQ_transcript/144728	0.912956370965565	-0.815317523044467	1.40573427459538	0.169266398229131	0.865205522006938	-4.95035069578359
a_HQ_transcript/90887	-1.21351640381019	0.492302823298361	-1.40556867090391	0.169315190264143	0.865378825259309	-4.88940211530157
a_HQ_transcript/67205	0.880185449790297	-0.517390211079367	1.4054943553945	0.169337089529111	0.865414659763336	-4.87716170527493
a_HQ_transcript/137372	1.58478045885809	1.92289679161679	1.40520912800194	0.169421160864199	0.865531306443242	-4.92970119475008
a_HQ_transcript/44874	1.97971280183176	1.59186976443832	1.40520402829095	0.169422664312058	0.865531306443242	-4.90607036511566
a_HQ_transcript/42656	1.15066647499952	1.42999668738026	1.40513170991958	0.169443985647369	0.865531306443242	-4.81518211483261
a_HQ_transcript/5076	-0.558231500591306	3.71444838776044	-1.40511581217297	0.169448672998538	0.865531306443242	-5.18968819079271
a_HQ_transcript/87782	-3.19197931747413	0.472866557694222	-1.40511581065055	0.169448673447418	0.865531306443242	-4.95233130989553
a_HQ_transcript/148281	-1.54655257408291	1.26320865279524	-1.40506453364342	0.169463792845749	0.865531306443242	-4.80064106547464
a_HQ_transcript/139368	1.52497898642235	0.876905164579478	1.40506334064456	0.169464144622738	0.865531306443242	-4.80744554985508
a_HQ_transcript/62973	-2.04635585672514	1.96233357858156	-1.40498720788903	0.169486594908531	0.865554143987185	-5.00238843155988
a_HQ_transcript/45211	-2.27259425066453	1.9019471303236	-1.40481291057617	0.169538001080693	0.865554143987185	-4.95413674414588
a_HQ_transcript/100276	-1.62885387018614	-0.0535675851580619	-1.40476737803362	0.16955143218272	0.865554143987185	-4.89959803555024
a_HQ_transcript/14679	-2.06619212234953	1.08847923551304	-1.40476737703676	0.169551432476782	0.865554143987185	-4.84585118518848
a_HQ_transcript/140228	7.10331070476828	2.69244834723305	1.40475145345546	0.16955612978129	0.865554143987185	-5.06307290582375
a_HQ_transcript/130199	-1.26988511093234	-0.0675706103354809	-1.40465613272753	0.169584250621546	0.865554143987185	-4.85872708897074
a_HQ_transcript/13371	1.22901792566306	1.31993558491909	1.40465507976475	0.16958456127954	0.865554143987185	-4.7814376681985
a_HQ_transcript/22609	1.25125693794545	1.37185819226672	1.40457138768159	0.169609254572562	0.865554143987185	-4.91074211159871
a_HQ_transcript/28247	0.869754289123391	4.08613010382624	1.40448391411799	0.16963506660413	0.865554143987185	-5.27843638916452
a_HQ_transcript/147900	2.47791077432875	2.11784089017274	1.40445976529023	0.169642193074873	0.865554143987185	-4.95259586902169
a_HQ_transcript/74439	1.2718126982057	-0.550592666425895	1.40444696530225	0.169645970526973	0.865554143987185	-4.94112094571076
a_HQ_transcript/98685	-1.62667097709144	0.0604878671219628	-1.40440752663531	0.16965760983372	0.865554143987185	-4.93168459791976
a_HQ_transcript/150628	-3.19539978891904	1.12703711282297	-1.40439199982314	0.169662192344309	0.865554143987185	-4.96769049337721
a_HQ_transcript/97669	-1.92655849372706	2.98588380459102	-1.40410477794818	0.169746979177988	0.865910698170957	-5.03668145031392

a_HQ_transcript/104164	-0.834237632886842	2.32274654211133	-1.40392408353814	0.169800336531273	0.866070959571492	-4.97416150266638
a_HQ_transcript/2666	2.03310854693645	0.287103043782784	1.40384937515983	0.169822401054393	0.866070959571492	-4.89548741658439
a_HQ_transcript/99535	-3.29047748387491	2.56171965068511	-1.40384702966635	0.169823093813582	0.866070959571492	-5.27768055886862
a_HQ_transcript/95203	1.67517714404655	0.496511284164328	1.4037396545729	0.169854810231223	0.866156716301478	-4.86992911917011
a_HQ_transcript/49425	1.04306106182914	-0.333942510047033	1.40368694731369	0.169870380583284	0.866160130047819	-4.9339457114152
a_HQ_transcript/92162	1.0592537412328	-0.441620227324116	1.40360778925141	0.169893766921078	0.8662033929996	-4.97698073459063
a_HQ_transcript/6237	1.12702948640071	-0.279386139182927	1.403395921385	0.169956373268577	0.8664465940498	-4.85852547254496
a_HQ_transcript/92742	-1.07306104341968	1.46683598012785	-1.40328622678687	0.169988794817673	0.866535882238156	-4.9543386230179
a_HQ_transcript/7792	-3.11415345016555	1.68549908487493	-1.40308810185216	0.170047365303128	0.866758440050881	-4.93897187907854
a_HQ_transcript/23460	1.15964359925596	3.17676548142768	1.40299869283207	0.170073801937973	0.866817181854494	-5.19740622686883
a_HQ_transcript/40181	0.350310208156388	3.29929453857557	1.40288595370482	0.170107141459721	0.866891038815016	-5.19882669394454
a_HQ_transcript/150758	-7.65352674402883	5.31575646722471	-1.40283553712644	0.170122052446176	0.866891038815016	-5.14942173231299
a_HQ_transcript/124242	0.547386988796814	3.43889918499316	1.40272441905913	0.170154919858642	0.866891038815016	-5.16957226244883
a_HQ_transcript/146815	1.95809874366781	0.144730718395742	1.40272211831239	0.170155600444987	0.866891038815016	-4.9788992292523
a_HQ_transcript/131981	1.29039643019561	1.94352457824088	1.4026331317978	0.17018192527889	0.866891038815016	-4.98310066724147
a_HQ_transcript/5901	-1.22603751832182	-0.632437662224519	-1.40263029256349	0.170182765260664	0.866891038815016	-4.90569429158167
a_HQ_transcript/75545	0.987633278354032	1.86163404395401	1.40259675532829	0.170192687430446	0.866891038815016	-4.90688094775047
a_HQ_transcript/20500	0.863864304396923	4.21974806371062	1.40254376342236	0.170208366293029	0.866894936991253	-5.32823533481353
a_HQ_transcript/82238	-1.12081470125624	-0.460672573052789	-1.40233121658009	0.170271264501922	0.86713930812754	-4.91278435046946
a_HQ_transcript/63531	1.27717530727627	-0.512978271126925	1.4021132315747	0.170335790937732	0.867303389738302	-4.94332291319558
a_HQ_transcript/93587	0.836338107460024	2.21407015007239	1.40209123358907	0.17034230369655	0.867303389738302	-4.92668408617538
a_HQ_transcript/145615	-2.82943281318361	2.20989772085089	-1.40207116548148	0.170348245262906	0.867303389738302	-4.84655870748643
a_HQ_transcript/90350	1.33469526948501	-0.410052383245813	1.40201531486692	0.170364781814685	0.867304526600039	-4.96992828510666
a_HQ_transcript/101216	0.532618306685179	2.86306856121936	1.40195566017502	0.170382446088494	0.867304526600039	-5.12048566537217
a_HQ_transcript/11420	2.24602355618923	3.10589472383352	1.40187892217383	0.170405170990839	0.867304526600039	-5.08172264665519
a_HQ_transcript/75052	1.16057910202015	2.25417217995485	1.40186885997772	0.170408150947892	0.867304526600039	-4.98356788041116
a_HQ_transcript/130363	-1.90647755779367	3.19969116222604	-1.40173693797049	0.170447223924051	0.867371047351898	-5.16787194174221
a_HQ_transcript/21000	1.36754856992537	-0.0069814242570682	1.40160982007991	0.170484880650619	0.867371047351898	-4.94199871964218
a_HQ_transcript/91979	1.88524093255109	0.670145993824576	1.4016069620981	0.170485727358891	0.867371047351898	-4.87557208148944
a_HQ_transcript/123496	1.30095061169865	0.106077102998668	1.40158247343443	0.170492982527667	0.867371047351898	-4.83477733779418
a_HQ_transcript/127043	1.75298440587502	-0.0517409015647482	1.40156039558654	0.170499523660174	0.867371047351898	-4.92398520664004
a_HQ_transcript/2310	-1.36043099330176	2.26740650593996	-1.40152250085276	0.170510751412231	0.867371047351898	-4.9655820429064
a_HQ_transcript/122152	0.384344530690708	4.03398316654784	1.4014627992192	0.170528441461425	0.867385128217509	-5.31019592657699
a_HQ_transcript/97600	-1.70480232579303	0.813218014556642	-1.40133422825169	0.170566542905399	0.867428235543231	-4.88808723887628
a_HQ_transcript/145515	1.48475246945218	-0.179137860467435	1.40133348953411	0.170566761840406	0.867428235543231	-4.96874781638282
a_HQ_transcript/123827	-1.60843036226357	0.541074190247341	-1.40120549928954	0.170604697856577	0.867457378703875	-4.79116956667336
a_HQ_transcript/16298	2.39890070067064	2.83493795400572	1.40105415608976	0.170649564170096	0.867457378703875	-5.02070754024651
a_HQ_transcript/64527	1.4688023841364	2.37704193208397	1.40103181787208	0.170656187209847	0.867457378703875	-4.9648650092707
a_HQ_transcript/113071	-1.78743107821726	0.649011102283242	-1.40098627327044	0.170669691315499	0.867457378703875	-4.86252069006859
a_HQ_transcript/77276	-1.26802675496054	3.03936234171416	-1.40095772371933	0.170678156766813	0.867457378703875	-5.11026961169011
a_HQ_transcript/101201	0.677192032346543	2.8448603319158	1.40091846609626	0.17068979789003	0.867457378703875	-5.04084518524966
a_HQ_transcript/120776	1.58777661922372	-0.282006737202131	1.40091226264854	0.17069163746485	0.867457378703875	-4.99999907742141
a_HQ_transcript/82412	-2.01176222910354	4.41072769952256	-1.40091145074783	0.170691878227617	0.867457378703875	-5.34158733018867
a_HQ_transcript/26573	0.566682240582741	3.20940724593858	1.4007340237098	0.170744499205698	0.867570564444123	-5.14579524593098
a_HQ_transcript/135800	0.720221397297439	6.69615175566386	1.40071748092279	0.170749406082439	0.867570564444123	-5.5465048856041
a_HQ_transcript/59425	1.29381012135954	2.476759729972	1.40068538835241	0.170758925609091	0.867570564444123	-4.85795584758034
a_HQ_transcript/7210	1.23247520391779	-0.256208313851038	1.40056781557778	0.170793804436245	0.867671933601647	-4.82414236290154
a_HQ_transcript/108917	0.963035389537707	1.92852712711837	1.40038974144535	0.170846642069292	0.867819528421471	-4.83829075956151
a_HQ_transcript/124292	2.24154506209943	2.14152353913111	1.40032792543186	0.170864986930533	0.867819528421471	-5.22379576640964
a_HQ_transcript/146894	-0.86733947176952	1.75459370010873	-1.40031896707924	0.170867645588765	0.867819528421471	-4.82626050583548

a_HQ_transcript/17220	-0.466293017948702	2.92841637256374	-1.40016854256508	0.17091229339172	0.86797045171011	-5.06991667932397
a_HQ_transcript/112110	1.61097583907581	0.981520704525948	1.40009711433516	0.170933497350009	0.867972414141326	-4.82902992782965
a_HQ_transcript/115946	1.85682232662614	3.75177043916984	1.40003534979156	0.170951834239118	0.867972414141326	-5.32749205919282
a_HQ_transcript/109726	1.67149777376634	4.49493942622609	1.40001634730468	0.170957476078724	0.867972414141326	-5.29206965145697
a_HQ_transcript/131336	-0.504892974266691	3.21004351482599	-1.39995544577193	0.170975558731099	0.867988408552082	-5.1885621944372
a_HQ_transcript/76362	1.29689468997945	0.580697482011154	1.3998839880914	0.170996777586067	0.868020320279705	-4.81881814465547
a_HQ_transcript/141459	2.23496857087102	0.483972443940351	1.39974064854973	0.171039347475353	0.868153256384346	-5.01082434819513
a_HQ_transcript/125637	0.353983486282242	-0.822749805660405	1.39969523671599	0.171052835903006	0.868153256384346	-4.96449230156848
a_HQ_transcript/150168	-0.629456839789968	3.06798413617272	-1.39949787199842	0.171111467745881	0.868375012845518	-5.12599695501265
a_HQ_transcript/64128	0.580861009574688	3.19763956765559	1.39942413271168	0.171133377780435	0.868410387046803	-5.13803796578794
a_HQ_transcript/74291	-1.4416441568889	-0.230209241004843	-1.39928396558626	0.171175031471835	0.868535006717638	-4.92548666955851
a_HQ_transcript/95341	2.05193741752529	0.0132592841800972	1.39924093847644	0.171187819501169	0.868535006717638	-4.82606686715041
a_HQ_transcript/89234	-1.38988830302091	0.899001439940127	-1.39910232436833	0.171229021903928	0.868545683103023	-4.83548311872778
a_HQ_transcript/35445	0.545713980017651	2.5759830707575	1.39904921911969	0.171244809252111	0.868545683103023	-5.09252414458753
a_HQ_transcript/19004	-0.901196786672696	2.1448785677909	-1.39898693644481	0.171263326356724	0.868545683103023	-4.91848199204583
a_HQ_transcript/89650	1.20320755788688	-0.639451685724669	1.39898130212576	0.171265001559256	0.868545683103023	-4.94243056517673
a_HQ_transcript/74752	-1.95203082061363	0.230295606940149	-1.39896049114871	0.171271189215322	0.868545683103023	-4.87479002663596
a_HQ_transcript/7810	0.934656549855188	-0.663050904611035	1.39880587012038	0.171317167638024	0.868545683103023	-4.92648719983119
a_HQ_transcript/135225	1.39943580233085	-0.149933395742835	1.39880211517033	0.171318284337855	0.868545683103023	-4.99193346400596
a_HQ_transcript/10381	1.84227443294569	1.87823495151421	1.39873445268397	0.171338407736488	0.868545683103023	-4.96571168657777
a_HQ_transcript/100928	-2.2358667616598	1.21700254677957	-1.39869121827148	0.171351266987976	0.868545683103023	-4.9091285222805
a_HQ_transcript/98225	1.54328207320537	-0.566706252164287	1.39864738338776	0.171364305610123	0.868545683103023	-4.93753997605433
a_HQ_transcript/61238	1.70291664937187	4.77941619292099	1.39863301123608	0.17136858075485	0.868545683103023	-5.38763614030215
a_HQ_transcript/26288	1.02554853675653	2.37518146165653	1.39858224465636	0.171383682466379	0.868545683103023	-4.9946538011616
a_HQ_transcript/69742	1.65007266206082	0.254341562355892	1.39856574364502	0.17138859130429	0.868545683103023	-5.02399878850948
a_HQ_transcript/114729	1.33168773008755	-0.44266224393028	1.39853038265064	0.171399111114691	0.868545683103023	-4.92951422023771
a_HQ_transcript/80626	-0.504336897281761	3.1404514925142	-1.39837511881691	0.171445307717157	0.868696651706221	-5.15416918867903
a_HQ_transcript/74354	1.22915304718465	0.295938275754711	1.39820852431618	0.171494886449956	0.868696651706221	-4.88090259872997
a_HQ_transcript/80569	0.903871553829677	1.06694612169615	1.39815247804912	0.171511568414056	0.868696651706221	-4.97515605981319
a_HQ_transcript/93367	-0.460664271173424	3.2396899515485	-1.39814960691764	0.17151242302995	0.868696651706221	-5.14260836349614
a_HQ_transcript/57501	1.14447596660347	-0.129597904227836	1.3981264620272	0.171519312418725	0.868696651706221	-4.95295243511267
a_HQ_transcript/84808	-2.42585998631078	2.10894402473564	-1.39809329945565	0.171529184083211	0.868696651706221	-5.06457742677801
a_HQ_transcript/7323	-0.549864934205707	2.39412625053257	-1.3980095863835	0.171554105341602	0.868696651706221	-4.94319892029875
a_HQ_transcript/36664	0.784204920462914	1.27211276644048	1.39799007875246	0.171559913142111	0.868696651706221	-4.95280259558738
a_HQ_transcript/115078	-1.55862122427491	3.41734252606719	-1.39788406640384	0.171591477769313	0.868696651706221	-5.19146184995643
a_HQ_transcript/101406	-2.46741768641114	2.84005963626766	-1.39784948659493	0.171601774711995	0.868696651706221	-5.04304438084483
a_HQ_transcript/47907	1.95518116551491	0.169310976847064	1.39783073021751	0.171607360062239	0.868696651706221	-4.92590417441684
a_HQ_transcript/29407	0.917997010271758	3.82438475006308	1.39782778242461	0.171608237880927	0.868696651706221	-5.01199400377669
a_HQ_transcript/35415	-1.72063884455915	1.69980611322852	-1.39775589879223	0.171629645085321	0.868729363420371	-4.85250402835197
a_HQ_transcript/735	-1.84550068650586	0.985968600054534	-1.39769528427344	0.171647697932603	0.868745092331417	-4.79728380664215
a_HQ_transcript/135063	-0.487709473223739	5.88552566961524	-1.39749587725056	0.171707097903801	0.868930241493242	-5.47673955879639
a_HQ_transcript/49895	0.867363966770914	1.94581678537612	1.39747211597574	0.171714177058094	0.868930241493242	-4.97129535625189
a_HQ_transcript/129962	-1.56534318300878	0.229489077017216	-1.39732669597093	0.171757506761627	0.869073846887175	-4.80768430951994
a_HQ_transcript/145137	-1.72926270856524	-0.119087886377793	-1.39718568510242	0.171799530885131	0.869210821767858	-4.94338559101348
a_HQ_transcript/75281	2.26865668850813	3.93063464845501	1.39712259836208	0.171818334631323	0.869230300735382	-5.02953068294905
a_HQ_transcript/143588	-1.40255398523066	-0.236613362210086	-1.39705228175758	0.171839295223105	0.869260686861774	-4.95376398968139
a_HQ_transcript/139976	2.49248450703538	0.290684473061038	1.3967672287512	0.171924286843402	0.869579028086861	-4.96515265332753
a_HQ_transcript/114078	-1.59804461062168	0.530212898390164	-1.39673498559262	0.171933902559935	0.869579028086861	-4.76866538793373
a_HQ_transcript/80507	2.63251096730329	-0.0804073324578045	1.39668598197121	0.171948517472191	0.869579028086861	-4.94415813107588
a_HQ_transcript/2731	-1.1045590475928	2.50469838872116	-1.3966405594087	0.171962065232908	0.869579028086861	-4.89095066546107

a_HQ_transcript/71069	1.47512725357514	4.07816774386378	1.39640794621681	0.172031457683727	0.869749550666297	-5.05039189691648
a_HQ_transcript/128484	0.733507257363182	3.57612593175716	1.39640607133645	0.172032017081323	0.869749550666297	-5.27454900023762
a_HQ_transcript/114394	-1.19663237931869	-0.230453256509771	-1.39637705532932	0.172040674607093	0.869749550666297	-4.83344378314531
a_HQ_transcript/80890	3.19978829062086	2.12893937961699	1.39622542668045	0.172085921699503	0.869796694831624	-4.98946015512987
a_HQ_transcript/112633	2.76965227586765	4.52671025480559	1.39617623081398	0.172100604105859	0.869796694831624	-5.34634868386582
a_HQ_transcript/108844	-0.37121731323491	3.51305817201702	-1.3961563855086	0.172106527174534	0.869796694831624	-5.24233291070463
a_HQ_transcript/74792	2.4810072710401	0.951327559430223	1.3961324130064	0.172113682267293	0.869796694831624	-4.95725817296508
a_HQ_transcript/138674	0.495648856915673	-0.699197037210167	1.39609510758463	0.172124817310903	0.869796694831624	-5.00550708804287
a_HQ_transcript/37942	-2.75672662328167	-0.000726222413121632	-1.39595004340479	0.172168121899968	0.869939898278974	-4.94738191776892
a_HQ_transcript/91994	-1.31421288083213	-0.0593066031949102	-1.39584691626871	0.17219891262101	0.870019851615303	-4.98149357930603
a_HQ_transcript/69724	-1.9433122322398	0.397325330132121	-1.3957065149483	0.172240839243813	0.870057591664548	-4.97688150882744
a_HQ_transcript/68206	1.62222063269572	0.222616359444145	1.39567309483978	0.172250820329409	0.870057591664548	-4.94864310507692
a_HQ_transcript/89644	1.31844443811906	-0.494828965791419	1.39567153494481	0.172251286211045	0.870057591664548	-4.92100305915513
a_HQ_transcript/76972	0.683969585590776	2.37745017689799	1.39559750119407	0.172273398427042	0.870093674842915	-4.91466919109178
a_HQ_transcript/87182	1.13595637459833	-0.481334463932418	1.39548187226106	0.172307938633843	0.870123645519902	-4.91339977263596
a_HQ_transcript/73375	-1.63723423904802	0.186099438301198	-1.39543540416815	0.172321820921419	0.870123645519902	-4.87745250587873
a_HQ_transcript/41335	0.981279250914363	-0.0728526932801248	1.39542730799385	0.172324239733435	0.870123645519902	-4.84674301242582
a_HQ_transcript/12228	-1.55870984148281	1.56678908406831	-1.39522008441322	0.172386158870561	0.870276933494408	-4.80880526312415
a_HQ_transcript/141466	-1.71597583525557	0.764966404184139	-1.39516586007512	0.172402364168558	0.870276933494408	-5.02370780157638
a_HQ_transcript/9843	0.970868164848886	2.40549830380206	1.39515630953001	0.172405218534917	0.870276933494408	-4.88337703691866
a_HQ_transcript/15253	1.37433110903277	-0.48573392359088	1.39511855079307	0.172416503830846	0.870276933494408	-4.9386582555296
a_HQ_transcript/51222	-2.13822386157209	1.26339772860248	-1.39507521593677	0.172429456424702	0.870276933494408	-4.99887329803341
a_HQ_transcript/144536	0.828383454209348	3.04373382616707	1.39490572020388	0.172480125253204	0.870457086362063	-5.12445695455364
a_HQ_transcript/145410	2.30133847834442	1.72503859351123	1.39480836264785	0.172509234460172	0.870528412249406	-4.84825028602484
a_HQ_transcript/127318	-1.90297871467452	0.425292732134133	-1.39475511042152	0.172525158118398	0.870533193672416	-4.88479822289846
a_HQ_transcript/49684	4.81984632205121	2.63591682689308	1.39460753719261	0.172569291968548	0.870680305836971	-5.23038219029462
a_HQ_transcript/16886	1.12422014546337	-0.0175337087947618	1.39455683523272	0.17258445714214	0.87068124672438	-4.83810402805181
a_HQ_transcript/115548	2.09063965934449	2.17349317946957	1.39445961136761	0.172613540134592	0.870752396159294	-4.98982992926181
a_HQ_transcript/96247	-3.53379844061553	0.707208551170726	-1.39439531875401	0.172632774366998	0.870773855293725	-5.01613222758642
a_HQ_transcript/17428	1.84174250486277	0.315351631009981	1.39433532823613	0.172650723066717	0.870788826934676	-4.82470257030473
a_HQ_transcript/79016	0.767353471372972	2.81316895293547	1.39425948036712	0.172673418253867	0.87081527784913	-5.08277224516499
a_HQ_transcript/116102	-1.45632819415504	-0.098660980163363	-1.39421767094011	0.172685929461996	0.87081527784913	-4.87299842910657
a_HQ_transcript/41314	1.38860652820083	1.20377249217411	1.3940681550876	0.172730676945282	0.870916066860901	-4.89751212792499
a_HQ_transcript/9941	1.51582790612392	1.11768268182061	1.39405076608776	0.172735881758177	0.870916066860901	-4.89417566553044
a_HQ_transcript/79353	-0.545994208245023	2.49989431298388	-1.39396420089933	0.172761793965247	0.870948289713332	-5.05282901825236
a_HQ_transcript/108539	0.624539459076649	3.11441073868893	1.39392930877803	0.172772239345658	0.870948289713332	-5.04513389310661
a_HQ_transcript/9052	0.92865298534572	-0.697652755298317	1.39387127016929	0.172789615002921	0.870960348672373	-4.95410861085625
a_HQ_transcript/19557	-2.59734990245989	1.40829735715469	-1.39378694120523	0.172814863929991	0.871012087966924	-5.02355534548054
a_HQ_transcript/120308	0.405501018100976	3.97243511001342	1.39369939806638	0.172841078266795	0.871055008223989	-5.30524341394529
a_HQ_transcript/145155	-2.46412246291661	1.82141969747434	-1.39365842052764	0.172853349846265	0.871055008223989	-4.98293143575197
a_HQ_transcript/149732	4.36195658816656	1.39246501907035	1.39345149660806	0.172915327947166	0.871233634295121	-5.02077981533764
a_HQ_transcript/81169	1.60249946574221	-0.262091367115348	1.39328249780361	0.172965959569951	0.871233634295121	-4.93949295815497
a_HQ_transcript/46690	1.39837296700496	0.769507543402497	1.39323800606624	0.172979291112738	0.871233634295121	-4.8210921460182
a_HQ_transcript/1951	-0.564664752137519	4.70563565693025	-1.39316832264558	0.17300017272888	0.871233634295121	-5.3858190578958
a_HQ_transcript/107072	1.53385186273714	-0.455910809515848	1.39309849395871	0.173021099854306	0.871233634295121	-4.96409738942914
a_HQ_transcript/115369	-1.68044101522286	0.915032482562687	-1.39308425836547	0.173025366396156	0.871233634295121	-4.85636331935788
a_HQ_transcript/152794	-1.69970919138765	2.44091998834086	-1.3930340204299	0.173040423838055	0.871233634295121	-5.02599462326063
a_HQ_transcript/127158	0.775284657175196	3.02504252699461	1.39302851831957	0.173042073006849	0.871233634295121	-5.13570569541414
a_HQ_transcript/86358	1.33691162603736	4.38021468468335	1.39293319692539	0.173070646008505	0.871233634295121	-5.3133971686869
a_HQ_transcript/65904	0.534838686431606	2.74299516767879	1.39289436648687	0.173082286658951	0.871233634295121	-5.12825700279015

a_HQ_transcript/29344	0.715971420119549	3.51373819627871	1.39274005756859	0.173128551684464	0.871233634295121	-5.29403495583927
a_HQ_transcript/63550	1.38073808919369	-0.538200096786007	1.39271435527565	0.17313625870663	0.871233634295121	-4.93460592876154
a_HQ_transcript/143004	1.46953522449112	0.182297504870102	1.39269881641321	0.173140918279348	0.871233634295121	-5.0054192037953
a_HQ_transcript/93879	2.63476164174693	1.36808309163983	1.39269622949802	0.173141694016149	0.871233634295121	-5.05790951581705
a_HQ_transcript/120209	0.843926461346105	4.92747606153071	1.39260416433218	0.173169303315749	0.871233634295121	-5.42118102151667
a_HQ_transcript/93292	-0.897876986608357	2.60057161565162	-1.39254964486185	0.173185654710337	0.871233634295121	-5.06490204544452
a_HQ_transcript/117849	1.50333634227351	-0.163949536302518	1.3924445162127	0.17321718814065	0.871233634295121	-4.91932590505494
a_HQ_transcript/105960	-1.13721049817312	2.8507685155621	-1.39228562410835	0.173264856489569	0.871233634295121	-5.04151779115425
a_HQ_transcript/110860	1.56497630571636	-0.185019002947307	1.39227738131786	0.173267329643861	0.871233634295121	-4.94496745976399
a_HQ_transcript/106184	1.84946244853476	1.1326188643209	1.39212508558091	0.173313029192685	0.871233634295121	-4.87888883832002
a_HQ_transcript/140355	1.57329191977654	6.34695284327255	1.39192726004416	0.173372404993855	0.871233634295121	-5.56033627447631
a_HQ_transcript/143017	1.72872799431757	1.55325730453464	1.39186624405259	0.1733907216805	0.871233634295121	-4.93982280809453
a_HQ_transcript/75655	-3.96901758034512	2.65997088175933	-1.39184649245483	0.173396651332736	0.871233634295121	-4.96980334105645
a_HQ_transcript/55504	-1.76747645482958	2.33922475676734	-1.39181200256572	0.173407005966374	0.871233634295121	-4.93145026553044
a_HQ_transcript/54380	-0.416436550790343	2.74227899015129	-1.39180001295628	0.173410605627699	0.871233634295121	-4.99157455828868
a_HQ_transcript/55423	-1.13959232037528	2.96889388844321	-1.39179185277946	0.173413055605219	0.871233634295121	-5.11381958630974
a_HQ_transcript/2858	-1.00929087800331	-0.745156456762254	-1.3917539511976	0.173424435373249	0.871233634295121	-4.89104315556455
a_HQ_transcript/65673	0.695085386374456	2.79168609120473	1.39175205525001	0.173425004637747	0.871233634295121	-5.00033974655478
a_HQ_transcript/128711	-1.61005348055752	1.55683239172644	-1.39174503033285	0.173427113904973	0.871233634295121	-4.87353782866282
a_HQ_transcript/98786	-1.00740328326715	0.245788158900032	-1.3916489780751	0.173455956098086	0.871233634295121	-4.8392638480374
a_HQ_transcript/89774	1.1989119152926	4.22058886244716	1.39152150184814	0.173494239949262	0.871233634295121	-5.37454038227409
a_HQ_transcript/115372	-1.52655680603327	1.30084534148273	-1.39145948325424	0.173512867855367	0.871233634295121	-4.94817534831075
a_HQ_transcript/6904	-1.21165998759819	1.99712086817475	-1.39145141682419	0.173515290803397	0.871233634295121	-4.88549176020394
a_HQ_transcript/4484	-1.58257343804032	-0.0259282709954253	-1.39141809367305	0.173525300500978	0.871233634295121	-4.92727557480247
a_HQ_transcript/12238	1.9952942047208	1.10921646207408	1.39131467693926	0.173556367972247	0.871233634295121	-4.92354062332445
a_HQ_transcript/138745	-2.02882618704651	0.83856307387533	-1.39128638011583	0.173564869392549	0.871233634295121	-5.00745737757107
a_HQ_transcript/10854	-1.42964606852	2.14321056736702	-1.39124898472298	0.173576104861701	0.871233634295121	-5.06574707312323
a_HQ_transcript/75955	1.35071976772131	0.568124920089115	1.39116033032649	0.173602743402877	0.871233634295121	-4.94782631282142
a_HQ_transcript/27523	1.58784715481836	0.35670021896522	1.39113641225782	0.17360993076214	0.871233634295121	-4.84209516618983
a_HQ_transcript/82490	1.53550897942415	0.420187621937413	1.39107372564509	0.173628769139904	0.871233634295121	-4.8718567584524
a_HQ_transcript/60026	0.353983486282242	-0.870292808778577	1.39104749922133	0.173636651094028	0.871233634295121	-5.01874100413204
a_HQ_transcript/117783	-2.61792855821945	0.583983566707131	-1.39100733930432	0.173648721090548	0.871233634295121	-4.94039460339891
a_HQ_transcript/114578	-1.45238345397032	0.40505555177367	-1.3909463544273	0.173667051248795	0.871233634295121	-4.95974847905583
a_HQ_transcript/23874	1.72606841050506	0.45935318212753	1.39088374040266	0.173685872652183	0.871233634295121	-4.88800746648021
a_HQ_transcript/56117	-2.01003083911875	0.396895594505658	-1.39086305158552	0.173692091938096	0.871233634295121	-4.90729094364827
a_HQ_transcript/12253	1.56883128432888	1.13747472126721	1.3908267435208	0.173703006962495	0.871233634295121	-4.91934036844779
a_HQ_transcript/77148	-2.21145074177247	0.725088758490385	-1.39075231799094	0.173725382634274	0.871233634295121	-4.86574961899767
a_HQ_transcript/103224	0.454012283126514	3.65183951864789	1.39070841158741	0.173738583936732	0.871233634295121	-5.28422532657932
a_HQ_transcript/41926	-3.52154330425209	3.01976936225722	-1.39063684559384	0.173760103306569	0.871233634295121	-5.21417830790479
a_HQ_transcript/141367	1.13429606307799	4.47779966973919	1.39053061629806	0.173792049519622	0.871233634295121	-5.24733177452786
a_HQ_transcript/112938	-1.79165436887985	-0.0857461353236131	-1.39052627363472	0.173793355581425	0.871233634295121	-5.00438348674545
a_HQ_transcript/99659	2.64662913854802	3.82057166440933	1.39051956040906	0.173795374608112	0.871233634295121	-5.18755566240577
a_HQ_transcript/143806	1.2919643411253	0.274537534845442	1.39046974374476	0.173810357721473	0.871233634295121	-5.01225735407625
a_HQ_transcript/28493	1.98701585953913	0.271894122878999	1.39044311714351	0.173818366487622	0.871233634295121	-5.04157963375633
a_HQ_transcript/85518	-1.08322906611709	-0.703002169759201	-1.39041770382027	0.173826010592312	0.871233634295121	-4.92290659701256
a_HQ_transcript/112728	2.37316315842475	1.952714333289	1.3904043289966	0.173830033727494	0.871233634295121	-4.90717076674702
a_HQ_transcript/94464	3.52110326749694	1.70404344060691	1.39040111460432	0.173831000624711	0.871233634295121	-4.91034600603754
a_HQ_transcript/31890	0.967617036441819	0.976839210699928	1.3903854065004	0.173835725722033	0.871233634295121	-4.84397009848744
a_HQ_transcript/132858	-4.44807649626134	2.31863936821569	-1.39036046545197	0.173843228353926	0.871233634295121	-5.03926775227082
a_HQ_transcript/121259	1.40972517034502	-0.460518852993442	1.39024516012774	0.173877917171142	0.871233634295121	-4.96115265492395

a_HQ_transcript/51060	1.61034635056819	-0.0995957321350629	1.3901962806439	0.173892623862159	0.871233634295121	-4.89272401613239
a_HQ_transcript/134945	0.688534535856396	2.8735444877218	1.39018640078985	0.173895596596809	0.871233634295121	-5.04948641026401
a_HQ_transcript/121166	0.38301983720805	3.69198684978616	1.39012654831836	0.173913606367614	0.871233634295121	-5.25330401045936
a_HQ_transcript/18514	2.029778299888	0.920386084396828	1.39011342077813	0.173917556674998	0.871233634295121	-4.88724863959017
a_HQ_transcript/97607	1.82204648795802	0.161562806585935	1.39006823209287	0.173931155280224	0.871233634295121	-4.98829802004683
a_HQ_transcript/13702	-0.827491905389481	2.65699985234929	-1.38993265748221	0.173971958657917	0.871233634295121	-5.04425101873867
a_HQ_transcript/104326	2.10753028919833	1.484002817733	1.38992669139286	0.173973754421128	0.871233634295121	-4.88337414655633
a_HQ_transcript/124582	-1.67144656474913	1.67973504300945	-1.38988109688249	0.173987478620377	0.871233634295121	-4.84391853830017
a_HQ_transcript/75282	-1.44161747523841	1.65103496425721	-1.38981271733841	0.174008062826822	0.871233634295121	-5.01434231519617
a_HQ_transcript/52615	1.30764949656915	-0.565985677228682	1.38980731211433	0.174009690035674	0.871233634295121	-4.94036463846046
a_HQ_transcript/14318	-0.383608380824183	3.68330859830242	-1.38979135652532	0.174014493435177	0.871233634295121	-5.29776788286671
a_HQ_transcript/5469	-2.22445449339724	1.89444700847964	-1.3897670327199	0.174021816269625	0.871233634295121	-4.987915966235
a_HQ_transcript/67231	1.39071664071062	1.61202658225478	1.38970551284862	0.174040338288427	0.871233634295121	-4.95634454866948
a_HQ_transcript/145196	1.1509143743148	1.5787443723994	1.38963689318794	0.174060999685376	0.871233634295121	-4.82524821049588
a_HQ_transcript/151052	-3.58972584442037	0.423790373389752	-1.38963269101675	0.174062265022464	0.871233634295121	-5.02606738617223
a_HQ_transcript/21173	1.27546041443733	0.84791553061527	1.38962937221174	0.174063264369761	0.871233634295121	-4.91363056722754
a_HQ_transcript/54458	1.56467272678333	0.380587272015337	1.38959647088889	0.174073171747007	0.871233634295121	-4.95814390546684
a_HQ_transcript/61058	1.48355422268847	-0.54258111718112	1.38959360748485	0.174074034007473	0.871233634295121	-4.94500228576371
a_HQ_transcript/76182	1.25059719372795	3.15195063392827	1.38955530880563	0.174085567257934	0.871233634295121	-5.16401197103045
a_HQ_transcript/64253	2.02833387632551	1.35976570068398	1.38950251233705	0.174101467346625	0.871233634295121	-4.93406692035879
a_HQ_transcript/23778	-0.932238649460042	2.97161919298981	-1.3894979538876	0.174102840214219	0.871233634295121	-5.22928355320616
a_HQ_transcript/114585	1.27517803173428	0.250839075366862	1.38940364880223	0.174131243962076	0.871241199681604	-4.88492940324546
a_HQ_transcript/115180	-1.0918880080464	3.30472821771389	-1.38937445124211	0.174140038709163	0.871241199681604	-5.17478888001282
a_HQ_transcript/106641	0.90012330730282	4.36891785813602	1.38934364959102	0.174149317009024	0.871241199681604	-5.36319252005627
a_HQ_transcript/150280	1.24546177471486	-0.560439200803707	1.38924147215938	0.174180098413124	0.871320203146132	-4.9489508040251
a_HQ_transcript/130693	1.74587946376026	2.84940255069381	1.38908993379335	0.174225757846793	0.871473612608072	-5.24868954917706
a_HQ_transcript/132240	-1.61829097163818	4.83618130560851	-1.38897067455376	0.174261697959879	0.871533277638961	-5.39860565541941
a_HQ_transcript/39995	-1.80268107515794	1.69933440075567	-1.38895085016003	0.174267672825448	0.871533277638961	-5.03663841226649
a_HQ_transcript/123621	1.49800223468902	-0.11015967545715	1.38889180395069	0.174285469685813	0.871547297134974	-4.93723842414437
a_HQ_transcript/50539	1.30909991747657	0.17648649702217	1.38877744678752	0.174319941618578	0.871567655948989	-4.8549272799908
a_HQ_transcript/45959	0.456083145940974	3.92479438225347	1.3887203860186	0.174337144063819	0.871567655948989	-5.34196227644792
a_HQ_transcript/122528	1.27685210393719	0.00155813031709255	1.38871093498775	0.174339993449665	0.871567655948989	-4.88827539285676
a_HQ_transcript/44639	-1.03532988234168	-0.717497905489007	-1.38867934871084	0.174349516642149	0.871567655948989	-4.92770308974057
a_HQ_transcript/21833	-1.21670967614893	-0.364046981674108	-1.38862151855877	0.174366953358244	0.871579866266058	-4.89660699659447
a_HQ_transcript/96784	1.55838034282533	1.31964940525154	1.38845273873781	0.174417850966684	0.871731695944655	-4.98246135485935
a_HQ_transcript/38011	1.47965932976794	0.121108131830956	1.38841314576974	0.174429792388795	0.871731695944655	-4.95656388619538
a_HQ_transcript/9925	0.978894155580429	1.65252590402755	1.38832761566366	0.174455590844542	0.871731695944655	-4.89819369203106
a_HQ_transcript/131396	-1.1523985674582	3.26757357782676	-1.38830541170667	0.174462288714073	0.871731695944655	-5.13524951525246
a_HQ_transcript/131042	-0.880323493437957	2.6013453245345	-1.38826202862822	0.174475375889176	0.871731695944655	-5.10029621401269
a_HQ_transcript/118692	1.5366021323188	1.18079000510472	1.38822247427777	0.17448730873789	0.871731695944655	-4.98399252678403
a_HQ_transcript/14838	-1.49934018277078	1.66237721414546	-1.38810765591847	0.174521951020411	0.871810385574351	-4.82429756486732
a_HQ_transcript/91876	0.713735302637958	3.15204313302992	1.38807085461811	0.174533055617409	0.871810385574351	-5.1060697513657
a_HQ_transcript/81302	1.39599624802492	1.80482623212517	1.38797255315605	0.17456272026839	0.871834148367718	-4.95796729768742
a_HQ_transcript/103771	-0.526226500238586	2.58313805182533	-1.38795568872035	0.174567809882533	0.871834148367718	-5.05506194763417
a_HQ_transcript/16834	2.65067915797749	1.3904789456377	1.38775252838218	0.174629131909806	0.872065479351648	-4.97732988916606
a_HQ_transcript/99479	1.11642197478733	-0.257687005101737	1.38756589966715	0.174685478852078	0.872271928074358	-4.88835828036166
a_HQ_transcript/141286	1.65336310250924	-0.567424137490193	1.38748564909699	0.174709712470847	0.872318000902373	-4.96251260323673
a_HQ_transcript/132698	4.43858293292266	2.53446069547133	1.38741728690112	0.17473035817617	0.87234615305887	-5.02866612155365
a_HQ_transcript/142404	0.319427100953697	10.3807776381082	1.38721813340572	0.174790514333677	0.872363664233162	-5.9867402665562
a_HQ_transcript/19427	2.07542535106606	1.66255118291081	1.38711592186398	0.174821394557781	0.872363664233162	-5.01770766465353

a_HQ_transcript/16203	-1.7623437705205	3.62675692235534	-1.38710572549645	0.174824475325377	0.872363664233162	-5.08433884491413
a_HQ_transcript/105655	2.47837296305095	3.43439591805058	1.38708359498979	0.174831162063105	0.872363664233162	-5.35190024213688
a_HQ_transcript/139847	1.51079770785928	-0.257940568085796	1.38708059584151	0.174832068271827	0.872363664233162	-4.95210186601748
a_HQ_transcript/126819	2.70828323303332	2.08172953048406	1.38706739021273	0.174836058466954	0.872363664233162	-5.02078657965615
a_HQ_transcript/144823	4.8553508231229	2.18672468537099	1.38705792310087	0.174838919080587	0.872363664233162	-4.92601854409141
a_HQ_transcript/117922	1.4224455567445	3.28444321982277	1.38689395596253	0.174888469730012	0.872530964894218	-5.19669595080045
a_HQ_transcript/113094	-2.75235658307467	1.04913468742002	-1.38676680511123	0.174926902097047	0.872530964894218	-5.03393340739764
a_HQ_transcript/79834	0.649635030119927	3.46030974051274	1.38669825316279	0.174947625211268	0.872530964894218	-5.20568929611211
a_HQ_transcript/138037	2.81406250632162	1.40249107206476	1.38665814672072	0.174959750195935	0.872530964894218	-5.04607705031443
a_HQ_transcript/117187	0.387512682294605	3.92284443651921	1.38665777249176	0.174959863335974	0.872530964894218	-5.28485818649829
a_HQ_transcript/47104	1.72604277647283	0.855422238491507	1.38663198715897	0.174967659111614	0.872530964894218	-4.86213404719424
a_HQ_transcript/883	-0.866376887900585	-0.749176288644335	-1.38659233909316	0.174979646588203	0.872530964894218	-4.93521521752033
a_HQ_transcript/70532	1.56372228805316	0.911275846617891	1.38654971846746	0.174992533524924	0.872530964894218	-4.90608681918369
a_HQ_transcript/137147	2.44102621460917	0.343887567621781	1.38636570043923	0.175048182434669	0.872534801892989	-4.92658896026685
a_HQ_transcript/113836	1.04617494014195	2.37485451235482	1.3862868930333	0.175072018816232	0.872534801892989	-5.01851020490279
a_HQ_transcript/17911	-0.839400281358297	2.44403009163672	-1.38626655613911	0.175078170400446	0.872534801892989	-4.8951093926889
a_HQ_transcript/1197	-1.08704911159304	0.870552852608976	-1.38624457916239	0.17508481827308	0.872534801892989	-4.83559186557472
a_HQ_transcript/63756	1.63865163801555	1.16442849889291	1.38622183200605	0.175091699326948	0.872534801892989	-4.80405608283892
a_HQ_transcript/2265	1.26431654108992	0.579217626309218	1.38607529732529	0.175136031381869	0.872534801892989	-4.82963237725406
a_HQ_transcript/77709	1.72475150325422	0.278471754918514	1.38602151776482	0.17515230384779	0.872534801892989	-4.96984051845826
a_HQ_transcript/98063	-1.78033405935449	0.658412028823889	-1.38596588287674	0.175169138935858	0.872534801892989	-4.98482939571739
a_HQ_transcript/108117	-2.28081200507517	0.86016692886693	-1.38594026003661	0.175176892818852	0.872534801892989	-4.93983579321263
a_HQ_transcript/19732	-0.690639330795098	2.04919436873277	-1.38585921091616	0.175201421349275	0.872534801892989	-4.89525296185048
a_HQ_transcript/140893	2.22224056552512	2.35843309235547	1.38574623624619	0.175235616233291	0.872534801892989	-5.03706779999125
a_HQ_transcript/6272	1.60850301577635	0.324212069922417	1.38571693341248	0.175244486391083	0.872534801892989	-4.91085193885332
a_HQ_transcript/147963	-2.05967581095604	0.751923884450834	-1.38561702674638	0.175274731422118	0.872534801892989	-5.00293210884247
a_HQ_transcript/92155	1.37590840936675	-0.000860409726834573	1.38558065872814	0.175285742228194	0.872534801892989	-4.84733523274432
a_HQ_transcript/143631	-1.31646733947983	-0.620958284550402	-1.38550596729095	0.175308357552395	0.872534801892989	-4.95654515369322
a_HQ_transcript/136675	-1.78976819459955	0.683722060672376	-1.38550176757992	0.17530962922265	0.872534801892989	-4.83047234557122
a_HQ_transcript/148223	-3.19360285223293	0.940974610773069	-1.38549591980348	0.175311399938229	0.872534801892989	-5.0152502259585
a_HQ_transcript/103636	-1.35862040037434	-0.142411708296401	-1.38548944096197	0.175313361757572	0.872534801892989	-4.93086739781956
a_HQ_transcript/21523	-1.77163529847319	1.94847805645623	-1.38548527681277	0.175314622687798	0.872534801892989	-5.06149515977542
a_HQ_transcript/47569	2.02334287500365	1.34152057656209	1.38542073155048	0.175334168299363	0.872534801892989	-4.93243236434985
a_HQ_transcript/17741	1.91508930612554	-0.0841679095170523	1.38541920878051	0.175334629445468	0.872534801892989	-4.98107219255944
a_HQ_transcript/97959	1.26916656373459	0.176934344060685	1.38538338759729	0.175345477581001	0.872534801892989	-4.89160158828462
a_HQ_transcript/76222	1.67941833375377	1.22031568587298	1.38536567341815	0.175350842360591	0.872534801892989	-4.93083321233798
a_HQ_transcript/17835	2.52903729486403	2.44995716662683	1.38516808042696	0.175410692536486	0.872534801892989	-5.04670618738138
a_HQ_transcript/25951	-2.42253237217971	2.7079501675649	-1.3850515140468	0.175446007534409	0.872534801892989	-5.15797744820395
a_HQ_transcript/82379	1.38481562189919	-0.576214259122645	1.38500653318101	0.175459636439887	0.872534801892989	-4.95203665567682
a_HQ_transcript/134627	-3.59080596994336	3.10606184101086	-1.38474938166645	0.175537567521172	0.872534801892989	-4.98848163730618
a_HQ_transcript/135504	2.90062146881751	1.20418397551052	1.38466983779162	0.175561679170042	0.872534801892989	-5.06871670594907
a_HQ_transcript/50916	-0.656808401994551	2.24956244779922	-1.38462125062212	0.175576408373765	0.872534801892989	-5.00023647035833
a_HQ_transcript/34266	1.40611261518326	1.1720528490791	1.38446685198745	0.175623220733448	0.872534801892989	-4.90447748858701
a_HQ_transcript/19687	1.33487206289924	1.57024961026022	1.38446228725594	0.175624604869678	0.872534801892989	-4.90609185791863
a_HQ_transcript/113958	-1.62880958257018	0.243835514946103	-1.38445784820795	0.175625950903814	0.872534801892989	-4.90321220129044
a_HQ_transcript/81277	1.81135450630936	0.603851148964179	1.38442635775066	0.17563549985592	0.872534801892989	-4.80256748155478
a_HQ_transcript/67865	0.350620455298663	4.17794435526037	1.38439302657376	0.175645607416792	0.872534801892989	-5.32476771966887
a_HQ_transcript/72431	1.99548597611682	-0.121777948392113	1.3843792349804	0.175649789801234	0.872534801892989	-4.9506944267795
a_HQ_transcript/129234	3.18563024872288	2.73072143961504	1.38427916071165	0.175680140259446	0.872534801892989	-5.14173226182445
a_HQ_transcript/49068	1.07741087390354	-0.591929621864264	1.38422370572227	0.175696960374531	0.872534801892989	-4.95756583991333

a_HQ_transcript/120321	-1.5317701495647	1.06059041109602	-1.38420032582384	0.175704052134434	0.872534801892989	-4.92683529123543
a_HQ_transcript/27275	2.52416172645724	0.989132993911539	1.38419013235736	0.175707144160357	0.872534801892989	-4.88389861415943
a_HQ_transcript/7400	-0.554555124237258	2.48134014418571	-1.38412988463051	0.175725420217328	0.872534801892989	-5.00509444484954
a_HQ_transcript/127070	0.611247896179888	1.87279889904446	1.38402336848717	0.175757735358902	0.872534801892989	-4.94566535722687
a_HQ_transcript/122294	-1.35321895651073	0.436725803379502	-1.3840206581645	0.175758557684072	0.872534801892989	-4.89283319750117
a_HQ_transcript/151282	1.64522546046508	1.35515300398495	1.38397990109435	0.175770923940822	0.872534801892989	-4.8558837907087
a_HQ_transcript/10586	1.73253594561112	1.84693687959877	1.38394804749484	0.175780589234627	0.872534801892989	-4.97283826624634
a_HQ_transcript/141797	0.971370056687993	1.11445431355833	1.3839282388882	0.175786599942018	0.872534801892989	-4.78430434497093
a_HQ_transcript/143389	1.18251218427532	3.23823917920543	1.38388312546503	0.175800289720895	0.872534801892989	-5.13025147846457
a_HQ_transcript/132934	0.387239352794429	5.58618025698835	1.3837807432844	0.175831360931438	0.872534801892989	-5.47969272268236
a_HQ_transcript/125006	3.41223317186078	2.87115035351919	1.38377064724142	0.175834425136964	0.872534801892989	-5.06370534666467
a_HQ_transcript/102424	-0.813414087613218	3.24256342396162	-1.38373994387892	0.175843744035327	0.872534801892989	-5.20446481678681
a_HQ_transcript/102766	1.84973338690403	-0.325857958042713	1.3836655790434	0.175866316396464	0.872534801892989	-4.98573079082617
a_HQ_transcript/74083	-0.685178550164093	2.01104119662445	-1.38361539733221	0.175881549600675	0.872534801892989	-4.88966376683843
a_HQ_transcript/26256	0.665385718222009	2.24819114628877	1.38358226555156	0.175891607677375	0.872534801892989	-4.96785702507001
a_HQ_transcript/33750	2.26987371201628	0.85855587725562	1.38348496452072	0.175921148715667	0.872534801892989	-5.01524627752007
a_HQ_transcript/56393	-1.0141609892187	1.38368523703877	-1.38347887769707	0.175922996831853	0.872534801892989	-4.86578928697072
a_HQ_transcript/149487	1.6472400983314	-0.446179229956867	1.38347339419645	0.175924661776627	0.872534801892989	-4.95357156009064
a_HQ_transcript/14365	-1.37067618259753	-0.262837627334711	-1.38338876540159	0.175950359014559	0.872534801892989	-4.89012928398637
a_HQ_transcript/32418	1.83995738912673	1.02702048802939	1.3833668946753	0.175957000460467	0.872534801892989	-4.88183636766334
a_HQ_transcript/149207	0.871046934157671	3.67852571786336	1.3833602235031	0.175959026322811	0.872534801892989	-5.23327384694817
a_HQ_transcript/36410	2.1730958388032	3.19330959456691	1.38315188804978	0.176022301568179	0.872534801892989	-5.23617164844562
a_HQ_transcript/139406	-2.17823291448321	0.492883556319748	-1.38313289136532	0.176028072088008	0.872534801892989	-4.96373110476833
a_HQ_transcript/12442	1.94996466467113	4.06829543987479	1.38301664066276	0.176063388149172	0.872534801892989	-5.10535497178249
a_HQ_transcript/95702	2.43015328595109	2.06842145247486	1.38299779111093	0.176069115017566	0.872534801892989	-5.09574307010531
a_HQ_transcript/60659	-1.57482195721599	0.312492894691756	-1.38294015089678	0.17608662816174	0.872534801892989	-4.92813800723835
a_HQ_transcript/92736	-1.46771208721662	2.36902243037547	-1.38288233476772	0.176104196120494	0.872534801892989	-5.03200342345866
a_HQ_transcript/83094	-2.25202143414167	1.70323110565298	-1.38285725536548	0.176111817151129	0.872534801892989	-4.96872716552139
a_HQ_transcript/28592	1.51026376011663	1.25507090014543	1.38282601043112	0.176121312099529	0.872534801892989	-4.8598530165246
a_HQ_transcript/118085	1.44276300288005	1.26881393959147	1.38282214382315	0.176122487141554	0.872534801892989	-4.96040580503277
a_HQ_transcript/124688	-1.65590151553438	-0.0167099563062456	-1.38275600013463	0.176142588811273	0.872534801892989	-4.88765072363313
a_HQ_transcript/73537	1.84034364371493	0.246708011314026	1.382677608624	0.176166415023609	0.872534801892989	-4.83943672144707
a_HQ_transcript/98457	-1.45914607198117	1.63955976960796	-1.38267713201279	0.176166559891882	0.872534801892989	-4.99695640820279
a_HQ_transcript/36486	1.23869581866443	0.673768116589253	1.38260746930127	0.17618773520814	0.872534801892989	-4.82193017903507
a_HQ_transcript/112220	1.59037554420546	0.128594740124412	1.38259489490334	0.176191557648944	0.872534801892989	-4.95667557880106
a_HQ_transcript/123703	-0.541658926157975	2.7203957371025	-1.38254962132063	0.176205320719633	0.872534801892989	-5.05227326442785
a_HQ_transcript/3964	-2.13343586010335	3.07266481953011	-1.3825354368624	0.176209632936641	0.872534801892989	-5.12515959747752
a_HQ_transcript/85217	-0.607487016427504	4.45407216706445	-1.38252691222041	0.176212224552581	0.872534801892989	-5.31720163073478
a_HQ_transcript/100571	1.00812876731448	2.14302298566596	1.38248420171716	0.176225209618443	0.872534801892989	-5.00627497056488
a_HQ_transcript/68332	-0.724790419420096	2.67518058953047	-1.38247119365642	0.176229164543965	0.872534801892989	-5.04340299320219
a_HQ_transcript/110157	-1.35509962244569	0.731631613389661	-1.38246918839717	0.176229774222169	0.872534801892989	-5.0161597365037
a_HQ_transcript/52794	-0.975491735658321	3.7458693894052	-1.38241270412076	0.176246948354518	0.872534801892989	-5.31663418280539
a_HQ_transcript/150027	0.804696619513415	4.08123989886155	1.38241062342799	0.176247581017334	0.872534801892989	-5.27710771679511
a_HQ_transcript/9552	1.85695312976551	-0.226610772890618	1.38240060204232	0.176250628180185	0.872534801892989	-4.87227706126169
a_HQ_transcript/141329	1.38080975398098	0.0439583348031519	1.38238734114864	0.176254660430519	0.872534801892989	-4.89816557494767
a_HQ_transcript/32871	1.4569476519995	1.29829240094371	1.38238161133728	0.176256402721015	0.872534801892989	-4.78445526489464
a_HQ_transcript/62428	1.10950765600448	-0.425425670063991	1.38231508727117	0.176276631988006	0.872534801892989	-4.91428963859733
a_HQ_transcript/65000	-1.31350756941086	5.51746161734925	-1.38230383045583	0.176280055246012	0.872534801892989	-5.5254873889934
a_HQ_transcript/148390	0.421722963285295	2.74648868803253	1.38228139264063	0.176286878860843	0.872534801892989	-5.14128203610831
a_HQ_transcript/129518	1.67239916266806	1.18731138113725	1.38224080285686	0.1762992232355	0.872534801892989	-4.80855300784271

a_HQ_transcript/132946	-1.31054475015076	-0.411885804040057	-1.38215262778916	0.176326041815978	0.872593236734136	-4.98897279312101
a_HQ_transcript/36424	0.683433172842719	2.6730904029607	1.38207519378787	0.176349596109298	0.872635508865351	-5.11897837849236
a_HQ_transcript/119046	2.56828823032444	1.2383245231511	1.38200880518062	0.176369792509131	0.872661159258662	-4.96157434363133
a_HQ_transcript/130667	3.06135542356534	2.41271754479007	1.38181507165302	0.176428739439031	0.872780275997791	-5.06322335956264
a_HQ_transcript/91495	0.856664434846147	1.69021497941296	1.38179139188051	0.176435945491967	0.872780275997791	-4.8968561264388
a_HQ_transcript/114675	0.826998504859062	2.86053512671548	1.38177885779289	0.176439759865569	0.872780275997791	-5.15183762462823
a_HQ_transcript/128422	0.966481611671842	-0.794758041986842	1.3816456214338	0.176480310330984	0.872780275997791	-4.97884785070781
a_HQ_transcript/138208	0.86289155316256	1.87991094610202	1.38161235059669	0.176490437439292	0.872780275997791	-4.92662241965129
a_HQ_transcript/152331	3.70965998235994	5.68643684058132	1.38153746549912	0.176513232918153	0.872780275997791	-5.60744987104852
a_HQ_transcript/122332	-1.70833690904004	0.317010269305917	-1.38153596411915	0.176513689970823	0.872780275997791	-4.94217275707326
a_HQ_transcript/49385	-2.01724762853907	0.055760848558294	-1.38149334450904	0.17652666469075	0.872780275997791	-4.86180157954087
a_HQ_transcript/69565	1.27030029352118	1.61951533662984	1.38145217261516	0.176539199387701	0.872780275997791	-4.94076262444261
a_HQ_transcript/25222	-1.64476196606862	1.36846996303488	-1.38138748141834	0.176558895889502	0.872780275997791	-5.09488347930469
a_HQ_transcript/105467	1.73819586114386	1.91643052207687	1.38125201990426	0.17660014535232	0.872780275997791	-4.96052723356026
a_HQ_transcript/80638	2.65959314192083	1.78125147643485	1.38121870048535	0.176610292619183	0.872780275997791	-4.91045407333648
a_HQ_transcript/11981	-0.891105730634541	1.51095193675773	-1.38119081814875	0.176618784398555	0.872780275997791	-4.85448958005505
a_HQ_transcript/106779	1.06295750301407	1.62894561184136	1.38112554160552	0.176638666115629	0.872780275997791	-4.90819460927572
a_HQ_transcript/24303	-1.60640685567571	1.34772626051035	-1.38107463964787	0.176654170879368	0.872780275997791	-4.93295447773512
a_HQ_transcript/34907	1.33354422559819	-0.464878589157472	1.38099014952725	0.176679908958579	0.872780275997791	-4.9652847023188
a_HQ_transcript/147400	5.25648761107458	1.63733240209682	1.38097281629049	0.176685189513988	0.872780275997791	-5.07941491827065
a_HQ_transcript/28141	0.387122573721967	3.99870158881263	1.38091593429518	0.17670251942876	0.872780275997791	-5.3100624393502
a_HQ_transcript/134272	1.70888056308652	2.21850975169726	1.38071722278043	0.176763070142296	0.872780275997791	-4.91933032206076
a_HQ_transcript/96010	1.06528121138817	-0.668419384931801	1.38064977802593	0.176783625361493	0.872780275997791	-4.9828770316797
a_HQ_transcript/101273	1.34803298428193	2.70005658247475	1.38059271747361	0.17680101723035	0.872780275997791	-5.2106481683123
a_HQ_transcript/118908	1.6113087225108	0.122121485263452	1.3805338221172	0.176818969740653	0.872780275997791	-4.95938208907589
a_HQ_transcript/18212	1.98182864825833	1.38330114027282	1.38045157850432	0.176844041655828	0.872780275997791	-4.92038790769113
a_HQ_transcript/42754	1.26481852010006	0.937480812403343	1.38044682779942	0.176845489989999	0.872780275997791	-4.80231101284225
a_HQ_transcript/137231	1.96417360170492	0.633355282971678	1.38043538869428	0.176848977436065	0.872780275997791	-4.81088050920113
a_HQ_transcript/57139	-1.70200228860754	3.98313458251575	-1.38043335648844	0.176849597001352	0.872780275997791	-5.4302091600264
a_HQ_transcript/124436	-1.42251741696609	-0.36124089881637	-1.38039781793102	0.176860432031941	0.872780275997791	-4.94780420539411
a_HQ_transcript/142562	-1.34118268687417	-0.743928067227296	-1.38039407315301	0.176861573773712	0.872780275997791	-4.94950977571172
a_HQ_transcript/15476	-1.59765365473501	0.322663561407832	-1.38039047001375	0.176862672336807	0.872780275997791	-4.85420249761719
a_HQ_transcript/77806	0.477229261831279	3.97595575182488	1.38037006038369	0.176868895140478	0.872780275997791	-5.32357395669545
a_HQ_transcript/104173	1.46174756161129	-0.299914795057687	1.38031904718026	0.176884449581529	0.872780275997791	-4.91291293779829
a_HQ_transcript/118422	1.55672645624733	2.90151816772973	1.38030382794758	0.176889090285873	0.872780275997791	-4.99486982610656
a_HQ_transcript/75160	2.50647775157993	0.470747740039329	1.3803029595938	0.176889355070362	0.872780275997791	-4.93433105743105
a_HQ_transcript/124127	4.04534240668367	4.4557510224376	1.38025290706844	0.176904617957593	0.872781499969356	-5.34419377935823
a_HQ_transcript/145831	-0.962505284884826	2.32174019601116	-1.38006916834155	0.176960655571654	0.872983873976842	-4.89563529939081
a_HQ_transcript/106167	-1.69465256746296	0.411580263540514	-1.37966893798678	0.177082767858972	0.873394036132028	-4.95081252231227
a_HQ_transcript/7764	-2.60614050963191	0.568265207056493	-1.37966230423931	0.177084792401865	0.873394036132028	-4.8760732260724
a_HQ_transcript/29464	1.3707036045295	2.31888659240052	1.3796489280257	0.177088874722201	0.873394036132028	-4.9831590460846
a_HQ_transcript/63357	2.08606860534155	1.92281184367721	1.37947277996218	0.177142640638157	0.873585086537268	-4.99796080438216
a_HQ_transcript/152461	-2.12393816617309	1.47359126313257	-1.37940254211264	0.177164082981965	0.873598239384101	-4.98747212097823
a_HQ_transcript/79752	1.1821796042394	1.11754854927703	1.37936558716389	0.177175365471015	0.873598239384101	-4.85115528143651
a_HQ_transcript/142631	-1.23247527048153	2.54671142079626	-1.37927310262264	0.177203603814635	0.873647973965268	-5.08155349219245
a_HQ_transcript/110	-1.67273997491823	5.93231067841261	-1.37922760935	0.1772174955818	0.873647973965268	-5.43946934221495
a_HQ_transcript/133733	-0.532663109992923	3.88504841612386	-1.37910913553609	0.177253676576952	0.873647973965268	-5.27222169258838
a_HQ_transcript/55124	1.10576137576949	-0.613861335635316	1.37910366098737	0.177255348601355	0.873647973965268	-4.96574362640414
a_HQ_transcript/110497	0.523350392887873	4.05690543182256	1.37908646438762	0.177260600828282	0.873647973965268	-5.38870148354808
a_HQ_transcript/7871	-0.961387121874667	1.89028736047723	-1.37902702205103	0.177278756784282	0.873663380615241	-5.00794505431677

a_HQ_transcript/49606	-1.98670721729012	3.69621484905869	-1.37891904762547	0.177311739998358	0.873737683302689	-5.18113478154988
a_HQ_transcript/7035	1.17129755423345	0.12749951259895	1.37887925616591	0.177323896399701	0.873737683302689	-4.8849131959159
a_HQ_transcript/120497	1.39553362616413	-0.27272477195315	1.37879068561306	0.177350957281581	0.873773560820902	-4.9905596362843
a_HQ_transcript/125659	-1.43104297190145	0.372632893748261	-1.37875702812	0.177361241469272	0.873773560820902	-4.90154305874679
a_HQ_transcript/132644	2.52975573733447	2.90863768415295	1.37866834724645	0.177388340506526	0.873833004827402	-5.25461885572767
a_HQ_transcript/140189	2.01828529586563	1.24185192895972	1.37847486654486	0.177447475399868	0.873836911270919	-5.1397500583858
a_HQ_transcript/145766	-2.57797804080786	5.12886695302286	-1.37847325979321	0.177447966547203	0.873836911270919	-5.41564496325936
a_HQ_transcript/76009	2.44936793220482	0.425926284260466	1.37844200654264	0.177457520163843	0.873836911270919	-4.95375238105341
a_HQ_transcript/86198	1.01576098431281	2.57876953545241	1.37843198723795	0.177460582989186	0.873836911270919	-5.17403792840411
a_HQ_transcript/29883	-0.604918934921467	2.92604783684589	-1.37834968844538	0.177485742663964	0.873836911270919	-5.02541142888352
a_HQ_transcript/106156	-3.28186269651894	1.27980577417518	-1.37834391409139	0.177487508053861	0.873836911270919	-4.98447165263025
a_HQ_transcript/112563	2.23242062415138	1.73968780032559	1.37827810864488	0.17750762768145	0.873836911270919	-5.1294811096656
a_HQ_transcript/25363	1.50668905731498	3.75336199525883	1.37827000118211	0.1775101066134	0.873836911270919	-5.0894868715868
a_HQ_transcript/15300	-1.02984671529604	1.71617950410698	-1.37822315658303	0.177524430312384	0.873836911270919	-4.87841481330181
a_HQ_transcript/18822	0.676846593853182	2.90810909621119	1.37804164192463	0.177579940656708	0.873978074461215	-5.1229969805295
a_HQ_transcript/138246	2.43025288692816	2.92689121251428	1.37791676248929	0.177618138816403	0.873978074461215	-5.07056164902349
a_HQ_transcript/98138	0.515718705840022	2.62081573957249	1.37788275256862	0.177628542890481	0.873978074461215	-5.04527810206498
a_HQ_transcript/48516	-0.967038283753002	2.17099890613946	-1.37787636667467	0.177630496470886	0.873978074461215	-4.93865833835277
a_HQ_transcript/138212	-1.31952926821257	-0.520580484836308	-1.37786202228975	0.177634884783515	0.873978074461215	-4.93828199599128
a_HQ_transcript/135206	-0.390196209955534	2.9324112765033	-1.37783444776306	0.1776433207707	0.873978074461215	-5.10371809183052
a_HQ_transcript/1167	0.896349111924824	-0.353025762489197	1.37770607365067	0.177682598895084	0.874097334848803	-4.91689126971642
a_HQ_transcript/78874	-1.44226222845626	-0.120218358387138	-1.37755492486397	0.177728853971018	0.874220494418843	-4.9275597281242
a_HQ_transcript/1261	0.742073743667045	4.26285850708449	1.37752597828228	0.177737713374654	0.874220494418843	-5.34821662276488
a_HQ_transcript/119842	-2.96452353654473	4.3331279073457	-1.37743775287624	0.177764717804113	0.874279339751037	-5.16767690775533
a_HQ_transcript/94791	-2.03302602383649	0.117154511513463	-1.37733175448593	0.177797166495897	0.874348343523891	-4.87475931614391
a_HQ_transcript/117223	1.51412679628075	2.16911194882544	1.37729365209095	0.177808831695498	0.874348343523891	-4.87546237939502
a_HQ_transcript/144070	1.60718918484547	0.0784296008123026	1.37724018114166	0.177825203044576	0.874354880948664	-4.95655352105375
a_HQ_transcript/115939	1.23183667986366	0.275421654231067	1.37711648880661	0.177863078770701	0.874467143938367	-4.89890100247669
a_HQ_transcript/78232	-1.52579188372222	-0.00738136778392456	-1.37700567644065	0.177897015869259	0.874560025240889	-4.97291209121459
a_HQ_transcript/136034	1.78403755107039	0.346581324879427	1.3769488550604	0.177914419789459	0.874571618902469	-4.91077221904799
a_HQ_transcript/14842	1.10748969658003	2.51394008481635	1.3767966131267	0.177961056776055	0.874650125987782	-5.05472214091001
a_HQ_transcript/103055	2.17488795396319	0.218008998449115	1.37679088411971	0.177962811955583	0.874650125987782	-4.9587648605556
a_HQ_transcript/89091	0.86156533398001	1.97185551280364	1.37672310382392	0.177983578634951	0.874650125987782	-4.93456114150295
a_HQ_transcript/139412	1.6324797413885	0.62880189882686	1.37669827479564	0.177991186280814	0.874650125987782	-4.89338633239782
a_HQ_transcript/47749	0.749844244238506	-0.850149373006588	1.37665115236017	0.17800562535156	0.874650125987782	-4.98807991006642
a_HQ_transcript/138287	1.41635407595172	-0.0492450166137098	1.37659443884317	0.178023004493514	0.87466158441374	-5.00730672903377
a_HQ_transcript/147448	2.79268147360378	1.31675077313561	1.37628023126195	0.178119313389889	0.874800668031822	-5.06941952199038
a_HQ_transcript/61439	1.17647578536075	0.516068263643863	1.3762538585419	0.178127398832877	0.874800668031822	-4.83634672724927
a_HQ_transcript/94945	1.7707427753198	-0.25923902016233	1.3762417548271	0.178131109728824	0.874800668031822	-4.95309022847239
a_HQ_transcript/22392	2.07040209013839	-0.107204436070163	1.37620278423026	0.178143058191185	0.874800668031822	-4.92386349918428
a_HQ_transcript/108644	-1.64945378103163	2.16165409990041	-1.37619939477401	0.178144097434706	0.874800668031822	-5.01350092380085
a_HQ_transcript/53309	-1.46675352209415	0.968918947525131	-1.37614290229724	0.178161419327248	0.874800668031822	-4.87898781511489
a_HQ_transcript/69485	1.68457220842253	0.273119860668823	1.37612506681048	0.178166888370099	0.874800668031822	-4.98330940364159
a_HQ_transcript/59453	0.724717624028039	0.491440521129966	1.37610934581001	0.178171709138947	0.874800668031822	-4.92164520591187
a_HQ_transcript/133201	0.442950659082081	4.21400161615667	1.37601095199498	0.178201883434694	0.874803493272621	-5.3864547586328
a_HQ_transcript/41815	1.52236774429412	0.447959580249976	1.37599022286048	0.17820824091735	0.874803493272621	-4.88821220527879
a_HQ_transcript/80532	2.06695457993166	1.57069292630465	1.37579842160471	0.178267073419161	0.874803493272621	-5.19210023850362
a_HQ_transcript/142699	1.76080330550657	-0.0840297738641648	1.37571115816768	0.178293845331724	0.874803493272621	-5.01498071694404
a_HQ_transcript/134806	-2.05690831731315	1.08581902032703	-1.3754981040454	0.178359222245613	0.874803493272621	-4.95466067456258
a_HQ_transcript/101315	1.57017305118463	-0.262516701510288	1.37547157871972	0.178367363004804	0.874803493272621	-5.0061989064654

a_HQ_transcript/68065	1.0538575468509	5.23449115610711	1.37545695235256	0.178371852036045	0.874803493272621	-5.45737404466176
a_HQ_transcript/10201	0.987781623729323	2.07879093735101	1.37536826832003	0.178399072257063	0.874803493272621	-5.05386070532591
a_HQ_transcript/152835	1.42317385764921	-0.300341091892895	1.37536312283934	0.178400651683727	0.874803493272621	-4.99175199148099
a_HQ_transcript/86472	-0.564580411613079	2.82594636705039	-1.3753520509577	0.178404050280696	0.874803493272621	-5.15457290922086
a_HQ_transcript/140950	-2.16007157386363	5.7966382431603	-1.37532273417987	0.178413049528636	0.874803493272621	-5.4342543830405
a_HQ_transcript/83450	1.93015635202338	1.65901061319747	1.37527007865169	0.178429213862159	0.874803493272621	-5.15152478244513
a_HQ_transcript/95651	1.02736233008322	0.532566089359937	1.37525791086662	0.17843294932325	0.874803493272621	-4.8169472338858
a_HQ_transcript/148593	3.38916280985647	0.87608566932416	1.37523310830268	0.178440563799221	0.874803493272621	-5.06371564669503
a_HQ_transcript/13819	-1.12612081285203	2.02194714960889	-1.37521099508306	0.178447352850204	0.874803493272621	-4.93095755552392
a_HQ_transcript/45887	-0.753803225249787	3.1209313925554	-1.3751974523597	0.17845151074475	0.874803493272621	-5.07641991557275
a_HQ_transcript/71295	-1.0914803297642	3.57286369554707	-1.3751749558468	0.178458417803535	0.874803493272621	-5.17495840904999
a_HQ_transcript/135525	-0.661630475365969	3.52437922661058	-1.37516834566452	0.178460447353976	0.874803493272621	-5.28677354303122
a_HQ_transcript/79385	0.886380249189155	2.62219944749419	1.37510983165253	0.178478413926503	0.874803493272621	-5.13313552512508
a_HQ_transcript/87130	-3.32669724293496	2.07713721598707	-1.37497055039214	0.178521185534908	0.874803493272621	-5.02302019961189
a_HQ_transcript/110904	-2.01780410909833	-0.196021474805	-1.37496177462008	0.178523880736734	0.874803493272621	-4.97391766194402
a_HQ_transcript/64452	1.61905480090887	1.25361066726221	1.37495469338504	0.178526055538016	0.874803493272621	-5.01992117026814
a_HQ_transcript/10453	1.97159119692851	2.59818601115917	1.37494748493952	0.178528269429587	0.874803493272621	-4.95537332185291
a_HQ_transcript/95097	1.66309564956895	0.387424248441956	1.37493053551214	0.178533475101382	0.874803493272621	-4.91079442007831
a_HQ_transcript/131130	2.74369089086438	0.943606452374772	1.37476214913582	0.178585197970582	0.874858307452465	-4.96617917516959
a_HQ_transcript/32258	1.01525528293401	2.06329392318475	1.37475499255335	0.178587396500549	0.874858307452465	-4.97018660235945
a_HQ_transcript/14959	1.50350151120334	1.57366139648257	1.37474712505759	0.178589813450161	0.874858307452465	-4.93549094875798
a_HQ_transcript/4366	-1.71614763269341	0.351207282686548	-1.37459124711262	0.178637705493812	0.874936475890131	-4.97281759451149
a_HQ_transcript/32190	-0.945904860286694	2.54608559142074	-1.37453500974193	0.178654986355204	0.874936475890131	-4.95148239132651
a_HQ_transcript/122806	1.83050186760904	1.74893323761237	1.37452429925197	0.178658277668198	0.874936475890131	-4.96357708622001
a_HQ_transcript/22454	1.14746761650383	1.94742864417747	1.37449924168873	0.178665977993667	0.874936475890131	-4.95964298982628
a_HQ_transcript/82007	1.70380399132423	1.35604017525712	1.37434671347189	0.178712856317601	0.875037738220974	-4.99290615196604
a_HQ_transcript/34866	-1.71716067119064	1.28296003483458	-1.37420455963263	0.178756554779671	0.875037738220974	-4.84822712094424
a_HQ_transcript/123855	1.0150705543028	-0.561374702396732	1.37419709558649	0.178758849476793	0.875037738220974	-4.96578465998941
a_HQ_transcript/132836	1.84504108083231	-0.208148448433514	1.37412982006998	0.178779533252717	0.875037738220974	-4.9989877650339
a_HQ_transcript/126133	1.54215124945941	-0.182868180803957	1.37399966159964	0.178819555605273	0.875037738220974	-5.01739510434825
a_HQ_transcript/63575	1.51777493845039	3.08047792824527	1.37397262751257	0.178827869175662	0.875037738220974	-5.09041009328638
a_HQ_transcript/60417	-1.0883043185586	-0.0558928941056255	-1.3739582991889	0.178832275569144	0.875037738220974	-4.85915483135382
a_HQ_transcript/100926	1.16731931606274	0.12517790688293	1.37392937201264	0.178841171809493	0.875037738220974	-5.00125515317164
a_HQ_transcript/76598	0.967903980676178	0.882900206348756	1.37380868955357	0.178878290111614	0.875037738220974	-4.89041297872643
a_HQ_transcript/44153	-1.1280594244962	0.614523815397307	-1.37376554448866	0.178891561693574	0.875037738220974	-4.93094953680834
a_HQ_transcript/142373	-1.56850345113967	1.17098040776648	-1.37375714375415	0.1788941458798	0.875037738220974	-4.8550500164921
a_HQ_transcript/12810	1.60041101644476	0.905124583878944	1.37372433011371	0.178904240103864	0.875037738220974	-4.84524692769012
a_HQ_transcript/33840	-1.73464344983329	0.330265821800037	-1.37368789420442	0.17891544913985	0.875037738220974	-4.96652267174842
a_HQ_transcript/2474	0.908178030691356	-0.564514532688824	1.3736719645068	0.178920349876911	0.875037738220974	-4.961701734872
a_HQ_transcript/111516	-1.71345928780657	1.9980929152938	-1.37360752697966	0.178940175009064	0.875037738220974	-5.02935028921728
a_HQ_transcript/129209	0.39818248191686	5.53439569445463	1.37353893475425	0.178961280272512	0.875037738220974	-5.48220665507276
a_HQ_transcript/95992	-1.48011572802508	0.657157231352019	-1.37353597754562	0.178962190224838	0.875037738220974	-4.88588883581829
a_HQ_transcript/47391	1.42599637471923	-0.114639972602692	1.37345067979987	0.178988438445922	0.875037738220974	-4.96602038354342
a_HQ_transcript/6472	0.324685355379162	5.03485394582842	1.37331759078454	0.179029399204443	0.875037738220974	-5.46259052551545
a_HQ_transcript/68033	-0.524256826408532	4.07615878815187	-1.37323855907435	0.179053726217595	0.875037738220974	-5.36493296991388
a_HQ_transcript/17660	1.97087188889801	2.48655104594624	1.37316969178336	0.179074926584603	0.875037738220974	-5.0733526743447
a_HQ_transcript/4471	1.22559610115589	1.0547386420038	1.37313867027487	0.179084477001103	0.875037738220974	-4.91215729485969
a_HQ_transcript/25540	1.50414116669348	0.71888784029018	1.37301187087244	0.179123518137906	0.875037738220974	-4.94740115068223
a_HQ_transcript/129116	-1.10858364547485	-0.385950486591754	-1.37298847463803	0.179130722488562	0.875037738220974	-4.89039439135513
a_HQ_transcript/139685	1.54532617974458	0.78456772369193	1.37291029088474	0.179154799074374	0.875037738220974	-4.86229108057775

a_HQ_transcript/81735	1.55399692974296	0.745617521756406	1.37282674803203	0.179180528770912	0.875037738220974	-4.87625483990409
a_HQ_transcript/32734	-1.69630198335129	-0.070735890203596	-1.37276139433998	0.179200658537469	0.875037738220974	-4.95313564688181
a_HQ_transcript/68464	1.42806559279015	0.463748903868417	1.37274368973876	0.179206112082257	0.875037738220974	-4.79832887758098
a_HQ_transcript/84506	-1.70104232467466	0.621245283422551	-1.37272568500378	0.179211658209629	0.875037738220974	-4.92997226916378
a_HQ_transcript/104037	-3.09337963412597	3.85472369035077	-1.37267783865871	0.179226397310798	0.875037738220974	-5.47313140683929
a_HQ_transcript/7534	1.47958176824275	0.713166543771773	1.37267352126885	0.179227727332259	0.875037738220974	-4.8403378365384
a_HQ_transcript/16247	-0.826644577335618	2.09403091916927	-1.37261421297719	0.179245998709095	0.875037738220974	-4.95007526826044
a_HQ_transcript/108907	1.60555520885446	1.07946136195611	1.37252047484455	0.17927488000024	0.875037738220974	-4.88046378989666
a_HQ_transcript/63393	-0.685969342725282	2.64452425794822	-1.37248552088637	0.179285650455013	0.875037738220974	-5.10988221714938
a_HQ_transcript/120101	0.278115070053304	6.44655949597563	1.37245885045377	0.179293868823964	0.875037738220974	-5.58325689784774
a_HQ_transcript/5562	1.72205681708381	1.43559529377207	1.37244623747716	0.179297755555749	0.875037738220974	-4.89542196008198
a_HQ_transcript/38007	-1.19509536026025	0.390064448588892	-1.37243738668258	0.179300482997445	0.875037738220974	-4.88285409064609
a_HQ_transcript/106293	1.96022655946042	0.539043900102711	1.37239376633467	0.179313925421021	0.875037738220974	-4.88422350501198
a_HQ_transcript/35683	-0.473271869619026	3.60657598283023	-1.3723660960727	0.17932245293466	0.875037738220974	-5.2619696762397
a_HQ_transcript/95226	-1.24183843488726	4.03082695606475	-1.37232757852168	0.17933432392734	0.875037738220974	-5.36431278849321
a_HQ_transcript/96780	2.28356224759443	1.1810364389908	1.3723063277035	0.179340873626925	0.875037738220974	-4.80943569164634
a_HQ_transcript/12419	1.24999192090737	0.735468596934087	1.37221537001804	0.179368909733749	0.875037738220974	-4.93275872234951
a_HQ_transcript/28761	-2.26116817082029	1.63059150805092	-1.37221130647983	0.179370162327504	0.875037738220974	-4.97646770974373
a_HQ_transcript/24751	-1.713065757243	0.778371154670545	-1.37219937013861	0.179373841767839	0.875037738220974	-4.85104575862046
a_HQ_transcript/128040	-1.03379373875309	-0.328443363207528	-1.37215433394673	0.179387724941031	0.875037738220974	-4.93038538633363
a_HQ_transcript/71948	2.13210964685653	1.21917460031699	1.37211055613336	0.179401220998891	0.875037738220974	-5.02252885601255
a_HQ_transcript/127265	-1.77452484877238	1.49938959160661	-1.37205120884401	0.179419518159189	0.875037738220974	-4.90701981597918
a_HQ_transcript/4257	-1.57280361785413	2.03856735125286	-1.37199979964318	0.179435369128007	0.875037738220974	-4.975179029159
a_HQ_transcript/140212	1.53422799564648	0.743701246781249	1.3719458616984	0.179452000954106	0.875037738220974	-5.03295545833905
a_HQ_transcript/1390	-1.98214228427185	0.797668461431192	-1.3718834948112	0.179471233349173	0.875037738220974	-5.05796310377153
a_HQ_transcript/120061	2.09170660508464	2.30575409715193	1.37185026607108	0.179481480919975	0.875037738220974	-5.14770771095761
a_HQ_transcript/133418	-1.3808779148169	0.258650289160927	-1.37185001347144	0.179481558822135	0.875037738220974	-4.90540961979509
a_HQ_transcript/76847	1.11374505442903	0.282274097332392	1.3718404802183	0.179484498912893	0.875037738220974	-4.9112367658514
a_HQ_transcript/61902	-1.4876969780986	1.39125245587367	-1.37167512527469	0.179535500954251	0.875125521669839	-5.02507946157269
a_HQ_transcript/150700	2.79783679576232	-0.286103324785431	1.37166974555194	0.179537160464061	0.875125521669839	-5.05344377985319
a_HQ_transcript/16763	-1.18438524764405	1.49539298069223	-1.371552027585	0.179573476497743	0.875125521669839	-4.84393111522604
a_HQ_transcript/141054	0.410394786815681	2.95730563274839	1.37152084297759	0.179583097917282	0.875125521669839	-5.14713175118433
a_HQ_transcript/274	-0.977485727129527	3.02886576874993	-1.37151255749596	0.179585654313022	0.875125521669839	-5.21243545494912
a_HQ_transcript/78827	0.810644554814584	1.80751714889432	1.37142254693552	0.179613427922062	0.875125521669839	-4.92175188401473
a_HQ_transcript/82285	2.45376388054216	1.99503896880666	1.37140332044678	0.179619360869894	0.875125521669839	-5.02760110154015
a_HQ_transcript/9	-0.567628020920617	4.34063350640377	-1.37139170294305	0.179622945896003	0.875125521669839	-5.37587291317168
a_HQ_transcript/136134	1.16819872332295	4.46777441882475	1.37126650689153	0.179661583468704	0.875218179419768	-5.43684431948031
a_HQ_transcript/105301	-1.82280628228292	1.90776406046752	-1.37123250625915	0.179672077742501	0.875218179419768	-4.98016407961979
a_HQ_transcript/116815	1.82016765042128	0.609583955732865	1.37117979055096	0.179688349355352	0.875224096809778	-4.8588623962539
a_HQ_transcript/76816	-2.65225142393216	1.80502979989098	-1.37108964422185	0.179716177231011	0.875232965846625	-5.05248682409561
a_HQ_transcript/84558	-0.916507240344205	-0.722544337809106	-1.37100625356659	0.17974192263685	0.875232965846625	-4.93451970566231
a_HQ_transcript/23128	-0.48166174527069	2.79247291721504	-1.37098282248636	0.179749157089147	0.875232965846625	-5.11125204251927
a_HQ_transcript/138992	-1.82712531803853	0.776051536601994	-1.37090981220115	0.179771700798133	0.875232965846625	-5.09426740963789
a_HQ_transcript/136138	3.21954131920258	3.63555505292918	1.37089768468582	0.179775445677693	0.875232965846625	-5.24207785026927
a_HQ_transcript/90786	-1.76754927246817	1.32666648405741	-1.37082473998511	0.179797971696791	0.875232965846625	-4.9175724261991
a_HQ_transcript/122238	0.42686197504162	3.04267948082525	1.3706238512927	0.179860019387864	0.875232965846625	-5.22116966911193
a_HQ_transcript/143737	0.42463200690737	3.91206331837182	1.37061173539891	0.179863762108154	0.875232965846625	-5.2812521732105
a_HQ_transcript/38755	-0.772833352873092	3.81837747813703	-1.37060281930714	0.179866516416415	0.875232965846625	-5.14782536833932
a_HQ_transcript/90368	1.57086131120707	2.19362248420426	1.37059115436039	0.179870119934816	0.875232965846625	-4.97482283627236
a_HQ_transcript/12719	-0.307239971176706	3.94223590132333	-1.37057617684681	0.179874746848872	0.875232965846625	-5.31151996577952

a_HQ_transcript/10842	1.24826242790876	2.34890942334526	1.37057546569758	0.17987496654227	0.875232965846625	-5.0203050185815
a_HQ_transcript/103873	-2.36441248945849	3.24099671812955	-1.37054003879246	0.179885911143849	0.875232965846625	-5.24962951753763
a_HQ_transcript/7034	2.10476763596446	2.17700727296066	1.37042094378666	0.179922707524823	0.875338729745806	-4.93606474511631
a_HQ_transcript/127643	-1.70710911358204	0.303998479349079	-1.3703191308733	0.179954168956779	0.875355143074037	-4.93368606719511
a_HQ_transcript/133202	1.16158892626252	-0.113814546951903	1.37031256053818	0.179956199417402	0.875355143074037	-4.9794673435258
a_HQ_transcript/137068	-1.65690456144746	0.287482328804464	-1.37022694972834	0.179982657744796	0.875370761598921	-4.91561876554395
a_HQ_transcript/54311	1.26169710187931	0.449623238810593	1.37020471806148	0.179989529015798	0.875370761598921	-4.92533089534103
a_HQ_transcript/7581	-0.716770659164154	2.82433785388758	-1.36992382242843	0.180076364640252	0.875710394336074	-5.24614282217807
a_HQ_transcript/144014	-1.36782900015282	1.59074908507874	-1.36988136374679	0.180089493082395	0.875710394336074	-4.89652774353056
a_HQ_transcript/20922	-0.377594642904133	4.56517980024536	-1.36972959720528	0.180136426159848	0.875741217991853	-5.41777960201592
a_HQ_transcript/151963	5.32752789861913	6.14037778913465	1.36971982691891	0.180139447899989	0.875741217991853	-5.44128693911192
a_HQ_transcript/1195	1.54060905794899	-0.0382947299208034	1.36968992578817	0.180148695923645	0.875741217991853	-4.92811433333554
a_HQ_transcript/61	-0.635047390286917	2.30276578529221	-1.36966600391842	0.180156094906871	0.875741217991853	-5.01169085214017
a_HQ_transcript/78891	1.13777944029839	-0.330623504766931	1.36955141554336	0.180191540123124	0.875788665864967	-5.03385588698825
a_HQ_transcript/35693	-0.53379709181229	3.85115208723287	-1.36952812462588	0.180198745283119	0.875788665864967	-5.30197066940017
a_HQ_transcript/111178	2.84552332632985	0.446817399406371	1.36948833321291	0.180211055470872	0.875788665864967	-4.9951943963888
a_HQ_transcript/87049	2.08178480279271	0.284629363810795	1.36937790393522	0.180245222174842	0.875881480904465	-4.98373650631158
a_HQ_transcript/30190	1.73727878074968	-0.0249605168536455	1.3691118394367	0.180327562938611	0.875899309271415	-5.03038134534452
a_HQ_transcript/75403	-1.54054953361979	0.153414625982413	-1.36903725620478	0.180350649957933	0.875899309271415	-4.95723661446438
a_HQ_transcript/72746	-1.51062900202638	2.21497639366606	-1.36902076750538	0.180355754296295	0.875899309271415	-5.16736566750345
a_HQ_transcript/18587	0.845605274098319	2.81764883470359	1.36897039625468	0.180371348211247	0.875899309271415	-4.92488171502525
a_HQ_transcript/129060	1.27410101310554	3.55707706630925	1.36893809675186	0.180381348032024	0.875899309271415	-5.233508431928
a_HQ_transcript/151784	-4.37722955582724	0.431659760561373	-1.36893336069374	0.180382814336406	0.875899309271415	-4.99159537964951
a_HQ_transcript/63958	-1.83494782615187	1.12163275990477	-1.36892141707113	0.180386512175536	0.875899309271415	-5.04415631051995
a_HQ_transcript/151198	1.70761323002663	0.701398113727775	1.36889339761877	0.180395187448774	0.875899309271415	-5.12410897926176
a_HQ_transcript/9876	-1.35472950286798	-0.00246910917071473	-1.36888822814228	0.180396788037302	0.875899309271415	-4.953228214217
a_HQ_transcript/152095	1.32077583836398	-0.147703809511619	1.3688792251324	0.180399575602074	0.875899309271415	-4.95748165170333
a_HQ_transcript/25771	1.68968837932209	-0.386011545013957	1.36881884877203	0.180418270550433	0.87591691560641	-4.96135060461786
a_HQ_transcript/47230	-1.65757641853645	0.488673835679288	-1.36870890070926	0.180452318758876	0.875941497112708	-4.90902682027504
a_HQ_transcript/141027	1.28343000418621	3.17830731305982	1.36863313636707	0.180475784017081	0.875941497112708	-5.05745476710247
a_HQ_transcript/33730	1.95171139931924	1.04332789086069	1.36859327530403	0.180488130488341	0.875941497112708	-5.15338027385216
a_HQ_transcript/110378	-1.6610011486127	1.42493408195161	-1.3685482148417	0.180502088199751	0.875941497112708	-4.9689881915161
a_HQ_transcript/3862	0.915361539260248	3.29719579478044	1.36848781774227	0.180520797829753	0.875941497112708	-5.21367072376738
a_HQ_transcript/87176	2.27751238600923	0.893199498350448	1.36847588127155	0.180524495651776	0.875941497112708	-4.95500155649892
a_HQ_transcript/110927	-1.82357053517125	1.03314366493348	-1.36846192893754	0.180528818046557	0.875941497112708	-5.11516547778882
a_HQ_transcript/125232	-1.29293400908412	1.11813131965062	-1.36828515178646	0.180583590089106	0.876097084034257	-4.99644126819446
a_HQ_transcript/145668	0.845998488337133	3.0851048402082	1.36824826396577	0.180595020916908	0.876097084034257	-5.23458191840918
a_HQ_transcript/66304	-0.683691812039773	1.89678110371012	-1.3682125142402	0.180606099607461	0.876097084034257	-4.90452026144267
a_HQ_transcript/122481	0.791810503806275	2.9186787953794	1.36806454366592	0.180651960687787	0.876104947592954	-5.16275675257331
a_HQ_transcript/51019	1.55393370472003	-0.274769027171621	1.36800704370542	0.180669784309825	0.876104947592954	-4.99159257548874
a_HQ_transcript/6765	1.55181003533553	0.00314194687112437	1.367989300962	0.180675284414773	0.876104947592954	-5.01420838793642
a_HQ_transcript/86570	-1.28842740489297	2.09240594768194	-1.36796665742389	0.18068230391488	0.876104947592954	-4.87030613857872
a_HQ_transcript/99839	-1.42603094583715	-0.370617997978791	-1.36796415188539	0.180683080645202	0.876104947592954	-4.94195434250449
a_HQ_transcript/72215	-1.40143012432162	-0.0362673011158108	-1.3675755808077	0.180803571165075	0.876616063448451	-4.87472425744535
a_HQ_transcript/17755	1.33577568631551	0.286278806765132	1.36741232873192	0.180854211999824	0.876725610718177	-4.83596358473585
a_HQ_transcript/2268	-0.438782653575883	2.99938607830141	-1.3674054990026	0.180856330823728	0.876725610718177	-5.08222082872846
a_HQ_transcript/89705	1.98527950128096	1.37452643721022	1.3672856025499	0.180893530090906	0.876799057865747	-5.00670662358855
a_HQ_transcript/32040	-2.21651823335008	2.93640936409707	-1.36725943449492	0.180901649824248	0.876799057865747	-5.22239918028693
a_HQ_transcript/58185	-0.629511445461929	2.51590337105439	-1.36711546748383	0.180946326674664	0.876838649331067	-4.95157892464978
a_HQ_transcript/57141	0.906670999713923	-0.55603521331935	1.36704853723976	0.180967099855371	0.876838649331067	-4.93953639000617

a_HQ_transcript/37490	1.61293232159431	-0.494570553935074	1.36703584784568	0.180971038479294	0.876838649331067	-4.94418660756703
a_HQ_transcript/144556	-0.527186072619373	2.84708703497151	-1.36694082475042	0.181000534536735	0.876838649331067	-5.09751934747261
a_HQ_transcript/1642	-0.914768081356138	2.91435175058677	-1.36690713346383	0.181010993524054	0.876838649331067	-5.04353020198499
a_HQ_transcript/144285	1.71199644243827	2.73367087508716	1.36689441661671	0.181014941412918	0.876838649331067	-5.15525233071914
a_HQ_transcript/13845	-1.91296455833861	2.03344084536077	-1.36680776551321	0.181041843645105	0.876838649331067	-5.08529812942424
a_HQ_transcript/143231	3.88543992511369	1.2202152735259	1.36669984164946	0.181075354692201	0.876838649331067	-4.98954179901996
a_HQ_transcript/39921	1.57262609820832	1.59255267991155	1.3666875614297	0.181079168083991	0.876838649331067	-5.089609985855
a_HQ_transcript/131120	-0.879181134844662	3.34066774070598	-1.36667990906903	0.181081544412542	0.876838649331067	-5.1872429872456
a_HQ_transcript/144418	1.64600353854403	-0.377424418412933	1.36662813524728	0.1810976226512	0.876838649331067	-4.9738196219871
a_HQ_transcript/114116	-0.859167992514847	1.64571336256785	-1.36652466070661	0.181129759749769	0.876838649331067	-4.95413335380814
a_HQ_transcript/123045	2.92720893270821	1.74253917042039	1.36650675211548	0.181135322245509	0.876838649331067	-5.0188907298961
a_HQ_transcript/38314	-1.06334205377893	1.83116510080633	-1.36650622367669	0.181135486383187	0.876838649331067	-4.94435344315409
a_HQ_transcript/150606	0.826829078975816	3.14585548926043	1.36643978632709	0.181156123321813	0.876838649331067	-5.1635283520518
a_HQ_transcript/139179	0.511460540796026	3.22749864730814	1.36642316077468	0.181161287878923	0.876838649331067	-5.21771170215285
a_HQ_transcript/152379	1.74847503565037	-0.00609621231022329	1.36637171496054	0.181177269717279	0.876838649331067	-4.87221849521141
a_HQ_transcript/43856	-2.27069695451386	3.7187457108039	-1.36631352431406	0.181195348183516	0.876838649331067	-5.30383860495913
a_HQ_transcript/55020	-1.5533845903699	1.15574679104412	-1.36624713675993	0.181215974947544	0.876838649331067	-5.0077413662284
a_HQ_transcript/24866	2.679493101886	0.964609235470442	1.3662287705307	0.181221681698817	0.876838649331067	-5.06781261601873
a_HQ_transcript/82444	1.2285609820883	1.29643409362828	1.36621295720185	0.181226595325204	0.876838649331067	-4.92848939188097
a_HQ_transcript/141255	2.76628822741382	1.21546825103302	1.36605191255854	0.181276642119082	0.876902329530132	-5.04811341465431
a_HQ_transcript/94241	-1.88152060149544	1.80808551525513	-1.36604803215644	0.181277848138779	0.876902329530132	-4.99535752174551
a_HQ_transcript/134854	1.28500640570693	-0.569897793762357	1.36602497620163	0.181285014002952	0.876902329530132	-4.98332766824564
a_HQ_transcript/43638	3.21554892967182	4.45091902498793	1.36563154290856	0.181407328237015	0.877420966530304	-4.99834625442097
a_HQ_transcript/27065	-1.11297336003032	3.173540909605	-1.36556269954837	0.181428737494226	0.877434784303537	-5.1861282563091
a_HQ_transcript/49969	-1.70563042876952	1.32783464257522	-1.36546285963328	0.181459789703667	0.877434784303537	-4.94208856834878
a_HQ_transcript/49436	0.839258020375512	-0.847402804271347	1.36544336337436	0.181465853911599	0.877434784303537	-4.99101791255021
a_HQ_transcript/82198	1.64135992387836	0.91430441181182	1.36542821911216	0.18147056456264	0.877434784303537	-4.79750024055679
a_HQ_transcript/152101	-1.60499847576922	1.81861292461302	-1.36523469535817	0.181530768848576	0.877558122541278	-5.06940917189212
a_HQ_transcript/50955	1.20240272815295	-0.18656371149619	1.36519717481664	0.181542443099025	0.877558122541278	-5.01804368593069
a_HQ_transcript/142960	1.7081242622932	-0.127096726424703	1.36515498293971	0.181555571498765	0.877558122541278	-4.9501078677865
a_HQ_transcript/147266	1.22845464336066	1.20354953631334	1.3651112040066	0.181569194505338	0.877558122541278	-4.88178002786303
a_HQ_transcript/138545	-2.1174384304299	2.52794904856781	-1.36510360802748	0.181571558281791	0.877558122541278	-5.21298124249883
a_HQ_transcript/145038	-1.60191509240753	-0.271145875916947	-1.36490900049997	0.181632125929939	0.877743659345465	-4.9630589314519
a_HQ_transcript/21447	1.22076989278971	1.03913769910086	1.36488323135068	0.181640147231396	0.877743659345465	-4.86689471634469
a_HQ_transcript/141364	1.30106381612098	-0.378656128134101	1.36483073661355	0.181656488400983	0.877749655675174	-4.97418461441527
a_HQ_transcript/67558	1.47768301368868	-0.326018579957937	1.36466605523843	0.181707759733901	0.877924416741102	-5.03361578854308
a_HQ_transcript/121377	-1.51902635452809	2.99275792336528	-1.36460465193862	0.181726879701601	0.877943821749887	-5.09113284085013
a_HQ_transcript/106413	-3.28900171481494	3.8936810783637	-1.36444014081631	0.181778113431645	0.8780876693761	-5.33944442115819
a_HQ_transcript/3332	1.6708799421527	0.677930027492042	1.36437550154168	0.181798247121799	0.8780876693761	-4.94503450191476
a_HQ_transcript/130208	-1.77960688519166	4.38984142939096	-1.36436353941247	0.181801973247684	0.8780876693761	-5.30672362610106
a_HQ_transcript/96126	-3.45253294998064	1.11749478550431	-1.36424073339757	0.181840229956103	0.878199475480923	-5.00176017102339
a_HQ_transcript/140372	2.09081806289206	-0.644096381279233	1.36400144395795	0.181914791703904	0.878372616608327	-4.99032112119717
a_HQ_transcript/96272	1.14198134995153	-0.000434963754272583	1.36395197436012	0.181930209216763	0.878372616608327	-4.94863917944905
a_HQ_transcript/50822	-2.39193866668899	0.0600755801886579	-1.36389830181778	0.181946937751111	0.878372616608327	-4.96877864359392
a_HQ_transcript/26938	-0.867408977000616	2.76240644972506	-1.36387477002654	0.18195427246211	0.878372616608327	-5.1346673940169
a_HQ_transcript/25622	-1.36511899500366	-0.514022517998782	-1.36384771215165	0.181962706514852	0.878372616608327	-4.96276601050481
a_HQ_transcript/14884	-1.51216737450658	3.7740373198098	-1.36380669853575	0.181975491209048	0.878372616608327	-5.24738686153362
a_HQ_transcript/143306	1.62248491159315	-0.496750990896718	1.36375683028653	0.181991036993731	0.878372616608327	-4.96549665804082
a_HQ_transcript/141572	-1.47457385506259	3.2297783304556	-1.3637035564871	0.182007645553739	0.878372616608327	-5.18382930413923
a_HQ_transcript/97472	2.18715333510645	-0.170202984219962	1.36368933420589	0.18201207966982	0.878372616608327	-5.03573048725561

a_HQ_transcript/111875	1.55782482435198	0.234846379994352	1.36361887058521	0.182034049525474	0.878392715957596	-5.01643395963534
a_HQ_transcript/107655	-0.908890672977836	2.59558914225997	-1.36357904625945	0.182046467264333	0.878392715957596	-5.12931880873127
a_HQ_transcript/36365	-2.25140556228447	0.239809401547237	-1.36337545622933	0.182109959536963	0.878626139439291	-4.93384295581731
a_HQ_transcript/66553	-1.49254882678196	0.360991223959367	-1.36332170036839	0.182126726888799	0.878634109103833	-4.86956705371984
a_HQ_transcript/58272	-0.467214450041775	2.62132463505905	-1.36322446552617	0.18215705910581	0.878648221528123	-5.07914049306358
a_HQ_transcript/24789	-0.430198455082581	2.7301808056248	-1.36321541158551	0.182159883664248	0.878648221528123	-4.99727687040487
a_HQ_transcript/136940	-1.83559991254886	0.457093179261529	-1.363088318633	0.182199536448468	0.878760109088064	-4.97547903943319
a_HQ_transcript/4338	0.905904496457701	2.24344066829675	1.36304416026175	0.182213315352987	0.878760109088064	-4.96659034456385
a_HQ_transcript/107991	0.74021066999792	3.45037274994405	1.36291182728378	0.182254612558677	0.87887329969669	-5.26934294533313
a_HQ_transcript/103082	-0.629792943513994	2.1368743223244	-1.3628511798526	0.182273541254091	0.87887329969669	-5.14069881656378
a_HQ_transcript/76846	-0.458944946756934	6.12031461739548	-1.36282361640656	0.182282144597153	0.87887329969669	-5.57794389098161
a_HQ_transcript/105536	-0.715812768064467	1.81816343379516	-1.36271106727656	0.182317277683403	0.878969786611995	-4.92155331417043
a_HQ_transcript/120120	2.09070639001075	2.80730439255038	1.36247311133539	0.182391574780894	0.879255055470545	-5.08457969074433
a_HQ_transcript/1692	1.72371795733954	0.742356310297357	1.36233503798435	0.182434696259779	0.879362743583561	-5.01320103636853
a_HQ_transcript/85545	1.75436412772869	0.23036761312748	1.36225926068556	0.182458365515008	0.879362743583561	-4.90806911828287
a_HQ_transcript/109347	-0.696416333873741	2.18533592581732	-1.36225627679381	0.182459297590857	0.879362743583561	-5.01459058301565
a_HQ_transcript/36218	1.05808023664163	4.92330725647186	1.362150573034	0.182492318572467	0.879379299188363	-5.45066006606524
a_HQ_transcript/3068	-1.10184163714137	1.55376840168148	-1.36214842589128	0.18249298937017	0.879379299188363	-4.83147321355319
a_HQ_transcript/31843	3.12838127097225	2.00987527763309	1.36201950111083	0.182533270798301	0.879424677543275	-4.92525789034841
a_HQ_transcript/55183	3.43671360416247	0.553406209774167	1.36197271895946	0.18254788918096	0.879424677543275	-4.97913333787937
a_HQ_transcript/85697	1.90038106436481	1.62407626028644	1.36191339157646	0.182566428976077	0.879424677543275	-5.06047940238243
a_HQ_transcript/118805	1.37751357456273	0.0561454634532075	1.36185123775869	0.18258585359841	0.879424677543275	-5.02563908054791
a_HQ_transcript/86127	0.464003076346038	2.80230523881436	1.36180885056069	0.182599101577383	0.879424677543275	-5.10646266665351
a_HQ_transcript/559	-1.53582052679965	0.443650703755811	-1.36174607888758	0.182618722026819	0.879424677543275	-4.92141434571224
a_HQ_transcript/123543	-1.87296472638809	0.928672662843972	-1.36174314803332	0.182619638159585	0.879424677543275	-5.02108231615736
a_HQ_transcript/2686	-0.98844185014123	3.71357927658542	-1.36173098753832	0.182623439351515	0.879424677543275	-5.30262399489622
a_HQ_transcript/30539	1.56299362898778	-0.383473990601276	1.36147002537638	0.182705027080132	0.879729240859653	-4.9851813153494
a_HQ_transcript/119094	-2.71192546890694	1.25378593636177	-1.36132497338288	0.182750388648639	0.879729240859653	-5.00854029329337
a_HQ_transcript/120728	0.754085004290528	5.58098965218001	1.36130966704176	0.182755175853123	0.879729240859653	-5.55123686954649
a_HQ_transcript/43613	1.45661543526821	0.359644645145072	1.36127516729535	0.1827659663353	0.879729240859653	-4.88943268778383
a_HQ_transcript/107962	0.457736769217951	6.46816980714019	1.36115126228978	0.182804724166264	0.879729240859653	-5.5855833462251
a_HQ_transcript/107526	-0.559347014596558	4.74260506763066	-1.36115062746243	0.182804922758444	0.879729240859653	-5.43178950646438
a_HQ_transcript/112098	1.10793416158115	2.78750811786577	1.36108295900381	0.182826092350273	0.879729240859653	-5.12107645403332
a_HQ_transcript/78443	-1.5008041674691	-0.035004163731102	-1.36106010000353	0.182833244054205	0.879729240859653	-4.99947052474474
a_HQ_transcript/149455	-0.571779640597924	4.32756660828808	-1.36105908910545	0.182833560330431	0.879729240859653	-5.35392056148244
a_HQ_transcript/95215	-2.18810187240763	2.06596316696428	-1.36101253583016	0.182848125754225	0.879729240859653	-4.99728839852793
a_HQ_transcript/131758	-0.917095051407273	2.75372761997017	-1.36099643510227	0.18285316350238	0.879729240859653	-5.07654389754458
a_HQ_transcript/57071	1.11769261380402	2.246972322032	1.36091920325928	0.182877330029348	0.879772692207725	-5.01638322145617
a_HQ_transcript/121998	1.38894182605786	-0.158554825847291	1.36080147364681	0.182914173433195	0.879877116296323	-4.91960027427288
a_HQ_transcript/127624	3.71225846915917	1.35638277322324	1.36059368708002	0.182979214148309	0.880004472024442	-4.98724433905633
a_HQ_transcript/19919	-1.70370513489567	2.26010542548357	-1.36059252135105	0.182979579091815	0.880004472024442	-5.17250611588057
a_HQ_transcript/123868	-1.16634565670597	2.47774791028718	-1.3605381793236	0.182996592051868	0.880004472024442	-5.15187100657678
a_HQ_transcript/24137	-0.936153937640509	1.31737651678806	-1.36050307654964	0.183007582395068	0.880004472024442	-4.83696281526294
a_HQ_transcript/120580	-1.53616890014204	0.826697426439643	-1.36047509245552	0.183016344314332	0.880004472024442	-5.00502759519806
a_HQ_transcript/124803	2.18561566917876	3.77318035494924	1.36039129813879	0.183042582558196	0.8800578361 40847	-5.26867348147638
a_HQ_transcript/128546	2.93808177235995	2.01200616000454	1.36030091944414	0.183070885817176	0.880115048500803	-5.08361337453342
a_HQ_transcript/8591	-0.573234441939397	3.47313364923976	-1.3602132578142	0.183098341434571	0.880115048500803	-5.21600520323538
a_HQ_transcript/17550	-1.03494434412393	2.05170828776976	-1.36020826569116	0.183099905063312	0.880115048500803	-4.96729039714981
a_HQ_transcript/130460	-0.774885409996441	3.77690948298329	-1.36010162617841	0.183133309078615	0.880202827031728	-5.27186807898943
a_HQ_transcript/86559	-1.09746999719796	2.46657791423146	-1.3600475011047	0.183150265153728	0.880211543022396	-5.13691956579892

a_HQ_transcript/135561	-2.02933346503715	0.863202774569922	-1.35996277586119	0.18317680996558	0.880266336779035	-4.96298847478587
a_HQ_transcript/71368	-1.56535408819034	0.117845790334836	-1.35988141408353	0.183202303795973	0.880316071344327	-4.91802811218174
a_HQ_transcript/84198	-0.947004935897402	2.51576555347716	-1.35983189521724	0.183217821342342	0.880317864026094	-5.09067603395035
a_HQ_transcript/90393	-4.39344491779411	3.69778239701167	-1.35974554077221	0.18324488435794	0.880375125048213	-5.35026686557591
a_HQ_transcript/12078	2.24054578899742	2.00701012360341	1.35966230494946	0.18327097294789	0.880397849773437	-5.11537082919071
a_HQ_transcript/120797	1.29622928729351	0.019403049660842	1.35963380587056	0.183279906071228	0.880397849773437	-5.006868288683
a_HQ_transcript/74774	1.97750993597349	0.905409034069335	1.35945045846827	0.183337384947785	0.880536343327511	-5.01466604667166
a_HQ_transcript/22679	1.47768241443006	0.804673874527323	1.35944519922005	0.183339033912966	0.880536343327511	-4.81799071727126
a_HQ_transcript/80751	1.86947530228675	1.45505154454468	1.3593053380681	0.183382889679009	0.880674207804149	-5.08767898329686
a_HQ_transcript/36235	2.33257885392808	2.58230376249504	1.35896844728203	0.183488560720797	0.881079507816737	-4.93224978367235
a_HQ_transcript/115846	-1.56305986451762	1.03079447263899	-1.35890688419325	0.183507876042744	0.881079507816737	-4.99770433886351
a_HQ_transcript/104911	-2.11687066519215	0.561354804610627	-1.35889132466163	0.183512758070762	0.881079507816737	-4.93363739529182
a_HQ_transcript/152846	2.54610978674238	0.462861511714641	1.35870336861109	0.18357173995393	0.88112430185572	-5.12546022420142
a_HQ_transcript/96103	-1.5217958779716	0.457316280660575	-1.35858363214083	0.183609321732239	0.88112430185572	-4.93962881551534
a_HQ_transcript/118831	2.17047642592785	-0.213733121400707	1.35854730247415	0.183620725731996	0.88112430185572	-4.99968653781247
a_HQ_transcript/110508	1.19670339203983	1.8819160063689	1.35850886324884	0.183632792526811	0.88112430185572	-4.9814996004021
a_HQ_transcript/139800	-1.63530087345637	0.820531992973043	-1.35846478713349	0.183646629603771	0.88112430185572	-4.93205990597609
a_HQ_transcript/140171	1.65026531249996	2.02923716657306	1.35845121581383	0.183650890291577	0.88112430185572	-5.06972585843427
a_HQ_transcript/147235	-2.3805170798809	3.49365690180363	-1.35844040112459	0.183654285595809	0.88112430185572	-5.13094098830108
a_HQ_transcript/22607	-1.59855824131717	0.487245818230735	-1.35843451269969	0.183656134304983	0.88112430185572	-4.93074977010725
a_HQ_transcript/144960	1.51575312321903	-0.297823029384298	1.35842693638586	0.183658512959054	0.88112430185572	-4.98773368032947
a_HQ_transcript/134015	1.75399657314168	1.10202529484415	1.3581768780146	0.183737034507941	0.881428167031135	-4.89414053545867
a_HQ_transcript/7852	2.51693724427936	6.35468419065313	1.35812866593037	0.183752176714893	0.881428167031135	-5.48734385937581
a_HQ_transcript/38089	2.69333474705005	2.60486859570335	1.35795209987784	0.183807639942847	0.881552955451217	-5.09006540990472
a_HQ_transcript/5976	-0.632058119589432	3.26990676822608	-1.35790436467407	0.183822636833867	0.881552955451217	-5.20802110369126
a_HQ_transcript/80802	-1.63511662461009	0.208172685059429	-1.35787258093684	0.183832622804604	0.881552955451217	-5.00463755128793
a_HQ_transcript/92408	1.38615469276538	-0.130575092368744	1.35783049868948	0.183845845058687	0.881552955451217	-4.9913841649398
a_HQ_transcript/109415	-1.56802723478187	2.21676878941582	-1.35773559812453	0.183875665548377	0.881552955451217	-4.8953433606488
a_HQ_transcript/34464	0.959010052453582	-0.3912452183532	1.35766050423933	0.183899264865644	0.881552955451217	-5.02477065387459
a_HQ_transcript/125334	0.913193442809753	1.72867816592732	1.3576310674543	0.18390851643275	0.881552955451217	-4.92015079553977
a_HQ_transcript/88559	-1.38369521161671	1.91191612558016	-1.35761627513232	0.183913165587454	0.881552955451217	-4.99385860608073
a_HQ_transcript/106026	1.30592859288813	-0.67121262282833	1.35761144735479	0.183914682954117	0.881552955451217	-5.00766330339591
a_HQ_transcript/214	0.766847512069236	-0.74732568650748	1.35753920261751	0.183937390576822	0.881589102856983	-4.95022722730292
a_HQ_transcript/12193	-2.53787485146273	3.82910626531025	-1.3574361601524	0.183969782154424	0.881671654470471	-5.2761399424898
a_HQ_transcript/85111	-1.33888079862271	0.37325246817371	-1.35726876730463	0.18402241180922	0.881844003009084	-4.95638874262252
a_HQ_transcript/107187	1.68595458218767	2.43258626888533	1.35722528186901	0.184036085888095	0.881844003009084	-4.88504255877745
a_HQ_transcript/149631	1.67153198945235	-0.0368690553677105	1.35706286662299	0.184087164636563	0.882016048960942	-4.95150913658426
a_HQ_transcript/12476	0.374092332590867	3.29468158987888	1.3569172114555	0.184132981770026	0.882141517602742	-5.23608174075582
a_HQ_transcript/90409	1.42516106642259	0.97444436124776	1.3568831317735	0.184143703113674	0.882141517602742	-4.96535177065077
a_HQ_transcript/118565	1.63124202529693	1.63084234071285	1.35682726192183	0.184161280611653	0.882153021787736	-4.93989235917668
a_HQ_transcript/63714	0.907032231535526	-0.428439060845704	1.35676944206285	0.18417947298017	0.882167469132445	-4.96537413132139
a_HQ_transcript/136787	3.48916226092802	0.688733411851872	1.35664203865389	0.184219563944476	0.882207724474365	-5.00415553161668
a_HQ_transcript/138159	-2.33821615849642	0.7372858103448	-1.35658611777971	0.184237163110705	0.882207724474365	-5.07176314123719
a_HQ_transcript/122574	-1.19426140895869	2.33655663112062	-1.35650323726237	0.18426324929058	0.882207724474365	-4.88680091433904
a_HQ_transcript/79219	0.6272384314671	3.01019823235641	1.356501630438	0.18426375505785	0.882207724474365	-5.15403381668258
a_HQ_transcript/147710	-1.21504760818533	-0.540157183246541	-1.3564807977075	0.184270312507354	0.882207724474365	-4.97245359823596
a_HQ_transcript/6186	0.565322236202611	3.69886756562722	1.35636889541715	0.184305538714	0.882207724474365	-5.30137726228861
a_HQ_transcript/14185	1.1508569466491	1.17277762532139	1.35632667396401	0.184318831143534	0.882207724474365	-5.01706418233869
a_HQ_transcript/34621	-2.52416715296964	2.98352589420461	-1.35628252101737	0.184332732452678	0.882207724474365	-5.22323671650236
a_HQ_transcript/151893	0.545869393627821	5.32942346218829	1.35625690824529	0.184340796863431	0.882207724474365	-5.48293534524246

a_HQ_transcript/62913	1.50388434485855	-0.0932249368916077	1.35624321206328	0.184345109341189	0.882207724474365	-4.95595636479946
a_HQ_transcript/142228	-3.16931676349853	3.4764054799628	-1.3561814082156	0.184364570314449	0.882207724474365	-5.38406533891494
a_HQ_transcript/145847	-1.76635862700264	0.985042691112837	-1.35616416140686	0.184370001323193	0.882207724474365	-4.9815705526345
a_HQ_transcript/109021	1.37809230715635	3.50278222677694	1.35607761827806	0.184397255568873	0.882265509235942	-5.07547166736226
a_HQ_transcript/29563	-4.0583586775683	3.56052286646906	-1.35601776161073	0.184416107513988	0.882278421934194	-5.33013968226511
a_HQ_transcript/128738	-2.9154284600287	1.83858080393418	-1.35597266806499	0.184430310778324	0.882278421934194	-5.13344344602479
a_HQ_transcript/111391	-1.8636531136804	0.407310761733774	-1.35585040927962	0.18446882329828	0.882382731086079	-4.96362547912005
a_HQ_transcript/49984	-2.37043555326062	0.959097304711553	-1.35576786490138	0.184494828976828	0.882382731086079	-5.05217775840185
a_HQ_transcript/113400	-0.571481511794287	2.72007434649163	-1.3557168601264	0.184510899495188	0.882382731086079	-5.14756810199223
a_HQ_transcript/113165	0.411802857285472	4.2671985817271	1.35571071615218	0.184512835403786	0.882382731086079	-5.38185093497833
a_HQ_transcript/4777	1.8768446151177	1.39856245227951	1.35562076781972	0.184541179080127	0.882445677654627	-4.99084574302358
a_HQ_transcript/5602	0.989674567724737	2.00646363741168	1.35543620842929	0.184599346265608	0.882599009689311	-5.01729680902071
a_HQ_transcript/124303	-0.508252398251474	3.48931177731277	-1.35540666531749	0.184608658622808	0.882599009689311	-5.21113142939464
a_HQ_transcript/12307	1.15528023697563	2.31756288495309	1.35537450699484	0.184618795740647	0.882599009689311	-4.97233113488856
a_HQ_transcript/139649	1.58267496335348	-0.311917848584634	1.3552613294184	0.184654475607972	0.882678743483129	-4.98219793020667
a_HQ_transcript/116441	-0.767598074493401	1.9846932990513	-1.35522527104415	0.184665844334887	0.882678743483129	-4.92830273704284
a_HQ_transcript/141165	-1.89488942394826	0.903698255264532	-1.35513359270816	0.184694751738857	0.882693168678887	-5.08743243476005
a_HQ_transcript/115059	-1.62168249567614	1.51256019889694	-1.35511938189793	0.18469923291084	0.882693168678887	-5.05777882708183
a_HQ_transcript/136171	2.39457157855343	-0.0232821189897912	1.3550276850269	0.184728150207849	0.882714378421967	-4.94859537430295
a_HQ_transcript/61140	0.703624383536885	0.597118289761457	1.35500900239438	0.184734042346257	0.882714378421967	-4.84889006835664
a_HQ_transcript/140683	0.789626240200702	2.49882044534306	1.3549528614451	0.184751748983969	0.882726423223751	-5.09297775903695
a_HQ_transcript/152119	-0.638954252253719	3.60402095706653	-1.35484290889185	0.184786431400534	0.882819568048843	-5.31235869457442
a_HQ_transcript/30621	-1.16387217117271	1.72228250840689	-1.3546161689538	0.184857968067956	0.883073799825433	-4.87486130872481
a_HQ_transcript/53589	1.15061582699788	1.36089526875283	1.35457794644125	0.184870029419139	0.883073799825433	-4.94960537294235
a_HQ_transcript/30260	1.15004927808211	5.27823812223774	1.35449153481128	0.184897299391571	0.883131488827709	-5.50375310311407
a_HQ_transcript/62989	-2.75416548378626	2.74443305310382	-1.35429260404917	0.184960090238103	0.883315985177209	-5.12695687889833
a_HQ_transcript/132190	1.3279910968597	2.27076744314879	1.35424716200066	0.184974435960343	0.883315985177209	-4.97503997164071
a_HQ_transcript/96432	-0.388947401793612	2.63514666260211	-1.3542247400656	0.184981514718589	0.883315985177209	-5.1056346699875
a_HQ_transcript/103798	1.90527187431876	2.48505995707553	1.3540927747396	0.185023181324475	0.883440716956771	-5.13974381593989
a_HQ_transcript/67746	1.65333180433592	1.32171292960279	1.35404574538663	0.185038032083483	0.883440716956771	-4.91677190546527
a_HQ_transcript/126148	0.70402601036941	2.0176785777899	1.35387576103015	0.18509171682476	0.883571726398622	-5.00309212498856
a_HQ_transcript/53964	1.6796710458199	0.844989650393287	1.35376414797729	0.185126973201653	0.883571726398622	-4.87114563663138
a_HQ_transcript/6882	1.76339848984899	0.1826151613224	1.35375261873112	0.185130615360891	0.883571726398622	-4.94864678230887
a_HQ_transcript/114393	1.36434344944682	-0.612609668608968	1.35372177219184	0.18514036024368	0.883571726398622	-5.00322623378899
a_HQ_transcript/67820	1.53263916553487	1.38686989694584	1.35367912965494	0.185153832312318	0.883571726398622	-4.94616458385469
a_HQ_transcript/18552	-1.09784213751263	-0.657177621738323	-1.35359677062077	0.18517985417334	0.883571726398622	-4.97079483079209
a_HQ_transcript/128496	1.69486610879247	-0.265421053556517	1.35351067701208	0.185207059023368	0.883571726398622	-4.93509863033977
a_HQ_transcript/50848	1.10442637952176	-0.644185622302844	1.35350897188064	0.185207597861682	0.883571726398622	-5.00066360242693
a_HQ_transcript/76572	1.70775795195145	-0.182641217055418	1.35344592122417	0.185227523341605	0.883571726398622	-4.98151387123115
a_HQ_transcript/130696	-1.19356927172107	1.65115958343535	-1.35340800065561	0.185239507925947	0.883571726398622	-4.95079710845505
a_HQ_transcript/7186	2.30709575487435	0.319119141775651	1.35329307310835	0.185275833802996	0.883571726398622	-4.93251195165799
a_HQ_transcript/133384	1.44453687287682	0.66285909497263	1.35328072299016	0.185279737711075	0.883571726398622	-4.91915274639691
a_HQ_transcript/151519	1.00770850341157	-0.567181240926872	1.35322980937314	0.185295832324975	0.883571726398622	-5.01338996385868
a_HQ_transcript/37285	1.28113846685313	0.618355846039319	1.35316537983235	0.185316201091132	0.883571726398622	-4.83640115523892
a_HQ_transcript/150002	1.17881292894049	0.148291212253282	1.35310938775043	0.185333903845037	0.883571726398622	-4.99088796726157
a_HQ_transcript/51137	0.76312480434702	2.55486427609876	1.35310563838824	0.18533508931005	0.883571726398622	-5.19553941140269
a_HQ_transcript/55970	1.90755400091404	0.552820152334095	1.35308816656244	0.185340613590545	0.883571726398622	-4.95674538333489
a_HQ_transcript/103436	-1.19493588930997	4.08248899055004	-1.35306040812506	0.185349390576714	0.883571726398622	-5.41494308530515
a_HQ_transcript/139588	0.472672771983775	4.94621415653191	1.35302424724307	0.185360824828231	0.883571726398622	-5.46284511963048
a_HQ_transcript/1049	1.80230103255129	2.86436939248921	1.35299687255608	0.185369481204087	0.883571726398622	-5.20442444068066

a_HQ_transcript/135879	-2.25438756002097	0.800675622131739	-1.35291151805624	0.185396473864133	0.883597721701808	-5.05304492083275
a_HQ_transcript/105682	-1.29219012259521	-0.240588105053796	-1.3528697619408	0.185409680001028	0.883597721701808	-4.96895518040634
a_HQ_transcript/145210	-1.14631043983261	2.91536153810748	-1.35274245489723	0.185449947680722	0.883597721701808	-5.2093919435253
a_HQ_transcript/101192	1.7454721417146	0.205231186034477	1.35272066443327	0.185456840761866	0.883597721701808	-5.04273116731025
a_HQ_transcript/73451	1.06423821859203	0.641319797354382	1.35266741535878	0.185473686129286	0.883597721701808	-4.84719350568444
a_HQ_transcript/138108	1.34433410165683	0.129438102686293	1.35259393162211	0.185496934685309	0.883597721701808	-4.86147352893913
a_HQ_transcript/29962	1.00334685012757	1.51616501400163	1.3525611229681	0.185507315313415	0.883597721701808	-4.89534789825602
a_HQ_transcript/131355	4.64252649624751	5.63570440498509	1.35251903308818	0.185520633171211	0.883597721701808	-5.46888158311021
a_HQ_transcript/14029	0.498228598402513	2.93748727755126	1.35246136358909	0.185538881851338	0.883597721701808	-5.21296224393556
a_HQ_transcript/28543	-2.22081164500824	2.37512385244557	-1.35245294472266	0.18554154599623	0.883597721701808	-5.23505053257509
a_HQ_transcript/148800	-3.60955577174151	0.931502807478584	-1.35240878793699	0.185555519867607	0.883597721701808	-5.0647070071023
a_HQ_transcript/35604	1.47334648634772	-0.215210683896044	1.35235961333892	0.185571082636099	0.883597721701808	-4.98824736744236
a_HQ_transcript/148687	-2.16530478605606	3.93611214474914	-1.35226804330461	0.185600065396768	0.883597721701808	-5.26557495951554
a_HQ_transcript/110511	-2.73591106353539	1.79252783040039	-1.35223805557479	0.185609557551265	0.883597721701808	-5.12913150107237
a_HQ_transcript/64675	1.28305140404682	-0.254507881568091	1.3522177450966	0.185615986734021	0.883597721701808	-4.95184094322074
a_HQ_transcript/128081	-0.748085072600797	2.81453207486144	-1.35219693885684	0.185622573026367	0.883597721701808	-5.15630635697289
a_HQ_transcript/10320	1.52402978596403	0.677137808861049	1.35213754869898	0.185641374195903	0.883597721701808	-4.84502874208413
a_HQ_transcript/148967	-1.76366012022963	1.43235822644943	-1.35210218460316	0.185652570123883	0.883597721701808	-5.02475983562643
a_HQ_transcript/93285	1.96321098901019	1.09729281171228	1.35202046758088	0.185678442935037	0.883597721701808	-4.87177915845682
a_HQ_transcript/117793	0.980682771810198	1.11569422168073	1.3519440169055	0.185702650869286	0.883597721701808	-4.95158678075807
a_HQ_transcript/42898	1.58099140932225	-0.327918128736029	1.35193848575146	0.185704402391487	0.883597721701808	-4.99010968202617
a_HQ_transcript/9472	1.06524603128215	1.54330907140338	1.35191351520029	0.185712309847071	0.883597721701808	-4.89583811825181
a_HQ_transcript/149816	-4.06849323653525	0.887316575568546	-1.35187484681057	0.185724555528363	0.883597721701808	-5.0914651130359
a_HQ_transcript/41025	-1.95060897719762	1.22594264093019	-1.35181708522996	0.185742848891732	0.883612433126983	-5.05295109944612
a_HQ_transcript/112851	-0.75792632426432	4.17741417181883	-1.35170113717382	0.18577957439275	0.883650941822358	-5.3891506132569
a_HQ_transcript/50486	1.81296409849467	0.60511489596076	1.35166676347409	0.185790463031714	0.883650941822358	-4.96668437835271
a_HQ_transcript/17725	-1.03772204542342	2.07703580834578	-1.35155067483857	0.185827240355778	0.883650941822358	-4.97823552881256
a_HQ_transcript/19953	1.54893301909144	0.140677158854216	1.35153904565237	0.185830924836832	0.883650941822358	-4.88390010400124
a_HQ_transcript/108162	1.41253169267731	0.299570616611769	1.35152995987412	0.185833803527963	0.883650941822358	-4.93470190943203
a_HQ_transcript/74651	-0.876413951599922	2.66667281985613	-1.35142964439482	0.185865589260517	0.883650941822358	-4.99593709742588
a_HQ_transcript/83912	1.27820265046878	2.40764623522084	1.35142198289791	0.185868017037723	0.883650941822358	-5.01718128257952
a_HQ_transcript/151165	1.92754211796116	0.233759487985062	1.35115910132025	0.185951333886468	0.883650941822358	-5.02195964235795
a_HQ_transcript/149718	1.36779188541833	1.23036692297079	1.3510979140325	0.185970730534643	0.883650941822358	-4.95742765659206
a_HQ_transcript/114358	1.81771731642594	0.0718838919937185	1.3510955655932	0.185971475031757	0.883650941822358	-4.92244751178204
a_HQ_transcript/141825	-3.25686083071362	0.935658975549261	-1.35109219445559	0.185972543746494	0.883650941822358	-5.05022556611452
a_HQ_transcript/71078	-0.694542199863444	3.50460512666715	-1.35096835188565	0.186011807489634	0.883650941822358	-5.24806249737552
a_HQ_transcript/149116	2.02568667803399	0.418757425905351	1.35090498173642	0.186031901197227	0.883650941822358	-5.10339383454302
a_HQ_transcript/44405	1.76028930729688	3.56429581413411	1.35090308882125	0.186032501437575	0.883650941822358	-5.19547206086825
a_HQ_transcript/19809	-0.821724991104773	1.68331613039969	-1.35083780438746	0.186053203943111	0.883650941822358	-4.96373900681219
a_HQ_transcript/102205	1.99965607980889	0.320408327643915	1.35077029609334	0.18607461353654	0.883650941822358	-4.91092224544104
a_HQ_transcript/96062	0.714432437363193	-0.708052804011744	1.35073345484224	0.186086298181758	0.883650941822358	-5.01093632036983
a_HQ_transcript/85397	1.87080544707573	1.65436431251888	1.35072919584048	0.186087649011909	0.883650941822358	-4.88526571518597
a_HQ_transcript/79197	1.33693811573661	1.92845707007363	1.35064396248434	0.186114684121579	0.883650941822358	-5.09729583405711
a_HQ_transcript/38586	0.813703367477041	2.63518367347526	1.35062460640133	0.186120824087526	0.883650941822358	-5.086323646281
a_HQ_transcript/137649	-1.65477856016394	3.50398071813365	-1.35058527603059	0.186133300603481	0.883650941822358	-5.30556880206335
a_HQ_transcript/80830	-1.02047987130947	1.70896409367067	-1.35056767707612	0.186138883614051	0.883650941822358	-4.86103695601692
a_HQ_transcript/103163	1.60530319959515	-0.474184684650079	1.35047797411348	0.186167342577894	0.883650941822358	-4.99555009967346
a_HQ_transcript/46145	-2.18333947861096	0.501954420166889	-1.3504718484701	0.186169286108735	0.883650941822358	-5.0320506496934
a_HQ_transcript/77673	1.78481430979844	1.4296379908161	1.35041494873962	0.186187339881653	0.883650941822358	-4.98997183424343
a_HQ_transcript/124947	1.23021158178683	-0.348065628887935	1.35040811247939	0.186189509056679	0.883650941822358	-5.00655359716181

a_HQ_transcript/78307	1.9640318817832	0.950027972252479	1.35038959737215	0.186195384078671	0.883650941822358	-4.98697991966945
a_HQ_transcript/95466	0.44750643243012	2.82418884546462	1.35038298665477	0.186197481757753	0.883650941822358	-5.13043654157225
a_HQ_transcript/56589	-1.82873074884005	-0.240028201256862	-1.35034570414331	0.186209312396119	0.883650941822358	-4.98098538364817
a_HQ_transcript/67430	1.3901172254763	0.0371618447954422	1.35033669818399	0.186212170290984	0.883650941822358	-4.91838218658662
a_HQ_transcript/116323	1.4948880822813	0.356847401425806	1.35030509307753	0.186222199926436	0.883650941822358	-4.97749542208969
a_HQ_transcript/135994	1.97186916893784	0.796770375369736	1.35011841417144	0.186281449551908	0.883775215489513	-4.91705598142106
a_HQ_transcript/47923	2.03438699662119	1.12794317032228	1.35008964150842	0.186290582943474	0.883775215489513	-5.04209597070272
a_HQ_transcript/29772	1.78788196895654	0.48858400944863	1.35005360161044	0.186302023683506	0.883775215489513	-4.9365895667438
a_HQ_transcript/16915	1.11178933106378	1.48730598376625	1.35001865412361	0.186313118160334	0.883775215489513	-4.91478225391447
a_HQ_transcript/90841	-1.51740946194323	0.139925526202358	-1.34985521612611	0.186365010192221	0.883775215489513	-4.87191251172554
a_HQ_transcript/141528	1.89052362528817	-0.0531910747525072	1.34976332283874	0.186394191482206	0.883775215489513	-5.02707545007463
a_HQ_transcript/32022	-1.97761504812901	2.63622522242799	-1.34975747217669	0.186396049516286	0.883775215489513	-5.15576303628751
a_HQ_transcript/145480	-2.25897698421832	0.997317107544713	-1.34971733676692	0.186408795974321	0.883775215489513	-5.1410042886203
a_HQ_transcript/33224	2.27574869181119	-0.332666406340562	1.34966421577339	0.186425667512836	0.883775215489513	-4.97961286507261
a_HQ_transcript/117748	1.01840159014794	1.37879018770677	1.34963424920306	0.186435185591921	0.883775215489513	-4.97860377662413
a_HQ_transcript/4600	1.57461703616915	2.87081716583173	1.34961448135712	0.186441464524844	0.883775215489513	-5.22437789673312
a_HQ_transcript/118632	-2.12983347409641	1.34335143933771	-1.34957852534418	0.186452885783439	0.883775215489513	-4.93490615575539
a_HQ_transcript/115983	1.60229472341905	-0.050810158265195	1.34944020290704	0.186496828299767	0.883775215489513	-5.02504413082042
a_HQ_transcript/99673	-1.99600905665699	1.3370406720944	-1.34934622525818	0.186526687853488	0.883775215489513	-4.94985850851209
a_HQ_transcript/119525	-1.23785462945798	-0.21290341850231	-1.34933629370165	0.186529843626632	0.883775215489513	-4.9168892424486
a_HQ_transcript/10327	2.42209777480709	2.86666122854075	1.3492713743416	0.186550472907818	0.883775215489513	-5.12725482319486
a_HQ_transcript/88526	0.860190070531957	5.96789716871769	1.34923289844346	0.186562700141243	0.883775215489513	-5.57331964911531
a_HQ_transcript/45511	0.588477049014559	2.9783042118368	1.34908880759706	0.186608496193492	0.883775215489513	-5.22122217562691
a_HQ_transcript/67519	1.50680877708093	0.828332183174316	1.34907160910936	0.186613962928648	0.883775215489513	-4.79944944748625
a_HQ_transcript/44699	1.04952748534649	-0.641503974330325	1.34905069222505	0.186620611765372	0.883775215489513	-4.94762565236022
a_HQ_transcript/20367	-1.03567106233119	1.59505204129257	-1.34904236698475	0.186623258155172	0.883775215489513	-4.84300641961087
a_HQ_transcript/105207	3.28972738645244	1.74653829574864	1.3490417206162	0.186623463621101	0.883775215489513	-5.15125236366233
a_HQ_transcript/138704	0.404224708778903	5.54091573894038	1.34901213376235	0.186632868799407	0.883775215489513	-5.52877046049533
a_HQ_transcript/80629	1.40803549586805	-0.52259104461087	1.3489640877093	0.186648142636804	0.883775215489513	-5.00405439691043
a_HQ_transcript/144666	0.524005524597797	2.75699262642636	1.34893242970093	0.186658207243932	0.883775215489513	-5.19275955570977
a_HQ_transcript/58792	-0.598743823062427	1.93235584960298	-1.34885772673428	0.186681958222033	0.883775215489513	-4.92339633591158
a_HQ_transcript/138185	1.80396232326958	0.195315722903003	1.3488295052517	0.186690931536834	0.883775215489513	-5.02274408984307
a_HQ_transcript/81758	-1.52387355110929	-0.0607307594609719	-1.34879336049995	0.186702424624748	0.883775215489513	-4.92723252366593
a_HQ_transcript/70446	-1.98741561795658	1.47271128150537	-1.34879058075341	0.186703308534263	0.883775215489513	-5.00669109364825
a_HQ_transcript/44452	-0.860594011455988	2.32852331898783	-1.34877160856619	0.186709341435732	0.883775215489513	-4.99088158880931
a_HQ_transcript/114763	1.07111633043294	1.22457745577073	1.34873899633905	0.186719712039408	0.883775215489513	-4.89940711975001
a_HQ_transcript/111794	2.71380506388481	1.50874372709417	1.34862214732226	0.186756873379976	0.883879134980559	-5.01346756412413
a_HQ_transcript/127966	1.82452618836088	1.27675672787792	1.34855338955215	0.186778742995861	0.883910671079005	-4.8197377981576
a_HQ_transcript/105335	-0.799379556202219	3.58048769797344	-1.34832168856707	0.186852454138191	0.884127631595685	-5.2679390444328
a_HQ_transcript/8190	1.66484149837367	0.338237213848854	1.34830485090327	0.186857811588295	0.884127631595685	-4.94455980439409
a_HQ_transcript/32922	1.4889959385867	0.273352588622723	1.34826585814403	0.186870218858116	0.884127631595685	-4.84565861313066
a_HQ_transcript/117075	1.40859496671782	0.0750591703508597	1.34817443714919	0.186899310985164	0.884193305847285	-4.96195993267366
a_HQ_transcript/143279	-0.597662598537284	3.97325678871271	-1.34807321583798	0.18693152586736	0.884269759314951	-5.31674376682377
a_HQ_transcript/72351	1.51035567790803	-0.245281511066058	1.34802806667396	0.186945896508076	0.884269759314951	-5.00355158126446
a_HQ_transcript/130121	0.560114747735116	3.69162983983649	1.34786834690085	0.186996740968995	0.88439115769884	-5.22753320340952
a_HQ_transcript/135065	-1.22507589652101	4.53930552113421	-1.34782291846243	0.187011204402062	0.88439115769884	-5.20362824108062
a_HQ_transcript/75736	3.54519212873614	3.23092698751549	1.34780407089811	0.187017205313001	0.88439115769884	-5.04988235295567
a_HQ_transcript/45048	1.63312228232164	-0.00525815537677294	1.34767796657588	0.187057359730997	0.884488048652986	-4.89207829452599
a_HQ_transcript/27323	0.981628906289111	1.69404010788017	1.34764415656358	0.187068126722746	0.884488048652986	-4.89612093746365
a_HQ_transcript/92076	-0.782195979868529	1.82340290009634	-1.34732347904667	0.187170272127969	0.884851302191582	-4.99515786192841

a_HQ_transcript/28239	2.18563906748972	1.87540955206238	1.34727660838596	0.187185205448491	0.884851302191582	-4.91325727741835
a_HQ_transcript/6041	1.21168641220408	1.57690352298574	1.34706227169002	0.187253506352018	0.884851302191582	-4.90671723524925
a_HQ_transcript/27011	-1.13350854645675	1.41665123100019	-1.34705226074925	0.187256696926452	0.884851302191582	-4.81861239964467
a_HQ_transcript/150682	-1.17947381667974	-0.64078468443921	-1.3470345420983	0.187262344118545	0.884851302191582	-4.98154563314444
a_HQ_transcript/136502	-0.522553283202863	2.75300834327698	-1.34703287550414	0.187262875293143	0.884851302191582	-5.1688508194408
a_HQ_transcript/89714	1.19783911530666	3.04191747344767	1.34703219863718	0.187263091023585	0.884851302191582	-5.03592294792492
a_HQ_transcript/43934	1.00919527282758	-0.246656585781914	1.34702076837237	0.187266734096491	0.884851302191582	-5.03808011445946
a_HQ_transcript/4787	1.57495830964701	-0.00986216738530979	1.34676240747405	0.187349093918752	0.885006545868131	-5.04590663550417
a_HQ_transcript/135537	-1.75546069010168	-0.258378238587433	-1.34668310181636	0.187374380446693	0.885006545868131	-4.96836009267309
a_HQ_transcript/82124	1.32562592572857	-0.356295642855153	1.34667270581883	0.187377695395368	0.885006545868131	-4.99043207223732
a_HQ_transcript/23584	2.27602901349639	2.26489985737769	1.34666143629158	0.187381288935714	0.885006545868131	-5.06895411421077
a_HQ_transcript/150584	1.54259415762765	0.579325960361695	1.34665406023497	0.187383640984855	0.885006545868131	-4.94462332942399
a_HQ_transcript/20452	2.02171576601659	0.352476579806867	1.34663117054843	0.187390940107091	0.885006545868131	-4.93296792185633
a_HQ_transcript/112699	1.43635126323572	-0.266513228748389	1.34650750322157	0.187430379262575	0.885120894124214	-5.00086218596467
a_HQ_transcript/130127	-1.88520822424037	0.34515791571951	-1.3463194816603	0.187490354126061	0.885275437970002	-4.97919229284491
a_HQ_transcript/109636	-3.96807102499374	3.83719824603977	-1.34628664115745	0.187500831066061	0.885275437970002	-5.0937184876567
a_HQ_transcript/63859	1.57315027031526	-0.00268067906090002	1.3462616810928	0.18750879425211	0.885275437970002	-5.02189905507702
a_HQ_transcript/68961	-0.657866696989682	2.61294874144219	-1.34615743953155	0.187542053999174	0.885292356261553	-5.09338628057527
a_HQ_transcript/120718	-0.434548679081462	3.30361476957312	-1.34615498317624	0.187542837789099	0.885292356261553	-5.20753049886705
a_HQ_transcript/137361	2.48628220206473	1.88552970729385	1.346030921142	0.187582427606224	0.885407336735251	-5.11401622473883
a_HQ_transcript/99725	-1.65522836900579	2.93184541045502	-1.34587948395007	0.18763076194368	0.885563570173939	-5.21326292915133
a_HQ_transcript/141582	2.26154130548487	2.92618313388851	1.34581161574364	0.187652426618545	0.885572400533663	-4.97419357035074
a_HQ_transcript/65831	-1.61360955599234	2.34346894417618	-1.34577817357853	0.187663102631669	0.885572400533663	-5.01779265387119
a_HQ_transcript/137522	-1.88976968300163	-0.115895480940449	-1.34563624066455	0.187708418246069	0.885576920241272	-4.99967591004818
a_HQ_transcript/127053	-1.80939382662928	1.81637231994225	-1.34560482586353	0.187718449345284	0.885576920241272	-4.91131674210061
a_HQ_transcript/65615	1.50277533925403	0.782640441091961	1.34557289670362	0.187728645109349	0.885576920241272	-4.99789217627025
a_HQ_transcript/83766	1.43692365395476	1.74254661741701	1.34556415679395	0.187731436051481	0.885576920241272	-4.90653269681594
a_HQ_transcript/102028	1.366360348899	0.784655455699061	1.34551035675588	0.187748616888331	0.885576920241272	-4.89123118462039
a_HQ_transcript/107068	1.41305459272302	-0.0303798714903996	1.34548889773305	0.187755470084184	0.885576920241272	-5.0307619091668
a_HQ_transcript/3812	1.11006137710588	2.83783838589396	1.34543037098226	0.187774162284843	0.885593225581611	-5.13051261481859
a_HQ_transcript/137980	-0.989389194960082	3.86560064392272	-1.34532100830036	0.187809094253881	0.885686113158332	-5.36062712989488
a_HQ_transcript/135435	0.842997589807635	1.53633935713034	1.34524468420829	0.187833476209119	0.885729237047428	-4.93297502817056
a_HQ_transcript/91066	-1.53498250554354	0.654578745965316	-1.34517799874208	0.187854781078047	0.885757845109079	-5.03286892639211
a_HQ_transcript/1388	0.791966954548377	-0.796236232767435	1.34506564535037	0.187890680291126	0.885814604610628	-5.02604129022727
a_HQ_transcript/110530	1.24807305649635	0.438807048163815	1.34501426691973	0.187907098518794	0.885814604610628	-4.95419262723915
a_HQ_transcript/6480	1.34280753218878	-0.161151720213049	1.34490588974968	0.187941734604607	0.885814604610628	-5.02435740111031
a_HQ_transcript/113348	2.38829790904169	1.59328148650865	1.34478255551706	0.187981156791706	0.885814604610628	-4.88444887071476
a_HQ_transcript/118014	-1.943284777992	3.55383499964235	-1.34476536593318	0.187986651726124	0.885814604610628	-5.48142557653319
a_HQ_transcript/105131	-1.12320778386948	-0.488980957991474	-1.34474202031079	0.187994114738559	0.885814604610628	-4.98970847337661
a_HQ_transcript/51855	1.55407877413886	-0.184127420882378	1.34473678144002	0.187995789506137	0.885814604610628	-4.88876138010886
a_HQ_transcript/59623	-1.36298292069498	2.26091823818411	-1.34473264784376	0.187997110946559	0.885814604610628	-5.02082327108187
a_HQ_transcript/135427	1.49028552550575	1.73409807503321	1.34471119192699	0.188003970153477	0.885814604610628	-4.95828937826289
a_HQ_transcript/129568	0.405547749428469	3.60803098991527	1.34461143640483	0.188035863371112	0.885841779139183	-5.32380335457709
a_HQ_transcript/22257	1.96389358593524	-0.286674677791781	1.34453633596031	0.188059876776511	0.885841779139183	-4.9902660116109
a_HQ_transcript/134455	-1.7884590954423	0.713788732765429	-1.34448505850272	0.18807627413005	0.885841779139183	-4.96309485469246
a_HQ_transcript/34867	-1.44918487710786	0.944434087512702	-1.34441903084712	0.188097389884198	0.885841779139183	-4.95714581643019
a_HQ_transcript/37397	-1.73922366073414	3.41966510503424	-1.34439741010884	0.188104304630113	0.885841779139183	-5.11948713129227
a_HQ_transcript/437	-1.36005471295154	-0.0860963111790064	-1.34434867574749	0.188119891576531	0.885841779139183	-4.97414710342501
a_HQ_transcript/1232	0.660769782999518	3.33613671098008	1.34429674431153	0.188136502156321	0.885841779139183	-5.24354016041272
a_HQ_transcript/118586	0.739600183091693	2.86283697731357	1.34421982514154	0.18816110729287	0.885841779139183	-5.17074460372309

a_HQ_transcript/4430	1.7663259499503	3.1042194043628	1.34414960336667	0.188183572215867	0.885841779139183	-5.24932777990417
a_HQ_transcript/42615	-1.1243671824566	-0.101866006807817	-1.34409100979836	0.188202318698582	0.885841779139183	-4.93526975916144
a_HQ_transcript/63846	-0.797808174844975	3.38291800881439	-1.34406717186003	0.188209945844156	0.885841779139183	-5.24092940806896
a_HQ_transcript/111554	1.23784437266981	0.196721456710692	1.34405662568937	0.188213320254847	0.885841779139183	-4.84356793370951
a_HQ_transcript/140473	-0.447407720975932	3.55651309441075	-1.34403087682828	0.188221559198117	0.885841779139183	-5.25099244273502
a_HQ_transcript/152375	1.64578741836361	2.86296527423946	1.34400189750612	0.188230832135115	0.885841779139183	-5.25583551819661
a_HQ_transcript/23803	-1.78491887043534	0.167589149593882	-1.34396662207739	0.18824212020527	0.885841779139183	-4.95854684608101
a_HQ_transcript/122658	1.35221524143719	-0.034859454785209	1.34388359728543	0.188268690043498	0.885841779139183	-4.93668825010398
a_HQ_transcript/89851	1.76088966043392	0.78888562886912	1.34388322505939	0.18826880917089	0.885841779139183	-5.04559652481214
a_HQ_transcript/25461	2.31000486050477	-0.106170903166736	1.3438258479352	0.188287172867768	0.885856477900251	-5.0582013921074
a_HQ_transcript/121495	2.76794421853608	2.30962462192341	1.34352909144661	0.188382172620956	0.886223322025662	-4.97612797757311
a_HQ_transcript/110915	1.76052520682079	2.81748294501489	1.34342677734677	0.188414934699298	0.886223322025662	-5.07492868083007
a_HQ_transcript/55843	1.71887676883858	0.282230462100388	1.34337693940148	0.188430894939876	0.886223322025662	-4.95934892761918
a_HQ_transcript/82138	1.38339471151958	0.0288554527396487	1.34337548289234	0.18843136139206	0.886223322025662	-4.89502154642851
a_HQ_transcript/113166	0.321186862946424	5.34497581370607	1.3433107846312	0.188452082137364	0.886223322025662	-5.51285351482009
a_HQ_transcript/34666	1.5280721261888	0.763551786242053	1.3432532991564	0.188470494337781	0.886223322025662	-4.97466399251057
a_HQ_transcript/25062	-1.37671968105846	1.71392189987471	-1.34323452269154	0.188476508610589	0.886223322025662	-4.87027447983538
a_HQ_transcript/110991	-1.35052411874444	-0.264712980458547	-1.34320141569929	0.188487113442803	0.886223322025662	-4.92049782083952
a_HQ_transcript/111957	1.74799533074825	0.276279198291636	1.34304559739753	0.188537031348495	0.886386099244321	-4.90770353785941
a_HQ_transcript/53884	-1.79007162617924	0.721278966166324	-1.34299815634046	0.188552231577829	0.886386099244321	-4.98211365681349
a_HQ_transcript/136361	-0.490219929100313	3.00471333023731	-1.34292756811818	0.188574849965523	0.886410544523865	-5.13622675217733
a_HQ_transcript/116114	1.41690443498164	-0.0175235391041975	1.34288675000261	0.188587930156321	0.886410544523865	-4.99235100435911
a_HQ_transcript/93257	-1.98191506924412	2.76313037114032	-1.34271982020569	0.188641430202537	0.886511812069132	-5.34454960347236
a_HQ_transcript/114058	-0.576411447583243	3.76665065176294	-1.34263612383626	0.188668258819251	0.886511812069132	-5.3493957615201
a_HQ_transcript/4057	1.35075474181383	-0.0960665039365929	1.34262016103255	0.188673375981794	0.886511812069132	-4.99972249168244
a_HQ_transcript/67572	-1.18820548705328	0.645660202581641	-1.34260302476766	0.188678869437544	0.886511812069132	-4.96455800554142
a_HQ_transcript/125279	2.25561124414862	2.0147944969212	1.34258162312875	0.188685730438331	0.886511812069132	-5.14234832988116
a_HQ_transcript/132544	1.41726305445084	-0.208759522734908	1.34247581872935	0.188719652354987	0.886584298999911	-5.00295923375215
a_HQ_transcript/151384	-2.7024196809646	1.90257474256868	-1.3424383598777	0.188731663154158	0.886584298999911	-5.0898862431744
a_HQ_transcript/101870	1.22645645235591	1.99423166728725	1.34236408573099	0.188755480147113	0.886624529292233	-4.97850870051315
a_HQ_transcript/150882	-1.30593691775524	1.11453174235315	-1.34226002423497	0.188788852750944	0.886688332969915	-4.95155973720615
a_HQ_transcript/103403	-1.4928688935585	-0.194740132748429	-1.34211265145627	0.188836123107295	0.886688332969915	-4.98929819156541
a_HQ_transcript/40757	2.23177922858977	1.04591412521508	1.3421022924234	0.188839446148404	0.886688332969915	-4.93646720005629
a_HQ_transcript/146158	2.07975068464435	0.249585168494023	1.34207954540419	0.18884674325046	0.886688332969915	-4.9854770575012
a_HQ_transcript/136044	0.664096743545931	4.92644064653974	1.34206112859641	0.188852651407058	0.886688332969915	-5.39313462894363
a_HQ_transcript/80489	-1.01524994992623	1.3929270028071	-1.34200936977036	0.188869256527675	0.886688332969915	-4.89295231741
a_HQ_transcript/137934	-0.734215809201642	3.18550417666229	-1.34193055394455	0.188894544162556	0.886688332969915	-5.18965462868767
a_HQ_transcript/102355	1.70446940531714	2.73239709790264	1.34186641776454	0.1889151238397	0.886688332969915	-5.01115538330153
a_HQ_transcript/134699	-2.75064874199962	2.20162429401216	-1.3418610797693	0.188916836745274	0.886688332969915	-5.05511703162766
a_HQ_transcript/37881	-1.21823788605826	-0.0586744669819779	-1.34184622323228	0.188921604112173	0.886688332969915	-4.95070163768766
a_HQ_transcript/120323	-1.3180917826048	0.804275162685147	-1.34166524528558	0.188979686221033	0.886774553089676	-4.89247950824137
a_HQ_transcript/67600	-0.801268539389234	3.12888040047134	-1.3416469461252	0.188985559824058	0.886774553089676	-5.02011822349395
a_HQ_transcript/22264	-1.06120449449421	1.26634504624331	-1.34164638109978	0.18898574118626	0.886774553089676	-4.86496703120913
a_HQ_transcript/6856	1.26067252601651	1.58544149643749	1.34145141192972	0.189048330527078	0.886987980637584	-4.90182525754971
a_HQ_transcript/137869	-5.00546358756255	3.12360183884402	-1.34140963338647	0.189061744427809	0.886987980637584	-5.2044496137051
a_HQ_transcript/194	-0.735110192211677	1.78776662753343	-1.3411650109306	0.189140300470928	0.887067559597203	-4.99525875613784
a_HQ_transcript/10847	-1.47850820089467	0.305961415637776	-1.34115595243562	0.1891432099249	0.887067559597203	-4.87313455125433
a_HQ_transcript/131149	1.36141470431091	0.480579476380144	1.34114279142	0.189147437109061	0.887067559597203	-4.87820944172457
a_HQ_transcript/85901	1.8980395310529	0.492583826311798	1.3410679500049	0.18917147679489	0.887067559597203	-4.89405714570917
a_HQ_transcript/25999	-1.88607806078842	0.656592352426434	-1.34097254104418	0.189202126365131	0.887067559597203	-4.91339501025029

a_HQ_transcript/24269	2.62078759016309	0.744448156486119	1.34092375894003	0.189217798810532	0.887067559597203	-5.0178593992655
a_HQ_transcript/130571	3.40864607985574	2.92235152762685	1.34087321518929	0.189234038284359	0.887067559597203	-4.98221775888985
a_HQ_transcript/25623	-3.13286290420732	1.80287952455142	-1.34086365922796	0.18923710869147	0.887067559597203	-5.15001622112301
a_HQ_transcript/146111	2.61155299085009	1.60087262371332	1.34084919012993	0.189241757802004	0.887067559597203	-5.10156636272911
a_HQ_transcript/67414	0.90226905937966	1.76974604651627	1.34080646420085	0.189255486716321	0.887067559597203	-4.86747026935192
a_HQ_transcript/13113	0.827474762438893	1.52919576352409	1.34075705292224	0.189271364759369	0.887067559597203	-4.88727742780494
a_HQ_transcript/90265	0.928528121094986	0.197947145107574	1.34072841492596	0.189280567892562	0.887067559597203	-4.84774420978246
a_HQ_transcript/117571	-1.05429380034386	1.995370609333	-1.3405739082605	0.189330226253563	0.887067559597203	-4.92519493958749
a_HQ_transcript/45417	0.353983486282242	-0.837154897483859	1.34055856593397	0.189335157817646	0.887067559597203	-5.08432230581149
a_HQ_transcript/26513	-1.23837618664944	1.50000603291513	-1.34036432591128	0.189397601969788	0.887067559597203	-4.93296984865531
a_HQ_transcript/134834	-0.780711369639199	1.39550410193315	-1.34036101481407	0.189398666556772	0.887067559597203	-4.96652239361003
a_HQ_transcript/151621	1.5224530481007	2.88168201496393	1.34026203860052	0.189430491609902	0.887067559597203	-5.06950435923447
a_HQ_transcript/25225	0.728516430337562	2.18095250199921	1.34024686291303	0.189435371602121	0.887067559597203	-4.9250288624558
a_HQ_transcript/81220	1.41473557199678	-0.312587983094832	1.34019272359337	0.189452781781854	0.887067559597203	-4.92958788480269
a_HQ_transcript/111852	-0.624530904224641	2.2406383859041	-1.3400503581053	0.189498569715409	0.887067559597203	-5.0264161910787
a_HQ_transcript/115839	0.76265460454423	2.68027834786393	1.34002035127559	0.189508221673652	0.887067559597203	-5.02407983896391
a_HQ_transcript/9734	-0.400840080248497	2.65701508484993	-1.33987130134971	0.189556170499957	0.887067559597203	-5.13122803365268
a_HQ_transcript/104841	0.478608531980562	4.94627463556936	1.33986029001354	0.189559713178374	0.887067559597203	-5.48751104054444
a_HQ_transcript/132811	-0.960912843228721	1.20002123575097	-1.33985506293674	0.189561394904249	0.887067559597203	-4.93316884746157
a_HQ_transcript/151744	2.16843134738493	1.87607726890472	1.33981800752462	0.189573317202722	0.887067559597203	-5.10703354405065
a_HQ_transcript/135569	-1.08771545907318	3.09380731167604	-1.33981266957995	0.189575034693677	0.887067559597203	-5.16398086358965
a_HQ_transcript/55929	1.54149297761986	0.150452007392986	1.33974049297368	0.189598258792937	0.887067559597203	-5.01415668878348
a_HQ_transcript/124910	1.72327599126492	0.0967474497864423	1.33973842176213	0.189598925274257	0.887067559597203	-5.04871152068244
a_HQ_transcript/98526	-0.451714724318849	3.97030182132983	-1.33971483654699	0.189606514730405	0.887067559597203	-5.3783123325958
a_HQ_transcript/89616	0.82126201751506	2.75492167778467	1.33971248950881	0.189607269993627	0.887067559597203	-5.10465418932845
a_HQ_transcript/11091	1.35521975188353	-0.30303783293389	1.33969962660091	0.189611409243707	0.887067559597203	-4.96781198617702
a_HQ_transcript/46820	2.62824683143633	0.0246219891461049	1.33966195141559	0.189623533420635	0.887067559597203	-4.96264941643869
a_HQ_transcript/30868	2.44044305942348	-0.113956027035064	1.33965856370872	0.189624623641178	0.887067559597203	-5.01242014221715
a_HQ_transcript/52927	2.160757139491	0.0159042721188725	1.33964293977053	0.189629651747497	0.887067559597203	-5.02578461118007
a_HQ_transcript/43185	0.932991025189539	3.67470772312011	1.33963236457519	0.189633055122337	0.887067559597203	-5.32083006443068
a_HQ_transcript/71881	1.40207418155321	2.49101452122616	1.3396295550513	0.189633959308627	0.887067559597203	-5.02922051949378
a_HQ_transcript/15485	-1.76883424786523	0.739340695303085	-1.33960038110951	0.189643348525916	0.887067559597203	-4.89877667413689
a_HQ_transcript/42673	1.24120618333713	-0.416936104661324	1.33950064610922	0.189675449521548	0.887083044573714	-4.96967997804366
a_HQ_transcript/63456	1.82526240877653	2.68702750075323	1.33949526779005	0.18967718072197	0.887083044573714	-5.03962462691507
a_HQ_transcript/22958	-1.09474874850058	3.07574190606032	-1.33942913086732	0.189698470203359	0.887111237005699	-5.24498618098005
a_HQ_transcript/26691	-0.496422356546617	3.01038705153516	-1.3393601576818	0.189720674640674	0.88713225493383	-5.18398039152819
a_HQ_transcript/104938	2.06397359180509	-0.417304051783172	1.33926168405248	0.189752379585931	0.88713225493383	-5.00526799714498
a_HQ_transcript/16652	-1.22712631537124	2.20544255416631	-1.33924135488439	0.189758925351127	0.88713225493383	-4.95400749768347
a_HQ_transcript/151746	-1.7978181691795	1.51675479104018	-1.33922555935691	0.189764011454845	0.88713225493383	-5.00477949851567
a_HQ_transcript/144346	-1.71318253179518	-0.488104565730699	-1.33911578604599	0.189799360974402	0.887226156390834	-5.02953371157311
a_HQ_transcript/27474	0.538893835939492	2.52641025364278	1.33904478633026	0.189822227212199	0.887261693743224	-5.11698209704317
a_HQ_transcript/90694	1.86557293546706	0.900216984783846	1.33898065300461	0.189842883879418	0.887286898298853	-4.8961755780108
a_HQ_transcript/144039	2.77454936020275	1.38637474145152	1.33887413637697	0.189877195590245	0.887375914557789	-5.16770278709648
a_HQ_transcript/109583	1.56426850637507	0.62652913020278	1.33869052052719	0.189936354142557	0.887468602123352	-4.90046502914322
a_HQ_transcript/18373	-1.60927026213078	1.03527012263603	-1.33867284069273	0.189942051096828	0.887468602123352	-5.09629379454175
a_HQ_transcript/64616	-1.30993723124666	2.37533069601072	-1.33867042045797	0.189942830976752	0.887468602123352	-5.1394447973329
a_HQ_transcript/144680	1.51386754407303	-0.131599545753576	1.33854780099823	0.189982346265086	0.887581885846079	-5.01627843106334
a_HQ_transcript/38966	1.121419164504	2.6906402429259	1.33840920755898	0.190027016949354	0.887687069571032	-5.26886662035564
a_HQ_transcript/38601	-1.29308562179369	0.0801256863453949	-1.33833341657168	0.19005144891284	0.887687069571032	-4.92833377764603
a_HQ_transcript/6788	1.21625012016579	-0.162015263369426	1.33827469750088	0.190070379242273	0.887687069571032	-4.93661954141138

a_HQ_transcript/143026	-2.40723721623836	1.13561947732961	-1.33819502276112	0.190096067753952	0.887687069571032	-5.05210820372012
a_HQ_transcript/121384	-1.51487852760804	1.38734545567623	-1.33817830392007	0.190101458536637	0.887687069571032	-4.94846356447138
a_HQ_transcript/143107	2.68855223252557	0.236414220748932	1.33815365488037	0.190109406528329	0.887687069571032	-4.91457313788734
a_HQ_transcript/18732	-1.47393663470268	1.43605430543502	-1.33814635922876	0.190111759033336	0.887687069571032	-5.12402389854153
a_HQ_transcript/111999	2.42164386591533	0.861126568600772	1.33790746308321	0.190188804218264	0.887897350228637	-5.05819443931741
a_HQ_transcript/30130	1.46786556472087	0.727136390997906	1.33785204710123	0.190206679583898	0.887897350228637	-4.94919417693308
a_HQ_transcript/73599	-2.54219766742858	1.68363929597233	-1.33784473827735	0.19020903726575	0.887897350228637	-4.89843641228027
a_HQ_transcript/146998	-0.839665358049115	3.18911217521534	-1.33778845348925	0.190227194378462	0.887897350228637	-5.13155168329453
a_HQ_transcript/49702	1.38366864518073	0.356055587136379	1.33776993636542	0.190233168176222	0.887897350228637	-4.91268431272636
a_HQ_transcript/108281	0.827995136478541	-0.217380792298459	1.33750246247131	0.190319473890891	0.888228854141285	-4.91980605363712
a_HQ_transcript/97390	-1.27233900406753	-0.312785603082406	-1.33736994928756	0.190362243053482	0.888357134249585	-4.97588203095295
a_HQ_transcript/93481	-1.48850371481517	0.512205748909371	-1.33726828736737	0.190395059867291	0.888434856676943	-5.0363979537619
a_HQ_transcript/147701	-1.34338978856699	-0.377011290737092	-1.33719242492469	0.190419551362256	0.888434856676943	-4.98203335042254
a_HQ_transcript/131319	-0.318615750696737	3.58914092685077	-1.33717632249967	0.190424750195053	0.888434856676943	-5.29521126847623
a_HQ_transcript/93196	1.43673672277843	1.49166784847624	1.33693942625615	0.190501247214581	0.88864790809292	-4.96723291648739
a_HQ_transcript/98445	1.49563740096929	0.0326112469195318	1.33683556723768	0.190534792169712	0.88864790809292	-4.91623524988469
a_HQ_transcript/31351	-2.09782550052097	0.561367099629106	-1.33674548326486	0.190563891670791	0.88864790809292	-4.88513490395759
a_HQ_transcript/51772	1.81667901845546	2.38898238994301	1.33672645932872	0.190570037342218	0.88864790809292	-5.03960462790646
a_HQ_transcript/132398	-1.28016003913873	1.41210373851352	-1.33665150415194	0.190594253054354	0.88864790809292	-5.02551972880326
a_HQ_transcript/142428	0.900651260594861	1.08873488197141	1.3366455276551	0.190596183978706	0.88864790809292	-5.05803467395659
a_HQ_transcript/142055	-4.16337359983262	3.5672768463426	-1.3366112900157	0.190607245982014	0.88864790809292	-5.01699105997731
a_HQ_transcript/146197	-2.59204163765284	2.88315585726221	-1.33658936450437	0.19061433026056	0.88864790809292	-5.1356515137926
a_HQ_transcript/134892	1.7163277813927	1.15103033458296	1.33656051797311	0.190623651075701	0.88864790809292	-5.14270929103567
a_HQ_transcript/139903	-2.60800486542139	4.84081208052371	-1.33651910608371	0.190637032590144	0.88864790809292	-5.44936134515203
a_HQ_transcript/6934	1.4242914093679	0.192569382133062	1.33651431554595	0.190638580613796	0.88864790809292	-5.07570691993648
a_HQ_transcript/131371	1.34433816693361	0.327018627894508	1.33622034606969	0.190733593012545	0.888949504219931	-4.99652166668472
a_HQ_transcript/113872	1.41845114940076	-0.46254907559993	1.33621949874367	0.190733866925251	0.888949504219931	-5.01183281334511
a_HQ_transcript/149647	-3.20823767365809	7.46565556492541	-1.33613024756941	0.190762720599133	0.888991702905448	-5.87858656636228
a_HQ_transcript/99852	0.354909924431237	3.24249716776606	1.33605043254946	0.190788526542418	0.888991702905448	-5.24554046282525
a_HQ_transcript/101252	2.03870997389741	2.43811388438907	1.33604941985238	0.190788853986828	0.888991702905448	-5.06461752317413
a_HQ_transcript/107276	-1.69192102716864	1.28163695401554	-1.33596187604966	0.190817161944576	0.888991702905448	-5.02674826829227
a_HQ_transcript/88817	0.367604480084941	2.69106861383027	1.33592583907552	0.190828815715631	0.888991702905448	-5.1677881618607
a_HQ_transcript/140283	2.99135723412025	1.22365256371888	1.33590769548384	0.190834683265452	0.888991702905448	-5.09365991798137
a_HQ_transcript/8931	-1.21838539474382	-0.479113130196731	-1.33585721586398	0.190851008861649	0.888996509584895	-4.99074632901904
a_HQ_transcript/86705	0.589036908365575	3.9248486745835	1.33575274428961	0.190884799394148	0.889082661793514	-5.26548126254786
a_HQ_transcript/121507	-2.57287782585217	2.37876622754389	-1.33551141516525	0.190962873073116	0.889178137410342	-5.07612780263658
a_HQ_transcript/138687	0.76795359470735	6.62114446308323	1.33550950174137	0.190963492193399	0.889178137410342	-5.65543398137893
a_HQ_transcript/110116	1.86690881488003	0.573931617010864	1.33550783978656	0.190964029947917	0.889178137410342	-4.97864400757798
a_HQ_transcript/12514	-1.64088714805333	1.66089855802234	-1.33548300873593	0.190972064607085	0.889178137410342	-5.05005283557202
a_HQ_transcript/115830	1.35639281058347	-0.362132867184893	1.33542120774169	0.190992062874679	0.889178137410342	-5.02325246636921
a_HQ_transcript/127333	1.08132218335104	-0.160728892781262	1.33536760721955	0.191009408846462	0.889178137410342	-4.94708329641424
a_HQ_transcript/7554	1.13175383545798	2.93258506268845	1.33532083019979	0.191024547617035	0.889178137410342	-5.26016947346849
a_HQ_transcript/126650	-3.14876313285882	3.61122145202855	-1.33531117328583	0.191027673065998	0.889178137410342	-5.0119100277872
a_HQ_transcript/141213	1.33168478522487	3.52203179808798	1.33523836310616	0.19105123926384	0.889216625504721	-5.3229003560758
a_HQ_transcript/746	0.502261502298858	2.67458271308923	1.33508658702092	0.191100371258617	0.889334475847508	-5.07752727041962
a_HQ_transcript/1057	-1.26610210654931	1.09966646894468	-1.33506562106341	0.191107158990697	0.889334475847508	-4.9167285837786
a_HQ_transcript/115193	2.41205383202105	-0.141401596990441	1.33491903966521	0.191154619928674	0.889352293657422	-4.99977421166995
a_HQ_transcript/141251	0.462994013805423	2.85630707931284	1.33491645960884	0.191155455394917	0.889352293657422	-5.08508614048282
a_HQ_transcript/23511	-0.756648439798169	-0.641476980565025	-1.33491203703547	0.191156887506121	0.889352293657422	-5.03660635700504
a_HQ_transcript/24763	-2.21803183589198	1.84705793180526	-1.33483377908854	0.191182230246064	0.889399013985049	-5.02532227842592

a_HQ_transcript/150309	0.449830458029015	9.81869186704857	1.33473103289718	0.19121550708929	0.889403080652626	-5.97588992576313
a_HQ_transcript/136033	-1.93391131461418	0.204280160546033	-1.33466574466213	0.191236654583344	0.889403080652626	-5.01407893663933
a_HQ_transcript/35359	0.662997550030261	4.52919715929073	1.33459502578058	0.191259563148464	0.889403080652626	-5.2938959268037
a_HQ_transcript/69009	2.09518966229108	-0.0510547697740134	1.33453792061848	0.191278063248427	0.889403080652626	-5.08390661791469
a_HQ_transcript/27218	0.984807152710028	3.31569406349635	1.33444687623911	0.191307561332079	0.889403080652626	-5.30634468873582
a_HQ_transcript/29220	-3.36618363151893	1.95300516019165	-1.33444031203399	0.191309688248621	0.889403080652626	-5.01454478513048
a_HQ_transcript/131487	-1.763398310088	-0.337701220786923	-1.3343250356685	0.191347042762034	0.889403080652626	-5.10673751114272
a_HQ_transcript/48412	-1.3941517501354	-0.258244751592659	-1.33430680838924	0.191352949700678	0.889403080652626	-4.95100843186308
a_HQ_transcript/10145	-0.910397959886104	2.93116317378367	-1.3342911586255	0.191358021452459	0.889403080652626	-5.12005644159508
a_HQ_transcript/110050	-2.19240966966354	0.797573241705123	-1.33426874734906	0.191365284645299	0.889403080652626	-5.01188056932194
a_HQ_transcript/116782	3.9813028805837	2.39220284203198	1.33426718607692	0.191365790640481	0.889403080652626	-5.19473473650996
a_HQ_transcript/22273	1.53890815165234	0.286124292712828	1.33413887033721	0.191407380214958	0.889403080652626	-4.98474421327002
a_HQ_transcript/35824	1.24344415849519	0.0778633617317859	1.33413009060728	0.191410226146954	0.889403080652626	-4.87542809896989
a_HQ_transcript/132878	1.85149167237517	3.04607056734691	1.33409355432294	0.191422069660746	0.889403080652626	-5.16033024546722
a_HQ_transcript/11414	2.07151319346383	1.0372705459908	1.33408148889515	0.191425980884631	0.889403080652626	-5.03664897150371
a_HQ_transcript/84895	1.76597629439639	2.76281225158193	1.3340446308903	0.191437929446202	0.889403080652626	-5.10318805065981
a_HQ_transcript/102935	0.744255356985731	3.60194700924884	1.33402831936453	0.191443217470507	0.889403080652626	-5.29925171067694
a_HQ_transcript/133153	0.979670596392736	3.44539152083469	1.33384704579866	0.191501992014856	0.889605034111686	-5.21762853813141
a_HQ_transcript/133451	1.03355146236835	3.86279553349111	1.33374298935354	0.19153573663536	0.889690690358014	-5.39530330989037
a_HQ_transcript/136703	1.47472659947837	2.26256829972142	1.3336064188545	0.191580032233454	0.889825338686871	-4.98508365527247
a_HQ_transcript/8560	-0.530217853019677	2.37524501381583	-1.33348871534984	0.191618214792763	0.889852694115517	-5.08314818220011
a_HQ_transcript/100534	-0.712567232533605	2.37888653858279	-1.33348335743779	0.191619953017991	0.889852694115517	-5.0020616593782
a_HQ_transcript/91268	-1.56617810999024	0.0105980479464562	-1.3334394161673	0.191634208996432	0.889852694115517	-5.0373174498622
a_HQ_transcript/123901	-1.67126492245735	1.99763641705659	-1.33330576790093	0.191677573862222	0.889852694115517	-5.22893939021074
a_HQ_transcript/10919	-1.20643554310318	2.9878130859824	-1.33328731906178	0.191683560552093	0.889852694115517	-5.31048883275293
a_HQ_transcript/54015	-1.70139113613433	1.14680351749482	-1.33326686740665	0.191690197328479	0.889852694115517	-4.96847422675969
a_HQ_transcript/143339	-3.26742491012529	0.23610877070743	-1.3332579801188	0.19169308140164	0.889852694115517	-5.02099378563251
a_HQ_transcript/117636	1.99553807277521	0.258537367391739	1.33302674548751	0.19176813263739	0.890130001912177	-4.99264181495838
a_HQ_transcript/150467	1.58673431193973	2.97809616067142	1.3328174601261	0.191836079350673	0.890292049310598	-5.14763809531837
a_HQ_transcript/22417	-0.829130294583812	3.73275341672905	-1.33278047056299	0.191848090331412	0.890292049310598	-5.19441624558362
a_HQ_transcript/6380	0.498816994934021	3.20304012558925	1.33277769367285	0.191848992046252	0.890292049310598	-5.27770788087186
a_HQ_transcript/73001	1.44158987099742	-0.325422772915431	1.33268486076328	0.191879138728603	0.890360866609424	-5.01506194238671
a_HQ_transcript/139417	-2.37941252306882	2.45503159699529	-1.33260418607835	0.191905340093645	0.890411367254423	-5.19425078588629
a_HQ_transcript/60914	-1.87346385092654	1.49441134468098	-1.3325394799106	0.191926357224997	0.890437807708087	-4.99840179094651
a_HQ_transcript/141550	2.12358559682532	1.63076963154214	1.33233783004503	0.191991866233626	0.890670646482698	-5.06697584078969
a_HQ_transcript/31343	1.6109212675552	1.69841740320915	1.33214663381936	0.192053995109693	0.890881769974803	-4.96939763802338
a_HQ_transcript/22076	-2.32819364854573	1.3460523685173	-1.33210345489067	0.192068028167565	0.890881769974803	-5.08456691389394
a_HQ_transcript/51570	1.16597220624129	-0.780491638748991	1.33195933923364	0.19211487115395	0.891027944661038	-5.0264844596829
a_HQ_transcript/17520	0.835874601897839	-0.682499536403061	1.33181256040569	0.192162588805846	0.89111229576905	-5.03255626471063
a_HQ_transcript/139510	1.4500644839313	0.81358306745207	1.33176737102756	0.192177281663856	0.89111229576905	-4.89181411453263
a_HQ_transcript/16755	-0.566588497299807	2.08936983307348	-1.33176193666996	0.192179048647138	0.89111229576905	-5.02939763067415
a_HQ_transcript/117037	1.79817955765294	3.3658109178839	1.33161486520069	0.192226873734005	0.891142974356705	-5.42235773333993
a_HQ_transcript/42117	-2.29790253835859	1.27383816337594	-1.33154262073125	0.19225036973395	0.891142974356705	-5.06692251058516
a_HQ_transcript/141897	2.1384750616908	0.402279447932202	1.33152982085585	0.192254532855638	0.891142974356705	-5.06998402663179
a_HQ_transcript/146283	0.729403233404413	4.49381911694275	1.33147225319061	0.192273257426263	0.891142974356705	-5.4530482312437
a_HQ_transcript/133981	-0.365396968968111	4.72386474250735	-1.33146283176579	0.192276321990797	0.891142974356705	-5.45673208480293
a_HQ_transcript/71748	1.18916417520995	-0.294446832695263	1.33145504412757	0.192278855151927	0.891142974356705	-4.94691729832079
a_HQ_transcript/5810	0.9366267951933	1.48904701080726	1.33141162262828	0.19229297975771	0.891142974356705	-4.90449848007198
a_HQ_transcript/122602	-2.45544964430546	0.589688042440894	-1.33132226488749	0.192322049502912	0.891206640107242	-5.14182394364329
a_HQ_transcript/19892	1.8833192810459	0.263311464689568	1.33109860823353	0.192394824018456	0.891428331573487	-5.05458843349434

a_HQ_transcript/112430	0.664263860866969	0.278043380943017	1.33104692647169	0.192411643500165	0.891428331573487	-5.05762419760276
a_HQ_transcript/89272	1.83330997261527	-0.298249982294081	1.33098943728362	0.192430354298542	0.891428331573487	-5.03008429223658
a_HQ_transcript/136532	-2.51376223923613	1.23217604977428	-1.33095233757616	0.192442429749357	0.891428331573487	-5.11358728978528
a_HQ_transcript/45736	-3.06208220842073	0.899688863121195	-1.33091562542485	0.192454379629696	0.891428331573487	-4.967292668531
a_HQ_transcript/80892	-1.25261871105099	0.0595376613643239	-1.33088905861209	0.192463027537565	0.891428331573487	-4.90435447605666
a_HQ_transcript/56287	0.300183997560645	3.94347985738044	1.33084540010507	0.192477239705113	0.891428331573487	-5.36401412174577
a_HQ_transcript/120413	1.76881593014316	1.14694910261816	1.33071263626604	0.192520463319501	0.891557480229122	-5.14874613660095
a_HQ_transcript/51654	1.18727335447415	1.44045469842912	1.33058048321558	0.1925634954951	0.891596287858501	-4.87013429837952
a_HQ_transcript/138789	1.61253502712914	-0.1081797262507	1.33053156684491	0.192579425704422	0.891596287858501	-4.95124784734053
a_HQ_transcript/108349	2.42143839059865	1.76926108304004	1.33044407094669	0.19260792233273	0.891596287858501	-5.10447683384987
a_HQ_transcript/141918	-0.789785273093598	2.63636853566423	-1.33042696013513	0.192613495549514	0.891596287858501	-5.17193581609433
a_HQ_transcript/26655	-0.697793640134703	1.92016381606554	-1.33037722783533	0.192629694718981	0.891596287858501	-4.93030111087603
a_HQ_transcript/719	0.999665387757753	-0.340666803723455	1.33036316195345	0.19263427655128	0.891596287858501	-4.97502402696272
a_HQ_transcript/150343	0.717366914790922	10.5285485894655	1.33034921835098	0.192638818634909	0.891596287858501	-6.06518978297626
a_HQ_transcript/141762	-3.15896381423716	1.76377313745603	-1.33031013200178	0.192651551326431	0.891596287858501	-5.14010288922746
a_HQ_transcript/56530	-1.50670746370748	2.58863531730799	-1.33023058127231	0.192677467613339	0.891645238231684	-5.20620904119203
a_HQ_transcript/118059	-0.58942255090115	2.76455248250519	-1.3301831474794	0.192692922019605	0.891645770669927	-5.02545963489969
a_HQ_transcript/126118	-1.73967463308014	0.731002739512962	-1.33008729493481	0.192724154651215	0.89166510265061	-4.90529433354401
a_HQ_transcript/139770	-1.29137031716409	1.80292765587709	-1.33004107509547	0.192739216333399	0.89166510265061	-4.9219019149724
a_HQ_transcript/92017	1.48436263321426	0.105208505813213	1.33002909976055	0.192743118889432	0.89166510265061	-5.03763320803873
a_HQ_transcript/81097	-1.33210748993626	0.869998086266419	-1.32988343281551	0.19279059411105	0.891778465624104	-4.95383899277064
a_HQ_transcript/142485	2.11108913239948	2.38863738971211	1.32985977079341	0.192798306797036	0.891778465624104	-5.12097913395426
a_HQ_transcript/128641	0.545291803076986	3.20066846996651	1.32966467045692	0.192861909206935	0.891966200130679	-5.2308013312373
a_HQ_transcript/76692	1.47168646488838	0.362120200814082	1.32964113187255	0.192869583839845	0.891966200130679	-4.99767973390801
a_HQ_transcript/93078	-3.45176699203313	0.799175115490642	-1.32949371437693	0.192917653887869	0.892117532632781	-5.12337029757575
a_HQ_transcript/141236	-1.59602197419203	2.46135421279924	-1.32935122803701	0.192964124734205	0.892261446388504	-5.11904516985802
a_HQ_transcript/73878	2.98211563052323	3.14361252788683	1.32924084847233	0.193000130113632	0.892296011369808	-5.1182849905297
a_HQ_transcript/3651	-1.6730014238789	4.84905753625056	-1.32923419390341	0.193002300972898	0.892296011369808	-5.47037474195488
a_HQ_transcript/56419	-1.0823353183613	0.635363875878597	-1.32909320410422	0.193048299200538	0.892381029452396	-4.94013028263089
a_HQ_transcript/118826	0.855017295130123	2.91114608859374	1.32906915281337	0.193056146827789	0.892381029452396	-5.10739081693094
a_HQ_transcript/92988	1.48421544684664	-0.327707129926295	1.32897596279398	0.193086555850269	0.892381029452396	-5.01209342607796
a_HQ_transcript/133442	2.52971510607795	3.69110640076658	1.32894144887356	0.193097819091417	0.892381029452396	-5.28264283229517
a_HQ_transcript/130013	1.43773090787561	0.587727738814991	1.32880001359197	0.193143980227664	0.892381029452396	-5.07658778953213
a_HQ_transcript/125989	1.20462867053426	1.18498938455768	1.3287919969611	0.193146596920715	0.892381029452396	-4.95898185247712
a_HQ_transcript/2980	-1.44150160026482	0.490414966539441	-1.32879196733469	0.193146606591063	0.892381029452396	-4.91913867764482
a_HQ_transcript/104694	1.414915569604	0.964140614791495	1.32876479596529	0.193155475748509	0.892381029452396	-4.96153758207325
a_HQ_transcript/116879	0.410615170845255	3.48468718777605	1.32866218124017	0.193188973614207	0.892381029452396	-5.31365552151983
a_HQ_transcript/40260	-2.74292305253075	0.249128728535425	-1.32864354424655	0.193195058010755	0.892381029452396	-5.04055650702717
a_HQ_transcript/150968	-1.53370547725806	0.447817359974162	-1.32859148390898	0.193212054866059	0.892381029452396	-5.00728405229943
a_HQ_transcript/57321	-1.53862319561152	0.687447959558557	-1.32859006489133	0.193212518168384	0.892381029452396	-4.98288846173661
a_HQ_transcript/128201	0.500284510272587	5.63888366940269	1.32856633419324	0.193220266251855	0.892381029452396	-5.55465817363746
a_HQ_transcript/45318	-1.33134997085433	2.02011408772357	-1.32845333677978	0.193257163233953	0.892407851250446	-5.14727259591397
a_HQ_transcript/75800	-1.31992370683167	0.499270597175571	-1.32841800210964	0.193268702155523	0.892407851250446	-5.00841085969235
a_HQ_transcript/121038	2.17640934679146	0.097700475457516	1.32836517654221	0.193285953907892	0.892407851250446	-5.0647004559555
a_HQ_transcript/51823	0.817239382829768	3.60893466929184	1.32835269054011	0.193290031754342	0.892407851250446	-5.2765875413438
a_HQ_transcript/9960	-0.618372657877462	3.00865003527574	-1.3282906915436	0.193310281199685	0.892407851250446	-5.17654300295212
a_HQ_transcript/152682	2.27269623384637	3.3469357535011	1.32826290952066	0.193319355594209	0.892407851250446	-5.17276499951867
a_HQ_transcript/118987	1.82096780513803	1.16195707631874	1.32819569502391	0.193341311104366	0.892407851250446	-4.88814694439266
a_HQ_transcript/86777	1.09085146951648	2.09428066913813	1.32817248381931	0.193348893453209	0.892407851250446	-4.99668451349861
a_HQ_transcript/77304	2.31265836089172	0.380610829185937	1.32801494128202	0.193400363538497	0.892431739708622	-4.99308916502033

a_HQ_transcript/50677	2.04306005329789	0.974329389044285	1.32799869522699	0.193405671820359	0.892431739708622	-5.02175393675993
a_HQ_transcript/144377	1.45292006935558	-0.0715829128865861	1.32786998813912	0.193447729895237	0.892431739708622	-5.05695955866829
a_HQ_transcript/139658	1.76735880325912	1.36424687673179	1.32767821463071	0.193510409448704	0.892431739708622	-4.92317499930005
a_HQ_transcript/15917	1.46327382614831	0.684588885526996	1.3276532343202	0.193518575201284	0.892431739708622	-4.87277155084985
a_HQ_transcript/150780	-0.457535339356941	4.74374916607959	-1.32761386889638	0.193531443805817	0.892431739708622	-5.47394099622444
a_HQ_transcript/75475	-0.421698276096595	3.12115891307962	-1.32749653577853	0.193569804044905	0.892431739708622	-5.22068890394157
a_HQ_transcript/58983	1.53207329894052	-0.160231269501334	1.32744657376731	0.193586140121531	0.892431739708622	-5.10282219354826
a_HQ_transcript/9415	2.53738379530478	0.494384287692606	1.32743655190843	0.193589417095857	0.892431739708622	-5.00179869857308
a_HQ_transcript/119609	0.412164802218225	3.15612378946079	1.3274057826134	0.193599478388922	0.892431739708622	-5.22911803911585
a_HQ_transcript/73292	0.785536891218667	-0.541713363622129	1.32738894383628	0.193604984693369	0.892431739708622	-5.02186439207189
a_HQ_transcript/122448	1.00532396901055	-0.443821370079126	1.32735102165163	0.193617385743243	0.892431739708622	-5.03591003000938
a_HQ_transcript/38439	1.50263271226773	-0.241842793759766	1.32733238230046	0.193623481278358	0.892431739708622	-5.06013783982112
a_HQ_transcript/7121	1.57738990082109	1.88930267597549	1.32732868078966	0.193624691782733	0.892431739708622	-4.95044908100462
a_HQ_transcript/39007	0.448595552378056	2.75209612744565	1.32720505479724	0.193665124508371	0.892431739708622	-5.10934824468436
a_HQ_transcript/132947	0.368963166357019	4.04976459600232	1.32718266179525	0.193672448986669	0.892431739708622	-5.4246253067491
a_HQ_transcript/108936	1.08149130384031	0.492943494534378	1.32715241783455	0.193682341755854	0.892431739708622	-4.9271939887347
a_HQ_transcript/68394	1.78152135028201	1.80653366376851	1.32710873044059	0.1936966325441	0.892431739708622	-4.98009802214749
a_HQ_transcript/32679	1.32011566647798	1.6774139793937	1.32704409977289	0.193717775670726	0.892431739708622	-4.92915435883917
a_HQ_transcript/151856	-0.955004130715596	4.52786678909078	-1.32703152069616	0.193721890966845	0.892431739708622	-5.49246751244612
a_HQ_transcript/133491	0.473691042504629	3.02853568618182	1.32691930738969	0.193758604978972	0.892431739708622	-5.18608805804696
a_HQ_transcript/97909	2.65802728221696	1.29447214337005	1.3267318557256	0.193819947443335	0.892431739708622	-5.02141792285623
a_HQ_transcript/152776	-1.47793396522772	0.51316918149801	-1.32666592878604	0.193841525196329	0.892431739708622	-4.97152314103451
a_HQ_transcript/129581	1.24775609536601	-0.56663259151781	1.32658912114114	0.193866666509626	0.892431739708622	-5.02576723803469
a_HQ_transcript/17475	-1.21471554571896	2.3291396467404	-1.32654717860401	0.193880396545121	0.892431739708622	-5.13809806886982
a_HQ_transcript/7834	0.646385819441225	4.85816504042717	1.32654695131316	0.193880470951606	0.892431739708622	-5.44208032868651
a_HQ_transcript/151215	-1.045194281078	6.16108319095365	-1.32648144976168	0.193901914613078	0.892431739708622	-5.54422019646278
a_HQ_transcript/149242	1.4978187759154	1.60343844764988	1.32647008968065	0.193905633820801	0.892431739708622	-4.9005213968888
a_HQ_transcript/55869	1.43865260617356	1.97821154977946	1.32641674295275	0.193923099883619	0.892431739708622	-4.99570812772575
a_HQ_transcript/138319	-1.91789717259031	-0.125508228967079	-1.32636223542477	0.193940947248225	0.892431739708622	-4.99177588585837
a_HQ_transcript/94509	-1.55582762905192	2.39312343229219	-1.32635165228619	0.19394441262472	0.892431739708622	-5.22567299634402
a_HQ_transcript/29913	-1.62144948054259	-0.175204613665934	-1.32632412679926	0.193953425880297	0.892431739708622	-5.00295859248549
a_HQ_transcript/138829	-2.14907357343829	1.94047616860744	-1.32628308886496	0.193966864401403	0.892431739708622	-5.135532239395
a_HQ_transcript/99964	-1.50682286838762	1.66808035303489	-1.32626626826047	0.193972372781243	0.892431739708622	-4.97853819743481
a_HQ_transcript/92259	-1.27397000242301	0.197021232166895	-1.32624002672932	0.193980966548355	0.892431739708622	-4.89094582565156
a_HQ_transcript/40724	-1.54861915189659	-0.0177663621051401	-1.32622576191329	0.193985638216567	0.892431739708622	-5.01477207414601
a_HQ_transcript/71561	2.04952133003017	0.0204431109340693	1.32617769123622	0.194001381800736	0.892431739708622	-5.01622826976027
a_HQ_transcript/35312	-1.5076299533596	-0.0255242874890567	-1.32614355451486	0.194012562483606	0.892431739708622	-5.03286088294349
a_HQ_transcript/106707	-1.49745777485108	1.84118028112842	-1.32609491551862	0.194028493901733	0.892431739708622	-5.05847861175236
a_HQ_transcript/110682	0.507081571333165	5.24386364879033	1.32607540454016	0.194034884890772	0.892431739708622	-5.53132633089653
a_HQ_transcript/101055	0.722854844713557	2.06787080082022	1.32606092959817	0.194039626387174	0.892431739708622	-5.04344619476237
a_HQ_transcript/115462	-1.70378058539428	3.14930547889874	-1.3260274845312	0.19405058218873	0.892431739708622	-5.13123610809687
a_HQ_transcript/32493	-1.29124066409625	1.09583946855897	-1.32601284605343	0.194055377552354	0.892431739708622	-4.91380009001798
a_HQ_transcript/25007	1.63016271824366	2.80947254742153	1.3259551783512	0.194074269582691	0.892431739708622	-5.12279722311598
a_HQ_transcript/137607	-0.993023587228969	0.564078984231839	-1.32594804101129	0.194076607884762	0.892431739708622	-4.86623142332973
a_HQ_transcript/112874	-1.9793156483776	0.448182497149222	-1.32593703521095	0.194080213596245	0.892431739708622	-4.96411467655456
a_HQ_transcript/137348	2.58094025228466	0.792257237765684	1.32592072674825	0.194085556656334	0.892431739708622	-5.00061329261538
a_HQ_transcript/21061	-1.53122180890736	0.306220482810535	-1.32590409126115	0.194091006974202	0.892431739708622	-4.91303846923434
a_HQ_transcript/103402	3.87180148472553	1.96925091932178	1.32573598528754	0.194146090467058	0.892531613778604	-5.1465562775982
a_HQ_transcript/98058	0.460267271010096	4.36874021547441	1.32569836397782	0.194158419532415	0.892531613778604	-5.42138342457229
a_HQ_transcript/92020	-0.272135100989665	3.88626040237105	-1.32568382004853	0.194163185956543	0.892531613778604	-5.38825055877457

a_HQ_transcript/116043	0.322260991487389	4.7534639457479	1.32565037685732	0.194174146501587	0.892531613778604	-5.48110020746776
a_HQ_transcript/90444	-2.9255248758046	1.76998064800005	-1.32548383948525	0.194228733905799	0.892667068358798	-5.09882209200541
a_HQ_transcript/36305	-1.08347929914558	2.38181425717931	-1.32546677155023	0.194234329077245	0.892667068358798	-5.07704361233194
a_HQ_transcript/131106	-2.61986871882405	0.947490942429505	-1.32540410910283	0.194254871999067	0.892690900431793	-5.08799177122211
a_HQ_transcript/119025	-1.72986130254921	2.24625115600605	-1.32513681351203	0.194342519497458	0.892715991882619	-4.97342566104427
a_HQ_transcript/142396	-1.90896581195016	0.749902292289813	-1.32508060525115	0.19436095431823	0.892715991882619	-5.08760163773656
a_HQ_transcript/14011	-2.10052658903053	2.14933598761103	-1.32496671707711	0.194398310739749	0.892715991882619	-5.14173886638969
a_HQ_transcript/7620	1.47054687134812	-0.456272972959257	1.32494783366969	0.194404505212697	0.892715991882619	-5.03889317273524
a_HQ_transcript/22084	0.647535958100708	3.48370493589453	1.32494350111973	0.194405926474581	0.892715991882619	-5.28411804685258
a_HQ_transcript/132903	-2.95003940095433	3.59406361708493	-1.32492143964258	0.194413163707404	0.892715991882619	-5.27877606079459
a_HQ_transcript/131256	1.50657597038572	1.75792853835814	1.32487125074596	0.194429628866593	0.892715991882619	-5.12808984979884
a_HQ_transcript/101822	-1.66947360807321	3.356720987135	-1.32483809545171	0.194440506505119	0.892715991882619	-5.28433564732578
a_HQ_transcript/10591	0.778556257660465	0.521744038499089	1.32482918690635	0.194443429313651	0.892715991882619	-4.98637252571167
a_HQ_transcript/112614	-1.64055769011719	-0.0366207426231856	-1.32481446108155	0.194448260789913	0.892715991882619	-4.92338075999082
a_HQ_transcript/38424	0.735188607805072	2.5220339251236	1.32473657783249	0.194473815463458	0.892715991882619	-5.02284741700007
a_HQ_transcript/60169	-1.64392475200464	0.548447678251514	-1.32471628864824	0.194480473075478	0.892715991882619	-5.08805737083193
a_HQ_transcript/8944	-1.85510963702085	2.1443879471567	-1.32469839639731	0.194486344312573	0.892715991882619	-5.03499940173056
a_HQ_transcript/110538	-4.50159842793956	2.61528028486823	-1.32468862302249	0.194489551445617	0.892715991882619	-5.08191191130114
a_HQ_transcript/22819	0.488801618994087	2.83863730275056	1.32468512759743	0.194490698479244	0.892715991882619	-5.12536093231163
a_HQ_transcript/129675	1.39059553522012	1.36709784514957	1.32462399569318	0.194510759926492	0.892737580182164	-4.9383391910835
a_HQ_transcript/27688	-2.114649262486	1.64552623753354	-1.32452457852063	0.194543388711967	0.89279722141066	-5.1451301449964
a_HQ_transcript/101847	-0.495536583711829	4.42154049583076	-1.32441452783471	0.194579512324709	0.89279722141066	-5.41638435115696
a_HQ_transcript/125616	0.848017853181712	4.43747324707585	1.32437964568551	0.194590963298645	0.89279722141066	-5.40394634571928
a_HQ_transcript/121949	1.11976285074412	4.45216476810398	1.32435967710979	0.194597518736554	0.89279722141066	-5.47286630504527
a_HQ_transcript/99326	-0.95764983927797	-0.527082377466372	-1.32430560836313	0.194615269692476	0.89279722141066	-4.9980411340012
a_HQ_transcript/105780	2.59977809042812	1.5641272269663	1.32430365933797	0.194615909587367	0.89279722141066	-5.15040892045875
a_HQ_transcript/136631	1.42010801760923	-0.330200324460498	1.32422973346739	0.194640181778666	0.892806433764292	-5.0355330635028
a_HQ_transcript/63605	-2.65840117277956	2.35697727891563	-1.32420398495119	0.194648636373088	0.892806433764292	-5.04435137269436
a_HQ_transcript/71228	0.524101341282982	3.53880533413117	1.32410193926903	0.194682146119783	0.892889678842573	-5.31298172748207
a_HQ_transcript/122676	2.0414614348809	0.916759081443619	1.32400237611584	0.19471484492244	0.892969191767382	-4.97337766148652
a_HQ_transcript/34180	1.14558817217808	-0.618587034242759	1.32380382255147	0.194780067014469	0.893097911343604	-5.03675629720255
a_HQ_transcript/25232	-1.93293391481799	0.959540924495471	-1.32377071588486	0.194790943726432	0.893097911343604	-5.01457741498532
a_HQ_transcript/7437	-0.853780657469846	0.0913737440397669	-1.32374443980136	0.194799576681915	0.893097911343604	-4.98031771110278
a_HQ_transcript/121231	2.58931731640924	1.12428233856993	1.32368957666034	0.194817602805677	0.893097911343604	-4.96312238741319
a_HQ_transcript/135117	3.21741547830427	1.49276809002197	1.32368308961674	0.194819734307681	0.893097911343604	-5.02817207363008
a_HQ_transcript/101065	1.40424693789008	0.565611679892464	1.3236165444296	0.194841600648411	0.893125119571935	-5.04655578320191
a_HQ_transcript/127183	1.45721130181236	0.0459364824523656	1.32349076308246	0.194882936774166	0.893125119571935	-4.93406916325987
a_HQ_transcript/82466	2.32772733604219	2.30641375938072	1.32345906739269	0.19489335414209	0.893125119571935	-5.01091333226378
a_HQ_transcript/2873	-1.0745158586937	1.98405466691969	-1.32340819273036	0.194910075923634	0.893125119571935	-4.9786553384085
a_HQ_transcript/61900	1.34867692002806	-0.253256370224992	1.32339992000606	0.194912795155131	0.893125119571935	-4.95795265325901
a_HQ_transcript/94310	-2.73592841522588	1.77022888645535	-1.32338451664423	0.194917858293586	0.893125119571935	-4.98741135591248
a_HQ_transcript/141663	-0.589804168677631	2.71998775090903	-1.32321839896689	0.194972468165475	0.893294886641011	-5.11878018773994
a_HQ_transcript/32064	-1.09005617908319	-0.713570682829846	-1.32317832438544	0.194985644125054	0.893294886641011	-4.96980458780155
a_HQ_transcript/111384	-0.480159408536552	3.8211282802714	-1.3230867905803	0.195015741717889	0.89336236382516	-5.30966403447352
a_HQ_transcript/67448	-0.4217269270301	2.89779354167273	-1.32302292556463	0.195036743538285	0.893388166146055	-5.10681046243114
a_HQ_transcript/127916	1.85360408189145	0.0699360242075914	1.32295782136981	0.195058154645744	0.893415839039304	-5.05941030969314
a_HQ_transcript/39842	1.04021677671072	1.42485403503191	1.32285626992462	0.195091555924761	0.893468405377904	-4.98479895129366
a_HQ_transcript/125076	-1.30102879350579	1.86706596892086	-1.32281937524799	0.19510369203739	0.893468405377904	-4.97017433844428
a_HQ_transcript/56623	-0.435982835761533	3.3199410373319	-1.32274126525062	0.195129387404472	0.893468405377904	-5.30307529849264
a_HQ_transcript/124175	1.3195101389531	0.193288852192821	1.32256871496027	0.195186159421198	0.893468405377904	-4.98980692774807

a_HQ_transcript/983	0.395150604643344	3.88283353933193	1.32253226528728	0.195198153612692	0.893468405377904	-5.34158063350247
a_HQ_transcript/151814	1.74002867792851	-0.21736013650412	1.32250791426424	0.19520616691697	0.893468405377904	-5.01528113579238
a_HQ_transcript/22455	-1.28705571479903	1.76480511601105	-1.3224829593639	0.195214379203857	0.893468405377904	-5.06257380084203
a_HQ_transcript/149405	-1.99368228651858	0.592438180670828	-1.3224629437153	0.195220966247994	0.893468405377904	-5.05060038255623
a_HQ_transcript/41455	-1.72052012487587	0.663616197018449	-1.32246216413809	0.195221222806184	0.893468405377904	-5.05311850731039
a_HQ_transcript/102924	0.480986116432052	2.73062053684296	1.32245573561439	0.195223338437668	0.893468405377904	-5.16432193410441
a_HQ_transcript/68347	0.778184086516221	3.05147123275718	1.32235716014176	0.19525578189553	0.893546535193149	-5.24773309466995
a_HQ_transcript/151968	-2.30023277472263	2.19162791734179	-1.32230404846817	0.195273263885276	0.893556190122278	-5.11567360459551
a_HQ_transcript/115239	1.23625892991477	1.76537594229583	1.32222706948478	0.195298604058836	0.89359712686044	-4.94528387378836
a_HQ_transcript/15678	2.77611375012269	0.511551999991801	1.32216157805776	0.195320164710427	0.89359712686044	-5.01027420062124
a_HQ_transcript/2685	1.314998573231	1.27669885449674	1.32187216689197	0.195415464596598	0.89359712686044	-4.93271255279215
a_HQ_transcript/61976	1.39332199962998	0.746419106581953	1.32185442143551	0.195421309139143	0.89359712686044	-5.03470246535168
a_HQ_transcript/108325	-1.53197038279336	1.6155303070858	-1.32185170386664	0.195422204194001	0.89359712686044	-4.88551891982502
a_HQ_transcript/55742	-0.521045568105883	2.61574302173143	-1.32178453356108	0.195444328315908	0.89359712686044	-5.03692706728178
a_HQ_transcript/59949	1.31437285099079	-0.174428834308468	1.32168881596091	0.195475858481766	0.89359712686044	-5.04228007931716
a_HQ_transcript/51665	-0.726398397261766	4.19138723853578	-1.32160384281228	0.195503852606624	0.89359712686044	-5.37764225542477
a_HQ_transcript/117135	-0.566939356929967	2.31499960867876	-1.3215546618486	0.195520056518827	0.89359712686044	-5.021083916986
a_HQ_transcript/83308	1.3618706852466	-0.0433436299213536	1.32151228869605	0.195534018251747	0.89359712686044	-4.87252850715701
a_HQ_transcript/47774	-1.15006058264312	-0.248193905700948	-1.32142056215305	0.19556424428948	0.89359712686044	-4.99373885228125
a_HQ_transcript/90261	-0.740693268817721	4.89993244250419	-1.32141580350881	0.195565812471668	0.89359712686044	-5.49069706211336
a_HQ_transcript/65572	1.18138514911177	3.34978558400154	1.32135574742478	0.195585604417	0.89359712686044	-5.2731322038754
a_HQ_transcript/93609	0.926078216235299	1.91880328564943	1.32135469159074	0.195585952388991	0.89359712686044	-4.92854602244989
a_HQ_transcript/140543	0.29595104363401	4.45818384719245	1.32135316579078	0.195586455248825	0.89359712686044	-5.46753524519484
a_HQ_transcript/45014	1.45442749609273	0.459709934126124	1.32134022923918	0.195590718804471	0.89359712686044	-4.87034766866655
a_HQ_transcript/149241	0.380643694762356	5.26645151187995	1.32130007657672	0.195603952546359	0.89359712686044	-5.54059737008916
a_HQ_transcript/53663	-1.15280317688278	3.00724747644938	-1.32129631793226	0.195605191376844	0.89359712686044	-5.32010029464951
a_HQ_transcript/125585	2.68175816673385	3.96608936202745	1.32126732497235	0.195614747514231	0.89359712686044	-5.31099707103094
a_HQ_transcript/115352	3.4902495605907	0.970707050249307	1.32123053563612	0.195626873868328	0.89359712686044	-4.88213819093369
a_HQ_transcript/15083	1.73404186446607	1.60382378006398	1.32119407887112	0.195638891170465	0.89359712686044	-5.17159733365924
a_HQ_transcript/95422	0.345008818135499	5.67611144978673	1.32115581983353	0.195651503167578	0.89359712686044	-5.60060380810467
a_HQ_transcript/59878	-0.582765706227464	3.22661330346036	-1.32111606458329	0.195664609048218	0.89359712686044	-5.30052407365845
a_HQ_transcript/359	1.00434555422953	2.02737706028792	1.32105098518603	0.195686064845656	0.89359712686044	-5.12432333395599
a_HQ_transcript/51658	1.46296005938714	-0.012423041667348	1.32105067408501	0.195686167415788	0.89359712686044	-4.93850349397034
a_HQ_transcript/145370	-0.446267622959368	5.03510252960938	-1.32097967915464	0.19570957555211	0.89359712686044	-5.47153399683073
a_HQ_transcript/132475	-1.09381010550473	1.6492309356791	-1.32097755905551	0.195710274614899	0.89359712686044	-4.96716727504912
a_HQ_transcript/39635	-0.848463280901604	1.87532488058109	-1.32094863976951	0.195719810396479	0.89359712686044	-4.9223270048939
a_HQ_transcript/99077	1.79115783948646	0.673038257281093	1.32085144940697	0.195751860343721	0.89359712686044	-5.08879855983835
a_HQ_transcript/128071	1.39979261494789	0.525282244113217	1.32083290661205	0.195757975559511	0.89359712686044	-4.94318976514699
a_HQ_transcript/116361	1.61496526521228	0.67411609885418	1.32082238110941	0.195761446823097	0.89359712686044	-5.01156059222842
a_HQ_transcript/99418	1.47379605357863	0.616282484524272	1.3207298288113	0.195791972188935	0.89359712686044	-4.97137258689349
a_HQ_transcript/16823	-1.43737742781014	0.575775503590471	-1.3207176389955	0.195795992875346	0.89359712686044	-4.94920358787746
a_HQ_transcript/145675	-0.940361949401145	4.5093839080929	-1.32062173491846	0.195827628066057	0.89359712686044	-5.49196378022283
a_HQ_transcript/29257	0.860624504210184	2.01482575791034	1.32050181604069	0.195867190362458	0.89359712686044	-4.97558246480003
a_HQ_transcript/121758	-0.438600772012308	2.85324626326831	-1.32045780479974	0.195881711596963	0.89359712686044	-5.18226110118523
a_HQ_transcript/30969	1.58670201321149	3.7135978653457	1.32045152317228	0.195883784248101	0.89359712686044	-5.46636562168465
a_HQ_transcript/147270	1.61605531384996	0.315437570696267	1.32044286799193	0.195886640091209	0.89359712686044	-5.06748929871936
a_HQ_transcript/123438	-0.594679380720626	1.65127405807596	-1.32041782213837	0.195894904342495	0.89359712686044	-4.97456175533633
a_HQ_transcript/144985	-1.79143150376164	0.0489472342181504	-1.32041108678983	0.195897126816381	0.89359712686044	-5.06721059023428
a_HQ_transcript/129293	0.948684892297884	2.20199515166109	1.32028402032545	0.195939058767488	0.893718268050578	-5.0237263508159
a_HQ_transcript/28431	0.60633195446959	4.24927870429393	1.32011425594316	0.195995091778446	0.893884663968709	-5.42166029962668

a_HQ_transcript/72400	1.02305326693406	4.82455035543946	1.32008031719101	0.196006295192423	0.893884663968709	-5.51962200200423
a_HQ_transcript/27199	-0.977594436116808	1.68652458413701	-1.31991825188793	0.196059800828086	0.894045617956921	-5.06582333674915
a_HQ_transcript/70454	2.76388163092362	0.952116298914895	1.31984907043002	0.196082644401379	0.894045617956921	-5.11180366454725
a_HQ_transcript/136445	0.867499945834395	-0.576446699058396	1.31980904667587	0.196095861093503	0.894045617956921	-5.09808159939044
a_HQ_transcript/128631	2.41320143553644	0.669994336201497	1.31978709323511	0.19610311087515	0.894045617956921	-4.99731694791198
a_HQ_transcript/20848	1.49693481380933	2.35626895785844	1.31971821763945	0.196125857300982	0.894072002531164	-5.134319586806
a_HQ_transcript/17352	1.07318720928547	2.28578804252531	1.31962049798102	0.196158133058789	0.894072002531164	-5.13018970489555
a_HQ_transcript/88269	-1.76260762348982	3.43891225859966	-1.31960312468355	0.196163871699455	0.894072002531164	-5.15743358310153
a_HQ_transcript/97113	-1.30058172552734	3.42873574928492	-1.3195832930476	0.196170422520686	0.894072002531164	-5.20626206658408
a_HQ_transcript/119099	0.357414135733767	3.01536069341039	1.31935306513084	0.196246484092219	0.894221662522477	-5.27245301453947
a_HQ_transcript/146800	1.19064773185627	3.33595379926384	1.31926270907381	0.196276341661349	0.894221662522477	-5.32048717638652
a_HQ_transcript/90411	1.88378333590629	1.21232875132805	1.31925439814952	0.196279088126911	0.894221662522477	-5.02378777952797
a_HQ_transcript/2460	1.39923012724167	0.925297666115456	1.31922230126947	0.196289695283423	0.894221662522477	-4.90983502472215
a_HQ_transcript/44668	-1.77871544663828	0.811048822888781	-1.31915471923031	0.196312030768457	0.894221662522477	-5.07481166212681
a_HQ_transcript/95262	1.74660145758047	0.771813800184563	1.31911913840731	0.196323790813894	0.894221662522477	-4.95843398902274
a_HQ_transcript/122973	-0.766253887917496	3.67800110140499	-1.31908382328664	0.196335463574392	0.894221662522477	-5.33917140012545
a_HQ_transcript/2964	0.370591458450274	2.45153317824708	1.31899345007117	0.196365337193558	0.894221662522477	-5.12785433812753
a_HQ_transcript/141651	1.91510807754633	-0.0657283010047666	1.31891843003907	0.196390138336852	0.894221662522477	-5.04422035836757
a_HQ_transcript/70950	1.51899546779208	2.78248888054731	1.31889848838532	0.196396731324141	0.894221662522477	-5.2558123203397
a_HQ_transcript/4708	1.47974162440001	-0.416875417173892	1.31876007523847	0.196442497306005	0.894221662522477	-5.02045837437599
a_HQ_transcript/31874	1.43046590693529	0.31604912637511	1.31859806924355	0.196496074591245	0.894221662522477	-4.9580022643829
a_HQ_transcript/110267	1.27012405196446	0.701477537011889	1.31853773394952	0.19651603104388	0.894221662522477	-4.94288394438025
a_HQ_transcript/84632	-0.735034159853381	1.69858903701948	-1.31853762750017	0.196516066254356	0.894221662522477	-4.95623973296523
a_HQ_transcript/126905	-1.39933606292372	1.06769770229747	-1.31849406675686	0.196530475337949	0.894221662522477	-4.93228806379309
a_HQ_transcript/151631	-2.54032777835149	0.599282684469373	-1.31846474537917	0.196540174760265	0.894221662522477	-5.02756342270044
a_HQ_transcript/129694	-1.28488331672605	1.09978628104281	-1.31845524140812	0.196543318723639	0.894221662522477	-5.06674679814235
a_HQ_transcript/136686	-0.736163092424192	1.25099698331625	-1.31842542289538	0.196553183093957	0.894221662522477	-5.06459615488085
a_HQ_transcript/16433	-2.26565850600573	-0.164061076632931	-1.31831970676404	0.196588158488223	0.894221662522477	-5.02995947777295
a_HQ_transcript/97516	1.54898321908661	2.01988797890938	1.31829998990971	0.196594682190321	0.894221662522477	-5.0299217720517
a_HQ_transcript/111489	2.44227168485651	1.25020200768121	1.31828009285362	0.196601265683857	0.894221662522477	-5.14667542103035
a_HQ_transcript/7512	0.603794706373491	2.25968320839593	1.31826794273333	0.196605285971653	0.894221662522477	-5.01154745434994
a_HQ_transcript/13171	1.55340685503439	-0.0206994478578764	1.31825149469139	0.196610728475958	0.894221662522477	-4.95790854034404
a_HQ_transcript/127176	1.8942485291144	1.9505743371521	1.31819105430498	0.196630728630211	0.894221662522477	-5.03168959737055
a_HQ_transcript/128465	1.00341612931201	3.37819502192589	1.31806312615602	0.196673066108672	0.894221662522477	-5.28767249197876
a_HQ_transcript/139228	4.87911456877041	5.50461816463766	1.3180081974499	0.196691246760682	0.894221662522477	-5.47187173091161
a_HQ_transcript/47147	-1.50193710920936	1.12862978816744	-1.31792146804941	0.196719955630866	0.894221662522477	-5.10751742743247
a_HQ_transcript/148413	0.623858466694019	4.11649022845944	1.31788011543175	0.196733645163813	0.894221662522477	-5.41417320664019
a_HQ_transcript/150187	-1.44834292678917	1.48305588939987	-1.31773655338023	0.196781176175943	0.894221662522477	-4.95365543736997
a_HQ_transcript/127912	2.02328026294358	1.58152498742741	1.31766364675925	0.196805317716991	0.894221662522477	-4.93473130870604
a_HQ_transcript/2306	1.21005154475823	1.7988941644485	1.31758987205366	0.196829749016804	0.894221662522477	-4.97844635214871
a_HQ_transcript/137597	1.17081047931814	-0.266159500365097	1.31758967360864	0.196829814737174	0.894221662522477	-5.1020527345328
a_HQ_transcript/117229	1.21529564945326	0.309445698778631	1.31757150170609	0.196835832919507	0.894221662522477	-5.10723782324401
a_HQ_transcript/91722	0.960685593335347	1.83715285274071	1.3175628373549	0.196838702434531	0.894221662522477	-5.05838582879188
a_HQ_transcript/108472	1.58664504146125	0.269968425251405	1.3175494939868	0.196843121638881	0.894221662522477	-4.99105632551297
a_HQ_transcript/57133	1.40740397716667	0.329460640964551	1.31754009954819	0.196846233038653	0.894221662522477	-4.94535445499984
a_HQ_transcript/118609	1.00968027340217	-0.659737034182292	1.31743618414016	0.196880651912211	0.894221662522477	-5.03979804514781
a_HQ_transcript/120110	-1.83252387844363	2.94912536622713	-1.31743030736383	0.196882598556518	0.894221662522477	-5.22004080040627
a_HQ_transcript/23482	1.49829316186678	-0.110933432111156	1.31742919163882	0.196882968134905	0.894221662522477	-4.99179606027069
a_HQ_transcript/140624	1.84379230658901	0.168524817023602	1.31740274592762	0.196891728301512	0.894221662522477	-5.16225766414791
a_HQ_transcript/116298	0.481455600571252	4.33390866592274	1.31738119879288	0.196898866030887	0.894221662522477	-5.44139269691925

a_HQ_transcript/2300	1.34429526529059	0.333711016666749	1.31737292467115	0.196901606978936	0.894221662522477	-4.87993355337055
a_HQ_transcript/75821	-3.11124832183402	0.732248829032141	-1.31721870448014	0.196952700475188	0.894221662522477	-5.00446969624018
a_HQ_transcript/67492	-0.423025290091622	2.42765081036711	-1.31719642950177	0.19696008106516	0.894221662522477	-5.07810497124718
a_HQ_transcript/151567	-0.8046274466043	3.42925295345686	-1.31715921625145	0.196972411773992	0.894221662522477	-5.3858061142374
a_HQ_transcript/131621	-0.535752596603333	3.02337109368074	-1.31710263006213	0.196991162890855	0.894221662522477	-5.26602785226516
a_HQ_transcript/146830	1.66289926759205	0.599648136590569	1.3170201490879	0.19701849728149	0.894221662522477	-5.179009351798
a_HQ_transcript/123535	1.58768458635798	0.547713193284786	1.31700568485724	0.197023291061337	0.894221662522477	-4.9193981451536
a_HQ_transcript/129865	0.389604587393468	3.03772508449018	1.31697507389448	0.19703343653553	0.894221662522477	-5.24966608327309
a_HQ_transcript/151916	4.16609524746396	0.292989580162169	1.31695331192183	0.197040649408417	0.894221662522477	-5.03086639275974
a_HQ_transcript/150435	1.83077122082332	0.387186431392066	1.3169372910362	0.197045959562366	0.894221662522477	-4.98914926400242
a_HQ_transcript/68857	1.1398638440897	2.87282654655318	1.31692479229473	0.197050102370864	0.894221662522477	-5.13288398365631
a_HQ_transcript/131464	0.474959426691117	2.56093088984449	1.31689332250612	0.197060533581221	0.894221662522477	-5.11401704196976
a_HQ_transcript/11709	0.929432459476674	1.30746468391573	1.31686549538632	0.197069757717899	0.894221662522477	-4.92698514247841
a_HQ_transcript/151114	-2.93584441811802	2.98423487747295	-1.31682622838007	0.197082774512101	0.894221662522477	-5.23731446846775
a_HQ_transcript/144742	-1.32312022419796	3.50940085591877	-1.31678776784489	0.197095524604168	0.894221662522477	-5.15628750129924
a_HQ_transcript/145655	-2.18111780821246	3.52618192386269	-1.3167635952125	0.197103538422096	0.894221662522477	-5.37552392158569
a_HQ_transcript/6693	-0.690230963073348	2.3352462932767	-1.31671774769866	0.197118738678259	0.894221662522477	-5.02026351963257
a_HQ_transcript/6726	0.642304582153275	2.3977727974598	1.31669682172739	0.197125676760205	0.894221662522477	-5.04039914978868
a_HQ_transcript/127876	1.43354409818449	1.31660711636213	1.31669020159336	0.197127871728698	0.894221662522477	-4.90969998961805
a_HQ_transcript/107558	-1.55901045700426	1.97617716943557	-1.31662093057821	0.197150840316538	0.894221662522477	-5.2642763063116
a_HQ_transcript/72376	1.45207822417377	-0.412691308579743	1.31658500628735	0.197162752747019	0.894221662522477	-5.03937630447719
a_HQ_transcript/22688	-1.91416650651288	1.4829950327203	-1.31649138997937	0.19719379833774	0.894221662522477	-5.04210161908911
a_HQ_transcript/51038	1.61341113905401	1.55210572419349	1.31646991494939	0.197200920541528	0.894221662522477	-5.05168506627497
a_HQ_transcript/109717	-2.53750963721265	1.66020339741961	-1.31646304810663	0.197203197974739	0.894221662522477	-5.07577414424117
a_HQ_transcript/76475	1.53649017759822	1.77000014773476	1.31638596761726	0.19722876360445	0.894267829235526	-4.92016312695018
a_HQ_transcript/60606	-1.16638488119552	1.78127772412791	-1.31630446498938	0.197255798706041	0.894320651055671	-4.99140033267125
a_HQ_transcript/133283	1.27870215219613	3.16512034778346	1.31616879613595	0.197300807491895	0.894410621015186	-5.18147411010409
a_HQ_transcript/111790	-4.75034000212899	4.22909105825763	-1.31615188992894	0.197306416757462	0.894410621015186	-5.26380642553111
a_HQ_transcript/103768	-1.80513845046279	2.9556347972115	-1.31598309967869	0.197362425924642	0.894594751571083	-5.08961574645605
a_HQ_transcript/149700	-1.09985722271106	2.54453018658189	-1.31591425358808	0.197385274414558	0.894628556036602	-5.10809154957153
a_HQ_transcript/75710	1.38566265721473	-0.442959747525705	1.31573407002764	0.197445082930215	0.894829859704315	-5.03145520901518
a_HQ_transcript/111097	-1.18110337487666	1.96310448284122	-1.31563317295601	0.197478579861187	0.894845451498338	-5.01979081660071
a_HQ_transcript/93032	1.80056159429034	0.0951992494225343	1.31563096760467	0.19747931206683	0.894845451498338	-5.09418835162581
a_HQ_transcript/105791	2.30947577057934	0.248419224407375	1.31555922049109	0.197503134186141	0.894883637381869	-5.15061638610535
a_HQ_transcript/127888	0.298176179469402	5.25191537710898	1.31536062571227	0.197569084880306	0.895112686354046	-5.53480161013375
a_HQ_transcript/13638	1.01904069363555	4.03875477015112	1.31529080172909	0.197592276506484	0.895147989148852	-5.43589774735507
a_HQ_transcript/12041	0.523945803060446	2.56873652822338	1.31517911577684	0.197629376674118	0.8951850154971	-5.13399254833156
a_HQ_transcript/152075	1.35488158385259	-0.651136149813819	1.31517347656162	0.197631250066651	0.8951850154971	-5.04227571958533
a_HQ_transcript/18704	1.1435457293453	2.58309310763141	1.31487236735251	0.197731300711155	0.895568415750195	-5.04457729920898
a_HQ_transcript/116219	-1.16383616222496	-0.703575579517257	-1.31478964192642	0.197758794977347	0.895588703293428	-5.01865892836981
a_HQ_transcript/16858	2.76302588318532	1.53299852086195	1.31475241998729	0.197771166855473	0.895588703293428	-5.01028660359176
a_HQ_transcript/30405	-1.08288372037941	-0.693817568839308	-1.31466542580725	0.197800084408966	0.895588703293428	-5.01849769802248
a_HQ_transcript/13	1.3321807173412	0.108763408576911	1.3145452813703	0.197840026692738	0.895588703293428	-4.99570229166333
a_HQ_transcript/105972	-0.997746360187513	0.623512101897246	-1.31444857530588	0.19787218132319	0.895588703293428	-4.84872478060199
a_HQ_transcript/60672	0.757182865895754	1.94826054035835	1.31443496897265	0.197876705730914	0.895588703293428	-4.9912305961488
a_HQ_transcript/34497	-1.49076116162313	0.243725220395074	-1.31428939217563	0.197925118203335	0.895588703293428	-4.91596940437394
a_HQ_transcript/145483	2.44959481985933	1.33605627219795	1.31428769084303	0.197925684045711	0.895588703293428	-5.04119629145428
a_HQ_transcript/22045	1.61865525622479	-0.140825940817048	1.31422811190084	0.197945500052904	0.895588703293428	-4.96426540098355
a_HQ_transcript/12321	1.48678639222177	-0.148035293993926	1.31418101948487	0.197961164105459	0.895588703293428	-5.07100829608334
a_HQ_transcript/68322	-1.25640551138655	0.579785201321388	-1.31415773560438	0.197968909225963	0.895588703293428	-5.08165594355058

a_HQ_transcript/12295	-1.67004166012942	0.92138946641629	-1.31411068929423	0.197984559358857	0.895588703293428	-4.97854850429498
a_HQ_transcript/128678	1.48472914739841	0.271166770674745	1.31402989044056	0.198011439612976	0.895588703293428	-4.95477072199175
a_HQ_transcript/54759	-1.43141545629357	0.745729490545817	-1.31401553262015	0.198016216481239	0.895588703293428	-4.89214874357106
a_HQ_transcript/77657	1.15039530397015	0.187603343537405	1.31399149419689	0.198024214297996	0.895588703293428	-5.00913228677639
a_HQ_transcript/48864	1.46123443963142	1.376911180094	1.3139784379525	0.198028558340866	0.895588703293428	-4.98878055507849
a_HQ_transcript/33024	1.56423586936015	1.26468912011495	1.31392251658504	0.198047165191817	0.895588703293428	-4.92751710251956
a_HQ_transcript/151350	-1.53505318720458	0.385080285974069	-1.31391605798602	0.198049314263195	0.895588703293428	-5.0149275130787
a_HQ_transcript/64137	0.895624225632073	2.11189874076233	1.31386068526934	0.198067740031942	0.895588703293428	-4.95256391246684
a_HQ_transcript/115677	1.16910807653965	-0.417560276702869	1.31385695903706	0.198068980016321	0.895588703293428	-5.07563161598759
a_HQ_transcript/151351	1.81345016676221	4.71390784008016	1.31385518408662	0.198069570671556	0.895588703293428	-5.4303729082346
a_HQ_transcript/49209	3.22392945058001	1.2646858628762	1.31383965620992	0.198074737984109	0.895588703293428	-4.97749975887663
a_HQ_transcript/134548	1.16956090844558	-0.31073965936276	1.31372833172938	0.19811178717766	0.895686549355452	-4.9667606690745
a_HQ_transcript/147561	-2.42250566556127	0.0315022107026291	-1.31363778046637	0.198141926880457	0.895721833476953	-5.04304478647507
a_HQ_transcript/5390	1.3279902086999	1.11229009450436	1.31356688445832	0.19816552684306	0.895721833476953	-4.89524408265058
a_HQ_transcript/145823	1.64256307669825	0.847228597456057	1.3135276910019	0.198178574539244	0.895721833476953	-5.04984358231898
a_HQ_transcript/26754	-1.85014396660202	2.55863770666311	-1.31351971652744	0.198181229361875	0.895721833476953	-5.22576193056372
a_HQ_transcript/19363	1.51503489111303	0.868770138712806	1.31341334917124	0.19821664325957	0.895812240661819	-4.968384672986
a_HQ_transcript/44573	2.2443110170544	1.38310594213915	1.31335123033808	0.198237327314891	0.895826210859078	-5.08819762802922
a_HQ_transcript/116684	0.444311410859617	2.9632713682327	1.31321454085859	0.198282847392199	0.895826210859078	-5.20250724357365
a_HQ_transcript/49505	-1.89129673843942	0.75858984288498	-1.3131914701761	0.198290531137127	0.895826210859078	-5.08045049163311
a_HQ_transcript/31108	1.71324634361007	0.426606982977704	1.31318973335161	0.198291109599821	0.895826210859078	-4.90622129617599
a_HQ_transcript/141386	1.3699956609884	-0.468956551644893	1.31307868130799	0.198328099014555	0.895826210859078	-5.03915723116529
a_HQ_transcript/149296	-1.21172494341791	-0.487545894068208	-1.31301725172005	0.198348562360632	0.895826210859078	-5.01512815703293
a_HQ_transcript/55246	-0.312822441308727	3.84614843071048	-1.31299339479101	0.198356509986038	0.895826210859078	-5.33104195296106
a_HQ_transcript/59380	1.31668088539977	1.00084669137852	1.31292484124724	0.198379349063207	0.895826210859078	-4.91245508909469
a_HQ_transcript/149646	2.84715968083771	1.82213785084234	1.31283332061752	0.198409842917146	0.895826210859078	-5.0639914870438
a_HQ_transcript/20103	1.23778186538501	0.0225761129222815	1.31283323397671	0.198409871786787	0.895826210859078	-5.06356684262477
a_HQ_transcript/127747	-1.46597612853761	0.50420626052874	-1.31280465233203	0.198419395670787	0.895826210859078	-4.95514980718245
a_HQ_transcript/120836	-1.73876992986278	1.18814850559459	-1.31279680237869	0.198422011468302	0.895826210859078	-5.00403806258932
a_HQ_transcript/137187	0.76109212900576	2.03723307053119	1.31275183130537	0.198436997444587	0.895826210859078	-4.99039676746511
a_HQ_transcript/16739	-1.42944532135732	0.0592750624525914	-1.31272101920861	0.198447265641497	0.895826210859078	-5.01963696600608
a_HQ_transcript/136937	-1.82580423483044	1.86601436347611	-1.31271010333959	0.198450903475646	0.895826210859078	-5.17770857319085
a_HQ_transcript/150113	-0.577028904212652	3.93264441341233	-1.31260907302464	0.198484575367953	0.895884547055255	-5.37265410267506
a_HQ_transcript/33701	-0.734260513662524	2.36343408486273	-1.31255872339457	0.198501357778452	0.895884547055255	-5.035717452515
a_HQ_transcript/117805	-2.09878811236484	3.71195467687438	-1.31247008288085	0.198530905847153	0.895884547055255	-5.2629206199004
a_HQ_transcript/73033	1.54049811151183	1.13817702426017	1.31235266193091	0.198570052976031	0.895884547055255	-4.90522632655266
a_HQ_transcript/18524	1.74690354192195	0.269851138851406	1.312289458138	0.198591127018823	0.895884547055255	-5.02811989097085
a_HQ_transcript/63968	-1.4518807542472	2.27141991540948	-1.31228297989942	0.198593287154813	0.895884547055255	-5.10244480580091
a_HQ_transcript/12271	0.726230005148516	0.352387835855431	1.31227258842773	0.198596752176202	0.895884547055255	-4.98797332165963
a_HQ_transcript/90538	-1.44586014359555	1.3547309947947	-1.31225797504589	0.198601625065762	0.895884547055255	-5.06644767447143
a_HQ_transcript/74238	1.3280021226757	0.49095584746686	1.31225524012799	0.198602537045039	0.895884547055255	-4.85294629149591
a_HQ_transcript/83396	-1.26263539544775	0.0923177408538135	-1.3120826104414	0.198660108217718	0.895974549479968	-5.01486553456684
a_HQ_transcript/130415	-1.96828159275445	1.25657610836387	-1.31203279380331	0.198676724201343	0.895974549479968	-5.08057595129251
a_HQ_transcript/27184	1.14914485160412	-0.341428928902571	1.3119978953762	0.198688364955617	0.895974549479968	-5.00367835981197
a_HQ_transcript/45467	-0.426914828486178	3.77348158005136	-1.31199200555574	0.198690329621489	0.895974549479968	-5.39568610355317
a_HQ_transcript/98474	0.638000134766471	3.44337086352243	1.31177736034991	0.198761938907591	0.895974549479968	-5.33908838566911
a_HQ_transcript/91913	-1.25009841830823	-0.471098413747637	-1.31174145948252	0.19877391797373	0.895974549479968	-5.01123050526598
a_HQ_transcript/98534	-1.33962202491626	1.65755411636735	-1.31167752620868	0.198795251998977	0.895974549479968	-5.00928170920339
a_HQ_transcript/78276	1.79261575762866	2.4534215185953	1.31160059863856	0.198820924439346	0.895974549479968	-5.15594222831304
a_HQ_transcript/119070	1.15251879175181	0.274808384190966	1.31154518351376	0.198839419269868	0.895974549479968	-4.97873668918756

a_HQ_transcript/96678	1.03168456142954	-0.653603549016796	1.31153876631236	0.198841561099637	0.895974549479968	-5.05537966586909
a_HQ_transcript/108501	-1.63007681171526	1.61094688009544	-1.31153116372628	0.198844098590781	0.895974549479968	-5.1057860826594
a_HQ_transcript/142416	1.36854840632567	1.03427962563392	1.31149407413179	0.1988564782228	0.895974549479968	-5.02907615524732
a_HQ_transcript/109779	1.29488578307913	0.526666943999625	1.31148028414746	0.198861081144729	0.895974549479968	-4.9498649054656
a_HQ_transcript/63615	-1.13611918372604	-0.253776939045367	-1.31146155006203	0.19886733447549	0.895974549479968	-5.03647139268633
a_HQ_transcript/4903	1.41030971362452	2.84135191681048	1.31145965417506	0.198867967320118	0.895974549479968	-5.09721325685137
a_HQ_transcript/129829	2.90150875825161	2.06143828245827	1.31145623034351	0.19886911019458	0.895974549479968	-5.04358123030806
a_HQ_transcript/8097	-1.47747738099498	0.597674567253952	-1.31138489769481	0.19889292216485	0.895977172777864	-4.97791440556402
a_HQ_transcript/87048	2.70244267491625	1.31620240169368	1.3113621379475	0.198900520188645	0.895977172777864	-5.07669733477794
a_HQ_transcript/125183	1.76504067956872	2.06301428533397	1.31118375093081	0.198960079892763	0.896176018907908	-5.13729168034531
a_HQ_transcript/43158	0.972333574255085	-0.517312725340133	1.31109489171576	0.198989753220789	0.896192838822853	-5.01164679463981
a_HQ_transcript/101078	-3.75176351686988	3.56908276126262	-1.31108023117599	0.198994649234217	0.896192838822853	-5.24407906514257
a_HQ_transcript/107519	1.11340646103084	0.977956927374403	1.31075856388796	0.199102095983035	0.896341751100786	-4.9399001548206
a_HQ_transcript/78615	1.76006818035398	0.614523003417421	1.31075499470275	0.199103288449305	0.896341751100786	-5.00040185533126
a_HQ_transcript/67832	-1.76916154128491	1.32154715459149	-1.31072752047689	0.199112467782479	0.896341751100786	-4.91078972516509
a_HQ_transcript/55855	-2.12204472831072	1.64068621495157	-1.31068371621892	0.19912710376425	0.896341751100786	-4.91824799323218
a_HQ_transcript/2048	1.71118110587751	2.40159380200972	1.31056875739709	0.199165517989264	0.896341751100786	-5.13500801661239
a_HQ_transcript/94821	0.841331981551674	1.69904497916729	1.31054372320706	0.199173884075242	0.896341751100786	-5.01130466166519
a_HQ_transcript/24660	1.77015711829345	1.40770430017751	1.31053649873223	0.199176298446484	0.896341751100786	-4.99361319556611
a_HQ_transcript/133638	1.60331617658888	0.164554008613372	1.31053298025737	0.199177474305389	0.896341751100786	-5.01669535464189
a_HQ_transcript/134521	1.58463172460087	2.87744772731253	1.31048591152828	0.199193204979259	0.896341751100786	-5.09955705188384
a_HQ_transcript/118614	0.871241430518119	6.56095346514935	1.31042821492159	0.199212488854646	0.896341751100786	-5.71728071006099
a_HQ_transcript/147198	-0.480851148318724	3.61391696550927	-1.31039677508273	0.199222997559393	0.896341751100786	-5.3799097208757
a_HQ_transcript/6647	-1.52804624437265	0.80967970424601	-1.31038753033408	0.199226087679226	0.896341751100786	-5.01890937284385
a_HQ_transcript/5812	0.998258643566783	2.68287784068143	1.31036551744781	0.199233445783052	0.896341751100786	-5.07059445442383
a_HQ_transcript/37819	0.513587468043089	2.27064615731028	1.31033515164076	0.199243596304247	0.896341751100786	-5.01397940292892
a_HQ_transcript/8070	-1.15693324483327	1.16234635276023	-1.31024891931279	0.199272423746289	0.896402062182655	-4.96780806328415
a_HQ_transcript/68943	1.41009081775074	-0.344964829698768	1.30998075622927	0.199362090967313	0.896736022885394	-5.03891232734754
a_HQ_transcript/12612	-1.66972486824054	-0.359860227343158	-1.30993359551507	0.199377863553873	0.896737577581208	-5.04092719595479
a_HQ_transcript/145097	-1.95090636541533	4.73353828297172	-1.30988368764883	0.199394555946191	0.89674326870717	-5.56772328799487
a_HQ_transcript/150847	4.86989104931238	3.1394765002914	1.30980122456642	0.199422139232714	0.896797935116759	-5.05491495044322
a_HQ_transcript/48017	-1.09044651267174	2.2090332581772	-1.309571718913	0.199498922521849	0.896821370607053	-5.11910953249302
a_HQ_transcript/34707	2.44161156526278	1.00223455602859	1.30954851861089	0.199506685660271	0.896821370607053	-5.11909373581071
a_HQ_transcript/70883	-1.00570713744031	2.08113415444231	-1.30945284358242	0.199538702273454	0.896821370607053	-4.99249199320488
a_HQ_transcript/113411	3.68895460237747	2.0261566998967	1.30938766278608	0.199560516571203	0.896821370607053	-5.20366453161889
a_HQ_transcript/17374	1.58255236065896	0.7151026318195	1.3092979780889	0.199590534659877	0.896821370607053	-4.95826380267177
a_HQ_transcript/114184	1.78735948108605	2.76077657870458	1.30924185370439	0.19960932163557	0.896821370607053	-5.0149853309915
a_HQ_transcript/91689	-0.427499732103234	2.69798972900796	-1.30919493422306	0.199625028415904	0.896821370607053	-5.24469946906572
a_HQ_transcript/42292	2.17444773947979	2.68773845855599	1.30918250377502	0.199629189794903	0.896821370607053	-5.19294463172939
a_HQ_transcript/61919	0.678097844989709	2.20608080372494	1.30916243196827	0.199635909434853	0.896821370607053	-5.10148623503765
a_HQ_transcript/30266	-0.667711649760286	3.56907955591128	-1.30915750612014	0.19963755853683	0.896821370607053	-5.3123597582198
a_HQ_transcript/123217	-1.95754101574529	1.51679706558978	-1.30914399561694	0.199642081709339	0.896821370607053	-5.05012820160637
a_HQ_transcript/23314	-1.59369808849074	-0.539509625235265	-1.3091438772281	0.199642121345006	0.896821370607053	-5.05977067807398
a_HQ_transcript/148069	1.69038731847578	0.551660820482438	1.30910583879721	0.199654856629046	0.896821370607053	-5.03032732908471
a_HQ_transcript/142071	1.4200873620969	1.82722048758263	1.30907983655353	0.199663562549442	0.896821370607053	-5.05384900876926
a_HQ_transcript/99816	-1.63118451863369	0.296859600554217	-1.30907623480897	0.199664768487542	0.896821370607053	-5.04882708599676
a_HQ_transcript/18608	-1.05002672098958	2.96457567868553	-1.30902522769549	0.19968184731518	0.896821370607053	-5.26735174024682
a_HQ_transcript/77234	1.60337567382132	-0.0215106310228293	1.30900197565505	0.199689633219225	0.896821370607053	-4.99780076820616
a_HQ_transcript/1327	-2.12082641667915	0.160396672876686	-1.30887349140861	0.19973266012267	0.896945308066331	-5.104842111056
a_HQ_transcript/31236	0.771921931119928	1.07096377096591	1.30868605921672	0.199795440267897	0.89703081961916	-4.88059657707704

a_HQ_transcript/8886	1.82100234725472	1.44240924913049	1.30861329960052	0.199819815060941	0.89703081961916	-4.94900103115935
a_HQ_transcript/46924	-1.46059114086062	0.323301407970013	-1.30859298793221	0.199826619965388	0.89703081961916	-5.11078444167031
a_HQ_transcript/30208	1.11120955001556	2.67443390776655	1.30852422546558	0.199849658384143	0.89703081961916	-5.18908531867952
a_HQ_transcript/99958	1.19293949650539	1.88555390772948	1.30848557217111	0.199862609811196	0.89703081961916	-5.00323936184804
a_HQ_transcript/108582	1.41468291532681	2.684504929103	1.30844350925923	0.199876704415916	0.89703081961916	-5.23123469685143
a_HQ_transcript/54546	1.74944317219289	0.277306562189952	1.30835823065615	0.199905282234426	0.89703081961916	-5.06351824392597
a_HQ_transcript/136663	0.512349107568754	5.04080764730515	1.30828979087505	0.199928219432981	0.89703081961916	-5.53043050620714
a_HQ_transcript/1366	-0.905515396947914	3.81328020924935	-1.30828441973989	0.199930019623117	0.89703081961916	-5.21527570577597
a_HQ_transcript/145457	2.19573010393545	4.74029971292167	1.30822083790746	0.199951330656962	0.89703081961916	-5.55837381142297
a_HQ_transcript/145426	0.826490941597808	2.4348284269821	1.30816841532693	0.199968902695412	0.89703081961916	-5.1132310722661
a_HQ_transcript/2913	-2.06718422017443	1.8222197836443	-1.30814983037587	0.199975132650615	0.89703081961916	-5.221165529643
a_HQ_transcript/38272	1.68751394016717	0.089417884080742	1.30814979065365	0.199975145966257	0.89703081961916	-4.93418179560604
a_HQ_transcript/12180	-1.1187545741838	2.26447506449327	-1.30814833910836	0.199975632552346	0.89703081961916	-5.17766263773968
a_HQ_transcript/36655	1.27544003695637	0.640043591621509	1.30799516619962	0.200026984162816	0.89703081961916	-4.94140042812909
a_HQ_transcript/30412	1.81970270944174	1.10670035512324	1.30791039726428	0.200055407496867	0.89703081961916	-5.0514320436984
a_HQ_transcript/105483	1.06767070280121	3.41647004372662	1.30787957350822	0.200065743580123	0.89703081961916	-5.31744239089226
a_HQ_transcript/70656	1.65209573818878	2.25660617546724	1.30785955107184	0.200072457891901	0.89703081961916	-4.93469955579064
a_HQ_transcript/136246	-1.36900725513402	-0.158429483350039	-1.30776219300668	0.200105108341101	0.89703081961916	-5.04468801581114
a_HQ_transcript/142762	-0.951958042272549	2.18057427589665	-1.30775661884817	0.200106977839796	0.89703081961916	-4.95152921753578
a_HQ_transcript/14990	1.47487600711007	3.37933195693441	1.30771720705204	0.200120196413273	0.89703081961916	-5.39523646340345
a_HQ_transcript/121472	3.10213504766144	0.866916197002612	1.30762843289307	0.200149973388202	0.89703081961916	-5.06034165051033
a_HQ_transcript/90262	3.01722731383106	1.80873583369877	1.30762810359803	0.200150083847932	0.89703081961916	-5.19115526706853
a_HQ_transcript/129009	-1.68335289127082	2.3875515227153	-1.30760115249744	0.200159124564973	0.89703081961916	-5.17024513457012
a_HQ_transcript/25674	0.795114296741962	2.8958612110733	1.3075703796931	0.200169447649855	0.89703081961916	-5.33216135196614
a_HQ_transcript/95965	-1.45419689192045	2.22390374281397	-1.30752907044102	0.20018330594473	0.89703081961916	-5.09876820226246
a_HQ_transcript/48675	2.12557702371155	0.936303887689468	1.30750209285323	0.200192356694701	0.89703081961916	-5.08644110888169
a_HQ_transcript/36232	-1.97878101510479	1.61900262101274	-1.30747200841771	0.200202450134252	0.89703081961916	-5.17660763499141
a_HQ_transcript/31019	-0.462917261335403	4.29280403479888	-1.30745601614822	0.200207815758178	0.89703081961916	-5.43009062061835
a_HQ_transcript/135285	1.12292555549911	-0.609880343667429	1.30743561233904	0.200214661673186	0.89703081961916	-5.04531930772876
a_HQ_transcript/144440	4.07996300379615	1.04483527713024	1.30729945284921	0.200260350677592	0.897105329524313	-5.13220799453803
a_HQ_transcript/53876	2.02005011732066	3.11429253341735	1.3072720382248	0.200269550756556	0.897105329524313	-5.32458097735263
a_HQ_transcript/143055	1.3060748797601	-0.608134242706672	1.30720251150164	0.200292884690585	0.897105329524313	-5.06776745085405
a_HQ_transcript/35114	-1.25795323179865	1.53285143156294	-1.30717658664731	0.200301585888831	0.897105329524313	-4.89224512610729
a_HQ_transcript/1452	1.09959768963612	3.93534997101132	1.30715611079382	0.200308458434766	0.897105329524313	-5.43901451404958
a_HQ_transcript/121422	1.54652165108318	0.0755760700298481	1.30709972844501	0.200327383621206	0.897120967267602	-5.08651468078356
a_HQ_transcript/127140	0.562090000509008	2.24396617108221	1.3070230319852	0.200353129594382	0.897124868466962	-5.11310306056735
a_HQ_transcript/137925	-1.79977707037688	-0.017725671691684	-1.30696293780524	0.200373304170995	0.897124868466962	-5.03825031910687
a_HQ_transcript/113045	-1.51869653161347	1.36890146172238	-1.30695921034673	0.200374555589502	0.897124868466962	-5.00533428940782
a_HQ_transcript/127112	1.4126951549515	0.299996032804911	1.30689348257812	0.200396623334053	0.897154568789419	-5.0252557779599
a_HQ_transcript/142604	-0.287764507496063	5.77982131532718	-1.30674542741324	0.200446338859948	0.897281422218081	-5.60236298714761
a_HQ_transcript/96518	-1.83935694707636	0.786749770318045	-1.30667334325228	0.200470547450263	0.897281422218081	-5.00481776714725
a_HQ_transcript/91966	0.897495511699296	3.24423549543692	1.30658897827186	0.200498883241125	0.897281422218081	-5.2524188219744
a_HQ_transcript/136526	1.02938264400886	1.35485160014354	1.30656755370968	0.200506079627625	0.897281422218081	-4.98359150841342
a_HQ_transcript/144306	1.30174931352669	-0.256041210546305	1.30654773033576	0.20051273835998	0.897281422218081	-5.06859457356829
a_HQ_transcript/97919	0.605652802237307	2.42249343273993	1.30649158014962	0.200531600297266	0.897281422218081	-5.10334695755737
a_HQ_transcript/92919	2.40208457249757	0.874985987410848	1.30635196586547	0.200578505319186	0.897281422218081	-5.09398142866784
a_HQ_transcript/23918	-1.25186630233417	0.0658721903220403	-1.30633374733372	0.20058462666217	0.897281422218081	-4.99398486144851
a_HQ_transcript/53482	1.2927074371259	0.665488229168304	1.30632122494251	0.200588834210838	0.897281422218081	-4.94836015112734
a_HQ_transcript/118899	-1.87446773186356	0.800918827700399	-1.30630887768633	0.200592982979786	0.897281422218081	-5.01192748878256
a_HQ_transcript/20241	-0.944866591181069	-0.809051916455492	-1.30630359564191	0.200594757805601	0.897281422218081	-5.02826666715706

a_HQ_transcript/111607	-1.07804414872122	2.07611631990269	-1.30615097553752	0.200646045042382	0.897354555518459	-5.16768196515179
a_HQ_transcript/101556	-0.460191341049723	2.94588476372299	-1.30613971261001	0.200649830290651	0.897354555518459	-5.20151214945685
a_HQ_transcript/124463	1.29840026321506	-0.223369421712981	1.30607183191729	0.200672644810375	0.897354555518459	-5.07957799666218
a_HQ_transcript/90116	1.01369390627463	1.41921291953751	1.30598102700639	0.20070316719636	0.897354555518459	-4.88978961802543
a_HQ_transcript/128016	1.00008486172363	1.76036217279686	1.3059647158027	0.200708650280456	0.897354555518459	-5.0292296628345
a_HQ_transcript/92833	-1.71898226909333	0.06962484344599	-1.30572863148989	0.200788023889484	0.897354555518459	-4.97092989759193
a_HQ_transcript/16320	-2.32514897108372	3.42522548415027	-1.30570175630361	0.200797061081459	0.897354555518459	-5.43342176671574
a_HQ_transcript/54350	0.91402945149444	2.33262471184772	1.30569642487661	0.20079885389216	0.897354555518459	-5.14074212456817
a_HQ_transcript/26341	-1.66678535639348	3.01914464044543	-1.30567662639136	0.200805511679389	0.897354555518459	-5.27027962719704
a_HQ_transcript/56655	-0.976217735317777	1.47535112049001	-1.3056500698671	0.200814442308499	0.897354555518459	-4.96094944724881
a_HQ_transcript/141344	-2.96256566797523	4.02859574145625	-1.30562758990892	0.20082200227613	0.897354555518459	-5.28855683943566
a_HQ_transcript/150863	-1.35895009146219	1.02807588356213	-1.30560847662359	0.200828430208962	0.897354555518459	-5.01661305532661
a_HQ_transcript/48408	1.2330107998928	0.680550664690703	1.30560610688889	0.200829227178426	0.897354555518459	-4.85451781917065
a_HQ_transcript/152400	0.842695732288591	-0.819337251522003	1.30558321318487	0.20083692672289	0.897354555518459	-5.07247100650264
a_HQ_transcript/100087	1.21683266518665	1.82130478694448	1.3055297662655	0.200854902713963	0.897354555518459	-5.01910488813672
a_HQ_transcript/14476	-0.875385012838234	1.76965619699488	-1.30550368827169	0.200863674063647	0.897354555518459	-4.94149677851266
a_HQ_transcript/115969	0.340580700298709	3.48413143701511	1.30547410595541	0.2008736244485	0.897354555518459	-5.34383076495391
a_HQ_transcript/152566	1.71174840990378	1.1196746119126	1.3054284460649	0.200888983466861	0.897354555518459	-4.99347654501479
a_HQ_transcript/115750	-1.49541513245535	1.54359125822496	-1.30525959263162	0.200945789968936	0.897508440412317	-5.09985434295365
a_HQ_transcript/123603	-2.94172787790094	2.09765898243689	-1.30515527889411	0.200980889837899	0.897508440412317	-5.29671029182062
a_HQ_transcript/12456	1.29957371394535	0.37784696515237	1.30507180826311	0.201008979713375	0.897508440412317	-5.07523148748649
a_HQ_transcript/123431	-2.61626519184127	0.562326952470956	-1.30503713633706	0.201020648531734	0.897508440412317	-5.05428817677468
a_HQ_transcript/136772	1.66955770969287	0.693253878685163	1.3050148189003	0.201028159726842	0.897508440412317	-4.96841687611558
a_HQ_transcript/135803	1.29788591898571	-0.327489907691278	1.30501012199282	0.20102974055344	0.897508440412317	-5.05089456781201
a_HQ_transcript/74569	1.51794383385617	0.284661034374463	1.30499666033077	0.201034271364511	0.897508440412317	-4.98596666159758
a_HQ_transcript/42493	-0.385297736995005	3.40948696706737	-1.30491994527359	0.201060092950411	0.897508440412317	-5.30781708850809
a_HQ_transcript/16323	-1.14582013253433	2.44345677451243	-1.30479830793422	0.201101040155521	0.897508440412317	-5.01267503004798
a_HQ_transcript/105615	1.98830950912065	0.907843877522666	1.30477804025737	0.201107863553559	0.897508440412317	-5.11326487384247
a_HQ_transcript/152406	2.55929055405778	0.244655675136225	1.3045982553248	0.201168398413935	0.897508440412317	-5.1307285370787
a_HQ_transcript/138574	-1.50552412797785	1.610783066609	-1.30456857088526	0.201178394712843	0.897508440412317	-5.09691264901481
a_HQ_transcript/14183	1.15009089429671	0.99922519491059	1.30453570888026	0.201189461506015	0.897508440412317	-4.89423721509323
a_HQ_transcript/140582	4.00326058893318	1.95421447543733	1.30452551617653	0.201192894152531	0.897508440412317	-5.09020228148534
a_HQ_transcript/114621	0.735876143852704	1.68419220933642	1.30450384340925	0.201200193144611	0.897508440412317	-5.00502534276443
a_HQ_transcript/139909	-3.31553026775489	2.48828630923567	-1.30444950118817	0.201218495500737	0.897508440412317	-5.25657690340639
a_HQ_transcript/7930	1.78565952399757	3.39205623118882	1.30444619709343	0.201219608354511	0.897508440412317	-5.31470660822801
a_HQ_transcript/51280	1.2048768179935	1.06336249538929	1.30443913907083	0.201221985586311	0.897508440412317	-5.09851282461006
a_HQ_transcript/78071	-1.78110843220189	1.56974900819572	-1.30440771356054	0.201232570358191	0.897508440412317	-5.13666302013606
a_HQ_transcript/136863	-1.18707377288919	3.58855245394434	-1.30439874427564	0.201235591479685	0.897508440412317	-5.38771779276973
a_HQ_transcript/112004	-4.251662896956	1.43675482923485	-1.30436286286254	0.201247677751413	0.897508440412317	-5.03581280083871
a_HQ_transcript/48582	0.395451842635623	2.88147733436674	1.30428720385303	0.201273164498736	0.897553241732452	-5.20583451240013
a_HQ_transcript/140315	-1.02611485074951	1.20677602287617	-1.30422306704205	0.201294771769178	0.897580737450044	-4.91408292261748
a_HQ_transcript/142888	0.617896464010735	5.40036745282934	1.30411584707135	0.201330897427256	0.897672962004413	-5.56268152430069
a_HQ_transcript/50245	-2.61890852745789	3.03378880309541	-1.30395916398766	0.201383697606755	0.897833030762933	-5.18266249477634
a_HQ_transcript/137539	1.74420981238522	1.08716446528297	1.30387513014535	0.201412020282332	0.897833030762933	-4.94886126476572
a_HQ_transcript/43222	-0.812592682863313	2.14656861756797	-1.30372474112966	0.201462714822756	0.897833030762933	-5.02624032433307
a_HQ_transcript/120274	0.831868688100755	2.13310176929739	1.30369768185579	0.201471837250304	0.897833030762933	-5.03520831624683
a_HQ_transcript/123803	3.46413544901883	3.75918016479302	1.30366649045163	0.201482353124082	0.897833030762933	-5.16603649279588
a_HQ_transcript/74190	1.43021133351142	0.147403736303746	1.30365052877296	0.201487734608391	0.897833030762933	-4.9565107480185
a_HQ_transcript/140129	2.18311872760728	0.562500695373098	1.3036214079848	0.201497552972679	0.897833030762933	-5.0628896427309
a_HQ_transcript/85718	-1.59561870735392	1.58955939218876	-1.30361377926868	0.201500125130793	0.897833030762933	-5.00203161456388

a_HQ_transcript/119896	2.22718750291537	0.902797207759412	1.30355052560305	0.201521453200987	0.897833030762933	-5.05528228251302
a_HQ_transcript/44316	1.59451750945241	2.32311837574767	1.30351862849433	0.201532209021988	0.897833030762933	-5.10984983363207
a_HQ_transcript/93610	1.05789610950915	1.51809805054374	1.3035053061033	0.201536701510369	0.897833030762933	-4.895456096976
a_HQ_transcript/63061	-2.00018849274464	0.77800649859917	-1.30343960663496	0.201558857367311	0.89786292137689	-4.96630985772482
a_HQ_transcript/2698	-1.18239954732368	3.45461434910933	-1.30329346777593	0.201608146496443	0.897936697164393	-5.44530376801212
a_HQ_transcript/25032	0.688624228718484	2.18632445723945	1.30324957659066	0.20162295173557	0.897936697164393	-5.05281163296258
a_HQ_transcript/71282	-1.38229489068357	1.50034298466228	-1.30323990815448	0.201626213174121	0.897936697164393	-4.97033357631848
a_HQ_transcript/97447	-3.16275375766526	3.29270330316506	-1.30316834910292	0.201650353329444	0.897936697164393	-5.35159045878207
a_HQ_transcript/15012	-0.769773291438854	2.01130412470873	-1.30316152070374	0.201652656977429	0.897936697164393	-5.07340305826696
a_HQ_transcript/136073	1.30221207698673	4.46204515491744	1.30304427983294	0.20169221282035	0.898020614821454	-5.47996872947898
a_HQ_transcript/143918	-0.44327091987237	4.50301810497814	-1.30301408652077	0.201702400686569	0.898020614821454	-5.46754041095132
a_HQ_transcript/82082	1.04420928388661	-0.659754807415831	1.30287184312	0.201750401920286	0.898165532880629	-4.98313481031554
a_HQ_transcript/88253	0.871908820404157	2.8086891658835	1.30267693212189	0.201816190432205	0.898249264585544	-5.33114427148581
a_HQ_transcript/67067	-1.76852495524654	1.30445771605037	-1.30267679923213	0.201816235292206	0.898249264585544	-5.05101768646884
a_HQ_transcript/95647	-0.831380638035417	0.80433313791122	-1.30260027783693	0.201842068116629	0.898249264585544	-4.9577418745779
a_HQ_transcript/148786	1.60732571772394	0.123725061098267	1.30257477427654	0.201850678412959	0.898249264585544	-5.08183973997863
a_HQ_transcript/49492	-2.10608899080959	0.616527736989008	-1.3024759932217	0.201884030681828	0.898249264585544	-5.06974253908863
a_HQ_transcript/101412	-0.674624161881488	4.10831411426265	-1.30244855700956	0.201893294944259	0.898249264585544	-5.46709818964722
a_HQ_transcript/47816	1.7144688362444	-0.0835553995671984	1.30242294680992	0.201901942917931	0.898249264585544	-5.05856881344125
a_HQ_transcript/8056	-0.92780526931725	2.21159897848557	-1.30239840336187	0.201910230939385	0.898249264585544	-5.08486442975502
a_HQ_transcript/4641	-0.49609730657807	2.52305402297236	-1.30239578035107	0.201911116713322	0.898249264585544	-5.07792581739865
a_HQ_transcript/130040	-1.54987950357354	3.27229251734013	-1.30220345313617	0.201976072459169	0.898249264585544	-5.35045224909665
a_HQ_transcript/35197	1.33648480211576	3.24793429855681	1.30218173002945	0.201983410127327	0.898249264585544	-5.12804901013734
a_HQ_transcript/9827	-0.556870253894951	3.71056942549111	-1.30215444562241	0.201992626589127	0.898249264585544	-5.38903081893673
a_HQ_transcript/148124	2.68794398892625	1.48994974003494	1.3020430007087	0.202030275150932	0.898249264585544	-5.07612008650332
a_HQ_transcript/75300	2.90309598946890	2.74505501576897	1.30195244076943	0.202060872247451	0.898249264585544	-5.08385524012835
a_HQ_transcript/86201	1.11847261825443	5.00487060120125	1.30193567042164	0.202066538760484	0.898249264585544	-5.53195792846441
a_HQ_transcript/7974	1.43025182921505	0.0926946975877198	1.30190283996776	0.202077632154745	0.898249264585544	-5.13804608569932
a_HQ_transcript/66425	1.81239115015549	0.623756266128541	1.30185572143654	0.202093554295145	0.898249264585544	-4.96217487279695
a_HQ_transcript/127565	2.93092179754441	0.26576711046623	1.30184916282109	0.202095770636956	0.898249264585544	-5.05859705587092
a_HQ_transcript/131589	-2.67296997544497	0.578944080791894	-1.30172199000843	0.202138749595398	0.898249264585544	-5.11656588939182
a_HQ_transcript/65848	1.28350471577455	0.992470223244927	1.30169495688079	0.202147886531991	0.898249264585544	-4.91546309566647
a_HQ_transcript/30427	-1.07473230092009	1.24311418036003	-1.30155178104674	0.202196283836445	0.898249264585544	-4.93327737782991
a_HQ_transcript/123622	-1.30477000549485	1.36910078291251	-1.30149325065252	0.202216071237474	0.898249264585544	-5.03326555699699
a_HQ_transcript/109799	2.00568986077922	0.817973796357133	1.30140995761605	0.202244232704261	0.898249264585544	-5.09394289986849
a_HQ_transcript/148305	-2.13205561668984	2.2819058062231	-1.30136961787638	0.202257872689465	0.898249264585544	-5.30274226801674
a_HQ_transcript/105547	-1.49073597458611	2.44196263885476	-1.30127030762844	0.202291455229338	0.898249264585544	-5.30520383424519
a_HQ_transcript/50540	1.50941642372935	1.31414624689713	1.30113231439051	0.202338125785452	0.898249264585544	-5.06481052718634
a_HQ_transcript/31712	0.827400920543515	2.15705491218813	1.30109958840302	0.202349195213733	0.898249264585544	-5.07284632550508
a_HQ_transcript/73342	0.741657308222927	2.78158433583561	1.30100957684484	0.20237964362847	0.898249264585544	-5.15985735100632
a_HQ_transcript/43048	0.753702331145377	1.87241703198786	1.30100898866882	0.202379842603603	0.898249264585544	-4.99192983920688
a_HQ_transcript/135574	1.56791097572828	0.124561229076589	1.30100719184898	0.202380450453965	0.898249264585544	-5.04491864236442
a_HQ_transcript/120787	2.41528107096932	6.39686225369395	1.30098605179258	0.202387602077352	0.898249264585544	-5.59087093478932
a_HQ_transcript/122882	-1.55872378394754	1.65772492373416	-1.30090647281289	0.202414525157223	0.898249264585544	-5.008771526823
a_HQ_transcript/23901	1.56258334923281	0.664144671009781	1.30090349845086	0.20241553149332	0.898249264585544	-4.97558657307734
a_HQ_transcript/95682	1.56834394934463	0.253692031744809	1.30089930100266	0.202416951650956	0.898249264585544	-5.10777464692413
a_HQ_transcript/134760	1.73129268122012	1.53093018348092	1.30089207850769	0.202419395315661	0.898249264585544	-5.04160869820085
a_HQ_transcript/140075	-1.6016894673558	0.551665541619014	-1.300859309264	0.20243048277028	0.898249264585544	-5.11904116429254
a_HQ_transcript/102848	-1.87150121328476	2.12925626654833	-1.30084503639506	0.202435312132	0.898249264585544	-5.22767987598066
a_HQ_transcript/12540	0.360976305153799	4.18003955004794	1.30083957768197	0.202437159163011	0.898249264585544	-5.45617535248817

a_HQ_transcript/38650	1.14903341125014	0.506332426511308	1.30079476325768	0.202452323228596	0.898249264585544	-5.02853165224327
a_HQ_transcript/53198	1.36520595691217	2.62609560366862	1.30078054126757	0.20245713577072	0.898249264585544	-5.29170212074251
a_HQ_transcript/116158	1.59843862063544	-0.0364323377885591	1.30076671377156	0.20246181490465	0.898249264585544	-5.0131020954215
a_HQ_transcript/61880	-1.05143298278415	1.54200204989139	-1.30076467819992	0.202462503735703	0.898249264585544	-4.95522962462713
a_HQ_transcript/71209	1.97390833347822	-0.0289108723768626	1.30075760306634	0.202464897952593	0.898249264585544	-5.10942645763379
a_HQ_transcript/137994	1.40528882025996	0.0579951156907531	1.30071799618555	0.202478301273221	0.898249264585544	-4.96882237940421
a_HQ_transcript/151536	1.33043422474646	2.67962120616006	1.30059445547994	0.202520112893064	0.898249264585544	-5.17678456059649
a_HQ_transcript/141872	0.410630428642005	3.55822533655885	1.30056595443911	0.202529759836007	0.898249264585544	-5.39142724150599
a_HQ_transcript/107141	1.66957304754788	0.378470657413181	1.30050702369691	0.202549707640089	0.898249264585544	-5.01793837274768
a_HQ_transcript/76784	1.94544521936686	-0.275555623306557	1.30042943659475	0.202575972826657	0.898249264585544	-5.05554244146905
a_HQ_transcript/90526	0.767191511490027	-0.758064309467471	1.30042772353699	0.202576552769118	0.898249264585544	-5.07922440259404
a_HQ_transcript/139364	2.11826094682512	2.85984523402383	1.30040813453447	0.20258318456448	0.898249264585544	-5.23312794620989
a_HQ_transcript/60937	1.22797559154056	2.07716083908258	1.30031518553493	0.202614654412942	0.898249264585544	-5.06864545418946
a_HQ_transcript/11354	-1.58632956386478	-0.348859812157557	-1.30029792217589	0.202620499698433	0.898249264585544	-5.02976838565596
a_HQ_transcript/23048	1.31772452950578	2.64610807893644	1.30029390963928	0.20262185834132	0.898249264585544	-5.12202142471554
a_HQ_transcript/19650	-1.2759498253958	2.62121880577042	-1.30027556571054	0.202628069674861	0.898249264585544	-5.09461197493842
a_HQ_transcript/96859	-1.44990954116106	0.968418030472607	-1.30025845728237	0.202633862793402	0.898249264585544	-5.0354719213778
a_HQ_transcript/149152	-4.69672838965858	2.40642174934899	-1.30025635242577	0.202634575531762	0.898249264585544	-5.08311129129936
a_HQ_transcript/3662	-2.04468428522422	0.960021057035715	-1.30010937144111	0.202684350388381	0.898288523727227	-5.10517876535394
a_HQ_transcript/21303	1.23014631120756	1.2183416050912	1.30008189252224	0.202693657110967	0.898288523727227	-4.92346969086685
a_HQ_transcript/62048	-1.7243903661486	1.16717661830729	-1.30003150282388	0.202710724238183	0.898288523727227	-5.05354502453187
a_HQ_transcript/48457	3.3807695179272	1.57447235419062	1.29998966939935	0.202724894164605	0.898288523727227	-5.1556437485271
a_HQ_transcript/41524	-1.26668808526156	1.744770744886	-1.29998030661067	0.202728065656167	0.898288523727227	-5.1582273500759
a_HQ_transcript/95048	1.63626822899941	1.97419958650765	1.29968164599896	0.202829251902812	0.898288523727227	-5.11131585149305
a_HQ_transcript/17367	1.56708654343447	0.745337430213878	1.29966016130384	0.202836532403882	0.898288523727227	-5.07590116115905
a_HQ_transcript/128860	1.84839198018185	1.8218438520812	1.2996372196282	0.202844306850681	0.898288523727227	-5.15145255683887
a_HQ_transcript/124546	0.730036971311023	2.09182665976761	1.29960973469266	0.202853621210876	0.898288523727227	-5.04569960665866
a_HQ_transcript/103924	2.29096723680546	0.304300514543794	1.29956795341447	0.202867781078137	0.898288523727227	-5.06258641359287
a_HQ_transcript/126979	-1.81576479030735	1.40893085128995	-1.29951179671941	0.20288681402711	0.898288523727227	-4.97133735458166
a_HQ_transcript/46193	-2.40659997077753	2.71803160381589	-1.29949391143048	0.202892876097527	0.898288523727227	-5.25499346581924
a_HQ_transcript/36983	-0.845613750276416	2.515951733983	-1.29948471833658	0.202895992074215	0.898288523727227	-5.01888382659532
a_HQ_transcript/13773	0.823306932821229	-0.843217253350119	1.2994678997041	0.202901692802963	0.898288523727227	-5.06906624994624
a_HQ_transcript/136986	1.12315436756408	0.492724278223226	1.29945334613558	0.20290662587964	0.898288523727227	-5.06670159199339
a_HQ_transcript/52491	-1.41396632091332	2.14176314824224	-1.29942019163629	0.202917864268003	0.898288523727227	-5.01178767669394
a_HQ_transcript/66318	1.12612515709184	1.00856374783736	1.29940852648847	0.202921818518717	0.898288523727227	-4.85962628874915
a_HQ_transcript/1061	1.59707878477678	0.665148708814696	1.29935361829061	0.202940432083368	0.898288523727227	-5.01085819656367
a_HQ_transcript/144217	-1.33793471025052	-0.117189593476557	-1.29930304134519	0.202957578529678	0.898288523727227	-4.92076195805961
a_HQ_transcript/55419	2.03956287462446	0.97383703568424	1.29928479688821	0.202963763982468	0.898288523727227	-4.93767805340591
a_HQ_transcript/78866	2.35613421262001	0.338470468053523	1.29922704380621	0.202983345066497	0.898288523727227	-5.00262461712401
a_HQ_transcript/5497	1.22992179410604	-0.249190500755787	1.29922684708662	0.202983411766397	0.898288523727227	-5.13577922591854
a_HQ_transcript/147059	1.52185062164404	0.393010252873527	1.29914322553289	0.203011766070416	0.898345610394423	-4.98890775854832
a_HQ_transcript/41210	1.75132364955321	1.29830258160887	1.29908152435976	0.203032689571417	0.898369808891475	-4.93990779092415
a_HQ_transcript/70105	1.35502063483672	-0.168431636030959	1.2990215687726	0.203053022700751	0.898391391653924	-5.06238679714075
a_HQ_transcript/34724	1.75414363353601	2.7488385335308	1.29879886373666	0.203128563701179	0.898657215223544	-5.21530526661446
a_HQ_transcript/110486	-2.21220794326287	1.66414360053868	-1.29870816291431	0.203159335333825	0.898667959470751	-5.14308137972448
a_HQ_transcript/105252	-0.338151702652829	3.32802592053665	-1.2986221108064	0.203188533103296	0.898667959470751	-5.31779407146948
a_HQ_transcript/99035	1.68532129897326	1.19121691850083	1.29861109754674	0.203192270170128	0.898667959470751	-5.08884549961833
a_HQ_transcript/30068	1.66981897690806	1.89385749509328	1.29859094129078	0.203199109813601	0.898667959470751	-5.17795616046194
a_HQ_transcript/96215	1.11495275320588	1.71003778917842	1.29854331233031	0.203215272495809	0.898667959470751	-5.11162215232535
a_HQ_transcript/10398	-1.37441775693703	-0.561872004504613	-1.29851803573342	0.203223850397793	0.898667959470751	-5.06893428396923

a_HQ_transcript/146807	0.340633133503709	4.83579792917407	1.29847276750649	0.203239213377417	0.898667959470751	-5.5378087287085
a_HQ_transcript/148395	0.340803478349562	8.30318231276867	1.2983766559295	0.203271834337483	0.898743834058659	-5.87373347414696
a_HQ_transcript/101366	1.49364295302845	0.585242591101603	1.29832967783286	0.203287780492732	0.898745976915238	-4.96695683875127
a_HQ_transcript/111011	1.85793124634951	0.437260707061828	1.29817614504237	0.203339902017544	0.898797518105846	-5.00917173420172
a_HQ_transcript/68410	1.51240739276766	0.580902755400193	1.29811886487007	0.2033593501778	0.898797518105846	-5.0797356052859
a_HQ_transcript/48818	0.982349943775295	0.0909719829681331	1.29811780125159	0.203359711318304	0.898797518105846	-5.03550225897417
a_HQ_transcript/19459	-1.04984329095239	2.53087463005178	-1.29811303025054	0.203361331267706	0.898797518105846	-5.18165143490532
a_HQ_transcript/118494	2.75446906006653	2.40039907353544	1.29802546822751	0.203391063891205	0.898797518105846	-5.06256780401853
a_HQ_transcript/120204	-1.7683058374478	2.51862706575873	-1.2977957401351	0.20346908627129	0.898797518105846	-5.17940480574761
a_HQ_transcript/15715	-0.744557869664906	1.56184472512206	-1.2977424941887	0.203487173406864	0.898797518105846	-4.93107831060484
a_HQ_transcript/126495	1.04357849262346	0.256349053571515	1.29771422800853	0.203496775653964	0.898797518105846	-4.99464137863108
a_HQ_transcript/114170	1.26420121912836	1.06149733378128	1.2976707133513	0.203511558605245	0.898797518105846	-4.9754537716385
a_HQ_transcript/101395	1.4743706697416	0.599788706355394	1.29766734857984	0.203512701731228	0.898797518105846	-5.06119037610513
a_HQ_transcript/145804	1.29967648521291	-0.471625630905747	1.29761664736142	0.203529927231831	0.898797518105846	-5.04553882690678
a_HQ_transcript/147168	-3.60499923499102	1.91105686689145	-1.29759094988795	0.203538658251771	0.898797518105846	-5.24565545291428
a_HQ_transcript/28918	1.1597222978789	-0.555436534358371	1.29747126476212	0.203579326445227	0.898797518105846	-5.066977397346
a_HQ_transcript/114658	-1.33026958687518	-0.0738555755055369	-1.29743230761712	0.203592565153959	0.898797518105846	-5.04547840546191
a_HQ_transcript/46417	-0.923094622576172	4.39445074616062	-1.29741201600123	0.203599461062575	0.898797518105846	-5.49643969308891
a_HQ_transcript/106209	1.72409744528286	1.58328799290006	1.297410083085	0.20360011795467	0.898797518105846	-5.07118580138742
a_HQ_transcript/148383	0.478881975036316	3.62085736226946	1.2973949537344	0.203605259645979	0.898797518105846	-5.37911180158763
a_HQ_transcript/6676	1.65301342598676	-0.00341309238520405	1.29734666247549	0.203621672033048	0.898797518105846	-5.00450604929084
a_HQ_transcript/2241	1.25354405083998	1.93149092216366	1.29732292772048	0.203629738955149	0.898797518105846	-5.03035695109996
a_HQ_transcript/40022	1.20275655672912	-0.245584189399378	1.2972477514425	0.203655291328146	0.898797518105846	-5.06840989045938
a_HQ_transcript/93989	0.298547725522759	3.47058377313439	1.29724019920272	0.203657858465043	0.898797518105846	-5.3534390606488
a_HQ_transcript/146720	2.00181171999082	2.15414422420352	1.29717063811315	0.203681504641373	0.898797518105846	-5.24600446351066
a_HQ_transcript/98642	0.952798971806785	3.10627160678489	1.29709865581831	0.203705976069288	0.898797518105846	-5.23910398207113
a_HQ_transcript/152394	1.99563771899571	-0.312589501037051	1.29696954948021	0.203749873256901	0.898797518105846	-4.99665558705016
a_HQ_transcript/136312	-1.66169924989259	2.70684055352925	-1.2969505589596	0.203756330793611	0.898797518105846	-5.18828926138444
a_HQ_transcript/45168	-0.997103250845667	2.9567181461947	-1.29692254754234	0.203765856080605	0.898797518105846	-5.31159456530057
a_HQ_transcript/48492	1.21788874643929	4.84569735822793	1.29686657386542	0.203784890954536	0.898797518105846	-5.53041520588852
a_HQ_transcript/104814	1.48972002527252	3.26753348837057	1.29679534838634	0.203809114441229	0.898797518105846	-5.37278532207154
a_HQ_transcript/70228	1.47760378174971	0.114768500669283	1.29678879899729	0.203811341971071	0.898797518105846	-5.01638987656513
a_HQ_transcript/68214	0.711440586522259	3.28767649016503	1.29677938224982	0.203814544758184	0.898797518105846	-5.34877544380467
a_HQ_transcript/96634	1.41571116444434	-0.254750764632462	1.29676796581596	0.203818427722172	0.898797518105846	-5.01822088989493
a_HQ_transcript/140250	0.856417268290235	1.65828767059379	1.29659603210215	0.203876912728811	0.898797518105846	-4.92774883738453
a_HQ_transcript/68635	-2.04735233209562	3.8829499184688	-1.29658318207665	0.203881284309333	0.898797518105846	-5.36239283155999
a_HQ_transcript/94999	0.295780020830148	3.74589753464316	1.29655891127266	0.203889541435091	0.898797518105846	-5.35744953001673
a_HQ_transcript/106041	1.02447054398438	-0.628405110474851	1.29649055768873	0.203912797251277	0.898797518105846	-5.04157751282355
a_HQ_transcript/13888	0.738013377362697	3.38111173072177	1.29648667899156	0.203914116953962	0.898797518105846	-5.26604835221255
a_HQ_transcript/26913	-0.770268127954403	2.13364537378911	-1.29648383545378	0.203915084454213	0.898797518105846	-5.06169887320754
a_HQ_transcript/9306	2.6958395562254	1.68436434479717	1.29647292182675	0.203918797796523	0.898797518105846	-5.18532656858442
a_HQ_transcript/4858	-1.81206812829645	-0.016526747485236	-1.29642996646493	0.203933413782708	0.898797518105846	-5.00323244993233
a_HQ_transcript/39566	1.07231411739304	1.1849534598648	1.2964072317213	0.203941149826669	0.898797518105846	-4.9155679036753
a_HQ_transcript/109502	-1.41925049086416	2.06430443318594	-1.29634648464147	0.203961821579703	0.898797518105846	-5.02557681948777
a_HQ_transcript/143137	1.65062157569187	1.37877366348946	1.29632720609472	0.203968382250929	0.898797518105846	-5.00631404778111
a_HQ_transcript/14560	3.72420484207675	1.31525708804402	1.29630299116467	0.203976623047639	0.898797518105846	-4.98429161777589
a_HQ_transcript/121969	-0.512327093693505	3.1378260502526	-1.29626828107213	0.20398843598798	0.898797518105846	-5.19966974066773
a_HQ_transcript/38088	1.1814319224186	2.47436017596561	1.29616596845548	0.204023259232662	0.898797518105846	-5.03900845441582
a_HQ_transcript/7026	1.24193644867233	-0.461138300953048	1.29614043959828	0.204031948970712	0.898797518105846	-5.03947601181607
a_HQ_transcript/105224	1.03713447236595	-0.504925341223318	1.29609048828339	0.204048952655998	0.898797518105846	-4.98835389624137

a_HQ_transcript/139211	1.42233942765984	-0.271072989284582	1.29604189289651	0.204065495811892	0.898797518105846	-5.00581181199789
a_HQ_transcript/4965	1.25086846243117	0.114674635870261	1.29602716028493	0.204070511384552	0.898797518105846	-5.10224674215585
a_HQ_transcript/60811	2.13459297515246	1.17631330962035	1.29596189672897	0.204092730849002	0.898797518105846	-5.13763276835856
a_HQ_transcript/56909	-1.05300336912006	1.6387395980484	-1.29588248939651	0.20411976815187	0.898797518105846	-4.97784861343351
a_HQ_transcript/28907	1.79617402906161	2.75678281082805	1.29587615472703	0.20412192515298	0.898797518105846	-5.14228777759453
a_HQ_transcript/11065	-1.54124062013402	1.91102143462258	-1.29586552939011	0.204125543196584	0.898797518105846	-5.1472528514292
a_HQ_transcript/30736	-1.49150447453548	-0.304449735904779	-1.29583949789603	0.204134407411805	0.898797518105846	-5.04086754856241
a_HQ_transcript/34326	-1.04987165073551	3.57562204936087	-1.29576173258965	0.204160889712487	0.898846034780537	-5.3653739611025
a_HQ_transcript/114821	-1.48477269054509	1.17862523535359	-1.29570690542764	0.204179562199229	0.898848124131754	-5.1011618937175
a_HQ_transcript/9825	0.74654870059803	2.33633098305486	1.29565841287477	0.20419607839171	0.898848124131754	-5.01100595175995
a_HQ_transcript/137516	2.26803822481512	1.10303278248162	1.29562413423531	0.204207754047687	0.898848124131754	-5.02096079769496
a_HQ_transcript/25975	1.2513034388551	1.88768920563581	1.29552902368193	0.204240152338817	0.898860437338871	-4.9849488569
a_HQ_transcript/136780	1.11139778384338	-0.339705458009537	1.29552513105745	0.204241478398909	0.898860437338871	-5.06775399669004
a_HQ_transcript/13780	0.983344538460388	2.16274871105422	1.29545778577268	0.204264421257749	0.898893351417248	-5.13963159612932
a_HQ_transcript/149527	0.89699495749813	3.1000856169117	1.29523134160886	0.204341579527197	0.899054935339854	-5.28455680125744
a_HQ_transcript/93982	-0.517606048176264	2.79541638429436	-1.29520500890872	0.204350553534754	0.899054935339854	-5.26903848446257
a_HQ_transcript/151795	-4.47382633591073	0.57873191711702	-1.29515759280581	0.204366713381053	0.899054935339854	-5.09361802020839
a_HQ_transcript/143201	1.56083823072217	0.0747967858029725	1.29493261170396	0.204443402279731	0.899054935339854	-5.079907530567
a_HQ_transcript/90827	-1.35769616404066	3.55436367146693	-1.29485378798919	0.204470275957231	0.899054935339854	-5.20291120660778
a_HQ_transcript/35543	1.47967503388745	3.97191904123358	1.29485239287106	0.204470751624527	0.899054935339854	-5.41094832759083
a_HQ_transcript/34879	-0.668760589927761	1.75600197479659	-1.2948389346679	0.204475340259247	0.899054935339854	-4.93024483569686
a_HQ_transcript/7303	-1.13610176193492	2.42713788753315	-1.29480279717952	0.204487661885784	0.899054935339854	-5.10026326057315
a_HQ_transcript/152763	1.37875709706654	-0.285582105212466	1.29477448355738	0.204497316240953	0.899054935339854	-5.09269829858948
a_HQ_transcript/50239	-0.498425899021661	2.31135673448596	-1.29469600041492	0.204524079170897	0.899054935339854	-5.05396677852137
a_HQ_transcript/37824	-1.19560424577641	-0.462008427483362	-1.29465753459358	0.204537197076879	0.899054935339854	-5.0631265873957
a_HQ_transcript/107845	-0.922410518730264	2.38378015635074	-1.29461743429631	0.204550873066022	0.899054935339854	-5.21739536667416
a_HQ_transcript/45768	1.46418447626001	0.99130362481366	1.29449951261299	0.204591093649993	0.899054935339854	-5.00405734362735
a_HQ_transcript/145153	2.61242797089693	1.78348586448752	1.29449469237557	0.204592737858741	0.899054935339854	-5.16570654324655
a_HQ_transcript/64217	0.861764427322859	1.91689575862634	1.29444940426943	0.204608186364936	0.899054935339854	-5.09700236631227
a_HQ_transcript/103230	-3.94240979765883	0.738917155107151	-1.29444331721103	0.204610262826785	0.899054935339854	-5.11182853481405
a_HQ_transcript/19597	1.5091044047305	0.110355917180993	1.2944136097162	0.204620397094691	0.899054935339854	-4.97116378372078
a_HQ_transcript/58850	1.34918270745674	3.08090979575161	1.29440737781224	0.204622523064074	0.899054935339854	-5.17578182164078
a_HQ_transcript/136511	3.61920733742359	2.1442077394279	1.29438800755107	0.204629131197206	0.899054935339854	-5.07838199267915
a_HQ_transcript/86947	-0.488620593084643	3.93618028422474	-1.2943759125053	0.204633257484794	0.899054935339854	-5.40841258712093
a_HQ_transcript/47434	-1.38637908210439	0.509512769725062	-1.29433525125317	0.204647129746123	0.899054935339854	-5.09037146880181
a_HQ_transcript/139492	-1.17106335538488	2.43478925779151	-1.29431055772141	0.204655554703816	0.899054935339854	-5.07623920505524
a_HQ_transcript/32765	-0.570666573394125	3.72688611049068	-1.29430668425417	0.204656876280225	0.899054935339854	-5.33479382878247
a_HQ_transcript/84984	0.971027606436663	1.24343642147653	1.29425376980619	0.204674930648889	0.899066301576257	-4.97236433356306
a_HQ_transcript/15016	1.02920003312919	0.261501790089752	1.29414224332589	0.204712987367325	0.899076873194348	-5.02692284830357
a_HQ_transcript/152089	-1.86308642561131	0.433711289609492	-1.2941029099496	0.204726410569379	0.899076873194348	-5.09450260357602
a_HQ_transcript/86582	1.65610197815853	0.620316347823768	1.29408098437464	0.204733893345763	0.899076873194348	-4.98105597925713
a_HQ_transcript/46338	1.31300937345585	-0.210086648747651	1.29406541772248	0.20473920607063	0.899076873194348	-5.01711843298786
a_HQ_transcript/13083	0.394974214958665	3.60420628012809	1.2940157951037	0.204756142417579	0.899083324252079	-5.34442241714214
a_HQ_transcript/1998	1.40194281441635	0.865079505948292	1.29387276130475	0.204804966242991	0.89918272052435	-4.92834342170845
a_HQ_transcript/29392	1.34320967682508	-0.352736854756727	1.29384240459017	0.204815329485374	0.89918272052435	-5.06754838301857
a_HQ_transcript/121931	1.30118986388005	-0.436864807667906	1.29375262646328	0.204845980476109	0.89918272052435	-5.06493610455391
a_HQ_transcript/37376	1.02172045813034	2.0297085275822	1.29364501640908	0.204882724032517	0.89918272052435	-5.10877217035697
a_HQ_transcript/10637	-0.547428372215545	2.21329205218585	-1.29359548595147	0.204899637940155	0.89918272052435	-5.08976517436377
a_HQ_transcript/25753	1.03687874643312	1.25531564708305	1.29356062461914	0.204911543198804	0.89918272052435	-5.02617755599184
a_HQ_transcript/18014	-1.30718343915089	1.82702648100769	-1.29355724909905	0.204912695977867	0.89918272052435	-5.01803548961092

a_HQ_transcript/78233	1.48198023065478	0.567965933913477	1.29355524213272	0.204913381382369	0.89918272052435	-4.92526277160292
a_HQ_transcript/97803	1.99136917028068	0.110908064763437	1.29354171852913	0.204917999910303	0.89918272052435	-5.09611354893201
a_HQ_transcript/31533	-1.32069830557537	1.98876507487701	-1.29342364532094	0.204958327172913	0.899283559886146	-5.04266157883724
a_HQ_transcript/142337	1.14884543954453	-0.101842280104472	1.29338384519553	0.204971922050157	0.899283559886146	-5.03583730659593
a_HQ_transcript/144253	-1.49804506733314	0.162618013295422	-1.29329513066311	0.205002227518695	0.89934864009107	-5.0424286510166
a_HQ_transcript/26802	1.07500211099979	2.76109302370551	1.2930774637633	0.205076598408694	0.899494614892563	-5.20744813616101
a_HQ_transcript/137365	2.34925590857501	0.961125042355962	1.29306027995091	0.205082470526515	0.899494614892563	-5.14845418642439
a_HQ_transcript/42360	0.803799987409472	2.42810699835606	1.29304980893287	0.205086048785405	0.899494614892563	-5.17450164112651
a_HQ_transcript/132963	1.78485913237752	0.0283994481375807	1.29301659500806	0.205097399287538	0.899494614892563	-5.05307399968947
a_HQ_transcript/81014	-1.88953025677022	0.388455271300138	-1.29274537376431	0.205190104121096	0.899703323860621	-4.98741282027942
a_HQ_transcript/134369	1.85636219496146	0.951319580983581	1.29272441289035	0.205197269983716	0.899703323860621	-5.28813122906805
a_HQ_transcript/148787	-1.32243496527559	-0.311333877637211	-1.29271343948786	0.205201021520324	0.899703323860621	-5.04917179267943
a_HQ_transcript/149467	1.79387337397545	2.48637747120648	1.29269624577202	0.205206899734106	0.899703323860621	-5.22423561063219
a_HQ_transcript/56667	-2.11194005837864	0.743592234647509	-1.29252358158543	0.205265937531729	0.899888149864282	-5.04488891826609
a_HQ_transcript/20790	-0.395978065076752	2.73611525553352	-1.29247169996159	0.205283679558551	0.899888149864282	-5.19139189032474
a_HQ_transcript/5437	1.44311011474165	-0.32780480498812	1.29241135686312	0.205304316634264	0.899888149864282	-5.06508905975524
a_HQ_transcript/146774	-1.32780877666157	0.649933436605141	-1.29237067822399	0.205318229441344	0.899888149864282	-5.0583686153011
a_HQ_transcript/148672	-0.857821991695414	3.16516081022307	-1.29234661122478	0.205326461114265	0.899888149864282	-5.32967432396495
a_HQ_transcript/102572	-0.710358043132037	1.62955994599921	-1.29227756219379	0.205350079454249	0.899923810201792	-4.9151357787287
a_HQ_transcript/138747	1.39117584559039	-0.268326518871947	1.29217295083355	0.205385865886988	0.900012786451477	-5.0736873183969
a_HQ_transcript/88125	-2.02049980529449	2.36897483013838	-1.29206983303114	0.205421146031609	0.900099530870297	-5.1579077636543
a_HQ_transcript/54058	-1.76566289963389	2.15965378589346	-1.29197397930845	0.205453945018288	0.900132027939445	-5.2188790145943
a_HQ_transcript/7527	-0.626079034508961	2.56330700331221	-1.29193206686676	0.205468287764032	0.900132027939445	-5.08610130035831
a_HQ_transcript/28255	1.27782108092612	1.83506004151446	1.29187322697343	0.205488424493111	0.900132027939445	-5.14088355520934
a_HQ_transcript/148513	3.26164140981348	1.21206554730129	1.29186715115512	0.205490503900985	0.900132027939445	-5.1326429391419
a_HQ_transcript/147425	1.21145757433808	1.87419045038179	1.29167720237517	0.205555520661782	0.900348979355592	-5.01589330898725
a_HQ_transcript/110320	1.32931304233188	2.24282855521852	1.2914851682545	0.205621267106174	0.90043711009608	-5.16106860505988
a_HQ_transcript/140552	0.414556254526495	4.18105777981257	1.29141554849094	0.205645106673173	0.90043711009608	-5.48398173183443
a_HQ_transcript/71040	0.65992110884689	4.35591265548621	1.29130437408405	0.205683179958296	0.90043711009608	-5.41597042348459
a_HQ_transcript/56030	0.756864392954989	1.15936687163406	1.29129846822653	0.205685202654624	0.90043711009608	-4.90097569710882
a_HQ_transcript/52918	1.34199815878138	-0.106037762870202	1.29124923633194	0.205702064667975	0.90043711009608	-5.03240956821459
a_HQ_transcript/150420	0.776781481332715	2.29794762735265	1.29121823951308	0.205712681673966	0.90043711009608	-5.22273203230354
a_HQ_transcript/149402	2.06652601703954	-0.192510474630078	1.29111199913428	0.205749074206328	0.90043711009608	-5.07667129845405
a_HQ_transcript/56377	1.33166944803352	0.951259771970177	1.29110757179865	0.205750590891832	0.90043711009608	-4.92525428381191
a_HQ_transcript/21953	2.04119715405912	2.1327766876045	1.29107265431809	0.205762552974667	0.90043711009608	-5.21547181709178
a_HQ_transcript/146528	-0.806019880016907	2.12271901685603	-1.29104878242346	0.205770731348442	0.90043711009608	-5.01254496192344
a_HQ_transcript/34401	1.5798898692287	2.13722050037225	1.29104685009039	0.20577139336545	0.90043711009608	-5.09927472417263
a_HQ_transcript/69202	-1.95630229660162	0.983692952360652	-1.29102050432246	0.205780419582964	0.90043711009608	-5.00183170172826
a_HQ_transcript/63209	-1.27984282004213	0.670518463334685	-1.29096329150658	0.205800022072256	0.90043711009608	-4.97960350454617
a_HQ_transcript/97641	0.626102640971183	2.91192748439092	1.29095109086135	0.205804202490946	0.90043711009608	-5.22189915749135
a_HQ_transcript/138613	-1.62379550455883	0.545317721006776	-1.29093464345265	0.205809838119239	0.90043711009608	-4.95888279882662
a_HQ_transcript/127374	0.347162195557197	3.86143065479793	1.2908868049186	0.205826230437955	0.90043711009608	-5.39453231102957
a_HQ_transcript/148297	-1.1215608747145	-0.348929046032065	-1.29083644495596	0.205843487820123	0.90043711009608	-5.08234600690573
a_HQ_transcript/52065	0.964021435680959	0.799475006755642	1.29066175874246	0.205903357922588	0.90043711009608	-4.88367985347324
a_HQ_transcript/19418	1.34058652056342	1.02785152686821	1.29062169664992	0.20591709024582	0.90043711009608	-5.04116803343693
a_HQ_transcript/75027	1.80703576108949	0.50297108103315	1.29053469683775	0.205946914089886	0.90043711009608	-5.04650203278378
a_HQ_transcript/148722	-1.60987459356166	6.98487311965005	-1.29052445096397	0.205950426627867	0.90043711009608	-5.82749306680495
a_HQ_transcript/109833	-2.89992504368355	-0.362983403854704	-1.29052131410644	0.205951502029024	0.90043711009608	-5.09537588056183
a_HQ_transcript/130183	1.74962465194002	0.10273225940938	1.29051176805574	0.205954774704383	0.90043711009608	-5.0578565224711
a_HQ_transcript/61634	1.46286805976287	1.14310946058298	1.29048577151805	0.205963687305448	0.90043711009608	-4.94335983024312

a_HQ_transcript/62533	1.3024388160209	0.401738321725626	1.29032505236734	0.206018794447801	0.90043711009608	-5.06471711975327
a_HQ_transcript/11248	0.309090324491247	3.84519435984097	1.29032148687261	0.206020017106378	0.90043711009608	-5.38590147721713
a_HQ_transcript/103560	-0.591503690047474	3.97314020210079	-1.29031107140941	0.206023588747633	0.90043711009608	-5.45419896658241
a_HQ_transcript/58811	1.08225569855963	-0.452578443777018	1.29029690341237	0.206028447272761	0.90043711009608	-5.12892038813266
a_HQ_transcript/56207	0.916081542211746	2.47448965561262	1.29027934499009	0.206034468571878	0.90043711009608	-5.01412569784021
a_HQ_transcript/22108	1.13831502761376	0.854050611198531	1.2902418325027	0.206047333154638	0.90043711009608	-4.98238794956064
a_HQ_transcript/24625	-1.81820639280368	1.13309400725074	-1.29021699929887	0.206055849822771	0.90043711009608	-5.02031709712607
a_HQ_transcript/77669	-2.04765685163808	4.41263695821996	-1.29008664137774	0.206100561095504	0.900564791051602	-5.54506456702551
a_HQ_transcript/115237	-0.67503332989725	4.41605468824467	-1.28993691159497	0.206151925793906	0.900630337238739	-5.48128766398332
a_HQ_transcript/136441	1.10054033573012	-0.625886814176595	1.2898686656257	0.206175340754302	0.900630337238739	-5.0867425031958
a_HQ_transcript/142176	0.430862894723557	4.79730110876872	1.28986429617048	0.206176839968407	0.900630337238739	-5.49706634942519
a_HQ_transcript/58660	1.50925733864302	0.897120933562607	1.28986226333338	0.20617753746277	0.900630337238739	-5.12289916952596
a_HQ_transcript/91359	1.51464572173402	-0.0348969369597631	1.28969925879141	0.206233472400534	0.900802088218528	-5.00476583564185
a_HQ_transcript/68023	0.444903556715312	3.04802808406425	1.28965736692953	0.206247849437455	0.900802088218528	-5.24195528004932
a_HQ_transcript/151154	-0.391416360412487	4.1271780555834	-1.28942735996242	0.206326800028273	0.901079206013783	-5.43025456733424
a_HQ_transcript/138086	1.12230637399719	3.11511042045501	1.28933820577601	0.206357408649736	0.901112878392694	-5.22163609462313
a_HQ_transcript/108077	3.01663982780852	4.80586185040467	1.28931459691217	0.206365514677325	0.901112878392694	-5.43386792954998
a_HQ_transcript/37886	1.61627517787723	1.95117413134737	1.28918792490888	0.206409011235084	0.901228659685353	-5.20596306417965
a_HQ_transcript/139500	2.1832737353822	-0.113746427469561	1.2891390662661	0.206425790148702	0.901228659685353	-5.07376483090483
a_HQ_transcript/115249	-1.52103436650343	-0.0106150559799826	-1.28906314182915	0.206451865986755	0.901228659685353	-5.12141750151333
a_HQ_transcript/67028	-1.55328790229176	0.198801811174191	-1.28905679233501	0.206454046799652	0.901228659685353	-5.04257595087365
a_HQ_transcript/69926	1.70596430256142	0.638972987961417	1.28899608103915	0.206474899734759	0.90125200659173	-4.91417721587468
a_HQ_transcript/115577	-1.79701896001651	0.0309742506467234	-1.28869163493605	0.206579494112211	0.901541626537988	-5.08713508661915
a_HQ_transcript/89508	-1.83226704811322	1.34562719565818	-1.28864323457826	0.206596126068283	0.901541626537988	-5.02538907909905
a_HQ_transcript/26460	-0.927887096141593	0.314299680232353	-1.2886139534873	0.206606188508866	0.901541626537988	-4.95693729910702
a_HQ_transcript/152824	1.01188634503566	2.61247920459536	1.28859259556166	0.206613528390252	0.901541626537988	-5.16681724360037
a_HQ_transcript/19645	1.4732557107669	-0.108459781692324	1.28853053164386	0.206634858448978	0.901541626537988	-5.10965311469376
a_HQ_transcript/107279	0.465207166290598	3.26752639179081	1.28850725281856	0.206642859321372	0.901541626537988	-5.30232995689931
a_HQ_transcript/121744	1.67202918544587	0.315629629540437	1.28848700628519	0.206649818194195	0.901541626537988	-5.07170287864771
a_HQ_transcript/113410	1.1583237238724	-0.190029072967984	1.28840235277624	0.206678916116164	0.90160090326457	-5.05790654391331
a_HQ_transcript/122501	-3.18237274081011	0.544251292383506	-1.28826859631711	0.206724898523143	0.901647301619782	-5.08923859587388
a_HQ_transcript/15581	-1.29497263326614	0.86059005000225	-1.28825341839361	0.206730116836548	0.901647301619782	-4.91749203674456
a_HQ_transcript/50956	1.79703797164	1.26663779329148	1.28820034032386	0.206748366364955	0.901647301619782	-4.99662265559378
a_HQ_transcript/89771	1.21608215101035	-0.534920035885414	1.28818505138315	0.20675362330081	0.901647301619782	-5.09113438454727
a_HQ_transcript/58661	1.12527576206848	-0.500942122362604	1.28814583084401	0.206767109320666	0.901647301619782	-5.07953575504343
a_HQ_transcript/14707	-1.16351798302086	0.59125291254313	-1.28804263946849	0.206802594965775	0.901701546080702	-5.08364548386541
a_HQ_transcript/144259	1.80022374941853	0.206256305055641	1.28799774447071	0.206818034986537	0.901701546080702	-4.9649529223417
a_HQ_transcript/144061	-1.24083993192063	0.654510389971243	-1.28793611859958	0.206839230415769	0.901701546080702	-4.99490277681849
a_HQ_transcript/34661	1.24951863127138	0.199918528868736	1.28790229235125	0.206850865222114	0.901701546080702	-4.97197342788782
a_HQ_transcript/123511	1.21059441563658	0.932837083527661	1.28785036162414	0.206868728174955	0.901701546080702	-4.96306777362143
a_HQ_transcript/7638	1.73227887881611	2.14769929897145	1.28783903969977	0.206872622807119	0.901701546080702	-5.00443598211914
a_HQ_transcript/94209	1.50883399087148	0.632371177382871	1.28765666213501	0.206935366595139	0.901907399673257	-5.08059115671953
a_HQ_transcript/24208	1.03470525143989	2.83529617279518	1.28753680451661	0.206976609367519	0.901975149053291	-5.16010842643092
a_HQ_transcript/63279	-1.35144400837205	2.16854554249919	-1.28750138097921	0.206988799732705	0.901975149053291	-5.30888245578707
a_HQ_transcript/105060	0.619154656236135	2.61482228212613	1.2874762097142	0.206997462296499	0.901975149053291	-5.11704006420751
a_HQ_transcript/125751	1.0227936053851	1.76374273523928	1.28741526545169	0.207018437097202	0.901998928984797	-5.0392659019119
a_HQ_transcript/6603	1.06113360089491	-0.0345550255060407	1.2871380715898	0.207113857520357	0.902263549005718	-4.97053905929316
a_HQ_transcript/148220	-2.40652836897689	1.70370567692149	-1.2871114307664	0.207123030040369	0.902263549005718	-5.17248486055862
a_HQ_transcript/96737	1.57890227189816	0.187562611509848	1.28704779017133	0.207144942947863	0.902263549005718	-4.99018132603035
a_HQ_transcript/39687	0.353983486282242	-0.836411532628433	1.28698505372415	0.207166546259803	0.902263549005718	-5.14870644720654

a_HQ_transcript/98936	-2.46839794704935	4.40359064953388	-1.28696264112419	0.207174264459226	0.902263549005718	-5.37297185780079
a_HQ_transcript/88443	1.54510167728712	-0.377412643751586	1.28695472186557	0.207176991656767	0.902263549005718	-5.05831235758681
a_HQ_transcript/16126	1.35142302963203	0.0647118843602403	1.28692326666801	0.207187824320952	0.902263549005718	-4.96050734908081
a_HQ_transcript/99336	1.44318691780473	-0.324741202605375	1.28686509000282	0.207207860562271	0.902283206140063	-5.03705555405137
a_HQ_transcript/98177	0.973867131618378	1.26149442687713	1.28675796058773	0.207244760146463	0.902376285977051	-4.92791460929981
a_HQ_transcript/59597	2.22007915245245	0.930535240833012	1.28655255819292	0.207315522764821	0.902496382606928	-4.93590911066201
a_HQ_transcript/89078	-1.50657784290355	-0.47234622886336	-1.28653758435514	0.207320682078175	0.902496382606928	-5.06230714008438
a_HQ_transcript/137873	1.82285522221361	0.72083500218298	1.28652593782938	0.207324695016341	0.902496382606928	-5.1968509564945
a_HQ_transcript/94053	2.22866570338784	0.12403922603055	1.28645555727584	0.20734894666297	0.902496382606928	-5.07228456679358
a_HQ_transcript/27364	0.982446079727676	1.01554278124403	1.28639295948353	0.207370518344857	0.902496382606928	-4.98528557456297
a_HQ_transcript/7331	0.924511311944484	2.48096863654116	1.28638825833414	0.207372138465969	0.902496382606928	-5.08802825463391
a_HQ_transcript/41461	-1.19380034464784	2.55366637246514	-1.28619043497075	0.207440321527936	0.902496382606928	-5.00849414542123
a_HQ_transcript/125086	0.838014093815558	2.80687235901213	1.28617084703432	0.207447073757009	0.902496382606928	-5.24742992397359
a_HQ_transcript/24654	1.42460839848759	1.70572276285648	1.28612633027759	0.207462419911272	0.902496382606928	-5.09229004128007
a_HQ_transcript/40860	1.49417687013814	-0.252974318874376	1.28611854844321	0.207465102612781	0.902496382606928	-4.96134978275579
a_HQ_transcript/131594	1.24340115611368	0.209539927318823	1.28611823726496	0.207465209888595	0.902496382606928	-4.94850424725798
a_HQ_transcript/114436	-1.20926836428489	4.365692518221	-1.28610926227914	0.207468303949814	0.902496382606928	-5.51229391383851
a_HQ_transcript/137648	-2.02157028127357	2.47048387515265	-1.28604641746342	0.207489970225374	0.902496382606928	-5.1020466809648
a_HQ_transcript/139738	0.829567336284096	3.35888509312809	1.2859814606984	0.207512366418392	0.902496382606928	-5.32611424578848
a_HQ_transcript/6007	-1.39960066218045	1.36212389518277	-1.28595520329647	0.207521420127798	0.902496382606928	-4.9516786962549
a_HQ_transcript/75083	1.26175497911737	2.79065431242404	1.28590187446368	0.207539809151671	0.902496382606928	-5.08437603702155
a_HQ_transcript/25607	-0.430626567351836	2.93389022277607	-1.28584000660658	0.207561144180028	0.902496382606928	-5.22573255643336
a_HQ_transcript/14994	1.3091822765664	0.626227578903609	1.28582333309968	0.207566894296162	0.902496382606928	-5.01758973790377
a_HQ_transcript/53458	1.28293044594875	-0.0718463394083057	1.28571288340905	0.207604987630012	0.902496382606928	-5.02724812107005
a_HQ_transcript/81560	1.67670624650205	0.512106243954418	1.28569487760262	0.207611198211092	0.902496382606928	-5.15495854200746
a_HQ_transcript/12815	1.82442507808506	1.67676224961725	1.28568774691544	0.207613657773957	0.902496382606928	-5.02457854156429
a_HQ_transcript/128869	0.920083326897239	2.18305119381373	1.28567164166648	0.207619212982269	0.902496382606928	-5.01957073223146
a_HQ_transcript/1117	1.13602179190939	0.275251109533763	1.28562428884078	0.207635547118916	0.902496382606928	-5.09064718217039
a_HQ_transcript/147994	1.41044984861476	4.47510759770209	1.2855969828336	0.207644966642325	0.902496382606928	-5.57950431198244
a_HQ_transcript/82000	-1.25536145942261	2.08378916154801	-1.28551813799887	0.207672166901686	0.902526453064432	-5.05710043209428
a_HQ_transcript/3591	1.29695004282953	1.74007253885441	1.28542551857858	0.207704122636449	0.902526453064432	-4.93296036642008
a_HQ_transcript/121988	-1.61554659187645	0.719930186707914	-1.28542411616741	0.207704606527799	0.902526453064432	-5.00575381709289
a_HQ_transcript/2576	1.65784424027388	0.482724920022071	1.28536669657335	0.207724419458356	0.902526453064432	-5.02334477453417
a_HQ_transcript/118152	-1.40874203267322	0.641189732514169	-1.28531665189249	0.207741688806591	0.902526453064432	-4.98854130739897
a_HQ_transcript/133903	-2.5389661559685	1.0004282805483	-1.28530692806071	0.207745044419249	0.902526453064432	-5.04718156464601
a_HQ_transcript/856	1.8674103580636	-0.257917826355271	1.28506255326226	0.207829389618812	0.902624987169804	-5.06111177590012
a_HQ_transcript/24600	0.588570988146904	2.43187789357697	1.28505351253138	0.207832510497351	0.902624987169804	-5.22974621108065
a_HQ_transcript/96645	0.64116124252082	-0.477149614258165	1.28500817737753	0.207848160820305	0.902624987169804	-5.1346387237437
a_HQ_transcript/10423	1.25263524024692	-0.133000211071116	1.28497463671068	0.207859740098645	0.902624987169804	-5.08967799342201
a_HQ_transcript/120232	-1.01706055087569	2.17303592522601	-1.28497023935235	0.207861258239411	0.902624987169804	-4.95602811926637
a_HQ_transcript/72596	0.763462396905223	2.23643940902021	1.2849339284415	0.207873794513271	0.902624987169804	-5.14395945264117
a_HQ_transcript/7114	-0.895070972895017	2.29336405120807	-1.28492631596853	0.207876422778044	0.902624987169804	-5.07762713295683
a_HQ_transcript/21879	-1.69333108894749	0.661702200127725	-1.28479522869134	0.207921685623907	0.902754088881571	-5.02128593157141
a_HQ_transcript/4833	0.63528343258667	3.29869933534726	1.28463346129519	0.207977552251901	0.902929207356675	-5.29107523963966
a_HQ_transcript/1986	1.14892938582933	1.62700977856019	1.28443189074569	0.208047180901572	0.903054137649876	-5.02797602771891
a_HQ_transcript/96746	-1.55401170347025	2.69011625847256	-1.28439011520961	0.208061613665563	0.903054137649876	-5.14804070170315
a_HQ_transcript/82574	1.66301258294733	0.0471357929024405	1.28431336153291	0.208088132784877	0.903054137649876	-4.99427320264
a_HQ_transcript/87216	0.428472588377499	3.77456782988712	1.28426536713601	0.208104716604983	0.903054137649876	-5.41055279915424
a_HQ_transcript/66982	2.69950020394972	1.55413760879174	1.28424933985525	0.208110254840238	0.903054137649876	-5.17360205157862
a_HQ_transcript/83232	1.43423706802763	0.00078477384110432	1.28422228484326	0.208119603967511	0.903054137649876	-5.14888133667912

a_HQ_transcript/150227	-1.1081546257369	-0.483769136112264	-1.28420387871404	0.208125964569859	0.903054137649876	-5.05880870307743
a_HQ_transcript/45711	-1.4074250846964	0.934037000642573	-1.28410591548376	0.208159820185152	0.903054137649876	-4.98272362466438
a_HQ_transcript/64677	-1.14780837604687	0.163256568877621	-1.28408274767937	0.208167827476449	0.903054137649876	-4.97145717222172
a_HQ_transcript/110462	1.50924344363016	0.899644057478257	1.28403687766576	0.208183681828745	0.903054137649876	-5.02724062243124
a_HQ_transcript/133388	1.59984674541015	0.549343135747581	1.28402124964781	0.208189083651861	0.903054137649876	-4.93096265523715
a_HQ_transcript/35821	-1.22479637907008	4.57462853630021	-1.2839791930084	0.208203621053978	0.903054137649876	-5.56207817236415
a_HQ_transcript/143203	-1.40011375630211	1.64257435162421	-1.28396568267458	0.208208291232859	0.903054137649876	-5.0036126996325
a_HQ_transcript/104803	0.779815436466494	0.158911907262897	1.2837315492323	0.20828923783471	0.903308623623466	-4.95684960592437
a_HQ_transcript/129328	-1.55953385963433	3.04650878727569	-1.28370607761514	0.208298045538276	0.903308623623466	-5.3236642446916
a_HQ_transcript/31333	-1.1505001117934	0.029281962930186	-1.28357826921002	0.208342244035616	0.903321462552544	-4.99429652761556
a_HQ_transcript/120760	1.75172054197787	1.57548119672565	1.28355662434617	0.208349729932086	0.903321462552544	-5.21314266919683
a_HQ_transcript/102400	-2.255172226316	0.916476919166632	-1.28352328137234	0.208361262030128	0.903321462552544	-5.10231497453771
a_HQ_transcript/135309	1.29851236899608	0.0507329209858035	1.28351777370473	0.208363166974685	0.903321462552544	-4.92813284073986
a_HQ_transcript/139950	-1.81990371299462	3.90107968968949	-1.2832371835785	0.208460232549454	0.903674874907501	-5.44683183014499
a_HQ_transcript/65163	1.19049113818096	-0.42644009009704	1.28316802183791	0.208484163193178	0.903709244683508	-5.08534291102526
a_HQ_transcript/117907	1.50777109492155	0.774895026082006	1.28306081495195	0.20852126195047	0.903709244683508	-5.0716672448529
a_HQ_transcript/93255	0.93289413630735	-0.106118020633297	1.28304006523407	0.208528442932751	0.903709244683508	-5.15289289795574
a_HQ_transcript/146325	0.550420827695493	3.15192152170368	1.28303455905075	0.208530348522913	0.903709244683508	-5.26486961581078
a_HQ_transcript/121961	1.54093998136438	1.53289056155971	1.28290837743062	0.208574021312292	0.903831125006777	-4.8988368740482
a_HQ_transcript/105303	0.977873699727172	-0.687137438162286	1.28283220924948	0.208600387280516	0.903850066395227	-5.08691319383824
a_HQ_transcript/138495	2.08619890014525	0.60516122187774	1.28280591147432	0.208609490964051	0.903850066395227	-5.06105116083623
a_HQ_transcript/5174	1.26108277781526	2.96180004042072	1.28256837614386	0.208691733873185	0.904139010701385	-5.14860545986121
a_HQ_transcript/51582	-1.32571351375858	1.00306724006262	-1.28249139171864	0.208718393807059	0.904187121420236	-4.97879931874188
a_HQ_transcript/97225	-1.54825448871081	0.958284313113442	-1.28242755592765	0.208740502288776	0.904215509131974	-4.98574751002036
a_HQ_transcript/88996	1.62060798230159	0.453196884655931	1.28231004361584	0.208781205398864	0.904275180770028	-5.09694264606692
a_HQ_transcript/136311	1.25788625230217	3.28394296815317	1.28226936191736	0.208795297849475	0.904275180770028	-5.31436200331462
a_HQ_transcript/36749	0.791294861680166	1.52993705359285	1.28225305324568	0.208800947500264	0.904275180770028	-4.95121949881298
a_HQ_transcript/132134	1.48753171147019	1.42439751277253	1.28215974293732	0.208833274290613	0.904347803618025	-5.01049136095828
a_HQ_transcript/95738	-2.48322579007277	0.662575870296396	-1.2820374215591	0.208875657538463	0.904423198572967	-5.12693663247242
a_HQ_transcript/117236	1.65757316377364	1.2458230582353	1.28201968703305	0.208881802932186	0.904423198572967	-5.05435997996199
a_HQ_transcript/89086	2.02277638797405	1.35738257447583	1.2818567288365	0.208938277860898	0.904467955177592	-5.15734450347157
a_HQ_transcript/133675	5.77904091293081	3.97824030428825	1.281841796346	0.208943453456264	0.904467955177592	-5.16331931155628
a_HQ_transcript/67177	0.951526740813225	0.760326349372394	1.28178871349481	0.208961852738712	0.904467955177592	-4.93823271489316
a_HQ_transcript/113973	1.34282868898535	-0.161894811463455	1.28177138201013	0.208967860347485	0.904467955177592	-5.01997925111382
a_HQ_transcript/145882	1.41284672031069	-0.24888748637726	1.28172681561022	0.208983308990336	0.904467955177592	-5.07845228615775
a_HQ_transcript/110965	1.28540461882598	-0.2160764299594	1.28167319816335	0.209001896265313	0.904467955177592	-5.08395553319678
a_HQ_transcript/12212	-1.18166784614584	1.20105671812401	-1.28167096264287	0.209002671268519	0.904467955177592	-5.12587029082733
a_HQ_transcript/143466	-1.16692368042919	-0.416818799172642	-1.28156492853344	0.209039433342618	0.904467955177592	-5.06593979743147
a_HQ_transcript/123837	1.78404966180898	0.501868380455546	1.28154913776447	0.209044908428791	0.904467955177592	-5.01002927600421
a_HQ_transcript/69161	0.97208453886816	0.656743371500826	1.28154097394036	0.209047739089784	0.904467955177592	-5.06979768957781
a_HQ_transcript/80759	1.72800008369403	1.75365560049898	1.28145755952456	0.209076663222085	0.904525772534485	-5.03917736163333
a_HQ_transcript/136544	0.692762485271711	2.6196518224143	1.28137534142682	0.209105175497426	0.904581799606636	-5.13047120312885
a_HQ_transcript/152230	-2.449328007164	3.01988413384161	-1.28125397891661	0.209147267984351	0.904596522759318	-5.49642761869612
a_HQ_transcript/87352	-1.31133584180446	-0.157971069248291	-1.28122453042115	0.209157482653844	0.904596522759318	-5.08030926278075
a_HQ_transcript/121153	1.26388320398707	-0.473258126535066	1.28120946844014	0.209162707282825	0.904596522759318	-5.0725494642956
a_HQ_transcript/74845	0.994100725756572	2.51818788094876	1.28118605931668	0.209170827525599	0.904596522759318	-5.17498690820089
a_HQ_transcript/119779	-1.35636038456577	0.297543679140671	-1.28087835258204	0.209277588186108	0.904939526561788	-5.04877714293913
a_HQ_transcript/20607	1.18697526442746	-0.101551968778051	1.28083009489213	0.209294335221443	0.904939526561788	-5.06795541440235
a_HQ_transcript/108810	-1.50469985352718	0.993068380849542	-1.2807994745789	0.209304962022861	0.904939526561788	-5.05940027223634
a_HQ_transcript/62185	1.09617630486788	0.905574782204681	1.2806795452947	0.209346587503846	0.904939526561788	-4.91799097293252

a_HQ_transcript/88340	1.66024703600262	1.03050148208334	1.28066169967017	0.209352781962708	0.904939526561788	-5.07679097004796
a_HQ_transcript/98971	3.97114625380363	2.07471323518069	1.28064723563849	0.209357802726652	0.904939526561788	-5.1877471664043
a_HQ_transcript/48914	-0.826786239948135	2.72741069445872	-1.2805903201268	0.209377560161664	0.904939526561788	-5.10905415274287
a_HQ_transcript/103589	0.804234244295468	3.03266781280982	1.28057659004796	0.209382326580762	0.904939526561788	-5.11520679325511
a_HQ_transcript/132826	4.16942494775505	1.03244794259018	1.28051188593279	0.209404789826099	0.904939526561788	-5.17092712288962
a_HQ_transcript/116437	1.50896585629611	0.616501436347049	1.2805089153898	0.209405821149577	0.904939526561788	-5.05210877143927
a_HQ_transcript/140834	1.26797530891924	-0.0142686565140414	1.28041874616566	0.209437128247425	0.904960091233996	-5.14464455770668
a_HQ_transcript/111561	-1.73188164100228	1.01808922731208	-1.28040553162755	0.20944171668337	0.904960091233996	-5.09413021287093
a_HQ_transcript/26863	1.99266228144203	0.716179873141489	1.2802993329171	0.20947859444133	0.905052158293862	-5.10158135654452
a_HQ_transcript/97221	1.42006456134137	-0.160937897775763	1.28020302501748	0.209512041846111	0.905129391930935	-5.12385705516613
a_HQ_transcript/143663	1.37376282395121	1.80945668724404	1.28005336398274	0.209564026643876	0.905286692981213	-5.05678397400484
a_HQ_transcript/104854	0.594051057957001	2.6127411647475	1.27998336902629	0.209588342763988	0.905289652118035	-5.15747883614158
a_HQ_transcript/26844	-1.2965683734601	-0.409220712123289	-1.27994173042385	0.2096028089527	0.905289652118035	-5.0166699859166
a_HQ_transcript/23182	2.05922737978473	2.46838465203144	1.27991690602675	0.209611433867613	0.905289652118035	-5.19395560054165
a_HQ_transcript/151634	2.55111758094345	0.85409138780503	1.27975699061634	0.209667000850074	0.905448706147513	-5.14702840232319
a_HQ_transcript/4805	1.24534334196261	1.32105087636548	1.27966704071253	0.209698261308509	0.905448706147513	-4.95985301942741
a_HQ_transcript/18049	-0.849226453068165	2.82464117393489	-1.27966675621324	0.209698360186681	0.905448706147513	-5.19772266140061
a_HQ_transcript/74032	1.04885136914267	2.15528162304723	1.27959158591176	0.209724486980317	0.905448706147513	-5.16003972174949
a_HQ_transcript/37553	0.895971861350038	1.36860349146214	1.27952709124483	0.209746905230519	0.905448706147513	-4.93538128995591
a_HQ_transcript/94228	1.06190792018578	1.4354946540256	1.27947679754054	0.209764388507229	0.905448706147513	-4.92941503777423
a_HQ_transcript/105857	-1.62550342259239	1.78080736563181	-1.27945818482109	0.209770859007012	0.905448706147513	-5.1118214788593
a_HQ_transcript/16609	1.61314984440623	0.383843602740163	1.27945238344441	0.209772875820406	0.905448706147513	-4.97034338159092
a_HQ_transcript/59416	2.89853457767884	1.24293762404005	1.27925564909395	0.209841278020376	0.905591822080518	-5.14654462407901
a_HQ_transcript/103726	-1.08097234822223	0.774814578780964	-1.27924194823699	0.209846042275138	0.905591822080518	-4.97145070229338
a_HQ_transcript/127762	2.21848524151843	1.10579698264571	1.27922259988312	0.209852770496569	0.905591822080518	-5.12676117870727
a_HQ_transcript/37269	-3.66378615149887	0.338376066565413	-1.27913542721066	0.209883086060645	0.905642945657197	-5.17355559417936
a_HQ_transcript/22240	-0.622587494324921	2.63978499778783	-1.27906374566889	0.209908016844545	0.905642945657197	-5.17929146907596
a_HQ_transcript/32462	-2.48636609545713	-0.208804281695133	-1.27905414013818	0.209911357811028	0.905642945657197	-4.97747063730848
a_HQ_transcript/89426	-1.62063846055407	1.2636848843175	-1.27900398551804	0.209928803092826	0.905650991997908	-5.05305343589709
a_HQ_transcript/134576	1.22141950974525	-0.308376006231096	1.27887891087677	0.209972312590733	0.905653537462444	-5.07386005190198
a_HQ_transcript/22424	1.06121907533552	1.22778620062141	1.2787234152797	0.210026414098723	0.905653537462444	-4.98493317839922
a_HQ_transcript/67924	0.600092780617482	5.32174090331825	1.27868432942764	0.210040014880921	0.905653537462444	-5.63143404552742
a_HQ_transcript/88389	1.14486915318561	-0.448307075725006	1.27868191783607	0.210040854069118	0.905653537462444	-5.01262513038545
a_HQ_transcript/51380	-1.17956720509779	2.42051046729347	-1.27856899474835	0.210080152009072	0.905653537462444	-5.23653651816249
a_HQ_transcript/35205	1.91880822102426	0.422242329376435	1.27856637987627	0.210081062066861	0.905653537462444	-5.03163317263684
a_HQ_transcript/88968	1.13426680661129	-0.555667750645341	1.2785486705149	0.210087225560629	0.905653537462444	-5.09906674010395
a_HQ_transcript/109093	1.38606368949343	0.283362393647725	1.27854162593255	0.210089677366363	0.905653537462444	-5.08126498235232
a_HQ_transcript/98056	2.06984767328931	-0.212172877709915	1.2783982415574	0.210139585763578	0.905653537462444	-5.07488124207183
a_HQ_transcript/85328	-0.798520937200134	2.85103306652228	-1.27837958156268	0.210146081486055	0.905653537462444	-5.1704945821617
a_HQ_transcript/101618	-1.51753526869368	0.558044696742432	-1.27836413069993	0.210151460194126	0.905653537462444	-4.99013089194778
a_HQ_transcript/138076	1.11693020168433	1.8619050798109	1.27833388146731	0.210161990768306	0.905653537462444	-5.05106909960353
a_HQ_transcript/14548	-2.06250439103876	3.14455298447815	-1.2783304481619	0.210163186019822	0.905653537462444	-5.22141366867172
a_HQ_transcript/23831	-1.73395539381445	0.568037418555581	-1.27826888560609	0.210184618933537	0.905653537462444	-5.09921596344896
a_HQ_transcript/136822	1.78495534730842	-0.143314029725462	1.27826690784864	0.210185307514345	0.905653537462444	-5.09185220949539
a_HQ_transcript/147796	1.21961202865418	3.43336734181775	1.27821781731452	0.210202399540717	0.905653537462444	-5.25384573600626
a_HQ_transcript/116356	3.04794093384102	2.1200895724728	1.2781972754162	0.210209551999128	0.905653537462444	-5.18159441065713
a_HQ_transcript/108163	-0.696231438812808	2.82685287841479	-1.27815493240642	0.210224295941404	0.905653537462444	-5.14456591637963
a_HQ_transcript/28396	-1.87310480048416	2.57742995695842	-1.27815170594327	0.210225419436085	0.905653537462444	-5.29992621142538
a_HQ_transcript/73178	-2.03350868297099	0.520517326924662	-1.2780728702849	0.210252872407865	0.905670407916831	-5.06107456173014
a_HQ_transcript/119041	-1.60949993792747	-0.056861413885256	-1.27802573044286	0.210269289230366	0.905670407916831	-4.99395613959364

a_HQ_transcript/22146	-0.40116089627942	3.4144241384933	-1.27794834638538	0.210296240944721	0.905670407916831	-5.3009159076956
a_HQ_transcript/110476	-0.906521730704323	-0.272715081845862	-1.27790916036001	0.210309889851368	0.905670407916831	-5.01652572251448
a_HQ_transcript/27115	-0.968636447343797	1.88068625415573	-1.27788936144381	0.210316786278165	0.905670407916831	-5.07349753769787
a_HQ_transcript/79311	0.611795193189459	3.64443980794035	1.27770241339199	0.210381913111922	0.905670407916831	-5.38365233486669
a_HQ_transcript/87778	-2.87964111146416	2.91614132707885	-1.27763352286327	0.210405916260327	0.905670407916831	-5.2985659225481
a_HQ_transcript/51995	0.952413679996443	-0.563886123235126	1.27759220480061	0.210420313480729	0.905670407916831	-5.09712639253577
a_HQ_transcript/82581	-2.96862154893352	1.52554660462942	-1.27752274263977	0.210444519153492	0.905670407916831	-5.14774166281589
a_HQ_transcript/115580	1.75378443888576	0.217679811855277	1.27752272407323	0.210444525623707	0.905670407916831	-4.94681820216619
a_HQ_transcript/66800	1.93194493387336	3.03643448243066	1.2774697934679	0.21046297191512	0.905670407916831	-5.19681368466811
a_HQ_transcript/120190	-1.68740105218024	0.885631398190727	-1.27746516464904	0.21046458511456	0.905670407916831	-4.98979216959028
a_HQ_transcript/126015	1.91099425679641	1.09547895108325	1.27745751007298	0.210467252847577	0.905670407916831	-5.09310567144284
a_HQ_transcript/58696	0.773534586337133	2.58888726171281	1.27745329548171	0.210468721705929	0.905670407916831	-5.21071149681111
a_HQ_transcript/92264	1.94158480553521	4.25327361793786	1.27738655997881	0.210491981223785	0.905670407916831	-5.28970384694846
a_HQ_transcript/90839	-0.361997494393073	2.6874234693297	-1.27730849329305	0.210519192501615	0.905670407916831	-5.27376944701079
a_HQ_transcript/105128	1.26528776983419	2.19816252115904	1.27721738148804	0.210550954213565	0.905670407916831	-5.14530134394526
a_HQ_transcript/40522	-0.685972608357601	3.52858573738933	-1.27714681294722	0.210575557006058	0.905670407916831	-5.3138566847453
a_HQ_transcript/93361	0.989043346636739	3.52141621853563	1.27705961010605	0.210605962134947	0.905670407916831	-5.42885039566679
a_HQ_transcript/132061	-1.15989845195789	3.75053965378948	-1.27698237715072	0.210632893826152	0.905670407916831	-5.20769258031982
a_HQ_transcript/9712	1.25878141629607	-0.166129590361494	1.27687652195983	0.210669810534079	0.905670407916831	-4.97743808370213
a_HQ_transcript/80411	-1.44582143678673	1.02703280352203	-1.27687254960488	0.210671195977344	0.905670407916831	-4.94301664966248
a_HQ_transcript/59408	-1.46852737722041	2.64826109555142	-1.27681255243421	0.210692122105351	0.905670407916831	-5.18288836430077
a_HQ_transcript/59056	2.20571784384115	1.89685407385498	1.27680870275812	0.210693464869302	0.905670407916831	-5.09039029085232
a_HQ_transcript/137381	1.29628819729659	1.41043451983274	1.27678943498094	0.210700185551971	0.905670407916831	-5.02304835207203
a_HQ_transcript/13058	0.523771664170021	3.60022987372201	1.27677077807845	0.210706693313606	0.905670407916831	-5.32612917598905
a_HQ_transcript/76434	2.49499397896743	0.680702803782396	1.27671100335548	0.210727544511584	0.905670407916831	-5.13382361047283
a_HQ_transcript/81987	-1.97846748691077	1.50365600495622	-1.27669901526928	0.210731726500007	0.905670407916831	-5.21273942759809
a_HQ_transcript/6562	-1.1003234431449	3.39285305109511	-1.2766839778905	0.21073697230887	0.905670407916831	-5.41827464040886
a_HQ_transcript/85733	-1.27694698352971	1.50223881617691	-1.27660147830604	0.21076575415362	0.905670407916831	-5.00338107187337
a_HQ_transcript/48960	-0.882497605659757	2.11284192762243	-1.27655784713947	0.210780977075249	0.905670407916831	-4.99838382260667
a_HQ_transcript/85078	-1.78920700884942	1.38729530566378	-1.27648735536489	0.210805573426319	0.905670407916831	-5.09609823806158
a_HQ_transcript/134168	-0.617814154116575	2.1222753907129	-1.27648723671946	0.210805614826523	0.905670407916831	-5.0328033694142
a_HQ_transcript/65654	1.68799479845422	0.815696664018626	1.27645014803841	0.210818556873281	0.905670407916831	-5.22000418547664
a_HQ_transcript/19307	1.90356324234777	0.299697639853394	1.27630711620718	0.2108684732761	0.905670407916831	-5.08552638869635
a_HQ_transcript/50170	-1.42990365641556	0.952688888322057	-1.2763015513696	0.210870415518817	0.905670407916831	-4.96757402911319
a_HQ_transcript/87985	1.68321616944582	0.333634423050095	1.27628112825564	0.210877543721491	0.905670407916831	-5.02266085984837
a_HQ_transcript/82067	-1.60706415028274	1.80134672380377	-1.27626930072103	0.210881671924821	0.905670407916831	-5.11843953554965
a_HQ_transcript/33252	1.27704207738792	0.20050958753742	1.27615841712919	0.210920376964799	0.905670407916831	-5.02943509108963
a_HQ_transcript/13936	-0.544478100296868	3.08440504477386	-1.27613653213973	0.210928016777179	0.905670407916831	-5.32963961421499
a_HQ_transcript/63168	1.51544683049425	1.70358526567324	1.27613588531498	0.210928242579869	0.905670407916831	-4.96683518519489
a_HQ_transcript/70680	-1.31381560407324	2.07736338887052	-1.27607637063727	0.210949019584258	0.905670407916831	-5.08436692687889
a_HQ_transcript/150297	1.485240858793	0.0334889055036157	1.27606512665351	0.21095294511427	0.905670407916831	-5.13013319330971
a_HQ_transcript/132926	1.68453972636924	1.02651337245177	1.27605825642375	0.210955343694673	0.905670407916831	-4.99789491839883
a_HQ_transcript/141262	0.77978580299	2.22298129849185	1.27601871819025	0.210969147947495	0.905670407916831	-5.18495860076344
a_HQ_transcript/135659	2.97405780294219	-0.0955805183539466	1.27599889619077	0.210976068794509	0.905670407916831	-5.16720955460866
a_HQ_transcript/89872	0.932254337367281	-0.753032460034858	1.27594747980558	0.210994021615703	0.905670407916831	-5.10577319693801
a_HQ_transcript/33327	-0.417117966966726	3.86882245726716	-1.2759360076894	0.210998027439166	0.905670407916831	-5.36000227230096
a_HQ_transcript/87532	-1.68033323963692	1.25774571009198	-1.27589522742957	0.211012267520494	0.905670407916831	-4.96772540734484
a_HQ_transcript/104309	1.54205810768306	0.314298581592883	1.27588630395785	0.211015383609477	0.905670407916831	-5.00520533236321
a_HQ_transcript/109925	1.33705939373419	-0.322080942906925	1.27586178101828	0.211023947234131	0.905670407916831	-5.07379966265541
a_HQ_transcript/10346	1.20588737399954	-0.284037355339948	1.27564233204666	0.211100592440184	0.905695269093576	-5.10445268913122

a_HQ_transcript/149139	1.68488882619119	2.44676976055879	1.27563912289986	0.211101713429769	0.905695269093576	-5.0601099779557
a_HQ_transcript/14470	1.19126182525114	-0.335447478215175	1.27561691270538	0.211109471813704	0.905695269093576	-5.0203290814109
a_HQ_transcript/20818	-1.36521262582045	-0.073645087256531	-1.27553230590092	0.211139028329963	0.905695269093576	-5.02912219983318
a_HQ_transcript/2242	-1.00447856424895	0.335824683022306	-1.27546810476092	0.211161458427	0.905695269093576	-4.95535773875886
a_HQ_transcript/11504	-2.39424717705473	3.91506750534635	-1.27546306764365	0.211163218332304	0.905695269093576	-5.28093812946008
a_HQ_transcript/63470	-1.3820353032333	0.628841608914343	-1.27538342045205	0.211191047532794	0.905695269093576	-4.91923014621064
a_HQ_transcript/152674	1.88592525906786	4.22434813892564	1.27524725940048	0.211238629438454	0.905695269093576	-5.35241563534153
a_HQ_transcript/54943	2.15992980722283	1.07681589257388	1.27520789959941	0.211252385355402	0.905695269093576	-5.284540647668
a_HQ_transcript/20244	1.10024610678527	1.31546716282737	1.27514680021199	0.211273740416348	0.905695269093576	-4.90144046326247
a_HQ_transcript/33015	-4.43170406041711	3.21530531662996	-1.27513772054877	0.211276914020645	0.905695269093576	-5.1851910640272
a_HQ_transcript/46229	0.705387032933929	3.18630157289689	1.27509818303297	0.211290733943852	0.905695269093576	-5.38143942516549
a_HQ_transcript/113184	-1.3638684956207	0.56662986209195	-1.2750624270529	0.211303232659968	0.905695269093576	-4.96494476593735
a_HQ_transcript/30794	1.78938963772467	0.113234119845796	1.27493755860599	0.211346885558571	0.905695269093576	-5.11059920380458
a_HQ_transcript/25913	-1.6474300122766	2.05758620306804	-1.27491356474132	0.211355274381767	0.905695269093576	-5.03621067783583
a_HQ_transcript/39316	1.39186002837421	0.947888868524085	1.27488933701169	0.211363745225311	0.905695269093576	-5.07002472838603
a_HQ_transcript/88531	2.25762062581187	1.77674416690422	1.27487593915914	0.211368429683441	0.905695269093576	-5.15156632668786
a_HQ_transcript/73155	-2.14346401655332	0.849423142175165	-1.2748091706004	0.211391775977784	0.905695269093576	-5.03017588292336
a_HQ_transcript/128681	-1.47478474691134	-0.0281752695185338	-1.2747492417746	0.211412732352488	0.905695269093576	-5.05488954050421
a_HQ_transcript/29973	2.40346220126273	0.799384921710141	1.27465898796449	0.211444295967954	0.905695269093576	-5.14866403711993
a_HQ_transcript/99874	1.82491663181039	0.474409144394075	1.27452653708308	0.211490623222923	0.905695269093576	-5.10878542590539
a_HQ_transcript/150471	1.68698290386119	0.408411742290966	1.27445433833517	0.211515879363518	0.905695269093576	-5.10733950528611
a_HQ_transcript/14308	0.47436984699327	4.45038773557907	1.27438653376934	0.211539600434772	0.905695269093576	-5.51113611322228
a_HQ_transcript/106381	2.10307114215439	3.03223550245398	1.27434490492855	0.211554165063132	0.905695269093576	-5.08240419007535
a_HQ_transcript/140876	-1.35582649475673	2.11377923483584	-1.27433902636431	0.211556221849879	0.905695269093576	-5.07751039771914
a_HQ_transcript/116098	1.46540292186029	0.0413673501292712	1.27433351396506	0.21155815053689	0.905695269093576	-5.13471075149533
a_HQ_transcript/10712	-0.417507139119256	2.85427720924555	-1.2743227115748	0.211561930132939	0.905695269093576	-5.2286583704105
a_HQ_transcript/69599	2.01361674910214	0.657793731617991	1.27430911944751	0.211566685889149	0.905695269093576	-4.97065914121877
a_HQ_transcript/33227	1.55758526836813	1.32450905228616	1.27427253121103	0.21157948816734	0.905695269093576	-5.03795003757235
a_HQ_transcript/84494	2.26268414933755	1.36877044140674	1.27422315587384	0.211596765600045	0.905695269093576	-5.05214016055869
a_HQ_transcript/100732	1.51544866666825	2.0220111190955	1.27420626190894	0.211602677386326	0.905695269093576	-5.1975063258932
a_HQ_transcript/34894	1.50547914448844	2.3705040313781	1.27414161490547	0.211625300776576	0.905695269093576	-5.2039898805146
a_HQ_transcript/81825	2.06594150869517	1.04491079916031	1.27413610330051	0.211627229661987	0.905695269093576	-5.08340015253445
a_HQ_transcript/109136	-1.30182057202944	-0.59073665068097	-1.2741343502177	0.211627843187674	0.905695269093576	-5.05252382038794
a_HQ_transcript/116271	1.52667122671742	1.69387633202665	1.27403147259532	0.211663849584065	0.905695269093576	-5.00633693311194
a_HQ_transcript/94782	1.9806594079745	0.609597844659707	1.27401724536524	0.211668829372616	0.905695269093576	-5.0231519004645
a_HQ_transcript/22138	1.40923231955653	0.0626737892625771	1.27401258576881	0.211670460335135	0.905695269093576	-5.09144086163857
a_HQ_transcript/93054	-1.74230852469034	1.24636641239229	-1.27397339261392	0.211684179188422	0.905695269093576	-5.14569340594946
a_HQ_transcript/8320	0.83473484982417	-0.782074505569095	1.27392637948455	0.211700636169207	0.905695269093576	-5.10916358308724
a_HQ_transcript/8440	1.17934797070123	2.1348567103511	1.27384188143652	0.211730217206265	0.905695269093576	-4.99164748566649
a_HQ_transcript/41185	-0.59100128106401	2.53648973429378	-1.27382141855849	0.211737381311221	0.905695269093576	-5.2013449746573
a_HQ_transcript/147509	0.353983486282242	-0.753623941859451	1.27381751108125	0.211738749349658	0.905695269093576	-5.17613416688104
a_HQ_transcript/78476	1.3759310127278	5.26479959795498	1.27375058791132	0.211762180714252	0.905695269093576	-5.65150561582991
a_HQ_transcript/94419	-0.477985781806822	4.26146665103121	-1.2737357960586	0.211767359951944	0.905695269093576	-5.39218770321168
a_HQ_transcript/107110	3.44189067977759	3.25082835305566	1.27368608539753	0.211847766406599	0.905695269093576	-5.14161028992288
a_HQ_transcript/136501	0.72322708036267	3.74618106682007	1.27364075545586	0.211800639872103	0.905695269093576	-5.38390610276784
a_HQ_transcript/75669	1.37368140817762	0.638903651156126	1.27360418662047	0.211813446062683	0.905695269093576	-4.98787613436724
a_HQ_transcript/118207	2.57513426513464	1.47198447833359	1.27359601663335	0.21181630722404	0.905695269093576	-5.08290803301925
a_HQ_transcript/21735	0.865829685473599	1.47088500638611	1.2735613899235	0.21182843395737	0.905695269093576	-4.9860977486353
a_HQ_transcript/50600	-0.811837844737575	1.71925383967205	-1.2735064557242	0.211847673720286	0.905695269093576	-4.9605849329191
a_HQ_transcript/62664	1.3385010871486	1.71628526333997	1.27347742176473	0.211857842901392	0.905695269093576	-5.08363320895971

a_HQ_transcript/101208	1.0083674336037	2.69347819568195	1.27336342588803	0.211897773672014	0.905695269093576	-5.23818404911054
a_HQ_transcript/26568	1.5520283472048	1.6680634354842	1.27334977287725	0.211902556465586	0.905695269093576	-4.99710922914529
a_HQ_transcript/79513	1.04452159331226	-0.60908966888234	1.27334138276406	0.211905495651167	0.905695269093576	-5.09773982233733
a_HQ_transcript/46452	-1.29837832458872	0.421221054030421	-1.27324416622809	0.211939554345905	0.905695269093576	-5.05233442111905
a_HQ_transcript/135567	1.65688271767971	2.20859098116884	1.27319776756371	0.211955811043361	0.905695269093576	-5.16465458204802
a_HQ_transcript/53090	-2.58380148239221	2.81107383220689	-1.27314382532597	0.211974711967567	0.905695269093576	-5.36722873271532
a_HQ_transcript/9813	1.0933343982018	0.269170978126876	1.27313599562351	0.211977455537964	0.905695269093576	-5.23502461125354
a_HQ_transcript/74426	-1.02625300803548	-0.566117378849527	-1.27309680689182	0.211991187886584	0.905695269093576	-5.01003383201489
a_HQ_transcript/103918	0.353983486282242	-0.824998489467312	1.27308888752682	0.211993963038479	0.905695269093576	-5.16846636891234
a_HQ_transcript/116964	1.47589067619026	-0.412198112966537	1.27303390697381	0.212013230414608	0.905695269093576	-5.08903201908883
a_HQ_transcript/64703	-1.32133320192588	0.569064309224765	-1.27303275739302	0.212013633287645	0.905695269093576	-5.05508567692022
a_HQ_transcript/138996	-1.29274303827805	-0.275030671662941	-1.27301769468721	0.212018912098499	0.905695269093576	-5.08840823096408
a_HQ_transcript/117161	1.96394725482687	-0.0773774138387717	1.27288920916258	0.212063944620527	0.905695269093576	-5.07394148452086
a_HQ_transcript/144500	0.384361798198786	4.15635274552445	1.27285648899593	0.212075413772977	0.905695269093576	-5.44344213121127
a_HQ_transcript/147896	1.1892812469852	-0.24825446736334	1.27284787889827	0.212078431883309	0.905695269093576	-5.05270536630918
a_HQ_transcript/85692	1.4378192459085	0.842176655197743	1.27282765726993	0.212085520328468	0.905695269093576	-5.01143793212805
a_HQ_transcript/89285	-0.657185376680709	3.90086327865537	-1.27279177331514	0.212098099452123	0.905695269093576	-5.4336662221637
a_HQ_transcript/126844	0.759632190331051	2.96736884105764	1.27277256714875	0.212104832407288	0.905695269093576	-5.22275426543388
a_HQ_transcript/39190	1.06961649400911	1.14670834120432	1.27268825786367	0.212134389964091	0.905710707729402	-4.90617926268564
a_HQ_transcript/5612	1.77996131280543	1.16844167794417	1.27266956290398	0.212140944555288	0.905710707729402	-4.98136552254419
a_HQ_transcript/32794	-0.484555089975912	6.11529094123217	-1.27252973868542	0.212189972816527	0.905710707729402	-5.67218688505141
a_HQ_transcript/126926	-1.23297189692471	0.191989981561672	-1.27249877975104	0.212200829481408	0.905710707729402	-4.97408164981622
a_HQ_transcript/5159	-1.78820312861199	1.89761684612155	-1.27248563509089	0.212205439171798	0.905710707729402	-5.07907396597769
a_HQ_transcript/118274	-0.642372807787418	4.33359480858886	-1.27246057264244	0.212214228511655	0.905710707729402	-5.495714063217
a_HQ_transcript/3060	-1.38494448071411	2.73431217011756	-1.27230357374023	0.212269293908173	0.905710707729402	-5.38674760310078
a_HQ_transcript/77545	0.643738843672191	1.53402107398937	1.27224469952769	0.212289946084734	0.905710707729402	-4.99591953410968
a_HQ_transcript/133619	0.424792539959827	5.68292445619626	1.27216088725597	0.212319348772794	0.905710707729402	-5.62371976368806
a_HQ_transcript/26626	-1.55034128865639	-0.280373971333134	-1.27214931862231	0.212323407476661	0.905710707729402	-5.07123340212461
a_HQ_transcript/130250	1.52737686120546	2.62494164045139	1.27213416460032	0.212328724155885	0.905710707729402	-5.15136775251226
a_HQ_transcript/31879	-0.455845307939295	4.5067600885856	-1.27211193596121	0.212336523095539	0.905710707729402	-5.49068742150027
a_HQ_transcript/111288	2.67403862254153	0.0597098304482281	1.27210047591306	0.212340543949181	0.905710707729402	-5.01457862901165
a_HQ_transcript/9066	-1.25501932693303	-0.254363766459476	-1.27204669864	0.212359412923528	0.905710707729402	-4.99344509505659
a_HQ_transcript/229	0.717974324401288	-0.567183874088521	1.27193888817758	0.212397244483855	0.905710707729402	-5.06733326657275
a_HQ_transcript/7347	1.9547246287523	1.13765463330494	1.27191662948762	0.212405055872057	0.905710707729402	-4.94783442273437
a_HQ_transcript/138464	-0.54791817657778	2.73197873679214	-1.27185753289136	0.212425796082228	0.905710707729402	-5.13426757243884
a_HQ_transcript/140687	1.57741113293427	0.304557660337576	1.27185075015682	0.212428176610652	0.905710707729402	-5.1742862752327
a_HQ_transcript/149115	-1.84522841759724	0.113594627313434	-1.27173398562815	0.212469160482646	0.905710707729402	-5.0642264610168
a_HQ_transcript/142706	2.03298364137448	0.173315975819492	1.27170477806372	0.212479413152736	0.905710707729402	-5.20349386199407
a_HQ_transcript/37967	-1.97442204536814	0.0651716375438077	-1.27168396146572	0.212486720587712	0.905710707729402	-5.11374353614817
a_HQ_transcript/98047	2.32082569515778	1.14434192542304	1.27166706419991	0.212492652324434	0.905710707729402	-4.95791392082262
a_HQ_transcript/12022	0.677397978753535	1.90919001930605	1.27165991437217	0.212495162288652	0.905710707729402	-5.05350642794609
a_HQ_transcript/143671	-2.88760271290588	2.63323901199767	-1.2715842904329	0.212521711626358	0.905710707729402	-5.26984084908543
a_HQ_transcript/63576	4.93465333781908	2.96249322240049	1.27151901312796	0.212544630581547	0.905710707729402	-5.1086677096
a_HQ_transcript/58030	-1.7792909732547	3.2538191578184	-1.27144715572766	0.212569861977663	0.905710707729402	-5.37497892639897
a_HQ_transcript/12708	0.882075073266263	1.20202887720328	1.27143307689282	0.212574805765149	0.905710707729402	-4.98090195226163
a_HQ_transcript/54627	1.20706145976426	1.15552302663866	1.27139635067332	0.212587702598228	0.905710707729402	-4.94319395375954
a_HQ_transcript/142969	1.3332134930979	-0.340753960661768	1.27137548723279	0.212595029297976	0.905710707729402	-5.06074306717748
a_HQ_transcript/76743	0.955113552048714	3.60573402632845	1.27125738519576	0.212636507271278	0.905710707729402	-5.26106336380795
a_HQ_transcript/138756	-1.454460864689	-0.161449076427192	-1.2712071464591	0.212654153198466	0.905710707729402	-5.06576170501202
a_HQ_transcript/63655	0.975779496891884	1.92457586446053	1.27114315032795	0.212676632895729	0.905710707729402	-5.00897400742073

a_HQ_transcript/77670	-3.32628813482101	2.6452896255168	-1.27114070690192	0.21267749122504	0.905710707729402	-5.2052766528316
a_HQ_transcript/111997	1.33836980059311	1.80928836126312	1.27114007385573	0.212677713602609	0.905710707729402	-5.01455131385028
a_HQ_transcript/139061	1.36017879185048	-0.19648611206044	1.27107588785351	0.212700261885637	0.905710707729402	-5.13939172215003
a_HQ_transcript/55694	-0.853203471789502	2.27868840605012	-1.27103915084498	0.212713168261079	0.905710707729402	-5.06819952115777
a_HQ_transcript/116577	-1.83122760506803	1.2526796545306	-1.27091941724885	0.212755236946411	0.905710707729402	-5.11644335632842
a_HQ_transcript/152568	3.14039971217519	3.34572932017389	1.27086501008307	0.212774355111676	0.905710707729402	-5.35267619964262
a_HQ_transcript/88250	1.69756061050465	0.775429547690563	1.27084063861204	0.212782919437891	0.905710707729402	-5.07225035395058
a_HQ_transcript/52412	-1.51689266061889	3.91707130786019	-1.27076213247893	0.212810508880309	0.905710707729402	-5.40192066570774
a_HQ_transcript/93379	1.08556031997798	2.2147001961862	1.27072617647588	0.212823145815642	0.905710707729402	-5.06540897598557
a_HQ_transcript/11807	-1.29748522297789	-0.322695484927604	-1.27069161421738	0.212835293446252	0.905710707729402	-4.97629734042265
a_HQ_transcript/71320	1.1834758428392	-0.0624072283098435	1.27068548593459	0.21283744741445	0.905710707729402	-5.10737767987987
a_HQ_transcript/63869	1.52592454782644	-0.412354751259425	1.27065609210481	0.212847778983658	0.905710707729402	-5.08076616666948
a_HQ_transcript/37667	0.774774035502333	2.2507125013009	1.27053506356262	0.212890323018406	0.905710707729402	-5.11056645599127
a_HQ_transcript/60176	-0.547898584477476	3.10631918092151	-1.27049161580119	0.212905597372034	0.905710707729402	-5.24064701842762
a_HQ_transcript/69778	-2.08781310430692	1.62845636336593	-1.27047090387289	0.212912879081597	0.905710707729402	-5.17872586784332
a_HQ_transcript/46998	1.30855429872304	-0.379318149265681	1.27039264582129	0.212940394027485	0.905710707729402	-5.11034202629646
a_HQ_transcript/19293	0.327851542542001	3.84034967385077	1.27031539381147	0.212967557891547	0.905710707729402	-5.39301195296885
a_HQ_transcript/112402	1.42107337137922	0.358916495664597	1.27026729278748	0.212984472815166	0.905710707729402	-5.09870953701888
a_HQ_transcript/802	-0.849055381782345	1.41710632302091	-1.27026440990059	0.212985486626481	0.905710707729402	-5.05992740423482
a_HQ_transcript/82319	1.72027817210745	0.293387231650037	1.27021980356978	0.213001173590305	0.905710707729402	-5.01018812233855
a_HQ_transcript/53917	1.3282922464131	1.24300938326344	1.27018577168457	0.213013142365315	0.905710707729402	-5.0213299254102
a_HQ_transcript/55672	-1.62060314241954	1.21646789200972	-1.27010427785268	0.213041805238808	0.905710707729402	-5.11857914277099
a_HQ_transcript/93491	1.20296771662235	2.31717461978055	1.27008827644382	0.213047433569643	0.905710707729402	-5.15218089307472
a_HQ_transcript/46009	1.91514324263353	-0.172947322376647	1.27005093733838	0.213060567652493	0.905710707729402	-5.08505128446215
a_HQ_transcript/72109	1.30612173606012	2.40326467372391	1.27002034461472	0.213071329141965	0.905710707729402	-5.10996688396931
a_HQ_transcript/44476	0.660588293109499	2.78799121965884	1.26989921055123	0.213113944056373	0.905710707729402	-5.25799391011675
a_HQ_transcript/64623	-3.11243990634694	2.79865031050764	-1.26984865905888	0.213131729954934	0.905710707729402	-5.04459403771593
a_HQ_transcript/103932	2.06946823044197	1.75193113982575	1.26983698314339	0.213135838136453	0.905710707729402	-5.11169213548508
a_HQ_transcript/86634	2.3092490528455	0.161710882355548	1.26976833145241	0.21315999450647	0.905710707729402	-5.14105167014553
a_HQ_transcript/33671	0.443860432442687	3.79538629421075	1.2696655393244	0.213196167688713	0.905710707729402	-5.42629054643402
a_HQ_transcript/212	-0.56013794772699	2.43119351509256	-1.26964342351419	0.213203950984508	0.905710707729402	-5.12743134843719
a_HQ_transcript/141988	2.22053372641866	1.2030936239251	1.26960638606723	0.21321698618575	0.905710707729402	-5.14264690522157
a_HQ_transcript/131609	1.37807095265117	1.49520478360605	1.26952610908816	0.213245241457112	0.905710707729402	-5.08028926872927
a_HQ_transcript/18885	-0.764297337211054	1.98024241873331	-1.26947884881552	0.21326187708565	0.905710707729402	-4.9639455984625
a_HQ_transcript/3608	0.805775978498584	2.60381353465572	1.26938319921149	0.213295548771305	0.905710707729402	-5.22554703141377
a_HQ_transcript/131731	1.68143269907959	0.00132281637567658	1.2693693786301	0.213300414385059	0.905710707729402	-5.07612563847126
a_HQ_transcript/12399	1.55666177280272	0.746355108659412	1.26935849229465	0.21330424703998	0.905710707729402	-5.13268636186294
a_HQ_transcript/2629	1.30024262032147	0.81803563681344	1.26934168468956	0.213310164445579	0.905710707729402	-5.08599436080787
a_HQ_transcript/5600	-0.587799833673438	2.23537991561243	-1.2692858306119	0.2133298297221	0.905710707729402	-5.06095914400545
a_HQ_transcript/33269	1.43357729339634	2.44877605164584	1.26916943306894	0.21337081572944	0.905710707729402	-5.23939533282994
a_HQ_transcript/42994	0.786930670547844	2.9413991601604	1.26903640594391	0.213417664642423	0.905710707729402	-5.27991269112167
a_HQ_transcript/42373	-1.32908594052222	1.35223693786322	-1.26903451242543	0.21341833154958	0.905710707729402	-5.07956369857725
a_HQ_transcript/18860	-1.08468093191126	-0.368042319843438	-1.26899934862964	0.213430716709218	0.905710707729402	-5.08144875993089
a_HQ_transcript/93450	1.37305697345463	3.68083671515505	1.26897508055908	0.213439264565141	0.905710707729402	-5.29137016596107
a_HQ_transcript/137084	1.29373334941341	0.274585905487068	1.26893326499016	0.213453993720356	0.905710707729402	-5.18500294536677
a_HQ_transcript/136131	0.476973119423695	4.06648854861138	1.26889886878399	0.213466110047685	0.905710707729402	-5.47906312033949
a_HQ_transcript/76048	1.36078625035106	-0.11722834616131	1.26889751141	0.213466588203731	0.905710707729402	-5.08334457873359
a_HQ_transcript/21726	1.16320935302223	1.33784770605191	1.26883487825773	0.21348865258314	0.905710707729402	-4.96445608785955
a_HQ_transcript/7113	1.09611616702923	1.31456456451732	1.26877776453644	0.21350877408112	0.905710707729402	-5.00007582213304
[truncated: 770,628 more chars]
