# Supplementary material for: Transcriptomics as an Early Warning of Domoic Acid Exposure in Pacific Razor Clams (Siliqua patula)
Source: Toxins (Basel). 2025 Apr 11;17(4):194. doi: 10.3390/toxins17040194 (PMC12031274; doi:10.3390/toxins17040194)
Supplement: Supplementary file 1 [file Supplementary File S2. Pre-bloom vs post-bloom DEG.pdf › Supplementary File S2. Pre-bloom vs post-bloom DEG.pdf]

Supplementary File S2. Differential Gene Expression values (Transcript ID, log FC, Average Expression, t value, P value, adjusted P value, and B value) for razor clam pre-bloom vs. post-bloom samples.

Transcript_ID	logFC	AveExpr	t	P.Value	adj.P.Val	B
a_HQ_transcript/72650	-7.63266682067715	-0.66302502980542	-18.2793399924549	1.07585072260383e-18	6.25370508035157e-14	25.1779916592714
a_HQ_transcript/79367	5.39453951783017	-0.635081666507411	10.2252239185814	1.06637225018526e-11	3.09930430793844e-07	14.5923028417071
a_HQ_transcript/75321	4.61674787412999	-0.718949747060596	9.88414134890438	2.47914086479896e-11	4.80358333963447e-07	13.8957615005269
a_HQ_transcript/27296	-5.50779386765853	-0.218201885112703	-9.32806851529099	1.0134794407032e-10	1.47278832322989e-06	13.4025665009985
a_HQ_transcript/122962	5.47870284247239	-0.568058149532234	9.14565439869371	1.62274093905055e-10	1.88653370610261e-06	12.3028054324136
a_HQ_transcript/131580	5.03348999496581	-0.663421011542513	9.05966534571026	2.02895901314177e-10	1.96565549193175e-06	12.2617431401786
a_HQ_transcript/45368	-5.82179005647393	-0.488353363103958	-8.93085840837026	2.84048567994477e-10	2.35873930862614e-06	12.2159818564456
a_HQ_transcript/95382	-4.42753264137073	-0.714632152041304	-7.88034194702214	4.77974583729165e-09	3.47296332537612e-05	9.76253312153841
a_HQ_transcript/23807	-7.05323923962788	1.55057015126014	-7.44715115216807	1.59184255378562e-08	0.000102811804407167	8.38388136220417
a_HQ_transcript/22218	-4.31205122095105	0.00279066033138447	-7.39561263437335	1.83939090233609e-08	0.000106920114370992	8.19991545907504
a_HQ_transcript/60848	4.54100040842793	-0.531145402215251	7.10341456214137	4.19647219051526e-08	0.000214480869464128	7.92911756141276
a_HQ_transcript/1070	-7.36909140379594	3.33529560635928	-7.08451509151571	4.42776361404062e-08	0.000214480869464128	8.00381774077555
a_HQ_transcript/129123	-6.03714068007311	-0.514907986309261	-7.02479258479342	5.24702922076905e-08	0.000234614857342203	7.78281475147119
a_HQ_transcript/125280	-4.86534395543304	6.34754323276643	-6.94753735312678	6.53897685683813e-08	0.000269945371516073	8.2141615625232
a_HQ_transcript/133860	4.19417897157442	-0.702438422772455	6.92146269756116	7.04417896507984e-08	0.000269945371516073	7.52564269528917
a_HQ_transcript/92691	-4.5676627119782	-0.626509396534239	-6.90277582890363	7.43037080969098e-08	0.000269945371516073	7.48329442609837
a_HQ_transcript/73314	-9.8831114749931	-0.02466991112433	-6.81827420497979	9.46246792378725e-08	0.000323549609102297	7.26491320697307
a_HQ_transcript/109357	-4.11789836040386	-0.611091536041889	-6.67559856017051	1.42529279062489e-07	0.000460274551852465	6.87158339644487
a_HQ_transcript/53710	-3.70834722237064	2.16555175186338	-6.64123044357795	1.57350988067472e-07	0.000481394643915053	7.02995334089325
a_HQ_transcript/146424	-4.9135565757166	-0.605999607407108	-6.60927568586907	1.72526399936397e-07	0.000501430728775145	6.76496030998157
a_HQ_transcript/50722	-3.89959570104292	-0.573123525699976	-6.56476164983397	1.96162925947568e-07	0.000542978979022867	6.49414877346889
a_HQ_transcript/35228	-7.48756256707121	2.63594498020381	-6.41516806798849	3.02338107924995e-07	0.000798832251702915	6.2677888586331
a_HQ_transcript/145194	5.82051216250908	-0.355350496250831	6.39560618377222	3.19973178648334e-07	0.000808669605585667	6.31480151665775
a_HQ_transcript/40414	-4.21214379370636	-0.537391021096956	-6.33502994981605	3.81441884665702e-07	0.000923852244660331	5.9493809938106
a_HQ_transcript/108352	3.32791495198284	-0.721329883259728	6.27165876260101	4.58541334755005e-07	0.00106616362826556	5.78933329078517
a_HQ_transcript/75035	-3.53580547163854	-0.556200754805242	-6.10801719337635	7.38462166930463e-07	0.00165097418612823	5.4668088875887
a_HQ_transcript/48432	6.57426554827348	-0.357750321668499	6.08445815507781	7.91000167121378e-07	0.00170293547090487	5.44449709860684
a_HQ_transcript/147165	5.59458157016112	-0.117843247084957	6.01615089254462	9.6558216966007e-07	0.00200454858421431	5.09363262178384
a_HQ_transcript/33295	4.63174751421387	-0.180984139802306	5.98183979464085	1.06741319646228e-06	0.00213953773392964	5.32886141744065
a_HQ_transcript/29225	-2.55036894241117	3.07877041461385	-5.88577713763203	1.41372243076314e-06	0.0026524058962162	5.12605292446582
a_HQ_transcript/148700	-4.70062647961182	-0.255055296959226	-5.8855787825446	1.41454346928679e-06	0.0026524058962162	4.96235098854794
a_HQ_transcript/86802	4.88271527372105	-0.275290269662923	5.86420279466681	1.50589040037807e-06	0.00273544991228677	4.83669164518807
a_HQ_transcript/83269	-5.77411718254589	-0.12086933747366	-5.84736938696044	1.58197773674088e-06	0.00278658187519012	4.90120067142321
a_HQ_transcript/20368	-3.80159698363125	-0.0658947246698745	-5.76467017878463	2.01572528380042e-06	0.0034461788028456	4.56124102364851
a_HQ_transcript/19744	-4.83629404060098	4.73831268997463	-5.73113044877268	2.22400635533185e-06	0.00369362975493514	4.85664501978245
a_HQ_transcript/55144	-4.8015631016955	3.41384697766839	-5.60092607520322	3.25865115989046e-06	0.00522638116412228	4.44035852077879
a_HQ_transcript/101587	-3.71872844053248	-0.580560243978355	-5.59388214714612	3.3267289958802e-06	0.00522638116412228	4.21897345398733
a_HQ_transcript/16345	-4.25358017338773	-0.417836165390691	-5.56925009784137	3.57619366936143e-06	0.00547044698980634	4.20519237703443
a_HQ_transcript/66970	-5.26870871571287	2.79129939436016	-5.55189711792614	3.76311010101928e-06	0.00560877087056535	4.18171945869865
a_HQ_transcript/120470	-3.52505627932635	-0.721650623992468	-5.46637292373933	4.83745613857605e-06	0.00702979126057872	3.90771758007409
a_HQ_transcript/119398	-5.45426053735849	-0.476370427079186	-5.44485941996856	5.15297023223155e-06	0.00730565496729648	3.91891447475929
a_HQ_transcript/143639	-4.23005500407825	-0.616215406764874	-5.41639861935307	5.60222936353009e-06	0.00767117796809566	3.78708058640639
a_HQ_transcript/130597	-3.5347970577803	-0.198006455453038	-5.41202078205622	5.6747290914553e-06	0.00767117796809566	3.75904837609261
a_HQ_transcript/63237	-4.51550152132498	-0.204288980477183	-5.37633360240792	6.30183350238109e-06	0.00813061401048515	3.77328166160228

a_HQ_transcript/76697	-4.82361127235424	0.0438519363553065	-5.37596136483507	6.30872710043499e-06	0.00813061401048515	3.753105363426
a_HQ_transcript/75195	-4.21930736854406	0.281986565190474	-5.36925530799183	6.43421835401729e-06	0.00813061401048515	3.36359373301378
a_HQ_transcript/107478	7.56563659044234	0.777411498044478	5.34100625771119	6.9908317538095e-06	0.00864602272734976	3.6774552741463
a_HQ_transcript/111917	3.91792598608973	-0.0586630950864065	5.26711095697403	8.68511635852631e-06	0.0105176759101754	3.3784944230456
a_HQ_transcript/9456	-4.2276788341526	-0.624426768003963	-5.25495237657994	9.00078871793763e-06	0.0106775070733934	3.36093669682529
a_HQ_transcript/49343	3.7906784304685	-0.33365300045546	5.24218378034517	9.34463836261618e-06	0.0108637027748431	3.21609113191889
a_HQ_transcript/152513	3.91515233881098	-0.417952952153582	5.20776430121807	1.03382579806497e-05	0.0115868132080084	3.2880336900264
a_HQ_transcript/6210	4.47021845464728	-0.502949709856226	5.20687432813869	1.0365302209201e-05	0.0115868132080084	3.21266899829529
a_HQ_transcript/127496	-4.49902064691225	-0.0677914706248925	-5.18554990820405	1.10348395046482e-05	0.0121025122778527	3.10026189993507
a_HQ_transcript/95521	-4.93912522867177	0.525293476488716	-5.17274778175998	1.14573484625188e-05	0.0123331991005425	3.01610429064927
a_HQ_transcript/71185	6.04317082084188	-0.101749658524449	5.13671565096003	1.27351400684654e-05	0.0134594222163592	3.1193280318565
a_HQ_transcript/113447	5.11866700743885	-0.5146548048098	5.11302615143591	1.36516860669499e-05	0.0141379411279479	3.00816861334872
a_HQ_transcript/91960	5.69489824812079	-0.213604041511221	5.1061031200385	1.39317487203412e-05	0.0141379411279479	3.03958792149866
a_HQ_transcript/111966	6.80818318545316	1.22661426339283	5.10184581405484	1.41068088601186e-05	0.0141379411279479	2.96455908158166
a_HQ_transcript/14699	4.257327273387	-0.0116256591485831	5.0889586382594	1.46502039697225e-05	0.0144336789212208	2.95555014571199
a_HQ_transcript/114092	4.81461534018676	-0.522692033621738	5.0806946309952	1.50095865424029e-05	0.0145412874422799	2.91564541505614
a_HQ_transcript/61033	3.63899174985615	-0.456581982394408	5.06002774195687	1.59472604654434e-05	0.0151964320710704	2.78912174863583
a_HQ_transcript/33824	5.09898847843063	-0.505250522603711	5.04646502148824	1.65940984685761e-05	0.0155577702545386	2.81668324120577
a_HQ_transcript/118361	-2.95209503075377	-0.728115560277924	-5.03915746217924	1.69533785762954e-05	0.0156423172997286	2.80995350890069
a_HQ_transcript/20087	-4.73497867985648	0.623236489598867	-5.02852554079944	1.74899813457042e-05	0.0158852755572358	2.60225631512747
a_HQ_transcript/72041	3.90022786079584	-0.535814678882066	5.02002912637493	1.79309346394503e-05	0.016035221057261	2.77561993369489
a_HQ_transcript/57741	8.08100801016262	1.11130072342566	5.01407972933319	1.82462730597569e-05	0.0160699903093568	2.83140122349144
a_HQ_transcript/143164	-6.87209656404756	0.257146267670322	-5.00715028671198	1.8620523723309e-05	0.0161548328804254	2.79407218827794
a_HQ_transcript/6797	4.84363008989685	-0.0144285293754142	4.97779585972768	2.02925358991775e-05	0.0173465371580499	2.59648201763576
a_HQ_transcript/113694	4.2493465076586	1.5857894636148	4.95156108274671	2.1912845320515e-05	0.018460143083926	2.57676898921024
a_HQ_transcript/44871	-4.38638855008408	-0.511377712982897	-4.91476282181793	2.4404615689838e-05	0.0202655928688415	2.49770384665278
a_HQ_transcript/41294	-6.03449363332029	0.385736485827615	-4.89394856383102	2.593676532837e-05	0.0212345393663027	2.49271809421297
a_HQ_transcript/73205	-3.36082754661439	1.65185211463057	-4.88259426875146	2.68124835632971e-05	0.0216466117301019	2.26747166214855
a_HQ_transcript/112264	-4.56329624598341	-0.651681458310621	-4.87388583580224	2.75039926467406e-05	0.0218841213100925	2.40721386228279
a_HQ_transcript/15918	4.55435335321476	0.0525427977209765	4.86949143719838	2.78596369554577e-05	0.0218841213100925	2.28424192892717
a_HQ_transcript/143229	-7.25646443441474	0.110124546713616	-4.82817626503128	3.14348877037822e-05	0.024081511113245	2.28564938595141
a_HQ_transcript/146734	-4.90975015992615	-0.522746687591436	-4.8276244754804	3.14855980698909e-05	0.024081511113245	2.29020204766464
a_HQ_transcript/142801	8.71810358939722	0.743722052962233	4.82067737867638	3.21310404448634e-05	0.0242560145321951	2.32813731370277
a_HQ_transcript/115495	-5.48276432076006	0.311045192354119	-4.80602932777416	3.35353361089319e-05	0.0249915643248717	2.22102068913103
a_HQ_transcript/64014	5.89892425272189	0.0649184940469495	4.78249357122676	3.59203528816726e-05	0.0260363612582068	2.22716675401082
a_HQ_transcript/128228	4.47160237321357	-0.392981869522164	4.77964661857376	3.62200329289169e-05	0.0260363612582068	2.13153957312998
a_HQ_transcript/132155	-4.24614513695455	-0.446660281614639	-4.77906976019935	3.62810566665764e-05	0.0260363612582068	2.15703083603567
a_HQ_transcript/43082	5.22344750749009	-0.216920690320758	4.77292224861647	3.69377506387282e-05	0.0261843605991219	2.13085988962513
a_HQ_transcript/32209	-4.07928478448035	-0.311560507037132	-4.76622149308535	3.7666983739608e-05	0.0263795955519992	2.12391040112987
a_HQ_transcript/47331	-4.88496296430698	0.104962827405953	-4.75722242281544	3.86688590730364e-05	0.0264202694604494	2.07604754777412
a_HQ_transcript/29459	4.74179812743092	-0.10497610809266	4.75653625833647	3.87463263663523e-05	0.0264202694604494	2.11672914078966
a_HQ_transcript/49759	-3.75763208134643	-0.521863386120216	-4.7535206241845	3.90886177676619e-05	0.0264202694604494	2.08361640278318
a_HQ_transcript/90500	3.58255306766684	2.34105549149531	4.74253496030989	4.03611127008478e-05	0.0266758887969464	2.00413924948297
a_HQ_transcript/116742	-4.16054924697298	-0.427624327060777	-4.74233511645179	4.0384637595157e-05	0.0266758887969464	2.05672461616928
a_HQ_transcript/72167	-2.30112733795494	2.06753636714097	-4.71739175722273	4.34302204771604e-05	0.0283653017516447	2.02520877007987
a_HQ_transcript/22375	-2.86416054045425	1.61402234152631	-4.71310465473956	4.39761435406535e-05	0.0284027252414567	1.82519291544804
a_HQ_transcript/119950	5.30528370619737	0.351434841996755	4.69152518107041	4.68291141992815e-05	0.0299129972546795	1.98601238893875
a_HQ_transcript/138347	5.09796668214611	-0.311340340399027	4.67993432924039	4.84364733287007e-05	0.0305842032470915	1.91529082454111
a_HQ_transcript/86278	-2.84653956780105	-0.642130100431394	-4.67643640840359	4.89321996624606e-05	0.0305842032470915	1.92081144850215
a_HQ_transcript/95376	-3.74941393400373	-0.388206485839146	-4.65272812219739	5.24272205093573e-05	0.0324201007847651	1.80999447073614

a_HQ_transcript/125181	6.488135595961	-0.160414790893547	4.63447127590829	5.52861264299195e-05	0.0334652994469529	1.79606614574909
a_HQ_transcript/102601	-3.97354574416821	-0.304569844725887	-4.63374690072435	5.54026885780984e-05	0.0334652994469529	1.79212553848095
a_HQ_transcript/19170	3.33251821481908	-0.481267269716107	4.63101437313494	5.58445851629926e-05	0.0334652994469529	1.75942071293743
a_HQ_transcript/149683	-2.04457271627367	-0.788865929691825	-4.61374724974968	5.87187950734596e-05	0.0348286338778578	1.75886753419553
a_HQ_transcript/42140	-5.07417874851201	2.06610850683591	-4.6094199387551	5.94617548741897e-05	0.0349130594679485	1.74125477634509
a_HQ_transcript/2649	-4.35497991246716	0.186629385024458	-4.59578592833741	6.18639634891994e-05	0.0358240517098492	1.69193848444196
a_HQ_transcript/44579	6.80132691225939	-0.251261063420382	4.59366664429484	6.22458922153656e-05	0.0358240517098492	1.67555815841378
a_HQ_transcript/107942	4.63580251883669	0.155423523123545	4.57535016224089	6.56455396343894e-05	0.0368870507326175	1.63706854374544
a_HQ_transcript/52827	-3.55151155518807	-0.671153230360708	-4.57512195693584	6.56890340166647e-05	0.0368870507326175	1.58784071861239
a_HQ_transcript/145556	-7.24932501145564	0.976569919211234	-4.57351221851752	6.59966500858832e-05	0.0368870507326175	1.69962888685893
a_HQ_transcript/20607	-4.8385430425804	-0.101551968778051	-4.56870708733131	6.69233968095487e-05	0.0370487924737661	1.604822768026
a_HQ_transcript/137713	3.58429074363442	0.200099652703703	4.56255496779355	6.8128744052949e-05	0.0373602607010361	1.61546153816926
a_HQ_transcript/61868	4.62693874039742	0.0881895119364266	4.54847781820539	7.09681623139125e-05	0.0383267375240523	1.52245339017815
a_HQ_transcript/148822	3.90664586368554	2.70020191173479	4.54730530525288	7.12098756640113e-05	0.0383267375240523	1.62896314391418
a_HQ_transcript/110145	-5.15581274572	-0.0345427820034334	-4.54237587663405	7.22350130668395e-05	0.0384144680328499	1.57431207907672
a_HQ_transcript/116838	-7.47718730210352	0.0703369666994108	-4.540188361948	7.26945961260233e-05	0.0384144680328499	1.58875887484497
a_HQ_transcript/131754	4.38746172824372	0.623697667602495	4.51727250526928	7.76862673312323e-05	0.0406824085354043	1.48853099105062
a_HQ_transcript/38749	5.61199575796885	0.30483119501361	4.51220395088373	7.88352756991321e-05	0.0409155080878496	1.41451354378119
a_HQ_transcript/122843	-5.69355664929599	1.28451579277317	-4.49357390957613	8.32047267310575e-05	0.0426359687117797	1.47465901727319
a_HQ_transcript/84204	2.38683053182633	-0.758590781835724	4.49186547713117	8.36171971019627e-05	0.0426359687117797	1.45402316418527
a_HQ_transcript/99445	4.9488165049367	-0.325311152969272	4.47390407333329	8.80777826057166e-05	0.044259579417865	1.3960866835428
a_HQ_transcript/69307	-4.31955123188123	-0.0083280149920451	-4.47293805107021	8.83242363830226e-05	0.044259579417865	1.40474887047845
a_HQ_transcript/140232	4.21698520026021	6.46484002591952	4.46434412544925	9.05469302766402e-05	0.0445688810535697	1.33699333530652
a_HQ_transcript/74543	-2.5882674125079	-0.888814213200297	-4.46271145921231	9.0975396019427e-05	0.0445688810535697	1.35260461514565
a_HQ_transcript/39600	3.98725310303512	0.183337286467848	4.46170057607989	9.12416880913639e-05	0.0445688810535697	1.32936148798764
a_HQ_transcript/148697	-4.34000061546739	-0.284172888585488	-4.45686749948495	9.25255155536378e-05	0.0446855404210323	1.34038096751453
a_HQ_transcript/54564	2.43148925410437	2.46616957699966	4.4550310447075	9.30180014957492e-05	0.0446855404210323	1.34986047764118
a_HQ_transcript/101676	-2.67572012501441	-0.882206117452529	-4.44172009569234	9.66658154305435e-05	0.0460572993389068	1.30088717312571
a_HQ_transcript/6100	-5.58633968824305	0.266768774627693	-4.43676929239584	9.80582878907439e-05	0.0463409118578306	1.28466459637759
a_HQ_transcript/134578	-8.0686420539107	1.03439243756549	-4.42494826206743	0.000101463659198029	0.047494000417745	1.26070843115386
a_HQ_transcript/17025	-5.13974107475713	0.791000148918188	-4.42267304419071	0.000102132363959161	0.047494000417745	1.27700908286103
a_HQ_transcript/81914	-4.28168921707329	1.27361481309715	-4.41071026332691	0.00010572050298205	0.0487723920423859	1.19107767269464
a_HQ_transcript/100461	-2.63464828858407	2.41314506498858	-4.40024890645863	0.000108960031551946	0.0498710922366261	1.20305008269996
a_HQ_transcript/65150	-7.50227758993034	2.00349253089693	-4.3903574579564	0.000112113042061941	0.0509133352263791	1.1898593210665
a_HQ_transcript/109565	-2.62568495604453	-0.870236189510941	-4.38291692355964	0.000114543929897923	0.0512388488119589	1.1528906963546
a_HQ_transcript/145048	-4.32508962805251	0.270821540287345	-4.38276893881718	0.000114592801155289	0.0512388488119589	1.08955949587971
a_HQ_transcript/118932	2.51324204771948	-0.788995815682508	4.37731949724767	0.000116406841454408	0.0513597582889242	1.15687340440607
a_HQ_transcript/40062	4.7639027047328	-0.279792072277588	4.3766539165007	0.000116630334677573	0.0513597582889242	1.11946017985253
a_HQ_transcript/138678	-5.94169047559025	1.20825488892041	-4.37229542435611	0.000118104349268364	0.0516178166486574	1.13213611462533
a_HQ_transcript/61942	-5.91002638665085	1.54596713068303	-4.36822768952826	0.000119496591283268	0.0517336024932031	1.16362135417484
a_HQ_transcript/139434	5.92921925288029	-0.0666270334310415	4.36633682654689	0.000120149262602918	0.0517336024932031	1.09021274444717
a_HQ_transcript/75138	6.20970239359227	0.256703529367295	4.35398654844846	0.000124499482817347	0.053212543655932	1.10575460174336
a_HQ_transcript/133525	-2.86975597460161	-0.876851438224917	-4.35081281344367	0.000125642229954149	0.0532591984207302	1.07515984569973
a_HQ_transcript/86979	4.32245249424397	-0.12388922946703	4.3473937098519	0.00012688489149741	0.0532591984207302	1.07426547457578
a_HQ_transcript/45953	4.12017827472377	-0.285465669061069	4.34292517444843	0.00012852723627511	0.0532591984207302	1.05568520046186
a_HQ_transcript/45163	-5.04632640931738	0.16741692693703	-4.34269099131351	0.000128613882046851	0.0532591984207302	1.06382321231209
a_HQ_transcript/118161	6.97004368386878	1.17063089540699	4.3411382538157	0.000129189839274067	0.0532591984207302	1.05893346996605
a_HQ_transcript/31679	-2.89702528901534	-0.875716897863781	-4.33595809005045	0.000131129758483824	0.0536782436700542	1.03825921380653
a_HQ_transcript/107243	-1.98754956967659	4.25315396078619	-4.32184258094049	0.000136562687138826	0.0555112998461936	1.00842354740764
a_HQ_transcript/16078	4.26541031993491	-0.539909101766448	4.30677407434054	0.000142607114798884	0.0575657386738163	0.972183512564436

a_HQ_transcript/119510	-5.07861989822489	0.465451068305749	-4.3035318367692	0.000143941708112495	0.0577037490287112	0.952169300057835
a_HQ_transcript/109681	-7.51708008103726	3.62000190231591	-4.29680907924597	0.000146748279118083	0.0584259175929859	0.975625282032069
a_HQ_transcript/137581	1.90799436671301	-0.797475100847019	4.29018448219682	0.000149566541989345	0.0589609849433729	0.937614460577585
a_HQ_transcript/51541	-3.40531048663883	1.87901076571752	-4.28766041318719	0.000150654299463833	0.0589609849433729	0.93252715766678
a_HQ_transcript/118243	8.79716897492036	0.410821550500586	4.28655023392814	0.000151135197436047	0.0589609849433729	0.947397463294903
a_HQ_transcript/125986	-4.79482918867867	-0.099068243213256	-4.27908449212214	0.000154408611956875	0.0598364253055282	0.857867665974944
a_HQ_transcript/27555	3.2740775800987	-0.436792145593458	4.27312816617624	0.000157070157125377	0.0604647290952577	0.88442333091709
a_HQ_transcript/141724	-3.296183030175	1.42201509332357	-4.26395195639818	0.000161259059662781	0.0616688593426192	0.716420505070683
a_HQ_transcript/142002	4.67390602857699	0.609717661685521	4.2532486038958	0.000166284082672595	0.063084208924599	0.650419889672311
a_HQ_transcript/146783	-3.94036402325059	-0.396779107413189	-4.25147685693863	0.000167130611312762	0.063084208924599	0.827897526001889
a_HQ_transcript/146453	-2.60057107404735	-0.885153926664406	-4.24577709499453	0.000169882769524258	0.063365126030077	0.811651948586039
a_HQ_transcript/109694	-3.83995354969572	0.160800153552211	-4.24542333591807	0.000170055045084847	0.063365126030077	0.743403378457807
a_HQ_transcript/126837	-2.98864879519988	-0.860763001149198	-4.2341137720064	0.000175654091615507	0.0650345289008036	0.784991491757098
a_HQ_transcript/104572	-1.70479979316806	-0.793241695694724	-4.22995196371189	0.000177759779387535	0.0651565830398294	0.787464118576279
a_HQ_transcript/129704	-4.63424758326494	-0.335017603279034	-4.22729640436897	0.000179116320670632	0.0651565830398294	0.768760612829588
a_HQ_transcript/130342	-3.36778012867576	-0.718947017572328	-4.22579542564302	0.000179887562757361	0.0651565830398294	0.784399344762203
a_HQ_transcript/110068	3.02457801412117	-0.740420798614906	4.22417090143172	0.000180725964213199	0.0651565830398294	0.761576024922905
a_HQ_transcript/152158	-1.23619526344864	6.78005724023949	-4.2221206304496	0.000181789573829166	0.0651565830398294	0.597995846986694
a_HQ_transcript/5731	-5.804147007423	1.33950772237088	-4.2203573042927	0.000182709245724818	0.0651565830398294	0.765058332223548
a_HQ_transcript/147702	4.20484769492941	-0.216569611951782	4.21668106732974	0.00018464133542242	0.0654440947892344	0.71771940984725
a_HQ_transcript/93341	4.00796970047031	0.285831909147395	4.20509936032342	0.000190860444873184	0.0671042994162228	0.605118444632178
a_HQ_transcript/25770	-3.80498275420212	-0.404238487919126	-4.20233353521463	0.000192375810330638	0.0671042994162228	0.667893241307976
a_HQ_transcript/136804	-4.01577719483611	0.930346664274878	-4.20040236832781	0.000193440877527976	0.0671042994162228	0.674867867380141
a_HQ_transcript/143334	-2.68887715946497	-0.880998221971209	-4.19949543485219	0.000193943061896598	0.0671042994162228	0.697417905957471
a_HQ_transcript/46806	-6.06457495330604	0.113185752005185	-4.19568045946985	0.000196069540012382	0.0674386403659155	0.683121444297071
a_HQ_transcript/124841	-2.64093853433606	-0.839701320761343	-4.19069474414169	0.000198883148241759	0.0676710384899108	0.674213456287736
a_HQ_transcript/118905	-4.73646914503127	-0.561341677503681	-4.19035985140777	0.000199073554599758	0.0676710384899108	0.68030683802774
a_HQ_transcript/134114	5.13495953133301	0.14912714193675	4.18471237116907	0.000202311581765662	0.0679972751644466	0.680446221083399
a_HQ_transcript/130548	-2.00171596509643	4.47666628917577	-4.18277901219189	0.000203431929827692	0.0679972751644466	0.627082193512493
a_HQ_transcript/15608	-4.93566438204803	-0.157865066472164	-4.18210894843878	0.00020382164021021	0.0679972751644466	0.673383039826687
a_HQ_transcript/87409	-2.56427584723326	-0.802768020303534	-4.17841549964181	0.000205982951901536	0.0679972751644466	0.641852441632136
a_HQ_transcript/27376	-5.50820258491284	2.6456179670852	-4.17740549533693	0.000206577888943385	0.0679972751644466	0.658155710101084
a_HQ_transcript/104293	6.5154915806682	-0.0257753145920844	4.17660268390601	0.000207051983624192	0.0679972751644466	0.644312663114552
a_HQ_transcript/105936	3.91482338460437	0.199938039768482	4.17134342056813	0.000210184337309749	0.0682266078357339	0.624697119508354
a_HQ_transcript/105334	2.83726214511011	0.709553808988061	4.16748838457514	0.000212509855858833	0.0682266078357339	0.598603859827219
a_HQ_transcript/119315	-1.9413615398788	3.74893756098523	-4.16616770511042	0.000213312339658049	0.0682266078357339	0.605462601705566
a_HQ_transcript/52370	1.74505358537453	-0.780932968893372	4.1658015478628	0.000213535353864723	0.0682266078357339	0.627279245135645
a_HQ_transcript/141952	-6.37585719186038	2.05671243288502	-4.16239872677409	0.000215618877376454	0.0682266078357339	0.628673049831266
a_HQ_transcript/107647	4.79492792556575	-0.0819326611295031	4.16189798668438	0.000215927156727775	0.0682266078357339	0.578805469282189
a_HQ_transcript/42438	3.53175543420623	-0.527335423789143	4.16183426763565	0.000215966416215508	0.0682266078357339	0.606958485662028
a_HQ_transcript/96375	-2.76101765109935	-0.877712411959244	-4.15819311107332	0.000218221536270356	0.0685663862720176	0.595542732538469
a_HQ_transcript/139239	5.8189176493662	-0.11610175092083	4.15145156734522	0.000222458031890174	0.0688809355807592	0.56993641497352
a_HQ_transcript/79095	2.74129730010551	-0.784961027564212	4.14982345817606	0.000223493202605917	0.0688809355807592	0.575045998067782
a_HQ_transcript/131155	6.24283765326534	-0.0610866038466513	4.14870621539554	0.000224206292138495	0.0688809355807592	0.568755420832757
a_HQ_transcript/77729	-4.51343966485889	-0.215786496649932	-4.147352510814	0.000225073297605283	0.0688809355807592	0.58558254463356
a_HQ_transcript/152542	3.91468654497554	-0.135957256306587	4.1472367882612	0.000225147566755165	0.0688809355807592	0.569905682042815
a_HQ_transcript/141870	-6.30028387399122	1.01874512524987	-4.14392860178497	0.000227280916041651	0.0691079136992035	0.56132518093681
a_HQ_transcript/70148	-4.14842384735586	1.1211720284485	-4.13786665513722	0.000231241677251571	0.0691079136992035	0.551632640941063
a_HQ_transcript/28658	-2.78956665643982	-0.876350377177891	-4.13712399989948	0.000231731546730634	0.0691079136992035	0.543582953088102
a_HQ_transcript/143270	-6.50827373850891	0.0826024480176038	-4.13522964458159	0.000232985715059788	0.0691079136992035	0.540990869513409

a_HQ_transcript/146023	2.55272542491439	-0.883790641179142	4.1347730965701	0.000233288969658518	0.0691079136992035	0.536657034041269
a_HQ_transcript/42181	4.62090059824061	-0.0859438030715466	4.13182903318687	0.000235253836996439	0.0691079136992035	0.525075328876787
a_HQ_transcript/119826	5.40709946113157	0.371406008741976	4.13060938292287	0.000236072579198237	0.0691079136992035	0.544448361866965
a_HQ_transcript/105284	-3.36467622727219	-0.0869311327277208	-4.12984395860355	0.000236587829800831	0.0691079136992035	0.529600281100614
a_HQ_transcript/145987	-2.79920893169581	-0.875889823283982	-4.12984147067001	0.000236589506367697	0.0691079136992035	0.525631016977678
a_HQ_transcript/119311	4.35620117765009	0.117533386564774	4.12619169077129	0.000239061608730097	0.0694087817545405	0.494686780043604
a_HQ_transcript/67721	-5.25339826644815	-0.404106717081654	-4.12480474281911	0.000240007657801105	0.0694087817545405	0.510140142427749
a_HQ_transcript/114684	-1.61010593607622	-0.798920394961093	-4.11482988112563	0.000246920612639743	0.0710036142503161	0.499105274489869
a_HQ_transcript/142310	-4.80778382096009	1.58378247800509	-4.11334618342741	0.000247965415854909	0.0710036142503161	0.505977166801526
a_HQ_transcript/7777	3.12616932786547	-0.510399116877064	4.10602689886827	0.000253183349868026	0.0721423615741598	0.468815840474921
a_HQ_transcript/31614	-6.9252346479014	1.28071931667823	-4.10309945860145	0.000255300336748284	0.0723907218268501	0.475433939860184
a_HQ_transcript/121500	2.63345559508281	-0.880155257123607	4.10097787604088	0.000256845401695928	0.0724752888824316	0.453701575964326
a_HQ_transcript/141706	2.74408336848183	-0.87608748586883	4.09785463247998	0.000259136630896169	0.0727685704383212	0.446546811554881
a_HQ_transcript/52003	-3.65699886644493	-0.555686089717925	-4.09320615931741	0.000262583925092815	0.073295281467612	0.410859795079515
a_HQ_transcript/121155	-3.21188351467529	-0.86215626748681	-4.09193533519413	0.00026353416299771	0.073295281467612	0.433620928947657
a_HQ_transcript/99370	-3.32303588695209	1.79484417953802	-4.08924018607519	0.00026556058415455	0.0735071696939794	0.412866652934722
a_HQ_transcript/149075	2.71499822130376	-0.801961837119902	4.08444628663976	0.000269202815284263	0.0741621860039983	0.415044212378421
a_HQ_transcript/147293	-3.96890408701667	-0.290458089692357	-4.07722035836029	0.000274785501843283	0.0748609021589606	0.391577588482589
a_HQ_transcript/123569	-4.15188669379494	3.16507356939311	-4.07678816788793	0.000275122980178572	0.0748609021589606	0.404366553695045
a_HQ_transcript/132199	3.81223837261643	-0.34278539044225	4.07607899194046	0.000275677624302606	0.0748609021589606	0.40834605911481
a_HQ_transcript/151643	2.7843182569618	-0.874308408868962	4.07362478062417	0.000277605523954467	0.0748609021589606	0.386993882641796
a_HQ_transcript/55373	9.86902602271094	1.19849670702052	4.07144936793611	0.000279325453710011	0.0748609021589606	0.380323146257442
a_HQ_transcript/112044	4.58260643378411	0.725525924865289	4.07127183216787	0.000279466277327526	0.0748609021589606	0.39901287762296
a_HQ_transcript/133278	3.01137698786296	-0.752533068621888	4.06945257613781	0.000280913348497561	0.074897026355741	0.388908613906369
a_HQ_transcript/18767	4.66155215427648	0.0200001211249429	4.06715160990918	0.00028275409821366	0.074897026355741	0.372430102441313
a_HQ_transcript/78367	2.60651154617828	-0.856930620891761	4.06408640747113	0.000285224594323066	0.074897026355741	0.362579086014284
a_HQ_transcript/152746	-3.89575762732435	5.25306092881334	-4.06401093746002	0.000285285687693532	0.074897026355741	0.353639395294663
a_HQ_transcript/123601	-4.41145870762718	6.13080723064274	-4.06307603920204	0.000286043556478367	0.074897026355741	0.329945334574415
a_HQ_transcript/111235	-4.21785475899355	0.387229493201896	-4.05777300570286	0.000290379888570491	0.0753068617815882	0.312661689159163
a_HQ_transcript/69200	2.71791411135585	-0.876361181855213	4.05742069042143	0.000290670249525272	0.0753068617815882	0.346799445063455
a_HQ_transcript/107895	2.77574957254924	2.14955326631116	4.05642137077947	0.000291495387779682	0.0753068617815882	0.321961710997146
a_HQ_transcript/119977	3.53273226374722	1.20816070268976	4.05326531917657	0.000294116431555536	0.0755358384287101	0.287682104393472
a_HQ_transcript/24311	-4.97866952655557	4.33189734978172	-4.05230730005124	0.000294980651722358	0.0755358384287101	0.323891545924486
a_HQ_transcript/142430	-3.25204642549984	-0.68652001911849	-4.04682632878442	0.000299535675112431	0.0760698647247717	0.335068402531374
a_HQ_transcript/91820	2.69540911555506	-0.863764969796631	4.04665236040309	0.000299683440372501	0.0760698647247717	0.320163011663936
a_HQ_transcript/126115	-2.91751886570683	-0.870421968053441	-4.04018959568134	0.000305223647428638	0.0771393051205734	0.305073180993018
a_HQ_transcript/37869	6.08162189792057	4.09317717885938	4.0371833067986	0.000307834841099651	0.077462440014894	0.230849566686334
a_HQ_transcript/41587	-2.73821636639271	-0.55820472020299	-4.035461010606	0.000309340628147817	0.0775058277283461	0.267527370446059
a_HQ_transcript/137388	2.97983679283674	-0.668039416878115	4.03093437257978	0.000313332686747582	0.0781691090783839	0.294488384436349
a_HQ_transcript/56727	3.98850603480051	1.50013072772772	4.02421627873743	0.000319350511844307	0.0790751494635091	0.256634622398897
a_HQ_transcript/62043	3.55065518089667	1.81826454339242	4.02384631314571	0.000319685179671151	0.0790751494635091	0.278875806283216
a_HQ_transcript/175	-2.93924812795605	3.44212541272994	-4.02092787761773	0.000322337232737093	0.0793932994260243	0.25164885311978
a_HQ_transcript/56168	4.40894223824675	0.271553830687574	4.01889495645018	0.000324197307267675	0.0795145193116261	0.214693566964839
a_HQ_transcript/74225	2.84993474461061	-0.863793117187421	4.01598065015585	0.000326882161926807	0.079836160960006	0.245388830164994
a_HQ_transcript/120743	-3.29755343407298	-0.858384678604183	-4.01209940076577	0.000330491634617409	0.0803799905315512	0.237094528781608
a_HQ_transcript/55669	-4.61474134268747	-0.32917065106844	-4.00819556222912	0.00033416144248597	0.0809339013701019	0.229813051947033
a_HQ_transcript/9777	4.32254012235032	1.43026494270847	4.00638690478335	0.000335875157983536	0.0810114156982033	0.210296711820789
a_HQ_transcript/115401	5.84769261197395	1.75153043099196	4.00072678041895	0.000341293904467299	0.081978231730889	0.21810534197206
a_HQ_transcript/65869	4.20066129967395	0.065538301923929	3.99394966651867	0.000347894567306555	0.0829907535478056	0.187482719009867
a_HQ_transcript/108929	2.95098829391983	0.590367307439213	3.99347176480265	0.000348364710047904	0.0829907535478056	0.177715202164047

a_HQ_transcript/82339	-8.5166910578801	0.861045261241506	-3.98732551462662	0.000354466834725015	0.0840997884444721	0.18167872687801
a_HQ_transcript/117467	-2.61561157153517	-0.833472074669643	-3.98578821845794	0.00035600936098969	0.08412240705532	0.152034512813915
a_HQ_transcript/109372	-6.92635917030413	2.84932034164968	-3.98289571829138	0.000358929514642764	0.0844690478832169	0.175959095391381
a_HQ_transcript/140778	8.14399031136672	3.3416648885653	3.97258985108468	0.000369525510109574	0.0866120114985861	0.131489604058652
a_HQ_transcript/69121	-2.72761419442733	0.9237838550546	-3.96667448715218	0.00037574488703217	0.0876815753973209	0.093545582824099
a_HQ_transcript/118088	4.32675479355703	-0.316337857585814	3.96249188476528	0.000380204073033774	0.0876815753973209	0.111666712477799
a_HQ_transcript/136852	-6.66691574860177	1.39327453310257	-3.96229732370302	0.000380412753840324	0.0876815753973209	0.121584716901305
a_HQ_transcript/149006	-4.08513343512826	0.19716484757054	-3.96141804333685	0.000381357242413484	0.0876815753973209	0.116224535971322
a_HQ_transcript/125574	-7.17213995379274	0.766003979127754	-3.96016845054608	0.000382703449629937	0.0876815753973209	0.11770959614553l
a_HQ_transcript/43654	-4.80297190704355	0.963659035774034	-3.95976481774505	0.000383139281429251	0.0876815753973209	0.109722327117313
a_HQ_transcript/56023	-4.65449229389991	0.943106949907201	-3.95755188280981	0.000385537381334534	0.0878843800086816	0.106490507246485
a_HQ_transcript/28374	-4.91505554035764	2.09226758989569	-3.95607508391782	0.000387145903104767	0.0879063166237262	0.0480870887611733
a_HQ_transcript/151861	3.92467838010097	-0.0229913321702823	3.95439269460039	0.000388986343752376	0.087980537702872	0.0921833451054281
a_HQ_transcript/38556	2.49327757960845	1.72528745064609	3.95054502475559	0.000393227671419059	0.0885951088536709	0.0409639189442474
a_HQ_transcript/108238	-4.66097893450647	-0.049194369924669	-3.94661068334088	0.00039761124829881	0.0891231983738371	0.0741976778016138
a_HQ_transcript/122717	2.88710285455718	-0.868785322522082	3.94569525132728	0.00039863803291353	0.0891231983738371	0.0733093183267099
a_HQ_transcript/101113	3.01150296254609	4.53421760940636	3.94250491714145	0.000402236720263512	0.0895832033543196	0.0181610035205937
a_HQ_transcript/25939	-7.3900941358716	0.28225937281033	-3.93507955923428	0.000410735815752522	0.0911269141147428	0.0525459193672599
a_HQ_transcript/120260	5.34216040623626	0.602240498487232	3.93180377587652	0.00041454081004924	0.0916214000248753	0.0482237143693318
a_HQ_transcript/26423	-3.31356163856666	1.25124144735293	-3.92710233528114	0.000420061994981359	0.0924900138040773	-0.0582022608412034
a_HQ_transcript/57974	5.4654550232375	0.199180183405051	3.91705206096714	0.000432106509389387	0.0940905207625942	0.00691240632621515
a_HQ_transcript/151112	-4.18207479200501	0.120500264839809	-3.91544634124899	0.000434061825272588	0.0940905207625942	0.0055938133154747
a_HQ_transcript/152607	-5.40568404469428	0.636220246978371	-3.91543199909367	0.000434079328848603	0.0940905207625942	0.00193291390349248
a_HQ_transcript/42850	-4.95202643380046	1.71160882654669	-3.91507983795683	0.000434509332579603	0.0940905207625942	-0.0281240051488529
a_HQ_transcript/137786	4.22355556329648	3.0230995802081	3.91362515773127	0.000436289969290022	0.0940905207625942	-0.00375339093080029
a_HQ_transcript/108656	6.85414319820823	0.0708451691603729	3.91301164527658	0.000437043087770101	0.0940905207625942	-0.00364072275820071
a_HQ_transcript/129977	-2.7184052905217	-0.827378549294857	-3.9083050933485	0.000442862982623636	0.0949916585016484	-0.01893584998622
a_HQ_transcript/18636	3.76781739498515	-0.553810800190859	3.90396874170352	0.000448292009274598	0.0958026393938009	-0.0285796328502581
a_HQ_transcript/64913	-4.66347985097643	1.53596929064485	-3.89657638750025	0.000457697182480745	0.0974542923928231	-0.040518327540644
a_HQ_transcript/105029	2.69361594340829	-0.722773300856166	3.89239665969027	0.000463099897001978	0.097980800889658	-0.0521975301620037
a_HQ_transcript/47652	-3.40095911669316	0.0729726134505664	-3.89132371217754	0.000464496793519151	0.097980800889658	-0.0978040049329989
a_HQ_transcript/82877	-4.19909321295644	-0.129191425092112	-3.89076428184988	0.000465226758972365	0.097980800889658	-0.0654541491273681
a_HQ_transcript/15379	-2.4330830790283	3.32489770332158	-3.88556240645951	0.000472068158759321	0.0990627362179127	-0.0788218352978038
a_HQ_transcript/96805	-3.22903412090386	-0.21561935870827	-3.88073219227244	0.000478508561129854	0.100053041875382	-0.107994642960576
a_HQ_transcript/43732	-3.78691828868806	1.03362136772686	-3.87755100158115	0.000482796945843697	0.100246552729555	-0.13343700371692
a_HQ_transcript/96470	-3.25740709369226	1.41420746860752	-3.87568564697623	0.000485328933185075	0.100246552729555	-0.162478670235399
a_HQ_transcript/88430	-1.94396950253859	5.63849689264028	-3.87532670374489	0.000485817637482122	0.100246552729555	-0.234873890685906
a_HQ_transcript/54233	4.14677187735338	-0.175603719944248	3.87494902553544	0.000486332367701185	0.100246552729555	-0.103162746684606
a_HQ_transcript/137567	-2.41896072464718	3.9984318101332	-3.86695693258352	0.000497350165271082	0.102155372462464	-0.151877003978673
a_HQ_transcript/119403	-2.89457886985211	-0.44293288142643	-3.85774107305306	0.000510357282459521	0.104172373094812	-0.14757059693686
a_HQ_transcript/8048	2.73606274226936	-0.342240039111848	3.8574633867199	0.000510754306565189	0.104172373094812	-0.186399825685212
a_HQ_transcript/140624	-6.06209394043737	0.168524817023602	-3.85448788673744	0.00051502748972453	0.104676636093383	-0.148295152505487
a_HQ_transcript/150932	-4.10762321891095	-0.268361630494411	-3.85193211005083	0.000518725700388446	0.104831088489882	-0.155308596932977
a_HQ_transcript/126185	-4.34965714965957	-0.396778210492611	-3.84883405134658	0.000523243302551305	0.104831088489882	-0.162557129570465
a_HQ_transcript/125971	-3.66628604605887	4.8411034197085	-3.84858175935033	0.000523612879444364	0.104831088489882	-0.274218464583432
a_HQ_transcript/91594	-4.65701004842264	-0.0144787346276181	-3.84711041909756	0.000525773285649475	0.104831088489882	-0.16267283495856
a_HQ_transcript/120428	-3.39525239208488	-0.340395945071971	-3.84691430706254	0.000526061897134717	0.104831088489882	-0.180571135590468
a_HQ_transcript/136035	-6.84306531483552	0.710105818063827	-3.84654342228733	0.00052660813788614	0.104831088489882	-0.163314740075791
a_HQ_transcript/1384	3.55937491049305	0.0628052698247346	3.83995068205969	0.000536410686903811	0.106065929252231	-0.188231338372119
a_HQ_transcript/36072	-3.98177252415468	-0.520070604993403	-3.83991739757107	0.00053646062483065	0.106065929252231	-0.189956291902487

a_HQ_transcript/146476	-4.22783502216585	3.59476302767228	-3.83651191690736	0.000541594000566064	0.106717884965777	-0.217917885075935
a_HQ_transcript/152361	-6.20203865785573	0.220810969713391	-3.83498158549287	0.000543916355291389	0.106813411825601	-0.196945199259645
a_HQ_transcript/195	3.29008146337	-0.147946484747776	3.83049123563513	0.000550786817142772	0.107797079271063	-0.219427461614821
a_HQ_transcript/115362	3.47692080655818	0.00907514567694103	3.82849522664938	0.000553867881266349	0.107797079271063	-0.21166994435169
a_HQ_transcript/39793	2.57955781861954	-0.833006015604848	3.82809426653301	0.000554488829859067	0.107797079271063	-0.218206951115664
a_HQ_transcript/88101	-4.07642238763654	1.02926255670406	-3.81878833704539	0.000569092461990255	0.110267355435232	-0.25965008508778
a_HQ_transcript/136417	-3.79228532376578	-0.428262314214382	-3.81702010392514	0.00057190935191576	0.110445006007174	-0.244067391036662
a_HQ_transcript/65289	-2.64480404181417	-0.822989536888153	-3.81448396020289	0.000575973276824139	0.110550088865571	-0.247809154269122
a_HQ_transcript/119340	5.02174876199019	3.38379709151133	3.81430744310843	0.000576257172554843	0.110550088865571	-0.293626659372713
a_HQ_transcript/76282	-3.20266011003963	1.13117050408028	-3.81206192359029	0.000579880594854739	0.110593447751362	-0.256894179851379
a_HQ_transcript/46906	5.79558283177984	2.9771898666903	3.81181008004236	0.000580288356113496	0.110593447751362	-0.249493285164868
a_HQ_transcript/128550	-5.11928663864096	-0.0740824631336174	-3.79398588700351	0.000609867571408915	0.115850922192344	-0.29772383015257
a_HQ_transcript/102599	4.9785061611234	0.025661574298661	3.79108377091827	0.000614820519354276	0.116194169098702	-0.305822141136175
a_HQ_transcript/42287	-4.92148971187829	1.15854607170766	-3.79058692812872	0.000615672379617399	0.116194169098702	-0.308851933407745
a_HQ_transcript/117321	-2.9645944238559	-0.808857481850397	-3.7878694627184	0.000620351957609417	0.116282651746701	-0.310718538314294
a_HQ_transcript/100922	3.3832941459538	0.0561281292904957	3.78719181655266	0.000621524269727742	0.116282651746701	-0.31974623096787
a_HQ_transcript/129016	4.49327638244741	2.32696929419178	3.78568286197019	0.000624142474444045	0.116282651746701	-0.313044994619753
a_HQ_transcript/134682	-4.37607836379481	0.0774193187218544	-3.78485904644002	0.000625576411219998	0.116282651746701	-0.315743030932665
a_HQ_transcript/78650	5.78401187863463	0.519833239093352	3.7845337470818	0.000626143510816088	0.116282651746701	-0.321374423697674
a_HQ_transcript/124985	3.30040734890081	-0.333708127300771	3.78264021495498	0.000629454477214596	0.116355119698111	-0.322924028114151
a_HQ_transcript/69742	-5.05500414364214	0.254341562355892	-3.78202314649924	0.000630537137092366	0.116355119698111	-0.323091552035584
a_HQ_transcript/69985	-3.78876569933051	1.90051554929719	-3.77567239705173	0.000641785440147215	0.118056025521764	-0.337318180124463
a_HQ_transcript/7572	4.82342896495009	-0.238591195329866	3.77392242230633	0.000644919099384553	0.118258225265064	-0.352042619927027
a_HQ_transcript/147589	-3.22836563160858	-0.856273540432773	-3.76968245166443	0.000652573393764282	0.119285491297894	-0.353981718670016
a_HQ_transcript/7649	-3.20606793893908	2.64518665178	-3.76629475323322	0.000658752527408307	0.119712086342547	-0.371472155519498
a_HQ_transcript/108973	5.09766465910367	1.76235025563015	3.76415913135544	0.000662677080804029	0.119712086342547	-0.373481041269446
a_HQ_transcript/34861	-7.64122534561301	1.09568562163505	-3.76397124008617	0.000663023447601992	0.119712086342547	-0.369187359432628
a_HQ_transcript/124802	3.65619075911884	1.8020535602303	3.76377028841507	0.000663394085297273	0.119712086342547	-0.366285266141844
a_HQ_transcript/112122	2.45382348451246	-0.672820666855959	3.76279031776503	0.000665204443446235	0.119712086342547	-0.373564567413246
a_HQ_transcript/34663	4.21959238406249	0.532055989067777	3.76067171207974	0.000669134701504745	0.120047722003296	-0.398071564154208
a_HQ_transcript/12110	-3.87654594799251	0.486421716078627	-3.75734384476242	0.000675353900691826	0.120790681659737	-0.383233052248905
a_HQ_transcript/29553	-4.11277819006278	0.575733965990473	-3.75436904026877	0.000680960824953593	0.121419910530376	-0.393707713226743
a_HQ_transcript/8882	-4.16481574609574	-0.149787867818838	-3.75320612765641	0.000683164966698456	0.121440407291278	-0.40636623456713
a_HQ_transcript/31601	5.37503706624507	1.65850027848808	3.74340886868852	0.000702011676492534	0.123882174637698	-0.42580730813118
a_HQ_transcript/282	4.85272664562569	0.550933128355009	3.74298002624676	0.000702848067425535	0.123882174637698	-0.425819783288958
a_HQ_transcript/15461	5.52351352361398	0.0814126112958593	3.74275119838436	0.00070329475692335	0.123882174637698	-0.420930434524559
a_HQ_transcript/152866	3.66879291740376	-0.333295194286172	3.73885566683829	0.000710941587022865	0.124557932287551	-0.427494335152886
a_HQ_transcript/144363	7.19962716239754	3.38179706872706	3.73861494360249	0.000711416761620334	0.124557932287551	-0.495863838048112
a_HQ_transcript/43802	-5.96208103798382	0.538063116775328	-3.73690648283454	0.000714798047800956	0.124774116884607	-0.433267311356724
a_HQ_transcript/87009	6.71341771204888	0.994590469481474	3.73551522064272	0.000717563082537669	0.124881757071107	-0.448824124540523
a_HQ_transcript/105824	-3.26975681909624	-0.854419860356411	-3.733093824235	0.000722400235323959	0.125253733102028	-0.442174630620008
a_HQ_transcript/152697	-3.1247713907122	-0.783975506009343	-3.73183743354825	0.000724922557186935	0.125253733102028	-0.445507225109026
a_HQ_transcript/119850	-2.06310933725845	3.69629107233797	-3.73069716543222	0.000727219156113806	0.125253733102028	-0.5181392169187
a_HQ_transcript/19615	-3.24292003709579	1.33946405555936	-3.73002503894191	0.000728576186252896	0.125253733102028	-0.469478196270313
a_HQ_transcript/36045	3.23099642475979	-0.854567457313395	3.72908690911719	0.000730474393090893	0.125253733102028	-0.451824540176286
a_HQ_transcript/61459	3.02229867525333	1.34202999446122	3.7275655751942	0.000733562867691814	0.125382842448936	-0.542897341036696
a_HQ_transcript/30049	-4.16667091916237	2.5608819730413	-3.72659425754725	0.000735541378941081	0.125382842448936	-0.489815019572499
a_HQ_transcript/76968	-4.11741031486294	0.595985166136834	-3.72381492222663	0.000741231380745676	0.125983326608142	-0.491275148672892
a_HQ_transcript/146850	-1.28645303165004	3.16598710466506	-3.72145526184326	0.000746095735246189	0.126295809936227	-0.503806836417384
a_HQ_transcript/21797	2.37496886236556	-0.470318427587378	3.72081771021734	0.000747415335433215	0.126295809936227	-0.470725553297437

a_HQ_transcript/107157	-3.28640271816392	-0.853692027618665	-3.71919271390814	0.000750789000754516	0.126352332382396	-0.475612490960046
a_HQ_transcript/30536	5.16575756624636	0.159223135273645	3.7175981480397	0.000754113861298653	0.126352332382396	-0.481247482510618
a_HQ_transcript/111482	-5.1621254835853	4.14753987456663	-3.71752299917499	0.000754270907939226	0.126352332382396	-0.546574040222715
a_HQ_transcript/1901	5.80783556870037	1.7922706867953	3.71272611313988	0.000764361453013672	0.127674719944766	-0.491862421885672
a_HQ_transcript/50359	-4.81679910705182	1.81267662600033	-3.70804616492627	0.000774332254569784	0.128742213197883	-0.529201292641381
a_HQ_transcript/27964	-2.87464995728164	-0.811177587767155	-3.70668932736398	0.000777246579804513	0.128742213197883	-0.506249028222427
a_HQ_transcript/141252	-2.45709784950193	-0.829189796537663	-3.70661955731016	0.000777396725028503	0.128742213197883	-0.506972084095737
a_HQ_transcript/94626	4.20321398057205	0.94642413602658	3.70210379984674	0.000787174751163857	0.129991175953559	-0.518868592399576
a_HQ_transcript/29039	5.07882817626285	1.37198573853799	3.6981781093228	0.000795772003563866	0.130967820266144	-0.529044651131923
a_HQ_transcript/7254	-4.93611662616345	0.0853285677118825	-3.69554784521077	0.000801583182321934	0.130967820266144	-0.53365965694861
a_HQ_transcript/137931	-4.90596148744652	0.160158846572208	-3.69522856249472	0.000802291386780467	0.130967820266144	-0.532848625189581
a_HQ_transcript/88622	-3.4870104986103	0.0219502891583466	-3.69444047983391	0.000804042041384438	0.130967820266144	-0.534645371729887
a_HQ_transcript/83330	5.38694317550397	0.148526215655769	3.69430004775348	0.000804354387472705	0.130967820266144	-0.548818785822878
a_HQ_transcript/12988	-3.41903405173257	-0.412305449765883	-3.69257467838506	0.000808201540487569	0.131226645657713	-0.557686315607045
a_HQ_transcript/102370	-3.64083711110667	-0.843227935667024	-3.690144161538	0.000813651305154373	0.131743518289731	-0.54525534868715
a_HQ_transcript/143741	-3.13627654130104	-0.707387567842847	-3.6882213961005	0.000817987820875318	0.132077766810668	-0.550184403916372
a_HQ_transcript/62428	-3.41081474810453	-0.425425670063991	-3.6855345854169	0.00082408510630552	0.13208074635771	-0.567885989804247
a_HQ_transcript/121864	-4.54322770256979	0.541494369129625	-3.68438679625852	0.000826703249574349	0.13208074635771	-0.559263074741527
a_HQ_transcript/105479	-3.04174981655535	-0.242657667434745	-3.68436423820284	0.00082675478597575	0.13208074635771	-0.569419880259639
a_HQ_transcript/77962	-5.62985325401254	2.88837176608068	-3.68421525523968	0.000827095232490479	0.13208074635771	-0.577271658386131
a_HQ_transcript/146759	-4.21372291363382	0.978754117287962	-3.68276017797957	0.000830427441984696	0.132249551637497	-0.564796040622646
a_HQ_transcript/53566	-5.69799809965989	2.27582138164364	-3.67954775751829	0.000837830242531504	0.13253422923148	-0.585217525205851
a_HQ_transcript/24735	2.05186708453447	3.42115179030048	3.67894141482345	0.000839234680295266	0.13253422923148	-0.605406028745801
a_HQ_transcript/49756	-4.83156669897683	0.073966798296595	-3.67739312915872	0.000842831255460895	0.13253422923148	-0.582473114055642
a_HQ_transcript/55801	-4.497706589724	2.62032139251116	-3.67737347954906	0.000842876996257372	0.13253422923148	-0.584345281054857
a_HQ_transcript/146751	-0.938691160939063	8.97542690122055	-3.67705649697423	0.000843615208086422	0.13253422923148	-1.07581205729104
a_HQ_transcript/145977	4.06806540560512	2.1772554177296	3.67478116802404	0.000848932593499216	0.132669448832348	-0.58182655474346
a_HQ_transcript/135092	-6.52184048644121	1.03380324910033	-3.67473507322302	0.000849040651074069	0.132669448832348	-0.606134473870839
a_HQ_transcript/146673	-3.55545293841521	-0.783467619293198	-3.67246442491062	0.000854380154340163	0.133145870272078	-0.587683624761339
a_HQ_transcript/135476	-3.6596050930732	-0.842346989646206	-3.67106822449975	0.000857679517149016	0.133302660355182	-0.59099166105046
a_HQ_transcript/52365	-5.28365216109982	0.318309393791696	-3.66837188209831	0.000864086251265844	0.133940281636216	-0.599413163658998
a_HQ_transcript/22469	3.8163823939859	-0.00323118941052627	3.66564728179331	0.000870607254500723	0.134425720328197	-0.603813419600323
a_HQ_transcript/19691	3.46275805194542	-0.103838174791805	3.66513314686418	0.000871843114570092	0.134425720328197	-0.605017860446841
a_HQ_transcript/135253	-3.84208570105641	-0.129419206022182	-3.66413107049712	0.000874256761764823	0.134441262031391	-0.616746713538554
a_HQ_transcript/145625	7.87283301644453	2.56640321911931	3.66021996850486	0.000883739355961142	0.135540900483666	-0.669498924658125
a_HQ_transcript/128422	-2.96889226787862	-0.794758041986842	-3.65864787677085	0.000887578971076318	0.135771553765064	-0.621388583591688
a_HQ_transcript/95347	4.3799462468599	0.985999193667398	3.65752056126908	0.00089034223244819	0.135836780282804	-0.624618314462097
a_HQ_transcript/109916	2.01980995332178	2.05587840600166	3.65438701715258	0.000898067010547707	0.1363369817818	-0.632425859344456
a_HQ_transcript/132171	-4.69243003263956	0.898427952410692	-3.65340000926286	0.000900513595998353	0.1363369817818	-0.646166088744586
a_HQ_transcript/68700	-3.38083262811337	-0.377677852469744	-3.65334216191423	0.000900657187658461	0.1363369817818	-0.63869644468822
a_HQ_transcript/81764	-6.27852568120138	3.93287216330815	-3.65238984436107	0.000903024271358238	0.136340246351978	-0.702972297434801
a_HQ_transcript/78693	4.1470283897648	8.58323559268003	3.65142669020254	0.0009054244226948	0.136348473684983	-1.04398931604372
a_HQ_transcript/42794	6.6833323919977	0.0890347243303459	3.64903693350728	0.000911406355491086	0.13689464762787	-0.646118562259709
a_HQ_transcript/39549	-4.09043179207105	-0.0554268016864907	-3.6455746987622	0.000920140853160312	0.137850380186862	-0.655668448446547
a_HQ_transcript/125031	3.43469958698177	1.39654384779449	3.6442519583898	0.000923499219758766	0.137997847419377	-0.696767724629881
a_HQ_transcript/50054	3.71861866155093	-0.209424592366275	3.64219698344496	0.000928740260842995	0.138425163800722	-0.66008433776282
a_HQ_transcript/17401	6.71408719694857	1.42398384638313	3.62963149568229	0.000961419864445468	0.142755509756676	-0.693003795712102
a_HQ_transcript/7911	-3.02260199262467	1.85041702022246	-3.62875749269951	0.000963733890268858	0.142755509756676	-0.695115773248684
a_HQ_transcript/129041	-2.57327271777395	-0.352626353425384	-3.62821925082221	0.00096516163181898	0.142755509756676	-0.708200102904645
a_HQ_transcript/131565	-7.94534824470051	0.177342289608439	-3.62662432557059	0.000969404372480137	0.143019130364278	-0.697272567998889

a_HQ_transcript/71767	2.22926215923011	-0.847325487626962	3.62016669378788	0.00098676798970953	0.14458164553401	-0.714366549321371
a_HQ_transcript/30634	3.78724339098763	0.71042500244662	3.62009194618343	0.000986970727396192	0.14458164553401	-0.727722361275942
a_HQ_transcript/36505	-3.24918894976631	-0.495265775267423	-3.61991264129897	0.000987457219876855	0.14458164553401	-0.71327861024868
a_HQ_transcript/75725	4.49596297841483	1.61903168780119	3.6170454194329	0.000995268218004027	0.14535917330688	-0.720081256884791
a_HQ_transcript/29740	7.10944070598366	1.57832609147501	3.61339460144555	0.00100530052847788	0.146337145425265	-0.741825904180053
a_HQ_transcript/95204	-3.15318850026656	-0.431338734944487	-3.6127798843465	0.0010069993491967	0.146337145425265	-0.755041936667506
a_HQ_transcript/89776	-2.60858982251754	-0.777803086525496	-3.60993648518803	0.00101489353598244	0.146868027014093	-0.745212700451089
a_HQ_transcript/78667	-2.58353105027318	-0.801397677904819	-3.60964513160449	0.00101570580201736	0.146868027014093	-0.73794050445448
a_HQ_transcript/88736	4.39822203526672	0.532985712734508	3.60853695759064	0.00101880103761593	0.146895245491488	-0.763484308595731
a_HQ_transcript/136987	-3.73135322469181	-0.839168363971427	-3.60777011730256	0.00102094823800167	0.146895245491488	-0.742175930439616
a_HQ_transcript/22656	7.07525455290359	2.24934982467585	3.6066505888253	0.00102409085383958	0.146962984708463	-0.77270080082801
a_HQ_transcript/143204	-4.64402358576423	2.65081583786864	-3.60429963153175	0.00103072070110298	0.146962984708463	-0.76766673305317
a_HQ_transcript/139895	4.60395439433005	-0.0714859597313601	3.6041577262831	0.00103112220932201	0.146962984708463	-0.750884435667884
a_HQ_transcript/123183	2.53980337384555	-0.619278992475098	3.60401291446563	0.00103153209745824	0.146962984708463	-0.75223146305956
a_HQ_transcript/88979	-3.42520831405358	-0.474879982808844	-3.60191587075913	0.00103748545882235	0.147124357531809	-0.756120548331439
a_HQ_transcript/27637	-4.0212844386881	0.926467775906919	-3.60155934695958	0.00103850090649832	0.147124357531809	-0.770618245059525
a_HQ_transcript/85998	5.16363377735425	0.889270662601552	3.60094326399258	0.00104025789543032	0.147124357531809	-0.772307869346633
a_HQ_transcript/83780	5.22626603487287	0.579285150063156	3.59863374330311	0.00104686998736136	0.147433643711143	-0.767103745344284
a_HQ_transcript/129792	-7.41182318144605	0.241866277032892	-3.59840834886736	0.00104751745893032	0.147433643711143	-0.766480753447364
a_HQ_transcript/8232	-1.84600953726329	1.66765863074368	-3.59570289231278	0.00105531949641053	0.14817297509022	-0.770874234725818
a_HQ_transcript/94048	-2.85559427680512	-0.767114739607287	-3.59421753432813	0.00105962687120696	0.148419254866309	-0.774719830504002
a_HQ_transcript/145598	-2.37723074315466	-0.765016816291138	-3.59202681177774	0.00106601078215994	0.148592853057154	-0.785789863662646
a_HQ_transcript/73806	1.81541017586912	2.20466576876339	3.59144355348782	0.00106771669742941	0.148592853057154	-0.794345430314435
a_HQ_transcript/140916	5.21569987053156	1.14991905290724	3.59116403357835	0.00106853517371818	0.148592853057154	-0.781680197373162
a_HQ_transcript/5666	3.50954806049953	1.90316690938374	3.58810515786202	0.00107753177685869	0.149431010560321	-0.793590753333659
a_HQ_transcript/125801	-2.41582247544205	-0.839386169757834	-3.58737035378243	0.00107970383352833	0.149431010560321	-0.790786290314999
a_HQ_transcript/53437	4.2349838786718	1.0522497424478	3.5862970355587	0.00108288413937713	0.149515176374617	-0.80721116390905
a_HQ_transcript/54847	-5.06620431512671	0.93977761474199	-3.58321737371281	0.00109205973709514	0.150424759236649	-0.800570139599346
a_HQ_transcript/22328	-4.48785165988352	-0.273496053923391	-3.57853488070538	0.00110615506847342	0.152006103593907	-0.811581871488268
a_HQ_transcript/62833	2.44293466504935	-0.819317632208447	3.5776128994652	0.00110895108959413	0.152030917301715	-0.814064587861377
a_HQ_transcript/3685	-1.48623517626011	3.38412587007607	-3.57509646234682	0.00111661731830828	0.152721721126173	-0.879231875822857
a_HQ_transcript/125817	2.6319163510802	-0.803144596641732	3.57165324367281	0.00112719002148899	0.153805872227962	-0.82788997767005
a_HQ_transcript/139578	2.25430667111947	-0.828893916546048	3.56512697948691	0.00114749565630635	0.156209900491277	-0.843354234081961
a_HQ_transcript/150936	5.13212994210325	9.05452295499229	3.56405810467395	0.00115085484418444	0.156301145754096	-1.32923829766081
a_HQ_transcript/86109	-5.04021857910903	1.01474094255629	-3.55272355978647	0.00118706609629879	0.16084330546773	-0.888473282137151
a_HQ_transcript/29420	-1.666008396659	3.8045949439081	-3.55147757704378	0.00119111324289566	0.161016350193113	-0.950040193963705
a_HQ_transcript/152558	-4.42909475569846	-0.149017378297626	-3.54901033436749	0.00119916660316094	0.161728900947887	-0.881495476278688
a_HQ_transcript/66281	-1.87526827858927	-0.676253847040456	-3.546926160837	0.00120601054598584	0.162275419020984	-0.889321130595583
a_HQ_transcript/152451	-6.20487688597942	0.434744359286356	-3.54338673579019	0.00121771973620455	0.162922272410682	-0.894854152303403
a_HQ_transcript/127881	-1.3418152768531	4.584964767493	-3.54316872175227	0.00121844455321709	0.162922272410682	-1.0579469425253
a_HQ_transcript/498	-1.26074415211716	-0.83312967523569	-3.54181562937703	0.00122295241868931	0.162922272410682	-0.900158439518995
a_HQ_transcript/123925	-5.79435919088684	0.500396225875997	-3.54143237366059	0.0012242321706248	0.162922272410682	-0.902157063046213
a_HQ_transcript/145294	-2.93612100818807	-0.80336018614418	-3.54053358203167	0.00122723845461318	0.162922272410682	-0.901292414367881
a_HQ_transcript/15084	-2.93259112739535	0.97098754255567	-3.54041524669579	0.00122763479417628	0.162922272410682	-0.906055400277881
a_HQ_transcript/72581	2.44379512737356	0.928406688220449	3.53582320756557	0.00124311070722128	0.164383653497135	-0.918032275135106
a_HQ_transcript/90347	1.78447432379251	3.03391787008253	3.53528162098511	0.00124494831397668	0.164383653497135	-0.939509358373886
a_HQ_transcript/77822	4.06101415354542	-0.017571516146904	3.53463953358925	0.00124713031916179	0.164383653497135	-0.916740986069614
a_HQ_transcript/128440	-2.81239649942291	-0.747222589592234	-3.52929383722756	0.00126544051048128	0.166419742066189	-0.931060674126412
a_HQ_transcript/52755	-8.07673168269689	0.17124997889033	-3.52844662088266	0.00126836613857108	0.166427961406005	-0.930816669454731
a_HQ_transcript/74466	-4.55563268737962	0.277019970777107	-3.52693146646085	0.00127361459638012	0.16674024607744	-0.935367943457019

a_HQ_transcript/139029	4.14297967565752	1.34353117773185	3.52533250109711	0.00127917610949607	0.166994485315973	-0.939516932656828
a_HQ_transcript/8680	4.52795817285858	0.763810850700416	3.52472298061372	0.00128130230613343	0.166994485315973	-0.944822956617858
a_HQ_transcript/103448	3.91164836349355	0.610891167759765	3.52352974703132	0.00128547455596042	0.167163456350933	-0.949121076334076
a_HQ_transcript/62001	4.58024389951297	0.258551123649382	3.518728263994	0.00130239632132838	0.168671870813058	-0.977036769636747
a_HQ_transcript/4049	-3.38069198499298	-0.468357015095217	-3.51776874720776	0.00130580360578441	0.168671870813058	-0.954754456396353
a_HQ_transcript/113934	5.82411090545654	1.16542862343765	3.51731933193865	0.00130740245511326	0.168671870813058	-0.967846295223898
a_HQ_transcript/59949	-4.00900710294248	-0.174428834308468	-3.51696030942765	0.00130868107859705	0.168671870813058	-0.956768403465441
a_HQ_transcript/37150	1.24629326949084	2.68135257390769	3.51506945021504	0.00131543512817376	0.169167285686912	-0.967763159546717
a_HQ_transcript/92367	3.66437382661617	-0.211866673887687	3.51363216959689	0.00132059150838566	0.169455503751527	-0.964441396042319
a_HQ_transcript/125531	4.82018343126937	-0.0597066764795738	3.51202550700401	0.00132637865026746	0.169489312605701	-0.97173346107368
a_HQ_transcript/15392	-3.64781278715331	1.66363492369513	-3.51143323013906	0.00132851817760845	0.169489312605701	-0.981093770717003
a_HQ_transcript/67722	-3.62423337337139	3.77363754073229	-3.51079780305451	0.00133081728252494	0.169489312605701	-0.975883946702674
a_HQ_transcript/138234	-3.30933210859856	1.53452389582318	-3.50984106233542	0.00133428620901967	0.169489312605701	-0.976534281656897
a_HQ_transcript/116803	-5.84123321667656	0.540164976468185	-3.50952503763223	0.00133543395908015	0.169489312605701	-0.978277992925026
a_HQ_transcript/96791	-6.04258252080268	1.3717907742256	-3.50866953381249	0.00133854579082118	0.169514138842818	-0.976954270938136
a_HQ_transcript/111564	3.35074582950966	-0.435313314734951	3.50723115360539	0.00134379356825345	0.169617671239356	-0.997837005407264
a_HQ_transcript/128603	1.72126242879946	3.24776159174336	3.50650261194571	0.00134645914245059	0.169617671239356	-1.01914252118425
a_HQ_transcript/70520	3.36568618180744	-0.391152302624007	3.50554770764766	0.00134996065349188	0.169617671239356	-0.983467932139955
a_HQ_transcript/99560	-4.0018850446595	2.78498519042116	-3.50525510901603	0.00135103533209162	0.169617671239356	-1.03963135962158
a_HQ_transcript/80813	-3.49899143827159	1.95058266940181	-3.49937449080184	0.00137280990673708	0.171979944523303	-1.01047941223093
a_HQ_transcript/136081	3.06006308011151	0.802509157848773	3.49687363776742	0.00138217222510557	0.172780445378358	-1.00414914983712
a_HQ_transcript/113258	4.89756337571685	-0.182465943052866	3.49574930882879	0.00138640135656599	0.172937206125468	-1.00754862896735
a_HQ_transcript/126877	-2.38923554726923	8.82247935388001	-3.49360743898508	0.001394492496209	0.173574003896439	-1.50510496352475
a_HQ_transcript/110928	4.44667039007888	0.320656902043874	3.49020970409125	0.00140742128982777	0.174115986873182	-1.02014266242811
a_HQ_transcript/136429	-5.77549110633878	0.634466712040432	-3.49007987910551	0.00140791757512819	0.174115986873182	-1.01949775759735
a_HQ_transcript/54209	-4.34156055127746	0.0814943145698933	-3.48936455209367	0.00141065510171412	0.174115986873182	-1.02115110924476
a_HQ_transcript/51635	5.40877225164889	0.226554793858516	3.48931932894292	0.00141082834119991	0.174115986873182	-1.02420738474194
a_HQ_transcript/124274	3.94508571581006	0.585931350155499	3.48786749145098	0.00141640090964359	0.17443337304187	-1.02741399424088
a_HQ_transcript/51325	-3.06328682804343	-0.780460582378798	-3.48598160602649	0.00142367117213389	0.174958050515431	-1.02918498854144
a_HQ_transcript/146667	-4.92597702144521	0.0151471690480264	-3.48450685767306	0.00142938151731171	0.175289216958429	-1.04324883125761
a_HQ_transcript/144352	-3.32091749876198	0.296942873443391	-3.48271445156053	0.00143635158939731	0.175588490859807	-1.03711160492913
a_HQ_transcript/31904	-4.65139866013459	0.988415811882765	-3.48232679399471	0.00143786336445892	0.175588490859807	-1.04067542556258
a_HQ_transcript/31215	-3.63100585046728	0.788988908032018	-3.48132731416095	0.00144176818375755	0.175612490378974	-1.04297907132131
a_HQ_transcript/142861	-2.7987790184983	1.85324684561038	-3.48073115323081	0.00144410216076847	0.175612490378974	-1.04597764716714
a_HQ_transcript/134180	4.29087323753717	0.283452749081214	3.47782860242484	0.00145551780903758	0.176631188316778	-1.04830487338192
a_HQ_transcript/149007	-5.70264729515302	0.374989139880222	-3.47658280182049	0.00146044413925453	0.176859785263724	-1.05230539649517
a_HQ_transcript/143458	7.12738151839364	1.56483717964473	3.47541243799875	0.00146508680291266	0.176892871336827	-1.10588933231343
a_HQ_transcript/138280	-2.43462654689053	-0.80900336519753	-3.47498053890502	0.00146680367437983	0.176892871336827	-1.05502362490587
a_HQ_transcript/39711	3.25098096599661	1.5636834604793	3.47074016551932	0.00148376315996425	0.178307312224171	-1.06899878868855
a_HQ_transcript/135376	-4.78990430353556	0.318010677378739	-3.4705154244185	0.0014846672707903	0.178307312224171	-1.07169592813265
a_HQ_transcript/79495	3.9943350216292	1.41917803470647	3.46912698554588	0.00149026460179707	0.17851333875919	-1.09190308850846
a_HQ_transcript/132916	8.00322736158363	0.924409572822535	3.46856775407914	0.00149252481827976	0.17851333875919	-1.11631323461665
a_HQ_transcript/3541	5.82302501310775	1.34760474197475	3.46775637371746	0.0014958100050979	0.178538899335381	-1.09339843438697
a_HQ_transcript/40904	4.28944420462005	1.7619805837851	3.46569130158403	0.00150420270843707	0.17917273572957	-1.07762056661028
a_HQ_transcript/44846	-2.93694892061668	-0.648017646406018	-3.46066732996636	0.00152481060569219	0.181256014085226	-1.08789639152686
a_HQ_transcript/72517	3.66633680774539	0.311077956466138	3.45968102779886	0.00152888814566123	0.181369816593869	-1.0946415972702
a_HQ_transcript/132148	0.993138963664673	3.28275841282999	3.45723260877684	0.0015390557721667	0.182204142412436	-1.16466263667166
a_HQ_transcript/142605	4.23410425472072	1.84822459191581	3.45547976891792	0.00154637481384219	0.182698526786623	-1.1065956296035
a_HQ_transcript/130013	-4.2632120997975	0.587727738814991	-3.45346402519004	0.00155483306098635	0.183325225494959	-1.1110503700562
a_HQ_transcript/72747	3.84088814768597	2.43858244399204	3.45077859785004	0.00156617060165428	0.184288187718543	-1.1469581937329

a_HQ_transcript/129089	3.65976576001837	-0.63883822963645	3.4491577406519	0.00157305213992289	0.184441334017657	-1.12430017896279
a_HQ_transcript/83136	-2.58201863960977	-0.808197005257047	-3.44897773837701	0.00157381815429325	0.184441334017657	-1.11585160254891
a_HQ_transcript/135292	4.78097765798273	0.142754091367529	3.44697027034442	0.00158238547357465	0.185072239050195	-1.1294574674915
a_HQ_transcript/104854	-1.81170102332927	2.6127411647475	-3.44575583280188	0.0015875901379057	0.185274409741562	-1.13526503799553
a_HQ_transcript/143962	-2.59238184366973	-0.80424851740115	-3.44492449474056	0.00159116246732758	0.185274409741562	-1.12522772049247
a_HQ_transcript/22664	-3.47551308106281	1.50610831401627	-3.44434060650959	0.00159367610911748	0.185274409741562	-1.12699353138308
a_HQ_transcript/66368	4.83879330962877	0.224129828302898	3.44319242846826	0.00159863017591691	0.185479390949497	-1.13257917229841
a_HQ_transcript/67920	-2.94995724214494	-0.456034339028469	-3.44092751455012	0.00160844614691827	0.185953288819326	-1.13673727132021
a_HQ_transcript/45943	-2.69318548355866	-0.795240072019572	-3.44070710705097	0.00160940446866967	0.185953288819326	-1.13442902874259
a_HQ_transcript/44489	4.07312456605371	1.19342613386663	3.4400392235822	0.00161231175276872	0.185953288819326	-1.14254004203808
a_HQ_transcript/7955	4.42972921172816	1.15738840287475	3.43763358408125	0.00162282540967786	0.186795238443079	-1.14119454099765
a_HQ_transcript/78061	-3.75208976863537	0.346381734791037	-3.43533366882524	0.00163293868703495	0.187441063337064	-1.15512991221198
a_HQ_transcript/26167	-2.67735746825546	-0.19992517526354	-3.43489252787757	0.00163488540998987	0.187441063337064	-1.15068352525597
a_HQ_transcript/138416	6.42400935136471	1.75773477101597	3.43263899681013	0.00164486497733067	0.18821399882338	-1.17205903975823
a_HQ_transcript/146674	3.40533620153465	0.317521968374333	3.4287306525763	0.00166231189614312	0.189190039563081	-1.16186936383271
a_HQ_transcript/77790	3.26860812602093	-0.340162408323341	3.42871126009449	0.00166239890688285	0.189190039563081	-1.16241764238494
a_HQ_transcript/141133	-2.75212682779732	2.72758720643891	-3.42831505244968	0.00166417758231708	0.189190039563081	-1.18075318849916
a_HQ_transcript/149548	4.99640910078344	2.86450177748427	3.42781750728558	0.00166641378090245	0.189190039563081	-1.18103390856933
a_HQ_transcript/14178	-4.04595600018375	1.80736105943482	-3.424267289776	0.00168245422612575	0.190638789973173	-1.17646938146655
a_HQ_transcript/135436	-1.23199942023098	3.86608375591895	-3.42167066967799	0.00169428011058491	0.191605280677198	-1.29850338717324
a_HQ_transcript/148532	-4.52892034193005	1.72583729986732	-3.41973629547417	0.00170314183321603	0.192233453361518	-1.19041056212965
a_HQ_transcript/34210	-3.07809151349017	-0.79609940849901	-3.41611454674007	0.0017198537522457	0.193217825638755	-1.19256599071686
a_HQ_transcript/14301	3.62418442840207	-0.355046255815741	3.41606794890739	0.00172006979395512	0.193217825638755	-1.19156734197566
a_HQ_transcript/116770	-4.54513692897134	0.0692960727368458	-3.41568739140923	0.00172183515140509	0.193217825638755	-1.19359539788049
a_HQ_transcript/31849	-4.67652735822885	0.569152847172796	-3.41239388876326	0.00173718625640632	0.194564860717508	-1.21847301331798
a_HQ_transcript/93097	2.6998386816056	-0.789028286237927	3.41043026717336	0.00174640128713664	0.195220796189766	-1.20478091072934
a_HQ_transcript/44260	4.26320634420938	1.60094976554653	3.40970790208206	0.00174980306802336	0.19522562905578	-1.21664915695955
a_HQ_transcript/23908	-2.99810419118384	-0.749882024674045	-3.40655638751209	0.00176471894214105	0.19651261047658	-1.21503925040701
a_HQ_transcript/53291	-2.47854939798396	-0.777420883063801	-3.40461088928424	0.00177398777943693	0.197069579317373	-1.21756362785411
a_HQ_transcript/117954	3.23371340074311	1.57715281039298	3.40408502278527	0.00177650116230222	0.197069579317373	-1.22494101032387
a_HQ_transcript/89892	-3.85362869460835	0.0282561785295225	-3.40330260201763	0.00178024708517733	0.197108957270834	-1.22274396574877
a_HQ_transcript/99985	-5.55911351371303	1.92897681117961	-3.39978174363579	0.00179719773709483	0.198607433577658	-1.27632428861817
a_HQ_transcript/146652	3.1316042925366	-0.406322598983237	3.39605146253802	0.00181532584129569	0.200230095830809	-1.23734273570113
a_HQ_transcript/38011	-4.14213398784593	0.121108131830956	-3.39363733779211	0.00182715133196814	0.201152751183038	-1.24400698397872
a_HQ_transcript/56013	3.77058083093339	0.27464976114522	3.39261630188214	0.00183217507979611	0.201324523702058	-1.2458608166539
a_HQ_transcript/22098	3.63663864004756	-0.836500119813226	3.39104805542923	0.00183991709738878	0.20177261435554	-1.25219727425947
a_HQ_transcript/145193	9.29845173071091	1.1848572621725	3.39038591658034	0.00184319533138576	0.20177261435554	-1.27663730687251
a_HQ_transcript/133781	-1.65658737992365	2.62626082348301	-3.38954313111165	0.00184737606136347	0.201850142283714	-1.31084855081219
a_HQ_transcript/27727	-5.76228360446902	0.450450434423831	-3.3886820325385	0.00185165704548215	0.20193831283262	-1.26427750500138
a_HQ_transcript/30000	-2.82628772576698	-0.777679543276653	-3.38686832802827	0.00186070514910547	0.202545072859931	-1.25974756143711
a_HQ_transcript/123498	-2.60285286973448	-0.489926026290252	-3.38525287197342	0.00186879999746247	0.203045992995324	-1.26233502646639
a_HQ_transcript/79479	-2.67447876976274	1.33955958066004	-3.38436141736797	0.00187328144007574	0.203153178262542	-1.26460198667958
a_HQ_transcript/635	-5.18010884161393	0.264258329896411	-3.3832776662779	0.00187874348439083	0.203366110355066	-1.26763908641769
a_HQ_transcript/101655	6.61984897281441	0.583369508604704	3.38257958765198	0.00188226985898927	0.203369112199495	-1.27651137084063
a_HQ_transcript/52838	-5.81115772373511	0.755430501775978	-3.38056270992435	0.00189249396298298	0.20385408118677	-1.28525785249646
a_HQ_transcript/104909	-2.74354946709503	-0.647581263541366	-3.38011257364876	0.0018947830972319	0.20385408118677	-1.28345093028826
a_HQ_transcript/152154	6.00393019345876	0.72759034167581	3.37962229145258	0.00189727941649536	0.20385408118677	-1.27735879751821
a_HQ_transcript/132233	1.27565811284816	2.90605645120205	3.37849405627596	0.00190303593953416	0.204095337810409	-1.31623965942524
a_HQ_transcript/139492	3.61931563508205	2.43478925779151	3.37624465484787	0.00191456302186625	0.204946118963817	-1.28267002257928
a_HQ_transcript/126277	3.37124319731254	0.145256510362652	3.37512411639588	0.0019203302267996	0.204946118963817	-1.28868911839474

a_HQ_transcript/13280	1.80953206484301	1.87854514745969	3.37488828053723	0.00192154615392376	0.204946118963817	-1.28712524079299
a_HQ_transcript/76662	2.88290303612778	2.95682209167084	3.37405498742986	0.00192584838720993	0.205028782146042	-1.3411078908129
a_HQ_transcript/53920	-4.53803679446754	0.678723619739504	-3.37064930642119	0.00194352802888591	0.206532718945302	-1.29611568725019
a_HQ_transcript/90451	4.29897115052204	0.153440535138475	3.36781534174696	0.00195835846259318	0.207728942908059	-1.30932898900537
a_HQ_transcript/144883	3.01731841140367	4.07731482825447	3.36588372525333	0.0019685290153475	0.208108033839202	-1.40305317828408
a_HQ_transcript/110687	4.39351290429001	0.4215115470501	3.36577695533752	0.0019690926681042	0.208108033839202	-1.3060722323331
a_HQ_transcript/137233	2.45310570642166	-0.549165156623605	3.3634477883915	0.0019814272800642	0.209031587904849	-1.33615232132535
a_HQ_transcript/10914	-2.95601018525213	-0.764637153812481	-3.36266776407784	0.00198557462302739	0.209089640737928	-1.31527848793521
a_HQ_transcript/114723	-1.79259795679394	4.13856618085362	-3.36186353465106	0.00198985938735943	0.209161928514338	-1.44510191867349
a_HQ_transcript/104320	4.07336384934591	-0.173232446788346	3.35989954660045	0.00200036043476557	0.209886193776269	-1.32239438369481
a_HQ_transcript/116166	-4.43451558385094	1.34683347324834	-3.35877384959499	0.00200640326857516	0.210140917469796	-1.32498078543741
a_HQ_transcript/3864	1.41171085902577	4.0799346235528	3.35758166671257	0.00201282210592276	0.210434034843666	-1.48714239083871
a_HQ_transcript/95397	-3.41399685532434	1.3285377347503	-3.35436405358582	0.00203024459518877	0.211561046584904	-1.33450006678161
a_HQ_transcript/108175	-3.30223428177135	-0.435001571710735	-3.35395629833372	0.00203246278222383	0.211561046584904	-1.33604717933631
a_HQ_transcript/148586	-1.20614489866461	8.75711854410776	-3.35349383654343	0.00203498138722402	0.211561046584904	-1.84725757505728
a_HQ_transcript/31341	-4.72605719226966	3.32297193605578	-3.35291090054438	0.00203816037172354	0.211561046584904	-1.42858252782175
a_HQ_transcript/29397	3.40680111087789	1.69940315056492	3.35185329023874	0.00204394011575828	0.211782800443489	-1.3379724771932
a_HQ_transcript/61318	3.02897261219447	0.158559243749244	3.35112573806405	0.00204792524929832	0.211818147493261	-1.33935286390738
a_HQ_transcript/63538	2.82999472475926	0.587424808885737	3.35030699806519	0.00205241877541179	0.211905858929194	-1.34183433169849
a_HQ_transcript/79361	-2.98412289540747	2.64913086051059	-3.34426016255417	0.00208590003967284	0.21474580276438	-1.39080892526634
a_HQ_transcript/1181	4.42363131777967	-0.064032413772081	3.34400687995676	0.00208731383432898	0.21474580276438	-1.35544235579884
a_HQ_transcript/90742	-6.55808600493479	1.91829889194847	-3.34048492180527	0.00210706851131789	0.215880590972643	-1.44090687378201
a_HQ_transcript/10476	3.50604291411413	-0.266176560242797	3.33954560109125	0.00211236738045871	0.215880590972643	-1.36565863147119
a_HQ_transcript/152928	5.35936316877549	2.91015857689139	3.33942197258315	0.00211306574108945	0.215880590972643	-1.45172510157004
a_HQ_transcript/112292	4.58780587390393	0.562993772657303	3.33939831115962	0.00211319942649728	0.215880590972643	-1.37611365840815
a_HQ_transcript/133459	-6.12324924128079	1.3046478550521	-3.33757775300699	0.00212350981262093	0.21632490269715	-1.37314463231766
a_HQ_transcript/70788	1.92652606768096	2.7490922180653	3.33731677978606	0.00212499173272902	0.21632490269715	-1.4282513191366
a_HQ_transcript/71709	-4.7639574503208	2.07542927189809	-3.33556643884741	0.00213495659744546	0.216839663997308	-1.46660549704027
a_HQ_transcript/35908	-2.9766872979705	1.04161151333636	-3.33489489725332	0.00213879163194217	0.216839663997308	-1.38316848641647
a_HQ_transcript/83385	4.63329284045687	-0.164373784926324	3.33382135099722	0.00214493615635556	0.216839663997308	-1.38896622981569
a_HQ_transcript/121843	8.02755461728073	2.63006152514151	3.33358383995066	0.00214629785396589	0.216839663997308	-1.61069604877711
a_HQ_transcript/123862	2.31215842756765	-0.609327696419562	3.33312918853648	0.00214890677248702	0.216839663997308	-1.38320373816516
a_HQ_transcript/31915	-5.51636152202547	0.264660441104203	-3.33172264773496	0.00215699717066269	0.216839663997308	-1.38573216934429
a_HQ_transcript/70407	-3.15777496798348	0.938251211080487	-3.33087666910443	0.00216187728181975	0.216839663997308	-1.38519757276545
a_HQ_transcript/142327	-1.52970104439087	2.9666210775651	-3.33078043391284	0.00216243309414885	0.216839663997308	-1.42259767070624
a_HQ_transcript/108058	4.37157022438544	0.338842201706913	3.33013773506283	0.00216614855565665	0.216839663997308	-1.387125187214
a_HQ_transcript/59731	3.31922313572956	-0.197683350866958	3.32992976744056	0.00216735213292107	0.216839663997308	-1.38731326510816
a_HQ_transcript/148072	-7.80237442878485	1.50351870543307	-3.32737942653528	0.00218216403626057	0.217946445188581	-1.42977600961299
a_HQ_transcript/27457	3.17641349450647	1.62949725006422	3.32578734206622	0.0021914597118383	0.218499434184797	-1.39836984483775
a_HQ_transcript/48533	-1.46734628364366	4.93112120982809	-3.32414437213871	0.00220109228406344	0.219084062137054	-1.62828483750778
a_HQ_transcript/120	-3.95425961370189	1.26793292274613	-3.32050162995475	0.00222259427765671	0.220684106779359	-1.41266572730311
a_HQ_transcript/50138	-4.36729456110787	0.288094591202809	-3.32013649215771	0.0022247606415618	0.220684106779359	-1.41208828399949
a_HQ_transcript/49824	5.09093745915519	0.963662071168108	3.318576440216	0.00223403925026806	0.220746109338808	-1.41909952491987
a_HQ_transcript/131086	-3.33536474737686	-0.268599860730385	-3.31499608846541	0.00225547427627505	0.220746109338808	-1.42280158783146
a_HQ_transcript/126094	-3.11288048846018	1.6791615053488	-3.31454707583266	0.00225817631363337	0.220746109338808	-1.42216747962888
a_HQ_transcript/131570	-4.68275271552451	3.66497033409771	-3.31440825025114	0.00225901235622495	0.220746109338808	-1.54498941419067
a_HQ_transcript/85762	5.0399475152085	1.15143260813397	3.31428554416834	0.00225975157005942	0.220746109338808	-1.42410045141855
a_HQ_transcript/115568	5.65071545789621	1.28113685750319	3.31349542040521	0.002264517038353	0.220746109338808	-1.42795511558241
a_HQ_transcript/148615	-2.61029926501388	-0.797115838153484	-3.31318654686891	0.00226638256189282	0.220746109338808	-1.4298057541045
a_HQ_transcript/35753	3.51923740458267	-0.745722149456915	3.31315282234218	0.00226658633924776	0.220746109338808	-1.43204302528653

a_HQ_transcript/81169	-4.35895749657579	-0.262091367115348	-3.31290055581757	0.00226811119344461	0.220746109338808	-1.42842307923476
a_HQ_transcript/95805	-4.60910932943695	0.449961237326262	-3.3126239025589	0.00226978458608739	0.220746109338808	-1.42783445719359
a_HQ_transcript/3769	1.09957349022147	3.12274499544304	3.31260166978772	0.00226991911679567	0.220746109338808	-1.49245870065655
a_HQ_transcript/130093	-2.22518381500954	3.4798064035318	-3.31243023283422	0.00227095674003247	0.220746109338808	-1.53998709657639
a_HQ_transcript/14005	-3.53832076281828	0.151950178122771	-3.31063517824622	0.00228184864508673	0.221426484840011	-1.4425026417066
a_HQ_transcript/60196	3.7307162829675	1.49344705256849	3.31002297154193	0.00228557478158558	0.221426484840011	-1.44819531729638
a_HQ_transcript/122789	1.1445729368357	3.43097094308035	3.30846814698878	0.00229506428950515	0.221975868586282	-1.54901416632655
a_HQ_transcript/48201	3.37147717057686	0.392705067265923	3.30630402588799	0.00230833534396817	0.222888566236183	-1.44191140372194
a_HQ_transcript/149230	2.55499711232806	-0.594709988670314	3.30514172200089	0.00231549325767138	0.223208942092739	-1.45502914880823
a_HQ_transcript/117907	-4.4413335862292	0.774895026082006	-3.30333094139887	0.00232668711827044	0.223570546630886	-1.45035742184405
a_HQ_transcript/99578	-5.55849188479564	1.03582942441181	-3.30329065115243	0.00232693677249666	0.223570546630886	-1.47033349972944
a_HQ_transcript/4932	4.83597504196512	0.05718276491831	3.30170485410248	0.00233678339861111	0.223785871735873	-1.45431995634322
a_HQ_transcript/76160	3.57168335415698	0.134208428810266	3.30168970559158	0.00233687765179733	0.223785871735873	-1.45172130193541
a_HQ_transcript/13985	-6.31393277213953	0.541711591782797	-3.30047940015448	0.00234441988181988	0.22413887975399	-1.45937859212368
a_HQ_transcript/112035	-4.04858018849388	0.29205492340718	-3.29811848097637	0.0023591995056721	0.224880503801156	-1.46576629211892
a_HQ_transcript/62537	4.98028960182735	-0.0890696367580293	3.29792922912229	0.00236038809884317	0.224880503801156	-1.4604542698474
a_HQ_transcript/141928	-4.598230711733	0.333436338131405	-3.29738915728127	0.00236378316512707	0.224880503801156	-1.49894508643149
a_HQ_transcript/103055	-5.93418155664923	0.218008998449115	-3.29634688202239	0.002370348469029	0.225136627136793	-1.46641742024791
a_HQ_transcript/65457	2.54292587071585	2.67765197919184	3.29469780913778	0.00238077167826608	0.225757742437603	-1.52465628211089
a_HQ_transcript/80062	2.46201466278017	-0.816947819637428	3.29363734735021	0.00238749765293528	0.226026813631632	-1.4743484575502
a_HQ_transcript/129872	4.00074296195585	1.88657354635019	3.29176678103434	0.00239940608328621	0.226691276554079	-1.53817960483328
a_HQ_transcript/150229	-7.43230542942819	0.382481134639897	-3.29131103998	0.00240231603284669	0.226691276554079	-1.48079577918428
a_HQ_transcript/43910	-3.41070103757942	1.87152884960909	-3.29040820868594	0.00240809067471466	0.226856675453142	-1.50144127537538
a_HQ_transcript/868	2.79615397316656	-0.773108731430832	3.28852395597008	0.00242018546871261	0.226856675453142	-1.48477416846866
a_HQ_transcript/45565	-1.17446038664423	2.85057089276291	-3.2884662059685	0.00242055707597487	0.226856675453142	-1.52986873144263
a_HQ_transcript/124728	2.96008678797704	-0.776738743017324	3.28807000198465	0.00242310802572818	0.226856675453142	-1.48625979923201
a_HQ_transcript/149557	2.88481540102102	-0.779194839352485	3.28799637409146	0.00242358236059044	0.226856675453142	-1.48846419294179
a_HQ_transcript/145954	10.5970634858207	7.58845806475111	3.28545189599422	0.00244002943496002	0.228002629180869	-1.93482541820855
a_HQ_transcript/74393	3.67130977149658	0.204572252607067	3.28485384378638	0.00244391061949647	0.228002629180869	-1.49939120649059
a_HQ_transcript/78750	-4.50149072708827	0.689477855901712	-3.28428738490153	0.00244759222076903	0.228002629180869	-1.49654563470049
a_HQ_transcript/79692	-3.65977916956645	-0.579818567022227	-3.28289535518977	0.00245666203934057	0.228481361636462	-1.49892024524294
a_HQ_transcript/148573	4.37668306069518	2.22888309804538	3.28083278711833	0.00247015993343465	0.229369738994712	-1.57529561040733
a_HQ_transcript/108292	-3.56888962982753	-0.042342693037054	-3.27840790955665	0.00248611962905111	0.230403751049126	-1.50671951318012
a_HQ_transcript/145282	-2.10540470784944	3.04277288418757	-3.27793811830958	0.00248922301917925	0.230403751049126	-1.52583703734736
a_HQ_transcript/115995	5.55940318341356	0.423782004498697	3.27636236191523	0.00249965938523822	0.230727516292715	-1.513693079509
a_HQ_transcript/65760	-4.4379373907386	2.35914392265274	-3.27560207602996	0.00250470978221605	0.230727516292715	-1.57774038232584
a_HQ_transcript/88608	-2.39942724622595	-0.601142155600073	-3.27526169935231	0.00250697398532912	0.230727516292715	-1.51605312533094
a_HQ_transcript/134700	-5.67909264873016	0.399478396271799	-3.27501772814984	0.00250859809897117	0.230727516292715	-1.52412376717178
a_HQ_transcript/404	3.0661732088587	-0.746964000510233	3.27303140025531	0.0025218585740178	0.231298199753021	-1.5231741756524
a_HQ_transcript/127112	-4.04371906967863	0.299996032804911	-3.27225435004118	0.00252706428907378	0.231298199753021	-1.51882923166540
a_HQ_transcript/62282	-3.108014971241	0.289510889051061	-3.27211040062468	0.00252802978146501	0.231298199753021	-1.51846054003529
a_HQ_transcript/128752	1.9763866548431	6.6694006296886	3.27170967670328	0.00253071936145956	0.231298199753021	-1.8638361238404
a_HQ_transcript/52685	-2.50186116631913	-0.469953048440234	-3.27040436799138	0.00253949932921536	0.231736290437411	-1.52238218367363
a_HQ_transcript/74818	-3.59058489705927	1.75350184225093	-3.26976368098979	0.00254381946984031	0.231766674205137	-1.52831496022746
a_HQ_transcript/130704	-1.53315789527552	3.75551825930941	-3.26875660897368	0.00255062435045222	0.232022992555691	-1.67376034068771
a_HQ_transcript/127450	-3.506069357285	2.30166945943985	-3.26720934862084	0.00256111327073212	0.232613112814244	-1.54546406694567
a_HQ_transcript/17439	5.67010882102143	0.676229366946339	3.26488066767901	0.00257697726490856	0.233549816184884	-1.54507178101524
a_HQ_transcript/364	-3.6473754855294	3.09627074170538	-3.26448040357691	0.00257971348680513	0.233549816184884	-1.59555649782918
a_HQ_transcript/134068	-3.08113198930591	-0.0419182055518551	-3.26217690987513	0.00259551434857402	0.233549816184884	-1.54462893250072
a_HQ_transcript/105405	4.18026719546921	0.257196868134334	3.26156508894663	0.00259972667641541	0.233549816184884	-1.5423896733263

a_HQ_transcript/12688	4.93274665943715	1.49302288135061	3.2615487529613	0.00259983923764496	0.233549816184884	-1.57462179145148
a_HQ_transcript/117426	-5.9392652594879	1.01152019375696	-3.26050110508141	0.00260706767570373	0.233549816184884	-1.58323516392293
a_HQ_transcript/61854	-4.88903487075319	1.18685074957755	-3.26034652860855	0.00260813582984684	0.233549816184884	-1.56124691803259
a_HQ_transcript/148956	2.99697180615619	-0.697768816311487	3.25988286562895	0.00261134234386394	0.233549816184884	-1.5511344205664
a_HQ_transcript/119143	4.0089835183786	0.0745587347787094	3.25975160307856	0.00261225078932341	0.233549816184884	-1.56179072486072
a_HQ_transcript/28483	-2.62163824369868	-0.651263535842848	-3.25948882972422	0.00261407030640263	0.233549816184884	-1.55244799583171
a_HQ_transcript/119741	2.45434233712422	-0.773156312739671	3.25915694210477	0.00261637012201715	0.233549816184884	-1.55291974165243
a_HQ_transcript/98745	5.47604332474628	0.864401807082216	3.25842192096575	0.00262147034025436	0.233549816184884	-1.54875911203654
a_HQ_transcript/44364	4.82744820419772	0.739655262409535	3.25789712249456	0.0026251176650517	0.233549816184884	-1.55776846379428
a_HQ_transcript/148744	2.68219281577323	-0.727629427237804	3.25752935778539	0.00262767650331878	0.233549816184884	-1.55500375762916
a_HQ_transcript/43359	4.07892894444504	1.08047502645325	3.2554220188961	0.00264238501731927	0.234498559216389	-1.55773060594033
a_HQ_transcript/44082	4.11000694718944	0.700849288515537	3.25352019397059	0.00265572665397595	0.2353232910706	-1.58175422396567
a_HQ_transcript/16437	3.86163457614575	0.0310460918161647	3.25234639460579	0.00266399317390575	0.235613694173785	-1.56341375679875
a_HQ_transcript/27576	4.09579026450684	1.06493024577057	3.25190462733393	0.0026671106999441	0.235613694173785	-1.56528253386092
a_HQ_transcript/97054	-2.74233851079289	0.791442402933982	-3.25022155103776	0.00267902005315268	0.235991777596783	-1.58039370103686
a_HQ_transcript/107979	3.93210808219714	0.2823382477611	3.25015242444017	0.00267951027411706	0.235991777596783	-1.58269495367702
a_HQ_transcript/76316	-2.638582634666	-0.767659222892327	-3.24768886253207	0.0026970370496165	0.236091747184542	-1.57908519509888
a_HQ_transcript/12670	2.27701952084704	4.20614031187183	3.24768397018216	0.00269707196453499	0.236091747184542	-1.73776908576122
a_HQ_transcript/89389	2.65660821993509	2.78601821127637	3.24764172568475	0.00269737346608779	0.236091747184542	-1.60050679955778
a_HQ_transcript/148954	1.70508572613933	8.55605777873445	3.24748264762017	0.00269850910498141	0.236091747184542	-2.09231941124634
a_HQ_transcript/106829	-4.40293658134778	6.30538178863086	-3.2466761683796	0.00270427349565817	0.236091747184542	-1.67301270935902
a_HQ_transcript/112988	1.03041041229275	3.85758663216415	3.24630958038287	0.00270689760831196	0.236091747184542	-1.72667595604858
a_HQ_transcript/54009	-2.68516411739167	1.41946558868645	-3.24600545585008	0.00270907644116586	0.236091747184542	-1.5814720981151
a_HQ_transcript/89652	3.82943975558425	0.983524905479177	3.24502263808172	0.00271612908657238	0.236352023269879	-1.58979888680981
a_HQ_transcript/152914	-3.74881295646989	0.157070934269514	-3.24433160899245	0.00272109837394437	0.236430502661642	-1.59268047165642
a_HQ_transcript/11782	3.48028741760582	2.0529592334603	3.24311655058791	0.00272985710441779	0.2364966882727	-1.59232555133797
a_HQ_transcript/36543	-5.73680307308802	1.58761098373215	-3.2425316933651	0.00273408263691388	0.2364966882727	-1.62041467699301
a_HQ_transcript/117078	-3.45237459587295	1.14585059901852	-3.2420508789474	0.00273756115109949	0.2364966882727	-1.58421879729244
a_HQ_transcript/52540	3.87165536727077	0.903297454699314	3.24197171052333	0.0027381343106167	0.2364966882727	-1.5845961981438
a_HQ_transcript/142727	-4.45714816282938	0.0842244391532996	-3.24109491987817	0.0027444897262204	0.236693915142046	-1.60330271074816
a_HQ_transcript/785	-6.15295818984754	1.80738442471296	-3.23875093494508	0.00276154937412126	0.237390234495383	-1.65144062688312
a_HQ_transcript/33246	-2.94651302664948	0.677585357042339	-3.23833818267893	0.00276456387857571	0.237390234495383	-1.60948031586164
a_HQ_transcript/111178	-7.65358736591723	0.446817399406371	-3.23800906464764	0.00276696981927033	0.237390234495383	-1.60468064587891
a_HQ_transcript/119847	3.27691598065893	1.38077402899344	3.23774531979418	0.00276889930821411	0.237390234495383	-1.59808344685554
a_HQ_transcript/135561	5.63499783046379	0.863202774569922	3.23608728004465	0.00278105856768864	0.237891413779824	-1.5987143128105
a_HQ_transcript/20068	2.52099509989629	-0.679623651024172	3.23583269137114	0.00278293010890242	0.237891413779824	-1.60996443968198
a_HQ_transcript/21492	4.09679797837751	0.78176797585637	3.23364087298804	0.00279909252096407	0.238044433797215	-1.60758297749764
a_HQ_transcript/147669	-1.06776122382245	3.41502198644025	-3.2335440301156	0.00279980870204671	0.238044433797215	-1.69818180342245
a_HQ_transcript/95859	-3.79639770493884	0.331497685763707	-3.23227021391146	0.00280924524847069	0.238044433797215	-1.61055873413589
a_HQ_transcript/34998	2.65808708418197	3.99063501632861	3.23224429522592	0.00280943757142888	0.238044433797215	-1.73277443395529
a_HQ_transcript/25035	-5.21424114276369	1.61957698650174	-3.23202194997874	0.00281108794404564	0.238044433797215	-1.67584958875027
a_HQ_transcript/23328	-3.42142503715736	0.132345250149798	-3.23154158144508	0.0028146566737214	0.238044433797215	-1.61097120490415
a_HQ_transcript/111757	4.02684323237864	0.827612673298925	3.23140541664653	0.00281566904921573	0.238044433797215	-1.60790231732105
a_HQ_transcript/84922	1.20797217942229	5.3443511505529	3.23116174477875	0.00281748160013219	0.238044433797215	-1.87840912666578
a_HQ_transcript/122760	1.00221018950844	4.21994917811763	3.22969649446281	0.00282840437033334	0.238620448822549	-1.8109476277372
a_HQ_transcript/138718	-6.68719679505281	1.85306254424795	-3.22854635885915	0.00283700642212507	0.23899928884824	-1.64022930175156
a_HQ_transcript/66242	2.76532621046419	3.57604687805596	3.22778842925469	0.00284268875166713	0.239131420777	-1.6655066885551
a_HQ_transcript/128712	7.00404671769864	4.85867424823625	3.22711468043329	0.00284774908653735	0.239210923269137	-1.89873316438979
a_HQ_transcript/35282	3.0415984417592	-0.753309118222675	3.22578152648656	0.00285778738535009	0.239707741898456	-1.63059085904923
a_HQ_transcript/132228	4.78716447668906	0.197734663073961	3.22160405772202	0.00288946189701367	0.241501141507674	-1.64928453636983

a_HQ_transcript/143192	-3.86747055755299	0.139360774866297	-3.22083014286337	0.00289536657439248	0.241501141507674	-1.6527634380811
a_HQ_transcript/52592	-5.47238634692892	0.70150852556652	-3.22050891513106	0.00289782080175396	0.241501141507674	-1.64056278404992
a_HQ_transcript/133795	4.91796155792651	0.462785373230853	3.22031129429066	0.00289933164075036	0.241501141507674	-1.64507321616895
a_HQ_transcript/63536	2.29849310446998	3.245282097087	3.22023155746085	0.00289994145286878	0.241501141507674	-1.66970860915108
a_HQ_transcript/59181	5.70446871635901	2.20477747731532	3.21849251117979	0.00291327184375823	0.242264185599397	-1.67693807084381
a_HQ_transcript/26913	-2.14317513230696	2.13364537378911	-3.21752553551636	0.00292070933018955	0.242488922066785	-1.64912332371265
a_HQ_transcript/130747	-4.98207503540784	2.09231014507276	-3.21512856918397	0.00293922380457897	0.242488922066785	-1.73306236412352
a_HQ_transcript/145014	-6.37805298626652	1.80718996513434	-3.21438246798068	0.00294500960942545	0.242488922066785	-1.70523691207019
a_HQ_transcript/56657	-1.59394705454192	4.60827831208551	-3.21422187170524	0.00294625640922925	0.242488922066785	-1.85531400742341
a_HQ_transcript/108402	-2.86122989003963	2.57216513940914	-3.21386223147149	0.00294905032748517	0.242488922066785	-1.73277291362287
a_HQ_transcript/102871	-4.54927905289433	0.624364834038615	-3.2135395365202	0.00295155938299791	0.242488922066785	-1.65739887693777
a_HQ_transcript/16214	-3.9458411805607	0.754207926979024	-3.21352689940211	0.00295165768203792	0.242488922066785	-1.69787771127207
a_HQ_transcript/50700	6.49063304424473	2.29903416046067	3.21280583072267	0.00295727176235992	0.242488922066785	-1.68559547039748
a_HQ_transcript/63275	6.06096581840962	1.2588793620646	3.21202581320192	0.00296335627892884	0.242488922066785	-1.74303608353809
a_HQ_transcript/43095	-3.06201544443274	-0.764308702507113	-3.21201150745827	0.00296346798218195	0.242488922066785	-1.65859961385953
a_HQ_transcript/28124	-4.9111214160726	0.296688827762339	-3.21122501769994	0.00296961530123793	0.242488922066785	-1.65746279804978
a_HQ_transcript/44064	4.77089851267532	0.691155060532953	3.21102925886071	0.00297114727127061	0.242488922066785	-1.65275293095514
a_HQ_transcript/101808	-3.08592501398459	3.3334838902275	-3.21087134143423	0.00297238365143197	0.242488922066785	-1.75127027128944
a_HQ_transcript/53424	-2.90971457601791	1.15830198286288	-3.21051890923469	0.00297514471348099	0.242488922066785	-1.65605679651236
a_HQ_transcript/78256	-5.89334657449284	2.81204794614674	-3.21008481584098	0.00297854889821918	0.242488922066785	-1.79514201870815
a_HQ_transcript/130338	-2.51808747696491	-0.550121236179938	-3.20943729940344	0.00298363366635022	0.242563157702945	-1.66017062542958
a_HQ_transcript/1516	3.72775640444946	2.45863296268301	3.20811981329505	0.00299400510466799	0.242835933003179	-1.68100211416603
a_HQ_transcript/56592	-3.05329834242508	1.28767985586707	-3.20795003329705	0.00299534413644507	0.242835933003179	-1.66460574344658
a_HQ_transcript/115274	-4.10438672200832	3.48003330174103	-3.20458748495745	0.00302198211667179	0.244654284788159	-1.76324026253518
a_HQ_transcript/110935	-1.47086057188478	3.04322715720587	-3.20334592178811	0.00303187474394742	0.245113790147671	-1.76848652323845
a_HQ_transcript/1079	4.45091546924687	1.99202170063483	3.2020276595979	0.00304241231174756	0.245624087301753	-1.77261260397117
a_HQ_transcript/131410	-2.57264726324196	2.9244264386331	-3.19826014152017	0.00307272101855158	0.247726945029634	-1.72965542524127
a_HQ_transcript/143224	-3.53357006146367	0.0438791854841852	-3.19750613418694	0.00307882129897735	0.247874964635672	-1.69023292984768
a_HQ_transcript/95223	10.0626443954122	6.04498388058583	3.19639699498373	0.00308781576229604	0.248255262283187	-1.90435837704184
a_HQ_transcript/3376	-2.14819146661501	-0.758504783069723	-3.19510734554333	0.00309830556612953	0.248754566226488	-1.68966320361578
a_HQ_transcript/130319	-3.95063810601252	1.86737185602378	-3.19100009355644	0.00313194022470913	0.250770213450399	-1.71057557532193
a_HQ_transcript/87146	3.18819193063296	-0.181742534385022	3.19083055903955	0.00313333601319367	0.250770213450399	-1.69780584560715
a_HQ_transcript/38109	-2.42661600312801	-0.65703482547267	-3.18996349223458	0.00314048390796136	0.250770213450399	-1.71057817239591
a_HQ_transcript/149456	-1.06416109306925	4.1761541170187	-3.18994125713894	0.00314066741315528	0.250770213450399	-1.86176109492272
a_HQ_transcript/97634	3.19224805234914	0.811690148392656	3.18833679304369	0.00315393599761732	0.251484213538408	-1.70416982450307
a_HQ_transcript/148659	-4.02400143799496	-0.581082453410463	-3.18403861688235	0.00318974487049116	0.25399108196152	-1.74273214220286
a_HQ_transcript/121392	-3.48265763876716	4.35399889487758	-3.18271207007774	0.00320087460521459	0.254135309200779	-1.93335537760459
a_HQ_transcript/88022	-3.32298091700831	0.860555003205861	-3.18268422614239	0.0032011086121305	0.254135309200779	-1.71914509741327
a_HQ_transcript/87571	-3.64128895478829	0.595525130990341	-3.18224796718679	0.00320477716382763	0.254135309200779	-1.728741641933
a_HQ_transcript/106148	-3.94540234037437	0.240956377144189	-3.18174114449072	0.00320904412595259	0.254135309200779	-1.72885430952267
a_HQ_transcript/38079	2.12311191910778	2.3528179076074	3.18107066809211	0.00321469721590242	0.25423662553194	-1.73683370125655
a_HQ_transcript/48061	4.10062181497302	1.49481999694248	3.17885013181582	0.00323348740806273	0.255100482219633	-1.73608470777734
a_HQ_transcript/141110	3.8722939948476	-0.825002274992967	3.17836092625012	0.00323764111550237	0.255100482219633	-1.73982763815396
a_HQ_transcript/112543	2.95056835137814	-0.744493232091076	3.17712799538692	0.00324813215436627	0.255100482219633	-1.73879198513346
a_HQ_transcript/113281	-3.18358286443166	1.12383681413056	-3.17681251085098	0.00325082182050071	0.255100482219633	-1.72793518434414
a_HQ_transcript/138548	-2.78474918629194	-0.783605461115532	-3.17564748256663	0.00326077268036353	0.255100482219633	-1.74082837416967
a_HQ_transcript/139093	-7.17594443668276	1.17473672390671	-3.1754223818041	0.00326269867699539	0.255100482219633	-1.73079780282579
a_HQ_transcript/84948	-3.16220635182124	1.21233368766258	-3.17472938742191	0.00326863485076473	0.255100482219633	-1.7419319182729
a_HQ_transcript/107447	-3.23110501882606	0.0274407726346091	-3.17372599704288	0.00327724810922463	0.255100482219633	-1.74557866717486
a_HQ_transcript/130602	-2.99829010913344	-0.5564320027074	-3.17359706243076	0.00327835647059833	0.255100482219633	-1.74479258571991

a_HQ_transcript/150406	-8.68138180449703	4.711927238303	-3.17337671741095	0.00328025145055574	0.255100482219633	-1.90291828246796
a_HQ_transcript/52254	-5.35134055997233	1.51316812305583	-3.17274557793567	0.0032856850640622	0.255100482219633	-1.81980873231168
a_HQ_transcript/16947	2.66345288339303	-0.684979810829969	3.17248084940973	0.00328796671987673	0.255100482219633	-1.74917119741452
a_HQ_transcript/65168	3.18616869826533	-0.517632249459021	3.17229877924885	0.00328953683156389	0.255100482219633	-1.74764809703723
a_HQ_transcript/123472	5.12492917298345	0.257331419978486	3.17165190793714	0.00329512101106299	0.255100482219633	-1.75521411399264
a_HQ_transcript/59005	2.24733649690509	2.16644824116437	3.17159334888224	0.00329562697333937	0.255100482219633	-1.7441449271706
a_HQ_transcript/111728	-4.19831128908891	0.609617049298682	-3.17156894576304	0.00329583784315553	0.255100482219633	-1.74994454147506
a_HQ_transcript/143562	-3.7990864929654	1.19059010165034	-3.17064235124422	0.00330385415681604	0.255380896844951	-1.74329742100813
a_HQ_transcript/43109	3.49114267924941	-0.76427473671085	3.16974233344365	0.00331165832609148	0.255644190144815	-1.75857837311357
a_HQ_transcript/85815	-3.63601708328155	-0.0257647199527481	-3.16808189316789	0.00332610232292673	0.256418668205683	-1.76406705247348
a_HQ_transcript/116627	3.6503174498181	0.219453825855275	3.16700985775106	0.00333545970522398	0.25647687272695	-1.75287069657804
a_HQ_transcript/50773	-3.37982412452444	0.600558110481188	-3.16697302216499	0.0033357816739795	0.25647687272695	-1.75138710060224
a_HQ_transcript/67694	-2.32818912647612	3.30476661850616	-3.16647996966311	0.00334009414833301	0.25647687272695	-1.87413708241665
a_HQ_transcript/140995	2.57853003152991	-0.769265294877837	3.16529821232842	0.00335045199349038	0.25693281461426	-1.76534762914784
a_HQ_transcript/152385	-1.99816299506972	8.28490852979101	-3.16417037897993	0.00336036573183766	0.257353543162398	-2.28779656157401
a_HQ_transcript/36388	-3.41906364176804	2.02453862789728	-3.16344695598614	0.00336673937372233	0.257502403047015	-1.82200393584516
a_HQ_transcript/99741	3.98856991507247	0.32743726509201	3.1621744117194	0.00337797893846283	0.257881363552345	-1.76635864045442
a_HQ_transcript/82042	4.37492619635217	0.948327357452205	3.16188196153288	0.00338056700775679	0.257881363552345	-1.80270608354159
a_HQ_transcript/67900	-3.46724032411722	1.49227304563357	-3.15956661695478	0.00340112365375607	0.258819560602026	-1.76603110772706
a_HQ_transcript/45664	4.58467473509633	0.844679359160174	3.15907919518553	0.00340546633856354	0.258819560602026	-1.76702882011135
a_HQ_transcript/119615	3.85050331572935	0.101590718869024	3.15816957566938	0.00341358471875601	0.258819560602026	-1.76903697378479
a_HQ_transcript/42862	3.71350833239468	1.29873530674588	3.15801959895549	0.00341492503359727	0.258819560602026	-1.77018839666208
a_HQ_transcript/143277	-3.42931963778511	0.137368579616224	-3.15799681060456	0.00341512873282675	0.258819560602026	-1.77072170288014
a_HQ_transcript/147286	10.286206460211	6.72942489380438	3.15698288643439	0.00342420365877153	0.25916941442327	-2.23177367334766
a_HQ_transcript/133645	4.3293903791452	0.304624606805914	3.15621993552121	0.00343104743197524	0.259349707575886	-1.77525817210888
a_HQ_transcript/96010	-2.83734926040238	-0.668419384931801	-3.15476995657351	0.00344408984818212	0.25979268517564	-1.78845610820187
a_HQ_transcript/147395	2.56347154552371	-0.798032775120454	3.15423128862401	0.00344894712623737	0.25979268517564	-1.79016434374676
a_HQ_transcript/143124	3.13013666281547	-0.371094221103905	3.15407964167328	0.00345031573347773	0.25979268517564	-1.81117391137634
a_HQ_transcript/117684	-2.66853174261615	-0.646443469778656	-3.15314419822842	0.00345876950826591	0.260092307860907	-1.7793925463204
a_HQ_transcript/132951	3.92615411550895	3.83636338046881	3.15196586889701	0.00346944635115439	0.26055811046499	-1.89672617568096
a_HQ_transcript/19582	-5.83796222360306	0.842814072287399	-3.15115569021003	0.00347680557398459	0.26065766394266	-1.80262336402207
a_HQ_transcript/9677	3.62635855739152	0.539537039389047	3.15078602160832	0.00348016837699612	0.26065766394266	-1.79194903051575
a_HQ_transcript/44190	3.30254361536167	1.21101629167013	3.1500892480051	0.00348651520910227	0.26065766394266	-1.78803179471221
a_HQ_transcript/15265	5.29039804338788	2.43968406366117	3.14984871482359	0.00348870875563222	0.26065766394266	-1.8917517639137
a_HQ_transcript/23292	2.26499541371454	-0.493739043040551	3.14761042974103	0.00350918391488904	0.261513746086967	-1.8015692174906
a_HQ_transcript/116659	3.92080675928132	-0.822837088852058	3.14750152601846	0.00351018304539021	0.261513746086967	-1.80938751089401
a_HQ_transcript/131090	3.97014589479471	1.41721874597731	3.14700971392872	0.0035146985181555	0.261513746086967	-1.79267076215497
a_HQ_transcript/150274	-3.98113380083503	0.290382263026716	-3.14663283524812	0.00351816249380691	0.261513746086967	-1.81470068273328
a_HQ_transcript/90724	-2.41356942142767	3.15180918088798	-3.14606066160822	0.00352342768005315	0.261554763886455	-1.91939200476705
a_HQ_transcript/152395	-9.23874432401869	0.93266260430085	-3.14554590901957	0.00352817088027253	0.261554763886455	-1.83092678426096
a_HQ_transcript/129383	-2.57280781566363	-0.589832203254061	-3.14510773710006	0.00353221321309639	0.261554763886455	-1.80872180679169
a_HQ_transcript/152826	-6.27233761821541	0.737495331052499	-3.14457755191458	0.00353711030247744	0.261584157331309	-1.81945722766206
a_HQ_transcript/83405	-3.44695288014793	1.85057502715787	-3.1431557024027	0.00355027522214102	0.262224139914375	-1.80889016373744
a_HQ_transcript/86264	-3.81395135658094	0.104670068846586	-3.14249018730415	0.0035564532399991	0.262347098901863	-1.91074044028518
a_HQ_transcript/136075	-5.36941291914277	1.32834327497863	-3.14167226074734	0.00356406011483671	0.262575014391924	-1.84153380677059
a_HQ_transcript/18083	4.46445795950205	-0.123484844773696	3.14051850876124	0.00357481655469558	0.262682327523026	-1.82317674225567
a_HQ_transcript/112040	-4.66788243563425	5.61818207381944	-3.14009981712658	0.00357872765300481	0.262682327523026	-2.1305261676392
a_HQ_transcript/97927	3.12728479292437	1.27537728386044	3.13967801176675	0.00358267195374645	0.262682327523026	-1.84235162347108
a_HQ_transcript/138992	4.92209990342624	0.776051536601994	3.13881274447052	0.00359077601350995	0.262682327523026	-1.85508668228992
a_HQ_transcript/78843	-2.22919902282345	2.20212480121043	-3.1387308476382	0.00359154395884519	0.262682327523026	-1.81585124995898

a_HQ_transcript/87918	5.37348883793857	1.72912693078601	3.13861495764418	0.00359263092452528	0.262682327523026	-1.90845100121
a_HQ_transcript/24432	5.47921841782926	0.298704299574108	3.13684307118724	0.00360928897979283	0.263568781177635	-1.82367117078304
a_HQ_transcript/4855	2.42465679664201	2.29303248148948	3.13583885317583	0.00361876254000992	0.263655182875511	-1.83673615151034
a_HQ_transcript/146571	-3.61022482858042	0.412952069073622	-3.13536119774976	0.0036232769272383	0.263655182875511	-1.82682495858171
a_HQ_transcript/110355	4.16501946411586	0.665867321541563	3.13527634265384	0.00362407946458735	0.263655182875511	-1.82118986103378
a_HQ_transcript/136613	3.24322500218589	0.755624670129229	3.13400191941215	0.00363615298480585	0.264037130659519	-1.82114867865096
a_HQ_transcript/17252	-3.20618296752838	-0.475435302430722	-3.1337636820101	0.00363841421790315	0.264037130659519	-1.82317628160117
a_HQ_transcript/134690	-2.6705911549166	1.48714486476901	-3.13315751948523	0.00364417364902002	0.26412534397785	-1.82593188013992
a_HQ_transcript/98167	-2.78638704401139	-0.259237443116802	-3.13218879375936	0.00365339596685819	0.264464010911	-1.83201393390747
a_HQ_transcript/129124	-2.69010858839152	-0.561819950136774	-3.13049512916678	0.00366957309143481	0.265304657535973	-1.84347656693848
a_HQ_transcript/117826	6.5472939448158	0.132942237446205	3.12928115011577	0.00368121036393598	0.265815398801082	-1.84323270120817
a_HQ_transcript/149628	3.11306821000481	-0.738520425868969	3.1274284192537	0.00369903842216361	0.266771346654499	-1.8524399647183
a_HQ_transcript/13375	-2.71729176020518	-0.755339991945063	-3.12645239387573	0.00370846329556263	0.267119646151753	-1.85262765490411
a_HQ_transcript/25007	-4.16216574830994	2.80947254742153	-3.12471596575339	0.003725287379994	0.267999387158776	-1.90581522233832
a_HQ_transcript/73511	-2.86295983446688	-0.297244553124765	-3.12390593665226	0.00373316047235886	0.268233809564	-1.84936253173704
a_HQ_transcript/136755	-2.27553516583256	1.56985253724765	-3.1233697277871	0.00373838086314263	0.268277287423154	-1.84454199971501
a_HQ_transcript/113538	-5.45970053723778	0.66054716329608	-3.11983097477132	0.00377300767786781	0.270348793277681	-1.90114807309698
a_HQ_transcript/55643	3.71214710852216	0.0571160620531449	3.11947083972781	0.00377654865368629	0.270348793277681	-1.89038033105826
a_HQ_transcript/85803	-4.64259593487388	0.300279750382835	-3.11874178843789	0.00378372661756781	0.270529472110679	-1.87658932953778
a_HQ_transcript/136353	-3.81322679220898	-0.245686538218871	-3.11805533100291	0.00379049707035774	0.270680606518126	-1.86669346993792
a_HQ_transcript/48471	-4.40410329966258	1.06948726585597	-3.11697399759087	0.0038011855013652	0.270977436228806	-1.86730787828595
a_HQ_transcript/102124	1.07558234039536	3.99068264513943	3.11669203915785	0.0038039772220394	0.270977436228806	-2.07011817929599
a_HQ_transcript/129439	-3.27402951460381	-0.310714876461813	-3.11599096435909	0.00381092713737648	0.271140235791212	-1.87435604360217
a_HQ_transcript/91837	-3.33771508738235	0.372159655634834	-3.11427566658447	0.00382798219540376	0.27202072011544	-1.86463486676346
a_HQ_transcript/127245	-4.23575974402952	0.431905619334908	-3.11338622276743	0.003836854391751	0.272318280932481	-1.88034234766735
a_HQ_transcript/5052	1.47319914729001	2.6048941624431	3.11240167345804	0.00384669805065167	0.272683980839366	-1.88570005217067
a_HQ_transcript/76988	-3.66512229219639	-0.57682612645423	-3.11111729727364	0.00385957549620237	0.272832513486002	-1.88803815002578
a_HQ_transcript/138170	-5.08318703412253	3.68367330422776	-3.11045612622189	0.00386622050675256	0.272832513486002	-2.01051977690541
a_HQ_transcript/12947	-5.52697841231628	0.310309473601198	-3.11045541764225	0.00386622763406446	0.272832513486002	-1.88434975895917
a_HQ_transcript/97067	-2.80755232527205	-0.423409576565664	-3.1102933987592	0.00386785764336689	0.272832513486002	-1.87555207606057
a_HQ_transcript/73253	-5.83326436706199	1.92236191270791	-3.10985597830684	0.00387226162307239	0.272832513486002	-1.95112923832291
a_HQ_transcript/114596	-2.68371058834774	-0.683118022354977	-3.108437153661	0.00388657926159911	0.273509781256941	-1.87899207588682
a_HQ_transcript/11374	-2.76273261553696	-0.280724874191789	-3.10736297283444	0.00389745247 06186	0.273815793450155	-1.88557667164759
a_HQ_transcript/46196	3.36081519372925	0.950784109022629	3.10707731698167	0.00390034883320824	0.273815793450155	-1.88641290086925
a_HQ_transcript/118954	-5.75778807595991	0.875719589952646	-3.10505434201142	0.00392091906281228	0.274804240057411	-1.88769100614609
a_HQ_transcript/29244	5.94302491675738	1.36581677164864	3.10476360425899	0.00392388382961139	0.274804240057411	-1.94523046820718
a_HQ_transcript/82825	2.9940174254413	-0.739917451590094	3.1040100941442	0.00393157758217244	0.274984727238271	-1.90412176703863
a_HQ_transcript/138749	-2.75319195263103	1.20676072626497	-3.10358519740028	0.00393592232766036	0.274984727238271	-1.88921611527731
a_HQ_transcript/77199	-4.82673954421472	5.40257874885834	-3.102190331855	0.00395021748943693	0.275326553714942	-2.23137240984874
a_HQ_transcript/9198	-1.10967795848207	3.67514698907487	-3.10153113847745	0.00395699032359606	0.275326553714942	-2.05903425367125
a_HQ_transcript/15535	-3.40403700295392	0.552007146021166	-3.10120522479873	0.00396034297295929	0.275326553714942	-1.94540448860572
a_HQ_transcript/108725	4.64884192863821	0.460386400027293	3.10108372106943	0.00396159356288697	0.275326553714942	-1.91883903910954
a_HQ_transcript/69468	-6.73828848054809	0.56191049921305	-3.10080169863677	0.00396449775425624	0.275326553714942	-1.90337366149031
a_HQ_transcript/66764	-2.75510953081501	-0.738160226703164	-3.10006917195373	0.00397205056735261	0.275521903793642	-1.9089053260157
a_HQ_transcript/149411	-1.60958831910088	6.63209692462184	-3.09863599340735	0.00398686708408213	0.276065264614575	-2.2669641801258
a_HQ_transcript/75296	5.4820919830182	0.0397218295723804	3.09839318964102	0.0039893824366268	0.276065264614575	-1.9112167755746
a_HQ_transcript/111610	2.98314305095941	-0.450797280017876	3.09627520571518	0.00401138792522177	0.276074883316196	-1.93355025154774
a_HQ_transcript/143301	-3.1561876253788	-0.595085311450375	-3.09618375585408	0.00401234066457351	0.276074883316196	-1.92091936102532
a_HQ_transcript/43934	-2.65610146938423	-0.246656585781914	-3.09569958807202	0.0040173883835662	0.276074883316196	-1.92866869250659
a_HQ_transcript/47799	-3.17587884944282	1.26427451111994	-3.09543544980738	0.0040201447136978	0.276074883316196	-1.90392863419211

a_HQ_transcript/1498	3.82121584360231	1.183407233432	3.09474575890654	0.00402735022985789	0.276074883316196	-1.93482174369429
a_HQ_transcript/136168	4.88849776002618	2.1663022807837	3.09441677514088	0.00403079159174671	0.276074883316196	-2.01796354925073
a_HQ_transcript/9338	-2.21351073875551	1.93138858730496	-3.09440561296602	0.00403090840355937	0.276074883316196	-1.93598682679675
a_HQ_transcript/126701	3.43726027158379	1.23807356792577	3.09423678100215	0.00403267561682529	0.276074883316196	-1.91743217945996
a_HQ_transcript/512	3.54097731300432	0.839333299274555	3.09343309792523	0.0040410980914732	0.276074883316196	-1.90917043001213
a_HQ_transcript/93745	3.36577236348594	-0.151646885264961	3.09341118218872	0.00404132799877109	0.276074883316196	-1.9091346450489
a_HQ_transcript/148211	3.18334310918851	0.369271619304699	3.09321053948347	0.00404343342085593	0.276074883316196	-1.93074361316727
a_HQ_transcript/44113	-3.6132848764781	1.5554428809958	-3.09226900713967	0.00405332720340177	0.276074883316196	-1.91064842950723
a_HQ_transcript/117975	3.57405965380863	-0.151570975886364	3.09193805072372	0.00405681040602344	0.276074883316196	-1.91605843758663
a_HQ_transcript/115727	-6.60829114841717	3.31305442469567	-3.09164233642115	0.00405992510126071	0.276074883316196	-2.02492671037198
a_HQ_transcript/70934	4.0131898611835	-0.818757598656869	3.09156283219034	0.00406076288940524	0.276074883316196	-1.93464520295519
a_HQ_transcript/146268	5.10338823593062	0.0914783564368897	3.09003505480709	0.00407689399516857	0.27660517045201	-1.9301721304395
a_HQ_transcript/46118	-5.25884423088963	1.17971122146005	-3.08987100873834	0.00407862969493284	0.27660517045201	-1.92372215194981
a_HQ_transcript/145110	3.79908523406871	0.753363834766854	3.08947349738812	0.0040828384986207	0.27660517045201	-1.94044622804635
a_HQ_transcript/28360	-2.9762458709777	-0.755150778268922	-3.08761202348252	0.00410260248185861	0.277276524949771	-1.93674843380418
a_HQ_transcript/57004	2.9811892664627	0.742478898862105	3.08743047320994	0.00410453492370288	0.277276524949771	-1.93188821511887
a_HQ_transcript/55391	5.81981388966057	1.10956001273927	3.08719352251446	0.00410705835366351	0.277276524949771	-1.9577062630849
a_HQ_transcript/4458	2.6574411215964	2.57332725442811	3.08619456588107	0.00411771304041235	0.277673345258804	-1.98788310751962
a_HQ_transcript/54892	-3.54296224442429	-0.343187693612142	-3.08407272417317	0.00414043131651454	0.278477491561635	-1.94882945103808
a_HQ_transcript/90538	4.07823524247592	1.3547309947947	3.08377671924155	0.00414361004070466	0.278477491561635	-1.94089588700886
a_HQ_transcript/60903	-5.6161195049336	1.95866094136533	-3.08373946293316	0.00414401029109576	0.278477491561635	-2.00619296655932
a_HQ_transcript/106005	-4.21502564503743	2.42941690598602	-3.08293458582758	0.00415266617641904	0.278736927832432	-2.02263518122342
a_HQ_transcript/62548	4.70669276030798	0.782132978150693	3.08083469416103	0.0041753298759192	0.279406607779161	-1.98343566622322
a_HQ_transcript/136088	3.15376330819457	1.19739143782485	3.08018345516773	0.00418238237566	0.279406607779161	-1.94244505081701
a_HQ_transcript/8791	-4.52811177771523	1.12078614088619	-3.07977497909312	0.00418681167569033	0.279406607779161	-1.93977573182096
a_HQ_transcript/58642	-3.52968034776937	0.57584648305023	-3.07921866400412	0.00419285122343153	0.279406607779161	-1.94798935556232
a_HQ_transcript/68310	2.24575682535502	-0.322396723306934	3.07886203441468	0.00419672726389776	0.279406607779161	-1.94185074753113
a_HQ_transcript/17576	-3.13996212276949	0.588507925175651	-3.0787434486451	0.00419801687026291	0.279406607779161	-1.94088906467615
a_HQ_transcript/80044	-2.56415129687704	-0.767928620826765	-3.07872116473726	0.00419825924711423	0.279406607779161	-1.95357596358259
a_HQ_transcript/53376	4.73979000977099	1.24460157325091	3.07821527273742	0.00420376528952273	0.279406607779161	-2.08331114316967
a_HQ_transcript/30175	-3.59683909471418	0.0347952492959307	-3.07801894808321	0.00420590389840982	0.279406607779161	-1.98264682461859
a_HQ_transcript/39827	4.10176185994711	-0.815790878479751	3.07755628845179	0.00421094783783456	0.279422346937954	-1.96589522174395
a_HQ_transcript/110402	-3.26391442467531	-0.694520319537776	-3.0757946324509	0.00423020608058228	0.280380181359278	-1.96335501105246
a_HQ_transcript/129702	-4.33404985039045	4.06768001591179	-3.07452645148722	0.00424412139790166	0.280982105486592	-2.0965062888686
a_HQ_transcript/74532	3.11002735289904	0.650336493168585	3.07399431626315	0.00424997325968606	0.281049426210502	-1.96282340576394
a_HQ_transcript/82917	3.37555230095173	-0.726245162972704	3.07247152699676	0.00426676160866894	0.28148187258264	-1.97562992391537
a_HQ_transcript/11721	-4.66963508591567	1.16677286780246	-3.07141397013157	0.00427845788557764	0.28148187258264	-1.96096454132089
a_HQ_transcript/17220	-1.17330128782513	2.92841637256374	-3.07135714471937	0.0042790872185559	0.28148187258264	-2.02375475727412
a_HQ_transcript/65866	5.20706462232522	0.192397405266128	3.07103122132974	0.00428269846785333	0.28148187258264	-2.00357780738442
a_HQ_transcript/95314	2.3233300202718	2.38947472843058	3.07087520350744	0.00428442817686869	0.28148187258264	-1.97607008089049
a_HQ_transcript/139267	-1.9827888431329	1.55244648790259	-3.07077248660169	0.00428556732100944	0.28148187258264	-1.9565992995824
a_HQ_transcript/10554	6.33848768434617	1.76816328903359	3.07017526991336	0.00429219622609179	0.281599077009327	-1.99145993754505
a_HQ_transcript/3730	3.70281991247972	-0.249204436374758	3.06920761395005	0.00430295753068644	0.281986826768592	-1.9836069192551
a_HQ_transcript/15884	4.51066461648899	0.110399530723596	3.06723277675935	0.00432499914872559	0.283112106438199	-1.99339630725378
a_HQ_transcript/9070	4.51855543815918	1.35244051928223	3.06659692570329	0.00433211878809442	0.283259168632568	-1.97591126775514
a_HQ_transcript/70663	2.35866403775149	-0.353155425650744	3.06602839390735	0.00433849406191855	0.283357284080002	-1.97410085472313
a_HQ_transcript/94849	2.98982706625627	0.849246790775635	3.0628919755637	0.00437382473983708	0.28524536756486	-1.98649226827445
a_HQ_transcript/141130	-3.45586559863792	-0.697277904255367	-3.06259210167059	0.00437721696717339	0.28524536756486	-1.9932944418732
a_HQ_transcript/118121	-3.09567693067905	-0.592852251180869	-3.06205083367535	0.00438334620327088	0.285324913889955	-1.98560222226174
a_HQ_transcript/99697	3.04108286428294	2.57335201721565	3.06089714278246	0.00439643758825185	0.285856962113986	-2.0881138212886

a_HQ_transcript/103918	-1.00288436377638	-0.824998489467312	-3.06034632149784	0.00440270103960073	0.285944364279231	-2.01453175218273
a_HQ_transcript/1747	-3.41719824993758	-0.681108089000797	-3.05956850513992	0.00441156008579529	0.286199960565969	-2.00398547923809
a_HQ_transcript/56832	-4.92595784490401	1.62806150966244	-3.05841490407496	0.00442473030570586	0.286734362553032	-1.98869084544342
a_HQ_transcript/99581	-3.45909989937598	0.576424236207968	-3.05693117004095	0.00444172432694221	0.287319610323755	-2.05941224166865
a_HQ_transcript/114529	2.24281505593331	-0.76417512809254	3.05676361671719	0.00444364729013652	0.287319610323755	-1.99077467894117
a_HQ_transcript/31735	3.92501232431272	2.21261940476414	3.05551590212545	0.0044579918019666	0.287926830516349	-2.00704662020026
a_HQ_transcript/110966	-3.19904004692666	-0.394331397727207	-3.0548760627137	0.00446536481119038	0.288082936453801	-2.00847236138645
a_HQ_transcript/82997	-3.77700454171898	1.19128614018504	-3.05383448811105	0.00447739182751646	0.288420526314208	-2.01608140271644
a_HQ_transcript/9102	2.57777800497755	-0.757528161896897	3.05307214147089	0.0044862140616693	0.288420526314208	-2.01057064748664
a_HQ_transcript/13620	4.2614246140543	0.0725581244498658	3.05273245871152	0.00449015033365761	0.288420526314208	-2.03258533622047
a_HQ_transcript/1197	2.77930687373204	0.870552852608976	3.05239689028936	0.00449404214195088	0.288420526314208	-1.99588117298148
a_HQ_transcript/129334	-4.09548925866478	0.872875552882875	-3.05227930309583	0.00449540663433582	0.288420526314208	-2.00023981557011
a_HQ_transcript/81201	5.71911103341624	1.43520138656057	3.05068129169827	0.00451398906724249	0.28929344707902	-2.00897393816251
a_HQ_transcript/35954	3.97220622358882	1.64802628861551	3.04913557979132	0.00453203257068	0.289855649368379	-2.04049354157502
a_HQ_transcript/120286	-5.30073889577614	2.28019836918494	-3.04907557476996	0.00453273440124994	0.289855649368379	-2.10311707100153
a_HQ_transcript/27550	-2.34178273866375	5.20321347563069	-3.04843056146231	0.00454028510997057	0.290019442716889	-2.33098993598116
a_HQ_transcript/58382	3.00978288369953	1.17122313074093	3.04639394997103	0.00456420455708866	0.291227313385784	-2.0083952264985
a_HQ_transcript/113878	-4.71083366646518	2.23300532374605	-3.04344951175372	0.00459899733407727	0.292893577432759	-2.0910226516594
a_HQ_transcript/97919	-1.57577929685068	2.42249343273993	-3.04333156194709	0.00460039630119923	0.292893577432759	-2.05948368203085
a_HQ_transcript/13728	-3.57277510005016	-0.648881210685292	-3.04215933760399	0.00461432162823917	0.293458739175368	-2.03940135314965
a_HQ_transcript/152723	2.70653693614891	2.24327771954958	3.04147898454689	0.00462242211858953	0.293504305327558	-2.03963474591125
a_HQ_transcript/84000	-3.65668932974388	0.113989035226732	-3.04092883461336	0.00462898221830595	0.293504305327558	-2.03638965538288
a_HQ_transcript/123868	3.14906460978365	2.47774791028718	3.04070324745646	0.00463167471380931	0.293504305327558	-2.08772117980785
a_HQ_transcript/120453	4.06713838286421	1.40379907176582	3.03997745357311	0.00464034749344197	0.293504305327558	-2.07108863012421
a_HQ_transcript/132231	4.30324034343219	0.792164466409801	3.03971639897013	0.00464347069071558	0.293504305327558	-2.03694452705463
a_HQ_transcript/142417	-5.22090049877774	1.13085156801539	-3.03956075206747	0.00464533376172161	0.293504305327558	-2.04472000397312
a_HQ_transcript/7628	-2.49294255065075	1.8366457545208	-3.03832943993997	0.00466009736303287	0.294117415329397	-2.03095673706068
a_HQ_transcript/135079	-2.93118465427366	-0.698926827342119	-3.03752538293665	0.00466976208724372	0.294280200846301	-2.05080845695057
a_HQ_transcript/117661	-2.42376777966489	-0.661707524741731	-3.03727281633554	0.00467280184044068	0.294280200846301	-2.04243702445587
a_HQ_transcript/68245	2.50972370914977	-0.694773313358569	3.03579992753539	0.00469056608167226	0.295079248047019	-2.04370645504334
a_HQ_transcript/30504	4.89883060040606	0.376238627288023	3.03515943410578	0.00469831088881304	0.295246935508027	-2.07084830653902
a_HQ_transcript/86167	-4.75535382513345	0.320854257853156	-3.03359670172827	0.00471725819607806	0.295810317696455	-2.05470305957314
a_HQ_transcript/60775	-2.81695828714221	0.576219228680916	-3.03358058557302	0.00471745397234747	0.295810317696455	-2.04039321975312
a_HQ_transcript/29011	-4.66819690586132	0.677116955852764	-3.03245232036654	0.00473117908046383	0.296351268953881	-2.06927627358613
a_HQ_transcript/14085	-4.00809413529705	-0.0545010265250945	-3.03163313775756	0.0047411679442693	0.296357188766779	-2.06733397048035
a_HQ_transcript/100669	-2.57339684758205	-0.770056798740355	-3.03160836723841	0.00474147029922076	0.296357188766779	-2.06146370123485
a_HQ_transcript/148643	2.72236780686919	-0.761956891745828	3.02920698134754	0.004770869017039	0.297874408402194	-2.06823408275034
a_HQ_transcript/34398	-2.84236721783194	-0.0305854626627458	-3.02812187005546	0.00478420990573761	0.298386859872013	-2.05008066531658
a_HQ_transcript/131358	7.59004437244079	0.435842246217098	3.02714501163452	0.00479625007704686	0.29870976445443	-2.07105317956692
a_HQ_transcript/83409	3.13508582870465	1.4958070173702	3.02686838021625	0.00479966487751923	0.29870976445443	-2.04951316493771
a_HQ_transcript/112664	-4.01240998038899	3.72185050258964	-3.0263644873018	0.0048058909592779	0.298777357947493	-2.17937556932765
a_HQ_transcript/67536	2.30630904891829	2.42787933601621	3.02493408772355	0.0048236065971099	0.299558337902568	-2.07754261049148
a_HQ_transcript/88098	-3.64908212293397	0.536745504768313	-3.02391911245679	0.00483621463203621	0.299729802854848	-2.07183940143725
a_HQ_transcript/147451	4.69469468505485	6.56721775999634	3.02388166951128	0.00483668034471937	0.299729802854848	-2.49177657252299
a_HQ_transcript/37206	5.51997683211365	6.36510373909123	3.02222270592936	0.00485735706461646	0.300064073501948	-2.30747691773644
a_HQ_transcript/53054	-3.22178258405099	-0.101379060047665	-3.02205785980527	0.00485941620943346	0.300064073501948	-2.07230360251175
a_HQ_transcript/4198	-3.70128383846392	0.903401542778868	-3.02203135006622	0.0048597474280174	0.300064073501948	-2.10361343647176
a_HQ_transcript/10610	-2.86614272998327	-0.72249308037301	-3.02160020532954	0.00486513724634508	0.300064073501948	-2.08493873233751
a_HQ_transcript/139410	-5.64852301972196	0.459435776122367	-3.02138057692158	0.00486788503496313	0.300064073501948	-2.09876164401028
a_HQ_transcript/51369	-2.80748513315707	1.37108467994942	-3.02019884313805	0.00488269502664894	0.30065815308162	-2.06592326617712

a_HQ_transcript/58891	3.24068717108768	0.220121989189751	3.01654483417608	0.00492875880222191	0.302920124859633	-2.07637183194268
a_HQ_transcript/37243	-2.31267676592142	1.09262330374909	-3.01633065827612	0.00493147149760662	0.302920124859633	-2.07408464464452
a_HQ_transcript/39679	3.76228254476778	0.12535427985625	3.01604724722221	0.00493506327831806	0.302920124859633	-2.07491524007296
a_HQ_transcript/23696	-3.87075428933733	2.44697001140233	-3.0135406990094	0.00496693766630629	0.303734058002837	-2.0878467747821
a_HQ_transcript/134256	-4.67470354185117	0.770678732192022	-3.01353216310804	0.00496704654460908	0.303734058002837	-2.1095097644402
a_HQ_transcript/152144	-4.09537994800443	-0.0348314503961712	-3.01345874131498	0.00496798315758728	0.303734058002837	-2.09805649972621
a_HQ_transcript/150185	4.38396191884608	1.14244837085918	3.01336144171712	0.00496922462773015	0.303734058002837	-2.08193715618022
a_HQ_transcript/122014	-0.624431483800766	6.46884583457073	-3.01238058246734	0.00498175605886053	0.304180164064543	-2.49246139719323
a_HQ_transcript/149412	-3.50788424021409	-0.73632633213325	-3.01166770549265	0.00499088250576812	0.304417647739024	-2.11010327702952
a_HQ_transcript/94729	5.79394895200154	0.212282668069997	3.01074283649261	0.00500274650058798	0.304821434576707	-2.1343185817633
a_HQ_transcript/152276	5.43116348129662	1.20279203933125	3.00951130456302	0.00501858570220591	0.305466334762121	-2.15083122511084
a_HQ_transcript/550	4.23348104600913	0.90820946034979	3.00893325923574	0.00502603650711848	0.305599843185966	-2.08760148603889
a_HQ_transcript/118545	-4.05825872894293	1.28117480704031	-3.0078715197362	0.00503974922425897	0.306113419966275	-2.11117668379833
a_HQ_transcript/19176	-4.29361038174346	2.82646480988638	-3.00674311669124	0.005054361700089	0.306526296619166	-2.12032450159356
a_HQ_transcript/6725	-4.12029235105949	1.32792057245465	-3.0065325208332	0.00505709328478151	0.306526296619166	-2.10222546808756
a_HQ_transcript/87007	-1.70907519721905	2.58964417737117	-3.00584766859484	0.00506598598097067	0.306745451147774	-2.11490680286713
a_HQ_transcript/86237	-5.51621470688253	0.525092242709218	-3.00231119415723	0.00511214232310064	0.309218115460139	-2.1446998292938
a_HQ_transcript/20373	2.78732795141801	0.446270954903448	3.00077309770597	0.00513234061843159	0.310117147056332	-2.12057903597979
a_HQ_transcript/92918	-2.33775572343769	-0.800275804536595	-2.99983240222578	0.00514473099326563	0.31054301472123	-2.13007788696935
a_HQ_transcript/36027	3.19357142197805	1.16924445344976	2.99879512703173	0.00515842624510441	0.310778013802064	-2.11291755159898
a_HQ_transcript/24323	2.27947262553401	-0.68890582963637	2.99872774548286	0.00515931708159564	0.310778013802064	-2.12898908324018
a_HQ_transcript/30500	5.87349950224311	0.680660031179973	2.99700815990899	0.00518210060889165	0.311583473979323	-2.13312397061476
a_HQ_transcript/103995	5.22697664177237	2.10839249724967	2.99659044261443	0.00518764946261896	0.311583473979323	-2.20984070996125
a_HQ_transcript/2033	2.80600598051826	-0.649320766496729	2.99650616545165	0.00518876966026673	0.311583473979323	-2.13865057855049
a_HQ_transcript/23392	-2.86523921519677	1.17672496500373	-2.99577709389071	0.00519846990267142	0.311674540135382	-2.14048113754584
a_HQ_transcript/142470	3.75856515236389	0.341390527536779	2.99558639915443	0.00520100990798446	0.311674540135382	-2.16324904583013
a_HQ_transcript/108676	2.29823408918253	-0.601546981187379	2.99438866761435	0.00521699022478385	0.311791321503171	-2.14118340781828
a_HQ_transcript/62625	-4.37089231631396	0.0906253604836256	-2.99420396328343	0.00521945870379313	0.311791321503171	-2.12819277227479
a_HQ_transcript/133866	-3.38619762920661	-0.193929722061226	-2.99384378392854	0.0052242754919402	0.311791321503171	-2.14337420826229
a_HQ_transcript/111569	3.12810163243162	1.30754999866852	2.99349509052017	0.00522894267664401	0.311791321503171	-2.13808823921441
a_HQ_transcript/88641	-4.75535952325606	1.36791976480677	-2.9934327094035	0.00522977804957322	0.311791321503171	-2.13039798634668
a_HQ_transcript/81064	2.00857661466001	1.70857729356917	2.99270877025738	0.00523948186530585	0.312049796994363	-2.13429275161397
a_HQ_transcript/81677	2.86295786104048	0.729070151949158	2.99128633208946	0.00525859807084604	0.312867746839446	-2.14894254152971
a_HQ_transcript/144359	-4.65020089487634	1.04357052616772	-2.98980911585548	0.00527852018583847	0.313579672285351	-2.15437326607068
a_HQ_transcript/128487	-2.64142784771357	-0.745396880360686	-2.98959947656129	0.00528135320615468	0.313579672285351	-2.15412131972497
a_HQ_transcript/143692	-3.37322549706241	-0.101784592285428	-2.98870176810182	0.00529350089974659	0.313828172235059	-2.1520689475272
a_HQ_transcript/73345	-2.84263865098831	-0.327344983006315	-2.98849251296723	0.00529633630888029	0.313828172235059	-2.14508471663848
a_HQ_transcript/144950	2.30729046580446	-0.525486406515749	2.98732217118692	0.00531222092318157	0.314448857253257	-2.15620849176418
a_HQ_transcript/114484	2.71224956627954	-0.764841418875178	2.98643663229301	0.00532426991235996	0.314841466394364	-2.16188521571699
a_HQ_transcript/75469	-3.68095818182689	1.12665545834694	-2.98574515982551	0.0053336963026114	0.314927874305022	-2.13735078224275
a_HQ_transcript/110116	-4.69522375554082	0.573931617010864	-2.98528591619067	0.00533996557924309	0.314927874305022	-2.15299401572209
a_HQ_transcript/55891	-0.89013993573316	2.78088471824576	-2.98513812110382	0.00534198465566942	0.314927874305022	-2.22281766568573
a_HQ_transcript/119446	3.78697430846743	-0.235676445748675	2.98450694739453	0.00535061544207224	0.315117096673531	-2.15782107605869
a_HQ_transcript/93257	5.24536088607332	2.76313037114032	2.98332317440886	0.00536683810138135	0.31575259631285	-2.32567152250746
a_HQ_transcript/25039	-2.32265516287076	2.8003185065826	-2.98179645544962	0.00538782915958521	0.316667071171253	-2.27522281513298
a_HQ_transcript/52617	-4.09191896991895	0.878139103882067	-2.98085524763711	0.00540080855919055	0.316792407676108	-2.17394108119736
a_HQ_transcript/26204	-4.63139406293593	0.567336930014417	-2.98085514145596	0.00540086147823808	0.316792407676108	-2.15011689132666
a_HQ_transcript/115926	3.6185606546597	1.15136973431188	2.98015168434376	0.00541053008501013	0.317039609658739	-2.19896949580819
a_HQ_transcript/27217	4.00743230115051	0.17269200902319	2.97816359592907	0.00543809000863075	0.318169493504769	-2.17389690938619
a_HQ_transcript/52451	4.07567053267225	0.332780888030809	2.97759207917852	0.00544603717127236	0.318169493504769	-2.1625085904884

a_HQ_transcript/151232	-3.17604583326086	1.99795527131784	-2.9772945757882	0.00545017841036676	0.318169493504769	-2.19313096573008
a_HQ_transcript/115754	-4.00824547829884	-0.543130637781946	-2.97672423115514	0.00545812590764081	0.318169493504769	-2.1835686074568
a_HQ_transcript/5703	-2.40895719107435	0.53077994033045	-2.97649486515524	0.00546132510428114	0.318169493504769	-2.15542914773734
a_HQ_transcript/16022	-3.17738099975699	-0.28845896403512	-2.97639962397584	0.00546265404826864	0.318169493504769	-2.18432031200524
a_HQ_transcript/8269	-2.88084867950187	0.887922980966595	-2.97561121846191	0.0054736667640446	0.318220609643309	-2.15935377499251
a_HQ_transcript/11935	-3.52460899477044	0.918243334968316	-2.9755530133725	0.00547448062282048	0.318220609643309	-2.17791404200617
a_HQ_transcript/114194	3.36396398072022	0.585923255406958	2.97316481845175	0.00550797249270533	0.319736511387269	-2.16526483611431
a_HQ_transcript/69630	4.38788477433842	1.60393904112072	2.97290978831253	0.00551156042544116	0.319736511387269	-2.23861222061411
a_HQ_transcript/76273	-3.75100231651255	1.63269811318871	-2.9719806965339	0.00552465017212612	0.320108954451288	-2.20476526020789
a_HQ_transcript/47077	-4.3430450620497	1.65927248996608	-2.97132024766899	0.00553397291331351	0.320108954451288	-2.20537780724088
a_HQ_transcript/50900	1.59534627578291	3.51971714256878	2.97128283742247	0.00553450143172901	0.320108954451288	-2.31503389748027
a_HQ_transcript/53982	-4.24506956315373	3.04052907290638	-2.97052687212486	0.00554519165034055	0.320155411381279	-2.18709007083381
a_HQ_transcript/115447	3.46819193596907	2.06587243849543	2.97044714795434	0.00554632017721146	0.320155411381279	-2.18272202207043
a_HQ_transcript/86339	4.84950486721403	6.75112138855578	2.96892689938447	0.00556788141600322	0.321081161656185	-2.56513884339006
a_HQ_transcript/109407	2.74911960111809	3.42580472426079	2.96751179028721	0.00558802256425238	0.321911549980054	-2.28565533990341
a_HQ_transcript/140075	4.27109769848724	0.551665541619014	2.96713778946592	0.00559335716831569	0.321911549980054	-2.21653228831029
a_HQ_transcript/108407	6.51496120437592	1.90528705993624	2.96658319928356	0.00560127647785295	0.321941645304526	-2.31068104079081
a_HQ_transcript/38305	4.02972083423078	-0.490128921082445	2.9663256982281	0.00560495707831304	0.321941645304526	-2.22809447974316
a_HQ_transcript/3880	-1.69188888131142	-0.708805398595513	-2.96505077601645	0.00562321387289233	0.322671447190015	-2.21430516419827
a_HQ_transcript/137074	-4.40543182902242	0.261417045346599	-2.96387062680207	0.00564016352576243	0.322692466034965	-2.2188182854712
a_HQ_transcript/15500	-5.75024449716229	0.544103458002948	-2.96337054881095	0.00564736032621018	0.322692466034965	-2.2383130147558
a_HQ_transcript/128790	-1.7035657697756	2.33179493965385	-2.96332040044318	0.00564808250705979	0.322692466034965	-2.22246260519591
a_HQ_transcript/114821	3.95707388122635	1.17862523535359	2.96310229062663	0.00565122449512167	0.322692466034965	-2.21703014316402
a_HQ_transcript/49941	-2.10682356127513	2.68230468178607	-2.96283153038082	0.00565512723507822	0.322692466034965	-2.21782549744277
a_HQ_transcript/122146	4.10168034072877	0.492373305042377	2.96244643890177	0.00566068232688813	0.322692466034965	-2.21830014625586
a_HQ_transcript/143298	-7.72364892837783	2.27051379923657	-2.96194658629582	0.00566790057079039	0.322692466034965	-2.31625222575623
a_HQ_transcript/149928	-3.90194470045176	1.2678241063111	-2.96173495565204	0.00567095929090893	0.322692466034965	-2.18669998954594
a_HQ_transcript/89208	-2.45137667135997	-0.647791258325127	-2.96155628315289	0.0056735428758556	0.322692466034965	-2.19316552850235
a_HQ_transcript/16606	-3.28773177660249	-0.206838608764943	-2.95930092763718	0.00570625069610804	0.324235523424602	-2.20734265190973
a_HQ_transcript/106986	-5.65875916614435	1.34866638181154	-2.95881434060606	0.00571333061306169	0.32432078308208	-2.26372522798229
a_HQ_transcript/135853	-3.27609457202481	1.29312663114987	-2.95791246280767	0.0057264749936864	0.324749793593174	-2.21719072159424
a_HQ_transcript/104522	4.73579153927372	0.215060120018818	2.95726424842933	0.00573593998115798	0.324969511914962	-2.2219228349989
a_HQ_transcript/95812	3.32487781868749	-0.356230955498091	2.95616617457735	0.00575200733090227	0.325072631862066	-2.21987021409752
a_HQ_transcript/152917	-4.33902122903033	3.98826695740773	-2.95611025438841	0.00575282670701265	0.325072631862066	-2.35211857046986
a_HQ_transcript/112495	3.33411954377096	1.68434470692855	2.95599354231098	0.00575453719698022	0.325072631862066	-2.23213562944386
a_HQ_transcript/6471	-3.07029085456675	-0.606324647889482	-2.95529002043427	0.00576485792387217	0.325339477086254	-2.20667175712809
a_HQ_transcript/145769	3.62652560772465	0.192923080805033	2.95471264221447	0.00577334115333099	0.325502206169567	-2.20834790464998
a_HQ_transcript/38774	-3.20446254269548	0.149357367532575	-2.95327214171496	0.00579455731469572	0.326247769795449	-2.20799016700258
a_HQ_transcript/128085	-2.28668661447368	10.3668466371729	-2.95305308032971	0.0057977901561846	0.326247769795449	-3.02775599787746
a_HQ_transcript/390	1.27005504371991	4.06781683669398	2.95216326537274	0.005810939270464	0.326549357262659	-2.44437364603177
a_HQ_transcript/16069	1.80512019773408	2.76266781925944	2.9519303873983	0.0058143852320199	0.326549357262659	-2.33521650678914
a_HQ_transcript/134625	2.32127395584319	-0.717505884689717	2.95143343610073	0.00582174520639976	0.326636864173602	-2.23051918708889
a_HQ_transcript/97558	-2.92061396365296	-0.617438718124486	-2.95106672809544	0.00582718187703044	0.326636864173602	-2.2495381820016
a_HQ_transcript/65667	-2.67316099271849	-0.270911025906503	-2.95025090304409	0.00583929416076224	0.327000473002686	-2.23232085681091
a_HQ_transcript/115944	-1.43671098317983	4.80607776856552	-2.94801917907266	0.00587254923257727	0.328114751306012	-2.50446433143335
a_HQ_transcript/80069	3.538017638307	0.400450957123498	2.94790170074809	0.00587430472300793	0.328114751306012	-2.24918518507305
a_HQ_transcript/70059	4.12851186972376	0.900827796883273	2.94749202430305	0.0058804304312296	0.328114751306012	-2.22812332318864
a_HQ_transcript/143891	-3.40956706053889	1.5534977496834	-2.94740243901433	0.00588177076212608	0.328114751306012	-2.26119602012894
a_HQ_transcript/95091	4.4445906686525	5.6347741804641	2.94603798712392	0.00590222065225971	0.328939867760837	-2.59889521178512
a_HQ_transcript/1117	-3.00812819138901	0.275251109533763	-2.94527532440114	0.00591368033851162	0.329133575603042	-2.24999627535408

a_HQ_transcript/129256	1.00670278550042	3.3507313232318	2.94480558164545	0.00592074907620756	0.329133575603042	-2.37929914611387
a_HQ_transcript/131746	2.30370331714709	2.75660515046323	2.94453640766302	0.00592480322319536	0.329133575603042	-2.31486089737993
a_HQ_transcript/71772	4.68267524113255	1.11412859734153	2.9443013767344	0.00592834526659071	0.329133575603042	-2.31923229879814
a_HQ_transcript/20141	4.96601432067132	1.06141431127037	2.94374255839653	0.00593677497841893	0.329287076283908	-2.22364975342391
a_HQ_transcript/45143	-2.68609923573151	-0.683573633469731	-2.9416279085801	0.00596877661731804	0.330108459433646	-2.25379052223177
a_HQ_transcript/108617	-4.39035071305156	0.0321400953659346	-2.94156382027701	0.00596974901891328	0.330108459433646	-2.24141252646289
a_HQ_transcript/110974	-2.88319275532526	-0.338394613200412	-2.94144850084417	0.0059714991183304	0.330108459433646	-2.26392785258583
a_HQ_transcript/7130	3.53443734778181	0.355211510367422	2.94126402095315	0.00597429980945837	0.330108459433646	-2.24332567558558
a_HQ_transcript/11386	2.43071807963547	-0.621299295694767	2.93931508110487	0.00600396346462746	0.331361897938121	-2.28129499006593
a_HQ_transcript/149338	2.7369401958328	-0.528797599602068	2.9390166495158	0.00600851795064592	0.331361897938121	-2.28010336761669
a_HQ_transcript/104027	-5.14471401223687	3.1527067457826	-2.93865208118237	0.00601408619468616	0.331361897938121	-2.35229895937015
a_HQ_transcript/91187	-3.10527948789058	0.291968446377952	-2.93783951950097	0.00602651437966489	0.331732223353372	-2.25259436281606
a_HQ_transcript/152547	-1.73968871768053	4.1156350718436	-2.93495315305655	0.00607085749637486	0.333760960062264	-2.48982655755479
a_HQ_transcript/73166	-2.21377004880676	0.335370150320638	-2.93469401036015	0.0060748536978027	0.333760960062264	-2.24378608895704
a_HQ_transcript/16815	3.53487597805673	2.66170963316283	2.93424111798946	0.00608184363581403	0.333829468236636	-2.31371900664513
a_HQ_transcript/73894	2.20813496601303	3.0989372971136	2.93353054934271	0.00609282582098101	0.334116772945268	-2.33596477783814
a_HQ_transcript/124151	4.35677422058972	0.928142531153043	2.93297179903511	0.00610147469877872	0.334261005969127	-2.30403581210312
a_HQ_transcript/55408	-3.43475827523161	0.152551052695155	-2.9326180169048	0.00610695685967542	0.334261005969127	-2.27271691826013
a_HQ_transcript/5323	-4.33294117388536	-0.0966837397841608	-2.93136413869658	0.00612642418106247	0.335011086356349	-2.30655542680694
a_HQ_transcript/57527	4.27358252018375	0.119991273788033	2.93079862432431	0.00613522329580509	0.33517693584451	-2.2554025040075
a_HQ_transcript/85607	-2.93221392203286	-0.491123540530764	-2.92978624802455	0.00615100509292631	0.335723590649409	-2.27958835866947
a_HQ_transcript/100535	-2.5320606283618	-0.696304868316902	-2.92889111735194	0.00616499100915057	0.336171292101223	-2.28504454426869
a_HQ_transcript/38496	3.31271197371498	0.81822932127722	2.92752095038707	0.0061864571289755	0.336582548082971	-2.28203907762207
a_HQ_transcript/140701	-3.74214856282497	-0.686048299954526	-2.9273263125968	0.00618951218287235	0.336582548082971	-2.29425701085454
a_HQ_transcript/70828	-2.83900563524501	-0.687586071292362	-2.92725494667747	0.00619063270532721	0.336582548082971	-2.28797866138328
a_HQ_transcript/76546	-3.85706697200316	1.26786160911847	-2.92687955787822	0.00619652986343081	0.336582548082971	-2.30974808108471
a_HQ_transcript/113667	-5.38678030591611	2.47212323452815	-2.92632444374451	0.0062052601081412	0.336582548082971	-2.45680576749856
a_HQ_transcript/11493	3.36262423673693	-0.0134207465722158	2.92619641966031	0.00620727517796837	0.336582548082971	-2.25907021022979
a_HQ_transcript/41003	-4.23346468783227	0.491918967442702	-2.92344122926496	0.00625079087066415	0.338317778973427	-2.28970523180635
a_HQ_transcript/18995	-3.49228745546303	1.0884400938637	-2.92343327919524	0.00625091684932323	0.338317778973427	-2.26685101930255
a_HQ_transcript/59822	1.27008393800704	3.24185451470696	2.92267589008728	0.00626292958311791	0.338652624006956	-2.40543843102243
a_HQ_transcript/152515	8.76390844301658	0.522870009136609	2.92192138647542	0.00627491816167854	0.338824894184013	-2.28194127726972
a_HQ_transcript/67273	-2.39405859629405	-0.244480737682176	-2.92155350172969	0.00628077144756548	0.338824894184013	-2.29723450148787
a_HQ_transcript/69074	-2.03138951012549	-0.460199331243254	-2.9213360914986	0.00628423300229269	0.338824894184013	-2.29859732898994
a_HQ_transcript/61164	3.01075759180712	-0.583478406853472	2.92100981510302	0.0062894312693461	0.338824894184013	-2.29469597368541
a_HQ_transcript/96660	4.85108834106914	1.35971621640836	2.92015850627117	0.00630301347240986	0.339242191781704	-2.30205187414691
a_HQ_transcript/145060	1.14170895320387	2.68976964240836	2.91811719415983	0.00633569415650944	0.340685689111546	-2.36793536268507
a_HQ_transcript/148712	5.65443586802879	1.46061387678441	2.91739455973844	0.00634730145873953	0.340994398515353	-2.30736219547628
a_HQ_transcript/147040	-2.12235196435995	2.71865064684595	-2.91628621565458	0.00636514311821381	0.341449584969807	-2.34922233384576
a_HQ_transcript/129080	2.51236109217876	-0.504087414325286	2.91606385158699	0.00636872832289645	0.341449584969807	-2.32874301868982
a_HQ_transcript/115908	-3.29241284436786	-0.225490262667858	-2.91577448670641	0.00637339663659924	0.341449584969807	-2.2995792584883
a_HQ_transcript/77262	3.81746975289042	1.246675433673	2.91491452325844	0.00638728941441258	0.341688597007741	-2.32806661338925
a_HQ_transcript/143935	-5.50471847144292	1.78928508235506	-2.91477077951436	0.00638961438458944	0.341688597007741	-2.33905477463405
a_HQ_transcript/42228	-2.87251277527451	0.297103516099806	-2.91345150607142	0.00641099008782333	0.342516573368561	-2.29227949798905
a_HQ_transcript/8454	-2.79058548269202	-0.342962153930449	-2.9114775785674	0.00644309862761047	0.343106844468896	-2.29949024373446
a_HQ_transcript/21912	2.16702572080027	1.06402653040122	2.91130881188343	0.00644585085185825	0.343106844468896	-2.30088569468044
a_HQ_transcript/50091	2.7389043706828	0.792275924635634	2.91040384757235	0.00646062776845186	0.343106844468896	-2.30562336805352
a_HQ_transcript/138844	-3.17489223807215	1.81611532871927	-2.91015104603487	0.00646476138883572	0.343106844468896	-2.33229606301977
a_HQ_transcript/149563	-2.44582892761764	-0.736876584238887	-2.91000758959401	0.006467108187226	0.343106844468896	-2.32097904550642
a_HQ_transcript/13402	-3.98013188705197	1.78087178769425	-2.90994105843967	0.00646819683989491	0.343106844468896	-2.30582436529913

a_HQ_transcript/135162	2.17867107209064	-0.766781582216687	2.90938039654527	0.00647737783107684	0.343106844468896	-2.32533159189907
a_HQ_transcript/56959	-2.27680676227669	0.383733568108556	-2.90918361850487	0.00648060303111051	0.343106844468896	-2.29625653456596
a_HQ_transcript/119438	0.773742400607229	4.43970809822411	2.90915953658833	0.00648099783845616	0.343106844468896	-2.56258807320442
a_HQ_transcript/125759	-3.97048871693772	-0.57041398166112	-2.9091554727244	0.00648106446509166	0.343106844468896	-2.33239413068696
a_HQ_transcript/148106	2.45240706668288	-0.772773870793106	2.90854045436475	0.00649115506098197	0.343328354308244	-2.32285482347245
a_HQ_transcript/120771	4.26848813809575	0.76266362555649	2.90780682285619	0.00650321107389053	0.343616950369471	-2.33183053846077
a_HQ_transcript/11903	4.00959085802295	1.08774767311427	2.90748938367983	0.00650843418587923	0.343616950369471	-2.33388847951373
a_HQ_transcript/150448	4.21993344028044	5.41505048216556	2.90705180922853	0.00651564046179683	0.343685252961276	-2.59463776774672
a_HQ_transcript/149857	-4.15750855355149	0.440440883945696	-2.9057577302322	0.00653699619422189	0.344499106779447	-2.31453986818792
a_HQ_transcript/145137	4.19046366353216	-0.119087886377793	2.90346734490275	0.00657495511809502	0.345973649359811	-2.33250341323062
a_HQ_transcript/88152	-2.60253132465066	2.20459348835865	-2.90335153005601	0.00657688003273107	0.345973649359811	-2.35477097457385
a_HQ_transcript/136005	-3.61235687058664	0.135634204161888	-2.90278623725052	0.00658628316066176	0.346139176422161	-2.3350960618491
a_HQ_transcript/148499	-3.28691383074281	1.75297415941788	-2.90244675487375	0.00659193621489355	0.346139176422161	-2.33302437571536
a_HQ_transcript/110971	2.40497417030183	-0.39060104116296	2.9013330144947	0.00661051426204541	0.346611265373604	-2.33359065956153
a_HQ_transcript/58311	-3.70760068981504	0.232029053879415	-2.90089571075508	0.0066178222803883	0.346611265373604	-2.34206819047321
a_HQ_transcript/135291	4.99854193762442	0.62800284307466	2.90083631555419	0.00661881545149842	0.346611265373604	-2.35954179121353
a_HQ_transcript/101149	2.59414052845902	-0.710271524478767	2.89936297815059	0.00664349663413747	0.347183852318576	-2.3466742827071
a_HQ_transcript/98825	3.69678672190204	-0.729808049629882	2.89870930666767	0.00665447456197343	0.347183852318576	-2.35637895674444
a_HQ_transcript/112137	-3.22151880451042	0.155332427366725	-2.89796583713375	0.00666698131646135	0.347183852318576	-2.32036663185308
a_HQ_transcript/97484	-3.96832384087064	0.972132186421288	-2.89776928659275	0.00667029141117072	0.347183852318576	-2.34516801080882
a_HQ_transcript/131745	-6.37719895652676	1.99429544391821	-2.89764519708948	0.00667238199008981	0.347183852318576	-2.43126579939335
a_HQ_transcript/89636	-3.00296022092126	1.53720038327049	-2.89740059484747	0.00667650469335198	0.347183852318576	-2.36532399881884
a_HQ_transcript/114729	-3.18978278907071	-0.44266224393028	-2.89734732366208	0.00667740288206732	0.347183852318576	-2.34354473444619
a_HQ_transcript/31316	4.27732403799897	-0.0256365247256823	2.89733970016313	0.00667753142878075	0.347183852318576	-2.35251496932157
a_HQ_transcript/150433	4.55266587180598	0.551178809990915	2.8966983050576	0.00668835490965761	0.347435830374064	-2.3668990420275
a_HQ_transcript/138447	-3.84312570877062	6.99676799795951	-2.8961041347284	0.00669839619432072	0.347646762485245	-2.87018857033385
a_HQ_transcript/26106	4.28056540364011	1.33394708998716	2.89531827432557	0.00671169875187709	0.347942437569323	-2.32217369558061
a_HQ_transcript/82277	1.5859474322757	1.83055779541106	2.89481753291073	0.00672018794401179	0.347942437569323	-2.33029669423245
a_HQ_transcript/118958	-5.03766796470128	0.711249191335802	-2.89440364318434	0.00672721233795114	0.347942437569323	-2.39297348947096
a_HQ_transcript/58332	-2.35155660685588	2.19800681053743	-2.89435511465446	0.00672803639946185	0.347942437569323	-2.39400636243096
a_HQ_transcript/133561	-5.35562099646729	0.536191667180035	-2.89397337333902	0.00673452204776971	0.347968264526896	-2.37925092478121
a_HQ_transcript/125644	0.712868915441768	3.27165095420767	2.89324454082712	0.00674692097876146	0.34829930963894	-2.49646797861741
a_HQ_transcript/62014	1.00072839513819	4.69499385967626	2.89154877932836	0.0067758524539319	0.349427586593684	-2.6336887191061
a_HQ_transcript/137173	-2.63875909479422	-0.743523802510781	-2.89119405642331	0.00678191913124865	0.349427586593684	-2.36277184723133
a_HQ_transcript/152478	5.12686314787866	2.46404801788615	2.8904475233901	0.00679470345759296	0.349427586593684	-2.45619664791326
a_HQ_transcript/122932	6.91197470789317	1.84330832698626	2.88983302675702	0.00680524366393615	0.349427586593684	-2.51316829508228
a_HQ_transcript/46251	3.10319537875097	0.827958399604647	2.88966350360285	0.00680815412805807	0.349427586593684	-2.35176756466598
a_HQ_transcript/61272	-2.93248221625691	3.66048066855307	-2.88950194660181	0.00681092891380598	0.349427586593684	-2.57434979320017
a_HQ_transcript/121318	-3.7701714305627	0.157475160584278	-2.88949573919951	0.00681103554884414	0.349427586593684	-2.37149385772815
a_HQ_transcript/100059	-2.57410542706125	-0.676646386587778	-2.88859294606631	0.00682656107380138	0.349427586593684	-2.36755371967302
a_HQ_transcript/85653	-3.43655135382785	0.24558238362248	-2.88836101304011	0.00683055504236928	0.349427586593684	-2.34804527090238
a_HQ_transcript/20124	1.01741791221468	3.36613631109945	2.88823139084959	0.00683278813885695	0.349427586593684	-2.48787652745549
a_HQ_transcript/99504	-2.74364935111219	3.9118048458859	-2.88810874393286	0.0068349016989578	0.349427586593684	-2.50177759201539
a_HQ_transcript/83544	-3.89976715427435	0.176678663216373	-2.88721393603302	0.00685034042527884	0.349909128506686	-2.36027219024083
a_HQ_transcript/152379	-4.23763047774254	-0.00609621231022329	-2.88677086586237	0.00685799714152285	0.349992675893275	-2.34266527198343
a_HQ_transcript/71882	6.00499595227289	0.933363190751042	2.8860069088584	0.00687121801096262	0.350359789948452	-2.36289595062559
a_HQ_transcript/144545	-4.39442181491806	-0.0163704087983611	-2.88527693742189	0.00688387310833688	0.35069743737196	-2.38630492039281
a_HQ_transcript/95	-3.03693404887645	0.430186286519354	-2.88415193365418	0.00690341955280574	0.351235796274322	-2.37976056858643
a_HQ_transcript/72519	-3.6298969462832	1.74762928446893	-2.88397344436447	0.00690652551509685	0.351235796274322	-2.42272641211401
a_HQ_transcript/132074	-4.8997593712243	1.75104777612156	-2.88354114170343	0.00691405362616032	0.351240127398077	-2.40753269825741

a_HQ_transcript/144662	-4.94349900354262	2.07387589975011	-2.88325014864658	0.00691912531532462	0.351240127398077	-2.41903655201042
a_HQ_transcript/38480	-2.15556830722634	-0.803435007005032	-2.88236486793121	0.00693457628800433	0.351240127398077	-2.3825639406663
a_HQ_transcript/137776	-4.92607730801511	2.044696943138	-2.88224416971379	0.00693668536652482	0.351240127398077	-2.36386332129253
a_HQ_transcript/136354	-4.44460462130735	1.14281215188766	-2.8817021139448	0.00694616467293462	0.351240127398077	-2.4101617320545
a_HQ_transcript/125454	5.91283872619139	0.785331537291449	2.88139022576415	0.00695162439335499	0.351240127398077	-2.40641363895508
a_HQ_transcript/93296	3.69222875251032	0.106086560586247	2.8807293402918	0.00696320676958166	0.351240127398077	-2.35942736078277
a_HQ_transcript/131287	-2.62842579859751	-0.664563356446184	-2.88037583995961	0.00696940949472104	0.351240127398077	-2.38604030403081
a_HQ_transcript/120010	-3.75770555309849	0.0934687100237585	-2.88031373950778	0.00697049968175943	0.351240127398077	-2.38538566674566
a_HQ_transcript/55117	-3.01258442468601	-0.665098422642123	-2.88021119809277	0.00697230016946024	0.351240127398077	-2.38937850327318
a_HQ_transcript/15113	3.33551542512409	2.53369907693457	2.87990133698748	0.00697774356415035	0.351240127398077	-2.40371100842951
a_HQ_transcript/135600	6.08000121227602	3.36873112703673	2.87968989904911	0.00698146022993052	0.351240127398077	-2.55340612239953
a_HQ_transcript/64642	-4.81109239984551	4.23671679403691	-2.87947932506065	0.00698516355753126	0.351240127398077	-2.50629955914105
a_HQ_transcript/136291	3.87674068762974	0.944695117227876	2.87877405668334	0.00699758043393373	0.35156037637312	-2.44480297317853
a_HQ_transcript/58603	-1.57444392496601	3.82312019417367	-2.87629210799419	0.00704144241992212	0.353267155323857	-2.57991814529232
a_HQ_transcript/15477	-2.88894748657362	1.39023231298026	-2.87585636379191	0.00704916966481541	0.353267155323857	-2.3627190133531
a_HQ_transcript/95486	3.5324611692227	5.43387684080703	2.8758216861764	0.00704978496035773	0.353267155323857	-2.6431720067595
a_HQ_transcript/100750	4.8243761030427	2.02213584194682	2.87503620915517	0.00706373543863769	0.353659362891662	-2.52143925953974
a_HQ_transcript/85715	3.06675392652027	0.94172295093625	2.87469631689028	0.00706978013487667	0.353659362891662	-2.37916956919659
a_HQ_transcript/146738	4.26280380505573	3.4028531101974	2.87381069547621	0.00708555299058799	0.353857981370032	-2.53289577690382
a_HQ_transcript/41287	4.61551967115289	1.56117288606057	2.87378978963866	0.0070859257210762	0.353857981370032	-2.40991279363694
a_HQ_transcript/151582	6.65793573326202	1.75094302404589	2.87283461815542	0.00710297514141635	0.354404926197639	-2.47733840552652
a_HQ_transcript/98685	3.87118999851099	0.0604878671219628	2.87238751199881	0.00711096903374991	0.354499492275999	-2.39824057470069
a_HQ_transcript/22090	-2.84483423262048	-0.64914236930044	-2.87092317818073	0.00713720931066522	0.354845984193031	-2.41154804871569
a_HQ_transcript/36257	-3.58848813078295	0.35721176758185	-2.87078835432391	0.00713962986609135	0.354845984193031	-2.40970532092551
a_HQ_transcript/83645	-2.6101991838303	-0.32542408467109	-2.87003296318689	0.00715320601916259	0.354845984193031	-2.40697409000886
a_HQ_transcript/35779	-2.60595842271395	-0.673762563378447	-2.86941433395369	0.00716434228992589	0.354845984193031	-2.39142032249699
a_HQ_transcript/63882	2.75713649799783	-0.66046449911364	2.86918490809884	0.00716847644206926	0.354845984193031	-2.4149901213281
a_HQ_transcript/62244	-5.07925977793996	2.42503298418515	-2.86915516167927	0.00716901262336144	0.354845984193031	-2.40496642777659
a_HQ_transcript/131167	3.74787989365597	0.0938292755168527	2.86894526510854	0.00717279709413548	0.354845984193031	-2.41111605214582
a_HQ_transcript/36380	-5.45553226982435	1.2389472673176	-2.86894260210782	0.00717284512053798	0.354845984193031	-2.50665558209585
a_HQ_transcript/56208	1.95883375754198	3.24887425233202	2.86894175288629	0.00717286043605167	0.354845984193031	-2.5387619028856
a_HQ_transcript/118645	-2.26001176501813	2.99298878162158	-2.86830774272941	0.00718430323475855	0.355039087483693	-2.47700869603563
a_HQ_transcript/8220	-3.06896517113982	-0.374931143675493	-2.86737407051288	0.00720118562882059	0.355039087483693	-2.41395094064228
a_HQ_transcript/138062	-4.25844262516439	0.629116074676367	-2.86714001759356	0.00720542354159936	0.355039087483693	-2.39005140127987
a_HQ_transcript/4192	-4.15965149363733	0.951508952665395	-2.86689761616539	0.00720981508598138	0.355039087483693	-2.42247128268844
a_HQ_transcript/116126	4.02791515865548	1.01403326624572	2.86673330705148	0.00721279327428681	0.355039087483693	-2.42338933482318
a_HQ_transcript/144210	-4.00204896554643	3.22788119461188	-2.86640172887431	0.00721880681882555	0.355039087483693	-2.62853724271123
a_HQ_transcript/144294	4.05321121130171	6.71094525681258	2.86630603927794	0.00722054313263673	0.355039087483693	-2.87223064446194
a_HQ_transcript/1637	4.78500908887513	0.236235894477849	2.86602599304479	0.00722562690086031	0.355039087483693	-2.41519386194823
a_HQ_transcript/50	-2.36234478657015	3.57312076063362	-2.86390026481519	0.00726432561569989	0.355956017895501	-2.46743482524389
a_HQ_transcript/90711	-5.30824965164362	1.48387900807987	-2.86377725543218	0.00726657093595467	0.355956017895501	-2.50611261648267
a_HQ_transcript/78124	-3.18061973142915	0.834681624739248	-2.86346185148664	0.00727233105793295	0.355956017895501	-2.44347189743148
a_HQ_transcript/129492	2.28295662185462	-0.713046890841299	2.86345325087225	0.00727248818822977	0.355956017895501	-2.42403447757471
a_HQ_transcript/24658	3.93973779190817	-0.714196890302528	2.86332092007128	0.00727490622866527	0.355956017895501	-2.43286352632314
a_HQ_transcript/118733	-3.5380350940554	-0.18431676655758	-2.86260474211645	0.00728800582926552	0.356297058741418	-2.46782957494975
a_HQ_transcript/138739	-4.16064431511102	4.48907482281911	-2.86197624952836	0.0072995198142198	0.356560073748713	-2.70584098075838
a_HQ_transcript/36652	6.09092941170805	3.63833278801564	2.86123589252638	0.00731310505626875	0.356828011115355	-2.62434739578283
a_HQ_transcript/11469	-1.43354416621172	-0.732912813749768	-2.86100850061792	0.00731728236391245	0.356828011115355	-2.44484538270593
a_HQ_transcript/14272	-3.73505116274721	0.145345056653371	-2.85967703507997	0.00734178705774221	0.357405716740603	-2.41551107793446
a_HQ_transcript/124565	-5.83121952251424	2.24261327626163	-2.85937375097243	0.00734737954864799	0.357405716740603	-2.52821066738625

a_HQ_transcript/146230	-8.41377935694014	3.70731856472836	-2.85931729059787	0.00734842110657326	0.357405716740603	-2.68493558518785
a_HQ_transcript/150826	3.55414261722529	-0.464157797257034	2.85902997908017	0.00735372345894855	0.357405716740603	-2.46244366882698
a_HQ_transcript/135856	-5.50506637948184	0.14459731325457	-2.85798108459085	0.0073731113679877	0.358048636255964	-2.46503049865513
a_HQ_transcript/116601	2.06420270835657	-0.579751141832203	2.85713665412311	0.00738875477634849	0.358508796026365	-2.43288119433799
a_HQ_transcript/121721	3.30500360380972	2.15744937214186	2.85568804585922	0.00741566337648222	0.359326463020677	-2.4388438027634
a_HQ_transcript/2119	-1.98046378719205	2.60217580017302	-2.85556410396827	0.0074179699219793	0.359326463020677	-2.47826357172894
a_HQ_transcript/116957	3.824617792842	4.52222487765172	2.8543112023079	0.00744132411277086	0.359912780999963	-2.67942594484158
a_HQ_transcript/121190	-7.29556547179504	1.38521296942136	-2.85405175718676	0.0074461688044813	0.359912780999963	-2.5491013072164
a_HQ_transcript/50069	3.73308318551207	0.166001918445635	2.85391899195883	0.00744864911132252	0.359912780999963	-2.43270138914425
a_HQ_transcript/11065	3.96465092057971	1.91102143462258	2.8516151317054	0.00749181321139341	0.361375274041117	-2.47746842619791
a_HQ_transcript/127080	-6.37530725438114	0.8860383860516	-2.85117869785566	0.00750001643045259	0.361375274041117	-2.42903052372007
a_HQ_transcript/98914	1.22763069156831	3.2962949989103	2.85107038750392	0.00750205353842673	0.361375274041117	-2.5758271310766
a_HQ_transcript/101563	-4.85782652687375	0.355130980199058	-2.85097840334065	0.00750378398994681	0.361375274041117	-2.44370070021446
a_HQ_transcript/100929	2.2317475560367	1.04590010966358	2.84967922316995	0.00752826483086298	0.362254120934109	-2.45629384567958
a_HQ_transcript/130871	3.25211880953559	-0.11194380161293	2.84907728542552	0.00753963270018976	0.362347924602933	-2.44305032094134
a_HQ_transcript/109415	3.8847684160835	2.21676878941582	2.84891599513404	0.00754268147484086	0.362347924602933	-2.41926186260835
a_HQ_transcript/96043	1.12697654567306	3.96745718664858	2.84818575088376	0.00755649932199613	0.362663042753607	-2.64325619663462
a_HQ_transcript/73342	-1.84747199902108	2.78158433583561	-2.84791022243941	0.00756171909952814	0.362663042753607	-2.50665262768307
a_HQ_transcript/90657	-3.40856436646745	-0.221549354888588	-2.84696974520371	0.00757956155017186	0.363219088036595	-2.46365567167605
a_HQ_transcript/67108	-1.75981474516935	2.72419445416944	-2.84638104290112	0.00759075031655717	0.363455629654724	-2.58181198472632
a_HQ_transcript/45014	-3.58373915437843	0.459709934126124	-2.84582013719844	0.00760142519305453	0.363667196396604	-2.42119167337662
a_HQ_transcript/125218	-2.39540640758594	-0.650986979244385	-2.84543297377981	0.00760880170347118	0.3637207445883	-2.46821208600973
a_HQ_transcript/60314	2.42318436356337	0.137906351513572	2.84383534241937	0.00763931195728983	0.364603957582762	-2.42923950440335
a_HQ_transcript/51735	-3.91145273076111	1.76028028294517	-2.84380864318202	0.00763982281062147	0.364603957582762	-2.47260846913319
a_HQ_transcript/38874	-4.6246572651844	-0.783249590441018	-2.84285526020208	0.00765808549424195	0.36496817479252	-2.47752016977058
a_HQ_transcript/148820	-5.85744845583192	0.989387168333997	-2.8426698217272	0.00766164244284142	0.36496817479252	-2.48738939199335
a_HQ_transcript/105496	5.85175405800785	2.00785678589973	2.84242761436793	0.00766629062451257	0.36496817479252	-2.47902385141667
a_HQ_transcript/93894	-4.33973142258655	-0.322851568903329	-2.84151904694156	0.00768375043834723	0.365500037217879	-2.44686200239456
a_HQ_transcript/76999	2.11330677781858	-0.586262797810553	2.84020849554012	0.00770900079978708	0.366165137945393	-2.46853275219919
a_HQ_transcript/92849	-3.80239506885604	0.536930312033151	-2.84013955918875	0.0077103311458361	0.366165137945393	-2.44241649924684
a_HQ_transcript/135021	-2.29940785900592	-0.644770677030238	-2.83898511419638	0.00773264183614142	0.366613253416016	-2.47597095407982
a_HQ_transcript/106010	7.31113441696798	2.22410936206418	2.83892894010466	0.00773372899355731	0.366613253416016	-2.55710809109697
a_HQ_transcript/50614	-2.62738672109702	-0.689832405219106	-2.8386727938238	0.00773868810111223	0.366613253416016	-2.46220292904837
a_HQ_transcript/53365	2.84616065040584	0.103221595360788	2.83781060309103	0.00775540241348642	0.366881621312457	-2.44844030248968
a_HQ_transcript/135867	3.04212321121379	2.99653694879985	2.83754120514947	0.00776063185953169	0.366881621312457	-2.61854628252131
a_HQ_transcript/51480	3.48785568134878	0.403807290552986	2.83714038477822	0.0077684185428475	0.366881621312457	-2.45686351640452
a_HQ_transcript/41658	-2.71147677886767	-0.507213044614683	-2.83707963122089	0.0077695994329004	0.366881621312457	-2.46823100353555
a_HQ_transcript/147204	4.19134169221324	-0.0647763182087915	2.8350958465268	0.00780825157883163	0.368194925448035	-2.48696849884292
a_HQ_transcript/84899	-3.89552378226299	0.252886619101655	-2.83500222138219	0.00781008022084756	0.368194925448035	-2.46170717723101
a_HQ_transcript/40571	-3.59101926220545	0.86778775546886	-2.83404712907603	0.0078287575754728	0.368776353603795	-2.51543033181872
a_HQ_transcript/68147	2.46654039469732	-0.721307025838766	2.83340030484433	0.00784143034200109	0.369074220987724	-2.4688568954855
a_HQ_transcript/6065	1.18988265115213	3.87445044736122	2.83187463078428	0.00787139797212101	0.369870807859099	-2.67539737782331
a_HQ_transcript/143403	-1.95829205213762	4.62380015103236	-2.83167557934482	0.00787531568918261	0.369870807859099	-2.79427666591125
a_HQ_transcript/8953	4.83321739427396	0.302924118079694	2.83096596285525	0.0078892971848428	0.369870807859099	-2.53489496603242
a_HQ_transcript/132651	4.24308023417753	0.32076135746216	2.83073684609139	0.00789381641177134	0.369870807859099	-2.49043228027371
a_HQ_transcript/146140	-4.06541879955046	0.778242290550657	-2.83073517767223	0.00789384932949559	0.369870807859099	-2.45213914408289
a_HQ_transcript/12224	2.07615429655169	1.84298640276824	2.83054361539389	0.00789762968716683	0.369870807859099	-2.46986658090455
a_HQ_transcript/78683	-3.30520581706201	-0.324344934009399	-2.8302768943075	0.00790289608039156	0.369870807859099	-2.47176186445606
a_HQ_transcript/106471	4.01536410221926	0.610087550992049	2.82981513710124	0.00791202123064667	0.369999975941295	-2.48139548583253
a_HQ_transcript/89584	-2.9822034112398	-0.0723458286591956	-2.82884074937242	0.00793130928156415	0.370603815047235	-2.48302501314087

a_HQ_transcript/150131	3.45286899598626	5.19120876994731	2.82831552815244	0.00794172432332109	0.370792410816071	-2.82014556366021
a_HQ_transcript/50375	2.40453883436395	-0.642217403243245	2.82770460230396	0.00795385499085717	0.371060740697067	-2.49686075539703
a_HQ_transcript/97579	-0.834755782155192	4.80036887168172	-2.82677724014636	0.00797230205790461	0.371619509504946	-2.77308777689892
a_HQ_transcript/151417	-8.73074834573574	5.94916469651856	-2.82604251159097	0.00798694570748348	0.371619509504946	-2.89892915928833
a_HQ_transcript/98719	-2.37930428609258	-0.60800649043256	-2.82581135513427	0.00799155802370247	0.371619509504946	-2.49261969509218
a_HQ_transcript/152524	-3.12145342610992	-0.505656098365158	-2.82551172984297	0.00799754022662185	0.371619509504946	-2.52926111725594
a_HQ_transcript/101242	5.37964171887391	3.718121030832	2.82535885252543	0.00800059413191798	0.371619509504946	-2.72680308570736
a_HQ_transcript/130615	2.26943762213765	-0.757092072938776	2.82514212257545	0.0080049254409987	0.371619509504946	-2.5029691882814
a_HQ_transcript/17465	-3.68992260669792	0.191253276981692	-2.82485912909792	0.00801058432097607	0.371619509504946	-2.51569196955917
a_HQ_transcript/92964	2.30966096656908	-0.767407270870732	2.82409832594201	0.00802581630606087	0.372029226665635	-2.5075966206396
a_HQ_transcript/149104	9.10345805795662	3.35571278426289	2.82319710282364	0.00804389472694138	0.372426313431909	-2.67796836671204
a_HQ_transcript/148209	-5.7779322959008	1.62041905022058	-2.82296844177824	0.00804848770039004	0.372426313431909	-2.51723513703345
a_HQ_transcript/93742	6.0094177132727	0.791699198198789	2.82189576072923	0.00807006678759331	0.372426313431909	-2.49197965460739
a_HQ_transcript/97765	2.85567663617352	0.424808591523723	2.82151167897095	0.00807780651124528	0.372426313431909	-2.48576290071398
a_HQ_transcript/145910	-4.25225666754134	1.51756958829892	-2.82105731357299	0.00808697151279241	0.372426313431909	-2.57882753907523
a_HQ_transcript/139821	-2.33338045629157	2.15632496137276	-2.82095551584112	0.00808902620895111	0.372426313431909	-2.55432647011968
a_HQ_transcript/144915	4.72027010199007	2.67838676507957	2.82086755167404	0.00809080208068732	0.372426313431909	-2.61968487539371
a_HQ_transcript/124153	3.93459178581163	0.78502195599118	2.82065252188547	0.00809514476420239	0.372426313431909	-2.48846118062136
a_HQ_transcript/140828	3.49827371606259	0.148954925165702	2.82055888460982	0.00809703651941886	0.372426313431909	-2.53146174184967
a_HQ_transcript/36155	-1.32405004543302	2.07203015982514	-2.82048879913769	0.00809845272808169	0.372426313431909	-2.50310803806911
a_HQ_transcript/17856	2.57288945460513	2.74835255163253	2.8193886002251	0.00812071473549044	0.373154866517461	-2.57953810979412
a_HQ_transcript/122676	-4.97016452781074	0.916759081443619	-2.81848618361008	0.00813901747253228	0.373604633251409	-2.4968025070351
a_HQ_transcript/82829	-4.46599824544682	0.880930800362315	-2.81786736337036	0.00815159064783248	0.373604633251409	-2.54153302346653
a_HQ_transcript/118033	1.15488679919299	3.18180650872016	2.81750354018512	0.00815899128514494	0.373604633251409	-2.62322366349365
a_HQ_transcript/151038	-4.63482448312677	1.60803509533166	-2.81739431932097	0.00816121420709593	0.373604633251409	-2.51976718382222
a_HQ_transcript/70785	-5.9757627243762	0.941266525698555	-2.81671452174088	0.00817506256640082	0.373604633251409	-2.56843026380775
a_HQ_transcript/72461	-4.1410904561801	0.607391008998397	-2.81669390589005	0.00817548288154518	0.373604633251409	-2.51920373250509
a_HQ_transcript/127656	2.09529188107087	0.593975046542683	2.81669337889997	0.00817549362606304	0.373604633251409	-2.50195058949946
a_HQ_transcript/124547	-2.87516641177018	3.34850842045206	-2.81626324911067	0.008184267721621	0.373711794283099	-2.57437079151059
a_HQ_transcript/133389	-3.57617780431861	0.671838200834238	-2.81500980810143	0.00820988660970293	0.37436754653251	-2.5127125905136
a_HQ_transcript/147164	4.39952996613598	0.0109127327344784	2.81443944443078	0.00822156901974097	0.37436754653251	-2.5335951821317
a_HQ_transcript/100192	0.845159141282661	4.90507862793733	2.81385889146793	0.00823347610985812	0.37436754653251	-2.81340936566277
a_HQ_transcript/129099	2.84249396441282	0.518181973656472	2.81366576071283	0.0082374407814056	0.37436754653251	-2.49956863815459
a_HQ_transcript/144355	4.34989921791485	0.299625718619977	2.81352713433854	0.00824028766508197	0.37436754653251	-2.52317203147191
a_HQ_transcript/47942	-3.83890742853281	0.278050982803833	-2.81338093075603	0.00824329115498037	0.37436754653251	-2.51935865501707
a_HQ_transcript/32498	-2.26072678143174	1.95086635104178	-2.81314031271122	0.00824823645137641	0.37436754653251	-2.51136953211114
a_HQ_transcript/73662	4.50315796630457	1.04886862873141	2.81303566776325	0.00825038803040205	0.37436754653251	-2.48797253630938
a_HQ_transcript/25646	-4.10517654030397	1.6796663130408	-2.81249806823926	0.00826144976600956	0.37436754653251	-2.53987368018206
a_HQ_transcript/133897	-1.11142896087132	5.08035718521087	-2.81227987164841	0.0082659433738424	0.37436754653251	-2.85887566575234
a_HQ_transcript/10734	-4.10376717155389	0.333440898582171	-2.81206378965639	0.00827039568738706	0.37436754653251	-2.5375535883775
a_HQ_transcript/40268	-3.87048805706022	0.434698049694918	-2.81155193158851	0.00828095134784185	0.37436754653251	-2.53652519332697
a_HQ_transcript/52873	3.00088939222762	5.40109411582448	2.81068742315785	0.00829880808605417	0.37436754653251	-2.88260795676756
a_HQ_transcript/130026	-5.97470219081459	0.497421121679785	-2.8101140938557	0.00831067027985194	0.37436754653251	-2.53016168934131
a_HQ_transcript/87336	-1.42195570282001	2.58747213474798	-2.80988393836656	0.00831543666315967	0.37436754653251	-2.52467454091184
a_HQ_transcript/97884	-2.70052516097664	1.48031578415603	-2.80961981615285	0.0083209096294802	0.37436754653251	-2.51158220220683
a_HQ_transcript/145176	-7.09038964387247	3.91424539970164	-2.80938862043765	0.00832570308119846	0.37436754653251	-2.8285074470064
a_HQ_transcript/17356	2.57610926221954	-0.570307471132133	2.8091951965902	0.00832971538237463	0.37436754653251	-2.54732393680049
a_HQ_transcript/127782	5.66169524746913	3.15961786144705	2.80911517178314	0.00833137591169506	0.37436754653251	-2.73723361483446
a_HQ_transcript/14470	-3.03862758486448	-0.335447478215175	-2.80906043860163	0.00833251181356114	0.37436754653251	-2.51089157857342
a_HQ_transcript/138268	-4.03972481176013	1.66347098067858	-2.80889948820183	0.00833585292796597	0.37436754653251	-2.54700138237995

a_HQ_transcript/130402	-2.89852204375066	-0.555584172576024	-2.80868451443688	0.00834031745044729	0.37436754653251	-2.55149011221594
a_HQ_transcript/138314	2.5169849477778	-0.708985630750343	2.80681831700541	0.00837916830961737	0.37582121566469	-2.54224949489698
a_HQ_transcript/116517	2.78705321670432	2.62552932942181	2.80624809173796	0.00839107310326606	0.375898915769752	-2.5789146671815
a_HQ_transcript/96323	1.12504450336076	2.72398882307808	2.80611594808006	0.00839383417060861	0.375898915769752	-2.56732110998927
a_HQ_transcript/81900	-2.29692923817178	4.43442588005627	-2.80565086625147	0.0084035585555978	0.375981105764032	-2.82659133647258
a_HQ_transcript/12643	2.99499138274646	-0.080270026604455	2.80540967380724	0.00840860579227295	0.375981105764032	-2.56064169336853
a_HQ_transcript/54959	-2.75548281870781	-0.544210635832854	-2.8049349914398	0.00841854736574341	0.375988642320917	-2.53309619453199
a_HQ_transcript/131764	4.52050406734685	0.871579699293071	2.80478405059639	0.00842171091903788	0.375988642320917	-2.51127409435221
a_HQ_transcript/136897	-2.34590425097009	-0.713207397746442	-2.8031263918081	0.00845652686846867	0.377253256953451	-2.54739927473404
a_HQ_transcript/23821	-2.56432186439163	-0.608060845643662	-2.80130491941189	0.00849493864412144	0.378344730469992	-2.54228647839376
a_HQ_transcript/144391	4.44287008921941	1.7981613750526	2.80086696703765	0.00850419862600746	0.378344730469992	-2.60211694782272
a_HQ_transcript/134476	5.46490387486738	1.49429585787687	2.80055632998929	0.00851077239914075	0.378344730469992	-2.59170736759999
a_HQ_transcript/66300	-3.92759402605582	0.920926942895672	-2.80051336890587	0.00851168192556115	0.378344730469992	-2.51951788114928
a_HQ_transcript/35683	1.12685413575462	3.60657598283023	2.80027607132431	0.00851670737601407	0.378344730469992	-2.71843275333067
a_HQ_transcript/119318	3.3990113972083	-0.109638241464251	2.8001184818211	0.00852004631477462	0.378344730469992	-2.53656352593134
a_HQ_transcript/116570	-2.99667461253181	0.733964244077734	-2.79978678649731	0.00852707813381163	0.378367937223055	-2.53038553057386
a_HQ_transcript/38923	-3.46252760934937	0.923019651595772	-2.79942118858954	0.00853483496348145	0.378423254582189	-2.54456198332656
a_HQ_transcript/117343	4.1324695873754	1.29415797285373	2.79818414626218	0.00856113005311292	0.379085475843773	-2.57867420500112
a_HQ_transcript/150702	8.82942557294292	4.03780736655348	2.79810046524637	0.00856291154493404	0.379085475843773	-2.76374926506552
a_HQ_transcript/26782	-3.3849882423845	-0.695510736562858	-2.79751386039471	0.00857540955898928	0.379085475843773	-2.56324179633253
a_HQ_transcript/150518	-2.28761844607963	-0.771909796476643	-2.79735077794348	0.00857888717047257	0.379085475843773	-2.55924402930665
a_HQ_transcript/82625	-2.96988905817663	-0.378000190697008	-2.79718712342174	0.00858237830667501	0.379085475843773	-2.58613789820728
a_HQ_transcript/23891	-3.54650876718316	1.22055022373851	-2.79658637715687	0.00859520503143506	0.379185290144133	-2.57344674689451
a_HQ_transcript/152827	-3.51621286857546	-0.499717163297113	-2.79636598590016	0.0085999151682169	0.379185290144133	-2.56690791067596
a_HQ_transcript/101523	4.69449139040668	2.06379977499674	2.79609187576654	0.00860577673672353	0.379185290144133	-2.68785926923744
a_HQ_transcript/121286	2.19598360617622	2.53902201145367	2.79586032173797	0.00861073119650179	0.379185290144133	-2.63079748023642
a_HQ_transcript/110536	2.12787535051278	4.55316086848424	2.79459792855817	0.00863778900816298	0.379311965769134	-2.82475560640037
a_HQ_transcript/147793	-2.83655141050459	-0.653282701227247	-2.79444256804123	0.0086411244541393	0.379311965769134	-2.57351724312735
a_HQ_transcript/58266	3.16429056882505	1.74447181642648	2.79435742440242	0.00864295291976308	0.379311965769134	-2.61771283574042
a_HQ_transcript/22197	-2.98888020194888	0.487503976185695	-2.79404059058705	0.00864976012634129	0.379311965769134	-2.52815436587852
a_HQ_transcript/117852	4.6919372336444	4.01536123011467	2.79376725724053	0.00865563674823635	0.379311965769134	-2.73119426494217
a_HQ_transcript/9728	2.46268966051025	0.0814958939403977	2.79376430180361	0.00865570031003730	0.379311965769134	-2.55214964546722
a_HQ_transcript/24370	-4.27693357196828	2.2593259394145	-2.79359760903712	0.00865928603385015	0.379311965769134	-2.60919982497065
a_HQ_transcript/144365	-2.22585095008462	-0.480079117962015	-2.79298758916262	0.00867241999979814	0.37960122722008	-2.59390758634616
a_HQ_transcript/61271	-1.5296899361421	-0.483844077709226	-2.79187292929516	0.00869646718165665	0.379942002278636	-2.58815576306202
a_HQ_transcript/90352	-2.74631741924029	3.33294336446017	-2.79174095507294	0.00869931845901055	0.379942002278636	-2.73293262014243
a_HQ_transcript/100521	-2.45872448324716	0.847749817232479	-2.79164491856271	0.00870139385843766	0.379942002278636	-2.54055073518779
a_HQ_transcript/61731	-6.62534476881059	3.89283626321406	-2.79141563867833	0.00870635058896131	0.379942002278636	-2.66121391256381
a_HQ_transcript/144925	-2.77977049902438	-0.652195007984853	-2.79058982923917	0.00872422538940975	0.380136998284872	-2.58116451370241
a_HQ_transcript/80768	-3.62502376750246	1.09581543884856	-2.79055631993392	0.00872495142836648	0.380136998284872	-2.63192600696902
a_HQ_transcript/145155	5.78068465378509	1.82141969747434	2.79023285732629	0.00873196272817004	0.380136998284872	-2.58538960866151
a_HQ_transcript/141421	-3.24231003430712	-0.629764724341763	-2.79000165110996	0.00873697752732915	0.380136998284872	-2.57945394414863
a_HQ_transcript/141313	-4.77518071904312	1.31882339334331	-2.78858344732314	0.00876779692565105	0.381188085226593	-2.58138590824859
a_HQ_transcript/127940	3.04859973030124	2.33899485497814	2.78828704617925	0.00877425092955515	0.381188085226593	-2.63603727670333
a_HQ_transcript/32998	2.89262525766203	-0.558917697613199	2.78694128191089	0.00880361023926632	0.38207851709034	-2.56817301591257
a_HQ_transcript/22295	2.16744346542647	-0.756223183863271	2.78674531375418	0.0088078931478987	0.38207851709034	-2.5852165081169
a_HQ_transcript/31967	-3.66827166866047	0.449924458495863	-2.78592713244498	0.00882579564834874	0.382569611817461	-2.55197951391164
a_HQ_transcript/54563	1.43910632885045	3.09376052321606	2.78432016138848	0.00886105656278140	0.383656341715177	-2.71599899084873
a_HQ_transcript/128576	1.25987021673628	3.00633115967974	2.78418325693859	0.00886406666190964	0.383656341715177	-2.64733871716228
a_HQ_transcript/142781	2.81931684195468	-0.731095132534237	2.78324743728653	0.00888466800059058	0.383907249984677	-2.58972275071425

a_HQ_transcript/43715	3.08127649846036	0.108470564374149	2.78302542551963	0.0088895619777879	0.383907249984677	-2.56922297960822
a_HQ_transcript/63382	-2.37913599395183	-0.528935620528106	-2.78302019820801	0.00888967723780922	0.383907249984677	-2.5813988746594
a_HQ_transcript/98138	-1.18184624624089	2.62081573957249	-2.78237225618018	0.00890397490533705	0.38423923778577	-2.63707584783911
a_HQ_transcript/52597	2.20255784801469	2.00531205122072	2.7810307968937	0.00893364422997827	0.385060089662304	-2.62088680914275
a_HQ_transcript/66229	-1.40298087314136	3.09934017995	-2.78074474557267	0.00893998282077723	0.385060089662304	-2.698400102762
a_HQ_transcript/83036	3.39456188710823	0.990078987950022	2.78050950349129	0.00894519868145975	0.385060089662304	-2.61750176278936
a_HQ_transcript/149833	6.38935231801248	8.2884183037794	2.78026037597965	0.00895072551376289	0.385060089662304	-3.28501594551226
a_HQ_transcript/132403	-4.65795361785178	0.666047414484463	-2.78001743178652	0.00895611824290247	0.385060089662304	-2.59215254494486
a_HQ_transcript/134402	3.66949567114706	1.0064524672045	2.77891343411463	0.00898066237727376	0.385829965015646	-2.60658332451103
a_HQ_transcript/102034	-4.1600066242487	0.891433921346606	-2.778360952848	0.00899296874652887	0.386073328876093	-2.56409298809786
a_HQ_transcript/104160	-4.03897598467689	-0.0262881152918367	-2.77734007394884	0.00901575000152734	0.386765694530466	-2.58412711610768
a_HQ_transcript/97223	-4.53418355158373	-0.784249181043406	-2.77607985558314	0.00904394653963045	0.387571680757587	-2.60489579857712
a_HQ_transcript/148736	-3.49484303561363	2.47499763948427	-2.77590465027577	0.00904787315558846	0.387571680757587	-2.62635527563655
a_HQ_transcript/91896	-4.16861264404331	0.79638924571459	-2.77534858546671	0.00906034595776418	0.387820169243679	-2.68042001754606
a_HQ_transcript/152035	2.75121642655292	2.04997274635871	2.77265782586465	0.00912092817232864	0.389863212379949	-2.60110921522459
a_HQ_transcript/46431	3.46732309335966	-0.180371638952356	2.77263295191543	0.00912148996760134	0.389863212379949	-2.61168679321253
a_HQ_transcript/57137	4.00007360671889	1.30497893806424	2.77199986260655	0.00913579960741734	0.390187920337954	-2.60437786579972
a_HQ_transcript/117685	1.06009252101024	2.30962434290382	2.77136300943279	0.00915021546841743	0.390516684837128	-2.6590816320763
a_HQ_transcript/60507	-3.00837839796436	1.94232282470609	-2.77028492599181	0.00917466747541083	0.391152271710726	-2.61283402031079
a_HQ_transcript/58771	-6.07430116598823	0.627950794464158	-2.76962118821456	0.00918975203852419	0.391152271710726	-2.62122423252134
a_HQ_transcript/137088	-5.56240945331473	0.933174244077058	-2.7691538236653	0.00920038757732146	0.391152271710726	-2.68308021783904
a_HQ_transcript/21918	4.37161258051075	2.09861926404866	2.7690970679805	0.00920167991509976	0.391152271710726	-2.71764088521551
a_HQ_transcript/121730	-1.11757630333627	2.90552000637959	-2.76897229117126	0.00920452170275586	0.391152271710726	-2.69671503704283
a_HQ_transcript/56768	2.55069257551558	1.8322146181347	2.76893009694382	0.00920548286024417	0.391152271710726	-2.59045977374759
a_HQ_transcript/3277	-2.35919630842399	0.166797906163605	-2.76802218810528	0.00922618715574702	0.391265662493936	-2.61862569545376
a_HQ_transcript/54126	2.01630386624414	1.57580793005986	2.76796188581474	0.00922756385035131	0.391265662493936	-2.58583316592472
a_HQ_transcript/20623	3.58109156489896	-0.125838880917933	2.76792768466639	0.00922834474399921	0.391265662493936	-2.62026394909789
a_HQ_transcript/148338	2.5378419670153	-0.745085973132692	2.76734266410133	0.00924171171292248	0.391546806449532	-2.62852657414832
a_HQ_transcript/72215	3.27584394291913	-0.0362673011158108	2.7665947447261	0.00925882703507821	0.391720605551459	-2.58760451134705
a_HQ_transcript/51450	-4.31035321736782	2.29162345949878	-2.76647162709468	0.00926164728288385	0.391720605551459	-2.7202590160263
a_HQ_transcript/76948	4.37032358704266	1.16689199157399	2.76625755163867	0.00926655300528028	0.391720605551459	-2.62844031447454
a_HQ_transcript/107761	4.68739757735156	1.32190334800668	2.76597714014654	0.0092729825441878	0.391720605551459	-2.62927834240041
a_HQ_transcript/86956	3.57549474696403	5.23132355362299	2.7656927063339	0.00927950856462219	0.391720605551459	-2.96283848935984
a_HQ_transcript/32370	2.53374842509063	-0.665738584476301	2.76466078988257	0.00930322075718095	0.392154516607624	-2.62816330665926
a_HQ_transcript/144451	2.02907749184402	-0.806579878445917	2.76465820092618	0.00930328031932826	0.392154516607624	-2.6243901718167
a_HQ_transcript/145126	2.45459471989058	-0.684650925603416	2.76312713878495	0.00933856671006562	0.393356670813547	-2.63656417967204
a_HQ_transcript/90232	-2.59745960289321	-0.765566995938051	-2.76188722817081	0.00936723442617708	0.394160802630268	-2.62515287865832
a_HQ_transcript/69175	3.41472470159888	0.0908807915530408	2.76156642361105	0.00937466504382678	0.394160802630268	-2.59755201499788
a_HQ_transcript/80707	3.43993639467055	0.362855490134841	2.76142251508489	0.00937800010386837	0.394160802630268	-2.6400040333472
a_HQ_transcript/32622	-2.30503079597764	1.42874634560535	-2.76111878203865	0.00938504270963804	0.394171793804798	-2.61448862584378
a_HQ_transcript/123343	2.61003830644629	-0.592308387415585	2.75980455282863	0.00941557243009593	0.395051707254583	-2.63868306347943
a_HQ_transcript/111255	3.9345972116272	1.26588679223136	2.7596320956127	0.00941958550534772	0.395051707254583	-2.6124431046203
a_HQ_transcript/91194	-6.4947286604826	1.77713716381877	-2.75923522192157	0.00942882680982006	0.395115752560869	-2.70047301358238
a_HQ_transcript/77550	4.62744657210295	0.88299637646722	2.75898287046549	0.00943470727626076	0.395115752560869	-2.6536198434035
a_HQ_transcript/77401	6.73680846031196	3.70426911256664	2.75843486988751	0.00944748894680411	0.395216681078586	-2.95727723800282
a_HQ_transcript/96464	-3.64252423748726	1.44379345934332	-2.75829664705409	0.00945071543316877	0.395216681078586	-2.70559834438915
a_HQ_transcript/133826	3.80180794259093	0.083481601107979	2.75724266197439	0.00947535206051964	0.395962088119256	-2.62944499609032
a_HQ_transcript/97781	3.6079002090352	0.549464575845843	2.756641319294	0.00948943512119007	0.396029671256712	-2.65996603676671
a_HQ_transcript/104071	-3.81474631396028	1.19598715606934	-2.75652000598829	0.00949227856717848	0.396029671256712	-2.61463126106659
a_HQ_transcript/133061	3.09288151728137	1.84484951484263	2.7563012269884	0.00949740850763585	0.396029671256712	-2.65703592536169

a_HQ_transcript/144006	-3.43392418294313	-0.408364312661305	-2.75592486274888	0.0095062395664406	0.396112975308539	-2.68861046276187
a_HQ_transcript/21707	1.78378373614485	0.553529294652608	2.75563546425328	0.00951303525892377	0.396112975308539	-2.67000986406526
a_HQ_transcript/144910	-4.45230259745309	-0.678470751681214	-2.75413399778619	0.00954836569355475	0.396707924822404	-2.6583950061055
a_HQ_transcript/135855	3.2288253106791	1.25709281507988	2.75405309625874	0.0095502728272721	0.396707924822404	-2.61645441696151
a_HQ_transcript/150312	4.155997932265	0.83002446420306	2.75393453169121	0.00955306845332905	0.396707924822404	-2.68590698363517
a_HQ_transcript/147604	2.98630212773905	-0.722207984417764	2.75364207354864	0.00955996756840554	0.396707924822404	-2.654354486169
a_HQ_transcript/1643	-4.13160667903715	1.28659352657651	-2.75290907151115	0.00957727958144536	0.396707924822404	-2.63390363854528
a_HQ_transcript/86597	2.46262868731355	0.197455234146169	2.75286393140329	0.00957834665394344	0.396707924822404	-2.62370906985086
a_HQ_transcript/7402	-5.36491953418508	2.29638861400905	-2.75277879761219	0.00958035944376972	0.396707924822404	-2.78735655168509
a_HQ_transcript/52398	-1.61635087797528	3.18579134681974	-2.75258102811621	0.00958503676477876	0.396707924822404	-2.76099701548752
a_HQ_transcript/144942	5.37018087285923	2.37061821126714	2.75224576641709	0.00959297069264232	0.396707924822404	-2.71164151788058
a_HQ_transcript/3711	2.81348673649322	3.02693241156319	2.75213594863144	0.00959557084882157	0.396707924822404	-2.77427481128542
a_HQ_transcript/86660	2.56322556307871	5.45706332630578	2.75173457233172	0.00960507982975072	0.396719953376992	-2.85155429586107
a_HQ_transcript/15341	3.21509453451488	-0.0710068119565169	2.75154762991834	0.00960951167001797	0.396719953376992	-2.62114673901062
a_HQ_transcript/128864	2.78723874590414	1.68290467865895	2.74996081328352	0.00964720717229622	0.397651925373702	-2.67468825002969
a_HQ_transcript/78155	2.29983501776752	-0.734644806932157	2.74987461731017	0.00964925873770532	0.397651925373702	-2.65649457042485
a_HQ_transcript/82290	2.85596677395031	-0.485871356324725	2.74973388590169	0.00965260918494174	0.397651925373702	-2.69636224164614
a_HQ_transcript/103149	2.4700976220994	-0.685195230823262	2.74883969949922	0.00967392278524456	0.398247722139303	-2.66643381401601
a_HQ_transcript/141198	7.65304541595498	3.89965245748788	2.74808821141938	0.00969186899412145	0.398666964867857	-2.78999618183742
a_HQ_transcript/148863	-3.07151172653259	-0.431669485558567	-2.74733142075257	0.00970997320182826	0.398666964867857	-2.65458746954804
a_HQ_transcript/7888	-2.9902444324876	1.46538089320661	-2.74730921376672	0.0097105049209166	0.398666964867857	-2.61511958723486
a_HQ_transcript/142183	-0.896297171561255	4.77170822508089	-2.74726596939463	0.00971154043237141	0.398666964867857	-2.95358180266133
a_HQ_transcript/112437	1.84473558842444	-0.649740804654916	2.74666425473047	0.00972595952453332	0.398823511079057	-2.68332231999762
a_HQ_transcript/146777	-5.53145409820392	2.29508187124985	-2.74653430579761	0.00972907615452282	0.398823511079057	-2.81577964259172
a_HQ_transcript/55765	-2.78422411748229	0.557673593346675	-2.74574683511866	0.00974798238253384	0.399165258051422	-2.65559005472314
a_HQ_transcript/72865	-2.8783455222139	2.20575422715454	-2.74561516810829	0.00975114689019095	0.399165258051422	-2.65062029792417
a_HQ_transcript/143331	-5.78703984808697	1.85553995507328	-2.74451388906799	0.00977765275167885	0.399741708864874	-2.7223891534082
a_HQ_transcript/151697	1.96571016358261	-0.489196100510387	2.74422120459677	0.00978470844431151	0.399741708864874	-2.66538838911264
a_HQ_transcript/72014	4.55208296066865	0.615828501857216	2.74417346750343	0.00978585968405442	0.399741708864874	-2.64274004018105
a_HQ_transcript/54442	-4.20713127923645	0.25195545131165	-2.74177489293596	0.00984386713694539	0.401563368621573	-2.68111978051716
a_HQ_transcript/4675	-1.2657960829489	2.65260333575863	-2.74175822859798	0.00984427126833439	0.401563368621573	-2.70072839207358
a_HQ_transcript/138853	-6.09979303863336	2.11998331345421	-2.74138514545834	0.00985332305861142	0.401650745267156	-2.75020258672831
a_HQ_transcript/101970	-4.84032762670304	2.74763897912145	-2.73968868044426	0.00989458082805409	0.402516093221052	-2.65188233927429
a_HQ_transcript/116792	2.77265073806217	1.72994717395375	2.73956043982881	0.00989770615647758	0.402516093221052	-2.69794645005589
a_HQ_transcript/108202	5.62922696343006	1.01263282569813	2.73943341171975	0.00990080284210361	0.402516093221052	-2.67372653324814
a_HQ_transcript/12944	-3.15422316436212	2.82893496715228	-2.73909634432574	0.00990902423334566	0.402516093221052	-2.73161977111366
a_HQ_transcript/104197	3.36180942722979	0.0758592754364378	2.7386226465072	0.00992058892286682	0.402516093221052	-2.6609068578331
a_HQ_transcript/124870	4.48198193284989	2.24874223444668	2.73855542975443	0.00992223094733091	0.402516093221052	-2.77325749292401
a_HQ_transcript/65780	2.14615523964446	-0.726898710329288	2.73852295377076	0.00992302438731365	0.402516093221052	-2.6855885879661
a_HQ_transcript/116540	2.07156336889659	2.12896993742476	2.73766894244173	0.00994391047658937	0.403082028021748	-2.66997316758782
a_HQ_transcript/122696	-5.70476723704347	0.78753852378154	-2.73730255722607	0.00995288351123227	0.403164608181818	-2.67799183143636
a_HQ_transcript/91900	-2.58435820199679	-0.599107165716799	-2.73600179538025	0.00998480107252714	0.403962129575214	-2.68676187702068
a_HQ_transcript/12626	2.68120683293464	0.372512460058695	2.73593385045995	0.00998647089525844	0.403962129575214	-2.63896216594885
a_HQ_transcript/115349	7.70371917280323	3.21427592878087	2.73540195667207	0.00999955177641321	0.404209976119156	-2.82564752836829
a_HQ_transcript/98628	-2.70421668575273	2.18530008396637	-2.73490513118782	0.0100117846400423	0.404423222763293	-2.69498498749084
a_HQ_transcript/40826	4.37062068734802	-0.389488759925219	2.73381801063902	0.0100386004284817	0.404904063757409	-2.73026070657446
a_HQ_transcript/89891	-2.38921771101418	-0.689059853363097	-2.73373705145434	0.0100406001075001	0.404904063757409	-2.69659697650066
a_HQ_transcript/105631	-3.31181933464694	-0.406954761589802	-2.733575747468	0.0100445853966795	0.404904063757409	-2.70794475028832
a_HQ_transcript/133095	2.36365088720776	2.32642059087934	2.73284097816671	0.0100627577734378	0.405355498166592	-2.72481220988299
a_HQ_transcript/129542	-3.4009732240547	0.287190340256811	-2.73242848756259	0.0100729729444962	0.405485991217227	-2.67484089829359

a_HQ_transcript/54927	-2.04645654811688	1.80264920706811	-2.73199419411562	0.0100837384934275	0.405638443699621	-2.67808206510565
a_HQ_transcript/147471	0.723897831355971	8.90900833668764	2.73114356724517	0.0101048554477866	0.406206803228866	-3.35413090606359
a_HQ_transcript/108088	3.06468847044416	0.440143358068668	2.73065134946401	0.0101170936434423	0.406417705118187	-2.66644365907861
a_HQ_transcript/104441	-3.68237420718969	0.125278280299143	-2.72982234834084	0.010137736621185	0.406965714306798	-2.70046594619521
a_HQ_transcript/109347	-1.5657621651177	2.18533592581732	-2.72866140596425	0.0101667112201604	0.407847197933391	-2.72781320125986
a_HQ_transcript/109757	-4.27524785960172	0.134444918523548	-2.72793449893712	0.0101848924844562	0.408294779542392	-2.68230829463063
a_HQ_transcript/89757	2.7579233144505	1.47586671449569	2.72680688643053	0.0102131560471744	0.408987206382824	-2.66463563736863
a_HQ_transcript/95155	5.474175427715	1.17843430560758	2.72668414387363	0.0102162369885057	0.408987206382824	-2.70960231118767
a_HQ_transcript/148637	-6.60392637105203	2.67123051319356	-2.72623775550027	0.0102274490106113	0.409154271224233	-2.79985533304131
a_HQ_transcript/112742	1.54223668650228	2.49628585847689	2.72548896582644	0.0102462822381915	0.409625786754881	-2.70717357283008
a_HQ_transcript/30492	7.42948834181911	4.7560596477979	2.72490072705873	0.0102611000037086	0.409925418748482	-3.08828302633163
a_HQ_transcript/137144	-5.13374092594599	1.22791177520327	-2.72430597803407	0.01027610204624	0.409925418748482	-2.6943480507707
a_HQ_transcript/75084	2.51422658137572	-0.511101459840549	2.72429640793474	0.0102763436107878	0.409925418748482	-2.7161610307448
a_HQ_transcript/8352	-2.78062847924816	2.47089371016473	-2.72407294687267	0.0102819856271554	0.409925418748482	-2.69822930193885
a_HQ_transcript/150986	5.2022272725469	1.10901520667484	2.72318738347235	0.0103043729890718	0.410021628886818	-2.80133601826794
a_HQ_transcript/143908	-3.23606939847555	0.0208320200542809	-2.7228300241807	0.0103134200060647	0.410021628886818	-2.72607508812576
a_HQ_transcript/128159	-5.40476383267978	2.91249869976496	-2.72274910345778	0.0103154696464041	0.410021628886818	-2.87275063253042
a_HQ_transcript/36309	5.38199540725062	1.37323091670793	2.72243000225638	0.010323555854972	0.410021628886818	-2.78730854731756
a_HQ_transcript/21454	2.77474194206355	0.575451472458327	2.72221026808753	0.0103291274760611	0.410021628886818	-2.66465255815216
a_HQ_transcript/78823	1.38501030978144	2.76233598355347	2.72216968837546	0.0103301567290857	0.410021628886818	-2.76877801173357
a_HQ_transcript/116529	-5.7284322457672	0.594042673816807	-2.72202705405576	0.0103337752256948	0.410021628886818	-2.7181805118273
a_HQ_transcript/8893	-2.35793509812692	3.4062465547851	-2.72059692682483	0.0103701214733894	0.411183097547873	-2.84898736739614
a_HQ_transcript/131933	-1.10597164847682	3.79974146344773	-2.72001432301271	0.0103849622573107	0.411490856232417	-2.94415755499452
a_HQ_transcript/19939	5.68316980182709	1.80126001450058	2.71954981134197	0.0103968090188807	0.411679642131811	-2.71514211435578
a_HQ_transcript/77408	-3.30551154861334	0.667706138547425	-2.71852169844707	0.0104230744374829	0.412282354212632	-2.72859969422865
a_HQ_transcript/41455	4.14515028982417	0.663616197018449	2.71839890453194	0.0104262156050882	0.412282354212632	-2.72735158383507
a_HQ_transcript/62446	-4.60871862717806	1.93067745727761	-2.71677479364486	0.0104678446507256	0.413609098828289	-2.75959960466724
a_HQ_transcript/24756	-3.78029846006589	0.757627386204967	-2.71653521240578	0.0104739986491061	0.413609098828289	-2.76704856399053
a_HQ_transcript/108547	3.15983277375434	0.996175342671398	2.71603306825001	0.0104869078895352	0.413837733742634	-2.71617717451252
a_HQ_transcript/83396	3.01320348727308	0.0923177408538135	2.71525347293866	0.0105069792512306	0.414348500621121	-2.71710758207198
a_HQ_transcript/138402	2.55022823465936	-0.684173955435153	2.71421875362185	0.0105336742154304	0.414959208017452	-2.73881267331059
a_HQ_transcript/63202	2.50809838925644	1.57270866745961	2.71407146467957	0.0105374792842131	0.414959208017452	-2.77702561636355
a_HQ_transcript/139852	-2.97942802453977	1.32909525901659	-2.71382375115694	0.01054388161027	0.414959208017452	-2.81547798149925
a_HQ_transcript/115308	-3.0607059194314	0.230371617994469	-2.71258471583617	0.0105759596929668	0.415818384116539	-2.71884670977737
a_HQ_transcript/121291	-3.73622723151965	0.17225842065858	-2.71225643617818	0.0105844739128022	0.415818384116539	-2.72972477411323
a_HQ_transcript/142692	-3.32011039618078	0.959382926677772	-2.71203073086115	0.0105903314766015	0.415818384116539	-2.69855005542987
a_HQ_transcript/51438	-6.1879944922126	2.28766283233763	-2.71183023435757	0.0105955373439777	0.415818384116539	-2.78317043561004
a_HQ_transcript/149615	-0.753389352726588	7.49034356046908	-2.71104526928105	0.0106159417826471	0.415818384116539	-3.27067464111098
a_HQ_transcript/121472	-7.31730960347232	0.866916197002612	-2.71082933113163	0.0106215613024234	0.415818384116539	-2.74928519635696
a_HQ_transcript/128879	-1.26429976196199	3.29813315515279	-2.71065733919696	0.0106260391567681	0.415818384116539	-2.85021804608596
a_HQ_transcript/95866	-3.24158852132193	2.45288815887821	-2.71063083081867	0.0106267294654047	0.415818384116539	-2.74112854930563
a_HQ_transcript/34959	3.43989472597501	0.536311399038827	2.7105016432992	0.0106300942540114	0.415818384116539	-2.72723622324702
a_HQ_transcript/152972	-4.6211589862982	0.279549487072639	-2.70965066828167	0.0106522833336509	0.416234762795601	-2.74415267878602
a_HQ_transcript/53152	-6.19515495120796	1.61683394044111	-2.70954429667704	0.0106550599889873	0.416234762795601	-2.78819388052117
a_HQ_transcript/88898	5.08952198612588	0.927391584489085	2.7089797288851	0.0106698083732003	0.416454827792462	-2.7567837010812
a_HQ_transcript/141897	-4.94716849912991	0.402279447932202	-2.70878031542766	0.0106750222510798	0.416454827792462	-2.7735794715117
a_HQ_transcript/94494	-1.19077481356262	3.46022605324345	-2.70843022072041	0.0106841815842587	0.416532600355324	-2.87802865882369
a_HQ_transcript/7993	4.03729734463916	0.398430373880175	2.70719660800488	0.0107165141997004	0.417411630302597	-2.74092644001635
a_HQ_transcript/42985	-5.17651964286498	3.1989223280056	-2.70702227428762	0.0107210907659265	0.417411630302597	-2.90140218288115
a_HQ_transcript/83951	-3.1258958398068	3.17314003779149	-2.70666516811852	0.0107304711060553	0.417497205122343	-2.86818073527099

a_HQ_transcript/118518	1.59880783638605	0.314340185239928	2.70632359986261	0.0107394504434997	0.41756707383261	-2.72943415711314
a_HQ_transcript/73908	2.58344031855175	0.830560795652762	2.70600613815485	0.0107478023199637	0.41761246875324	-2.78148832371341
a_HQ_transcript/18490	-3.30153584485175	0.950280283434654	-2.70515771371669	0.0107701525740628	0.418201355260603	-2.73658707342801
a_HQ_transcript/56042	4.43289294400178	1.94335109374715	2.70486906371978	0.0107777664010795	0.418217627077402	-2.80963247204737
a_HQ_transcript/82306	-3.11818656372376	-0.614330413666867	-2.7038953785338	0.0108034865888925	0.418732643757229	-2.76426791010458
a_HQ_transcript/87150	-3.26699917841174	-0.396757825002415	-2.70368090569887	0.0108091596189768	0.418732643757229	-2.73692049470403
a_HQ_transcript/34193	3.96272336945485	1.51165180513175	2.70354901502901	0.0108126496400977	0.418732643757229	-2.81081631775617
a_HQ_transcript/91491	-2.95116457101037	-0.683839906177028	-2.70160510261651	0.0108642101027363	0.420449270873404	-2.76308407411621
a_HQ_transcript/75652	3.23303390249707	0.739255875400429	2.70127809939497	0.0108729059876778	0.420505841152185	-2.74835487952364
a_HQ_transcript/88021	-4.30389802822101	3.77448482305959	-2.70055240372999	0.010892227285898	0.420972997124122	-3.03671525183546
a_HQ_transcript/67981	3.21123387143599	0.222930413943468	2.69993182007329	0.0109087753427919	0.421332420681598	-2.74571582522617
a_HQ_transcript/35112	-2.92700570565	1.11939377793594	-2.69832786542628	0.0109516536247924	0.422707650665295	-2.71854955634579
a_HQ_transcript/113367	1.76897187665534	2.869178206175	2.69786814775025	0.0109639720458418	0.42290230065076	-2.93134409622583
a_HQ_transcript/91640	2.86302755423304	0.750000624802697	2.69753498601735	0.0109729073695569	0.422966286192047	-2.7133257932822
a_HQ_transcript/20725	-3.19615931476868	-0.0907881127586346	-2.69682938526078	0.0109918537782241	0.423306515181332	-2.75717478607343
a_HQ_transcript/14996	2.77239861864865	1.38664722038436	2.6966640197996	0.0109962984779076	0.423306515181332	-2.74062745249583
a_HQ_transcript/145423	-2.7981521953001	-0.557407975220064	-2.69598537231375	0.0110145566941487	0.423568918328968	-2.77650992865271
a_HQ_transcript/48500	3.98986268596923	0.265292440603483	2.6958447475741	0.0110183435606833	0.423568918328968	-2.78484895228328
a_HQ_transcript/3953	-2.09028020565251	-0.748755589754154	-2.69530711496382	0.0110328325634116	0.423568918328968	-2.77364799575264
a_HQ_transcript/136441	-2.68325404985188	-0.625886814176595	-2.69497681923101	0.0110417426919702	0.423568918328968	-2.77503338512648
a_HQ_transcript/8839	1.89460457682876	-0.801903134029476	2.69496979569897	0.011041932232874	0.423568918328968	-2.77397422480799
a_HQ_transcript/105713	-2.77620828407907	-0.487040500334307	-2.69469971181007	0.0110492231597114	0.423568918328968	-2.77376886215434
a_HQ_transcript/90128	-4.67115075237371	2.29655010526429	-2.69451830402735	0.0110541227825668	0.423568918328968	-2.98538152419391
a_HQ_transcript/126423	-6.63266055065549	2.39996326931631	-2.69416368229035	0.0110637065541529	0.423656873899735	-2.95292229360524
a_HQ_transcript/44539	2.28565939325929	-0.740948433511741	2.69371663643581	0.0110757991214373	0.423840718453526	-2.77654807189881
a_HQ_transcript/144129	3.97110831833451	-0.675996446488209	2.69313733053307	0.0110914875875445	0.423994041447685	-2.78209034736103
a_HQ_transcript/91240	-2.44231868482318	-0.697027632392059	-2.69293094660644	0.0110970817586635	0.423994041447685	-2.75632207089191
a_HQ_transcript/139810	-2.41278138417445	-0.702532309729273	-2.69257925149448	0.0111066207256074	0.423994041447685	-2.77768418189998
a_HQ_transcript/132902	-3.60461148448623	-0.676174801597679	-2.69214530829359	0.0111184009940848	0.423994041447685	-2.79116291296314
a_HQ_transcript/109664	-3.39551735910892	0.530104149394459	-2.69211555648133	0.0111192090925329	0.423994041447685	-2.75156814316641
a_HQ_transcript/147477	-3.00930741126574	-0.376894921261579	-2.69156126104979	0.0111342744708409	0.423994041447685	-2.77980512550516
a_HQ_transcript/123533	-4.10323957734073	-0.645974358897549	-2.69148527359349	0.0111363412366097	0.423994041447685	-2.79378853584182
a_HQ_transcript/33833	1.02751603343395	2.96508655629757	2.69134791502569	0.0111400781260664	0.423994041447685	-2.89877827341836
a_HQ_transcript/140089	-4.36034644654576	0.26174301474601	-2.69115042124721	0.0111454530575981	0.423994041447685	-2.74253932591092
a_HQ_transcript/8604	-1.25772907951953	2.53883420095243	-2.6901127854476	0.0111737326522346	0.424791845395092	-2.83859128403497
a_HQ_transcript/107872	3.22160912217258	0.0815440819830914	2.68982775680517	0.011181512450194	0.424809774970509	-2.79206767747412
a_HQ_transcript/111427	-5.76889447922619	2.56085719430445	-2.68936637716616	0.0111941163793512	0.424866920737531	-3.00496461785377
a_HQ_transcript/111380	-2.28842843528251	-0.665586909837699	-2.68923766361913	0.0111976349189702	0.424866920737531	-2.78418597345435
a_HQ_transcript/142218	2.20844452300593	-0.593839856970367	2.68879947913177	0.0112096209232974	0.425044256379275	-2.80194646596311
a_HQ_transcript/45318	3.30038971024325	2.02011408772357	2.68787500885735	0.0112349477948293	0.425407353698419	-2.82417222452252
a_HQ_transcript/27701	-3.63211360296336	2.65833647952929	-2.68787018075391	0.011235080205416	0.425407353698419	-2.85423529460813
a_HQ_transcript/109187	-5.20948032349618	2.54169827406811	-2.6874327946621	0.0112470815214495	0.425407353698419	-3.02946834854852
a_HQ_transcript/148371	-5.06899068479008	0.477231578228574	-2.68715603736872	0.0112546815388134	0.425407353698419	-2.78931746590631
a_HQ_transcript/116082	-9.26329085978403	2.45949429016248	-2.68704269383319	0.0112577954363488	0.425407353698419	-2.93357430844326
a_HQ_transcript/88615	1.99721149134399	2.90585516053538	2.68670581198264	0.0112670553477743	0.425407353698419	-2.88067106845707
a_HQ_transcript/62880	-2.75032142786128	2.53767242778872	-2.6862295371065	0.0112801588440903	0.425407353698419	-2.85118144225265
a_HQ_transcript/142181	3.41351776743778	2.21390146541602	2.68597944363121	0.0112870451989838	0.425407353698419	-2.84977301389561
a_HQ_transcript/118139	-3.02429311851453	0.211863343642185	-2.68571372406910	0.0112943660984632	0.425407353698419	-2.83920820542059
a_HQ_transcript/1738	2.80656933343328	0.323478390254349	2.68548417952238	0.0113006938823939	0.425407353698419	-2.77100955082228
a_HQ_transcript/143936	-4.24245980249559	0.289451125376854	-2.68531858471901	0.0113052608278349	0.425407353698419	-2.77295784442852

a_HQ_transcript/108694	-4.93495328498558	1.10554849225545	-2.68525487527524	0.0113070183296184	0.425407353698419	-2.77989342474357
a_HQ_transcript/112662	-6.40530003165183	2.88430744369129	-2.68427465128618	0.0113340909978305	0.425774623559098	-2.92219360916863
a_HQ_transcript/67277	3.75300693169162	0.390597484736971	2.68379574932912	0.0113473396036083	0.425774623559098	-2.85725423876913
a_HQ_transcript/63798	4.26169965719114	0.677415840957072	2.68370033955146	0.0113499807894433	0.425774623559098	-2.74950169928815
a_HQ_transcript/108857	-1.27243421393654	4.43749559898376	-2.68347367142314	0.011356257830554	0.425774623559098	-3.06969289863109
a_HQ_transcript/136846	4.93067555474438	-0.778860236040724	2.68343488269093	0.0113573323163368	0.425774623559098	-2.81200392567534
a_HQ_transcript/25219	3.6128871125378	-0.513089429378068	2.68331229426735	0.011360728756196	0.425774623559098	-2.80075459046349
a_HQ_transcript/78564	4.38827127902954	0.893736575997255	2.68170421908184	0.0114053695827014	0.426927010708788	-2.80127379235876
a_HQ_transcript/121462	-3.29963586543734	1.88191245781842	-2.6816755644309	0.011406166522687	0.426927010708788	-2.79619214282158
a_HQ_transcript/90855	3.62744643662665	4.60530027582086	2.6806308634637	0.0114352569634427	0.427740422632559	-3.0851482609883
a_HQ_transcript/51887	5.21695910557837	1.33292673339376	2.67986524782762	0.0114566198017038	0.427778559058985	-2.83534557977077
a_HQ_transcript/111722	2.39319886496126	-0.521840439892193	2.67982258192327	0.0114578113902748	0.427778559058985	-2.81342390191124
a_HQ_transcript/8930	2.75650876658494	-0.578372956993074	2.67980314533055	0.0114583542605085	0.427778559058985	-2.82991864797471
a_HQ_transcript/6910	-2.26637096708784	3.47024869851292	-2.67873346742776	0.011488267554607	0.428584360269583	-2.97448562884642
a_HQ_transcript/38768	2.24733086306577	-0.720775257972862	2.67834437617049	0.0114991663722259	0.428584360269583	-2.80624433485482
a_HQ_transcript/131115	-2.13844478036806	-0.66747634651296	-2.67794669577066	0.0115103156987624	0.428584360269583	-2.80216864790567
a_HQ_transcript/102270	3.2858345486347	0.496372516826892	2.67773335139756	0.0115163011348645	0.428584360269583	-2.80668466529344
a_HQ_transcript/42249	-2.26726553265666	0.177372487906686	-2.67764806872475	0.0115186945724058	0.428584360269583	-2.75578189043396
a_HQ_transcript/139600	4.79371228532552	1.80303607048016	2.67745278492352	0.0115241769044412	0.428584360269583	-2.84092148914641
a_HQ_transcript/10895	-1.31820314473234	3.14427327581207	-2.67715766052092	0.0115324667248322	0.428618430806294	-2.96744846187744
a_HQ_transcript/30351	2.97421230658421	0.432439863029899	2.67667668695676	0.0115459887704904	0.428846795687583	-2.75922976215415
a_HQ_transcript/150955	5.81251295765197	8.79669584568106	2.67582927153239	0.0115698487436053	0.429458600107464	-3.45929851726137
a_HQ_transcript/242	-3.66581764190512	0.0482999139127171	-2.67525611616683	0.0115860125419629	0.429784133400905	-2.78225822675844
a_HQ_transcript/48004	7.32266896405176	3.2096931140786	2.67427802757985	0.0116136444477408	0.430316613019761	-2.91756825040283
a_HQ_transcript/149017	5.49906084697265	7.02540367071015	2.67422399064771	0.0116151728225297	0.430316613019761	-3.27675773865374
a_HQ_transcript/119095	2.60373482733641	-0.473872687188901	2.67319969857092	0.0116441791204616	0.431116461091841	-2.83231792373172
a_HQ_transcript/110894	-0.981272418130362	3.82505988794504	-2.67151920582433	0.0116919136266963	0.432495001360023	-3.0422995661511
a_HQ_transcript/5102	3.2826070902605	0.177433683050915	2.67080527278599	0.0117122478502717	0.432495001360023	-2.79428462152827
a_HQ_transcript/27393	2.17199943810176	2.96813958371041	2.67039384142383	0.0117239811287047	0.432495001360023	-2.84680017780963
a_HQ_transcript/123630	-3.70609938368191	0.376729756509856	-2.67028393922161	0.0117271171861564	0.432495001360023	-2.84716711638084
a_HQ_transcript/140398	3.728487681222	-0.380283703893148	2.67002270133293	0.0117345747292305	0.432495001360023	-2.82401575279095
a_HQ_transcript/102803	-5.20304553588136	2.18504642833	-2.66995657460297	0.0117364631430097	0.432495001360023	-2.89244502324988
a_HQ_transcript/8818	-2.62087662747464	-0.633631224745273	-2.6698541419547	0.0117393889196026	0.432495001360023	-2.81925249270497
a_HQ_transcript/52957	2.79391682638486	1.56068402347532	2.6697999970353	0.0117409357305621	0.432495001360023	-2.80936146895631
a_HQ_transcript/74685	-2.10389733799341	-0.393290783400103	-2.66919154109423	0.0117583310957412	0.432647585969182	-2.86374769450712
a_HQ_transcript/67871	-3.18876899583702	0.994705962070114	-2.66913446911519	0.0117599639731508	0.432647585969182	-2.80642857235228
a_HQ_transcript/74898	-1.78142538819432	3.83830972112154	-2.66886072474635	0.0117677989553806	0.432661997266515	-2.98192704076507
a_HQ_transcript/102662	5.09791341111175	2.11671210261185	2.6685806977378	0.0117758187674159	0.432683181613371	-2.84578907149042
a_HQ_transcript/104545	-3.933869363665	1.89916650886968	-2.66751702950395	0.0118063278479807	0.433307508187379	-2.8512907842599
a_HQ_transcript/24055	4.91235778063329	-0.778873639096903	2.66746858745149	0.0118077190505231	0.433307508187379	-2.84483217600111
a_HQ_transcript/20776	-2.4257497617169	-0.396992528146668	-2.66550605870057	0.0118642087758172	0.435105821905807	-2.83439706466475
a_HQ_transcript/61545	-2.5981836323671	1.06265268844535	-2.66467404777249	0.0118882330909884	0.435369440958247	-2.80680629270902
a_HQ_transcript/135842	0.817732864202023	3.59230349495471	2.66452840408166	0.0118924431905803	0.435369440958247	-3.02637002403789
a_HQ_transcript/19271	-2.61664239025889	-0.0770434685478883	-2.66447917690367	0.0118938665056719	0.435369440958247	-2.86632226605855
a_HQ_transcript/146662	-8.64029178024359	1.06600648894558	-2.66378379564678	0.011913989088634	0.435831565603599	-2.89481875003321
a_HQ_transcript/68767	-2.58732645113047	-0.0573856004481497	-2.66310781511927	0.0119335805269515	0.435881096368348	-2.81119587554042
a_HQ_transcript/120441	-3.56187733038185	0.0369570091803907	-2.66302069305203	0.0119361076932554	0.435881096368348	-2.83813360334113
a_HQ_transcript/134951	-3.67980485370164	-0.577761021405809	-2.66296101747478	0.0119378390004543	0.435881096368348	-2.85094244880247
a_HQ_transcript/18571	-1.48371822857771	3.29650193401714	-2.66230361207921	0.0119569270604564	0.436057931841572	-2.905284389957
a_HQ_transcript/135482	3.40540985063014	0.298789855601824	2.66225067222829	0.0119584654235957	0.436057931841572	-2.84643371385545

a_HQ_transcript/61465	-3.33367128757087	1.06329768101138	-2.66201942926588	0.0119651871952812	0.436057931841572	-2.80581101959096
a_HQ_transcript/81857	2.24260231504833	-0.772289353007589	2.66117667881971	0.0119897139359841	0.436678002300053	-2.8451458736751
a_HQ_transcript/145000	-7.8708184069202	2.22121204479135	-2.65955211279097	0.0120371256335428	0.438130268520087	-2.91727736827911
a_HQ_transcript/130250	-3.60697931615805	2.62494164045139	-2.65856277070582	0.0120660839255888	0.438909465848951	-2.88719078915987
a_HQ_transcript/76176	2.53073954717242	-0.665495967260262	2.6576012423795	0.012094289986704	0.439246199243624	-2.85307410591175
a_HQ_transcript/134743	-0.966219577160127	5.4479304548287	-2.65758544478303	0.0120947539131094	0.439246199243624	-3.19387546395238
a_HQ_transcript/151674	-4.3263307936196	-0.65430053936811	-2.65747456124173	0.0120980106831311	0.439246199243624	-2.86438030197674
a_HQ_transcript/142397	2.24337556385347	-0.716856504500977	2.65690361127416	0.0121147929791163	0.43938394582777	-2.85385502204923
a_HQ_transcript/12544	2.42808804012884	0.205876109105444	2.65668632338382	0.0121211855285698	0.43938394582777	-2.87663872175259
a_HQ_transcript/37498	1.37609919977788	4.26632714611838	2.65657433890982	0.0121244813017435	0.43938394582777	-3.11143196562077
a_HQ_transcript/99667	-2.41716237226395	-0.688967181841301	-2.65617142760283	0.0121363461067894	0.439539891897478	-2.82939921797743
a_HQ_transcript/93870	6.71222893119532	2.32363391561787	2.65558435361666	0.0121536533454696	0.439892628683349	-3.02763715462591
a_HQ_transcript/93054	4.32001837773481	1.24636641239229	2.65472286760987	0.0121790918147633	0.440539047298422	-2.86993861310996
a_HQ_transcript/68028	3.19226065634058	0.39296882374819	2.65427320351506	0.0121923893587991	0.440745776522557	-2.8386735657942
a_HQ_transcript/75999	1.96786822353275	-0.145930526787466	2.65324846409203	0.0122227434261495	0.44156844616235	-2.83810273550473
a_HQ_transcript/149486	-4.08020131822752	1.58070537396393	-2.65227427564097	0.0122516650426055	0.44164490488413	-2.86551709828409
a_HQ_transcript/27126	2.27367607716107	2.75504439164601	2.65214248259611	0.0122555825695134	0.44164490488413	-2.98845752438837
a_HQ_transcript/117682	3.88635502421989	0.360963540366927	2.65204536466855	0.0122584701271187	0.44164490488413	-2.85712834503682
a_HQ_transcript/146925	-3.63895742466023	-0.240302830278157	-2.65193265570098	0.0122618220356421	0.44164490488413	-2.87410772641853
a_HQ_transcript/127441	-1.46210463152554	2.71517192837155	-2.65187371730992	0.0122635751722401	0.44164490488413	-2.91730683811646
a_HQ_transcript/127466	2.18628519266908	-0.788795502624355	2.6516149701721	0.0122712744169295	0.44164490488413	-2.86419893197196
a_HQ_transcript/66666	4.94211178121929	2.77014082830756	2.651387576033	0.0122780444242491	0.44164490488413	-2.99174216404217
a_HQ_transcript/80242	2.76501816426063	0.601060938925951	2.6508410510576	0.0122943297952433	0.441957206145889	-2.84778984621576
a_HQ_transcript/75429	3.47754629045657	1.18791072838767	2.64945783111206	0.0123356365214075	0.442555818791357	-2.8129973621349
a_HQ_transcript/49330	-2.55022186195047	2.25109000438016	-2.64943802941697	0.0123362287867404	0.442555818791357	-2.83803588420057
a_HQ_transcript/136573	-3.0408565302412	-0.634606773045694	-2.64937218095699	0.0123381984924929	0.442555818791357	-2.87466265010002
a_HQ_transcript/58574	2.50855484362161	0.544710978861163	2.64926397576405	0.0123414358357554	0.442555818791357	-2.83368363350752
a_HQ_transcript/83026	4.25119548430362	2.26811317508045	2.64819144518924	0.0123735669969449	0.443434465103831	-2.86589311122931
a_HQ_transcript/96435	-4.66800178965538	0.785851319556454	-2.64780222958287	0.0123852463792309	0.44356957951979	-2.85599210880993
a_HQ_transcript/143344	-6.90482457716121	0.828335429445497	-2.64754310787222	0.0123930276335635	0.44356957951979	-2.87972660039971
a_HQ_transcript/5488	3.31581386171713	0.477905539845376	2.64710316057984	0.012406249335689	0.44356957951979	-2.82956825033722
a_HQ_transcript/148839	1.19616036139119	8.56304991382983	2.64683165287375	0.0124144154561297	0.44356957951979	-3.51330747246448
a_HQ_transcript/151758	-1.34311863175788	8.33430812595402	-2.64679587970688	0.0124154917746817	0.44356957951979	-3.47420959991775
a_HQ_transcript/47983	1.90342155881989	4.49829751302651	2.6446930683912	0.0124789118380589	0.445082441893055	-3.04833293513531
a_HQ_transcript/152145	-4.52256279442042	1.93280863622713	-2.64468351980085	0.0124792005034951	0.445082441893055	-2.98764017895735
a_HQ_transcript/95840	-0.677257175300639	5.30563991863783	-2.64463036557413	0.0124808075331283	0.445082441893055	-3.22887215110841
a_HQ_transcript/17080	-2.48306380287098	-0.708510299721758	-2.64372575251729	0.0125081864146302	0.445617630543939	-2.88203798267062
a_HQ_transcript/103251	3.97771046129061	1.0348512770704	2.64362803160866	0.0125111473480544	0.445617630543939	-2.83980809391732
a_HQ_transcript/118449	-6.26398756901551	0.732546329211251	-2.64272882580258	0.0125384236808634	0.446315671599036	-2.87906295743727
a_HQ_transcript/38899	-4.09012240538929	1.86839251046517	-2.64132681692369	0.0125810618531902	0.447202115253737	-2.86240704610472
a_HQ_transcript/140372	-4.62265383370235	-0.644096381279233	-2.64108501120945	0.0125884292604049	0.447202115253737	-2.88937422095611
a_HQ_transcript/149242	-3.39175239117512	1.60343844764988	-2.64099976006627	0.0125910276697129	0.447202115253737	-2.83395668411503
a_HQ_transcript/122384	-2.96975527464852	1.10044634051138	-2.64089897099878	0.0125941003074313	0.447202115253737	-2.98671819486467
a_HQ_transcript/72976	3.64912917696507	2.53088100867085	2.64010896784891	0.0126182082683377	0.447548894185265	-2.9852531829248
a_HQ_transcript/138357	0.99047537084751	4.15463951148769	2.64007437045631	0.0126192650283796	0.447548894185265	-3.13401865837167
a_HQ_transcript/66517	2.90587725286836	-0.321905981843119	2.63950291297674	0.0126367317724843	0.447895088092055	-2.86469121453996
a_HQ_transcript/130445	-3.49593548534238	1.62566685022421	-2.63830469586356	0.0126734282945063	0.448922023097538	-2.85713287848981
a_HQ_transcript/117972	4.74927594619297	0.47984709754711	2.63757245427014	0.0126959024306101	0.44901609858879	-2.88978875804185
a_HQ_transcript/26690	-3.31929976963113	1.1700354384191	-2.63750523932952	0.0126979627559088	0.44901609858879	-2.84662272559613
a_HQ_transcript/84939	-2.72553829567839	-0.566703469300192	-2.63687310386082	0.0127174015100118	0.44901609858879	-2.88664795896539

a_HQ_transcript/110483	7.56728578938322	2.09531501421882	2.63668306822901	0.0127232493115847	0.44901609858879	-2.95312154830083
a_HQ_transcript/131808	-3.60550363617559	-0.592463817140974	-2.63665006171254	0.0127242652461965	0.44901609858879	-2.90483656808411
a_HQ_transcript/150258	6.88989210408477	0.911777518379109	2.63657748443208	0.0127264994259286	0.44901609858879	-2.89977317476481
a_HQ_transcript/88661	-1.8489303046511	-0.810965828763441	-2.63582711007126	0.012749619843403	0.44901609858879	-2.88714388500158
a_HQ_transcript/113991	-2.27675701280477	1.86970199392141	-2.63535781611999	0.0127640994144661	0.44901609858879	-2.86129622749604
a_HQ_transcript/106366	-5.42957352844475	-0.749057209849471	-2.63529462568328	0.0127660502514496	0.44901609858879	-2.91096003049977
a_HQ_transcript/149707	-7.20780115932688	3.36702860251179	-2.63489788834995	0.0127782769599898	0.44901609858879	-2.99021540763968
a_HQ_transcript/46719	-5.2183401772	0.466415386796401	-2.6348167422903	0.0127808125722864	0.44901609858879	-2.87733179691732
a_HQ_transcript/29612	4.03493957792081	1.29175645624373	2.63480640371254	0.0127811321164067	0.44901609858879	-2.91784737836163
a_HQ_transcript/147174	5.26122245669765	7.03271319983847	2.63471339011393	0.012784007307555	0.44901609858879	-3.38941152430366
a_HQ_transcript/75231	0.83687206085796	4.52712774576599	2.63470623028923	0.0127842286533933	0.44901609858879	-3.17844990182177
a_HQ_transcript/139935	-5.70900793732272	2.73017311710595	-2.63398430043629	0.0128065653233081	0.449292887227853	-3.02922676007961
a_HQ_transcript/74083	1.53257746733726	2.01104119662445	2.63395192008377	0.0128075680246448	0.449292887227853	-2.85892550968845
a_HQ_transcript/63958	4.1193593405711	1.12163275990477	2.63192161125087	0.0128705846102447	0.451000783939547	-2.9314767183111
a_HQ_transcript/145114	4.51315388236258	1.21655531588386	2.63188347798677	0.0128717709289105	0.451000783939547	-2.87686616453435
a_HQ_transcript/17299	-2.39120414734905	-0.524228974524795	-2.63054027165554	0.0129136224490959	0.451437033955953	-2.8932200604092
a_HQ_transcript/111327	3.19793423665859	-0.107973092992403	2.63051450744462	0.0129144264380216	0.451437033955953	-2.89976400429432
a_HQ_transcript/1090	6.12750706893027	2.77324132860989	2.63050516192977	0.0129147180823281	0.451437033955953	-3.09007435285594
a_HQ_transcript/37355	-1.96452565829711	2.55419830096779	-2.63048694038377	0.0129152867373512	0.451437033955953	-2.95879280006211
a_HQ_transcript/150123	3.5725870803498	-0.637144969495944	2.62799918904632	0.0129931419835297	0.453263826894664	-2.92243810504372
a_HQ_transcript/98783	-4.71785837911423	3.06002452691006	-2.6278203109885	0.0129987567518315	0.453263826894664	-2.97802111687246
a_HQ_transcript/67811	-1.68252419106738	3.21777366107947	-2.62773599967796	0.0130014039614522	0.453263826894664	-3.0456527703934
a_HQ_transcript/15653	7.33207615656013	2.30992341047602	2.62737424250935	0.0130127680894282	0.453263826894664	-2.98505104233788
a_HQ_transcript/4069	-3.87338126180283	1.38299764992943	-2.62735584071803	0.0130133464031998	0.453263826894664	-2.93005334718943
a_HQ_transcript/143978	-4.24132030047567	2.22115303116636	-2.6270153841213	0.0130240502362189	0.453263826894664	-2.97910148228961
a_HQ_transcript/75977	-4.05065414591995	-0.659893708215418	-2.62692454978446	0.0130269074114658	0.453263826894664	-2.91674634311423
a_HQ_transcript/138376	-5.05856032384279	1.3087051565628	-2.62682843098458	0.0130299314399426	0.453263826894664	-2.92452374506816
a_HQ_transcript/152305	4.73114230015302	0.204483964775227	2.62638643355863	0.0130438456440137	0.453415214690885	-2.88409150376618
a_HQ_transcript/84886	1.42215718905054	2.40877157509551	2.6261947570009	0.0130498839488345	0.453415214690885	-2.92816239696268
a_HQ_transcript/134893	3.02498419786176	-0.460710685956443	2.62557541069503	0.0130694126351413	0.453822471717737	-2.94702074597497
a_HQ_transcript/101618	3.61168080033772	0.558044696742432	2.62529926069993	0.0130781286676724	0.45385400787729	-2.87171808260507
a_HQ_transcript/70540	-3.40090861094103	0.748268739203552	-2.62405255642495	0.0131175449718841	0.454209078951698	-2.86501403890893
a_HQ_transcript/85485	-2.46330723105403	-0.418783856430265	-2.6238160927852	0.0131250334894537	0.454209078951698	-2.90863798903733
a_HQ_transcript/94607	-2.36720687388135	2.7814466484269	-2.62371944848206	0.0131280952359921	0.454209078951698	-2.98307415192543
a_HQ_transcript/72439	3.97076203953894	0.0499798878155138	2.62353650030697	0.0131338929475802	0.454209078951698	-2.89819519237088
a_HQ_transcript/118019	3.30037858294723	-0.166192552017838	2.6232927837484	0.0131416201164466	0.454209078951698	-2.92846218145662
a_HQ_transcript/79757	-1.50611583408782	1.28686959779687	-2.62329070819354	0.0131416859411223	0.454209078951698	-2.86919773758543
a_HQ_transcript/2155	3.8667564742903	0.118347119128845	2.62294152380189	0.0131527644067551	0.454209078951698	-2.88428732633629
a_HQ_transcript/91409	5.47149167679692	1.51039693254723	2.62285985576169	0.0131553567097339	0.454209078951698	-2.97040791177179
a_HQ_transcript/148529	-4.77962725752558	-0.657396490758961	-2.62275499735141	0.0131586858132855	0.454209078951698	-2.93588475094627
a_HQ_transcript/104275	-6.44460614434868	3.9395492799531	-2.62241023098725	0.0131696371430589	0.454317310297763	-3.16153586224603
a_HQ_transcript/113201	2.26256091666636	-0.415547323160588	2.62188270788631	0.0131864099678713	0.454626120173443	-2.93375381419908
a_HQ_transcript/17361	3.858803245126	0.498828633815917	2.6215692625611	0.0131963854532274	0.45470035188216	-2.91113106340646
a_HQ_transcript/117659	-3.26029954625008	0.124688933938592	-2.62100480059439	0.0132143672183214	0.455050200039447	-2.8836409125979
a_HQ_transcript/137571	-1.01654276138018	3.95481924418907	-2.62024247103346	0.0132386883181165	0.455265447314215	-3.18643349198764
a_HQ_transcript/65626	-2.98477009863307	-0.471546892991542	-2.62015681988553	0.0132414234883278	0.455265447314215	-2.92770498993574
a_HQ_transcript/112755	-2.92347600784602	0.179416458016655	-2.62006681089956	0.013244298384234	0.455265447314215	-2.89471741439455
a_HQ_transcript/53186	3.21823439611193	0.403988776192424	2.61973233031715	0.0132549867862184	0.455265447314215	-2.86746036117603
a_HQ_transcript/787	-3.12653120198501	-0.554432484835257	-2.61949867087644	0.0132624581582462	0.455265447314215	-2.93278148839065
a_HQ_transcript/135128	-3.02072296918414	-0.631489903413311	-2.61916039852186	0.013273281478844	0.455265447314215	-2.92768920930634

a_HQ_transcript/76072	-5.49175571781626	2.36877393044239	-2.61909288224913	0.0132754426988301	0.455265447314215	-2.9302209688897
a_HQ_transcript/9852	-2.45715327314693	2.24912841409349	-2.61882861576298	0.0132839050934663	0.455287049099651	-2.96486928660352
a_HQ_transcript/115993	-2.38991048336014	-0.463502715976688	-2.61853375792719	0.0132933529783959	0.455342381808012	-2.88042931939632
a_HQ_transcript/64906	4.2208502909325	0.370419964790798	2.61749924201807	0.0133265502812032	0.456158383448771	-2.93362650312588
a_HQ_transcript/19363	-3.44076427004735	0.868770138712806	-2.61730255889767	0.0133328704493439	0.456158383448771	-2.89901484621364
a_HQ_transcript/3316	-7.28501194402481	0.503684795053971	-2.61598453972668	0.0133752949282818	0.457160701617272	-2.98901468963116
a_HQ_transcript/38020	4.1729035681665	1.23850206549858	2.61575157443913	0.013382806596524	0.457160701617272	-3.00179321743836
a_HQ_transcript/118185	1.98595424932548	-0.662701549025665	2.6151098214277	0.0134035192788298	0.457160701617272	-2.92522992848026
a_HQ_transcript/94377	-2.45251313331903	-0.0517714637308622	-2.6148756960959	0.0134110830875447	0.457160701617272	-2.91353937835306
a_HQ_transcript/83845	3.3504799329914	0.898763519474452	2.61484832496896	0.0134119676149702	0.457160701617272	-2.90572146448082
a_HQ_transcript/2727	-3.00487780476656	6.36552354839434	-2.61480902914178	0.0134132375962192	0.457160701617272	-3.39489441192066
a_HQ_transcript/142828	3.94729455822925	-0.660209991775647	2.61434186744908	0.0134283440698288	0.457160701617272	-2.94269837299847
a_HQ_transcript/120627	3.43166802878562	0.177308895776029	2.61433108776444	0.0134286928349816	0.457160701617272	-2.88802586205149
a_HQ_transcript/57218	-2.82179196700751	3.96171491981199	-2.61407487062263	0.0134369849316047	0.457160701617272	-3.203342877579
a_HQ_transcript/9857	-5.18174816847129	2.79605598769456	-2.61395660420483	0.0134408140494068	0.457160701617272	-3.08039348279183
a_HQ_transcript/152199	-3.89147989402898	3.46604370049432	-2.61361986486404	0.0134517222018908	0.457264156813749	-3.0933588454708
a_HQ_transcript/42465	2.07594844767301	1.45575793458519	2.61273103447246	0.0134805538149521	0.457976406870564	-2.90228143582375
a_HQ_transcript/102837	-5.23317796121577	-0.768069738622407	-2.61164806575089	0.0135157600269708	0.458556653196759	-2.95890301799278
a_HQ_transcript/108348	3.45165942678143	0.115449158335491	2.61109236413273	0.0135338582965489	0.458556653196759	-2.92188391779363
a_HQ_transcript/26848	2.62759092714094	1.54337998475625	2.61087992290372	0.0135407830674859	0.458556653196759	-2.88688337178341
a_HQ_transcript/136461	-3.16708412037257	-0.0121902484987342	-2.61074433537884	0.0135452044137117	0.458556653196759	-2.90880319245825
a_HQ_transcript/7186	-5.08968135704233	0.319119141775651	-2.6106106227275	0.0135495659292952	0.458556653196759	-2.91571857480806
a_HQ_transcript/123685	4.24899472717406	1.07461897637536	2.61041360018953	0.0135559948883076	0.458556653196759	-2.93231032419439
a_HQ_transcript/24413	-2.619740474585	1.54643240734154	-2.61021585146326	0.0135624503780497	0.458556653196759	-2.89604926317242
a_HQ_transcript/113924	3.81644117786209	1.39669790522292	2.61010032579794	0.013566223017864	0.458556653196759	-2.88619474127159
a_HQ_transcript/150523	-3.41345839155451	-0.338738288777271	-2.60976593856974	0.0135771483319635	0.458556653196759	-2.97791356341576
a_HQ_transcript/105064	-4.3894887082489	1.31364490921068	-2.60964449617509	0.0135811181882775	0.458556653196759	-2.99825717528655
a_HQ_transcript/40126	-4.34051439167257	0.38890036730625	-2.60954383536888	0.0135844095238924	0.458556653196759	-2.96568451749721
a_HQ_transcript/75137	-2.59912794038471	1.7702614030128	-2.60889105988508	0.0136057714102483	0.459011190095714	-2.96916701430715
a_HQ_transcript/102588	2.09132474827493	-0.717461647316218	2.60813835912285	0.0136304418557538	0.459576754171262	-2.95144153041564
a_HQ_transcript/102815	2.92661032287843	-0.0742093958959827	2.6073184959846	0.0136573606392033	0.460217425643833	-3.00416923694204
a_HQ_transcript/96529	2.31389910882164	-0.125007768619524	2.60688453623173	0.0136716288288258	0.460431309711463	-2.92085599530752
a_HQ_transcript/44674	2.16080766383072	-0.662205238309182	2.60640550593556	0.0136873948885403	0.460695361946189	-2.95547210186066
a_HQ_transcript/74134	-6.1174057674455	1.54358401099273	-2.60550648986099	0.0137170290656124	0.461425616623794	-3.05008267825469
a_HQ_transcript/133322	-3.70096367227373	0.487741442749459	-2.60493888086378	0.0137357696211089	0.461788789205216	-2.96753418523327
a_HQ_transcript/51312	3.18953471617334	3.66533573462972	2.60398070611627	0.0137674590332145	0.462586623516008	-3.13123604201072
a_HQ_transcript/130713	5.15672970110677	-0.768780242325043	2.6031019293212	0.0137965819060255	0.463297350105979	-2.9761857140706
a_HQ_transcript/97041	-3.0706147991183	-0.142106036831846	-2.60277081493761	0.0138075698691177	0.463398626646695	-2.95670506006515
a_HQ_transcript/138833	5.1610013063633	-0.768590797750592	2.60175121289457	0.0138414559465622	0.464114464733627	-2.97892004608386
a_HQ_transcript/78647	-1.60836835096306	0.00555427382192739	-2.60147551353331	0.0138506318923928	0.464114464733627	-2.90676112037107
a_HQ_transcript/7159	0.897952287609404	3.28779169983435	2.60130595531977	0.0138562779925237	0.464114464733627	-3.08594182450351
a_HQ_transcript/84126	4.00909071039606	1.63256765754457	2.6011691017282	0.0138608366153588	0.464114464733627	-2.99114313141545
a_HQ_transcript/37483	3.8298802327499	0.610765622798402	2.60029234060377	0.0138900745993164	0.464825708871078	-2.90393257320904
a_HQ_transcript/102799	-2.09188514075028	4.43159502631022	-2.59970563451813	0.0139096717105615	0.465213692285109	-3.1691676067001
a_HQ_transcript/112110	-3.44663203897618	0.981520704525948	-2.59892142382864	0.0139359057325756	0.465710383675642	-2.91764708380396
a_HQ_transcript/108155	-5.94199201874587	1.50858660807382	-2.59872238925858	0.0139425712572199	0.465710383675642	-2.98558336096866
a_HQ_transcript/136932	-2.28265791140859	-0.642712002260118	-2.59836363181263	0.0139545932228961	0.465710383675642	-2.96598960697652
a_HQ_transcript/137285	5.08307768487304	1.24451618585278	2.59830467495788	0.0139565697832881	0.465710383675642	-3.07312791276631
a_HQ_transcript/107865	3.89080668656002	1.58224784190577	2.59788244721828	0.0139707327538525	0.46576023322048	-2.98878357785524
a_HQ_transcript/12957	2.77081977962909	1.04031797890753	2.59778244775459	0.0139740890231303	0.46576023322048	-2.95520238891338

a_HQ_transcript/136121	-8.23036318919625	1.49568331810429	-2.59690336404493	0.0140036256560272	0.466381727429963	-2.98213850642892
a_HQ_transcript/64826	-2.94658567879073	0.936725046730775	-2.59675006579709	0.0140087822751981	0.466381727429963	-2.9797869750776
a_HQ_transcript/135232	-3.79775257510506	2.14778956910835	-2.59606189160823	0.0140319525652655	0.46666740769083	-2.99565647350051
a_HQ_transcript/4658	-3.92744082317239	1.0382412237039	-2.59601834773879	0.0140334198431663	0.46666740769083	-2.92849194066643
a_HQ_transcript/109226	6.53085822779002	2.1913205295142	2.59571556164006	0.0140436266062555	0.466739809816136	-3.05238884049752
a_HQ_transcript/34339	-3.05346164216442	0.454629555043872	-2.59529401584703	0.0140578481115769	0.466945482874137	-2.91937212718083
a_HQ_transcript/81165	2.75928718285886	-0.372235287233741	2.59454739655009	0.0140830691141613	0.467516071655037	-2.97686301934006
a_HQ_transcript/10595	-2.20414670548232	-0.583462064035262	-2.59391841378499	0.0141043487361996	0.467523712815462	-2.99850998806776
a_HQ_transcript/60223	1.35687188866502	-0.707275136896085	2.59385885239144	0.0141063653434244	0.467523712815462	-3.00418981533264
a_HQ_transcript/22030	2.51501900865839	-0.340167057099634	2.59369423640094	0.0141119402341627	0.467523712815462	-2.97867792305273
a_HQ_transcript/127542	-2.82051221164795	-0.573376227912825	-2.59356793219177	0.0141162190357171	0.467523712815462	-2.98281180564745
a_HQ_transcript/55843	-3.8124983084902	0.282230462100388	-2.59335266747801	0.0141235143081467	0.467523712815462	-2.95507586516763
a_HQ_transcript/114556	2.86206086342055	0.211298328309799	2.59259817971128	0.0141491112137973	0.468104460236545	-2.95120057368796
a_HQ_transcript/121568	-2.98196189497954	-0.137431607145365	-2.59227192538656	0.0141601930414553	0.468204608142044	-2.96558647524386
a_HQ_transcript/125987	-1.48913731753219	4.57335137418242	-2.5917530338295	0.0141778346522045	0.468521417091155	-3.30843959756955
a_HQ_transcript/91314	-2.56315945104158	-0.304400276068844	-2.59112585102688	0.0141991850985615	0.468960358755217	-2.94339605601078
a_HQ_transcript/135883	4.09229720541627	0.144518335323342	2.5902535450276	0.014228929328321	0.469675868254765	-3.06514200228441
a_HQ_transcript/89234	3.04147091353166	0.899001439940127	2.58924698585981	0.0142633228340038	0.470480530789194	-2.93212129340497
a_HQ_transcript/79577	-2.35091879962829	2.85389516685434	-2.5889014420974	0.0142751475305022	0.470480530789194	-3.12420766044088
a_HQ_transcript/145179	-2.33698811014724	-0.503956483007915	-2.58883014849603	0.0142775883620998	0.470480530789194	-2.9577403901955
a_HQ_transcript/14714	-3.4632804438299	2.83701256751713	-2.58830525570534	0.0142955706271413	0.470806192302816	-2.98812232115149
a_HQ_transcript/67425	-2.86868495376472	2.05490596258633	-2.58769423963172	0.0143165297082907	0.471229467091462	-2.99594566528703
a_HQ_transcript/79054	2.28492315507344	-0.742363185883263	2.58718652413874	0.0143339669380496	0.47134840052542	-2.99498018154132
a_HQ_transcript/67697	-3.45216215578714	4.24263551815038	-2.58711687138278	0.0143363606545717	0.47134840052542	-3.16193140520024
a_HQ_transcript/71884	-2.63884209261428	0.318979648048633	-2.58613302186034	0.014370211475 1	0.472194263778754	-2.95572537050099
a_HQ_transcript/57189	-2.51808674342985	-0.533236895104699	-2.58482872540675	0.0144152014480942	0.472679882334092	-2.99829320116228
a_HQ_transcript/76796	-2.80980896518044	-0.196729001594342	-2.58481102224825	0.0144158129879462	0.472679882334092	-3.00921699884132
a_HQ_transcript/108706	-3.65564880879202	0.348945288664174	-2.58439741171321	0.0144301076098062	0.472679882334092	-3.00826888566741
a_HQ_transcript/69098	-3.85465515552129	1.55357606627967	-2.58437170652473	0.0144309964276156	0.472679882334092	-2.93567512705839
a_HQ_transcript/123551	3.78853031035029	1.32686526375944	2.58434749386464	0.0144318336839127	0.472679882334092	-2.95213175473464
a_HQ_transcript/90839	0.852076294877797	2.6874234693297	2.58395865457621	0.014445285605855	0.472679882334092	-3.1083115165676
a_HQ_transcript/138582	-1.94336250069869	2.50280227741473	-2.58393637794612	0.0144460566176293	0.472679882334092	-3.05782069441724
a_HQ_transcript/90614	-2.19240049574912	-0.532345950573252	-2.58382119245226	0.0144500438843188	0.472679882334092	-3.01192685976599
a_HQ_transcript/17640	3.14908284871741	1.77618286099601	2.58308071767681	0.0144757004469797	0.47325282091228	-2.97537621188834
a_HQ_transcript/103345	-2.67360421083246	2.74371297034214	-2.58276898488971	0.0144865141785279	0.47334013275406	-3.00368243221517
a_HQ_transcript/121464	-2.48754548826437	2.6051151062584	-2.58246460477272	0.0144970800423756	0.473419252080453	-3.12840383188832
a_HQ_transcript/34395	-3.90657550726952	2.90044756275914	-2.58203070311869	0.0145121542420039	0.473422192975976	-3.10902762837431
a_HQ_transcript/9222	-2.78920628691355	1.16086720517653	-2.5815097549854	0.0145302716275056	0.473422192975976	-2.94552518135153
a_HQ_transcript/101115	2.94283184959761	1.84682639958814	2.58114217905183	0.0145430676296551	0.473422192975976	-3.00164557305898
a_HQ_transcript/116213	-2.89348930701315	1.20446000537703	-2.58108572007886	0.0145450339921524	0.473422192975976	-2.98948795497467
a_HQ_transcript/72640	3.34382908527386	1.9684577658587	2.5808518161264	0.0145531830506735	0.473422192975976	-3.03232752501442
a_HQ_transcript/5698	3.69473917540144	1.33579883631299	2.58078971488011	0.0145553473242242	0.473422192975976	-2.97121184921968
a_HQ_transcript/152751	-4.04464282821862	-0.647854154052178	-2.58063472009305	0.0145607503003084	0.473422192975976	-3.01960739668575
a_HQ_transcript/103431	-1.73870745130355	1.91520920461171	-2.58058953041248	0.0145623259193684	0.473422192975976	-2.97790016290172
a_HQ_transcript/41636	-1.85978811593882	-0.0392623948241094	-2.58029285740116	0.0145726738603376	0.47349378767675	-3.0026176304048
a_HQ_transcript/876	-3.38999867418805	0.995563035906294	-2.57957745453589	0.0145976549808356	0.473973306898497	-2.94902036152118
a_HQ_transcript/103591	2.50468449258828	-0.104715613268163	2.57911866389358	0.014613696250094	0.473973306898497	-2.99675344872501
a_HQ_transcript/141387	-3.41053662058827	1.32262447464168	-2.57909048766077	0.0146146819405178	0.473973306898497	-3.03194938001433
a_HQ_transcript/29720	-2.71471704306558	0.0183018713504833	-2.57886252896917	0.0146226588847117	0.473973306898497	-2.97564454216505
a_HQ_transcript/150687	2.98102503320928	1.59647247867103	2.5787041963319	0.014628201771537	0.473973306898497	-2.99969806808922

a_HQ_transcript/143055	-2.98631646044803	-0.608134242706672	-2.57761871700149	0.0146662542873401	0.474526810141875	-3.01602809116418
a_HQ_transcript/32606	1.76868727106577	0.933896184390461	2.57757919252356	0.0146676415765295	0.474526810141875	-2.97984329188877
a_HQ_transcript/117782	-3.37992919532874	0.492289649091897	-2.57751841873274	0.0146697749419376	0.474526810141875	-2.96809262675682
a_HQ_transcript/2157	1.1953127229019	2.60780991866699	2.57725160656626	0.0146791443365098	0.474565796436397	-3.07614546448284
a_HQ_transcript/146530	-4.18436205108797	-0.534282181932746	-2.57692448176098	0.0146906392038701	0.474673416143726	-3.0271554081595
a_HQ_transcript/123386	-3.37963122116404	-0.514412602350607	-2.57639523474631	0.0147092540396825	0.474979330718466	-3.02467119893813
a_HQ_transcript/116901	4.57453590794643	4.09625116349415	2.57588789699596	0.0147271186909613	0.474979330718466	-3.2585067767929
a_HQ_transcript/141868	0.819414758102936	8.34903964183895	2.57578787769051	0.0147306429847256	0.474979330718466	-3.65046707544555
a_HQ_transcript/49138	3.58661455667103	-0.159729452482646	2.57572689960187	0.0147327919984413	0.474979330718466	-3.05288502099235
a_HQ_transcript/145690	-2.53344133411724	-0.623193699958573	-2.57526733743833	0.0147489973693382	0.475238203483864	-2.98619880753216
a_HQ_transcript/115624	-5.56347673406244	1.91060122893203	-2.57419042908052	0.0147870363902141	0.476064847051316	-3.00724562475896
a_HQ_transcript/138770	-4.17949786718637	0.563280507756389	-2.57407745638981	0.0147910320976926	0.476064847051316	-2.98326534042326
a_HQ_transcript/50747	-3.73920157985865	0.473127191833019	-2.57346901625581	0.0148125690114257	0.476494195625984	-3.04127745910917
a_HQ_transcript/60733	-2.88243563916569	0.164508054001157	-2.57218002800082	0.0148582907681883	0.477700622662195	-2.98477569030446
a_HQ_transcript/16936	3.56877643031316	0.903308961830179	2.57185585165505	0.0148698100961213	0.477806700534737	-2.97782546767489
a_HQ_transcript/60021	3.55028926555207	1.56868767940592	2.57047847900114	0.0149188457001881	0.479117493293112	-2.97342889945405
a_HQ_transcript/117314	-1.85267021771172	-0.821663802471935	-2.56973594722519	0.014945342232616	0.479703397734679	-3.02652950333751
a_HQ_transcript/28858	2.8674728015011	0.396652326074448	2.56910125903401	0.0149680248429515	0.479857570441584	-3.00810932046163
a_HQ_transcript/7698	2.93003440201068	-0.0135009974995303	2.56907399271282	0.0149690000024464	0.479857570441584	-2.99015926377232
a_HQ_transcript/119361	3.8884818138188	0.513459422380718	2.56890874805605	0.0149749111061973	0.479857570441584	-3.00128622297975
a_HQ_transcript/42870	-2.75464218987924	2.34523288291204	-2.5677545615329	0.0150162584931294	0.480917395971695	-3.01337045580023
a_HQ_transcript/102855	4.09129767820086	-0.19243221402358	2.56705729178282	0.0150412883183105	0.481453748549975	-3.1103744100599
a_HQ_transcript/57990	-2.85494521315289	-0.512057485034929	-2.56618996603738	0.0150724762773608	0.481912192505186	-3.03313493215049
a_HQ_transcript/10854	3.21621632283803	2.14321056736702	2.56535462288276	0.0151025705012763	0.481912192505186	-3.07514189428645
a_HQ_transcript/133321	-2.5250745011347	0.857407141052573	-2.56502919970764	0.0151143092325651	0.481912192505186	-3.0200898380093
a_HQ_transcript/141659	-2.56008521057426	-0.0542672513177952	-2.56486111859378	0.0151203755805166	0.481912192505186	-3.05770965977037
a_HQ_transcript/6894	3.715155425785	-0.58766196375786	2.56484682492565	0.0151208915680383	0.481912192505186	-3.04345392682133
a_HQ_transcript/97108	1.84084906330346	-0.810236414071117	2.56469155698861	0.015126497634923	0.481912192505186	-3.02941331790521
a_HQ_transcript/69487	-3.50675046230731	-0.589001981214473	-2.56409473598583	0.015148064141664	0.481912192505186	-3.02994620973935
a_HQ_transcript/5861	-4.19016292346436	1.6681180218688	-2.56388156291562	0.0151557741496705	0.481912192505186	-3.13077435261343
a_HQ_transcript/59481	2.45991188681763	2.33631015069537	2.56367744851451	0.0151631599130811	0.481912192505186	-3.03233831460926
a_HQ_transcript/77912	-2.34625381356292	1.19930923673607	-2.5636135590371	0.0151654723987213	0.481912192505186	-2.99207010847768
a_HQ_transcript/85257	1.62024425849939	2.19861723758798	2.56358882774228	0.0151663676374814	0.481912192505186	-2.98675683998106
a_HQ_transcript/152804	-5.38955731716414	-0.761168514563148	-2.56339387924561	0.015173426208755	0.481912192505186	-3.05623565741453
a_HQ_transcript/137955	3.82224137524848	0.24625442848388	2.56336739600604	0.015174385330511	0.481912192505186	-3.02930179436651
a_HQ_transcript/139674	-5.07180903783257	1.30621775053285	-2.56316919829784	0.0151815650674897	0.481912192505186	-3.06003136298597
a_HQ_transcript/142765	-6.58639192921012	4.81157083637022	-2.56304834206612	0.0151859446355022	0.481912192505186	-3.22879827614089
a_HQ_transcript/96864	-2.91003278255968	1.46864724776545	-2.56261976321972	0.0152014847876219	0.481912192505186	-3.03424701057325
a_HQ_transcript/128260	3.07240098733381	0.512359852587246	2.56254273908068	0.0152042792143489	0.481912192505186	-2.99681536861951
a_HQ_transcript/140311	-2.83391695380831	-0.430622477777253	-2.56245953919397	0.0152072982280903	0.481912192505186	-3.05575447009015
a_HQ_transcript/42161	-4.21825368990807	0.960752525915691	-2.56202036379244	0.015223243418663	0.481912192505186	-3.04542546944534
a_HQ_transcript/17216	-2.14478033420411	2.48273702964593	-2.56105640534636	0.0152582959778419	0.481912192505186	-3.04918830464923
a_HQ_transcript/100414	2.71040175638313	-0.624236268506902	2.56102318058562	0.0152595054588315	0.481912192505186	-3.04125697258016
a_HQ_transcript/24829	-1.52894352794144	2.02657249865904	-2.56090213072215	0.0152639127840767	0.481912192505186	-3.00908782046658
a_HQ_transcript/144349	-2.29300224660968	-0.667055149070424	-2.56073913530501	0.0152698491644296	0.481912192505186	-3.0471811791119
a_HQ_transcript/87598	3.93568887984496	-0.602427853241716	2.56058023456555	0.0152756384613561	0.481912192505186	-3.04647509424883
a_HQ_transcript/102792	3.5345438683213	-0.196932567396272	2.56039082116814	0.015282542079784	0.481912192505186	-3.04727395492768
a_HQ_transcript/84115	-2.5588796076564	0.476535188074803	-2.56036159411898	0.0152836075844623	0.481912192505186	-2.98227189807452
a_HQ_transcript/126046	-1.54079178169189	2.82523570105232	-2.56031326530294	0.0152853696154674	0.481912192505186	-3.07733638972714
a_HQ_transcript/22349	-1.76034558733537	3.27008453558206	-2.56024810197163	0.0152877457159985	0.481912192505186	-3.15473386213603

a_HQ_transcript/144870	-4.33703129566599	1.50614367164937	-2.55963484689453	0.0153101239774735	0.482356036077279	-3.05550520819698
a_HQ_transcript/144894	-7.31933893080614	2.85889768908941	-2.55932491245925	0.015321445252741	0.482434591485562	-3.21294852103744
a_HQ_transcript/127984	-3.47662056960287	2.35052042095976	-2.55911228958457	0.0153292163926822	0.482434591485562	-3.0927993026676
a_HQ_transcript/15219	2.45542124102296	0.11718502219162	2.55837412854532	0.0153562235781303	0.483023032548462	-2.98109174620684
a_HQ_transcript/51528	-3.19899876641443	1.49425087288644	-2.55753096100455	0.0153871262552617	0.483345270218244	-3.01847915980539
a_HQ_transcript/142127	4.21320370489218	2.54109435423432	2.55752643765182	0.0153872921936074	0.483345270218244	-3.19665554653067
a_HQ_transcript/80504	-2.4233339940293	1.49244930389979	-2.55723643956593	0.0153979341589087	0.483345270218244	-3.10992382754385
a_HQ_transcript/89644	-2.80945991332358	-0.494828965791419	-2.55718755078923	0.0153997288818502	0.483345270218244	-3.03402782702831
a_HQ_transcript/35087	2.21757518665716	-0.604611057411235	2.55681606145894	0.0154133726703278	0.483512426649118	-3.05910834875836
a_HQ_transcript/93121	-4.29614949410743	2.29207774649158	-2.55623510177529	0.015434732048276	0.483770059636374	-3.13655345000384
a_HQ_transcript/84768	-3.10638082609092	-0.47160897066105	-2.55614001784805	0.0154382304676829	0.483770059636374	-3.05379793181444
a_HQ_transcript/68694	-6.05703016231101	1.04958762958984	-2.55534514277434	0.0154675048522238	0.48442625110456	-3.06263143489725
a_HQ_transcript/17129	-1.57443101937898	1.71579548344687	-2.5548518163845	0.0154856992205407	0.484608071482251	-3.00484059699953
a_HQ_transcript/80416	-3.03463673825943	0.468333100685497	-2.55473571230157	0.0154899841180502	0.484608071482251	-3.07150245602107
a_HQ_transcript/149320	-2.38959725457782	1.62961320304258	-2.55411384940834	0.0155129529627175	0.485065588927834	-3.01583526096334
a_HQ_transcript/60603	2.85184300304206	0.736253798397588	2.55368162035194	0.0155289360441635	0.485150525321756	-3.03896471316189
a_HQ_transcript/43241	2.71214309102452	-0.675041634001185	2.55358903081456	0.0155323618157134	0.485150525321756	-3.05851801707803
a_HQ_transcript/151009	4.10601078604665	8.82301547187735	2.55265552942341	0.0155669398375831	0.485969430117632	-3.7029127301965
a_HQ_transcript/110460	-1.52595868490866	2.11560433682352	-2.5519499700699	0.0155931216125402	0.486330931949719	-3.05084541848524
a_HQ_transcript/98743	1.83596513471129	-0.731960347920069	2.55149490429698	0.0156100296100239	0.486330931949719	-3.05905203243372
a_HQ_transcript/145699	2.1701831842121	2.01361135440244	2.55145831198594	0.0156113899324088	0.486330931949719	-3.01536327695717
a_HQ_transcript/144663	3.94246825242929	2.05056120936663	2.55067839324199	0.0156404094386748	0.486330931949719	-3.23487689627838
a_HQ_transcript/108195	2.5273255014574	0.150635929561083	2.55052736833112	0.0156460345642963	0.486330931949719	-2.99872958687418
a_HQ_transcript/34372	-2.33356561560126	-0.687577242152585	-2.55023578124384	0.0156569003852137	0.486330931949719	-3.04880678637038
a_HQ_transcript/64123	3.07263861363482	1.62464887179992	2.55020723903895	0.0156579643667497	0.486330931949719	-3.05462744414265
a_HQ_transcript/35421	-3.12255974049456	-0.581059005926901	-2.54980783805	0.0156728600124192	0.486330931949719	-3.06845968525948
a_HQ_transcript/67931	2.83633202284373	1.65369332055986	2.54969901057715	0.015676920989397	0.486330931949719	-3.08378959262939
a_HQ_transcript/57940	2.19571746454287	-0.704052791909039	2.5494590666064	0.0156858780961896	0.486330931949719	-3.06200691432619
a_HQ_transcript/112214	2.34844695752444	-0.366405067396208	2.54945634069997	0.0156859798813096	0.486330931949719	-3.07202990439161
a_HQ_transcript/118592	-2.46322922662803	5.37164368589543	-2.54933812814126	0.0156903945132324	0.486330931949719	-3.47943879959076
a_HQ_transcript/2375	2.63101562729464	1.46085334197296	2.54924903272621	0.0156937225249953	0.486330931949719	-3.0206992840297
a_HQ_transcript/41463	-3.40928450320977	0.503674312999761	-2.54919740033914	0.0156956514646585	0.486330931949719	-3.02937581043931
a_HQ_transcript/112776	4.36909432298571	0.482381170696598	2.54880145360637	0.0157104509330155	0.486530150151479	-3.08486336220108
a_HQ_transcript/124362	-2.50453648669367	3.61096539215592	-2.54856004380897	0.0157194805095673	0.486550566059705	-3.21554727487653
a_HQ_transcript/53174	-2.06043092520158	1.25152065808733	-2.54797441459653	0.0157414049530338	0.486969870734405	-3.00052967952352
a_HQ_transcript/25982	-5.85076253603545	0.78947151477184	-2.5477216438049	0.0157508767287155	0.487003703450411	-3.09143135046408
a_HQ_transcript/89031	-1.9637961861134	3.20665865721753	-2.54659077418999	0.0157933166941463	0.488056306643985	-3.21262662338621
a_HQ_transcript/85177	2.5380135629289	2.20383515593091	2.54596685174943	0.0158167766505997	0.488521569153061	-3.03932391695601
a_HQ_transcript/130371	-1.04693421349006	5.22607692757961	-2.54548127441916	0.0158350569082126	0.488826440765046	-3.45088541631029
a_HQ_transcript/28969	2.62009496223848	2.44007947466599	2.54471538722682	0.0158639293456176	0.489457794587081	-3.15939778735634
a_HQ_transcript/3725	2.99444369188233	-0.325651741114005	2.5437978896641	0.0158985809529159	0.489904306455662	-3.08814356586086
a_HQ_transcript/104417	-4.00662079432129	4.95556242995464	-2.54376728140976	0.0158997381500381	0.489904306455662	-3.34890318436
a_HQ_transcript/137477	2.8468313850551	0.342001009331522	2.54366289011682	0.0159036854232355	0.489904306455662	-3.07378916115259
a_HQ_transcript/45968	-5.11001181437474	0.260372111532548	-2.54273613547191	0.0159387676783547	0.490724940470023	-3.08062732075342
a_HQ_transcript/47970	-4.59172661393887	1.88093421027159	-2.54221175284816	0.0159586496799582	0.490891857135745	-3.06696959532302
a_HQ_transcript/87955	-2.41747534523337	0.299930606429552	-2.54213721684077	0.01596147756856	0.490891857135745	-3.04112103051909
a_HQ_transcript/118741	2.49536886044897	-0.597210403833437	2.54192519476323	0.0159695241853099	0.490891857135745	-3.04382728628087
a_HQ_transcript/40641	3.56207318554247	0.547043086256648	2.54158888577555	0.0159822953657421	0.490982087779997	-3.10266677868885
a_HQ_transcript/10398	3.09823372145562	-0.561872004504613	2.54127168012959	0.0159943497135621	0.490982087779997	-3.08631843832977
a_HQ_transcript/28766	-2.53754263179307	0.485244513163294	-2.54109936580847	0.0160009014519973	0.490982087779997	-3.04905221626081

a_HQ_transcript/45291	3.72395358886332	-0.311038726047612	2.5409588543518	0.0160062458082696	0.490982087779997	-3.11711186141005
a_HQ_transcript/127153	3.87643025389235	1.14382817775341	2.53982652683986	0.0160493738696878	0.492045360916251	-3.05412178753296
a_HQ_transcript/68481	-3.09687673718906	-0.27239725212033	-2.5391948454261	0.0160734797489793	0.4923325617768	-3.08996021115206
a_HQ_transcript/38006	-2.24267149298594	-0.594052633827088	-2.5389923473084	0.0160812144158664	0.4923325617768	-3.08425590976495
a_HQ_transcript/141416	2.630249076484	4.21270912140351	2.53879638034877	0.0160887028758374	0.4923325617768	-3.24806085888162
a_HQ_transcript/115146	4.9235832942801	2.0124337842809	2.53869388237117	0.0160926208948514	0.4923325617768	-3.19275317026151
a_HQ_transcript/138057	-2.95472545324684	2.33409663213129	-2.53826500887696	0.0161090242437672	0.492362880793011	-3.18712306398539
a_HQ_transcript/136010	2.02971789400976	2.75872769589248	2.53822507073564	0.016110552561043	0.492362880793011	-3.2156122249772
a_HQ_transcript/67940	-2.63494326072512	1.96774472270081	-2.53725782613591	0.0161476069575993	0.493235994341216	-3.06581497026247
a_HQ_transcript/127890	-3.04551478900163	-0.158390696333444	-2.53625217686651	0.0161862156862951	0.49366174061508	-3.14568533362457
a_HQ_transcript/112017	-2.51141255075875	-0.594397071316739	-2.53624600707708	0.0161864528176168	0.49366174061508	-3.08415558594705
a_HQ_transcript/95669	2.2371772127384	-0.311165502435681	2.53623117006592	0.0161870230803114	0.49366174061508	-3.09907765788149
a_HQ_transcript/144029	-0.982080135455007	3.09079876237071	-2.53573244275243	0.0162062024867938	0.493793308849472	-3.24754734882781
a_HQ_transcript/120899	-3.85341630574802	1.75665693425437	-2.53567723120812	0.0162083270245801	0.493793308849472	-3.13654425203134
a_HQ_transcript/143114	2.64252868720712	-0.165672947922781	2.53544912752069	0.016217107158939	0.493801993156002	-3.19520774742745
a_HQ_transcript/104987	-3.30990465194408	0.278168486400325	-2.53416456378053	0.0162666341031406	0.495050736726363	-3.09666374516197
a_HQ_transcript/2411	-6.14002171025935	1.75109708667985	-2.53314957506296	0.0163058657547713	0.495985015485791	-3.19246559019593
a_HQ_transcript/117529	1.14969869815212	4.07290029913846	2.5328666722941	0.0163168160873769	0.496058517535064	-3.35891043534735
a_HQ_transcript/16358	-4.65033934409657	1.51357065793279	-2.53257734267868	0.0163280221755101	0.496139714071118	-3.20231299765497
a_HQ_transcript/131355	-8.96994196807673	5.63570440498509	-2.53201616073768	0.0163497776041847	0.496493284237445	-3.54008972688473
a_HQ_transcript/142986	3.19690432933784	-0.231410036912938	2.53168208393796	0.0163627414602161	0.496493284237445	-3.09996092858832
a_HQ_transcript/22694	5.23063666134954	1.29104317199553	2.53154418447396	0.0163680954055514	0.496493284237445	-3.15142000053945
a_HQ_transcript/30996	2.42856224251705	1.14800072506876	2.53139668812697	0.0163738237318191	0.496493284237445	-3.08007048632719
a_HQ_transcript/28419	-2.67099408157212	-0.40210984445131	-2.53084168820379	0.0163953948181504	0.496781165108337	-3.1094538975836
a_HQ_transcript/12417	3.26340112709162	0.746269165601295	2.53063771312486	0.0164033292435065	0.496781165108337	-3.0869761841406
a_HQ_transcript/141879	5.57466685110513	1.42291846050767	2.5304930987849	0.0164089567335537	0.496781165108337	-3.14969632402219
a_HQ_transcript/2672	-2.37254134105105	-0.595101779907216	-2.53005717924976	0.0164259307455683	0.496895043863244	-3.10325354400937
a_HQ_transcript/123083	2.46111738495755	-0.450826984848117	2.52970450584653	0.0164396750925009	0.496895043863244	-3.09923232672842
a_HQ_transcript/63159	-3.19140260171035	0.439548527744002	-2.52932661483516	0.0164544139458	0.496895043863244	-3.09619982890725
a_HQ_transcript/104786	6.80122211987287	1.60860918746114	2.52928404334066	0.0164560751192647	0.496895043863244	-3.17336418857978
a_HQ_transcript/85897	-2.97552067644007	0.739491722454655	-2.52907671597314	0.0164641674005993	0.496895043863244	-3.04119821914745
a_HQ_transcript/36571	-1.61145296178575	-0.818711849868178	-2.52904974140048	0.0164652205252387	0.496895043863244	-3.10673292898298
a_HQ_transcript/56015	4.55110356003158	-0.374416338369184	2.528861889324	0.0164725562469803	0.496895043863244	-3.11552984776201
a_HQ_transcript/129461	-3.0938744883748	-0.493881721542097	-2.52858731869115	0.0164832837766495	0.496915121516243	-3.10692777551133
a_HQ_transcript/121931	-2.95507243810289	-0.436864807667906	-2.5284073387175	0.0164903191130752	0.496915121516243	-3.10265856480268
a_HQ_transcript/79679	-2.5489747361224	-0.0170441478597083	-2.52802722177474	0.0165051867822294	0.497029724143306	-3.09766484980773
a_HQ_transcript/104408	-3.55745305873325	0.582439012804069	-2.52787297374869	0.016511223460651	0.497029724143306	-3.06415928047724
a_HQ_transcript/52407	5.08235275285007	1.21293296680322	2.52694828624565	0.0165474547508519	0.497770882674939	-3.18775025556036
a_HQ_transcript/27688	4.73992685963088	1.64552623753354	2.52674758574668	0.0165553282796062	0.497770882674939	-3.17065451124101
a_HQ_transcript/18791	-2.15218976321454	2.42235861019788	-2.52608288445401	0.016581429254493	0.497770882674939	-3.13949204037058
a_HQ_transcript/21357	-2.04798363055925	-0.367766822227598	-2.52607467900858	0.0165817516956627	0.497770882674939	-3.13584576878485
a_HQ_transcript/58694	-3.32684595012243	1.31086259051868	-2.52564720311167	0.0165985577572463	0.497770882674939	-3.08982195642792
a_HQ_transcript/15573	3.40277080938075	0.26276487502683	2.52542300143008	0.0166073784205839	0.497770882674939	-3.09112252614127
a_HQ_transcript/21543	2.55923147685258	1.54368811092045	2.52494301655842	0.0166262767236363	0.497770882674939	-3.12373544666699
a_HQ_transcript/23314	3.56458487783108	-0.539509625235265	2.52472177634575	0.016634994199382	0.497770882674939	-3.11780191415732
a_HQ_transcript/111155	4.69820152260314	0.499578371146977	2.52461206843737	0.0166393185512506	0.497770882674939	-3.07362823794848
a_HQ_transcript/88453	3.71421379314062	2.17777808501954	2.52450376847521	0.0166435884190142	0.497770882674939	-3.10616339882878
a_HQ_transcript/130292	6.19832316951021	3.05337989937755	2.52443025477751	0.0166464873662513	0.497770882674939	-3.34673433668127
a_HQ_transcript/4777	-4.00453935772994	1.39856245227951	-2.52442855955005	0.0166465542215086	0.497770882674939	-3.11600859521643
a_HQ_transcript/101631	-3.68710397696401	-0.289103395691303	-2.52429851658707	0.0166516835049446	0.497770882674939	-3.14901906385905

a_HQ_transcript/2829	-2.54517283625733	-0.648101848149093	-2.52419591086522	0.0166557316061581	0.497770882674939	-3.11496402087864
a_HQ_transcript/148405	-1.00096413321543	3.46502797855922	-2.52316992019129	0.0166962597353997	0.498640823686888	-3.25567289053371
a_HQ_transcript/111420	2.94026108434286	1.40422472745218	2.52299810499031	0.0167030555347925	0.498640823686888	-3.07108586256463
a_HQ_transcript/58011	-4.48622560994322	0.339200345438532	-2.52241982250727	0.0167259469878294	0.498640823686888	-3.10557911977343
a_HQ_transcript/54398	1.67762683221791	3.58243109277881	2.52241061049652	0.0167263118805781	0.498640823686888	-3.24668641817173
a_HQ_transcript/106797	6.62260717317389	0.466427660888843	2.5223747599829	0.0167277320084887	0.498640823686888	-3.1264469538702
a_HQ_transcript/138704	0.875329573838169	5.54091573894038	2.52166469828417	0.0167558821445439	0.49897865782119	-3.51654768635867
a_HQ_transcript/66267	-2.71852748859465	1.10100463056259	-2.52165584294173	0.0167562334858754	0.49897865782119	-3.04328515529264
a_HQ_transcript/5930	2.39943790413924	-0.567820891073395	2.52140873273737	0.016766040469259	0.499015053966763	-3.15181820891855
a_HQ_transcript/112253	-4.28797728888498	0.126337533714104	-2.52087154561472	0.0167873778302895	0.499054910047221	-3.1203089794092
a_HQ_transcript/152817	-2.81600240015151	0.150576255214654	-2.52086303660878	0.0167877160131284	0.499054910047221	-3.12496944583586
a_HQ_transcript/150006	-1.55357686902378	6.36051810596725	-2.52072668831081	0.0167931359078648	0.499054910047221	-3.56417402283099
a_HQ_transcript/32328	2.26301581436919	0.669735171232379	2.51929511277206	0.0168501386465829	0.500493029764216	-3.11061760033158
a_HQ_transcript/130762	2.36720224169705	-0.673028673706079	2.51894562463541	0.01686408158616	0.500651345475132	-3.11215608549088
a_HQ_transcript/90404	-2.88973445830477	-0.317669877181151	-2.51866787563547	0.0168751700246969	0.500704895915096	-3.12177210489243
a_HQ_transcript/4497	4.553071255674	0.76217041008235	2.51846901731983	0.016883113060721	0.500704895915096	-3.105011940815
a_HQ_transcript/52704	5.79561974852202	1.35663020631247	2.51798972059166	0.0169022718128814	0.500859192782491	-3.1336478166145
a_HQ_transcript/122371	-5.10186896423156	1.04966295428599	-2.51790779168224	0.0169055487241819	0.500859192782491	-3.10177078183531
a_HQ_transcript/117693	4.95104002540173	0.968303330156231	2.51753064652515	0.0169206409266869	0.501050950477053	-3.19242584063396
a_HQ_transcript/152394	-4.40915639925723	-0.312589501037051	-2.51707388449506	0.0169389357174185	0.501337299074391	-3.09965341859178
a_HQ_transcript/89459	-3.67846075189513	-0.263157456671603	-2.51622963736946	0.0169727983473588	0.501826599029197	-3.16347569153136
a_HQ_transcript/50355	1.20087356576654	3.60927371593982	2.51614453026171	0.0169762154271946	0.501826599029197	-3.23086344095679
a_HQ_transcript/147258	-4.13332058646653	0.590774484487097	-2.51601624465364	0.0169813673322741	0.501826599029197	-3.146453129443
a_HQ_transcript/146567	3.39728052990364	-0.036936194463213	2.51521340121381	0.017013641856444	0.502229458792438	-3.14336163760598
a_HQ_transcript/116979	-3.34278786555262	2.15564938001401	-2.51516954264202	0.0170154066040042	0.502229458792438	-3.19725220746645
a_HQ_transcript/89133	-2.12118745549967	-0.345104392386808	-2.51483117214123	0.0170290273467357	0.502229458792438	-3.12606282137976
a_HQ_transcript/84930	-4.41901995336232	3.12269310291254	-2.51481794503143	0.0170295599931168	0.502229458792438	-3.23262378530086
a_HQ_transcript/144341	-2.9721665609732	-0.193229553860638	-2.51449023012066	0.0170427617258602	0.502363921704262	-3.14487637244988
a_HQ_transcript/130135	-2.3744855115181	0.324767026973859	-2.51410960762636	0.0170581065898355	0.502561388674079	-3.08478118702892
a_HQ_transcript/140415	-2.93033496629935	1.68769228586744	-2.51335772712849	0.0170884560625777	0.502787069792014	-3.31326868816496
a_HQ_transcript/5442	-3.34138055148377	-0.452708576815586	-2.51330700740655	0.017090505135144	0.502787069792014	-3.14283771062986
a_HQ_transcript/124494	3.93828961692207	0.759761025524173	2.51296773237734	0.0171042176190648	0.502787069792014	-3.15680640473292
a_HQ_transcript/29074	-1.98081159462841	-0.516734881227048	-2.51290282236226	0.0171068422386415	0.502787069792014	-3.17281742384846
a_HQ_transcript/10309	2.44884437313	-0.200356676938866	2.51284909420762	0.0171090150022124	0.502787069792014	-3.13245142290908
a_HQ_transcript/138130	-2.44004916995928	-0.489409059511013	-2.51229689207079	0.0171313607095059	0.503016950093805	-3.10769375093839
a_HQ_transcript/128383	-2.05289509769689	-0.761898213484859	-2.5122004219109	0.0171352672700772	0.503016950093805	-3.14207152980968
a_HQ_transcript/59328	2.58509149868827	-0.651452270826099	2.51201304487245	0.0171428574424204	0.503016950093805	-3.14191560337233
a_HQ_transcript/52877	-3.03560299498457	-0.507685491247672	-2.51180096755257	0.0171514518835316	0.503016950093805	-3.14652086430894
a_HQ_transcript/42286	-2.44231289859109	-0.361951275951097	-2.51123586691318	0.0171743719177377	0.503435144142339	-3.08883962959673
a_HQ_transcript/58235	-2.68995490686457	-0.16056666116243	-2.51021760666979	0.0172157427162073	0.50433716582213	-3.11636595401787
a_HQ_transcript/18620	1.46487240867567	1.85952054632219	2.51004568616649	0.0172227366748776	0.50433716582213	-3.07996515511384
a_HQ_transcript/70128	-3.75146133643947	1.59306208629897	-2.50981782248787	0.0172320104955859	0.50433716582213	-3.09644776117299
a_HQ_transcript/95467	-6.14163473927873	1.49244773632017	-2.50931411261875	0.0172525272393314	0.50433716582213	-3.19628084085878
a_HQ_transcript/53090	6.21402426584654	2.81107383220689	2.50916462894467	0.0172586202110836	0.50433716582213	-3.29579403267906
a_HQ_transcript/25690	2.34081480171939	1.20082173460952	2.50899083342592	0.0172657066186448	0.50433716582213	-3.08492319456927
a_HQ_transcript/116210	-1.93673695546072	1.18308956737686	-2.50898662955616	0.0172658780619674	0.50433716582213	-3.06879750269651
a_HQ_transcript/116680	2.69340425270598	-0.462686974531439	2.50820886417408	0.0172976239859383	0.504817936832887	-3.1166868127252
a_HQ_transcript/79121	3.54255076083845	0.659723000978268	2.50789765240938	0.0173103416614691	0.504817936832887	-3.13429669451521
a_HQ_transcript/65031	-2.57921273448913	-0.52341363073699	-2.50786052880874	0.0173118592907943	0.504817936832887	-3.14279482540406
a_HQ_transcript/150310	5.04059049391676	0.607206036726857	2.50773295430929	0.0173170755237541	0.504817936832887	-3.20193007129518

a_HQ_transcript/109232	1.96043567292174	-0.542941485577148	2.50716812418319	0.0173401874809558	0.505167619342875	-3.10909102662128
a_HQ_transcript/137406	-6.12633512311301	1.3346955759343	-2.50701514272531	0.0173464521092826	0.505167619342875	-3.18381073560236
a_HQ_transcript/50171	4.15189269351741	2.76526983222277	2.50542515748619	0.0174116854413579	0.506655264299787	-3.2990976431027
a_HQ_transcript/64328	-2.02207675738006	0.46648660196084	-2.50513373092379	0.0174236663591952	0.506655264299787	-3.13889617878794
a_HQ_transcript/38388	-2.88466881033105	1.14408400657839	-2.50513331460088	0.0174236834801692	0.506655264299787	-3.12205669435236
a_HQ_transcript/111875	-3.29756938625894	0.234846379994352	-2.5049208621035	0.0174324224467538	0.506655925992453	-3.17266493925236
a_HQ_transcript/150113	1.29031671854301	3.93264441341233	2.50428632694454	0.0174585471954338	0.506798634853804	-3.37426680135057
a_HQ_transcript/148969	5.15816077063701	9.47893496617673	2.5040982177275	0.0174662988276167	0.506798634853804	-4.0570536279947
a_HQ_transcript/63198	-3.16170507043848	1.96547625823202	-2.50407975235479	0.0174670599215305	0.506798634853804	-3.17925311434226
a_HQ_transcript/81572	4.86018448864806	1.45883376978244	2.50392663478043	0.0174733721957439	0.506798634853804	-3.18491209305235
a_HQ_transcript/149358	3.16250490780106	6.01515810266172	2.50374346945743	0.0174809259372742	0.506798634853804	-3.52979025055466
a_HQ_transcript/104960	3.84297967923042	0.990565014566847	2.50306690483898	0.0175088534464354	0.507355250814755	-3.14538772453272
a_HQ_transcript/2576	-3.69948504178956	0.482724920022071	-2.50165442938455	0.0175672901192367	0.508577144929925	-3.13347265719714
a_HQ_transcript/66207	-2.87938941235492	0.141717397957962	-2.5016247579787	0.0175685195950194	0.508577144929925	-3.08022481026105
a_HQ_transcript/141466	3.56264545652087	0.764966404184139	2.50026900773913	0.0176247812290543	0.509951858278979	-3.20863453115236
a_HQ_transcript/39364	-4.05912983638946	1.65718091857334	-2.49975345052461	0.0176462194166081	0.510318130471938	-3.23589715817344
a_HQ_transcript/97428	-2.98413866497946	2.32277526917009	-2.49733193274597	0.0177472325658397	0.512984154444121	-3.21808970374392
a_HQ_transcript/119848	2.56711907486489	3.04273952491753	2.49675040103038	0.0177715698595621	0.513432312523173	-3.19541466158691
a_HQ_transcript/79235	3.10753797689298	1.36175415846684	2.4961195067108	0.0177980075924212	0.513940678257457	-3.11419084380565
a_HQ_transcript/92094	4.77345175394894	2.93459473140427	2.49568683886083	0.017816159447199	0.514209392426406	-3.26790720148342
a_HQ_transcript/82238	2.26227628245999	-0.460672573052789	2.49528449909833	0.0178330541633779	0.514422046583101	-3.16540223587529
a_HQ_transcript/9745	-2.86770711029522	-0.412330935641259	-2.49508560727893	0.017841411285864	0.514422046583101	-3.17403593360068
a_HQ_transcript/123126	-3.31748110029367	1.26495141793489	-2.49455012879819	0.0178639290981494	0.514422046583101	-3.21862607337425
a_HQ_transcript/50045	-0.750290996604012	3.8495152449089	-2.494195388463	0.0178788608946241	0.514422046583101	-3.37765503324722
a_HQ_transcript/73373	-1.48703494856792	0.486691448841674	-2.49419405806843	0.0178789169153657	0.514422046583101	-3.14959468887553
a_HQ_transcript/152492	-6.71897230055776	1.31374863946525	-2.49395501638489	0.017888985181905	0.514422046583101	-3.22389882113949
a_HQ_transcript/60099	3.99907923970077	0.427148111282185	2.49374600728869	0.0178977927550621	0.514422046583101	-3.17824850324905
a_HQ_transcript/79823	-2.81014409004333	2.95580685789775	-2.49374026308806	0.0178980348698534	0.514422046583101	-3.21605360777022
a_HQ_transcript/113338	-1.51081765676623	2.63744994921689	-2.49353214183358	0.0179068090873256	0.514422046583101	-3.23397884758234
a_HQ_transcript/152510	-3.84685642268273	-0.588336833731125	-2.4934084530736	0.0179120255691611	0.514422046583101	-3.1923540745728
a_HQ_transcript/144587	2.91025881443305	2.28816822069427	2.49240146614396	0.0179545463491193	0.515221885991569	-3.16742235874041
a_HQ_transcript/95328	-1.08233179661299	4.90241789662895	-2.49232916456863	0.0179576028939396	0.515221885991569	-3.55473281009926
a_HQ_transcript/97166	-7.83590084684374	1.8734770717251	-2.49168898406298	0.017984687297724	0.515744402177585	-3.26798307226818
a_HQ_transcript/118634	-1.0990335173682	3.39614303969894	-2.49145809756448	0.0179944646971209	0.51577033723582	-3.32007494163891
a_HQ_transcript/106746	2.90401582433626	0.824465693397302	2.49103916928321	0.0180122175802571	0.516024733122318	-3.17455074256296
a_HQ_transcript/43144	-3.33565974538801	-0.181785719798278	-2.49064477249725	0.0180289455599763	0.516249530793253	-3.17489892744768
a_HQ_transcript/150304	3.35400769466565	2.21050203368628	2.49020576418319	0.0180475824373567	0.516299344960043	-3.27857136892126
a_HQ_transcript/93245	-3.12462169818028	0.75701281110823	-2.48984016258083	0.0180631165065792	0.516299344960043	-3.1517195837484
a_HQ_transcript/103916	2.67586601983986	-0.332011754401766	2.48977353184795	0.0180659489033845	0.516299344960043	-3.18174813046734
a_HQ_transcript/120822	4.59371353180227	1.12659863940501	2.48955798799855	0.0180751142153121	0.516299344960043	-3.25522512344058
a_HQ_transcript/77545	-1.43849372422133	1.53402107398937	-2.48917418697891	0.0180914446719022	0.516299344960043	-3.13865000819429
a_HQ_transcript/76778	4.39399302674679	0.631110242360386	2.48917012604668	0.0180916175338884	0.516299344960043	-3.15819642810131
a_HQ_transcript/85730	2.59077497439652	-0.469604461996592	2.4890983991077	0.0180946709889295	0.516299344960043	-3.21848069051574
a_HQ_transcript/146398	2.1021114749819	2.91542212992675	2.48893233821346	0.0181017421041248	0.516299344960043	-3.21773498042006
a_HQ_transcript/100631	-3.85955431968944	0.887908795847023	-2.48809672560194	0.018137362144529	0.517061592318384	-3.12214075994538
a_HQ_transcript/16126	-3.02418458047605	0.0647118843602403	-2.48762049942966	0.01815769116975	0.517172666993144	-3.12924588278508
a_HQ_transcript/139378	-3.07971607785921	0.23896130188987	-2.48738678538183	0.0181676755310488	0.517172666993144	-3.11605925352453
a_HQ_transcript/1875	4.1616699543393	0.958732063581219	2.48738036694128	0.0181679498004404	0.517172666993144	-3.18391248209858
a_HQ_transcript/151506	6.3357547273261	0.320209851081572	2.48715015727573	0.0181777895056589	0.517199485259393	-3.19897254634274
a_HQ_transcript/141278	-2.26122589130032	2.03144021532007	-2.48669005140923	0.0181974701480531	0.517506137361072	-3.18893101655167

a_HQ_transcript/93584	-1.44061845905952	2.6389516773219	-2.48579063242697	0.0182359984205133	0.517892179095564	-3.27901165195686
a_HQ_transcript/43832	-5.44059127969871	2.73140438994761	-2.48575806012394	0.0182373951161722	0.517892179095564	-3.31982986399562
a_HQ_transcript/99606	-3.06032301998385	-0.514657464104425	-2.48541027022068	0.0182523144067695	0.517892179095564	-3.16382971788763
a_HQ_transcript/152750	4.51282949378538	5.50538472424339	2.48520857258269	0.0182609718362677	0.517892179095564	-3.6297352703081
a_HQ_transcript/143504	2.3905579042028	-0.492349980492204	2.48506655192236	0.0182670700185023	0.517892179095564	-3.18827810170698
a_HQ_transcript/133350	1.72468127711104	3.18683857091284	2.48493148232879	0.018272871461433	0.517892179095564	-3.3557994240495
a_HQ_transcript/54478	-2.59142692947709	-0.444952883555465	-2.48491891285284	0.0182734114252168	0.517892179095564	-3.19492980329324
a_HQ_transcript/114471	-4.30965321191466	2.86085180153908	-2.48447084932961	0.018292669037121	0.51815510561308	-3.23398003722918
a_HQ_transcript/113467	-2.3337106161631	0.0861984489018578	-2.48428838416691	0.0183005166498702	0.51815510561308	-3.14211811093717
a_HQ_transcript/130354	3.00696235731751	2.7784745816421	2.48379831608452	0.0183216091677081	0.518499755453035	-3.3002290394036
a_HQ_transcript/75745	1.95113152925182	-0.714903084580798	2.48284940078867	0.0183625137611356	0.51940447684053	-3.19826574893747
a_HQ_transcript/95102	5.10624417283866	0.738364266379904	2.48233418991363	0.0183847578080111	0.519700050067489	-3.27182639910676
a_HQ_transcript/113472	-3.58083588220566	0.0529091811526048	-2.48219331342498	0.0183908443949357	0.519700050067489	-3.19367668925074
a_HQ_transcript/122214	2.63264438170861	1.23054766802449	2.48072706341201	0.0184543035502543	0.521239920684732	-3.2238665268365
a_HQ_transcript/22982	1.73534625598643	4.80568182169869	2.4803115346864	0.0184723240178066	0.521495507774193	-3.53329215714059
a_HQ_transcript/144272	-3.88186328043881	0.0586566559579777	-2.47972619875924	0.0184977359425221	0.521959414983943	-3.20493401883838
a_HQ_transcript/72889	-2.23285333588153	1.66599753823238	-2.47916294968859	0.0185222191957392	0.522396679966001	-3.18166875605938
a_HQ_transcript/107879	-2.84596872752612	-0.365743471752019	-2.47843322146849	0.0185539830632147	0.52298714960771	-3.18463960084713
a_HQ_transcript/128090	-3.07935244297375	1.75164984497749	-2.47810608806761	0.0185682388229578	0.52298714960771	-3.15620174264776
a_HQ_transcript/72435	3.4931957749673	1.70346930948166	2.4780623287686	0.0185701465178625	0.52298714960771	-3.16464866269467
a_HQ_transcript/97386	1.96440640471647	1.62655930617356	2.47756439078798	0.0185918668581166	0.523317934859395	-3.20102023086347
a_HQ_transcript/145156	2.48385851189439	-0.446039030406076	2.47738041890816	0.018599897698519	0.523317934859395	-3.21398367456232
a_HQ_transcript/1373	2.85573908806863	0.194630082295701	2.4768135411218	0.0186246633260939	0.523761214232795	-3.17645529167434
a_HQ_transcript/36208	-2.53283460253666	0.158840720473849	-2.47610864344637	0.0186555008349362	0.524006492242396	-3.24639872526722
a_HQ_transcript/10504	-3.66808290154055	1.52592518622471	-2.47604866177581	0.0186581270392141	0.524006492242396	-3.19660946705575
a_HQ_transcript/50421	-3.83099787100568	3.50070604839223	-2.47595773109839	0.0186621089430006	0.524006492242396	-3.36237806649909
a_HQ_transcript/123789	-3.68075246150332	0.603847352931344	-2.47579027254697	0.0186694440791702	0.524006492242396	-3.15915221847569
a_HQ_transcript/138828	5.93452201422198	0.765919238029248	2.47520025917021	0.0186953092882927	0.524235405754205	-3.18357919174815
a_HQ_transcript/64703	2.96962852896643	0.569064309224765	2.47519278513615	0.0186956371478198	0.524235405754205	-3.19044627490537
a_HQ_transcript/119076	3.17724991968025	-0.540475090097882	2.4747639067043	0.0187144593495083	0.524510170235398	-3.22053661112419
a_HQ_transcript/66503	-2.91415782904967	3.70490624485292	-2.47435755064574	0.0187323091096417	0.524750250723393	-3.40330772069623
a_HQ_transcript/91303	2.86356640026744	-0.421664363280881	2.47415177825554	0.0187413538919007	0.524750250723393	-3.21000812588832
a_HQ_transcript/87407	-1.08516283043086	5.61435625214797	-2.47395270811211	0.0187501078783458	0.524750250723393	-3.63971847400928
a_HQ_transcript/58138	4.25617261662985	0.451555933081312	2.47346416437979	0.0187716071488314	0.525038448048022	-3.22767409319246
a_HQ_transcript/148812	-3.24004375019521	0.598185666769481	-2.47330831111661	0.0187784705046077	0.525038448048022	-3.16102370609639
a_HQ_transcript/31302	3.43979489022527	1.27616151976115	2.47284265483319	0.0187989904287461	0.525131018537345	-3.19008881854239
a_HQ_transcript/126455	-1.03021917777787	4.28835171146259	-2.47282317101578	0.0187998494628443	0.525131018537345	-3.50087115140761
a_HQ_transcript/152577	5.81104627937697	0.623437980306829	2.47253131144233	0.0188127217400912	0.525238179302603	-3.25326464963982
a_HQ_transcript/84047	-1.95212712933962	-0.721683335055216	-2.47219258781771	0.0188276710367389	0.525314400007455	-3.21891426731966
a_HQ_transcript/129448	-3.3401487959487	-0.524383855633056	-2.47205998815034	0.0188335261769807	0.525314400007455	-3.22633546242027
a_HQ_transcript/133640	-3.57897131797684	1.2781560204706	-2.47151062758594	0.018857801783337	0.525720081182883	-3.31154571571419
a_HQ_transcript/22307	-3.35589477310859	2.38302809950501	-2.4713216526409	0.0188661589827191	0.525720081182883	-3.21363455195061
a_HQ_transcript/12698	-2.27183752468294	1.31350524742562	-2.47089469800316	0.0188850530380099	0.525994424050521	-3.19682967645872
a_HQ_transcript/17728	-2.5723021068171	0.386952259158405	-2.47036127780635	0.0189086828465758	0.5264003431541	-3.20776846282982
a_HQ_transcript/26760	4.05022807380007	-0.579097339447317	2.4699126152597	0.0189285789441427	0.526701980308822	-3.22528066144125
a_HQ_transcript/117561	3.08358554898536	0.710572965973324	2.46913877702791	0.0189629400799911	0.527307049402638	-3.21065101553703
a_HQ_transcript/137096	-1.65983895363132	-0.816346411195265	-2.46901443013556	0.0189684668369962	0.527307049402638	-3.22415683029798
a_HQ_transcript/598	-3.68438216879856	1.2716160103525	-2.46852470405023	0.0189902476726472	0.527563656574162	-3.25510020455517
a_HQ_transcript/90585	-2.84322516087155	-0.106552913184427	-2.46839883666865	0.0189958493877257	0.527563656574162	-3.20031329097029
a_HQ_transcript/144722	3.13107940468575	0.39298786092591	2.46802906558704	0.0190123147561036	0.527768783258258	-3.29549575389378

a_HQ_transcript/117052	-2.93896288090836	-0.544018741753275	-2.46757862950085	0.0190323896564121	0.527943852758025	-3.22452457551968
a_HQ_transcript/144509	3.0954804774449	4.50825948087657	2.46748003610199	0.0190367863229566	0.527943852758025	-3.53609661502184
a_HQ_transcript/72714	-6.59603706211208	2.55395872504783	-2.46715942798284	0.0190510899200985	0.528088581247251	-3.2475488981352
a_HQ_transcript/125029	2.92941499432158	0.772688828461351	2.46498592812788	0.0191483178386749	0.529136959420222	-3.22111110630335
a_HQ_transcript/124720	1.74914560459601	1.95512403161831	2.46485837913582	0.0191540376035951	0.529136959420222	-3.2728287029141
a_HQ_transcript/85010	4.66758299912947	0.876337717048836	2.46469959592432	0.0191611602087149	0.529136959420222	-3.17745749653674
a_HQ_transcript/26511	-3.12595964273329	0.215825044890951	-2.46444785425878	0.0191724576544313	0.529136959420222	-3.19980553868179
a_HQ_transcript/132452	2.59326137101052	-0.313650202064226	2.46438983708742	0.0191750621625981	0.529136959420222	-3.2395597429299
a_HQ_transcript/21098	-2.6412935816817	-0.344141968730723	-2.46413324327054	0.0191865850582428	0.529136959420222	-3.21118822390447
a_HQ_transcript/137852	1.10531930415815	3.87274083687125	2.4640985678024	0.0191881427200706	0.529136959420222	-3.48790065645176
a_HQ_transcript/37724	-3.73948796015844	-0.301306520441993	-2.46389789144249	0.0191971596038015	0.529136959420222	-3.23291021597879
a_HQ_transcript/82384	3.58477606609406	0.0190298512698235	2.46373839602494	0.019204328889535	0.529136959420222	-3.24012831917388
a_HQ_transcript/110376	2.54832399964724	1.44362166308155	2.46372797957663	0.0192047971918684	0.529136959420222	-3.19895122730489
a_HQ_transcript/90925	2.49003642972339	2.10521635433205	2.46322334496776	0.0192274970482883	0.529136959420222	-3.23667725130302
a_HQ_transcript/147086	-0.695012057262564	4.78122170784228	-2.46312082000757	0.0192321119011875	0.529136959420222	-3.59357538894502
a_HQ_transcript/52333	-3.78049832088824	0.734897520717604	-2.46276228242426	0.0192482583575212	0.529136959420222	-3.21317670739591
a_HQ_transcript/93708	1.40250036037504	0.350063935987119	2.46268800929496	0.019251604738483	0.529136959420222	-3.17240086641984
a_HQ_transcript/78834	2.03708186505369	-0.791036598708112	2.4623993806552	0.0192646139723763	0.529136959420222	-3.23900871410235
a_HQ_transcript/127972	3.21952778555316	0.790081127016935	2.46222120036595	0.0192726490275253	0.529136959420222	-3.16893942083775
a_HQ_transcript/13480	2.37550976856977	0.873076272687057	2.46206289468412	0.0192797904059428	0.529136959420222	-3.17482425325398
a_HQ_transcript/86807	-3.26110635953557	1.27357365742178	-2.46164946197193	0.0192984523130123	0.529136959420222	-3.22466482837465
a_HQ_transcript/145601	-4.53071739659818	1.05591221398892	-2.46143018364268	0.0193083570015976	0.529136959420222	-3.18321817105723
a_HQ_transcript/121188	-1.95803803777565	0.629595405218607	-2.46135235451427	0.0193118736203868	0.529136959420222	-3.17268751308387
a_HQ_transcript/1835	-2.34360893442207	1.8772807697444	-2.46134150552299	0.0193123638659564	0.529136959420222	-3.22617997961591
a_HQ_transcript/152336	-5.34320408431816	7.31814874649568	-2.46132691126563	0.0193130233710244	0.529136959420222	-3.81572804944685
a_HQ_transcript/149692	-2.14180924650545	-0.530162692939273	-2.46132635712915	0.0193130484124998	0.529136959420222	-3.22419495378498
a_HQ_transcript/138646	2.74422085235265	0.262773102283705	2.46128637160399	0.0193148554401959	0.529136959420222	-3.19092584064068
a_HQ_transcript/23283	-3.32911806928061	1.77827220647798	-2.4612503206232	0.0193164847902854	0.529136959420222	-3.21248427974384
a_HQ_transcript/300	3.09906970715492	0.964276120304356	2.46077162195278	0.0193381318372283	0.529480418009612	-3.17784335234488
a_HQ_transcript/44477	-1.54524924002347	3.03422183475246	-2.45995393538192	0.0193751594248741	0.530153216191113	-3.35879944596584
a_HQ_transcript/109720	2.13415480462395	-0.492538918940593	2.45952251422914	0.0193947217276922	0.530153216191113	-3.23766302398564
a_HQ_transcript/54863	2.08740610817246	-0.443125857152645	2.45926971577007	0.0194061929746841	0.530153216191113	-3.22922470927456
a_HQ_transcript/72038	-2.8813828455939	-0.556256967932395	-2.45910298757478	0.0194137620011053	0.530153216191113	-3.24651060279416
a_HQ_transcript/12679	5.3280709201183	0.74803627364528	2.45882613188379	0.0194263364903283	0.530153216191113	-3.20087367115269
a_HQ_transcript/62262	-2.57776268666466	-0.47965970712981	-2.45871986303588	0.0194311650845546	0.530153216191113	-3.21217167833351
a_HQ_transcript/140743	-3.46084950643133	0.393713566330395	-2.45870888271429	0.0194316640657999	0.530153216191113	-3.21770027246819
a_HQ_transcript/129974	-1.40627669915367	3.18250030914066	-2.4580731968901	0.0194605716548669	0.530153216191113	-3.37714329464834
a_HQ_transcript/136786	-4.09626808069902	-0.117732202550871	-2.45761782456859	0.0194813037083839	0.530153216191113	-3.2209802878269
a_HQ_transcript/127614	-3.96145685187215	-0.524888982420477	-2.45748226611174	0.0194874792707641	0.530153216191113	-3.25165583283032
a_HQ_transcript/107518	-1.19696333913661	3.77729979878912	-2.45746436487215	0.0194882949214563	0.530153216191113	-3.45312893097768
a_HQ_transcript/84808	5.02032110938636	2.10894402473564	2.45709734844263	0.0195050245085681	0.530153216191113	-3.31550873482601
a_HQ_transcript/101273	-2.7296147735389	2.70005658247475	-2.45694296063206	0.0195120658375439	0.530153216191113	-3.43051715345058
a_HQ_transcript/108296	-4.70531256671311	6.79311603890329	-2.45688369238751	0.0195147695644035	0.530153216191113	-3.80833507488463
a_HQ_transcript/29557	3.38028239967372	0.022293748950214	2.45680655992993	0.0195182887421454	0.530153216191113	-3.2691129722286
a_HQ_transcript/139173	2.14026846879178	3.19781440419837	2.45668797436378	0.0195237003543076	0.530153216191113	-3.42760992725072
a_HQ_transcript/61011	2.63534894128312	0.082231068146705	2.4564846438075	0.0195329824667313	0.530153216191113	-3.31100067158226
a_HQ_transcript/11063	2.38956430224513	4.08763831201567	2.45626783431062	0.0195428843402622	0.530153216191113	-3.51276939822773
a_HQ_transcript/144641	5.75577695048386	1.19647549172545	2.45590781357948	0.0195593369256945	0.530153216191113	-3.29987417016116
a_HQ_transcript/9854	1.87516667808971	-0.461490585522705	2.45589786225105	0.019559791871115	0.530153216191113	-3.23970380089171
a_HQ_transcript/120649	-2.35453947013037	-0.286891505514449	-2.45582411207242	0.0195631638134295	0.530153216191113	-3.28038035319352

a_HQ_transcript/126054	-4.11537669514237	1.24698897696453	-2.45581994992017	0.0195633541276138	0.530153216191113	-3.19954852851345
a_HQ_transcript/3740	1.38333061327012	3.09943870432845	2.45551772168166	0.0195771780199682	0.53026345034394	-3.32384050583282
a_HQ_transcript/53631	-3.58681634432509	1.94070277559647	-2.45533223473405	0.0195856665959338	0.53026345034394	-3.4026874967021
a_HQ_transcript/78287	-3.99466761940323	-0.444394209578571	-2.45489730765686	0.0196055836749881	0.530555571629288	-3.25856525505084
a_HQ_transcript/139076	-2.71616300461468	0.325231249324219	-2.45444436960138	0.0196263452214495	0.530732369505509	-3.21973993998532
a_HQ_transcript/151211	-3.0610132597605	2.13415953216544	-2.45424048727073	0.019635697232006	0.530732369505509	-3.29599789790584
a_HQ_transcript/89147	-0.757424450451113	3.84029266842845	-2.4539876947561	0.0196472983894963	0.530732369505509	-3.50360963127304
a_HQ_transcript/118447	-2.92496508402354	0.161422953863837	-2.45395850198783	0.0196486385077046	0.530732369505509	-3.2665190435627
a_HQ_transcript/34494	-2.63174426062549	-0.334735936120642	-2.45344038248706	0.0196724371120544	0.531128390362052	-3.2422696514051
a_HQ_transcript/92626	-2.24472186036819	3.09764016105777	-2.45321503327185	0.0196827962158372	0.531161364175573	-3.4118911053505
a_HQ_transcript/125201	-3.95028957021298	0.774191601919058	-2.45301535838335	0.0196919792577661	0.531162584823863	-3.33190148893247
a_HQ_transcript/104204	2.36912627252808	1.34673671538785	2.45234981031282	0.0197226160673677	0.531576345638632	-3.23224045139384
a_HQ_transcript/81566	1.28101082330975	4.09149539328459	2.45212402251841	0.0197330195214759	0.531576345638632	-3.48207573494425
a_HQ_transcript/81797	2.70239781414213	1.61760878563577	2.4518133829373	0.0197473408209485	0.531576345638632	-3.20478303669684
a_HQ_transcript/128396	2.86745408577004	0.0131284043112554	2.45181260219595	0.0197473768271314	0.531576345638632	-3.24442222200571
a_HQ_transcript/80666	2.26094285269619	0.610407325505851	2.45168974713217	0.0197530433969764	0.531576345638632	-3.24135651958278
a_HQ_transcript/90645	-0.888425412777201	3.70956472606378	-2.45101587642059	0.0197841514533644	0.532062029637447	-3.49834476527776
a_HQ_transcript/16628	2.72282667754803	1.31135918270748	2.4507191078019	0.0197978654435805	0.532062029637447	-3.24854989901559
a_HQ_transcript/92835	3.02837204952364	-0.212845356277189	2.45070427829762	0.0197985509583299	0.532062029637447	-3.21232599271685
a_HQ_transcript/15310	-2.90531190211883	-0.212684579020603	-2.45030029016843	0.0198172342222917	0.532318017963664	-3.21983670126362
a_HQ_transcript/44792	-2.35458330743564	1.88761221297807	-2.45002025289891	0.0198301945725296	0.532401296450255	-3.25557044679305
a_HQ_transcript/142978	-4.14193851803828	0.157848987723306	-2.4498375848573	0.0198386527682227	0.532401296450255	-3.25983663241308
a_HQ_transcript/149757	-2.69742534619658	2.52063158846627	-2.44938618182138	0.0198595685079254	0.532690720145922	-3.33383116338546
a_HQ_transcript/138500	3.73849249738151	0.647481953712932	2.4492093898677	0.0198677656426569	0.532690720145922	-3.27307411685308
a_HQ_transcript/152579	4.56174071048185	1.74666491723619	2.44886966406535	0.0198835260425296	0.532694332870456	-3.30491494878562
a_HQ_transcript/32806	-2.6131357139141	1.70637110946347	-2.44838247015703	0.0199061476432399	0.532694332870456	-3.26022533286347
a_HQ_transcript/116403	-3.00848265765806	1.44271053746806	-2.44837821370297	0.0199063453843117	0.532694332870456	-3.25103442734045
a_HQ_transcript/45367	-1.92004602149775	0.61972695333025	-2.44804183612503	0.0199219780713743	0.532694332870456	-3.23104695903038
a_HQ_transcript/31951	4.4786680371976	1.50995958420648	2.44797464309604	0.0199251021185126	0.532694332870456	-3.26929830415544
a_HQ_transcript/37596	-3.38508962390363	0.419999920901145	-2.44785072863502	0.0199308645236996	0.532694332870456	-3.23107247752482
a_HQ_transcript/132035	2.41136423517198	-0.559591713332741	2.44763027772528	0.0199411199344004	0.532694332870456	-3.26463191789974
a_HQ_transcript/120671	2.39246290025905	-0.47363898365041	2.44754289911315	0.0199451861344986	0.532694332870456	-3.27084880702209
a_HQ_transcript/53024	-5.59810814670382	2.04352596227901	-2.44743135830445	0.0199503778326965	0.532694332870456	-3.31338303927952
a_HQ_transcript/2186	-3.44067950094162	0.768974399469725	-2.4467391988303	0.0199826221892925	0.533036844906941	-3.28809149715084
a_HQ_transcript/121239	3.13517289257447	1.22588718599705	2.44666988755829	0.0199858536856442	0.533036844906941	-3.20997693276467
a_HQ_transcript/113405	1.19612435290956	3.05746394149887	2.44656562325209	0.0199907156946245	0.533036844906941	-3.34525625513384
a_HQ_transcript/134035	2.74147888877441	1.4149104505645	2.44553536135431	0.0200388165286693	0.533838452957579	-3.29501636451743
a_HQ_transcript/56667	4.6769651048003	0.743592234647509	2.44538535391065	0.020045828878421	0.533838452957579	-3.24885161274405
a_HQ_transcript/113237	-1.23987256813615	3.63274344154733	-2.44533185567992	0.0200483302849985	0.533838452957579	-3.46323677144403
a_HQ_transcript/94708	-0.853263037674493	3.94941803817688	-2.44456099775848	0.0200844047951593	0.53455415839424	-3.53454237171852
a_HQ_transcript/127566	-4.1956324577208	-0.480950862980693	-2.443996939448	0.0201108390622738	0.535012747373845	-3.28947799878575
a_HQ_transcript/14939	2.34992426582859	2.29526218714786	2.44343886021939	0.0201370243802468	0.535061524057369	-3.36211960209405
a_HQ_transcript/151210	-2.77008935793659	-0.489913662306943	-2.44337448746352	0.0201400467790898	0.535061524057369	-3.24073593696948
a_HQ_transcript/14914	-2.28447313295424	-0.246803188305135	-2.44299830868688	0.0201577172279587	0.535061524057369	-3.25701433838422
a_HQ_transcript/1661	4.06223828701496	2.07866704921133	2.44276599527005	0.0201686368796196	0.535061524057369	-3.33486528069345
a_HQ_transcript/32223	2.49671556769466	0.369332467438836	2.44260464992217	0.0201762239279242	0.535061524057369	-3.2335798546235
a_HQ_transcript/77767	-4.21709223742566	0.798423829009866	-2.44249994069105	0.0201811491329036	0.535061524057369	-3.27603678642106
a_HQ_transcript/130719	-2.01175006170131	-0.681876094955002	-2.4424673683605	0.0201826814604387	0.535061524057369	-3.25114180503503
a_HQ_transcript/104367	-2.91390142539363	2.00642429511413	-2.44239021175984	0.0201863116270611	0.535061524057369	-3.2943825459907
a_HQ_transcript/138033	-2.62526874929834	-0.426773227646861	-2.44179526796746	0.0202143233595589	0.535559794095006	-3.26662517994101

a_HQ_transcript/115752	-3.46186307094754	1.50920222628192	-2.44152274710485	0.0202271663016316	0.535655910150906	-3.29904216913819
a_HQ_transcript/26030	-3.88986902538171	-0.47042450117866	-2.44130406793847	0.0202374772611508	0.535684917229586	-3.27753277120639
a_HQ_transcript/81768	2.92317558599221	-0.214572703021379	2.44070006118795	0.0202659817648587	0.536058308891924	-3.27796692899493
a_HQ_transcript/66549	-2.41316351781088	1.12877776122509	-2.44061439381928	0.0202700275761156	0.536058308891924	-3.21066853855244
a_HQ_transcript/152122	2.99781285205785	-0.687543345576771	2.43910385609247	0.0203414869183477	0.53770347957695	-3.29346216030135
a_HQ_transcript/146158	-4.32546996695623	0.249585168494023	-2.43842199876561	0.0203738189458171	0.538110043855843	-3.27101630002029
a_HQ_transcript/148386	4.54085731175329	-0.273173319829959	2.43828391906439	0.0203803720591294	0.538110043855843	-3.34663012723641
a_HQ_transcript/123296	3.34313799223665	0.830465906470887	2.43811728946184	0.0203882826810015	0.538110043855843	-3.24189342452541
a_HQ_transcript/10310	-3.82472731774609	2.17469320602361	-2.43799907164351	0.0203938966868707	0.538110043855843	-3.40339361983658
a_HQ_transcript/107017	3.82888098331962	2.3433469010318	2.43726535858789	0.0204287712950043	0.538785670524504	-3.38156236025597
a_HQ_transcript/138214	4.35947384563151	1.00651556976152	2.43705057182413	0.0204389907610718	0.538810727872825	-3.24209118126313
a_HQ_transcript/86179	-3.4121815768361	1.03008922511011	-2.43619271834922	0.0204798536153908	0.539223391315014	-3.24479176937317
a_HQ_transcript/144499	-3.00111464925433	2.75252324850099	-2.43605563071334	0.0204863905291054	0.539223391315014	-3.44898384447727
a_HQ_transcript/141759	2.26644494995869	-0.65482526323979	2.43590016634847	0.0204938060259521	0.539223391315014	-3.29220651783671
a_HQ_transcript/13175	-3.10475369343529	0.930726973172367	-2.43587283876305	0.0204951097778733	0.539223391315014	-3.31932229855241
a_HQ_transcript/50231	-2.4159590853167	1.16997377607412	-2.43574883038445	0.0205010269544141	0.539223391315014	-3.2064531231717
a_HQ_transcript/145939	-5.44934428117783	6.25459029273808	-2.43532413696553	0.0205213034121732	0.539512584686931	-3.70092271377893
a_HQ_transcript/139990	3.16021610920678	-0.341120882504842	2.43469336271966	0.0205514527114505	0.539801841008891	-3.31127087707081
a_HQ_transcript/104349	1.13387104583809	2.43716217725469	2.43467418866351	0.0205523698118975	0.539801841008891	-3.39250707157379
a_HQ_transcript/143631	2.64180213424463	-0.620958284550402	2.43439373776955	0.0205657881237727	0.539801841008891	-3.29400679586023
a_HQ_transcript/107899	-4.23099772871459	0.669000749733951	-2.43400839302054	0.0205842381690695	0.539801841008891	-3.231560513864
a_HQ_transcript/58840	0.842330800114194	2.87002488440717	2.43398253072173	0.0205854769789457	0.539801841008891	-3.38352096232904
a_HQ_transcript/34679	-2.66467709572529	-0.107774000539669	-2.43392935365213	0.0205880243861256	0.539801841008891	-3.29127092270293
a_HQ_transcript/1101	-1.7201007464887	4.36529489149479	-2.4328973040911	0.0206375209203758	0.540401236889916	-3.51591388326043
a_HQ_transcript/64473	-3.91546783337764	0.674871072837607	-2.43284933595578	0.0206398240835928	0.540401236889916	-3.34674325484937
a_HQ_transcript/47850	1.08806603732498	4.55116718284259	2.43260312641124	0.0206516493878754	0.540401236889916	-3.5700090260604
a_HQ_transcript/30355	3.00390608707167	-0.33122829634063	2.43250264084479	0.0206564774279923	0.540401236889916	-3.3260210235132
a_HQ_transcript/109693	4.20535312345034	1.45347460451569	2.43248408585662	0.0206573690539739	0.540401236889916	-3.31056545263677
a_HQ_transcript/144070	-3.27727368141597	0.0784296008123026	-2.43168542244872	0.0206957806526141	0.54072524841631	-3.2878477240182
a_HQ_transcript/130957	-4.97890512019191	2.29401209263809	-2.43165804060529	0.0206970987326203	0.54072524841631	-3.27332298088163
a_HQ_transcript/17438	3.4167036597771	1.99632486867837	2.43164634654611	0.0206976616729681	0.54072524841631	-3.27761756191696
a_HQ_transcript/116175	-3.57144029417099	0.735086567472764	-2.431226926765	0.0207178613579349	0.540993449322174	-3.26461067558378
a_HQ_transcript/52482	-3.08024068673101	1.5505104527191	-2.43088935622893	0.0207341321569518	0.540993449322174	-3.26862854499717
a_HQ_transcript/150168	1.30538028118027	3.06798413617272	2.43080878124343	0.0207380175696431	0.540993449322174	-3.40468616107674
a_HQ_transcript/140786	4.82385417385553	0.742961655955636	2.43041241178321	0.0207571406019688	0.540993449322174	-3.31504402845379
a_HQ_transcript/62306	-2.47217790134072	0.310133848174582	-2.42999378512845	0.0207773549037064	0.540993449322174	-3.27456506398368
a_HQ_transcript/1862	-2.10376657292935	-0.426170941521809	-2.42981329958484	0.02078607557931	0.540993449322174	-3.32257001074778
a_HQ_transcript/2078	-2.0685421161461	1.77104337945318	-2.42975621419444	0.0207888345180852	0.540993449322174	-3.25386248054411
a_HQ_transcript/99976	-2.07754570537437	2.10009626197243	-2.42960063345127	0.0207963554358648	0.540993449322174	-3.33793042464661
a_HQ_transcript/24274	0.90230359232584	3.79887554386539	2.42932203922054	0.0208098291399168	0.540993449322174	-3.48169450238718
a_HQ_transcript/57810	4.07083703681412	0.991148589812909	2.42931143503796	0.0208103421491824	0.540993449322174	-3.28893496963521
a_HQ_transcript/129761	-7.5120117796264	2.41416844530383	-2.42924556211289	0.0208135292086438	0.540993449322174	-3.35896881116376
a_HQ_transcript/50258	-2.86329058139133	0.0498500761282084	-2.42911988460096	0.020819610964315	0.540993449322174	-3.25793538587642
a_HQ_transcript/151855	2.31641234950839	-0.232719196215268	2.42868378800936	0.0208407270145121	0.541217156535197	-3.25484744624982
a_HQ_transcript/69485	-3.42107622166939	0.273119860668823	-2.42843662204814	0.0208527036029453	0.541217156535197	-3.31136251826413
a_HQ_transcript/17013	-2.22884339994003	3.57316203403736	-2.42836547036198	0.0208561524676376	0.541217156535197	-3.49195873082325
a_HQ_transcript/82419	-2.79020651610798	0.498307700220609	-2.42804433178126	0.020871725170061	0.541379580850203	-3.23690023890994
a_HQ_transcript/146088	-3.09777518301307	-0.165507696850349	-2.42750406175451	0.0208979479529306	0.541817983322011	-3.30622809161351
a_HQ_transcript/108248	-2.29657266696978	-0.480089821228142	-2.4273044276311	0.0209076450803715	0.541827727700326	-3.30615616315374
a_HQ_transcript/123928	1.32118958735325	2.24570608013888	2.42706322226784	0.0209193669829121	0.541889912648268	-3.30366829221683

a_HQ_transcript/118006	4.31640700505514	1.93260532989153	2.42647440769174	0.0209480068866772	0.542263667501823	-3.34772095806618
a_HQ_transcript/25236	1.50348368295645	2.23036307573375	2.42624384062258	0.0209592313906411	0.542263667501823	-3.30936300288554
a_HQ_transcript/54292	-2.27380500883799	-0.470716183630807	-2.42612901030907	0.0209648236240299	0.542263667501823	-3.3018747038869
a_HQ_transcript/114676	0.822936647902427	2.85925782790038	2.42599994488682	0.0209711107305274	0.542263667501823	-3.41703535091292
a_HQ_transcript/19023	2.90615231390395	1.42443946770675	2.4257365683878	0.0209839458057469	0.542354291594688	-3.27603468527752
a_HQ_transcript/127584	-4.18410416264355	1.68202350070716	-2.42528752916782	0.0210058452601591	0.542679010347791	-3.30135741878004
a_HQ_transcript/130323	5.54812073069697	0.277758515482276	2.42404297407841	0.0210666506289523	0.544008115397485	-3.34491167569122
a_HQ_transcript/91439	-2.90696404773723	-0.481635946497242	-2.42335743070361	0.0211002127857084	0.544306764412549	-3.33861952772976
a_HQ_transcript/149515	4.32351165762517	3.02307462791344	2.42329750079423	0.0211031490903622	0.544306764412549	-3.38398694875816
a_HQ_transcript/35612	-5.34745023728709	2.02558795365268	-2.42323304458729	0.021106307579581	0.544306764412549	-3.38974652873722
a_HQ_transcript/106300	4.10173096469734	2.2459923848052	2.42268888183394	0.0211329898733259	0.544606646515843	-3.44813128372151
a_HQ_transcript/138365	-6.8787173493079	2.02179363541605	-2.42252239545046	0.0211411594504788	0.544606646515843	-3.41537212490715
a_HQ_transcript/72438	-2.66586760014407	0.464027140492563	-2.42242289765366	0.0211460432353815	0.544606646515843	-3.30582786418804
a_HQ_transcript/148902	-2.32427151005625	3.97422518193172	-2.42192128230904	0.0211706803620802	0.544698114580202	-3.55250311472027
a_HQ_transcript/80907	-5.02695444321482	1.71225744792562	-2.42145101219302	0.0211938017061109	0.544698114580202	-3.39181753659993
a_HQ_transcript/123217	4.22356147652545	1.51679706558978	2.42131424955793	0.0212005301085459	0.544698114580202	-3.3103296389631
a_HQ_transcript/68627	3.38603229302826	1.42196754797642	2.42110364833376	0.0212108950041222	0.544698114580202	-3.27612124572813
a_HQ_transcript/122910	-3.95056170138424	1.58418832593498	-2.4209837292204	0.0212167989754775	0.544698114580202	-3.49710175892072
a_HQ_transcript/118787	-0.866911347937302	2.59352358424269	-2.42078224439689	0.0212267220390931	0.544698114580202	-3.41139842036646
a_HQ_transcript/151818	-1.1648514689751	7.41556075948469	-2.42058033618471	0.0212366701968632	0.544698114580202	-3.89406480106651
a_HQ_transcript/15440	-2.45305605669448	1.42721144284445	-2.42043139017904	0.021244011592848	0.544698114580202	-3.33962408371786
a_HQ_transcript/64534	2.3879212502289	-0.462261669940228	2.42025329283635	0.021252792864194	0.544698114580202	-3.31919326355848
a_HQ_transcript/62973	4.28753221575256	1.96233357858156	2.42024880644504	0.0212530141130021	0.544698114580202	-3.33443137787342
a_HQ_transcript/137555	2.60437792395579	1.68654042764864	2.42015961675596	0.0212574129862734	0.544698114580202	-3.30409175741717
a_HQ_transcript/144563	-1.18770565988889	3.44729377340236	-2.42005068239217	0.0212627868007311	0.544698114580202	-3.5239344441881
a_HQ_transcript/134705	-2.05033340249498	2.99777937910483	-2.41987586151449	0.0212714134340947	0.544698114580202	-3.4745924113212
a_HQ_transcript/35315	-2.66703736864247	-0.0681301961753518	-2.41940428450024	0.021294699570601	0.544939900484322	-3.28103697098104
a_HQ_transcript/58151	-2.31809941589091	-0.452362998595771	-2.41884108022938	0.021322540629802	0.544939900484322	-3.28911632598834
a_HQ_transcript/121002	-3.86049462029839	1.84743199251584	-2.4185409696379	0.0213373896348816	0.544939900484322	-3.38873044931135
a_HQ_transcript/69283	-2.26218620831268	1.44234827013376	-2.41852445120629	0.0213382072146706	0.544939900484322	-3.31182228247341
a_HQ_transcript/42025	-2.49089414482433	2.13901404348271	-2.41851994719206	0.0213384301458115	0.544939900484322	-3.32429092348399
a_HQ_transcript/8111	3.92321042599723	0.670955592150052	2.41848032547049	0.0213403913579689	0.544939900484322	-3.32769123326582
a_HQ_transcript/74431	2.28826036982167	-0.570711855622705	2.41835735230126	0.0213464793800372	0.544939900484322	-3.34114828623753
a_HQ_transcript/18002	-1.92941940555102	1.1448451064391	-2.41802330328729	0.0213630251040462	0.545122881144863	-3.26072321555713
a_HQ_transcript/62285	-6.1152594958927	3.20284587002936	-2.41752386487726	0.0213877844959179	0.54551519841102	-3.49594815486399
a_HQ_transcript/81719	-3.93615693519748	0.29744601193527	-2.41705675353448	0.021410964947169	0.545690705237028	-3.3499173487709
a_HQ_transcript/66809	1.1032851517481	2.46879632873061	2.41700688554176	0.0214134410034004	0.545690705237028	-3.33484502006808
a_HQ_transcript/124222	-3.12051857978348	0.222534482947569	-2.41656220079081	0.0214355321226625	0.545802779478233	-3.36215873632395
a_HQ_transcript/22985	2.30438353317446	-0.206997450037532	2.41637562172691	0.0214448072081698	0.545802779478233	-3.25769370367096
a_HQ_transcript/94156	1.88053102721577	2.59141378263488	2.416351473411	0.0214460079192177	0.545802779478233	-3.38355233436424
a_HQ_transcript/63417	3.26931696959855	0.0834072361751882	2.41596712125795	0.0214651270583058	0.546050286934442	-3.35381423248729
a_HQ_transcript/144599	-5.81351017848835	3.25380102544542	-2.41515182919668	0.021505734196207	0.546793463525344	-3.55778765735687
a_HQ_transcript/135316	1.72892716201423	3.66464374902575	2.41495931579821	0.021515332890409	0.546793463525344	-3.53246938593864
a_HQ_transcript/124412	-2.14673410428042	-0.152161195930278	-2.4148143925014	0.0215225613223573	0.546793463525344	-3.34716628102346
a_HQ_transcript/128740	-2.27305128236915	-0.582010572896599	-2.41370731246344	0.0215778528907514	0.547957696780946	-3.32661571355015
a_HQ_transcript/125502	3.41691223706456	0.00867652400238915	2.41344243907146	0.0215911007870995	0.547957696780946	-3.40147595113947
a_HQ_transcript/149966	-5.91750268489431	4.95683361570384	-2.41319851956578	0.021603307206053	0.547957696780946	-3.4516414242067
a_HQ_transcript/14298	-3.47571474353483	-0.0714842355487935	-2.4127345229298	0.0216265442540836	0.547957696780946	-3.32012002852549
a_HQ_transcript/71238	-5.99055459543371	3.16472832749053	-2.41273243369128	0.0216266489350389	0.547957696780946	-3.51783290988831
a_HQ_transcript/131634	2.89142909668273	1.25078432335752	2.4122172809346	0.0216524746687271	0.547957696780946	-3.32665342058751

a_HQ_transcript/125598	-8.47681939140219	1.59451196669191	-2.41205075163936	0.0216608291510355	0.547957696780946	-3.31728904986683
a_HQ_transcript/91181	3.29969969222747	1.13469250229113	2.4120491100982	0.0216609115188816	0.547957696780946	-3.29257394722737
a_HQ_transcript/140444	-2.56353131602397	-0.438711098521835	-2.41202531244258	0.0216621056492947	0.547957696780946	-3.33222163333223
a_HQ_transcript/128309	-5.6161453010428	-0.618323926083788	-2.41201437267223	0.0216626546105597	0.547957696780946	-3.35373840246807
a_HQ_transcript/78501	-2.47612837087509	-0.352057173549227	-2.4115167867786	0.0216876370301818	0.548351007085866	-3.29034142381133
a_HQ_transcript/35066	-2.04387420257266	2.1542426828925	-2.41130831859257	0.0216981114405823	0.548377313833985	-3.39951967434267
a_HQ_transcript/141411	4.03185432559949	0.0643904678890125	2.41082351105651	0.0217224882388458	0.548754800672591	-3.45667898870829
a_HQ_transcript/129096	2.6644251162704	1.03522429430439	2.41024604815366	0.0217515564166586	0.548900046543868	-3.29566549536047
a_HQ_transcript/7035	-2.35715567133405	0.12749951259895	-2.41022790210231	0.0217524704215626	0.548900046543868	-3.29058856678274
a_HQ_transcript/25843	2.70177191908414	0.823591557871212	2.41008473312931	0.02175968297683	0.548900046543868	-3.26798360727649
a_HQ_transcript/66203	-2.09841687135693	-0.413887960029502	-2.40977512270822	0.0217752879526697	0.548900046543868	-3.27113286230862
a_HQ_transcript/126469	3.13942972763808	0.645547684759291	2.4097415347423	0.0217769814648862	0.548900046543868	-3.32436550561607
a_HQ_transcript/97611	1.99456371677934	-0.756496863771032	2.40944682140823	0.0217918461153324	0.548900046543868	-3.33555463503335
a_HQ_transcript/555	2.35445373490675	-0.258712683810787	2.40907398470816	0.0218106643604909	0.548900046543868	-3.3297053163487
a_HQ_transcript/128928	3.5332023493573	-0.0673526500751648	2.40857851609601	0.0218356951191894	0.548900046543868	-3.43326834968454
a_HQ_transcript/110185	2.80198310427337	3.24863602713894	2.40835442856097	0.0218470244649036	0.548900046543868	-3.44017618719905
a_HQ_transcript/44319	-2.14291163621199	1.99728393597538	-2.40815036824296	0.0218573459359294	0.548900046543868	-3.37706012262761
a_HQ_transcript/53537	4.35965594461476	3.05404711787023	2.40807858651531	0.0218609777462168	0.548900046543868	-3.48423131413922
a_HQ_transcript/144088	2.21018048801325	3.48180664877014	2.4080475293293	0.0218625492607556	0.548900046543868	-3.48226049923809
a_HQ_transcript/111994	-2.00534404600465	-0.399786359636599	-2.40795660085426	0.0218671508941946	0.548900046543868	-3.40039296929038
a_HQ_transcript/121052	-3.00430419216428	-0.312118313455542	-2.40775365721963	0.0218774244787688	0.548900046543868	-3.38816647028283
a_HQ_transcript/127787	-3.0618393072252	1.2469000909732	-2.40773598435357	0.0218783193375031	0.548900046543868	-3.3052680211703
a_HQ_transcript/115750	3.26361503018417	1.54359125822496	2.40768061835781	0.0218811229889798	0.548900046543868	-3.35723023081625
a_HQ_transcript/79915	0.737218748654343	4.2918209366785	2.40746708946278	0.0218919388370804	0.548900046543868	-3.62768335901569
a_HQ_transcript/97960	2.74314156257175	0.0442629828539688	2.40712443238652	0.0219093055675608	0.548900046543868	-3.2761507283703
a_HQ_transcript/132562	-3.23551608107572	-0.588388739728969	-2.40700591155676	0.0219153154224561	0.548900046543868	-3.35580160621642
a_HQ_transcript/145480	4.73774563924336	0.997317107544713	2.40697078477511	0.0219170968901101	0.548900046543868	-3.42610384328787
a_HQ_transcript/145364	-2.58675306628387	-0.575233880536675	-2.4062163209039	0.0219553916365825	0.549503938870467	-3.3444024362763
a_HQ_transcript/80156	-2.67093442560201	-0.328843722105017	-2.40608889912022	0.021961865263816	0.549503938870467	-3.34804185143788
a_HQ_transcript/83987	-2.6458745317111	-0.0514809906709088	-2.40578450777477	0.021977336811751	0.549503938870467	-3.34628130260547
a_HQ_transcript/85246	1.70820116463615	-0.442149394999655	2.40556727096701	0.0219883845501689	0.549503938870467	-3.33089106260369
a_HQ_transcript/137540	-1.06150151212582	3.90962350653617	-2.40556546339232	0.0219884764969155	0.549503938870467	-3.61320838235901
a_HQ_transcript/140194	1.36183935280233	3.10205722291377	2.40517607745055	0.0220082917327101	0.54959754821434	-3.45199923105107
a_HQ_transcript/141589	2.54882236042776	-0.507427408608671	2.40512028613851	0.0220111321952068	0.54959754821434	-3.33971220595315
a_HQ_transcript/56703	-2.66042557092625	0.26165121811352	-2.40483949604837	0.0220254329226618	0.549604665619039	-3.32743282762264
a_HQ_transcript/152216	6.47117034921175	3.96461019879218	2.40474343277509	0.0220303273963041	0.549604665619039	-3.71496110732976
a_HQ_transcript/11982	2.08441396083703	0.821744858578161	2.4035985982508	0.0220887335469734	0.550549865541824	-3.27253800616623
a_HQ_transcript/70015	-3.01787251234562	-0.225281556240259	-2.40356433272314	0.022090483845083	0.550549865541824	-3.34643959682937
a_HQ_transcript/63344	-2.32752815384101	1.53282501809882	-2.40321286839484	0.0221084440840809	0.550549865541824	-3.34114820455209
a_HQ_transcript/110122	-4.5052206580461	1.65050899786144	-2.4028648697355	0.0221262403139675	0.550549865541824	-3.37804438708273
a_HQ_transcript/102860	3.07263701989075	0.814592485553589	2.40277334491532	0.022130922946849	0.550549865541824	-3.30706400865686
a_HQ_transcript/113674	3.17880818395406	0.578514764979022	2.4026647542549	0.022136479879328	0.550549865541824	-3.31077832398502
a_HQ_transcript/19499	-2.57058672414733	-0.372550490919853	-2.40255700480125	0.0221419950194398	0.550549865541824	-3.28851706400057
a_HQ_transcript/102461	-5.9270647770408	4.18364818703685	-2.40233550571006	0.0221533363452062	0.550549865541824	-3.54066279024307
a_HQ_transcript/89454	-3.66294479873593	0.291749757087303	-2.40233315420943	0.0221534567764644	0.550549865541824	-3.34504920398831
a_HQ_transcript/129233	-3.07233574188582	1.58376361245055	-2.40199266444829	0.0221709011262234	0.550718689449328	-3.34372643146343
a_HQ_transcript/61460	2.83820356567815	1.64066624509235	2.40183079610497	0.0221791985274029	0.550718689449328	-3.44204945868622
a_HQ_transcript/110454	1.5430804126728	-0.827111306995701	2.40127542901822	0.0222076882277543	0.550968838228027	-3.3544054229626
a_HQ_transcript/128436	-2.92593316403734	-0.418096355770586	-2.40126487603766	0.0222082299058675	0.550968838228027	-3.35431229150638
a_HQ_transcript/23319	-2.86241028918579	0.992604459537876	-2.40071587777115	0.0222364262396282	0.55143301384689	-3.26589867212017

a_HQ_transcript/91220	-2.79112221103776	-0.221936794681573	-2.40015402927523	0.0222653162740444	0.551913989073626	-3.35599299897238
a_HQ_transcript/149836	2.31393041777725	-0.639111026629591	2.39949837001261	0.0222990731940224	0.552486965295941	-3.3576694685935
a_HQ_transcript/65507	0.862798650225968	2.6734816864723	2.39933598976224	0.0223074406060689	0.552486965295941	-3.46725170301138
a_HQ_transcript/137906	-3.42104100994175	0.875260001061074	-2.39912745158194	0.0223181907135737	0.552517798040295	-3.33411135736118
a_HQ_transcript/99466	-1.73246014415798	-0.715536621565615	-2.39876967249416	0.0223366451429277	0.552700337370471	-3.33962481583319
a_HQ_transcript/51647	-2.51299154424806	-0.41455382036416	-2.39860027417503	0.0223453876363066	0.552700337370471	-3.36471722087698
a_HQ_transcript/107337	-4.02178951707512	-0.330885395630523	-2.39841255576437	0.0223550792485147	0.552700337370471	-3.40407536993848
a_HQ_transcript/115992	-3.75958732656658	-0.468531567159919	-2.39802042396973	0.0223753367453064	0.552700337370471	-3.37709240151575
a_HQ_transcript/92279	-4.26717752522349	1.3633451952527	-2.39790714583532	0.0223811917921323	0.552700337370471	-3.36036701147004
a_HQ_transcript/140989	-3.8125828401812	1.41264603962786	-2.39787963179472	0.022382614130369	0.552700337370471	-3.38310839691339
a_HQ_transcript/137310	-4.19400254528549	4.07287165812792	-2.39747993369815	0.0224032857965602	0.552975879737771	-3.7223595853748
a_HQ_transcript/86920	-2.25765263673277	2.86576473758325	-2.3972934001655	0.0224129389166829	0.552979335037751	-3.48038655201146
a_HQ_transcript/10024	4.41185660451858	1.23153725421106	2.39682734906399	0.0224370736212167	0.553245705843391	-3.29624419589042
a_HQ_transcript/22651	-2.72172425214232	2.70147319024985	-2.39671740292962	0.0224427706850178	0.553245705843391	-3.41410523253993
a_HQ_transcript/53070	2.49963186573265	2.07096970169187	2.39620753721878	0.0224692075275331	0.553662609224435	-3.41699351885481
a_HQ_transcript/68976	-2.35634794047014	1.44796495599854	-2.39586765661997	0.0224868462690648	0.553862457596695	-3.34891960396225
a_HQ_transcript/50443	2.78173693413211	0.358375348349827	2.39505151353295	0.0225292529803698	0.554671925981761	-3.31318655783424
a_HQ_transcript/6157	-3.73428924967759	0.84406358340252	-2.39465794844138	0.0225497285611756	0.554940991449624	-3.32053774096591
a_HQ_transcript/46101	3.08426786558221	0.305538440669788	2.39447228817512	0.0225593935778348	0.554941027174779	-3.32836111161077
a_HQ_transcript/46011	-1.80312951073098	2.00737583786518	-2.3942401987522	0.0225714808796703	0.554941027174779	-3.3091329473986
a_HQ_transcript/33726	3.79834845356076	3.28171800320214	2.39380995797169	0.0225939035544037	0.554941027174779	-3.530671870976
a_HQ_transcript/122938	-2.12887877816299	1.54902013923196	-2.39375191538403	0.0225969300851109	0.554941027174779	-3.32975101551488
a_HQ_transcript/62734	2.85364487841736	2.32099041786265	2.39374166884155	0.0225974644116898	0.554941027174779	-3.35245197950377
a_HQ_transcript/71445	-3.21625769150889	3.25801572162164	-2.3933050953454	0.0226202410901526	0.555163146554014	-3.50944186466504
a_HQ_transcript/131865	-0.990354104913659	6.56014016437708	-2.39320223272475	0.0226256106211543	0.555163146554014	-3.8924501640309
a_HQ_transcript/16220	-2.45034100591264	3.15684640792999	-2.39265041385895	0.0226544359034452	0.555635886158422	-3.56887430459752
a_HQ_transcript/99065	-2.95524641047707	4.32873514967222	-2.39206171049012	0.0226852247228949	0.555816846834642	-3.71634039449354
a_HQ_transcript/95258	5.61201157493089	2.47080255356199	2.39192555674847	0.0226923508930623	0.555816846834642	-3.49377151502793
a_HQ_transcript/106419	4.7283204398896	1.35038960540431	2.39179489422159	0.022699191571336	0.555816846834642	-3.42536460803688
a_HQ_transcript/2561	0.899769818240253	2.63640730990556	2.39172892674953	0.022702645930519	0.555816846834642	-3.42864839425576
a_HQ_transcript/150694	-2.78325729076494	-0.45556957778694	-2.39159569939679	0.0227096237825536	0.555816846834642	-3.36593487674052
a_HQ_transcript/151534	-3.8241961782475	0.793990860919684	-2.39069506233983	0.0227568462809444	0.556035007181965	-3.392593427047
a_HQ_transcript/123834	-3.34424678989602	-0.079479929835608	-2.39066903244095	0.0227582124153874	0.556035007181965	-3.37445101762449
a_HQ_transcript/45593	1.80504782813173	1.45840467570655	2.39047268604127	0.0227685197205305	0.556035007181965	-3.32486692938623
a_HQ_transcript/7901	-5.74641261544281	1.63960294831458	-2.39044223806587	0.0227701184826328	0.556035007181965	-3.4142634826437
a_HQ_transcript/121519	5.66076439775031	1.13599840643931	2.3900470323136	0.0227908792040366	0.556035007181965	-3.37611383019993
a_HQ_transcript/74982	0.982918551382558	4.57757748280559	2.39003116490344	0.0227917131008615	0.556035007181965	-3.71675057788378
a_HQ_transcript/131291	4.02704633411021	3.01745000790377	2.38997178169817	0.0227948341755152	0.556035007181965	-3.39623557313233
a_HQ_transcript/65848	-2.72628719150385	0.992470223244927	-2.38996742823759	0.0227950630008709	0.556035007181965	-3.29844707138961
a_HQ_transcript/10694	1.2719130210827	1.79182993410767	2.38975161373728	0.0228064091988224	0.556069146259475	-3.33352537046343
a_HQ_transcript/32596	-3.06425480140121	0.177105670387754	-2.38954659269789	0.0228171926974349	0.556069146259475	-3.35428938879832
a_HQ_transcript/23385	-2.18350696044844	0.41397891739302	-2.38939514412351	0.0228251614191974	0.556069146259475	-3.28734186096092
a_HQ_transcript/147222	1.57877479508406	8.99195633281886	2.38912407932935	0.0228394302653311	0.5561836625317	-4.09764875419138
a_HQ_transcript/152882	1.91906312734052	-0.776886493688434	2.38894180300123	0.0228490298465105	0.556184425007522	-3.37921028891436
a_HQ_transcript/108506	-1.05118484801401	4.29027558894803	-2.38831763914886	0.0228819292149607	0.556752106072515	-3.66814337551274
a_HQ_transcript/44280	1.10934884039091	2.56204577851133	2.38770645545639	0.0229141861181508	0.557265456507529	-3.39184883760168
a_HQ_transcript/105402	-4.73474375525405	1.29566481486263	-2.38755471354538	0.022922201116665	0.557265456507529	-3.35436855575941
a_HQ_transcript/145751	-4.24205621811107	2.0508016064191	-2.38732516297458	0.0229343308062818	0.557327249626903	-3.37444055508697
a_HQ_transcript/51479	2.61763754022683	3.04452872444192	2.38705004699131	0.0229488759039627	0.557397105728179	-3.49326151953978
a_HQ_transcript/54658	-2.5917857383145	1.42765091904815	-2.38690810156695	0.0229563836896721	0.557397105728179	-3.3649595872646

a_HQ_transcript/53393	-3.30566803372428	0.652892160473604	-2.38643376308266	0.0229814886230854	0.557699576461456	-3.32490585463584
a_HQ_transcript/144407	8.94382765710788	1.68727403390583	2.38631025345914	0.0229880296105431	0.557699576461456	-3.38412825595175
a_HQ_transcript/39367	-2.99012425021598	0.49018067288121	-2.38606700281325	0.0230009169498973	0.557779432817534	-3.35116820444219
a_HQ_transcript/82084	-3.3786548369935	1.33938256892012	-2.38566220944129	0.0230223773227105	0.557861590913708	-3.33903820191288
a_HQ_transcript/81402	-2.6834890998393	-0.497140689006551	-2.38544484200266	0.0230339086993233	0.557861590913708	-3.38551983264494
a_HQ_transcript/80765	-1.60837757579758	-0.818406931380882	-2.38528626645665	0.0230423244673687	0.557861590913708	-3.38478804403702
a_HQ_transcript/121928	-3.26955106680411	1.06372116378454	-2.38527931668841	0.0230426933626447	0.557861590913708	-3.35951949777303
a_HQ_transcript/80135	-1.80415750515867	3.22093653577328	-2.38497794072296	0.0230586956300425	0.557966288384259	-3.55708186887791
a_HQ_transcript/143461	2.14963927505764	1.6418920520206	2.38483637685387	0.023066215782194	0.557966288384259	-3.38184914761912
a_HQ_transcript/100695	-2.05728715290501	-0.159368922312758	-2.38407271518988	0.0231068214430036	0.558716021979582	-3.38338655066639
a_HQ_transcript/96706	-1.97493943965284	-0.618435398856843	-2.38387554018581	0.0231173162384015	0.558737363120915	-3.41036462356989
a_HQ_transcript/3964	4.55441374417426	3.07266481953011	2.38328404991067	0.0231488247607057	0.559266369779842	-3.45940594075546
a_HQ_transcript/98190	-2.54461718725207	0.37987364724929	-2.38281195119309	0.0231740013189036	0.559641521868395	-3.36629251309235
a_HQ_transcript/149266	3.57548116457109	0.535570854214642	2.38257482790527	0.0231866562517912	0.559641521868395	-3.37383178688048
a_HQ_transcript/80628	2.59798123512148	0.226762340143361	2.38245158427192	0.0231932360683485	0.559641521868395	-3.31298892323953
a_HQ_transcript/19742	1.76835059370344	5.12495142553808	2.3821470272473	0.0232095032452231	0.559801661675655	-3.75615521158076
a_HQ_transcript/117679	6.16406876454684	1.23697998503373	2.38151076709132	0.0232435209894371	0.560389625912069	-3.41667740876215
a_HQ_transcript/69228	2.81775953308181	0.923221904218514	2.38111981826735	0.0232644455830541	0.560621889790588	-3.33194126161046
a_HQ_transcript/89283	-2.62940921878779	0.533445176556833	-2.38097046414847	0.023272443917986	0.560621889790588	-3.33722306537904
a_HQ_transcript/151173	0.769829933128196	8.67598951121785	2.38070162783712	0.0232868471559948	0.560736475345346	-4.07643734816203
a_HQ_transcript/22387	-1.80438714531727	1.5534409541615	-2.38011291191253	0.0233184166217415	0.560970108350902	-3.36257485221314
a_HQ_transcript/83554	3.37407450835553	1.85995427218576	2.38000222597088	0.0233243564145225	0.560970108350902	-3.39920088637296
a_HQ_transcript/115105	-2.51815347802923	2.72467865519196	-2.37949890800575	0.0233513835426146	0.560970108350902	-3.50030799818819
a_HQ_transcript/77823	2.70326138781298	-0.460807091824741	2.37947601189495	0.0233526136922232	0.560970108350902	-3.38900448455008
a_HQ_transcript/84195	4.61884821680777	3.86118421508516	2.37938835382721	0.0233573238805196	0.560970108350902	-3.57672535331181
a_HQ_transcript/128809	-1.9120403514143	0.754739946927692	-2.37931987579632	0.0233610040555936	0.560970108350902	-3.31581861827389
a_HQ_transcript/82934	2.45916558589562	-0.554704366867666	2.37926220347695	0.0233641039140781	0.560970108350902	-3.39131928318943
a_HQ_transcript/77688	4.19885705470459	2.19064790664998	2.37908098334841	0.023373846837048	0.560972324089152	-3.44696137603334
a_HQ_transcript/88670	-1.77662521530741	2.91789975722518	-2.37852965939385	0.0234035103128612	0.561452433952125	-3.44459688016741
a_HQ_transcript/122052	4.30981014504589	0.969033302563539	2.37775012347494	0.0234455108636923	0.561983703378735	-3.42083161516867
a_HQ_transcript/6242	-4.81541380300353	1.35618128676304	-2.37774216091866	0.0234459402305026	0.561983703378735	-3.38363590079
a_HQ_transcript/150467	-3.04856712668665	2.97809616067142	-2.37758048632941	0.0234546597921279	0.561983703378735	-3.5282490240858
a_HQ_transcript/93343	9.68692888745081	1.29484886356033	2.37669351860831	0.0235025488452047	0.562899117953878	-3.44032599477993
a_HQ_transcript/9999	-2.44137583689146	1.28355790255735	-2.37626822380023	0.0235255427857885	0.562921651860747	-3.39424945503666
a_HQ_transcript/42525	-2.27920483818583	-0.446317281745951	-2.37620531169313	0.0235289459182065	0.562921651860747	-3.42575329152989
a_HQ_transcript/151757	-2.87300400495744	-0.546682475603371	-2.37605741512518	0.0235369479130851	0.562921651860747	-3.4123548380508
a_HQ_transcript/7377	-2.39494958430056	-0.60985684555936	-2.37569293523341	0.0235566787719792	0.562921651860747	-3.40259186582305
a_HQ_transcript/108686	-3.11031861742362	-0.295033570225604	-2.37560436339225	0.0235614758127997	0.562921651860747	-3.44394794740932
a_HQ_transcript/99966	3.83902117956108	1.30720118353614	2.37560216776835	0.023561594738804	0.562921651860747	-3.40795006159576
a_HQ_transcript/109349	2.96347232615299	1.96363245132116	2.37426176725472	0.0236342993553696	0.564190100984976	-3.43002370979809
a_HQ_transcript/74002	4.06688214886833	0.964433586981989	2.37411155440509	0.0236424597226321	0.564190100984976	-3.37847526024934
a_HQ_transcript/148977	2.3425041448953	-0.298946869681516	2.37405347926404	0.0236456153611378	0.564190100984976	-3.42493626068323
a_HQ_transcript/24678	-2.28941176064306	0.695750706728861	-2.37379738281623	0.023659535476915	0.564190100984976	-3.39921559457869
a_HQ_transcript/20317	4.39967719061141	1.67507786475159	2.37354920251958	0.0236730324072984	0.564190100984976	-3.39382336645367
a_HQ_transcript/122666	-2.51141390488169	0.779278889574818	-2.37337914007175	0.0236822850451752	0.564190100984976	-3.37061380917371
a_HQ_transcript/32971	4.08034494457943	1.20705409506832	2.3733728232113	0.0236826287916897	0.564190100984976	-3.40236486738275
a_HQ_transcript/134761	-3.41870022327409	0.36317861333738	-2.37292998051721	0.0237067383878606	0.564445544073173	-3.37193082663236
a_HQ_transcript/91809	0.784729461305275	3.11197366733153	2.3726347389837	0.0237228245386403	0.564445544073173	-3.54245949709007
a_HQ_transcript/96569	-2.45996195707497	1.42955139137318	-2.37255161663627	0.0237273552229562	0.564445544073173	-3.31965124584881
a_HQ_transcript/110195	-3.8297574004181	1.36710925345617	-2.37246364885213	0.0237321508690276	0.564445544073173	-3.44380014955536

a_HQ_transcript/143381	-2.19000505571831	-0.367706227623942	-2.37205164553245	0.0237546233265359	0.564747952852712	-3.40026790984175
a_HQ_transcript/41645	2.92274675309966	0.861803283412776	2.37158128755229	0.0237803023104757	0.564961873297013	-3.34240748782071
a_HQ_transcript/88771	-1.49435132916701	2.24351240260759	-2.37153080522448	0.0237830598671516	0.564961873297013	-3.41880773871212
a_HQ_transcript/127628	-3.07134150916519	-0.260249039905781	-2.37120839093224	0.0238006783357113	0.565149440481302	-3.45360558202065
a_HQ_transcript/79878	3.34218983267155	2.14196980223325	2.37093006745057	0.0238158969677726	0.565192127822743	-3.4332123043594
a_HQ_transcript/37490	-3.23500483458077	-0.494570553935074	-2.37081991442764	0.0238219225358815	0.565192127822743	-3.38762790810942
a_HQ_transcript/2229	1.66825926967951	3.93329564011625	2.37016863929916	0.0238575767342226	0.565510134027825	-3.72223703888773
a_HQ_transcript/150609	7.78340449259138	2.37593363408106	2.37012979460138	0.0238597048251149	0.565510134027825	-3.53121410544994
a_HQ_transcript/22215	-3.73711665879145	2.970770110382	-2.36999816497207	0.0238669173814509	0.565510134027825	-3.55256352581118
a_HQ_transcript/129355	-3.40102158909192	-0.263735171930602	-2.36986454912729	0.0238742407945273	0.565510134027825	-3.43557134192529
a_HQ_transcript/64386	-2.30038886863204	0.355670735829018	-2.369497817231	0.0238943516538685	0.565575759676232	-3.38484448098883
a_HQ_transcript/122780	1.75565218474211	1.98631084421424	2.36945918633957	0.0238964709909996	0.565575759676232	-3.40060925674793
a_HQ_transcript/123324	3.60727517540426	0.567712083983244	2.36926336854747	0.0239072164142404	0.565599786620663	-3.47959946912552
a_HQ_transcript/116011	-2.90835030200368	1.15902542838406	-2.36852928332618	0.0239475380844174	0.566323227734342	-3.34505089953122
a_HQ_transcript/152689	3.87168015509726	-0.351315428012166	2.36764682810051	0.0239960910538402	0.567220514247181	-3.45490201619453
a_HQ_transcript/98524	3.43480875460776	1.45146682505346	2.36734741424864	0.0240125851810112	0.567220514247181	-3.4789216794839
a_HQ_transcript/129720	-1.46629699916224	3.23374701292839	-2.36730803778319	0.0240147551190874	0.567220514247181	-3.49873851165242
a_HQ_transcript/91604	-1.60268721532144	4.00754014393216	-2.36712054266379	0.0240250899444811	0.567234130094557	-3.6211074200828
a_HQ_transcript/38213	-3.32088313930545	0.35863045631877	-2.36687493285558	0.0240386341854424	0.567323478656678	-3.40179622209952
a_HQ_transcript/150084	-0.594574589921806	9.44685449199647	-2.36633794821287	0.0240682705269396	0.567792381976438	-4.21560874857209
a_HQ_transcript/86122	-2.4768193002743	-0.639698081335546	-2.36573499560135	0.024101587159947	0.568347691048032	-3.42013340067708
a_HQ_transcript/86985	1.9395315715882	1.82222424123302	2.36524199993146	0.0241288591137142	0.568760065921322	-3.39135718057527
a_HQ_transcript/123019	3.70249973971084	0.79981528247918	2.3650226916379	0.024140999989565	0.568815584675085	-3.37857737670031
a_HQ_transcript/148623	-0.965579279334654	6.72338247788822	-2.36472497202046	0.0241574905720468	0.568973505661239	-3.94103732213805
a_HQ_transcript/67331	-3.75123638995293	0.427275233881322	-2.36357227772822	0.0242214342966193	0.570248494448718	-3.42829841058049
a_HQ_transcript/89358	-2.73098326994751	3.89844921640393	-2.36324461283552	0.0242396389391803	0.570446045448046	-3.50522726110081
a_HQ_transcript/126420	-2.50561167586548	-0.297682946559749	-2.36276567398045	0.0242662704944156	0.570841671913959	-3.46528886027418
a_HQ_transcript/111898	1.55793083061563	-0.316119298463646	2.36208416350207	0.0243042118558403	0.5715029234451	-3.42152358675783
a_HQ_transcript/144130	-7.13470438332014	3.05627650433596	-2.36100412714301	0.0243644503205177	0.572371087366573	-3.61693022619844
a_HQ_transcript/27888	-5.03183648463413	0.613738918108806	-2.3608846146472	0.0243711243799537	0.572371087366573	-3.42034969578076
a_HQ_transcript/27999	-3.11530062625752	3.90703017984542	-2.36075038801151	0.0243786221125944	0.572371087366573	-3.7044595432073
a_HQ_transcript/109169	-2.36864909947881	-0.300974060888023	-2.36030345503482	0.0244036023101355	0.572371087366573	-3.41791633522822
a_HQ_transcript/3582	-2.543338133062	2.58984396592596	-2.36009868459567	0.0244150551907073	0.572371087366573	-3.47641145292707
a_HQ_transcript/91694	-2.45579611766004	-0.0136817735997739	-2.36004829010874	0.0244178745187051	0.572371087366573	-3.39353289316022
a_HQ_transcript/43063	-3.35722046878989	1.21234601895602	-2.3597197464512	0.0244362621847271	0.572371087366573	-3.40794552693876
a_HQ_transcript/9190	-2.04242561663268	1.42963589577846	-2.35947859147609	0.0244497669397505	0.572371087366573	-3.44700808622495
a_HQ_transcript/152042	-5.40135590574345	1.58571579908669	-2.35940742231332	0.0244537537283299	0.572371087366573	-3.48333928016504
a_HQ_transcript/77201	1.49269941809743	3.09274053403729	2.35931158651947	0.0244591232353589	0.572371087366573	-3.60053963582179
a_HQ_transcript/30819	-3.55169649766708	1.69716660369479	-2.359265188419	0.0244617232214166	0.572371087366573	-3.4006704228439
a_HQ_transcript/132295	-1.52470098804736	2.8156257561832	-2.35920001177843	0.0244653759138890	0.572371087366573	-3.49308210279799
a_HQ_transcript/97667	-2.51437877546951	-0.492623856517986	-2.3590106823969	0.0244759892992697	0.572371087366573	-3.42446207658084
a_HQ_transcript/73535	2.42753461034991	-0.51211409579031	2.35892119780742	0.0244810070594579	0.572371087366573	-3.4373523316311
a_HQ_transcript/140854	-2.72356712052287	-0.557342861885293	-2.35865278766976	0.0244960634877672	0.572371087366573	-3.43505454961211
a_HQ_transcript/83849	3.42834514646501	0.131867154195574	2.35857690632534	0.0245003215627608	0.572371087366573	-3.58947682052202
a_HQ_transcript/78925	-2.39922457759085	-0.424466829822596	-2.35843072086299	0.024508526638718	0.572371087366573	-3.45824393053256
a_HQ_transcript/62682	-2.51938748653643	1.66484132051806	-2.35757112864575	0.0245568241114983	0.573111554679137	-3.38348840803841
a_HQ_transcript/38492	-2.97284553677609	0.323749650404317	-2.3572423796413	0.0245753181612509	0.573111554679137	-3.40290383080361
a_HQ_transcript/101291	5.18766076372266	1.22184712810844	2.35717283814219	0.024579231893751	0.573111554679137	-3.43595588147388
a_HQ_transcript/27894	1.82501102638117	1.66339021511977	2.35704130369263	0.0245866360782109	0.573111554679137	-3.39865962906156
a_HQ_transcript/121943	-0.978513359699223	2.56832988810242	-2.35680775341323	0.024599787801178	0.573111554679137	-3.56824741249483

a_HQ_transcript/17253	3.96831206589903	1.11091015162209	2.35670346137444	0.0246056627714057	0.573111554679137	-3.36115134632713
a_HQ_transcript/104715	-2.72983066487754	-0.53174171713333	-2.35663980554202	0.0246092492512924	0.573111554679137	-3.4474867835159
a_HQ_transcript/71347	3.75387651488699	2.45235127185772	2.35621815418686	0.0246330177856712	0.573435345552861	-3.54047005210724
a_HQ_transcript/20585	2.15854446610093	-0.42508908268694	2.35560581263953	0.02466757259779	0.573517129921825	-3.41909792521038
a_HQ_transcript/47615	1.28205146319058	3.23307544921148	2.35544289571435	0.0246767734966352	0.573517129921825	-3.62285779137498
a_HQ_transcript/2634	2.46398186484238	0.255660539754181	2.35540858746208	0.024678711486453	0.573517129921825	-3.40559383577996
a_HQ_transcript/129408	0.87945613537404	4.31462823885376	2.35531894103717	0.0246837760463198	0.573517129921825	-3.71681127343648
a_HQ_transcript/7808	3.13822471146488	-0.465021633301609	2.35522549124816	0.0246890564793316	0.573517129921825	-3.41168727757155
a_HQ_transcript/120542	6.75745754503255	2.69286791552641	2.35510741862562	0.0246957297033156	0.573517129921825	-3.49484133642072
a_HQ_transcript/95360	7.54369536288676	3.56326566119684	2.35470776655095	0.0247183293562168	0.573730632270111	-3.52241537401745
a_HQ_transcript/8616	2.33204908159212	2.03026061903155	2.35459581486847	0.0247246633952076	0.573730632270111	-3.52578772681605
a_HQ_transcript/143369	2.25281767587405	-0.674983002640402	2.35435567893966	0.0247382548460861	0.573816950396366	-3.43868743150739
a_HQ_transcript/101542	4.65514140008021	0.285614888714687	2.35410206825465	0.024752616305422	0.573826477125017	-3.45422923739057
a_HQ_transcript/147524	2.94069043894131	1.08743130624998	2.35399981021634	0.0247584091079952	0.573826477125017	-3.3735990652833
a_HQ_transcript/78018	-2.7227111636831	0.316064098534126	-2.35312265063105	0.0248081496969165	0.574266958178782	-3.4821195402986
a_HQ_transcript/92976	-2.33729674388139	-0.503788168800383	-2.35307260218566	0.0248109904943786	0.574266958178782	-3.40926696762315
a_HQ_transcript/49429	-3.43108765192531	0.814612440437343	-2.35279364664119	0.0248268296776099	0.574266958178782	-3.46669776366316
a_HQ_transcript/151139	-8.27905139978249	0.691183134464802	-2.35242138239461	0.0248479812296601	0.574266958178782	-3.46882840761695
a_HQ_transcript/119622	-2.65076707017624	0.151690385739905	-2.35229885530838	0.0248549466239369	0.574266958178782	-3.42892402718476
a_HQ_transcript/143347	-3.47396970273428	1.03312764898453	-2.35226773593164	0.0248567159734773	0.574266958178782	-3.40779691060647
a_HQ_transcript/143993	-3.33313583452148	-0.359143973977998	-2.35217888379706	0.0248617684535116	0.574266958178782	-3.41815689692721
a_HQ_transcript/139896	-1.76003879205888	5.07486905759985	-2.35208367622771	0.0248671833611263	0.574266958178782	-3.83630846000214
a_HQ_transcript/10842	-2.37623593645361	2.34890942334526	-2.35172015434656	0.0248878684110364	0.574266958178782	-3.49851013856124
a_HQ_transcript/88754	4.78852321057375	1.32126155693212	2.35156726689911	0.0248965726381165	0.574266958178782	-3.37326008801763
a_HQ_transcript/131950	2.45699046191161	1.28054182264216	2.35153336183047	0.0248985033037116	0.574266958178782	-3.38001145679919
a_HQ_transcript/66261	-2.31257386709841	2.9204824362025	-2.35141537340704	0.0249052230081638	0.574266958178782	-3.54275866999905
a_HQ_transcript/147019	-2.9117215528062	-0.542573314951019	-2.35140444046573	0.024905845746778	0.574266958178782	-3.44601821147434
a_HQ_transcript/128488	-3.1160314080237	1.73904181057238	-2.35073633498564	0.0249439277116074	0.574916982561583	-3.46297992444598
a_HQ_transcript/61587	4.07615857128596	3.14343636802164	2.35030908703539	0.0249683084596116	0.575250826056403	-3.42518684382746
a_HQ_transcript/91689	0.904429474709034	2.69798972900796	2.35012254241022	0.0249789603293245	0.575256316532082	-3.57583237216562
a_HQ_transcript/95032	-1.93317930294475	-0.43857465711974	-2.34995834579008	0.0249883395135478	0.575256316532082	-3.41033581784604
a_HQ_transcript/148756	1.25923752946972	3.18312151696325	2.34975395597976	0.0250000190568114	0.575297350647004	-3.5749974768691
a_HQ_transcript/152867	-2.94511548332355	-0.514552976101572	-2.34929201523783	0.0250264341741846	0.575677311308668	-3.4637734011093
a_HQ_transcript/140568	-2.94019840061716	0.986182790987519	-2.34892488116923	0.0250474459979451	0.575773732285816	-3.40538025219126
a_HQ_transcript/137495	2.33279606903083	-0.646790444213152	2.34887265277084	0.0250504364325425	0.575773732285816	-3.45776305140842
a_HQ_transcript/152879	-2.36144603555715	-0.448291515423673	-2.3483107469741	0.0250826298609473	0.575834223711654	-3.44606821069081
a_HQ_transcript/22398	-1.02404302797427	2.65013379990409	-2.34786372242664	0.0251082681245591	0.575834223711654	-3.52426298022006
a_HQ_transcript/3908	2.72136796527988	0.761503481260489	2.34772592482246	0.0251161760350488	0.575834223711654	-3.36890297509422
a_HQ_transcript/4000	2.44111147574934	-0.610306062683739	2.34766012319368	0.0251199530458345	0.575834223711654	-3.45324150810658
a_HQ_transcript/120159	4.01288110900786	0.665168267487417	2.34763229848997	0.0251215503374322	0.575834223711654	-3.38016399109733
a_HQ_transcript/145461	2.7774649386137	0.429031721047584	2.34762893682385	0.0251217433218532	0.575834223711654	-3.51970517503904
a_HQ_transcript/127348	2.90718869785943	-0.551555158932669	2.34761728108579	0.0251224124575549	0.575834223711654	-3.45666637858688
a_HQ_transcript/41597	-2.00308764506831	3.47750638137227	-2.34742142597192	0.0251336585745923	0.575864921412654	-3.55865031875604
a_HQ_transcript/102848	4.06875355813762	2.12925626654833	2.34706227189158	0.025154293262542	0.576110622050843	-3.5473240957556
a_HQ_transcript/80886	2.77986804378327	1.00597001605888	2.34644903587767	0.0251895613325397	0.576388773707131	-3.37461812193342
a_HQ_transcript/58931	-2.94780090753484	1.89979907200259	-2.34638018659265	0.0251935237469618	0.576388773707131	-3.44852289993728
a_HQ_transcript/7970	0.723349386190474	4.10379068860631	2.34633394160131	0.0251961855558392	0.576388773707131	-3.73393455293047
a_HQ_transcript/48214	-2.54946207925076	-0.437408489101725	-2.34603574332873	0.0252133556244542	0.576487095851494	-3.4583997277642
a_HQ_transcript/620	3.42625329165456	1.01689353086475	2.34591486588611	0.0252203186889339	0.576487095851494	-3.48209982386247
a_HQ_transcript/13881	2.67118508836424	0.325598068773211	2.34544872656741	0.0252471866416585	0.576782962924487	-3.45342268430441

a_HQ_transcript/102274	2.84327971397679	0.644407070385194	2.34534606206072	0.0252531076356114	0.576782962924487	-3.47591373760737
a_HQ_transcript/115064	3.14912445032545	0.268670963777692	2.34491411086302	0.0252780334341266	0.577046202301098	-3.40938071884392
a_HQ_transcript/37426	2.07771737573529	0.534056435357573	2.34470331551188	0.0252902054930283	0.577046202301098	-3.3935988161889
a_HQ_transcript/151702	-0.9597213510276	2.93163435105767	-2.34463044620196	0.0252944144553949	0.577046202301098	-3.55331052746713
a_HQ_transcript/86246	-5.59083959825831	1.41997403499951	-2.34430330146787	0.0253133182910449	0.577250908443256	-3.43998077977174
a_HQ_transcript/50369	-4.76598214288727	1.37238392267478	-2.34403821542357	0.0253286454885034	0.577373923512049	-3.42667310931155
a_HQ_transcript/149087	0.985066921508783	5.4289187885461	2.34380574931189	0.0253420935257679	0.577454022918791	-3.87961088695257
a_HQ_transcript/151148	3.64819198438668	0.661722453466075	2.34318626313684	0.0253779620344057	0.577988624140197	-3.42302290760642
a_HQ_transcript/71104	-2.6262033675817	0.343283259163886	-2.34282996781294	0.0253986124957007	0.577988624140197	-3.40171684687579
a_HQ_transcript/119717	-2.1571165712796	3.33721161173915	-2.34277856265914	0.0254015931338431	0.577988624140197	-3.60094667834283
a_HQ_transcript/85954	-2.231335975123	-0.27731392280671	-2.34256728654959	0.0254138469359995	0.577988624140197	-3.44615765690556
a_HQ_transcript/137711	-6.21974926274306	2.65114000985389	-2.34239468685863	0.0254238615160375	0.577988624140197	-3.58305415208379
a_HQ_transcript/32714	-2.63403706211226	1.02915076754486	-2.3423713602291	0.0254252152478407	0.577988624140197	-3.42882880974752
a_HQ_transcript/51421	2.25152842834888	5.04998381393315	2.34206247718124	0.0254431470434356	0.578048417478998	-3.88665472574186
a_HQ_transcript/63764	2.1234429299933	-0.647444736487184	2.34198349105128	0.0254477343161429	0.578048417478998	-3.4659426358108
a_HQ_transcript/88665	2.30035147575503	-0.500272561211111	2.34159106996916	0.0254705360333501	0.578340358807255	-3.46151261430919
a_HQ_transcript/17090	2.70931751356207	-0.521216918737897	2.3413774486993	0.0254829563188846	0.578348231273159	-3.46764698536096
a_HQ_transcript/109417	0.630025267068058	3.76068999687094	2.34109025415318	0.0254996629131567	0.578348231273159	-3.72891225285429
a_HQ_transcript/77915	-1.48799618463023	1.57549475006789	-2.34064113580244	0.0255258087650652	0.578348231273159	-3.383364616309
a_HQ_transcript/144869	-5.19542823203458	-0.210840703713859	-2.33990248901956	0.0255688625650243	0.578348231273159	-3.50927950178036
a_HQ_transcript/129855	-2.35603047422106	-0.159556156726112	-2.33990066272553	0.0255689690963575	0.578348231273159	-3.43390782549482
a_HQ_transcript/145832	6.38114572451379	3.0107979137527	2.33969848116404	0.0255807652298686	0.578348231273159	-3.687148363397
a_HQ_transcript/27115	2.1156843688112	1.88068625415573	2.33964679708988	0.0255837814892788	0.578348231273159	-3.44616619402867
a_HQ_transcript/83424	3.65745209846937	1.92896947511625	2.33957525730775	0.0255879570500323	0.578348231273159	-3.55840276386367
a_HQ_transcript/126254	-4.29126836833889	4.22383541593198	-2.33929833308528	0.0256041260948233	0.578348231273159	-3.58414264629915
a_HQ_transcript/42336	2.48398622362275	-0.504896669949059	2.33925971567591	0.0256063816216872	0.578348231273159	-3.43639773466764
a_HQ_transcript/65201	-4.29251145113447	1.93430862963759	-2.33918303416367	0.0256108608914968	0.578348231273159	-3.56743772664413
a_HQ_transcript/16452	-2.72602623062527	0.120529587499614	-2.33917643676821	0.0256112463045351	0.578348231273159	-3.45609981681237
a_HQ_transcript/53305	-2.50095622120082	-0.150883214059801	-2.33883730534137	0.0256310650839361	0.578348231273159	-3.43701021162029
a_HQ_transcript/32334	2.50669261438349	0.915076913074819	2.33882190772931	0.0256319652469053	0.578348231273159	-3.40928646345192
a_HQ_transcript/37884	0.864457056469818	3.70674648022258	2.33881594360461	0.0256323139245142	0.578348231273159	-3.67394842424627
a_HQ_transcript/40166	-2.74441385069926	0.920477650573593	-2.33875509866976	0.0256358713164761	0.578348231273159	-3.41018645110188
a_HQ_transcript/115715	-2.83183711091462	1.561199555305	-2.33868405902162	0.0256400253232682	0.578348231273159	-3.43768857474498
a_HQ_transcript/48620	2.85814681263402	1.95412182326401	2.3382380604674	0.0256661187786016	0.578570371560019	-3.48837014963849
a_HQ_transcript/3304	-2.59614646920404	0.535168580083523	-2.3380903474457	0.0256747661275118	0.578570371560019	-3.46845351760231
a_HQ_transcript/23745	-1.90550885135472	1.3037367900571	-2.33790939781566	0.0256853627918842	0.578570371560019	-3.41746180089336
a_HQ_transcript/69368	2.47312742621793	1.37860147001553	2.33779930315886	0.0256918120258925	0.578570371560019	-3.40124778736824
a_HQ_transcript/103040	-1.2641371875433	3.78548154518546	-2.33763964156217	0.0257011674446882	0.578570371560019	-3.7339283870547
a_HQ_transcript/78894	-3.13517334210248	-0.0161457503307691	-2.33749588018163	0.0257095938229344	0.578570371560019	-3.53310082131419
a_HQ_transcript/140976	-2.64672644560659	-0.495889190716008	-2.33727164492316	0.0257227420590116	0.578642240869284	-3.47442454812775
a_HQ_transcript/28045	1.67072610522014	-0.665497000221312	2.33665489176137	0.0257589373233978	0.579087163415759	-3.50351555204342
a_HQ_transcript/113872	-2.88556553297904	-0.46254907559993	-2.33652554526599	0.0257665340975703	0.579087163415759	-3.4677730264062
a_HQ_transcript/42326	-2.48745725841019	1.96164326276999	-2.33631356302545	0.0257789886151888	0.579087163415759	-3.434160422075
a_HQ_transcript/65952	2.61177128389274	1.50460835791818	2.33625603327987	0.0257823695795483	0.579087163415759	-3.44287744067427
a_HQ_transcript/149914	3.11425132205362	0.0943318467145509	2.33576792470095	0.0258110713680648	0.579507901306633	-3.4645851228334
a_HQ_transcript/118580	2.78289271089019	1.51918556916448	2.33526145149006	0.0258408835917763	0.579953236070568	-3.42699591273576
a_HQ_transcript/139074	-3.14998685151496	2.20535134786335	-2.33429477663382	0.0258978708691265	0.581007888028013	-3.61098992506046
a_HQ_transcript/141218	0.855413338197579	3.60940193035952	2.33338807822041	0.0259514257250517	0.581889378333172	-3.61685289532649
a_HQ_transcript/126857	3.7137315906933	1.04860860077643	2.33314018486094	0.0259660851696993	0.581889378333172	-3.45638316032769
a_HQ_transcript/22666	-1.18811410237439	3.00401784469259	-2.33312144110889	0.0259671939064865	0.581889378333172	-3.55318579888282

a_HQ_transcript/95338	-2.59995283103671	0.0808400733324763	-2.33285034750502	0.0259832345166483	0.582024453172922	-3.48375821512727
a_HQ_transcript/37076	3.49225721457829	1.21381585628411	2.33251724212729	0.0260029566357386	0.582241857982363	-3.46368020001214
a_HQ_transcript/32539	-3.2047791052126	3.17770333165576	-2.33233385526989	0.0260138201713842	0.582260815911522	-3.68385511669221
a_HQ_transcript/116366	-1.83849129737013	1.67669222309636	-2.33206174767222	0.0260299469420753	0.582376686157634	-3.4129133224676
a_HQ_transcript/38848	2.60296693060257	0.561407479365972	2.33190847725891	0.0260390346704461	0.582376686157634	-3.42302394833635
a_HQ_transcript/107184	2.87665459498386	0.549014497306021	2.33096410394829	0.0260950919070438	0.583371106845154	-3.50580078873042
a_HQ_transcript/2272	1.56891748878471	1.85559474029028	2.33069287022257	0.0261112122839842	0.583371106845154	-3.43642351689057
a_HQ_transcript/96656	2.41085396666447	0.555842927022215	2.33062356924539	0.0261153325296752	0.583371106845154	-3.44232373900614
a_HQ_transcript/30987	2.69306109749882	0.479632029580979	2.33048385771779	0.0261236407775588	0.583371106845154	-3.43075953709232
a_HQ_transcript/54877	-2.76390998493724	3.82137476035654	-2.33028998999375	0.0261351735003171	0.58340451813611	-3.7510641736754
a_HQ_transcript/35342	-2.58119076213734	-0.0880638268667408	-2.3298620055781	0.0261606495623807	0.583638861692621	-3.46447074959693
a_HQ_transcript/108769	3.28953592491354	1.11082735409154	2.32928725184133	0.0261948974954055	0.583638861692621	-3.50709696146845
a_HQ_transcript/3606	-4.24224960406515	-0.312620096263822	-2.32920420615494	0.0261998493012628	0.583638861692621	-3.48263374665558
a_HQ_transcript/128030	2.50624997357555	0.838804582773654	2.32920199240038	0.0261999813134728	0.583638861692621	-3.42515574255888
a_HQ_transcript/87279	-3.01729250891871	0.890446207697108	-2.32896350684058	0.0262142063794906	0.583638861692621	-3.44853732235141
a_HQ_transcript/119094	5.44048407249774	1.25378593636177	2.3289390253051	0.0262156670368987	0.583638861692621	-3.4953534129599
a_HQ_transcript/152463	-2.18641577976609	-0.657255764106463	-2.3289341887323	0.0262159556131199	0.583638861692621	-3.485244569411
a_HQ_transcript/119011	-4.61154733306462	1.57648851915794	-2.32869588825886	0.0262301774721517	0.583731912749324	-3.43820410230406
a_HQ_transcript/110329	-1.9310407911408	-0.374176017708981	-2.32809149083525	0.0262662794189196	0.583999143453234	-3.50140242325466
a_HQ_transcript/64557	1.94114557699959	2.17500853076425	2.32803984292275	0.0262693665414382	0.583999143453234	-3.4883971310687
a_HQ_transcript/43999	1.76060265784147	1.79889486139231	2.32776506884781	0.0262857959782088	0.583999143453234	-3.42577374331529
a_HQ_transcript/139592	2.87678132337995	1.19485290062288	2.32774171957281	0.0262871925185686	0.583999143453234	-3.46512343719665
a_HQ_transcript/139479	-2.29224837977593	0.286689001471076	-2.32765433834451	0.0262924194607954	0.583999143453234	-3.49195738693385
a_HQ_transcript/50009	2.22162269442918	1.65525219361494	2.32739724389638	0.0263078037036782	0.584117652287016	-3.47143367089776
a_HQ_transcript/150007	5.0897486293613	9.17979232096698	2.32714781931728	0.0263227367672999	0.584226056819247	-4.27064374017826
a_HQ_transcript/122772	4.35157779878209	-0.607618682397647	2.32695920454093	0.0263340342325952	0.584253718271867	-3.51379365632615
a_HQ_transcript/48076	-4.40045566309507	2.43849386826082	-2.32672226747244	0.0263482322715237	0.584345686943582	-3.49169096782741
a_HQ_transcript/18921	3.23401050612114	0.748793399011386	2.32638576065045	0.0263684087469883	0.584570123434377	-3.43062143295617
a_HQ_transcript/104233	3.03347685133216	0.0153319629915913	2.32577280185521	0.0264051967885967	0.584735090493772	-3.49562762289409
a_HQ_transcript/118149	1.98776377722507	1.76340671985157	2.32567231605409	0.0264112320846503	0.584735090493772	-3.46786316102288
a_HQ_transcript/146118	-2.89543460556449	-0.545694469251741	-2.32565380749054	0.0264123438668453	0.584735090493772	-3.49621067203746
a_HQ_transcript/48415	-2.12746778260344	1.29736005661618	-2.32548021069354	0.0264227736305021	0.584735090493772	-3.42996797521332
a_HQ_transcript/134067	-2.74213744302405	-0.472936091139405	-2.32542407351838	0.0264261471705054	0.584735090493772	-3.47537462371301
a_HQ_transcript/32487	-3.06700758179492	0.926860204543274	-2.325060010841	0.0264480348157715	0.584872553167841	-3.45346901225287
a_HQ_transcript/13504	-2.94756065746342	1.38286604437988	-2.32498605338841	0.0264524831798488	0.584872553167841	-3.44850341239617
a_HQ_transcript/23487	-1.65096919964865	-0.103762205004484	-2.32470496295855	0.0264693962678506	0.585023979565636	-3.45356267763577
a_HQ_transcript/118435	2.30406969487977	0.976478675296653	2.32428183916288	0.0264948738717085	0.585173803124508	-3.43262457965632
a_HQ_transcript/125292	1.3831725363708	4.07034500054567	2.32417870578739	0.0265010872126734	0.585173803124508	-3.67390812913345
a_HQ_transcript/34866	-3.31571483978691	1.28296003483458	-2.32409093615931	0.0265063759913782	0.585173803124508	-3.4295020007626
a_HQ_transcript/97044	5.81106528785141	1.48696339022163	2.32373954960097	0.0265275592240339	0.585236332788547	-3.61120964775519
a_HQ_transcript/150864	3.55600359299831	-0.603556328978074	2.32369918807313	0.0265299933859543	0.585236332788547	-3.50652889109819
a_HQ_transcript/49150	3.22387109772166	0.404751003228548	2.32354303762444	0.0265394125590182	0.585236332788547	-3.44192639488847
a_HQ_transcript/123100	-1.61572705915595	-0.792665525855541	-2.32335496136051	0.0265507615459777	0.585264568503826	-3.46315515147595
a_HQ_transcript/50167	0.69747505979708	3.06830696523158	2.32279889931999	0.0265843413628567	0.585782636368512	-3.6294418488842
a_HQ_transcript/59638	3.47079777843872	0.284447067263958	2.32248034432787	0.0266035957447301	0.585984772053684	-3.5324206919755
a_HQ_transcript/58774	-2.92362254388928	-0.44577958656491	-2.32209704183412	0.0266267803562965	0.586273291117728	-3.50638034466289
a_HQ_transcript/124643	3.47936527457164	1.44723767807197	2.32170655400837	0.026650418356388	0.586392825271019	-3.52709161362192
a_HQ_transcript/142271	-3.28772002735212	-0.235191603665719	-2.321674077572	0.0266523851563108	0.586392825271019	-3.50839621655285
a_HQ_transcript/89310	1.64291210805246	1.03033847525477	2.32144928855719	0.0266660021615883	0.586434251694154	-3.41275240478104
a_HQ_transcript/151852	-1.83857690265688	-0.627069792878019	-2.32100694962391	0.0266928160082702	0.586434251694154	-3.50600191006533

a_HQ_transcript/152192	-2.76638269196643	-0.689319999677433	-2.32086175594813	0.0267016227154704	0.586434251694154	-3.50595641253113
a_HQ_transcript/41824	-1.71006191132965	2.04163862869895	-2.32076908380827	0.0267072451069015	0.586434251694154	-3.52243274814595
a_HQ_transcript/24471	2.41784692153405	-0.527348962136672	2.3205579051154	0.0267200612522523	0.586434251694154	-3.52329725403073
a_HQ_transcript/2980	2.92775856869505	0.490414966539441	2.32047057579719	0.0267253627727398	0.586434251694154	-3.43858177013622
a_HQ_transcript/101100	-3.43510267878784	-0.227030586959791	-2.32007126879875	0.0267496157017285	0.586434251694154	-3.51252131037335
a_HQ_transcript/4074	-3.72775621573082	2.02276535649875	-2.31984792459674	0.0267631897514144	0.586434251694154	-3.56024921540567
a_HQ_transcript/96937	4.21172961368953	1.32012330422051	2.31975637090618	0.0267687558504503	0.586434251694154	-3.53385272653565
a_HQ_transcript/38042	-2.57414751729474	0.505682281386816	-2.31967579177974	0.0267736556050743	0.586434251694154	-3.52782914063783
a_HQ_transcript/69939	2.94040590283346	2.01366232710562	2.31962783076705	0.0267765723429239	0.586434251694154	-3.4723845234871
a_HQ_transcript/137413	-2.51631861730819	-0.642773396874071	-2.31949502396042	0.0267846504561247	0.586434251694154	-3.5049248610531
a_HQ_transcript/1449	2.18229960300623	0.0866458561703417	2.31921970744228	0.0268014038854139	0.586434251694154	-3.46411053385213
a_HQ_transcript/623	-0.986822054523398	3.06903483690151	-2.31919981558579	0.0268026147017934	0.586434251694154	-3.5779184963025
a_HQ_transcript/150642	1.90364704242188	-0.630061059315265	2.31908869581526	0.0268093794661852	0.586434251694154	-3.50708578703668
a_HQ_transcript/105081	3.27420381993615	-0.491794226999283	2.31898511310352	0.0268156867775093	0.586434251694154	-3.51547322799949
a_HQ_transcript/108810	3.14105960091957	0.993068380849542	2.31877716777045	0.0268283529370795	0.586490597791108	-3.49436283745251
a_HQ_transcript/86986	2.67661919695487	1.10485404253718	2.31772518050529	0.026892513409191	0.587672187762952	-3.56254013496364
a_HQ_transcript/33840	-3.29399575781532	0.330265821800037	-2.31735141025523	0.0269153429245771	0.587950038902599	-3.5201175166461
a_HQ_transcript/51957	-2.69287703563246	0.211924874938696	-2.31714323249082	0.0269280658043633	0.588006990637127	-3.48508010094108
a_HQ_transcript/13203	-1.95619535409392	-0.712034673286735	-2.31697745301517	0.0269382013783025	0.588007423852035	-3.51414088252297
a_HQ_transcript/150572	1.93402951670549	-0.706562099611611	2.3166421432969	0.0269587123777362	0.58823424665655	-3.49358680515367
a_HQ_transcript/150707	1.78932025574088	-0.754831056511801	2.31639936871092	0.0269735717769366	0.588259652122722	-3.51486237676098
a_HQ_transcript/5531	-1.79726139814694	-0.719974248982908	-2.31629247278212	0.0269801168552019	0.588259652122722	-3.51466667294883
a_HQ_transcript/150105	8.28325263587437	3.24900942266772	2.31589238882497	0.0270046261316367	0.588573268758823	-3.68623979353016
a_HQ_transcript/126394	1.33367780351973	2.9111481712814	2.31564119990264	0.0270200243223759	0.588688146106097	-3.58216212435782
a_HQ_transcript/15011	-2.51824575017749	0.506960292532472	-2.31531762268013	0.0270398716915419	0.588899835775926	-3.51347859893445
a_HQ_transcript/99489	2.96487659611108	2.56255594137132	2.31511923505711	0.0270520467721857	0.588944335121203	-3.5515783391206
a_HQ_transcript/87882	1.95371857760157	-0.225267978392436	2.31479112401362	0.0270721938653806	0.589162293151196	-3.52343042709971
a_HQ_transcript/123371	2.24632792097582	4.21084674279766	2.31446320130621	0.0270923429313948	0.589317825129744	-3.79387063540705
a_HQ_transcript/130359	4.12855102493692	2.42410505751085	2.31434486846406	0.0270996171650806	0.589317825129744	-3.62561601321161
a_HQ_transcript/87819	5.1991766759347	1.49473130620506	2.31409272620733	0.0271151229015456	0.589380640475073	-3.47783006104109
a_HQ_transcript/108457	-2.20522851799939	2.16279043021906	-2.31396818845378	0.0271227844286888	0.589380640475073	-3.49255728171929
a_HQ_transcript/125940	-4.0790476368578	0.510574150193007	-2.31377154785139	0.0271348856826515	0.589423256711947	-3.47500698679932
a_HQ_transcript/28203	-2.19248801570114	0.683625259649417	-2.31334823636735	0.0271609527980794	0.589737803974932	-3.4505378913795
a_HQ_transcript/107858	0.693342396393637	5.71467933201407	2.31306841334249	0.0271781964427816	0.589737803974932	-3.96454795997172
a_HQ_transcript/30565	-1.80903857655535	-0.720780067805859	-2.31303968398731	0.0271799674025788	0.589737803974932	-3.51652368714523
a_HQ_transcript/147147	-9.87465184743217	2.86849957392484	-2.31287780073924	0.0271899482977708	0.589737803974932	-3.65771577081679
a_HQ_transcript/37168	-9.44451733066287	1.93742346561916	-2.31265050409244	0.0272039678345941	0.589821798690521	-3.55069839781925
a_HQ_transcript/106458	-3.3585029067449	0.251947311234388	-2.31231372950826	0.0272247519117649	0.590002969055717	-3.51578571705174
a_HQ_transcript/80815	-3.23975468025524	0.181020044465639	-2.3121777828432	0.0272331459347458	0.590002969055717	-3.45611702034727
a_HQ_transcript/55189	2.0097098342558	3.99524760514706	2.31195779268969	0.0272467341705148	0.590002969055717	-3.72051728453604
a_HQ_transcript/134573	1.37826312020456	-0.740896550773956	2.31185761215581	0.027252924097072	0.590002969055717	-3.55008835631529
a_HQ_transcript/76229	3.35880565271311	-0.41864004581994	2.31071629008311	0.0273235334568848	0.590681932188425	-3.5344515640306
a_HQ_transcript/5257	-1.78525619526506	2.57298501670768	-2.3104526116008	0.0273398697317231	0.590681932188425	-3.58326436413198
a_HQ_transcript/37584	2.34946412640021	-0.389409733075264	2.31031009279495	0.0273487031947517	0.590681932188425	-3.54440175227582
a_HQ_transcript/123289	3.23687141744802	1.19043435862797	2.31001579354887	0.0273669523199433	0.590681932188425	-3.52478835663525
a_HQ_transcript/56458	-3.04444611404071	0.167473673822414	-2.31000622838989	0.0273675456277812	0.590681932188425	-3.49431522717359
a_HQ_transcript/89347	-3.18003971102531	0.224687943400289	-2.30998341815026	0.0273689605485576	0.590681932188425	-3.5632190289699
a_HQ_transcript/80612	2.30809583708457	1.00018022976907	2.30989933388439	0.0273741768718673	0.590681932188425	-3.4889837185905
a_HQ_transcript/23027	2.63632190711462	-0.157332823361366	2.30958121651882	0.0273939199934405	0.590681932188425	-3.52110972601774
a_HQ_transcript/115872	-3.21507448975951	0.501012549243406	-2.30935899071431	0.0274077194806421	0.590681932188425	-3.53635412753717

a_HQ_transcript/9351	3.01348895951319	0.655237881260596	2.30935871028137	0.027407736898563	0.590681932188425	-3.59002588770481
a_HQ_transcript/62970	2.23545512165576	1.54890789989143	2.30933179861101	0.0274094084510657	0.590681932188425	-3.46474363920515
a_HQ_transcript/134972	1.68137415066138	-0.772739677508907	2.30925759376535	0.0274140179800077	0.590681932188425	-3.52768061936359
a_HQ_transcript/126620	-1.00530156299744	5.05693086645082	-2.30921943100638	0.0274163888839177	0.590681932188425	-3.92441416365845
a_HQ_transcript/116778	0.758139553921592	4.59140614566697	2.30859330719848	0.0274553139590983	0.590938118723922	-3.83693750487177
a_HQ_transcript/1898	-3.20618670259355	0.181113874656385	-2.30854431045715	0.0274583621083153	0.590938118723922	-3.56596983171952
a_HQ_transcript/91487	4.34150923754925	-0.293003025594205	2.30846287810907	0.0274634287932836	0.590938118723922	-3.53612593416654
a_HQ_transcript/9300	2.09184731853765	0.815994606439592	2.30837424696127	0.0274689443433808	0.590938118723922	-3.45731293671192
a_HQ_transcript/14751	-1.27849092029585	2.47613363213737	-2.30708660613152	0.0275491875650812	0.592445125705897	-3.58119794617926
a_HQ_transcript/77974	-2.23759937268461	-0.193595045697913	-2.30655684024755	0.027582262898228	0.592531123106447	-3.49005348516334
a_HQ_transcript/27273	3.15893184570166	1.27205473963219	2.30652790390902	0.0275840705374242	0.592531123106447	-3.50119840427918
a_HQ_transcript/134104	1.33650197767375	2.83451563242037	2.30637089263747	0.0275938808201552	0.592531123106447	-3.70443983987175
a_HQ_transcript/23298	3.48672407334212	0.35112970702295	2.30636961364841	0.0275939607460974	0.592531123106447	-3.54057038202193
a_HQ_transcript/1068	-1.68523142410778	2.79271028118127	-2.30585879879267	0.0276258990394374	0.592857723838901	-3.66358811863405
a_HQ_transcript/13348	0.877624593328396	3.33508109151075	2.30568206790606	0.0276369567619865	0.592857723838901	-3.66584284459717
a_HQ_transcript/141711	-5.11360917763477	0.346082766202544	-2.30556892121028	0.0276440382367994	0.592857723838901	-3.52165757746585
a_HQ_transcript/12444	-2.50771708351896	0.078464546488336	-2.30533543518457	0.0276586565248268	0.592857723838901	-3.47140797872122
a_HQ_transcript/59092	2.58899400248999	2.19528235210796	2.3053113270175	0.0276601663062741	0.592857723838901	-3.61654964298339
a_HQ_transcript/146432	-2.68737541146861	3.21680148329397	-2.30483093831726	0.0276902663052633	0.592947416667529	-3.70904202320658
a_HQ_transcript/120581	-2.7290323049847	4.84377636971681	-2.30481987792094	0.02769095967097	0.592947416667529	-3.93485143781482
a_HQ_transcript/40611	3.42949041414907	0.454776337353309	2.30466653385491	0.0277005742785781	0.592947416667529	-3.49427891210103
a_HQ_transcript/67045	-3.03983727685964	0.450135660931728	-2.30448737186507	0.027711811474166	0.592947416667529	-3.51492007479937
a_HQ_transcript/138045	-0.990670892334664	5.05391494424227	-2.30439814132509	0.0277174096252324	0.592947416667529	-3.90514609757177
a_HQ_transcript/108936	-2.13276130262854	0.492943494534378	-2.30394359496782	0.0277459428222558	0.592947416667529	-3.48094236613325
a_HQ_transcript/119250	0.747085463350947	4.94816547204359	2.30381454431887	0.0277540485351837	0.592947416667529	-3.90807327226828
a_HQ_transcript/42635	-4.57330226502051	1.99008861049719	-2.30378735482713	0.0277557565883772	0.592947416667529	-3.66493389736592
a_HQ_transcript/20582	-2.08336332429766	2.91477723734012	-2.30375472263799	0.0277578066788947	0.592947416667529	-3.66396805283302
a_HQ_transcript/118921	10.9648932079945	5.4218378045665	2.30353934201246	0.0277713412167011	0.592947416667529	-3.98467539405971
a_HQ_transcript/98088	-1.89854275018198	3.94214008057241	-2.30345633621866	0.0277765588973589	0.592947416667529	-3.80799294428708
a_HQ_transcript/140688	-2.82535604051678	0.0861485889251987	-2.30307174710836	0.0278007454172483	0.59323787719235	-3.5433329236743
a_HQ_transcript/96826	-4.75972184828675	-0.38050673444415	-2.3029155029221	0.0278105769224668	0.59323787719235	-3.51732432836336
a_HQ_transcript/10454	-2.6740358073328	-0.517221237601543	-2.30266692530207	0.0278262248786805	0.593353925072612	-3.53491119678246
a_HQ_transcript/67358	-3.60984372819411	1.86598297903468	-2.30200640922977	0.0278678429495759	0.593888578380825	-3.480861622176
a_HQ_transcript/19528	5.83839510300877	-0.709882890545854	2.30194473085546	0.0278717320709966	0.593888578380825	-3.53767112256798
a_HQ_transcript/141797	-1.84984127671693	1.11445431355833	-2.30139896452166	0.0279061666294859	0.594294234557517	-3.44325562886046
a_HQ_transcript/31952	-1.10892014036397	2.75890179840991	-2.30131895986531	0.0279112176634672	0.594294234557517	-3.59604100929081
a_HQ_transcript/98890	-1.72210961763664	-0.6518998328228	-2.300416634077	0.0279682424470395	0.5952108574624	-3.53902088886924
a_HQ_transcript/8520	-1.61548573081056	1.92285130547645	-2.30023300072126	0.0279798604859786	0.5952108574624	-3.54035370191212
a_HQ_transcript/60045	2.21600819023689	1.08998156899078	2.30013334992346	0.0279861669729688	0.5952108574624	-3.47834815710734
a_HQ_transcript/6272	-3.09124178828575	0.324212069922417	-2.299990244233	0.0279952257827932	0.5952108574624	-3.51140168582812
a_HQ_transcript/77868	-1.81482858591921	1.73390188736211	-2.29963074176402	0.0280179945077003	0.59547714250223	-3.53873400225844
a_HQ_transcript/62592	-2.512410322943	0.34701950075289	-2.29908840471863	0.02805237447517	0.595608707713735	-3.5116287374067
a_HQ_transcript/144551	-1.57599924098262	4.72291902652378	-2.29893828323282	0.0280618977346969	0.595608707713735	-3.78417099918594
a_HQ_transcript/117767	-1.93686213120711	-0.740115122849258	-2.29878097351691	0.0280718801220913	0.595608707713735	-3.5417478530325
a_HQ_transcript/137856	2.3969930820225	-0.425850001435116	2.29874620584017	0.0280740868031981	0.595608707713735	-3.54525599988295
a_HQ_transcript/2004	3.76151233223756	1.08518371601392	2.29872524357104	0.0280754173399331	0.595608707713735	-3.51487843771672
a_HQ_transcript/126737	3.04494451327873	1.17563259076899	2.29838473985784	0.0280970380748431	0.595849919450741	-3.50428340471805
a_HQ_transcript/101924	-1.9743240338671	-0.545581917078771	-2.29791695231626	0.0281267653458408	0.596262806718831	-3.51360728676529
a_HQ_transcript/148569	-2.61582420552259	-0.612300652842578	-2.29726090645506	0.0281685039562844	0.596929929993037	-3.55717203250087
a_HQ_transcript/78712	1.65839577564503	0.943564621026005	2.29665326653784	0.0282072127155526	0.597244105293245	-3.52901365899554

a_HQ_transcript/151922	0.942589928708263	6.99949695676221	2.29654787440406	0.0282139314363326	0.597244105293245	-4.09627833278498
a_HQ_transcript/7117	-1.41701313840116	3.0630633543895	-2.29654439183251	0.0282141534739756	0.597244105293245	-3.68779030748548
a_HQ_transcript/78492	-2.66401041065209	0.0982944184261384	-2.2963567226754	0.0282261209903115	0.597279927529969	-3.52014194109672
a_HQ_transcript/36230	-3.35281811432781	1.13388534839408	-2.29592051310112	0.0282539554186597	0.597380376725991	-3.54072139832468
a_HQ_transcript/144393	-3.73753090443468	-0.0865007362554283	-2.29589123675731	0.0282558244209476	0.597380376725991	-3.57922221456375
a_HQ_transcript/151586	6.03086135965703	2.15845515115922	2.2957992287355	0.0282616989402091	0.597380376725991	-3.60841910289435
a_HQ_transcript/68630	-3.80134401073775	2.15424724975807	-2.29552900157029	0.0282789587441624	0.59752792216673	-3.55934184727052
a_HQ_transcript/146349	-3.45840274447871	0.242185631535912	-2.29526614280155	0.0282957570343771	0.597665612243559	-3.5437625707952
a_HQ_transcript/131556	-3.4254567874573	-0.0530827573095823	-2.29491731258817	0.0283180633171631	0.597919500363262	-3.56929343451267
a_HQ_transcript/82039	-2.11329888807636	3.15219153955614	-2.29464063474915	0.0283357670179982	0.597971686961945	-3.71397017843507
a_HQ_transcript/132735	-2.06814637209847	0.566691010200148	-2.29449906086236	0.028344829721286	0.597971686961945	-3.48703856888761
a_HQ_transcript/58328	2.609787674831	0.760037500356818	2.29431570684988	0.0283565708183397	0.597971686961945	-3.53838391938176
a_HQ_transcript/9025	-2.60993370072937	0.490140431580227	-2.29423588574183	0.028361683542425	0.597971686961945	-3.53155366257917
a_HQ_transcript/89856	-4.7458028561221	2.10716248964099	-2.29361186666179	0.0284016820356316	0.598517189931188	-3.70303808515632
a_HQ_transcript/138038	-2.2096462198207	-0.423003902488576	-2.29351103827802	0.0284081497216513	0.598517189931188	-3.54861966512747
a_HQ_transcript/125920	-5.20157055386209	0.941818325760653	-2.29256398070501	0.0284689640148747	0.599581137774144	-3.52798588839729
a_HQ_transcript/56974	3.05172026734087	1.01370147304507	2.29229961481128	0.0284859609234337	0.599721816934935	-3.5837272775003
a_HQ_transcript/127921	-5.19139196459098	5.88204378450867	-2.29163361915487	0.0285288204091321	0.600406688175971	-4.08492506218209
a_HQ_transcript/23823	-2.63977249985987	-0.225269199069934	-2.29037317589766	0.0286100940687818	0.60155379439728	-3.57547788440753
a_HQ_transcript/40612	-2.30362038542367	0.42736965152369	-2.29036042982763	0.0286109170026333	0.60155379439728	-3.4958214768932
a_HQ_transcript/72373	5.34932470870418	1.43359432331092	2.29029886126563	0.0286148923991222	0.60155379439728	-3.63286364236112
a_HQ_transcript/107917	5.00576778068076	2.3823699870474	2.28981801291484	0.0286459572352118	0.60155379439728	-3.64045264833594
a_HQ_transcript/67495	-2.54434645123688	1.68794315589473	-2.2896754656859	0.0286551722261108	0.60155379439728	-3.53663355975663
a_HQ_transcript/68328	2.42513434887747	0.318927575192671	2.28950030616151	0.028666499101854	0.60155379439728	-3.5554541301159
a_HQ_transcript/132897	5.62343874548727	1.19151060096132	2.28945587855406	0.0286693727013079	0.60155379439728	-3.62032704993725
a_HQ_transcript/2362	3.85681206515319	1.07819002973255	2.28936553812329	0.0286752167651432	0.60155379439728	-3.55731815885215
a_HQ_transcript/116849	-2.91316379450768	-0.322512158270011	-2.28934624264496	0.0286764651162067	0.60155379439728	-3.58533351909578
a_HQ_transcript/16112	1.34966428375289	3.16854800955927	2.28878088602688	0.0287130634855501	0.602104240363656	-3.74630859653643
a_HQ_transcript/135199	2.658551471143	1.42842990399676	2.2880024375691	0.0287635252186985	0.602944895027951	-3.54302355257376
a_HQ_transcript/32295	-1.07515071840648	2.37745263439506	-2.28763284447503	0.0287875114851609	0.603207384479644	-3.59201684591371
a_HQ_transcript/139508	-2.88056033551656	-0.318436012594818	-2.28748976973273	0.0287968017466799	0.603207384479644	-3.57291773482843
a_HQ_transcript/93088	5.26116774062695	2.39401593179033	2.28676167334163	0.0288441210302822	0.603631860802144	-3.69801915142331
a_HQ_transcript/99659	-4.661631485285	3.82057166440933	-2.28662277569956	0.0288531559960551	0.603631860802144	-3.80254218954177
a_HQ_transcript/21209	-2.6951549312138	1.31135407295006	-2.28656757435536	0.0288567474217958	0.603631860802144	-3.59728966615167
a_HQ_transcript/99630	2.95645573304146	-0.0837582757603922	2.28653903851283	0.02885860413517	0.603631860802144	-3.52699136547084
a_HQ_transcript/103962	-2.27130337364398	1.39050106097083	-2.28623293102164	0.0288785280971319	0.603717574295672	-3.50767418037065
a_HQ_transcript/112923	-2.22735166861578	-0.515191241269518	-2.28615697337428	0.0288834739560326	0.603717574295672	-3.55726492486771
a_HQ_transcript/109396	-2.47444035648499	-0.107906220141482	-2.28575176248171	0.0289098714988059	0.603758945322411	-3.52700956316379
a_HQ_transcript/110477	2.34789872055878	-0.421105562821171	2.28565789729426	0.02891598946363	0.603758945322411	-3.59063834189181
a_HQ_transcript/92119	-5.72214464663197	1.60171335871845	-2.28564832550733	0.0289166134010734	0.603758945322411	-3.58495952159857
a_HQ_transcript/101873	-2.8916355237288	0.691636867950768	-2.28507229883393	0.0289541840594458	0.604238276875875	-3.53849171837305
a_HQ_transcript/10980	2.6689587304864	-0.168010087130682	2.2849405068546	0.0289627862128926	0.604238276875875	-3.56954412406646
a_HQ_transcript/120617	2.29783755019487	-0.554162249218701	2.28456813569982	0.0289871035750439	0.604238276875875	-3.57204456060836
a_HQ_transcript/76213	-3.96641478534366	2.93345274339458	-2.28446857636352	0.0289936083220695	0.604238276875875	-3.56436155111409
a_HQ_transcript/82820	1.08393662746284	4.21668165101441	2.28439192996704	0.0289986169389784	0.604238276875875	-3.84993398558536
a_HQ_transcript/33694	-2.65525442482617	0.169241416653147	-2.28434107769302	0.029001940415698	0.604238276875875	-3.5283445115197
a_HQ_transcript/107916	-3.67955111118427	0.678945342630294	-2.28265683625206	0.0291122088816131	0.605840070877419	-3.56497042595064
a_HQ_transcript/40065	2.18852981845112	1.4905387526311	2.28261095911957	0.0291152177639978	0.605840070877419	-3.51615342196361
a_HQ_transcript/140773	-2.87662990491841	1.40360453643606	-2.28243732049799	0.0291266085046284	0.605840070877419	-3.49197298008395
a_HQ_transcript/3778	-2.50630617975343	0.22991486887352	-2.28233952963149	0.0291330253780393	0.605840070877419	-3.55561680021939

a_HQ_transcript/62828	1.75729060953	0.863575916743593	2.28226306408611	0.0291380438062104	0.605840070877419	-3.53926357392405
a_HQ_transcript/59041	-2.12731031863165	1.8810999886369	-2.28221257752783	0.0291413576619403	0.605840070877419	-3.57989468075675
a_HQ_transcript/37259	2.29322413801215	0.434339966970085	2.28171427651277	0.0291740835389365	0.606165865272421	-3.48107144101709
a_HQ_transcript/27174	-1.34013056196748	1.50630430349466	-2.28143361966435	0.0291925302164663	0.606165865272421	-3.49001125199531
a_HQ_transcript/20103	-2.51133806929249	0.0225761129222815	-2.2813401776454	0.0291986741913779	0.606165865272421	-3.58394017456265
a_HQ_transcript/137071	-4.85659173323386	0.112481756356176	-2.28113997694163	0.0292118416542901	0.606165865272421	-3.57564820058957
a_HQ_transcript/138812	-0.992894225426211	3.01002710633842	-2.28111630763798	0.029213398768764	0.606165865272421	-3.70677751219201
a_HQ_transcript/68209	7.22360760485185	2.2044130622775	2.28102209589945	0.0292195973453985	0.606165865272421	-3.67857708844884
a_HQ_transcript/67838	4.11512318238427	1.2771759572754	2.28057199305204	0.0292492278020814	0.606564079086475	-3.55531236033317
a_HQ_transcript/112444	3.10668664565366	1.28416884420163	2.27949907302431	0.029319967686349	0.607792576311257	-3.65693651603987
a_HQ_transcript/62664	-2.73201092513801	1.71628526333997	-2.27915006439253	0.0293430117360185	0.607792576311257	-3.58325912157181
a_HQ_transcript/38072	-1.0620496701925	3.16617062994591	-2.27911589411121	0.0293452687786973	0.607792576311257	-3.76662489680172
a_HQ_transcript/118348	-2.8350854727822	0.922430832150221	-2.27903985706292	0.029350291799231	0.607792576311257	-3.4984395106517
a_HQ_transcript/152273	-4.19901895270914	4.03527471129475	-2.27853077904039	0.0293839415212901	0.608272703970639	-3.77794996020548
a_HQ_transcript/138180	-2.99535738583264	0.743535509020958	-2.27756167919004	0.0294480943629792	0.608899845695478	-3.54906332406375
a_HQ_transcript/38931	1.37661732176617	2.6033896305044	2.27723698142634	0.0294696170051922	0.608899845695478	-3.63556456375992
a_HQ_transcript/75862	2.32931317419394	-0.546348455402347	2.27720209768029	0.0294719301217074	0.608899845695478	-3.59168552044546
a_HQ_transcript/103271	1.24091698224104	2.8417801815589	2.27701113822724	0.0294845954035319	0.608899845695478	-3.72337494842917
a_HQ_transcript/140401	2.70725613013405	-0.529928421230428	2.27689650221152	0.0294922009274921	0.608899845695478	-3.59445043461347
a_HQ_transcript/79618	-0.896624667901329	3.36311568159143	-2.27686829665662	0.0294940724950296	0.608899845695478	-3.78005306863513
a_HQ_transcript/115585	-1.9931709396102	-0.408680587076914	-2.27669038700383	0.0295058800774683	0.608899845695478	-3.5893800197269
a_HQ_transcript/85396	3.59913332851697	1.15392432168611	2.27613274090516	0.0295429177354173	0.608899845695478	-3.60178062082872
a_HQ_transcript/71431	-1.11129296405072	4.61661994730666	-2.27609818572138	0.0295452141925628	0.608899845695478	-3.93410031260946
a_HQ_transcript/136037	-4.98711116747278	4.12383187894652	-2.27609258316495	0.0295455865405656	0.608899845695478	-3.9572835211146
a_HQ_transcript/133256	-3.11616189124901	-0.551968606300375	-2.27594507281679	0.029555391652969	0.608899845695478	-3.5894468624248
a_HQ_transcript/85227	2.90988872802252	0.714490586365199	2.27583915766033	0.02956243371077	0.608899845695478	-3.52958481417704
a_HQ_transcript/57645	-2.56204529605363	0.502297488804511	-2.27581250858034	0.0295642057851865	0.608899845695478	-3.50650400114174
a_HQ_transcript/134740	-1.90769139731002	0.879023481761993	-2.27580415405641	0.0295647613526791	0.608899845695478	-3.5068874075262
a_HQ_transcript/39205	-3.16429721095921	1.58549399319179	-2.27570487084358	0.0295713643063297	0.608899845695478	-3.66908459473162
a_HQ_transcript/152137	1.59852979022202	-0.624573458217603	2.27542188799987	0.0295901917143405	0.609071764862317	-3.60865092794313
a_HQ_transcript/44835	-0.992635003311044	4.15876340379775	-2.27512697005864	0.0296098246650666	0.609240691876395	-3.9031047012218
a_HQ_transcript/32009	2.5646032755	0.141289207531396	2.27498378813709	0.0296193606393251	0.609240691876395	-3.63369344412521
a_HQ_transcript/117374	-2.90521124599996	0.878303086685953	-2.27458671968688	0.0296458866818412	0.609368481440702	-3.58136618837799
a_HQ_transcript/36396	2.04432907782749	0.906401997878708	2.27457592257425	0.0296465398003425	0.609368481440702	-3.50477543881082
a_HQ_transcript/134985	-0.807842060842144	3.61984322330954	-2.27433276951881	0.0296627535978815	0.609426211892694	-3.83533007335911
a_HQ_transcript/90868	-3.0182145431749	0.972750894810144	-2.27409847373669	0.0296783843265333	0.609426211892694	-3.62508793264543
a_HQ_transcript/49484	1.82965260353677	2.24434856180544	2.27406225770124	0.02968080109187	0.609426211892694	-3.544079776698
a_HQ_transcript/21329	1.97016537817058	-0.386441285690747	2.27362446233687	0.029710030024195	0.609644888141235	-3.58533627807614
a_HQ_transcript/118830	-4.40255637708023	1.41189778316441	-2.27348800693455	0.029719145613054	0.609644888141235	-3.63418845667743
a_HQ_transcript/62269	2.43762203517788	1.20269137620464	2.27342522728049	0.0297233403082738	0.609644888141235	-3.64129427195438
a_HQ_transcript/28663	-1.79030528896786	-0.09792258254897	-2.27327465496187	0.0297334031427264	0.609644888141235	-3.54757723442619
a_HQ_transcript/62081	1.43554798054087	1.82646297927549	2.27303017416191	0.0297497484629662	0.609748943978079	-3.53512809915424
a_HQ_transcript/142848	4.59300691337432	4.09366985092154	2.27283513759558	0.0297627938723842	0.609748943978079	-3.82538917683789
a_HQ_transcript/36959	-3.04528281974606	0.682090936957191	-2.27272822064288	0.0297699474093343	0.609748943978079	-3.59423847028904
a_HQ_transcript/151004	-2.02959539910304	-0.688340395866289	-2.27254536308351	0.029782185520737	0.609784741088201	-3.59097941608436
a_HQ_transcript/39015	5.04353481201798	0.732796624431484	2.2721916198764	0.0298058733456501	0.61005486120984	-3.62980116646218
a_HQ_transcript/66649	-6.56379237419131	0.376177123213011	-2.27192529281439	0.0298237186754034	0.610202097033106	-3.62017433728029
a_HQ_transcript/84360	0.865666872022896	5.49985063541004	2.27174321762338	0.0298359242120847	0.610202097033106	-4.00951814957421
a_HQ_transcript/145083	-5.03699135319514	2.76664147888609	-2.27116980266529	0.0298743928436584	0.610202097033106	-3.68620368606405
a_HQ_transcript/18142	-2.0309774083646	0.716927144914125	-2.271152457902	0.0298755571448492	0.610202097033106	-3.51732923182545

a_HQ_transcript/49109	0.751240923870782	3.00377360252896	2.27106093487816	0.0298817014828907	0.610202097033106	-3.68571116822241
a_HQ_transcript/144845	-2.21271549418352	2.71765371179409	-2.27083089802568	0.029897149874479	0.610202097033106	-3.67619571701817
a_HQ_transcript/60686	3.35153632009052	0.196093112463644	2.27078177632205	0.0299004496300196	0.610202097033106	-3.57847264277253
a_HQ_transcript/105363	3.80588090637781	-0.290573530380092	2.27072177107203	0.0299044809333754	0.610202097033106	-3.5473702697998
a_HQ_transcript/152536	3.78085252372792	2.84135456099642	2.27067616817492	0.0299075449774174	0.610202097033106	-3.64742745597894
a_HQ_transcript/139534	-1.1354921417372	2.38758837239161	-2.27046690625244	0.0299216088398849	0.610274834612221	-3.61603320628511
a_HQ_transcript/114476	-4.75075351578634	1.58053310905582	-2.26990922781796	0.0299591177764899	0.610825534237744	-3.63476787860881
a_HQ_transcript/146366	-3.14556368826815	2.99073211077185	-2.26937289058437	0.0299952312082916	0.611318457431959	-3.7973208952834
a_HQ_transcript/94260	-3.621047554381	2.06284422561531	-2.26923788633161	0.0300043276743287	0.611318457431959	-3.68149872391606
a_HQ_transcript/94066	1.82528068332453	1.85555045556781	2.26905534973774	0.0300166307717115	0.611347966829734	-3.58024763289495
a_HQ_transcript/126702	5.2371240561035	0.800706033671388	2.26889366007099	0.0300275325600442	0.611347966829734	-3.61483176659982
a_HQ_transcript/37262	-0.929452327355217	4.46603208234263	-2.26859349389175	0.0300477804501582	0.611347966829734	-3.9147454709112
a_HQ_transcript/56438	-3.32165465423806	1.43820050843166	-2.26859253545607	0.030047845121672	0.611347966829734	-3.61611881715822
a_HQ_transcript/126763	-2.48587892560505	0.352800762231213	-2.26830299899557	0.0300673876475376	0.611385428687198	-3.55076575652574
a_HQ_transcript/78316	5.13028076134836	2.17195181727864	2.26825361197673	0.0300707222098937	0.611385428687198	-3.62202139677935
a_HQ_transcript/69385	-1.89737717357005	1.38831389866985	-2.26802811892723	0.0300859515017825	0.611405799740338	-3.51831967755018
a_HQ_transcript/70694	-2.15746102365931	-0.667771364114377	-2.26782308311576	0.0300998051775224	0.611405799740338	-3.60584984044786
a_HQ_transcript/91724	3.33585495069878	0.866697657922337	2.26777168422233	0.0301032789508816	0.611405799740338	-3.65495322427598
a_HQ_transcript/151482	2.90455090364969	0.527800225691152	2.26722622432103	0.0301401658538712	0.611766172019564	-3.72037944229883
a_HQ_transcript/116265	-2.0011340631817	-0.744491373585718	-2.26719806509197	0.0301420712335541	0.611766172019564	-3.6063239470554
a_HQ_transcript/144413	3.32023539910967	0.532897783097525	2.26698868473843	0.0301562422437508	0.611838132067747	-3.59298172930282
a_HQ_transcript/96543	-3.76832002436796	0.921244985367351	-2.26672914650273	0.0301738162889359	0.611838132067747	-3.53134039486396
a_HQ_transcript/59275	3.56824172380732	1.33620195233802	2.26663863055981	0.0301799475367645	0.611838132067747	-3.51784556117931
a_HQ_transcript/16467	1.1905863246314	2.91490914604922	2.26643608467233	0.0301936713767078	0.611838132067747	-3.65870151893881
a_HQ_transcript/145089	-2.00084605827499	2.95443367540165	-2.26631207034623	0.0302020769466173	0.611838132067747	-3.70129060932057
a_HQ_transcript/72761	3.42719505105245	2.30767590484104	2.26621333005229	0.030208770971553	0.611838132067747	-3.55799488465233
a_HQ_transcript/114967	2.80724853902825	-0.21856779235816	2.26599588784683	0.030223517007594	0.611923579455737	-3.58242484362761
a_HQ_transcript/53663	2.44027298966836	3.00724747644938	2.26539445430338	0.0302643374650489	0.612306453945945	-3.77783096513752
a_HQ_transcript/72942	-2.35875876603646	-0.243271427770931	-2.26526595627166	0.0302730652979613	0.612306453945945	-3.61909387313657
a_HQ_transcript/95938	-3.75593715026929	3.78812627067057	-2.26515524333595	0.0302805869474864	0.612306453945945	-3.74428730807568
a_HQ_transcript/22204	-4.00835387945523	1.4816119435072	-2.26509673468261	0.0302845626048478	0.612306453945945	-3.60789496111727
a_HQ_transcript/98551	0.578819204489761	3.36829681245224	2.26447166247209	0.0303270654939281	0.612864479312806	-3.80216084408076
a_HQ_transcript/149022	-1.25800145340625	4.33555978425929	-2.26423445799803	0.0303432086603327	0.612864479312806	-3.91745182274388
a_HQ_transcript/1573	-2.64036969147241	1.90886788749912	-2.26422588104141	0.0303437925175863	0.612864479312806	-3.60813604860832
a_HQ_transcript/100527	2.0476886987671	2.0696010572988	2.26318369511934	0.030414812211583	0.614085517275059	-3.59588703280337
a_HQ_transcript/104222	-1.79821924296194	-0.727986254860409	-2.26289701085059	0.0304343744752896	0.614242694574415	-3.61313737443934
a_HQ_transcript/37904	3.43632539468258	1.24774391050617	2.26274377948969	0.0304448350476423	0.614242694574415	-3.60783405122439
a_HQ_transcript/92929	-5.0332766499435	2.44374495156454	-2.26245317578242	0.0304646824279202	0.614242694574415	-3.64635543332519
a_HQ_transcript/82056	2.65193811842107	-0.551735509044621	2.26245049940385	0.030464865270748	0.614242694574415	-3.61116868694433
a_HQ_transcript/27690	-2.19614794734668	3.44431850670494	-2.26214125821868	0.0304859984198547	0.614242741800323	-3.84247368833371
a_HQ_transcript/87387	-2.78770055394635	3.148517972013	-2.26214120941078	0.0304860017563641	0.614242741800323	-3.73111953451852
a_HQ_transcript/91041	2.36860545503538	0.918780917248114	2.26183572761194	0.0305068909299639	0.614450643096654	-3.54411664765536
a_HQ_transcript/22951	-2.75174261484433	0.386380536944902	-2.26165773324075	0.0305190683061984	0.6144829935929	-3.55145928556657
a_HQ_transcript/142529	3.23513201007317	-0.239363159845521	2.26122240533883	0.0305488694064078	0.614796087207703	-3.62734759317786
a_HQ_transcript/2067	-4.33263217817044	1.80356975173372	-2.2611216329462	0.0305557716753209	0.614796087207703	-3.6685626707021
a_HQ_transcript/80046	1.94579438756302	-0.524167998445032	2.26085295407829	0.0305741813208824	0.614879048690573	-3.59826686821087
a_HQ_transcript/318	-2.42743758133182	0.149855571175508	-2.2606664034624	0.0305869694708071	0.614879048690573	-3.56486117470436
a_HQ_transcript/142624	6.44521912794685	2.4786658620406	2.26059070221531	0.0305921602047157	0.614879048690573	-3.75734178665812
a_HQ_transcript/47140	3.44578761014032	2.06939796316537	2.2604442134994	0.0306022069890901	0.614879048690573	-3.56742765535002
a_HQ_transcript/104010	3.87093762988101	1.50458209658351	2.26016944885003	0.0306210594477714	0.615045246572239	-3.56859226837636

a_HQ_transcript/152633	-2.36130368683659	-0.573631729295061	-2.25975811822533	0.0306493016278457	0.615167661371066	-3.61078665093128
a_HQ_transcript/140499	-0.832270046387246	4.74055011144543	-2.25975121529313	0.0306497757864594	0.615167661371066	-3.99864560618953
a_HQ_transcript/86305	-4.47523977237272	3.47461570511698	-2.25961835713816	0.030658903024222	0.615167661371066	-3.82925873582585
a_HQ_transcript/64016	7.71559764580115	3.46384003880643	2.2590034925235	0.0307011754896187	0.61550208796668	-3.72432190465479
a_HQ_transcript/61769	2.98587347735572	0.29561830924877	2.25892260669105	0.0307067403550111	0.61550208796668	-3.61689194049875
a_HQ_transcript/35127	-2.53491392887788	1.7928544725605	-2.25867642378979	0.0307236830721638	0.61550208796668	-3.55956960229838
a_HQ_transcript/137597	-2.36317437524904	-0.266159500365097	-2.25844511694804	0.0307396096459951	0.61550208796668	-3.65198762813141
a_HQ_transcript/120871	-3.46339374299365	1.39031512980528	-2.25838136112643	0.0307440008406064	0.61550208796668	-3.59286856220097
a_HQ_transcript/16058	3.62647563896189	0.425013206643033	2.25829525645239	0.0307499322112217	0.61550208796668	-3.71786669076012
a_HQ_transcript/13294	2.27760490048224	-0.00264673599133258	2.25812226219373	0.0307618521290124	0.61550208796668	-3.61131296661607
a_HQ_transcript/84783	1.13595472425763	2.72831206294589	2.25792465886857	0.0307754727771489	0.61550208796668	-3.65829068302151
a_HQ_transcript/50376	4.89671307431471	0.967773442125067	2.25782800806222	0.030782136817075	0.61550208796668	-3.64829830531861
a_HQ_transcript/120393	-3.75603618667336	0.61359168888991	-2.25780769635776	0.0307835374669484	0.61550208796668	-3.63622727156169
a_HQ_transcript/150773	-2.91890922653515	2.9368849460426	-2.25766496916545	0.0307933812307206	0.61550208796668	-3.77916432521967
a_HQ_transcript/136522	3.16721395726867	2.40691235565888	2.25753083217044	0.0308026351138018	0.61550208796668	-3.7581390997143
a_HQ_transcript/47035	-2.35735212650592	0.660523225015372	-2.25735872695449	0.0308145120185534	0.615527819455146	-3.6453092903999
a_HQ_transcript/66670	3.95151858881362	1.06568092840193	2.25692359819124	0.0308445584113054	0.615560391456084	-3.57740273105437
a_HQ_transcript/135935	-1.85053251620113	1.38901958942482	-2.25673654119022	0.0308574831064218	0.615560391456084	-3.65743649736909
a_HQ_transcript/97069	-4.19684622061693	2.85193281977576	-2.25646510067453	0.0308762469278547	0.615560391456084	-3.82617637781636
a_HQ_transcript/137407	-2.89169917733935	-0.071152452247391	-2.25643663444444	0.0308782153016075	0.615560391456084	-3.67269405282843
a_HQ_transcript/87170	-2.66404776051489	-0.346065657753227	-2.2563909821786	0.0308813722851684	0.615560391456084	-3.66204221616501
a_HQ_transcript/137553	0.620258632762341	4.96026494727655	2.25620021964769	0.0308945671916804	0.615560391456084	-4.00144889074555
a_HQ_transcript/141743	-0.972510802594741	3.35008028098062	-2.25606729921052	0.0309037641955102	0.615560391456084	-3.8248339731488
a_HQ_transcript/123200	-2.20284182058581	-0.386913149537856	-2.25605765940689	0.0309044312864647	0.615560391456084	-3.66755993410183
a_HQ_transcript/54026	4.29771413549281	1.24165586077777	2.25591202272901	0.0309145111683076	0.615560391456084	-3.6532891311958
a_HQ_transcript/27193	3.8188831211139	4.38427231176813	2.25559137867971	0.030936714159585	0.615560391456084	-3.96605267480776
a_HQ_transcript/136509	-2.41201210684703	1.68820608756144	-2.25551498078702	0.0309420064424722	0.615560391456084	-3.55249656602873
a_HQ_transcript/119006	-1.7741048125019	2.11528634084465	-2.25534607943521	0.0309537095691268	0.615560391456084	-3.62345745025705
a_HQ_transcript/107746	-2.60758017805941	1.63983162437805	-2.25480314725832	0.0309913561177734	0.615560391456084	-3.65232543337539
a_HQ_transcript/116647	2.34757366316168	1.76673049441246	2.25467858255305	0.0309999991468522	0.615560391456084	-3.66004264263928
a_HQ_transcript/148802	-2.53964513402809	-0.630701992367902	-2.25462515084001	0.031003707215509	0.615560391456084	-3.63458368468312
a_HQ_transcript/126850	2.22948370355281	-0.404836428906828	2.25461357958876	0.0310045102928411	0.615560391456084	-3.58801982402419
a_HQ_transcript/62618	-2.55091415513784	-0.539030270048903	-2.25460842153458	0.0310048682823057	0.615560391456084	-3.59132890502031
a_HQ_transcript/139118	-2.1761922986746	-0.66977525463138	-2.25458119532293	0.0310067579511322	0.615560391456084	-3.63049454177816
a_HQ_transcript/75711	-2.1047050477116	-0.355481268335414	-2.2538999783057	0.0310540723389901	0.616289217111922	-3.66439070560502
a_HQ_transcript/3700	2.02782738458395	3.19049729116483	2.25372100293369	0.0310665139437284	0.616325707345066	-3.73927704240456
a_HQ_transcript/519	3.03663905998932	1.10982159929043	2.25332756787938	0.0310938796067642	0.616617068106167	-3.56004675235912
a_HQ_transcript/87391	1.57454090378855	4.90738718901139	2.25316528254938	0.0311051737856413	0.616617068106167	-3.97337339015029
a_HQ_transcript/90003	1.40460808832308	4.08204561847904	2.25297924904592	0.0311181252399893	0.616617068106167	-3.88976365749201
a_HQ_transcript/141451	2.73101187481849	-0.554993351203678	2.25290017227175	0.0311236319471424	0.616617068106167	-3.59790906730293
a_HQ_transcript/85340	2.60428166304374	0.654515941809549	2.25243593705306	0.0311559777666643	0.617047589649288	-3.5272535314936
a_HQ_transcript/5625	1.94560575845957	-0.144004583653139	2.25140085354255	0.0312282064170442	0.618267432769055	-3.58650998678007
a_HQ_transcript/103037	3.68022479122643	0.222489750391095	2.25076503089381	0.0312726489397447	0.618699223240769	-3.58543404420811
a_HQ_transcript/44558	3.74575658434077	2.20469086586676	2.25066315955145	0.0312797747842393	0.618699223240769	-3.69672654907171
a_HQ_transcript/148357	4.8459380590544	1.60001816536017	2.25033041631684	0.031303060147705	0.618699223240769	-3.71450015465233
a_HQ_transcript/25486	0.648434562111877	4.12741469041592	2.25029531841457	0.031305517205036	0.618699223240769	-3.86228238580868
a_HQ_transcript/83979	-2.43268923507835	-0.261497470923843	-2.25026348272893	0.0313077460379572	0.618699223240769	-3.6597190391492
a_HQ_transcript/4575	1.01090140265656	3.16881014033055	2.25017590339626	0.031313878247563	0.618699223240769	-3.78395386179246
a_HQ_transcript/5851	-2.82717354216522	0.925001744816603	-2.24985342403229	0.0313364671968185	0.618869187853703	-3.5626787003117
a_HQ_transcript/145818	-3.63609307485753	1.52850596392333	-2.24974915840735	0.0313437738962514	0.618869187853703	-3.68939335431338

a_HQ_transcript/142699	-3.31913874756529	-0.0840297738641648	-2.2493840302241	0.0313693733103608	0.61916432318664	-3.66894145223014
a_HQ_transcript/62474	-4.37105095586292	1.12171576524495	-2.24895691248345	0.0313993426740651	0.619349689274013	-3.69112090739953
a_HQ_transcript/19603	-2.36163867421881	0.676991606432592	-2.24894648629492	0.0314000745645905	0.619349689274013	-3.54648238876444
a_HQ_transcript/77214	3.45163245421374	0.577788729758982	2.24879369887902	0.0314108015885824	0.619351110834843	-3.58868537676052
a_HQ_transcript/137154	-2.05825304881415	7.53283000657261	-2.24819834254612	0.0314526322384918	0.619466667125542	-4.30284929947283
a_HQ_transcript/18372	-1.75784248227989	2.31965987652431	-2.24778704189547	0.0314815600469457	0.619466667125542	-3.72016887043881
a_HQ_transcript/115249	3.04106005323353	-0.0106150559799826	2.24769869178374	0.0314877770468082	0.619466667125542	-3.67698836178704
a_HQ_transcript/142384	4.13244063031859	-0.0901315232971941	2.24761737110027	0.0314935003752929	0.619466667125542	-3.6832755285707
a_HQ_transcript/1995	-1.34006311555537	3.21630088699664	-2.24758679998427	0.0314956522039	0.619466667125542	-3.76165961256504
a_HQ_transcript/139051	-1.80169142986808	2.75069587792049	-2.24744307300448	0.0315057705747886	0.619466667125542	-3.67288837513026
a_HQ_transcript/109745	-2.49260952821533	-0.505735013354522	-2.24735588689796	0.0315119098929533	0.619466667125542	-3.59065298670923
a_HQ_transcript/84219	-2.41305665124724	-0.624794074320455	-2.24735336898406	0.0315120872109421	0.619466667125542	-3.64648248654387
a_HQ_transcript/6195	1.0225456302895	2.85926241210284	2.24683766381756	0.0315484233760749	0.619466667125542	-3.73673050510565
a_HQ_transcript/105615	-3.90925001686085	0.907843877522666	-2.24675856261174	0.0315540001070286	0.619466667125542	-3.68872268650089
a_HQ_transcript/144715	-2.25311763676247	3.87964226607826	-2.24658031171745	0.031566570254089	0.619466667125542	-3.8366177536759
a_HQ_transcript/139237	3.14899355146865	1.04169780419536	2.24638666059736	0.0315802315082886	0.619466667125542	-3.56813977489969
a_HQ_transcript/77047	2.6606250533722	0.842997389260516	2.24635898997062	0.0315821839851996	0.619466667125542	-3.63245050387951
a_HQ_transcript/144370	1.5856512144491	6.04793452237314	2.24630162943262	0.0315862317674786	0.619466667125542	-4.04109751179256
a_HQ_transcript/17562	-1.02827248068285	4.55143620865382	-2.24609904250605	0.0316005315196444	0.619466667125542	-3.98909780660807
a_HQ_transcript/68912	-2.84652854196738	0.946128686444296	-2.24605559876601	0.031603598785354	0.619466667125542	-3.60662734343316
a_HQ_transcript/115000	3.7861493992547	-0.313020116912041	2.24593197280539	0.0316123286316807	0.619466667125542	-3.60262629252843
a_HQ_transcript/80391	-2.93549163260395	2.15440760873981	-2.24588714187611	0.0316154949019812	0.619466667125542	-3.72254178334584
a_HQ_transcript/7576	2.90622550507464	-0.205985008880765	2.24567702337557	0.0316303387163389	0.619466667125542	-3.6764243567987
a_HQ_transcript/43355	-5.95674266469233	1.30767364288065	-2.24551148136588	0.0316420378297283	0.619466667125542	-3.58687869350772
a_HQ_transcript/55048	5.56885982883838	3.29718216237249	2.24540421919024	0.0316496202885371	0.619466667125542	-3.82890569014825
a_HQ_transcript/46924	2.85564918557551	0.323301407970013	2.24532058258955	0.0316555337655413	0.619466667125542	-3.69410037011767
a_HQ_transcript/61573	3.54505001114448	1.79300952258823	2.24523237160749	0.0316617717456301	0.619466667125542	-3.69765966109061
a_HQ_transcript/119525	2.35251367514942	-0.21290341850231	2.2447589082813	0.0316952723089884	0.619627151677747	-3.59384673738516
a_HQ_transcript/105373	3.75748240951651	0.71828720734364	2.2446861642262	0.0317004222343216	0.619627151677747	-3.65818880970175
a_HQ_transcript/111525	3.57848637755256	1.41974764100528	2.24463452068812	0.0317040788013211	0.619627151677747	-3.57720543667259
a_HQ_transcript/93815	-3.21846836408772	1.30228110859018	-2.24441050158104	0.0317199446223763	0.619627151677747	-3.64725069653322
a_HQ_transcript/141838	-3.66367794386089	1.67144839072512	-2.24433300947134	0.0317254345440937	0.619627151677747	-3.61510710406435
a_HQ_transcript/138386	-3.49715632231543	1.00944987500597	-2.24421308112727	0.0317339325375835	0.619627151677747	-3.66747333631949
a_HQ_transcript/135126	-1.19487510796489	2.25103253492732	-2.24383869626604	0.0317604741856394	0.619782587668752	-3.68369614244631
a_HQ_transcript/93851	-2.31559535707011	-0.449354000192066	-2.24354497638432	0.0317813111077445	0.619782587668752	-3.64166891575448
a_HQ_transcript/44494	-1.97750084608623	3.18193341189943	-2.2435343533102	0.0317820649540419	0.619782587668752	-3.74563389105595
a_HQ_transcript/67345	0.792246189065962	4.52131555282536	2.24330678665643	0.0317982176425737	0.619782587668752	-3.98138761522702
a_HQ_transcript/8670	2.71512132648791	-0.381637009202384	2.24312229558653	0.0318113182295117	0.619782587668752	-3.6432507051364
a_HQ_transcript/3055	-2.54643097287021	0.521172007648386	-2.24300424032383	0.0318197037944975	0.619782587668752	-3.58779641272468
a_HQ_transcript/2936	1.36950447749421	4.2325567466441	2.24291668547943	0.0318259241695525	0.619782587668752	-3.95347683689349
a_HQ_transcript/61170	3.07664768673753	0.997264016943635	2.24287142890049	0.0318291398720724	0.619782587668752	-3.59606281623865
a_HQ_transcript/69880	-2.17305218261116	-0.312790799751123	-2.24274483303894	0.0318381366786523	0.619782587668752	-3.65171758163057
a_HQ_transcript/100505	-4.79713202102702	2.16175000072361	-2.24254493737839	0.0318523473374284	0.619782587668752	-3.66262831228046
a_HQ_transcript/126997	-2.67805137351818	0.829641457552925	-2.24244886394201	0.0318591792587777	0.619782587668752	-3.62119890773083
a_HQ_transcript/49584	3.06972881358257	-0.246798880002253	2.24219040404454	0.0318775652416088	0.619932791021825	-3.65532860164929
a_HQ_transcript/13611	-2.47225321149517	-0.479492621035706	-2.2417033171626	0.0319122408628152	0.620399577549739	-3.64762289785387
a_HQ_transcript/134316	2.22378821926226	-0.714440618837706	2.24133434652839	0.0319385303268655	0.620703072831841	-3.64303573205704
a_HQ_transcript/132616	3.11590695031283	1.01848843849149	2.24019179797231	0.0320200611037855	0.622079582834507	-3.58192183884539
a_HQ_transcript/136246	2.6764695573992	-0.158429483350039	2.23984145200155	0.0320450986999926	0.622327529257483	-3.65500707890644
a_HQ_transcript/1064	1.14219370981102	3.0988927190838	2.23971365859531	0.0320542358690632	0.622327529257483	-3.8401555059784

a_HQ_transcript/98756	-1.39576882418643	-0.638095651479996	-2.2395142805431	0.0320684959729188	0.62237845052664	-3.66934190082854
a_HQ_transcript/45853	2.68142123538156	1.75885869695841	2.23937763233904	0.0320782727390898	0.62237845052664	-3.6588814115371
a_HQ_transcript/95126	-2.69688811617906	1.14480404914649	-2.23906141233917	0.0321009075745478	0.622400131263828	-3.590933203005
a_HQ_transcript/43920	1.42729132572762	2.0627101959258	2.2390353895833	0.0321027709052156	0.622400131263828	-3.61017112936168
a_HQ_transcript/15297	-1.21356861642822	1.79840446708659	-2.23891332617582	0.0321115124150189	0.622400131263828	-3.63721507304504
a_HQ_transcript/131847	1.72987313967347	4.991895179529	2.23854692451179	0.0321377649070146	0.622701332838315	-3.99672087036582
a_HQ_transcript/103674	3.17479048901059	-0.317513675834476	2.23822422329241	0.0321609022208135	0.622941994099116	-3.65484921690067
a_HQ_transcript/136997	-3.03248598101502	1.27324887205253	-2.23796765342959	0.0321793086474997	0.623090890427003	-3.59732627823973
a_HQ_transcript/114731	-6.55595567540267	1.72504151883642	-2.2377144821912	0.032197480513074	0.623120126990149	-3.70905554632707
a_HQ_transcript/75475	0.82940047292829	3.12115891307962	2.23764794127326	0.0322022581454446	0.623120126990149	-3.79590475128971
a_HQ_transcript/133467	0.570545294326503	3.76623892788093	2.2373306640433	0.0322250473657717	0.623353595100692	-3.90441656701453
a_HQ_transcript/58695	-3.29142936030472	1.87374141647946	-2.23680322818318	0.032262963746845	0.623879426705458	-3.6891846474739
a_HQ_transcript/138270	-2.92554688403595	-0.352891154705707	-2.23640277470929	0.0322917783061099	0.624070071455156	-3.63113841091148
a_HQ_transcript/115858	-2.01006188483327	0.783595866918316	-2.23635338137664	0.0322953339924571	0.624070071455156	-3.63312663686528
a_HQ_transcript/152190	7.58535084489563	1.47826121616819	2.23621870076279	0.0323050310523081	0.624070071455156	-3.79712134115479
a_HQ_transcript/106508	-2.71248204257277	1.74385298422196	-2.23602357158189	0.0323190850642148	0.624134145054046	-3.66593224992426
a_HQ_transcript/121335	8.51092500058802	1.53215400098661	2.23559289269617	0.0323501237413906	0.624526068694637	-3.64797956042021
a_HQ_transcript/66819	1.44598269790758	2.79061478924892	2.23474768503856	0.0324111148544914	0.625214383703104	-3.69497209959172
a_HQ_transcript/44880	2.67340889715225	-0.00829268312730107	2.2345973534587	0.0324219737361885	0.625214383703104	-3.6996023024552
a_HQ_transcript/17811	3.18983429265741	0.90136592012086	2.23451272278163	0.0324280882874777	0.625214383703104	-3.61658972473126
a_HQ_transcript/99839	2.66506054471402	-0.370617997978791	2.2345028538367	0.0324288013842702	0.625214383703104	-3.6420497803228
a_HQ_transcript/111749	-4.48075751879694	-0.12901935670224	-2.23389109373147	0.0324730325611242	0.625745975718701	-3.71956399466489
a_HQ_transcript/114251	2.84293570998954	0.312733584526554	2.23354598000087	0.0324980086510898	0.625745975718701	-3.68132296815824
a_HQ_transcript/57638	-2.67061393108133	0.368343022332242	-2.23352945189148	0.03249920523227	0.625745975718701	-3.61252247206575
a_HQ_transcript/130951	-4.60091302225965	3.12626873048155	-2.2333831693498	0.0325097973322017	0.625745975718701	-3.73130237296543
a_HQ_transcript/65098	2.14066865936156	1.74643742622342	2.23332175013007	0.0325142455280989	0.625745975718701	-3.66459390606757
a_HQ_transcript/79498	2.65432798863643	1.01572218027843	2.23322899861566	0.0325209639527628	0.625745975718701	-3.56866449230218
a_HQ_transcript/141178	2.51212805546878	0.321459417479086	2.23283729223369	0.0325493507957549	0.625875218653178	-3.61818705076062
a_HQ_transcript/152063	-1.72235021726979	3.20004437510535	-2.23273355760236	0.0325568721310256	0.625875218653178	-3.85281684015835
a_HQ_transcript/112944	-2.95008792614692	0.089381059110321	-2.23269066588337	0.0325599824732867	0.625875218653178	-3.6638311456971
a_HQ_transcript/28679	2.38146975629684	2.69289333627166	2.23209542670323	0.0326031744236324	0.626477615358879	-3.77351797444984
a_HQ_transcript/131669	4.46631615008778	4.00865944906187	2.23182413337773	0.032622877130284	0.626477615358879	-3.87331519890526
a_HQ_transcript/57745	2.11846066596077	1.47332719922873	2.23112971223153	0.0326733581539451	0.626477615358879	-3.56867090091452
a_HQ_transcript/95474	-1.44848238090452	1.70362264475859	-2.231083727604	0.0326767034811765	0.626477615358879	-3.61146456953704
a_HQ_transcript/74687	2.8940615472455	-0.172137230808596	2.23102530144094	0.0326809543580623	0.626477615358879	-3.63850510079833
a_HQ_transcript/42811	2.42450230222296	1.59294254660272	2.23095636705783	0.0326859704122823	0.626477615358879	-3.60911876330273
a_HQ_transcript/120122	3.54944136115243	0.440712041156907	2.23092120968633	0.0326885289263825	0.626477615358879	-3.67264447405664
a_HQ_transcript/137410	1.37393645465716	2.03573885380575	2.23086829790668	0.0326923798236488	0.626477615358879	-3.6501753616018
a_HQ_transcript/15937	-3.37619392407045	1.91440067693248	-2.2307599750388	0.0327002647846762	0.626477615358879	-3.74916124304743
a_HQ_transcript/114704	-1.7245293753742	1.77227629978347	-2.2307530424318	0.0327007694760746	0.626477615358879	-3.6226175491293
a_HQ_transcript/138545	4.23778947700295	2.52794904856781	2.23062799456479	0.0327098741159888	0.626477615358879	-3.80702678835662
a_HQ_transcript/139522	-2.85831407970773	0.99740581774405	-2.23047147172322	0.0327212736253734	0.626489523483433	-3.66455358405993
a_HQ_transcript/120666	-2.32795573912095	-0.465399648182035	-2.22971763916349	0.0327762248788832	0.627335001567244	-3.66688104004431
a_HQ_transcript/123827	2.94619259606736	0.541074190247341	2.22956390043384	0.0327874419363641	0.627343128662597	-3.57995146637766
a_HQ_transcript/18971	-2.80435273311962	1.04480303780884	-2.22900149816955	0.0328285051356306	0.627611451603588	-3.61818568251645
a_HQ_transcript/15082	2.90241348246901	0.279114414163112	2.22898015588423	0.0328300643266501	0.627611451603588	-3.64630591114899
a_HQ_transcript/99412	3.34443898764305	0.702822439477692	2.2289282489892	0.0328338567355924	0.627611451603588	-3.59541950465719
a_HQ_transcript/44455	-2.06281184553605	1.1392675259475	-2.22857289674453	0.0328598299359351	0.627901444614081	-3.60056551735246
a_HQ_transcript/5145	-2.33351861582021	1.56114573819308	-2.22828168158709	0.0328811289853403	0.628101960453455	-3.60507089253883
a_HQ_transcript/140829	1.93322211484799	3.22693381476949	2.22805386829982	0.0328977995348894	0.628198409650717	-3.85015385353737

a_HQ_transcript/51344	-3.39437933862959	1.47794272141203	-2.22791735905297	0.0329077924130614	0.628198409650717	-3.61883676761357
a_HQ_transcript/77833	-2.16351949440447	-0.668975732492567	-2.22764605528661	0.0329276607179413	0.628221996706063	-3.67936004724919
a_HQ_transcript/147117	-3.50771628742659	0.641990392980446	-2.22760534277372	0.0329306431317674	0.628221996706063	-3.66306397235562
a_HQ_transcript/20684	-2.52947502689256	0.234461246884105	-2.22617285759504	0.0330357346338704	0.629877353787838	-3.71782725932615
a_HQ_transcript/122414	-4.0034511668564	2.20465888305685	-2.2261272286423	0.0330390870440944	0.629877353787838	-3.66169478626067
a_HQ_transcript/18269	3.11530236329329	0.773386427104649	2.22547648319499	0.0330869312071194	0.630582667936864	-3.61664581530992
a_HQ_transcript/149503	-3.36190302761392	0.836656310818502	-2.22517297930389	0.0331092666551907	0.630595360040454	-3.77607499270164
a_HQ_transcript/45225	-2.16287537932475	1.34762424010829	-2.22444091040342	0.0331631966131888	0.630595360040454	-3.58882141571654
a_HQ_transcript/139588	-0.892362195370681	4.94621415653191	-2.22436818467362	0.033168558455669	0.630595360040454	-4.06965779670701
a_HQ_transcript/19144	1.29478372553468	3.03294385761118	2.22420587560559	0.033180527799231	0.630595360040454	-3.84400738328974
a_HQ_transcript/82146	3.27474483783029	0.442506496824395	2.22418436365178	0.0331821144701084	0.630595360040454	-3.63864251213666
a_HQ_transcript/149463	1.23223991607553	4.3552118891031	2.22386552022808	0.0332056395744025	0.630595360040454	-3.96817249301467
a_HQ_transcript/125653	-1.8890847734088	-0.379434266351144	-2.22372316948933	0.0332161474063415	0.630595360040454	-3.6754278272832
a_HQ_transcript/136975	-2.39519740945039	3.57891992347847	-2.22354719820386	0.0332291411038833	0.630595360040454	-3.91886926994091
a_HQ_transcript/151617	-0.597817080835637	5.25600235339034	-2.2231370763811	0.03325944211198	0.630595360040454	-4.08630832165055
a_HQ_transcript/123939	-2.94949467088964	1.15650551356615	-2.22298314012701	0.0332708217593232	0.630595360040454	-3.6705129688361
a_HQ_transcript/6539	-4.04980794155372	1.05241142837153	-2.22295593828159	0.0332728330021652	0.630595360040454	-3.69773532035917
a_HQ_transcript/43189	-2.60796352860964	1.23790442874233	-2.22286616201923	0.0332794716281228	0.630595360040454	-3.60166956643455
a_HQ_transcript/72441	3.40626565975058	-0.376839574521693	2.22282272561053	0.0332826840171775	0.630595360040454	-3.70223348034641
a_HQ_transcript/56355	1.36326615758912	3.05588259649955	2.22278574007463	0.0332854195434123	0.630595360040454	-3.81273240654723
a_HQ_transcript/1215	-3.37363071240527	1.79853917173315	-2.22269750560099	0.0332919463592877	0.630595360040454	-3.66829303211839
a_HQ_transcript/131450	-4.05356712891688	-0.46607779916297	-2.22261997467597	0.0332976823657757	0.630595360040454	-3.68381587583797
a_HQ_transcript/103851	-4.50830813739139	0.940526436434151	-2.22252805196203	0.0333044842730025	0.630595360040454	-3.69717583936035
a_HQ_transcript/145489	3.49177561625868	5.75213802014804	2.22250120745118	0.0333064708923575	0.630595360040454	-4.18385087548124
a_HQ_transcript/117683	2.2909025549431	2.26518723005924	2.22229978202456	0.0333213807007024	0.630595360040454	-3.71310433878324
a_HQ_transcript/113001	2.99932185580855	1.00855828816017	2.22211604045195	0.0333349867348961	0.630595360040454	-3.73790540727912
a_HQ_transcript/67322	2.55698658116615	-0.0916279981609078	2.22210788325991	0.0333355908889505	0.630595360040454	-3.65084970337687
a_HQ_transcript/152835	-2.6855893168179	-0.300341091892895	-2.22201878083451	0.0333421908069401	0.630595360040454	-3.69440676388234
a_HQ_transcript/70783	2.23365035068995	1.31855075470162	2.22195130214465	0.0333471898085754	0.630595360040454	-3.60905338105464
a_HQ_transcript/125606	1.97596405066866	-0.0217864854050835	2.2219305954417	0.033348723950981	0.630595360040454	-3.68607395770372
a_HQ_transcript/149426	2.67507906247924	0.162303922932735	2.22179452350015	0.0333588069798444	0.630595360040454	-3.67389493820666
a_HQ_transcript/45630	-5.43363677999073	1.13658944022223	-2.22151453571782	0.0333795628614274	0.630782584528301	-3.7542017755679
a_HQ_transcript/108611	3.23524350740813	1.81845873934183	2.22126586079272	0.0333980071699517	0.630926019101382	-3.76278638284355
a_HQ_transcript/23918	2.42874912411289	0.0658721903220403	2.22111197101413	0.0334094257991829	0.630936680589638	-3.65369539374129
a_HQ_transcript/53307	4.16390608978284	1.52339109018395	2.22086676274857	0.0334276274849473	0.630978606561336	-3.64715710275959
a_HQ_transcript/84461	3.47130500961631	0.571948507116084	2.22067811318335	0.0334416368875665	0.630978606561336	-3.69135007417356
a_HQ_transcript/27917	-1.12158838977615	2.14599500206448	-2.2205053008882	0.0334544748022107	0.630978606561336	-3.72669328357113
a_HQ_transcript/148088	4.18494677589389	-0.603292306337257	2.2203788694231	0.033463869962365	0.630978606561336	-3.71021668715676
a_HQ_transcript/100176	2.83021940686342	-0.238089185790301	2.22035127540618	0.0334659207959778	0.630978606561336	-3.68921800284618
a_HQ_transcript/23249	2.67921402385551	0.114806900131452	2.22020043422393	0.0334771335539555	0.630979996435476	-3.65075962049713
a_HQ_transcript/114457	-2.71339859523153	-0.46976549559084	-2.22005826808771	0.0334877045314383	0.630979996435476	-3.69105244728249
a_HQ_transcript/130547	2.20463713614292	-0.37698030115995	2.21960480553277	0.0335214423884745	0.63122003094613	-3.71922502341516
a_HQ_transcript/83984	-2.57179917120323	0.599886581688963	-2.21936560665571	0.0335392511656908	0.63122003094613	-3.66374455703041
a_HQ_transcript/94129	3.20754416826761	0.855670155471819	2.21919991620426	0.0335515920747962	0.63122003094613	-3.70571468141297
a_HQ_transcript/90554	-6.17788005342583	1.65125830338042	-2.21917156921506	0.03355370381424	0.63122003094613	-3.77363614267848
a_HQ_transcript/62513	-2.46005537481834	-0.350444214793393	-2.21915766771327	0.0335547394650348	0.63122003094613	-3.69508665358761
a_HQ_transcript/82858	3.2490416388494	-0.208674211160044	2.2189150347828	0.0335728200323725	0.631355833983095	-3.66701873715684
a_HQ_transcript/52802	-2.47739736013654	0.431673465957053	-2.21819350224313	0.0336266388577771	0.632147772803823	-3.6323389843703
a_HQ_transcript/9555	-1.89232221069505	2.42082724554466	-2.21782042844968	0.0336544965517322	0.632147772803823	-3.69851375119968
a_HQ_transcript/142701	7.53375120009354	3.97941272837811	2.21767338214138	0.0336654822801321	0.632147772803823	-4.02849260398923

a_HQ_transcript/120098	2.40432042571382	2.31893137151174	2.21762947640931	0.0336687630691556	0.632147772803823	-3.6530193671816
a_HQ_transcript/152028	2.15259621043425	-0.675996445669783	2.21762219124384	0.0336693074697329	0.632147772803823	-3.69854657277096
a_HQ_transcript/11661	-1.46546265630244	2.50720070666002	-2.21742928573603	0.0336837256388962	0.632214273147484	-3.73747461034248
a_HQ_transcript/145654	-4.60542182506125	3.83077085666528	-2.2167507387649	0.0337344855908421	0.632533294897217	-4.00168435609141
a_HQ_transcript/130661	3.42304500697049	1.38536483379135	2.21670029598645	0.0337382617871109	0.632533294897217	-3.66074113281259
a_HQ_transcript/136185	1.91550641391586	-0.648220031216103	2.21667546206084	0.0337401210182629	0.632533294897217	-3.69703945648662
a_HQ_transcript/101080	-2.37875153941286	-0.329782383218423	-2.21658290038991	0.0337470516018902	0.632533294897217	-3.64603178790621
a_HQ_transcript/5608	2.60208795866584	0.70388442036252	2.2164750111788	0.0337551314473432	0.632533294897217	-3.67696416365705
a_HQ_transcript/54942	-2.160952551721	1.39270748940955	-2.21584100521507	0.0338026472649429	0.633219555338899	-3.65236783459344
a_HQ_transcript/127306	-4.69246220275446	-0.326706226869007	-2.2155652172631	0.0338233349672851	0.633402968743025	-3.71982070193035
a_HQ_transcript/111237	-2.43608059841943	-0.0606050779370009	-2.21525935285475	0.0338462920435675	0.633628748440738	-3.66707012332127
a_HQ_transcript/24127	0.966905983741055	3.69834104606649	2.2150642228998	0.0338609450742529	0.633698974654274	-3.98147218672517
a_HQ_transcript/10069	2.38249088513628	3.65630109042618	2.21490472686869	0.0338729264364269	0.633719172158552	-3.92902595751061
a_HQ_transcript/3650	1.99858779337826	2.10286951231202	2.21424321793944	0.0339226595640209	0.634269371020804	-3.68552756022102
a_HQ_transcript/53886	1.67605777967916	1.75430958591772	2.21422329626943	0.0339241583144729	0.634269371020804	-3.69143571375991
a_HQ_transcript/113995	0.906606113612105	4.19716829686885	2.21399522616637	0.033941320744116	0.634355938459753	-4.01233254018419
a_HQ_transcript/15182	1.36655018804205	3.21060751524987	2.21365881408192	0.0339666501578421	0.634355938459753	-3.8840550332868
a_HQ_transcript/51544	-4.3282159815879	0.302887301080408	-2.21356081693871	0.0339740318181075	0.634355938459753	-3.67814392875541
a_HQ_transcript/68832	-1.32897933246993	1.99584605828898	-2.21334368349312	0.0339903925631971	0.634355938459753	-3.65963381521175
a_HQ_transcript/109336	-1.51480125763832	2.76826242249826	-2.213315722923	0.0339924998707369	0.634355938459753	-3.83448963330966
a_HQ_transcript/76589	-1.62630501199549	2.74783026480027	-2.21308100765987	0.0340101942913823	0.634355938459753	-3.74722396382759
a_HQ_transcript/102073	1.45201803966546	3.39528607123582	2.21307852833152	0.0340103812438471	0.634355938459753	-3.89049316056094
a_HQ_transcript/149713	-3.71919102073574	-0.60721086068847	-2.21300278454108	0.0340160931079523	0.634355938459753	-3.71463891771516
a_HQ_transcript/84654	-3.21678088752409	-0.56705604800118	-2.2119521206338	0.0340954125559799	0.635631219067991	-3.70175603657897
a_HQ_transcript/70670	-2.64316656248779	-0.45886762193096	-2.21162945997265	0.0341198048692895	0.635857764357401	-3.71411040787994
a_HQ_transcript/143775	-3.39045002408038	0.815665745966376	-2.21141221751597	0.0341362366277083	0.635857764357401	-3.68706512667871
a_HQ_transcript/107809	-1.94431449578929	1.24488369516838	-2.21085323038132	0.0341785497632186	0.635857764357401	-3.68355570954232
a_HQ_transcript/27553	4.55219989667282	1.53619183392965	2.21081583686732	0.0341813819785914	0.635857764357401	-3.63721381611884
a_HQ_transcript/78122	2.15690478112354	2.64760205786475	2.21050739959763	0.0342047512756505	0.635857764357401	-3.9022631513928
a_HQ_transcript/140806	1.62703267502377	-0.723943832181478	2.21049575128228	0.0342056341108189	0.635857764357401	-3.70711211664313
a_HQ_transcript/107050	1.63733048755661	2.58433423009994	2.2101583741803	0.0342312130347565	0.635857764357401	-3.73045197246507
a_HQ_transcript/131619	-1.81804781987303	-0.472666100034499	-2.21013694979498	0.0342328379450216	0.635857764357401	-3.70872107650284
a_HQ_transcript/91571	4.24491937243354	2.23753223714663	2.21012689776882	0.0342336003542811	0.635857764357401	-3.86442460096667
a_HQ_transcript/25745	4.2285371682734	0.708571749186362	2.21012440279087	0.0342337895915452	0.635857764357401	-3.68515449112717
a_HQ_transcript/88555	-3.58182584806434	1.1749715205589	-2.20999258667829	0.0342437888138995	0.635857764357401	-3.72732028243549
a_HQ_transcript/146519	0.469914110309635	10.202290870328	2.20994670746012	0.034247269702865	0.635857764357401	-4.6106171102734
a_HQ_transcript/107376	2.24544555468065	1.39910619044551	2.20958128188906	0.0342750060982412	0.635857764357401	-3.61887045218902
a_HQ_transcript/88587	-2.55867325889534	0.144954596197415	-2.2092173157398	0.0343026516832351	0.635857764357401	-3.69727725866274
a_HQ_transcript/104420	-5.19568999608534	3.95059770590013	-2.20904463403672	0.0343157749479979	0.635857764357401	-4.19375699585832
a_HQ_transcript/148893	2.97760027548301	1.0967310697741	2.2090281712904	0.0343170262991218	0.635857764357401	-3.70721298345065
a_HQ_transcript/105434	3.57111451878665	-0.193752266051229	2.20897787273875	0.0343208497990785	0.635857764357401	-3.76270247693965
a_HQ_transcript/45990	3.20451399468275	2.21397948368985	2.20893709218736	0.0343239500573004	0.635857764357401	-3.63320531630983
a_HQ_transcript/107287	1.6467110816187	1.2712172105889	2.20893514334566	0.0343240982202825	0.635857764357401	-3.64948949312886
a_HQ_transcript/103797	-2.51667571712954	-0.414048171016027	-2.20882150294146	0.0343327388545122	0.635857764357401	-3.71307932170774
a_HQ_transcript/53969	-3.55681640305815	1.58433047577263	-2.20848546286692	0.0343583009987281	0.635857764357401	-3.69804691942694
a_HQ_transcript/6809	3.51410787582698	1.06594448147422	2.20844837210446	0.034361123488575	0.635857764357401	-3.70825244221292
a_HQ_transcript/16704	2.96113842703087	0.686303078107872	2.20817040511645	0.0343822824965659	0.635857764357401	-3.67418670259144
a_HQ_transcript/65936	-2.06429081151427	0.521685055896909	-2.20799201370244	0.0343958678897289	0.635857764357401	-3.61163314193388
a_HQ_transcript/50678	-3.20332804223219	1.28333368102803	-2.20785566577369	0.0344062546977104	0.635857764357401	-3.77413402405833
a_HQ_transcript/115548	-3.70802501196129	2.17349317946957	-2.20780970132545	0.0344097568415101	0.635857764357401	-3.76069293443123

a_HQ_transcript/118808	2.59064112572902	-0.263673404127113	2.20776933357015	0.034412832822398	0.635857764357401	-3.66652740873929
a_HQ_transcript/105100	3.94852898747922	1.13554372239948	2.20766448260904	0.0344208235047622	0.635857764357401	-3.78019901069761
a_HQ_transcript/100118	2.50518233569303	0.251315893553101	2.20761203122816	0.0344248214421544	0.635857764357401	-3.63760946266417
a_HQ_transcript/3105	3.15748683941706	0.698495002998386	2.20740875801636	0.0344403192090803	0.635857764357401	-3.69172098731893
a_HQ_transcript/149709	-3.4595055102813	-0.481933624815232	-2.20732546788319	0.034446671138891	0.635857764357401	-3.70081264036203
a_HQ_transcript/150912	-9.867053821842	1.40775866311219	-2.2071133200382	0.0344628548355202	0.635903816887	-3.7255130402436
a_HQ_transcript/151513	3.74019275724469	-0.374873167589601	2.20659769009788	0.0345022179985299	0.635903816887	-3.72759818440442
a_HQ_transcript/56764	1.01382591704595	5.83799959428579	2.20656597230751	0.0345046406445052	0.635903816887	-4.14865449294929
a_HQ_transcript/44406	-1.87981173029193	2.58859363094468	-2.20648181827189	0.0345110691758074	0.635903816887	-3.79838808059458
a_HQ_transcript/133082	2.07306668816553	-0.646877898782514	2.20647453802437	0.0345116253646146	0.635903816887	-3.71784801143937
a_HQ_transcript/8476	-1.08309024105228	3.41876051161205	-2.20635955332286	0.0345204109093109	0.635903816887	-3.93704696890597
a_HQ_transcript/117866	3.56819075396267	0.712592719716804	2.20628976715291	0.0345257439804461	0.635903816887	-3.70919412881913
a_HQ_transcript/31027	2.78732920337934	0.204735609539924	2.20585394134646	0.0345590665266859	0.636315938886031	-3.67106430036502
a_HQ_transcript/7172	2.45746103388257	3.23303512497091	2.2053036924976	0.0346011787291036	0.63688958744944	-3.94596104054154
a_HQ_transcript/78553	3.67342982193204	5.04550120020124	2.20469253637994	0.0346480060996889	0.637523141008727	-4.14774211293972
a_HQ_transcript/151438	0.711232187491436	5.62455495411025	2.20456827808639	0.0346575338148152	0.637523141008727	-4.16668843212874
a_HQ_transcript/74486	1.22880411732176	2.58014426557693	2.20424185584579	0.0346825739477616	0.637781922947007	-3.79507348812956
a_HQ_transcript/140073	-0.562119957048397	3.83455004048985	-2.20397294340421	0.0347032145979872	0.637958033834796	-3.97957608762403
a_HQ_transcript/120867	0.96826716857257	4.2492516449248	2.20376654731311	0.0347190641863701	0.637958033834796	-4.03095493064277
a_HQ_transcript/83059	-2.56050556597362	0.981810032769953	-2.203688282159	0.0347250760227996	0.637958033834796	-3.72741949989972
a_HQ_transcript/135580	-2.89394332481855	0.513988339757563	-2.20340438410665	0.0347468910913318	0.637978944020479	-3.66843781744428
a_HQ_transcript/128544	3.69322914055671	-0.493462618384114	2.2033878102021	0.0347481650283656	0.637978944020479	-3.72622182210732
a_HQ_transcript/39460	3.55367954197895	2.1887310601244	2.20305170631149	0.0347740083326457	0.638251833394388	-3.90676277135485
a_HQ_transcript/137648	-3.9071828493966	2.47048387515265	-2.20262043250292	0.0348071944853776	0.638470805934851	-3.75194442393925
a_HQ_transcript/68413	3.37662091073702	0.556677424698185	2.20254134237295	0.0348132834731221	0.638470805934851	-3.71027601875986
a_HQ_transcript/105189	-3.0251979805825	-0.0740190948791222	-2.20204451074191	0.0348515552978012	0.638470805934851	-3.69068762204034
a_HQ_transcript/145624	-0.887542633544491	4.46879432151069	-2.20204299289638	0.034851672277715	0.638470805934851	-4.06864343345521
a_HQ_transcript/40482	2.75667322306911	1.33911619806352	2.20183128212889	0.0348679922021504	0.638470805934851	-3.71286602482772
a_HQ_transcript/120309	-3.00348695913252	1.8219116315657	-2.20174265027023	0.0348748264998455	0.638470805934851	-3.6940398322224
a_HQ_transcript/68348	2.82267494918416	-0.369272070889632	2.20155124031061	0.0348895899783135	0.638470805934851	-3.76565086125352
a_HQ_transcript/59762	-2.28172625195956	0.128022035228718	-2.20147130374326	0.034895757150119	0.638470805934851	-3.65559405669131
a_HQ_transcript/100597	-1.16385337971414	4.37862975549881	-2.20125593801369	0.0349123776396743	0.638470805934851	-3.9286835589354
a_HQ_transcript/122658	-2.58371682028775	-0.034859454785209	-2.20123904615998	0.0349136815392351	0.638470805934851	-3.66957507520409
a_HQ_transcript/26395	-1.80688727250791	1.39073466467913	-2.2011996263245	0.0349167245660982	0.638470805934851	-3.6555218743889
a_HQ_transcript/126386	-1.78278165141592	-0.560054155019834	-2.20118640576883	0.034917745184195	0.638470805934851	-3.74268903148372
a_HQ_transcript/59695	-2.73302119724062	1.29471301871918	-2.20038136460216	0.0349799440687302	0.6394069776186	-3.65174993839247
a_HQ_transcript/150186	-3.06881839242289	0.459723098532325	-2.2000078582338	0.0350088354181495	0.639573777599822	-3.67842619932828
a_HQ_transcript/67052	-1.56089435738558	1.92013515961653	-2.19992659754337	0.0350151238923641	0.639573777599822	-3.7146718070571
a_HQ_transcript/147714	-1.75002727012571	5.13174724477326	-2.19983675413606	0.035022077726745	0.639573777599822	-4.06135598484028
a_HQ_transcript/105440	4.86925863260814	4.00148518036877	2.1996671920497	0.0350352051075749	0.639579702920072	-3.91958014570544
a_HQ_transcript/86973	3.47356518406046	0.759841478660926	2.19954835389104	0.0350444080959336	0.639579702920072	-3.74554672563251
a_HQ_transcript/83444	-2.49362870706114	-0.425142376172006	-2.19929012311778	0.0350644132814982	0.639687783615054	-3.7257532126785
a_HQ_transcript/74125	2.72988386123097	0.205759475839591	2.1990325887247	0.035084374676351	0.639687783615054	-3.69953242068838
a_HQ_transcript/88955	0.873590378309547	5.33291513232428	2.19883164561404	0.0350999567502413	0.639687783615054	-4.14534502478321
a_HQ_transcript/106163	4.20164583146442	3.95770543697506	2.19874202172458	0.0351069086010769	0.639687783615054	-3.94109007018348
a_HQ_transcript/94969	-2.57391852080418	-0.401967017898982	-2.19859284142536	0.0351184827873134	0.639687783615054	-3.72082879186106
a_HQ_transcript/103347	2.78913074652652	0.508075970634319	2.19853371001191	0.035123071454029	0.639687783615054	-3.72932647547453
a_HQ_transcript/23547	-3.2566701790361	1.08238361911267	-2.1984784028353	0.035127363840133	0.639687783615054	-3.67907542109228
a_HQ_transcript/71225	3.35862086648832	1.77907493187251	2.1977598403131	0.0351831740249361	0.640503457476194	-3.72604297745715
a_HQ_transcript/8835	-2.31543779205251	-0.299541727958256	-2.19727691549442	0.0352207268935863	0.640986353434685	-3.70781009969283

a_HQ_transcript/147470	0.558160570188884	6.39395028697279	2.19708250671617	0.0352358544775628	0.641060954326062	-4.23760627890294
a_HQ_transcript/116645	-0.712153413061035	5.07335621431316	-2.19654060306852	0.0352780524064091	0.641460749525434	-4.15176666499126
a_HQ_transcript/118784	-2.79155758554853	0.0765927142367714	-2.19651689201565	0.0352798998113269	0.641460749525434	-3.72228775553638
a_HQ_transcript/12532	-3.01314262147221	0.539527213086485	-2.19597890133565	0.0353218396291151	0.641978590252385	-3.63413429795468
a_HQ_transcript/124450	2.82455249144111	-0.269641091465026	2.19575357951027	0.0353394181301418	0.641978590252385	-3.73391167758579
a_HQ_transcript/61340	2.08217235524664	0.605426099159833	2.19572672940952	0.0353415133637426	0.641978590252385	-3.67548108892428
a_HQ_transcript/143956	-0.529105600765065	7.3060084648285	-2.19538894566528	0.0353678816178507	0.642256864318159	-4.31680137611334
a_HQ_transcript/34292	-8.05153584263761	3.13604226451909	-2.19511671893951	0.035389145105513	0.642442294407639	-3.80729215751724
a_HQ_transcript/109106	-3.037750454129	3.62931139784078	-2.19491409580364	0.0354049793012427	0.642529077996451	-3.87881212183441
a_HQ_transcript/20701	-2.98050185876429	1.83245252103779	-2.19455292814066	0.0354332188092244	0.642647047077251	-3.77936307968648
a_HQ_transcript/72445	2.94001326339918	0.0739193969753997	2.19454816793164	0.0354335911416631	0.642647047077251	-3.70383928617404
a_HQ_transcript/98753	4.4320924797877	1.88744644669972	2.19376259904118	0.0354950843767586	0.643531616372638	-3.85072078054843
a_HQ_transcript/105718	-0.645157402243722	4.08410180254148	-2.19364235306981	0.0355045054656456	0.643531616372638	-4.03668511955457
a_HQ_transcript/41806	-1.64871174384335	0.0458510919377994	-2.19345697542417	0.0355190338951946	0.643594265043602	-3.67446415462128
a_HQ_transcript/84669	2.31381583588916	0.773911995894484	2.19239910211917	0.0356020432061326	0.644725854846338	-3.66372745848343
a_HQ_transcript/125132	2.65322729731258	0.744607309338867	2.19237842192985	0.0356036676654408	0.644725854846338	-3.67352412940781
a_HQ_transcript/53079	-2.90833559396289	1.22738861284311	-2.19213047920132	0.0356231490836464	0.644877735887323	-3.64429955999592
a_HQ_transcript/36068	-2.29919015456484	-0.482948057604366	-2.19151417077907	0.0356716150450252	0.645236267990725	-3.70008409644414
a_HQ_transcript/11973	2.42147818074942	-0.635519696361625	2.19140188190656	0.0356804516797787	0.645236267990725	-3.74055752211792
a_HQ_transcript/84974	-2.21143498421075	1.75985900785646	-2.19139010366279	0.0356813786883635	0.645236267990725	-3.78051912796343
a_HQ_transcript/136850	3.85688407496586	0.713055533321236	2.19127785751519	0.0356902141181169	0.645236267990725	-3.77971478788347
a_HQ_transcript/131890	1.93952403221145	0.592562659448276	2.19099690865042	0.0357123375036267	0.645236267990725	-3.681250313064
a_HQ_transcript/7347	-3.86893175435375	1.13765463330494	-2.19079913121178	0.0357279188744631	0.645236267990725	-3.6522697284182
a_HQ_transcript/122947	2.74771423582129	-0.117794276811854	2.19067746891861	0.0357375067289656	0.645236267990725	-3.7347278430213
a_HQ_transcript/92978	-5.80410603759141	0.703889244836468	-2.19061015655415	0.0357428124088449	0.645236267990725	-3.69379567002985
a_HQ_transcript/109839	-2.41488008077056	0.795988982579104	-2.19030810895641	0.035766628990662	0.645236267990725	-3.68981314078029
a_HQ_transcript/121219	-2.53783560718268	0.107021100012403	-2.19017506533955	0.0357771240283872	0.645236267990725	-3.75896827493214
a_HQ_transcript/33538	1.41514042295919	3.49117332640019	2.18991913457602	0.0357973206467248	0.645236267990725	-3.96226013197223
a_HQ_transcript/61204	-3.0934740151074	2.33069852023104	-2.18954600345332	0.0358267842874271	0.645236267990725	-3.79899278551807
a_HQ_transcript/127214	-2.20199364221514	4.40049004811699	-2.18952214649642	0.0358286688456951	0.645236267990725	-4.09587019456453
a_HQ_transcript/59933	-2.17617897120756	-0.607497737594396	-2.18948531519433	0.0358315784740628	0.645236267990725	-3.74077247997717
a_HQ_transcript/113613	1.96887317744532	-0.348089802844148	2.18940254547365	0.0358381179512837	0.645236267990725	-3.76188919638489
a_HQ_transcript/85170	-4.07118396215875	1.10294627367149	-2.18938705022751	0.0358393423193601	0.645236267990725	-3.73321885500274
a_HQ_transcript/5931	-2.77621597329578	0.738198727297892	-2.18937431236608	0.0358403488386408	0.645236267990725	-3.68220008967841
a_HQ_transcript/3166	2.81332288518888	0.363205681362415	2.18934380912019	0.0358427592441173	0.645236267990725	-3.6719268627355
a_HQ_transcript/40519	-3.49510832489726	0.92075146322517	-2.18907527514597	0.0358639853843757	0.645248729334204	-3.76220191631953
a_HQ_transcript/61461	0.737927929743364	3.44399826183278	2.18879265862556	0.0358863367852382	0.645248729334204	-3.92175646069928
a_HQ_transcript/150352	-2.19137327193218	2.39538644655544	-2.18878594160803	0.0358868681677449	0.645248729334204	-3.80042369075704
a_HQ_transcript/118085	-2.63334180227656	1.26881393959147	-2.18877348798005	0.0358878533914375	0.645248729334204	-3.74536551760795
a_HQ_transcript/139807	-2.58097853531878	-0.609240651205264	-2.18812085267158	0.0359395180523516	0.645977830966943	-3.74582475639675
a_HQ_transcript/27739	-3.37663877264395	0.0804643526006884	-2.18793462828052	0.0359542723233562	0.645998884399945	-3.78257551932445
a_HQ_transcript/149373	1.18400039556638	4.973460659752	2.18774846311199	0.0359690273031049	0.645998884399945	-4.15211558870521
a_HQ_transcript/105085	-4.31602467604106	0.776527417506888	-2.1874848895389	0.0359899267149495	0.645998884399945	-3.71066976796255
a_HQ_transcript/47577	1.50707434520219	2.90925006122325	2.18719557469359	0.0360128796879561	0.645998884399945	-3.89892281802311
a_HQ_transcript/72792	2.24859828314683	-0.504789881503428	2.18709651064083	0.0360207419967801	0.645998884399945	-3.70558613580517
a_HQ_transcript/58336	-0.837031939007176	3.11861714951177	-2.18699318196246	0.0360289444029571	0.645998884399945	-3.90692069848105
a_HQ_transcript/30830	2.95039922780476	-0.39823426843053	2.18698900403452	0.0360292760890239	0.645998884399945	-3.75341749128656
a_HQ_transcript/150469	-7.00697577989384	1.9615333318556	-2.18698496862154	0.0360295964634018	0.645998884399945	-3.82759853374144
a_HQ_transcript/79008	2.53117074297619	0.164236437868298	2.18676845131832	0.0360467896563299	0.646001077992751	-3.79116083034886
a_HQ_transcript/109105	2.28397135250564	1.10433446831563	2.1867035387607	0.0360519456545638	0.646001077992751	-3.70625084839825

a_HQ_transcript/84354	-3.07352588699585	-0.106508571419595	-2.18645170757679	0.0360719548086419	0.646160428079118	-3.75059480354013
a_HQ_transcript/130204	2.60929989401088	1.33801731745899	2.1862897818554	0.0360848257890942	0.646191852578085	-3.68307984033983
a_HQ_transcript/81052	2.56590915915982	-0.217681541021621	2.1858492315317	0.0361198645233635	0.64662010625626	-3.78044455397337
a_HQ_transcript/700	1.13915752726338	2.97461574355975	2.18525108404268	0.0361674861628521	0.647273286845525	-3.91080265869223
a_HQ_transcript/17518	-2.06941171823767	-0.63150653165782	-2.18472113101856	0.0362097252622098	0.647829766094715	-3.73553569804013
a_HQ_transcript/86947	0.963845026561269	3.93618028422474	2.1843967317872	0.0362356027114425	0.647884235987821	-4.01808636644716
a_HQ_transcript/124735	-1.77258309623766	1.78671798316128	-2.18433120865849	0.0362408315177602	0.647884235987821	-3.79193297308298
a_HQ_transcript/101342	4.53369659034008	4.75856194393878	2.18403199848688	0.0362647173193365	0.647884235987821	-4.24598024387806
a_HQ_transcript/96222	1.842294408913	-0.0208134362081223	2.18388275314053	0.0362766367492229	0.647884235987821	-3.70272813053323
a_HQ_transcript/142662	1.09145568438006	2.51146922568697	2.18363634715473	0.0362963235268236	0.647884235987821	-3.80788384621304
a_HQ_transcript/53317	3.25073303751672	0.318517429443706	2.18341951127817	0.0363136556600604	0.647884235987821	-3.71487306580897
a_HQ_transcript/110903	1.48362006597104	1.94884305515654	2.18326472208635	0.0363260327896329	0.647884235987821	-3.74702945258876
a_HQ_transcript/141100	-4.82790317979728	0.0425955442364651	-2.18284145470224	0.0363598969668847	0.647884235987821	-3.77544756686296
a_HQ_transcript/109851	3.34701927859888	-0.0988523954306431	2.18280992115007	0.0363624209849725	0.647884235987821	-3.68268183604279
a_HQ_transcript/149722	3.08585794175194	2.21571638952948	2.18249922652258	0.0363872980520198	0.647884235987821	-3.80469714952168
a_HQ_transcript/100633	3.27408225613189	-0.481903368511459	2.18218270103917	0.0364126575947422	0.647884235987821	-3.77451278627487
a_HQ_transcript/149740	-4.43064644929293	0.180534298690711	-2.18201997615286	0.0364257009983831	0.647884235987821	-3.88205243997448
a_HQ_transcript/140292	4.43488596677111	0.383656662126916	2.18198408317067	0.0364285786030695	0.647884235987821	-3.75833026506356
a_HQ_transcript/75879	0.815128860236325	3.80325082693569	2.18192463455933	0.0364333451505983	0.647884235987821	-4.02461420950138
a_HQ_transcript/39336	3.17634130062878	0.469484265220687	2.18190600811798	0.0364348387198687	0.647884235987821	-3.74646997485746
a_HQ_transcript/116936	-7.31356659028003	2.84112861031696	-2.18181999902303	0.0364417361044525	0.647884235987821	-3.8994275434424
a_HQ_transcript/55313	-1.70711351555786	-0.633784602852899	-2.18176430291086	0.0364462032008232	0.647884235987821	-3.72133629894434
a_HQ_transcript/131102	-1.12783551458704	3.11838211335706	-2.1817497527971	0.0364473702704304	0.647884235987821	-3.88899403386487
a_HQ_transcript/48720	2.5956715355389	3.14704858901068	2.18153988892826	0.036464207229692	0.647884235987821	-3.87218282240798
a_HQ_transcript/148858	6.36418463826943	3.58266802683121	2.18146337251393	0.0364703477136788	0.647884235987821	-3.8289924317348
a_HQ_transcript/146724	-2.6827542434618	0.16528589357629	-2.18145826142915	0.036470757913865	0.647884235987821	-3.67155271280944
a_HQ_transcript/9214	2.09493658877333	0.402623668930942	2.18074254862082	0.0365282394882538	0.647884235987821	-3.74063101845142
a_HQ_transcript/150262	2.55304568355021	2.95830231305712	2.18068620833337	0.0365327678148705	0.647884235987821	-3.86870532710477
a_HQ_transcript/55720	-2.61536218125791	1.27727275733974	-2.1805997629127	0.036539716801743	0.647884235987821	-3.71208659321549
a_HQ_transcript/102521	-3.79179738976911	1.97152320333159	-2.18030399268686	0.0365635014502753	0.647884235987821	-3.71838419874505
a_HQ_transcript/18458	2.47037001186231	1.32278676269424	2.18021164625149	0.0365709304048106	0.647884235987821	-3.71258101034236
a_HQ_transcript/151883	-1.26300608090942	6.72492548443099	-2.18017709178463	0.0365737105392669	0.647884235987821	-4.30422961027623
a_HQ_transcript/65136	-2.09335173302441	1.59630523686723	-2.18000575992313	0.0365874980991416	0.647884235987821	-3.68981422503326
a_HQ_transcript/72828	-3.42488217116018	0.0209828037709706	-2.17996160734179	0.0365910519330937	0.647884235987821	-3.75496241683597
a_HQ_transcript/144612	2.51507240606583	-0.288994854032686	2.17985568621159	0.0365995787618485	0.647884235987821	-3.81206823355621
a_HQ_transcript/144082	2.02777633244419	0.731578647291187	2.17982598399391	0.0366019701581775	0.647884235987821	-3.66128501247905
a_HQ_transcript/65281	-1.71845813731279	-0.789959956411567	-2.17961397633859	0.0366190434452114	0.647884235987821	-3.76322603792269
a_HQ_transcript/70686	0.558133231832413	3.34199885540899	2.17947577549334	0.0366301767833541	0.647884235987821	-3.9607810749522
a_HQ_transcript/31346	4.05282917327349	0.131878122227794	2.17945640024131	0.0366317378776823	0.647884235987821	-3.69416779973459
a_HQ_transcript/134750	-4.43867285531018	1.76423801484448	-2.17915817011222	0.0366557742276089	0.647884235987821	-3.75066590675969
a_HQ_transcript/23552	-1.35515957282378	3.93651193451488	-2.17886829521268	0.0366791506454219	0.647884235987821	-4.02191345296877
a_HQ_transcript/146369	3.62177923482443	0.230282496953846	2.17883761932065	0.0366816252225204	0.647884235987821	-3.78818270243041
a_HQ_transcript/141929	1.37750397478427	4.38689523046534	2.17881020959247	0.0366838364490741	0.647884235987821	-4.10180985517715
a_HQ_transcript/130942	-1.4522608409527	-0.815991822480582	-2.17877354317764	0.0366867946265973	0.647884235987821	-3.76472231463296
a_HQ_transcript/28180	2.35961003697252	0.485165135962046	2.17873011974888	0.0366902982223157	0.647884235987821	-3.69049007866855
a_HQ_transcript/74216	-1.49217207887847	0.836112995578303	-2.1786095497015	0.0367000279146055	0.647884235987821	-3.67411319683427
a_HQ_transcript/137718	5.72620097190968	1.27012849199517	2.17850276313424	0.0367086472357924	0.647884235987821	-3.78212824120937
a_HQ_transcript/145407	2.42067170238161	0.712040556119144	2.17849083726213	0.0367096099493971	0.647884235987821	-3.74522009229652
a_HQ_transcript/43004	2.18295248143331	2.54358847983472	2.17833387452034	0.0367222828320992	0.647884235987821	-3.82430423471856
a_HQ_transcript/152673	1.44585727234724	3.54421130798714	2.17816350897736	0.0367360422449133	0.647884235987821	-4.01694934168027

a_HQ_transcript/86658	2.35732561347124	0.0947601318261153	2.17813893369973	0.0367380274232462	0.647884235987821	-3.69175894862222
a_HQ_transcript/27526	1.07775007696478	2.65334849679766	2.1781178108084	0.0367397337959876	0.647884235987821	-3.79697264203012
a_HQ_transcript/139343	-4.1119596476664	1.39375736956158	-2.17789965993175	0.0367573608332691	0.647884235987821	-3.82850862709002
a_HQ_transcript/102793	3.12741675157811	3.79065363913691	2.17788043033213	0.0367589149856839	0.647884235987821	-4.02608247479369
a_HQ_transcript/100867	-3.18618786975221	2.42859206407651	-2.17758819041042	0.0367825412708072	0.647981058190275	-3.92722643711922
a_HQ_transcript/112096	7.22339678119659	1.66319174943408	2.17753672592066	0.0367867033448236	0.647981058190275	-3.75569678418504
a_HQ_transcript/136469	-1.49114905574153	2.79669434985221	-2.17729766141623	0.0368060426484971	0.648125309624914	-3.83369448510391
a_HQ_transcript/132946	2.33135438559087	-0.411885804040057	2.17698395864811	0.0368314336155733	0.648376006422183	-3.79910535971104
a_HQ_transcript/70173	2.1515697225709	1.10903772758364	2.17642339864861	0.0368768439693127	0.648978863532609	-3.71093244277795
a_HQ_transcript/226	3.61451047063938	2.17901788644749	2.17619289282279	0.036895531465309	0.649111214593064	-3.94483012805805
a_HQ_transcript/148861	2.13032003592654	-0.413264610516915	2.17565098530089	0.0369394980633396	0.649688091808111	-3.77935456931057
a_HQ_transcript/140157	-2.35777294332981	-0.311155037888674	-2.1753270260715	0.0369658041461081	0.649720854484964	-3.74106077087499
a_HQ_transcript/110662	-2.56835956428394	-0.551524600213387	-2.17523848094724	0.0369729970762424	0.649720854484964	-3.77171122934838
a_HQ_transcript/130640	-2.157078387256	-0.517373305919415	-2.17521514330013	0.0369748931089365	0.649720854484964	-3.77368251340929
a_HQ_transcript/135103	2.80896523256465	0.920170496677374	2.17504327265593	0.0369888591541568	0.64976984131545	-3.73378130432191
a_HQ_transcript/60795	2.23015418297611	-0.560622885384447	2.17392303703574	0.0370800036400588	0.651015306286796	-3.77755478688245
a_HQ_transcript/71436	1.95620177282263	-0.704187940970099	2.17373850995255	0.0370950362897688	0.651015306286796	-3.73391328396194
a_HQ_transcript/87980	-2.91562225059493	1.96008545058441	-2.17367973927471	0.0370998252308754	0.651015306286796	-4.01309083479951
a_HQ_transcript/70065	-2.383287427621	1.04211093427202	-2.17354263862958	0.0371109990469511	0.651015306286796	-3.72146089771995
a_HQ_transcript/80686	2.85445165382285	1.45020072340337	2.1734842701418	0.0371157570367885	0.651015306286796	-3.7161766885949
a_HQ_transcript/42246	2.44459228088077	-0.287611970860425	2.17314495225938	0.0371434277771573	0.651063854893805	-3.76779045342808
a_HQ_transcript/152748	-2.23649744503849	-0.456337398924677	-2.17313861641349	0.0371439446282887	0.651063854893805	-3.78834055229173
a_HQ_transcript/52403	-2.51695100405616	3.88238056746065	-2.17276088724141	0.0371747697322539	0.651063854893805	-3.93535336392181
a_HQ_transcript/109784	4.37543889535172	1.92182484656357	2.17272168508809	0.0371779701821779	0.651063854893805	-3.83280701970265
a_HQ_transcript/50579	1.89184399706866	0.252252022796872	2.17266364923388	0.0371827086593881	0.651063854893805	-3.71208802971291
a_HQ_transcript/16712	-2.56369570254385	-0.514214954657902	-2.17262667220869	0.0371857280182946	0.651063854893805	-3.7633927745583
a_HQ_transcript/88036	-2.33248413809816	0.237961864136053	-2.17226875063897	0.0372149653928732	0.651079067944602	-3.80370115190366
a_HQ_transcript/6784	1.70427463922423	1.95003724415522	2.17213300588678	0.0372260592660874	0.651079067944602	-3.83252458955531
a_HQ_transcript/95972	2.58618439409227	-0.424515539530379	2.1720545124301	0.0372324755649222	0.651079067944602	-3.75854781834628
a_HQ_transcript/105739	-3.19544211086896	0.219365254352211	-2.17176330690072	0.0372562882155061	0.651079067944602	-3.72266178382754
a_HQ_transcript/143514	-4.27113096409785	1.37246839193102	-2.17175997585152	0.0372565606827074	0.651079067944602	-3.79891748950857
a_HQ_transcript/103865	2.79872781071329	0.473961423027968	2.17174067829452	0.0372581391846614	0.651079067944602	-3.74000076886954
a_HQ_transcript/33916	-1.9961368320825	-0.620673861178604	-2.17153372001989	0.0372750717069801	0.651079067944602	-3.80215043453567
a_HQ_transcript/76388	2.51513625611228	-0.24711097130456	2.17151989360959	0.0372762031743675	0.651079067944602	-3.77552523567402
a_HQ_transcript/9522	4.53876560331125	1.77270977566333	2.17116208803942	0.0373054943959922	0.651394946906048	-3.82964710245301
a_HQ_transcript/22417	-1.51615697416864	3.73275341672905	-2.17007274740661	0.0373947978908733	0.652333202164184	-3.93188287573457
a_HQ_transcript/82388	2.85230065831712	1.88075310031518	2.16987875740716	0.0374107210291231	0.652333202164184	-3.79461044984107
a_HQ_transcript/79218	3.36160201715861	1.4897112844108	2.16984982452087	0.0374130964232422	0.652333202164184	-3.73688637720971
a_HQ_transcript/103313	-6.24092187609947	1.43691710476751	-2.16984235972899	0.0374137093055223	0.652333202164184	-3.81766800517989
a_HQ_transcript/142455	2.28468674799603	-0.691742109521582	2.16974304567899	0.037421864143998	0.652333202164184	-3.77918191093704
a_HQ_transcript/23373	3.72325021244456	0.907415431852431	2.1696858344152	0.0374265625725563	0.652333202164184	-3.67265641836031
a_HQ_transcript/122462	2.8372683666475	1.16521395716714	2.16930591286689	0.0374577766492365	0.652576597673826	-3.75697958604515
a_HQ_transcript/84676	0.976366342248339	2.69926198863822	2.16916740161125	0.0374691623974294	0.652576597673826	-3.86724779446411
a_HQ_transcript/69886	2.51037913429259	-0.533112375489588	2.1691060488837	0.0374742066308015	0.652576597673826	-3.77601352052847
a_HQ_transcript/101444	-2.3300228485539	-0.196293619720219	-2.1683996790333	0.037532325765997	0.652775179883191	-3.72156477261336
a_HQ_transcript/116179	2.38358499172185	-0.591930173973574	2.1682794716288	0.0375422242517964	0.652775179883191	-3.77579289785899
a_HQ_transcript/92499	-2.70153574776665	-0.351807459488523	-2.16814298730431	0.0375534658790677	0.652775179883191	-3.75332471213048
a_HQ_transcript/95281	0.612301123727639	2.9382225512462	2.16801267733808	0.0375642017475121	0.652775179883191	-3.8809347867554
a_HQ_transcript/76927	2.14689198457716	-0.668652838997862	2.16799830305429	0.0375653861712916	0.652775179883191	-3.78733799182738
a_HQ_transcript/1669	-2.21774798344383	1.18074189683312	-2.16797637376423	0.0375671931825984	0.652775179883191	-3.67783307526136

a_HQ_transcript/45785	4.74119778719207	0.930482306792881	2.16776287334557	0.0375847900298828	0.652775179883191	-3.79532962500637
a_HQ_transcript/44008	2.42467218652971	0.802755720542514	2.16764804390534	0.0375942573835369	0.652775179883191	-3.76149451496517
a_HQ_transcript/33058	-2.26106485696262	-0.531930325295441	-2.16762007633467	0.0375965635499547	0.652775179883191	-3.78782595578026
a_HQ_transcript/119151	2.08788176598379	1.84477445037187	2.16758496651578	0.0375994588343765	0.652775179883191	-3.73060444868016
a_HQ_transcript/141969	-2.74566140106657	1.1108772080366	-2.16736867835581	0.0376172991274007	0.652775179883191	-3.74298804650195
a_HQ_transcript/151827	-3.95403546733339	1.43276383883925	-2.16733146068466	0.0376203697462271	0.652775179883191	-3.85760285119418
a_HQ_transcript/83519	3.78282916040497	-0.302223057513567	2.1668848849804	0.0376572315785302	0.653039785027076	-3.79277005537157
a_HQ_transcript/148468	2.64142998876092	-0.042991271022057	2.16666413030548	0.0376754652704013	0.653039785027076	-3.75022278604283
a_HQ_transcript/21536	-1.86085580383439	2.32543531761116	-2.16651384216249	0.0376878831284442	0.653039785027076	-3.79378359186187
a_HQ_transcript/72820	-2.12653516809522	0.426572950550689	-2.1664547078864	0.0376927702148601	0.653039785027076	-3.67529945118585
a_HQ_transcript/137153	6.88444640516444	3.8808917723251	2.16635945623898	0.0377006433673558	0.653039785027076	-4.05350062389273
a_HQ_transcript/81667	5.991401797181	2.01234785585903	2.16633062912612	0.0377030263995126	0.653039785027076	-3.92074553184869
a_HQ_transcript/98801	-1.98717277160052	-0.263936956312425	-2.16606395171408	0.0377250780056237	0.65322708796869	-3.79185284419551
a_HQ_transcript/40244	-4.6676211242275	1.68899421143775	-2.16577799655783	0.0377487364463797	0.653442094149839	-4.01896030067365
a_HQ_transcript/104778	4.02049006179333	-0.373914742086823	2.16521140091458	0.0377956526298136	0.654059451046682	-3.80899602217313
a_HQ_transcript/3330	1.1050641021572	3.20233963817324	2.16491791727769	0.037819974541365	0.654285559565615	-4.00503376236452
a_HQ_transcript/140893	-3.89267628577649	2.35843309235547	-2.16456556783735	0.037849193247365	0.654421601641679	-3.86733839217385
a_HQ_transcript/36486	-2.21308975654578	0.673768116589253	-2.16447738039393	0.0378565093659186	0.654421601641679	-3.69997507111265
a_HQ_transcript/132985	-1.88497417653428	1.02556072816034	-2.16441586968708	0.0378616131007598	0.654421601641679	-3.733338433992
a_HQ_transcript/108957	-3.13249489410321	1.21080254722372	-2.16344596003969	0.0379421705161682	0.655432878624781	-3.82128393323842
a_HQ_transcript/132042	3.09021852431888	1.55112030283093	2.16335931976058	0.0379493739803768	0.655432878624781	-3.89323088153176
a_HQ_transcript/144090	-2.83998864715362	-0.406177744111164	-2.16330431706172	0.0379539476577727	0.655432878624781	-3.73721136103448
a_HQ_transcript/121838	-1.48487400210311	1.82049013160003	-2.16286837489597	0.0379902152113369	0.655766185124114	-3.7692039904246
a_HQ_transcript/133245	0.818842031977902	3.90418940656703	2.16266283786788	0.0380073252501896	0.655766185124114	-4.01435647778942
a_HQ_transcript/68104	3.028483278607	1.14836816182948	2.16262991664608	0.0380100664317443	0.655766185124114	-3.6955091035572
a_HQ_transcript/39995	3.41461236669356	1.69933440075567	2.16221998214765	0.0380442143140424	0.655766185124114	-3.82333708637159
a_HQ_transcript/50391	-2.90744246965946	3.01721602532419	-2.16212555313254	0.038052084195972	0.655766185124114	-3.80663821839769
a_HQ_transcript/71528	-2.38776058856561	-0.0406272598124384	-2.16191261877489	0.038069835839769	0.655766185124114	-3.78661094889489
a_HQ_transcript/80209	-1.00797794428317	2.20221211875912	-2.16169481364157	0.0380880011667025	0.655766185124114	-3.76206729375158
a_HQ_transcript/56152	2.67926687677198	1.59057844551805	2.16168802720218	0.0380885672914157	0.655766185124114	-3.70779528633001
a_HQ_transcript/9155	1.00738450860247	2.59528271683349	2.16153837004361	0.0381010535933112	0.655766185124114	-3.7803332471236
a_HQ_transcript/152260	-3.947679575947	0.730938616088351	-2.16153482680019	0.0381013492598282	0.655766185124114	-3.7529882409145
a_HQ_transcript/60925	3.17519069773375	-0.0349360306656246	2.16126292944787	0.0381240438674356	0.655766185124114	-3.79771228786647
a_HQ_transcript/72275	-1.09087500854059	3.06384851154465	-2.1611395042269	0.0381343498371389	0.655766185124114	-3.85972084271506
a_HQ_transcript/132740	-4.12908177947162	0.221821819040951	-2.16111704762316	0.0381362252234389	0.655766185124114	-3.7876634216597
a_HQ_transcript/120824	0.720829758788298	3.27523328307016	2.1609553938323	0.038149727607139	0.655766185124114	-3.98226553195304
a_HQ_transcript/114720	-5.17111162281314	2.537537973308	-2.16091920554751	0.0381527508723251	0.655766185124114	-3.90223328234537
a_HQ_transcript/125696	2.63007720403944	0.893892585267514	2.16090723465466	0.038153750999342	0.655766185124114	-3.7546732364215
a_HQ_transcript/10556	-2.16040175933019	2.43230133156403	-2.16046059936899	0.0381910825173312	0.656213787930071	-3.89342991462193
a_HQ_transcript/145589	2.14829124009299	0.605964241021311	2.16014558108177	0.038217432511703	0.656401940725275	-3.71481987026964
a_HQ_transcript/57883	2.77110457166141	0.386953612839071	2.15997050557981	0.0382320838536323	0.656401940725275	-3.83142635932213
a_HQ_transcript/67641	-2.74103822139499	0.799243221020422	-2.15992479612226	0.0382359099108138	0.656401940725275	-3.72590419630823
a_HQ_transcript/53380	1.38693310539421	4.04721392466098	2.15963427033333	0.0382602359983521	0.656625626841514	-3.99307428666425
a_HQ_transcript/151903	-3.01651162284034	2.71147617346608	-2.15908339040558	0.038306399665619	0.657101302244465	-3.87675785229033
a_HQ_transcript/86425	-1.70485734762019	3.8152277502738	-2.15889968961032	0.0383218047800885	0.657101302244465	-4.0169832872738
a_HQ_transcript/8291	1.14325942542371	4.50485717363069	2.15848567799615	0.0383565439221007	0.657101302244465	-4.1260775876427
a_HQ_transcript/14267	-1.65261178125812	-0.734428032363965	-2.15840835851183	0.038363034796073	0.657101302244465	-3.80074680512416
a_HQ_transcript/148409	3.88036473797052	-0.257478587461242	2.1583940362724	0.0383642372373885	0.657101302244465	-3.85515513099671
a_HQ_transcript/33366	-2.37537398979129	1.17423736954056	-2.15838408813952	0.0383650724649095	0.657101302244465	-3.73905038379455
a_HQ_transcript/119795	-2.52352776913429	0.920961532366374	-2.15836013814426	0.0383670833301974	0.657101302244465	-3.77336245093972

a_HQ_transcript/32793	-3.18012664722588	1.26524392514353	-2.15821380541156	0.0383793716073474	0.657118148097758	-3.79927741169801
a_HQ_transcript/33550	-1.5992148148993	-0.107901570582276	-2.15795758815848	0.0384008958566577	0.657191804056294	-3.78656365658965
a_HQ_transcript/77768	-3.06467861465524	-0.0984335372177345	-2.15789345275239	0.0384062854111484	0.657191804056294	-3.80303779485364
a_HQ_transcript/93871	2.76902132664638	3.55500335489835	2.15733090814678	0.0384535871176601	0.657668843526035	-3.97785814795685
a_HQ_transcript/132579	2.38672420600637	0.98163580149066	2.15729281700767	0.0384567918928054	0.657668843526035	-3.79573485582329
a_HQ_transcript/48675	-4.00872634657191	0.936303887689468	-2.15707335557523	0.0384752607756696	0.657791164225919	-3.8331367351696
a_HQ_transcript/142053	-2.27637205558508	3.90597866165678	-2.15688753966194	0.0384909043695635	0.657865124726253	-4.09777427770886
a_HQ_transcript/104378	-2.53500312444424	-0.187550092926853	-2.15652503571853	0.0385214393743355	0.658193482642968	-3.76526581794965
a_HQ_transcript/146485	-3.78363517986461	0.71515750848661	-2.1563802257331	0.0385336432575107	0.658208526380423	-3.74782796670442
a_HQ_transcript/44198	3.19136751412375	0.625721982235535	2.15580223032015	0.0385823881604064	0.65864964967746	-3.80800238390821
a_HQ_transcript/71099	-2.14810754656179	0.543165737958226	-2.15571606949355	0.0385896591767529	0.65864964967746	-3.74291547001683
a_HQ_transcript/8117	-3.03127533205672	1.27038507070754	-2.15567102282897	0.0385934610996667	0.65864964967746	-3.84082836017256
a_HQ_transcript/133509	3.92782049495034	4.20136491530117	2.15521958186769	0.0386315809577553	0.659106703232287	-4.14671650990301
a_HQ_transcript/8042	2.33954866617409	-0.253523966951059	2.15481810477908	0.0386655099660956	0.659492008013264	-3.74217064458478
a_HQ_transcript/143366	-3.33275082838732	2.66312583080572	-2.15421820035318	0.0387162575275371	0.660163865520879	-3.79565530577132
a_HQ_transcript/61425	-2.66806740374507	3.12255018115699	-2.15401466304842	0.0387334887546591	0.660263998337485	-3.95930652807632
a_HQ_transcript/142912	-2.58623591721927	0.316338126226614	-2.15374536280821	0.038756297871474	0.660329322495038	-3.78818098342828
a_HQ_transcript/60651	2.31108540405687	1.28191615155089	2.15355245439661	0.0387726441136838	0.660329322495038	-3.71884058197333
a_HQ_transcript/67538	4.50795527099914	0.820624101104741	2.15323269357671	0.0387997527910997	0.660329322495038	-3.83615923274507
a_HQ_transcript/111763	-1.14788315243294	4.21343587694435	-2.15316539105208	0.0388054607092034	0.660329322495038	-4.04051542367151
a_HQ_transcript/91503	-1.07779016124281	2.89774333798709	-2.15304090169949	0.0388160206017669	0.660329322495038	-3.91175300452042
a_HQ_transcript/111547	-3.0215809286163	0.6754567831008	-2.15300059501148	0.0388194401908831	0.660329322495038	-3.89780735916264
a_HQ_transcript/143834	2.04815159507164	2.2915354920565	2.15289805873378	0.0388281404987253	0.660329322495038	-3.86770835416903
a_HQ_transcript/82309	-3.742718043967	1.16279365804103	-2.15265718570925	0.0388485856394093	0.660329322495038	-3.77989246272028
a_HQ_transcript/29924	1.56092205889515	3.04661661825094	2.15257307912757	0.0388557268032531	0.660329322495038	-3.95639971215804
a_HQ_transcript/112535	1.19924809488721	3.98981807268411	2.15256008998733	0.0388568297648085	0.660329322495038	-4.09761052544311
a_HQ_transcript/6979	-2.53022053723267	0.527980015803487	-2.15249590945806	0.0388622800071484	0.660329322495038	-3.75276544268783
a_HQ_transcript/150774	2.34136875168832	2.52754096484596	2.15222049797246	0.0388856758018827	0.660533770605446	-3.81303061774477
a_HQ_transcript/112752	3.35049445720232	1.14163436255611	2.15159817424286	0.0389385873824367	0.661239324383955	-3.90324111917271
a_HQ_transcript/64029	-1.56983704825779	2.11065847675797	-2.15124248338329	0.0389688578726565	0.661560096501688	-3.77930139883815
a_HQ_transcript/117937	-1.38435529515945	4.44204974248415	-2.15107390102475	0.0389832121028228	0.661610555653396	-4.11219724857046
a_HQ_transcript/97514	2.92838582287456	-0.288790228644965	2.15090993782242	0.0389971775345012	0.66161134643555	-3.81435315736963
a_HQ_transcript/27656	-3.73691384608802	1.16556604791309	-2.15080611750701	0.0390060226437282	0.66161134643555	-3.8054417773319
a_HQ_transcript/70703	2.66262366609222	2.17453574902256	2.15046686598403	0.0390349380590315	0.66190865796248	-3.80487648432462
a_HQ_transcript/103050	1.78506219124394	1.61829745670356	2.14996778566891	0.0390775107806781	0.662437313111478	-3.88764911527252
a_HQ_transcript/73014	3.1149720949709	0.163032938761181	2.14959589332729	0.039109260891447	0.662617294108437	-3.80393952881566
a_HQ_transcript/117779	-3.71646657370386	0.425085787332985	-2.14938256962567	0.0391274836324777	0.662617294108437	-3.81864601087783
a_HQ_transcript/106011	-2.3601198193693	2.50562198646943	-2.14929764966107	0.0391347398459924	0.662617294108437	-3.87216005374423
a_HQ_transcript/79350	-4.78374283680508	1.65924085886268	-2.14906164462518	0.0391549122068967	0.662617294108437	-3.92162075974985
a_HQ_transcript/147131	-1.21451386376423	8.94492047340245	-2.14891057618666	0.0391678295223125	0.662617294108437	-4.56001594324227
a_HQ_transcript/79621	2.08941694688095	0.466849877785373	2.14888461391396	0.0391700498435147	0.662617294108437	-3.7905140592161
a_HQ_transcript/91069	2.53060614482979	-0.505531069947037	2.1486701002223	0.0391883995624834	0.662617294108437	-3.81934761294538
a_HQ_transcript/107673	3.33811329794863	1.92941278335576	2.14820763697783	0.039227985121401	0.662617294108437	-3.80519515886125
a_HQ_transcript/40603	4.67961160935003	2.54045037035366	2.14816987053624	0.0392312193915679	0.662617294108437	-4.04701968754878
a_HQ_transcript/139678	-4.50080136333208	1.76507275516078	-2.14813592169625	0.0392341269293698	0.662617294108437	-3.93391041729368
a_HQ_transcript/152741	3.67668452155138	0.0952180496158362	2.14794243046671	0.0392507020806116	0.662617294108437	-3.8981041691208
a_HQ_transcript/26584	-1.60740133786711	2.54815888470345	-2.14787941738536	0.0392561013494127	0.662617294108437	-3.82970053105174
a_HQ_transcript/115492	-2.44867118588599	0.0855028061920126	-2.14774872150682	0.0392673021146863	0.662617294108437	-3.73394808667026
a_HQ_transcript/43265	1.22463843791973	0.75081015387653	2.14768551052971	0.0392727203761449	0.662617294108437	-3.73696783050686
a_HQ_transcript/55058	2.40260474663641	-0.538949095849856	2.14763386173282	0.0392771480541334	0.662617294108437	-3.81689503514595

a_HQ_transcript/109047	5.36829716006192	0.912256417946951	2.14754476424866	0.0392847871247325	0.662617294108437	-3.8636959937795
a_HQ_transcript/119469	-2.57429075531616	-0.290991802386907	-2.14726081190267	0.0393091415350785	0.662617294108437	-3.8474456488459
a_HQ_transcript/60114	2.31265344182674	-0.611293657501486	2.14713284689142	0.0393201213954698	0.662617294108437	-3.79426968275519
a_HQ_transcript/132972	3.5055270206382	2.3557005656242	2.14688803557781	0.0393411346828566	0.662617294108437	-3.8847385133715
a_HQ_transcript/21706	2.90889197228444	-0.588163643584057	2.14633247470558	0.0393888580517493	0.662617294108437	-3.81574552712027
a_HQ_transcript/78563	3.17728868392309	2.38868793102987	2.14630439011122	0.039391271918175	0.662617294108437	-3.87533175832913
a_HQ_transcript/29208	1.92932614788747	5.2306703070263	2.14630238263073	0.0393914444658364	0.662617294108437	-4.21002220090598
a_HQ_transcript/10507	0.772418160552929	3.99475411901705	2.14597574224913	0.0394195289167971	0.662617294108437	-4.11932930986624
a_HQ_transcript/99974	1.36827593915011	-0.785363418402318	2.14588581675586	0.0394272638076686	0.662617294108437	-3.79195846777616
a_HQ_transcript/8207	0.909073109301225	3.1766933029362	2.14567888588323	0.0394450679710822	0.662617294108437	-3.91217525474887
a_HQ_transcript/8120	-0.858096542874431	2.8508449323891	-2.14566381150936	0.0394463652369123	0.662617294108437	-3.91979713875709
a_HQ_transcript/72427	2.38404363641582	-0.339112891259619	2.14539287497733	0.0394696878717359	0.662617294108437	-3.83840656616546
a_HQ_transcript/9169	4.11740802759008	2.05658483720519	2.14531944544249	0.0394760109085131	0.662617294108437	-3.87743492993194
a_HQ_transcript/109016	-1.97190956894118	2.31486663785163	-2.14531664493571	0.0394762520787068	0.662617294108437	-3.94162558159347
a_HQ_transcript/143687	-2.59975121961421	-0.244549109955946	-2.14526668090725	0.0394805550321683	0.662617294108437	-3.84686791725573
a_HQ_transcript/114152	-3.33331819771767	2.8612552048955	-2.14495095886566	0.0395077549728483	0.662617294108437	-4.02328996788545
a_HQ_transcript/90941	-0.805751273417185	3.60013147568893	-2.14485910457172	0.0395156714866175	0.662617294108437	-4.07437367894815
a_HQ_transcript/83481	2.51140662659623	-0.370596453645783	2.14484937715901	0.0395165099316223	0.662617294108437	-3.79379520949836
a_HQ_transcript/46446	1.76334326677128	1.74099782460607	2.14484757151884	0.0395166655687829	0.662617294108437	-3.77936225144781
a_HQ_transcript/130457	2.84531871046656	-0.0207809343751706	2.14483037118251	0.0395181481791228	0.662617294108437	-3.80075003541008
a_HQ_transcript/78323	3.05731316470831	-0.0836876662430947	2.14477574731207	0.0395228568980588	0.662617294108437	-3.86321149276324
a_HQ_transcript/147961	3.98943742762403	3.53510704870855	2.14473428603767	0.0395264312998038	0.662617294108437	-4.17337531307022
a_HQ_transcript/118769	-2.71981606321708	-0.481629922778407	-2.14468142797442	0.0395309886419753	0.662617294108437	-3.83461285369075
a_HQ_transcript/107782	-2.15931329376277	2.34591884440474	-2.14466158220913	0.0395326998342978	0.662617294108437	-3.91248663076639
a_HQ_transcript/132421	-3.90303435019926	0.524019308501227	-2.14413488042771	0.0395781385092362	0.662910182311956	-3.80933981643928
a_HQ_transcript/80273	2.74224706278867	-0.496453109625841	2.14395811208677	0.0395933987433722	0.662910182311956	-3.82772418535341
a_HQ_transcript/8230	0.882130690987576	4.99476016186289	2.14387653819091	0.0396004426980677	0.662910182311956	-4.2290069578932
a_HQ_transcript/143357	-2.95137079069802	-0.289320930057272	-2.14384642815636	0.0396030429987387	0.662910182311956	-3.7831392258722
a_HQ_transcript/33977	-2.92769794898749	-0.153607465377769	-2.14379834751654	0.039607195554112	0.662910182311956	-3.78358799959708
a_HQ_transcript/83594	-3.21776427893169	-0.327575295934594	-2.14350862120246	0.0396322263803325	0.663138185099588	-3.82060740377555
a_HQ_transcript/142404	0.572082877193324	10.3807776381082	2.14261559478316	0.0397094675536558	0.663693400558884	-4.7576973984159
a_HQ_transcript/511	0.637386658895994	5.02870677803857	2.14260495533471	0.039710388604255	0.663693400558884	-4.22048078129929
a_HQ_transcript/80932	3.28566684442005	0.00754596993867624	2.14248765317674	0.0397205446384311	0.663693400558884	-3.86618509287073
a_HQ_transcript/106634	2.53005715303612	-0.326647609011828	2.14247787629475	0.0397213912260975	0.663693400558884	-3.81969131058139
a_HQ_transcript/139996	-1.8515465693648	2.18665432894062	-2.14246509952031	0.039722497600887	0.663693400558884	-3.75925883221929
a_HQ_transcript/79973	2.85870026342874	0.960075138188981	2.14230945642442	0.0397359773463671	0.66372784229587	-3.79959053180929
a_HQ_transcript/182	2.45237632750184	3.2185115067077	2.14181620794402	0.0397787228371813	0.664115030622636	-4.0040527833188
a_HQ_transcript/118047	-3.38629934273341	0.482470231060095	-2.14167218103077	0.0397912120765518	0.664115030622636	-3.80949600228166
a_HQ_transcript/81465	3.02528901443078	0.10738088457597	2.14157211685068	0.0397998911535474	0.664115030622636	-3.81773945806167
a_HQ_transcript/149178	-2.34822424297126	0.223597358567106	-2.14144892291301	0.0398105787006992	0.664115030622636	-3.79991296344077
a_HQ_transcript/110216	-1.10442118764683	2.70278873149344	-2.14108639586442	0.0398420440967081	0.664115030622636	-3.93739889465185
a_HQ_transcript/38581	1.61716327975476	3.34709386635787	2.14098305279964	0.0398510177613122	0.664115030622636	-4.03984745563998
a_HQ_transcript/103557	2.78776805290365	1.60770464674389	2.14092521235781	0.0398560410461972	0.664115030622636	-3.76035424898925
a_HQ_transcript/73155	4.18990338721542	0.849423142175165	2.14092218753187	0.0398563037595604	0.664115030622636	-3.78470908696678
a_HQ_transcript/7719	2.24997010834854	1.19828892735158	2.1408568034734	0.0398619828970957	0.664115030622636	-3.75464361511494
a_HQ_transcript/27242	-1.51699823206666	-0.599473348695707	-2.14030662171502	0.0399097991217304	0.66451311334807	-3.83310891703663
a_HQ_transcript/96505	1.57076849606918	1.37007499858007	2.14022253125687	0.0399171118964418	0.66451311334807	-3.75692280799972
a_HQ_transcript/33693	-2.47124963600061	-0.0486282541272242	-2.140182817496	0.0399205659453772	0.66451311334807	-3.84404023190772
a_HQ_transcript/131982	-1.56616397775558	3.6927534707821	-2.13991650120987	0.0399437352883933	0.66451311334807	-3.9872797952679
a_HQ_transcript/152769	-3.40434242799646	-0.414975838732561	-2.13984704758621	0.0399497796703517	0.66451311334807	-3.83958217941089

a_HQ_transcript/133307	2.07509920003589	-0.253253112573559	2.13952113963148	0.0399781535010383	0.66451311334807	-3.75208281719455
a_HQ_transcript/87291	1.73281868890489	-0.781677531931105	2.13950395810049	0.0399796498363418	0.66451311334807	-3.8345748854654
a_HQ_transcript/141427	2.52879197805784	0.141076500756023	2.1392900142168	0.0399982863203292	0.66451311334807	-3.76297260758735
a_HQ_transcript/77744	2.50635890500612	-0.343815961183006	2.13926379237454	0.0400005710148221	0.66451311334807	-3.82213364924032
a_HQ_transcript/25292	0.969283166736073	5.04696238399645	2.13916390020345	0.0400092756251344	0.66451311334807	-4.2466567416196
a_HQ_transcript/129640	-0.888201955627129	2.82753694088765	-2.1391369112761	0.0400116277304956	0.66451311334807	-3.94936126034821
a_HQ_transcript/3400	2.48085795225542	0.904292186731356	2.13883809650517	0.040037677866025	0.664622122078127	-3.75503097340369
a_HQ_transcript/119452	1.52844332688137	2.08724267578518	2.13879932618263	0.0400410588961877	0.664622122078127	-3.84173391550413
a_HQ_transcript/96264	0.87417259501465	4.88788558710405	2.13853048000751	0.0400645110438735	0.664821552371762	-4.22811594717419
a_HQ_transcript/94149	1.1334958877378	4.43365014573056	2.13827406933811	0.0400868897674877	0.665003061759283	-4.16691170152731
a_HQ_transcript/142307	-1.97160832099268	-0.179736297671087	-2.13779110726241	0.0401290713195729	0.665512883784346	-3.86087729400208
a_HQ_transcript/117630	-1.86981861111726	0.742022819801113	-2.13741825459994	0.0401616629347757	0.665863417875825	-3.72146183483446
a_HQ_transcript/79644	1.40162947371375	3.53969705777479	2.13706276735482	0.0401927584844848	0.666097694931595	-4.05246308207092
a_HQ_transcript/117601	0.66128217024244	4.61754550400634	2.1369947374478	0.0401987117021063	0.666097694931595	-4.20594362633154
a_HQ_transcript/146397	-2.66449691661866	0.938618741283487	-2.13657031169157	0.040235870375278	0.666523417832476	-3.85665868331553
a_HQ_transcript/112402	-2.72356772751688	0.358916495664597	-2.1361286042773	0.0402745744278178	0.666597321760921	-3.84902404770749
a_HQ_transcript/117157	-1.69093465747814	-0.77065720540329	-2.13601940856258	0.0402841476551871	0.666597321760921	-3.82715792530147
a_HQ_transcript/147993	-2.75242938125831	-0.67692687035723	-2.1359536688363	0.0402899120541202	0.666597321760921	-3.84702745598728
a_HQ_transcript/104091	-0.770632156271058	4.04848473812143	-2.13589087836286	0.0402954185306594	0.666597321760921	-4.11408341879163
a_HQ_transcript/149869	-1.4659574627769	2.31263336061119	-2.13586520172314	0.0402976704629073	0.666597321760921	-3.90416982221026
a_HQ_transcript/55689	-2.41757329335439	2.2905682087708	-2.13558215817617	0.0403225017852082	0.66680158319576	-3.99865758339826
a_HQ_transcript/58648	1.9490631082265	-0.749026213097668	2.13544382698009	0.0403346424759458	0.66680158319576	-3.83032449261546
a_HQ_transcript/115929	2.71338579613332	0.254275729726157	2.13532778451303	0.0403448294891152	0.66680158319576	-3.80534492991042
a_HQ_transcript/126912	-2.55848464340809	0.115481108673288	-2.13520166538753	0.0403559036898342	0.66680158319576	-3.82503697966462
a_HQ_transcript/95039	-1.22284220878003	3.27091395585408	-2.13470442492776	0.0403995913951666	0.667333744989555	-4.12589690738641
a_HQ_transcript/106614	-1.56013022793335	2.97339990992508	-2.13415805877815	0.0404476436746238	0.667937679408674	-4.02506966401846
a_HQ_transcript/713	0.990554806896783	3.14427675675474	2.13389135954152	0.0404711179780145	0.668135514293107	-4.04769942259471
a_HQ_transcript/123554	-2.9503857793393	1.99741454334814	-2.13366193618962	0.040491321005457	0.66827924684986	-3.75924067057882
a_HQ_transcript/7554	-1.99383027295366	2.93258506268845	-2.13253458377493	0.0405907257343413	0.669727423423522	-4.07335797694598
a_HQ_transcript/46349	-1.6993782519132	2.08366454543129	-2.13213095136535	0.0406263687617942	0.669727423423522	-3.79473686405157
a_HQ_transcript/120442	1.24902767368336	2.63549711267329	2.1320906055103	0.0406299330548321	0.669727423423522	-3.95431320882477
a_HQ_transcript/148015	-0.786272722032561	4.37109701151011	-2.1320341545393	0.040634920595275	0.669727423423522	-4.17070932677502
a_HQ_transcript/64417	1.8707510199823	1.11897319176841	2.13197732637568	0.0406399420097796	0.669727423423522	-3.76343929553915
a_HQ_transcript/127129	3.11456974841277	1.02761070575567	2.13182833482564	0.040653109720487	0.669727423423522	-3.83931572898075
a_HQ_transcript/73230	-4.53345642183451	-0.430547160219809	-2.13175358089297	0.0406597178169146	0.669727423423522	-3.82771011614603
a_HQ_transcript/147194	1.58665657941474	0.537885817767293	2.13157934167283	0.0406751239095085	0.669791388841902	-3.77816568313989
a_HQ_transcript/152347	10.2653088142101	2.13895876855995	2.13138037368327	0.0406927228310852	0.66989141680128	-3.84607827438463
a_HQ_transcript/125151	4.37327243435896	-0.400424007084422	2.13104294199313	0.0407225844455104	0.670115080945516	-3.86069847330837
a_HQ_transcript/51999	2.43163555332895	-0.393720947291738	2.13096634317683	0.0407293658990591	0.670115080945516	-3.86894588390821
a_HQ_transcript/133643	3.8177180704077	0.610848364044119	2.13080760951904	0.0407434221100575	0.670156661124341	-3.85655446062642
a_HQ_transcript/45660	-1.06230315240656	2.56937562130185	-2.13025876766508	0.0407920564075242	0.670635095908883	-3.95335327303122
a_HQ_transcript/21655	-2.80372928146275	1.36949934953786	-2.13014486286531	0.0408021562546689	0.670635095908883	-3.81031769912519
a_HQ_transcript/126201	-2.9057561950837	-0.157670533853444	-2.13008887934022	0.0408071210815737	0.670635095908883	-3.83921861569768
a_HQ_transcript/4475	1.53115381529474	2.22280633543428	2.12975512286856	0.0408367309626547	0.670834357972163	-3.89411518046806
a_HQ_transcript/55583	-2.40967443409759	3.08529937260257	-2.12967782872288	0.0408435909854615	0.670834357972163	-4.06550609400742
a_HQ_transcript/60098	-2.04091025635247	0.608536075259422	-2.12954084217882	0.0408557513480612	0.670834357972163	-3.7619064356089
a_HQ_transcript/140897	3.37103652618043	3.69661287267889	2.12943208078587	0.0408654084361999	0.670834357972163	-4.0884207320529
a_HQ_transcript/106726	3.50784092367246	-0.353190226240157	2.12898106374913	0.0409054765207225	0.67091855478658	-3.85779109363469
a_HQ_transcript/16105	3.60991943764609	1.25629535117842	2.12897019335113	0.0409064426701178	0.67091855478658	-3.87578258161066
a_HQ_transcript/57908	-3.0736559024525	0.21404782806651	-2.12882658670659	0.0409192081749601	0.67091855478658	-3.86481635359837

a_HQ_transcript/113213	2.22865643751913	-0.488351165083381	2.12881812178589	0.0409199607502644	0.67091855478658	-3.84245016859981
a_HQ_transcript/134414	-4.39807444198854	2.49840410326149	-2.12871692087087	0.0409289589862639	0.67091855478658	-4.01578655897814
a_HQ_transcript/7686	-2.69943800418448	1.75749524428698	-2.1285951331829	0.04093979001218	0.67091855478658	-3.74703046128303
a_HQ_transcript/127002	-2.95635305795932	0.432313109461977	-2.12808368565712	0.0409853026645299	0.671475105209638	-3.88628948976751
a_HQ_transcript/127269	-3.19129880280148	2.20249208344668	-2.12776156724622	0.0410139903284257	0.671661816289048	-3.91640050874315
a_HQ_transcript/40942	-0.740566852689372	3.03355448908741	-2.12769625874325	0.0410198088326817	0.671661816289048	-4.02606296838108
a_HQ_transcript/14052	2.27727762876835	2.04992831500673	2.12713310388044	0.0410700121299841	0.672122348799741	-3.80645712404514
a_HQ_transcript/26542	-3.52073530692454	1.68735891206339	-2.12695500118998	0.0410859006976604	0.672122348799741	-3.89850481309988
a_HQ_transcript/129801	4.16492685821412	2.43590553841378	2.12691128679257	0.0410898012982715	0.672122348799741	-3.92506480690475
a_HQ_transcript/105441	5.7295876736244	1.42934471297954	2.12670991426952	0.041107773846046	0.672122348799741	-3.77368824371122
a_HQ_transcript/150171	-2.74367952575279	-0.487835910899262	-2.12662308136706	0.0411155258540704	0.672122348799741	-3.8667000854598
a_HQ_transcript/59425	-2.22734082982575	2.476759729972	-2.12660308382132	0.0411173113186739	0.672122348799741	-3.80561260749057
a_HQ_transcript/129020	-3.03038931505216	0.103675580208609	-2.12646152607097	0.0411299521524693	0.672139965903496	-3.91405162260318
a_HQ_transcript/16544	2.58736012673246	-0.0313892337400529	2.12567980485434	0.0411998203994502	0.672842158449614	-3.78910800006375
a_HQ_transcript/44476	-1.25538665081938	2.78799121965884	-2.12563718583789	0.0412036326003769	0.672842158449614	-3.99530023859086
a_HQ_transcript/33069	-3.38134337563373	-0.476679738648667	-2.12559231407873	0.0412076466432808	0.672842158449614	-3.87182922564962
a_HQ_transcript/44772	-2.30292877087065	1.03089629742714	-2.1252316471124	0.0412399230157534	0.673180074434068	-3.81339905473264
a_HQ_transcript/32519	-2.74557907397862	2.86361603909581	-2.12502644325774	0.0412582968695814	0.673290926567946	-3.99677866471348
a_HQ_transcript/145573	3.1161046540125	0.825964733620034	2.12472923781535	0.0412849213638529	0.673322476913597	-3.75701566197235
a_HQ_transcript/127015	1.06228983480904	3.45751045933538	2.12471994277441	0.0412857542850832	0.673322476913597	-4.02379512518689
a_HQ_transcript/126939	-3.05150784688425	0.453819277948035	-2.12448136614339	0.0413071380443808	0.673322476913597	-3.84259579736076
a_HQ_transcript/2220	1.17520148255898	2.58048602164746	2.12439459255122	0.0413149180446096	0.673322476913597	-3.86764328326955
a_HQ_transcript/96535	-6.22105091600477	2.41277216279462	-2.1237738100387	0.0413706144295426	0.673322476913597	-3.98625309635393
a_HQ_transcript/7623	2.87159826500585	-0.300049407750817	2.12370489675743	0.0413768014054694	0.673322476913597	-3.84873128527118
a_HQ_transcript/45525	1.86487270933523	2.74129069867792	2.12368874660766	0.0413782514707937	0.673322476913597	-3.8531134928299
a_HQ_transcript/3679	3.2879327277046	0.702827163399797	2.1236080758668	0.0413854952880085	0.673322476913597	-3.81450204656224
a_HQ_transcript/16163	-2.6422442853165	0.803593339870809	-2.1234955985008	0.0413955970519611	0.673322476913597	-3.81445460620479
a_HQ_transcript/122807	-0.96829205497798	5.17828529136965	-2.12328615235074	0.0414144135526763	0.673322476913597	-4.29388182267091
a_HQ_transcript/144876	3.00732768204089	-0.342057105398419	2.12321623142087	0.041420696888888	0.673322476913597	-3.91067400173088
a_HQ_transcript/67891	2.77872175109132	-0.398997253424558	2.12314186553984	0.0414273805906622	0.673322476913597	-3.82160535029745
a_HQ_transcript/78099	-2.31328446075062	2.13444685308689	-2.12297229606736	0.0414426243763202	0.673322476913597	-3.91493561492437
a_HQ_transcript/14244	3.08232275668926	0.973958770162475	2.12297222868706	0.0414426304345934	0.673322476913597	-3.85968260297831
a_HQ_transcript/16335	1.64624616137094	-0.231145357222281	2.12296758070197	0.0414430483445016	0.673322476913597	-3.831640138106
a_HQ_transcript/28135	2.67745986831497	2.27263763697253	2.1227176372731	0.0414655267724489	0.673322476913597	-3.95269528255002
a_HQ_transcript/105580	2.95618937096026	1.98757102169005	2.12270460259149	0.0414666993306229	0.673322476913597	-3.92528464895931
a_HQ_transcript/119948	-2.64566524907188	1.49818416067174	-2.12257193393787	0.0414786354486897	0.673322476913597	-3.8648785443287
a_HQ_transcript/53289	-1.90946313962196	0.539173811381803	-2.12255326106933	0.0414803156796654	0.673322476913597	-3.75404058181943
a_HQ_transcript/2629	2.48208752743166	0.81803563681344	2.12230659295868	0.0415025171478678	0.673494784134913	-3.862109996209
a_HQ_transcript/32653	-2.03243770136586	-0.511220175408555	-2.12203569975676	0.0415269111344629	0.67362482428003	-3.85878462816447
a_HQ_transcript/83562	2.7878124277933	1.94646457031956	2.12196024784539	0.0415337078554161	0.67362482428003	-3.88070636467873
a_HQ_transcript/54037	-2.63887258335757	-0.0311788733421319	-2.12156016347659	0.0415697639931553	0.67375611504126	-3.85706501079773
a_HQ_transcript/104721	-4.10365564093089	2.57408077948333	-2.12154293355033	0.0415713173997372	0.67375611504126	-4.00182959315675
a_HQ_transcript/141007	-2.6886622305847	-0.389329836361584	-2.12117224212309	0.0416047504582335	0.67375611504126	-3.85582727457446
a_HQ_transcript/140056	-0.929222804149709	4.29204094147781	-2.12109073650262	0.0416121047313332	0.67375611504126	-4.20361828540866
a_HQ_transcript/104220	2.92022144328197	0.683033745340974	2.12087023285919	0.0416320066040355	0.67375611504126	-3.79510164561861
a_HQ_transcript/133773	2.84718743955533	-0.23002993252905	2.12078543932131	0.041639662010755	0.67375611504126	-3.85983493565308
a_HQ_transcript/34757	2.68424766722589	1.88759330384458	2.12059109891265	0.0416572123311249	0.67375611504126	-3.97364677270725
a_HQ_transcript/3983	-1.36265862040364	1.95828399306043	-2.12055216705481	0.0416607289423142	0.67375611504126	-3.83952270519805
a_HQ_transcript/4158	2.97366087208579	0.610968097992939	2.12052329502355	0.0416633370459328	0.67375611504126	-3.86008844511369
a_HQ_transcript/736	-1.88820738132089	3.89145009281112	-2.12048159325533	0.0416671043563395	0.67375611504126	-3.97919096304697

a_HQ_transcript/30318	-2.03181590995622	3.00964883299019	-2.12044633688389	0.0416702896292126	0.67375611504126	-4.03468473740541
a_HQ_transcript/114747	-2.25411902355581	-0.488139944046153	-2.12032898219864	0.0416808937119524	0.67375611504126	-3.8788725221284
a_HQ_transcript/21704	-2.46665894093841	-0.305959847338087	-2.11987001726279	0.0417223884017471	0.674180695624835	-3.86688324529391
a_HQ_transcript/2778	-2.82111996574704	0.101039640053987	-2.11978193366703	0.0417303561598224	0.674180695624835	-3.84814727304979
a_HQ_transcript/93833	4.32722490828967	1.51706048909455	2.1195094925665	0.0417550088346677	0.674377372250341	-3.89497433944717
a_HQ_transcript/67995	-2.70748023577315	1.9863706976354	-2.11934629714749	0.0417697822531604	0.674377372250341	-3.95445737903562
a_HQ_transcript/122721	4.14528524522198	1.2420854341082	2.11922313476761	0.0417809347082676	0.674377372250341	-3.92754689237843
a_HQ_transcript/8889	2.67589771124428	0.213664310986209	2.11913478589016	0.0417889363963276	0.674377372250341	-3.85963068247323
a_HQ_transcript/96950	1.93567190342104	2.54042950793367	2.11885932276339	0.0418138935875714	0.67442237071337	-3.98485979892217
a_HQ_transcript/110995	-2.51047612936295	3.29047768429239	-2.11874970377164	0.0418238288316027	0.67442237071337	-4.04732285286715
a_HQ_transcript/113962	3.11375332362008	7.27000634878741	2.11871988384529	0.0418265319023827	0.67442237071337	-4.48088408892218
a_HQ_transcript/58272	0.850771600027619	2.62132463505905	2.1185641935518	0.0418406471891604	0.674462878483504	-3.93262129424752
a_HQ_transcript/62629	-2.57855539492913	0.80712547709377	-2.11807700920069	0.0418848438465313	0.674890635851672	-3.85827968611719
a_HQ_transcript/130422	-2.42196659106912	0.262401148162928	-2.11787560606733	0.0419031269214031	0.674890635851672	-3.79472435617741
a_HQ_transcript/110847	2.38794767467431	0.384901478436248	2.11780699635682	0.0419093568217975	0.674890635851672	-3.79270550664354
a_HQ_transcript/69863	4.16174620761836	2.09306122108951	2.1177406209216	0.0419153846260244	0.674890635851672	-3.97160050071515
a_HQ_transcript/1076	3.74765629311218	2.0283332957484	2.11755044545094	0.0419326594306815	0.674890635851672	-3.94163027175822
a_HQ_transcript/140417	-3.06142342066823	0.0447957174671151	-2.11750436808239	0.0419368458693958	0.674890635851672	-3.86187154316592
a_HQ_transcript/86796	-2.81016952059119	0.0375719000140587	-2.11725795935347	0.0419592400413074	0.674928612467415	-3.79272026967831
a_HQ_transcript/26067	3.13965943662451	0.0456557467500629	2.11715847532046	0.041968284371041	0.674928612467415	-3.82568513909163
a_HQ_transcript/142887	1.95517285930693	2.88300696489522	2.1170287773939	0.0419800781097765	0.674928612467415	-3.98945589627132
a_HQ_transcript/129375	-2.06857532128612	-0.358544737493789	-2.11689544978011	0.0419922049633426	0.674928612467415	-3.90484770451343
a_HQ_transcript/152337	-3.48578018898521	-0.253831618131511	-2.11683987096938	0.0419972610668635	0.674928612467415	-3.87849234504449
a_HQ_transcript/55703	2.83059876331498	4.19664481806814	2.1166395026114	0.0420154934084745	0.675034991942456	-4.13728298489173
a_HQ_transcript/149955	-3.25243599381494	2.53013252463184	-2.11609941404089	0.0420646731995154	0.675638387328387	-3.98433773945102
a_HQ_transcript/133756	2.5508441354826	2.06960165529143	2.11568895431701	0.0421020832278372	0.675913274739588	-3.93829183661608
a_HQ_transcript/37261	1.20264832429363	2.67433547508239	2.11565648838342	0.0421050434873391	0.675913274739588	-4.0146911913619
a_HQ_transcript/101683	-3.28021234803351	-0.221230874822995	-2.11523000988467	0.0421439471150673	0.675987875998758	-3.87389449294176
a_HQ_transcript/360	-0.76071334236072	2.6175731107823	-2.11516036068648	0.0421503035791302	0.675987875998758	-3.91455712440651
a_HQ_transcript/133433	2.10663127090905	2.97865803777079	2.11512870389208	0.0421531929851873	0.675987875998758	-4.0588374234635
a_HQ_transcript/120018	-3.33752606258923	0.191120633238206	-2.11507889399163	0.0421577396320845	0.675987875998758	-3.97361218373185
a_HQ_transcript/146889	-2.70007669845187	0.548023007194594	-2.11480869859578	0.0421824106288815	0.675987875998758	-3.80696410295853
a_HQ_transcript/142844	-0.706444403306791	7.40346684706942	-2.1146012761832	0.0422013586219092	0.675987875998758	-4.49268256756925
a_HQ_transcript/72431	-3.50632138066196	-0.121777948392113	-2.11458712793213	0.0422026513360584	0.675987875998758	-3.87168121528925
a_HQ_transcript/120412	3.22164670491588	0.373235580605419	2.11445345094858	0.0422148670231917	0.675987875998758	-3.9208106548023
a_HQ_transcript/135448	-2.40344557079378	-0.370615254689041	-2.11440179095096	0.0422195886621154	0.675987875998758	-3.89745169241258
a_HQ_transcript/43692	-1.73998011294271	1.0179248095189	-2.114227146314	0.0422355543567374	0.675987875998758	-3.769962067368
a_HQ_transcript/116098	-2.82987074552459	0.0413673501292712	-2.11407925062945	0.0422490788897511	0.675987875998758	-3.90549175954444
a_HQ_transcript/108172	3.19062495413512	1.97172048280626	2.11407746448276	0.0422492422499224	0.675987875998758	-3.90935680464359
a_HQ_transcript/71594	0.589903559499828	4.28533178013704	2.11365601432831	0.0422878035245054	0.676333953742044	-4.24007407225173
a_HQ_transcript/147402	2.51223592421519	-0.336603140553103	2.11349398399665	0.0423026370445774	0.676333953742044	-3.88400788448039
a_HQ_transcript/16295	3.35735660382529	0.670360893262775	2.11345968245062	0.0423057778661931	0.676333953742044	-3.93010382271623
a_HQ_transcript/61710	-2.23458958266536	0.978713998875843	-2.11292562273267	0.0423547056831003	0.67682470965492	-3.82486865571365
a_HQ_transcript/78696	-2.21813734035452	0.612334480918938	-2.11287045382273	0.0423597628290084	0.67682470965492	-3.83755442095121
a_HQ_transcript/108683	-2.7202238717478	-0.105560211668779	-2.11255065642519	0.0423890880949284	0.676941511781037	-3.87792445008892
a_HQ_transcript/95018	-1.94078057173177	1.17138041066297	-2.11241855406426	0.0424012070601298	0.676941511781037	-3.9208009809292
a_HQ_transcript/42545	1.18056148065678	2.50872496744071	2.11240980149231	0.0424020101223981	0.676941511781037	-3.91577780183266
a_HQ_transcript/125643	-3.66691273604956	0.00499846208325837	-2.11197837647844	0.0424416107345406	0.677246989252767	-3.91236691631855
a_HQ_transcript/76204	-0.921870395117055	2.96375272339473	-2.11194749698413	0.0424444464259536	0.677246989252767	-4.02705777309583
a_HQ_transcript/141207	-2.41219543618244	-0.0544095583178016	-2.1118030322447	0.0424577149785452	0.677272792610559	-3.85643014200631

a_HQ_transcript/57904	0.707458611784157	3.46092752305227	2.11106509892704	0.0425255487047495	0.67791324591213	-4.04459718502456
a_HQ_transcript/63265	2.32820414024883	0.0443818432494827	2.1109704911089	0.0425342523585504	0.67791324591213	-3.78645027425052
a_HQ_transcript/68367	3.09885096158643	1.17845437920354	2.11081481808971	0.0425485772702076	0.67791324591213	-3.85769248057169
a_HQ_transcript/32350	1.88503667922729	0.462663268940183	2.11080445053924	0.0425495314358362	0.67791324591213	-3.79089401371701
a_HQ_transcript/152416	-4.31444923156716	1.33566502687697	-2.11073225259458	0.0425561766159745	0.67791324591213	-3.91385009418356
a_HQ_transcript/149094	3.4057021367368	-0.522376814176943	2.11039898597639	0.0425868627548612	0.678003713590857	-3.85431127078742
a_HQ_transcript/121562	-2.68168904117215	2.53165328558056	-2.11031839907282	0.0425942858815185	0.678003713590857	-3.98864718516087
a_HQ_transcript/105841	4.89889254023616	2.52757855909328	2.11025850788306	0.0425998033989589	0.678003713590857	-3.97464738272694
a_HQ_transcript/120007	2.45930729873651	2.13207669053902	2.1101639959847	0.0426085116595685	0.678003713590857	-3.89260570772586
a_HQ_transcript/129354	4.42280605127404	1.03983628715035	2.10984689843325	0.0426377403155616	0.678283133295831	-3.88521955669938
a_HQ_transcript/93588	2.31347467133023	2.48901874217388	2.10919024015521	0.0426983245937472	0.679061070310626	-3.97975651513193
a_HQ_transcript/19307	-3.61646805706453	0.299697639853394	-2.10892265206515	0.0427230345046991	0.679268202869023	-3.88684624201006
a_HQ_transcript/109917	-3.24303472450888	0.48650879851166	-2.10860288801607	0.042752579095066	0.679383280078833	-3.9938170990971
a_HQ_transcript/126879	-2.90084177076068	0.311398268901875	-2.10859132512684	0.0427536477864088	0.679383280078833	-3.8995596179352
a_HQ_transcript/113922	2.84509500789514	-0.561814975706351	2.10823176617767	0.0427868915469742	0.679562859719497	-3.87862991258522
a_HQ_transcript/63593	-2.66649745019332	-0.4899709934294	-2.10809917092491	0.0427991566829085	0.679562859719497	-3.88262943794413
a_HQ_transcript/126381	4.97906050372204	1.0972335453248	2.10808982665518	0.0428000211504452	0.679562859719497	-3.94599650628887
a_HQ_transcript/117918	3.57915266196881	3.00714057818735	2.10782568186754	0.0428244643987334	0.67975868653712	-3.9272957833084
a_HQ_transcript/123977	0.858837599142272	2.83728107252823	2.10770384522631	0.0428357429945202	0.67975868653712	-4.01922729059899
a_HQ_transcript/47929	-4.03749679186487	0.815218767487174	-2.10756993596617	0.0428481422010458	0.679769871687334	-3.88319411300374
a_HQ_transcript/42673	-2.29158196230268	-0.416936104661324	-2.10710322248445	0.0428913819296987	0.680270190671085	-3.84819379109374
a_HQ_transcript/97131	-2.48158600436153	-0.456476624880258	-2.10655605045156	0.0429421250668954	0.68076419715124	-3.8923023590274
a_HQ_transcript/81403	2.10413528496876	0.231576904849993	2.10644266239155	0.0429526469833778	0.68076419715124	-3.86818697519704
a_HQ_transcript/145365	-2.47003855452771	-0.43731166174469	-2.10615365548905	0.0429794758739363	0.68076419715124	-3.89168658328208
a_HQ_transcript/31205	1.64272777490481	2.12857264091923	2.1058412843497	0.0430084903620826	0.68076419715124	-3.89920342666893
a_HQ_transcript/97240	-4.20225421729903	1.37967231006771	-2.105834174031	0.0430091510032243	0.68076419715124	-4.0730171058953
a_HQ_transcript/140542	2.67449137206627	-0.526737740144954	2.10573160822401	0.0430186816999465	0.68076419715124	-3.85060644365982
a_HQ_transcript/28242	3.70157641589409	-0.110164302806877	2.10558700455383	0.0430321218432782	0.68076419715124	-3.87474814121077
a_HQ_transcript/6918	2.09911582397313	0.524504621955279	2.10549494844991	0.0430406799036333	0.68076419715124	-3.82512480822051
a_HQ_transcript/84082	-2.38491707620063	1.05255560489433	-2.10538789912189	0.0430506337123315	0.68076419715124	-3.82939664976352
a_HQ_transcript/141107	1.38383711823479	0.967925269374051	2.10538054357133	0.0430513177310451	0.68076419715124	-3.78368026333849
a_HQ_transcript/14348	-2.15202658733564	1.35834513835377	-2.10519310959717	0.0430687511235454	0.68076419715124	-3.85617306281742
a_HQ_transcript/19343	2.64221807407998	0.662391729840892	2.10500741726192	0.0430860286803547	0.68076419715124	-3.82915100902975
a_HQ_transcript/117936	2.25644939872595	-0.447025853179761	2.10491843346967	0.0430943102599083	0.68076419715124	-3.81797054013043
a_HQ_transcript/8694	1.86603076380801	2.52121207364241	2.10491411922678	0.0430947118153838	0.68076419715124	-3.96874878411431
a_HQ_transcript/68592	2.31182656120355	1.04457347273767	2.10487663023668	0.043098201306024	0.68076419715124	-3.84994073973099
a_HQ_transcript/50656	-2.62254394018541	2.46168454241736	-2.10443258587398	0.0431395521480644	0.680922993184792	-4.01302958966911
a_HQ_transcript/133209	2.21969875159024	0.129828653688717	2.10433538738736	0.0431486082628818	0.680922993184792	-3.85252255851245
a_HQ_transcript/41832	-2.71820378939666	1.3224943167321	-2.10426672153941	0.0431550069662373	0.680922993184792	-3.77859168884713
a_HQ_transcript/74814	2.29715153694117	-0.445295008577697	2.1042656024945	0.0431551112526282	0.680922993184792	-3.88184829069098
a_HQ_transcript/82711	2.90761762625793	0.720655682461605	2.10387372603501	0.0431916448337408	0.681027592859557	-3.83834878892241
a_HQ_transcript/124568	3.9749570320406	0.463520630808769	2.10384265601809	0.0431945425769282	0.681027592859557	-3.91964316753639
a_HQ_transcript/73962	-1.63939269995962	-0.513002270801835	-2.10377473844976	0.0432008775032483	0.681027592859557	-3.8653496514702
a_HQ_transcript/60765	1.91433544950333	2.04512709722072	2.10369190821635	0.0432086045015491	0.681027592859557	-3.8644494871579
a_HQ_transcript/138157	-3.59811054273086	1.67811226478688	-2.10352994483653	0.0432237171380991	0.681081114069782	-3.92619888086861
a_HQ_transcript/76104	1.71718912632766	1.0083407268604	2.10328788647558	0.0432463120663379	0.681252473656393	-3.82932799400589
a_HQ_transcript/41635	-4.01942284483456	0.818237051496689	-2.10301019318798	0.0432722461963318	0.68130822521045	-3.84431675654575
a_HQ_transcript/141639	-2.68478741739555	0.581495536810327	-2.10299898896063	0.0432732928619079	0.68130822521045	-3.86904482408569
a_HQ_transcript/152728	-4.31326239907993	3.3586327554744	-2.10283261891805	0.0432888372900067	0.681320235165212	-3.9425316988825
a_HQ_transcript/11495	-3.11246710681186	2.01095904615647	-2.10271735622022	0.0432996095074335	0.681320235165212	-3.83636700888586

a_HQ_transcript/95437	-1.70583038588636	0.603168799267268	-2.10261455847689	0.0433092187746948	0.681320235165212	-3.85102098504907
a_HQ_transcript/8478	-2.57727664952118	0.716497203707844	-2.10240732075725	0.0433285965522816	0.681427003257837	-3.78173253224049
a_HQ_transcript/146368	-4.27846191010264	1.88165036753745	-2.102291268213	0.0433394514011187	0.681427003257837	-3.879041843307
a_HQ_transcript/51740	3.44443818505343	3.02811736773854	2.10204612608018	0.0433623884070161	0.681603275641707	-4.17783582511464
a_HQ_transcript/99335	-1.76556858882363	-0.617021364058213	-2.10150431385956	0.0434131217628728	0.681755894045344	-3.86787904059251
a_HQ_transcript/41770	1.56791239076102	2.77345937720583	2.1011540511618	0.0434459470234097	0.681755894045344	-3.90485093982917
a_HQ_transcript/26362	1.18952809790528	1.44600227319971	2.10115080327055	0.0434462515057987	0.681755894045344	-3.79061914238564
a_HQ_transcript/144816	-1.55415071441533	3.42273883946457	-2.10113575325319	0.0434476624351817	0.681755894045344	-4.09431097760854
a_HQ_transcript/124611	-2.56129033010582	-0.516112990648442	-2.10107223475009	0.0434536176998979	0.681755894045344	-3.90801432165876
a_HQ_transcript/110189	4.74731621245394	1.43229831545873	2.10088940648519	0.0434707630428455	0.681755894045344	-3.95131117226214
a_HQ_transcript/71221	3.12282469015333	1.77769708946851	2.1007807253581	0.0434809578188926	0.681755894045344	-3.92269168945808
a_HQ_transcript/113013	-4.3078579837013	1.98022440622955	-2.10077659791987	0.0434813450327149	0.681755894045344	-3.83955547035037
a_HQ_transcript/52179	1.08178082970018	4.0912413154792	2.1007230940426	0.0434863647515241	0.681755894045344	-4.19534999410276
a_HQ_transcript/48818	-1.85475689433506	0.0909719829681331	-2.10069092592723	0.0434893830016538	0.681755894045344	-3.87470381576973
a_HQ_transcript/123166	4.12741333237846	2.7266340018366	2.10040642032344	0.0435160854796428	0.681951168445729	-4.13330436675189
a_HQ_transcript/69377	-1.76761834044238	2.07820173269661	-2.10030824261916	0.0435253033810497	0.681951168445729	-3.89606194651443
a_HQ_transcript/57210	3.07924043108314	0.419442577463185	2.10018104854988	0.043537248194118	0.681954503645295	-3.88156857438905
a_HQ_transcript/123903	1.62572787272034	-0.395751348662387	2.09999594284261	0.0435546366732947	0.682036804124333	-3.85683541544171
a_HQ_transcript/10205	-0.626826365648425	3.69973050272349	-2.09970371956864	0.0435821000666468	0.682036804124333	-4.17562018920489
a_HQ_transcript/9687	1.52056670540645	4.35034479092834	2.09959558474913	0.0435922665481726	0.682036804124333	-4.22156537093712
a_HQ_transcript/110303	-3.29952746602472	1.86423154314952	-2.09955225091995	0.0435963412410367	0.682036804124333	-4.0250701104543
a_HQ_transcript/92358	2.71223735258005	0.523952476576765	2.09950091067747	0.0436011692149398	0.682036804124333	-3.87203775102294
a_HQ_transcript/7864	1.2462626393167	2.79727960171783	2.0990491189181	0.0436436755396304	0.682518044597158	-3.96142875824772
a_HQ_transcript/59788	-2.7522791974646	0.362932180746212	-2.09882321467444	0.04366494321842	0.682666976707993	-3.82388837525027
a_HQ_transcript/8040	3.75327301768942	1.80426274080028	2.09855651331646	0.0436900635155451	0.682682599565243	-3.86919775044423
a_HQ_transcript/395	1.45798820293127	3.98325958864277	2.09848030080849	0.0436972442304161	0.682682599565243	-4.18399269958622
a_HQ_transcript/137999	-4.26884105384511	2.48360876907967	-2.09843857667799	0.0437011759045945	0.682682599565243	-4.04242821381324
a_HQ_transcript/8700	-2.74848791929959	-0.300750343132206	-2.09827341434464	0.0437167422561195	0.682742287443233	-3.92492076306153
a_HQ_transcript/100709	-3.45660381218354	0.790179919916592	-2.09811281806117	0.0437318829607519	0.682795297540314	-3.93670830229818
a_HQ_transcript/124068	2.60249465505617	1.42484465151688	2.09784309779988	0.0437573220869237	0.683009027462058	-3.89163810117098
a_HQ_transcript/129590	-3.20769408175438	1.02843812416118	-2.0975238560608	0.0437874488112393	0.68315639417636	-3.89353902205003
a_HQ_transcript/106604	2.97769505416361	0.929995934743698	2.09749398728189	0.0437902684541205	0.68315639417636	-3.88442699341974
a_HQ_transcript/28307	3.99797667983245	1.55331973072912	2.09717231768317	0.0438206445569749	0.683446854523166	-3.91237788014239
a_HQ_transcript/115559	4.80934509338348	1.00267545391119	2.09703334115706	0.0438337742323998	0.683468248009908	-4.00185913483443
a_HQ_transcript/19886	2.58774300265168	-0.198683403240647	2.09673765155483	0.0438617207913889	0.683720596986285	-3.90323626841671
a_HQ_transcript/131819	2.670447458455	0.314521880964084	2.09630392337895	0.0439027422909572	0.683757732139593	-3.84144860220747
a_HQ_transcript/95682	-2.92758626655171	0.253692031744809	-2.09603366447017	0.0439283201952981	0.683757732139593	-3.94305380451818
a_HQ_transcript/3513	-2.42833985405118	-0.412298013154571	-2.09594966430363	0.0439362728390993	0.683757732139593	-3.93913568135314
a_HQ_transcript/146526	2.30983760290716	0.156108569912969	2.09593956310438	0.0439372292471433	0.683757732139593	-3.92826845227532
a_HQ_transcript/134117	-1.11188255751291	4.08773010066833	-2.09587005528225	0.0439438109287846	0.683757732139593	-4.18447652420007
a_HQ_transcript/113357	-3.95670320230499	1.92609728154741	-2.09581149169849	0.0439493569787133	0.683757732139593	-4.0310524090323
a_HQ_transcript/40330	-2.55460785945288	2.25566057277778	-2.09538523666887	0.043989742533087	0.683757732139593	-3.97747422915615
a_HQ_transcript/125782	-3.92676062295576	0.843076412902013	-2.09535568597354	0.0439925435298829	0.683757732139593	-3.97987209379681
a_HQ_transcript/46819	-1.91360285073921	1.45326452697762	-2.09528580988147	0.0439991674426945	0.683757732139593	-3.81735064066418
a_HQ_transcript/151203	-2.99122875941904	0.273318096002014	-2.09526913532028	0.0440007482399798	0.683757732139593	-3.84315176468004
a_HQ_transcript/109759	-2.27875844678435	-0.597666413714811	-2.09516360230742	0.0440107542425337	0.683757732139593	-3.91552393037913
a_HQ_transcript/16911	3.50824309857894	1.04639510797581	2.09503787252092	0.0440226778047053	0.683757732139593	-3.87759209888245
a_HQ_transcript/42968	-2.22848216039987	1.37739262758108	-2.0945454559923	0.0440694035144631	0.683757732139593	-3.89391658480828
a_HQ_transcript/29785	2.93739120000163	1.68089097215819	2.09450266414565	0.0440734661272972	0.683757732139593	-3.88492547283217
a_HQ_transcript/88932	-2.78563595616379	1.75269682471472	-2.09450021505211	0.0440736986516641	0.683757732139593	-3.89451918583308

a_HQ_transcript/62327	-1.64396566935896	1.69752416642329	-2.09447015692596	0.0440765525495667	0.683757732139593	-3.8393363817021
a_HQ_transcript/82914	2.68447738856937	0.297477077575616	2.09434780427048	0.044088171125413	0.683757732139593	-3.89107710618978
a_HQ_transcript/150623	-3.22942028174483	-0.416357062166328	-2.09418795562742	0.0441033543857201	0.683757732139593	-3.92790271612911
a_HQ_transcript/44803	4.92684619054477	2.37422458857298	2.09418098505219	0.044104016592489	0.683757732139593	-4.01071626936637
a_HQ_transcript/99721	-3.39056496193277	1.64862551639285	-2.09410535744229	0.04411201802968	0.683757732139593	-3.94829296531451
a_HQ_transcript/151658	-3.4142371273283	1.3269527140854	-2.09400252389555	0.0441209734473947	0.683757732139593	-3.91807515678786
a_HQ_transcript/136878	2.50881771604841	-0.50366731263493	2.09386055532824	0.0441344669955273	0.683757732139593	-3.9047685189985
a_HQ_transcript/119851	1.01909131106839	2.74325648280907	2.09379592527241	0.0441406110326446	0.683757732139593	-3.97518552644481
a_HQ_transcript/114873	1.90671651650884	3.24609390358565	2.09362001122541	0.0441573380757418	0.683757732139593	-4.11476980676832
a_HQ_transcript/133735	2.50622649082409	-0.561845534666325	2.09361118752722	0.0441581772373388	0.683757732139593	-3.9142393502975
a_HQ_transcript/127612	0.685231721197877	4.43193585019165	2.09305161265783	0.04421142335189	0.684197508600895	-4.27062913752833
a_HQ_transcript/17913	-1.70261764548857	2.63178090636733	-2.0929485848775	0.0442212330958127	0.684197508600895	-3.97018922068362
a_HQ_transcript/1879	-1.13906595009607	1.73042806798541	-2.09287367903892	0.0442283664283424	0.684197508600895	-3.82728830386842
a_HQ_transcript/151416	1.64695416192906	5.13117127363224	2.09275038228424	0.044240110272636	0.684197508600895	-4.14587305999654
a_HQ_transcript/13179	3.60771997984318	0.736421284937319	2.09269452641499	0.0442454313726735	0.684197508600895	-3.86344492197295
a_HQ_transcript/146927	-5.83519303555025	3.12181083868296	-2.0923757997404	0.0442758056308506	0.684485114284597	-4.16039895427488
a_HQ_transcript/137066	2.48724956786147	1.52319024180695	2.09202889365439	0.0443088862769358	0.684814395508035	-3.84232816564796
a_HQ_transcript/145149	-3.63646663855921	0.374183112909356	-2.09185976932104	0.0443250217301252	0.684881675472811	-3.93514693794997
a_HQ_transcript/117093	-2.05673038214283	0.880112423026989	-2.09117703775322	0.0443902110828283	0.685706667505353	-3.81502945356909
a_HQ_transcript/149116	-3.57746627552376	0.418757425905351	-2.09102159104962	0.0444050654471465	0.685753890624797	-4.00628071696825
a_HQ_transcript/75025	-2.7708216962056	4.36187553185684	-2.09088182015536	0.0444184255903574	0.685778019313758	-4.28321379795065
a_HQ_transcript/111933	-3.00274845197177	-0.490389701975732	-2.09063753476389	0.0444417843797751	0.68581979223893	-3.93278857227179
a_HQ_transcript/142610	-2.38959679811502	-0.417867086433815	-2.09059390215202	0.044445957710779	0.68581979223893	-3.84991720658251
a_HQ_transcript/67027	3.74274529915715	-0.104631732276996	2.09048341967534	0.044456526581962	0.68581979223893	-3.96324322954446
a_HQ_transcript/93399	2.26116357897649	0.158293364325304	2.09018730910474	0.0444848637767093	0.685898939212208	-3.89530854698452
a_HQ_transcript/122839	3.24179168939689	0.114397903495959	2.09017284759351	0.0444862481229751	0.685898939212208	-3.98227507525575
a_HQ_transcript/91321	-1.82660982482517	-0.171952004420277	-2.09005995210784	0.0444970564920389	0.685898939212208	-3.86182345087851
a_HQ_transcript/32111	2.05069288214501	-0.681892719295461	2.08984401304843	0.0445177364872766	0.686035786461403	-3.91963192095028
a_HQ_transcript/139447	3.20429010469858	0.258773715056512	2.08957518880964	0.0445434930316572	0.686249719957481	-3.84073457750156
a_HQ_transcript/149066	-4.31369915549031	-0.0226661309213103	-2.08945272808313	0.0445552305794029	0.686249719957481	-3.99156759929444
a_HQ_transcript/136394	3.14870904201785	-0.458202486547369	2.08912709700423	0.044586454769304	0.686548726577511	-3.92625787295927
a_HQ_transcript/101789	2.16190467533658	-0.581946943304431	2.08893652976206	0.0446047368840539	0.686648343643084	-3.91586647459392
a_HQ_transcript/107251	-1.09609218850456	3.36340422865929	-2.08867558578232	0.0446297813415384	0.686851980360324	-4.09339819283741
a_HQ_transcript/87698	-2.06415941943443	0.718824765287859	-2.08852894324071	0.0446438610078845	0.686886805893676	-3.82325390614356
a_HQ_transcript/54614	-1.85093712804028	-0.688523838561086	-2.08828571154476	0.0446672231832445	0.687064395129833	-3.91153877286667
a_HQ_transcript/48924	2.01644563538128	-0.675493909511763	2.08796190342972	0.0446983413915768	0.687361160954914	-3.91773371412248
a_HQ_transcript/19682	-3.28197525931175	0.633284140739309	-2.08757659920254	0.0447353943512005	0.68769755783458	-3.9163291335539
a_HQ_transcript/58277	1.16267604007651	2.94438692736297	2.08745295020618	0.0447472908582895	0.68769755783458	-4.06080760005505
a_HQ_transcript/148172	2.71606934525215	1.30335440300363	2.08723900833491	0.0447678812107522	0.68769755783458	-3.96770613166952
a_HQ_transcript/36013	2.5456586912408	0.0709081704749045	2.08686531299283	0.0448038667336669	0.68769755783458	-3.92246485192148
a_HQ_transcript/133975	-2.56220614615383	1.72818247306424	-2.0865820319406	0.0448311626912877	0.68769755783458	-3.95144974413394
a_HQ_transcript/35957	-0.814108314858682	3.62423337171974	-2.08633944352043	0.0448545493140999	0.68769755783458	-4.15921925203849
a_HQ_transcript/4621	-2.60320323376833	1.28723280594266	-2.086180328002	0.0448698946165929	0.68769755783458	-3.92461714951464
a_HQ_transcript/123942	2.12183205276974	4.48964525550157	2.08611536492233	0.0448761610700476	0.68769755783458	-4.27311978829144
a_HQ_transcript/81373	-1.33492852385316	1.1173622137901	-2.08611532430347	0.0448761649884562	0.68769755783458	-3.8526832569732
a_HQ_transcript/72896	-1.99362825925433	0.169494191890684	-2.08592939834745	0.0448941040041106	0.68769755783458	-3.83645549354809
a_HQ_transcript/152360	2.70551350480957	3.5162859997962	2.08585634602218	0.0449011541692929	0.68769755783458	-4.12532077805456
a_HQ_transcript/13081	3.14744350565501	0.531486940153619	2.08576695591011	0.0449097823976749	0.68769755783458	-3.86151156601609
a_HQ_transcript/143932	2.26160014227498	1.10983003014443	2.08552355082722	0.0449332840745801	0.68769755783458	-3.96282917443057
a_HQ_transcript/80892	2.24528304969595	0.0595376613643239	2.08545291933736	0.0449401058417847	0.68769755783458	-3.84629047595774

a_HQ_transcript/151294	-2.83405219104207	1.04313835022644	-2.08532850326644	0.0449521244818346	0.68769755783458	-3.8815221451152
a_HQ_transcript/143710	1.33016096595434	1.91428735164175	2.08519372156526	0.0449651476458471	0.68769755783458	-3.91189672005678
a_HQ_transcript/55504	3.12832262117999	2.33922475676734	2.08514225605524	0.0449701213339166	0.68769755783458	-3.89993523785691
a_HQ_transcript/1627	1.0383846536116	3.04747791604517	2.08487024511903	0.0449964168572631	0.68769755783458	-4.02897741462182
a_HQ_transcript/83393	-1.9435488121923	0.988436856878114	-2.08482832407876	0.0450004706052866	0.68769755783458	-3.83642713224742
a_HQ_transcript/95181	3.1914699077441	0.750739985348277	2.08477100201408	0.0450060141477863	0.68769755783458	-3.83879875382969
a_HQ_transcript/86869	-2.26021612820293	0.0531943786340672	-2.08473326490099	0.0450096639839041	0.68769755783458	-3.86456922913298
a_HQ_transcript/70204	1.51570860258756	3.33911010230122	2.08457669697634	0.0450248096186144	0.68769755783458	-4.0715971168793
a_HQ_transcript/21145	-2.64664791159352	0.362161423048678	-2.08450144044256	0.0450320911768373	0.68769755783458	-3.96421165847038
a_HQ_transcript/139248	-3.32038956537289	0.546193250855446	-2.0845007561592	0.0450321573904605	0.68769755783458	-3.87852030838242
a_HQ_transcript/83143	2.5293370321695	-0.0953408812418874	2.08437553727288	0.0450442754488371	0.68769755783458	-3.94094406188545
a_HQ_transcript/51082	1.79266892623219	-0.474441384740978	2.08429104030657	0.0450524542687299	0.68769755783458	-3.88950991470093
a_HQ_transcript/86665	4.0655373210415	2.83333080818181	2.08406526722331	0.0450743142307944	0.68769755783458	-4.01806588865468
a_HQ_transcript/108722	-2.88769025390626	0.0514297333452406	-2.0838856728841	0.0450917097231119	0.68769755783458	-3.96241591498427
a_HQ_transcript/142325	3.88870772802634	0.0149026860478106	2.08368412201464	0.0451112389755038	0.68769755783458	-3.90327957724796
a_HQ_transcript/144138	2.56606691191653	-0.464279977947102	2.08362231777364	0.0451172289861672	0.68769755783458	-3.92776803294826
a_HQ_transcript/93779	2.50241384134688	0.379332319482912	2.08353194867796	0.0451259887394767	0.68769755783458	-3.90787916328527
a_HQ_transcript/96700	0.70708554007065	3.84614544855332	2.08335037165036	0.0451435940929036	0.68769755783458	-4.20959486812663
a_HQ_transcript/7723	1.80479335673411	2.02002024709495	2.0831935114503	0.045158807828607	0.68769755783458	-3.92027429737984
a_HQ_transcript/119641	-1.99028501386395	5.37654286329173	-2.08317501072919	0.0451606024957456	0.68769755783458	-4.34604234046131
a_HQ_transcript/133267	-3.51048080437195	3.6607443612888	-2.08311708291114	0.0451662222053074	0.68769755783458	-4.0864870348506
a_HQ_transcript/152784	-2.23013239045874	-0.604155252293912	-2.08308326500302	0.0451695032427599	0.68769755783458	-3.91969004963238
a_HQ_transcript/86638	-2.06972879508543	2.67987187711438	-2.08306148793443	0.0451716161806964	0.68769755783458	-3.99904375296546
a_HQ_transcript/72775	-3.45046061026195	0.842259594587394	-2.08295851464035	0.0451816084298238	0.68769755783458	-3.85036628019127
a_HQ_transcript/54941	2.40760158090181	-0.0817457473602735	2.08292561725442	0.0451848011138251	0.68769755783458	-3.88511356647753
a_HQ_transcript/101002	-2.02313209609053	1.12106448315585	-2.08283654330436	0.0451934467197924	0.68769755783458	-3.84733487026948
a_HQ_transcript/883	1.46823627289129	-0.749176288644335	2.08247357185385	0.0452286921782405	0.688053760517342	-3.92861371162275
a_HQ_transcript/109339	7.24574140242763	2.34776720587179	2.08225327623495	0.0452500952681901	0.688079143299194	-3.96094731944773
a_HQ_transcript/43484	-2.56166058740323	-0.260456107059235	-2.08221273251797	0.0452540353157311	0.688079143299194	-3.88771589354274
a_HQ_transcript/152521	-2.56604473266349	-0.283715997452754	-2.08203995932136	0.0452708288462061	0.688140949612724	-3.95664970832665
a_HQ_transcript/112642	3.6613963389406	1.22042447214335	2.08192735447747	0.0452817769795739	0.688140949612724	-3.83546363645548
a_HQ_transcript/152749	2.72968964131562	-0.159230718641586	2.08159421279565	0.0453141807314495	0.688159036860254	-3.97195799579266
a_HQ_transcript/9024	1.93314395698031	1.45714054022461	2.08157290269037	0.0453162542010413	0.688159036860254	-3.86980356812465
a_HQ_transcript/142458	-1.05296241840777	2.30224678234992	-2.08151776301905	0.0453216196699771	0.688159036860254	-3.95018840659609
a_HQ_transcript/90262	-5.4372684132167	1.80873583369877	-2.08124431511721	0.0453482363094308	0.688159036860254	-4.05819704483783
a_HQ_transcript/23778	1.7005789199553	2.97161919298981	2.08122431156483	0.0453501839392116	0.688159036860254	-4.12418348329967
a_HQ_transcript/91985	-1.74598333911964	1.25798533703541	-2.0811851274675	0.0453539992810975	0.688159036860254	-3.80765850768592
a_HQ_transcript/128666	-2.2213536747299	0.0942836465689907	-2.08085388683767	0.0453862633781455	0.68831865744342	-3.93932514391563
a_HQ_transcript/74593	4.02578293994566	1.05315125331915	2.08073628661676	0.0453977229545819	0.68831865744342	-3.88682439970344
a_HQ_transcript/120438	-1.80743900656192	3.51817124087899	-2.0807016920722	0.0454010945123804	0.68831865744342	-4.179721163942
a_HQ_transcript/51256	-0.904100940761242	3.09762380453083	-2.08059098933427	0.0454118850002669	0.68831865744342	-4.01475327996355
a_HQ_transcript/24457	-1.45567304840231	3.37449243728278	-2.07999833226243	0.0454696913191964	0.689015176486508	-4.12860218912998
a_HQ_transcript/15715	1.39969837883286	1.56184472512206	2.07967750375134	0.0455010112076459	0.689310080656252	-3.8375063985317
a_HQ_transcript/81684	-3.07504876974609	0.582547351421933	-2.0792632129553	0.0455414832023419	0.689737243525881	-3.87863111961486
a_HQ_transcript/54819	2.7354994662395	1.74166190012684	2.07914599786286	0.0455529396830418	0.689737243525881	-4.00277216275415
a_HQ_transcript/19340	2.96447189505795	0.785861385435171	2.07880690846952	0.0455860962354444	0.69005953176404	-3.8470900163028
a_HQ_transcript/118976	-1.07050392407428	3.06217770187204	-2.07867839028376	0.045598668451392	0.690070137917863	-4.15814588160343
a_HQ_transcript/117184	-2.55021128805274	-0.244925247550908	-2.07821446800428	0.0456440768130149	0.690577536956515	-3.95049766728221
a_HQ_transcript/4955	3.42983946558994	-0.318125463605675	2.07800703700013	0.0456643928966292	0.690705134086719	-3.94456802388096
a_HQ_transcript/135321	-1.67392429915104	4.36820937047271	-2.07778118006271	0.045686522707	0.690815703820756	-4.2850460942665

a_HQ_transcript/31804	-1.87302665954828	-0.138679500611644	-2.0776898735991	0.0456954717380747	0.690815703820756	-3.85924601843278
a_HQ_transcript/79663	1.792852982621	-0.673599117287409	2.07743004746524	0.0457209459939214	0.690898223737482	-3.9277765278113
a_HQ_transcript/84061	2.69353503883953	0.421633362725639	2.07739175061519	0.04572470180839	0.690898223737482	-3.87732342313293
a_HQ_transcript/12366	2.16125033934627	-0.250368650219673	2.07719533741231	0.0457439685431962	0.690940203420941	-3.93490108965672
a_HQ_transcript/59574	2.64295684630798	5.02477331203964	2.07712109408646	0.0457512531476604	0.690940203420941	-4.33919175692165
a_HQ_transcript/13606	-2.6453843995639	1.70261903832328	-2.07653062515146	0.0458092251765555	0.691458845002259	-3.90627513892445
a_HQ_transcript/95845	1.31113202526394	3.05636313290432	2.07641152417204	0.0458209263120993	0.691458845002259	-4.07306576522105
a_HQ_transcript/152833	-4.44382551079657	-0.576654442153546	-2.07640790579462	0.0458212818426352	0.691458845002259	-3.96607379767353
a_HQ_transcript/57843	-1.64027584325451	3.46519195366236	-2.07594930723918	0.0458663620009944	0.691824527356662	-4.04348035880554
a_HQ_transcript/92217	3.80645939705558	0.618089762674387	2.07573783765107	0.0458871625899326	0.691824527356662	-3.96468568698838
a_HQ_transcript/100020	-3.69649638623341	-0.550070703401013	-2.07551917554101	0.0459086793926793	0.691824527356662	-3.96094743755707
a_HQ_transcript/139145	-3.4894960782686	0.133838357825497	-2.07546332740106	0.0459141763902428	0.691824527356662	-3.91344822951559
a_HQ_transcript/47107	-3.20562580389667	0.5223948897301	-2.07543795733081	0.0459166736966027	0.691824527356662	-3.84401164844529
a_HQ_transcript/151480	-4.14350995915897	-0.0142192255032215	-2.07543540264103	0.045916925174477	0.691824527356662	-4.06355506699755
a_HQ_transcript/137273	-4.36680632153582	1.61591802492476	-2.07515626173424	0.0459444104863093	0.692059262178851	-4.02065046311991
a_HQ_transcript/40053	0.91299278383618	2.61976433514298	2.07499744263705	0.0459600549100641	0.692062367181273	-3.99649591110861
a_HQ_transcript/114232	-4.04937752380257	0.429589400798106	-2.07491245730415	0.0459684282907875	0.692062367181273	-3.8947225437704
a_HQ_transcript/118041	-1.47451422953254	2.64078291988987	-2.07448840570881	0.046010229068942	0.692321290457927	-4.0036230191388
a_HQ_transcript/71931	3.46341983128607	0.507069999865243	2.07447288766548	0.0460117593912312	0.692321290457927	-3.90573755200591
a_HQ_transcript/7370	-3.19705645513953	2.15636774118681	-2.07437557002868	0.0460213574581859	0.692321290457927	-4.10954292541586
a_HQ_transcript/135646	-2.91284413708077	3.5208497629061	-2.07411164190966	0.0460473965621868	0.692371101779136	-4.09470027376996
a_HQ_transcript/69119	-3.58755075861751	0.0544245581550281	-2.0741005526825	0.0460484909076201	0.692371101779136	-3.98137520655356
a_HQ_transcript/148056	2.0068222663891	-0.608203756171949	2.0738826223464	0.0460700021133483	0.692515408028113	-3.94033977677003
a_HQ_transcript/7738	-1.68860474117824	-0.675016167030631	-2.07354858717034	0.0461029908433635	0.692685980167409	-3.92441424677426
a_HQ_transcript/136986	-2.11511170468141	0.492724278223226	-2.07352640094387	0.046105182653243	0.692685980167409	-3.93854797274199
a_HQ_transcript/23143	2.73701101742571	0.594452122215151	2.07339919413239	0.0461177513733321	0.692695775666421	-3.88417820684796
a_HQ_transcript/84116	1.27672832102663	-0.696367500162709	2.07263819877189	0.0461930048964181	0.693268731505272	-3.98069589546527
a_HQ_transcript/50854	-1.59795638377353	-0.432330391813001	-2.07263079323346	0.0461937377486062	0.693268731505272	-3.93596107010338
a_HQ_transcript/116446	-5.03742686281754	0.485082136825846	-2.07254666490029	0.0462020638088404	0.693268731505272	-3.92861615495884
a_HQ_transcript/3587	2.53359200025609	1.14584599019673	2.07253110870888	0.0462036035275843	0.693268731505272	-3.85179629620367
a_HQ_transcript/56611	-3.24514469903835	-0.126424273785484	-2.07227959091049	0.0462285044994768	0.693463357302087	-3.95912638229619
a_HQ_transcript/141776	3.57731536999346	0.517633190531589	2.07196702224364	0.0462594661551408	0.693501194761114	-3.94786458363816
a_HQ_transcript/62622	3.23147687439489	-0.0699216155537796	2.07191326843749	0.0462647926076045	0.693501194761114	-3.96035600485922
a_HQ_transcript/117042	1.42555391257429	3.92072020911019	2.07188976215055	0.0462671220100366	0.693501194761114	-4.22308991383406
a_HQ_transcript/114216	-2.30964106874658	0.147673883289648	-2.07177244616139	0.0462787492168725	0.693501194761114	-3.89107956782634
a_HQ_transcript/86139	0.791753735186064	2.22200763818176	2.07158312490712	0.046297518306141	0.693603645386434	-3.93541541441148
a_HQ_transcript/3760	1.6808185430058	3.78480346798495	2.07121916661834	0.0463336195458533	0.693799660902918	-4.11661221096174
a_HQ_transcript/62658	2.25511152093051	1.68603480579062	2.07121055902832	0.046334473637922	0.693799660902918	-3.93023694862723
a_HQ_transcript/149009	4.97217695227179	-0.0305395313654294	2.0709830083343	0.0463570574899189	0.693884187701715	-3.99771681691788
a_HQ_transcript/101145	-2.4574096297473	0.544030452275104	-2.07091314703512	0.0463639929987865	0.693884187701715	-3.87062779615572
a_HQ_transcript/122482	2.35282834155026	1.54576817156782	2.07048818989236	0.0464062004844187	0.69433709697768	-3.90933890822712
a_HQ_transcript/106164	4.72583657240497	0.761351003515011	2.07026005209028	0.04642887348804	0.694497570281212	-3.93516893504899
a_HQ_transcript/143836	2.30892500424333	-0.252312890569294	2.07010444302516	0.0464443439697826	0.694550251164272	-3.9083802368515
a_HQ_transcript/135267	7.50057520236686	2.26269188961221	2.06960950777817	0.046493580076695	0.695074142378993	-4.03039041470664
a_HQ_transcript/78956	-3.19450451978199	-0.377097103491489	-2.06944326478066	0.0465101282233401	0.695074142378993	-3.96301462892618
a_HQ_transcript/47419	-3.58676052654787	2.01874901661013	-2.06939182846196	0.0465152493437634	0.695074142378993	-4.0058326770189
a_HQ_transcript/59995	-2.74377690962973	-0.179098717284957	-2.06907861297282	0.0465464445323029	0.69536153373778	-3.95125398888465
a_HQ_transcript/43668	-2.78411736162794	2.23579610689711	-2.06892013769895	0.0465622351442821	0.695418706183666	-4.09048411384388
a_HQ_transcript/24458	-1.8982972565262	3.24247853348712	-2.06866117974764	0.0465880480890135	0.695615852369006	-4.1773695341937
a_HQ_transcript/131426	2.73000027732358	2.65016763780943	2.06843294960467	0.0466108085305634	0.695615852369006	-4.1480540341676

a_HQ_transcript/105657	2.55162392619113	2.26671026253742	2.06842766054332	0.0466113361026919	0.695615852369006	-3.96054900308763
a_HQ_transcript/69676	1.15722927571472	2.39265636730816	2.06776641482435	0.0466773353331228	0.696422009302814	-3.98516961423226
a_HQ_transcript/100686	-1.81001154818279	-0.376175085617226	-2.06755158315931	0.0466987954804863	0.696563403564205	-3.89765454551641
a_HQ_transcript/32276	-1.60250348128233	2.27769474424603	-2.06732384556941	0.0467215543230275	0.696724091762171	-3.94283540110294
a_HQ_transcript/13311	-1.49896444921172	2.20628647601907	-2.0667799417869	0.0467759485949434	0.697130978223785	-3.94002085026842
a_HQ_transcript/132334	1.24880051390087	4.5456102460942	2.06675448779281	0.0467784955417151	0.697130978223785	-4.17095832309298
a_HQ_transcript/72144	-3.00406793685819	1.1976101314283	-2.06669129909935	0.0467848187801229	0.697130978223785	-3.98849535018791
a_HQ_transcript/3373	-2.73935949853408	2.25194119913419	-2.06639852459709	0.0468141262956324	0.697387654159973	-3.93137013534809
a_HQ_transcript/65155	2.06435944291549	-0.650927599529833	2.06619363289242	0.0468346461192954	0.697387654159973	-3.95680958021815
a_HQ_transcript/87600	2.80728049861712	0.186153895143635	2.06606448785157	0.0468475840124525	0.697387654159973	-3.90157033347001
a_HQ_transcript/25173	-1.9739116509384	-0.651726174201465	-2.06595912919978	0.0468581412892468	0.697387654159973	-3.96298564340169
a_HQ_transcript/71118	2.35573857591209	-0.553643742207612	2.06592030952338	0.0468620316740444	0.697387654159973	-3.95634805773497
a_HQ_transcript/103924	-4.16680932358616	0.304300514543794	-2.06557468332599	0.0468966817727857	0.697724678292421	-3.96739434796263
a_HQ_transcript/61369	-2.06762609237189	0.0612372711838409	-2.06541350088411	0.0469128485228754	0.697786606688256	-3.91666261333151
a_HQ_transcript/115090	3.11388127513159	3.19678698718981	2.06517107588334	0.0469371732163247	0.69792813050917	-4.2321584943051
a_HQ_transcript/102905	-2.03398970312472	1.72978551304256	-2.06507937993225	0.0469463767941586	0.69792813050917	-3.90057895842285
a_HQ_transcript/28492	2.46434120263476	1.43123204074163	2.06475710909782	0.0469787359157542	0.698162579931547	-3.96546092233627
a_HQ_transcript/122297	1.99998188859345	0.322591038508375	2.06468311239608	0.046986168674171	0.698162579931547	-3.86438173166022
a_HQ_transcript/54044	2.09589066877391	0.306300499249557	2.06440124476005	0.047014490959271	0.698404888954894	-3.90945175815947
a_HQ_transcript/57832	2.77066515549276	-0.323734252085203	2.06406097938815	0.0470487011136891	0.698644810209755	-3.94439119506516
a_HQ_transcript/119900	3.45266563931521	2.81746082181614	2.06400153499707	0.0470546798783923	0.698644810209755	-4.26485275371806
a_HQ_transcript/104063	-2.10500424651791	-0.409440278156475	-2.06334865578095	0.0471203888151002	0.699424337901051	-3.93847111027449
a_HQ_transcript/45873	-2.59540914703791	-0.170912445938632	-2.06324084578342	0.0471312471022298	0.699424337901051	-3.94020189843905
a_HQ_transcript/145873	2.26898501646524	0.370329415911763	2.06268164702015	0.0471876031998946	0.699689690678721	-3.94640772040862
a_HQ_transcript/11230	-1.00191724425424	3.62351455649568	-2.06250402551906	0.0472055163149029	0.699689690678721	-4.20942977829497
a_HQ_transcript/26145	-1.87571609153662	4.91100154627316	-2.06240530536474	0.0472154748219865	0.699689690678721	-4.32337757580103
a_HQ_transcript/149653	-0.820104042107046	3.58995457919049	-2.06220177942248	0.0472360115660668	0.699689690678721	-4.20074003399117
a_HQ_transcript/147792	-2.73401427275327	0.0894647298318924	-2.06212350281539	0.0472439121439616	0.699689690678721	-3.94832893671459
a_HQ_transcript/52649	-1.72706275505523	0.459688186679673	-2.06205078568049	0.0472512526376415	0.699689690678721	-3.91362299641279
a_HQ_transcript/127867	3.93162462823625	3.4918796412463	2.06204561470615	0.0472517746643103	0.699689690678721	-4.16843511963983
a_HQ_transcript/117571	1.90727620904874	1.995370609333	2.06200504791012	0.0472558701903113	0.699689690678721	-3.89347782019222
a_HQ_transcript/147648	-2.04204459190341	0.0351258419603259	-2.06198928598288	0.0472574615607738	0.699689690678721	-3.94842827845015
a_HQ_transcript/141582	-3.90177937818481	2.92618313388851	-2.06168532229221	0.0472881598523506	0.699745127741582	-3.95740725716908
a_HQ_transcript/108040	3.50021810110922	2.90639199232152	2.06164298065189	0.0472924374657549	0.699745127741582	-4.09689365177792
a_HQ_transcript/68134	1.37842825678681	0.776822290398232	2.06151870640839	0.0473049943818058	0.699745127741582	-3.89045313517933
a_HQ_transcript/136579	-2.47768579507132	1.84699134632832	-2.06147552868348	0.0473093578314137	0.699745127741582	-3.90986005547232
a_HQ_transcript/69558	2.59703557215708	0.760762610618789	2.06129252764684	0.0473278554608805	0.69984064671332	-3.86995951282793
a_HQ_transcript/108623	1.18470917666505	3.05539664649406	2.06102467018212	0.0473549417984796	0.699947851422942	-4.08765148180304
a_HQ_transcript/124308	8.96680657122252	3.01942957774379	2.06084813354007	0.0473728010290693	0.699947851422942	-3.97287878562352
a_HQ_transcript/137548	1.54661252650727	2.10795359708449	2.06065365353932	0.0473924823492717	0.699947851422942	-3.95094572403998
a_HQ_transcript/147120	2.5470260851163	0.90056554360572	2.06062929254513	0.0473949481818188	0.699947851422942	-3.8816784421049
a_HQ_transcript/37865	-2.16204024259012	1.71436215724612	-2.06062569029423	0.0473953128131142	0.699947851422942	-3.89280763606955
a_HQ_transcript/131628	2.8742893751865	2.5610517364115	2.0603500720161	0.0474232190918028	0.700040273529239	-4.04959129039408
a_HQ_transcript/88640	-1.14606786070772	2.42559997920461	-2.06032599942656	0.047425657121493	0.700040273529239	-4.0448477317814
a_HQ_transcript/150141	-2.86311825488072	0.0433756164896809	-2.05999399706741	0.0474592929880924	0.700070644003598	-4.00970843083555
a_HQ_transcript/39665	3.99658025046462	1.27774634073439	2.05996898553454	0.047461827809668	0.700070644003598	-4.02822369883109
a_HQ_transcript/17367	-2.88633545686103	0.745337430213878	-2.05977662991942	0.0474813262836973	0.700070644003598	-3.97826324917959
a_HQ_transcript/144085	-2.45113410791523	2.11739090238732	-2.05963484278535	0.0474957033036339	0.700070644003598	-4.01353960680027
a_HQ_transcript/3058	2.3504865759091	0.20044655836019	2.05954891706801	0.0475044179164196	0.700070644003598	-3.91839250117232
a_HQ_transcript/65276	-3.4584244596722	1.28931857892991	-2.05931613898616	0.047528033408573	0.700070644003598	-3.93757873611023

a_HQ_transcript/43028	1.31685688779056	3.07187965272963	2.05931490485237	0.047528158639788	0.700070644003598	-4.06500770529485
a_HQ_transcript/132866	2.73169038283798	2.23593831852443	2.05928862594617	0.0475308253072148	0.700070644003598	-3.93091531913459
a_HQ_transcript/78457	-3.88588872035179	1.94947875768377	-2.05908405842071	0.0475515884173458	0.700070644003598	-4.01272802481169
a_HQ_transcript/44850	-2.56634170178441	0.048236947643987	-2.05874815376673	0.0475856992355205	0.700070644003598	-3.89941186058565
a_HQ_transcript/95170	2.29738861968998	2.61385601802962	2.05870150692086	0.0475904378827423	0.700070644003598	-4.09469110965622
a_HQ_transcript/11524	-0.891906519914713	2.71426328044936	-2.05859479028607	0.0476012803157521	0.700070644003598	-4.12537658083279
a_HQ_transcript/145075	1.75997790969961	4.62686445388876	2.05857733885827	0.0476030535912594	0.700070644003598	-4.37638276888931
a_HQ_transcript/87312	-2.02857422036373	-0.665436918868233	-2.05852917137018	0.0476079482904073	0.700070644003598	-3.9697949158628
a_HQ_transcript/143979	-0.986263114607194	2.6276219802134	-2.05846598335735	0.0476143700213013	0.700070644003598	-4.08649847686646
a_HQ_transcript/32056	-2.11860298614177	-0.416158529867605	-2.05838490003968	0.0476226115492098	0.700070644003598	-3.96404056698639
a_HQ_transcript/13225	-3.72376070978952	1.5981836658241	-2.05828806362841	0.047632455908241	0.700070644003598	-3.97295744586634
a_HQ_transcript/133406	2.97694774887992	0.536770036920525	2.0580052944898	0.0476612123747773	0.700316216613007	-3.94471563574876
a_HQ_transcript/54755	-2.21136251545772	-0.0704517152088152	-2.05779392704251	0.0476827175525103	0.700335879762017	-3.94879884788495
a_HQ_transcript/126175	-2.60092880476626	-0.0435908192375133	-2.05765206444022	0.0476971558780482	0.700335879762017	-4.00650738865742
a_HQ_transcript/76698	2.4486810621476	0.668182686204592	2.0576369433014	0.0476986950863237	0.700335879762017	-3.87541647825002
a_HQ_transcript/148781	2.67586978684709	2.13732238177466	2.05738620898448	0.0477242241579743	0.700533763094628	-3.91711865224341
a_HQ_transcript/132843	-2.54877577134158	1.08485817792135	-2.05708195907779	0.0477552181752778	0.70081174503725	-3.96092666809709
a_HQ_transcript/71427	3.01094432205644	-0.242360135880415	2.05690627572183	0.0477731231453964	0.700890367890726	-3.97216922216755
a_HQ_transcript/14175	-2.40764260162055	-0.21878963979904	-2.0567928012531	0.0477846911634832	0.700890367890726	-3.97880951319495
a_HQ_transcript/94182	2.91247569261936	1.78428958225689	2.05655795719831	0.0478086398826015	0.701064737410661	-3.87965229257832
a_HQ_transcript/109860	2.61762725486666	0.00616637740217527	2.05618621244687	0.0478465709176237	0.701262577867292	-3.97818804524811
a_HQ_transcript/148779	0.565730868497828	6.05804455077159	2.0559998988243	0.0478655914110927	0.701262577867292	-4.46568715343419
a_HQ_transcript/8010	2.99740062432852	1.79244398508142	2.05582993066533	0.0478829490184683	0.701262577867292	-3.98331548317119
a_HQ_transcript/22471	-0.709474217828934	2.8934526580736	-2.05576154775031	0.0478899340276045	0.701262577867292	-4.10232276477522
a_HQ_transcript/63080	-3.00712489120108	0.189181413884378	-2.055759754018	0.0478901172614008	0.701262577867292	-4.05884557326193
a_HQ_transcript/92592	-2.27397817920666	1.13100390238556	-2.05571669385511	0.0478945161390922	0.701262577867292	-4.0008262602536
a_HQ_transcript/12942	-2.30531902768193	-0.00310009261138849	-2.05524972489789	0.0479422428992819	0.701765890972416	-3.90715774150053
a_HQ_transcript/130572	3.98100562811348	-0.277535906654173	2.0550117424659	0.0479665820768424	0.701765890972416	-3.98527808775151
a_HQ_transcript/134455	3.17915709131524	0.713788732765429	2.05494825025596	0.0479730774513186	0.701765890972416	-3.94110718885306
a_HQ_transcript/90745	-2.81352467705359	0.688780057008294	-2.05441780195508	0.048027373552395	0.701765890972416	-3.98420620693249
a_HQ_transcript/53696	5.59168823900234	1.25420042260304	2.05432482791001	0.0480368958354293	0.701765890972416	-3.97530697186584
a_HQ_transcript/129780	3.06552789688441	1.70772847574288	2.05418349050179	0.0480513746118689	0.701765890972416	-4.01434312771064
a_HQ_transcript/53883	-4.79512637731742	1.72944673668355	-2.05415656787992	0.0480541330330529	0.701765890972416	-3.92149232673296
a_HQ_transcript/107544	3.38855829032719	-0.414622210851763	2.05405401911454	0.048064641185476	0.701765890972416	-3.97667676814451
a_HQ_transcript/80890	-5.36432009138547	2.12893937961699	-2.05399786814496	0.04807039582046	0.701765890972416	-4.02430500449253
a_HQ_transcript/61372	4.80413955552434	3.28447417887481	2.05389602873456	0.0480808343831144	0.701765890972416	-4.02546664611885
a_HQ_transcript/27893	2.88849227057279	0.519219766543825	2.05377019441228	0.0480937351819459	0.701765890972416	-3.88157621528257
a_HQ_transcript/131618	-2.06772196130615	-0.511702349951913	-2.05368164485733	0.0481028152921792	0.701765890972416	-3.99624287793558
a_HQ_transcript/100180	3.99873631772039	1.17652373037157	2.05365986347388	0.0481050490449262	0.701765890972416	-3.98426199993271
a_HQ_transcript/103082	1.12213677116819	2.13687432232444	2.05360042697168	0.0481111449182709	0.701765890972416	-4.09072436375376
a_HQ_transcript/102287	-2.33127599855218	0.338606299869194	-2.05345795664924	0.0481257595967487	0.701765890972416	-3.91995748423017
a_HQ_transcript/103212	2.24782133363665	1.87521884301834	2.05342367239913	0.0481292770751507	0.701765890972416	-3.99720574640859
a_HQ_transcript/18762	0.815123899578865	3.96692844143605	2.05337639238965	0.0481341282567269	0.701765890972416	-4.24963696070838
a_HQ_transcript/13237	3.22772633643257	-0.376139411269499	2.05315287882164	0.0481570677625649	0.701924281570304	-3.99455308952246
a_HQ_transcript/126544	1.382333603658	3.50007456721518	2.05291194140952	0.0481818062638665	0.702108807848091	-4.19903613854407
a_HQ_transcript/46882	-3.62011563650668	0.0516840864968971	-2.052544839873	0.0482195202751491	0.702325293145161	-4.01907991791727
a_HQ_transcript/147102	-1.14735566867517	3.39412417536799	-2.05252840773942	0.0482212090295653	0.702325293145161	-4.24200068000552
a_HQ_transcript/124355	2.6456093908311	-0.470349323964793	2.05241457107932	0.0482329096173184	0.702325293145161	-3.97882287920475
a_HQ_transcript/34591	4.10314783263965	1.33550189214887	2.05226506204973	0.048248280544919	0.70233178391642	-3.98750562076641
a_HQ_transcript/109033	2.97003164351831	1.09999387392943	2.05212636443813	0.0482625438070703	0.70233178391642	-3.85463109203837

a_HQ_transcript/79027	2.68207066114407	-0.362447234482062	2.05205773443319	0.0482696028892461	0.70233178391642	-3.94038953303089
a_HQ_transcript/42568	2.4349890081299	-0.41468135189173	2.05190863452759	0.0482849419981385	0.702379156273223	-3.97435042616478
a_HQ_transcript/111730	4.46579988712525	1.24066141636095	2.05168575485152	0.048307879415245	0.702537006417153	-4.04126156980472
a_HQ_transcript/139506	4.99738812226071	1.16030709638778	2.05154084931328	0.0483227973461625	0.702578180124496	-3.97406131811895
a_HQ_transcript/84534	0.829139609869971	3.85886807040692	2.05128840317866	0.0483487961825676	0.702780401225379	-4.24725041480339
a_HQ_transcript/32808	-2.61237699399185	-0.375633269254182	-2.05100704522395	0.0483777870645963	0.702861871106104	-3.98013440607038
a_HQ_transcript/13276	-1.11104232537994	2.11954418380454	-2.05077574793692	0.0484016311787284	0.702861871106104	-4.02047314185925
a_HQ_transcript/151178	1.83408799977242	-0.663240788343752	2.05076979214315	0.0484022452895524	0.702861871106104	-3.95351855389485
a_HQ_transcript/62885	2.83357425427958	1.5887509951477	2.05070225876608	0.0484092092363272	0.702861871106104	-4.02057735629604
a_HQ_transcript/63697	2.15974604703338	0.0998772354785428	2.0504962398054	0.0484304590619037	0.702861871106104	-3.9113992061385
a_HQ_transcript/116122	-2.25733774127506	-0.394495838837306	-2.05048666458167	0.0484314468976596	0.702861871106104	-3.94271083584295
a_HQ_transcript/41345	-3.16342135542067	0.047679767387389	-2.05041304625971	0.0484390423832069	0.702861871106104	-4.02250924846284
a_HQ_transcript/77462	2.96855956282651	0.87763879349684	2.04984091873911	0.0484981066672169	0.703499806902789	-4.06346308127082
a_HQ_transcript/135921	4.46194564404211	4.06832950147649	2.04975277304774	0.0485072121192261	0.703499806902789	-4.1308713378537
a_HQ_transcript/21231	-2.11941158935134	0.631354461222883	-2.04917966430233	0.048566450908121	0.703981179795478	-3.86541350446635
a_HQ_transcript/142163	1.87096730221898	-0.64464921090581	2.04906064971035	0.0485787606950853	0.703981179795478	-3.99180820722885
a_HQ_transcript/106282	1.36070486806772	-0.828357019739899	2.04895777726347	0.0485894030941095	0.703981179795478	-3.98048463432534
a_HQ_transcript/6010	-0.798603992881507	3.70861275239415	-2.04892176082379	0.0485931295644609	0.703981179795478	-4.23487500302899
a_HQ_transcript/105483	-1.89414176350282	3.41647004372662	-2.04881598724747	0.048604074965528	0.703981179795478	-4.22522684565686
a_HQ_transcript/98937	1.96035126619726	4.15147025600199	2.04872909002491	0.0486130686708479	0.703981179795478	-4.39082776857054
a_HQ_transcript/118394	2.0823040454711	0.00349530673583749	2.0486013747986	0.0486262896193247	0.703997251056564	-4.06036039315905
a_HQ_transcript/151736	-4.08802843411898	-0.311699604312983	-2.04823625053592	0.0486641043329151	0.704236687837389	-4.01349975255283
a_HQ_transcript/113469	-2.53674831947107	1.21716632918845	-2.04819335960486	0.0486685481001532	0.704236687837389	-3.9022151446113
a_HQ_transcript/10627	-2.29556445229254	1.90058159514268	-2.04809081552326	0.0486791737498388	0.704236687837389	-3.96343212706725
a_HQ_transcript/118422	-2.57517295938934	2.90151816772973	-2.04789786843622	0.0486991725123182	0.704350709080874	-4.03708713168504
a_HQ_transcript/54028	2.86935438075744	1.44508989179865	2.04746598778551	0.04874396264771	0.704721045721084	-3.95052619529357
a_HQ_transcript/1184	3.20272411543139	0.99160928237451	2.04725522089515	0.0487658343174029	0.704721045721084	-4.01361520782352
a_HQ_transcript/15684	-2.16094589103203	2.60331565872445	-2.04724014058231	0.0487673995597568	0.704721045721084	-3.99475426881013
a_HQ_transcript/137840	-0.824002785434804	3.89681086650182	-2.04718356439266	0.048773272208504	0.704721045721084	-4.29135215546787
a_HQ_transcript/127316	-1.53870372474179	3.02923440440016	-2.04694751329641	0.0487977811836556	0.704899956422349	-4.12554907659313
a_HQ_transcript/67617	2.34856262667922	1.75312998125872	2.04667295412828	0.0488263020264544	0.705136716569874	-3.99613653841657
a_HQ_transcript/1083	-0.93312380006321	2.82363305005953	-2.04602844439776	0.0488933103774729	0.705603960337382	-4.16513594695288
a_HQ_transcript/44081	1.75005504227036	2.65836063864998	2.04580629326589	0.0489164256843742	0.705603960337382	-4.07386049595939
a_HQ_transcript/54920	3.10444280136671	-0.474495467961454	2.04572445708996	0.0489249433346846	0.705603960337382	-3.94613543565619
a_HQ_transcript/34389	1.80000138020557	1.88067424693258	2.04567747461122	0.0489298339406434	0.705603960337382	-3.95065168895412
a_HQ_transcript/137841	1.03256984171391	5.97668367175026	2.04566508014067	0.0489311242053515	0.705603960337382	-4.50304527878297
a_HQ_transcript/26783	1.67919890982612	0.873869669230686	2.04565340856737	0.0489323392437545	0.705603960337382	-3.89064176464752
a_HQ_transcript/61128	1.06617206180117	2.87377873468814	2.04554498839548	0.0489436273066392	0.705603960337382	-4.04108483712657
a_HQ_transcript/97417	1.04772374622774	2.79186703769617	2.04526719205151	0.0489725602456125	0.705846015858409	-4.07996427034832
a_HQ_transcript/116991	2.74211056505083	-0.495719350427132	2.04502249663222	0.0489980581112115	0.706038453616386	-3.98603602761702
a_HQ_transcript/5674	1.58428754842611	-0.774033476179821	2.04465279485683	0.0490366040608215	0.706161326147443	-3.99797859809314
a_HQ_transcript/76008	-2.41986449223469	1.27855075009491	-2.0445044127219	0.0490520822073399	0.706161326147443	-3.98026143334621
a_HQ_transcript/70986	1.05013604806608	3.70862683849851	2.04448174167697	0.0490544474638573	0.706161326147443	-4.20930750092188
a_HQ_transcript/79959	-2.62635089502385	-0.0167369470835119	-2.04408860410403	0.049095479200477	0.706161326147443	-3.96352488125372
a_HQ_transcript/133131	-3.23961980355317	1.18013651993393	-2.04407255681109	0.0490971546951378	0.706161326147443	-3.9810006463507
a_HQ_transcript/45449	-2.52348618108156	0.581465348785548	-2.04387993167095	0.0491172705665655	0.706161326147443	-3.89870785186821
a_HQ_transcript/139612	-4.50599804156673	2.5004335458924	-2.0437893232056	0.0491267353242374	0.706161326147443	-4.1909872675748
a_HQ_transcript/111396	-3.69841006655828	1.5068720396868	-2.04371150585796	0.049134865228601	0.706161326147443	-4.17807220357049
a_HQ_transcript/89986	-2.44777475686793	0.110202440340646	-2.04360381345293	0.0491461182540652	0.706161326147443	-3.98148790990479
a_HQ_transcript/98285	-1.94170873663482	4.24124410638125	-2.04354807962089	0.049151942898068	0.706161326147443	-4.27858393292242

a_HQ_transcript/123747	2.90895202965668	0.820629770436398	2.04351798595511	0.0491550881857297	0.706161326147443	-3.99879739850844
a_HQ_transcript/60730	1.89660167404993	-0.366920023560177	2.04325754255568	0.0491823162282744	0.706161326147443	-3.96325698770997
a_HQ_transcript/102766	-3.15212894936472	-0.325857958042713	-2.04323752351767	0.0491844096655812	0.706161326147443	-4.0191992367779
a_HQ_transcript/117882	-2.26481374435576	2.62190136199732	-2.04306807938595	0.0492021319658428	0.706161326147443	-4.06026220205164
a_HQ_transcript/114001	3.972816413304	0.916867412214576	2.04300951512839	0.0492082585530585	0.706161326147443	-3.96513043880039
a_HQ_transcript/3244	1.13510252848275	3.19992163600208	2.04300504316596	0.0492087264063343	0.706161326147443	-4.15309238982297
a_HQ_transcript/63288	-2.01275433729486	2.25656917244131	-2.04296316521606	0.049213107834835	0.706161326147443	-4.07417701540089
a_HQ_transcript/129167	2.19140874207182	2.46231671534875	2.04281143607343	0.0492289851766157	0.706214819927522	-4.08328924778522
a_HQ_transcript/141549	-2.57401970453471	1.12275308105283	-2.0426189915229	0.0492491295564454	0.706329485037517	-3.95661713349913
a_HQ_transcript/64196	-1.34704428926904	1.08924063107357	-2.04204517424837	0.0493092375802754	0.706931757205647	-3.91624028246597
a_HQ_transcript/151126	3.33011830884339	1.09343272518341	2.04198593598774	0.0493154465226551	0.706931757205647	-4.07135733920357
a_HQ_transcript/152747	-4.05699800752212	-0.446835237633686	-2.04152269922143	0.0493640234508879	0.707156182312517	-4.02486668974991
a_HQ_transcript/120303	-0.480947729880344	4.90964150008061	-2.04150379713391	0.0493660064937926	0.707156182312517	-4.39994737756664
a_HQ_transcript/140830	4.13711499613	1.0445413741962	2.04148861919295	0.0493675988821943	0.707156182312517	-4.04515353260087
a_HQ_transcript/90791	1.46904561117494	1.27144819832103	2.0413685266809	0.0493801999362348	0.707162419781586	-3.92586080492028
a_HQ_transcript/61094	-1.76949411977951	-0.682125314122075	-2.0411385424313	0.0494043395795473	0.707333854945795	-4.00416585112126
a_HQ_transcript/19192	-2.13807036340593	1.1120885985029	-2.04100685010816	0.0494181669534865	0.707357598786571	-3.88449021227987
a_HQ_transcript/137692	-4.6376120884936	0.13413157169292	-2.04075364506694	0.0494447624124974	0.707393284141273	-4.07055572954878
a_HQ_transcript/140026	-5.12441091758495	0.171907590132829	-2.04075139124718	0.0494449991994562	0.707393284141273	-4.115237054169
a_HQ_transcript/1182	0.700662373603938	4.51213358548113	2.04056094167957	0.0494650114822773	0.70750545950832	-4.34567685796526
a_HQ_transcript/117953	5.46295003483339	2.7938354854439	2.04017940024005	0.0495051249296548	0.707905018920289	-4.28409262489169
a_HQ_transcript/51913	-2.30336301496691	-0.0830188148802705	-2.03999715201586	0.0495242957127762	0.70794160279906	-3.9711506328756
a_HQ_transcript/108614	2.60367420198465	2.60806149651768	2.03992353536783	0.0495320413326414	0.70794160279906	-4.15637262049412
a_HQ_transcript/8122	-1.36653526772712	3.99865904954498	-2.03963856384608	0.0495620348072647	0.707986561295988	-4.32899146048979
a_HQ_transcript/112762	-3.46262884077021	1.80876400603194	-2.03956557673248	0.049569719321392	0.707986561295988	-4.04945895378402
a_HQ_transcript/116532	-0.864137635373522	5.00182526181631	-2.0394895962429	0.0495777201066559	0.707986561295988	-4.44352533902604
a_HQ_transcript/123680	3.44363715301065	0.864510778475135	2.03943085798342	0.0495839060527795	0.707986561295988	-4.02527226768726
a_HQ_transcript/88026	1.04106321552665	2.7126838158155	2.03929869697056	0.0495978269007604	0.707996941942947	-4.12579202945551
a_HQ_transcript/16896	1.47642848691156	3.51403316925606	2.03919271246527	0.0496089929901875	0.707996941942947	-4.1599435994092
a_HQ_transcript/130496	-2.70063676551396	2.44917656032309	-2.03850315853486	0.0496816954255865	0.708679625891813	-3.99873091218346
a_HQ_transcript/24472	-2.93001620687591	0.520321248083648	-2.03841481662314	0.0496910164206141	0.708679625891813	-3.91626621450834
a_HQ_transcript/15034	3.72416024392126	0.957685591812828	2.03838594688174	0.0496940628127363	0.708679625891813	-4.01750187792054
a_HQ_transcript/52423	2.40291420452092	1.03127796297847	2.03827212111548	0.0497060755293063	0.708679625891813	-3.9649075133737
a_HQ_transcript/1912	-1.03266495004526	2.20238299338931	-2.03816117438258	0.0497177868563655	0.708679625891813	-4.0465261370165
a_HQ_transcript/12115	-1.84335487069168	1.48174332832027	-2.03772666229832	0.0497636764422556	0.708961292475279	-3.97630293498875
a_HQ_transcript/77898	-1.61394361167946	3.50759280362782	-2.03771341273686	0.0497650763354754	0.708961292475279	-4.28922449954083
a_HQ_transcript/57046	1.70019592392116	-0.652907687935045	2.0375258852893	0.0497848934065485	0.708961292475279	-4.00268700475296
a_HQ_transcript/39348	-3.24969374676109	1.28959348308251	-2.03750398298268	0.0497872083969288	0.708961292475279	-4.055518545269
a_HQ_transcript/95013	-1.18220675137387	2.24374346822697	-2.03730352791116	0.0498084001227104	0.708961292475279	-3.97817023543292
a_HQ_transcript/137877	-3.07816145908567	0.272219163425219	-2.03728152560911	0.049810726645834	0.708961292475279	-4.02812014105262
a_HQ_transcript/18445	3.37194405336364	0.857953495104792	2.03702560750608	0.0498377944275193	0.709013273803185	-4.03330315989016
a_HQ_transcript/149139	-3.04478958165514	2.44676976055879	-2.03701635119403	0.0498387736849679	0.709013273803185	-3.99970735087337
a_HQ_transcript/150438	-2.44453969972987	0.0609900580414412	-2.03677997317573	0.0498637866583899	0.709195544624147	-4.00175829652407
a_HQ_transcript/10265	1.47734174913624	-0.365616268627999	2.03651910680366	0.0498914037160082	0.709412566129928	-3.98856683025635
a_HQ_transcript/8618	-2.049154696149	1.68661164055048	-2.03636470563334	0.0499077559767002	0.709412566129928	-3.94830170399075
a_HQ_transcript/121846	-2.18878110450451	0.324249307998864	-2.03629010469927	0.0499156584687484	0.709412566129928	-3.996436491367
a_HQ_transcript/102130	-0.553356086172693	4.24119998597757	-2.03579573142663	0.0499680552760014	0.709936539561613	-4.36633009903646
a_HQ_transcript/130734	2.13503734416853	3.36644152439859	2.03562195227153	0.0499864849478798	0.709936539561613	-4.15331272024608
a_HQ_transcript/84946	-3.39631428277685	-0.0827424040570974	-2.03559667401185	0.049989166261108	0.709936539561613	-3.98273205657965
a_HQ_transcript/16374	-2.44641701838857	1.94076648298809	-2.0354123226661	0.0500087245755155	0.710040826117627	-3.95600815571461

a_HQ_transcript/144809	-2.24494215189199	0.375240607009989	-2.03477584783572	0.0500763014495453	0.710826679037648	-4.01297063300551
a_HQ_transcript/115509	-2.3337005069086	0.901331872127735	-2.03466032244084	0.0500885757904163	0.710827327525712	-3.93674811694294
a_HQ_transcript/2253	-1.64186743534268	3.27959481007656	-2.03449798111195	0.0501058286896932	0.710837000364537	-4.20139252055826
a_HQ_transcript/56266	-3.1960972283509	0.8531003003129	-2.03432401981282	0.050124322291692	0.710837000364537	-3.92425049145335
a_HQ_transcript/82607	2.59339690611189	1.26283326806427	2.03430876913938	0.050125943856562	0.710837000364537	-4.02653830108461
a_HQ_transcript/9827	1.01060275549527	3.71056942549111	2.03400392478674	0.0501583668252798	0.711123304102406	-4.28902663614077
a_HQ_transcript/31349	3.42763984295679	2.17501463816149	2.03385931801779	0.050173753500964	0.711167994026831	-4.23577487227987
a_HQ_transcript/73105	2.61505215947783	-0.199803518225837	2.03355360085092	0.0502062965246328	0.711312405715096	-3.95091102675715
a_HQ_transcript/76614	0.87681607611593	1.19786766524299	2.03352865908695	0.0502089523455429	0.711312405715096	-3.99991826882643
a_HQ_transcript/19758	-2.59375406622791	1.36670270468419	-2.03330567872242	0.050232700965199	0.711312405715096	-3.95218185486694
a_HQ_transcript/69330	-2.11150606641144	0.749649098455955	-2.03330390492934	0.0502328899232808	0.711312405715096	-3.93105272306061
a_HQ_transcript/25801	2.59897858654559	0.29674191315499	2.03273247425147	0.0502937955693502	0.71200140011086	-4.04765780584512
a_HQ_transcript/95236	-1.76860341104125	2.67524979521482	-2.03212709410916	0.0503583903330837	0.712564875999029	-4.07494722837286
a_HQ_transcript/7096	-1.2559738769287	1.91508314323112	-2.03204852735295	0.0503667788279525	0.712564875999029	-3.95539631206769
a_HQ_transcript/7482	-1.19232015156061	2.76091885929134	-2.03201486236506	0.0503703735803745	0.712564875999029	-4.11551748575875
a_HQ_transcript/58481	-1.87794972168546	0.342933942793215	-2.03156608765181	0.0504183153030881	0.713049548531235	-3.98797276384894
a_HQ_transcript/91600	1.81525291110712	3.49652815549307	2.03146454659226	0.0504291682839924	0.713049548531235	-4.19059684865031
a_HQ_transcript/133539	3.6758798952635	-0.377526139142721	2.03115475382332	0.0504622924248025	0.713123178531488	-4.03053226902713
a_HQ_transcript/102183	-4.07210980571537	2.67142926221611	-2.0311491110786	0.0504628959439166	0.713123178531488	-4.08352290132177
a_HQ_transcript/93859	2.56651135935583	1.12828546181033	2.03105452224788	0.0504730136236302	0.713123178531488	-3.96605575303463
a_HQ_transcript/151128	1.1069950057741	2.64451425764022	2.03095698777184	0.0504834482462337	0.713123178531488	-4.0606024145343
a_HQ_transcript/2807	0.84571458766807	2.2372717988717	2.03050221155388	0.0505321269701197	0.713637384965772	-3.9738012530067
a_HQ_transcript/119832	-2.55211593799652	-0.316129074981095	-2.0302019379211	0.0505642904647682	0.713918162772904	-4.01887599355494
a_HQ_transcript/16217	-2.77052018243145	1.06692618460278	-2.02992154026309	0.0505943411676608	0.714168980911556	-3.97016422400199
a_HQ_transcript/136532	4.49118854569967	1.23217604977428	2.02974717846027	0.0506130357176949	0.714259417382415	-4.08370739619304
a_HQ_transcript/114814	1.79571296106171	2.59442807449828	2.02947900643825	0.0506418001365001	0.714334045899443	-4.14325185706417
a_HQ_transcript/137778	1.3887367399161	2.74083447638107	2.02946873705903	0.0506429019259497	0.714334045899443	-4.13008478943829
a_HQ_transcript/34314	-3.60554020059009	0.389615399800877	-2.0288406833892	0.050710325005265	0.714992845013414	-4.05886784327032
a_HQ_transcript/7182	2.12199133239017	1.27735079374155	2.02880453236403	0.0507142082987597	0.714992845013414	-4.03375842431953
a_HQ_transcript/140764	-2.58864467240618	0.0202074660993775	-2.02842770215898	0.050754702403167	0.715223383945882	-4.00678210064599
a_HQ_transcript/115162	-1.99662501831885	2.67744757396401	-2.02842336241309	0.0507551689164734	0.715223383945882	-4.0997892091666
a_HQ_transcript/62518	2.8756475458195	0.104387864440377	2.02801646579569	0.0507989261222922	0.71566649966956	-3.93835262359755
a_HQ_transcript/125046	-2.92179214679865	-0.0547398797857036	-2.02782864688419	0.0508191351074917	0.715675205748453	-4.06093883666036
a_HQ_transcript/63676	-2.74729504858299	0.886756273732504	-2.02767574564429	0.0508355922148709	0.715675205748453	-4.02671602248035
a_HQ_transcript/138541	3.05337975095846	0.00178615249058889	2.02746457558119	0.0508583286073043	0.715675205748453	-4.05959045322234
a_HQ_transcript/37380	1.6279548175941	-0.641006711705974	2.02744078146145	0.0508608910462474	0.715675205748453	-4.00365249627963
a_HQ_transcript/118485	4.00098610908447	0.734048370700332	2.02743880062139	0.0508611043721934	0.715675205748453	-3.98033425144141
a_HQ_transcript/26724	4.70539529513051	1.45069487344794	2.02707621659765	0.0509001659544438	0.716051511761836	-3.99058068093166
a_HQ_transcript/25603	-0.905996436712346	3.63572749167741	-2.02684027824037	0.0509255979741064	0.716196636170465	-4.29364989151751
a_HQ_transcript/93969	-2.2974146128044	0.173892250601355	-2.02670451101427	0.0509402374990313	0.716196636170465	-4.08317217542957
a_HQ_transcript/144506	3.96457449908882	3.73046379738692	2.02659250441295	0.0509523177363732	0.716196636170465	-4.28056746499531
a_HQ_transcript/22025	2.32263264304436	0.205846402053418	2.02652345508705	0.0509597661574636	0.716196636170465	-4.04097776076457
a_HQ_transcript/7705	-2.37535872564734	0.251031402826934	-2.02628586515953	0.0509854025148109	0.716383726705566	-4.00990216863822
a_HQ_transcript/98932	3.035039920464	2.56815566438678	2.02587783875119	0.0510294556267891	0.716829433705653	-4.10429787439723
a_HQ_transcript/134768	-2.57880138933022	-0.37988733462734	-2.02573394917941	0.0510449988074006	0.716874532659237	-3.98709807649626
a_HQ_transcript/30236	2.17624018642208	-0.290137157633144	2.02559213661193	0.0510603216852006	0.716916516646701	-3.99299614468622
a_HQ_transcript/121873	-1.48062302805431	1.10301534061649	-2.02537221845797	0.0510840918586865	0.717000063498254	-3.94454735714902
a_HQ_transcript/144709	-1.89772993425502	-0.573124281209846	-2.02527551689458	0.0510945470562077	0.717000063498254	-4.01109183318818
a_HQ_transcript/124119	-1.5507059374217	-0.530022609450423	-2.02519478928157	0.0511032766149406	0.717000063498254	-3.97816766896863
a_HQ_transcript/35686	-3.65875764931733	1.74449775118929	-2.02469882375538	0.0511569370073534	0.717099322167048	-4.02567693333228

a_HQ_transcript/137802	3.10650363043495	3.18325721184707	2.02463770714739	0.0511635528600557	0.717099322167048	-4.21989808367968
a_HQ_transcript/43224	-3.5631015658301	1.23845568512298	-2.02463500572355	0.0511638453055948	0.717099322167048	-4.02657495353428
a_HQ_transcript/42184	3.34150178986032	2.60189636496932	2.0245644393482	0.0511714850630824	0.717099322167048	-4.19388576580384
a_HQ_transcript/94739	-2.39989725254395	-0.0394594203583412	-2.02446521979729	0.0511822286018963	0.717099322167048	-4.0094760674883
a_HQ_transcript/30381	2.14145320638959	-0.332471334197819	2.02444544107714	0.0511843704870472	0.717099322167048	-4.01195770162348
a_HQ_transcript/133098	1.94749280660578	-0.566140932606392	2.02426091093729	0.0512043574834695	0.717147789177389	-4.01413319721651
a_HQ_transcript/109217	-1.92961698349703	-0.498525058060323	-2.02418570983472	0.0512125046943873	0.717147789177389	-4.0208428818775
a_HQ_transcript/73390	-3.43214968479349	1.77844359096226	-2.02401426054245	0.0512310835820676	0.717149550135002	-3.97819198119881
a_HQ_transcript/118667	-1.57396581099683	-0.599267106049554	-2.02395068406278	0.0512379744680682	0.717149550135002	-4.02786345535762
a_HQ_transcript/40840	0.836442216446981	3.42894953842879	2.0236666035701	0.0512687751192623	0.717149550135002	-4.24268377667326
a_HQ_transcript/81112	-4.08605441637992	1.49248337875138	-2.02364966036327	0.0512706126525537	0.717149550135002	-4.00841875827412
a_HQ_transcript/60734	-1.67765702944654	-0.737837141933198	-2.02361550053708	0.0512743175468117	0.717149550135002	-4.02720376224827
a_HQ_transcript/130619	-3.38760733594332	-0.219677554897174	-2.0229324898035	0.0513484445170835	0.717593096968027	-4.03777573702264
a_HQ_transcript/69426	-3.16294784651091	0.183521305052353	-2.02284047924392	0.051358437568775	0.717593096968027	-3.99307310811457
a_HQ_transcript/22335	1.0813937842706	1.96125599942356	2.02274584709879	0.0513687171215284	0.717593096968027	-3.95058797737102
a_HQ_transcript/105221	2.35670466930686	0.507149609737778	2.02270272835762	0.0513734015543276	0.717593096968027	-4.07517575259941
a_HQ_transcript/42182	1.61110626318821	2.40591541964657	2.02270129378444	0.0513735574132134	0.717593096968027	-4.12530257952496
a_HQ_transcript/61879	2.24552558548819	1.41625754093683	2.02262547100775	0.0513817957514275	0.717593096968027	-4.06036383476049
a_HQ_transcript/125793	-2.02978713989027	2.79565275351674	-2.02239300969359	0.0514070604785541	0.717593096968027	-4.12821358224627
a_HQ_transcript/62818	-0.875022145216202	2.42262909894626	-2.0223566832271	0.0514110095516605	0.717593096968027	-4.09934926701831
a_HQ_transcript/123576	-3.09372725085064	0.811247783318681	-2.02230033765289	0.0514171354402669	0.717593096968027	-4.05008170264834
a_HQ_transcript/43736	-2.18649202428714	-0.355578899337846	-2.02214938941594	0.0514335496770202	0.71764987413006	-4.04672739099587
a_HQ_transcript/9475	1.97703130606395	-0.571225380370428	2.02189753057702	0.0514609472329776	0.717859836995086	-4.02938050369234
a_HQ_transcript/120235	3.48437239384784	1.68477152626506	2.02176235071359	0.0514756575573623	0.717892759715537	-4.0691790624283
a_HQ_transcript/63224	-3.40848865220473	1.853053345856	-2.02131336777674	0.0515245424823902	0.718402160090279	-4.07408428481235
a_HQ_transcript/107245	2.57048303038405	2.14680587881222	2.02118993046591	0.0515379893668967	0.718417325160425	-4.07346387877544
a_HQ_transcript/107861	-2.95053758486714	0.824051128825894	-2.02100704062083	0.0515579184790052	0.718482888356861	-4.03892750576018
a_HQ_transcript/36771	2.06238629268647	0.0021699720978112	2.02091992667112	0.0515674134706996	0.718482888356861	-4.02379198645732
a_HQ_transcript/85100	3.26461776211746	1.52921734727883	2.02075539769006	0.0515853504966115	0.718560568815489	-4.0683413640031
a_HQ_transcript/151135	3.52249978758181	1.046231552482	2.02054286215022	0.0516085293065388	0.718711210237299	-4.1215386030795
a_HQ_transcript/56475	-3.34858066722722	0.035681169315658	-2.02039705663355	0.0516244359129414	0.71876052952035	-4.08036916463316
a_HQ_transcript/150740	-2.34668233617049	-0.302188129946341	-2.02013800060685	0.0516527081369256	0.718901343023823	-4.03941350134514
a_HQ_transcript/114573	-2.42665621033989	0.754900289824269	-2.02007775652613	0.0516592848508551	0.718901343023823	-3.96554183755332
a_HQ_transcript/115817	-2.97748626661834	0.020591875187918	-2.0196641489843	0.0517044572767789	0.719357753131786	-4.07590420107654
a_HQ_transcript/141224	-5.18547284606866	8.83558270524221	-2.01937073246957	0.0517365239241224	0.719595280447171	-4.51559065347141
a_HQ_transcript/71596	2.09550824580253	-0.583878343985227	2.01928141392952	0.0517462887467171	0.719595280447171	-4.04415002230475
a_HQ_transcript/119105	-5.36334298267887	2.27455886884017	-2.01862010426748	0.0518186371789179	0.720383494611022	-4.1580624619342
a_HQ_transcript/92134	1.00515192208711	2.78951315762434	2.01853681407276	0.0518277555474693	0.720383494611022	-4.14104983404037
a_HQ_transcript/79255	2.06631048359012	-0.626530470848353	2.01832717806923	0.0518507121050472	0.720530287650534	-4.03767786602466
a_HQ_transcript/151053	-2.34633615747964	-0.268665727159311	-2.01812532667563	0.0518728246062296	0.720665284108727	-4.00860012450131
a_HQ_transcript/6904	2.08262875533577	1.99712086817475	2.01780770742272	0.0519076360073891	0.720886991692657	-3.97721492615054
a_HQ_transcript/127137	-3.00955448140016	-0.0975799954455979	-2.01775343517183	0.0519135863478093	0.720886991692657	-4.02099265990099
a_HQ_transcript/116311	-3.62945539249629	1.60615571704865	-2.01737202869907	0.0519554201114424	0.721295595948871	-4.09647794055486
a_HQ_transcript/105278	-3.54132057699082	0.471236923373596	-2.01714203113291	0.0519806611677045	0.721473704001033	-4.07361355048002
a_HQ_transcript/89228	2.18035443011981	1.30307029456199	2.01660149251237	0.0520400247728256	0.721891304437704	-4.07051035434292
a_HQ_transcript/71527	-1.1263716470233	3.81986474097417	-2.01645907983081	0.0520556748413141	0.721891304437704	-4.2793878863863
a_HQ_transcript/82507	4.0193028507223	-0.270795546260376	2.01625912913407	0.0520776548505383	0.721891304437704	-4.04823609940591
a_HQ_transcript/89744	1.84413638898974	-0.702468576916694	2.01608472430706	0.0520968333066992	0.721891304437704	-4.03076815818847
a_HQ_transcript/75473	-1.76059161158607	-0.607397475585192	-2.01604169871471	0.0521015655735947	0.721891304437704	-4.04453538701488
a_HQ_transcript/144306	-2.36295349395566	-0.256041210546305	-2.01599619001754	0.0521065713597496	0.721891304437704	-4.04567749448797

a_HQ_transcript/148722	2.95780128799491	6.98487311965005	2.01598443561623	0.052107864368238	0.721891304437704	-4.70592156939024
a_HQ_transcript/16908	-3.2908288806	0.57606760648576	-2.01593634692498	0.0521131545162974	0.721891304437704	-4.15663214252745
a_HQ_transcript/146059	2.20126762340536	0.981399023592242	2.01576176791804	0.0521323635807436	0.721891304437704	-4.03023214445328
a_HQ_transcript/149780	2.43784594629629	0.687236759814965	2.01573837181069	0.0521349383434744	0.721891304437704	-4.00106121590762
a_HQ_transcript/46894	3.12849159014157	1.99218246876723	2.01539748981471	0.0521724653390832	0.722238881931466	-4.17516340992484
a_HQ_transcript/6119	2.34079935975809	5.80429167714634	2.01527061275428	0.0521864390079053	0.72226031586941	-4.52239999125081
a_HQ_transcript/10492	2.15683516754876	2.24199347603064	2.01510093081578	0.0522051321321322	0.722347041317919	-4.01814278421124
a_HQ_transcript/121240	1.09181749302221	5.12737269666531	2.01471483915489	0.0522476880125276	0.722541673384377	-4.48959600070864
a_HQ_transcript/99421	-4.24823370333545	3.13756387944106	-2.01459334192404	0.0522610859844823	0.722541673384377	-4.22683658332834
a_HQ_transcript/24189	2.0530683929408	2.71621326730081	2.01453941286574	0.05226703391462	0.722541673384377	-4.1412763941067
a_HQ_transcript/89745	2.88033922570351	0.465486224915493	2.01449907205249	0.0522714835606889	0.722541673384377	-4.02605289131791
a_HQ_transcript/96847	-2.02962252793824	0.647091918998343	-2.0143831765428	0.0522842688355662	0.722541673384377	-4.03957808848569
a_HQ_transcript/130570	-0.730138483968273	4.13228932423885	-2.0142969798856	0.0522937795886332	0.722541673384377	-4.40560275554808
a_HQ_transcript/125933	-3.07897970823726	2.90867312447976	-2.01416868954791	0.0523079376705147	0.722565542041749	-4.21442612420688
a_HQ_transcript/95145	6.62998231069285	2.2241469616493	2.01387943832992	0.052339871650939	0.722713664548452	-4.14904805305212
a_HQ_transcript/13734	1.10056213378751	2.73626741124912	2.01382574642599	0.0523458012335982	0.722713664548452	-4.1077148897739
a_HQ_transcript/92771	2.07170324829974	0.129030005074074	2.01372130972289	0.0523573366123746	0.722713664548452	-4.03082334192631
a_HQ_transcript/20910	2.01951412552747	-0.382417416363455	2.01362122745154	0.0523683931165373	0.722713664548452	-4.06904406012404
a_HQ_transcript/54460	2.10044593149007	-0.247483416250609	2.01347803108206	0.0523842161663144	0.722760436106224	-4.04326424782141
a_HQ_transcript/122608	2.42315796673428	0.0887358820915003	2.01324040675342	0.0524104826365524	0.722951242215832	-3.96906890542025
a_HQ_transcript/103145	1.91792176417954	0.311484111781858	2.01301911383594	0.0524349542291418	0.723117205084592	-4.02275367828293
a_HQ_transcript/47774	2.00263485193641	-0.248193905700948	2.01286820430492	0.0524516482338906	0.723175855915463	-4.02386769064561
a_HQ_transcript/127059	1.53638279760805	1.96954050256708	2.0124332796091	0.052499786746544	0.723521431101008	-4.01028101044215
a_HQ_transcript/442	-2.90172046490495	2.80608330953152	-2.01238307027197	0.0525053465243796	0.723521431101008	-4.09607418095716
a_HQ_transcript/29133	-3.0865180251414	1.58946283455316	-2.0123044460307	0.0525140537746895	0.723521431101008	-3.99995494122687
a_HQ_transcript/106780	2.94407016405603	0.465065713924073	2.01214871125446	0.0525313043684815	0.723543781743847	-4.03329001546231
a_HQ_transcript/62015	2.5022928725069	0.666057501589139	2.01203793229703	0.0525435782655532	0.723543781743847	-4.03577128881942
a_HQ_transcript/30945	-1.40691520358793	1.68787358614159	-2.01195274616181	0.0525530182790139	0.723543781743847	-3.98478068734843
a_HQ_transcript/127837	2.10704074784497	-0.104661342586664	2.01173594810746	0.0525770497323554	0.723703231551587	-4.01407533540514
a_HQ_transcript/145205	-2.27613122256408	0.0526743551856996	-2.01157247928754	0.0525951761458694	0.723781344461907	-4.05073142073002
a_HQ_transcript/63481	-3.47537568560312	2.37221371809165	-2.01145840241824	0.0526078289184447	0.723784113460675	-4.12957139767845
a_HQ_transcript/83760	-2.2407558359553	0.160231270578209	-2.01111176563563	0.0526462922814682	0.723840437123859	-4.07670202777576
a_HQ_transcript/54515	2.53146674559676	-0.0198095449828432	2.01097180172356	0.0526618298665975	0.723840437123859	-3.96383442770935
a_HQ_transcript/68305	-1.67911603937198	-0.525737913902719	-2.01079707692716	0.0526812319331368	0.723840437123859	-4.06235135598281
a_HQ_transcript/47838	3.74791356284538	-0.191566429805338	2.01075651001997	0.0526857375217922	0.723840437123859	-4.05515749051645
a_HQ_transcript/124652	-2.31823734340137	1.00568530852813	-2.01072511428207	0.0526892247401843	0.723840437123859	-3.92324565261263
a_HQ_transcript/114247	4.31102544322296	2.84193526560243	2.01032414045713	0.0527337798559584	0.723840437123859	-4.11243871571214
a_HQ_transcript/132312	-3.96778618037855	5.25553573187262	-2.01028813607959	0.0527377821756569	0.723840437123859	-4.36255618939876
a_HQ_transcript/116888	-2.94973038490414	2.08288744410811	-2.01027664978482	0.0527390590713921	0.723840437123859	-4.04547422825313
a_HQ_transcript/18825	-2.5897965954107	-0.315711967453383	-2.0101664947878	0.0527513060334673	0.723840437123859	-4.0235506670773
a_HQ_transcript/104966	2.06669814045249	-0.236410765526742	2.01010145070928	0.0527585387602741	0.723840437123859	-3.98121802925397
a_HQ_transcript/124094	-4.03495955652765	1.79331805336969	-2.01003789652506	0.052765606652465	0.723840437123859	-4.213275986238
a_HQ_transcript/70820	-1.24750902480755	2.60752725044777	-2.00996608520822	0.052773593820807	0.723840437123859	-4.22291128792505
a_HQ_transcript/150915	1.51826020802521	2.59023795682203	2.00989379957368	0.0527816348125748	0.723840437123859	-4.10459198318565
a_HQ_transcript/108465	0.623746311958413	4.80531580081801	2.00985224190698	0.0527862581366646	0.723840437123859	-4.42131972509469
a_HQ_transcript/10911	1.03902851872092	2.02697939210488	2.00969854198229	0.0528033604548647	0.723904183141598	-3.98597360562699
a_HQ_transcript/113398	-2.37342532107134	0.25767298685335	-2.00957957365823	0.0528166014855192	0.723914975512913	-3.97263446087104
a_HQ_transcript/72351	-2.62931026190086	-0.245281511066058	-2.00941846533552	0.0528345372752203	0.723990094939652	-4.04886120313275
a_HQ_transcript/125164	-1.53750098983918	1.9318702499417	-2.00916287052617	0.0528630029380123	0.724209435489224	-4.06076364805893
a_HQ_transcript/124707	-1.57607305812874	2.06306268811048	-2.00826126925381	0.0529635216229606	0.724957558079642	-4.17968838726527

a_HQ_transcript/148596	-2.2819328837261	-0.478212191817621	-2.00825577702749	0.0529641344578877	0.724957558079642	-4.06328043968306
a_HQ_transcript/89653	4.66364678283059	1.0965461355523	2.00824762761077	0.0529650437995014	0.724957558079642	-4.18277561596318
a_HQ_transcript/104864	2.02444602406884	-0.35847133068242	2.00810736200931	0.0529806972858782	0.724957558079642	-4.07323025947922
a_HQ_transcript/43705	1.41296523666454	2.14212011076914	2.00806915353825	0.0529849620099779	0.724957558079642	-4.12972017411039
a_HQ_transcript/18860	1.93797629233979	-0.368042319843438	2.00790517244244	0.0530032685356519	0.724957558079642	-4.06065804429761
a_HQ_transcript/4536	1.70649721407353	2.11610990458006	2.00786299944671	0.0530079775394455	0.724957558079642	-4.12365474562698
a_HQ_transcript/93221	-1.88644673207265	0.138187524546686	-2.00777243312576	0.0530180913402955	0.724957558079642	-4.06266686949735
a_HQ_transcript/149611	0.398024153863375	8.83248967944437	2.00766709243816	0.0530298571592802	0.724957558079642	-4.82075782821923
a_HQ_transcript/126961	-4.02633695318922	1.34343211379627	-2.00754380697752	0.0530436301830893	0.724975343353542	-3.94119514581782
a_HQ_transcript/88434	2.38548572583391	-0.267674839680281	2.00718583718699	0.0530836390496682	0.724994028725724	-4.09081789827201
a_HQ_transcript/104920	-2.18532098297595	-0.331639372709696	-2.00711589095974	0.0530914597428071	0.724994028725724	-4.04366900866406
a_HQ_transcript/96741	1.74103464279695	-0.592773477193327	2.00699364475242	0.0531051305192752	0.724994028725724	-4.06988763787223
a_HQ_transcript/128201	-0.869967325150539	5.63888366940269	-2.0069796850551	0.0531066918260398	0.724994028725724	-4.52762971449063
a_HQ_transcript/106827	-4.68901803003536	1.64397273585048	-2.00695014384965	0.0531099959614911	0.724994028725724	-4.07663171850197
a_HQ_transcript/39430	0.651759003483898	3.21711732856201	2.00686221654413	0.0531198315500767	0.724994028725724	-4.23283822810108
a_HQ_transcript/87115	2.42107397439169	-0.234820754088946	2.00642115380991	0.0531691930583625	0.725151776121609	-4.01541724816844
a_HQ_transcript/144637	-4.60633061525986	1.60345076123123	-2.00636150853179	0.0531758713325662	0.725151776121609	-4.05168878626952
a_HQ_transcript/131219	-4.34419556685519	-0.124410911733095	-2.00635719181553	0.0531763546887136	0.725151776121609	-4.08088769494569
a_HQ_transcript/38373	3.58669040451178	0.50729201259339	2.00621885778844	0.0531918464136275	0.725151776121609	-4.04072532534327
a_HQ_transcript/26418	-3.58088693275805	2.30638850967947	-2.00606255139749	0.0532093555709831	0.725151776121609	-4.19387216215816
a_HQ_transcript/3688	-2.02072088792324	0.99738753501119	-2.00603966703893	0.0532119194578831	0.725151776121609	-4.00128706734338
a_HQ_transcript/45166	1.43290454694718	2.51350571627733	2.00597901531414	0.0532187151963733	0.725151776121609	-4.22168071920635
a_HQ_transcript/28735	2.17680673479507	0.027903257354879	2.00572640938527	0.0532470266536937	0.725367510036538	-4.0259614771006
a_HQ_transcript/35823	-3.01745270174298	0.486456104652222	-2.00520265929261	0.0533057692331371	0.725816725464495	-4.0348458703571
a_HQ_transcript/111136	-3.14911401227797	0.80627967479442	-2.0051720766406	0.0533092010609772	0.725816725464495	-4.15253838304157
a_HQ_transcript/59199	2.06901264192498	0.153097140197262	2.00509846858909	0.0533174617694294	0.725816725464495	-3.98159897212503
a_HQ_transcript/128945	-2.55280415060725	0.624465133797752	-2.00464370549774	0.0533685226449805	0.726219420751279	-3.98332280664283
a_HQ_transcript/125387	-4.21324271449402	-0.097489680498439	-2.00452138702837	0.0533822638738742	0.726219420751279	-4.06723247387577
a_HQ_transcript/84814	-3.04239175314563	1.2760568217997	-2.00421628165182	0.05341655281749	0.726219420751279	-3.97152286502454
a_HQ_transcript/152837	-0.624854513196437	5.7776709924514	-2.00414263205798	0.0534248327319897	0.726219420751279	-4.5707242617623
a_HQ_transcript/71667	-2.05362023071515	-0.386524399619667	-2.00390392387009	0.0534516767515067	0.726219420751279	-4.08496689112583
a_HQ_transcript/136815	-1.02808871529018	2.4156700456645	-2.00350783929969	0.0534962445952859	0.726219420751279	-4.08133893149544
a_HQ_transcript/44611	-1.2536283254966	3.09809502236469	-2.00336578156557	0.0535122369871608	0.726219420751279	-4.12990258857974
a_HQ_transcript/136038	-2.19118621298322	1.41799879131922	-2.0033515127193	0.0535138435577244	0.726219420751279	-4.10986898868263
a_HQ_transcript/107882	1.533798575189	-0.681151625532997	2.00325199642994	0.0535250495558272	0.726219420751279	-4.07789048117463
a_HQ_transcript/108905	-4.64769265406783	0.921030928603009	-2.00325096215326	0.0535251660309667	0.726219420751279	-4.08995331035642
a_HQ_transcript/111495	-0.7360218503225	3.0704284218593	-2.00324185831042	0.0535261912704145	0.726219420751279	-4.20877801152473
a_HQ_transcript/140060	-3.44032096635961	2.3379269475581	-2.00323925504091	0.0535264844436783	0.726219420751279	-4.12980105410016
a_HQ_transcript/50271	-3.97976334713936	3.03879156104108	-2.00320012799664	0.0535308909959826	0.726219420751279	-4.28907670813374
a_HQ_transcript/135077	-2.41242334378478	1.15225310893463	-2.00307685227081	0.0535447765842811	0.726219420751279	-4.07236885408539
a_HQ_transcript/19446	1.03484016020116	2.83840858805504	2.00306549766653	0.053546055707725	0.726219420751279	-4.20907692731654
a_HQ_transcript/45938	2.47971537433062	1.8369431061807	2.00286592887378	0.0535685419703457	0.726219420751279	-4.08457478374196
a_HQ_transcript/52746	-3.96911107665329	0.195774932798505	-2.00276459009139	0.0535799633990016	0.726219420751279	-4.07200054714582
a_HQ_transcript/93518	2.79697706221863	-0.127051254465074	2.00255878290036	0.0536031655303715	0.726219420751279	-4.1222685169762
a_HQ_transcript/116362	3.0958031991886	2.79180605683045	2.00249916320832	0.0536098885285173	0.726219420751279	-4.10204775952486
a_HQ_transcript/83838	-2.67578122826751	-0.494463704054312	-2.00244679375225	0.0536157945635423	0.726219420751279	-4.06599704686367
a_HQ_transcript/120764	2.27363568841431	-0.506957107586893	2.00238606188116	0.0536226443928805	0.726219420751279	-4.06068581279076
a_HQ_transcript/148786	-2.8683286979582	0.123725061098267	-2.00237760288988	0.0536235985266734	0.726219420751279	-4.08350411898594
a_HQ_transcript/10793	2.18382416207113	-0.0803394432176864	2.0022733930684	0.0536353541135063	0.726219420751279	-4.02828736954619
a_HQ_transcript/121498	-2.54645187649845	0.230109913985306	-2.00217113931399	0.0536468912308938	0.726219420751279	-4.05550378228843

a_HQ_transcript/76037	1.49503685045404	0.782513544144112	2.00193481194132	0.0536735639429934	0.726219420751279	-3.99692883719552
a_HQ_transcript/51961	3.80794575878668	2.3820545300167	2.00175389004968	0.0536939912326226	0.726219420751279	-4.20455487969432
a_HQ_transcript/99280	3.02447261667292	0.467524283558182	2.00173676509437	0.0536959251062256	0.726219420751279	-4.10496001910275
a_HQ_transcript/27218	-1.68246376285052	3.31569406349635	-2.00172848773089	0.0536968598676885	0.726219420751279	-4.32332205610284
a_HQ_transcript/143308	-2.48167493625072	2.57852163282187	-2.00147353623756	0.053725658465967	0.726317784337493	-4.17253745624171
a_HQ_transcript/44230	-2.99313312246188	-0.400562169272397	-2.00142203823827	0.0537314771716803	0.726317784337493	-4.07719796388672
a_HQ_transcript/81464	-1.51781730852918	1.72571832065335	-2.00129157423155	0.0537462206288981	0.726317784337493	-3.98606765983903
a_HQ_transcript/116099	3.90168905104209	4.39141779389259	2.00122174371478	0.0537541134774961	0.726317784337493	-4.35269777855747
a_HQ_transcript/51682	-3.75296247387302	0.872874696414875	-2.00091342803844	0.0537889740854647	0.726447783566486	-4.17031665701541
a_HQ_transcript/16135	-3.59433955424613	2.53246747184438	-2.00084400319379	0.0537968265307439	0.726447783566486	-4.13889658033335
a_HQ_transcript/24663	3.3166933644308	1.44103980898159	2.0008051039951	0.0538012267453503	0.726447783566486	-4.11517957664502
a_HQ_transcript/33860	-1.67910137845788	-0.698404741116803	-2.00002889580789	0.0538890959106009	0.727465250137345	-4.04129249758731
a_HQ_transcript/152152	-3.53135961589492	3.13016596462634	-1.9999137702356	0.0539021391493203	0.727472380884999	-4.19982826255487
a_HQ_transcript/147250	-2.23769350464509	-0.49056332182314	-1.99947025350873	0.0539524134872878	0.72798186889254	-4.05461359469163
a_HQ_transcript/18067	2.34671987959818	0.149393823907545	1.9991542427869	0.0539882595056391	0.728223309278831	-4.10833943934932
a_HQ_transcript/22590	-2.00731703590113	1.9372016601997	-1.9990916412929	0.0539953630434861	0.728223309278831	-4.03823760874925
a_HQ_transcript/70550	-2.72354741097019	0.29226345495674	-1.99873042134226	0.0540363674605239	0.728341543893103	-4.05699617330675
a_HQ_transcript/116469	3.97613790486405	1.54980798598113	1.99859615706488	0.0540516155992494	0.728341543893103	-4.1144050317262
a_HQ_transcript/130421	2.56400331350816	-0.353722333122149	1.9984656108865	0.0540664450831285	0.728341543893103	-4.07812786423119
a_HQ_transcript/20182	-1.04237052225609	1.8098721933722	-1.99845426950524	0.0540677335829367	0.728341543893103	-4.04394829706352
a_HQ_transcript/129713	-2.50823470465914	0.559875701347952	-1.99838393069502	0.0540757254080333	0.728341543893103	-3.96296734997625
a_HQ_transcript/4821	-4.22808815711926	0.430890666369609	-1.99835238868809	0.0540793095142209	0.728341543893103	-4.03254485339264
a_HQ_transcript/2376	-1.06758431017044	2.43055277588557	-1.99809842622586	0.0541081747312879	0.728394474673118	-4.1008503636414
a_HQ_transcript/48645	2.67420618913109	-0.138192559601994	1.99808861024112	0.05410929068006	0.728394474673118	-4.09678131918281
a_HQ_transcript/141818	1.79833012290919	2.93926571429653	1.99795817413983	0.0541241214626458	0.728394474673118	-4.21171593235289
a_HQ_transcript/5925	1.50933552569627	3.39615067662252	1.99782810168297	0.0541389144327715	0.728394474673118	-4.32572162142487
a_HQ_transcript/48895	-2.16848308219799	2.00450901101108	-1.99776674233498	0.0541458939764407	0.728394474673118	-4.12949213869674
a_HQ_transcript/138687	-1.31847107683095	6.62114446308323	-1.99725549259496	0.0542040785004876	0.72884759153641	-4.65134439923039
a_HQ_transcript/66971	-3.357898320175	2.30362635454698	-1.99718281985467	0.0542123536999606	0.72884759153641	-4.08652593928195
a_HQ_transcript/14386	-2.33836956803693	0.0607668023177811	-1.99713936253141	0.0542173026851645	0.72884759153641	-4.00294054978874
a_HQ_transcript/18986	-3.18667455192427	2.20941161888151	-1.996890904176	0.054245605070635	0.72884759153641	-4.10570273306653
a_HQ_transcript/150595	0.415062077190797	8.44087315536933	1.99684890859858	0.0542503901445473	0.72884759153641	-4.80406200932829
a_HQ_transcript/30736	-2.5870241870253	-0.304449735904779	-1.99666737604168	0.054271078626553	0.72884759153641	-4.07355001985531
a_HQ_transcript/6132	2.24017124091559	1.7300547513822	1.99656615942972	0.0542826168375822	0.72884759153641	-4.00462220562356
a_HQ_transcript/25245	0.924980382431863	3.24299059882502	1.99654189805665	0.0542853828365696	0.72884759153641	-4.2063685708445
a_HQ_transcript/87751	-2.11442204216378	3.25251521813348	-1.99638945229329	0.0543027657415133	0.72884759153641	-4.17606289035585
a_HQ_transcript/142973	-2.26484728516022	0.219388412262241	-1.99634174833401	0.0543082062702082	0.72884759153641	-4.09755290706585
a_HQ_transcript/72643	3.19711265321541	0.0130493088322419	1.99620701971958	0.0543235743339172	0.72884759153641	-4.04613584952879
a_HQ_transcript/128757	-2.27661085094218	-0.448364850204068	-1.99606873457276	0.0543393520279132	0.72884759153641	-4.07601480215113
a_HQ_transcript/12836	-0.877868527482925	2.40896413143138	-1.99604045131368	0.054342579509338	0.72884759153641	-4.12331095373744
a_HQ_transcript/113985	2.91042229831364	1.12023949843177	1.99584055211853	0.0543653953343551	0.728985397922812	-4.06451462728855
a_HQ_transcript/88061	2.25947173396291	-0.170542523162896	1.99553620726426	0.0544001482945467	0.72901331017369	-4.02333591223783
a_HQ_transcript/127234	2.32461355250248	0.834229822162191	1.99545353320213	0.0544095921459465	0.72901331017369	-4.07914243866421
a_HQ_transcript/23194	-2.55076852830172	2.13706906078221	-1.99539449088524	0.0544163374209255	0.72901331017369	-4.01577659210101
a_HQ_transcript/6380	-0.858308898804172	3.20304012558925	-1.99538306362924	0.0544176430092837	0.72901331017369	-4.29967421613388
a_HQ_transcript/27697	-2.70296995421437	0.397857570072561	-1.99525304522158	0.0544324998150449	0.729013823425864	-4.02436012799993
a_HQ_transcript/109829	1.94659063391397	0.957249406720603	1.99511402509955	0.0544483891357295	0.729013823425864	-3.96375481966491
a_HQ_transcript/132147	-2.6887426337103	0.115747322465976	-1.9949407027379	0.054468204704891	0.729013823425864	-4.03579539838668
a_HQ_transcript/75417	-2.56943896134913	-0.332381160770458	-1.99490439772507	0.0544723561753992	0.729013823425864	-4.07812546382236
a_HQ_transcript/137167	-2.18040479127606	-0.571634755349373	-1.99470644657654	0.0544949967058062	0.729013823425864	-4.07635208194749

a_HQ_transcript/94303	-1.91297187362506	-0.597267032878308	-1.99466284944733	0.0544999842018657	0.729013823425864	-4.05685022167548
a_HQ_transcript/131256	-2.61413784981367	1.75792853835814	-1.99453495156733	0.0545146179726602	0.729013823425864	-4.16081674512848
a_HQ_transcript/136703	-2.49747268390278	2.26256829972142	-1.99450527898166	0.0545180135293186	0.729013823425864	-4.06432767509951
a_HQ_transcript/78768	4.447327972936	1.83755999854612	1.99423923259768	0.0545484665652846	0.72925328070535	-4.13258059468893
a_HQ_transcript/6308	-2.98486635628252	2.08282474471598	-1.99373404685659	0.0546063335836872	0.729815826626224	-4.10534077684115
a_HQ_transcript/147063	-3.0710175672019	1.29476919828296	-1.99354734440994	0.0546277331611422	0.729815826626224	-4.03851859760361
a_HQ_transcript/69513	-2.32206246563975	-0.347111591259179	-1.99352788987419	0.0546299634342325	0.729815826626224	-4.07098671284234
a_HQ_transcript/79543	2.55334090323818	-0.458388714913652	1.99329105345689	0.0546571207959217	0.729815826626224	-4.07934081780923
a_HQ_transcript/40381	-1.46756364558457	3.00311397351843	-1.99312083696395	0.0546766463151416	0.729815826626224	-4.16435213075767
a_HQ_transcript/151254	-5.57181493760685	-0.282026957381066	-1.99301912167178	0.0546883169727255	0.729815826626224	-4.09454850756059
a_HQ_transcript/1947	-3.4376399330755	0.0609610543556014	-1.99297272085813	0.0546936416533339	0.729815826626224	-4.04897399810402
a_HQ_transcript/75519	-1.28249644321485	2.47033470713607	-1.99282169653438	0.0547109754365281	0.729815826626224	-4.02277594006534
a_HQ_transcript/134888	-3.60370197852138	0.0865336558370112	-1.99268245376826	0.0547269612412175	0.729815826626224	-4.15407093431659
a_HQ_transcript/55390	-3.19720269621853	0.32768332643504	-1.99262317965897	0.0547337674586007	0.729815826626224	-4.2100417095359
a_HQ_transcript/78079	1.60119604392848	0.373892626201289	1.99256660609753	0.0547402642713855	0.729815826626224	-4.02185875020829
a_HQ_transcript/30658	-2.04012835730959	1.23896844640398	-1.99252148925448	0.0547454458961579	0.729815826626224	-4.10770853976875
a_HQ_transcript/132931	2.21326984010621	0.299481193605551	1.99228198917847	0.0547729594096402	0.729815826626224	-3.99516651365407
a_HQ_transcript/136760	-4.97676929459715	2.17847644582343	-1.99227333227858	0.05477395413097	0.729815826626224	-4.17663172024468
a_HQ_transcript/25789	2.8179542968551	-0.382175842570364	1.99205298388567	0.0547992785766231	0.729815826626224	-4.09890982734094
a_HQ_transcript/143405	-7.82518231030066	2.23024230326818	-1.9919858858273	0.0548069921258756	0.729815826626224	-4.1546223268679
a_HQ_transcript/1869	3.17115139761293	-0.125827074143418	1.99190844445854	0.0548158959130219	0.729815826626224	-4.08618781198341
a_HQ_transcript/140136	2.44677997484113	1.8920704908044	1.99186327350226	0.0548210900065123	0.729815826626224	-4.06080070610744
a_HQ_transcript/133661	-4.0488394665566	-0.122402217444768	-1.99168630537904	0.0548414432582591	0.729815826626224	-4.15902517083259
a_HQ_transcript/123466	-1.23132512045878	2.29567593614603	-1.99168449320683	0.0548416517117972	0.729815826626224	-4.07677453387654
a_HQ_transcript/57146	-1.71682604025218	1.74923115535218	-1.99144991947244	0.0548686404837095	0.729849013630807	-4.03213423758925
a_HQ_transcript/79327	-3.43021983536592	1.1465958084715	-1.99141563202839	0.0548725863801655	0.729849013630807	-4.0372512212734
a_HQ_transcript/137269	1.88734321172647	-0.356251571476699	1.99132092728208	0.0548834865577228	0.729849013630807	-4.09481446939172
a_HQ_transcript/149145	-3.38108200432764	2.76837587643156	-1.99122639204409	0.0548943691094462	0.729849013630807	-4.12814299890673
a_HQ_transcript/35780	-1.33280533520268	1.52131358396802	-1.99105879407031	0.0549136670041177	0.729938631515059	-4.18217121548173
a_HQ_transcript/95711	-1.65131891249302	-0.739552893139189	-1.99086393385804	0.0549361114216911	0.729944002445844	-4.0696123815632
a_HQ_transcript/117161	-3.53666155993996	-0.0773774138387717	-1.99083724505313	0.0549391861185757	0.729944002445844	-4.07487082299559
a_HQ_transcript/62910	5.27835781490721	0.303807879405221	1.99057683954504	0.054969194142478	0.730175803728053	-4.09077910421093
a_HQ_transcript/70001	2.53973003737088	0.817354220253708	1.99039800546787	0.0549898105020743	0.730254207814866	-3.96636578253236
a_HQ_transcript/45977	1.24730984491853	2.20319809935135	1.99030771183953	0.0550002222992961	0.730254207814866	-4.13960661883612
a_HQ_transcript/14622	2.50282607028773	2.05805756980969	1.99013289808885	0.0550203850360757	0.730355090517699	-4.05261915498448
a_HQ_transcript/39423	3.69010214825097	0.063469833699537	1.98968118092774	0.0550725152256979	0.730639405117056	-4.15354784547692
a_HQ_transcript/100665	1.75295996895581	-0.185107856719741	1.9895454518125	0.0550881873905345	0.730639405117056	-4.106777287173
a_HQ_transcript/71368	2.67577801027173	0.117845790334836	1.98954266286775	0.0550885094609859	0.730639405117056	-4.02716358694563
a_HQ_transcript/98325	-1.6052200460002	4.42541661342317	-1.98951173213902	0.0550920814861695	0.730639405117056	-4.43317420244585
a_HQ_transcript/94573	2.55337654307647	1.00844153449072	1.98921928510041	0.0551258646239967	0.730920679485329	-4.00277912975008
a_HQ_transcript/103432	-2.86704038216074	0.118300896043117	-1.98835829942873	0.0552254296018618	0.731912880814267	-4.08247577109704
a_HQ_transcript/62450	3.26484244843784	0.95106953757536	1.98835441622969	0.0552258790127198	0.731912880814267	-4.06842683151506
a_HQ_transcript/20381	-2.85849331029953	3.74660072230884	-1.98807539102253	0.0552581795410583	0.732174027892098	-4.37544263501261
a_HQ_transcript/63619	2.4589342129414	-0.354521079344449	1.9879222412469	0.0552759154744162	0.732227371320975	-4.05239034679104
a_HQ_transcript/6106	0.931158784230761	2.37419550773845	1.987513307717	0.0553232974800462	0.732227371320975	-4.10451771375875
a_HQ_transcript/144402	-4.34319074854042	1.9835142676474	-1.98742362524511	0.0553336934760963	0.732227371320975	-4.1775052774409
a_HQ_transcript/147011	0.875983997734293	-0.657856863548859	1.98733496290504	0.0553439728929571	0.732227371320975	-4.15162297471788
a_HQ_transcript/82538	-1.67260916466111	-0.432916801652044	-1.98727423118542	0.0553510150244959	0.732227371320975	-4.0605282514356
a_HQ_transcript/18023	-3.68197248413161	0.202774963057296	-1.98720844861778	0.0553586437074019	0.732227371320975	-4.06713357474539
a_HQ_transcript/136215	-1.1534449820629	3.1248885060203	-1.98717925533724	0.0553620294919528	0.732227371320975	-4.1814480929647

a_HQ_transcript/85572	-1.81335907647314	2.27180003419456	-1.98711509667994	0.0553694711329673	0.732227371320975	-3.99913502870575
a_HQ_transcript/94576	-2.63918488932713	2.83666673768181	-1.98706246241498	0.0553755767328483	0.732227371320975	-4.27576946000268
a_HQ_transcript/121948	-2.38487466555045	0.130456657211493	-1.98668910836395	0.0554189028351987	0.73227582989013	-3.99960815021383
a_HQ_transcript/119630	2.55383421137098	0.135469986865228	1.98656713557652	0.0554330636469934	0.73227582989013	-4.12760837477355
a_HQ_transcript/136078	3.77720993830173	2.28738127009322	1.98653129695204	0.0554372250437132	0.73227582989013	-4.10685034230452
a_HQ_transcript/119070	-2.00481741603641	0.274808384190966	-1.98637428089414	0.0554554601517273	0.73227582989013	-4.04485057679737
a_HQ_transcript/85785	-1.51754744596748	0.915982187619943	-1.98632266070405	0.0554614562205089	0.73227582989013	-4.04380324445211
a_HQ_transcript/90858	-3.30553847319165	0.994808787387487	-1.9862806063935	0.0554663415591162	0.73227582989013	-4.07514615790132
a_HQ_transcript/55329	-2.62373605379647	0.809010355772599	-1.98627128048648	0.0554674249760226	0.73227582989013	-4.08716689516336
a_HQ_transcript/20859	2.4081013471692	0.384710931478421	1.98611406822022	0.0554856915455809	0.732350653533498	-4.08223801230015
a_HQ_transcript/28396	3.50545398908278	2.57742995695842	1.98600437554923	0.0554984398965217	0.7323526252678	-4.24190858513022
a_HQ_transcript/82505	-1.40626974245949	2.88246638218475	-1.98568878656479	0.0555351315141531	0.732670477679231	-4.20230890714987
a_HQ_transcript/41465	-2.71284077273942	0.946964743601899	-1.98535526102657	0.0555739314902495	0.733015994931977	-3.95822830828176
a_HQ_transcript/138056	2.81261974744241	3.34143418395562	1.98507107481153	0.0556070103271887	0.733156381483865	-4.30337507954581
a_HQ_transcript/100710	-2.02372827734325	-0.404056074607594	-1.98503633070968	0.0556110556643216	0.733156381483865	-4.08999289668925
a_HQ_transcript/39151	-2.49417410595673	1.31840952978758	-1.98483891035266	0.0556340466495633	0.733156381483865	-4.06783515402145
a_HQ_transcript/73466	-2.33307836345287	0.931066860682216	-1.98475238653295	0.0556441255664723	0.733156381483865	-4.0020602639184
a_HQ_transcript/11748	-3.33089621147364	1.93545732306058	-1.98472222889273	0.0556476389193987	0.733156381483865	-4.07546179265841
a_HQ_transcript/109936	2.0406140734695	0.336959925815619	1.98450372825725	0.0556730999318847	0.733181268436705	-4.04481045638695
a_HQ_transcript/42705	-1.63344825298736	1.8578706458359	-1.98448953372213	0.0556747543159857	0.733181268436705	-4.10730678412852
a_HQ_transcript/50996	-3.13653919401611	2.73865714626332	-1.98431891700641	0.0556946431803226	0.733239197052042	-4.3283597547315
a_HQ_transcript/42058	-3.44225492287884	1.21472032090667	-1.98414421581709	0.0557150145906348	0.733239197052042	-4.07571001988835
a_HQ_transcript/8193	-1.91674245018095	0.398134356931899	-1.98412722810438	0.0557169958260884	0.733239197052042	-3.99087313302876
a_HQ_transcript/134558	3.0071501767847	-0.29539249741256	1.9839853162864	0.0557335490543437	0.733256628810091	-4.08946782729871
a_HQ_transcript/139607	2.65930238897874	0.112943666612831	1.98389960005444	0.0557435494548547	0.733256628810091	-4.03395183937282
a_HQ_transcript/58200	1.93954764096385	-0.500959694110783	1.98370968688389	0.0557657119474019	0.733377557174723	-4.09672780375799
a_HQ_transcript/36409	-2.48385403067028	-0.207079445792696	-1.98360462423171	0.0557779758510434	0.733377557174723	-4.11387930919944
a_HQ_transcript/12625	-3.70344267742524	0.27791738526289	-1.98344633893961	0.0557964568418081	0.733454645703442	-4.05373672054203
a_HQ_transcript/16408	-2.98334823121597	0.450743597224919	-1.9829646132559	0.0558527347567414	0.73377102551495	-3.98417260423952
a_HQ_transcript/120608	4.66875786570555	0.96681052202602	1.98290876030999	0.0558592630126878	0.73377102551495	-3.99473550947185
a_HQ_transcript/121038	-3.72419993302156	0.097700475457516	-1.98281957519173	0.0558696886093223	0.73377102551495	-4.13511143898254
a_HQ_transcript/67738	2.73117689236321	0.822262979691473	1.98280820042089	0.055871018423637	0.73377102551495	-4.14597972376583
a_HQ_transcript/125444	-3.43608759660093	0.577762331641321	-1.98250920080208	0.055905984096525	0.734064387522657	-4.17126482033111
a_HQ_transcript/37696	1.89621772922939	-0.0926499259257768	1.9822598205878	0.0559351617512687	0.734233452678277	-4.12292858598591
a_HQ_transcript/6026	2.44527033279629	0.475607202361756	1.98218325483372	0.0559441226588234	0.734233452678277	-4.11478270318765
a_HQ_transcript/144182	4.22892744988162	1.51692774022362	1.98185083059664	0.0559830425873135	0.734298983746202	-4.17131839657364
a_HQ_transcript/106812	-1.52291822268852	2.47126792386326	-1.98171180676333	0.0559993263675742	0.734298983746202	-4.21086248000601
a_HQ_transcript/89829	-1.7375444353896	2.70546976230837	-1.98158487506782	0.0560141974070855	0.734298983746202	-4.23839958183736
a_HQ_transcript/41223	2.72556324379001	0.486858476900331	1.98145512972099	0.0560294016441346	0.734298983746202	-4.05493041379295
a_HQ_transcript/446	-0.896006027985682	3.83949594409522	-1.98124030583007	0.0560545837354833	0.734298983746202	-4.36021134934111
a_HQ_transcript/30558	-2.34333497366655	2.74171901244724	-1.98105232777154	0.0560766269936111	0.734298983746202	-4.09257573770933
a_HQ_transcript/137479	0.625103819953621	-0.741514840004745	1.9809026087757	0.0560941892036366	0.734298983746202	-4.16805154716494
a_HQ_transcript/12317	2.65791551114717	-0.273929345102581	1.98089394089447	0.0560952061027907	0.734298983746202	-4.09898047310463
a_HQ_transcript/36301	3.8854007269122	0.74542477411487	1.98084957380253	0.056100411416206	0.734298983746202	-4.03200829966372
a_HQ_transcript/131615	1.57757973026224	-0.729238481918009	1.98063729377387	0.056125322728783	0.734298983746202	-4.10360920721915
a_HQ_transcript/116326	-4.66778076457699	2.59659225407687	-1.98060336126343	0.0561293056423372	0.734298983746202	-4.25563579970923
a_HQ_transcript/117539	3.645721508415	0.97581887375375	1.98055001999763	0.0561355672050794	0.734298983746202	-4.13775960869024
a_HQ_transcript/3737	-2.28428279122014	1.0351367762343	-1.9805128296851	0.0561399332184919	0.734298983746202	-4.03379284898492
a_HQ_transcript/124464	1.18548357812897	4.66486362557367	1.98022425103105	0.0561738214049804	0.734298983746202	-4.49101617352428
a_HQ_transcript/79945	-3.63625228425368	0.896434346311078	-1.98017369629668	0.0561797599508573	0.734298983746202	-3.99996821214813

a_HQ_transcript/33912	2.31995818378153	-0.490242236559101	1.98011783282194	0.0561863227381149	0.734298983746202	-4.10278862013041
a_HQ_transcript/89364	-3.21826612823082	2.25238818092009	-1.98009923798054	0.0561885073903469	0.734298983746202	-4.24357878657731
a_HQ_transcript/75494	2.65050643962644	0.380660117915499	1.98005836238124	0.0561933100022472	0.734298983746202	-4.05237085676804
a_HQ_transcript/120484	-3.23861720487882	0.552598809002208	-1.97992684048865	0.0562087653792782	0.734298983746202	-4.15141109404772
a_HQ_transcript/126505	-2.39454472786173	3.26374106084673	-1.97984711516994	0.0562181358493171	0.734298983746202	-4.31657303445946
a_HQ_transcript/32222	-1.62465931039285	0.834507854817817	-1.97959651861851	0.0562475984348614	0.734298983746202	-4.18526638671786
a_HQ_transcript/48412	2.36098610567743	-0.258244751592659	1.9795088981501	0.0562579031288517	0.734298983746202	-4.05852139200566
a_HQ_transcript/4858	3.19931168588722	-0.016526747485236	1.97943142339263	0.0562670159961544	0.734298983746202	-4.05774042962207
a_HQ_transcript/134176	5.18064565378956	-0.390954216998913	1.97929293014746	0.0562833092874264	0.734298983746202	-4.13254554124082
a_HQ_transcript/131609	-2.44399604707812	1.49520478360605	-1.97922410372383	0.05629140802668	0.734298983746202	-4.10344017712121
a_HQ_transcript/143910	2.8088300383844	3.38684074934367	1.97919099684435	0.0562953040430178	0.734298983746202	-4.36022534649238
a_HQ_transcript/144500	-0.687871246355869	4.15635274552445	-1.97903155497497	0.0563140704460499	0.734298983746202	-4.41051683468547
a_HQ_transcript/79673	-2.97461254498208	1.64540471972758	-1.97902543428065	0.0563147909640192	0.734298983746202	-4.10772586580083
a_HQ_transcript/149783	-2.42219595260869	-0.300638359355644	-1.97901977864905	0.0563154567427155	0.734298983746202	-4.1122912316055
a_HQ_transcript/53903	2.09492429734023	0.325473429918817	1.97890140522425	0.056329393188728	0.734315982793088	-4.04313773481441
a_HQ_transcript/23704	-3.03588061140768	1.25149522340914	-1.97866077232444	0.056357732857813	0.734520694071515	-4.00434893319677
a_HQ_transcript/6212	2.59064535994824	0.802757571358186	1.97844989580116	0.0563825782871593	0.734679782711499	-4.15318706025469
a_HQ_transcript/147798	-0.549940926494395	4.07554926236654	-1.97832257859318	0.0563975833933136	0.734710606787659	-4.40170545485969
a_HQ_transcript/91367	1.87487309579288	-0.65297093216425	1.97820748537255	0.0564111508215341	0.734722692125058	-4.10423145571502
a_HQ_transcript/57923	-2.2974980190538	1.60510789626953	-1.97806909497379	0.05642746833375	0.734770582281411	-4.08984227255294
a_HQ_transcript/123904	3.19889207648015	1.84764233187798	1.97785041667114	0.0564532609153687	0.734821100487434	-4.08854707360445
a_HQ_transcript/144543	-3.4831926243354	4.60024506770236	-1.97782185212104	0.0564566307937118	0.734821100487434	-4.41921965896699
a_HQ_transcript/141290	0.612652960295769	9.03237978091885	1.97743255234072	0.0565025755699587	0.734861569137695	-4.92162681769511
a_HQ_transcript/135884	3.77317141058634	-0.246663647919154	1.97742316179633	0.0565036842348527	0.734861569137695	-4.0854222169468
a_HQ_transcript/4726	1.90560314438916	3.83308522750713	1.97737458532556	0.0565094195645727	0.734861569137695	-4.41603050220636
a_HQ_transcript/39446	0.750383801687999	3.45229162614807	1.97726630826388	0.056522205451909	0.734861569137695	-4.34795225287819
a_HQ_transcript/120643	-1.29322322858126	3.62658286932305	-1.97707198106613	0.0565451588805788	0.734861569137695	-4.34468957949077
a_HQ_transcript/96493	1.35840376493545	3.07105239859256	1.97700402883929	0.056553187138173	0.734861569137695	-4.27912289779892
a_HQ_transcript/68426	-1.08548875133005	1.58513740376548	-1.97696999358979	0.056557208626736	0.734861569137695	-4.01382878676365
a_HQ_transcript/42687	-2.21568549391807	0.201570987963576	-1.97693322229126	0.0565615536773571	0.734861569137695	-4.07359009737213
a_HQ_transcript/68356	-1.86372281886033	-0.234973821593236	-1.97683197348351	0.0565735191627302	0.734861569137695	-4.07207620294133
a_HQ_transcript/86648	-1.53496455450769	4.42172033174396	-1.9766662269327	0.0565931116864553	0.734951831123832	-4.29515695930891
a_HQ_transcript/102061	3.07954005838013	0.955825665324959	1.97647677292777	0.0566155138153117	0.735078532309369	-4.10390017293593
a_HQ_transcript/26393	-3.4026289034938	0.110525405590337	-1.97618060053861	0.0566505505027311	0.735369182586591	-4.09955255187615
a_HQ_transcript/49690	-1.69039066357319	-0.600818543261577	-1.97594678846848	0.0566782233771546	0.735564136741961	-4.1130390015529
a_HQ_transcript/24506	2.80964882006241	-0.0438025298072756	1.97543411193224	0.0567389425144986	0.735918021737606	-4.07081072857418
a_HQ_transcript/138632	-3.24717283655946	-0.101850840820791	-1.97540509918767	0.0567423803480443	0.735918021737606	-4.11519608369685
a_HQ_transcript/143858	-1.60865447373918	2.43899834945004	-1.97530473110423	0.0567542747537067	0.735918021737606	-4.18259379813066
a_HQ_transcript/39695	-3.08702847358866	0.586680336977718	-1.97528905349309	0.056756132869696	0.735918021737606	-4.14727533678091
a_HQ_transcript/114616	4.77825369820991	2.03149708934392	1.97517420744236	0.0567697460792797	0.735930374686969	-4.14220673617302
a_HQ_transcript/69932	2.610872014705	0.345628327956422	1.97491059133554	0.0568010044130836	0.736077513500279	-4.10982597293769
a_HQ_transcript/56053	2.87414875802312	-0.0848513599925524	1.97475015558889	0.0568200354486286	0.736077513500279	-4.09745706083592
a_HQ_transcript/118352	2.31532387756229	2.67912558548757	1.97473462596643	0.0568218778813482	0.736077513500279	-4.28126990892769
a_HQ_transcript/76289	-2.50010030906236	0.421494604191572	-1.97454608822999	0.056844250127067	0.736077513500279	-4.06193112688974
a_HQ_transcript/127444	-2.19176499052828	-0.154005813747165	-1.97433665507882	0.0568691108526028	0.736077513500279	-4.11172149295472
a_HQ_transcript/24751	2.83401754294066	0.778371154670545	1.97422952054709	0.0568818318975329	0.736077513500279	-4.02179621780882
a_HQ_transcript/256	1.008742919321	5.55555938049792	1.97420894988708	0.0568842747201714	0.736077513500279	-4.60855067978215
a_HQ_transcript/152680	2.42664246600267	-0.2267666216518	1.97409280430938	0.0568980690416564	0.736077513500279	-4.11981968007664
a_HQ_transcript/78271	-2.02862161350371	-0.482632843320837	-1.97402491256584	0.0569061337237706	0.736077513500279	-4.13993448023243
a_HQ_transcript/52610	1.85451456559692	2.58484029133474	1.97401150207459	0.0569077268385331	0.736077513500279	-4.19497352746039

a_HQ_transcript/24660	-3.01641063392854	1.40770430017751	-1.97379752265345	0.0569331520231774	0.736148676547059	-4.08452500566289
a_HQ_transcript/121988	2.85703338967509	0.719930186707914	1.97375204363903	0.0569385571455336	0.736148676547059	-4.06177373529244
a_HQ_transcript/9695	2.49297843116002	1.88111715021269	1.97289709223657	0.0570402501804023	0.737181597799002	-4.07008920009133
a_HQ_transcript/149696	-2.49405844306601	0.550473433689368	-1.97269271957775	0.0570645828865548	0.737181597799002	-4.08825076708279
a_HQ_transcript/28798	-2.31125232207833	-0.201194448715242	-1.97262470155476	0.0570726831483358	0.737181597799002	-4.05204045231701
a_HQ_transcript/142712	0.837027896595428	5.02327108957353	1.97259101175749	0.0570766956346216	0.737181597799002	-4.55172899054527
a_HQ_transcript/109655	-2.83579095900262	0.339463422095052	-1.97247909122845	0.0570900272368028	0.737181597799002	-3.99379455179475
a_HQ_transcript/19699	-1.13628099303286	2.87448512681658	-1.97244119206392	0.0570945422737942	0.737181597799002	-4.28753259655931
a_HQ_transcript/114662	3.14277182924992	-0.389133128075177	1.97226453192757	0.0571155924072731	0.737289619242721	-4.11599564624858
a_HQ_transcript/11077	0.924317897499199	2.47961439809584	1.97200665125353	0.0571463325902131	0.737445153233642	-4.17235492760915
a_HQ_transcript/13653	-2.43514633330925	0.505768835151545	-1.97154331400887	0.0572016000166277	0.737445153233642	-4.07686499762808
a_HQ_transcript/149302	2.33999740180591	1.4274341392218	1.97151929445071	0.0572044663663215	0.737445153233642	-4.08130673642823
a_HQ_transcript/61654	-2.69507926732326	1.69604617639462	-1.97150804835353	0.0572058084509236	0.737445153233642	-4.10828711911439
a_HQ_transcript/85356	-2.56458950333205	0.187407703182447	-1.9713854665702	0.0572204388696145	0.737445153233642	-4.07264226652397
a_HQ_transcript/85798	2.89039066262922	-0.381964005579539	1.97133061279801	0.0572269868484048	0.737445153233642	-4.08481723943109
a_HQ_transcript/124196	-2.3117921359132	-0.463508294528316	-1.97129595655212	0.0572311241536741	0.737445153233642	-4.10688829811564
a_HQ_transcript/126668	0.921926502746981	3.22627338353864	1.97125330385152	0.0572362164428566	0.737445153233642	-4.2992085683543
a_HQ_transcript/55653	-1.14060402407784	2.3807549754649	-1.97106733498228	0.0572584238008651	0.737445153233642	-4.20869937512607
a_HQ_transcript/118226	1.72398717509318	2.0551536314725	1.97103359903205	0.0572624531619548	0.737445153233642	-4.14033214557351
a_HQ_transcript/43336	2.66162652939039	1.20136577744164	1.97099391334816	0.0572671934643659	0.737445153233642	-4.02873301861475
a_HQ_transcript/116102	2.35079211605064	-0.098660980163363	1.97069348042813	0.0573030901006696	0.737743969296062	-4.06750498980102
a_HQ_transcript/11132	2.17216665629889	3.46683117263584	1.97049118896607	0.0573272715268019	0.737745354980618	-4.4301073866001
a_HQ_transcript/40880	1.89868931276506	1.64358833167504	1.97039368818508	0.0573389297022973	0.737745354980618	-4.07736699588175
a_HQ_transcript/5229	2.37067449780311	0.0928110090605126	1.97036202552672	0.0573427160523767	0.737745354980618	-4.02007667481068
a_HQ_transcript/118542	-2.44991909069546	-0.17814370963138	-1.9701892083308	0.0573633860734341	0.737745354980618	-4.10184635568812
a_HQ_transcript/130347	-0.825292327073403	4.39477586500528	-1.97012701907479	0.0573708258847657	0.737745354980618	-4.47135160201544
a_HQ_transcript/67143	-1.46001167415535	1.53072094155377	-1.97005579014038	0.0573793481603939	0.737745354980618	-4.11166093330038
a_HQ_transcript/10355	-5.08044143570383	1.77043928538111	-1.96967464085451	0.0574249699665626	0.737978711884752	-4.02477570955879
a_HQ_transcript/115948	-2.32523079913737	-0.340605695356762	-1.96950002920379	0.0574458807120291	0.737978711884752	-4.11083253848768
a_HQ_transcript/8056	1.64046545795581	2.21159897848557	1.96945526022283	0.0574512431208665	0.737978711884752	-4.13609759911852
a_HQ_transcript/108544	-2.18237519209653	0.816974212346731	-1.96945167063999	0.0574516730983787	0.737978711884752	-4.05220421009071
a_HQ_transcript/44856	2.93467781902068	0.472994095239852	1.96933531463013	0.0574656122976936	0.737978711884752	-4.03013450539855
a_HQ_transcript/95023	-2.53772387081393	0.383576657570526	-1.96926804505331	0.0574736723902813	0.737978711884752	-3.9955477777733
a_HQ_transcript/126133	-2.58212199201675	-0.182868180803957	-1.96913647521943	0.0574894396411855	0.738018142107516	-4.15223364936249
a_HQ_transcript/117962	1.2709000070851	1.91017995833489	1.96895548111002	0.0575111360170774	0.73813365299198	-4.07863232188655
a_HQ_transcript/119444	3.45374444655377	0.661052769985011	1.96874071809945	0.0575368896260357	0.738151287768738	-4.31231324001111
a_HQ_transcript/14826	-1.54661972493976	2.50567782223808	-1.96873223194865	0.0575379074607788	0.738151287768738	-4.1074402500425
a_HQ_transcript/151608	1.90371462049044	3.43808123524323	1.96860353584649	0.0575533452796296	0.738186419773678	-4.24568036645835
a_HQ_transcript/12599	-2.90620734282997	2.09928778164374	-1.96829140449664	0.0575908021528665	0.73833142714111	-4.15674923733093
a_HQ_transcript/4703	1.75865662461445	1.99995329733808	1.96823491292268	0.0575975836087125	0.73833142714111	-4.10412949585355
a_HQ_transcript/89797	-1.92743270031645	0.536374050519993	-1.96806626763999	0.057617832539666	0.73833142714111	-4.09019681869984
a_HQ_transcript/138370	-2.16774147924432	-0.648057619495043	-1.96795482171334	0.057631217046327	0.73833142714111	-4.12039118755409
a_HQ_transcript/22114	-2.78385933177247	-0.17583294572924	-1.96789556979911	0.0576383342230815	0.73833142714111	-4.11593069668836
a_HQ_transcript/110157	-2.16018979337975	0.731631613389661	-1.96782838747854	0.057646404903276	0.73833142714111	-4.19041419416766
a_HQ_transcript/78274	-3.93252404885109	0.559328908656693	-1.96776880317015	0.0576535636490761	0.73833142714111	-4.2470297844198
a_HQ_transcript/10297	-1.92142341925	1.16806502564723	-1.96715384113102	0.0577274933372521	0.738856535453756	-4.05241014422422
a_HQ_transcript/126692	6.15745562279243	1.25856655374676	1.9671149439221	0.0577321722648163	0.738856535453756	-4.16165835857602
a_HQ_transcript/98144	-4.98324196712302	3.30750130987089	-1.96711055711115	0.0577326999730071	0.738856535453756	-4.22074682814077
a_HQ_transcript/78596	1.94976471935588	1.97341457767642	1.96688369930038	0.0577599953850713	0.73904314588233	-4.11781508470114
a_HQ_transcript/123332	0.86481724329104	2.90997944452315	1.96653900838807	0.0578014898963786	0.739243144345248	-4.19143136903903

a_HQ_transcript/102697	2.65798537959214	0.592677603467126	1.96653229264151	0.0578022986074268	0.739243144345248	-4.05232869601818
a_HQ_transcript/143117	-2.16687378809594	2.9480068884302	-1.96641671360838	0.0578162181912484	0.739243144345248	-4.28994617892124
a_HQ_transcript/26990	0.664528009687753	4.12104067088365	1.96633138645185	0.0578264963070782	0.739243144345248	-4.47214899579933
a_HQ_transcript/122147	1.67475706967468	-0.65338095662675	1.9661920967395	0.0578432779177819	0.739295087687956	-4.12959839436384
a_HQ_transcript/1496	2.56292671679883	1.08121831184046	1.96597273257605	0.0578697154778072	0.739342650387626	-4.02945241988808
a_HQ_transcript/132338	-8.78353971076517	3.82732979860029	-1.96585473016105	0.057883941362676	0.739342650387626	-4.16902198809307
a_HQ_transcript/41219	-6.39711245873394	2.34128251033164	-1.9658446483269	0.0578851569280568	0.739342650387626	-4.23483216196784
a_HQ_transcript/147096	3.17085038614449	3.01900527533441	1.96561034548654	0.0579134130432147	0.739541053026358	-4.18664217302688
a_HQ_transcript/131108	-2.3832942895699	0.744113340831173	-1.96516499634593	0.0579671536816703	0.739955186245776	-4.04568669865047
a_HQ_transcript/74513	-2.32656764974795	-0.214525562835842	-1.96512025318694	0.0579725552709832	0.739955186245776	-4.10077729934587
a_HQ_transcript/22722	-3.11209948792424	0.0136014758779772	-1.96494261139505	0.0579940052814918	0.739955186245776	-4.12656456214971
a_HQ_transcript/41570	2.4272485344813	0.825553478726591	1.9649197786254	0.0579967628085562	0.739955186245776	-4.00543872345209
a_HQ_transcript/57708	-1.4261366966559	3.86333882242668	-1.96462623061488	0.0580322249311427	0.740160091743478	-4.38504034315535
a_HQ_transcript/6980	4.26110655453114	0.851365870423389	1.96450116299248	0.0580473394752776	0.740160091743478	-4.20523590675001
a_HQ_transcript/43141	-4.04799876535534	1.41187989669233	-1.96447068834276	0.0580510228849869	0.740160091743478	-4.21255515924258
a_HQ_transcript/138913	3.35173346710678	-0.0466444892199533	1.96390090964799	0.058119928320642	0.740804368435309	-4.13694816866713
a_HQ_transcript/26432	3.43167582908995	1.61407735299821	1.96383904962796	0.058127413558079	0.740804368435309	-4.20974705338786
a_HQ_transcript/31039	1.65416314534009	2.63466223235567	1.96373680876494	0.0581397868290992	0.740804368435309	-4.15829638982537
a_HQ_transcript/113174	1.90494432757717	1.01369953973911	1.96355368211651	0.0581619546863114	0.740867711648927	-4.03237468362223
a_HQ_transcript/91592	2.64894679869511	1.068940088098	1.96321907065188	0.0582024790721416	0.740867711648927	-4.16755830212352
a_HQ_transcript/112041	3.41086533660553	-0.13208231055892	1.96318667769133	0.0582064034468544	0.740867711648927	-4.18709671624335
a_HQ_transcript/139608	1.04811313372694	2.54299428383934	1.96318028696574	0.0582071777040229	0.740867711648927	-4.21105917101629
a_HQ_transcript/15524	-3.05710099036974	0.257284407852112	-1.96304348154772	0.0582237542722724	0.740867711648927	-4.12846906279293
a_HQ_transcript/78041	-2.02144666052734	-0.439063470300438	-1.96299456417163	0.0582296825351144	0.740867711648927	-4.14865820130703
a_HQ_transcript/8404	-2.66578152935567	0.297531006732399	-1.96293190188267	0.0582372773009801	0.740867711648927	-4.16043782240113
a_HQ_transcript/149102	4.12028138508047	4.01539911146996	1.9627676641195	0.0582571872569346	0.740867711648927	-4.52250511185911
a_HQ_transcript/99666	2.08812072387665	2.03883755475562	1.96274885984402	0.058259467209387	0.740867711648927	-4.05525945287373
a_HQ_transcript/111319	1.26587922978753	5.99429101624175	1.96249702198036	0.058290009143222	0.740971465053857	-4.6521058930312
a_HQ_transcript/32408	2.84252152607795	-0.00716096089064427	1.96247137310337	0.0582931205218016	0.740971465053857	-4.12534603728065
a_HQ_transcript/48556	-1.23539333546348	2.388577105334	-1.96195422981451	0.0583558842316813	0.741336320879828	-4.16052581863999
a_HQ_transcript/49357	2.31737051241464	2.42922270173997	1.96172187973443	0.0583841027936306	0.741336320879828	-4.15410571027843
a_HQ_transcript/26061	2.39513598384588	0.571164262747944	1.96171886192419	0.0583844693800766	0.741336320879828	-4.06018884824026
a_HQ_transcript/137696	9.42215798993958	5.39526423573331	1.96168883433994	0.0583881170694343	0.741336320879828	-4.26654690836764
a_HQ_transcript/116235	1.25353645999615	2.79951245472743	1.9616691208836	0.0583905119273891	0.741336320879828	-4.25120361109821
a_HQ_transcript/152674	-3.03979990834641	4.22434813892564	-1.96160464511047	0.0583983452606099	0.741336320879828	-4.4366462997794
a_HQ_transcript/6138	-2.23254744133872	1.77457115114707	-1.96120190594973	0.0584472957865727	0.741587129057099	-4.15795397275968
a_HQ_transcript/98935	-2.21016006322974	0.266659469624052	-1.96118334845449	0.0584495521978382	0.741587129057099	-4.16491739939282
a_HQ_transcript/106981	-4.22238947173075	1.29579407356421	-1.96112723079688	0.0584563760208441	0.741587129057099	-4.16152554610024
a_HQ_transcript/71282	2.44742695154679	1.50034298466228	1.9608874675849	0.0584855386668546	0.741795197823898	-4.05963225510246
a_HQ_transcript/150923	2.30697358331327	0.0119192922641623	1.96039184267346	0.0585458621184153	0.742053756771264	-4.1796868760342
a_HQ_transcript/67488	-6.75724245750157	2.85657947214823	-1.96037412259324	0.0585480198638723	0.742053756771264	-4.16472089098948
a_HQ_transcript/135756	4.16848171942174	4.72284993366432	1.96032798933221	0.0585536377604861	0.742053756771264	-4.52660176647309
a_HQ_transcript/144254	2.65410443933694	0.343448139685959	1.96030048181833	0.0585569877220924	0.742053756771264	-4.1301850005511
a_HQ_transcript/36860	-3.30106426632902	3.28603491883311	-1.95984775875808	0.058612145857506	0.742590848824131	-4.28135380819337
a_HQ_transcript/66125	-2.74553989888711	-0.000610533291240097	-1.95971279362272	0.0586285982534878	0.742637428476517	-4.11411903422623
a_HQ_transcript/88795	-2.5063870975564	-0.125413138962578	-1.95945530799745	0.0586599971605663	0.742873271230805	-4.18342440353888
a_HQ_transcript/13529	2.34316854759575	1.05586833894269	1.95929143598915	0.0586799880274538	0.742958672044366	-4.03449178439048
a_HQ_transcript/134890	2.79249030136423	1.07076512793641	1.95915016566176	0.0586972264495019	0.742958672044366	-4.02957942375775
a_HQ_transcript/50160	2.60976617450888	-0.57924121627074	1.95902372194804	0.058712659394845	0.742958672044366	-4.15092201241386
a_HQ_transcript/75135	-2.08390802399926	1.19738618461994	-1.95878582170747	0.0587417055981303	0.742958672044366	-4.10629287885928

a_HQ_transcript/129085	-2.58896421994116	0.363434933897824	-1.95869178701545	0.0587531901165387	0.742958672044366	-4.23942424376131
a_HQ_transcript/45503	1.42570710755876	0.600557395831578	1.95863025985486	0.0587607055246093	0.742958672044366	-4.05905018847474
a_HQ_transcript/129234	-5.08073576533582	2.73072143961504	-1.95856236990734	0.0587689991024076	0.742958672044366	-4.31403601502865
a_HQ_transcript/36331	-2.4688897544174	2.27300950013975	-1.95851177306666	0.0587751807795399	0.742958672044366	-4.17103408545431
a_HQ_transcript/36770	1.2911728636465	2.02478927243067	1.95829533599891	0.0588016303902277	0.742958672044366	-4.18676186037527
a_HQ_transcript/118421	-2.89238389148316	1.91882324928064	-1.95802159449113	0.0588350976778665	0.742958672044366	-4.27683094791745
a_HQ_transcript/67861	2.38052688412135	2.13308215136444	1.95801437767087	0.0588359802209443	0.742958672044366	-4.25677863174997
a_HQ_transcript/106751	-1.04208568123342	3.61028859005415	-1.95797857584323	0.0588403585872053	0.742958672044366	-4.26400082123723
a_HQ_transcript/23946	1.97293735031522	-0.645314337225476	1.95779503791382	0.0588628087099636	0.742958672044366	-4.10105628188596
a_HQ_transcript/48676	-2.46190532540689	0.0471736281515415	-1.95779490729276	0.0588628246900134	0.742958672044366	-4.1235945677702
a_HQ_transcript/94945	-2.92751923837048	-0.25923902016233	-1.95762720794547	0.0588833439679122	0.742958672044366	-4.1244862972515
a_HQ_transcript/128641	-0.921956504429508	3.20066846996651	-1.95759735122809	0.0588869978136412	0.742958672044366	-4.32016350353178
a_HQ_transcript/5187	3.7296980516824	0.83458958602469	1.95758477129947	0.0588885373961307	0.742958672044366	-4.07486332524459
a_HQ_transcript/161	4.0511903740709	1.6961025034457	1.9575172103863	0.0588968063718077	0.742958672044366	-4.04206232509467
a_HQ_transcript/104911	3.53674617625903	0.561354804610627	1.95738167628557	0.0589133978239568	0.742992012455978	-4.09433456489452
a_HQ_transcript/151355	-0.732743257665778	7.88413013677182	-1.95728680961573	0.0589250133743128	0.742992012455978	-4.79200309452216
a_HQ_transcript/152123	-1.60595645346753	1.58108971441515	-1.95708992180819	0.0589491268226778	0.743026220174328	-4.05165276043534
a_HQ_transcript/29756	-1.10932742824482	-0.609270745421011	-1.95695726130634	0.0589653789905879	0.743026220174328	-4.1692395251134
a_HQ_transcript/141938	-2.46119817543291	0.941895841650398	-1.9569515883183	0.0589660740721197	0.743026220174328	-4.09005659148664
a_HQ_transcript/17313	2.45129090858957	0.692968444945782	1.95670721915113	0.0589960221041521	0.743242473530592	-4.04859048618752
a_HQ_transcript/137924	3.21647653141765	2.04451100772627	1.95648966874015	0.0590226945479946	0.743417375663235	-4.11159668129138
a_HQ_transcript/110531	-1.04085709824991	2.96287024670242	-1.95597723463504	0.0590855621618464	0.744047997691466	-4.25545653536253
a_HQ_transcript/133075	0.576227816443822	4.35943898494488	1.95554386177605	0.059138775585448	0.74445538240097	-4.47955597472322
a_HQ_transcript/16433	3.91096406709714	-0.164061076632931	1.95550517989623	0.0591435273177759	0.74445538240097	-4.13321281423003
a_HQ_transcript/94110	2.07716594837536	-0.291441489510181	1.95505960425433	0.0591982863195632	0.744579474120572	-4.17175570635677
a_HQ_transcript/90596	0.747574462225921	4.83779040222296	1.95505081593176	0.0591993668027448	0.744579474120572	-4.5782114014802
a_HQ_transcript/49622	5.6374997736237	1.45548594260961	1.95493439495342	0.0592136818340383	0.744579474120572	-4.22696812173234
a_HQ_transcript/93299	-2.04196297986806	1.22466588976121	-1.95491756285277	0.0592157517445007	0.744579474120572	-4.07915348424212
a_HQ_transcript/66167	-1.72941937872284	2.39360933802142	-1.95482363559839	0.0592273035067446	0.744579474120572	-4.21547583061215
a_HQ_transcript/22140	2.21993607995068	0.916919509042779	1.95479974788684	0.0592302416792858	0.744579474120572	-4.15884237418644
a_HQ_transcript/38586	-1.33956213591693	2.63518367347526	-1.95433486938966	0.0592874466284246	0.74490938072633	-4.22870735591892
a_HQ_transcript/131980	1.42438895179979	2.29539505798904	1.95431435100206	0.0592899725939149	0.74490938072633	-4.19952384142067
a_HQ_transcript/141782	-2.34904076528639	-0.113746555864611	-1.95427408236038	0.0592949302336349	0.74490938072633	-4.14924052696835
a_HQ_transcript/96747	-2.2221383392956	-0.216209763373321	-1.95383490490838	0.0593490225679017	0.745326265162914	-4.13272807470647
a_HQ_transcript/17973	-1.31530753824614	3.11292317110978	-1.95379646924134	0.0593537586264645	0.745326265162914	-4.32904680156849
a_HQ_transcript/126686	-5.13724751214317	-0.111317635701946	-1.95354900344216	0.0593842593216315	0.745342425606194	-4.15454703820713
a_HQ_transcript/36297	-0.987903981052795	2.01127563775571	-1.95353424290466	0.0593860790197927	0.745342425606194	-4.16975990027844
a_HQ_transcript/135198	1.86200095310934	-0.505662372024515	1.95343739929416	0.0593980192262627	0.745342425606194	-4.10006896777805
a_HQ_transcript/33211	2.43964177216668	0.245336596110922	1.95336996004353	0.059406335291658	0.745342425606194	-4.10411201326814
a_HQ_transcript/136929	-0.635602689585014	3.66772252481553	-1.95301583473622	0.0594500197654688	0.745729553070171	-4.42787703531024
a_HQ_transcript/104287	-2.06326331744586	1.00831957727315	-1.95281457524438	0.0594748593221436	0.745762950716535	-4.07563140121396
a_HQ_transcript/89551	0.800295600363872	3.11154783381167	1.95278636648306	0.0594783415827459	0.745762950716535	-4.33077251049748
a_HQ_transcript/148278	-2.37466583772065	0.403184635257333	-1.95264508418989	0.0594957849893829	0.745820787117285	-4.0698700662311
a_HQ_transcript/70602	0.841727453840638	2.77712367467732	1.95252843310079	0.0595101906534486	0.745840526585524	-4.27753644031011
a_HQ_transcript/69996	-1.95108033320048	-0.639276018184256	-1.95227572780483	0.0595414085280166	0.745882142581005	-4.08261287188539
a_HQ_transcript/59014	2.26888974696552	0.0865051655211927	1.95221282079918	0.0595491819360421	0.745882142581005	-4.09699366754313
a_HQ_transcript/90970	-2.86530720133555	0.347317444647803	-1.9520116647406	0.0595740446689357	0.745882142581005	-4.05726190501669
a_HQ_transcript/135061	-3.61868354275694	2.18967698527701	-1.95200603713051	0.059574740366733	0.745882142581005	-4.33249284947363
a_HQ_transcript/151454	-1.91207500706765	2.4738039077447	-1.95190626504103	0.0595870755874024	0.745882142581005	-4.19934384795423
a_HQ_transcript/33320	2.53900468819288	1.23658549659824	1.95187855824663	0.0595905014820084	0.745882142581005	-4.04942717712858

a_HQ_transcript/102282	-1.59081677722016	2.99715192042923	-1.95167720688181	0.0596154033475883	0.746033189620799	-4.23363503772459
a_HQ_transcript/100416	-3.63642763667609	2.45486828172719	-1.95148544334946	0.0596391278477906	0.746068650260144	-4.29680283682729
a_HQ_transcript/122737	1.50487373464009	3.86539137372811	1.95138734622806	0.0596512673441143	0.746068650260144	-4.48802377275404
a_HQ_transcript/55881	1.85513909177162	0.281357436885037	1.95134311391874	0.0596567417838073	0.746068650260144	-4.11162772807995
a_HQ_transcript/117027	4.86243374435689	1.26300371536889	1.95108671985114	0.0596884831482435	0.746101927014063	-4.1356038693475
a_HQ_transcript/46761	1.69776985026951	0.927395316806322	1.95105882783606	0.0596919370395459	0.746101927014063	-4.03294005538798
a_HQ_transcript/42017	-2.98659497581814	1.54031709835705	-1.95101060323603	0.059697909140903	0.746101927014063	-4.0734176384552
a_HQ_transcript/8218	-2.15247369731684	-0.114600406027651	-1.95081564079746	0.0597220584420348	0.746243296027213	-4.22740828245499
a_HQ_transcript/18978	2.12408120202176	-0.522904521751839	1.95049269755792	0.0597620789221986	0.74643092101341	-4.14575358260566
a_HQ_transcript/143668	4.6768743989133	3.19810757072404	1.95046870238058	0.0597650534338932	0.74643092101341	-4.25514357315226
a_HQ_transcript/59960	2.06458950632561	-0.385190587210363	1.95028822287244	0.0597874303145228	0.74643092101341	-4.09382429971923
a_HQ_transcript/31102	-2.38322689354052	-0.48260181364275	-1.95019111108824	0.0597994737969256	0.74643092101341	-4.15272380411651
a_HQ_transcript/35075	2.91103541106529	1.56594241501066	1.95017654900805	0.0598012799194785	0.74643092101341	-4.18309528286526
a_HQ_transcript/50574	-2.95822499000689	-0.00722484586325681	-1.94977285164052	0.059851369015586	0.746833378935683	-4.21100126121478
a_HQ_transcript/97523	2.85614096647164	4.04775203809965	1.94954072495289	0.059880186815719	0.746833378935683	-4.29240449094746
a_HQ_transcript/26814	-1.21065312966859	3.54507498989255	-1.94952725570375	0.0598818593502925	0.746833378935683	-4.4211484478072
a_HQ_transcript/90474	2.90420290017828	1.56293914973958	1.94936576476308	0.0599019155316691	0.746833378935683	-4.13429862684295
a_HQ_transcript/138554	-2.09012515027248	-0.0379604082589036	-1.94899415324513	0.059948089536952	0.746833378935683	-4.19524343109495
a_HQ_transcript/9009	-4.47459475816486	2.15603019935202	-1.9489843419267	0.0599493090452551	0.746833378935683	-4.18410916461472
a_HQ_transcript/64738	-2.5016847908365	0.0485702022861031	-1.94895920672976	0.059952433349619	0.746833378935683	-4.13516913282246
a_HQ_transcript/34580	-0.803975087173081	3.51904471548074	-1.94894525883001	0.0599541671341688	0.746833378935683	-4.40917124549063
a_HQ_transcript/9433	-2.23789958345347	3.55078085052514	-1.9488307947967	0.0599683971545329	0.746833378935683	-4.4040446304002
a_HQ_transcript/53097	1.32421039937898	1.98899632695819	1.94881769051127	0.0599700264492691	0.746833378935683	-4.08957048823676
a_HQ_transcript/23466	2.39626623222499	2.97064492043483	1.94874278572547	0.0599793403210809	0.746833378935683	-4.3284676236867
a_HQ_transcript/24672	-0.869365523380698	2.679627945465	-1.94867556056566	0.0599877003552626	0.746833378935683	-4.21763958491323
a_HQ_transcript/147621	2.93812160845957	1.71966652952519	1.94847963754906	0.060012070856358	0.746976799729845	-4.17794905837956
a_HQ_transcript/101265	3.16045677536301	-0.258521490856796	1.94808742404347	0.0600608833801281	0.747340074473034	-4.15327161558972
a_HQ_transcript/146967	-1.50055169667454	2.21789235151664	-1.94803853742256	0.0600669699273674	0.747340074473034	-4.13738175791871
a_HQ_transcript/150226	-4.12057783618675	-0.111803720747003	-1.94785549813989	0.0600897636811963	0.747463681416772	-4.17089807793689
a_HQ_transcript/11139	0.669414778235881	3.22925681917085	1.9476333553771	0.0601174370439275	0.747587750462115	-4.35639872944396
a_HQ_transcript/41351	3.19351098681445	-0.351328695867622	1.94746768646861	0.0601380823915211	0.747587750462115	-4.13453407636011
a_HQ_transcript/112029	0.642552112469321	4.22499347762541	1.94744881076804	0.0601404350360806	0.747587750462115	-4.47874917644297
a_HQ_transcript/101325	2.67064150612998	1.12527360955516	1.94716785830731	0.06017546205196	0.747587750462115	-4.11596620410447
a_HQ_transcript/12008	-2.47183388061442	0.958492226171536	-1.94707689510976	0.0601868064434255	0.747587750462115	-4.06517064720253
a_HQ_transcript/146796	-1.72737389763487	-0.619113600450239	-1.94705865534987	0.0601890814215863	0.747587750462115	-4.11946818977608
a_HQ_transcript/125577	-3.58482807124324	3.02438019905823	-1.9470531731514	0.0601897652106162	0.747587750462115	-4.40884631818719
a_HQ_transcript/102001	0.609553659841177	3.32897510357616	1.94668257537649	0.0602360051216283	0.747746001656814	-4.37121385313952
a_HQ_transcript/102820	-2.3526631265026	-0.0318140441458757	-1.94656643826449	0.0602505020227433	0.747746001656814	-4.2231786641825
a_HQ_transcript/132023	2.48731282760797	0.210676269154587	1.94650793417383	0.0602578059854497	0.747746001656814	-4.13536322001753
a_HQ_transcript/36235	-3.77590264146086	2.58230376249504	-1.94650050993742	0.0602587329216358	0.747746001656814	-4.12604762757012
a_HQ_transcript/134506	-1.40368841453844	-0.813357642183609	-1.94627076044893	0.0602874238868764	0.747746001656814	-4.16254765434697
a_HQ_transcript/27820	2.94514096514967	2.05665425033071	1.94625532156663	0.0602893523091696	0.747746001656814	-4.24990997925589
a_HQ_transcript/150815	2.08162850999644	0.906697661713194	1.94602033614812	0.0603187102049905	0.747746001656814	-4.13355136060103
a_HQ_transcript/119328	-1.313912818185	2.30404686464547	-1.94595937368081	0.0603263285743398	0.747746001656814	-4.13875768614222
a_HQ_transcript/45262	2.58833582980432	-0.362431216311369	1.94580661708951	0.0603454219552732	0.747746001656814	-4.15470258203444
a_HQ_transcript/95764	3.49258211297941	2.37104742110528	1.94579326697933	0.0603470908638125	0.747746001656814	-4.1781147713619
a_HQ_transcript/1992	1.80480619277594	2.27194021685822	1.94575575140548	0.0603517809316727	0.747746001656814	-4.16645563520235
a_HQ_transcript/38007	1.93621267510014	0.390064448588892	1.94563671004177	0.060366665166297	0.747746001656814	-4.09479095055029
a_HQ_transcript/135313	-2.20823245181667	0.720559592254162	-1.9456121571595	0.0603697355108628	0.747746001656814	-4.12884659247053
a_HQ_transcript/115295	3.69104336609946	2.12453020260629	1.94550749445892	0.0603828251291612	0.747748798276073	-4.16760997526568

a_HQ_transcript/42060	-1.34002371052718	-0.447416941742959	-1.94526559979286	0.0604130870530785	0.747964201112108	-4.16704169485511
a_HQ_transcript/139864	3.28171936710833	0.878130858141547	1.94512980715706	0.0604300809816199	0.74797258632111	-4.05067383421306
a_HQ_transcript/123148	4.58700646120722	0.404767546001654	1.9450392013545	0.0604414222568126	0.74797258632111	-4.1907138753077
a_HQ_transcript/676	-0.642673296527296	3.45111514352141	-1.94495177447519	0.0604523673709155	0.74797258632111	-4.3906003590897
a_HQ_transcript/88808	2.17213792549688	1.02967036901022	1.94480359527484	0.0604709220931003	0.748042936673279	-4.04684405121496
a_HQ_transcript/56543	-3.38078889434548	0.326977535936994	-1.9447002440284	0.0604838664662949	0.748043870202721	-4.17739443596858
a_HQ_transcript/98286	4.37771084353122	1.54499766391581	1.94455413637489	0.0605021700259184	0.748111069829097	-4.19488355413767
a_HQ_transcript/23266	2.6438879326867	2.94098428945108	1.9442775418587	0.0605368334129314	0.74838048758547	-4.37064050242549
a_HQ_transcript/68788	-2.43768556994861	0.642841379653834	-1.94386107898083	0.0605890578735271	0.74879935190857	-4.13652956665215
a_HQ_transcript/8651	-2.32722749414461	0.264051727158337	-1.94380192187122	0.0605964793452022	0.74879935190857	-4.11167401149594
a_HQ_transcript/147835	-0.82283505327039	5.08896738985364	-1.943402221873	0.0606466438023982	0.748955659004005	-4.60762436833518
a_HQ_transcript/105060	-1.07123186289888	2.61482228212613	-1.94335065725085	0.0606531180559489	0.748955659004005	-4.20421888704597
a_HQ_transcript/82978	-2.09404994148859	1.30863303137167	-1.94331060655674	0.0606581470789285	0.748955659004005	-4.08817179229976
a_HQ_transcript/87328	-2.59146785209579	1.39301253778397	-1.94329054041453	0.0606606668488655	0.748955659004005	-4.17287543724066
a_HQ_transcript/21407	0.945702325028798	2.22887922855942	1.94296357344718	0.0607017379308765	0.749195860420934	-4.13873405588602
a_HQ_transcript/180	-2.2472948932282	-0.135788297081046	-1.94284249660545	0.0607169527907087	0.749195860420934	-4.15530599827643
a_HQ_transcript/100542	-1.6862515288632	-0.678598992833297	-1.94279351553627	0.0607231088302774	0.749195860420934	-4.1677361821163
a_HQ_transcript/2947	2.47891606738384	0.549718765029425	1.94272535291392	0.060731676546646	0.749195860420934	-4.07597596325743
a_HQ_transcript/85089	2.30028284589952	-0.234897647769858	1.9422025292605	0.0607974278415868	0.749669358297216	-4.13497700163825
a_HQ_transcript/74666	1.40005566786275	-0.746060138456965	1.94195795370185	0.060828207309383	0.749669358297216	-4.12087689699447
a_HQ_transcript/119273	-2.51907806559752	0.40409616816894	-1.94187590675988	0.0608385358196685	0.749669358297216	-4.08228337324604
a_HQ_transcript/127627	-4.7459927316542	1.89936946938091	-1.94186054750872	0.0608404694937376	0.749669358297216	-4.12782857092221
a_HQ_transcript/8699	-2.44819741361056	3.00863772291015	-1.94185065014042	0.0608417155647714	0.749669358297216	-4.27727091768596
a_HQ_transcript/104094	2.50492564912877	1.8573647168098	1.94180517852566	0.0608474406903087	0.749669358297216	-4.19167596083743
a_HQ_transcript/59363	3.03544964281497	2.61115075199114	1.94155351597661	0.060879134830177	0.749755006573762	-4.31088964406562
a_HQ_transcript/1424	-2.44697720504055	1.618646400829	-1.94154514679105	0.0608801890831984	0.749755006573762	-4.10299566270717
a_HQ_transcript/54676	2.70352603174923	1.37679106872257	1.94129116025794	0.060912190892367	0.749966973105571	-4.28848539203318
a_HQ_transcript/58750	-2.12666551515963	4.20687058593668	-1.94120377391768	0.0609232047723043	0.749966973105571	-4.49615997256536
a_HQ_transcript/133442	-3.93501011830368	3.69110640076658	-1.94106571008238	0.0609406093911619	0.750022388881952	-4.45449234508519
a_HQ_transcript/21011	-1.59352501712828	-0.413473774425375	-1.94063365729767	0.0609951027278646	0.750468715065045	-4.11505545695312
a_HQ_transcript/110993	-2.86599252709774	0.346911598795595	-1.9405734849611	0.0610026954081052	0.750468715065045	-4.17477632786952
a_HQ_transcript/126644	-2.12433784881379	0.184771466965305	-1.94005157699974	0.0610685852975221	0.750752468729411	-4.10023099420306
a_HQ_transcript/89365	-3.13991760467943	2.65879833229916	-1.93996172048193	0.0610799357341276	0.750752468729411	-4.30166726406968
a_HQ_transcript/144677	-0.52726746932677	4.16402916607151	-1.93986193035997	0.0610925430999003	0.750752468729411	-4.50979902475201
a_HQ_transcript/50588	1.04517264665018	2.95539421610021	1.93972942618137	0.0611092870062663	0.750752468729411	-4.27720547983128
a_HQ_transcript/73493	2.21022438996044	-0.296898527120081	1.93963298948574	0.0611214757413008	0.750752468729411	-4.15262123348854
a_HQ_transcript/89092	-1.91828057232535	1.10281726773852	-1.93962744363013	0.0611221767518654	0.750752468729411	-4.07346359529382
a_HQ_transcript/152127	5.41085653541098	6.01762385203351	1.9395090248456	0.061137146856932	0.750752468729411	-4.8376302212637
a_HQ_transcript/132544	-2.4035221760616	-0.208759522734908	-1.93941559319561	0.0611489604147619	0.750752468729411	-4.15535613973408
a_HQ_transcript/116665	2.8114873044694	0.180970058872825	1.93909295608762	0.0611897700695568	0.750752468729411	-4.15041186132096
a_HQ_transcript/92080	-2.66111367922844	-0.33708177695144	-1.93905307928091	0.0611948156371634	0.750752468729411	-4.15499347574513
a_HQ_transcript/104826	2.09316876479457	-0.646315812206742	1.93904559120069	0.0611957631357566	0.750752468729411	-4.12561459600906
a_HQ_transcript/8684	-2.76019295479789	0.94490103720069	-1.93881568079715	0.0612248608636509	0.750752468729411	-4.12582124212778
a_HQ_transcript/122952	2.18293160784368	-0.277380738295974	1.93881176915962	0.0612253560287512	0.750752468729411	-4.15294436366819
a_HQ_transcript/117931	3.29442309866051	0.265517092121972	1.93878368143871	0.0612289116899557	0.750752468729411	-4.17012725812485
a_HQ_transcript/112807	-1.82265173943522	-0.625582263720228	-1.93874256629505	0.0612341168322601	0.750752468729411	-4.13873876740417
a_HQ_transcript/101778	-2.46332242657821	0.7502048282285	-1.93870308192392	0.0612391158810672	0.750752468729411	-4.03298064189197
a_HQ_transcript/126317	1.71109003403577	1.66088671197882	1.93863903029222	0.0612472261009361	0.750752468729411	-4.06349877660057
a_HQ_transcript/150489	2.16572019368104	-0.522769801051443	1.93841839009555	0.0612751706958179	0.750752468729411	-4.15817169547253
a_HQ_transcript/62853	3.09110759700369	0.652106897627906	1.93837050257642	0.0612812372205714	0.750752468729411	-4.16222190821539

a_HQ_transcript/9130	-0.925902939072226	2.51601473127905	-1.9383481369216	0.0612840707425174	0.750752468729411	-4.24195933398956
a_HQ_transcript/122465	-1.43112880730726	2.12076150072366	-1.9380067649387	0.0613273335125563	0.750995604727465	-4.13417739272203
a_HQ_transcript/133812	-2.67090217484784	1.58554413479526	-1.93792598738869	0.0613375744875952	0.750995604727465	-4.189587168247
a_HQ_transcript/137803	-4.11654998227465	2.65318775021481	-1.93788574440571	0.0613426770445569	0.750995604727465	-4.3489970384759
a_HQ_transcript/95209	2.20834230081197	0.141884868831878	1.93776289876344	0.0613582553737733	0.751028146634384	-4.08860717058707
a_HQ_transcript/10560	-1.49601948344253	2.10047323872895	-1.93749053870262	0.0613928061838008	0.751039873659255	-4.0900682848285
a_HQ_transcript/50585	4.76424920426868	4.71470180748057	1.93740531916658	0.0614036203494389	0.751039873659255	-4.36466013508533
a_HQ_transcript/60189	-1.79363058797201	0.236766281007195	-1.93731039324568	0.0614156681762554	0.751039873659255	-4.06959608224716
a_HQ_transcript/63990	-1.84711899316915	3.20687018052841	-1.93724003660294	0.0614245990359864	0.751039873659255	-4.30902176030608
a_HQ_transcript/110576	2.1727817432978	-0.208708427378784	1.93715543703054	0.0614353393400212	0.751039873659255	-4.15590395120225
a_HQ_transcript/124282	1.68598247369542	0.791426357733742	1.93711848781671	0.0614400307227662	0.751039873659255	-4.08494420937533
a_HQ_transcript/136832	-2.75260077880388	0.0612151111380979	-1.93704268226132	0.0614496566047932	0.751039873659255	-4.12324352281156
a_HQ_transcript/91253	1.60310185735905	1.27069231942894	1.93687491453589	0.0614709646010005	0.751142365004616	-4.20080009465964
a_HQ_transcript/91619	-2.73694003505459	1.55692684685957	-1.93666015912073	0.0614982497934003	0.751317836063634	-4.13837887836728
a_HQ_transcript/129775	-3.12614139093776	4.73839471709319	-1.93636954427226	0.0615351898144152	0.751611160649786	-4.42301305255446
a_HQ_transcript/91049	-2.51501652708761	2.60437344918384	-1.93625973655889	0.0615491524632068	0.751623767727161	-4.2833564752677
a_HQ_transcript/68548	1.71444659919175	-0.556486459050539	1.93590671652327	0.0615940594790305	0.751725111361776	-4.15675954699886
a_HQ_transcript/114655	2.84591242254601	-0.039172403234931	1.93582063942074	0.0616050134884026	0.751725111361776	-4.1265350524773
a_HQ_transcript/107464	-2.55227343628839	-0.345176316592838	-1.93563466204022	0.0616286863799356	0.751725111361776	-4.16952994853629
a_HQ_transcript/95523	-1.28825167479941	1.75567673843433	-1.93559150115921	0.061634181415568	0.751725111361776	-4.1238890949967
a_HQ_transcript/106990	-1.1819651674048	3.07998650195695	-1.93550460372471	0.0616452460660369	0.751725111361776	-4.39241211606535
a_HQ_transcript/151626	-0.828235758791189	6.09147724228339	-1.9354307077433	0.0616546565950416	0.751725111361776	-4.69045487153003
a_HQ_transcript/109067	0.912688131097133	3.39661784593359	1.93540724789241	0.0616576444278205	0.751725111361776	-4.33463908449667
a_HQ_transcript/66957	-1.94572538212774	1.74663803167487	-1.93537684186828	0.0616615171077356	0.751725111361776	-4.17930591622814
a_HQ_transcript/110143	-3.05219011782716	2.72990868218191	-1.93522762167599	0.0616805256734253	0.751725111361776	-4.37855290043796
a_HQ_transcript/10131	3.01158219989831	0.0321415385946196	1.93516506430233	0.0616884961177262	0.751725111361776	-4.17481535873183
a_HQ_transcript/93750	2.75906836944722	0.502043068502341	1.93503329694766	0.0617052875344563	0.751725111361776	-4.15023603377926
a_HQ_transcript/130884	2.92693125005542	-0.156508939145355	1.93486992738702	0.0617261115893185	0.751725111361776	-4.18501737821985
a_HQ_transcript/27654	2.34827498374782	-0.257914988069408	1.93482205476309	0.0617322148713797	0.751725111361776	-4.16204766575367
a_HQ_transcript/125621	-2.8481568219051	0.489557606256586	-1.93477273933456	0.0617385026431516	0.751725111361776	-4.13106642648353
a_HQ_transcript/72835	1.55093310422377	-0.776630713969123	1.93461198793504	0.061759002475645	0.751774857286276	-4.1737212015312
a_HQ_transcript/152669	2.37828765395889	0.807779107096263	1.93453710553949	0.0617685538704003	0.751774857286276	-4.10610816190945
a_HQ_transcript/38268	2.4937180109361	1.56305048024702	1.93443650630731	0.0617813875112947	0.751774857286276	-4.15019839871724
a_HQ_transcript/27341	-3.54468364456914	1.40813637648282	-1.93431650758994	0.0617966990025519	0.751803792302289	-4.19582544151109
a_HQ_transcript/51089	2.27260345073242	0.981194533632832	1.93410475563378	0.0618237259593735	0.751975212924558	-4.1368165399159
a_HQ_transcript/67902	1.81339834017338	0.882934088339669	1.93378605119822	0.0618644230910144	0.752113103498122	-4.07747962644189
a_HQ_transcript/22372	1.87986131232754	1.10099513851271	1.93367944922695	0.0618780408628464	0.752113103498122	-4.09671354832357
a_HQ_transcript/25864	-1.07546152376828	3.86686601618741	-1.93359715135173	0.0618885557054745	0.752113103498122	-4.52207675778447
a_HQ_transcript/131185	-3.05616888444271	-0.132467846013268	-1.93344072380526	0.0619085460401842	0.752113103498122	-4.18147373375509
a_HQ_transcript/3925	2.40591254945851	1.4984280585787	1.93342603730608	0.0619104231580493	0.752113103498122	-4.13260704974368
a_HQ_transcript/82717	-1.73273890670629	-0.141662695378957	-1.93340825423029	0.0619126961230132	0.752113103498122	-4.13819597434129
a_HQ_transcript/122426	-4.82986736532387	1.6089370023794	-1.93310124880651	0.061951947779534	0.752181951654569	-4.2307254266131
a_HQ_transcript/74402	2.36358453831912	-0.523101196913319	1.93300762160744	0.061963922612772	0.752181951654569	-4.19015648174827
a_HQ_transcript/120787	4.21427176355934	6.39686225369395	1.93287505538409	0.0619808811394143	0.752181951654569	-4.64229687078939
a_HQ_transcript/143050	-2.55900268959602	0.551237663903008	-1.93281761951181	0.061988229867798	0.752181951654569	-4.14235763927374
a_HQ_transcript/50842	1.7474838294388	1.01860913659302	1.93270368721741	0.0620028093575535	0.752181951654569	-4.09744800466256
a_HQ_transcript/50777	2.97416302191084	1.70233650387832	1.93268337855006	0.0620054084935472	0.752181951654569	-4.09619886078439
a_HQ_transcript/129027	-3.13877128417443	1.20145021134146	-1.93261029476815	0.0620147626543942	0.752181951654569	-4.24692557605171
a_HQ_transcript/67705	-2.02381877899764	-0.289306798530789	-1.93255465946513	0.0620218843634797	0.752181951654569	-4.16177392261539
a_HQ_transcript/139351	1.36426966556232	2.54282895694462	1.93196602509426	0.0620972770815545	0.752834069299987	-4.23954157578853

a_HQ_transcript/42052	2.10375512479508	0.588307930330303	1.93193262021752	0.0621015579805505	0.752834069299987	-4.13672562114655
a_HQ_transcript/112375	-3.63197244598977	0.0534203431796645	-1.9314811588018	0.0621594386909915	0.753246154255319	-4.2405378903327
a_HQ_transcript/4684	-2.95753859335086	0.576107504396232	-1.93146533841426	0.0621614678289768	0.753246154255319	-4.13609616206
a_HQ_transcript/50766	2.17294937391453	-0.074092508689054	1.93132247759529	0.062179793890811	0.753311183677586	-4.23447158248079
a_HQ_transcript/113728	-2.43300583393116	-0.115586297836178	-1.93108771068503	0.0622099197444482	0.753459524383235	-4.17092600986674
a_HQ_transcript/106695	2.14020819253751	-0.630383613320588	1.93091256071817	0.0622324036374699	0.753459524383235	-4.16863346394429
a_HQ_transcript/108246	2.53575711225825	3.12472756550396	1.93082487770665	0.0622436620934036	0.753459524383235	-4.40729419372456
a_HQ_transcript/130856	-3.15943424033246	1.31081480326624	-1.93067531648667	0.0622628697495206	0.753459524383235	-4.19641181877332
a_HQ_transcript/69386	-1.38479662491993	1.59003963566578	-1.93065804495528	0.0622650882059761	0.753459524383235	-4.16615306492688
a_HQ_transcript/26697	2.48477861225382	0.214726086843511	1.93059973973657	0.0622725777744571	0.753459524383235	-4.22539442762207
a_HQ_transcript/140535	-1.8603839632291	4.54388911648751	-1.93050227897737	0.0622850987899902	0.753459524383235	-4.5287950183557
a_HQ_transcript/73672	3.44769668698135	4.37549198849128	1.93030971412714	0.0623098444607717	0.753459524383235	-4.49275983573733
a_HQ_transcript/86424	-1.80792028950955	1.43852638897699	-1.93026527467121	0.0623155563901977	0.753459524383235	-4.06744008200838
a_HQ_transcript/41157	2.01452800830216	0.769204695517541	1.9300739825666	0.0623401488819978	0.753459524383235	-4.18247544452027
a_HQ_transcript/60210	2.97365121557902	0.647341057141781	1.93002429684821	0.0623465378465711	0.753459524383235	-4.22368380864248
a_HQ_transcript/147544	2.77610634268305	0.647317272729491	1.93001616829072	0.0623475831317671	0.753459524383235	-4.19810523116793
a_HQ_transcript/18798	0.81435532590122	2.99726629580265	1.92991444913646	0.062360664905734	0.75346097061744	-4.37803787942707
a_HQ_transcript/119245	-1.87047621002696	2.23979962827697	-1.92946109918478	0.0624189976807363	0.753986451817096	-4.28013784748007
a_HQ_transcript/101593	1.8007390054879	-0.613385548341171	1.92937486389804	0.0624300989642802	0.753986451817096	-4.17462740490224
a_HQ_transcript/73012	-3.43092205551523	0.0114242517270853	-1.9291073201405	0.0624645514143092	0.754133941304355	-4.21593901071964
a_HQ_transcript/88765	3.93229889875208	4.27503542558406	1.92883305110948	0.0624998869773013	0.754133941304355	-4.51860896698044
a_HQ_transcript/65143	1.88148506533617	1.76488317255316	1.9287698567616	0.0625080310985804	0.754133941304355	-4.08982130952562
a_HQ_transcript/105188	-2.39019508528127	0.601294807225117	-1.92870350621662	0.0625165829605418	0.754133941304355	-4.1706503355628
a_HQ_transcript/134602	-2.21484953396147	-0.483501619234203	-1.92861903231116	0.0625274721911739	0.754133941304355	-4.18571333531052
a_HQ_transcript/112506	-3.26489851746375	-0.231524091309711	-1.92857330904869	0.0625333668988824	0.754133941304355	-4.20174608679273
a_HQ_transcript/57532	-0.931961760062257	3.46367226044111	-1.92851396375901	0.0625410184947488	0.754133941304355	-4.44956101937441
a_HQ_transcript/141707	6.26594705662824	4.28014832274043	1.92847455136718	0.0625461005200298	0.754133941304355	-4.61574807886759
a_HQ_transcript/121349	1.42670926612307	2.53835913857047	1.92806868997253	0.0625984550509834	0.754608667607541	-4.17811977863569
a_HQ_transcript/83902	0.717401658844332	3.24936202608326	1.92787649302383	0.0626232609326476	0.754751173852984	-4.40670519330496
a_HQ_transcript/31236	-1.29860113490043	1.07096377096591	-1.92775072230641	0.0626394981224693	0.754790370411048	-4.06738508476675
a_HQ_transcript/150804	0.870405681885519	7.99337570408627	1.92763100920513	0.0626549566485773	0.754820169962384	-4.86947543767342
a_HQ_transcript/100202	-1.51682601180196	1.16937369509272	-1.92738464097151	0.0626867805106184	0.754893622350784	-4.08034061754421
a_HQ_transcript/108426	1.07001874265384	3.73884989465672	1.92738273188444	0.0626870271656901	0.754893622350784	-4.51575367211414
a_HQ_transcript/146458	-2.85851965893423	0.59918408120985	-1.92715256312179	0.0627167712472188	0.75500922520273	-4.14768117448634
a_HQ_transcript/64563	-4.52790996541086	3.52417043529185	-1.92710743549407	0.0627226043989812	0.75500922520273	-4.34048126528276
a_HQ_transcript/280	4.3979788296019	2.59225564856288	1.9269043296107	0.0627488634717935	0.755013001853742	-4.37571067016517
a_HQ_transcript/152096	-3.2097297206919	0.355206858330728	-1.9269040800206	0.0627488957465495	0.755013001853742	-4.2166571773344
a_HQ_transcript/148410	3.18593458049743	3.0705676396329	1.9267839291559	0.062764434247777	0.755043674245609	-4.27696809190526
a_HQ_transcript/144555	-2.60197268602934	-0.472032364655782	-1.92645822464099	0.0628065727029015	0.755283932552093	-4.18529627527984
a_HQ_transcript/128835	-2.22504102542372	0.176622854001452	-1.92642870467428	0.0628103930972477	0.755283932552093	-4.11206458278847
a_HQ_transcript/52247	3.10610088045405	-0.153429781766781	1.92614080931266	0.0628476622793414	0.755575783448512	-4.11263882382694
a_HQ_transcript/51983	2.57162514835686	0.410888896462619	1.92582033663305	0.0628891712211042	0.755785587491434	-4.16990736249157
a_HQ_transcript/21133	-2.75774280011048	-0.418086113374812	-1.9258053135931	0.0628911176489139	0.755785587491434	-4.175334920596
a_HQ_transcript/139770	2.2086350642202	1.80292765587709	1.92532180882443	0.0629537897361992	0.756382366636169	-4.10101861093534
a_HQ_transcript/145961	3.26923484917925	-0.475499696555987	1.92502000287331	0.0629929373261414	0.756696313472608	-4.20398429777084
a_HQ_transcript/136166	0.633517972701915	3.99261321051105	1.92481799793326	0.0630191513759583	0.756761202607389	-4.47599458448211
a_HQ_transcript/12911	2.72347964799804	2.47423991491149	1.9244774433255	0.0630633662876963	0.756761202607389	-4.20945548266651
a_HQ_transcript/11598	1.927804883035	-0.731927929568291	1.92439673187275	0.0630738491551734	0.756761202607389	-4.18866276361016
a_HQ_transcript/87491	-1.70197337866287	2.06273914229169	-1.92435415009656	0.0630793803172895	0.756761202607389	-4.21088613354802
a_HQ_transcript/150958	-1.45059874471957	2.60210760870979	-1.924349641574	0.0630799659766292	0.756761202607389	-4.29514568503177

a_HQ_transcript/69057	-1.2404701309236	1.03594338794517	-1.9242604291526	0.0630915556821047	0.756761202607389	-4.07934024818113
a_HQ_transcript/145076	0.702849832618192	4.01756203175483	1.92406878493948	0.0631164586665579	0.756761202607389	-4.46447565997282
a_HQ_transcript/149211	-2.20065316359869	-0.409305568873601	-1.92405188703054	0.0631186548534519	0.756761202607389	-4.19017684946328
a_HQ_transcript/128169	2.74778457863396	1.60538166923048	1.92399377025642	0.0631262086770346	0.756761202607389	-4.12466064054099
a_HQ_transcript/51923	1.44294122055298	-0.750344633588228	1.92384923037515	0.0631449988767409	0.756761202607389	-4.19852420987567
a_HQ_transcript/72268	-1.38315081265371	-0.816951844123457	-1.92363243455742	0.0631731914239907	0.756761202607389	-4.18760873445783
a_HQ_transcript/61472	2.87165516027511	0.013194157215661	1.92356536095658	0.0631819160092413	0.756761202607389	-4.18174868439733
a_HQ_transcript/67138	-2.21520150199537	-0.033977148640444	-1.92339057510449	0.0632046561396897	0.756761202607389	-4.16124684897966
a_HQ_transcript/61480	2.75883176677668	-0.273955826672597	1.92316986988715	0.0632333806191859	0.756761202607389	-4.18894794209784
a_HQ_transcript/67375	-2.78113971302981	0.205582097634002	-1.92303900070864	0.0632504183929198	0.756761202607389	-4.19069325173149
a_HQ_transcript/150968	2.53215772252808	0.447817359974162	1.92294719693885	0.0632623726352573	0.756761202607389	-4.19003931535617
a_HQ_transcript/134238	2.04422255863498	-0.166508152136872	1.92269595881936	0.0632950976361008	0.756761202607389	-4.20449207446293
a_HQ_transcript/27890	1.57139253909234	-0.514445534321511	1.92265983757028	0.0632998038097721	0.756761202607389	-4.09752662779585
a_HQ_transcript/44836	2.98835940721328	2.16992675269354	1.922639436137	0.0633024620101412	0.756761202607389	-4.23396689650547
a_HQ_transcript/151270	-1.75518101983927	-0.70181981425715	-1.92262789952105	0.0633039652137604	0.756761202607389	-4.18731202251107
a_HQ_transcript/22438	-2.94940892193035	1.04278371521647	-1.92248849112104	0.0633221323576352	0.756761202607389	-4.16751637899539
a_HQ_transcript/58452	1.85137120792682	2.24359329374864	1.92228540462154	0.0633486058429942	0.756761202607389	-4.2675054717459
a_HQ_transcript/138660	-3.83589551943628	3.64802304718523	-1.92223101273763	0.0633556977602812	0.756761202607389	-4.4022416184375
a_HQ_transcript/12476	-0.609005657038459	3.29468158987888	-1.92216444406062	0.0633643782899524	0.756761202607389	-4.41487325217466
a_HQ_transcript/134805	-3.79117771498591	-0.217379334153529	-1.9221367212074	0.0633679936425471	0.756761202607389	-4.16089811546148
a_HQ_transcript/87919	2.28320679149212	3.49320055677023	1.92210175392333	0.0633725539997158	0.756761202607389	-4.4528121080877
a_HQ_transcript/38693	-3.25128919947654	1.35422543821997	-1.92203612952559	0.0633811133569781	0.756761202607389	-4.20465146234051
a_HQ_transcript/32970	-2.043202860771	-0.347807473959719	-1.9219909435658	0.0633870075200699	0.756761202607389	-4.13093985273244
a_HQ_transcript/139501	-1.10549714674801	2.37190496303458	-1.92189446766033	0.0633995936506629	0.756761202607389	-4.19206855260832
a_HQ_transcript/10590	2.02699712590041	1.91768389637997	1.92164846251643	0.0634316969653556	0.756761202607389	-4.22132155206599
a_HQ_transcript/68853	-4.75026466573585	2.09994903859367	-1.92156567715462	0.0634425034951146	0.756761202607389	-4.37204975916672
a_HQ_transcript/144810	3.21107671222857	0.893190569774442	1.92151343499138	0.0634493238349555	0.756761202607389	-4.33310063249161
a_HQ_transcript/69757	-2.37944328943976	0.0505137229317867	-1.92138742322194	0.0634657775825405	0.756761202607389	-4.22765852742406
a_HQ_transcript/124910	-2.8753267681623	0.0967474497864423	-1.92134920597981	0.063470768455335	0.756761202607389	-4.22502926735091
a_HQ_transcript/11114	3.11341568738378	1.30316185978487	1.92128239435939	0.0634794943452712	0.756761202607389	-4.08044920101906
a_HQ_transcript/90403	-2.77056626244687	0.0754214099114051	-1.92127113975251	0.0634809643482296	0.756761202607389	-4.30647652850091
a_HQ_transcript/106133	2.59930727673581	1.34688590392745	1.92125034180196	0.0634836809176035	0.756761202607389	-4.06557746093129
a_HQ_transcript/143473	3.01321671929802	-0.144028653929507	1.92117856902368	0.0634930564463983	0.756761202607389	-4.1750435665044
a_HQ_transcript/150073	-2.02786533405388	0.714671592643198	-1.92101028401485	0.063515043860851	0.756868074937177	-4.22851271654763
a_HQ_transcript/146249	3.88418650527634	3.5896344853477	1.92083175166414	0.0635383773453946	0.756990940424902	-4.43664716595825
a_HQ_transcript/72250	1.92058267392828	-0.0793209897459025	1.92065436329308	0.063561568654858	0.757112062042948	-4.21559221322585
a_HQ_transcript/69011	2.58589348395096	0.368885204005859	1.92019579575048	0.0636215545319091	0.757460629238145	-4.18540268198091
a_HQ_transcript/55861	-1.84947877092022	1.92526487222268	-1.92011556685879	0.0636320544161208	0.757460629238145	-4.17364578258323
a_HQ_transcript/139847	-2.45386069852313	-0.257940568085796	-1.92005870850986	0.0636394966083925	0.757460629238145	-4.18954195708684
a_HQ_transcript/62250	2.0227253272506	0.0165417327649797	1.91999855767532	0.0636473705732238	0.757460629238145	-4.19548350537962
a_HQ_transcript/4304	1.86713735726124	2.2668216510835	1.91993274745119	0.0636559863375368	0.757460629238145	-4.26801543340664
a_HQ_transcript/106142	-0.866089798771172	2.54434205973694	-1.91946004848759	0.0637179009237243	0.757662494060417	-4.25299297122907
a_HQ_transcript/94310	4.66566727737125	1.77022888645535	1.91944267304132	0.0637201777685878	0.757662494060417	-4.15656088951156
a_HQ_transcript/88984	2.82051274951434	1.59599631652382	1.9193975099478	0.0637260961819106	0.757662494060417	-4.208735740002
a_HQ_transcript/99738	-2.50125792836347	0.977405095579938	-1.91928769099996	0.0637404894264247	0.757662494060417	-4.17028694193972
a_HQ_transcript/94102	1.90200705582566	2.07029467732091	1.91913176903014	0.0637609299160918	0.757662494060417	-4.17493558031096
a_HQ_transcript/1650	2.74397870277433	0.842748951674011	1.91910809187296	0.0637640343530742	0.757662494060417	-4.13402369922698
a_HQ_transcript/63150	-3.9365744826447	1.99929170708833	-1.91910689369626	0.0637641914558141	0.757662494060417	-4.3524577990763
a_HQ_transcript/59330	-2.44168545415433	0.78515892343325	-1.91882811201952	0.0638007538950501	0.757942003354072	-4.14524105139067
a_HQ_transcript/46796	2.19035704728883	0.209437143339249	1.91870531584877	0.0638168644578335	0.757978483286667	-4.18850947336039

a_HQ_transcript/24615	-2.79877822839645	2.1165161723625	-1.91843725654711	0.0638520454089714	0.758171265767746	-4.25906555118365
a_HQ_transcript/123108	2.40549382911215	2.56640463159248	1.91838289710777	0.0638591817574815	0.758171265767746	-4.36220490773823
a_HQ_transcript/53026	2.97240378229443	1.37608192449595	1.91819095530213	0.0638843855373668	0.758182999566745	-4.44272713603347
a_HQ_transcript/11087	3.2622002254053	1.34742275698106	1.91817670757597	0.0638862567416377	0.758182999566745	-4.17471981176011
a_HQ_transcript/58057	2.67053921518776	1.43956932889089	1.91801657118075	0.0639072912786941	0.758277817401088	-4.16580562364245
a_HQ_transcript/14885	2.17491386515233	1.55924721249358	1.91776482406124	0.0639403713392074	0.758515490858254	-4.17245606179773
a_HQ_transcript/73814	1.32860821084809	2.50297684418385	1.9172713319886	0.0640052600874085	0.759076148344325	-4.3232406569314
a_HQ_transcript/117091	2.19296846214062	-0.01045945127067	1.91716673756524	0.064019020402277	0.759076148344325	-4.15430778800395
a_HQ_transcript/75294	0.93834120508601	10.3573429765978	1.91704093560645	0.0640355741405357	0.759076148344325	-5.23232034588451
a_HQ_transcript/55440	-2.03885800215305	0.710870917795877	-1.91689611357125	0.064054635227904	0.759076148344325	-4.13201642306351
a_HQ_transcript/141542	-1.26809661942255	3.32688433711686	-1.9166297700306	0.0640897034476056	0.759076148344325	-4.43319266878154
a_HQ_transcript/15712	0.761424979969703	5.09456813269985	1.91641491726966	0.0641180042007135	0.759076148344325	-4.60767416905949
a_HQ_transcript/116436	2.25513697100452	-0.156377404505594	1.91637449104717	0.0641233304147916	0.759076148344325	-4.16043736432526
a_HQ_transcript/97599	5.27750651638458	5.26016146973112	1.91633605369251	0.0641283949471028	0.759076148344325	-4.6892447581827
a_HQ_transcript/100037	0.513600694879415	3.83670398337231	1.91631785753358	0.0641307926060462	0.759076148344325	-4.50504448956567
a_HQ_transcript/147935	2.01766802255315	1.90498072439576	1.91619070575828	0.0641475492151508	0.759076148344325	-4.24076930488755
a_HQ_transcript/107845	1.61534631746013	2.38378015635074	1.91618630341866	0.0641481294421852	0.759076148344325	-4.31883041898598
a_HQ_transcript/109597	0.73429796513376	3.83626028715716	1.91606510111454	0.0641641056501217	0.759076148344325	-4.48367761038693
a_HQ_transcript/115619	2.09284268626044	-0.627379251706235	1.91604681689228	0.0641665160721104	0.759076148344325	-4.19642977784229
a_HQ_transcript/114456	1.50581964812336	4.71873562844405	1.91589796529029	0.0641861421951763	0.759076148344325	-4.46779572755429
a_HQ_transcript/139259	1.89940301393132	-0.405636158600091	1.91580131821371	0.0641988879141562	0.759076148344325	-4.21246293507828
a_HQ_transcript/148260	-1.13847924461849	3.6825187713731	-1.9156276483622	0.0642217968134322	0.759076148344325	-4.52906925947272
a_HQ_transcript/87357	-3.08647293437128	0.421929249751209	-1.91558071869573	0.0642279885470564	0.759076148344325	-4.12762122259003
a_HQ_transcript/20177	2.02741154663322	-0.529978410036488	1.91555169249137	0.0642318184195365	0.759076148344325	-4.19804291004856
a_HQ_transcript/119325	1.89203166181594	2.49746708420461	1.91552191034424	0.0642357482401895	0.759076148344325	-4.30586534325314
a_HQ_transcript/22011	-2.3751614458431	-0.524175599546264	-1.91534309885668	0.0642593471814438	0.759200677431497	-4.16216260211284
a_HQ_transcript/25225	-1.18758299681707	2.18095250199921	-1.91507756129024	0.0642944057581864	0.759460519795135	-4.14764231393781
a_HQ_transcript/60169	2.84649685904186	0.548447678251514	1.91489871286989	0.0643180281909535	0.759551151713909	-4.23390880586535
a_HQ_transcript/122186	-0.695473265557322	4.42850256041152	-1.91482162580747	0.0643282122193706	0.759551151713909	-4.59147084822574
a_HQ_transcript/72226	2.09567275507458	0.964579661868693	1.91426589042716	0.0644016720507137	0.760073411041538	-4.15467150174254
a_HQ_transcript/143919	7.77311207282838	6.65213656825509	1.91418647286438	0.0644121757685907	0.760073411041538	-4.59294482542415
a_HQ_transcript/17462	-4.8292132762175	1.70343097682907	-1.91409808307836	0.0644238678868895	0.760073411041538	-4.23561836230823
a_HQ_transcript/97100	2.61935046853465	1.09087444339198	1.91409143740464	0.0644247470444821	0.760073411041538	-4.22271453790774
a_HQ_transcript/26058	-2.17282182644703	-0.400719698488461	-1.9139523965767	0.0644431431615735	0.760136165928561	-4.14427592989668
a_HQ_transcript/28536	1.68157143079701	1.24164461633627	1.9137433031174	0.0644708162942962	0.760189682290464	-4.07328328072962
a_HQ_transcript/81274	-2.76523741401327	2.06151164892272	-1.91371711452748	0.0644742830288316	0.760189682290464	-4.2205806513456
a_HQ_transcript/62765	1.92914604863773	-0.0607066503059222	1.91350733702633	0.0645020582953924	0.760189682290464	-4.1657097158723
a_HQ_transcript/21879	2.93373050211861	0.661702200127725	1.91340343890582	0.0645158185916873	0.760189682290464	-4.16422053375902
a_HQ_transcript/151831	-0.82169916056554	6.82555666048539	-1.91338453455889	0.0645183225612793	0.760189682290464	-4.78962141761388
a_HQ_transcript/25127	-1.5954390190816	1.96554817062092	-1.91329259226773	0.0645305019465248	0.760189682290464	-4.20293941823965
a_HQ_transcript/32734	2.75252145379315	-0.070735890203596	1.91313590655108	0.0645512623203075	0.760189682290464	-4.19203726601895
a_HQ_transcript/3965	0.978908450756816	3.12225380934197	1.91312805293747	0.06455230305164	0.760189682290464	-4.32422541072628
a_HQ_transcript/148485	-0.960216885361484	8.56456400247503	-1.91295329537484	0.0645754650151312	0.760308412072017	-4.95273893693308
a_HQ_transcript/131398	-2.77767637825769	2.83986168244286	-1.91273431296165	0.0646044985682492	0.760398402946715	-4.46571122533275
a_HQ_transcript/114162	-2.00896950432043	1.36643319544364	-1.91265683828777	0.0646147731655127	0.760398402946715	-4.21239647730815
a_HQ_transcript/21826	2.6093119180751	0.559160968555227	1.91254348132018	0.0646298089715789	0.760398402946715	-4.22820200695486
a_HQ_transcript/47418	1.57144272916553	5.89533640747235	1.91250107914352	0.0646354340242175	0.760398402946715	-4.70277499991289
a_HQ_transcript/58249	-1.71531885150394	-0.665313932951299	-1.91220665369432	0.0646745040242902	0.7605894134135	-4.20236380135411
a_HQ_transcript/85114	2.16955212817353	2.98629075354882	1.91200722920009	0.0647009790625354	0.7605894134135	-4.34362318417017
a_HQ_transcript/85109	2.07667729883125	-0.109492178255256	1.91185144727575	0.064721666744523	0.7605894134135	-4.11376737096293

a_HQ_transcript/128397	3.65237157002502	2.20934742445947	1.91164554408873	0.0647490192422335	0.7605894134135	-4.2622361107633
a_HQ_transcript/134842	4.53691374223628	5.85488561644723	1.91109316911153	0.0648224468928541	0.7605894134135	-4.81801665926039
a_HQ_transcript/130936	-2.83572794756801	0.784097713401682	-1.91105426656032	0.0648276209489563	0.7605894134135	-4.08235419891525
a_HQ_transcript/134611	4.30529381033119	5.98370131833396	1.91101478732522	0.0648328720687582	0.7605894134135	-4.70909885059538
a_HQ_transcript/114034	2.74813692709434	0.131166822340349	1.91085565808156	0.0648540415190669	0.7605894134135	-4.19899336886426
a_HQ_transcript/9819	1.87914631935719	0.552753113160226	1.91077742353165	0.064864451488539	0.7605894134135	-4.16749110281503
a_HQ_transcript/12010	1.3321482375769	1.4987336691651	1.91065994286126	0.0648800862964212	0.7605894134135	-4.15127647178763
a_HQ_transcript/80885	3.12317080050883	2.38525304428453	1.91065332188919	0.0648809675392824	0.7605894134135	-4.40977804475185
a_HQ_transcript/143014	-3.84774629899609	2.15013116033621	-1.91060192027645	0.0648878093792789	0.7605894134135	-4.18151674283368
a_HQ_transcript/133546	6.0250780770571	4.66609560935125	1.91045334359023	0.0649075892630522	0.7605894134135	-4.30405558745068
a_HQ_transcript/67681	2.12190600993113	2.58621083406899	1.91043755492917	0.064909691499541	0.7605894134135	-4.2439721569759
a_HQ_transcript/26743	-2.10748640746234	0.625283378306207	-1.91042444810319	0.0649114366983663	0.7605894134135	-4.11455219338816
a_HQ_transcript/150457	-2.65040092708207	0.0957420844224082	-1.91039167292678	0.0649158009527706	0.7605894134135	-4.26576911179885
a_HQ_transcript/16045	1.39900145963766	0.438462723336944	1.91018678652041	0.0649430888171522	0.7605894134135	-4.13965560942304
a_HQ_transcript/50582	2.32079001419395	-0.246828069510248	1.91017266358171	0.064944970150179	0.7605894134135	-4.23178486963846
a_HQ_transcript/114849	-1.15000811944317	3.29906317151843	-1.91003910699132	0.0649627637044369	0.7605894134135	-4.36360460628912
a_HQ_transcript/55938	0.768825252871446	3.5682922094892	1.90998612306326	0.0649698238434281	0.7605894134135	-4.48446449206644
a_HQ_transcript/59794	2.77051751801118	2.0929997514729	1.9099423389675	0.0649756585992366	0.7605894134135	-4.14914697007425
a_HQ_transcript/98364	1.60590248021731	0.119107103970461	1.90991959305175	0.0649786899437894	0.7605894134135	-4.17218157923379
a_HQ_transcript/16958	-1.79388160507062	-0.199108715109774	-1.90990046942714	0.0649812386406396	0.7605894134135	-4.19975785287696
a_HQ_transcript/146482	4.13945565386809	5.36452725470014	1.90973912291832	0.0650027454963823	0.7605894134135	-4.77857591301991
a_HQ_transcript/150046	1.06507577981715	-0.471157675707782	1.90960011528051	0.0650212795949593	0.7605894134135	-4.23682685507974
a_HQ_transcript/35156	2.49233901525288	2.08209220490128	1.90918453378631	0.0650767169085847	0.7605894134135	-4.16883355406982
a_HQ_transcript/119643	-1.19783205872942	2.2181408541143	-1.90916141919186	0.0650798015227571	0.7605894134135	-4.26760381167261
a_HQ_transcript/139517	-3.98492485057388	2.27275109986324	-1.90912792785906	0.0650842711237657	0.7605894134135	-4.19786999672976
a_HQ_transcript/82496	-1.18699370510414	1.72865095865796	-1.90905828750703	0.0650935658567287	0.7605894134135	-4.22333678637112
a_HQ_transcript/147794	3.1288984913287	2.26358242943357	1.90903634174549	0.0650964951429637	0.7605894134135	-4.3618742170351
a_HQ_transcript/117923	-2.80447193642845	0.244954642403825	-1.90902960795249	0.0650973939820247	0.7605894134135	-4.2490414119621
a_HQ_transcript/139533	-3.11862340142233	0.769540411713427	-1.90890381448156	0.0651141870948074	0.7605894134135	-4.26536401979917
a_HQ_transcript/139543	2.34585035184569	0.505011570416178	1.90888276235672	0.0651169978658369	0.7605894134135	-4.19940883328377
a_HQ_transcript/54644	-2.3951158958622	0.675662412962508	-1.90876521500214	0.0651326941065386	0.7605894134135	-4.16586639122531
a_HQ_transcript/17725	1.74078679024641	2.07703580834578	1.90875072780021	0.0651346288261639	0.7605894134135	-4.18647386825258
a_HQ_transcript/90108	-2.84333474147313	0.262051993527204	-1.90872154060886	0.0651385268331242	0.7605894134135	-4.2005672422199
a_HQ_transcript/137148	-4.05575283872624	1.76466224765153	-1.90867814828766	0.0651443223353269	0.7605894134135	-4.19166205958634
a_HQ_transcript/125451	2.69449751547436	1.93694251732008	1.90852129043685	0.0651652760677979	0.7605894134135	-4.21513370495363
a_HQ_transcript/43773	2.56882018328398	0.719526215561103	1.90850634912422	0.0651672722946498	0.7605894134135	-4.24319184996313
a_HQ_transcript/151576	-3.5023750146584	3.53321365148537	-1.90828121716128	0.0651973573305952	0.7605894134135	-4.48061470121953
a_HQ_transcript/45655	-2.39033829779753	-0.127318661034853	-1.90827697185865	0.0651979247577951	0.7605894134135	-4.25329132012297
a_HQ_transcript/131093	-2.38105755108236	2.21907200777635	-1.90820672513521	0.0652073145543865	0.7605894134135	-4.16585841165719
a_HQ_transcript/67432	-1.44405844380745	1.33388031619442	-1.90802219605178	0.0652319858984445	0.7605894134135	-4.13002685453897
a_HQ_transcript/90529	-1.99637591010984	1.90596078148505	-1.90798463148727	0.0652370092278127	0.7605894134135	-4.21402348507525
a_HQ_transcript/8576	-3.0285530700902	1.38697286341177	-1.90795865350162	0.0652404833347047	0.7605894134135	-4.22735673673681
a_HQ_transcript/89682	-4.73439192093854	1.32842950566585	-1.90776870867861	0.0652658900256247	0.760733036978045	-4.23131832666019
a_HQ_transcript/140321	-2.79245580791426	0.864500159854934	-1.90754706418011	0.0652955476037574	0.760926140960547	-4.18031183442971
a_HQ_transcript/133563	-2.47154745644233	1.0138448999136	-1.90731191509437	0.0653270249022034	0.761127447019867	-4.19117980938847
a_HQ_transcript/10100	2.05216486290545	0.595896378299769	1.90713396627964	0.0653508540159288	0.761127447019867	-4.1508045407024
a_HQ_transcript/48423	-2.2479891803049	0.00734165314015853	-1.90706479291015	0.0653601190412654	0.761127447019867	-4.26241114393362
a_HQ_transcript/63086	2.21243006782461	0.0384230054054866	1.90702687839085	0.0653651977622347	0.761127447019867	-4.20567877590144
a_HQ_transcript/110657	-3.58651452279697	3.74554787458954	-1.90679627474902	0.0653960948866359	0.76133188695729	-4.67113044285437
a_HQ_transcript/126017	-1.79052601806078	-0.583672977538363	-1.9067003573463	0.0654089499632656	0.76133188695729	-4.21148965714548

a_HQ_transcript/103617	-3.23189193567003	-0.108512215618742	-1.90576305876219	0.065534683718113	0.762268384451042	-4.23206220202368
a_HQ_transcript/46784	2.81223405938588	1.36101129172828	1.90575481478383	0.065535790529519	0.762268384451042	-4.19998761824298
a_HQ_transcript/443	1.46460662063715	-0.553271063603864	1.90563708687982	0.0655515980785478	0.762268384451042	-4.21350648661511
a_HQ_transcript/111789	-3.05832893755592	0.916401461505288	-1.90560428529021	0.0655560029960607	0.762268384451042	-4.18401478513802
a_HQ_transcript/22082	1.95632733017164	1.90047589313594	1.90553636042816	0.0655651254211703	0.762268384451042	-4.15731866820277
a_HQ_transcript/147730	2.60917515952725	-0.165329386042347	1.90541803986767	0.065581018689896	0.762268384451042	-4.26472899925185
a_HQ_transcript/8513	1.58367240518034	3.15325170395346	1.9054166649787	0.0655812033897547	0.762268384451042	-4.30578473675103
a_HQ_transcript/455	1.09881874281137	3.61085667272332	1.90522036374077	0.0656075787149767	0.762306172290837	-4.50093514436053
a_HQ_transcript/7220	3.21841923149913	3.50590183430058	1.90519726442424	0.0656106829750044	0.762306172290837	-4.48479616806923
a_HQ_transcript/141484	-3.71017049127501	-0.0447159844464023	-1.90497408066081	0.0656406826155157	0.76250231796057	-4.23238998388548
a_HQ_transcript/118098	-0.972559980083565	4.96338606839472	-1.90435294673664	0.0657242357240345	0.763133542628594	-4.63702560482587
a_HQ_transcript/33473	0.847305233402505	2.89181441653776	1.90426987846387	0.065735416774975	0.763133542628594	-4.39403017130387
a_HQ_transcript/92271	-4.07781325992941	1.22050046588366	-1.90412740135375	0.0657545981194356	0.763133542628594	-4.31975422975241
a_HQ_transcript/12058	-3.1280945719315	0.862611770282056	-1.90409543435829	0.0657589024207407	0.763133542628594	-4.1474314268078
a_HQ_transcript/104516	10.1443555965074	2.62510745837559	1.90405105516852	0.0657648784056558	0.763133542628594	-4.24012992305979
a_HQ_transcript/81818	1.99721453583016	-0.115599890417931	1.90398485842038	0.0657737931559533	0.763133542628594	-4.10693273175383
a_HQ_transcript/146197	4.44353152107911	2.88315585726221	1.90388363150503	0.0657874274524153	0.763139409889043	-4.3003087085562
a_HQ_transcript/46090	-2.56268697153073	2.83084394699086	-1.90356346642543	0.0658305666713424	0.763487465976016	-4.50843752712611
a_HQ_transcript/133833	2.59996049942645	0.321562067127512	1.90322433500147	0.0658762880299609	0.763647825979197	-4.180389097461
a_HQ_transcript/117043	5.56975768285486	1.1495855437743	1.90318757093033	0.0658812461700228	0.763647825979197	-4.23967099635825
a_HQ_transcript/138059	-3.34559568912064	0.264540465367205	-1.9031685945589	0.0658838055203288	0.763647825979197	-4.27999008013282
a_HQ_transcript/147389	-1.9759334079601	1.02998612496236	-1.90277296596317	0.065937183627727	0.764026887087603	-4.2169301914606
a_HQ_transcript/95612	-2.78990475611002	2.10445076424261	-1.90271719979932	0.0659447105819756	0.764026887087603	-4.21276258625358
a_HQ_transcript/89333	3.42086012992918	0.835288888255824	1.90263400781387	0.0659559406724056	0.764026887087603	-4.37284656349461
a_HQ_transcript/35431	-2.08459844798814	1.63948456399714	-1.90244533720147	0.0659814154391512	0.764057996778998	-4.14844355278377
a_HQ_transcript/27617	-2.24203556915608	1.0502156728195	-1.90241942314417	0.0659849150810379	0.764057996778998	-4.17047441884886
a_HQ_transcript/1208	0.70637136520901	3.55986280146011	1.90223264933604	0.0660101432465417	0.764197890188204	-4.49161891280347
a_HQ_transcript/22207	-2.39508188041942	-0.0518319259238654	-1.90183595174664	0.0660637541147231	0.764323142207182	-4.24994408081347
a_HQ_transcript/81390	1.16267101891223	1.6871191133278	1.90174100980653	0.0660765904112464	0.764323142207182	-4.13531517897736
a_HQ_transcript/142034	-3.0427780947144	0.922181086834761	-1.90168516091262	0.0660841422721959	0.764323142207182	-4.21484901007459
a_HQ_transcript/19752	-3.53765488947906	3.26030058532699	-1.90163663151616	0.066090704998405	0.764323142207182	-4.34556213258438
a_HQ_transcript/136973	1.6406419817414	1.29367120790224	1.90162779215402	0.0660919004233126	0.764323142207182	-4.17978873310637
a_HQ_transcript/113333	2.62299917024439	2.50331902495896	1.90156896873073	0.0660998561085106	0.764323142207182	-4.16032128557153
a_HQ_transcript/135437	4.4358549228531	0.526997031264531	1.90123537044976	0.066144989859617	0.764692913794713	-4.32487292898768
a_HQ_transcript/37738	-2.15088784230799	0.339791389690696	-1.90095610658217	0.0661827929337902	0.764888116135537	-4.16707142361747
a_HQ_transcript/134720	-6.01963431812474	0.681573370703485	-1.90082064873068	0.0662011361457743	0.764888116135537	-4.26715237802652
a_HQ_transcript/149784	-2.31659676690913	1.39200935168581	-1.90072582102736	0.0662139799780402	0.764888116135537	-4.15743933499099
a_HQ_transcript/26880	2.14451233493857	0.0327345888327179	1.90070405430252	0.0662169284508675	0.764888116135537	-4.24773596193741
a_HQ_transcript/63683	0.702611097984775	2.21431957153847	1.90062477763167	0.0662276680517162	0.764888116135537	-4.24928393349417
a_HQ_transcript/89062	-1.70446740119777	-0.239868813523857	-1.90028566166891	0.0662736250003799	0.765266840290442	-4.22056596782071
a_HQ_transcript/89818	1.90105897125046	0.257352284508456	1.9001858380835	0.0662871583149466	0.765271090075713	-4.12447333807873
a_HQ_transcript/132697	1.81083665829483	1.36273768765831	1.8999694222124	0.0663165064963549	0.765450408255388	-4.14086935093135
a_HQ_transcript/7764	4.17037958353093	0.568265207056493	1.89978638956552	0.0663413363161254	0.765450408255388	-4.15943244085039
a_HQ_transcript/65640	4.38971233993577	4.03817164493704	1.89978005503552	0.0663421957884435	0.765450408255388	-4.52488200504514
a_HQ_transcript/135347	-2.44723259791572	0.140742759812704	-1.89963775078757	0.0663615062324086	0.765521261019537	-4.24112641573837
a_HQ_transcript/127352	-2.4670694360808	0.768714918393745	-1.89922485358938	0.0664175631025999	0.765789309489785	-4.1623541334955
a_HQ_transcript/55964	2.39963987047987	0.685336646638993	1.89921207084916	0.0664192991987855	0.765789309489785	-4.29078063219505
a_HQ_transcript/98513	2.45559929279464	-0.164563781348122	1.89916013462303	0.0664263533534492	0.765789309489785	-4.17777417851422
a_HQ_transcript/92020	0.451851344648503	3.88626040237105	1.89907852188433	0.0664374395774323	0.765789309489785	-4.55077854692471
a_HQ_transcript/101123	-2.21728247415213	0.934843695688683	-1.89854391541836	0.0665100996321834	0.766474835729491	-4.20068242826466

a_HQ_transcript/29771	2.06392806714844	-0.325665676389079	1.89820864473042	0.0665557022642916	0.766486701385152	-4.25829741582407
a_HQ_transcript/88318	2.52480123152427	0.108230834913301	1.89816643023934	0.0665614460772109	0.766486701385152	-4.22882318451939
a_HQ_transcript/21173	-1.96482555890072	0.84791553061527	-1.89816031284972	0.0665622784605118	0.766486701385152	-4.22010495579882
a_HQ_transcript/127375	-2.09061265409856	1.21792480970057	-1.89813152425102	0.0665661957989623	0.766486701385152	-4.13580883912855
a_HQ_transcript/124354	-2.1345482598695	0.327061457485172	-1.89803270999436	0.0665796432195825	0.766486701385152	-4.15226078089947
a_HQ_transcript/46122	3.60515276995764	1.94713004847616	1.89795480798691	0.0665902463871975	0.766486701385152	-4.40754414680319
a_HQ_transcript/21247	1.27791364030103	2.66868889288421	1.89769712723577	0.066625329435086	0.766738695189602	-4.32274510763831
a_HQ_transcript/126762	1.98791868023951	-0.479560583342819	1.89729455600127	0.0666801710957905	0.766982540309999	-4.23329088401842
a_HQ_transcript/120814	5.25222671535557	2.47406569997059	1.8972483081623	0.0666864738580785	0.766982540309999	-4.40742147668765
a_HQ_transcript/135800	-1.12129083651427	6.69615175566386	-1.89722591901335	0.0666895252874001	0.766982540309999	-4.80295674809118
a_HQ_transcript/108211	-2.19680554729452	-0.467340568076011	-1.89715422663377	0.0666992970903359	0.766982540309999	-4.23425286204231
a_HQ_transcript/92374	8.8057443915811	2.38645187417782	1.89703004754991	0.0667162258465857	0.767025469938753	-4.30799121526759
a_HQ_transcript/67606	-2.41070720539161	1.34191806379266	-1.89686471882089	0.066738770087729	0.767132930128438	-4.23502210670864
a_HQ_transcript/28537	2.24002392742314	-0.0607053413711691	1.89659397466318	0.0667757029621705	0.767393029816265	-4.25336955923826
a_HQ_transcript/149549	-4.15274032804068	0.287886779954342	-1.89641739730035	0.0667997997994238	0.767393029816265	-4.33552310261897
a_HQ_transcript/118955	0.549354565472984	4.42710913954761	1.89631465113529	0.0668138246231442	0.767393029816265	-4.59794617597922
a_HQ_transcript/83758	-1.53819828976936	3.02192151271898	-1.89631186277146	0.0668142052694075	0.767393029816265	-4.31708151089192
a_HQ_transcript/65574	2.75182364106142	0.108674241448677	1.89561492163879	0.0669094049932667	0.768128211036411	-4.1932068637005
a_HQ_transcript/33160	2.77170779866555	2.46732717213901	1.89560024508997	0.0669114110141958	0.768128211036411	-4.35597200562741
a_HQ_transcript/29527	2.66590235605427	0.360660369167881	1.895553078535	0.0669178581869045	0.768128211036411	-4.16154321029595
a_HQ_transcript/110989	2.36003280855519	2.0229994874179	1.89536657489514	0.0669433565175367	0.768269186110834	-4.28326369202377
a_HQ_transcript/152309	5.26324865207238	0.049399048559747	1.8951651189427	0.0669709084948985	0.768338369439497	-4.34778940176546
a_HQ_transcript/111117	1.69217730866795	2.06050804463266	1.89512920758334	0.0669758209116077	0.768338369439497	-4.26551597296207
a_HQ_transcript/68322	2.17525209645526	0.579785201321388	1.894324427333	0.0670859905307468	0.769242014927804	-4.24988392464288
a_HQ_transcript/149635	-2.97631125591812	0.124661139114543	-1.89416845287213	0.0671073605520897	0.769242014927804	-4.25802081927936
a_HQ_transcript/106515	-0.939822956862485	3.17373785129028	-1.89393872825527	0.0671388457402448	0.769242014927804	-4.41297569274631
a_HQ_transcript/99648	-0.67632051014533	2.4141083512573	-1.89390075502903	0.0671440514323131	0.769242014927804	-4.32915192818103
a_HQ_transcript/136483	-1.71190284751581	-0.588784093036893	-1.893899134054	0.067144273657061	0.769242014927804	-4.24688742911178
a_HQ_transcript/148456	-2.65536779314226	1.07733205879485	-1.89366117181208	0.0671769035487886	0.769242014927804	-4.30695894206113
a_HQ_transcript/129875	2.5507686554351	-0.44951493522275	1.89359253906932	0.0671863171543222	0.769242014927804	-4.2514929452039
a_HQ_transcript/7854	-1.68012390622825	0.970519806744218	-1.89355296811336	0.0671917451875162	0.769242014927804	-4.17728103160376
a_HQ_transcript/9983	0.924554205540192	3.10740552686225	1.89339850338528	0.0672129370612529	0.769242014927804	-4.40125618038359
a_HQ_transcript/12150	-2.40492308038807	0.93029028761209	-1.89330119933124	0.0672262896995521	0.769242014927804	-4.26193104651956
a_HQ_transcript/61658	-2.88122401591689	-0.110834568941942	-1.89321798522188	0.0672377106443349	0.769242014927804	-4.18099122176497
a_HQ_transcript/126503	0.704542732691317	4.94797070884017	1.89317926521217	0.067243025446052	0.769242014927804	-4.65048709691003
a_HQ_transcript/87352	2.19298321449091	-0.157971069248291	1.89310808100003	0.0672527973062588	0.769242014927804	-4.25790418564458
a_HQ_transcript/119773	-3.85397039082494	3.03150785866098	-1.89292347162109	0.0672781453801862	0.769242014927804	-4.37712780117878
a_HQ_transcript/79866	-4.12063526664244	-0.0522005876646947	-1.89286692156495	0.0672859117182615	0.769242014927804	-4.23937254223661
a_HQ_transcript/359	1.72533399450371	2.02737706028792	1.8928133657	0.0672932675589409	0.769242014927804	-4.29555055242456
a_HQ_transcript/115556	-2.56030793457832	0.0133084603674778	-1.89280630778516	0.067294237007623	0.769242014927804	-4.19871495762634
a_HQ_transcript/46319	-2.14482426397159	-0.413925386462388	-1.89279749652316	0.0672954473063675	0.769242014927804	-4.23188135701989
a_HQ_transcript/148734	-2.70507687568127	0.981022543926408	-1.8926948168361	0.0673095525813333	0.769242014927804	-4.25457643052312
a_HQ_transcript/139073	-2.47165296349444	0.63776422855721	-1.89261351781242	0.0673207225657201	0.769242014927804	-4.27611918464531
a_HQ_transcript/109685	-5.68483838515059	1.59253411115504	-1.89252783367739	0.0673324967649441	0.769242014927804	-4.27324865578306
a_HQ_transcript/40112	-2.27074176096965	0.32607811429388	-1.89221636411489	0.0673753119809467	0.769383397243455	-4.14309717353195
a_HQ_transcript/29682	4.64334794299534	1.38818320292633	1.89221395327771	0.0673756434707507	0.769383397243455	-4.15124947690077
a_HQ_transcript/92538	-4.11753334448639	0.808655143281689	-1.89211243661547	0.0673896032734743	0.769383397243455	-4.21236394776859
a_HQ_transcript/67121	2.36391033787388	2.05793740728447	1.89205272026261	0.0673978161774648	0.769383397243455	-4.20713206835162
a_HQ_transcript/140827	-2.07273742538695	1.54834165091336	-1.89170551773435	0.0674455846779301	0.769629262687618	-4.18782648103061
a_HQ_transcript/45685	1.87882491321992	-0.436567449623597	1.89170370293466	0.0674458344366007	0.769629262687618	-4.28998121581856

a_HQ_transcript/143317	-4.55916723690502	4.68209371654317	-1.89139679356202	0.0674880837643503	0.769819404506513	-4.53982364265946
a_HQ_transcript/80978	-3.51233368711466	0.139130010600133	-1.8913902529067	0.0674889844027868	0.769819404506513	-4.30296764521967
a_HQ_transcript/36628	-3.15193454982734	0.599004417723978	-1.89124421953977	0.0675090956734244	0.769897726761784	-4.17456363459588
a_HQ_transcript/130320	-6.15074273505294	4.2975837197053	-1.89112096893714	0.067526073385837	0.769940289088257	-4.53905669600167
a_HQ_transcript/148490	-5.1418775644897	1.44766957557793	-1.890819327445	0.0675676398663914	0.770071949278204	-4.28230801877828
a_HQ_transcript/50230	2.62269602141517	-0.446136023464938	1.89077615609244	0.0675735907207636	0.770071949278204	-4.20403583177834
a_HQ_transcript/119515	1.44835508150005	5.21861358076489	1.89074878414129	0.0675773639772247	0.770071949278204	-4.7138257789426
a_HQ_transcript/98223	4.32464708495288	0.679138636643169	1.89062058324683	0.0675950390393539	0.77008757949083	-4.34993803464858
a_HQ_transcript/140663	-1.8070693144166	-0.355317718054626	-1.8903640995651	0.06763041239978	0.77008757949083	-4.26441641073773
a_HQ_transcript/58564	2.51615394581172	1.29459101759432	1.89027121297927	0.0676432269328393	0.77008757949083	-4.23177310378954
a_HQ_transcript/139519	-2.47902158485553	1.71080510234899	-1.89014770089526	0.067660269763909	0.77008757949083	-4.26752206248003
a_HQ_transcript/75467	-1.20541037603369	4.2223167334538	-1.89001533692007	0.0676785381272646	0.77008757949083	-4.55445586715401
a_HQ_transcript/13345	-2.07062101623316	1.46826601484447	-1.89000015620151	0.0676806335826316	0.77008757949083	-4.19063017842634
a_HQ_transcript/64799	2.72774358619295	0.617933496982932	1.88997743447246	0.0676837700582214	0.77008757949083	-4.27057462575727
a_HQ_transcript/147241	-4.31787013586842	5.83342024543679	-1.88993468813721	0.0676896710414433	0.77008757949083	-4.70966682723318
a_HQ_transcript/11354	2.63757636624868	-0.348859812157557	1.88979814299427	0.0677085235911904	0.77008757949083	-4.23273438685073
a_HQ_transcript/54017	-1.78271243315706	-0.468746443599262	-1.88957241243917	0.0677396997267927	0.77008757949083	-4.24771282073005
a_HQ_transcript/84517	2.54702622515391	1.45408221748562	1.88953928056936	0.0677442766807392	0.77008757949083	-4.20158797881821
a_HQ_transcript/35446	1.52347406522131	1.30474323761076	1.88951906631258	0.0677470692805673	0.77008757949083	-4.20330050832763
a_HQ_transcript/116644	2.68090860081088	3.62012593155829	1.88926614274461	0.0677820190513389	0.77008757949083	-4.40243918315366
a_HQ_transcript/43783	2.73731450341088	0.920810588451568	1.88910641684735	0.0678040984696435	0.77008757949083	-4.26534921136783
a_HQ_transcript/138700	-1.83891365660418	2.01895644839367	-1.8890908102008	0.0678062561579101	0.77008757949083	-4.34508783539312
a_HQ_transcript/150077	0.613799121372484	8.36702070078066	1.88903113052689	0.0678145076832106	0.77008757949083	-4.99902828769413
a_HQ_transcript/45326	2.14514372090006	-0.232722000962992	1.88902184581813	0.067815791497955	0.77008757949083	-4.23426531538723
a_HQ_transcript/150026	-6.76518132144847	3.24675568212054	-1.88901164487159	0.0678172020267953	0.77008757949083	-4.42982431788811
a_HQ_transcript/132692	5.17796545491369	3.59627924745295	1.88869612331552	0.0678608430226426	0.770432633441439	-4.42789663211106
a_HQ_transcript/98256	0.839146689746463	2.77813543029702	1.88836132988787	0.0679071760111599	0.770755488675459	-4.29789599747528
a_HQ_transcript/19711	4.20074353887339	1.55870915835074	1.88829903744263	0.067915799838214	0.770755488675459	-4.23394579805762
a_HQ_transcript/107223	-3.44673763566023	2.46236246256863	-1.88786900435492	0.0679753597544612	0.771280833848784	-4.35372427669327
a_HQ_transcript/13976	-3.16716154317704	2.50637190342546	-1.88759156905891	0.0680138086059533	0.771545928020232	-4.2316088154836
a_HQ_transcript/2635	-2.91897514968441	0.201763709295546	-1.88750889488306	0.0680252697685055	0.771545928020232	-4.21422895148838
a_HQ_transcript/122065	-5.34573180106917	2.30747461299283	-1.88730525125606	0.0680535080754407	0.771715629615531	-4.25929493934196
a_HQ_transcript/137372	-2.38676211797321	1.92289679161679	-1.88691160146218	0.0681081222402038	0.772026655930849	-4.27182816605671
a_HQ_transcript/111516	2.80446143972018	1.9980929152938	1.88679809528672	0.0681238768568474	0.772026655930849	-4.28343821024865
a_HQ_transcript/23698	1.21940706969836	3.3776792411478	1.88677152886122	0.0681275647200141	0.772026655930849	-4.43746372818431
a_HQ_transcript/103932	-3.46906876936513	1.75193113982575	-1.88672472848808	0.0681340618105776	0.772026655930849	-4.28563436618997
a_HQ_transcript/121549	4.55224474563611	0.836104129531169	1.88629858767368	0.0681932456012577	0.772546673223525	-4.21285503325594
a_HQ_transcript/41492	2.58471213707037	1.58072067938967	1.88593918898116	0.0682431944091725	0.772822145902264	-4.15877849201777
a_HQ_transcript/46664	1.51743508553775	1.95609670017497	1.88593230007685	0.068244152128343	0.772822145902264	-4.25592152215098
a_HQ_transcript/25559	2.78158221928443	1.51691806411468	1.88571091138943	0.0682749365023046	0.772923762016482	-4.17525444557769
a_HQ_transcript/78973	-2.68512857452628	1.54241471709272	-1.88564129927802	0.0682846186218126	0.772923762016482	-4.31180004567847
a_HQ_transcript/7830	-1.91443928496574	0.765128724247838	-1.88558093007475	0.068293016131927	0.772923762016482	-4.17823763335398
a_HQ_transcript/39539	-2.42031524330081	1.10021254081448	-1.88500097124624	0.0683737351555783	0.773686680382219	-4.27739976582257
a_HQ_transcript/84084	2.28233069021356	-0.0555616454294507	1.8846756557157	0.0684190486864359	0.773865588438248	-4.25629248591607
a_HQ_transcript/89590	1.07560939138647	2.02854350524053	1.88461243188991	0.0684278581945725	0.773865588438248	-4.20946633649292
a_HQ_transcript/119016	-3.77974291452294	2.65328803721689	-1.88453400488243	0.0684387874457883	0.773865588438248	-4.40771767009568
a_HQ_transcript/128167	-1.28939385782329	3.59329787223115	-1.88450522486564	0.0684427984819886	0.773865588438248	-4.5307194204822
a_HQ_transcript/26620	1.55140704537025	1.61509928940417	1.88434404637793	0.0684652654703923	0.773969068701471	-4.13916529615688
a_HQ_transcript/147649	1.20711366854047	1.71111089363632	1.88420133804715	0.0684851631612282	0.774043469616153	-4.21965451690061
a_HQ_transcript/124614	1.23847826760963	3.92210807694707	1.8840847431827	0.0685014235670677	0.77407673971744	-4.48036627824155

a_HQ_transcript/122622	1.49474464061811	0.129786312409673	1.88385379500595	0.0685336415726638	0.774191802879438	-4.19848046155392
a_HQ_transcript/49677	2.8308407586412	2.30741404246765	1.88357750268853	0.0685722023505472	0.774191802879438	-4.31203724550631
a_HQ_transcript/129509	3.79366139286508	3.95323591859034	1.88347024641316	0.0685871766151775	0.774191802879438	-4.49105066764658
a_HQ_transcript/131442	-3.02989287157548	1.13274770873863	-1.88344298419139	0.0685909831970666	0.774191802879438	-4.26366524342717
a_HQ_transcript/133043	-2.81396072274282	2.073693143638	-1.88326650802004	0.0686156286869722	0.774191802879438	-4.25976489929895
a_HQ_transcript/29308	3.72020656629415	0.390020322813127	1.88319053031368	0.0686262415735985	0.774191802879438	-4.19637005714946
a_HQ_transcript/6993	-1.01930260897641	2.64778381321849	-1.88300651016945	0.0686519521338938	0.774191802879438	-4.43221209726769
a_HQ_transcript/60840	-2.17782331686892	1.86227202706297	-1.88276137170816	0.0686862147677676	0.774191802879438	-4.34703992599132
a_HQ_transcript/84715	1.56005360497488	0.419343577774182	1.88270289911848	0.068694389565264	0.774191802879438	-4.18988507955317
a_HQ_transcript/149390	-1.24101371130541	6.83752663077475	-1.88270060645834	0.0686947101091134	0.774191802879438	-4.85050318744347
a_HQ_transcript/104046	0.460950809457152	4.18490260048105	1.88268540685601	0.0686968352446839	0.774191802879438	-4.61873461549316
a_HQ_transcript/148842	1.68437043701035	-0.666534176114775	1.88266251516729	0.0687000359576009	0.774191802879438	-4.25114281148703
a_HQ_transcript/330	0.699489884963425	3.66336723523695	1.88257181544596	0.0687127188413352	0.774191802879438	-4.5146576431839
a_HQ_transcript/28233	2.29191898789833	0.97330657070785	1.882560584258	0.0687142894807401	0.774191802879438	-4.252154497228
a_HQ_transcript/23479	-0.764171159409088	3.52962372972417	-1.88252157718577	0.0687197447129142	0.774191802879438	-4.51749225887747
a_HQ_transcript/105041	2.70781160686235	1.02728999666439	1.88248610525226	0.0687247058708006	0.774191802879438	-4.21259549359363
a_HQ_transcript/19919	2.91649681291094	2.26010542548357	1.88224572462427	0.0687583339820422	0.774420545961664	-4.37266403993788
a_HQ_transcript/15384	-1.77344571952829	-0.53443530678791	-1.88184465681007	0.0688144729655773	0.774902689760379	-4.25190165754038
a_HQ_transcript/31838	-1.88011533683826	0.491006521952623	-1.88162056778299	0.0688458567142745	0.775105938231134	-4.16573668156392
a_HQ_transcript/146340	2.2532175598283	-0.142422588291945	1.88146036625998	0.0688683005376566	0.775146053413256	-4.20357332941232
a_HQ_transcript/41707	1.7940963537434	1.66060236944816	1.88137495875077	0.0688802684816527	0.775146053413256	-4.21053655531007
a_HQ_transcript/50955	-1.96151656914167	-0.18656371149619	-1.88130962128333	0.0688894252672186	0.775146053413256	-4.27839701437321
a_HQ_transcript/5658	2.61669934354244	-0.153383133669694	1.88100634603422	0.0689319417878964	0.775326980164247	-4.24453946577293
a_HQ_transcript/77051	2.39600244559198	-0.353248709313288	1.8810046381594	0.068932181280774	0.775326980164247	-4.28807938256422
a_HQ_transcript/90199	-2.25797756955388	0.0332788057139667	-1.88084994319372	0.0689538769046842	0.775420962800442	-4.20890860698177
a_HQ_transcript/117806	-3.72062609975644	0.528705026942139	-1.88033131570921	0.0690266560873398	0.775926080806321	-4.26168907876749
a_HQ_transcript/152614	-3.47917573718195	6.30554349137351	-1.88028480390596	0.0690331863308524	0.775926080806321	-4.85190475092111
a_HQ_transcript/74367	-0.811290183250625	2.95189817607514	-1.8802445387722	0.0690388399726516	0.775926080806321	-4.42657496146092
a_HQ_transcript/102028	-2.2345490037873	0.784655455699061	-1.88004077091074	0.0690674571947272	0.77601082923945	-4.16682006951078
a_HQ_transcript/2417	1.04849181890689	3.11396345231709	1.88000073802764	0.0690730806235362	0.77601082923945	-4.39908690437897
a_HQ_transcript/95629	-0.816121598623718	2.68884657665128	-1.87977442014287	0.0691048789536467	0.776218049046874	-4.36844548778499
a_HQ_transcript/43083	2.05384021387598	-0.545867836311523	1.8796552013987	0.0691216345979268	0.776256255005466	-4.27261652414395
a_HQ_transcript/109185	2.06893698812073	2.55324759299188	1.87947249252215	0.0691473202638219	0.776279817039546	-4.32662838910596
a_HQ_transcript/37799	-3.42715370504194	-0.0333474309753951	-1.87941031008201	0.0691560638960197	0.776279817039546	-4.2124248970593
a_HQ_transcript/3064	0.723549435300482	2.8399346069405	1.87935532228036	0.0691637966633603	0.776279817039546	-4.43702413841077
a_HQ_transcript/38399	-3.07065560490845	0.60216739020475	-1.87887689293714	0.0692311080938849	0.776772967488517	-4.19348771179732
a_HQ_transcript/140270	-2.51931469045858	1.18122433657944	-1.87885307249022	0.0692344609234449	0.776772967488517	-4.16786110778814
a_HQ_transcript/125175	2.58428142900216	-0.390992475009373	1.87858912237184	0.069271622370319	0.777039919942474	-4.28106952649459
a_HQ_transcript/134806	3.2455329763866	1.08581902032703	1.87829907570772	0.0693124777071532	0.777193343998299	-4.25633487535251
a_HQ_transcript/64289	2.09996037913462	-0.181562621512004	1.87822101235991	0.069323477074928	0.777193343998299	-4.20245689052566
a_HQ_transcript/135891	-2.295461232439	-0.378929691437713	-1.87804687384582	0.0693480191368029	0.777193343998299	-4.26258094029118
a_HQ_transcript/95943	1.98791099338543	-0.376177010105878	1.8780153908669	0.0693524569602289	0.777193343998299	-4.25522900320715
a_HQ_transcript/14476	1.47955750636383	1.76965619699488	1.87795805183295	0.069360540065949	0.777193343998299	-4.17123266171295
a_HQ_transcript/115116	-0.887027724114432	4.06579324828378	-1.87781629548095	0.0693805269882955	0.777193343998299	-4.6126067586093
a_HQ_transcript/100332	2.74475410807613	0.464155404467543	1.87777009180195	0.0693870425406135	0.777193343998299	-4.25931564783433
a_HQ_transcript/31148	-2.53903660421935	-0.108191014326451	-1.87764523240344	0.0694046525987407	0.777193343998299	-4.26654012921894
a_HQ_transcript/137109	2.08203833143825	-0.530574887692111	1.87763828026364	0.0694056332351908	0.777193343998299	-4.25859399802457
a_HQ_transcript/53406	2.50934194797695	0.449123872127786	1.87751511107369	0.069423008881135	0.777238185719397	-4.205787444082
a_HQ_transcript/72070	-2.14998610993396	0.772499897903609	-1.87738395880683	0.0694415148199242	0.777295662132208	-4.12233680298352
a_HQ_transcript/126902	-1.70804192923011	2.64175703764689	-1.87728413921304	0.0694556024743735	0.777303669740159	-4.40073260144754

a_HQ_transcript/140601	2.35647883210111	0.403540776279884	1.87717193320502	0.0694714411693028	0.777331267043163	-4.18003064891337
a_HQ_transcript/120376	-2.29587924478947	0.0740993541569013	-1.87703102653575	0.06949133556699	0.777373389569852	-4.27855878869423
a_HQ_transcript/144580	-3.77555020428514	1.09756505991919	-1.87691605464817	0.0695075718986458	0.777373389569852	-4.26147114217672
a_HQ_transcript/141985	-3.09501158195286	0.248731607518871	-1.87686115371304	0.0695153261592363	0.777373389569852	-4.31930951235644
a_HQ_transcript/101828	2.92104234176428	-0.0702191184856751	1.876557222858	0.0695582670915311	0.777703971820835	-4.27726940903474
a_HQ_transcript/146047	-3.61561854597587	0.0613866671928728	-1.87642205688364	0.0695773713633849	0.777751765442891	-4.34864419465272
a_HQ_transcript/18309	-2.24784515134701	0.791311273479238	-1.87624375248457	0.0696025796876839	0.777751765442891	-4.13300814822976
a_HQ_transcript/66190	-1.39064095222191	2.47832533745837	-1.87624303079977	0.0696026817339994	0.777751765442891	-4.29572519830035
a_HQ_transcript/54848	-3.02202800448308	1.52848180615779	-1.8760630448584	0.0696281357806764	0.7777771472623	-4.25365666793341
a_HQ_transcript/132163	2.79642895693026	3.34396597241376	1.87603774738814	0.0696317140509394	0.7777771472623	-4.46471402153632
a_HQ_transcript/723	2.60395237564679	2.36628107428662	1.87582018652165	0.0696624940680025	0.77783298588594	-4.3187163212951
a_HQ_transcript/54129	1.89155978482892	-0.559590310874772	1.87581324836171	0.0696634758553916	0.77783298588594	-4.25769337190045
a_HQ_transcript/32771	1.71333413012789	0.875329242487047	1.87555267072188	0.0697003576167866	0.778043380202023	-4.1352728718176
a_HQ_transcript/124303	0.813195292971147	3.48931177731277	1.87547311621274	0.0697116209822071	0.778043380202023	-4.4635838257
a_HQ_transcript/21962	1.32142338810016	3.98091756900235	1.87539647062875	0.0697224739793617	0.778043380202023	-4.56917018476305
a_HQ_transcript/147407	-2.67495611190836	2.87208107000027	-1.87513965153608	0.0697588500840002	0.778299892069628	-4.459775680736
a_HQ_transcript/123037	3.23164851068381	0.868704295640679	1.87477972469236	0.0698098579021372	0.778719520271624	-4.32431551944944
a_HQ_transcript/66047	-2.40821957598095	0.642734111602202	-1.8746276460819	0.0698314196798403	0.778810583873707	-4.13689199951111
a_HQ_transcript/96097	-2.51851483781187	-0.293156870171059	-1.8744340710378	0.0698588731154726	0.778826036205065	-4.31009348334893
a_HQ_transcript/84229	3.14327288853236	0.414056364871276	1.87423675015166	0.0698868673385986	0.778826036205065	-4.2310550161642
a_HQ_transcript/145269	0.761536710991711	5.49627656868691	1.87419313079548	0.0698930569851113	0.778826036205065	-4.75274699919375
a_HQ_transcript/3926	-2.17122449118599	-0.452117929463157	-1.87408822218482	0.0699079455888737	0.778826036205065	-4.24779259613954
a_HQ_transcript/137117	2.20037630645781	-0.0172667612181004	1.87401985704454	0.069917649418275	0.778826036205065	-4.27190931089157
a_HQ_transcript/115588	1.05079590652558	2.83519047441483	1.87395946466684	0.0699262225465602	0.778826036205065	-4.31558620468982
a_HQ_transcript/6326	2.36326637658699	0.67186745961936	1.87395684488876	0.0699265944631543	0.778826036205065	-4.18651183583697
a_HQ_transcript/132475	1.80845595013048	1.6492309356791	1.87359674809668	0.0699777317441118	0.7792157063006	-4.21578107678577
a_HQ_transcript/28592	-2.32660676965563	1.25507090014543	-1.87346482899327	0.0699964735883698	0.7792157063006	-4.2028088967398
a_HQ_transcript/152729	-2.79835920868552	-0.15291978244625	-1.87342736894218	0.0700017963511859	0.7792157063006	-4.30022500726761
a_HQ_transcript/127941	-4.68195973908856	2.5253068992574	-1.87326519963764	0.0700248432750948	0.779223748721915	-4.45593686446919
a_HQ_transcript/104529	0.998500672539171	2.04108457227286	1.87323363794285	0.0700293294681269	0.779223748721915	-4.26042471664629
a_HQ_transcript/139000	-2.87094644584034	-0.287176290811828	-1.87304274369158	0.0700564685211032	0.779376536305204	-4.28048919286999
a_HQ_transcript/115517	2.27190010110211	-0.0105371432104519	1.87265688582173	0.0701113527390772	0.779821312598157	-4.30039317373664
a_HQ_transcript/95225	2.8225406407975	1.43084374924268	1.8725730690278	0.0701232796750373	0.779821312598157	-4.33844486469616
a_HQ_transcript/129490	-2.43892896477063	3.59523176787444	-1.87233048025498	0.0701578093148121	0.780022091788516	-4.63989097184861
a_HQ_transcript/86108	2.13748960675898	2.12396297138214	1.87225769504642	0.0701681722743282	0.780022091788516	-4.25561994662403
a_HQ_transcript/91128	-2.85467207011329	-0.116363091281767	-1.87183610531261	0.0702282229276798	0.780192321488971	-4.28324079312813
a_HQ_transcript/86183	4.44475309380616	2.13544119680013	1.87183144186397	0.0702288874294543	0.780192321488971	-4.34310949632838
a_HQ_transcript/66498	3.60776452502158	2.10266520582304	1.87163872130923	0.0702563531911628	0.780192321488971	-4.3787666434226
a_HQ_transcript/149781	-2.67482966535321	0.363007112190076	-1.87160774590572	0.070260768541745	0.780192321488971	-4.26072964361519
a_HQ_transcript/20875	-2.4801321229243	-0.0991808254505686	-1.87120212793461	0.0703186088422803	0.780192321488971	-4.31420576094001
a_HQ_transcript/47086	1.95025731387748	-0.29431642124993	1.87117180634524	0.0703229342810706	0.780192321488971	-4.21793951933216
a_HQ_transcript/127629	-2.20720043221672	1.35903453753951	-1.87117112256192	0.0703230318268384	0.780192321488971	-4.20632311760328
a_HQ_transcript/6165	-1.59595074022958	0.130158119282058	-1.87114159536102	0.0703272441689613	0.780192321488971	-4.16383738160889
a_HQ_transcript/117137	1.81452990229497	-0.666466011080466	1.87108696627226	0.0703350381104001	0.780192321488971	-4.28419748556927
a_HQ_transcript/93142	1.90886976345867	4.42718874342068	1.87099508702013	0.0703481482113163	0.780192321488971	-4.70272362727029
a_HQ_transcript/23817	2.88678906551432	0.00592733309426866	1.87083272908663	0.0703713199292192	0.780192321488971	-4.30988731408102
a_HQ_transcript/102242	-0.769532201195253	5.80058899474801	-1.87075985269943	0.0703817229746496	0.780192321488971	-4.75043974329931
a_HQ_transcript/3686	5.73132615426203	3.02457781500588	1.8706572676168	0.0703963691494619	0.780192321488971	-4.39393019140228
a_HQ_transcript/29342	1.35613199789155	3.01064550807641	1.87059105902511	0.0704058232053992	0.780192321488971	-4.44458818380449
a_HQ_transcript/99827	1.63266087902923	-0.711990503392109	1.87055211061298	0.0704113852354397	0.780192321488971	-4.27879981449202

a_HQ_transcript/63302	2.30052526545851	1.10319541515979	1.87038928775329	0.0704346412468847	0.780192321488971	-4.20674973276899
a_HQ_transcript/31862	2.10219345769629	-0.145133213814707	1.87035343200065	0.0704397634147134	0.780192321488971	-4.26884171866661
a_HQ_transcript/99831	-2.00281970527132	1.86403568826557	-1.87032047204374	0.0704444721861488	0.780192321488971	-4.27624331946407
a_HQ_transcript/104017	-1.53749852593062	-0.322970522405934	-1.87026520185986	0.0704523688788376	0.780192321488971	-4.27699754078793
a_HQ_transcript/67021	-2.16405931409524	1.50437758935717	-1.87004079730923	0.0704844383432753	0.780192321488971	-4.21974768316876
a_HQ_transcript/15121	1.40015464957233	2.53135176542927	1.86999839666071	0.0704904991912709	0.780192321488971	-4.2821772459894
a_HQ_transcript/3138	1.98182534459406	0.680999143366163	1.86997225211968	0.0704942365761405	0.780192321488971	-4.21013960819211
a_HQ_transcript/114784	-3.08487643030922	0.726589355011649	-1.86987688683276	0.0705078705685127	0.780192321488971	-4.21670592863549
a_HQ_transcript/119517	-3.30747355263143	1.75065023263517	-1.86976789719144	0.0705234551514888	0.780192321488971	-4.4043858886204
a_HQ_transcript/127476	-1.45223517981459	2.49586060140667	-1.86975530094101	0.0705252564977384	0.780192321488971	-4.32951423835939
a_HQ_transcript/58944	3.417171548742	-0.160475691886083	1.86967385842037	0.0705369042643373	0.780192321488971	-4.28248967612546
a_HQ_transcript/51796	-2.77935208611889	1.81824585250421	-1.86961111544703	0.0705458787803817	0.780192321488971	-4.25056552009906
a_HQ_transcript/24414	2.90148566092346	0.971639889203855	1.86944618394609	0.0705694746285483	0.780304816665067	-4.22118106016081
a_HQ_transcript/94998	-2.76433849172113	4.22993876631477	-1.86927172139748	0.0705944414033115	0.78043242485578	-4.57406113862685
a_HQ_transcript/102385	3.3083766586615	-0.0229737352895876	1.86889729578604	0.0706480498439962	0.780876562337291	-4.22864377464176
a_HQ_transcript/133135	-1.0889477364246	3.42011860138682	-1.86861697664018	0.0706882074433755	0.781095313481609	-4.45036234874601
a_HQ_transcript/90136	-2.49000775958668	1.79252907776588	-1.86842680205597	0.0707154623803501	0.781095313481609	-4.23260659514779
a_HQ_transcript/142590	-2.84083895784234	1.33714319035963	-1.86833981201493	0.0707279323940442	0.781095313481609	-4.29506564931787
a_HQ_transcript/138400	-0.726628373898911	4.13032987362556	-1.86833284377945	0.0707289313715313	0.781095313481609	-4.61898814482888
a_HQ_transcript/23526	2.18343922854722	0.644253757446559	1.86829031667364	0.070735028388508	0.781095313481609	-4.21790025135534
a_HQ_transcript/112447	1.67096284712935	1.47368312689649	1.86810129244976	0.0707621338305556	0.781246213732676	-4.18800102495387
a_HQ_transcript/84730	0.96047491664413	2.44233750864842	1.86777530660442	0.0708089000508648	0.781437334289667	-4.35496761121358
a_HQ_transcript/81146	1.35192170077305	3.67572156478525	1.86773982332496	0.0708139921139124	0.781437334289667	-4.57082572716187
a_HQ_transcript/54243	-2.14081013091587	2.00932907655678	-1.86764641607459	0.0708273981158369	0.781437334289667	-4.36438412857888
a_HQ_transcript/96563	5.33413264451756	2.78929073926372	1.86760586813599	0.070833218317717	0.781437334289667	-4.46891966092333
a_HQ_transcript/8875	-1.44504136208247	1.80321291133336	-1.8670517743959	0.0709127933888347	0.78209720602454	-4.23535532798862
a_HQ_transcript/73001	-2.36994664842024	-0.325422772915431	-1.86700202548073	0.0709199417312715	0.78209720602454	-4.27175764938628
a_HQ_transcript/110288	-1.01153887617498	7.35268426005776	-1.86657963994163	0.0709806585482132	0.782618308059662	-4.94960979725543
a_HQ_transcript/24816	-2.92407777706572	1.8786743114442	-1.86640042068282	0.0710064343202832	0.782754032651132	-4.18861861600356
a_HQ_transcript/44310	1.63137270435587	1.67300836361757	1.86610283543501	0.0710492515278785	0.783077529922738	-4.18176449458131
a_HQ_transcript/101917	-2.41214392679344	1.79190200618445	-1.86596806887088	0.0710686493172184	0.783142833651426	-4.2982056789387
a_HQ_transcript/630	2.31947941944741	2.30160505278334	1.86567252969658	0.0711112040136258	0.783346946540173	-4.25919584762421
a_HQ_transcript/29103	2.5021353761974	1.20032792006417	1.86565225175105	0.0711141246368788	0.783346946540173	-4.22991630875773
a_HQ_transcript/146111	-4.10935037179734	1.60087262371332	-1.8645234228657	0.0712768717188689	0.78490140269479	-4.3831190144188
a_HQ_transcript/6444	2.22260458817912	1.4252474317252	1.86440447592698	0.0712940392905451	0.78490140269479	-4.20001045456789
a_HQ_transcript/145882	-2.41238171489333	-0.24888748637726	-1.8643554188713	0.0713011207117256	0.78490140269479	-4.27893831246948
a_HQ_transcript/834	2.4457606649845	2.01135146476126	1.86427454024368	0.0713127969167389	0.78490140269479	-4.43368580825171
a_HQ_transcript/135719	2.31761581515229	1.22516446665694	1.86420555898247	0.0713227568303379	0.78490140269479	-4.41176339174148
a_HQ_transcript/92870	0.441528297773076	4.46224153140557	1.86410733028685	0.0713369417166527	0.784908905566078	-4.65571611577143
a_HQ_transcript/10782	2.11860050408071	0.215404895883916	1.8638486042144	0.0713743150859487	0.784998161104323	-4.24032751637595
a_HQ_transcript/22432	-1.70749178549013	-0.423934955406425	-1.86380317857661	0.0713808786200236	0.784998161104323	-4.25019344968856
a_HQ_transcript/140777	-2.85967253672097	0.0431214347004114	-1.86357159362918	0.0714143482922407	0.784998161104323	-4.29902502498403
a_HQ_transcript/12554	-2.77300625874408	0.668802480008546	-1.86343128413647	0.0714346329815306	0.784998161104323	-4.24277973803211
a_HQ_transcript/135042	2.35307418812996	0.0863653670902134	1.86341738965428	0.0714366419901056	0.784998161104323	-4.32100872315047
a_HQ_transcript/34582	3.00550010510498	-0.540575476719659	1.86338519174341	0.0714412976847665	0.784998161104323	-4.25874505457629
a_HQ_transcript/20549	-1.63733094633134	-0.125647477983195	-1.86337675737761	0.0714425173047811	0.784998161104323	-4.23332754910149
a_HQ_transcript/27642	1.18614452381714	5.19708705659374	1.86330363982448	0.0714530909441744	0.784998161104323	-4.73889237865213
a_HQ_transcript/26568	-2.58411342726323	1.6680634354842	-1.8629794276323	0.0714999919257387	0.785091223465199	-4.22722513624793
a_HQ_transcript/139976	-3.81207456943512	0.290684473061038	-1.86287790593297	0.0715146836203926	0.785091223465199	-4.31707469545138
a_HQ_transcript/21155	1.73709195227393	1.84641703819199	1.86277837173204	0.0715290902074549	0.785091223465199	-4.30255347042631

a_HQ_transcript/148080	-1.61062124405904	0.956896858409739	-1.86263937377704	0.0715492129449104	0.785091223465199	-4.21514210308097
a_HQ_transcript/74037	-2.04130435166845	-0.166578989589136	-1.86256322013623	0.0715602397673975	0.785091223465199	-4.28437771059388
a_HQ_transcript/46944	-1.90942542847759	-0.426663207735474	-1.86234572013624	0.0715917411494831	0.785091223465199	-4.2790554852657
a_HQ_transcript/68610	2.0804764519614	-0.653521508517036	1.86233111648665	0.0715938566794837	0.785091223465199	-4.30038214665635
a_HQ_transcript/125510	-2.48807515382047	2.2229370778088	-1.86224126409394	0.0716068741553619	0.785091223465199	-4.40063564694851
a_HQ_transcript/11303	-1.71009727145608	2.16735469858825	-1.8622037337717	0.0716123120077391	0.785091223465199	-4.32668155638029
a_HQ_transcript/85886	2.19531575213476	-0.232294369891387	1.8621864304952	0.0716148192374384	0.785091223465199	-4.31980950026219
a_HQ_transcript/37607	-0.708777507706342	2.1792426632203	-1.86211142680589	0.0716256880768795	0.785091223465199	-4.30142885824935
a_HQ_transcript/146104	3.16050751323619	-0.360623827793128	1.86206648145759	0.0716322018177845	0.785091223465199	-4.29043660674146
a_HQ_transcript/115481	0.924175751423744	2.95182276435839	1.86182694316191	0.0716669256654175	0.785091223465199	-4.45351740367203
a_HQ_transcript/116216	1.85303211031999	0.447777393377418	1.86180064511821	0.0716707387501172	0.785091223465199	-4.18916165346538
a_HQ_transcript/98517	1.54748348719768	-0.589765886551079	1.86175020779981	0.0716780523954976	0.785091223465199	-4.28797724263052
a_HQ_transcript/119433	0.694659500497597	3.9225613711441	1.86172706027261	0.0716814091086241	0.785091223465199	-4.60035529970378
a_HQ_transcript/147803	2.43702151502284	0.4381578257683	1.86165976922394	0.0716911680111698	0.785091223465199	-4.32426794186866
a_HQ_transcript/113730	-3.55629454098832	2.17339719122656	-1.86140485733202	0.0717281470079215	0.785096893769355	-4.54980300799177
a_HQ_transcript/120730	-2.65345359841407	1.87315946851261	-1.86136338502607	0.0717341647673174	0.785096893769355	-4.36781058670956
a_HQ_transcript/147848	-2.21836385367318	0.95468308982312	-1.861312017533	0.0717416189488775	0.785096893769355	-4.24668589295993
a_HQ_transcript/57811	-2.30743714999173	0.993662776632895	-1.86119352287623	0.0717588168055152	0.785096893769355	-4.20661700936823
a_HQ_transcript/7604	3.46765542488833	0.544660254961578	1.86118112774365	0.071760615991325	0.785096893769355	-4.32049111666365
a_HQ_transcript/19163	2.02744645935551	-0.535128281472241	1.86109771980686	0.0717727238764511	0.785096893769355	-4.29341730497189
a_HQ_transcript/117035	2.82493243856948	1.1065156496999	1.86085339286894	0.0718082015932343	0.785337185740644	-4.37863436738071
a_HQ_transcript/112747	-3.41193951908887	3.13505994509713	-1.86027693830994	0.071891965782942	0.786105377545307	-4.51516225955965
a_HQ_transcript/144686	2.42282878489477	0.0663544109047683	1.85967365029172	0.0719797187975974	0.786916888144958	-4.27328608201077
a_HQ_transcript/33050	-2.3732152628068	-0.358982536700932	-1.85933115257396	0.0720295786408353	0.787098366451132	-4.3316123103197
a_HQ_transcript/50986	-1.70693060019241	0.702963931057844	-1.85929522262571	0.0720348109354848	0.787098366451132	-4.22549812480056
a_HQ_transcript/132011	2.17298293528203	3.1676450198037	1.85928059583958	0.0720369410528492	0.787098366451132	-4.38969647537663
a_HQ_transcript/773	2.15617166242958	1.15626629334052	1.85909140873747	0.0720644974747012	0.787251477017371	-4.15415639017093
a_HQ_transcript/131990	4.80016677596512	1.50474407524593	1.85892802569557	0.0720883026239875	0.78736355785929	-4.27689096704963
a_HQ_transcript/126297	-3.33402238106669	1.93062733401891	-1.85859184833044	0.0721373053584297	0.787505730317041	-4.22174121183793
a_HQ_transcript/76824	1.75684934974576	-0.0275505859216729	1.858504900129	0.0721499839668401	0.787505730317041	-4.31776524003887
a_HQ_transcript/134980	2.28785517148795	-0.212024881602281	1.85848721116841	0.0721525635685323	0.787505730317041	-4.2588810910949
a_HQ_transcript/143361	-3.60242520771753	-0.111431530677297	-1.85845143248366	0.0721577814573232	0.787505730317041	-4.32421467461959
a_HQ_transcript/33224	-3.60179325788544	-0.332666406340562	-1.85832695671302	0.0721759372635776	0.787505730317041	-4.28595146697473
a_HQ_transcript/4035	-0.889415222082223	2.49927341059141	-1.85818695020452	0.0721963630319501	0.787505730317041	-4.30109399178997
a_HQ_transcript/150397	-1.3982007281488	4.38523513916074	-1.85816014985762	0.0722002735412126	0.787505730317041	-4.55671786072798
a_HQ_transcript/127747	2.3715381381527	0.50420626052874	1.8580955396208	0.0722097017373697	0.787505730317041	-4.23431739823356
a_HQ_transcript/108863	3.40526703870592	1.82650749249356	1.85781982656845	0.0722499467967443	0.787796831251389	-4.37337647986001
a_HQ_transcript/76741	3.85865461105331	0.959376320950637	1.85758809715062	0.0722837865512811	0.788017994120943	-4.29288364380878
a_HQ_transcript/150950	-3.2332705457327	0.574089548160673	-1.85739456120267	0.0723120592686689	0.788168844315733	-4.25302460027977
a_HQ_transcript/62398	-2.05650190859935	-0.0997225112245534	-1.85730776336029	0.0723247422168339	0.788168844315733	-4.2538283483125
a_HQ_transcript/97173	-0.957345444721659	2.77967776108481	-1.85676273512578	0.0724044256043657	0.788580568533379	-4.40113682953643
a_HQ_transcript/12176	-2.32367408217404	1.26297863912162	-1.85665223370102	0.0724205901319644	0.788580568533379	-4.22846165386183
a_HQ_transcript/90195	2.71320325051726	0.633530900858422	1.85662385072175	0.0724247425905553	0.788580568533379	-4.26347921193517
a_HQ_transcript/27442	2.71162930599572	-0.228644089849897	1.85634978557692	0.0724648490930918	0.788580568533379	-4.30654836281236
a_HQ_transcript/116327	2.6503285488505	0.912773064743037	1.85621913633818	0.0724839749074076	0.788580568533379	-4.42986839545947
a_HQ_transcript/48583	-1.86897589268193	2.06479169092308	-1.85620379743685	0.0724862206612703	0.788580568533379	-4.30392736369914
a_HQ_transcript/11477	-1.58973495245243	-0.688949572188333	-1.85615539779954	0.0724933071965953	0.788580568533379	-4.29075483227789
a_HQ_transcript/82581	-4.90276555954256	1.52554660462942	-1.85614362340336	0.0724950312594766	0.788580568533379	-4.36790297039978
a_HQ_transcript/135440	-2.53298432808043	-0.563339325655181	-1.85609491856284	0.0725021632261434	0.788580568533379	-4.28993471761983
a_HQ_transcript/15670	1.33674386726649	3.39003092303943	1.85600653097498	0.0725151075677663	0.788580568533379	-4.59743475448553

a_HQ_transcript/129618	1.98112429530282	1.39926709498147	1.85586299952357	0.0725361319328049	0.788580568533379	-4.21577372715975
a_HQ_transcript/135230	-2.1343590064151	3.65736027168642	-1.85566801956451	0.0725647007954676	0.788580568533379	-4.43850394926956
a_HQ_transcript/145095	-3.46801468162403	1.05011304990521	-1.85546015666769	0.0725951679061375	0.788580568533379	-4.33680183435547
a_HQ_transcript/37434	-4.9547011974492	0.831441009290971	-1.85530774156417	0.0726175148232255	0.788580568533379	-4.31146490819332
a_HQ_transcript/94248	-2.07147360511722	-0.606205998905064	-1.85529298069305	0.0726196793570973	0.788580568533379	-4.29148146019796
a_HQ_transcript/29200	5.6278479187476	1.24182648061781	1.85526145549021	0.0726243023973111	0.788580568533379	-4.2458370239428
a_HQ_transcript/131557	4.00920153733672	1.17796609321912	1.85522253729164	0.0726300099373499	0.788580568533379	-4.26832915102213
a_HQ_transcript/137847	-2.43211505720762	1.36424294592736	-1.85521924636293	0.0726304925854083	0.788580568533379	-4.27868942435866
a_HQ_transcript/95976	1.63034913375211	-0.570927468345521	1.85515541596619	0.0726398545035688	0.788580568533379	-4.35116683729629
a_HQ_transcript/139154	-1.4122846908107	3.613547540097	-1.85501186823534	0.0726609122313669	0.788580568533379	-4.55946542309425
a_HQ_transcript/85542	2.68684255762753	4.26666243333708	1.85495256642365	0.0726696130338717	0.788580568533379	-4.63344183879463
a_HQ_transcript/32791	3.71572229745493	1.67468141607662	1.8549518139296	0.072669723446049	0.788580568533379	-4.32133647039772
a_HQ_transcript/62108	2.12260834448668	-0.362629508454308	1.85491893642295	0.0726745476471462	0.788580568533379	-4.26110299406238
a_HQ_transcript/138296	-0.847622610010208	2.33572510999481	-1.85447199900376	0.0727401551363685	0.788768386733379	-4.30973975972864
a_HQ_transcript/90823	-2.18490727301331	-0.416570410221567	-1.8543434587604	0.0727590333923436	0.788768386733379	-4.29027659405915
a_HQ_transcript/41434	-2.48661531046404	1.81904834857962	-1.85431937201038	0.0727625713958242	0.788768386733379	-4.33025831086429
a_HQ_transcript/125172	2.45443379809219	2.21422595807006	1.85429313145191	0.0727664259310347	0.788768386733379	-4.39985235249074
a_HQ_transcript/64170	2.58234902208324	0.290531597562439	1.85421896553095	0.0727773212771224	0.788768386733379	-4.31959190597271
a_HQ_transcript/121963	-1.50523514930237	-0.540317457798132	-1.85415913394844	0.0727861118550267	0.788768386733379	-4.34658449960751
a_HQ_transcript/141473	-1.65287939228798	-0.429430841449651	-1.8541042933538	0.0727941699454777	0.788768386733379	-4.25524437331945
a_HQ_transcript/93682	3.0373310419872	0.115895955727972	1.85406181025337	0.0728004127928809	0.788768386733379	-4.35471228466624
a_HQ_transcript/70641	-0.992311730886116	3.20038600006201	-1.85380961032366	0.0728374827504952	0.789022958874541	-4.49909915404789
a_HQ_transcript/126067	2.05530444731639	0.0433054760464275	1.8536165019385	0.072865877980725	0.789159473485598	-4.2368872001474
a_HQ_transcript/13918	1.79670991144954	1.33382754408442	1.85353926782785	0.0728772373670295	0.789159473485598	-4.19486240728913
a_HQ_transcript/133936	-2.70971598499708	3.48432015321296	-1.85336579106208	0.0729027573935812	0.789209754510537	-4.55931021559059
a_HQ_transcript/149633	-3.15034365254105	0.522233269025889	-1.85332312647877	0.072909034918139	0.789209754510537	-4.28951475843426
a_HQ_transcript/128963	-1.05005950261758	4.00833479864423	-1.85319888134645	0.0729273185692913	0.789260691453316	-4.67351293001365
a_HQ_transcript/85823	-1.30871270310811	2.41414947395998	-1.85279445721117	0.0729868599604014	0.789610262621665	-4.31852816353886
a_HQ_transcript/97277	-2.52470334329992	0.534029403441528	-1.85264396952574	0.073009026150449	0.789610262621665	-4.33798948979631
a_HQ_transcript/51588	-2.15956188815027	1.38378222761996	-1.85241475480794	0.0730427995694458	0.789610262621665	-4.36696807965205
a_HQ_transcript/836	2.22172390058235	0.933918619680509	1.85221424550809	0.0730723543837428	0.789610262621665	-4.24142966287517
a_HQ_transcript/36026	2.98461149693459	2.28750752502365	1.85211630906244	0.0730867938116906	0.789610262621665	-4.42400218782456
a_HQ_transcript/140706	2.35528271711513	-0.285466502419808	1.85205549849698	0.0730957607509611	0.789610262621665	-4.3623923968755
a_HQ_transcript/9061	-1.9407881164865	-0.141267177512099	-1.85205328202072	0.0730960876035339	0.789610262621665	-4.33772796017256
a_HQ_transcript/46709	2.09275828124552	0.530311178182569	1.85198012570646	0.0731068762983512	0.789610262621665	-4.1813619715975
a_HQ_transcript/123689	2.71579366700618	0.395853682526477	1.8518379373052	0.0731278493690802	0.789610262621665	-4.27163349879719
a_HQ_transcript/118215	0.498139915455474	3.93848247161733	1.85181463972931	0.0731312862987275	0.789610262621665	-4.63366992187345
a_HQ_transcript/4355	-1.61377291870561	1.51433328635401	-1.85179216187774	0.0731346024312054	0.789610262621665	-4.28398253853223
a_HQ_transcript/132022	3.38541911943799	0.191008639107823	1.85170983638739	0.0731467489184761	0.789610262621665	-4.3676627035972
a_HQ_transcript/78341	1.41859167008426	-0.121503916810932	1.85165185392891	0.0731553048167278	0.789610262621665	-4.21113903292228
a_HQ_transcript/31088	-1.90315788217932	-0.452223692357865	-1.85161787550568	0.0731603190748819	0.789610262621665	-4.30779281477115
a_HQ_transcript/4565	-0.950342238633357	2.90261009052255	-1.85152135673869	0.0731745641310095	0.789610262621665	-4.43846293924612
a_HQ_transcript/149906	2.68941456910405	5.61845073686553	1.85146902034625	0.073182289371277	0.789610262621665	-4.85787962833818
a_HQ_transcript/79782	2.72960858430682	0.0043630717411881	1.85130222186766	0.0732069147239659	0.789610262621665	-4.29755332455097
a_HQ_transcript/134272	-2.59208894725246	2.21850975169726	-1.85126946311057	0.0732117519079494	0.789610262621665	-4.28779103441092
a_HQ_transcript/151825	-1.34238207179755	3.83817649034749	-1.85116745150889	0.0732268167742951	0.789610262621665	-4.59981048768734
a_HQ_transcript/3724	-0.80706700618583	2.44214682733915	-1.85093414336971	0.0732612812212576	0.789610262621665	-4.39050745104826
a_HQ_transcript/114862	2.08188149864121	-0.424567764682951	1.85077705041373	0.0732844949271562	0.789610262621665	-4.31873273766968
a_HQ_transcript/45696	3.92117350625934	2.84470571773712	1.85057804122218	0.073313911656008	0.789610262621665	-4.31448321237163
a_HQ_transcript/95511	1.03892641374128	3.50933375695765	1.85051113827665	0.0733238032468216	0.789610262621665	-4.61058052645619

a_HQ_transcript/127636	-6.19391103684306	0.0630042975180938	-1.85038792282739	0.0733420236222527	0.789610262621665	-4.32971695100879
a_HQ_transcript/73156	-1.98227610338965	0.473967764217351	-1.85033582943054	0.0733497280525914	0.789610262621665	-4.24409555213116
a_HQ_transcript/91330	2.05752766681619	-0.310725947152405	1.85033176560654	0.0733503291070055	0.789610262621665	-4.25171793165885
a_HQ_transcript/123331	3.83576918446581	0.0907010414005655	1.85031273749173	0.0733531434907742	0.789610262621665	-4.30886155217944
a_HQ_transcript/78286	-2.60681112437143	0.299114116275726	-1.85020313542802	0.0733693561587879	0.789610262621665	-4.19599496236497
a_HQ_transcript/9624	1.07643468234798	4.53847644256171	1.85016496119404	0.0733750037267442	0.789610262621665	-4.64610002538151
a_HQ_transcript/126823	-2.36934293234899	-0.428859556960383	-1.85008194187155	0.0733872870458218	0.789610262621665	-4.32154756290679
a_HQ_transcript/30957	2.33127796026195	1.06580641284352	1.85005877607719	0.0733907149094362	0.789610262621665	-4.19522528507688
a_HQ_transcript/16850	-2.16196918204306	0.307211351666047	-1.85003450471269	0.0733943065122636	0.789610262621665	-4.29004960317026
a_HQ_transcript/94779	-1.67605665626867	-0.0948749866139183	-1.84992580298943	0.0734103937087688	0.789634941520578	-4.25339504537973
a_HQ_transcript/35604	-2.36576169477293	-0.215210683896044	-1.84983543953711	0.0734237692492211	0.789634941520578	-4.2957311767459
a_HQ_transcript/38348	-2.47131713606589	0.510597516398661	-1.84937828965963	0.0734914682516022	0.789946707152077	-4.28977638080634
a_HQ_transcript/120390	-1.23876704870254	4.44931174133831	-1.84936631721508	0.073493241960018	0.789946707152077	-4.66870004443915
a_HQ_transcript/59408	2.58412892206012	2.64826109555142	1.84924963644069	0.0735105300449613	0.789946707152077	-4.36151536835587
a_HQ_transcript/139656	3.01434621493577	0.383084528807095	1.84919317424139	0.0735188970538532	0.789946707152077	-4.32326684780156
a_HQ_transcript/70098	1.61602591254686	-0.310396284375499	1.84909596412023	0.0735333043168658	0.789946707152077	-4.3352248145615
a_HQ_transcript/68465	0.950654597766232	3.48700595758053	1.8489994631927	0.0735476088616566	0.789946707152077	-4.56287560868227
a_HQ_transcript/121235	-2.37645493630228	-0.384527622755277	-1.84899758656822	0.0735478870614341	0.789946707152077	-4.34413839793184
a_HQ_transcript/54346	-2.45292434436356	1.56757915480925	-1.84863182622184	0.0736021263159689	0.790383225289977	-4.34521298174638
a_HQ_transcript/33705	-1.3920260775847	-0.155974097178756	-1.84847528341742	0.0736253507848089	0.79040418573421	-4.26491122252993
a_HQ_transcript/34324	-5.4016449345161	3.41533974673005	-1.84843536858767	0.0736312734955744	0.79040418573421	-4.57109595426159
a_HQ_transcript/80225	-4.4780143460768	0.102042404097478	-1.84830815699656	0.0736501523443131	0.790460866962746	-4.31690737138567
a_HQ_transcript/6320	-1.4697473307271	1.70396065607489	-1.84813531787787	0.0736758091869342	0.790574819517534	-4.28626611678199
a_HQ_transcript/9041	-1.6071933891181	1.76193865348637	-1.84802051133807	0.0736928556972977	0.790574819517534	-4.2584230731242
a_HQ_transcript/132305	2.10864474167453	8.68382316203961	1.84772343352657	0.0737369815664704	0.790574819517534	-5.04122531983302
a_HQ_transcript/29333	3.14923044429957	0.959809912720518	1.84770202301152	0.0737401626083721	0.790574819517534	-4.20591413524954
a_HQ_transcript/96181	-3.08393670532937	0.9682447073559	-1.84763092207859	0.0737507271875661	0.790574819517534	-4.30056042616251
a_HQ_transcript/84085	1.93723782130004	0.587973695144415	1.8476155538907	0.0737530108501242	0.790574819517534	-4.25420091842056
a_HQ_transcript/128527	7.79341351202801	4.53550735420552	1.84750976426073	0.0737687324854409	0.790574819517534	-4.65578776784654
a_HQ_transcript/112834	2.73507233031627	3.66018365035946	1.84750409958962	0.0737695744058475	0.790574819517534	-4.57777236280383
a_HQ_transcript/119896	-3.54282205461297	0.902797207759412	-1.8471346005175	0.0738245095296029	0.790920707410725	-4.33714660516837
a_HQ_transcript/48284	2.15916225827907	0.173390724305537	1.84710398579382	0.0738290627307079	0.790920707410725	-4.27595942895626
a_HQ_transcript/68065	-1.62403531685018	5.23449115610711	-1.84672326578053	0.0738857057149114	0.790965929427366	-4.7722129686231
a_HQ_transcript/75083	-2.04175642057378	2.79065431242404	-1.84662287931985	0.0739006472621467	0.790965929427366	-4.34282150309146
a_HQ_transcript/138329	-2.30496693810859	1.72086168821843	-1.84661642349913	0.073901608236651	0.790965929427366	-4.33643088957865
a_HQ_transcript/123980	-1.14498095911231	1.74717099057693	-1.84658777536739	0.07390587275358	0.790965929427366	-4.26463166805556
a_HQ_transcript/129947	5.94964676432568	1.33609086450432	1.84656649400646	0.0739090408005759	0.790965929427366	-4.34247044730106
a_HQ_transcript/53113	1.96176827456636	-0.0550368808102647	1.84651291205511	0.0739170177851601	0.790965929427366	-4.29620318356046
a_HQ_transcript/27373	2.81848734521299	1.78401142135207	1.84643555746252	0.073928535208142	0.790965929427366	-4.29076549381246
a_HQ_transcript/144952	1.55401966821729	2.83059057072333	1.84622133847421	0.0739604385559665	0.791161643794851	-4.38411844282143
a_HQ_transcript/74957	-3.36924709166124	-0.275483885016736	-1.84546609127204	0.0740730104762082	0.792166900227673	-4.34175407928433
a_HQ_transcript/69045	-3.89175561607023	0.45492374049643	-1.84524370106528	0.0741061863369006	0.792166900227673	-4.23826096548059
a_HQ_transcript/48732	-1.92593261120936	0.752805025176136	-1.84522705280793	0.0741086704118845	0.792166900227673	-4.24956626637123
a_HQ_transcript/84075	-2.59715620226867	1.01275566080647	-1.84522534531406	0.0741089251898927	0.792166900227673	-4.34897110296031
a_HQ_transcript/77320	-2.58215697722224	0.447632516309249	-1.845077872594	0.0741309326668163	0.792186892723532	-4.26781796605064
a_HQ_transcript/13474	1.93735636278326	-0.347797794826955	1.84503017263306	0.0741380521679055	0.792186892723532	-4.3646608718897
a_HQ_transcript/145555	5.02653929444648	3.29238120800905	1.84476065820829	0.0741782897755972	0.792383500048621	-4.44671054734256
a_HQ_transcript/140807	-2.17652677825573	0.912435301804739	-1.84466657537385	0.074192340426933	0.792383500048621	-4.20899941593049
a_HQ_transcript/87541	3.78446019805602	3.86962874950905	1.8444677653378	0.0742220388820084	0.792383500048621	-4.4553410594598
a_HQ_transcript/69338	2.30646224045331	2.80314023887137	1.84446072534777	0.0742230907095596	0.792383500048621	-4.34876714935952

a_HQ_transcript/118424	2.30171680787106	-0.275399938110489	1.84445055354346	0.0742246104762721	0.792383500048621	-4.31666781774624
a_HQ_transcript/12483	0.489520236876053	5.06795720846974	1.84434774197295	0.0742399730208694	0.792401974248457	-4.74808860492815
a_HQ_transcript/118650	-2.14788301268492	-0.182239664657366	-1.84410419044863	0.0742763763812564	0.792510692177649	-4.30053633399248
a_HQ_transcript/93267	3.50956190226186	0.895913169009124	1.84409716590246	0.074277426558351	0.792510692177649	-4.48241786872195
a_HQ_transcript/110565	-2.44615512132531	1.48576933957646	-1.84391667808614	0.0743044140325326	0.792653143491109	-4.34962936145863
a_HQ_transcript/5282	1.74742014304241	1.6776032126959	1.84353590850804	0.0743613761772552	0.793115243014952	-4.31983134212287
a_HQ_transcript/111297	2.32110595283184	1.53552108120892	1.8433715474264	0.0743859757045154	0.793161244115875	-4.23626081877157
a_HQ_transcript/129388	0.523451249936598	4.85517822656805	1.84332476134077	0.0743929793373203	0.793161244115875	-4.73694851617496
a_HQ_transcript/50126	-2.27429130267833	0.649635052797815	-1.84311005618865	0.0744251268085467	0.793334908821688	-4.18346815364879
a_HQ_transcript/131958	-2.4832053201926	-0.179573100664744	-1.84303368902746	0.0744365640089713	0.793334908821688	-4.35670181297236
a_HQ_transcript/83949	-3.79031594150784	1.73956355618852	-1.84283222366452	0.0744667438717407	0.793511070169852	-4.43141083502716
a_HQ_transcript/24013	-1.09981257230802	2.38487769310868	-1.84271168187483	0.0744848062436978	0.79355806769312	-4.38111991071391
a_HQ_transcript/121684	3.15048634871572	3.7992646596411	1.84250225704448	0.0745161960446011	0.793681141266176	-4.58043907853278
a_HQ_transcript/35035	2.12533959770763	0.439243251493033	1.84243642125852	0.0745260662278062	0.793681141266176	-4.19831899893429
a_HQ_transcript/117508	-1.65439553630111	2.12101350935405	-1.84236136427878	0.0745373202272925	0.793681141266176	-4.37859151881684
a_HQ_transcript/135390	-1.81143654096636	1.05911351985422	-1.84216242704915	0.0745671557872499	0.793818163557991	-4.29422500014598
a_HQ_transcript/74477	2.35847348342884	0.474124892719801	1.84208541764915	0.0745787079928153	0.793818163557991	-4.23332041697433
a_HQ_transcript/42426	1.50157416240852	-0.575778002948638	1.84200243735522	0.0745911576065536	0.793818163557991	-4.30971074036759
a_HQ_transcript/85347	-2.2785390139976	-0.0970546393445749	-1.84156596715159	0.0746566709046769	0.794369937094464	-4.32999123043125
a_HQ_transcript/81439	4.01945857700802	1.11106702163153	1.84137724580366	0.0746850128257154	0.794428570212903	-4.45811333866523
a_HQ_transcript/126514	3.27892111556601	1.43774902855521	1.84128780144165	0.0746984486729877	0.794428570212903	-4.24733686531317
a_HQ_transcript/128129	-2.11248227419256	-0.62442886603827	-1.84125629411011	0.074703182025594	0.794428570212903	-4.31336547911607
a_HQ_transcript/124292	-3.21687501912065	2.14152353913111	-1.84104879071708	0.0747343617053907	0.794487996854244	-4.63218658307614
a_HQ_transcript/3393	-3.70141351645981	3.01843984244799	-1.84103718479228	0.0747361059523638	0.794487996854244	-4.42737374372018
a_HQ_transcript/139497	-1.05955967616417	4.36713467543155	-1.84090219001977	0.0747563967860947	0.794558389537778	-4.68632925934892
a_HQ_transcript/26821	-2.31480966303489	0.124576763576425	-1.84061806669001	0.0747991182624343	0.794561552729613	-4.23103829318816
a_HQ_transcript/67013	-2.67489742726334	0.251960282892938	-1.84044511310075	0.0748251342009082	0.794561552729613	-4.40738135286203
a_HQ_transcript/8273	-2.18473523942661	2.8098858542665	-1.84023721886323	0.0748564161981857	0.794561552729613	-4.48247002785168
a_HQ_transcript/120571	2.41194688705764	1.07425216185584	1.84023130142005	0.0748573067637349	0.794561552729613	-4.22744869473062
a_HQ_transcript/83006	1.8053239004746	-0.492262320507917	1.84015297237825	0.0748690960099956	0.794561552729613	-4.30538759110996
a_HQ_transcript/146265	2.46628751950998	0.325889569709035	1.83998477683272	0.0748944163590704	0.794561552729613	-4.3709292179974
a_HQ_transcript/6882	-2.77323785416608	0.1826151613224	-1.83995575940268	0.0748987854161204	0.794561552729613	-4.28862893276311
a_HQ_transcript/30042	-1.96550695321341	-0.530671384820124	-1.83987751829174	0.0749105670030042	0.794561552729613	-4.29455260103427
a_HQ_transcript/36108	2.98075510714396	1.59356526915112	1.83975619407206	0.0749288391985099	0.794561552729613	-4.24710814540055
a_HQ_transcript/127724	2.3653267326314	1.39666995009099	1.83964585533919	0.0749454602169903	0.794561552729613	-4.25274833990822
a_HQ_transcript/41261	1.94975411417161	-0.376098801677523	1.83960732274295	0.074951265366915	0.794561552729613	-4.30245852670484
a_HQ_transcript/89003	3.33580376081435	-0.376222185830141	1.8395042312187	0.0749667985676161	0.794561552729613	-4.33216582616597
a_HQ_transcript/126096	-2.92609289330299	0.6498719630606	-1.83942394522843	0.0749788974751054	0.794561552729613	-4.31355928701947
a_HQ_transcript/59588	-2.52200662772656	0.437764445838993	-1.83941132548819	0.0749807993916376	0.794561552729613	-4.36723424656419
a_HQ_transcript/21075	-2.64855568699286	1.2680437558095	-1.83931288148035	0.0749956372679326	0.794561552729613	-4.26954062629341
a_HQ_transcript/38715	0.867874440079705	3.29713453711963	1.83918442225224	0.0750150029317899	0.794561552729613	-4.54548016787195
a_HQ_transcript/129601	-2.08369127540812	-0.565590737843609	-1.83898782417606	0.0750446490315144	0.794561552729613	-4.31427008589045
a_HQ_transcript/88939	2.22503660518014	-0.0956514135076594	1.83893539724143	0.0750525564664184	0.794561552729613	-4.31415695908321
a_HQ_transcript/54502	-1.95928170123408	0.901952126315316	-1.83888386615621	0.0750603294761621	0.794561552729613	-4.28533958508897
a_HQ_transcript/85284	-2.37847613357565	1.75153827009762	-1.83884547245551	0.0750661212744592	0.794561552729613	-4.29374154540778
a_HQ_transcript/30001	2.41434785306379	0.695017344427105	1.83881779567681	0.0750702966316139	0.794561552729613	-4.25741834414741
a_HQ_transcript/82926	-2.51487664926601	0.828704198518089	-1.83879644438155	0.0750735178531656	0.794561552729613	-4.27913968492231
a_HQ_transcript/93166	-2.68428505456554	-0.00603744877733002	-1.83878975270425	0.0750745274356677	0.794561552729613	-4.27923254955685
a_HQ_transcript/104099	0.802759699502717	3.84970174874135	1.83872197043904	0.075084754492564	0.794561552729613	-4.53393058160365
a_HQ_transcript/150776	-2.63518563316521	-0.157801461778229	-1.83822221550067	0.0751601947723326	0.795215107704068	-4.33241243761838

a_HQ_transcript/149050	2.73771019468832	1.83417517436507	1.83807941525987	0.0751817630097709	0.795298547812914	-4.33664952337342
a_HQ_transcript/67257	-2.46289525906105	0.32453177171993	-1.83792822293613	0.0752046045318383	0.79535925051371	-4.33630143216562
a_HQ_transcript/151755	2.12327628664697	0.501037067798893	1.83786030524106	0.0752148671909212	0.79535925051371	-4.34603053664626
a_HQ_transcript/101208	-1.61988864015118	2.69347819568195	-1.83760578102629	0.0752533375503819	0.795361093765605	-4.48907212865666
a_HQ_transcript/108050	-2.11941646080753	2.76577279373866	-1.83744449160009	0.0752777245286936	0.795361093765605	-4.35151581929581
a_HQ_transcript/146993	0.932502672353283	3.69343408766778	1.83712598682088	0.075325902314414	0.795361093765605	-4.60519428625017
a_HQ_transcript/28353	-2.44129029195235	1.39361237085918	-1.83712586409933	0.0753259208826393	0.795361093765605	-4.20318147202624
a_HQ_transcript/139856	-1.33263549912083	2.07057066749106	-1.83700532844732	0.0753441602627177	0.795361093765605	-4.31596192141958
a_HQ_transcript/38458	2.58321266603518	0.137728962812619	1.83694746103453	0.0753529180639419	0.795361093765605	-4.42664476921838
a_HQ_transcript/134886	2.90309199187974	1.48981372756077	1.83692758357156	0.0753559265703108	0.795361093765605	-4.30947211734714
a_HQ_transcript/52990	-2.37505849441989	1.91853862450885	-1.836897885182	0.0753604216909422	0.795361093765605	-4.35871504347912
a_HQ_transcript/52180	-2.07563748753156	-0.423919137681579	-1.83686617224923	0.0753652219838307	0.795361093765605	-4.27514135152669
a_HQ_transcript/63357	-3.11274464221436	1.92281184367721	-1.83686048042743	0.0753660835657152	0.795361093765605	-4.3841842715718
a_HQ_transcript/20675	3.73825648398325	1.15464768884449	1.83677402582402	0.0753791713978261	0.795361093765605	-4.36631410112938
a_HQ_transcript/78597	2.88803725171205	0.54187029456893	1.83677359509518	0.0753792366080842	0.795361093765605	-4.37410394856263
a_HQ_transcript/47754	-2.02779301471345	-0.322565873110154	-1.83618397523963	0.0754685473847069	0.796050646935741	-4.24307190011986
a_HQ_transcript/141996	-2.31737575912655	-0.0753892214561332	-1.83603432747551	0.0754912291744907	0.796050646935741	-4.33966804024614
a_HQ_transcript/27405	-1.00347885118576	2.67195968036383	-1.8360122488493	0.0754945760771887	0.796050646935741	-4.36146543898929
a_HQ_transcript/2396	-1.32592226195699	-0.669677733126735	-1.83598064454736	0.0754993671992283	0.796050646935741	-4.33449443696267
a_HQ_transcript/30169	1.47050092455866	2.16609395742188	1.83568219730022	0.0755446237568524	0.796204050871625	-4.32202035631784
a_HQ_transcript/25862	0.574267532297893	3.92548137298624	1.8355777024028	0.0755604748440962	0.796204050871625	-4.54301457382073
a_HQ_transcript/89153	1.98919010276552	0.108337104079301	1.83554130724013	0.075565996382462	0.796204050871625	-4.2302996561547
a_HQ_transcript/136302	-2.28506838744534	3.40651598352463	-1.83552344694174	0.0755687061082224	0.796204050871625	-4.35678493435083
a_HQ_transcript/11558	1.88855127296315	-0.197490989039548	1.83523281896428	0.0756128112177073	0.796345968447964	-4.25096787156368
a_HQ_transcript/109870	-2.56891861610648	2.31536974360599	-1.83522836731948	0.0756134869611486	0.796345968447964	-4.38891136401885
a_HQ_transcript/145383	-2.535206750963	1.92540376541875	-1.8351638878604	0.0756232752861402	0.796345968447964	-4.22899052675598
a_HQ_transcript/125473	-3.15609980101381	-0.341494232552242	-1.8348082783071	0.0756772781504677	0.796718967783101	-4.35747328356171
a_HQ_transcript/75521	3.03264811815299	1.21929634081557	1.83464631806906	0.0757018843432026	0.796718967783101	-4.38775922149442
a_HQ_transcript/98736	-2.02965745283479	-0.307476043578701	-1.83457592111181	0.0757125816975046	0.796718967783101	-4.29415173875074
a_HQ_transcript/3599	1.95211364064091	2.26903309507395	1.83456973685035	0.0757135215048487	0.796718967783101	-4.33728780363124
a_HQ_transcript/18276	2.4261251538711	1.76252850245348	1.8344347777964	0.0757340333885513	0.79678262568364	-4.27875503392677
a_HQ_transcript/98056	-3.4236383134801	-0.212172877709915	-1.83434957289621	0.0757469857818572	0.79678262568364	-4.33160631318497
a_HQ_transcript/137736	-1.29064066986324	4.8017057811822	-1.83416344145277	0.0757752870376705	0.796784941911019	-4.76066477130046
a_HQ_transcript/124695	2.68544778827771	0.385561835966832	1.83396396239643	0.075805627880396	0.796784941911019	-4.32220969963226
a_HQ_transcript/137362	1.67654816399484	-0.661250214073888	1.83393061397665	0.0758107011949503	0.796784941911019	-4.33583249692027
a_HQ_transcript/23085	-2.31470434012817	0.0358873948466706	-1.83381024274208	0.0758290157527595	0.796784941911019	-4.29172014827945
a_HQ_transcript/124926	2.30573582944764	0.998494361173447	1.83371252726087	0.0758438860015076	0.796784941911019	-4.28060678612667
a_HQ_transcript/16349	-1.78710789969433	1.37978090354198	-1.83364117709029	0.0758547455750193	0.796784941911019	-4.30290109904391
a_HQ_transcript/40186	-2.23009934681322	-0.293707482912988	-1.83364026146635	0.0758548849426059	0.796784941911019	-4.28562768259876
a_HQ_transcript/55348	-1.69459038606643	-0.554658615926436	-1.83339917469883	0.0758915884897188	0.796784941911019	-4.32653474666628
a_HQ_transcript/2587	-4.2429672902352	0.422916879632008	-1.83334056266461	0.0759005139975629	0.796784941911019	-4.30307507653443
a_HQ_transcript/37111	-1.71872417422779	1.74705240826609	-1.83327528523586	0.0759104555741479	0.796784941911019	-4.43362755866803
a_HQ_transcript/147642	-2.23519254258054	1.81322375767255	-1.83326969105841	0.0759113076040198	0.796784941911019	-4.31979998833228
a_HQ_transcript/125749	-2.48939700683036	1.16477926726462	-1.83306941839712	0.0759418158207853	0.796784941911019	-4.35891890320264
a_HQ_transcript/44668	2.98583642103481	0.811048822888781	1.83304142886133	0.0759460803956593	0.796784941911019	-4.34228820882203
a_HQ_transcript/62111	-0.935394840826204	3.76777027217547	-1.83299414971782	0.0759532844609714	0.796784941911019	-4.52054345060185
a_HQ_transcript/145078	2.03471229091607	0.926894582709514	1.8329652499921	0.0759576882863811	0.796784941911019	-4.2552155419876
a_HQ_transcript/46554	-1.57399061693195	0.778019571174448	-1.83290726606652	0.0759665247053204	0.796784941911019	-4.25064867244092
a_HQ_transcript/66730	-1.67403498112525	2.24041355373368	-1.83249342567864	0.0760296171012832	0.79730282930965	-4.43203862400944
a_HQ_transcript/83774	2.04216540844013	1.04819523178772	1.83223472551649	0.0760690801752039	0.797373484276195	-4.29923465956134

a_HQ_transcript/63621	1.94438663203214	-0.620725562093172	1.83217379837434	0.07607837676882	0.797373484276195	-4.32876410875569
a_HQ_transcript/149801	-3.03782304290637	0.22299672010304	-1.83211479202485	0.0760873812018778	0.797373484276195	-4.29567748398047
a_HQ_transcript/86716	-2.08052135672999	0.0390785193766907	-1.8320896063762	0.0760912248362244	0.797373484276195	-4.28093226372899
a_HQ_transcript/128233	-1.4062761388983	2.3000203452608	-1.83192991659972	0.0761155992829062	0.797387097986773	-4.39197469584305
a_HQ_transcript/140141	2.8345523276448	-0.19613584041014	1.83181879617737	0.076132564218016	0.797387097986773	-4.34082070143156
a_HQ_transcript/90081	-1.88483672087677	2.25488059731006	-1.83179650341995	0.0761359680780991	0.797387097986773	-4.37197012291754
a_HQ_transcript/5159	3.0439471128176	1.89761684612155	1.83172167129091	0.0761473950750856	0.797387097986773	-4.3170993069844
a_HQ_transcript/132144	-2.39707437351407	0.347402694127183	-1.83149079062782	0.0761826601818189	0.797426183441074	-4.32265173653533
a_HQ_transcript/72983	1.70079602072522	2.13240937364527	1.83145862540218	0.0761875742593646	0.797426183441074	-4.2998167262659
a_HQ_transcript/110978	1.0030016282751	2.39980565408252	1.83122557370637	0.0762231870689261	0.797426183441074	-4.35682866839245
a_HQ_transcript/48658	-2.17912428546266	0.598994090581827	-1.8309786893441	0.0762609291549669	0.797426183441074	-4.28391654341951
a_HQ_transcript/24761	-2.27072514465561	-0.504047370631669	-1.83072756495293	0.0762993357899941	0.797426183441074	-4.29844138793195
a_HQ_transcript/117566	2.53899982789017	-0.327324054662147	1.83071650968747	0.0763010269470568	0.797426183441074	-4.37566590270389
a_HQ_transcript/112978	-1.77279159309642	0.623744481060647	-1.83054234552222	0.0763276735827341	0.797426183441074	-4.24301457283277
a_HQ_transcript/136238	2.27694239808893	0.234789408220427	1.83036000987507	0.0763555789409738	0.797426183441074	-4.30942380267759
a_HQ_transcript/88924	-2.96008031628695	-0.00382939790364518	-1.83027154929285	0.0763691204294432	0.797426183441074	-4.31774452507671
a_HQ_transcript/80012	1.68365477703991	-0.680335572542431	1.83015428205791	0.076387074780535	0.797426183441074	-4.34775158088383
a_HQ_transcript/87429	-1.75095064956667	-0.353403415047638	-1.83003216238001	0.0764057758995606	0.797426183441074	-4.38623553529891
a_HQ_transcript/31502	1.08764312037561	1.91568617324937	1.83001370821505	0.0764086022665568	0.797426183441074	-4.36801425369658
a_HQ_transcript/56852	-1.81065000812244	-0.42203151898157	-1.83000088763111	0.0764105658690606	0.797426183441074	-4.35277072171145
a_HQ_transcript/118225	4.39120322921773	3.27777799469833	1.82992541925962	0.0764221254885921	0.797426183441074	-4.55731544192832
a_HQ_transcript/1749	-1.08416125600313	2.72556299608438	-1.82984776596497	0.0764340213341307	0.797426183441074	-4.42717374196178
a_HQ_transcript/141643	2.70996048079846	-0.31818272873409	1.82984140416846	0.0764349959790085	0.797426183441074	-4.34016809807305
a_HQ_transcript/45606	1.78188526170179	0.794475610865787	1.8297915786248	0.0764426297573334	0.797426183441074	-4.30629222000411
a_HQ_transcript/145724	-2.32313354270982	0.338741074017626	-1.82966096739717	0.0764626438090979	0.797426183441074	-4.25896166177619
a_HQ_transcript/57971	2.09545606579817	-0.25560537490175	1.8296138970994	0.0764698576645329	0.797426183441074	-4.36277808195427
a_HQ_transcript/81817	-2.01300300560495	1.07208774493222	-1.82956889098061	0.0764767557130273	0.797426183441074	-4.27143448461008
a_HQ_transcript/122397	2.27949248110202	0.967058814496732	1.82955339714399	0.076479130562672	0.797426183441074	-4.32422264618132
a_HQ_transcript/73245	-2.18464642327057	-0.148211783907938	-1.82955148260823	0.0764794240214419	0.797426183441074	-4.36223458027119
a_HQ_transcript/31950	-2.12508785339117	0.64169194860371	-1.82948805823847	0.0764891462098668	0.797426183441074	-4.2437605898481
a_HQ_transcript/124840	4.4620051031783	-0.153990668333672	1.82946777776045	0.0764922551842391	0.797426183441074	-4.36140411499039
a_HQ_transcript/144321	-2.46812018580208	-0.431376556510013	-1.82943833736238	0.0764967685558707	0.797426183441074	-4.34287543847225
a_HQ_transcript/142039	3.63251349087652	1.28557203123939	1.82936633841421	0.0765078073398512	0.797426183441074	-4.34631002153432
a_HQ_transcript/14330	2.34850776049197	0.457296964342908	1.82886811439417	0.0765842316396055	0.798079637279848	-4.37720851051867
a_HQ_transcript/88249	1.51452457209066	3.29362751385905	1.82862778870418	0.076621119304864	0.798320921841393	-4.51060246857824
a_HQ_transcript/141200	-2.36584480676201	-0.220309746680218	-1.82827845607384	0.0766747655083815	0.79873669703785	-4.33760779391994
a_HQ_transcript/150200	2.72128809621039	-0.2099606169301	1.82816974407927	0.0766914666936265	0.798767528394038	-4.35185573493377
a_HQ_transcript/149489	1.60457110056226	1.6326636314881	1.82806037721065	0.0767082716170008	0.798799428977611	-4.2726949861607
a_HQ_transcript/107723	-2.04189097421665	-0.302492740177638	-1.82790267506919	0.0767325090965121	0.798908702984427	-4.34772220903714
a_HQ_transcript/91865	3.12377914412339	0.489262993971222	1.82773856330093	0.0767577386172756	0.799028264746597	-4.27039164649372
a_HQ_transcript/142622	4.22379420700491	3.9392125821872	1.82750162099112	0.0767941771346458	0.799192176237008	-4.59642937443237
a_HQ_transcript/54060	1.66112878807921	-0.622182019374416	1.82745738117133	0.0768009822539899	0.799192176237008	-4.3468927199789
a_HQ_transcript/144817	-4.40960363021148	3.07880319083177	-1.82727737569761	0.0768286766118644	0.799315037099943	-4.51730340964917
a_HQ_transcript/125251	2.49605494693192	1.29369529085165	1.82719667865004	0.0768410948488415	0.799315037099943	-4.31574665550049
a_HQ_transcript/78687	1.77409821934155	-0.592793779914574	1.82695963441931	0.0768775828061335	0.799315037099943	-4.34533693262316
a_HQ_transcript/74467	-2.34204430167159	2.0868862131922	-1.82692814690359	0.0768824307564216	0.799315037099943	-4.35860103589405
a_HQ_transcript/145784	-3.09462600503064	4.03496996096659	-1.82686511362831	0.0768921364079042	0.799315037099943	-4.60199832443009
a_HQ_transcript/87209	2.36248275622296	-0.058267045597491	1.8265486067788	0.0769408868656214	0.799315037099943	-4.25800619801813
a_HQ_transcript/49377	-2.87836548353591	0.157672128192129	-1.82654681921876	0.0769411622721662	0.799315037099943	-4.36821775443073
a_HQ_transcript/107419	-4.78704958527187	-0.200889199320306	-1.8264331128533	0.0769586825595941	0.799315037099943	-4.37828915150744

a_HQ_transcript/58293	1.25002352767641	3.46953284359753	1.82637661492642	0.0769673892294215	0.799315037099943	-4.67055000391135
a_HQ_transcript/42619	-1.87556273816749	1.05315484425885	-1.82625282020287	0.0769864696767131	0.799315037099943	-4.26771121645143
a_HQ_transcript/79693	2.15646033668145	1.56673595978843	1.82618043207952	0.0769976287075269	0.799315037099943	-4.26785315571263
a_HQ_transcript/151030	3.57670095526857	-0.382542019198185	1.82613436646507	0.0770047306957377	0.799315037099943	-4.36265744639367
a_HQ_transcript/120403	3.2373706363401	0.432242290016894	1.82613107771935	0.0770052377467717	0.799315037099943	-4.36844597405387
a_HQ_transcript/135324	-2.14446788168202	4.71153270609897	-1.82603661470164	0.0770198030468352	0.799315037099943	-4.73207995775954
a_HQ_transcript/9969	0.647033776070003	3.73810211752688	1.82581071557115	0.0770546440743312	0.799315037099943	-4.62224854042297
a_HQ_transcript/38551	-2.51013852507843	-0.0828760673050801	-1.82579183286433	0.0770575570133243	0.799315037099943	-4.34374724046589
a_HQ_transcript/111017	2.90997520619798	0.509659500332355	1.82575457612336	0.0770633046963933	0.799315037099943	-4.28379814948755
a_HQ_transcript/135633	3.2527865553106	0.843747716783671	1.82561862092871	0.0770842819180281	0.799315037099943	-4.38586370814843
a_HQ_transcript/96338	1.12409658728247	2.6483967490009	1.82560156544741	0.0770869138390845	0.799315037099943	-4.3064657839505
a_HQ_transcript/113921	-2.60436447510974	3.47050321599351	-1.82559577178932	0.0770878079063838	0.799315037099943	-4.4777666192675
a_HQ_transcript/128310	-1.74962782728676	1.34758796619019	-1.8253218258271	0.0771300928684605	0.799568610218756	-4.23894843802098
a_HQ_transcript/105846	1.52726358821826	2.73957334786262	1.82521213759995	0.0771470293520806	0.799568610218756	-4.42325918938666
a_HQ_transcript/1798	3.00636205740152	-0.044506677021253	1.82517004831894	0.0771535290172895	0.799568610218756	-4.34335879774103
a_HQ_transcript/70394	2.20683217361118	1.00279569899215	1.82505550041777	0.0771712205177136	0.799588945962416	-4.22153706445951
a_HQ_transcript/122181	-1.94311223215894	0.372252255336103	-1.8249792268214	0.0771830026113941	0.799588945962416	-4.36681246074547
a_HQ_transcript/7675	3.40998194817662	-0.0556828742896421	1.82434147016668	0.077281577852412	0.800395574006842	-4.33630465411058
a_HQ_transcript/144137	-2.59082170734737	0.23154039490066	-1.82429733139338	0.0772884041580719	0.800395574006842	-4.35905713103856
a_HQ_transcript/61439	-1.8115178037885	0.516068263643863	-1.82402505612182	0.0773305244032957	0.80041675939782	-4.2410135574716
a_HQ_transcript/140901	2.37247402532471	0.184266614260073	1.82386089731706	0.0773559287795093	0.80041675939782	-4.34870878936917
a_HQ_transcript/130523	2.82948471713484	3.55500417740416	1.82361172170862	0.0773945035167786	0.80041675939782	-4.606651297133
a_HQ_transcript/145518	-2.26708288551517	2.04264054487158	-1.8236091491334	0.0773949018612243	0.80041675939782	-4.37397706681359
a_HQ_transcript/25389	-2.66234790643161	0.429077106745102	-1.82359940490319	0.0773964106998092	0.80041675939782	-4.30711117904427
a_HQ_transcript/86930	-2.22541107777913	1.11899581608043	-1.82350752032777	0.0774106397364175	0.80041675939782	-4.24332793542404
a_HQ_transcript/144901	-3.09641057291053	-0.15368551131684	-1.82331717759871	0.0774401228736164	0.80041675939782	-4.42367518548227
a_HQ_transcript/2118	-1.58627164803954	-0.29903459639157	-1.82329207107663	0.0774440124628408	0.80041675939782	-4.34564317108721
a_HQ_transcript/119509	-1.65051905403548	-0.407983484253209	-1.82327074444885	0.0774473165885797	0.80041675939782	-4.33141141740225
a_HQ_transcript/140504	0.791001735071447	4.04246398747441	1.82315331405216	0.0774655121845775	0.80041675939782	-4.66779838631611
a_HQ_transcript/6437	2.7715575827663	-0.222248815506227	1.82310964716215	0.0774722792078472	0.80041675939782	-4.3576129511956
a_HQ_transcript/128575	-3.86624742208666	0.13488649381735	-1.82309595650846	0.0774744009413311	0.80041675939782	-4.36511860658302
a_HQ_transcript/109214	-2.98506820864436	0.927260206494384	-1.82294671959651	0.0774975324109261	0.80041675939782	-4.3066010471834
a_HQ_transcript/8081	-2.97142483230341	-0.250456764150659	-1.82285432887093	0.077511855767688	0.80041675939782	-4.38834974718889
a_HQ_transcript/98383	2.4233786746381	1.11857209316573	1.82275292352957	0.0775275792599373	0.80041675939782	-4.31346898494874
a_HQ_transcript/83374	-2.44229530427425	1.34410949305648	-1.82271383832745	0.0775336403753307	0.80041675939782	-4.35844920935024
a_HQ_transcript/69480	-2.45369623193978	1.51668825814021	-1.82266888970257	0.0775406112569795	0.80041675939782	-4.47338915024913
a_HQ_transcript/100988	-2.01404047795669	0.839887937641553	-1.82263162125052	0.0775463914599729	0.80041675939782	-4.26311298617915
a_HQ_transcript/67772	-2.23733686370735	0.61032292603965	-1.82251585842798	0.0775643481999256	0.80041675939782	-4.23821791288264
a_HQ_transcript/146624	-2.0388416362517	1.60617774876464	-1.82237330722206	0.0775864651328715	0.80041675939782	-4.39224255449008
a_HQ_transcript/91074	2.9671657784877	1.70910732008756	1.82236627490259	0.077587556341937	0.80041675939782	-4.34962827675999
a_HQ_transcript/87217	-4.42130494967913	1.42972748683204	-1.8223286961363	0.0775933876824717	0.80041675939782	-4.35494676852603
a_HQ_transcript/127804	-2.63999224170551	0.320880979513164	-1.82204548445964	0.0776373474894777	0.800479090756072	-4.379421709256
a_HQ_transcript/90118	-1.66124874972667	2.81151432002053	-1.82181401441396	0.0776732917785369	0.800479090756072	-4.42904897327118
a_HQ_transcript/3995	-2.92117257998819	0.337332934072017	-1.82177862427382	0.0776787886570084	0.800479090756072	-4.34833139015781
a_HQ_transcript/149695	2.9625527662568	2.7439247427802	1.82175498682749	0.0776824602645381	0.800479090756072	-4.33590510805739
a_HQ_transcript/1221	2.27033348460725	2.79261181787914	1.8217089734841	0.0776896079471634	0.800479090756072	-4.52459499328223
a_HQ_transcript/81688	-4.01072397024172	-0.11103778786561	-1.82169659139635	0.0776915314682676	0.800479090756072	-4.30671957174344
a_HQ_transcript/125314	-1.37258012336152	2.35320867663319	-1.82164939001466	0.0776988644370716	0.800479090756072	-4.46047389736609
a_HQ_transcript/128526	2.12965227563513	2.01637333561888	1.82145000382882	0.077729846578698	0.800479090756072	-4.53412103903173
a_HQ_transcript/117377	1.80930602922036	-0.508145212703932	1.82131749059622	0.0777504433154302	0.800479090756072	-4.33098436641365

a_HQ_transcript/36812	1.29295122863198	1.71494243208297	1.82126869672178	0.0775580285942955	0.800479090756072	-4.31231852196691
a_HQ_transcript/121715	0.973424913768739	1.98893609004277	1.82117989372948	0.07777183513104	0.800479090756072	-4.31304742355629
a_HQ_transcript/3930	-0.78107996884057	5.96884583713672	-1.82117798278371	0.0777721322559581	0.800479090756072	-4.89120158305431
a_HQ_transcript/152759	2.76350481597836	-0.313518101115085	1.8210766040045	0.0777878966027929	0.800479090756072	-4.36431856956451
a_HQ_transcript/30127	0.587406567172288	2.94347193320591	1.82102981004907	0.0777951739566831	0.800479090756072	-4.52500185382509
a_HQ_transcript/112457	-0.887625123689232	3.729969682685	-1.82096023840296	0.0778059947490332	0.800479090756072	-4.61503999877478
a_HQ_transcript/149886	-2.82896695469031	2.23094684760744	-1.82071336533192	0.077844402383485	0.800732511369176	-4.40306971758924
a_HQ_transcript/26398	-1.76133470282602	0.680434841828755	-1.82032472223482	0.0779048988287146	0.801155221774471	-4.31674028303988
a_HQ_transcript/1752	-2.61843429428036	-0.0149438384318723	-1.82012797447454	0.0779355399868401	0.801155221774471	-4.28213345405781
a_HQ_transcript/77192	-3.023410660937	1.2931436570815	-1.82008079973514	0.0779428884256908	0.801155221774471	-4.35505458960619
a_HQ_transcript/30018	1.87198612698378	-0.452245556401205	1.82002420007878	0.0779517057688123	0.801155221774471	-4.33044975182808
a_HQ_transcript/65005	0.828508145874242	2.56447588142834	1.81997956068096	0.0779586604897443	0.801155221774471	-4.439864128769
a_HQ_transcript/115980	2.54104381182885	0.3318886673703	1.81991838471839	0.0779681924301229	0.801155221774471	-4.35503438128018
a_HQ_transcript/145627	2.86387479132949	0.261568126923952	1.81979500413004	0.0779874196095939	0.801211157134407	-4.39777937649965
a_HQ_transcript/102550	-2.02600299194588	0.879496909032203	-1.81966177456634	0.0780081861533838	0.801223686599965	-4.36724598449061
a_HQ_transcript/148210	2.81434406286658	-0.0726701528624422	1.81960134998022	0.0780176061098127	0.801223686599965	-4.35580191869256
a_HQ_transcript/98293	0.777172063861298	2.70626853493531	1.8192948512628	0.0780654029863639	0.801223686599965	-4.40055237491463
a_HQ_transcript/47173	-1.70386522663097	-0.558508110036621	-1.81928025789672	0.0780676793677179	0.801223686599965	-4.3604889455498
a_HQ_transcript/30810	-2.25566801256421	2.58337607058456	-1.8191158053906	0.078093335797371	0.801223686599965	-4.42765656522983
a_HQ_transcript/37292	0.705818899360594	5.86376762994772	1.81908557924516	0.0780980521953631	0.801223686599965	-4.85798448965443
a_HQ_transcript/92016	-0.810390114262154	3.09567141031294	-1.81908402252122	0.0780982951085142	0.801223686599965	-4.5387055690876
a_HQ_transcript/138169	-1.26823290506852	2.1515821310974	-1.81901808688275	0.0781085843790441	0.801223686599965	-4.27442808010753
a_HQ_transcript/11044	-1.95415468719519	-0.20187829457883	-1.81889332396727	0.0781280568186237	0.801223686599965	-4.32875528056682
a_HQ_transcript/128427	-2.38452414070999	2.35641625850375	-1.81879653835318	0.0781431655339131	0.801223686599965	-4.4697061363994
a_HQ_transcript/135381	-3.98876206086857	1.79422725941235	-1.81863419236182	0.0781685141386521	0.801223686599965	-4.39207315386364
a_HQ_transcript/140185	-2.08614892424569	2.39420250571877	-1.8185964874109	0.0781744023690329	0.801223686599965	-4.31585995329759
a_HQ_transcript/84744	6.95518073871925	2.71955762505698	1.81855456512815	0.0781809496467147	0.801223686599965	-4.43677186603605
a_HQ_transcript/103767	-1.413415566682	3.02749309557518	-1.8185503233525	0.0781816121386424	0.801223686599965	-4.45777509795534
a_HQ_transcript/137796	3.92677198126556	0.204772389993267	1.81795955976824	0.078273925905536	0.801536146172928	-4.3670428062632
a_HQ_transcript/4265	4.85736923743616	3.47005260556592	1.81795264585325	0.0782750068355651	0.801536146172928	-4.45336697514442
a_HQ_transcript/146846	3.34866003877072	2.96445488055571	1.81793455324319	0.0782778355167921	0.801536146172928	-4.55780143803732
a_HQ_transcript/85630	-1.76407567255263	-0.621895080148256	-1.81792976079169	0.078278584805116	0.801536146172928	-4.34279250373532
a_HQ_transcript/145657	-0.76322490605581	4.46820075793039	-1.81791401265835	0.0782810470310988	0.801536146172928	-4.75728099178282
a_HQ_transcript/11858	-2.15135991021514	-0.0986033434166181	-1.81781361269659	0.0782967461500424	0.801555699226782	-4.30387195151549
a_HQ_transcript/81975	2.66897366695494	0.965937744483734	1.81719596659926	0.078393383869256	0.8022299581527	-4.37336323167254
a_HQ_transcript/128667	-1.34662546534386	2.38224261124177	-1.81714791743112	0.0784009059618778	0.8022299581527	-4.47596181757118
a_HQ_transcript/120218	-1.59009681957967	1.22008191729605	-1.81712807991172	0.0784040117028883	0.8022299581527	-4.24835735055178
a_HQ_transcript/28292	2.32473956889679	0.816405139883744	1.81684658739242	0.0784480931663251	0.802539732413261	-4.29929482284337
a_HQ_transcript/116269	2.09246027065868	0.114035498783948	1.81674250868266	0.0784643971379218	0.802565278344733	-4.33321723642108
a_HQ_transcript/13519	1.63091604125577	1.37010808717863	1.8164804193197	0.0785054663242722	0.802717833616455	-4.26378176961078
a_HQ_transcript/121575	2.56571664645175	0.291308088941995	1.81644072985681	0.0785116872258841	0.802717833616455	-4.3573641270036
a_HQ_transcript/37334	-2.00028705231764	1.20396813358925	-1.8163829747931	0.0785207404683313	0.802717833616455	-4.28985847378762
a_HQ_transcript/130698	2.52730322140028	2.85544114130561	1.81620826831955	0.0785481315318367	0.802854335037334	-4.55335364122913
a_HQ_transcript/30088	2.04814684543389	2.27680648116109	1.81612163941913	0.0785617165168654	0.802854335037334	-4.3624399608642
a_HQ_transcript/133221	1.81164508994173	1.12140340361889	1.81603026332263	0.0785760481164866	0.802859645792781	-4.26172616844684
a_HQ_transcript/14745	-1.93115548541074	2.14362243350804	-1.81519947487402	0.0787064527063657	0.803978769128344	-4.3775348890659
a_HQ_transcript/18173	-2.39579463156943	0.496589997673425	-1.81515627045809	0.0787132393185626	0.803978769128344	-4.36623825069434
a_HQ_transcript/26981	2.07947660721488	0.34087780804192	1.81503222761374	0.078732726916762	0.804036533769772	-4.25170386271579
a_HQ_transcript/83236	1.54743129032324	1.86470524103511	1.81489317309131	0.078754577789228	0.804118408173589	-4.30226766583369
a_HQ_transcript/62287	2.26568194343107	0.992495135582673	1.81470567881689	0.0787840486037937	0.80427804307013	-4.28943413908694

a_HQ_transcript/84070	1.98647011248977	-0.113574799908669	1.81451669870501	0.0788137624658599	0.804312635675134	-4.34614142628943
a_HQ_transcript/42276	1.76681695213443	-0.652498094801829	1.81444488306442	0.0788250567375968	0.804312635675134	-4.37272060987902
a_HQ_transcript/108728	-2.90996760272705	1.25774674693178	-1.81418312652722	0.0788662342173094	0.804312635675134	-4.41353660885919
a_HQ_transcript/47648	-2.84711763334868	0.41610282933015	-1.81403289890851	0.0788898751138786	0.804312635675134	-4.28786089464861
a_HQ_transcript/141006	-3.41653165711937	1.32373535624253	-1.81402770884083	0.0788906919679794	0.804312635675134	-4.375140529874
a_HQ_transcript/41046	-1.600319954374	1.25131219464718	-1.81390549506121	0.0789099290248491	0.804312635675134	-4.31446739827016
a_HQ_transcript/143087	-2.65274298292082	3.17941076392655	-1.81382502626761	0.0789225973931583	0.804312635675134	-4.56003563909006
a_HQ_transcript/134919	-4.2134192208395	3.97300876670335	-1.8137190759599	0.0789392800090697	0.804312635675134	-4.62245008120597
a_HQ_transcript/2630	-0.963870484436564	5.06783333991372	-1.81371670451555	0.0789396534438865	0.804312635675134	-4.81890606673595
a_HQ_transcript/128429	3.62875428645255	4.76402907584414	1.81365437600023	0.0789494689456514	0.804312635675134	-4.62035643099169
a_HQ_transcript/127333	-1.71344658557012	-0.160728892781262	-1.81352203976708	0.078970312715291	0.804312635675134	-4.30481997712841
a_HQ_transcript/22107	1.88832772132609	1.09564104805572	1.81349521386439	0.0789745385306812	0.804312635675134	-4.26536480468009
a_HQ_transcript/10652	-1.54252853186576	0.0975541521949087	-1.81349274505482	0.0789749274455695	0.804312635675134	-4.28450540391094
a_HQ_transcript/151504	2.68189929915529	0.190676286135363	1.81342519181364	0.0789855698309659	0.804312635675134	-4.39897665425626
a_HQ_transcript/143394	-2.72852725908607	-0.153346421040824	-1.8133319919942	0.0790002546001746	0.804312635675134	-4.43036550465636
a_HQ_transcript/25115	-2.35969949137999	-0.0447709007416408	-1.8132219065584	0.0790176028976552	0.804312635675134	-4.2809504462451
a_HQ_transcript/37764	-1.81420476164541	1.68326609412666	-1.81318978929385	0.0790226648489659	0.804312635675134	-4.28527080358667
a_HQ_transcript/9569	1.33679825349679	4.09692808790998	1.81301609660685	0.07905004506145	0.804346571320297	-4.6927590237326
a_HQ_transcript/134511	3.39111929799664	1.93009426288828	1.81275945971832	0.0790905150403651	0.804346571320297	-4.55360881308861
a_HQ_transcript/112567	-1.82452800138303	0.336903385936481	-1.81243774519398	0.0791412722474244	0.804346571320297	-4.27281062531829
a_HQ_transcript/136087	-0.731585108094347	2.63089269859537	-1.81237538645861	0.0791511138512359	0.804346571320297	-4.50159516498037
a_HQ_transcript/99787	-3.58190282713541	0.123956765996024	-1.8123662024278	0.0791525633847911	0.804346571320297	-4.35873300910631
a_HQ_transcript/148241	-0.731660673665003	4.80549466754517	-1.81233985803326	0.0791567214974788	0.804346571320297	-4.78230503866325
a_HQ_transcript/42064	1.07972560751198	2.43146028832245	1.81227590449048	0.0791668164879599	0.804346571320297	-4.35245764396494
a_HQ_transcript/98092	1.97559013724307	-0.557423684914346	1.81213101574158	0.0791896910569944	0.804346571320297	-4.36869116216161
a_HQ_transcript/21553	1.65980169258799	-0.653892250828849	1.8118813594601	0.0792291191819516	0.804346571320297	-4.36405366679713
a_HQ_transcript/82959	2.88727002803915	0.0543227739926211	1.81184806046447	0.0792343793425443	0.804346571320297	-4.35847867416103
a_HQ_transcript/12520	2.18929243728539	0.0211726726475276	1.81181382190723	0.0792397882332456	0.804346571320297	-4.27905243715769
a_HQ_transcript/64039	1.41432129188923	0.0847835269673523	1.81165912890058	0.0792642300245394	0.804346571320297	-4.30352996917751
a_HQ_transcript/92873	-2.45509212580824	-0.417635227473943	-1.81165085234257	0.0792655379173334	0.804346571320297	-4.37302063778061
a_HQ_transcript/98710	2.33474044632088	-0.166181544139596	1.8114650592395	0.0792949024748183	0.804346571320297	-4.37311016278449
a_HQ_transcript/112237	2.50833316733949	1.56112257782606	1.81135092702189	0.0793129456348591	0.804346571320297	-4.35618107378945
a_HQ_transcript/131519	2.8540139837536	0.376398290418633	1.81134412653098	0.0793140208346012	0.804346571320297	-4.41166438286125
a_HQ_transcript/28001	-1.48211986986823	0.414857552994847	-1.81129664627445	0.0793215281028831	0.804346571320297	-4.31877751097905
a_HQ_transcript/53602	2.69183968386553	0.969543823450721	1.81128403419413	0.079323522344338	0.804346571320297	-4.30533770749276
a_HQ_transcript/17414	2.48963874944659	-0.308835196008169	1.81125314769559	0.0793284063449526	0.804346571320297	-4.35843403150706
a_HQ_transcript/72974	-3.32919959616556	0.783687025197991	-1.81119908357249	0.0793369559781629	0.804346571320297	-4.30655408995604
a_HQ_transcript/149352	0.533936616660055	8.78461794121947	1.81111917630524	0.0793495938497746	0.804346571320297	-5.15091037431199
a_HQ_transcript/44496	-1.82041375508445	0.610396028415389	-1.81099256715576	0.0793696214450925	0.804346571320297	-4.24752731766155
a_HQ_transcript/95671	-2.17055662326376	-0.330427402753755	-1.81089580215434	0.0793849310605538	0.804346571320297	-4.3693315655677
a_HQ_transcript/59075	5.21397110143733	2.1763702529228	1.81084575226203	0.0793928506594392	0.804346571320297	-4.51510601992283
a_HQ_transcript/73505	-2.02379142400336	0.558808025714522	-1.81084432196636	0.0793930769908435	0.804346571320297	-4.39020292488513
a_HQ_transcript/37669	-1.09940623698395	2.13330646787314	-1.81081743705304	0.0793973313883203	0.804346571320297	-4.37437572076723
a_HQ_transcript/69422	-1.23863985679564	1.73789714350332	-1.81072942390279	0.0794112603677271	0.804346571320297	-4.31655031216032
a_HQ_transcript/62736	3.8034230955146	0.1771173958998	1.81071559475445	0.079413449160597	0.804346571320297	-4.3469652944222
a_HQ_transcript/112850	-1.80355478676451	-0.5724367726203	-1.81037572307758	0.0794672581010547	0.80469940666891	-4.35736582917075
a_HQ_transcript/121167	2.46455598739708	1.11700186514682	1.81032070291098	0.0794759718842247	0.80469940666891	-4.25347608624252
a_HQ_transcript/131132	-4.33053538071609	1.07836163878245	-1.81001515309533	0.0795243779309633	0.804845528484947	-4.47915825741376
a_HQ_transcript/97275	4.35223548029333	1.23584031531763	1.81000138727857	0.0795265593400245	0.804845528484947	-4.42833159694924
a_HQ_transcript/108970	-3.66635115547212	2.32887664373873	-1.80996742226335	0.0795319418458839	0.804845528484947	-4.41286925557619

a_HQ_transcript/24894	1.16553351793909	2.36418444305489	1.80973201706084	0.0795692555106867	0.80508297377288	-4.44644446624528
a_HQ_transcript/135017	3.14049678270283	-0.00117346866317596	1.80938559317829	0.0796241936333303	0.805498629919635	-4.43255141052197
a_HQ_transcript/75777	-1.19713951039578	3.62920493254333	-1.80926000847783	0.0796441176280815	0.805559991210218	-4.66698197298769
a_HQ_transcript/44580	3.54038996517344	0.0722415862888714	1.80903868739419	0.0796792405083353	0.805775033449638	-4.3660629995538
a_HQ_transcript/152310	-2.43200114833763	-0.35340165201717	-1.80888194261573	0.0797041233238806	0.805886463832063	-4.41086386859089
a_HQ_transcript/12389	2.56853434327189	0.839880994674173	1.80784266753414	0.079869272713067	0.807415840741766	-4.32321718883528
a_HQ_transcript/32851	2.16242455831297	0.820398790445305	1.80772231248998	0.0798884169117297	0.807430666340338	-4.27684229991122
a_HQ_transcript/152182	-3.7195918068315	1.77115484432246	-1.80745289591349	0.0799312856333879	0.807430666340338	-4.50550354844191
a_HQ_transcript/142371	-1.80218403520172	-0.115914068144346	-1.80741602245993	0.0799371543433787	0.807430666340338	-4.51346530611164
a_HQ_transcript/14379	-1.67435423624329	1.88673736123953	-1.80740965959177	0.0799381670826576	0.807430666340338	-4.35883270721925
a_HQ_transcript/148575	0.652537413339035	9.42971850783569	1.80739356677673	0.0799407285274847	0.807430666340338	-5.25254244524098
a_HQ_transcript/124789	-4.24367032992085	0.515561715369153	-1.80730967388804	0.0799540826358207	0.807430666340338	-4.32056535868685
a_HQ_transcript/23116	3.3655579925264	2.99447234190436	1.80710866907035	0.0799860863920808	0.807613553899405	-4.62514913049443
a_HQ_transcript/126273	2.22423053439516	1.86901505512242	1.8069969608106	0.0800038771614857	0.807629048882995	-4.43274457888702
a_HQ_transcript/62433	2.4979179384214	0.247129270434581	1.80692456386511	0.0800154089684346	0.807629048882995	-4.25992160489636
a_HQ_transcript/37814	2.43849080151957	0.054095128696102	1.80670774351953	0.0800499538305085	0.807642940399894	-4.35443952106382
a_HQ_transcript/144554	-4.34930556762897	1.43383157661797	-1.8066773901108	0.0800547908935574	0.807642940399894	-4.38710133136522
a_HQ_transcript/64415	-2.95598518587093	0.591959090127803	-1.80661735248819	0.080064359109916	0.807642940399894	-4.48024476600802
a_HQ_transcript/99549	-2.83724499009193	1.42126569599282	-1.80647773156785	0.0800866143035541	0.807642940399894	-4.39233895837456
a_HQ_transcript/82706	1.50614193838671	0.996502855471899	1.80646419220418	0.0800887727208976	0.807642940399894	-4.24732217225784
a_HQ_transcript/81889	0.695986255091956	3.41450733461406	1.80639282623153	0.0801001505540427	0.807642940399894	-4.60842067087244
a_HQ_transcript/10418	2.16891726589808	-0.0519823613903587	1.80614662584044	0.0801394126800775	0.807733722802211	-4.32143992216873
a_HQ_transcript/18447	1.68586191354949	1.91916855643727	1.80599090612821	0.0801642540963827	0.807733722802211	-4.32053668211491
a_HQ_transcript/38354	-1.71174238000558	-0.340210189671687	-1.80596533875341	0.0801683333950921	0.807733722802211	-4.36195950387761
a_HQ_transcript/131379	-2.21224005923683	3.6735510842647	-1.80590071726016	0.0801786446018833	0.807733722802211	-4.72407562918052
a_HQ_transcript/44509	-2.27319029772827	1.55754151490414	-1.80588779953199	0.0801807059300495	0.807733722802211	-4.34715054906279
a_HQ_transcript/4680	1.94842539028091	0.156111127716591	1.80581371456691	0.0801925288035294	0.807733722802211	-4.41070024658549
a_HQ_transcript/25790	1.00035012025031	2.16554395251482	1.80565257087526	0.0802182500903468	0.807758829357755	-4.37311470592463
a_HQ_transcript/71476	2.52200902767721	0.748044234904434	1.80562398371108	0.0802228138226384	0.807758829357755	-4.32565334150869
a_HQ_transcript/130186	-3.19361621004436	4.94882128812722	-1.80532018913589	0.0802713260487898	0.808107315650165	-4.78504489035834
a_HQ_transcript/7147	-1.96330783373986	-0.308120214894032	-1.80505238760445	0.0803141113113145	0.808398036762613	-4.41024107689403
a_HQ_transcript/146192	-2.04673758750331	2.27001836738817	-1.80495018022529	0.080330445564764	0.808422461874758	-4.3823290584287
a_HQ_transcript/82703	-2.39585571012277	0.715689705155463	-1.80459142468701	0.0803878023684296	0.808859646195616	-4.4244009814557
a_HQ_transcript/30664	-2.1687267726849	0.069436493822335	-1.8042835442469	0.0804370531593886	0.809119376880606	-4.32865140304907
a_HQ_transcript/80637	1.25393814305548	2.19206716135474	1.80424925626724	0.0804425397004207	0.809119376880606	-4.32299234824577
a_HQ_transcript/52859	2.75092413610866	1.50001682727023	1.80416905445678	0.080455374318227	0.809119376880606	-4.39386315332427
a_HQ_transcript/139356	3.78876082342775	1.95227007459746	1.80407682283661	0.0804701362063747	0.809127845944326	-4.52646128661894
a_HQ_transcript/65095	1.68321408680362	0.508986626712512	1.80394177966894	0.0804917543363313	0.809205239720212	-4.23126837597448
a_HQ_transcript/22257	-3.04951582674223	-0.286674677791781	-1.8038058379518	0.080513521293986	0.809274757281571	-4.36991826731868
a_HQ_transcript/152762	-3.88449958142793	-0.294301970460072	-1.8036459025829	0.080539136514707	0.809274757281571	-4.41180000495384
a_HQ_transcript/10863	2.72389423236289	1.08281174025483	1.80344095263999	0.0805719713856276	0.809274757281571	-4.39403395415487
a_HQ_transcript/131732	-0.817762126800441	7.63408651488206	-1.80339155219392	0.0805798874939622	0.809274757281571	-5.06177544497418
a_HQ_transcript/103159	-2.55689181504532	1.97697205245796	-1.80338537830474	0.0805808768670304	0.809274757281571	-4.34755200205018
a_HQ_transcript/73880	2.00804096241862	0.650089451172316	1.80319555459198	0.0806113013808374	0.809274757281571	-4.33187548972441
a_HQ_transcript/127657	-2.70635216734969	0.887613960418976	-1.80316980224413	0.0806154296606053	0.809274757281571	-4.34003357893167
a_HQ_transcript/146229	-0.785262072500129	4.21137356916324	-1.80314560456711	0.0806193088795877	0.809274757281571	-4.77628887019707
a_HQ_transcript/29487	2.47579401405703	1.17442256756307	1.80308818236127	0.0806285150803786	0.809274757281571	-4.42732979760999
a_HQ_transcript/76653	0.725654420464026	4.68442088322553	1.8029056519022	0.0806577851641803	0.809274757281571	-4.76350613898867
a_HQ_transcript/109599	1.97097763602305	0.228963717332875	1.80277455595557	0.080678812921534	0.809274757281571	-4.3547952136267
a_HQ_transcript/55366	2.7881448853187	-0.155475563563733	1.80277447108916	0.080678826535595	0.809274757281571	-4.38687111367609

a_HQ_transcript/17458	-1.19453658330045	3.83924449457823	-1.80276928174151	0.0806796590016292	0.809274757281571	-4.64735390314833
a_HQ_transcript/52211	-2.22354977580073	0.376863613976231	-1.80267082419784	0.0806954547708744	0.809293546397755	-4.42446306636463
a_HQ_transcript/31905	1.34357776715727	3.93115491966586	1.80247186979593	0.0807273815015336	0.809368313678912	-4.67312318409999
a_HQ_transcript/31368	4.11785935853173	2.28273594165859	1.802363157761	0.0807448313389728	0.809368313678912	-4.47235443958801
a_HQ_transcript/109511	-3.94311374056367	0.943362187322438	-1.80234531207411	0.080747696133334	0.809368313678912	-4.31415040984257
a_HQ_transcript/113253	2.16487842472848	0.144846793395641	1.80211030421467	0.0807854303485618	0.809368313678912	-4.40230775562367
a_HQ_transcript/111662	-2.21993390740596	0.917530874921372	-1.80204789632731	0.0807954534356212	0.809368313678912	-4.28448204684858
a_HQ_transcript/25667	1.60836822864754	1.36189233044805	1.80203836777154	0.0807969838727234	0.809368313678912	-4.27629739964335
a_HQ_transcript/119121	-1.93532284397144	2.46757394393899	-1.80200282823042	0.0808026923036602	0.809368313678912	-4.45335735459619
a_HQ_transcript/59090	1.91881504574472	-0.23089425173386	1.80193056096981	0.0808143010699216	0.809368313678912	-4.31495995037193
a_HQ_transcript/70859	0.863602261466535	3.49092124066996	1.8016341468937	0.0808619308428712	0.809705825328927	-4.53703207607499
a_HQ_transcript/36889	-2.27686646838098	-0.09058725571334	-1.80140211169336	0.080899232432808	0.809884639236382	-4.33265656461771
a_HQ_transcript/84863	0.697355975925549	3.7739653469365	1.80129593680551	0.0809163058042812	0.809884639236382	-4.69390209906311
a_HQ_transcript/40946	-1.30043252751605	1.1784274569775	-1.80126310088286	0.0809215865793578	0.809884639236382	-4.27040607413151
a_HQ_transcript/129082	-1.02784614494961	2.79754996854269	-1.80101229748877	0.0809619312196969	0.810148930614312	-4.59334691687763
a_HQ_transcript/101522	2.134070554385	2.70019058419783	1.80079879814261	0.0809962885333581	0.810230324600987	-4.37030218015397
a_HQ_transcript/96382	-1.74785354277904	0.0476319587760003	-1.80078852062834	0.0809979427514508	0.810230324600987	-4.2900644623963
a_HQ_transcript/26388	-1.9462209719172	0.104920371437984	-1.80060162826827	0.0810280290319982	0.810275505428038	-4.3982586561708
a_HQ_transcript/76412	-3.34322688304099	0.158732465123049	-1.80054037790215	0.0810378912952943	0.810275505428038	-4.31101040372785
a_HQ_transcript/74522	1.86158209173976	3.1985404774842	1.80044126142979	0.0810538527517624	0.810275505428038	-4.57360701703321
a_HQ_transcript/101691	-2.74236269330548	-0.199353465153517	-1.80041416066751	0.081058217452244	0.810275505428038	-4.40335903079319
a_HQ_transcript/57452	-2.081669767866	0.682377431637706	-1.80030700518215	0.0810754772837597	0.810308690431635	-4.29863078691817
a_HQ_transcript/141640	-1.82318853173849	2.86577584138531	-1.80008005935608	0.081112042385378	0.810534777338362	-4.5960736783096
a_HQ_transcript/123843	-2.51979399234678	-0.136274706665812	-1.79986401012703	0.0811468648605443	0.810619345955696	-4.39780269509329
a_HQ_transcript/28934	0.892974390550436	3.05968492031727	1.79985451106003	0.081148396196604	0.810619345955696	-4.43621785864744
a_HQ_transcript/122604	-2.53083715390073	-0.376963278827957	-1.79962090230212	0.0811860637796175	0.810694978103866	-4.37382433474799
a_HQ_transcript/92728	-2.51261463414198	1.74580772534216	-1.79953435661433	0.0812000223634996	0.810694978103866	-4.38053321847104
a_HQ_transcript/98356	-6.89010534740163	2.20012748358969	-1.79950178373461	0.0812052764318928	0.810694978103866	-4.35755083556765
a_HQ_transcript/92208	1.06565438015747	3.70448069219683	1.79946162592806	0.0812117543610448	0.810694978103866	-4.63363073819274
a_HQ_transcript/118461	-3.1505891628403	0.509069042722821	-1.7992228823716	0.0812502755817844	0.810940250518194	-4.41645728916176
a_HQ_transcript/88484	1.16393212920237	2.06322519118911	1.79905444865762	0.0812774616595982	0.811072324695129	-4.44550340316219
a_HQ_transcript/14599	-2.00446893605111	0.260664484775824	-1.7988129660774	0.0813164516813968	0.811322125529734	-4.27493896949054
a_HQ_transcript/118963	5.71590784637404	2.06868349112142	1.79872466050386	0.0813307135543808	0.81132516174516	-4.53134410146355
a_HQ_transcript/123028	1.77077794673173	3.13030727379031	1.79830506279619	0.0813985100984906	0.811748442276855	-4.55610684158931
a_HQ_transcript/149547	1.97818798310037	-0.302096956774502	1.79827611879752	0.0814031884969831	0.811748442276855	-4.35793118917558
a_HQ_transcript/126478	-1.43675487489293	3.90288107204326	-1.79816545727711	0.0814210775120292	0.811748442276855	-4.67173340212524
a_HQ_transcript/12821	-1.91232484345262	1.91850436776259	-1.79787130941081	0.0814686442870857	0.811748442276855	-4.46606956350871
a_HQ_transcript/48062	-2.31436237874899	-0.217650659493161	-1.79786960181644	0.0814689204917605	0.811748442276855	-4.36696568769264
a_HQ_transcript/84888	-4.07346474811935	0.0951511113386983	-1.79785049209896	0.0814720115571871	0.811748442276855	-4.40297288965778
a_HQ_transcript/73679	2.52420112728524	-0.202425258592733	1.79777824810479	0.0814836981825373	0.811748442276855	-4.38640813990813
a_HQ_transcript/141619	-3.09157753824375	-0.146525223013046	-1.79772022627143	0.081493085175772	0.811748442276855	-4.39391598890006
a_HQ_transcript/41590	-2.23142422570538	1.87188818991494	-1.79768472842935	0.081498828604592	0.811748442276855	-4.4180419964737
a_HQ_transcript/152110	-1.89273765798017	-0.0723277877716991	-1.79720102999243	0.0815771236031065	0.811861669792274	-4.32241570138233
a_HQ_transcript/11360	2.35790450445154	0.415643580552086	1.797169668134	0.0815822022687172	0.811861669792274	-4.33137807264583
a_HQ_transcript/30949	1.78096691262667	0.264217032126878	1.79707052669711	0.0815982587654852	0.811861669792274	-4.34298589454893
a_HQ_transcript/105983	-2.30034831772486	0.339101881386242	-1.79704213258701	0.0816028578407754	0.811861669792274	-4.3416342630314
a_HQ_transcript/89371	-2.43055303773881	2.36082638570602	-1.79695632771505	0.0816167572388861	0.811861669792274	-4.47095159342016
a_HQ_transcript/66822	-2.55688380179153	-0.21617752493418	-1.79691403692559	0.0816236085976848	0.811861669792274	-4.39667708104955
a_HQ_transcript/100171	2.89048767548991	0.0211543439860478	1.7968401037929	0.0816355873767715	0.811861669792274	-4.4317913748086
a_HQ_transcript/112529	0.847799499600378	2.8348293652178	1.79678677895842	0.0816442280887089	0.811861669792274	-4.52998342905387

a_HQ_transcript/100793	-1.21196493385146	3.0779860385725	-1.79660715546108	0.0816733398440364	0.811861669792274	-4.58245396605369
a_HQ_transcript/126926	2.04807541487241	0.191989981561672	1.79655406406613	0.0816819461052281	0.811861669792274	-4.28996100237464
a_HQ_transcript/1453	0.717109535660667	4.91108927372037	1.79642869567244	0.0817022717222027	0.811861669792274	-4.80740947966061
a_HQ_transcript/145425	2.25341930716769	-0.0597320726558474	1.79634953048019	0.0817151087585685	0.811861669792274	-4.38332441661246
a_HQ_transcript/41670	-2.3717292661035	-0.23542238775605	-1.79624878079911	0.0817314483050058	0.811861669792274	-4.38733792679152
a_HQ_transcript/54224	-2.83610561537655	0.438598717257511	-1.79597990366591	0.0817750682783241	0.811861669792274	-4.48891256275845
a_HQ_transcript/73247	2.21698794462003	1.81802540181115	1.7959643594013	0.0817775906305759	0.811861669792274	-4.26618341800589
a_HQ_transcript/30821	1.73882527508463	1.88850711723041	1.79590401701636	0.0817873829547587	0.811861669792274	-4.39747460234207
a_HQ_transcript/22935	-2.03973872016469	-0.109600217189273	-1.79584062951143	0.0817976705110006	0.811861669792274	-4.34412615689587
a_HQ_transcript/51696	-2.38819641882215	0.102808367905956	-1.79574996605699	0.0818123867614117	0.811861669792274	-4.3924196668098
a_HQ_transcript/4130	2.53794392881124	0.718221877590833	1.79571954430221	0.0818173252431379	0.811861669792274	-4.45304223247545
a_HQ_transcript/151713	-0.308746514621966	11.0267166558663	-1.79571653103481	0.0818178144123211	0.811861669792274	-5.4542372300204
a_HQ_transcript/13324	2.45211541789507	0.750180574790541	1.79568723470479	0.0818225704627717	0.811861669792274	-4.33425117779704
a_HQ_transcript/93104	-2.18285936327297	0.839208717158304	-1.79567596096341	0.0818244007368883	0.811861669792274	-4.34611332947524
a_HQ_transcript/126341	1.31078206872241	3.88709149235641	1.79552458260756	0.081848980140265	0.811861669792274	-4.71665917432738
a_HQ_transcript/148358	2.19918197830333	1.8189038554047	1.7954957183254	0.081853667564117	0.811861669792274	-4.48252277935995
a_HQ_transcript/18466	-2.05087940597973	-0.429951944172726	-1.79546062821128	0.0818593663407053	0.811861669792274	-4.34495882364258
a_HQ_transcript/134239	-4.29956933906109	0.74222841163798	-1.79505547429129	0.0819251893720974	0.812375879873981	-4.44773004167278
a_HQ_transcript/110945	1.6444196931578	-0.0410003526258824	1.79491876867858	0.0819474092815808	0.81245730673254	-4.32446923838517
a_HQ_transcript/15286	2.43882118885623	0.440481495934482	1.79470609805319	0.0819819865891068	0.81245730673254	-4.39102364180662
a_HQ_transcript/82511	-2.00912258212301	-0.225964250554154	-1.79470188054241	0.081982672423322	0.81245730673254	-4.41491505578379
a_HQ_transcript/6915	1.69786079842621	1.38371453385205	1.79461185122401	0.08199731378133	0.81245730673254	-4.28240152575307
a_HQ_transcript/72269	4.59064992192583	2.45259940077485	1.79457513107185	0.0820032861718932	0.81245730673254	-4.57442921943109
a_HQ_transcript/130768	1.25279190718581	2.39026242676459	1.79437437714523	0.0820359445474391	0.812600098978917	-4.36404851196331
a_HQ_transcript/150749	-2.36702965249713	0.0673067229988002	-1.79429020681673	0.0820496405455998	0.812600098978917	-4.41203109056032
a_HQ_transcript/30022	3.09889168082379	3.36586892973451	1.79406764675419	0.0820858643452743	0.812600098978917	-4.57697119708549
a_HQ_transcript/146503	-8.02905830876347	3.18005719012344	-1.79406248319114	0.0820867049260851	0.812600098978917	-4.47073875084714
a_HQ_transcript/28622	2.14185906201956	0.157744552283701	1.79400699646717	0.0820957381179668	0.812600098978917	-4.4849248928633
a_HQ_transcript/76935	2.63142303313747	2.21601612921126	1.79397114281462	0.0821015755109961	0.812600098978917	-4.39900322534306
a_HQ_transcript/53489	1.67688797351756	0.513235113986492	1.79366049807642	0.0821521668470658	0.812659463497158	-4.28468857611757
a_HQ_transcript/135724	1.50719215869579	3.12476818188629	1.7936288100444	0.0821573290193346	0.812659463497158	-4.57705251312122
a_HQ_transcript/57711	-2.56275811223869	-0.270085446707623	-1.79345192060213	0.0821861504373597	0.812659463497158	-4.4029813266935
a_HQ_transcript/69647	1.96185004468233	0.975066521604285	1.79344234155864	0.0821877114402249	0.812659463497158	-4.32640810391091
a_HQ_transcript/119751	3.16026093679779	1.24138519206622	1.79343835897482	0.0821883604502941	0.812659463497158	-4.32489222087792
a_HQ_transcript/17309	1.55039480339975	2.26168424693289	1.79341936048965	0.0821914565424545	0.812659463497158	-4.36045802488809
a_HQ_transcript/43475	2.60463897353908	0.813249431133057	1.79322198821455	0.0822236272119565	0.812821577720625	-4.40197410430996
a_HQ_transcript/143905	-1.87671023578912	0.736335846311116	-1.79314720549908	0.0822358192020196	0.812821577720625	-4.32116510878473
a_HQ_transcript/136349	0.554633527106035	4.57929114847909	1.79300579976268	0.0822588770816046	0.812911255865269	-4.78653937998809
a_HQ_transcript/41882	0.835777004626623	3.06433536201208	1.79276151678565	0.0822987232365512	0.813166782983894	-4.53506839337427
a_HQ_transcript/8927	-2.05390953093187	-0.0872267372792493	-1.7924771122856	0.0823451344331494	0.81346092968995	-4.35970980466421
a_HQ_transcript/67867	1.08632330880423	3.965050818952	1.792364393198	0.0823635348975945	0.81346092968995	-4.68138962811605
a_HQ_transcript/91495	-1.26950951097412	1.69021497941296	-1.79232187802793	0.0823704760555162	0.81346092968995	-4.3539664784561
a_HQ_transcript/136391	3.53015206112987	-0.222359561996215	1.79216545220424	0.0823960188904844	0.813485913347222	-4.41092978406823
a_HQ_transcript/23820	-2.3861614775248	-0.0101013547918207	-1.79210403693721	0.0824060492506901	0.813485913347222	-4.3866012651214
a_HQ_transcript/73045	-0.930101322206059	1.74403206856954	-1.79204929811017	0.0824149900857038	0.813485913347222	-4.36845167129779
a_HQ_transcript/92363	-1.22303935362236	2.28409203579742	-1.79174212239741	0.082465178283751	0.813610392524358	-4.4550538819511
a_HQ_transcript/123142	6.11503983293517	2.20199198519319	1.79146781737078	0.0825100177839651	0.813610392524358	-4.50345573047542
a_HQ_transcript/140032	-3.60270443279607	2.7077921129609	-1.79141096436726	0.0825193138946113	0.813610392524358	-4.47537200651622
a_HQ_transcript/85231	5.46066229721047	0.216694855860309	1.79139856587226	0.0825213413071057	0.813610392524358	-4.41380201616922
a_HQ_transcript/75890	1.78350823836405	0.476637047689253	1.79123009828482	0.0825488934586345	0.813610392524358	-4.38350951283409

a_HQ_transcript/96630	-2.34404988549333	3.31934582063491	-1.79117220139337	0.0825583640483608	0.813610392524358	-4.63314258107033
a_HQ_transcript/22852	-1.92411133855873	-0.387802974362868	-1.79107448590926	0.0825743501273898	0.813610392524358	-4.34594290977327
a_HQ_transcript/135111	-2.77129567362481	0.18826810489919	-1.79100041469074	0.0825864697960875	0.813610392524358	-4.39741026323813
a_HQ_transcript/8931	1.84752949865343	-0.479113130196731	1.79096256280547	0.0825926637735038	0.813610392524358	-4.39629694083806
a_HQ_transcript/147235	3.84687528137083	3.49365690180363	1.79091579144354	0.0826003178535408	0.813610392524358	-4.48681874198653
a_HQ_transcript/149026	-3.14488970268053	-0.256987159676876	-1.79077679354304	0.0826230682522665	0.813610392524358	-4.4109354531414
a_HQ_transcript/6925	2.85873155655452	0.545727282323292	1.79075844065979	0.0826260725466741	0.813610392524358	-4.29237666676417
a_HQ_transcript/69694	1.94295788258754	4.33106882115978	1.79074106468177	0.0826289170109438	0.813610392524358	-4.80474805535311
a_HQ_transcript/117707	-2.84955055385576	0.56079685516195	-1.79070050657294	0.0826355567354413	0.813610392524358	-4.31992483874861
a_HQ_transcript/98557	2.3004541555057	0.113254850580886	1.79068830506872	0.0826375543191545	0.813610392524358	-4.29904977914513
a_HQ_transcript/101712	-2.07335345196407	0.780506811057556	-1.79054257647017	0.0826614156150645	0.813707496506768	-4.48240141932891
a_HQ_transcript/6959	1.20861158455601	0.0540719342739949	1.79044666180443	0.08267712367027	0.813724321826186	-4.33659142912864
a_HQ_transcript/1248	1.48302532114408	2.25000996905717	1.79009307862153	0.0827350522649426	0.814069801208965	-4.41828669925659
a_HQ_transcript/74921	-5.33590176792168	3.0509083424848	-1.78997840550705	0.0827538468961122	0.814069801208965	-4.67440295621337
a_HQ_transcript/133298	-3.24729334880524	0.733671729253914	-1.7899641438647	0.0827561845960764	0.814069801208965	-4.46343283986613
a_HQ_transcript/54943	-3.40717318388764	1.07681589257388	-1.78989057432063	0.0827682446522327	0.814069801208965	-4.60241012208608
a_HQ_transcript/103326	3.13287262587865	0.837928335216741	1.78957351998127	0.0828202355650133	0.814151109429794	-4.33661580415085
a_HQ_transcript/113077	-1.5512259624782	3.16315876490974	-1.7894075168837	0.0828474679762733	0.814151109429794	-4.43591785938287
a_HQ_transcript/70908	-1.42182858316056	0.66301173681143	-1.78923366665237	0.0828759958306091	0.814151109429794	-4.26801700791772
a_HQ_transcript/137493	-2.56378177779418	0.667085709053389	-1.78918676547932	0.0828836934783471	0.814151109429794	-4.35858040339668
a_HQ_transcript/81256	-2.90304260610885	0.359345601169261	-1.78914034350022	0.0828913130754359	0.814151109429794	-4.40944103578925
a_HQ_transcript/11995	-1.17801367538986	2.67782041464019	-1.78908662774129	0.082900130598069	0.814151109429794	-4.49596733368177
a_HQ_transcript/76318	-3.0196772405245	2.29485905802874	-1.7890668632515	0.0829033751689472	0.814151109429794	-4.43462448217531
a_HQ_transcript/150109	-2.6520040119998	0.151608648204952	-1.78900829697676	0.0829129901363948	0.814151109429794	-4.42625160101097
a_HQ_transcript/11842	-2.1487593756353	0.162144514042308	-1.78894092385353	0.0829240521150102	0.814151109429794	-4.31784451236755
a_HQ_transcript/118360	1.52033625808697	1.34028411231825	1.78890780625829	0.0829294901445807	0.814151109429794	-4.36881478841021
a_HQ_transcript/136187	-2.5652336270176	0.769660058060805	-1.7889011729601	0.082930579392613	0.814151109429794	-4.31308056215109
a_HQ_transcript/80074	2.456106848159	-0.026202219726076	1.78876981825076	0.0829521515319901	0.814188864613483	-4.35383724133778
a_HQ_transcript/103967	-0.776981500574805	2.8021652691844	-1.78852757881211	0.0829919465417217	0.814188864613483	-4.49308250974748
a_HQ_transcript/121345	-2.30598222306199	0.946884489436708	-1.78830642614314	0.0830282915573786	0.814188864613483	-4.35499377947282
a_HQ_transcript/115806	3.49129994704377	2.0067009913538	1.78828791580671	0.0830313342237777	0.814188864613483	-4.46475925993765
a_HQ_transcript/131356	2.525195438654	-0.0337229533527816	1.78823055257233	0.0830407639983528	0.814188864613483	-4.28871271403196
a_HQ_transcript/76645	-1.00024608809785	3.20221669592716	-1.78793066600207	0.0830900762576348	0.814188864613483	-4.63810117867473
a_HQ_transcript/23764	-2.59173306123216	-0.109295233485123	-1.78791890774517	0.083092010248395	0.814188864613483	-4.40711134213426
a_HQ_transcript/120244	1.69168253399708	4.08202899907347	1.78785755981977	0.0831021013358902	0.814188864613483	-4.75612611243763
a_HQ_transcript/35665	2.13083642011542	0.610551619432594	1.78773670126978	0.0831219843337663	0.814188864613483	-4.35944651059402
a_HQ_transcript/125827	-2.57822967046904	-0.0278851597265993	-1.787650337821	0.0831361948537852	0.814188864613483	-4.3016665576847
a_HQ_transcript/76190	-1.91827571853438	-0.525562754596542	-1.78751023885855	0.0831592515685843	0.814188864613483	-4.40354802112245
a_HQ_transcript/147997	2.29412924711912	0.0523915143191722	1.78742516770444	0.0831732547550492	0.814188864613483	-4.46821230272333
a_HQ_transcript/7333	2.67994023465333	0.4634982736397	1.78740126463965	0.0831771896925433	0.814188864613483	-4.34658624470249
a_HQ_transcript/28213	-2.925323309241	2.47278566469272	-1.7873356817352	0.0831879868018329	0.814188864613483	-4.45459171106023
a_HQ_transcript/47491	-1.41815843099438	2.94248126632885	-1.78722343162952	0.0832064696287335	0.814188864613483	-4.51644271652169
a_HQ_transcript/22095	-0.87224066200832	3.68106571965844	-1.78706374749015	0.0832327688254214	0.814188864613483	-4.74396493747324
a_HQ_transcript/115897	-5.59788649031042	0.166254040857548	-1.78701067762524	0.0832415107324305	0.814188864613483	-4.41790746559121
a_HQ_transcript/37500	2.25180802001734	0.356817099082885	1.78694190384347	0.0832528406170938	0.814188864613483	-4.39437150413601
a_HQ_transcript/117805	3.32003784526266	3.71195467687438	1.78690531359674	0.0832588690759183	0.814188864613483	-4.60194641624788
a_HQ_transcript/7430	1.0705590825819	1.68867090411726	1.78681216022105	0.0832742183137742	0.814188864613483	-4.35115209126355
a_HQ_transcript/138044	-5.14116645306848	3.44559784517235	-1.78676474663104	0.083282031753324	0.814188864613483	-4.58955771002931
a_HQ_transcript/131849	-0.790271061856068	2.90666905730667	-1.78673864845664	0.0832863328214303	0.814188864613483	-4.59254524005533
a_HQ_transcript/19817	-2.49740247207967	-0.335869423967888	-1.78667455176728	0.0832968969733756	0.814188864613483	-4.42924952692406

a_HQ_transcript/68585	0.544302160865299	3.69443253606139	1.78662207836575	0.0833055462684434	0.814188864613483	-4.70051279288177
a_HQ_transcript/108221	-1.06084608817224	2.76600004413368	-1.7864454663773	0.0833346631665427	0.814188864613483	-4.56560280643491
a_HQ_transcript/151937	-2.91597557461196	0.104264213723681	-1.78638820883769	0.0833441047051879	0.814188864613483	-4.40489044959775
a_HQ_transcript/121448	-2.57213867699396	0.179106503731198	-1.78633258749534	0.0833532773095949	0.814188864613483	-4.40354228955412
a_HQ_transcript/62814	-2.0502965738304	1.57039112506541	-1.78611305454956	0.0833894891917477	0.814188864613483	-4.41266999268039
a_HQ_transcript/67351	-1.93183980843509	2.00116999331468	-1.78611006560153	0.0833899823096611	0.814188864613483	-4.42030311159411
a_HQ_transcript/29199	-2.03547590957078	-0.472235735129731	-1.78602505222608	0.0834040088872227	0.814188864613483	-4.40195049346276
a_HQ_transcript/65271	3.0952988918495	0.514667629056737	1.78599465999843	0.0834090238649577	0.814188864613483	-4.50318562511204
a_HQ_transcript/104155	-2.79700305401913	4.15315632189296	-1.78596422258949	0.0834140465541241	0.814188864613483	-4.80442557890792
a_HQ_transcript/141251	-0.711136215676578	2.85630707931284	-1.78583434670826	0.0834354811597457	0.814188864613483	-4.47609732925525
a_HQ_transcript/151823	-2.64915949131861	2.86243014545373	-1.78579082833954	0.0834426644385552	0.814188864613483	-4.49890731387713
a_HQ_transcript/81632	2.69162490742539	0.846907710242548	1.78574950220058	0.0834494863461968	0.814188864613483	-4.37550886777924
a_HQ_transcript/10659	-2.24239793624643	1.5017424529981	-1.78574089502814	0.0834509072336714	0.814188864613483	-4.45881038823101
a_HQ_transcript/112117	1.20228803065354	3.27788511509787	1.78573016979202	0.0834526778035909	0.814188864613483	-4.68160658934783
a_HQ_transcript/37450	-2.28545252494642	1.2369560440637	-1.78543378740619	0.0835016185297639	0.814297230696113	-4.29298537434903
a_HQ_transcript/21475	1.60018342506712	2.30181212648682	1.78543192427251	0.0835019262603012	0.814297230696113	-4.44146415623289
a_HQ_transcript/93011	2.4239690279012	1.36091897613247	1.78535062959142	0.0835153544967367	0.814297230696113	-4.32634392216572
a_HQ_transcript/126407	0.606767270943291	5.01474663883018	1.78532359862935	0.0835198198701181	0.814297230696113	-4.83929903475351
a_HQ_transcript/6765	-2.37723999420913	0.00314194687112437	-1.78508306041565	0.0835595644348786	0.814548106904347	-4.43441163933255
a_HQ_transcript/105547	2.5105803152075	2.44196263885476	1.78499698517933	0.0835737906952731	0.814550185368013	-4.58755724794927
a_HQ_transcript/136627	-4.2819882933199	0.0130021381555378	-1.78480325907298	0.0836058166809221	0.814596117971266	-4.42906660249781
a_HQ_transcript/19948	2.64410445571985	1.24397711135916	1.78451899845464	0.0836528282757268	0.814596117971266	-4.38836383327788
a_HQ_transcript/33252	-2.10006405562252	0.20050958753742	-1.78451707270703	0.0836531468361226	0.814596117971266	-4.35544489814055
a_HQ_transcript/47944	-2.72966457043216	1.75961603383632	-1.78450673574607	0.0836548568110852	0.814596117971266	-4.44313713004863
a_HQ_transcript/102423	-2.65741634217962	2.50139157962257	-1.78446395012666	0.0836619348676253	0.814596117971266	-4.60747233698455
a_HQ_transcript/25440	-2.75304780200018	2.08708384715397	-1.78430384745867	0.0836884252721144	0.814596117971266	-4.46706997861495
a_HQ_transcript/47220	-1.93399417575195	0.182536309562278	-1.78429344248051	0.0836901471138272	0.814596117971266	-4.30924535444036
a_HQ_transcript/130569	-1.70375055462743	-0.198712794824846	-1.78429062053209	0.0836906141020576	0.814596117971266	-4.34557098125643
a_HQ_transcript/136046	-2.64641702038275	2.28274857894008	-1.78408707727917	0.0837243031432155	0.814658850334806	-4.53951611307621
a_HQ_transcript/124980	-2.32511069987643	0.317979539041	-1.78408233025422	0.0837250889743347	0.814658850334806	-4.39724382394213
a_HQ_transcript/46193	3.95893662605002	2.71803160381589	1.78386554884919	0.0837609820207036	0.814715035249252	-4.56112636124647
a_HQ_transcript/136416	-2.25593143017136	-0.0170101266857262	-1.78380885828407	0.0837703705712684	0.814715035249252	-4.39469086824877
a_HQ_transcript/136946	-3.31271822762114	4.17542110290432	-1.7837462282693	0.0837807437933723	0.814715035249252	-4.65622754173715
a_HQ_transcript/152469	-1.3701992044922	3.36694482475944	-1.78367585074861	0.0837924015102019	0.814715035249252	-4.64575763071897
a_HQ_transcript/2065	-3.40284385348039	0.860889840138474	-1.7835488863968	0.0838134360546809	0.814715035249252	-4.28577997271663
a_HQ_transcript/113307	1.46614048688165	2.20837062548719	1.78353969765388	0.083814958553374	0.814715035249252	-4.46613900407582
a_HQ_transcript/99299	-2.28368182508557	-0.224218672815325	-1.78316148002246	0.0838776464195709	0.815073037672213	-4.39231109348469
a_HQ_transcript/122149	3.94890433257427	0.154169986993424	1.78314829415396	0.0838798326341037	0.815073037672213	-4.39834113095725
a_HQ_transcript/49371	1.54585097500065	-0.555718804696506	1.78294877095069	0.0839129194357565	0.815210231396634	-4.4135481227842
a_HQ_transcript/59821	-0.705985989060839	3.35144874434806	-1.78289402321668	0.0839220001492818	0.815210231396634	-4.64464455255063
a_HQ_transcript/116823	-1.68344774773486	3.09374051874895	-1.78255057021453	0.0839789858529474	0.815627483652486	-4.61281584684761
a_HQ_transcript/70571	2.94356451946379	3.36809070729812	1.78232676995803	0.0840161364143158	0.815819919935602	-4.67297419210383
a_HQ_transcript/148467	-5.83669903773382	2.00214788316395	-1.78223007639731	0.084032191729832	0.815819919935602	-4.47945344213699
a_HQ_transcript/49490	-2.83761311227806	0.804757973953536	-1.78217761173754	0.0840409042212769	0.815819919935602	-4.38622419024011
a_HQ_transcript/128418	-2.91078927965282	-0.333260917749699	-1.78195687699331	0.0840775686982981	0.815912490126968	-4.41158544025145
a_HQ_transcript/151840	0.776676462182673	2.82241466420774	1.78195119173528	0.0840785132098221	0.815912490126968	-4.52876141318626
a_HQ_transcript/58343	3.04926543345152	0.308516831095748	1.78161272082418	0.0841347607454899	0.816260219657806	-4.39381824620142
a_HQ_transcript/18525	-2.67085403184985	0.873569210467296	-1.78150817188531	0.0841521412649125	0.816260219657806	-4.31377046799937
a_HQ_transcript/144643	-2.58089294409571	-0.277123784955234	-1.78147630878987	0.084157438883043	0.816260219657806	-4.4278273677581
a_HQ_transcript/131443	2.34021578825978	3.92263000708611	1.78139766206428	0.0841705160444001	0.816260219657806	-4.52377751588495

a_HQ_transcript/128559	6.37902500159051	3.42097402086587	1.78116012286036	0.0842100238591355	0.81650713375877	-4.53102417272014
a_HQ_transcript/14147	1.04790234026984	2.48551379704803	1.780798719513	0.0842701630338204	0.816861464116683	-4.42254229683761
a_HQ_transcript/126890	2.60938540027647	-0.0214045547713802	1.78077162505879	0.0842746731404443	0.816861464116683	-4.38459860689877
a_HQ_transcript/109116	-2.2471973549912	1.07669451438704	-1.78028807808032	0.084355198105569	0.817425324244722	-4.38394207707663
a_HQ_transcript/129694	-1.9442590280691	1.09978628104281	-1.78025013608551	0.0843615193327856	0.817425324244722	-4.46805288353755
a_HQ_transcript/141173	3.11998592817627	0.794197096500966	1.78013583048794	0.0843805653434862	0.817425324244722	-4.47944109236061
a_HQ_transcript/136011	1.6230434731353	-0.508415838818459	1.77996763156017	0.0844085978755188	0.817425324244722	-4.41428337097827
a_HQ_transcript/33015	7.3494641976086	3.21530531662996	1.77996158777775	0.0844096052969577	0.817425324244722	-4.48632950727883
a_HQ_transcript/55002	-3.44376037056793	3.13090833457177	-1.77987636379665	0.0844238121314935	0.817425324244722	-4.60481309482123
a_HQ_transcript/7319	2.34457990979356	0.352045714590014	1.77983154946558	0.084431283491008	0.817425324244722	-4.32592314862896
a_HQ_transcript/19599	-4.80021781803861	6.43905002766991	-1.77969722965585	0.0844536803862957	0.817506000581947	-5.06416177888295
a_HQ_transcript/98880	2.19622000384132	1.69370267577196	1.77956392389832	0.0844759131685777	0.817585061715465	-4.41256749229729
a_HQ_transcript/138578	-2.9929955973685	3.55193940880153	-1.77927188221398	0.0845246373057043	0.817920445697683	-4.77898756797599
a_HQ_transcript/101201	-0.985254113774999	2.8448603319158	-1.77898433625963	0.0845726346204012	0.817968494365236	-4.52217634785083
a_HQ_transcript/97057	3.50354347240623	3.53849517689094	1.77890806734502	0.0845853693368024	0.817968494365236	-4.5990493106203
a_HQ_transcript/128024	2.02690225108946	1.4003002383585	1.77887667056148	0.0845906121688111	0.817968494365236	-4.29656008781558
a_HQ_transcript/144333	-2.2703070874155	0.333360619324234	-1.77882764497457	0.0845987993192351	0.817968494365236	-4.37541109635764
a_HQ_transcript/92527	-2.18577628268243	1.39176720283432	-1.77871923393189	0.0846169060740553	0.817968494365236	-4.4139373708058
a_HQ_transcript/42971	2.73882465193332	1.31099093242154	1.7785986878907	0.084637043461815	0.817968494365236	-4.41597289318784
a_HQ_transcript/88126	-0.674743148099626	5.08702207851551	-1.77858582071031	0.084639193182219	0.817968494365236	-4.86430401276429
a_HQ_transcript/114872	2.45109581194372	0.0150705399187037	1.77847707442347	0.0846573632753139	0.817968494365236	-4.3917997145832
a_HQ_transcript/151912	-1.58218633892701	1.36819710768687	-1.77845954737185	0.0846602921271109	0.817968494365236	-4.37561414242729
a_HQ_transcript/85362	0.744217900070442	2.91891427403534	1.77830212326247	0.0846866022727452	0.817968494365236	-4.55811165258341
a_HQ_transcript/104833	-2.54318647110751	-0.0110332380014351	-1.77822132761714	0.0847001082615857	0.817968494365236	-4.43069911226343
a_HQ_transcript/133431	-0.879711061342617	3.47027222105413	-1.77821241785033	0.0847015977506323	0.817968494365236	-4.65188404629825
a_HQ_transcript/126982	1.00777925443931	3.89503341761078	1.77814698745167	0.0847125367478447	0.817968494365236	-4.70082161090956
a_HQ_transcript/152258	-6.17937548185272	5.31736898622923	-1.77805018277897	0.0847287232541316	0.817973829285351	-5.00627781483992
a_HQ_transcript/24203	-1.64712591958249	-0.525481656050368	-1.77794313809554	0.0847466250190055	0.817973829285351	-4.42517719987971
a_HQ_transcript/54652	-2.53756434470946	0.0984538883955576	-1.77789124202694	0.0847553050816417	0.817973829285351	-4.44019423377691
a_HQ_transcript/132215	4.10552395663277	2.76961477717823	1.77766067813469	0.0847938779691673	0.818123699871755	-4.39305777071316
a_HQ_transcript/137247	-1.16208298499466	1.39094600228725	-1.77759867997795	0.0848042526738377	0.818123699871755	-4.54710405093103
a_HQ_transcript/53458	-2.05550343868349	-0.0718463394083057	-1.77745170442365	0.0848288516970326	0.818123699871755	-4.38237144136415
a_HQ_transcript/144126	-0.97584740674314	2.99002327919351	-1.77740120392619	0.0848373052645761	0.818123699871755	-4.60334944079647
a_HQ_transcript/148660	-3.00960283087091	0.204515174958971	-1.77737789872561	0.0848412066960319	0.818123699871755	-4.44181144080409
a_HQ_transcript/78589	1.48516502631377	-0.676101442466491	1.77713063211282	0.0848826099749092	0.818207244185025	-4.41602117311429
a_HQ_transcript/126658	-2.20365233779969	1.4088201746978	-1.77700042562925	0.0849044191248509	0.818207244185025	-4.35080566892149
a_HQ_transcript/143324	-3.3238508853701	0.723089607721181	-1.77694197705032	0.0849142106081373	0.818207244185025	-4.41804838093691
a_HQ_transcript/104084	2.4175803740586	0.263857497973649	1.77688001774189	0.0849245912629101	0.818207244185025	-4.38652402946307
a_HQ_transcript/146330	-4.66632653361653	-0.071228663190527	-1.77680105110827	0.0849378228793918	0.818207244185025	-4.45578080817122
a_HQ_transcript/55199	1.76882775261401	0.374936255338636	1.77675529883783	0.0849454899091152	0.818207244185025	-4.36258631575736
a_HQ_transcript/148042	-2.92456880422425	-0.0869481250843262	-1.77673486075498	0.0849489150516433	0.818207244185025	-4.4611677435497
a_HQ_transcript/49237	-1.90955078342021	1.23265521367774	-1.77659280859688	0.0849727242754157	0.818207244185025	-4.32787272626764
a_HQ_transcript/147879	3.68800020158652	0.195432724725776	1.77656996235761	0.0849765540384151	0.818207244185025	-4.45845487936094
a_HQ_transcript/84583	2.03367961130987	1.84869125052314	1.77634970250823	0.085013484151967	0.818248296985005	-4.38512884688564
a_HQ_transcript/149405	3.08388392277242	0.592438180670828	1.77626140328344	0.0850282927532157	0.818248296985005	-4.44497427196503
a_HQ_transcript/112070	1.89711033767495	1.21704191774088	1.77613985810924	0.0850486805796414	0.818248296985005	-4.3682001376703
a_HQ_transcript/97521	-2.3286117962182	2.93774251222441	-1.77608174079525	0.0850584305626122	0.818248296985005	-4.48502161087218
a_HQ_transcript/148772	3.1152192500721	0.390639013071874	1.77606287004226	0.0850615965957423	0.818248296985005	-4.41100172011599
a_HQ_transcript/22713	2.94605472524794	1.33333894081028	1.77587397492475	0.0850932938947094	0.818248296985005	-4.37868732047575
a_HQ_transcript/42447	4.26359126202804	2.10176325968354	1.77576631640992	0.0851113638623855	0.818248296985005	-4.37330182043331

a_HQ_transcript/141009	-2.13300457821401	-0.167387828476352	-1.77576411221524	0.085111733859853	0.818248296985005	-4.44447114284799
a_HQ_transcript/126611	1.68835949492118	-0.637080097042369	1.77574667702895	0.0851146605888553	0.818248296985005	-4.4201965231328
a_HQ_transcript/121054	2.24442900072394	0.776257115096915	1.77570543296887	0.0851215842944592	0.818248296985005	-4.37364314962742
a_HQ_transcript/46049	3.69777325183307	1.57136861270436	1.77545177673075	0.085164176447043	0.818522362518802	-4.60658178080384
a_HQ_transcript/114196	-0.540313068018354	3.87440183092125	-1.77486639513235	0.0852625383914282	0.819273257453343	-4.7245026293758
a_HQ_transcript/141572	2.23562492220345	3.2297783304556	1.77478921664082	0.0852755138979054	0.819273257453343	-4.60446317405592
a_HQ_transcript/42218	3.24130328796938	0.725855515662497	1.77462583912009	0.0853029869851288	0.819273257453343	-4.3863274674945
a_HQ_transcript/73599	3.98975214415057	1.68363929597233	1.77445017611158	0.0853325343172222	0.819273257453343	-4.31788402597354
a_HQ_transcript/135342	-0.761259480338479	4.90062789550114	-1.77444369037481	0.0853336254139178	0.819273257453343	-4.8691695575498
a_HQ_transcript/18524	-2.68843620725867	0.269851138851406	-1.77440786296891	0.0853396528777038	0.819273257453343	-4.42572552328699
a_HQ_transcript/80362	-1.1803934972272	1.90322934155586	-1.77440006801594	0.0853409643180566	0.819273257453343	-4.39524090802988
a_HQ_transcript/151897	-1.90842238463825	-0.174208031767182	-1.7742875412319	0.0853598979758632	0.819310204239982	-4.42050966471952
a_HQ_transcript/107773	1.46300299162314	-0.76425781461234	1.77409837663939	0.0853917346516492	0.819310204239982	-4.42708705562886
a_HQ_transcript/99382	-2.16615816431745	1.44408607321885	-1.77393708264189	0.0854188885988222	0.819310204239982	-4.39178255497094
a_HQ_transcript/57637	-4.15796902094065	2.93943581392476	-1.77382655878954	0.0854374995766796	0.819310204239982	-4.70099559888391
a_HQ_transcript/8567	-1.20117990851406	1.68378309317158	-1.77366044458847	0.0854654777976201	0.819310204239982	-4.35393481169674
a_HQ_transcript/35691	-1.90471947862476	-0.2325859751365	-1.77365319127791	0.0854666996320764	0.819310204239982	-4.44097888309959
a_HQ_transcript/148750	3.85936569812515	3.27251121411838	1.77364742302044	0.0854676713169613	0.819310204239982	-4.74829427267154
a_HQ_transcript/13190	1.66182259465689	0.688690257722628	1.77360305337132	0.0854751458652947	0.819310204239982	-4.3135154855229
a_HQ_transcript/52041	0.977138624850195	3.3766510785498	1.77350284132391	0.0854920297055709	0.819310204239982	-4.6602423859992
a_HQ_transcript/25503	-2.81902704431993	2.16597476287565	-1.77343443006023	0.0855035573335516	0.819310204239982	-4.50925409105992
a_HQ_transcript/71395	-2.36770047873837	1.83583041602312	-1.77341164449678	0.0855073971027769	0.819310204239982	-4.34229835179128
a_HQ_transcript/123440	5.20442345182656	0.075901730012406	1.77337274835789	0.0855139521250339	0.819310204239982	-4.45544552473076
a_HQ_transcript/65981	1.85229143945183	-0.476065030324734	1.77322130451659	0.0855394784440155	0.819419710447221	-4.42287356819618
a_HQ_transcript/133457	-1.67895052674081	1.88423556447053	-1.77311881441525	0.0855567571178773	0.819450185821053	-4.41190953472198
a_HQ_transcript/98126	-0.523316427946189	4.33481528705301	-1.77296104884305	0.0855833603812965	0.819514825739736	-4.82010793002185
a_HQ_transcript/60178	1.1116124466925	2.69168551696466	1.77291158358082	0.0855917029153926	0.819514825739736	-4.48935921225106
a_HQ_transcript/128343	2.74543857439549	-0.142586899494275	1.77261207491274	0.085642231061047	0.819671600321608	-4.43026482452869
a_HQ_transcript/23359	-1.31207681813734	2.83046466107876	-1.7725848798708	0.0856468202074458	0.819671600321608	-4.49987127374252
a_HQ_transcript/23332	2.13744702181598	3.57756422520264	1.77256378438061	0.0856503802015113	0.819671600321608	-4.71061874643068
a_HQ_transcript/142959	1.16971194896896	5.50389499654671	1.77216122774167	0.0857183380993176	0.820053206664765	-4.8950458059593
a_HQ_transcript/144423	-0.602075282086177	2.96839325283945	-1.77216044060988	0.0857184710242071	0.820053206664765	-4.60785965873169
a_HQ_transcript/12969	-1.6036145535636	4.30951981640329	-1.77192002314959	0.0857590790730582	0.820306688885754	-4.78498243197713
a_HQ_transcript/39075	2.04357179836557	0.835918694487472	1.77175387311409	0.0857871523830652	0.820440209562819	-4.38485270194794
a_HQ_transcript/47437	-2.71472323889297	1.31423934060117	-1.77157266026096	0.085817779620187	0.820598107215369	-4.58814153954707
a_HQ_transcript/112285	-7.37060802285739	3.19660996029999	-1.77115015551782	0.085889224112483	0.821083102780353	-4.49235080805333
a_HQ_transcript/44639	1.49182387237151	-0.717497905489007	1.771105660106	0.0858967511011445	0.821083102780353	-4.42772841919777
a_HQ_transcript/87133	2.03285455061897	1.25452698198417	1.77094234378852	0.0859243830013428	0.821132153904278	-4.40902536726472
a_HQ_transcript/106642	-2.82100288900525	0.247632535259857	-1.77075532033309	0.0859560352025769	0.821132153904278	-4.4631996182891
a_HQ_transcript/118522	2.29886350703514	1.79709904419478	1.77066863905982	0.0859707086471371	0.821132153904278	-4.45262994060433
a_HQ_transcript/81336	-1.45391229875205	1.39658398729016	-1.77065946976093	0.0859722609539885	0.821132153904278	-4.4403965745126
a_HQ_transcript/142706	-3.23917733375772	0.173315975819492	-1.77053417926757	0.0859934742510063	0.821132153904278	-4.54117321606576
a_HQ_transcript/22147	-1.585132464144	-0.520479504663325	-1.77048970061015	0.0860010061258202	0.821132153904278	-4.40947238511253
a_HQ_transcript/4602	-1.94741889061724	2.49262941267055	-1.77039424635174	0.0860171719300327	0.821132153904278	-4.60287984411127
a_HQ_transcript/117929	-3.10132569461376	0.504707169922867	-1.77032690831371	0.0860285776133585	0.821132153904278	-4.51661320157352
a_HQ_transcript/26518	-5.45731029798306	2.54794774157995	-1.77032430247604	0.0860290190145378	0.821132153904278	-4.53059779519001
a_HQ_transcript/129659	2.55635739704925	2.24381668556735	1.76984696035812	0.0861099079959107	0.82145073063257	-4.45628430153388
a_HQ_transcript/13242	-1.66744835562207	-0.621379545492329	-1.76979854631679	0.0861181156858856	0.82145073063257	-4.42242240228966
a_HQ_transcript/121968	1.80614191220831	1.05840711141287	1.76973695943828	0.0861285575382976	0.82145073063257	-4.30743540842423
a_HQ_transcript/85782	2.54661876932973	1.30322926982843	1.76970047368832	0.0861347440818466	0.82145073063257	-4.47481309824563

a_HQ_transcript/746	0.771998589479573	2.67458271308923	1.76961607994395	0.086149055370215	0.82145073063257	-4.48740087070717
a_HQ_transcript/105261	-0.877743272753156	3.5933035141666	-1.76927833965548	0.0862063486755701	0.82145073063257	-4.6607552100319
a_HQ_transcript/132133	1.94542300799213	-0.486880436684693	1.76915722531902	0.086226902012305	0.82145073063257	-4.42168668315094
a_HQ_transcript/101521	1.29660256648013	2.4514264978075	1.76911025425226	0.0862348742076053	0.82145073063257	-4.40099070144757
a_HQ_transcript/87964	2.02702789118803	0.490930660559461	1.76909777393399	0.0862369925426128	0.82145073063257	-4.34523809055797
a_HQ_transcript/73033	-2.36539126593894	1.13817702426017	-1.76906549695795	0.0862424712485518	0.82145073063257	-4.32938308545539
a_HQ_transcript/119393	2.12832555698712	1.61762635836231	1.76903976013184	0.0862468400378171	0.82145073063257	-4.40072672624426
a_HQ_transcript/122810	1.24558207076428	3.92995339918096	1.76895594524524	0.0862610687909438	0.82145073063257	-4.814425502684
a_HQ_transcript/77686	1.47245001649311	-0.398145178210539	1.76855963045026	0.0863283756620174	0.82145073063257	-4.45794652849048
a_HQ_transcript/48958	2.26274769756675	1.0159073525791	1.76855125861147	0.0863297979453806	0.82145073063257	-4.30921654486543
a_HQ_transcript/41951	2.61955835044344	1.98300255441828	1.76852860370534	0.0863336468641524	0.82145073063257	-4.58680228326354
a_HQ_transcript/405	-1.10681174901898	2.77869169544669	-1.76842543450926	0.0863511764625558	0.82145073063257	-4.67294025844697
a_HQ_transcript/146861	-2.6581216576074	-0.108415891106335	-1.76837199565412	0.0863602575032604	0.82145073063257	-4.42508289304728
a_HQ_transcript/69855	4.0221356604543	3.8754283237171	1.76830496481877	0.08637164941505	0.82145073063257	-4.73675665581027
a_HQ_transcript/78347	2.40168929016054	1.81310440597561	1.76829265792679	0.086373741113239	0.82145073063257	-4.40125292789893
a_HQ_transcript/28002	-0.72873079650091	3.22099990645944	-1.76807653452714	0.0864104807544822	0.82145073063257	-4.63222776013706
a_HQ_transcript/760	-3.68594535260765	2.59758710045528	-1.76805714851861	0.0864137769013715	0.82145073063257	-4.4762242382731
a_HQ_transcript/1421	1.91776162516088	2.09839669518698	1.76799561286987	0.0864242403334648	0.82145073063257	-4.36011490569441
a_HQ_transcript/65482	2.23568152168373	-0.298752578869118	1.76797063513803	0.0864284878160993	0.82145073063257	-4.38727278709063
a_HQ_transcript/143876	-1.98734291912773	0.540132130312899	-1.76790616934556	0.0864394510892194	0.82145073063257	-4.36589554230625
a_HQ_transcript/17881	-2.8340075808539	0.36979281375966	-1.76764159062257	0.0864844585616483	0.82145073063257	-4.38510158202336
a_HQ_transcript/109998	1.19572089684366	2.6822699513581	1.76755834491913	0.0864986235787055	0.82145073063257	-4.53908403249259
a_HQ_transcript/118214	1.50108327041398	-0.535024885192997	1.76752838358794	0.0865037222527217	0.82145073063257	-4.43217688154708
a_HQ_transcript/52998	-4.06085828500387	1.58713471911095	-1.76752437328233	0.0865044047263483	0.82145073063257	-4.36578505415221
a_HQ_transcript/16602	-2.46445908082231	0.955256715405652	-1.76752024910628	0.0865051065831887	0.82145073063257	-4.34505691133932
a_HQ_transcript/132242	-1.77246935971953	0.613189899073672	-1.76748180278647	0.0865116496519136	0.82145073063257	-4.35718315105206
a_HQ_transcript/764	-3.5739540767171	1.07184534176958	-1.76745935792758	0.0865154696713034	0.82145073063257	-4.5078989141151
a_HQ_transcript/7298	-4.43181292508063	1.25813525405132	-1.7673981502778	0.0865258876769446	0.82145073063257	-4.46338583762555
a_HQ_transcript/136916	2.72535775109154	0.0570403294116725	1.76735912376865	0.0865325308378429	0.82145073063257	-4.41193982595083
a_HQ_transcript/39016	1.95063407756814	0.686639793077406	1.76729835638761	0.0865428756260986	0.82145073063257	-4.34449886119833
a_HQ_transcript/7026	-1.95170169804314	-0.461138300953048	-1.76713071588085	0.0865714194723765	0.821576569885127	-4.4219136477962
a_HQ_transcript/144898	3.54060988541847	2.88817893723937	1.76697578976636	0.0865978055343242	0.821576569885127	-4.40625899745037
a_HQ_transcript/122016	2.22840123741215	0.180563095795652	1.76694359290989	0.0866032899574257	0.821576569885127	-4.43638770418513
a_HQ_transcript/46019	-1.94223780236096	1.52805503910536	-1.7668885366317	0.0866126689419223	0.821576569885127	-4.38763218333474
a_HQ_transcript/128745	0.901719170986956	2.8265383801107	1.7666849334493	0.0866473607451213	0.821607620729382	-4.58340328350867
a_HQ_transcript/112684	-2.39078409607405	0.0678187781493435	-1.76611368536269	0.0867447579890532	0.821607620729382	-4.39545571877698
a_HQ_transcript/48049	-2.18512944447548	-0.35579583714181	-1.76609951152669	0.086747175789776	0.821607620729382	-4.4417891189168
a_HQ_transcript/91925	1.81077106888473	-0.556721621894393	1.76609328791722	0.0867482374432655	0.821607620729382	-4.4428133332471
a_HQ_transcript/118692	-2.17898520705516	1.18079000510472	-1.76599812878372	0.0867644715168385	0.821607620729382	-4.49541076456478
a_HQ_transcript/20958	1.91164618205372	1.00263722939112	1.76592894094239	0.0867762765196868	0.821607620729382	-4.48154297456063
a_HQ_transcript/147577	-3.56841371721902	1.33414532939171	-1.76581568005412	0.0867956043048971	0.821607620729382	-4.46777140412843
a_HQ_transcript/107814	1.65082961689324	1.93384712606588	1.76574381601202	0.0868078696717459	0.821607620729382	-4.43511728877401
a_HQ_transcript/152419	-3.39335490662495	1.21504130108472	-1.76559494334767	0.0868332831130473	0.821607620729382	-4.43210884571329
a_HQ_transcript/134685	-2.58000916896355	1.80908198747337	-1.7655235677156	0.0868454695829084	0.821607620729382	-4.46637430162888
a_HQ_transcript/145899	2.85331733179322	-0.0835685453344769	1.76547755627834	0.0868533262090253	0.821607620729382	-4.42943677319501
a_HQ_transcript/45156	-2.75429214998159	0.477609631631276	-1.76538085400233	0.0868698404436586	0.821607620729382	-4.46095145748696
a_HQ_transcript/89473	1.64719794395706	1.58100306992262	1.76528115991867	0.0868868683779494	0.821607620729382	-4.42171560107994
a_HQ_transcript/91776	-1.6987709642795	2.80209619261732	-1.76519658339665	0.0869013164151337	0.821607620729382	-4.53748284368913
a_HQ_transcript/4519	-2.01482525071866	-0.315107617536113	-1.76518742702123	0.0869028807020728	0.821607620729382	-4.47275800549574
a_HQ_transcript/151460	-0.511481806046101	9.23318742311974	-1.76513202884541	0.086912345504875	0.821607620729382	-5.28173336167121

a_HQ_transcript/1537	2.99584929036836	1.69829012921712	1.76509160939305	0.0869192517355595	0.821607620729382	-4.42886501564941
a_HQ_transcript/16014	-0.96373638566451	2.27685320133065	-1.76504347056596	0.0869274775345351	0.821607620729382	-4.40716941648241
a_HQ_transcript/105320	1.34506732906759	2.01403519029571	1.76503593160134	0.0869287658267071	0.821607620729382	-4.45592662754644
a_HQ_transcript/110799	-4.49136657365633	2.3587789983906	-1.76502391170323	0.0869308198742968	0.821607620729382	-4.46897206460866
a_HQ_transcript/27493	1.34924506299537	1.16386241821569	1.76494724141487	0.0869439228154353	0.821607620729382	-4.34060912180784
a_HQ_transcript/35761	0.861195022014092	3.47675206327251	1.76493877552348	0.086945369737308	0.821607620729382	-4.65562562249526
a_HQ_transcript/152307	-0.312592356760056	11.3015658412455	-1.76488479297568	0.0869545964773096	0.821607620729382	-5.54680420622877
a_HQ_transcript/56294	-1.70107052286563	-0.0659281037922126	-1.76455616055321	0.0870107844366995	0.821607620729382	-4.41468507969229
a_HQ_transcript/60228	-0.928264370099536	2.53476009408459	-1.76450751572377	0.0870191040959554	0.821607620729382	-4.44673629202062
a_HQ_transcript/72305	-2.41382781035297	0.51657549614576	-1.76438006383007	0.0870409052069245	0.821607620729382	-4.37711145318178
a_HQ_transcript/81249	1.72807188519867	0.853648794267912	1.76436984326988	0.0870426536707743	0.821607620729382	-4.42581271517731
a_HQ_transcript/46009	-3.08301570426938	-0.172947322376647	-1.7643408679084	0.0870476107395951	0.821607620729382	-4.43602085183478
a_HQ_transcript/7684	-1.01188129787976	1.93985547569602	-1.76432132718202	0.087050953877385	0.821607620729382	-4.50545243030636
a_HQ_transcript/85595	-2.385428921523	0.0504596968056281	-1.76424169148219	0.0870645795240126	0.821607620729382	-4.35760665520629
a_HQ_transcript/118392	-0.792348331699679	2.96658698176932	-1.76423320564018	0.0870660315554665	0.821607620729382	-4.66430874548355
a_HQ_transcript/115282	-1.7805961859242	-0.463550391266327	-1.76419071588448	0.0870733023804277	0.821607620729382	-4.4647523736374
a_HQ_transcript/104493	1.91146989974706	0.479347694908165	1.76412060805573	0.0870853003168174	0.821607620729382	-4.43359997347281
a_HQ_transcript/29913	2.45932387827935	-0.175204613665934	1.76404193783964	0.0870987652437835	0.821607620729382	-4.43249411382295
a_HQ_transcript/10061	-1.34867161178727	-0.642546543643175	-1.76397251817877	0.0871106483373793	0.821607620729382	-4.43599260885071
a_HQ_transcript/103324	-1.79370312630195	0.713793984347844	-1.76388222560576	0.0871261064553362	0.821620103185558	-4.30373513786672
a_HQ_transcript/134279	2.66946357791666	2.23059646261584	1.76378175452116	0.0871433098607463	0.821642417255743	-4.57124337788838
a_HQ_transcript/99310	1.87619511866363	-0.498096561690576	1.76367408706223	0.0871617486669551	0.821642417255743	-4.4356669577347
a_HQ_transcript/100021	-2.35654846034105	-8.81395862485356e-05	-1.76362078730767	0.087170877842282	0.821642417255743	-4.452542199499
a_HQ_transcript/16903	1.91112723282702	-0.0905811422345653	1.76347178336022	0.0871964035039005	0.821714188123383	-4.46649372381065
a_HQ_transcript/45519	-2.28034867592901	0.421070582191546	-1.76341131043307	0.0872067648385141	0.821714188123383	-4.37678380485113
a_HQ_transcript/70689	1.58398587484319	2.74577681119517	1.76330113083283	0.087225645507794	0.821758885263703	-4.56076435705345
a_HQ_transcript/96171	0.809398468029251	6.79875447250096	1.76308115892713	0.0872633508037493	0.82198088729871	-5.0374534507763
a_HQ_transcript/20137	-4.54789262698331	-0.333287367725155	-1.7626238380195	0.0873417840475251	0.822404999496793	-4.47178486353483
a_HQ_transcript/31922	1.87878643954162	-0.235616682358937	1.76241675498674	0.0873773195881805	0.822404999496793	-4.50447651772559
a_HQ_transcript/125425	-2.20410654867527	-0.386680244581316	-1.76239559286581	0.0873809517049226	0.822404999496793	-4.44236278895552
a_HQ_transcript/67746	2.46211925155201	1.32171292960279	1.76230680406186	0.0873961921766034	0.822404999496793	-4.3866083377842
a_HQ_transcript/44623	-2.11069821885376	0.30230191054582	-1.76228109848268	0.0874006049215463	0.822404999496793	-4.40820674845968
a_HQ_transcript/12530	1.38277563304185	1.26500519398302	1.76226442174096	0.0874034678328748	0.822404999496793	-4.49057447044698
a_HQ_transcript/149246	-2.34426561596298	1.25169148184509	-1.76224144310837	0.0874074127252131	0.822404999496793	-4.46574168330911
a_HQ_transcript/112974	-2.59645545073481	2.08913164493901	-1.76201027475191	0.0874471072745314	0.822581876864546	-4.58939156542915
a_HQ_transcript/122548	-2.02692080461083	-0.559617816848079	-1.76196714923555	0.0874545141588028	0.822581876864546	-4.45422322648987
a_HQ_transcript/52720	2.28681550164	-0.0747930222401644	1.7617735316728	0.0874877748475533	0.822649982887501	-4.45718063119444
a_HQ_transcript/137649	2.64948704993301	3.50398071813365	1.7617043372385	0.0874996640375797	0.822649982887501	-4.68032344971625
a_HQ_transcript/48038	-1.43562537709963	-0.724371776757971	-1.76158454533735	0.0875202502570341	0.822649982887501	-4.4532024220726
a_HQ_transcript/47368	-2.15269505723946	-0.367402882282156	-1.76148317076536	0.0875376746553486	0.822649982887501	-4.43016504088068
a_HQ_transcript/37063	1.98110864511605	-0.237459334225681	1.76142131792407	0.0875483074436489	0.822649982887501	-4.459252701935
a_HQ_transcript/141098	-1.21859093979298	2.57714628922278	-1.76135297990314	0.0875600563318995	0.822649982887501	-4.45230463179346
a_HQ_transcript/152066	4.69776838970184	0.066468696045427	1.76134017934529	0.0875622571922057	0.822649982887501	-4.47298337134493
a_HQ_transcript/59497	2.27251063217354	0.333009096773766	1.76121400788183	0.0875839529459529	0.822649982887501	-4.47010689685686
a_HQ_transcript/113126	-3.01799439095157	3.13648009931417	-1.76113721290659	0.0875971604106558	0.822649982887501	-4.74923553153518
a_HQ_transcript/94951	-2.57255516165289	0.231458516409774	-1.76085395015387	0.08764589145422	0.822649982887501	-4.37498070583648
a_HQ_transcript/25478	-2.03625928780562	2.04714223468818	-1.76084047661831	0.0876482099408565	0.822649982887501	-4.41828301758663
a_HQ_transcript/117980	2.7186449956938	0.311365437619437	1.76080775952753	0.0876538400177346	0.822649982887501	-4.49840317819634
a_HQ_transcript/120707	-1.06795515138441	3.4613612592732	-1.76071102736048	0.0876704878290754	0.822649982887501	-4.73551567672618
a_HQ_transcript/124460	2.90500689910503	3.84963964703177	1.76069053644682	0.0876740147013571	0.822649982887501	-4.73974491113983

a_HQ_transcript/102023	3.71361433031527	1.48863996242339	1.76069038478133	0.0876740408062908	0.822649982887501	-4.45414412886859
a_HQ_transcript/66920	-2.00479477830667	1.03540165694218	-1.76040998008657	0.0877223157826962	0.822832761976492	-4.33163248958597
a_HQ_transcript/69771	-1.9805353886166	-0.44137897848395	-1.76031049510192	0.0877394486876365	0.822832761976492	-4.39798607435513
a_HQ_transcript/132824	2.15957809315462	1.84446761812974	1.76029822952512	0.0877415612116204	0.822832761976492	-4.50349712850293
a_HQ_transcript/28690	-3.48344112419624	2.20221054464498	-1.76024840723657	0.0877501426419673	0.822832761976492	-4.4163153024713
a_HQ_transcript/19076	1.94862209642846	-0.23928975177084	1.76007540347802	0.0877799464467763	0.822908609727364	-4.45303935441515
a_HQ_transcript/3937	2.08910383529028	1.33486200694294	1.75996538881209	0.0877989034011953	0.822908609727364	-4.50249803364461
a_HQ_transcript/109113	0.528868181125028	4.45103010181569	1.75990081196087	0.0878100324397895	0.822908609727364	-4.81670970081307
a_HQ_transcript/140954	-1.62303153477678	2.13041567985067	-1.75981026242109	0.087825639561942	0.822908609727364	-4.41370792154946
a_HQ_transcript/96837	2.88265868158448	1.13588270901695	1.75976551002884	0.0878333539503911	0.822908609727364	-4.50114983124792
a_HQ_transcript/136885	-2.7551868205919	1.57259773393659	-1.75969262426397	0.0878459191755431	0.822908609727364	-4.45469883579387
a_HQ_transcript/133184	1.90613919912425	2.68855982781093	1.75962644694768	0.087857329203964	0.822908609727364	-4.5682501210744
a_HQ_transcript/44571	1.17922286067282	2.61824600631353	1.75951998635402	0.0878756873384937	0.822925735707358	-4.42266967975688
a_HQ_transcript/54717	-3.77154997074248	1.93204712734942	-1.75945165636892	0.0878874719114933	0.822925735707358	-4.44812667779884
a_HQ_transcript/5499	-1.47352573691584	1.35667091357065	-1.75917390219951	0.0879353886854129	0.823029202230033	-4.37154505024045
a_HQ_transcript/111152	-2.07854014593924	1.71206239352925	-1.75916068662586	0.0879376691200525	0.823029202230033	-4.36631933488157
a_HQ_transcript/1324	1.08606991882531	2.20797276959926	1.75914139130431	0.0879409987450237	0.823029202230033	-4.53826206825802
a_HQ_transcript/119400	2.06949679251469	1.46834125649274	1.75894330161531	0.0879751875038419	0.82321662897993	-4.41971730820511
a_HQ_transcript/45165	-2.63415289009943	0.488168331556759	-1.75869513301309	0.0880180353251961	0.823414721652992	-4.35082310997166
a_HQ_transcript/104640	1.40512408260702	-0.367386107214806	1.75857765153108	0.0880383253592408	0.823414721652992	-4.41777233741134
a_HQ_transcript/82462	2.24659701976314	-0.230676478949589	1.75857459196931	0.0880388538238602	0.823414721652992	-4.4447340272348
a_HQ_transcript/119594	-2.00578929773003	1.36337078314256	-1.75849249585764	0.0880530349228947	0.823414867116799	-4.5512164243483
a_HQ_transcript/9953	-2.60540921509915	0.532102989153525	-1.75827567057586	0.0880904981056769	0.82363269645919	-4.41918132667448
a_HQ_transcript/141104	2.81957241579963	0.672984011839098	1.75783891029997	0.0881660025987404	0.824062425042815	-4.51066900431123
a_HQ_transcript/28240	1.59677321086665	-0.419434831700478	1.75775788303421	0.088180016108365	0.824062425042815	-4.44309550905498
a_HQ_transcript/35466	-0.770400003564648	2.74537344543548	-1.75769260045607	0.0881913079714515	0.824062425042815	-4.56343671660138
a_HQ_transcript/130930	-2.5159522016658	1.28875124074521	-1.75766874761083	0.0881954340788266	0.824062425042815	-4.43306644060354
a_HQ_transcript/80956	2.0493104765858	0.75875240573706	1.75759991045546	0.0882073425649669	0.824062425042815	-4.43583176128058
a_HQ_transcript/36121	2.74607840045157	2.2315089770229	1.75747779298511	0.0882284716188841	0.824118386970939	-4.64796564373575
a_HQ_transcript/50869	-1.70142191624258	1.37345793446755	-1.757390044555	0.0882436566962743	0.824118386970939	-4.31736192756635
a_HQ_transcript/149481	-0.94394363994225	2.78160417278657	-1.75726878463773	0.0882646446458916	0.824118386970939	-4.54693206065661
a_HQ_transcript/134046	1.68886782758452	1.51102226151268	1.7572375972888	0.0882700433058262	0.824118386970939	-4.31739441530977
a_HQ_transcript/136333	-0.947805160369704	5.5528826080712	-1.75675886066409	0.0883529495714308	0.824333400438031	-4.95273429634302
a_HQ_transcript/136862	0.909005478650263	2.95415259076675	1.75672393809164	0.088358999934237	0.824333400438031	-4.65074004105669
a_HQ_transcript/66916	0.959613339067824	3.17465818295458	1.75657140296017	0.0883854308614433	0.824333400438031	-4.67824268087207
a_HQ_transcript/16634	1.95259595896278	0.320764257252031	1.75651016662245	0.0883960436252861	0.824333400438031	-4.41768933545281
a_HQ_transcript/113351	0.998986342529347	1.93743857577923	1.7565015436527	0.0883975381436006	0.824333400438031	-4.46746060364079
a_HQ_transcript/111854	-2.48136899135247	-0.386056817216666	-1.75647211007066	0.0884026396820542	0.824333400438031	-4.45929159401143
a_HQ_transcript/4395	-2.20500011437596	-0.20203476254547	-1.75637118322048	0.088420134586722	0.824333400438031	-4.47194445440728
a_HQ_transcript/30166	2.21881728172241	1.81148276819452	1.75634304205072	0.0884250131658703	0.824333400438031	-4.43545902370871
a_HQ_transcript/146058	1.67849381659906	0.663307810083907	1.75630013360921	0.0884324522498868	0.824333400438031	-4.36919276608846
a_HQ_transcript/151063	1.81669679625938	-0.477378608868673	1.75628609331562	0.0884348865457536	0.824333400438031	-4.46190891017529
a_HQ_transcript/86092	2.80496579149153	1.54557294571152	1.75588655315415	0.088504182219777	0.82451950296782	-4.36111272277176
a_HQ_transcript/122997	2.35272982018185	0.973520021582817	1.75578771656608	0.0885213313601442	0.82451950296782	-4.40671145605281
a_HQ_transcript/17286	1.82554708229383	2.51343055311026	1.75578279668166	0.0885221850827079	0.82451950296782	-4.53043946514597
a_HQ_transcript/59630	1.25727910494922	0.587451187511501	1.75576479295954	0.0885253092363947	0.82451950296782	-4.31460345238446
a_HQ_transcript/7451	-2.65518383218838	1.95250952410166	-1.75575072888168	0.0885277498153553	0.82451950296782	-4.46472028480463
a_HQ_transcript/127158	1.17119725019479	3.02504252699461	1.75568037707922	0.0885399590133005	0.82451950296782	-4.60194689510737
a_HQ_transcript/130171	1.98884649847686	-0.302790318628675	1.75526497055275	0.0886120796579373	0.825058940630559	-4.49681991430187
a_HQ_transcript/14121	-2.39647627134108	0.901483590515258	-1.75506632621229	0.0886465847247683	0.825248026406363	-4.40175444353234

a_HQ_transcript/137483	-2.12441760337858	0.958435774364552	-1.75477421332028	0.0886973461168769	0.825588364304535	-4.3493824101038
a_HQ_transcript/77834	1.15757355518632	3.18037982837398	1.75464772630152	0.0887193337743634	0.825660812301665	-4.65667758289403
a_HQ_transcript/29653	2.42000202793736	-0.202284743234197	1.75446868175565	0.0887504655383931	0.825818322525326	-4.45887322927145
a_HQ_transcript/130281	6.77284782366357	5.49923769105598	1.75428955997704	0.0887816199482936	0.825975993014471	-4.84099882430321
a_HQ_transcript/16706	-0.777363598528833	4.03921057133988	-1.75397242430671	0.0888368015447266	0.826204767295591	-4.81763079429794
a_HQ_transcript/128465	-1.51649439399889	3.37819502192589	-1.75393143519532	0.0888439357586787	0.826204767295591	-4.70167898120109
a_HQ_transcript/148528	-2.20249588153939	1.0298226185375	-1.75384342022533	0.0888592565234188	0.826204767295591	-4.37565912571109
a_HQ_transcript/14545	-1.63247336881046	-0.626743674837841	-1.75367673123948	0.0888882781772927	0.826204767295591	-4.46472353132162
a_HQ_transcript/134696	-3.9204804314972	2.01799933849918	-1.75358954036484	0.0889034618677374	0.826204767295591	-4.53632321990852
a_HQ_transcript/145817	3.70879219768682	1.11229291017649	1.75358210227311	0.0889047572614163	0.826204767295591	-4.44389014149482
a_HQ_transcript/73779	3.11734677278304	-0.0564743821796887	1.75348380258409	0.0889218783079709	0.826204767295591	-4.46838055122146
a_HQ_transcript/105887	1.0715500059222	2.54925503488762	1.75341762528425	0.0889334061015426	0.826204767295591	-4.41240910368998
a_HQ_transcript/131753	2.53809854653049	0.683240385488976	1.75339300009444	0.0889376960225605	0.826204767295591	-4.41288986567796
a_HQ_transcript/46885	-1.13210834651002	3.49369988177921	-1.75332916336766	0.088948817744745	0.826204767295591	-4.67320743986649
a_HQ_transcript/113959	-2.906573147118	3.7896094928735	-1.7532031661559	0.0889707725929219	0.826204767295591	-4.77870565774442
a_HQ_transcript/117256	2.35631128623736	0.398335039757577	1.75316873657939	0.0889767726959537	0.826204767295591	-4.43035984453541
a_HQ_transcript/109380	2.25071896207258	1.77328945972304	1.752921268207	0.0890199094841297	0.826473294760181	-4.46889105135439
a_HQ_transcript/28429	0.832564476080225	2.82426281593925	1.7526618161725	0.0890651541019523	0.826761302720902	-4.48348377600687
a_HQ_transcript/95690	-2.33546453587349	-0.375337555856065	-1.75256450551002	0.0890821286477781	0.826786839220509	-4.45497941382019
a_HQ_transcript/24487	-1.60848734896582	-0.711367728974882	-1.75239658771413	0.0891114260782117	0.826861289071959	-4.44456677921438
a_HQ_transcript/118123	-1.18713796861229	3.61451805678138	-1.75231936014848	0.0891249030717235	0.826861289071959	-4.69497055842526
a_HQ_transcript/122555	-1.69182442349708	-0.448692106573679	-1.75227397091788	0.0891328247544195	0.826861289071959	-4.45859834786943
a_HQ_transcript/98217	-2.41050662416035	0.175793598700298	-1.7520811347339	0.0891664866471697	0.827020321436726	-4.45722528522068
a_HQ_transcript/152531	-2.34616210617004	-0.225386475151423	-1.75201277038573	0.089178423045097	0.827020321436726	-4.47572188831962
a_HQ_transcript/50565	2.68745084343315	1.69500217638288	1.75181910898441	0.0892122435885216	0.827077458453074	-4.45256135856074
a_HQ_transcript/118646	-2.45537538869039	5.62123620819215	-1.75178529283402	0.0892181502650817	0.827077458453074	-4.95235433264451
a_HQ_transcript/970	-1.42470376837575	3.21230693008654	-1.75173308624479	0.0892272698520373	0.827077458453074	-4.56908245247628
a_HQ_transcript/47433	2.23446993916432	-0.0674644903193485	1.75136366129249	0.0892918244454794	0.827311458353892	-4.4904771245708
a_HQ_transcript/151666	2.16093710843712	-0.316457624479493	1.75130180884891	0.0893026366012481	0.827311458353892	-4.45682018349276
a_HQ_transcript/94464	-5.0475065221823	1.70404344060691	-1.75129147556832	0.0893044430244977	0.827311458353892	-4.43902008839842
a_HQ_transcript/117107	2.05592469338408	0.079610926586194	1.75124366096795	0.0893128021847942	0.827311458353892	-4.36867684949524
a_HQ_transcript/142723	2.93187108423599	0.186736883294039	1.75118146083318	0.0893236772747906	0.827311458353892	-4.47533774833682
a_HQ_transcript/131206	-6.24415243272922	4.89240772262785	-1.75074431612555	0.0894001392713706	0.827455297608181	-5.05472277293766
a_HQ_transcript/64308	-2.61884329111844	1.48126781602572	-1.7507288704541	0.0894028419182017	0.827455297608181	-4.42640002250628
a_HQ_transcript/68858	-3.01674502216424	0.523385718573935	-1.75060182589852	0.0894250744844872	0.827455297608181	-4.42013528583191
a_HQ_transcript/151087	1.00761832897567	5.54001234378765	1.75059832895545	0.089425686509046	0.827455297608181	-4.97095117233484
a_HQ_transcript/127945	1.82483969181681	-0.243278221674152	1.75059684182032	0.0894259467840758	0.827455297608181	-4.3961539399565
a_HQ_transcript/131802	4.37508081077715	2.10242984237806	1.7504193916588	0.0894570082932661	0.827455297608181	-4.57237055595878
a_HQ_transcript/79998	-1.73238439744334	-0.344439008009498	-1.75029687717615	0.0894784589700995	0.827455297608181	-4.47230301313548
a_HQ_transcript/134576	-1.95969380657645	-0.308376006231096	-1.7502246649738	0.0894911044083859	0.827455297608181	-4.44847305989355
a_HQ_transcript/57142	-2.88377420171188	-0.058261261702949	-1.75010790141633	0.0895115546422571	0.827455297608181	-4.4987185225876
a_HQ_transcript/136920	-2.45913294857621	-0.15087329392727	-1.75008542920073	0.089515490928803	0.827455297608181	-4.39568246870951
a_HQ_transcript/139753	2.01788407020153	0.252150316270012	1.74986853627644	0.0895534899052403	0.827455297608181	-4.47144072646726
a_HQ_transcript/84036	-2.0527294933801	0.6791017885778	-1.74984577535138	0.0895574783374936	0.827455297608181	-4.39263622587981
a_HQ_transcript/115845	0.496216557402638	3.80309577953753	1.74963467744193	0.0895944764836198	0.827455297608181	-4.78886346871188
a_HQ_transcript/148390	-0.610832905023629	2.74648868803253	-1.74938278431754	0.0896386414675364	0.827455297608181	-4.64036166639191
a_HQ_transcript/96552	-2.3419734473508	0.597560253576111	-1.74936567376471	0.0896416421641393	0.827455297608181	-4.38231768023198
a_HQ_transcript/27014	2.22604892655923	1.10252107883701	1.74929730982968	0.0896536320677119	0.827455297608181	-4.33741327482453
a_HQ_transcript/144831	-1.70218245499617	-0.216212386806322	-1.74927484336316	0.0896575726093334	0.827455297608181	-4.5326277271603
a_HQ_transcript/32474	-2.11419006165103	0.691291259419584	-1.74918602540637	0.0896731524048401	0.827455297608181	-4.40050074508025

a_HQ_transcript/60491	2.06227789582494	0.0168251980735682	1.74909706773251	0.0896887589946541	0.827455297608181	-4.47702232321405
a_HQ_transcript/55795	-2.29106044867956	1.77013703953306	-1.74894595982125	0.0897152743659185	0.827455297608181	-4.46866761799122
a_HQ_transcript/114336	0.97770833819696	2.08151587304553	1.74892894783706	0.0897182599248027	0.827455297608181	-4.41551173900654
a_HQ_transcript/14877	-1.42100467134306	-0.76268454941428	-1.74891670696819	0.0897204082171641	0.827455297608181	-4.46493045290821
a_HQ_transcript/71788	2.26008511338637	1.11599647782309	1.74886298884576	0.0897298363472501	0.827455297608181	-4.3410204422737
a_HQ_transcript/21305	-2.15187644688447	-0.260617672712192	-1.74877693871556	0.0897449408449145	0.827455297608181	-4.47890941867357
a_HQ_transcript/47879	2.02641777445141	2.86408395277892	1.74875752357928	0.0897483491066208	0.827455297608181	-4.49601651533223
a_HQ_transcript/54245	1.36210949339168	1.09459580074367	1.74874090094539	0.0897512672405909	0.827455297608181	-4.38025693273913
a_HQ_transcript/108188	1.05244013943654	3.08902011224912	1.74867686428451	0.0897625097413388	0.827455297608181	-4.58553475734076
a_HQ_transcript/16082	-1.06797170974744	2.60210799098079	-1.74859968656589	0.0897760609087302	0.827455297608181	-4.53998758576054
a_HQ_transcript/97194	-0.886844300569965	3.32516353109743	-1.7485382369798	0.089786851701049	0.827455297608181	-4.66427961521484
a_HQ_transcript/84945	2.19760891267441	1.17962171192319	1.74849130358008	0.0897950941290694	0.827455297608181	-4.47838547671754
a_HQ_transcript/130309	1.88171056631727	-0.537968155877072	1.74846965454857	0.089798896339579	0.827455297608181	-4.45790230320607
a_HQ_transcript/8623	2.80528291668314	1.7749669719136	1.74846251338662	0.0898001505686657	0.827455297608181	-4.53857178029376
a_HQ_transcript/49631	0.895085542692084	3.96812702417571	1.74841233372392	0.0898089642273949	0.827455297608181	-4.76409416102483
a_HQ_transcript/151407	2.82271493484291	1.01464383144003	1.7482071688536	0.0898450073859923	0.827479719691694	-4.42551600044404
a_HQ_transcript/149772	-2.42413740560864	0.293051140878907	-1.74812396936013	0.0898596272616372	0.827479719691694	-4.48041344006942
a_HQ_transcript/40038	-3.31335747717238	1.88758434982596	-1.7480787945373	0.0898675662538231	0.827479719691694	-4.54107358068184
a_HQ_transcript/150303	3.45220543131144	5.30617940553946	1.74807315824847	0.0898685568127867	0.827479719691694	-4.90598849076317
a_HQ_transcript/17770	1.2198226634891	2.45045950809545	1.74773748480627	0.0899275668865952	0.827745143707934	-4.47766987116805
a_HQ_transcript/16941	0.873380222553003	2.47371621199746	1.74764099065046	0.0899445362186319	0.827745143707934	-4.49717194886782
a_HQ_transcript/102471	6.25121004033218	1.49678690336823	1.74746760115897	0.0899750350378595	0.827745143707934	-4.51391190866644
a_HQ_transcript/140035	2.41436851734389	0.247284992596669	1.74725707081474	0.09001207855721	0.827745143707934	-4.42488760365624
a_HQ_transcript/112244	-1.47785679918916	2.11979483840904	-1.74725125088386	0.0900131027759665	0.827745143707934	-4.43570356921941
a_HQ_transcript/149142	-4.088053509929	3.38091583738938	-1.74721136950438	0.0900201215524241	0.827745143707934	-4.712382459813
a_HQ_transcript/34519	-1.93363369174217	1.42537608641246	-1.7471798765672	0.0900256643615779	0.827745143707934	-4.39858720326335
a_HQ_transcript/128191	-1.17778498973084	4.10926399041395	-1.74714591211422	0.0900316424837592	0.827745143707934	-4.72262852175512
a_HQ_transcript/83010	-4.3383062583951	0.456143976620644	-1.74710763552876	0.0900383799899972	0.827745143707934	-4.43611847436072
a_HQ_transcript/121331	-1.46216223028048	1.97140382354981	-1.74696913845057	0.0900627620168391	0.827745143707934	-4.53749932636375
a_HQ_transcript/72954	1.17965746396459	2.29155765001173	1.74695300126437	0.0900656032854084	0.827745143707934	-4.53492022906636
a_HQ_transcript/50043	-3.49860903390715	0.312050570099192	-1.74691871002178	0.0900716411827967	0.827745143707934	-4.48419202341112
a_HQ_transcript/20276	-1.96353641173872	3.83615432784143	-1.74684710062684	0.0900842510458212	0.827745143707934	-4.8169463370097
a_HQ_transcript/14960	2.31463050888431	-0.26109956440206	1.74663532665881	0.0901215515107286	0.827745143707934	-4.47633095853978
a_HQ_transcript/125378	1.90242382997748	-0.0384194441246309	1.74661825446506	0.0901245590603643	0.827745143707934	-4.51106173040471
a_HQ_transcript/112023	3.13856688468313	4.0263962617824	1.74653463462967	0.0901392912999532	0.827745143707934	-4.77192736395641
a_HQ_transcript/123727	2.64108706591316	0.804883811449191	1.74653365481992	0.09013946393599	0.827745143707934	-4.44314342249347
a_HQ_transcript/50457	3.43254591074848	1.82724100907884	1.74633886531523	0.0901737900967086	0.827929564167032	-4.48486660135924
a_HQ_transcript/145414	2.58241000695095	0.351231213902075	1.74617458971264	0.0902027475975611	0.82803979782229	-4.54622214978077
a_HQ_transcript/132906	-2.66228135070737	0.736847074336697	-1.74610914260331	0.0902142863956882	0.82803979782229	-4.35712549446461
a_HQ_transcript/118827	-0.749739332579868	4.08121222550326	-1.74602159311984	0.0902297239454119	0.828050741000774	-4.81614227945221
a_HQ_transcript/131181	3.37862992136437	-0.242215215618569	1.74584924524732	0.0902601204455514	0.828084898095602	-4.42862428801485
a_HQ_transcript/138850	-1.36916013470347	2.10998110370147	-1.74583894300831	0.0902619376949789	0.828084898095602	-4.55010400903369
a_HQ_transcript/33274	-1.33100361032966	1.29682429056596	-1.74569969816707	0.0902865026214972	0.82808673661022	-4.51746726668565
a_HQ_transcript/80019	-0.579608679004638	3.45819847025369	-1.74561184677932	0.0903020038501127	0.82808673661022	-4.70899410206539
a_HQ_transcript/88775	-1.46466970764147	-0.749630087172027	-1.74559557145596	0.0903048758493701	0.82808673661022	-4.47541969058573
a_HQ_transcript/125669	-0.543065799862043	5.85971605410927	-1.74538242193723	0.0903424960425551	0.828179574799226	-5.00985792285445
a_HQ_transcript/57834	2.82276696487998	0.167134166581598	1.74537676240767	0.0903434951108225	0.828179574799226	-4.45772076948159
a_HQ_transcript/68126	-2.49400478403755	0.10104954283512	-1.74524777913932	0.0903662668556992	0.828247491669055	-4.42214612010984
a_HQ_transcript/116335	-2.39207341246717	0.292437849037435	-1.74517339563557	0.0903794013153182	0.828247491669055	-4.43087576305089
a_HQ_transcript/65775	-2.50142138308829	0.827042573336295	-1.74505761380658	0.0903998489892805	0.828286939432277	-4.47982028279866

a_HQ_transcript/146285	-2.28097546677784	0.434763791090599	-1.74493991046315	0.0904206400048023	0.828286939432277	-4.46416027958492
a_HQ_transcript/75849	1.24169305366934	1.09532206598962	1.74490700004795	0.0904264539918324	0.828286939432277	-4.36442925258923
a_HQ_transcript/139449	3.02975888878067	-0.0805172720367076	1.74470677155058	0.0904618333420839	0.82848045509826	-4.48325675534631
a_HQ_transcript/25726	-2.05592138099721	-0.0644998234899911	-1.74435239714203	0.0905244780484394	0.828732779246609	-4.41305267836415
a_HQ_transcript/121891	0.617577135967253	2.99718633326458	1.7443273673643	0.0905289040798559	0.828732779246609	-4.62119046751883
a_HQ_transcript/72875	2.69887704764568	1.49305722978942	1.74430534852889	0.0905327978350516	0.828732779246609	-4.51477852319316
a_HQ_transcript/106640	5.10305998495025	0.951160275055079	1.74422836361148	0.0905464127614095	0.828732779246609	-4.62453079585657
a_HQ_transcript/126092	2.4376367440083	0.572516309926773	1.7438370435247	0.0906156450987171	0.828996022287898	-4.51347320670585
a_HQ_transcript/104339	-1.08087438234095	1.85353421159934	-1.74359048513709	0.0906592890781813	0.828996022287898	-4.45034376565214
a_HQ_transcript/12480	2.33968462556042	-0.251639742367006	1.74357761687399	0.0906615674106131	0.828996022287898	-4.48712580291834
a_HQ_transcript/113229	4.89964640980023	2.52078378185681	1.7434896864142	0.0906771368323577	0.828996022287898	-4.53673348643918
a_HQ_transcript/132766	-2.49591437366654	0.597473323572453	-1.74346981563941	0.0906806555661875	0.828996022287898	-4.46388477926677
a_HQ_transcript/15553	-3.56557398403262	2.650062921905	-1.74324624166468	0.0907202541588826	0.828996022287898	-4.63567349479053
a_HQ_transcript/25769	-2.90801242161466	1.51169012903723	-1.74318453237336	0.0907311864420993	0.828996022287898	-4.60851132120283
a_HQ_transcript/50987	-2.55207095967903	1.38983329715092	-1.74314433336355	0.0907383086072244	0.828996022287898	-4.49053914622823
a_HQ_transcript/3571	1.55170830847707	1.54187617630211	1.74312911626825	0.0907410047831055	0.828996022287898	-4.38769597217191
a_HQ_transcript/59393	1.50704454434338	2.79716559436869	1.74311970434183	0.0907426724287406	0.828996022287898	-4.65886520095883
a_HQ_transcript/7427	-2.49248748879458	1.12297199437941	-1.74311364868959	0.0907437454089074	0.828996022287898	-4.41356544412118
a_HQ_transcript/122340	5.51181028157318	0.798114116884968	1.7430991572903	0.0907463131333934	0.828996022287898	-4.43783813452375
a_HQ_transcript/87794	1.61327935321643	0.226410842981287	1.74277737009099	0.0908033462184875	0.829386692172885	-4.49369856140424
a_HQ_transcript/80505	-2.00346147844428	2.08248626849674	-1.74265014012564	0.0908259045886298	0.829462400931323	-4.44230869907372
a_HQ_transcript/87532	2.71369183251976	1.25774571009198	1.74249133467654	0.0908540680325195	0.829589265880348	-4.36402272947158
a_HQ_transcript/37330	-3.10994879050741	1.34588096100415	-1.74232563438456	0.0908834620806283	0.829727325871331	-4.46971775420839
a_HQ_transcript/128851	3.38121619482501	-0.269972054698071	1.74220103712674	0.0909055700149886	0.829785927342037	-4.50180309268072
a_HQ_transcript/20355	2.01438561793127	0.867819665239528	1.74212856476226	0.0909184312421111	0.829785927342037	-4.38468062554606
a_HQ_transcript/4745	-2.17361837158429	0.766275675216375	-1.74198667274958	0.0909436163831241	0.829867196054141	-4.46431906305948
a_HQ_transcript/144680	-2.31440038851908	-0.131599545753576	-1.74178517297247	0.0909793916994835	0.829867196054141	-4.47010017376599
a_HQ_transcript/54873	3.93281191271887	2.1727996183773	1.7417796204994	0.0909803776820013	0.829867196054141	-4.74951173829927
a_HQ_transcript/34266	-1.99838899304653	1.1720528490791	-1.74167953704233	0.090998151581693	0.829867196054141	-4.44649913208185
a_HQ_transcript/68780	2.70707657713127	0.721446589402905	1.74165497035742	0.0910025148451217	0.829867196054141	-4.48991656081599
a_HQ_transcript/24742	-1.76884177041923	3.19635975346136	-1.74159596735932	0.0910129950255496	0.829867196054141	-4.69280462819379
a_HQ_transcript/50782	-1.66523653187079	0.988970204889564	-1.74147166933389	0.0910350763070229	0.829938349368668	-4.40805557693953
a_HQ_transcript/130322	-1.07332138640858	3.97527337406648	-1.74135734172582	0.0910553903445177	0.829993371482849	-4.82207288656396
a_HQ_transcript/28544	3.8260437762873	1.83998870707641	1.74118926247996	0.091085262042994	0.829996066208931	-4.52387274536786
a_HQ_transcript/129192	2.1268541371244	0.939680506937446	1.74112751015194	0.0910962389782447	0.829996066208931	-4.41324715853715
a_HQ_transcript/15799	-2.48678273477507	1.82447008541428	-1.74097045939569	0.0911241609321928	0.829996066208931	-4.60338987081064
a_HQ_transcript/61938	-2.28885302541113	1.45494278839841	-1.74089107097104	0.091138278089269	0.829996066208931	-4.394883273169
a_HQ_transcript/21302	1.61567845364693	0.639597818371374	1.74075488494041	0.0911624995021067	0.829996066208931	-4.35335719948984
a_HQ_transcript/135980	-1.72085547611424	0.478209090477746	-1.74056045816396	0.0911970887273068	0.829996066208931	-4.40580764078997
a_HQ_transcript/143058	1.98220404219552	0.222310148714732	1.74051134155933	0.0912058284952071	0.829996066208931	-4.50175754476548
a_HQ_transcript/94342	2.02896410505238	-0.270299771420781	1.74048020250038	0.0912113697184883	0.829996066208931	-4.42422652281922
a_HQ_transcript/136135	-3.21933926968219	5.86238143275761	-1.7404478840815	0.0912171211094266	0.829996066208931	-4.98920057476469
a_HQ_transcript/1291	2.31733418211544	4.76127890486055	1.74043839230377	0.091218810325651	0.829996066208931	-4.75421207286351
a_HQ_transcript/81652	2.59231893833862	0.627263469307177	1.74041657758753	0.091222692708951	0.829996066208931	-4.50227613951274
a_HQ_transcript/77224	3.09225598998419	1.27665171451689	1.74038974279776	0.0912274687096088	0.829996066208931	-4.44133134106334
a_HQ_transcript/114331	-1.76820544568683	1.83192008322488	-1.74031198022219	0.0912413099207794	0.829996066208931	-4.44424838023974
a_HQ_transcript/91617	-2.90836185454704	0.708653487841122	-1.74013614112148	0.0912726146023246	0.830150921859478	-4.46664467501689
a_HQ_transcript/39931	-2.52696007676761	0.440477946942933	-1.73992765399134	0.0913097433278075	0.830351165056518	-4.59186574821287
a_HQ_transcript/90298	2.9702824656472	0.657357383928328	1.73985210623699	0.0913232004921263	0.830351165056518	-4.46482365536962
a_HQ_transcript/109135	-2.19302678290332	0.610579200239717	-1.73947874570261	0.0913897309252105	0.830473132332572	-4.41544587027919

a_HQ_transcript/58731	-3.87531447959205	1.51852257722783	-1.7394740766605	0.0913905631763548	0.830473132332572	-4.68646198231855
a_HQ_transcript/125936	-1.79397880758448	-0.609368528507659	-1.73944180166794	0.0913963163293282	0.830473132332572	-4.47199472706941
a_HQ_transcript/84891	-2.4721745462718	1.83536351820559	-1.73943519862473	0.0913974933870489	0.830473132332572	-4.39485640784992
a_HQ_transcript/61732	0.931965700597018	2.53543370975688	1.7393759843402	0.0914080494884359	0.830473132332572	-4.5216790961839
a_HQ_transcript/141664	-2.11771102056304	-0.0635113629808532	-1.73920283985704	0.09143892175358	0.830623791794358	-4.44149929796472
a_HQ_transcript/12212	-1.80719508102592	1.20105671812401	-1.73908561500107	0.0914598283324789	0.830683890829739	-4.54122604267138
a_HQ_transcript/139182	-0.781802887079306	2.73792469724412	-1.7386542308003	0.0915367985822631	0.831181165320376	-4.58278540824511
a_HQ_transcript/126638	1.48218296513641	0.74215525025657	1.73861849360787	0.0915431774769654	0.831181165320376	-4.43120184442108
a_HQ_transcript/146228	-2.09335315217132	1.00182011147277	-1.73849704052501	0.0915648589866814	0.831209595462329	-4.42302896461188
a_HQ_transcript/86717	2.02139477784276	-0.449282679451032	1.7383785160603	0.0915860218500406	0.831209595462329	-4.49062325284543
a_HQ_transcript/119617	2.59410767360912	1.02616493569164	1.73825912095761	0.0916073443268091	0.831209595462329	-4.47603513859081
a_HQ_transcript/4822	1.8429250082709	-0.5120606322092	1.73815021132389	0.0916267978701644	0.831209595462329	-4.46587264070626
a_HQ_transcript/91649	1.70088967111221	0.0350396997620821	1.73803592340254	0.091647215823855	0.831209595462329	-4.34704393005198
a_HQ_transcript/73290	1.21749258990003	2.00703549726116	1.73793214454345	0.091665759609667	0.831209595462329	-4.41547135975345
a_HQ_transcript/81715	2.82587814832133	-0.323070956537824	1.73787376085185	0.0916761933203054	0.831209595462329	-4.48066334837949
a_HQ_transcript/95311	2.66796057150486	3.10135842060481	1.73787341384359	0.0916762553369072	0.831209595462329	-4.66283297452531
a_HQ_transcript/135157	-3.29123296919219	0.713942149337866	-1.73779240821412	0.0916907334612412	0.831209595462329	-4.44564456444251
a_HQ_transcript/148118	3.71426033784197	1.96234765666265	1.73778482701616	0.0916920885460306	0.831209595462329	-4.64623100855043
a_HQ_transcript/142614	2.38931426612315	0.475324397143792	1.73772040203493	0.0917036047292168	0.831209595462329	-4.46324080012025
a_HQ_transcript/149621	-1.87640525749879	1.43988269856707	-1.73738976825632	0.091762725773936	0.831615797285212	-4.49986586363688
a_HQ_transcript/17140	2.93004911167208	0.523028079389908	1.73714107052022	0.0918072168474781	0.831819392735046	-4.40713832201102
a_HQ_transcript/16938	1.04450871071388	2.81305843413508	1.73707193001879	0.0918195890402435	0.831819392735046	-4.48290925264963
a_HQ_transcript/47449	-2.55162526937889	0.0504145630576124	-1.73702425228808	0.0918281214420037	0.831819392735046	-4.56365008763788
a_HQ_transcript/143772	1.35483857548482	4.2689070765556	1.73683290561171	0.0918623715300745	0.831937243342753	-4.8180664191613
a_HQ_transcript/8608	1.74945951056902	1.04936983548342	1.73679165941454	0.091869755797845	0.831937243342753	-4.34274761246678
a_HQ_transcript/110103	-2.12580300840878	-0.379917316601088	-1.73650745127006	0.091920650881439	0.832268472653627	-4.44630015319175
a_HQ_transcript/144197	-2.63057992214896	2.82252807587397	-1.73606176605415	0.0920005104916353	0.832612541590356	-4.4452055158838
a_HQ_transcript/6282	1.3070755048301	0.245420584738939	1.73601796190756	0.0920083626359501	0.832612541590356	-4.34709448653671
a_HQ_transcript/131751	-2.44090771540685	3.82380084873431	-1.73600575311789	0.0920105512318536	0.832612541590356	-4.77372859535677
a_HQ_transcript/39822	-1.96926048416399	-0.51362146928483	-1.73595641868803	0.0920193955625202	0.832612541590356	-4.48140870271157
a_HQ_transcript/66394	2.13086021154796	0.851004981997051	1.73584540546437	0.0920392998482526	0.832612541590356	-4.47209173988245
a_HQ_transcript/56793	2.08598882248515	2.2220219219809	1.73581587841828	0.0920445945544252	0.832612541590356	-4.60675710524885
a_HQ_transcript/148780	-1.57537366772444	3.99122721998074	-1.73566799463399	0.0920711165078039	0.832722866090808	-4.84003583115618
a_HQ_transcript/122282	2.27846509948609	0.229128325259678	1.73550514946703	0.0921003291148809	0.832857487677317	-4.47441556985059
a_HQ_transcript/107936	-0.605584713146786	3.59524746886781	-1.73518511174127	0.0921577630155577	0.833097262102656	-4.81141129051702
a_HQ_transcript/98321	-1.8948637552809	-0.237732960841936	-1.73516083976023	0.092162120087858	0.833097262102656	-4.42801952078989
a_HQ_transcript/82714	-2.42102578944752	0.191460580200035	-1.73504699974593	0.0921825578614166	0.833097262102656	-4.42524836268427
a_HQ_transcript/14831	-2.97205576749805	-0.246821091983913	-1.73503800595422	0.0921841726851824	0.833097262102656	-4.4691925732584
a_HQ_transcript/13002	-1.41267330935353	-0.400485032296154	-1.73462376617121	0.0922585746770937	0.833640048007167	-4.42375120095167
a_HQ_transcript/139210	2.16679049794792	-0.250462080270759	1.73446625762505	0.0922868781734033	0.833766188135465	-4.48184357842185
a_HQ_transcript/147276	-3.94230133473776	3.64585256394266	-1.73436889326893	0.0923043777095866	0.833794695804639	-4.87122235887543
a_HQ_transcript/63425	2.35497117939764	0.532484451843951	1.73402959821038	0.092365381841335	0.834119727924033	-4.51475495766701
a_HQ_transcript/129307	-2.41916407349105	0.800331619921043	-1.73400914987868	0.0923690594661265	0.834119727924033	-4.48353775808838
a_HQ_transcript/95736	-0.758790149943659	2.50640732685282	-1.73368300803596	0.0924277325887682	0.834414250597741	-4.55193170441928
a_HQ_transcript/54768	-1.60808093460355	3.01318517313236	-1.73366827409463	0.0924303839732806	0.834414250597741	-4.69879121873262
a_HQ_transcript/49374	-6.88920144145067	3.4549892088425	-1.73338596279655	0.0924811984622239	0.834630022145504	-4.65338345549631
a_HQ_transcript/20184	-1.7259933506672	-0.237030078501904	-1.73337594213871	0.0924830025571015	0.834630022145504	-4.50988195049806
a_HQ_transcript/134959	2.75318843703697	0.333132569435454	1.73304638104643	0.0925423524161418	0.835035992121311	-4.47616016958223
a_HQ_transcript/69644	-0.820908666638913	3.06253434602959	-1.73272567568654	0.0926001381873438	0.835427725058811	-4.63279659990705
a_HQ_transcript/147645	0.669368169683467	9.46719154977675	1.73262405703331	0.0926184545018911	0.835463302806631	-5.41219005907135

a_HQ_transcript/141729	-1.17585033249215	3.70810115858051	-1.73249296770918	0.0926420872709089	0.835502901674009	-4.81623102533402
a_HQ_transcript/96592	-1.15756996426645	1.89483154227997	-1.73239135513353	0.0926604094602258	0.835502901674009	-4.52858232370664
a_HQ_transcript/14923	2.49062172684525	4.2757239952679	1.73236054875143	0.0926659648894222	0.835502901674009	-4.81197563083549
a_HQ_transcript/26883	-2.00554149199404	0.0328517754298889	-1.73213703953548	0.092706279516096	0.835545835311743	-4.41343597856688
a_HQ_transcript/145310	-3.32666762799421	1.1304834666567	-1.73209803126745	0.092713316994354	0.835545835311743	-4.5382431495762
a_HQ_transcript/77140	2.44130394047654	0.815770057561414	1.73207908732517	0.0927167348312357	0.835545835311743	-4.43096400490467
a_HQ_transcript/138089	1.71439619295949	0.0820680001100358	1.7320154129627	0.0927282236374218	0.835545835311743	-4.53772334372663
a_HQ_transcript/105390	-2.27074595131162	-0.0843694544056848	-1.73186483186722	0.0927553978396725	0.835661154002554	-4.49007379029607
a_HQ_transcript/8179	1.86012065024868	2.01206975096411	1.73156672914226	0.0928092138567879	0.835987348622977	-4.48833691705759
a_HQ_transcript/140604	2.42997746735595	2.77855174512829	1.73150496152853	0.092820367946819	0.835987348622977	-4.53251168950752
a_HQ_transcript/134469	1.03152146343528	4.81749870681815	1.7312472430913	0.0928669192871364	0.836277038624736	-4.83602504756317
a_HQ_transcript/91328	-1.88423596746491	-0.34258857458769	-1.73094822465263	0.0929209551660844	0.836634027554856	-4.48355960479663
a_HQ_transcript/26079	2.56478718800884	0.747013896800184	1.73063739196214	0.0929771539999608	0.83701037752977	-4.37506107020628
a_HQ_transcript/11397	1.56032526916415	-0.0157148262469473	1.73050608523678	0.0930009029508148	0.837012219289741	-4.40017198710112
a_HQ_transcript/2246	-2.64636871759194	0.02822437618888	-1.73043383983949	0.093013971875156	0.837012219289741	-4.50526811052663
a_HQ_transcript/129907	2.26860879082135	0.122308574962214	1.73032387856078	0.0930338664228442	0.837012219289741	-4.41478874735814
a_HQ_transcript/9029	2.87366291651101	1.24819917776426	1.73021899123116	0.0930528463053227	0.837012219289741	-4.46263574507576
a_HQ_transcript/53123	3.07453905229394	2.52984618269279	1.7301423574943	0.0930667156158577	0.837012219289741	-4.62988608859589
a_HQ_transcript/147436	-2.65453253342647	-0.0497936181486589	-1.73014202631999	0.0930667755561445	0.837012219289741	-4.53316728150148
a_HQ_transcript/111801	-5.1134118526375	1.81523105482368	-1.73007915812715	0.0930781548563324	0.837012219289741	-4.44611205448337
a_HQ_transcript/108971	3.42836420965071	0.577350645024405	1.72989107062106	0.0931122061427382	0.837188912399859	-4.55273595919334
a_HQ_transcript/29748	1.50871824705572	2.03653543378805	1.72937712882601	0.0932053033071846	0.837532825563437	-4.46392924081636
a_HQ_transcript/52781	-2.94999588209467	1.15843918206982	-1.72932027441164	0.0932156069089781	0.837532825563437	-4.43347302891898
a_HQ_transcript/142591	2.67899485441337	4.34451530933901	1.72920977374917	0.0932356354394626	0.837532825563437	-4.73706158781263
a_HQ_transcript/139212	-3.88405569154584	5.97295520303772	-1.7291903713602	0.0932391525454853	0.837532825563437	-5.22608858174117
a_HQ_transcript/41056	-1.77650786919491	2.04424986343131	-1.72914062077991	0.0932481714319076	0.837532825563437	-4.51845857955203
a_HQ_transcript/653	1.73696613382566	2.19099432816652	1.72913568684739	0.0932490659051574	0.837532825563437	-4.50231522864259
a_HQ_transcript/110915	-2.53731473728387	2.81748294501489	-1.72904471485011	0.0932655595210863	0.837532825563437	-4.5815935214668
a_HQ_transcript/12236	-2.5850929898445	1.79539835896754	-1.72898943407344	0.0932755833610575	0.837532825563437	-4.49809379181445
a_HQ_transcript/143946	-2.61656141764036	0.460643480082171	-1.72890058258029	0.0932916963387855	0.837532825563437	-4.38875781017907
a_HQ_transcript/115454	-1.60351155639552	0.973998675022948	-1.72881132184647	0.0933078858843537	0.837532825563437	-4.35333134892193
a_HQ_transcript/38862	2.24469889271812	2.21711646601366	1.72880546170001	0.0933089488430502	0.837532825563437	-4.45489410971023
a_HQ_transcript/121906	1.92066001386045	0.209471793144847	1.72857221482971	0.0933512652209904	0.837783286207461	-4.45609764482469
a_HQ_transcript/81495	2.18289549295158	0.33891360880515	1.72845255070605	0.0933729813139336	0.837848820286559	-4.42972261721948
a_HQ_transcript/122304	-1.4785444958045	-0.534599711709012	-1.72799680003448	0.0934557276830589	0.83846188281538	-4.54791477410629
a_HQ_transcript/94118	1.48078185971181	-0.718142334407181	1.72772554788663	0.0935050056043478	0.838774531754557	-4.49978551456647
a_HQ_transcript/129278	1.04396500370018	3.59542103828763	1.72746173783719	0.0935529524528838	0.83883338459669	-4.81729936145218
a_HQ_transcript/28397	2.50498915879842	1.89160326279153	1.72744593906209	0.0935558244975102	0.83883338459669	-4.46354714903738
a_HQ_transcript/9444	2.82883757405879	1.37574883797283	1.72739715069338	0.093564694156854	0.83883338459669	-4.54735302222005
a_HQ_transcript/148964	-0.440630705488502	6.71831945073112	-1.72737187458037	0.0935692895975251	0.83883338459669	-5.09952153079053
a_HQ_transcript/26385	2.48045668978291	0.0606649692251064	1.7271376105591	0.0936118900675798	0.839027004214082	-4.51230084360272
a_HQ_transcript/113083	-2.37723901865013	0.405814352852638	-1.72709436743339	0.0936197555280164	0.839027004214082	-4.43998813328449
a_HQ_transcript/9682	0.569552329536048	3.02222126598611	1.72673032148043	0.0936859935738358	0.839491045850895	-4.65545686880983
a_HQ_transcript/115801	-3.4579935951992	1.27923102879646	-1.72650401886053	0.093727189086165	0.839491045850895	-4.58013395597062
a_HQ_transcript/150752	2.24734941980514	0.0975356589527258	1.72640888891323	0.0937445108207126	0.839491045850895	-4.48531157222567
a_HQ_transcript/109048	2.52475727981783	-0.445408473269345	1.72624881004324	0.0937736648428379	0.839491045850895	-4.50155868001602
a_HQ_transcript/95531	2.17322100325964	0.868865045056293	1.72616343238738	0.0937892171771545	0.839491045850895	-4.47771822988304
a_HQ_transcript/66628	-3.11385391236686	-0.0859733873838788	-1.72598959871982	0.0938208892875376	0.839491045850895	-4.50048815199931
a_HQ_transcript/122818	-2.28893228933765	1.85292139523844	-1.72589691869346	0.0938377790517877	0.839491045850895	-4.49664515263374
a_HQ_transcript/140479	3.23361658696983	0.625842975769823	1.72589083399492	0.0938388880004993	0.839491045850895	-4.52396218424524

a_HQ_transcript/26763	4.40521623895744	1.49395249740992	1.72572078012775	0.0938698851134588	0.839491045850895	-4.41502884385855
a_HQ_transcript/193	-0.602576411370388	4.47317307101538	-1.72570100650223	0.0938734899719184	0.839491045850895	-4.88917643651422
a_HQ_transcript/96579	-1.5042888996275	-0.673047151631017	-1.72567573218563	0.0938780978106991	0.839491045850895	-4.50284087294533
a_HQ_transcript/38793	-1.86680398982616	-0.17200958253709	-1.72559771298275	0.0938923229299911	0.839491045850895	-4.45817938151898
a_HQ_transcript/60504	-2.33907082111481	0.824764645193428	-1.72543683018702	0.0939216621492968	0.839491045850895	-4.44233439283426
a_HQ_transcript/107580	-2.54056925267247	1.13742231412643	-1.72541992122947	0.0939247461800037	0.839491045850895	-4.51894677685068
a_HQ_transcript/152287	-1.08687749055597	3.33549260412988	-1.72541183538102	0.0939262209907765	0.839491045850895	-4.64481463674247
a_HQ_transcript/46679	0.858819934064757	3.63992968054932	1.72520578556614	0.0939638098116102	0.839491045850895	-4.79605476603034
a_HQ_transcript/117793	-1.45734530079798	1.11569422168073	-1.72519552117729	0.0939656826318878	0.839491045850895	-4.45803681309795
a_HQ_transcript/41295	1.81554453678141	-0.419233664933487	1.72514144380857	0.0939755499998316	0.839491045850895	-4.52484004025453
a_HQ_transcript/10392	1.47906703340468	-0.43176919249608	1.72510253207552	0.0939826506684332	0.839491045850895	-4.41196355787234
a_HQ_transcript/80557	-2.09151576433803	1.39709937955837	-1.72490850886128	0.0940180630205804	0.839491045850895	-4.39768679145418
a_HQ_transcript/97865	-0.835755721404156	3.08507641487974	-1.72483931071928	0.0940306955008604	0.839491045850895	-4.72241929198207
a_HQ_transcript/17434	-0.898063416946827	3.24644711551636	-1.72479950254203	0.0940379633356745	0.839491045850895	-4.67478061056966
a_HQ_transcript/140941	-3.16422962114953	4.41902747819788	-1.72479312414046	0.0940391278932145	0.839491045850895	-4.92078320217036
a_HQ_transcript/148544	2.33418462964226	0.103628965654171	1.72477582838078	0.0940422857840279	0.839491045850895	-4.52582004894284
a_HQ_transcript/3494	2.06926723526435	-0.25390183467562	1.72474054951231	0.0940487273389237	0.839491045850895	-4.49488976549614
a_HQ_transcript/134147	2.66453494538441	1.89817846104657	1.72461784538326	0.0940711347212713	0.839491045850895	-4.43546950118568
a_HQ_transcript/7479	-1.63108550564042	0.0148092957522299	-1.72445520644219	0.0941008416266335	0.839491045850895	-4.39038716242339
a_HQ_transcript/28538	1.97143804149007	0.561701306816976	1.7243566860476	0.0941188407512388	0.839491045850895	-4.47403325417553
a_HQ_transcript/59044	-3.30935029290874	2.73779978380385	-1.72432503009245	0.0941246247301538	0.839491045850895	-4.62106886288205
a_HQ_transcript/134903	3.17299288676582	1.25474145666844	1.72429605366391	0.0941299193840729	0.839491045850895	-4.51406484224474
a_HQ_transcript/50255	2.60959516026366	-0.227472366529212	1.72423808590171	0.0941405121653515	0.839491045850895	-4.45747006216039
a_HQ_transcript/107170	-3.46751719886099	1.40470988392381	-1.72423357100806	0.0941413372397106	0.839491045850895	-4.58231293668734
a_HQ_transcript/4870	-1.44110038260088	2.85924058324688	-1.72417860856519	0.094151381839252	0.839491045850895	-4.61644547773796
a_HQ_transcript/113770	-2.20610775052726	1.69490201676837	-1.72405397762045	0.0941741619586659	0.839491045850895	-4.54751135209437
a_HQ_transcript/350	-2.41494303258902	2.82507482000489	-1.72403840962984	0.0941770078102409	0.839491045850895	-4.46694153115497
a_HQ_transcript/44405	-2.46744020350743	3.56429581413411	-1.72387579379311	0.0942067385372792	0.839564174244616	-4.7268171515998
a_HQ_transcript/146101	5.14506815981056	5.26065097951432	1.72380242911344	0.0942201542316174	0.839564174244616	-4.82427201537836
a_HQ_transcript/55934	-2.16842285669087	0.739591902819552	-1.72375656601886	0.0942285417143524	0.839564174244616	-4.41156733819154
a_HQ_transcript/1415	-3.2484162608026	0.062679553220344	-1.72344676337095	0.0942852150998604	0.839940380586158	-4.52029807063371
a_HQ_transcript/87533	-2.70498993844414	1.70266503043342	-1.72317742675999	0.0943345091101447	0.840132987806126	-4.468835450623
a_HQ_transcript/142718	-2.69099440277142	-0.140744534466919	-1.72303301651724	0.0943609479869482	0.840132987806126	-4.50854637688459
a_HQ_transcript/23182	-3.179877182949	2.46838465203144	-1.72297915787401	0.0943708101115902	0.840132987806126	-4.61241021415071
a_HQ_transcript/140992	-3.23764695354549	0.237366992797498	-1.72287778028087	0.0943893758368218	0.840132987806126	-4.56119176193489
a_HQ_transcript/88626	2.48117065120246	1.88506828276499	1.72287227008803	0.0943903850304868	0.840132987806126	-4.46802496771368
a_HQ_transcript/150027	1.17644619424681	4.08123989886155	1.72275191600923	0.0944124301781316	0.840132987806126	-4.79002049238113
a_HQ_transcript/20618	-2.21851577433095	1.38977737343074	-1.72268684682054	0.0944243506409274	0.840132987806126	-4.50376046007198
a_HQ_transcript/65189	1.88721540087673	-0.0344933877162737	1.72263484512572	0.0944338780890548	0.840132987806126	-4.41695406787011
a_HQ_transcript/89040	-2.15283809817812	1.17481278811358	-1.72261545281591	0.0944374312416862	0.840132987806126	-4.53410439062481
a_HQ_transcript/74196	-1.77373962163991	-0.669476602596543	-1.72215159889604	0.0945224542198409	0.840132987806126	-4.45245462650447
a_HQ_transcript/92123	3.03205429143095	1.00138748586692	1.7221199464065	0.0945282583616176	0.840132987806126	-4.66667403206371
a_HQ_transcript/54552	2.20382086895172	1.32738956039472	1.72207243304149	0.0945369714856256	0.840132987806126	-4.49282932124815
a_HQ_transcript/128271	0.718537842610695	3.61544217530541	1.72199743254607	0.0945507266415721	0.840132987806126	-4.74490658193381
a_HQ_transcript/26598	-2.6712835988093	-0.491710314814768	-1.72195045285363	0.0945593436128611	0.840132987806126	-4.52189586625878
a_HQ_transcript/143807	3.46822812132039	0.0401840201625209	1.7218741616233	0.0945733382821724	0.840132987806126	-4.50818090270885
a_HQ_transcript/59071	-3.62254472121229	-0.186404009709262	-1.7217709577881	0.0945922724953591	0.840132987806126	-4.45682266672177
a_HQ_transcript/2524	-2.75064973976615	2.61145186985671	-1.72171443553901	0.0946026436540348	0.840132987806126	-4.62038060033729
a_HQ_transcript/59577	-1.34593410468149	1.14555924848026	-1.72169821850541	0.09460561946235	0.840132987806126	-4.39961461729995
a_HQ_transcript/61415	0.831911432547688	4.13385501997232	1.72163226413271	0.0946177228280059	0.840132987806126	-4.80163493130144

a_HQ_transcript/119798	2.88370874965712	0.674216008054479	1.72159459419088	0.0946246362665875	0.840132987806126	-4.43207515676529
a_HQ_transcript/59560	-3.10573804368192	0.600991433519152	-1.72157585337553	0.094628075863613	0.840132987806126	-4.60761637945899
a_HQ_transcript/28293	3.0265073167872	0.768407142889785	1.7215307558277	0.0946363532753347	0.840132987806126	-4.46716729193493
a_HQ_transcript/12996	-0.919152482681665	2.3440542133422	-1.72145490300273	0.0946502770217922	0.840132987806126	-4.55479131886111
a_HQ_transcript/115770	-4.97182489809271	3.56297289445278	-1.7213028670834	0.0946781903121553	0.840132987806126	-4.70734640365965
a_HQ_transcript/61935	-1.61824189981566	0.858948301006139	-1.7211490420938	0.0947064390946234	0.840132987806126	-4.38874853302686
a_HQ_transcript/151814	-2.64187521228938	-0.21736013650412	-1.72114698806144	0.0947068163497626	0.840132987806126	-4.48799512472398
a_HQ_transcript/87057	-2.1260510609862	0.0645589060236818	-1.72113120196933	0.0947097157543059	0.840132987806126	-4.51350502378466
a_HQ_transcript/73085	-2.95857607733757	1.05013410492702	-1.7211213568143	0.0947115240347774	0.840132987806126	-4.41924055461648
a_HQ_transcript/78573	2.1664255194783	0.0602088294091241	1.72101230544356	0.0947315556673259	0.840182463812988	-4.51423599050104
a_HQ_transcript/44598	-1.71126249322627	0.60338441228302	-1.72058398725183	0.0948102677627535	0.840276717937716	-4.37852828877169
a_HQ_transcript/137199	-1.13515094193796	1.78890892269934	-1.72032581096417	0.0948577393387174	0.840276717937716	-4.46108228593738
a_HQ_transcript/151625	2.51680572362375	0.359031700789036	1.72026921220374	0.0948681489694763	0.840276717937716	-4.52059487334363
a_HQ_transcript/57690	2.72748189098471	3.30370772822892	1.72025892815993	0.0948700405112211	0.840276717937716	-4.6907551262739
a_HQ_transcript/11963	0.757257816920442	3.32108495997154	1.72014507775281	0.0948909831023767	0.840276717937716	-4.74148709897927
a_HQ_transcript/140241	-0.993851573548098	5.42930379714942	-1.72007866726664	0.0949032009894724	0.840276717937716	-5.02881923882899
a_HQ_transcript/69093	2.34169338745388	2.04172374932394	1.72002646567655	0.094912805716837	0.840276717937716	-4.67297451018132
a_HQ_transcript/133369	-3.57247304269752	1.91889465320594	-1.72000302940741	0.0949171180914469	0.840276717937716	-4.6609748370465
a_HQ_transcript/71346	3.18448297425992	0.726970786993971	1.71984950655825	0.0949453710203705	0.840276717937716	-4.53555178978169
a_HQ_transcript/76596	-1.65277682381074	-0.35082921831043	-1.71982833559848	0.0949492676815971	0.840276717937716	-4.44209512396809
a_HQ_transcript/33943	-2.87317250609553	-0.0170990825774727	-1.71962704250077	0.0949863237628963	0.840276717937716	-4.50762274986943
a_HQ_transcript/50736	2.8177955079431	2.3312684834724	1.71955265046395	0.0950000216736774	0.840276717937716	-4.67190198249401
a_HQ_transcript/111167	-1.72225151022797	2.40749798736419	-1.71952520693733	0.0950050753070831	0.840276717937716	-4.52796440261204
a_HQ_transcript/31639	2.09531506666158	1.40987421024036	1.71951381749841	0.0950071727002388	0.840276717937716	-4.49270180786581
a_HQ_transcript/97201	3.52313036285598	1.23656974605078	1.7194434522832	0.0950201314881686	0.840276717937716	-4.50574975920809
a_HQ_transcript/127772	1.93571259825339	0.758940195294112	1.71940559043252	0.0950271049172018	0.840276717937716	-4.39081116395015
a_HQ_transcript/4927	-1.86072673397616	1.46144495204969	-1.71936498140836	0.0950345847998833	0.840276717937716	-4.4442231238026
a_HQ_transcript/94353	0.617055997716993	3.72604451492987	1.71931181753303	0.0950443779395293	0.840276717937716	-4.80825904546899
a_HQ_transcript/95965	2.29815283390806	2.22390374281397	1.71924914028336	0.0950559245925663	0.840276717937716	-4.52784870655014
a_HQ_transcript/2502	1.23499783153605	2.43029819739599	1.7191640085454	0.0950716097842206	0.840276717937716	-4.5958298604582
a_HQ_transcript/148221	2.34533764818987	0.999387391654286	1.71912859954439	0.0950781343937765	0.840276717937716	-4.49677855595051
a_HQ_transcript/61779	-2.29000389279296	1.26698440608734	-1.71910069189511	0.0950832770375946	0.840276717937716	-4.5012529301982
a_HQ_transcript/105947	-2.11659305029185	-0.523607688534183	-1.71907376938897	0.0950882383664682	0.840276717937716	-4.46207218909779
a_HQ_transcript/46859	-2.27821986355886	1.19212193190395	-1.71901244719885	0.0950995397422992	0.840276717937716	-4.44415104322419
a_HQ_transcript/18655	1.8290073390758	4.70718110865845	1.71893322967004	0.095114140807013	0.840276717937716	-4.92971282872436
a_HQ_transcript/136859	-3.16226956806095	0.828572167391505	-1.71887120175046	0.0951255748618949	0.840276717937716	-4.42359045950888
a_HQ_transcript/51559	2.89856415771038	0.653846959352898	1.71876734229542	0.0951447226057392	0.840276717937716	-4.57844832829962
a_HQ_transcript/31555	-2.47898119263077	1.18009610224335	-1.71875531331911	0.0951469405014115	0.840276717937716	-4.41374649940349
a_HQ_transcript/136726	3.11962026697864	3.3530264290691	1.71848600032823	0.0951966076212572	0.840587636003104	-4.84480870007879
a_HQ_transcript/139812	0.438188569397827	4.3154625448982	1.71838869023284	0.0952145590435795	0.840618452017799	-4.87268874854907
a_HQ_transcript/101716	2.12965654088435	-0.321253666345212	1.71822006353141	0.0952456734192052	0.840657527904623	-4.50237154034919
a_HQ_transcript/131473	1.02790226969826	2.81628598402902	1.71801938673954	0.095282712663358	0.840657527904623	-4.69085534282605
a_HQ_transcript/61851	-2.36985999261752	1.53997759261504	-1.71793618025939	0.0952980737595102	0.840657527904623	-4.45343634377126
a_HQ_transcript/26473	-2.94293458323136	0.678977490769778	-1.71790528998641	0.0953037770701869	0.840657527904623	-4.51951621800931
a_HQ_transcript/126475	10.073748655093	2.6591553766609	1.7178768607321	0.0953090262526022	0.840657527904623	-4.61841819827715
a_HQ_transcript/122049	-2.56251038150869	1.45475411418616	-1.71785185177357	0.0953136441113333	0.840657527904623	-4.60328532147514
a_HQ_transcript/148424	2.59497170255875	-0.108889115949597	1.71778696899452	0.0953256254728102	0.840657527904623	-4.50049211132134
a_HQ_transcript/121352	-2.14408006427414	2.13309184245676	-1.71773792696429	0.0953346824928997	0.840657527904623	-4.44885018536047
a_HQ_transcript/127423	-1.37838367238415	-0.71389838402033	-1.71739451893591	0.0953981228594041	0.840762781915807	-4.51875802595317
a_HQ_transcript/43831	-1.59801218945068	1.28953958335892	-1.71736473537869	0.0954036266683006	0.840762781915807	-4.51223562139131

a_HQ_transcript/74186	-4.21423546378631	1.54399122841447	-1.71735487330804	0.0954054491738606	0.840762781915807	-4.45221351476296
a_HQ_transcript/105116	-1.86515096993341	0.908843691879919	-1.71730663807462	0.0954143634403781	0.840762781915807	-4.40829694755859
a_HQ_transcript/6480	-1.97411716266455	-0.161151720213049	-1.71712834482199	0.0954473195530165	0.840762781915807	-4.53467683043748
a_HQ_transcript/122120	-2.6685273512444	2.45460154498485	-1.71712704746803	0.0954475593936964	0.840762781915807	-4.61178173120817
a_HQ_transcript/149758	-2.24161653657588	1.22648557756043	-1.71706560209279	0.0954589193242311	0.840762781915807	-4.50362964117233
a_HQ_transcript/44994	2.31722127801651	0.797031825917045	1.716892402533	0.0954909463014333	0.840762781915807	-4.47246444547831
a_HQ_transcript/36102	-2.70727334107955	0.750444810268838	-1.71686078654587	0.0954967935032906	0.840762781915807	-4.45775994394892
a_HQ_transcript/37510	-1.75770070626136	-0.365185321179931	-1.71686046409055	0.0954968531411635	0.840762781915807	-4.51617950137875
a_HQ_transcript/377	-0.83288536509662	2.79888737117498	-1.71677194973265	0.0955132249860939	0.840762781915807	-4.61416311403981
a_HQ_transcript/65914	1.45715454771133	1.84363213679103	1.71669504632514	0.0955274511487444	0.840762781915807	-4.50914885473873
a_HQ_transcript/72892	-1.76143359682923	1.22784375600999	-1.71665613096566	0.0955346506770215	0.840762781915807	-4.42451448568021
a_HQ_transcript/648	1.11321166865034	2.02018475138328	1.71656020802751	0.095552398822395	0.840791680101147	-4.47423154368249
a_HQ_transcript/95692	2.1428470994861	1.63250786671629	1.71590717821839	0.0956732991236193	0.841490160411346	-4.50060709613081
a_HQ_transcript/23921	0.433751919644669	3.89975174561777	1.71589670087981	0.0956752399159017	0.841490160411346	-4.82985501577619
a_HQ_transcript/100847	-1.8786539367813	1.69582186800167	-1.71582682366269	0.0956881846156277	0.841490160411346	-4.38179965512926
a_HQ_transcript/103665	-2.6234855863784	1.47127889812879	-1.71581872948337	0.0956896841508222	0.841490160411346	-4.42777091061135
a_HQ_transcript/139107	-2.65450685520744	0.282473973595071	-1.71572896934657	0.0957063145169893	0.841509098509084	-4.51951851464831
a_HQ_transcript/147784	5.0727823828837	8.17867936642705	1.71560626950305	0.0957290517252516	0.841581717889508	-5.27878811916328
a_HQ_transcript/1242	0.709812424467301	3.57140981932085	1.7154419976203	0.0957594996309924	0.841661970565913	-4.77390109234122
a_HQ_transcript/58802	2.36442463812689	3.02600449018478	1.71540078710132	0.0957671393015922	0.841661970565913	-4.65747285566818
a_HQ_transcript/44362	1.62041936177441	1.53532585443303	1.71530454484365	0.0957849828306645	0.841691531667554	-4.49814104539252
a_HQ_transcript/127609	-1.82150063944907	-0.225362285336843	-1.71515749337505	0.0958122518790947	0.841803896195286	-4.47970868351291
a_HQ_transcript/35590	-0.667978182363572	2.92657445766215	-1.71466527975085	0.0959035747008809	0.842426471169146	-4.65120531810259
a_HQ_transcript/124750	3.26366822937406	0.172297304106027	1.7145538586271	0.0959242573283259	0.842426471169146	-4.45080707337519
a_HQ_transcript/78745	0.925837379936197	2.04785180988839	1.71437381813661	0.0959576853712888	0.842426471169146	-4.53243240586721
a_HQ_transcript/105781	2.27095824366986	-0.0134332063077519	1.7143640034775	0.0959595079353598	0.842426471169146	-4.47142567918496
a_HQ_transcript/72608	-1.57642340531535	1.81797034050188	-1.71422140995176	0.0959859905604262	0.842426471169146	-4.53266902612852
a_HQ_transcript/137196	2.39893933178467	-0.174332479664338	1.71420705587912	0.0959886567535159	0.842426471169146	-4.50905315161569
a_HQ_transcript/85567	0.773036262194885	4.21149009577714	1.71420450305739	0.0959891309331503	0.842426471169146	-4.88154397324468
a_HQ_transcript/63875	-3.82988633744359	2.34718744037041	-1.7141510891682	0.0959990528665088	0.842426471169146	-4.59985488715252
a_HQ_transcript/84824	1.46848916450684	3.99614553101241	1.71390455623579	0.0960448588910208	0.842701216244114	-4.88003400018627
a_HQ_transcript/131611	3.96080623317956	0.313787900043514	1.71380029862903	0.0960642355477257	0.842744021116541	-4.55879757109235
a_HQ_transcript/48306	-2.44531098868276	1.20991961876279	-1.7134379585392	0.0961316032374912	0.843037908837549	-4.48753120876217
a_HQ_transcript/54751	-5.58894385316946	5.09541307376595	-1.7133993399705	0.0961387856855789	0.843037908837549	-5.14500607399805
a_HQ_transcript/133195	-3.62581643906073	3.72472323973785	-1.71338611652028	0.0961412451432032	0.843037908837549	-4.78882996043201
a_HQ_transcript/95515	-1.97132513639381	0.358175493753355	-1.71282025612196	0.0962465400996255	0.843827412053498	-4.42577106085795
a_HQ_transcript/132798	-2.69662219708511	0.342473824628986	-1.71271651135862	0.0962658553324043	0.843827412053498	-4.48113173245198
a_HQ_transcript/74649	3.07123991531145	2.5702506927036	1.71256402704573	0.0962942508007924	0.843827412053498	-4.64020758294261
a_HQ_transcript/10306	0.911268501787996	3.76311048000667	1.71254690534185	0.0962974396243691	0.843827412053498	-4.76094825771755
a_HQ_transcript/5996	1.62201584553854	1.87963284532523	1.7125124083234	0.0963038647736531	0.843827412053498	-4.43088041547093
a_HQ_transcript/3881	-3.02237608564754	2.17672158915108	-1.7122397679048	0.0963546573100803	0.844145217802614	-4.51358759629174
a_HQ_transcript/7204	3.17172031883378	0.213224417394738	1.71203266645685	0.0963932550137282	0.84435610720886	-4.56362937955055
a_HQ_transcript/96672	-2.49492106706513	-0.367349740689345	-1.71191446364761	0.0964152903910555	0.844383557252359	-4.51307806318619
a_HQ_transcript/132542	-4.16828559032934	0.612284315713056	-1.71184538960297	0.0964281691321946	0.844383557252359	-4.55247782615491
a_HQ_transcript/2641	-2.05706298688155	-0.0572110164840745	-1.71178211629684	0.0964399675990643	0.844383557252359	-4.58907260162019
a_HQ_transcript/50624	0.857736769066587	2.69504799123924	1.71156471362302	0.0964805155199418	0.84457382253463	-4.53326407155369
a_HQ_transcript/10253	1.95841906309585	2.8619614272525	1.71132567773291	0.0965251147382796	0.84457382253463	-4.4895858312525
a_HQ_transcript/31937	-1.50947353242532	1.55787361345677	-1.71124973768409	0.0965392872132472	0.84457382253463	-4.46300357055903
a_HQ_transcript/89812	1.52457978859445	0.769016167406981	1.71124408869458	0.0965403415376681	0.84457382253463	-4.40590824309907
a_HQ_transcript/135497	-3.85113566857631	0.33532653175844	-1.71122276679854	0.0965443211319477	0.84457382253463	-4.4503481956504

a_HQ_transcript/123137	-2.52821105552301	3.90778992694562	-1.71119836483355	0.0965488757697257	0.84457382253463	-4.82992774201353
a_HQ_transcript/47470	-2.10522125641964	-0.232224257710461	-1.71104459623903	0.096577580885282	0.844697806454961	-4.51661031811095
a_HQ_transcript/47481	-2.28410947282858	-0.391551257287422	-1.71073654994468	0.0966351076454901	0.844845721200398	-4.52184987483928
a_HQ_transcript/48418	2.24327602448228	0.905825357545327	1.7106567521422	0.0966500143320821	0.844845721200398	-4.47259154513246
a_HQ_transcript/12907	-1.19683817330712	4.78869461724495	-1.71063591949128	0.0966539063074129	0.844845721200398	-4.80361714670182
a_HQ_transcript/88157	7.47359009406732	2.6535867977166	1.71037200548154	0.0967032223275683	0.844845721200398	-4.5399426507401
a_HQ_transcript/20829	-2.45701823120431	0.998683669677542	-1.71036753485959	0.0967040579071089	0.844845721200398	-4.44790114405209
a_HQ_transcript/115267	1.97763770991086	2.1928718053521	1.71035667032276	0.096706088563557	0.844845721200398	-4.47659472761342
a_HQ_transcript/8167	-0.927483196866899	1.37315566724357	-1.71028198776828	0.0967200482110634	0.844845721200398	-4.39672766337794
a_HQ_transcript/83589	2.45395387585748	0.524681741205464	1.71021037646321	0.0967334353651478	0.844845721200398	-4.50137785571278
a_HQ_transcript/108151	-1.244055140437	4.19294835798415	-1.71005986373481	0.0967615775570658	0.844845721200398	-4.89767948697581
a_HQ_transcript/3146	-1.69384113824397	1.65718426934542	-1.71003006416445	0.0967671501590181	0.844845721200398	-4.50435032899831
a_HQ_transcript/140433	1.34880696568372	1.74830557242684	1.70999495152781	0.0967737166637283	0.844845721200398	-4.57051665562864
a_HQ_transcript/151930	-3.38027009636244	1.99143100382673	-1.70997039203824	0.0967783098188332	0.844845721200398	-4.58132543542376
a_HQ_transcript/96539	2.97386609800168	0.187414428729205	1.7099265355491	0.0967865123835258	0.844845721200398	-4.48015418659652
a_HQ_transcript/123431	3.99106693245073	0.562326952470956	1.70985084144915	0.0968006709673583	0.844845721200398	-4.52227501881631
a_HQ_transcript/20624	2.6337708098364	0.092513527203493	1.70975335143568	0.0968189090313795	0.844845721200398	-4.51718769374952
a_HQ_transcript/126667	2.85563287608812	0.264501290341513	1.70970989174771	0.0968270402325394	0.844845721200398	-4.48299597244658
a_HQ_transcript/56427	-2.06164423902248	0.140661840012172	-1.70950520204716	0.0968653448588764	0.845053094095268	-4.4879086623318
a_HQ_transcript/117339	-0.627308506863137	3.6201476172473	-1.70930144822949	0.0969034869417974	0.845215117517671	-4.79031023173349
a_HQ_transcript/39986	-1.81086180278546	-0.312622444316044	-1.70923338770138	0.0969162304615236	0.845215117517671	-4.55588242138401
a_HQ_transcript/34882	2.22787967773498	1.39920745052259	1.7091729986363	0.0969275387657031	0.845215117517671	-4.46609405161562
a_HQ_transcript/59292	1.3940045747881	0.960648891957569	1.70888189464903	0.0969820656552732	0.845563748674024	-4.36242309351648
a_HQ_transcript/71885	1.13319502999847	2.49280940937427	1.70871560074062	0.0970132257997967	0.845708576678252	-4.66112162255381
a_HQ_transcript/122977	2.50585764339091	0.40236438219044	1.70860976866437	0.0970330609708698	0.845754651089327	-4.38510537185139
a_HQ_transcript/135539	3.55440215953667	0.613288804841466	1.70826514931705	0.0970976734464339	0.846010943744498	-4.5413223555492
a_HQ_transcript/45507	-0.990145888930013	1.9634576734501	-1.70823402746972	0.0971035102347629	0.846010943744498	-4.5214877976828
a_HQ_transcript/62674	-2.0857162756578	0.21102098153105	-1.70815160570359	0.0971189695529004	0.846010943744498	-4.50396719244746
a_HQ_transcript/71523	-3.8230507695213	1.78164799407977	-1.7081086214939	0.0971270326403059	0.846010943744498	-4.66130629087164
a_HQ_transcript/123003	3.14677715883446	0.461067762188379	1.70797250587123	0.0971525692499425	0.846010943744498	-4.57597050395814
a_HQ_transcript/59806	-2.46035012139974	1.80657440848804	-1.70792535377894	0.0971614167491511	0.846010943744498	-4.43999848211429
a_HQ_transcript/36383	1.31472517107732	2.30110816889954	1.70776617816649	0.0971912890368092	0.846010943744498	-4.55017980032546
a_HQ_transcript/110681	1.82319138669685	-0.122615424541069	1.70775376549514	0.097193618830186	0.846010943744498	-4.55613473657061
a_HQ_transcript/120618	-4.21266275929311	2.0740561295719	-1.70769862187659	0.0972039695633069	0.846010943744498	-4.59573741505381
a_HQ_transcript/51561	2.55048377735481	1.07587750804526	1.70767710801394	0.0972080080730372	0.846010943744498	-4.56432533623028
a_HQ_transcript/139116	2.23764699358322	-0.107084541091375	1.70736601179044	0.0972664216983827	0.846289973439033	-4.51299696555865
a_HQ_transcript/135264	-2.01253682631046	0.237930633644416	-1.70735128739512	0.0972691871825313	0.846289973439033	-4.4807426746357
a_HQ_transcript/112559	-3.68324591621895	0.67353936155614	-1.70721712206989	0.0972943886723168	0.846382553837837	-4.56380640770777
a_HQ_transcript/143500	-2.86548187229373	-0.275207257899909	-1.70712061294713	0.0973125202334436	0.846387946048174	-4.53402204145995
a_HQ_transcript/78397	-1.90430230691529	-0.066884769131911	-1.70698924071463	0.0973372062110426	0.846387946048174	-4.43452934261591
a_HQ_transcript/101055	-1.05928928914362	2.06787080082022	-1.70691873872724	0.0973504563033661	0.846387946048174	-4.55477430545741
a_HQ_transcript/104998	2.41326976669175	-0.109224458008297	1.70685631454235	0.097362189518638	0.846387946048174	-4.57846004923764
a_HQ_transcript/70080	2.43978650786795	0.604924580630203	1.70676765180171	0.0973788565514613	0.846387946048174	-4.55027305404901
a_HQ_transcript/61438	0.664728202636067	3.47911321371688	1.70668695307211	0.0973940285650591	0.846387946048174	-4.77616021982588
a_HQ_transcript/152783	-1.56352445389768	2.26867940902496	-1.70667150126696	0.097396933854876	0.846387946048174	-4.64253348663917
a_HQ_transcript/4205	-1.57737342568566	3.51549789525026	-1.70638146140763	0.0974514813784919	0.84673538259626	-4.68775482551374
a_HQ_transcript/56310	-1.95067974562486	-0.436876549924856	-1.70609567996298	0.0975052530148259	0.847075974778927	-4.56451882473375
a_HQ_transcript/110250	0.867870646463621	2.45094470368161	1.70598209038535	0.0975266325226581	0.847135100908109	-4.52229959214311
a_HQ_transcript/88920	-1.79595345637162	0.841761895747416	-1.70580856571953	0.0975593004117433	0.847292247771375	-4.40989159484219
a_HQ_transcript/101474	1.73769606221727	-0.453364544201419	1.70568488451341	0.0975825903176128	0.847367913053809	-4.5269245446276

a_HQ_transcript/50269	2.84775344495672	1.55812394615427	1.70560022868142	0.0975985341939777	0.847379775299109	-4.54831280608958
a_HQ_transcript/138495	-3.20244535618732	0.60516122187774	-1.7054050290035	0.097635305936184	0.847542670413088	-4.51787769141836
a_HQ_transcript/126299	-3.70099635357931	4.49105651388584	-1.70503434385133	0.0977051675467534	0.847542670413088	-4.80232781168608
a_HQ_transcript/103792	1.83474678309257	1.96711325665511	1.70503303517435	0.0977054142621172	0.847542670413088	-4.46957051307595
a_HQ_transcript/86283	-1.3834260073835	3.53448480025058	-1.70502847383745	0.0977062741818975	0.847542670413088	-4.81090357455471
a_HQ_transcript/98674	2.85661726770102	1.43642650018963	1.70494134342212	0.097722701538964	0.847542670413088	-4.60991162843659
a_HQ_transcript/2081	0.962880452957856	3.13570158845192	1.70493408875499	0.0977240694204453	0.847542670413088	-4.66935943478033
a_HQ_transcript/145054	2.08167684847692	-0.257818467931748	1.70491576039865	0.0977275253387034	0.847542670413088	-4.53123833140165
a_HQ_transcript/65862	1.73038983442919	1.38682937878035	1.70488173681638	0.0977339409540829	0.847542670413088	-4.45043424240368
a_HQ_transcript/90489	2.31233704642698	-0.234574914247891	1.70460720750857	0.0977857201247314	0.847608129977668	-4.52368789027546
a_HQ_transcript/124906	2.20876479820171	1.22325927375149	1.70454822200544	0.0977968484160536	0.847608129977668	-4.38371276373438
a_HQ_transcript/27444	-1.72712213359287	3.01535295855122	-1.70454643901589	0.0977971848139578	0.847608129977668	-4.69002307733511
a_HQ_transcript/114414	2.65155895944852	0.0400073941874542	1.70453249115028	0.0977998164010498	0.847608129977668	-4.52458357559995
a_HQ_transcript/83132	2.37948057417909	0.885028118782299	1.70434654530136	0.0978349050378461	0.84763768739495	-4.49310110130496
a_HQ_transcript/28699	2.57634586425792	0.857035823384998	1.70429806748393	0.097844054702359	0.84763768739495	-4.47940739764886
a_HQ_transcript/136784	2.25413213793805	0.614457521214286	1.70418835386866	0.0978647646041715	0.84763768739495	-4.46899585122718
a_HQ_transcript/59524	1.88387839360109	0.863627233100816	1.70418069332427	0.0978662107705233	0.84763768739495	-4.41455663233425
a_HQ_transcript/20035	2.56409201342147	1.60545087910481	1.70412810922202	0.0978761381398794	0.84763768739495	-4.49658975405471
a_HQ_transcript/118322	-3.01587432079476	0.19081060952832	-1.70396196675364	0.0979075097617526	0.84778306680041	-4.52469733522113
a_HQ_transcript/139593	2.27409687586076	2.4002149295978	1.70387897002782	0.0979231846521733	0.847792504834902	-4.59913674709387
a_HQ_transcript/75306	1.25931318273188	0.38170591366696	1.70357373018333	0.0979808507703763	0.848165434635954	-4.46024296377376
a_HQ_transcript/41457	-2.19820330930466	0.886234495350028	-1.70328561484628	0.0980353077568521	0.84841122215129	-4.51818578434564
a_HQ_transcript/125884	7.39195358879418	4.6495004822402	1.70326907093192	0.0980384355076764	0.84841122215129	-4.91137684841218
a_HQ_transcript/27358	-2.45601015529011	1.05925158717743	-1.7029765737616	0.0980937480628163	0.848520481841334	-4.66441664049851
a_HQ_transcript/105806	-2.12028670457578	0.610529890976357	-1.70297611696877	0.0980938344648299	0.848520481841334	-4.51436098846584
a_HQ_transcript/114314	2.94977464481735	3.34571469812371	1.70297073009824	0.0980948533920618	0.848520481841334	-4.67153546509092
a_HQ_transcript/30715	1.76755894851594	2.23805512901125	1.70284023762996	0.0981195387616508	0.848521404482067	-4.5527475158252
a_HQ_transcript/30827	-2.63270094785863	2.11888290736939	-1.70274086113898	0.0981383413783792	0.848521404482067	-4.47029041812668
a_HQ_transcript/115278	0.640992090197703	2.80094909074636	1.70273868870866	0.0981387524486123	0.848521404482067	-4.67239196969954
a_HQ_transcript/47259	-2.75430357898545	-0.0847979388854693	-1.70258264094377	0.098168283784684	0.848609683448112	-4.52016244418182
a_HQ_transcript/73482	-1.24609550680338	1.67700942349182	-1.7025304590412	0.0981781606315124	0.848609683448112	-4.45126143921716
a_HQ_transcript/78034	-2.45807216443283	1.67815673091364	-1.7023907262513	0.0982046129591965	0.848712123415428	-4.46738225933376
a_HQ_transcript/14837	-1.8819208155055	1.32859760643186	-1.70214866660088	0.0982504505256509	0.848934230628242	-4.47010734632661
a_HQ_transcript/2536	-2.56467999014607	1.57183946483506	-1.70206687180977	0.0982659436176853	0.848934230628242	-4.47777332798966
a_HQ_transcript/113626	2.86666241416702	3.03005384898116	1.70188509246256	0.0983003825121166	0.848934230628242	-4.6545417850026
a_HQ_transcript/69397	-2.51102081439725	0.8628232677243	-1.70182860232798	0.098311086869988	0.848934230628242	-4.67281080691477
a_HQ_transcript/139285	-0.518626302090719	3.55766812489787	-1.70179518657134	0.0983174193052558	0.848934230628242	-4.79454667674306
a_HQ_transcript/6658	1.80924345631315	1.28036008261189	1.70164832286917	0.0983452546748273	0.848934230628242	-4.43240854271744
a_HQ_transcript/76578	-3.82894000884596	0.665901727354201	-1.70158278192775	0.0983576789049773	0.848934230628242	-4.55100813494775
a_HQ_transcript/33600	1.17155482554966	1.31294523698433	1.70145871896032	0.0983812004200616	0.848934230628242	-4.49294552772938
a_HQ_transcript/22611	2.60094463702797	1.04201580261829	1.70144556360688	0.0983836948633738	0.848934230628242	-4.57224080458097
a_HQ_transcript/140424	1.96751577410941	-0.455259121296591	1.70135202021433	0.0984014335536301	0.848934230628242	-4.53354541915166
a_HQ_transcript/108975	2.32682215663419	0.1659133313992	1.70133505575869	0.0984046508199238	0.848934230628242	-4.44565304145502
a_HQ_transcript/23099	0.714809816748497	2.24311658084314	1.70127648223999	0.0984157598159497	0.848934230628242	-4.57013367502589
a_HQ_transcript/57450	-1.84882593831291	-0.283343355404473	-1.70125294306139	0.0984202245286359	0.848934230628242	-4.49598743272082
a_HQ_transcript/28142	3.01227152172011	4.14395513353017	1.70117622494527	0.0984347769480173	0.848934230628242	-4.85172290949887
a_HQ_transcript/139353	-2.71598672037915	1.3668751403995	-1.70089643447845	0.0984878647629886	0.849019316034197	-4.54510687141188
a_HQ_transcript/111042	-3.18962832591968	1.96815135140799	-1.70078869161735	0.0985083144158026	0.849019316034197	-4.45189156883692
a_HQ_transcript/137742	3.73719734209729	3.80060749183699	1.70075238803299	0.0985152056546569	0.849019316034197	-4.90996956781117
a_HQ_transcript/140878	2.51609474696558	0.128289069397668	1.70071526084278	0.0985222536490862	0.849019316034197	-4.56969946801675

a_HQ_transcript/130046	2.74489269363572	1.86439461377727	1.70069429052811	0.0985262347091295	0.849019316034197	-4.65098052074309
a_HQ_transcript/82343	-2.33267624444165	0.0201470063115376	-1.70063458676996	0.0985375697647056	0.849019316034197	-4.54113623505445
a_HQ_transcript/13484	2.4913498522739	1.29370183412445	1.70057639656965	0.0985486185122803	0.849019316034197	-4.46244407515335
a_HQ_transcript/145662	-0.95826068912784	3.4090795780642	-1.70043578440792	0.098575321237227	0.849019316034197	-4.80756217467174
a_HQ_transcript/72101	1.67944775285867	1.53273993761807	1.70037669591355	0.0985865441458926	0.849019316034197	-4.41787170504556
a_HQ_transcript/107592	2.12554576820787	3.11557481743981	1.70035480108644	0.0985907029870428	0.849019316034197	-4.77472768679464
a_HQ_transcript/68890	2.71664115755218	0.743757286174508	1.70010043415542	0.0986390297858195	0.849042122387119	-4.58160452675423
a_HQ_transcript/58321	-2.20465893747873	-0.245925314273231	-1.69996846928224	0.0986641093939625	0.849042122387119	-4.52670939961165
a_HQ_transcript/151171	-5.1304347184546	1.17575529876669	-1.69981803787462	0.0986927050202487	0.849042122387119	-4.69747409570407
a_HQ_transcript/65049	-2.09630851714344	-0.284397396055055	-1.69978866680261	0.0986982889985766	0.849042122387119	-4.5269974991741
a_HQ_transcript/18176	2.15232678150548	0.900518487069887	1.69978562104943	0.098698868067102	0.849042122387119	-4.56969112889712
a_HQ_transcript/29101	-1.81954311338659	0.387446021315336	-1.6997558384306	0.0987045305852809	0.849042122387119	-4.41245167976621
a_HQ_transcript/81659	-0.860829150927354	2.29813351236473	-1.69974209186712	0.0987071442873323	0.849042122387119	-4.51048030978448
a_HQ_transcript/93333	-1.79159108761718	1.22421044612566	-1.69967442431611	0.0987200110960646	0.849042122387119	-4.44966788673024
a_HQ_transcript/88749	-1.5544219512399	0.299649246541361	-1.69964090899975	0.0987263844650921	0.849042122387119	-4.44844979117459
a_HQ_transcript/37177	2.60925606898843	-0.126260578761233	1.6995723886896	0.0987394155542411	0.849042122387119	-4.49467937164065
a_HQ_transcript/83375	2.60221646913967	3.33485463627418	1.69930922393327	0.0987894771877579	0.849283192124783	-4.82601607919842
a_HQ_transcript/29458	2.00150766279598	-0.00411397232217531	1.69927141173135	0.0987966719162501	0.849283192124783	-4.53236199612419
a_HQ_transcript/134986	-2.02765111850699	-0.426166870999769	-1.69918125171841	0.0988138289069049	0.84930507861904	-4.58201053060023
a_HQ_transcript/87553	-2.2205898013305	0.14227155842723	-1.69893639569877	0.0988604363169298	0.849375129972458	-4.51683825613216
a_HQ_transcript/98154	2.33470629181328	1.00761121135489	1.69891364558067	0.0988647676473517	0.849375129972458	-4.38031680937628
a_HQ_transcript/13370	1.98316134270634	0.701986656661164	1.69874634999688	0.0988966234475843	0.849375129972458	-4.44504900877024
a_HQ_transcript/89424	-1.89967288782437	0.280378574653439	-1.69871685845558	0.0989022400055418	0.849375129972458	-4.40196355685439
a_HQ_transcript/143737	-0.605795017935661	3.91206331837182	-1.69868713044894	0.0989079018671126	0.849375129972458	-4.83270736871226
a_HQ_transcript/63787	-1.67275042909193	1.84798470332013	-1.69857878218795	0.0989285396779243	0.849375129972458	-4.49304381888583
a_HQ_transcript/49390	-3.58018323233837	0.410279198770906	-1.69852401751974	0.0989389724353155	0.849375129972458	-4.54852940838446
a_HQ_transcript/131089	2.2088181322495	-0.193536218883334	1.69842204381038	0.098958401039583	0.849375129972458	-4.55960529253323
a_HQ_transcript/101923	-2.16597265786501	1.00745503275378	-1.6983018703778	0.0989813012476345	0.849375129972458	-4.40527839683969
a_HQ_transcript/10919	1.88765847269645	2.9878130859824	1.69829514445389	0.0989825830681678	0.849375129972458	-4.76251228311979
a_HQ_transcript/125216	-0.625478532698647	5.15234435827715	-1.69828246139148	0.0989850002325205	0.849375129972458	-4.98149913840913
a_HQ_transcript/130932	3.36162788336238	0.630855056782185	1.69821779651691	0.0989973249649636	0.849375129972458	-4.63288649250816
a_HQ_transcript/134015	2.53295850763133	1.10202529484415	1.69804854046658	0.0990295902081193	0.849455772406536	-4.45892264304736
a_HQ_transcript/147284	1.79096719319429	0.745500226546861	1.69801517775846	0.0990359511698165	0.849455772406536	-4.5823540770322
a_HQ_transcript/43430	2.47608277420398	0.323628740033065	1.69776293624003	0.0990840547911964	0.849668194057932	-4.62304024741478
a_HQ_transcript/152231	2.12670410200802	-0.224643514082393	1.69755072245508	0.0991245400512237	0.849668194057932	-4.49853129178213
a_HQ_transcript/39120	1.09665797401915	2.69918813950892	1.69749695898632	0.0991347990142841	0.849668194057932	-4.75470936119655
a_HQ_transcript/119496	-2.52804006436886	0.117444416250573	-1.69747381289064	0.0991392159469067	0.849668194057932	-4.5032749936851
a_HQ_transcript/48384	0.944962083810596	3.64785328164059	1.69739798990853	0.0991536862769479	0.849668194057932	-4.8435966411753
a_HQ_transcript/142101	1.65050695746792	1.64784207373599	1.69733839920539	0.0991650600183496	0.849668194057932	-4.46732402476552
a_HQ_transcript/56207	-1.37810598574234	2.47448965561262	-1.69727486901271	0.0991771868677641	0.849668194057932	-4.50254104654374
a_HQ_transcript/40131	2.31005540471184	-0.324992688547269	1.69727241967763	0.0991776544295876	0.849668194057932	-4.54024421062793
a_HQ_transcript/1943	2.08982994682055	1.82393399972389	1.6970301480991	0.099223911563426	0.849929073746764	-4.54989720945485
a_HQ_transcript/131930	-2.6188856789927	-0.469626405971471	-1.69690298862189	0.0992481974468728	0.849929073746764	-4.47806844541984
a_HQ_transcript/116389	2.26851432381843	1.07365080551945	1.69679160750016	0.0992694739376346	0.849929073746764	-4.46678983702516
a_HQ_transcript/77678	0.949066638107084	5.47334096466054	1.69670326214556	0.0992863527474985	0.849929073746764	-5.02292817883347
a_HQ_transcript/97752	-2.8120953944327	1.14581086876328	-1.69666283243438	0.0992940778397342	0.849929073746764	-4.5135570878772
a_HQ_transcript/148291	-4.02073202178247	0.106024904280979	-1.69649654015362	0.0993258573537506	0.849929073746764	-4.55894072971329
a_HQ_transcript/43887	-1.88995836490492	0.240558352107315	-1.69646287949946	0.099332291152523	0.849929073746764	-4.40283332709998
a_HQ_transcript/64236	2.40804950224707	0.94944821391528	1.69643849477777	0.0993369521940849	0.849929073746764	-4.42211263155929
a_HQ_transcript/75645	2.51181294782294	2.55374900412848	1.69629845533999	0.0993637237056915	0.849929073746764	-4.61933806648481

a_HQ_transcript/135947	2.5051436711463	1.40659355799878	1.69622088155797	0.0993785561734963	0.849929073746764	-4.56080525187497
a_HQ_transcript/104085	2.62405424288172	0.802115217097987	1.69614958335592	0.0993921903514692	0.849929073746764	-4.53066635619271
a_HQ_transcript/9122	-1.62761539230331	0.545021330246302	-1.69610055122632	0.0994015675499935	0.849929073746764	-4.43906841408844
a_HQ_transcript/2046	2.89046565271551	1.18652047776094	1.69605879277577	0.0994095542688868	0.849929073746764	-4.49459943326301
a_HQ_transcript/115452	2.45126866126963	-0.166260256940338	1.69604177528586	0.0994128091866957	0.849929073746764	-4.47400895408933
a_HQ_transcript/134646	1.39323430694021	3.46363273740151	1.69590763715418	0.0994384687641834	0.850023428283007	-4.81626328822826
a_HQ_transcript/99153	0.593107999479114	3.95595034651626	1.69565051137974	0.0994876703754171	0.850236233337302	-4.84246186885752
a_HQ_transcript/980	1.08335317291189	2.56077511946321	1.69557597301396	0.0995019372631186	0.850236233337302	-4.69765547680395
a_HQ_transcript/69663	1.6887923968759	1.83427706505019	1.69554824841512	0.0995072442780359	0.850236233337302	-4.48686375848785
a_HQ_transcript/16929	-1.7230132145683	1.67367077007408	-1.69520014251769	0.0995738984712205	0.850571641688412	-4.42155730329864
a_HQ_transcript/131279	4.25761012742811	4.95100364078432	1.69509541180876	0.0995939592608881	0.850571641688412	-5.02248324611662
a_HQ_transcript/145005	-4.02672981208028	3.14295466276577	-1.69505859221769	0.0996010127222565	0.850571641688412	-4.91973636950441
a_HQ_transcript/151581	0.801203791333222	3.66725730656163	1.69497834603172	0.0996163867812853	0.850571641688412	-4.86393737459716
a_HQ_transcript/130924	1.19680186722656	3.72385778088096	1.69496124994566	0.0996196624107953	0.850571641688412	-4.80902229789812
a_HQ_transcript/142423	-4.51761545625691	1.96961385403646	-1.69471537175857	0.0996667828965287	0.850848987547278	-4.63089020130037
a_HQ_transcript/25974	-1.32985318010832	2.85382850674359	-1.69452931194187	0.0997024520586574	0.851028507087465	-4.67358623504135
a_HQ_transcript/29610	-1.86493744108143	1.52399752122451	-1.69439437714656	0.0997283268081576	0.851124384188017	-4.54008218936577
a_HQ_transcript/131717	2.08505010626684	2.08178951568077	1.69429884709642	0.0997466487932972	0.851130360641179	-4.55563362431422
a_HQ_transcript/14928	-1.23738508160557	2.23238014707147	-1.69423804441762	0.0997583117782885	0.851130360641179	-4.55263921437582
a_HQ_transcript/148434	-2.08028630781596	0.557339780705214	-1.69374624134176	0.0998526897891814	0.851162399983898	-4.44021347152822
a_HQ_transcript/9602	1.03538269578741	2.08066413020042	1.69360112859094	0.0998805514539591	0.851162399983898	-4.60851059080514
a_HQ_transcript/130952	0.897745029035368	3.15105352326957	1.69352161885676	0.0998958200830016	0.851162399983898	-4.72057347927904
a_HQ_transcript/14668	1.48659530398418	-0.377321485103048	1.6934582391666	0.0999079925787728	0.851162399983898	-4.52364183157523
a_HQ_transcript/88563	2.26205277458642	-0.172463582261222	1.69344368251562	0.0999107884562758	0.851162399983898	-4.48002096525526
a_HQ_transcript/63003	-1.40299144793314	1.92179313427874	-1.69333436532663	0.0999317869565336	0.851162399983898	-4.56054753391776
a_HQ_transcript/103291	-6.06628256421542	3.89487895951334	-1.69329285725037	0.0999397611169142	0.851162399983898	-4.80212955127262
a_HQ_transcript/101525	0.840976135099581	3.17384512877427	1.69327053347377	0.0999440499807917	0.851162399983898	-4.59878657380221
a_HQ_transcript/145170	-1.89858323625037	-0.0835554273774659	-1.69322485997144	0.0999528252933759	0.851162399983898	-4.50923274265376
a_HQ_transcript/56161	-2.91903022679165	1.39083732817625	-1.69316556983097	0.0999642177483246	0.851162399983898	-4.61504149496216
a_HQ_transcript/123670	-0.740168760277474	2.76211850269151	-1.69313562465215	0.0999699720527323	0.851162399983898	-4.66847346762381
a_HQ_transcript/150401	1.62626865042927	4.54339001997534	1.69311037470126	0.099974824330837	0.851162399983898	-4.95438964842686
a_HQ_transcript/123043	2.97371474897408	-0.0288539399716599	1.6931018635032	0.0999764599703832	0.851162399983898	-4.58442551671056
a_HQ_transcript/142232	-0.68084519774274	4.04699589965257	-1.69307748845634	0.0999811443687274	0.851162399983898	-4.87475934039079
a_HQ_transcript/151408	2.9072313765261	0.0812620686170097	1.69303909728427	0.0999885227584675	0.851162399983898	-4.58757324752116
a_HQ_transcript/90260	1.97409292507754	-0.465721081949983	1.69298532364391	0.0999988582648229	0.851162399983898	-4.59217879441718
a_HQ_transcript/51506	2.49636318980704	2.58388963603593	1.69287053696215	0.100020923701523	0.851162399983898	-4.70715836620465
a_HQ_transcript/63841	1.66187139609663	2.97828636478094	1.69282430180506	0.100029812630579	0.851162399983898	-4.71306847407738
a_HQ_transcript/127832	2.94870591280849	1.6642780696462	1.69261825485489	0.100069434152139	0.851162399983898	-4.42310987937292
a_HQ_transcript/97955	-2.02436401706005	-0.505024330095	-1.69259622374256	0.100073671370094	0.851162399983898	-4.52921579370666
a_HQ_transcript/94674	-2.73761482172312	0.119123987886488	-1.69237037705129	0.100117116832509	0.851162399983898	-4.50216907242221
a_HQ_transcript/103262	-0.66856869816388	2.77197676737304	-1.6923615010508	0.100118824603897	0.851162399983898	-4.69032666611875
a_HQ_transcript/2203	-1.18289735561981	2.76723446910247	-1.6923492438155	0.100121182975824	0.851162399983898	-4.63386778214252
a_HQ_transcript/124588	-1.86899394315478	-0.49052229600994	-1.69233644634841	0.100123645341326	0.851162399983898	-4.55313039531999
a_HQ_transcript/135512	-2.16785409457464	-0.267374933097688	-1.69229335349026	0.100131937226046	0.851162399983898	-4.46794968349969
a_HQ_transcript/100732	-2.36122301175658	2.0220111190955	-1.69198643510454	0.10019101072821	0.851162399983898	-4.63903553113643
a_HQ_transcript/13249	0.539022757767037	2.75974475433054	1.69195414346271	0.10019722768747	0.851162399983898	-4.65465278169107
a_HQ_transcript/149088	2.32883587585563	-0.161894422243161	1.69182612175616	0.100221878277074	0.851162399983898	-4.5776450628957
a_HQ_transcript/100662	1.34250421941835	-0.729376683982948	1.69173946031968	0.100238567814855	0.851162399983898	-4.55696331249592
a_HQ_transcript/65406	-2.78482332216951	3.10060583264447	-1.6917144298958	0.100243388685797	0.851162399983898	-4.62942551969861
a_HQ_transcript/2392	-2.65643899876306	2.3282133260145	-1.691703299488	0.100245532469493	0.851162399983898	-4.52814064090037

a_HQ_transcript/102906	2.20582197463544	-0.0690988957754444	1.69131184961851	0.100320952405236	0.851162399983898	-4.54114999839044
a_HQ_transcript/107285	-1.1753077977636	2.05004338847223	-1.69127193070077	0.100328646169517	0.851162399983898	-4.57297010709777
a_HQ_transcript/124997	-3.30637455490626	0.360126574341577	-1.69111134079496	0.100359602404471	0.851162399983898	-4.5928634850072
a_HQ_transcript/148879	5.46837618770183	5.60834464214816	1.69111092383876	0.100359682789718	0.851162399983898	-4.76881349815279
a_HQ_transcript/77934	-3.43525207870983	-0.0353747363865818	-1.69109861802394	0.100362055259645	0.851162399983898	-4.60322635005281
a_HQ_transcript/14820	-0.574397810762532	3.08987875486225	-1.69099491576486	0.100382050145218	0.851162399983898	-4.80223571228343
a_HQ_transcript/149623	-2.61535147200151	0.716097814358953	-1.69091780090934	0.100396920853947	0.851162399983898	-4.55045228747501
a_HQ_transcript/38784	-2.43848865276847	0.60501415650542	-1.6908874901821	0.100402766430467	0.851162399983898	-4.4853531447928
a_HQ_transcript/26462	2.07595214921148	1.58523232638591	1.69084282394328	0.100411381057166	0.851162399983898	-4.46994303189206
a_HQ_transcript/84644	2.66752584404441	2.89262145109136	1.69079885746902	0.100419861324732	0.851162399983898	-4.68275344854748
a_HQ_transcript/80125	1.8565561994748	6.28087139602431	1.69078606394018	0.100422329056855	0.851162399983898	-5.13637861210525
a_HQ_transcript/48972	-1.86898171766978	-0.414968100908574	-1.69078057638381	0.100423387562104	0.851162399983898	-4.50057692734436
a_HQ_transcript/145490	1.78632306447409	-0.146085731929062	1.69072218728521	0.100434650923385	0.851162399983898	-4.47378162446049
a_HQ_transcript/50587	1.41736627915526	1.11059476429443	1.69065804192327	0.100447025892733	0.851162399983898	-4.38991516265608
a_HQ_transcript/34115	2.26959463875876	-0.010464007134053	1.69043283396273	0.100490483245526	0.851162399983898	-4.58661251431254
a_HQ_transcript/104762	-0.761021051387416	3.83616950891888	-1.69031848483952	0.100512554675911	0.851162399983898	-4.87626005658531
a_HQ_transcript/2673	1.2162586436745	-0.771087355176967	1.6902615956838	0.100523536808648	0.851162399983898	-4.50902459309452
a_HQ_transcript/67579	-2.19783878499937	0.276852491903591	-1.69026013695788	0.100523818420672	0.851162399983898	-4.53623423665679
a_HQ_transcript/29126	-1.73352375839968	-0.346717818885535	-1.69010571428406	0.100553633967436	0.851162399983898	-4.53034888606804
a_HQ_transcript/61630	-1.71479738182283	-0.0456777648154555	-1.6900392583593	0.10056646738227	0.851162399983898	-4.5197958552926
a_HQ_transcript/150021	-1.67998218360026	0.700859806078041	-1.69001997953385	0.100570190604117	0.851162399983898	-4.45194027285028
a_HQ_transcript/117730	2.52249524036557	3.91683200556376	1.69000772809435	0.100572556722272	0.851162399983898	-4.82558948611216
a_HQ_transcript/51731	-1.75839638135398	-0.325780305542713	-1.6898516780743	0.10060269869451	0.851162399983898	-4.51529587992986
a_HQ_transcript/48525	-0.537066137583652	3.95767082049738	-1.68972144516097	0.100627859708741	0.851162399983898	-4.89473524615204
a_HQ_transcript/66070	-1.49649482511375	1.75207653017252	-1.68971027533307	0.100630017965138	0.851162399983898	-4.46394221658559
a_HQ_transcript/3231	-1.56910468415896	1.45528518426933	-1.6896749604432	0.100636841832468	0.851162399983898	-4.52277747229356
a_HQ_transcript/120991	2.86188014924327	1.92086306468019	1.6896037563542	0.100650601713846	0.851162399983898	-4.74573903622524
a_HQ_transcript/118702	3.17664247207267	-0.034958716644138	1.6895219584419	0.10066641074199	0.851162399983898	-4.54185698283822
a_HQ_transcript/100870	2.2764228449396	2.10126066896065	1.6894642579048	0.100677563731907	0.851162399983898	-4.54075279164182
a_HQ_transcript/34188	-2.16948283831728	0.116166166681536	-1.68945685930943	0.100678993887744	0.851162399983898	-4.47149236196255
a_HQ_transcript/85302	0.493768899692933	3.20167359924056	1.68930416627362	0.100708513386226	0.851162399983898	-4.75419772222521
a_HQ_transcript/105498	4.38158034712066	3.45432450928123	1.68929235694681	0.100710796734059	0.851162399983898	-4.75702106261097
a_HQ_transcript/40596	-1.64686731968594	0.605742467200801	-1.68913417919113	0.100741384759034	0.851162399983898	-4.54685592846108
a_HQ_transcript/123070	1.30941003535187	2.81100996155371	1.68911508190207	0.10074507826982	0.851162399983898	-4.76247242233457
a_HQ_transcript/134665	2.2598340360832	0.499896930725568	1.68908567553883	0.100750765827679	0.851162399983898	-4.48368501914652
a_HQ_transcript/127337	3.02392719857723	4.79966583224622	1.68893968793802	0.10077900561677	0.851162399983898	-5.03835352581907
a_HQ_transcript/25379	-1.98308034933768	1.61358941587067	-1.68891975505055	0.100782861939418	0.851162399983898	-4.46141439414559
a_HQ_transcript/137381	-1.94281377153875	1.41043451983274	-1.68891242408097	0.100784280258813	0.851162399983898	-4.51522504791361
a_HQ_transcript/57117	1.71066120369248	-0.180661778948997	1.68889800531784	0.100787069899002	0.851162399983898	-4.6153071184326
a_HQ_transcript/130739	3.37417614284819	1.90211020176091	1.68879613359253	0.10080678115346	0.851202698530785	-4.72800342192739
a_HQ_transcript/147243	-2.62354215730742	0.59000732582629	-1.68872199211536	0.100821128877382	0.851202698530785	-4.50679364581475
a_HQ_transcript/48489	2.98688312603616	1.35665414630971	1.68848715605207	0.100866585163487	0.851210506540156	-4.68604519635246
a_HQ_transcript/152908	4.83030988239642	7.89509680223918	1.68848208016047	0.100867567872337	0.851210506540156	-5.34110132267629
a_HQ_transcript/74031	1.0756113400955	2.91389004160603	1.68846507886399	0.100870859435863	0.851210506540156	-4.67669834934534
a_HQ_transcript/30785	2.63246540496664	1.60394698828866	1.68833781848115	0.100895500732438	0.851210506540156	-4.44735757389301
a_HQ_transcript/17604	-0.854366755812738	3.96283722942871	-1.6883279671719	0.100897408440547	0.851210506540156	-4.88757886816305
a_HQ_transcript/82568	-0.850134525640363	3.84093135081959	-1.68822672651037	0.100917015458606	0.851210506540156	-4.70921577197135
a_HQ_transcript/62228	-1.19518483575784	-0.832501794147861	-1.68818777582733	0.100924559783147	0.851210506540156	-4.55034494063076
a_HQ_transcript/29065	-2.5212003011687	0.923392562800427	-1.68803596902119	0.10095396760143	0.851335010697215	-4.4713809164185
a_HQ_transcript/5672	1.12461965467997	2.61751709246539	1.68776875411734	0.101005749481764	0.851648129659991	-4.58862420153135

a_HQ_transcript/24341	1.48196345921693	2.43691993208073	1.68757401547256	0.10104350059428	0.851842872015125	-4.53289416355801
a_HQ_transcript/78433	1.8745199340292	1.44385239818872	1.68739301843596	0.101078598369204	0.852015192286117	-4.54704245899462
a_HQ_transcript/30130	-2.17461791093712	0.727136390997906	-1.68701488926875	0.101151955525534	0.852354627009633	-4.49103394356106
a_HQ_transcript/8644	-2.1372966485782	0.388276796078713	-1.68701454404635	0.101152022519039	0.852354627009633	-4.51823883960338
a_HQ_transcript/3142	2.31452585226506	4.6899072180121	1.68688328358142	0.101177497470902	0.852354627009633	-4.90550666493701
a_HQ_transcript/98221	2.25138448726415	-0.200998209524505	1.68688316362911	0.101177520753621	0.852354627009633	-4.57822364624841
a_HQ_transcript/104993	0.583877873254541	4.26662048534874	1.68645936593319	0.101259807678985	0.852924228483414	-4.92058798747449
a_HQ_transcript/93665	2.5599405795495	1.44531712220985	1.68621602349646	0.101307081607627	0.852940276951901	-4.52762606764377
a_HQ_transcript/12667	-3.01642424729299	2.06931140919883	-1.68615503272087	0.101318933116222	0.852940276951901	-4.57495299816646
a_HQ_transcript/139007	-3.08370180778715	0.647025824101145	-1.68615456691842	0.101319023633727	0.852940276951901	-4.61567101990548
a_HQ_transcript/127477	1.01558930438761	2.35992871077377	1.68604092832954	0.101341108574983	0.852940276951901	-4.69962624892045
a_HQ_transcript/10905	2.97724711318002	2.51504501521746	1.68594107924278	0.101360516924739	0.852940276951901	-4.7729521309716
a_HQ_transcript/67196	2.05124265164484	1.46165034612238	1.68592098570988	0.101364423016511	0.852940276951901	-4.5363125996512
a_HQ_transcript/145337	-0.606894436498082	3.80250282894864	-1.68590775584427	0.101366994910943	0.852940276951901	-4.88179023313396
a_HQ_transcript/101481	-0.78294306382639	3.33787251375495	-1.68577069629442	0.101393642576239	0.852940276951901	-4.79875371873772
a_HQ_transcript/145838	-4.58594987642388	7.76841745821765	-1.68577001875267	0.101393774321113	0.852940276951901	-5.02745300746875
a_HQ_transcript/38382	2.1014659454942	0.155764579569061	1.68546100147567	0.101453876178908	0.853322372236658	-4.42759156320467
a_HQ_transcript/78855	-1.40255385669869	1.86611918793828	-1.6852886146116	0.101487417204774	0.853425238409722	-4.4927947693171
a_HQ_transcript/102995	1.83743199346583	-0.344992393542821	1.68521468536468	0.101501804326344	0.853425238409722	-4.62266979152402
a_HQ_transcript/133080	-1.71231085808707	-0.126629692551105	-1.68517179576372	0.101510151705973	0.853425238409722	-4.56086841531261
a_HQ_transcript/12813	1.85253062278935	0.180537590769815	1.68505089554532	0.101533684964614	0.853479953823174	-4.44152391174946
a_HQ_transcript/104353	-2.3866194661982	0.180548837852313	-1.68498750722233	0.101546025334453	0.853479953823174	-4.48513190615972
a_HQ_transcript/1012	2.42708149530953	0.41060029460558	1.68473584404759	0.101595031178486	0.853687263864301	-4.58638164265606
a_HQ_transcript/2372	2.80647132974218	0.949411517299303	1.6846663814443	0.101608560955587	0.853687263864301	-4.65692491561608
a_HQ_transcript/138622	2.06908706217551	0.396207128302872	1.68459069258108	0.101623305179343	0.853687263864301	-4.50098474065293
a_HQ_transcript/24081	2.00272690567709	-0.176827716024667	1.68445534388176	0.101649675609271	0.853687263864301	-4.56503478700754
a_HQ_transcript/22344	-2.4326098169731	0.996423858559408	-1.68441047493284	0.101658418831557	0.853687263864301	-4.47771817920704
a_HQ_transcript/18580	3.69672153519575	0.436414732020479	1.68431379308515	0.101677260512901	0.853687263864301	-4.50657991502525
a_HQ_transcript/35211	1.76547077824548	-0.0553501145008862	1.68426404262748	0.101686957181943	0.853687263864301	-4.49545608319137
a_HQ_transcript/40544	2.70464584224685	0.807997583560352	1.68425776126297	0.101688181513151	0.853687263864301	-4.59470726243307
a_HQ_transcript/106360	4.37650459166506	0.546867321884537	1.68417896157339	0.101703541786092	0.853692920858046	-4.61881457322961
a_HQ_transcript/66995	0.799515648474485	5.14444337143786	1.68388571372999	0.101760721010687	0.853796313162804	-5.05378478863973
a_HQ_transcript/4989	2.61590649847336	3.82820356606992	1.68384226422564	0.101769195333368	0.853796313162804	-4.77137657644561
a_HQ_transcript/124467	-0.598587427815607	5.04603649476227	-1.68370856048712	0.101795276387427	0.853796313162804	-4.95593456857429
a_HQ_transcript/16849	-0.720999199583131	2.35989993160063	-1.6836630130498	0.101804162415425	0.853796313162804	-4.54711541275496
a_HQ_transcript/42119	3.33107986593912	2.78622045618985	1.68365615841804	0.101805499768329	0.853796313162804	-4.73645201332485
a_HQ_transcript/81704	1.80910874205088	-0.427796843787991	1.6835720038338	0.101821919695433	0.853796313162804	-4.52680849818023
a_HQ_transcript/107142	2.28746952693642	2.12598764879472	1.68357107437986	0.10182210105932	0.853796313162804	-4.59522682271321
a_HQ_transcript/138375	-1.10798578781338	3.13585670688977	-1.68345782579326	0.101844201213865	0.853796313162804	-4.80559602154518
a_HQ_transcript/143156	2.7947198612376	3.99791807165411	1.68343808910518	0.101848053183851	0.853796313162804	-4.99331235929926
a_HQ_transcript/119073	-2.97971060255274	0.174126936088528	-1.68273275592281	0.101985791337129	0.854827697021579	-4.62361202355722
a_HQ_transcript/48858	3.48289393055156	4.00522983174438	1.68253187165068	0.102025048588626	0.855015197376841	-4.94955977241878
a_HQ_transcript/138063	2.34352025107947	0.307679974716699	1.68246776269593	0.102037579552077	0.855015197376841	-4.52997820858719
a_HQ_transcript/3085	-2.18822189708873	1.07575171020261	-1.68221646716821	0.10208671102689	0.855089324119074	-4.46681242492102
a_HQ_transcript/132166	3.96577574817498	1.64984325187205	1.68216127538757	0.102097504360973	0.855089324119074	-4.4764036850143
a_HQ_transcript/129610	-3.23883902904253	0.941750001488154	-1.68213295272898	0.102103043524176	0.855089324119074	-4.6373985478552
a_HQ_transcript/1544	-1.39746683774407	-0.530746439857906	-1.68207400273522	0.102114573386959	0.855089324119074	-4.57767615019679
a_HQ_transcript/150667	2.38751360731071	0.311462057643867	1.68196368531128	0.102136152973519	0.855089324119074	-4.51483633602277
a_HQ_transcript/115369	2.40425754848667	0.915032482562687	1.68195334183199	0.102138176493284	0.855089324119074	-4.4692341601576
a_HQ_transcript/39199	0.724565458566611	3.04521529286248	1.6817861955228	0.102170880357408	0.855089324119074	-4.69858580931158

a_HQ_transcript/112479	1.83309152783878	1.69170044704253	1.68175376367752	0.102177226985657	0.855089324119074	-4.5550910296016
a_HQ_transcript/98558	3.00142234642782	0.407894864247736	1.68172642416109	0.102182577344745	0.855089324119074	-4.55030305111196
a_HQ_transcript/119793	-1.87017359171358	1.73809117529804	-1.68160611287712	0.102206125104671	0.855089324119074	-4.57254037373118
a_HQ_transcript/60578	0.585699504023671	2.78005104500349	1.68155480138325	0.102216169350455	0.855089324119074	-4.64128164117906
a_HQ_transcript/39791	4.39708938177894	0.937567169096084	1.68152015784301	0.102222951302358	0.855089324119074	-4.56542869968923
a_HQ_transcript/41126	-2.06785871026709	1.31998152739272	-1.68133511954654	0.102259181452592	0.85523269944559	-4.4852833503599
a_HQ_transcript/31200	-1.08724255169294	2.51562641850537	-1.6812428733387	0.102277247079998	0.85523269944559	-4.62510108527903
a_HQ_transcript/58643	-2.54702103520268	0.738522759771301	-1.68112005488295	0.102301304144085	0.85523269944559	-4.47857433564607
a_HQ_transcript/7886	-1.14361421943339	1.87346195987196	-1.6810854141101	0.102308090257005	0.85523269944559	-4.52564697616401
a_HQ_transcript/21611	2.28025147750051	2.91902688943847	1.68105700468738	0.102313655930784	0.85523269944559	-4.6702469344307
a_HQ_transcript/140673	0.822764802871877	4.16411617776077	1.68089032592181	0.102346314951339	0.855382688064908	-4.90352677866942
a_HQ_transcript/124272	3.64010939603929	1.60246377160297	1.68051180083466	0.102420515358696	0.855879775268872	-4.63970435168464
a_HQ_transcript/128172	-2.5199789052433	-0.338271999478593	-1.68030235291582	0.102461591645888	0.856099956761847	-4.49065784903436
a_HQ_transcript/140015	2.45976584584448	0.414891627496306	1.67982795560279	0.102554679714333	0.856670262731756	-4.6603117301271
a_HQ_transcript/87414	-2.64796240738588	2.51803065162413	-1.67979048245086	0.102562035839888	0.856670262731756	-4.65527014242593
a_HQ_transcript/27754	-2.16896939580745	2.33284992290508	-1.67972922844267	0.102574061185883	0.856670262731756	-4.62522093322868
a_HQ_transcript/71514	-3.21743983494729	2.86004811070452	-1.6794165332945	0.102635467579348	0.856838386990096	-4.77008201067445
a_HQ_transcript/57250	1.97540992504172	-0.273973587429086	1.67929451167711	0.102659438218191	0.856838386990096	-4.52010454483824
a_HQ_transcript/15481	-1.5880747533785	1.16204302311059	-1.67919590770193	0.102678811962858	0.856838386990096	-4.46260907190126
a_HQ_transcript/18130	-1.60194930332441	0.988185160424477	-1.67917307759399	0.102683298064572	0.856838386990096	-4.47435417735425
a_HQ_transcript/27385	-1.70588030681095	1.74124203100788	-1.67909026919769	0.102699571230939	0.856838386990096	-4.53356386277353
a_HQ_transcript/37871	-3.77486049860873	1.9753499042813	-1.67907267545092	0.102703028958357	0.856838386990096	-4.56167760778709
a_HQ_transcript/66425	-2.66436776030825	0.623756266128541	-1.67898580260103	0.102720103640114	0.856838386990096	-4.49696859009987
a_HQ_transcript/95107	-1.76951914278558	0.896353294791331	-1.678973136821	0.102722593270734	0.856838386990096	-4.46855333520627
a_HQ_transcript/148714	1.64322080577305	-0.266291388600003	1.6789514482284	0.102726856574009	0.856838386990096	-4.56111470931225
a_HQ_transcript/81512	3.21712872392163	4.39785093243081	1.67875118306931	0.102766229439132	0.85695246399528	-4.91753232154328
a_HQ_transcript/72580	2.56325121632927	0.767735102284865	1.67849126375611	0.102817349258279	0.85695246399528	-4.5328022689777
a_HQ_transcript/38724	1.7322642388698	0.202118513605677	1.67845199462017	0.102825074383978	0.85695246399528	-4.51301217633188
a_HQ_transcript/32891	0.918038743057787	2.16945124998186	1.67839983156825	0.102835336781095	0.85695246399528	-4.54703648811723
a_HQ_transcript/3836	0.943131844031933	2.63907030556091	1.67839084657392	0.102837104547079	0.85695246399528	-4.56895084449422
a_HQ_transcript/28909	2.60163734421939	0.553085900349228	1.67835913822007	0.102843343256117	0.85695246399528	-4.5734345420994
a_HQ_transcript/60663	2.47805806554728	0.609752876071242	1.67835716814651	0.102843730884102	0.85695246399528	-4.50959985643419
a_HQ_transcript/148328	2.73809181837942	1.13764566616209	1.67827652009186	0.102859600088015	0.856961850926778	-4.60572146453006
a_HQ_transcript/88097	-2.4060432346237	2.14807720799444	-1.67814768514163	0.102884955306888	0.85703579457629	-4.69845341886735
a_HQ_transcript/59810	-2.26139493943091	0.509979747967914	-1.67808159920672	0.102897963293902	0.85703579457629	-4.53285505365803
a_HQ_transcript/146119	-3.02138031507981	0.932156397129126	-1.67794207726053	0.102925430511018	0.857129839307911	-4.64274494202249
a_HQ_transcript/5246	1.56049202026422	1.68718735489138	1.67775186202483	0.102962887375222	0.857129839307911	-4.51100357694655
a_HQ_transcript/151916	-6.04981273805023	0.292989580162169	-1.677686308301	0.102975798732205	0.857129839307911	-4.57558675354154
a_HQ_transcript/68119	-2.23129201369369	-0.211880060070026	-1.67765881177226	0.102981214805755	0.857129839307911	-4.52781230229443
a_HQ_transcript/112740	-1.79528348685613	-0.0775119563130578	-1.67752037706064	0.103008486306223	0.857129839307911	-4.50720161720211
a_HQ_transcript/108210	1.90135737238164	3.42293702240084	1.67747043005644	0.103018327291521	0.857129839307911	-4.68199911391434
a_HQ_transcript/152883	-2.92315558886216	-0.00116062530307504	-1.67729653101069	0.103052596470271	0.857129839307911	-4.60916572342377
a_HQ_transcript/98290	-0.9213605560007	2.75572406969802	-1.67721518082903	0.10306863088935	0.857129839307911	-4.59720400197424
a_HQ_transcript/100942	2.31865124790362	0.582690050665024	1.67720704671508	0.10307023426719	0.857129839307911	-4.63538200203469
a_HQ_transcript/150992	0.974129992284645	5.62734299745421	1.67719930864992	0.103071759596065	0.857129839307911	-5.06416623102749
a_HQ_transcript/58940	1.83893242510392	2.38204345029677	1.67697995088197	0.103115007254581	0.857129839307911	-4.56915132379055
a_HQ_transcript/66514	-0.491280930378324	4.59873054207734	-1.67692423052422	0.103125995251261	0.857129839307911	-4.97937244120393
a_HQ_transcript/76816	3.82570783860212	1.80502979989098	1.67687976519992	0.103134764465926	0.857129839307911	-4.6315171217475
a_HQ_transcript/104674	2.62773264152322	1.7487225945133	1.6767130885027	0.103167641079048	0.857129839307911	-4.55938899432681
a_HQ_transcript/72888	2.50838764164816	-0.0271816591154505	1.67643628999278	0.103222258241889	0.857129839307911	-4.59741497437241

a_HQ_transcript/12848	2.0647320193082	-0.425573662807446	1.67637394157112	0.10323456398939	0.857129839307911	-4.54248610738634
a_HQ_transcript/143198	2.4912015215502	-0.00741557641387336	1.67629026449775	0.103251081304563	0.857129839307911	-4.58000169998776
a_HQ_transcript/21399	1.68622148506515	1.57516870234136	1.67623530184175	0.103261931775038	0.857129839307911	-4.4754738514078
a_HQ_transcript/15885	-1.75535245115815	-0.646564174532705	-1.67620540792614	0.103267833690307	0.857129839307911	-4.57953706987187
a_HQ_transcript/12760	-1.70774690651712	1.16144885455498	-1.67608722368648	0.103291169392509	0.857129839307911	-4.52787714916684
a_HQ_transcript/91498	4.78008650789475	2.3681079885344	1.67604041742018	0.103300412591182	0.857129839307911	-4.66632259510198
a_HQ_transcript/54780	2.03526803682292	0.108532062208204	1.67602598427614	0.10330326295557	0.857129839307911	-4.59155135620578
a_HQ_transcript/151562	-3.50333411491702	1.32104156250595	-1.67593505250649	0.10332122234086	0.857129839307911	-4.6298262699757
a_HQ_transcript/142366	-3.66250374874246	2.3111321389917	-1.67589559887744	0.103329015397044	0.857129839307911	-4.6143625663112
a_HQ_transcript/150807	-2.34836390001555	0.437795040857892	-1.67576976934866	0.103353873071505	0.857129839307911	-4.45496311220851
a_HQ_transcript/33599	2.96509404250024	1.98121333943784	1.67553132406945	0.103400991677989	0.857129839307911	-4.70513791464283
a_HQ_transcript/50521	-1.82387318452319	0.57618863962881	-1.6755251441485	0.103402213115035	0.857129839307911	-4.57536573061726
a_HQ_transcript/140696	-0.486751886859764	5.60644926768583	-1.67551485908123	0.103404245944702	0.857129839307911	-5.07528342142456
a_HQ_transcript/151477	-2.09289604809459	-0.0680149610395041	-1.67548083254879	0.103410971479834	0.857129839307911	-4.58172224121319
a_HQ_transcript/70952	1.17911558107967	3.68544418747974	1.67543701011438	0.103419633766045	0.857129839307911	-4.82005635964102
a_HQ_transcript/122220	-2.80199729030207	1.09931220106482	-1.67543601362749	0.10341983074648	0.857129839307911	-4.51584066610712
a_HQ_transcript/75829	-1.03057037966799	3.95016207221909	-1.675428218905	0.103421371578148	0.857129839307911	-4.87761011769388
a_HQ_transcript/117009	-1.65164339453433	4.49182306686875	-1.6753560518958	0.103435638190181	0.857129839307911	-4.89797095809335
a_HQ_transcript/109179	0.615265353590372	5.70888789158556	1.67527145924303	0.103452363297222	0.857129839307911	-5.05036716280355
a_HQ_transcript/37120	0.613210237761703	3.82825584292501	1.67520526755017	0.103465451855247	0.857129839307911	-4.90503487365438
a_HQ_transcript/60279	-2.19114645372245	2.5834051469259	-1.67518838671858	0.103468790043529	0.857129839307911	-4.56557595333277
a_HQ_transcript/143569	-2.7325597113019	0.503849889574024	-1.67511913771264	0.103482484987712	0.857129839307911	-4.58305836649447
a_HQ_transcript/83060	-3.25990508435831	-0.226673773783846	-1.67505209634699	0.103495744776945	0.857129839307911	-4.5810689340023
a_HQ_transcript/75032	1.91039264823107	1.29549922965707	1.6748667783408	0.103532405286355	0.857129839307911	-4.58744906727412
a_HQ_transcript/123763	-0.387052901209238	5.51096420936184	-1.67483414402032	0.103538862284708	0.857129839307911	-5.08579909873881
a_HQ_transcript/149911	2.7106622799439	0.743218935051046	1.67478132967784	0.103549312794347	0.857129839307911	-4.50186972324242
a_HQ_transcript/101154	1.57642344262275	-0.615091718663536	1.67473848194415	0.103557791830061	0.857129839307911	-4.52955285288676
a_HQ_transcript/6397	-1.63580244573507	0.303368239372727	-1.67469438035139	0.103566519591986	0.857129839307911	-4.48645719617958
a_HQ_transcript/3337	1.78926025509439	1.62187728562624	1.6745412296188	0.103596833070081	0.857129839307911	-4.49711013105993
a_HQ_transcript/81561	1.51876523128915	2.45307917491919	1.67443819697248	0.103617230711393	0.857129839307911	-4.55186976114836
a_HQ_transcript/18090	1.33641032215085	2.06414585203924	1.67443454894613	0.103617952981889	0.857129839307911	-4.53081461949302
a_HQ_transcript/19756	-2.15850842446661	-0.173757452990773	-1.67438320935033	0.103628118121036	0.857129839307911	-4.56451451705014
a_HQ_transcript/135049	2.5719439678731	1.24806997312569	1.67427752691645	0.103649045646113	0.857129839307911	-4.54193863284152
a_HQ_transcript/27449	-1.80076473773581	0.379842330909118	-1.67424963242036	0.103654569976887	0.857129839307911	-4.53989917602712
a_HQ_transcript/85647	2.31437037359597	1.53241843694464	1.67422828972299	0.103658796930181	0.857129839307911	-4.59272830688991
a_HQ_transcript/137453	2.63543035430679	0.781837813358143	1.67420685008673	0.103663043226691	0.857129839307911	-4.57390651783889
a_HQ_transcript/37693	-1.87392496488504	-0.332680236762443	-1.67413569482121	0.103677137149719	0.857129839307911	-4.52668723046131
a_HQ_transcript/127739	-2.15700530898569	1.44145168552888	-1.67406687921897	0.10369076916483	0.857129839307911	-4.45174654807134
a_HQ_transcript/94317	-1.95957843201864	2.55284490419537	-1.67396629379317	0.103710697299371	0.857172673484694	-4.66582772811756
a_HQ_transcript/103915	-2.31760023180793	2.1490154687184	-1.67383211118784	0.103737286715422	0.857206489148245	-4.66913373004319
a_HQ_transcript/13234	-1.62798271432608	3.0817678172758	-1.67379681211936	0.103744282465557	0.857206489148245	-4.81991614251918
a_HQ_transcript/12890	1.14649248017727	2.6707969677158	1.67369668084228	0.10376412912957	0.857248621097733	-4.64911117288462
a_HQ_transcript/151984	-2.09415600060416	1.93822170762174	-1.67337993623848	0.103826930740361	0.857401068884618	-4.53490480317411
a_HQ_transcript/36061	-2.55379269881547	1.77494727206138	-1.67334125420265	0.103834602473078	0.857401068884618	-4.61197907168476
a_HQ_transcript/135852	2.20380251385913	-0.205541623174927	1.67331099196773	0.103840604651709	0.857401068884618	-4.57483522286028
a_HQ_transcript/95361	-1.41267302399374	0.294295765842848	-1.67330606040845	0.103841582799128	0.857401068884618	-4.45089473555756
a_HQ_transcript/63498	0.881825034256703	3.36243492764815	1.67311761609718	0.10387896542261	0.857531990131522	-4.81935099654693
a_HQ_transcript/150565	2.93040824846063	1.08310886418762	1.67306834554974	0.103888741308118	0.857531990131522	-4.52586701197972
a_HQ_transcript/122224	-1.03700450442997	2.75269378219343	-1.67300305787327	0.103901696368296	0.857531990131522	-4.68024061363704
a_HQ_transcript/118705	-2.67006397790519	0.0206954222550013	-1.67283864181372	0.103934327464494	0.857679526810916	-4.5829949236086

a_HQ_transcript/76954	-2.24319131602053	0.315778614659567	-1.67261425913527	0.103978873679604	0.857788311149675	-4.57258198418544
a_HQ_transcript/127742	-1.02366308753853	2.34588521148509	-1.67258277838065	0.103985124756284	0.857788311149675	-4.63141451712469
a_HQ_transcript/114170	-1.85440624719146	1.06149733378128	-1.67248067986613	0.104005400424048	0.857788311149675	-4.51468146485614
a_HQ_transcript/87593	1.51022629741395	1.22227954973011	1.67244995659627	0.104011502378563	0.857788311149675	-4.46838520803879
a_HQ_transcript/68461	-1.85639808505081	2.71277490517133	-1.67240065663199	0.104021294475882	0.857788311149675	-4.74621801629711
a_HQ_transcript/20333	-1.915964090713	0.220768385864672	-1.67219119279968	0.104062907316269	0.858009741344697	-4.61293160724271
a_HQ_transcript/62671	-2.31085170414086	5.1039873215165	-1.67209377169862	0.104082266058026	0.858047647343771	-5.13848509863479
a_HQ_transcript/128390	-2.33050076284513	3.69458725911195	-1.67182448265435	0.104135792597619	0.85828249702492	-4.78519280187975
a_HQ_transcript/104041	2.14678619990242	0.318777282726211	1.67180026022563	0.104140608407337	0.85828249702492	-4.46020409730966
a_HQ_transcript/145911	-0.795428028841458	3.04730547934874	-1.6716587259795	0.104168751400779	0.85828249702492	-4.71363461659137
a_HQ_transcript/9119	-2.28439550617272	1.12864425212357	-1.67148563903371	0.104203176997911	0.85828249702492	-4.56750363601464
a_HQ_transcript/2008	-0.807724810578775	2.52137123216739	-1.67138537398898	0.104223123231681	0.85828249702492	-4.68734991196433
a_HQ_transcript/40622	-1.3186718912106	2.1766744527241	-1.67130925399999	0.104238268286202	0.85828249702492	-4.67053970958398
a_HQ_transcript/32187	2.86062511571867	0.675415132752468	1.67128639992168	0.104242815757165	0.85828249702492	-4.49804194007172
a_HQ_transcript/76855	-1.60698428557568	-0.070287980803659	-1.67109171657826	0.104281560248662	0.85828249702492	-4.5024151324553
a_HQ_transcript/127649	0.592187891158282	3.19305129170125	1.67097898392447	0.104304000969407	0.85828249702492	-4.69113320163352
a_HQ_transcript/147870	-2.49856617502902	0.67324559517378	-1.67090417630378	0.104318894494941	0.85828249702492	-4.56808575008216
a_HQ_transcript/144128	-2.15531336030052	0.222478610077948	-1.67089995434688	0.104319735101336	0.85828249702492	-4.57230845106526
a_HQ_transcript/114149	-2.1001253898664	3.1285662620755	-1.67088877908675	0.104321960161914	0.85828249702492	-4.70339093440703
a_HQ_transcript/99782	-2.22266872529789	1.92972341591556	-1.67088501305683	0.104322710009695	0.85828249702492	-4.70188786359621
a_HQ_transcript/108638	-2.6555894415975	0.847472220337037	-1.67086667596132	0.104326361141452	0.85828249702492	-4.66538434140277
a_HQ_transcript/151845	-2.26431474355945	-0.146467123130673	-1.6708369149419	0.104332287137109	0.85828249702492	-4.6028293943646
a_HQ_transcript/52162	-2.25714619874995	0.46328329688667	-1.67076303217932	0.104346999836139	0.85828249702492	-4.51527054127296
a_HQ_transcript/123517	-1.71997115029633	-0.564886723383649	-1.67053350463162	0.10439271797526	0.858537055810116	-4.57689107554593
a_HQ_transcript/15875	1.47410223351686	1.08572486288823	1.6702512290992	0.104448965475131	0.858866868749784	-4.45418781179804
a_HQ_transcript/148648	-1.85173439600837	7.52609342417568	-1.6701839674934	0.104462372042062	0.858866868749784	-5.14755485594157
a_HQ_transcript/150921	1.03580329444194	2.56950023683543	1.66991661933533	0.104515673950758	0.85887543656064	-4.58597918539312
a_HQ_transcript/90368	-2.14383163626268	2.19362248420426	-1.6697790667049	0.104543106986288	0.85887543656064	-4.60692065664176
a_HQ_transcript/126833	1.27022710134309	3.44708062412921	1.66970625121759	0.104557631484849	0.85887543656064	-4.83600800350118
a_HQ_transcript/75462	-1.00000509668161	3.05629200717185	-1.66966871297534	0.104565119889583	0.85887543656064	-4.73446483889301
a_HQ_transcript/107232	-1.73800899953819	-0.354151849330891	-1.66962744786656	0.104573352271276	0.85887543656064	-4.6004519774024
a_HQ_transcript/72283	0.501422966230826	2.9992802347832	1.66961993768475	0.104574850609105	0.85887543656064	-4.73649411243407
a_HQ_transcript/102616	-2.57507035473454	-0.210931557999157	-1.66954112971704	0.104590574467917	0.85887543656064	-4.59746599205888
a_HQ_transcript/149386	3.1442441116678	3.22806311637093	1.66952362837161	0.104594066623591	0.85887543656064	-4.99255043550073
a_HQ_transcript/132591	-2.13817084010345	0.00353899262118547	-1.66951196256923	0.104596394429755	0.85887543656064	-4.5606955206883
a_HQ_transcript/39987	-2.66227337486983	0.752137862209444	-1.66937306848772	0.104624112804584	0.858981699026114	-4.55345163626908
a_HQ_transcript/63606	1.38583447761164	1.04738524529702	1.6690962069486	0.104679382840904	0.859296336364697	-4.51492626255141
a_HQ_transcript/135118	-2.50296400504272	0.106146620422849	-1.66903301471643	0.104692001344185	0.859296336364697	-4.58387670631218
a_HQ_transcript/99326	1.36593334878591	-0.527082377466372	1.6688404106638	0.104730469166052	0.859490711800689	-4.57134440943202
a_HQ_transcript/89540	-2.81767914597901	3.20745323080748	-1.66875504162081	0.104747523243786	0.859495456153176	-4.73840965387579
a_HQ_transcript/136631	-2.1100978575286	-0.330200324460498	-1.66868786582144	0.104760944499668	0.859495456153176	-4.57682309623642
a_HQ_transcript/113353	1.69478014034245	1.23657017132593	1.66855311561422	0.104787870957853	0.859495456153176	-4.47946185793003
a_HQ_transcript/22451	2.42223359284756	-0.191086723124763	1.66852831469062	0.10479282742849	0.859495456153176	-4.59031771524697
a_HQ_transcript/8134	2.03774077290074	-0.00730323213752028	1.66846751767409	0.10480497855102	0.859495456153176	-4.52710195554555
a_HQ_transcript/151596	-2.06823718621822	1.69964705411811	-1.66808675298042	0.104881106251862	0.859889325803227	-4.5301439196206
a_HQ_transcript/109069	2.00335851053206	-0.0102249239186283	1.66807932301463	0.104882592209346	0.859889325803227	-4.56546012331177
a_HQ_transcript/32193	-1.83336887536715	-0.475745192215621	-1.66791340790129	0.104915778991289	0.860040107348139	-4.57716059783239
a_HQ_transcript/42321	-4.15612342314362	3.04245016474318	-1.66772222570076	0.104954030579181	0.860127215588749	-4.763484520151
a_HQ_transcript/30283	4.28902144093418	0.0251281737603764	1.66771238645534	0.104955999521246	0.860127215588749	-4.60237113803635
a_HQ_transcript/60548	2.25198417736203	0.553224573943673	1.66744656044525	0.105009205865216	0.860441939460566	-4.50551427066378

a_HQ_transcript/113296	-2.07523518867665	-0.399231003426075	-1.66719250497036	0.105060077215701	0.860478163052662	-4.53368039777138
a_HQ_transcript/82761	2.31565821261949	1.24708907182009	1.66716619303144	0.10506534701354	0.860478163052662	-4.61132260821643
a_HQ_transcript/58492	-2.51539557807333	0.526963973355411	-1.66709382476705	0.105079842179314	0.860478163052662	-4.63150238254854
a_HQ_transcript/84614	-1.55281179855349	2.11278911725692	-1.66704705965732	0.105089209986526	0.860478163052662	-4.70844682648919
a_HQ_transcript/131409	-1.11938030105687	2.64577351474154	-1.66703542069265	0.105091541567028	0.860478163052662	-4.66087405841411
a_HQ_transcript/85923	1.55410330740159	2.33009968481145	1.66697374289114	0.105103897915715	0.860478163052662	-4.65516965340163
a_HQ_transcript/83012	-2.54875450452383	-0.0166935523157701	-1.66690710775295	0.105117248758549	0.860478163052662	-4.54258993105681
a_HQ_transcript/16200	1.83347027664511	2.470371713209	1.66673736137716	0.105151265064548	0.860607067261609	-4.54958912926739
a_HQ_transcript/20787	1.85098246301824	-0.57806841596985	1.66653575762	0.105191677302834	0.860607067261609	-4.58308167500358
a_HQ_transcript/42070	-3.17813521816194	1.81782221288768	-1.66645446077867	0.10520797721214	0.860607067261609	-4.62763105115535
a_HQ_transcript/84811	1.89831015460578	2.99859075466337	1.66637718015944	0.105223473818788	0.860607067261609	-4.71109488267286
a_HQ_transcript/93821	-1.93869124489442	1.8984500051061	-1.6663355540147	0.105231821638687	0.860607067261609	-4.4730525579456
a_HQ_transcript/147976	-2.37749674161622	-0.0151728008277802	-1.66629812438137	0.1052393283484	0.860607067261609	-4.57381274442133
a_HQ_transcript/150471	-2.50917545468093	0.408411742290966	-1.66619018387077	0.105260978875511	0.860607067261609	-4.61833824192439
a_HQ_transcript/82954	-1.30123577950444	3.10362866530065	-1.66613942341197	0.105271161602044	0.860607067261609	-4.66558195994314
a_HQ_transcript/123920	-1.14110439443582	4.18407755597806	-1.66609497548447	0.105280078684316	0.860607067261609	-4.98943682076865
a_HQ_transcript/17587	1.9176755029071	2.06799551710153	1.66608715461971	0.10528164776037	0.860607067261609	-4.63581368370224
a_HQ_transcript/81457	2.55985959213784	0.145441177515624	1.66595596669168	0.105307970486009	0.860607067261609	-4.62440440982041
a_HQ_transcript/64096	1.85732890558617	2.25150347908747	1.66594256201166	0.105310660429257	0.860607067261609	-4.51152584083582
a_HQ_transcript/114271	1.50189304726873	-0.560295828407614	1.66581671032846	0.105335918114191	0.860664645991763	-4.58196261963949
a_HQ_transcript/143387	-2.59704366933455	3.78909264569332	-1.66575796632429	0.105347709408548	0.860664645991763	-4.91032648686266
a_HQ_transcript/138725	0.445782517251404	4.33480276935746	1.66568615392116	0.105362125324755	0.860664645991763	-4.96837299651798
a_HQ_transcript/31536	1.94887328406641	2.63984088472601	1.66554322586247	0.105390822163527	0.860738527061033	-4.71120057434563
a_HQ_transcript/61509	-2.11570119130262	2.71468111376604	-1.66542168872681	0.105415229276408	0.860738527061033	-4.6815356459487
a_HQ_transcript/16966	-1.20366144502353	1.84559321400349	-1.66541987906297	0.105415592728934	0.860738527061033	-4.54211840970509
a_HQ_transcript/115085	2.72758034383642	1.57761866504064	1.66519157504923	0.105461453607849	0.86099204709509	-4.60466888797077
a_HQ_transcript/60068	-2.14710647882841	1.3514424678806	-1.66506660641996	0.105486563865534	0.861076110711386	-4.49917194147093
a_HQ_transcript/109879	-3.11493875333961	1.22262437784751	-1.66479645595244	0.105540862834848	0.86135062539768	-4.63275695260622
a_HQ_transcript/151266	-1.7711978760494	1.79794000850786	-1.66460714020434	0.105578928233535	0.86135062539768	-4.49897460511296
a_HQ_transcript/27854	-0.847672878977697	3.10838868119125	-1.66459996661159	0.105580370839841	0.86135062539768	-4.6600566457919
a_HQ_transcript/20800	1.88944986948137	-0.0684608408515279	1.66459316229426	0.105581739200145	0.86135062539768	-4.47665831774789
a_HQ_transcript/125555	2.55773177139916	-0.3568697478138	1.66453078396449	0.105594284279243	0.86135062539768	-4.5794639122917
a_HQ_transcript/116740	-3.9157766380079	1.95931419633396	-1.66424901280338	0.105650967479962	0.861692077686997	-4.66076872649413
a_HQ_transcript/84267	1.46368129124281	1.04079419103755	1.66405067642867	0.105690881458122	0.861896683136608	-4.44974828456892
a_HQ_transcript/16006	-1.14631392431935	1.99894350164393	-1.66393226042466	0.105714717919129	0.8619662129808	-4.56076532900978
a_HQ_transcript/42729	-1.35836465130928	1.51138919845632	-1.66386099623478	0.105729065141637	0.8619662129808	-4.59728381830383
a_HQ_transcript/142078	-1.86281955573351	-0.383277090327833	-1.66369479704986	0.105762531378103	0.862118135457349	-4.58992588086722
a_HQ_transcript/149816	5.9040070891582	0.887316575568546	1.66342722470895	0.105816428810284	0.862436535878325	-4.6653580893116
a_HQ_transcript/63962	-2.40601894098449	0.910318610142244	-1.66332743449183	0.105836535514418	0.862479480776968	-4.51416846852393
a_HQ_transcript/143914	3.22164033396893	0.211464091757464	1.66321714655947	0.105858761089164	0.862522375736927	-4.63484599497561
a_HQ_transcript/82608	0.944198256351121	2.89967886374079	1.66315406168059	0.105871475896005	0.862522375736927	-4.83092880930484
a_HQ_transcript/117075	-2.04686274330591	0.0750591703508597	-1.66299805514516	0.105902924578599	0.862657679358854	-4.54540831416379
a_HQ_transcript/94625	-5.06010786108284	2.47760556888768	-1.6628175325799	0.105939325017313	0.862752464553386	-4.6761779966755
a_HQ_transcript/83776	-1.86850305221174	0.01947615128605	-1.66274005719401	0.105954950281758	0.862752464553386	-4.49613151292457
a_HQ_transcript/150866	3.6662295023615	0.00545000320841995	1.66261076507408	0.105981030223267	0.862752464553386	-4.6001262033929
a_HQ_transcript/123462	3.18861851429215	1.30402728181938	1.66259852561305	0.105983499361465	0.862752464553386	-4.64880175448596
a_HQ_transcript/47201	1.44900424283769	2.91823813029078	1.66257238915728	0.105988772181663	0.862752464553386	-4.71818726125594
a_HQ_transcript/53947	-1.64598038084242	-0.306410553052956	-1.6624987309398	0.106003633307188	0.862752617877377	-4.61551898773619
a_HQ_transcript/30438	-1.76374466537828	1.02683794790049	-1.6621170479701	0.106080668550357	0.863258729034738	-4.55659884668726
a_HQ_transcript/23482	-2.22133940353336	-0.110933432111156	-1.66188999501939	0.106126516780871	0.863404358690356	-4.54295867461495

a_HQ_transcript/22027	-2.00834583848023	0.654265455660596	-1.66178805455227	0.106147106710224	0.863404358690356	-4.53131938570693
a_HQ_transcript/112595	-3.24773555703612	0.771050821314317	-1.66175055355082	0.106154681994116	0.863404358690356	-4.71153130987141
a_HQ_transcript/113400	0.81237124420442	2.72007434649163	1.66170372774148	0.10616414153923	0.863404358690356	-4.73751657999163
a_HQ_transcript/123949	2.07802043792575	-0.566102528619723	1.66154270809695	0.106196675361098	0.863404358690356	-4.5324417072174
a_HQ_transcript/140912	1.9013244735901	-0.0545610107224195	1.66152248063701	0.106200762879375	0.863404358690356	-4.58642040219898
a_HQ_transcript/13006	-2.05871482193102	0.82905733284955	-1.66151369344531	0.106202538615401	0.863404358690356	-4.65543685964892
a_HQ_transcript/113817	-2.8880315916182	2.22591072849517	-1.66135872739805	0.106233858560944	0.86344478896974	-4.60493831472766
a_HQ_transcript/125469	-2.17384846886191	0.742966494733377	-1.66134209723382	0.10623722011271	0.86344478896974	-4.49230902584308
a_HQ_transcript/80266	-2.11702128934969	2.02713032386558	-1.66123641888282	0.106258583554401	0.863462666398427	-4.63861168410248
a_HQ_transcript/63079	-1.07740951688126	2.56469901168959	-1.66118015828518	0.106269958387724	0.863462666398427	-4.71559773698676
a_HQ_transcript/94989	3.41756050712859	0.178089970324233	1.66111079738839	0.106283983245265	0.863462666398427	-4.55087285732656
a_HQ_transcript/121796	1.95480969939732	-0.243098408686153	1.66092967331998	0.106320613992468	0.863607283695796	-4.63211457399737
a_HQ_transcript/84511	2.71468558041954	0.773019297406557	1.66065859064107	0.106375457643885	0.863607283695796	-4.57616355359571
a_HQ_transcript/120731	-2.57372423623214	-0.0511679480863851	-1.66065603846202	0.106375974095335	0.863607283695796	-4.56022665196254
a_HQ_transcript/48770	-1.14782358576083	1.34484743783459	-1.66062324091071	0.106382611096517	0.863607283695796	-4.57444097093213
a_HQ_transcript/136689	1.89481208419651	-0.259542197763816	1.66061196271629	0.106384893461813	0.863607283695796	-4.55645485292061
a_HQ_transcript/837	0.993480868127159	2.20320499906332	1.6605434713014	0.106398754924183	0.863607283695796	-4.55191452850615
a_HQ_transcript/120229	-2.48875171980996	0.00101429650775558	-1.66050874644207	0.106405783199651	0.863607283695796	-4.54652900642176
a_HQ_transcript/89523	2.25451938251813	-0.33894202591334	1.66033142431802	0.106441679018242	0.863778014515202	-4.58771635128852
a_HQ_transcript/110363	1.92288622121824	1.76198931201179	1.66020063471638	0.106468161563201	0.863865030081891	-4.65070090194816
a_HQ_transcript/1649	2.85318592947349	1.24415853109078	1.66013168604391	0.106482124630759	0.863865030081891	-4.67464579392919
a_HQ_transcript/89461	2.08149147769496	1.13195945258643	1.66003548473938	0.106501609274484	0.863902531943514	-4.50533248298542
a_HQ_transcript/23105	2.13637954198935	0.73218737080711	1.65992154628361	0.106524690232099	0.863969191267119	-4.59731631504264
a_HQ_transcript/86978	-2.36404102392582	1.09035356114954	-1.65979942539957	0.106549433342613	0.864049311012749	-4.48251155466989
a_HQ_transcript/35401	1.93933714946896	2.95139046306616	1.65949558876202	0.106611014861479	0.864117513948571	-4.62452863926213
a_HQ_transcript/64715	-2.53934150518954	2.33635658247784	-1.65937725587908	0.106635006520601	0.864117513948571	-4.67855676250607
a_HQ_transcript/115176	2.07100391890444	0.30900765937712	1.65933515623252	0.106643543185369	0.864117513948571	-4.555002418325
a_HQ_transcript/139638	-3.10584067732781	1.5994313878646	-1.65929675210972	0.106651330992633	0.864117513948571	-4.69524641773117
a_HQ_transcript/77373	-2.13213677629966	-0.53413227001472	-1.65914280214179	0.106682554581283	0.864117513948571	-4.59554072283886
a_HQ_transcript/99971	2.53821170590725	0.896046975543571	1.65910837112141	0.106689538796462	0.864117513948571	-4.62392412881847
a_HQ_transcript/54951	1.42181471087238	1.91032895318548	1.65909758937682	0.10669172591458	0.864117513948571	-4.61392500553259
a_HQ_transcript/63209	-1.87169165665036	0.670518463334685	-1.65908394458309	0.10669449386647	0.864117513948571	-4.53070474905725
a_HQ_transcript/127512	2.25740199409856	0.664970647518051	1.65904694198426	0.106702000429425	0.864117513948571	-4.55713908830554
a_HQ_transcript/89144	2.23242107847327	0.468393584682981	1.65902475593882	0.106706501429997	0.864117513948571	-4.53324168805373
a_HQ_transcript/2312	-0.792732578566785	3.3362759101453	-1.65885236594019	0.106741480467607	0.864156632999686	-4.75454154962743
a_HQ_transcript/18932	-1.84604349578498	-0.353126620985478	-1.65870853847667	0.106770671259159	0.864156632999686	-4.50536697124528
a_HQ_transcript/116025	1.96930810615189	0.177149203119848	1.65865033504743	0.10678248593433	0.864156632999686	-4.54299643408041
a_HQ_transcript/14263	-2.36373381331788	1.49845427788419	-1.65847490491591	0.106818102937867	0.864156632999686	-4.56990730946981
a_HQ_transcript/12157	1.60672956773607	0.101678304006131	1.6583152737419	0.106850520890806	0.864156632999686	-4.48839114213058
a_HQ_transcript/118288	1.94028534848012	1.64285518581532	1.65831124857472	0.106851338428557	0.864156632999686	-4.5433280709467
a_HQ_transcript/108418	-0.727779271882648	4.7210072918481	-1.65828394042177	0.106856885029803	0.864156632999686	-5.00666238555045
a_HQ_transcript/37845	4.45940388847342	4.25005154725776	1.65827113079912	0.106859486894361	0.864156632999686	-5.17701037100138
a_HQ_transcript/87813	-1.28139485193033	0.364879292045229	-1.65824461761739	0.106864872364923	0.864156632999686	-4.51771569262973
a_HQ_transcript/7941	1.62876007039565	-0.48937363784316	1.65824276713963	0.10686524825026	0.864156632999686	-4.59506927256028
a_HQ_transcript/88817	-0.524067101158592	2.69106861383027	-1.65814761219065	0.10688457843907	0.864156632999686	-4.74648472244282
a_HQ_transcript/9311	-0.916463668565909	1.74863331476284	-1.65812225837549	0.106889729412121	0.864156632999686	-4.57985560126556
a_HQ_transcript/64146	-2.42899940575444	2.67041293464053	-1.65790194201617	0.106934498348833	0.864314721925918	-4.59334778031966
a_HQ_transcript/136289	-1.12725109288892	4.12163183611276	-1.65787968374726	0.106939022159565	0.864314721925918	-4.97772479054794
a_HQ_transcript/27818	1.90815906707716	1.07851650066199	1.65748556104418	0.107019150652281	0.864782499744097	-4.51878557077332
a_HQ_transcript/13083	-0.57725957771729	3.60420628012809	-1.65736954662898	0.107042746848896	0.864782499744097	-4.87223355205301

a_HQ_transcript/43162	-2.11175911312947	1.05065316562452	-1.65734171683052	0.107048407798929	0.864782499744097	-4.50941499159105
a_HQ_transcript/17147	0.907698109469231	4.71048671673406	1.65730239047832	0.107056407723619	0.864782499744097	-4.97688051076311
a_HQ_transcript/126916	3.82165845378524	3.11648719563555	1.65716839007935	0.107083670345402	0.864797353447828	-4.80432871825314
a_HQ_transcript/110849	1.79870686611941	0.270826757354276	1.65714710441611	0.107088001481514	0.864797353447828	-4.60082760368339
a_HQ_transcript/110374	-1.95057947298111	0.232552986233476	-1.65702249541925	0.107113359420355	0.864881977550547	-4.45300737570031
a_HQ_transcript/148541	-3.30609966522438	1.62092727041598	-1.6565240165949	0.107214849684332	0.865581219784839	-4.74847500300801
a_HQ_transcript/134883	-2.87278154820728	-0.0528103771621639	-1.65639119580707	0.107241905435624	0.865679416631296	-4.59640458795714
a_HQ_transcript/53433	-1.69213186573631	1.28459518754864	-1.65605972727729	0.107309450652636	0.865924679891146	-4.64052827340079
a_HQ_transcript/26923	3.63602626690756	-0.379622403584119	1.65605727018569	0.107309951479721	0.865924679891146	-4.61441418947306
a_HQ_transcript/10496	1.2837331303898	1.57193094557838	1.65602279043791	0.107316979664461	0.865924679891146	-4.45733507296634
a_HQ_transcript/55827	-2.18856115643153	0.786327790452804	-1.65584223891627	0.107353788641886	0.866101460954274	-4.55612523302963
a_HQ_transcript/111070	-2.20239734681167	0.723797680367711	-1.65570190004264	0.10738240670768	0.866117522572095	-4.59428985710355
a_HQ_transcript/130274	-0.611131934569511	4.22994180298005	-1.65568634134779	0.107385579844087	0.866117522572095	-4.98766298828865
a_HQ_transcript/97806	3.42530601909369	4.49721464793364	1.65536161994852	0.107451823271919	0.866531573688972	-4.89405353996048
a_HQ_transcript/125246	-2.96160616201838	2.63308930050861	-1.65500604328465	0.107524400040958	0.866798581701142	-4.70597732238251
a_HQ_transcript/40139	2.2656828634545	3.48708231851768	1.65495838274131	0.107534131123395	0.866798581701142	-4.81313512944405
a_HQ_transcript/14679	2.82980570247981	1.08847923551304	1.65455077561765	0.107617384044743	0.866798581701142	-4.522840793621
a_HQ_transcript/126807	-3.73033167549079	0.440511742293385	-1.65453940666499	0.107619706895476	0.866798581701142	-4.57772921594336
a_HQ_transcript/18996	-0.835002394876737	2.44731596102868	-1.65452494252999	0.1076226621993	0.866798581701142	-4.65503743124052
a_HQ_transcript/66243	2.18296035122503	2.5139292151939	1.65444925404181	0.107638127924758	0.866798581701142	-4.81610114878168
a_HQ_transcript/142460	-0.696028711568265	3.76774719623692	-1.65436596379721	0.107655149075155	0.866798581701142	-4.82764844908782
a_HQ_transcript/29807	-1.68148400727794	3.07495449107258	-1.65431893489031	0.107664760862863	0.866798581701142	-4.71660731581339
a_HQ_transcript/117849	-2.10318856189375	-0.163949536302518	-1.65428414209805	0.10767187228595	0.866798581701142	-4.5677549448093
a_HQ_transcript/4895	1.62121278321607	-0.320980955271136	1.6542490886738	0.107679037374126	0.866798581701142	-4.60553579635406
a_HQ_transcript/21102	-2.12303000069531	0.449450123358231	-1.65418053655999	0.107693050897046	0.866798581701142	-4.54714625117407
a_HQ_transcript/38374	-1.98881485706142	0.771434138360567	-1.65408678487894	0.107712218198573	0.866798581701142	-4.57922018939164
a_HQ_transcript/53167	-2.95656967095453	1.6162264492744	-1.65403336383042	0.107723141265204	0.866798581701142	-4.66142557394624
a_HQ_transcript/83146	-2.60089906758668	2.05972612560551	-1.65396422206107	0.107737280129344	0.866798581701142	-4.70303733079342
a_HQ_transcript/112526	2.0676495062859	1.25768617833983	1.65395098405814	0.10773998735613	0.866798581701142	-4.49361097043526
a_HQ_transcript/22146	0.600915469849627	3.4144241384933	1.65393945460115	0.107742345224072	0.866798581701142	-4.80610272386487
a_HQ_transcript/101674	2.36920162197708	-0.274777210376121	1.65384398116869	0.107761871962204	0.866798581701142	-4.60256185821699
a_HQ_transcript/96605	-3.27932553245308	1.65513754387481	-1.65382931814659	0.107764871181797	0.866798581701142	-4.73271119346649
a_HQ_transcript/125509	-3.14966517408829	0.303575208135964	-1.65381275646718	0.107768258841766	0.866798581701142	-4.5877386523446
a_HQ_transcript/111208	-3.0497827205104	1.05311772786056	-1.65364225995594	0.107803138708051	0.866959165304591	-4.55160280623867
a_HQ_transcript/20457	0.598755917648433	5.2418752026487	1.65325602432013	0.107882188685565	0.867362799619634	-5.0793320307975
a_HQ_transcript/79139	3.59538901755277	0.389701994047183	1.65322995253988	0.107887526467814	0.867362799619634	-4.63984147969507
a_HQ_transcript/131770	-1.40163818311128	-0.606022464131843	-1.65317834013712	0.107898093931489	0.867362799619634	-4.61315477717236
a_HQ_transcript/125288	-1.83390492111397	0.693744510030941	-1.65305254530691	0.107923853586387	0.867449911679963	-4.46268524352857
a_HQ_transcript/34732	1.36521730266263	-0.781021268462057	1.65295078340386	0.107944695622466	0.867497479212318	-4.61300521324787
a_HQ_transcript/148077	2.66135889365089	1.22486673912913	1.65280966085219	0.107973604700888	0.867609855412392	-4.67425129986669
a_HQ_transcript/121521	-2.41077911033099	-0.0480863161273283	-1.65268936364014	0.107998252753553	0.867687966283142	-4.6090280791416
a_HQ_transcript/120296	2.50137633563393	-0.0183251016377907	1.65255404016722	0.108025985151592	0.867790832627383	-4.5510282115049
a_HQ_transcript/130040	2.35768722408312	3.27229251734013	1.65241803519872	0.108053863154427	0.867880513532352	-4.85348552007852
a_HQ_transcript/46851	-1.32996763322262	2.90462711236681	-1.65208477918144	0.108122198439642	0.867880513532352	-4.8114845575026
a_HQ_transcript/148395	-0.499553851189756	8.30318231276867	-1.65196198146873	0.10814738755331	0.867880513532352	-5.39233223230848
a_HQ_transcript/92819	-1.40299408524326	1.74680171763632	-1.6516878915346	0.108203628302254	0.867880513532352	-4.57158595254526
a_HQ_transcript/83880	-1.59565928768662	-0.692896874168484	-1.65162409388643	0.10821672247107	0.867880513532352	-4.61474356499798
a_HQ_transcript/1203	2.8139753964317	0.213997096420145	1.65158861640015	0.10822400462693	0.867880513532352	-4.52370909040904
a_HQ_transcript/61706	2.61030344878126	0.611555043988983	1.65156943892557	0.108227941189758	0.867880513532352	-4.54260252006102
a_HQ_transcript/54519	-1.03704997392606	2.89466977336696	-1.65144513253275	0.1082534604556	0.867880513532352	-4.82511494838242

a_HQ_transcript/59470	-1.08648720465397	3.1147031017378	-1.65143236531205	0.10825608176225	0.867880513532352	-4.87421481264093
a_HQ_transcript/22607	2.30887717092061	0.487245818230735	1.65143058689087	0.108256446903625	0.867880513532352	-4.53932161505395
a_HQ_transcript/59394	1.93834483845874	2.0819859489007	1.65141200484685	0.108260262187467	0.867880513532352	-4.58892011624276
a_HQ_transcript/95984	-2.50112893706043	1.7135038412829	-1.65138545863597	0.108265712875176	0.867880513532352	-4.54876828076936
a_HQ_transcript/108636	-1.55035394210691	2.34547163691003	-1.65129960590325	0.108283342424813	0.867880513532352	-4.66192206206584
a_HQ_transcript/29214	2.9429691017883	0.550719206726326	1.65128515130759	0.108286310857813	0.867880513532352	-4.68765987235574
a_HQ_transcript/112523	-2.26582332712933	0.872106982810442	-1.65126630533366	0.108290181216778	0.867880513532352	-4.54904265407677
a_HQ_transcript/141192	3.67751632555032	0.738211422804587	1.65124589714978	0.108294372532787	0.867880513532352	-4.57524324501372
a_HQ_transcript/81497	-1.22709429129829	3.76567764262651	-1.65123858164265	0.108295874982482	0.867880513532352	-4.76109561000312
a_HQ_transcript/104554	-0.787535116736473	3.51000711697555	-1.65111005778664	0.108322273866881	0.867880513532352	-4.92483824542945
a_HQ_transcript/145551	-1.42740789324408	3.02258833604433	-1.65101628830265	0.108341537542538	0.867880513532352	-4.72717193450165
a_HQ_transcript/152228	6.12477321129772	2.13679523443554	1.65101363394603	0.108342082885587	0.867880513532352	-4.77724057596908
a_HQ_transcript/78164	-4.81839646300547	1.55195634750721	-1.65097174368065	0.108350689628136	0.867880513532352	-4.53651674387756
a_HQ_transcript/142383	2.84576433767451	0.361163196839169	1.6508251138133	0.108380820550356	0.867881313536781	-4.55696840090163
a_HQ_transcript/39479	2.10435846775782	0.198675604979196	1.65075470220794	0.108395291873161	0.867881313536781	-4.54756014828286
a_HQ_transcript/151992	-2.23148868499465	1.02424541471892	-1.65075329518578	0.108395581067249	0.867881313536781	-4.61737055108008
a_HQ_transcript/90768	2.01892898116909	4.34178092225845	1.65062682462268	0.108421577964803	0.867969905514129	-5.02524365766854
a_HQ_transcript/129862	-1.40726904987952	3.61225661014971	-1.65044109088807	0.108459766174583	0.86799164406167	-4.805794951685
a_HQ_transcript/47961	2.728629492952	2.57398746617517	1.65044028546479	0.10845993179973	0.86799164406167	-4.68255226715144
a_HQ_transcript/135435	-1.17945985009339	1.53633935713034	-1.65037973665537	0.108472383498948	0.86799164406167	-4.55577294171981
a_HQ_transcript/112803	1.82225107838496	1.56660296024889	1.65031289914997	0.108486129825899	0.86799164406167	-4.53292050625142
a_HQ_transcript/60274	2.21503385906502	0.129533381255477	1.65010967879957	0.108527934581302	0.86799164406167	-4.59710743040945
a_HQ_transcript/51654	-1.67950194036397	1.44045469842912	-1.65006268325456	0.108537604002177	0.86799164406167	-4.4850311714881
a_HQ_transcript/91237	3.28127487075066	0.296501417963508	1.64988556620632	0.108574052574527	0.86799164406167	-4.65447788596128
a_HQ_transcript/85692	-2.11233399487429	0.842176655197743	-1.64978731952017	0.108594274937889	0.86799164406167	-4.55656347052825
a_HQ_transcript/93176	1.73426047911612	-0.56671936073502	1.64967543727454	0.108617307740242	0.86799164406167	-4.60401262124999
a_HQ_transcript/59277	3.58488588243001	1.43199618631731	1.64966929831037	0.108618571664129	0.86799164406167	-4.59806834251889
a_HQ_transcript/108569	2.78694279199226	2.49558186831902	1.64964959659727	0.108622628044	0.86799164406167	-4.74215279541997
a_HQ_transcript/131904	-1.06708906608417	3.58254136556136	-1.64963947699538	0.108624711614586	0.86799164406167	-4.84763910055911
a_HQ_transcript/13858	3.85279602795388	3.97501803158377	1.64961719884794	0.108629298679683	0.86799164406167	-4.9064673669459
a_HQ_transcript/15591	2.36158637340006	2.32933698817448	1.64959412884812	0.108634048956006	0.86799164406167	-4.56623767149284
a_HQ_transcript/29952	1.81524544762172	-0.516370426004091	1.64952502140811	0.108648279696406	0.86799164406167	-4.56322274971054
a_HQ_transcript/123496	-1.75942686096023	0.106077102998668	-1.64936305680887	0.108681637813192	0.868138826824958	-4.52403504345748
a_HQ_transcript/29535	-1.70481997293376	0.388663838116577	-1.64917713471106	0.108719940651844	0.868325461694198	-4.60453427857153
a_HQ_transcript/93481	2.1999889829775	0.512205748909371	1.64907630194594	0.108740718440111	0.868372095272258	-4.60819077132248
a_HQ_transcript/46451	-2.1830160068133	0.845321324778417	-1.64886398687983	0.10878447922743	0.868602226446708	-4.5051042824924
a_HQ_transcript/52038	-4.51138874538991	2.32674411546245	-1.64862455738633	0.108833846124619	0.868645056829393	-4.70041525040437
a_HQ_transcript/135412	-1.61403924478885	-0.583332538471518	-1.64862193896243	0.108834386108227	0.868645056829393	-4.63861066287441
a_HQ_transcript/83185	1.66686792421667	-0.644954776272465	1.6486205414035	0.108834674320267	0.868645056829393	-4.60162648238912
a_HQ_transcript/111660	1.39607510978599	2.24535787247676	1.648434047846	0.108873139681255	0.868832765429982	-4.63521693391269
a_HQ_transcript/102946	1.59781746378395	0.519330422967351	1.64821005887967	0.108919353556803	0.869082248943011	-4.50678913841498
a_HQ_transcript/35442	-1.87955004586777	3.27459086691719	-1.64796805046901	0.108969303488331	0.869285815069288	-4.73225980441019
a_HQ_transcript/54454	-1.25395313681516	2.60659893877085	-1.6479072016745	0.108981865509698	0.869285815069288	-4.69766783231278
a_HQ_transcript/120355	1.99802446882131	-0.0980161955223339	1.64785666123668	0.108992300317908	0.869285815069288	-4.6215819550385
a_HQ_transcript/54429	-2.25889944091266	1.6098684853261	-1.64779668385463	0.109004684593312	0.869285815069288	-4.55490652116131
a_HQ_transcript/144307	2.58876468062324	0.349357718222562	1.64765535057721	0.109033872036863	0.869304412628944	-4.69264626508533
a_HQ_transcript/138679	-2.953918582661	0.668982647796167	-1.64764056113315	0.109036926652863	0.869304412628944	-4.786945628968
a_HQ_transcript/111334	-1.38312510934047	-0.805599406559592	-1.6475202885088	0.109061770424908	0.869330900567037	-4.6213947457463
a_HQ_transcript/144113	-1.29646016373277	1.75519892521365	-1.64747967859193	0.109070159954504	0.869330900567037	-4.5667806430778
a_HQ_transcript/21897	2.0599886773642	-0.0460949534217994	1.64738513298495	0.109089694030258	0.869367388893722	-4.53490723057818

a_HQ_transcript/34660	1.79251497541592	0.472349703974333	1.6469577503831	0.109178031710752	0.86994213453424	-4.56223451121822
a_HQ_transcript/107450	3.58800294195918	1.52523532730169	1.64685555560899	0.109199163586552	0.86994213453424	-4.66276402743675
a_HQ_transcript/5037	3.6186396068581	2.81736908271666	1.64677620588657	0.109215573887538	0.86994213453424	-4.75571215185224
a_HQ_transcript/32571	2.53027214276028	0.0879201926078694	1.64674669289836	0.109221677983603	0.86994213453424	-4.56237610110894
a_HQ_transcript/67734	1.31795855328661	2.69453201724753	1.64635101403396	0.109303542499072	0.87047490319031	-4.60712315461719
a_HQ_transcript/83025	-2.25738963788384	1.01993926312834	-1.64607246143186	0.109361204461903	0.87073131316061	-4.54187460800311
a_HQ_transcript/95869	2.42687670647195	2.26067894269097	1.64602515989632	0.109370998646261	0.87073131316061	-4.75328071891501
a_HQ_transcript/99371	2.32705914934534	0.0671358637280912	1.64593346193062	0.109389987555235	0.87073131316061	-4.60614905425857
a_HQ_transcript/63231	-2.09077083409083	0.163292872803815	-1.64590608409315	0.109395657514656	0.87073131316061	-4.54113803138378
a_HQ_transcript/34712	0.976570791379168	2.34064259022632	1.64552177100969	0.10947527454838	0.870739009269251	-4.62851012396424
a_HQ_transcript/144700	1.97085695084794	0.348640157148137	1.64551931300779	0.109475783919775	0.870739009269251	-4.58731682428975
a_HQ_transcript/75809	-2.89186242873689	0.883241001609913	-1.64549856985182	0.109480082599232	0.870739009269251	-4.56594657753109
a_HQ_transcript/104164	1.14479462416566	2.32274654211133	1.64545742070584	0.109488610499254	0.870739009269251	-4.64795166051026
a_HQ_transcript/61379	-1.25025008931759	1.14627900239879	-1.64541693293126	0.109497001870564	0.870739009269251	-4.49543685731641
a_HQ_transcript/114394	-1.60728446072839	-0.230453256509771	-1.64541179533549	0.109498066710855	0.870739009269251	-4.52779757071593
a_HQ_transcript/1046	-0.944534398528995	3.73655815389481	-1.64526331958485	0.109528844137527	0.870739009269251	-4.93600488479576
a_HQ_transcript/111607	1.63007057003358	2.07611631990269	1.64525175518487	0.109531241613655	0.870739009269251	-4.69767784822633
a_HQ_transcript/137229	-2.3699832335919	1.83188182626662	-1.64523673700209	0.109534355176319	0.870739009269251	-4.63735953610032
a_HQ_transcript/124285	1.39433582568901	8.46277563469901	1.6451347827374	0.109555494222633	0.870739009269251	-5.42266728724263
a_HQ_transcript/58782	1.27897401986778	2.42635696138933	1.64510629737196	0.109561400939225	0.870739009269251	-4.67301369787916
a_HQ_transcript/131806	0.524064060983854	4.32847191532142	1.64496812047107	0.109590056997013	0.870750134648566	-5.01255199232018
a_HQ_transcript/97699	2.3512534590784	1.24108548679662	1.64485747002046	0.109613008903644	0.870750134648566	-4.56789981651633
a_HQ_transcript/108579	2.19014894047103	-0.119558692530757	1.64477520362198	0.109630075764272	0.870750134648566	-4.63172301308269
a_HQ_transcript/140632	2.86761225406775	0.974652815687038	1.64476043998723	0.109633138837857	0.870750134648566	-4.66828061324831
a_HQ_transcript/59655	-1.85348462143932	1.14342188445966	-1.64472075566869	0.109641372661705	0.870750134648566	-4.47166476055853
a_HQ_transcript/4054	3.75526224093009	2.05617540720728	1.64466626182515	0.109652680044514	0.870750134648566	-4.55626340918905
a_HQ_transcript/99623	1.69180149835952	0.616991129880467	1.64453791242345	0.10967931614523	0.870762962377399	-4.47855531065265
a_HQ_transcript/24572	2.15215442661097	0.103560768805581	1.6444429296156	0.109699031187176	0.870762962377399	-4.57500107869424
a_HQ_transcript/122874	2.15325809660358	2.23056740254719	1.64443172052664	0.10970135798745	0.870762962377399	-4.79654917025235
a_HQ_transcript/63099	-2.26397010921243	1.16106135947482	-1.64436978302385	0.109714215807392	0.870762962377399	-4.5550419386214
a_HQ_transcript/113639	-2.08650266105283	0.448581292436654	-1.64417860538535	0.109753910894268	0.870792922600374	-4.61489530818967
a_HQ_transcript/138246	-3.25527789443604	2.92689121251428	-1.64382441690224	0.109827484054646	0.870792922600374	-4.74235810544888
a_HQ_transcript/140272	0.760196509047892	2.82467461210914	1.64381657641594	0.109829113166757	0.870792922600374	-4.68603387332418
a_HQ_transcript/146921	2.16662888749234	0.763895913736801	1.6437931376496	0.109833983440228	0.870792922600374	-4.51227817502683
a_HQ_transcript/71126	1.77748681463875	-0.218411114718043	1.6437646016544	0.109839913093224	0.870792922600374	-4.48632446979197
a_HQ_transcript/91948	2.04462985226274	1.88677211645213	1.6436809282744	0.109857301575971	0.870792922600374	-4.72626429120137
a_HQ_transcript/132586	2.73061769044818	0.86340682148775	1.64367865815408	0.109857773370007	0.870792922600374	-4.62872978118579
a_HQ_transcript/127358	1.82829265433073	0.0206332458928981	1.64365546054608	0.109862594573128	0.870792922600374	-4.73934397871722
a_HQ_transcript/68452	2.16826044781374	3.18652588006052	1.64363348767546	0.109867161398095	0.870792922600374	-4.9118894853254
a_HQ_transcript/114099	3.26220850526027	0.0202312631879612	1.64363043039348	0.109867796833731	0.870792922600374	-4.62774027257096
a_HQ_transcript/54015	2.4012194260007	1.14680351749482	1.64354696755083	0.109885145202051	0.87081168647646	-4.58222569438554
a_HQ_transcript/130960	-1.06865730831717	2.29197055994387	-1.64337726207432	0.109920426724458	0.870972541526623	-4.63041794082023
a_HQ_transcript/103359	0.600735516555152	2.81630436886298	1.6429772717226	0.110003621197251	0.871503141076191	-4.76346733970493
a_HQ_transcript/101492	-1.95987714530244	2.94443489901011	-1.64288347147189	0.110023138358224	0.871503141076191	-4.68258401329295
a_HQ_transcript/148558	1.90555330234476	-0.0058459020536934	1.64283911284386	0.110032369122594	0.871503141076191	-4.62715816867181
a_HQ_transcript/74890	2.03757457234201	1.30099744978619	1.6425606686178	0.11009032632138	0.871693510211353	-4.62762641187542
a_HQ_transcript/106464	-1.07210650716546	2.70807945408998	-1.64237772810604	0.110128418490758	0.871693510211353	-4.71682919035757
a_HQ_transcript/76173	2.00715137791331	2.32842913222287	1.6422915394713	0.110146368603606	0.871693510211353	-4.6607068232367
a_HQ_transcript/136068	2.01393999152897	0.793943807946583	1.64226577818075	0.110151734259298	0.871693510211353	-4.5420537501189
a_HQ_transcript/2890	-2.17743057386511	0.0632432562637629	-1.64226236333901	0.110152445531229	0.871693510211353	-4.45358971754962

a_HQ_transcript/105205	1.40481410907269	0.863569588662337	1.64210324413778	0.11018559242626	0.871693510211353	-4.48252851482249
a_HQ_transcript/76911	-2.2959619703328	1.63044693249395	-1.64203332182636	0.11020016089089	0.871693510211353	-4.5890766549842
a_HQ_transcript/22970	-3.74041249369031	1.87870427662998	-1.64194210717281	0.110219168054532	0.871693510211353	-4.57645483736551
a_HQ_transcript/111613	-0.883563787017095	2.08443532129624	-1.64192233098319	0.110223289342769	0.871693510211353	-4.65979222241548
a_HQ_transcript/50802	2.09409319109623	3.56503356936678	1.64190191788741	0.110227543493617	0.871693510211353	-4.92851830313548
a_HQ_transcript/97957	2.62125249903585	-0.281395896174854	1.64178441298733	0.11025203450942	0.871693510211353	-4.62917536354882
a_HQ_transcript/101913	-1.61153250438744	-0.256419474606808	-1.64175590202321	0.11025797759904	0.871693510211353	-4.59421020724269
a_HQ_transcript/49532	1.38428725201968	0.373067606968261	1.64175443665352	0.110258283061435	0.871693510211353	-4.47239420529049
a_HQ_transcript/1033	1.35294289181266	2.64354259809031	1.64171574001357	0.110266349789844	0.871693510211353	-4.68756137631409
a_HQ_transcript/107105	2.14740244018481	0.911203230254982	1.64141255269037	0.110329569316492	0.871999614676252	-4.6339297191718
a_HQ_transcript/59946	1.39290961007335	2.99691955034095	1.64138616148384	0.110335073732863	0.871999614676252	-4.79465870792368
a_HQ_transcript/120624	-2.4118975922415	0.817980968448091	-1.64096074854643	0.110423833473348	0.872198462643438	-4.6896065969394
a_HQ_transcript/149809	-2.07164333183834	1.24980594916696	-1.64085755120993	0.110445373842581	0.872198462643438	-4.51263704006963
a_HQ_transcript/60156	-2.52010154651907	0.295044310793663	-1.64082186929254	0.110452822533412	0.872198462643438	-4.55647512049064
a_HQ_transcript/152202	-1.43545606543702	11.4617719975372	-1.64077522847155	0.110462559549245	0.872198462643438	-5.7496342801422
a_HQ_transcript/99359	-2.24339319428002	0.74967486425489	-1.64065474885423	0.110487714875618	0.872198462643438	-4.67222739283254
a_HQ_transcript/87363	1.83420104469261	1.23803388163305	1.64065131732434	0.110488431425108	0.872198462643438	-4.60493891142792
a_HQ_transcript/60680	-1.12464190113915	2.0070997860339	-1.64053189249056	0.110513371325861	0.872198462643438	-4.58239194349594
a_HQ_transcript/137607	1.41985613399904	0.564078984231839	1.64051378986476	0.110517152165813	0.872198462643438	-4.48245252100299
a_HQ_transcript/98900	-1.9579566391936	-0.418170855210408	-1.64050953554865	0.110518040720285	0.872198462643438	-4.62793941073778
a_HQ_transcript/76084	1.82913859781007	0.363146619319133	1.64043980322081	0.110532605823205	0.872198462643438	-4.51650681589207
a_HQ_transcript/46945	-2.41075313055059	0.614773888036405	-1.64041758920762	0.110537246032852	0.872198462643438	-4.49610283914445
a_HQ_transcript/85726	0.50870817041603	4.16151914653626	1.64040300928055	0.110540291671728	0.872198462643438	-4.98571622540748
a_HQ_transcript/46690	-1.8941743785786	0.769507543402497	-1.64027842814324	0.110566318577579	0.872262452248026	-4.51443155297622
a_HQ_transcript/134341	-2.08862912808225	-0.398396126102439	-1.64021803439213	0.110578937578801	0.872262452248026	-4.64121655307164
a_HQ_transcript/121545	-2.41397948696206	-0.53652781125651	-1.6401487331275	0.110593419231144	0.872262452248026	-4.63800785523167
a_HQ_transcript/38940	-2.89365958564158	0.816843301658136	-1.63986869680911	0.110651953442725	0.872575915511067	-4.76828538631649
a_HQ_transcript/5076	0.75352335868448	3.71444838776044	1.63974785348346	0.110677220461223	0.872575915511067	-4.87236350744592
a_HQ_transcript/78368	-1.14763899112646	2.77941121296286	-1.63974318433237	0.110678196825335	0.872575915511067	-4.7154903643534
a_HQ_transcript/115455	-0.848344523060395	2.59393974161571	-1.63962271896171	0.110703389746362	0.872656175640973	-4.77918332522376
a_HQ_transcript/6162	2.00682066381294	1.68849988051018	1.63954933797176	0.110718738233837	0.872658822516135	-4.58868780285106
a_HQ_transcript/28926	-1.16685019304179	2.90716977670242	-1.63941178503613	0.11074751376737	0.87276728311682	-4.73294246447385
a_HQ_transcript/134745	0.935983488320611	3.48629781846978	1.63927544392574	0.110776041889663	0.872873764804436	-4.76536244497314
a_HQ_transcript/127840	-1.95671145885847	-0.529600349772625	-1.6390750597047	0.11081798146322	0.872999305963509	-4.62009425754663
a_HQ_transcript/65744	2.3070437271828	1.57222962803216	1.63898012754121	0.110837854944592	0.872999305963509	-4.58633092849588
a_HQ_transcript/31344	-3.25985596194548	0.798621035917436	-1.63862001565662	0.110913268989352	0.872999305963509	-4.52182704980863
a_HQ_transcript/44363	2.04669847475015	1.10655505165635	1.63854759440308	0.110928440449188	0.872999305963509	-4.50391781911176
a_HQ_transcript/103794	-1.58491074821114	0.366277986675388	-1.63854495552617	0.110928993297388	0.872999305963509	-4.47777063401107
a_HQ_transcript/41324	1.25091870928211	3.25551108324599	1.63848967625057	0.110940574901928	0.872999305963509	-4.87137858338867
a_HQ_transcript/117843	-1.71269107713957	2.51095310014753	-1.63846575465787	0.110945587043311	0.872999305963509	-4.76593043882408
a_HQ_transcript/915	0.743678404412511	2.59588034073892	1.63843444391844	0.110952147668971	0.872999305963509	-4.73955955536105
a_HQ_transcript/9089	1.78621849100885	2.50062026474014	1.63840325686784	0.110958682696369	0.872999305963509	-4.51874663815208
a_HQ_transcript/130635	-1.15668046881345	1.9929759977101	-1.63830848342493	0.110978543757089	0.872999305963509	-4.63832007558361
a_HQ_transcript/138087	1.25049737040183	1.78172741802147	1.63827457280709	0.110985650900486	0.872999305963509	-4.55286975114843
a_HQ_transcript/141554	2.19189733193017	0.12487150098652	1.63817760312622	0.11100597632753	0.872999305963509	-4.62313537216592
a_HQ_transcript/62762	1.4920435452203	1.34129604944571	1.63817607728393	0.111006296177803	0.872999305963509	-4.50866996321708
a_HQ_transcript/152208	0.799016143705537	4.56247820307743	1.63815839831305	0.111010002136405	0.872999305963509	-5.04871203083149
a_HQ_transcript/87587	3.1011267910336	1.05225208094889	1.63812381111674	0.111017252781487	0.872999305963509	-4.74834307128074
a_HQ_transcript/111706	-2.08811470224729	-0.3821367208011	-1.63781169148695	0.111082701298721	0.873258349160675	-4.62394518294748
a_HQ_transcript/142534	-3.93687625136459	2.68105745572132	-1.63767743402038	0.111110863614076	0.873258349160675	-4.69552447824572

a_HQ_transcript/150311	-2.19129271167533	1.11600190800818	-1.63763012936219	0.111120787809459	0.873258349160675	-4.55379467548604
a_HQ_transcript/91344	2.29654074459171	0.82639994043169	1.63760267147971	0.111126548622186	0.873258349160675	-4.64997105246407
a_HQ_transcript/77789	-2.78710950257204	2.19827255190029	-1.63757944957931	0.111131420895429	0.873258349160675	-4.74931459063304
a_HQ_transcript/47786	-1.45945925605139	-0.390906979272733	-1.63751484989632	0.111144975723225	0.873258349160675	-4.56732631390337
a_HQ_transcript/148121	-0.536558626388275	4.28384683902413	-1.63741446262623	0.111166042501842	0.873258349160675	-4.98636732476163
a_HQ_transcript/135356	-2.60481430972728	0.577582326781802	-1.63733809900748	0.111182070003771	0.873258349160675	-4.67252732598033
a_HQ_transcript/11593	-0.937161812265649	3.98021880366499	-1.63732222516713	0.111185401908515	0.873258349160675	-4.96934895113382
a_HQ_transcript/20427	-1.25371443585081	-0.548113427558271	-1.63720758680673	0.111209466845306	0.873292195335224	-4.62392055266588
a_HQ_transcript/23330	-2.26594644688961	0.487238530483852	-1.63708243747213	0.111235743162172	0.873292195335224	-4.5306966142961
a_HQ_transcript/21651	-2.31197400189934	0.92669285061479	-1.63703003533304	0.111246747022567	0.873292195335224	-4.70734340245179
a_HQ_transcript/118142	-1.25804766789857	-0.580895999169772	-1.63700398492189	0.111252217650744	0.873292195335224	-4.57971638622398
a_HQ_transcript/31082	1.80270675565522	-0.450859009649355	1.63690781314733	0.11127241580212	0.873292195335224	-4.62120092501642
a_HQ_transcript/1629	4.08015291626857	2.16343736333104	1.63679594514801	0.111295914310226	0.873292195335224	-4.60672591460541
a_HQ_transcript/131106	-3.66468407032107	0.947490942429505	-1.63678589535301	0.111298025527311	0.873292195335224	-4.70144256447485
a_HQ_transcript/150147	2.5246461291705	1.47793823433295	1.63672937287843	0.111309900138293	0.873292195335224	-4.67401347659072
a_HQ_transcript/151780	-2.27206209511818	1.83685131561519	-1.63656363023805	0.111344726468327	0.87338315437885	-4.67048954514403
a_HQ_transcript/75282	2.02134223291834	1.65103496425721	1.63642463677263	0.111373939120184	0.87338315437885	-4.66942033675978
a_HQ_transcript/102486	1.07962659196376	3.78944367918769	1.63642145229278	0.11137460848553	0.87338315437885	-4.88729750682867
a_HQ_transcript/36917	1.76466845345296	-0.54329307379248	1.6363059438502	0.11139889015913	0.87338315437885	-4.6204175289011
a_HQ_transcript/82253	-0.815920422787747	2.84112180028446	-1.63603957842296	0.111454900984923	0.87338315437885	-4.66137926301113
a_HQ_transcript/200	-1.09245711454534	1.92376453339007	-1.63602844206269	0.111457243224378	0.87338315437885	-4.59776535543294
a_HQ_transcript/91890	2.94567145823053	0.35988210158718	1.63596912487511	0.111469719714032	0.87338315437885	-4.56515633828018
a_HQ_transcript/68107	1.44457132364836	-0.490389990268122	1.63595130849201	0.111473467350882	0.87338315437885	-4.61320687548953
a_HQ_transcript/126722	-2.95602345971101	0.433959939416472	-1.63594296537189	0.111475222343546	0.87338315437885	-4.58392151977725
a_HQ_transcript/27930	-3.37367833923814	0.713921270411004	-1.63590542101845	0.111483120158702	0.87338315437885	-4.52653289269406
a_HQ_transcript/14606	0.569557621655565	2.89190676653843	1.63588806809077	0.111486770669747	0.87338315437885	-4.77162735092954
a_HQ_transcript/90235	2.19884494026556	1.81904345487573	1.63547898926882	0.111572856601535	0.873939766680234	-4.59699673140216
a_HQ_transcript/123446	-1.10057623993254	4.09603070445834	-1.63534996919048	0.111600018774809	0.874031922980819	-5.00839348640801
a_HQ_transcript/34181	1.83819261233465	0.708255013111016	1.63528027022099	0.111614694541127	0.874031922980819	-4.59849835710462
a_HQ_transcript/57221	-1.27033373273207	4.95010964224422	-1.63505334759904	0.11166248624911	0.874288389101328	-5.0804954453863
a_HQ_transcript/81014	2.74336511803696	0.388455271300138	1.63487623882647	0.111699798502207	0.874375995929102	-4.56754845262804
a_HQ_transcript/57321	2.22778840071192	0.687447959558557	1.63472918490655	0.11173078679339	0.874375995929102	-4.58406436719159
a_HQ_transcript/64807	-2.21359859580977	1.02847748733959	-1.63460536459615	0.111756884629805	0.874375995929102	-4.57829694980563
a_HQ_transcript/99051	2.22946907736811	0.521435585698073	1.63452755788889	0.1117732866683	0.874375995929102	-4.66915980918556
a_HQ_transcript/82011	-1.48741678778493	1.51440243986526	-1.63449674373037	0.111779782994559	0.874375995929102	-4.53140718711177
a_HQ_transcript/137278	-2.20041322198359	1.51477883145937	-1.63430512818247	0.111820186910959	0.874375995929102	-4.75918274257481
a_HQ_transcript/88400	2.13747785373358	-0.365432277782208	1.63426258330716	0.111829159528078	0.874375995929102	-4.63209421470923
a_HQ_transcript/1733	2.05340708291704	-0.089296850421037	1.63421014415746	0.111840219642804	0.874375995929102	-4.65464184153398
a_HQ_transcript/54702	-1.66255821529783	0.199513291175266	-1.63417864368138	0.111846863946643	0.874375995929102	-4.53897060725212
a_HQ_transcript/130269	-1.32920376658738	2.76036756852007	-1.63406454670244	0.111870932818248	0.874375995929102	-4.69804130794637
a_HQ_transcript/107473	-3.22988992228383	3.22219703659754	-1.63404026722914	0.111876055149037	0.874375995929102	-4.84486306840391
a_HQ_transcript/35949	-1.59516556992355	1.88879284856708	-1.63400521124898	0.111883451381127	0.874375995929102	-4.72870613587564
a_HQ_transcript/31445	-1.92864126420766	1.08617793704973	-1.63397218270222	0.111890420227569	0.874375995929102	-4.47670161044107
a_HQ_transcript/149527	-1.29731737780474	3.1000856169117	-1.63388124407258	0.111909609642642	0.874375995929102	-4.847595257082
a_HQ_transcript/53218	2.36762571482128	0.861032058810976	1.63387897317381	0.111910088871113	0.874375995929102	-4.49369815492787
a_HQ_transcript/89911	2.73675803347389	0.861041382350506	1.63385877555996	0.111914351254344	0.874375995929102	-4.6152376679367
a_HQ_transcript/13537	2.87698582522248	1.01094777441073	1.63377316192567	0.111932420129855	0.874399639471601	-4.64536185585509
a_HQ_transcript/15469	1.99459592754092	0.519516366995944	1.63369358479021	0.111949217153143	0.874413342472173	-4.56008274853832
a_HQ_transcript/104028	-3.05511365701933	3.42362737725806	-1.63345000527077	0.112000644486403	0.874645919222211	-4.69455101879292
a_HQ_transcript/4140	-2.17268521617717	1.58115260873017	-1.63330711120621	0.112030823012231	0.874645919222211	-4.56184887221004

a_HQ_transcript/80473	-2.13212782799314	0.921132347442323	-1.63308662838936	0.112077401068561	0.874645919222211	-4.63554672069979
a_HQ_transcript/52650	1.10894531102868	2.96354358675371	1.63307599875347	0.112079647034078	0.874645919222211	-4.84941282185703
a_HQ_transcript/110452	-1.76481670150482	4.6392429340876	-1.63304362107129	0.112086488434046	0.874645919222211	-5.04996248841594
a_HQ_transcript/63886	-2.63637648708035	0.903313644667535	-1.63302912547161	0.112089551464032	0.874645919222211	-4.66162739026039
a_HQ_transcript/33419	-1.50614995115355	1.91372008704817	-1.63301620516361	0.112092281681183	0.874645919222211	-4.5541036373217
a_HQ_transcript/111352	-2.38699195053169	3.87515393892367	-1.63292239982111	0.11211210552892	0.874645919222211	-5.06530900282072
a_HQ_transcript/145849	-1.27286846944931	3.38914659977856	-1.63291147006204	0.11211441549898	0.874645919222211	-4.91532549614862
a_HQ_transcript/41400	-2.12740344724726	0.313079135359694	-1.63283548249861	0.112130476316337	0.87465382814225	-4.67466042725097
a_HQ_transcript/6677	1.68208723115841	1.12337334092593	1.63271873068383	0.112155156811757	0.874728962183523	-4.52212925250616
a_HQ_transcript/116623	-2.72904948248665	1.05045297707385	-1.63257682873106	0.112185159901098	0.874845582872419	-4.58484569288214
a_HQ_transcript/138374	2.42712742557659	2.45931715674237	1.63243146376204	0.112215902062005	0.874902541003052	-4.76836871949364
a_HQ_transcript/127287	2.02595661545663	2.94456247858864	1.63239995514113	0.112222566503557	0.874902541003052	-4.71546555629842
a_HQ_transcript/14363	3.11532989301903	2.64636716667761	1.63222555360777	0.112259460373232	0.875072805763072	-4.67857447974421
a_HQ_transcript/45939	-1.80200882998848	0.777738135377136	-1.63212028547787	0.112281734221821	0.875129075736932	-4.64927812838082
a_HQ_transcript/65004	-1.99056651034891	3.29372485397798	-1.63199831929412	0.112307545797889	0.875140983840184	-4.8149117247041
a_HQ_transcript/15564	-1.80172223957038	-0.230374679458189	-1.6319042581	0.112327455213858	0.875140983840184	-4.55465746907314
a_HQ_transcript/136438	2.56279783189894	1.36849879377636	1.63189966113127	0.112328428303599	0.875140983840184	-4.7241709927035
a_HQ_transcript/65262	2.19559946109273	1.09945944741777	1.63160956039064	0.112389851113939	0.875502179784377	-4.64821601734424
a_HQ_transcript/11291	-2.10857603395492	1.31941322345397	-1.63139348184176	0.112435619266605	0.875727416945689	-4.52527505276356
a_HQ_transcript/86606	-1.23906923849191	1.88397722163977	-1.63133081281503	0.112448896230433	0.875727416945689	-4.77291266958248
a_HQ_transcript/114436	1.82062957001525	4.365692518221	1.63119574199225	0.112477516525911	0.875813059044034	-5.02916364765086
a_HQ_transcript/107010	-1.19237486764273	1.91174192721907	-1.63113670811887	0.11249002716114	0.875813059044034	-4.60770529285295
a_HQ_transcript/476	-2.63392193655661	1.25994194582345	-1.63085784154361	0.112549140921819	0.876155947971544	-4.82228121293258
a_HQ_transcript/77667	1.51771536485173	2.40926889896506	1.63041468409412	0.112643133558615	0.876303127032857	-4.63880011191251
a_HQ_transcript/52843	2.29271118780588	0.333113423804893	1.63036441371508	0.112653799873874	0.876303127032857	-4.70663180112329
a_HQ_transcript/120597	1.37373226768125	0.64244250622831	1.63032716696454	0.112661703386879	0.876303127032857	-4.52472303835843
a_HQ_transcript/274	1.39193194995019	3.02886576874993	1.63020937089068	0.112686701941774	0.876303127032857	-4.83792633373034
a_HQ_transcript/114788	2.70699346743414	0.634153404110305	1.63009643309037	0.11271067377515	0.876303127032857	-4.57927525157275
a_HQ_transcript/47602	-2.11173696179905	1.10948461446244	-1.63002557772545	0.112725715466167	0.876303127032857	-4.56813696634662
a_HQ_transcript/96717	1.87458722169073	3.50460020521286	1.62995427370226	0.112740854073046	0.876303127032857	-4.8173757514292
a_HQ_transcript/147536	3.43787663825005	1.67413082133193	1.62989869706992	0.112752654749438	0.876303127032857	-4.73794175671468
a_HQ_transcript/105360	-1.51069663339002	4.78607759426205	-1.62986232659526	0.112760377902744	0.876303127032857	-5.01103194296029
a_HQ_transcript/118249	2.29967755589758	1.36488890549475	1.62984740798968	0.11276354594684	0.876303127032857	-4.67942870439955
a_HQ_transcript/111102	-3.72225444632363	0.767482464908693	-1.62971954587007	0.112790701149157	0.876303127032857	-4.52064878331891
a_HQ_transcript/137274	-1.19589788789408	-0.526489569185173	-1.62970375238546	0.112794055724531	0.876303127032857	-4.69183525931846
a_HQ_transcript/150068	1.99430390719144	-0.113136565434525	1.62964980275463	0.112805515380805	0.876303127032857	-4.66923575231343
a_HQ_transcript/107992	1.60664679999807	-0.203408621705311	1.62953253306734	0.112830428415029	0.876303127032857	-4.52851933647141
a_HQ_transcript/144060	-2.1115428405854	-0.057170569989021	-1.62951877758404	0.112833350957943	0.876303127032857	-4.52855649276055
a_HQ_transcript/32844	0.893735225828903	3.61385767307604	1.62945607955006	0.112846672814345	0.876303127032857	-4.91898947488354
a_HQ_transcript/49576	-1.47779430147711	1.1325008021992	-1.62944814909324	0.112848357942158	0.876303127032857	-4.48689083458016
a_HQ_transcript/16610	-1.74166272116926	1.86670107327543	-1.62937461454872	0.112863984147242	0.876303127032857	-4.63410054991221
a_HQ_transcript/130209	2.10100507585965	0.948640280872086	1.62922988973259	0.112894743607918	0.876303127032857	-4.57796398725716
a_HQ_transcript/26137	1.3120326784915	2.81857718545178	1.62908715796117	0.112925086242639	0.876303127032857	-4.76140800745705
a_HQ_transcript/112846	-1.41597981253045	-0.581735955909585	-1.62899573800318	0.11294452428816	0.876303127032857	-4.66950869644348
a_HQ_transcript/148496	2.64753550753924	-0.00542500917528891	1.62897358369661	0.112949235233101	0.876303127032857	-4.64046269648407
a_HQ_transcript/81138	3.21956196520612	1.84963496183622	1.62892515652135	0.112959533468148	0.876303127032857	-4.92652716848413
a_HQ_transcript/98678	-2.86105293431521	1.58890375486939	-1.62874802795417	0.11299720717127	0.876303127032857	-4.77999161182731
a_HQ_transcript/7207	-0.665774692479989	3.12486440549141	-1.62870159458784	0.113007084857645	0.876303127032857	-4.85798935373684
a_HQ_transcript/152683	-3.32938141091495	0.714939034051753	-1.62868548611292	0.113010511750843	0.876303127032857	-4.68255737047377
a_HQ_transcript/130415	2.940869443845	1.25657610836387	1.62865835507544	0.113016283761452	0.876303127032857	-4.64691675159215

a_HQ_transcript/3114	-1.94221377792447	-0.16456392647198	-1.62861033109366	0.113026501251368	0.876303127032857	-4.65715256187357
a_HQ_transcript/85385	-2.44053555702601	0.229058286330286	-1.62852056908723	0.113045600885442	0.876303127032857	-4.64593626271539
a_HQ_transcript/89571	-1.95585723301516	1.27301495656927	-1.62851772271657	0.113046206582079	0.876303127032857	-4.52201009617963
a_HQ_transcript/152285	1.9587526907839	0.63131629950459	1.6284220040939	0.113066576691915	0.876303127032857	-4.55939135227125
a_HQ_transcript/63506	1.10035949266508	2.56097926805553	1.62834658536748	0.113082628863865	0.876303127032857	-4.63105845865842
a_HQ_transcript/4587	1.80004332773757	-0.227174065622876	1.62834135988331	0.113083741129	0.876303127032857	-4.66482151579404
a_HQ_transcript/110783	2.96451281899848	0.704109263947042	1.62829273060873	0.113094092496533	0.876303127032857	-4.52537203605626
a_HQ_transcript/32068	0.896446829300943	1.22330755524084	1.62828524300433	0.11309568639899	0.876303127032857	-4.55976818253578
a_HQ_transcript/5600	-0.865667759227761	2.23537991561243	-1.62809028799223	0.113137193411791	0.876364218760547	-4.61904128697044
a_HQ_transcript/149243	-2.88857220104243	1.1136798826195	-1.62804408825441	0.11314703143513	0.876364218760547	-4.63678902816998
a_HQ_transcript/9636	1.64218051755778	4.17664335959922	1.62801226951396	0.113153807500878	0.876364218760547	-4.99759858067804
a_HQ_transcript/50916	-0.887047755523425	2.24956244779922	-1.62796498983283	0.113163876720628	0.876364218760547	-4.68895709105597
a_HQ_transcript/142319	2.35866499326373	-0.11739128010832	1.62785246409739	0.113187844455221	0.876433065471308	-4.66018227764253
a_HQ_transcript/125313	-2.94000147738106	0.275149547595889	-1.62775628668783	0.113208333349495	0.876474960167746	-4.69654778497591
a_HQ_transcript/8692	1.85201797571233	0.435253706244728	1.62762542853915	0.113236215271586	0.876574073952154	-4.53101282858283
a_HQ_transcript/120274	-1.17210062108356	2.13310176929739	-1.62727327172782	0.113311277378929	0.876831219924014	-4.64458653672375
a_HQ_transcript/109083	-3.2912676514646	2.29034943356447	-1.62725938856362	0.113314237410993	0.876831219924014	-4.67331015589688
a_HQ_transcript/138723	-2.20130467371866	0.260398089440757	-1.62720706134048	0.113325394681062	0.876831219924014	-4.64311401175372
a_HQ_transcript/6093	1.75562814346163	1.56423376807042	1.62715292246336	0.113336939187245	0.876831219924014	-4.51447163098566
a_HQ_transcript/112385	3.57574452056933	2.03829972061552	1.6270524236708	0.113358371993796	0.876831219924014	-4.70766842836154
a_HQ_transcript/41659	1.40997016720527	2.8920874053653	1.62687472285829	0.113396277413416	0.876831219924014	-4.75647599547027
a_HQ_transcript/134932	1.2239819195566	4.58990836978397	1.62667431895346	0.11343903817241	0.876831219924014	-5.03452867179692
a_HQ_transcript/68131	2.36587154811772	2.29321595110466	1.62640119818067	0.113497336138821	0.876831219924014	-4.73958346908998
a_HQ_transcript/81947	-1.56066881496726	0.131622079057636	-1.62640035446437	0.11349751626936	0.876831219924014	-4.6254454070538
a_HQ_transcript/145937	3.32108770728582	2.1259722193205	1.62624981964525	0.113529658709387	0.876831219924014	-4.74587823100581
a_HQ_transcript/132768	-3.35692100018789	2.50954181587095	-1.62599475543022	0.113584137562222	0.876831219924014	-4.83914136851826
a_HQ_transcript/97542	-0.955157100755443	1.9338217097439	-1.62591087276591	0.113602058664991	0.876831219924014	-4.57715710727845
a_HQ_transcript/69936	1.37216282334727	0.41620956011069	1.62590411290558	0.113603502976084	0.876831219924014	-4.4993119355192
a_HQ_transcript/137788	-1.19632192553443	2.0047957913755	-1.62588761004738	0.113607029038712	0.876831219924014	-4.79046337548629
a_HQ_transcript/142637	-4.35303758476906	1.89495225830159	-1.62584941336306	0.113615190631646	0.876831219924014	-4.71919384227665
a_HQ_transcript/151077	2.40687670642744	0.0170560184465977	1.62561406306584	0.113665489253471	0.876831219924014	-4.688849532248
a_HQ_transcript/121954	3.65767758441308	1.77344646542786	1.62561346235145	0.113665617660493	0.876831219924014	-4.66378492915752
a_HQ_transcript/91429	1.34110940367775	2.11488619819445	1.62560100508323	0.113668280518153	0.876831219924014	-4.67292910619978
a_HQ_transcript/127299	1.03308798078013	3.30019777251091	1.6255668490356	0.113675581956697	0.876831219924014	-4.86334647149072
a_HQ_transcript/107641	-1.51393281383283	0.159675214362947	-1.62542622197117	0.113705647465245	0.876831219924014	-4.54668662095821
a_HQ_transcript/20881	-2.31991465361029	2.04341939204589	-1.62540961165375	0.113709199117905	0.876831219924014	-4.63307580295666
a_HQ_transcript/9831	-1.7733967093533	1.75558677023352	-1.62538957650686	0.113713483196375	0.876831219924014	-4.57809187625618
a_HQ_transcript/135617	-1.39790006972289	1.71759767873664	-1.6252336374045	0.113746831913418	0.876831219924014	-4.59745069834554
a_HQ_transcript/19912	-0.838273046625833	3.90244953051415	-1.62523312270332	0.113746941999371	0.876831219924014	-4.98778651397847
a_HQ_transcript/18107	1.93586726927994	3.67466384680543	1.62520165557948	0.11375367245637	0.876831219924014	-4.78072754621166
a_HQ_transcript/35997	2.2721722121778	-0.048325035550865	1.6251741035007	0.113759565799584	0.876831219924014	-4.59047359324377
a_HQ_transcript/35632	-2.32112711765154	0.499084683779068	-1.62514985342506	0.113764753058066	0.876831219924014	-4.53112690142129
a_HQ_transcript/19999	1.9421109210545	3.67791493099589	1.62509341381512	0.113776826634734	0.876831219924014	-5.05661484910535
a_HQ_transcript/140091	3.11287348710194	1.62573113425608	1.6250905008544	0.113777449804733	0.876831219924014	-4.66655306544689
a_HQ_transcript/2153	1.74941349410601	0.693279448381218	1.62507910569761	0.113779887598873	0.876831219924014	-4.66210756169065
a_HQ_transcript/139658	-2.46028043092732	1.36424687673179	-1.62503877426637	0.113788516147694	0.876831219924014	-4.56553198329822
a_HQ_transcript/109843	2.86545099915062	2.96089177315376	1.62502882128858	0.11379064558118	0.876831219924014	-4.71626164900322
a_HQ_transcript/52082	2.56660049085117	0.802899761788318	1.62499775345557	0.113797292736201	0.876831219924014	-4.55846569501937
a_HQ_transcript/132347	2.08192880981917	-0.377327630693752	1.62493814996857	0.11381004616664	0.876831219924014	-4.65053944679145
a_HQ_transcript/41965	-1.43258613891407	1.83649305989851	-1.62490198192547	0.113817785660868	0.876831219924014	-4.618516593253

a_HQ_transcript/76519	2.41730174127519	0.922063586439385	1.62488762639016	0.113820857680294	0.876831219924014	-4.60815518254812
a_HQ_transcript/131968	-2.28792558599229	0.81351287666267	-1.62485630936251	0.11382755961923	0.876831219924014	-4.71907067203644
a_HQ_transcript/123962	2.50317990607745	-0.370014908466153	1.62455422760084	0.113892222729745	0.876878556489698	-4.62332666129169
a_HQ_transcript/56833	-1.49609377897746	1.11213198845094	-1.62454969128861	0.113893193995685	0.876878556489698	-4.5327823467166
a_HQ_transcript/59117	2.43472563366229	0.574188424343755	1.62444265191965	0.113916114084075	0.876878556489698	-4.65905437820193
a_HQ_transcript/110318	8.65544189401341	5.37201710556306	1.6242757682384	0.113951856075665	0.876878556489698	-5.01152202996183
a_HQ_transcript/118727	2.16986698935767	-0.142674265807218	1.62421915822712	0.113963982514498	0.876878556489698	-4.65862747583547
a_HQ_transcript/63846	1.13339114607348	3.38291800881439	1.62419270534537	0.113969649354661	0.876878556489698	-4.85654884429173
a_HQ_transcript/89013	1.70982207658566	1.51069602650196	1.62407972761123	0.11399385450151	0.876878556489698	-4.63589415803827
a_HQ_transcript/21642	-0.828519028571436	3.13514213064704	-1.62405332067725	0.113999512719979	0.876878556489698	-4.90126953980164
a_HQ_transcript/28381	-2.08821382485893	0.891566845211338	-1.62394903282931	0.114021860757182	0.876878556489698	-4.62454821015946
a_HQ_transcript/144865	-2.69199238279795	-0.0070576176323995	-1.62388577795924	0.114035417519558	0.876878556489698	-4.63652770149759
a_HQ_transcript/23197	-3.85756098823361	1.4564639442208	-1.62375753544681	0.114062906486654	0.876878556489698	-4.62100121597467
a_HQ_transcript/44730	2.10155529616703	1.70570540187034	1.62359990822986	0.114096701585738	0.876878556489698	-4.67144282503557
a_HQ_transcript/48059	-2.88624135590525	0.491228142797462	-1.62353532429514	0.114110550691157	0.876878556489698	-4.58453146382682
a_HQ_transcript/90008	3.71416861347824	3.40235154416641	1.62298368726738	0.114228897835366	0.876878556489698	-4.9200647211226
a_HQ_transcript/147780	-2.78921538492169	0.402277807184098	-1.62295634810358	0.114234765755368	0.876878556489698	-4.66149109081806
a_HQ_transcript/1967	1.19254553869964	0.428428309581109	1.62294513111904	0.114237173375887	0.876878556489698	-4.56766509956568
a_HQ_transcript/37274	2.77600962882607	0.815459939247835	1.62293856845271	0.11423858201004	0.876878556489698	-4.58157622235393
a_HQ_transcript/1263	2.32937903395991	2.132789910374	1.62289351208322	0.114248253456506	0.876878556489698	-4.68932745698417
a_HQ_transcript/119837	1.16307977841889	4.00938172355641	1.62283962078228	0.114259822225725	0.876878556489698	-4.95135276419347
a_HQ_transcript/110688	-1.75147540932858	-0.563192345521748	-1.62278219620364	0.114272150540509	0.876878556489698	-4.60052268707413
a_HQ_transcript/43290	2.92188336535323	0.708521067002386	1.62262818790348	0.114305219541472	0.876878556489698	-4.58325685408915
a_HQ_transcript/113328	3.37548480489534	0.33273690644299	1.62252038377651	0.114328372170161	0.876878556489698	-4.72212769223494
a_HQ_transcript/137404	1.31768391516229	0.721745533659628	1.62247821454031	0.114337429726523	0.876878556489698	-4.55128600133233
a_HQ_transcript/146616	-1.53526100433241	2.65791973296935	-1.62240410680447	0.114353348804956	0.876878556489698	-4.71505189342478
a_HQ_transcript/146521	-2.04944128288945	0.159553980896696	-1.62237979066314	0.114358572551595	0.876878556489698	-4.71698807205486
a_HQ_transcript/100157	-1.7355120231416	2.7358732816287	-1.62229612534816	0.114376547562993	0.876878556489698	-4.78162673908357
a_HQ_transcript/90922	-3.30006526283209	1.39589634938861	-1.62221615230799	0.114393731485484	0.876878556489698	-4.70444952929297
a_HQ_transcript/66359	2.84771966638538	2.54402081306917	1.62218814201081	0.11439975060119	0.876878556489698	-4.80119252531159
a_HQ_transcript/27213	-1.58057204189697	0.663443009961167	-1.62199715440525	0.11444079876434	0.876878556489698	-4.61419338652051
a_HQ_transcript/91716	5.30548734719162	1.99396209747072	1.62193722569338	0.11445368149036	0.876878556489698	-4.75995859848883
a_HQ_transcript/63087	2.62873327217441	0.138503016051352	1.62193440830584	0.114454287166537	0.876878556489698	-4.68308848771929
a_HQ_transcript/62609	-1.51434483508591	-0.0119898659641267	-1.62190875988378	0.114459801132955	0.876878556489698	-4.62473379425586
a_HQ_transcript/119810	3.30196716953112	1.38430756214621	1.62185615811248	0.114471110286925	0.876878556489698	-4.72687479024556
a_HQ_transcript/72644	-1.82867109463476	0.0923837809035997	-1.62174710582846	0.114494558989063	0.876878556489698	-4.58493986957767
a_HQ_transcript/66942	1.77256531075476	-0.330164771000001	1.62167481456012	0.114510105423303	0.876878556489698	-4.62887480776646
a_HQ_transcript/29394	-2.59087385325195	0.950627852183608	-1.62163507114986	0.114518653092495	0.876878556489698	-4.51896195393216
a_HQ_transcript/53164	-2.50674005037271	1.23657661617342	-1.62155757222919	0.114535322401779	0.876878556489698	-4.70700758965433
a_HQ_transcript/83016	1.80813184739253	1.76434814937613	1.62144707609568	0.114559092565071	0.876878556489698	-4.70388322205731
a_HQ_transcript/110778	1.03137355687559	3.69704284389502	1.62136074688057	0.114577666716043	0.876878556489698	-4.88632386832354
a_HQ_transcript/17621	2.11321179691988	1.1649175166954	1.62132343491754	0.114585695334043	0.876878556489698	-4.59994604340374
a_HQ_transcript/126529	-3.35983321677669	0.404236091043041	-1.62120158313452	0.114611918071642	0.876878556489698	-4.65545563575935
a_HQ_transcript/132783	-0.808494328834705	2.59533278743518	-1.62109304080785	0.114635280749735	0.876878556489698	-4.74321387873867
a_HQ_transcript/135064	3.00271446844797	0.394928801217724	1.62099949691188	0.114655418307585	0.876878556489698	-4.77575508049605
a_HQ_transcript/38433	-1.7309207924415	-0.361628419598989	-1.62082546496275	0.114692890591464	0.876878556489698	-4.64592800395089
a_HQ_transcript/91035	-2.01913024518912	0.570738366264804	-1.62077396511355	0.114703981390784	0.876878556489698	-4.58371867181903
a_HQ_transcript/81825	-3.0400387080976	1.04491079916031	-1.62075589693908	0.11470787268903	0.876878556489698	-4.64931274202244
a_HQ_transcript/23076	-2.48114544042252	0.0363342759248267	-1.62074185833847	0.11471089622291	0.876878556489698	-4.60109805429474
a_HQ_transcript/140580	1.24845105435148	-0.59376797385175	1.62071885532934	0.114715850589136	0.876878556489698	-4.68658262425794

a_HQ_transcript/145771	-3.09897577746315	1.36966705092731	-1.62071745296662	0.114716152634382	0.876878556489698	-4.722625356454
a_HQ_transcript/223	2.29631176666942	1.21960937374946	1.6206882640808	0.114722439576087	0.876878556489698	-4.63574369012272
a_HQ_transcript/19882	-1.98509218325386	1.25024858183914	-1.62067038847489	0.114726289910807	0.876878556489698	-4.48950748625255
a_HQ_transcript/144692	-0.740147732723856	4.38473607409065	-1.6206206712183	0.114736999370456	0.876878556489698	-5.02770131236147
a_HQ_transcript/67252	-3.73423332984615	0.834699131612249	-1.62054323419752	0.114753681508834	0.876878556489698	-4.75110610793141
a_HQ_transcript/33490	1.50011361883292	0.618028231657653	1.6205212160505	0.114758425209015	0.876878556489698	-4.62286713895863
a_HQ_transcript/5137	-0.853309603516373	3.60399687944668	-1.62051654438934	0.114759431715919	0.876878556489698	-4.9163154021599
a_HQ_transcript/118082	-4.69598790489845	0.473466930750321	-1.6204956229388	0.114763939320437	0.876878556489698	-4.66980045578141
a_HQ_transcript/5261	-1.31994777137464	1.73269427222476	-1.62041110697365	0.114782150084063	0.876878556489698	-4.58607906225506
a_HQ_transcript/143039	-1.8726167028764	1.86172372825556	-1.62035307321756	0.114794656069685	0.876878556489698	-4.69647306294858
a_HQ_transcript/114506	-2.81683537455224	1.80949972852643	-1.62033707810328	0.114798103134404	0.876878556489698	-4.77996614719957
a_HQ_transcript/147109	-0.709924803134401	3.23968232272404	-1.6203155573851	0.114802741141844	0.876878556489698	-4.92347122878158
a_HQ_transcript/91835	-5.1600700912864	3.21821332286065	-1.62026079505624	0.114814543864189	0.876878556489698	-4.92726747099323
a_HQ_transcript/139646	1.91062532980753	-0.0840176365189769	1.62024564227239	0.114817809864135	0.876878556489698	-4.65430225938875
a_HQ_transcript/130003	2.47251367919824	1.37201502748247	1.62021351924388	0.114824733848739	0.876878556489698	-4.52608891070911
a_HQ_transcript/128431	-2.15719334037278	-0.0608284189160115	-1.62013870266931	0.114840861581495	0.876878556489698	-4.66189435392868
a_HQ_transcript/57568	1.97406282099793	0.456201003156744	1.62010503933787	0.11484811877986	0.876878556489698	-4.59227595731001
a_HQ_transcript/60409	2.31348252646714	-0.348388772227251	1.6199812584436	0.114874806922506	0.876878556489698	-4.59133409769217
a_HQ_transcript/9839	1.30752724756066	2.32349998832526	1.61981590338703	0.114910466749588	0.876878556489698	-4.66169378567802
a_HQ_transcript/145638	-4.08686589682619	0.675276647940323	-1.61976010763627	0.114922501499744	0.876878556489698	-4.67162943254251
a_HQ_transcript/10262	-1.00716849956835	2.25988426787526	-1.61974399160838	0.114925977806486	0.876878556489698	-4.69637399214593
a_HQ_transcript/10868	-5.02994536738613	2.12208518526806	-1.61967337380789	0.114941211435193	0.876878556489698	-4.62152635619545
a_HQ_transcript/18816	-0.512342676041852	3.80457169950954	-1.61956816088863	0.114963910984803	0.876878556489698	-4.92335812107837
a_HQ_transcript/106173	-2.04761927618095	-0.169112218059155	-1.61952095760714	0.114974096230088	0.876878556489698	-4.54727415894489
a_HQ_transcript/127457	1.1630797551021	3.13786045195822	1.61932078187854	0.115017297220736	0.876878556489698	-4.84993308086617
a_HQ_transcript/60199	-2.42542738771082	0.853032223653933	-1.61924901370409	0.115032789146457	0.876878556489698	-4.66410146181413
a_HQ_transcript/148759	-0.986663132829949	3.00873225500057	-1.61907004485696	0.115071428962345	0.876878556489698	-4.78854280871407
a_HQ_transcript/58076	2.3982922686061	0.534421501765992	1.61906864927498	0.115071730313912	0.876878556489698	-4.6231572204346
a_HQ_transcript/138451	-2.26873389130922	-0.105029999901926	-1.61886769323343	0.115115130044422	0.876878556489698	-4.59802210615428
a_HQ_transcript/106189	-1.59250937917256	2.36621209514592	-1.61869876145091	0.115151624036406	0.876878556489698	-4.64132782325943
a_HQ_transcript/144312	-1.77159281786878	-0.604455172716741	-1.61867002646154	0.11515783254563	0.876878556489698	-4.65263014044861
a_HQ_transcript/60783	2.1661765445593	1.13267807771694	1.61864848343624	0.115162487332926	0.876878556489698	-4.57242368010182
a_HQ_transcript/93409	2.4412959079558	2.78921099072459	1.61837789518145	0.115220966347325	0.876878556489698	-4.83511285468756
a_HQ_transcript/55419	-2.91490310371272	0.97383703568424	-1.61832324789603	0.115232779579438	0.876878556489698	-4.55315781122479
a_HQ_transcript/15485	2.50702554939193	0.739340695303085	1.61831907892941	0.115233680835668	0.876878556489698	-4.54542950483924
a_HQ_transcript/106775	-2.23686947715314	1.61371830438827	-1.61828233714831	0.115241624004307	0.876878556489698	-4.61490187432779
a_HQ_transcript/89413	2.37396286307235	0.410734879750651	1.61827019329266	0.115244249471301	0.876878556489698	-4.73171871143462
a_HQ_transcript/122418	2.37183589503284	1.73687677593081	1.61802423219878	0.115297436154737	0.876878556489698	-4.59626205031758
a_HQ_transcript/19247	-1.94912750179269	0.115312146971972	-1.61790937372664	0.115322280095042	0.876878556489698	-4.60814754216185
a_HQ_transcript/28056	0.784123294172447	2.76975982954506	1.61789533967561	0.115325315968717	0.876878556489698	-4.71516906745952
a_HQ_transcript/45310	-1.47106295433996	0.604952451194409	-1.61778270547013	0.115349683604846	0.876878556489698	-4.51723949199314
a_HQ_transcript/106901	-2.64973570919048	-0.0136549975302517	-1.61756157384827	0.115397536235357	0.876878556489698	-4.69868356716497
a_HQ_transcript/9040	1.48704866088428	1.47327382046803	1.61750334558569	0.115410139481462	0.876878556489698	-4.62549216813317
a_HQ_transcript/34756	-2.35325544742939	1.09299382727104	-1.61748595429171	0.11541390396864	0.876878556489698	-4.58023418948688
a_HQ_transcript/11955	-1.60316330787888	2.51245309349605	-1.61747562763259	0.115416139305892	0.876878556489698	-4.63305315195424
a_HQ_transcript/18069	2.0522460866373	1.38158464461576	1.61745944123081	0.11541964313096	0.876878556489698	-4.64569656975531
a_HQ_transcript/69017	-2.63082392803313	0.230181106206148	-1.61734104684339	0.115445274293931	0.876878556489698	-4.57612457062169
a_HQ_transcript/135358	2.43002523034922	0.183982076945318	1.61733467176347	0.115446654565807	0.876878556489698	-4.68469919122046
a_HQ_transcript/92539	0.686508327677949	3.9090386923528	1.61724908738653	0.115465185798622	0.876878556489698	-4.96414532908248
a_HQ_transcript/142463	1.83434351991778	-0.386020019426743	1.61722117056493	0.115471231042428	0.876878556489698	-4.68691560946917

a_HQ_transcript/145412	-1.93343334355559	0.00743542128710528	-1.61714954731673	0.115486741878721	0.876878556489698	-4.66473223407696
a_HQ_transcript/51501	-2.02242300499451	0.378805633914356	-1.61713636338852	0.115489597196471	0.876878556489698	-4.52343231348847
a_HQ_transcript/22859	-2.1366974181457	0.0970795835209534	-1.61704680547126	0.115508994797566	0.876878556489698	-4.68844718516119
a_HQ_transcript/132834	3.06673888842149	0.141200226418389	1.61698278778548	0.115522862210943	0.876878556489698	-4.66916219385948
a_HQ_transcript/3313	1.82571282466675	-0.330054592015814	1.61693668637919	0.115532849475554	0.876878556489698	-4.5332237747381
a_HQ_transcript/82461	0.713426902532252	4.03231144641882	1.61665044925177	0.115594874891222	0.876878556489698	-4.99928802471109
a_HQ_transcript/126941	-0.78095949589319	4.03046613309927	-1.61658325883795	0.115609438513939	0.876878556489698	-4.98560177845098
a_HQ_transcript/100052	2.07743957482968	0.0474981756615917	1.6165507673591	0.115616481632038	0.876878556489698	-4.64049129337722
a_HQ_transcript/62374	2.06607214789539	0.334044910936513	1.6164444919105	0.11563952123112	0.876878556489698	-4.61757513256913
a_HQ_transcript/23106	-1.04034678393445	4.81101894557035	-1.61643120767167	0.115642401404877	0.876878556489698	-5.09591770394113
a_HQ_transcript/91677	-2.82594586330215	1.18053683012962	-1.61638550846403	0.115652309962494	0.876878556489698	-4.64433681741719
a_HQ_transcript/64911	2.43591131591445	0.162586226090179	1.61638488293317	0.115652445595699	0.876878556489698	-4.56845383081017
a_HQ_transcript/64588	0.778572802012254	2.28356011105681	1.61627377738115	0.115676538572649	0.876878556489698	-4.71164677963812
a_HQ_transcript/131734	-2.14919562076333	0.785010257613222	-1.61623955915404	0.115683959546073	0.876878556489698	-4.66721918112605
a_HQ_transcript/108844	0.49925142794191	3.51305817201702	1.6162136580949	0.115689577019522	0.876878556489698	-4.94319143248458
a_HQ_transcript/124636	2.48951863836865	-0.267242887102307	1.61611099012813	0.115711846060871	0.876878556489698	-4.66220272216774
a_HQ_transcript/11873	0.523472077147453	3.65816938024294	1.61606322020055	0.115722208727324	0.876878556489698	-4.95455230742936
a_HQ_transcript/100113	0.717817152748195	5.34745746760825	1.61602960069477	0.115729502219397	0.876878556489698	-5.17446882006593
a_HQ_transcript/144383	-0.798028757159424	4.49609719379715	-1.616016300477	0.115732387703285	0.876878556489698	-5.06933620628875
a_HQ_transcript/141219	1.65172101485723	2.26634066938022	1.6159622935849	0.115744105113248	0.876878556489698	-4.7234795201029
a_HQ_transcript/57085	-1.6859567648246	-0.244725734057333	-1.61590200494164	0.115757186573324	0.876878556489698	-4.56969022005095
a_HQ_transcript/5756	2.4347353980727	0.55264305819264	1.61587528144656	0.115762985439699	0.876878556489698	-4.66200839859145
a_HQ_transcript/32727	0.834732841702342	3.52490048825979	1.61587104826668	0.115763904040737	0.876878556489698	-4.96323280212211
a_HQ_transcript/94542	1.82136661703886	2.47570233575999	1.61582617564909	0.11577364177805	0.876878556489698	-4.67720368675062
a_HQ_transcript/109900	3.3081952241519	3.94938684804425	1.61582355678899	0.115774210113636	0.876878556489698	-4.99489961743675
a_HQ_transcript/143237	-1.24597229651219	3.21727325441572	-1.61572302840591	0.115796028156141	0.876878556489698	-4.74592881243449
a_HQ_transcript/5719	1.76223299574536	-0.215912151586278	1.61571230382479	0.115798355950823	0.876878556489698	-4.61084891485142
a_HQ_transcript/68775	2.27736529154315	0.485045517436016	1.61554426025332	0.115834835216423	0.876878556489698	-4.57886043518991
a_HQ_transcript/122939	-1.21111373379472	2.43173769147519	-1.61549937959656	0.115844579606633	0.876878556489698	-4.69038661662026
a_HQ_transcript/117412	-1.78582791181715	3.69810001257382	-1.61548216168008	0.11584831810201	0.876878556489698	-4.83527867417548
a_HQ_transcript/42151	-0.872372562285786	-0.673916225694375	-1.61543970499321	0.115857537070449	0.876878556489698	-4.69820663440465
a_HQ_transcript/55559	-1.97877737541838	-0.41375592083413	-1.61535343781292	0.115876270829067	0.876878556489698	-4.64806722332633
a_HQ_transcript/91385	-4.3232520934352	1.52250263985688	-1.61523604729915	0.115901767324579	0.876878556489698	-4.74952337272988
a_HQ_transcript/20451	2.74077562717364	1.20776660858362	1.6151481267295	0.115920866151827	0.876878556489698	-4.71735694468213
a_HQ_transcript/6656	-2.23971349049835	0.645103451739703	-1.61513379994006	0.115923978579781	0.876878556489698	-4.56705393690218
a_HQ_transcript/55326	-2.29759228974946	1.2859808274215	-1.61512172994468	0.115926600783129	0.876878556489698	-4.66997710204742
a_HQ_transcript/59997	-2.38338182913482	1.1123037821928	-1.61511925819013	0.115927137777177	0.876878556489698	-4.61648524340758
a_HQ_transcript/140475	-3.42308836482438	0.390406505397004	-1.61511228998446	0.115928651646021	0.876878556489698	-4.62587505459841
a_HQ_transcript/99990	-2.25525720559868	1.32406903032711	-1.61501174545683	0.115950497132038	0.876878556489698	-4.58805239098975
a_HQ_transcript/150012	-3.93125544455048	0.374167435353493	-1.61494800290498	0.115964348341641	0.876878556489698	-4.66447673789101
a_HQ_transcript/86859	2.01954131268895	0.676194102275048	1.61493708919698	0.115966720018793	0.876878556489698	-4.64695627364021
a_HQ_transcript/64601	-1.52258962738946	2.22907859793577	-1.61484937090022	0.115985783685558	0.876878556489698	-4.72308178913385
a_HQ_transcript/19478	-2.39706435772087	2.02705387028828	-1.61484898985388	0.115985866503328	0.876878556489698	-4.6674801077096
a_HQ_transcript/107030	-2.24481861864619	0.787763358688189	-1.6148404669233	0.115987718915645	0.876878556489698	-4.66182051317587
a_HQ_transcript/21135	-1.84910867762288	-0.278830181164654	-1.6147596253745	0.116005290593766	0.876878556489698	-4.60726623116621
a_HQ_transcript/113131	-1.97128060516023	-0.250890226625857	-1.61475641475359	0.116005988497897	0.876878556489698	-4.66545717879276
a_HQ_transcript/78869	-3.94993661896861	2.24760092091778	-1.61442950552124	0.11607706800222	0.877205469245504	-4.81313415379199
a_HQ_transcript/28818	1.10609146777638	4.0459163009382	1.61425976135091	0.116113989408814	0.877205469245504	-4.94393318483179
a_HQ_transcript/90084	-1.73540650408415	-0.464631822604745	-1.61425593937036	0.116114820846885	0.877205469245504	-4.64119078383986
a_HQ_transcript/140722	2.76614096597545	4.06856163155479	1.6142170657042	0.116123277747346	0.877205469245504	-5.07541818160106

a_HQ_transcript/123849	-2.56375427582962	-0.000990432621037916	-1.61421056591299	0.116124691815376	0.877205469245504	-4.66629544170182
a_HQ_transcript/124095	-1.7906478542575	-0.508327345969579	-1.61404344628799	0.116161054541061	0.877267592610206	-4.66853823738263
a_HQ_transcript/56030	-1.08386182648509	1.15936687163406	-1.61394523697694	0.11618242778263	0.877267592610206	-4.52004702723993
a_HQ_transcript/61355	-2.18399824498888	0.1444979856627	-1.61384963117965	0.11620323752891	0.877267592610206	-4.60766719307568
a_HQ_transcript/97590	-2.00697047329241	0.280829272980828	-1.61384837479129	0.116203511017277	0.877267592610206	-4.6828068085203
a_HQ_transcript/5588	1.26259386976984	2.24064000280528	1.61382602711874	0.116208375707036	0.877267592610206	-4.76678122640466
a_HQ_transcript/108740	-2.15047103607067	-0.357298056768788	-1.6136988149408	0.116236070719135	0.877273825471836	-4.66290825676475
a_HQ_transcript/85949	-1.1662849079751	0.468735870004323	-1.61368359047478	0.116239385559181	0.877273825471836	-4.56848207380939
a_HQ_transcript/64599	1.88476054888176	0.0561974732974745	1.61360825598615	0.116255789365711	0.877283723776455	-4.60272088074418
a_HQ_transcript/116364	-1.12538611728716	2.57532480471126	-1.61329133436293	0.116324818725128	0.877690688324795	-4.71762996976449
a_HQ_transcript/67699	2.65425845879738	1.37472423642164	1.61307175746674	0.116372664985734	0.877804424215788	-4.70694617479714
a_HQ_transcript/12980	-1.59530523027449	1.01827576930241	-1.61305865206375	0.116375521191769	0.877804424215788	-4.60731352945426
a_HQ_transcript/148873	-2.67864689900548	1.06342369243905	-1.61298500197547	0.116391573645572	0.877804424215788	-4.55970254467557
a_HQ_transcript/99714	3.46537977426185	0.457221400804516	1.61294256810002	0.116400823175515	0.877804424215788	-4.69583092750092
a_HQ_transcript/136452	-1.1781772762683	3.95933196220023	-1.61287570505599	0.116415398883146	0.877804424215788	-4.95754220421098
a_HQ_transcript/56486	-1.33080479352019	2.39429951414285	-1.61275470889542	0.116441779071574	0.87788945964623	-4.77393155807996
a_HQ_transcript/82283	4.78457584800282	1.99704917470893	1.61258485741496	0.116478819232278	0.877902290119613	-4.76918482519588
a_HQ_transcript/106744	-2.06100706562042	1.22120514764403	-1.61253210615812	0.116490324871201	0.877902290119613	-4.63418967303486
a_HQ_transcript/116890	1.81782111198726	2.70264262299447	1.61230285189465	0.116540338642614	0.877902290119613	-4.67653594244691
a_HQ_transcript/13607	2.5656125825427	5.86712876239904	1.61225771819287	0.116550187023529	0.877902290119613	-5.16083395672299
a_HQ_transcript/109893	1.65409853613347	-0.435031208666633	1.61220716047209	0.116561219762635	0.877902290119613	-4.61343278098176
a_HQ_transcript/99134	-5.89139610599369	1.31617156997329	-1.6121716013951	0.11656898000203	0.877902290119613	-4.66336680135987
a_HQ_transcript/15642	-1.35511580579907	0.869766252551321	-1.61215075284075	0.116573530086249	0.877902290119613	-4.52627979299307
a_HQ_transcript/25232	2.69576454668708	0.959540924495471	1.61213428738629	0.116577123685799	0.877902290119613	-4.65824107361999
a_HQ_transcript/128890	3.65792162121499	2.0113990958438	1.61212382514507	0.116579407126227	0.877902290119613	-4.80595483694154
a_HQ_transcript/17687	2.98880433573192	0.00563135791070086	1.61186637359383	0.11663560889638	0.878211745327564	-4.6734682206021
a_HQ_transcript/101524	-1.69435443293389	1.08727135579143	-1.61146590150385	0.1167230763647	0.878460017333963	-4.77777853019074
a_HQ_transcript/126161	-2.30358531709629	1.23605340564791	-1.61145484163408	0.116725492725533	0.878460017333963	-4.62312563227188
a_HQ_transcript/120554	-3.24406456556718	1.54387446951701	-1.6114130855369	0.116734615970094	0.878460017333963	-4.66940485246588
a_HQ_transcript/133450	0.804887610170066	4.25072381959048	1.61139124720032	0.116739387638052	0.878460017333963	-5.03336664199862
a_HQ_transcript/136607	2.26044998988511	0.70472190167576	1.61128094455678	0.116763491171916	0.878460017333963	-4.60066778547949
a_HQ_transcript/147579	0.679586956055956	6.07786749088071	1.61124756957252	0.11677078514	0.878460017333963	-5.2168631930391
a_HQ_transcript/18974	-1.33474448627519	-0.711110311617193	-1.61123116897004	0.116774369562681	0.878460017333963	-4.67324132320225
a_HQ_transcript/141353	5.77064834572407	4.70210017356223	1.61112411964334	0.116797767877878	0.878522340994472	-4.80516875645758
a_HQ_transcript/5905	-1.10648954486745	2.23067098161516	-1.61101787109364	0.116820994972839	0.87858336082044	-4.72177817131468
a_HQ_transcript/68309	-2.76990524556563	0.0425618602234954	-1.61080889935688	0.116866689554857	0.878637904546582	-4.67550458429319
a_HQ_transcript/26816	-2.18939958961879	0.817551392609156	-1.6107981441379	0.116869041730712	0.878637904546582	-4.63890732941603
a_HQ_transcript/123020	-1.93744131566175	0.191752821741473	-1.61077608904779	0.116873865320015	0.878637904546582	-4.66469711402425
a_HQ_transcript/133639	1.38123083568363	2.38907130778713	1.6107082203132	0.116888709672769	0.878637904546582	-4.69758432091372
a_HQ_transcript/2750	4.00308921759235	4.32418961827038	1.61063512006434	0.116904700003291	0.878644479155847	-5.01627263453862
a_HQ_transcript/145823	2.29018923257558	0.847228597456057	1.61054386804165	0.116924663470088	0.878680909914582	-4.68711338900496
a_HQ_transcript/39249	-2.0799765817082	1.07304693492351	-1.6103464707146	0.116967858233906	0.878884528732799	-4.57160255376099
a_HQ_transcript/132748	-2.01221077930276	-0.00802964635363598	-1.61021599818086	0.116996415615906	0.878884528732799	-4.68710674046005
a_HQ_transcript/50524	-1.91025669351599	2.52183546373254	-1.61017769296685	0.117004800817917	0.878884528732799	-4.81768690906866
a_HQ_transcript/90010	1.91243071941964	1.02124607514548	1.60991211296133	0.117062951183996	0.878884528732799	-4.59390393636828
a_HQ_transcript/10795	-1.10098551678858	1.97625766141901	-1.60980421062536	0.11708658384266	0.878884528732799	-4.60765906241216
a_HQ_transcript/15353	-2.98181223881984	1.79469968185304	-1.60979553602517	0.117088483914889	0.878884528732799	-4.87554090060352
a_HQ_transcript/12852	-1.31559217931862	1.58432649918997	-1.60963963267902	0.117122637083889	0.878884528732799	-4.67090655340205
a_HQ_transcript/111400	-1.77401263046541	0.742689124256442	-1.60956251463992	0.117139534072574	0.878884528732799	-4.49724357091669
a_HQ_transcript/42362	2.38552519748403	1.33631286749825	1.60954399368712	0.117143592413948	0.878884528732799	-4.66826780391063

a_HQ_transcript/148666	-2.41930829483012	0.589602422831583	-1.60954233380759	0.117143956135097	0.878884528732799	-4.77885789046709
a_HQ_transcript/53027	1.86693276131897	-0.463013512270076	1.60953817436723	0.117144867576658	0.878884528732799	-4.64308009919394
a_HQ_transcript/1053	-1.9825238692229	1.58427291493143	-1.60950498494135	0.117152140451777	0.878884528732799	-4.55220765143786
a_HQ_transcript/32546	-1.42185753801898	0.980084306325518	-1.60944257725467	0.117165816996041	0.878884528732799	-4.59880466826665
a_HQ_transcript/144480	2.19583957372189	-0.380480481011244	1.60934825455199	0.117186490154144	0.878884528732799	-4.6850513365111
a_HQ_transcript/10412	1.34666301653033	2.27745309564987	1.60925021771087	0.11720798053358	0.878884528732799	-4.70640951331208
a_HQ_transcript/149922	-4.47434116038423	0.234319418234537	-1.60918449558888	0.117222389107208	0.878884528732799	-4.67055802368223
a_HQ_transcript/110433	-2.00657796415844	0.293535390705193	-1.60917250963982	0.117225017000746	0.878884528732799	-4.59473302130713
a_HQ_transcript/46787	-3.652707741663	3.35664322891275	-1.60912841158598	0.117234685820735	0.878884528732799	-5.06405692168636
a_HQ_transcript/62816	3.63499729967424	1.52758386345109	1.60905269664853	0.117251288401151	0.878884528732799	-4.82204822676756
a_HQ_transcript/152225	-2.6512560638091	1.03807536135695	-1.60903962473031	0.1172541549739	0.878884528732799	-4.57964710359724
a_HQ_transcript/81317	2.69857504416645	0.0940868251311863	1.60884225226859	0.11729744425865	0.879095647223671	-4.71213800036955
a_HQ_transcript/14063	-1.45017128877209	0.298455219304335	-1.60866530521373	0.117336264841956	0.879273224536964	-4.6302951823601
a_HQ_transcript/138939	-1.75406197715028	0.712871385688222	-1.608546939908	0.117362239009869	0.879354502341538	-4.53893033609744
a_HQ_transcript/147177	-1.54474538344782	0.20263994031587	-1.60843899768339	0.117385930046479	0.879381032338087	-4.58388972351271
a_HQ_transcript/107103	2.63983929391139	1.74981758138141	1.60839295552567	0.117396036521875	0.879381032338087	-4.82374128875039
a_HQ_transcript/79759	-0.659700649886873	2.30845209925511	-1.60817995752518	0.117442799910156	0.879385304694475	-4.73037802121341
a_HQ_transcript/53055	-3.28974302702971	2.06386513537291	-1.60817423172774	0.117444057211447	0.879385304694475	-4.68044984124289
a_HQ_transcript/48118	2.62347943769775	0.849290699478444	1.6081218183593	0.117455566933602	0.879385304694475	-4.64049481396613
a_HQ_transcript/41341	-1.92045801758125	0.777063792711122	-1.60800859391649	0.117480433635778	0.879385304694475	-4.57004591670908
a_HQ_transcript/120444	-2.50360184070637	2.39535685336323	-1.60793088741498	0.11749750227977	0.879385304694475	-4.64709832828788
a_HQ_transcript/137094	-1.19262291805081	2.84239586733738	-1.60789246009233	0.117505943797011	0.879385304694475	-4.83168280397236
a_HQ_transcript/117046	1.56623367147979	1.72418555841398	1.60786064140044	0.117512933941083	0.879385304694475	-4.64961391125346
a_HQ_transcript/53300	0.539335302534615	3.60165867734997	1.60781833454958	0.117522228723317	0.879385304694475	-4.94615222620332
a_HQ_transcript/67438	-1.75516601900575	4.57819306153933	-1.60777039045478	0.117532762733474	0.879385304694475	-5.12768859920349
a_HQ_transcript/140641	2.21753121479201	0.0599325239316833	1.60758638162855	0.117573199330009	0.879571631876287	-4.71115854846943
a_HQ_transcript/57006	-2.08199581641862	0.746041339667155	-1.60723329752532	0.117650822816892	0.879571631876287	-4.57494489395192
a_HQ_transcript/96071	-2.5089135852649	0.850367959887618	-1.60715197849245	0.117668706294456	0.879571631876287	-4.55037629487849
a_HQ_transcript/47836	2.34934544580676	1.17158956514481	1.6069200828539	0.11771971646056	0.879571631876287	-4.69256213529806
a_HQ_transcript/57876	-1.68946965239552	-0.211803234875671	-1.60691083049795	0.117721752081224	0.879571631876287	-4.64976494829102
a_HQ_transcript/29196	2.12161560676627	-0.20157955444166	1.60679720909754	0.117746752403209	0.879571631876287	-4.67408224224798
a_HQ_transcript/39384	1.54569923555416	-0.562742287485647	1.60669492174045	0.117769262603879	0.879571631876287	-4.66791943240617
a_HQ_transcript/139716	3.95173626656774	5.13992192694377	1.60668770947335	0.117770849928297	0.879571631876287	-5.09990377110347
a_HQ_transcript/30920	0.729632949104777	3.46450840753255	1.60668481486328	0.117771486998556	0.879571631876287	-4.89352461651417
a_HQ_transcript/123599	0.617679705819322	2.52870866744904	1.60646855439426	0.117819091434568	0.879571631876287	-4.74944421521975
a_HQ_transcript/54048	-3.41411292054063	1.61399508130138	-1.60643629398999	0.117826194125012	0.879571631876287	-4.6046687020197
a_HQ_transcript/27629	-1.96103016195527	1.02730474533069	-1.60642127166611	0.117829501671488	0.879571631876287	-4.61299594683661
a_HQ_transcript/149771	-5.23808178729229	5.87602990721514	-1.60640400008045	0.11783330454433	0.879571631876287	-5.28750671530239
a_HQ_transcript/144724	-1.7729891739584	1.0745057789303	-1.60637249880485	0.117840240782257	0.879571631876287	-4.65026708384285
a_HQ_transcript/141029	-1.91626164575134	0.758940585769276	-1.60636094780878	0.117842784269833	0.879571631876287	-4.53796017722565
a_HQ_transcript/89925	-1.75838999029606	0.706233151347492	-1.60607302719888	0.117906197912775	0.879571631876287	-4.57616313747216
a_HQ_transcript/41008	-2.84973644629667	0.18665740174294	-1.60601476271218	0.117919033897343	0.879571631876287	-4.67553225662103
a_HQ_transcript/35150	1.59956316823885	1.18292179314482	1.60600133363865	0.117921992558249	0.879571631876287	-4.5407199633414
a_HQ_transcript/87872	0.603359054128204	3.33930699240139	1.6059297354355	0.11793776792938	0.879571631876287	-4.96796843305409
a_HQ_transcript/137767	-1.19027155114323	2.32057108601235	-1.60582661088476	0.117960492606719	0.879571631876287	-4.6926024527905
a_HQ_transcript/134513	-4.37449990476703	2.5368138132366	-1.60576593948359	0.117973863925185	0.879571631876287	-4.90120710162726
a_HQ_transcript/116970	2.4833779292096	2.17436110918456	1.6057456865564	0.117978327727572	0.879571631876287	-4.61600434861195
a_HQ_transcript/45451	2.06780235568838	0.937313766016739	1.60569357629661	0.117989813613429	0.879571631876287	-4.60371999885166
a_HQ_transcript/104000	3.98041793845641	-0.118038466197681	1.60564893782922	0.117999653333708	0.879571631876287	-4.66205949214788
a_HQ_transcript/115667	-1.69845566513624	1.41580856318174	-1.60562132524551	0.118005740351675	0.879571631876287	-4.56210940809996

a_HQ_transcript/127638	0.758276743011507	2.71084027360202	1.60562112938055	0.118005783529777	0.879571631876287	-4.72715879716947
a_HQ_transcript/57927	-4.1358915701194	2.19261944731036	-1.60551850883438	0.118028407840625	0.879571631876287	-4.7282118002554
a_HQ_transcript/14029	-0.679340689270852	2.93748727755126	-1.60538068823658	0.118058798157728	0.879571631876287	-4.88632860841002
a_HQ_transcript/129804	-2.19819115404183	-0.225821321268955	-1.60535984471029	0.118063394846789	0.879571631876287	-4.65244103934438
a_HQ_transcript/15501	-1.96356327206122	0.0813500792035888	-1.60535410604564	0.118064660438525	0.879571631876287	-4.65298065459317
a_HQ_transcript/84558	1.21649132940671	-0.722544337809106	1.605284197719	0.118080078752241	0.879571631876287	-4.65281808404149
a_HQ_transcript/72253	-1.86787443713416	-0.521662784492202	-1.60516816768833	0.118105672877228	0.879571631876287	-4.56737353543124
a_HQ_transcript/116146	2.49863455077374	0.44823132361362	1.60514483582946	0.118110820012928	0.879571631876287	-4.65037896615814
a_HQ_transcript/128496	-2.35643315217954	-0.265421053556517	-1.60513935349573	0.118112029472454	0.879571631876287	-4.61105133481038
a_HQ_transcript/137734	-3.16045856485963	0.39976804579679	-1.60510658590213	0.118119258554496	0.879571631876287	-4.71238515338477
a_HQ_transcript/114203	-11.0969666498106	3.99362619219236	-1.60508189095558	0.11812470691487	0.879571631876287	-4.77408300169974
a_HQ_transcript/99392	-1.54044783816426	-0.583888324918495	-1.60504660599467	0.118132492071312	0.879571631876287	-4.66846497751682
a_HQ_transcript/86910	4.30683426043577	1.69427356063325	1.60504580829334	0.118132668078347	0.879571631876287	-4.76140307508464
a_HQ_transcript/106154	2.16681898603576	1.96703132788347	1.60479751551008	0.11818746254901	0.879781987335467	-4.70843110984617
a_HQ_transcript/120311	3.23789732564806	2.33194967766962	1.60475353516134	0.118197170522821	0.879781987335467	-4.87061626389823
a_HQ_transcript/49946	1.55812741665087	-0.497710696112356	1.60467264344736	0.118215027811913	0.879781987335467	-4.63890103557643
a_HQ_transcript/52301	3.00039113198318	3.864181815419	1.6045845404686	0.118234479546388	0.879781987335467	-4.84863081002013
a_HQ_transcript/7071	3.74450046877816	0.455873766851788	1.60454898781372	0.118242329750224	0.879781987335467	-4.65014061166233
a_HQ_transcript/94036	1.21532504853974	3.15322071562494	1.6045064095031	0.118251731816887	0.879781987335467	-4.97804871651142
a_HQ_transcript/29021	-1.92022293826075	1.98703190740726	-1.60430677777752	0.118295822321662	0.87978357032732	-4.70142905332862
a_HQ_transcript/145650	-4.15651136327684	0.360322706952353	-1.60430671654795	0.118295835846846	0.87978357032732	-4.69869283754355
a_HQ_transcript/83922	2.52858080758517	-0.243450746800601	1.60428517447073	0.118300594419797	0.87978357032732	-4.68818866950777
a_HQ_transcript/98464	-1.76984194595234	-0.181058730966246	-1.60423134567311	0.118312485708241	0.87978357032732	-4.6820350152425
a_HQ_transcript/135461	2.18761398952007	-0.213524423080118	1.60395352397227	0.118373874736768	0.880028660888301	-4.65945172155825
a_HQ_transcript/94068	-1.78918018706024	1.63106324275817	-1.60379042533314	0.118409926095231	0.880028660888301	-4.67229443089533
a_HQ_transcript/125705	-1.79106357617176	-0.371091161333573	-1.60367963041152	0.118434421367568	0.880028660888301	-4.5990306697826
a_HQ_transcript/47566	-1.92722592663703	0.473083308624622	-1.60361037799773	0.118449734259901	0.880028660888301	-4.66354318097703
a_HQ_transcript/109270	2.23491525708893	0.589663427688607	1.60353204638356	0.118467056676701	0.880028660888301	-4.63897339915341
a_HQ_transcript/93927	3.33710365726706	0.639639095020463	1.60339727532523	0.118496865095088	0.880028660888301	-4.80055312427244
a_HQ_transcript/142575	2.52758485145757	1.5503004241636	1.60334679849154	0.118508031053756	0.880028660888301	-4.65631155665398
a_HQ_transcript/147337	-3.47744191637492	-0.125961047086258	-1.60334219758193	0.118509048861924	0.880028660888301	-4.69706225171287
a_HQ_transcript/86653	2.82563051901977	0.605647331900215	1.60334085631281	0.118509345577354	0.880028660888301	-4.60047672627026
a_HQ_transcript/40160	1.13207853573003	2.83732774537767	1.6032148586049	0.118537221497085	0.880028660888301	-4.75435121525809
a_HQ_transcript/73369	-1.05931123921716	1.35756939942018	-1.60319641314308	0.118541302849846	0.880028660888301	-4.52739940159911
a_HQ_transcript/32365	-0.749243806826649	3.32720814474608	-1.60312567047712	0.118556956862657	0.880028660888301	-4.90962166271931
a_HQ_transcript/151536	-1.87440119116644	2.67962120616006	-1.60307609715691	0.118567927510716	0.880028660888301	-4.79867308369075
a_HQ_transcript/146217	-2.03467251888656	0.141450178192561	-1.60299904176042	0.118584981635917	0.880028660888301	-4.62434994305254
a_HQ_transcript/85680	-0.916461090499416	3.69250424412203	-1.60290789875179	0.118605156270712	0.880028660888301	-5.04115962927677
a_HQ_transcript/131293	1.48940849972591	-0.6408444156416	1.60288972149454	0.118609180169301	0.880028660888301	-4.68375190163847
a_HQ_transcript/61505	1.73777719598374	1.37706963373051	1.60288149920179	0.118611000374656	0.880028660888301	-4.78557313500637
a_HQ_transcript/113064	3.66070946519533	1.12855996413891	1.60283515796134	0.118621259568852	0.880028660888301	-4.64637244150196
a_HQ_transcript/65826	-1.33213641987593	2.63943036075383	-1.6027317921336	0.118644145698969	0.880028660888301	-4.75672416828849
a_HQ_transcript/148935	-1.31330003557449	3.17852167429758	-1.60264635524104	0.118663064935064	0.880028660888301	-4.7162514704116
a_HQ_transcript/110997	-1.99676584184904	0.538608703896695	-1.60251831912696	0.118691422021934	0.880028660888301	-4.69440899326337
a_HQ_transcript/77740	-2.09385995739396	-0.414166002776908	-1.60250830788843	0.118693639517876	0.880028660888301	-4.6616163196423
a_HQ_transcript/35973	-5.14934663535527	3.01484229288453	-1.60244001239834	0.118708767920844	0.880028660888301	-4.89820690888232
a_HQ_transcript/46372	-1.34511082293284	2.99681772587773	-1.60243989837016	0.118708793181	0.880028660888301	-4.77905987002897
a_HQ_transcript/24917	3.6430784197591	4.27815097342687	1.60219295617534	0.118763507530069	0.880271025061777	-4.94016798499883
a_HQ_transcript/151933	-2.72812734820485	0.710536419892569	-1.60215565753513	0.118771773492285	0.880271025061777	-4.65610281520143
a_HQ_transcript/147379	-0.719248893346348	5.43129280559725	-1.60202250003194	0.118801287128416	0.880377513794052	-5.1731071377855

a_HQ_transcript/72937	3.9670957691103	1.97482229617905	1.60183919807883	0.118841924849972	0.880486700977329	-4.6771725280987
a_HQ_transcript/117660	-2.31259913815613	2.58179414839193	-1.6018193943245	0.118846315990024	0.880486700977329	-4.76885160802219
a_HQ_transcript/145684	2.10087460423338	0.0106232121791131	1.60169313145684	0.118874315726926	0.880504466546448	-4.70362144018402
a_HQ_transcript/74633	1.07410603286008	3.1733755844721	1.60162311131449	0.118889845550397	0.880504466546448	-4.93919261020133
a_HQ_transcript/62337	1.68813750216671	0.180014710023556	1.60160367339597	0.118894156997025	0.880504466546448	-4.60990569437733
a_HQ_transcript/13708	-1.96272958547799	-0.274489237031263	-1.60148425062663	0.118920648496316	0.880588465706227	-4.61351980300187
a_HQ_transcript/47272	-2.20555133274918	1.98557492976093	-1.60122367403381	0.118978468892834	0.880904399414425	-4.64346480400686
a_HQ_transcript/49242	-2.80464263393029	0.720504985998713	-1.60107844365183	0.119010704656775	0.880970434879312	-4.60708329392741
a_HQ_transcript/94924	-2.12055414604349	2.26769798233371	-1.60092175370663	0.119045492052339	0.880970434879312	-4.72145341562907
a_HQ_transcript/82646	-1.63917016408791	1.14839266285099	-1.60081209327758	0.119069843187072	0.880970434879312	-4.54075524249828
a_HQ_transcript/40070	-1.63999037505335	1.05455294263262	-1.60074441666395	0.119084873457224	0.880970434879312	-4.54609243938047
a_HQ_transcript/149425	-3.00303614408028	0.590229697969001	-1.60071102589219	0.119092289772659	0.880970434879312	-4.7859522478545
a_HQ_transcript/53395	-1.64176415408787	0.0546073679779756	-1.60062227142139	0.119112004580321	0.880970434879312	-4.71886749448919
a_HQ_transcript/140789	-1.22634505778053	6.11774383359658	-1.60057759566412	0.11912192930674	0.880970434879312	-5.27451475217386
a_HQ_transcript/35869	-2.12134396159266	1.03735613596363	-1.60054965194187	0.119128137353727	0.880970434879312	-4.58653897372818
a_HQ_transcript/19646	0.618827557373495	3.86142093199902	1.60044546128598	0.119151286948577	0.880970434879312	-5.01003241306579
a_HQ_transcript/139527	4.07444363659065	2.99816175898467	1.60034519056364	0.119173569076674	0.880970434879312	-4.86832189464165
a_HQ_transcript/5592	-1.56585908929626	1.9415141838608	-1.60013646296337	0.119219963418022	0.880970434879312	-4.71071474556353
a_HQ_transcript/727	-3.18730493825503	1.01806404722503	-1.60005209121447	0.119238721114575	0.880970434879312	-4.78203181354663
a_HQ_transcript/85204	-1.22522804680621	4.26763706249703	-1.59998176123973	0.119254358865108	0.880970434879312	-4.97328379099681
a_HQ_transcript/591	-0.953947903940951	2.70527389582549	-1.59992741420139	0.119266443988678	0.880970434879312	-4.86345341823883
a_HQ_transcript/8223	2.35440801302524	2.01142491691373	1.59978224200006	0.119298730782189	0.880970434879312	-4.67045163168085
a_HQ_transcript/123705	0.505666660525931	4.13968325898724	1.59975101882316	0.119305675859794	0.880970434879312	-5.02912201186966
a_HQ_transcript/116447	2.02282951416872	2.20694044312533	1.5997271268227	0.119310990462745	0.880970434879312	-4.61758499845896
a_HQ_transcript/143656	1.61857102120199	2.96784928856183	1.59967215422622	0.119323219456984	0.880970434879312	-4.7131265451405
a_HQ_transcript/89737	2.83955879202469	0.677479127659922	1.5996169378625	0.119335503713338	0.880970434879312	-4.66948077296186
a_HQ_transcript/136122	-0.769112550632534	3.00011382098621	-1.59958361114346	0.119342918574441	0.880970434879312	-4.8314393264331
a_HQ_transcript/41379	-1.82382172025154	-0.145028535806915	-1.59954677000673	0.119351115797881	0.880970434879312	-4.61708538322782
a_HQ_transcript/90732	-3.96306821600829	2.80667473464366	-1.59952081420451	0.119356891290752	0.880970434879312	-4.86665352543857
a_HQ_transcript/132346	-1.78579290304226	0.846476086265734	-1.59943959251244	0.119374965620615	0.880970434879312	-4.54750226241497
a_HQ_transcript/131632	-1.73571156389813	2.08075461070677	-1.59942301134577	0.11937865571707	0.880970434879312	-4.72421119987595
a_HQ_transcript/130895	-2.02369822150763	1.8585819038239	-1.59932869308816	0.119399647784444	0.880970434879312	-4.77945215225964
a_HQ_transcript/72426	2.02547545175606	1.59726328322029	1.59931277837369	0.119403190162262	0.880970434879312	-4.58439819160748
a_HQ_transcript/7301	2.10593835700107	-0.0923153534556899	1.59917226255691	0.119434470624233	0.880970434879312	-4.58540439934225
a_HQ_transcript/114047	2.51967140273077	0.971521470020533	1.59912928080672	0.119444040205593	0.880970434879312	-4.67957176777654
a_HQ_transcript/6245	0.958700799626175	2.72944275671105	1.59911509026158	0.119447199767699	0.880970434879312	-4.70938049848822
a_HQ_transcript/68330	0.6023529549447	2.43632876978065	1.5990927503771	0.119452173940434	0.880970434879312	-4.65464760382615
a_HQ_transcript/120804	3.00951932941096	0.718130145378451	1.59900821093029	0.119470998932833	0.880970434879312	-4.66745177693844
a_HQ_transcript/137272	-2.32900473380498	2.5686355299407	-1.59898265477181	0.119476690180885	0.880970434879312	-4.67880554401853
a_HQ_transcript/119182	-1.61965527290194	0.898980503103232	-1.59893400024086	0.119487525952871	0.880970434879312	-4.6309294735369
a_HQ_transcript/135574	-2.264539071175	0.124561229076589	-1.59860278330439	0.119561312154264	0.881275413204588	-4.66205780938606
a_HQ_transcript/148923	-4.19284674417077	0.890439685132065	-1.59849435609909	0.119585474925493	0.881275413204588	-4.82047842411572
a_HQ_transcript/127757	0.848712107028564	2.88986030999307	1.59842044804828	0.119601947471007	0.881275413204588	-4.87378279280738
a_HQ_transcript/81205	1.71095926644733	1.71030539227539	1.59840775806761	0.11960477598711	0.881275413204588	-4.7273777282933
a_HQ_transcript/52009	-1.55165646567501	-0.330810709796633	-1.59836461851566	0.119614391929346	0.881275413204588	-4.69765368346418
a_HQ_transcript/149290	-3.18150381101344	1.07875803623958	-1.59834010498665	0.119619856354669	0.881275413204588	-4.7637554193704
a_HQ_transcript/124195	-2.14078374384472	0.536592424115956	-1.59821422146717	0.119647920862078	0.881308652399189	-4.63206245527099
a_HQ_transcript/132128	2.43633149009132	0.848023361386732	1.5981507317384	0.11966207732755	0.881308652399189	-4.66584963430144
a_HQ_transcript/140361	-1.18912591102029	5.43367530893259	-1.59798924939746	0.119698089640765	0.881308652399189	-5.22440043978436
a_HQ_transcript/55424	2.20598587223642	3.32985399306993	1.59798532361216	0.119698965244057	0.881308652399189	-4.92996509985152

a_HQ_transcript/74299	2.86562286124762	0.753214143157932	1.59792579657764	0.119712242738668	0.881308652399189	-4.71330619820595
a_HQ_transcript/120931	-0.519207326695875	4.06813386100051	-1.5979012115588	0.119717726775047	0.881308652399189	-5.05514212191879
a_HQ_transcript/148958	-1.54642126900827	1.4610011130767	-1.59784395843602	0.119730498692479	0.881308652399189	-4.60355328480111
a_HQ_transcript/39190	-1.55200192781917	1.14670834120432	-1.59759334104069	0.119786419087879	0.881498165334886	-4.52363994706088
a_HQ_transcript/91977	2.48497336017988	1.37132050995905	1.59759264424704	0.11978657459366	0.881498165334886	-4.61964789057219
a_HQ_transcript/114466	-1.48056209090473	-0.692322939919181	-1.59751435277329	0.119804048218817	0.881515153780173	-4.67784869570612
a_HQ_transcript/36447	3.38234328610323	1.48437686292107	1.5973708960409	0.119836071291927	0.881628530945331	-4.61917871871849
a_HQ_transcript/34513	-2.54047417669634	1.3000839807205	-1.59730944418347	0.119849791004851	0.881628530945331	-4.56798762684608
a_HQ_transcript/65099	-3.82445980435698	1.94376364876104	-1.5972136747117	0.11987117501868	0.881674258064764	-4.78071600158924
a_HQ_transcript/150402	-1.82280874925047	3.29247763366722	-1.59709950702436	0.119896671188127	0.881705434536973	-4.80831546554708
a_HQ_transcript/148429	-1.06246959097362	6.42519768143398	-1.59705885654009	0.119905750413136	0.881705434536973	-5.24632192874396
a_HQ_transcript/152357	3.43426018608666	3.74296884193189	1.59691548430413	0.119937776886304	0.881803988165118	-5.06869592932196
a_HQ_transcript/75644	2.48198173840702	0.878613277984616	1.59686304245404	0.119949493091481	0.881803988165118	-4.61984877972851
a_HQ_transcript/107730	3.01012243691896	0.920868220753145	1.59665468672446	0.119996051792418	0.881981234465	-4.71318297538091
a_HQ_transcript/135975	3.01459599720436	1.45545865254423	1.59654396337498	0.120020799807495	0.881981234465	-4.55607600272769
a_HQ_transcript/135235	1.59030954816236	-0.155765040308593	1.59653556068142	0.120022678082421	0.881981234465	-4.70541493899637
a_HQ_transcript/97696	2.5417331592569	1.30107190682555	1.5964835898209	0.12003429579295	0.881981234465	-4.58721677352585
a_HQ_transcript/41277	3.13154463612755	-0.0744640390307229	1.59633324526811	0.120067909420696	0.882116713701495	-4.68711841829042
a_HQ_transcript/99399	-2.28059520555457	2.39967186501445	-1.59616040619537	0.120106561842274	0.882289173103463	-4.69883824079201
a_HQ_transcript/143782	1.2727789333099	8.48470182740188	1.59606074078327	0.120128854894689	0.882341430037713	-5.51663725740677
a_HQ_transcript/145588	1.94361736744632	0.951490838808311	1.59568276664777	0.120213430555561	0.882798171105028	-4.72990829326961
a_HQ_transcript/76702	1.6161642222238	-0.157486886910069	1.59564710171562	0.120221413474873	0.882798171105028	-4.69919224304528
a_HQ_transcript/148609	-1.42582727595849	-0.737513066967342	-1.59550640417685	0.12025291018956	0.882917918845361	-4.68035367401712
a_HQ_transcript/92411	2.48615905285897	0.88715005994781	1.59541436746614	0.120273517292044	0.882924633678174	-4.66751659282267
a_HQ_transcript/86888	0.726534071198302	3.65262710255058	1.59525072785015	0.120310163494248	0.882924633678174	-4.97341206916126
a_HQ_transcript/11416	1.41026817568293	0.337825808548543	1.59516802477408	0.120328687877911	0.882924633678174	-4.56572748973392
a_HQ_transcript/41759	-1.40657200933783	1.60941573786818	-1.59514547833967	0.120333738383183	0.882924633678174	-4.60363418980976
a_HQ_transcript/108817	-1.92872775950393	0.0993090850147481	-1.59512169994638	0.120339065041078	0.882924633678174	-4.65367576681605
a_HQ_transcript/110567	-1.10263997736948	1.28622606130589	-1.59504240571918	0.120356829337126	0.882924633678174	-4.57061510719327
a_HQ_transcript/104186	1.2942896257235	-0.771151886667276	1.59502758453724	0.120360149966726	0.882924633678174	-4.68404220441185
a_HQ_transcript/126029	-2.63191714260835	0.65642215546484	-1.59477689446542	0.120416327497917	0.883141221576086	-4.73098195010714
a_HQ_transcript/2688	2.81746547708787	0.651222798580948	1.59470228177157	0.120433051720536	0.883141221576086	-4.72884214560887
a_HQ_transcript/80470	2.10252463453728	0.132579829638921	1.5946418011636	0.120446609672593	0.883141221576086	-4.62946758703732
a_HQ_transcript/73274	-2.40494684105785	2.58621044129308	-1.59462468261422	0.120450447368828	0.883141221576086	-4.77522580811932
a_HQ_transcript/142832	-2.04807633607086	0.0506896104133269	-1.59437107579215	0.120507313566918	0.883219975870962	-4.67164705809407
a_HQ_transcript/62856	-1.52007202128092	1.42734683939377	-1.59435129049081	0.12051175094495	0.883219975870962	-4.66258844678046
a_HQ_transcript/44488	2.85228062775834	-0.380403131763708	1.59424431665077	0.120535744980862	0.883219975870962	-4.68357081507493
a_HQ_transcript/150451	-2.74444032563967	5.93530341810208	-1.59423344949652	0.120538182682532	0.883219975870962	-5.26176415674005
a_HQ_transcript/126368	0.886792097495581	2.09165370930781	1.59422182611793	0.120540790063861	0.883219975870962	-4.67895043395417
a_HQ_transcript/25278	-1.39276933797466	1.71600788202821	-1.59405683774289	0.120577805593587	0.883219975870962	-4.74438784829944
a_HQ_transcript/90604	-0.454318692645579	4.31989671751869	-1.59404884908173	0.12057959810477	0.883219975870962	-5.04834811430286
a_HQ_transcript/139341	2.33475629544624	0.484543064130164	1.59403483017527	0.120582743746765	0.883219975870962	-4.85485443275654
a_HQ_transcript/4443	1.97521121276448	0.99564359741522	1.5938496333432	0.120624305574782	0.88338892984379	-4.62896809242733
a_HQ_transcript/41692	-2.18462701550587	-0.0301080957854994	-1.59379661982747	0.120636205014795	0.88338892984379	-4.55569508626918
a_HQ_transcript/55205	-0.760045353582846	2.95809362230201	-1.5936066194671	0.120678860487405	0.883452794615633	-4.81250747514425
a_HQ_transcript/119234	-1.36546121687167	1.78089710106505	-1.59357480964874	0.120686003064159	0.883452794615633	-4.71278941461467
a_HQ_transcript/124998	2.40619664613271	-0.105917346043018	1.59355468673253	0.120690521642629	0.883452794615633	-4.6965537470354
a_HQ_transcript/127644	-0.74674118597296	3.18417121126658	-1.59330837968469	0.120745840844021	0.883644642561877	-4.93621475590365
a_HQ_transcript/113781	-1.58402772498194	0.884578146513134	-1.59319622314447	0.120771037462329	0.883644642561877	-4.62289991074442
a_HQ_transcript/142778	1.9654686217322	-0.282407128948127	1.59317436606306	0.120775948283915	0.883644642561877	-4.70489084930921

a_HQ_transcript/103484	-1.88206541958187	2.07935424050108	-1.59311827540438	0.120788551410884	0.883644642561877	-4.7054918066829
a_HQ_transcript/44556	0.953831586392192	5.47181680254108	1.59299173555544	0.120816987868597	0.883644642561877	-5.18897662129503
a_HQ_transcript/137520	-4.52336613110325	0.300311535528435	-1.59281854247199	0.120855917283042	0.883644642561877	-4.6981333816049
a_HQ_transcript/51222	2.95288049689812	1.26339772860248	1.59279195734911	0.12086189385392	0.883644642561877	-4.71141658896726
a_HQ_transcript/18853	-0.821639681343222	0.845515121217939	-1.59277835257438	0.12086495242128	0.883644642561877	-4.57874087774731
a_HQ_transcript/6349	-1.3762180936366	-0.68702567463596	-1.59275163687492	0.120870958715178	0.883644642561877	-4.69863793481871
a_HQ_transcript/124947	-1.71818815962545	-0.348065628887935	-1.59273301462848	0.120875145562209	0.883644642561877	-4.68324156560335
a_HQ_transcript/13599	-2.07523756596369	-0.0860143341882213	-1.59266773908193	0.120889822426828	0.883644642561877	-4.63518755062833
a_HQ_transcript/12449	0.650133196897862	3.0566013536645	1.59261333383598	0.120902056276214	0.883644642561877	-4.83264973271179
a_HQ_transcript/80355	2.82603447849178	1.16623106034036	1.5924341264996	0.120942360948327	0.883644642561877	-4.7157526649681
a_HQ_transcript/140189	-2.68343769015038	1.24185192895972	-1.59238957998124	0.120952381401518	0.883644642561877	-4.86436899014052
a_HQ_transcript/126435	-1.55365556101131	-0.526576535795619	-1.5923564864464	0.120959826019692	0.883644642561877	-4.67831714348714
a_HQ_transcript/39040	-2.58630966314908	0.452646919551224	-1.59233391654992	0.120964903488251	0.883644642561877	-4.64453991638843
a_HQ_transcript/86294	-1.86971696427339	0.610903871702727	-1.59228833028673	0.120975159398352	0.883644642561877	-4.67216041852715
a_HQ_transcript/128475	-2.72840826214362	-0.0939815349542585	-1.59206425773481	0.121025581162061	0.883712583104726	-4.70504329776284
a_HQ_transcript/94823	-1.60630759826196	-0.0476725727585803	-1.59205719941844	0.121027169734075	0.883712583104726	-4.58972287679077
a_HQ_transcript/137271	2.09521931541694	0.527468504701366	1.59203705499026	0.121031703610971	0.883712583104726	-4.55737020834708
a_HQ_transcript/107765	-1.68375445007931	-0.209127168833638	-1.59193107442484	0.121055558791596	0.883712583104726	-4.7170574821812
a_HQ_transcript/90359	-2.15053030930132	0.54650339735732	-1.5918219694148	0.121080121274994	0.883712583104726	-4.59040970603373
a_HQ_transcript/138158	1.42094797176497	2.53729115729537	1.59179692067169	0.12108576099919	0.883712583104726	-4.8478494202958
a_HQ_transcript/115637	2.35849761542578	2.96046790321327	1.59174951429416	0.121096435133143	0.883712583104726	-4.75854427733926
a_HQ_transcript/63279	2.04901014584361	2.16854554249919	1.59170666536599	0.121106083763629	0.883712583104726	-4.86800560162187
a_HQ_transcript/67512	-1.93808457798409	0.678318426132075	-1.59141861567555	0.121170962517423	0.88395337507044	-4.66383310621755
a_HQ_transcript/5795	-1.22426333841739	2.30440152010587	-1.59133359247242	0.121190118121298	0.88395337507044	-4.67507200286036
a_HQ_transcript/100534	-0.976446850236724	2.37888653858279	-1.59123510802635	0.121212309622215	0.88395337507044	-4.68439469743844
a_HQ_transcript/120153	2.46018399699254	0.700841667659994	1.591206051854	0.121218857485154	0.88395337507044	-4.76601158181328
a_HQ_transcript/10954	1.39974054585005	2.30617346805849	1.59119719596384	0.121220853234183	0.88395337507044	-4.70819970133674
a_HQ_transcript/2858	1.30927496706581	-0.745156456762254	1.59101833850021	0.121261166019235	0.88395337507044	-4.65447467373112
a_HQ_transcript/60538	-1.37336577998084	2.8270695044551	-1.59097679962277	0.121270530060418	0.88395337507044	-4.83974931592621
a_HQ_transcript/74995	-1.62085107179766	-0.519908882899662	-1.59097058613325	0.121271930808083	0.88395337507044	-4.65656594779266
a_HQ_transcript/45887	1.01369530342621	3.1209313925554	1.59095277746605	0.121275945606021	0.88395337507044	-4.79377517696638
a_HQ_transcript/11885	-2.9229491574272	0.717537476239345	-1.59079489825845	0.121311542758654	0.884101975611215	-4.712116538715
a_HQ_transcript/141422	-3.43462977459794	2.60387227623004	-1.59058601236812	0.121358653571776	0.884237833792227	-4.91350850147274
a_HQ_transcript/21389	0.490929183508308	2.89031487727696	1.59057734677736	0.121360608278186	0.884237833792227	-4.78773316407083
a_HQ_transcript/132939	1.73310312761921	-0.0986004962364047	1.59042464917384	0.121395056664901	0.884326923025654	-4.69746794116547
a_HQ_transcript/111581	-1.7935296915125	-0.0394653735417921	-1.59033294527172	0.12141574879557	0.884326923025654	-4.68991734777056
a_HQ_transcript/93210	4.65105212273057	1.73392730521228	1.5903208597528	0.121418475995523	0.884326923025654	-4.65978784196138
a_HQ_transcript/115827	-2.12074393792252	0.941262393675149	-1.59008269566292	0.121472229974025	0.884607590068921	-4.55718202012294
a_HQ_transcript/75499	-1.94269715311154	0.211715273586239	-1.58998888041039	0.121493409557941	0.884650997217088	-4.59281477483625
a_HQ_transcript/152569	-4.33867302104427	2.06279649041577	-1.58976039448486	0.121545004824485	0.884915836728165	-4.80761563520028
a_HQ_transcript/63797	1.61267850141772	0.782052824911131	1.58958908422845	0.121583700798624	0.885004922119139	-4.63361002628116
a_HQ_transcript/60091	3.21531013948526	1.75041523422256	1.58949837487185	0.121604194528897	0.885004922119139	-4.71606776819323
a_HQ_transcript/147931	2.28448787482499	3.6125921034305	1.5894693274173	0.121610757742642	0.885004922119139	-5.06018236896805
a_HQ_transcript/12041	-0.714617325527539	2.56873652822338	-1.58939083199859	0.121628495074124	0.885004922119139	-4.80400010884677
a_HQ_transcript/69969	2.01711832642976	0.0081132791767844	1.58936927584491	0.121633366412225	0.885004922119139	-4.68107422432206
a_HQ_transcript/133700	-0.50395456914273	4.08579457158567	-1.58922881818504	0.12166511145492	0.885029794353988	-5.10005111959801
a_HQ_transcript/139680	1.90576062374217	0.150968016362795	1.5891413580175	0.121684881880971	0.885029794353988	-4.70134588861902
a_HQ_transcript/83214	-1.24868294860607	2.30093391258104	-1.58912589528938	0.121688377513861	0.885029794353988	-4.63550475751281
a_HQ_transcript/87109	2.00213442416405	2.03011180211146	1.58908471737224	0.121697686936957	0.885029794353988	-4.66958570218451
a_HQ_transcript/39033	-2.55912688072597	0.483342205388406	-1.58893478892371	0.121731587391357	0.885091990429315	-4.58785131678221

a_HQ_transcript/59463	-2.72295702451863	0.495475138415933	-1.58891221384478	0.121736692531695	0.885091990429315	-4.66402365584565
a_HQ_transcript/114475	-0.71622772483519	4.81085679186866	-1.58868296453551	0.12178854501452	0.885274104360853	-5.15155009302481
a_HQ_transcript/15196	0.85000626420994	3.50946595552153	1.58866680735487	0.121792200188786	0.885274104360853	-4.81197737394858
a_HQ_transcript/26028	2.33339380144559	0.796886467527509	1.58831930994263	0.12187083486202	0.885664641843034	-4.67578677498754
a_HQ_transcript/98940	-2.16489968254605	0.514622425062495	-1.58829471687653	0.121876401563832	0.885664641843034	-4.68296279294111
a_HQ_transcript/149053	2.05750434838061	-0.498263049193199	1.58798349229772	0.121946865997536	0.885959353579929	-4.68871641728104
a_HQ_transcript/76830	1.67918180012766	-0.215798557592847	1.58797419762947	0.121948970918076	0.885959353579929	-4.60591280511612
a_HQ_transcript/126013	0.445605431465453	4.56717944716269	1.58781684804442	0.121984609661829	0.885959353579929	-5.06457985465932
a_HQ_transcript/9043	2.4914647629773	0.59511638164853	1.58776460030285	0.121996445348077	0.885959353579929	-4.57151946795113
a_HQ_transcript/118509	1.9300706948836	0.072221685379986	1.58776004474835	0.121997477362828	0.885959353579929	-4.70952243521952
a_HQ_transcript/44006	1.19023986458917	3.24720115498598	1.58771180501258	0.122008406024761	0.885959353579929	-4.76239904391382
a_HQ_transcript/133882	-2.67484182856707	1.02976633518631	-1.58751947970866	0.122051985083373	0.886165099790947	-4.75619142529009
a_HQ_transcript/40425	-1.95062659768046	2.63723854619173	-1.58734215953081	0.122092175405194	0.8861747312059	-4.83582131355566
a_HQ_transcript/75542	3.16062690632991	0.406483888516223	1.58723380387491	0.122116739972445	0.8861747312059	-4.74328876884928
a_HQ_transcript/125785	-1.72290004341519	-0.59606655680236	-1.58721456130279	0.122121102746589	0.8861747312059	-4.69158317738684
a_HQ_transcript/106404	-0.852543619945689	3.92237554810683	-1.58717641471499	0.1221297519125	0.8861747312059	-4.98907340758345
a_HQ_transcript/129304	2.06342144081838	0.449964025185221	1.58707633298227	0.122152446324032	0.8861747312059	-4.68629219214462
a_HQ_transcript/125268	2.8753300691058	1.08112696396047	1.58701709955866	0.122165879647569	0.8861747312059	-4.80953879571763
a_HQ_transcript/2972	-2.17708431573935	0.963476785034479	-1.58686606187202	0.122200138380209	0.8861747312059	-4.6310592436272
a_HQ_transcript/60938	-2.78128052524482	2.27659235296098	-1.58673245541223	0.122230449868886	0.8861747312059	-4.94483305606902
a_HQ_transcript/46586	-2.10101870698406	0.672956771175686	-1.58655916550179	0.122269773558335	0.8861747312059	-4.6147031627762
a_HQ_transcript/3638	2.37172217331907	1.51360578017456	1.58651650918159	0.122279454900076	0.8861747312059	-4.81299930495608
a_HQ_transcript/56327	-2.09858764806026	0.597204977728217	-1.58648135281143	0.12228743451327	0.8861747312059	-4.62751990324183
a_HQ_transcript/125983	4.89418189595105	3.63656703943421	1.58643531771742	0.122297883967486	0.8861747312059	-4.812275536484
a_HQ_transcript/144054	2.25470170267006	0.216644662192025	1.58628149021539	0.122332806395933	0.8861747312059	-4.73068971903683
a_HQ_transcript/23104	-2.09497567613698	-0.421161585652339	-1.58613247166805	0.12236664485558	0.8861747312059	-4.69484855286689
a_HQ_transcript/130222	-3.04998058838528	1.39572625941964	-1.58607748166952	0.12237913366964	0.8861747312059	-4.8477334514732
a_HQ_transcript/27359	-2.89616277170819	-0.105974231113559	-1.58603699989186	0.122388328178354	0.8861747312059	-4.70041160759442
a_HQ_transcript/133688	2.36078150524635	0.741318193016965	1.58594372496305	0.122409515592566	0.8861747312059	-4.63953934967147
a_HQ_transcript/81852	0.939043600157204	2.63077232827883	1.58583222857099	0.12243484595015	0.8861747312059	-4.78983907669465
a_HQ_transcript/117008	0.748867280753376	4.34324469505386	1.58581121956513	0.12243961937038	0.8861747312059	-5.07473827844282
a_HQ_transcript/51059	-3.43942159304207	1.22915627514281	-1.58573625688266	0.122456652753235	0.8861747312059	-4.67678807308439
a_HQ_transcript/149381	-1.54833262273502	-0.0536695899125616	-1.58566702804725	0.122472384984546	0.8861747312059	-4.66184525385965
a_HQ_transcript/83798	-1.60481718539752	1.70250797880813	-1.58565138392796	0.122475940334701	0.8861747312059	-4.60261495940663
a_HQ_transcript/31238	-2.36547405565456	0.419870338212533	-1.58553983787426	0.122501293219053	0.8861747312059	-4.67265335633423
a_HQ_transcript/141947	-2.31299405842475	0.575583258628797	-1.58547529545021	0.122515964784297	0.8861747312059	-4.57583615189634
a_HQ_transcript/72846	-2.11761607023295	1.22379794886517	-1.58544658090867	0.122522492538388	0.8861747312059	-4.76991799591977
a_HQ_transcript/134693	-3.14567163507734	3.58834102592962	-1.58534139853746	0.122546406358917	0.8861747312059	-4.79718031348582
a_HQ_transcript/8253	-4.09409002134941	2.75309155680594	-1.58529354614346	0.122557287140429	0.8861747312059	-4.85739248136711
a_HQ_transcript/2796	1.52455914346196	-0.606673892778942	1.58523010545218	0.122571713640645	0.8861747312059	-4.705115117409
a_HQ_transcript/25884	-1.00088520928502	2.87928374989524	-1.58521191577163	0.122575850255051	0.8861747312059	-4.76410686994869
a_HQ_transcript/79455	-2.18966943670753	2.02822886797236	-1.58517588686347	0.122584044122393	0.8861747312059	-4.66925499521268
a_HQ_transcript/14365	1.7920616142235	-0.262837627334711	1.58501655499155	0.122620285511324	0.8861747312059	-4.64837697091471
a_HQ_transcript/145616	3.03678143012344	0.997512170589359	1.58488966439663	0.122649154121549	0.8861747312059	-4.68367159767785
a_HQ_transcript/69404	-1.96355131633446	-0.0929934007130288	-1.58474261299205	0.122682616425333	0.8861747312059	-4.64152996533307
a_HQ_transcript/28333	0.888985121010044	3.06391696264687	1.5846302211972	0.122708196788339	0.8861747312059	-4.86884897362151
a_HQ_transcript/48187	-2.18387923446254	1.50213354357803	-1.58462205658246	0.122710055223828	0.8861747312059	-4.68307895616498
a_HQ_transcript/98136	-2.91648838334877	0.539454774100361	-1.5845762119291	0.12272049084419	0.8861747312059	-4.68077784157809
a_HQ_transcript/50995	-1.82172436183974	0.897975635663978	-1.58447554526305	0.122743408147149	0.8861747312059	-4.62326651531312
a_HQ_transcript/43186	-3.6969645777699	1.61367847173691	-1.58439257270206	0.12276229992282	0.8861747312059	-4.86276921935035

a_HQ_transcript/4722	-1.25038109172832	1.78600451707577	-1.58438163697654	0.122764790022913	0.8861747312059	-4.5997115772686
a_HQ_transcript/2579	1.49365513325823	-0.307808791692925	1.58436953995618	0.122767544601699	0.8861747312059	-4.70973693011296
a_HQ_transcript/147172	-2.93704876701376	0.32731585644991	-1.58430185135344	0.122782958717042	0.8861747312059	-4.69621192914358
a_HQ_transcript/45335	1.32862693951641	-0.307597445910407	1.58422025113499	0.122801542902305	0.8861747312059	-4.64225179346209
a_HQ_transcript/11166	-1.6695635821602	1.44092474781313	-1.58416446219481	0.122814249975925	0.8861747312059	-4.61251882267766
a_HQ_transcript/150343	-0.980247657679798	10.5285485894655	-1.58416071750347	0.122815102944557	0.8861747312059	-5.7293694660601
a_HQ_transcript/84794	1.79334317280964	1.6165450919791	1.58415897562328	0.122815499712997	0.8861747312059	-4.70862382926651
a_HQ_transcript/139981	-2.92512613230623	2.22583900748364	-1.58415578391259	0.12281622672902	0.8861747312059	-4.86358905348091
a_HQ_transcript/89689	-1.64768693998129	0.27532155808726	-1.58409913307	0.122829131388156	0.8861747312059	-4.64517767877994
a_HQ_transcript/47917	1.03642988175213	1.51464188267168	1.58406056980927	0.122837916458759	0.8861747312059	-4.58430808715824
a_HQ_transcript/146553	1.78307972367679	-0.387187007820612	1.58402529025922	0.12284595391928	0.8861747312059	-4.69666050182777
a_HQ_transcript/8743	2.06884739720033	0.620657661673575	1.58395413377005	0.122862166246599	0.8861747312059	-4.67844419693348
a_HQ_transcript/99328	2.80726113251487	1.02660833275273	1.58392900504083	0.122867892005694	0.8861747312059	-4.80680156748488
a_HQ_transcript/152370	5.44399065563025	6.3334176157001	1.5839240922557	0.122869011444102	0.8861747312059	-5.12013711389269
a_HQ_transcript/91033	-1.01586580788458	5.41534580517157	-1.58377432271718	0.122903142273313	0.8861747312059	-5.1860832829331
a_HQ_transcript/105590	2.07099283558018	1.942576961063	1.58376632437877	0.122904965224543	0.8861747312059	-4.67698449828358
a_HQ_transcript/25230	4.25167476062492	3.16657475433351	1.58371426554203	0.122916830819	0.8861747312059	-4.6963746830546
a_HQ_transcript/111112	2.11951024249441	-0.34232335788988	1.58371236229735	0.122917264636857	0.8861747312059	-4.68900944363609
a_HQ_transcript/82432	2.31832000917903	0.743992127979762	1.58369031659902	0.12292228973495	0.8861747312059	-4.67899045795812
a_HQ_transcript/118778	-1.22012859352182	1.26105188963871	-1.58361976829999	0.122938371647818	0.886180762294687	-4.6394409994621
a_HQ_transcript/42639	-2.39962360768866	1.14652956047681	-1.58350017301911	0.122965638046319	0.886185366020595	-4.6585262360762
a_HQ_transcript/1410	0.980175300307837	1.83681229018877	1.58348323055081	0.122969501140967	0.886185366020595	-4.6656929813626
a_HQ_transcript/20071	1.50802427432942	-0.381349266146401	1.58340369914249	0.122987636622329	0.886206190849482	-4.68744400605406
a_HQ_transcript/14838	1.98890223103738	1.66237721414546	1.58315942822712	0.123043351184963	0.886426006539942	-4.58233359209484
a_HQ_transcript/36669	-1.48164590718573	-0.630365097411213	-1.58313623742038	0.123048641734978	0.886426006539942	-4.70340160737427
a_HQ_transcript/31551	1.66262694806411	2.59674129427664	1.58275918607075	0.123134685053426	0.88643638939923	-4.78794475246284
a_HQ_transcript/1731	-1.21559811628074	2.14175903733624	-1.58265700877548	0.123158010431893	0.88643638939923	-4.65027720530403
a_HQ_transcript/10787	2.00730074079959	1.81470224607429	1.58259454689064	0.123172271220346	0.88643638939923	-4.76789939493999
a_HQ_transcript/102555	-2.55860847551845	4.17613236357826	-1.58257461479052	0.123176822238974	0.88643638939923	-5.02621749528674
a_HQ_transcript/71342	2.97540913660604	2.24940512274212	1.58250196203384	0.123193411922589	0.88643638939923	-4.73770999760592
a_HQ_transcript/83070	1.95596913415001	0.655550338011446	1.58235555173017	0.123226849106826	0.88643638939923	-4.58338659800967
a_HQ_transcript/83221	-3.71290919642245	1.63598064724749	-1.58227666287127	0.123244868818066	0.88643638939923	-4.73387568834644
a_HQ_transcript/149735	-0.94325951979036	3.98184466546056	-1.58227622852506	0.123244955078966	0.88643638939923	-5.02041411924414
a_HQ_transcript/7587	-3.66518377114412	3.46632845631937	-1.5822244902229	0.123256787223022	0.88643638939923	-4.97045431345492
a_HQ_transcript/149883	3.74447983702553	1.46251377755405	1.58216564032441	0.123270232120134	0.88643638939923	-4.91416644154246
a_HQ_transcript/90092	-2.36645381556037	1.54844942146448	-1.58206300464871	0.123293683219911	0.88643638939923	-4.72802582657693
a_HQ_transcript/58105	0.87496730802851	2.6275255992312	1.5819630056239	0.123316535380385	0.88643638939923	-4.88089760997493
a_HQ_transcript/30636	1.23283485769421	1.78510129933886	1.58183784445046	0.123345142567794	0.88643638939923	-4.65806704386702
a_HQ_transcript/125858	-2.10928994726533	-0.419704356014774	-1.58169971477928	0.123376720164899	0.88643638939923	-4.71567726251982
a_HQ_transcript/54803	1.11395728752581	3.51563682352722	1.58169288639614	0.123378281361652	0.88643638939923	-5.02983742868826
a_HQ_transcript/32064	1.51375183643272	-0.713570682829846	1.58165536292827	0.123386860769386	0.88643638939923	-4.648461477049
a_HQ_transcript/129695	-1.65732884543431	-0.241166044745305	-1.58159252176889	0.123401229934116	0.88643638939923	-4.68512845396048
a_HQ_transcript/48727	2.00101072469228	1.15598690231175	1.5815866786523	0.123402566081808	0.88643638939923	-4.75509027426694
a_HQ_transcript/109669	1.83546005586441	-0.0160447787985919	1.58149450851808	0.123423644225223	0.88643638939923	-4.71161962687517
a_HQ_transcript/5006	1.46570495343222	1.9545372120337	1.58134852025418	0.123457035912741	0.88643638939923	-4.77257621113797
a_HQ_transcript/111594	-1.40058951295533	1.89525173296024	-1.58124843683721	0.123479932113885	0.88643638939923	-4.63529085601185
a_HQ_transcript/151585	5.08970093052155	5.58204170375838	1.58124342922179	0.123481077803082	0.88643638939923	-5.07293683781631
a_HQ_transcript/7852	3.50844672623828	6.35468419065313	1.5810867776847	0.123516922395254	0.88643638939923	-5.15899213081686
a_HQ_transcript/146634	-2.53926504278792	0.126654866766497	-1.58104899288291	0.12352556948634	0.88643638939923	-4.68765656472767
a_HQ_transcript/23369	-1.53904663852682	-0.509473703043475	-1.58090856847324	0.123557710092236	0.88643638939923	-4.66069876282127

a_HQ_transcript/61514	-1.9941274870563	-0.171150543798135	-1.58082431680854	0.123576997052109	0.88643638939923	-4.64175280324489
a_HQ_transcript/98063	2.42141193359121	0.658412028823889	1.58078519328724	0.123585954076661	0.88643638939923	-4.71485972591892
a_HQ_transcript/59370	-1.88462281312266	-0.129155840567156	-1.58073262984526	0.123597988900911	0.88643638939923	-4.5998325160922
a_HQ_transcript/63834	1.99993654949716	-0.209142852046313	1.58070963752523	0.123603253479086	0.88643638939923	-4.69064204454431
a_HQ_transcript/125951	-0.525278306951055	4.09578306947437	-1.58070519713071	0.123604270222471	0.88643638939923	-5.03586101830123
a_HQ_transcript/3333	2.52629115709699	0.562129101316944	1.58066554232864	0.123613350519869	0.88643638939923	-4.63079638326321
a_HQ_transcript/17825	2.03303684959287	1.96505183683361	1.58061500923623	0.1236249225564	0.88643638939923	-4.71509138624911
a_HQ_transcript/120613	-4.53363838922494	2.49144308360528	-1.58051780620768	0.123647184457944	0.88643638939923	-4.86798598455957
a_HQ_transcript/66117	2.06912727521588	1.35043957633615	1.58046954798634	0.12365823800244	0.88643638939923	-4.68935492959182
a_HQ_transcript/26110	2.53656188621936	3.29196912206786	1.58045714013129	0.12366108015179	0.88643638939923	-4.93038573937856
a_HQ_transcript/136102	0.998704827144761	1.7553412196784	1.58042642576186	0.123668115829796	0.88643638939923	-4.66183840577012
a_HQ_transcript/126574	2.2789874646265	0.680156492196583	1.58035740323958	0.123683927871252	0.88643638939923	-4.69554143805667
a_HQ_transcript/77107	-1.58020618312842	0.780597776745338	-1.58030825926823	0.123695187036371	0.88643638939923	-4.64444039510491
a_HQ_transcript/149576	2.09001202011795	1.73953444482882	1.5802287047415	0.123713415208237	0.88643638939923	-4.67345315513324
a_HQ_transcript/142104	-0.714128327443899	2.59762569237869	-1.58022715938139	0.123713769315273	0.88643638939923	-4.80372083381794
a_HQ_transcript/103903	1.61921138063303	-0.475914656645656	1.58017972470679	0.123724638998189	0.88643638939923	-4.65268715000901
a_HQ_transcript/8384	3.45784649182044	3.16862472746167	1.5801662599437	0.12372772459851	0.88643638939923	-4.84041023944728
a_HQ_transcript/113744	-2.89997926272096	0.837476512967434	-1.58006330844522	0.123751319157701	0.88643638939923	-4.81732510043922
a_HQ_transcript/121204	-3.54653729730338	0.107979767719582	-1.57991815866831	0.123784591016333	0.88643638939923	-4.77316027452643
a_HQ_transcript/46133	2.37835098564825	1.04323074143524	1.57990177687638	0.123788346580179	0.88643638939923	-4.76628952288066
a_HQ_transcript/54539	-1.41381996066933	1.89876739122747	-1.57979801609291	0.123812136142164	0.88643638939923	-4.60414949539944
a_HQ_transcript/8211	1.89437362858252	1.93740868205173	1.57976974974562	0.123818617503813	0.88643638939923	-4.69867666805672
a_HQ_transcript/149303	10.2516535991671	3.4898801661032	1.57968982566512	0.123836945278813	0.88643638939923	-4.85057531444312
a_HQ_transcript/116799	1.32714996674469	2.00807713089212	1.57967865739567	0.123839506504769	0.88643638939923	-4.65801300217341
a_HQ_transcript/87567	-2.9637503324096	0.14433760425997	-1.57966554531201	0.12384251356202	0.88643638939923	-4.76841362681953
a_HQ_transcript/147167	2.16874173247481	-0.150405948011478	1.57963568702228	0.1238493613298	0.88643638939923	-4.68862158459073
a_HQ_transcript/136434	-1.36046216727658	2.25106665638602	-1.57963143562449	0.123850336380145	0.88643638939923	-4.70528482884266
a_HQ_transcript/134749	1.76757698023425	1.1217707140776	1.57959663188012	0.123858318791298	0.88643638939923	-4.66237835393717
a_HQ_transcript/603	-2.21159486778949	2.14112855978006	-1.57948408039826	0.123884135907522	0.886512009360143	-4.66301199684452
a_HQ_transcript/4884	-1.63733247237095	3.78641371953244	-1.57934492160456	0.12391606229801	0.886569786383365	-5.03320050338794
a_HQ_transcript/12017	2.31638997763132	0.999205670583432	1.57913921358572	0.123963268995752	0.886569786383365	-4.59897048359761
a_HQ_transcript/134116	-0.865668497079408	2.54669441912012	-1.579061809426	0.123981035813526	0.886569786383365	-4.84050259992917
a_HQ_transcript/55645	-1.22596510578545	-0.744590845026679	-1.57896122894699	0.124004125470342	0.886569786383365	-4.71672342649243
a_HQ_transcript/83872	1.05623294780849	-0.778682307654447	1.57889081214962	0.12402029272136	0.886569786383365	-4.70502605565994
a_HQ_transcript/69202	2.763663444618	0.983692952360652	1.57872529528931	0.124058301122366	0.886569786383365	-4.65177099659001
a_HQ_transcript/146880	-1.63448270963664	3.82165171159876	-1.57871971648713	0.124059582374147	0.886569786383365	-5.0783696978133
a_HQ_transcript/50322	0.973480279747303	2.8354516601259	1.57870919492358	0.124061998831231	0.886569786383365	-4.9213301137006
a_HQ_transcript/46137	2.05608445822364	2.24151530788943	1.57870152272897	0.124063760906217	0.886569786383365	-4.8235362874709
a_HQ_transcript/73747	1.53180828023917	0.501405753711913	1.57869502942754	0.124065252240458	0.886569786383365	-4.72464563242124
a_HQ_transcript/63278	-2.11181637630734	0.397491192620078	-1.57869135404638	0.124066096381754	0.886569786383365	-4.82396070576357
a_HQ_transcript/11249	-1.68379469790369	0.434004048826827	-1.57865156950234	0.124075234176794	0.886569786383365	-4.67189643403117
a_HQ_transcript/122012	-1.71008381721873	-0.262518824873725	-1.57845749916519	0.124119816525316	0.886682876840802	-4.67052545942502
a_HQ_transcript/73710	1.96922648355747	2.429067205912	1.57844987122205	0.124121569103592	0.886682876840802	-4.73483738646437
a_HQ_transcript/57166	-1.51045649977401	3.14834118593396	-1.57836749186393	0.124140497678562	0.88670912374778	-4.82987901509652
a_HQ_transcript/139218	-1.81851323415855	-0.368224795687198	-1.57806371510023	0.124210317830455	0.886808487602719	-4.70034157685257
a_HQ_transcript/132653	-2.04856473532887	-0.227796703729233	-1.5780395851242	0.124215865246281	0.886808487602719	-4.73881157793353
a_HQ_transcript/110534	1.81884495543235	1.7102482827245	1.57800286087648	0.124224308439064	0.886808487602719	-4.68650901223558
a_HQ_transcript/5341	2.07579580701297	1.65034002547189	1.57784887969691	0.124259715021583	0.886808487602719	-4.77219814554509
a_HQ_transcript/14345	-1.12216411336732	2.63239712649101	-1.57780373590193	0.124270096991385	0.886808487602719	-4.77322324829234
a_HQ_transcript/142685	3.6229833414858	0.732576011431756	1.57779288150648	0.124272593342718	0.886808487602719	-4.83151018521003

a_HQ_transcript/21112	3.27156355778007	1.28290204625647	1.57774498934707	0.124283608323903	0.886808487602719	-4.82220526940776
a_HQ_transcript/19892	2.54713229396785	0.263311464689568	1.57770323469575	0.124293212356179	0.886808487602719	-4.75455485633381
a_HQ_transcript/62301	-1.64682094640857	-0.509317054773498	-1.57741676515559	0.124359119861394	0.886808487602719	-4.69794616838812
a_HQ_transcript/143941	3.05016821817357	0.611282521690809	1.57736143127099	0.124371853710352	0.886808487602719	-4.79164162582277
a_HQ_transcript/85461	2.13212684444127	-0.201829814482522	1.5773158727236	0.124382338784209	0.886808487602719	-4.65668856716072
a_HQ_transcript/88705	4.46758929070957	2.13360857449125	1.57730761202588	0.124384240020116	0.886808487602719	-4.83705061075984
a_HQ_transcript/906	-0.576073456938669	2.98179553476025	-1.57730633949106	0.124384532901711	0.886808487602719	-4.86509125309737
a_HQ_transcript/105510	-2.12639018364423	0.176984326206732	-1.57723388789385	0.124401209003905	0.886808487602719	-4.57868925657172
a_HQ_transcript/128065	2.32365636415634	0.953973954764458	1.57721858006105	0.124404732623982	0.886808487602719	-4.65863919451088
a_HQ_transcript/95028	4.65701363599018	3.28071474372262	1.57713917654272	0.124423011360555	0.886808487602719	-5.08227610414083
a_HQ_transcript/33501	-1.98373894253499	1.49752185867797	-1.57712012585848	0.124427397165314	0.886808487602719	-4.81483390400033
a_HQ_transcript/104843	-2.87694041653289	1.34790571548318	-1.57705316775203	0.12444281310794	0.886808487602719	-4.65140216709029
a_HQ_transcript/114669	-4.3937084108114	2.42681751029669	-1.57704681726541	0.124444275278272	0.886808487602719	-4.77085578737552
a_HQ_transcript/81346	2.51258089712047	1.1529450701397	1.57696618983402	0.124462840592718	0.88681438022984	-4.63468759112175
a_HQ_transcript/141539	6.06790640742619	3.23052140480719	1.57691071899965	0.124475614648625	0.88681438022984	-5.05517877670451
a_HQ_transcript/71869	-2.38544156787621	4.93556572527605	-1.57669455109352	0.12452540490949	0.886942384431339	-5.19982500800024
a_HQ_transcript/67896	1.70149414982638	-0.18402334014433	1.57659993134838	0.124547203923884	0.886942384431339	-4.66843495192412
a_HQ_transcript/29811	-1.60073060456573	1.94063201862318	-1.57658863565505	0.12454980649558	0.886942384431339	-4.68970050321174
a_HQ_transcript/4965	-1.80370931536275	0.114674635870261	-1.57642973354147	0.12458642286507	0.886942384431339	-4.73370809335339
a_HQ_transcript/145675	1.30539335906507	4.5093839080929	1.57634410620574	0.12460615791374	0.886942384431339	-5.15013722459253
a_HQ_transcript/49789	1.87266554292178	1.07641941579378	1.57629475088402	0.124617534291534	0.886942384431339	-4.59472013158803
a_HQ_transcript/66705	-2.98724543175909	0.985820288284867	-1.57624728560026	0.124628475816052	0.886942384431339	-4.75726638727836
a_HQ_transcript/37808	2.83899264710528	0.789653744789801	1.57618194081564	0.124643540140655	0.886942384431339	-4.74699862133143
a_HQ_transcript/99583	-1.61039953084439	0.462524796884099	-1.57605693008763	0.124672363753299	0.886942384431339	-4.62426242930758
a_HQ_transcript/148684	5.09901495516236	5.67327625764842	1.57600883562559	0.124683454299519	0.886942384431339	-4.905201628397
a_HQ_transcript/119919	-4.68142471696731	1.66874667997725	-1.5759132556744	0.124705497353185	0.886942384431339	-4.66267117567466
a_HQ_transcript/76560	-0.568568365338634	2.0403216729478	-1.57579086322049	0.124733728663241	0.886942384431339	-4.71727121989804
a_HQ_transcript/34843	3.60257693120319	0.0103673806219472	1.57564244033837	0.124767971205339	0.886942384431339	-4.73162340772867
a_HQ_transcript/120963	-2.76973112155185	0.639073024312658	-1.57547279207096	0.12480712003579	0.886942384431339	-4.73010240458365
a_HQ_transcript/152115	4.64705321870731	5.21279852026122	1.57535372933087	0.12483460149781	0.886942384431339	-5.22606523760275
a_HQ_transcript/138560	3.03759833904883	2.59328962039631	1.57533829603198	0.124838164095586	0.886942384431339	-4.90660750552131
a_HQ_transcript/126721	-1.26233769410555	-0.693477317485058	-1.57530235407897	0.124846461199508	0.886942384431339	-4.71191638655055
a_HQ_transcript/131171	-3.11198291927239	1.26234523283762	-1.57530077236321	0.124846826344704	0.886942384431339	-4.72973274271735
a_HQ_transcript/42452	-1.37130207302504	1.75209319346439	-1.57521660539109	0.124866257872486	0.886942384431339	-4.62274665243059
a_HQ_transcript/19131	2.06626660248832	-0.0502913366864484	1.57514088926419	0.124883740472164	0.886942384431339	-4.75204516624492
a_HQ_transcript/2725	-2.45655251389578	0.861887794305561	-1.57513192713808	0.124885809929195	0.886942384431339	-4.82438380252769
a_HQ_transcript/3318	-2.35797198495947	2.20368268821476	-1.575118925492	0.124888812207151	0.886942384431339	-4.66768190491363
a_HQ_transcript/139503	2.47342973038659	0.588303996493135	1.5751008906642	0.124892976820866	0.886942384431339	-4.70645417422432
a_HQ_transcript/115720	-1.66242554564868	2.31940237086977	-1.57510001643837	0.12489317870052	0.886942384431339	-4.66981812632442
a_HQ_transcript/31519	-1.49877778948766	-0.315365073088334	-1.5750089931365	0.124914199615167	0.886942384431339	-4.68053472732194
a_HQ_transcript/122433	2.03804056541763	0.170739520017643	1.57496266779729	0.124924899093967	0.886942384431339	-4.7426618525414
a_HQ_transcript/133479	-2.48788093150375	-0.315657964976555	-1.57484702416632	0.124951611855554	0.886942384431339	-4.71720255870481
a_HQ_transcript/104277	-2.38167693195456	1.74933949001013	-1.57471626728535	0.124981821271383	0.886942384431339	-4.65111343995482
a_HQ_transcript/147520	2.05470784549097	2.25186909350814	1.57460648178923	0.125007190164872	0.886942384431339	-4.66216691246373
a_HQ_transcript/148309	3.19944174994108	1.29988436046971	1.57459200575027	0.125010535556727	0.886942384431339	-4.8932887873819
a_HQ_transcript/137517	-2.21158869719194	2.08918828989946	-1.57458148122045	0.125012967806335	0.886942384431339	-4.77278898518986
a_HQ_transcript/88694	-1.87222794666313	0.907626168101765	-1.57456619771128	0.125016499938725	0.886942384431339	-4.66209438992378
a_HQ_transcript/121563	-2.87747238959406	0.302563178735408	-1.57455936289876	0.125018079540964	0.886942384431339	-4.77515517007949
a_HQ_transcript/117330	-2.2615492801144	-0.276449340028984	-1.57450894433888	0.12502973234173	0.886942384431339	-4.71816478818933
a_HQ_transcript/142499	-2.16325016202544	-0.0659162628647517	-1.57445853216145	0.125041384553275	0.886942384431339	-4.67445523359851

a_HQ_transcript/80753	1.74094417378559	-0.297432159264518	1.57437933690567	0.125059691440778	0.886942384431339	-4.74979122643001
a_HQ_transcript/47149	3.17152485484804	1.95711485002738	1.57411242783483	0.125121406622643	0.886942384431339	-4.82646712331083
a_HQ_transcript/43500	0.870701116967654	1.96288083400511	1.57410108656687	0.125124029520595	0.886942384431339	-4.62923651391407
a_HQ_transcript/77317	1.70721178291749	0.0394463985819019	1.57406247225364	0.125132960199057	0.886942384431339	-4.79189522098288
a_HQ_transcript/51086	5.06821954299385	0.246490370248389	1.57396930214889	0.125154510625477	0.886942384431339	-4.73969244217838
a_HQ_transcript/110002	-3.21832273321735	3.63874301210618	-1.57382531653675	0.125187820732021	0.886942384431339	-4.92645566353045
a_HQ_transcript/141440	-0.877777317660329	3.21297428992551	-1.57381334250758	0.125190591169101	0.886942384431339	-4.95775976599104
a_HQ_transcript/4490	-1.77609110859651	0.638181585193844	-1.57379079290125	0.125195808618496	0.886942384431339	-4.58868860107955
a_HQ_transcript/127722	2.68737147214289	0.198211563464383	1.57375548645569	0.125203978057188	0.886942384431339	-4.71711197938023
a_HQ_transcript/87753	2.55610692775186	0.500353868195895	1.57374031240036	0.125207489263981	0.886942384431339	-4.73178941495297
a_HQ_transcript/108309	3.11699752764297	0.0470338778096844	1.57373927418254	0.125207729505757	0.886942384431339	-4.73795391245861
a_HQ_transcript/990	2.84859414266514	1.46918997842587	1.57367173445698	0.125223358887243	0.886942384431339	-4.89757899933801
a_HQ_transcript/150281	-2.18603919361543	2.92932942102104	-1.57358113477315	0.125244327077367	0.886942384431339	-4.90152199629207
a_HQ_transcript/114338	2.04193235269119	1.25338335266648	1.57354562747416	0.125252545589064	0.886942384431339	-4.72926204288868
a_HQ_transcript/96312	1.85265903963184	0.367397566219923	1.57340336720417	0.125285477531923	0.886942384431339	-4.69043344798574
a_HQ_transcript/7952	-2.50374793545882	0.228762162102394	-1.5733739363466	0.125292291386075	0.886942384431339	-4.66917712738348
a_HQ_transcript/102108	1.82262609725531	1.00660808330823	1.57335786403556	0.125296012587142	0.886942384431339	-4.61238054189836
a_HQ_transcript/21522	1.800032918449	1.05373390586419	1.57324280464646	0.125322654768972	0.886942384431339	-4.67436927864461
a_HQ_transcript/118690	-3.38306371713463	0.240005456371613	-1.57321664182624	0.125328713455691	0.886942384431339	-4.70939618550983
a_HQ_transcript/41865	1.95670533699441	1.01805773147739	1.57308847841549	0.12535839650401	0.886942384431339	-4.65039438776448
a_HQ_transcript/52491	2.02637108348822	2.14176314824224	1.57305767778207	0.125365530881877	0.886942384431339	-4.66045481150902
a_HQ_transcript/136101	2.36419650087443	0.901722205423454	1.57299548015193	0.12537993878329	0.886942384431339	-4.61916627703801
a_HQ_transcript/1915	1.47335146001188	2.00193025035765	1.57290991844873	0.125399761111369	0.886942384431339	-4.7026207623755
a_HQ_transcript/81992	-1.755251150696	0.616006193222686	-1.57278835253924	0.125427929033601	0.886942384431339	-4.5897869374416
a_HQ_transcript/104092	-2.17710221607305	0.480563378250118	-1.57272551651202	0.125442490732944	0.886942384431339	-4.72597556666316
a_HQ_transcript/138778	-2.97377357536062	0.0996703014345067	-1.57267949277083	0.125453157206825	0.886942384431339	-4.71322309305612
a_HQ_transcript/47413	-2.38528026086613	0.21329583586844	-1.57255988395089	0.125480881236044	0.886942384431339	-4.69848438535698
a_HQ_transcript/5088	2.30763576705172	1.08430877668989	1.57226328643105	0.125549650885407	0.886942384431339	-4.60709840143799
a_HQ_transcript/125751	1.45044479252026	1.76374273523928	1.57221301994232	0.125561308811494	0.886942384431339	-4.68745504735522
a_HQ_transcript/141861	-2.36550280460008	1.53008546613489	-1.57220611497745	0.12556291029666	0.886942384431339	-4.58508324850631
a_HQ_transcript/128255	2.99246839072561	0.957731332198651	1.57220093484464	0.1255641117483	0.886942384431339	-4.755124946028
a_HQ_transcript/141506	-2.54254010203271	0.656279206068236	-1.57213995903107	0.12557825484923	0.886942384431339	-4.67838653328882
a_HQ_transcript/137171	0.535170128994755	4.38535435501627	1.57208786257902	0.125590339446045	0.886942384431339	-5.12270558989356
a_HQ_transcript/31791	2.16270619493592	2.18535519532083	1.57207429201059	0.125593487509905	0.886942384431339	-4.68855913558013
a_HQ_transcript/5312	-2.00194895359563	0.61271173214904	-1.57206406374013	0.12559586027902	0.886942384431339	-4.62140913239475
a_HQ_transcript/145173	1.81179360402292	-0.494872040419969	1.57203452467143	0.125602713000519	0.886942384431339	-4.72771661509779
a_HQ_transcript/92302	1.51408692149634	0.0361929203954213	1.57198486217684	0.125614234810691	0.886942384431339	-4.65000850830471
a_HQ_transcript/135190	3.94330993570072	0.236940270462337	1.57196165791817	0.125619618546162	0.886942384431339	-4.78753436495103
a_HQ_transcript/144843	4.17066584783975	1.23080314569208	1.57194835140021	0.125622705942458	0.886942384431339	-4.87800469635675
a_HQ_transcript/108480	-2.75125523093684	0.875183620270951	-1.57164983503748	0.125691984377221	0.887323739115751	-4.76241176897629
a_HQ_transcript/108658	-2.46619037579992	3.18538659930576	-1.57143532442516	0.125741786339216	0.887442277887804	-4.89506710557789
a_HQ_transcript/151659	0.719237864795514	3.05430065346527	1.57133113810459	0.125765980611452	0.887442277887804	-4.8502776665552
a_HQ_transcript/109804	2.1172488918539	0.335372567226255	1.57130630041706	0.125771749009231	0.887442277887804	-4.63383320131104
a_HQ_transcript/147761	-4.27560650599783	4.67748374219848	-1.57118016462536	0.125801046588179	0.887442277887804	-5.02678025197944
a_HQ_transcript/151809	2.02287167469731	1.26088967362927	1.57117582510226	0.125802054628843	0.887442277887804	-4.78314863830156
a_HQ_transcript/127021	-1.15323896033819	3.96748440849327	-1.57107223358468	0.125826120163933	0.887442277887804	-5.01636401413418
a_HQ_transcript/69205	-1.24424305450594	3.31760617603291	-1.57100438371048	0.12584188452641	0.887442277887804	-4.94605391314404
a_HQ_transcript/4830	1.4598238926909	3.37130454429256	1.57079444986574	0.125890671133834	0.887442277887804	-4.95575780942711
a_HQ_transcript/34275	-1.28390876856481	1.76461211492882	-1.5707736172903	0.125895513264905	0.887442277887804	-4.64053565996666
a_HQ_transcript/76222	-2.235902079539	1.22031568587298	-1.57065669230132	0.125922693044849	0.887442277887804	-4.6885494919402

a_HQ_transcript/71652	2.47193916357085	0.181904408652769	1.57061081698388	0.125933358290988	0.887442277887804	-4.70310798654699
a_HQ_transcript/6218	0.746607830094356	2.96027265653227	1.57046164540091	0.125968043285998	0.887442277887804	-4.83777532430837
a_HQ_transcript/139599	-4.53518666707853	3.15880747526634	-1.57041240666282	0.125979493860697	0.887442277887804	-4.88564734285681
a_HQ_transcript/109808	-2.02186932743549	0.297166403478063	-1.57029363369767	0.126007118261459	0.887442277887804	-4.60356803709047
a_HQ_transcript/119454	0.700069209724885	2.73002459475492	1.57028566240373	0.126008972414177	0.887442277887804	-4.84244159746845
a_HQ_transcript/138424	2.21247568179401	-0.248535604472406	1.57028335349677	0.126009509478706	0.887442277887804	-4.70660815296495
a_HQ_transcript/111781	-1.88129825955848	1.49899483487689	-1.5701683488864	0.126036262548145	0.887442277887804	-4.67918937633987
a_HQ_transcript/47094	-1.90215164913831	0.424330574444049	-1.56999538024587	0.126076508279083	0.887442277887804	-4.79069059610311
a_HQ_transcript/113933	-2.31875265747795	1.88602241223367	-1.56996227154555	0.1260842130885	0.887442277887804	-4.71214359726196
a_HQ_transcript/134245	2.27662312944145	-0.207102991418372	1.56990719231775	0.126097031565209	0.887442277887804	-4.7114901635684
a_HQ_transcript/131329	-5.3155389461589	1.3912784876929	-1.56988054504766	0.126103233511433	0.887442277887804	-4.8524656067578
a_HQ_transcript/132211	3.53993143962686	5.0415786072604	1.56980466004794	0.12612089652089	0.887442277887804	-4.83914345624738
a_HQ_transcript/143367	2.59304951144363	0.0598913027576327	1.56977461448821	0.126127890489274	0.887442277887804	-4.73236356436412
a_HQ_transcript/95114	4.37124445749979	0.734534136226097	1.5696241890257	0.126162911096217	0.887442277887804	-4.80169563011788
a_HQ_transcript/2605	2.44516714198792	-0.21585441570388	1.56953662903881	0.126183299617496	0.887442277887804	-4.69097242276283
a_HQ_transcript/6391	3.44396646303706	2.01469752620178	1.56948700825925	0.126194855109086	0.887442277887804	-4.68268338829083
a_HQ_transcript/79879	-2.13424865409122	0.568985847824471	-1.56926054376927	0.126247604217084	0.887442277887804	-4.76320618797838
a_HQ_transcript/98384	-0.79442561214921	2.97488240232765	-1.56923579301574	0.12625337036083	0.887442277887804	-4.88125051020329
a_HQ_transcript/17275	1.92547153809207	0.0550935807621195	1.56916525214184	0.126269805333691	0.887442277887804	-4.73640919926832
a_HQ_transcript/109977	-1.85096796078979	0.412388840027432	-1.56915199605683	0.126272893998629	0.887442277887804	-4.64487381003895
a_HQ_transcript/22677	-1.79809963438366	0.362938585668125	-1.56911390774286	0.126281768910129	0.887442277887804	-4.63594831296497
a_HQ_transcript/17622	-1.68702153126546	1.43135315160908	-1.56911195234937	0.126282224547694	0.887442277887804	-4.60938372968347
a_HQ_transcript/146877	-2.12834647049052	1.59554543785626	-1.56903477237845	0.126300209769852	0.887442277887804	-4.6769396543346
a_HQ_transcript/14134	-1.83609436492397	0.971935730552381	-1.56899443529359	0.1263096103374	0.887442277887804	-4.56547605819328
a_HQ_transcript/36619	-2.26738603093152	2.29389518575314	-1.5689840960663	0.126312019988663	0.887442277887804	-4.72103785780281
a_HQ_transcript/94247	-1.98313093529874	-0.22297205739585	-1.56884496585154	0.126344449198495	0.887442277887804	-4.71048307082896
a_HQ_transcript/50771	-0.810270508341318	2.71522344467255	-1.5687547187659	0.126365488096183	0.887442277887804	-4.78596741136451
a_HQ_transcript/81446	-2.71483451737083	-0.143676427111863	-1.56868085890631	0.126382708837628	0.887442277887804	-4.60039138552977
a_HQ_transcript/115	2.91033234350725	1.67368995039856	1.56866920789124	0.126385425496115	0.887442277887804	-4.63648026181611
a_HQ_transcript/4472	-1.39556118107471	1.8404693794822	-1.56866684616078	0.126385976184844	0.887442277887804	-4.67657226290869
a_HQ_transcript/53064	-1.92114035157259	-0.455379137623036	-1.56847929390214	0.126429714308624	0.887442277887804	-4.68504603227412
a_HQ_transcript/15792	2.25324779028289	2.3166151418975	1.56837206909813	0.12645472520909	0.887442277887804	-4.65741496477417
a_HQ_transcript/139130	-2.12027739265431	0.907143636819031	-1.56836957755206	0.126455306426746	0.887442277887804	-4.62927995927908
a_HQ_transcript/13215	-1.57998493706603	1.11472342707225	-1.56834858918422	0.12646020259357	0.887442277887804	-4.63530646511218
a_HQ_transcript/33065	-1.78826632512907	0.78620616096015	-1.56833819985681	0.126462626273206	0.887442277887804	-4.59458788327249
a_HQ_transcript/28707	2.56868734390416	0.271385466938732	1.56832184647683	0.126466441356612	0.887442277887804	-4.72102934400308
a_HQ_transcript/59598	-2.46565372498661	1.30022483804235	-1.56816859581281	0.126502197792905	0.887442277887804	-4.68229315974399
a_HQ_transcript/137251	-3.45059743478923	3.27238346141791	-1.56801231839993	0.126538668911956	0.887442277887804	-4.95876646077493
a_HQ_transcript/42162	-2.24660122768078	0.0576981787066309	-1.56801097610758	0.126538982205485	0.887442277887804	-4.7259250650131
a_HQ_transcript/140599	-3.87376870258127	1.76974611434952	-1.56795734693183	0.126551499871817	0.887442277887804	-4.75575806813299
a_HQ_transcript/85587	-2.0427423607869	2.48164554334261	-1.56789124440013	0.126566930350939	0.887442277887804	-4.95531005748432
a_HQ_transcript/46507	-2.85568863963077	0.546463743457361	-1.56787508536773	0.126570702627382	0.887442277887804	-4.75262329939173
a_HQ_transcript/106002	1.66743052006404	1.27799432470824	1.56779292433573	0.126589884284636	0.887442277887804	-4.68183688009856
a_HQ_transcript/112462	-1.46697521792012	1.74110154286603	-1.56770543833939	0.126610311733656	0.887442277887804	-4.6371737323667
a_HQ_transcript/139167	-8.33006670004115	3.15084092211091	-1.56768336062468	0.126615467170332	0.887442277887804	-4.8022056224185
a_HQ_transcript/30096	-1.75132939384135	3.69701689415695	-1.56762155367944	0.126629900814264	0.887442277887804	-5.0280774714889
a_HQ_transcript/138921	1.07633974620998	3.53976672499316	1.56760564296282	0.126633616626872	0.887442277887804	-5.0360550564669
a_HQ_transcript/7268	-2.68512279443408	0.472368120707194	-1.56755034050818	0.126646532735957	0.887442277887804	-4.74493506014661
a_HQ_transcript/6510	0.991210088039389	2.35208340812857	1.56742320668757	0.126676229416801	0.887442277887804	-4.79393550418793
a_HQ_transcript/99030	1.52727178600725	1.69843875622511	1.56738973660695	0.126684048502313	0.887442277887804	-4.61883369894274

a_HQ_transcript/74706	-2.31182747114324	0.552416755061036	-1.5671978388838	0.126728886121272	0.887442277887804	-4.66554493562048
a_HQ_transcript/77168	2.28715407106193	1.62018467411517	1.56713411503537	0.126743778294977	0.887442277887804	-4.68085181669479
a_HQ_transcript/76686	0.921937432204745	5.76962762778558	1.56709182335869	0.126753662587251	0.887442277887804	-5.26415697290756
a_HQ_transcript/51922	2.26773481099116	0.836326064941326	1.56706265519459	0.126760480054762	0.887442277887804	-4.59487813692374
a_HQ_transcript/65171	-2.45700671358438	0.678797234851465	-1.56703778036483	0.126766294278306	0.887442277887804	-4.77434585678087
a_HQ_transcript/134965	-1.21634654724881	2.00893387294255	-1.56695569289719	0.126785482881029	0.887442277887804	-4.62988742837079
a_HQ_transcript/88858	-1.73049658971709	1.15482683099232	-1.56695009438602	0.12678679166401	0.887442277887804	-4.66396801564486
a_HQ_transcript/53470	0.428845013665758	5.387070923974	1.56694970962556	0.126786881611176	0.887442277887804	-5.25061405274718
a_HQ_transcript/135034	-2.45691697982643	0.299626191289301	-1.5669324340767	0.126790920246645	0.887442277887804	-4.74580234650042
a_HQ_transcript/136699	-3.30362382988877	0.456271758975082	-1.5669209929971	0.126793594971871	0.887442277887804	-4.65429298782777
a_HQ_transcript/137038	-6.61809626750398	1.24560021937625	-1.5668823834704	0.126802621546284	0.887442277887804	-4.64660170299717
a_HQ_transcript/58238	-1.52956298907119	0.708848157024624	-1.56677191739754	0.126828450452745	0.887442277887804	-4.60380660597706
a_HQ_transcript/48605	-2.16113847520974	-0.337762179835681	-1.56677099231468	0.126828666771445	0.887442277887804	-4.73675877941277
a_HQ_transcript/17221	-2.25865987518091	1.45467462297295	-1.56667896852075	0.126850186851047	0.887442277887804	-4.74532231263787
a_HQ_transcript/126501	3.44886435527462	1.52599207984137	1.56649140193542	0.126894059148678	0.887442277887804	-4.83340892023336
a_HQ_transcript/31333	1.59734829242569	0.029281962930186	1.56640605178393	0.126914026855315	0.887442277887804	-4.66428103891452
a_HQ_transcript/98479	0.898961419898481	2.3434235858774	1.56633133792468	0.126931508297043	0.887442277887804	-4.7701892046426
a_HQ_transcript/7061	-1.67231744069477	1.86540221446362	-1.56631022314783	0.126936449057685	0.887442277887804	-4.76921036550402
a_HQ_transcript/96273	1.35450456385757	2.02877267892261	1.56623587712412	0.126953846931878	0.887442277887804	-4.74843675621165
a_HQ_transcript/112906	-1.93407658194026	1.21609320939706	-1.56614792584654	0.126974431108585	0.887442277887804	-4.58183645012045
a_HQ_transcript/134407	-2.91775079395101	1.17374606697539	-1.56611256662325	0.126982707371204	0.887442277887804	-4.75906441671522
a_HQ_transcript/134270	2.50074806721604	-0.0442598983384216	1.5661099419585	0.126983321723898	0.887442277887804	-4.76252496597836
a_HQ_transcript/85981	2.03274178123581	0.0806075901902628	1.56600306439515	0.127008340506157	0.887442277887804	-4.74573854309933
a_HQ_transcript/13455	-0.965397005155917	4.59910348927421	-1.56598707593161	0.127012083562838	0.887442277887804	-5.16224203822728
a_HQ_transcript/95022	-1.350972544224	1.25916277185322	-1.56594906130078	0.127020983522797	0.887442277887804	-4.80513812586269
a_HQ_transcript/46496	1.68082648672638	-0.279550486390243	1.56594582661703	0.127021740848241	0.887442277887804	-4.71267413245562
a_HQ_transcript/45152	2.51903968185983	1.76974172709932	1.56569955011868	0.127079411520928	0.887678295684209	-4.6754615685669
a_HQ_transcript/67780	-1.28159735457783	2.48046041565139	-1.56561154874636	0.127100024009857	0.887678295684209	-4.84514671710357
a_HQ_transcript/119782	2.88750355545649	0.761597895934134	1.56555991344886	0.127112119770391	0.887678295684209	-4.81916111116609
a_HQ_transcript/38089	-3.49765022327675	2.60486859570335	-1.56554075892518	0.12711660702717	0.887678295684209	-4.84683173938486
a_HQ_transcript/42387	4.66320887497032	2.51742790889226	1.56513937925158	0.127210666378616	0.887813663229718	-4.77737565852433
a_HQ_transcript/101834	-1.58159934399662	1.19939845164293	-1.56510753074335	0.127218132187182	0.887813663229718	-4.70592771872024
a_HQ_transcript/109087	-1.84698154072591	1.77114636053384	-1.56510198208653	0.127219432919119	0.887813663229718	-4.72189419237699
a_HQ_transcript/131202	-1.80154236767318	-0.139314328939395	-1.56506784596461	0.127227435444291	0.887813663229718	-4.78388838740303
a_HQ_transcript/103375	-3.55768913881344	0.620571041102916	-1.56491731055473	0.127262730323603	0.887813663229718	-4.73024568787073
a_HQ_transcript/142264	2.37235426634623	0.762184677884667	1.56486978028834	0.127273876035253	0.887813663229718	-4.70531914976349
a_HQ_transcript/45278	1.20015257973392	3.18355389339305	1.5648321937674	0.127282690529746	0.887813663229718	-4.95790772900876
a_HQ_transcript/46602	-1.98152510659874	0.158275649280206	-1.56478940569638	0.127292725455694	0.887813663229718	-4.69289243214981
a_HQ_transcript/86536	1.52342397141701	1.72052483689186	1.56466521199394	0.127321855787468	0.887813663229718	-4.69582513605829
a_HQ_transcript/54202	2.30158984012601	0.372674477751269	1.56462964439216	0.127330199368288	0.887813663229718	-4.73370204398503
a_HQ_transcript/27071	0.625721770213068	4.206273741439	1.56455821368617	0.127346957194693	0.887813663229718	-5.09531760974735
a_HQ_transcript/68297	1.17528166263102	0.597602534038179	1.56428054794386	0.127412115344648	0.887813663229718	-4.59550248199845
a_HQ_transcript/145824	-2.72318545325588	1.08554329002772	-1.56422887313447	0.127424244556411	0.887813663229718	-4.79578328462298
a_HQ_transcript/16891	2.96871430239269	0.416564297590061	1.56413150153833	0.127447102361211	0.887813663229718	-4.7520084809873
a_HQ_transcript/140	-1.45415418094894	2.6931580483649	-1.56412270376266	0.12744916778743	0.887813663229718	-4.87439891321651
a_HQ_transcript/139008	2.36658766944121	0.104991737346957	1.56409471743793	0.127455738230377	0.887813663229718	-4.77290238997661
a_HQ_transcript/30214	-2.13622556414346	1.60795059202728	-1.56403832216315	0.127468979175102	0.887813663229718	-4.59848048949882
a_HQ_transcript/26076	-1.98625855753745	-0.00561565616262188	-1.56401234128082	0.1274750795563	0.887813663229718	-4.72680894686724
a_HQ_transcript/7485	2.56533207791521	1.03533731991228	1.563760106072	0.127534317417902	0.887813663229718	-4.62443960631267
a_HQ_transcript/115380	-1.62048555542484	3.33964721335689	-1.56369833517933	0.12754882783077	0.887813663229718	-4.98676758781755

a_HQ_transcript/2360	3.04190031993232	1.84622554168093	1.56368276296417	0.127552486064644	0.887813663229718	-4.96742643613583
a_HQ_transcript/48609	-4.10775092542169	0.918817818737535	-1.56367500532122	0.127554308526524	0.887813663229718	-4.86573618208254
a_HQ_transcript/120422	0.667241369446053	4.24218842788882	1.56365754307456	0.127558410916993	0.887813663229718	-5.13836462062116
a_HQ_transcript/151611	-0.286970726376016	10.5795535332608	-1.56364524879397	0.127561299266095	0.887813663229718	-5.76933064211937
a_HQ_transcript/102107	-2.4562834136385	2.34602074485553	-1.56358080285137	0.127576440704169	0.887813663229718	-4.83737993513504
a_HQ_transcript/144578	-0.650145170253922	3.72012057210396	-1.56357479470297	0.12757785238049	0.887813663229718	-5.02553502244822
a_HQ_transcript/21428	-1.19569928239708	1.04564094219885	-1.56357003515425	0.12757897069449	0.887813663229718	-4.63745009309445
a_HQ_transcript/85638	-3.04030665896126	-0.19202246145561	-1.56356772271867	0.1275795140323	0.887813663229718	-4.7093459767767
a_HQ_transcript/27975	-2.01990850809355	3.43987050296386	-1.56353734272681	0.127586652394524	0.887813663229718	-4.99068390741803
a_HQ_transcript/83403	-2.34448406205612	0.877018824420372	-1.56334275469716	0.127632382297416	0.887813663229718	-4.70678494969267
a_HQ_transcript/102846	2.48485506224758	2.89369284512156	1.56333234832671	0.127634828261994	0.887813663229718	-4.89336362337611
a_HQ_transcript/97756	1.24847877981312	2.43589083088938	1.56328083266649	0.127646937318498	0.887813663229718	-4.68657878962005
a_HQ_transcript/122507	2.0660033992612	1.09637678250901	1.56325113599779	0.127653918118598	0.887813663229718	-4.66991854674224
a_HQ_transcript/145030	-1.32497280977117	3.88467405851438	-1.56324530740881	0.127655288282308	0.887813663229718	-4.94177506458079
a_HQ_transcript/9649	-0.918036968483831	2.20208682569083	-1.56304596580478	0.127702155994422	0.887947455411362	-4.78228101376787
a_HQ_transcript/142752	3.3363728429357	3.02668656958315	1.56303354308237	0.127705077195826	0.887947455411362	-4.90338060517525
a_HQ_transcript/70900	2.22372813303748	1.15670203581274	1.56294349986686	0.127726252470895	0.887988470712616	-4.6597026698087
a_HQ_transcript/62572	2.07072036758358	0.851348069155644	1.56285384773651	0.127747338614193	0.888028856609163	-4.74872496930735
a_HQ_transcript/61864	-3.16910778269829	1.69246554385398	-1.56278620653776	0.127763249670689	0.888033262807346	-4.6986219709265
a_HQ_transcript/149619	-1.58339568760132	-0.205579166641376	-1.56263350021527	0.12779917629706	0.888176771855031	-4.71310858984844
a_HQ_transcript/85529	0.673600542276899	2.78643455408107	1.56233150683803	0.127870249317982	0.888519508798573	-4.81456980368927
a_HQ_transcript/104179	-1.9188690385396	1.29474273211924	-1.56228936219445	0.127880170460578	0.888519508798573	-4.66643249568724
a_HQ_transcript/120741	4.40046502629074	1.62355263924459	1.56214921530637	0.127913166518941	0.888519508798573	-4.79744437371646
a_HQ_transcript/28860	-2.94036134916054	0.153182507244147	-1.56200320620023	0.127947550140957	0.888519508798573	-4.77503120558092
a_HQ_transcript/95986	2.07152445840782	0.977086206967642	1.56181365500322	0.127992198690094	0.888519508798573	-4.61111484873051
a_HQ_transcript/7603	1.92608397732255	0.01406718778328	1.56178533636732	0.127998870197969	0.888519508798573	-4.72451104706233
a_HQ_transcript/92259	1.71010093288975	0.197021232166895	1.56164168421423	0.128032717163413	0.888519508798573	-4.6187044627322
a_HQ_transcript/123530	-2.65814156381222	0.647398186186129	-1.5615898287354	0.128044937011929	0.888519508798573	-4.73855138437753
a_HQ_transcript/13014	4.44916690286784	0.129486159142363	1.56151190090159	0.12806330264819	0.888519508798573	-4.75853013842651
a_HQ_transcript/36472	-2.66770744826627	3.63123810851312	-1.56141066096164	0.128087165564082	0.888519508798573	-5.08149431094278
a_HQ_transcript/2992	-1.96241429310854	1.72523573222657	-1.56139886412756	0.128089946390336	0.888519508798573	-4.70078916280737
a_HQ_transcript/52988	-1.96813084047057	2.83530860387285	-1.56137591182049	0.128095356997806	0.888519508798573	-4.74591817336011
a_HQ_transcript/30670	-0.625796109901342	2.99869474429836	-1.56134702576916	0.128102166646618	0.888519508798573	-4.92583932867673
a_HQ_transcript/96184	-2.51515919365545	0.439221260588465	-1.56134349166449	0.128102999802923	0.888519508798573	-4.69343706232365
a_HQ_transcript/114184	-2.41022031559882	2.76077657870458	-1.56131600731218	0.128109479320444	0.888519508798573	-4.72202696079693
a_HQ_transcript/103384	2.0219284134589	0.790677950117539	1.56116925442273	0.128144081262146	0.888519508798573	-4.71677377738027
a_HQ_transcript/39885	-1.63613129262622	3.60029206837541	-1.56113988001064	0.128151008183398	0.888519508798573	-5.07567138851025
a_HQ_transcript/110872	0.550676527531985	3.70847320343463	1.56109642527105	0.128161256012982	0.888519508798573	-5.04709770113317
a_HQ_transcript/151744	-2.80196441772383	1.87607726890472	-1.56102419585126	0.128178291183132	0.888519508798573	-4.86041997841278
a_HQ_transcript/38623	-1.89146866631679	2.051893070401	-1.56095127282691	0.128195491807256	0.888519508798573	-4.74789699847095
a_HQ_transcript/44711	1.24814627513106	2.47057328569779	1.56085546202551	0.128218093910473	0.888519508798573	-4.73234766157229
a_HQ_transcript/142790	-0.520725860892376	2.90726238688884	-1.56085122828075	0.128219092740456	0.888519508798573	-4.89494322684417
a_HQ_transcript/139068	-2.61970392129217	1.43275374750605	-1.56075026542234	0.128242913890761	0.888519508798573	-4.82691067198904
a_HQ_transcript/89625	-0.742250200347354	5.28132495450083	-1.56064131851268	0.128268622834379	0.888519508798573	-5.23243711495932
a_HQ_transcript/37109	-2.45981582021188	1.06449676498224	-1.56055301024539	0.12828946461341	0.888519508798573	-4.77928651532543
a_HQ_transcript/134335	-0.902011219773498	2.00923774261476	-1.56048885026181	0.128304608844123	0.888519508798573	-4.68696221328843
a_HQ_transcript/83217	2.75435460379896	2.15079448066799	1.56044126981882	0.128315840602876	0.888519508798573	-4.76421052162355
a_HQ_transcript/146129	-1.93073618342388	0.642480332119093	-1.56033385505757	0.128341199690267	0.888519508798573	-4.82240721633121
a_HQ_transcript/14849	3.02890372383451	0.335930207568416	1.56029193455151	0.128351097627494	0.888519508798573	-4.80968014232501
a_HQ_transcript/47979	-1.90779723102521	-0.292669784699284	-1.56027190803118	0.128355826349715	0.888519508798573	-4.72504167525044

a_HQ_transcript/35508	-1.69811694154271	1.28874439544704	-1.56024101205549	0.128363121878382	0.888519508798573	-4.56956332832806
a_HQ_transcript/118150	2.52799354997029	0.734822297642397	1.56014019303401	0.128386930822133	0.888519508798573	-4.60556826183519
a_HQ_transcript/149691	1.18484732056317	3.60096589306861	1.56002542140802	0.128414039118966	0.888519508798573	-5.04975794745889
a_HQ_transcript/146811	-3.34750118220457	1.1546941461468	-1.55990582931724	0.128442290930365	0.888519508798573	-4.8292075076002
a_HQ_transcript/138122	-1.58316412447668	1.75338657108221	-1.55989779101483	0.12844419003828	0.888519508798573	-4.69358096514066
a_HQ_transcript/117768	-4.98640235627867	3.09263040709023	-1.55988408443343	0.128447428371362	0.888519508798573	-4.97309474495236
a_HQ_transcript/146746	-2.19798786662804	-0.124761115864323	-1.55983495687424	0.128459035851975	0.888519508798573	-4.78481994364898
a_HQ_transcript/113912	-1.68676912229903	-0.137829220770729	-1.55980100621135	0.128467057951136	0.888519508798573	-4.72414314230493
a_HQ_transcript/118107	-2.47171671281717	0.188455869193393	-1.55973567390375	0.128482496270388	0.888519508798573	-4.67394818057228
a_HQ_transcript/99846	-2.35000413681841	1.11635963041812	-1.55972153386604	0.128485837823762	0.888519508798573	-4.75239624315727
a_HQ_transcript/119943	-0.459646591352305	4.62578340821037	-1.5596342873697	0.128506457353864	0.888519508798573	-5.1603636640457
a_HQ_transcript/29094	1.79354753761136	0.632120278965259	1.55962956238122	0.128507574117841	0.888519508798573	-4.70723970242848
a_HQ_transcript/835	-2.61058160119079	1.17285419959405	-1.55953036858575	0.128531020665497	0.888519508798573	-4.76454131380824
a_HQ_transcript/115670	-2.81908510414349	3.44292521177721	-1.55951439832473	0.128534795898984	0.888519508798573	-5.11201510980431
a_HQ_transcript/104938	-2.79961090944447	-0.417304051783172	-1.55950785351749	0.128536343061643	0.888519508798573	-4.72709266073032
a_HQ_transcript/144878	1.40939218851183	3.71985551373187	1.55916510941804	0.128617387374923	0.888855308789035	-4.89736937143021
a_HQ_transcript/74281	1.53037665172521	2.21928107210851	1.55913957946472	0.128623425779024	0.888855308789035	-4.68974138050607
a_HQ_transcript/62048	2.47109779243694	1.16717661830729	1.55910842381068	0.128630795099322	0.888855308789035	-4.71134712377812
a_HQ_transcript/41757	-2.09413560712309	0.578887041887552	-1.55900569049247	0.128655097285999	0.888917567459947	-4.71497722439896
a_HQ_transcript/111018	0.629139776959079	2.90936076281591	1.55888361315229	0.128683980272156	0.88895587281782	-4.83313818083441
a_HQ_transcript/46284	2.55007374830377	-0.183323849560636	1.55885008383176	0.128691914091542	0.88895587281782	-4.74175373390545
a_HQ_transcript/27965	1.59526013579537	2.19659701015651	1.55873873602508	0.128718264433864	0.88895587281782	-4.82344920312553
a_HQ_transcript/29072	1.55027726331635	2.63673587648911	1.55871206982521	0.12872457561336	0.88895587281782	-4.87450690599089
a_HQ_transcript/67695	1.89815809813399	1.41739583734724	1.55864024294215	0.128741576375794	0.88895587281782	-4.60077038748402
a_HQ_transcript/136369	-1.80180155435759	0.792633352768934	-1.55853371079839	0.128766794916186	0.88895587281782	-4.70818855765953
a_HQ_transcript/59844	0.953548617402	5.22347591223744	1.55849359192473	0.128776292991698	0.88895587281782	-5.15188089749281
a_HQ_transcript/56744	1.71858745865853	-0.0409225522990093	1.55846532318398	0.128782985910385	0.88895587281782	-4.6746633836173
a_HQ_transcript/82206	2.06695781384653	-0.173134684342686	1.55832915222083	0.128815229766393	0.88900256985768	-4.71485889485282
a_HQ_transcript/120746	3.31964212954155	4.0432010167652	1.55830757906866	0.128820338664864	0.88900256985768	-5.09069410862183
a_HQ_transcript/97039	-1.07114075711997	4.34466860196544	-1.55807845219162	0.128874610070752	0.889223755460278	-5.11025728748461
a_HQ_transcript/116033	-1.96362647935996	0.753790333289235	-1.55803666899408	0.128884508918169	0.889223755460278	-4.62222106479597
a_HQ_transcript/62784	-2.34598229430944	0.538583663805872	-1.55797003499468	0.128900296442144	0.889223755460278	-4.6563821156888
a_HQ_transcript/50310	2.23698361323653	1.08318730231303	1.55789158236787	0.128918033072016	0.889223755460278	-4.6640403177582
a_HQ_transcript/55171	2.09917620026336	0.594450607126709	1.55782756956689	0.128934055901688	0.889223755460278	-4.73713234494315
a_HQ_transcript/137758	2.01261908828637	1.44205627731073	1.55778487052229	0.128944175522548	0.889223755460278	-4.658479696731
a_HQ_transcript/139390	3.91509082025819	2.27233013070547	1.55760326165351	0.128987223814176	0.889415106271698	-4.8189132558959
a_HQ_transcript/94171	0.979164310328929	2.49728929782011	1.55745406964321	0.129022596803523	0.889517617308455	-4.85076073193764
a_HQ_transcript/3830	2.04974923650158	1.98312909952497	1.55741148083494	0.129032695932165	0.889517617308455	-4.6993997998801
a_HQ_transcript/74395	2.62555322863174	2.20189202098139	1.55729404928121	0.129060545928147	0.889604104554886	-4.79559024335087
a_HQ_transcript/152894	3.13982564010208	5.94684830194602	1.557024100584	0.129124585324526	0.889888910527204	-5.12656149356144
a_HQ_transcript/134024	0.975029109906435	2.93551809655586	1.55693554975138	0.129145597688893	0.889888910527204	-4.89020429104438
a_HQ_transcript/105501	5.87196038632014	5.92466837426912	1.55692630340806	0.129147791928287	0.889888910527204	-5.13233439260118
a_HQ_transcript/856	-2.63957328652792	-0.257917826355271	-1.55665875117441	0.129211297575596	0.890032419963967	-4.72274065881086
a_HQ_transcript/37484	-0.53072649936674	0.176600139320739	-1.55657026182479	0.129232306810109	0.890032419963967	-4.78272136950361
a_HQ_transcript/52513	-2.45135484009208	0.780880050253032	-1.55654741962598	0.129237730479823	0.890032419963967	-4.7838099951118
a_HQ_transcript/128808	-1.63849514981418	1.50822557864057	-1.556546269307	0.129238003617361	0.890032419963967	-4.72553689022517
a_HQ_transcript/93366	-2.10962741778263	-0.0162606145136589	-1.55651605870885	0.129245177142097	0.890032419963967	-4.63316641198142
a_HQ_transcript/5222	1.88396331716887	3.73341035479188	1.5560277997015	0.129361159425096	0.89059850249799	-4.83281881013594
a_HQ_transcript/90231	1.91246747432215	0.627213153489634	1.55602493999801	0.129361838975391	0.89059850249799	-4.64008432576165
a_HQ_transcript/86268	2.29956629115599	1.69880147724731	1.55593616821224	0.129382935225244	0.89059850249799	-4.64958527304037

a_HQ_transcript/87074	2.9423114074978	4.91500390574573	1.55591205719096	0.129388665593097	0.89059850249799	-5.19553534767409
a_HQ_transcript/64078	1.52338929910947	2.43418195260805	1.55567579008049	0.129444829145006	0.890879591349856	-4.88064672740192
a_HQ_transcript/7548	-2.31149201914006	0.122477850104119	-1.55540331009143	0.129509625532973	0.891220020478352	-4.69502827166952
a_HQ_transcript/112125	-1.43532833157363	2.04058395893588	-1.55515657144603	0.129568323305007	0.891304725409007	-4.70966387888353
a_HQ_transcript/115420	-4.30570281645704	2.80397684453284	-1.55514169688204	0.129571862573033	0.891304725409007	-4.90398191167001
a_HQ_transcript/149218	-0.467661381713359	5.81966654845799	-1.5550371365664	0.129596743971833	0.891304725409007	-5.28564404289984
a_HQ_transcript/19774	-1.45150642974756	2.15094661844966	-1.55498202485512	0.129609860036096	0.891304725409007	-4.84683819073786
a_HQ_transcript/79718	1.53547845978008	2.22741334959063	1.55492178402519	0.129624198015685	0.891304725409007	-4.75926477645166
a_HQ_transcript/23114	1.78045289049998	1.1595119368012	1.55491504786849	0.12962580137512	0.891304725409007	-4.72562711067795
a_HQ_transcript/145058	-2.36806814980099	0.675384766812415	-1.55490047963555	0.129629269003023	0.891304725409007	-4.67945212632268
a_HQ_transcript/54866	-1.63008313302023	-0.283049544439868	-1.55468347856478	0.129680929967393	0.89133141880357	-4.76360919452574
a_HQ_transcript/151705	5.24038219257027	0.337660803774755	1.55464758098788	0.129689477636245	0.89133141880357	-4.85305763648534
a_HQ_transcript/80169	3.1062814926066	1.80181033778322	1.55458993550689	0.129703204714898	0.89133141880357	-4.70315406772293
a_HQ_transcript/22958	1.48091700853515	3.07574190606032	1.55457583219597	0.129706563306331	0.89133141880357	-4.96228103736058
a_HQ_transcript/117895	-1.7714957742531	0.219751196219486	-1.55456215310347	0.129709820941016	0.89133141880357	-4.68493730187337
a_HQ_transcript/117391	-2.35044400087482	0.656403173556134	-1.55448308244989	0.129728652681688	0.891355451900846	-4.64309319970855
a_HQ_transcript/119857	-0.876236922203343	4.53781041057526	-1.55430739489266	0.129770502989307	0.891537619402253	-5.13610675193586
a_HQ_transcript/50067	1.22054689063524	2.90278612434882	1.55396640381819	0.129851761337924	0.891990449426946	-4.80869767094312
a_HQ_transcript/53209	-2.18382221313835	0.283958853934879	-1.55368577194431	0.129918667059534	0.892269943764317	-4.61608165700613
a_HQ_transcript/87884	-1.8980533395617	4.58872241815586	-1.55366697699633	0.129923148981922	0.892269943764317	-5.18530057323677
a_HQ_transcript/52631	-1.13107413122476	2.29349693682178	-1.55340206920562	0.129986333369556	0.892461286951969	-4.76729706860825
a_HQ_transcript/7655	-0.871076554006566	2.5402357101061	-1.55338199336416	0.129991122768979	0.892461286951969	-4.84356467246091
a_HQ_transcript/7049	0.921977720534016	2.82400073306975	1.55335706276506	0.1299970705447	0.892461286951969	-4.96094810354363
a_HQ_transcript/105577	-2.15000735490035	1.53472079778822	-1.55299983362078	0.13008232016451	0.892536789904484	-4.78898135586711
a_HQ_transcript/49066	1.51318865704186	2.39834363425322	1.55299822735093	0.130082703589316	0.892536789904484	-4.67827204645386
a_HQ_transcript/19518	-2.94502828099752	1.09555371414691	-1.55291727869209	0.130102027635786	0.892536789904484	-4.76918275028529
a_HQ_transcript/23048	-1.78085881449447	2.64610807893644	-1.55288341814203	0.130110111510574	0.892536789904484	-4.82469745488806
a_HQ_transcript/15630	2.28007026277723	1.60303129927746	1.55283602649671	0.130121426486892	0.892536789904484	-4.85752598616312
a_HQ_transcript/131411	3.66268451688021	0.755927417894936	1.5527193585205	0.130149284915521	0.892536789904484	-4.76410965134147
a_HQ_transcript/69122	-1.55568366585127	2.94520969431091	-1.55271506349005	0.130150310591867	0.892536789904484	-4.93050344427053
a_HQ_transcript/124213	2.56108844800953	2.2746019602541	1.5526455520306	0.130166911214665	0.892536789904484	-4.76786194046712
a_HQ_transcript/90303	2.04090596051616	0.430349244946234	1.55262389014539	0.130172084825114	0.892536789904484	-4.63086134833673
a_HQ_transcript/16906	2.5798862813709	3.00844633784792	1.5525562809864	0.130188233314148	0.892536789904484	-4.94390201510776
a_HQ_transcript/139500	-3.04849228571271	-0.113746427469561	-1.55253245890441	0.13019392362061	0.892536789904484	-4.74906301705609
a_HQ_transcript/77561	1.804464109352	-0.335154917655406	1.55248238307528	0.130205885736335	0.892536789904484	-4.72835237916644
a_HQ_transcript/130740	-1.98440242116338	0.57395561328663	-1.5524748754633	0.130207679231868	0.892536789904484	-4.6052842791312
a_HQ_transcript/150078	-1.72269914564905	0.733725428151407	-1.55236905991479	0.130232959672067	0.892604820164827	-4.77528429170636
a_HQ_transcript/58660	-2.02345070645836	0.897120933562607	-1.55224993525232	0.130261424567878	0.892694657779017	-4.82684190880227
a_HQ_transcript/43447	-1.12171906335361	-0.772481279214916	-1.55176563856327	0.130377199520708	0.893021642185254	-4.75151290354331
a_HQ_transcript/17050	0.873329724348415	5.07132632845341	1.55176031305342	0.130378473090225	0.893021642185254	-5.21538338344265
a_HQ_transcript/80057	2.29640499920866	0.239695850855608	1.55169976682446	0.130392953133687	0.893021642185254	-4.69365964297018
a_HQ_transcript/81741	1.90450197040717	0.320254508648731	1.55164411717361	0.130406263278708	0.893021642185254	-4.75925605480392
a_HQ_transcript/39090	1.19102369976117	2.44934018517624	1.55162886956766	0.130409910355687	0.893021642185254	-4.71212932048392
a_HQ_transcript/7401	1.5677238361964	1.10010566283008	1.55160913445425	0.130414630923161	0.893021642185254	-4.65397741045763
a_HQ_transcript/83241	1.91591354189513	-0.394979587680414	1.55155496592195	0.130427588552434	0.893021642185254	-4.76129888379171
a_HQ_transcript/122417	-1.49169021807274	0.584906377550206	-1.55151466207081	0.13043723029601	0.893021642185254	-4.64028981851436
a_HQ_transcript/92313	2.11554971604747	0.957086425799921	1.55147213256463	0.130447405102446	0.893021642185254	-4.78294390771041
a_HQ_transcript/84029	2.01149635581816	-0.570153445637739	1.55116478319098	0.130520954865936	0.893226385325798	-4.73868993994628
a_HQ_transcript/22333	1.24530532819281	0.75005034393725	1.55116368565131	0.130521217571273	0.893226385325798	-4.72829685072471
a_HQ_transcript/123325	3.29653676827653	1.77265892056229	1.55112010304787	0.130531649780396	0.893226385325798	-4.87613688932609

a_HQ_transcript/98799	2.07044847114042	0.0168100945128375	1.55109032112895	0.130538778959239	0.893226385325798	-4.7692007650821
a_HQ_transcript/137455	-1.21740828720897	3.70644393875956	-1.55090169403024	0.130583939748648	0.89323470712476	-5.05597030734422
a_HQ_transcript/49472	-1.41007978472007	0.344676489878873	-1.55089435637641	0.130585696773907	0.89323470712476	-4.63456289350546
a_HQ_transcript/67764	-3.61562548174308	1.28723303096227	-1.55089269253439	0.130586095189	0.89323470712476	-4.95578973735407
a_HQ_transcript/139121	1.9512497219246	2.63654396901188	1.55063413194541	0.130648020777106	0.893495500325555	-4.88132312260567
a_HQ_transcript/128461	-2.45531298349735	0.146272905332206	-1.55060514742333	0.130654964092472	0.893495500325555	-4.85990587935444
a_HQ_transcript/7930	2.63925300648807	3.39205623118882	1.55046651214674	0.130688178665539	0.893544409983097	-4.93984575715967
a_HQ_transcript/104823	2.2891643246334	0.991056352575901	1.55044697434243	0.130692860130682	0.893544409983097	-4.68239563770616
a_HQ_transcript/98415	-1.75782601051717	0.428416889016993	-1.5503210633298	0.130723033010251	0.893623893672254	-4.71237953223725
a_HQ_transcript/83963	-2.92182379517746	0.633235425962697	-1.55024853457487	0.130740416116705	0.893623893672254	-4.72142428549617
a_HQ_transcript/70503	1.16029276623917	4.45465496279268	1.55017720210073	0.130757514338303	0.893623893672254	-5.13909662165634
a_HQ_transcript/119596	2.41766407232878	0.244939411465218	1.55006348461005	0.130784775898969	0.893623893672254	-4.74892196739158
a_HQ_transcript/141615	2.39905480245468	0.685691842240189	1.55002411605668	0.130794214820279	0.893623893672254	-4.71209236009333
a_HQ_transcript/130818	3.85505166976268	1.30753931694096	1.5499457395989	0.130813007840576	0.893623893672254	-4.66780495076127
a_HQ_transcript/123786	1.73086311065823	0.0770005430567046	1.54994098561224	0.130814147816731	0.893623893672254	-4.69345657455483
a_HQ_transcript/87280	0.692665786384526	3.76373537879897	1.5498441269967	0.130837375660401	0.893623893672254	-4.98464038674404
a_HQ_transcript/35359	0.887608130965973	4.52919715929073	1.5498198215689	0.130843204915351	0.893623893672254	-5.01986662601364
a_HQ_transcript/47452	-3.69805355600986	2.27844905256097	-1.54975722141681	0.130858219497286	0.893623893672254	-4.73676147894827
a_HQ_transcript/44572	-1.48973153421038	-0.233644890237043	-1.54967695127078	0.130877474255175	0.893650396276851	-4.67678163591122
a_HQ_transcript/56373	-0.665733871305879	4.35884139522829	-1.54942586861568	0.130937717416945	0.893789073814707	-5.06939103994121
a_HQ_transcript/139510	-1.92590428728978	0.81358306745207	-1.54931554220811	0.130964195543394	0.893789073814707	-4.64061539500403
a_HQ_transcript/110748	0.861126720840612	1.88142955475713	1.5493033634261	0.130967118694034	0.893789073814707	-4.66401759962477
a_HQ_transcript/99945	-2.55987204130706	1.68500875761334	-1.54927662329608	0.130973537044446	0.893789073814707	-4.73515949831928
a_HQ_transcript/129241	-1.46829440046393	1.62750734102875	-1.54927192385734	0.130974665062512	0.893789073814707	-4.81340144691962
a_HQ_transcript/997	1.96007494341827	1.98041399064732	1.5490023165423	0.131039392784354	0.893905200831748	-4.67600136845845
a_HQ_transcript/129070	-2.41779330852569	0.86254187422527	-1.54887146709569	0.13107081666759	0.893905200831748	-4.79989713318217
a_HQ_transcript/24480	1.47178859662416	-0.321265189671818	1.54870288198464	0.131111311896749	0.893905200831748	-4.67684730143732
a_HQ_transcript/130111	-2.6914328229209	0.855375755023548	-1.54864666869264	0.131124816948153	0.893905200831748	-4.76674630269906
a_HQ_transcript/148049	2.21058588587336	0.126914346788645	1.5486404360547	0.13112631438764	0.893905200831748	-4.79239807831388
a_HQ_transcript/141092	-1.47962425902659	3.4156225572682	-1.54849152857277	0.131162094688457	0.893905200831748	-5.06272506612101
a_HQ_transcript/86213	-2.72963385671033	1.56637671024067	-1.54845597440171	0.131170639009792	0.893905200831748	-4.65720149948531
a_HQ_transcript/67434	-1.3350319001487	1.96287262141887	-1.54840045953894	0.131183981155644	0.893905200831748	-4.71050061302294
a_HQ_transcript/49520	-1.81228093784741	1.44584888309096	-1.54838409817576	0.131187913568434	0.893905200831748	-4.63694832074617
a_HQ_transcript/142636	2.18398192587787	0.329598103777389	1.54835767192678	0.131194265253393	0.893905200831748	-4.60628913374016
a_HQ_transcript/139888	0.521147741468728	5.85025734984081	1.5483267586827	0.131201695727384	0.893905200831748	-5.29037351818778
a_HQ_transcript/68370	-2.05243568015881	0.206339050810335	-1.54831302025825	0.131204998078738	0.893905200831748	-4.61025018585351
a_HQ_transcript/75006	-2.36386160704714	-0.0508493445833776	-1.54830949069902	0.131205846501665	0.893905200831748	-4.7263979922398
a_HQ_transcript/22672	-2.07149952567222	1.26980894663905	-1.54830250565706	0.131207525554396	0.893905200831748	-4.67049867959711
a_HQ_transcript/55451	-2.49416794554232	0.604694479429572	-1.54813099128551	0.131248759362763	0.893905200831748	-4.69228829098232
a_HQ_transcript/141062	-4.05602953269226	0.741081362314393	-1.54807019999234	0.13126337673448	0.893905200831748	-4.70193892061347
a_HQ_transcript/76945	3.25323711011542	3.13507991506643	1.54806630823522	0.131264312559202	0.893905200831748	-5.00039499700064
a_HQ_transcript/152709	-3.91083772234358	2.52362291417862	-1.54804893549954	0.131268490130398	0.893905200831748	-4.85471265847493
a_HQ_transcript/89154	3.3471049990829	2.34600322595342	1.54780939290557	0.131326103239269	0.893972048219094	-4.70623820123905
a_HQ_transcript/145181	1.68950209328801	-0.363911454725055	1.54774010088888	0.131342772695149	0.893972048219094	-4.79184645351894
a_HQ_transcript/127793	-2.37138257822434	0.488401353288689	-1.54767679338046	0.131358003969166	0.893972048219094	-4.81853995271462
a_HQ_transcript/148661	3.73593573032215	3.17736677452516	1.54760586004886	0.131375071656707	0.893972048219094	-4.94024665723266
a_HQ_transcript/138407	-2.10843349271569	3.71961188841607	-1.5475435544776	0.131390064852	0.893972048219094	-5.22496675455548
a_HQ_transcript/103060	-2.04244665585725	1.01049577022077	-1.54754148152051	0.131390563711676	0.893972048219094	-4.73796711800041
a_HQ_transcript/70762	2.50653104606854	0.396475680218804	1.54753800465414	0.131391400427296	0.893972048219094	-4.6700227496672
a_HQ_transcript/72026	1.45802880173733	-0.402747895908987	1.54740525517153	0.131423350118662	0.893972048219094	-4.79432015293129

a_HQ_transcript/25174	2.1639488029858	-0.198754437277795	1.54739343052721	0.131426196339273	0.893972048219094	-4.74336112916402
a_HQ_transcript/135174	2.025210739104	0.37110545715957	1.54730079236296	0.131448496300852	0.893972048219094	-4.66603897136491
a_HQ_transcript/72968	-2.74168769845052	1.30716845742642	-1.54725589335466	0.131459305544169	0.893972048219094	-4.85192027324956
a_HQ_transcript/28437	-1.78763613936474	-0.466459348557252	-1.54724113378809	0.131462859003868	0.893972048219094	-4.747429240706
a_HQ_transcript/152002	-4.18430379826156	0.465277577698674	-1.54686833115715	0.131552639454236	0.894257141384249	-4.77523437812677
a_HQ_transcript/133232	-3.62799574172182	1.84041998132949	-1.54681261923798	0.131566060585971	0.894257141384249	-4.83765688361792
a_HQ_transcript/15452	0.858932512832119	2.43147095418884	1.54678479611176	0.131572763658014	0.894257141384249	-4.84446370349792
a_HQ_transcript/16289	-3.29176699139044	0.561012981068761	-1.54663940513282	0.131607795363778	0.894257141384249	-4.74060768100476
a_HQ_transcript/64744	3.59873817756033	2.77499867156176	1.54656550713571	0.131625603858367	0.894257141384249	-4.83670881439602
a_HQ_transcript/81306	-2.3892282935672	0.807059429965735	-1.54656249051821	0.131626330867035	0.894257141384249	-4.6359286511607
a_HQ_transcript/127322	-2.16783031523775	1.77871476724336	-1.54652714241671	0.131634850047311	0.894257141384249	-4.64471809621242
a_HQ_transcript/132099	-3.81802109820426	0.0592994438179858	-1.54646840466391	0.13164900731296	0.894257141384249	-4.78646095621971
a_HQ_transcript/26841	-1.88165539770179	3.81517758920621	-1.54637416469336	0.131671724079967	0.894257141384249	-5.0399886119567
a_HQ_transcript/14662	-2.47530690273889	1.74548562247953	-1.54636968006742	0.131672805188858	0.894257141384249	-4.61890757380929
a_HQ_transcript/10105	-1.45208955350706	2.0044600132999	-1.54636468092346	0.131674010341106	0.894257141384249	-4.63954580366976
a_HQ_transcript/91946	1.45735563261543	1.98866110984088	1.54625694460561	0.131699984694804	0.894272212955874	-4.85433189793409
a_HQ_transcript/114592	-0.982424591643782	3.98099841836153	-1.54622785527808	0.131706998608506	0.894272212955874	-5.03739262247926
a_HQ_transcript/83305	2.18657088437739	1.3878772127784	1.5461619188745	0.131722898077818	0.894275708884301	-4.78352654504462
a_HQ_transcript/112388	-0.441987370863139	3.9880154559337	-1.54601561979137	0.131758181231914	0.894410785781703	-5.10143780895157
a_HQ_transcript/130273	2.05168827818551	0.779732970718434	1.54592863274733	0.131779163651867	0.894421973650438	-4.68425181832835
a_HQ_transcript/84991	2.8754968227677	1.05258324833154	1.54588121088705	0.131790603569984	0.894421973650438	-4.67715603581444
a_HQ_transcript/72504	1.7951726898517	1.33250621845276	1.54572039384212	0.131829404624728	0.894513407755339	-4.75457373096389
a_HQ_transcript/63097	-0.648744356152657	2.95068771647399	-1.54568268507388	0.131838504130167	0.894513407755339	-4.91919016252832
a_HQ_transcript/91821	-1.96175617234937	1.01607011870786	-1.54553529479522	0.131874075774512	0.894513407755339	-4.81600354466509
a_HQ_transcript/111282	2.23574476191818	2.12662086021042	1.54550778848293	0.131880715098127	0.894513407755339	-4.87994622417556
a_HQ_transcript/123455	-1.61728427863136	-0.0978609898894483	-1.54544699040633	0.131895391167875	0.894513407755339	-4.69221919018927
a_HQ_transcript/54168	-1.89866293008864	-0.324519977512031	-1.54544277723963	0.131896408234775	0.894513407755339	-4.76030640307106
a_HQ_transcript/6013	1.81436762669214	0.372243640911743	1.54534809755098	0.131919265779002	0.894547558257479	-4.6597141897333
a_HQ_transcript/20075	-1.08067136045837	2.75974108030686	-1.54525714575637	0.131941226360729	0.894547558257479	-4.88749974299538
a_HQ_transcript/22446	-0.593849276609675	5.19957451761121	-1.54523070318075	0.131947611555526	0.894547558257479	-5.25029911937414
a_HQ_transcript/139381	3.84702181985214	4.12155428985189	1.54492297108381	0.132021939285805	0.894947088840265	-5.09136544868402
a_HQ_transcript/13166	-0.973894115456496	2.6928299341246	-1.54482083483562	0.132046616153125	0.895009993440865	-4.76292759915235
a_HQ_transcript/133086	2.37280670271056	0.395675630299121	1.5446667038791	0.132083862403339	0.895149951482677	-4.82644788960459
a_HQ_transcript/107530	2.02886817594485	0.977381550667329	1.54460794327691	0.132098064337641	0.895149951482677	-4.74921549813138
a_HQ_transcript/48455	-3.0027864598728	0.187552588207781	-1.54423034531952	0.13218935607106	0.89532058218416	-4.72038775274561
a_HQ_transcript/93310	4.91457483366135	3.74574077152344	1.54416375987767	0.132205459714817	0.89532058218416	-5.07660794059612
a_HQ_transcript/133392	2.22078369155636	2.67818299570582	1.54410931411506	0.13221862856723	0.89532058218416	-4.88457753124665
a_HQ_transcript/4923	-1.27965051068618	1.57224240378742	-1.54409513693592	0.132222057790568	0.89532058218416	-4.72502986334878
a_HQ_transcript/75618	0.849217356803431	2.58052482175018	1.54405967653078	0.132230635387237	0.89532058218416	-4.78896573334265
a_HQ_transcript/437	1.77292309806618	-0.0860963111790064	1.54397394627282	0.132251374734524	0.89532058218416	-4.74316766129001
a_HQ_transcript/53538	3.93867591101081	2.69721343950039	1.54396816548369	0.132252773283079	0.89532058218416	-5.01155274441214
a_HQ_transcript/24546	-2.20246395229939	0.158224702601487	-1.543966961583	0.132253064544747	0.89532058218416	-4.68005723689619
a_HQ_transcript/43885	-5.00343499426697	1.87746309038145	-1.54374532668087	0.132306693899942	0.89532058218416	-4.89815895043241
a_HQ_transcript/19273	-2.04490182480408	2.00033047799763	-1.54366708002944	0.132325631578257	0.89532058218416	-4.88700124480627
a_HQ_transcript/21509	0.490909235201986	3.29438367897563	1.54364982937234	0.132329806971287	0.89532058218416	-5.01225285209647
a_HQ_transcript/54088	2.63718661983859	2.07634883337338	1.5436170457114	0.132337742306494	0.89532058218416	-4.75281464010133
a_HQ_transcript/131937	-5.39916426251811	4.83940204495105	-1.54356915995986	0.132349333818944	0.89532058218416	-4.95344117786693
a_HQ_transcript/5901	1.52602160738433	-0.632437662224519	1.54354040819125	0.132356294040028	0.89532058218416	-4.74835066973067
a_HQ_transcript/69006	-3.24502591492875	0.820720126032477	-1.54352506170487	0.132360009235125	0.89532058218416	-4.68414866258176
a_HQ_transcript/58676	-2.0023909500963	-0.0922694644145703	-1.54340310126042	0.132389537359845	0.89532058218416	-4.74867774799263

a_HQ_transcript/386	-0.690281726306684	2.87322182597542	-1.5433837620663	0.132394220107182	0.89532058218416	-4.87792985619642
a_HQ_transcript/29182	3.4561448929366	0.361740317687581	1.54335786605163	0.132400490717985	0.89532058218416	-4.75173353382463
a_HQ_transcript/91469	0.428512301251652	4.16559467864873	1.54327083844924	0.132421565848936	0.895348361598525	-5.13680584723049
a_HQ_transcript/138346	-0.598222517166148	3.41881729883972	-1.54321369781939	0.132435404848337	0.895348361598525	-5.00845874299279
a_HQ_transcript/77166	1.77057637330228	0.48548662394336	1.54311434599974	0.132459469848136	0.895406915145067	-4.65303145564805
a_HQ_transcript/136110	-1.88773316256673	0.0140849919280341	-1.54298200164082	0.132491531806219	0.895519507073477	-4.77456670518563
a_HQ_transcript/20531	2.32964333650381	0.572680882302538	1.54289037624071	0.132513732807946	0.895562018132822	-4.78457156306849
a_HQ_transcript/106692	-2.26595337794314	0.210270834327766	-1.54282888170557	0.132528634736763	0.895562018132822	-4.78331867926807
a_HQ_transcript/152459	2.14207540673152	0.654107926043731	1.54251448412143	0.132604843711798	0.89597284148313	-4.70723803160886
a_HQ_transcript/105453	-2.29614844473793	0.483265637659881	-1.54238370027959	0.132636555756535	0.89601984970078	-4.77418571012136
a_HQ_transcript/12497	0.536483072666943	3.18659211359676	1.54235865163392	0.13264263017264	0.89601984970078	-4.94491088223108
a_HQ_transcript/68755	-1.34236009065745	1.03631360317218	-1.54213875747015	0.132695965233086	0.896242291320862	-4.66122536929995
a_HQ_transcript/56918	-2.43095519927338	-0.113592155376505	-1.54203803594465	0.132720400918058	0.896242291320862	-4.75434771666
a_HQ_transcript/82313	2.21603239132255	0.369196012103878	1.54199100249433	0.132731812782073	0.896242291320862	-4.75461161473932
a_HQ_transcript/128701	3.66763386445434	1.05812472021135	1.54196866403722	0.132737233105927	0.896242291320862	-4.77333768108035
a_HQ_transcript/90555	2.76442844069019	0.197362350507062	1.54189160980243	0.132755931337864	0.89624252972605	-4.80029597131429
a_HQ_transcript/34549	1.75161747813536	-0.250056683629826	1.54184144649819	0.132768105275788	0.89624252972605	-4.71959838566374
a_HQ_transcript/355	-2.96391488514911	0.824051378211227	-1.54146011455369	0.132860678823854	0.896747360991074	-4.81248415028365
a_HQ_transcript/35663	1.82404728659666	2.47734171279989	1.54140631134187	0.132873744498626	0.896747360991074	-4.8562857342463
a_HQ_transcript/118011	-0.808069410207051	3.43506076441939	-1.54121099501969	0.132921184236508	0.896836442307921	-5.0091252311629
a_HQ_transcript/26700	2.31420279002931	0.643463563509442	1.54084235012436	0.133010760569907	0.896836442307921	-4.59404779552019
a_HQ_transcript/142831	2.53436537264701	0.0247773685450287	1.54083118141684	0.133013475196283	0.896836442307921	-4.81216572479534
a_HQ_transcript/68272	-1.44394923188329	3.02505413216115	-1.54078907024732	0.133023710991192	0.896836442307921	-4.84817261128853
a_HQ_transcript/117694	2.72183739925376	0.452218676332012	1.54074836537084	0.133033605570485	0.896836442307921	-4.73681283897337
a_HQ_transcript/129745	-2.06211923997397	0.708647610748624	-1.54073025601034	0.133038007802324	0.896836442307921	-4.65349272170898
a_HQ_transcript/140289	2.28618403186362	-0.366970378521874	1.54070933708316	0.133043093162862	0.896836442307921	-4.7355017770787
a_HQ_transcript/26911	1.18645534408704	2.53473900847347	1.54066854059764	0.133053011181693	0.896836442307921	-4.83448132369151
a_HQ_transcript/125157	-0.671492917892398	4.19226204574851	-1.54056448337478	0.133078311207522	0.896836442307921	-5.11131741665115
a_HQ_transcript/126480	2.75012278827432	0.317144968067302	1.54054601272834	0.133082802488318	0.896836442307921	-4.77789688168176
a_HQ_transcript/53896	-1.92099873986499	2.62177507774845	-1.54045895490819	0.133103972925012	0.896836442307921	-4.86061147654411
a_HQ_transcript/54111	-2.08863275092227	1.47336997385508	-1.54043669997138	0.133109385245724	0.896836442307921	-4.71864485083168
a_HQ_transcript/35200	1.79137015851804	-0.0355244392464686	1.54037392048014	0.133124653953268	0.896836442307921	-4.6905311456889
a_HQ_transcript/65902	-1.90774328987323	1.56564724603675	-1.54035729435435	0.133128697859383	0.896836442307921	-4.71733994893578
a_HQ_transcript/68347	-1.00940165321151	3.05147123275718	-1.54033944556269	0.133133039260374	0.896836442307921	-5.00285266927116
a_HQ_transcript/114574	0.691814018245914	2.87079055315171	1.54024221483905	0.13315669091775	0.896836442307921	-4.85421272622131
a_HQ_transcript/49492	2.95468405837393	0.616527736989008	1.54020205341959	0.133166461293441	0.896836442307921	-4.75956903808505
a_HQ_transcript/29069	1.90045948635581	0.292936455431092	1.53985647120479	0.133250557719402	0.896836442307921	-4.69778321937309
a_HQ_transcript/115210	-2.25726731172617	0.177106683093261	-1.53983461633591	0.13325587748389	0.896836442307921	-4.75756384882681
a_HQ_transcript/64946	-2.09622670545912	2.01489415828827	-1.53970079576767	0.133288454933513	0.896836442307921	-4.84546096983064
a_HQ_transcript/52652	2.79036694375362	0.66734951007453	1.53969283038559	0.133290394239649	0.896836442307921	-4.79174905446365
a_HQ_transcript/21198	-1.98694927218467	-0.164509774791029	-1.53961411101984	0.133309561021099	0.896836442307921	-4.75885020792907
a_HQ_transcript/47645	0.943081370323357	3.9102983769396	1.53946648513693	0.133345511345872	0.896836442307921	-5.07646915409916
a_HQ_transcript/52892	-1.85701536863495	0.467639143503414	-1.53945397855948	0.133348557348128	0.896836442307921	-4.64019961957745
a_HQ_transcript/134783	-2.70708164206576	1.98287264276559	-1.53940526132045	0.133360423067563	0.896836442307921	-4.87003914884764
a_HQ_transcript/174	1.69697249765774	4.28593451715157	1.53920998286463	0.133407994267438	0.896836442307921	-5.01595143019177
a_HQ_transcript/39210	-2.47837187762598	1.57286623656718	-1.53916315602983	0.133419403654874	0.896836442307921	-4.71397840607769
a_HQ_transcript/14949	1.18164947624007	2.12037992944408	1.53907288524535	0.133441400417499	0.896836442307921	-4.82756109581928
a_HQ_transcript/69937	-2.37794709666235	0.419372431305169	-1.53906632087847	0.133443000106259	0.896836442307921	-4.72924743145954
a_HQ_transcript/40724	2.05015015212848	-0.0177663621051401	1.53906086250767	0.133444330283592	0.896836442307921	-4.76438100075795
a_HQ_transcript/18292	2.17235576899788	3.28948834915637	1.53905022588909	0.133446922404546	0.896836442307921	-4.99126529332389

a_HQ_transcript/62455	3.32764495885204	1.26647900777304	1.53904464695154	0.133448281995865	0.896836442307921	-4.70237822316834
a_HQ_transcript/53181	-1.06975794844752	2.42954868979849	-1.53903083533666	0.133451647945167	0.896836442307921	-4.90948743073978
a_HQ_transcript/148646	-1.9130488877642	3.10136987816714	-1.53902842255899	0.133452235956402	0.896836442307921	-4.87019108384917
a_HQ_transcript/137644	3.48814396604188	1.78455218783669	1.53898237782064	0.133463457791684	0.896836442307921	-4.95665500566317
a_HQ_transcript/118197	2.41125551244689	2.55200268269475	1.53887310885827	0.133490091434472	0.896836442307921	-4.97487726130151
a_HQ_transcript/93939	-0.827622745389512	2.19295572924008	-1.53875268933802	0.133519447942407	0.896836442307921	-4.70114724123122
a_HQ_transcript/118255	-2.19578604151421	0.536141251648712	-1.53868905999234	0.133534961951769	0.896836442307921	-4.70599314506481
a_HQ_transcript/49296	-3.05514211869934	0.30128816864833	-1.53867911110761	0.133537387805513	0.896836442307921	-4.81855123285124
a_HQ_transcript/70237	-2.47904625628648	1.17327428460709	-1.53862602808233	0.133550331734348	0.896836442307921	-4.64758055291655
a_HQ_transcript/77917	-2.13629922528732	0.296949127438041	-1.53862438689712	0.133550731942207	0.896836442307921	-4.74507794898561
a_HQ_transcript/112034	-2.97552963004749	4.30117076807174	-1.53860727944557	0.133554903702604	0.896836442307921	-5.09659462257257
a_HQ_transcript/126565	-1.79461524850959	0.186051356216734	-1.53857806400703	0.133562028316265	0.896836442307921	-4.7914662285478
a_HQ_transcript/127769	2.78026719543424	0.392705525347372	1.53856257997404	0.133565804449829	0.896836442307921	-4.82833144028805
a_HQ_transcript/102177	2.52353518792776	6.3587245196311	1.53836626958461	0.133613686698187	0.897054328989628	-5.44707998058834
a_HQ_transcript/118217	-1.71068446563901	-0.352490295491532	-1.53826647331044	0.13363803342744	0.897114171044028	-4.73985763780802
a_HQ_transcript/129302	2.37304698650495	0.233839137295849	1.5380677796018	0.133686518295883	0.897336020265947	-4.6761271203314
a_HQ_transcript/113629	0.508160867309625	3.57298115698901	1.53792973626803	0.13372021175631	0.897458546238401	-5.05817332844522
a_HQ_transcript/85724	-2.56996537893703	0.851230005550868	-1.53744428304331	0.133838755230662	0.897970366444905	-4.73941857347604
a_HQ_transcript/115874	-0.508600574816192	7.7249795492416	-1.53724867541121	0.133886544959123	0.897970366444905	-5.43818414506164
a_HQ_transcript/135272	-1.57069734072687	0.267069951494761	-1.5372236523255	0.133892659451494	0.897970366444905	-4.67066874608706
a_HQ_transcript/135687	0.58576941639958	3.55301572120173	1.5371666824419	0.133906581116469	0.897970366444905	-5.05162253451927
a_HQ_transcript/75602	1.19100384614797	2.19158847767254	1.53706605322936	0.133931174624096	0.897970366444905	-4.84341705726164
a_HQ_transcript/104422	-3.38815086503697	1.61473497997097	-1.53702152015288	0.133942059554598	0.897970366444905	-4.82911408857503
a_HQ_transcript/92175	2.3174646899578	2.09598973696247	1.53701461523343	0.133943747343306	0.897970366444905	-4.6714541242571
a_HQ_transcript/147017	0.554531461698362	4.83996920046611	1.53698008648139	0.133952187560694	0.897970366444905	-5.21095472608383
a_HQ_transcript/93999	-1.82958267506934	1.23797355566917	-1.53697144135541	0.13395430084498	0.897970366444905	-4.71389761419191
a_HQ_transcript/75859	2.18252896688625	-0.145649496971647	1.53696652226966	0.133955503318003	0.897970366444905	-4.73994147964212
a_HQ_transcript/126004	1.64151109594847	1.55998723997273	1.53692193932304	0.133966402040501	0.897970366444905	-4.83453189845381
a_HQ_transcript/105729	3.10798791882913	0.609759922474314	1.53675055610136	0.134008304970829	0.898086291900417	-4.71669749058945
a_HQ_transcript/13571	-1.33890825708047	-0.354580790068677	-1.53660762668293	0.13404325910261	0.898086291900417	-4.74539199643659
a_HQ_transcript/79353	0.697590353377218	2.49989431298388	1.53650630121758	0.134068043241038	0.898086291900417	-4.87197110663615
a_HQ_transcript/48697	1.97063794795169	1.38197626264564	1.53647501439437	0.134075696725599	0.898086291900417	-4.74480585797147
a_HQ_transcript/88857	0.390830480172361	4.67959995697953	1.53641825168201	0.134089583107823	0.898086291900417	-5.20455999470076
a_HQ_transcript/77158	1.12141989636232	2.47939099955213	1.5364180688708	0.134089627832483	0.898086291900417	-4.78469906407302
a_HQ_transcript/103246	1.78243220496979	1.12866774089828	1.5363983751149	0.134094445969636	0.898086291900417	-4.73823742340692
a_HQ_transcript/73728	1.61480190949513	0.888646413205813	1.53634584656737	0.134107297923473	0.898086291900417	-4.63282306403374
a_HQ_transcript/98211	2.18830840601159	1.00517571211054	1.53622453385942	0.134136982837803	0.898110462329943	-4.64790359649625
a_HQ_transcript/7737	-2.09050739922298	0.773065201505833	-1.53620481572236	0.134141808318686	0.898110462329943	-4.63018671259341
a_HQ_transcript/137123	0.911316249353128	4.1477319464006	1.53595110602145	0.134203909441391	0.898302915265819	-5.17460175852862
a_HQ_transcript/128227	1.35240623407852	2.0453676204138	1.53590405067017	0.134215429848103	0.898302915265819	-4.84684352694086
a_HQ_transcript/72476	1.30353198211115	2.67180687686222	1.53589798589963	0.13421691472412	0.898302915265819	-4.82841947037593
a_HQ_transcript/117613	-2.06909563697503	0.921076264732279	-1.535776549298	0.134246649611238	0.898398485908592	-4.75643151674955
a_HQ_transcript/91	1.47184154900192	4.17785586444477	1.53550637810865	0.134312822674954	0.898725695080818	-5.21238135826679
a_HQ_transcript/112721	4.26613529429775	0.0588886099802605	1.53545068694424	0.134326466399363	0.898725695080818	-4.86443973070391
a_HQ_transcript/45083	-2.78891358246887	0.0194655273636749	-1.53535158023907	0.134350749228579	0.898784710686939	-4.75144031049734
a_HQ_transcript/105567	-2.02313659432092	0.068430459414481	-1.53520260304961	0.134387257857365	0.898815631876159	-4.72757103187364
a_HQ_transcript/112288	-2.76817295523255	4.85127072863174	-1.53510068569681	0.134412238538266	0.898815631876159	-5.2703823885221
a_HQ_transcript/25830	1.38704355163228	1.72707245536127	1.53509837149486	0.134412805809535	0.898815631876159	-4.72479340873048
a_HQ_transcript/52640	1.5224801960534	0.224073755077186	1.53508035524448	0.134417222128741	0.898815631876159	-4.67880091278989
a_HQ_transcript/88031	-2.86994228490696	2.71215631303026	-1.53485579328444	0.134472278800998	0.899024471096363	-4.94513338164658

a_HQ_transcript/41427	2.68125096020825	0.272014144691746	1.53477414600209	0.134492301084165	0.899024471096363	-4.77777123078488
a_HQ_transcript/151551	-2.16719368005494	0.287511020510894	-1.53472558636022	0.134504210461391	0.899024471096363	-4.76676679788891
a_HQ_transcript/118402	2.28435126280236	1.03793263709327	1.53466853794682	0.134518202821604	0.899024471096363	-4.61991762778923
a_HQ_transcript/86456	2.07830242456416	0.0775811887141668	1.53435694600851	0.134594648282199	0.899024471096363	-4.80708856785622
a_HQ_transcript/51964	1.48235631985267	2.5242134540841	1.53433727145454	0.134599476384687	0.899024471096363	-4.92535024873547
a_HQ_transcript/45261	0.469454957501992	4.99709722520086	1.53433592207964	0.134599807524154	0.899024471096363	-5.23246959389229
a_HQ_transcript/125724	-3.54798283254792	2.8802748005837	-1.534320720617	0.134603538040914	0.899024471096363	-4.86273689708045
a_HQ_transcript/76218	-1.9527396363807	0.944384754293781	-1.5343107146324	0.134605993606408	0.899024471096363	-4.745806312434
a_HQ_transcript/2597	1.34325033261716	2.69631939813288	1.53424286900354	0.134622644536472	0.899024471096363	-4.81288944992766
a_HQ_transcript/85687	2.2290901721323	2.47651443885017	1.5342186852972	0.134628580195667	0.899024471096363	-4.81102014064512
a_HQ_transcript/58857	-2.05930019218133	0.138058256315642	-1.53419640301962	0.134634049354766	0.899024471096363	-4.68974465950544
a_HQ_transcript/25627	0.944986851389885	2.74620709012943	1.5340546497552	0.134668846737204	0.89910361539558	-4.80113204536785
a_HQ_transcript/22579	-1.16794959041284	2.47543159789268	-1.53396036918276	0.134691994621319	0.89910361539558	-4.79030218816273
a_HQ_transcript/40888	1.57026526497692	1.18249461070385	1.53395910666331	0.134692304618509	0.89910361539558	-4.64549792064307
a_HQ_transcript/144086	-2.22568606756429	0.141078343875732	-1.53386230198517	0.134716075617159	0.899159035879462	-4.79778488773145
a_HQ_transcript/82145	-2.51455577878537	-0.0672245663216132	-1.53356467478124	0.134789181107338	0.899246992657619	-4.65907860775594
a_HQ_transcript/86037	-1.24297549114494	2.3523751159576	-1.53353622868747	0.134796169935983	0.899246992657619	-4.71455179636157
a_HQ_transcript/91076	4.65138653382093	4.05596054745991	1.53343705829444	0.134820537080662	0.899246992657619	-4.90391593336069
a_HQ_transcript/136158	0.464492140458699	5.48972887110069	1.53342523750156	0.134823441803799	0.899246992657619	-5.28589873134962
a_HQ_transcript/115202	2.85456234727616	2.08909422480667	1.53342007961539	0.134824709266898	0.899246992657619	-4.89350539349485
a_HQ_transcript/151023	-1.45174024766048	2.91591540240912	-1.53332767854604	0.134847416896245	0.899246992657619	-4.89961105693531
a_HQ_transcript/2560	-0.821944894175875	2.19074190170351	-1.53326885306822	0.134861874911142	0.899246992657619	-4.76022295148419
a_HQ_transcript/68864	-2.11764620489513	2.05480616677532	-1.53325896279344	0.134864305847317	0.899246992657619	-4.73548718366365
a_HQ_transcript/46515	1.0676140394716	2.78624401007919	1.53324196121511	0.134868484757589	0.899246992657619	-4.75134297562559
a_HQ_transcript/138284	-1.69562158829695	0.604922566324384	-1.53307243069977	0.134910160312385	0.899417240518126	-4.67953762028138
a_HQ_transcript/130052	2.50699488234304	0.800414716295491	1.5329406270808	0.134942568689276	0.899417240518126	-4.64179295907426
a_HQ_transcript/23856	1.26525937220722	1.91372217734272	1.53289255525275	0.134954390340607	0.899417240518126	-4.82117188674789
a_HQ_transcript/126547	1.67267889405264	3.09378611903616	1.53288637345205	0.134955910607609	0.899417240518126	-5.00349976950147
a_HQ_transcript/144593	-0.942730382082428	4.30202449387303	-1.53274971367816	0.134989522369396	0.899538112608996	-5.16618041806438
a_HQ_transcript/39187	3.4259870024035	2.99698723167835	1.53257808200751	0.135031745132948	0.899573946673808	-4.92755167363415
a_HQ_transcript/93167	-2.47820012683513	0.899184089017597	-1.53250794725196	0.135049001917054	0.899573946673808	-4.88378399115577
a_HQ_transcript/82618	5.49006517890477	3.57343169307903	1.532495781444	0.135051995517714	0.899573946673808	-5.00491161212276
a_HQ_transcript/76657	1.33108101926046	4.93767340035128	1.53247624543978	0.135056802790778	0.899573946673808	-5.12530416152831
a_HQ_transcript/40081	1.86399004476143	-0.244651842250642	1.53219177432165	0.135126818983284	0.899738318967002	-4.71944396501982
a_HQ_transcript/150522	-4.69153716153533	6.43135670055883	-1.53182043195937	0.135218260744979	0.899738318967002	-5.23402218350877
a_HQ_transcript/25942	2.18332916869907	0.708022092289421	1.53180405523363	0.135222294608161	0.899738318967002	-4.72052371437594
a_HQ_transcript/52393	-1.54008577434125	0.272427466950051	-1.53179752045428	0.135223904261455	0.899738318967002	-4.77839429322577
a_HQ_transcript/3045	-0.7923786453883	5.25502632641612	-1.53178012971883	0.135228188040039	0.899738318967002	-5.24370839674353
a_HQ_transcript/138245	2.72471012475318	0.287381604578482	1.53173428912001	0.135239480266272	0.899738318967002	-4.84587240184209
a_HQ_transcript/152550	-0.244695493362234	11.1182790651046	-1.53172645841296	0.135241409333795	0.899738318967002	-5.88249402962347
a_HQ_transcript/95912	-2.07346075494716	0.351116030289387	-1.53159629113162	0.135273478850622	0.899738318967002	-4.66033266173697
a_HQ_transcript/128695	2.89918259213618	1.92569179196296	1.53152304926552	0.135291526266561	0.899738318967002	-4.95734850484897
a_HQ_transcript/46000	-2.00308846626576	0.604500260636121	-1.53140419781553	0.135320816412392	0.899738318967002	-4.74815933095586
a_HQ_transcript/57850	-1.30890295158278	2.50252409013383	-1.53131317024769	0.13534325301491	0.899738318967002	-4.78982033247048
a_HQ_transcript/142836	-2.19151970366641	-0.0106869008916845	-1.53129909066515	0.135346723638691	0.899738318967002	-4.77629983095735
a_HQ_transcript/47660	-0.676295630166654	2.70485054770752	-1.5312855120463	0.135350070842836	0.899738318967002	-4.8930370986823
a_HQ_transcript/132914	-1.18828205345501	4.6776494055095	-1.53120843056747	0.135369073119021	0.899738318967002	-5.21742646007984
a_HQ_transcript/36330	-1.71941754246369	2.01542600958707	-1.53115180197752	0.135383034683383	0.899738318967002	-4.86065235174963
a_HQ_transcript/111815	-0.797663203495166	4.66931044676028	-1.53106883099032	0.135403492968209	0.899738318967002	-5.20748137988721
a_HQ_transcript/99574	-1.95361994079239	0.628241766847581	-1.53104025443629	0.135410539711968	0.899738318967002	-4.76294541952502

a_HQ_transcript/132974	-0.710993686527307	3.73719206935877	-1.53102515402111	0.135414263470167	0.899738318967002	-5.06500092797408
a_HQ_transcript/149778	-2.3498690161132	0.547604391428203	-1.53098863486132	0.13542326942703	0.899738318967002	-4.89853227563518
a_HQ_transcript/147327	-2.28911592387927	0.663523048953136	-1.53093107055572	0.135437466291298	0.899738318967002	-4.74366837229067
a_HQ_transcript/85658	0.712725631354227	2.92020634396188	1.53092417953501	0.135439165877976	0.899738318967002	-4.94321287313008
a_HQ_transcript/77782	1.7993298042926	0.415030636655255	1.53091615696144	0.135441144570162	0.899738318967002	-4.63153563450003
a_HQ_transcript/18732	2.03631330606938	1.43605430543502	1.53080485958323	0.135468597430722	0.899738318967002	-4.84949247527173
a_HQ_transcript/151219	-0.739280644681352	3.50485329140379	-1.53079278985479	0.135471574847289	0.899738318967002	-5.0262382117646
a_HQ_transcript/52717	1.71462612617844	0.907271349748163	1.53076929896491	0.135477369840573	0.899738318967002	-4.68904368232772
a_HQ_transcript/59877	0.519318843230728	4.59162797399868	1.53068954014702	0.135497047125625	0.899738318967002	-5.19891453776605
a_HQ_transcript/79063	-1.8059481127942	0.741524046040948	-1.5306517990977	0.135506359018964	0.899738318967002	-4.65405079379934
a_HQ_transcript/121495	-3.56241283441685	2.30962462192341	-1.5305574181717	0.135529647999717	0.899738318967002	-4.76553394942012
a_HQ_transcript/43351	1.91087568716387	0.2253665763453	1.53055453607718	0.135530359222321	0.899738318967002	-4.75652236272482
a_HQ_transcript/119482	-1.85859935010231	0.284765296835224	-1.53038950458014	0.13557108954327	0.899802300218002	-4.66661188178988
a_HQ_transcript/149884	-1.40160793952009	-0.613410516879167	-1.53036590574202	0.135576914623237	0.899802300218002	-4.7838778196921
a_HQ_transcript/148592	4.53192147712068	6.6712568731393	1.53032630864994	0.135586689127901	0.899802300218002	-5.53154116329978
a_HQ_transcript/152760	-3.54364386653191	3.7283844634585	-1.53022896316587	0.135610721189699	0.899802300218002	-5.04292729184162
a_HQ_transcript/121019	1.4888931258483	1.78425967058925	1.53013525760774	0.135633857900593	0.899802300218002	-4.88814376948871
a_HQ_transcript/71200	2.30461989339313	2.3026491801184	1.53000197619654	0.135666771726972	0.899802300218002	-4.73491427054207
a_HQ_transcript/51946	2.39051975921203	-0.205892341771133	1.52994381230341	0.135681137315493	0.899802300218002	-4.76940923355213
a_HQ_transcript/58780	-0.995971304937801	0.865385227906271	-1.52990016949621	0.135691917226033	0.899802300218002	-4.87285714998491
a_HQ_transcript/79534	-1.39370669315436	-0.23199707250814	-1.52988181556357	0.135696450911828	0.899802300218002	-4.7163925691269
a_HQ_transcript/37326	-1.49453654546966	1.43646681776084	-1.52985120046682	0.135704013554219	0.899802300218002	-4.67649512478008
a_HQ_transcript/120077	2.19839801193942	1.0162997538876	1.52982586082628	0.135710273293614	0.899802300218002	-4.78068991036482
a_HQ_transcript/139880	1.95311765085319	0.833573585054389	1.52967299174107	0.135748042025062	0.899875174709197	-4.66090981580959
a_HQ_transcript/46923	-3.74107909549674	2.82720968346209	-1.52965605821986	0.135752226242516	0.899875174709197	-5.0562503518976
a_HQ_transcript/16313	2.00160106417549	2.77006935569572	1.52942597481328	0.135809089426022	0.900144599623065	-4.88634773255562
a_HQ_transcript/54075	-3.35044831841335	2.56555479605918	-1.52936629504679	0.135823841922893	0.900144599623065	-4.96232668939406
a_HQ_transcript/29973	-3.31424005681108	0.799384921710141	-1.52920593329204	0.135863488846064	0.900209070530332	-4.84199090490874
a_HQ_transcript/113711	-3.19171516022264	0.725941153776448	-1.52920166861318	0.135864543348517	0.900209070530332	-4.84058690340029
a_HQ_transcript/56061	-1.87395681939266	-0.369983295655863	-1.52899983835954	0.135914456310156	0.900350534442498	-4.78359911329658
a_HQ_transcript/40035	2.17627046376003	-0.0933839821929366	1.52896621540069	0.135922772764049	0.900350534442498	-4.75185802386023
a_HQ_transcript/147363	-2.10537173636855	2.75690284803654	-1.52885909924681	0.135949270096968	0.900350534442498	-5.05579421846407
a_HQ_transcript/137361	-3.12219692127446	1.88552970729385	-1.5288326398861	0.135955815994252	0.900350534442498	-4.92190115653048
a_HQ_transcript/49701	-1.43026798217226	1.79599919345939	-1.52874218513188	0.135978195916031	0.900350534442498	-4.82499659393555
a_HQ_transcript/148017	-0.873041093066611	2.54413840720135	-1.52855420926702	0.136024713606791	0.900350534442498	-4.90188615623882
a_HQ_transcript/35016	4.31864261821158	0.394667751315809	1.52849655278575	0.136038984218878	0.900350534442498	-4.77659681710708
a_HQ_transcript/99158	1.84633060456986	-0.0235974236577168	1.52847410606842	0.136044540354961	0.900350534442498	-4.72427620016395
a_HQ_transcript/19487	-2.05193575927451	1.05788257669529	-1.52839344865282	0.136064506639233	0.900350534442498	-4.70715832242035
a_HQ_transcript/22706	1.1612919694729	2.30270183313332	1.52838330893003	0.136067016837419	0.900350534442498	-4.7985864706297
a_HQ_transcript/70865	1.51821924061507	0.864669194587741	1.52834911950395	0.136075481075866	0.900350534442498	-4.72211857804165
a_HQ_transcript/115886	2.72726326264473	1.46501873853389	1.52833045537128	0.136080101914064	0.900350534442498	-4.77602807777244
a_HQ_transcript/24821	-1.93400800692906	0.13396749653646	-1.52825644875703	0.136098425609437	0.900350534442498	-4.7869572067556
a_HQ_transcript/145297	1.11739318719832	2.67027977386218	1.52810376363465	0.136136236038043	0.900350534442498	-4.73665956500372
a_HQ_transcript/13256	2.34309854491218	2.31278971419166	1.52806039803268	0.136146976496655	0.900350534442498	-4.84028615867712
a_HQ_transcript/15336	-2.0547155544341	0.784795143482524	-1.52805696328613	0.136147827217507	0.900350534442498	-4.7916931446765
a_HQ_transcript/66175	1.14905204580594	2.7622114237102	1.52793194303579	0.136178795258954	0.900350534442498	-4.87505594348445
a_HQ_transcript/19872	-1.27039533826723	0.91099140237208	-1.52788842311646	0.136189576660956	0.900350534442498	-4.60287719230164
a_HQ_transcript/27119	2.25226648678805	-0.439250164268803	1.52774780631723	0.136224417060239	0.900350534442498	-4.77904476765759
a_HQ_transcript/64996	2.28872979250752	0.887515475403709	1.52773542312961	0.136227485567726	0.900350534442498	-4.66764485742842
a_HQ_transcript/31052	1.83343795486333	0.452283796814242	1.52772882624286	0.136229120274334	0.900350534442498	-4.62424659805981

a_HQ_transcript/83581	-1.96064534023093	1.02514560497433	-1.527664914192	0.13624495848229	0.900350534442498	-4.74354526390148
a_HQ_transcript/24368	-0.989792453104744	2.7922816464707	-1.52759172610728	0.136263097234678	0.900350534442498	-4.84787924109245
a_HQ_transcript/137290	-1.23355141630397	3.49272830086382	-1.52754696978813	0.136274190490332	0.900350534442498	-5.02701843344219
a_HQ_transcript/77418	-2.18174490164623	-0.271991566696504	-1.52749994289718	0.136285847313532	0.900350534442498	-4.75938945497519
a_HQ_transcript/2009	-2.03362799084983	2.49509941989426	-1.52738919988117	0.136313300996991	0.900350534442498	-4.81801860123553
a_HQ_transcript/72754	4.68417769747896	2.45778678321369	1.52731998738765	0.136330461348447	0.900350534442498	-4.91680222285081
a_HQ_transcript/86851	0.693786458507038	4.43826039585982	1.52729666148971	0.136336245099252	0.900350534442498	-5.16363138599548
a_HQ_transcript/23901	-2.07691059060079	0.664144671009781	-1.52728610943542	0.136338861588929	0.900350534442498	-4.72062525139298
a_HQ_transcript/45906	-1.98054117168951	-0.401701884183757	-1.5272389048963	0.136350566933273	0.900350534442498	-4.77329890847378
a_HQ_transcript/72661	-1.04216808347276	3.0482499981909	-1.52703116787389	0.136402089282315	0.900506040968062	-5.03473677882873
a_HQ_transcript/106006	-2.05635099038917	3.43361107792808	-1.52682981108976	0.136452044228733	0.900506040968062	-4.96504561562646
a_HQ_transcript/16275	-0.939353520457291	1.73774983441762	-1.52681270927523	0.136456287727615	0.900506040968062	-4.71171522857004
a_HQ_transcript/65798	-1.58857838139576	-0.368559649278373	-1.52673489002668	0.136475598480363	0.900506040968062	-4.78724918074085
a_HQ_transcript/84455	-1.63667472067487	-0.511418592385738	-1.52663605186954	0.136500128231602	0.900506040968062	-4.78672714585074
a_HQ_transcript/146727	-2.20180530958553	0.345087854299635	-1.52652522897523	0.136527636601017	0.900506040968062	-4.79093923799964
a_HQ_transcript/18245	-2.03188624486479	2.30031306053013	-1.5264162434904	0.136554693256182	0.900506040968062	-4.81623967007449
a_HQ_transcript/141524	-1.98650532153397	-0.0905367146458994	-1.52638079165378	0.136563495436006	0.900506040968062	-4.76726554097503
a_HQ_transcript/21833	1.51669376521144	-0.364046981674108	1.52632238697674	0.136577997474321	0.900506040968062	-4.7438980805062
a_HQ_transcript/59779	0.904819720868206	3.53557060057598	1.52615120769461	0.136620508914355	0.900506040968062	-4.95578156612581
a_HQ_transcript/39431	-1.10746450314038	4.45607633738552	-1.52614316257425	0.136622507138933	0.900506040968062	-5.23220249382588
a_HQ_transcript/144279	1.31977993742558	1.06822498088786	1.52611399276312	0.136629752453253	0.900506040968062	-4.75927958988833
a_HQ_transcript/102387	-1.97155196381679	-0.00466661434291933	-1.52605131570518	0.136645321481083	0.900506040968062	-4.76099441506189
a_HQ_transcript/33523	2.1882718800646	0.615983994378151	1.52590807938147	0.136680906868334	0.900506040968062	-4.85398159400797
a_HQ_transcript/84959	2.18490673759068	2.12581007814557	1.52589564125745	0.136683997327668	0.900506040968062	-4.71902271133232
a_HQ_transcript/108949	-4.46327708998649	1.81167779000671	-1.52589304235846	0.136684643074589	0.900506040968062	-4.80213333130639
a_HQ_transcript/136576	-0.97093665007131	3.41737344944288	-1.52584679252132	0.136696135156718	0.900506040968062	-5.09844728070291
a_HQ_transcript/108842	-2.11089931500758	0.983092415143156	-1.52579021505782	0.136710194491111	0.900506040968062	-4.73168337174412
a_HQ_transcript/99039	2.64309910569691	0.841605982224129	1.52576451632129	0.136716580937668	0.900506040968062	-4.65504323257818
a_HQ_transcript/65821	-0.697896553053823	3.5110931239941	-1.52572068156648	0.136727474959803	0.900506040968062	-4.97801935696093
a_HQ_transcript/117197	2.15206481703724	0.840991018459057	1.52567439128931	0.136738980000954	0.900506040968062	-4.79856325429034
a_HQ_transcript/148266	-4.83261230881816	1.77279345595996	-1.52560594788544	0.136755992439149	0.900506040968062	-4.92135243765544
a_HQ_transcript/23131	2.41353108133255	1.34576731646455	1.52553996915586	0.136772393870398	0.900506040968062	-4.76712280077011
a_HQ_transcript/14557	1.98581866036623	0.63394390432472	1.52544502815254	0.136795997723146	0.900506040968062	-4.73122616739786
a_HQ_transcript/45843	-1.86445259126972	1.65700438920129	-1.52544036101522	0.13679715813298	0.900506040968062	-4.83382564101685
a_HQ_transcript/2660	-2.27255140329585	0.854618947522825	-1.52542032813367	0.136802139082204	0.900506040968062	-4.74780757423379
a_HQ_transcript/116085	-1.03192746710157	4.32319516939188	-1.52541728796122	0.136802894999465	0.900506040968062	-5.13360702906905
a_HQ_transcript/142440	3.90832505673851	2.52056140975403	1.52532508039152	0.136825823358301	0.900506040968062	-4.902138171499
a_HQ_transcript/109840	2.6449417460819	0.745567114270578	1.52524153647032	0.136846600090902	0.900506040968062	-4.77053563597435
a_HQ_transcript/145139	-3.12512013628432	1.75536123941604	-1.52520839706958	0.1368548423113	0.900506040968062	-4.86357451231035
a_HQ_transcript/61763	1.77994877453493	0.146558060790788	1.5251756125963	0.136862996650773	0.900506040968062	-4.73395394224985
a_HQ_transcript/99516	2.66294671874128	2.976934729635	1.52514804397925	0.136869853976618	0.900506040968062	-4.93281312924941
a_HQ_transcript/148504	-0.58626639736084	9.93605626803255	-1.52503570865005	0.136897798759635	0.900587963592132	-5.73923764868754
a_HQ_transcript/18552	1.39782622657514	-0.657177621738323	1.52450892644593	0.137028903707008	0.901348434387345	-4.77957867804772
a_HQ_transcript/80336	-2.09090826962966	0.970775633542672	-1.52437646598441	0.137061886255918	0.901463376814212	-4.68274428706538
a_HQ_transcript/139957	0.651941526988019	4.30650164656858	1.52419957446493	0.137105942095349	0.901580463808916	-5.16431067572396
a_HQ_transcript/45128	-2.53044160009566	2.70391894064445	-1.52418043679564	0.137110709125909	0.901580463808916	-4.90239065767036
a_HQ_transcript/35512	-2.39937523765609	1.46109552115715	-1.52386126968535	0.137190230659239	0.901998424143933	-4.83415333515655
a_HQ_transcript/69923	3.35336242064862	5.26843793509369	1.52376141369947	0.137215117749564	0.901998424143933	-5.12795716556493
a_HQ_transcript/135422	3.06690573011323	8.09327300492858	1.52373851972469	0.137220824124429	0.901998424143933	-5.54738910639079
a_HQ_transcript/138830	2.59629445761153	1.19497602319793	1.52363293161725	0.137247144677175	0.902049210279556	-4.71328879653116

a_HQ_transcript/111761	4.13023202182101	5.7510091200295	1.5235392581592	0.137270498607671	0.902049210279556	-5.38347100719377
a_HQ_transcript/78936	-1.55757825447711	2.06097508867048	-1.52352078253421	0.137275105183955	0.902049210279556	-4.6906737195465
a_HQ_transcript/118737	-1.5343358870949	1.33134920285777	-1.52315741712797	0.137365729396872	0.902065386429331	-4.66635489995046
a_HQ_transcript/110269	4.58314174291551	1.25297792832432	1.52315678542249	0.137365886987778	0.902065386429331	-4.79415757124499
a_HQ_transcript/73357	1.8451737893626	0.0423298957421033	1.52312044481895	0.137374953085861	0.902065386429331	-4.82772873620508
a_HQ_transcript/30576	2.08644119809616	-0.105834953929802	1.52305080534693	0.137392327792229	0.902065386429331	-4.74986970702032
a_HQ_transcript/89810	1.90385541418294	0.155644056103164	1.52299763103367	0.137405595716254	0.902065386429331	-4.73005955223853
a_HQ_transcript/143433	3.37933526172987	4.50262666910903	1.52299336037806	0.137406661364602	0.902065386429331	-5.03765566683797
a_HQ_transcript/40661	2.42923095108629	0.352251327468157	1.52298436746606	0.137408905370171	0.902065386429331	-4.87472104986572
a_HQ_transcript/42378	2.12601091290131	3.1892711437821	1.52297224530608	0.137411930265261	0.902065386429331	-4.96020979572602
a_HQ_transcript/39635	1.14237038006714	1.87532488058109	1.5229509899951	0.137417234324796	0.902065386429331	-4.67692776031474
a_HQ_transcript/57624	0.792528002299375	2.65307599041892	1.52279069534726	0.137457239641966	0.902185729926253	-4.75816738790952
a_HQ_transcript/139139	2.29746358457861	0.0748795583904516	1.52269476985122	0.137481184610643	0.902185729926253	-4.75434384724812
a_HQ_transcript/107056	-2.45466498800863	0.300032415785839	-1.52269098674587	0.13748212902021	0.902185729926253	-4.76998878990852
a_HQ_transcript/80626	0.636679639012361	3.1404514925142	1.52261359412066	0.137501450364886	0.902210667886906	-4.9955112930615
a_HQ_transcript/37567	-1.98837586252853	0.257991333410494	-1.52247443678016	0.137536197002383	0.902336801281547	-4.77261508493822
a_HQ_transcript/83616	2.06026400171162	0.00557779692846106	1.5223650197219	0.137563522669525	0.902362478971228	-4.7495520981267
a_HQ_transcript/129162	2.88378359355006	2.86311646175727	1.52230806556949	0.137577748055381	0.902362478971228	-4.93700759100831
a_HQ_transcript/39201	1.8580652995034	0.189634902690413	1.52227229919209	0.137586681997007	0.902362478971228	-4.79494202230615
a_HQ_transcript/17062	-1.74811002040516	1.34193195999912	-1.52205007800152	0.137642200236459	0.902509289636615	-4.68717394072363
a_HQ_transcript/100276	2.03009915742858	-0.0535675851580619	1.52170714821439	0.137727910960852	0.902509289636615	-4.76181702619411
a_HQ_transcript/4656	1.43978181995026	-0.222467439232058	1.52168017358673	0.137734654735338	0.902509289636615	-4.74283191320773
a_HQ_transcript/23819	-1.99057075597106	1.80957797433648	-1.52162137488868	0.137749355589315	0.902509289636615	-4.76110214700099
a_HQ_transcript/75529	-1.93417043412732	0.883685450863718	-1.52149527914442	0.137780886322735	0.902509289636615	-4.7675328166572
a_HQ_transcript/151469	-0.976644806965999	-0.739171655545849	-1.52141956349968	0.137799822112911	0.902509289636615	-4.74475218253552
a_HQ_transcript/123488	1.30877579603309	2.51311498112183	1.5214040641712	0.137803698612033	0.902509289636615	-4.8630403248805
a_HQ_transcript/100959	-1.96977430630527	-0.130085080227543	-1.52136007963792	0.137814699955076	0.902509289636615	-4.75473355380608
a_HQ_transcript/20241	1.24485068024357	-0.809051916455492	1.52128385088033	0.137833767851236	0.902509289636615	-4.78499819298835
a_HQ_transcript/141834	-1.75507088900588	0.1883931805465	-1.52118626868788	0.137858180211086	0.902509289636615	-4.83065632939383
a_HQ_transcript/8231	1.26551076194982	1.89109719592557	1.52106316791647	0.137888981587044	0.902509289636615	-4.69742069735609
a_HQ_transcript/131267	2.44868842049102	2.89250294524364	1.52095138018595	0.137916957105571	0.902509289636615	-4.91445067878091
a_HQ_transcript/98267	2.17367944455701	0.835291004983656	1.52093607768656	0.137920786999723	0.902509289636615	-4.6505117927146
a_HQ_transcript/145836	-1.26229682148925	3.30851305883523	-1.52079930077508	0.137955023197761	0.902509289636615	-5.0231199037409
a_HQ_transcript/17131	1.30174144925191	1.25029413437615	1.52077740878931	0.137960503548097	0.902509289636615	-4.75180528962024
a_HQ_transcript/67131	1.03504120968732	2.74749617542037	1.52076567469312	0.137963441086111	0.902509289636615	-4.84466466791005
a_HQ_transcript/136367	-3.63970445081115	0.00611152129903543	-1.52073473716124	0.137971186293958	0.902509289636615	-4.78964738828578
a_HQ_transcript/152264	2.24807282135361	-0.130613721906544	1.52071195229656	0.137976890706678	0.902509289636615	-4.77270945675004
a_HQ_transcript/135106	-3.89996155759403	3.29089199768731	-1.52066988202721	0.137987423908111	0.902509289636615	-4.94004109216655
a_HQ_transcript/57747	-2.44880940155976	0.114459926301721	-1.52066494970359	0.137988658864432	0.902509289636615	-4.80014626086442
a_HQ_transcript/90969	1.05340376015522	3.55546290450187	1.52065158572122	0.137992004986211	0.902509289636615	-5.0840285170174
a_HQ_transcript/52497	-3.67572122301894	2.94486000562934	-1.52061687749773	0.138000695661235	0.902509289636615	-4.98528109854899
a_HQ_transcript/144892	3.10976307078067	2.29647393147118	1.52046532189323	0.138038649203637	0.902509289636615	-4.93937456237282
a_HQ_transcript/91458	-0.505468276760298	2.9329468366244	-1.52043993173426	0.138045008395991	0.902509289636615	-4.94675691406175
a_HQ_transcript/111872	-1.40899643782017	-0.637191911244907	-1.52032926256541	0.138072729238314	0.902509289636615	-4.78981498044895
a_HQ_transcript/23160	1.78538360932002	1.42601066025115	1.52028780502666	0.138083114839111	0.902509289636615	-4.66824568329007
a_HQ_transcript/123799	-3.01129576266555	0.662052281904856	-1.52015877243747	0.138115443051438	0.902509289636615	-4.70655007590124
a_HQ_transcript/126385	1.99471663843478	0.440387729590841	1.51998017358509	0.13816019981512	0.902509289636615	-4.76644818913622
a_HQ_transcript/79067	-2.33482493612379	0.938986010876421	-1.5199334449595	0.138171911910454	0.902509289636615	-4.81606344983784
a_HQ_transcript/8855	1.93302624035289	1.23773069100572	1.5198904689637	0.138182684150814	0.902509289636615	-4.80620698213109
a_HQ_transcript/110984	1.54967340238136	-0.281495129442241	1.51988165741527	0.138184892912038	0.902509289636615	-4.79076402091399

a_HQ_transcript/94170	1.96287260125906	0.462632879854646	1.5198703152678	0.138187736051928	0.902509289636615	-4.77788322407501
a_HQ_transcript/136901	3.41708083343813	1.17569677938192	1.51977925766656	0.138210563200785	0.902509289636615	-4.79654577898999
a_HQ_transcript/133078	1.37882721012961	1.50167189531116	1.51977809501746	0.138210854683911	0.902509289636615	-4.70328877686623
a_HQ_transcript/147984	-1.8483860369029	0.156404940498709	-1.51961518828474	0.138251701283107	0.902509289636615	-4.75497376176794
a_HQ_transcript/143217	-2.3424757331965	4.55140205081586	-1.51959650601951	0.13825638622358	0.902509289636615	-5.02958327171043
a_HQ_transcript/151865	-2.55691754142213	1.51829379052625	-1.51953501315425	0.138271807656758	0.902509289636615	-4.82404256797225
a_HQ_transcript/90890	1.60978892053976	-0.452757452910146	1.51952181592075	0.138275117493926	0.902509289636615	-4.71870640252026
a_HQ_transcript/72585	-2.91118732866932	0.313437609544926	-1.51940126433643	0.138305354523589	0.902509289636615	-4.78630188128623
a_HQ_transcript/35913	1.18361885779502	1.62797553952287	1.51937077429177	0.138313002952411	0.902509289636615	-4.76486435708485
a_HQ_transcript/76065	-0.747054699378066	3.0678433230789	-1.51936581063957	0.138314248117027	0.902509289636615	-4.98831994441813
a_HQ_transcript/38666	-1.54229302140535	1.37387798157028	-1.51936173229089	0.138315271204228	0.902509289636615	-4.67536338349638
a_HQ_transcript/63463	-2.64341290822914	1.95874035662975	-1.51935073024536	0.138318031187982	0.902509289636615	-4.79613549649603
a_HQ_transcript/127974	2.48699339260848	0.329574032550537	1.51930460983654	0.138329601481471	0.902509289636615	-4.85700488261706
a_HQ_transcript/148813	-4.17770530599437	3.08152084048171	-1.51929855908161	0.138331119500992	0.902509289636615	-4.99476775824417
a_HQ_transcript/13390	-0.637472187937864	2.71920645450688	-1.51921965373289	0.138350916584465	0.902509289636615	-4.92428612881381
a_HQ_transcript/118933	2.01547205907484	0.178304376149705	1.5191406539856	0.13837073964066	0.902509289636615	-4.7484013234766
a_HQ_transcript/31121	1.78792596235285	-0.0612348181357098	1.51912381123111	0.138374966214489	0.902509289636615	-4.79556085562454
a_HQ_transcript/36041	0.671540610898325	1.7773906473075	1.51906986226245	0.1383885050392	0.902509289636615	-4.71851795352294
a_HQ_transcript/98630	-0.538013019547107	3.03110529226747	-1.51901790469593	0.138401545118918	0.902509289636615	-5.01948167049514
a_HQ_transcript/49698	-1.52508257934043	2.13679117522343	-1.51896696244247	0.138414331341677	0.902509289636615	-4.72526917393452
a_HQ_transcript/66316	0.912088345914275	3.77843022232442	1.51895859635483	0.138416431274264	0.902509289636615	-4.997900632239
a_HQ_transcript/150742	-0.746378881867767	4.44179479124496	-1.51877810883381	0.13846174085882	0.902563692167364	-5.20039031046481
a_HQ_transcript/66894	1.70949093165616	0.0101005579170068	1.51876467340463	0.138465114166891	0.902563692167364	-4.7902495616522
a_HQ_transcript/106527	1.50772686983428	2.10153047121495	1.51873981199934	0.138471356433191	0.902563692167364	-4.83928017675067
a_HQ_transcript/7520	1.98523844603973	0.275964724400947	1.51854859846245	0.138519374408467	0.902775445186158	-4.74127792604928
a_HQ_transcript/140727	-3.9040599377689	0.671872302258707	-1.51844590137659	0.138545169473013	0.902788933091593	-4.94925139701625
a_HQ_transcript/60721	-2.52979527930113	1.27747273194077	-1.51839915030893	0.138556913511486	0.902788933091593	-4.73327821636179
a_HQ_transcript/123897	2.83464545675808	0.354251910906369	1.51835487206135	0.138568037108505	0.902788933091593	-4.79853344118909
a_HQ_transcript/11263	2.17127385575468	-0.0836398551051731	1.5182702942245	0.138589286783471	0.902826186501132	-4.76539720803842
a_HQ_transcript/144502	1.15262231251799	1.80200771524185	1.51818280369581	0.13861127101888	0.90286821624669	-4.65536045892124
a_HQ_transcript/65698	1.6271665611257	-0.183167292132864	1.51811669284621	0.138627884927113	0.902875259948823	-4.79706178658751
a_HQ_transcript/132529	2.1412124047535	0.196026083057179	1.51801059289452	0.138654551604223	0.902947767829969	-4.72036602584831
a_HQ_transcript/37160	1.31463137045923	-0.582709523020873	1.5179320126838	0.138674304260482	0.902975238944021	-4.748036836967
a_HQ_transcript/88992	2.34593477453918	0.668121574609608	1.51775249430932	0.138719438186937	0.903142928945618	-4.78603688202595
a_HQ_transcript/149337	-2.6645543390113	0.395509388211589	-1.51770599225693	0.138731131512445	0.903142928945618	-4.78721170641411
a_HQ_transcript/21675	0.662036864375129	2.58924185502541	1.51748533251694	0.138786629058139	0.903403042988967	-4.83588117832146
a_HQ_transcript/141569	-1.78784428572053	2.34729840528891	-1.51721589415646	0.138854419047835	0.903537997785097	-4.91390435754518
a_HQ_transcript/149929	-0.659884976814829	3.74440766852493	-1.5171250068048	0.138877292087002	0.903537997785097	-5.08911806300896
a_HQ_transcript/84516	-1.16695562630531	2.12203318874168	-1.5170956413837	0.138884682943942	0.903537997785097	-4.82065369252696
a_HQ_transcript/152036	2.46266573344112	1.65419010243262	1.51701626606756	0.138904662163398	0.903537997785097	-4.76495494118438
a_HQ_transcript/131811	-3.04061457559865	2.29283333213472	-1.51687975520283	0.13893902814319	0.903537997785097	-4.80614631438643
a_HQ_transcript/3122	1.63008399637169	-0.18468746666035	1.51683449740844	0.138950423096178	0.903537997785097	-4.71875209347335
a_HQ_transcript/126922	-0.892656090029969	2.25836891567585	-1.51681044154453	0.138956480159163	0.903537997785097	-4.79139101999774
a_HQ_transcript/58981	0.483750503380619	3.61212687364949	1.5167453033349	0.138972882477328	0.903537997785097	-5.08520796813378
a_HQ_transcript/151682	1.99391256830627	-0.472711653012268	1.51668001647881	0.138989323791374	0.903537997785097	-4.78179559457928
a_HQ_transcript/104621	4.1458413327485	0.163397554361199	1.51667581833583	0.138990381071398	0.903537997785097	-4.8282936369361
a_HQ_transcript/134020	-1.96850702609273	0.14735942890206	-1.51667019585093	0.138991797074525	0.903537997785097	-4.79883119102107
a_HQ_transcript/148970	-0.817796767945431	3.6550660219483	-1.51666188982987	0.138993888938108	0.903537997785097	-5.04174698915677
a_HQ_transcript/4071	-2.69706765907117	0.103870617331435	-1.51646124353079	0.139044429228751	0.903762514518603	-4.79791426121079
a_HQ_transcript/18654	0.6166295859058	1.86430508364078	1.51640133383027	0.13905952260278	0.903762514518603	-4.76263425588785

a_HQ_transcript/148728	-1.68603539241305	-0.0734744236562399	-1.51633468262407	0.139076315952074	0.903770608570391	-4.80250731918391
a_HQ_transcript/54678	-3.15636452905774	0.720014517544243	-1.51626796225304	0.139093128364689	0.903778824679482	-4.8317303537793
a_HQ_transcript/131006	2.55342067223756	2.76322804376612	1.51603503758018	0.139151834307311	0.904051165563358	-4.99436311687844
a_HQ_transcript/141222	-1.17113195719343	4.39347191229227	-1.51597825931996	0.139166147630418	0.904051165563358	-5.20871115043857
a_HQ_transcript/118685	4.59438486873428	1.66268637626098	1.51564982078857	0.139248967507998	0.904414723103373	-4.84292297590512
a_HQ_transcript/100293	-1.69995463529372	0.612410370226977	-1.51560426359021	0.139260458459861	0.904414723103373	-4.74122107925622
a_HQ_transcript/1151	3.92100443959848	0.419223092267782	1.51557123677039	0.139268789335575	0.904414723103373	-4.87290858775369
a_HQ_transcript/114595	0.453170464140855	4.02967988003907	1.51545028516239	0.139299302295242	0.90451182348278	-5.11020519347052
a_HQ_transcript/131889	1.04063162405794	2.10171328996572	1.51534390420067	0.139326143909439	0.904585065695059	-4.77004433840525
a_HQ_transcript/129617	-2.22655648628277	0.794664483107428	-1.51508195691359	0.139392255151572	0.904913223972589	-4.6698809075296
a_HQ_transcript/137235	-1.98386174039757	3.2732124072776	-1.51498197663697	0.139417495212441	0.904924297511296	-5.0496384460418
a_HQ_transcript/24421	-1.60216539629346	1.92671903996275	-1.51495186952725	0.139425096485535	0.904924297511296	-4.78906266599606
a_HQ_transcript/44333	-2.54824070759056	0.739470248632821	-1.51487020342895	0.139445716761924	0.904957086517484	-4.68236652056302
a_HQ_transcript/128406	-1.05955226122256	4.18755801800769	-1.51473199037337	0.139480620455513	0.905082552560624	-5.15353625499227
a_HQ_transcript/41134	-2.30382202111265	-0.0946321884576493	-1.51457321599457	0.139520725296739	0.905188021888055	-4.75539316108278
a_HQ_transcript/92665	0.370444197212024	6.34821341968385	1.51447774259539	0.139544845406578	0.905188021888055	-5.39693556935266
a_HQ_transcript/58509	-1.41385701569036	2.2978631344905	-1.51443987836499	0.139554412240921	0.905188021888055	-4.91008533851558
a_HQ_transcript/77268	-1.43279841574425	0.186768055126192	-1.51429834473616	0.139590177021887	0.905188021888055	-4.74566970665443
a_HQ_transcript/76215	2.85668675345739	0.626088294717829	1.51413455871854	0.139631574074944	0.905188021888055	-4.79446519532509
a_HQ_transcript/84480	-3.67965443639081	0.0552419811005722	-1.51410567160196	0.139638876343462	0.905188021888055	-4.82650516113214
a_HQ_transcript/12385	1.93475702520486	0.867679064525484	1.51410490359934	0.139639070488231	0.905188021888055	-4.7464702426905
a_HQ_transcript/42295	1.74282835065713	0.462740029853352	1.51403783950031	0.139656024581323	0.905188021888055	-4.74236181791742
a_HQ_transcript/142263	2.27818486610193	1.82634915729	1.51394271243999	0.139680075955385	0.905188021888055	-4.95804134552826
a_HQ_transcript/110538	6.05175855018703	2.61528028486823	1.51390521846411	0.1396895566312	0.905188021888055	-4.83735364768836
a_HQ_transcript/11278	-1.49370071513054	1.33105859254703	-1.51386142553509	0.139700630707885	0.905188021888055	-4.76159280086884
a_HQ_transcript/124776	-3.17816509344775	0.517318750061817	-1.51385416378904	0.139702467080264	0.905188021888055	-4.82339035586665
a_HQ_transcript/140335	2.77335750425069	1.62688615387438	1.51379948397993	0.139716295300653	0.905188021888055	-4.95064081915356
a_HQ_transcript/82519	-0.872275100917849	1.59290344339319	-1.51375869706023	0.139726610804141	0.905188021888055	-4.75519872287324
a_HQ_transcript/42734	1.77370057718814	1.27416722023583	1.51367430486124	0.139747956555651	0.905188021888055	-4.83706558863389
a_HQ_transcript/33534	0.637734723984268	4.39029894169361	1.51366650581035	0.139749929342123	0.905188021888055	-5.21360936408009
a_HQ_transcript/101599	2.01264964323845	-0.0912726539339546	1.51362035541715	0.139761603641021	0.905188021888055	-4.76531200343265
a_HQ_transcript/131281	-2.63319986895166	1.39070167773125	-1.51349278024987	0.13979387940532	0.905229598311329	-4.78267297430386
a_HQ_transcript/151241	1.96954280418621	0.328927325569736	1.51331464287391	0.139838957151499	0.905229598311329	-4.74559366617411
a_HQ_transcript/86351	2.05533766598931	0.735291280836185	1.51327501909353	0.139848985558782	0.905229598311329	-4.65710897762933
a_HQ_transcript/127630	-2.51953000113468	2.38643081587587	-1.51324419136349	0.139856788168736	0.905229598311329	-4.91336353798613
a_HQ_transcript/27164	1.96611959966252	0.479078858466831	1.51323215323944	0.139859835156631	0.905229598311329	-4.62504406177784
a_HQ_transcript/27985	-1.20314276199434	3.41179596508746	-1.51322572871451	0.139861461299788	0.905229598311329	-5.03895318521792
a_HQ_transcript/122485	-0.74173770762354	5.13815496779848	-1.51307602629626	0.13989935752716	0.905374065279308	-5.30633978964911
a_HQ_transcript/79312	0.771462495330324	3.04743061451989	1.51299254924368	0.139920492809289	0.905398843032803	-4.90271549612546
a_HQ_transcript/82027	1.00718239122786	3.03701247692733	1.51292632678621	0.139937261285566	0.905398843032803	-4.88746105348089
a_HQ_transcript/147773	1.13965057167216	4.9100992700293	1.51287636237218	0.139949914062926	0.905398843032803	-5.28119806843017
a_HQ_transcript/135868	-0.852688685214763	2.60504840199494	-1.51263327281149	0.140011486180602	0.905429411701175	-4.87934382251117
a_HQ_transcript/12657	-1.36658088486874	1.42835331652967	-1.51237679690353	0.140076472566744	0.905429411701175	-4.7714141130132
a_HQ_transcript/121668	0.954334889683848	1.76190803831063	1.51237549510842	0.140076802480163	0.905429411701175	-4.75590968872437
a_HQ_transcript/128468	1.97592922728413	1.02455200789511	1.512366825713	0.140078999577562	0.905429411701175	-4.81509664463019
a_HQ_transcript/77148	2.76499313993005	0.725088758490385	1.51233418565739	0.140087271842839	0.905429411701175	-4.72523351325397
a_HQ_transcript/103956	0.532063230937707	3.45958367889247	1.51208826063697	0.140149611496056	0.905429411701175	-5.09111173370419
a_HQ_transcript/92755	-1.43859252996285	-0.390943404831654	-1.5120833103867	0.140150866566664	0.905429411701175	-4.7715909241533
a_HQ_transcript/97469	-0.951574798248161	-0.847011578710125	-1.51204999052638	0.140159314612799	0.905429411701175	-4.81596503344018
a_HQ_transcript/109248	1.74536481687205	-0.315962350883414	1.51204394623411	0.140160847149943	0.905429411701175	-4.78649403842302

a_HQ_transcript/8601	-1.35484226616043	0.474212707862684	-1.51199726001756	0.140172684947471	0.905429411701175	-4.69143014182189
a_HQ_transcript/76922	-1.69055959812268	-0.0516269031121331	-1.51191152839805	0.140194425227213	0.905429411701175	-4.75061863351546
a_HQ_transcript/98034	-2.92220972166957	3.22791522468532	-1.51177091130544	0.140230089533923	0.905429411701175	-4.97240569912448
a_HQ_transcript/139642	-1.7780285430975	-0.267642075120498	-1.51168834309055	0.140251034474537	0.905429411701175	-4.79570034326507
a_HQ_transcript/70272	2.53858587920998	4.98567111730866	1.51167593343322	0.140254182629166	0.905429411701175	-5.19597367127159
a_HQ_transcript/54631	1.53950373913208	-0.128131965120884	1.51161832262533	0.140268798421692	0.905429411701175	-4.82380345473501
a_HQ_transcript/130763	3.67721219547464	1.9015876155058	1.51161822187174	0.140268823983828	0.905429411701175	-4.87423465794286
a_HQ_transcript/18822	-0.847512246952742	2.90810909621119	-1.51160476739219	0.140272237546165	0.905429411701175	-4.96749694803735
a_HQ_transcript/134137	1.81056742875644	0.00274324943958659	1.51158347684252	0.140277639349808	0.905429411701175	-4.84250066705497
a_HQ_transcript/148939	-1.6060731049039	2.20538922878236	-1.51153130377802	0.140290877322807	0.905429411701175	-4.86726064072977
a_HQ_transcript/97847	-1.20678731749587	0.834885194690524	-1.51140225611285	0.140323625157176	0.905429411701175	-4.64502754180265
a_HQ_transcript/6416	0.733119425972503	3.37150829275057	1.51131477686526	0.140345827863737	0.905429411701175	-5.06397720303218
a_HQ_transcript/60177	-1.73893972191753	2.39789807198038	-1.51123431122998	0.140366252974053	0.905429411701175	-4.87118865532736
a_HQ_transcript/3426	1.28266014356008	0.98211668783695	1.51110658759066	0.140398678800589	0.905429411701175	-4.71060891993985
a_HQ_transcript/2657	2.63422493299676	3.38551868056531	1.51107760069785	0.140406038684506	0.905429411701175	-5.00446636803489
a_HQ_transcript/133138	0.575956703737397	4.178271924616	1.51101605368789	0.140421666737396	0.905429411701175	-5.1860040674214
a_HQ_transcript/120740	-3.46601261184818	1.45868184996823	-1.51082477916494	0.140470244212892	0.905429411701175	-4.85795249713381
a_HQ_transcript/126427	-1.79971539994318	1.61210620789774	-1.51081718184957	0.140472173961843	0.905429411701175	-4.79467542049338
a_HQ_transcript/137974	-0.949785580641981	4.62031747521172	-1.51075199662008	0.140488732151677	0.905429411701175	-5.25760451849112
a_HQ_transcript/61885	0.74008516857987	3.12738398035482	1.51065026914333	0.140514575850641	0.905429411701175	-4.97577694337282
a_HQ_transcript/82814	-2.44360134750138	0.591559710299137	-1.51061702323936	0.140523022747582	0.905429411701175	-4.76762202040322
a_HQ_transcript/13828	0.754466345752687	2.90103193568752	1.51055480643813	0.140538831474269	0.905429411701175	-4.96888056056028
a_HQ_transcript/93230	-1.04532115263285	1.67308099706723	-1.51052914008944	0.140545353478399	0.905429411701175	-4.68841623652425
a_HQ_transcript/32165	-2.61760772585289	0.678478468393407	-1.51051410974331	0.140549172910725	0.905429411701175	-4.68299161766297
a_HQ_transcript/39344	1.2104123067452	3.33918697704434	1.51047601443098	0.140558853864828	0.905429411701175	-5.0323831626305
a_HQ_transcript/26424	-0.686058596419278	3.69392623701668	-1.5103598722831	0.140588371747732	0.905429411701175	-5.10320953219835
a_HQ_transcript/134432	-1.43735180264678	5.5821146196501	-1.5103541610311	0.140589823408605	0.905429411701175	-5.32943771316177
a_HQ_transcript/8038	2.00667867988471	0.830660467034009	1.51025290164581	0.140615563075885	0.905429411701175	-4.71457707267921
a_HQ_transcript/68166	-3.41028724257767	1.08875554252346	-1.51016031233123	0.140639102170156	0.905429411701175	-4.86273205762486
a_HQ_transcript/54490	-3.12991118525738	2.8983145601334	-1.51011007366822	0.140651875733596	0.905429411701175	-4.9960266948777
a_HQ_transcript/81954	1.13926622172242	3.62984566729178	1.51010415265904	0.140653381256858	0.905429411701175	-5.08087931052936
a_HQ_transcript/67162	1.62403644985979	1.43177337525574	1.51010203475628	0.140653919774942	0.905429411701175	-4.73337955831897
a_HQ_transcript/604	0.689721348047867	3.3056631717835	1.51006492427278	0.140663356108383	0.905429411701175	-5.08791149752644
a_HQ_transcript/80437	-2.56806824307564	-0.311781381197973	-1.5100491803909	0.140667359565527	0.905429411701175	-4.78094666849815
a_HQ_transcript/109586	2.16912237646632	0.0535809886569237	1.50992960714921	0.140697768423189	0.905429411701175	-4.79466220349582
a_HQ_transcript/115209	-5.35459391552537	4.45961077410244	-1.50991499035433	0.140701486005885	0.905429411701175	-5.17497875929146
a_HQ_transcript/101595	-2.96703806462408	0.184926110697261	-1.50989167206412	0.140707416859021	0.905429411701175	-4.84348503874569
a_HQ_transcript/140432	2.01829845251504	1.96683378270934	1.5098650049696	0.14071419970498	0.905429411701175	-4.80343077291165
a_HQ_transcript/102788	1.20332005034775	1.84074446784248	1.50982419285515	0.140724580883556	0.905429411701175	-4.74707774495388
a_HQ_transcript/27139	-2.01679598842607	0.712065941908922	-1.50976858179177	0.140738727390531	0.905429411701175	-4.68231777568026
a_HQ_transcript/99934	2.35628286950775	1.00527303872824	1.50975184599916	0.140742984914994	0.905429411701175	-4.70830984871072
a_HQ_transcript/82285	-3.03816370164097	1.99503896880666	-1.50966841665972	0.140764210579942	0.905429411701175	-4.87997247911499
a_HQ_transcript/85733	1.78516685122263	1.50223881617691	1.50964713980837	0.140769624139568	0.905429411701175	-4.71422855808829
a_HQ_transcript/19079	0.449952841238881	4.87878009896113	1.50960560545149	0.140780192384999	0.905429411701175	-5.24423908848658
a_HQ_transcript/148698	-2.90467602042648	2.42616528254522	-1.50953841919252	0.140797289002364	0.905439187424427	-4.85994847147744
a_HQ_transcript/90450	-1.98734088047491	0.257793203950276	-1.50922484763742	0.140877104389058	0.905759129241145	-4.7722648154391
a_HQ_transcript/2075	1.85648854397018	0.517912721727799	1.50918342835474	0.140887649826825	0.905759129241145	-4.71058100841016
a_HQ_transcript/142036	-2.88807608004941	0.514511457892196	-1.50909815050483	0.14090936374919	0.905759129241145	-4.77820590116325
a_HQ_transcript/91333	2.52990591896372	0.201621629217519	1.50909261159322	0.140910774190903	0.905759129241145	-4.80820273757683
a_HQ_transcript/45602	-0.546282751322626	3.57262873077833	-1.50894693254234	0.140947874330995	0.905759129241145	-5.12271998149378

a_HQ_transcript/63868	0.932025999590359	4.00751154392073	1.50889207456008	0.140961847071012	0.905759129241145	-5.14315438143224
a_HQ_transcript/105131	1.42319187293199	-0.488980957991474	1.50887041503637	0.140967364221168	0.905759129241145	-4.80930137498214
a_HQ_transcript/50735	-2.63278232220755	0.235738063859073	-1.50885340302331	0.140971697671426	0.905759129241145	-4.80536854555981
a_HQ_transcript/110164	-2.58333570599783	1.17292946254659	-1.50876612535938	0.140993931494235	0.905801862278612	-4.84124841206251
a_HQ_transcript/79057	1.36005163120831	1.25575455558913	1.50860094555276	0.141036018464476	0.905885134011292	-4.77846870333886
a_HQ_transcript/6426	2.41809435094637	2.17371761662559	1.50855878483797	0.141046762417776	0.905885134011292	-4.81239514703831
a_HQ_transcript/35644	0.364666650962076	3.82042802945869	1.5084749779764	0.14106812115036	0.905885134011292	-5.14873386081129
a_HQ_transcript/83770	-1.93516363485044	0.549325022627976	-1.50843014474195	0.141079548264589	0.905885134011292	-4.7691311499397
a_HQ_transcript/98469	-1.78157247998859	0.939469290505171	-1.50840948271981	0.141084814860381	0.905885134011292	-4.72221944914883
a_HQ_transcript/1072	-1.11088103258224	2.28320195604016	-1.50830200926888	0.141112211594451	0.905949807038128	-4.79741523727163
a_HQ_transcript/92921	1.38706857877712	1.1239423807001	1.5082121842136	0.141135112747542	0.905949807038128	-4.73207796765343
a_HQ_transcript/27	0.610033762263044	0.478376606968738	1.5080862582618	0.14116722295966	0.905949807038128	-4.89438038058259
a_HQ_transcript/93889	1.54148370283232	-0.218738730489577	1.50794347298972	0.141203639285455	0.905949807038128	-4.78705052901806
a_HQ_transcript/73610	2.10529088317085	0.41642887545505	1.50790156029025	0.141214330243242	0.905949807038128	-4.71579762914468
a_HQ_transcript/77332	2.06458391646326	-0.0570517090134995	1.50784737287034	0.141228153162498	0.905949807038128	-4.77135062644783
a_HQ_transcript/90675	-1.90184469885276	3.54825417044263	-1.50782970212171	0.141232661110686	0.905949807038128	-5.13955419822516
a_HQ_transcript/60185	-1.84747711151519	4.93192988917329	-1.50781310980621	0.141236894047373	0.905949807038128	-5.17506701979496
a_HQ_transcript/146664	-1.23599868345651	2.31597813697148	-1.50777855464069	0.141245709891198	0.905949807038128	-4.82198378964964
a_HQ_transcript/149908	1.26176407925894	3.70699003851118	1.50773722716299	0.141256254093934	0.905949807038128	-5.17041408989537
a_HQ_transcript/89131	-1.23596626968871	1.03817935193488	-1.50769774955407	0.14126632691635	0.905949807038128	-4.63803320514597
a_HQ_transcript/10937	-4.8295113251365	0.4754738430208	-1.50740171713264	0.141341878825693	0.906188803339917	-4.80058965905567
a_HQ_transcript/139071	3.24627910600015	2.02030885329401	1.50737565767254	0.141348531146186	0.906188803339917	-4.79133942696226
a_HQ_transcript/102807	-2.32092088262956	0.296226148842566	-1.50730984541505	0.14136533247042	0.906188803339917	-4.82827046602019
a_HQ_transcript/146160	-0.411285508428503	6.43192468000786	-1.50730741805392	0.141365952186319	0.906188803339917	-5.41838890169538
a_HQ_transcript/45868	2.1489042480931	0.671982112848051	1.5071329165948	0.141410508895155	0.906374469187073	-4.88024715831111
a_HQ_transcript/64956	1.42836020180699	2.25481512478611	1.50702508391344	0.141438048231848	0.906397683986686	-4.78859700690963
a_HQ_transcript/138030	-1.59160491393025	1.51786008947502	-1.50695268515161	0.141456540536504	0.906397683986686	-4.80052450070782
a_HQ_transcript/143588	1.70253807429317	-0.236613362210086	1.50693557863361	0.141460910217575	0.906397683986686	-4.84167077825215
a_HQ_transcript/76427	0.732770633914633	2.7254504412185	1.50681146624063	0.141492616680534	0.906500917271697	-4.85264990441874
a_HQ_transcript/106124	-2.60113074763503	0.324780949208506	-1.50673368592163	0.1415124897989	0.906528323454974	-4.79837306318378
a_HQ_transcript/74905	2.36056592283846	1.76556339940348	1.50666236546949	0.141530714373196	0.906545164196707	-4.90671479948551
a_HQ_transcript/133204	2.87995895789419	2.80168694317167	1.50654007030984	0.141561968949054	0.90662302919073	-5.03875495337612
a_HQ_transcript/239	-3.26615855631555	2.82841571156142	-1.50649274680185	0.141574064753032	0.90662302919073	-4.84019407984679
a_HQ_transcript/93729	1.88754895486019	1.23251742245646	1.50639720042385	0.141598488768862	0.906640780559186	-4.71594713673482
a_HQ_transcript/125677	-3.69745145900211	0.260894777229435	-1.50635987356573	0.141608031356607	0.906640780559186	-4.83663174869612
a_HQ_transcript/135514	-2.34183216077034	1.60396632814449	-1.50616417798231	0.141658069270616	0.906768162073032	-4.72900829032708
a_HQ_transcript/103133	2.27181266938045	1.09737623605412	1.50616004552391	0.141659126062916	0.906768162073032	-4.76031646354303
a_HQ_transcript/118484	2.85263732360584	0.112636721992396	1.50594697264037	0.141713623714227	0.907017123900085	-4.84103360654137
a_HQ_transcript/136502	0.684560051337774	2.75300834327698	1.50586278435331	0.141735161197278	0.907055097443069	-4.96084602989113
a_HQ_transcript/121306	1.95025433350788	1.83392875121542	1.50572756086644	0.141769760276621	0.907148520397065	-4.79048458733432
a_HQ_transcript/66221	2.96181803577696	4.14345738168167	1.50568375000211	0.14178097143902	0.907148520397065	-5.07273447640505
a_HQ_transcript/121688	1.73208990253741	-0.0630056969207112	1.50558223676586	0.1418069513275	0.907201298267428	-4.7231180700714
a_HQ_transcript/50977	3.80589021948099	1.099245053711	1.50550524574898	0.14182665789126	0.907201298267428	-4.7221716181567
a_HQ_transcript/92926	-1.45764744297204	0.0228509413771624	-1.50546858967094	0.141836041127415	0.907201298267428	-4.70132673992563
a_HQ_transcript/68531	2.68501008092512	2.71390263980006	1.50522972411599	0.141897198240213	0.907487523596762	-4.83802320075023
a_HQ_transcript/45979	-0.967013591589431	3.09344580466891	-1.50514093599243	0.141919936201978	0.907487523596762	-5.07486906994667
a_HQ_transcript/42000	1.82296977413988	-0.301997396454165	1.5050621224885	0.141940122193181	0.907487523596762	-4.79101732132687
a_HQ_transcript/53589	-1.46928271050027	1.36089526875283	-1.50504995628849	0.141943238448626	0.907487523596762	-4.77405521127903
a_HQ_transcript/88525	0.544713017966357	4.61889906814683	1.50484059988178	0.141996871699594	0.907592545646473	-5.22903790715431
a_HQ_transcript/51088	2.41946488926063	2.5789299335198	1.504743630035	0.142021719104883	0.907592545646473	-5.01014246500811

a_HQ_transcript/122046	-2.26396406579621	3.00844249237092	-1.50471618026403	0.142028753426797	0.907592545646473	-4.82211343993455
a_HQ_transcript/144422	1.72986027214943	-0.216990958509965	1.50468142979155	0.142037659039709	0.907592545646473	-4.76129135471334
a_HQ_transcript/51614	-2.233096516309	1.99145160890256	-1.50464465898424	0.142047082898863	0.907592545646473	-4.80759064823243
a_HQ_transcript/91066	1.93115334305576	0.654578745965316	1.5046041071741	0.142057476360415	0.907592545646473	-4.86055951887572
a_HQ_transcript/3714	-0.769234903205546	3.96710280613771	-1.50455930019983	0.14206896113469	0.907592545646473	-5.1770667593154
a_HQ_transcript/138996	1.72147045340535	-0.275030671662941	1.50402830084848	0.142205121946221	0.908218573703272	-4.83162296284579
a_HQ_transcript/83210	-1.21771359065907	1.45055927167087	-1.50399581199149	0.14221345626192	0.908218573703272	-4.73219097009794
a_HQ_transcript/104238	5.04351845847755	1.11478267534418	1.50399435863381	0.142213829098665	0.908218573703272	-4.97377541824525
a_HQ_transcript/39175	1.68150134111912	0.60441385389127	1.50380388302091	0.142262699509421	0.908226572799582	-4.70872653845948
a_HQ_transcript/52694	1.68252050078157	-0.297494802743985	1.50378770842169	0.142266850054903	0.908226572799582	-4.7791607380953
a_HQ_transcript/74801	3.51310880922359	2.87829083759658	1.50375860043084	0.142274319668391	0.908226572799582	-5.01881704701483
a_HQ_transcript/37167	-2.12017706182539	-0.0764416365610908	-1.50370674078252	0.142287628531238	0.908226572799582	-4.82007619614863
a_HQ_transcript/99079	-3.33320595717029	1.13008657955642	-1.50364150452477	0.142304371685297	0.908226572799582	-4.79776581319598
a_HQ_transcript/123379	-1.91112936068151	0.795655256978204	-1.5036241376861	0.142308829222726	0.908226572799582	-4.74472310202827
a_HQ_transcript/36272	-3.05531361149211	1.35205719042051	-1.50354282389282	0.14232970148406	0.908240978723988	-4.90046697901522
a_HQ_transcript/137373	-2.69846947508893	0.299090062590909	-1.50342421595148	0.142360151109976	0.908240978723988	-4.72807364375643
a_HQ_transcript/134730	-1.46613926854369	-0.0338178215729663	-1.50333954877318	0.142381890494384	0.908240978723988	-4.81426291779341
a_HQ_transcript/52844	-2.13875016931799	4.08108887317493	-1.50332647944967	0.142385246448514	0.908240978723988	-5.22117089354474
a_HQ_transcript/94671	-1.91426158864967	1.67183732090428	-1.50328426176169	0.142396087583433	0.908240978723988	-4.69683736232997
a_HQ_transcript/9416	-1.4897165110366	1.11860598189693	-1.5030274104552	0.142462059055939	0.908240978723988	-4.79464596862091
a_HQ_transcript/151786	0.703600105081724	3.27021571131966	1.50302183974234	0.14246349014851	0.908240978723988	-5.07733970710401
a_HQ_transcript/67458	-1.42041389612019	2.0118950152475	-1.50287912712458	0.142500156354491	0.908240978723988	-4.72621609055282
a_HQ_transcript/118929	-3.12671274749569	1.20236604295484	-1.50278114254742	0.142525335271006	0.908240978723988	-4.79043388732863
a_HQ_transcript/143086	1.87834658620335	-0.154857970458295	1.50273112213684	0.142538190301788	0.908240978723988	-4.84347491954887
a_HQ_transcript/105719	-1.15266128026063	3.05048453810323	-1.50267140133806	0.142553539510576	0.908240978723988	-4.90413940849855
a_HQ_transcript/21309	-2.54598524090667	1.67725433994293	-1.50266307976824	0.14255567839367	0.908240978723988	-4.79127523369009
a_HQ_transcript/87714	-0.605552480884856	2.53187299946865	-1.50257375477754	0.14257863911236	0.908240978723988	-4.82980287211236
a_HQ_transcript/76191	-2.02619081866243	0.547519660627467	-1.50257292231448	0.142578853108442	0.908240978723988	-4.7106922318165
a_HQ_transcript/40799	2.03366013697083	1.17501999309375	1.50249314501753	0.142599362157416	0.908240978723988	-4.81359326278816
a_HQ_transcript/58985	2.19626265704195	1.34038623671241	1.50247714207723	0.142603476458894	0.908240978723988	-4.78005732319611
a_HQ_transcript/17597	-1.01625784578344	2.47177712974778	-1.50236632971183	0.142631968557152	0.908240978723988	-4.8210974207005
a_HQ_transcript/144253	-1.96266555063228	0.162618013295422	-1.50227603988639	0.142655187276442	0.908240978723988	-4.81162503196354
a_HQ_transcript/3301	-1.20158181695115	2.22467476042346	-1.50226538489428	0.142657927489443	0.908240978723988	-4.94401716976866
a_HQ_transcript/129072	-3.36275628109123	1.04007340245522	-1.5022518959818	0.142661396580652	0.908240978723988	-4.89873535579871
a_HQ_transcript/68643	-1.95057627496543	-0.428466088906847	-1.50224637919677	0.142662815412144	0.908240978723988	-4.81181297328519
a_HQ_transcript/112004	5.72946892376052	1.43675482923485	1.50223564615514	0.142665575816358	0.908240978723988	-4.79056746854346
a_HQ_transcript/77234	-2.16285880984087	-0.0215106310228293	-1.50216975626897	0.142682522812015	0.908240978723988	-4.75674332006509
a_HQ_transcript/138099	2.14713665449021	-0.284885554181567	1.50213837976762	0.14269059347217	0.908240978723988	-4.80729774834443
a_HQ_transcript/65791	-2.35865398436032	2.24133568358328	-1.50209517348212	0.142701707588188	0.908240978723988	-4.90008952905691
a_HQ_transcript/150039	3.97618722804452	1.76240402581215	1.50152348184262	0.142848831465582	0.909077827395594	-4.98016235947453
a_HQ_transcript/2100	-1.83564461416424	3.04049744070339	-1.50145328554403	0.14286690475876	0.909093315798269	-5.01502964205985
a_HQ_transcript/128688	-1.4919372934446	1.74218567206504	-1.50138608298348	0.142884208981078	0.909103907580136	-4.75006524748706
a_HQ_transcript/72961	-2.33447175921104	0.454681390232523	-1.50118870071426	0.142935043364162	0.90918532501683	-4.77482991028345
a_HQ_transcript/2163	1.96523761101577	0.019532822287445	1.50118662841624	0.142935577146635	0.90918532501683	-4.83978676805722
a_HQ_transcript/144618	2.28895571359866	0.853533759492508	1.50115420622206	0.142943928663102	0.90918532501683	-4.77316127690727
a_HQ_transcript/89907	-2.42208938482216	0.920802788774923	-1.50105333193006	0.142969915015662	0.909212399800959	-4.79072307001538
a_HQ_transcript/65084	1.25643720744318	-0.116103808696729	1.50101625063606	0.142979468527742	0.909212399800959	-4.7231378773224
a_HQ_transcript/18634	1.86318610673496	-0.129147735949315	1.50091165149662	0.143006419895246	0.909239661072261	-4.78812248410109
a_HQ_transcript/23710	1.57380885398825	1.32853568104832	1.50087290904297	0.14301640344197	0.909239661072261	-4.90494931791562
a_HQ_transcript/124944	2.58631156100694	0.819116974172698	1.50081750451105	0.143030681613762	0.909239661072261	-4.84284088590452

a_HQ_transcript/25436	-1.54511245040635	2.46237416171617	-1.50069411261884	0.143062484761855	0.909299007319159	-4.89922838672963
a_HQ_transcript/13874	2.30361645093802	0.390183636039797	1.50065990160845	0.14307130334677	0.909299007319159	-4.84482051855583
a_HQ_transcript/88631	2.47647536587902	2.77500427904117	1.5005874999093	0.14308996779912	0.909318207961875	-5.0448930851615
a_HQ_transcript/137376	-1.68520378363922	-0.20390415194191	-1.50046929626322	0.143120443748431	0.909412456734674	-4.83925237771715
a_HQ_transcript/30342	-2.04416649036305	0.561595459812922	-1.50039468945248	0.143139681991636	0.909435286349311	-4.83623085794943
a_HQ_transcript/194	0.954680262223775	1.78776662753343	1.50022319184025	0.143183912539138	0.90950169439037	-4.80148511550353
a_HQ_transcript/139699	3.28720766023839	0.291174052116592	1.50019621920009	0.143190869988132	0.90950169439037	-4.83219036473448
a_HQ_transcript/90196	1.36056062866852	-0.186200577370463	1.50017216906699	0.14319707382089	0.90950169439037	-4.69207825489698
a_HQ_transcript/84404	0.86288112886042	2.89815167153523	1.4999762620862	0.143247616887551	0.909723311967612	-4.98493070944324
a_HQ_transcript/82068	-1.7831782738968	0.0719464930994318	-1.49983513712684	0.143284035328123	0.909803171618316	-4.73765664231111
a_HQ_transcript/15045	-1.891228286675	0.948051571062369	-1.49975091826924	0.143305772228791	0.909803171618316	-4.72495967984097
a_HQ_transcript/103340	-3.39736538332893	2.03290582942919	-1.49974559220989	0.143307146974561	0.909803171618316	-4.8494349253569
a_HQ_transcript/100335	-1.88620262463241	2.86566136479183	-1.49959366670875	0.143346365967975	0.909839762379076	-4.81677672175574
a_HQ_transcript/86389	-2.18812880839564	0.210538989259816	-1.49958588962094	0.143348373825574	0.909839762379076	-4.80005573828028
a_HQ_transcript/76460	-0.908514542982623	0.935149039648768	-1.49951510225656	0.143366650463761	0.909839762379076	-4.8951696211677
a_HQ_transcript/46391	-3.79261253711783	0.0997137118930856	-1.49940951698631	0.143393915073078	0.909839762379076	-4.83661420069228
a_HQ_transcript/136182	3.26032843257099	1.49857825260041	1.49933554204617	0.143413019628433	0.909839762379076	-4.76754194940079
a_HQ_transcript/68374	-1.57364965036377	3.40416477289215	-1.499333805608	0.143413468100506	0.909839762379076	-5.10696157702941
a_HQ_transcript/135951	-0.869070370038257	3.67290861229949	-1.49929892501527	0.143422476993523	0.909839762379076	-5.13724551135856
a_HQ_transcript/24299	2.23309559824871	4.0948866297012	1.49909119219691	0.143476139239212	0.909887025162942	-5.20019069290006
a_HQ_transcript/12349	-1.7095285511768	0.561038854462856	-1.49902345523163	0.143493640765622	0.909887025162942	-4.9555483303243
a_HQ_transcript/111000	3.31561697465578	1.27871084856124	1.49900776506083	0.143497694955151	0.909887025162942	-4.8201290276332
a_HQ_transcript/50576	1.91928812690438	2.67909985437621	1.49886263515014	0.143535199489486	0.909887025162942	-4.91079981536718
a_HQ_transcript/112338	-2.29700157087352	1.50510887513862	-1.49884343114005	0.143540162787446	0.909887025162942	-4.881257495984
a_HQ_transcript/38797	-5.63797379790815	5.31694740791498	-1.49877292387489	0.143558386650585	0.909887025162942	-5.06605529172026
a_HQ_transcript/125949	1.00216179849932	3.49793696736888	1.4987463034731	0.143565267652228	0.909887025162942	-5.14648113174826
a_HQ_transcript/12607	0.510104453819157	5.01533962583512	1.49863810598014	0.143593237915842	0.909887025162942	-5.28915498423963
a_HQ_transcript/148971	-2.52736673174802	1.31587553792312	-1.49860818625943	0.143600973270605	0.909887025162942	-4.82014605470383
a_HQ_transcript/35880	3.15208575233349	1.60555638045015	1.498577318346	0.143608954118772	0.909887025162942	-4.65416860752265
a_HQ_transcript/141905	-2.29296718257586	1.22618091566471	-1.49854827745185	0.143616462919114	0.909887025162942	-4.789814287626
a_HQ_transcript/114562	-1.2608310992834	2.24567419936	-1.49854324087736	0.143617765205581	0.909887025162942	-4.80246333264271
a_HQ_transcript/16015	-0.996535945880682	-0.779178252898625	-1.49844134847061	0.143644113143809	0.909954774283273	-4.83319856431308
a_HQ_transcript/72137	2.24448850219542	-0.0449382208439816	1.49831599991452	0.143676531832809	0.909983048819028	-4.7924836268816
a_HQ_transcript/76710	-1.33780594071677	1.19897622847017	-1.49830303157196	0.143679886148862	0.909983048819028	-4.67404559606839
a_HQ_transcript/117073	-1.22188464180592	-0.620987889772015	-1.49809994484583	0.143732423579052	0.910042902997427	-4.82880368511369
a_HQ_transcript/138708	-1.69227516946084	4.63500136692889	-1.49809671569798	0.143733259066515	0.910042902997427	-5.28669036217435
a_HQ_transcript/127432	-4.09256399581496	2.50314265243979	-1.49808494630023	0.143736304232373	0.910042902997427	-5.00584135019806
a_HQ_transcript/151283	-3.35843437192152	6.28151362175281	-1.49796372797437	0.143767670779193	0.910082611073498	-5.21407459157866
a_HQ_transcript/113946	2.47622528628416	0.469546608787631	1.49793917641153	0.143774024429275	0.910082611073498	-4.89164946081832
a_HQ_transcript/3068	1.41991589923619	1.55376840168148	1.49787920411676	0.143789545487528	0.910082611073498	-4.66635440284028
a_HQ_transcript/30985	2.02958901187052	0.453433905960879	1.49770567024613	0.143834464288349	0.910216682672227	-4.69263613682903
a_HQ_transcript/81264	-1.18553151584272	3.88458174083366	-1.49767638445666	0.143842045950782	0.910216682672227	-5.17089919044903
a_HQ_transcript/36655	-1.67280056001935	0.640043591621509	-1.49757565437467	0.143868125943712	0.910282619446621	-4.72658330464565
a_HQ_transcript/43714	-2.41017737654741	2.62229549327159	-1.49733720697813	0.14392987740355	0.910289474921517	-4.86519135782082
a_HQ_transcript/54605	1.30658893133813	-0.452212707933053	1.49732586116499	0.143932816193825	0.910289474921517	-4.86508120382334
a_HQ_transcript/147710	1.51503169724783	-0.540157183246541	1.49731073101435	0.143936735277363	0.910289474921517	-4.82123087607199
a_HQ_transcript/140017	0.3271653725231	5.81140752985089	1.49729747379643	0.143940169295345	0.910289474921517	-5.37227809968204
a_HQ_transcript/15131	-2.24384737020174	1.07343648082282	-1.49726913596585	0.143947509865789	0.910289474921517	-4.81310028866464
a_HQ_transcript/103622	2.39552265571588	0.340735567476047	1.49710596412816	0.143989783390528	0.910369419718143	-4.90506766663543
a_HQ_transcript/150688	-3.70022709493438	5.08690389849805	-1.49709943639786	0.143991474760677	0.910369419718143	-5.36895091963153

a_HQ_transcript/126294	-1.90548392563639	2.17263256738102	-1.49700825647037	0.144015101638324	0.910419774663675	-4.91932870465803
a_HQ_transcript/97345	2.02820584897101	-0.199843509757448	1.49686770657236	0.144051527536472	0.910551021383214	-4.85500206667253
a_HQ_transcript/37322	0.760196841118814	3.12910455047163	1.4967538723561	0.144081035028325	0.910638513007117	-5.04162030624638
a_HQ_transcript/16609	-2.17144224999647	0.383843602740163	-1.49654147074197	0.144136105594573	0.910831613424074	-4.72184232926692
a_HQ_transcript/14434	1.37688607082183	-0.422717256388713	1.49640344095862	0.144171902407518	0.910831613424074	-4.88505632772588
a_HQ_transcript/721	3.02023200706076	1.99669926756955	1.49625130614484	0.144211365498087	0.910831613424074	-4.9040900840974
a_HQ_transcript/74291	1.74162824595141	-0.230209241004843	1.49623074971895	0.144216698407509	0.910831613424074	-4.82764406933333
a_HQ_transcript/87015	-1.63484198214839	1.9686833969692	-1.496070505792	0.144258275568591	0.910831613424074	-4.80927673247071
a_HQ_transcript/142562	1.64116677593668	-0.743928067227296	1.49604253206115	0.144265534665881	0.910831613424074	-4.82917347768708
a_HQ_transcript/14118	1.32525728737818	1.54274581142368	1.49603932131087	0.144266367864468	0.910831613424074	-4.77655289659837
a_HQ_transcript/121171	1.90857436973641	0.571769595621682	1.49600943154285	0.144274124527247	0.910831613424074	-4.80943686588564
a_HQ_transcript/111672	-3.12268930660669	0.755956762760062	-1.49589302657579	0.144304335848105	0.910831613424074	-4.88218604229077
a_HQ_transcript/150459	-3.87715734177197	2.4390859574833	-1.49582586572537	0.14432176883872	0.910831613424074	-4.9086876138773
a_HQ_transcript/129160	-2.25077193404359	-0.0632134938500911	-1.49582156264017	0.144322885851244	0.910831613424074	-4.89032895952569
a_HQ_transcript/73478	2.25646149760239	1.86481707941029	1.49568776397752	0.144357621317503	0.910831613424074	-4.87780655957202
a_HQ_transcript/103636	1.65860448943685	-0.142411708296401	1.49564429080364	0.14436890882986	0.910831613424074	-4.81627037192955
a_HQ_transcript/149841	-2.33442155577281	0.0748655455372147	-1.49562644703351	0.144373542047673	0.910831613424074	-4.86144429945871
a_HQ_transcript/58744	-1.40010146875242	-0.0761002157205512	-1.49560377608267	0.1443794288374	0.910831613424074	-4.70077252999588
a_HQ_transcript/87184	-2.6201743105335	2.19337223515455	-1.49540654483894	0.144430650465772	0.910831613424074	-4.90038335127165
a_HQ_transcript/83019	1.70910277915429	0.978473390203434	1.49537876149477	0.144437867066218	0.910831613424074	-4.68519249015126
a_HQ_transcript/11361	-2.44349612911901	0.00912311864445382	-1.49537822658944	0.144438006008343	0.910831613424074	-4.81082356074914
a_HQ_transcript/150682	1.47945790574224	-0.64078468443921	1.49537218224472	0.144439576039593	0.910831613424074	-4.82142282161216
a_HQ_transcript/35525	-2.00694224760034	0.480690644161237	-1.49535496913587	0.144444047255797	0.910831613424074	-4.75889413615385
a_HQ_transcript/138212	1.61951335727508	-0.520580484836308	1.49528449804649	0.144462353733967	0.910831613424074	-4.81484376544066
a_HQ_transcript/63470	1.88618989736026	0.628841608914343	1.49525959029145	0.144468824538382	0.910831613424074	-4.66494499030263
a_HQ_transcript/43690	4.59065740743597	2.10757391813881	1.49524742972769	0.144471983824834	0.910831613424074	-4.71053290942847
a_HQ_transcript/127274	-1.60269694682006	3.25004095665784	-1.49512708634986	0.144503251744574	0.910869062924606	-4.939125691277
a_HQ_transcript/15050	1.46177333436411	2.90370986586982	1.49510394885577	0.14450926400858	0.910869062924606	-5.13106427147125
a_HQ_transcript/92827	-2.35859332830531	1.04660723956784	-1.49489980160955	0.144562320242465	0.911104689477826	-4.89546481300913
a_HQ_transcript/108508	-1.72176459698856	0.579071354865839	-1.49483497050797	0.144579172594118	0.911112114543678	-4.74870607177534
a_HQ_transcript/100109	1.43632623443649	1.72725278868646	1.49467298232677	0.144621287075375	0.911181340566758	-4.78055594640074
a_HQ_transcript/124496	2.27497684469521	0.0600618368236152	1.49462566530377	0.144633590643128	0.911181340566758	-4.79884055093841
a_HQ_transcript/139538	0.531268161891844	3.71402117457265	1.49461184673371	0.144637183963141	0.911181340566758	-5.10997821174287
a_HQ_transcript/89192	3.08176306302472	1.67241015820953	1.49449798731552	0.144666794189052	0.911269117102429	-4.70274087766553
a_HQ_transcript/22534	-1.95384667459542	1.12903569450181	-1.49439852377739	0.144692664606767	0.911333319781355	-4.79529019693662
a_HQ_transcript/96858	0.545895371223719	3.53475232385408	1.49431729986268	0.144713793658752	0.911367648731955	-5.09790508450801
a_HQ_transcript/4413	0.853279305901711	1.05690322169006	1.49416451257637	0.144753545436553	0.911519238342105	-4.71291032292934
a_HQ_transcript/102542	-0.815315607270688	3.11712215116553	-1.4940384798495	0.144786342875618	0.911552910592224	-4.91032299075312
a_HQ_transcript/102535	-1.71573197991075	-0.487722118378217	-1.49402344267291	0.144790256391034	0.911552910592224	-4.83111195096991
a_HQ_transcript/5286	3.0055999811277	2.17109150307286	1.4938715090703	0.144829802780241	0.911703137969447	-4.84834609097124
a_HQ_transcript/109021	-1.71263451252488	3.50278222677694	-1.4937516923655	0.144860995655392	0.911800753162602	-4.92753795417508
a_HQ_transcript/16329	-1.77270920442333	-0.232122877720751	-1.49352802894856	0.144919238216337	0.911841929865373	-4.80423857100866
a_HQ_transcript/35171	-1.45664517554543	1.15019860034291	-1.49347982357322	0.14493179348563	0.911841929865373	-4.67106698546348
a_HQ_transcript/57196	-1.76297619866179	1.56177041993148	-1.49330324724222	0.144977790895054	0.911841929865373	-4.8677522306345
a_HQ_transcript/123663	-0.482939214894009	3.38548805787136	-1.49315354466329	0.145016796968156	0.911841929865373	-5.09779587653543
a_HQ_transcript/56947	1.00548374230016	1.90744280969478	1.49304852184545	0.145044166431964	0.911841929865373	-4.79027039942766
a_HQ_transcript/13314	-1.73229189998543	1.38943826463836	-1.49303798310181	0.145046913109065	0.911841929865373	-4.73234532644172
a_HQ_transcript/41685	-0.458625101434416	4.30495659402203	-1.49301202102244	0.145053679695845	0.911841929865373	-5.18839996156271
a_HQ_transcript/141136	3.7726863984619	1.7677919125526	1.49300444931781	0.145055653183142	0.911841929865373	-5.01838572170047
a_HQ_transcript/112087	1.79503104813118	1.69446956121657	1.49294969997239	0.14506992367996	0.911841929865373	-4.79795974510924

a_HQ_transcript/88007	-3.34539449504993	1.52617993085616	-1.49294773776909	0.145070435152003	0.911841929865373	-4.87823633323993
a_HQ_transcript/84728	-2.4229534449723	1.7676899574181	-1.4929029053871	0.145082121648877	0.911841929865373	-4.79206517709247
a_HQ_transcript/100425	1.30840149143581	-0.314971020880148	1.49287969842264	0.145088171325335	0.911841929865373	-4.84449237580863
a_HQ_transcript/8929	-0.822116480028071	3.68259592126841	-1.4928749018204	0.145089421746383	0.911841929865373	-4.99869144441769
a_HQ_transcript/22854	1.72271510183996	0.873090890077586	1.49280566640613	0.145107471613825	0.911841929865373	-4.7033668596731
a_HQ_transcript/114683	2.06429097577741	0.669608426710097	1.49278131578094	0.14511382031819	0.911841929865373	-4.75065825770999
a_HQ_transcript/97362	-1.92383596784208	0.511996393953507	-1.49270894524023	0.145132690107522	0.911841929865373	-4.74326700347503
a_HQ_transcript/15447	2.2852480788966	0.941942496500209	1.49262904770085	0.145153524759642	0.911841929865373	-4.81842532781889
a_HQ_transcript/150584	-1.98910219399361	0.579325960361695	-1.4925931075948	0.145162897539665	0.911841929865373	-4.76780985231867
a_HQ_transcript/5583	0.631576114074761	4.71064269593531	1.4925827966125	0.145165586618741	0.911841929865373	-5.28313590211037
a_HQ_transcript/106150	1.22304607576393	1.06687365333197	1.49239968188235	0.145213349145376	0.911982071426924	-4.76823163209094
a_HQ_transcript/152289	-1.00273065606191	3.02895447743652	-1.49237696399597	0.145219275618078	0.911982071426924	-5.02907693201096
a_HQ_transcript/566	1.84453984592652	0.465418426629939	1.49226833456852	0.145247616732288	0.912061517274976	-4.75844543342809
a_HQ_transcript/24946	2.4266205098504	0.395611643667369	1.49206784415186	0.145299935762498	0.912183154191847	-4.8257820953802
a_HQ_transcript/128004	-2.04039362398228	2.97272332138713	-1.49203507386873	0.145308488776352	0.912183154191847	-4.94964724278332
a_HQ_transcript/109468	1.75855311808535	1.4830121645469	1.49201370731314	0.145314065645068	0.912183154191847	-4.7541673474528
a_HQ_transcript/43012	-1.83151316952938	0.355709823670061	-1.49173495555643	0.145386838150121	0.912541424035224	-4.76345584217161
a_HQ_transcript/86827	-0.989809287894953	1.6353130253845	-1.49146988954992	0.145456064861968	0.912877363236501	-4.704674190284
a_HQ_transcript/102149	-2.30732361154322	1.70270280441457	-1.49140603209284	0.145472746322469	0.912883493277822	-4.71794100548667
a_HQ_transcript/129744	-2.42363164988632	0.878460753679567	-1.49128190131781	0.145505177346179	0.91289609200512	-4.7658719414146
a_HQ_transcript/15174	-1.68533414795506	0.0672512210231763	-1.49127812578031	0.14550616385266	0.91289609200512	-4.83318241486709
a_HQ_transcript/131162	-1.62367812046389	1.24885009155821	-1.49112835643494	0.145545301260621	0.91304308997166	-4.74806920531085
a_HQ_transcript/136188	-1.84787930010945	4.35192905319126	-1.49106095595619	0.145562916962686	0.913055059588544	-5.27395065724263
a_HQ_transcript/126855	3.34259098821802	0.230413135211008	1.4909672102947	0.145587421047972	0.91306420675898	-4.88410868444305
a_HQ_transcript/30700	1.44428646334037	2.25063442852647	1.49093519258257	0.145595790883034	0.91306420675898	-4.88724202856944
a_HQ_transcript/77829	1.70359968818139	1.17665345767813	1.49067374203107	0.145664151793486	0.913221915906486	-4.76093523490378
a_HQ_transcript/22783	1.43411426565049	1.57000482469007	1.49062238902703	0.145677581973373	0.913221915906486	-4.85942462482242
a_HQ_transcript/11129	1.74660768938864	-0.0613386141203917	1.49044015259791	0.145725249665648	0.913221915906486	-4.76812511114788
a_HQ_transcript/41904	-1.73249030646579	1.77672631496635	-1.49041416256509	0.145732048912134	0.913221915906486	-4.88253887227125
a_HQ_transcript/6186	-0.705776071172385	3.69886756562722	-1.49024806170174	0.145775508523028	0.913221915906486	-5.14981702063871
a_HQ_transcript/67546	-2.08738300845125	-0.0817389529088166	-1.49024047577413	0.145777493597815	0.913221915906486	-4.80984375137321
a_HQ_transcript/96317	5.45023289050507	1.98943966466175	1.49005212363772	0.1458267882618	0.913221915906486	-4.79003839898845
a_HQ_transcript/99501	1.71739177942208	0.868911055182601	1.49003443236639	0.145831419026718	0.913221915906486	-4.71552170544655
a_HQ_transcript/77551	-1.33228637096128	0.581697803152543	-1.49000836892871	0.145838241456102	0.913221915906486	-4.67223483125688
a_HQ_transcript/148469	5.55163393645088	0.825104074000343	1.48993603622838	0.145857176780536	0.913221915906486	-4.87181646525396
a_HQ_transcript/150643	-2.61174099941314	0.999592891943006	-1.48990194922613	0.145866100791711	0.913221915906486	-4.86317055102674
a_HQ_transcript/65803	-0.745774825421961	3.67756437813626	-1.48981081958848	0.145889960769264	0.913221915906486	-5.15646191502986
a_HQ_transcript/107863	-1.79303436687205	0.17514248957239	-1.48973422012163	0.145910018809344	0.913221915906486	-4.77659185277148
a_HQ_transcript/17649	1.66427803626982	1.08601016997101	1.48957159156828	0.14595261141639	0.913221915906486	-4.68247956223579
a_HQ_transcript/74098	-1.01336754385334	4.92396992772878	-1.48941891984058	0.145992605381159	0.913221915906486	-5.31290361685447
a_HQ_transcript/58157	1.51528625463535	0.957152802324929	1.48936226645265	0.146007448564674	0.913221915906486	-4.75163487233933
a_HQ_transcript/102197	-1.68333121953155	0.669244876997083	-1.48933536406552	0.146014497411376	0.913221915906486	-4.70907935794011
a_HQ_transcript/70776	0.910646570745349	2.12949062452039	1.48929685358674	0.146024588234186	0.913221915906486	-4.82594134977305
a_HQ_transcript/103648	0.463967900384347	5.13925821941938	1.48917521843561	0.146056463714611	0.913221915906486	-5.28506190944906
a_HQ_transcript/129540	-4.65018877865399	2.3674207167769	-1.48906704725614	0.146084815532983	0.913221915906486	-5.04594476638497
a_HQ_transcript/11528	-2.02091384146658	0.493718071197221	-1.48903641897749	0.146092844048542	0.913221915906486	-4.79206201893231
a_HQ_transcript/9616	-1.30070167288621	3.40469091236069	-1.4889690615527	0.146110501528141	0.913221915906486	-5.12471777449643
a_HQ_transcript/35865	-2.11062767505353	0.249253675235485	-1.48893991553412	0.146118142570142	0.913221915906486	-4.69840140291141
a_HQ_transcript/60593	2.23516463563887	0.205184681582898	1.48893672950809	0.146118977851439	0.913221915906486	-4.88173656525257
a_HQ_transcript/114941	-2.41280093504007	1.71096832839802	-1.48876815360058	0.14616317889625	0.913221915906486	-4.90843078070235

a_HQ_transcript/110855	3.63929230320057	0.389529180634787	1.48866389985632	0.146190519859366	0.913221915906486	-4.93169401219034
a_HQ_transcript/121675	-1.83269666189894	0.743228920892295	-1.48865496423136	0.146192863453439	0.913221915906486	-4.88512605691521
a_HQ_transcript/18390	-1.3522078712082	1.20699484549898	-1.48860772839582	0.14620525274815	0.913221915906486	-4.73836014918421
a_HQ_transcript/112101	-1.61333928128236	0.841474366008782	-1.48857218668269	0.146214575393474	0.913221915906486	-4.72439062138495
a_HQ_transcript/83083	4.1446765837113	2.47952433714028	1.48844436870055	0.146248106178479	0.913221915906486	-5.01271541653352
a_HQ_transcript/75906	-0.5427624649965	3.27549215389703	-1.48843239610673	0.146251247291892	0.913221915906486	-5.09358063034505
a_HQ_transcript/75746	-2.30033239623861	0.0837647154774381	-1.48841237679511	0.146256499651915	0.913221915906486	-4.8317558517221
a_HQ_transcript/7234	-2.33907612683182	0.217402420146285	-1.48834776868088	0.146273451568595	0.913221915906486	-4.84651024559979
a_HQ_transcript/14653	-2.39884312808237	0.327434968301847	-1.48826802377108	0.146294377256302	0.913221915906486	-4.95188356844869
a_HQ_transcript/91789	-1.43600563080384	0.285344370110135	-1.48821616814458	0.146307985864148	0.913221915906486	-4.73180952563983
a_HQ_transcript/41211	-3.06241808147685	0.813804745910398	-1.48821430147552	0.146308475757895	0.913221915906486	-4.83171118331429
a_HQ_transcript/103449	-2.03552538082757	2.66374252376617	-1.48817423604849	0.146318990955113	0.913221915906486	-4.90555716382884
a_HQ_transcript/100117	2.88861916485662	1.08797550294073	1.48811432973089	0.146334714535659	0.913221915906486	-4.88424710068813
a_HQ_transcript/151206	-2.22611538796476	0.432763461140039	-1.4880786912219	0.146344069198692	0.913221915906486	-4.86193047379227
a_HQ_transcript/54833	-1.38116615627568	1.42591232383828	-1.48805486169396	0.146350324418952	0.913221915906486	-4.89061787481701
a_HQ_transcript/133867	1.71024840622742	-0.279514830980527	1.48803114986449	0.146356548956004	0.913221915906486	-4.74517149371234
a_HQ_transcript/62788	-1.10724917411431	-0.390467289465256	-1.48802653983503	0.146357759148695	0.913221915906486	-4.8124164305934
a_HQ_transcript/148960	2.05894387296504	4.01302611861099	1.48794224959525	0.146379887845483	0.913221915906486	-5.10542002298226
a_HQ_transcript/102990	-1.88147178609163	1.58406719719416	-1.48790101923962	0.146390713016622	0.913221915906486	-4.71722951010967
a_HQ_transcript/6617	0.467952688531274	3.24203410409604	1.48786642183388	0.146399797178611	0.913221915906486	-5.05498968569362
a_HQ_transcript/94430	2.05149429730539	0.0508937651766698	1.48783839906924	0.146407155382889	0.913221915906486	-4.82973033767555
a_HQ_transcript/128181	-4.18875384815367	4.04015288301931	-1.48780723658866	0.146415338360006	0.913221915906486	-4.99484038617758
a_HQ_transcript/145558	3.32243512074622	0.944023026730841	1.48775049549755	0.146430238982354	0.913221915906486	-4.90494512979304
a_HQ_transcript/72471	1.4336923201708	1.17197265627676	1.48774858264086	0.146430741333556	0.913221915906486	-4.75308339070603
a_HQ_transcript/150002	-1.53415207342317	0.148291212253282	-1.48774299643982	0.146432208380169	0.913221915906486	-4.81311540388122
a_HQ_transcript/36365	2.82191755760827	0.239809401547237	1.48767385469138	0.1464503673417	0.913221915906486	-4.79202203777309
a_HQ_transcript/143245	2.53077483655636	0.907791788278549	1.48763972010173	0.14645933290409	0.913221915906486	-4.88587657472674
a_HQ_transcript/108501	2.2195768181948	1.61094688009544	1.4875935832847	0.146471451589363	0.913221915906486	-4.86463554894982
a_HQ_transcript/116903	-1.8634955172991	1.01174107588586	-1.48758370579941	0.146474046197243	0.913221915906486	-4.86519167579537
a_HQ_transcript/47187	1.50925373132928	-0.547038046377044	1.48731856645516	0.146543706484039	0.913221915906486	-4.83576584851063
a_HQ_transcript/132257	-1.91793894541596	0.294025188481043	-1.48731492218745	0.146544664130349	0.913221915906486	-4.80567272057305
a_HQ_transcript/43146	-1.28166261801569	-0.468870664730845	-1.48728479620785	0.146552580873792	0.913221915906486	-4.77370845705557
a_HQ_transcript/143544	0.613026535452825	4.46978445891338	1.48725695188386	0.146559898330164	0.913221915906486	-5.22852466336454
a_HQ_transcript/39467	-2.53758403840323	0.0544156877214783	-1.48724499195197	0.146563041476326	0.913221915906486	-4.86397712481812
a_HQ_transcript/152403	1.87838128397221	0.178147781936791	1.48724151904608	0.146563954188189	0.913221915906486	-4.80233515150097
a_HQ_transcript/145402	-0.410379386981701	4.2347447063323	-1.48718320056099	0.14657928150646	0.913221915906486	-5.22160418215278
a_HQ_transcript/57260	-1.25584815209352	-0.421957814646102	-1.48699104123335	0.146629794078572	0.91330922331769	-4.72216175430558
a_HQ_transcript/136447	0.661843668338439	4.26348169577361	1.48695882353127	0.146638264450959	0.91330922331769	-5.24121596128289
a_HQ_transcript/103488	3.83969529781716	1.29032504327298	1.48692946958039	0.146645982254063	0.91330922331769	-4.87677564360238
a_HQ_transcript/127734	-3.48903264896469	0.690570118061136	-1.48681680088089	0.146675608366967	0.91330922331769	-4.88227921965443
a_HQ_transcript/14796	0.802824253781542	2.09412128672166	1.4867409584549	0.146695553748748	0.91330922331769	-4.74070378518243
a_HQ_transcript/148749	2.94215062742034	-0.0135577494373698	1.48670116758	0.146706018999976	0.91330922331769	-4.8581959438564
a_HQ_transcript/134995	-2.96340766644988	2.10335341227778	-1.48668788214195	0.146709513287097	0.91330922331769	-4.96307070037432
a_HQ_transcript/121211	2.30750536942474	2.77580910279824	1.48660044019516	0.146732513608974	0.91330922331769	-4.9023481641945
a_HQ_transcript/26062	-1.36142971102459	0.0362425854162211	-1.48654715455647	0.146746531030075	0.91330922331769	-4.73814770472887
a_HQ_transcript/21067	-3.61881905493066	0.581100432605234	-1.48653238932322	0.146750415389954	0.91330922331769	-4.90852713989313
a_HQ_transcript/97092	1.80755060083784	2.55096065840562	1.48638702460555	0.146788661568542	0.913449450771461	-4.82028028976384
a_HQ_transcript/103820	1.17168338883155	-0.722212201429076	1.48615963017704	0.146848506171574	0.913608447197947	-4.8633198180085
a_HQ_transcript/93402	-0.605263068275545	4.64551165911486	-1.48595317333411	0.146902857429519	0.913608447197947	-5.261985494746
a_HQ_transcript/109866	3.09258995457467	1.17236291659406	1.48592901919208	0.146909217233977	0.913608447197947	-4.70541547380572

a_HQ_transcript/3262	-0.563933276939455	3.1857138531386	-1.48588754337838	0.146920138362134	0.913608447197947	-5.04169579312782
a_HQ_transcript/80544	-4.0664138837853	0.44385613461061	-1.48587357840876	0.146923815668819	0.913608447197947	-4.92429960807144
a_HQ_transcript/27898	1.76146652911354	1.00419192488302	1.485869020338	0.146925015932563	0.913608447197947	-4.86094955127932
a_HQ_transcript/7	0.730471970000924	3.90910938342379	1.48576520056696	0.146952356621004	0.913608447197947	-5.19562920530884
a_HQ_transcript/99021	2.60595563415718	2.40303574235721	1.48567210784585	0.146976875828523	0.913608447197947	-5.10704366130179
a_HQ_transcript/34172	2.77630743814577	2.14945137079223	1.48566500574449	0.14697874654845	0.913608447197947	-4.92971256398006
a_HQ_transcript/43388	0.961742176210536	2.71210759128647	1.4856266113589	0.146988860102443	0.913608447197947	-4.91324616822633
a_HQ_transcript/63138	-6.25208231317481	1.8930634424991	-1.48562574118001	0.146989089324732	0.913608447197947	-4.79900188485465
a_HQ_transcript/2194	-2.33088653675018	1.96840468334031	-1.48557362592287	0.147002818033347	0.913608447197947	-4.88785121595658
a_HQ_transcript/30405	1.38286780944192	-0.693817568839308	1.4852677075985	0.147083426708477	0.914003193026778	-4.83316836352725
a_HQ_transcript/32377	1.23733478065344	2.11333675372075	1.48521324193136	0.147097781977111	0.914003193026778	-4.81232784485852
a_HQ_transcript/151532	2.79719394133191	2.54408956364955	1.48496427980461	0.147163414069065	0.914286347904723	-5.03353659940847
a_HQ_transcript/79963	3.15815396371964	2.77897802799269	1.48481877540749	0.147201783192092	0.914286347904723	-5.09557155261362
a_HQ_transcript/4131	-2.35306505652959	0.381763350527546	-1.48464075394294	0.147248737865606	0.914286347904723	-4.72022363709884
a_HQ_transcript/133448	2.13902231461368	0.919764402923981	1.4846267638823	0.147252428370732	0.914286347904723	-4.69281344183408
a_HQ_transcript/39731	0.502208572289325	2.74170681343145	1.48461361844621	0.147255896135732	0.914286347904723	-4.95253212697835
a_HQ_transcript/5398	-1.02264539583957	2.6218745577163	-1.48460910992432	0.147257085498496	0.914286347904723	-4.87663526509694
a_HQ_transcript/52194	-1.96361466977978	0.645598774094993	-1.48454976586605	0.14727274137078	0.914286347904723	-4.68106081914507
a_HQ_transcript/65657	-2.31034467196527	-0.121374968932985	-1.48453570110466	0.147276452065635	0.914286347904723	-4.8190327659831
a_HQ_transcript/108164	-1.28058268744429	1.92453361180913	-1.48444622720283	0.147300059645728	0.914286347904723	-4.80658843173428
a_HQ_transcript/79955	0.578416822007996	3.72765185108864	1.48444402476315	0.147300640794931	0.914286347904723	-5.1139913086054
a_HQ_transcript/23347	2.15750166340606	1.43936854723822	1.4843385923734	0.14732846297579	0.914361402504453	-4.73237195785162
a_HQ_transcript/24747	1.82939791837026	0.367046462284817	1.48393250598223	0.147435662960006	0.914672743683475	-4.71732854878602
a_HQ_transcript/61963	-1.70502056111873	0.703635819636561	-1.48388537977709	0.147448107525927	0.914672743683475	-4.84342835650404
a_HQ_transcript/71879	1.01305349272931	2.52859874408016	1.48382447295072	0.147464192370241	0.914672743683475	-4.95562575314689
a_HQ_transcript/64272	-1.92937428285484	0.728286879767083	-1.48368896448789	0.147499983747469	0.914672743683475	-4.77366512625979
a_HQ_transcript/110713	-0.813368728757957	4.26287158521976	-1.48363536681161	0.147514142232144	0.914672743683475	-5.25319755120015
a_HQ_transcript/110696	-1.99662641339113	0.938202034803248	-1.48361484996868	0.147519562295964	0.914672743683475	-4.75929490789872
a_HQ_transcript/37614	1.29372067592281	0.64426697940891	1.48357441157233	0.147530245628601	0.914672743683475	-4.6578781004551
a_HQ_transcript/111667	2.97430332871312	0.0620402048609022	1.48346031163243	0.147560392781802	0.914672743683475	-4.82564133390949
a_HQ_transcript/33156	-1.68984767989097	2.05737189248796	-1.48340436024824	0.14757517789369	0.914672743683475	-4.86112187560817
a_HQ_transcript/114057	0.571326376645079	3.13287167562734	1.48339935895807	0.147576499538605	0.914672743683475	-5.01849199689442
a_HQ_transcript/14352	-0.546058674066371	3.80340413379275	-1.48337888466127	0.147581910191259	0.914672743683475	-5.17928595519285
a_HQ_transcript/53434	3.00327930616067	2.01964964248412	1.48335577376002	0.147588017798432	0.914672743683475	-4.9518275870047
a_HQ_transcript/103409	-2.46217069387114	0.567425572018782	-1.48319693916617	0.147629999099556	0.914672743683475	-4.8733807687277
a_HQ_transcript/56731	2.09485891878339	0.973556438956069	1.48318039551231	0.147634372273677	0.914672743683475	-4.73972517717454
a_HQ_transcript/23418	1.73583339753237	-0.171662980760044	1.48311361384908	0.147652026491323	0.914672743683475	-4.7841460429214
a_HQ_transcript/12711	1.60002659035448	0.990529211777317	1.48307532226661	0.14766214991079	0.914672743683475	-4.74469049154094
a_HQ_transcript/97493	-1.83032567675388	3.99245164595474	-1.48304992811323	0.147668863850835	0.914672743683475	-5.05740317609896
a_HQ_transcript/41145	-3.47930018113005	0.952430841428155	-1.48302614119731	0.147675153076089	0.914672743683475	-4.89998913731742
a_HQ_transcript/88423	1.80572605009016	-0.28557443060918	1.48301687589104	0.147677602867283	0.914672743683475	-4.81742106249544
a_HQ_transcript/78828	2.05566786124995	1.23877974187594	1.4829347044598	0.147699330808173	0.914709855233057	-4.80551770932967
a_HQ_transcript/62718	1.54604487567295	4.13851755665055	1.48287134748831	0.147716085535955	0.914716162781934	-4.99522893121971
a_HQ_transcript/89156	1.96528004826409	1.37847858767385	1.48279105573432	0.147737320844365	0.91475021155105	-4.70973627263342
a_HQ_transcript/75900	-2.81830467938155	0.106017306341778	-1.48261324375744	0.14778435668161	0.91485917808501	-4.89981165454287
a_HQ_transcript/29012	1.36437737507586	1.23079000646922	1.482561084806	0.147798156335987	0.91485917808501	-4.81563188716552
a_HQ_transcript/97361	2.50307433532904	1.84711760545854	1.48249564629123	0.147815470811704	0.91485917808501	-4.78477619064683
a_HQ_transcript/40028	-1.75770347324782	2.42952118993044	-1.48248656279414	0.147817874356152	0.91485917808501	-5.08565198154258
a_HQ_transcript/143335	2.09059594104716	-0.235676857557704	1.48238492290139	0.14784477097511	0.914928228174297	-4.85728079982217
a_HQ_transcript/11145	-1.50717043168745	-0.521917890464658	-1.48231948023689	0.14786209091568	0.914938005189127	-4.84125941018249

a_HQ_transcript/74418	-1.59235358211906	0.314955861053437	-1.48218812989648	0.147896858762075	0.914938781998234	-4.6885526283763
a_HQ_transcript/149255	-2.59640951368959	1.03241956281322	-1.48206222343879	0.147930191772871	0.914938781998234	-4.92494815841093
a_HQ_transcript/106112	-1.85120333167607	-0.511622219074972	-1.48204885746827	0.147933730689378	0.914938781998234	-4.84808383601785
a_HQ_transcript/5695	0.440424702162646	3.14457087709527	1.48203599321443	0.147937136829968	0.914938781998234	-5.04536188549898
a_HQ_transcript/133011	-2.31208857377107	0.942659552254015	-1.48202171734884	0.147940916804318	0.914938781998234	-4.8838183548579
a_HQ_transcript/61211	1.16384980042324	2.96785205411986	1.48185688636233	0.147984566469504	0.914944120078051	-4.97194427821208
a_HQ_transcript/91379	-1.4876017509657	3.01021023849434	-1.48184161572736	0.147988610878952	0.914944120078051	-5.06652520041582
a_HQ_transcript/101456	1.01425984254473	2.04905134544456	1.48181673655548	0.147995200287213	0.914944120078051	-4.75660875275157
a_HQ_transcript/80684	1.49517768872829	4.41611891403234	1.48177815673431	0.148005418864242	0.914944120078051	-5.27051350293491
a_HQ_transcript/22743	2.46233529719204	1.16543506383047	1.48168509788847	0.148030069536929	0.914944120078051	-4.79127035461518
a_HQ_transcript/107868	-1.95027322661506	0.836059773118845	-1.4816618777308	0.148036220914775	0.914944120078051	-4.8140679947655
a_HQ_transcript/134378	-1.46155414718456	-0.546168033448426	-1.48155238334735	0.148065230419484	0.915026123094167	-4.86146043700521
a_HQ_transcript/146327	-1.98723477187484	-0.367096509285919	-1.48140599567865	0.148104021551953	0.91511240808451	-4.79043949566375
a_HQ_transcript/39159	1.63496032226646	-0.273712943834418	1.48132363327829	0.14812585025925	0.91511240808451	-4.8207092950387
a_HQ_transcript/146	-0.883202232918801	4.19268524619269	-1.48129423284554	0.148133642950349	0.91511240808451	-5.21358929836758
a_HQ_transcript/28720	0.79258056082794	1.88397695981455	1.48126208254332	0.148142164879444	0.91511240808451	-4.75936381497145
a_HQ_transcript/1746	-1.47802599650337	-0.280353572090327	-1.48108035207851	0.148190342686349	0.915312744625662	-4.76099216063855
a_HQ_transcript/125340	1.13559207885708	-0.786998956291035	1.48101004948728	0.148208983673103	0.915330620797933	-4.83801977584005
a_HQ_transcript/150614	1.98517185699951	6.50853962991745	1.48088971389301	0.148240895441925	0.915390886630181	-5.50907978139447
a_HQ_transcript/44652	-1.99538714835983	0.897233359587977	-1.48073083563973	0.148283036742383	0.915390886630181	-4.65853099681681
a_HQ_transcript/981	-2.3748769127625	0.289628897707207	-1.48073021654308	0.148283200971993	0.915390886630181	-4.7014616955023
a_HQ_transcript/10713	-2.12536311472631	2.99143654208251	-1.48071639008187	0.148286868796336	0.915390886630181	-4.98690203135383
a_HQ_transcript/32281	2.60399579019461	0.95850161203468	1.48063107576382	0.148309502217062	0.915390886630181	-4.95251520349119
a_HQ_transcript/83005	-3.46063845188372	1.4967020542521	-1.48054208161458	0.148333114819235	0.915390886630181	-4.80736007217435
a_HQ_transcript/42672	1.37786097756683	0.0205799293202235	1.48051107071096	0.148341343573147	0.915390886630181	-4.78363292811738
a_HQ_transcript/125721	-1.6754445934263	1.62170067294747	-1.48049832932991	0.148344724608731	0.915390886630181	-5.00401870089035
a_HQ_transcript/53745	-1.81210274692856	-0.555936035666805	-1.4801699988255	0.148431871191239	0.915773547974233	-4.83710306170577
a_HQ_transcript/80596	-2.49327376881084	0.717690417319192	-1.4801459870023	0.148438246095053	0.915773547974233	-4.82683276321707
a_HQ_transcript/94817	3.0186139560009	-0.0147721715269731	1.48003761219561	0.148467021267069	0.915786270592994	-4.80817515127394
a_HQ_transcript/150881	-3.2863622279258	0.169535241957707	-1.48001954954659	0.14847181761059	0.915786270592994	-4.8551214497799
a_HQ_transcript/125330	-1.06181928098763	2.9366508969025	-1.47986999106566	0.148511536025515	0.915903412549513	-4.98485771989064
a_HQ_transcript/34179	1.91844016445181	0.504990820797476	1.4798293801583	0.148522322575896	0.915903412549513	-4.80044026233448
a_HQ_transcript/16395	0.528002874827912	3.22371627564038	1.47962004814658	0.148577932601916	0.916149153101112	-5.0194799486659
a_HQ_transcript/56433	1.90743548521929	1.66896664254161	1.47955937530575	0.148594053735131	0.916151374153129	-4.78626919523662
a_HQ_transcript/100908	-1.95285057640478	1.00054691814103	-1.47942631113231	0.148629414568603	0.916227631975725	-4.73904916377314
a_HQ_transcript/18985	3.36558229625027	0.725384730945609	1.47914413511056	0.148704423036495	0.916227631975725	-4.73263878931241
a_HQ_transcript/142577	-2.69010528431198	0.439514950652884	-1.47912568325351	0.148709328991185	0.916227631975725	-4.87991683845749
a_HQ_transcript/151693	1.17069152503669	2.75484153005954	1.47912154819459	0.14871042843277	0.916227631975725	-4.97721488569487
a_HQ_transcript/138108	-1.72431675318051	0.129438102686293	-1.47905580469688	0.148727909379182	0.916227631975725	-4.70715009556033
a_HQ_transcript/109755	-2.54151602313223	0.340191950275498	-1.47903947538171	0.148732251536624	0.916227631975725	-4.83189469267262
a_HQ_transcript/98243	2.09131617303967	0.949479778367986	1.47900963301877	0.148740187234505	0.916227631975725	-4.82139485034982
a_HQ_transcript/14066	1.45396629299446	0.53159757693661	1.47896724701449	0.148751459126013	0.916227631975725	-4.75546483317297
a_HQ_transcript/88125	2.8012880821406	2.36897483013838	1.47895761140671	0.148754021659638	0.916227631975725	-4.8966570960577
a_HQ_transcript/133601	2.53147956795495	2.56825767451865	1.47891992414885	0.148764044704564	0.916227631975725	-5.01339389995466
a_HQ_transcript/502	2.47650595436201	2.05452009165807	1.47869306066615	0.148824391163292	0.91623383963487	-4.93886161693082
a_HQ_transcript/149378	-2.71071364316634	3.33464313820998	-1.47869252729839	0.148824533063974	0.91623383963487	-5.12094862048996
a_HQ_transcript/5467	1.46702230726375	0.261318679130252	1.47862086408953	0.148843599800353	0.91623383963487	-4.7517684638782
a_HQ_transcript/6672	-2.05480692384332	3.38924451751856	-1.47861689256947	0.148844656521329	0.91623383963487	-4.90350024229936
a_HQ_transcript/25937	-2.75750809873458	1.38374764723688	-1.47857749575378	0.14885513934155	0.91623383963487	-4.79474913443456
a_HQ_transcript/127838	1.6847984114486	-0.184708785890516	1.47856063187571	0.148859626711941	0.91623383963487	-4.82445617779336

a_HQ_transcript/143260	3.56995765298086	2.24741410116324	1.47829703829244	0.148929781330954	0.916568589645918	-4.99466347696951
a_HQ_transcript/121337	-3.20023990599114	2.69705617056406	-1.4782373217935	0.148945678362037	0.916569383001111	-5.03263548509251
a_HQ_transcript/83805	-1.08207095295499	2.41264734958519	-1.47795767921804	0.149020139590439	0.916656638873677	-4.97895392531415
a_HQ_transcript/4736	-1.22783844764837	1.57875394689803	-1.47794818346951	0.149022668572474	0.916656638873677	-4.84638070014351
a_HQ_transcript/115851	1.96239807896169	0.441457771659166	1.47791786512643	0.149030743420259	0.916656638873677	-4.81687523715378
a_HQ_transcript/52666	-0.947359325907349	3.7075295645612	-1.47789377163287	0.149037160619541	0.916656638873677	-5.07093309408133
a_HQ_transcript/148850	5.26024045577233	1.70888269098595	1.47773490248297	0.149079480266569	0.916656638873677	-4.8555898981555
a_HQ_transcript/120548	1.46536599122527	0.66934430791517	1.47768800048934	0.149091975879108	0.916656638873677	-4.7875663271173
a_HQ_transcript/30809	-0.875982825652018	4.30076378676811	-1.47766692583842	0.149097590852549	0.916656638873677	-5.18385943344411
a_HQ_transcript/14936	-1.25972436353412	-0.435547813429978	-1.47749989978832	0.149142098006982	0.916656638873677	-4.85320739818947
a_HQ_transcript/123866	-6.60361807165118	5.75036555498643	-1.47743621284892	0.149159071354342	0.916656638873677	-5.21737738187123
a_HQ_transcript/31719	-1.6891138323528	0.635163071782403	-1.47737732592033	0.149174766812963	0.916656638873677	-4.83612631165645
a_HQ_transcript/85510	1.18522853408588	1.02730384365508	1.47732134243522	0.149189689623874	0.916656638873677	-4.71071507840153
a_HQ_transcript/106416	-1.25526319207362	1.60520874067975	-1.47729939916663	0.149195539089223	0.916656638873677	-4.7863102894017
a_HQ_transcript/129091	-1.85876402970241	2.51170706033887	-1.47720717787265	0.149220124724953	0.916656638873677	-4.85291861781848
a_HQ_transcript/135187	-0.858422938522268	1.8470717256633	-1.47717106371804	0.149229753420414	0.916656638873677	-4.75293350479494
a_HQ_transcript/96806	-3.28722174642066	0.419447092123279	-1.47705125994485	0.149261698855424	0.916656638873677	-4.83689254115986
a_HQ_transcript/152507	-1.92613885146144	2.2545007175749	-1.47702295653281	0.149269246700778	0.916656638873677	-4.88827478192973
a_HQ_transcript/40203	-2.1331029241697	2.1686069533138	-1.47701491171078	0.14927139211869	0.916656638873677	-4.99154510649505
a_HQ_transcript/26965	2.21345842894359	3.28986148196777	1.47695760348898	0.149286675964324	0.916656638873677	-5.26605415825134
a_HQ_transcript/140952	-1.50042207008871	-0.236644674788963	-1.47685696118207	0.149313519840616	0.916656638873677	-4.84887902107754
a_HQ_transcript/92209	1.64776479862107	-0.468321143718077	1.47683254116509	0.149320033864582	0.916656638873677	-4.86634350841889
a_HQ_transcript/131096	2.31085219652654	0.480039865755538	1.47676175327361	0.149338917771966	0.916656638873677	-4.82350202482027
a_HQ_transcript/152545	3.56516829660407	2.16033929816137	1.47675742461672	0.149340072578727	0.916656638873677	-4.98639199727415
a_HQ_transcript/53414	-1.40891020396781	1.65668296355981	-1.47673063411757	0.149347219954256	0.916656638873677	-4.87622789514293
a_HQ_transcript/107418	-2.05467585521425	1.44540867286061	-1.47671226818346	0.149352119917315	0.916656638873677	-4.76271017659712
a_HQ_transcript/6688	-1.04590669135124	2.12726848822015	-1.47670485293826	0.149354098313594	0.916656638873677	-4.87582980954316
a_HQ_transcript/1824	-0.9913100679531	2.71010539692785	-1.4764692227956	0.149416975616443	0.916868728278245	-4.90317076172436
a_HQ_transcript/16867	-3.25127374048835	1.5141944052239	-1.4764356189053	0.149425944450888	0.916868728278245	-4.75161917394968
a_HQ_transcript/130953	2.16048575451291	1.15774266769093	1.47639804044238	0.14943597460274	0.916868728278245	-4.86758310159632
a_HQ_transcript/53099	1.67334230213134	2.04776978185861	1.47624071178899	0.149477973383897	0.916886640310497	-4.80639256400166
a_HQ_transcript/116145	3.74845540359241	4.25397913461628	1.47618277294665	0.149493442494379	0.916886640310497	-5.28414168832145
a_HQ_transcript/138840	1.58799494438103	-0.598220405165222	1.47606978794159	0.149523612074657	0.916886640310497	-4.80360561893054
a_HQ_transcript/59665	-4.81271094932612	1.48897342459841	-1.47606328271499	0.14952534926693	0.916886640310497	-4.89546113348512
a_HQ_transcript/85868	2.94132416536107	1.20267146346865	1.47604780923527	0.149529481456681	0.916886640310497	-4.83713646980773
a_HQ_transcript/43209	2.09782808304221	1.43079041158321	1.47600121989471	0.149541923681581	0.916886640310497	-4.81304012049957
a_HQ_transcript/147418	2.75173446874867	0.272339070266929	1.47597356716416	0.149549309055598	0.916886640310497	-4.84775138419358
a_HQ_transcript/108670	1.27685969936813	-0.117710458861698	1.47574729266895	0.149609752429978	0.91710544655844	-4.84322661281511
a_HQ_transcript/82466	-2.92209125655181	2.30641375938072	-1.47572184154515	0.149616552259044	0.91710544655844	-4.85126013347868
a_HQ_transcript/105989	1.56709137521381	2.78146700688413	1.47549190128271	0.149677997057026	0.917290879781922	-5.04971155637678
a_HQ_transcript/86821	-1.27428264648692	3.44262604510778	-1.47543777186982	0.149692464485696	0.917290879781922	-5.14403176228351
a_HQ_transcript/7575	-1.5644518289302	1.13089865260167	-1.47543148293869	0.149694145430968	0.917290879781922	-4.77987929579407
a_HQ_transcript/106690	1.6339352018023	2.08672066647173	1.4752913202362	0.149731612914913	0.917338150721748	-4.9032286545395
a_HQ_transcript/123504	-0.967934763656456	2.53882210811838	-1.47518703824583	0.149759493830931	0.917338150721748	-4.88371805279541
a_HQ_transcript/129149	-3.5832684169713	3.06521147599523	-1.47517025059912	0.149763982576798	0.917338150721748	-4.923315510703
a_HQ_transcript/88930	-1.55734259251929	2.79654459353891	-1.4751665015039	0.149764985039041	0.917338150721748	-5.06575276662115
a_HQ_transcript/4284	2.23025226977394	0.514674623192567	1.47503789331679	0.149799376540176	0.917389115703877	-4.68529292710227
a_HQ_transcript/27545	-1.94929953870943	0.917895032436008	-1.4750173523337	0.149804870049911	0.917389115703877	-4.81164216145754
a_HQ_transcript/92985	-1.54287729250053	2.9183974428757	-1.47478989012101	0.149865713618681	0.91744599284007	-4.96402784570652
a_HQ_transcript/79513	-1.43233837082077	-0.60908966888234	-1.47466431515937	0.149899311950317	0.91744599284007	-4.84244473024569

a_HQ_transcript/89259	4.42138662249894	3.44546048400954	1.47465493488845	0.149901821939188	0.91744599284007	-5.17834223721568
a_HQ_transcript/111980	-1.98129371380875	0.667729453690847	-1.47458497326165	0.149920543448629	0.91744599284007	-4.78661142714788
a_HQ_transcript/142958	2.6822374631734	0.939558017318495	1.47455231297881	0.149929283875688	0.91744599284007	-4.84255835341555
a_HQ_transcript/151632	2.18152268664078	0.279509953202152	1.4745512216775	0.149929575932829	0.91744599284007	-4.855552068754
a_HQ_transcript/105055	-0.611929665545099	3.31282337083336	-1.47452107510222	0.149937644024058	0.91744599284007	-5.08682995912076
a_HQ_transcript/6189	1.11407792147697	2.7507458968984	1.4744424035895	0.149958700417358	0.91744599284007	-4.92496702610005
a_HQ_transcript/147867	-2.22991074654217	1.0773435947306	-1.47435032848184	0.149983347274064	0.91744599284007	-4.80276771673827
a_HQ_transcript/9792	1.64376373979889	0.971406413440874	1.47420629849644	0.150021908005054	0.91744599284007	-4.69686209697479
a_HQ_transcript/7468	-1.12263509633794	2.05207300822614	-1.47418586351351	0.150027379644763	0.91744599284007	-4.80713498431893
a_HQ_transcript/17083	-1.67468545486769	2.9377679997492	-1.4740802991413	0.1500556479359	0.91744599284007	-4.99633648894571
a_HQ_transcript/1490	2.74188501553403	3.54692741167281	1.47407971300034	0.150055804906056	0.91744599284007	-5.13952539796802
a_HQ_transcript/150910	-1.41667647561956	-0.687663622906817	-1.47401690004419	0.150072627147408	0.91744599284007	-4.80818981588366
a_HQ_transcript/6919	1.56292786638193	1.73634074973622	1.47387590400376	0.150110393459424	0.91744599284007	-4.75466502865425
a_HQ_transcript/13323	-1.1071044953124	3.28528679003224	-1.47383994230731	0.150120027146971	0.91744599284007	-5.16180975282105
a_HQ_transcript/53506	-1.66508399293944	-0.445988865000586	-1.47382492522877	0.150124050180042	0.91744599284007	-4.79920635199489
a_HQ_transcript/143456	-0.649942035460448	5.20487243505611	-1.47354106098873	0.150200112796704	0.91744599284007	-5.31057005578715
a_HQ_transcript/62124	4.03267114449754	1.04204337877354	1.47343226396731	0.150229273566973	0.91744599284007	-4.92027572155017
a_HQ_transcript/49298	-1.93346562410297	0.659201357235274	-1.47341581320622	0.150233683243215	0.91744599284007	-4.80115847073434
a_HQ_transcript/85308	-1.1346958756838	1.65111908083047	-1.47341463906771	0.15023399797854	0.91744599284007	-4.84154301440933
a_HQ_transcript/545	-2.40033474765107	2.51494338490656	-1.47337046873943	0.150245838499323	0.91744599284007	-5.16392778618518
a_HQ_transcript/59803	-1.7043670197087	-0.437667810577913	-1.47335571836053	0.150249792726076	0.91744599284007	-4.84602566260209
a_HQ_transcript/73425	-0.88593951522351	3.365884927259	-1.47334273192599	0.150253274149918	0.91744599284007	-5.1237572561859
a_HQ_transcript/57709	1.52429242941669	-0.346311449242461	1.47331054787775	0.150261902377741	0.91744599284007	-4.79093896721361
a_HQ_transcript/20670	-2.47463788137957	0.676136860088826	-1.47328811942525	0.150267915459742	0.91744599284007	-4.77138484036883
a_HQ_transcript/42632	1.45460465618133	0.342412765193469	1.47323219630312	0.150282909319637	0.91744599284007	-4.8026221154386
a_HQ_transcript/143434	1.11636672926724	0.216861323637011	1.47318441174166	0.150295722050486	0.91744599284007	-4.78164960464668
a_HQ_transcript/135913	2.0222586027165	0.0996957767947406	1.47310924877764	0.150315877663111	0.91744599284007	-4.87538777431845
a_HQ_transcript/26144	-1.27656220016269	5.23952913652593	-1.47304225482888	0.150333844499966	0.91744599284007	-5.30797298392879
a_HQ_transcript/141340	-2.15936617735774	2.1167950610227	-1.47303794261642	0.150335001033523	0.91744599284007	-4.86038751850253
a_HQ_transcript/20550	-1.95313567975435	-0.0957618465934552	-1.47298685832328	0.150348702359524	0.91744599284007	-4.82098755810614
a_HQ_transcript/67575	1.47470518993659	3.13835985441696	1.47296096759715	0.150355646895542	0.91744599284007	-5.11559379230248
a_HQ_transcript/132272	-2.75139882198573	1.37418319962505	-1.4729394450477	0.150361419972845	0.91744599284007	-4.88663827693689
a_HQ_transcript/69994	-0.732712675038249	2.16280825226098	-1.47290605138902	0.150370377634121	0.91744599284007	-4.81351789399673
a_HQ_transcript/20064	0.770837796754827	4.95808285882517	1.47286141008136	0.15038235307907	0.91744599284007	-5.31324583460159
a_HQ_transcript/107094	-1.9763010091845	0.856757977855529	-1.47263551404023	0.150442963455876	0.917656850689146	-4.79704950547367
a_HQ_transcript/13616	1.90581604060795	0.28881356794008	1.47258578884692	0.150456307885623	0.917656850689146	-4.80559153088163
a_HQ_transcript/124519	-3.9879693877055	0.940870630834382	-1.47255609848212	0.150464276147782	0.917656850689146	-4.85894581605944
a_HQ_transcript/147398	2.41162067989785	0.316886813675408	1.47243401502897	0.150497044287347	0.917705193868728	-4.87920506982002
a_HQ_transcript/14481	2.62316166477398	0.698719999391414	1.47240660419235	0.150504402349694	0.917705193868728	-4.93366736535446
a_HQ_transcript/95738	3.29688359749299	0.662575870296396	1.47235011963668	0.150519565757371	0.917705193868728	-4.90091548580313
a_HQ_transcript/133167	3.40886962148265	1.58041255162563	1.47219919640337	0.150560087422056	0.917855979199715	-4.73198642348288
a_HQ_transcript/25622	1.66510308406617	-0.514022517998782	1.47195179365498	0.150626531879261	0.917943607531064	-4.85322730120703
a_HQ_transcript/124914	2.1347665635535	1.13037216442918	1.47172814343643	0.15068661729547	0.917943607531064	-4.87596224689598
a_HQ_transcript/75734	-1.44150406285247	-0.452618491950639	-1.47167507189516	0.15070087819488	0.917943607531064	-4.82439306140382
a_HQ_transcript/85148	-2.09706810106947	0.480547416266248	-1.47161699063931	0.15071648649273	0.917943607531064	-4.68498881907929
a_HQ_transcript/109271	-0.731415959515867	2.87825989868537	-1.47161214322225	0.150717789207592	0.917943607531064	-4.91390619478767
a_HQ_transcript/152785	-3.39153316855228	2.30783270868884	-1.47159166008869	0.15072329402858	0.917943607531064	-5.15098944018631
a_HQ_transcript/141429	-1.45273964741913	2.69884958079547	-1.47157013803232	0.150729078231773	0.917943607531064	-4.98123214642068
a_HQ_transcript/75884	1.80516499517379	4.26635693102661	1.47153678848376	0.150738041505963	0.917943607531064	-5.36340019228545
a_HQ_transcript/122066	2.19653539179161	2.25797797073343	1.47149696326394	0.150748745786985	0.917943607531064	-4.88613423099625

a_HQ_transcript/30860	-1.83545318399414	0.818253154342403	-1.4714436154286	0.150763085646481	0.917943607531064	-4.70973040280695
a_HQ_transcript/78897	-2.70763950611568	0.531585524198126	-1.4713452041661	0.150789541377203	0.917943607531064	-4.86690773195253
a_HQ_transcript/14925	-3.67548002406146	0.821673059687566	-1.47128687441293	0.150805223812229	0.917943607531064	-4.74163319229183
a_HQ_transcript/146299	-2.42514049562764	1.32639067418168	-1.47127612289305	0.150808114588966	0.917943607531064	-4.84381652837036
a_HQ_transcript/151312	-2.42855403390683	0.087087515922344	-1.47125592957941	0.150813544113939	0.917943607531064	-4.87449003848161
a_HQ_transcript/58031	-1.87233229202203	1.8084906180218	-1.47122631455961	0.15082150720443	0.917943607531064	-4.89215549597235
a_HQ_transcript/104098	1.94635252557607	0.0817647780502556	1.47120540502661	0.150827129705636	0.917943607531064	-4.84973310056826
a_HQ_transcript/9928	2.1641656745586	-0.396666867960618	1.47106929052434	0.150863734511363	0.918070263785231	-4.82743433289627
a_HQ_transcript/26457	-1.40589285376565	-0.566075359156563	-1.47099325635696	0.150884185202992	0.91809859912902	-4.78998705399753
a_HQ_transcript/108956	2.83335421176667	3.92812962593513	1.4708806069617	0.150914488251525	0.918183667830451	-5.25906008534719
a_HQ_transcript/102334	-4.48849367280179	2.06666317058761	-1.47081810960795	0.150931302333264	0.918183667830451	-4.94302939096862
a_HQ_transcript/70735	1.6733931608798	-0.090845362393761	1.47074773147019	0.150950238423482	0.918183667830451	-4.84539745703612
a_HQ_transcript/13796	1.39509193328403	0.719937872928496	1.47067079055567	0.150970942476041	0.918183667830451	-4.73654948544267
a_HQ_transcript/92304	0.738043839840606	3.11701129360023	1.47064774141689	0.150977145216134	0.918183667830451	-5.02710328150426
a_HQ_transcript/102703	-2.31216840934207	0.607150278379082	-1.47055974716898	0.151000827165016	0.918231622706148	-4.90738406557609
a_HQ_transcript/98982	3.14068981248093	2.83624413956404	1.47021527353785	0.151093564073705	0.918699444819697	-4.92435517812505
a_HQ_transcript/24041	1.24896215467998	0.539794968587385	1.4701467745957	0.151112010317112	0.918715504205952	-4.76417995462942
a_HQ_transcript/121656	2.14685283941288	0.528193100084911	1.46991100263594	0.151175515637476	0.918903057294148	-4.91934630884842
a_HQ_transcript/37290	-2.17989400186916	1.60745807183516	-1.46989396923964	0.15118010441716	0.918903057294148	-4.7482680786492
a_HQ_transcript/19300	2.39367007264077	1.48102732457716	1.46983674460416	0.151195521489184	0.918903057294148	-4.81408481820696
a_HQ_transcript/29886	-2.19249457546331	1.2711172244548	-1.46978571262748	0.151209271234553	0.918903057294148	-4.78806719283052
a_HQ_transcript/63515	0.685273952591875	3.11041731592787	1.46973884167337	0.15122190073761	0.918903057294148	-5.09083305257495
a_HQ_transcript/20671	-2.02592568770319	0.487043797415194	-1.46955945628852	0.1512702443749	0.91901512154802	-4.90087720801306
a_HQ_transcript/63615	1.43610327278854	-0.253776939045367	1.46950995922889	0.151283585801425	0.91901512154802	-4.86903610506196
a_HQ_transcript/104260	-2.36573672895584	3.35184224475695	-1.46949442346408	0.151287773501462	0.91901512154802	-5.0981529286883
a_HQ_transcript/55324	-6.08714526156573	4.71179233760729	-1.46909641073371	0.151395090235102	0.91952896807266	-5.01335403106737
a_HQ_transcript/17790	-0.61122791606693	3.09561326200665	-1.46901205253454	0.151417843647486	0.91952896807266	-5.11223675338098
a_HQ_transcript/102322	2.11990822638569	1.55628706856569	1.46900472662043	0.151419819749372	0.91952896807266	-4.88749729403147
a_HQ_transcript/80863	-1.64434746437624	1.02577446126878	-1.46877022315614	0.151483085889909	0.919722590686357	-4.82413817306743
a_HQ_transcript/78874	1.74224631751877	-0.120218358387138	1.46876778062924	0.15148374496438	0.919722590686357	-4.83708809130027
a_HQ_transcript/3696	1.40621860673567	1.23164964907997	1.46871061461948	0.151499170895642	0.919722590686357	-4.78006923796825
a_HQ_transcript/137090	-0.690836447240874	4.31886499121493	-1.46854167149469	0.151544766580708	0.919824877348672	-5.20457076334666
a_HQ_transcript/95197	3.17792598083064	3.03346499331634	1.46850751157114	0.151553987255919	0.919824877348672	-4.9301824126417
a_HQ_transcript/37511	-1.53662369077848	-0.450486829540931	-1.4684423370073	0.151571580850891	0.919824877348672	-4.84217522205052
a_HQ_transcript/144453	-2.27671611219664	3.13079558576605	-1.46835719335124	0.151594567469355	0.919824877348672	-4.97079498346198
a_HQ_transcript/88774	-1.67517827502218	1.78676103127649	-1.46835507107315	0.151595140465873	0.919824877348672	-4.86729444928666
a_HQ_transcript/140590	-0.573886863670111	3.26169411534177	-1.46811097884799	0.151661054748483	0.920128774701998	-5.05447694505132
a_HQ_transcript/123205	-1.56500331789449	2.99171581256869	-1.46802119974322	0.15168530430232	0.920145197618568	-5.04819602920892
a_HQ_transcript/139325	3.15377802004615	0.212655449012124	1.46795240292772	0.15170388858007	0.920145197618568	-4.86639926237383
a_HQ_transcript/126797	1.42421188941749	0.355477566365049	1.46785621265384	0.151729875772137	0.920145197618568	-4.87977368329204
a_HQ_transcript/143073	-2.98079026576931	0.849777912230619	-1.46784484146323	0.151732948098019	0.920145197618568	-4.87367763988622
a_HQ_transcript/102331	-0.855400879054884	4.34005424463446	-1.46780797258042	0.151742909860508	0.920145197618568	-5.2457926469126
a_HQ_transcript/74932	2.19772261956959	0.700424292719347	1.46773939401277	0.151761440785895	0.920161576092885	-4.82075674517203
a_HQ_transcript/1845	-3.87517443380266	0.722855442042226	-1.46760547167493	0.15179763374691	0.920285028623321	-4.87043101670225
a_HQ_transcript/74003	2.44483239795954	4.11762013979938	1.46739378021158	0.151854858103929	0.920466200582308	-5.17387176443401
a_HQ_transcript/69393	2.43864812073449	0.67745729379058	1.46733670089476	0.15187029070544	0.920466200582308	-4.88113468506898
a_HQ_transcript/126357	-3.64000587435904	0.277614844211689	-1.4673135237933	0.151876557482155	0.920466200582308	-4.91925441949539
a_HQ_transcript/36559	1.75915349260263	0.478227640871611	1.46721998445361	0.151901851356285	0.920466200582308	-4.74946944642407
a_HQ_transcript/94093	-1.51338710769598	-0.275528889471857	-1.46720208024357	0.151906693197531	0.920466200582308	-4.70764439429461
a_HQ_transcript/122265	1.42872306748147	2.26250883150254	1.46712585618756	0.151927307869574	0.920495158624412	-4.91080643678316

a_HQ_transcript/61976	-1.82965483395959	0.746419106581953	-1.4670434836152	0.151949587903812	0.920534199653233	-4.84652390541219
a_HQ_transcript/110028	2.65201826999576	1.83049549018621	1.46670154974197	0.152042101600325	0.920853071179498	-4.93681924297159
a_HQ_transcript/140225	-2.24687836945282	0.374618836653124	-1.46668376861151	0.152046913693732	0.920853071179498	-4.81742079076893
a_HQ_transcript/143584	-2.4425165307262	1.47772577231819	-1.46667329418043	0.1520497484376	0.920853071179498	-4.79422232322263
a_HQ_transcript/45255	-0.650785712579859	2.6081573067345	-1.46653847282644	0.152086239525081	0.920921934636191	-5.07412736901342
a_HQ_transcript/58943	2.12361296296905	0.0813945410454975	1.46641896159856	0.152118592568509	0.920921934636191	-4.86859874764581
a_HQ_transcript/98581	-1.72046237234029	-0.438574783759589	-1.46640391785939	0.152122665467642	0.920921934636191	-4.86145093875378
a_HQ_transcript/8157	2.71833682165396	1.26644425548877	1.46639717491762	0.152124491060706	0.920921934636191	-4.92304484222327
a_HQ_transcript/109183	0.735130844832252	3.51466719147254	1.46617668233322	0.15218419713478	0.921104784063787	-5.14459322533595
a_HQ_transcript/42615	1.3925384557735	-0.101866006807817	1.46616859383579	0.152186387733082	0.921104784063787	-4.80716919153257
a_HQ_transcript/56156	-2.1956933383027	0.192129963484496	-1.4660775977582	0.152211033825294	0.921158039999654	-4.84789940396465
a_HQ_transcript/88355	-2.47294242028521	0.609573036781878	-1.46570223944458	0.152312732428075	0.921658367634874	-4.78569208545097
a_HQ_transcript/5389	1.23974198455863	-0.515409351937059	1.46562423117432	0.152333874576761	0.921658367634874	-4.84683768331938
a_HQ_transcript/127053	2.30733847900452	1.81637231994225	1.46559693018685	0.152341274364091	0.921658367634874	-4.76488377912674
a_HQ_transcript/19424	-1.73362441597818	-0.0904443852109445	-1.46542946523979	0.152386671111129	0.921792535786954	-4.8442306967623
a_HQ_transcript/64223	-0.88052011098502	2.9721085449705	-1.465397220944	0.152395413198963	0.921792535786954	-4.98354573229671
a_HQ_transcript/21484	-0.963175899162571	2.11106749440459	-1.46533964206313	0.152411025004274	0.921792535786954	-4.81913917499669
a_HQ_transcript/77237	3.47624476830086	0.321927507728791	1.46511246243337	0.152472634394355	0.922069214739396	-4.87973687000421
a_HQ_transcript/114345	0.728474390457707	3.39299866043634	1.46497963199314	0.152508666188653	0.922191173225216	-5.15821787191366
a_HQ_transcript/41531	-1.91320081508419	1.61392170077451	-1.46488187228611	0.152535188972319	0.92225561312492	-4.82123118863251
a_HQ_transcript/126038	1.7472967582137	0.162734136042053	1.46450952466641	0.1526362427133	0.922770620534445	-4.7268845833115
a_HQ_transcript/99690	1.29819418590859	1.42257274846433	1.46444984212917	0.152652445285224	0.92277221310616	-4.84457800316658
a_HQ_transcript/15482	-0.677829839625691	3.89096456593363	-1.46435249570827	0.152678875759636	0.92277221310616	-5.23335684015769
a_HQ_transcript/1290	1.4218798142939	1.23509607782075	1.46426761156144	0.152701925582619	0.92277221310616	-4.84540749737197
a_HQ_transcript/92054	2.60917908326343	1.88017648365779	1.46424547639192	0.152707936721557	0.92277221310616	-5.02510814933516
a_HQ_transcript/28562	1.3113993824264	0.513883230101042	1.46421622635104	0.152715880300049	0.92277221310616	-4.81046598765993
a_HQ_transcript/37678	0.911610288932764	3.15911833216796	1.46409190282897	0.152749647125994	0.922866040833737	-5.05787947321241
a_HQ_transcript/120661	0.531277196735159	3.88943393201886	1.46404215183791	0.152763161383537	0.922866040833737	-5.19141634264493
a_HQ_transcript/145408	-2.4134994774996	2.17935890876989	-1.46389001548582	0.152804493298019	0.923008756899274	-4.96376090341006
a_HQ_transcript/20636	3.20188292360314	0.3960316469922	1.46378313617412	0.152833535249683	0.923008756899274	-4.85495397597778
a_HQ_transcript/136633	0.68607216160461	2.74512699412907	1.46375618529812	0.152840859213252	0.923008756899274	-4.92757343303677
a_HQ_transcript/70017	-2.55648461820134	1.69759053135629	-1.4637214428943	0.152850300955003	0.923008756899274	-4.86778738707176
a_HQ_transcript/126113	-2.69804638054773	0.552782407399945	-1.46364505207154	0.152871062879243	0.923038240681897	-4.90967877436269
a_HQ_transcript/116219	1.46382025128746	-0.703575579517257	1.46339215884282	0.152939811632568	0.923205919156167	-4.8620863538566
a_HQ_transcript/137780	2.48691592798378	1.14341632619465	1.46314862069481	0.153006040479181	0.923205919156167	-4.90341004352654
a_HQ_transcript/7514	1.22420565564134	-0.75061956831523	1.46314680260871	0.153006534983437	0.923205919156167	-4.80221388569429
a_HQ_transcript/57595	2.39240749435554	2.18493836307502	1.46309579675035	0.153020408670044	0.923205919156167	-4.83678026270553
a_HQ_transcript/127723	-2.30586897502413	2.07084697910006	-1.46298884311704	0.153049503505023	0.923205919156167	-4.81795029275641
a_HQ_transcript/48756	-2.00984401368506	1.85225781957017	-1.46298218190118	0.153051315716063	0.923205919156167	-4.80400125043371
a_HQ_transcript/54599	1.75262555266617	2.02873670358849	1.46285596022378	0.153085658068421	0.923205919156167	-4.97160803546041
a_HQ_transcript/63193	1.42983195443394	-0.39066842452055	1.46274852319218	0.153114894332745	0.923205919156167	-4.85441111146075
a_HQ_transcript/115686	-1.54573266289952	0.833289103212594	-1.46272154992592	0.153122235121231	0.923205919156167	-4.85317394827326
a_HQ_transcript/12084	-1.92163391181316	-0.715708731338152	-1.46266730366485	0.153136999117673	0.923205919156167	-4.87378780182245
a_HQ_transcript/35533	2.71069595872327	0.793555864596406	1.4626496951607	0.153141791800174	0.923205919156167	-4.82775257332142
a_HQ_transcript/104286	-0.408385647760924	5.57644027094245	-1.46253487539076	0.153173046371603	0.923205919156167	-5.3976006185198
a_HQ_transcript/61017	-1.38126751218835	3.17438201610147	-1.46251948549006	0.153177235972849	0.923205919156167	-5.0609969344817
a_HQ_transcript/89127	-1.94707212317251	0.050580029441478	-1.46249313122337	0.153184410621387	0.923205919156167	-4.8826322539219
a_HQ_transcript/33508	3.22871268880881	0.462748191854473	1.46238911832007	0.153212729559031	0.923205919156167	-4.88096535313613
a_HQ_transcript/59981	1.80538443551349	2.8818019724502	1.46231554067645	0.1532327645965	0.923205919156167	-4.92984915665189
a_HQ_transcript/67868	1.92175394358864	0.672244938242408	1.46221131973419	0.153261147308214	0.923205919156167	-4.86580300131045

a_HQ_transcript/9188	-2.66462177977027	1.30692807094721	-1.46219435342511	0.153265768175369	0.923205919156167	-5.02575545842362
a_HQ_transcript/149418	-5.65645586561274	1.45234833565804	-1.46218829321409	0.153267418733892	0.923205919156167	-4.89781458160677
a_HQ_transcript/137516	-2.91080213806473	1.10303278248162	-1.46210913286585	0.153288980138425	0.923205919156167	-4.8397074908052
a_HQ_transcript/124856	1.87860805975656	-0.157697864934119	1.46208225003933	0.153296302932919	0.923205919156167	-4.85378177573196
a_HQ_transcript/6738	-2.08804171584946	0.453936028517697	-1.46207986835681	0.153296951708916	0.923205919156167	-4.84442071677961
a_HQ_transcript/16652	1.5697404982618	2.20544255416631	1.46198062787486	0.153323986995311	0.923205919156167	-4.80253635700699
a_HQ_transcript/134080	-1.64739783845426	1.5569836491451	-1.46196706078487	0.153327683263336	0.923205919156167	-4.85652150900411
a_HQ_transcript/36229	-1.53813622697194	0.83326095314644	-1.46196546683084	0.153328117530704	0.923205919156167	-4.7514857463649
a_HQ_transcript/9510	-3.64623661252442	3.3001137875712	-1.4619246837605	0.153339229072797	0.923205919156167	-4.82649054887997
a_HQ_transcript/104605	-2.36430091026758	0.218929505265873	-1.46188832983422	0.153349134413658	0.923205919156167	-4.85174114311748
a_HQ_transcript/142176	-0.561894809363337	4.79730110876872	-1.46188482374872	0.153350089742402	0.923205919156167	-5.28921388153056
a_HQ_transcript/56209	2.3348114469396	1.55059604065341	1.46185058298964	0.15335941982129	0.923205919156167	-4.77205783912524
a_HQ_transcript/131995	1.65711330825625	-0.305845968822618	1.4617249344936	0.15339366096947	0.923224544035102	-4.89057031712417
a_HQ_transcript/124570	-2.2701386320497	3.04158833045383	-1.46147707941036	0.153461223129578	0.923224544035102	-5.11691948970542
a_HQ_transcript/8826	3.04804577273501	2.33173637786308	1.46140013145681	0.15348220298436	0.923224544035102	-4.96937827024243
a_HQ_transcript/122272	0.676569923602421	5.31910377034489	1.46138530935891	0.153486244490575	0.923224544035102	-5.35253954234174
a_HQ_transcript/78232	1.82577597278472	-0.00738136778392456	1.46137651380337	0.153488642793839	0.923224544035102	-4.89464916648019
a_HQ_transcript/99930	1.77391760674324	1.60943073768863	1.46136877756473	0.153490752275509	0.923224544035102	-4.79385805775128
a_HQ_transcript/12032	1.8116495505704	1.01386871347265	1.46135172537884	0.153495402067926	0.923224544035102	-4.79591004872099
a_HQ_transcript/113621	2.15794146754137	-0.326289981268711	1.46129831565664	0.153509966563701	0.923224544035102	-4.85716702895133
a_HQ_transcript/15600	-2.17614574018212	0.367573019572641	-1.46126499006009	0.15351905480166	0.923224544035102	-4.79396694206014
a_HQ_transcript/96634	-1.88127517196416	-0.254750764632462	-1.46115696253893	0.15354851797726	0.923224544035102	-4.81312702701793
a_HQ_transcript/93040	2.36234241610969	1.22373039652025	1.46108091289335	0.153569262279628	0.923224544035102	-4.79610072228989
a_HQ_transcript/52867	-3.38501953705738	1.20102664316804	-1.46091641212203	0.153614141303552	0.923224544035102	-4.85450608264445
a_HQ_transcript/108495	-3.72480582396659	3.45843226540331	-1.46087972255615	0.153624152356939	0.923224544035102	-5.07418925310598
a_HQ_transcript/4480	2.0822443642218	0.716992214231219	1.46086620752021	0.153627840177789	0.923224544035102	-4.86255507676091
a_HQ_transcript/2827	-2.13936531677417	1.35624543561546	-1.46078762615422	0.153649283913445	0.923224544035102	-4.92917771791446
a_HQ_transcript/70215	-1.6242308602795	0.0823943096572706	-1.4606410092806	0.15368929993668	0.923224544035102	-4.71557631128793
a_HQ_transcript/4847	1.39863394892644	2.38384500936569	1.46048786650258	0.153731105922136	0.923224544035102	-4.84917734091528
a_HQ_transcript/120243	2.04556019677861	-0.191234945087836	1.46041206974289	0.15375180079779	0.923224544035102	-4.84338095721166
a_HQ_transcript/124696	2.34726088556565	0.865852318198184	1.46040209497171	0.153754524385941	0.923224544035102	-4.93466463675587
a_HQ_transcript/123814	1.55446391418491	-0.497273506883342	1.46037659579251	0.153761487052188	0.923224544035102	-4.83294557572459
a_HQ_transcript/39716	2.62703056609799	0.664315394380024	1.46026870215253	0.153790950674764	0.923224544035102	-4.87594035018209
a_HQ_transcript/673	-2.34001358041818	2.06986430624282	-1.46015201862341	0.153822819697269	0.923224544035102	-4.93578298618075
a_HQ_transcript/40253	-1.49391231579014	1.0762049193282	-1.46011761104661	0.153832218220499	0.923224544035102	-4.77780338825629
a_HQ_transcript/144285	-1.96420416504374	2.73367087508716	-1.45993572841636	0.153881907545581	0.923224544035102	-5.10668342810003
a_HQ_transcript/25592	-1.05470137781136	1.94437147582787	-1.45975779327902	0.153930530790916	0.923224544035102	-4.89368794818545
a_HQ_transcript/127174	2.02478232592511	1.40199889388109	1.45974900616471	0.153932932308444	0.923224544035102	-4.73071984243008
a_HQ_transcript/49542	-2.84356860652939	1.27108707107487	-1.4597318002303	0.153937634775037	0.923224544035102	-4.89633710873962
a_HQ_transcript/122168	2.07707393442096	3.31369681260257	1.459695928042	0.153947439188166	0.923224544035102	-5.03999918559538
a_HQ_transcript/71845	-3.25481761278956	0.868460398945144	-1.45959337272967	0.153975471850246	0.923224544035102	-4.90544649299172
a_HQ_transcript/22072	-2.03528702760634	0.491348421570578	-1.45947715180389	0.154007244804207	0.923224544035102	-4.78388627272175
a_HQ_transcript/124803	-2.62782948492683	3.77318035494924	-1.45947581173784	0.154007611187374	0.923224544035102	-5.17641849651232
a_HQ_transcript/26122	-1.52293269771128	0.121510828189824	-1.45931759023813	0.154050874894152	0.923224544035102	-4.89758017975068
a_HQ_transcript/24990	2.81101498667329	1.49626816283011	1.4593010292772	0.154055403842286	0.923224544035102	-4.97231228182967
a_HQ_transcript/106502	6.00065227084149	2.49321978900586	1.45927789714335	0.154061729995121	0.923224544035102	-4.96726490714242
a_HQ_transcript/51408	1.18185719243216	1.59802283587412	1.45926740632216	0.154064599082508	0.923224544035102	-4.80679226223806
a_HQ_transcript/148591	-2.12712913196544	0.139944455650292	-1.4592551154941	0.154067960499712	0.923224544035102	-4.89989063130388
a_HQ_transcript/28765	-1.76370786383735	0.471383265125632	-1.45918510719415	0.154087108174235	0.923224544035102	-4.81067227286879
a_HQ_transcript/116525	-1.93500474557939	1.66443486292661	-1.45912809719996	0.154102702135087	0.923224544035102	-4.71457412984883

a_HQ_transcript/20380	-0.620560344553428	2.50787598485267	-1.45910638264759	0.154108642053178	0.923224544035102	-4.91713782127481
a_HQ_transcript/124598	-1.99547504844284	3.03226177576112	-1.45901651941288	0.154133225672662	0.923224544035102	-5.16355546773904
a_HQ_transcript/74726	-1.21370387759691	2.94473991327943	-1.45881673338922	0.154187891718798	0.923224544035102	-5.05702439438064
a_HQ_transcript/152268	1.87358674705944	-0.428527099163299	1.45872215978022	0.154213774607208	0.923224544035102	-4.84310226862013
a_HQ_transcript/29961	-2.56807216797276	3.03580807342239	-1.45871551763901	0.154215592557069	0.923224544035102	-5.10807130218577
a_HQ_transcript/105158	1.68345241307082	4.170049967397	1.45866717867977	0.154228823413059	0.923224544035102	-5.30232155503299
a_HQ_transcript/151554	-0.960103870282806	3.59993879609815	-1.45862309146799	0.154240891310336	0.923224544035102	-5.07205008178843
a_HQ_transcript/151061	-2.34002083705629	1.33024071619975	-1.45855090135613	0.154260653375133	0.923224544035102	-4.88163074600109
a_HQ_transcript/59586	-0.716063863630452	3.55033326843369	-1.45854789509522	0.154261476383628	0.923224544035102	-5.17377921153197
a_HQ_transcript/22487	-1.19950214590509	3.8665385704275	-1.45848193281376	0.154279535414883	0.923224544035102	-5.2756329620454
a_HQ_transcript/54305	-2.15189128006708	0.259507192052313	-1.45843197529812	0.154293213809685	0.923224544035102	-4.85815077083625
a_HQ_transcript/116899	1.51672620454829	0.698551932909752	1.45841421049846	0.154298078053853	0.923224544035102	-4.78645780176383
a_HQ_transcript/6931	-1.89695156000926	0.375666969234335	-1.45840178775241	0.154301479643414	0.923224544035102	-4.83652295765701
a_HQ_transcript/85126	1.78838205649311	1.65760694513175	1.45833169604065	0.154320673235105	0.923224544035102	-4.82061553418006
a_HQ_transcript/144189	-2.53323883448269	1.70758659249826	-1.45830034855234	0.154329257901345	0.923224544035102	-4.87176517963816
a_HQ_transcript/128676	-3.48179855327744	2.77005343808886	-1.45824496100753	0.154344426985296	0.923224544035102	-5.11431504881744
a_HQ_transcript/103494	-1.78380825660032	-0.140908147405144	-1.45814392106974	0.154372102023972	0.923224544035102	-4.85408910241401
a_HQ_transcript/103419	-2.2104499653713	0.508893488655147	-1.45810219890066	0.154383530959474	0.923224544035102	-4.94771841573593
a_HQ_transcript/109883	0.85340250151777	5.11078048533244	1.45808916213542	0.154387102252811	0.923224544035102	-5.36605920106362
a_HQ_transcript/55622	-1.47530983660331	0.61057031741995	-1.45808202391451	0.154389057725899	0.923224544035102	-4.73809560437287
a_HQ_transcript/90806	-1.9493076100641	-0.0952967225724233	-1.45805915609687	0.154395322360888	0.923224544035102	-4.81250456308411
a_HQ_transcript/137480	2.12554346081241	0.227519936091176	1.45794831047177	0.154425691366399	0.923224544035102	-4.91024625383304
a_HQ_transcript/115494	3.52530346639346	2.33185020932678	1.45792672461789	0.154431605917843	0.923224544035102	-5.02103177060576
a_HQ_transcript/40769	1.93101442647156	-0.173006412540631	1.45786110237017	0.154449587603285	0.923224544035102	-4.85827878124017
a_HQ_transcript/138608	0.529674820891645	3.620927963373	1.45785303811495	0.154451797470245	0.923224544035102	-5.19285705945466
a_HQ_transcript/98281	0.649116670627041	2.65882364092681	1.45784344110476	0.154454427394441	0.923224544035102	-4.95605451129097
a_HQ_transcript/102791	1.55683377713745	0.406176379630317	1.45761091233588	0.154518159479897	0.923224544035102	-4.83196691714141
a_HQ_transcript/152433	2.01914787741688	3.13639070762751	1.4575220109137	0.154542531335081	0.923224544035102	-5.08211755518132
a_HQ_transcript/44962	1.17574117360239	0.510290161829307	1.45749063265493	0.154551134248573	0.923224544035102	-4.79731238728341
a_HQ_transcript/147166	6.34828731439591	3.21453550882074	1.45737217775555	0.154583614219234	0.923224544035102	-4.84492212713672
a_HQ_transcript/107721	-5.84408897777218	1.92779821884845	-1.45734264851756	0.154591711890754	0.923224544035102	-5.06942447906932
a_HQ_transcript/129374	-0.390073331931587	4.11965269420456	-1.45731561901971	0.15459912436525	0.923224544035102	-5.21847206469948
a_HQ_transcript/36662	-2.30203945715004	1.04904344507066	-1.45730250012716	0.154602722146079	0.923224544035102	-4.85828543817
a_HQ_transcript/88015	-1.97645440744835	0.307172484734614	-1.45721770438198	0.15462597849696	0.923224544035102	-4.87693382431186
a_HQ_transcript/23973	0.727338912298607	2.69520468428676	1.45720763019832	0.154628741659437	0.923224544035102	-5.01969996552934
a_HQ_transcript/45677	1.95730488249412	3.04197693316191	1.45718077493123	0.15463610775488	0.923224544035102	-5.04983623767249
a_HQ_transcript/108035	1.9621822006289	-0.0728394613254496	1.45716087916204	0.154641565118784	0.923224544035102	-4.80154083964261
a_HQ_transcript/136175	2.45869708567575	2.1489509644539	1.4571524524854	0.154643876583035	0.923224544035102	-4.97541996621205
a_HQ_transcript/135990	1.97240521930099	-0.043008577565054	1.45714983989429	0.154644593230678	0.923224544035102	-4.84550776496491
a_HQ_transcript/61092	-1.61014857414385	-0.188426539953682	-1.4571289266908	0.154650329928814	0.923224544035102	-4.82149744693229
a_HQ_transcript/61721	-1.84619108914314	-0.306842956215753	-1.45711045922939	0.154655395876401	0.923224544035102	-4.84092846535768
a_HQ_transcript/42555	2.03759954889306	2.28256741344806	1.45706425449722	0.154668071220263	0.923224544035102	-5.07012872114216
a_HQ_transcript/7590	-2.04892045648312	1.15475904808784	-1.45703212601185	0.154676885514163	0.923224544035102	-4.84599829372136
a_HQ_transcript/51297	0.380359814433252	3.66605500586074	1.45702728850728	0.154678212694629	0.923224544035102	-5.2117949443848
a_HQ_transcript/67842	-1.80989515250404	2.20818370246505	-1.45691864559581	0.154708021510903	0.923224544035102	-4.86311460648071
a_HQ_transcript/50749	1.21191935907461	2.25684487736715	1.45687552672096	0.15471985348511	0.923224544035102	-4.88415744773084
a_HQ_transcript/99879	-2.31073709425476	-0.162709487706148	-1.45685243535789	0.154726190133841	0.923224544035102	-4.85622802975109
a_HQ_transcript/17264	-1.17224295719561	2.4912184973824	-1.45679708593898	0.154741379761955	0.923224544035102	-4.91158013191286
a_HQ_transcript/134110	0.798228268177306	2.78424575559744	1.45675960881967	0.154751665339719	0.923224544035102	-5.02883514278094
a_HQ_transcript/48019	-2.07462149006632	0.0537021489089865	-1.45670935688026	0.15476545781367	0.923224544035102	-4.89123350924158

a_HQ_transcript/112233	-3.16428257577126	1.82896719539163	-1.4566707080212	0.154776066295453	0.923224544035102	-4.79464659628498
a_HQ_transcript/70657	-2.23686211542762	0.979055207172547	-1.45660078098333	0.154795261596272	0.92324430187442	-4.94683017093323
a_HQ_transcript/21120	-2.04088924284392	0.264721490915476	-1.45653982161727	0.154811996772649	0.923249384261879	-4.78894380081541
a_HQ_transcript/146351	-0.684823416239216	3.11427755279154	-1.45627430008677	0.154884907178705	0.92351281564526	-5.121286237176
a_HQ_transcript/17370	1.40459507015952	-0.0626602546703621	1.45621226123949	0.154901946556572	0.92351281564526	-4.76313063774824
a_HQ_transcript/25911	-2.3474171948224	2.7872073480748	-1.45620539646363	0.154903832103999	0.92351281564526	-5.1230470768264
a_HQ_transcript/9376	-1.69879702693402	-0.47438439050982	-1.45607239674659	0.154940366725675	0.92363589755205	-4.8781273697344
a_HQ_transcript/100678	1.33100851190749	0.490795453024843	1.45590057617831	0.154987575455585	0.923822578556425	-4.81595202222621
a_HQ_transcript/82890	1.68859131319793	2.29381429270874	1.45577781658311	0.155021311389639	0.923917335193073	-4.92544476069784
a_HQ_transcript/134063	-2.02452923669073	-0.188578039262519	-1.4557244414647	0.155035981386701	0.923917335193073	-4.89136995808158
a_HQ_transcript/111709	-1.40162853740852	1.05245664503141	-1.45566923402675	0.155051156152085	0.923917335193073	-4.8106503313705
a_HQ_transcript/151978	-0.502512090132097	5.78890044074433	-1.45554276264162	0.155085923554402	0.924013761035627	-5.40006088475434
a_HQ_transcript/55220	-1.49027378578186	1.34708441877727	-1.45549472585198	0.155099130650024	0.924013761035627	-4.75446010704724
a_HQ_transcript/24304	-4.54851077763631	1.65275826222037	-1.45526490255062	0.155162329968347	0.924066903744668	-4.79634605547283
a_HQ_transcript/137099	0.869087273537049	3.96061431181234	1.4552602400471	0.155163612326158	0.924066903744668	-5.1234192800706
a_HQ_transcript/4850	-0.761683849294521	3.89950875299013	-1.45523959596297	0.155169290300277	0.924066903744668	-5.08355269373082
a_HQ_transcript/6257	0.622045808654347	3.97031562893073	1.45509621644835	0.155208730132249	0.924066903744668	-5.21027719076431
a_HQ_transcript/87299	3.87800945738685	3.61458473995517	1.45506880100529	0.155216272287157	0.924066903744668	-5.08472181981294
a_HQ_transcript/39217	-1.88870597180865	-0.239682179403647	-1.45506497426471	0.155217325069895	0.924066903744668	-4.86577869781375
a_HQ_transcript/13013	1.14277616773568	3.75599053788033	1.45497163058364	0.155243006805481	0.924066903744668	-5.26401051799314
a_HQ_transcript/115021	2.47834045004535	0.852477790175648	1.45495814069609	0.155246718570236	0.924066903744668	-4.77897614058393
a_HQ_transcript/108472	-2.04799463791005	0.269968425251405	-1.45491935770323	0.155257390167271	0.924066903744668	-4.82446162371898
a_HQ_transcript/10857	-3.6325022825306	1.5523956464355	-1.45488435563844	0.15526702189778	0.924066903744668	-4.96142979644964
a_HQ_transcript/151761	2.11323439180107	1.44042158341334	1.45474306814662	0.155305905659701	0.924174704961693	-4.88116127860041
a_HQ_transcript/54051	-1.6696884702366	0.21052970258562	-1.4547030037636	0.155316933195203	0.924174704961693	-4.8959647609444
a_HQ_transcript/47129	2.23137227585009	0.937147644001904	1.45452256689758	0.15536660531483	0.924307495130839	-4.95490934502772
a_HQ_transcript/125090	0.426048611120429	3.92603092876437	1.45442430802184	0.15539366011956	0.924307495130839	-5.21971578674653
a_HQ_transcript/128881	-0.64367542611418	3.19424299644019	-1.45440767321607	0.155398240752245	0.924307495130839	-5.12738957989358
a_HQ_transcript/108073	-1.78558208395368	1.11696114131825	-1.45439091707448	0.155402854904929	0.924307495130839	-4.71324629305565
a_HQ_transcript/113852	1.78083341815993	0.563545169280385	1.45412699394362	0.155475545995925	0.924480721451133	-4.83042895100107
a_HQ_transcript/58188	-0.755670925562298	3.5114935925627	-1.45401526616854	0.155506326780854	0.924480721451133	-5.0382593454605
a_HQ_transcript/115719	1.22119240804967	3.14601813873125	1.45393325761453	0.155528923058377	0.924480721451133	-4.97294112958431
a_HQ_transcript/37650	0.686939909045064	3.92436522598121	1.45380301821847	0.155564814010399	0.924480721451133	-5.15868718981476
a_HQ_transcript/42468	-2.37887412502421	1.03752444512714	-1.45368992039361	0.155595986480077	0.924480721451133	-4.72951905677019
a_HQ_transcript/80636	2.31923998800052	1.68733618670243	1.45366640111548	0.1556024695796	0.924480721451133	-4.89731797248003
a_HQ_transcript/106669	1.598239933769	1.03565030286035	1.45364748441967	0.155607684130928	0.924480721451133	-4.80424427047445
a_HQ_transcript/106958	-1.05897469366096	2.34809011002422	-1.45364402846401	0.155608636810084	0.924480721451133	-4.90718438215198
a_HQ_transcript/21941	1.4736974207628	3.24142999214112	1.45360347409232	0.155619816496591	0.924480721451133	-5.10926199126078
a_HQ_transcript/19658	-1.28518103689491	4.5706362853069	-1.45360004841695	0.155620760887047	0.924480721451133	-5.28942450480309
a_HQ_transcript/80309	-1.69743779997233	1.62772654658261	-1.45354061382854	0.155637146543679	0.924480721451133	-4.7922563680604
a_HQ_transcript/97869	-2.46057479711321	0.862915597926885	-1.45350008946126	0.155648319584143	0.924480721451133	-4.93087222317184
a_HQ_transcript/19643	-0.964647077078519	3.00939640665401	-1.45348029061694	0.155653778587965	0.924480721451133	-4.98138117895319
a_HQ_transcript/89334	2.22784844239211	0.296738308011342	1.45338951346531	0.1556788099159	0.924480721451133	-4.8679662517211
a_HQ_transcript/718	-0.684425290613106	3.46417104165158	-1.45335214293625	0.155689115574037	0.924480721451133	-5.14434322953493
a_HQ_transcript/12537	2.05698199985066	0.483674618681412	1.45329058363334	0.155706092943481	0.924480721451133	-4.9335478415641
a_HQ_transcript/8171	0.772592103611709	2.25029184445416	1.4531646493696	0.155740828789891	0.924480721451133	-4.85963596221871
a_HQ_transcript/133340	-2.03016007216928	1.21476008809233	-1.45312821281892	0.155750880057377	0.924480721451133	-4.77358608162756
a_HQ_transcript/79612	-2.38079007363218	0.626652225575474	-1.4531107776558	0.155755689846573	0.924480721451133	-4.8632226807374
a_HQ_transcript/3755	0.787188662644772	2.6879906099062	1.45307925295508	0.155764386776161	0.924480721451133	-5.01876483780282
a_HQ_transcript/75655	4.8990632275595	2.65997088175933	1.45307352159773	0.155765967965394	0.924480721451133	-4.88419262500144

a_HQ_transcript/125106	-1.88582915934327	1.36471322807095	-1.45281390542667	0.155837605254488	0.924811466894626	-4.73248434529522
a_HQ_transcript/143409	-1.07908123780239	3.8192363442651	-1.45274870314691	0.155855600980684	0.924823843793916	-5.19516157031205
a_HQ_transcript/73741	-1.26515350333829	0.00154347013577636	-1.45267621903386	0.155875608418208	0.924848154142452	-4.81457867210761
a_HQ_transcript/59310	-2.08836923462363	0.403532883697712	-1.45250667080269	0.155922415957215	0.924979069799775	-4.752154082118
a_HQ_transcript/32246	-3.16429671257242	3.36491657318385	-1.45248101863267	0.155929498778007	0.924979069799775	-5.16038150660054
a_HQ_transcript/82044	1.3670690714328	0.745185203146968	1.45221685271473	0.156002452493143	0.925317403930759	-4.86386716660076
a_HQ_transcript/25029	2.23735258683594	3.22926006047772	1.45203264312604	0.156053341003184	0.925524804186625	-4.90574263007336
a_HQ_transcript/1294	1.26722457122466	2.32414229486502	1.45190413437289	0.1560888497761	0.925569556561418	-4.8207426717015
a_HQ_transcript/120864	-5.19456045696747	0.473569805672207	-1.45189008341784	0.156092732641267	0.925569556561418	-4.92565300009575
a_HQ_transcript/148713	-3.05686452402841	0.156741315850588	-1.45175655261952	0.156129636599774	0.925644573016355	-4.89478296257693
a_HQ_transcript/47041	-0.990073475302924	3.37408527197916	-1.45172907200292	0.156137232287802	0.925644573016355	-5.05430111818943
a_HQ_transcript/98553	1.81055221849924	2.62607204644473	1.45163637636778	0.156162855678887	0.925702067601706	-4.97479381479067
a_HQ_transcript/102453	3.80165182850276	4.05309452493722	1.45137841345357	0.156234180649306	0.925718301207711	-5.25535306539031
a_HQ_transcript/147	1.26575881285321	0.918760392924651	1.45131688702547	0.156251196102085	0.925718301207711	-4.78326024152008
a_HQ_transcript/1350	-0.977166685488903	3.42578342152063	-1.45131053688965	0.156252952348825	0.925718301207711	-5.12337834508193
a_HQ_transcript/83739	2.37755234038329	1.25546531783349	1.45126868752951	0.156264526950226	0.925718301207711	-4.84750029474792
a_HQ_transcript/68661	-1.31913426180118	0.426724583399161	-1.4512504611243	0.156269568180922	0.925718301207711	-4.84760446443981
a_HQ_transcript/145594	2.88496145882022	0.510921814493547	1.45121927375162	0.156278194577422	0.925718301207711	-4.90737850252065
a_HQ_transcript/33108	-2.87720909700695	1.07721001298572	-1.45109801210785	0.15631173902052	0.925718301207711	-4.92462619974863
a_HQ_transcript/64421	2.20970127572446	0.988813263864863	1.45106610798498	0.15632056556409	0.925718301207711	-4.87824823032546
a_HQ_transcript/6848	0.837128258199697	2.9675010382606	1.45085145883061	0.156379960340636	0.925718301207711	-5.02065565393577
a_HQ_transcript/149120	-3.52020063093384	3.57867232228586	-1.45082013668057	0.156388628874743	0.925718301207711	-5.22516233581926
a_HQ_transcript/121631	-1.97224780555152	0.451139579527504	-1.45080397567689	0.156393101648271	0.925718301207711	-4.85388241729626
a_HQ_transcript/4269	-2.87228715150601	0.420725806234878	-1.45080224429479	0.156393580837401	0.925718301207711	-4.80423082641532
a_HQ_transcript/91741	1.5171564283908	0.274953780955009	1.45077319569885	0.15640162069896	0.925718301207711	-4.7408570233744
a_HQ_transcript/79300	1.23096777942336	1.67115522689403	1.45069744200842	0.156422588804032	0.925718301207711	-4.81422669629844
a_HQ_transcript/34777	-0.757532147987899	2.46443575030925	-1.45062692830413	0.156442108521566	0.925718301207711	-4.91614729242901
a_HQ_transcript/151981	-1.92333440232785	2.64459259784663	-1.45060531538613	0.156448091831511	0.925718301207711	-5.0933935680253
a_HQ_transcript/1483	1.3724908335971	1.96239688520978	1.45051147830025	0.156474071751882	0.925718301207711	-4.86802946567617
a_HQ_transcript/96154	0.895160994243187	2.20837451934962	1.45046153763937	0.15648789981757	0.925718301207711	-4.98038519879758
a_HQ_transcript/148823	2.55560219327995	1.29649498828088	1.45037057645541	0.156513088544599	0.925718301207711	-4.92246738318465
a_HQ_transcript/77477	2.20168402933646	0.103759838801826	1.45034491933062	0.156520194028536	0.925718301207711	-4.8550930825372
a_HQ_transcript/127682	-1.2322457536044	1.5356342019864	-1.45030018734463	0.156532582716622	0.925718301207711	-4.79036516453829
a_HQ_transcript/69514	2.24629564227533	0.080578961363366	1.45028508857933	0.156536764550878	0.925718301207711	-4.89540274204913
a_HQ_transcript/86582	-2.1226089520563	0.620316347823768	-1.45023143137001	0.156551626454052	0.925718301207711	-4.81060122946378
a_HQ_transcript/146637	-2.03082064717403	1.52639759061213	-1.45022142192814	0.156554398980002	0.925718301207711	-4.9177422799278
a_HQ_transcript/151291	-3.73113568090722	0.34369981598192	-1.45015318990823	0.156573299677942	0.925718301207711	-4.97501696074499
a_HQ_transcript/20643	-1.81873607950256	1.6861266468583	-1.45009568842902	0.156589229354753	0.925718301207711	-4.89456825178379
a_HQ_transcript/89265	2.16939802518727	0.563946526335137	1.4499957220883	0.156616926164669	0.925718301207711	-4.85280658910062
a_HQ_transcript/47752	-0.53561674301228	3.40549736671222	-1.44999127690611	0.156618157843145	0.925718301207711	-5.13968595295079
a_HQ_transcript/109531	2.78036763213717	2.61335988984719	1.44995779918823	0.156627434151835	0.925718301207711	-5.07739783955256
a_HQ_transcript/150722	-0.540607987557566	10.2053006740432	-1.44985709143535	0.15665534179483	0.925789112225484	-5.87769973086216
a_HQ_transcript/94755	-1.85566349509393	-0.435885510653462	-1.44975924743367	0.156682459627972	0.925855241766264	-4.88298185749154
a_HQ_transcript/134440	2.14389847112369	-0.337797439876503	1.44965465108515	0.156711453018643	0.92588659935012	-4.87217756470505
a_HQ_transcript/40680	-1.56035653550689	-0.22746915596958	-1.44957905386979	0.156732410698616	0.92588659935012	-4.85956661764571
a_HQ_transcript/35186	-1.04998386478327	1.91366911231742	-1.44955873074506	0.156738045221188	0.92588659935012	-4.85899585108246
a_HQ_transcript/51652	-3.31394074305375	1.95450714960246	-1.4493422814674	0.156798065076258	0.92588659935012	-5.1654985595717
a_HQ_transcript/90348	1.77385122469718	1.64583196980688	1.44917881770153	0.156843404508233	0.92588659935012	-4.82575263309108
a_HQ_transcript/1237	-2.84881657753414	1.61685414573075	-1.44905620426535	0.15687742023919	0.92588659935012	-4.83787049703379
a_HQ_transcript/20759	0.950302387456904	2.41785860988972	1.44905354758206	0.156878157327793	0.92588659935012	-4.8789924275728

a_HQ_transcript/117219	-5.57638518212386	5.23820247972375	-1.44904304253618	0.156881071947359	0.92588659935012	-5.25211277490613
a_HQ_transcript/131424	-0.433998294204707	4.51542698072222	-1.44883619173117	0.156938471348413	0.92588659935012	-5.30748742635244
a_HQ_transcript/50513	2.31263649113462	3.91229247076646	1.44880918996341	0.156945965346625	0.92588659935012	-5.04713426528128
a_HQ_transcript/1663	1.9998926003238	-0.263102721141939	1.44879826032187	0.156948998810739	0.92588659935012	-4.87157786669664
a_HQ_transcript/68672	-0.658900514062581	5.24449962850401	-1.44875054692109	0.156962241957124	0.92588659935012	-5.40179372308825
a_HQ_transcript/83376	-0.761956092587955	2.05068732979166	-1.44874023623713	0.156965103867033	0.92588659935012	-4.8789233404081
a_HQ_transcript/18601	0.781790027761408	3.38177889351494	1.44867716043236	0.15698261255644	0.92588659935012	-5.10537962721121
a_HQ_transcript/100035	1.41506567746218	2.47246697367909	1.44865403987077	0.156989030789358	0.92588659935012	-4.91500382096236
a_HQ_transcript/139120	3.03553518973703	1.13947238490327	1.4486519419227	0.156989613186871	0.92588659935012	-4.87322094511698
a_HQ_transcript/149296	1.51170903248041	-0.487545894068208	1.44860982125478	0.157001306390032	0.92588659935012	-4.87509708935124
a_HQ_transcript/65645	-1.73561303165522	0.114586032799107	-1.44854636225779	0.157018924674138	0.92588659935012	-4.87459899253436
a_HQ_transcript/101306	1.33376281196032	0.209936910185161	1.44850807730364	0.157029554582653	0.92588659935012	-4.80207552771957
a_HQ_transcript/97242	-1.39872035405556	1.45445693867029	-1.4484445365549	0.157047198082486	0.92588659935012	-4.91048040194819
a_HQ_transcript/91456	-2.21181672068744	0.847005739152392	-1.44840759225254	0.157057457211333	0.92588659935012	-4.84646192440328
a_HQ_transcript/137845	1.40243060101739	0.258434653120023	1.44838237891554	0.157064459052808	0.92588659935012	-4.97487560594585
a_HQ_transcript/81302	-1.64018337403631	1.80482623212517	-1.44833851901281	0.157076639707908	0.92588659935012	-4.91110942020748
a_HQ_transcript/115343	4.11876129366003	4.78239139774949	1.44818646321479	0.157118874040176	0.92588659935012	-5.34568615612137
a_HQ_transcript/60628	1.88041416715645	0.469241510994596	1.44813472844949	0.157133245708634	0.92588659935012	-4.95461217549828
a_HQ_transcript/140737	-2.23063845925432	-0.267995792055314	-1.44810814460407	0.157140630978314	0.92588659935012	-4.83635287864713
a_HQ_transcript/99816	2.04053473341761	0.296859600554217	1.44805141541127	0.157156391860729	0.92588659935012	-4.90423713218454
a_HQ_transcript/103824	2.59803030001003	2.62441489821239	1.44803901838564	0.157159836251941	0.92588659935012	-5.07999129598248
a_HQ_transcript/71585	-0.324923214585828	5.06989246620394	-1.4480150258639	0.157166502507383	0.92588659935012	-5.3870842312786
a_HQ_transcript/119590	-1.7697603028149	0.33348416428126	-1.44794365737513	0.157186333369324	0.92588659935012	-4.77181049975427
a_HQ_transcript/108806	-2.9681379332152	2.37583703525127	-1.44793178777154	0.157189631718651	0.92588659935012	-4.93197541791052
a_HQ_transcript/112672	-2.89029325243112	1.97247177218988	-1.4479035619699	0.157197475381681	0.92588659935012	-5.00916971977459
a_HQ_transcript/74426	1.32623709709798	-0.566117378849527	1.44779017616301	0.157228987271819	0.92597837610297	-4.823402454698
a_HQ_transcript/25469	-2.3957856060388	2.56100228201043	-1.44749922558873	0.157309870425264	0.926360870031378	-4.8476189966703
a_HQ_transcript/117657	4.13367397268609	2.55390142087291	1.44718967197878	0.15739596137902	0.926717384501073	-5.09298156437163
a_HQ_transcript/140280	1.75451570681992	1.05596859930005	1.44716689549711	0.157402297295264	0.926717384501073	-4.80805612403473
a_HQ_transcript/109815	-3.05748209167803	2.25216263278437	-1.44693074144101	0.157468002080176	0.926831521240361	-4.79098417959722
a_HQ_transcript/33364	-1.90129337789616	3.79169405097958	-1.44690361396389	0.157475551109454	0.926831521240361	-4.99465874186203
a_HQ_transcript/60408	3.47501811058781	3.1896524370115	1.44689671622569	0.157477470656516	0.926831521240361	-5.30694572476775
a_HQ_transcript/152157	-3.51693755556713	1.48523618987949	-1.44681706377884	0.157499638197204	0.926831521240361	-4.96577930358417
a_HQ_transcript/79049	-2.37461647603073	0.514299776876211	-1.44681070968839	0.157501406668254	0.926831521240361	-4.86893404053503
a_HQ_transcript/21678	3.07190179493926	0.217913634918007	1.44671011539734	0.157529406178625	0.926842581488398	-4.83920469427417
a_HQ_transcript/139056	1.91286477870156	1.4051606217776	1.44668938816424	0.157535175906712	0.926842581488398	-4.8986183755141
a_HQ_transcript/150933	-1.37097123680633	1.8000785321644	-1.44654410738481	0.157575621637095	0.926986715364948	-4.83004386783061
a_HQ_transcript/57897	-2.48942312509037	0.396051218181883	-1.44642476947269	0.15760885111047	0.927088372530805	-4.83929069149922
a_HQ_transcript/55930	2.31699611084411	1.68495322426984	1.44625821739553	0.157655236589156	0.927221301256632	-4.96192212509623
a_HQ_transcript/48293	1.7105760614378	0.985027116317139	1.44622908110567	0.157663352284967	0.927221301256632	-4.79524547669755
a_HQ_transcript/134581	2.90809272150473	4.49973415494999	1.44615681527803	0.157683482820787	0.927239344218056	-5.2327441178332
a_HQ_transcript/86856	1.17846753226087	3.43487954895347	1.44608506982122	0.157703470416643	0.927239344218056	-5.20347807248383
a_HQ_transcript/68483	-1.40503367520779	2.96701715478669	-1.44602579530315	0.157719985243834	0.927239344218056	-5.26068022845814
a_HQ_transcript/125848	-4.36150338863025	2.53118316095028	-1.44598903833837	0.157730227009843	0.927239344218056	-5.00879495876398
a_HQ_transcript/145241	1.54548256090086	0.0780845741238634	1.44591271862881	0.157751494013436	0.927252722470773	-4.83584927604508
a_HQ_transcript/105202	1.13937528153174	3.15797803767654	1.4458663840286	0.157764406572322	0.927252722470773	-5.0379517094945
a_HQ_transcript/141320	-2.05711197068064	0.830795303582174	-1.44572295381031	0.157804383117562	0.927295481232747	-4.80203222318057
a_HQ_transcript/143764	-0.461196363397228	4.61246411032917	-1.44566691793467	0.157820003491079	0.927295481232747	-5.31181642126287
a_HQ_transcript/8165	1.3457164717252	4.18509566269972	1.44559813713224	0.157839178273344	0.927295481232747	-5.26943118102662
a_HQ_transcript/147701	1.6433738776295	-0.377011290737092	1.44543662040229	0.157884213332343	0.927295481232747	-4.87390733211467

a_HQ_transcript/78046	3.45904318779932	0.424056448439236	1.44541430245588	0.157890436956272	0.927295481232747	-4.88753620461217
a_HQ_transcript/74101	-1.25739543953898	2.28288512412012	-1.44534864075626	0.157908748627319	0.927295481232747	-4.86262184908399
a_HQ_transcript/97652	2.05662955984171	-0.247749608817616	1.44532718540065	0.157914732440946	0.927295481232747	-4.88097034514912
a_HQ_transcript/120558	1.41595033619375	4.54039773254993	1.44530579373212	0.157920698671432	0.927295481232747	-5.37104458865591
a_HQ_transcript/117466	-2.54385248125967	-0.0505498735009722	-1.44529319893973	0.15792421149802	0.927295481232747	-4.94165649178086
a_HQ_transcript/69320	-1.54568894329699	-0.278995632672421	-1.44521431692763	0.157946213970455	0.927295481232747	-4.85256281899394
a_HQ_transcript/97225	2.06127686376644	0.958284313113442	1.44519104663086	0.157952705192707	0.927295481232747	-4.78616633412913
a_HQ_transcript/24947	-1.4193401436036	2.47107482158794	-1.44512581415497	0.157970902843278	0.927295481232747	-4.96224182355253
a_HQ_transcript/53357	2.94077713764195	2.0008578622973	1.44509655377462	0.157979066037846	0.927295481232747	-5.06771966795738
a_HQ_transcript/104075	-1.76298217231483	-0.322394563014124	-1.4448680457597	0.158042827749312	0.927507092660741	-4.87214189060838
a_HQ_transcript/41293	1.88641942804837	0.11039934761765	1.44484871146779	0.158048223627316	0.927507092660741	-4.87019610074801
a_HQ_transcript/15198	-0.943478600715657	1.76978249530437	-1.444795817607	0.158062986166689	0.927507092660741	-4.84798166429899
a_HQ_transcript/105883	6.92220518236272	4.44081906406021	1.44473837416085	0.158079019721407	0.927507546014531	-4.9100376345434
a_HQ_transcript/23002	-2.41494563616836	1.01991392406634	-1.44443256021042	0.158164399890493	0.927887391432631	-4.9001521189147
a_HQ_transcript/98329	1.52762952246654	2.73901770936781	1.44439215254062	0.158175684037055	0.927887391432631	-5.08529842610015
a_HQ_transcript/45756	-2.91215039229731	1.7842659554648	-1.4442380747519	0.15821871727615	0.927995120057102	-4.96394147225846
a_HQ_transcript/146965	2.10240309552389	0.196443192005734	1.44415101426786	0.158243036982857	0.927995120057102	-4.91944202353131
a_HQ_transcript/70598	2.41561184337607	2.00408841313082	1.44403466939522	0.158275541692696	0.927995120057102	-4.92121807299694
a_HQ_transcript/108120	2.26162363874741	0.420592695575438	1.44396679799323	0.15829450621125	0.927995120057102	-4.92588253248833
a_HQ_transcript/150395	2.94299954591892	1.44461942058349	1.44391503427753	0.158308971156129	0.927995120057102	-4.81598665285137
a_HQ_transcript/145653	-0.71701101372388	2.49367856414649	-1.44381767995314	0.158336178859157	0.927995120057102	-4.94750737396271
a_HQ_transcript/543	-2.02847943915545	1.16938506032476	-1.44377334542205	0.158348570299314	0.927995120057102	-4.79363414123499
a_HQ_transcript/129962	1.85921015192345	0.229489077017216	1.44374347578329	0.158356919255289	0.927995120057102	-4.76312915056751
a_HQ_transcript/110991	1.65050820780695	-0.264712980458547	1.44373786255567	0.158358488264997	0.927995120057102	-4.81901437117056
a_HQ_transcript/471	-2.39819402927962	1.0738348161309	-1.44362127815525	0.15839107872806	0.927995120057102	-4.83711296519776
a_HQ_transcript/145437	2.65820491829511	0.0235329092119564	1.44361753466654	0.158392125285708	0.927995120057102	-4.88980466331482
a_HQ_transcript/85991	-2.20369459350877	1.4307929493323	-1.44360361727537	0.158396016183529	0.927995120057102	-4.89267673855065
a_HQ_transcript/152040	-2.29895610482909	0.309396212795853	-1.44358368331133	0.158401589272064	0.927995120057102	-4.91784463004001
a_HQ_transcript/146982	3.55045616520135	-0.0123514512816906	1.44345668713009	0.158437098199275	0.928109608397407	-4.88057405709033
a_HQ_transcript/70891	-1.63425242055996	-0.157383919539337	-1.44331436498504	0.158476899855831	0.928170233356696	-4.8785643988745
a_HQ_transcript/47677	1.36895440624088	1.92980649444044	1.44324924721647	0.158495113261488	0.928170233356696	-4.80198377975953
a_HQ_transcript/24345	0.972980521998891	-0.468407723101323	1.44324839891508	0.158495350541883	0.928170233356696	-4.85518867957844
a_HQ_transcript/48236	1.53739078045244	2.66212337797813	1.44314744823044	0.158523589707074	0.928242089502649	-5.04745045900706
a_HQ_transcript/6842	1.50035970527214	0.140469607611086	1.44286492864239	0.15860264074295	0.928611432424071	-4.86034623832499
a_HQ_transcript/131057	-1.53104510612883	1.06571208786344	-1.44274727358783	0.158635570692101	0.928680715546509	-4.96403500359524
a_HQ_transcript/150819	1.56952368784418	3.27235322015137	1.44256416857698	0.158686829911077	0.928680715546509	-4.96402633568487
a_HQ_transcript/73415	1.50573317969902	0.567319061607399	1.44251366110783	0.158700971501682	0.928680715546509	-4.92216583397292
a_HQ_transcript/15692	2.38863844206433	1.66442494403012	1.4425063596579	0.158703015917857	0.928680715546509	-4.83917603344105
a_HQ_transcript/132915	-1.65414231223312	0.380183103415612	-1.44248879336445	0.158707934590015	0.928680715546509	-4.83914833956557
a_HQ_transcript/139481	-2.79777250852515	0.484544448769385	-1.44247666155437	0.158711331643294	0.928680715546509	-4.93128688266424
a_HQ_transcript/1890	-1.75701360145865	1.5244700734865	-1.44242317456132	0.15872630933379	0.928680715546509	-4.86584591462305
a_HQ_transcript/35381	0.481650404403253	3.11895533978701	1.44236072600168	0.158743797902827	0.928689561644074	-5.10591516984879
a_HQ_transcript/132717	2.46706909324237	0.518356648580056	1.4422565517431	0.15877297505017	0.928766780086169	-4.94541274535919
a_HQ_transcript/19296	-1.90563775674766	1.85967686713218	-1.44219154031549	0.158791185616348	0.928779838750965	-4.94203367132238
a_HQ_transcript/133573	-1.12183315519742	2.55807676969135	-1.44200722831321	0.158842822854977	0.928914658408158	-5.04241494111013
a_HQ_transcript/134217	2.39661407500309	0.938480279550486	1.44196751165149	0.158853951700058	0.928914658408158	-4.80487834736642
a_HQ_transcript/129482	-2.576651792351	1.2031335004124	-1.44189474510068	0.158874342922977	0.928914658408158	-4.96477013915279
a_HQ_transcript/135144	2.29399750098242	0.272249214915805	1.44186124870436	0.158883730246923	0.928914658408158	-4.94841245305376
a_HQ_transcript/114578	-1.69272735864205	0.40505555177367	-1.44177995432846	0.158906514722751	0.928914658408158	-4.92609282187902
a_HQ_transcript/104566	3.04418959797614	0.160781265831719	1.4417670973013	0.158910118414718	0.928914658408158	-4.89072748597055

a_HQ_transcript/123172	-3.78389785074161	3.8521368618077	-1.441456819597	0.158997105681448	0.929329689195698	-5.04123513731692
a_HQ_transcript/25377	1.62593261728932	1.39940864389048	1.44131077446256	0.15903806291382	0.929389594152989	-4.75492774943868
a_HQ_transcript/87915	-1.42577262114746	2.74186308545445	-1.44129216991742	0.159043281015006	0.929389594152989	-5.08892122161697
a_HQ_transcript/93009	0.766262268104919	1.95912008608858	1.44124924557636	0.159055320716934	0.929389594152989	-4.92662044508677
a_HQ_transcript/3986	-1.03991933735776	3.33625077881429	-1.44116122956927	0.159080010283545	0.929440429969034	-5.01288798897052
a_HQ_transcript/118631	1.92111313110722	1.92396096038258	1.44085643540584	0.159165532226055	0.929618779983949	-4.88253926956081
a_HQ_transcript/123463	-1.47961166596301	0.448824965226119	-1.44080828862773	0.159179045019628	0.929618779983949	-4.90424876706893
a_HQ_transcript/31762	1.06250692590603	5.02492518777504	1.44080811559239	0.159179093585075	0.929618779983949	-5.37206096266502
a_HQ_transcript/127621	-2.85858975506942	2.18479169902341	-1.44077252933168	0.159189081751209	0.929618779983949	-4.92771187202714
a_HQ_transcript/5589	-3.02811960037965	0.915696947899038	-1.44071897697892	0.15920411348166	0.929618779983949	-5.0226290840431
a_HQ_transcript/122366	-0.98825922594726	2.11087207014857	-1.44062988777359	0.159229122621149	0.929618779983949	-4.89961664339079
a_HQ_transcript/133642	-0.756265525212651	5.53342367468793	-1.44051452770264	0.159261511144742	0.929618779983949	-5.40646055616453
a_HQ_transcript/110266	2.52911996443818	0.511273060563335	1.44050004991529	0.159265576300448	0.929618779983949	-4.86935227932197
a_HQ_transcript/24171	2.48219948425015	0.606368918506853	1.44040134515443	0.159293293375473	0.929618779983949	-4.89075187338737
a_HQ_transcript/62494	1.98576346831626	2.9289244275534	1.44036244587316	0.159304217649338	0.929618779983949	-5.04167042979445
a_HQ_transcript/98240	-2.6027913836736	1.38398689944578	-1.44029135686549	0.159324183452654	0.929618779983949	-4.8569145487199
a_HQ_transcript/62687	4.38858719089682	8.16304646476127	1.44027732506142	0.159328124608833	0.929618779983949	-5.41866108621445
a_HQ_transcript/38587	2.56386086990666	0.23904001887993	1.44014512205037	0.159365260666485	0.929618779983949	-4.88017583246222
a_HQ_transcript/89667	2.07601549534737	0.0378280289101553	1.44009296208975	0.159379914375861	0.929618779983949	-4.90905476423651
a_HQ_transcript/129367	1.66494241754519	-0.450646754177573	1.4400531732958	0.159391093272924	0.929618779983949	-4.89857058055921
a_HQ_transcript/86261	-1.54057936444976	-0.444091313528755	-1.43998794645029	0.159409420483887	0.929618779983949	-4.89142770621917
a_HQ_transcript/129846	-1.8879497315239	-0.0500937703115347	-1.43985614511376	0.159446458653616	0.929618779983949	-4.87162068273115
a_HQ_transcript/23805	0.901238705740784	2.54769445769172	1.43985209469247	0.159447596991163	0.929618779983949	-4.96389765485212
a_HQ_transcript/123174	-0.90481446313956	3.79999607837522	-1.43985097309712	0.159447912207439	0.929618779983949	-5.25683273020442
a_HQ_transcript/145787	-3.50684274558186	0.610111006208055	-1.43975068907404	0.159476098301882	0.929618779983949	-4.89706171428976
a_HQ_transcript/59314	-1.95704532625782	1.72097662279734	-1.43973383655473	0.159480835302877	0.929618779983949	-4.81430997964303
a_HQ_transcript/96989	-1.1803217590573	1.86054418039436	-1.43973130019577	0.159481548246574	0.929618779983949	-4.80060653630344
a_HQ_transcript/146705	-1.29004298204608	-0.669845651669025	-1.43967723229275	0.159496746762796	0.929618779983949	-4.89857162915954
a_HQ_transcript/60037	-1.81965457631706	-0.42131018926776	-1.43963844282851	0.15950765120807	0.929618779983949	-4.80088531337657
a_HQ_transcript/141470	2.17192962267891	0.0560940440072982	1.43962883765828	0.159510351492567	0.929618779983949	-4.87293973709722
a_HQ_transcript/148150	-0.637591712990072	3.6313113359068	-1.43944894302164	0.159560931638508	0.929732651709156	-5.19968584652842
a_HQ_transcript/140930	2.21875051303817	0.176712885739091	1.43939565808965	0.159575915954062	0.929732651709156	-4.89841956489365
a_HQ_transcript/130506	2.5075671127865	0.510161736106231	1.43938869509751	0.159577874107182	0.929732651709156	-4.99433361661647
a_HQ_transcript/17614	-2.40567178556604	0.266595101304378	-1.43927647218692	0.159609436387444	0.929735089769266	-4.94046262728086
a_HQ_transcript/23448	1.72141776999919	-0.515110994449607	1.43927346645717	0.159610281805799	0.929735089769266	-4.86801683471019
a_HQ_transcript/32984	-1.2968424148003	-0.65872826560397	-1.43916790528241	0.159639975131354	0.929750568244209	-4.90063734407261
a_HQ_transcript/40209	-2.54059534250129	1.40968563403494	-1.439150296247	0.159644928806177	0.929750568244209	-4.89381301745792
a_HQ_transcript/17823	1.39804358112201	1.58567906170581	1.43890924644267	0.159712751813713	0.929904106103696	-4.82720825512263
a_HQ_transcript/101555	-2.4784958127673	1.26900897165094	-1.43889183656342	0.159717651229351	0.929904106103696	-4.8742006492076
a_HQ_transcript/879	-1.37177308372545	1.98688224120463	-1.43888603131015	0.159719284945969	0.929904106103696	-4.93074740997034
a_HQ_transcript/71475	-2.09842336807697	0.510070266833211	-1.43880612574658	0.159741773344379	0.92994189293561	-4.9209257717083
a_HQ_transcript/96138	-0.802544217453761	2.67137698004891	-1.43861489605727	0.159795602667933	0.930090244363958	-5.05276465440188
a_HQ_transcript/111410	2.08494377768508	1.09210145199018	1.43860191218161	0.159799258024753	0.930090244363958	-4.84924273529274
a_HQ_transcript/152688	-1.00418038898026	6.6534416893525	-1.43847579841329	0.159834766338062	0.930203774299048	-5.49544880087992
a_HQ_transcript/71896	2.04724423405415	0.165491642429417	1.43832706997948	0.159876650009719	0.930251572001	-4.91182742806992
a_HQ_transcript/133918	-2.47126901158807	0.032378031907916	-1.43824921355926	0.159898578753839	0.930251572001	-4.89612238612217
a_HQ_transcript/94966	-0.419555166428809	4.48207539399352	-1.43823862578144	0.159901561051025	0.930251572001	-5.30218419867448
a_HQ_transcript/3360	-1.07329541657319	1.6787006265171	-1.43821934051019	0.159906993315338	0.930251572001	-4.87399091337995
a_HQ_transcript/31430	1.6155745316681	2.26725266171652	1.43764669612193	0.160068362017054	0.930689145142153	-4.91426026539762
a_HQ_transcript/20788	-2.98054264292544	0.940166956584167	-1.43760232300235	0.160080871546046	0.930689145142153	-4.85735535297572

a_HQ_transcript/25900	1.77178785000362	0.673309131180954	1.43757582613429	0.160088341827787	0.930689145142153	-4.75769263223563
a_HQ_transcript/125160	-0.435148481210061	4.42274847948691	-1.4375581426872	0.160093327488645	0.930689145142153	-5.32446554064755
a_HQ_transcript/54058	2.38556080761377	2.15965378589346	1.43742662795977	0.160130410529085	0.930689145142153	-5.0068473104239
a_HQ_transcript/23558	1.19318253164708	3.68371576452967	1.43742013485162	0.160132241558556	0.930689145142153	-5.28570805089919
a_HQ_transcript/97113	1.70812350191791	3.42873574928492	1.4373602166969	0.160149139010451	0.930689145142153	-5.03253142753256
a_HQ_transcript/8944	2.36897350119686	2.1443879471567	1.43724370189121	0.160182001259127	0.930689145142153	-4.89210701074599
a_HQ_transcript/18785	1.1064355834069	4.45753574572338	1.43721207049878	0.160190923609769	0.930689145142153	-5.31564690204732
a_HQ_transcript/134232	2.30516103407512	2.10129293595579	1.43720937846947	0.16019168297576	0.930689145142153	-4.99833122302444
a_HQ_transcript/138969	1.00526875394598	6.04632678607175	1.43715287278879	0.160207622721019	0.930689145142153	-5.41652971426924
a_HQ_transcript/130643	-1.49196613680326	1.06021593871242	-1.43714964532791	0.160208533196636	0.930689145142153	-4.82601602308646
a_HQ_transcript/112844	-1.65562847670595	-0.38346313523534	-1.43709849928142	0.16022296218388	0.930689145142153	-4.89456865618308
a_HQ_transcript/37824	1.49558833483892	-0.462008427483362	1.43709204436827	0.160224783274704	0.930689145142153	-4.91854970501479
a_HQ_transcript/138911	3.35339449070421	1.52814976458239	1.43704678105177	0.160237553636991	0.930689145142153	-5.07230664379431
a_HQ_transcript/147574	-2.12287969219308	0.57958623422573	-1.4370438270685	0.16023838708682	0.930689145142153	-4.87658509193119
a_HQ_transcript/33585	-2.04720140083848	-0.0657842708927099	-1.43695184098594	0.160264342160666	0.930746895905206	-4.83047207423707
a_HQ_transcript/98039	5.20018287614149	3.85894623967961	1.436773227879	0.160314749678207	0.930830788613941	-5.05258441818568
a_HQ_transcript/111171	-2.238184430274	5.94458880209509	-1.43668436487625	0.160339832931104	0.930830788613941	-5.50933059231564
a_HQ_transcript/68071	-0.504898541621185	3.28629523353112	-1.43666054245172	0.160346557785846	0.930830788613941	-5.16263370640808
a_HQ_transcript/50294	-1.3086891863009	2.117902280804	-1.43665219860048	0.160348913232253	0.930830788613941	-4.92873335514871
a_HQ_transcript/97525	-1.7129430913729	0.075992980858504	-1.43661698267856	0.160358854892307	0.930830788613941	-4.87736117378698
a_HQ_transcript/12809	-0.647608083195406	3.67800303609686	-1.43630193532926	0.1604478162877	0.931114340692875	-5.19254049622747
a_HQ_transcript/80314	2.6409900361406	2.12966223476265	1.43625918119681	0.160459891979722	0.931114340692875	-5.00874728288529
a_HQ_transcript/14973	2.64242929493245	0.57000025572646	1.43624636809229	0.160463511117341	0.931114340692875	-4.9265743164682
a_HQ_transcript/35142	-1.45949135057162	0.239176682418321	-1.43608439308571	0.160509267488552	0.931114340692875	-4.91345979561063
a_HQ_transcript/136996	-2.56586816740513	0.478741502428626	-1.43603650671614	0.160522796900452	0.931114340692875	-4.92452006481089
a_HQ_transcript/106292	-1.56150108012427	-0.257661975025739	-1.43602289702096	0.16052664223379	0.931114340692875	-4.89440853725656
a_HQ_transcript/107139	-0.703576054730564	4.73425542885136	-1.43596247382335	0.160543715305993	0.931114340692875	-5.3107054492198
a_HQ_transcript/24240	2.8715700060907	1.98351475998946	1.43571755338336	0.160612934301817	0.931114340692875	-4.88093467086263
a_HQ_transcript/150931	-0.738651811132514	8.38587891934274	-1.43569350245489	0.160619732806461	0.931114340692875	-5.68473608357847
a_HQ_transcript/112633	-3.1066934210089	4.52671025480559	-1.43562822063002	0.160638187244922	0.931114340692875	-5.35701485196004
a_HQ_transcript/19317	-0.779420697504297	2.57991114979388	-1.43561259902548	0.16064260354563	0.931114340692875	-4.96239051616107
a_HQ_transcript/152745	4.68297004569383	1.71527581036851	1.4356079838261	0.160643908302466	0.931114340692875	-4.95560745107705
a_HQ_transcript/43977	1.93204652081611	0.101969668088473	1.43553738842922	0.160663867277052	0.931114340692875	-4.83282876660758
a_HQ_transcript/118104	1.74634566722389	1.87694684285516	1.43550801782789	0.160672171612562	0.931114340692875	-4.99552922584407
a_HQ_transcript/130312	1.51874915093973	0.646420004244482	1.43547683761104	0.160680987975839	0.931114340692875	-4.80017564532185
a_HQ_transcript/125729	2.38144532028082	2.76218219279704	1.43544903342758	0.160688850071333	0.931114340692875	-5.14062420266593
a_HQ_transcript/45180	-1.25811977906374	1.46928591561255	-1.43542588584784	0.160695395666681	0.931114340692875	-4.81580956637509
a_HQ_transcript/111990	-1.35105578198609	-0.618831823223748	-1.43542362842152	0.160696034025442	0.931114340692875	-4.90850600850723
a_HQ_transcript/147749	1.16297637202812	1.59747096087907	1.43532943315902	0.160722672505767	0.931175870369304	-4.83564376722326
a_HQ_transcript/142462	-0.896569968252739	4.8329310766839	-1.43520741492175	0.160757184532029	0.931283000047618	-5.40497185151646
a_HQ_transcript/29078	2.03616400129588	0.126716292547488	1.43508901119362	0.160790679820659	0.931384218895393	-4.94139940571337
a_HQ_transcript/138243	1.84344034664043	1.86371193961641	1.43500303430613	0.160815005322523	0.931424563283459	-4.91523698519683
a_HQ_transcript/45814	1.61685384152099	-0.0832598590940802	1.43481643856854	0.160867808997265	0.931424563283459	-4.87622450949174
a_HQ_transcript/120836	-2.14805395728574	1.18814850559459	-1.43478285144175	0.160877315084344	0.931424563283459	-4.88183321091923
a_HQ_transcript/11271	-2.45482892925017	1.49538994782464	-1.43471884047116	0.160895433191558	0.931424563283459	-4.85895733846517
a_HQ_transcript/76879	1.71384506436255	1.26851362501486	1.43457289378293	0.160936748982387	0.931424563283459	-4.84941474888584
a_HQ_transcript/124700	-2.75090770670357	1.23009773945877	-1.43446915633286	0.160966120933659	0.931424563283459	-4.78265858231006
a_HQ_transcript/96487	1.83116235660334	-0.354286255554859	1.43444880571488	0.16097188345179	0.931424563283459	-4.88465270492288
a_HQ_transcript/49870	2.53597378055414	2.37554471761493	1.43443331148789	0.160976270934736	0.931424563283459	-4.96544723273948
a_HQ_transcript/6200	0.518750161740323	2.75138207001731	1.43440233978211	0.160985041441421	0.931424563283459	-4.99262912341006

a_HQ_transcript/76280	-2.20728816089172	1.94162846897917	-1.43435539468416	0.160998335982088	0.931424563283459	-4.90195295722438
a_HQ_transcript/67036	1.77980275842214	1.15987672789928	1.43421220630508	0.16103889134316	0.931424563283459	-4.86688021471869
a_HQ_transcript/47977	-2.38163867945489	0.554628513268114	-1.43419406892067	0.161044028985503	0.931424563283459	-4.88311563349278
a_HQ_transcript/108	-2.25093549677836	2.10718167322463	-1.43419347394225	0.161044197522821	0.931424563283459	-5.1099281437778
a_HQ_transcript/1606	-2.19409696808225	3.6474255176679	-1.43415977865225	0.161053742489517	0.931424563283459	-4.9106092369441
a_HQ_transcript/588	1.75332653412323	1.77892468372022	1.43411861391781	0.161065403955208	0.931424563283459	-4.84017847792622
a_HQ_transcript/147082	2.3690558288607	1.32336178470194	1.43405983392272	0.161082056765755	0.931424563283459	-4.92389463481786
a_HQ_transcript/73293	0.503129849596366	2.96979879422402	1.43397803243557	0.16110523400145	0.931424563283459	-5.11296821340246
a_HQ_transcript/114621	-0.92519969416676	1.68419220933642	-1.43396085474169	0.161110101380607	0.931424563283459	-4.8657041272619
a_HQ_transcript/114801	1.86776268285877	-0.272799584700094	1.43393036040332	0.161118742379022	0.931424563283459	-4.89164391030988
a_HQ_transcript/65616	-0.736250877307936	3.24815379027432	-1.43381269701524	0.161152087383945	0.931424563283459	-5.06887431594163
a_HQ_transcript/144628	1.70506483839621	1.92332712767031	1.43379231375043	0.161157864417019	0.931424563283459	-4.83789947176079
a_HQ_transcript/15351	-1.43830181270985	-0.398526665705605	-1.43377256652544	0.161163461339547	0.931424563283459	-4.89200754942878
a_HQ_transcript/141008	-1.24451181665747	3.26872412963789	-1.43370792131059	0.161181784698018	0.931424563283459	-5.12013706800917
a_HQ_transcript/114932	2.04788506484077	0.0363524337110618	1.43368422796417	0.161188500869299	0.931424563283459	-4.89528339957019
a_HQ_transcript/12229	-3.145337838807	2.22010983141901	-1.43355451311025	0.161225274065051	0.931424563283459	-5.05817152825736
a_HQ_transcript/33586	2.17321717800274	1.42059258290314	1.43352908255705	0.16123248421498	0.931424563283459	-4.83174562056418
a_HQ_transcript/137751	-2.78536973843259	4.35353351013668	-1.43350161507566	0.161240272167438	0.931424563283459	-5.32185473586916
a_HQ_transcript/102361	-1.87285801808078	1.14392136496456	-1.43348032857496	0.161246307808998	0.931424563283459	-4.86358765601774
a_HQ_transcript/84349	-1.87512033811461	0.972569023306484	-1.43339901489834	0.16126936538928	0.931453584899068	-4.86440283225064
a_HQ_transcript/25855	-0.917237717191824	0.66869062585677	-1.43333738041877	0.161286844406244	0.931453584899068	-4.79612265991187
a_HQ_transcript/107965	-1.79937500455421	0.869297165929791	-1.4332470008266	0.161312478001412	0.931453584899068	-4.8666084108976
a_HQ_transcript/2835	1.28994749751281	-0.0355142451150212	1.43323659792365	0.16131542869493	0.931453584899068	-4.81540183124687
a_HQ_transcript/15530	0.692649912830394	2.61916061392968	1.43308602673996	0.161358141686895	0.931607673815639	-4.98217204833785
a_HQ_transcript/147176	2.23647043123308	1.25706820256094	1.43293462920695	0.161401098102881	0.931763137404338	-4.93756156182081
a_HQ_transcript/4728	-2.36200111172602	0.892313175684055	-1.43278722352093	0.161442930584134	0.931825103908271	-4.76745512714162
a_HQ_transcript/126217	2.9872886943963	2.12359443211788	1.43277461389437	0.16144650948679	0.931825103908271	-4.94306435172099
a_HQ_transcript/14376	-2.17389391136831	1.12507626176029	-1.43269357082723	0.161469512873568	0.931825103908271	-4.78524040380842
a_HQ_transcript/142935	-3.98055641960535	2.63495873363528	-1.43267087871188	0.161475954302023	0.931825103908271	-5.08146414857566
a_HQ_transcript/94704	0.875406038081223	2.64549191598335	1.43229869574786	0.161581631868665	0.931995934354797	-4.92039654287798
a_HQ_transcript/151185	-1.94243324521194	0.281720812487314	-1.43228230216683	0.161586287915455	0.931995934354797	-4.95541508411579
a_HQ_transcript/10518	-1.85671495971684	0.549525790966602	-1.43225151926244	0.161595031053288	0.931995934354797	-4.81697744190367
a_HQ_transcript/144731	-1.90955362478095	0.00499936879139644	-1.43211566095713	0.161633622777101	0.931995934354797	-4.78664112925612
a_HQ_transcript/150570	0.302086602291036	10.5286636010698	1.43210760480623	0.161635911424957	0.931995934354797	-5.95301730871299
a_HQ_transcript/54087	-1.37543466334245	0.377597630028734	-1.43206777963448	0.161647225615225	0.931995934354797	-4.93209991292272
a_HQ_transcript/37953	1.38840146010884	1.26567522897442	1.43205328765621	0.161651342890206	0.931995934354797	-4.73830141694832
a_HQ_transcript/38205	-1.36328107942331	0.196365453831296	-1.43198132948607	0.161671787949642	0.931995934354797	-4.77863818939321
a_HQ_transcript/17618	2.68621503341674	1.22626067486162	1.43190192365583	0.161694351440771	0.931995934354797	-4.82653815990244
a_HQ_transcript/47633	-1.7874731329258	-0.537385416692197	-1.43184077051934	0.161711730050145	0.931995934354797	-4.91242291011516
a_HQ_transcript/78427	-2.17239271177045	0.935874610220855	-1.43179930151083	0.161723515627883	0.931995934354797	-4.90493830323071
a_HQ_transcript/696	1.40303347428327	1.37652048155235	1.43172189059066	0.161745517786705	0.931995934354797	-4.79752848860394
a_HQ_transcript/135007	-1.41306780464041	0.899372677781787	-1.43171918078659	0.161746288025005	0.931995934354797	-4.84758309536637
a_HQ_transcript/13910	-0.771569541690914	2.60972116045723	-1.43164317925601	0.161767891981166	0.931995934354797	-5.03124558058514
a_HQ_transcript/116267	0.714834506333255	2.5381246930627	1.43157027156149	0.161788618635458	0.931995934354797	-4.97504225857034
a_HQ_transcript/73863	0.934483548971191	3.96511987545827	1.43148228889181	0.161813633691111	0.931995934354797	-5.26183248118728
a_HQ_transcript/69161	-1.28173264934573	0.656743371500826	-1.43143722792484	0.161826446517686	0.931995934354797	-4.88243927530546
a_HQ_transcript/63569	-1.86452072709336	-0.128780493038936	-1.43139648931304	0.161838030997269	0.931995934354797	-4.92773285219433
a_HQ_transcript/26261	0.482135039096409	3.57362719627107	1.43129564963342	0.161866708704888	0.931995934354797	-5.20996690852584
a_HQ_transcript/98055	3.1106554169983	1.36962273089809	1.43128209154456	0.161870564783956	0.931995934354797	-4.86670100817574
a_HQ_transcript/104654	-4.29333990469525	0.328604323629288	-1.43120647695286	0.161892071789214	0.931995934354797	-4.79857856236509

a_HQ_transcript/35767	1.15276373047082	2.18314976210048	1.4311958105413	0.161895105810826	0.931995934354797	-4.85702022543832
a_HQ_transcript/144163	0.991033733536919	-0.839053769298879	1.43118926092707	0.161896968846806	0.931995934354797	-4.90810994581847
a_HQ_transcript/105277	-1.84338013841483	1.39384468282916	-1.43117374904274	0.161901381265624	0.931995934354797	-4.85521589166403
a_HQ_transcript/52312	-1.78694884351986	2.62822318739655	-1.43109947632353	0.161922509760122	0.931995934354797	-5.25760375387502
a_HQ_transcript/85551	-1.97947165137057	0.308842779046536	-1.43109934575226	0.161922546905883	0.931995934354797	-4.91328150470202
a_HQ_transcript/97854	-1.36114473523626	4.7817902168669	-1.43104340361669	0.161938462307037	0.931995934354797	-5.42599641158954
a_HQ_transcript/31480	-1.03767758997283	2.68845669270485	-1.43088596347577	0.161983260275457	0.932161464537348	-5.05863642344034
a_HQ_transcript/95243	-2.71942483135836	0.861207918917033	-1.43071053026235	0.162033189516885	0.932217117780117	-4.93560480040662
a_HQ_transcript/33558	1.97162033669314	2.30089398382027	1.43066159662183	0.162047118463553	0.932217117780117	-4.9661426806059
a_HQ_transcript/91872	-1.59283792227383	0.451153463438364	-1.43060320967735	0.162063739527847	0.932217117780117	-4.84157049522566
a_HQ_transcript/117350	-0.527764906424749	4.39059760267423	-1.43058111633054	0.162070029211996	0.932217117780117	-5.32416293441629
a_HQ_transcript/995	1.57339596134484	0.127433704377783	1.43057026745612	0.162073117813891	0.932217117780117	-4.8005217290493
a_HQ_transcript/53206	-1.50583404388543	1.94436011090057	-1.43039300562054	0.162123589656768	0.93226894432668	-4.95756072278859
a_HQ_transcript/57938	1.68991348938996	0.0606725440382681	1.43035583085773	0.162134176018286	0.93226894432668	-4.8014661902245
a_HQ_transcript/12706	-1.86274573371204	0.483525376907232	-1.4302694272068	0.162158783537462	0.93226894432668	-4.85593414476657
a_HQ_transcript/21113	2.56907931373829	0.709460670195994	1.43025889053248	0.162161784554726	0.93226894432668	-4.87788544968421
a_HQ_transcript/89688	-1.7723292565421	-0.328741056021778	-1.43014081543531	0.162195417274314	0.93226894432668	-4.88734785394492
a_HQ_transcript/92005	2.2117221362202	2.03502281615914	1.43013192041971	0.162197951169403	0.93226894432668	-5.12177245642136
a_HQ_transcript/56214	0.421834084878473	3.47261383391939	1.43012781119611	0.16219912176155	0.93226894432668	-5.17422276770429
a_HQ_transcript/33619	1.04369321778432	-0.681883366595907	1.4300522198559	0.162220656613051	0.93226894432668	-4.9516416908773
a_HQ_transcript/128950	2.33363051774176	3.63558573885266	1.42997851201156	0.16224165705861	0.93226894432668	-5.17630119025928
a_HQ_transcript/148761	0.483597416185965	5.34834300861372	1.42980314445611	0.162291630476667	0.93226894432668	-5.37956630080113
a_HQ_transcript/30116	0.357271451024468	4.2505947579804	1.42971089759142	0.162317922378646	0.93226894432668	-5.29997156844573
a_HQ_transcript/81672	-1.47145783450353	0.962876970009321	-1.42971087253878	0.162317929519523	0.93226894432668	-4.86863494310365
a_HQ_transcript/19232	-2.84794551576135	2.65900009801457	-1.42970788164457	0.162318782030805	0.93226894432668	-4.96333344464707
a_HQ_transcript/39526	1.58990967108862	1.18037082687592	1.42966025633924	0.16233235741392	0.93226894432668	-4.86066987651868
a_HQ_transcript/108901	2.01313548843846	0.115004009557374	1.42964932137707	0.16233547450318	0.93226894432668	-4.8589670551325
a_HQ_transcript/148982	-7.81189357450215	1.94824587346387	-1.42963786743205	0.162338739582897	0.93226894432668	-4.94714068057906
a_HQ_transcript/66502	-1.87048968940826	0.306659102047347	-1.4294776973607	0.162384403350002	0.932357954293245	-4.90997235789298
a_HQ_transcript/115672	-2.02590637439585	0.0265459935906671	-1.42935116024559	0.162420485683072	0.932357954293245	-4.92859764632751
a_HQ_transcript/74044	-1.00950326467635	2.43735101496307	-1.42930585224985	0.162433406895438	0.932357954293245	-4.95204198454313
a_HQ_transcript/42932	1.34389369834853	0.809618216735802	1.42930111038132	0.16243475925753	0.932357954293245	-4.74217724181292
a_HQ_transcript/141953	-1.22126805569611	0.974827006967532	-1.42927366118641	0.162442587833687	0.932357954293245	-4.75208965015088
a_HQ_transcript/94709	1.99257857028206	1.85426087447811	1.42924599854959	0.162450477585363	0.932357954293245	-4.91629521865692
a_HQ_transcript/72043	2.76564329859182	3.53740196043236	1.42915024811154	0.162477789218954	0.932422641101723	-4.96308905793557
a_HQ_transcript/4975	1.10979995795546	1.19232004603915	1.42908606918621	0.162496097496453	0.932435652050725	-4.82236341243686
a_HQ_transcript/12448	-1.50251401954013	-0.486622999390389	-1.42896145645766	0.162531650334449	0.932533049257333	-4.89192534064439
a_HQ_transcript/107795	-2.47030822423482	1.79031537453018	-1.4288301023242	0.162569133188125	0.932533049257333	-4.87375330285068
a_HQ_transcript/1802	1.74908283642987	-0.351358574875878	1.42875077440786	0.162591773288745	0.932533049257333	-4.84081332222542
a_HQ_transcript/146822	1.07409050879459	2.01896209979841	1.42872591114858	0.162598869748069	0.932533049257333	-4.88975222104153
a_HQ_transcript/30427	1.36235040147369	1.24311418036003	1.42869876768375	0.162606617301724	0.932533049257333	-4.79280400075688
a_HQ_transcript/82671	-1.13047040994624	-0.451590332058733	-1.42865083144168	0.162620300445347	0.932533049257333	-4.8591950491207
a_HQ_transcript/79604	2.15831380332579	1.37653050886668	1.42862027688896	0.162629022553132	0.932533049257333	-4.87313760521621
a_HQ_transcript/29832	-1.84126491648097	0.526258261350571	-1.42857687403404	0.162641412974313	0.932533049257333	-4.96385135736349
a_HQ_transcript/107116	1.55855124166841	1.07766758208665	1.42844224876363	0.162679849836513	0.932611912207923	-4.81991641742475
a_HQ_transcript/92694	1.65629508526381	1.16941281199553	1.42828817325246	0.162723848736049	0.932611912207923	-4.81237644207564
a_HQ_transcript/9711	-2.53409653438317	1.11323523133167	-1.42818116082647	0.162754413493321	0.932611912207923	-4.796126419617
a_HQ_transcript/61780	3.35778850465531	0.791310459964849	1.42811520047408	0.162773255265206	0.932611912207923	-4.98062306010943
a_HQ_transcript/151911	3.06753864759475	1.33466298233831	1.42811433468596	0.162773502591619	0.932611912207923	-4.96340577952221
a_HQ_transcript/12802	-1.50406177412375	1.40250264542816	-1.42803024935269	0.162797524343615	0.932611912207923	-5.00461303132382

a_HQ_transcript/56901	1.77917145195587	-0.345762910319715	1.4280059700417	0.162804461051111	0.932611912207923	-4.90832040503313
a_HQ_transcript/45823	-1.69791081611774	0.410530438550853	-1.42785818328442	0.162846689419105	0.932611912207923	-4.80989536488474
a_HQ_transcript/60696	-2.13051271774423	1.54891333772218	-1.42779562888138	0.162864566224927	0.932611912207923	-4.8157288593971
a_HQ_transcript/8483	-1.90484456457062	0.563081858507025	-1.42778609003776	0.162867292373053	0.932611912207923	-4.82150157248295
a_HQ_transcript/152206	2.25017566398559	0.963965668861792	1.42772374984393	0.16288510973841	0.932611912207923	-4.93692322700326
a_HQ_transcript/37507	1.60931458621209	-0.147039948684697	1.42763970883182	0.162909131819698	0.932611912207923	-4.84804406003616
a_HQ_transcript/141347	-2.28001693893119	0.78372483588251	-1.42760735686889	0.162918379972477	0.932611912207923	-4.82587679807902
a_HQ_transcript/53147	-1.11279258361845	2.22813758487626	-1.42757605471711	0.162927328419959	0.932611912207923	-4.85892556110806
a_HQ_transcript/118389	0.416042897903902	4.57893564589166	1.42757098367134	0.162928778132742	0.932611912207923	-5.35522431241071
a_HQ_transcript/28716	2.98035007842061	2.73322354329665	1.4275371798489	0.162938442244412	0.932611912207923	-5.08112362029458
a_HQ_transcript/95452	7.16579476256768	3.45968253149863	1.42752821291632	0.162941005859938	0.932611912207923	-4.84836262455261
a_HQ_transcript/16415	-2.0189111974501	-0.150963279700288	-1.42746355291076	0.162959492877991	0.932611912207923	-4.88730630769928
a_HQ_transcript/82031	-2.13002254121653	2.31183807660998	-1.42746176049747	0.162960005372555	0.932611912207923	-4.89370837715849
a_HQ_transcript/46014	0.400103451076024	4.44211702214338	1.42740437180375	0.162976414868567	0.932614003099043	-5.3215804077462
a_HQ_transcript/82049	2.38200932203415	2.93563830091343	1.42726201693471	0.163017124885751	0.932745644761555	-5.12017611333138
a_HQ_transcript/143466	1.46690776949169	-0.416818799172642	1.4272117134766	0.163031512365425	0.932745644761555	-4.91784906986369
a_HQ_transcript/119961	1.99260171481695	0.569057905362259	1.42700466291248	0.163090742220188	0.932824167641097	-4.97430262419647
a_HQ_transcript/129834	-0.863511327845335	3.00532759504465	-1.42696368249175	0.16310246728415	0.932824167641097	-5.17100868616857
a_HQ_transcript/34878	-3.15971577794763	1.57269009666547	-1.42693474644855	0.163110746686109	0.932824167641097	-4.94775386817121
a_HQ_transcript/16943	2.33497196842681	-0.0460169819932792	1.42689473887815	0.163122194504341	0.932824167641097	-4.89969077155422
a_HQ_transcript/145736	2.29390878163178	1.77908172251193	1.42684715870491	0.163135809982334	0.932824167641097	-4.91432459364184
a_HQ_transcript/10272	1.05174026236262	1.69164530713532	1.42679917198723	0.163149542704784	0.932824167641097	-4.82584915580632
a_HQ_transcript/123875	-2.2644081511369	0.838229840089592	-1.42677111839735	0.163157571435574	0.932824167641097	-4.8387822861185
a_HQ_transcript/3783	-0.623203173046326	3.58574714094217	-1.42661976478155	0.163200893103929	0.932954129757461	-5.19477502986294
a_HQ_transcript/109665	3.75594024544895	3.23894743246655	1.42643675021308	0.163253289150585	0.932954129757461	-5.1117932202748
a_HQ_transcript/138851	-4.2613117414189	2.25671765146966	-1.42643262246282	0.163254471055412	0.932954129757461	-5.02868541922658
a_HQ_transcript/71354	-2.03744764457517	0.845518995046851	-1.42626763020228	0.163301719061329	0.932954129757461	-4.87384872125712
a_HQ_transcript/61997	1.47376515000341	1.48617867324849	1.4262666516995	0.163301999302439	0.932954129757461	-4.79475632427294
a_HQ_transcript/77831	-1.95028621586438	0.847499853781728	-1.4262198492722	0.163315403860886	0.932954129757461	-4.9034880822237
a_HQ_transcript/62087	1.30089474934618	0.199380641006867	1.42619760641534	0.163321774682532	0.932954129757461	-4.87536956195317
a_HQ_transcript/67096	-1.40632425472195	1.77478092462786	-1.42617727633896	0.163327597815152	0.932954129757461	-4.85979164253007
a_HQ_transcript/11064	1.81223532206148	1.5902454063155	1.42615596804944	0.163333701312633	0.932954129757461	-4.84623032822908
a_HQ_transcript/65079	0.47112695436371	3.65303881438552	1.42613117680504	0.16334080268617	0.932954129757461	-5.22400080879945
a_HQ_transcript/147088	-1.10276498190854	1.19422347992937	-1.42602226531215	0.16337200292441	0.933040654941059	-4.8133146603289
a_HQ_transcript/29384	-2.19944761993093	1.11103382714864	-1.4259097317555	0.16340424572587	0.933108400328423	-4.90358665537904
a_HQ_transcript/21926	1.68530407304094	3.60600587712128	1.42586881546329	0.163415970192392	0.933108400328423	-5.19078497114674
a_HQ_transcript/150983	0.318905790332898	7.8405698058878	1.4258112018349	0.16343248036612	0.933111012545113	-5.64548355980172
a_HQ_transcript/99661	1.99273057695751	0.500818915217343	1.4257173531815	0.163459377125432	0.933156625852681	-4.81996517584365
a_HQ_transcript/68333	1.84653072192738	0.465383693609404	1.42567130248781	0.163472576401353	0.933156625852681	-4.96494068366967
a_HQ_transcript/109243	-2.21507696901657	2.0051643406224	-1.42557851373114	0.163499174524121	0.933187180360734	-5.00308281558534
a_HQ_transcript/79898	-0.384434261335184	4.00178586448399	-1.42546428852665	0.163531922144514	0.933187180360734	-5.25486719279119
a_HQ_transcript/38730	1.44641065803681	1.63990892766345	1.42545285490945	0.163535200372761	0.933187180360734	-4.85277773649905
a_HQ_transcript/74981	-1.48803665119502	1.66106159622717	-1.42540385717197	0.163549249511924	0.933187180360734	-4.83968391296156
a_HQ_transcript/148942	4.12210774311432	0.194597486438111	1.42520416000542	0.163606518600596	0.933187180360734	-4.94075888967343
a_HQ_transcript/100676	-2.01402287771102	0.482873562500523	-1.42509742866583	0.163637133464902	0.933187180360734	-4.84453222729612
a_HQ_transcript/103403	1.792852982621	-0.194740132748429	1.42508062013302	0.163641955243632	0.933187180360734	-4.91313088520177
a_HQ_transcript/43269	2.54713649597037	1.6158924558669	1.42507536523657	0.163643462712168	0.933187180360734	-5.0791883229777
a_HQ_transcript/88424	1.45396822781699	1.31936759346883	1.42497703364769	0.163671673047704	0.933187180360734	-4.78376526477755
a_HQ_transcript/83754	1.60816670415741	1.7186242843692	1.42496198110992	0.163675991806617	0.933187180360734	-4.80554421918105
a_HQ_transcript/16380	2.03262537449238	1.88370240707426	1.42496167761991	0.163676078882571	0.933187180360734	-4.93505471038811

a_HQ_transcript/85147	0.966359834786626	2.62674783798106	1.42490380626389	0.163692683733036	0.933187180360734	-5.07842655508902
a_HQ_transcript/20030	2.27083587444191	1.11599380424299	1.42486802740838	0.163702950314766	0.933187180360734	-4.80259515745809
a_HQ_transcript/55231	-1.53292724412701	-0.336674681807989	-1.42476530719991	0.163732428238678	0.933187180360734	-4.83514430674219
a_HQ_transcript/125989	-1.49474672057755	1.18498938455768	-1.42476037460809	0.163733843864563	0.933187180360734	-4.85122287580024
a_HQ_transcript/152003	0.629687826225761	4.35055714857601	1.42475706717005	0.163734793085933	0.933187180360734	-5.2918643454161
a_HQ_transcript/94452	-2.12973988098741	1.67190969369385	-1.42468396243436	0.16375577495819	0.933215263408793	-4.9716527728707
a_HQ_transcript/97602	2.4302707669842	1.4560805372787	1.42457298148206	0.163787631778807	0.93325662422126	-4.90398698865383
a_HQ_transcript/31529	-2.5029622398712	-0.0597793620006147	-1.42454681632395	0.16379514313765	0.93325662422126	-4.92108102219821
a_HQ_transcript/148634	1.99521012649074	-0.026688603607148	1.4243372619287	0.163855310734854	0.933366423808521	-4.89609790155479
a_HQ_transcript/70963	0.557413635742334	2.71003781643252	1.42432091955063	0.163860003717412	0.933366423808521	-4.99320602269912
a_HQ_transcript/120154	2.70936149426188	1.15767232705569	1.42431193006108	0.163862585242326	0.933366423808521	-5.12668505552216
a_HQ_transcript/21337	2.35657337211457	0.0427320774530305	1.42418418155444	0.163899274434922	0.933398840822894	-4.95437165136067
a_HQ_transcript/120990	1.91835481294005	1.63715871751106	1.42413155767186	0.163914389824135	0.933398840822894	-4.97549561803472
a_HQ_transcript/135047	-1.48752340632462	0.462520160341094	-1.42406255143414	0.163934212455003	0.933398840822894	-4.94971863042461
a_HQ_transcript/100508	-3.17267549033446	2.05401464184941	-1.42405459754445	0.163936497399206	0.933398840822894	-5.04727547704896
a_HQ_transcript/70181	5.00311830762188	3.23628907185677	1.42401259297024	0.163948564629813	0.933398840822894	-5.09744306727936
a_HQ_transcript/57198	-2.49461612276997	-0.255180275663654	-1.42392258763784	0.16397442405539	0.933454639260769	-4.90956852548977
a_HQ_transcript/126748	-1.76028544152882	2.73067610642157	-1.42380928679264	0.164006981085356	0.9335485503848	-4.95696330616653
a_HQ_transcript/132746	2.29712246690305	0.633679591816119	1.42369677491535	0.164039316448658	0.933564078994858	-4.91762626447454
a_HQ_transcript/140338	1.50670237627324	-0.3489921211063	1.42364716251325	0.164053576407202	0.933564078994858	-4.91288000826368
a_HQ_transcript/91917	1.50967861717625	0.503381172770987	1.42363215337163	0.164057890636741	0.933564078994858	-4.79652874356798
a_HQ_transcript/152177	1.68613780968524	1.43660049644963	1.42355509598193	0.164080041430975	0.933598732214146	-4.88643092296957
a_HQ_transcript/117941	1.01205734407693	0.987725407569382	1.42328855214953	0.164156679880514	0.933683073961483	-4.91564935415779
a_HQ_transcript/28334	-0.476809254428615	2.91551176725466	-1.4232666127886	0.16416298928782	0.933683073961483	-5.04418846681605
a_HQ_transcript/137232	2.23592201546839	1.49824678633197	1.42318702302697	0.164185879627842	0.933683073961483	-4.809124547801
a_HQ_transcript/151804	-0.426973709063828	6.11766164083328	-1.42311247991948	0.164207320811427	0.933683073961483	-5.47373726985256
a_HQ_transcript/58153	1.04607489854807	2.27549290627511	1.4230554505004	0.164223725940994	0.933683073961483	-5.01951659393829
a_HQ_transcript/44874	-2.3005268271019	1.59186976443832	-1.42297345708219	0.164247314499198	0.933683073961483	-4.89818178940413
a_HQ_transcript/1602	-0.914821419633186	2.2479890400682	-1.42295606379734	0.164252318689474	0.933683073961483	-4.94549595700218
a_HQ_transcript/136969	2.37638986566589	-0.188321177875287	1.42293627422215	0.164258012458242	0.933683073961483	-4.90579657583739
a_HQ_transcript/72446	-0.868275822499214	4.38406251876546	-1.42293406450373	0.164258648238276	0.933683073961483	-5.22426652908195
a_HQ_transcript/133903	3.31337681173838	1.0004282805483	1.42289569516345	0.164269688171581	0.933683073961483	-4.87671453659769
a_HQ_transcript/120362	3.9974733687155	2.42407892242667	1.42288921625947	0.164271552391345	0.933683073961483	-4.96372870765341
a_HQ_transcript/10381	-2.11982425786423	1.87823495151421	-1.42256145612532	0.164365882841106	0.93397429062315	-4.96159795554404
a_HQ_transcript/151777	0.57111842822543	3.23426098515779	1.42252606369332	0.16437607145366	0.93397429062315	-5.17477610089705
a_HQ_transcript/90633	2.33486455109329	0.805324829884637	1.4223740254379	0.164419845198578	0.93397429062315	-4.91922273056157
a_HQ_transcript/142402	-2.74280175376364	0.767844859219825	-1.42236397971781	0.16442273781256	0.93397429062315	-4.89739000641974
a_HQ_transcript/132601	-0.730130633969126	3.38856506260451	-1.42236114164221	0.164423555029253	0.93397429062315	-5.15277520233296
a_HQ_transcript/127733	3.03160177278529	1.46382042119887	1.42228242750561	0.164446221841607	0.93397429062315	-5.00200871521515
a_HQ_transcript/82447	1.46882662863	2.98372289176988	1.42227293231185	0.164448956278991	0.93397429062315	-5.11200236958512
a_HQ_transcript/101815	-1.71906076484402	2.61452063708618	-1.42226469251881	0.164451329213597	0.93397429062315	-5.08983984074764
a_HQ_transcript/104217	-0.436512369640546	4.05862838721985	-1.42211905917091	0.164493273848394	0.934072684299337	-5.30035114727615
a_HQ_transcript/145803	-0.427265508107626	4.12908907250581	-1.42209295706034	0.164500792547005	0.934072684299337	-5.30306426656657
a_HQ_transcript/149071	1.49811134094051	-0.228021243060459	1.4219776034466	0.164534023335566	0.934091848599493	-4.87339990654823
a_HQ_transcript/122459	-0.661304424170397	2.73722467956177	-1.42196374934506	0.164538014747281	0.934091848599493	-5.02782154047799
a_HQ_transcript/37469	2.92775210682033	2.39237526448175	1.42191215418118	0.16455288015311	0.934091848599493	-4.98254162440947
a_HQ_transcript/125694	-3.16631817966617	0.468860772144985	-1.42175973699493	0.16459680020505	0.934091848599493	-4.92610988375786
a_HQ_transcript/70358	-0.910124392267412	-0.718457495972559	-1.42174079886402	0.164602258001837	0.934091848599493	-4.94934937578406
a_HQ_transcript/34979	1.51384362468808	1.59016391814984	1.42167318266053	0.164621745539275	0.934091848599493	-4.87801354277249
a_HQ_transcript/1523	1.95596447140924	2.25835973902075	1.42164175859112	0.164630802829382	0.934091848599493	-4.9306836766637

a_HQ_transcript/149564	-1.9961277974537	0.353252449740693	-1.42163509281573	0.164632724141581	0.934091848599493	-4.95355103331593
a_HQ_transcript/54187	-2.94318085830842	0.959555730814553	-1.42155874514546	0.164654731500389	0.934125535101956	-5.03206140289798
a_HQ_transcript/66648	-1.27494690940398	1.77674303904293	-1.42149287701708	0.164673719974367	0.934142089847759	-4.7821499898308
a_HQ_transcript/101922	-0.607549080315992	4.34166439967443	-1.42131326059454	0.164725508592349	0.934344688081194	-5.29774680947282
a_HQ_transcript/333	-0.473123684881443	2.81816180664298	-1.42109001605205	0.164789894348638	0.934567641016755	-5.04594086657379
a_HQ_transcript/149233	-2.38546844605269	0.472023844593346	-1.42106423562356	0.164797330935675	0.934567641016755	-4.91506960953354
a_HQ_transcript/49335	-0.756790913743684	2.99544015685282	-1.4209922271492	0.164818103802172	0.934567641016755	-5.1489153297276
a_HQ_transcript/66979	-3.30539983347221	0.902111748402594	-1.42094564007098	0.164831544252214	0.934567641016755	-5.03224949109194
a_HQ_transcript/150562	-2.91564948157003	8.85930605346144	-1.42089829562094	0.164845204090022	0.934567641016755	-5.76487489862921
a_HQ_transcript/56890	2.1672595049031	0.331239281246685	1.42077135312874	0.164881833985414	0.934661358538625	-4.97084004283164
a_HQ_transcript/120120	-2.4368506712217	2.80730439255038	-1.42060819510221	0.164928923473358	0.934661358538625	-5.04817316021823
a_HQ_transcript/73704	1.53491106372386	0.203808004992877	1.42059127527178	0.164933807358564	0.934661358538625	-4.91219006250015
a_HQ_transcript/127224	-2.07995806565049	1.62032940135039	-1.42057421426073	0.164938732110724	0.934661358538625	-4.81054404751358
a_HQ_transcript/86112	2.03665926698277	2.0307562447178	1.42053711598614	0.164949441126092	0.934661358538625	-4.94018533219843
a_HQ_transcript/324	0.712426333641978	2.28738466774533	1.42046474668333	0.164970333269233	0.934661358538625	-4.89578258251544
a_HQ_transcript/150849	1.71556084070895	-0.142395888063735	1.42045104102064	0.164974290163197	0.934661358538625	-4.85986955545505
a_HQ_transcript/46092	0.426985308611137	3.12464307101978	1.42031112432723	0.165014689096353	0.934714595385885	-5.12141986189179
a_HQ_transcript/139623	-1.74712825143987	1.85255334574929	-1.42030711307332	0.165015847403144	0.934714595385885	-4.94587332100204
a_HQ_transcript/49780	-2.06708724470701	0.892638913405245	-1.42013028044608	0.165066916712283	0.9349127676753	-4.84532902210342
a_HQ_transcript/30170	1.03390160600945	1.94368747828778	1.42004873350395	0.165090471684792	0.934955079705144	-4.94755773561096
a_HQ_transcript/150727	-2.35189565332794	0.0941433589341146	-1.41987474970119	0.165140736052096	0.934976861752505	-4.93322980078693
a_HQ_transcript/25034	2.65672842678344	1.92913238056256	1.41987249014103	0.165141388924114	0.934976861752505	-4.94666153490064
a_HQ_transcript/100080	2.87125177121111	2.87886677020747	1.41986736177095	0.16514287071089	0.934976861752505	-5.03457786580795
a_HQ_transcript/8949	0.633783696106128	3.08751014116509	1.41972605578661	0.165183703658778	0.934976861752505	-5.06199705756643
a_HQ_transcript/63066	2.38062826777517	0.690503535644102	1.41964959181461	0.165205802637309	0.934976861752505	-4.96656809882023
a_HQ_transcript/66061	1.63440367992333	1.27957207829758	1.41963756427094	0.165209278949468	0.934976861752505	-4.79683110438183
a_HQ_transcript/99402	1.74741347614019	0.627690397325939	1.41963494642407	0.165210035591472	0.934976861752505	-4.84740587585228
a_HQ_transcript/109355	-0.976823222006298	3.42930394738541	-1.41958214596392	0.165225297206597	0.934976861752505	-5.1074499551795
a_HQ_transcript/34533	-1.95701242780632	0.69862326611033	-1.41942213965332	0.165271552727419	0.934976861752505	-4.79125789096838
a_HQ_transcript/104326	-2.44125885101085	1.484002817733	-1.41932006177829	0.165301067299882	0.934976861752505	-4.86959958129446
a_HQ_transcript/69587	-2.62258050596355	0.403368762218898	-1.41931543445121	0.165302405333412	0.934976861752505	-4.93044779490686
a_HQ_transcript/45492	-2.41882161693361	2.03177767394687	-1.41920291800182	0.165334943109225	0.934976861752505	-5.05250389059001
a_HQ_transcript/125523	1.44007685250331	1.8194861338702	1.41919638740878	0.165336831796602	0.934976861752505	-4.86185080860565
a_HQ_transcript/20519	-1.40180551662659	2.66387901635959	-1.41916836107304	0.165344937376924	0.934976861752505	-5.00544563265257
a_HQ_transcript/33792	5.04242365051182	1.10626653226398	1.41915244960259	0.165349539320119	0.934976861752505	-4.93410983081193
a_HQ_transcript/132860	-3.2134760999981	3.4115167411744	-1.4191069363242	0.165362703306692	0.934976861752505	-5.0366729838256
a_HQ_transcript/132492	2.17024180984698	0.572238466045329	1.41902596959538	0.16538612367594	0.934976861752505	-4.94107493653782
a_HQ_transcript/135023	1.79618006663174	1.89229096478769	1.41901831620865	0.165388337623403	0.934976861752505	-5.0168934533522
a_HQ_transcript/13871	-1.42106110067283	2.72487367735945	-1.4189176119969	0.165417471192789	0.934976861752505	-4.9966193243113
a_HQ_transcript/131822	-1.55809708815997	0.270055758896437	-1.41890379036285	0.165421470085074	0.934976861752505	-4.81926244162265
a_HQ_transcript/26471	0.914397785503928	3.54370774116481	1.41886705586313	0.165432098526089	0.934976861752505	-5.13475312213686
a_HQ_transcript/78625	2.16956750549999	0.354354461982844	1.41877860795818	0.165457691480716	0.935030594049294	-4.95091229228711
a_HQ_transcript/50550	-1.27374206116541	-0.038722161830108	-1.41866430034444	0.165490771722248	0.935054752921323	-4.87273725900892
a_HQ_transcript/140744	-2.04186485357477	1.25145410122607	-1.41865266665989	0.165494138763669	0.935054752921323	-4.97133485752037
a_HQ_transcript/146650	-1.97349659459299	0.0379169289763571	-1.41855809129138	0.165521512934827	0.935090663267303	-4.91129423141291
a_HQ_transcript/5197	-1.17922047027072	1.35906182898719	-1.41851955507709	0.165532667991683	0.935090663267303	-4.77779894241623
a_HQ_transcript/18587	-0.998928788467451	2.81764883470359	-1.41823272654709	0.165615714690621	0.935360490796667	-4.88341202929194
a_HQ_transcript/42257	-2.02501233303629	-0.0562154915400094	-1.41809902185764	0.165654437993425	0.935360490796667	-4.94579474465027
a_HQ_transcript/132755	-0.905511179090151	2.07881825521814	-1.41792634922623	0.165704457666343	0.935360490796667	-4.90094913799535
a_HQ_transcript/57110	-1.38854951470174	2.72314751337131	-1.4179116391782	0.16570871941116	0.935360490796667	-5.04793314984147

a_HQ_transcript/41628	0.780528092859926	2.99182695169576	1.41773887051345	0.165758779816833	0.935360490796667	-5.03806073895874
a_HQ_transcript/92792	-1.35868406547083	-0.719778698154759	-1.41770741301545	0.165767896032766	0.935360490796667	-4.92297270581938
a_HQ_transcript/108379	-1.51359496172114	0.59898762563239	-1.41762710127495	0.165791171739021	0.935360490796667	-4.80748835176398
a_HQ_transcript/130492	-2.19207752365086	0.297279209779752	-1.41759115678892	0.165801589894899	0.935360490796667	-4.89588913871988
a_HQ_transcript/117774	1.005586603727	4.72516800680333	1.41754106213809	0.165816110196561	0.935360490796667	-5.32924794094018
a_HQ_transcript/62873	2.09972672550517	0.255212160739168	1.41751098293755	0.165824829354271	0.935360490796667	-4.90219738227521
a_HQ_transcript/93916	0.541599433331219	2.61435259617954	1.4174308406551	0.165848062225649	0.935360490796667	-4.93447348622963
a_HQ_transcript/89862	-0.780546124590722	2.55432665589084	-1.41742024112386	0.165851135171839	0.935360490796667	-5.03971309205393
a_HQ_transcript/92801	2.12039314879446	-0.141568951365109	1.41732405494496	0.165879022883338	0.935360490796667	-4.8506785028469
a_HQ_transcript/142747	-3.09593029685774	0.917045379552369	-1.41730225399437	0.165885344248656	0.935360490796667	-5.0203740037689
a_HQ_transcript/84258	1.89588411826492	-0.0331279452495091	1.41729822436931	0.165886512692421	0.935360490796667	-4.95199284258496
a_HQ_transcript/88446	-1.55021903668935	1.39813170059888	-1.41723253741325	0.165905560418817	0.935360490796667	-4.89801181342403
a_HQ_transcript/150363	-0.70329910622656	5.45373339118736	-1.41695505600869	0.165986042726391	0.935360490796667	-5.43607694777386
a_HQ_transcript/137078	-3.0196268484472	0.774312691315072	-1.41694226882875	0.165989752334436	0.935360490796667	-4.92325386987898
a_HQ_transcript/103450	-0.607796568026509	4.65565422874132	-1.41692914450722	0.165993559816361	0.935360490796667	-5.32741199782123
a_HQ_transcript/136863	1.50208815104685	3.58855245394434	1.41678646750065	0.166034956115649	0.935360490796667	-5.24833295426444
a_HQ_transcript/138745	-2.34488932090709	0.83856307387533	-1.41675829416039	0.16604313128771	0.935360490796667	-4.99919639328021
a_HQ_transcript/144081	-2.13353055732656	0.374412716799744	-1.41674854598167	0.166045960029922	0.935360490796667	-4.86628868787093
a_HQ_transcript/127575	-0.358831839543762	4.91783192017632	-1.41666795560985	0.166069347326899	0.935360490796667	-5.37899495593686
a_HQ_transcript/142811	3.22243025413411	1.44958607270735	1.41665710470222	0.166072496454428	0.935360490796667	-4.95836709950211
a_HQ_transcript/72695	-2.18842765676804	2.22015328668806	-1.41665237629395	0.166073868737806	0.935360490796667	-4.88368765981853
a_HQ_transcript/134984	2.0842875838466	-0.00656504099254856	1.41662444650464	0.166081974731259	0.935360490796667	-4.87443296504772
a_HQ_transcript/120207	0.723409530177048	3.48416315413743	1.41656629064186	0.166098854162266	0.935360490796667	-5.21599316657126
a_HQ_transcript/85483	1.15730260375944	1.58361589281731	1.41656619684614	0.166098881387065	0.935360490796667	-4.89442645608776
a_HQ_transcript/122521	-1.9500104661691	1.9631659497466	-1.41653045663613	0.166109255464806	0.935360490796667	-4.94976650332883
a_HQ_transcript/139752	3.20870289849238	2.88326615651716	1.4165234030128	0.166111302934313	0.935360490796667	-5.06972293690595
a_HQ_transcript/146841	2.87554795988809	-0.171032106715774	1.41648751981597	0.166121719129951	0.935360490796667	-4.97908236904789
a_HQ_transcript/109308	2.06555440862191	1.07550357478655	1.41639169374215	0.16614953809884	0.935360490796667	-4.84291073894414
a_HQ_transcript/105812	1.6654455318653	0.991068346220051	1.41635004503759	0.16616163014746	0.935360490796667	-4.86754277814789
a_HQ_transcript/54546	-2.22979622410737	0.277306562189952	-1.41617270195559	0.166213126685241	0.935360490796667	-4.92733681002868
a_HQ_transcript/66586	-1.83566948408005	1.27411714755365	-1.41616936251249	0.166214096506672	0.935360490796667	-4.86641536932232
a_HQ_transcript/49636	-1.0713116017341	-0.752565714924125	-1.41615092973733	0.166219449724987	0.935360490796667	-4.9251270688068
a_HQ_transcript/146959	-0.967618439114696	1.67269132953447	-1.41613506528974	0.166224057162183	0.935360490796667	-4.79816195776599
a_HQ_transcript/42913	-0.954958442379263	1.61280959513926	-1.4161134739416	0.166230327997752	0.935360490796667	-5.02064951205358
a_HQ_transcript/1434	-0.935298626897763	3.69829623745857	-1.41603224233663	0.166253921987509	0.935360490796667	-5.24014861550595
a_HQ_transcript/139938	1.97562079591738	1.15554451498135	1.41595665639178	0.166275878543372	0.935360490796667	-4.98491022971129
a_HQ_transcript/100153	-3.43152501296775	-0.211251175202921	-1.4159491723476	0.166278052667388	0.935360490796667	-4.88180092376843
a_HQ_transcript/96884	1.6402679491352	2.13083125385254	1.41591587839878	0.166287724871477	0.935360490796667	-4.9659491624271
a_HQ_transcript/1728	-1.79900715572559	1.73414851500615	-1.41590870888504	0.16628980774033	0.935360490796667	-4.95956120063641
a_HQ_transcript/32649	2.11642264616871	1.08198482047351	1.41586979474686	0.166301113334661	0.935360490796667	-5.03827491762762
a_HQ_transcript/116653	-1.86118920410904	1.39428750384018	-1.41581300763143	0.16631761259058	0.935360490796667	-4.80331406970192
a_HQ_transcript/145599	-4.25469897079738	1.04226017836159	-1.41580259601981	0.166320637779974	0.935360490796667	-4.99244515737135
a_HQ_transcript/26853	2.13899906038837	0.76655411080994	1.41571147210858	0.166347116517301	0.935367125191805	-4.97676141039863
a_HQ_transcript/39783	1.64434629764246	-0.482864549635882	1.41568778349312	0.166354000485702	0.935367125191805	-4.91835732932489
a_HQ_transcript/33315	0.701242021605335	2.28015247747973	1.41555541993993	0.16639246977706	0.935368764035886	-4.89506267186681
a_HQ_transcript/122287	-3.11016352477618	0.165107625089803	-1.41542991398906	0.166428952483828	0.935368764035886	-4.92311123563082
a_HQ_transcript/133916	-3.69572176691136	2.03987231089101	-1.415275364685	0.166473886306551	0.935368764035886	-4.93420111950051
a_HQ_transcript/7527	0.791331018501219	2.56330700331221	1.41526060170428	0.166478179010045	0.935368764035886	-4.94745734539038
a_HQ_transcript/7974	-1.84344367086583	0.0926946975877198	-1.415241586933	0.166483708155622	0.935368764035886	-4.98798062970288
a_HQ_transcript/49455	2.30729639557596	1.55584315811561	1.41520793852294	0.166493492848121	0.935368764035886	-4.85546226312095

a_HQ_transcript/107924	-3.9516720845853	1.08034191704949	-1.41519118214119	0.166498365640648	0.935368764035886	-4.87143702094827
a_HQ_transcript/105764	-4.53862315741546	2.2463505411859	-1.41515275932046	0.16650953950473	0.935368764035886	-5.07445955470139
a_HQ_transcript/34575	1.15268629083332	2.07077884904912	1.41512682161264	0.166517082866527	0.935368764035886	-4.86925474283111
a_HQ_transcript/146914	2.04127537661786	4.06884772776466	1.41512131017996	0.166518685769504	0.935368764035886	-5.0870315586928
a_HQ_transcript/34013	-2.28624829631149	0.560208679325498	-1.4150779427654	0.166531298840617	0.935368764035886	-4.94240462340523
a_HQ_transcript/120942	2.06122142889186	2.24078289531698	1.41498281040693	0.166558969971025	0.93539323066578	-5.09150923451007
a_HQ_transcript/74752	2.25201490967614	0.230295606940149	1.41495232250679	0.166567838745897	0.93539323066578	-4.87339761727617
a_HQ_transcript/134941	1.06153641966923	-0.58354089907344	1.41482524781194	0.16660480812881	0.935433686404321	-4.95362124531608
a_HQ_transcript/59488	1.01110224592873	3.88947115341386	1.41477653454431	0.166618981815597	0.935433686404321	-5.29740843829402
a_HQ_transcript/587	-1.50194102252917	2.29735875475506	-1.41476162276898	0.16662332075816	0.935433686404321	-4.98254241402575
a_HQ_transcript/131474	2.85325083185562	0.952934551847613	1.41469157071124	0.166643705292377	0.935457778970091	-4.92536612034394
a_HQ_transcript/87200	-1.30525358607274	2.00669343506717	-1.41460757975049	0.16666814851121	0.935490328566882	-4.8291078554417
a_HQ_transcript/136910	-1.0627146400198	3.37243429991654	-1.41454222766946	0.166687169356604	0.935490328566882	-5.12491521550462
a_HQ_transcript/114696	-1.5116968510614	1.95242028336638	-1.41446653170685	0.166709202942415	0.935490328566882	-4.87138876759609
a_HQ_transcript/134744	1.71158694767421	-0.475737859917385	1.41437021632657	0.166737241741778	0.935490328566882	-4.90498106093739
a_HQ_transcript/125196	1.792215499192	0.408615585476605	1.41434736051126	0.166743895944528	0.935490328566882	-4.86564621639254
a_HQ_transcript/12816	-1.27113616330012	0.351082724077416	-1.41429552143018	0.166758989059588	0.935490328566882	-4.88453103910598
a_HQ_transcript/117617	-3.22922757997922	0.690854530482832	-1.41422706002923	0.16677892346321	0.935490328566882	-5.02693570256734
a_HQ_transcript/143676	-3.05247201131008	2.08564689199778	-1.41407122480372	0.166824306127398	0.935490328566882	-4.7679880464691
a_HQ_transcript/127676	0.694417239088432	2.38716720839226	1.41398109882323	0.166850557239056	0.935490328566882	-5.04149736181876
a_HQ_transcript/100989	-0.917276991307353	0.628712719359201	-1.41395545023376	0.166858028530943	0.935490328566882	-4.89142636852422
a_HQ_transcript/24940	2.20786112713929	0.115172321924083	1.41394776295325	0.16686026784438	0.935490328566882	-4.94660036446468
a_HQ_transcript/85017	1.4376524038621	1.7941800975675	1.41390293591928	0.166873326480463	0.935490328566882	-4.93467674386628
a_HQ_transcript/93507	-2.21896925501724	0.129088937033285	-1.41383011412717	0.166894542028509	0.935490328566882	-4.92837833416703
a_HQ_transcript/152064	-1.78398390625792	0.25694051828671	-1.41382936352598	0.166894760716069	0.935490328566882	-4.89844936252122
a_HQ_transcript/142519	-2.44971306193641	4.7230161722151	-1.41381124370581	0.166900039991697	0.935490328566882	-5.39942072310881
a_HQ_transcript/56379	1.24240250009137	2.85268855821299	1.41372023864684	0.166926556633578	0.935490328566882	-5.02342548793383
a_HQ_transcript/96662	-1.29224678855606	0.0719411831704314	-1.41367535528227	0.16693963576503	0.935490328566882	-4.84858185379219
a_HQ_transcript/98477	1.0949061736027	1.92195948795288	1.41363880314234	0.166950287750959	0.935490328566882	-5.06631388761287
a_HQ_transcript/123975	-2.26368411107894	1.47868806813061	-1.41361775377787	0.166956422177014	0.935490328566882	-4.94073281952581
a_HQ_transcript/96238	1.30097400379985	1.61987648943608	1.41356644365104	0.166971376253809	0.935490328566882	-4.8980973244426
a_HQ_transcript/25905	-2.1691309507392	0.360230080088813	-1.4132926838772	0.167051179958126	0.935696654179095	-4.88041951197345
a_HQ_transcript/61406	-1.68668020106088	3.29174362524743	-1.41325263233325	0.167062857897354	0.935696654179095	-5.15405875622128
a_HQ_transcript/27081	1.09437503692413	3.72675919171301	1.41319521156739	0.167079601348495	0.935696654179095	-5.27588833164029
a_HQ_transcript/122514	-0.611660546262021	4.03748663252243	-1.41318078731126	0.167083807557373	0.935696654179095	-5.28599537843084
a_HQ_transcript/17318	-1.32334121261905	-0.369320179766611	-1.41316405043409	0.167088688246267	0.935696654179095	-4.95982965500648
a_HQ_transcript/108710	2.08490246315993	0.990957663808016	1.41298909291155	0.167139714824327	0.935857735426751	-4.96079462766838
a_HQ_transcript/42029	2.4391567166272	1.34094691226827	1.41283573327945	0.167184452427844	0.935857735426751	-4.98803215429123
a_HQ_transcript/12090	-1.15736711389238	3.12698881056101	-1.41277999043152	0.167200715894204	0.935857735426751	-5.08616515260406
a_HQ_transcript/62133	0.871226775700683	1.16123999663355	1.4126306908943	0.167244281475684	0.935857735426751	-4.77875588827151
a_HQ_transcript/23700	1.85209991337451	0.975375597693328	1.4126191893019	0.167247638008768	0.935857735426751	-4.83792792754134
a_HQ_transcript/151888	-2.25662508468901	0.859691124024649	-1.41252545702197	0.16727499406522	0.935857735426751	-4.98278558541089
a_HQ_transcript/127488	-0.489110229342229	5.84702349091583	-1.4125237862547	0.167275481715739	0.935857735426751	-5.49626750427591
a_HQ_transcript/40731	2.15312672862305	0.164486364270246	1.41251547573913	0.167277907340804	0.935857735426751	-4.98535665539233
a_HQ_transcript/131119	2.36364817773307	1.84263568875609	1.41248274521303	0.1672874608058	0.935857735426751	-4.89702805089193
a_HQ_transcript/59266	1.31079439599652	0.645980358579258	1.412403907044	0.167310474042012	0.935857735426751	-4.7887689769651
a_HQ_transcript/135807	-3.5260238519338	1.17144030543829	-1.41230790301041	0.167338501436552	0.935857735426751	-5.09562946032475
a_HQ_transcript/32215	2.71174707810258	0.126077094257717	1.41229468892497	0.16734235944247	0.935857735426751	-4.9396381126076
a_HQ_transcript/123171	-3.04147845816893	2.55560919464699	-1.41228218484163	0.16734601021941	0.935857735426751	-4.89689577920922
a_HQ_transcript/117490	-1.60073413570214	0.574680546576574	-1.41228022156173	0.167346583437606	0.935857735426751	-4.88409457553673

a_HQ_transcript/52119	2.16491933234134	1.32259800528745	1.41223786048324	0.16735895196396	0.935857735426751	-4.80989025098655
a_HQ_transcript/21000	-1.62029328071558	-0.0069814242570682	-1.41213589233309	0.167388727423881	0.935934200432413	-4.92472606488331
a_HQ_transcript/127556	2.74494240871257	8.12041150220442	1.41202650439468	0.167420674147356	0.935938138847357	-5.73396518042209
a_HQ_transcript/56280	3.61714557751804	1.41925612454823	1.41200642377623	0.167426539208259	0.935938138847357	-5.0022027226042
a_HQ_transcript/145561	-2.80408694838879	0.99339385920596	-1.41189965610617	0.167457726163918	0.935938138847357	-4.86636406515356
a_HQ_transcript/71290	-1.04948732168262	1.78823219787866	-1.41180290395204	0.167485991520266	0.935938138847357	-4.86197442535914
a_HQ_transcript/73828	-1.46723306075328	0.539572102095909	-1.41179631459382	0.167487916684177	0.935938138847357	-4.95933775990073
a_HQ_transcript/77809	-0.543150506717167	2.99863261004014	-1.41179555190355	0.167488139514856	0.935938138847357	-5.09579849137284
a_HQ_transcript/18982	-1.57485907263699	0.217085117860685	-1.41174762976068	0.167502141109776	0.935938138847357	-4.87807804558832
a_HQ_transcript/77631	-1.45559093389637	0.590767987498677	-1.41155674631527	0.167557921384505	0.935962309332302	-4.80218240572872
a_HQ_transcript/82605	-2.57583281776823	0.0426378672992066	-1.41151950375378	0.16756880616926	0.935962309332302	-4.94219019411304
a_HQ_transcript/152468	-1.21253588761354	5.58492321539534	-1.41150592348191	0.167572775377613	0.935962309332302	-5.47918002263963
a_HQ_transcript/90649	-2.33374646801151	1.34481265255896	-1.4114805077266	0.167580204030444	0.935962309332302	-4.86564797539469
a_HQ_transcript/60478	1.06160967248478	-0.7836000454811	1.4114143811466	0.167599533073624	0.935962309332302	-4.94120905213552
a_HQ_transcript/102355	-2.02173928929787	2.73239709790264	-1.41135243578221	0.167617641521705	0.935962309332302	-4.95415641316348
a_HQ_transcript/87390	-1.58245742338012	0.184654436324626	-1.41118983063053	0.167665183096629	0.935962309332302	-4.93130824111123
a_HQ_transcript/15945	-1.34527003633091	-0.67126173130852	-1.41114091724637	0.167679486188699	0.935962309332302	-4.9304738985149
a_HQ_transcript/135663	-1.95460848866892	0.622000355437609	-1.41111828949108	0.16768610324744	0.935962309332302	-4.8924546364192
a_HQ_transcript/77959	-1.53357540129919	-0.49541932448132	-1.41111786938335	0.167686226101933	0.935962309332302	-4.91810904149867
a_HQ_transcript/145084	-3.32372668164412	0.559929555038554	-1.41107898473564	0.167697597667076	0.935962309332302	-4.9935350662038
a_HQ_transcript/40852	1.62231622175524	1.12745693695928	1.41100747741065	0.167718511106488	0.935962309332302	-4.85308603577568
a_HQ_transcript/150387	-2.28927041590673	0.615465068477514	-1.41097443440791	0.167728175743625	0.935962309332302	-4.83412562674397
a_HQ_transcript/91268	1.86616219905275	0.0105980479464562	1.41096173153837	0.167731891279841	0.935962309332302	-4.97188185187582
a_HQ_transcript/124253	-0.579167214512946	5.84909949114129	-1.41088819804336	0.167753400790668	0.935992482353615	-5.49979149975442
a_HQ_transcript/129734	-3.78031293562473	1.36633484300347	-1.41081308715274	0.167775373948368	0.936025236286661	-5.07987301744028
a_HQ_transcript/67791	-1.86410446290214	0.300399143526933	-1.41055544200061	0.16785076339504	0.936334604602611	-4.79650729448855
a_HQ_transcript/118026	-1.99330673979136	0.535413294151305	-1.41029111512014	0.167928135642848	0.936334604602611	-4.89145733528178
a_HQ_transcript/74232	-2.31554395175074	-0.0768611338992859	-1.41021922604729	0.167949183446617	0.936334604602611	-4.91886547033635
a_HQ_transcript/118815	-1.48937774362451	-0.046408644484034	-1.41019692839691	0.16795571221141	0.936334604602611	-4.83292603443836
a_HQ_transcript/14223	-1.81467111363849	2.18941944417697	-1.41018754019457	0.167958461142088	0.936334604602611	-5.02020056067391
a_HQ_transcript/21214	1.44745480186507	3.35089581576658	1.41012488058878	0.167976809214119	0.936334604602611	-5.0895043736411
a_HQ_transcript/35312	1.80761404242211	-0.0255242874890567	1.41010084061664	0.167983849049877	0.936334604602611	-4.95882383302598
a_HQ_transcript/139191	3.3140295007832	0.0761142887872751	1.40999938342659	0.168013562201936	0.936334604602611	-4.96170357487715
a_HQ_transcript/122079	2.57550012654155	1.18861631295236	1.40997391539744	0.168021021517584	0.936334604602611	-4.92345832993065
a_HQ_transcript/17076	0.675281547140958	2.66789425798638	1.40986614406862	0.168052589477227	0.936334604602611	-4.96388906460703
a_HQ_transcript/19542	2.26497853879864	1.51269753481324	1.40981451822211	0.168067713170689	0.936334604602611	-4.88071686184362
a_HQ_transcript/76495	-0.733106325228557	4.09721251747423	-1.40981134725229	0.16806864213512	0.936334604602611	-5.3621878650756
a_HQ_transcript/46790	1.37448835919166	3.78599786161025	1.40973634483094	0.168090615952399	0.936334604602611	-5.2232512554699
a_HQ_transcript/70850	-2.6710170868799	0.469365617334156	-1.40969334513057	0.168103214797757	0.936334604602611	-4.97572264869485
a_HQ_transcript/25054	1.39607709407476	2.38111728923344	1.4096547040746	0.168114537200001	0.936334604602611	-5.05496987247577
a_HQ_transcript/127147	-2.56777404563716	0.714644921662456	-1.40963441420687	0.168120482671934	0.936334604602611	-5.03008644427474
a_HQ_transcript/33476	-1.45637465082603	1.29549634681603	-1.40953338622891	0.168150089020848	0.936334604602611	-4.91100259877505
a_HQ_transcript/58627	-3.77317063436447	2.18753524609707	-1.40951860285524	0.168154421646655	0.936334604602611	-4.93518413510404
a_HQ_transcript/78522	2.4939117832776	1.32139782526111	1.40946324016264	0.168170647804225	0.936334604602611	-4.95725511504182
a_HQ_transcript/68023	-0.551496127605542	3.04802808406425	-1.40945671343954	0.168172560791471	0.936334604602611	-5.1193424479881
a_HQ_transcript/19639	0.379942887754791	3.65531655388676	1.40933832549277	0.168207263351565	0.936334604602611	-5.23507003124679
a_HQ_transcript/143995	-0.585872713175904	3.98053073922471	-1.40933322240111	0.168208759325787	0.936334604602611	-5.31804179148056
a_HQ_transcript/89851	2.08098071704213	0.78888562886912	1.40928878374801	0.168221786984041	0.936334604602611	-4.99420316689316
a_HQ_transcript/104109	-1.26492110312394	2.75725471514925	-1.40927918124022	0.168224602164273	0.936334604602611	-5.10297652303313
a_HQ_transcript/79584	1.89948720911778	1.44436121537224	1.40918901034708	0.168251039492887	0.936334604602611	-4.87204698717112

a_HQ_transcript/103894	1.10948353891435	1.92667879464795	1.40916587607404	0.168257822788412	0.936334604602611	-4.95604085915381
a_HQ_transcript/76501	-2.08947110169606	2.45829073327587	-1.40899970987747	0.168306551377115	0.936334604602611	-4.95306904027388
a_HQ_transcript/136848	-3.00551746970968	0.996618865394951	-1.40890328841011	0.168334832258659	0.936334604602611	-5.00230676736794
a_HQ_transcript/149823	-0.581115299567369	4.18876381761363	-1.40882522854356	0.168357730328242	0.936334604602611	-5.2692503593828
a_HQ_transcript/131042	1.05471656330805	2.6013453245345	1.4087911116314	0.168367738946469	0.936334604602611	-5.06638572046195
a_HQ_transcript/122364	3.8801439781086	0.597772763980281	1.4087830769647	0.168370096083332	0.936334604602611	-4.9698516085393
a_HQ_transcript/123518	2.43017143122609	3.28365461433786	1.40876521365528	0.168375336750171	0.936334604602611	-5.04692305491671
a_HQ_transcript/42817	1.89861143718318	0.705458764144648	1.40875937342849	0.168377050160527	0.936334604602611	-4.7636832650811
a_HQ_transcript/84851	1.82718852979058	1.04475691148897	1.40861193379592	0.168420310653972	0.936334604602611	-4.99818211106147
a_HQ_transcript/48492	1.56053219547333	4.84569735822793	1.40859458477522	0.168425401631321	0.936334604602611	-5.37976243584896
a_HQ_transcript/140399	2.30266766803325	2.9254848829785	1.40859006227031	0.168426728756063	0.936334604602611	-5.1347019618357
a_HQ_transcript/119441	0.528181887497954	3.22804286557401	1.4085855188775	0.168428062018611	0.936334604602611	-5.17764278190224
a_HQ_transcript/75870	3.01323640348432	1.19718288797536	1.40853483572318	0.16844293559609	0.936334604602611	-4.99369635257322
a_HQ_transcript/104705	2.27310379958872	0.481044357010301	1.40833537998254	0.168501478286009	0.936363479392524	-4.93371001268844
a_HQ_transcript/60440	2.271143733815	0.252895869614786	1.40820622740323	0.168539394664743	0.936363479392524	-4.94966387183188
a_HQ_transcript/88530	1.48869745774349	-0.242584641114385	1.40815569413817	0.16855423195225	0.936363479392524	-4.99094875356361
a_HQ_transcript/103819	-0.820303894626056	2.38361548442576	-1.40811223351457	0.168566993431839	0.936363479392524	-4.95819200098196
a_HQ_transcript/32965	-1.85131746042378	0.650046295272	-1.40808401347564	0.168575280176928	0.936363479392524	-4.81847967642226
a_HQ_transcript/44831	-0.84732698648978	2.0049372718255	-1.40804508928368	0.168586710695226	0.936363479392524	-4.85756459322382
a_HQ_transcript/65568	-2.56211572401852	1.07522496113893	-1.40799761985103	0.168600651441502	0.936363479392524	-5.00183972102646
a_HQ_transcript/86514	4.72689209535756	2.2172827973178	1.40794779079748	0.168615286131355	0.936363479392524	-4.9941950863965
a_HQ_transcript/138658	-3.22462435573442	1.72126725553959	-1.4078872321298	0.168633073429583	0.936363479392524	-4.95535757193017
a_HQ_transcript/129984	-2.39142341723919	1.9400733291618	-1.4078570910461	0.168641927021059	0.936363479392524	-4.90328505365139
a_HQ_transcript/74963	-1.73639165636308	0.0265567290511605	-1.40777522107849	0.168665977210655	0.936363479392524	-4.8991430399087
a_HQ_transcript/143957	2.70713433585821	0.390207892826308	1.40771143600678	0.16868471663283	0.936363479392524	-4.95176747346652
a_HQ_transcript/24	0.72873383476074	2.00740638360863	1.40768924755257	0.168691235764144	0.936363479392524	-4.8382882040622
a_HQ_transcript/76601	-1.86198116200776	0.42887263419554	-1.40768164698666	0.168693468911897	0.936363479392524	-4.91911819535446
a_HQ_transcript/27478	0.737536311445123	4.68146805233027	1.40767577599473	0.168695193903609	0.936363479392524	-5.40999605852148
a_HQ_transcript/64702	0.596395301829032	3.66439554966653	1.40761636957932	0.168712649241678	0.936363479392524	-5.25522113129252
a_HQ_transcript/127892	3.99266960686946	2.50256257448187	1.40750306386886	0.168745945697409	0.936363479392524	-4.98711762199427
a_HQ_transcript/100392	1.10272948681119	3.50083075273493	1.40733246885562	0.168796087142678	0.936363479392524	-5.15155268697836
a_HQ_transcript/78243	1.70389994872127	3.05716420964579	1.40729682944328	0.168806563789125	0.936363479392524	-4.99869489847735
a_HQ_transcript/136735	-2.62065451929922	0.133366058652762	-1.40725991311773	0.168817416338353	0.936363479392524	-4.88501099264669
a_HQ_transcript/127106	1.69379959148058	-0.114052396389633	1.40720541536792	0.168833438422573	0.936363479392524	-4.92573205670851
a_HQ_transcript/47015	-4.28089177815662	-0.0892447037575164	-1.40716035882013	0.16884668573903	0.936363479392524	-4.95685205672946
a_HQ_transcript/73878	-3.51651622260672	3.14361252788683	-1.40714122828035	0.168852310658107	0.936363479392524	-5.06144254889355
a_HQ_transcript/27243	-1.76287275108233	0.233363169335368	-1.40713577226434	0.168853914907894	0.936363479392524	-4.91771691471289
a_HQ_transcript/70692	-1.37689722049236	2.12660039860865	-1.40712153630878	0.168858100807658	0.936363479392524	-5.00132104499135
a_HQ_transcript/131594	-1.58329036825409	0.209539927318823	-1.40705266638579	0.168878352273851	0.936363479392524	-4.81177164594016
a_HQ_transcript/103966	1.85946602791849	-0.150913443535516	1.40700580272961	0.168892133802773	0.936363479392524	-4.92653641519196
a_HQ_transcript/48829	-1.46231735430077	0.307740795521341	-1.4069818700515	0.16889917219637	0.936363479392524	-4.9257607625528
a_HQ_transcript/40816	-1.0511218137994	1.67547381706016	-1.40673981625037	0.168970371069021	0.936668866059515	-4.92484198960439
a_HQ_transcript/151234	-2.17200432355719	0.504850755854112	-1.40663091347781	0.169002411940398	0.93670439159367	-4.86068007553662
a_HQ_transcript/135500	-0.847453701460494	3.23523183968106	-1.40660530296029	0.169009947641431	0.93670439159367	-5.11528862605331
a_HQ_transcript/122820	-2.79237466113912	0.790702002267205	-1.40650289671597	0.169040082540333	0.93670439159367	-4.97035288100161
a_HQ_transcript/137616	-1.20961713840513	-0.341734860811061	-1.40629803872446	0.169100378386967	0.93670439159367	-4.9449269026882
a_HQ_transcript/150488	-2.77117889680632	8.87045508572379	-1.40627955176631	0.169105820483247	0.93670439159367	-5.84686559979921
a_HQ_transcript/36970	0.707148152583082	2.41351705833387	1.40627271914767	0.169107831869406	0.93670439159367	-4.9002675423601
a_HQ_transcript/137860	-1.96996765803619	-0.260291193304328	-1.40626042216827	0.16911145190131	0.93670439159367	-4.91316619713518
a_HQ_transcript/115983	1.8798298178375	-0.050810158265195	1.40621699864438	0.16912423557129	0.93670439159367	-4.98497113091499

a_HQ_transcript/32378	1.46948828733914	0.884962762933488	1.40620083363312	0.16912899466415	0.93670439159367	-4.87542931050262
a_HQ_transcript/136742	-2.92104499481526	3.6391235437474	-1.40617050189714	0.16913792482396	0.93670439159367	-5.15732989413746
a_HQ_transcript/2226	-0.909694549610229	2.66183702185736	-1.4060967430017	0.169159642195092	0.936735417882855	-5.04562935115552
a_HQ_transcript/81080	2.07477049659685	-0.0422383688476553	1.4059713005208	0.169196582169118	0.936780702871939	-4.85430158548447
a_HQ_transcript/36342	2.45782234858992	-0.168362726640085	1.40595265731659	0.169202072711585	0.936780702871939	-4.9245043875782
a_HQ_transcript/53946	-0.859607162546118	2.48046593428065	-1.4058981789569	0.169218117737171	0.936780702871939	-4.94118271932289
a_HQ_transcript/41095	4.49861125500016	0.961684163056334	1.40582216464688	0.169240507549904	0.936780702871939	-4.98514846335853
a_HQ_transcript/132493	0.80469879548053	2.07743777312935	1.40579537447719	0.169248399077228	0.936780702871939	-4.93166078992453
a_HQ_transcript/54173	2.42660201769348	1.32342067771007	1.40561573779544	0.169301321760845	0.936984407437339	-4.8982359892698
a_HQ_transcript/12402	3.5931799353475	2.6979746152163	1.40532651126881	0.16938655794985	0.937366892660783	-4.89122604135317
a_HQ_transcript/58869	-2.26793974493991	1.43701537393067	-1.40507856547335	0.169459655353475	0.937682136733633	-4.97735630430026
a_HQ_transcript/278	2.06341903661719	1.69245626571382	1.40491092080182	0.169509093045127	0.937720365790703	-4.89068964117353
a_HQ_transcript/100432	-2.87363434624864	2.18434702574186	-1.40490384598845	0.169511179625987	0.937720365790703	-5.07874522426221
a_HQ_transcript/92877	2.15942490563111	0.533905392011458	1.40483736691647	0.169530787341013	0.937720365790703	-4.8325637792647
a_HQ_transcript/110066	-0.580160385595768	2.8761022162478	-1.40481681850623	0.169536848365886	0.937720365790703	-5.01128197876701
a_HQ_transcript/132624	0.518832995284177	3.36741135900709	1.40478164336109	0.169547224133985	0.937720365790703	-5.1591508764517
a_HQ_transcript/103537	-1.48160567947509	1.57033252283093	-1.40465383181932	0.169584929464283	0.937839670811516	-4.87347920060182
a_HQ_transcript/6197	0.520548431627817	4.314501603831	1.40454274124793	0.169617707331684	0.937931705838672	-5.34855250834652
a_HQ_transcript/20077	-1.57941712868261	2.12717091288073	-1.40444946696611	0.169645232246448	0.937994678970945	-5.16828060156363
a_HQ_transcript/116268	-2.14297280185634	1.09477849536114	-1.40436564654068	0.169669970350906	0.938042232885433	-4.91239941072252
a_HQ_transcript/116093	-1.67707672350053	-0.142424211313242	-1.40430959998886	0.16968651307056	0.938044472826011	-4.97311552144232
a_HQ_transcript/84671	-1.4070806163323	1.39176402786314	-1.40408176448925	0.169753774104051	0.938210266684803	-4.92407693682757
a_HQ_transcript/60650	0.601960624490605	3.48418827491451	1.40400816762476	0.169775505657739	0.938210266684803	-5.17423466956978
a_HQ_transcript/94473	-1.66710225372259	2.05974886544277	-1.40379124924916	0.16983956963383	0.938210266684803	-5.08657873212449
a_HQ_transcript/140677	1.77191378688634	1.59233609642763	1.40377388523478	0.169844698685739	0.938210266684803	-4.81047649942746
a_HQ_transcript/106601	-0.693572109226881	2.82643084234102	-1.40369829963298	0.169867026878366	0.938210266684803	-4.99829168954471
a_HQ_transcript/4938	2.5472064941869	0.268779159519092	1.40364910175796	0.169881561301754	0.938210266684803	-4.87339463592311
a_HQ_transcript/137444	-2.37992162519893	1.27154295211404	-1.40363349051662	0.169886173501413	0.938210266684803	-4.90225960811738
a_HQ_transcript/136481	1.63426505973689	-0.341917506377315	1.40354347673437	0.169912769173022	0.938210266684803	-4.97371350038943
a_HQ_transcript/114238	-0.998159607046513	2.18845753829035	-1.40350007516184	0.169925593864824	0.938210266684803	-4.9453810882718
a_HQ_transcript/145668	-0.990530680715709	3.0851048402082	-1.40343961580713	0.169943460210999	0.938210266684803	-5.21053824793001
a_HQ_transcript/129199	2.90202324801711	0.399570930756641	1.40328412909902	0.16998941486165	0.938210266684803	-4.90487192984654
a_HQ_transcript/149957	-5.11215362307185	0.725392669700699	-1.40314933960686	0.170029260263311	0.938210266684803	-5.02840251189003
a_HQ_transcript/110888	0.554314080736277	5.62113501284667	1.40309525194331	0.170045251289312	0.938210266684803	-5.47266875638705
a_HQ_transcript/135159	2.18289528322199	0.924798205585642	1.40309233654232	0.170046113261565	0.938210266684803	-4.94322544746353
a_HQ_transcript/35721	1.46937103415458	1.96779691916153	1.40305720451366	0.170056500724436	0.938210266684803	-4.92012261409869
a_HQ_transcript/117361	2.05069559096051	2.11449547363999	1.40304073207821	0.17006137128953	0.938210266684803	-4.96385122964778
a_HQ_transcript/137400	-2.7060602716287	0.266281110692703	-1.40293824524149	0.170091677026758	0.938210266684803	-4.96619115794197
a_HQ_transcript/120737	2.66550634895369	0.397695624341684	1.40280349358435	0.170131530036676	0.938210266684803	-4.95458712826819
a_HQ_transcript/152335	-3.33656205370167	1.16520380587822	-1.40276551119827	0.170142764708752	0.938210266684803	-4.99198510858621
a_HQ_transcript/38616	-1.6392834891122	-0.32746578607507	-1.40269843100169	0.170162607537747	0.938210266684803	-4.91487441454269
a_HQ_transcript/135490	1.33955325785835	2.89749303505933	1.40261217796958	0.170188124503479	0.938210266684803	-5.087851370321
a_HQ_transcript/88570	2.4474929013694	1.56438204741432	1.40253158118814	0.17021197084468	0.938210266684803	-4.91800675245697
a_HQ_transcript/4614	2.26196555609928	-0.252333457718533	1.40246480415941	0.170231730291098	0.938210266684803	-4.92435876716368
a_HQ_transcript/40412	-1.94514099220357	0.975349272384559	-1.40245297689071	0.170235230189314	0.938210266684803	-4.8794036359238
a_HQ_transcript/16013	2.15510241346022	0.344505615478758	1.40244352039801	0.17023802857353	0.938210266684803	-4.91917161199933
a_HQ_transcript/150114	-3.06110062232806	0.294717934370291	-1.40240258332799	0.170250143169204	0.938210266684803	-5.003070133637119
a_HQ_transcript/123380	1.37849842062835	1.95675111895764	1.40235879962746	0.170263100922616	0.938210266684803	-4.86874822329259
a_HQ_transcript/18882	-0.970671303952146	0.355838327028482	-1.4023532955907	0.170264729892896	0.938210266684803	-4.88255640922465
a_HQ_transcript/94810	-1.05066155971928	2.33518430089603	-1.40222383038251	0.170303049836374	0.938210266684803	-5.11229836413318

a_HQ_transcript/104435	-3.83266721741059	1.37010596135513	-1.40220957235272	0.170307270432949	0.938210266684803	-5.05645203578179
a_HQ_transcript/33560	1.39806248988979	0.421805105771076	1.40214136753858	0.170327461243836	0.938210266684803	-4.92775086880852
a_HQ_transcript/119005	0.73053021610652	3.85606377728256	1.40213317458497	0.170329886746955	0.938210266684803	-5.28066149197256
a_HQ_transcript/69778	2.78370267780859	1.62845636336593	1.40212411727366	0.170332568172499	0.938210266684803	-4.99347237910707
a_HQ_transcript/136643	2.99979395612113	1.65266608560263	1.40211823909982	0.170334308429116	0.938210266684803	-5.00191911905131
a_HQ_transcript/125111	-2.37741941221441	0.105555201169463	-1.40196104005865	0.170380852999181	0.938210266684803	-4.99251219513706
a_HQ_transcript/147963	2.35965990001855	0.751923884450834	1.4018605960113	0.170410598383051	0.938210266684803	-5.00824772141818
a_HQ_transcript/1595	2.70701900668668	2.39918542470554	1.40184324453824	0.170415737240624	0.938210266684803	-5.10612379144217
a_HQ_transcript/260	-3.09127162076515	2.50366152123911	-1.40182122019982	0.170422260201199	0.938210266684803	-4.99398649132253
a_HQ_transcript/101816	-3.11517458156821	4.64927802582751	-1.40170006993887	0.170458144841787	0.938210266684803	-5.30659098768888
a_HQ_transcript/87574	2.65075731697268	2.65411168846087	1.40163529936105	0.170477332281497	0.938210266684803	-5.0566550613634
a_HQ_transcript/64603	2.08898255549655	0.0504897710077966	1.40162121314766	0.170481505362847	0.938210266684803	-4.94448465443863
a_HQ_transcript/1504	1.50306526163246	0.269416018367837	1.40147350542638	0.170525269024735	0.938210266684803	-4.91572114658514
a_HQ_transcript/139514	4.08777049887803	0.792425930993205	1.40145856656815	0.170529695682822	0.938210266684803	-4.87883708223953
a_HQ_transcript/38535	-1.16592869606754	1.36962434868645	-1.40137860402597	0.170553391584566	0.938210266684803	-4.77112807062961
a_HQ_transcript/86285	2.16109746169926	0.0849573723650878	1.40137169435499	0.17055543930052	0.938210266684803	-4.8916403176917
a_HQ_transcript/108595	-1.46176490532629	1.11098733790739	-1.40136984252678	0.170555988102453	0.938210266684803	-4.89239039792697
a_HQ_transcript/92557	-1.69069490035833	-0.434580553657851	-1.40136017582614	0.170558852917669	0.938210266684803	-4.95031703701293
a_HQ_transcript/138065	-1.84795879330201	0.385957779962725	-1.4013408823065	0.170564570841597	0.938210266684803	-4.88110736698663
a_HQ_transcript/10529	-1.47236195253054	1.03612017758071	-1.40133756287463	0.170565554620173	0.938210266684803	-4.83124069909651
a_HQ_transcript/138353	-2.77262458760968	1.95414610442271	-1.4013248460297	0.170569323547124	0.938210266684803	-4.93487540001378
a_HQ_transcript/122264	2.55908053960583	0.634236873310743	1.40131577183601	0.170572012931241	0.938210266684803	-4.89724661190853
a_HQ_transcript/80065	2.91376492790758	0.992082254363872	1.40130063936164	0.170576497925569	0.938210266684803	-4.99301049422606
a_HQ_transcript/4632	1.33413013414302	2.35748616291592	1.40128444646792	0.170581297311594	0.938210266684803	-4.93911418071376
a_HQ_transcript/152236	1.92977587405772	0.34049007522852	1.40126153972946	0.170588086784195	0.938210266684803	-4.82754192519615
a_HQ_transcript/19488	2.52591291087955	1.7194750408923	1.40115743773151	0.170618944900784	0.938220654310221	-4.98419911160629
a_HQ_transcript/6952	1.00283743189834	1.66609775084567	1.40112181342484	0.170629505731946	0.938220654310221	-4.88859738764061
a_HQ_transcript/93783	1.66247913126315	1.1022535270708	1.40107279399386	0.170644038387467	0.938220654310221	-4.85135114932831
a_HQ_transcript/150989	3.42945253465644	1.30082790853477	1.40102362797416	0.170658615476413	0.938220654310221	-5.17689345305588
a_HQ_transcript/131039	1.52798128660825	-0.201489895010707	1.40098294402291	0.170670678479842	0.938220654310221	-4.92607416698508
a_HQ_transcript/52549	1.29007525796321	-0.0129687526270566	1.40088454617864	0.170699856716476	0.938262970087603	-5.01211235801069
a_HQ_transcript/145098	1.47691156278871	-0.463120455148262	1.40084409131753	0.170711854063886	0.938262970087603	-4.94019944935952
a_HQ_transcript/126538	1.55847333660337	4.16544072615198	1.40077167094089	0.170733332797837	0.938262970087603	-5.30811449494519
a_HQ_transcript/145672	-2.5832270930449	1.21639670682043	-1.40068982704978	0.170757608946292	0.938262970087603	-4.90286722697058
a_HQ_transcript/78303	1.99832200050403	0.364486425014393	1.40068485869413	0.170759082723589	0.938262970087603	-4.97727888043081
a_HQ_transcript/96085	1.71050349087771	-0.435102692498912	1.4004904818439	0.170816749100769	0.93841748224739	-4.92871191836293
a_HQ_transcript/132852	1.09015635685846	1.52527803581637	1.40045709707916	0.170826654997286	0.93841748224739	-4.8677909157313
a_HQ_transcript/117393	-1.02919575182666	0.961110224585175	-1.40042683372495	0.170835635100844	0.93841748224739	-4.76783439832179
a_HQ_transcript/37044	-1.31958962035585	0.617386798681843	-1.4002917894674	0.170875711553774	0.938504164866523	-4.83826677676448
a_HQ_transcript/20990	1.57680698549966	1.00653561236433	1.40026485274752	0.170883706319627	0.938504164866523	-4.88391735884659
a_HQ_transcript/119377	-3.13223056116378	1.36945542229341	-1.40010370165975	0.170931541771378	0.938678191789007	-4.99519606560304
a_HQ_transcript/89494	0.854094223697502	2.59754548672912	1.39985919260401	0.171004140913704	0.93877429623547	-4.94651819523975
a_HQ_transcript/18283	-2.39344848918023	1.19117604938879	-1.39984096234792	0.171009554772951	0.93877429623547	-4.94409342980701
a_HQ_transcript/51794	0.65100118478935	2.658357433338	1.39983953877395	0.171009977538936	0.93877429623547	-5.0684018842947
a_HQ_transcript/138763	3.49450694782028	1.13238999435176	1.39980945451186	0.171018912006457	0.93877429623547	-5.0743677867504
a_HQ_transcript/124417	0.722385488282436	3.07566065586028	1.39977281814464	0.171029792821594	0.93877429623547	-5.1342298780552
a_HQ_transcript/48516	-1.1282795860599	2.17099890613946	-1.39966040469103	0.171063182433039	0.938849230242088	-4.92561674796703
a_HQ_transcript/110798	2.15542920171888	1.66201046024602	1.39961810631793	0.171075747431947	0.938849230242088	-4.98397743849183
a_HQ_transcript/131127	1.56855868675899	0.181879313084941	1.39947563717242	0.171118074112142	0.938992864343492	-4.80834435371838
a_HQ_transcript/92895	-1.93230869854765	1.30028982458091	-1.39924906444672	0.171185404335154	0.939203158816314	-5.02868922682457

a_HQ_transcript/106684	2.33262652342909	1.26519356941787	1.39922972763744	0.171191151582457	0.939203158816314	-4.82920747274148
a_HQ_transcript/121735	1.66370284113119	0.564781175384692	1.39917184862157	0.171208355169922	0.939203158816314	-4.97958354626678
a_HQ_transcript/73870	-2.09553829902853	0.338652384001869	-1.39910526347607	0.171228148187117	0.939203158816314	-4.87057345172524
a_HQ_transcript/15562	-2.23699311868903	1.11567446777386	-1.39895314007759	0.171273374925823	0.939203158816314	-5.00487103918352
a_HQ_transcript/30066	-1.21329790823995	0.141205829861967	-1.39893453098768	0.171278908106041	0.939203158816314	-4.87956273895397
a_HQ_transcript/133991	-1.99379555778367	0.521320482011611	-1.39884701039839	0.171304933137356	0.939203158816314	-4.84942619792493
a_HQ_transcript/140500	-1.02614957094566	3.8922019195014	-1.39870843297345	0.171346146712578	0.939203158816314	-5.28660278514263
a_HQ_transcript/88151	2.18227847457862	4.23940169767936	1.39870209901097	0.17134803064832	0.939203158816314	-5.37703023330535
a_HQ_transcript/91774	2.44592998056112	2.16555073542108	1.39864047899327	0.171366359383034	0.939203158816314	-5.09311624771619
a_HQ_transcript/3392	0.500713509144792	3.35816538237806	1.39858387518212	0.171383197411991	0.939203158816314	-5.19347591768959
a_HQ_transcript/138777	-2.63172796108435	4.99737975487056	-1.39856208322162	0.171389680247935	0.939203158816314	-5.39214290582275
a_HQ_transcript/64314	1.81560183275446	-0.36317199889706	1.3984873676176	0.171411908665054	0.939203158816314	-4.89847647072299
a_HQ_transcript/102562	-4.10601175959854	0.317665349835494	-1.3983128504217	0.17146383755557	0.939203158816314	-4.96694616819998
a_HQ_transcript/120345	-2.1823574688749	-0.218572708663378	-1.39829160153359	0.171470161161915	0.939203158816314	-4.94120307986926
a_HQ_transcript/41507	2.03621340204489	1.90137218205415	1.39821442200355	0.171493131099037	0.939203158816314	-4.92847809803613
a_HQ_transcript/127383	0.95144214836252	5.52297552837276	1.39820012665281	0.171497385902441	0.939203158816314	-5.51325009212546
a_HQ_transcript/21381	-1.97796548691462	1.75397960716097	-1.3981659916081	0.171507546036913	0.939203158816314	-4.89508698463722
a_HQ_transcript/129333	1.83624178755123	0.649576915468302	1.39806108324202	0.171538774466899	0.939203158816314	-5.03325719020994
a_HQ_transcript/114387	2.375108485609	-0.00362319319872035	1.39804257348811	0.171544284790513	0.939203158816314	-4.93783673213369
a_HQ_transcript/142178	-1.75719590090964	0.81295150942173	-1.39795612004095	0.171570023677257	0.939203158816314	-4.90794270764747
a_HQ_transcript/54557	-1.69486104640965	1.01913204899677	-1.39793350777407	0.171576756291642	0.939203158816314	-4.846556968779
a_HQ_transcript/129995	-0.596004248454018	3.20435667231065	-1.39791719131771	0.171581614509347	0.939203158816314	-5.20820317809307
a_HQ_transcript/135457	-3.08890536877735	1.54974309331816	-1.39788637926214	0.171590789079211	0.939203158816314	-5.07380091584815
a_HQ_transcript/54529	-0.90778026878534	2.50766082299417	-1.39786892715149	0.171595985774388	0.939203158816314	-5.08855731014525
a_HQ_transcript/84985	-0.394702497728058	3.82600337786398	-1.39785178974221	0.171601088881259	0.939203158816314	-5.31064059813584
a_HQ_transcript/132461	-2.6586055854326	0.150201009947751	-1.39778853222815	0.171619926471442	0.939203158816314	-5.00007528953686
a_HQ_transcript/141054	-0.480511984420585	2.95730563274839	-1.39768344771878	0.171651223392158	0.939203158816314	-5.12893968947017
a_HQ_transcript/105108	0.372642860509369	4.75535529927611	1.39761755512831	0.171670850212635	0.939203158816314	-5.380643553536
a_HQ_transcript/121272	0.517428697851832	3.1566357757432	1.39754591856273	0.17169218993239	0.939203158816314	-5.15729464702478
a_HQ_transcript/18235	0.420954755502725	4.87054139892282	1.39750005550972	0.171705853106123	0.939203158816314	-5.41258855084267
a_HQ_transcript/131221	-2.63746011763343	2.00194152413984	-1.39748583061142	0.171710091053217	0.939203158816314	-4.92999560035551
a_HQ_transcript/15208	1.47790859035837	1.60228411731743	1.39747532215657	0.171713221833007	0.939203158816314	-4.96479404132862
a_HQ_transcript/146919	3.38565893123427	0.948806360890375	1.39741499164874	0.171731196942463	0.939203158816314	-5.02705057803927
a_HQ_transcript/136687	3.39944551105956	2.92351127474032	1.39728288839496	0.171770561453309	0.939203158816314	-5.19117877270901
a_HQ_transcript/41388	-1.00599604537479	4.91869720426584	-1.39708059219166	0.171830855959169	0.939203158816314	-5.40443190795355
a_HQ_transcript/137040	2.3755290552814	-0.00861820066225263	1.39690314422446	0.171883758077928	0.939203158816314	-4.94813655147706
a_HQ_transcript/22118	1.46753552180564	-0.45600202238825	1.39688456676585	0.171889297266677	0.939203158816314	-4.95533244596504
a_HQ_transcript/131142	1.44949247937912	-0.289098596924117	1.3968479779607	0.171900207256907	0.939203158816314	-4.97044654177621
a_HQ_transcript/16054	0.950101946476648	2.53130682462069	1.39682289780507	0.171907685930039	0.939203158816314	-4.98054619064
a_HQ_transcript/46441	1.36815483211017	-0.34939415889331	1.39680527028766	0.17191294244705	0.939203158816314	-4.82407814176888
a_HQ_transcript/63969	-1.71712475023989	-0.118178220844351	-1.3965302995997	0.171994954866638	0.939203158816314	-4.88525389103192
a_HQ_transcript/68496	1.60265068487656	2.22727820718747	1.39645306178425	0.172017997233031	0.939203158816314	-4.98369152396133
a_HQ_transcript/68301	-2.37453965033631	5.07193748300905	-1.3963252077959	0.172056145240274	0.939203158816314	-5.41997019544172
a_HQ_transcript/109166	1.76420858919104	0.676513507965448	1.39630045164957	0.172063532538263	0.939203158816314	-4.92388193231891
a_HQ_transcript/115263	1.89659088889514	0.39507082039941	1.39624079122791	0.17208133638253	0.939203158816314	-5.01278450004814
a_HQ_transcript/144778	-4.2606805433993	2.5954246714919	-1.39623994944635	0.172081587597035	0.939203158816314	-5.09487137483418
a_HQ_transcript/120876	2.67142278474553	0.0722782753072783	1.39611618944406	0.172118524659636	0.939203158816314	-4.94707880084323
a_HQ_transcript/114679	-1.52646244072167	-0.201806188942463	-1.39605988024419	0.172135332599797	0.939203158816314	-4.92948910906017
a_HQ_transcript/96202	1.77391144729959	0.391847955823043	1.39605358686186	0.172137211214471	0.939203158816314	-4.96123897469028
a_HQ_transcript/144732	-1.00357516913232	3.96334972188447	-1.39604418816751	0.172140016814429	0.939203158816314	-5.28633665763625

a_HQ_transcript/142913	2.52489032108916	1.79684243306771	1.3959503195339	0.172168039461745	0.939203158816314	-5.12789162935915
a_HQ_transcript/104696	3.10579740027278	0.4491101653835	1.39583938759372	0.172201160630141	0.939203158816314	-4.9719316740661
a_HQ_transcript/137243	3.9691172947869	1.30442749593114	1.39581171300124	0.172209424265264	0.939203158816314	-5.07706878279202
a_HQ_transcript/88529	2.08594004656291	0.336631529850778	1.39569919588945	0.172243025079288	0.939203158816314	-4.93222402279847
a_HQ_transcript/148999	-2.50024859669393	0.617529323188511	-1.39568989045586	0.17224580417654	0.939203158816314	-5.04265317791917
a_HQ_transcript/147771	-1.94847255751713	1.55438595038482	-1.39566359476174	0.172253657658742	0.939203158816314	-4.88696862015604
a_HQ_transcript/35020	1.17202230215269	2.07922335917898	1.3956291329022	0.172263950475487	0.939203158816314	-4.96916428283909
a_HQ_transcript/146802	1.87703878034701	0.934372044186599	1.39557840572481	0.172279102174645	0.939203158816314	-4.87383381456623
a_HQ_transcript/136822	-2.29027264176667	-0.143314029725462	-1.39554910323919	0.172287855008602	0.939203158816314	-4.94818910370934
a_HQ_transcript/51868	2.53467086580986	0.648717724707937	1.39553145194412	0.172293127727781	0.939203158816314	-4.95403684866093
a_HQ_transcript/43613	-1.75619152568793	0.359644645145072	-1.39545637713242	0.172315555164847	0.939203158816314	-4.84474778377548
a_HQ_transcript/115489	1.67127893321915	0.53984974297718	1.39544771867617	0.172318141891506	0.939203158816314	-4.79927618560238
a_HQ_transcript/146900	0.817377693298883	2.42667018186562	1.39542021411381	0.172326359121988	0.939203158816314	-4.90598992408481
a_HQ_transcript/129	1.90264960884139	1.42571579678276	1.39538490462724	0.172336908592037	0.939203158816314	-4.93852598070511
a_HQ_transcript/372	-0.710875898565995	4.1326347118506	-1.39534656177617	0.172348364917644	0.939203158816314	-5.36100460888903
a_HQ_transcript/141352	-1.34566855473708	3.97038591103966	-1.3953245738118	0.17235493489313	0.939203158816314	-5.30153138652535
a_HQ_transcript/151515	-2.31467918344827	0.264419601351787	-1.39529182642621	0.172364720133255	0.939203158816314	-4.95562960489499
a_HQ_transcript/108315	2.37754205274789	1.2649956249152	1.39526960685392	0.172371359807099	0.939203158816314	-4.94746429246309
a_HQ_transcript/146646	2.24224390597913	0.639267714939169	1.39524388281471	0.172379046939161	0.939203158816314	-5.03674912779
a_HQ_transcript/14378	-1.38158790241866	1.95396888887165	-1.39522746638362	0.172383952812651	0.939203158816314	-4.85706746214417
a_HQ_transcript/7965	-1.36011835159909	-0.0677700565455411	-1.39514413535212	0.172408857078643	0.939203158816314	-4.88503325346483
a_HQ_transcript/38262	1.79055086460045	0.895957377800508	1.39514154772753	0.172409630459778	0.939203158816314	-4.87675852193039
a_HQ_transcript/146417	-1.93335431753783	0.573293822001006	-1.39513459501951	0.17241170847683	0.939203158816314	-4.89856513171543
a_HQ_transcript/133326	-1.30797014071849	2.86059877382051	-1.39501125251104	0.172448576197034	0.939203158816314	-5.0260862867528
a_HQ_transcript/117313	-1.70102360278213	-0.0522789262931396	-1.3950104968889	0.172448802075455	0.939203158816314	-4.85613238250654
a_HQ_transcript/6555	-1.03496569388451	1.44382162619504	-1.39499710587448	0.172452805095038	0.939203158816314	-4.79157270142321
a_HQ_transcript/60711	-0.94748079106017	-0.380986380086654	-1.39494444014881	0.17246854933757	0.939203158816314	-4.88579831204655
a_HQ_transcript/134658	0.666043021027517	2.73949873033606	1.3949242092746	0.172474597588833	0.939203158816314	-5.06628193003607
a_HQ_transcript/129158	-0.867342980541869	1.98436068409307	-1.39489595732357	0.172483044110551	0.939203158816314	-4.89819521089499
a_HQ_transcript/12354	-2.39985409782752	1.98705544050582	-1.39487997908108	0.172487821289353	0.939203158816314	-5.03310463003518
a_HQ_transcript/7281	-1.35339761682986	2.25258947711757	-1.39483541403467	0.172501145904913	0.939203158816314	-5.01863765954225
a_HQ_transcript/124485	-3.68921205232957	1.37005609451573	-1.39477757549922	0.172518440395698	0.939203158816314	-4.99630990446315
a_HQ_transcript/115846	1.87858377927874	1.03079447263899	1.39468097939291	0.172547326941031	0.939203158816314	-4.9529381472299
a_HQ_transcript/41150	1.66765616940521	-0.24805514361505	1.39462168874563	0.172565059364541	0.939203158816314	-4.9617261098422
a_HQ_transcript/134502	-0.762870159535436	4.37335907716909	-1.39461563937897	0.172566868666518	0.939203158816314	-5.31115024388748
a_HQ_transcript/26522	-2.05279042089311	3.79524556948255	-1.39456158849031	0.172583035378109	0.939203158816314	-5.30588178131735
a_HQ_transcript/86662	2.90983358435955	0.311981640096428	1.39455853771382	0.172583947905819	0.939203158816314	-4.95046283994263
a_HQ_transcript/59800	-1.07912168783814	2.83517506968785	-1.39445740735062	0.17261419947612	0.939203158816314	-5.05758274824288
a_HQ_transcript/22474	1.76859407788599	1.40318594157463	1.3944158112981	0.172626643490662	0.939203158816314	-4.80731861780713
a_HQ_transcript/101693	-0.988845954999624	3.62380646134551	-1.39441552286263	0.172626729782437	0.939203158816314	-5.2265992384109
a_HQ_transcript/70019	0.57930965241162	3.57642933143897	1.39431487105842	0.172656844030229	0.939221322316563	-5.24028118263105
a_HQ_transcript/112599	-1.76868568217084	1.19933555102082	-1.39428285436469	0.17266642404122	0.939221322316563	-4.79109838423497
a_HQ_transcript/494	1.71809047166047	0.657355233742151	1.39419134991589	0.172693806226849	0.939221322316563	-4.90571689851974
a_HQ_transcript/145701	-3.32601956933627	2.25254827015627	-1.39407706178395	0.172728011072489	0.939221322316563	-4.97108889066681
a_HQ_transcript/98767	0.815331474404709	3.12229657684319	1.39401040236571	0.17274796375536	0.939221322316563	-5.14579690762922
a_HQ_transcript/40654	-1.60511257420586	-0.331767585952744	-1.39398961517337	0.172754186204606	0.939221322316563	-4.9718869774799
a_HQ_transcript/97728	1.97683537504271	-0.013400361514818	1.39397458476597	0.172758685524032	0.939221322316563	-4.85030588680422
a_HQ_transcript/100048	-2.84172558753386	0.0591015591804077	-1.39391619459844	0.17277616536241	0.939221322316563	-4.93812600551355
a_HQ_transcript/108723	-2.09195805587455	0.891320581177213	-1.39382379239148	0.172803829964725	0.939221322316563	-4.82506770791796
a_HQ_transcript/27731	2.10522684800048	0.216096452172612	1.39381764491311	0.172805670601566	0.939221322316563	-4.89352135600975

a_HQ_transcript/130696	-1.40998351041803	1.65115958343535	-1.39379019605734	0.172813889341471	0.939221322316563	-4.91649408701899
a_HQ_transcript/113650	1.8610180208438	1.39432754876699	1.39371524359999	0.17283633317371	0.939221322316563	-4.87820143620663
a_HQ_transcript/88184	1.39933185312516	-0.349196165442129	1.39360045254906	0.172870710731955	0.939221322316563	-4.97485684912828
a_HQ_transcript/15595	2.22311883623424	1.2537324047342	1.39355924073026	0.172883054128959	0.939221322316563	-4.83939328919462
a_HQ_transcript/146454	2.27202410523526	-0.160719423307683	1.39334794997468	0.172946348885749	0.939221322316563	-4.94400937520136
a_HQ_transcript/21163	1.8740154107378	0.987085576549454	1.39332552314677	0.172953068183358	0.939221322316563	-4.87804497102649
a_HQ_transcript/141543	3.45313227782537	4.39334235201381	1.39311995686395	0.173014667374336	0.939221322316563	-5.0552571275963
a_HQ_transcript/56393	1.17816121243075	1.38368523703877	1.3930560220446	0.173033829331944	0.939221322316563	-4.86400335201016
a_HQ_transcript/51100	2.01173155323617	2.63772427928975	1.39295121717381	0.173065244077921	0.939221322316563	-5.18760802177858
a_HQ_transcript/21992	2.78627373858037	1.60780835160761	1.39293275511405	0.173070778451966	0.939221322316563	-4.92335149751785
a_HQ_transcript/89324	3.31228376601057	0.682628779322501	1.39277028816977	0.173119487159124	0.939221322316563	-5.00462179770933
a_HQ_transcript/122386	2.57175602841538	2.85952152231625	1.39269332147731	0.173142566045928	0.939221322316563	-5.12628290660231
a_HQ_transcript/74667	1.74992913700268	0.845752823115286	1.39265582704925	0.17315380982853	0.939221322316563	-4.78769249079409
a_HQ_transcript/40850	1.8885995165245	1.21027609368743	1.3926521435484	0.173154914463008	0.939221322316563	-4.94702534920834
a_HQ_transcript/131862	-0.835466140964429	3.14551297302813	-1.39259902195671	0.173170845557402	0.939221322316563	-5.05657845738421
a_HQ_transcript/141194	-2.25803942552578	0.734545994501245	-1.39253196710201	0.1731909568558	0.939221322316563	-4.83161972877395
a_HQ_transcript/148841	-1.85800098918449	2.2385922445404	-1.3924951128894	0.173202011062648	0.939221322316563	-4.9996501881445
a_HQ_transcript/109963	3.60908521757292	1.34804817242477	1.39243334931754	0.173220537924463	0.939221322316563	-5.00323777166518
a_HQ_transcript/34220	1.89349033831943	0.220355097463779	1.3923659378245	0.173240760729748	0.939221322316563	-4.75796236564406
a_HQ_transcript/123482	-2.41060218817635	0.596049839317729	-1.39234400420508	0.173247341004641	0.939221322316563	-4.93462263346208
a_HQ_transcript/147532	-1.98890686219977	1.7742521846163	-1.39231474425717	0.173256119545587	0.939221322316563	-4.91740243893549
a_HQ_transcript/92315	1.49214330498391	0.511409139110674	1.39227467532848	0.17326814155081	0.939221322316563	-4.89383869677276
a_HQ_transcript/17961	1.2538196459064	2.33422365683605	1.39218147701943	0.173296106652914	0.939221322316563	-5.00174160247211
a_HQ_transcript/36855	-1.65013972967191	-0.249672736078228	-1.39215316657693	0.173304602188612	0.939221322316563	-4.92295092322198
a_HQ_transcript/141663	0.724374354518537	2.71998775090903	1.39204929884876	0.173335774113148	0.939221322316563	-5.03568761977841
a_HQ_transcript/99709	-3.50324728419677	2.52875253396748	-1.39201852921172	0.173345009284327	0.939221322316563	-5.11417089071949
a_HQ_transcript/40123	-1.92778661807421	0.489192634060879	-1.39201145749206	0.17334713183829	0.939221322316563	-4.91547510628691
a_HQ_transcript/46449	2.25167452393931	0.714875632609817	1.39198484245349	0.173355120438916	0.939221322316563	-4.8423007583656
a_HQ_transcript/149507	5.69635752876435	1.05922504322584	1.39186686548141	0.173390535123139	0.939221322316563	-5.06591710870762
a_HQ_transcript/123853	-4.06231942930267	2.01962061087069	-1.39182015274208	0.173404559058095	0.939221322316563	-5.11421729644299
a_HQ_transcript/144168	2.08358183108105	1.12089848680951	1.39179643560755	0.173411679672659	0.939221322316563	-4.93129794076108
a_HQ_transcript/22384	1.58050649174089	0.431452509701821	1.39173408311164	0.173430400903996	0.939221322316563	-4.9148492780587
a_HQ_transcript/53718	-2.33078515129028	2.65452011307611	-1.39168751604794	0.17344438361704	0.939221322316563	-5.05909117755629
a_HQ_transcript/145307	-2.78998109424031	3.54800395231369	-1.39161687867413	0.173465595615817	0.939221322316563	-5.09693496901284
a_HQ_transcript/148232	2.35227609226138	1.43525930757951	1.39159555082169	0.17347200064648	0.939221322316563	-4.97084483651441
a_HQ_transcript/138271	0.829360767398992	3.5526449533981	1.39159378146163	0.173472532016559	0.939221322316563	-5.22598095583971
a_HQ_transcript/53142	1.28689370331398	0.614110415041601	1.3915904726706	0.173473525708364	0.939221322316563	-4.94334394388419
a_HQ_transcript/109799	2.45586509140389	0.817973796357133	1.39155962438932	0.173482790238167	0.939221322316563	-5.00163919716484
a_HQ_transcript/111561	2.19886288007573	1.01808922731208	1.39154407144719	0.17348746133217	0.939221322316563	-4.96234554330756
a_HQ_transcript/64403	0.75244238394743	2.47903056993978	1.39147723860136	0.173507534699171	0.939221322316563	-4.91816000916468
a_HQ_transcript/10565	-1.90350271558067	1.95906934920273	-1.3914258602919	0.173522967502806	0.939221322316563	-4.90093966909052
a_HQ_transcript/30310	-1.60327639121025	0.419530540696657	-1.39141011842182	0.173527696195312	0.939221322316563	-4.86776999523289
a_HQ_transcript/36693	2.40113142788267	0.970764771382781	1.39136780848398	0.17354040615584	0.939221322316563	-5.04347789014319
a_HQ_transcript/88070	1.69701703813992	0.799388446862948	1.39132857501078	0.173552192592857	0.939221322316563	-4.89223659167245
a_HQ_transcript/111843	-0.578609018879587	2.87552034528971	-1.39132845172741	0.173552229630374	0.939221322316563	-5.11370394258206
a_HQ_transcript/63714	-1.09307487588472	-0.428439060845704	-1.39125664426294	0.17357380350182	0.939221322316563	-4.91994447267566
a_HQ_transcript/131656	1.59706757433353	1.09979813792896	1.39111422033353	0.173616599624576	0.939221322316563	-4.82449675775714
a_HQ_transcript/88340	-2.12586889511447	1.03050148208334	-1.39104198810334	0.173638307412575	0.939221322316563	-4.9395308273759
a_HQ_transcript/143454	2.03751971728417	1.98022018979404	1.39103615480194	0.173640060573997	0.939221322316563	-4.96479872460372
a_HQ_transcript/139033	0.745167696376709	4.45344377167583	1.39103033730999	0.173641808997779	0.939221322316563	-5.23174344367202

a_HQ_transcript/38471	1.65507213850525	1.53791648254761	1.39099803629831	0.173651517187174	0.939221322316563	-4.90742880988087
a_HQ_transcript/4723	-0.889554315511183	1.95625577777819	-1.39098404851234	0.173655721400042	0.939221322316563	-4.93712889380469
a_HQ_transcript/97992	-1.98872399040783	1.10936113593844	-1.3909606866827	0.173662743282368	0.939221322316563	-4.85023239624041
a_HQ_transcript/26129	2.3261454177665	0.0859243094606487	1.3909559458873	0.173664168253826	0.939221322316563	-4.92557388156303
a_HQ_transcript/129312	1.24926433930152	2.92026129096486	1.39088231368557	0.173686301533438	0.939253636202038	-5.0249255165751
a_HQ_transcript/41044	-2.02454933130072	1.32485424381683	-1.39063175024455	0.173761635520225	0.939535187978459	-4.87425576527176
a_HQ_transcript/124200	1.98645984860835	3.21208389771515	1.39056999153041	0.17378020771608	0.939535187978459	-5.19710360789785
a_HQ_transcript/86004	1.45889415694914	2.86824408648658	1.39054788643155	0.173786855579831	0.939535187978459	-5.03396910052516
a_HQ_transcript/105795	1.98012842046204	1.27441026021954	1.39045546602133	0.173814652148382	0.939596244759482	-4.98521306869653
a_HQ_transcript/114614	2.50675678353403	1.23739052372041	1.39027723754715	0.173868266355929	0.939596244759482	-4.89515473685479
a_HQ_transcript/1116	-0.635270183957689	3.09315481758	-1.39026878197122	0.173870810260599	0.939596244759482	-5.18142413828275
a_HQ_transcript/148033	3.22095357387114	3.58512982744703	1.39025650946714	0.173874502560026	0.939596244759482	-5.39542783699395
a_HQ_transcript/124217	-0.628695751408426	4.15390723164688	-1.39011713121624	0.173916440131903	0.939596244759482	-5.29421965780159
a_HQ_transcript/12783	1.19497625741846	3.6771122812083	1.39007051479334	0.173930468328545	0.939596244759482	-5.25133062912689
a_HQ_transcript/56673	-2.14631401765678	0.88863532462307	-1.3900460393966	0.173937834019955	0.939596244759482	-4.81513833934021
a_HQ_transcript/72864	-3.92822825371106	2.85277834958364	-1.39003520091457	0.173941095859645	0.939596244759482	-4.99038533596775
a_HQ_transcript/146997	0.574496681614339	2.80691156998977	1.38996196945186	0.173963136111366	0.939596244759482	-5.12080744429456
a_HQ_transcript/138886	-1.09970358184446	2.76228264432072	-1.38993185162157	0.173972201217116	0.939596244759482	-5.07172811874767
a_HQ_transcript/50604	-1.89834568044267	1.19201100808004	-1.3899193764375	0.173975956206068	0.939596244759482	-4.96215430800694
a_HQ_transcript/96608	-3.13391020615573	1.35546110536442	-1.38978456609056	0.174016537713992	0.939613008083169	-5.01394518159952
a_HQ_transcript/47055	1.00187442900817	5.56146712880707	1.38963866811731	0.174060465229547	0.939613008083169	-5.49905208592011
a_HQ_transcript/45116	1.44931216923019	-0.340898544166373	1.38950796946185	0.174099823836223	0.939613008083169	-4.91967610349659
a_HQ_transcript/103769	2.18174627187868	0.292450462304277	1.3894868923252	0.174106171658626	0.939613008083169	-4.98077083970176
a_HQ_transcript/4384	1.76421972106508	1.21876985101814	1.38938222986622	0.174137695635908	0.939613008083169	-4.92822167465384
a_HQ_transcript/119655	-1.42481230394539	0.173070308006813	-1.3893343657722	0.174152113624981	0.939613008083169	-4.92100059056874
a_HQ_transcript/39200	2.8386523787236	0.804090085777877	1.38930276619027	0.174161632804416	0.939613008083169	-4.92209039311759
a_HQ_transcript/117278	2.6482037638946	-0.109364948847676	1.38929379818383	0.174164334435203	0.939613008083169	-4.95257680011057
a_HQ_transcript/125781	-3.47625873261103	1.82450015854557	-1.38917139087784	0.174201213168036	0.939613008083169	-4.84161557908917
a_HQ_transcript/110547	-2.08742228351913	2.52276711651382	-1.38916210644452	0.174204010620447	0.939613008083169	-4.99489366086047
a_HQ_transcript/128378	1.24324056425951	1.40726636538831	1.38903384752835	0.17424265935191	0.939613008083169	-4.88267536058206
a_HQ_transcript/4513	-1.40371914010855	-0.533294230790127	-1.38895963477101	0.174265025215538	0.939613008083169	-5.00260712547293
a_HQ_transcript/122601	-1.64087213771359	0.645722960449492	-1.38893008006471	0.174273932887343	0.939613008083169	-4.87143627497468
a_HQ_transcript/92967	1.95557065152931	1.25842599390708	1.38888714126572	0.174286875105854	0.939613008083169	-4.92361159618773
a_HQ_transcript/80694	2.40269761283195	0.184342145173007	1.38879758885427	0.174313869588142	0.939613008083169	-4.98530532708766
a_HQ_transcript/108412	2.3050535023783	0.757234395264759	1.38854286461205	0.174390670968574	0.939613008083169	-4.99338803488736
a_HQ_transcript/89900	-1.22978741275958	-0.340163729705521	-1.38844643625735	0.174419751781161	0.939613008083169	-4.93509452069422
a_HQ_transcript/133154	-3.2874015203758	2.754865288589	-1.38843393621997	0.174423521813294	0.939613008083169	-5.03867756814407
a_HQ_transcript/135537	2.05544477916419	-0.258378238587433	1.38842686514998	0.174425654487998	0.939613008083169	-4.94016788483745
a_HQ_transcript/100149	1.47274714649097	-0.449097818829452	1.38833545460251	0.174453226260419	0.939613008083169	-4.94667699984738
a_HQ_transcript/41524	1.61732796345463	1.744770744886	1.38825544988712	0.174477360534932	0.939613008083169	-5.0327364992006
a_HQ_transcript/128092	-1.93131241810262	-0.0534237260838728	-1.38820504641801	0.174492566617644	0.939613008083169	-4.94142838636239
a_HQ_transcript/146834	2.83878588697238	4.09755559802163	1.3881652968794	0.174504559275901	0.939613008083169	-5.1324557199315
a_HQ_transcript/130672	-2.02225731765893	2.19474229117596	-1.38815746766576	0.174506921469383	0.939613008083169	-5.05572737060163
a_HQ_transcript/143279	0.711738456332578	3.97325678871271	1.38815484085043	0.174507714025335	0.939613008083169	-5.27556771850727
a_HQ_transcript/131225	-0.405220842841791	4.15483966700982	-1.38814405578187	0.17451096809791	0.939613008083169	-5.36449635581837
a_HQ_transcript/105967	5.34899691063662	0.963557396544602	1.38814390244744	0.17451101436234	0.939613008083169	-5.03975062098246
a_HQ_transcript/68105	1.47736993382847	2.37444463005282	1.38813631063007	0.174513304995572	0.939613008083169	-5.00756185249361
a_HQ_transcript/90764	-1.003406408213	2.69322612024194	-1.38799953196108	0.174554578422179	0.939613008083169	-5.18059558043623
a_HQ_transcript/4	1.800011124726	2.65852225925075	1.3879221692324	0.174577926264014	0.939613008083169	-4.93336247631553
a_HQ_transcript/129381	-2.3863539574574	0.505217919158584	-1.38791027870721	0.174581515005526	0.939613008083169	-4.9460840545969

a_HQ_transcript/13509	-1.7733568926469	0.159524488143822	-1.38781456219869	0.174610405804287	0.939613008083169	-5.01490919379452
a_HQ_transcript/146361	4.04745191432918	2.85449288375217	1.38769532700554	0.174646400640365	0.939613008083169	-5.04242981756926
a_HQ_transcript/143495	-2.49439739354124	1.34427982056725	-1.38764177166406	0.174662569865379	0.939613008083169	-5.00593314228378
a_HQ_transcript/75633	0.902347098875629	2.19105626865575	1.38757822719638	0.174681756486362	0.939613008083169	-4.93183226353237
a_HQ_transcript/17925	1.23421541998078	-0.527779481970323	1.38746371788763	0.174716335591809	0.939613008083169	-4.95576087335815
a_HQ_transcript/30882	1.9452965122476	0.562818851839024	1.38742617187178	0.174727674765411	0.939613008083169	-4.92478045483873
a_HQ_transcript/142261	-1.74392168360556	0.469119886799229	-1.38740780448356	0.174733222061469	0.939613008083169	-4.84317615941451
a_HQ_transcript/65759	-1.28100201049946	2.52194023084793	-1.38738948776318	0.174738754191892	0.939613008083169	-5.02755784774105
a_HQ_transcript/119788	2.2911320085496	-0.448793751573736	1.38735195012748	0.174750091970862	0.939613008083169	-4.96101456663163
a_HQ_transcript/114585	-1.50028196783534	0.250839075366862	-1.38732439090755	0.174758416258479	0.939613008083169	-4.88208767278688
a_HQ_transcript/96609	-1.6807196263106	1.19729222567563	-1.38732098902599	0.174759443821395	0.939613008083169	-4.87282450490287
a_HQ_transcript/53980	1.13842188034576	0.213957789289534	1.38726960981238	0.174774963858222	0.939613008083169	-4.90203547488453
a_HQ_transcript/3070	-1.19464568785661	1.62708350010085	-1.38718939182117	0.174799197331128	0.939613008083169	-4.82989298848637
a_HQ_transcript/120718	0.519142029547493	3.30361476957312	1.38717736201616	0.174802831704203	0.939613008083169	-5.16340578539262
a_HQ_transcript/151350	1.83503727626709	0.385080285974069	1.38712531176634	0.174818557496016	0.939613008083169	-4.95200408270708
a_HQ_transcript/111944	-1.85911232332482	0.72074913179118	-1.38709599908957	0.174827414136872	0.939613008083169	-4.875018297961
a_HQ_transcript/152094	-5.04184115479696	0.473084402354112	-1.38703905867714	0.174844619325861	0.939613008083169	-4.98213953723878
a_HQ_transcript/99170	-1.78910211544476	0.50408426652577	-1.38703246559852	0.17484661158449	0.939613008083169	-4.86511144297911
a_HQ_transcript/52139	2.33917978623419	-0.122196101117461	1.38695362593473	0.174870436275049	0.939613008083169	-4.92874507201923
a_HQ_transcript/19460	1.19605439662054	1.94364235995916	1.38691623887425	0.174881735220012	0.939613008083169	-4.88729273568905
a_HQ_transcript/48406	2.08853426654091	2.18093767348949	1.38688621229666	0.174890810126665	0.939613008083169	-5.05432174225005
a_HQ_transcript/70286	1.3027359009873	3.79724731903754	1.3868685222118	0.174896156757937	0.939613008083169	-5.23011422706537
a_HQ_transcript/95119	2.46690132098063	1.17191708580391	1.3867832605602	0.174921927933734	0.939613008083169	-5.04758881184229
a_HQ_transcript/114877	1.27559934003507	0.198643901324474	1.38677648313895	0.174923976603451	0.939613008083169	-4.87528410108967
a_HQ_transcript/132438	0.690222193890019	4.31014415266335	1.38675627356453	0.174930085637489	0.939613008083169	-5.31974211969099
a_HQ_transcript/122253	0.526899729421373	4.99359149158137	1.38670725399022	0.174944904170146	0.939613008083169	-5.41400542851259
a_HQ_transcript/39873	1.82171764934164	0.994087210862973	1.38665249260647	0.17496145960126	0.939613008083169	-5.06878809229937
a_HQ_transcript/50090	0.851810712131457	1.71847052113164	1.38662392299623	0.174970097235643	0.939613008083169	-4.93179647090001
a_HQ_transcript/137457	0.829030005105436	2.57731986152048	1.3866046140048	0.174975935236067	0.939613008083169	-5.02593165521279
a_HQ_transcript/13844	1.48638073365197	0.507694805163296	1.38658275341183	0.174982544887164	0.939613008083169	-4.8534940697088
a_HQ_transcript/106449	1.44879298807745	-0.215434218446499	1.38654667555953	0.174993453618646	0.939613008083169	-4.83877533448445
a_HQ_transcript/66337	2.56640455772342	0.201026056666855	1.38653353568285	0.174997426808223	0.939613008083169	-4.9674047782204
a_HQ_transcript/24831	1.60243427772275	-0.0919233656939604	1.386448492882	0.175023143454051	0.939664291373147	-4.93404441537937
a_HQ_transcript/81749	-2.11081706572924	0.15411095481935	-1.38624582551513	0.175084441255337	0.939906575664041	-4.82844185062262
a_HQ_transcript/102751	-1.46639055356819	-0.286336998041854	-1.38602785458749	0.175150386408713	0.939952850476307	-4.93774756058699
a_HQ_transcript/25345	-1.1866314057997	0.435909734431057	-1.38597304164852	0.175166972624426	0.939952850476307	-4.90207351572885
a_HQ_transcript/138811	-2.7716196942666	0.82613981232681	-1.38596595126276	0.175169118241506	0.939952850476307	-5.05685759701845
a_HQ_transcript/138604	4.53247483352625	4.86448471321981	1.38592840893511	0.175180479243174	0.939952850476307	-5.36955189213858
a_HQ_transcript/119620	1.85186653412347	2.43325624017027	1.38589892636108	0.175189401617893	0.939952850476307	-5.00340684167748
a_HQ_transcript/93155	1.96654919164937	1.64878595209398	1.38589480478033	0.175190648969	0.939952850476307	-4.87585215382939
a_HQ_transcript/152411	-1.83671606244461	1.2928783795998	-1.38584090270694	0.175206962477402	0.939952850476307	-4.91662557925784
a_HQ_transcript/33068	-3.25832832076871	1.21060768655458	-1.38578981788013	0.175222424438502	0.939952850476307	-4.84949145025354
a_HQ_transcript/117621	-1.22413467955454	-0.0825397140568681	-1.38554800032053	0.175295630370263	0.940191195627889	-4.94359868923115
a_HQ_transcript/82773	-2.64519534369206	2.6898043965593	-1.38553619501051	0.175299204827537	0.940191195627889	-5.03264458846791
a_HQ_transcript/31290	1.25653290757493	0.237962860489915	1.38545031719634	0.175325208955913	0.940237983625262	-4.87690600993505
a_HQ_transcript/30237	2.51826584064807	1.86821846322237	1.38540055313052	0.175340279082333	0.940237983625262	-4.94351742523615
a_HQ_transcript/13337	2.19683077125454	1.11006906855493	1.38521038307782	0.175397877880705	0.940333133693234	-4.90286921278439
a_HQ_transcript/109136	1.60180466109195	-0.59073665068097	1.38515822691444	0.1754136775459	0.940333133693234	-4.94674167012991
a_HQ_transcript/79990	2.49839856848555	1.98070184701118	1.38514188463782	0.175418628339935	0.940333133693234	-4.89698889560584
a_HQ_transcript/146975	-2.01936503377477	1.83549567260586	-1.38512834260309	0.175422730900245	0.940333133693234	-5.01332301583354

a_HQ_transcript/8716	1.79751369778431	0.463059490989746	1.38491965324284	0.175485962827661	0.940477650273004	-4.84071651445759
a_HQ_transcript/61764	2.18744995291489	1.10373312165502	1.38486594444418	0.175502239228021	0.940477650273004	-4.85501024401584
a_HQ_transcript/56589	2.12871483790256	-0.240028201256862	1.3848607466028	0.175503814491449	0.940477650273004	-4.9620691962677
a_HQ_transcript/85929	-1.49605524912386	-0.0629176221095496	-1.38477688215289	0.175529232068078	0.940477650273004	-4.9913933820641
a_HQ_transcript/63779	-1.52919126665161	3.18328995614183	-1.38477240808174	0.175530588147052	0.940477650273004	-5.27529643196737
a_HQ_transcript/74210	-1.94145386453851	1.90052311260331	-1.38452617390667	0.175605233677099	0.9405229086991	-4.97361344139587
a_HQ_transcript/48912	-3.15491976472008	0.0822981210425819	-1.38450927240136	0.175610358252337	0.9405229086991	-4.97119203639811
a_HQ_transcript/135526	2.5908009429682	1.93148068487242	1.38434993272164	0.17565867614718	0.9405229086991	-5.13839646494749
a_HQ_transcript/81089	2.2967395615771	3.26032912993843	1.38426046370435	0.175685811129273	0.9405229086991	-5.16474207620102
a_HQ_transcript/132534	0.655935872029247	2.70654430747397	1.38423557281295	0.17569336084823	0.9405229086991	-5.14215923910237
a_HQ_transcript/98143	1.77848897206209	2.99480861831176	1.38419659602056	0.175705183506007	0.9405229086991	-5.04411114158906
a_HQ_transcript/50170	1.79072852228275	0.952688888322057	1.38411023851458	0.175731380157713	0.9405229086991	-4.85261244381407
a_HQ_transcript/118615	1.24522821535208	2.71008521218416	1.38404814075025	0.175750219472133	0.9405229086991	-5.10165142357811
a_HQ_transcript/82490	-1.77158081534273	0.420187621937413	-1.38401758422659	0.175759490335521	0.9405229086991	-4.88655736966698
a_HQ_transcript/66354	2.08230861978995	0.944882384984255	1.38398720865528	0.175768706676171	0.9405229086991	-4.95377252403673
a_HQ_transcript/60032	1.20143563507183	0.568808945605934	1.38396480548277	0.175775504329737	0.9405229086991	-4.86943603005592
a_HQ_transcript/142429	2.68875988586131	0.592522775280233	1.38387376770986	0.175803129458838	0.9405229086991	-5.00453504076999
a_HQ_transcript/10617	1.49480595676247	1.17460418526679	1.38378314630435	0.175830631607579	0.9405229086991	-4.87472730660058
a_HQ_transcript/13640	1.32320543516052	3.41209779600843	1.383769278788	0.175834840473438	0.9405229086991	-5.20712894166713
a_HQ_transcript/68464	-1.65179748550885	0.463748903868417	-1.38371380394961	0.175851678171884	0.9405229086991	-4.79810506283466
a_HQ_transcript/45929	-1.08344397133917	1.82980060895148	-1.3836578242889	0.175868670369268	0.9405229086991	-4.97721644358153
a_HQ_transcript/150756	3.00834771688102	0.954055372666516	1.38352297193428	0.17590960903019	0.9405229086991	-5.0211118666731
a_HQ_transcript/74493	2.16881843473926	2.15225215402308	1.38334058118446	0.175964991292323	0.9405229086991	-4.91310778653833
a_HQ_transcript/66378	1.52528125901843	1.18328256851458	1.38333001202452	0.17596820099483	0.9405229086991	-4.80764612756036
a_HQ_transcript/83855	-2.29967418041544	0.551220379065492	-1.38330697446646	0.175975197329591	0.9405229086991	-4.89372126872152
a_HQ_transcript/20227	1.38473572788212	1.11034424789454	1.38327481671302	0.175984963762715	0.9405229086991	-4.80264077355275
a_HQ_transcript/124085	-1.85147387575864	2.83305933896867	-1.38327462554283	0.175985021823097	0.9405229086991	-5.1013207984935
a_HQ_transcript/18022	-2.30907389261215	1.27346136038792	-1.38327097005151	0.175986132036897	0.9405229086991	-4.88871856696823
a_HQ_transcript/80977	2.0213228648972	0.473590433436209	1.38326783578439	0.175987083953401	0.9405229086991	-4.84868939983042
a_HQ_transcript/97287	-1.60640139567655	-0.495459789661207	-1.38318543979965	0.176012110095626	0.9405229086991	-4.96003107804432
a_HQ_transcript/21276	1.42094385250787	1.75700536305682	1.38318424330205	0.176012473528437	0.9405229086991	-4.9458775723984
a_HQ_transcript/75373	3.21787744218498	2.11828291063244	1.38312133456738	0.17603158270607	0.9405229086991	-4.94206079783712
a_HQ_transcript/4889	2.11315013100257	1.29845798374218	1.38308811849185	0.176041673084934	0.9405229086991	-4.79746037715663
a_HQ_transcript/94791	2.33301011289899	0.117154511513463	1.38296672895346	0.176078552635327	0.9405229086991	-4.88459039770897
a_HQ_transcript/41025	2.25059306626012	1.22594264093019	1.38296528844672	0.176078990314181	0.9405229086991	-5.04373813236306
a_HQ_transcript/37183	2.00135645478452	1.39198017319705	1.38295382714837	0.176082472707806	0.9405229086991	-4.99788226035022
a_HQ_transcript/1254	0.959986478017047	4.34998572551421	1.38294124715535	0.176086295066775	0.9405229086991	-5.3950186618731
a_HQ_transcript/147676	-3.34292253252938	2.55622129548302	-1.38293386313595	0.176088538689011	0.9405229086991	-5.15377337213431
a_HQ_transcript/106526	3.01130984529812	0.583187422535334	1.38293181138493	0.176089162114167	0.9405229086991	-4.87138414287035
a_HQ_transcript/24082	1.34718267491493	1.71504201950906	1.38278563960048	0.176133580886158	0.940614900653235	-4.88516128444451
a_HQ_transcript/146039	1.49170989756377	1.30778037528389	1.38273034328502	0.176150386643508	0.940614900653235	-5.04566668060109
a_HQ_transcript/101104	-3.58042089821586	0.101919539144372	-1.38271539277526	0.176154930644631	0.940614900653235	-4.94918953006979
a_HQ_transcript/99790	-1.82809974453371	1.16837889998805	-1.38265767158582	0.17617247506175	0.940622176025478	-4.91027927931663
a_HQ_transcript/58936	-0.486616593613596	3.57662977171904	-1.38246065790023	0.176232367849369	0.940703067635971	-5.24158437746738
a_HQ_transcript/139597	-2.18290000799521	3.35332859054029	-1.38245758580854	0.176233301900788	0.940703067635971	-5.33998291080791
a_HQ_transcript/6611	0.581220308683395	2.87432428609697	1.38241243187558	0.176247031134251	0.940703067635971	-5.0415862613388
a_HQ_transcript/54455	1.93963204703813	2.02481307079849	1.38239491066314	0.176252358753498	0.940703067635971	-4.93746594150491
a_HQ_transcript/136512	-0.769861376916278	3.91709509960608	-1.38227818101435	0.176287855572649	0.940806139251466	-5.37197783015513
a_HQ_transcript/42736	-0.618141674312281	3.23180888014774	-1.38215988118384	0.176323835564868	0.940911770285011	-5.16467857759535
a_HQ_transcript/63998	2.12104240664331	1.14566168328468	1.38206335795091	0.176353196614448	0.940959623372355	-5.10214548100264

a_HQ_transcript/98145	1.72170800383782	0.425438706531371	1.38202397155581	0.176365178513656	0.940959623372355	-4.96038605835737
a_HQ_transcript/39215	1.73438776947273	0.356957404895668	1.3819172567549	0.176397645858656	0.941000325458696	-4.85912378849918
a_HQ_transcript/85745	-1.650322181289	1.82736610251856	-1.38187761156575	0.176409708859923	0.941000325458696	-4.98732674260923
a_HQ_transcript/49164	1.28839472281765	1.92360635146837	1.38178806890089	0.176436956734875	0.941000325458696	-4.87983523405308
a_HQ_transcript/125200	2.37556603642576	-0.235761606167579	1.38176693657392	0.176443387790663	0.941000325458696	-4.95245543625414
a_HQ_transcript/56837	1.75880323887349	2.07770304061165	1.38168429595777	0.176468538996862	0.941000325458696	-5.09862693201518
a_HQ_transcript/11689	2.01474686737523	1.13861774919713	1.38151099888844	0.176521290064523	0.941000325458696	-4.89206019029894
a_HQ_transcript/14573	-0.96239105858813	1.22685682515109	-1.38150166486315	0.176524131660665	0.941000325458696	-4.85827794622242
a_HQ_transcript/136327	-1.97952239817464	1.28250581145471	-1.38149393680756	0.17652648437177	0.941000325458696	-4.91578372607481
a_HQ_transcript/46705	-1.78672918461517	1.95431988880385	-1.38143130603355	0.176545552439882	0.941000325458696	-4.96360009631651
a_HQ_transcript/99330	-1.83014935022559	3.52535706169609	-1.38141474620264	0.176550594383018	0.941000325458696	-5.07893444182174
a_HQ_transcript/144822	0.488542546096967	3.03758147947217	1.38141380830923	0.176550879945165	0.941000325458696	-5.15773097854411
a_HQ_transcript/7534	-1.74651800060499	0.713166543771773	-1.38135770870546	0.176567961350906	0.941005084570045	-4.82813728167176
a_HQ_transcript/43427	-1.77524847947307	3.45313411929058	-1.38128434715119	0.176590300681202	0.941037861935912	-5.14749372160796
a_HQ_transcript/94851	1.52070651936467	-0.168195789217506	1.38095829063681	0.176689614836457	0.941332776797808	-4.93240209419481
a_HQ_transcript/18725	-2.47037293313383	1.99467783947593	-1.38095786558297	0.17668974433281	0.941332776797808	-4.94577501369035
a_HQ_transcript/62945	-1.53776688079102	1.15091273369429	-1.3809041667708	0.176706104739119	0.941332776797808	-4.85705389225527
a_HQ_transcript/69277	-1.17128680253046	-0.722632840168168	-1.38087319958458	0.176715540042569	0.941332776797808	-4.97008155852528
a_HQ_transcript/113298	3.02571615899764	1.04796931424	1.38078966821483	0.176740992936018	0.941332776797808	-4.84553374391378
a_HQ_transcript/111988	-1.70858827289352	-0.383603047700416	-1.38078371287691	0.176742807699754	0.941332776797808	-4.96074836222551
a_HQ_transcript/117352	-1.2822011182023	2.9519127873224	-1.38057495855099	0.176806430363837	0.941585358148339	-5.16279926714118
a_HQ_transcript/150578	-3.13036010364968	0.308947717980709	-1.38051304940494	0.176825302027672	0.941599592915399	-4.92094086212211
a_HQ_transcript/152240	1.97846908108919	3.92776035999548	1.38039569219553	0.176861080144688	0.941703844155944	-5.38182966121557
a_HQ_transcript/7540	-2.54633498192158	5.01603232765493	-1.3802060657144	0.176918902568677	0.941898618430174	-5.53264533155569
a_HQ_transcript/133206	2.07932739471482	0.967026159131582	1.38016945342836	0.176930068377358	0.941898618430174	-4.96744365078523
a_HQ_transcript/126301	-2.39053275676682	1.32717819610642	-1.38005609660708	0.176964642786011	0.941996406214767	-4.97633087992074
a_HQ_transcript/101605	-2.5808723439949	0.676029504994761	-1.37989054099537	0.177015147556147	0.942026813937833	-4.88260339968712
a_HQ_transcript/46310	1.99590616162984	1.62900608558052	1.37988409177035	0.177017115198726	0.942026813937833	-4.97905187262028
a_HQ_transcript/127317	-0.794800848431141	4.04436451643101	-1.37987800114532	0.177018973448991	0.942026813937833	-5.31507591281052
a_HQ_transcript/111982	-2.45318807757693	1.86375937390384	-1.37974275257312	0.177060241719591	0.942160173075467	-4.88707750649925
a_HQ_transcript/141468	-1.80590152533902	0.202646180253816	-1.37953361627983	0.177124070046838	0.942175092494968	-4.95522221802499
a_HQ_transcript/19597	-1.89111191453701	0.110355917180993	-1.37947397936383	0.177142274500116	0.942175092494968	-4.86847416925659
a_HQ_transcript/151866	-6.33871614018261	3.1605069808111	-1.37945662445097	0.177147572444079	0.942175092494968	-5.16138379462215
a_HQ_transcript/43051	1.6632040702484	1.15311191256335	1.37942310984324	0.177157803816713	0.942175092494968	-4.8415957161111
a_HQ_transcript/25152	-0.773889805603609	2.09303155561905	-1.37941631116453	0.177159879380283	0.942175092494968	-4.916587169351
a_HQ_transcript/120439	0.958476772173362	1.1318086118932	1.37938375170017	0.177169819697716	0.942175092494968	-4.88547786358004
a_HQ_transcript/107079	-0.984755006123391	3.19737571804957	-1.37928603636428	0.177199654538908	0.942175092494968	-5.20735049593906
a_HQ_transcript/107626	2.90447175835333	3.24025864155069	1.37926605694284	0.177205755218797	0.942175092494968	-5.30747003510046
a_HQ_transcript/147711	-3.587170862328	0.408847122894755	-1.37920790992841	0.177223511235188	0.942175092494968	-4.95893535013232
a_HQ_transcript/34373	1.52247425829324	1.28731071835149	1.37916056232849	0.177237970521391	0.942175092494968	-4.99089227128504
a_HQ_transcript/7241	-1.16770007298486	0.725039676113147	-1.3790470089992	0.17727265184651	0.942175092494968	-4.91788253547107
a_HQ_transcript/104240	-2.78806216481068	0.763853707772122	-1.37902561452564	0.17727918671377	0.942175092494968	-4.88252690542389
a_HQ_transcript/17635	-0.954442440400895	2.36444201542169	-1.37891344871458	0.177313450441906	0.942175092494968	-4.96788078373282
a_HQ_transcript/30719	2.44214218250342	0.981163301877334	1.37890163134996	0.177317060638933	0.942175092494968	-4.97913287908159
a_HQ_transcript/42221	1.16214834470149	0.903481772536565	1.37884001345559	0.177335885792835	0.942175092494968	-4.81884423600134
a_HQ_transcript/20300	-2.98339356149825	0.296243128374104	-1.37878852749893	0.177351616688429	0.942175092494968	-4.96885680708751
a_HQ_transcript/96021	1.76633778073623	-0.300551923349657	1.37878204992189	0.177353595908788	0.942175092494968	-4.96100424764601
a_HQ_transcript/144868	-1.80587051709887	0.220193304977505	-1.37877810652456	0.177354800820258	0.942175092494968	-4.9978295798616
a_HQ_transcript/127280	2.30797783448951	2.18449176344617	1.37867788608907	0.177385425479017	0.942251668851714	-5.06165584399678
a_HQ_transcript/43354	1.3054806111592	2.96869181428215	1.37861982804116	0.177403168335899	0.94225981076655	-5.01543341074082

a_HQ_transcript/36075	-1.15605055887821	2.63866011203063	-1.3784580565184	0.177452613894015	0.942288024797736	-5.01954116723745
a_HQ_transcript/68033	0.611104222313147	4.07615878815187	1.37837047734895	0.177479386997848	0.942288024797736	-5.36471201596338
a_HQ_transcript/61046	2.4808332322735	0.565457973524256	1.37821269937596	0.177527627941346	0.942288024797736	-4.99072196279212
a_HQ_transcript/121794	2.38447643316122	2.02765818582812	1.3781814589914	0.177537180965724	0.942288024797736	-4.87205955313177
a_HQ_transcript/146539	-2.689315344713	0.451222512600605	-1.37817514207168	0.17753911267079	0.942288024797736	-5.00525254002853
a_HQ_transcript/77556	-3.05166432584576	1.05844871323824	-1.37813467994347	0.177551486320509	0.942288024797736	-4.96025004159198
a_HQ_transcript/14041	0.541819363462568	2.76395953962886	1.37812507390934	0.177554424023013	0.942288024797736	-5.06585798758675
a_HQ_transcript/47617	1.5029657730838	-0.481147389377458	1.37812335969052	0.177554948266743	0.942288024797736	-4.97166886655839
a_HQ_transcript/75752	0.855098633481593	3.15102046703255	1.37807819929051	0.177568759689834	0.942288024797736	-5.18934371833261
a_HQ_transcript/74736	2.44383465503714	1.22927891098165	1.37807222781648	0.177570586010776	0.942288024797736	-4.96021140461041
a_HQ_transcript/33075	0.818992576736679	2.78261618224555	1.37793390361494	0.177612895304611	0.942403964682612	-5.12220816324145
a_HQ_transcript/7215	1.3224534990297	1.67761302044347	1.37788339125272	0.177628347504387	0.942403964682612	-5.03310461611179
a_HQ_transcript/8639	-1.82984803529188	0.728929934525684	-1.37784179773164	0.177641072134382	0.942403964682612	-4.97744908767171
a_HQ_transcript/142835	-1.38767465465703	6.82567734507107	-1.37776712730232	0.177663917704653	0.94243915024056	-5.43438888288023
a_HQ_transcript/151483	2.23514882025623	1.52425418222368	1.37768127738795	0.177690186490175	0.9424924865682	-5.04195923677788
a_HQ_transcript/152165	4.91311678944663	3.30878009697309	1.37751803832647	0.177740143541186	0.942592966300392	-5.16404005639059
a_HQ_transcript/80220	1.28001470211149	1.2383420443253	1.37743651195658	0.177765097653186	0.942592966300392	-4.82284395694282
a_HQ_transcript/70392	-1.65573881704993	2.25859868926018	-1.37743242844212	0.177766347633153	0.942592966300392	-5.02820319979565
a_HQ_transcript/89725	2.85614983728496	2.28943914220879	1.37739240847073	0.177778598267502	0.942592966300392	-4.89621079114084
a_HQ_transcript/8323	2.43651778827833	4.26361837110787	1.37733198544211	0.177797095789563	0.942592966300392	-5.26010654945387
a_HQ_transcript/86276	-1.02207410021495	2.09290425973842	-1.37730151282065	0.177806425053052	0.942592966300392	-4.97770191467625
a_HQ_transcript/106329	0.714248950515647	4.46423091876836	1.37711940725682	0.177862185042123	0.942604139081852	-5.39356437476017
a_HQ_transcript/110328	-0.988377781699644	4.14606063949308	-1.3771077696812	0.177865748883881	0.942604139081852	-5.32542694614125
a_HQ_transcript/48668	5.43530981815491	3.42607801397602	1.37710491777169	0.177866622249032	0.942604139081852	-4.99401559812771
a_HQ_transcript/125076	1.59886445028284	1.86706596892086	1.37707929665196	0.177874468578054	0.942604139081852	-4.89919484822227
a_HQ_transcript/81552	-2.5170539085974	-0.0484885042861136	-1.37699248407873	0.177901056462704	0.942604139081852	-4.98689935507163
a_HQ_transcript/74644	-1.87857408423763	-0.0816296815750951	-1.3769769032774	0.177905828686124	0.942604139081852	-5.03332429603821
a_HQ_transcript/136564	5.29677430055015	2.77793370903717	1.37668247902633	0.177996026256463	0.942996082230741	-5.31112713588316
a_HQ_transcript/122176	-2.21634982527867	1.49785736271436	-1.3765798862815	0.178027464155562	0.943076682441858	-4.9167136711082
a_HQ_transcript/145477	-2.15227600609674	2.89483487820815	-1.37652025798685	0.178045738276215	0.943087540962261	-5.13426230716956
a_HQ_transcript/139666	0.532437607176951	4.15530140700492	1.37635211253461	0.178097277223152	0.943231168218978	-5.37987988860033
a_HQ_transcript/149466	-2.44932238549202	1.49972371606277	-1.37624840153681	0.178129071896741	0.943231168218978	-4.93561459635045
a_HQ_transcript/38196	0.578546487616646	3.24901123973057	1.37619620860547	0.17814507435235	0.943231168218978	-5.2150588227041
a_HQ_transcript/140569	-1.71099795880073	4.79387549944428	-1.37618744375612	0.178147761782233	0.943231168218978	-5.40601700896242
a_HQ_transcript/138387	-4.81783540500953	0.285553384334919	-1.37612615833316	0.178166553663715	0.943231168218978	-5.01146936162976
a_HQ_transcript/120065	2.00706598774939	1.54373070711714	1.37604925163981	0.178190137662317	0.943231168218978	-4.89655524202677
a_HQ_transcript/6370	-1.28764791553704	1.74089502033902	-1.37604525861054	0.178191362220267	0.943231168218978	-4.9832188856699
a_HQ_transcript/47104	-1.94837277062178	0.855422238491507	-1.37596183222454	0.178216948416314	0.943231168218978	-4.89486055111053
a_HQ_transcript/70304	1.75358604603218	-0.426050389955963	1.37595548602227	0.178218894862184	0.943231168218978	-4.96927621363729
a_HQ_transcript/20260	-2.11494372319308	0.755567360864122	-1.3758255730629	0.178258744149052	0.943356179888573	-4.87609531234441
a_HQ_transcript/128402	-0.630414564736865	4.54816846261352	-1.37569452850308	0.178298947570823	0.943483042730708	-5.41362951055053
a_HQ_transcript/73374	-0.944298544832435	7.32306534329671	-1.37542262970975	0.178382386467921	0.943689990938373	-5.67777933415035
a_HQ_transcript/97011	2.33619174484665	1.12432743862589	1.37538707704327	0.178393298934006	0.943689990938373	-4.85563052975152
a_HQ_transcript/64331	-2.32280451300214	2.15627363655041	-1.37528153436731	0.178425697059176	0.943689990938373	-5.0502262320704
a_HQ_transcript/125298	1.56401971412475	1.30563432150054	1.37526592117854	0.178430490182849	0.943689990938373	-4.95406248603461
a_HQ_transcript/17540	-0.820403942049158	3.28068984548465	-1.37521004914058	0.178447643271547	0.943689990938373	-5.18031078825251
a_HQ_transcript/9402	1.41277574571341	1.44796855267603	1.37513796548167	0.178469775332939	0.943689990938373	-4.89508154419749
a_HQ_transcript/136071	2.424370352205	0.705852117413942	1.37509091084225	0.178484223812549	0.943689990938373	-4.97127470302167
a_HQ_transcript/113550	2.71591693559377	1.56806397347312	1.3750661050553	0.178491840981911	0.943689990938373	-4.92961174624321
a_HQ_transcript/87923	-0.560927061255997	3.37175329340858	-1.3750482995046	0.178497308728868	0.943689990938373	-5.27678131825545

a_HQ_transcript/5105	2.44537611391392	0.65465984348705	1.37503822190509	0.178500403426359	0.943689990938373	-4.94009232132203
a_HQ_transcript/145251	2.51652145891264	0.243773475336237	1.37491892809155	0.178537040152654	0.943780360034057	-5.0329487299754
a_HQ_transcript/136325	-1.82576040221022	-0.477754451909488	-1.37482823314252	0.178564897702363	0.943780360034057	-4.97202028480051
a_HQ_transcript/103621	-1.78322699001633	1.53699885000212	-1.37479489980894	0.17857513710529	0.943780360034057	-4.84079113170354
a_HQ_transcript/29251	-1.54470122517168	1.44889222818252	-1.37477112085698	0.178582441852715	0.943780360034057	-4.88455795895518
a_HQ_transcript/116715	-0.657364139484407	2.53681829105058	-1.37461071461908	0.178631723737827	0.943896890678582	-5.08051296259902
a_HQ_transcript/96039	-3.14334880302675	2.99605709504554	-1.37459364621117	0.178636968317422	0.943896890678582	-5.24950593328122
a_HQ_transcript/48245	1.15755673970036	2.17825034350917	1.37446761784035	0.178675696543078	0.94401036629137	-4.98338117279377
a_HQ_transcript/140310	-0.535282372450246	4.20925985378599	-1.37431680039687	0.178722050984212	0.94401036629137	-5.41664724683036
a_HQ_transcript/127581	2.03128435705735	1.38479670468927	1.37431347631921	0.17872307276034	0.94401036629137	-4.83856734022996
a_HQ_transcript/12748	0.716653363878679	2.83411000576485	1.3743123957376	0.178723404917364	0.94401036629137	-5.13605530877418
a_HQ_transcript/70799	-1.41259814554916	2.18838676951629	-1.37421005737347	0.178754864605146	0.94404380073491	-4.96673483476211
a_HQ_transcript/12440	-0.929976382626432	2.11957259259371	-1.37412775794799	0.178780167279048	0.94404380073491	-4.9641306821763
a_HQ_transcript/29632	-1.03802710012804	2.26111679645595	-1.37408203790512	0.178794224952776	0.94404380073491	-4.94083790094714
a_HQ_transcript/135601	6.10986819223143	3.19090664561918	1.37408049962402	0.178794697947472	0.94404380073491	-5.2440323797241
a_HQ_transcript/42459	0.385532174237306	4.96948424285446	1.37402273313259	0.178812460844489	0.9440518368727	-5.45480770270924
a_HQ_transcript/77953	1.54885509493621	0.549605061034995	1.37396392658867	0.178830544963203	0.944061567307334	-4.97275974125676
a_HQ_transcript/142900	-1.15345564938603	-0.22829479542303	-1.37386876489369	0.178859811990692	0.944130326134665	-4.94740402018507
a_HQ_transcript/59603	-1.69880516279403	3.28983177005905	-1.3738007666814	0.178880727159028	0.94415499031145	-5.21699632232166
a_HQ_transcript/5029	-1.21474384462342	1.70788852241403	-1.37370454103842	0.178910327888976	0.944217785982914	-4.87683253846689
a_HQ_transcript/98342	-2.44938933002376	1.16461197314164	-1.37364711087766	0.178927996250895	0.944217785982914	-4.97880366502102
a_HQ_transcript/146511	-2.39469133526279	0.848203207206494	-1.37360058087229	0.178942312180998	0.944217785982914	-4.98917149600015
a_HQ_transcript/134075	3.90547667547763	2.96121116509157	1.37352728989267	0.178964863492984	0.944217785982914	-5.19918101006244
a_HQ_transcript/111814	2.35025084088795	1.4857737887013	1.37349452682129	0.178974945261297	0.944217785982914	-4.96806502901039
a_HQ_transcript/120943	-1.52776126726757	-0.152608024651007	-1.37341123830679	0.179000576579559	0.944217785982914	-4.91546283153774
a_HQ_transcript/39498	-1.75729703250957	4.4414346286155	-1.37337235620097	0.17901254318863	0.944217785982914	-5.35599775543053
a_HQ_transcript/88337	1.08082857351777	1.93429040067382	1.37333580466831	0.179023793093132	0.944217785982914	-4.88218812812342
a_HQ_transcript/133252	-2.68857750394645	2.70152765711661	-1.37311154804156	0.179092827294564	0.944217785982914	-5.03322816602308
a_HQ_transcript/144470	-2.0972212243924	-0.0336071731706952	-1.37311046024496	0.179093162207702	0.944217785982914	-5.01151959593679
a_HQ_transcript/127819	0.609332969363534	6.00914807371057	1.37310519327607	0.17909478382021	0.944217785982914	-5.541994178119
a_HQ_transcript/124072	-1.35161796635366	2.67931301146457	-1.37303523959865	0.179116322482874	0.944217785982914	-4.96544931691824
a_HQ_transcript/148282	3.90819301335691	0.806632160950571	1.37302115133657	0.179120660487596	0.944217785982914	-5.04127335348685
a_HQ_transcript/9891	-1.77670560616204	0.0756583766126505	-1.3729217852938	0.179151259228064	0.944217785982914	-4.99141163769167
a_HQ_transcript/46120	-2.1044317469827	-0.0810208219307	-1.37291235543127	0.179154163267569	0.944217785982914	-4.94682874019053
a_HQ_transcript/865	-0.941233706975282	1.90308213011008	-1.37287507422018	0.179165644823305	0.944217785982914	-5.01629506701453
a_HQ_transcript/133556	-0.987301505517484	2.87757562598143	-1.37281312383088	0.179184725052596	0.944217785982914	-5.17007479376432
a_HQ_transcript/136704	3.56303743651588	1.23601733135384	1.37277909187351	0.179195207298806	0.944217785982914	-4.95680050207035
a_HQ_transcript/11309	0.342934270017069	3.48786398871885	1.37275945434777	0.179201256106584	0.944217785982914	-5.25286498766943
a_HQ_transcript/112415	2.70078899792338	6.28841633987902	1.37266547311255	0.179230206679614	0.944269637948085	-5.61154250717158
a_HQ_transcript/17188	3.27706334993211	0.630301166419818	1.37262204327109	0.179243586311574	0.944269637948085	-4.94113958016613
a_HQ_transcript/148389	0.893416678869657	3.1216998538734	1.37244181861926	0.179299117257396	0.944422805441387	-5.10111869789563
a_HQ_transcript/100835	-1.24863225167779	1.69165539896024	-1.37242222412463	0.179305155533498	0.944422805441387	-4.96176935195709
a_HQ_transcript/130857	-6.01755821166009	0.468939468507364	-1.3723335774416	0.179332475038176	0.944454047807635	-5.01287082987005
a_HQ_transcript/126076	0.747633447075934	4.77606815421184	1.37229753790895	0.179343582777675	0.944454047807635	-5.40498108535642
a_HQ_transcript/65514	5.43367600927529	4.0318232877971	1.37220379438294	0.179372477964267	0.944520644905054	-5.22807560995444
a_HQ_transcript/21298	-1.17536382654191	2.2622591899474	-1.37207415174857	0.179412444538427	0.944634922097792	-5.03516699337281
a_HQ_transcript/81763	-1.37262661780615	0.424542758950942	-1.37201611312468	0.179430339083798	0.944634922097792	-4.84690227594602
a_HQ_transcript/35242	-1.21073600428544	0.546649519953217	-1.37197526907873	0.179442933006534	0.944634922097792	-4.87406422249541
a_HQ_transcript/117312	-2.73441373325473	0.966804138435233	-1.37179664744918	0.179498017584243	0.944702686096756	-4.88290224319176
a_HQ_transcript/151080	-2.03253570375012	3.02590358189079	-1.37176395380398	0.179508101301507	0.944702686096756	-5.13720969651528

a_HQ_transcript/43217	-1.58265658281367	0.217856069441881	-1.37174832882911	0.179512920675913	0.944702686096756	-4.9004795670504
a_HQ_transcript/97718	-3.1122708043437	1.04483784831372	-1.37172273876567	0.179520813904225	0.944702686096756	-5.01428354249981
a_HQ_transcript/124603	-0.9357807908856	2.7681740009139	-1.37164500450249	0.17954479261217	0.944743342532833	-5.13843406929422
a_HQ_transcript/5264	1.53373803586001	0.324029657816734	1.37147248007997	0.179598020165855	0.944917347084788	-4.84045515117752
a_HQ_transcript/11916	-1.52950029931659	0.614139246374474	-1.37143244665485	0.179610373106589	0.944917347084788	-4.80007562095376
a_HQ_transcript/103598	1.9108346671039	2.69838867777259	1.37116625252744	0.179692528284857	0.945184381648195	-5.05913003852693
a_HQ_transcript/23709	1.47010790741455	-0.410441376559637	1.37106046350353	0.179725185941009	0.945184381648195	-4.95800073242551
a_HQ_transcript/146891	0.309349937967522	6.4218262452562	1.37100206271351	0.179743216565118	0.945184381648195	-5.57575802755281
a_HQ_transcript/56101	-2.14052695758314	1.78096195940341	-1.37096306424681	0.179755257711229	0.945184381648195	-5.08719811762629
a_HQ_transcript/140245	-2.5340514367859	1.43082679859219	-1.3708606366769	0.179786886181781	0.945184381648195	-5.05959437560281
a_HQ_transcript/31471	0.864471894305155	-0.84642700045084	1.37085139648975	0.179789739659481	0.945184381648195	-4.99582207479398
a_HQ_transcript/121150	-1.27818514074379	-0.569281542542265	-1.37084150647165	0.179792793851595	0.945184381648195	-4.9717444059293
a_HQ_transcript/99687	1.13877849405658	-0.699702585696396	1.37082134059365	0.179799021514817	0.945184381648195	-4.97819092583196
a_HQ_transcript/58862	-1.95612928094524	0.0631027072241708	-1.37079396893112	0.179807474749961	0.945184381648195	-5.01027463314953
a_HQ_transcript/78801	-1.32147934416063	0.545940151494545	-1.3706194802955	0.179861369625459	0.945215614432385	-4.81613570342877
a_HQ_transcript/147830	3.42974381286538	1.83420082459543	1.37058985956915	0.179870519923307	0.945215614432385	-5.06288633251067
a_HQ_transcript/101288	2.09268472218791	0.762419996970395	1.37056760557486	0.179877394762505	0.945215614432385	-5.04353648435283
a_HQ_transcript/4331	-0.983383393208476	3.01424450714767	-1.37050609974902	0.179896396578975	0.945215614432385	-5.05378183192728
a_HQ_transcript/1482	2.08749657963103	0.716541502948906	1.3704916473428	0.179900861780394	0.945215614432385	-4.8910959907809
a_HQ_transcript/12253	-1.74635664538971	1.13747472126721	-1.37045889271401	0.179910981937791	0.945215614432385	-4.9704708884507
a_HQ_transcript/112471	1.76033923980436	0.634106475459472	1.37027225389534	0.179968655952919	0.94543317064901	-4.93002883191351
a_HQ_transcript/3363	-0.820085074158803	2.17628219307295	-1.37008665926003	0.180026021556392	0.945649067506772	-4.93340806485457
a_HQ_transcript/142502	-1.50261007069052	1.63049141908581	-1.36989507036672	0.180085254843866	0.94583414956526	-4.92172340373682
a_HQ_transcript/150628	3.61971744870751	1.12703711282297	1.36984136733132	0.180101860859921	0.94583414956526	-5.01610926749286
a_HQ_transcript/99479	-1.29665536750564	-0.257687005101737	-1.36979530167163	0.180116106200504	0.94583414956526	-4.9054576085446
a_HQ_transcript/94760	1.49370404987937	0.927098972844646	1.36973616304098	0.180134395509753	0.94583414956526	-5.03936629810555
a_HQ_transcript/96794	-1.82986817340987	0.0508621910405105	-1.36967105531469	0.180154532501114	0.94583414956526	-4.93951292078205
a_HQ_transcript/101197	1.68780976975729	1.99569814325239	1.36940917976488	0.180235544954451	0.94583414956526	-4.95326798947294
a_HQ_transcript/111958	1.61687057717897	0.499532885537989	1.3694068042716	0.180236279954255	0.94583414956526	-5.12985407011197
a_HQ_transcript/142671	-2.05083618428723	-0.355868376952384	-1.36940092967994	0.180238097617781	0.94583414956526	-4.97618212781861
a_HQ_transcript/105136	-1.22036244646927	1.91336297270955	-1.3693828954489	0.180243677697024	0.94583414956526	-4.87631251296396
a_HQ_transcript/31351	2.53964067375215	0.561367099629106	1.36928034402415	0.180275411299835	0.94583414956526	-4.84022219760457
a_HQ_transcript/55878	1.1921041417257	0.00976529784669765	1.36926486444138	0.180280201692767	0.94583414956526	-4.86900800926628
a_HQ_transcript/39810	-1.53893413187116	-0.160934817949195	-1.3692182953371	0.180294613808892	0.94583414956526	-5.01744838410328
a_HQ_transcript/143042	1.85664329725321	-0.17424586400574	1.36921497103853	0.18029564264062	0.94583414956526	-4.972117923668
a_HQ_transcript/73292	-0.950140333864412	-0.541713363622129	-1.36921212416412	0.180296523718754	0.94583414956526	-4.96976011887214
a_HQ_transcript/104133	1.32337886541848	2.38070456174955	1.36918367116087	0.180305329812356	0.94583414956526	-5.10089787710264
a_HQ_transcript/17453	-0.899735436537643	2.46414965611957	-1.36904340338724	0.180348747028862	0.945919537214423	-5.02956482287724
a_HQ_transcript/34224	-1.23005617498904	2.08748343693862	-1.36902593877293	0.180354153436338	0.945919537214423	-4.88922424329103
a_HQ_transcript/132358	-1.51773889505361	1.21979906131857	-1.3689097989234	0.180390109301633	0.9459357649929	-4.896370533705
a_HQ_transcript/133016	1.90326587738528	1.61774602781453	1.36890632804528	0.180391183940399	0.9459357649929	-4.93683946424847
a_HQ_transcript/80272	2.34283850595428	1.92461175962384	1.36885825869264	0.180406067484023	0.9459357649929	-4.9243220594267
a_HQ_transcript/3341	-1.76682919500484	1.80701817555749	-1.36875044178553	0.18043945392318	0.945944419571325	-4.96357773972477
a_HQ_transcript/99964	1.84046726570097	1.66808035303489	1.36874782270165	0.180440265004935	0.945944419571325	-4.91944331452129
a_HQ_transcript/65953	1.62960927295901	0.828142003346869	1.36844802098145	0.180533126773239	0.946244968415791	-4.85973592665481
a_HQ_transcript/79481	-2.4699632435736	0.931066544068073	-1.36841220012124	0.180544224551645	0.946244968415791	-5.06247402487486
a_HQ_transcript/5957	-1.18436667479482	1.95378222874113	-1.36840507826423	0.180546431060755	0.946244968415791	-4.97801001351813
a_HQ_transcript/132073	-2.71280485700604	2.54635227706611	-1.36833246510811	0.18056892942179	0.946259291488394	-5.07741579282562
a_HQ_transcript/948	1.94340262766955	0.80548711065572	1.36829118125536	0.180581721725859	0.946259291488394	-4.97860951144184
a_HQ_transcript/5477	-2.17817463564853	2.9007948421299	-1.36815391877377	0.180624259239995	0.946396875888085	-5.08391852379574

a_HQ_transcript/77172	-1.45776075824522	1.30307051777917	-1.36787768582191	0.180709887176886	0.946649593778011	-4.98071917684131
a_HQ_transcript/40453	-2.29416561215788	2.97577737632969	-1.3678475848554	0.180719219916074	0.946649593778011	-5.1370229761326
a_HQ_transcript/55693	-1.07558476229812	2.75428891627981	-1.36784071987557	0.18072134844059	0.946649593778011	-5.12983871319462
a_HQ_transcript/134379	-4.5381907938324	2.5797749172915	-1.3676999944249	0.180764985428787	0.946691464481507	-5.19196481917823
a_HQ_transcript/148086	-0.85000017126772	4.43836375135613	-1.36767806364113	0.180771786594563	0.946691464481507	-5.37530344805504
a_HQ_transcript/22563	1.82497132244436	0.123292072785299	1.36765738124245	0.180778200793847	0.946691464481507	-4.99182747563578
a_HQ_transcript/78593	-2.80614550624385	0.777942932810726	-1.36760396343152	0.180794767992535	0.946692935219354	-4.98047965459899
a_HQ_transcript/122594	1.84777906756613	0.603612338970971	1.36754880955278	0.180811874859671	0.946697231295526	-4.85640513983946
a_HQ_transcript/151822	2.2229123803807	2.62702700194268	1.36744915963294	0.180842786082465	0.94672615248225	-5.10319128231246
a_HQ_transcript/88214	-1.35127359056663	-0.38434946004077	-1.36742599458633	0.180849972425731	0.94672615248225	-4.94570306482886
a_HQ_transcript/148928	1.64168211441381	-0.236385928989078	1.36734429444157	0.180875319507006	0.946734420907793	-4.96414587287273
a_HQ_transcript/139204	-1.18986341173529	2.55871937016849	-1.36723275956538	0.180909927128087	0.946734420907793	-5.03617335678976
a_HQ_transcript/104128	-0.552394820800525	4.9228188412514	-1.36716683457578	0.180930385095603	0.946734420907793	-5.47812411187115
a_HQ_transcript/148989	-3.0918526563933	0.674704587450603	-1.36709102945449	0.180953911306	0.946734420907793	-4.96205535413893
a_HQ_transcript/83161	0.839785987325166	1.75576406550918	1.3670886303895	0.180954655897499	0.946734420907793	-4.90001029564364
a_HQ_transcript/40702	2.48336837612772	1.22730257510605	1.36707157960587	0.180959947973002	0.946734420907793	-5.14560403587749
a_HQ_transcript/88365	2.64154900580743	0.853737190047065	1.36701313055011	0.180978089800584	0.946734420907793	-4.88523048625813
a_HQ_transcript/144985	2.09141559282415	0.0489472342181504	1.36700102164886	0.180981848422918	0.946734420907793	-5.03997541564904
a_HQ_transcript/5060	-1.30762003188201	-0.471753690783468	-1.3668881175924	0.181016896941892	0.94673732509559	-4.95633370249845
a_HQ_transcript/95891	1.50053319788419	1.49717758432143	1.36688615892273	0.181017505013263	0.94673732509559	-4.91686446223609
a_HQ_transcript/148253	3.08474683431994	0.527931154880322	1.3668418379407	0.181031264940089	0.94673732509559	-4.98327700794479
a_HQ_transcript/36253	-1.23276696363431	0.725509775248728	-1.36672082259221	0.181068839598518	0.946766668455755	-4.80910451681743
a_HQ_transcript/122099	-2.23969553909667	1.59906754679833	-1.36671246325817	0.181071435353493	0.946766668455755	-4.93087253734082
a_HQ_transcript/95691	1.70421534961471	2.21558828687733	1.36665923520619	0.181087964499052	0.946766668455755	-4.98112896082356
a_HQ_transcript/36983	1.02871408717085	2.515951733983	1.36661302596625	0.181102315011956	0.946766668455755	-4.9481555409527
a_HQ_transcript/33987	-1.44483435157218	1.65843707918171	-1.36656151208628	0.181118313949009	0.946766668455755	-4.99455822942392
a_HQ_transcript/29742	-1.72321132178637	0.957001208610526	-1.36646596537328	0.181147991301815	0.946768338488043	-4.98144823380251
a_HQ_transcript/9712	-1.57355163995233	-0.166129590361494	-1.36645560756409	0.181151208723232	0.946768338488043	-4.87566964993688
a_HQ_transcript/7778	0.622961125821813	2.58188679801796	1.36615999417754	0.181243053114424	0.947163192613077	-5.06449556531719
a_HQ_transcript/86927	2.44582428156703	1.7436548472362	1.36604612672646	0.181278440344144	0.947225328419088	-5.03277441790157
a_HQ_transcript/26803	-3.2216584252134	1.99326722430483	-1.36583712019203	0.1813434084488	0.947225328419088	-5.12800628308696
a_HQ_transcript/45773	2.01499921127049	1.28275509591032	1.36582540932979	0.181347049216956	0.947225328419088	-4.99145411854033
a_HQ_transcript/111367	-1.60695270356015	1.26199904015543	-1.36579455414389	0.181356642000091	0.947225328419088	-4.99567710280795
a_HQ_transcript/42118	1.72089857397168	2.17463002166529	1.36576647264292	0.181365372795606	0.947225328419088	-5.03129544280438
a_HQ_transcript/63132	-3.73368183382389	2.59657922356742	-1.36574502985093	0.18137203977558	0.947225328419088	-5.04101066958043
a_HQ_transcript/61541	-1.85676446686687	0.251233172168464	-1.3656267774719	0.181408810153456	0.947225328419088	-4.98809430306976
a_HQ_transcript/3609	0.624536353323504	4.23268442740676	1.36557434487354	0.181425115834071	0.947225328419088	-5.33719478308965
a_HQ_transcript/6777	0.917997694986596	2.02021094733035	1.36553809901773	0.181436388367852	0.947225328419088	-4.88338488045754
a_HQ_transcript/15162	-2.045691755522	1.41823060169094	-1.36542709729192	0.1814709135107	0.947225328419088	-4.97413561406229
a_HQ_transcript/6346	-2.03246165186559	0.180999060129279	-1.36538754752629	0.181483216005383	0.947225328419088	-4.91426411049265
a_HQ_transcript/89774	1.40218177174097	4.22058886244716	1.3653489839682	0.181495212350413	0.947225328419088	-5.39131995969283
a_HQ_transcript/69356	-0.521400931518168	2.37311291917174	-1.36529735044673	0.181511275461657	0.947225328419088	-5.04106883364009
a_HQ_transcript/137244	0.80274209492284	3.26219394790416	1.3652041939616	0.18154025909751	0.947225328419088	-5.21841298356153
a_HQ_transcript/114239	-3.2543139436663	3.7671912588306	-1.36508170592577	0.181578374083645	0.947225328419088	-5.06345939352684
a_HQ_transcript/8109	2.63610807587615	2.38009019071501	1.36498605328802	0.181608142935718	0.947225328419088	-4.91258687272924
a_HQ_transcript/103117	1.57016462947267	1.12904764021098	1.36493178642135	0.181625033462987	0.947225328419088	-4.93369606105356
a_HQ_transcript/47462	2.16759188046347	0.756312932442722	1.36492980966051	0.181625648751548	0.947225328419088	-4.94775667008519
a_HQ_transcript/134653	-1.77436111295625	1.21614333185521	-1.36490829750884	0.181632344750114	0.947225328419088	-4.85495069812592
a_HQ_transcript/26123	-2.12010670301239	0.374147555093268	-1.36489121052766	0.181637663480739	0.947225328419088	-5.04878290657655
a_HQ_transcript/34706	1.74955886248389	-0.364959736075313	1.36483390951108	0.181655500672289	0.947225328419088	-4.97103887067472

a_HQ_transcript/45594	-1.50364126353619	3.84898781143982	-1.36472327669285	0.181689943329447	0.947225328419088	-5.37850454735649
a_HQ_transcript/93529	-1.688014619327	1.99744061072624	-1.36459854115137	0.181728782584715	0.947225328419088	-4.93946968894956
a_HQ_transcript/47848	1.23310659900889	0.692834475264699	1.36459687913568	0.181729300134716	0.947225328419088	-4.84217086288927
a_HQ_transcript/882	2.24520773162152	0.0761432115326869	1.36456861831217	0.181738100701684	0.947225328419088	-4.97098593550572
a_HQ_transcript/130543	1.07954962613729	1.94523001832585	1.36447622260644	0.181766875512914	0.947225328419088	-4.93111466335762
a_HQ_transcript/10349	-1.68859780944538	1.45355514062068	-1.36439675603898	0.181791626629625	0.947225328419088	-4.9328776020794
a_HQ_transcript/117997	-1.54924299103177	-0.64727257217849	-1.36435418125883	0.181804888293354	0.947225328419088	-4.98397095891353
a_HQ_transcript/37549	-1.15623230927071	-0.101049569373955	-1.36432201266204	0.181814909022068	0.947225328419088	-4.84255778077571
a_HQ_transcript/6419	-0.725277736262631	3.13363787091159	-1.36427566635299	0.181829346954999	0.947225328419088	-5.15998007135809
a_HQ_transcript/33758	1.12020357372426	0.182518093992233	1.36425970983672	0.181834317979938	0.947225328419088	-4.99109061998612
a_HQ_transcript/12762	-1.41551349312121	2.12830922642173	-1.36425453399688	0.181835930461641	0.947225328419088	-5.01204758737425
a_HQ_transcript/96610	-3.12124610045885	1.8095864906727	-1.36420566662095	0.181851155156429	0.947225328419088	-4.96497543540064
a_HQ_transcript/2854	1.32929294048647	2.45670915719422	1.36419506403108	0.181854458537798	0.947225328419088	-5.11489060415725
a_HQ_transcript/95234	1.57386800515496	2.17980990472377	1.36415537422304	0.181866824852002	0.947225328419088	-4.95336444534119
a_HQ_transcript/18183	1.74917052285998	0.145262169908328	1.364118946249	0.181878175437941	0.947225328419088	-4.83126382383103
a_HQ_transcript/103317	-1.01102496756854	1.38359125806235	-1.36408349410171	0.18188922249425	0.947225328419088	-4.91727280022691
a_HQ_transcript/110092	-0.961080078141182	3.07841326415651	-1.3640493726563	0.181899855389509	0.947225328419088	-5.32660967369097
a_HQ_transcript/86570	1.49175509116758	2.09240594768194	1.36388555164937	0.181950911869744	0.947225328419088	-4.88017656749729
a_HQ_transcript/36936	-0.616444830025136	3.72033734025833	-1.36382231256992	0.181970623946826	0.947225328419088	-5.29837996517667
a_HQ_transcript/13513	2.15268465708985	0.737039406985139	1.36381032903616	0.18197435948754	0.947225328419088	-4.93124131773474
a_HQ_transcript/121406	2.2747119404178	0.431073304294355	1.36362298973304	0.182032765159531	0.947225328419088	-4.92861100010742
a_HQ_transcript/113725	2.43779411045686	0.73570104584178	1.36353656732983	0.182059713467668	0.947225328419088	-5.19250817877259
a_HQ_transcript/151653	-2.57866968282749	0.544844475578194	-1.36350281077722	0.182070240305917	0.947225328419088	-4.97413185572031
a_HQ_transcript/129867	1.8960514946494	-0.0615594378429797	1.363497567964	0.182071875297446	0.947225328419088	-4.90590675823073
a_HQ_transcript/89181	-1.49776352798349	-0.15921076446934	-1.36349365920014	0.182073094267953	0.947225328419088	-4.9588252219899
a_HQ_transcript/25753	-1.26516866577034	1.25531564708305	-1.36349199543814	0.18207361312363	0.947225328419088	-4.95186768561333
a_HQ_transcript/19578	1.40561017731933	3.78031579469906	1.36345099795605	0.182086398834299	0.947225328419088	-5.13167502572428
a_HQ_transcript/89529	-2.13538523924332	0.842647761429124	-1.36341581371836	0.182097372148795	0.947225328419088	-4.96045970923943
a_HQ_transcript/40772	1.17809551590932	2.46100705110548	1.36337703652863	0.182109466633319	0.947225328419088	-4.9730251411161
a_HQ_transcript/114439	0.330058758120672	4.54469569024699	1.36327147698619	0.182142393484161	0.947225328419088	-5.41326543748862
a_HQ_transcript/38959	-3.08206011100465	1.45632878199672	-1.36326269425365	0.182145133262073	0.947225328419088	-4.86821659319947
a_HQ_transcript/125443	3.99811440092224	1.68004930174484	1.36324946547292	0.18214926004737	0.947225328419088	-5.20284562179092
a_HQ_transcript/13018	-1.53619202211678	3.09172939883903	-1.36317920124784	0.182171180545413	0.947225328419088	-5.15638534140465
a_HQ_transcript/120996	0.911629576452678	2.26283925642681	1.36317852342545	0.182171392017254	0.947225328419088	-4.99950383664937
a_HQ_transcript/113735	-2.1351304503261	0.219374625872814	-1.36313648997276	0.18218450628439	0.947225328419088	-4.90387215809137
a_HQ_transcript/144670	-0.596891917255729	4.99729979506153	-1.36306227280482	0.182207663529462	0.947225328419088	-5.44412404338766
a_HQ_transcript/1646	-0.832785287558464	3.52801342393589	-1.36304944946631	0.182211664899742	0.947225328419088	-5.25714564752966
a_HQ_transcript/9213	-3.14562730495042	1.37221153782415	-1.36301554172427	0.182222245735973	0.947225328419088	-5.04816468324632
a_HQ_transcript/53861	0.618155759874639	2.99401180465888	1.36296270128874	0.182238735428074	0.947225328419088	-5.10090428730667
a_HQ_transcript/59111	1.57812193645455	-0.00700294481198637	1.36292990999353	0.182248969051732	0.947225328419088	-4.91629379909118
a_HQ_transcript/141597	2.35556349812154	1.06525808584757	1.36280607564424	0.182287619736886	0.947341507381824	-5.06716064071501
a_HQ_transcript/73823	1.46088294114579	0.756431850031206	1.36266942089289	0.182330279257888	0.947376421021688	-4.92885758774449
a_HQ_transcript/47963	-0.946739902462444	2.30353103226572	-1.36260946576736	0.182348997897591	0.947376421021688	-4.94736894713809
a_HQ_transcript/134164	-2.34892957751956	1.96516169230983	-1.36258923089951	0.182355315779124	0.947376421021688	-5.02467351271483
a_HQ_transcript/30395	0.861678688462539	2.83167695221487	1.36245590270373	0.182396948751065	0.947376421021688	-5.1805589907353
a_HQ_transcript/120304	-0.65302354303084	4.55625452358121	-1.36244676670561	0.182399801821636	0.947376421021688	-5.4039336979142
a_HQ_transcript/140918	1.42363517258673	3.42226640302392	1.36243313616145	0.182404058553312	0.947376421021688	-5.37714996090647
a_HQ_transcript/150862	3.66654358658592	1.98471488338381	1.36240124894592	0.182414017027677	0.947376421021688	-5.16518479205011
a_HQ_transcript/97642	-5.8496172630879	2.43644777499758	-1.36236697072919	0.182424722689509	0.947376421021688	-5.02762375997877
a_HQ_transcript/21859	1.00769405777008	2.19896540715246	1.36222926680206	0.182467734848207	0.9475151412593	-4.99353777269727

a_HQ_transcript/97068	-2.77222752078243	2.81099542894655	-1.36209150468568	0.182510773056133	0.947628897060248	-5.09619537707121
a_HQ_transcript/22187	-2.55988300741909	1.31966948275537	-1.36203853196568	0.182527324338988	0.947628897060248	-4.8633525675682
a_HQ_transcript/101383	-0.602472177874112	2.55068909112172	-1.36199054092995	0.182542320104136	0.947628897060248	-5.00165184706979
a_HQ_transcript/132194	-3.58587368715671	0.55151538608013	-1.36195044000598	0.182554851178101	0.947628897060248	-4.99552315152063
a_HQ_transcript/133732	3.08588324669906	1.0066170199027	1.36182159818911	0.182595117269737	0.947638911884216	-5.00850575345806
a_HQ_transcript/118119	1.62804769520135	-0.449489624801157	1.36159524919646	0.182665873328108	0.947638911884216	-4.98516945876157
a_HQ_transcript/73986	1.86877017194216	0.197766426815307	1.361580926321	0.182670351334171	0.947638911884216	-4.95084537119342
a_HQ_transcript/145306	1.29920475938721	0.614375082887236	1.36153133297759	0.182685857209165	0.947638911884216	-4.92339464799732
a_HQ_transcript/107354	2.10952316613398	0.532801698042918	1.36149639986835	0.18269678002229	0.947638911884216	-4.99181192451721
a_HQ_transcript/30631	3.52896611658699	1.97625486623423	1.36149634801236	0.18269679623689	0.947638911884216	-5.0659054254247
a_HQ_transcript/65462	1.6513317932659	0.6822604132761	1.36139124970016	0.182729661219458	0.947638911884216	-4.793374661187
a_HQ_transcript/92213	-3.31052292437776	0.534339484276083	-1.36139012663615	0.182730012434249	0.947638911884216	-4.98575307233478
a_HQ_transcript/6150	-1.77322906964743	0.927171667083451	-1.36136583491133	0.182737609291537	0.947638911884216	-4.88439390560334
a_HQ_transcript/151036	2.30612901322121	2.33440656949699	1.3612886209646	0.182761758373752	0.947638911884216	-4.98975428308546
a_HQ_transcript/71294	3.3575599620804	2.48883529627494	1.36120191020642	0.182788880586474	0.947638911884216	-5.16275825719145
a_HQ_transcript/146677	-1.33824594512407	2.38150129166709	-1.36113899785509	0.18280856086235	0.947638911884216	-5.03993587724353
a_HQ_transcript/151442	0.360448119032242	10.1447130126896	1.36112279809969	0.182813628744726	0.947638911884216	-6.012262703
a_HQ_transcript/113799	3.53085685530924	2.68023478862049	1.36110889530821	0.182817978138767	0.947638911884216	-5.39173244749987
a_HQ_transcript/152702	-4.45723390046173	1.81813855834359	-1.36108341165893	0.182825950734051	0.947638911884216	-5.05749223653957
a_HQ_transcript/87724	-1.51453172293818	-0.229056323339271	-1.36097214595132	0.182860763525572	0.947638911884216	-4.91858229208214
a_HQ_transcript/111727	0.792075622394765	3.09731947894897	1.36093199790507	0.182873326300277	0.947638911884216	-5.12450140488429
a_HQ_transcript/145345	1.41194405385594	0.243558084739105	1.36091748845962	0.182877866633447	0.947638911884216	-4.85186300636023
a_HQ_transcript/84459	4.11438001184528	0.180811194392388	1.36090783503749	0.182880887455686	0.947638911884216	-5.01372389780435
a_HQ_transcript/73669	1.6178447058879	-0.345709523835293	1.36078643984307	0.182918878666574	0.947638911884216	-4.89444792848024
a_HQ_transcript/22928	3.07621752337614	2.29789386451223	1.36078148979778	0.182920427936978	0.947638911884216	-4.90707037439775
a_HQ_transcript/124703	-0.356716717535827	4.26992007842198	-1.36075057611887	0.182930103563115	0.947638911884216	-5.39489614340975
a_HQ_transcript/136477	-0.902736081470338	1.90391825479852	-1.360672161975	0.182954648069245	0.947638911884216	-4.9567314520041
a_HQ_transcript/149770	1.62717339862316	0.140818727364926	1.36066562286019	0.18295669500087	0.947638911884216	-4.97837273395606
a_HQ_transcript/121910	1.25126263013109	3.69023971911425	1.36064118132101	0.182964346065176	0.947638911884216	-5.32914725886787
a_HQ_transcript/104086	2.63071440875612	2.0511359925758	1.36052636422376	0.18300029118961	0.947740638477338	-5.10184494465683
a_HQ_transcript/140140	5.10574082871456	3.14649615932208	1.36044617879289	0.183025397626536	0.947786219441897	-5.03818236345546
a_HQ_transcript/59824	1.7791659012612	0.980050363087719	1.36027715698958	0.183078327901105	0.947843635162092	-4.97794782001399
a_HQ_transcript/36644	-2.08176422509863	2.33112904412425	-1.3602220951529	0.183095573435879	0.947843635162092	-4.99193289823085
a_HQ_transcript/61320	-1.17887735989908	-0.535973456059809	-1.36020639626414	0.183100490606398	0.947843635162092	-4.92844827117763
a_HQ_transcript/92228	1.97525249931762	1.55116445087841	1.36019022441994	0.183105556022033	0.947843635162092	-5.11951214870862
a_HQ_transcript/95537	-2.20243799469128	1.63312755964677	-1.36014423314391	0.183119962204278	0.947843635162092	-4.95518631994815
a_HQ_transcript/99070	-1.78825114848299	1.13833653001786	-1.36009839289648	0.183134321953369	0.947843635162092	-4.92323870776426
a_HQ_transcript/36789	-0.596456082465082	3.07219722444391	-1.35999920244961	0.183165396980855	0.947879983015522	-5.23247024810055
a_HQ_transcript/129819	0.762562763323071	6.05129773628018	1.3599079525699	0.183193987946827	0.947879983015522	-5.55123455800291
a_HQ_transcript/151392	3.63111743917067	3.29885916457944	1.35980972481317	0.183224769131235	0.947879983015522	-5.19423658330781
a_HQ_transcript/83218	1.08601128963977	1.59700461899384	1.3598077127261	0.183225399691702	0.947879983015522	-4.91580999880902
a_HQ_transcript/149252	-1.5907302065019	0.0833925436924598	-1.35979379874896	0.183229760187197	0.947879983015522	-4.94918620994777
a_HQ_transcript/143547	-0.527783833224388	2.89506644096248	-1.35976372454718	0.183239185403685	0.947879983015522	-5.15543497924165
a_HQ_transcript/137349	-1.65472118105022	0.617782306169766	-1.3596959253781	0.183260434955899	0.947880481445073	-4.95000421224364
a_HQ_transcript/89557	1.9200775134006	1.27543663419178	1.3596092403456	0.183287606481006	0.947880481445073	-4.95566015177724
a_HQ_transcript/5444	-1.1167120987163	-0.428895698306195	-1.35957389236706	0.183298687242898	0.947880481445073	-4.9304484652219
a_HQ_transcript/68323	0.83884470111727	2.97386329407398	1.35955532183959	0.183304508875655	0.947880481445073	-5.05223459767356
a_HQ_transcript/47219	2.15054568463773	0.750996332625519	1.35933271220499	0.183374305427746	0.947974754523082	-5.00468407625158
a_HQ_transcript/27544	1.55568465325054	1.7543298885347	1.35932614957027	0.183376363374939	0.947974754523082	-4.94656810024344
a_HQ_transcript/91341	-1.59376559437383	1.60783515056225	-1.3592946061923	0.183386255171262	0.947974754523082	-4.97961382439523

a_HQ_transcript/66361	-1.44386065082622	-0.533614524374919	-1.35927408792569	0.183392689787088	0.947974754523082	-4.94616474754471
a_HQ_transcript/87894	-1.78766300035826	1.04493035101751	-1.35923712567969	0.183404281746604	0.947974754523082	-4.8702677984046
a_HQ_transcript/119993	1.90790695090092	1.33861123092126	1.35908078921775	0.183453317678124	0.948073414495806	-4.98594466298942
a_HQ_transcript/113931	-1.56083359505774	2.94124115850824	-1.35898516564011	0.183483315634471	0.948073414495806	-5.27684372808566
a_HQ_transcript/136149	-4.53096752988928	1.7412193616163	-1.35897069140164	0.183487856661585	0.948073414495806	-5.00603170650951
a_HQ_transcript/148819	-1.67259068506109	0.325998830366483	-1.35895742824288	0.183492017811128	0.948073414495806	-4.95180607455763
a_HQ_transcript/126306	0.466249917708083	3.97969492564475	1.3588662129258	0.183520637453349	0.948073414495806	-5.33992268090285
a_HQ_transcript/67609	-1.32617909200182	1.73401673755653	-1.35883006296102	0.18353198079375	0.948073414495806	-4.97571295657954
a_HQ_transcript/8290	1.06496785981409	3.32098093147553	1.35881234657156	0.18353754014109	0.948073414495806	-5.19368831514775
a_HQ_transcript/50050	-1.94026715320722	0.514370353043316	-1.35868170700719	0.183578538456417	0.948142389616384	-5.05707910283958
a_HQ_transcript/129396	2.17548245163235	0.658220894125399	1.35866584905058	0.183583515605773	0.948142389616384	-5.05069221794543
a_HQ_transcript/10501	-1.50220210260708	1.08161986303445	-1.35855461582105	0.183618430004636	0.948238459426929	-4.88983206143069
a_HQ_transcript/39188	-0.624237800667937	2.70901972421936	-1.35832315845175	0.183691097408841	0.948529458131038	-5.10173480664203
a_HQ_transcript/97937	-1.73875991920383	1.04631360581599	-1.3582279138858	0.183721006474091	0.948599632645757	-4.83164504641428
a_HQ_transcript/117437	4.4252218029702	3.60668988843055	1.35815969906694	0.1837424298738	0.948625984874697	-5.00314625820712
a_HQ_transcript/17045	-2.10015714689568	1.33435887428821	-1.35809563742755	0.18376255069784	0.948645608078514	-5.01801780599339
a_HQ_transcript/75438	-0.765999143419495	2.87338668886128	-1.35802381064984	0.183785112468994	0.948677827688277	-5.20289499032447
a_HQ_transcript/119254	1.54298610107036	1.85084768693225	1.35786633714088	0.183834584560505	0.948765419859257	-5.03531675498751
a_HQ_transcript/149329	-0.905524299645068	2.65847156253492	-1.35786018413835	0.183836517805637	0.948765419859257	-5.11271878353604
a_HQ_transcript/25286	0.475009095855031	4.47830278313964	1.35781394159763	0.183851047503693	0.948765419859257	-5.42961593351249
a_HQ_transcript/148521	-0.779267540398268	6.7015336824995	-1.35770626960822	0.183884882170785	0.948779648833592	-5.64000075797221
a_HQ_transcript/75757	-1.64734442945842	1.03447036025162	-1.35770128297131	0.183886449280196	0.948779648833592	-4.87241110323927
a_HQ_transcript/28921	1.55931565597565	1.58482194472887	1.35760182089141	0.183917708572908	0.948856711096653	-4.89458014792222
a_HQ_transcript/124436	1.61376254174714	-0.36124089881637	1.35748376240585	0.183954817754199	0.94891209787102	-4.98614742954203
a_HQ_transcript/117528	-1.77029855382715	0.41399161402044	-1.35744992661297	0.183965454387859	0.94891209787102	-5.12818932159655
a_HQ_transcript/75961	1.70159459952497	5.24349396846887	1.35741134552863	0.183977583334826	0.94891209787102	-5.47920910153367
a_HQ_transcript/148250	-3.83709338831855	3.89454435326676	-1.35735994823538	0.183993742346275	0.94891209787102	-5.32819632084996
a_HQ_transcript/81132	0.498733088781921	3.62507107477577	1.35725430054813	0.184026960821234	0.948999217407441	-5.27830881547088
a_HQ_transcript/124455	3.07342126522215	0.5982948291954	1.3571654583215	0.184054898804291	0.949059093204633	-4.95742764047986
a_HQ_transcript/15835	0.992609615490698	1.34249990476913	1.35702440129628	0.184099263390065	0.949203652859473	-4.90224470062968
a_HQ_transcript/110885	1.40469259975748	1.02914076722672	1.35675397517565	0.184184339698366	0.949501133322634	-4.85028128848816
a_HQ_transcript/25903	-3.26990021000527	0.97639858141889	-1.35673716427996	0.18418962942723	0.949501133322634	-5.00866517857593
a_HQ_transcript/83477	-1.51499612941793	0.467299220094385	-1.35663353622467	0.184222239706828	0.949574423060829	-5.09787703072044
a_HQ_transcript/82074	1.38237076718703	1.44249105796949	1.35658816616321	0.184236518429673	0.949574423060829	-4.93715545746426
a_HQ_transcript/66467	3.04023190160061	2.46542675030789	1.35645610070118	0.184278086536351	0.949599323754166	-4.94908983905575
a_HQ_transcript/54458	1.72173097397449	0.380587272015337	1.35643024979161	0.184286224058753	0.949599323754166	-5.02747896137468
a_HQ_transcript/122128	2.8521958317679	0.325237774281901	1.35641711534955	0.184290358713025	0.949599323754166	-4.92677787549328
a_HQ_transcript/74358	3.95189287701622	2.37993921080954	1.3562988121659	0.184327603180818	0.949696215763988	-5.14929443851311
a_HQ_transcript/5576	1.30457727576619	2.14089899768103	1.35623915836715	0.184346385731916	0.949696215763988	-5.01775892470354
a_HQ_transcript/137524	0.957429568571162	1.51737357133777	1.35620171220746	0.18435817675958	0.949696215763988	-4.87592384796895
a_HQ_transcript/76504	0.938758628279564	2.27820191726501	1.35612076538203	0.184383667251148	0.949743359324299	-5.0227725768384
a_HQ_transcript/102736	2.61985486801749	1.29738807411361	1.3560257610806	0.184413587982799	0.949813312268665	-5.09252610667081
a_HQ_transcript/117718	-2.04068946439815	0.0370627838341967	-1.35593184731097	0.184443168957924	0.949881503073111	-4.90684272705393
a_HQ_transcript/119757	3.27798157977854	1.73817293988602	1.3557397212564	0.184503696305919	0.950109041359894	-4.96893623299832
a_HQ_transcript/70207	-2.84533638321679	0.695964168879693	-1.35563275264981	0.184537402337963	0.950198434148384	-5.05906418463895
a_HQ_transcript/16056	-1.30427192344524	0.277272806741332	-1.35549197837266	0.184581767875748	0.950342692921299	-5.02089716542193
a_HQ_transcript/92953	1.61996905075194	1.54732214328773	1.35519026525864	0.184676881713104	0.950462908707066	-4.89424758585172
a_HQ_transcript/43658	1.2723189819552	0.148678986464039	1.35516757365045	0.184684036685125	0.950462908707066	-4.91422809639766
a_HQ_transcript/63352	-1.69738848836553	0.273184647710274	-1.35516494602177	0.184684865225979	0.950462908707066	-4.96950575023965
a_HQ_transcript/106785	2.75840881831499	0.892372263613771	1.35514160571999	0.184692224989435	0.950462908707066	-4.92623828306929

a_HQ_transcript/104319	-1.44699550775054	0.799888440301432	-1.35501975716624	0.18473065048291	0.950462908707066	-4.90140605465505
a_HQ_transcript/69000	1.55754029580815	1.99755697725459	1.35501890278823	0.184730919936742	0.950462908707066	-5.02060524876865
a_HQ_transcript/106592	0.941632204954829	4.30880527053964	1.3548025493708	0.18479916329951	0.950462908707066	-5.38939919603893
a_HQ_transcript/40499	0.97430768713624	2.67479267655113	1.35479861274319	0.184800405192656	0.950462908707066	-5.03528319071871
a_HQ_transcript/110581	-3.42578486035909	4.76525489470056	-1.35476806045055	0.184810043784737	0.950462908707066	-5.56721734225706
a_HQ_transcript/147408	-1.88360418252815	-0.126679766382955	-1.35476647233574	0.184810544811491	0.950462908707066	-4.98156967652028
a_HQ_transcript/120651	1.78136573495202	0.907118322478121	1.35476433974871	0.18481121761284	0.950462908707066	-4.94685073291804
a_HQ_transcript/47601	-1.45123157724745	1.7093872523028	-1.35470019578484	0.184831455021785	0.950462908707066	-4.86158030116884
a_HQ_transcript/23546	-1.32475133914906	-0.117726351378729	-1.35465519690236	0.184845653185168	0.950462908707066	-4.92723245563084
a_HQ_transcript/88040	-1.24102950548275	2.81288919153401	-1.35463498395576	0.184852031101049	0.950462908707066	-5.13660436330763
a_HQ_transcript/85367	-2.01994854739936	1.14040099176444	-1.3546028635832	0.184862166591131	0.950462908707066	-5.03998358511275
a_HQ_transcript/35288	2.89889514490924	2.5185870649091	1.35450387044536	0.18489340629444	0.950462908707066	-5.04529734364559
a_HQ_transcript/32482	-2.06153885635955	-0.0898722604520945	-1.35450141637228	0.184894180789079	0.950462908707066	-4.92458063748517
a_HQ_transcript/2506	-0.946651105557659	5.287738279938	-1.35448475636225	0.184899438681178	0.950462908707066	-5.48116945557445
a_HQ_transcript/145976	-0.558799504882672	4.66314367078111	-1.35443185276132	0.184916135802723	0.950464686704456	-5.43702149779853
a_HQ_transcript/122415	-1.67635720737233	-0.0287005140072506	-1.35419931919469	0.184989540515084	0.950653732995174	-4.95589012387719
a_HQ_transcript/17995	-3.15703201425729	1.19264008761927	-1.35411929171559	0.185014808296529	0.950653732995174	-5.04962724485428
a_HQ_transcript/137809	-0.946920836567049	2.97221980313882	-1.35408549599043	0.185025479721207	0.950653732995174	-5.28106437134415
a_HQ_transcript/22830	0.574652305594325	3.46463196882178	1.35407895888167	0.185027543950402	0.950653732995174	-5.22279320915604
a_HQ_transcript/87844	0.884125032012996	2.38841069235478	1.35405633565631	0.185034687845916	0.950653732995174	-5.00565239471562
a_HQ_transcript/50377	-3.02344255831666	0.334271239344256	-1.35366878781101	0.185157099728312	0.951146835724849	-4.89639007408981
a_HQ_transcript/112173	-1.96954259451866	0.860674697468529	-1.35364887536936	0.185163391017451	0.951146835724849	-4.86388525383565
a_HQ_transcript/90508	2.9010059136189	0.917549343055956	1.35351373568394	0.185206092456005	0.951161447663371	-5.15126695823569
a_HQ_transcript/28918	-1.43225085860114	-0.555436534358371	-1.35345027054114	0.185226148802584	0.951161447663371	-4.99132382985743
a_HQ_transcript/33154	-1.21531997689177	1.56126889791562	-1.35344081020503	0.185229138615719	0.951161447663371	-4.94178349192038
a_HQ_transcript/19434	2.32877436678573	1.25912379673304	1.35339575415949	0.185243378491013	0.951161447663371	-4.9624529658438
a_HQ_transcript/141607	-1.54207325889791	1.54349459198913	-1.35331326621657	0.185269450840243	0.951161447663371	-4.94922622025071
a_HQ_transcript/100575	1.43088793120131	1.24782706153412	1.35321404615788	0.185300815550443	0.951161447663371	-4.9770630574218
a_HQ_transcript/120369	-2.95704678779375	0.756602952258488	-1.3532064861893	0.185303205520223	0.951161447663371	-5.01246111048015
a_HQ_transcript/69449	0.89363190577509	3.42159444170955	1.35319297291713	0.185307477596379	0.951161447663371	-5.30422640929711
a_HQ_transcript/149236	-1.82242535035694	0.482623431420615	-1.35317390896653	0.185313504589659	0.951161447663371	-4.91949535482558
a_HQ_transcript/108675	-2.09938323688887	0.0420027919652074	-1.35308541317665	0.185341484173773	0.951221065870834	-4.91480636985036
a_HQ_transcript/139300	4.58200469283306	0.787728008759555	1.35294108697344	0.185387122592381	0.951243952461361	-4.85596362514202
a_HQ_transcript/97021	-1.73070257705246	0.713140218481523	-1.35293855126911	0.185387924503285	0.951243952461361	-5.01991749718217
a_HQ_transcript/55232	-1.79869782562207	0.612700505714811	-1.35291605992335	0.18539503745931	0.951243952461361	-4.94609982487927
a_HQ_transcript/11608	-2.12301312473573	0.99482778855473	-1.3527302907212	0.185453795607953	0.951315874158022	-4.95184821868783
a_HQ_transcript/18598	-0.879309914489563	3.31019212174635	-1.35272529084229	0.185455377251309	0.951315874158022	-5.23735082428321
a_HQ_transcript/59410	3.32179952241516	3.17857567887373	1.35267294590388	0.18547193648388	0.951315874158022	-5.08798130880088
a_HQ_transcript/38088	-1.40880623106767	2.47436017596561	-1.35266478480761	0.185474518335963	0.951315874158022	-4.99045574001761
a_HQ_transcript/138267	1.84965793503018	0.660180741698093	1.35259793138738	0.185495669193983	0.951340414585129	-4.87464711182465
a_HQ_transcript/61333	1.92748485507062	0.667438489397306	1.35242099071615	0.185551658090541	0.951366773272849	-4.9222162013107
a_HQ_transcript/9980	3.72073343986755	1.87432984762585	1.35241452017312	0.185553705796217	0.951366773272849	-5.17822929812559
a_HQ_transcript/3332	-1.87043199314382	0.677930027492042	-1.35237052815569	0.185567628229517	0.951366773272849	-4.98600414042775
a_HQ_transcript/32180	2.32571832202834	3.31419870550931	1.3522956033196	0.18559134202931	0.951366773272849	-5.19765666574334
a_HQ_transcript/130928	3.15111755108232	0.948336008192557	1.3522795846649	0.18559641225685	0.951366773272849	-5.07398417790938
a_HQ_transcript/99465	1.94312986367907	-0.0804869433015673	1.35220647413097	0.185619554577535	0.951366773272849	-5.01704175741816
a_HQ_transcript/63599	-0.648185443269973	2.35590678853901	-1.35218578386001	0.185626104256446	0.951366773272849	-5.03608241624187
a_HQ_transcript/45441	-1.75701937753705	1.28454731365735	-1.35216797245921	0.185631742748084	0.951366773272849	-4.93205440184446
a_HQ_transcript/100869	1.97339808857936	0.680798876324461	1.35209928355134	0.185653488591795	0.951394338787258	-4.95917926623669
a_HQ_transcript/50426	-2.31449218235364	1.44925243743421	-1.35183626760524	0.18573677358894	0.95145251380103	-5.07453769595428

a_HQ_transcript/120359	-1.74024694228511	-0.0259792169014603	-1.35179775622422	0.185748970789775	0.95145251380103	-4.92837987246507
a_HQ_transcript/149754	-3.61068730997342	2.0051428396037	-1.35178032027059	0.185754493252785	0.95145251380103	-5.15265939569253
a_HQ_transcript/73288	-1.49185069122279	1.41774195789395	-1.35166551753657	0.185790857719591	0.95145251380103	-4.93562828225882
a_HQ_transcript/46655	-1.57150328167266	-0.166100166352891	-1.35164383841173	0.185797725332942	0.95145251380103	-4.96851264641968
a_HQ_transcript/409	-1.02844451916411	4.85705309178553	-1.35148114122553	0.185849271567566	0.95145251380103	-5.45497263415007
a_HQ_transcript/146601	3.54926450068151	1.25346054574905	1.35141655401268	0.185869737359188	0.95145251380103	-5.03327196241752
a_HQ_transcript/126991	1.94363122201968	-0.110059191828273	1.35131749589456	0.185901129356377	0.95145251380103	-4.90003492991482
a_HQ_transcript/42359	1.17286414115931	2.14214936840898	1.35129684325726	0.185907674793815	0.95145251380103	-5.047239597093
a_HQ_transcript/149063	2.34234973787285	1.00985531466352	1.35128429140634	0.185911652937269	0.95145251380103	-5.05245949920088
a_HQ_transcript/135129	-0.963454804134385	2.87946205442747	-1.35127863908254	0.185913444388184	0.95145251380103	-5.10477240038364
a_HQ_transcript/150966	1.99703015359351	0.573860294259519	1.35121413029895	0.185933890786944	0.95145251380103	-5.01358338555501
a_HQ_transcript/122868	-1.67751975285217	3.8651022984597	-1.35114002399455	0.185957381317463	0.95145251380103	-5.35695529363581
a_HQ_transcript/11921	1.96244821352672	0.603121197193178	1.35102626332818	0.185993446122884	0.95145251380103	-5.00099324854204
a_HQ_transcript/145708	-0.489279472767558	2.718776954135	-1.351020910981	0.185995143076021	0.95145251380103	-5.13138291214158
a_HQ_transcript/31843	-3.56027205961132	2.00987527763309	-1.35101507552329	0.185996993212226	0.95145251380103	-4.95264722324464
a_HQ_transcript/8558	-4.31532092211768	1.97601239184044	-1.35097925907681	0.186008349155065	0.95145251380103	-4.99634579016711
a_HQ_transcript/126476	0.460086858799032	3.95062682255978	1.35091534035165	0.186028616523569	0.95145251380103	-5.30842238783749
a_HQ_transcript/98025	0.827166041942925	3.68747413261732	1.35089075879726	0.186036411304966	0.95145251380103	-5.29397169265104
a_HQ_transcript/14287	-3.70604207410069	2.60573464914761	-1.35086162515021	0.186045649876632	0.95145251380103	-4.98441969504502
a_HQ_transcript/53378	-1.09659198830768	-0.514530130793858	-1.35085868620535	0.186046581865229	0.95145251380103	-4.96309331161761
a_HQ_transcript/149002	-3.77867371463265	-0.00834697745337384	-1.35084092099371	0.186052215588018	0.95145251380103	-5.02025871102354
a_HQ_transcript/28204	1.90077750877171	1.31715356816783	1.35079384186719	0.186067146008868	0.95145251380103	-5.0971307317057
a_HQ_transcript/145279	-2.34414900142962	0.908139815880834	-1.3507593113792	0.186078097411164	0.95145251380103	-5.19204277691365
a_HQ_transcript/79244	-1.02675431174171	1.88158005077028	-1.35074924076067	0.18608129141866	0.95145251380103	-4.97633259008499
a_HQ_transcript/21044	1.7599391324041	2.07208513966919	1.350701547305	0.186096418498997	0.95145251380103	-5.03373989188209
a_HQ_transcript/151968	-2.64137819993213	2.19162791734179	-1.35063836200758	0.186116460639316	0.95145251380103	-5.11369201131979
a_HQ_transcript/22130	1.20306910745465	2.9275311016298	1.35045875298348	0.186173441068947	0.95145251380103	-5.21645602384997
a_HQ_transcript/19996	1.98044621451438	1.29126939428993	1.35045341405288	0.186175135035302	0.95145251380103	-4.95213325911143
a_HQ_transcript/65172	-0.42890041832672	2.77016085889943	-1.35042162102053	0.186185222756829	0.95145251380103	-5.15587372967784
a_HQ_transcript/52493	-0.522710300913873	3.04033964943725	-1.35041097930435	0.186188599398304	0.95145251380103	-5.23363995741082
a_HQ_transcript/141	-2.76128906344348	1.36032519040798	-1.3504109013039	0.186188624148203	0.95145251380103	-5.08477660591386
a_HQ_transcript/83311	1.88132884195887	0.980155235188931	1.35007623833058	0.186294837672114	0.951538482980714	-4.83651647229129
a_HQ_transcript/31434	1.29315443211631	0.688704871776833	1.35002771183488	0.186310242637911	0.951538482980714	-4.89510186879454
a_HQ_transcript/128264	2.05422653741165	0.328481823776095	1.34994067773668	0.186337874492324	0.951538482980714	-5.02813330142712
a_HQ_transcript/92140	-1.45684216525628	0.229239363976126	-1.34989834280318	0.186351316263462	0.951538482980714	-4.9788111124867
a_HQ_transcript/87417	-1.32638737410284	2.93533349409463	-1.34987954936788	0.186357283610264	0.951538482980714	-5.205693849737
a_HQ_transcript/34963	3.12084963193662	2.07137242211148	1.34986976819745	0.186360389414707	0.951538482980714	-5.17270391598019
a_HQ_transcript/39753	-1.10964301596223	0.144955722922369	-1.34978659701554	0.186386800287226	0.951538482980714	-4.99509340570689
a_HQ_transcript/6625	0.9225031485772	1.35743684285933	1.34976238692442	0.186394488705847	0.951538482980714	-4.86887668255493
a_HQ_transcript/133328	1.87646333486719	0.974821116287461	1.34973055853703	0.186404596846251	0.951538482980714	-4.91993221039905
a_HQ_transcript/135963	-0.945592297031733	2.71336278354349	-1.3497281231009	0.186405370315723	0.951538482980714	-5.1888599279178
a_HQ_transcript/7891	-1.56916028683811	0.41390251998667	-1.34967109641501	0.186423482114214	0.951538482980714	-4.84519281002152
a_HQ_transcript/132240	-1.80089770722034	4.83618130560851	-1.34966384568923	0.186425785057872	0.951538482980714	-5.46860254796732
a_HQ_transcript/122501	3.87891151184404	0.544251292383506	1.34964376296817	0.186432163758337	0.951538482980714	-5.02085421168644
a_HQ_transcript/144410	2.00826362790655	0.292456241929359	1.34963601950911	0.186434623291139	0.951538482980714	-4.98010898924845
a_HQ_transcript/47111	-1.73492656987696	0.572786949568349	-1.34958258498035	0.186451596231987	0.951541561525282	-4.96732617717674
a_HQ_transcript/140869	-1.37129470205238	-0.529849063390411	-1.34936833092354	0.186519663879653	0.951805374593669	-4.99387186454026
a_HQ_transcript/91332	-1.14185673001975	2.51148289251483	-1.34904340588709	0.186622927912003	0.951936553771603	-4.99748460760068
a_HQ_transcript/71411	0.779112982969683	2.85407216699774	1.34902431627156	0.186628996134035	0.951936553771603	-5.14973595039437
a_HQ_transcript/95412	1.02059478638743	1.74066115617993	1.34901890123151	0.186630717499008	0.951936553771603	-4.902194989879

a_HQ_transcript/139868	-0.571393812562722	4.37596672356086	-1.3489795290007	0.186643233745809	0.951936553771603	-5.44262784346751
a_HQ_transcript/146668	-0.229665827464054	10.1690936090227	-1.34896199067912	0.186648809304583	0.951936553771603	-6.0183974935221
a_HQ_transcript/90694	2.14624086294534	0.900216984783846	1.34894605503598	0.186653875471989	0.951936553771603	-4.90161942758307
a_HQ_transcript/128699	-1.63886714231748	0.735455774490825	-1.34892051540455	0.186661995105475	0.951936553771603	-4.93066808404789
a_HQ_transcript/61797	2.06780781601535	1.92948113808185	1.34887429712495	0.186676689648818	0.951936553771603	-5.05554877276048
a_HQ_transcript/112691	-0.911922812540239	2.37307411036231	-1.34877939500957	0.186706865432779	0.951936553771603	-4.99868834696058
a_HQ_transcript/35404	1.45553391657961	0.374435595250822	1.34876484435898	0.186711492399485	0.951936553771603	-5.02964149577778
a_HQ_transcript/59125	-1.69717875856591	2.6074766590924	-1.34871136247614	0.186728499884694	0.951936553771603	-5.06521729003135
a_HQ_transcript/42713	-0.916631419214956	3.07020359610922	-1.34866926166065	0.186741888980484	0.951936553771603	-5.11856046700733
a_HQ_transcript/8072	-1.41660189929739	1.73047332504286	-1.3485302319828	0.186786109112998	0.952066429136348	-4.96078307788351
a_HQ_transcript/49751	1.33769538373697	0.380221483040163	1.3484544546168	0.186810214462592	0.952066429136348	-4.87635043342204
a_HQ_transcript/64901	-1.92394066608879	0.200592954089358	-1.34830530890019	0.186857665860115	0.952066429136348	-5.04283425127656
a_HQ_transcript/104007	-1.92303533906031	1.37066288238958	-1.3482731164512	0.186867909258642	0.952066429136348	-5.18766771283066
a_HQ_transcript/18123	1.38803154789232	1.53525064169615	1.34817578773024	0.186898881175744	0.952066429136348	-4.9412147034545
a_HQ_transcript/135879	2.55437164908348	0.800675622131739	1.34813884462361	0.186910638239144	0.952066429136348	-5.08155364205284
a_HQ_transcript/10129	1.65801618538676	2.56321902003708	1.34812603934149	0.186914713625766	0.952066429136348	-5.01263164725051
a_HQ_transcript/12001	0.779726476093632	2.62730738938584	1.34811564782328	0.186918020862602	0.952066429136348	-5.23949889820018
a_HQ_transcript/113887	-1.90305125122547	0.685037465499265	-1.34805062887009	0.186938715023318	0.952066429136348	-4.82243184592998
a_HQ_transcript/68034	1.5216686502407	0.0310962332086883	1.34803340955647	0.186944195864051	0.952066429136348	-4.95248670065454
a_HQ_transcript/87627	-1.34023228816684	1.86077441033897	-1.34802292407862	0.18694753341182	0.952066429136348	-5.00892110769119
a_HQ_transcript/152254	0.46801569667652	10.8157297725339	1.34790835193052	0.186984004947975	0.952098844566817	-6.10487798966802
a_HQ_transcript/48080	-1.01004169432026	3.1187864966233	-1.34784638338258	0.187003733570616	0.952098844566817	-5.19696437303805
a_HQ_transcript/67309	1.31820542368978	0.804767230483739	1.3477677464098	0.187028771153975	0.952098844566817	-4.8755095437109
a_HQ_transcript/70898	-1.53539766497757	-0.0158473040017255	-1.34775112034861	0.187034065132984	0.952098844566817	-4.89575811721977
a_HQ_transcript/21552	-1.65345812665722	0.376268015715767	-1.34774452417329	0.187036165482956	0.952098844566817	-4.82153329062258
a_HQ_transcript/1850	2.03631734235082	0.456995657531959	1.34765217829603	0.187065572112838	0.952098844566817	-5.06947469320731
a_HQ_transcript/12108	-0.773733225725663	4.16345573477088	-1.34764281504646	0.187068553946422	0.952098844566817	-5.44801626332159
a_HQ_transcript/138319	2.21788126165281	-0.125508228967079	1.34756364492827	0.187093768041666	0.952106672297821	-4.98740511046755
a_HQ_transcript/90225	-1.68682390713999	0.989775819200305	-1.34753512751516	0.187102850909338	0.952106672297821	-4.94165192776892
a_HQ_transcript/38734	-1.15051015660553	2.09846307332787	-1.34718724605484	0.187213679463646	0.952119995763386	-5.06526477155325
a_HQ_transcript/63467	1.78481284313602	1.69641370321942	1.3471261517159	0.187233148217952	0.952119995763386	-4.98480619532034
a_HQ_transcript/70627	-0.945173382421986	2.4080036039543	-1.34698125439522	0.187279328478549	0.952119995763386	-5.09162065578088
a_HQ_transcript/88354	1.21666260860039	2.53114801099533	1.34697939022484	0.187279922666084	0.952119995763386	-4.99577511895145
a_HQ_transcript/73786	-1.69775716286177	2.38512710490362	-1.34685799373086	0.187318619842625	0.952119995763386	-5.14805713926911
a_HQ_transcript/67820	-1.74561305103312	1.38686989694584	-1.34683249740057	0.187326748012211	0.952119995763386	-4.97044093445146
a_HQ_transcript/100634	-1.27187200872787	0.505541043038119	-1.34682578605695	0.187328887617939	0.952119995763386	-4.91245494649299
a_HQ_transcript/140459	2.92934138254328	2.21996591702187	1.34682438582051	0.187329334021826	0.952119995763386	-4.95621093501302
a_HQ_transcript/138159	2.63820024755893	0.7372858103448	1.34677423221033	0.187345323839555	0.952119995763386	-5.10714904353188
a_HQ_transcript/59672	-1.56602028918091	0.910345630259085	-1.34673042026048	0.187359292691844	0.952119995763386	-4.94279542615953
a_HQ_transcript/137895	1.40342715263609	-0.170141699429326	1.34672434429367	0.187361229995866	0.952119995763386	-4.97796766075503
a_HQ_transcript/62426	-2.16244782765734	1.52264310923667	-1.34669128099921	0.187371772400647	0.952119995763386	-5.01145362131295
a_HQ_transcript/57660	1.60674001059448	0.568701434118452	1.34669040075032	0.187372053078886	0.952119995763386	-4.88233032874656
a_HQ_transcript/91021	-1.83528950627311	0.512618947793574	-1.34665786453018	0.187382427882684	0.952119995763386	-4.94189909394025
a_HQ_transcript/5613	0.818289095396772	1.96738211276259	1.3466051454431	0.18739923932662	0.952119995763386	-4.92461252023569
a_HQ_transcript/92833	2.01896635815584	0.06962484344599	1.34660034799245	0.18740076923024	0.952119995763386	-4.94313964576591
a_HQ_transcript/147925	2.1046305169021	1.74402467353789	1.34650899330614	0.187429904016616	0.952119995763386	-5.10441847792164
a_HQ_transcript/129783	-1.54634933492964	-0.199027030294642	-1.34647362186635	0.187441185596514	0.952119995763386	-5.01166189613876
a_HQ_transcript/41023	-2.28615964554723	1.7192296926777	-1.34646434503162	0.187444144492856	0.952119995763386	-5.087453025052
a_HQ_transcript/144063	2.37575061208001	0.445558369622503	1.34646305475509	0.187444556036355	0.952119995763386	-5.02673426997332
a_HQ_transcript/134574	1.97996378559626	1.22865432834158	1.34633486958952	0.187485445148221	0.952119995763386	-4.87992607209729

a_HQ_transcript/119044	-3.5478407847063	3.23355003060392	-1.34630372723017	0.187495380126009	0.952119995763386	-5.25458293645542
a_HQ_transcript/123968	2.42220963165054	2.81111041768318	1.34627626710343	0.187504140742197	0.952119995763386	-5.02899239428518
a_HQ_transcript/145863	5.61892171008694	1.26583422287795	1.34626355777837	0.187508195510751	0.952119995763386	-4.93117295077347
a_HQ_transcript/99513	-1.75469996626039	0.0910663354989083	-1.34622874628421	0.187519302076973	0.952119995763386	-4.90734682767495
a_HQ_transcript/80593	-2.47145029162894	1.2450158642126	-1.3461770161191	0.187535807463991	0.952119995763386	-5.10329122004416
a_HQ_transcript/47666	-1.41473600878142	-0.316933412510985	-1.34613967827672	0.187547721433574	0.952119995763386	-5.00340752869081
a_HQ_transcript/30884	-1.80288861532395	0.158225334273627	-1.34605177399754	0.187575772736048	0.95217924352467	-4.92166478166258
a_HQ_transcript/90042	4.52667382260643	1.9902927850966	1.34599548213298	0.187593737842746	0.952187285480542	-5.02365796023792
a_HQ_transcript/106636	4.03027571213434	0.85278075919858	1.34569699118337	0.18768902109681	0.952536839335268	-5.0553789585271
a_HQ_transcript/78228	1.49985477815625	-0.15103217122874	1.3456247985446	0.187712071795693	0.952536839335268	-4.90783332881584
a_HQ_transcript/87403	1.40996742194831	-0.507955899036149	1.34553378138074	0.187741136174505	0.952536839335268	-5.00032979091434
a_HQ_transcript/124491	2.47969242204359	3.46939555102798	1.34553057200246	0.187742161084053	0.952536839335268	-5.28666423441342
a_HQ_transcript/2482	1.35201929263115	-0.42956116188209	1.34552312561904	0.187744539090699	0.952536839335268	-4.94255801880213
a_HQ_transcript/143147	1.15941969807525	0.847981190675364	1.34544599744692	0.18776917138341	0.95257866941655	-4.89192329590441
a_HQ_transcript/43024	-0.393573329698979	4.22637602645428	-1.34536835744443	0.187793969659468	0.952621334179732	-5.39629620250201
a_HQ_transcript/151174	2.54784014935804	3.12719804649579	1.3452423708935	0.187834215242488	0.952742344120013	-5.1180806786086
a_HQ_transcript/82977	1.3201329256878	-0.267590149924607	1.34509859114417	0.187880152885296	0.952892201982068	-4.98625924961395
a_HQ_transcript/133886	1.48383003754619	-0.214834496528099	1.34485721239846	0.18795729292558	0.952959920302625	-4.94669587857307
a_HQ_transcript/47209	-1.19178798130386	0.152833923179862	-1.34480859067955	0.187972834458176	0.952959920302625	-4.92826181794976
a_HQ_transcript/26585	2.55456859102609	1.49958678013029	1.34464057491666	0.188026546954686	0.952959920302625	-5.02280637585465
a_HQ_transcript/137925	2.09976115943938	-0.017725671691684	1.34460356346698	0.188038380635896	0.952959920302625	-5.01777061336496
a_HQ_transcript/10631	-1.20697829949252	-0.425808099204007	-1.34458577288382	0.188044069028705	0.952959920302625	-4.94702480463779
a_HQ_transcript/111567	-4.36256600123376	1.27135602517689	-1.34456841693764	0.188049618577959	0.952959920302625	-5.02338816929745
a_HQ_transcript/125210	-1.07273174718513	4.57791916661106	-1.34453963086908	0.188058823178091	0.952959920302625	-5.47663098132963
a_HQ_transcript/108401	-0.750651430979086	4.92203019411813	-1.34446782779016	0.188081784364093	0.952959920302625	-5.503681924101
a_HQ_transcript/119631	3.4956169607394	0.653589093351756	1.34442537367801	0.188095361356848	0.952959920302625	-5.08506130698231
a_HQ_transcript/140635	2.97878116211575	0.44009527885154	1.34442026394108	0.188096995521923	0.952959920302625	-5.07278070082145
a_HQ_transcript/124755	-0.763665253210924	2.81936442318046	-1.34438494035509	0.188108292796806	0.952959920302625	-5.1843877287414
a_HQ_transcript/38638	1.38871093698335	1.86355558923005	1.34436452705899	0.188114821665505	0.952959920302625	-4.86776212573103
a_HQ_transcript/54165	2.51337250927685	0.9554509063927	1.34435673588134	0.188117313596127	0.952959920302625	-5.04516977446296
a_HQ_transcript/37694	-1.355801798842	1.92907540894599	-1.34430663648489	0.188133337998312	0.952959920302625	-4.96612283210131
a_HQ_transcript/16202	0.906163480427323	3.35332470296159	1.34428438260008	0.188140456290383	0.952959920302625	-5.09092725149469
a_HQ_transcript/15314	2.35629708639594	0.200475261531015	1.34420276857559	0.188166563735276	0.952959920302625	-4.97369531207297
a_HQ_transcript/66532	-2.85672898644965	1.09905507618588	-1.3441851328916	0.188172205567601	0.952959920302625	-5.06986171793614
a_HQ_transcript/107082	-2.90495175417438	1.32387452325997	-1.34409328465745	0.18820159085107	0.953025705461364	-5.02908673801934
a_HQ_transcript/120930	2.0808450779724	0.321046045513953	1.344026271714	0.188223032740226	0.953051136761772	-4.91273660070763
a_HQ_transcript/59103	-1.91096218726074	0.252473586194856	-1.34397510875186	0.188239404437825	0.953051136761772	-4.95129908154647
a_HQ_transcript/136584	2.58239121934421	2.0273120157343	1.34382506474714	0.188287423538697	0.953121633606753	-5.20931547198911
a_HQ_transcript/133872	-2.24474238030548	1.08228794969688	-1.34377385308455	0.188303815148408	0.953121633606753	-5.15152960942506
a_HQ_transcript/119082	3.1713874939699	2.9949615856096	1.34367622524497	0.188335066501192	0.953121633606753	-5.19206264254699
a_HQ_transcript/121463	3.0113087119991	1.15340189879572	1.34365745387326	0.188341075807197	0.953121633606753	-5.09616800079976
a_HQ_transcript/79228	4.3307006578861	0.712804112597982	1.34361706498061	0.188354006062896	0.953121633606753	-5.03791595766064
a_HQ_transcript/110125	-0.894569703239157	4.43120901255724	-1.34358989756027	0.188362703931008	0.953121633606753	-5.42151281133273
a_HQ_transcript/37942	3.05671071234418	-0.000726222413121632	1.34353668970333	0.188379739752629	0.953121633606753	-5.02300005040963
a_HQ_transcript/17125	1.45806311695403	-0.211280814059994	1.34340797674909	0.188420955331789	0.953121633606753	-5.01771423379768
a_HQ_transcript/15299	1.17341838536084	0.691753779624604	1.34339649055349	0.188424633700827	0.953121633606753	-4.90744916100467
a_HQ_transcript/141998	1.81473130363359	1.37731763215691	1.34337574423454	0.188431277696236	0.953121633606753	-4.97579129167474
a_HQ_transcript/1712	1.4190618670093	2.27908189271751	1.34336819669541	0.188433694835687	0.953121633606753	-5.06894963649176
a_HQ_transcript/38271	-1.6719303975582	1.55635364987144	-1.3432484456707	0.188472048939284	0.953232686047395	-4.87281944755855
a_HQ_transcript/60977	1.16336867581475	2.96524859172315	1.34312631826665	0.188511170360732	0.953347599680583	-5.24949995383228

a_HQ_transcript/69376	1.94067225318063	0.0186959110111834	1.34298596738572	0.188556137101108	0.95349205197157	-4.89836465753655
a_HQ_transcript/89385	-3.12179394529456	2.29477388280732	-1.34290492039939	0.188582107354773	0.953540425914946	-4.92477971442277
a_HQ_transcript/107655	1.0453531595084	2.59558914225997	1.3427851861372	0.188620479399748	0.953651494002655	-5.1562345392584
a_HQ_transcript/43796	1.80607738432653	0.111130653208773	1.34269762270958	0.188648545255509	0.953710439955839	-4.95683114225761
a_HQ_transcript/147431	2.0790485931349	1.87318128321181	1.34250388760626	0.188710652742174	0.953900803477825	-4.98485649845134
a_HQ_transcript/97876	-1.70896938113359	1.31457678202672	-1.34229765829497	0.188776782952683	0.953900803477825	-4.99668392955854
a_HQ_transcript/57549	-2.1776289544109	0.709342015904264	-1.34218257297483	0.188813694386609	0.953900803477825	-5.16127097608479
a_HQ_transcript/20855	0.917929015583045	2.34984576763622	1.34218031862495	0.188814417482192	0.953900803477825	-4.97225193953026
a_HQ_transcript/142485	2.58058686139426	2.38863738971211	1.34217273508694	0.188816849960953	0.953900803477825	-5.07767311453564
a_HQ_transcript/37921	2.09237263209171	0.726241826661783	1.34215884868779	0.188821304194099	0.953900803477825	-5.04630175065377
a_HQ_transcript/4184	-1.29530322935364	1.6883502722714	-1.34213911122005	0.188827635368915	0.953900803477825	-5.0194862827569
a_HQ_transcript/130575	1.7800019693878	4.66366604949601	1.34212874379136	0.188830960987959	0.953900803477825	-5.38426628036992
a_HQ_transcript/79043	-1.41206915458163	1.75586207791324	-1.34211960281293	0.188833893229069	0.953900803477825	-4.91114301818199
a_HQ_transcript/26626	1.8503253777189	-0.280373971333134	1.34192631343648	0.188895904780879	0.95398982310566	-5.01570893406822
a_HQ_transcript/146989	-2.32687770565766	0.263432022711509	-1.34189952169323	0.188904501410692	0.95398982310566	-5.06550363246631
a_HQ_transcript/132637	2.48975154030214	1.95476191799109	1.34185225073071	0.188919669915617	0.95398982310566	-5.02172531453424
a_HQ_transcript/5151	0.72537198299252	1.54658880326067	1.34182587339456	0.188928134394493	0.95398982310566	-4.91577287341131
a_HQ_transcript/114953	1.81619220650632	2.72634658457266	1.3418089198657	0.188933574931055	0.95398982310566	-5.16634924104625
a_HQ_transcript/4098	-1.6661425695011	0.708051401866538	-1.34156181658168	0.189012886270739	0.954180960666441	-4.93608264782563
a_HQ_transcript/90065	-2.19428818907037	0.769486367016125	-1.3415557957683	0.189014819057585	0.954180960666441	-4.98541125521597
a_HQ_transcript/56011	-1.66424408599547	1.56916438491468	-1.34151613196323	0.189027552216776	0.954180960666441	-4.90135166655915
a_HQ_transcript/106293	-2.23335460911299	0.539043900102711	-1.34148642442022	0.18903708957877	0.954180960666441	-4.92317102270004
a_HQ_transcript/119918	-1.74286314195706	3.57009459338694	-1.34138867792518	0.189068472906311	0.954256507171836	-5.258068214932
a_HQ_transcript/106202	0.378840981751005	3.27439894024108	1.34128345148053	0.18910226230321	0.954344183292326	-5.26198395004684
a_HQ_transcript/31336	-0.735390532393816	2.17557450384973	-1.34117535495061	0.189136978167638	0.954436519396514	-5.0132728166246
a_HQ_transcript/116172	-0.474228721700983	4.69193855650714	-1.34103860014429	0.189180904857915	0.954575315762228	-5.47384089020228
a_HQ_transcript/146438	-0.863944707611531	3.23612851929688	-1.34091543271722	0.18922047391353	0.954601170921337	-5.24875611541781
a_HQ_transcript/146068	-1.13156247571548	-0.0638341855221259	-1.3408438603115	0.189243470364615	0.954601170921337	-4.96690194799916
a_HQ_transcript/119720	-1.81430601222216	1.30050194529255	-1.34081777556505	0.189251852016082	0.954601170921337	-5.02524443105824
a_HQ_transcript/54653	-1.52190866202237	4.09514135168185	-1.34079063884464	0.189260571995793	0.954601170921337	-5.2898848311621
a_HQ_transcript/113236	1.57545624747885	1.14343690140482	1.34074332187153	0.189275777339557	0.954601170921337	-5.02031907938631
a_HQ_transcript/23346	-0.478545101584574	4.69153581087554	-1.34071598245886	0.189284563309237	0.954601170921337	-5.46840361771727
a_HQ_transcript/40483	-2.74572129368274	0.772207017768508	-1.34064575742964	0.189307132178582	0.954632168878785	-5.00738273741165
a_HQ_transcript/43300	1.88975625149136	3.48965791419604	1.34058657567678	0.18932615457451	0.954645273517275	-5.27068125046385
a_HQ_transcript/136737	-2.11495698000893	0.640468392829598	-1.34031362732218	0.189413903131922	0.954819495824313	-5.00123308904271
a_HQ_transcript/139826	1.28257019751382	-0.342574855097507	1.34029730951451	0.18941915004122	0.954819495824313	-5.10651982663307
a_HQ_transcript/20945	-0.746872817414428	2.57858187818493	-1.34024552858722	0.189435800681197	0.954819495824313	-5.09829943575836
a_HQ_transcript/73308	1.41562025277327	0.00384495877608024	1.34023817544675	0.189438165243608	0.954819495824313	-5.03806924530939
a_HQ_transcript/115613	-1.54701705312513	-0.156693740393301	-1.340223646907	0.189442837278795	0.954819495824313	-4.90737649528029
a_HQ_transcript/96325	0.962694196107165	2.50939627355591	1.34014874098225	0.189466926664981	0.954858116280734	-5.07493624300808
a_HQ_transcript/24086	1.12133402604144	3.21160868491415	1.34009400506935	0.189484530965895	0.954864049933726	-5.32387643427898
a_HQ_transcript/124306	-2.90102574656403	2.01583989256549	-1.33986754464981	0.189557379137697	0.955148347305484	-5.11987289959087
a_HQ_transcript/142954	-1.95572986833022	1.1158506756194	-1.33979046050353	0.189582180623039	0.955190517054348	-5.02920898018364
a_HQ_transcript/135004	3.58653681882712	1.3345869100224	1.33966790276355	0.189621618189351	0.955257619208298	-5.21127521346059
a_HQ_transcript/64758	1.58967290921194	-0.304611629803321	1.33963087740879	0.189633533735211	0.955257619208298	-5.00018237896892
a_HQ_transcript/3847	-1.43312653933291	-0.275693799205111	-1.33959587169181	0.189644799849707	0.955257619208298	-4.98638304302917
a_HQ_transcript/139338	1.45918406296353	2.7091515888502	1.33947529168265	0.189683610838539	0.955269427236931	-5.10391373636314
a_HQ_transcript/13385	1.58662149354215	-0.002479268558855	1.33945791798844	0.189689203400031	0.955269427236931	-5.00793706746845
a_HQ_transcript/141346	-2.72056526413765	1.13803511340995	-1.33943541969254	0.189696445750686	0.955269427236931	-4.92189409907109
a_HQ_transcript/66355	1.61617278051337	0.79443187436139	1.33929393667674	0.189741994958158	0.955303860825739	-4.90634971904223

a_HQ_transcript/82911	-1.08030459408806	1.30299383700808	-1.33925990876332	0.189752951202286	0.955303860825739	-5.05574099531455
a_HQ_transcript/143288	-2.17837640885306	1.10445449740734	-1.33919522779114	0.189773778405944	0.955303860825739	-5.00619771778157
a_HQ_transcript/125543	2.22892704958343	0.675912840049056	1.33915304757368	0.189787361340176	0.955303860825739	-4.99589026923853
a_HQ_transcript/107513	2.57047213957231	1.94007354665401	1.33910908459104	0.189801519160759	0.955303860825739	-5.1395681600396
a_HQ_transcript/121488	1.21899693529444	2.00940454036104	1.33910793166262	0.189801890460302	0.955303860825739	-4.98494684293846
a_HQ_transcript/72537	-2.33932032377786	2.01746810203364	-1.33892975818644	0.189859277796459	0.955391042741229	-5.03437816145309
a_HQ_transcript/147747	-0.65855909400563	3.83886632557933	-1.33887868490664	0.189875730295613	0.955391042741229	-5.32982144560333
a_HQ_transcript/144621	3.29616356710863	2.21209359517447	1.33884188915316	0.189887584182729	0.955391042741229	-5.19922876385439
a_HQ_transcript/100951	1.74854126329579	0.226227909168117	1.33881515883297	0.189896195811314	0.955391042741229	-5.01647053002343
a_HQ_transcript/121116	0.906775060404415	3.31602046049399	1.33875656252488	0.189915074664872	0.955391042741229	-5.29138539356976
a_HQ_transcript/1374	-0.909215833090048	2.71326378750447	-1.33874801746025	0.189917827877699	0.955391042741229	-5.09727582589966
a_HQ_transcript/115731	3.20764992872177	2.98051935553651	1.33865561960853	0.189947600355131	0.955458126812308	-5.1029184305481
a_HQ_transcript/23582	2.43709740996102	0.949736191004581	1.33855450360605	0.189980186122179	0.955539349217794	-4.97110061342873
a_HQ_transcript/568	1.56740718381654	1.54079248611749	1.3383155841456	0.190057197721061	0.955764942656122	-5.01888265745426
a_HQ_transcript/6855	1.73164329953805	0.161052856501887	1.33831333312575	0.190057923413194	0.955764942656122	-4.90509101556382
a_HQ_transcript/76453	-1.29872407424468	1.6209351094222	-1.3380699936818	0.190136384682415	0.955793422288881	-4.88243469722001
a_HQ_transcript/128753	-1.24644753500752	-0.562674980203101	-1.33798557502725	0.190163610083648	0.955793422288881	-5.0261930227246
a_HQ_transcript/3100	-1.28229554406562	2.83633551807177	-1.33794852219122	0.19017556074111	0.955793422288881	-5.01246915791879
a_HQ_transcript/81007	1.8362900840969	0.453407294429459	1.33783155649084	0.190213289505998	0.955793422288881	-5.01540017356688
a_HQ_transcript/89285	0.801075209515476	3.90086327865537	1.33771123326957	0.190252107301063	0.955793422288881	-5.36160111230419
a_HQ_transcript/138668	0.347529081089289	6.07911171496528	1.3377109173471	0.190252209229687	0.955793422288881	-5.59991120067012
a_HQ_transcript/41624	2.56472506544847	2.32909214897755	1.33763755703749	0.190275879200206	0.955793422288881	-5.17534033210301
a_HQ_transcript/65909	1.63093955249547	1.00689343055779	1.33760049402689	0.190287838578212	0.955793422288881	-4.90378196459249
a_HQ_transcript/58493	1.82614502455823	0.647070610168674	1.33755674004737	0.190301957723046	0.955793422288881	-4.89922371912632
a_HQ_transcript/104336	2.35945276421718	0.803717083345397	1.3375508642506	0.190303853868655	0.955793422288881	-4.95999687644395
a_HQ_transcript/49648	-1.34268615720567	2.88437781711272	-1.3374873747098	0.190324343152944	0.955793422288881	-5.05055320705284
a_HQ_transcript/76217	1.28227785062634	2.72477512884885	1.33745497050903	0.190334801261083	0.955793422288881	-5.24200064390436
a_HQ_transcript/43365	-1.64781206219419	0.234906153218409	-1.33731017284091	0.190381538565857	0.955793422288881	-5.03818105052875
a_HQ_transcript/17212	1.72181172018034	0.0728085843269847	1.33730392791874	0.190383554479774	0.955793422288881	-4.99167898020636
a_HQ_transcript/143766	1.54339596025203	3.81247106259999	1.33727350270458	0.190393376232612	0.955793422288881	-5.2027830614323
a_HQ_transcript/141087	3.71844936731121	0.359260867821007	1.33722392971348	0.190409380034937	0.955793422288881	-5.0283609426927
a_HQ_transcript/138606	-0.569060494370691	5.00845780247951	-1.33713416458582	0.190438361827248	0.955793422288881	-5.47345600021522
a_HQ_transcript/11438	-0.954947886970354	3.18168550660528	-1.33712565287506	0.190441110115782	0.955793422288881	-5.30034099185826
a_HQ_transcript/795	0.976015951834868	3.79260264367776	1.33703517707181	0.190470325123016	0.955793422288881	-5.35853933965523
a_HQ_transcript/101513	1.45522250829934	1.62021533453881	1.33701504647186	0.190476825845796	0.955793422288881	-4.90668282717472
a_HQ_transcript/128054	-1.86350552921989	2.29223378641952	-1.33694282086669	0.190500150881463	0.955793422288881	-5.02492867367861
a_HQ_transcript/25780	1.42749848942603	0.291372134409216	1.33692356281703	0.190506370580638	0.955793422288881	-4.98353963005549
a_HQ_transcript/128105	-3.75484158992221	1.53878957210558	-1.33687606475845	0.190521711517147	0.955793422288881	-4.87220054053757
a_HQ_transcript/148932	-5.54968805327362	1.96051737028821	-1.33678770909813	0.190550251185721	0.955793422288881	-5.12214630273406
a_HQ_transcript/92273	2.49301217961307	0.996359714380088	1.33667746816064	0.190585864613379	0.955793422288881	-5.1163805170753
a_HQ_transcript/73460	-2.25957193408724	3.49446428343798	-1.33665550326407	0.190592961004361	0.955793422288881	-5.18912223811015
a_HQ_transcript/20463	1.47376477515726	0.0990110383111511	1.33652035174753	0.190636630065509	0.955793422288881	-4.97187138201705
a_HQ_transcript/17189	1.22340099804554	1.35601574529029	1.3364549340712	0.190657770062635	0.955793422288881	-4.9714339022242
a_HQ_transcript/102237	2.25164368956395	2.70619094464507	1.3364306640391	0.190665613484848	0.955793422288881	-5.25735774969753
a_HQ_transcript/46455	-1.85849842771569	2.3374606164877	-1.3363886992787	0.190679175954295	0.955793422288881	-5.09660363245997
a_HQ_transcript/100872	1.37859709402125	0.181353117453211	1.33624400315612	0.190725945574912	0.955793422288881	-4.95094791855409
a_HQ_transcript/148853	-2.33598729412957	2.00412687900697	-1.33620793828317	0.190737604073166	0.955793422288881	-5.09094863876445
a_HQ_transcript/132125	2.78457779604255	0.963553017051055	1.33617521660193	0.190748182310769	0.955793422288881	-5.09393699290295
a_HQ_transcript/7933	-0.824374062371316	3.61235595768947	-1.33616037767324	0.190752979576107	0.955793422288881	-5.27524928612817
a_HQ_transcript/104562	-2.80132426451567	3.77277624728762	-1.33615242552743	0.190755550457188	0.955793422288881	-5.29951594231474

a_HQ_transcript/56769	0.400645425129536	3.77097763413591	1.33613834701791	0.190760102020404	0.955793422288881	-5.33923601689272
a_HQ_transcript/82465	-3.09559622521848	0.133089203015777	-1.33613041004277	0.190762668070682	0.955793422288881	-5.01518362955406
a_HQ_transcript/95975	-1.51151885093948	1.28130986674784	-1.33609144065481	0.190775267388314	0.955793422288881	-4.95270206959879
a_HQ_transcript/47813	-1.66589906779128	-0.118877414706966	-1.33603241810983	0.190794351377091	0.955793422288881	-5.00996683740153
a_HQ_transcript/143662	0.505335041577503	3.00369620990477	1.33602303134936	0.190797386570355	0.955793422288881	-5.14912781014489
a_HQ_transcript/104044	1.59440387016833	-0.0623282567448448	1.33600679498912	0.190802636658035	0.955793422288881	-5.00583087502026
a_HQ_transcript/9331	0.629563304491609	3.68393711987337	1.33590213329021	0.19083648207981	0.955793422288881	-5.34421447734968
a_HQ_transcript/53205	-1.84655408802207	1.16478995090616	-1.33585573847742	0.190851486678925	0.955793422288881	-5.01132669139919
a_HQ_transcript/138275	0.681746689467597	2.42393912038802	1.33570266370754	0.190900999209719	0.955793422288881	-5.04261139675359
a_HQ_transcript/111674	1.32485414445898	2.61924841529286	1.33566373365104	0.190913592840731	0.955793422288881	-5.14730388630105
a_HQ_transcript/99618	-2.9028521722674	5.2032925972733	-1.33566282119431	0.190913888022479	0.955793422288881	-5.66485597906138
a_HQ_transcript/115161	2.45433447144256	3.56463705128085	1.33559718828459	0.190935121334474	0.955793422288881	-5.11411939143002
a_HQ_transcript/81625	0.448035065122925	3.57336221223891	1.33549737592941	0.190967415738507	0.955793422288881	-5.25222725430007
a_HQ_transcript/84780	0.529223616085425	3.13322618848877	1.3354969990046	0.190967537700942	0.955793422288881	-5.22723077581602
a_HQ_transcript/10025	-1.36395009006749	0.623474889544284	-1.33530485650955	0.191029717504594	0.955793422288881	-5.01942077564065
a_HQ_transcript/62573	-1.80341866833476	-0.234781749847793	-1.33520541621837	0.191061903795274	0.955793422288881	-5.01566568416403
a_HQ_transcript/64729	2.38964579720503	0.870390658828161	1.33495384947336	0.191143348194195	0.955793422288881	-5.021467680895
a_HQ_transcript/80378	-1.94134761207803	0.0194046640907668	-1.33492922962648	0.191151320272964	0.955793422288881	-4.99809610751598
a_HQ_transcript/43568	1.67140982408038	0.711666318785363	1.33488477922527	0.191165714272518	0.955793422288881	-4.95678675720238
a_HQ_transcript/113453	-2.45666365969859	0.0865227200574304	-1.33487112651622	0.191170135482013	0.955793422288881	-4.91965932874326
a_HQ_transcript/70454	-3.22767173616009	0.952116298914895	-1.3348145816812	0.191188447452712	0.955793422288881	-5.10422684474324
a_HQ_transcript/59306	-1.05612253609551	2.22624279646699	-1.33469321163971	0.191227757542827	0.955793422288881	-5.0611229214896
a_HQ_transcript/135554	-2.53704868653327	0.230429558273229	-1.33466970866796	0.191235370551967	0.955793422288881	-5.01804816336634
a_HQ_transcript/6818	2.08359618827273	0.498961805048575	1.3346339368089	0.191246958108663	0.955793422288881	-4.95075595563451
a_HQ_transcript/23518	-1.31462344711405	2.19256908110852	-1.3346181467654	0.191252073139909	0.955793422288881	-5.00824612683521
a_HQ_transcript/140759	-1.66399211837076	0.138266906383264	-1.33460959913057	0.191254842107151	0.955793422288881	-4.94749599435594
a_HQ_transcript/83788	-2.07565837894319	1.11412591389878	-1.33460016133762	0.191257899472908	0.955793422288881	-4.88335298311232
a_HQ_transcript/13116	-1.86007236568469	1.36034250420509	-1.33451798221485	0.191284522927013	0.955793422288881	-5.02107521393381
a_HQ_transcript/74032	-1.25190395413773	2.15528162304723	-1.33446494670529	0.191301706281038	0.955793422288881	-5.11262350516015
a_HQ_transcript/46688	-1.49010674239149	-0.295417126525946	-1.33443739266926	0.191310634179378	0.955793422288881	-4.95797315952877
a_HQ_transcript/144652	1.74077729405643	4.41309956186419	1.33443501955607	0.191311403116702	0.955793422288881	-5.34198234365038
a_HQ_transcript/134823	-1.92742673167721	0.89087530402209	-1.3343742293262	0.191331101208721	0.955793422288881	-5.00474161594486
a_HQ_transcript/34680	-1.94634561058533	0.452014699042237	-1.33434008968499	0.191342164292743	0.955793422288881	-4.92762329340244
a_HQ_transcript/95218	2.52182916330482	2.96859435753798	1.33433933401153	0.191342409177249	0.955793422288881	-5.3511741029541
a_HQ_transcript/113882	-1.58071279087614	0.25541042766934	-1.33433492509749	0.191343837940373	0.955793422288881	-5.03648710293871
a_HQ_transcript/23129	1.79127002868283	1.18469813664145	1.33433485279916	0.191343861369611	0.955793422288881	-4.86163287546887
a_HQ_transcript/143570	-1.68665874236137	0.865283214031922	-1.33426319428974	0.191367084349846	0.955793422288881	-4.94045173875892
a_HQ_transcript/121762	1.37544553092985	2.39881600180633	1.33426296268097	0.191367159412778	0.955793422288881	-5.02902976642077
a_HQ_transcript/79573	-6.91955341393189	2.5747039551606	-1.33418359174172	0.191392884366239	0.955793422288881	-4.93178374695235
a_HQ_transcript/339	2.45085179709137	1.19588152965769	1.33416530635527	0.19139881122901	0.955793422288881	-4.98756714936634
a_HQ_transcript/116560	1.2287350531648	1.57342071377926	1.33413731471661	0.191407884463907	0.955793422288881	-4.91857759088718
a_HQ_transcript/139483	2.06713347256613	1.05988316283033	1.33411033829775	0.191416628937903	0.955793422288881	-5.05267087205592
a_HQ_transcript/138175	2.41530790510205	0.953132395936989	1.33404550760659	0.191437645227231	0.955793422288881	-5.04062315178527
a_HQ_transcript/124138	-2.64847800887123	0.177466698830553	-1.33404251779505	0.191438614483013	0.955793422288881	-5.01449180392674
a_HQ_transcript/70617	-1.68673445528277	0.517957995648527	-1.3339836364734	0.191457703767662	0.955793422288881	-4.98763543500138
a_HQ_transcript/18461	0.862740471170275	2.47421008437014	1.33397898404131	0.191459212145468	0.955793422288881	-5.02725383873943
a_HQ_transcript/111556	-2.03902222389336	2.04965540313721	-1.33397124693257	0.191461720635034	0.955793422288881	-5.16591851187126
a_HQ_transcript/142628	-0.488599941220932	3.70664320191621	-1.33396427411698	0.191463981350692	0.955793422288881	-5.32731718406764
a_HQ_transcript/67260	2.23202589281579	0.30516328418651	1.33394412301431	0.191470514826047	0.955793422288881	-5.01694222270018
a_HQ_transcript/20754	1.97014623658442	0.465165264085008	1.33387364482099	0.191493366912599	0.955793422288881	-5.00200369701979

a_HQ_transcript/118020	-1.20733561992091	1.56388328436919	-1.33385636240539	0.191498970941751	0.955793422288881	-4.91272224782824
a_HQ_transcript/94909	-1.10548297257831	2.27105728378546	-1.33379553900081	0.191518694659406	0.955793422288881	-5.0445633858293
a_HQ_transcript/135525	0.744503483316019	3.52437922661058	1.33378982754862	0.19152054684043	0.955793422288881	-5.34630834974842
a_HQ_transcript/16492	1.08534512339968	1.51253387621381	1.33369205692042	0.191552255258969	0.955793422288881	-4.94276903924286
a_HQ_transcript/89380	-1.59710993126643	0.754357566691815	-1.33365518837522	0.191564213306814	0.955793422288881	-5.01651479021344
a_HQ_transcript/17769	-0.830512488395582	2.14784548611724	-1.33362815569006	0.191572981529247	0.955793422288881	-5.01414207795576
a_HQ_transcript/119566	0.42301274436515	6.5499082247568	1.33358365661076	0.191587415759984	0.955793422288881	-5.67119681474531
a_HQ_transcript/13068	1.0565158943647	1.82394306967957	1.33356278438638	0.191594186402039	0.955793422288881	-4.98417349455588
a_HQ_transcript/55897	0.819098329776892	1.46793381277573	1.33345673694873	0.191628589473662	0.955793422288881	-4.990974087865
a_HQ_transcript/34056	-0.577472331454898	3.31581585723849	-1.33342340105291	0.191639405023833	0.955793422288881	-5.31280495128511
a_HQ_transcript/148593	-3.76495515556955	0.87608566932416	-1.33340705481839	0.191644708593417	0.955793422288881	-5.13203918155165
a_HQ_transcript/82522	1.79873086856591	1.21974871673869	1.33336439899522	0.191658548895527	0.955793422288881	-4.90817126390968
a_HQ_transcript/109950	1.94733561819576	0.596693531571049	1.33328800661501	0.191683337436941	0.955835038048769	-4.96785497435657
a_HQ_transcript/107110	-4.05260067824848	3.25082835305566	-1.33322665002833	0.191703248805045	0.955852328576057	-5.10299047910097
a_HQ_transcript/113820	0.878026447433844	1.3802360856184	1.33300478790021	0.191775260531224	0.955963254157739	-4.89314929335678
a_HQ_transcript/86314	0.876067233119812	3.76516372038463	1.33297818119929	0.191783897898197	0.955963254157739	-5.32438207475236
a_HQ_transcript/82286	1.7134555120322	3.64885446636673	1.33296204582094	0.191789136092326	0.955963254157739	-5.3265355979872
a_HQ_transcript/64666	2.48872458843943	0.903393216558214	1.33292602042057	0.19180083178664	0.955963254157739	-5.01440983518844
a_HQ_transcript/64735	1.3357709274385	-0.248254636573797	1.33280177248581	0.191841173255958	0.955963254157739	-5.0207984230961
a_HQ_transcript/136686	0.837869010475787	1.25099698331625	1.33268157969472	0.191880204293867	0.955963254157739	-5.0779098272977
a_HQ_transcript/69049	3.34113149962471	2.62106772728102	1.33267319794931	0.191882926384073	0.955963254157739	-5.07415506461714
a_HQ_transcript/2728	1.64687645981863	3.34426717629939	1.33263192622076	0.191896330394311	0.955963254157739	-5.26955574489781
a_HQ_transcript/72748	0.672541140279378	2.83397302538561	1.33256422468121	0.191918319697096	0.955963254157739	-5.10952555523454
a_HQ_transcript/116047	-1.38181467648721	-0.281124291169653	-1.33248284737846	0.191944753422226	0.955963254157739	-4.99644255155252
a_HQ_transcript/91850	1.85938687633677	0.333842277346711	1.33247782153209	0.191946386055803	0.955963254157739	-4.99353703123655
a_HQ_transcript/11610	-1.60526099926606	0.251633478912411	-1.33244621550934	0.191956653437872	0.955963254157739	-4.97762421520473
a_HQ_transcript/122160	0.942648759679046	2.41267046974729	1.33243703173462	0.191959636914447	0.955963254157739	-5.14239969559824
a_HQ_transcript/22084	0.75975168854672	3.48370493589453	1.33235640168741	0.19198583223543	0.955963254157739	-5.27708217963889
a_HQ_transcript/138294	3.83393220519695	2.20437826376469	1.3323497851472	0.191987981958114	0.955963254157739	-5.21039413870382
a_HQ_transcript/99098	-2.11819381550123	0.576956396196883	-1.33230169948672	0.192003605609427	0.955963254157739	-4.96452314361489
a_HQ_transcript/34404	1.65830824382507	0.238542933500199	1.33217845843057	0.192043652685348	0.955963254157739	-4.9041461736125
a_HQ_transcript/136940	2.06349111159821	0.457093179261529	1.33214230452284	0.192055402086055	0.955963254157739	-5.02546979666502
a_HQ_transcript/113434	-1.94899904594778	2.74586132290357	-1.33196606311988	0.192112685443596	0.955963254157739	-5.13344851623088
a_HQ_transcript/116504	1.60958887257437	-0.132620620790177	1.33194132856394	0.192120725914084	0.955963254157739	-5.09408259637829
a_HQ_transcript/105149	-1.39995300672781	1.49783110280653	-1.33191809772996	0.192128277804955	0.955963254157739	-4.92402078557123
a_HQ_transcript/107195	-1.94315793809778	0.745681223466963	-1.33190693185915	0.192131907692976	0.955963254157739	-5.02015534764834
a_HQ_transcript/3842	-1.48316816318071	1.33371214365546	-1.33188833652121	0.192137952927	0.955963254157739	-4.8367860511019
a_HQ_transcript/87331	-0.99388017230139	2.81399298702555	-1.33188780616376	0.192138125345205	0.955963254157739	-5.11122645323401
a_HQ_transcript/61783	1.84664291120667	1.03329382937044	1.33185584632857	0.192148515647076	0.955963254157739	-4.9765436586759
a_HQ_transcript/18047	0.811769605617931	2.57245279329482	1.33184178450912	0.19215308735169	0.955963254157739	-5.0955726215112
a_HQ_transcript/97645	1.21419718898656	-0.577472296148527	1.33177146806395	0.192175949519302	0.955995172756353	-5.00318910807283
a_HQ_transcript/99327	2.90216493600016	0.600125962535536	1.33172074143938	0.192192443697362	0.955995410511744	-4.95888656429363
a_HQ_transcript/32462	2.97909522614471	-0.208804281695133	1.33166155446909	0.192211690203063	0.956009337565128	-4.92945876286215
a_HQ_transcript/1839	2.38081035654783	1.46540280010841	1.33154359513045	0.19225005281633	0.956118332486966	-4.96117516596036
a_HQ_transcript/32938	1.59198299626307	0.522692143997124	1.33142285080573	0.192289327260555	0.956214506786328	-4.86220925339053
a_HQ_transcript/40264	2.01039983823313	0.459051629170573	1.3313345903853	0.192318039586838	0.956214506786328	-5.09487396469003
a_HQ_transcript/150778	-0.231704060893074	11.0631424002664	-1.33128049662158	0.192335638660837	0.956214506786328	-6.15232391950231
a_HQ_transcript/38154	-1.59477879351729	1.36910305237528	-1.33125554758795	0.192343756093277	0.956214506786328	-5.00495853340843
a_HQ_transcript/76097	-1.3421026125271	0.537736710304807	-1.33119698553047	0.192362810914534	0.956214506786328	-4.99497612316806
a_HQ_transcript/13340	1.84758039983132	0.277361108796127	1.33118075621557	0.192368091838001	0.956214506786328	-5.00639454487349

a_HQ_transcript/89252	-2.02924233268746	1.20541887955857	-1.33108550675757	0.192399087698649	0.956286803740663	-4.90216855329087
a_HQ_transcript/139042	-2.15528800077291	0.568802938592098	-1.33093037961325	0.192449577042595	0.956383342320124	-4.99917754428678
a_HQ_transcript/2133	0.793479149772796	1.97263257614999	1.33092472763295	0.192451416789129	0.956383342320124	-4.96288937414625
a_HQ_transcript/140811	0.483815900761488	4.03414680555194	1.3307607921055	0.192504784471988	0.956566773105466	-5.40162285168078
a_HQ_transcript/113121	-1.86926120384403	0.574633604941905	-1.33052433256793	0.192581781720488	0.956598170149043	-5.06519062603648
a_HQ_transcript/87167	-1.78598911807151	0.735515800101079	-1.33047753932928	0.192597021598083	0.956598170149043	-4.94382221866038
a_HQ_transcript/30495	2.63805082756746	2.71055297188442	1.33047244740055	0.19259868002121	0.956598170149043	-5.05673590385437
a_HQ_transcript/2879	2.44880113047662	0.569542428426289	1.33045118556617	0.192605605043927	0.956598170149043	-4.95224807557104
a_HQ_transcript/34036	4.45869545320986	1.7000398958094	1.33043631372857	0.192610448945514	0.956598170149043	-4.92354315819244
a_HQ_transcript/71134	1.03583095571155	0.483952757098916	1.33039569938247	0.192623677908849	0.956598170149043	-5.00934921403528
a_HQ_transcript/84900	0.59962578362835	2.93442422927318	1.33038764873172	0.192626300261398	0.956598170149043	-5.17076107943711
a_HQ_transcript/33730	-2.22290751662067	1.04332789086069	-1.33028113004119	0.192660999365836	0.956620298286648	-5.19932592581389
a_HQ_transcript/4387	2.03688653496208	1.06311088813889	1.33027293121107	0.192663670383323	0.956620298286648	-5.08926540622454
a_HQ_transcript/21034	-0.601248942549759	3.60048168351697	-1.33012517258082	0.19271181211861	0.956664860872935	-5.4008785775517
a_HQ_transcript/9668	1.10644527267509	1.09734770308677	1.33009880858333	0.192720402833637	0.956664860872935	-4.93318414361591
a_HQ_transcript/130266	-1.3939875687957	2.21812153374792	-1.33001990149466	0.192746116487619	0.956664860872935	-5.09737409553092
a_HQ_transcript/152029	-3.15014670812875	0.687558668243577	-1.33001324459482	0.192748285909203	0.956664860872935	-5.02746542932708
a_HQ_transcript/105432	0.694926985299647	2.74678666557585	1.32999284271542	0.19275493480842	0.956664860872935	-5.13843399796944
a_HQ_transcript/9949	2.50223339974354	1.46782236353287	1.32982294387805	0.192810311079746	0.956858000720862	-5.19194234458089
a_HQ_transcript/18962	-2.70531768491203	1.99939356720489	-1.3297227599851	0.19284297042348	0.9569383801243	-4.96141518329084
a_HQ_transcript/12915	1.84056342469993	1.48369419656129	1.32951727777081	0.192909969693744	0.957071873412715	-4.87785444249118
a_HQ_transcript/124605	1.70806864181213	2.68030631788022	1.32951127991186	0.19291192561684	0.957071873412715	-5.24223653243449
a_HQ_transcript/90268	2.68074351587475	0.694536275044636	1.32943048694938	0.192938273974893	0.957071873412715	-5.12729602529809
a_HQ_transcript/89088	1.39757658662004	2.4304829732417	1.32940196252655	0.192947577076033	0.957071873412715	-5.07551685034927
a_HQ_transcript/102144	2.89250998125878	1.64547726396929	1.3293561740972	0.192962511469748	0.957071873412715	-5.039702661035
a_HQ_transcript/129079	-1.50133703800719	-0.554610817690906	-1.32931942695545	0.192974497592133	0.957071873412715	-5.0254782244341
a_HQ_transcript/71018	-1.68382464051284	1.83892718104872	-1.3292746280196	0.192989110809841	0.957071873412715	-4.95955781973108
a_HQ_transcript/83556	0.998461554377839	1.9442264452328	1.32923636927815	0.193001591318192	0.957071873412715	-5.00844942047259
a_HQ_transcript/77889	3.27209449957243	0.59544248345795	1.32918322127788	0.193018929929822	0.957076205660724	-5.02682049865631
a_HQ_transcript/132412	0.851613273420411	2.91398034422252	1.32904582714363	0.193063757928469	0.957123506216133	-5.20853959152809
a_HQ_transcript/149293	-4.09592512542117	2.96978768156816	-1.3290284411794	0.193069431069938	0.957123506216133	-5.25743653857256
a_HQ_transcript/67426	-2.47956458657543	2.97944962025206	-1.32893724773046	0.193099190123085	0.957123506216133	-5.18561240980996
a_HQ_transcript/10807	-1.58106168285391	-0.0625858172944164	-1.32892556154461	0.193103003917547	0.957123506216133	-4.96132945234849
a_HQ_transcript/7150	1.26201137381066	4.13195206286764	1.32890167877917	0.193110798253902	0.957123506216133	-5.4412474244716
a_HQ_transcript/82184	-1.2965985790316	-0.498916620797701	-1.32885119904876	0.193127273523763	0.957123553192027	-5.01168568369114
a_HQ_transcript/23460	-1.23977935342688	3.17676548142768	-1.32872417524265	0.193168735567864	0.957146627214195	-5.32266883387431
a_HQ_transcript/1135	1.89043724819601	-0.0568810678266666	1.32872253420072	0.193169271267758	0.957146627214195	-5.0783661810142
a_HQ_transcript/126938	-0.613271833162161	2.39298297445089	-1.328685601193	0.193181327939666	0.957146627214195	-5.04059653075164
a_HQ_transcript/35981	0.51219751833878	3.12058924010302	1.32852399847028	0.193234089477344	0.957159332181641	-5.22708533583998
a_HQ_transcript/95493	1.46803455033097	2.56392797978562	1.32843451657479	0.193263309111059	0.957159332181641	-5.09458023093205
a_HQ_transcript/132419	1.13061671680176	3.02232213369538	1.32830356404443	0.193306076787803	0.957159332181641	-5.2122313748688
a_HQ_transcript/77490	0.594522480776312	4.33509461854803	1.32828629517061	0.193311717157652	0.957159332181641	-5.44653388090499
a_HQ_transcript/152583	-1.26001789910358	5.35768319062571	-1.32827233331792	0.193316277478771	0.957159332181641	-5.54885017881084
a_HQ_transcript/136729	3.67997435352626	1.66161509726296	1.32826159849509	0.193319783820333	0.957159332181641	-5.20274149018816
a_HQ_transcript/113732	-0.962073850626371	1.86745229765909	-1.32824882725212	0.193323955386484	0.957159332181641	-5.01363500453564
a_HQ_transcript/59530	-3.44538826575903	1.09263458964971	-1.32822653402818	0.193331237353954	0.957159332181641	-5.06612031709999
a_HQ_transcript/59197	-1.22378249536398	2.7072764890147	-1.32818136545732	0.19334599208271	0.957159332181641	-5.12856212925675
a_HQ_transcript/100781	1.52277693104733	1.12602047398136	1.32812708963605	0.193363722923365	0.957159332181641	-4.93219394425324
a_HQ_transcript/142137	-2.1170250407678	0.188966502836137	-1.32812311113367	0.193365022670813	0.957159332181641	-4.96498312426705
a_HQ_transcript/4404	-1.74632397425138	0.346891142999825	-1.32805824178073	0.193386215958333	0.957182728306029	-4.96722510761242

a_HQ_transcript/115059	1.90772405010992	1.51256019889694	1.3280039097225	0.19340396800918	0.957189089181576	-5.069762595755
a_HQ_transcript/97609	-1.82033367403796	-0.391840342825664	-1.32794800729492	0.193422234458041	0.957197994600458	-5.03918288943128
a_HQ_transcript/93163	-1.46071438301806	0.836901174198557	-1.32776033216992	0.193483568113245	0.95739966340128	-4.88899616349795
a_HQ_transcript/122783	-1.99814298598251	0.455969883329381	-1.32769285913199	0.193505622466636	0.95739966340128	-4.92491218049598
a_HQ_transcript/117951	0.393092084084891	3.26697376770157	1.3276721326171	0.193512397558864	0.95739966340128	-5.28214998779944
a_HQ_transcript/143371	-1.97992853537865	1.87149124744145	-1.32751917007848	0.193562403657526	0.957537751400081	-5.02083885484786
a_HQ_transcript/22099	-1.47042499905964	2.47586513712794	-1.3274859837869	0.193573254140902	0.957537751400081	-5.04500849213167
a_HQ_transcript/37412	2.14548512664569	1.0521938632066	1.32736011257165	0.193614412837993	0.957544044981884	-4.9266388840905
a_HQ_transcript/144752	1.09844311349632	0.931637739824341	1.32717289865696	0.193675642456964	0.957544044981884	-4.90669961040925
a_HQ_transcript/76820	-1.56927591462127	-0.300250226323426	-1.327164610444	0.19367835351883	0.957544044981884	-5.07848270563569
a_HQ_transcript/152634	-2.59829303467044	0.46223465312441	-1.32705258246787	0.193715000560483	0.957544044981884	-5.12442426616028
a_HQ_transcript/117917	-2.12237591814156	0.328745777336435	-1.32692563062233	0.193756535998063	0.957544044981884	-5.01930986024225
a_HQ_transcript/118616	-1.37279841657366	2.45297063602717	-1.32683602749948	0.193785855995871	0.957544044981884	-5.01476817893243
a_HQ_transcript/138073	1.07682134195188	2.78886733556631	1.32682581425157	0.193789198198555	0.957544044981884	-5.17693934712692
a_HQ_transcript/105981	1.05652374070228	3.19717536202984	1.32682471509401	0.193789557891605	0.957544044981884	-5.29523405851626
a_HQ_transcript/58841	-0.96526792910393	2.47130295576763	-1.32678470942781	0.193802649867134	0.957544044981884	-5.08721232815162
a_HQ_transcript/14448	-1.54281489771346	0.794548996721775	-1.32678418090456	0.193802822832515	0.957544044981884	-4.89526711648983
a_HQ_transcript/80935	4.97368983873378	2.50744970844943	1.32674652057921	0.193815147917544	0.957544044981884	-5.06813394125093
a_HQ_transcript/131861	1.4817859081922	3.10909160519338	1.32669928450084	0.193830607708178	0.957544044981884	-5.17772888323532
a_HQ_transcript/92214	-0.576287936179647	3.36615733071853	-1.32667979426708	0.19383698689938	0.957544044981884	-5.31023797498785
a_HQ_transcript/6912	2.09897616886503	1.10171246585553	1.32667027373884	0.193840103045325	0.957544044981884	-5.00798516263176
a_HQ_transcript/71569	2.00979481815844	2.78327610810762	1.32662865334479	0.193853726189048	0.957544044981884	-5.22904431192396
a_HQ_transcript/104057	1.19332029056068	0.994581507565257	1.32662539566038	0.193854792521934	0.957544044981884	-4.94921078027151
a_HQ_transcript/100968	-1.71582330853903	1.39687323704113	-1.3265966334175	0.193864207418698	0.957544044981884	-5.03049986270652
a_HQ_transcript/112383	-1.49500443439774	-0.535416182243163	-1.32629571503228	0.193962729686896	0.957544044981884	-5.02720698748651
a_HQ_transcript/82779	-0.625861021725146	4.8195586235255	-1.32627355506673	0.193969986496915	0.957544044981884	-5.4217107466969
a_HQ_transcript/62164	-1.66407051373223	0.235255158956885	-1.32626484998099	0.193972837242224	0.957544044981884	-4.91063502172755
a_HQ_transcript/141271	-1.69233610578097	3.76746641708699	-1.32612659403762	0.194018117674403	0.957544044981884	-5.49367622614895
a_HQ_transcript/36107	-2.24158639714189	1.31450072362838	-1.32611824613652	0.194020851969186	0.957544044981884	-4.99745699162563
a_HQ_transcript/134563	-2.66010345053118	0.66236311167769	-1.32611279037848	0.194022638979366	0.957544044981884	-5.02399346350213
a_HQ_transcript/92183	-1.55552850211898	-0.549124561737227	-1.32607213472591	0.194035955962413	0.957544044981884	-5.01588378991807
a_HQ_transcript/112517	-0.909649968808446	2.17493669860927	-1.32604218153004	0.194045767745983	0.957544044981884	-4.97566166613013
a_HQ_transcript/130207	2.37324625990911	1.27155368423896	1.32603070442683	0.194049527407499	0.957544044981884	-5.07425227279008
a_HQ_transcript/135340	-1.43995287247203	1.64033152202325	-1.32599185665884	0.194062253547655	0.957544044981884	-4.89527552849564
a_HQ_transcript/23802	-2.93999606949931	2.03840876894043	-1.32598440688133	0.194064694093496	0.957544044981884	-5.00422636727105
a_HQ_transcript/151723	0.490594913021211	4.22146201173491	1.32597828616083	0.194066699258133	0.957544044981884	-5.42838251025483
a_HQ_transcript/15316	-1.2947909312481	0.983052802702731	-1.32597212645932	0.194068717209117	0.957544044981884	-4.9148274520062
a_HQ_transcript/127648	1.16145252172247	1.95979367077046	1.32587757572149	0.194099694556464	0.957550859990122	-5.02657800517416
a_HQ_transcript/103642	-1.52879073745432	1.60027959348263	-1.325867350877	0.194103044716206	0.957550859990122	-4.93305858758738
a_HQ_transcript/141039	-2.71261869913527	0.870990562620012	-1.32571669572351	0.194152411870308	0.957713119246205	-5.09862059199459
a_HQ_transcript/110431	-1.17668089956954	3.72836764530199	-1.32559968056879	0.194190762417716	0.957821012967076	-5.33843037809611
a_HQ_transcript/37947	-5.15471790737862	0.991659653719262	-1.32550882905338	0.194220542105994	0.957826982372884	-5.01552097940994
a_HQ_transcript/144283	3.33461149066635	5.72303798523426	1.32549544813668	0.194224928454947	0.957826982372884	-5.5550874397027
a_HQ_transcript/151965	-2.3725356874309	0.617571156998799	-1.32533502759463	0.194277521245253	0.957888660469173	-5.01338030874306
a_HQ_transcript/97447	3.90185065469135	3.29270330316506	1.3252776425782	0.194296337190757	0.957888660469173	-5.2923655757105
a_HQ_transcript/141069	1.57657784633654	0.0655907522450529	1.32518191010972	0.194327729978766	0.957888660469173	-4.94790275633293
a_HQ_transcript/39080	-5.37899146744596	0.456103442529954	-1.32514414708257	0.194340114379593	0.957888660469173	-5.03594840462343
a_HQ_transcript/138567	1.74089403042678	2.42247489606467	1.32512604810912	0.1943460501605	0.957888660469173	-5.0061783193764
a_HQ_transcript/1202	1.00746107623767	2.80291765214225	1.32506806330617	0.194365067927232	0.957888660469173	-5.25582810174048
a_HQ_transcript/140431	1.63980011317154	2.33546103305198	1.3249820647786	0.19439327622394	0.957888660469173	-5.05568883173306

a_HQ_transcript/133486	-2.10305086966836	0.263591189478081	-1.32495724793427	0.19440141695835	0.957888660469173	-5.0660854442287
a_HQ_transcript/137502	1.5773662568056	-0.286699485918215	1.32493318577322	0.194409310382002	0.957888660469173	-5.02656278027434
a_HQ_transcript/45445	2.01978810715285	0.805765277922468	1.32492426844334	0.19441223571221	0.957888660469173	-4.98131564204505
a_HQ_transcript/111002	0.874831167188125	2.63632639204131	1.32490455194516	0.194418703829743	0.957888660469173	-5.10484734498273
a_HQ_transcript/76107	2.88326037864631	3.51093612883987	1.32478511135055	0.19445789057837	0.957985945145996	-5.20528890772294
a_HQ_transcript/94595	1.57220921172051	1.78753566246143	1.32474390696342	0.194471410554685	0.957985945145996	-4.9632662521894
a_HQ_transcript/127521	2.37550621716984	2.7040420240995	1.32463641675304	0.194506683621282	0.958045603930584	-5.19413286937883
a_HQ_transcript/135486	-2.53154715220206	5.2196709833448	-1.32449810312288	0.19455207866458	0.958045603930584	-5.50717541939208
a_HQ_transcript/139222	1.20861963131099	2.35045567927724	1.32444943959392	0.194568052148561	0.958045603930584	-5.10133500737784
a_HQ_transcript/24630	0.372482101014231	3.19973113995251	1.3243153471168	0.19461207233428	0.958045603930584	-5.25917588801657
a_HQ_transcript/17845	2.38348066363601	0.732991095850656	1.32429283838243	0.194619462302638	0.958045603930584	-4.96019272580103
a_HQ_transcript/76132	1.69292449595381	-0.155516614818123	1.32426651897435	0.194628103647826	0.958045603930584	-5.05327540493246
a_HQ_transcript/5299	0.921124237731819	2.29163726183364	1.32422185630676	0.194642768235574	0.958045603930584	-4.96676434045007
a_HQ_transcript/58441	-1.9066005470401	1.09819871209895	-1.32415316255098	0.19466532487212	0.958045603930584	-5.15097052813617
a_HQ_transcript/100684	-1.63773918405006	2.2621453479865	-1.32410283323059	0.194681852541583	0.958045603930584	-4.92508027836595
a_HQ_transcript/92138	1.7210644974883	-0.401131566584028	1.32410138661126	0.194682327613521	0.958045603930584	-5.00611435096105
a_HQ_transcript/77178	0.543653456168845	2.52150809305231	1.32409308881027	0.194685052640887	0.958045603930584	-5.09859451726299
a_HQ_transcript/113057	2.01575943312513	0.426204305439342	1.32408973839033	0.194686152939015	0.958045603930584	-4.91751456771476
a_HQ_transcript/101097	1.87340981661568	0.280440667087838	1.32405432619096	0.1946977828109	0.958045603930584	-4.93519833637025
a_HQ_transcript/29314	0.488131470931221	3.89028335264418	1.3239803913416	0.194722065790301	0.958083988510124	-5.31671062543981
a_HQ_transcript/74174	-2.39386385182307	1.00535902325659	-1.3238943656493	0.194750322782745	0.958141918130801	-5.00333867848732
a_HQ_transcript/76514	2.86372696071015	0.509726126027291	1.32368415392131	0.194819384599029	0.958225619497126	-5.08747770049758
a_HQ_transcript/65854	1.32692208818003	0.0085788922300125	1.32367024360384	0.194823955282075	0.958225619497126	-4.98819139998333
a_HQ_transcript/86766	-1.68692569122918	-0.413381681930311	-1.32365173858799	0.194830035828923	0.958225619497126	-4.98801361996027
a_HQ_transcript/97730	2.01685038918647	1.1122949003159	1.32364188160024	0.194833274787306	0.958225619497126	-4.95548698575866
a_HQ_transcript/64945	2.22189929434485	1.64470121183349	1.32348125326031	0.1948860623062	0.958309317991015	-5.11355396465346
a_HQ_transcript/120092	1.30343494958503	1.04113672637803	1.32347107694889	0.194889406931585	0.958309317991015	-4.89308752843403
a_HQ_transcript/31343	-1.81428829723766	1.69841740320915	-1.32339603597485	0.194914071840141	0.958309317991015	-5.00326289737204
a_HQ_transcript/54423	-3.07209062285515	1.21469835693093	-1.32337313359115	0.194921600007478	0.958309317991015	-5.06690150016671
a_HQ_transcript/109174	-1.81265634357215	-0.264876917393089	-1.32333929338705	0.194932723918349	0.958309317991015	-4.97946027118247
a_HQ_transcript/14839	-1.44180637688691	3.04301077979833	-1.32303444819715	0.195032954226524	0.958692826011058	-5.19859348335526
a_HQ_transcript/106424	-1.17980683905667	0.797295703095596	-1.32292883684057	0.195067687497712	0.958692826011058	-4.97410117077562
a_HQ_transcript/138026	1.77079445174315	-0.254377904300675	1.32291585209126	0.195071958226185	0.958692826011058	-5.0302767286306
a_HQ_transcript/12738	1.7839075738715	3.18694490934425	1.32290141824868	0.195076705650612	0.958692826011058	-5.09217441445062
a_HQ_transcript/11089	1.08424968185673	3.01061736738244	1.32278949521637	0.195113521181552	0.95872098118395	-5.21331224002866
a_HQ_transcript/148829	-3.83268481460993	2.70230524062756	-1.3227363335953	0.195131009828054	0.95872098118395	-5.27088269813669
a_HQ_transcript/85570	1.47859876028929	2.20847353727035	1.32273358357004	0.195131914540107	0.95872098118395	-5.08159594172759
a_HQ_transcript/151138	-3.12658522822769	1.19306817077633	-1.32252950679864	0.195199061348687	0.958758616002037	-5.18757127820983
a_HQ_transcript/81791	-2.5363991518953	1.18269688684642	-1.32245885990048	0.19522231023081	0.958758616002037	-4.91586492709997
a_HQ_transcript/136331	-1.35917492222037	2.3393802753783	-1.32243476524914	0.195230239921313	0.958758616002037	-5.18660386661263
a_HQ_transcript/6386	1.22131962040051	0.769178845854581	1.32240223715911	0.195240945497815	0.958758616002037	-4.90157779087414
a_HQ_transcript/136197	2.04488190173377	3.88521197322154	1.32238807648106	0.1952456061711	0.958758616002037	-5.42414352301698
a_HQ_transcript/63019	-1.2542424641984	0.12092552823248	-1.32234623169273	0.195259378954496	0.958758616002037	-4.97395865521285
a_HQ_transcript/9267	0.630658242173805	3.0286708021075	1.32233940674303	0.195261625387482	0.958758616002037	-5.1898595942185
a_HQ_transcript/134417	0.361634444334676	3.9645638211905	1.32230932858156	0.195271525854117	0.958758616002037	-5.42267216375203
a_HQ_transcript/12749	-1.62574234530652	0.743697146834176	-1.32213820859172	0.195327858692574	0.958904775132856	-4.86245032728439
a_HQ_transcript/95893	2.22547021536655	0.402313636400597	1.32209136811418	0.195343280789088	0.958904775132856	-5.06009803038184
a_HQ_transcript/11637	1.92987479388884	2.92880518758502	1.32206858146974	0.19535078356598	0.958904775132856	-5.20144618932521
a_HQ_transcript/27434	1.90428678093397	-0.155285729270751	1.32195312305689	0.195388803036349	0.95895186367023	-5.02804247451681
a_HQ_transcript/86650	3.17538296268783	3.58583811467539	1.32193925191883	0.195393371065755	0.95895186367023	-5.27638226072414

a_HQ_transcript/109519	-2.56587555974894	1.4884701000963	-1.32183785304193	0.195426766132024	0.959017988504096	-5.00452669639295
a_HQ_transcript/22855	2.19974067586719	0.2529610897374	1.32179815613882	0.195439841243799	0.959017988504096	-4.95270523802918
a_HQ_transcript/66576	1.6446801250948	0.216942570353902	1.32161372277541	0.1955005975272	0.959180456640146	-5.06197882852193
a_HQ_transcript/106896	-3.35835256010592	2.78346413819668	-1.32156881956807	0.195515391794513	0.959180456640146	-4.99674556489384
a_HQ_transcript/104036	2.22289721888699	1.02880482197737	1.32153514911038	0.195526485769365	0.959180456640146	-4.96261587438706
a_HQ_transcript/114489	3.45558866804343	2.17208641452846	1.32149730464602	0.195538955601186	0.959180456640146	-5.28454077579661
a_HQ_transcript/103158	1.7893955491569	1.4739574511983	1.32129930694131	0.195604206213863	0.959209739488042	-5.00420016288447
a_HQ_transcript/11503	1.7365302651415	2.73230059381296	1.32126583303531	0.195615239269341	0.959209739488042	-5.2575588736548
a_HQ_transcript/126801	-0.502557261235266	3.19229899545897	-1.32126014380646	0.195617114496161	0.959209739488042	-5.30205438343874
a_HQ_transcript/10279	0.854348987130772	2.10459518499765	1.32120161157969	0.195636408105198	0.959209739488042	-5.15514154908154
a_HQ_transcript/147139	2.00760784904753	1.90965001127253	1.32119942764585	0.195637128009794	0.959209739488042	-5.05850251761299
a_HQ_transcript/119558	-1.98146753994863	1.49134185476154	-1.32110001551631	0.195669900040983	0.959209739488042	-4.89292240770086
a_HQ_transcript/96871	1.7683578730176	1.53784978511835	1.32094028625711	0.195722564931571	0.959209739488042	-4.97025840316628
a_HQ_transcript/143032	-3.40717561428491	2.50219977773123	-1.32092596149169	0.195727288531103	0.959209739488042	-5.12650887944601
a_HQ_transcript/76865	-2.00286012597023	1.16318975140537	-1.32089097427406	0.195738825953575	0.959209739488042	-5.07615421240543
a_HQ_transcript/126496	3.80812753188294	2.98729009888003	1.32085675006973	0.195750112268665	0.959209739488042	-5.20200008375179
a_HQ_transcript/42258	-2.18427468490059	1.72658333750728	-1.32082786768794	0.1957596373682	0.959209739488042	-4.99952934099321
a_HQ_transcript/102899	0.811123863415595	2.93078376969528	1.32077436630127	0.195777282490502	0.959209739488042	-5.1735976965188
a_HQ_transcript/106098	6.62790796718701	2.58985834721346	1.320764848506	0.195780421652118	0.959209739488042	-5.10650787352983
a_HQ_transcript/130036	-2.12124667392743	2.5904844036949	-1.32048864878452	0.195871534729967	0.959209739488042	-5.15659250449048
a_HQ_transcript/5055	-0.558446033922527	2.31934869917123	-1.32043609782329	0.195888873982512	0.959209739488042	-5.05995242986768
a_HQ_transcript/110690	1.99250557048626	0.221905492583469	1.32043445034704	0.195889417588303	0.959209739488042	-5.01695214358275
a_HQ_transcript/137588	-2.23678222734727	1.03687446183074	-1.32034335249336	0.195919478287162	0.959209739488042	-5.05294128731403
a_HQ_transcript/125398	4.54349234431142	2.60023253476519	1.32026136968841	0.19594653420371	0.959209739488042	-5.04439201055657
a_HQ_transcript/136956	-1.57682971582407	1.69425928795999	-1.32022311796023	0.195959158996613	0.959209739488042	-4.88813623122555
a_HQ_transcript/150495	-1.0540724607418	3.17639370515853	-1.32010335138547	0.195998691393902	0.959209739488042	-5.21239531938752
a_HQ_transcript/109593	3.82055508619042	4.41745740301548	1.32004594671767	0.196017641621008	0.959209739488042	-5.44296253336243
a_HQ_transcript/81031	-1.69919100296445	0.877561232054431	-1.32000958943543	0.196029644485907	0.959209739488042	-5.07022629299161
a_HQ_transcript/98375	1.42025780666987	3.0629619153542	1.31995307911401	0.196048301722855	0.959209739488042	-5.0598458080636
a_HQ_transcript/119108	-2.66703816585169	1.36132777673259	-1.31980985586915	0.196095593873962	0.959209739488042	-5.164845521434
a_HQ_transcript/33316	-1.311887782686	-0.568731120900188	-1.31973374311097	0.196120729778718	0.959209739488042	-5.0467177443214
a_HQ_transcript/42919	-1.51546247753542	-0.0257197989467906	-1.31970289281521	0.196130918657252	0.959209739488042	-4.99683377464172
a_HQ_transcript/109399	1.86156288007064	-0.227721926491921	1.31962847101271	0.196155499499135	0.959209739488042	-5.03324582883672
a_HQ_transcript/101297	1.67086123191005	0.0809751939826598	1.31962543069584	0.196156503738257	0.959209739488042	-5.0296278467741
a_HQ_transcript/56684	-1.6102382296947	0.150049904770365	-1.31957515280408	0.196173111468948	0.959209739488042	-4.98616574881612
a_HQ_transcript/16968	-2.45280340715982	3.09092627557025	-1.31956051631693	0.196177946377788	0.959209739488042	-5.21452548871032
a_HQ_transcript/44596	-1.46648936026265	0.214143531861773	-1.31950973223386	0.196194722724398	0.959209739488042	-4.94239768690453
a_HQ_transcript/113168	-1.14703712026917	0.701107884183317	-1.31947780110953	0.19620527162437	0.959209739488042	-4.94101162760061
a_HQ_transcript/146721	0.582956714081986	2.45667397160291	1.31944594155666	0.196215797313102	0.959209739488042	-5.0701110756042
a_HQ_transcript/136858	2.43491554805129	2.74136435915916	1.31940712666848	0.196228621475168	0.959209739488042	-5.15201780958737
a_HQ_transcript/102734	-1.40070933823048	1.6172826963313	-1.31939424610006	0.196232877265062	0.959209739488042	-4.9490957226928
a_HQ_transcript/70010	-1.4323742680416	0.764237565358496	-1.31939214386502	0.196233571858412	0.959209739488042	-5.03135852045397
a_HQ_transcript/47153	-1.53472329647682	0.306291701032842	-1.31937551550122	0.196239066054027	0.959209739488042	-5.1218087242577
a_HQ_transcript/87169	1.5786964845499	1.04142462945415	1.31923611943026	0.196285128698661	0.959209739488042	-4.9823477230375
a_HQ_transcript/75868	2.86042733982466	0.933604683066648	1.31921058649447	0.196293566811287	0.959209739488042	-5.19640233623233
a_HQ_transcript/53215	0.463827326519381	3.610995912602	1.31918194330598	0.19630303312974	0.959209739488042	-5.24326862672894
a_HQ_transcript/149168	0.342471825411357	7.97118717664432	1.31914863297721	0.196314042336617	0.959209739488042	-5.78216156732931
a_HQ_transcript/138117	1.33352776626047	2.63646522569752	1.31910535489471	0.196328346635954	0.959209739488042	-5.18278331498377
a_HQ_transcript/145178	2.63543125790638	3.94948748091464	1.31910356231693	0.196328939137133	0.959209739488042	-5.23841066739068
a_HQ_transcript/91484	-1.88074347681931	0.586475996192355	-1.31909383952266	0.196332152838629	0.959209739488042	-5.13268352556218

a_HQ_transcript/141781	2.22755961407129	3.02269888709745	1.31901039656107	0.196359735124729	0.959209739488042	-5.33926307197254
a_HQ_transcript/143633	-0.887684572177949	3.07551740520585	-1.31900623793363	0.196361109847463	0.959209739488042	-5.20656399731116
a_HQ_transcript/103029	-1.71437676508226	0.400072124415375	-1.31900243117936	0.196362368257503	0.959209739488042	-5.01740633774519
a_HQ_transcript/48073	1.64926792542651	0.403932361291517	1.31889593294997	0.196397576198772	0.959209739488042	-4.9535517829091
a_HQ_transcript/50387	-1.89700483779741	0.326349120774084	-1.31889461139167	0.196398013131711	0.959209739488042	-4.95682757377976
a_HQ_transcript/21953	-2.27105444418695	2.1327766876045	-1.31886556909681	0.196407615268688	0.959209739488042	-5.24269027266121
a_HQ_transcript/81958	-2.60723046280233	2.3074871234484	-1.31885234190475	0.196411988641199	0.959209739488042	-5.21564799315852
a_HQ_transcript/43429	0.535455354838444	3.13409043750916	1.3187872503938	0.196433511259825	0.959209739488042	-5.26527299836246
a_HQ_transcript/137347	-1.79175986718033	1.76406959408175	-1.31877952561803	0.196436065590053	0.959209739488042	-5.03249417598123
a_HQ_transcript/95086	-0.758802604860873	4.98362761641345	-1.31867062194577	0.196472079175797	0.959209739488042	-5.47637221554966
a_HQ_transcript/101660	-1.33135076820346	1.71406825342548	-1.31860834051105	0.196492677429924	0.959209739488042	-5.02626196534783
a_HQ_transcript/112581	0.724908966816397	3.74428436817936	1.3185902767656	0.196498651937531	0.959209739488042	-5.32267518280134
a_HQ_transcript/72344	1.94019459666227	1.60158912506018	1.31857989829378	0.19650208463629	0.959209739488042	-5.16387264375452
a_HQ_transcript/123035	-2.82987009082265	1.68391652039015	-1.31847963062819	0.196535250719433	0.959209739488042	-4.8751584644033
a_HQ_transcript/59690	1.64374211951537	0.163800216800478	1.31839117763266	0.196564512361637	0.959209739488042	-5.06081619195804
a_HQ_transcript/27073	-3.47429158121313	2.55335408188323	-1.31838912276098	0.1965651921852	0.959209739488042	-5.01023203533131
a_HQ_transcript/96784	-1.75257233840208	1.31964940525154	-1.31836313004129	0.1965737916433	0.959209739488042	-5.05748211069019
a_HQ_transcript/34191	0.654880106727247	1.72188303626007	1.31833725378085	0.196582352858322	0.959209739488042	-4.99150252979069
a_HQ_transcript/47208	1.31395254597009	0.0500302724768438	1.31831077506764	0.196591113691942	0.959209739488042	-5.10879595167687
a_HQ_transcript/116322	-1.01950214224873	2.66276245179215	-1.3182371611976	0.196615471390079	0.959209739488042	-5.12487068512143
a_HQ_transcript/111918	-1.64703388326675	-0.200723333263471	-1.31823092635459	0.196617534510047	0.959209739488042	-4.94616264083063
a_HQ_transcript/5371	0.933292352518748	2.35380048609493	1.31813759043608	0.196648421504819	0.959279913161475	-5.01028580183103
a_HQ_transcript/104889	-2.82113839662413	0.573200295731263	-1.31785254325918	0.196742773169761	0.959317537232192	-5.03851879249955
a_HQ_transcript/1312	1.62511408404977	-0.271449249175143	1.3178309354946	0.196749926833753	0.959317537232192	-5.02951453718047
a_HQ_transcript/87957	-0.667114112912634	3.2301953828726	-1.31778073714059	0.196766546729002	0.959317537232192	-5.29680325260175
a_HQ_transcript/102686	2.02348282260772	1.10566136606925	1.31769771863509	0.19679403522752	0.959317537232192	-5.02477657074607
a_HQ_transcript/107904	-1.38204021401139	1.14462419608873	-1.31766344653656	0.196805384019776	0.959317537232192	-4.98612831881342
a_HQ_transcript/136318	1.69617184474736	0.375484508395243	1.31762970103684	0.196816558925207	0.959317537232192	-4.97182021341012
a_HQ_transcript/144556	0.59034923984041	2.84708703497151	1.31758482956872	0.196831418975637	0.959317537232192	-5.16349136658737
a_HQ_transcript/42957	1.67514368322214	1.43106024708469	1.31758185473921	0.19683240417808	0.959317537232192	-5.01051878532049
a_HQ_transcript/52598	0.779088129050376	2.65710232489199	1.3175620831484	0.196838952218962	0.959317537232192	-5.16524815090682
a_HQ_transcript/86997	2.17783687980591	1.78951950485895	1.3175392383538	0.196846518264611	0.959317537232192	-4.979509182254
a_HQ_transcript/62040	-1.60308286682558	-0.0732513383284976	-1.31753062485492	0.196849371056615	0.959317537232192	-5.09168785571283
a_HQ_transcript/15123	-1.50718417191862	3.33471255980739	-1.317500559427	0.196859328978729	0.959317537232192	-5.26520473644185
a_HQ_transcript/7393	-0.578150566354633	2.70546808880517	-1.31746047392063	0.196872606235341	0.959317537232192	-5.19136300026726
a_HQ_transcript/105372	-2.35768646161092	1.28751156595072	-1.31741646506489	0.19688718378716	0.959317537232192	-4.9888314079116
a_HQ_transcript/2384	1.42149724202252	0.421607255233921	1.31735546646808	0.196907390411053	0.959335578728833	-4.97891098788371
a_HQ_transcript/120299	-1.63924812576151	2.11393669571928	-1.31727284113725	0.196934763721899	0.959349200446553	-5.22415207020734
a_HQ_transcript/31106	-0.650241756088934	1.87336876615837	-1.31706854880094	0.197002457149424	0.959349200446553	-4.98161668675085
a_HQ_transcript/128014	-1.44523753059521	-0.072804351217475	-1.31703272750417	0.197014328576699	0.959349200446553	-5.0150044076773
a_HQ_transcript/108283	-0.919192135429976	2.77751759332127	-1.31697400940796	0.197033789348379	0.959349200446553	-5.05565853387911
a_HQ_transcript/151549	-1.81186146255805	2.85836896203801	-1.31693655135865	0.197046204732587	0.959349200446553	-5.12578647461901
a_HQ_transcript/105788	-1.47708892664524	-0.0840103181677689	-1.31693042007112	0.197048236990806	0.959349200446553	-4.89358681583512
a_HQ_transcript/149903	-2.56480176340082	1.19458992383883	-1.31681650966977	0.197085996312208	0.959349200446553	-5.18637941641882
a_HQ_transcript/143992	1.60653987589131	0.346182595674551	1.31680352916199	0.197090299477104	0.959349200446553	-4.88484184782886
a_HQ_transcript/53720	1.17408389004486	1.0195595202325	1.31679821996653	0.197092059547908	0.959349200446553	-4.92955505662303
a_HQ_transcript/74343	1.36916330222168	-0.213513414213193	1.31674568899634	0.197109474930967	0.959349200446553	-5.03025950348713
a_HQ_transcript/125384	1.44067376702267	0.569954422503625	1.31666569834468	0.197135996177085	0.959349200446553	-5.03852309090362
a_HQ_transcript/13104	-2.80369180147502	0.825490435791986	-1.31654919496474	0.197174628266234	0.959349200446553	-5.13166414580747
a_HQ_transcript/15505	1.69202486838528	1.53877687221272	1.31654026015908	0.197177591254115	0.959349200446553	-4.97044788988674

a_HQ_transcript/98952	-2.34470772358402	0.846205726491448	-1.31652135961312	0.197183859224007	0.959349200446553	-4.99820971339191
a_HQ_transcript/127130	2.68038780100538	2.51814377445327	1.3165070663479	0.197188599387173	0.959349200446553	-5.15252465638623
a_HQ_transcript/113727	-2.42757621168308	2.33115684867159	-1.3164241967477	0.197216083660918	0.959349200446553	-5.04933215031371
a_HQ_transcript/83828	1.61714874685566	1.2060261712892	1.31634814136875	0.19724131053365	0.959349200446553	-4.99933311350429
a_HQ_transcript/75736	-3.90378490116387	3.23092698751549	-1.31629508882937	0.197258909038595	0.959349200446553	-5.11730376521263
a_HQ_transcript/149474	-2.08927335550555	1.36049321271737	-1.31627648765706	0.197265079675008	0.959349200446553	-5.01622987146571
a_HQ_transcript/100496	-3.16917260419257	2.24948763722496	-1.3162692372107	0.197267484932726	0.959349200446553	-5.23405273161732
a_HQ_transcript/140938	-2.52136027837515	1.27077621990248	-1.31623785081172	0.19727789729265	0.959349200446553	-5.04830221695229
a_HQ_transcript/30270	0.462274963001412	3.43539599995894	1.31622267494629	0.197282931999306	0.959349200446553	-5.33262763411141
a_HQ_transcript/25073	-1.83882391240274	1.25788327362415	-1.31620203330116	0.197289780177162	0.959349200446553	-5.05013973540701
a_HQ_transcript/105384	1.94937887301169	1.0434894855827	1.31614665919697	0.197308152272886	0.959358258667288	-5.09858677091595
a_HQ_transcript/83308	-1.57647991321116	-0.0433436299213536	-1.31609693343822	0.19732465146962	0.959358258667288	-4.88286826113491
a_HQ_transcript/133417	2.04662180341525	0.418529881803557	1.31604635860616	0.197341433476118	0.959359609023983	-5.0370052953806
a_HQ_transcript/75964	-4.10200184762954	3.95612410507667	-1.31596389091456	0.197368800683485	0.959374606533051	-5.4591308821215
a_HQ_transcript/39979	1.65659548277311	-0.0218906698248618	1.31593759552276	0.197377527517354	0.959374606533051	-4.97739405016308
a_HQ_transcript/61362	1.37475594058629	1.83106674606743	1.31582958562252	0.19741337661154	0.959458995233031	-5.03707159480737
a_HQ_transcript/129347	-2.84556994079174	1.07267972251922	-1.31577794931889	0.197430516753364	0.959458995233031	-5.13295001863049
a_HQ_transcript/4861	2.12621179395439	0.657179642807184	1.31568701253081	0.197460705059363	0.959458995233031	-5.02474486722632
a_HQ_transcript/116865	-0.620142996025365	2.55744455293844	-1.31563060399469	0.197479432790324	0.959458995233031	-5.08579674502641
a_HQ_transcript/95687	1.26909826403176	1.95591714596586	1.31552903242114	0.197513158157317	0.959458995233031	-5.02200267331635
a_HQ_transcript/121128	1.49490800499455	1.72319389197534	1.31548688361996	0.197527154349984	0.959458995233031	-4.97884790415387
a_HQ_transcript/137759	-1.98735607822214	1.26045182289491	-1.3154840984566	0.197528079235379	0.959458995233031	-5.06976952633328
a_HQ_transcript/34677	1.19188022185009	1.85254725200316	1.31544896324058	0.197539747075714	0.959458995233031	-4.94532612924639
a_HQ_transcript/71621	-1.94138987379703	0.125443368590605	-1.31533087384049	0.197578966535001	0.959458995233031	-4.94447535674088
a_HQ_transcript/54497	-4.28205039050618	2.92848870510424	-1.31527437452739	0.197597733008027	0.959458995233031	-5.1405879810257
a_HQ_transcript/64645	1.59779417076505	-0.0450456183968633	1.31526384005768	0.197601232225288	0.959458995233031	-4.96050553843624
a_HQ_transcript/135364	-2.1665886545849	0.0851872939370602	-1.31524960314813	0.197605961351225	0.959458995233031	-4.98684641250142
a_HQ_transcript/102302	4.53482278449763	1.37972246565639	1.31523905000743	0.197609466882223	0.959458995233031	-5.13968885578206
a_HQ_transcript/147445	1.37939302266999	1.99368483246071	1.31510185022893	0.197655046092241	0.959600143593899	-5.02076735322302
a_HQ_transcript/127507	-2.03769528597002	-0.0696238948331257	-1.31501043538228	0.197685419534699	0.959626512861752	-5.03289342127957
a_HQ_transcript/107800	0.670039575917373	4.28975077837813	1.31495913372542	0.197702466558211	0.959626512861752	-5.40639106698611
a_HQ_transcript/67712	-1.23132160054517	1.84195004011983	-1.31487143460976	0.197731610697202	0.959626512861752	-5.05001310369474
a_HQ_transcript/45815	-2.88469527804392	3.22769689537664	-1.31478658188664	0.197759812055123	0.959626512861752	-5.19932811524659
a_HQ_transcript/68075	-1.60813624842676	1.20739050444492	-1.31477330833424	0.19776422388569	0.959626512861752	-4.9146148717
a_HQ_transcript/75901	-1.19772059960233	-0.713599521119521	-1.31471532169492	0.197783498225387	0.959626512861752	-5.01719113780521
a_HQ_transcript/151055	4.21593673423202	5.98229149515026	1.31468842893908	0.197792437671193	0.959626512861752	-5.75888957433757
a_HQ_transcript/115339	0.605289757784108	2.55913257124746	1.31464880983374	0.197805608058181	0.959626512861752	-5.07347120251345
a_HQ_transcript/40597	0.962730169199105	2.32888913542613	1.31463843446315	0.197809057203233	0.959626512861752	-5.01361334654197
a_HQ_transcript/122602	2.75543373336797	0.589688042440894	1.31426711297038	0.197932528092087	0.960145372021766	-5.1862303703559
a_HQ_transcript/27263	-1.46800807247019	0.210987501875198	-1.31409999294117	0.197988117673274	0.960146102731104	-4.91203017013474
a_HQ_transcript/56707	-1.752652388633	1.21410796321423	-1.31409579585115	0.197989513916358	0.960146102731104	-5.08909758207145
a_HQ_transcript/56799	0.96101316456231	3.97304551338702	1.31409204884497	0.197990760436722	0.960146102731104	-5.36664679295883
a_HQ_transcript/117158	1.04597017597672	-0.559502706695347	1.31406803320411	0.197998749887107	0.960146102731104	-4.99722532350953
a_HQ_transcript/97797	1.78954731711104	0.876094336881274	1.31385507246532	0.198069607816132	0.960341475362994	-5.02001020075399
a_HQ_transcript/46161	2.85834181017029	0.979629621262304	1.31379599105389	0.198089269275248	0.960341475362994	-5.02071243722434
a_HQ_transcript/3255	0.636151383021858	2.8534311363413	1.31367856310717	0.198128352072505	0.960341475362994	-5.10227795541592
a_HQ_transcript/46426	1.66589672442268	-0.491489831719617	1.31363856004319	0.198141667385792	0.960341475362994	-5.04916427068173
a_HQ_transcript/3512	1.38375779921371	2.00740837640407	1.3135641609549	0.19816643348961	0.960341475362994	-4.96702385301178
a_HQ_transcript/108340	1.44681084589203	1.83969819181655	1.31334931566032	0.198237964882097	0.960341475362994	-4.95312968436148
a_HQ_transcript/128478	1.33430291143419	0.721088520919708	1.31328317095045	0.198259991326277	0.960341475362994	-4.85874343740411

a_HQ_transcript/141625	1.94389974371586	2.95637438990255	1.31325330762455	0.198269936543005	0.960341475362994	-5.27125185089857
a_HQ_transcript/107593	2.118163412988	0.259361959830404	1.3132133512318	0.198283243594619	0.960341475362994	-4.98467272914152
a_HQ_transcript/115663	2.32238227602957	1.96887063658992	1.31318155893948	0.198293832167712	0.960341475362994	-5.10788972149272
a_HQ_transcript/146297	-1.77715645659245	3.30833379209911	-1.3131724667014	0.198296860460115	0.960341475362994	-5.39439639560121
a_HQ_transcript/73213	-2.13053814262683	0.853726814549452	-1.31313042458082	0.198310863615074	0.960341475362994	-4.92756393791265
a_HQ_transcript/125283	-1.37968674485454	-0.401622542879485	-1.31310725740882	0.198318580330873	0.960341475362994	-5.05144676922398
a_HQ_transcript/134678	-1.19138017150069	-0.583627129264956	-1.31300058648219	0.198354114142064	0.960341475362994	-5.0632825589062
a_HQ_transcript/121981	0.990458191021527	1.70344956956742	1.31296694589041	0.198365321377893	0.960341475362994	-5.11859898164307
a_HQ_transcript/36347	0.838632203432323	2.0068030884525	1.31293676517985	0.198375376379772	0.960341475362994	-5.01352439985375
a_HQ_transcript/69446	1.05153082129568	-0.272505843736039	1.31292896452701	0.198377975307761	0.960341475362994	-5.12579387734652
a_HQ_transcript/146825	2.77380413990518	0.0164264117632212	1.31292790474232	0.198378328396131	0.960341475362994	-5.03886140959429
a_HQ_transcript/32923	-1.73356968063355	1.00638132475654	-1.31292738974629	0.198378499977485	0.960341475362994	-5.04527466294859
a_HQ_transcript/84249	0.620669590674471	4.1191312012454	1.31290707312373	0.198385268962213	0.960341475362994	-5.40787910110743
a_HQ_transcript/122336	-0.903388389062675	4.52039882246986	-1.31285988217885	0.198400992473436	0.960341475362994	-5.44900249331228
a_HQ_transcript/43375	-1.70008368872534	0.920341642941524	-1.31285534449071	0.198402504432188	0.960341475362994	-5.1900971014364
a_HQ_transcript/142205	-1.6409448733336	0.750137318999234	-1.3126092469976	0.198484517381611	0.960597389649324	-4.94530003147795
a_HQ_transcript/65977	-0.711463790011778	1.47671608586117	-1.31259751922954	0.198488426353531	0.960597389649324	-4.92225791469754
a_HQ_transcript/59358	0.928446593872555	4.43412649699541	1.31249226530417	0.198523511082331	0.960676487470328	-5.45013602487189
a_HQ_transcript/63384	-2.40551189437497	1.50696613993957	-1.31244933020294	0.198537824180791	0.960676487470328	-5.21320171487162
a_HQ_transcript/66251	-3.34424865322935	0.697373985065519	-1.31237729260018	0.198561840834502	0.960678969703877	-5.07090092169555
a_HQ_transcript/135503	-2.33775909915916	1.9206946877058	-1.31234864861547	0.198571391085055	0.960678969703877	-5.17520526049811
a_HQ_transcript/58637	-2.20160852763974	0.340983330202873	-1.31216539416331	0.198632498650269	0.960689240230906	-4.97711985163215
a_HQ_transcript/60442	1.28617344104528	-0.439974236942863	1.31211012018528	0.198650932994915	0.960689240230906	-5.03324399911134
a_HQ_transcript/129464	-2.68638902405476	0.203537640909244	-1.31202994607254	0.198677674073716	0.960689240230906	-4.98441260779258
a_HQ_transcript/118326	-2.39262369440638	1.00403993441683	-1.31200319925856	0.198686595753684	0.960689240230906	-5.10632615237595
a_HQ_transcript/111011	-2.20547721302539	0.437260707061828	-1.31195132674903	0.198703899246214	0.960689240230906	-4.99010118480849
a_HQ_transcript/11941	2.63678906424627	0.924314060185083	1.31193481374767	0.198709407850808	0.960689240230906	-5.05135840993041
a_HQ_transcript/13133	-1.44265126197388	0.0288278264337394	-1.31185635380478	0.198735583053794	0.960689240230906	-4.97705556912296
a_HQ_transcript/29260	-2.7599004612521	2.67505153677901	-1.31181480160684	0.198749446446985	0.960689240230906	-5.25445209539374
a_HQ_transcript/5022	-1.02418426291541	2.83324274989217	-1.3118028791743	0.198753424360403	0.960689240230906	-5.17896568504235
a_HQ_transcript/16739	1.61588149698365	0.0592750624525914	1.31179933352002	0.198754607377857	0.960689240230906	-5.04657771687916
a_HQ_transcript/116180	1.66961749735439	3.71493367980062	1.31178849455321	0.198758223862821	0.960689240230906	-5.32400341117198
a_HQ_transcript/143294	1.38402571005791	3.15953497298546	1.31174768818219	0.198771839599799	0.960689240230906	-5.36241426291573
a_HQ_transcript/88804	-1.91635507577854	3.3928562269807	-1.31169313021487	0.198790044904576	0.96069735036691	-5.28960894800625
a_HQ_transcript/126110	-1.63179326341678	-0.106158034825965	-1.31155896958641	0.198834818037033	0.960794836608748	-5.0185666546613
a_HQ_transcript/57822	-1.2870583084748	-0.0184881204152634	-1.3115336315286	0.198843274917479	0.960794836608748	-5.04128557848175
a_HQ_transcript/148890	-1.71730676664671	0.146357014864116	-1.3113841576121	0.198893169227635	0.960956041963591	-5.06392168449118
a_HQ_transcript/83592	-1.06212772620856	1.40658832618357	-1.31131241379644	0.198917120674463	0.96097573087107	-4.87800038282855
a_HQ_transcript/121003	1.65452727084963	0.853694969297724	1.31120282465122	0.198953710924114	0.96097573087107	-4.9100821644
a_HQ_transcript/103316	-1.42979592870845	0.0395102812355435	-1.31112792923595	0.19897872039435	0.96097573087107	-4.91150217279185
a_HQ_transcript/32141	1.52578808525233	0.644468942055247	1.31109845121555	0.198988564509103	0.96097573087107	-4.93994801073151
a_HQ_transcript/147251	-2.00331712589329	0.998371109790904	-1.31108599638995	0.198992723880405	0.96097573087107	-4.99424720083435
a_HQ_transcript/109746	4.11503039370037	1.99288435050742	1.31107487888757	0.198996436699957	0.96097573087107	-5.14579626558762
a_HQ_transcript/33448	1.21012540322157	-0.181428223707839	1.31094985024522	0.199038195121481	0.961072524910266	-4.99675397436646
a_HQ_transcript/77308	-1.56536694685454	0.571442391522014	-1.31091586206317	0.199049548021516	0.961072524910266	-5.02609481467925
a_HQ_transcript/101678	1.64952169341908	2.6535240082803	1.31077031837349	0.199098168843127	0.961161592526719	-5.05737257277275
a_HQ_transcript/19334	-1.4810188108651	0.698145017984481	-1.31069483747262	0.199123387826865	0.961161592526719	-5.01481750851498
a_HQ_transcript/31038	3.41827423973788	0.62944595841492	1.31065102381218	0.199138027564287	0.961161592526719	-4.92361578952107
a_HQ_transcript/148236	-2.16542618830835	1.15459782679057	-1.31058584719977	0.199159806963888	0.961161592526719	-5.05267142963465
a_HQ_transcript/149348	-4.05039751998271	2.19538183443063	-1.31048157429617	0.199194654558104	0.961161592526719	-5.22146340725531

a_HQ_transcript/569	1.02268061263203	2.39716903842851	1.31046252141794	0.199201022458863	0.961161592526719	-5.05546075190125
a_HQ_transcript/72011	-1.25779227552168	2.67552046933298	-1.31037134481782	0.199231497885888	0.961161592526719	-5.17838848367527
a_HQ_transcript/117394	-2.02680665725132	1.01488359417231	-1.3103488161418	0.199239028558257	0.961161592526719	-5.00045368106001
a_HQ_transcript/96516	-1.54464944368021	0.861595428862106	-1.31033428551593	0.19924388583367	0.961161592526719	-5.02841759346917
a_HQ_transcript/146435	-1.69527934385885	0.218387360385348	-1.31032890451585	0.199245684609129	0.961161592526719	-4.90113250827179
a_HQ_transcript/76272	1.92962386000073	0.617324727344923	1.3103159380984	0.199250019109718	0.961161592526719	-4.9751644240482
a_HQ_transcript/43953	1.24311267393227	2.67407943586636	1.31026688337119	0.199266418103831	0.961161592526719	-5.28247022292397
a_HQ_transcript/20966	1.15467641418735	2.86987933311032	1.31017690516493	0.199296500498666	0.961209261837884	-5.34844971509555
a_HQ_transcript/499	1.86811430209251	1.78538180540161	1.31005962349645	0.199335716467203	0.961209261837884	-4.96989480311498
a_HQ_transcript/25697	1.67809044706193	-0.349641592162513	1.31004248371196	0.199341448063696	0.961209261837884	-5.07889340586454
a_HQ_transcript/135880	-1.03677890345061	3.87297980322519	-1.31003950207187	0.199342445146155	0.961209261837884	-5.43040997307112
a_HQ_transcript/149798	-1.26285472788527	1.91673129594463	-1.30993615185184	0.199377008579622	0.961296180716346	-4.95047046957197
a_HQ_transcript/7849	1.975309495374	2.36913940207305	1.30975793201714	0.199436621435612	0.961452272756381	-5.10677203757593
a_HQ_transcript/41111	-1.6227300498194	1.88333840431332	-1.30972977650637	0.199446040435133	0.961452272756381	-5.01646980896149
a_HQ_transcript/104177	0.71228378260953	3.0965297352099	1.30960664084647	0.199487237600683	0.961452272756381	-5.25071162842542
a_HQ_transcript/19064	-1.80483701406758	1.05434908579747	-1.30960073476448	0.199489213746094	0.961452272756381	-4.8990925381593
a_HQ_transcript/14896	-1.94867390463333	0.964221672185769	-1.30955400147413	0.199504850998396	0.961452272756381	-5.03754932848711
a_HQ_transcript/49338	1.48849877288669	-0.114035866406357	1.30951402366965	0.199518228566397	0.961452272756381	-5.03474397336238
a_HQ_transcript/89855	2.66350790808508	0.570459439409332	1.3094932967709	0.199525164572327	0.961452272756381	-5.07186274398249
a_HQ_transcript/146325	-0.643943393201864	3.15192152170368	-1.30939390116822	0.19955842866993	0.961486168528612	-5.25049967075825
a_HQ_transcript/138762	1.98371037048617	2.58198094880041	1.30937342904511	0.199565280472366	0.961486168528612	-5.11003931343564
a_HQ_transcript/133183	0.567395349669333	4.6742964324646	1.30929410592195	0.199591830778675	0.961534389151569	-5.52520729831425
a_HQ_transcript/77018	-1.10525534985952	-0.206680847871355	-1.30899162495566	0.199693099206994	0.961942526784135	-4.99115301858717
a_HQ_transcript/131261	-3.16404615794617	0.991362307118693	-1.30892160313227	0.199716547601709	0.961975760605912	-5.18559341024225
a_HQ_transcript/116737	-0.902441855610984	1.87555680561418	-1.30862382312136	0.19981628949436	0.962376441770499	-5.06473527299607
a_HQ_transcript/129591	0.340225344516694	3.41950212410938	1.30833904792949	0.199911711011955	0.962523064277952	-5.35459354584183
a_HQ_transcript/92735	-0.521338490389318	2.80243754802381	-1.3083163189984	0.199919328446491	0.962523064277952	-5.20331785430797
a_HQ_transcript/130743	-2.09383924687861	1.14834597707339	-1.30828392971206	0.199930183861504	0.962523064277952	-5.0576705188352
a_HQ_transcript/60373	-2.7017304342892	1.98622546745002	-1.30827913228763	0.199931791778182	0.962523064277952	-5.13600769334613
a_HQ_transcript/94367	1.26227218798131	2.44297676809739	1.3082737847807	0.199933584073527	0.962523064277952	-5.06644394524468
a_HQ_transcript/64062	0.67901806495622	3.26427603166847	1.30810555672887	0.199989974440713	0.962523064277952	-5.28224971914096
a_HQ_transcript/135046	-2.76899135172941	2.30008837095061	-1.30803509063303	0.200013598423283	0.962523064277952	-5.2531259845329
a_HQ_transcript/142248	1.72493941826579	-0.241532011428634	1.30801743686857	0.200019517237957	0.962523064277952	-5.04172092957795
a_HQ_transcript/95059	3.37279244594582	1.91338247041181	1.30800067240643	0.200025138018272	0.962523064277952	-5.05443371885838
a_HQ_transcript/33540	-0.587919312686779	5.05523784511141	-1.30798640767719	0.200029920784463	0.962523064277952	-5.53320242868
a_HQ_transcript/50733	-2.12019495712822	0.536898639927961	-1.30798101074908	0.200031730322512	0.962523064277952	-5.08492900492054
a_HQ_transcript/132355	-1.55835608132401	-0.392080515811703	-1.3078618898559	0.200071673596578	0.962523064277952	-5.04995961880582
a_HQ_transcript/133190	1.73484195216101	0.101155430676996	1.3078342800856	0.200080932495037	0.962523064277952	-5.03450885986051
a_HQ_transcript/136136	2.37634398874903	0.356015815428472	1.30778344524738	0.200097980744414	0.962523064277952	-4.96488464707537
a_HQ_transcript/151089	-6.85412422901456	5.60830125463	-1.30773359210221	0.200114700845845	0.962523064277952	-5.36287197127682
a_HQ_transcript/114481	-0.69496762038889	3.66878782307674	-1.30769211005109	0.20012861420407	0.962523064277952	-5.35464224602122
a_HQ_transcript/116254	1.92124072844921	1.22653163628714	1.30765388397139	0.200141436142027	0.962523064277952	-4.99679867245639
a_HQ_transcript/34695	1.68311063202797	1.8325526328154	1.3075706081232	0.200169371018898	0.962523064277952	-4.92205530744172
a_HQ_transcript/140974	4.84861707410951	2.92272092563928	1.30749149139069	0.200195913480288	0.962523064277952	-5.28395021145779
a_HQ_transcript/129486	1.20711735120679	-0.514859792601151	1.30746458959522	0.200204939236438	0.962523064277952	-5.1047579151555
a_HQ_transcript/114816	1.38246667192538	1.88771507931643	1.30741674711392	0.200220991519416	0.962523064277952	-5.09186174609819
a_HQ_transcript/128646	-3.55623073945506	2.46393366176151	-1.30735436981844	0.200241922052841	0.962523064277952	-5.1247089396812
a_HQ_transcript/128648	1.86171242449174	0.366202881781293	1.30733683970169	0.200247804537185	0.962523064277952	-4.98915604310081
a_HQ_transcript/130102	-0.763923859927629	2.83990234419754	-1.30733027977825	0.200250005848368	0.962523064277952	-5.21995579646385
a_HQ_transcript/136868	-3.50696837430007	6.24812304757655	-1.30723465095629	0.200282098077497	0.962523064277952	-5.43357013179245

a_HQ_transcript/150630	1.85743866067602	0.924828395441957	1.30723205190309	0.200282970352756	0.962523064277952	-5.12340376855797
a_HQ_transcript/18279	-0.512642672147495	3.53536450822337	-1.30713411624667	0.20031584091798	0.962523064277952	-5.33878279983681
a_HQ_transcript/143697	1.41686282755423	0.45499510639156	1.30712864149859	0.200317678552835	0.962523064277952	-5.11048863733828
a_HQ_transcript/19425	1.26677939178775	0.325008989154227	1.30710106730036	0.200326934207863	0.962523064277952	-4.93575395813376
a_HQ_transcript/120774	2.13144160752977	2.34220807835596	1.3070039989953	0.200359519105354	0.962532364415984	-4.97921224904703
a_HQ_transcript/22022	-1.30593536711851	1.74492108632003	-1.30686165901574	0.200407308590891	0.962532364415984	-4.94013571173435
a_HQ_transcript/144438	1.49539420894921	2.06015728095939	1.30685295146416	0.20041023236282	0.962532364415984	-4.95965178567996
a_HQ_transcript/125814	-2.18042709339776	0.333246139283335	-1.3067964705988	0.20042919797632	0.962532364415984	-5.10317846405823
a_HQ_transcript/105303	-1.17426630711186	-0.687137438162286	-1.30678497473595	0.200433058320428	0.962532364415984	-5.05296903323776
a_HQ_transcript/122915	-0.689421428720724	2.53343606321176	-1.30673638833419	0.200449374402445	0.962532364415984	-5.16249271753727
a_HQ_transcript/124739	-2.33527298063271	1.16602750110079	-1.30671243797886	0.200457417683821	0.962532364415984	-4.99554630190608
a_HQ_transcript/148442	2.56413589000051	4.1958349936015	1.30670075718022	0.200461340552228	0.962532364415984	-5.3249964320346
a_HQ_transcript/145738	-0.403238784393427	3.50210700706238	-1.3065371356158	0.200516297228307	0.962581129684084	-5.36710431385377
a_HQ_transcript/13118	0.647951199399392	2.29906865825242	1.30649594424981	0.20053013426274	0.962581129684084	-5.15825070261798
a_HQ_transcript/136057	-2.86723854416826	4.26419917722415	-1.30648522813288	0.200533734148293	0.962581129684084	-5.51515995370413
a_HQ_transcript/145696	3.99654406605028	2.77968976337315	1.30647331755041	0.200537735350851	0.962581129684084	-5.13057284150839
a_HQ_transcript/101025	2.42806023131213	0.639260166798716	1.30631674271867	0.200590340263456	0.962663424784326	-5.13976702272122
a_HQ_transcript/136456	2.35561930304504	0.616812738036579	1.30628392299686	0.20060136813919	0.962663424784326	-5.1031867722743
a_HQ_transcript/17028	-0.619727841660911	2.58256870704884	-1.30621572822594	0.200624283996322	0.962663424784326	-5.2276208965425
a_HQ_transcript/126849	-1.72798588472185	1.54114755835969	-1.3061557806456	0.200644430156725	0.962663424784326	-5.07655120616661
a_HQ_transcript/108881	0.833060628043004	4.75124589282682	1.30613567444603	0.200651187451369	0.962663424784326	-5.48484007049802
a_HQ_transcript/139629	-0.339205314447254	5.21643964974978	-1.30612657175249	0.200654246743168	0.962663424784326	-5.56788785197839
a_HQ_transcript/130504	-2.96161880815883	1.44952630715556	-1.3060568437964	0.200677682544825	0.962696404305156	-5.05671808572208
a_HQ_transcript/81200	0.715464159247261	1.86389404930954	1.30593165837306	0.200719763034193	0.9627574744771	-5.00524325440791
a_HQ_transcript/82616	0.6258869368946	2.69306941290779	1.30584124222374	0.200750160187181	0.9627574744771	-5.17053894739653
a_HQ_transcript/9103	0.528462738320498	2.9389827687656	1.30581366131133	0.20075943335891	0.9627574744771	-5.23221309771883
a_HQ_transcript/3475	-1.65195798017251	1.0513148091249	-1.30577952259809	0.200770911827814	0.9627574744771	-5.0624702424263
a_HQ_transcript/62344	-1.23653778139118	3.45368589099806	-1.3057333727681	0.200786429594481	0.9627574744771	-5.32299428942247
a_HQ_transcript/18549	-0.49286832887179	-0.725878713811591	-1.3057233818424	0.200789789139243	0.9627574744771	-5.12382458984911
a_HQ_transcript/73456	-0.668557897531661	2.43433263150761	-1.30560691041282	0.20082895694367	0.962865853614453	-5.1327145701813
a_HQ_transcript/46055	-1.39123801753529	1.72465082371309	-1.3054810461511	0.200871289992432	0.962894176973747	-4.9681272671591
a_HQ_transcript/126639	-1.42752160380406	1.88731437635761	-1.30546146972978	0.200877874916944	0.962894176973747	-4.98916908391916
a_HQ_transcript/82527	-1.78317706782991	-0.498803191086704	-1.30544159704515	0.2008845596642	0.962894176973747	-5.01282760551699
a_HQ_transcript/62322	-1.56544970621999	-0.00250384838950068	-1.30535843623709	0.200912535029017	0.962948865119284	-5.01375884836419
a_HQ_transcript/67280	-2.04345510533404	1.68729110703905	-1.30528429450064	0.200937478878907	0.962981732691907	-5.17323776144293
a_HQ_transcript/68859	-0.410655014573162	3.04764347325274	-1.30523957336266	0.20095252576302	0.962981732691907	-5.25863249810856
a_HQ_transcript/52020	1.58893835833166	0.486306603757846	1.3050759410558	0.201007588858679	0.963029589069209	-4.92122870637252
a_HQ_transcript/71590	-1.42821560652829	0.922160643261113	-1.30506512376926	0.201011229336002	0.963029589069209	-4.952726081805
a_HQ_transcript/126727	1.81340624185803	0.0581587369999169	1.30489841640508	0.201067339825447	0.963029589069209	-4.96016184855835
a_HQ_transcript/144437	1.53277124281053	1.39458828540901	1.30484372061093	0.201085751981848	0.963029589069209	-5.07003447641656
a_HQ_transcript/102379	1.41678385540211	1.79434335926733	1.30469500253501	0.201135821214462	0.963029589069209	-5.05576293804597
a_HQ_transcript/16763	1.31980511388583	1.49539298069223	1.30466624095234	0.201145505536226	0.963029589069209	-4.91967426329627
a_HQ_transcript/135057	-2.32927293031841	1.11674376511767	-1.30461462180556	0.201162887128478	0.963029589069209	-4.94436327888064
a_HQ_transcript/18988	3.94904734575691	3.23946130923866	1.30456249153475	0.201180441993995	0.963029589069209	-5.15437737156246
a_HQ_transcript/132825	2.71412010981917	3.65253664181961	1.30451887710521	0.201195130048986	0.963029589069209	-5.16036545457997
a_HQ_transcript/130427	2.01127611189635	1.09832204921883	1.30451443957868	0.201196624523075	0.963029589069209	-5.03888676430174
a_HQ_transcript/111942	2.62235022595827	0.861827805726458	1.30449082418305	0.201204577879331	0.963029589069209	-5.06853361653325
a_HQ_transcript/22559	-1.71517877750294	0.659717785194048	-1.30448861588425	0.201205321617744	0.963029589069209	-4.92610586597369
a_HQ_transcript/46408	-1.74427462179375	2.17063924658173	-1.30437876522121	0.20124232114295	0.963029589069209	-5.10484951538778
a_HQ_transcript/68514	-1.61303354017514	0.697458239530535	-1.30432583420721	0.201260151040433	0.963029589069209	-5.04044916806682

a_HQ_transcript/146052	3.18264301955611	3.13868680197526	1.30429776779634	0.201269605745431	0.963029589069209	-5.33086333586691
a_HQ_transcript/118586	-0.822017546997541	2.86283697731357	-1.30425881446333	0.201282728479298	0.963029589069209	-5.23710453995747
a_HQ_transcript/14465	-4.20142069713221	3.8294315887855	-1.30424264396815	0.201288176243601	0.963029589069209	-5.3417597374092
a_HQ_transcript/68551	-3.77502218354461	1.53231370657459	-1.30416417039931	0.201314615220811	0.963029589069209	-5.09523964359713
a_HQ_transcript/77120	1.64921127186525	1.75784546813622	1.3041576613859	0.201316808328652	0.963029589069209	-4.93934236726424
a_HQ_transcript/135811	-1.61547539800185	2.63377286553622	-1.30410989154609	0.201332904169296	0.963029589069209	-5.06852298133499
a_HQ_transcript/76056	-0.524050796206381	2.71693467457305	-1.30406356338618	0.201348515180384	0.963029589069209	-5.17377780628784
a_HQ_transcript/91398	-1.72734346923198	0.911710689357241	-1.30399509404297	0.201371588707435	0.963029589069209	-5.14655975505563
a_HQ_transcript/35301	-2.23061974686506	0.939597700572542	-1.30399390050741	0.201371990935631	0.963029589069209	-4.99222276860622
a_HQ_transcript/35531	0.517654404234705	5.04346327951393	1.30398320977306	0.201375593800569	0.963029589069209	-5.53994897878445
a_HQ_transcript/71715	1.41777815975904	1.17963990887966	1.30394771377387	0.201387556595569	0.963029589069209	-5.06594638861238
a_HQ_transcript/146894	-0.923631788941932	1.75459370010873	-1.3039307775503	0.201393264600972	0.963029589069209	-4.95493420166066
a_HQ_transcript/131521	-0.801148783569063	6.00659872343087	-1.30387191265001	0.201413104763917	0.963045237617584	-5.67031972596065
a_HQ_transcript/34811	0.509075374607171	3.42574527300062	1.303682347641	0.201477006976992	0.963206875188386	-5.3005472148518
a_HQ_transcript/127426	1.87281055562689	0.44427619050669	1.30367331919994	0.201480050843235	0.963206875188386	-5.14144992036516
a_HQ_transcript/70514	-1.57516380572562	3.91043838472511	-1.30362372677523	0.201496771155972	0.963207591591642	-5.45006680747736
a_HQ_transcript/76731	2.05753381495206	0.0518794689039533	1.30356516553029	0.201516516710989	0.963222768142124	-5.06142442093677
a_HQ_transcript/136728	0.826305401308078	3.30208772892754	1.30346864131258	0.201549065762621	0.963244313327735	-5.3158710650246
a_HQ_transcript/132833	1.23929219583862	4.10590424396573	1.30345351652603	0.201554166374299	0.963244313327735	-5.29522488077937
a_HQ_transcript/96033	-3.07129484712597	2.79557746305666	-1.30330724321193	0.201603499979949	0.96335066941313	-5.22637797245932
a_HQ_transcript/141367	-1.21791041711069	4.47779966973919	-1.30328925736081	0.201609566704699	0.96335066941313	-5.3753340407861
a_HQ_transcript/54536	0.660248449287453	1.65600588795156	1.30317170106622	0.20164922250901	0.963460957258239	-4.95395181527557
a_HQ_transcript/88604	2.83926359947071	2.0617954594189	1.3029783968843	0.201714443635991	0.963693365634329	-5.19508809043893
a_HQ_transcript/47255	-1.0025716478024	2.03519725210551	-1.30291666731554	0.201735274677468	0.963713679031218	-5.0173934338129
a_HQ_transcript/91935	-1.43638894653682	0.855251271260855	-1.30275981494288	0.201788212907462	0.963787567513117	-4.99282321846849
a_HQ_transcript/64721	-1.43105518959472	0.526207290906311	-1.3027425124615	0.201794053205096	0.963787567513117	-5.02411187390778
a_HQ_transcript/97087	-1.5094068815671	0.994445461027506	-1.3027234635488	0.201800483144133	0.963787567513117	-4.89747831859736
a_HQ_transcript/77390	1.51743543617213	0.784973973698339	1.30242867782819	0.201900007660713	0.964082097560342	-4.93573369881238
a_HQ_transcript/152159	-2.33734072958383	0.547869049471275	-1.3024231576996	0.201901871704195	0.964082097560342	-5.09443236400662
a_HQ_transcript/11235	-2.06344843276016	1.10270220105971	-1.30236672789835	0.201920927738661	0.964082097560342	-4.97215998481366
a_HQ_transcript/112698	1.77312826929042	2.97396223175635	1.30227685362035	0.201951280622418	0.964082097560342	-5.22736809155258
a_HQ_transcript/8016	-1.68041128312471	1.73981086292269	-1.30226391204667	0.201955651615491	0.964082097560342	-5.10115741965809
a_HQ_transcript/68980	-1.75297137721315	0.411012101421713	-1.30224610607323	0.201961665668736	0.964082097560342	-4.94356219362575
a_HQ_transcript/7845	-1.99458532295029	1.0401950392626	-1.30194366299961	0.202063838142623	0.964160621125267	-4.96507972381077
a_HQ_transcript/52378	-1.41297455853215	-0.649393347414973	-1.30188263754433	0.202084458774597	0.964160621125267	-5.05796410232383
a_HQ_transcript/15458	1.89011263975581	0.652468461907832	1.30185693876576	0.202093142927184	0.964160621125267	-4.92910119934522
a_HQ_transcript/150083	4.48492955905849	8.10789564608642	1.3018436269381	0.202097641382947	0.964160621125267	-5.63618083705068
a_HQ_transcript/45012	-3.39587746059714	2.62016524055457	-1.30182461712927	0.202104065485557	0.964160621125267	-5.17880597797808
a_HQ_transcript/141584	1.79863142385439	-0.313357321863077	1.30181732335751	0.202106530356437	0.964160621125267	-5.02318233047352
a_HQ_transcript/140527	2.3986144180334	1.94664402082564	1.30176300615475	0.202124887134814	0.964160621125267	-4.98191305351356
a_HQ_transcript/123911	4.51898812683782	2.09474582845381	1.30169318016082	0.20214848705725	0.964160621125267	-5.37037677399925
a_HQ_transcript/135958	1.67062781206081	-0.175495171191437	1.30168903930102	0.202149886658944	0.964160621125267	-4.99076137818915
a_HQ_transcript/116485	-3.01053577946011	1.43097169497082	-1.30167551175636	0.202154458991423	0.964160621125267	-5.1032840957677
a_HQ_transcript/124688	1.78309800063313	-0.0167099563062456	1.30160561943323	0.202178083968931	0.964160621125267	-4.99948196104552
a_HQ_transcript/141560	3.3583520215534	1.22371117319435	1.30158847596006	0.202183879121063	0.964160621125267	-5.13444688713862
a_HQ_transcript/48913	2.96600770744155	1.75623105588191	1.3015111936093	0.202210005095703	0.964160621125267	-5.03105019113671
a_HQ_transcript/45728	1.25250399306538	0.0716351035881281	1.30150695476705	0.202211438147884	0.964160621125267	-4.96134612968218
a_HQ_transcript/109018	1.94277942411592	0.241553303070051	1.3014032083297	0.202246514776361	0.964160621125267	-5.07146602509898
a_HQ_transcript/27677	-1.67967997697539	-0.0115369657041828	-1.30139853532079	0.202248094827995	0.964160621125267	-4.98209882682948
a_HQ_transcript/141186	1.73365582698853	2.43163650767014	1.30132462036476	0.202273088424373	0.964160621125267	-5.21893113830832

a_HQ_transcript/7968	3.58082427808412	2.75361974865489	1.301310826666	0.202277752885196	0.964160621125267	-5.32412528540201
a_HQ_transcript/50125	0.61095654251145	3.30606005660071	1.30126480531835	0.202293316003011	0.964160621125267	-5.28260218543898
a_HQ_transcript/102253	1.8913244346375	0.995639986147885	1.30113159864634	0.202338367877981	0.964160621125267	-5.07102028458045
a_HQ_transcript/58989	-0.917821522945311	2.83787542952023	-1.30112700795482	0.202339920633742	0.964160621125267	-5.23462389318445
a_HQ_transcript/131417	-2.28822260568482	5.58291656588878	-1.30106318087152	0.202361510451535	0.964160621125267	-5.57436078218706
a_HQ_transcript/52288	1.01035223701636	2.41639946490398	1.30102253129399	0.202375261282231	0.964160621125267	-5.12003346847176
a_HQ_transcript/95795	-1.89300867461431	-0.434957407134503	-1.30087463412672	0.202425297554212	0.964160621125267	-5.00231271387919
a_HQ_transcript/29303	1.03406802585345	1.86788183816926	1.30076041925724	0.202463944954315	0.964160621125267	-4.97116376865617
a_HQ_transcript/112594	1.54020015494884	1.8994823319498	1.30071378383	0.202479726811599	0.964160621125267	-5.04857997052137
a_HQ_transcript/8586	-2.73851773457164	2.099048323154	-1.30070988523879	0.202481046173189	0.964160621125267	-5.3474178883631
a_HQ_transcript/22949	1.5304150713281	1.80093784608874	1.30069952790662	0.202484551334319	0.964160621125267	-5.0564078183868
a_HQ_transcript/53690	2.53048980733959	0.981755350999566	1.30069052159022	0.202487599318023	0.964160621125267	-5.01557057963668
a_HQ_transcript/21378	1.70900514533399	0.227830781924068	1.30066902984026	0.202494872855159	0.964160621125267	-4.99989806213293
a_HQ_transcript/2919	1.12690489493184	3.2429245201114	1.30064727078184	0.202502237061494	0.964160621125267	-5.26805467575413
a_HQ_transcript/118574	-0.684631235913765	2.40370924138463	-1.30062760033897	0.202508894565758	0.964160621125267	-5.09963848675171
a_HQ_transcript/37257	1.27656847029725	-0.644275043668037	1.30057407282581	0.202527011914243	0.964167907334244	-5.07268043895925
a_HQ_transcript/22995	0.851590777588335	2.46611639637039	1.30042881867698	0.202576182017796	0.964215259243837	-5.17869014989317
a_HQ_transcript/92018	2.23938486512747	-0.0173496915440443	1.30030243610599	0.202618971292267	0.964215259243837	-5.05036985216362
a_HQ_transcript/35168	1.03330956978461	2.69766282901684	1.30026503102829	0.202631636829418	0.964215259243837	-5.22614909147669
a_HQ_transcript/8812	-0.95073056783276	3.28599761269791	-1.30024434947445	0.202638639961009	0.964215259243837	-5.1767627993913
a_HQ_transcript/127763	2.54789049228523	0.249096017515536	1.30024289740562	0.202639131663547	0.964215259243837	-5.06530968388413
a_HQ_transcript/36052	-1.26963760675347	1.85965850395021	-1.30019780368134	0.202654401846053	0.964215259243837	-5.15261657968008
a_HQ_transcript/65342	1.54512545296871	0.794373118358578	1.30016397184706	0.202665858967114	0.964215259243837	-4.85947379116195
a_HQ_transcript/136486	-3.34214839816889	0.692744564260596	-1.30010471008734	0.202685929100269	0.964215259243837	-5.12476367098461
a_HQ_transcript/127282	-2.08889290947	-0.0328289117525138	-1.3001037670266	0.20268624849815	0.964215259243837	-5.04148063397461
a_HQ_transcript/7885	-1.44449183734725	3.18646380169901	-1.29982165296907	0.202781812733976	0.964454440943496	-5.235494591309
a_HQ_transcript/53455	-1.29345899842837	-0.160717422521445	-1.29980772438715	0.20278653183688	0.964454440943496	-5.11305700011628
a_HQ_transcript/147228	-1.36023733323937	1.28630148506643	-1.2997724833471	0.202798472126491	0.964454440943496	-4.89262979831789
a_HQ_transcript/45403	1.21925968232123	3.06508511248954	1.29975943238903	0.202802894158621	0.964454440943496	-5.25841313936372
a_HQ_transcript/70531	1.55015802323412	1.00558711050451	1.2996094362645	0.202853722346881	0.964541879195214	-5.02187603059919
a_HQ_transcript/13271	2.48117130990344	0.792798729522991	1.29960723819372	0.202854467264683	0.964541879195214	-4.98795759589517
a_HQ_transcript/52874	3.16436222692991	1.67940639474202	1.2994354242703	0.202912700799581	0.96455976242223	-4.97723734781361
a_HQ_transcript/93721	-1.21078576966533	2.66535391933846	-1.2993969392332	0.202925746423567	0.96455976242223	-5.0584757105695
a_HQ_transcript/135053	-1.54866723186881	-0.139242510360374	-1.2993709141864	0.20293456873334	0.96455976242223	-4.98154959569586
a_HQ_transcript/32434	-0.940194152909045	1.61165202555666	-1.29934775799614	0.202942418766493	0.96455976242223	-5.00596693076189
a_HQ_transcript/145445	-2.36356601758836	0.832639267884033	-1.29930541391248	0.202956774164256	0.96455976242223	-5.06744632463374
a_HQ_transcript/150381	-1.45688452362552	4.62273275000762	-1.29926714550517	0.202969748501028	0.96455976242223	-5.30183048762368
a_HQ_transcript/11345	-2.21270633274341	1.93338990042009	-1.29925347228307	0.202974384357775	0.96455976242223	-4.93752947939056
a_HQ_transcript/116503	2.03302330421497	2.44061918124024	1.29905191076527	0.203042732428916	0.964737414180997	-5.25413853513991
a_HQ_transcript/101380	-1.90023852861107	1.59192620613085	-1.29892544938017	0.203085623535611	0.964737414180997	-4.91962972023452
a_HQ_transcript/152188	-0.628126542732341	7.87660009166364	-1.29887727621728	0.203101963938096	0.964737414180997	-5.79946040121563
a_HQ_transcript/33722	-1.6436052823659	0.158689595143802	-1.29886354088795	0.203106623164089	0.964737414180997	-4.96585942206715
a_HQ_transcript/85546	2.89929763618685	2.81776626889817	1.29885823338383	0.203108423569469	0.964737414180997	-5.1260411569542
a_HQ_transcript/145800	3.96361515078028	3.27001231364679	1.29884961044933	0.203111348657223	0.964737414180997	-5.27906152222447
a_HQ_transcript/24856	-1.66584332488137	1.39741993608732	-1.29874300589819	0.203147513901733	0.964797801910391	-4.98813636109837
a_HQ_transcript/33897	1.74393976292361	1.27979853750351	1.29871428550347	0.203157258040586	0.964797801910391	-5.05503574321419
a_HQ_transcript/101406	2.67960019648325	2.84005963626766	1.29860159178686	0.203195495747739	0.964900561786176	-5.16503969327366
a_HQ_transcript/21497	1.37403786526176	1.94345367130265	1.2984813130336	0.203236313157211	0.965000023273265	-5.14696054325962
a_HQ_transcript/8568	-0.736606970746696	2.53786627354824	-1.29843377987067	0.203252445580332	0.965000023273265	-5.18105043327412
a_HQ_transcript/118908	-1.79211466139779	0.122121485263452	-1.29839312325902	0.203266244924268	0.965000023273265	-5.048736254033

a_HQ_transcript/48610	2.08952164219624	-0.0222562060672633	1.29829444481633	0.203299740545964	0.965057514776316	-5.04719297942029
a_HQ_transcript/31782	-1.29180689808672	0.116117097425493	-1.29825962925061	0.203311559419742	0.965057514776316	-5.04079946882973
a_HQ_transcript/14568	-1.76733404373869	1.14873995261342	-1.29806226386913	0.20337856913323	0.965170924280102	-5.09471737336627
a_HQ_transcript/9286	0.828454014371789	4.0121746896797	1.29804633446137	0.203383978237252	0.965170924280102	-5.40712146720635
a_HQ_transcript/140118	-1.23189625777861	1.83856123643628	-1.29804254673072	0.203385264442385	0.965170924280102	-5.02264422655144
a_HQ_transcript/89532	1.19680523433328	0.85977758659288	1.29784900572216	0.203450993689516	0.96535428582371	-4.93298098095297
a_HQ_transcript/132120	1.42344728206204	-0.319258312286691	1.29782292302504	0.20345985297745	0.96535428582371	-4.98107536513213
a_HQ_transcript/94696	1.41393614218199	2.34259207498596	1.29778208286532	0.203473725397607	0.96535428582371	-4.97604827721458
a_HQ_transcript/12803	-1.16379974952167	1.13448790230809	-1.29766570701685	0.203513259427141	0.96537241641144	-4.89687805139639
a_HQ_transcript/83947	0.99266068117453	2.43680321432957	1.29763477804581	0.20352376728947	0.96537241641144	-5.08122649715559
a_HQ_transcript/151877	-2.88670046755902	2.24868003249295	-1.29753508427778	0.203557640236663	0.96537241641144	-5.10198743423617
a_HQ_transcript/95706	-1.44809707558309	1.09727208974498	-1.29753469191703	0.203557773557544	0.96537241641144	-4.93183052290991
a_HQ_transcript/125504	1.38100514845181	2.35568719263379	1.29752641677397	0.203560585396969	0.96537241641144	-5.09370799502904
a_HQ_transcript/44202	-2.16363068163913	3.9347879587753	-1.29720882624487	0.203668522939813	0.965697456437931	-5.55177296131555
a_HQ_transcript/45002	0.402486182369245	3.62481012598788	1.29718184848452	0.203677693714137	0.965697456437931	-5.38537133790602
a_HQ_transcript/17998	-1.09913529553147	2.19658937270005	-1.29714057725052	0.20369172399645	0.965697456437931	-5.04160254468321
a_HQ_transcript/15956	2.42995714686545	1.52253308933198	1.29712924301676	0.203695577232753	0.965697456437931	-5.07443047359217
a_HQ_transcript/148512	1.53168997537728	-0.156293443428745	1.29699220964467	0.203742168098454	0.965839565097613	-5.05073200585535
a_HQ_transcript/122509	1.77948592215025	1.77026937440066	1.2968960442664	0.203774868836154	0.965877457151154	-5.08262793023325
a_HQ_transcript/4064	-1.51272816889718	1.29579658497971	-1.29687097523593	0.203783394138827	0.965877457151154	-4.96821461115277
a_HQ_transcript/64685	-1.42309013870514	-0.380890401270036	-1.29680991999358	0.203804158520297	0.965887370264464	-5.01741189714367
a_HQ_transcript/40767	2.47970570008122	2.61445231054385	1.29676711015357	0.20381871875282	0.965887370264464	-5.22048832484727
a_HQ_transcript/84436	3.24789821816985	0.6433248129391	1.29664975979302	0.203858635333895	0.96590314339714	-5.04472135530749
a_HQ_transcript/105113	1.64898464718899	2.53821333750487	1.29663125044313	0.203864931810098	0.96590314339714	-5.07707698899898
a_HQ_transcript/36564	1.11454748031731	1.93116794883573	1.29652060707779	0.203902573353186	0.96590314339714	-4.98010541562231
a_HQ_transcript/140905	1.59260323977428	0.232230151712042	1.29646885043646	0.203920183093062	0.96590314339714	-5.08147339757776
a_HQ_transcript/83407	2.38689290979023	2.60142591381721	1.29638438046896	0.203948925740727	0.96590314339714	-5.25107046610202
a_HQ_transcript/1466	0.851014226166334	1.54123102163716	1.29637891207258	0.203950786581645	0.96590314339714	-5.05451406684687
a_HQ_transcript/103486	-1.58300532483972	1.99792708674113	-1.29636746231656	0.203954682861653	0.96590314339714	-5.16512723535785
a_HQ_transcript/99397	1.42137074448515	0.913899565159702	1.29636658385259	0.203954981799761	0.96590314339714	-4.96446226811303
a_HQ_transcript/113323	1.23120493214163	1.53780072098641	1.29624113730454	0.203997674232061	0.966026623850202	-4.94183145986061
a_HQ_transcript/86347	1.48075598338632	0.245317735087369	1.29614634033478	0.204029940400825	0.96610071485982	-4.90251887361155
a_HQ_transcript/75431	2.17810941902941	3.20373726547181	1.2959167135349	0.204108114883588	0.96630360639813	-5.14735085289626
a_HQ_transcript/103559	-1.78965835057694	1.58329830659027	-1.29590572458962	0.204111856547918	0.96630360639813	-4.98811974920169
a_HQ_transcript/7310	0.439781100161776	2.97012497756177	1.29587399651839	0.20412266004271	0.96630360639813	-5.30105422648953
a_HQ_transcript/80361	-1.71867212303439	0.927578026050995	-1.29572920958815	0.204171965914328	0.966446467075511	-5.10806760545472
a_HQ_transcript/79388	1.71679922849708	0.940721896338506	1.2956877379045	0.204186090389388	0.966446467075511	-5.03625807959089
a_HQ_transcript/120612	-0.992463623102932	1.83333814859462	-1.29559488764359	0.204217716138459	0.966463152673078	-5.05225910322386
a_HQ_transcript/130975	-0.601493742807075	3.92186108898315	-1.29553704498516	0.204237419825139	0.966463152673078	-5.41505096449627
a_HQ_transcript/21715	-0.598506993755951	3.4113988988542	-1.29550890939747	0.204247004534492	0.966463152673078	-5.286794871418
a_HQ_transcript/39740	0.571472072621725	2.9983729022981	1.29540461884109	0.204282535291216	0.966463152673078	-5.29614120383804
a_HQ_transcript/143688	1.76594715971828	0.0741680262095567	1.29539515555775	0.204285759570713	0.966463152673078	-5.02404441778785
a_HQ_transcript/16689	1.61656148369994	3.3304502879416	1.29538454605378	0.204289374430466	0.966463152673078	-5.26031030582171
a_HQ_transcript/19905	-2.44911037501986	0.605819258670054	-1.29532132944006	0.204310914542444	0.966486396526951	-5.06124967253486
a_HQ_transcript/81283	2.66808693994546	0.719150770181843	1.29522392205657	0.204344108030486	0.966564758043459	-5.10642857474424
a_HQ_transcript/41365	1.69141639530884	2.05229344612201	1.29514736164264	0.20437020038366	0.966601799614364	-5.15573298519955
a_HQ_transcript/87385	1.85316443816722	0.763228989853746	1.29507128517693	0.204396130315927	0.966601799614364	-4.93617808725047
a_HQ_transcript/61865	0.625565422314573	4.1670692606364	1.29493470960488	0.204442687070896	0.966601799614364	-5.50731412694004
a_HQ_transcript/121559	3.64853116425594	5.23623354098026	1.29491819361112	0.204448317695611	0.966601799614364	-5.43082010780766
a_HQ_transcript/112624	0.462532058440226	3.92534710352383	1.29489682342364	0.204455603384917	0.966601799614364	-5.44646886767036

a_HQ_transcript/29404	1.74765357840686	0.319874827767441	1.29488350397288	0.204460144454881	0.966601799614364	-5.00659473969955
a_HQ_transcript/9876	1.48613617606502	-0.00246910917071473	1.2947956108317	0.204490112247792	0.966601799614364	-5.04724785359835
a_HQ_transcript/49136	-2.05778025971502	0.145068876184101	-1.29479284144713	0.20449105654353	0.966601799614364	-5.0844984851135
a_HQ_transcript/133201	0.483564825863984	4.21400161615667	1.29476192446246	0.204501598741699	0.966601799614364	-5.49618422326347
a_HQ_transcript/26005	-5.46324063367638	0.861822364485525	-1.2945694801156	0.204567228493379	0.966833389532739	-5.15265829472128
a_HQ_transcript/56083	2.68153846614209	4.29799802451409	1.29442615346152	0.204616117936836	0.966914007743025	-5.44974576438788
a_HQ_transcript/29561	-1.36549634245951	0.980682208250109	-1.29442194229367	0.204617554521865	0.966914007743025	-5.04466167068532
a_HQ_transcript/86539	-1.63955968673883	0.360697275194367	-1.294318397128	0.204652880020596	0.966933496969558	-4.89246585466638
a_HQ_transcript/80757	-1.24253677841992	4.78326587501315	-1.29430997576216	0.204655753260295	0.966933496969558	-5.55409148428214
a_HQ_transcript/13297	-0.669131606946861	2.94849192641359	-1.29423772926035	0.204680403910122	0.966933496969558	-5.23189400141789
a_HQ_transcript/121752	-1.74775830158125	0.471383027692868	-1.29418109433922	0.204699729437595	0.966933496969558	-4.98693457817649
a_HQ_transcript/134947	2.94706538024685	4.09184052191978	1.29416608414167	0.204704851598324	0.966933496969558	-5.36620142228314
a_HQ_transcript/62878	1.53667672050893	1.12579201267168	1.2939569782832	0.204776218153597	0.967085537693175	-4.97308549361337
a_HQ_transcript/96327	2.45435197144471	1.96566737852121	1.29392321322909	0.204787743736114	0.967085537693175	-5.06502447112015
a_HQ_transcript/133210	2.51190954948037	0.828479568115177	1.29381458107137	0.204824828290255	0.967085537693175	-5.03261217887267
a_HQ_transcript/103115	-1.32508163603939	2.04699550842191	-1.2937784621123	0.20483715961542	0.967085537693175	-5.18903177581103
a_HQ_transcript/145016	-3.06376405660448	2.39130147114509	-1.29377034664374	0.204839930384333	0.967085537693175	-5.30044291696226
a_HQ_transcript/128398	-2.06621869577082	3.26368237146973	-1.29376048299411	0.204843298052531	0.967085537693175	-5.28153864391465
a_HQ_transcript/139706	-0.538126419646513	2.23125877058614	-1.29370584255794	0.204861954269381	0.967085537693175	-5.07526448134432
a_HQ_transcript/1599	-0.587762472816952	3.77069083520793	-1.29364271654305	0.204883509378716	0.967085537693175	-5.42555962802256
a_HQ_transcript/62283	1.16955488747219	4.29662625548719	1.29362901434909	0.204888188379577	0.967085537693175	-5.4756924474798
a_HQ_transcript/103670	1.15781047408279	2.71180846778084	1.2935752900351	0.204906534847306	0.967085537693175	-5.23502480379638
a_HQ_transcript/143739	-0.722740313485294	3.27602449633738	-1.29353572066342	0.204920048303173	0.967085537693175	-5.27260296302903
a_HQ_transcript/108921	-1.13934991512237	2.05130197319933	-1.29335419412811	0.204982050670793	0.967216871377629	-5.05901386418812
a_HQ_transcript/91768	-2.08854137871691	1.19071603525623	-1.29332468213375	0.204992132163062	0.967216871377629	-5.02361204587882
a_HQ_transcript/92984	-2.1940362065038	0.851881943116425	-1.29330810415672	0.204997795475027	0.967216871377629	-5.1703560016179
a_HQ_transcript/126320	-1.84415264776042	-0.327676584770074	-1.293238221871	0.205021669731667	0.967251003827802	-5.07021958426268
a_HQ_transcript/48438	1.50056294778881	1.37269977983229	1.29312562255823	0.20506014208346	0.967310265000013	-4.96374799339358
a_HQ_transcript/52907	-1.91590010521753	1.83782789473671	-1.29310405149796	0.205067512998816	0.967310265000013	-5.05986008986342
a_HQ_transcript/107515	-2.29172682550913	2.50554185520402	-1.29298877787116	0.205106905863096	0.967335801207877	-5.2735857916939
a_HQ_transcript/19523	-1.66557609646431	1.64065408208671	-1.2929767758584	0.205111007684257	0.967335801207877	-4.98006124754984
a_HQ_transcript/94842	-1.72934574846975	-0.0390145160646089	-1.2929143433377	0.205132345698259	0.967335801207877	-5.04323081412229
a_HQ_transcript/348	2.51092690012847	0.432114413672333	1.29286460103369	0.205149347693857	0.967335801207877	-5.04295808477957
a_HQ_transcript/3377	-1.86857728944569	-0.181683706136391	-1.29283746144522	0.205158624498713	0.967335801207877	-5.05101790889106
a_HQ_transcript/129598	0.826491816564626	2.25528031332803	1.29272014706798	0.20519872835734	0.967335801207877	-5.03080863365415
a_HQ_transcript/37967	2.27440613443064	0.0651716375438077	1.29270129983777	0.20520517182911	0.967335801207877	-5.11481026243125
a_HQ_transcript/101460	3.77443141530544	1.4580218524326	1.29269870654119	0.205206058434736	0.967335801207877	-5.17873185834592
a_HQ_transcript/145410	-2.41997240038341	1.72503859351123	-1.29264325263602	0.205225017914035	0.967346727319737	-4.98972765225251
a_HQ_transcript/105568	-1.71535020904157	0.694957219826494	-1.29249451934163	0.205275875841593	0.967507995696112	-5.02882082453589
a_HQ_transcript/19847	1.97012042387678	1.15145686160801	1.29233996059758	0.205328735882825	0.967678673536311	-4.95687798793039
a_HQ_transcript/65118	0.769428785056535	1.40741501649825	1.2922776316033	0.205350055711567	0.967695953919074	-4.93633573505022
a_HQ_transcript/72703	0.997193144965991	1.59801400459069	1.29223190444653	0.205365697900249	0.967695953919074	-4.93386527632073
a_HQ_transcript/90827	1.55855775997598	3.55436367146693	1.29208908250253	0.205414559775915	0.967793897198623	-5.22014904349168
a_HQ_transcript/125430	-2.10669978989713	-0.513413449157013	-1.29207074405035	0.20542083432044	0.967793897198623	-5.08514468265671
a_HQ_transcript/94213	0.436314239579617	2.73557349339417	1.29196628881571	0.205456576704411	0.967793897198623	-5.19396818529505
a_HQ_transcript/141688	0.635826416239119	3.56821927281399	1.29190043274944	0.205479113695865	0.967793897198623	-5.36349434502293
a_HQ_transcript/15114	1.55100758944387	3.86774520803456	1.29176339340964	0.205526016760312	0.967793897198623	-5.43930089980637
a_HQ_transcript/86982	-1.63425780436174	0.324327578745011	-1.29165644112385	0.20556262789055	0.967793897198623	-4.94865770787987
a_HQ_transcript/54071	-3.06199614744003	1.98272649794721	-1.29158740631773	0.205586262017993	0.967793897198623	-5.09401291194157
a_HQ_transcript/17615	-1.31080963213743	-0.607161023709782	-1.29157934254875	0.205589022791269	0.967793897198623	-5.08399877793786

a_HQ_transcript/147037	1.80165480509026	-0.275865794922219	1.29156901513362	0.205592558604832	0.967793897198623	-5.07124727212873
a_HQ_transcript/23815	-0.918833247761355	2.08213041110498	-1.29155735048516	0.20559655230471	0.967793897198623	-5.08515463631617
a_HQ_transcript/103877	1.23390205589353	2.93606529390524	1.29154138645718	0.205602018106119	0.967793897198623	-5.09669612022848
a_HQ_transcript/73537	-2.01571525786226	0.246708011314026	-1.29149677902907	0.205617291487922	0.967793897198623	-4.94262015950258
a_HQ_transcript/46328	-2.02592986144564	1.38158336002604	-1.29149022453918	0.20561953578834	0.967793897198623	-5.00227396537824
a_HQ_transcript/137396	-2.7476247028508	0.56987566485498	-1.29146825945776	0.205627056912394	0.967793897198623	-5.10733950918315
a_HQ_transcript/40119	5.42473200463522	0.738641080545187	1.29137866330538	0.205657737944855	0.967793897198623	-5.08193980218124
a_HQ_transcript/142558	-1.29758780487662	1.79739323901202	-1.29137109689219	0.205660329123645	0.967793897198623	-5.05882426375585
a_HQ_transcript/37065	3.23265710241728	1.88195540368668	1.29134425179826	0.205669522641319	0.967793897198623	-5.09525779782021
a_HQ_transcript/10535	-1.7495965892565	1.11085132500321	-1.291239271029	0.205705477930249	0.967884735399831	-4.87908448945777
a_HQ_transcript/56927	-1.16464312149306	5.24573328217184	-1.29105820608174	0.2057675028278	0.96809821160456	-5.57068448069151
a_HQ_transcript/119541	2.60025325125348	4.04877436366662	1.29097086953724	0.205797425574786	0.968124884342299	-5.27924717632611
a_HQ_transcript/112590	-0.648330290262238	2.64427635678121	-1.2909182698803	0.205815448564396	0.968124884342299	-5.21421411139111
a_HQ_transcript/120875	3.12892020801237	1.22855774555215	1.29089583177254	0.205823137226502	0.968124884342299	-5.27212265401774
a_HQ_transcript/9373	-1.04877944179159	2.43799212300987	-1.29080444356442	0.205854454647762	0.968133899573457	-5.20130233211616
a_HQ_transcript/81379	0.55808684417738	2.58018503211063	1.29072196903387	0.205882720590146	0.968133899573457	-5.13846509361925
a_HQ_transcript/75208	-3.30181843135128	0.699850745792425	-1.29066398109842	0.205902596172716	0.968133899573457	-5.05866887283377
a_HQ_transcript/15520	1.26019892447609	2.02343185257895	1.29057318370343	0.205933720250884	0.968133899573457	-5.12745262301912
a_HQ_transcript/50902	-0.742586716365034	2.24155793409575	-1.29056236506408	0.205937428967724	0.968133899573457	-5.0630401923504
a_HQ_transcript/138599	1.79884821654851	0.683109565076142	1.29055478236033	0.205940028410003	0.968133899573457	-4.93772364769991
a_HQ_transcript/143582	1.20638560433178	0.909947546765524	1.29055008038923	0.205941640314922	0.968133899573457	-4.99746618238745
a_HQ_transcript/78239	-1.1004529943437	-0.0730394639843528	-1.29040051792452	0.205992917534601	0.96823274267356	-5.04713305715246
a_HQ_transcript/147554	-1.79612729576796	0.58909100501383	-1.29039158623533	0.205995980055118	0.96823274267356	-4.98862673087093
a_HQ_transcript/40445	-2.13658058662774	0.0126502580158503	-1.29027017840869	0.206037612115233	0.968234492559763	-5.01797218860369
a_HQ_transcript/15272	-1.34664172157956	0.457446422312225	-1.2902191764994	0.20605510313055	0.968234492559763	-5.03121292971886
a_HQ_transcript/26730	-0.56332327499797	2.08260873544572	-1.29021652231231	0.206056013410253	0.968234492559763	-5.16769452343434
a_HQ_transcript/79311	-0.709167591768098	3.64443980794035	-1.29017039316056	0.206071834347122	0.968234492559763	-5.38525647391717
a_HQ_transcript/104469	1.36522689248178	1.49654723902642	1.29014764381298	0.206079637041519	0.968234492559763	-5.07229716290216
a_HQ_transcript/70009	-2.31643836519788	1.69257923623279	-1.29005362999387	0.206111884791886	0.968280250383189	-5.14790110064645
a_HQ_transcript/34872	1.95938308540732	0.0524425958092859	1.28995697361327	0.20614504296795	0.968280250383189	-4.99730562490704
a_HQ_transcript/130437	-1.3430692484199	1.01339516269947	-1.28988758130821	0.206168850645363	0.968280250383189	-4.96454113469981
a_HQ_transcript/53270	1.82021807308962	1.08339662555706	1.28985913231277	0.206178611762508	0.968280250383189	-5.02661214233765
a_HQ_transcript/134940	-1.32257241239848	2.61436308774876	-1.28985643388375	0.20617953763705	0.968280250383189	-5.1297426501032
a_HQ_transcript/126043	-2.23739661311082	0.0267762990524093	-1.28980554762455	0.206196998125452	0.968280250383189	-5.04891505966293
a_HQ_transcript/91377	-2.03073790510014	1.97842526952398	-1.28977937172305	0.206205980241768	0.968280250383189	-4.97661930454106
a_HQ_transcript/151746	2.04410735125134	1.51675479104018	1.28965808150531	0.206247604192929	0.968339351452761	-5.05549127707877
a_HQ_transcript/119797	-0.472912769985439	4.86845252190691	-1.28961935675158	0.20626089496274	0.968339351452761	-5.55812501890953
a_HQ_transcript/72685	-2.67011802515356	2.38864882131623	-1.28949929789345	0.206302104646743	0.968339351452761	-5.10276459790234
a_HQ_transcript/25362	0.800711876117339	2.86558051257015	1.2894831843358	0.20630763603197	0.968339351452761	-5.1017013516185
a_HQ_transcript/134452	1.43170196141768	0.030209819576109	1.28946160169049	0.20631504499611	0.968339351452761	-5.01893534040872
a_HQ_transcript/151621	-1.66555920692931	2.88168201496393	-1.28945148219897	0.206318518919324	0.968339351452761	-5.15286972280243
a_HQ_transcript/95714	1.33782713842591	0.205764169457915	1.28937322973187	0.206345383732705	0.968387248959684	-5.04497822274299
a_HQ_transcript/14283	1.40899001850873	3.49957168163083	1.2890783607327	0.206446638935913	0.96875144633694	-5.23860001031118
a_HQ_transcript/14614	-1.42776270763613	-0.29100479277328	-1.28899005597786	0.206476969292161	0.96875144633694	-5.06365518611947
a_HQ_transcript/140228	-7.3920543290076	2.69244834723305	-1.28897732750935	0.20648134146486	0.96875144633694	-5.2314170057448
a_HQ_transcript/11007	-0.948932080474726	2.63209704598685	-1.28895278223827	0.206489772855664	0.96875144633694	-5.20958076861163
a_HQ_transcript/149349	-0.815596499707064	1.7236195065655	-1.2889046226713	0.206506316604064	0.96875144633694	-4.9822993893338
a_HQ_transcript/129774	-1.54628309036224	0.973192086422464	-1.28885353235422	0.206523868223082	0.968755601361468	-5.0177395902892
a_HQ_transcript/104898	-1.78954262914991	2.03770078474475	-1.28879245255396	0.206544853126783	0.96877585915869	-5.00247370791355
a_HQ_transcript/63168	-1.7416879479164	1.70358526567324	-1.28865792968285	0.206591076239092	0.968822011435749	-4.97273365701819

a_HQ_transcript/1390	2.17338529779333	0.797668461431192	1.28855942042056	0.206624929777988	0.968822011435749	-5.16006384006838
a_HQ_transcript/50475	1.45235240041417	1.9471305495624	1.28847197231073	0.206654985589844	0.968822011435749	-5.07398015351899
a_HQ_transcript/126347	-2.20076656536004	0.174317697078525	-1.28845867980241	0.206659554500026	0.968822011435749	-5.03988573323565
a_HQ_transcript/113764	-1.07910239704454	0.171352259719106	-1.28843445551889	0.206667881084931	0.968822011435749	-4.95671061754125
a_HQ_transcript/147561	2.72248975462378	0.0315022107026291	1.28840558174739	0.206677806165908	0.968822011435749	-5.08859360368968
a_HQ_transcript/149719	-2.18438050774186	0.0577590038577121	-1.28836547992141	0.206691591383232	0.968822011435749	-5.04869221620348
a_HQ_transcript/27596	2.52406824795277	0.361384142940002	1.28833731147016	0.206701274856769	0.968822011435749	-5.05637196389758
a_HQ_transcript/63851	2.29932377811014	0.870812984578963	1.28831965257371	0.206707345634527	0.968822011435749	-5.23655781466325
a_HQ_transcript/104973	1.66184685032027	0.774063092608493	1.2882788788931	0.206721363333292	0.968822011435749	-5.07743746981528
a_HQ_transcript/146130	-1.71850167586737	1.89325834959041	-1.28810918023513	0.206779712271539	0.968917662452789	-5.04669479878368
a_HQ_transcript/95489	-2.35145428365123	0.988485018285014	-1.28807780569991	0.206790501416081	0.968917662452789	-5.1350010270925
a_HQ_transcript/14400	0.573717283474152	2.65481407477658	1.28807409106977	0.206791778839618	0.968917662452789	-5.08913671154901
a_HQ_transcript/46848	-0.668574536919441	4.79522825741516	-1.28791661779706	0.206845937810745	0.969093308056982	-5.54743498230919
a_HQ_transcript/102572	0.817725781864908	1.62955994599921	1.2877131121618	0.206915944413567	0.969212380359087	-4.9288511095931
a_HQ_transcript/67169	2.07736600503333	2.11464323452827	1.28769811144403	0.206921105421544	0.969212380359087	-5.00761132550041
a_HQ_transcript/131369	1.61950611671647	-0.195717007791982	1.28769733019638	0.206921374213052	0.969212380359087	-5.1126009840321
a_HQ_transcript/64395	3.64690143985697	3.26722938261386	1.28763790446191	0.206941820661777	0.969230050070725	-5.25117155619831
a_HQ_transcript/134659	0.729743771447694	3.48320394349622	1.2874609756658	0.207002705151108	0.969298791762047	-5.36224570617926
a_HQ_transcript/38122	2.24999187209283	0.676787555941221	1.28741464937876	0.20701864913536	0.969298791762047	-4.91021508988709
a_HQ_transcript/5730	-1.68553420043568	-0.229295679675809	-1.28737237558929	0.207033199197117	0.969298791762047	-5.01123349197621
a_HQ_transcript/143532	-2.18325043436727	0.305204232668123	-1.28735825026247	0.207038061115061	0.969298791762047	-5.08716075624949
a_HQ_transcript/99447	1.892148900352	2.64999151710504	1.28735298322138	0.207039874045513	0.969298791762047	-5.22013768121641
a_HQ_transcript/104951	1.68314915437994	0.434240089379178	1.28725159316239	0.20707477514074	0.969384112859864	-5.07501641165429
a_HQ_transcript/93921	2.06644551795724	0.777789267645461	1.28712319310353	0.207118980192347	0.96942359123517	-5.03630054899082
a_HQ_transcript/41220	-2.32644367916546	2.69026202284128	-1.28706913741601	0.207137592408714	0.96942359123517	-5.27899996220057
a_HQ_transcript/18152	-1.18954421942244	3.64339486427758	-1.28701786590295	0.207155247164073	0.96942359123517	-5.32644880641641
a_HQ_transcript/107967	-1.68241023664238	0.358903426387338	-1.28701251038302	0.207157091341627	0.96942359123517	-5.10402414403177
a_HQ_transcript/74494	-1.69023020298991	3.96982019565322	-1.28698491137979	0.207166595278064	0.96942359123517	-5.32507114316259
a_HQ_transcript/96582	1.67446912624741	0.507273951296932	1.28682242973518	0.207222553838544	0.96954535687261	-5.10738989631852
a_HQ_transcript/12584	-1.07503009518208	2.33290340894137	-1.28681249516192	0.20722597567068	0.96954535687261	-5.02374640678807
a_HQ_transcript/105382	-1.73541355682464	0.874808286862067	-1.28669109037884	0.207267795405791	0.969657057232961	-5.03922081143777
a_HQ_transcript/87319	-1.81102138166134	0.94234713789013	-1.28664633762787	0.207283212792059	0.969657057232961	-4.9620625397648
a_HQ_transcript/6551	0.428674896248507	4.02071685290784	1.2863633263564	0.207380730743174	0.96984569768349	-5.4210310432926
a_HQ_transcript/148947	-3.16351079471438	0.852252531062424	-1.28625477302481	0.207418144496565	0.96984569768349	-5.12582440484391
a_HQ_transcript/132137	-1.61519241061209	0.707234257116737	-1.28622738824605	0.207427583683831	0.96984569768349	-5.06650092412569
a_HQ_transcript/133626	2.63606714427198	0.805037102747631	1.28618132243234	0.207443462723561	0.96984569768349	-5.12122259573745
a_HQ_transcript/88536	1.26654242756789	2.18919627355813	1.28609092203371	0.207474626725242	0.96984569768349	-5.03437088112738
a_HQ_transcript/127807	1.28370989561023	0.273834129294091	1.28585313468743	0.207556616843867	0.96984569768349	-5.06213990609107
a_HQ_transcript/126167	-1.68461693701002	0.143360043139759	-1.28575198384847	0.207591501552308	0.96984569768349	-4.98157701270328
a_HQ_transcript/140650	-0.966458772995453	1.56944904479388	-1.28573108218319	0.207598710633623	0.96984569768349	-5.00100971858868
a_HQ_transcript/29257	0.980720317182296	2.01482575791034	1.2855855233054	0.207648919835918	0.96984569768349	-5.01508013442866
a_HQ_transcript/14648	0.452129069155153	4.73901347879281	1.28550914255752	0.207675270362939	0.96984569768349	-5.53754908941887
a_HQ_transcript/30776	-1.02287523955905	1.59816444169887	-1.28544389934788	0.207697780570397	0.96984569768349	-4.96824556961282
a_HQ_transcript/80741	-5.30151995310532	1.62403640297794	-1.28541862024926	0.207706502860957	0.96984569768349	-5.15917568072637
a_HQ_transcript/150442	-0.313151951824923	8.02072652064514	-1.28541438402127	0.207707964554646	0.96984569768349	-5.85064919393538
a_HQ_transcript/48750	1.6794849643755	1.04981649568211	1.28540433876797	0.207711430660727	0.96984569768349	-4.95773740948223
a_HQ_transcript/32825	-0.836092737767631	4.71699585438065	-1.2853962118109	0.207714234892453	0.96984569768349	-5.55912337342217
a_HQ_transcript/131431	-1.70718451791153	0.875830077555639	-1.28536595806466	0.207724674293978	0.96984569768349	-5.04059659761029
a_HQ_transcript/135332	-2.18728379804933	0.556100319937954	-1.28532290137096	0.20773953218579	0.96984569768349	-5.02139092072534
a_HQ_transcript/30794	-2.12130297743158	0.113234119845796	-1.2853013050024	0.207746984908209	0.96984569768349	-5.09437116997248

a_HQ_transcript/146240	-1.59953045660641	1.8383969412722	-1.28523092407257	0.207771274174573	0.96984569768349	-4.99097822654887
a_HQ_transcript/2160	-1.24996915852775	1.52778231565382	-1.28514644152176	0.20780043291855	0.96984569768349	-4.94006392937151
a_HQ_transcript/10741	-0.704023792330304	2.56115201165085	-1.28507010361105	0.207826783250432	0.96984569768349	-5.15280345295762
a_HQ_transcript/133862	-3.27687124462849	0.278669701347833	-1.28503085477014	0.207840332158521	0.96984569768349	-5.07997395707062
a_HQ_transcript/137664	0.490006731198822	3.38224208283205	1.28495504330934	0.207866504574938	0.96984569768349	-5.28357378844546
a_HQ_transcript/127262	-1.3326675430778	1.1209908690067	-1.28491791042203	0.20787932488684	0.96984569768349	-4.93484894129404
a_HQ_transcript/149035	-1.12262263869572	2.01245129789552	-1.28491315536602	0.207880966636647	0.96984569768349	-5.0284065392276
a_HQ_transcript/40319	-3.50714884066227	1.70200663427424	-1.2848398884482	0.207906264311953	0.96984569768349	-5.14895304870451
a_HQ_transcript/76743	-1.08659583379184	3.60573402632845	-1.28476808788895	0.207931057948091	0.96984569768349	-5.27790562995794
a_HQ_transcript/130327	-3.59451149560561	0.539388131554614	-1.28475141902656	0.207936814236832	0.96984569768349	-5.08474771756339
a_HQ_transcript/129465	3.75319786915078	2.40843433396996	1.2847452779277	0.207938934984219	0.96984569768349	-5.30830826971799
a_HQ_transcript/127431	-1.1425628252271	0.630781786796705	-1.28473674585757	0.207941881449203	0.96984569768349	-4.97635438338606
a_HQ_transcript/111780	2.44174222724724	1.02915446383818	1.28470394398187	0.207953209543568	0.96984569768349	-5.05057457539842
a_HQ_transcript/147543	-1.34453578526488	3.45015478766516	-1.2846846567745	0.207959870579626	0.96984569768349	-5.40192938908653
a_HQ_transcript/149424	2.01905208230049	1.71606844775039	1.28455930604294	0.208003165693839	0.96984569768349	-5.07955334994964
a_HQ_transcript/37765	-1.93322570123306	0.834165478308319	-1.28451515524489	0.208018416645976	0.96984569768349	-5.05598392709908
a_HQ_transcript/144360	3.46558411050267	1.33365778833887	1.28450705166471	0.208021215947186	0.96984569768349	-5.17269977296354
a_HQ_transcript/78725	-1.94154287505337	2.2082194170751	-1.28442432695624	0.208049794010709	0.96984569768349	-5.26549224289
a_HQ_transcript/134635	2.67873819358083	0.974076681540256	1.28440650066072	0.208055952669827	0.96984569768349	-5.18986802866298
a_HQ_transcript/112324	2.01638185435138	0.760017018449048	1.28431647079345	0.208087058456189	0.96984569768349	-5.07495534620571
a_HQ_transcript/147191	-3.66740167991385	2.19866789724342	-1.28430420176366	0.208091297742853	0.96984569768349	-5.10996889887919
a_HQ_transcript/51965	2.00358762011925	0.581342793853305	1.28421295388073	0.208122828447909	0.96984569768349	-5.1604138446647
a_HQ_transcript/31699	-1.2101138111	1.19420272685254	-1.28420971964173	0.208123946105682	0.96984569768349	-5.00181065899039
a_HQ_transcript/95971	4.03672045903049	1.85392574842137	1.28417264623331	0.208136757909391	0.96984569768349	-5.22310741892628
a_HQ_transcript/130521	1.44374473012793	0.620084146355873	1.28408459476903	0.208167189073976	0.96984569768349	-5.00954854367884
a_HQ_transcript/10644	-2.00912633523104	1.54505644939653	-1.28404641389823	0.208180385683022	0.96984569768349	-5.0577957127857
a_HQ_transcript/120645	0.359730405034767	4.30071229809084	1.28397787458842	0.208204076794742	0.96984569768349	-5.48476340612174
a_HQ_transcript/91378	1.22168931471283	1.02617680978334	1.28388882885967	0.208234859154578	0.96984569768349	-4.96733658707827
a_HQ_transcript/100051	1.94551175232553	2.85179750205333	1.28388707102485	0.208235466858049	0.96984569768349	-5.11757480126228
a_HQ_transcript/55671	-0.829768431379857	1.16170227179701	-1.28382313588731	0.208257570876064	0.96984569768349	-5.02935758744927
a_HQ_transcript/144793	-1.95412350571254	-0.0702801089339706	-1.28380501795916	0.208263835032527	0.96984569768349	-5.05354920639174
a_HQ_transcript/133174	-1.74286908216299	0.692815575536616	-1.28370054831492	0.20829995752477	0.96984569768349	-5.17966367788564
a_HQ_transcript/49739	-1.79777819393716	0.357458348492381	-1.28363504588386	0.208322608739285	0.96984569768349	-4.99214392342359
a_HQ_transcript/36148	-2.11204069396021	2.27198066726327	-1.28362434751398	0.208326308490503	0.96984569768349	-5.07942663805515
a_HQ_transcript/120339	1.63738587127248	1.60472760936387	1.28358830621963	0.208338772795674	0.96984569768349	-5.23121067153978
a_HQ_transcript/49846	2.17798972722422	2.21361007521865	1.28357651556702	0.208342850527097	0.96984569768349	-5.15269396980666
a_HQ_transcript/138192	-1.65810267483699	1.04618839813168	-1.28357113534681	0.208344711266358	0.96984569768349	-4.98797589681116
a_HQ_transcript/36142	1.50113455541364	0.0983506406588748	1.28355536943374	0.208350163950983	0.96984569768349	-5.05816944935689
a_HQ_transcript/127479	-1.58245134706537	1.63090817284553	-1.28351185769231	0.20836521316874	0.96984569768349	-5.06854254770914
a_HQ_transcript/140875	1.91446864799344	0.268104979640919	1.28349158062297	0.208372226592658	0.96984569768349	-5.09661678159091
a_HQ_transcript/149895	1.71417493079833	1.72750165864445	1.28344220256262	0.208389306203697	0.96984569768349	-5.11674686449799
a_HQ_transcript/144335	1.3332185878773	0.670265986119918	1.2833721755221	0.208413530009216	0.96984569768349	-5.04573890882283
a_HQ_transcript/109045	0.765151427174525	1.86059621575708	1.28336022384603	0.2084176645552	0.96984569768349	-4.99761325779651
a_HQ_transcript/137857	-3.05662787283955	2.05576587112775	-1.28333059121207	0.208427915895225	0.96984569768349	-5.35058664000321
a_HQ_transcript/3687	1.93422698543375	0.270324753231844	1.28332369056546	0.208430303212595	0.96984569768349	-5.04620895942763
a_HQ_transcript/19029	0.477718428469229	3.90744533318953	1.28332099805515	0.208431234707254	0.96984569768349	-5.40678195528781
a_HQ_transcript/17988	-1.2011411333254	1.8576612463159	-1.28322698055376	0.208463762765526	0.96984569768349	-4.96939493478687
a_HQ_transcript/40503	-2.03023662702611	1.3879418161349	-1.28320428863717	0.208471614263438	0.96984569768349	-5.17700489289815
a_HQ_transcript/100756	2.35125211895228	0.124221363003479	1.28319619408474	0.208474415066666	0.96984569768349	-5.12777838362944
a_HQ_transcript/12158	-1.65114707525406	2.09746171315685	-1.28316906226117	0.208483803181044	0.96984569768349	-5.06121434450309

a_HQ_transcript/44510	-1.78290672899677	0.859065043955452	-1.28315348105925	0.208489194710966	0.96984569768349	-5.04006065720526
a_HQ_transcript/60503	1.70782622545263	1.08267940927835	1.28314692210216	0.208491464324472	0.96984569768349	-5.04845694238838
a_HQ_transcript/27209	-1.16333610603997	1.04808403019004	-1.28301946201082	0.208535573402322	0.969973258440439	-4.96623459199367
a_HQ_transcript/15774	-0.479432552767042	3.72730943326719	-1.28295020388399	0.208559543967112	0.970007134879206	-5.411340349263
a_HQ_transcript/105323	-2.50642037730168	1.38211742235879	-1.28288921272676	0.208580655025193	0.970027707440949	-5.14689350442913
a_HQ_transcript/102413	0.807297583950978	2.85922761663738	1.28283544461496	0.208599267292383	0.970036656733733	-5.17368929260752
a_HQ_transcript/142647	-1.44593519923266	1.75514515154685	-1.28271864978616	0.208639701107754	0.970054331571417	-5.06155488317874
a_HQ_transcript/118563	-1.48606977844023	0.507113958696127	-1.28270452607792	0.208644591071347	0.970054331571417	-5.03983306874431
a_HQ_transcript/114532	0.645050971002526	2.4673987593858	1.28267985534858	0.208653132872926	0.970054331571417	-5.02305888341547
a_HQ_transcript/124388	0.25589646130639	5.91143431112999	1.2824983328054	0.208715989982276	0.970268959188237	-5.65925453221886
a_HQ_transcript/42614	-0.664397531758765	2.03646749624894	-1.28241339709767	0.208745406208498	0.970274165306904	-5.04671626096081
a_HQ_transcript/68904	-1.08286732968048	3.00064040412857	-1.28239870784486	0.20875049393284	0.970274165306904	-5.26761524207576
a_HQ_transcript/24723	-1.20765659665702	1.43187656360792	-1.28228873455303	0.208788586930772	0.970293956531251	-4.98128508880062
a_HQ_transcript/118395	1.44657442757157	-0.377353145753273	1.28218246274156	0.208825402802729	0.970293956531251	-5.07314961194591
a_HQ_transcript/130938	0.49631192347772	3.03126077389764	1.28201489637119	0.20888346302281	0.970293956531251	-5.24990378568252
a_HQ_transcript/148917	1.70678537122869	-0.375046370066304	1.2819709708463	0.208898684841524	0.970293956531251	-5.04512276041151
a_HQ_transcript/4030	0.947885556678739	2.77887326046647	1.28190070165173	0.20892303746492	0.970293956531251	-5.14557005607453
a_HQ_transcript/38611	1.24641789504554	-0.0308499182144621	1.28187745603265	0.208931093983924	0.970293956531251	-5.04128047896126
a_HQ_transcript/117688	0.476191799373563	3.57010419475353	1.2818521261409	0.208939873143029	0.970293956531251	-5.34777861714258
a_HQ_transcript/116783	-1.37129960429813	3.34765276491251	-1.28182679099776	0.208948654402017	0.970293956531251	-5.30563003434719
a_HQ_transcript/17293	-2.73213341692902	2.78809181752306	-1.28181743375179	0.208951897730562	0.970293956531251	-5.35618596260766
a_HQ_transcript/148142	2.737835329498	1.9084375658841	1.28178866220022	0.208961870518751	0.970293956531251	-5.19492226222433
a_HQ_transcript/140669	1.78346126299298	0.628526524605325	1.28178525808092	0.208963050477628	0.970293956531251	-5.07313960500639
a_HQ_transcript/110716	-2.41781963816658	1.91769376237758	-1.28177607590208	0.208966233291808	0.970293956531251	-5.09738868073543
a_HQ_transcript/103893	1.88711788258949	1.88876052975236	1.28173155530323	0.20898166596648	0.970293956531251	-5.06524000913073
a_HQ_transcript/11534	-0.901477130857444	3.44642122969538	-1.28170801994232	0.208989824641832	0.970293956531251	-5.30835173265716
a_HQ_transcript/127374	-0.396651817213302	3.86143065479793	-1.28166384890699	0.209005137450588	0.970293956531251	-5.42020328871081
a_HQ_transcript/133776	-5.02178266675416	2.52512645146043	-1.28160024468807	0.209027188671252	0.970318832701049	-5.26857754352943
a_HQ_transcript/126905	1.60119813060213	1.06769770229747	1.28152756492064	0.209052388492239	0.970358319753804	-4.96931179060623
a_HQ_transcript/135550	-1.15744410107712	1.68399148691157	-1.28133351543313	0.209119681394356	0.970445903921427	-5.08323785525833
a_HQ_transcript/29288	0.379962029935774	3.0015926538614	1.28129409119729	0.20913335502319	0.970445903921427	-5.30098359155247
a_HQ_transcript/34105	1.28753774995894	-0.374079397055288	1.28123000145661	0.209155584911348	0.970445903921427	-5.07225884072305
a_HQ_transcript/130614	1.74071292218053	0.374460718623598	1.28122048520079	0.209158885831098	0.970445903921427	-5.03683336157179
a_HQ_transcript/104945	-2.1894918947585	1.83704718962368	-1.28119426903961	0.209167979679528	0.970445903921427	-5.25049650183165
a_HQ_transcript/62907	-2.35697451403416	2.35097722392499	-1.28116703309342	0.209177427587898	0.970445903921427	-5.23817119409371
a_HQ_transcript/3860	1.42319833126084	2.79905398420789	1.28113620389774	0.209188122353005	0.970445903921427	-5.20286321098148
a_HQ_transcript/152435	-3.71204117070896	0.802804657488567	-1.28103801320136	0.209222187841116	0.970495370838247	-5.09821271206693
a_HQ_transcript/8429	-2.70395589741634	6.30069186103152	-1.28082811795265	0.209295021307342	0.970495370838247	-5.3536356590128
a_HQ_transcript/73206	0.796146008425413	2.0064837897747	1.28080359637968	0.209303531525158	0.970495370838247	-5.02801751630049
a_HQ_transcript/30386	1.95474866048103	1.08561048768658	1.28078387985994	0.209310374338318	0.970495370838247	-5.10544341327992
a_HQ_transcript/63545	2.75533984439208	0.902741145890465	1.28077396972958	0.209313813811099	0.970495370838247	-5.28647522720931
a_HQ_transcript/69358	1.03387492716441	2.14751195172717	1.28071646509642	0.209333772579799	0.970495370838247	-5.09160945495435
a_HQ_transcript/138034	-6.54017288937952	5.5132517828742	-1.28071547631174	0.209334115780841	0.970495370838247	-5.40687422901012
a_HQ_transcript/118289	2.87686790695121	0.743452029096717	1.28068827764493	0.209343556436997	0.970495370838247	-5.10373017831206
a_HQ_transcript/141226	1.8402882141076	-0.0769950887947291	1.28067245715724	0.20934904787608	0.970495370838247	-5.02716894747817
a_HQ_transcript/128240	2.16100126475036	0.566821741470021	1.28049524551992	0.20941056715232	0.970703145728075	-5.12377145069625
a_HQ_transcript/112058	2.23110146117695	0.173085583853203	1.28030330963568	0.209477213427546	0.970858219474155	-5.08313840281553
a_HQ_transcript/91973	-1.94836233563911	0.205923015448902	-1.28030269899861	0.209477425485908	0.970858219474155	-5.06896761530221
a_HQ_transcript/135236	1.58048843532445	3.40165175154536	1.28020775567162	0.209510398811562	0.970933625298451	-5.21206506792872
a_HQ_transcript/78349	-1.41533348858487	1.1227377589368	-1.28003973669779	0.209568760570195	0.970942537172649	-4.94594418253209

a_HQ_transcript/20900	2.00386597067546	0.276547245803134	1.28003819192349	0.209569297208081	0.970942537172649	-5.10732403082473
a_HQ_transcript/114186	-2.35776159834806	0.056244371201838	-1.28002050008066	0.209575443237222	0.970942537172649	-5.01967829404946
a_HQ_transcript/92721	1.62748355287808	0.566023084520536	1.28000987052636	0.20957913593974	0.970942537172649	-4.98827821380181
a_HQ_transcript/57773	1.50906204020933	1.11609398999341	1.2799050608032	0.209615549431738	0.970962665341095	-5.00693365853858
a_HQ_transcript/40260	3.04290714159326	0.249128728535425	1.27983139637053	0.209641145147691	0.970962665341095	-5.10995303526541
a_HQ_transcript/119996	-2.13957915577539	0.745126189383996	-1.27980897449946	0.209648936403982	0.970962665341095	-5.14557508577699
a_HQ_transcript/140578	1.20663982558585	0.976774089243441	1.27980506154788	0.209650296117122	0.970962665341095	-4.94802561454606
a_HQ_transcript/107307	0.586774938303394	3.23832143691102	1.27974543367459	0.209671017057962	0.970981268287543	-5.28424260680952
a_HQ_transcript/141113	-1.4960992477946	1.57454727310499	-1.27965393654446	0.209702815719272	0.971051164831501	-5.11309304647162
a_HQ_transcript/87904	2.25603883999441	1.59140891239943	1.27958242627597	0.209727670745465	0.971088899561285	-5.11577217731642
a_HQ_transcript/117723	-2.28589844678816	0.487803852674428	-1.27938874754699	0.209794999418383	0.971224102708424	-5.07923187536644
a_HQ_transcript/147725	1.75683738859868	0.233086401770909	1.27925651038316	0.209840978523512	0.971224102708424	-5.00558252183366
a_HQ_transcript/108063	1.06166082833562	-0.337416637292572	1.27921898797538	0.209854026524109	0.971224102708424	-5.0733218470774
a_HQ_transcript/19039	-1.59795920221699	3.4254297585516	-1.27917043758438	0.20987091029559	0.971224102708424	-5.26760138236637
a_HQ_transcript/25988	-1.48369480330417	1.67224487155605	-1.27916468744891	0.209872910017536	0.971224102708424	-5.10074855934468
a_HQ_transcript/11455	-1.41100151445448	0.736054541709832	-1.27914680904147	0.209879127675215	0.971224102708424	-4.93784270586215
a_HQ_transcript/139405	4.4598740892646	3.2969255727711	1.27912620032265	0.209886295039248	0.971224102708424	-5.61070816398577
a_HQ_transcript/147933	-0.45524689567909	3.54152299508388	-1.27898612296567	0.209935016489752	0.971224102708424	-5.37861308383598
a_HQ_transcript/121859	-0.741967276028475	2.97326781144032	-1.2789812022911	0.209936728145556	0.971224102708424	-5.40717618500254
a_HQ_transcript/144826	3.2228128353753	4.83911915662096	1.2789119311912	0.209960825207766	0.971224102708424	-5.45747562592683
a_HQ_transcript/121101	-0.477400946743211	2.80242137188937	-1.2788926009268	0.209967549923932	0.971224102708424	-5.25446467781469
a_HQ_transcript/130662	-0.580523221101474	3.47755488834445	-1.2788443416042	0.209984339348331	0.971224102708424	-5.37492070764823
a_HQ_transcript/98049	-0.89851366605998	5.7675441061759	-1.27881092073267	0.209995967069742	0.971224102708424	-5.54507459552744
a_HQ_transcript/56752	-1.1686279422965	-0.228476776866862	-1.27877740828976	0.210007627140226	0.971224102708424	-5.02205955058109
a_HQ_transcript/66393	-1.76184415068182	1.82503263172972	-1.27877420229035	0.210008742637553	0.971224102708424	-5.17697584535492
a_HQ_transcript/56126	0.535779510126809	3.34705438721981	1.27871559524348	0.210029135198999	0.971224102708424	-5.30380134627219
a_HQ_transcript/95948	1.82322983916823	-0.0506631834790081	1.27868174827374	0.210040913073693	0.971224102708424	-5.01036762908761
a_HQ_transcript/5660	-2.12767288490764	1.72313508248072	-1.27854744857641	0.210087650844159	0.971362946887468	-4.95747678573193
a_HQ_transcript/6077	2.03390473295844	1.02476277438145	1.27840104267786	0.210138610679905	0.971518273212982	-4.94289666108248
a_HQ_transcript/41788	1.1033546209317	4.05516992707545	1.27834315275213	0.210158763141388	0.971518273212982	-5.44190333915729
a_HQ_transcript/53964	-1.86158371321058	0.844989650393287	-1.27830689645649	0.210171385316083	0.971518273212982	-4.95426198438357
a_HQ_transcript/80174	0.879207881563384	2.83564195345607	1.27820841179567	0.210205674413925	0.971599510363599	-5.19672227984904
a_HQ_transcript/28222	-1.17771136314541	1.38809911965027	-1.27815541093146	0.210224129313412	0.971607552574541	-5.1413221268703
a_HQ_transcript/96755	1.46874961086086	0.852001816941737	1.27806002474928	0.210257345870929	0.971683813069277	-4.99223896473619
a_HQ_transcript/21653	-2.00719123809851	-0.12446863735154	-1.27784256509018	0.210333087226279	0.971876680322767	-5.08735832606919
a_HQ_transcript/19250	1.74863958963846	0.608385893564569	1.27777642535724	0.210356127840146	0.971876680322767	-4.9653668116907
a_HQ_transcript/149646	-3.20087974119146	1.82213785084234	-1.27774914851267	0.210365630631163	0.971876680322767	-5.1147799842432
a_HQ_transcript/73550	-1.67670575974153	0.786574949504494	-1.27774030031135	0.210368713264587	0.971876680322767	-5.12951241735709
a_HQ_transcript/68827	-1.98556302640701	-0.100062155726743	-1.27770021991411	0.210382677341408	0.971876680322767	-5.07942364995453
a_HQ_transcript/56679	-1.19726976592655	2.53433356283256	-1.27754497457263	0.210436771700478	0.97192422100393	-5.13803899722863
a_HQ_transcript/21925	-2.04372250418859	0.0768159827708897	-1.27753759709082	0.210439342604308	0.97192422100393	-5.10455678644895
a_HQ_transcript/47936	-0.795944498075603	2.65233241114425	-1.27752672962531	0.210443129740495	0.97192422100393	-5.16079746613811
a_HQ_transcript/99356	-1.80037733380097	0.144647950248863	-1.27731458943245	0.210517067507765	0.972186370193879	-5.09894256634695
a_HQ_transcript/3291	-1.80785685422335	1.69874007519448	-1.2772481303903	0.210540234694502	0.972186370193879	-5.07318829883424
a_HQ_transcript/68069	-1.38027888086582	0.925661326249292	-1.27714354722924	0.210576695608322	0.972186370193879	-4.96485458858013
a_HQ_transcript/126246	1.2726493595072	3.01496241127309	1.27712974744842	0.210581506994313	0.972186370193879	-5.1372348272002
a_HQ_transcript/84027	0.556568846067839	2.86176174533076	1.27712035215256	0.210584782774752	0.972186370193879	-5.22723762632904
a_HQ_transcript/19455	-1.04207079200072	2.14776877560441	-1.27701137351087	0.210622782279557	0.972186370193879	-5.07256690935828
a_HQ_transcript/21295	0.607766022046667	2.0824552088165	1.27700362966163	0.210625482661574	0.972186370193879	-5.04458068756634
a_HQ_transcript/103943	1.30159403662173	-0.447976617507912	1.27698009336303	0.210633690239157	0.972186370193879	-5.06284224234034

a_HQ_transcript/125659	1.51014523648817	0.372632893748261	1.27681287328449	0.210692010193323	0.972223070205097	-5.04014104525839
a_HQ_transcript/95641	1.72272018764168	0.156332055205644	1.27679003429111	0.210699976507589	0.972223070205097	-4.98691148997877
a_HQ_transcript/146809	-1.03199494269103	-0.663599502871638	-1.27670889245977	0.210728280883233	0.972223070205097	-5.06336659275519
a_HQ_transcript/34308	0.687115486919086	2.42537605640859	1.27670530444691	0.210729532541647	0.972223070205097	-5.18547761177915
a_HQ_transcript/47928	-1.41997077509505	0.268842467192991	-1.27653355532092	0.210789452855672	0.972223070205097	-5.01305989096539
a_HQ_transcript/621	-1.06525679792094	-0.647343229937177	-1.2764640747023	0.210813697111607	0.972223070205097	-5.09736434902013
a_HQ_transcript/73748	-1.60264099987382	-0.230566227239029	-1.27645769431168	0.210815923562354	0.972223070205097	-5.10265387658416
a_HQ_transcript/64422	-2.25564149755224	0.173704598885763	-1.27643388740458	0.210824231189842	0.972223070205097	-4.98682140206355
a_HQ_transcript/52953	3.95037064647827	2.66626459678858	1.27634721750045	0.210854477500718	0.972223070205097	-5.26824007854804
a_HQ_transcript/82889	-1.20472825846865	2.47968297309157	-1.27633773440659	0.210857787137267	0.972223070205097	-5.30229793240274
a_HQ_transcript/86575	-1.30414088090164	-0.0412439118460666	-1.27630339856529	0.210869770808046	0.972223070205097	-5.03963391541743
a_HQ_transcript/100442	-1.40504782276797	1.07448208866662	-1.27625465451496	0.210886784023104	0.972223070205097	-5.06202484930185
a_HQ_transcript/32513	2.36023989901929	0.592661302697095	1.2762387056984	0.210892350890035	0.972223070205097	-5.10115301461582
a_HQ_transcript/57283	0.876905623121263	0.651609081932315	1.27621040896974	0.210902228017182	0.972223070205097	-4.96937407893363
a_HQ_transcript/13108	1.90225607302754	0.073151032587852	1.27617052229898	0.210916151271326	0.972223070205097	-5.01532082231774
a_HQ_transcript/113301	-2.02428324657166	0.211381679797434	-1.27616099030701	0.210919478710101	0.972223070205097	-5.08276086868312
a_HQ_transcript/8842	2.07287662750377	0.960709432322029	1.27614237781922	0.210925976093388	0.972223070205097	-5.09080744831625
a_HQ_transcript/144498	-1.67219212451701	-0.0300271562202203	-1.2760616702288	0.210954151841722	0.972275843502666	-5.08911228978227
a_HQ_transcript/51066	1.84853172411146	2.50797156425181	1.27598121493453	0.210982242346463	0.972328215580371	-5.19559338961437
a_HQ_transcript/114693	-1.64309563637473	-0.370229923861787	-1.27588546735512	0.211015675754124	0.972403375543896	-5.00538741458589
a_HQ_transcript/47147	1.74139559427233	1.12862978816744	1.27582914387139	0.211035344818806	0.972403375543896	-5.13770804044482
a_HQ_transcript/145122	2.18007372731017	0.393677627272771	1.27574653582098	0.211064195386015	0.972403375543896	-5.1098605031002
a_HQ_transcript/33306	0.940323224415384	1.96820377459585	1.27568767181084	0.211084755253024	0.972403375543896	-4.97972554518849
a_HQ_transcript/133734	2.00132839272935	1.67341816755314	1.27565076639779	0.211097646251855	0.972403375543896	-5.23631808739224
a_HQ_transcript/131195	-1.04385352523222	3.82100787078406	-1.27560740154236	0.211112794284385	0.972403375543896	-5.47529880256207
a_HQ_transcript/137486	-2.99856664697947	1.64132484095962	-1.27553172724854	0.211139230486996	0.972403375543896	-5.20513592266858
a_HQ_transcript/4535	-2.00600738239364	0.874960307175204	-1.27552323135805	0.211142198614048	0.972403375543896	-5.08519441394791
a_HQ_transcript/33569	-1.75287781378179	0.733478769570379	-1.27550345168773	0.211149108968398	0.972403375543896	-5.05897947227157
a_HQ_transcript/102673	1.92698742111735	5.89103407910502	1.27535567506825	0.211200742585998	0.972564110357199	-5.64812369546489
a_HQ_transcript/26876	1.70711151469201	0.548973820699737	1.27528598188732	0.211225096918857	0.972599210527514	-5.09830839345495
a_HQ_transcript/142604	0.324785575641197	5.77982131532718	1.27513514799477	0.211277813209061	0.972627527764829	-5.65175120749893
a_HQ_transcript/82849	-4.28659938681193	1.82393978441349	-1.27507079141166	0.211300308798346	0.972627527764829	-5.11540960074174
a_HQ_transcript/2017	1.186277630231	1.19961552863036	1.27499636378617	0.21132632694056	0.972627527764829	-4.93456624318919
a_HQ_transcript/117010	-2.20350163140068	2.52290190978961	-1.27491703346061	0.211354061619794	0.972627527764829	-5.24783744362331
a_HQ_transcript/140049	1.45551925805023	3.15716539439459	1.27489633852344	0.211361297230766	0.972627527764829	-5.34598426670635
a_HQ_transcript/87808	0.783995452373258	2.80104083004184	1.27485863586461	0.211374479766151	0.972627527764829	-5.17576288457728
a_HQ_transcript/47563	-0.940959762254382	2.05849446906282	-1.27483916388195	0.211381288285567	0.972627527764829	-5.0629454839376
a_HQ_transcript/96141	1.89688984189259	1.25415639833188	1.2748127810913	0.211390513483635	0.972627527764829	-5.1577218303315
a_HQ_transcript/141109	-2.06037094819338	2.47377108226347	-1.27479181267541	0.211397845669377	0.972627527764829	-5.17258645221712
a_HQ_transcript/46839	-2.07092171067916	-0.0449775986228986	-1.27474952802016	0.211412632252195	0.972627527764829	-5.02148016852048
a_HQ_transcript/33429	1.31914989972268	1.17440155060289	1.27474188685664	0.211415304385298	0.972627527764829	-5.04573189318213
a_HQ_transcript/92797	-2.13802407939454	1.77554395402741	-1.27461744457782	0.211458825743678	0.972688311944935	-5.02283038824355
a_HQ_transcript/106167	1.85370706945515	0.411580263540514	1.27457853623812	0.211472434598237	0.972688311944935	-5.06525371322878
a_HQ_transcript/122292	2.54044785533678	0.518334026931308	1.27456057399314	0.21147871742293	0.972688311944935	-5.21555771486848
a_HQ_transcript/36294	1.77910447655208	1.16043726236867	1.27447848107596	0.211507433638273	0.972722384862214	-4.92642292549558
a_HQ_transcript/110646	1.56004942841453	0.0218898903832494	1.2744370617961	0.211521923278925	0.972722384862214	-5.04298791008948
a_HQ_transcript/20511	1.89274110704461	2.30164642111751	1.27439588773145	0.211536327880595	0.972722384862214	-5.03045431637117
a_HQ_transcript/111786	1.66651633344027	0.928828748382251	1.2742003811348	0.211604735303182	0.972959978935562	-5.02259526274019
a_HQ_transcript/130530	3.1742074513593	0.624835019647174	1.27411337115569	0.211635185325966	0.97299479979381	-5.15605904552631
a_HQ_transcript/117599	-2.31453998390834	1.0650407886035	-1.27408308228715	0.211645785999741	0.97299479979381	-5.11204001952192

a_HQ_transcript/151466	5.6287132102704	6.70290119117215	1.27384383369132	0.211729533726538	0.973152539806301	-5.46907688788411
a_HQ_transcript/20194	-1.65736019629394	-0.255938532996744	-1.27380186177888	0.211744228360191	0.973152539806301	-5.0650507695569
a_HQ_transcript/59148	1.44139792229565	0.0930821759738088	1.27375617102194	0.211760225861869	0.973152539806301	-5.13573020539705
a_HQ_transcript/63721	2.64099979572337	1.47742048141915	1.27375201300585	0.211761681734943	0.973152539806301	-5.16825422374643
a_HQ_transcript/98494	1.37416895446245	1.73946384797212	1.27374594800545	0.211763805326347	0.973152539806301	-5.13500872777821
a_HQ_transcript/130192	-2.24334342406261	1.16756280859889	-1.27360700798925	0.21181245801512	0.973288275613188	-5.15600270334776
a_HQ_transcript/145392	-1.3144368079099	2.68863246312778	-1.27356596975539	0.211826830009332	0.973288275613188	-5.18385252997615
a_HQ_transcript/88250	-2.00602430612412	0.775429547690563	-1.27341183844725	0.211880814900254	0.97345937468558	-5.06290083631598
a_HQ_transcript/42133	1.52739375841913	2.00266068126673	1.27336081923972	0.211898686802083	0.973464543304474	-5.05304384910989
a_HQ_transcript/134055	2.44968152924192	2.78465386208932	1.27319008552236	0.211958502690744	0.973562257130487	-5.2589963975213
a_HQ_transcript/119376	-0.424568452809295	3.90999264577702	-1.27311934869885	0.21198328880029	0.973562257130487	-5.41652652434741
a_HQ_transcript/138677	1.66455880537448	1.32910739577777	1.27310475807021	0.211988401614087	0.973562257130487	-5.11058323114643
a_HQ_transcript/109344	-1.67774901194242	0.11284666395841	-1.27306444876863	0.212002527189481	0.973562257130487	-5.00639064939559
a_HQ_transcript/136860	-3.66741776308204	2.35251171281485	-1.27300715559172	0.212022605643505	0.973562257130487	-5.10494929470779
a_HQ_transcript/97901	-1.61205109473435	1.39558476099984	-1.27299869268417	0.212025571600229	0.973562257130487	-5.10713986408872
a_HQ_transcript/99522	-3.48270671804644	0.456596160261717	-1.27296552281893	0.212037196794522	0.973562257130487	-5.0933969255425
a_HQ_transcript/15829	0.853268782688102	2.2401997418409	1.2727728241699	0.21210474230433	0.973705235929316	-5.01694179711718
a_HQ_transcript/50281	-1.48930879190654	0.458364664017382	-1.2725992190356	0.212165609008115	0.973705235929316	-4.94569388332849
a_HQ_transcript/149342	2.60523447920827	0.431775360083614	1.27256598497401	0.212177262518369	0.973705235929316	-5.12952779678577
a_HQ_transcript/128221	0.601106643708097	3.48081292947157	1.27253526374729	0.212188035334531	0.973705235929316	-5.32758279880642
a_HQ_transcript/149776	-1.7946476107278	0.595953977119536	-1.27250244179226	0.212199545256576	0.973705235929316	-5.05684827228198
a_HQ_transcript/67231	-1.43582204976405	1.61202658225478	-1.27248700725521	0.212204957965046	0.973705235929316	-5.12482310624501
a_HQ_transcript/103709	-1.54605700851272	-0.193666682410654	-1.27245689562064	0.21221551805743	0.973705235929316	-4.98883516415565
a_HQ_transcript/58995	-0.86632451013257	3.1471530316632	-1.27240970945502	0.212232066954151	0.973705235929316	-5.3173889370952
a_HQ_transcript/47415	1.49828670153642	1.45097408331935	1.27237679106349	0.212243612506022	0.973705235929316	-5.00944001194732
a_HQ_transcript/10706	1.05448709680534	1.60161331147608	1.27236884219524	0.212246400504139	0.973705235929316	-4.93385447142332
a_HQ_transcript/1186	0.576937500100684	2.60087239829764	1.27235117141317	0.212252598480257	0.973705235929316	-5.26306673567338
a_HQ_transcript/81311	-0.613618872799605	4.14243683846386	-1.27222423895671	0.212297123696902	0.97375345118084	-5.50442262675713
a_HQ_transcript/104	2.4865486391833	2.82616516223845	1.27217971008545	0.212312745154518	0.97375345118084	-5.20188553881676
a_HQ_transcript/126313	-0.798352313714346	2.43494535931712	-1.2721505412725	0.212322978523057	0.97375345118084	-5.13759368474735
a_HQ_transcript/144336	3.59079411520196	0.877706053135617	1.27208312066736	0.212346633291725	0.97375345118084	-5.12516026955495
a_HQ_transcript/141840	-2.27831496237893	1.07193616438669	-1.27208245153316	0.21234686806992	0.97375345118084	-5.12264428731464
a_HQ_transcript/1522	-2.34736062291595	2.56359963841465	-1.2720268761302	0.212366368419789	0.97376605375921	-5.15999181722412
a_HQ_transcript/134051	1.30378310558774	1.92763641237906	1.27192742590614	0.212401266986903	0.97384925441037	-5.10812183212673
a_HQ_transcript/53417	-1.4908088625419	1.16314055821752	-1.27166906362253	0.212491950426328	0.974188192632036	-4.98008273842981
a_HQ_transcript/140350	-3.19808640880017	0.311320058334411	-1.27149080144576	0.212554536321674	0.974384600417646	-5.1065281186286
a_HQ_transcript/54279	3.26328988125696	1.45129227037792	1.27145155616103	0.212568316781863	0.974384600417646	-5.10671756764625
a_HQ_transcript/144536	0.890718927627284	3.04373382616707	1.27127531263803	0.212630210678199	0.974472385776561	-5.26871946661919
a_HQ_transcript/84559	-4.05384937634563	4.284264158463	-1.27119105834131	0.212659804246379	0.974472385776561	-5.32824284724208
a_HQ_transcript/50698	4.93280152687592	1.43290548008719	1.27117001278126	0.212667196796969	0.974472385776561	-5.23537486224807
a_HQ_transcript/120796	0.569098154851342	3.1896943394675	1.27111302405566	0.212687215867751	0.974472385776561	-5.32882581373687
a_HQ_transcript/9577	0.772764425241066	2.43637481165971	1.27101369380557	0.212722112126522	0.974472385776561	-5.12233838932417
a_HQ_transcript/131643	1.38549084724601	-0.175300932720315	1.27093016433606	0.212751460674797	0.974472385776561	-5.08481022847108
a_HQ_transcript/86468	0.506118918780496	5.61084209471287	1.27092466907842	0.212753391571703	0.974472385776561	-5.62716564454945
a_HQ_transcript/130126	-1.58295693302094	0.216872968044628	-1.27091999801581	0.212755032877378	0.974472385776561	-5.10551210781754
a_HQ_transcript/24608	-1.89673699438011	1.67507400938207	-1.27089273178107	0.212764613806067	0.974472385776561	-5.11427553584724
a_HQ_transcript/117446	-2.2673016673609	0.878070704715772	-1.27088093648224	0.21276875859073	0.974472385776561	-5.00128817055536
a_HQ_transcript/149645	1.71600670552478	0.33311023537489	1.27087206932118	0.212771874488648	0.974472385776561	-5.09680608396574
a_HQ_transcript/143959	-0.87952230998143	2.24691456101542	-1.27075577911949	0.212812741762238	0.974499320282363	-5.10171753904597
a_HQ_transcript/143747	1.58590434765493	-0.134082303771036	1.27071477951812	0.212827151457575	0.974499320282363	-5.02963031865467

a_HQ_transcript/133246	-2.55684411350058	1.23349102565978	-1.27071222393416	0.212828049665989	0.974499320282363	-5.06823903789168
a_HQ_transcript/141787	1.99691642410833	2.01795470475265	1.27049399076784	0.212904762415094	0.974773789356064	-5.19393244776546
a_HQ_transcript/39557	-0.834963169634383	2.00509369491967	-1.27040523151986	0.212935968808678	0.974792631703437	-5.16495452772503
a_HQ_transcript/143594	-2.7930391586833	2.53177575897612	-1.27038689130515	0.212942417378376	0.974792631703437	-5.41374811600224
a_HQ_transcript/105493	1.5342810514258	1.64264897113771	1.2701842938206	0.213013662131206	0.974932607548595	-5.04790128956808
a_HQ_transcript/69425	-2.50725255738761	0.405082216816757	-1.27017842762101	0.213015725287545	0.974932607548595	-5.07685744086781
a_HQ_transcript/59871	1.79876192250625	0.71988572855505	1.27009422837983	0.213045340024303	0.974932607548595	-5.0499040018223
a_HQ_transcript/31864	-1.20048507195969	2.15318874104276	-1.27002582265861	0.213069402120508	0.974932607548595	-5.07281771755498
a_HQ_transcript/33548	-1.14083116018073	2.16083952332257	-1.27002126647328	0.213071004857183	0.974932607548595	-5.08030379135263
a_HQ_transcript/107803	-1.62804823904811	1.72566412570958	-1.26991059434689	0.213109938967581	0.974932607548595	-5.04636710393583
a_HQ_transcript/98520	-1.53530181098646	2.55592599208981	-1.26990596951422	0.213111566086338	0.974932607548595	-5.16933892083427
a_HQ_transcript/134202	1.56182833587305	2.02073449606883	1.26986912232954	0.213124530078771	0.974932607548595	-5.13548671841825
a_HQ_transcript/149323	-2.22622740094291	1.37917282368365	-1.26983401993447	0.213136880753731	0.974932607548595	-5.13538800817481
a_HQ_transcript/149428	1.69071186383264	0.360729594398486	1.26982236241329	0.21314098253803	0.974932607548595	-5.08430442690745
a_HQ_transcript/83017	1.39916332377501	2.44657012783254	1.269773203025	0.21315828028518	0.974932607548595	-5.24699860333017
a_HQ_transcript/142469	0.967469863907819	2.41089257014248	1.26969856899635	0.213184543838372	0.974932607548595	-5.218404815821
a_HQ_transcript/104193	-1.40663122097296	1.35685382279232	-1.26963488216112	0.213206957030746	0.974932607548595	-4.95408478828954
a_HQ_transcript/134028	-1.29051720740623	-0.367875246260422	-1.26961222399965	0.213214931505677	0.974932607548595	-5.10807770627151
a_HQ_transcript/62179	1.08501644057307	-0.0170388247145403	1.26958481740212	0.213224577480135	0.974932607548595	-5.04880605311433
a_HQ_transcript/6903	-2.05080834087672	2.49344803302119	-1.26940256629753	0.213288730620307	0.975149231830835	-5.23400911244013
a_HQ_transcript/80	0.719288814185723	2.31085862190557	1.26921103647515	0.213356165617767	0.975195164218882	-5.16359279463428
a_HQ_transcript/118814	-2.86104298551308	3.91317162268653	-1.26919715511492	0.213361053678203	0.975195164218882	-5.38430807584755
a_HQ_transcript/37913	-1.45248373000236	1.7075937524044	-1.2691432636915	0.213380031332946	0.975195164218882	-4.9501716785305
a_HQ_transcript/116030	-2.65159812981273	0.659865948669701	-1.2691359517015	0.213382606319949	0.975195164218882	-5.0718542614023
a_HQ_transcript/40107	2.4059868615158	2.03558584196853	1.26913579764833	0.213382660571497	0.975195164218882	-5.02342638402704
a_HQ_transcript/100661	-0.63604616122021	4.22198316782431	-1.26897330799424	0.21343988891946	0.975317853543003	-5.52626741166343
a_HQ_transcript/50486	-2.00655749904316	0.60511489596076	-1.2689264954618	0.213456378297571	0.975317853543003	-5.05665017029026
a_HQ_transcript/37698	-1.73928741825564	0.789107215769553	-1.26891666072136	0.213459842636493	0.975317853543003	-5.00034712128754
a_HQ_transcript/67895	-1.7366915599724	1.5951249969928	-1.26864087837513	0.21355700570586	0.975557547852374	-5.04599445439866
a_HQ_transcript/51418	-2.0766509168786	1.19184207200992	-1.26859603860597	0.213572806717241	0.975557547852374	-5.15864001910094
a_HQ_transcript/145688	2.17340943807558	2.93662984756497	1.26859422239093	0.21357344674883	0.975557547852374	-5.1002248462837
a_HQ_transcript/27657	1.15799065648097	1.86687010003641	1.26857723188784	0.213579434248027	0.975557547852374	-5.02929094102113
a_HQ_transcript/19178	2.1169805503689	2.18428042461668	1.26851709647927	0.213600627146747	0.975573753151437	-4.98307254598973
a_HQ_transcript/135836	3.1528420730275	3.77840171315382	1.26845056743326	0.21362407513897	0.975573753151437	-5.49678867069322
a_HQ_transcript/44797	-1.99257329216103	1.14320381064376	-1.26842430534618	0.213633331679477	0.975573753151437	-5.10563013187269
a_HQ_transcript/23471	1.14644364518546	1.75134079084569	1.26831964284611	0.2136702248511	0.975594949334566	-5.00925590972778
a_HQ_transcript/44569	1.38093041628799	-0.0857923735133284	1.26826695131803	0.213688800254212	0.975594949334566	-5.0504106425347
a_HQ_transcript/42063	1.22989351408615	0.212232064693245	1.26826396861624	0.213689851785864	0.975594949334566	-4.95550546696442
a_HQ_transcript/95054	2.44887753668483	0.593641799074833	1.26822069650711	0.213705107519217	0.975594949334566	-4.97567233326376
a_HQ_transcript/4063	-1.40780878203968	2.43258297054226	-1.26805662561721	0.213762958755304	0.975707709627538	-5.08220680065417
a_HQ_transcript/125126	0.698078193118405	3.40674758631285	1.26805543449635	0.213763378786586	0.975707709627538	-5.35472769086383
a_HQ_transcript/10894	-0.95368889789793	-0.670295825064119	-1.26765410130393	0.213904938523565	0.976020583736875	-5.11601623840081
a_HQ_transcript/111381	-0.630871529056283	3.90724444263842	-1.26753429624652	0.21394721032252	0.976020583736875	-5.43953701314138
a_HQ_transcript/135031	-2.38421623018664	0.981415740285497	-1.2675226631743	0.213951315250473	0.976020583736875	-5.08427654722922
a_HQ_transcript/104236	-1.17691505736329	-0.472580401474345	-1.26747439489192	0.213968348172339	0.976020583736875	-5.08151791618385
a_HQ_transcript/57631	2.40691117719468	0.351110274229086	1.26741036264894	0.213990945463651	0.976020583736875	-5.0812147017439
a_HQ_transcript/21949	0.922710640886997	1.95031944339297	1.26738877828072	0.213998563098588	0.976020583736875	-5.0908790961471
a_HQ_transcript/113775	-1.87867851363684	2.16810567572029	-1.26738243827622	0.214000800675281	0.976020583736875	-5.19949513753038
a_HQ_transcript/132325	7.13866893099591	2.68793848876555	1.26730441571046	0.214028338611953	0.976020583736875	-4.99374470077619
a_HQ_transcript/79838	-1.91688880327356	0.0832715993291176	-1.26728042914625	0.214036805168017	0.976020583736875	-5.07769651040433

a_HQ_transcript/74859	-2.03271370809811	1.53649046179411	-1.26723427134073	0.214053098235537	0.976020583736875	-5.07793645746008
a_HQ_transcript/41249	-1.74216518633985	1.36846067798591	-1.26719324440362	0.214067580963259	0.976020583736875	-5.06587181083355
a_HQ_transcript/119819	-2.86617839645355	0.913653501977926	-1.26718585475293	0.214070189627994	0.976020583736875	-5.17908260122808
a_HQ_transcript/138103	-0.76676207782557	4.54144873361657	-1.26717882603581	0.214072670899675	0.976020583736875	-5.53632615763353
a_HQ_transcript/51515	-1.46333264245255	1.00048673424918	-1.26717380356166	0.214074443942486	0.976020583736875	-4.9253070631735
a_HQ_transcript/34950	-2.35815482297043	2.0589917638181	-1.26714733462475	0.214083788237083	0.976020583736875	-5.27621871959577
a_HQ_transcript/51477	-1.74367956006836	2.25271880611336	-1.26706122588285	0.214114189224872	0.976035583396548	-5.12230119364458
a_HQ_transcript/89288	3.14146612237665	1.46084807474811	1.26704289733607	0.214120660601995	0.976035583396548	-5.10364445734707
a_HQ_transcript/28746	2.09154158560412	0.244039520577015	1.26698198299173	0.214142169079398	0.976057084940583	-5.1281937103758
a_HQ_transcript/107920	2.12725501370256	2.47727830381614	1.26685126275336	0.214188331089713	0.976190944768924	-5.08798877587054
a_HQ_transcript/99206	-1.0391440334984	-0.325467274300546	-1.26680299872033	0.214205376751177	0.976192092496465	-5.05450002110696
a_HQ_transcript/56274	-4.4837640595742	0.321540659729316	-1.26672330879715	0.21423352349969	0.976243826747412	-5.11862277810895
a_HQ_transcript/151943	-1.48538159729715	0.143703059385668	-1.26654754780936	0.21429561273794	0.976299855413146	-5.08186163192932
a_HQ_transcript/80293	3.11799670631488	0.157699160449571	1.26643834750704	0.214334195620914	0.976299855413146	-5.10895580862763
a_HQ_transcript/56359	-1.79538799559229	0.52141443402353	-1.26641130070287	0.214343752665206	0.976299855413146	-5.06663958644271
a_HQ_transcript/58634	-1.42761534886683	0.225651131327529	-1.26638351318927	0.214353571775614	0.976299855413146	-5.14901323298287
a_HQ_transcript/40955	2.4291219139738	0.437811611473403	1.2663674561958	0.214359245895791	0.976299855413146	-5.08679158509611
a_HQ_transcript/5683	1.7734949123599	1.26140635601165	1.26626858416026	0.214394187176584	0.976299855413146	-5.30722924058624
a_HQ_transcript/48970	1.68685275543166	0.535608757216423	1.26621958406233	0.214411505356036	0.976299855413146	-5.12119509373321
a_HQ_transcript/64669	1.92690730344848	1.51235627244209	1.266150403964	0.214435957581033	0.976299855413146	-5.07443487537014
a_HQ_transcript/111201	-1.56836326465066	1.86608312494304	-1.26605531972895	0.214469569251073	0.976299855413146	-5.00268790069787
a_HQ_transcript/60658	-1.36136327800978	2.99382574338951	-1.26600908171503	0.214485915530306	0.976299855413146	-5.32795461328162
a_HQ_transcript/85202	1.73677494628429	1.11382047038515	1.26596125242933	0.21450282535251	0.976299855413146	-5.09255175878242
a_HQ_transcript/89888	2.27348766097162	3.06222170771559	1.26584851591388	0.214542686804522	0.976299855413146	-5.31931076412419
a_HQ_transcript/129399	2.51087395092822	2.14975221175197	1.26577366603104	0.214569155362843	0.976299855413146	-5.10286305249524
a_HQ_transcript/134058	-1.96753828445862	0.475888271749724	-1.26573806261326	0.214581746375976	0.976299855413146	-5.1450731083926
a_HQ_transcript/65553	3.10593800705196	3.66949137046929	1.26572320340315	0.214587001443925	0.976299855413146	-5.29948519740606
a_HQ_transcript/128389	1.45924958816245	3.0199259483781	1.26568689108321	0.214599843969079	0.976299855413146	-5.1799898628808
a_HQ_transcript/128643	1.95007522414385	1.11567232132137	1.26568129578638	0.214601822901095	0.976299855413146	-5.05246006195201
a_HQ_transcript/22758	1.0891881135736	-0.489126078802724	1.26563557426723	0.214617994101204	0.976299855413146	-5.09004625453709
a_HQ_transcript/41234	-2.14414271165051	0.558806367223461	-1.26562628271524	0.214621280533683	0.976299855413146	-5.04232931702211
a_HQ_transcript/8531	2.56702676072751	2.55626362424124	1.26553869637093	0.214652261788831	0.976299855413146	-5.18318848749355
a_HQ_transcript/11722	-0.610882240559535	2.76534180689381	-1.26552754856741	0.21465620525909	0.976299855413146	-5.26581448160747
a_HQ_transcript/126773	-1.58201865088309	0.940340026242792	-1.2654993302974	0.214666187551248	0.976299855413146	-5.08775697033643
a_HQ_transcript/80063	-5.0303658369866	1.21697664102328	-1.26549665938731	0.214667132411326	0.976299855413146	-5.19682541219064
a_HQ_transcript/18203	-0.564563350872977	2.52348223522752	-1.26547832697214	0.214673617763804	0.976299855413146	-5.13343894995968
a_HQ_transcript/9888	1.0012839524658	2.37352923755273	1.26545590296469	0.214681550776195	0.976299855413146	-5.13051872153405
a_HQ_transcript/42157	-0.864810727864896	4.01265613424517	-1.26545320080751	0.21468250674186	0.976299855413146	-5.44953646597174
a_HQ_transcript/86924	2.15681750206662	-0.118992590079326	1.26535366710277	0.214717721884729	0.976350592714481	-5.0937602481141
a_HQ_transcript/128999	-0.44147634073336	2.99095975953612	-1.26532671958901	0.214727256696634	0.976350592714481	-5.34551399936229
a_HQ_transcript/18105	-1.67087318604074	4.53667423048252	-1.26517610281553	0.214780555161085	0.976414694063176	-5.54779998395404
a_HQ_transcript/29498	-1.23100386048679	0.45427599726297	-1.26508369434054	0.214813260507534	0.976414694063176	-5.0754507863441
a_HQ_transcript/145636	-0.451692950060559	4.1825931896624	-1.26506713209885	0.214819122637984	0.976414694063176	-5.50769636164023
a_HQ_transcript/3591	-1.45636418564778	1.74007253885441	-1.26506467851483	0.214819991083202	0.976414694063176	-4.97306844988909
a_HQ_transcript/17835	-2.67321885542322	2.44995716662683	-1.2650495588753	0.21482534273283	0.976414694063176	-5.19999555485433
a_HQ_transcript/631	3.08382055352387	1.71099945811927	1.26492500698307	0.214869432139963	0.976485875543665	-5.09863424373065
a_HQ_transcript/141531	1.63143124818462	-0.0492346259228228	1.26487923915252	0.214885634945431	0.976485875543665	-5.1241379589575
a_HQ_transcript/74711	-1.15852885658359	1.79247479824448	-1.26483733511357	0.214900470692946	0.976485875543665	-4.96536660505951
a_HQ_transcript/101726	-1.74214876357942	0.064961050796563	-1.26481550643214	0.214908199247008	0.976485875543665	-5.1110753607825
a_HQ_transcript/146632	-0.511979749820017	3.61374611548472	-1.26464122282401	0.214969912750939	0.976524364604788	-5.44498120968791

a_HQ_transcript/82066	-1.64948527784679	1.46946954609766	-1.26463745256693	0.214971247939697	0.976524364604788	-5.01392550620689
a_HQ_transcript/123659	0.731556390838378	4.19483403881835	1.26457836672195	0.21499217325987	0.976524364604788	-5.50501310743459
a_HQ_transcript/149599	2.66391151516345	1.63748588492766	1.26457633546865	0.214992892657883	0.976524364604788	-5.14337610570563
a_HQ_transcript/62453	2.24115383553861	1.46079642679495	1.26452259033207	0.215011927942217	0.976524364604788	-5.19491108702963
a_HQ_transcript/123401	2.59786533312997	0.888477258759986	1.26449166257344	0.215022882415174	0.976524364604788	-5.04646092338238
a_HQ_transcript/7221	-0.859197049623377	1.93443795514576	-1.26433148525841	0.215079623234588	0.976524364604788	-5.05621339732901
a_HQ_transcript/30601	-2.22355774335511	1.33025072702739	-1.26430236807889	0.215089938845645	0.976524364604788	-5.16604894414871
a_HQ_transcript/119408	-0.84127762479483	2.36528217368895	-1.2642392360503	0.215112306491704	0.976524364604788	-5.13915203280867
a_HQ_transcript/53122	-1.9195917594274	2.37489218640707	-1.26409690957779	0.215162739124312	0.976524364604788	-5.21007742359166
a_HQ_transcript/101216	-0.550371526500451	2.86306856121936	-1.26407537598788	0.215170370214465	0.976524364604788	-5.30735309197496
a_HQ_transcript/100571	-1.05593505670587	2.14302298566596	-1.26407400969633	0.215170854408801	0.976524364604788	-5.16437848156955
a_HQ_transcript/69138	1.84596200115187	1.23663715386544	1.26406196945805	0.215175121333902	0.976524364604788	-5.0732769098635
a_HQ_transcript/26871	2.47674583609356	0.823701877442633	1.26404200047779	0.215182198256698	0.976524364604788	-4.96290425404346
a_HQ_transcript/78280	-2.01115254731932	0.425733884486632	-1.26403000552559	0.215186449301792	0.976524364604788	-5.14032898835344
a_HQ_transcript/49758	1.75185662284683	1.34192665295909	1.26401108990932	0.215193153178546	0.976524364604788	-5.03922541883744
a_HQ_transcript/133158	1.60950638213562	1.05472471267883	1.26398538801235	0.215202262430968	0.976524364604788	-4.93198500078845
a_HQ_transcript/128473	2.38925796947368	1.62425253827492	1.26391414136823	0.215227515148198	0.976562719579615	-5.22020255600121
a_HQ_transcript/111120	-1.72710880972507	-0.197754399190941	-1.26373637789607	0.215290531516982	0.976756460468252	-5.08414306252975
a_HQ_transcript/28696	1.98768825614282	2.48718264225684	1.26366098051811	0.215317263756993	0.976756460468252	-5.23296190943547
a_HQ_transcript/129939	3.47870962815693	1.63149989054615	1.26362251492084	0.215330902749405	0.976756460468252	-5.1891094948261
a_HQ_transcript/79805	-1.78628520192749	0.431260591843903	-1.26349091074874	0.215377571407723	0.976756460468252	-5.16257272279888
a_HQ_transcript/492	1.36481590941089	4.4441812319407	1.26347811403798	0.215382109704992	0.976756460468252	-5.35755676910401
a_HQ_transcript/129551	-1.79004286375548	3.10479465298978	-1.26345011145067	0.215392040951097	0.976756460468252	-5.48073852686786
a_HQ_transcript/147863	-2.41350280535997	1.91484686720581	-1.26330982775169	0.21544179840588	0.976756460468252	-5.25054824172825
a_HQ_transcript/115887	1.94542786946335	1.30900181887344	1.26327917450329	0.215452672010268	0.976756460468252	-5.08129346471595
a_HQ_transcript/147892	-1.49473288885632	1.50551263142685	-1.26318436136198	0.21548630762556	0.976756460468252	-5.02656784506737
a_HQ_transcript/137344	1.66914768375729	2.5748336105592	1.26310563176652	0.215514240500292	0.976756460468252	-5.11049344029777
a_HQ_transcript/571	1.36002235735915	0.4456118708347	1.26308293394548	0.215522294082877	0.976756460468252	-5.02581216314546
a_HQ_transcript/92388	-1.31743488291618	2.02093191757464	-1.2628643949897	0.215599847122381	0.976756460468252	-5.14760828899283
a_HQ_transcript/70581	1.85082484509949	0.620762646487716	1.26281012218089	0.215619110204262	0.976756460468252	-5.11250824089925
a_HQ_transcript/135510	1.85647026436364	0.977607435966113	1.2627710870926	0.215632965754572	0.976756460468252	-5.12992082750621
a_HQ_transcript/117434	1.59621448198278	5.01196365999034	1.26276822359377	0.215633982183257	0.976756460468252	-5.56506677226453
a_HQ_transcript/133023	4.47202069870403	2.77964056807486	1.26270949477124	0.215654829386073	0.976756460468252	-5.30831336088148
a_HQ_transcript/41968	1.59546663700725	-0.162541885242806	1.26269292369283	0.215660711962228	0.976756460468252	-5.21609683307605
a_HQ_transcript/114106	2.58170215667697	5.98448807737356	1.26266198910715	0.215671693771289	0.976756460468252	-5.68348046624079
a_HQ_transcript/84866	-1.3749063422497	2.57608356251219	-1.26254016200723	0.21571494661309	0.976756460468252	-5.06592406375761
a_HQ_transcript/21456	1.40694493198437	2.64421144761443	1.2625247865473	0.215720405898735	0.976756460468252	-5.23615767011275
a_HQ_transcript/56850	-1.76948599574441	0.203786937120384	-1.2624859436313	0.215734198117407	0.976756460468252	-5.15858673649297
a_HQ_transcript/94908	-1.01843799844187	1.75812976371678	-1.26248232732421	0.215735482218008	0.976756460468252	-5.11134758638851
a_HQ_transcript/142656	2.0114423827343	-0.0705699452444117	1.26247509902996	0.215738048902525	0.976756460468252	-5.09478509073722
a_HQ_transcript/114031	2.88738764042649	2.23365646925919	1.26246696947095	0.215740935643342	0.976756460468252	-5.16513177122914
a_HQ_transcript/17023	-0.688942384291194	2.26550530911076	-1.26243640547928	0.21575178893067	0.976756460468252	-5.14949397830987
a_HQ_transcript/152155	-2.94060235588129	0.343498951546732	-1.26241962390619	0.215757748249898	0.976756460468252	-5.15283362175405
a_HQ_transcript/27815	1.56707918965504	0.603395863951055	1.26241920308597	0.215757897689332	0.976756460468252	-4.9797675017868
a_HQ_transcript/84313	-1.84225809384009	0.510317950981134	-1.26235926092453	0.215779184827265	0.976756460468252	-4.99343073468779
a_HQ_transcript/103993	1.97455651080629	1.71652596712414	1.26235017711966	0.21578241087853	0.976756460468252	-5.24162352116212
a_HQ_transcript/116552	-1.92320685032844	0.439634597411826	-1.26231466384144	0.215795023525624	0.976756460468252	-4.9747550032476
a_HQ_transcript/25299	-1.06144060556422	3.3564656569818	-1.26228850945912	0.215804312688084	0.976756460468252	-5.25629731826136
a_HQ_transcript/108312	0.611085569732775	6.12807582016832	1.26227833131062	0.21580792770771	0.976756460468252	-5.70687706564026
a_HQ_transcript/146252	-2.55092067073641	1.05348299640817	-1.26210918229985	0.215868011818487	0.976944286350971	-5.0839511775315

a_HQ_transcript/2888	-0.632212435767777	3.82445043066171	-1.26198872584159	0.215910807333729	0.976944286350971	-5.38504002130333
a_HQ_transcript/85518	1.11364555271586	-0.703002169759201	1.26197828716227	0.215914516266614	0.976944286350971	-5.0989282034673
a_HQ_transcript/138115	-2.41906583904147	0.0548457365028505	-1.26197227145898	0.215916653708212	0.976944286350971	-5.03360625222384
a_HQ_transcript/43160	-2.1578733527696	1.7036820292392	-1.26183931976552	0.215963896890505	0.977081989294152	-4.94121669066108
a_HQ_transcript/29737	-1.50573604450565	0.320680177862489	-1.26166394988062	0.216026224906774	0.977287881773861	-4.98482414087798
a_HQ_transcript/120240	-2.40680120371897	1.02911690347709	-1.26161667098255	0.216043030566923	0.977287881773861	-5.17464137487635
a_HQ_transcript/81558	-1.64164958408927	0.789062010647942	-1.26151064722393	0.216080721136296	0.97738231719015	-5.03569484354226
a_HQ_transcript/51786	-1.93120652233691	0.960854378057146	-1.26136725789151	0.216131702739065	0.977394552790889	-5.08086340979236
a_HQ_transcript/22319	3.89346951235095	2.85389087231615	1.26135459709038	0.216136204680419	0.977394552790889	-5.32068077596425
a_HQ_transcript/124541	-2.66837761014129	2.34875249238368	-1.26129412524796	0.216157708298163	0.977394552790889	-5.25769379825238
a_HQ_transcript/85328	0.926829318917729	2.85103306652228	1.26129051895063	0.216158990738315	0.977394552790889	-5.18548666155452
a_HQ_transcript/48461	0.905292111269955	1.70820775524188	1.26122505688923	0.21618227078486	0.977394552790889	-4.94397493809494
a_HQ_transcript/27424	1.18082744527733	-0.316819489401721	1.26121616277825	0.216185433912546	0.977394552790889	-5.1128472992358
a_HQ_transcript/64677	1.32497371895847	0.163256568877621	1.26117203586737	0.216201127852072	0.977394552790889	-4.99328562152795
a_HQ_transcript/121780	-2.66940471066158	2.44489679030793	-1.26109840064431	0.216227318470233	0.977436936623196	-5.21350254257573
a_HQ_transcript/152388	2.07390900510391	0.881965877681668	1.26087212496524	0.216307815290885	0.977724781277493	-5.09953837922215
a_HQ_transcript/33861	-0.878164722477781	1.40118841410782	-1.2608015498308	0.21633292627843	0.977762255483757	-4.98929664386527
a_HQ_transcript/77328	-0.869391177506033	2.36383363574867	-1.26074425185588	0.216353315706989	0.977778380921773	-5.12596804828929
a_HQ_transcript/119090	3.04243156195475	1.51072130778947	1.26069630747012	0.216370377363921	0.977779467885407	-5.16755046244693
a_HQ_transcript/29464	-1.42176004098876	2.31888659240052	-1.26052639690613	0.216430850492499	0.977894634664463	-5.14808127154547
a_HQ_transcript/63647	-0.655314762601378	2.38419949972881	-1.26038850464807	0.2164799372946	0.977894634664463	-5.12291123706368
a_HQ_transcript/124785	-0.574247712966546	2.42764465778126	-1.26037626229234	0.2164842957262	0.977894634664463	-5.15320300066327
a_HQ_transcript/68611	0.375366948156351	3.83527846957906	1.26028688094826	0.21651611860425	0.977894634664463	-5.42695266760106
a_HQ_transcript/136300	2.34558672738002	-0.325993403498692	1.26027465681672	0.216520471095589	0.977894634664463	-5.12618201887767
a_HQ_transcript/118426	-6.33113836445111	3.23906460332189	-1.26025062705292	0.216529027260641	0.977894634664463	-5.2755593264664
a_HQ_transcript/105327	-1.70379459361908	1.17605217790286	-1.26019959674072	0.216547198228678	0.977894634664463	-5.01335235675288
a_HQ_transcript/47646	-1.29213698289786	1.08217074381151	-1.26019750969944	0.216547941410648	0.977894634664463	-4.98199879517097
a_HQ_transcript/51365	1.82560925716616	0.250680146672831	1.26014882278468	0.216565279052772	0.977894634664463	-5.09719520161038
a_HQ_transcript/121515	-0.311300870867745	5.01514068778759	-1.26003760139927	0.216604889439622	0.977894634664463	-5.61688767271757
a_HQ_transcript/140158	-0.551051201540454	5.21911565595274	-1.25989438026141	0.216655904255626	0.977894634664463	-5.61746207020201
a_HQ_transcript/86223	1.50619723254246	1.03719526984535	1.25989157366618	0.216656904044389	0.977894634664463	-4.98695811305288
a_HQ_transcript/91913	1.36026884166423	-0.471098413747637	1.25983161743249	0.216678262984052	0.977894634664463	-5.09534579669949
a_HQ_transcript/100751	2.2882577987786	2.19979068731669	1.2597264554655	0.216715729942676	0.977894634664463	-5.17050074890889
a_HQ_transcript/141115	-1.17739723692325	2.61794198098973	-1.25972004126562	0.216718015342694	0.977894634664463	-5.16863690852191
a_HQ_transcript/135388	-2.69283093965044	0.694770650470968	-1.25969653800467	0.21672638978516	0.977894634664463	-5.0732440122749
a_HQ_transcript/49265	-3.43489692330982	0.911928962355158	-1.25963518659254	0.216748251043499	0.977894634664463	-5.16660805646643
a_HQ_transcript/69279	1.73327616339474	0.916379358599418	1.25959942210676	0.216760995716182	0.977894634664463	-5.14708079955541
a_HQ_transcript/56848	-4.01332648211598	0.624230761295913	-1.25959768538205	0.21676161461231	0.977894634664463	-5.11742853299892
a_HQ_transcript/126400	1.27048691087485	-0.600761019573423	1.25955258786995	0.216777685947387	0.977894634664463	-5.07221709942369
a_HQ_transcript/141881	1.71420158453172	1.42239512863235	1.25954043758697	0.21678201607957	0.977894634664463	-4.9667817803664
a_HQ_transcript/94439	-1.8404574980631	0.689423716049023	-1.25941543571947	0.216826568174501	0.977894634664463	-5.21917771649644
a_HQ_transcript/143328	-0.555595854605503	3.47808631664116	-1.25940412672477	0.216830599169587	0.977894634664463	-5.34879496507865
a_HQ_transcript/141373	0.558452681780439	4.07693006217205	1.25940160438833	0.216831498242678	0.977894634664463	-5.50605713598855
a_HQ_transcript/94441	1.5391979275621	0.251999926561508	1.25940036324291	0.216831940643237	0.977894634664463	-5.11814846741323
a_HQ_transcript/131313	-1.43139447965341	1.98643519256594	-1.25939665174395	0.21683326359397	0.977894634664463	-5.02368512472368
a_HQ_transcript/105508	2.49442895335479	0.570014705327474	1.25907327280042	0.216948554215239	0.978237829098836	-5.01725460358069
a_HQ_transcript/107435	1.63811366174427	0.204855202812883	1.25903253186746	0.216963082390235	0.978237829098836	-4.98668530844348
a_HQ_transcript/23755	-1.73144609421269	1.54756317727294	-1.25902901256435	0.21696433740449	0.978237829098836	-5.01794677249418
a_HQ_transcript/130149	-1.82460903205168	0.194789606600886	-1.25895108386708	0.216992128859707	0.978237829098836	-5.03377693607085
a_HQ_transcript/73276	-1.83040301862401	1.72048400925149	-1.25894721999833	0.216993506888253	0.978237829098836	-5.18040318705654

a_HQ_transcript/14664	2.21490726254658	-0.197382718209962	1.25887814052205	0.217018144833472	0.978273030079878	-5.09615908878796
a_HQ_transcript/21687	0.537533215720527	3.44903170808204	1.25877757612141	0.217054015983377	0.978276183150701	-5.42626974110308
a_HQ_transcript/54216	-2.50099332622338	0.237758174571739	-1.25872945131488	0.217071183598709	0.978276183150701	-5.10937255908763
a_HQ_transcript/142061	-2.41451545364498	2.63090902599505	-1.25870025705223	0.21708159859837	0.978276183150701	-5.08244967775704
a_HQ_transcript/41828	-1.19209581732903	0.60071060234151	-1.25863881942567	0.217103517587799	0.978276183150701	-5.03547341318298
a_HQ_transcript/136049	1.64115251818956	4.44423632444524	1.25860162401648	0.217116788535417	0.978276183150701	-5.33677402144835
a_HQ_transcript/29138	-0.874951596528436	-0.73195433273718	-1.25854416060092	0.217137292104159	0.978276183150701	-5.0667665784031
a_HQ_transcript/152453	5.63221331150287	1.98813370547773	1.25853678051411	0.21713992550464	0.978276183150701	-5.23217757869766
a_HQ_transcript/75959	-1.356064198445	0.292781352292378	-1.25840562650193	0.217186728563035	0.978276183150701	-5.13151387968292
a_HQ_transcript/144286	-2.18554884493539	3.10176192640196	-1.25834909612203	0.217206904093763	0.978276183150701	-5.20398989622023
a_HQ_transcript/23498	0.572746297329587	5.39949998045505	1.25831598729676	0.217218721193778	0.978276183150701	-5.61515575372619
a_HQ_transcript/81662	-2.01958401130413	2.08047898207231	-1.2583008632482	0.217224119383656	0.978276183150701	-5.13798150154452
a_HQ_transcript/61512	1.21913028257166	-0.539944024974533	1.25828873567815	0.217228448120694	0.978276183150701	-5.1088889962622
a_HQ_transcript/139900	2.98285007908645	1.39736583995137	1.25826301119911	0.217237630266124	0.978276183150701	-5.28114615413417
a_HQ_transcript/60586	-1.10067366450208	-0.625720071294275	-1.25817586568051	0.217268738327688	0.978331229872088	-5.05735637027602
a_HQ_transcript/137601	0.344581590707034	3.84539691869063	1.25808573943725	0.217300913935949	0.978331229872088	-5.444556512475
a_HQ_transcript/117830	-1.61350614865558	1.23399927713086	-1.25808509368039	0.217301144487952	0.978331229872088	-5.05097955590923
a_HQ_transcript/21116	-1.25974355061532	0.0786444403121407	-1.2579182743282	0.217360709505106	0.978331229872088	-4.98974370613115
a_HQ_transcript/110500	-3.99164492895573	3.29481832785114	-1.25785300265163	0.217384018949719	0.978331229872088	-5.43009647862151
a_HQ_transcript/57713	3.49042714625841	1.26753558774057	1.25774273401281	0.217423401723518	0.978331229872088	-5.12454500084726
a_HQ_transcript/107587	-6.25515750892069	3.90517580325926	-1.25772934153179	0.217428185253782	0.978331229872088	-5.31185768712271
a_HQ_transcript/84505	-1.99916838787957	0.464186927329005	-1.25772513514285	0.217429687709351	0.978331229872088	-5.13086010387616
a_HQ_transcript/109683	-1.21277770353995	1.88998729804703	-1.25771313672256	0.21743397339815	0.978331229872088	-5.05786730096997
a_HQ_transcript/92393	-0.937959478115026	2.02760383444868	-1.25767264467903	0.217448437129544	0.978331229872088	-5.12040518302117
a_HQ_transcript/133027	3.48184292050337	0.467262557062705	1.25764724905934	0.217457508797698	0.978331229872088	-5.1145937720189
a_HQ_transcript/74473	-0.570487419049811	3.41093473872452	-1.25757573598703	0.217483055790842	0.978331229872088	-5.4179873169743
a_HQ_transcript/112147	-1.05315142177421	1.76283029940021	-1.25756300059104	0.217487605560639	0.978331229872088	-5.11054114353185
a_HQ_transcript/125409	-2.60914589578526	3.42899725085593	-1.25744288708324	0.217530520102495	0.978331229872088	-5.37280953465832
a_HQ_transcript/67211	1.13096414096992	3.38054595271494	1.25743528448087	0.217533236599229	0.978331229872088	-5.18789939887107
a_HQ_transcript/48828	-1.58189787467905	0.418845003552899	-1.25743010170233	0.217535088479881	0.978331229872088	-5.04998596288671
a_HQ_transcript/87981	1.80767406869163	2.01073211983695	1.25736163313167	0.217559554389776	0.978331229872088	-5.26492750271687
a_HQ_transcript/39710	0.919297868630881	3.0477009501213	1.25733585862353	0.217568764944572	0.978331229872088	-5.3392731063015
a_HQ_transcript/139669	-0.626755564843919	2.5941905011453	-1.25725759451349	0.217596734526682	0.978331229872088	-5.273242796523
a_HQ_transcript/39764	0.487503641619643	2.30514620694748	1.25723863516594	0.217603510517462	0.978331229872088	-5.10786812776416
a_HQ_transcript/3530	0.783372387394344	2.61147494748287	1.25719261036084	0.217619960249062	0.978331229872088	-5.14046885009268
a_HQ_transcript/48847	-0.805214503528469	1.08098579883103	-1.25719214093332	0.217620128032034	0.978331229872088	-4.9944579546927
a_HQ_transcript/67958	3.11010912330718	0.596117202848898	1.25714315482041	0.217637637203114	0.97833391591226	-5.15522685108614
a_HQ_transcript/77595	-1.72439498173536	0.439705603067987	-1.25701514676455	0.217683396295748	0.97833391591226	-5.09944570056922
a_HQ_transcript/68871	1.62471679824576	0.567003947512434	1.25699113332064	0.217691981197777	0.97833391591226	-5.00837411183443
a_HQ_transcript/83775	0.47661790376083	3.25170456336522	1.25695841836289	0.217703677335644	0.97833391591226	-5.35943381206657
a_HQ_transcript/119339	1.21870324951678	1.40029421231899	1.2569550574957	0.217704878927971	0.97833391591226	-4.98865035438209
a_HQ_transcript/33090	1.11691068122167	-0.489071318834197	1.25661556241709	0.217826282444298	0.97878919333043	-5.07830484147219
a_HQ_transcript/24006	-1.51237640259693	0.847659189761595	-1.25655022704331	0.217849652231663	0.97878919333043	-4.98617433168963
a_HQ_transcript/145272	0.780510139137042	2.17741825026674	1.25653050882946	0.217856705603308	0.97878919333043	-5.05797472161011
a_HQ_transcript/137289	-2.28584080855115	3.65523896474674	-1.25641618097131	0.217897605029709	0.978857915681913	-5.50928124379625
a_HQ_transcript/14621	0.855907199990518	4.40134402368385	1.25637862212601	0.217911042518636	0.978857915681913	-5.48990668089985
a_HQ_transcript/141995	-1.17010939300292	1.86996952647373	-1.25634654090755	0.217922520761761	0.978857915681913	-5.01059614556391
a_HQ_transcript/52578	-2.89059310983764	2.75946990175497	-1.25622265607284	0.217966849415212	0.978921443289946	-5.20803001351784
a_HQ_transcript/96292	1.66181655994094	-0.112906195505241	1.25619386161459	0.217977153661958	0.978921443289946	-5.14006321613614
a_HQ_transcript/31600	1.6648411811219	2.76193177169828	1.25606402358252	0.218023621428761	0.978921443289946	-5.25445454792024

a_HQ_transcript/128278	3.8465407561456	2.46275376493136	1.25604923489276	0.218028914629394	0.978921443289946	-5.08253808501724
a_HQ_transcript/116872	-2.62340185562877	0.926886150562701	-1.25604321147036	0.218031070573802	0.978921443289946	-5.26760623164822
a_HQ_transcript/135065	1.31431213130521	4.53930552113421	1.25601478660188	0.21804124481288	0.978921443289946	-5.3279532235754
a_HQ_transcript/140329	-0.686246422283967	5.3975742984769	-1.25597761763852	0.218054549403355	0.978921443289946	-5.62988633900219
a_HQ_transcript/98125	-0.504855439189994	3.81382091027913	-1.25588419376481	0.218087993074765	0.978995973546217	-5.46478812141768
a_HQ_transcript/8184	-1.64228240545461	1.78437200398203	-1.25577221511148	0.218128084028351	0.979043106802824	-5.04654720481569
a_HQ_transcript/145255	1.02001888062579	1.9712685608135	1.25576077944228	0.218132178574927	0.979043106802824	-5.0284861249612
a_HQ_transcript/143339	3.5674089991878	0.23610877070743	1.25557601993673	0.21819833981212	0.979087838038715	-5.11793313193033
a_HQ_transcript/136659	-0.68071331612989	3.15011944359441	-1.25550125836767	0.218225115756564	0.979087838038715	-5.28801012400557
a_HQ_transcript/37780	2.57634190546617	1.23495122403757	1.25549424825881	0.218227626562059	0.979087838038715	-5.05997339375174
a_HQ_transcript/86976	1.42551391244225	1.8380816565908	1.25546817366695	0.218236965869988	0.979087838038715	-5.16704146953447
a_HQ_transcript/97516	-1.66786256307301	2.01988797890938	-1.25546667109348	0.218237504065763	0.979087838038715	-5.12908778374075
a_HQ_transcript/2772	1.58956877400889	1.35402347323302	1.25531282137956	0.218292615650232	0.979087838038715	-5.06491768169937
a_HQ_transcript/722	1.78280901025978	2.65687631434425	1.25522246833612	0.218324986524979	0.979087838038715	-5.406536694677
a_HQ_transcript/145998	2.68764181978444	6.40322873272218	1.25520894276055	0.218329832657009	0.979087838038715	-5.81619590239709
a_HQ_transcript/51235	-0.581619979284028	2.97400513204559	-1.25518596558032	0.218338065427324	0.979087838038715	-5.2100722066885
a_HQ_transcript/57936	0.994481525029153	0.277340640640707	1.25516159712285	0.218346796950172	0.979087838038715	-4.96553243178695
a_HQ_transcript/88934	1.48429807702746	1.00098255996975	1.25513654038437	0.218355775366508	0.979087838038715	-5.10403818965284
a_HQ_transcript/3104	1.4150600252397	-0.0159796745169412	1.25511644503255	0.218362976202386	0.979087838038715	-4.94180343361427
a_HQ_transcript/114719	1.45817916322261	1.84334216364123	1.25501669027127	0.218398724309169	0.979087838038715	-5.16545942914725
a_HQ_transcript/136793	-0.879106889618497	1.92606186039761	-1.25499914845123	0.218405011049065	0.979087838038715	-5.04125051461067
a_HQ_transcript/91976	-1.8337400575255	-0.230949483244348	-1.25496818374079	0.218416108694902	0.979087838038715	-5.10661683536288
a_HQ_transcript/68749	2.47169899103677	1.81141404399596	1.25495605222221	0.218420456705078	0.979087838038715	-5.0498800063267
a_HQ_transcript/3955	-1.1040982892169	-0.475299098170859	-1.25493364753886	0.218428486851191	0.979087838038715	-5.05149569243115
a_HQ_transcript/144161	-2.07547442554798	2.49710345468933	-1.25477859951917	0.218484064273893	0.979182777534489	-5.23330643570369
a_HQ_transcript/43488	2.19921389845587	0.392864761180524	1.25469108849695	0.218515437553217	0.979182777534489	-5.1049694050063
a_HQ_transcript/141255	-2.8717575564479	1.21546825103302	-1.25465842186193	0.218527149625716	0.979182777534489	-5.20857367001803
a_HQ_transcript/13559	-1.33605705564647	1.2556508349951	-1.2546362366471	0.218535104030426	0.979182777534489	-5.26934461204061
a_HQ_transcript/96572	-0.865866690511151	1.82771639224016	-1.25458534178382	0.218553352967438	0.979182777534489	-5.07379367562296
a_HQ_transcript/90712	-2.55435446403397	1.89914118894802	-1.25457271958397	0.218557878979278	0.979182777534489	-5.19924301613099
a_HQ_transcript/148383	-0.532877560557562	3.62085736226946	-1.25444607740047	0.2186032936662	0.979182777534489	-5.44506905054674
a_HQ_transcript/23016	1.64177627183574	0.00695170291009151	1.25443691943025	0.218606578047216	0.979182777534489	-5.05258508823481
a_HQ_transcript/107624	-3.18315053928858	4.28799541638697	-1.25443004288107	0.218609044252101	0.979182777534489	-5.45559129877332
a_HQ_transcript/17109	-0.759430471793682	2.67512006945726	-1.2543544209675	0.218636166667176	0.979182777534489	-5.23635930664848
a_HQ_transcript/93948	0.708901913212924	2.2371638549821	1.25430929227054	0.218652353647071	0.979182777534489	-5.07269295887051
a_HQ_transcript/111554	-1.34221944171355	0.196721456710692	-1.25429555705297	0.218657280440347	0.979182777534489	-4.94747796979892
a_HQ_transcript/107055	-0.61493590520875	3.02865653277983	-1.25425101329618	0.218673258765191	0.979182777534489	-5.34058450401183
a_HQ_transcript/25068	-1.93631171981529	2.14049320725678	-1.25413330591136	0.218715485894985	0.979182777534489	-5.14031212729669
a_HQ_transcript/24998	1.67441243055086	-0.517478691621091	1.25412135796014	0.218719772525099	0.979182777534489	-5.12558792001906
a_HQ_transcript/18061	2.28235019293715	1.04298504151599	1.25407657243271	0.218735841029429	0.979182777534489	-5.01142623494011
a_HQ_transcript/66638	-0.591883740055399	2.4915426199989	-1.25407602588424	0.218736037129874	0.979182777534489	-5.22951132077047
a_HQ_transcript/73758	0.591496314078183	4.80655476560543	1.25397098578593	0.218773727750538	0.979185282083252	-5.55461199476749
a_HQ_transcript/2855	-1.03019423842271	2.75809470984456	-1.25390855106966	0.218796132967356	0.979185282083252	-5.2462514033511
a_HQ_transcript/68230	1.74193891357559	1.66027549556292	1.25382547239199	0.218825949115722	0.979185282083252	-5.05644496350101
a_HQ_transcript/139233	-1.31591455784967	-0.392774155030314	-1.25379502743573	0.218836876286003	0.979185282083252	-5.11991020532644
a_HQ_transcript/132038	1.60759295559849	0.272875378867208	1.25373623763981	0.21885797802257	0.979185282083252	-5.14849234887164
a_HQ_transcript/146139	2.60635348889342	0.479579970758576	1.25349929336595	0.218943041204581	0.979185282083252	-5.10222903023566
a_HQ_transcript/14688	-1.39503176583128	3.03936346684548	-1.25349827321642	0.218943407492787	0.979185282083252	-5.40468689303157
a_HQ_transcript/54466	1.68467540173314	2.56268870650709	1.25345710430297	0.218958189717321	0.979185282083252	-5.15056961277433
a_HQ_transcript/59498	-1.76732199927118	0.404908096482376	-1.25343532311077	0.218966010836151	0.979185282083252	-5.21379082206787

a_HQ_transcript/121610	2.89471004008481	5.99978482013418	1.25343052947008	0.21896773214907	0.979185282083252	-5.9172319674227
a_HQ_transcript/144110	2.22399752128819	3.52634106313963	1.25332977353166	0.219003914205737	0.979185282083252	-5.49922338535492
a_HQ_transcript/151784	4.67721364488975	0.431659760561373	1.25327855732103	0.219022307973952	0.979185282083252	-5.12914619434341
a_HQ_transcript/106702	-1.07627238066177	2.74506507879931	-1.25325652034356	0.219030222681949	0.979185282083252	-5.28890647813476
a_HQ_transcript/102965	1.92168173341392	4.95624146202181	1.25317545148685	0.219059340880816	0.979185282083252	-5.36049928765262
a_HQ_transcript/70713	0.778010082944839	3.00818257777734	1.25310985316115	0.219082904525179	0.979185282083252	-5.23872683982351
a_HQ_transcript/74019	-2.91611836711854	4.64814095774173	-1.25307035082854	0.219097095118939	0.979185282083252	-5.45967445826131
a_HQ_transcript/98343	2.16383416433951	1.36951558600893	1.25303232795191	0.219110754893728	0.979185282083252	-5.03906073592632
a_HQ_transcript/78567	0.52873994238473	2.85424647777733	1.25302681004753	0.219112737262228	0.979185282083252	-5.23584789434525
a_HQ_transcript/45298	1.18325718021462	-0.33271227720622	1.25298118705294	0.219129128346715	0.979185282083252	-5.09182673199452
a_HQ_transcript/129580	0.422473726343686	4.72467165154933	1.25291766758285	0.219151950676	0.979185282083252	-5.56000778241074
a_HQ_transcript/126716	2.97182181940921	3.65777382235604	1.25289020167319	0.219161819636924	0.979185282083252	-5.3904761641671
a_HQ_transcript/119593	-1.02499728627103	2.99675894282483	-1.25284741327197	0.219177194890827	0.979185282083252	-5.16252445066705
a_HQ_transcript/36236	-1.7392100955568	3.57966635068491	-1.25279604823745	0.219195653076431	0.979185282083252	-5.28820874006928
a_HQ_transcript/102585	0.716444377867878	4.97449343495639	1.25276954682921	0.219205176897038	0.979185282083252	-5.50269661020328
a_HQ_transcript/32957	1.97336034780171	1.65111231667342	1.25271540322356	0.219224635470595	0.979185282083252	-5.07339691251377
a_HQ_transcript/29395	-1.8965122860374	2.89697878954904	-1.252633018247	0.219254246149777	0.979185282083252	-5.25964156937383
a_HQ_transcript/34650	1.55537798119158	0.223035317545041	1.25252818362737	0.219291929989361	0.979185282083252	-5.05207925059
a_HQ_transcript/11412	-1.92216596658753	1.32650991903201	-1.25250702986923	0.219299534506685	0.979185282083252	-5.09085501088696
a_HQ_transcript/87375	1.5897573954001	-0.298678481311094	1.2525045616552	0.21930042181235	0.979185282083252	-5.10616353038401
a_HQ_transcript/41508	-1.45648635559176	0.82803937132844	-1.25244783939769	0.219320813810085	0.979185282083252	-5.01077548555036
a_HQ_transcript/97929	1.22226173220708	-0.22956092993361	1.25238811143498	0.219342287917029	0.979185282083252	-5.11298517238276
a_HQ_transcript/145871	-1.41648270691601	9.15116930151307	-1.25238785185822	0.219342381246599	0.979185282083252	-6.0556158560929
a_HQ_transcript/1110	3.28935635283857	1.88500299141356	1.25233180717066	0.219362532541662	0.979185282083252	-5.26923642368582
a_HQ_transcript/127912	-2.18187085547966	1.58152498742741	-1.25232095752525	0.219366433775144	0.979185282083252	-5.03045281813337
a_HQ_transcript/114544	2.88996154601741	2.45225384075492	1.25231925959311	0.219367044309466	0.979185282083252	-5.08677247443897
a_HQ_transcript/138557	-0.865091244010367	1.13324010900913	-1.25227278341206	0.219383756488174	0.979185282083252	-5.09110892225328
a_HQ_transcript/25493	1.77635527409143	-0.105548416415739	1.25226324454244	0.219387186649353	0.979185282083252	-5.04736336586143
a_HQ_transcript/119292	-0.573239851958267	2.88851402186841	-1.25221007842569	0.219406305832034	0.979185282083252	-5.27909618443923
a_HQ_transcript/29893	-1.73670485349368	0.284670615066585	-1.25219594917507	0.21941138709306	0.979185282083252	-5.10792200827802
a_HQ_transcript/136245	3.23634950071419	3.01414396587725	1.25218448773623	0.219415509001687	0.979185282083252	-5.39791871278963
a_HQ_transcript/4176	1.40607553080145	1.27621894906787	1.2521051399196	0.21944404666969	0.979185282083252	-5.03180003349332
a_HQ_transcript/2913	2.41550338249633	1.8222197836443	1.2520779478195	0.219453827027338	0.979185282083252	-5.25092375727635
a_HQ_transcript/149940	-2.87889567917695	3.41858518078094	-1.25205673655966	0.219461456445022	0.979185282083252	-5.37802719949828
a_HQ_transcript/136025	-1.91252358247121	0.998755588605106	-1.25197696093034	0.219490152498054	0.979185282083252	-5.05396459263293
a_HQ_transcript/130509	-3.88150241394837	6.78584847684677	-1.25191361956788	0.219512938996336	0.979185282083252	-5.60795689711301
a_HQ_transcript/70593	1.13867015409949	0.387641823140058	1.25191029563507	0.219514134801015	0.979185282083252	-5.16768662038203
a_HQ_transcript/75281	-2.32249503328212	3.93063464845501	-1.25173964782705	0.219575532943581	0.979185282083252	-5.22378590148758
a_HQ_transcript/112934	-1.07447637065514	1.36224237304856	-1.25173720889011	0.219576410553164	0.979185282083252	-5.09623071324792
a_HQ_transcript/123411	-1.97028206761598	0.704130394191729	-1.25170352817649	0.219588530249135	0.979185282083252	-5.28039041839817
a_HQ_transcript/46606	-2.51685047667455	1.81130435462855	-1.25168066148195	0.219596758905877	0.979185282083252	-5.28378063952901
a_HQ_transcript/77773	1.29407829629553	1.04497995665377	1.2515365707011	0.219648615784926	0.979185282083252	-4.99050570136746
a_HQ_transcript/122711	-1.45801171471855	1.67691662163242	-1.25143689866117	0.219684492169961	0.979185282083252	-5.19180924507616
a_HQ_transcript/40505	-0.795242280111383	1.73380489410338	-1.25136993081833	0.219708599336749	0.979185282083252	-5.01180631479645
a_HQ_transcript/89360	-1.947124464226	0.385479826408661	-1.25134937900171	0.219715998003509	0.979185282083252	-5.08121623670554
a_HQ_transcript/710	-1.13646554044313	-0.287483197969852	-1.25130754613348	0.219731058440177	0.979185282083252	-5.10776226262891
a_HQ_transcript/17428	-1.92209528317587	0.315351631009981	-1.25123681199996	0.219756525513519	0.979185282083252	-4.99203410657137
a_HQ_transcript/87360	-1.75168801741889	-0.130307847211469	-1.25118279795778	0.219775974188278	0.979185282083252	-5.15043378558116
a_HQ_transcript/71651	2.0643915354593	1.04657196772793	1.25115926426925	0.21978444829747	0.979185282083252	-5.12940653327244
a_HQ_transcript/52093	-3.40283523960668	1.49662822392908	-1.25113089908369	0.219794662478491	0.979185282083252	-5.14117794929982

a_HQ_transcript/5634	1.22237985807209	0.990568306798736	1.25108623606735	0.219810746160693	0.979185282083252	-4.98506229059123
a_HQ_transcript/138711	1.25063073174179	1.86378364944973	1.25106642141603	0.219817881934815	0.979185282083252	-5.06341232483599
a_HQ_transcript/137337	2.18967599357711	1.26188870922909	1.25106526698164	0.219818297682192	0.979185282083252	-5.14658564747254
a_HQ_transcript/72831	-1.8114047154389	0.531039543554323	-1.25100929871891	0.219838454285564	0.979185282083252	-5.15088476240786
a_HQ_transcript/113206	-1.76996481082833	0.653366475768906	-1.25091606214658	0.219872035908737	0.979185282083252	-5.04927174671263
a_HQ_transcript/139776	-1.9363858557452	3.80992917297494	-1.25090731579034	0.219875186338422	0.979185282083252	-5.44490198352802
a_HQ_transcript/98283	-2.1140356205026	1.72599974167	-1.25090489691876	0.219876057619783	0.979185282083252	-5.20776118788125
a_HQ_transcript/75449	1.75653004999141	0.606916007379865	1.25086073507524	0.219891965241306	0.979185282083252	-5.15127945909006
a_HQ_transcript/75062	-1.05008329033843	-0.714087577963356	-1.25084744139507	0.219896753952321	0.979185282083252	-5.13128824443164
a_HQ_transcript/45280	-1.42816543213832	0.657014757421874	-1.25079929177172	0.21991409928806	0.979185282083252	-4.97140551160014
a_HQ_transcript/86843	-2.86368423288393	0.692815088174836	-1.25079465512611	0.219915769639362	0.979185282083252	-5.10174251604154
a_HQ_transcript/98834	0.868974370833572	2.56085144850944	1.25036810669538	0.220069474441202	0.979679588303278	-5.09421749165849
a_HQ_transcript/117632	-3.03179117908971	0.868571702052689	-1.25036049744057	0.220072217133937	0.979679588303278	-5.02330556723923
a_HQ_transcript/50612	-1.29406140497275	1.73531410103086	-1.25034626366895	0.220077347647678	0.979679588303278	-4.99673145975918
a_HQ_transcript/126340	1.38894696401818	-0.27298166708343	1.25008474307774	0.220171627823356	0.979877194254137	-5.11060048051543
a_HQ_transcript/101884	2.51559490529457	1.50501506956836	1.24995201458156	0.220219489061121	0.979877194254137	-4.93155556423244
a_HQ_transcript/86580	0.516094239467347	3.41599211681195	1.24985904316244	0.22025301874539	0.979877194254137	-5.37942862522003
a_HQ_transcript/118973	0.966695049366907	3.07700944056492	1.24982823693098	0.220264129706282	0.979877194254137	-5.31288171412179
a_HQ_transcript/26147	-1.49262847607388	0.280366397072349	-1.24978491615545	0.220279755031106	0.979877194254137	-5.04363226520577
a_HQ_transcript/133106	0.459845065278299	5.41946577260022	1.2497415964481	0.220295380802695	0.979877194254137	-5.62511675905011
a_HQ_transcript/48814	0.982904255075249	1.89931674938132	1.24972962127753	0.220299700491143	0.979877194254137	-5.13848646038813
a_HQ_transcript/140039	1.40110038993898	3.18778403405478	1.24964002750253	0.220332020811886	0.979877194254137	-5.3521376616095
a_HQ_transcript/132501	-1.92030189687255	0.138869973988035	-1.24961885275555	0.22033965997212	0.979877194254137	-5.10892592948838
a_HQ_transcript/73725	-1.73578286502149	1.29917104607021	-1.24953286192084	0.220370684712475	0.979877194254137	-5.00443886194406
a_HQ_transcript/39767	-0.74136032576103	2.87641895457455	-1.24934562254074	0.220438250384724	0.979877194254137	-5.24929121416908
a_HQ_transcript/43034	-1.65166669119414	1.1473880686608	-1.24932204300197	0.220446760206584	0.979877194254137	-4.95339064995362
a_HQ_transcript/45195	1.10334188272872	2.70009656460137	1.24931576586167	0.220449025659106	0.979877194254137	-5.22214891776316
a_HQ_transcript/43394	0.763670455313898	3.51687477484787	1.24925650890133	0.220470412664849	0.979877194254137	-5.33788312083383
a_HQ_transcript/114287	0.657101285382545	2.67798060138778	1.24923482886339	0.22047823780725	0.979877194254137	-5.15597918720786
a_HQ_transcript/143608	-1.53262173463922	0.272585154540802	-1.24920753495696	0.220488089502681	0.979877194254137	-5.10672773023
a_HQ_transcript/18128	1.93402791253812	1.1416926024119	1.24918026118079	0.220497934262151	0.979877194254137	-4.95500527806876
a_HQ_transcript/114352	-3.53624906184545	1.36327906716664	-1.24914531296885	0.220510549673022	0.979877194254137	-5.28531506258041
a_HQ_transcript/61852	-1.68585101851623	0.321138839599153	-1.24909054047592	0.220530322232199	0.979877194254137	-5.00216709487314
a_HQ_transcript/70040	1.8747328458627	1.16012621817513	1.24908461215927	0.220532462400673	0.979877194254137	-5.15206739204931
a_HQ_transcript/99488	-1.99380274156791	0.481254507031388	-1.24906107452487	0.220540959823798	0.979877194254137	-5.0996792361224
a_HQ_transcript/128401	1.87518076660094	3.94766805104654	1.24881121392773	0.220631178227807	0.979877194254137	-5.53611679043356
a_HQ_transcript/1044	-2.03173229706025	2.65092851193155	-1.2487917668324	0.22063820124845	0.979877194254137	-5.30166256887282
a_HQ_transcript/115372	1.60474832825982	1.30084534148273	1.2487810185428	0.220642082900615	0.979877194254137	-5.11503827679546
a_HQ_transcript/91914	-1.61667897337295	-0.134621830226495	-1.24864699027063	0.220690490360012	0.979877194254137	-5.00072530259759
a_HQ_transcript/29061	1.03202310047922	1.84426059561181	1.24860697447212	0.220704944550598	0.979877194254137	-5.12840762579036
a_HQ_transcript/125369	1.54287373130889	0.965637733251679	1.24842945503974	0.220769075283584	0.979877194254137	-5.17565205253945
a_HQ_transcript/6069	1.07476709685305	3.3342194453352	1.24839308823807	0.220782214892735	0.979877194254137	-5.27293515978923
a_HQ_transcript/46774	1.67093112859401	1.16381981531942	1.24833753532465	0.220802287726564	0.979877194254137	-5.02715200024597
a_HQ_transcript/25681	1.48537301332345	-0.237181882924256	1.24829134402444	0.220818978988229	0.979877194254137	-5.12147031346048
a_HQ_transcript/140899	-0.519038898842777	3.93943108566224	-1.24826895647375	0.220827069087413	0.979877194254137	-5.49505325013245
a_HQ_transcript/133051	1.72872496358302	1.71901375605485	1.24824851396543	0.220834456508701	0.979877194254137	-5.31768766713336
a_HQ_transcript/32253	-1.34411960997581	1.86560586799241	-1.24824497020895	0.220835737154267	0.979877194254137	-5.07909866570822
a_HQ_transcript/78821	-1.35639902806474	1.77489022556468	-1.24815775916271	0.220867255308207	0.979877194254137	-5.14281190534281
a_HQ_transcript/68274	0.985529891616578	2.12269709473082	1.24808502114427	0.220893545477627	0.979877194254137	-5.07124384678545
a_HQ_transcript/5117	-0.59165854286031	4.07491767768441	-1.24798238615918	0.220930645493502	0.979877194254137	-5.50915109935581

a_HQ_transcript/114391	0.471386921553957	3.55844327919463	1.24791633261206	0.220954524694118	0.979877194254137	-5.39108244635188
a_HQ_transcript/73136	1.24462095152642	0.269298207488623	1.24791626450798	0.220954549315613	0.979877194254137	-5.01808361857274
a_HQ_transcript/130368	-1.19520531865231	3.02625788512965	-1.24791067294625	0.220956570825629	0.979877194254137	-5.37885856556717
a_HQ_transcript/43974	2.04232290714567	0.274039706081414	1.24783574037747	0.220983662438366	0.979877194254137	-4.94817931825186
a_HQ_transcript/9065	-1.85663197192227	0.952505198546096	-1.24771721720245	0.221026519173089	0.979877194254137	-5.05424043274437
a_HQ_transcript/12700	-2.23084305469847	1.49423116579857	-1.24771318603374	0.221027976910971	0.979877194254137	-5.03615379142786
a_HQ_transcript/101332	6.26741588530768	2.84893009507552	1.24768172468418	0.22103935410784	0.979877194254137	-5.15602227744562
a_HQ_transcript/36175	1.75904813517215	0.55499466429295	1.24765490896384	0.221049051677868	0.979877194254137	-5.11074526841528
a_HQ_transcript/38702	1.84399816588967	1.97872435936606	1.24758260768057	0.221075200123485	0.979877194254137	-5.06918754746614
a_HQ_transcript/140191	-2.00194809230937	0.0349815543566656	-1.24742840540074	0.221130976593837	0.979877194254137	-5.13196659909742
a_HQ_transcript/50981	1.11447008528022	-0.48427339377673	1.24741135873459	0.221137143187343	0.979877194254137	-5.14870250059587
a_HQ_transcript/142920	-2.54386324599095	5.68631686794343	-1.2473538965159	0.221157930967245	0.979877194254137	-5.72848033002951
a_HQ_transcript/19640	0.911559340651312	1.32080224460049	1.24733528680235	0.221164663611646	0.979877194254137	-4.95613703562235
a_HQ_transcript/25123	-2.06596483241223	1.73424525210207	-1.24720172697208	0.221212987557768	0.979877194254137	-5.1159045476328
a_HQ_transcript/14768	1.42321283912557	1.3412160222434	1.24718073813939	0.221220582351688	0.979877194254137	-4.98741092893174
a_HQ_transcript/39562	-0.440276855194885	2.90351901494939	-1.24717352441166	0.221223192679008	0.979877194254137	-5.28673401042439
a_HQ_transcript/148141	2.50413207882896	4.06101319069337	1.24715389771414	0.221230294825107	0.979877194254137	-5.39214021838532
a_HQ_transcript/120483	2.44807912539215	0.790299979733402	1.24710810548978	0.221246865932847	0.979877194254137	-5.17766779129687
a_HQ_transcript/100920	1.18733771147823	-0.576137169320092	1.24703377145457	0.221273767619623	0.979877194254137	-5.12276684146892
a_HQ_transcript/17183	-2.47892201389624	1.48532739659641	-1.24699588441582	0.221287479984445	0.979877194254137	-5.09335591394494
a_HQ_transcript/7967	1.58551084057981	0.00824530673987 09	1.24696179575258	0.221299818157039	0.979877194254137	-5.08342557831926
a_HQ_transcript/140932	-1.59687664230662	0.538274608385025	-1.24694961806715	0.2213042259171	0.979877194254137	-4.98646551788801
a_HQ_transcript/40419	1.633105746683	2.2289646255881	1.24693861650144	0.221308208032721	0.979877194254137	-5.2523373395723
a_HQ_transcript/141574	3.8524835183318	3.15358159087559	1.24693220147759	0.221310530033083	0.979877194254137	-5.33205108251591
a_HQ_transcript/133757	0.395360212531813	4.19245953946784	1.24691710898176	0.221315993028058	0.979877194254137	-5.5203239186573
a_HQ_transcript/83861	0.519748815909455	2.70415855582851	1.24690241114241	0.221321313267371	0.979877194254137	-5.28351332174091
a_HQ_transcript/15225	1.32012615994121	2.048956220524	1.24687463590182	0.221331367451035	0.979877194254137	-5.3237774845117
a_HQ_transcript/118861	2.31544799730618	2.54281082403736	1.24676480614495	0.221371127385391	0.979877194254137	-5.40935330513734
a_HQ_transcript/114825	0.920248293033851	2.73985796858859	1.24674688666216	0.22137761500132	0.979877194254137	-5.19060464913353
a_HQ_transcript/147480	-2.00412249851657	3.71854678260207	-1.24671727471472	0.221388336099915	0.979877194254137	-5.35926972733548
a_HQ_transcript/133802	-2.1014713626807	-0.146423240642895	-1.24669250311897	0.221397305033088	0.979877194254137	-5.12925092890253
a_HQ_transcript/129061	4.84390165904643	0.84859822022289	1.24666639967809	0.221406756475912	0.979877194254137	-5.24330756161008
a_HQ_transcript/25424	-0.34046136347817	4.22533867863457	-1.24658651728095	0.221435681895496	0.979877194254137	-5.52395350978373
a_HQ_transcript/106600	-3.0010419478893	0.36114127452667	-1.2464359766155	0.221490200379531	0.979877194254137	-5.14598102596684
a_HQ_transcript/9309	0.974219091263893	4.39813516309692	1.24636727424154	0.221515084372077	0.979877194254137	-5.44249206256834
a_HQ_transcript/73434	0.412131807275403	2.61017978308039	1.24625957142498	0.221554098539943	0.979877194254137	-5.18151092221745
a_HQ_transcript/92256	-0.77647660066503	1.68356621398877	-1.2462428874326	0.221560142594659	0.979877194254137	-4.99551385290986
a_HQ_transcript/101870	-1.29362926435312	1.99423166728725	-1.24620414232059	0.221574179135126	0.979877194254137	-5.11178354252622
a_HQ_transcript/103641	-2.49869262873839	0.686492952896974	-1.24610761209124	0.221609152910232	0.979877194254137	-5.05696443898934
a_HQ_transcript/25568	-1.32403473479339	2.46166703550353	-1.24608496520355	0.221617358681884	0.979877194254137	-5.19713827221085
a_HQ_transcript/93477	1.50388570999903	3.1005998396548	1.24591736246435	0.22167809418835	0.979877194254137	-5.2503550726991
a_HQ_transcript/77263	1.03105052025156	1.19953082111457	1.24589180033456	0.2216873584326	0.979877194254137	-4.95149013939469
a_HQ_transcript/132156	1.05103550619627	3.32368893139987	1.24584293192712	0.22170507016157	0.979877194254137	-5.4390011079229
a_HQ_transcript/143827	-0.687729348423433	2.7505228317886	-1.24584060851725	0.221705912278168	0.979877194254137	-5.23819220624834
a_HQ_transcript/104303	1.54043355896979	-0.119272346768072	1.24582640421957	0.221711060657771	0.979877194254137	-5.12230579941435
a_HQ_transcript/24632	1.22168951505279	2.50100919445222	1.2458096095255	0.221717148048007	0.979877194254137	-5.16746455137213
a_HQ_transcript/137113	-2.42233990045418	1.13889727224579	-1.24580073088566	0.221720366242733	0.979877194254137	-5.18864528538567
a_HQ_transcript/11792	-0.452143927456841	3.11108545149661	-1.24579048455236	0.22172408022177	0.979877194254137	-5.32591442395069
a_HQ_transcript/93648	-1.1007968805369	2.52276174577923	-1.24578612239905	0.221725661381635	0.979877194254137	-5.26928549771689
a_HQ_transcript/28511	-1.27393358251018	0.556549655527175	-1.24578281865791	0.221726858902004	0.979877194254137	-4.99458544255482

a_HQ_transcript/65355	1.39902313222147	-0.443949220978259	1.24570852535655	0.221753789575019	0.979877194254137	-5.10624120271948
a_HQ_transcript/6374	1.69320545618348	0.397111871048813	1.24565800961269	0.22177210249161	0.979877194254137	-4.98911603158283
a_HQ_transcript/75123	1.78458776143805	1.55799699664691	1.24564805010575	0.221775713135836	0.979877194254137	-5.20341104539288
a_HQ_transcript/92011	1.05826600543208	3.47890699145821	1.24561186249735	0.221788832688319	0.979877194254137	-5.30127646505965
a_HQ_transcript/11807	1.4499917136869	-0.322695484927604	1.24560173430554	0.221792504694319	0.979877194254137	-5.02266268731068
a_HQ_transcript/44776	-1.40089275032361	0.959947166866857	-1.24554557309449	0.221812866935108	0.979877194254137	-4.99184811367239
a_HQ_transcript/28543	2.4599876493501	2.37512385244557	1.24554081616885	0.221814591707046	0.979877194254137	-5.33973363017962
a_HQ_transcript/140134	1.99250150145996	2.87201339517755	1.24538822958315	0.221869922072412	0.979877194254137	-5.07848461848102
a_HQ_transcript/71155	2.1934175908691	1.49901541699686	1.24523615248143	0.221925077984551	0.979877194254137	-5.13350247476137
a_HQ_transcript/145088	0.316524083345344	10.3232301432275	1.24522879133272	0.221927748015239	0.979877194254137	-6.17710928175102
a_HQ_transcript/92311	1.5637028182348	-0.186353149985611	1.24521380012729	0.221933185689193	0.979877194254137	-5.06429904616905
a_HQ_transcript/111907	-1.11455450727295	-0.614792389986819	-1.24518801321881	0.221942539460239	0.979877194254137	-5.14000986722458
a_HQ_transcript/51644	1.52865995489588	1.84477378082494	1.24514070999441	0.221959698684315	0.979877194254137	-5.23016585172198
a_HQ_transcript/36238	1.5957436770507	2.92502402778227	1.24507423622007	0.221983813697614	0.979877194254137	-5.36308893839444
a_HQ_transcript/83573	1.75490103988405	1.39634963561248	1.24505872492496	0.221989441087899	0.979877194254137	-5.14472094897873
a_HQ_transcript/134250	2.39446150466696	2.17485344691184	1.24503923031718	0.221996513748038	0.979877194254137	-5.13434913145115
a_HQ_transcript/30791	-0.917267407880439	1.45565859990007	-1.24500336101278	0.222009527603205	0.979877194254137	-5.03025622301557
a_HQ_transcript/84185	-1.99135013970776	0.541080526714578	-1.24498735290939	0.222015335738099	0.979877194254137	-5.11937333551133
a_HQ_transcript/95239	1.43525455574608	-0.112436959238169	1.24497993802984	0.222018026077946	0.979877194254137	-5.08572542407573
a_HQ_transcript/8170	0.44712191619419	3.1319183142336	1.24492552551536	0.22203776931158	0.979877194254137	-5.38105605521595
a_HQ_transcript/143818	1.93650042235388	0.405189577263382	1.24491557472729	0.222041380033856	0.979877194254137	-5.10672690980695
a_HQ_transcript/56817	-1.01812760109752	1.62187484020721	-1.24486989193514	0.222057956961655	0.979877194254137	-5.08430862831223
a_HQ_transcript/136494	2.28963461810068	-0.00694334436653199	1.24477871715402	0.22209104435347	0.979877194254137	-5.12572530823704
a_HQ_transcript/61141	1.45781555686102	1.4840859030465	1.244694588074	0.222121578133093	0.979877194254137	-5.03872664478264
a_HQ_transcript/114429	-0.874647527651331	0.290260210920463	-1.24467912032542	0.222127192334873	0.979877194254137	-5.1712664300912
a_HQ_transcript/145572	0.425904810893097	2.81852200649664	1.24460298422883	0.222154828379199	0.979877194254137	-5.3013082213558
a_HQ_transcript/129191	1.17049722123427	3.2158734776353	1.24458204201505	0.222162430480924	0.979877194254137	-5.39463915442383
a_HQ_transcript/133387	-0.50714958273986	4.16570870608987	-1.24455755682834	0.222171318942264	0.979877194254137	-5.48506336933968
a_HQ_transcript/128654	3.43559746629773	3.0702212274957	1.24450496237127	0.222190412358485	0.979877194254137	-5.58282429751012
a_HQ_transcript/125033	-0.924818410770366	2.98295541598483	-1.24447587222319	0.222200973511024	0.979877194254137	-5.40371591170656
a_HQ_transcript/136836	-1.7754168843033	2.4218477880303	-1.24447443770874	0.22220149431996	0.979877194254137	-5.18011394004078
a_HQ_transcript/21805	2.55664982723315	0.868862048942137	1.24446686444913	0.222204243851711	0.979877194254137	-5.15154812141914
a_HQ_transcript/67492	0.465398872944651	2.42765081036711	1.24445237926095	0.222209502884777	0.979877194254137	-5.16579195785656
a_HQ_transcript/82324	-0.973031528135753	2.32657319697663	-1.24443494637457	0.222215832241102	0.979877194254137	-5.11971522155777
a_HQ_transcript/114925	-1.51799633849706	0.665493865203177	-1.24428494122775	0.222270300177892	0.979877194254137	-4.98965661052108
a_HQ_transcript/132382	2.13933295661453	1.17920025159236	1.24427346783861	0.222274466659015	0.979877194254137	-5.19046654646333
a_HQ_transcript/3656	-0.689627847898645	2.62125254489631	-1.24426421292525	0.2222778275588	0.979877194254137	-5.17206374421912
a_HQ_transcript/88223	-2.21992866016031	1.36319598242141	-1.24415911270191	0.222315997126854	0.979877194254137	-5.0768752054258
a_HQ_transcript/27836	-1.60783426247988	1.05559948634594	-1.24404909652066	0.222355957299569	0.979877194254137	-5.05280857279215
a_HQ_transcript/143457	4.10835180936032	3.64758345832929	1.24404464028751	0.222357576009847	0.979877194254137	-5.55237266865234
a_HQ_transcript/23624	-2.91558782107724	2.10500795871484	-1.24403140712229	0.222362382960405	0.979877194254137	-5.15329817918069
a_HQ_transcript/22867	2.09576148154046	2.72490523562451	1.24400219776562	0.222372993542108	0.979877194254137	-5.13648043393119
a_HQ_transcript/18349	-1.9216809580104	0.355984573410093	-1.24394490964894	0.222393805107025	0.979877194254137	-5.14019037825384
a_HQ_transcript/121611	2.54748370413035	3.15868308495442	1.24394294760717	0.222394517901279	0.979877194254137	-5.3989276338524
a_HQ_transcript/133841	-2.0968264152931	1.44576041267477	-1.24393560778014	0.222397184417553	0.979877194254137	-4.99984255601159
a_HQ_transcript/86252	1.37082773369755	1.5043704296341	1.24388839108699	0.222414338539026	0.979877194254137	-5.14095253270095
a_HQ_transcript/127493	0.54210198050538	2.85508250717096	1.24384758525293	0.222429164353697	0.979877194254137	-5.28906520006127
a_HQ_transcript/120600	3.06310987309966	0.914448981306943	1.24382648782225	0.22243682988618	0.979877194254137	-5.21913842552145
a_HQ_transcript/143522	-3.25115088284116	4.31980310367158	-1.24379679609839	0.222447618401199	0.979877194254137	-5.53267883359026
a_HQ_transcript/58888	-1.48578252375049	-0.0392041398306313	-1.24373365873086	0.222470560724633	0.979877194254137	-5.11193911975632

a_HQ_transcript/11251	-1.53961265642724	0.0100798246612541	-1.24372445801764	0.222473904150095	0.979877194254137	-5.10811941453771
a_HQ_transcript/137084	-1.50629808706452	0.274585905487068	-1.24368722182234	0.222487435707718	0.979877194254137	-5.20107656021288
a_HQ_transcript/79635	0.930693005845987	4.24175054815334	1.24366129375971	0.222496858277099	0.979877194254137	-5.37354935533864
a_HQ_transcript/134834	-0.832214135035949	1.39550410193315	-1.2436146091945	0.222513824762617	0.979877194254137	-5.09098635944567
a_HQ_transcript/79927	-1.59344180994401	-0.362706948305806	-1.24360876331664	0.222515949387524	0.979877194254137	-5.11110559623658
a_HQ_transcript/138435	3.61373194047392	1.33614771714873	1.24357988236638	0.222526446098451	0.979877194254137	-5.14250783254729
a_HQ_transcript/147107	-2.98879655046	0.845522782782414	-1.24354596732164	0.222538772912338	0.979877194254137	-5.1034400746226
a_HQ_transcript/110382	0.472493951488397	2.65820693207411	1.24349940778972	0.222555696344032	0.979877194254137	-5.2129218142743
a_HQ_transcript/58080	-0.976297796625825	1.63579376229983	-1.2434709601548	0.222566036948413	0.979877194254137	-5.14656802344185
a_HQ_transcript/71927	1.69001827143088	0.581444897719443	1.24341773422186	0.222585385334266	0.979888161065601	-5.12463779308302
a_HQ_transcript/24429	2.08923268537316	2.14970278861295	1.24329923911465	0.22262846452127	0.979938736139675	-5.09407726633372
a_HQ_transcript/142988	0.901911301725286	2.89627752064476	1.24326269459486	0.222641751631211	0.979938736139675	-5.32427350405009
a_HQ_transcript/124439	2.03689037447395	2.75469378497755	1.24320922463938	0.222661193678912	0.979938736139675	-5.15745420694458
a_HQ_transcript/4571	0.8498936876102	4.90036424039036	1.24320066312909	0.222664306821718	0.979938736139675	-5.61455757465015
a_HQ_transcript/79894	-2.05524743776954	-0.200033678526098	-1.24312649170852	0.222691278454266	0.979983241274098	-5.06673059658631
a_HQ_transcript/80832	1.23806564381888	3.08668451297814	1.24306356714674	0.222714162207386	0.980008586162649	-5.29052416639396
a_HQ_transcript/83345	-2.02231787646405	1.76150017082415	-1.24301793919811	0.222730756809021	0.980008586162649	-5.05902291087046
a_HQ_transcript/107422	1.81638219359374	1.36514405247102	1.24296501553723	0.222750005976069	0.980012284508295	-5.04377879184819
a_HQ_transcript/108749	-5.49310335374014	2.02636733091278	-1.24292292333687	0.222765316460365	0.980012284508295	-5.32065601802593
a_HQ_transcript/82923	2.10681561083931	0.956075468296916	1.24283307822791	0.222797999074008	0.980075010057803	-5.10254706883589
a_HQ_transcript/8204	1.4501100271517	1.43859244797946	1.24273632774084	0.222833197646831	0.980075010057803	-5.01390832591342
a_HQ_transcript/47823	-1.20009841509852	2.41431772134606	-1.24273331830727	0.222834292568744	0.980075010057803	-5.18881222873594
a_HQ_transcript/37131	1.1516819751743	3.41286894215395	1.24269834536962	0.22284701706465	0.980075010057803	-5.47562911767834
a_HQ_transcript/121014	-2.03998408900518	0.0892451741955036	-1.24261144475783	0.222878637195985	0.980132045621693	-5.08928360862253
a_HQ_transcript/60760	0.724953536431678	2.33466283254278	1.24257002691123	0.222893708902305	0.980132045621693	-5.14349816142218
a_HQ_transcript/96824	0.610523907292207	2.80841115433639	1.24241647137781	0.22294959350239	0.980303628676773	-5.21355612724241
a_HQ_transcript/27095	1.85818884954349	0.745499339513963	1.24222524136374	0.223019203924971	0.98031451814481	-5.09460118670384
a_HQ_transcript/16768	-1.71298925490772	0.876074105851237	-1.24220758237454	0.223025632866943	0.98031451814481	-5.10716669749942
a_HQ_transcript/71319	-1.18565076632401	1.07001707899922	-1.242183753356	0.22303430829341	0.98031451814481	-5.13880815770814
a_HQ_transcript/64312	1.94063005389936	1.53805650600405	1.24216295300493	0.223041881280028	0.98031451814481	-5.01011065154729
a_HQ_transcript/46712	-1.60578071185362	3.25704129975869	-1.24211616497629	0.223058916557521	0.98031451814481	-5.2961440549508
a_HQ_transcript/22943	-1.86177815263408	0.152106927457198	-1.24206120463175	0.223078928577095	0.98031451814481	-5.1616269280634
a_HQ_transcript/81498	1.05829642363127	-0.518387409316443	1.24203967527349	0.223086768158229	0.98031451814481	-5.14394410145372
a_HQ_transcript/89996	1.76226461716064	0.0586381216613285	1.24201774815981	0.223094752787874	0.98031451814481	-5.18487026012835
a_HQ_transcript/28079	1.51008509343821	1.1043861911795	1.24199275850263	0.223103852885661	0.98031451814481	-4.98174724336217
a_HQ_transcript/62168	1.52087651929805	0.803772884511133	1.24192377241388	0.223128975927727	0.980350802171346	-5.06724655267999
a_HQ_transcript/87668	1.28146520801586	-0.473444716561614	1.24186784741938	0.223149343994192	0.980366190589856	-5.05186369439982
a_HQ_transcript/70895	-2.63887634852743	3.61952077487084	-1.24179782098581	0.223174848814004	0.980404146764543	-5.18393766228511
a_HQ_transcript/114707	4.19394889571039	3.36478882543158	1.24175058543968	0.223192055711761	0.980405638510786	-5.1260380401345
a_HQ_transcript/138076	-1.23236292256064	1.8619050798109	-1.24169391157623	0.223212700897274	0.980422236493635	-5.11510648188136
a_HQ_transcript/49174	1.503752774606	0.0311371135041257	1.24162865973925	0.223236472642429	0.980452563789885	-5.09982179341913
a_HQ_transcript/150962	1.67398053115047	1.51286841992847	1.24140073638449	0.223319521744328	0.980612998869299	-5.10406672727286
a_HQ_transcript/147045	2.44959425408199	0.293375174576913	1.24140027599706	0.223319689520455	0.980612998869299	-5.20426721781618
a_HQ_transcript/150016	0.929696390349039	3.00523500172581	1.24137446048663	0.223329097457216	0.980612998869299	-5.20062626505106
a_HQ_transcript/141168	-1.38654189061859	-0.0785138436648508	-1.24134322438585	0.2233404812144	0.980612998869299	-5.07768764812524
a_HQ_transcript/111695	-2.17346601492621	0.755182029094413	-1.2412954182693	0.223357904627854	0.980615429018725	-5.19187871375592
a_HQ_transcript/95098	4.01732342079071	1.55468219544255	1.24110672815288	0.223426684547442	0.980782229909575	-5.21800702199886
a_HQ_transcript/76582	1.56680900294885	0.14461638787671	1.24097369347526	0.223475186889093	0.980782229909575	-5.04706647629958
a_HQ_transcript/22075	-2.21620459493764	0.934126000080309	-1.24095522245391	0.223481921754744	0.980782229909575	-5.28914649764951
a_HQ_transcript/77174	2.88618805334195	1.13814962794851	1.24094699590336	0.223484921351515	0.980782229909575	-5.3433346511733

a_HQ_transcript/72780	3.11246612980227	2.7007456954088	1.24090953291628	0.223498581632302	0.980782229909575	-5.33174559644146
a_HQ_transcript/133814	2.57833956914275	3.9602979956814	1.24083474973999	0.223525851993996	0.980782229909575	-5.50747675387952
a_HQ_transcript/93078	3.75175108109564	0.799175115490642	1.24083390753943	0.223526159124233	0.980782229909575	-5.2309546774521
a_HQ_transcript/80279	0.475020639628814	2.88356359043891	1.24073811529259	0.223561094307927	0.980782229909575	-5.32723347238115
a_HQ_transcript/11702	1.05864634533828	-0.518189501704993	1.24071802845883	0.223568420442365	0.980782229909575	-5.13449225373325
a_HQ_transcript/111841	-2.78419552238591	2.4365858525848	-1.24068198036531	0.223581568469277	0.980782229909575	-5.24784212058553
a_HQ_transcript/1843	0.553122544782589	2.34633020675687	1.24065494124963	0.223591430980148	0.980782229909575	-5.15548743521916
a_HQ_transcript/82684	1.22468523979864	0.551203734075862	1.24063591492692	0.223598371021912	0.980782229909575	-4.95877730725523
a_HQ_transcript/55123	-1.74298165454616	1.27449323810477	-1.24039434637076	0.223686499591649	0.98094207227784	-5.12247473636756
a_HQ_transcript/150827	3.52725133781448	0.341503969137557	1.24032684109953	0.223711131379849	0.98094207227784	-5.17103257990079
a_HQ_transcript/58147	-2.33741359990669	1.18819613580045	-1.24030983885393	0.223717335596317	0.98094207227784	-5.16355086211121
a_HQ_transcript/110827	-2.11509588248028	2.11132501624977	-1.24028503587458	0.223726386576005	0.98094207227784	-5.02437928682785
a_HQ_transcript/97734	-2.67119753303071	0.948661161561904	-1.24020867869992	0.223754252176338	0.98094207227784	-4.98693841370842
a_HQ_transcript/59491	1.659230104005	0.341099170809833	1.24018683290154	0.223762225006348	0.98094207227784	-5.02379164616191
a_HQ_transcript/123066	-1.29867102843661	2.06641907066298	-1.24017838963418	0.223765306513312	0.98094207227784	-5.20538195576709
a_HQ_transcript/1429	-1.39212221233488	1.27085951284104	-1.24015426104414	0.2237741128075	0.98094207227784	-4.96577247852181
a_HQ_transcript/12817	-1.02196384152974	0.417484598043108	-1.24001911488177	0.223823442360291	0.98094207227784	-5.00991543460128
a_HQ_transcript/133553	-2.30450357363008	0.301047759474357	-1.24001225830561	0.223825945288538	0.98094207227784	-5.06452489782725
a_HQ_transcript/34867	1.58086867019071	0.944434087512702	1.23993439890377	0.22385436859578	0.98094207227784	-5.06784111592127
a_HQ_transcript/9794	0.452286391622058	4.74722267771748	1.23990427544476	0.223865366172556	0.98094207227784	-5.60277768752876
a_HQ_transcript/99558	-2.06944879242824	1.71547792793793	-1.23984133109771	0.223888347417891	0.98094207227784	-5.18435910484075
a_HQ_transcript/115596	-1.89451247409941	3.2055355149892	-1.23982312727433	0.223894994039314	0.98094207227784	-5.26565907591166
a_HQ_transcript/73815	2.15752110229163	-0.494242508637561	1.23980140947968	0.223902923883078	0.98094207227784	-5.12659180130756
a_HQ_transcript/138345	-1.16305982019069	2.26873727358011	-1.23969752713081	0.2239408574735	0.98094207227784	-5.14435854215881
a_HQ_transcript/25016	0.885726447609741	1.64183212127804	1.23969550095537	0.223941597397814	0.98094207227784	-5.0014346054907
a_HQ_transcript/57907	0.691363987738766	3.43146884712874	1.23968606544453	0.223945043107683	0.98094207227784	-5.38229762211534
a_HQ_transcript/16579	1.47129648136813	1.84414006233248	1.23965757590532	0.223955447309373	0.98094207227784	-5.19093002909103
a_HQ_transcript/2856	-1.55682449067388	0.184959122254496	-1.23956447211678	0.223989450753451	0.981017088110049	-5.12944546886101
a_HQ_transcript/82866	1.5183851468597	0.762114721805017	1.2394722451884	0.224023137758315	0.981026621837745	-5.02221672438189
a_HQ_transcript/127929	-1.42193631110193	1.25357698325466	-1.23944135285648	0.224034422404942	0.981026621837745	-5.04663655946587
a_HQ_transcript/150085	3.30435556661967	6.33102449521867	1.23932594867937	0.224076582109149	0.981026621837745	-5.76831771782496
a_HQ_transcript/117970	1.40997506568455	1.99651165833697	1.23931834098983	0.224079361575326	0.981026621837745	-5.17146200726122
a_HQ_transcript/40986	-1.16955846265517	3.75812770932616	-1.23929745135805	0.224086993726163	0.981026621837745	-5.48968682420534
a_HQ_transcript/40714	0.519271307672108	3.3438897147519	1.23926944205202	0.224097227397415	0.981026621837745	-5.35670362865449
a_HQ_transcript/101889	1.03596952208426	3.18872029676735	1.2391937941575	0.224124868375979	0.981026621837745	-5.27215229325429
a_HQ_transcript/125365	1.96797305525859	0.192600535677557	1.23914056286008	0.224144320084638	0.981026621837745	-5.15333532946527
a_HQ_transcript/12337	-1.30012914804573	1.29570102927012	-1.23901463487694	0.224190341545774	0.981026621837745	-5.05589711538254
a_HQ_transcript/45221	-1.23122981267563	0.949842070379076	-1.23894010831456	0.224217581246459	0.981026621837745	-4.94087348919621
a_HQ_transcript/100056	1.27688735686166	4.33137951366096	1.23893094232339	0.224220931616987	0.981026621837745	-5.54214301815112
a_HQ_transcript/124011	1.25423355864366	-0.0398712128257771	1.23881201127817	0.224264406914415	0.981026621837745	-4.99617386376676
a_HQ_transcript/108294	1.57372213665124	0.85197856204414	1.23881018822748	0.224265073380487	0.981026621837745	-5.00215207537271
a_HQ_transcript/34615	-1.32182497585907	-0.20702415829134	-1.23880930837977	0.22426539503343	0.981026621837745	-5.12672099724037
a_HQ_transcript/44382	-2.45790299374627	2.13452506998557	-1.23880042364494	0.224268643116087	0.981026621837745	-5.12925461942988
a_HQ_transcript/112515	0.514035889272466	3.20144382101721	1.23878734054392	0.224273426102135	0.981026621837745	-5.35645233411973
a_HQ_transcript/66293	1.85993949716325	0.867723640932301	1.2387733617172	0.22427853663642	0.981026621837745	-5.07380794333705
a_HQ_transcript/55375	2.35727539404703	0.620987369515165	1.23861369238074	0.224336916501	0.981070886399494	-5.08823683970154
a_HQ_transcript/122270	1.61532294926646	2.97023999882845	1.23859669559383	0.22434313170182	0.981070886399494	-5.20230238422217
a_HQ_transcript/125232	1.38043707674579	1.11813131965062	1.23852790795361	0.224368286535976	0.981070886399494	-5.14141712975342
a_HQ_transcript/92726	2.02638254473896	0.357328055418196	1.23852027559323	0.224371077730775	0.981070886399494	-5.1753782315127
a_HQ_transcript/102410	1.63226475200892	1.22524442772823	1.23849398921494	0.224380690997926	0.981070886399494	-5.07039017102404

a_HQ_transcript/46600	2.9250217413851	0.633081807499363	1.23846874660036	0.224389922837209	0.9810708863994944	-5.15056838941484
a_HQ_transcript/40287	1.12952271224075	1.63605446543789	1.23836386613947	0.224428283220006	0.98116480497988	-4.9928516422023
a_HQ_transcript/43448	-1.92761665441647	5.50862558700548	-1.23828759165566	0.224456183950542	0.981202129655236	-5.63946826972701
a_HQ_transcript/101428	-1.41855184335148	2.59472761131262	-1.23824823662497	0.2244705807899	0.981202129655236	-5.19610970115143
a_HQ_transcript/75027	-2.04347731922751	0.50297108103315	-1.23799028758826	0.224564960687473	0.981540870354268	-5.09957859644876
a_HQ_transcript/124139	-1.57464926815411	0.220757612240221	-1.2379152565755	0.224592419042663	0.981587077752775	-4.96761677847253
a_HQ_transcript/108760	1.11046682348504	1.63217243481016	1.23778995097068	0.224638281489237	0.981678900256408	-5.00675656455642
a_HQ_transcript/152375	-1.73167821207674	2.86296527423946	-1.2376529670985	0.224688426249516	0.981678900256408	-5.40435433779211
a_HQ_transcript/100359	-0.559102100686889	3.15319287111085	-1.23761955838828	0.2247006572198	0.981678900256408	-5.38079230627894
a_HQ_transcript/112813	-1.39756389391072	1.95692121085108	-1.23760600330488	0.224705619894632	0.981678900256408	-5.22591301309403
a_HQ_transcript/34548	-2.28730212889318	6.65666861660119	-1.23759477689884	0.22470973007545	0.981678900256408	-5.85109962415448
a_HQ_transcript/73019	-1.38860655407045	0.340725081090051	-1.23752051558955	0.224736919837066	0.981678900256408	-5.0909175432782
a_HQ_transcript/139175	-1.28164357119998	4.86372366650707	-1.23750781976749	0.224741568484705	0.981678900256408	-5.59625784691966
a_HQ_transcript/106139	-1.25655909369482	0.25344191885569	-1.23748879581343	0.224748534348546	0.981678900256408	-5.10788837122156
a_HQ_transcript/114992	1.31906349670294	2.28086782836832	1.23737555551788	0.224790002070181	0.98169485648806	-5.14650357860799
a_HQ_transcript/14999	1.63282684844603	3.77996505293625	1.2373529319266	0.224798287340156	0.98169485648806	-5.53703798057657
a_HQ_transcript/127986	1.78870467602661	0.4627594669649	1.23734046550428	0.224802852923076	0.98169485648806	-5.08199594344998
a_HQ_transcript/3	1.07039894282629	3.24983325533897	1.23716790504923	0.224866056930615	0.981782036637987	-5.38996282042426
a_HQ_transcript/83815	1.98367391091485	2.67999347887278	1.23716596505308	0.224866767571775	0.981782036637987	-5.17316657629047
a_HQ_transcript/125066	2.82015914966048	0.664804746106023	1.23714762252991	0.224873486715493	0.981782036637987	-5.129674070864
a_HQ_transcript/134838	-1.4259994731525	2.04173472412388	-1.23701962702339	0.224920377584164	0.981913008502612	-5.102756629478
a_HQ_transcript/65021	1.48107716374001	1.08101884507871	1.2367584773745	0.225016071847851	0.982111473269281	-5.09707935570255
a_HQ_transcript/148040	-1.07199467473087	-0.442869068924025	-1.23674647965184	0.225020468959541	0.982111473269281	-5.11175956073735
a_HQ_transcript/135960	2.07083471740924	1.18260102469144	1.23674099960882	0.225022477392166	0.982111473269281	-5.10354022670796
a_HQ_transcript/147894	3.46492696832894	0.378866382096222	1.23671113965021	0.225033421285328	0.982111473269281	-5.14993077656503
a_HQ_transcript/71295	1.13384477677591	3.57286369554707	1.23665335691271	0.225054600211047	0.982130165245327	-5.35322688917643
a_HQ_transcript/147740	-2.02212597127374	-0.133518046795498	-1.23649002208557	0.225114474872009	0.982182011537573	-5.1213805589471
a_HQ_transcript/48046	-0.864536627566456	2.7713106869393	-1.23645193301951	0.2251284391292	0.982182011537573	-5.28535420635713
a_HQ_transcript/133612	-2.70475158372268	1.37940861159828	-1.23644373613397	0.225131444365461	0.982182011537573	-5.20546106103261
a_HQ_transcript/66663	1.4709488473777	1.2893259926043	1.23643657934841	0.225134068292847	0.982182011537573	-5.07959600164775
a_HQ_transcript/35340	-1.51955420305178	0.87705642093218	-1.23635097306167	0.22516545631766	0.982245226629115	-4.97808705003809
a_HQ_transcript/116049	1.83773991081111	1.33733895022393	1.23621940405222	0.225213703208795	0.982381970592889	-5.09862204056044
a_HQ_transcript/150062	3.87816670208567	1.95536833547979	1.23605081029199	0.225275538520489	0.982561953076101	-5.14616321028195
a_HQ_transcript/72662	-1.42350840746613	0.877516714844927	-1.23601473507307	0.225288771514559	0.982561953076101	-5.04294149723793
a_HQ_transcript/147423	-2.01326281066475	1.48964423449637	-1.23583896822698	0.225353254046154	0.982676970085689	-5.1860040917504
a_HQ_transcript/144020	2.81763170561764	0.361556527933695	1.23571476063523	0.225398829659103	0.982676970085689	-5.12099145210705
a_HQ_transcript/45369	2.44609188842544	0.807062498635462	1.23569363835772	0.225406580762244	0.982676970085689	-5.2004802022597
a_HQ_transcript/149392	-2.9833149472969	2.53128142672467	-1.23567438696907	0.225413645491118	0.982676970085689	-5.18644422386172
a_HQ_transcript/82843	2.49169793748496	1.94681962873321	1.23567127739798	0.225414786633498	0.982676970085689	-5.2261218801565
a_HQ_transcript/15281	-2.04907310425353	1.15448355304706	-1.23559935604307	0.225441181351519	0.982676970085689	-5.04921677127767
a_HQ_transcript/108536	1.37292229583098	1.40246698673831	1.23549053658059	0.225481121850639	0.982676970085689	-4.98283824141787
a_HQ_transcript/151395	3.77334615592486	2.84689912982509	1.23539799237904	0.225515092927439	0.982676970085689	-5.24082574589571
a_HQ_transcript/60083	1.83030933200573	2.67733864521929	1.23535820537662	0.225529699094814	0.982676970085689	-5.35924897287681
a_HQ_transcript/81315	3.95174848758273	1.84272475043166	1.23531577362262	0.225545276953239	0.982676970085689	-5.43328519125361
a_HQ_transcript/113977	2.47673202234103	1.34372059137259	1.23529624770195	0.225552445723331	0.982676970085689	-5.22698441244023
a_HQ_transcript/59799	-0.831814511190886	2.30882377728043	-1.23528282187218	0.225557374997455	0.982676970085689	-5.13734871818515
a_HQ_transcript/65478	1.37304718548012	1.36663170848755	1.2350640391173	0.225637712115145	0.982676970085689	-5.08020798033817
a_HQ_transcript/121078	-1.74177729978854	1.08309065287311	-1.23498965465634	0.225665030989147	0.982676970085689	-5.05455386171294
a_HQ_transcript/123311	-2.13797060685046	1.76858652345512	-1.23497756508584	0.22566947130926	0.982676970085689	-5.36343399997391
a_HQ_transcript/105956	-1.16028799070039	1.62400352453713	-1.23492470739236	0.225688885923482	0.982676970085689	-5.12948489519506

a_HQ_transcript/108427	3.38129623256856	2.75672532548572	1.23491243963416	0.225693392045626	0.982676970085689	-5.25972319485617
a_HQ_transcript/135131	-1.83035400625937	1.04455659155575	-1.234858742954	0.225713116391298	0.982676970085689	-5.19633101528245
a_HQ_transcript/141558	-0.500998982229417	2.51596542988431	-1.23480768587248	0.225731872330336	0.982676970085689	-5.29106058667768
a_HQ_transcript/109011	-1.08295584180901	-0.45154050099343	-1.23471157404038	0.225767182396875	0.982676970085689	-5.08141423229068
a_HQ_transcript/30803	-1.45944914829775	1.16240377524911	-1.23462057208637	0.225800618972576	0.982676970085689	-5.06237297015454
a_HQ_transcript/76138	-1.30897321887806	0.0613702787056159	-1.23458292899826	0.225814451141367	0.982676970085689	-5.01598035729317
a_HQ_transcript/137036	-0.568153561499444	2.45283126139332	-1.23457464022965	0.225817496982001	0.982676970085689	-5.22818742825684
a_HQ_transcript/134699	3.03157544876766	2.20162429401216	1.23451194751225	0.225840535417914	0.982676970085689	-5.16059259838889
a_HQ_transcript/134141	2.0504378357346	1.70075572810165	1.2345008745672	0.225844604706705	0.982676970085689	-5.21820369957698
a_HQ_transcript/42053	1.43091085506853	0.603856410200292	1.2344968921804	0.225846068240586	0.982676970085689	-5.07549945761205
a_HQ_transcript/9683	1.34549206149038	2.18059478610212	1.23442584893917	0.225872177942365	0.982676970085689	-5.13289854727736
a_HQ_transcript/124943	-2.16659928197654	1.36113047324256	-1.23438473106474	0.225887290549879	0.982676970085689	-5.15773128494386
a_HQ_transcript/115088	-1.24420768216472	-0.153681383484171	-1.2343835918648	0.225887709266167	0.982676970085689	-5.05494918857811
a_HQ_transcript/81496	-2.4899624180742	1.43166660212649	-1.23436964793232	0.225892834446518	0.982676970085689	-5.30716989739105
a_HQ_transcript/37174	-1.86325573354573	1.26251711860697	-1.23435094829996	0.225899707750712	0.982676970085689	-5.01310216806893
a_HQ_transcript/22766	-1.02050538151542	0.18279247686905	-1.23434741934487	0.225901004883671	0.982676970085689	-5.03356757727058
a_HQ_transcript/19004	0.923272740192588	2.1448785677909	1.23434683012347	0.225901221463479	0.982676970085689	-5.11669031071397
a_HQ_transcript/142886	-1.67261610045359	-0.272881044514826	-1.23429819111023	0.225919100215398	0.982676970085689	-5.12588745697217
a_HQ_transcript/69686	-2.05184148503048	0.0285023525149859	-1.23427572327364	0.225927359310463	0.982676970085689	-5.09888873770227
a_HQ_transcript/127720	-1.93461865771798	1.82646594625077	-1.23413673543844	0.225978455746319	0.982676970085689	-5.0529957100303
a_HQ_transcript/66541	2.02955777482405	3.52439378788151	1.23411778625671	0.225985422748635	0.982676970085689	-5.29178202212162
a_HQ_transcript/49469	-1.70255873002251	0.34394089254485	-1.23394483440373	0.226049018975579	0.982676970085689	-5.12336893670909
a_HQ_transcript/21583	2.19488124885123	1.69107318303427	1.23386227097881	0.226079383133951	0.982676970085689	-5.14850180516377
a_HQ_transcript/125740	-1.55030940809484	1.43646899855037	-1.23380459069225	0.226100597885848	0.982676970085689	-5.11209392744013
a_HQ_transcript/39566	-1.16620043020247	1.1849534598648	-1.23374861290926	0.226121187880165	0.982676970085689	-5.00045730366811
a_HQ_transcript/112077	-1.00754891754522	4.51398776713015	-1.23374520029341	0.226122443168819	0.982676970085689	-5.58923209822236
a_HQ_transcript/128884	-1.42238375435072	4.32914646049681	-1.23372033724667	0.22613158889102	0.982676970085689	-5.52892277894939
a_HQ_transcript/5139	1.6906204428649	0.831134047343854	1.23371555680872	0.22613334737803	0.982676970085689	-5.0108331699877
a_HQ_transcript/58518	-3.46205070496966	1.66840192963067	-1.23362311517201	0.226167354098537	0.982676970085689	-5.20357690793095
a_HQ_transcript/89694	2.38072952054989	2.66417069548884	1.23362190401486	0.226167799675089	0.982676970085689	-5.33912035968926
a_HQ_transcript/31252	-1.43421818758694	0.0898567103577346	-1.23358205504216	0.226182460209125	0.982676970085689	-4.99497394693002
a_HQ_transcript/142115	2.0348780169094	2.24488142732598	1.233558967726	0.226190954413609	0.982676970085689	-5.07804556347644
a_HQ_transcript/81855	1.33849355168557	1.36900111314081	1.2333984804264	0.226250006922144	0.982676970085689	-5.07717574636613
a_HQ_transcript/144328	-2.03304686012454	0.835111648208172	-1.23337786877233	0.226257591969381	0.982676970085689	-5.05612452736005
a_HQ_transcript/4403	-1.79107065822816	0.461785024288105	-1.23327819656713	0.226294273819668	0.982676970085689	-5.11197910172616
a_HQ_transcript/23625	1.87048176595444	0.580548469462106	1.23319637205762	0.226324390593035	0.982676970085689	-5.06513265461498
a_HQ_transcript/74500	-1.51591615671922	1.87123256781911	-1.23313376386098	0.226347436526901	0.982676970085689	-5.10323083638522
a_HQ_transcript/120505	-2.06313459072022	0.673845453380505	-1.23307959038135	0.226367379074715	0.982676970085689	-5.14830305294331
a_HQ_transcript/123512	-4.16553730814783	3.52196634967477	-1.23298108501095	0.226403644608634	0.982676970085689	-5.16464581612998
a_HQ_transcript/99383	0.485444912747325	4.80342249288936	1.23297880931116	0.226404482476837	0.982676970085689	-5.59833160061306
a_HQ_transcript/58910	1.36176661121492	-0.337861961198664	1.23297173189739	0.226407088257107	0.982676970085689	-5.04718996349648
a_HQ_transcript/146273	-0.721925673299977	2.07618169779151	-1.23293794665394	0.226419527702884	0.982676970085689	-5.11032033997873
a_HQ_transcript/109833	3.19990913274605	-0.362983403854704	1.23288668008465	0.22643840460214	0.982676970085689	-5.17109719562521
a_HQ_transcript/32501	0.464362584944731	2.61849638402291	1.232877484366	0.226441790688399	0.982676970085689	-5.22609934250537
a_HQ_transcript/58389	-2.36040661974222	0.284042180719098	-1.2328453453852	0.226453625336251	0.982676970085689	-5.1329435991577
a_HQ_transcript/48413	-1.05903654578724	2.15324445311538	-1.23280058718354	0.226470107572713	0.982676970085689	-5.19398427794811
a_HQ_transcript/75753	1.75685017488347	0.433608811384962	1.23279811366526	0.226471018473519	0.982676970085689	-5.04589489726549
a_HQ_transcript/122639	0.899803696358255	3.65795688865695	1.23275954304691	0.226485222889571	0.982676970085689	-5.41623927755617
a_HQ_transcript/64810	1.1755007636546	0.415492993247757	1.23265683175527	0.226523051659259	0.982676970085689	-5.05298985431113
a_HQ_transcript/136595	-2.43382000546539	0.617796443510946	-1.23259316711679	0.22654650183809	0.982676970085689	-5.19800896305772

a_HQ_transcript/85370	-0.665799449880614	2.51966260475278	-1.23256870466755	0.226555512797799	0.982676970085689	-5.30968893450397
a_HQ_transcript/1865	1.10473482941869	2.59544714058334	1.23255761437072	0.22655959809495	0.982676970085689	-5.13528384988371
a_HQ_transcript/99753	2.5113165220385	3.02454946978026	1.23255343913642	0.226561136126894	0.982676970085689	-5.16854534523398
a_HQ_transcript/36512	1.79264240972533	-0.295448229438398	1.23251657098389	0.226574717594374	0.982676970085689	-5.12149315799391
a_HQ_transcript/41290	0.485230049886103	2.91469654947614	1.23249231779827	0.226583652299471	0.982676970085689	-5.30129165289317
a_HQ_transcript/90943	1.02349036029686	-0.447316396080167	1.23244019317319	0.226602855539313	0.982676970085689	-5.10019019478773
a_HQ_transcript/13936	0.610181555923104	3.08440504477386	1.23243492286317	0.226604797242536	0.982676970085689	-5.38774609179549
a_HQ_transcript/54737	-0.890781404230052	1.77480139553245	-1.23243212242679	0.22660582899265	0.982676970085689	-5.14286149722554
a_HQ_transcript/125783	-1.50865738017147	1.35828473816364	-1.23233128132975	0.226642983686851	0.982676970085689	-5.14983716052168
a_HQ_transcript/15202	-1.18185795007112	0.485720753351176	-1.23223792931888	0.226677383095939	0.982676970085689	-5.00597214405367
a_HQ_transcript/66917	-1.51144868031667	-0.0966268127995251	-1.23222003854227	0.226683976137477	0.982676970085689	-5.13328952005002
a_HQ_transcript/129603	-0.515021132296287	3.51647021715605	-1.23216867685328	0.226702904548761	0.982676970085689	-5.44433078807717
a_HQ_transcript/112881	-1.63561145296932	-0.120452694370938	-1.23215199846604	0.226709051317142	0.982676970085689	-5.09577934774896
a_HQ_transcript/59473	-0.510585313550921	3.21844691150413	-1.23194226183498	0.226786359727015	0.982676970085689	-5.44631304315187
a_HQ_transcript/102052	1.85029269393709	0.365010339304706	1.23193060873141	0.226790655609436	0.982676970085689	-5.19400293303612
a_HQ_transcript/76401	1.93945354660262	1.08872973438412	1.23190247278731	0.226801028092992	0.982676970085689	-5.09719352037884
a_HQ_transcript/22006	-2.53157500260581	1.38484137104678	-1.23183046886624	0.226827574380317	0.982676970085689	-5.10793067604903
a_HQ_transcript/115700	3.23723614608876	1.07005761267086	1.23180560116313	0.226836743102985	0.982676970085689	-5.21853894296492
a_HQ_transcript/151886	3.6122391039018	1.80789057768651	1.23178143451809	0.226845653610541	0.982676970085689	-5.23126844102048
a_HQ_transcript/110797	0.373827217315539	3.36679309747887	1.23176240437575	0.226852670416958	0.982676970085689	-5.42240312172303
a_HQ_transcript/34917	-0.467331619729344	2.95764214473141	-1.23175937135788	0.226853788768239	0.982676970085689	-5.31136236991442
a_HQ_transcript/55908	-1.58521777583252	0.159618003288695	-1.23173165320225	0.226864009351951	0.982676970085689	-5.06515707087576
a_HQ_transcript/57770	-0.349987039876515	-0.877038658965845	-1.23164391179225	0.226896364723623	0.982676970085689	-5.2143320590388
a_HQ_transcript/39058	-2.28908246629573	1.66086359067245	-1.23163497962772	0.226899658726401	0.982676970085689	-5.11581194804806
a_HQ_transcript/29409	-2.93029228672678	1.66202667442049	-1.23159648224541	0.226913856195755	0.982676970085689	-5.27516112186386
a_HQ_transcript/15381	1.44865472992884	2.28768854631526	1.23159527977738	0.226914299665234	0.982676970085689	-5.32762106377497
a_HQ_transcript/76676	-1.10354919848246	0.582289201626283	-1.23153280660661	0.22693734062237	0.982676970085689	-5.04751474806866
a_HQ_transcript/127046	-0.638409525953783	3.7509478295438	-1.23145139757788	0.226967368002032	0.982676970085689	-5.48219159988778
a_HQ_transcript/13745	3.38415963279653	1.38651635481708	1.23143025801046	0.22697516572793	0.982676970085689	-5.17677223702669
a_HQ_transcript/33640	-1.92274957774679	1.25846896407828	-1.23135376041216	0.227003384972404	0.982676970085689	-5.11815971409152
a_HQ_transcript/94939	-0.963805180422244	1.44371394235738	-1.2313405538767	0.227008257004113	0.982676970085689	-5.14511000575606
a_HQ_transcript/104944	2.0966467661172	3.35189975354139	1.23133674576171	0.227009661872819	0.982676970085689	-5.19111679236039
a_HQ_transcript/149805	1.93328136211576	2.50483594872753	1.23131312870415	0.227018374691877	0.982676970085689	-5.34647083654474
a_HQ_transcript/116204	-1.33816550977969	0.0470302083826358	-1.23130765175545	0.227020395286725	0.982676970085689	-5.08390505609553
a_HQ_transcript/149164	-1.9550677432237	1.43724733141278	-1.23130170663067	0.227022588619611	0.982676970085689	-5.0642457684013
a_HQ_transcript/60219	-2.14831386801981	0.640135834406753	-1.23112803224952	0.227086669229077	0.982881154798793	-5.16753665950081
a_HQ_transcript/113202	2.38171229168593	4.52499951500709	1.23096766288206	0.22714585267411	0.983064114678031	-5.46923487933334
a_HQ_transcript/10810	1.54352962499461	0.685801333598665	1.23085670192243	0.227186808950107	0.983064638685316	-5.18257105707461
a_HQ_transcript/58696	-0.852209270817673	2.58888726171281	-1.23085245610569	0.22718837621292	0.983064638685316	-5.28361425906086
a_HQ_transcript/93532	0.974186561335427	3.11656835401296	1.2308190037311	0.227200724805424	0.983064638685316	-5.31869909232415
a_HQ_transcript/22464	1.18970175319696	-0.56568743861681	1.23078406658782	0.227213622019289	0.983064638685316	-5.15296118386367
a_HQ_transcript/6675	1.34770630560025	2.2249645517884	1.23071932723074	0.227237522307062	0.983094871737489	-5.18935338549574
a_HQ_transcript/54366	1.46220454355022	0.0436316671373895	1.23065300123623	0.227262010290159	0.983127642639455	-5.0493587001797
a_HQ_transcript/129970	1.94077328099482	1.09744217885565	1.23048285833541	0.227324837144696	0.983307288263451	-5.10673722671094
a_HQ_transcript/104959	2.20123800943201	1.12817488618214	1.23043561475061	0.227342284577657	0.983307288263451	-5.09328955585418
a_HQ_transcript/10692	0.946036815367698	4.29862372331571	1.23040311844203	0.227354286303688	0.983307288263451	-5.58888311737979
a_HQ_transcript/148513	-3.56877143396008	1.21206554730129	-1.23011070294435	0.227462304162103	0.98363313942814	-5.21802001993724
a_HQ_transcript/141520	-2.6118579967345	5.85419546050186	-1.23010754392553	0.227463471308028	0.98363313942814	-5.55876841120782
a_HQ_transcript/129795	1.94595327041142	0.941309439547688	1.23000638780699	0.227500847284288	0.983686360544341	-5.08864723460719
a_HQ_transcript/8878	-0.474282114451215	2.13334351404047	-1.22998263554448	0.227509624125346	0.983686360544341	-5.13969841105572

a_HQ_transcript/36643	-2.45360381827959	2.65286648373608	-1.22980261099154	0.227576154289764	0.98371670825085	-5.44824028748438
a_HQ_transcript/33584	-2.02504762962869	0.935808531547346	-1.22979864281923	0.227577620937364	0.98371670825085	-5.12122923213631
a_HQ_transcript/91451	-1.74187359892288	-0.196978935635198	-1.22978266217024	0.227583527501351	0.98371670825085	-5.13167018753691
a_HQ_transcript/122590	-2.02920064165439	-0.244967806590978	-1.22974377715176	0.227597900164322	0.98371670825085	-5.11195863383853
a_HQ_transcript/72746	1.60321614359448	2.21497639366606	1.22973468892215	0.227601259449244	0.98371670825085	-5.3258925930963
a_HQ_transcript/148066	-3.50713617630417	1.35519700920958	-1.22952228083668	0.227679782443415	0.983744159011311	-5.30424208960468
a_HQ_transcript/110893	0.916118206013159	3.31745138681316	1.22951575904619	0.227682193737751	0.983744159011311	-5.34758728294318
a_HQ_transcript/110825	1.33484449303825	2.48268868018467	1.22949461234989	0.227690012413221	0.983744159011311	-5.15071325229816
a_HQ_transcript/93318	2.31505669792077	0.264712066758571	1.22943424730145	0.227712332590934	0.983744159011311	-5.1287640256426
a_HQ_transcript/99069	2.16929992077452	1.781617336369	1.22943320541887	0.227712717844812	0.983744159011311	-5.07554474323003
a_HQ_transcript/70541	1.30894689454784	-0.400616664343289	1.22940816372657	0.227721977584472	0.983744159011311	-5.03938595989891
a_HQ_transcript/99804	-2.46814204793654	1.72828325701527	-1.22939707767292	0.227726076996563	0.983744159011311	-5.17184397941119
a_HQ_transcript/118136	-4.56136508480253	0.445037682915032	-1.22927775177505	0.22777020493155	0.983832353961213	-5.16638933296679
a_HQ_transcript/72700	1.73581014349645	2.96389967471315	1.22925033802323	0.227780343717486	0.983832353961213	-5.2565745078523
a_HQ_transcript/138529	-2.30982970426598	2.63973685318499	-1.22914529108328	0.227819197715172	0.983911171207224	-5.23254365224147
a_HQ_transcript/26018	1.35971560599876	-0.428602179998364	1.22910947827511	0.22783244502562	0.983911171207224	-5.12526506959854
a_HQ_transcript/29220	3.73652738927418	1.95300516019165	1.22897113135526	0.227883625536661	0.983936098747446	-5.1117146338384
a_HQ_transcript/24425	1.35045420565361	0.202210378613935	1.22892831169064	0.22789946812033	0.983936098747446	-4.98854457867281
a_HQ_transcript/114771	1.50734200794809	2.26163095152672	1.22885793680228	0.227925507475287	0.983936098747446	-5.14246681416457
a_HQ_transcript/151125	2.60655827256628	1.01728563445479	1.22885593500137	0.227926248192452	0.983936098747446	-5.20424426677365
a_HQ_transcript/29986	2.01806896223924	0.814672254459956	1.22880443455527	0.227945305282289	0.983936098747446	-5.23204522855562
a_HQ_transcript/30158	1.66683363155026	-0.374853861777576	1.22877095317389	0.227957695281961	0.983936098747446	-5.07398359962559
a_HQ_transcript/86935	-1.66433927687476	-0.160116062311579	-1.22872165322232	0.22797593995889	0.983936098747446	-5.10326801204969
a_HQ_transcript/140313	3.68331416613479	3.45827506445906	1.22870347536151	0.227982667404472	0.983936098747446	-5.45742369694682
a_HQ_transcript/67414	-0.945946287685797	1.76974604651627	-1.22868214775439	0.227990560728553	0.983936098747446	-5.00717729939765
a_HQ_transcript/151706	0.300487803398934	10.7682292690346	1.22836206645931	0.228109046942439	0.984332514954915	-6.25126733157962
a_HQ_transcript/139137	1.83775590669224	2.26987491465111	1.22829295335314	0.228134636937675	0.984332514954915	-5.23553019748746
a_HQ_transcript/103062	-1.28322854397729	-0.0252440126263736	-1.22826339049405	0.228145583610866	0.984332514954915	-5.11388179492749
a_HQ_transcript/150825	-2.89454613003083	2.63105870414508	-1.2282510561567	0.228150150942533	0.984332514954915	-5.17162280058875
a_HQ_transcript/150883	6.42439092824165	6.38736793869998	1.22792462890747	0.228271049782069	0.984781028776319	-5.6242490563316
a_HQ_transcript/31281	-0.966685763166876	2.12137830724418	-1.22783222667818	0.228305281460475	0.984855614154694	-5.09331078570886
a_HQ_transcript/124411	-0.720812229602422	2.68196282669565	-1.22773898765549	0.228339827018783	0.984931542367753	-5.22793227602693
a_HQ_transcript/119160	-0.747315669515116	4.83138855041306	-1.22762553533862	0.228381866965443	0.984994362342597	-5.63409669378597
a_HQ_transcript/83887	2.07725911711052	0.506187273258707	1.22760822646651	0.228388281304251	0.984994362342597	-5.1159841374413
a_HQ_transcript/61593	1.16286696008522	1.33548010133994	1.22751716615197	0.228422028736274	0.985048356605555	-5.09926049704713
a_HQ_transcript/147677	-3.21977048305186	2.50417194603885	-1.22742394229906	0.228456581836862	0.985048356605555	-5.26349449701186
a_HQ_transcript/69670	-1.54286976373568	-0.454841919929917	-1.22742050970772	0.228457854189393	0.985048356605555	-5.13473318267974
a_HQ_transcript/149368	-0.908591724828971	3.91372818636359	-1.22736304312453	0.228479156009947	0.985048356605555	-5.49153421145253
a_HQ_transcript/65707	1.9194667687864	4.80077090751914	1.22733801496875	0.228488433956264	0.985048356605555	-5.52907625715638
a_HQ_transcript/96536	-3.06748929732481	1.74324458210678	-1.2272876676983	0.228507098557962	0.985048356605555	-5.18267105885104
a_HQ_transcript/123885	-0.703071598076533	3.14948149244928	-1.22725442133411	0.228519424181563	0.985048356605555	-5.34477600884185
a_HQ_transcript/78845	-2.36225122517433	1.26378064963388	-1.22718936815082	0.228543543170787	0.985079273130023	-5.2791164572859
a_HQ_transcript/70721	1.69271877832222	1.39038473800917	1.22712715821156	0.228566609778917	0.985105649383028	-5.08002753109257
a_HQ_transcript/121920	-1.81311968212635	2.79352984536926	-1.22697522298094	0.228622952609954	0.985196341002342	-5.26428769976326
a_HQ_transcript/15857	-1.29806389628511	1.43431828024898	-1.22690432650842	0.228649247011653	0.985196341002342	-5.07395062397794
a_HQ_transcript/66632	-1.83546547777817	0.944863844167291	-1.22689575488326	0.228652426247121	0.985196341002342	-4.95365604197247
a_HQ_transcript/36572	-1.89807987495955	2.67446207311465	-1.22684513639079	0.228671201433917	0.985196341002342	-5.26755548147717
a_HQ_transcript/45362	-1.50939862508281	3.18989266816181	-1.22684191599535	0.228672395967582	0.985196341002342	-5.30209602964678
a_HQ_transcript/25205	-1.5324686530429	2.18209508672696	-1.22673208478178	0.228713138183262	0.985298843571975	-5.08234810841102
a_HQ_transcript/43931	1.38467037479848	-0.278302461166774	1.22666676383504	0.228737371747732	0.985330216759463	-5.14105850538878

a_HQ_transcript/116543	1.14487456903137	-0.199881605788457	1.22655382143816	0.228779277014357	0.985402170599464	-5.19800942946718
a_HQ_transcript/74927	-1.20552882015639	1.02413687011272	-1.22647026002058	0.228810284672703	0.985402170599464	-5.09335998679489
a_HQ_transcript/71479	-2.02705117353334	0.831195517304582	-1.22641171138978	0.22883201254511	0.985402170599464	-5.16637390877385
a_HQ_transcript/91901	1.1926550769901	1.84917616585603	1.2263858841913	0.228841597716817	0.985402170599464	-5.04434256395807
a_HQ_transcript/9353	-1.79161523357389	2.61543886885312	-1.2263234815205	0.228864758269749	0.985402170599464	-5.37439184594096
a_HQ_transcript/79320	-2.20923913649431	0.613777006350143	-1.22623845382582	0.228896318839115	0.985402170599464	-5.20690180000262
a_HQ_transcript/34326	1.16535190279503	3.57562204936087	1.22622249885219	0.228902241365552	0.985402170599464	-5.4451509977368
a_HQ_transcript/118819	-0.861605941104712	5.57002530930122	-1.22621505998358	0.228905002731355	0.985402170599464	-5.71725327582917
a_HQ_transcript/30969	-1.69042609425274	3.7135978653457	-1.22621063361982	0.228906645843734	0.985402170599464	-5.59797536644533
a_HQ_transcript/7893	1.26174673520976	1.15831513381038	1.22611725108093	0.228941312463953	0.985427736658608	-5.05122893001919
a_HQ_transcript/19348	-1.75824881730289	1.05249703493789	-1.22606434331478	0.228960955274374	0.985427736658608	-5.07718627283943
a_HQ_transcript/20225	-0.557416530895424	2.81348679570475	-1.22605764304246	0.228963442941632	0.985427736658608	-5.20829979389364
a_HQ_transcript/58699	1.50046486787916	2.63404650972271	1.22600111119666	0.228984432803821	0.985445110684867	-5.2494272996147
a_HQ_transcript/84681	0.531159492607982	3.4353027123205	1.2259061955172	0.229019677527186	0.985523824052433	-5.43658026484846
a_HQ_transcript/143286	-0.841763923802534	2.1828415912566	-1.22572879765307	0.229085560919394	0.985698463432232	-5.25222489088286
a_HQ_transcript/12220	2.74755859255243	1.04173729710237	1.22570560446346	0.229094175629119	0.985698463432232	-5.10932589647889
a_HQ_transcript/48385	2.28372426221018	1.20483620452455	1.22561728822853	0.229126981378095	0.98576664743882	-5.14835720001737
a_HQ_transcript/121346	-3.57886887918759	0.973450307055072	-1.2255077492232	0.229167675350442	0.985868756125703	-5.21630697129555
a_HQ_transcript/141390	-3.94827945253258	1.35968066411587	-1.22540658247189	0.229205263794024	0.98595749084726	-5.11882595895635
a_HQ_transcript/133031	-0.461427799101065	3.07628039111403	-1.22533340743496	0.229232454795184	0.985988704969062	-5.35656561754929
a_HQ_transcript/6850	1.71716434663121	0.127999429932602	1.22528606131007	0.229250049350047	0.985988704969062	-5.16818700744625
a_HQ_transcript/44424	0.915346468748332	1.72877212652998	1.22512751919465	0.229308973383419	0.985988704969062	-5.13189285106602
a_HQ_transcript/36792	0.941484094660738	-0.640285119786552	1.22510512597122	0.229317296996036	0.985988704969062	-5.16198998355965
a_HQ_transcript/120338	-2.25394512465378	0.418658675824143	-1.22510083227184	0.22931889299967	0.985988704969062	-5.20222142658301
a_HQ_transcript/98997	3.34469049844639	2.76688991726845	1.22509511387327	0.229321018588644	0.985988704969062	-5.32426761499909
a_HQ_transcript/124345	1.32970549232408	2.78147751992592	1.22503304680452	0.229344090515649	0.985988704969062	-5.39511626782611
a_HQ_transcript/134064	-2.32620938758546	1.05697643134849	-1.22502194075962	0.229348219100721	0.985988704969062	-5.18556956122603
a_HQ_transcript/70216	-1.64242460382695	0.702507154405005	-1.22477560807528	0.22943980557178	0.986305417374704	-5.06465142817866
a_HQ_transcript/109377	1.08946993252071	2.19666794632337	1.22473253113137	0.229455824373075	0.986305417374704	-5.17562089793925
a_HQ_transcript/73675	1.33585115615619	0.471893376690002	1.22436321787683	0.229593193166712	0.98656896458571	-5.04079547051891
a_HQ_transcript/97881	-1.51502946776	0.349523275042297	-1.22424660929044	0.229636579307792	0.98656896458571	-5.1339655263546
a_HQ_transcript/37000	-3.01184481594874	2.28498108182026	-1.22424457836745	0.229637335000144	0.98656896458571	-5.12104237321307
a_HQ_transcript/110699	1.44637223988315	1.55582693698249	1.22423020146186	0.229642684599906	0.98656896458571	-5.03248265733027
a_HQ_transcript/20107	-1.271092331199	0.0926983853603617	-1.22421455944533	0.229648505048712	0.98656896458571	-5.04148689832974
a_HQ_transcript/151997	2.16742675720905	10.7451779180568	1.22421401032107	0.229648709381758	0.98656896458571	-6.22131169285316
a_HQ_transcript/104314	-0.61357321982491	2.72163485071974	-1.2242117162306	0.229649563030607	0.98656896458571	-5.24859786116267
a_HQ_transcript/15171	1.99958537591769	0.351746349036252	1.22411795254022	0.229684455248268	0.98656896458571	-5.15427741389246
a_HQ_transcript/114973	-1.20855280750206	-0.546231412885321	-1.22398346768689	0.229734507893969	0.98656896458571	-5.12607081905125
a_HQ_transcript/135268	-0.985982280144952	1.04288832146996	-1.22396863344864	0.229740029405993	0.98656896458571	-5.07622133172152
a_HQ_transcript/148985	0.845864958800352	2.4414589092041	1.22395885156531	0.229743670414535	0.98656896458571	-5.29680527338429
a_HQ_transcript/2126	-2.52207609117892	0.90986913687943	-1.22394590520348	0.229748489369993	0.98656896458571	-5.23695050317226
a_HQ_transcript/143326	1.61874829359001	-0.088926338409162	1.22388125720943	0.229772554075241	0.98656896458571	-5.13538414594852
a_HQ_transcript/150704	-3.39478468069257	4.70293573798099	-1.22381082898494	0.229798772561063	0.98656896458571	-5.30770148263204
a_HQ_transcript/7018	1.01382289735278	-0.490162694291084	1.2238036153179	0.2298014581361	0.98656896458571	-5.13345413552378
a_HQ_transcript/116275	1.95016405592867	0.103684704582353	1.22379808879095	0.229803515621807	0.98656896458571	-5.13769963504723
a_HQ_transcript/37157	-1.97519022503194	0.615561665330626	-1.22371840469408	0.22983318295458	0.98656896458571	-5.03058123884281
a_HQ_transcript/133954	-2.1736308384801	2.02053456128972	-1.22370736406151	0.229837293737668	0.98656896458571	-5.24157954498536
a_HQ_transcript/67243	-2.24200721980889	1.59931425824466	-1.22367158207305	0.229850616900518	0.98656896458571	-5.22073637392271
a_HQ_transcript/4573	-2.40025204027966	0.533831837752114	-1.22359124861751	0.229880530571796	0.98656896458571	-5.05496515193926
a_HQ_transcript/117670	0.940578519912834	6.21484387942401	1.22340246830667	0.229950837872499	0.98656896458571	-5.71422068467935

a_HQ_transcript/17567	-0.926315646181745	-0.147408588439151	-1.22338563741646	0.229957106964484	0.98656896458571	-5.14338480416793
a_HQ_transcript/60257	1.89108262755198	0.424044624911647	1.22335567448933	0.229968267731529	0.98656896458571	-5.18312387412032
a_HQ_transcript/10035	-1.10183449157036	-0.347054398680213	-1.2232923066347	0.229991872690013	0.98656896458571	-5.12802231011341
a_HQ_transcript/45224	-1.80192882635004	0.32093120937292	-1.22326688400481	0.230001343300046	0.98656896458571	-5.11752339833373
a_HQ_transcript/92319	-3.16228163852946	2.21855695793933	-1.22322419375176	0.230017247214745	0.98656896458571	-5.28887319970577
a_HQ_transcript/34703	4.07472612082862	2.82509425989803	1.22307099378429	0.230074327384437	0.98656896458571	-5.40119905891754
a_HQ_transcript/88719	2.31862469650176	0.240687075665333	1.22302001716578	0.230093322898203	0.98656896458571	-5.14210130779315
a_HQ_transcript/76188	-1.47880538208133	2.06989062259293	-1.2229497538189	0.230119507175017	0.98656896458571	-5.12296299069423
a_HQ_transcript/101266	-1.44206626673904	0.945027980665597	-1.22291043826867	0.230134159440721	0.98656896458571	-5.07409431980712
a_HQ_transcript/34904	1.45578681989986	-0.154447707809609	1.22287675501218	0.230146713194403	0.98656896458571	-5.0864497831779
a_HQ_transcript/128444	2.44863205428329	2.85774411641567	1.22285138757293	0.230156167978418	0.98656896458571	-5.40584984218339
a_HQ_transcript/149004	-1.35048086712772	-0.198407247809835	-1.22283533658878	0.230162150544265	0.98656896458571	-5.18010881696572
a_HQ_transcript/150227	1.19700766627193	-0.483769136112264	1.22281252931654	0.230170651530963	0.98656896458571	-5.15188036133232
a_HQ_transcript/73844	-2.42788818039496	0.631205318965215	-1.22280064657562	0.230175080694292	0.98656896458571	-5.25120042088946
a_HQ_transcript/10618	0.959683030082695	-0.547035797896051	1.22279783627776	0.230176128211739	0.98656896458571	-5.18750978336057
a_HQ_transcript/37672	-0.764477346432093	1.66373862900446	-1.22276863751049	0.230187012043107	0.98656896458571	-5.02389373596206
a_HQ_transcript/149440	1.98303864255113	3.59105982753671	1.22274150985864	0.230197124210021	0.98656896458571	-5.19904967214828
a_HQ_transcript/13990	0.629310754750506	2.68483225378863	1.2227365369531	0.230198977957765	0.98656896458571	-5.25331534192581
a_HQ_transcript/15053	0.519263159747615	1.89694263299602	1.22260853985807	0.230246695199418	0.98656896458571	-5.14791725518667
a_HQ_transcript/38091	1.39934854788935	-0.264242218539328	1.22260600597976	0.230247639902045	0.98656896458571	-5.12296702705797
a_HQ_transcript/93	-0.588259342463678	3.30856577122004	-1.22258497609712	0.230255480557744	0.98656896458571	-5.45197781202288
a_HQ_transcript/130605	-1.21399695126256	2.32612023355604	-1.22258339402477	0.230256070416174	0.98656896458571	-5.22432885682255
a_HQ_transcript/146414	2.17333011237973	1.05887788995866	1.22255254303698	0.230267573095418	0.98656896458571	-5.12710542353189
a_HQ_transcript/96406	1.80043357264913	1.4039507285249	1.22251682125494	0.230280892366827	0.98656896458571	-5.09282255016915
a_HQ_transcript/131973	-3.62450459534519	4.10715474198152	-1.22237100458787	0.230335267710149	0.986680285956234	-5.40983493227114
a_HQ_transcript/106935	2.1119037190058	1.69597436110506	1.22228394677137	0.230367736307857	0.986680285956234	-5.21673080221162
a_HQ_transcript/167	0.355353742677006	4.0822635736061	1.22222405082063	0.230390076744827	0.986680285956234	-5.56246895888108
a_HQ_transcript/32182	2.39941568674859	0.761001709807452	1.22219659148936	0.230400319269183	0.986680285956234	-5.1768512510472
a_HQ_transcript/134641	1.68061928835023	1.88763914770666	1.22218680159513	0.230403971050745	0.986680285956234	-5.23982988439376
a_HQ_transcript/142367	-2.03808368975213	1.49327440938558	-1.2221740647431	0.230408722157479	0.986680285956234	-5.36472923541038
a_HQ_transcript/71736	2.12653365019242	2.01020902491316	1.22207610692633	0.230445264865864	0.986745371692274	-5.21364317598433
a_HQ_transcript/91530	-2.90065394823466	3.85786645288704	-1.22204231523744	0.230457871698567	0.986745371692274	-5.42142348125737
a_HQ_transcript/138078	-2.84810424414778	0.490220220566721	-1.22196474692302	0.230486812434365	0.98678527598434	-5.18679027403804
a_HQ_transcript/57958	2.25578688192184	2.39281543806567	1.22192633844614	0.230501143636722	0.98678527598434	-5.27502371217502
a_HQ_transcript/87350	2.26138623700524	1.00224800545021	1.22173578366056	0.230572254389408	0.986969771888472	-5.08981405404872
a_HQ_transcript/13822	-0.920713113566015	-0.797765690298651	-1.22162668958662	0.230612973184948	0.986969771888472	-5.12724636038829
a_HQ_transcript/139201	0.70997210961001	5.34115154935521	1.22161356054524	0.230617873891912	0.986969771888472	-5.65740161108456
a_HQ_transcript/27420	-2.82813275159701	2.21794468415715	-1.22154515562528	0.230643408804322	0.986969771888472	-5.38464259913429
a_HQ_transcript/139332	-1.28983376265493	1.6031912041878	-1.22149666406176	0.230661511522088	0.986969771888472	-5.13553849661928
a_HQ_transcript/113997	2.04087705410253	0.85139271465088	1.2214852907992	0.230665757505646	0.986969771888472	-5.19948340157094
a_HQ_transcript/51589	1.68412199398037	1.12104942499226	1.22145683346628	0.23067638174508	0.986969771888472	-5.06596006415052
a_HQ_transcript/11091	-1.45824744695381	-0.30303783293389	-1.2214326329105	0.230685417048885	0.986969771888472	-5.09495175873568
a_HQ_transcript/143167	2.84723781779792	2.1840503175496	1.22138599627589	0.230702829629513	0.986969771888472	-5.36508225551043
a_HQ_transcript/129006	-1.95176599689658	0.915410772772274	-1.22131374850654	0.230729806493066	0.986969771888472	-5.16733991351757
a_HQ_transcript/87510	-1.30603268101577	0.238790601853834	-1.22131052176731	0.23073101139197	0.986969771888472	-5.09417588924968
a_HQ_transcript/75952	-0.827601824937666	0.322884850060462	-1.22121761924236	0.230765704194458	0.987044335885462	-5.01970623087289
a_HQ_transcript/92220	-1.58146583151087	1.03372006047018	-1.2211729036698	0.230782403816049	0.987044335885462	-5.11339307597147
a_HQ_transcript/144614	1.38097998416257	0.0961443479549334	1.22100007059849	0.230846959061597	0.987151382173058	-5.08317765116615
a_HQ_transcript/113273	-0.511173223922625	3.56225754229499	-1.22094062382245	0.230869166256098	0.987151382173058	-5.49389902671003
a_HQ_transcript/19457	1.30400616383291	2.38368649668913	1.2209284033425	0.230873731588243	0.987151382173058	-5.23271667822917

a_HQ_transcript/101041	-1.96725788731618	2.20333557621359	-1.22092403917824	0.23087536197087	0.987151382173058	-5.18106682895844
a_HQ_transcript/126227	1.98008080190456	0.868421339911417	1.22085728487558	0.230900301387898	0.987185401520722	-5.12172216776598
a_HQ_transcript/64526	-1.32040660414419	2.60049328281766	-1.22080838887578	0.230918570211523	0.987190898672901	-5.11361870486575
a_HQ_transcript/117124	-0.418048675701999	-0.822349194091376	-1.22072542057971	0.230949571794976	0.98725082433434	-5.23146600540141
a_HQ_transcript/108616	1.63336425736882	0.200783148326411	1.22041400522239	0.231065961553271	0.987562552488801	-5.16284200703575
a_HQ_transcript/137532	2.12425014722431	0.744416988471747	1.22039745649768	0.231072147767294	0.987562552488801	-5.1266622944695
a_HQ_transcript/87215	2.4219244140443	1.25929029022207	1.22039393728398	0.231073463329208	0.987562552488801	-5.19715176492859
a_HQ_transcript/90786	1.85250783767587	1.32666648405741	1.22026219888535	0.231122714137211	0.987700421068064	-5.08441450997661
a_HQ_transcript/37478	-0.967145819211895	1.85133581617964	-1.22014542950139	0.231166375257284	0.987792067510858	-5.07627193596129
a_HQ_transcript/105972	1.07631795834157	0.623512101897246	1.22009134381058	0.231186600460048	0.987792067510858	-4.95404387652713
a_HQ_transcript/151041	-4.48096418390583	2.3365174335776	-1.22006850955381	0.231195139665656	0.987792067510858	-5.32274492483499
a_HQ_transcript/44171	-0.664954690354779	2.53840303652231	-1.2200039812863	0.23121927222643	0.987821793553143	-5.20683185494908
a_HQ_transcript/2273	-1.07448496441492	1.45919375969442	-1.21973995155353	0.231318034716539	0.987821793553143	-5.07219878051185
a_HQ_transcript/123813	-3.0340409499427	0.188878392581571	-1.21970893005729	0.231329640620392	0.987821793553143	-5.11190026596796
a_HQ_transcript/1103	1.73744433713396	0.455856712295926	1.21967799022475	0.231341216403002	0.987821793553143	-5.04189203052212
a_HQ_transcript/5344	1.53217376731429	-0.300772466671318	1.21966541445359	0.231345921606263	0.987821793553143	-5.08851295011693
a_HQ_transcript/116406	0.663112898462291	2.30435569246985	1.21965132772542	0.23135119221598	0.987821793553143	-5.17459742036735
a_HQ_transcript/38625	2.82758468610492	0.797217122429084	1.21961953656957	0.23136308734121	0.987821793553143	-5.09179553526256
a_HQ_transcript/141988	2.42722712774404	1.2030936239251	1.21961090125036	0.231366318450517	0.987821793553143	-5.22173190562978
a_HQ_transcript/42566	-1.06896525103943	0.330505658411838	-1.21946744970608	0.231419999152035	0.987821793553143	-5.13074785013958
a_HQ_transcript/13368	-0.744833631859482	2.44487529207951	-1.21945334940595	0.231425276095441	0.987821793553143	-5.20818218077624
a_HQ_transcript/126090	-0.896487605734564	3.06684060686672	-1.21941010491601	0.231441460615549	0.987821793553143	-5.42909698993103
a_HQ_transcript/85720	1.72445410858493	0.685877308432041	1.21934842992608	0.23146454432075	0.987821793553143	-5.09089030675495
a_HQ_transcript/57294	1.36074184605229	0.654608466527891	1.21923704676713	0.231506237127237	0.987821793553143	-4.95298934110204
a_HQ_transcript/133324	0.97803897810932	1.98313346147927	1.21920437985257	0.231518466024865	0.987821793553143	-5.11340914500646
a_HQ_transcript/142956	-3.24795078732056	1.25176175573055	-1.2191749230175	0.231529493637145	0.987821793553143	-5.18532393993507
a_HQ_transcript/55060	1.52377887945984	0.450941358874813	1.21913824260432	0.231543226051001	0.987821793553143	-5.19936895272756
a_HQ_transcript/28311	-2.06954242077543	0.45574709246517	-1.21912071389484	0.231549788663384	0.987821793553143	-5.24107202203063
a_HQ_transcript/73900	3.43095626435914	1.33985265689787	1.2190362794479	0.231581402199815	0.987821793553143	-5.05507554922327
a_HQ_transcript/16393	-0.667565440437672	0.665900362096393	-1.21903530648519	0.231581766510488	0.987821793553143	-5.13338129713398
a_HQ_transcript/132509	1.78476497977421	2.06171677973947	1.2189762227506	0.23160389028956	0.987821793553143	-5.20645852898014
a_HQ_transcript/129647	1.04228470890448	1.77886630877505	1.2189680428737	0.231606953350891	0.987821793553143	-5.09827754140842
a_HQ_transcript/42775	-0.978711261392104	3.68238148928656	-1.21895272112471	0.231612690860162	0.987821793553143	-5.43647259123064
a_HQ_transcript/250	-0.984208744741669	2.23860644792594	-1.21894305454351	0.231616310742719	0.987821793553143	-5.26895052963749
a_HQ_transcript/10917	-0.72904632604379	4.07513511855987	-1.21892571028203	0.231622805821706	0.987821793553143	-5.49769497251449
a_HQ_transcript/116223	1.43982107040036	0.151283993613452	1.21881682105773	0.23166358575059	0.987821793553143	-5.08460256328044
a_HQ_transcript/103226	-1.27368979383167	6.30586384588875	-1.21876454230518	0.231683166478106	0.987821793553143	-5.7512714525031
a_HQ_transcript/93413	-2.47121727355975	0.842825544685743	-1.21872249511549	0.231698915920773	0.987821793553143	-5.21180468649666
a_HQ_transcript/88039	1.27630389043197	0.529165799962121	1.21863033988383	0.231733436909052	0.987821793553143	-5.07830422082026
a_HQ_transcript/102893	-0.689970106030204	3.04679422291776	-1.21861537886035	0.231739041610349	0.987821793553143	-5.42949789476901
a_HQ_transcript/94563	-2.4278807330379	0.575370101392988	-1.21861475613015	0.231739274899831	0.987821793553143	-5.17144567095702
a_HQ_transcript/149362	0.431487674364783	4.61457723046833	1.21853505708636	0.231769133489814	0.987821793553143	-5.61110424040674
a_HQ_transcript/105533	-1.59360792519715	0.168985112788539	-1.21851519063443	0.231776576737406	0.987821793553143	-5.09846653197826
a_HQ_transcript/2830	0.449247166259057	3.16494540557819	1.21848370448711	0.231788373832286	0.987821793553143	-5.44505210326944
a_HQ_transcript/98560	-2.41193220460421	0.814076660915921	-1.21843706805115	0.231805848192299	0.987821793553143	-5.19850698765347
a_HQ_transcript/32512	-1.55196749933924	2.78150120809063	-1.21842239649467	0.231811345728599	0.987821793553143	-5.15247896836361
a_HQ_transcript/88603	-2.01763821842593	0.514127363213266	-1.2183929804483	0.231822368421704	0.987821793553143	-5.07105227516053
a_HQ_transcript/10019	1.00515009899667	1.63561743823803	1.21835307377307	0.231837322754492	0.987821793553143	-5.02665331975199
a_HQ_transcript/25577	-1.76299926731958	1.18084308903185	-1.21832494070619	0.231847865562991	0.987821793553143	-5.24371827027314
a_HQ_transcript/101076	-1.17437145273675	0.76099366225202	-1.21820391540257	0.231893223607135	0.987942634259423	-5.06664808053506

a_HQ_transcript/140400	2.78189894149611	1.76737573677567	1.21804590268045	0.231952453774276	0.98796131344948	-5.1915361244372
a_HQ_transcript/6871	-1.12308354065411	3.21019166919135	-1.21793022701456	0.23199582126295	0.98796131344948	-5.42049672383109
a_HQ_transcript/110111	-1.81616639989012	1.51277061845689	-1.21791314897227	0.232002224431914	0.98796131344948	-5.11911202505846
a_HQ_transcript/79819	-0.70218958866011	3.21292135605508	-1.21791168871742	0.232002771939784	0.98796131344948	-5.35491251968813
a_HQ_transcript/30280	-0.674278780940042	4.10876125129086	-1.21779834787456	0.232045270877849	0.98796131344948	-5.48063071077251
a_HQ_transcript/57952	1.58078285930364	1.47265946706594	1.21778006594503	0.232052126517541	0.98796131344948	-5.11403696228618
a_HQ_transcript/25924	1.53858468871619	-0.0886372202504551	1.21775810583015	0.232060361658784	0.98796131344948	-5.10989932644938
a_HQ_transcript/60906	-0.9713423981267	4.39101111762445	-1.21775231238031	0.232062534264565	0.98796131344948	-5.57044308188305
a_HQ_transcript/144647	-1.55670837822688	0.449908809017181	-1.21766873395069	0.232093878752825	0.98796131344948	-5.0535673020006
a_HQ_transcript/148771	1.80326062526385	0.982247556702134	1.21766078616549	0.232096859581096	0.98796131344948	-5.1384469253996
a_HQ_transcript/38129	-1.54292354470547	4.70835484033735	-1.21755824429317	0.232135320608261	0.98796131344948	-5.61272148533022
a_HQ_transcript/150164	-1.74881042501622	1.91236550406826	-1.21755357268364	0.232137072931077	0.98796131344948	-5.18779644748197
a_HQ_transcript/125485	1.52995978802807	2.54509400638619	1.21750623266883	0.232154830746055	0.98796131344948	-5.27458945965935
a_HQ_transcript/56512	-1.44098359619337	1.68568979942755	-1.2174852727243	0.232162693398295	0.98796131344948	-5.14946422802668
a_HQ_transcript/130199	1.25650488040374	-0.0675706103354809	1.21744424519025	0.232178084528389	0.98796131344948	-5.09226549230296
a_HQ_transcript/51067	-1.92980774986542	0.063963555904413	-1.21738238265914	0.23220129316321	0.98796131344948	-5.12873509084408
a_HQ_transcript/129733	-1.45805558398034	0.143492408824966	-1.21736740307617	0.232206913232352	0.98796131344948	-5.06013240271783
a_HQ_transcript/144904	-1.36940402029363	0.0959452762190691	-1.21726428782811	0.232245602953798	0.98796131344948	-5.07538968738249
a_HQ_transcript/49592	-1.26112549986432	1.9937854967881	-1.21725675105364	0.232248431003687	0.98796131344948	-5.20193403838013
a_HQ_transcript/107589	-1.12827272881315	2.4154372425833	-1.21716031604459	0.232284618902384	0.98796131344948	-5.24113021570541
a_HQ_transcript/24122	1.01579827290038	-0.542563444504159	1.21708603955524	0.232312494519535	0.98796131344948	-5.16963653790679
a_HQ_transcript/118511	-2.82664657441672	0.236432473913873	-1.21708024123379	0.232314670706849	0.98796131344948	-5.18489741983872
a_HQ_transcript/124105	-0.694560699411921	4.50953834766709	-1.21697313465521	0.23235487195619	0.98796131344948	-5.5584918161696
a_HQ_transcript/142810	-2.32097019840077	0.484616840414244	-1.21694076634809	0.232367022052702	0.98796131344948	-5.2245236997741
a_HQ_transcript/91657	0.550449373117773	4.06417663501191	1.21693852928116	0.232367861798297	0.98796131344948	-5.51724449328603
a_HQ_transcript/110964	-1.54268401806611	-0.0576127631451143	-1.21692484155072	0.232372999919558	0.98796131344948	-5.14111123927134
a_HQ_transcript/7976	-1.14949637389296	1.71894798869511	-1.21691473327991	0.232376794431937	0.98796131344948	-5.03533291555712
a_HQ_transcript/72109	-1.41873152498396	2.40326467372391	-1.21689264283436	0.232385087055838	0.98796131344948	-5.19486635618275
a_HQ_transcript/88111	-1.9533209303254	0.110250319956989	-1.21687822127683	0.232390500942656	0.98796131344948	-5.14175779846602
a_HQ_transcript/74553	-3.2258694863433	1.42797993082824	-1.21680405807577	0.232418343463248	0.988007420566892	-5.13575790758241
a_HQ_transcript/132306	-0.995559128855529	0.0776536576417909	-1.21673690426295	0.232443556640181	0.98804234445195	-5.05570237126953
a_HQ_transcript/61617	1.85562615001633	1.36039456413709	1.21655415256246	0.232512181807745	0.988103843262981	-5.17157495127266
a_HQ_transcript/122729	2.16291121693617	3.02486971685556	1.21652670035752	0.232522491696334	0.988103843262981	-5.21710845982987
a_HQ_transcript/119321	-1.19829618048397	0.0831782767128015	-1.21642085421896	0.232562246212761	0.988103843262981	-5.08157495648969
a_HQ_transcript/82925	0.919237736521567	2.62410370794114	1.21641919843349	0.232562868145646	0.988103843262981	-5.24936056198937
a_HQ_transcript/150029	-4.80720606503868	1.6053500710063	-1.21640943377705	0.232566535892846	0.988103843262981	-5.25139388803292
a_HQ_transcript/100307	-0.53304166072244	3.09395114310604	-1.21631868971897	0.232600622737243	0.988103843262981	-5.25677061590888
a_HQ_transcript/3060	1.55696478055195	2.73431217011756	1.21624965880714	0.232626555800365	0.988103843262981	-5.44738248903919
a_HQ_transcript/48873	2.19911390113447	3.01965651608501	1.21624828853743	0.232627070595691	0.988103843262981	-5.1604119745638
a_HQ_transcript/144811	0.196324620736965	9.21150231451548	1.21620005949765	0.232645190256744	0.988103843262981	-6.06273300081471
a_HQ_transcript/9731	0.780980692316619	1.56078077704834	1.2161280331233	0.232672252532804	0.988103843262981	-5.02567480122293
a_HQ_transcript/44145	-0.441501484852617	3.18531420170209	-1.21612257378087	0.232674303852156	0.988103843262981	-5.32595871502592
[truncated: 764,014 more chars]
