# Supplementary material for: Transcriptomics as an Early Warning of Domoic Acid Exposure in Pacific Razor Clams (Siliqua patula)
Source: Toxins (Basel). 2025 Apr 11;17(4):194. doi: 10.3390/toxins17040194 (PMC12031274; doi:10.3390/toxins17040194)
Supplement: Supplementary file 1 [file Supplementary File S3. Bloom vs post-bloom DEG.pdf › Supplementary File S3. Bloom vs post-bloom DEG.pdf]

Supplementary File S3. Differential Gene Expression values (Transcript ID, log FC, Average Expression, t value, P value, adjusted P value, and B value) for razor clam bloom vs. post-bloom samples.

Transcript_ID	logFC	AveExpr	t	P.Value	adj.P.Val	B
a_HQ_transcript/72650	-7.27868333439491	-0.66302502980542	-20.4922542821178	3.47049747606283e-20	2.0173307728858e-15	27.8078348687298
a_HQ_transcript/45368	-5.46780657019168	-0.488353363103958	-9.86150642308839	2.62330768272258e-11	7.62438144906489e-07	14.3828130914027
a_HQ_transcript/23807	-7.23643543304535	1.55057015126014	-9.02627087042354	2.21343179715591e-10	3.34801238879547e-06	11.9792894414735
a_HQ_transcript/27296	-4.49835824680734	-0.218201885112703	-9.01091856392416	2.30388961519094e-10	3.34801238879547e-06	12.9118331208454
a_HQ_transcript/1070	-7.17005188340003	3.33529560635928	-8.56307105394438	7.51175700816428e-10	8.17184295208986e-06	11.7531732054164
a_HQ_transcript/55144	-6.1619114021485	3.41384697766839	-8.47688756286148	9.45798429238207e-10	8.17184295208986e-06	11.842150197157
a_HQ_transcript/95382	-4.07354915508849	-0.714632152041304	-8.46207858239502	9.8408513392219e-10	8.17184295208986e-06	11.2331489229551
a_HQ_transcript/66970	-6.62441366150464	2.79129939436016	-8.30180893852801	1.51466181797779e-09	9.54229553388209e-06	11.0608407086643
a_HQ_transcript/102242	-2.93562876450097	5.80058899474801	-8.26227720140983	1.68548656654828e-09	9.54229553388209e-06	11.6903387933699
a_HQ_transcript/34261	-3.01614405286079	4.96248036288928	-8.2610573895616	1.69105868305983e-09	9.54229553388209e-06	11.5957340368297
a_HQ_transcript/125280	-4.89326307491467	6.34754323276643	-8.23681673833784	1.80576057790915e-09	9.54229553388209e-06	11.6549374649685
a_HQ_transcript/146424	-4.9054054384577	-0.605999607407108	-7.7691351875876	6.49592887639795e-09	2.73923819406549e-05	9.67793772186394
a_HQ_transcript/58336	-2.56631008872792	3.11861714951177	-7.76865373889652	6.50458291893455e-09	2.73923819406549e-05	10.0798031008477
a_HQ_transcript/129123	-5.68315719379087	-0.514907986309261	-7.75696637035473	6.71829295513135e-09	2.73923819406549e-05	9.6570779848088
a_HQ_transcript/73314	-9.52912798871086	-0.02466991112433	-7.73859989191922	7.06863695826148e-09	2.73923819406549e-05	9.59760754922239
a_HQ_transcript/22218	-3.71852149246816	0.00279066033138447	-7.48143976103131	1.44612729389717e-08	5.25378045872842e-05	8.5902587575861
a_HQ_transcript/92691	-4.21367922569596	-0.626509396534239	-7.43807258929412	1.63285894744811e-08	5.58322499395668e-05	8.88916116289458
a_HQ_transcript/79902	-3.65975632058919	-0.433956745393014	-7.41529860679767	1.74053070517426e-08	5.62075382390941e-05	8.86349222253581
a_HQ_transcript/35228	-7.10857045934023	2.63594498020381	-7.33159336527955	2.20203021163548e-08	6.73682169168143e-05	8.66816540841195
a_HQ_transcript/95521	-5.72794276749579	0.525293476488716	-7.18557640746774	3.32485518167839e-08	9.66335910003008e-05	8.01625550681129
a_HQ_transcript/88979	-5.60209441798176	-0.474879982808844	-7.03588894434546	5.08397322943253e-08	0.000140724378990693	7.9371038306208
a_HQ_transcript/98226	-2.6427842540514	5.14605637708665	-7.00568616470348	5.540293643822e-08	0.000146384631330948	8.35591498085202
a_HQ_transcript/29225	-2.57030882294555	3.07877041461385	-6.89452108560912	7.60772068338359e-08	0.000192270255601618	7.78058845880968
a_HQ_transcript/88608	-4.19079137373642	-0.601142155600073	-6.85908569626581	8.41900079354102e-08	0.000203908199219564	7.49067678369404
a_HQ_transcript/73205	-3.95844398549566	1.65185211463057	-6.8219587837267	9.36311684082138e-08	0.000213598970348289	7.05896295681399
a_HQ_transcript/42140	-6.27368010391205	2.06610850683591	-6.81491253938944	9.5540414758043e-08	0.000213598970348289	7.44342894554874
a_HQ_transcript/19744	-4.81751294263926	4.73831268997463	-6.7120984332537	1.28327762613799e-07	0.000276275414267218	7.55261903690399
a_HQ_transcript/62111	-2.91068195424938	3.76777027217547	-6.607186311288	1.73568662986497e-07	0.000360328544359968	7.12674955609781
a_HQ_transcript/141915	-4.94772494497006	-0.297641686820032	-6.50039105947719	2.36249588051231e-07	0.000470392573342335	6.61027106762156
a_HQ_transcript/87007	-3.17643401377809	2.58964417737117	-6.49097410422238	2.42770733558183e-07	0.000470392573342335	6.50696467990754
a_HQ_transcript/40414	-3.66403623959146	-0.537391021096956	-6.40801670518987	3.08668469070148e-07	0.000578783250648695	6.22942698429886
a_HQ_transcript/148700	-4.34664299332958	-0.255055296959226	-6.35777533651771	3.57075966102509e-07	0.000648628492425208	6.23092184816324
a_HQ_transcript/20368	-3.45284989445485	-0.0658947246698745	-6.15150157903445	6.50533587617287e-07	0.00114588534487932	5.61928633451981
a_HQ_transcript/48533	-2.28291250988413	4.93112120982809	-6.01536193984394	9.67810222843092e-07	0.00165461390098304	5.6448336558461
a_HQ_transcript/101587	-3.41398259708432	-0.580560243978355	-6.004790471271	9.98168334010934e-07	0.00165775796912536	5.33209397448722
a_HQ_transcript/119398	-5.10027705107625	-0.476370427079186	-5.98534076987331	1.05654616201446e-06	0.00170596986959936	5.37713947075488
a_HQ_transcript/25345	-4.42235727691155	0.435909734431057	-5.96517086055171	1.12071507885358e-06	0.00174014079069838	5.04467163352169
a_HQ_transcript/16345	-3.89959668710548	-0.417836165390691	-5.96006187202092	1.13758171701312e-06	0.00174014079069838	5.26788420850589
a_HQ_transcript/75035	-2.96073081366765	-0.556200754805242	-5.92228198012446	1.27048878624878e-06	0.00189361467095049	5.07789425528384
a_HQ_transcript/135178	5.33940052880972	0.484900470760079	5.89088925485149	1.39272654114549e-06	0.00202231375223204	5.10099757848085
a_HQ_transcript/107478	7.34219257297346	0.777411498044478	5.88272271284097	1.42641865953608e-06	0.00202231375223204	5.11037833036218
a_HQ_transcript/109357	-3.10246008378842	-0.611091536041889	-5.85952033851465	1.52667843773138e-06	0.00211292295782022	4.94172936730935
a_HQ_transcript/63237	-4.16151803504274	-0.204288980477183	-5.81120004253875	1.75878146084334e-06	0.00237754531990469	4.95724017579537
a_HQ_transcript/143639	-3.87607151779601	-0.616215406764874	-5.78636860811584	1.8915226908731e-06	0.00249887343125163	4.79074917373431

a_HQ_transcript/120470	-3.1710727930441	-0.721650623992468	-5.69602293418363	2.46519654402616e-06	0.00318437654913673	4.54598638312544
a_HQ_transcript/107243	-2.21720163145371	4.25315396078619	-5.64232288951331	2.88584927333645e-06	0.00364670970783699	4.61259603936184
a_HQ_transcript/72446	-2.96245356474423	4.38406251876546	-5.62114683748193	3.07087934363033e-06	0.0037979590316286	4.54731387362149
a_HQ_transcript/143164	-6.51811307776532	0.257146267670322	-5.60434623703681	3.22610137157537e-06	0.00390680876097778	4.39656418750258
a_HQ_transcript/36072	-4.82148217000239	-0.520070604993403	-5.59099206322611	3.35507158679477e-06	0.00398007349382054	4.2725462045533
a_HQ_transcript/70915	3.18248818131532	-0.448088062605346	5.48949210803729	4.51991061518007e-06	0.00525466728478375	4.10765176805145
a_HQ_transcript/76594	-4.70789273831472	1.83731124271816	-5.46117144886268	4.91192182202944e-06	0.0055984351308025	3.90868754187746
a_HQ_transcript/53566	-7.13583986718315	2.27582138164364	-5.45172143483749	5.0501581426195e-06	0.00564529985604204	3.82324037516839
a_HQ_transcript/101470	4.09978505404339	-0.628658497946083	5.40670743350355	5.76398326717681e-06	0.00632167583687648	3.80286396798083
a_HQ_transcript/136081	4.15982747107488	0.802509157848773	5.39632304137943	5.94249499409727e-06	0.00639676572253493	3.69424778661846
a_HQ_transcript/75195	-3.62101921074355	0.281986565190474	-5.37747325158932	6.28077473110293e-06	0.00663797951944638	3.4778211785957
a_HQ_transcript/111235	-4.67563850857206	0.387229493201896	-5.36852232642358	6.44808513703304e-06	0.00664914937389845	3.6256713833223
a_HQ_transcript/120428	-3.96293913566368	-0.340395945071971	-5.3625475538354	6.562237891246e-06	0.00664914937389845	3.64903204680286
a_HQ_transcript/29420	-2.15003414197515	3.8045949439081	-5.35881841683689	6.63450770172912e-06	0.00664914937389845	3.82811324641852
a_HQ_transcript/52838	-7.44278024777388	0.755430501775978	-5.33324708137364	7.15197200082715e-06	0.00704626827905222	3.69934338813348
a_HQ_transcript/115495	-5.12878083447781	0.311045192354119	-5.26084842865764	8.84630429058639e-06	0.00825684295294286	3.42715256020513
a_HQ_transcript/127496	-3.91864484664456	-0.0677914706248925	-5.25487731395161	9.00277272528654e-06	0.00825684295294286	3.33360467381699
a_HQ_transcript/1104	-1.68983145768796	4.75192415476932	-5.25342815308473	9.04116172486177e-06	0.00825684295294286	3.53182464398295
a_HQ_transcript/129720	-2.8138114161217	3.23374701292839	-5.25301476772759	9.0521424424098e-06	0.00825684295294286	3.4838113318634
a_HQ_transcript/112264	-4.20931275970117	-0.651681458310621	-5.25155831164149	9.09093636437419e-06	0.00825684295294286	3.41774243299491
a_HQ_transcript/26913	-2.91344326026137	2.13364537378911	-5.24016690266668	9.40013821103816e-06	0.00829186900696591	3.39676553576274
a_HQ_transcript/146734	-4.55576667364391	-0.522746687591436	-5.23963616186822	9.41479759255007e-06	0.00829186900696591	3.38877630832659
a_HQ_transcript/62043	4.00198085459474	1.81826454339242	5.20295311137307	1.04853022224211e-05	0.00901177022459679	3.3565059402767
a_HQ_transcript/52617	-5.90992532247918	0.878139103882067	-5.1966191511609	1.06820739125891e-05	0.00901177022459679	3.28536776974483
a_HQ_transcript/90430	2.03039232537639	5.01372702198626	5.19613417498903	1.06972912451345e-05	0.00901177022459679	3.36360904417803
a_HQ_transcript/145556	-6.8953415251734	0.976569919211234	-5.18356948160662	1.10991685484365e-05	0.00921674956262165	3.33562883969878
a_HQ_transcript/130548	-2.10339793505064	4.47666628917577	-5.16164206866454	1.18369193802482e-05	0.00966327609681656	3.27921187424343
a_HQ_transcript/140232	4.53955724142021	6.46484002591952	5.15785001689457	1.19693758424648e-05	0.00966327609681656	3.26230044077703
a_HQ_transcript/47331	-4.53097947802473	0.104962827405953	-5.14760277399027	1.23347536437605e-05	0.00982184328499326	3.10914074908808
a_HQ_transcript/131746	3.62564343557643	2.75660515046323	5.14246172448984	1.25222328011216e-05	0.00983638308464319	3.19013503915835
a_HQ_transcript/119340	5.99390648072297	3.38379709151133	5.13452802986096	1.28171425932863e-05	0.00993379819550061	3.21534719494542
a_HQ_transcript/116838	-7.12320381582128	0.0703369666994108	-5.10864551875261	1.3828239881822e-05	0.0105764201032967	3.09508487959454
a_HQ_transcript/15084	-3.51914779148899	0.97098754255567	-5.07627844011378	1.52052242136012e-05	0.0114785619881586	2.90356284293919
a_HQ_transcript/114019	3.72624104191444	1.66711044969896	5.04618845734506	1.66075567979379e-05	0.0123764623275709	2.77574164686939
a_HQ_transcript/41294	-5.27154593530313	0.385736485827615	-5.03733780099481	1.70440414414211e-05	0.0125409625431256	2.8995790133303
a_HQ_transcript/89892	-4.73174135303331	0.0282561785295225	-4.99990967170533	1.90197463992431e-05	0.0138197477336901	2.68880545630556
a_HQ_transcript/17220	-1.63959430577383	2.92841637256374	-4.99178530834144	1.94778470273666e-05	0.0139778801482317	2.80461222974385
a_HQ_transcript/80242	4.47475500303543	0.601060938925951	4.98265458946128	2.00058099480593e-05	0.0140410509535476	2.66126075802577
a_HQ_transcript/141107	-2.86324188628047	0.967925269374051	-4.98191859941482	2.00489820593251e-05	0.0140410509535476	2.4330322653454
a_HQ_transcript/49759	-3.36807130099483	-0.521863386120216	-4.94648058463237	2.2241192293299e-05	0.0153909050669629	2.60896921597232
a_HQ_transcript/60560	1.67754778393357	4.39688825615139	4.93691236062609	2.28729205732604e-05	0.0156418485539115	2.66546919787264
a_HQ_transcript/8232	-2.13160246581345	1.66765863074368	-4.92230679563682	2.38718076877987e-05	0.0161351213636787	2.57131039429073
a_HQ_transcript/57283	2.91819675654232	0.651609081932315	4.90531498153652	2.50885934000111e-05	0.0167626408868488	2.32976841588714
a_HQ_transcript/112742	2.43645559458674	2.49628585847689	4.87331911995214	2.75496030648529e-05	0.0181977650790201	2.39028840287475
a_HQ_transcript/76697	-3.70476012569914	0.0438519363553065	-4.84999041374737	2.94938185588259e-05	0.0192631088223307	2.41554149830845
a_HQ_transcript/115926	5.11344294358523	1.15136973431188	4.83392725253548	3.09111667295027e-05	0.0199644922183614	2.35719143919463
a_HQ_transcript/22375	-2.48680307629278	1.61402234152631	-4.81685761687897	3.24915082117584e-05	0.0207545757069571	2.14798737054288
a_HQ_transcript/135559	1.53056531931775	3.96075426711963	4.79921574075206	3.42091495408323e-05	0.0216142330924946	2.29958546830654
a_HQ_transcript/69307	-3.93328917515174	-0.0083280149920451	-4.78143277418844	3.60317273121542e-05	0.0225209918838807	2.221868285537
a_HQ_transcript/55390	-6.5227738970462	0.32768332643504	-4.77592342244808	3.66156965384471e-05	0.0226425234934771	2.23847965043605

a_HQ_transcript/52827	-3.19752806890583	-0.671153230360708	-4.76666735838978	3.76180203568146e-05	0.0230174767084307	2.10079891460978
a_HQ_transcript/107872	4.92555461146198	0.0815440819830914	4.74415426016135	4.01709951929034e-05	0.024323537589303	2.09393240636531
a_HQ_transcript/73908	3.85950679315134	0.830560795652762	4.73195564148419	4.16253506745868e-05	0.024944313237241	2.09404586746383
a_HQ_transcript/112755	-4.42457431482526	0.179416458016655	-4.72743139441531	4.21779182157045e-05	0.0250175309188007	2.00366766033288
a_HQ_transcript/7957	-5.7161310018325	0.484731296961551	-4.70790933796586	4.46468443370132e-05	0.0260887203464327	2.03040308642619
a_HQ_transcript/53982	-5.75139469517304	3.04052907290638	-4.69898192154918	4.58231140653504e-05	0.0260887203464327	1.96249449653718
a_HQ_transcript/120548	3.97508544078565	0.66934430791517	4.69438241903015	4.64410788888023e-05	0.0260887203464327	1.90507168772701
a_HQ_transcript/24742	-4.10655875943728	3.19635975346136	-4.69358433373091	4.65491432340437e-05	0.0260887203464327	2.02337474161479
a_HQ_transcript/32209	-3.44740680460777	-0.311560507037132	-4.69126094681836	4.68651611944345e-05	0.0260887203464327	1.96390283846218
a_HQ_transcript/104414	3.86392143864052	0.879895918565103	4.68977409271221	4.70685120641933e-05	0.0260887203464327	1.88671771188713
a_HQ_transcript/86278	-2.4925560815188	-0.642130100431394	-4.68354518430614	4.79299619966915e-05	0.0260887203464327	1.95545643404352
a_HQ_transcript/110329	-3.2496715008025	-0.374176017708981	-4.68205842311815	4.81378780537706e-05	0.0260887203464327	1.94968203379132
a_HQ_transcript/109347	-2.26217849899144	2.18533592581732	-4.68019356454633	4.83999331294578e-05	0.0260887203464327	1.95952250781777
a_HQ_transcript/101113	3.2372024675355	4.53421760940636	4.67534553848814	4.90878239803696e-05	0.0260887203464327	1.96744462934406
a_HQ_transcript/20087	-3.78563876135902	0.623236489598867	-4.67429105514081	4.92387234889864e-05	0.0260887203464327	1.80065946076394
a_HQ_transcript/62015	4.97769501280569	0.666057501589139	4.67337870718943	4.93696538347715e-05	0.0260887203464327	1.89173532108069
a_HQ_transcript/129305	-4.23645168436044	-0.0855682679723796	-4.66263497950843	5.09376699292763e-05	0.0266748187175583	1.85966104268862
a_HQ_transcript/24702	2.82477691814783	-0.721815363212724	4.65400095005331	5.2233454512361e-05	0.0270905937711787	1.85975712124806
a_HQ_transcript/7408	2.47606753953788	-0.810811751688042	4.65118083936725	5.26637265370078e-05	0.0270905937711787	1.85005219921176
a_HQ_transcript/50722	-2.37006621558856	-0.573123525699976	-4.63751543208251	5.47989277415291e-05	0.0279416848399965	1.78926840955716
a_HQ_transcript/17129	-2.44113868784926	1.71579548344687	-4.62799958493223	5.63361781825582e-05	0.0284757336121369	1.69317782979912
a_HQ_transcript/123687	4.75149157576666	1.79286045125112	4.62220787981207	5.7292629520328e-05	0.0287095342134278	1.73423113549784
a_HQ_transcript/109681	-6.50504497541626	3.62000190231591	-4.61640487548393	5.8267067523079e-05	0.0288469359823778	1.81785290867614
a_HQ_transcript/31904	-5.1233828532152	0.988415811882765	-4.61468293105151	5.85593594467483e-05	0.0288469359823778	1.73655019040615
a_HQ_transcript/143270	-6.15429025222667	0.0826024480176038	-4.58573175275233	6.36967217564423e-05	0.0311139751450292	1.69793306449156
a_HQ_transcript/113694	3.4345140592191	1.5857894636148	4.58043452326122	6.4683849675062e-05	0.0313328567826	1.66247207264884
a_HQ_transcript/112137	-4.3180238780961	0.155332427366725	-4.56661046724038	6.73317864806764e-05	0.0322360259811451	1.55501242885963
a_HQ_transcript/91821	-4.94847098477932	1.01607011870786	-4.56494729197277	6.7657500166868e-05	0.0322360259811451	1.65982875554031
a_HQ_transcript/107916	-6.25549210069108	0.678945342630294	-4.55879796399829	6.88753650217445e-05	0.0323041924201874	1.6084531871796
a_HQ_transcript/129704	-4.2802640969827	-0.335017603279034	-4.55861438582082	6.89120537452387e-05	0.0323041924201874	1.62565171608829
a_HQ_transcript/1462	1.89311212255721	4.65725224599906	4.54647495480766	7.13815444698082e-05	0.0331941313355281	1.58876362918915
a_HQ_transcript/38931	2.4424119615766	2.6033896305044	4.5408505566438	7.25551689192488e-05	0.0334721179280801	1.59017615227587
a_HQ_transcript/141208	4.31112747481756	-0.132645711253313	4.53365939058615	7.40835518021793e-05	0.0338785418809465	1.57108138225253
a_HQ_transcript/118905	-4.38248565874903	-0.561341677503681	-4.53125425663759	7.46017988019741e-05	0.0338785418809465	1.56028941002598
a_HQ_transcript/77421	-1.79294920931145	3.52413368284294	-4.51155248371761	7.89841718257305e-05	0.0355906351929152	1.52602206574373
a_HQ_transcript/98995	4.2025148939043	0.115050662994042	4.50104021867769	8.14255826741763e-05	0.036408509766804	1.41265328719258
a_HQ_transcript/24483	2.79771198773423	1.28331627015184	4.48889843568335	8.43383416729601e-05	0.0374230467539376	1.33816661009535
a_HQ_transcript/37869	6.09385264710897	4.09317717885938	4.48308948695368	8.57680511377979e-05	0.037769130882863	1.42424575094952
a_HQ_transcript/27975	-4.8463559466379	3.43987050296386	-4.47589159571738	8.75728554963505e-05	0.0379076465190731	1.43082897537022
a_HQ_transcript/126246	3.90241854869286	3.01496241127309	4.4741545016099	8.80140037063838e-05	0.0379076465190731	1.4114450899902
a_HQ_transcript/77729	-4.15945617857665	-0.215786496649932	-4.47356368132963	8.81645465926121e-05	0.0379076465190731	1.42840631730795
a_HQ_transcript/21477	-2.44394865496289	4.6205154870355	-4.47150473307845	8.86911630641678e-05	0.0379076465190731	1.3902600033653
a_HQ_transcript/106592	2.75569112911274	4.30880527053964	4.4603292839274	9.16041514397311e-05	0.0388669059480926	1.36908462571472
a_HQ_transcript/30556	2.68436138095605	2.75640322887719	4.45444052923129	9.31769104607448e-05	0.0392477351540737	1.3788356848489
a_HQ_transcript/66096	2.32991107178426	-0.822597907086957	4.44037381616867	9.70425288687141e-05	0.0404360173719802	1.31437540444505
a_HQ_transcript/150782	-5.17438766783523	0.35988805736438	-4.43913926576546	9.73892518592973e-05	0.0404360173719802	1.33013631165361
a_HQ_transcript/126879	-5.11874952623618	0.311398268901875	-4.43361051569184	9.89570569819848e-05	0.040670004701838	1.31259542550125
a_HQ_transcript/141639	-4.76923224703661	0.581495536810327	-4.42968013430587	0.000100086729083843	0.040670004701838	1.27379367943379
a_HQ_transcript/121318	-4.77012503975627	0.157475160584278	-4.42845452400438	0.000100441590493658	0.040670004701838	1.30375748939507
a_HQ_transcript/56727	3.82959387521232	1.50013072772772	4.42738780621767	0.000100751456734528	0.040670004701838	1.26978565068181

a_HQ_transcript/16534	-2.74892077166892	1.3445274787515	-4.42396333126341	0.000101752606306302	0.0407908655129153	1.12729163475088
a_HQ_transcript/81373	-2.41512720612395	1.1173622137901	-4.40618167805321	0.00010711102248277	0.0426448596909484	1.15502415800458
a_HQ_transcript/101676	-2.32173663873217	-0.882206117452529	-4.39778710240405	0.000109736487591847	0.043392942522033	1.20494267755168
a_HQ_transcript/93898	-2.6944783458149	3.05228316628448	-4.39530437934457	0.000110525067697658	0.0434094671292531	1.22681731418504
a_HQ_transcript/74543	-2.23428392622566	-0.888814213200297	-4.38930253518645	0.000112454574235322	0.043870869068126	1.18227846855069
a_HQ_transcript/144363	7.51670378620255	3.38179706872706	4.3867747173253	0.000113277137546562	0.0438971563420439	1.19293298922653
a_HQ_transcript/145282	-2.4244068576581	3.04277288418757	-4.38062633846117	0.000115302692634334	0.0443861915062821	1.18833365344458
a_HQ_transcript/67721	-4.75776435570563	-0.404106717081654	-4.3686195632078	0.00011936176562165	0.0450096818853957	1.13813104483445
a_HQ_transcript/133525	-2.51577248831937	-0.876851438224917	-4.36713531281279	0.000119873220697622	0.0450096818853957	1.13039386398788
a_HQ_transcript/118361	-2.2304866058028	-0.728115560277924	-4.36560115134065	0.000120404144820557	0.0450096818853957	1.12616902683629
a_HQ_transcript/130342	-3.01379664239352	-0.718947017572328	-4.36506255972927	0.000120591083239195	0.0450096818853957	1.14743541243588
a_HQ_transcript/43028	2.49545744653597	3.07187965272963	4.36125555846911	0.00012192062781361	0.0450096818853957	1.14013682167342
a_HQ_transcript/31679	-2.5430418027331	-0.875716897863781	-4.36007063018272	0.000122337388708717	0.0450096818853957	1.11292933633397
a_HQ_transcript/73739	3.5625225576562	-0.401085480706151	4.35901296336185	0.000122710575149208	0.0450096818853957	1.11495120444565
a_HQ_transcript/62856	-3.56182997493232	1.42734683939377	-4.35340231590872	0.000124709071870726	0.0450096818853957	1.07704099947069
a_HQ_transcript/25939	-6.9457493989695	0.28225937281033	-4.35237407681431	0.000125078789731214	0.0450096818853957	1.11125105186353
a_HQ_transcript/83389	3.06532977049448	1.40864473581528	4.35225071435872	0.000125123218941148	0.0450096818853957	1.08755848774051
a_HQ_transcript/140663	-3.48163350493662	-0.355317718054626	-4.35137276107356	0.000125439864874658	0.0450096818853957	1.10342506390191
a_HQ_transcript/82126	1.97254840007879	-0.756109324733384	4.34150280776508	0.000129054387364334	0.0460225363724785	1.07598178851871
a_HQ_transcript/146794	-7.69817140166646	0.640412469479781	-4.323780914052	0.000135803712651036	0.0473558756371832	1.02940314072314
a_HQ_transcript/55669	-4.26075785640523	-0.32917065106844	-4.32365527467048	0.000135852781896543	0.0473558756371832	1.03217576467121
a_HQ_transcript/109565	-2.27170146976229	-0.870236189510941	-4.32335410608038	0.000135970476277966	0.0473558756371832	1.0166446050431
a_HQ_transcript/102601	-3.18398965051675	-0.304569844725887	-4.322410169587	0.000136340010418326	0.0473558756371832	1.02575135784607
a_HQ_transcript/133136	5.70023475746135	0.602942397883645	4.32056495170045	0.000137065238753477	0.0473558756371832	0.999452693312966
a_HQ_transcript/99445	4.18702132089541	-0.325311152969272	4.31900483758433	0.000137681375287021	0.0473558756371832	1.01605853914988
a_HQ_transcript/7955	4.86686465612324	1.15738840287475	4.31598034911884	0.00013888361694714	0.0474883934464903	0.98634180136397
a_HQ_transcript/150936	5.63063839485402	9.05452295499229	4.31081074346462	0.000140962527907923	0.0475977141601753	0.68333152142471
a_HQ_transcript/8454	-3.46285967245078	-0.342962153930449	-4.30875468399965	0.000141797843933383	0.0475977141601753	0.958972534592615
a_HQ_transcript/56023	-4.30050880761767	0.943106949907201	-4.30806435194148	0.000142079396780934	0.0475977141601753	1.00383072895734
a_HQ_transcript/130610	-1.86697555718105	6.23511907957121	-4.30515300001667	0.000143272863257948	0.0475977141601753	0.828331500600339
a_HQ_transcript/66562	-2.52940977015975	2.01767661625053	-4.3050930317019	0.000143297549856019	0.0475977141601753	0.870925020962957
a_HQ_transcript/96011	3.14690492960313	0.539180317833389	4.29764023247532	0.000146398397967237	0.0483513981649975	0.851522465448975
a_HQ_transcript/128312	3.97771696596453	-0.286184926203821	4.29420359594591	0.000147850422798329	0.0485550812227189	0.943984073010482
a_HQ_transcript/149683	-1.69058922999142	-0.788865929691825	-4.29013308233663	0.000149588615666695	0.0486686540943144	0.943742742811939
a_HQ_transcript/109372	-6.15488492798564	2.84932034164968	-4.28945914968243	0.000149878332936601	0.0486686540943144	0.958126017576189
a_HQ_transcript/126837	-2.63466530891764	-0.860763001149198	-4.28295810331251	0.000152701581321833	0.0486686540943144	0.919781934068753
a_HQ_transcript/6100	-4.58186872604988	0.266768774627693	-4.28292187743579	0.000152717459061003	0.0486686540943144	0.922912096835394
a_HQ_transcript/99016	-4.02619290826832	0.0727762463134926	-4.28256292086764	0.000152874876939193	0.0486686540943144	0.914806192529269
a_HQ_transcript/52003	-3.30301538016269	-0.555686089717925	-4.28177754647491	0.000153219854446386	0.0486686540943144	0.894347221860425
a_HQ_transcript/134341	-4.61608275026928	-0.398396126102439	-4.27070237723704	0.000158166994330879	0.049967016556877	0.894351079244761
a_HQ_transcript/2579	3.38062839028922	-0.307808791692925	4.2546940378495	0.000165596588883036	0.0514271831473018	0.852260694316542
a_HQ_transcript/33840	-5.02863920764861	0.330265821800037	-4.2545243283945	0.000165677162957776	0.0514271831473018	0.858174476017687
a_HQ_transcript/53437	4.39082190776269	1.0522497424478	4.25266575921336	0.00016656209540458	0.0514271831473018	0.789509081789871
a_HQ_transcript/142801	6.7818054172061	0.743722052962233	4.2514532722152	0.000167141908469786	0.0514271831473018	0.85928329986343
a_HQ_transcript/147471	0.980083824100135	8.90900833668764	4.25130559798518	0.000167212661967383	0.0514271831473018	0.492618742693335
a_HQ_transcript/138704	1.27955428261707	5.54091573894038	4.24103826107639	0.000172204828787764	0.0526838015146062	0.696339773585535
a_HQ_transcript/152361	-5.84805517157348	0.220810969713391	-4.23691590703714	0.00017425017112442	0.0530304395137188	0.808746371275521
a_HQ_transcript/32810	-1.59149732198373	3.05735796254023	-4.22500916528082	0.000180292865739324	0.0545836651025805	0.790723536129262
a_HQ_transcript/15297	-1.94400696780198	1.79840446708659	-4.21484087587979	0.000185615996792406	0.0556308435498105	0.738804486527621
a_HQ_transcript/87409	-2.26658202780021	-0.802768020303534	-4.21474703541966	0.000185665834858644	0.0556308435498105	0.742962751673203

a_HQ_transcript/111482	-4.94835702636684	4.14753987456663	-4.21152625975391	0.00018738436224053	0.055857836965731	0.712963199261416
a_HQ_transcript/9456	-2.90297908847198	-0.624426768003963	-4.20958736945901	0.000188426434077917	0.0558818967351081	0.739368726109246
a_HQ_transcript/46521	3.3379420181966	-0.454764068876786	4.19752503782129	0.000195038522179718	0.0570093960804767	0.708166414960722
a_HQ_transcript/4205	-3.23482337834762	3.51549789525026	-4.19726361257731	0.00019518431992655	0.0570093960804767	0.713069777184941
a_HQ_transcript/124720	2.65372741174011	1.95512403161831	4.19503169936819	0.000196433432401497	0.0570093960804767	0.707198205817806
a_HQ_transcript/121155	-2.85790002839305	-0.86215626748681	-4.19413540979089	0.000196937256788157	0.0570093960804767	0.698725532322717
a_HQ_transcript/95347	4.38976857166652	0.985999193667398	4.19053297042813	0.00019897510383587	0.0570093960804767	0.682458550086267
a_HQ_transcript/10560	-2.76962195332239	2.10047323872895	-4.18942333922272	0.000199606968236553	0.0570093960804767	0.623089509427206
a_HQ_transcript/136035	-6.31012857634508	0.710105818063827	-4.1888514934572	0.000199933367172358	0.0570093960804767	0.698269103577228
a_HQ_transcript/115345	4.14883004070873	0.453872535710096	4.18625952247378	0.000201419401254182	0.0570093960804767	0.607396695930328
a_HQ_transcript/15535	-3.83900497481281	0.552007146021166	-4.18529277661298	0.000201976431083807	0.0570093960804767	0.689060239842682
a_HQ_transcript/146453	-2.24658758776511	-0.885153926664406	-4.1851899355537	0.000202035776090321	0.0570093960804767	0.671286912065622
a_HQ_transcript/31572	2.22494955597175	-0.487004151315449	4.17580214532637	0.000207525799929144	0.0582756507163347	0.595366663261259
a_HQ_transcript/143334	-2.33489367318273	-0.880998221971209	-4.16806513106673	0.000212160338180104	0.0592906545083321	0.629494552111077
a_HQ_transcript/147794	5.90207207539945	2.26358242943357	4.16394547082129	0.000214669354399444	0.0594336339335168	0.631878360426976
a_HQ_transcript/142430	-2.8980629392176	-0.68652001911849	-4.16243899509091	0.000215594105068699	0.0594336339335168	0.630355927963441
a_HQ_transcript/38899	-5.41608384054991	1.86839251046517	-4.1622029636676	0.000215739346957956	0.0594336339335168	0.606256924673109
a_HQ_transcript/96375	-2.4070341648171	-0.877712411959244	-4.14914684111743	0.00022392479282993	0.0606965201625363	0.582994787551055
a_HQ_transcript/67736	2.25684138790644	1.18298035457009	4.14797772104955	0.000224672461876412	0.0606965201625363	0.520837189993182
a_HQ_transcript/111728	-4.55849723243142	0.609617049298682	-4.14736583943637	0.000225064745022135	0.0606965201625363	0.586460054179677
a_HQ_transcript/111569	3.74998357853557	1.30754999866852	4.14686129103966	0.000225388721397107	0.0606965201625363	0.571093892224362
a_HQ_transcript/52009	-3.42467949405613	-0.330810709796633	-4.14599264114411	0.000225947564111017	0.0606965201625363	0.579240710410122
a_HQ_transcript/138089	3.44684999938695	0.0820680001100358	4.14294054044322	0.000227921925032683	0.0606965201625363	0.579686340598071
a_HQ_transcript/131005	-4.95508109235625	1.4941941449399	-4.14180683797473	0.000228659604989072	0.0606965201625363	0.577567326215999
a_HQ_transcript/104896	-4.22884538827284	0.423360613927291	-4.14151566878495	0.000228849441380503	0.0606965201625363	0.575627502412447
a_HQ_transcript/134578	-6.40011125833507	1.03439243756549	-4.13684665022334	0.000231914752843128	0.0606965201625363	0.558204878475052
a_HQ_transcript/28658	-2.43558317015757	-0.876350377177891	-4.13672318627416	0.000231996353898	0.0606965201625363	0.5522792967548
a_HQ_transcript/16295	5.70528971849627	0.670360893262775	4.13590383550394	0.000232538601827745	0.0606965201625363	0.561265373895364
a_HQ_transcript/145987	-2.44522544541357	-0.875889823283982	-4.13230848210995	0.000234932749807938	0.0606965201625363	0.541359417593914
a_HQ_transcript/120743	-2.94356994779073	-0.858384678604183	-4.13140182524699	0.000235540301048895	0.0606965201625363	0.542144857800447
a_HQ_transcript/118215	0.965534761933668	3.93848247161733	4.13080174868283	0.00023594326006426	0.0606965201625363	0.487948664310063
a_HQ_transcript/93493	-2.16758585722166	4.56167307165769	-4.13022149610546	0.000236333550129202	0.0606965201625363	0.481601314266464
a_HQ_transcript/1290	3.41000061683478	1.23509607782075	4.12586166381849	0.000239286390754309	0.0606965201625363	0.518704097904079
a_HQ_transcript/100942	4.8385952937312	0.582690050665024	4.12525343836205	0.000239701197587121	0.0606965201625363	0.535645030553737
a_HQ_transcript/145717	2.65097310578347	1.17551549956374	4.12485151178235	0.000239975696545228	0.0606965201625363	0.519858744960804
a_HQ_transcript/126185	-3.99567366337733	-0.396778210492611	-4.12241709910567	0.000241644899634356	0.0606965201625363	0.521182087153286
a_HQ_transcript/141672	-1.03720077243035	3.75101835340982	-4.12231715945987	0.000241713668027606	0.0606965201625363	0.471775841384302
a_HQ_transcript/39695	-5.44629674319611	0.586680336977718	-4.12153656298529	0.000242251456745603	0.0606965201625363	0.524517055078486
a_HQ_transcript/34866	-5.03287551097755	1.28296003483458	-4.11532363679636	0.00024657387379886	0.0615143610994856	0.436961368882475
a_HQ_transcript/102738	2.85244809221032	-0.231823745664619	4.10956115277777	0.000250650468130912	0.0622641470577506	0.439895266655794
a_HQ_transcript/151843	0.682130856960178	11.6823770235735	4.09870691796462	0.000258509409084648	0.0639431273671166	-0.240590503325532
a_HQ_transcript/83269	-3.43881809478798	-0.12086933747366	-4.09659598054366	0.000260065638927339	0.0640554892354592	0.46126866086278
a_HQ_transcript/5789	-2.76872562635923	2.55294562468485	-4.09489261978862	0.000261328086100855	0.0640948480543058	0.448905047398295
a_HQ_transcript/15265	6.06688757397708	2.43968406366117	4.09128589636989	0.000264021070770009	0.0644832638727692	0.434603846056155
a_HQ_transcript/120640	3.22777149628986	0.458293738239472	4.08734434899006	0.000266995162612339	0.06493679837795	0.409665296463622
a_HQ_transcript/104817	2.29235334017586	-0.164028662976738	4.07851051074707	0.000273780490085774	0.0661612656868181	0.407321382158019
a_HQ_transcript/149456	-1.16937354395454	4.1761541170187	-4.07628017489722	0.000275520168566992	0.0661612656868181	0.349504160312076
a_HQ_transcript/126115	-2.56353537942458	-0.870421968053441	-4.07570794300452	0.000275968256852639	0.0661612656868181	0.401328553592791
a_HQ_transcript/131565	-7.59136475841827	0.177342289608439	-4.07492500029697	0.000276582500032631	0.0661612656868181	0.40569060102999
a_HQ_transcript/51126	3.33307810342691	1.43526087387864	4.0708377858815	0.00027981086130714	0.066659203877301	0.32737447401442

a_HQ_transcript/69422	-2.37210664043747	1.73789714350332	-4.06832834804636	0.000281811248152512	0.0668617315616702	0.356786361784994
a_HQ_transcript/70407	-3.24264175474322	0.938251211080487	-4.0557637766988	0.000292039614268341	0.0690068239763827	0.339877178024812
a_HQ_transcript/122384	-3.64308905169363	1.10044634051138	-4.04713532367345	0.000299273396910127	0.0701488750962568	0.311887176305968
a_HQ_transcript/131462	1.45555625376116	3.31315568323478	4.04585858104827	0.00030035856699416	0.0701488750962568	0.321868598875602
a_HQ_transcript/142864	4.06531935927607	-0.139649813451738	4.04570047178071	0.00030049322011712	0.0701488750962568	0.334149632077939
a_HQ_transcript/145977	3.91455959507849	2.1772554177296	4.036634076395	0.000308314247035292	0.0715797016063967	0.307790408005952
a_HQ_transcript/23710	3.55359590381422	1.32853568104832	4.03389165214205	0.000310718980118969	0.0715797016063967	0.303523552263901
a_HQ_transcript/8000	2.15704008850404	2.51175953898311	4.03323813371782	0.000311294730278568	0.0715797016063967	0.301264154231003
a_HQ_transcript/3587	4.30679237686548	1.14584599019673	4.0329509826894	0.000311548040641659	0.0715797016063967	0.21308988866007
a_HQ_transcript/20607	-3.65156777815294	-0.101551968778051	-4.03018199262446	0.000314001082824917	0.0718592714269559	0.285764382756637
a_HQ_transcript/129702	-4.74388003832418	4.06768001591179	-4.02525664530822	0.000318411241552773	0.0725234742914417	0.229413757073707
a_HQ_transcript/116290	-3.3503540042829	0.601640809685978	-4.02416295078963	0.000319398730708249	0.0725234742914417	0.269279117287975
a_HQ_transcript/21522	3.90244181176469	1.05373390586419	4.02113347885152	0.000322149695049866	0.0728634921161814	0.251387100139694
a_HQ_transcript/136417	-3.43830183748354	-0.428262314214382	-4.01630760041048	0.000326579873242253	0.073579204929557	0.251035029526729
a_HQ_transcript/151706	0.851956398857999	10.7682292690346	4.01165466895967	0.00033090770685114	0.0742664215592396	-0.36834786780855
a_HQ_transcript/129792	-7.05783969516381	0.241866277032892	-4.00970893790015	0.000332734084287776	0.0743776593442397	0.233148409200745
a_HQ_transcript/104840	2.14978172163822	2.27342471435832	4.0084061706049	0.000333962446477542	0.0743776593442397	0.213834077653279
a_HQ_transcript/150824	-5.09025243936222	1.62737343351282	-4.0041431571401	0.000338013076555158	0.0748039753716713	0.227693533565686
a_HQ_transcript/14826	-2.68498356858231	2.50567782223808	-4.00368588440543	0.000338450411552945	0.0748039753716713	0.217323951425188
a_HQ_transcript/126548	2.75660618764068	-0.715021304056201	4.00042933845587	0.00034158101463888	0.0749869914930265	0.213995806040645
a_HQ_transcript/13199	4.30967535259728	1.42533272052827	4.00014206688035	0.000341858531957955	0.0749869914930265	0.219083382040274
a_HQ_transcript/137648	-5.92875313067018	2.47048387515265	-3.99713035709378	0.000344781287522024	0.0753437845153392	0.211781784243557
a_HQ_transcript/99697	3.45003753734103	2.57335201721565	3.99348647326523	0.000348350231121515	0.0758385851484322	0.182555259676702
a_HQ_transcript/92596	2.36526809844829	-0.736992740101095	3.99008329746001	0.000351716030995393	0.0760058439586748	0.186197361593474
a_HQ_transcript/8882	-3.8108322598135	-0.149787867818838	-3.98829340855018	0.000353499002578227	0.0760058439586748	0.171335095612866
a_HQ_transcript/76968	-3.7634268285807	0.595985166136834	-3.98754973113408	0.000354242402197044	0.0760058439586748	0.163279928425178
a_HQ_transcript/152723	3.14898222773579	2.24327771954958	3.986222534178	0.000355572903681175	0.0760058439586748	0.174550610105233
a_HQ_transcript/5728	3.07762034703847	0.0996769439171692	3.98532223668064	0.000356478223026302	0.0760058439586748	0.16886367078893
a_HQ_transcript/104441	-4.47288500946571	0.125278280299143	-3.98473376979907	0.000357071190004982	0.0760058439586748	0.178154612816948
a_HQ_transcript/28460	-3.06582362551113	3.69708598323494	-3.98354556479995	0.000358271422458658	0.0760058439586748	0.133275638546169
a_HQ_transcript/36469	3.08474552420516	-0.435034440019614	3.9801188102105	0.000361754998616162	0.0764657983984009	0.168596492949128
a_HQ_transcript/16056	-3.26189499929691	0.277272806741332	-3.97367275812677	0.000368397896133199	0.0775878003856182	0.150281927486462
a_HQ_transcript/56832	-5.41371131274763	1.62806150966244	-3.97006579603924	0.000372166880745469	0.0780986153212008	0.0545666680132957
a_HQ_transcript/52755	-7.72274819641465	0.17124997889033	-3.96820560447067	0.000374125335293706	0.0782271852156565	0.138642173922533
a_HQ_transcript/126004	3.71754084031989	1.55998723997273	3.96389261815798	0.000378704983639095	0.0788801027387224	0.12808063408095
a_HQ_transcript/27376	-4.30875895190935	2.6456179670852	-3.96271774749169	0.000379961959242401	0.0788801027387224	0.124320039311132
a_HQ_transcript/114608	2.71052874596244	0.2640813502785	3.95766895571863	0.000385410145851077	0.0796624724899709	0.0891612180668826
a_HQ_transcript/62554	1.41556189801509	3.63245379741918	3.95553830743973	0.000387732180628283	0.0796624724899709	0.0794070490788306
a_HQ_transcript/133941	-2.48683690089642	3.72042937018381	-3.95543784958337	0.000387841998944773	0.0796624724899709	0.0758186811941446
a_HQ_transcript/124841	-2.1812129567332	-0.839701320761343	-3.94639709238858	0.000397850589003608	0.0811577467345484	0.0787985180870026
a_HQ_transcript/50793	-4.31049632422824	1.66706734126596	-3.94614734341848	0.000398130625301011	0.0811577467345484	0.0838782051158056
a_HQ_transcript/150932	-3.6121613490904	-0.268361630494411	-3.94509703844628	0.000399310410922118	0.0811577467345484	0.0792164151667496
a_HQ_transcript/65457	2.77749986771477	2.67765197919184	3.94332382274229	0.000401309981373802	0.0811585403343991	0.0713324341051464
a_HQ_transcript/117467	-2.26162808525292	-0.833472074669643	-3.94252000544609	0.000402219626246661	0.0811585403343991	0.0581056052263067
a_HQ_transcript/21707	2.25796239240446	0.553529294652608	3.94138901732329	0.000403502927275002	0.0811585403343991	0.0705768104969886
a_HQ_transcript/22417	-2.34528726875245	3.73275341672905	-3.93958194472921	0.00040556166686038	0.081291339900897	0.0438406723918634
a_HQ_transcript/21471	4.97468919083753	0.716220666338817	3.93597211819564	0.000409704988275431	0.0816961576127168	0.0482382151900449
a_HQ_transcript/145676	4.19328625544575	-0.133950962721917	3.93537683798754	0.000410392203807344	0.0816961576127168	0.0559067292819329
a_HQ_transcript/1052	1.47692980638066	4.00163046838214	3.93198766147069	0.000414326308390426	0.0820729043105854	-0.0368977091004297
a_HQ_transcript/91220	-3.84934117087828	-0.221936794681573	-3.93131745818053	0.00041510862006799	0.0820729043105854	0.0448549020918669

a_HQ_transcript/28712	2.2696068120622	3.8552641319891	3.92893636444501	0.000417899694614259	0.0823446557577547	-0.038114493118897
a_HQ_transcript/8567	-2.27021304326295	1.68378309317158	-3.9274113391427	0.000419696919121178	0.0824194003874183	0.00517896231059822
a_HQ_transcript/74898	-2.22490217936122	3.83830972112154	-3.92237073827115	0.000425691066476664	0.0833150515560792	-0.0208211348490277
a_HQ_transcript/44082	4.3373795124615	0.700849288515537	3.92072532616306	0.000427665780385792	0.0834206593364608	0.0205612704069829
a_HQ_transcript/152451	-5.85089339969718	0.434744359286356	-3.91736168366549	0.000431730465909012	0.0838035662344735	0.0114859889785253
a_HQ_transcript/93333	-3.53941317572038	1.22421044612566	-3.91671831251587	0.000432512212192782	0.0838035662344735	-0.0293932496332854
a_HQ_transcript/52685	-2.59390646812844	-0.469953048440234	-3.91246534728646	0.000437714764885452	0.084192770667783	-0.0104766248536299
a_HQ_transcript/47652	-2.92814193339152	0.0729726134505664	-3.91110108923439	0.000439396532355016	0.084192770667783	-0.0297896480707518
a_HQ_transcript/22643	-2.02048384031049	3.48314055182867	-3.90953332555364	0.000441336956805777	0.084192770667783	-0.0323195847073778
a_HQ_transcript/59117	5.00124976463873	0.574188424343755	3.90942014120446	0.000441477368554654	0.084192770667783	-0.0095698216158393
a_HQ_transcript/119900	5.81028766942757	2.81746082181614	3.90919006914467	0.000441762920686654	0.084192770667783	-0.0845342987589879
a_HQ_transcript/49067	2.04490108850766	1.23465829233036	3.90354839882004	0.000448821710219696	0.0851656209967908	-0.08139772051815
a_HQ_transcript/68700	-3.12207186986105	-0.377677852469744	-3.90177268566718	0.000451066147723353	0.0851656209967908	-0.0360411994272356
a_HQ_transcript/44556	2.05668465535247	5.47181680254108	3.90161741483947	0.000451262924356792	0.0851656209967908	-0.198663263798751
a_HQ_transcript/136238	4.13432577455586	0.234789408220427	3.89466504053456	0.000460160114625843	0.0865637124367993	-0.0550474640002303
a_HQ_transcript/42415	2.20731122928431	1.91844245942307	3.89103530813453	0.000464872974176195	0.0865752166437751	-0.0661068512637755
a_HQ_transcript/152182	-6.77265826331044	1.77115484432246	-3.88930974104963	0.000467129939124503	0.0865752166437751	-0.0708815688780291
a_HQ_transcript/146707	1.11639956912543	8.51780477214996	3.88896065263435	0.000467587829445053	0.0865752166437751	-0.461241123274176
a_HQ_transcript/129977	-2.36442180423946	-0.827378549294857	-3.88765090953121	0.000469309692013103	0.0865752166437751	-0.0644933143849293
a_HQ_transcript/74003	6.10216933546962	4.11762013979938	3.88758047453062	0.000469402464902451	0.0865752166437751	-0.100583066960418
a_HQ_transcript/282	4.41426918197432	0.550933128355009	3.8869029056608	0.000470295833489728	0.0865752166437751	-0.0738252771848646
a_HQ_transcript/89636	-3.32231060352478	1.53720038327049	-3.88663691432334	0.000470646993865829	0.0865752166437751	-0.0651624358817608
a_HQ_transcript/27576	4.27458670381266	1.06493024577057	3.87996319637538	0.000479541784084458	0.0879331382500359	-0.124937121914929
a_HQ_transcript/132211	7.61478741502845	5.0415786072604	3.87830062218549	0.000481783061509986	0.088066307545448	-0.0894950421021008
a_HQ_transcript/105983	-4.26878923955909	0.339101881386242	-3.87585817448735	0.00048509420611379	0.0883935925171862	-0.109340816656579
a_HQ_transcript/147589	-2.87438214532633	-0.856273540432773	-3.87470607042071	0.000486663767501606	0.0884024733666667	-0.0947021787536437
a_HQ_transcript/10069	3.77651555361982	3.65630109042618	3.87069824211645	0.000492162477153861	0.0889354560595706	-0.147438554324561
a_HQ_transcript/63058	-4.11401661239011	0.483199468118627	-3.8703393109318	0.000492657873162361	0.0889354560595706	-0.122574054601865
a_HQ_transcript/10245	-2.99073760255144	3.97149335441607	-3.86290618684475	0.000503027023814363	0.0902703799567278	-0.170977477896423
a_HQ_transcript/102370	-3.28685362482443	-0.843227935667024	-3.86165013366522	0.000504800095925771	0.0902703799567278	-0.126420555486964
a_HQ_transcript/143812	-2.98917131611592	-0.160976892016935	-3.86043774565317	0.000506517306817583	0.0902703799567278	-0.140587032310052
a_HQ_transcript/67861	3.97687607473084	2.13308215136444	3.85876870137945	0.000508890637829399	0.0902703799567278	-0.147153948088053
a_HQ_transcript/78693	3.87628451052371	8.58323559268003	3.85860060773073	0.000509130262016492	0.0902703799567278	-0.51445141331904
a_HQ_transcript/107447	-3.33290049429968	0.0274407726346091	-3.85843220663891	0.000509370434658112	0.0902703799567278	-0.134501352529186
a_HQ_transcript/60343	4.14118556289292	4.98139121378699	3.85303644181972	0.000517124567022396	0.0912531763448423	-0.316989891077683
a_HQ_transcript/4675	-1.50735211663415	2.65260333575863	-3.8523937404632	0.000518055811206268	0.0912531763448423	-0.149536336239749
a_HQ_transcript/150829	0.656450077485071	10.3033934007669	3.84827547241611	0.000524061894183223	0.0915677297294584	-0.758716569949617
a_HQ_transcript/144545	-4.7924838157678	-0.0163704087983611	-3.8477226872798	0.000524873223608643	0.0915677297294584	-0.161672758510441
a_HQ_transcript/51365	4.71748359451937	0.250680146672831	3.84734747646341	0.000525424622268309	0.0915677297294584	-0.164147311682159
a_HQ_transcript/105824	-2.915773332814	-0.854419860356411	-3.84556008020975	0.000528059085096156	0.0915677297294584	-0.166239777215607
a_HQ_transcript/118139	3.77359669534494	0.211863343642185	3.84500120868799	0.000528885450086104	0.0915677297294584	-0.169176134208104
a_HQ_transcript/135476	-3.30562160679096	-0.842346989646206	-3.84472573647841	0.000529293235430396	0.0915677297294584	-0.168023085027741
a_HQ_transcript/147293	-3.18715220461403	-0.290458089692357	-3.84357299831954	0.000531002975501715	0.0915909227298626	-0.171472084972772
a_HQ_transcript/38696	-5.97963433014485	0.59449512282276	-3.84085487139425	0.000535055830094819	0.0918513944878184	-0.17765569089845
a_HQ_transcript/142404	0.891509978147021	10.3807776381082	3.8402958859387	0.00053589303197651	0.0918513944878184	-0.783792404860847
a_HQ_transcript/77974	-3.15392499772727	-0.193595045697913	-3.83938930599731	0.000537253546068302	0.0918513944878184	-0.193199915221312
a_HQ_transcript/145088	0.84651043775358	10.3232301432275	3.83611103796003	0.000542201419044956	0.0922901543025057	-0.797110449269111
a_HQ_transcript/110157	-3.51528941582544	0.731631613389661	-3.83464770186557	0.000544424328059851	0.0922901543025057	-0.198284265949311
a_HQ_transcript/107157	-2.93241923188167	-0.853692027618665	-3.83454344513644	0.000544583039598119	0.0922901543025057	-0.193245829229606
a_HQ_transcript/15174	-3.64875936231632	0.0672512210231763	-3.8320863602087	0.000548336570391436	0.0926561283828878	-0.199412494333226

a_HQ_transcript/109664	-4.07933882649793	0.530104149394459	-3.82986724680743	0.000551748215975674	0.0928923721325414	-0.214533451718023
a_HQ_transcript/146673	-3.20146945213297	-0.783467619293198	-3.8291011589615	0.000552930786503222	0.0928923721325414	-0.20645938704279
a_HQ_transcript/117321	-2.61061093757365	-0.808857481850397	-3.82573264090143	0.000558159951995371	0.0933426364296968	-0.215541232663407
a_HQ_transcript/41046	-2.86482230477943	1.25131219464718	-3.82530800183329	0.000558822555008507	0.0933426364296968	-0.228015588755134
a_HQ_transcript/101523	5.64946324061	2.06379977499674	3.8183496133606	0.000569790109565625	0.0949018896528099	-0.265129636866821
a_HQ_transcript/127656	2.4898524139544	0.593975046542683	3.81510514546664	0.000574975295362908	0.0954918970538717	-0.253838807384277
a_HQ_transcript/73511	-2.95806486151259	-0.297244553124765	-3.8107117947727	0.000582069861134934	0.0963947489688075	-0.254107478557435
a_HQ_transcript/152697	-2.77078790442995	-0.783975506009343	-3.80615727514102	0.000589514638296838	0.097350303678746	-0.263059737198398
a_HQ_transcript/130338	-2.53658355907978	-0.550121236179938	-3.80512560272511	0.000591213832056087	0.0973543275630488	-0.267259120454206
a_HQ_transcript/23328	-3.38094317512065	0.132345250149798	-3.80042594055538	0.000599014818943363	0.0983602638292086	-0.277622033177522
a_HQ_transcript/96466	-3.0833803794998	1.83368795991826	-3.7953864592037	0.000607491171222124	0.0993440751635547	-0.296930651874434
a_HQ_transcript/134030	2.99121076863189	-0.572066308817819	3.79483583820563	0.000608424352433001	0.0993440751635547	-0.29045476535194
a_HQ_transcript/63998	4.88722057450985	1.14566168328468	3.79008033316387	0.000616542142827166	0.0998335159739442	-0.320156407125761
a_HQ_transcript/136987	-3.37736973840957	-0.839168363971427	-3.78932147050384	0.000617847256712181	0.0998335159739442	-0.303861457785558
a_HQ_transcript/106154	4.42670493775974	1.96703132788347	3.7886565137874	0.000618993077725961	0.0998335159739442	-0.305488978830774
a_HQ_transcript/73927	4.17673146164179	1.51525980334215	3.78861797464975	0.000619059549835937	0.0998335159739442	-0.30873579400266
a_HQ_transcript/125827	-4.65588494795527	-0.0278851597265993	-3.78772943927649	0.000620594018440406	0.0998335159739442	-0.344488020074579
a_HQ_transcript/152129	0.62686940862507	11.2125170496601	3.78697574934537	0.000621898513937986	0.0998335159739442	-1.03009542866913
a_HQ_transcript/55367	-0.849776944925429	4.94534130083143	-3.78608467843922	0.000623444231670482	0.0998335159739442	-0.455011166673324
a_HQ_transcript/2629	3.78233014775313	0.81803563681344	3.78306679655358	0.00062870708800707	0.100156452283662	-0.319747309250194
a_HQ_transcript/115872	-4.34908144018792	0.501012549243406	-3.78284013799685	0.000629104095945796	0.100156452283662	-0.320434407595071
a_HQ_transcript/141853	3.83333453521024	-0.688145721857639	3.78151782904787	0.000631425076286338	0.100156452283662	-0.322983560311808
a_HQ_transcript/30736	-4.07852866156078	-0.304449735904779	-3.78099046748349	0.000632353048240157	0.100156452283662	-0.324250076852414
a_HQ_transcript/125574	-5.78891043481916	0.766003979127754	-3.7787094981857	0.000636382056924097	0.100520696208924	-0.334822159643293
a_HQ_transcript/151987	0.749564825482033	11.1700226035346	3.77561131274048	0.00064189457324479	0.100850508145667	-1.05458573182473
a_HQ_transcript/125606	2.84236761282091	-0.0217864854050835	3.77528922567324	0.000642470311849999	0.100850508145667	-0.33830599108554
a_HQ_transcript/65289	-2.29082055553192	-0.822989536888153	-3.77403918342875	0.000644709554123286	0.100850508145667	-0.342251554811019
a_HQ_transcript/62878	3.83782128467946	1.12579201267168	3.77364906405851	0.000645409940651464	0.100850508145667	-0.365528837530895
a_HQ_transcript/73426	-2.83894967419667	1.38941478634058	-3.7712715759382	0.000649694299956516	0.101247802326736	-0.365042775804548
a_HQ_transcript/78892	2.93580725839321	-0.626200737953787	3.76644439598607	0.000658478383284726	0.10234233011651	-0.359960845880074
a_HQ_transcript/143741	-2.7822930550188	-0.707387567842847	-3.76540335960333	0.000660387853095821	0.102365400332677	-0.36238134821754
a_HQ_transcript/34861	-6.50153149821921	1.09568562163505	-3.76432553828705	0.000662370466059298	0.10239965545504	-0.364920471819263
a_HQ_transcript/127245	-4.20199575121248	0.431905619334908	-3.76019557632596	0.000670021090243578	0.102613906401964	-0.375930630669388
a_HQ_transcript/35758	-2.59837870409065	0.853972297568522	-3.75970715541637	0.000670931535423358	0.102613906401964	-0.379467403402839
a_HQ_transcript/15477	-3.19275093283166	1.39023231298026	-3.75935374394951	0.000671591064942582	0.102613906401964	-0.404642883409275
a_HQ_transcript/132735	-2.87092832472289	0.566691010200148	-3.7593008691747	0.000671689792991579	0.102613906401964	-0.398826435313429
a_HQ_transcript/104572	-1.35081630688582	-0.793241695694724	-3.75805875842879	0.000674013131520415	0.102613906401964	-0.380211528160252
a_HQ_transcript/120577	2.85721460988398	-0.376502778528321	3.75787996584756	0.000674348201306605	0.102613906401964	-0.388176722830086
a_HQ_transcript/132155	-2.86083504114889	-0.446660281614639	-3.75661061879009	0.000676731706289167	0.102707730086623	-0.383751340713268
a_HQ_transcript/53393	-4.40919708735109	0.652892160473604	-3.75421852446554	0.000681245717155227	0.103123570434372	-0.407442582423474
a_HQ_transcript/145412	-3.78513663692748	0.00743542128710528	-3.74924096383928	0.000690732646033978	0.104288070775748	-0.401662302344127
a_HQ_transcript/116979	-4.17533088892531	2.15564938001401	-3.7476101407526	0.000693868729262475	0.104418074675027	-0.419837343543171
a_HQ_transcript/146521	-3.93286350975823	0.159553980896696	-3.7468295330882	0.000695374735417308	0.104418074675027	-0.415271553460705
a_HQ_transcript/27637	-3.53640162243531	0.926467775906919	-3.74599786579845	0.000696982744527774	0.104418074675027	-0.418097930359539
a_HQ_transcript/8157	6.03068874754584	1.26644425548877	3.7347058749133	0.00071917637738493	0.107466026901366	-0.438601206390919
a_HQ_transcript/80707	4.07786051922714	0.362855490134841	3.73364557740037	0.000721295235255003	0.107506280602315	-0.439965890355224
a_HQ_transcript/149411	-1.66537004729354	6.63209692462184	-3.73262521074719	0.000723340022247661	0.107535316657831	-0.711830503628022
a_HQ_transcript/86339	5.62243127557712	6.75112138855578	3.73055727921186	0.000727501385327242	0.107878062567097	-0.661301873915437
a_HQ_transcript/145269	1.31692567730893	5.49627656868691	3.72939228611022	0.000729855968193739	0.107951826257419	-0.665880491095644
a_HQ_transcript/46806	-4.58871086635699	0.113185752005185	-3.72702960118315	0.000734653972020384	0.108385700724875	-0.455614228360657

a_HQ_transcript/150765	-2.14872205343547	2.95595375052447	-3.72374448754516	0.00074137613231167	0.108584541335791	-0.497721391903966
a_HQ_transcript/19176	-4.3793533676773	2.82646480988638	-3.72205987954125	0.000744846391874246	0.108584541335791	-0.470613548480046
a_HQ_transcript/36505	-2.89520546348406	-0.495265775267423	-3.72201862579447	0.000744931571360013	0.108584541335791	-0.467809900852084
a_HQ_transcript/132231	4.60790007010836	0.792164466409801	3.72183627879103	0.000745308189255024	0.108584541335791	-0.470027589192674
a_HQ_transcript/27964	-2.5206664709994	-0.811177587767155	-3.721807015214	0.000745368647205326	0.108584541335791	-0.468330732644962
a_HQ_transcript/103797	-3.53811919523628	-0.414048171016027	-3.7209168968425	0.000747209890832584	0.108584541335791	-0.470877150812029
a_HQ_transcript/390	1.39847755947125	4.06781683669398	3.71814995034712	0.000752961672963746	0.108816776982855	-0.5954229986407
a_HQ_transcript/148954	1.70876233823786	8.55605777873445	3.71788974860857	0.000753504772899236	0.108816776982855	-0.913704925995853
a_HQ_transcript/136490	5.99428703523685	1.20715705353058	3.71744973377923	0.000754424049065691	0.108816776982855	-0.481428068411677
a_HQ_transcript/152136	0.739396177960361	10.7735831134425	3.71519124009857	0.000759159645561341	0.108865904459463	-1.16773191609431
a_HQ_transcript/110145	-3.59077516533685	-0.0345427820034334	-3.71416083222909	0.000761329774706735	0.108865904459463	-0.489355417607736
a_HQ_transcript/149373	1.76092270954401	4.973460659752	3.714065623996	0.000761530595279123	0.108865904459463	-0.674306959580631
a_HQ_transcript/137077	3.07054252384772	-0.630723316132006	3.71372186373765	0.000762256109190086	0.108865904459463	-0.487855608443013
a_HQ_transcript/141724	-2.43309197012294	1.42201509332357	-3.71110828169439	0.000767794123266578	0.109388080385391	-0.52916677438938
a_HQ_transcript/105479	-2.62462755665628	-0.242657667434745	-3.69401795892115	0.000804982159368576	0.114405875207278	-0.538784701151178
a_HQ_transcript/152607	-4.34519664139851	0.636220246978371	-3.69294920211645	0.00080736492966749	0.114459177102304	-0.539679188408856
a_HQ_transcript/41324	2.46373703013599	3.25551108324599	3.69208556915335	0.000809295378974795	0.114459177102304	-0.600409205259705
a_HQ_transcript/119403	-2.39874732035929	-0.44293288142643	-3.68796951843374	0.000818557552495061	0.115488139348138	-0.550523137320822
a_HQ_transcript/119272	-1.64293440977039	6.42720454116305	-3.6841805728065	0.000827174506121918	0.11588017124521	-0.850053153830348
a_HQ_transcript/49941	-2.18145457998612	2.68230468178607	-3.68354874295738	0.00082861997037626	0.11588017124521	-0.564245358396715
a_HQ_transcript/33959	-1.06396029684642	3.58230532132369	-3.68326349203859	0.000829273353680036	0.11588017124521	-0.619218330859737
a_HQ_transcript/128116	3.08242022008582	0.0177255591145662	3.68324735279367	0.000829310336464478	0.11588017124521	-0.565829011507102
a_HQ_transcript/150229	-7.07832194314594	0.382481134639897	-3.67874587198682	0.000839688092003575	0.117048895472383	-0.57311322191166
a_HQ_transcript/13607	5.51103756519061	5.86712876239904	3.67248752573398	0.000854325668397459	0.118804407781358	-0.755510957867906
a_HQ_transcript/84886	1.76382810946367	2.40877157509551	3.67096171524872	0.00085793171586943	0.11890593637896	-0.591070714563331
a_HQ_transcript/104197	3.91266195861366	0.0758592754364378	3.66992308039765	0.000860394827848496	0.11890593637896	-0.598529210140407
a_HQ_transcript/66184	2.18460416497285	-0.657415499671533	3.66958732788426	0.000861192527104704	0.11890593637896	-0.595598324366162
a_HQ_transcript/150570	0.671673783794248	10.5286636010698	3.66823662213807	0.000864408859530621	0.119067199494777	-1.26139369105982
a_HQ_transcript/3045	-1.63863431061533	5.25502632641612	-3.66508105083133	0.000871968436107324	0.119493517081315	-0.804360913323672
a_HQ_transcript/132534	1.51624918566018	2.70654430747397	3.66425866314036	0.000873949076719647	0.119493517081315	-0.637872673569938
a_HQ_transcript/13166	-2.00467812358122	2.6928299341246	-3.66408419609293	0.000874369824071402	0.119493517081315	-0.607545117999923
a_HQ_transcript/138567	4.22409929436494	2.42247489606467	3.66352213184169	0.00087572664252409	0.119493517081315	-0.612144576493194
a_HQ_transcript/146783	-2.91616755233618	-0.396779107413189	-3.66228860235939	0.000878711531828658	0.119620009185331	-0.612437508894739
a_HQ_transcript/61731	-7.21061233878461	3.89283626321406	-3.66072842842049	0.000882500963018334	0.119855177519462	-0.66026022587993
a_HQ_transcript/67273	-2.5895724245924	-0.244480737682176	-3.6571611254224	0.000891225028923577	0.120757875247715	-0.624154039617851
a_HQ_transcript/109916	1.8046129093779	2.05587840600166	3.6550814364366	0.000896349552983008	0.121077825435886	-0.6318949290439
a_HQ_transcript/103666	2.88121242087561	-0.490423080293469	3.6545141880133	0.000897752249567623	0.121077825435886	-0.630514092889351
a_HQ_transcript/150710	0.715605938390068	10.680696450313	3.65153563339423	0.000905152629727202	0.121793314955516	-1.32916020379632
a_HQ_transcript/39711	2.98189210211639	1.5636834604793	3.65063327036573	0.000907406244323652	0.121814573140982	-0.642577284952202
a_HQ_transcript/90500	2.43879314108202	2.34105549149531	3.64812430246328	0.000913700903992649	0.122070927540995	-0.650455325919471
a_HQ_transcript/150524	0.826099527257316	9.96042091396823	3.64744935924038	0.000915401454612988	0.122070927540995	-1.26106809534948
a_HQ_transcript/62242	1.03499449953811	5.43081131637773	3.64665742591148	0.0009174006778156	0.122070927540995	-0.875559999032098
a_HQ_transcript/89358	-3.50945935381593	3.89844921640393	-3.64653267515546	0.000917715994622465	0.122070927540995	-0.675986737986567
a_HQ_transcript/8129	-4.81090041809514	0.191409109266186	-3.64477952232496	0.000922158341917175	0.122235099260591	-0.654395798960206
a_HQ_transcript/151438	1.02148933419213	5.62455495411025	3.64438694930954	0.000923155941635689	0.122235099260591	-0.895498078496637
a_HQ_transcript/150751	0.535248808353164	11.3713790172458	3.64245036794654	0.000928092470673511	0.12260945258025	-1.43386414293948
a_HQ_transcript/58332	-2.51662549263797	2.19800681053743	-3.64030229671114	0.000933598026245082	0.122914966854636	-0.678427713144779
a_HQ_transcript/41587	-2.15702617436556	-0.55820472020299	-3.63989940088607	0.000934634175436091	0.122914966854636	-0.668712033596973
a_HQ_transcript/27985	-2.48796093781635	3.41179596508746	-3.63526625862691	0.00094662983622823	0.124211510429514	-0.7437580966148
a_HQ_transcript/88888	-4.25971957398247	6.26972418304058	-3.63110732855429	0.000957524632953846	0.125358089784552	-0.9686357892912

a_HQ_transcript/44489	3.77227104788201	1.19342613386663	3.62857029478611	0.00096423021971074	0.125855902085187	-0.696207677085683
a_HQ_transcript/152928	5.15135408415528	2.91015857689139	3.62803253895251	0.000965657382500571	0.125855902085187	-0.753094867931051
a_HQ_transcript/66998	6.17346176940252	0.0416359269929084	3.62532813709665	0.000972865727064542	0.126431819558215	-0.71442013691441
a_HQ_transcript/151009	5.18964298011588	8.82301547187735	3.62462294529953	0.000974753902078361	0.126431819558215	-1.1385161061376
a_HQ_transcript/135253	-3.27603584765983	-0.129419206022182	-3.62393421954236	0.00097660141380468	0.126431819558215	-0.705158345285785
a_HQ_transcript/785	-5.7989747035653	1.80738442471296	-3.62306394562609	0.000978940779571474	0.126453043633179	-0.749061764663645
a_HQ_transcript/67052	-2.16630983054483	1.92013515961653	-3.62133311327018	0.00098360953308451	0.126774401195424	-0.710063714962491
a_HQ_transcript/2725	-4.80559419196	0.861887794305561	-3.61794062134188	0.000992823073397984	0.127402411161007	-0.73861118465901
a_HQ_transcript/80069	3.77708999055017	0.400450957123498	3.61792502936628	0.000992865611339389	0.127402411161007	-0.719182665575248
a_HQ_transcript/47491	-2.45346693266753	2.94248126632885	-3.61688185429263	0.000995715607509694	0.127486689060184	-0.742625346945993
a_HQ_transcript/114676	1.07729120025678	2.85925782790038	3.61444691187525	0.00100239882053745	0.128022342519918	-0.755907384008133
a_HQ_transcript/72030	4.43959031226858	0.210780030500611	3.61329444792018	0.00100557712158865	0.128022342519918	-0.734555122061024
a_HQ_transcript/151422	3.58323216775567	-0.0859254712241455	3.61295811369817	0.00100650651203555	0.128022342519918	-0.730183343349993
a_HQ_transcript/141252	-2.10311436321969	-0.829189796537663	-3.61067023331911	0.00101285070068026	0.128548003338738	-0.735367239295002
a_HQ_transcript/130186	-5.47427569687125	4.94882128812722	-3.60880362939622	0.00101805536479035	0.128674502773835	-0.926163393887762
a_HQ_transcript/132595	3.37188202685997	-0.531118096860999	3.60616038723713	0.00102546983851467	0.128674502773835	-0.746391068092611
a_HQ_transcript/28365	2.96055435001976	-0.171940647580763	3.60611062135383	0.00102560993521822	0.128674502773835	-0.748993446554524
a_HQ_transcript/94048	-2.50161079052288	-0.767114739607287	-3.60577933796233	0.00102654300837299	0.128674502773835	-0.747052198513734
a_HQ_transcript/131778	-2.83989512014638	1.41413934590402	-3.60576658464054	0.00102657894504017	0.128674502773835	-0.747571144068781
a_HQ_transcript/90347	1.62799302836861	3.03391787008253	3.60452471018263	0.00103008417146924	0.128674502773835	-0.773567434289964
a_HQ_transcript/117954	3.02174692298914	1.57715281039298	3.6044809806424	0.00103020781022695	0.128674502773835	-0.763474094554504
a_HQ_transcript/27727	-5.25014955472523	0.450450434423831	-3.60400428238147	0.00103155653544948	0.128674502773835	-0.764347204227121
a_HQ_transcript/101094	4.82272812545543	-0.104853626551314	3.6008380039398	0.00104055837105117	0.129285345369229	-0.758836197834118
a_HQ_transcript/140160	-2.35802214585395	3.39591868965944	-3.60015090410136	0.00104252183242513	0.129285345369229	-0.815546661846674
a_HQ_transcript/31905	2.47513790461131	3.93115491966586	3.59963497001887	0.00104399852545849	0.129285345369229	-0.83821299692661
a_HQ_transcript/114684	-1.25612244979398	-0.798920394961093	-3.59916335872098	0.00104535012943053	0.129285345369229	-0.764700761159868
a_HQ_transcript/25770	-2.80763465237297	-0.404238487919126	-3.59683264816157	0.00105205467600317	0.129838289186225	-0.769795915125171
a_HQ_transcript/54209	-3.81163379724989	0.0814943145698933	-3.59532328594074	0.00105641869892391	0.130100648582731	-0.772540825751174
a_HQ_transcript/24480	2.89861080260815	-0.321265189671818	3.59452598916002	0.00105873098912149	0.130109756734997	-0.778486923014658
a_HQ_transcript/136253	2.25091864361485	-0.637015448373451	3.59159056093927	0.00106728648091756	0.130623801859406	-0.785407690381936
a_HQ_transcript/124802	3.04285354390908	1.8020535602303	3.59154895106042	0.00106740823498517	0.130623801859406	-0.786452913395498
a_HQ_transcript/134104	1.84791511807044	2.83451563242037	3.58733462499445	0.00107980955454434	0.131665588850221	-0.845858214657795
a_HQ_transcript/142181	4.00843031346956	2.21390146541602	3.58711774356722	0.00108045151874407	0.131665588850221	-0.80629208257321
a_HQ_transcript/121219	-3.44106611754518	0.107021100012403	-3.58423480880727	0.00108902008724384	0.132432133119896	-0.804921658940222
a_HQ_transcript/86597	2.75387666871721	0.197455234146169	3.58275465198761	0.00109344485957462	0.132692615443327	-0.80605702487152
a_HQ_transcript/25039	-2.39118192790552	2.8003185065826	-3.58095297749448	0.00109885424037698	0.1329255693987	-0.872592285558216
a_HQ_transcript/131848	0.630511365826169	10.5618012800022	3.58059303074689	0.00109993804845814	0.1329255693987	-1.49936793149194
a_HQ_transcript/145294	-2.58213752190583	-0.80336018614418	-3.57691252616759	0.00111107962365068	0.133993436438935	-0.816149075507881
a_HQ_transcript/60101	3.02882562792324	1.76004723917794	3.57551723625995	0.00111533188871501	0.134127222837839	-0.852875941108077
a_HQ_transcript/123935	3.52385760843854	-0.155568609390557	3.57371546553235	0.00112084622272593	0.134127222837839	-0.823426188379187
a_HQ_transcript/130774	3.10606288367925	-0.187970577702	3.57368168840544	0.00112094984918844	0.134127222837839	-0.823633547457749
a_HQ_transcript/6918	3.09741921277558	0.524504621955279	3.57282195323159	0.00112359059242196	0.134127222837839	-0.834755388932129
a_HQ_transcript/135092	-5.2687896801403	1.03380324910033	-3.57277785140346	0.00112372621666026	0.134127222837839	-0.887931879534561
a_HQ_transcript/38724	3.12401011508244	0.202118513605677	3.56903278124633	0.00113530116995645	0.135219450558827	-0.835736530490099
a_HQ_transcript/62244	-5.23600508860411	2.42503298418515	-3.56831606436163	0.0011375294406012	0.135219450558827	-0.838339704384072
a_HQ_transcript/89776	-2.25460633623529	-0.777803086525496	-3.56727937953327	0.00114075997221117	0.135326725846308	-0.84028624327183
a_HQ_transcript/23195	2.88166889676106	0.309653868986922	3.56601726828121	0.00114470495270347	0.135518145602336	-0.841941052589406
a_HQ_transcript/112125	-2.84336974534009	2.04058395893588	-3.5634072230656	0.00115290504513493	0.136211513137405	-0.847874312737213
a_HQ_transcript/79537	2.02790081834536	-0.750286793997967	3.56222131790782	0.00115664958645926	0.136376728522726	-0.850498765176466
a_HQ_transcript/142417	-5.00432275515418	1.13085156801539	-3.55897875203277	0.0011669482188538	0.137312481914035	-0.865950422062241

a_HQ_transcript/119510	-3.58164614753148	0.465451068305749	-3.5549734422114	0.00117979175869003	0.138109716413715	-0.869454646077104
a_HQ_transcript/94156	2.47803137249273	2.59141378263488	3.55485605844221	0.00118017021747528	0.138109716413715	-0.876817620514435
a_HQ_transcript/70540	-3.88007907030411	0.748268739203552	-3.5534867017719	0.00118459385625255	0.138109716413715	-0.881320028316821
a_HQ_transcript/78667	-2.22954756399094	-0.801397677904819	-3.5534073446716	0.00118485070596017	0.138109716413715	-0.8718513559934
a_HQ_transcript/145580	3.89415536655141	-0.161840114123825	3.55307859466853	0.00118591532195265	0.138109716413715	-0.874773983103193
a_HQ_transcript/117629	1.44405775664133	3.62016552498455	3.55180777622913	0.0011900394078984	0.138109716413715	-0.973930412912095
a_HQ_transcript/50900	1.67100630813078	3.51971714256878	3.55171063724204	0.00119035521475487	0.138109716413715	-0.948382725013328
a_HQ_transcript/40519	-4.70236067404676	0.92075146322517	-3.55096446786961	0.00119278377418746	0.138115807223045	-0.885265162532983
a_HQ_transcript/136102	1.95990261800064	1.7553412196784	3.54898937523812	0.00119923524095497	0.138229375988796	-0.88352397510378
a_HQ_transcript/61593	2.94910243610783	1.33548010133994	3.54811364143845	0.00120210652372726	0.138229375988796	-0.8855209880605
a_HQ_transcript/26059	-4.93799369670874	0.81431539840608	-3.54765542217519	0.00120361154018435	0.138229375988796	-0.888186615342041
a_HQ_transcript/129694	-3.22914234479515	1.09978628104281	-3.54640480257693	0.00120772845678268	0.138229375988796	-0.901608195089005
a_HQ_transcript/77040	3.24089476720299	0.272628737784213	3.5456594986258	0.00121018838941031	0.138229375988796	-0.914649269745247
a_HQ_transcript/57671	4.99222523930765	1.41657671200124	3.54521208674072	0.00121166743059418	0.138229375988796	-0.890377528986768
a_HQ_transcript/42818	2.23768972084129	1.73827308508658	3.54480603657556	0.00121301125071094	0.138229375988796	-0.895631099392987
a_HQ_transcript/92356	2.42113691896615	1.38883626638233	3.54429689223584	0.00121469829466511	0.138229375988796	-0.923286346854724
a_HQ_transcript/51325	-2.70930334176119	-0.780460582378798	-3.54349050239308	0.00121737489646959	0.138229375988796	-0.895388624370927
a_HQ_transcript/148033	7.27870166922405	3.58512982744703	3.54343939152328	0.00121754473758367	0.138229375988796	-1.07088139584236
a_HQ_transcript/108238	-3.57930950414481	-0.049194369924669	-3.54254912922017	0.001220506747728	0.138295548210396	-0.899678052732869
a_HQ_transcript/97980	-1.25857797900508	2.80902843530591	-3.54102585114518	0.00122559102769872	0.13832748089425	-0.909594034264435
a_HQ_transcript/150215	-9.41641707242703	2.32244581415272	-3.54070541562241	0.00122666315379762	0.13832748089425	-0.955875460929125
a_HQ_transcript/79008	3.49792629065579	0.164236437868298	3.53978878080888	0.00122973507161681	0.13832748089425	-0.914410000428179
a_HQ_transcript/98285	-2.87529227727346	4.24124410638125	-3.5396182519244	0.00123030738408904	0.13832748089425	-1.03514317370017
a_HQ_transcript/113882	-3.59408992151075	0.25541042766934	-3.53872299879667	0.00123331616824241	0.138398073798445	-0.907744128171609
a_HQ_transcript/100750	5.23902139559111	2.02213584194682	3.53756576800444	0.00123721594310859	0.138568185628162	-0.973181899407031
a_HQ_transcript/128440	-2.45841301314066	-0.747222589592234	-3.53601710047538	0.00124245346426498	0.138887182636143	-0.912548314603608
a_HQ_transcript/31215	-3.13542140770181	0.788988908032018	-3.53454309012214	0.00124745838177148	0.139179003484861	-0.924685683091691
a_HQ_transcript/148575	1.11019520970757	9.42971850783569	3.53281171876	0.00125336201916024	0.139306664460568	-1.49883063298247
a_HQ_transcript/113357	-5.57833823767714	1.92609728154741	-3.53280186239724	0.00125339570452926	0.139306664460568	-0.969213599970992
a_HQ_transcript/150813	0.63415080926119	10.8167481202588	3.53184128721199	0.00125668278925628	0.139405452621926	-1.65883471969885
a_HQ_transcript/120219	-1.20512772630866	5.76607998767452	-3.53034971366891	0.00126180345115561	0.139706878111949	-1.18677089412072
a_HQ_transcript/39343	3.83483653322742	1.95521892494558	3.52842760051565	0.00126843189505462	0.140173781740941	-0.932498105347237
a_HQ_transcript/148839	1.39119799551962	8.56304991382983	3.52712579230547	0.00127294028618383	0.14040507202143	-1.42226521825554
a_HQ_transcript/147146	1.57011235788286	4.33677018276846	3.51994838445057	0.00129807601369938	0.142678212372054	-1.07545559052376
a_HQ_transcript/26454	2.90714366938172	0.571011436923702	3.51984031033291	0.00129845813282439	0.142678212372054	-0.953944443128849
a_HQ_transcript/86648	-2.33261500901155	4.42172033174396	-3.5170377135119	0.00130840530953422	0.143036026027683	-1.02962695471272
a_HQ_transcript/77900	5.2603328793497	1.30830507409233	3.51703552946737	0.00130841308990397	0.143036026027683	-0.978234020159331
a_HQ_transcript/115734	-2.39783615547058	-0.772152411138274	-3.51684371512153	0.00130909657732465	0.143036026027683	-0.957113291743652
a_HQ_transcript/82581	-7.87138710847607	1.52554660462942	-3.51105371161313	0.00132989089176605	0.14503545545324	-0.988877357319409
a_HQ_transcript/140339	-2.71633632173773	-0.419677953494129	-3.50728899288416	0.00134358216528637	0.14625420244151	-0.979286335087387
a_HQ_transcript/128752	1.85184461858623	6.6694006296886	3.50509331252034	0.00135162994493866	0.146738450372999	-1.30399408982228
a_HQ_transcript/87058	2.58060689609696	2.15647375102104	3.50469915068701	0.00135307957266597	0.146738450372999	-0.985431473735398
a_HQ_transcript/22328	-3.76369897075529	-0.273496053923391	-3.50383011270685	0.00135628097560965	0.146811732868227	-0.992409137887344
a_HQ_transcript/119756	3.41743595975683	1.12048140427902	3.50195375308171	0.00136321813589112	0.147106658039709	-0.992057325875643
a_HQ_transcript/2649	-2.84157574852409	0.186629385024458	-3.50172477574566	0.00136406703625453	0.147106658039709	-0.997378614816605
a_HQ_transcript/24444	-6.69891666786392	1.31573898059689	-3.49914360781486	0.00137367168256716	0.147868125119007	-1.02814424374034
a_HQ_transcript/14714	-4.01907215637335	2.83701256751713	-3.49758877928303	0.00137948871716066	0.148219815436442	-1.00440214955066
a_HQ_transcript/1649	5.17907128655606	1.24415853109078	3.49403865994692	0.00139285986103861	0.149380365318178	-1.02818683362083
a_HQ_transcript/152170	0.66527572770149	11.026655230374	3.49278002361734	0.00139763032245284	0.149548343091922	-1.78429410568002
a_HQ_transcript/13175	-3.64996734001361	0.930726973172367	-3.49126551905055	0.00140339145824135	0.149548343091922	-1.03826335709526

a_HQ_transcript/39087	3.23701944912501	0.692830761187934	3.49125231779301	0.00140344177600944	0.149548343091922	-1.01807953519086
a_HQ_transcript/49002	-2.20657971688332	2.42177039829324	-3.49091788008969	0.00140471709551661	0.149548343091922	-1.02395841145364
a_HQ_transcript/2888	-1.50740798368049	3.82445043066171	-3.48801484226136	0.00141583436879812	0.15014090069459	-1.12168853607332
a_HQ_transcript/28118	1.62650893369402	2.56538280058768	3.48671108290544	0.00142085471473874	0.15014090069459	-1.02889542939969
a_HQ_transcript/145598	-2.02324725687242	-0.765016816291138	-3.48668521289292	0.00142095450504485	0.15014090069459	-1.02773023055514
a_HQ_transcript/1498	3.77908118479521	1.183407233432	3.48600104032808	0.00142359606796487	0.15014090069459	-1.03714640029634
a_HQ_transcript/124412	-2.58078124034428	-0.152161195930278	-3.48551493077285	0.00142547579372317	0.15014090069459	-1.0431371690325
a_HQ_transcript/112744	3.08737763942636	-0.765340094775649	3.48543615311677	0.00142578064243417	0.15014090069459	-1.03423381762506
a_HQ_transcript/7580	2.03739225857392	3.46640596188252	3.48469273692712	0.00142866056348555	0.150164721436099	-1.07924608922677
a_HQ_transcript/129016	3.62320337189447	2.32696929419178	3.48258451803899	0.00143685812935644	0.150164721436099	-1.05236889390894
a_HQ_transcript/151047	2.96153173927275	-0.632760287316694	3.48251416338823	0.00143713247564029	0.150164721436099	-1.04093731925633
a_HQ_transcript/125801	-2.0618389891598	-0.839386169757834	-3.48190472101862	0.00143951109578105	0.150164721436099	-1.04043696081259
a_HQ_transcript/34210	-2.72410802720793	-0.79609940849901	-3.48160837040382	0.00144066910688541	0.150164721436099	-1.04298143431899
a_HQ_transcript/72976	4.35304271415124	2.53088100867085	3.48096722373383	0.00144317750677156	0.150164721436099	-1.07564401848408
a_HQ_transcript/76988	-3.51937858605159	-0.57682612645423	-3.48073418780107	0.0014440902711736	0.150164721436099	-1.04706412580703
a_HQ_transcript/14722	4.43889914015016	-0.190323011064037	3.47931392426469	0.00144966525778161	0.150235566854801	-1.05104675873853
a_HQ_transcript/88640	-1.67373641906792	2.42559997920461	-3.47824768248989	0.00145386422321827	0.150235566854801	-1.06963051800923
a_HQ_transcript/75725	3.78281553247123	1.61903168780119	3.47806174494192	0.00145459766300577	0.150235566854801	-1.05198800689725
a_HQ_transcript/122433	3.80457673176561	0.170739520017643	3.47723713341944	0.00145785468363618	0.150235566854801	-1.05831748442337
a_HQ_transcript/41003	-4.19473576104734	0.491918967442702	-3.47667707069478	0.00146007080594629	0.150235566854801	-1.05590673476734
a_HQ_transcript/10905	5.29944969344752	2.51504501521746	3.47662450819165	0.00146027895804023	0.150235566854801	-1.12862533344485
a_HQ_transcript/10835	3.51964218676173	0.747018475757346	3.47499974308323	0.0014667272933714	0.150394148972159	-1.0582736538506
a_HQ_transcript/88430	-1.48613356608467	5.63849689264028	-3.47493245139335	0.00146699495023421	0.150394148972159	-1.30474303948643
a_HQ_transcript/79313	1.85935777518009	1.18138117865304	3.47147331451857	0.00148081746210129	0.151543939149689	-1.07984829426852
a_HQ_transcript/67920	-2.59597375586269	-0.456034339028469	-3.47063683508401	0.00148417878199861	0.151620991634474	-1.0712750339632
a_HQ_transcript/39873	3.81621301661271	0.994087210862973	3.46833810590883	0.00149345393158567	0.152300859886336	-1.10539118858099
a_HQ_transcript/30722	4.15507283795338	0.0677963240828536	3.46652217138203	0.00150082051247313	0.152784053851206	-1.08970150718642
a_HQ_transcript/124897	3.31332213368617	-0.157388788960999	3.46514743423672	0.00150642058764723	0.153086041816011	-1.10320702311957
a_HQ_transcript/143582	2.75058875936099	0.909947546765524	3.46375889624947	0.00151209731407278	0.153384659149724	-1.08159064049736
a_HQ_transcript/40160	2.15460525573246	2.83732774537767	3.46313941510176	0.00151463656674824	0.153384659149724	-1.09685092650897
a_HQ_transcript/95376	-2.39483003237283	-0.388206485839146	-3.46129188033327	0.00152223402770922	0.153885947065533	-1.08851250963705
a_HQ_transcript/120787	6.62955283452865	6.39686225369395	3.45847479865288	0.00153388917902693	0.154794982983468	-1.34516892578189
a_HQ_transcript/108175	-2.94825079548911	-0.435001571710735	-3.45773705080386	0.00153695564753217	0.154835628907712	-1.09914940633469
a_HQ_transcript/147222	1.99841539831427	8.99195633281886	3.4564079463119	0.00154249501239524	0.155124827128911	-1.63333800809705
a_HQ_transcript/151442	0.794710421064198	10.1447130126896	3.45560092459701	0.00154586784768013	0.155195520293524	-1.78339449024154
a_HQ_transcript/113651	-3.16333101455717	0.288451348748444	-3.45480421634836	0.00154920454592177	0.155262348009208	-1.10485370876518
a_HQ_transcript/113921	-4.17221441509464	3.47050321599351	-3.45332076417852	0.00155543589023411	0.155618549789206	-1.15378591827769
a_HQ_transcript/86638	-2.90814360821531	2.67987187711438	-3.45205079333315	0.00156078966196277	0.155885878815416	-1.13545157152203
a_HQ_transcript/22666	-1.51888059040689	3.00401784469259	-3.45124357842764	0.00156420182914027	0.155958703129101	-1.13745249280273
a_HQ_transcript/9578	2.23688144934426	-0.745170907631604	3.44976576758947	0.00157046728519882	0.156247805816543	-1.11713111434305
a_HQ_transcript/15524	-4.55357677409206	0.257284407852112	-3.44923819940587	0.0015727098572376	0.156247805816543	-1.11893620293533
a_HQ_transcript/61604	-3.50287702987947	0.0276477821066253	-3.44826229704486	0.00157686632555724	0.156247805816543	-1.11662044250665
a_HQ_transcript/23908	-2.64412070490159	-0.749882024674045	-3.44789319643055	0.00157844111796631	0.156247805816543	-1.12244075402018
a_HQ_transcript/128712	6.89317215176125	4.85867424823625	3.44691886432402	0.00158260544885141	0.156247805816543	-1.34377858890783
a_HQ_transcript/151327	0.69166745265116	10.5062821237869	3.44677309437422	0.00158322938387599	0.156247805816543	-1.83749811939747
a_HQ_transcript/20832	3.33183222010002	-0.149320404693588	3.44495728950619	0.00159102139896507	0.156736421611854	-1.12461665811108
a_HQ_transcript/141290	0.927985196494556	9.03237978091885	3.44436448482946	0.00159357323789922	0.156736421611854	-1.69515062911977
a_HQ_transcript/113174	2.88038634176945	1.01369953973911	3.44287213673264	0.00160001478762111	0.156826094715777	-1.13916468366481
a_HQ_transcript/101901	4.4863601493857	0.844929220539708	3.442265426459	0.00160264074348413	0.156826094715777	-1.14508054704383
a_HQ_transcript/45990	4.40485350546357	2.21397948368985	3.44220125978671	0.0016029187113469	0.156826094715777	-1.1413071971825

a_HQ_transcript/43034	-3.93469826396681	1.1473880686608	-3.44165520491883	0.00160528608136441	0.156826094715777	-1.15605561670955
a_HQ_transcript/129041	-2.0541959378456	-0.352626353425384	-3.43983212742682	0.00161321426474602	0.156826094715777	-1.13611240876507
a_HQ_transcript/40904	3.71952192006957	1.7619805837851	3.43934205973183	0.00161535188613416	0.156826094715777	-1.13725252033857
a_HQ_transcript/88771	-1.84107198560949	2.24351240260759	-3.43925797503506	0.00161571892825663	0.156826094715777	-1.14567049273549
a_HQ_transcript/116250	2.04195842632761	-0.637885894301649	3.43917790586426	0.0016160685166314	0.156826094715777	-1.13925071492526
a_HQ_transcript/108054	-0.898891600505511	3.79559736911045	-3.43763074524713	0.0016228378554716	0.157220531438088	-1.25620300913954
a_HQ_transcript/67746	4.11545105588792	1.32171292960279	3.435512880912	0.00163214847452369	0.157718760177818	-1.1464402516496
a_HQ_transcript/144479	1.80529851656063	5.90073901666059	3.43522745467121	0.00163340719837335	0.157718760177818	-1.45684421279352
a_HQ_transcript/29039	4.13196861788465	1.37198573853799	3.43383399654007	0.00163956574591364	0.15805087508867	-1.15185682856816
a_HQ_transcript/115585	-2.57337139416362	-0.408680587076914	-3.43301321024784	0.00164320375617014	0.158122639202121	-1.15745648909935
a_HQ_transcript/22173	-1.63094456093951	2.84064134667525	-3.43243962383656	0.00164575070047625	0.158122639202121	-1.19973058280224
a_HQ_transcript/11063	3.04989521777332	4.08763831201567	3.43073134480774	0.00165335864157947	0.158591470491306	-1.2885623280285
a_HQ_transcript/22509	2.84654789477272	0.112574421802472	3.4272426456379	0.00166900107560719	0.159828162311194	-1.16658731864164
a_HQ_transcript/114897	1.41173033344854	2.89895045831779	3.42494383460784	0.00167938607705642	0.160357610363023	-1.23501881780724
a_HQ_transcript/57417	-3.583914279441	0.0475932557141504	-3.42473969451613	0.00168031129036606	0.160357610363023	-1.179448763196
a_HQ_transcript/137781	-5.05377071801294	0.639985715983226	-3.42408927910852	0.00168326240498597	0.160357610363023	-1.19777160634161
a_HQ_transcript/12988	-2.7446943143445	-0.412305449765883	-3.4235826413242	0.00168556461484666	0.160357610363023	-1.17386352399039
a_HQ_transcript/95805	-3.99960351428622	0.449961237326262	-3.42207969048969	0.00169241200047348	0.160745955495952	-1.18246581652389
a_HQ_transcript/110048	2.78824852507656	-0.62182913577816	3.42040816221585	0.00170005884697019	0.161036813471843	-1.18660682053665
a_HQ_transcript/134148	3.90424354951236	-0.355089412784535	3.42019965179762	0.00170101506110156	0.161036813471843	-1.18799073095288
a_HQ_transcript/93969	-3.22980309978027	0.173892250601355	-3.41794715180506	0.00171137790399568	0.161585584525834	-1.21613461875093
a_HQ_transcript/127782	6.13898358402207	3.15961786144705	3.41773190943553	0.00171237131963794	0.161585584525834	-1.31729339804953
a_HQ_transcript/48996	2.86237811185919	0.268341850892705	3.41596930546383	0.00172052722104135	0.161780020071854	-1.19329767536
a_HQ_transcript/20135	3.78946423892461	0.949701101618371	3.415228705743	0.00172396525134392	0.161780020071854	-1.19587911640592
a_HQ_transcript/30492	8.58240731982947	4.7560596477979	3.414535833264	0.0017271877025609	0.161780020071854	-1.44124720896275
a_HQ_transcript/123705	0.938211755215537	4.13968325898724	3.41444903441114	0.0017275917997312	0.161780020071854	-1.35550075881267
a_HQ_transcript/151258	0.513737938780141	11.3877448166531	3.41428673147834	0.00172834765456616	0.161780020071854	-2.02245343720645
a_HQ_transcript/45943	-2.33920199727641	-0.795240072019572	-3.41350975792412	0.00173197047628428	0.161858488497512	-1.19992997979559
a_HQ_transcript/121128	3.40473477346186	1.72319389197534	3.41238344671383	0.0017372351357503	0.162089894014275	-1.20042713321674
a_HQ_transcript/136797	1.78622702791542	-0.838967395247612	3.41113443047158	0.00174309134408426	0.162375662898926	-1.20744433691384
a_HQ_transcript/78563	4.48497661823935	2.38868793102987	3.40933272329617	0.00175157238298352	0.162714853931303	-1.22137888705772
a_HQ_transcript/12212	-2.98886292717176	1.20105671812401	-3.40904173282273	0.00175294585658515	0.162714853931303	-1.23152782494346
a_HQ_transcript/22469	3.09465192801129	-0.00323118941052627	3.40713171055082	0.00176198689232654	0.162714853931303	-1.22324614607233
a_HQ_transcript/25236	1.85210159506109	2.23036307573375	3.40672728168445	0.00176390698809865	0.162714853931303	-1.21628208516217
a_HQ_transcript/53674	3.09521171493676	-0.60139242171414	3.4065505564976	0.00176474665285212	0.162714853931303	-1.21990911945752
a_HQ_transcript/69575	-3.33934704309171	0.513940713723866	-3.40543627554191	0.00177004973211974	0.162714853931303	-1.21537548507661
a_HQ_transcript/143192	-3.51348707127075	0.139360774866297	-3.40521719958185	0.00177109415778727	0.162714853931303	-1.23583251655685
a_HQ_transcript/47219	4.6861643481683	0.750996332625519	3.40462447084036	0.00177392291159517	0.162714853931303	-1.22189345626823
a_HQ_transcript/50700	6.05337726996171	2.29903416046067	3.40447597388202	0.00177463228239466	0.162714853931303	-1.24466773238564
a_HQ_transcript/79244	-2.22091596749563	1.88158005077028	-3.4044565596177	0.00177472504459892	0.162714853931303	-1.21890063457643
a_HQ_transcript/114471	-5.00562924977245	2.86085180153908	-3.40271176470234	0.00178308081256997	0.163050626115798	-1.24643120217053
a_HQ_transcript/122419	2.35877992548417	-0.683537671192475	3.40252085726888	0.00178399735428103	0.163050626115798	-1.22788526575797
a_HQ_transcript/130209	3.73546281203721	0.948640280872086	3.39950882947877	0.00179851810098844	0.164119717698989	-1.22891694438332
a_HQ_transcript/37206	5.50810942029618	6.36510373909123	3.39794929186835	0.00180608107953048	0.164551537603366	-1.4162134407211
a_HQ_transcript/127477	1.83508605207864	2.35992871077377	3.39729305835718	0.00180927261349194	0.164584348164412	-1.27195067682042
a_HQ_transcript/142727	-3.99576682139645	0.0842244391532996	-3.39644098627789	0.00181342468019378	0.1647042965786	-1.25455998902462
a_HQ_transcript/83136	-2.22803515332753	-0.808197005257047	-3.39475295917909	0.00182167733468644	0.165195725601643	-1.24522760894657
a_HQ_transcript/151534	-4.51965832151813	0.793990860919684	-3.39248057499912	0.00183284388812579	0.165655307649156	-1.26385448461197
a_HQ_transcript/50359	-3.73110691777486	1.81267662600033	-3.39188866649508	0.00183576332231563	0.165655307649156	-1.2464663288007
a_HQ_transcript/79538	-1.43772510046461	2.35834273155422	-3.39127906426064	0.00183877469690778	0.165655307649156	-1.25751584748017

a_HQ_transcript/148969	6.47258528941095	9.47893496617673	3.39077002970501	0.0018412929133455	0.165655307649156	-1.93990347266135
a_HQ_transcript/20520	3.8775760698915	1.05341636414443	3.39065106531052	0.00184188191301882	0.165655307649156	-1.28424789747373
a_HQ_transcript/5666	2.89667069203657	1.90316690938374	3.38980414589391	0.00184608029583244	0.165655307649156	-1.25159095843714
a_HQ_transcript/143962	-2.23839835738749	-0.80424851740115	-3.38934024540865	0.0018483838581068	0.165655307649156	-1.25753866995511
a_HQ_transcript/1184	4.59498951578345	0.99160928237451	3.38910681816585	0.00184954401775912	0.165655307649156	-1.26307093516645
a_HQ_transcript/148659	-3.67001795171272	-0.581082453410463	-3.38551455479371	0.00186748644513797	0.167005003204585	-1.28861195428682
a_HQ_transcript/61341	-2.70385424722787	0.755339495979422	-3.38380231423326	0.00187609738056511	0.167430592108148	-1.26490909566232
a_HQ_transcript/65118	-1.75371481788256	1.40741501649825	-3.38327667002132	0.0018787485124992	0.167430592108148	-1.27675546702531
a_HQ_transcript/65027	2.83716213323234	-0.199255447387923	3.38140745364102	0.00188820525055034	0.167430592108148	-1.30041813777284
a_HQ_transcript/135245	2.49953380096627	-0.505295647191211	3.38106893514593	0.00188992276618989	0.167430592108148	-1.27605059280626
a_HQ_transcript/113472	-4.03154510067232	0.0529091811526048	-3.37955150598657	0.00189764008840961	0.167430592108148	-1.28603078659015
a_HQ_transcript/117008	1.39767731974042	4.34324469505386	3.37947959904686	0.00189800654197754	0.167430592108148	-1.45454965650734
a_HQ_transcript/138280	-2.08064306060828	-0.80900336519753	-3.37842539650269	0.00190338679893169	0.167430592108148	-1.28246589963253
a_HQ_transcript/195	2.51645709012078	-0.147946484747776	3.37839863615511	0.00190352356423479	0.167430592108148	-1.27869910223498
a_HQ_transcript/105193	4.17483755421336	2.02678920745393	3.37833010019957	0.00190387387704562	0.167430592108148	-1.28911001040097
a_HQ_transcript/85929	-3.06859206285021	-0.0629176221095496	-3.37800620115619	0.00190553028374799	0.167430592108148	-1.29370974163722
a_HQ_transcript/150843	0.665582264976655	10.5923776373038	3.3771364037187	0.00190998524486102	0.167430592108148	-2.02753930558387
a_HQ_transcript/37937	-0.826823040236894	3.25866719936478	-3.37699770822053	0.0019106965447355	0.167430592108148	-1.37483133679727
a_HQ_transcript/30109	1.87066519988377	0.930068183851426	3.37672515633718	0.00191209506836154	0.167430592108148	-1.28670050876004
a_HQ_transcript/112214	2.64991504066498	-0.366405067396208	3.37663249452471	0.0019125707603876	0.167430592108148	-1.29177899098947
a_HQ_transcript/133350	2.08400299368992	3.18683857091284	3.37575785473163	0.00191706644591881	0.167571787020103	-1.36769286280876
a_HQ_transcript/83036	3.57718556710865	0.990078987950022	3.3733149285046	0.00192967701340501	0.168420818971781	-1.30462025659177
a_HQ_transcript/111908	4.89042604762491	0.437368509385275	3.37274030851717	0.00193265479639417	0.168427823095653	-1.29601629493589
a_HQ_transcript/109720	2.48415355404534	-0.492538918940593	3.37059119799587	0.00194383103057527	0.168696570924401	-1.30251644302582
a_HQ_transcript/16335	2.21200691978055	-0.231145357222281	3.37036981695296	0.00194498581977366	0.168696570924401	-1.29801029008592
a_HQ_transcript/97428	-3.40701887839063	2.32277526917009	-3.36967397731529	0.0019486198165082	0.168696570924401	-1.33066294489322
a_HQ_transcript/99581	-3.27882531508199	0.576424236207968	-3.36944669337135	0.00194980821031356	0.168696570924401	-1.35089484598171
a_HQ_transcript/138677	3.8866967592007	1.32910739577777	3.36936235315876	0.00195024937484857	0.168696570924401	-1.30284066280817
a_HQ_transcript/114394	-2.80391684004708	-0.230453256509771	-3.36822688931463	0.00195619806741596	0.168959704699487	-1.30142955943565
a_HQ_transcript/85803	-4.28861244859164	0.300279750382835	-3.36748595941402	0.00196008918628985	0.16904460566863	-1.31892832753094
a_HQ_transcript/95204	-2.54900080932709	-0.431338734944487	-3.36464324789008	0.00197508722728869	0.170085733848647	-1.3103834100777
a_HQ_transcript/359	2.72967954873324	2.02737706028792	3.36406057112378	0.00197817498439552	0.17009963830317	-1.323761031652
a_HQ_transcript/136786	-4.75452157495576	-0.117732202550871	-3.36033734226769	0.00199801503281675	0.171551872714287	-1.32073851917365
a_HQ_transcript/104909	-2.38956598081278	-0.647581263541366	-3.35965332048466	0.00200168070232906	0.171613120744813	-1.33800504870547
a_HQ_transcript/150006	-1.78656461956447	6.36051810596725	-3.35783322499801	0.00201146605138633	0.172198083409403	-1.65890686705836
a_HQ_transcript/140018	-4.90294812943485	0.598543786647138	-3.35413431273558	0.00203149409866943	0.173656895540378	-1.36322611410563
a_HQ_transcript/143229	-4.28201590343572	0.110124546713616	-3.35347984153288	0.00203505765187233	0.173706066355411	-1.34971666847794
a_HQ_transcript/94513	-4.64119881285495	3.06128801813286	-3.35235823565069	0.00204117867590716	0.173973070488462	-1.46759920467718
a_HQ_transcript/140616	1.88818817550609	1.21255212116124	3.35124143385804	0.0020472910327801	0.174022093144601	-1.34095699548613
a_HQ_transcript/27393	2.43093704557856	2.96813958371041	3.35109899237596	0.00204807188955282	0.174022093144601	-1.35112601034431
a_HQ_transcript/100955	1.07291322987394	4.68090034555448	3.35043682242674	0.00205170562419996	0.174022093144601	-1.57345065597004
a_HQ_transcript/82049	4.72148353327223	2.93563830091343	3.34993564729861	0.00205445999291788	0.174022093144601	-1.43735269309787
a_HQ_transcript/23820	-3.72769311101569	-0.0101013547918207	-3.34952441250812	0.00205672271522057	0.174022093144601	-1.35102533399
a_HQ_transcript/30996	2.77712820755999	1.14800072506876	3.34767741543057	0.0020669148991277	0.174104870439255	-1.34869761494337
a_HQ_transcript/54037	-3.49277768686552	-0.0311788733421319	-3.34759616574528	0.00206736436574534	0.174104870439255	-1.35735224555718
a_HQ_transcript/41685	-0.888468525486959	4.30495659402203	-3.34726884070578	0.00206917605111051	0.174104870439255	-1.5293584677966
a_HQ_transcript/746	1.27426009177843	2.67458271308923	3.34717750835359	0.00206968183101991	0.174104870439255	-1.36679719589362
a_HQ_transcript/141039	-5.85028827496322	0.870990562620012	-3.3452587971971	0.00208033472039316	0.174748116513025	-1.3772862259316
a_HQ_transcript/148822	2.61299113929067	2.70020191173479	3.34354023952578	0.00208992097530551	0.175300038171081	-1.40097467461966
a_HQ_transcript/67063	2.45924877713341	-0.607134042101055	3.34226046636374	0.00209708716569628	0.175647669693939	-1.3666311391588

a_HQ_transcript/23392	-2.66969920562894	1.17672496500373	-3.34117096286169	0.00210320649455659	0.175906744051202	-1.37880740946395
a_HQ_transcript/30000	-2.435455760736	-0.777679543276653	-3.3398376972733	0.00211071824811237	0.176281365411316	-1.37264127463001
a_HQ_transcript/31952	-1.37726770917594	2.75890179840991	-3.33909990750769	0.00211488608288892	0.176376037627213	-1.39874377536079
a_HQ_transcript/150274	-3.62715031455278	0.290382263026716	-3.33819168408761	0.0021200275506262	0.176551520720344	-1.38741750369398
a_HQ_transcript/148930	0.619466361356939	10.397755469937	3.33758655785233	0.00212345983214492	0.176584367843948	-2.10480184634555
a_HQ_transcript/100149	2.98878269822325	-0.449097818829452	3.33679059551046	0.00212798265355231	0.176707679550984	-1.37954217593935
a_HQ_transcript/45977	1.86309334197633	2.20319809935135	3.33608719019157	0.00213198723508718	0.176787666192793	-1.39055120491266
a_HQ_transcript/18246	1.39876342765711	3.74418287905434	3.33543034426385	0.00213573327216428	0.176846016587415	-1.48093484125889
a_HQ_transcript/31614	-4.76005114591085	1.28071931667823	-3.33427889517402	0.00214231530016377	0.177138696682674	-1.41353181815609
a_HQ_transcript/58695	-4.08884250407228	1.87374141647946	-3.33356800190099	0.00214638868609865	0.177195906780517	-1.40638082496284
a_HQ_transcript/103246	3.33170535413421	1.12866774089828	3.33309484866655	0.0021491039478438	0.177195906780517	-1.38264715107762
a_HQ_transcript/151112	-3.0481736565784	0.120500264839809	-3.33225443724425	0.0021539348989815	0.17721955810875 1	-1.40737316212207
a_HQ_transcript/131106	-6.28455278914513	0.947490942429505	-3.33198457442589	0.00215548836331694	0.17721955810875 1	-1.41790949162194
a_HQ_transcript/126763	-3.06821313486218	0.352800762231213	-3.3299044118236	0.0021674989180876	0.177823494312141	-1.38785705995579
a_HQ_transcript/53291	-2.12456591170172	-0.777420883063801	-3.32965344939091	0.00216895226856778	0.177823494312141	-1.38898718008732
a_HQ_transcript/14021	3.88248176560245	0.284610427046796	3.32800050305656	0.00217854802665869	0.178343668364966	-1.39289482029714
a_HQ_transcript/109062	1.63620012106146	3.50978533937334	3.32750487598771	0.00218143318551285	0.178343668364966	-1.51290222585612
a_HQ_transcript/76213	-4.9401787973226	2.93345274339458	-3.32681711179155	0.00218544288755	0.178420539561104	-1.40196104783823
a_HQ_transcript/138804	3.49655435910273	0.0332028519951948	3.32617888960479	0.00218917007660689	0.178474162991592	-1.3966000638308
a_HQ_transcript/139610	-6.18417548638364	9.91518478646703	-3.32154766332095	0.00221639934426438	0.180326578269608	-2.15174067959789
a_HQ_transcript/4721	2.35318649577803	1.2307348961038	3.321260843742	0.00221809632987149	0.180326578269608	-1.4120379324592
a_HQ_transcript/135805	2.46764818521021	-0.668870259307493	3.32023819578819	0.00222415703029392	0.180396852621134	-1.41633081451596
a_HQ_transcript/95891	3.09484490261122	1.49717758432143	3.31994004407419	0.00222592700589765	0.180396852621134	-1.41038101938573
a_HQ_transcript/144294	4.2348024872722	6.71094525681258	3.31916253284661	0.0022305490484213	0.180396852621134	-1.7880749739683
a_HQ_transcript/150958	-2.12919245732645	2.60210760870979	-3.31902383918119	0.00223137450169618	0.180396852621134	-1.46345822865871
a_HQ_transcript/61619	3.49231233548032	0.408944576751481	3.31656973802871	0.00224602891037002	0.181329400697206	-1.42474557063817
a_HQ_transcript/17156	-2.38080175489649	-0.0211199201034729	-3.3158605545776	0.00225028087291807	0.181339690610767	-1.42098674951663
a_HQ_transcript/147648	-2.75568752125236	0.0351258419603259	-3.31482241746014	0.00225651901411321	0.181339690610767	-1.42987288344995
a_HQ_transcript/85238	-4.1504508717457	1.22600069789421	-3.31471225503174	0.00225718194916232	0.181339690610767	-1.48093708759724
a_HQ_transcript/67495	-3.0797626375473	1.68794315589473	-3.31447090241279	0.00225863501242422	0.181339690610767	-1.42833921325854
a_HQ_transcript/150027	1.98114281376022	4.08123989886155	3.30689300145675	0.00230471630248574	0.184394637279447	-1.57870373321367
a_HQ_transcript/34115	3.76905587311464	-0.010464007134053	3.30637902788958	0.00230787418155798	0.184394637279447	-1.46204445847585
a_HQ_transcript/3277	-2.43851688960972	0.166797906163605	-3.30626228372453	0.0023085920409069	0.184394637279447	-1.4485738826174
a_HQ_transcript/134015	4.286955080773	1.10202529484415	3.30599440330928	0.00231024004493145	0.184394637279447	-1.44184527381815
a_HQ_transcript/12525	5.96391729148057	0.256062963365437	3.30561983448357	0.00231254628710289	0.184394637279447	-1.47393910687531
a_HQ_transcript/140343	-7.1637454296323	1.75386161090484	-3.30490811554914	0.00231693445959451	0.18449146064015	-1.47924288491985
a_HQ_transcript/116326	-6.59418371179096	2.59659225407687	-3.30231251929357	0.00233300554067103	0.185517026084987	-1.53607940924873
a_HQ_transcript/137999	-5.72017063057589	2.48360876907967	-3.29995916502555	0.00234766897689247	0.186412976380349	-1.53493349426193
a_HQ_transcript/69426	-4.32215600121627	0.183521305052353	-3.29934834223482	0.00235148933535735	0.186412976380349	-1.46054411231855
a_HQ_transcript/59181	5.14213860159702	2.20477747731532	3.29885799501053	0.00235456049305687	0.186412976380349	-1.49909555384276
a_HQ_transcript/135047	2.92900336575995	0.462520160341094	3.29797194158749	0.00236011979381068	0.186412976380349	-1.48045538300008
a_HQ_transcript/49756	-3.71361625191396	0.073966798296595	-3.29794206971365	0.00236030743558934	0.186412976380349	-1.46489261995375
a_HQ_transcript/82042	4.03113641073959	0.948327357452205	3.29609346722969	0.00237194736846145	0.187078095839793	-1.49953989894801
a_HQ_transcript/31424	3.12570197246061	0.58546482365684	3.29406859087411	0.00238476030064883	0.187655919894512	-1.47704061917233
a_HQ_transcript/44846	-2.42347954661326	-0.648017646406018	-3.29391573908724	0.00238573019546595	0.187655919894512	-1.47642999661485
a_HQ_transcript/54161	2.15022549724961	1.94774588175276	3.29214690269887	0.00239698155204012	0.188286140077011	-1.4725708551727
a_HQ_transcript/14995	-1.24293606794759	2.99402497635736	-3.29087895337648	0.00240507806636637	0.188667176574554	-1.50488297765701
a_HQ_transcript/84715	-2.38094249410978	0.419343577774182	-3.28962330233174	0.00241312184732853	0.189043054907699	-1.47821018785605
a_HQ_transcript/14005	-2.99506309199913	0.151950178122771	-3.28785498230717	0.00242449350174165	0.189678274924951	-1.48399968701674
a_HQ_transcript/63209	-3.15153447669249	0.670518463334685	-3.28689749678775	0.0024306722567695	0.189906071157927	-1.4847795625435

a_HQ_transcript/110974	-2.78945094304835	-0.338394613200412	-3.28632078032526	0.00243440113905507	0.189942106591937	-1.50244180840337
a_HQ_transcript/62828	2.22980111773118	0.863575916743593	3.28552803919067	0.00243953571137854	0.190087576180981	-1.48762507582194
a_HQ_transcript/127214	-2.8348258223165	4.40049004811699	-3.28035193546268	0.00247331691733496	0.192461801567398	-1.73952186041152
a_HQ_transcript/13313	2.63376173585487	-0.468389459814054	3.27862158560193	0.00248470933340746	0.192893983600981	-1.52351474795349
a_HQ_transcript/86786	-1.55946325386183	2.10655699011261	-3.27850060405726	0.00248550773666967	0.192893983600981	-1.50404164354471
a_HQ_transcript/69630	4.24868681197505	1.60393904112072	3.27705822990301	0.00249504544205701	0.193155161477686	-1.55421345026519
a_HQ_transcript/86167	-4.40137033885121	0.320854257853156	-3.2765756858368	0.00249824407688697	0.193155161477686	-1.52066771810628
a_HQ_transcript/80308	-0.859956800942388	3.39537095685775	-3.27648556556231	0.00249884189084813	0.193155161477686	-1.61926358301483
a_HQ_transcript/81071	3.0098008176045	-0.247338891132898	3.27373533523357	0.00251715153963409	0.193861595455234	-1.51454271869811
a_HQ_transcript/85998	4.12919271490502	0.889270662601552	3.27354325887995	0.00251843507094228	0.193861595455234	-1.56507361196194
a_HQ_transcript/151690	-4.51572896698685	1.30190485463116	-3.27325036254367	0.00252039352729167	0.193861595455234	-1.57338702782251
a_HQ_transcript/65667	-2.46484870661056	-0.270911025906503	-3.27311168156684	0.00252132132817501	0.193861595455234	-1.53203295488673
a_HQ_transcript/50916	-1.54385615751798	2.24956244779922	-3.2712872326952	0.00253355768615967	0.194269973991733	-1.5425682452593
a_HQ_transcript/116803	-4.66019985741644	0.540164976468185	-3.2708306369487	0.00253662889446848	0.194269973991733	-1.5231513106172
a_HQ_transcript/43095	-2.7080319581505	-0.764308702507113	-3.27082617424522	0.00253665892959891	0.194269973991733	-1.53195623861787
a_HQ_transcript/148468	3.42369795518899	-0.042991271022057	3.2700011582312	0.00254221734588543	0.194439486686353	-1.52515722726905
a_HQ_transcript/148615	-2.25631577873164	-0.797115838153484	-3.26840741496389	0.00255298795006223	0.195006680106725	-1.53753779404348
a_HQ_transcript/137802	4.59840880734247	3.18325721184707	3.26756949726592	0.00255866813572253	0.195184070069921	-1.60762184815135
a_HQ_transcript/27174	-1.65623346497347	1.50630430349466	-3.26541390820992	0.00257333632225155	0.195817525976308	-1.53697768094956
a_HQ_transcript/61587	4.98298372200598	3.14343636802164	3.26535921048481	0.00257370956932802	0.195817525976308	-1.5346381500606
a_HQ_transcript/130734	3.06893267114562	3.36644152439859	3.26355807231242	0.00258602916292786	0.195893919854272	-1.59991402571122
a_HQ_transcript/4413	1.63028400887656	1.05690322169006	3.26346533699103	0.00258666498678279	0.195893919854272	-1.53868906832081
a_HQ_transcript/109694	-2.51425305503374	0.160800153552211	-3.26336989588999	0.0025873195184545	0.195893919854272	-1.53730660199656
a_HQ_transcript/53174	-2.25500122989217	1.25152065808733	-3.2626071193756	0.00259255630678024	0.195893919854272	-1.54387639724932
a_HQ_transcript/106473	3.36314259793109	0.52076936125152	3.26223545763414	0.00259511159525888	0.195893919854272	-1.54237563366576
a_HQ_transcript/143355	-2.05177399270867	-0.791997407767235	-3.26206848447255	0.00259626037083992	0.195893919854272	-1.55054515776002
a_HQ_transcript/59844	1.73595409175536	5.22347591223744	3.2613172595793	0.0026014348172059	0.195893919854272	-1.77534018039332
a_HQ_transcript/27999	-3.63483537797817	3.90703017984542	-3.26086695154317	0.00260454127024833	0.195893919854272	-1.75002969248538
a_HQ_transcript/21687	1.21544644075468	3.44903170808204	3.26079411865414	0.0026050440415523	0.195893919854272	-1.69219361813498
a_HQ_transcript/135029	-2.6795687922175	0.177199938540398	-3.25774701867325	0.00262616177157216	0.197226784829388	-1.56595836255675
a_HQ_transcript/127158	1.94648190736999	3.02504252699461	3.25690001023191	0.00263206091924815	0.197358508888207	-1.60889406948395
a_HQ_transcript/116770	-3.6046948276587	0.0692960727368458	-3.25652078388063	0.00263470621554584	0.197358508888207	-1.55826343344996
a_HQ_transcript/26261	0.955719325202168	3.57362719627107	3.25560912176895	0.00264107592513785	0.197424902027799	-1.70583847429887
a_HQ_transcript/3730	3.4217238602447	-0.249204436374758	3.25542197589952	0.00264238531822233	0.197424902027799	-1.57226197270902
a_HQ_transcript/145823	3.93275230927383	0.847228597456057	3.25279203409715	0.00266085185113802	0.198075555205444	-1.5809388450322
a_HQ_transcript/135634	3.5850305650454	-0.0508273279567906	3.25273778110353	0.0026612340923305	0.198075555205444	-1.58426213264175
a_HQ_transcript/68370	-3.69287555577577	0.206339050810335	-3.25272607805831	0.00266131655338996	0.198075555205444	-1.56544479814718
a_HQ_transcript/80628	3.05299501283767	0.226762340143361	3.25201806070393	0.00266630987463865	0.198193427612526	-1.56477134098038
a_HQ_transcript/16054	1.93686211609305	2.53130682462069	3.25011246737724	0.00267979367433528	0.198941311241075	-1.58303812852556
a_HQ_transcript/110640	2.18253536462104	3.37409714938222	3.24946332364198	0.00268440181874452	0.199029220561201	-1.6296012576659
a_HQ_transcript/128550	-3.7424124683606	-0.0740824631336174	-3.24871325201848	0.00268973586190717	0.199121786140384	-1.5862349107428
a_HQ_transcript/118214	2.33006233069745	-0.535024885192997	3.24831773991301	0.00269255257564831	0.199121786140384	-1.58457928878624
a_HQ_transcript/68297	-2.12991631738486	0.597602534038179	-3.24767113642148	0.00269716355646102	0.199121786140384	-1.57770290206029
a_HQ_transcript/51751	-2.17079748554258	3.33477483802697	-3.24736453499245	0.0026993525921866	0.199121786140384	-1.6822876999063
a_HQ_transcript/25830	2.53740334440853	1.72707245536127	3.2458810695232	0.00270996806227844	0.199527937830668	-1.57841537784566
a_HQ_transcript/51312	3.58233550911918	3.66533573462972	3.24563627256005	0.00271172362521035	0.199527937830668	-1.689641262876
a_HQ_transcript/135861	5.03662034222255	1.27896436680093	3.24399164602337	0.00272354628264391	0.200144498505089	-1.65862719944149
a_HQ_transcript/4694	1.30750120777458	1.83488991084549	3.24272585286964	0.00273267916221756	0.200484996336591	-1.58510024955058
a_HQ_transcript/83683	-7.37417181208438	1.02196765157303	-3.24239407882905	0.00273507779546719	0.200484996336591	-1.62380824843937
a_HQ_transcript/104160	-4.03282096757995	-0.0262881152918367	-3.24148824541956	0.00274163696544639	0.200555606656499	-1.59075266719078

a_HQ_transcript/133344	-2.5513366843114	1.8598964743425	-3.24088891659373	0.00274598498412929	0.200555606656499	-1.59686665524684
a_HQ_transcript/28909	4.30533380917997	0.553085900349228	3.24083288685657	0.00274639180598977	0.200555606656499	-1.60144301415446
a_HQ_transcript/9246	4.50784442660681	2.30592799102776	3.23948269204975	0.00275621275948464	0.20102024502299	-1.59971917803742
a_HQ_transcript/1901	4.4503818196881	1.7922706867953	3.23625290588473	0.00277984165876003	0.202012973514426	-1.63342856141102
a_HQ_transcript/1180	-2.92771091347134	0.861323610679146	-3.23578668946583	0.0027832684080364	0.202012973514426	-1.60940990189385
a_HQ_transcript/16069	1.77454421365871	2.76266781925944	3.23539328278623	0.00278616313528068	0.202012973514426	-1.68593814351652
a_HQ_transcript/29498	-2.67782866428082	0.45427599726297	-3.23515685450635	0.00278790418239533	0.202012973514426	-1.6078552671973
a_HQ_transcript/139975	1.89836007602577	2.07452204796807	3.23461610149743	0.00279189017048143	0.202012973514426	-1.62061078134109
a_HQ_transcript/82339	-5.77173741559236	0.861045261241506	-3.2341508275336	0.00279532414521979	0.202012973514426	-1.70466797249273
a_HQ_transcript/37871	-6.17149495297677	1.9753499042813	-3.23312086823614	0.00280294016045313	0.202012973514426	-1.61783282189152
a_HQ_transcript/18934	2.55564729989071	1.37057443091926	3.23285590160732	0.00280490265421142	0.202012973514426	-1.6053660663287
a_HQ_transcript/110028	5.0754965226202	1.83049549018621	3.23213657761344	0.00281023699708547	0.202012973514426	-1.64221789805047
a_HQ_transcript/79692	-3.10448436825147	-0.579818567022227	-3.23213477003735	0.0028102504138546	0.202012973514426	-1.6238149527416
a_HQ_transcript/82706	2.36161793593134	0.996502855471899	3.23204704817124	0.00281090160466541	0.202012973514426	-1.61529220177806
a_HQ_transcript/125292	1.67797996923615	4.07034500054567	3.23196269410694	0.00281152793100005	0.202012973514426	-1.72099191214575
a_HQ_transcript/107374	4.07835609009049	0.704993160284647	3.22884593695323	0.00283476342557306	0.203278604266711	-1.66327542569889
a_HQ_transcript/149699	3.79595052032607	0.872795902799154	3.22866250730709	0.00283613659313788	0.203278604266711	-1.61595731949762
a_HQ_transcript/105812	3.21567088866103	0.991068346220051	3.22638561152729	0.00285323460453716	0.204252242724798	-1.62298233801874
a_HQ_transcript/66281	-1.52128479230703	-0.676253847040456	-3.22525120894787	0.00286178992293961	0.204612699434974	-1.64560920579349
a_HQ_transcript/70065	-2.97362018338631	1.04211093427202	-3.22348866914363	0.00287513107118505	0.204837111408311	-1.62868249194848
a_HQ_transcript/75519	-1.80138261317133	2.47033470713607	-3.22336025864982	0.00287610536271228	0.204837111408311	-1.62612824193021
a_HQ_transcript/135021	-2.27894498625363	-0.644770677030238	-3.22295867906345	0.00287915431032476	0.204837111408311	-1.64102626006161
a_HQ_transcript/63202	2.6465283329087	1.57270866745961	3.22282894756282	0.00288013994138187	0.204837111408311	-1.6609781269359
a_HQ_transcript/134331	3.23729617913053	1.37642886305719	3.22251215457809	0.00288254812021743	0.204837111408311	-1.62874943319744
a_HQ_transcript/108973	3.83988814760421	1.76235025563015	3.22168465867679	0.00288884760365007	0.205034106843677	-1.69222285817332
a_HQ_transcript/26517	-1.25158532854376	2.95116316853579	-3.22027817667023	0.00289958490315186	0.205545208841965	-1.66976255102277
a_HQ_transcript/14085	-3.65411064901481	-0.0545010265250945	-3.21864464764987	0.00291210333436497	0.206075469500064	-1.66081889096905
a_HQ_transcript/28483	-2.26765475741644	-0.651263535842848	-3.2182830853596	0.00291488110564032	0.206075469500064	-1.65214361461209
a_HQ_transcript/19271	-2.73308442239008	-0.0770434685478883	-3.21769065568114	0.00291943802771038	0.206075469500064	-1.67190280100002
a_HQ_transcript/130602	-2.6443066228512	-0.5564320027074	-3.21681437970102	0.00292619074253572	0.206075469500064	-1.65473808167921
a_HQ_transcript/119736	2.51122979731669	-0.361433480827093	3.21673597656329	0.00292679565503045	0.206075469500064	-1.645244642498
a_HQ_transcript/53886	2.13448909469283	1.75430958591772	3.21627075695623	0.00293038747195585	0.206075469500064	-1.65048284794996
a_HQ_transcript/54892	-3.18897875814205	-0.343187693612142	-3.21607739547443	0.0029318815936649	0.206075469500064	-1.66293831411915
a_HQ_transcript/127488	-0.962350188477182	5.84702349091583	-3.21494142499571	0.00294067403492208	0.206416448046683	-2.00323579970445
a_HQ_transcript/62474	-5.12795097062925	1.12171576524495	-3.21416701311241	0.00294668242296458	0.206416448046683	-1.70447466051536
a_HQ_transcript/65507	1.02198082341209	2.6734816864723	3.2139975858043	0.00294799851074739	0.206416448046683	-1.71586028513011
a_HQ_transcript/81403	2.73438290157084	0.231576904849993	3.21361955280608	0.00295093704112981	0.206416448046683	-1.65734951375257
a_HQ_transcript/19892	4.43045157501375	0.263311464689568	3.21288265707312	0.00295667312480108	0.206453871632148	-1.67920821142461
a_HQ_transcript/91837	-2.98373160110011	0.372159655634834	-3.21263856998256	0.00295857547257053	0.206453871632148	-1.65106665076657
a_HQ_transcript/129775	-4.44673274887704	4.73839471709319	-3.20955648859571	0.00298269708489713	0.207509033955133	-1.81235225511148
a_HQ_transcript/78061	-3.01518191337793	0.346381734791037	-3.2094785343805	0.00298330961265404	0.207509033955133	-1.69491895336454
a_HQ_transcript/94260	-4.21488955665698	2.06284422561531	-3.20933903829766	0.00298440600719947	0.207509033955133	-1.73326060542596
a_HQ_transcript/38259	3.07014516508028	0.460096601628088	3.2087303791619	0.00298919437340347	0.207593656555791	-1.67573655096117
a_HQ_transcript/129439	-2.92004602832157	-0.310714876461813	-3.20770123681227	0.00299730739275309	0.207734575268589	-1.67743633821771
a_HQ_transcript/123944	4.442992143872	0.43594842240801	3.20756651640554	0.00299837098558949	0.207734575268589	-1.69181717425286
a_HQ_transcript/76316	-2.28459914838376	-0.767659222892327	-3.20656386095889	0.00300629808670984	0.208035827600321	-1.67831893436142
a_HQ_transcript/109417	0.752205026711362	3.76068999687094	3.20464336053589	0.00302153763162332	0.208841782938169	-1.85386497885751
a_HQ_transcript/149050	4.10022838684881	1.83417517436507	3.2039864783849	0.0030267670138913	0.208954766013626	-1.68623479159115
a_HQ_transcript/61879	3.07254963638837	1.41625754093683	3.20106441072402	0.00305013415094759	0.210318147006265	-1.70076128286726
a_HQ_transcript/144883	2.60936101042819	4.07731482825447	3.2003359804007	0.0030559859886334	0.210471982875927	-1.79771606109547

a_HQ_transcript/114578	-3.14511081261238	0.40505555177367	-3.19855160484609	0.0030703660314359	0.211064457408857	-1.71393289276551
a_HQ_transcript/70185	3.19185616323258	0.0477023206483836	3.19836784494136	0.00307185058780438	0.211064457408857	-1.72312807309814
a_HQ_transcript/145496	-2.67502955320804	1.80951738329361	-3.19653738633526	0.00308667588773035	0.211681503730313	-1.76842267779086
a_HQ_transcript/130492	-4.18795198844972	0.297279209779752	-3.19636021898587	0.00308811442271033	0.211681503730313	-1.69668268590714
a_HQ_transcript/150262	3.32415271354261	2.95830231305712	3.1944332191104	0.00310380231957268	0.212506267646785	-1.75104823778763
a_HQ_transcript/69228	3.25797590892595	0.923221904218514	3.18959414152203	0.00314353346782326	0.214973309903095	-1.69987129108707
a_HQ_transcript/143366	-4.2084773936107	2.66312583080572	-3.18816874638832	0.0031553287962807	0.215118140230247	-1.71828449253149
a_HQ_transcript/68091	-2.86196609133785	0.321059816151912	-3.18812056032789	0.00315572827853819	0.215118140230247	-1.70524919478485
a_HQ_transcript/77002	2.4522821484405	0.0527144192578866	3.18735727440448	0.00316206266754039	0.215118140230247	-1.71155164323018
a_HQ_transcript/101563	-4.6455075327372	0.355130980199058	-3.18715102069213	0.00316377641358745	0.215118140230247	-1.72187384443566
a_HQ_transcript/104828	-1.5140948255292	4.01238821582006	-3.18677111778081	0.00316693531504282	0.215118140230247	-1.87094055967178
a_HQ_transcript/121054	3.43748778265298	0.776257115096915	3.18666047171677	0.00316785590484949	0.215118140230247	-1.71084859079059
a_HQ_transcript/111917	2.06964779422032	-0.0586630950864065	3.1861783200057	0.0031718704492377	0.215139422955997	-1.72413191079557
a_HQ_transcript/53026	4.28195965900379	1.37608192449595	3.181721224885	0.00320921194051315	0.217183330984584	-1.86394866848992
a_HQ_transcript/21673	-2.95540793605867	0.0935383243095188	-3.18168975970472	0.00320947703887554	0.217183330984584	-1.73862753149844
a_HQ_transcript/100534	-1.68901408277033	2.37888653858279	-3.18043327893837	0.00322008013160634	0.217444763660033	-1.73303638627466
a_HQ_transcript/147451	4.45887799925025	6.56721775999634	3.18034551359439	0.00322082200507997	0.217444763660033	-2.11792056588133
a_HQ_transcript/37434	-7.09928951673948	0.831441009290971	-3.17956411858658	0.00322743426002937	0.217638397525507	-1.74934756605828
a_HQ_transcript/25529	2.59957500045798	-0.311092424358277	3.17897623485507	0.0032324175260163	0.217721860894873	-1.73495928902319
a_HQ_transcript/5290	-2.75609795268047	4.55309536325384	-3.17814809888153	0.00323944975991257	0.2177506159415	-1.89868606366084
a_HQ_transcript/35950	-2.49163067978815	2.17551452180639	-3.17804378999225	0.00324033654674851	0.2177506159415	-1.77315008508718
a_HQ_transcript/138548	-2.43076570000969	-0.783605461115532	-3.17530382863967	0.00326371347177389	0.218780789371883	-1.74894413941007
a_HQ_transcript/140624	-4.21830163384836	0.168524817023602	-3.17502359551412	0.0032661134151073	0.218780789371883	-1.81857419702407
a_HQ_transcript/1747	-3.06321476365533	-0.681108089000797	-3.17492504188383	0.00326695783744142	0.218780789371883	-1.75744621057789
a_HQ_transcript/101491	-0.889837612769714	3.65255413343097	-3.17319518836869	0.00328181339485009	0.219133707208901	-1.92961013929966
a_HQ_transcript/128153	4.75125997069046	1.8514564486389	3.17311468264262	0.00328250632324962	0.219133707208901	-1.74480872339491
a_HQ_transcript/13728	-3.21473585463933	-0.648881210685292	-3.17299492618158	0.00328353734824788	0.219133707208901	-1.75907500153306
a_HQ_transcript/52339	3.32511985522072	1.71317071678482	3.17224291639728	0.00329001871715486	0.219196951346716	-1.73774403726706
a_HQ_transcript/110536	2.13901584221561	4.55316086848424	3.17201020282741	0.00329202688077489	0.219196951346716	-1.96633889628421
a_HQ_transcript/25982	-6.11052134613676	0.78947151477184	-3.17072666008403	0.0033031240022474	0.219427924410067	-1.78119807874079
a_HQ_transcript/76662	2.48590043775828	2.95682209167084	3.17053414561314	0.00330479149424961	0.219427924410067	-1.81647778282478
a_HQ_transcript/152254	0.956575488168751	10.8157297725339	3.17008362797666	0.00330869685281388	0.219427924410067	-2.57376483764783
a_HQ_transcript/143301	-2.80220413909656	-0.595085311450375	-3.16981230406542	0.00331105097287869	0.219427924410067	-1.7660370487628
a_HQ_transcript/95641	3.64138086481556	0.156332055205644	3.16943004571417	0.00331437031434144	0.219427924410067	-1.74446911995831
a_HQ_transcript/143772	2.17068373611442	4.2689070765556	3.16812308096851	0.00332574330984003	0.219930383520343	-1.94631626667326
a_HQ_transcript/110402	-2.90993093839307	-0.694520319537776	-3.16735641023077	0.00333243204335122	0.220122283881727	-1.76900025915698
a_HQ_transcript/8249	1.35332097923324	1.37706320643648	3.16523400660254	0.00335101561799008	0.220939145083409	-1.77437646029337
a_HQ_transcript/98006	2.05200503275929	3.6018534890693	3.16507631053546	0.00335240032279738	0.220939145083409	-1.93921724678269
a_HQ_transcript/136852	-4.48338362761176	1.39327453310257	-3.16312576265491	0.00336957290278588	0.221589611091805	-1.85353068237358
a_HQ_transcript/144253	-3.46071061796542	0.162618013295422	-3.16224062132995	0.0033773932714137	0.221589611091805	-1.78362072157052
a_HQ_transcript/9854	2.04666713482833	-0.461490585522705	3.16222267598549	0.00337755200009843	0.221589611091805	-1.77845627177735
a_HQ_transcript/30111	3.95743223398126	0.333263257237459	3.1619905356227	0.00337960594939285	0.221589611091805	-1.79349270239303
a_HQ_transcript/145301	0.911498542284269	4.17514827043528	3.16179571697967	0.00338133059865179	0.221589611091805	-1.96976405096399
a_HQ_transcript/135426	3.63640348138	1.30888200021859	3.16136203624237	0.00338517280567789	0.221591582036537	-1.79225821898425
a_HQ_transcript/152226	0.78911013812771	6.79940100706256	3.15723267104351	0.00342196588246318	0.223748068409246	-2.21162518946741
a_HQ_transcript/5600	-1.4534675929012	2.23537991561243	-3.15604879600877	0.00343258436382745	0.224190184157935	-1.78395335056908
a_HQ_transcript/80712	-1.40516712532998	5.64295105592227	-3.15295860959836	0.00346044904927576	0.225576935879238	-2.13832464810363
a_HQ_transcript/77201	1.76320298618368	3.09274053403729	3.15283386960746	0.00346157835817988	0.225576935879238	-1.89754276874694
a_HQ_transcript/150400	0.466757688553335	10.6730497226356	3.15114440644029	0.00347690817445267	0.226233868568645	-2.60370283896429
a_HQ_transcript/54564	1.54151279727791	2.46616957699966	3.15052046731481	0.00348258598304314	0.226233868568645	-1.8081976095601

a_HQ_transcript/119006	-2.11191417293181	2.11528634084465	-3.15034884282414	0.00348414930258028	0.226233868568645	-1.80774847851641
a_HQ_transcript/122807	-1.24001964633054	5.17828529136965	-3.15001115220249	0.00348722726117371	0.226233868568645	-2.11463758830638
a_HQ_transcript/118121	-2.76276479337841	-0.592852251180869	-3.14867866764707	0.0034993977698792	0.226770338425349	-1.80179262953007
a_HQ_transcript/89489	4.69713408690753	1.70448688992633	3.14757131755724	0.00350954271606105	0.227174497771934	-1.85446199837293
a_HQ_transcript/127375	-2.94232120027163	1.21792480970057	-3.14693983969467	0.0035153405025543	0.227230499595583	-1.79285289239596
a_HQ_transcript/50025	4.22444939802069	1.53601017689799	3.14562278573656	0.00352746211212075	0.227230499595583	-1.82020900530742
a_HQ_transcript/146759	-3.04733091970189	0.978754117287962	-3.14537066582448	0.00352978704894642	0.227230499595583	-1.82239384939413
a_HQ_transcript/92984	-4.59344153823123	0.851881943116425	-3.14449415398716	0.0035378812005868	0.227230499595583	-1.85446588190038
a_HQ_transcript/108617	-4.03636722676931	0.0321400953659346	-3.14396311662542	0.00354279365553603	0.227230499595583	-1.80979050235195
a_HQ_transcript/151173	0.88398782056303	8.67598951121785	3.14367745320842	0.00354543891966734	0.227230499595583	-2.38623214408018
a_HQ_transcript/110903	1.88136869148417	1.94884305515654	3.14364781475105	0.00354571348153843	0.227230499595583	-1.81302759849899
a_HQ_transcript/145082	0.652752042457459	5.2626179953192	3.14355030359131	0.00354661693876465	0.227230499595583	-2.13880639781753
a_HQ_transcript/82711	3.78856529990887	0.720655682461605	3.14336162807107	0.00354836567106262	0.227230499595583	-1.80273461287987
a_HQ_transcript/135856	-5.1510828931996	0.14459731325457	-3.14323936811396	0.00354949927113937	0.227230499595583	-1.85415060209824
a_HQ_transcript/53920	-3.63761480778258	0.678723619739504	-3.14267494552776	0.00355473709301002	0.227315465063241	-1.80828798142698
a_HQ_transcript/39557	-1.77846527456299	2.00509369491967	-3.14152115542163	0.00356546711662	0.227601184653749	-1.84734814385613
a_HQ_transcript/112567	-2.7025494598064	0.336903385936481	-3.14135271207281	0.00356703618255515	0.227601184653749	-1.80510483262154
a_HQ_transcript/70088	3.67245229173265	0.562154615525067	3.13982113424045	0.00358133315253385	0.228262865669394	-1.83325210195062
a_HQ_transcript/139093	-6.07919608650977	1.17473672390671	-3.13869734307938	0.00359185817561127	0.228418695672267	-1.8139912891211
a_HQ_transcript/66916	1.51616954775499	3.17465818295458	3.13831030575818	0.00359548983534921	0.228418695672267	-1.93763129937441
a_HQ_transcript/149412	-3.15390075393185	-0.73632633213325	-3.13830210817181	0.00359556679294185	0.228418695672267	-1.84139625824307
a_HQ_transcript/106352	2.34005297765047	-0.517634662030842	3.13647577477563	0.00361275122941933	0.229163829934338	-1.84104517853341
a_HQ_transcript/147993	-3.40349673892247	-0.67692687035723	-3.13591301665234	0.00361806209080753	0.229163829934338	-1.84826288584253
a_HQ_transcript/33476	-2.75826980824352	1.29549634681603	-3.13580066729394	0.00361912324318267	0.229163829934338	-1.83320724559751
a_HQ_transcript/42228	-2.58965048598777	0.297103516099806	-3.13534090641931	0.00362346882196528	0.229189331537756	-1.82532271385524
a_HQ_transcript/117684	-2.31454825633391	-0.646443469778656	-3.13448084697155	0.00363161128214236	0.229454674574316	-1.82193578663414
a_HQ_transcript/23346	-0.966814424536899	4.69153581087554	-3.13395192833746	0.00363662736483546	0.229522123195609	-2.10328264549281
a_HQ_transcript/136168	4.37012751835821	2.1663022807837	3.13207772081168	0.00365445480427043	0.230397124579861	-1.94326628102731
a_HQ_transcript/134046	2.62980928216834	1.51102226151268	3.13120247420934	0.00366280858170166	0.230673604807318	-1.83071284010507
a_HQ_transcript/26204	-4.13946765164421	0.567336930014417	-3.13033895923099	0.00367106818212683	0.230943562002888	-1.83413712553569
a_HQ_transcript/27242	-1.93675739818682	-0.599473348695707	-3.12947225768023	0.00367937607127464	0.231215970022759	-1.85047107450011
a_HQ_transcript/64277	1.32374486096425	4.96444768813339	3.12784827686866	0.00369499112687885	0.231469409164137	-2.10959537117749
a_HQ_transcript/113065	-3.81011066104284	5.49366064639289	-3.12783495796513	0.0036951194522217	0.231469409164137	-2.11910143705716
a_HQ_transcript/42286	-2.63097558254244	-0.361951275951097	-3.12745477449274	0.00369878424200253	0.231469409164137	-1.83572574531491
a_HQ_transcript/64123	3.32802597762333	1.62464887179992	3.12739742711027	0.0036993373436809	0.231469409164137	-1.84731521082716
a_HQ_transcript/41344	3.35204862920122	0.991263204919926	3.12681222406123	0.00370498598384005	0.231573575557693	-1.86857444284757
a_HQ_transcript/79879	-3.54865473880033	0.568985847824471	-3.12566132075715	0.00371611894788641	0.23198425679609	-1.88466850793253
a_HQ_transcript/150580	2.56288079479781	-0.232934730996775	3.12528797662706	0.00371973722275353	0.23198425679609	-1.85832482093489
a_HQ_transcript/78789	-1.78411574090703	1.76753305336128	-3.12489706866321	0.00372352930757556	0.23198425679609	-1.84344019112635
a_HQ_transcript/123925	-4.34189491868746	0.500396225875997	-3.12422401486139	0.00373006702103382	0.232082617342385	-1.89091202442462
a_HQ_transcript/45298	2.49926114544184	-0.33271227720622	3.1237653729816	0.00373452829321028	0.232082617342385	-1.85997554905575
a_HQ_transcript/9250	3.26938439039838	1.20839002257393	3.12328177598954	0.00373923780136429	0.232082617342385	-1.84404260497995
a_HQ_transcript/105804	1.60470515367764	3.03425239828393	3.12288635360801	0.0037430928166069	0.232082617342385	-1.99650654596835
a_HQ_transcript/118748	1.73500538147824	6.93060164441443	3.1222048906195	0.00374974534068497	0.232082617342385	-2.29950847537439
a_HQ_transcript/133431	-1.3335122358243	3.47027222105413	-3.12191371852919	0.00375259122379232	0.232082617342385	-1.99417208238471
a_HQ_transcript/28360	-2.62226238469546	-0.755150778268922	-3.12186614843799	0.00375305636357421	0.232082617342385	-1.86933262748959
a_HQ_transcript/102124	0.945218430112353	3.99068264513943	3.12104189699844	0.00376112459187234	0.232229100006349	-2.08028598301054
a_HQ_transcript/98364	2.22376945806742	0.119107103970461	3.12061914266219	0.00376526913918171	0.232229100006349	-1.85999913213178
a_HQ_transcript/107437	3.51486254613304	-0.493010656615711	3.12040088511831	0.00376741056471901	0.232229100006349	-1.87884753122199
a_HQ_transcript/146526	2.92354788568399	0.156108569912969	3.11899146434335	0.00378126694399675	0.232790807960958	-1.88927364201179

a_HQ_transcript/138745	-4.3737155079536	0.83856307387533	-3.11866000408913	0.00378453264387396	0.232790807960958	-1.91062594198354
a_HQ_transcript/40381	-1.98822848147851	3.00311397351843	-3.1181737717868	0.00378932808076772	0.23283938972396	-1.91671058961398
a_HQ_transcript/48120	-1.10717435981053	3.88811703708866	-3.11695269643551	0.00380139633988191	0.233334283468486	-2.05981093739166
a_HQ_transcript/95361	2.29694391199444	0.294295765842848	3.11610078859287	0.00380983762797422	0.233605740125407	-1.86050063720891
a_HQ_transcript/50009	2.62302424190606	1.65525219361494	3.1143280828851	0.00382745995193838	0.234408033107652	-1.87283731994486
a_HQ_transcript/71076	4.66470193733942	1.02157048577238	3.11397418067916	0.00383098732886508	0.234408033107652	-1.89523141559174
a_HQ_transcript/138116	-4.18625914936035	1.12901771047696	-3.11311652977071	0.00383954843622909	0.234460686929456	-1.93169607177777
a_HQ_transcript/119850	-1.46626136321594	3.69629107233797	-3.11307985691963	0.00383991491117606	0.234460686929456	-2.07050351981447
a_HQ_transcript/3376	-1.83927698106516	-0.758504783069723	-3.11137194396124	0.00385701909983661	0.235257928893287	-1.87867165283612
a_HQ_transcript/45677	3.67619117698988	3.04197693316191	3.11059350159604	0.00386483894002513	0.23548779654694	-1.96674988975399
a_HQ_transcript/13375	-2.36330827392294	-0.755339991945063	-3.10967299561303	0.00387410531866153	0.235805229280793	-1.89842150079163
a_HQ_transcript/35359	1.55060568099623	4.52919715929073	3.10824571362045	0.00388851496740928	0.236277899194645	-2.03796366858459
a_HQ_transcript/102093	1.15635049285287	4.63258836910597	3.10809885602065	0.00389000050800433	0.236277899194645	-2.13070769999169
a_HQ_transcript/129124	-2.33612510210927	-0.561819950136774	-3.10735693293732	0.00389751369005463	0.236487135465026	-1.90331167610402
a_HQ_transcript/140967	4.58087002301397	0.138599421447653	3.10570457104423	0.00391429612202693	0.237257773703004	-1.93558382660467
a_HQ_transcript/149358	3.72310345003573	6.01515810266172	3.10520461402003	0.00391938751453272	0.237318914004956	-2.14591367238611
a_HQ_transcript/11374	-2.40874912925472	-0.280724874191789	-3.1034424583214	0.00393738291707316	0.238160451824795	-1.90057658266
a_HQ_transcript/149969	2.08386683555702	-0.643921770659923	3.10266175030118	0.00394538068931038	0.238396142108351	-1.93720151093251
a_HQ_transcript/131015	-1.8250931024671	4.09510150459333	-3.10143688739995	0.00395795960014129	0.238907866705102	-2.17278967453947
a_HQ_transcript/149730	-5.0872574217756	3.87230559665635	-3.10029629248004	0.00396970735073462	0.239189295739467	-2.05798728848204
a_HQ_transcript/59351	3.53801652723361	0.385615895088874	3.09998778592581	0.00397289055171977	0.239189295739467	-1.90025892583667
a_HQ_transcript/91187	-2.72634142569645	0.291968446377952	-3.09978670590447	0.00397496661994779	0.239189295739467	-1.91885732653407
a_HQ_transcript/119517	-4.41015071216305	1.75065023263517	-3.09850333282038	0.00398824120944032	0.239739901781124	-2.01743278370562
a_HQ_transcript/70598	4.486042627831	2.00408841313082	3.09786937139946	0.00399481412346467	0.239886937364416	-1.93205811609071
a_HQ_transcript/112559	-5.64596993957393	0.67353936155614	-3.09711956482137	0.00400260139882846	0.239897131925591	-1.94456078013337
a_HQ_transcript/124502	2.87178738186689	1.5295967135313	3.09705833104705	0.00400323799146406	0.239897131925591	-1.90342538189784
a_HQ_transcript/105064	-4.21045395337489	1.31364490921068	-3.09593627242113	0.00401492006441857	0.240349406286841	-1.99326352627458
a_HQ_transcript/145170	-2.92696795992673	-0.0835554273774659	-3.09434461008349	0.00403154685361226	0.240869635331961	-1.92154094189828
a_HQ_transcript/127002	-3.5442790671241	0.432313109461977	-3.09370977072187	0.00403819671989459	0.240869635331961	-1.96528185861019
a_HQ_transcript/120836	-3.88682388714852	1.18814850559459	-3.09353789717316	0.00403999886535847	0.240869635331961	-1.93174168615087
a_HQ_transcript/145641	-1.82098361593303	2.56878910183902	-3.09352011529001	0.00404018535729187	0.240869635331961	-1.95738366408793
a_HQ_transcript/80956	3.10172157186147	0.75875240573706	3.09292361125608	0.00404644608007499	0.24093803330677	-1.92745947599019
a_HQ_transcript/115983	3.48212454125655	-0.050810158265195	3.0926213028443	0.00404962253201063	0.24093803330677	-1.95932141176983
a_HQ_transcript/102183	-5.17466993944558	2.67142926221611	-3.0897786661179	0.0040796070403917	0.241820299982334	-1.98663585104047
a_HQ_transcript/41581	2.66791307026095	0.32814960651285	3.08972364000905	0.00408018953716783	0.241820299982334	-1.92729188560596
a_HQ_transcript/104099	1.17444765508213	3.84970174874135	3.08953374844921	0.00408220030245019	0.241820299982334	-2.05089751051201
a_HQ_transcript/60765	2.48100107700813	2.04512709722072	3.08886982241405	0.00408923802405361	0.241820299982334	-1.92695615346487
a_HQ_transcript/150448	4.11616160439661	5.41505048216556	3.08872265697359	0.00409079955976178	0.241820299982334	-2.16291537294305
a_HQ_transcript/114596	-2.3297271020655	-0.683118022354977	-3.0884630181667	0.00409355590255694	0.241820299982334	-1.9272465316684
a_HQ_transcript/66764	-2.40112604453277	-0.738160226703164	-3.0884614667402	0.00409357237790078	0.241820299982334	-1.9427095673312
a_HQ_transcript/79067	-3.94190701778174	0.938986010876421	-3.08623107883917	0.0041173231384526	0.242519630932496	-1.96883396503958
a_HQ_transcript/100417	-2.40108076668743	0.449722779267622	-3.08584965369685	0.00412139789892358	0.242519630932496	-1.9383543223748
a_HQ_transcript/136859	-4.80615049867807	0.828572167391505	-3.08553275636968	0.00412478622010651	0.242519630932496	-1.92851384118425
a_HQ_transcript/8663	2.29265292193388	-0.417476823243301	3.08547056214576	0.00412545152181815	0.242519630932496	-1.93558831037708
a_HQ_transcript/23745	-2.16018378269136	1.3037367900571	-3.084782136102	0.00413282253302388	0.242519630932496	-1.93033695965205
a_HQ_transcript/82692	1.77795796413809	3.94211560171588	3.08456813927666	0.0041351163528455	0.242519630932496	-2.1113134782051
a_HQ_transcript/24458	-2.43246705739095	3.24247853348712	-3.0842656753702	0.00413836050712872	0.242519630932496	-2.09667535740527
a_HQ_transcript/104211	-3.34643490377677	2.62924701633141	-3.08422582743868	0.00413878808637895	0.242519630932496	-1.98549729643002
a_HQ_transcript/150131	3.34559440754711	5.19120876994731	3.08287924556466	0.00415326195187529	0.242895935641235	-2.24337510638245
a_HQ_transcript/126303	-3.94008614856036	2.29628648972237	-3.08285088535262	0.00415356730022343	0.242895935641235	-1.98830387130097

a_HQ_transcript/137713	2.11136358797488	0.200099652703703	3.08212204777019	0.0041614218515771	0.243049131117555	-1.9659208930571
a_HQ_transcript/125759	-3.61650523065548	-0.57041398166112	-3.08165794555429	0.00416643074057459	0.243049131117555	-1.96931367736992
a_HQ_transcript/89851	3.84187037747604	0.78888562886912	3.08144500959405	0.00416873079624625	0.243049131117555	-1.98975085795263
a_HQ_transcript/67931	3.03481009274111	1.65369332055986	3.08060467921514	0.00417781950002453	0.243334961821068	-1.96635937755173
a_HQ_transcript/143114	2.76513997923244	-0.165672947922781	3.07955044861618	0.0041892482615597	0.24375637932727	-2.03954796077118
a_HQ_transcript/118732	3.5375079486292	4.84275217841343	3.07913732477654	0.00419373496239494	0.243773425894093	-2.21183839991574
a_HQ_transcript/55468	2.2117801969916	-0.620896800382023	3.07845948442766	0.00420110647621081	0.243845777226972	-1.97550690137647
a_HQ_transcript/2830	0.98645116594408	3.16494540557819	3.07816947171397	0.00420426411814524	0.243845777226972	-2.10928244257066
a_HQ_transcript/151281	3.15517730902549	-0.315892417255363	3.07734124976068	0.0042132941539472	0.243845777226972	-1.96123955097612
a_HQ_transcript/114573	-3.14027381795675	0.754900289824269	-3.07713750162562	0.00421551842160041	0.243845777226972	-1.9475827743245
a_HQ_transcript/99797	3.02545032728673	0.0126551946477211	3.07709756315296	0.00421595455052827	0.243845777226972	-1.96679285659024
a_HQ_transcript/140701	-3.38816507654273	-0.686048299954526	-3.0759271983958	0.00422875398093749	0.244342953681844	-1.98257496298719
a_HQ_transcript/36388	-2.67814087782108	2.02453862789728	-3.0748352095285	0.00424072950765527	0.244791583734842	-2.0820841159508
a_HQ_transcript/49691	-2.70139673136404	1.09923200853759	-3.07441670252436	0.004245327675214	0.244813895937341	-1.94944409631996
a_HQ_transcript/125751	2.47323839790536	1.76374273523928	3.07395893603887	0.00425036260530344	0.244861325590762	-1.96233695929186
a_HQ_transcript/9292	1.26560201212345	2.18770281074178	3.07324724000931	0.00425820173289811	0.245070049831585	-1.98474196160738
a_HQ_transcript/150443	4.62386070535365	0.276198620085716	3.07241721923543	0.00426736149753477	0.245245109962353	-2.00001235967687
a_HQ_transcript/54458	3.28640373675782	0.380587272015337	3.07220725037278	0.00426968158687554	0.245245109962353	-1.99681323469852
a_HQ_transcript/134646	2.22982860953428	3.46363273740151	3.07044717260506	0.00428917699236232	0.245853924599848	-2.12587830092871
a_HQ_transcript/4580	2.41831725931559	-0.137905505164463	3.07006822429185	0.00429338542390121	0.245853924599848	-1.96959221453803
a_HQ_transcript/3711	2.87811163329573	3.02693241156319	3.06960267816103	0.00429856093328722	0.245853924599848	-2.06013390537481
a_HQ_transcript/21247	1.82734989104326	2.66868889288421	3.06934975749056	0.00430137515024973	0.245853924599848	-2.01935117760589
a_HQ_transcript/30658	-2.62355108364134	1.23896844640398	-3.06923274534605	0.00430267772100183	0.245853924599848	-2.00916776258465
a_HQ_transcript/42426	2.11444828150029	-0.575778002948638	3.06867222743548	0.00430892255568312	0.245853924599848	-1.98663370240331
a_HQ_transcript/109796	-3.76326602087867	0.541300536989546	-3.06858567239296	0.00430988764738586	0.245853924599848	-1.96689324202405
a_HQ_transcript/38874	-4.27067377890216	-0.783249590441018	-3.0678209520698	0.00431842321609106	0.245860540422478	-2.00480442352221
a_HQ_transcript/107584	-3.29426532778433	-0.481505184295761	-3.06781739955714	0.00431846290550767	0.245860540422478	-2.0153688141539
a_HQ_transcript/135079	-2.57720116799141	-0.698926827342119	-3.06701736746244	0.00432740984806331	0.246128845056971	-1.9961823890065
a_HQ_transcript/1090	6.33956911178451	2.77324132860989	3.06660166971602	0.0043320656281448	0.246152796513002	-2.13163492877533
a_HQ_transcript/35242	-2.3153702444931	0.546649519953217	-3.06502526819739	0.00434976438917343	0.246681556474306	-1.97106804531752
a_HQ_transcript/56959	-2.08474108686572	0.383733568108556	-3.06501687081041	0.00434985885263838	0.246681556474306	-1.97045325236293
a_HQ_transcript/91398	3.61379043357812	0.911710689357241	3.06453189786652	0.00435531768732497	0.246750396226926	-2.0191367850578
a_HQ_transcript/80505	-2.97461423733671	2.08248626849674	-3.06329983678468	0.0043692149406986	0.247296714774029	-1.99169733898041
a_HQ_transcript/126514	4.79550885849397	1.43774902855521	3.06249140604778	0.00437835661346001	0.247573067341637	-1.97582546806922
a_HQ_transcript/7852	6.02538397051764	6.35468419065313	3.06197959980218	0.00438415345040017	0.247659933687912	-2.27141802099503
a_HQ_transcript/135307	-3.20140490299881	0.300287317855744	-3.06118739298404	0.00439314051962107	0.247926671965566	-2.00234554643736
a_HQ_transcript/126172	1.50532944435661	1.56057941702021	3.06012326800377	0.00440523981178407	0.248368360600761	-1.98317683830389
a_HQ_transcript/101242	5.33718642608051	3.718121030832	3.05807833340905	0.00442857982133714	0.249442333192524	-2.18695674027165
a_HQ_transcript/142965	-1.35252681931393	2.6015994059614	-3.0559271799511	0.00445325865130611	0.250190361513774	-2.07033030136462
a_HQ_transcript/148532	-3.45634877559311	1.72583729986732	-3.05550840378072	0.00445807814017474	0.250190361513774	-2.04524444270523
a_HQ_transcript/6725	-3.49131083947447	1.32792057245465	-3.05548476058114	0.00445835038549911	0.250190361513774	-2.01290017216529
a_HQ_transcript/44623	-3.08871891920574	0.30230191054582	-3.05524396577266	0.00446112397299457	0.250190361513774	-2.00788975041889
a_HQ_transcript/10072	5.24952501195996	2.23849281565863	3.05504813070566	0.00446338089887462	0.250190361513774	-2.13714423213355
a_HQ_transcript/58266	3.00176105180031	1.74447181642648	3.05400106742994	0.00447546628330158	0.250626111864889	-2.06404650570502
a_HQ_transcript/77192	-4.28563020469953	1.2931436570815	-3.05231857811297	0.00449495083983933	0.251306199926349	-2.02775570230688
a_HQ_transcript/117802	-6.22959494614070	1.10101902274232	-3.05216041670196	0.00449678660188062	0.251306199926349	-2.10552929134723
a_HQ_transcript/115401	3.94610832826358	1.75153043099196	3.05183372843492	0.00450058068612938	0.251306199926349	-2.04516963267152
a_HQ_transcript/147324	-3.79514656006619	0.448000548911422	-3.05097925428924	0.00451051870580175	0.251619415864534	-2.02542791145242
a_HQ_transcript/101474	2.625460274926	-0.453364544201419	3.04954879117344	0.00452720236392072	0.252307976040253	-2.0336395858686
a_HQ_transcript/147174	5.54535399022564	7.03271319983847	3.04881915744137	0.00453573466935106	0.252541364808466	-2.4373111952398

a_HQ_transcript/150639	5.66426199704248	1.89183999388877	3.04771870640555	0.00454863212044136	0.252970515640997	-2.11359194742817
a_HQ_transcript/114084	4.21708894444061	3.17690189567947	3.047300520503	0.00455354242676116	0.252970515640997	-2.14105181843605
a_HQ_transcript/148072	-6.00489624215339	1.50351870543307	-3.04583239809181	0.00457082079849225	0.252970515640997	-2.11388975923753
a_HQ_transcript/143240	0.661457990682964	10.4576411735256	3.04547218052061	0.00457506968816767	0.252970515640997	-2.83547523892244
a_HQ_transcript/623	-1.12167988210711	3.06903483690151	-3.04521300584814	0.00457812905645182	0.252970515640997	-2.08338680726679
a_HQ_transcript/120275	3.95809417642511	-0.237137217558273	3.04511868527349	0.00457924292307041	0.252970515640997	-2.07521575717819
a_HQ_transcript/149637	-3.28638145264281	-0.701903737438539	-3.04462268192536	0.00458510463644655	0.252970515640997	-2.05179767135867
a_HQ_transcript/113786	-3.83512331945586	1.42958378725807	-3.04457137924584	0.0045857113313587	0.252970515640997	-2.0770023653196
a_HQ_transcript/147820	0.721574278309956	10.3391999451983	3.04450329190749	0.00458651663562256	0.252970515640997	-2.8295881979056
a_HQ_transcript/149561	-0.730706978412185	4.68727051004167	-3.04446564715012	0.00458696193720085	0.252970515640997	-2.26946280063867
a_HQ_transcript/102979	3.31324368760877	1.21455707546316	3.04337868910187	0.00459983729186722	0.253440134693515	-2.02598608065093
a_HQ_transcript/106613	3.170681258709	0.440799606535641	3.042614518167	0.0046089096191635	0.25367217676481	-2.0168036105202
a_HQ_transcript/62283	2.4643831501207	4.29662625548719	3.04228921333999	0.00461277681737552	0.25367217676481	-2.25718159098573
a_HQ_transcript/135271	5.51605605591136	2.15584027921643	3.04116340579528	0.00462618406620334	0.254169024007815	-2.15433498234407
a_HQ_transcript/55903	-1.64602931038162	2.33257479644826	-3.03976036207428	0.00464294458687504	0.254848992394592	-2.03194744182232
a_HQ_transcript/86956	3.52704302506111	5.23132355362299	3.03917813228576	0.00464991667505963	0.254984712115046	-2.34163053420671
a_HQ_transcript/123903	1.99332267788824	-0.395751348662387	3.03882164782664	0.0046541903997052	0.254984712115046	-2.0392540100853
a_HQ_transcript/103161	4.65560013096982	-0.421683997630085	3.03660497273786	0.00468084867050214	0.256203739660027	-2.07091436237544
a_HQ_transcript/151968	-4.94161097465476	2.19162791734179	-3.0346401799	0.00470459856448209	0.257224332532602	-2.12564340607569
a_HQ_transcript/61704	3.38987113052103	1.05688608542473	3.03433108434187	0.00470834520050042	0.257224332532602	-2.05993160313772
a_HQ_transcript/10610	-2.51215924370103	-0.72249308037301	-3.03220494996771	0.00473419333814668	0.25837099520226	-2.07161415950289
a_HQ_transcript/88566	-3.94881782160229	3.82036389215588	-3.03187440480649	0.00473822393486116	0.25837099520226	-2.158737793114
a_HQ_transcript/31915	-4.2686866566015	0.264660441104203	-3.03148071313097	0.00474302876561043	0.258390605517716	-2.07540215759487
a_HQ_transcript/142766	4.01058086895413	1.36047100243138	3.0304832272882	0.00475522329149312	0.258468461784708	-2.04648168376555
a_HQ_transcript/80894	2.38953359313113	-0.381476044123838	3.03037005969325	0.00475660866818255	0.258468461784708	-2.09271248977042
a_HQ_transcript/93894	-3.98574793630431	-0.322851568903329	-3.03000071136352	0.00476113282081171	0.258468461784708	-2.05602274684015
a_HQ_transcript/152673	1.77313946157239	3.54421130798714	3.02991004168758	0.00476224405744945	0.258468461784708	-2.23906309723794
a_HQ_transcript/48732	-2.67324312738672	0.752805025176136	-3.02930552754347	0.00476965918776101	0.258629430285608	-2.05373936562386
a_HQ_transcript/114766	-5.43570765383183	1.30444481133064	-3.0283336784968	0.00478160305869579	0.25892154454886	-2.08741652749469
a_HQ_transcript/41492	3.65729675564844	1.58072067938967	3.02799554323879	0.00478576532214514	0.25892154454886	-2.04744707839036
a_HQ_transcript/115754	-3.50450174180444	-0.543130637781946	-3.02750852831277	0.00479176624165737	0.25892154454886	-2.08753549341079
a_HQ_transcript/13257	-2.4267960478886	-0.3109342299502	-3.02722856791028	0.00479521909185091	0.25892154454886	-2.07802748387976
a_HQ_transcript/40262	1.57826631139211	-0.763648093304977	3.02705847579024	0.00479731804774158	0.25892154454886	-2.10028177720572
a_HQ_transcript/77398	3.17542175410455	0.299406421087548	3.02653813543899	0.00480374450673804	0.259027885610082	-2.06356378896614
a_HQ_transcript/32622	-2.122507521693	1.42874634560535	-3.02603548226337	0.00480996025717441	0.259122678247483	-2.07148801754214
a_HQ_transcript/76698	3.09229128300241	0.668182686204592	3.02484403257758	0.00482472400439643	0.259677367525514	-2.05428848365242
a_HQ_transcript/54252	3.51339212184177	0.971484266324871	3.02384435805081	0.00483714446421887	0.259800457712026	-2.08525680301805
a_HQ_transcript/134068	-2.39164832229219	-0.0419182055518551	-3.02372986207408	0.0048385689496806	0.259800457712026	-2.09849459511183
a_HQ_transcript/68069	-2.79565468380269	0.925661326249292	-3.02358118030901	0.00484041934527464	0.259800457712026	-2.05798021026206
a_HQ_transcript/133333	1.61481310689685	-0.76277114861345	3.02284747265465	0.00484956039919006	0.260050965760258	-2.10918769551987
a_HQ_transcript/55537	3.29409202036463	1.29525695647977	3.02143127342792	0.00486725063657363	0.260427106664165	-2.06458018616346
a_HQ_transcript/10604	2.05829909250585	0.593449459124728	3.02137521609164	0.00486795212309241	0.260427106664165	-2.0612880226792
a_HQ_transcript/63790	-3.41084023421495	0.512904263851015	-3.02121036594479	0.00487001556812461	0.260427106664165	-2.07487803875469
a_HQ_transcript/50233	4.00361665859287	0.570203659721079	3.01919297238407	0.00489533460665132	0.261139130838747	-2.08136847504615
a_HQ_transcript/74982	1.08157835721322	4.57757748280559	3.01912459883386	0.00489619489837784	0.261139130838747	-2.36797885273572
a_HQ_transcript/80190	3.67197280268776	-0.0896631257572083	3.01879285679357	0.00490037098408495	0.261139130838747	-2.10615100137002
a_HQ_transcript/58980	-2.32464719819999	2.38419453710309	-3.01871905890555	0.00490130043602175	0.261139130838747	-2.13473853564837
a_HQ_transcript/45335	-2.21194175407227	-0.307597445910407	-3.01686601317943	0.00492469347940962	0.261513116696161	-2.0790279751264
a_HQ_transcript/62081	1.66866195555283	1.82646297927549	3.01628608092353	0.00493203628013415	0.261513116696161	-2.07614713102996
a_HQ_transcript/150134	0.48033060257658	10.5355650427678	3.0162775905977	0.00493214385711288	0.261513116696161	-2.91806750519559

a_HQ_transcript/148283	2.35091257523612	-0.756287941503513	3.01608405252526	0.00493459669032511	0.261513116696161	-2.10776795442569
a_HQ_transcript/78033	-4.98993805473273	0.296660030552627	-3.01586681951871	0.00493735119930249	0.261513116696161	-2.13618867187106
a_HQ_transcript/73166	-1.92082343641297	0.335370150320638	-3.01555807333825	0.004941268595833	0.261513116696161	-2.08069206770214
a_HQ_transcript/113338	-1.53797224826197	2.63744994921689	-3.0154375824925	0.00494279819092666	0.261513116696161	-2.15921957239417
a_HQ_transcript/10380	5.43642249012833	3.6998279098717	3.01531844125149	0.00494431109360516	0.261513116696161	-2.29981447646003
a_HQ_transcript/16965	2.93191809462409	0.946092120811303	3.01419582827076	0.00495858800379954	0.261904434917857	-2.12121409644876
a_HQ_transcript/6797	2.56518771442212	-0.0144285293754142	3.01402838049535	0.00496072087194743	0.261904434917857	-2.09425700010438
a_HQ_transcript/113898	-2.00501246633267	2.45462886526022	-3.01337517562368	0.00496904937556262	0.262068824414943	-2.15265630664261
a_HQ_transcript/42626	-1.69425430864872	1.96490826162316	-3.01270763251837	0.00497757435834404	0.262068824414943	-2.08834505090418
a_HQ_transcript/4049	-2.4874475420627	-0.468357015095217	-3.0126261688329	0.00497861565202513	0.262068824414943	-2.11422835076429
a_HQ_transcript/148573	3.5496650648159	2.22888309804538	3.01234377895384	0.00498222684086978	0.262068824414943	-2.21326272657374
a_HQ_transcript/58771	-5.62128900842405	0.627950794464158	-3.01201948464765	0.00498637695779878	0.262068824414943	-2.1209677603401
a_HQ_transcript/25181	-0.82692296469797	3.32615235944944	-3.01042410282201	0.0050068413150751	0.262586769009186	-2.22609931120146
a_HQ_transcript/103976	2.09683451041252	-0.145583717635996	3.01001765312992	0.0050120676276244	0.262586769009186	-2.08927035149242
a_HQ_transcript/137173	-2.40233815492239	-0.743523802510781	-3.00960877882554	0.00501733032226343	0.262586769009186	-2.11631012561275
a_HQ_transcript/98801	-2.37621132204069	-0.263936956312425	-3.00952320790854	0.00501843238200886	0.262586769009186	-2.12323613749374
a_HQ_transcript/4797	-3.0391978042066	3.0849331886457	-3.00949320661688	0.00501881882000423	0.262586769009186	-2.1061693270666
a_HQ_transcript/117083	3.18223489639168	3.86937978608034	3.00791157034618	0.00503923131588778	0.263417660008925	-2.20882928859688
a_HQ_transcript/4387	3.93999314233093	1.06311088813889	3.00685621447549	0.00505289531370683	0.263674641403515	-2.15110144080647
a_HQ_transcript/37014	1.85714427083934	-0.751397935277389	3.00661432471473	0.00505603206191879	0.263674641403515	-2.14730829125592
a_HQ_transcript/38870	1.97987692544157	-0.745003828408128	3.00619805873411	0.00506143437572217	0.263674641403515	-2.14393298566003
a_HQ_transcript/48516	-2.0953178698129	2.17099890613946	-3.006132027294	0.00506229183536889	0.263674641403515	-2.1170802775701
a_HQ_transcript/6956	-3.12636910265441	1.24265945219111	-3.00565205536199	0.00506852870087114	0.26370881133241	-2.11661990338953
a_HQ_transcript/72226	2.89337138372971	0.964579661868693	3.00518582318185	0.0050745939848052	0.26370881133241	-2.10300286625635
a_HQ_transcript/55329	-3.30401360645115	0.809010355772599	-3.0050349689472	0.00507655793904773	0.26370881133241	-2.13654854272084
a_HQ_transcript/137493	-3.63608602646254	0.667085709053389	-3.00453655999568	0.00508305177971661	0.263810387367292	-2.1154053247841
a_HQ_transcript/36935	3.2112539600799	0.764290046586996	3.00290164644851	0.00510440847855785	0.264682476397512	-2.11514663451198
a_HQ_transcript/7993	3.94276809164843	0.398430373880175	3.00189054349446	0.0051176588328612	0.265133041565558	-2.12559699620885
a_HQ_transcript/22758	2.19144258979159	-0.489126078802724	3.00067269404508	0.00513366173977113	0.26566663257856	-2.13609029310218
a_HQ_transcript/133884	1.93721799623807	-0.7611246339105	3.00040952076756	0.0051371261256885	0.26566663257856	-2.15199095854641
a_HQ_transcript/73345	-2.48865516470607	-0.327344983006315	-2.99929543383841	0.00515181634599395	0.26566663257856	-2.13020037996019
a_HQ_transcript/55440	-2.69418723560086	0.710870917795877	-2.99876199106426	0.00515886431019993	0.26566663257856	-2.11799613459649
a_HQ_transcript/52540	3.1204904477936	0.903297454699314	2.99869993188312	0.00515968484141863	0.26566663257856	-2.1168583822015
a_HQ_transcript/64170	3.57351198237296	0.290531597562439	2.99816038908503	0.00516682374358112	0.26566663257856	-2.15219968753215
a_HQ_transcript/87960	0.707705390100379	5.00673689061273	2.99814327949644	0.00516705027990217	0.26566663257856	-2.44808027113407
a_HQ_transcript/149486	-3.82885539964015	1.58070537396393	-2.99808178996999	0.00516786449802327	0.26566663257856	-2.16247004265682
a_HQ_transcript/127972	3.42470472986564	0.790081127016935	2.99798913047432	0.00516909168466748	0.26566663257856	-2.11053223250653
a_HQ_transcript/77767	-4.28867375794524	0.798423829009866	-2.99626200848098	0.00519201624367749	0.266531422676454	-2.16263209798454
a_HQ_transcript/69742	-3.40493148158133	0.254341562355892	-2.99603109764269	0.00519508845809974	0.266531422676454	-2.20103122594358
a_HQ_transcript/123980	-1.60142998155025	1.74717099057693	-2.99546279320422	0.00520265693422828	0.26668434062859	-2.12853277558377
a_HQ_transcript/124438	2.47124952230359	1.06851711632149	2.99353028452307	0.00522847143393423	0.267771442741611	-2.15142370164327
a_HQ_transcript/99377	2.62954926135264	-0.297133392658959	2.99264302249602	0.00524036400286444	0.267997439766418	-2.14621899149618
a_HQ_transcript/85177	2.63407428470937	2.20383515593091	2.99243755751974	0.00524312163062008	0.267997439766418	-2.13118801399505
a_HQ_transcript/16220	-2.59548264266862	3.15684640792999	-2.99216992908257	0.00524671563539401	0.267997439766418	-2.32277495972063
a_HQ_transcript/130696	-2.6035527821391	1.65115958343535	-2.99121250073849	0.00525959209031318	0.26841928799449	-2.14542196003794
a_HQ_transcript/136429	-4.21886918987767	0.634466712040432	-2.99086381486162	0.00526428898007652	0.268423324415691	-2.16417332387511
a_HQ_transcript/84916	-5.8235150861259	1.4773522501974	-2.98970002906453	0.00527999418203479	0.26898816986268	-2.2356625572687
a_HQ_transcript/36652	5.68136830382768	3.63833278801564	2.98808014238227	0.0053019281347447	0.269690016596195	-2.34484456171584
a_HQ_transcript/111909	1.84544679384186	-0.764095423545154	2.98799746145244	0.00530304997538967	0.269690016596195	-2.16655329330508
a_HQ_transcript/75599	3.7521466345556	0.461101626126922	2.98639161340593	0.00532488314604465	0.270272145315605	-2.22263778698997

a_HQ_transcript/115960	4.42656073201447	0.449726872684215	2.98633591139695	0.00532564199433843	0.270272145315605	-2.1993158423452
a_HQ_transcript/36902	3.44177348518614	0.146222382114003	2.98613019156054	0.00532844547432705	0.270272145315605	-2.18355890348223
a_HQ_transcript/101074	3.11673551802465	0.26981376261343	2.98456858250588	0.00534977205217904	0.27102955746437	-2.15947435797194
a_HQ_transcript/97223	-4.18020006530149	-0.784249181043406	-2.98435441261797	0.00535270320618458	0.27102955746437	-2.1767901338826
a_HQ_transcript/108426	1.46870702345796	3.73884989465672	2.98363488542717	0.00536256185180507	0.271074209601638	-2.38178581158127
a_HQ_transcript/118733	-3.18405160777316	-0.18431676655758	-2.98335176351552	0.00536644576376037	0.271074209601638	-2.22258928940422
a_HQ_transcript/19798	1.31870931191507	2.20675114992614	2.98307059777814	0.00537030546988349	0.271074209601638	-2.17699068377273
a_HQ_transcript/128702	-5.53734166349851	4.08661807971754	-2.98284276370748	0.00537343498783338	0.271074209601638	-2.32309494617073
a_HQ_transcript/42704	5.14078701662719	0.274010226876503	2.9825905005069	0.00537690207250703	0.271074209601638	-2.2176039413067
a_HQ_transcript/125890	1.2434509631845	3.76551457730386	2.98181825915051	0.00538752883262601	0.271374589239935	-2.34935017108351
a_HQ_transcript/102860	3.29815277455016	0.814592485553589	2.98049486909899	0.00540578604754031	0.271872625256513	-2.15788547018997
a_HQ_transcript/77030	4.70481075560099	2.56918028248199	2.98042363123294	0.00540677048576467	0.271872625256513	-2.17732056846089
a_HQ_transcript/101030	1.87305428556384	-0.771425697156111	2.98006082345473	0.00541178676470584	0.271889663836492	-2.20318314555319
a_HQ_transcript/132240	-3.41918867885851	4.83618130560851	-2.97931051574591	0.00542217466920357	0.271959820358189	-2.48129412921968
a_HQ_transcript/123119	3.14425332677572	0.622421055295255	2.97928411987862	0.00542254045890347	0.271959820358189	-2.16023851503277
a_HQ_transcript/72070	-2.92069218202477	0.772499897903609	-2.9787589106992	0.00542982356999797	0.272090331445554	-2.15080556658477
a_HQ_transcript/100669	-2.21941336129981	-0.770056798740355	-2.97827358680983	0.00543656179973078	0.272193337032516	-2.18693491054768
a_HQ_transcript/1653	0.684835124449544	6.23636046779252	2.977324670009	0.00544975936449551	0.272612225108877	-2.57661288942229
a_HQ_transcript/53369	1.4951974082845	0.768503228333336	2.97699811881902	0.00545430804090324	0.272612225108877	-2.19505314697371
a_HQ_transcript/56768	2.41567298836511	1.8322146181347	2.97654462439566	0.00546063091211853	0.272693774621672	-2.16272254876416
a_HQ_transcript/146717	1.23325258857498	5.28535916722543	2.97568988788777	0.00547256694207199	0.273055254256447	-2.51556413778748
a_HQ_transcript/145809	0.496777401306517	10.5476474406513	2.97439443673169	0.00549070433213493	0.273725267082623	-3.01612281449103
a_HQ_transcript/74987	2.88408338159479	0.976571187075951	2.97254474941909	0.00551669987348696	0.274785544341088	-2.16694159552735
a_HQ_transcript/103117	-2.98071385633218	1.12904764021098	-2.97182943152408	0.00552678408368039	0.274816868834748	-2.17909377431804
a_HQ_transcript/77198	-3.65211589538807	0.383088496325852	-2.97182941390372	0.00552678433229803	0.274816868834748	-2.19071073986851
a_HQ_transcript/83127	1.93595805145385	-0.196465422451726	2.97089781830408	0.00553994361164264	0.27486377042632	-2.17113414597114
a_HQ_transcript/30518	4.15556931902279	0.817433740187083	2.97084819179079	0.00554064544137524	0.27486377042632	-2.17652181066118
a_HQ_transcript/65150	-4.27360485813216	2.00349253089693	-2.9707585530998	0.00554191334536964	0.27486377042632	-2.2343028979703
a_HQ_transcript/58144	-2.11994295698281	-0.42486379572096	-2.96925193828794	0.00556326484628366	0.275333748435559	-2.20246073362702
a_HQ_transcript/27457	2.46784304523808	1.62949725006422	2.96919139085687	0.0055641245353997	0.275333748435559	-2.18058077080509
a_HQ_transcript/94486	2.42746658896197	2.54177130285713	2.96907542146903	0.00556577148912541	0.275333748435559	-2.26794964143924
a_HQ_transcript/10297	-2.48596389970885	1.16806502564723	-2.96843124808089	0.00557492817706326	0.275333748435559	-2.18058508093262
a_HQ_transcript/151124	0.642193318177725	10.2629299833837	2.96841938360125	0.00557509695955003	0.275333748435559	-2.99219043542727
a_HQ_transcript/119688	3.0844233018035	-0.579400368946345	2.96808826798887	0.00557980931146931	0.275333748435559	-2.21643788004757
a_HQ_transcript/98524	3.74157736010372	1.45146682505346	2.96572804494737	0.00561350845464886	0.276761678924367	-2.25474976150976
a_HQ_transcript/95770	2.05647837273336	-0.652149674440607	2.96502408704516	0.00562359665603569	0.277024090188172	-2.2132861953874
a_HQ_transcript/60490	-3.00457500077114	0.286492943847691	-2.96351432695254	0.00564529027677683	0.277857267746387	-2.22500832006896
a_HQ_transcript/130924	1.83008935224588	3.72385778088096	2.96248474390981	0.00566012953199252	0.278351953837277	-2.37475112546935
a_HQ_transcript/143782	2.06487598206853	8.48470182740188	2.96163720647464	0.00567237259585379	0.278606086201933	-2.8505850431357
a_HQ_transcript/88898	4.9225391060026	0.927391584489085	2.96146362630178	0.00567488312109635	0.278606086201933	-2.22778311533862
a_HQ_transcript/114799	0.56388330655367	6.84531366996146	2.96084376983817	0.0056838567893426	0.278811162405829	-2.68778031898368
a_HQ_transcript/35590	-0.99638432123298	2.92657445766215	-2.95958502691329	0.0057021208351888	0.279471230950973	-2.30016917243267
a_HQ_transcript/18266	1.9701193420141	2.2699767417836	2.95826348932634	0.00572135558508719	0.280177723209729	-2.30807649248819
a_HQ_transcript/107376	2.62234031041468	1.39910619044551	2.957702803743	0.00572953474494653	0.28034208388405	-2.19456198318972
a_HQ_transcript/144910	-4.09831911117085	-0.678470751681214	-2.95666109637951	0.00574476023700846	0.280438966463659	-2.2448497439859
a_HQ_transcript/128487	-2.28744436143133	-0.745396880360686	-2.95634337791816	0.00574941156842068	0.280438966463659	-2.23654212640719
a_HQ_transcript/150207	0.618910426350087	10.661021378805	2.95630311049237	0.00575000132862843	0.280438966463659	-3.08186084481288
a_HQ_transcript/106960	3.1327900701376	0.96100396871538	2.95624770965934	0.00575081282728946	0.280438966463659	-2.23022007692598
a_HQ_transcript/110997	3.24834299379442	0.538608703896695	2.95485671705778	0.00577122320701741	0.28119837600797	-2.23711754353948
a_HQ_transcript/117661	-2.06978429338264	-0.661707524741731	-2.95205033218745	0.00581261013430207	0.282966224662813	-2.23512141092853

a_HQ_transcript/87734	-4.22821328324092	0.541375272803474	-2.95154044642215	0.00582015961465813	0.282966224662813	-2.25693556458379
a_HQ_transcript/98965	-2.36507556117092	-0.64853105390986	-2.9510111740259	0.0058280059175773	0.282966224662813	-2.24924397303026
a_HQ_transcript/146894	-1.79097126071145	1.75459370010873	-2.95097288646487	0.00582857390577886	0.282966224662813	-2.21484606336619
a_HQ_transcript/149109	-2.32950655858482	1.26863325598089	-2.9507523943794	0.00583184587713408	0.282966224662813	-2.24257121080999
a_HQ_transcript/96234	3.2645361275825	0.491394987571579	2.9493371078171	0.00585288916100217	0.283706964719139	-2.25848312595416
a_HQ_transcript/149596	1.27534044655159	-0.78001158982172	2.94854634662268	0.0058646778028027	0.283706964719139	-2.27790040583879
a_HQ_transcript/110918	-1.1698197938694	2.96985833465618	-2.94838343318621	0.00586710929057448	0.283706964719139	-2.38195937988604
a_HQ_transcript/90694	4.0118137984124	0.900216984783846	2.9483510732097	0.0058675923772987	0.283706964719139	-2.22036921289408
a_HQ_transcript/118708	2.84128030717935	-0.389740440764623	2.94800013762589	0.00587283373724122	0.283706964719139	-2.27644733881131
a_HQ_transcript/68491	3.82103513296676	0.576276095757148	2.94776174992209	0.00587639666807464	0.283706964719139	-2.26656904219916
a_HQ_transcript/96543	-4.14442830055538	0.921244985367351	-2.94739986257223	0.00588180931384235	0.283732623896289	-2.2224026002291
a_HQ_transcript/109799	4.46155495218311	0.817973796357133	2.94653649220986	0.00589474148727599	0.284120674272288	-2.27765468777271
a_HQ_transcript/141416	2.77211736462992	4.21270912140351	2.94585215086008	0.0059050110768687	0.284317440740774	-2.36599516012372
a_HQ_transcript/44962	2.021438710279	0.510290161829307	2.94561283714545	0.00590860632423023	0.284317440740774	-2.23392857219687
a_HQ_transcript/130230	2.36319278101425	1.22160017022118	2.94515885323989	0.00591543226650618	0.284410460535543	-2.22918691575861
a_HQ_transcript/44694	3.66759185944677	0.427737676811361	2.94409890533062	0.0059313982206907	0.284942409729181	-2.31808320046295
a_HQ_transcript/3986	-1.80612942378956	3.33625077881429	-2.94353998466471	0.00593983356673698	0.285057807259998	-2.32879096878663
a_HQ_transcript/145489	4.11112109544802	5.75213802014804	2.94324621880638	0.00594427167862365	0.285057807259998	-2.65249194146425
a_HQ_transcript/135829	-4.67107144465175	2.7018663863306	-2.94296572120881	0.00594851225238056	0.285057807259998	-2.41463517013902
a_HQ_transcript/3316	-6.93102845774257	0.503684795053971	-2.94224168412008	0.00595947145722503	0.285347740416455	-2.32350251523578
a_HQ_transcript/119011	-4.87799988504323	1.57648851915794	-2.94068262025085	0.00598313447594207	0.286071471684342	-2.24689050698177
a_HQ_transcript/15501	-3.02190358039469	0.0813500792035888	-2.94059747350578	0.00598442935535645	0.286071471684342	-2.26705292312001
a_HQ_transcript/113507	3.30419242059224	0.642129022926963	2.9395641740787	0.00600016444663834	0.286587969559732	-2.23339632371284
a_HQ_transcript/152319	0.445942571867747	11.2502111139063	2.93922149368038	0.006005391390146	0.286602127033175	-3.1844471478243
a_HQ_transcript/43802	-3.97203659945293	0.538063116775328	-2.93825591726166	0.00602014252790838	0.287070422364445	-2.32741846911089
a_HQ_transcript/125987	-1.45430404514857	4.57335137418242	-2.93726450813753	0.00603532383562637	0.287467585615395	-2.57682544844376
a_HQ_transcript/94405	1.00015419265597	5.58206649762996	2.93664986964035	0.00604475381577676	0.287467585615395	-2.63104093587044
a_HQ_transcript/82168	3.50834258793546	1.47587995426231	2.93651036972553	0.00604689600029224	0.287467585615395	-2.2576834973364
a_HQ_transcript/127633	-1.59022840396232	3.29827951970259	-2.93642201780904	0.0060482531173897	0.287467585615395	-2.41464359886041
a_HQ_transcript/103042	2.26232709565346	-0.508370409571849	2.9354360947645	0.00606341673154022	0.287952849486086	-2.27261822544638
a_HQ_transcript/139071	5.48693845870298	2.02030885329401	2.93487905565435	0.0060719998885942	0.28812506899935	-2.27196451873187
a_HQ_transcript/5152	4.01377870155705	2.19703334416937	2.9343035003921	0.00608088037640329	0.288311104828361	-2.2706679748988
a_HQ_transcript/21416	-2.44650068295608	3.02394013635331	-2.93361671224547	0.00609149313655802	0.288578902234592	-2.36810977838114
a_HQ_transcript/148478	4.63546148411501	1.5729994507375	2.93262673828235	0.00610682165859753	0.289069486458434	-2.37720669839448
a_HQ_transcript/146398	2.18490783541389	2.91542212992675	2.93195437085363	0.00611725314982492	0.289140469218242	-2.305507067904
a_HQ_transcript/41054	3.54838061240464	0.127879149986194	2.93188891074221	0.00611826963147601	0.289140469218242	-2.25679566505037
a_HQ_transcript/129160	-3.75702549361509	-0.0632134938500911	-2.93065076238802	0.00613752591449676	0.289814871127431	-2.32759706647619
a_HQ_transcript/113067	2.12571847343535	-0.445366114668533	2.92915653503554	0.00616084088531366	0.290679674497981	-2.27062750113525
a_HQ_transcript/152819	-3.72266262628082	2.01565964982939	-2.92881565485796	0.00616617143643518	0.290695225674861	-2.34835811932178
a_HQ_transcript/1661	4.25854153214266	2.07866704921133	2.92830318717605	0.00617419338119446	0.290718974404949	-2.33713870285106
a_HQ_transcript/3724	-1.09349340239209	2.44214682733915	-2.92814459483985	0.00617667790720672	0.290718974404949	-2.34395556689128
a_HQ_transcript/127352	-3.21045460508791	0.768714918393745	-2.92758560372973	0.00618544263802257	0.290895962510498	-2.2710694461328
a_HQ_transcript/40063	-2.17783580325077	2.26949685944383	-2.92688862059574	0.00619638743066381	0.291175107978679	-2.3062391254935
a_HQ_transcript/112100	3.99540822786661	0.255350152347765	2.92557767120305	0.00621702282693079	0.291908806852854	-2.303431162102
a_HQ_transcript/142505	1.37496076094008	-0.803922904146225	2.92520371333402	0.00622292107728361	0.29194992443934	-2.32029534406843
a_HQ_transcript/46554	-2.13581087479748	0.778019571174448	-2.92408544280007	0.00624059045505171	0.292542775783263	-2.27255171640675
a_HQ_transcript/152550	0.403725898120433	11.1182790651046	2.92202055215289	0.00627334125022402	0.293586998459705	-3.20899740674005
a_HQ_transcript/71431	-1.22700745722756	4.61661994730666	-2.92194034604828	0.00627461664174052	0.293586998459705	-2.59487508975044
a_HQ_transcript/137841	1.28862490164421	5.97668367175026	2.92168495649011	0.0062786793266857	0.293586998459705	-2.70437576510635
a_HQ_transcript/68679	2.31384851460344	-0.630323749397343	2.92130901318349	0.00628466426264859	0.293586998459705	-2.30865881855418

a_HQ_transcript/45143	-2.33211574944927	-0.683573633469731	-2.9209341343883	0.00629063760010874	0.293586998459705	-2.30877103046653
a_HQ_transcript/53164	-3.81217234108722	1.23657661617342	-2.9207752900098	0.00629317024636651	0.293586998459705	-2.34786244141696
a_HQ_transcript/123498	-1.96412957585502	-0.489926026290252	-2.92044565867111	0.0062984290172822	0.293596697607522	-2.29044254626747
a_HQ_transcript/150595	0.526726498170693	8.44087315536933	2.91890104713771	0.00632312612651674	0.294135067017069	-2.91257497625444
a_HQ_transcript/152972	-4.26717550001596	0.279549487072639	-2.91863758606016	0.00632734775023621	0.294135067017069	-2.32181396400698
a_HQ_transcript/120707	-1.51932346857691	3.4613612592732	-2.91844027563594	0.00633051113266519	0.294135067017069	-2.50921658164488
a_HQ_transcript/55117	-2.65860093840377	-0.665098422642123	-2.91837299809653	0.0063315901011931	0.294135067017069	-2.31996539642026
a_HQ_transcript/55570	3.32565682851479	0.545260338258817	2.9181430569477	0.00633527910654711	0.294135067017069	-2.30158148218851
a_HQ_transcript/124519	-6.72339667382663	0.940870630834382	-2.91762040921971	0.0063436716067959	0.294289659345437	-2.32509506120922
a_HQ_transcript/3582	-2.68178777063653	2.58984396592596	-2.91675461511302	0.00635759726773358	0.294591894542724	-2.35889101777433
a_HQ_transcript/47983	1.83210700454493	4.49829751302651	2.91658538784164	0.00636032252358793	0.294591894542724	-2.47518406567569
a_HQ_transcript/95091	4.0644238595711	5.6347741804641	2.91578529407267	0.0063732222238432	0.294777384339987	-2.65382745019955
a_HQ_transcript/143569	-3.97746133720871	0.503849889574024	-2.91537219927623	0.00637989207768993	0.294777384339987	-2.33769280515487
a_HQ_transcript/106614	-1.83095632172508	2.97339990992508	-2.91507218310201	0.00638474027516806	0.294777384339987	-2.45846383946616
a_HQ_transcript/77353	-4.85541357270477	2.57065504151086	-2.91492392067663	0.00638713744445563	0.294777384339987	-2.32983260726023
a_HQ_transcript/98736	-2.75212297572132	-0.307476043578701	-2.91455149827411	0.0063931626636008	0.294777384339987	-2.3087747149142
a_HQ_transcript/94439	3.79766331900131	0.689423716049023	2.91439435862221	0.00639570654488179	0.294777384339987	-2.36199756837051
a_HQ_transcript/149017	5.45253260305609	7.02540367071015	2.91378532272618	0.00640557502783819	0.294777384339987	-2.73089161229457
a_HQ_transcript/45326	2.8118448197804	-0.232722000962992	2.91351375422106	0.00640997999288118	0.294777384339987	-2.32407583507186
a_HQ_transcript/150955	5.72422283852207	8.79669584568106	2.91334382348591	0.00641273779686426	0.294777384339987	-2.91806724396995
a_HQ_transcript/127032	3.06471486139723	0.608385250138062	2.91320207582626	0.00641503907222137	0.294777384339987	-2.31963707076444
a_HQ_transcript/120441	-3.22249234431345	0.0369570091803907	-2.91228155233191	0.00643000270782711	0.295039573806423	-2.34719273273649
a_HQ_transcript/141576	6.80210803144775	0.730938873953121	2.91222664482417	0.00643089629804462	0.295039573806423	-2.40104076690179
a_HQ_transcript/145954	9.58198811707981	7.58845806475111	2.91189414685915	0.00643631002108682	0.295055070114933	-2.73837098262102
a_HQ_transcript/123786	2.75612067873609	0.0770005430567046	2.9109289923155	0.00645204894519227	0.295543499673866	-2.31405861097179
a_HQ_transcript/46761	2.19343910411067	0.927395316806322	2.90994938533449	0.00646806057705049	0.296043641907709	-2.29250652029184
a_HQ_transcript/106300	4.32815903488754	2.2459923848052	2.90827199388586	0.00649556432045451	0.297068578142707	-2.43891089294963
a_HQ_transcript/38473	1.14844532701329	5.41706047949323	2.90737608030992	0.00651029942362897	0.297508400076026	-2.68982117313729
a_HQ_transcript/67536	1.95649573004419	2.42787933601621	2.90694666702935	0.00651737313383445	0.29759769483388	-2.34203513779266
a_HQ_transcript/138936	-6.56468999642701	5.81225672245773	-2.90485770737647	0.00655188776260231	0.298767703814452	-2.71518649395593
a_HQ_transcript/25037	2.21380827902846	0.0286120303137619	2.90477390774477	0.00655327591459238	0.298767703814452	-2.3217114194573
a_HQ_transcript/970	-2.02430063743868	3.21230693008654	-2.90331593431482	0.00657747176253551	0.299635798285787	-2.41305335501911
a_HQ_transcript/136887	0.928540894094295	4.57611265093374	2.90265179781154	0.00658852129813919	0.299829839694496	-2.65369614963237
a_HQ_transcript/1749	-1.47840313143932	2.72556299608438	-2.90214093361553	0.00659703265200876	0.299829839694496	-2.40150058151295
a_HQ_transcript/81007	3.36675059595515	0.453407294429459	2.90185160429382	0.00660185766651973	0.299829839694496	-2.35632058173716
a_HQ_transcript/99538	-5.29891639037862	0.37541239995209	-2.90137601911449	0.00660979599874762	0.299829839694496	-2.35943411041357
a_HQ_transcript/29480	4.37175820707297	0.984006106966524	2.90111938755247	0.0066140833481381	0.299829839694496	-2.41526531040606
a_HQ_transcript/117980	3.83985103593177	0.311365437619437	2.90105696564577	0.00661512657911792	0.299829839694496	-2.38303408936083
a_HQ_transcript/49758	3.52418792422073	1.34192665295909	2.90089477362133	0.00661783794949143	0.299829839694496	-2.32594381952231
a_HQ_transcript/113351	1.45647282682433	1.93743857577923	2.898455884072	0.00665873519370345	0.301447787647659	-2.35755982218608
a_HQ_transcript/46906	3.87493306031298	2.9771898666903	2.89779726369889	0.00666982015625502	0.301714635052756	-2.40295778655089
a_HQ_transcript/114723	1.22621832406546	4.13856618085362	2.89589017457481	0.00670201552104035	0.302503325635838	-2.63219659924506
a_HQ_transcript/145060	1.00822880800329	2.68976964240836	2.89588636485949	0.0067020799824301	0.302503325635838	-2.42538777000176
a_HQ_transcript/100451	-3.03688732873149	2.36825931142983	-2.89583982337419	0.0067028675237228	0.302503325635838	-2.42371229026889
a_HQ_transcript/139599	-7.14679618513002	3.15880747526634	-2.89483608617342	0.00671987322597757	0.302927919700618	-2.46550069599612
a_HQ_transcript/67191	1.9307864366528	-0.853661660096046	2.89466956183946	0.00672269846569291	0.302927919700618	-2.37069113880778
a_HQ_transcript/4458	2.21914395114874	2.57332725442811	2.89435264283586	0.00672807837587952	0.302935507229376	-2.42491827180374
a_HQ_transcript/38691	2.14431634909426	-0.837377033406671	2.89396777673965	0.00673461717569465	0.302995222282337	-2.3724156768297
a_HQ_transcript/139334	1.91765788246373	1.42015863059112	2.89351344217284	0.00674234392086499	0.303102198184413	-2.33164769957048
a_HQ_transcript/56281	1.88941669987185	-0.856835735953374	2.8932099273307	0.00674751035858812	0.303102198184413	-2.37384075802389

a_HQ_transcript/116607	1.87819935208366	-0.857712885920764	2.89275346624943	0.00675528725945712	0.303102198184413	-2.37482865703693
a_HQ_transcript/108244	0.830495615628259	3.54543590252942	2.89212129296018	0.00676607175335403	0.303102198184413	-2.54615837346304
a_HQ_transcript/59005	1.81388912137328	2.16644824116437	2.89209395062913	0.00676653856194569	0.303102198184413	-2.35810762042366
a_HQ_transcript/92827	-3.82958167448847	1.04660723956784	-2.89198262000338	0.0067684395937452	0.303102198184413	-2.42156795543986
a_HQ_transcript/119203	2.0735897305733	1.26309231057046	2.89158911590396	0.00677516291749984	0.303102198184413	-2.3368180037078
a_HQ_transcript/92918	-1.98377223715545	-0.800275804536595	-2.89125923469995	0.00678080403170382	0.303102198184413	-2.37357424616332
a_HQ_transcript/122214	2.63688731335663	1.23054766802449	2.89107687020754	0.0067839244398211	0.303102198184413	-2.39714727719662
a_HQ_transcript/143465	8.7659861677523	2.08594905669036	2.8901307933012	0.00680013427984321	0.303362596941893	-2.43029930244593
a_HQ_transcript/81064	1.71680035485446	1.70857729356917	2.8901275253351	0.00680019033538548	0.303362596941893	-2.35928044616068
a_HQ_transcript/105795	3.4763484683106	1.27441026021954	2.88966858164374	0.00680806692854148	0.303481069342223	-2.39914990733506
a_HQ_transcript/56400	1.53173760940562	1.54419184751328	2.88912969003294	0.00681732656943053	0.303660964619048	-2.3470995742585
a_HQ_transcript/102201	2.08675120985983	-0.841499583145002	2.88856824807814	0.00682698627742536	0.303703988924289	-2.38396400336745
a_HQ_transcript/141414	1.7353671133277	-0.868988482003771	2.88820955052171	0.00683316446573526	0.303703988924289	-2.38478331837288
a_HQ_transcript/55411	4.65886597865797	0.614891981265066	2.8881629950353	0.00683396672022037	0.303703988924289	-2.4002959047768
a_HQ_transcript/95397	-2.43966973486841	1.3285377347503	-2.88762387510221	0.00684326340528243	0.303820370007859	-2.40240962526343
a_HQ_transcript/16058	4.04671957958624	0.425013206643033	2.88740512107814	0.00684703902956054	0.303820370007859	-2.45048048804345
a_HQ_transcript/106366	-5.07559004216251	-0.749057209849471	-2.88577705396995	0.00687520051348935	0.304766445094549	-2.40824121328805
a_HQ_transcript/99021	4.32774173192665	2.40303574235721	2.88556674447417	0.00687884626968154	0.304766445094549	-2.55964886778783
a_HQ_transcript/122696	-5.14496705108487	0.78753852378154	-2.88495637160882	0.00688943749309566	0.304946192825491	-2.3861706502461
a_HQ_transcript/152524	-2.76746993982767	-0.505656098365158	-2.88472849402698	0.00689339556449035	0.304946192825491	-2.4188106254772
a_HQ_transcript/124285	2.13540011014737	8.46277563469901	2.88435954534579	0.0068998084714134	0.304997769449671	-3.01517909494862
a_HQ_transcript/86979	2.5217666795864	-0.12388922946703	2.88396351593169	0.00690669832220538	0.305070334402093	-2.39976004802741
a_HQ_transcript/32701	1.96230645525391	-0.850929924303028	2.88252130145232	0.00693184366792824	0.305948525990382	-2.39686283259309
a_HQ_transcript/22090	-2.49085074633824	-0.64914236930044	-2.88187643164533	0.00694311493114078	0.306029635122742	-2.40089688131256
a_HQ_transcript/140809	-2.66109316344285	0.236601576032631	-2.88181378220391	0.00694421085753675	0.306029635122742	-2.38591968786016
a_HQ_transcript/8712	1.86568026040673	-0.858551598246018	2.88108348569749	0.00695699792654894	0.306257693714038	-2.39996508939187
a_HQ_transcript/50869	-2.38048145985053	1.37345793446755	-2.87983245560074	0.00697895416013441	0.306257693714038	-2.3544647927329
a_HQ_transcript/108905	-5.50215561289944	0.921030928603009	-2.87969655567011	0.00698134319119233	0.306257693714038	-2.43481722060194
a_HQ_transcript/149629	1.13652021051216	6.03065606061855	2.87966406326328	0.00698191450004823	0.306257693714038	-2.80010349095709
a_HQ_transcript/79495	2.89047864000883	1.41917803470647	2.87955285174493	0.00698387024705632	0.306257693714038	-2.35802355690765
a_HQ_transcript/143375	1.73658319361305	-0.8687971682936	2.8793347392627	0.00698770743099162	0.306257693714038	-2.40388096276093
a_HQ_transcript/132692	6.95475032339272	3.59627924745295	2.87911706758602	0.00699153883451634	0.306257693714038	-2.50572645307883
a_HQ_transcript/96660	4.18941381379617	1.35971621640836	2.87885385377003	0.00699617449571993	0.306257693714038	-2.40124472156345
a_HQ_transcript/75294	1.23586640795498	10.3573429765978	2.87881812854704	0.00699680390263286	0.306257693714038	-3.27030874202772
a_HQ_transcript/138588	4.21245706876964	2.8801419475661	2.87786421513901	0.00701362963297207	0.306708886212405	-2.47083690009591
a_HQ_transcript/4220	1.39677945789764	1.33566521412506	2.87763576689021	0.00701766478568846	0.306708886212405	-2.39767157931711
a_HQ_transcript/17603	3.26613798818749	0.0215896413151688	2.87681267043333	0.00703222148578446	0.307034941397916	-2.40407789340584
a_HQ_transcript/35533	4.62226669552306	0.793555864596406	2.87661682735748	0.00703568920214052	0.307034941397916	-2.38862752311868
a_HQ_transcript/137877	-3.58218427994576	0.272219163425219	-2.875116397737	0.00706231006044464	0.307902422152348	-2.44132604792632
a_HQ_transcript/99693	1.79963986247223	-0.863699492414651	2.87474179639536	0.00706897103892959	0.307902422152348	-2.41367038222814
a_HQ_transcript/62428	-2.30130709210005	-0.425425670063991	-2.87460199702332	0.00707145839480774	0.307902422152348	-2.39237692225743
a_HQ_transcript/86016	1.63853670677165	-0.87679167091489	2.87404178556831	0.00708143410843646	0.308105989412571	-2.41547151891794
a_HQ_transcript/94290	1.83113832353003	2.08840001385764	2.87307201136991	0.00709873417569334	0.308423083432551	-2.41620188824564
a_HQ_transcript/20077	-2.70506927753973	2.12717091288073	-2.87287177693487	0.00710231115281226	0.308423083432551	-2.56702508783145
a_HQ_transcript/151131	-1.38179167009311	2.60494055514926	-2.87274146706219	0.00710463991047662	0.308423083432551	-2.44332418674971
a_HQ_transcript/81502	3.31051716802408	0.397495179423371	2.87206317079129	0.00711677327576265	0.308592375712528	-2.43002546332809
a_HQ_transcript/60775	-2.26948259047179	0.576219228680916	-2.87121003573911	0.00713206178721054	0.308592375712528	-2.39991865825666
a_HQ_transcript/100535	-2.17807714207956	-0.696304868316902	-2.87110848266167	0.00713388371048757	0.308592375712528	-2.41941233648122
a_HQ_transcript/106184	3.68119785346168	1.1326188643209	2.87036488681565	0.00714723758910072	0.308592375712528	-2.40151026622683
a_HQ_transcript/48770	-1.70167754322836	1.34484743783459	-2.87031687875998	0.00714810054870185	0.308592375712528	-2.41149783021807

a_HQ_transcript/9879	-1.70149566185349	-0.65894024301331	-2.87002579992256	0.00715333487579363	0.308592375712528	-2.43336832372678
a_HQ_transcript/148986	5.13769886679094	2.83539306331607	2.86995730346869	0.00715456713671954	0.308592375712528	-2.52170257438842
a_HQ_transcript/84899	-3.39983970856105	0.252886619101655	-2.86938106757523	0.00716494159651373	0.308592375712528	-2.40181988993875
a_HQ_transcript/67512	-2.93656676456627	0.678318426132075	-2.86933714011165	0.00716573303876223	0.308592375712528	-2.41657825345012
a_HQ_transcript/45580	1.73493038779705	-0.868921009901111	2.86931975906698	0.00716604621615843	0.308592375712528	-2.42540030420825
a_HQ_transcript/53341	1.73441040518457	-0.868964119289551	2.86927033173759	0.00716693688432275	0.308592375712528	-2.4255073654427
a_HQ_transcript/47499	1.97867500609855	-0.836768805048501	2.86865440434627	0.00717804446371262	0.308834871231206	-2.42666110871759
a_HQ_transcript/50585	6.21117646485521	4.71470180748057	2.86836911755194	0.00718319477540239	0.308834871231206	-2.54349529018043
a_HQ_transcript/121286	2.04077430784442	2.53902201145367	2.86801769482286	0.00718954381515953	0.308856883379714	-2.47417585750144
a_HQ_transcript/129652	2.22916298107748	-0.830263469476569	2.86705063128233	0.00720704264544484	0.308856883379714	-2.43054063418835
a_HQ_transcript/498	-0.906760665834915	-0.83312967523569	-2.86694554843822	0.00720894650609576	0.308856883379714	-2.440380281341
a_HQ_transcript/120244	2.43175146448485	4.08202899907347	2.86663661603553	0.00721454638908579	0.308856883379714	-2.64830433184156
a_HQ_transcript/138169	-1.70745089493779	2.1515821310974	-2.86648213153833	0.00721734819361879	0.308856883379714	-2.393414625767
a_HQ_transcript/43359	3.13830966194285	1.08047502645325	2.86647319649955	0.00721751027498914	0.308856883379714	-2.42066511408521
a_HQ_transcript/87964	2.80555346625613	0.490930660559461	2.86603727437567	0.00722542204234046	0.308856883379714	-2.39319998308587
a_HQ_transcript/150727	-3.95369038227149	0.0941433589341146	-2.86572547347811	0.00723108606550176	0.308856883379714	-2.44886513179043
a_HQ_transcript/93518	3.44060096956519	-0.127051254465074	2.86505811722853	0.00724322294434262	0.308856883379714	-2.46878660995149
a_HQ_transcript/43555	2.49768793524723	-0.607704926427642	2.86479496660096	0.0072480139897156	0.308856883379714	-2.43260328261258
a_HQ_transcript/145048	-2.42890256968099	0.270821540287345	-2.8645318610902	0.0072528071875709	0.308856883379714	-2.40760029459981
a_HQ_transcript/143348	1.62889201950084	-0.877572790187168	2.86435502154042	0.00725603048312196	0.308856883379714	-2.4362863923132
a_HQ_transcript/7117	-1.52421862646609	3.0630633543895	-2.86425528891069	0.00725784892589944	0.308856883379714	-2.5421605419269
a_HQ_transcript/137767	-1.83232509061973	2.32057108601235	-2.86395073976039	0.00726340447266932	0.308856883379714	-2.44267928610119
a_HQ_transcript/126605	2.61684740961393	-0.138383955604883	2.86376426413038	0.00726680810738199	0.308856883379714	-2.45129259442184
a_HQ_transcript/123533	-3.74925609105849	-0.645974358897549	-2.86365949602479	0.00726872103742514	0.308856883379714	-2.45183986808027
a_HQ_transcript/129542	-3.04696837701542	0.287190340256811	-2.86249791870572	0.00728996163108623	0.309349347125415	-2.42084412810161
a_HQ_transcript/41314	2.72525959021452	1.20377249217411	2.86244369751923	0.00729095454104422	0.309349347125415	-2.43003071275105
a_HQ_transcript/152003	1.09951709415426	4.35055714857601	2.86118477196106	0.00731404397257817	0.30972312660619	-2.68208499386539
a_HQ_transcript/113207	2.56111623108931	2.59556477311472	2.86090902100549	0.00731911056019166	0.30972312660619	-2.46981262424068
a_HQ_transcript/84593	4.6932888983405	2.11069418372723	2.86061799423611	0.00732446139479229	0.30972312660619	-2.48868448771068
a_HQ_transcript/76448	1.90308539355521	-0.789151516059185	2.8603640405937	0.00732913360009641	0.30972312660619	-2.45235434237558
a_HQ_transcript/130292	6.2591129463104	3.05337989937755	2.86015337679494	0.00733301148691611	0.30972312660619	-2.63151361820236
a_HQ_transcript/76204	-1.0760856297459	2.96375272339473	-2.85990128157013	0.00733765457181919	0.30972312660619	-2.54929041411461
a_HQ_transcript/102016	1.66512219439681	-0.874597921445683	2.85951833910714	0.00734471287743584	0.30972312660619	-2.44657008145203
a_HQ_transcript/39907	3.03549155006732	1.25653815745028	2.85948445941298	0.0073453376467017	0.30972312660619	-2.39768871678873
a_HQ_transcript/16628	2.81442083211886	1.31135918270748	2.85935536649673	0.00734771868273356	0.30972312660619	-2.42942057287047
a_HQ_transcript/129834	-1.4880948299245	3.00532759504465	-2.85798176500056	0.00737309877566899	0.310248202309443	-2.60663403281297
a_HQ_transcript/10668	-3.2911262927747	1.50548204884336	-2.85736558769218	0.00738451061716011	0.310248202309443	-2.44731380798612
a_HQ_transcript/117686	1.58586899378934	-0.881134007372932	2.85729353725257	0.00738584610085544	0.310248202309443	-2.45153489982375
a_HQ_transcript/144006	-3.07994069666089	-0.408364312661305	-2.85674002190957	0.00739611328942547	0.310248202309443	-2.4930778481212
a_HQ_transcript/7930	4.42491253048564	3.39205623118882	2.8567352241536	0.00739620234179608	0.310248202309443	-2.53336228098022
a_HQ_transcript/25292	1.12784129699739	5.04696238399645	2.85671103910836	0.00739665126189934	0.310248202309443	-2.78045654515218
a_HQ_transcript/21591	2.63669087564076	-0.377388219441981	2.85651918486351	0.00740021334485225	0.310248202309443	-2.44672763818467
a_HQ_transcript/140146	2.06599468965721	-0.667295686141479	2.85630320785556	0.00740422523154856	0.310248202309443	-2.45321793138311
a_HQ_transcript/69237	2.23773898075766	-0.606796167688325	2.85608873098739	0.00740821127177104	0.310248202309443	-2.45404898918735
a_HQ_transcript/116389	3.26316403206624	1.07365080551945	2.85542753841052	0.00742051217200279	0.310539619535045	-2.42085282302102
a_HQ_transcript/26432	4.37066991300772	1.61407735299821	2.85420685879147	0.00744327219299246	0.311268004341199	-2.49853970135632
a_HQ_transcript/145306	2.30886958455102	0.614375082887236	2.8524770228978	0.00747563780434852	0.311679185487066	-2.43472033638001
a_HQ_transcript/56169	1.55702201975407	-0.883563897658248	2.85175008542129	0.00748927832282966	0.311679185487066	-2.46349704033001
a_HQ_transcript/117723	-4.28393317781398	0.487803852674428	-2.85174735790256	0.0074893295469166	0.311679185487066	-2.46393165180182
a_HQ_transcript/115663	4.28123750308218	1.96887063658992	2.85164298959233	0.00749128987986945	0.311679185487066	-2.49479100433562

a_HQ_transcript/102837	-4.87919447493353	-0.768069738622407	-2.85150416341245	0.00749389817528807	0.311679185487066	-2.48219093660735
a_HQ_transcript/13001	1.6064741869963	-0.879475596625017	2.85136833644198	0.00749645094536217	0.311679185487066	-2.46415941684086
a_HQ_transcript/77401	6.35346693405407	3.70426911256664	2.85131488287169	0.00749745579036307	0.311679185487066	-2.74334612792695
a_HQ_transcript/92068	-4.51096135377372	0.550569996174034	-2.85112379292163	0.00750104902108506	0.311679185487066	-2.47872254154967
a_HQ_transcript/45010	1.55368353106871	-0.88384596703697	2.85110714330612	0.00750136217479365	0.311679185487066	-2.4648837131776
a_HQ_transcript/133201	0.926515484946064	4.21400161615667	2.8505041680781	0.00751271148050589	0.3117708045573	-2.73579058826069
a_HQ_transcript/89310	1.75064844730046	1.03033847525477	2.85042014436417	0.00751429426756084	0.3117708045573	-2.41362347686496
a_HQ_transcript/47879	2.9325043510435	2.86408395277892	2.84954377555331	0.00753082141842021	0.312222497696569	-2.46914654596056
a_HQ_transcript/143453	1.54357819254946	-0.884700929961349	2.84916022672917	0.00753806535998071	0.312222497696569	-2.46908183462976
a_HQ_transcript/44581	0.793808509773841	4.74646357542234	2.84878171126754	0.00754522064507859	0.312222497696569	-2.77873036731587
a_HQ_transcript/74101	-2.14955687152416	2.28288512412012	-2.84870528539628	0.00754666613789705	0.312222497696569	-2.4527813164403
a_HQ_transcript/134738	4.2924100806596	0.783113888575242	2.84725457985817	0.00757415358925562	0.312926046289126	-2.44108342599176
a_HQ_transcript/151674	-3.97234730733736	-0.65430053936811	-2.84719046853368	0.00757537051149297	0.312926046289126	-2.48887687711317
a_HQ_transcript/26275	1.57822536719032	-0.881895462324548	2.84695605040188	0.00757982165522794	0.312926046289126	-2.47368203406204
a_HQ_transcript/6340	-2.73449978007061	0.780157505547146	-2.84601657628533	0.00759768506197672	0.313135719591242	-2.45736259563716
a_HQ_transcript/22613	2.91180506477634	0.147946901974443	2.8458447300976	0.00760095685838466	0.313135719591242	-2.47254845931005
a_HQ_transcript/23954	1.90516284576566	-0.783041332543945	2.84484637481014	0.0076199907909611	0.313135719591242	-2.47068012852312
a_HQ_transcript/36026	4.09757194471267	2.28750752502365	2.8444876493947	0.00762684090274557	0.313135719591242	-2.53031715150264
a_HQ_transcript/150082	-5.95553200201097	9.90310617504109	-2.84441861413951	0.00762815984139774	0.313135719591242	-2.84961966760139
a_HQ_transcript/12008	-3.0526125987651	0.958492226171536	-2.84381598347301	0.00763968236101633	0.313135719591242	-2.43843801047831
a_HQ_transcript/36871	1.8451587403949	-0.885880580069148	2.84374969877732	0.00764095074447542	0.313135719591242	-2.48069283015729
a_HQ_transcript/125680	1.09296716826369	4.66651624732539	2.84349640258183	0.00764579948489034	0.313135719591242	-2.75877527264645
a_HQ_transcript/48492	2.77842094191262	4.84569735822793	2.84343297677438	0.00764701406946807	0.313135719591242	-2.76515664023714
a_HQ_transcript/100059	-2.22012194077901	-0.676646386587778	-2.84331888822859	0.00764919928518069	0.313135719591242	-2.47460706350523
a_HQ_transcript/86	-1.51122846108807	1.76557906818888	-2.84330908146112	0.00764938714820871	0.313135719591242	-2.45594030813686
a_HQ_transcript/114004	2.01212658986862	-0.717656833048814	2.84320710316497	0.00765134094858274	0.313135719591242	-2.46950543076378
a_HQ_transcript/89774	2.60109368703357	4.22058886244716	2.84301975917907	0.00765493148808071	0.313135719591242	-2.72305010395964
a_HQ_transcript/23283	-3.20832427426516	1.77827220647798	-2.84193991173703	0.00767565810851408	0.313719966227103	-2.47337596449538
a_HQ_transcript/98551	0.634223654135737	3.36829681245224	2.84171362530403	0.00768000811899888	0.313719966227103	-2.62987360710675
a_HQ_transcript/113071	-3.52652604590877	0.649011102283242	-2.84024054764085	0.007708382325894	0.314278694741769	-2.46068193225515
a_HQ_transcript/90273	0.946365221929943	4.6323572975036	2.83994306299486	0.00771412434565354	0.314278694741769	-2.78208495826862
a_HQ_transcript/148529	-4.42564377124334	-0.657396490758961	-2.83972794049476	0.00771827911710121	0.314278694741769	-2.50648460188843
a_HQ_transcript/40829	2.77105461646352	0.678849997898723	2.83968476104149	0.00771911331681993	0.314278694741769	-2.47272991404614
a_HQ_transcript/142127	4.22442688091178	2.54109435423432	2.83953899135672	0.00772193011921708	0.314278694741769	-2.59539866919791
a_HQ_transcript/80127	3.4805577454943	0.663589861587873	2.83888531424911	0.0077345733992812	0.314278694741769	-2.49187686924869
a_HQ_transcript/137152	1.48954570402205	-0.889302766075328	2.83871986085402	0.00773777663869818	0.314278694741769	-2.49157068012822
a_HQ_transcript/60633	1.34459661958541	3.45691050072338	2.83842779993543	0.00774343408204708	0.314278694741769	-2.60323098424355
a_HQ_transcript/1995	-1.43986382120242	3.21630088699664	-2.83767635200406	0.00775800802937339	0.314278694741769	-2.59255935720627
a_HQ_transcript/110117	2.75230259014661	-0.790032072976621	2.83755470501223	0.0077603697269058	0.314278694741769	-2.49591208790079
a_HQ_transcript/115631	2.25997356332301	-0.680103006522589	2.83752447699298	0.00776095668883481	0.314278694741769	-2.49148253704631
a_HQ_transcript/100521	-2.10218948551954	0.847749817232479	-2.83742233313282	0.00776294040677168	0.314278694741769	-2.46219421834979
a_HQ_transcript/146372	-3.25485758414979	3.64286395974031	-2.83736918839352	0.00776397270935144	0.314278694741769	-2.63779049625818
a_HQ_transcript/115263	3.24353917727474	0.39507082039941	2.8366958555614	0.00777706291578123	0.314525717532104	-2.53462674652315
a_HQ_transcript/143561	2.88046564242372	-0.0131827047183621	2.83649883853233	0.00778089701712025	0.314525717532104	-2.49696545942988
a_HQ_transcript/131771	1.75407617684158	-0.836192980829042	2.83497224897974	0.00781066571242946	0.314945058580336	-2.49862750294267
a_HQ_transcript/130276	1.05449789146754	3.82344341642325	2.83490361422227	0.00781200660316933	0.314945058580336	-2.71230586559925
a_HQ_transcript/35779	-2.2519749364317	-0.673762563378447	-2.8348909916291	0.00781225322937961	0.314945058580336	-2.47248841954741
a_HQ_transcript/8241	3.13529392684904	-0.14134553082255	2.83477272670144	0.00781456430008579	0.314945058580336	-2.49506517051799
a_HQ_transcript/110208	2.78640113780157	0.567218945856792	2.83457848092136	0.00781836153886982	0.314945058580336	-2.45586898899259
a_HQ_transcript/128422	-2.00241065620678	-0.794758041986842	-2.83425102333191	0.00782476680072483	0.314984795424192	-2.50143694945417

a_HQ_transcript/148301	2.74288861764153	0.741576694160512	2.83337119669978	0.00784200108890282	0.315066944173291	-2.52487927973121
a_HQ_transcript/26385	3.47407594609754	0.0606649692251064	2.83323613369695	0.00784464988770566	0.315066944173291	-2.51022506727515
a_HQ_transcript/9186	1.36789530569464	-0.82433061207414	2.83314682467535	0.00784640183911812	0.315066944173291	-2.50729954495864
a_HQ_transcript/134087	-8.08155854273785	1.38665233936176	-2.83304048141366	0.00784848842490581	0.315066944173291	-2.53964851508746
a_HQ_transcript/43732	-2.34619509361688	1.03362136772686	-2.83208731026746	0.00786721404682051	0.315600702631872	-2.47310947047833
a_HQ_transcript/116957	3.41982925999034	4.52222487765172	2.83161254827571	0.0078765566432727	0.315757575558728	-2.73392552706226
a_HQ_transcript/14949	1.90241017521427	2.12037992944408	2.83128139473897	0.00788307939621994	0.31580126750067	-2.5301645165055
a_HQ_transcript/148830	0.575932179375559	10.1047952056659	2.83021109463373	0.00790419579721791	0.31642912761755	-3.29909183585958
a_HQ_transcript/118645	-1.91233140099281	2.99298878162158	-2.82923651680403	0.0079234697402171	0.31698241504428	-2.5899757703472
a_HQ_transcript/2411	-5.78603822397711	1.75109708667985	-2.82885856414243	0.00793095624236685	0.317063703202407	-2.60740840198961
a_HQ_transcript/82625	-2.61590557189439	-0.378000190697008	-2.82812917870855	0.00794542267741824	0.317220665356177	-2.53626974989964
a_HQ_transcript/130402	-2.54453855746841	-0.555584172576024	-2.82811032155354	0.00794579701277514	0.317220665356177	-2.5249102524663
a_HQ_transcript/139539	0.750864362321431	7.73803673678718	2.82714379681595	0.00796500573627405	0.317484024083879	-3.05667294656015
a_HQ_transcript/96950	2.34551571629921	2.54042950793367	2.82702259460295	0.00796741757751442	0.317484024083879	-2.57119205578872
a_HQ_transcript/6822	2.79005047007531	-0.35091477126042	2.82695418954888	0.00796877909335225	0.317484024083879	-2.48560176231616
a_HQ_transcript/136121	-7.61856854281462	1.49568331810429	-2.82643571695379	0.00797910572107017	0.317559735866425	-2.53157286692267
a_HQ_transcript/114275	5.09852649763285	-0.0623724401926017	2.82631029589526	0.00798160566509853	0.317559735866425	-2.51501677846847
a_HQ_transcript/135627	2.07775780964291	2.46811502562535	2.82588325554488	0.00799012311155779	0.317681173890993	-2.56691277688198
a_HQ_transcript/81332	-1.1282469383138	3.39871376652504	-2.82480546186039	0.00801165790063064	0.317848345282648	-2.61249002236461
a_HQ_transcript/105431	2.69564826045711	0.14691434026342	2.82461490649509	0.00801547093084661	0.317848345282648	-2.54901037167714
a_HQ_transcript/150021	-2.38430015923949	0.700859806078041	-2.8243895547306	0.00801998243378634	0.317848345282648	-2.47829997007576
a_HQ_transcript/22084	1.40728764664743	3.48370493589453	2.82414566906943	0.00802486766177983	0.317848345282648	-2.66158123380753
a_HQ_transcript/105148	-0.524672316234561	4.67365671077158	-2.82401797609263	0.00802742656736826	0.317848345282648	-2.81758583836848
a_HQ_transcript/40065	2.38627514939461	1.4905387526311	2.82391892305199	0.00802941206877492	0.317848345282648	-2.47875963339456
a_HQ_transcript/103405	2.82760658911745	-0.0184264271335771	2.82375971957206	0.00803260423926868	0.317848345282648	-2.57074047982117
a_HQ_transcript/76138	2.62451800238267	0.0613702787056159	2.82323486987507	0.00804313635970098	0.318048592052176	-2.47395710132681
a_HQ_transcript/63599	-1.16892704092042	2.35590678853901	-2.82216749211743	0.00806459525839782	0.31846834493125	-2.5375283313133
a_HQ_transcript/116792	2.45795193408654	1.72994717395375	2.82205653880011	0.00806682897254087	0.31846834493125	-2.54442851196891
a_HQ_transcript/29614	2.9026212745937	0.0192269427452337	2.82188975658545	0.00807018772508485	0.31846834493125	-2.51031298412117
a_HQ_transcript/58509	-2.23144288094566	2.2978631344905	-2.82146734848366	0.00807870027284289	0.318587984708149	-2.59664981049184
a_HQ_transcript/36027	2.6306892066846	1.16924445344976	2.82041090290895	0.00810002703991732	0.319198399802414	-2.49464710740171
a_HQ_transcript/152715	3.41719141841521	0.0644755307231715	2.81983590315533	0.00811165686748766	0.319198399802414	-2.54077101764041
a_HQ_transcript/121631	-3.22582646542976	0.451139579527504	-2.81973850886643	0.00811362829225157	0.319198399802414	-2.52328157843827
a_HQ_transcript/139705	2.13252522466131	-0.724440183411743	2.81961424354453	0.00811614428344288	0.319198399802414	-2.53166223701139
a_HQ_transcript/3294	2.21688906060772	4.56254403109587	2.81919136049362	0.00812471184084382	0.319281105874848	-2.71480809669219
a_HQ_transcript/77832	-4.12807417573048	3.11205107205735	-2.818903032575	0.00813055818256785	0.319281105874848	-2.67404497635666
a_HQ_transcript/30374	2.0987207788616	-0.602412310148363	2.81869763382925	0.00813472539568969	0.319281105874848	-2.52551310071583
a_HQ_transcript/60359	3.30511978996684	1.19556386562382	2.8178345269129	0.00815225832602747	0.319364303761019	-2.53207327584545
a_HQ_transcript/88924	-3.82236844524032	-0.00382939790364518	-2.81776082508791	0.00815375712423104	0.319364303761019	-2.52857440534624
a_HQ_transcript/5012	3.55378876261725	0.121275095849628	2.81759269205843	0.00815717723832804	0.319364303761019	-2.51189899322895
a_HQ_transcript/152143	0.409714455544591	11.4804402593886	2.81714127087698	0.0081663665643736	0.319364303761019	-3.5038468484948
a_HQ_transcript/132902	-3.25062799820398	-0.676174801597679	-2.81692148586843	0.00817084411085805	0.319364303761019	-2.54949778142138
a_HQ_transcript/92341	3.30564028392103	1.48413316748047	2.81675982875511	0.00817413891988473	0.319364303761019	-2.60258292916655
a_HQ_transcript/147679	5.59656404826859	0.185806621573895	2.81662580205586	0.00817687152361166	0.319364303761019	-2.58635535830108
a_HQ_transcript/79577	2.05677436331305	2.85389516685434	2.8164332738967	0.00818079838116154	0.319364303761019	-2.71047953003087
a_HQ_transcript/142405	1.396631978833	3.88131424735265	2.81597709236661	0.00819010983424336	0.319513224459663	-2.7476179834139
a_HQ_transcript/126628	0.560531849722432	7.41838005466559	2.81546729896708	0.00820052733700822	0.319705065758292	-3.06326513666633
a_HQ_transcript/18791	-2.06189349301363	2.42235861019788	-2.81389539185076	0.00823272701494736	0.320745278770014	-2.56802076942788
a_HQ_transcript/94066	1.98313276855648	1.85555045556781	2.813506028779	0.00824072117760919	0.320841688286716	-2.52422689644604
a_HQ_transcript/39704	1.63500463009388	-0.82957199904792	2.81212114848934	0.00826921360469563	0.321735507639724	-2.54770908845509

a_HQ_transcript/42967	-2.73704143155617	1.84336471481323	-2.8118523576463	0.00827475436178381	0.321735733472755	-2.54563737135646
a_HQ_transcript/39987	-3.75788944298036	0.752137862209444	-2.8112941162846	0.00828627285086121	0.321968227456458	-2.54034129489926
a_HQ_transcript/65869	2.58347838788136	0.065538301923929	2.81086992631817	0.00829503541833736	0.322016801552896	-2.54635570840642
a_HQ_transcript/6157	-3.6650014358615	0.84406358340252	-2.81052347525204	0.00830219855252661	0.322016801552896	-2.52497253419593
a_HQ_transcript/65792	-2.60197466311387	0.350633039980098	-2.81037610564623	0.0083052472836347	0.322016801552896	-2.53286126874534
a_HQ_transcript/115368	3.17743568995884	-0.23927366406866	2.81003928552345	0.00831221923539227	0.322016801552896	-2.54702626407239
a_HQ_transcript/139125	3.28375289624723	-0.186773090139543	2.8098943055919	0.0083152219090782	0.322016801552896	-2.51777472820442
a_HQ_transcript/17439	4.28180888875074	0.676229366946339	2.80936234951725	0.0083262479277228	0.322111353332494	-2.56345851324001
a_HQ_transcript/139521	2.3145833879219	1.03011588051256	2.80924190656351	0.00832874628507327	0.322111353332494	-2.5194161298026
a_HQ_transcript/110681	2.55131782661271	-0.122615424541069	2.80865697474816	0.00834088954960923	0.322311630443199	-2.57287256978196
a_HQ_transcript/56890	3.62596185632225	0.331239281246685	2.80845845954234	0.00834501451653274	0.322311630443199	-2.58669035977027
a_HQ_transcript/45240	2.22110321063371	-0.174952146282692	2.80796510825286	0.00835527417981039	0.322371099063577	-2.53278407467411
a_HQ_transcript/19779	3.51590663350178	0.399777064976197	2.80718187736172	0.00837158638385982	0.322371099063577	-2.54404468124791
a_HQ_transcript/37021	-1.63139511664436	2.77210606744151	-2.80669010094859	0.00838184374337489	0.322371099063577	-2.60976183265643
a_HQ_transcript/50614	-2.27340323481478	-0.689832405219106	-2.80665534629178	0.00838256909273602	0.322371099063577	-2.53938709133623
a_HQ_transcript/121873	-1.75556423291468	1.10301534061649	-2.80647594385881	0.00838631425721501	0.322371099063577	-2.51514196607315
a_HQ_transcript/59978	0.711987001744728	6.04078456857897	2.80598572128219	0.00839655601785065	0.322371099063577	-2.97429482192082
a_HQ_transcript/7254	-3.20142468757368	0.0853285677118825	-2.80586554487718	0.0083990685362346	0.322371099063577	-2.5671961051485
a_HQ_transcript/152804	-5.0355738308819	-0.761168514563148	-2.80583671476332	0.00839967138970093	0.322371099063577	-2.58053126544041
a_HQ_transcript/133802	-3.9633771350913	-0.146423240642895	-2.8056059400612	0.00840449847430985	0.322371099063577	-2.56992219415076
a_HQ_transcript/22985	-2.34958057157463	-0.206997450037532	-2.80552383873816	0.00840621640289032	0.322371099063577	-2.50960615393229
a_HQ_transcript/110110	3.10881201513512	0.0422064986075188	2.80529975375377	0.00841090693984368	0.322371099063577	-2.54691722375453
a_HQ_transcript/35035	2.77089343530093	0.439243251493033	2.80519479254445	0.00841310482520381	0.322371099063577	-2.50701860813508
a_HQ_transcript/149489	2.16467110720039	1.6326636314881	2.80448552637529	0.00842797093212578	0.32268213794683	-2.52010813867366
a_HQ_transcript/52156	2.89388789159094	1.78805417306076	2.80427803060544	0.00843232465492077	0.32268213794683	-2.63226851790279
a_HQ_transcript/147793	-2.48256792422234	-0.653282701227247	-2.80349206019422	0.00844883514892985	0.322777320754208	-2.56832932098943
a_HQ_transcript/139070	1.90851232177635	-0.875701159300155	2.80344848775602	0.00844975133788456	0.322777320754208	-2.56605675983797
a_HQ_transcript/16425	2.23656986658776	0.332671364763217	2.80336673510202	0.0084514705853961	0.322777320754208	-2.55177970899445
a_HQ_transcript/89013	2.57298578717991	1.51069602650196	2.80289090846084	0.00846148365361626	0.322947552079715	-2.55343227518597
a_HQ_transcript/38109	-1.87634374717205	-0.65703482547267	-2.80262590192791	0.0084670651346486	0.322948531592424	-2.56652440817778
a_HQ_transcript/141473	-2.16453587444657	-0.429430841449651	-2.8015089740414	0.00849062737336212	0.323634877350028	-2.53915223733034
a_HQ_transcript/107284	-2.23768605501689	1.96633691473115	-2.80113151183301	0.00849860401264672	0.323726640922103	-2.5206104620261
a_HQ_transcript/141133	-1.86546766661992	2.72758720643891	-2.80061433797071	0.00850954446001525	0.323931106988714	-2.64826028592083
a_HQ_transcript/16041	3.42590113077476	0.370699108227822	2.79986387057238	0.00852544349575864	0.324323939477394	-2.57519704165435
a_HQ_transcript/99805	3.08702242523456	-0.523731176519898	2.79908582208296	0.00854195618146795	0.324739587257272	-2.56805947727111
a_HQ_transcript/42862	2.86458456642238	1.29873530674588	2.79870214104049	0.00855011013222832	0.324837125337364	-2.52706819704219
a_HQ_transcript/67131	1.67617363122202	2.74749617542037	2.7978741873076	0.00856773053037167	0.32529395184157	-2.59490558060175
a_HQ_transcript/68410	3.0620956316411	0.580902755400193	2.79638370169554	0.00859953646228408	0.325679614830036	-2.61278630399196
a_HQ_transcript/139073	-3.02804341236622	0.63776422855721	-2.79616637572728	0.00860418326068042	0.325679614830036	-2.61495882105846
a_HQ_transcript/59392	-1.442627717093	1.93036126468611	-2.79595499883423	0.00860870511005094	0.325679614830036	-2.55527023308929
a_HQ_transcript/138770	-3.84223846761226	0.563280507756389	-2.79540862257979	0.00862040368189894	0.325679614830036	-2.55715450314803
a_HQ_transcript/127686	3.14722625521491	-0.175628743354277	2.79539586393969	0.00862067703723556	0.325679614830036	-2.58495946404958
a_HQ_transcript/42850	-3.02935635050337	1.71160882654669	-2.79534172825584	0.00862183699066837	0.325679614830036	-2.55839798036055
a_HQ_transcript/148863	-2.71752824025034	-0.431669485558567	-2.79527323568858	0.00862330477502141	0.325679614830036	-2.56826459171854
a_HQ_transcript/39526	2.65012810535267	1.18037082687592	2.79519999966564	0.00862487446931607	0.325679614830036	-2.56150228913388
a_HQ_transcript/26423	-2.0033904247311	1.25124144735293	-2.79503959123391	0.00862831349501541	0.325679614830036	-2.53311657615641
a_HQ_transcript/98546	3.62354340912645	-0.188568130884793	2.79326171016749	0.00866651576507884	0.326620447193224	-2.58426796526229
a_HQ_transcript/53305	-2.50035449627578	-0.150883214059801	-2.79281797312428	0.00867607520713868	0.326620447193224	-2.57162818100688
a_HQ_transcript/78596	2.44913465158	1.97341457767642	2.79273370183024	0.0086778917799531	0.326620447193224	-2.57280244615621
a_HQ_transcript/120826	4.88651653336682	3.7441499821414	2.79233852413581	0.00868641507227993	0.326620447193224	-2.70862576275867

a_HQ_transcript/145761	0.674019861321627	9.82268818421468	2.79225651946512	0.00868818474998896	0.326620447193224	-3.36058895116156
a_HQ_transcript/116210	-1.83981685920067	1.18308956737686	-2.79156215915078	0.00870318270409076	0.326620447193224	-2.53207228741606
a_HQ_transcript/139158	-3.35956902468181	4.77638293808847	-2.7915139478335	0.00870422495074093	0.326620447193224	-3.07219116387167
a_HQ_transcript/118233	2.25681956845698	1.36934305486987	2.7913298458814	0.00870820599529721	0.326620447193224	-2.56311470637617
a_HQ_transcript/85367	-3.50326284439243	1.14040099176444	-2.79131771479246	0.00870846837930817	0.326620447193224	-2.61836324569805
a_HQ_transcript/134951	-3.3258213674194	-0.577761021405809	-2.79127330090326	0.00870942907289942	0.326620447193224	-2.60375546334664
a_HQ_transcript/75977	-3.69667065963771	-0.659893708215418	-2.79047026450554	0.00872681623172908	0.32685860738259	-2.59946197783231
a_HQ_transcript/98479	1.40123384187732	2.3434235858774	2.79046059224798	0.00872702585084261	0.32685860738259	-2.60645555275446
a_HQ_transcript/20752	2.36691463663958	-0.522491581627566	2.7900587918718	0.008735737909551	0.326974226147058	-2.59293058489004
a_HQ_transcript/49406	-2.08665878876622	3.18101221601767	-2.78933901357819	0.00875136487106164	0.327348350852684	-2.7773395510466
a_HQ_transcript/144925	-2.42578701274214	-0.652195007984853	-2.78833999549818	0.00877309765586827	0.327560032589415	-2.59947218094805
a_HQ_transcript/17913	-1.90785826126014	2.63178090636733	-2.78817364288842	0.00877672141033089	0.327560032589415	-2.64821358430567
a_HQ_transcript/82258	2.39081810740373	-0.0896019965993885	2.78794609593837	0.00878168046189139	0.327560032589415	-2.67572296512069
a_HQ_transcript/148802	-2.69858228043451	-0.630701992367902	-2.78789605978832	0.00878277127748133	0.327560032589415	-2.60457069989549
a_HQ_transcript/2898	2.14131274206328	1.33831087172779	2.78778468583879	0.00878519974550816	0.327560032589415	-2.55874382142712
a_HQ_transcript/1731	-1.83626326647557	2.14175903733624	-2.78665220002666	0.00880992884245565	0.328108787116729	-2.5728244259079
a_HQ_transcript/134834	-1.61292550467515	1.39550410193315	-2.78659376544038	0.00881120659044201	0.328108787116729	-2.57937365260653
a_HQ_transcript/10500	4.13745804601606	1.79442765614388	2.78620810576432	0.00881964388367639	0.328212714257581	-2.60152305712625
a_HQ_transcript/141998	3.23127185174042	1.37731763215691	2.78534935374504	0.0088384584156453	0.328634816282795	-2.58401912863118
a_HQ_transcript/118472	-3.50813159229412	1.06218132615815	-2.78517450591732	0.00884229377694555	0.328634816282795	-2.62697865562977
a_HQ_transcript/152403	2.98027125923578	0.178147781936791	2.78480926158983	0.00885031058456416	0.328722590197793	-2.59221416635953
a_HQ_transcript/150007	5.49559216999166	9.17979232096698	2.78392831505774	0.00886967458013682	0.329231445717875	-3.28663195119902
a_HQ_transcript/98190	-2.55840116114404	0.37987364724929	-2.78267335623988	0.00889732807809761	0.329954496072609	-2.59190401810409
a_HQ_transcript/32251	-2.57320049761998	-0.155089035655244	-2.78252934185424	0.00890050663779677	0.329954496072609	-2.59155290159097
a_HQ_transcript/710	-2.14559742815813	-0.287483197969852	-2.78210684590268	0.00890983772241354	0.330000439824058	-2.60649427243993
a_HQ_transcript/103995	4.3715106675454	2.10839249724967	2.78175619403915	0.00891758902584392	0.330000439824058	-2.68710708707983
a_HQ_transcript/14779	1.5875698908854	-0.830372008786135	2.7814580175504	0.00892418529574721	0.330000439824058	-2.61236698000958
a_HQ_transcript/17313	2.9916915711231	0.692968444945782	2.78144585269126	0.00892445450391238	0.330000439824058	-2.56305146237371
a_HQ_transcript/136037	-4.98042077226734	4.12383187894652	-2.77966958164504	0.00896384489569011	0.331245992245978	-2.97870627345527
a_HQ_transcript/8757	0.963817062934398	2.80928349099445	2.77911714315386	0.00897612878502049	0.331245992245978	-2.69413764007917
a_HQ_transcript/111978	-3.39476319490002	1.86707324197169	-2.77903545273708	0.00897794656749925	0.331245992245978	-2.66584779245816
a_HQ_transcript/30915	4.58904697898923	3.21814482203445	2.77890126919137	0.00898093317815273	0.331245992245978	-2.72275073138596
a_HQ_transcript/113467	-2.22097760353983	0.0861984489018578	-2.77841581803596	0.00899174593532472	0.331434500779046	-2.57880671017471
a_HQ_transcript/82237	4.90586575484269	0.700244667983968	2.7776863308071	0.00900801712940261	0.331823840112747	-2.7110749040555
a_HQ_transcript/107729	-4.08335914407164	1.32953642423599	-2.77643450543718	0.00903600317100723	0.332643947007162	-2.68926089356124
a_HQ_transcript/152540	-3.26411332666028	0.414514433407318	-2.77573662007314	0.00905164046233996	0.333008706832214	-2.56638317619024
a_HQ_transcript/146925	-3.28497393837799	-0.240302830278157	-2.77504418944208	0.00906718050710964	0.333369429802194	-2.63858177405993
a_HQ_transcript/16022	-2.56790658141916	-0.28845896403512	-2.7745261310401	0.00907882345124938	0.33337930074782	-2.63242118861556
a_HQ_transcript/121796	2.76046916009415	-0.243098408686153	2.77452185958902	0.00907891950667146	0.33337930074782	-2.65296309259459
a_HQ_transcript/148135	0.439247882980678	10.8921069463397	2.774233192887	0.00908541317778025	0.333407132069451	-3.52659896754829
a_HQ_transcript/90891	4.24094845437791	-0.173154902717644	2.77284233275347	0.0091167619658061	0.333814313663422	-2.66039850741838
a_HQ_transcript/30949	2.33054059926628	0.264217032126878	2.77283197709101	0.00911699575188551	0.333814313663422	-2.6054346873035
a_HQ_transcript/113358	-3.27520912049133	0.912175570700357	-2.77280263018912	0.00911765830844393	0.333814313663422	-2.66079701166143
a_HQ_transcript/132694	3.02229493348081	0.723416853356966	2.77266122882848	0.00912085131655959	0.333814313663422	-2.60362375850866
a_HQ_transcript/62977	-0.877051719643983	4.30493649987136	-2.77239056151927	0.00912696621351173	0.333814313663422	-2.89542751711215
a_HQ_transcript/125744	5.99605897055308	4.43029332890046	2.77221363324283	0.00913096543360928	0.333814313663422	-2.82403977796485
a_HQ_transcript/146519	0.511826575171164	10.202290870328	2.77092311271917	0.00916018540634336	0.334498207932555	-3.45478960283491
a_HQ_transcript/106658	-3.32795155938688	0.0612286341481875	-2.77087919582903	0.00916118130726375	0.334498207932555	-2.67358210553618
a_HQ_transcript/143510	-2.56855397822072	2.5304793600296	-2.76981296489612	0.00918539121223845	0.335171638659759	-2.75431967412636
a_HQ_transcript/83580	4.30821818605386	1.06649049607976	2.76869521017372	0.00921083514338851	0.335889225354384	-2.64831398022039

a_HQ_transcript/63851	4.1714073885206	0.870812984578963	2.76805804892558	0.00922536854811865	0.33614519362831	-2.73987415599623
a_HQ_transcript/122252	1.11597112695863	3.27808426836721	2.76774768808005	0.00923245551143639	0.33614519362831	-2.81707777495058
a_HQ_transcript/42809	2.40715728782127	1.40582814145444	2.76762743090421	0.00923520290091542	0.33614519362831	-2.62059348738939
a_HQ_transcript/65515	-1.27701000830489	2.4207010520436	-2.76531346384311	0.00928821653442849	0.337786687014957	-2.63841024376756
a_HQ_transcript/115349	6.893505323093	3.21427592878087	2.76515213424304	0.00929192321320046	0.337786687014957	-2.77509994292325
a_HQ_transcript/132951	3.17332681291328	3.83636338046881	2.76298120092268	0.0093419366525911	0.339392558588634	-2.76575158291114
a_HQ_transcript/82306	-2.76420307744152	-0.614330413666867	-2.76209035190456	0.0093625324262471	0.339688005972173	-2.66022953218201
a_HQ_transcript/88654	2.3647452676273	0.409360990789653	2.76157943994071	0.00937436344678798	0.339688005972173	-2.58923644900779
a_HQ_transcript/1898	-3.26044348369483	0.181113874656385	-2.76141945353498	0.00937807106688723	0.339688005972173	-2.69027867157211
a_HQ_transcript/84955	-4.03392399403316	0.781520157667187	-2.76133941527479	0.00937992643461618	0.339688005972173	-2.6762900070461
a_HQ_transcript/147258	-3.80959954634044	0.590774484487097	-2.76091015292438	0.00938988301585148	0.339688005972173	-2.67944916978755
a_HQ_transcript/128665	2.45639920299019	0.230171950906254	2.76089128515214	0.00939032087302755	0.339688005972173	-2.63300206191768
a_HQ_transcript/8190	3.3222716528442	0.338237213848854	2.76086307676499	0.00939097552981836	0.339688005972173	-2.63378567407035
a_HQ_transcript/146162	3.65126304973125	1.88977242270798	2.76048610009735	0.00939972844677566	0.339793168628219	-2.80061659084074
a_HQ_transcript/67425	-2.53880988578847	2.05490596258633	-2.7597009478053	0.00941798312512962	0.340208037938482	-2.67956113005277
a_HQ_transcript/131808	-3.25152014989334	-0.592463817140974	-2.75895679382231	0.00943531512708897	0.340208037938482	-2.67256012615925
a_HQ_transcript/144235	5.00267109490603	1.61339922634816	2.75894576802707	0.00943557215117482	0.340208037938482	-2.66306396739152
a_HQ_transcript/23821	-2.21033837810939	-0.608060845643662	-2.75877625165123	0.00943952459706638	0.340208037938482	-2.64173547097098
a_HQ_transcript/110849	2.53099112558908	0.270826757354276	2.75873576939411	0.00944046871034221	0.340208037938482	-2.66432423639443
a_HQ_transcript/152089	-3.79736634581373	0.433711289609492	-2.7580074272703	0.00945746991399618	0.340609548426747	-2.69245577488078
a_HQ_transcript/74818	-2.55929205238539	1.75350184225093	-2.75729289403819	0.00947417654059534	0.340967895664408	-2.61705892631652
a_HQ_transcript/95437	-1.9320090939158	0.603168799267268	-2.75708034416478	0.00947915151723237	0.340967895664408	-2.63236350815707
a_HQ_transcript/131484	9.18211826375942	5.71428487628262	2.75677305155991	0.00948634836655111	0.341015743878097	-2.9057571676603
a_HQ_transcript/83872	1.56564138632354	-0.778682307654447	2.75625390877498	0.00949851836749941	0.34108910352329	-2.66439482599704
a_HQ_transcript/121721	2.79386807948044	2.15744937214186	2.75607707964976	0.00950266700738807	0.34108910352329	-2.66042450024208
a_HQ_transcript/200	-1.57753853655142	1.92376453339007	-2.75576516789399	0.00950998898124026	0.34108910352329	-2.64723822901172
a_HQ_transcript/132766	-3.28931240313321	0.597473323572453	-2.75564420588759	0.00951282992038868	0.34108910352329	-2.67014133903492
a_HQ_transcript/90711	-4.31725507885589	1.48387900807987	-2.75543570974671	0.00951772856308107	0.34108910352329	-2.7810270147262
a_HQ_transcript/66934	-2.38405024744859	2.21264381524972	-2.75511089651126	0.00952536477910283	0.341152436155077	-2.64367938806565
a_HQ_transcript/30293	2.43198218447618	2.90838210228072	2.75437802378271	0.00954261529360372	0.341183071906093	-2.71762510661886
a_HQ_transcript/29257	1.84134482139248	2.01482575791034	2.75411350588769	0.00954884872612199	0.341183071906093	-2.63140837555325
a_HQ_transcript/140502	2.68179917228672	0.659304178436377	2.75380174465961	0.00955620033608374	0.341183071906093	-2.63206945987013
a_HQ_transcript/125218	-2.0414229213037	-0.650986979244385	-2.75357307314778	0.00956159597178892	0.341183071906093	-2.67277399058839
a_HQ_transcript/145769	2.93964021975765	0.192923080805033	2.75339688589808	0.00956575514917006	0.341183071906093	-2.63853739878061
a_HQ_transcript/97244	3.91787954532645	-0.233535849549143	2.75327437188998	0.00956864827980878	0.341183071906093	-2.67250959090768
a_HQ_transcript/77915	-1.50507026142076	1.57549475006789	-2.75310569098821	0.00957263296288646	0.341183071906093	-2.61274060694996
a_HQ_transcript/74226	-2.67297251988576	0.60198115203413	-2.75286241406521	0.00957838252440902	0.341183071906093	-2.66633821816104
a_HQ_transcript/59731	2.3800275819227	-0.197683350866958	2.75258307267475	0.00958498839932686	0.341183071906093	-2.64989577858038
a_HQ_transcript/145375	0.613963858362776	9.29997164898242	2.75237081148405	0.00959001080019393	0.341183071906093	-3.38101214714717
a_HQ_transcript/59949	-2.69463425195169	-0.174428834308468	-2.75219844439663	0.00959409105573881	0.341183071906093	-2.68074155828011
a_HQ_transcript/120014	-2.31714875569347	1.82741839641218	-2.75166769537853	0.00960666506138073	0.341183071906093	-2.69221844107779
a_HQ_transcript/121237	-1.68480799994032	2.50931041331603	-2.75161260733196	0.00960797103550786	0.341183071906093	-2.70257816352133
a_HQ_transcript/139990	3.09204630841405	-0.341120882504842	2.75106173573882	0.00962103967606791	0.341183071906093	-2.69188797077781
a_HQ_transcript/119236	2.85344905097805	-0.15777014251827	2.75092513176566	0.00962428297411871	0.341183071906093	-2.64402504361397
a_HQ_transcript/46819	-2.16398573303908	1.45326452697762	-2.75088665144527	0.00962519677103887	0.341183071906093	-2.6218483628886
a_HQ_transcript/136289	-1.61408557580246	4.12163183611276	-2.75085275037	0.00962600189110227	0.341183071906093	-2.94940237816592
a_HQ_transcript/110688	-2.54008180390154	-0.563192345521748	-2.75016493715173	0.00964235040658737	0.341554262299885	-2.65168836466511
a_HQ_transcript/15469	2.85742905649232	0.519516366995944	2.74949920927131	0.00965819863549042	0.341739446610624	-2.64307382287459
a_HQ_transcript/78553	4.05102064783936	5.04550120020124	2.74936101525189	0.00966149150686926	0.341739446610624	-3.03422319165468
a_HQ_transcript/122737	1.84977835052636	3.86539137372811	2.74891168850463	0.00967220525060981	0.341739446610624	-2.93508468564115

a_HQ_transcript/120366	3.07430446243567	0.565590128698971	2.74886934099258	0.00967321555543317	0.341739446610624	-2.68321412991982
a_HQ_transcript/16209	-1.7410751034826	1.88315054491686	-2.74871185102119	0.00967697373247121	0.341739446610624	-2.62793208804418
a_HQ_transcript/146530	-3.83037856480573	-0.534282181932746	-2.74845326798839	0.00968314724233433	0.341749837827814	-2.69808821642162
a_HQ_transcript/115438	-1.23529544218053	5.27161981457304	-2.74720706007987	0.0097129512155805	0.342468106443436	-3.04701122725377
a_HQ_transcript/141198	6.76769608067232	3.89965245748788	2.74710975552826	0.00971528192136707	0.342468106443436	-2.80626429121301
a_HQ_transcript/56053	3.44351789093079	-0.0848513599925524	2.74615070364754	0.00973828170811626	0.342867881240093	-2.67520776429243
a_HQ_transcript/22095	-1.1565358704976	3.68106571965844	-2.74588436353154	0.00974467802326083	0.342867881240093	-2.93366787744697
a_HQ_transcript/67900	-2.51490181359585	1.49227304563357	-2.74572693204312	0.0097484606750538	0.342867881240093	-2.66665134903559
a_HQ_transcript/62615	-3.70931134657217	3.46022297555725	-2.74484450971611	0.00976968824350234	0.342867881240093	-2.93659414751776
a_HQ_transcript/98477	1.92140865181711	1.92195948795288	2.74462247200464	0.00977503636850315	0.342867881240093	-2.74329833373139
a_HQ_transcript/49502	2.90233256148393	1.31325806518117	2.74453800670933	0.0097770715635453	0.342867881240093	-2.6668404036753
a_HQ_transcript/97	-2.27675037813736	3.79603803342918	-2.74428692566193	0.00978312370514331	0.342867881240093	-2.87899107484647
a_HQ_transcript/87319	-3.2970352573762	0.94234713789013	-2.74408157545455	0.00978807613128889	0.342867881240093	-2.64084793757632
a_HQ_transcript/79300	2.04402542840331	1.67115522689403	2.74391650173626	0.00979205890463088	0.342867881240093	-2.65062619407384
a_HQ_transcript/95	-2.44697237366883	0.430186286519354	-2.7437242532794	0.00979669923364429	0.342867881240093	-2.70200569741576
a_HQ_transcript/141988	4.6477608541627	1.2030936239251	2.74367264192357	0.00979794533343952	0.342867881240093	-2.72856010577777
a_HQ_transcript/116204	-2.53011918331235	0.0470302083826358	-2.74352837801005	0.00980142921174072	0.342867881240093	-2.66630279504641
a_HQ_transcript/104854	-1.21764996537227	2.6127411647475	-2.74338350000789	0.00980492908198633	0.342867881240093	-2.75212275409933
a_HQ_transcript/28353	-3.09815596235888	1.39361237085918	-2.74320669207404	0.00980920187349082	0.342867881240093	-2.63177436778469
a_HQ_transcript/152751	-3.69065934193638	-0.647854154052178	-2.74292375921804	0.00981604292165213	0.342900807061175	-2.7095303714655
a_HQ_transcript/86594	2.56040452049675	0.267212692117154	2.7425389665881	0.0098253539798197	0.343019925608985	-2.675787068863
a_HQ_transcript/138147	3.33118748507228	0.294259101733174	2.74226984564051	0.00983187095252977	0.343041413402551	-2.68684782422859
a_HQ_transcript/129318	-3.10886203463056	-0.58475529689682	-2.74122519375784	0.00985720620239462	0.343719065466583	-2.70848091636856
a_HQ_transcript/142485	4.69167599379374	2.38863738971211	2.74007817712163	0.00988509411414979	0.344484862510371	-2.71440699941584
a_HQ_transcript/130317	2.66672809147647	-0.796474507062807	2.7392144699748	0.00990614231364366	0.344804101929894	-2.70332026547763
a_HQ_transcript/103894	1.89856811422135	1.92667879464795	2.73907488296986	0.00990954791140881	0.344804101929894	-2.700573716323
a_HQ_transcript/64588	1.16347469642713	2.28356011105681	2.73897234864522	0.00991205020514816	0.344804101929894	-2.7185531504656
a_HQ_transcript/74900	-2.06516814378311	1.22589478348748	-2.73806609220733	0.00993419251090445	0.345367668824075	-2.65208000732179
a_HQ_transcript/3882	0.937980920900172	-0.828582608888635	2.73733687999702	0.00995204260196043	0.345781429986106	-2.73495772385673
a_HQ_transcript/39549	-2.61963361827827	-0.0554268016864907	-2.73691412937978	0.00996240462467193	0.345874922274326	-2.69944938280476
a_HQ_transcript/144509	3.14546041891247	4.50825948087657	2.73674170117548	0.0099666338908873	0.345874922274326	-2.95330085901285
a_HQ_transcript/19340	3.39673299912264	0.785861385435171	2.73646815956373	0.00997334666810646	0.345901369405544	-2.64984749566943
a_HQ_transcript/11496	2.90296833892216	0.704096389186724	2.73599661905516	0.00998492827737469	0.34609654794707	-2.69237364180117
a_HQ_transcript/66062	-1.75033780286666	3.07531549084011	-2.735253889599	0.0100031960343205	0.346294052107264	-2.82613724372276
a_HQ_transcript/151658	-3.67971072702284	1.3269527140854	-2.73509519968644	0.010007103117531	0.346294052107264	-2.73163296886855
a_HQ_transcript/41211	-4.82208757004245	0.813804745910398	-2.73496383363798	0.0100103385391404	0.346294052107264	-2.70795852551668
a_HQ_transcript/119063	1.98143708556755	1.03471805969663	2.73479670957571	0.0100144560554692	0.346294052107264	-2.65521709260578
a_HQ_transcript/6437	3.57209242538584	-0.222248815506227	2.73426213505344	0.0100276372247889	0.346543695958697	-2.71176666371106
a_HQ_transcript/6119	2.78762122775471	5.80429167714634	2.73385086802458	0.0100377889621227	0.346609537890853	-3.09561175358062
a_HQ_transcript/133357	2.66058491545899	1.17515322169947	2.73370191165784	0.010041468170386	0.346609537890853	-2.69560906678181
a_HQ_transcript/148820	-4.78782938926998	0.989387168333997	-2.73342686022694	0.0100482652168753	0.346638314852539	-2.74725388501854
a_HQ_transcript/147477	-2.6553239249835	-0.376894921261579	-2.7322907890035	0.0100763851463741	0.347402203907732	-2.71154673504593
a_HQ_transcript/56469	3.18483663916343	0.731834626052807	2.73077620006215	0.0101139881299692	0.348262631312736	-2.66641289757647
a_HQ_transcript/90451	3.04960486136698	0.153440535138475	2.73058810322546	0.0101186671590001	0.348262631312736	-2.73629819354184
a_HQ_transcript/2727	-2.6831362265416	6.36552354839434	-2.73056203740514	0.0101193157219793	0.348262631312736	-3.18790418969517
a_HQ_transcript/44690	2.48970834969956	-0.601069754171882	2.72991521944142	0.0101354220858122	0.348445039910753	-2.71512849827434
a_HQ_transcript/30655	4.09510534178615	0.0393253874047292	2.72983770524359	0.0101373538621879	0.348445039910753	-2.72822485331804
a_HQ_transcript/146840	3.79947026577434	0.616982145254307	2.72931599328277	0.0101503646732669	0.348445039910753	-2.74844611992404
a_HQ_transcript/61128	-1.23779275936463	2.87377873468814	-2.72929184165424	0.0101509673596554	0.348445039910753	-2.74868788947063
a_HQ_transcript/38480	-1.8015848209441	-0.803435007005032	-2.7289696352678	0.0101590109754828	0.348445039910753	-2.7145210259596

a_HQ_transcript/69074	-1.67740602384325	-0.460199331243254	-2.72890670969114	0.0101605825531366	0.348445039910753	-2.71340365030708
a_HQ_transcript/107915	0.476863428724856	5.07076569333031	2.72761194098418	0.0101929699398202	0.349317256641912	-3.07841415742392
a_HQ_transcript/110497	1.04323834625433	4.05690543182256	2.72740979462041	0.0101980351039314	0.349317256641912	-3.01739890435762
a_HQ_transcript/93983	2.27927521837522	-0.199100364926456	2.72651059063893	0.0102205947976267	0.349686085866461	-2.6957461648949
a_HQ_transcript/148311	5.62854084683892	2.26402431480133	2.72650105173086	0.0102208343635962	0.349686085866461	-2.79025310464661
a_HQ_transcript/119726	5.1154282432736	0.0192904007761825	2.72553433850944	0.0102451401260063	0.350311473673232	-2.72991306127821
a_HQ_transcript/115851	3.0683041423103	0.441457771659166	2.72497679131472	0.0102591828185013	0.35058540792113	-2.71782049094932
a_HQ_transcript/75991	2.65986605127504	1.33713104686511	2.72415620282067	0.0102798832173362	0.350807846538981	-2.73025841048476
a_HQ_transcript/38717	3.08413601731915	-0.00828570757639579	2.72409733131391	0.0102813698208246	0.350807846538981	-2.74065242836548
a_HQ_transcript/112035	-2.87019086406396	0.29205492340718	-2.7240012161799	0.0102837973180296	0.350807846538981	-2.74494698183188
a_HQ_transcript/88525	0.858320176656187	4.61889906814683	2.7237023200708	0.0102913496902542	0.35085957465988	-3.0262848849999
a_HQ_transcript/132052	-5.21434523220493	1.7350679391696	-2.72346220223462	0.0102974206198018	0.35086076540905	-2.82187414026443
a_HQ_transcript/32714	-2.56185057224424	1.02915076754486	-2.72221907889055	0.0103289040138118	0.351425281623093	-2.71723241334351
a_HQ_transcript/130347	-0.982574155129699	4.39477586500528	-2.72206983100664	0.0103326898914053	0.351425281623093	-3.01754647690793
a_HQ_transcript/138427	2.10739910739407	7.72052838693783	2.72145887809557	0.0103482010229703	0.351425281623093	-3.31960315455146
a_HQ_transcript/136456	4.17339426620163	0.616812738036579	2.7213376324801	0.0103512818346502	0.351425281623093	-2.76496557435016
a_HQ_transcript/143461	2.15487612772099	1.6418920520206	2.72103916849442	0.0103588693457995	0.351425281623093	-2.727901479063
a_HQ_transcript/85828	1.25668042159975	3.2655233258784	2.72093633772537	0.010361484694668	0.351425281623093	-2.87806316604217
a_HQ_transcript/62632	2.45534978839756	3.36461290799178	2.72093496159191	0.0103615196987596	0.351425281623093	-2.92531475122167
a_HQ_transcript/149386	4.59799889883805	3.22806311637093	2.72090215045526	0.0103623543335739	0.351425281623093	-3.01409899891121
a_HQ_transcript/146357	2.61489368107583	0.279767919890269	2.71976139876691	0.010391411199714	0.35187942348664	-2.7589963717637
a_HQ_transcript/132382	3.95247274139324	1.17920025159236	2.71963181879562	0.0103947166122649	0.35187942348664	-2.78070925905374
a_HQ_transcript/123480	-4.21178118485587	1.4010076740184	-2.71903382605821	0.0104099832942628	0.35187942348664	-2.74592695946142
a_HQ_transcript/119795	-2.67027321416451	0.920961532366374	-2.71884541779145	0.0104147976563933	0.35187942348664	-2.7333882671314
a_HQ_transcript/147062	2.52388013964514	-0.380925902269248	2.71818711530613	0.0104316354150934	0.35187942348664	-2.75119414858343
a_HQ_transcript/33833	0.91036499706069	2.96508655629757	2.71816306436626	0.0104322510584642	0.35187942348664	-2.86052610831055
a_HQ_transcript/80046	1.98355231392108	-0.524167998445032	2.71797830773133	0.0104369814898931	0.35187942348664	-2.73734476407272
a_HQ_transcript/55801	-2.65615153696531	2.62032139251116	-2.71786025086518	0.0104400052137128	0.35187942348664	-2.86438462157223
a_HQ_transcript/137224	2.18514977051971	2.19467004310386	2.71783115620475	0.0104407505240287	0.35187942348664	-2.72574009314239
a_HQ_transcript/144536	1.71910238183663	3.04373382616707	2.71778763536612	0.0104418654784231	0.35187942348664	-2.81822369822394
a_HQ_transcript/139007	-4.16298961514883	0.647025824101145	-2.71772500790075	0.0104434701173365	0.35187942348664	-2.79124698668263
a_HQ_transcript/104046	0.57863119468868	4.18490260048105	2.71744296433268	0.0104506994731373	0.35187942348664	-3.01848425401244
a_HQ_transcript/100034	1.56200927428604	3.18488876394653	2.71729705299254	0.0104544413081721	0.35187942348664	-2.80282173504646
a_HQ_transcript/40126	-3.87120370019389	0.38890036730625	-2.71666454642765	0.0104706760954403	0.35222190976606	-2.77011026128417
a_HQ_transcript/12227	-1.15824253783525	2.99087774955726	-2.7150081018357	0.0105133039370685	0.353143695372673	-2.87157310683109
a_HQ_transcript/129872	2.91665931310457	1.88657354635019	2.71480499407479	0.0105185419227335	0.353143695372673	-2.85674123334971
a_HQ_transcript/39075	2.76304222628945	0.835918694487472	2.71475013380316	0.0105199571414695	0.353143695372673	-2.71445485777071
a_HQ_transcript/143840	-3.86194572139979	1.03213512981729	-2.71465624497216	0.0105223795827393	0.353143695372673	-2.8248616657607
a_HQ_transcript/105258	2.09498616024705	0.894799751157245	2.71371184301508	0.0105467751397906	0.353713489472703	-2.72832401403498
a_HQ_transcript/147286	8.86130550070955	6.72942489380438	2.71342846137551	0.0105541056427516	0.353713489472703	-3.18412843398456
a_HQ_transcript/72640	3.12988256638154	1.9684577658587	2.7132929552829	0.0105576125831809	0.353713489472703	-2.7681434265244
a_HQ_transcript/136346	3.1305715955538	-0.31419349287662	2.71179978770957	0.0105963280956894	0.354806082687921	-2.75040230776462
a_HQ_transcript/35127	-2.59244897910715	1.7928544725605	-2.71076454503774	0.0106232478189493	0.354876955588359	-2.7188686267259
a_HQ_transcript/126220	2.63535503226672	-0.195450391327354	2.71062856033945	0.0106267885932326	0.354876955588359	-2.71755017744754
a_HQ_transcript/59092	2.73944717117343	2.19528235210796	2.71056824521566	0.0106283594319642	0.354876955588359	-2.80682060804085
a_HQ_transcript/3650	2.1651744244837	2.10286951231202	2.71035254717338	0.0106339788096321	0.354876955588359	-2.73932574777295
a_HQ_transcript/43999	1.77638989809299	1.79889486139231	2.71017295671284	0.0106386596190144	0.354876955588359	-2.70884436567332
a_HQ_transcript/35341	2.50828023540417	-0.045950570887242	2.7101667535189	0.0106388213320449	0.354876955588359	-2.73725295685251
a_HQ_transcript/25074	-3.24259894002177	0.441055235411106	-2.71007627182626	0.0106411803879457	0.354876955588359	-2.76923303069009
a_HQ_transcript/124168	-4.39250779657147	2.33569595694673	-2.70916010367128	0.0106650943221754	0.355470529105166	-2.84561422203609

a_HQ_transcript/90235	3.20235395524264	1.81904345487573	2.70876450963587	0.0106754356117418	0.355516995913571	-2.73470916042735
a_HQ_transcript/150523	-3.05947490527227	-0.338738288777271	-2.70844119439038	0.0106838943752915	0.355516995913571	-2.79656853527649
a_HQ_transcript/129795	3.64057148726938	0.941309439547688	2.70836347800145	0.0106859285663356	0.355516995913571	-2.73965648024718
a_HQ_transcript/17125	2.56290106164344	-0.211280814059994	2.70817158285439	0.0106909528773899	0.355516995913571	-2.75883186268694
a_HQ_transcript/128964	2.38375914568101	0.916886398256021	2.70719941279348	0.0107164405839283	0.355976613203675	-2.82637710440421
a_HQ_transcript/79963	5.05535079073886	2.77897802799269	2.70717724912259	0.0107170223146579	0.355976613203675	-2.92681991526378
a_HQ_transcript/31039	2.01937660534349	2.63466223235567	2.70616376219706	0.0107436547453063	0.356657431773368	-2.77451753514659
a_HQ_transcript/147227	-4.34821741173344	5.64531015312621	-2.70588415518691	0.0107510130901122	0.356676128119069	-3.03343109115549
a_HQ_transcript/119273	-2.95928291116722	0.40409616816894	-2.705676154413	0.0107564900322173	0.356676128119069	-2.72591352218847
a_HQ_transcript/139004	2.40697767142122	0.136530231951554	2.70481672808397	0.0107791474133139	0.3568734448436	-2.76091661815721
a_HQ_transcript/137786	2.64739167732926	3.0230995802081	2.70478826037321	0.0107798986772014	0.3568734448436	-2.849148837671
a_HQ_transcript/123019	3.68964274794899	0.79981528247918	2.70475187528996	0.0107808589517162	0.3568734448436	-2.73294261636903
a_HQ_transcript/19615	-1.96498975632842	1.33946405555936	-2.70233694306263	0.0108447719445959	0.358784805688941	-2.7547557162216
a_HQ_transcript/50	-1.83325866473729	3.57312076063362	-2.70088137641522	0.0108834645943126	0.359860085289082	-2.8666801405884
a_HQ_transcript/90210	1.72170798477505	-0.479767685715695	2.70045050306881	0.0108949428964281	0.360034815624544	-2.75900181847242
a_HQ_transcript/23027	2.64304330595009	-0.157332823361366	2.69971741878084	0.0109144978503661	0.360476097185274	-2.78031588097949
a_HQ_transcript/27126	2.12139493357668	2.75504439164601	2.69932365236028	0.0109250149978835	0.36061855297954	-2.88120661294145
a_HQ_transcript/63506	1.59809528503313	2.56097926805553	2.69839204489546	0.0109499349197831	0.360808910706355	-2.76949479618992
a_HQ_transcript/91717	1.20111319994162	2.57673617489194	2.69838334403058	0.0109501679113811	0.360808910706355	-2.84028697841498
a_HQ_transcript/75509	1.78747667883212	1.40577820509167	2.6983369885189	0.0109514092960931	0.360808910706355	-2.71413004769693
a_HQ_transcript/145087	-3.2599165781529	3.02843369082459	-2.69818014416454	0.0109556105043476	0.360808910706355	-2.86764393773318
a_HQ_transcript/120052	2.08593936936974	0.610483867019228	2.69687914922129	0.0109905165473487	0.361753536729492	-2.72126419490391
a_HQ_transcript/150147	3.55011123427881	1.47793823433295	2.69570388981178	0.0110221379153064	0.362530178797235	-2.81904087827845
a_HQ_transcript/91946	2.26520558726677	1.98866110984088	2.6955388432847	0.0110265853996957	0.362530178797235	-2.83205727028258
a_HQ_transcript/139173	2.08491165046291	3.19781440419837	2.6947188048962	0.011048707594056	0.362856538988179	-2.94384527600946
a_HQ_transcript/6185	1.29520136786668	2.08078306216163	2.69470810218345	0.0110489965938803	0.362856538988179	-2.75429587192042
a_HQ_transcript/62446	-3.76967231185961	1.93067745727761	-2.69370730333838	0.0110760517120837	0.363539657775269	-2.853613326689
a_HQ_transcript/6471	-2.42938376108853	-0.606324647889482	-2.6931742811071	0.0110904862947426	0.363682782333278	-2.761324056437
a_HQ_transcript/145423	-2.44416870901786	-0.557407975220064	-2.69308427310989	0.0110929254933406	0.363682782333278	-2.79674095852099
a_HQ_transcript/91900	-2.23037471571455	-0.599107165716799	-2.69253836386486	0.0111077302081289	0.363962875726109	-2.78856947385493
a_HQ_transcript/54959	-2.30695290423893	-0.544210635832854	-2.69166852201091	0.0111313577106363	0.364482862759612	-2.77933168389262
a_HQ_transcript/131024	-2.18902606467387	-0.580123156285671	-2.69149266015544	0.011136140315529	0.364482862759612	-2.78781897704495
a_HQ_transcript/47107	-3.5221554333437	0.5223948897301	-2.69092509332801	0.0111515884576621	0.364783080397852	-2.74245456278483
a_HQ_transcript/127152	2.87905847295632	0.84521642209611	2.69054600550563	0.0111619176306991	0.36491560632018	-2.79450167027208
a_HQ_transcript/135107	-0.930612499240317	5.1300635825026	-2.68990085734025	0.0111795167064832	0.365128324141396	-3.15932409222275
a_HQ_transcript/120393	-3.73114525017157	0.61359168888991	-2.68962864675865	0.0111869501031909	0.365128324141396	-2.83047389615042
a_HQ_transcript/31168	4.56075800298396	1.2299339256535	2.68941259753307	0.0111928531406988	0.365128324141396	-2.77656911442488
a_HQ_transcript/66635	2.3800814767893	-0.435683419467123	2.68938710018992	0.0111935499865808	0.365128324141396	-2.79351145602729
a_HQ_transcript/136573	-2.68687304395895	-0.634606773045694	-2.68859177653901	0.0112153065383315	0.365443194883972	-2.81081673171345
a_HQ_transcript/3373	-2.98154921294373	2.25194119913419	-2.68840204139082	0.0112205026569654	0.365443194883972	-2.7975005925322
a_HQ_transcript/139266	-2.6651254234449	-0.119501721708087	-2.68834506616234	0.0112220634267116	0.365443194883972	-2.83706846381318
a_HQ_transcript/36102	-3.59452492158359	0.750444810268838	-2.68712456587325	0.0112555460779029	0.366271406738074	-2.77939742303556
a_HQ_transcript/146039	2.44672338980592	1.30778037528389	2.68695888388239	0.0112600984696005	0.366271406738074	-2.86286355451045
a_HQ_transcript/120438	-2.0100917687188	3.51817124087899	-2.68651140776611	0.0112724021784559	0.366466551358661	-3.01079552783188
a_HQ_transcript/95859	-2.70231392055575	0.331497685763707	-2.68625844190202	0.0112793631985923	0.366473298657646	-2.8082945354719
a_HQ_transcript/79523	-2.80070639944127	0.15318237505385	-2.68604575574021	0.0112852189065027	0.366473298657646	-2.79889399484392
a_HQ_transcript/4562	2.9099389222057	-0.105637262035856	2.68540700945951	0.0113028219452729	0.36683999666936	-2.81155936877634
a_HQ_transcript/44319	-2.0257481613713	1.99728393597538	-2.68492455052901	0.0113161348053783	0.36694806251932	-2.85384413230926
a_HQ_transcript/109	-2.62694383583639	3.48144866786496	-2.68470832699803	0.0113221059523324	0.36694806251932	-2.94447697232014
a_HQ_transcript/11038	2.28067590395185	1.91572087210986	2.68460031475408	0.011325089873377	0.36694806251932	-2.86594278646852

a_HQ_transcript/112338	-3.4981706836476	1.50510887513862	-2.68434993557547	0.0113320095992406	0.366967718097303	-2.85522508894465
a_HQ_transcript/17252	-2.38236312132657	-0.475435302430722	-2.68376108076374	0.0113482992518354	0.367174715063705	-2.78404072346763
a_HQ_transcript/141941	-3.23269258702757	-0.608265394411931	-2.68366226287008	0.0113510350084207	0.367174715063705	-2.8250448574164
a_HQ_transcript/140998	-3.05494833821536	1.98637309511075	-2.6830205220134	0.0113688164048503	0.36744207211876	-2.77561885840345
a_HQ_transcript/37243	-1.74546979093549	1.09262330374909	-2.68272447314835	0.0113770280618522	0.36744207211876	-2.75249114612135
a_HQ_transcript/2596	2.62767937070351	-0.438006059334472	2.68267993194265	0.0113782640003745	0.36744207211876	-2.81799134600712
a_HQ_transcript/22656	4.6480726290923	2.24934982467585	2.68221782964724	0.0113910938688603	0.367652140149424	-2.91959178367763
a_HQ_transcript/43166	-3.14879806028841	2.51722723373995	-2.68118159902267	0.0114199127654249	0.367869916322081	-2.85109686738369
a_HQ_transcript/23330	-3.13938203085527	0.487238530483852	-2.68101120028326	0.0114246582487967	0.367869916322081	-2.77286067406391
a_HQ_transcript/72644	-2.55635750928734	0.0923837809035997	-2.68101040420397	0.0114246804233337	0.367869916322081	-2.78882460139942
a_HQ_transcript/150698	0.446950319893892	10.4118500031519	2.68083658523864	0.0114295230528057	0.367869916322081	-3.68814626358811
a_HQ_transcript/137969	-2.47504547906676	-0.17180320395452	-2.68081884078179	0.0114300175239568	0.367869916322081	-2.83722656723446
a_HQ_transcript/48118	3.75225030269207	0.849290699478444	2.68056434061588	0.0114371116704517	0.367869916322081	-2.80385986681054
a_HQ_transcript/21428	-1.75668118824484	1.04564094219885	-2.68038398124287	0.0114421416307171	0.367869916322081	-2.77359664537137
a_HQ_transcript/146263	3.23498109077064	1.47201569912751	2.67996391685789	0.0114538645785971	0.368043250538802	-2.86939679417236
a_HQ_transcript/96505	1.72843066361284	1.37007499858007	2.67937612405498	0.0114702871284939	0.368277341952571	-2.76818246895253
a_HQ_transcript/10793	-2.55987941970267	-0.0803394432176864	-2.6792497430587	0.0114738209860327	0.368277341952571	-2.79228183688361
a_HQ_transcript/27701	-2.94858323033732	2.65833647952929	-2.67807349689675	0.011506759626157	0.369126183581225	-2.93410753694951
a_HQ_transcript/119810	4.72997414128758	1.38430756214621	2.67785216494714	0.0115129674310618	0.369126183581225	-2.86920337600821
a_HQ_transcript/63275	4.46986650600282	1.2588793620646	2.67681595364328	0.0115420719268586	0.369855323574661	-2.9604184285571
a_HQ_transcript/88026	1.21593189962586	2.7126838158155	2.67657880355582	0.01154874245705	0.369865179913721	-2.90423066793539
a_HQ_transcript/75321	1.09454732186139	-0.718949747060596	2.6762454845486	0.0115581240653597	0.369961803783716	-2.83574659947873
a_HQ_transcript/77004	2.75553892115056	-0.218949235915165	2.67589197667423	0.0115680816400821	0.369963146974869	-2.84971885141715
a_HQ_transcript/63224	-3.74391577460701	1.853053345856	-2.67579213946926	0.0115708952862701	0.369963146974869	-2.87800107270062
a_HQ_transcript/42568	2.71107998142085	-0.41468135189173	2.67555158938085	0.0115776771603497	0.369976480471032	-2.82875690981738
a_HQ_transcript/119262	3.43521427381752	-0.324033170759877	2.67513110367132	0.0115895408559104	0.370152104874924	-2.84156377271593
a_HQ_transcript/130013	-2.82548119192189	0.587727738814991	-2.67420958483059	0.0116155803066322	0.370780039573814	-2.8727569322509
a_HQ_transcript/120896	-2.87279191537434	0.355571978673334	-2.67270509182961	0.0116582096410496	0.371532852175265	-2.81276801301178
a_HQ_transcript/3133	2.45944155811408	2.37337934414054	2.67269858334178	0.0116583943720153	0.371532852175265	-2.85361948074968
a_HQ_transcript/9819	2.2700302387028	0.552753113160226	2.67238592562051	0.0116672717632405	0.371532852175265	-2.80576286466075
a_HQ_transcript/919	0.980040307995061	3.94245418057285	2.67232022185781	0.0116691381088062	0.371532852175265	-3.04902588639952
a_HQ_transcript/787	-2.77254771570277	-0.554432484835257	-2.67225038553206	0.0116711221454727	0.371532852175265	-2.84265432070182
a_HQ_transcript/38529	-2.40934844421449	0.113970899453848	-2.6715727339261	0.011690390361579	0.371942534722423	-2.82421300478925
a_HQ_transcript/127793	-3.39561011539542	0.488401353288689	-2.67011307671743	0.0117319942905895	0.37285964573071	-2.89998387548979
a_HQ_transcript/80813	-2.23205024270196	1.95058266940181	-2.67011131241265	0.011732044660774	0.37285964573071	-2.92138802227734
a_HQ_transcript/9695	2.97141290368153	1.88111715021269	2.66718778027633	0.0118157865206998	0.375315868237836	-2.81083697415541
a_HQ_transcript/1053	-2.78884680700011	1.58427291493143	-2.6667453994366	0.011828506307481	0.375514699421768	-2.79352045847645
a_HQ_transcript/69487	-3.15276697602507	-0.589001981214473	-2.666187698357	0.0118445600015793	0.375819095945307	-2.84321484483375
a_HQ_transcript/119415	1.93665926640761	-0.665536283228923	2.66494720037219	0.0118803408387641	0.376561839140942	-2.83171408632038
a_HQ_transcript/30096	-2.55607399336015	3.69701689415695	-2.66492697392459	0.0118809250788688	0.376561839140942	-3.07166599510037
a_HQ_transcript/35869	-2.99022160639766	1.03735613596363	-2.66454083116361	0.0118920839088891	0.376710110875153	-2.80828976504798
a_HQ_transcript/123386	-3.02564773488179	-0.514412602350607	-2.66412916719394	0.0119039909618299	0.376881909928785	-2.86651544063045
a_HQ_transcript/56410	2.67058127434895	-0.193281979807942	2.66329692821018	0.0119280965953706	0.377322326921708	-2.79617329218944
a_HQ_transcript/107113	2.75306620944809	-0.295074597376874	2.66255297974154	0.011949683228989	0.377322326921708	-2.85868874794295
a_HQ_transcript/85724	-3.73210512202784	0.851230005550868	-2.66238953116753	0.0119544307542681	0.377322326921708	-2.8515740388166
a_HQ_transcript/4664	1.32883159013262	5.22995236364373	2.66238468313617	0.0119545715968364	0.377322326921708	-3.23095331644954
a_HQ_transcript/152713	2.24509987482394	-0.337865482704764	2.66229062154374	0.0119573045315094	0.377322326921708	-2.86078868885608
a_HQ_transcript/59709	-2.30189753748632	-0.644064502100094	-2.66192387540393	0.0119679657784432	0.377322326921708	-2.85839597489811
a_HQ_transcript/99695	1.66009707298876	-0.76000884610994	2.66154274030931	0.0119790546505611	0.377322326921708	-2.82877118868915
a_HQ_transcript/123411	-3.43605423860909	0.704130394191729	-2.66150435240121	0.0119801720492377	0.377322326921708	-2.98663277945101

a_HQ_transcript/106148	-2.8355595019377	0.240956377144189	-2.66139563127519	0.0119833372381118	0.377322326921708	-2.87105182082609
a_HQ_transcript/36780	2.41100817782011	0.432513722207022	2.66105215858364	0.0119933418251088	0.377322326921708	-2.80737252850793
a_HQ_transcript/9538	1.0522226540759	2.78205900683531	2.66090779784025	0.011997549040362	0.377322326921708	-2.94855392669846
a_HQ_transcript/44772	-2.42813430882007	1.03089629742714	-2.66082668543711	0.0119999135615	0.377322326921708	-2.83862612898713
a_HQ_transcript/149022	-1.27657555732988	4.33555978425929	-2.66074525623024	0.0120022877525158	0.377322326921708	-3.13923153084842
a_HQ_transcript/33860	-1.94895121247889	-0.698404741116803	-2.6603888543598	0.012012684311683	0.377445034415952	-2.83765602403057
a_HQ_transcript/71953	-2.11026632886254	3.9583067007821	-2.66003478214032	0.0120230211817397	0.37744882801417	-3.1083926968279
a_HQ_transcript/20182	-1.19636567487621	1.8098721933722	-2.65993992501255	0.0120257918642004	0.37744882801417	-2.84304233384268
a_HQ_transcript/135633	4.10802449952565	0.843747716783671	2.65967174261349	0.0120336284092024	0.377490961775563	-2.88381112765231
a_HQ_transcript/66293	3.3910588378781	0.867723640932301	2.6593868705449	0.0120419578285165	0.377548503050705	-2.83717774649314
a_HQ_transcript/27544	2.67995746734219	1.7543298885347	2.6587749146566	0.0120598689835262	0.377743461400903	-2.83081939865934
a_HQ_transcript/12444	-2.46716322521943	0.078464546488336	-2.65855836951233	0.012066212893829	0.377743461400903	-2.82031360649522
a_HQ_transcript/144257	-3.33344623316455	1.02711281006204	-2.65833200137306	0.0120728478865859	0.377743461400903	-2.97329805013576
a_HQ_transcript/17990	-3.01066467047511	1.35222176664528	-2.65828690964936	0.012074169957385	0.377743461400903	-2.87511801526212
a_HQ_transcript/22056	2.75250502998984	0.653265181168525	2.65749271839044	0.0120974773322933	0.37826904915091	-2.84172386572943
a_HQ_transcript/24816	-3.51989864164085	1.8786743114442	-2.65652268992938	0.0121260016435027	0.378643262627587	-2.81786733808396
a_HQ_transcript/24750	2.71660162866261	-0.0133194430120313	2.65646122448769	0.0121278111733869	0.378643262627587	-2.8378855248687
a_HQ_transcript/125940	-3.91522415888198	0.510574150193007	-2.65620415248416	0.0121353820333327	0.378643262627587	-2.84728210099371
a_HQ_transcript/78589	1.87572344164281	-0.676101442466491	2.65614546968475	0.0121371108763255	0.378643262627587	-2.87281945828947
a_HQ_transcript/140567	1.56019575675511	-0.833255624428039	2.65595175685257	0.0121428194349182	0.378643262627587	-2.85521389798388
a_HQ_transcript/135128	-2.6667394829019	-0.631489903413311	-2.65575809949385	0.0121485288466909	0.378643262627587	-2.86975850364221
a_HQ_transcript/107816	2.17281743595516	0.627536628444355	2.65486630376503	0.0121748529250429	0.379260370218057	-2.86273113547914
a_HQ_transcript/116025	2.678085526746	0.177149203119848	2.65459984718662	0.0121827284594034	0.379302431648742	-2.84573188782194
a_HQ_transcript/111815	1.18454474426228	4.66931044676028	2.65394838684633	0.0122020032638275	0.379382101723894	-3.20458570591738
a_HQ_transcript/7159	-0.80616494204511	3.28779169983435	-2.65391256749127	0.0122030638730922	0.379382101723894	-2.99319776423323
a_HQ_transcript/84534	0.934287542997527	3.85886807040692	2.65385166578898	0.0122048673655326	0.379382101723894	-3.08647468296625
a_HQ_transcript/140769	10.65600225307	3.81597606125333	2.65327649325126	0.0122219122363758	0.379659353763345	-2.93568960169476
a_HQ_transcript/127234	2.63384933534959	0.834229822162191	2.65289617425352	0.01223319486216	0.379659353763345	-2.88320764130818
a_HQ_transcript/40714	0.966953757333656	3.3438897147519	2.65265946975227	0.0122402218623151	0.379659353763345	-3.01339692778459
a_HQ_transcript/65186	2.18600846001198	-0.525241227198211	2.65248345521115	0.0122454495966375	0.379659353763345	-2.88073204198778
a_HQ_transcript/74778	-0.807804375491336	3.04138830374502	-2.65244998709365	0.0122464438533284	0.379659353763345	-3.00403939937835
a_HQ_transcript/81031	-2.91664089728788	0.877561232054431	-2.65035443224383	0.0123088469249914	0.380483619150471	-2.90046256644573
a_HQ_transcript/5625	2.01407961647686	-0.144004583653139	2.65032164312331	0.0123098256824484	0.380483619150471	-2.84081969378286
a_HQ_transcript/94441	2.7318692128303	0.251999926561508	2.64964349651862	0.0123300845822961	0.380483619150471	-2.89506003967949
a_HQ_transcript/10734	-3.26018003218274	0.333440898582171	-2.64958537598107	0.01233182230747	0.380483619150471	-2.90668932640505
a_HQ_transcript/139819	-3.15878534518217	1.08487111031622	-2.64949946024197	0.0123343914871352	0.380483619150471	-2.90329934004134
a_HQ_transcript/38164	1.84569329315488	-0.612281017110095	2.64943380471451	0.0123363551502829	0.380483619150471	-2.90734361690952
a_HQ_transcript/11782	2.47467399488166	2.0529592334603	2.64934341636417	0.0123390590108129	0.380483619150471	-2.89609990436722
a_HQ_transcript/16336	1.64513568882598	-0.690883707000009	2.64923392237869	0.0123423351293448	0.380483619150471	-2.88762287308751
a_HQ_transcript/151139	-7.92506791350024	0.691183134464802	-2.64921172393816	0.0123429994168054	0.380483619150471	-2.91315340761516
a_HQ_transcript/139154	-1.74346694676221	3.613547540097	-2.64901413505851	0.0123489137163068	0.380483619150471	-3.08030781589666
a_HQ_transcript/128309	-5.26216181476056	-0.618323926083788	-2.64898951333237	0.0123496508864042	0.380483619150471	-2.91057557127556
a_HQ_transcript/146052	5.62134786356941	3.13868680197526	2.64892511713487	0.0123515790898868	0.380483619150471	-3.05981883942677
a_HQ_transcript/89891	-2.03523422473194	-0.689059853363097	-2.64706655037738	0.0124073501681077	0.381494943070598	-2.88650757479203
a_HQ_transcript/151376	0.461236887293096	10.7586027512166	2.64658837383774	0.0124217367746333	0.381494943070598	-3.80290970095989
a_HQ_transcript/97205	-3.83667719528455	2.95454819299974	-2.64658660118285	0.0124217901361637	0.381494943070598	-3.07385678800734
a_HQ_transcript/141197	2.87267732750162	2.79084033666984	2.64657704671235	0.0124220777542752	0.381494943070598	-2.94398177115227
a_HQ_transcript/149474	-3.54477502824064	1.36049321271737	-2.64644700632212	0.0124259929721034	0.381494943070598	-2.88119097053163
a_HQ_transcript/24695	1.54966516844627	-0.516373536563721	2.64617704487397	0.0124341245425389	0.381494943070598	-2.91478047815982
a_HQ_transcript/85168	-5.54908675823531	1.33291944140327	-2.64612460301608	0.0124357047274624	0.381494943070598	-2.95182290828982

a_HQ_transcript/22719	2.62939470497475	-0.316645016987076	2.64596708943889	0.0124404520658667	0.381494943070598	-2.86307148491181
a_HQ_transcript/130322	-1.40776566990159	3.97527337406648	-2.64586676398746	0.0124434766732359	0.381494943070598	-3.15608981992266
a_HQ_transcript/115929	2.88907387938667	0.254275729726157	2.64513124744379	0.0124656718082854	0.38187169987477	-2.86954147040361
a_HQ_transcript/128383	-1.90328369480469	-0.761898213484859	-2.64502421849686	0.0124689045961037	0.38187169987477	-2.88840371049089
a_HQ_transcript/51962	-2.13044965118106	2.06597886386716	-2.644676989467	0.0124793979269485	0.381991807634366	-2.87585993674835
a_HQ_transcript/118635	-3.75730172289569	0.411441758876412	-2.64423540831482	0.0124927544146661	0.382199383481953	-2.89273697232581
a_HQ_transcript/140955	-3.68283119199276	3.76986699946979	-2.64332904523243	0.0125202106368952	0.382837876855047	-3.13614709684534
a_HQ_transcript/77789	-3.64827563521978	2.19827255190029	-2.64264409241997	0.0125409968044145	0.383271851864882	-3.01407161681548
a_HQ_transcript/56160	-0.746719988318936	3.68059112865333	-2.64116743395325	0.0125859175313943	0.384442571868043	-3.10586888926556
a_HQ_transcript/79361	-2.01434950930748	2.64913086051059	-2.64039305718051	0.0126095340007392	0.384900032362433	-2.99611747712443
a_HQ_transcript/75458	2.9937602517476	0.253798467743441	2.64024227922919	0.0126141371051892	0.384900032362433	-2.86087765738612
a_HQ_transcript/89093	2.61002282364593	1.16668873657753	2.64000642794688	0.0126213405368489	0.384917776876157	-2.91278999923201
a_HQ_transcript/130077	2.86076664681152	2.22835991409693	2.63968375507384	0.012631201867529	0.385016519221671	-2.9183571020692
a_HQ_transcript/89459	-3.32447726561288	-0.263157456671603	-2.63873075423971	0.0126603685666417	0.385582280791151	-2.94008652373659
a_HQ_transcript/46894	3.55299193581371	1.99218246876723	2.63864391405726	0.0126630294183579	0.385582280791151	-3.00515855453802
a_HQ_transcript/141224	-5.74467319567458	8.83558270524221	-2.63741854657041	0.0127006308938242	0.385945991192256	-3.33663824874214
a_HQ_transcript/72968	-3.88908730625569	1.30716845742642	-2.63731944708085	0.012703676362654	0.385945991192256	-2.99053722764247
a_HQ_transcript/102361	-2.9533203638889	1.14392136496456	-2.63727229113409	0.0127051257696508	0.385945991192256	-2.88798559045656
a_HQ_transcript/116376	2.82958699298463	0.889301311610181	2.63726425022992	0.0127053729338815	0.385945991192256	-2.85860157401604
a_HQ_transcript/28557	2.7708433296622	0.067214033811376	2.63717319646611	0.0127081720881843	0.385945991192256	-2.90775239483343
a_HQ_transcript/3880	-1.33790539502918	-0.708805398595513	-2.63659856302983	0.0127258505163588	0.38612101374874	-2.91587522559811
a_HQ_transcript/20990	2.51563397981849	1.00653561236433	2.63655406895476	0.0127272203128025	0.38612101374874	-2.88301704833444
a_HQ_transcript/149459	1.19750535856535	5.01041935710776	2.63583087707535	0.0127495036780033	0.386405723680845	-3.25776104959775
a_HQ_transcript/52433	2.1329012296556	1.62411243994448	2.63581803042315	0.0127498998420703	0.386405723680845	-2.85734748580859
a_HQ_transcript/89424	-2.50933549955696	0.280378574653439	-2.63556876244495	0.0127575890050057	0.386437276541413	-2.84349306460469
a_HQ_transcript/76673	4.62614355307885	0.652598511590003	2.63465705815362	0.012785748904845	0.386815709925425	-2.85484965264238
a_HQ_transcript/150304	3.09674343172034	2.21050203368628	2.63454904532725	0.0127890889173849	0.386815709925425	-3.00438029428735
a_HQ_transcript/66207	-2.59439142290151	0.141717397957962	-2.63451809867265	0.0127900460101271	0.386815709925425	-2.83914433394227
a_HQ_transcript/52873	2.53570754261616	5.40109411582448	2.63402255860758	0.0128053807010303	0.387003668655636	-3.27732413541108
a_HQ_transcript/2203	-1.56101003998906	2.76723446910247	-2.63388706959736	0.0128095764260502	0.387003668655636	-2.98005787621915
a_HQ_transcript/90745	-2.99902528378619	0.688878057008294	-2.63341847712081	0.0128240972703178	0.387241104482613	-2.93801907998094
a_HQ_transcript/152510	-3.49287293640048	-0.588336833731125	-2.63307311950135	0.0128348090380586	0.38732315116876	-2.93662698145791
a_HQ_transcript/48073	2.80047220414557	0.403932361291517	2.63290131347737	0.0128401409355595	0.38732315116876	-2.87610337702379
a_HQ_transcript/64016	7.93093061201444	3.46384003880643	2.63264317569834	0.0128481559422341	0.387363904880802	-2.99859437916673
a_HQ_transcript/112529	1.08763571046018	2.8348293652178	2.63220063494545	0.0128619073144933	0.387577474534404	-3.00746928372674
a_HQ_transcript/130951	-4.4558266491459	3.12626873048155	-2.63182573738866	0.0128735674206929	0.387727837839397	-3.00420892137319
a_HQ_transcript/119175	0.589035107381205	4.1131097669535	2.63052145844587	0.0129142095229526	0.388589152500478	-3.2014442779671
a_HQ_transcript/150488	-4.40889246563421	8.87045508572379	-2.63047490007696	0.0129156625019888	0.388589152500478	-3.68958175972178
a_HQ_transcript/144822	0.81086068713676	3.03758147947217	2.63026482070093	0.0129222204752172	0.388589152500478	-3.0401488653948
a_HQ_transcript/98167	-1.95185709807314	-0.259237443116802	-2.62978412392508	0.0129372378103828	0.388839586060977	-2.92879078090137
a_HQ_transcript/91240	-2.08833519854093	-0.697027632392059	-2.62863128977439	0.0129733190696365	0.389722527586476	-2.89721714885909
a_HQ_transcript/145696	7.02611006140757	2.77968976337315	2.62750368744654	0.0130087006905795	0.3897635046622	-2.9635079643359
a_HQ_transcript/46784	3.42887139577044	1.36101129172828	2.62731987404798	0.013014476797726	0.3897635046622	-2.90284423452167
a_HQ_transcript/66125	-3.07074864209526	-0.000610533291240097	-2.62723882911309	0.0130170242876288	0.3897635046622	-2.92760510725764
a_HQ_transcript/3216	1.42543300406034	2.5003767791053	2.62717135991299	0.0130191454026422	0.3897635046622	-2.99812333700328
a_HQ_transcript/84939	-2.37155480939615	-0.566703469300192	-2.62691404431115	0.0130272378963659	0.3897635046622	-2.9214256527362
a_HQ_transcript/95223	7.45445515757585	6.04498388058583	2.62660397319169	0.0130369957176524	0.3897635046622	-3.20869651371332
a_HQ_transcript/51297	0.597062955026286	3.66605500586074	2.62655622232757	0.0130384990201215	0.3897635046622	-3.16808823286043
a_HQ_transcript/148750	5.0716965545974	3.27251121411838	2.6265514122858	0.0130386504597315	0.3897635046622	-3.15811072356587
a_HQ_transcript/151210	-2.56715283439056	-0.489913662306943	-2.62647572073885	0.0130410337507243	0.3897635046622	-2.90201002433561

a_HQ_transcript/144384	-2.16718390444212	0.54085168985131	-2.62645342771753	0.0130417357653451	0.3897635046622	-2.97055992371414
a_HQ_transcript/58931	-2.75956254474232	1.89979907200259	-2.6252762484063	0.013078855239907	0.390289185120345	-2.94040185050331
a_HQ_transcript/30874	2.26916627759763	-0.330191079462661	2.62490258644882	0.0130906581790017	0.390289185120345	-2.90313026492428
a_HQ_transcript/27792	1.26910618803456	2.36477910858433	2.6248607965657	0.0130919788173469	0.390289185120345	-2.93220845996426
a_HQ_transcript/79945	-4.06723626969268	0.896434346311078	-2.62477905025054	0.0130945625099385	0.390289185120345	-2.87531423644576
a_HQ_transcript/124614	1.5131191836963	3.92210807694707	2.62474910599223	0.0130955090530016	0.390289185120345	-3.09607147796329
a_HQ_transcript/146667	-3.1852550890698	0.0151471690480264	-2.62461935593036	0.0130996112057837	0.390289185120345	-2.89172476673209
a_HQ_transcript/83791	2.2719118505534	-0.558658300587977	2.6242565897076	0.0131110866613005	0.390430965905777	-2.93869733280703
a_HQ_transcript/138348	-2.1204557033345	1.06302112506454	-2.62272323366687	0.0131596944183106	0.391381421146135	-2.88283531750057
a_HQ_transcript/124861	-1.71796053066257	-0.220681268593106	-2.62268932045795	0.0131607713569799	0.391381421146135	-2.89296649290461
a_HQ_transcript/144810	3.80911333274083	0.893190569774442	2.62261274875752	0.0131632032469841	0.391381421146135	-3.03634830339011
a_HQ_transcript/71661	-3.05017679179272	0.711791547832827	-2.62227643053613	0.0131738895172779	0.391427852930725	-2.96967247395156
a_HQ_transcript/57651	-2.37780342949097	0.201451152801725	-2.62213981522815	0.0131782326621495	0.391427852930725	-2.92506862811891
a_HQ_transcript/50322	1.41817671233361	2.8354516601259	2.62120508005615	0.0132079844295081	0.391778967485448	-3.07769137064375
a_HQ_transcript/54563	1.18969394579718	3.09376052321606	2.62107111500504	0.0132122535011516	0.391778967485448	-3.08529671088677
a_HQ_transcript/70900	3.1974157365784	1.15670203581274	2.62095477002029	0.0132159621086107	0.391778967485448	-2.90217947417858
a_HQ_transcript/7370	-3.32874729844809	2.15636774118681	-2.62092179463588	0.0132170134055699	0.391778967485448	-3.10799047897684
a_HQ_transcript/35132	3.23911184190358	0.705438944220983	2.61987588108991	0.0132503986123159	0.392366979944553	-2.97154153435722
a_HQ_transcript/38553	-3.26801285436197	1.11155894342828	-2.61966926267193	0.0132570030206221	0.392366979944553	-2.9176219741877
a_HQ_transcript/34998	1.90809809220657	3.99063501632861	2.61960114774344	0.0132591809322014	0.392366979944553	-3.13149911031673
a_HQ_transcript/116730	2.24063385130634	-0.525205050649882	2.61945513290118	0.0132638507361521	0.392366979944553	-2.94880913393201
a_HQ_transcript/39440	2.68965340421675	0.426147083724693	2.61825539045791	0.0133022781781803	0.393303573723939	-2.96684368782824
a_HQ_transcript/79552	2.80891467756483	-0.271858683556855	2.61670516989226	0.0133520838013399	0.394227816219041	-2.96389433448015
a_HQ_transcript/137283	2.26323991095547	3.24735206085579	2.61638207551666	0.013362485895372	0.394227816219041	-3.12813703470698
a_HQ_transcript/6189	1.74032031278822	2.7507458968984	2.61588362546714	0.0133785483010257	0.394227816219041	-2.98689524004397
a_HQ_transcript/122465	-1.67458696858083	2.12076150072366	-2.61575076294596	0.0133828327689008	0.394227816219041	-2.92475113505111
a_HQ_transcript/82837	-2.68424003460256	-0.473104105084505	-2.61543820378827	0.013392916985093	0.394227816219041	-2.96283965563952
a_HQ_transcript/20141	3.86322226109539	1.06141431127037	2.61532415727162	0.0133965982607173	0.394227816219041	-2.89368464538866
a_HQ_transcript/76056	-0.907715471175413	2.71693467457305	-2.61503674673137	0.0134058796550863	0.394227816219041	-3.02177091653024
a_HQ_transcript/84126	3.52065890173552	1.63256765754457	2.61488632216382	0.0134107397092564	0.394227816219041	-2.98064129328999
a_HQ_transcript/79016	1.46413404545739	2.81316895293547	2.61486216790317	0.013411520258912	0.394227816219041	-3.03183987849454
a_HQ_transcript/8694	2.08255435522902	2.52121207364241	2.6148244518872	0.0134127391433373	0.394227816219041	-2.99491615769731
a_HQ_transcript/56703	-2.51057406090083	0.26165121811352	-2.61480615358821	0.0134133305340937	0.394227816219041	-2.93552643350195
a_HQ_transcript/68134	1.50549799702028	0.776822290398232	2.6147568971936	0.0134149225929202	0.394227816219041	-2.90263189703228
a_HQ_transcript/74569	2.91787037279361	0.284661034374463	2.61366745925528	0.0134501799584206	0.395064204458348	-2.93568464197387
a_HQ_transcript/130535	1.88366742243178	-0.555391781273905	2.61343613544301	0.0134576772837668	0.395084780379189	-2.9269694924943
a_HQ_transcript/108694	-4.11623908164779	1.10554849225545	-2.61310183114335	0.0134685190864509	0.395094043170842	-2.94697149387181
a_HQ_transcript/69368	2.37151861097153	1.37860147001553	2.61252613061701	0.0134872085218993	0.395094043170842	-2.90067691779533
a_HQ_transcript/132368	1.8309364962145	2.87442636825146	2.61225753087282	0.0134959364933801	0.395094043170842	-3.02254748262869
a_HQ_transcript/139810	-2.05879789789221	-0.702532309729273	-2.61202176780426	0.013503601760933	0.395094043170842	-2.95347515695278
a_HQ_transcript/50766	2.49383288262186	-0.074092508689054	2.61200808444936	0.0135040467657468	0.395094043170842	-3.0064017054996
a_HQ_transcript/40868	1.41028131223643	-0.758063873438954	2.61197918751061	0.0135049865854399	0.395094043170842	-2.97909001523594
a_HQ_transcript/81662	-3.59494100626237	2.08047898207231	-2.61193815473389	0.0135063212047797	0.395094043170842	-2.97543833365796
a_HQ_transcript/131889	1.57203852903796	2.10171328996572	2.61175227714805	0.0135123685284825	0.395094043170842	-2.93709131621562
a_HQ_transcript/106282	1.46518915905958	-0.828357019739899	2.61110305293411	0.0135335099690373	0.395179978092837	-2.96588693504881
a_HQ_transcript/112495	2.60826187146816	1.68434470692855	2.61106426514001	0.0135347740282888	0.395179978092837	-2.96693843241452
a_HQ_transcript/103616	-5.55752853151698	0.8596650680444	-2.61103576568971	0.0135357028692341	0.395179978092837	-3.01095690051802
a_HQ_transcript/95607	-2.80207809251565	0.386244995274354	-2.61017605946238	0.0135637497277477	0.395657399032588	-2.92164474144988
a_HQ_transcript/52543	3.16885195101811	1.09620446859304	2.61011564916588	0.0135657225577064	0.395657399032588	-2.91633540756574
a_HQ_transcript/116742	-1.96401410471554	-0.427624327060777	-2.60990892899473	0.0135724754622726	0.395657399032588	-2.97518555631047

a_HQ_transcript/129103	2.64344407355035	-0.348371074163964	2.60934913086786	0.0135907779255448	0.395793340141879	-2.96248967481078
a_HQ_transcript/16320	-4.49582753835619	3.42522548415027	-2.60912326475273	0.013598169019152	0.395793340141879	-3.26540553310383
a_HQ_transcript/110897	1.57966856110268	-0.38864911672269	2.6085983017321	0.0136153619147293	0.395793340141879	-2.93985208819822
a_HQ_transcript/82519	-1.28555241962118	1.59290344339319	-2.60856183347286	0.0136165570209103	0.395793340141879	-2.94385362352308
a_HQ_transcript/147639	3.1823879187075	-0.201051054121989	2.6083578939119	0.0136232421385165	0.395793340141879	-3.01722745338385
a_HQ_transcript/142734	0.472820990171697	9.12866256521016	2.60827465963836	0.0136259714207865	0.395793340141879	-3.69482719929791
a_HQ_transcript/97609	-3.01031024145672	-0.391840342825664	-2.60808306378247	0.0136322558393023	0.395793340141879	-2.98129399575045
a_HQ_transcript/104407	3.32193115040921	-0.247343513586262	2.60753011895376	0.0136504076666778	0.395793340141879	-2.94398314521187
a_HQ_transcript/130798	-2.86576446666076	1.65442505411626	-2.60734675071473	0.0136564321274495	0.395793340141879	-2.93107438233607
a_HQ_transcript/41636	1.6631053947786	-0.0392623948241094	2.60734212529814	0.0136565841246844	0.395793340141879	-2.95807538456072
a_HQ_transcript/2516	1.48212936679153	2.73609043273441	2.6070708286441	0.0136655020226032	0.395793340141879	-3.01199202650313
a_HQ_transcript/129586	1.19172821675497	6.45359702394071	2.60697347568035	0.0136687034614838	0.395793340141879	-3.45879931043181
a_HQ_transcript/42639	-3.31714370562682	1.14652956047681	-2.60683635913838	0.0136732136956189	0.395793340141879	-2.95283787872735
a_HQ_transcript/103448	2.5474881555286	0.610891167759765	2.60682345006349	0.0136736383902611	0.395793340141879	-2.92815811760263
a_HQ_transcript/90265	1.76941382211762	0.197947145107574	2.60645195042621	0.013685865551547	0.395793340141879	-2.90042721859212
a_HQ_transcript/106827	-4.98460523263802	1.64397273585048	-2.60604820574827	0.0136991654487176	0.395793340141879	-3.01184376296199
a_HQ_transcript/35421	-2.76857625421231	-0.581059005926901	-2.60567570643683	0.0137114466716452	0.395793340141879	-2.97552963351779
a_HQ_transcript/24413	-2.2334894291918	1.54643240734154	-2.60567304436214	0.0137115344762781	0.395793340141879	-2.92104211535423
a_HQ_transcript/50582	2.69668393053932	-0.246828069510248	2.60563575949757	0.0137127643174977	0.395793340141879	-2.98927490322591
a_HQ_transcript/149621	-2.39406839753328	1.43988269856707	-2.60560963896937	0.0137136259638033	0.395793340141879	-2.98907458347267
a_HQ_transcript/85923	2.15861055768323	2.33009968481145	2.60525160076762	0.0137254417289458	0.395793340141879	-3.00296200264376
a_HQ_transcript/148405	-0.879964982608207	3.46502797855922	-2.60520632228899	0.0137269366523195	0.395793340141879	-3.12559676775042
a_HQ_transcript/42602	4.93911009274039	1.60466872890311	2.60473695347467	0.0137424422924282	0.396043969050207	-3.00003284203114
a_HQ_transcript/138950	-2.41850841939936	4.71282213654656	-2.60453070498026	0.0137492608457184	0.396044120138711	-3.2619545502828
a_HQ_transcript/145241	2.37749451237019	0.0780845741238634	2.60373944195515	0.0137754489243953	0.396471715115917	-2.95027514354669
a_HQ_transcript/99131	2.06484204279406	-0.490763758980347	2.6035648857743	0.0137812323328776	0.396471715115917	-2.96424612789303
a_HQ_transcript/136478	-1.13335218113853	2.34423102909878	-2.60342544069102	0.0137858540494187	0.396471715115917	-2.98574684040877
a_HQ_transcript/107673	3.58571979248493	1.92941278335576	2.60325852548254	0.0137913881083881	0.396471715115917	-2.95807901466692
a_HQ_transcript/31302	3.16012941566742	1.27616151976115	2.60290788939073	0.0138030201012167	0.396609961662642	-2.94987380576569
a_HQ_transcript/144357	-2.28737426529171	-0.625519595756999	-2.60234123736861	0.0138218373600284	0.396636429674829	-2.98135742112312
a_HQ_transcript/95779	3.16906224962983	1.52071891007593	2.60219009514859	0.013826860460116	0.396636429674829	-2.90893656228251
a_HQ_transcript/118911	9.10580056070888	1.5690620564676	2.6020674227782	0.0138309386254175	0.396636429674829	-3.1956817038734
a_HQ_transcript/27166	2.7807979218151	0.172247485023454	2.6020585013547	0.0138312352558299	0.396636429674829	-2.9321973483377
a_HQ_transcript/127614	-3.60747336558991	-0.524888982420477	-2.60111065994339	0.0138627837459483	0.397175221226962	-2.99081741723065
a_HQ_transcript/131977	2.26001922329656	-0.205017464617057	2.60094250996671	0.0138683874817868	0.397175221226962	-2.94114308805151
a_HQ_transcript/33614	2.11165796692178	1.77213141487263	2.60067373407605	0.0138773490037687	0.397175221226962	-2.99334773462322
a_HQ_transcript/147599	-2.1542930558473	1.59941412657938	-2.60044065222654	0.0138851247454248	0.397175221226962	-2.93154959551166
a_HQ_transcript/117046	2.19714901796085	1.72418555841398	2.60036329013385	0.0138877064777024	0.397175221226962	-2.96705116753712
a_HQ_transcript/55810	2.97411317165169	0.265214078799466	2.6002640119624	0.0138910202441924	0.397175221226962	-3.01080734475734
a_HQ_transcript/129659	3.30074074661053	2.24381668556735	2.59987186206172	0.0139041168000779	0.397354228788067	-2.98909578216426
a_HQ_transcript/17275	2.69762285565748	0.0550935807621195	2.59938281581093	0.0139204653634089	0.397625951176526	-3.0010265429751
a_HQ_transcript/5442	-2.98739706520153	-0.452708576815586	-2.59822635474683	0.0139591959105075	0.398534584262177	-2.9944547574656
a_HQ_transcript/23081	-1.81233878579644	1.98978259500987	-2.59802385538651	0.0139659879600546	0.398534584262177	-2.94292179597596
a_HQ_transcript/55672	-3.20013962686849	1.21646789200972	-2.59770541198798	0.0139766750724711	0.39864385113474	-3.01959847499595
a_HQ_transcript/32529	-3.92314516992658	3.17675322103965	-2.59718269690425	0.0139942340133472	0.398948913549703	-3.06198311907864
a_HQ_transcript/5032	-2.46629673145222	1.19384621970805	-2.59657861573061	0.0140145515590216	0.399271827665943	-2.98134290962232
a_HQ_transcript/46058	2.07505800269588	-0.248078787719178	2.59627035503148	0.0140249300305547	0.399271827665943	-2.92915656452078
a_HQ_transcript/35315	-2.48658088112657	-0.0681301961753518	-2.59623360993901	0.0140261676316724	0.399271827665943	-2.95492616283677
a_HQ_transcript/74644	-2.99031191377983	-0.0816296815750951	-2.59490904167724	0.0140708474313045	0.400344142122036	-3.04079968559425
a_HQ_transcript/93361	2.04234405914251	3.52141621853563	2.59470884116257	0.0140776119339637	0.400344142122036	-3.2268922195673

a_HQ_transcript/137410	1.40380273873616	2.03573885380575	2.59423561093967	0.0140936136765394	0.400603215545174	-2.97343589431664
a_HQ_transcript/80416	-2.68065325197719	0.468333100685497	-2.59401870449286	0.0141009537325488	0.400615952378101	-3.01137800177966
a_HQ_transcript/70272	3.90115058388909	4.98567111730866	2.593245638353	0.014127142796264	0.401163925970313	-3.26779016585468
a_HQ_transcript/76327	-2.89220378211019	1.12895008155325	-2.59273084608855	0.0141446072465397	0.401463735364677	-3.04154173604579
a_HQ_transcript/72714	-5.8812950307097	2.55395872504783	-2.59200491580739	0.0141692684735287	0.401571245661123	-3.03088714281865
a_HQ_transcript/151403	0.564427863335183	4.6710686093679	2.59189577414727	0.0141729796528059	0.401571245661123	-3.34636358891224
a_HQ_transcript/47970	-3.89042547303421	1.88093421027159	-2.59184087958967	0.0141748465893665	0.401571245661123	-3.00510468869674
a_HQ_transcript/49636	-1.71145230098248	-0.752565714924125	-2.59180612488373	0.0141760286969554	0.401571245661123	-2.99409410558594
a_HQ_transcript/152042	-5.04737241946121	1.58571579908669	-2.59097363369111	0.0142043713275982	0.401601967378318	-3.0587625341484
a_HQ_transcript/101444	-2.40323669027594	-0.196293619720219	-2.59081885417346	0.0142096466460247	0.401601967378318	-2.95731422631951
a_HQ_transcript/126254	-3.84840258759466	4.22383541593198	-2.59054075195519	0.0142191296903842	0.401601967378318	-3.15129439828185
a_HQ_transcript/122332	-3.10867583713113	0.317010269305917	-2.59036703599001	0.0142250562176399	0.401601967378318	-3.00442331883941
a_HQ_transcript/143775	-3.29433775318448	0.815665745966376	-2.59033529774102	0.0142261392521318	0.401601967378318	-3.00781255917883
a_HQ_transcript/152750	4.27680759110666	5.50538472424339	2.59024962981392	0.0142290629603432	0.401601967378318	-3.40365800419859
a_HQ_transcript/9731	1.45143587642604	1.56078077704834	2.59017848364996	0.0142314914857171	0.401601967378318	-2.93623410690803
a_HQ_transcript/23547	-3.26234337970807	1.08238361911267	-2.59015232137266	0.0142323846132558	0.401601967378318	-2.97165683015144
a_HQ_transcript/122932	5.49832817501855	1.84330832698626	2.58981937268951	0.014243755331011	0.401727806832123	-3.17814096542275
a_HQ_transcript/82663	2.31031439001763	0.0860466320091622	2.58933590722927	0.0142602813588828	0.401998852972425	-3.07003005329189
a_HQ_transcript/139492	2.44825227969717	2.43478925779151	2.58902666177979	0.0142708613996923	0.402102099583767	-2.99175706012946
a_HQ_transcript/73806	1.14834032186448	2.20466576876339	2.58820317842202	0.0142990701121791	0.402701718740673	-2.96351152719645
a_HQ_transcript/25727	-2.66676752732969	0.222511014289561	-2.58774861005702	0.0143146635445481	0.402758432712827	-3.01172678747687
a_HQ_transcript/72889	-2.0174439739873	1.66599753823238	-2.58754657693737	0.0143215990885582	0.402758432712827	-2.98743176413834
a_HQ_transcript/99667	-2.0631788859817	-0.688967181841301	-2.58753867654105	0.0143218703622594	0.402758432712827	-2.97804360751017
a_HQ_transcript/37724	-3.3855044738762	-0.301306520441993	-2.58683835974472	0.0143459357918373	0.403240113978685	-3.01235433892503
a_HQ_transcript/104339	-1.38102768043577	1.85353421159934	-2.58625373276002	0.0143660542697496	0.403318419215601	-2.99590851663704
a_HQ_transcript/12790	2.54845682690165	0.0182557856007093	2.58619407805948	0.0143681086003608	0.403318419215601	-3.02745551029303
a_HQ_transcript/68481	-2.74289325090682	-0.27239725212033	-2.58615260437013	0.0143695369907017	0.403318419215601	-3.01445305365449
a_HQ_transcript/110830	2.2524708442951	0.746165988282681	2.58588031459066	0.0143789181446079	0.403386946867648	-2.93289946202458
a_HQ_transcript/93615	-3.27062763176421	0.155032642877145	-2.58500947334589	0.0144089590409763	0.403820671094553	-3.0249620224895
a_HQ_transcript/132635	4.09441031665199	0.156997299731403	2.58478673099826	0.0144166521465087	0.403820671094553	-2.97567487651159
a_HQ_transcript/80581	5.89018727076347	1.13224867819443	2.58474292910653	0.0144181654280132	0.403820671094553	-3.13706431164221
a_HQ_transcript/111567	-7.19696522652009	1.27135602517689	-2.58462712921781	0.0144221668248055	0.403820671094553	-3.0223252567415
a_HQ_transcript/127974	3.60576273248505	0.329574032550537	2.58427324887601	0.0144344013013977	0.403908322295207	-3.06333873270029
a_HQ_transcript/99618	-4.79682998034201	5.2032925972733	-2.58398068469303	0.0144445231634349	0.403908322295207	-3.53102391997221
a_HQ_transcript/56083	5.17168497622015	4.29799802451409	2.58376318642808	0.0144520522080464	0.403908322295207	-3.20773384713928
a_HQ_transcript/135602	-3.29643431210922	3.80680351221498	-2.58369740423617	0.0144543300750984	0.403908322295207	-3.13036731120122
a_HQ_transcript/125430	-3.51496604466048	-0.513413449157013	-2.58339901527174	0.0144646666718773	0.403908322295207	-3.03817409053852
a_HQ_transcript/4692	2.19922277422655	0.844226341375092	2.58332444122747	0.0144672510823824	0.403908322295207	-2.95100931355946
a_HQ_transcript/9009	-4.85027650526483	2.15603019935202	-2.58313156120144	0.0144739374370513	0.403908322295207	-3.06837223428074
a_HQ_transcript/85006	-2.5986795936113	-0.447226957694568	-2.5826423111138	0.014490910507415	0.404187929930431	-3.0515809352156
a_HQ_transcript/114225	2.26521750973603	2.80440027781913	2.58227770175739	0.0145035714779229	0.404347051735588	-3.08461116308948
a_HQ_transcript/37361	-2.94395576252663	1.53725459921451	-2.58154926919927	0.0145288966825743	0.40485891963791	-3.03459405937578
a_HQ_transcript/17080	-2.12908031658874	-0.708510299721758	-2.5809942102717	0.0145482216317905	0.405100844671984	-3.02211910650807
a_HQ_transcript/62722	1.28638022525478	2.98203501322877	2.5808996354115	0.0145515167161282	0.405100844671984	-3.14482012226166
a_HQ_transcript/91582	2.01788251029737	-0.650809156212179	2.58067511284717	0.0145593420677606	0.4051246700405589	-2.98108975221014
a_HQ_transcript/44345	1.84760804185428	-0.198932346456058	2.58008377979269	0.014579970551163	0.405394986960031	-3.02832060556426
a_HQ_transcript/90932	2.84469553641473	0.850720135055467	2.57984763684722	0.0145882158591169	0.405394986960031	-2.94384506746971
a_HQ_transcript/144059	-2.78997174892518	1.6973750004311	-2.57979715043631	0.014589979923067	0.405394986960031	-3.08983356054951
a_HQ_transcript/57317	2.84804588544392	1.58080082097111	2.57959334637153	0.0145970996258801	0.405399047803708	-3.05089094065924
a_HQ_transcript/152348	-3.52228223068317	2.6840528781738	-2.57784857704399	0.0146581887137751	0.406828118059023	-3.16681931708126

a_HQ_transcript/139852	-2.29965838816988	1.32909525901659	-2.5777241721577	0.0146625534567447	0.406828118059023	-3.16601936537804
a_HQ_transcript/150561	1.98196962982549	-0.670219011993511	2.57742858392664	0.0146729289712588	0.406921762996817	-3.0318837726321
a_HQ_transcript/26030	-3.53588553909947	-0.47042450117866	-2.5770010841134	0.0146879467230552	0.407093960691433	-3.03378658545881
a_HQ_transcript/133095	1.99352423204973	2.32642059087934	2.57673777242352	0.0146972037197844	0.407093960691433	-3.0524541190875
a_HQ_transcript/97263	-1.4041294815326	5.22911585048809	-2.57665404551639	0.014700148353484	0.407093960691433	-3.40779166796163
a_HQ_transcript/149114	1.88587769029727	-0.813280778903796	2.575564005141	0.0147385342065039	0.407962626836028	-3.00595569954163
a_HQ_transcript/57990	-2.50096172687065	-0.512057485034929	-2.57476858207921	0.014766603391031	0.408465858894064	-3.03207073936716
a_HQ_transcript/117566	-3.12991613874264	-0.327324054662147	-2.57415750050596	0.0147882009313156	0.408465858894064	-3.05164097542567
a_HQ_transcript/136218	2.56588431391286	2.53249883177152	2.57411802536915	0.0147895971074136	0.408465858894064	-3.0663141986039
a_HQ_transcript/92527	-2.64204018039696	1.39176720283432	-2.57346978485638	0.0148125417870968	0.408465858894064	-3.04634262874116
a_HQ_transcript/43144	-2.98167625910577	-0.181785719798278	-2.57342127636088	0.0148142600797749	0.408465858894064	-3.03167604701763
a_HQ_transcript/11044	-2.38937376237586	-0.20187829457883	-2.57341803230774	0.0148143749988354	0.408465858894064	-3.01518294342562
a_HQ_transcript/132916	5.25066897719761	0.924409572822535	2.57334651627991	0.0148169086293352	0.408465858894064	-3.1590541112259
a_HQ_transcript/12329	1.79871577038726	1.09165928591513	2.57293875576991	0.0148313621777282	0.408465858894064	-3.04585390567645
a_HQ_transcript/103557	2.88765305035963	1.60770464674389	2.57287271837019	0.0148337041731588	0.408465858894064	-2.98700222450141
a_HQ_transcript/89208	-1.86524969080814	-0.647791258325127	-2.57273042575394	0.0148387516943849	0.408465858894064	-2.99883625910414
a_HQ_transcript/59821	-0.877339367666227	3.35144874434806	-2.57267764126856	0.0148406245118181	0.408465858894064	-3.21773763541552
a_HQ_transcript/2005	1.90553383064689	2.73759972010444	2.57246107174872	0.014848310777588	0.408465858894064	-3.07441352493482
a_HQ_transcript/15359	-0.820495440605914	2.92876250674656	-2.57232986043661	0.0148529693813734	0.408465858894064	-3.14960198538723
a_HQ_transcript/74995	-2.26126647781315	-0.519908882899662	-2.57227007029994	0.0148550926524575	0.408465858894064	-3.01568909961177
a_HQ_transcript/121214	1.49540184678627	2.09011019002441	2.57173047712424	0.014874267379446	0.408760960396263	-3.04010906296498
a_HQ_transcript/24341	1.98099528283407	2.43691993208073	2.57141699315644	0.0148854176714376	0.408760960396263	-3.01814177652601
a_HQ_transcript/55964	2.76956847473224	0.685336646638993	2.57127418488279	0.0148904997640646	0.408760960396263	-3.09037674536956
a_HQ_transcript/150040	2.49188009072972	-0.344066140998727	2.57111228533696	0.0148962631886523	0.408760960396263	-3.02866903053726
a_HQ_transcript/42526	1.51828366744555	0.107700515075777	2.57097967944548	0.0149009853268594	0.408760960396263	-3.02235330562612
a_HQ_transcript/65351	-1.86175910826993	2.82875551038343	-2.57039913233446	0.0149216750421897	0.408845698760314	-3.08519262729869
a_HQ_transcript/133642	-1.16450016501756	5.53342367468793	-2.57034777363592	0.0149235066533744	0.408845698760314	-3.44337161163597
a_HQ_transcript/138674	0.897883887184363	-0.699197037210167	2.57030099778178	0.0149251750063547	0.408845698760314	-3.09059327702473
a_HQ_transcript/4736	-1.80914708867441	1.57875394689803	-2.56992079265678	0.0149387421496324	0.408851797561654	-3.05186634958172
a_HQ_transcript/67212	3.06324626702587	0.860316038858445	2.56990054614152	0.0149394649398045	0.408851797561654	-3.0256017173473
a_HQ_transcript/143224	-2.45130051529104	0.0438791854841852	-2.56946531485775	0.0149550102680703	0.409078509640737	-3.04727769436836
a_HQ_transcript/8042	2.38506865949056	-0.253523966951059	2.5690066648519	0.0149714081836039	0.409078509640737	-3.00141417168385
a_HQ_transcript/43004	2.30098378861813	2.54358847983472	2.568942887556	0.0149736896975354	0.409078509640737	-3.08083780982309
a_HQ_transcript/111017	3.53453831004606	0.509659500332355	2.56864029261613	0.0149845188348754	0.409078509640737	-3.00355131475046
a_HQ_transcript/30819	-3.21866926522951	1.69716660369479	-2.56862064967094	0.0149852220575409	0.409078509640737	-3.03395866827662
a_HQ_transcript/120453	3.00864737508673	1.40379907176582	2.56832196874928	0.0149959186618128	0.409078509640737	-3.0967783515646
a_HQ_transcript/88138	0.803629419033799	4.42999357900027	2.56787679209717	0.0150118747501781	0.409078509640737	-3.36808235086808
a_HQ_transcript/142234	-2.22785256351993	3.76450408359746	-2.56779268569866	0.0150148910608937	0.409078509640737	-3.34063229868497
a_HQ_transcript/152749	2.89115909870655	-0.159230718641586	2.5676856512998	0.0150187304468985	0.409078509640737	-3.08437386251584
a_HQ_transcript/141671	4.26014898278649	0.578156850337974	2.56763379374076	0.0150205909329541	0.409078509640737	-3.10540905666869
a_HQ_transcript/147932	-1.43491588979975	5.68046382332992	-2.5675064083067	0.0150251620231726	0.409078509640737	-3.50362876358599
a_HQ_transcript/151950	2.29155587033633	-0.026222945560434	2.56708877769294	0.0150401572433302	0.409259682207743	-2.9953056553671
a_HQ_transcript/106127	0.643616413355915	2.9724423477037	2.56692900256596	0.0150458976892022	0.409259682207743	-3.18957845692373
a_HQ_transcript/14831	-3.7015296445936	-0.246821091983913	-2.5662347556755	0.015070864239513	0.409476732642072	-3.05001115186593
a_HQ_transcript/104227	3.15095332976321	0.556269346900947	2.56616720932945	0.0150732953820062	0.409476732642072	-3.07845702843838
a_HQ_transcript/60663	3.24618417453926	0.609752876071242	2.56611956408429	0.0150750104571641	0.409476732642072	-3.01630783960829
a_HQ_transcript/19239	2.28965495761918	-0.107924494015846	2.56577714001669	0.01508734190996	0.409620275825387	-3.06073784255742
a_HQ_transcript/150540	0.516310105217315	9.84884710407886	2.56513494638785	0.0151104937950266	0.410057321810134	-3.87439307865498
a_HQ_transcript/38322	2.33775426103567	-0.196350522315628	2.5647265360832	0.0151252345210494	0.41026581065775	-3.00533575066491
a_HQ_transcript/15909	-1.51448905327923	1.86949355226226	-2.56448827916116	0.0151338400299459	0.410303014439809	-3.01718085241836

a_HQ_transcript/119505	-3.6286811921026	2.2081380886855	-2.56429779398578	0.0151407233342518	0.410303014439809	-3.03276601060297
a_HQ_transcript/42141	2.07466532445733	-0.640570222704122	2.56385157181301	0.0151568591529006	0.41044022141387	-3.06019960108191
a_HQ_transcript/83357	-1.77334780611781	1.69205794626349	-2.56351711651698	0.0151689637601768	0.41044022141387	-2.9899583711658
a_HQ_transcript/20945	-1.23132486692053	2.57858187818493	-2.56338458061214	0.0151737629633714	0.41044022141387	-3.10757923793398
a_HQ_transcript/144844	1.11720280121197	3.53185110559037	2.56337719771097	0.0151740303436968	0.41044022141387	-3.25524807502957
a_HQ_transcript/129672	-2.69982894921166	1.50945842186046	-2.56240213952718	0.0152093813699114	0.410858734111943	-3.03514889031789
a_HQ_transcript/11633	1.47376548660033	2.01825885990579	2.5624009222394	0.0152094255504197	0.410858734111943	-3.0306207277211
a_HQ_transcript/129388	0.63227546675144	4.85517822656805	2.56219588496561	0.0152168689051278	0.410858734111943	-3.40147975448646
a_HQ_transcript/7713	1.99529415300833	-0.626188353866006	2.56217091776315	0.0152177755048	0.410858734111943	-3.05767174561115
a_HQ_transcript/127566	-3.77045519042003	-0.480950862980693	-2.56106189311016	0.0152580962152687	0.411550546479018	-3.0851504534226
a_HQ_transcript/70820	-1.36337774652206	2.60752725044777	-2.56102095761554	0.0152595863847647	0.411550546479018	-3.20711117834885
a_HQ_transcript/96700	0.757646170993407	3.84614544855332	2.56088216781184	0.0152646397297131	0.411550546479018	-3.301627017086
a_HQ_transcript/110531	-1.17762547393687	2.96287024670242	-2.5603586847764	0.0152837136515353	0.411561566972105	-3.1523696401471
a_HQ_transcript/60178	1.41610046113353	2.69168551696466	2.56018810819632	0.0152899336158143	0.411561566972105	-3.09376037700342
a_HQ_transcript/139353	-3.44636272117233	1.3668751403995	-2.56011103819639	0.0152927446879085	0.411561566972105	-3.07940979074168
a_HQ_transcript/23937	-2.17397563926823	1.46948357523975	-2.55993714257654	0.0152990891526616	0.411561566972105	-3.08247788457767
a_HQ_transcript/56657	-1.09074472652737	4.60827831208551	-2.55989985708432	0.0153004498043407	0.411561566972105	-3.38051163058907
a_HQ_transcript/66092	5.88530571202643	2.38780127295686	2.55942232372689	0.0153178861865069	0.411840003815574	-3.16786373186984
a_HQ_transcript/30280	-1.22674106984503	4.10876125129086	-2.55756234732783	0.0153859748992701	0.413479403118249	-3.29305279587907
a_HQ_transcript/146668	0.376644212488599	10.1690936090227	2.55735389941429	0.0153936229567781	0.413493768591312	-3.92533685126314
a_HQ_transcript/73894	1.71152155113744	3.0989372971136	2.55664350502537	0.0154197139818869	0.414000164030066	-3.15043173488792
a_HQ_transcript/56186	2.45707309810848	2.08740475770228	2.556378134763	0.0154294708157352	0.414000164030066	-3.14128565180197
a_HQ_transcript/141855	2.87253222252201	0.855549122043187	2.5562593014248	0.015433841788005	0.414000164030066	-3.01993469600923
a_HQ_transcript/139116	2.85116385258551	-0.107084541091375	2.55603989067595	0.0154419152322093	0.414025668181671	-3.0675331819516
a_HQ_transcript/134250	4.1910463668262	2.17485344691184	2.55509608536292	0.0154766878761316	0.414623602763374	-3.08191078663466
a_HQ_transcript/81496	-4.42698282697323	1.43166660212649	-2.55494897589446	0.0154821143236713	0.414623602763374	-3.18570383413353
a_HQ_transcript/133064	4.2212228807376	0.175930412379207	2.55476303376592	0.0154889757032494	0.414623602763374	-3.13174635484093
a_HQ_transcript/42971	3.40905906981175	1.31099093242154	2.55460957102999	0.0154946406817568	0.414623602763374	-3.0684448258415
a_HQ_transcript/123197	-3.94301415553805	0.966176682221841	-2.55416491833144	0.0155110655231531	0.414623602763374	-3.11190714480645
a_HQ_transcript/129383	-1.8336916417256	-0.589832203254061	-2.55388561178668	0.0155213909063779	0.414623602763374	-3.07389067408022
a_HQ_transcript/52877	-2.68161950870232	-0.507685491247672	-2.5535066037233	0.0155354121666445	0.414623602763374	-3.08305606295889
a_HQ_transcript/50513	3.59749894086635	3.91229247076646	2.55346194936989	0.0155370649051857	0.414623602763374	-3.17505845418785
a_HQ_transcript/142887	2.11821818163805	2.88300696489522	2.55343151811666	0.0155381913137511	0.414623602763374	-3.15558872166796
a_HQ_transcript/997	2.82312915072262	1.98041399064732	2.55332922239466	0.0155419783266472	0.414623602763374	-3.02786636218018
a_HQ_transcript/94377	-2.0848760905329	-0.0517714637308622	-2.5533103048823	0.0155426787507121	0.414623602763374	-3.05039147863227
a_HQ_transcript/65652	2.05587681820101	-0.320298016171718	2.55311735909631	0.0155498242605222	0.414623937897079	-3.08894686323904
a_HQ_transcript/87882	-1.88934380879697	-0.225267978392436	-2.55285794291436	0.0155594361858155	0.414690007615353	-3.07963618155495
a_HQ_transcript/146476	-2.29480561982661	3.59476302767228	-2.5524674885488	0.0155739136679211	0.414885634137909	-3.29315328877313
a_HQ_transcript/2372	3.6536017266224	0.949411517299303	2.55216040212999	0.0155853086973503	0.414963863858428	-3.14959825578187
a_HQ_transcript/107337	-3.66780603079288	-0.330885395630523	-2.55168124753704	0.015603103975968	0.414963863858428	-3.13078862687207
a_HQ_transcript/119452	1.61339720917904	2.08724267578518	2.55160181160343	0.0156060559464718	0.414963863858428	-3.07751360014162
a_HQ_transcript/92726	3.526361276723	0.357328055418196	2.5510721869855	0.0156257508149954	0.414963863858428	-3.11574980583908
a_HQ_transcript/67446	2.95205571100491	0.0947335025673843	2.55090554032977	0.01563195254533	0.414963863858428	-3.11412923240983
a_HQ_transcript/38923	-2.61803466228993	0.923019651595772	-2.5508770227053	0.015633014051439	0.414963863858428	-3.08556572350711
a_HQ_transcript/133762	0.68541774554354	3.76613756752053	2.55085188307712	0.0156339498740552	0.414963863858428	-3.31729314010862
a_HQ_transcript/6968	-1.78977051494257	-0.781665089189109	-2.55067506878354	0.0156405332425564	0.414963863858428	-3.07737423843803
a_HQ_transcript/58382	2.18065773927976	1.17122313074093	2.55051225698154	0.015646597509268	0.414963863858428	-3.01918596246628
a_HQ_transcript/99421	-4.54191493176589	3.13756387944106	-2.55032878186293	0.0156534340179004	0.414963863858428	-3.25034207916917
a_HQ_transcript/151390	-2.14129885213092	2.00615954186997	-2.55027665082482	0.0156553769859884	0.414963863858428	-3.04137774286556
a_HQ_transcript/97797	3.02211612307536	0.876094336881274	2.54942181046877	0.0156872692898978	0.415619685179208	-3.06363844223398

a_HQ_transcript/139638	-3.88464271348889	1.5994313878646	-2.54909790811525	0.0156993690201949	0.415750761916123	-3.18875967774062
a_HQ_transcript/59214	2.27441646217048	-0.274459572144813	2.54855952309018	0.0157194999914444	0.416094305784463	-3.07870360860061
a_HQ_transcript/69468	-4.71320101888276	0.56191049921305	-2.5474092682076	0.0157625892486116	0.416978266299776	-3.09142128580962
a_HQ_transcript/23527	-2.19910517225798	-0.345373535967475	-2.54696772526887	0.0157791585671169	0.416978266299776	-3.05941590973358
a_HQ_transcript/94274	-2.12225271609838	1.30815485622036	-2.54668045731138	0.0157899471738437	0.416978266299776	-3.12483031718111
a_HQ_transcript/117907	-2.93356249130766	0.774895026082006	-2.54648059836789	0.0157974570595035	0.416978266299776	-3.09566814348296
a_HQ_transcript/100118	2.48608807336785	0.251315893553101	2.54634902917957	0.0158024026878104	0.416978266299776	-3.03345469762897
a_HQ_transcript/126998	3.48845187958755	1.6797063083143	2.5462285407708	0.0158069330440757	0.416978266299776	-3.04332463465834
a_HQ_transcript/54873	5.19222708275998	2.1727996183773	2.54615368750294	0.0158097481238056	0.416978266299776	-3.27386070666096
a_HQ_transcript/74687	2.84733398382532	-0.172137230808596	2.54599185694631	0.0158158358198396	0.416978266299776	-3.06772585776272
a_HQ_transcript/51983	2.94364071236291	0.410888896462619	2.54594879936543	0.015817455910938	0.416978266299776	-3.07524510616076
a_HQ_transcript/84000	-2.6379494222512	0.113989035226732	-2.54441016215789	0.015875449219588	0.418317367287493	-3.0948301013347
a_HQ_transcript/139351	-1.58706595400749	2.54282895694462	-2.54414961132431	0.0158852890765148	0.418386988418509	-3.1282392883811
a_HQ_transcript/6008	-1.64790197622902	1.58346698287136	-2.54373547261421	0.0159009408177635	0.418609550658947	-3.08355698898268
a_HQ_transcript/130436	1.48707927484809	-0.78736154593196	2.54307064351857	0.0159260966894883	0.419082004692883	-3.10279409584936
a_HQ_transcript/22611	3.32670787585506	1.04201580261829	2.542843756973	0.0159346900210554	0.419118398888646	-3.12646449203592
a_HQ_transcript/8775	3.32199739395983	0.0300667473396573	2.54242561928325	0.0159505381669677	0.419257343590655	-3.13332949650466
a_HQ_transcript/38011	-2.662474658078	0.121108131830956	-2.54232384059471	0.0159543979497407	0.419257343590655	-3.109203127237
a_HQ_transcript/29096	1.60921414902925	-0.650738163673073	2.54190920147273	0.0159701313105811	0.419293874236749	-3.11804502558125
a_HQ_transcript/136005	-2.72831950917563	0.135634204161888	-2.54189134413013	0.0159708092226828	0.419293874236749	-3.09950897722232
a_HQ_transcript/129767	-3.47929819917835	2.03898974855275	-2.54171703256768	0.0159774279423754	0.419293874236749	-3.20503486699866
a_HQ_transcript/115116	-1.0362756267407	4.06579324828378	-2.54082062091636	0.0160115051219494	0.419955625532253	-3.3910748263675
a_HQ_transcript/72051	0.808431360504751	4.11475035617702	2.54055707824856	0.0160215364309781	0.419955625532253	-3.3890533121795
a_HQ_transcript/152613	2.2562345226886	-0.12046654695854	2.540432908251	0.0160262647563162	0.419955625532253	-3.08214951861661
a_HQ_transcript/90114	-1.59081452577079	2.2686343186492	-2.5402699790328	0.0160324709583011	0.419955625532253	-3.14941951952647
a_HQ_transcript/121981	1.69953067309473	1.70344956956742	2.54010473226004	0.0160387676968346	0.419955625532253	-3.13129815924908
a_HQ_transcript/66576	2.66518180951619	0.216942570353902	2.53918971509999	0.0160736756659048	0.420465232459436	-3.12931762477023
a_HQ_transcript/4155	1.34947140381903	2.04027094177705	2.53917477559827	0.0160742461881977	0.420465232459436	-3.0828109410634
a_HQ_transcript/96805	-1.83148491247054	-0.21561935870827	-2.53896514829108	0.0160822535767851	0.420465232459436	-3.0758581112836
a_HQ_transcript/137071	-4.47952450693426	0.112481756356176	-2.53883664104015	0.0160871641375892	0.420465232459436	-3.12581963424836
a_HQ_transcript/140601	2.77221991439244	0.403540776279884	2.53857527147354	0.0160971559306829	0.420537294354488	-3.04488516628697
a_HQ_transcript/22694	4.60465876034847	1.29104317199553	2.5383589121321	0.0161054313627313	0.420564471811701	-3.15446047568103
a_HQ_transcript/97054	-1.81538258545711	0.791442402933982	-2.53813040831343	0.0161141755509863	0.420603860093278	-3.03545589131835
a_HQ_transcript/39316	2.69467519540695	0.947888868524085	2.53668030753658	0.0161697685972809	0.421865488789383	-3.11209976660805
a_HQ_transcript/150684	0.436858763450306	10.9785849140332	2.53611013898988	0.0161916756173927	0.422247519195964	-4.07261793861581
a_HQ_transcript/123862	-1.56384939124153	-0.609327696419562	-2.53561114190194	0.0162108704748638	0.42240807841236	-3.10481908431548
a_HQ_transcript/150309	0.859219744871468	9.81869186704857	2.53554415225966	0.0162134489490989	0.42240807841236	-3.91997647137124
a_HQ_transcript/133866	-2.48063887587982	-0.193929722061226	-2.53538352662493	0.0162196330686827	0.42240807841236	-3.1195956981697
a_HQ_transcript/118424	2.69505475350398	-0.275399938110489	2.53488577462233	0.016238810396353	0.422541884274192	-3.11724341698122
a_HQ_transcript/141825	-5.97860033790849	0.935658975549261	-2.53487283335489	0.0162393092738189	0.422541884274192	-3.15807956300928
a_HQ_transcript/121757	3.24277858659244	4.13571850860241	2.53447296150245	0.0162547310197805	0.422740220027437	-3.22656817179512
a_HQ_transcript/139957	0.944466377554053	4.30650164656858	2.5341462190008	0.0162673424004988	0.422740220027437	-3.40174568328236
a_HQ_transcript/88936	1.67241732705959	-0.817070176822501	2.53401796405612	0.0162722951538186	0.422740220027437	-3.12011592851665
a_HQ_transcript/18241	-1.43326136371732	2.09764494454233	-2.53392147598013	0.0162760220964321	0.422740220027437	-3.09271415170699
a_HQ_transcript/3953	-1.73629671937026	-0.748755589754154	-2.53358314093903	0.0162890968059816	0.422890852674451	-3.11039439417926
a_HQ_transcript/94609	2.79190817947977	-0.59193581451595	2.53301674058791	0.0163110065443783	0.423123926739743	-3.0985709619355
a_HQ_transcript/128519	-3.19319444439875	1.71797968990767	-2.53280864612019	0.0163190629448358	0.423123926739743	-3.14375284313815
a_HQ_transcript/123799	-4.24024136745281	0.662052281904856	-2.53278672121206	0.0163199119830461	0.423123926739743	-3.07603678440075
a_HQ_transcript/133844	2.01277010322567	-0.31814686311746	2.53208886203591	0.0163469576800672	0.423503353270886	-3.17325428968143
a_HQ_transcript/94300	1.68929464492136	-0.362235391711147	2.53186153794557	0.0163557765703565	0.423503353270886	-3.09831868116724

a_HQ_transcript/10556	-2.15616047570691	2.43230133156403	-2.53182159617209	0.0163573265370777	0.423503353270886	-3.21606398684359
a_HQ_transcript/6895	2.8336798351044	2.91875029819331	2.53165766786226	0.0163636892968348	0.423503353270886	-3.22702992663611
a_HQ_transcript/142034	-3.44626654857001	0.922181086834761	-2.5312214367136	0.0163806323788037	0.42362415720244	-3.11677028402175
a_HQ_transcript/115459	2.74395048463155	1.01812071455433	2.53116224556298	0.0163829325865518	0.42362415720244	-3.08340921629141
a_HQ_transcript/122341	2.99300921558808	0.0134321332391704	2.53061274601037	0.0164043006815151	0.423801863559853	-3.09939964587751
a_HQ_transcript/98959	-2.39685939420395	2.20783798256356	-2.53058085674579	0.0164055415282889	0.423801863559853	-3.24042310269555
a_HQ_transcript/85815	-2.50255627859888	-0.0257647199527481	-2.53030137701031	0.0164164200871546	0.423801863559853	-3.14667449899593
a_HQ_transcript/105041	3.12909752807116	1.02728999666439	2.53023593252104	0.0164189684272087	0.423801863559853	-3.09099089707263
a_HQ_transcript/67260	3.59925269177133	0.30516328418651	2.52945740221889	0.0164493114822725	0.424322024836424	-3.12555625778242
a_HQ_transcript/34191	1.08782515255078	1.72188303626007	2.52913788617516	0.0164617794606087	0.424322024836424	-3.09307498700639
a_HQ_transcript/5810	1.81216470658452	1.48904701080726	2.52904295255859	0.0164654855807691	0.424322024836424	-3.05683237664969
a_HQ_transcript/40850	3.00736781098107	1.21027609368743	2.52879902139331	0.0164750119431798	0.424322024836424	-3.11340154489993
a_HQ_transcript/103217	-1.22364717225593	3.39653355075538	-2.52878346885172	0.0164756194958679	0.424322024836424	-3.25198779275955
a_HQ_transcript/151659	1.01033507130945	3.05430065346527	2.52811995264849	0.0165015586262557	0.424801859976525	-3.21134692840447
a_HQ_transcript/120260	3.02216563740329	0.602240498487232	2.52699941165784	0.0165454496385602	0.425743203448529	-3.18589698549165
a_HQ_transcript/100461	-1.25202803439784	2.41314506498858	-2.52653885724326	0.016563520400101	0.425882757712128	-3.16923801202241
a_HQ_transcript/137567	-1.35495389356333	3.9984318101332	-2.52648776195585	0.0165655263416447	0.425882757712128	-3.33886839994998
a_HQ_transcript/94171	1.41611852957395	2.49728929782011	2.5257723050235	0.0165936378014667	0.425928813686721	-3.18984490541781
a_HQ_transcript/84730	1.13923025595576	2.44233750864842	2.52569576432862	0.0165966478028539	0.425928813686721	-3.17759184750996
a_HQ_transcript/149180	-0.687159203670967	3.82249972844515	-2.52567117129237	0.0165976150428674	0.425928813686721	-3.36161267754031
a_HQ_transcript/118535	7.34651864808216	2.10542040088084	2.52555433962614	0.0166022107202332	0.425928813686721	-3.2421243383757
a_HQ_transcript/32882	-1.22177470527999	3.00720807890917	-2.52551000612416	0.0166039549238596	0.425928813686721	-3.24107623647007
a_HQ_transcript/148734	-2.99206088448363	0.981022543926408	-2.52481710557062	0.0166312374497906	0.426065613699775	-3.16193722904506
a_HQ_transcript/12389	3.07735521889406	0.839880994674173	2.52479640303863	0.016632053232281	0.426065613699775	-3.09950487827518
a_HQ_transcript/127628	-2.83552137102187	-0.260249039905781	-2.52466637225637	0.0166371779309169	0.426065613699775	-3.17632869471794
a_HQ_transcript/141549	-2.67019021213712	1.12275308105283	-2.5245138705635	0.0166431900883992	0.426065613699775	-3.11490683412284
a_HQ_transcript/115992	-3.40560384028434	-0.468531567159919	-2.52444421799055	0.0166459367036917	0.426065613699775	-3.15597008349788
a_HQ_transcript/127617	2.46361912384407	1.43303874519912	2.52410538490409	0.0166593038754548	0.426169579329048	-3.10525654865719
a_HQ_transcript/149794	-4.55294258658618	2.85655294141077	-2.52391092912404	0.0166669797270377	0.426169579329048	-3.24655382524353
a_HQ_transcript/57087	-0.809617825495679	2.97659616252514	-2.52378396381867	0.0166719932458412	0.426169579329048	-3.2410199451142
a_HQ_transcript/18412	-4.42409488983574	2.05381531465982	-2.52304784169904	0.016701088039612	0.426607081927281	-3.13982033433999
a_HQ_transcript/72101	2.18358946313378	1.53273993761807	2.52297962084089	0.0167037867889226	0.426607081927281	-3.06078425960185
a_HQ_transcript/148670	-2.6246183792612	1.66199337508645	-2.52254069994816	0.0167211596441099	0.426760756194456	-3.22038311593326
a_HQ_transcript/119315	-1.00759524759163	3.74893756098523	-2.52229018327901	0.016731082740672	0.426760756194456	-3.3963900653136
a_HQ_transcript/107195	-3.13924442908457	0.745681223466963	-2.52209469684195	0.0167388298176238	0.426760756194456	-3.13911537463349
a_HQ_transcript/53602	3.27660293995136	0.969543823450721	2.52208609287175	0.0167391708664217	0.426760756194456	-3.08964348749098
a_HQ_transcript/9796	1.30143864962919	2.43596289555828	2.52161281815259	0.0167579406179912	0.427051982570186	-3.12823561009784
a_HQ_transcript/8099	-1.99618668654353	1.71762048875691	-2.52072097371027	0.0167933631004103	0.427767138606772	-3.11868105284783
a_HQ_transcript/125451	3.05391437382396	1.93694251732008	2.51988479082615	0.0168266372505521	0.428084115672251	-3.14352028777755
a_HQ_transcript/120314	3.80181076022377	0.965014429492961	2.51977916028017	0.0168308449017728	0.428084115672251	-3.08564845898947
a_HQ_transcript/129127	1.93304486653958	-0.740057562094781	2.51952285165025	0.0168410586294033	0.428084115672251	-3.14851698739239
a_HQ_transcript/98348	2.85104196362406	0.0865644545180643	2.51944314237505	0.0168442361508272	0.428084115672251	-3.15490325144972
a_HQ_transcript/2186	-3.061187542181	0.768974399469725	-2.51923770977013	0.016852428030686	0.428084115672251	-3.16928929022348
a_HQ_transcript/117782	-2.79727598478496	0.492289649091897	-2.51914357837712	0.0168561828587625	0.428084115672251	-3.10459530293991
a_HQ_transcript/87091	1.99342255657584	0.211544826830577	2.51911410714354	0.0168573586012555	0.428084115672251	-3.08375934401295
a_HQ_transcript/1375	3.03652624236275	0.349412451443475	2.51828116286274	0.0168906197206264	0.428741459877978	-3.16568435291985
a_HQ_transcript/141186	2.9957744971266	2.43163650767014	2.51779290265838	0.0169101449240064	0.429009382099181	-3.23070576411488
a_HQ_transcript/92271	-4.37736389827105	1.22050046588366	-2.51738294579221	0.0169265548311915	0.429009382099181	-3.25995377548261
a_HQ_transcript/122639	1.60148869930822	3.65795688865695	2.51729378714173	0.0169301256422131	0.429009382099181	-3.33503216161882
a_HQ_transcript/104096	-2.16002047300761	1.29564330362886	-2.51716067909626	0.0169354579170441	0.429009382099181	-3.08028003811018

a_HQ_transcript/9734	-0.744726073112722	2.65701508484993	-2.51709531717951	0.0169380768634328	0.429009382099181	-3.23551588857387
a_HQ_transcript/98929	2.86322966886097	-0.135026409446204	2.51596220671423	0.0169835379066095	0.429791807161362	-3.17764792065798
a_HQ_transcript/127080	-4.80661930714607	0.8860383860516	-2.51572289821218	0.0169931534167413	0.429791807161362	-3.12829950096358
a_HQ_transcript/20549	-1.88189533627672	-0.125647477983195	-2.51564292284455	0.0169963679726237	0.429791807161362	-3.11730417392548
a_HQ_transcript/95795	-3.09907456026787	-0.434957407134503	-2.5155119944474	0.0170016317561566	0.429791807161362	-3.12389257180702
a_HQ_transcript/101288	3.24543526379439	0.762419996970395	2.51540491427415	0.0170059378693767	0.429791807161362	-3.20359880577213
a_HQ_transcript/127681	-2.80797380967347	2.7830986046218	-2.51495531472469	0.0170240289577549	0.430062040528631	-3.35767064042066
a_HQ_transcript/120643	1.36027325106118	3.62658286932305	2.51460643445967	0.0170380794524989	0.430230009737122	-3.38768623534413
a_HQ_transcript/101910	1.76000525545017	2.32943314705266	2.51350047123788	0.0170826904226751	0.431076278118225	-3.14936151189956
a_HQ_transcript/53864	1.71970763202952	-0.790229652367296	2.51340799277446	0.0170864255571221	0.431076278118225	-3.16091458826026
a_HQ_transcript/59554	2.05069102574648	-0.457989605278142	2.5125232579142	0.0171221972304448	0.43170722730773	-3.16247386450971
a_HQ_transcript/143523	4.46389596689798	1.33654535618899	2.51226471156811	0.0171326637685778	0.43170722730773	-3.20624378596916
a_HQ_transcript/139744	2.56502529559545	0.0727132034481835	2.51223875701751	0.0171337147914763	0.43170722730773	-3.16708881274694
a_HQ_transcript/67430	2.52490741803137	0.0371618447954422	2.51184670179739	0.0171495981672312	0.431920208953559	-3.12216664071661
a_HQ_transcript/140437	2.14303456938747	0.00980434968363509	2.51050626148754	0.0172040057090371	0.432972399855637	-3.15986008420823
a_HQ_transcript/1483	2.09566688047603	1.96239688520978	2.51045048380119	0.0172062731156503	0.432972399855637	-3.14313617672618
a_HQ_transcript/86152	2.05062631569612	-0.679566524591489	2.50994174156901	0.0172269665463861	0.433275632717818	-3.16503004323928
a_HQ_transcript/107979	2.65501044567358	0.2823382477611	2.50978783753251	0.0172332311939787	0.433275632717818	-3.19285412125568
a_HQ_transcript/6211	1.12149031516898	2.51965174113409	2.5095118111242	0.0172444720568996	0.433349240292697	-3.16179798753921
a_HQ_transcript/32418	3.21428236402859	1.02702048802939	2.50899799885278	0.017265414399922	0.433349240292697	-3.13486221746167
a_HQ_transcript/121538	2.71295507085933	-0.442305859054625	2.50873883830662	0.017275986309904	0.433349240292697	-3.16985865920356
a_HQ_transcript/112682	-2.59020650396051	0.78554546911448	-2.50865265793728	0.0172795031727692	0.433349240292697	-3.18886002187233
a_HQ_transcript/151206	-3.11958820195344	0.432763461140039	-2.50862667949506	0.0172805634342116	0.433349240292697	-3.20405610734338
a_HQ_transcript/28419	-2.31701059528988	-0.40210984445131	-2.5086186930198	0.0172808893992305	0.433349240292697	-3.1695003374599
a_HQ_transcript/144352	-2.01223770934503	0.296942873443391	-2.50833465065378	0.0172924861518678	0.433436697118356	-3.19042486247768
a_HQ_transcript/93721	-2.02060787257995	2.66535391933846	-2.50816808007027	0.0172992901409749	0.433436697118356	-3.16045642165464
a_HQ_transcript/40483	-4.2850276436857	0.772207017768508	-2.50792490838054	0.0173092275029409	0.43348741591751	-3.17661136287918
a_HQ_transcript/8791	-3.17801657127547	1.12078614088619	-2.50775364691801	0.0173162293517833	0.43348741591751	-3.09317036049528
a_HQ_transcript/14298	-3.12173125725259	-0.0714842355487935	-2.50752948278555	0.0173253979985878	0.433530234550975	-3.15827007847345
a_HQ_transcript/90303	2.81423939765162	0.430349244946234	2.50730986312824	0.0173343850888253	0.433568475233752	-3.10401259438479
a_HQ_transcript/123569	-2.09989742301867	3.16507356939311	-2.50667671670041	0.0173603181163534	0.43399609148508	-3.36169270254481
a_HQ_transcript/149545	2.31042959639372	-0.299001022193618	2.50652801540509	0.0173664139277852	0.43399609148508	-3.15580008712776
a_HQ_transcript/15684	-2.28611784422016	2.60331565872445	-2.50548660539128	0.0174091602044859	0.434800636052707	-3.17577368517792
a_HQ_transcript/140399	3.68610492446226	2.9254848829785	2.50520417083703	0.0174207697916565	0.434800636052707	-3.28752435454983
a_HQ_transcript/280	5.03323838680685	2.59225564856288	2.50518346950723	0.0174216210075161	0.434800636052707	-3.31432471846249
a_HQ_transcript/122818	-2.81645019422076	1.85292139523844	-2.50501552501231	0.0174285281104254	0.434800636052707	-3.17839624235354
a_HQ_transcript/110195	-3.47577391413586	1.36710925345617	-2.50444362515119	0.0174520676505775	0.435201110421607	-3.21421038166691
a_HQ_transcript/118033	0.900363285119714	3.18180650872016	2.50413785142769	0.01746466533384	0.435328501940613	-3.30530253199928
a_HQ_transcript/15202	-2.05198483482068	0.485720753351176	-2.50388392020853	0.0174751334778037	0.435402725588414	-3.10702578786842
a_HQ_transcript/141711	-4.75962569135253	0.346082766202544	-2.50346694235831	0.0174923355945887	0.435644594448267	-3.17087788123577
a_HQ_transcript/75231	0.693406415227872	4.52712774576599	2.503151291421	0.0175053678705102	0.435782451210714	-3.48603454949664
a_HQ_transcript/138416	4.12303870195138	1.75773477101597	2.50290731029138	0.0175154472171567	0.435824216034866	-3.24232265554998
a_HQ_transcript/107094	-2.82967020553891	0.856757977855529	-2.50274777319618	0.0175220408903365	0.435824216034866	-3.15283706232861
a_HQ_transcript/97386	1.74384707620855	1.62655930617356	2.50239782533904	0.0175365122205152	0.435997597242988	-3.16613603185828
a_HQ_transcript/113735	-3.32568451814753	0.219374625872814	-2.50202037113033	0.0175521333025247	0.436131046021179	-3.13512100604
a_HQ_transcript/19732	-1.24228717876662	2.04919436873277	-2.50190560123773	0.0175568856263687	0.436131046021179	-3.14402597715038
a_HQ_transcript/46355	3.61479348826023	0.557246618913913	2.5015156168996	0.0175730426857297	0.436345931326824	-3.19120256129447
a_HQ_transcript/136501	1.44156954632575	3.74618106682007	2.50114618042014	0.0175883610224954	0.436512415558593	-3.38393052449606
a_HQ_transcript/118006	3.89445076361918	1.93260532989153	2.50070130870479	0.0176068234607669	0.436512415558593	-3.21992829087809
a_HQ_transcript/116234	2.35079016175894	1.65375752193476	2.50041088430755	0.0176188858302929	0.436512415558593	-3.13877567599282

a_HQ_transcript/14517	4.27586416866981	2.14901887466382	2.50025371409763	0.0176254168339142	0.436512415558593	-3.27606600010357
a_HQ_transcript/129568	0.762259479213024	3.60803098991527	2.5001451956065	0.017629927474076	0.436512415558593	-3.41084828940934
a_HQ_transcript/103731	1.98706610785652	-0.672210639515029	2.50013324817094	0.0176304241415175	0.436512415558593	-3.15212096874682
a_HQ_transcript/28766	-2.18355914551083	0.485244513163294	-2.50008778860021	0.0176323140608928	0.436512415558593	-3.14151347077928
a_HQ_transcript/98845	2.70988497594862	0.320352232638106	2.49909994150813	0.0176734283557254	0.437343994662242	-3.17521791894954
a_HQ_transcript/104204	2.10181442441623	1.34673671538785	2.49887113033531	0.0176829640291721	0.437393758760729	-3.15914854767471
a_HQ_transcript/91061	-7.34233427527103	4.39436037446021	-2.49775814469507	0.0177294148627988	0.438356200401858	-3.4515091288212
a_HQ_transcript/120286	-3.49528590993771	2.28019836918494	-2.49643177005844	0.0177849176412693	0.439425160287373	-3.37567905773768
a_HQ_transcript/15452	1.22720104101286	2.43147095418884	2.49636374646304	0.0177877684103391	0.439425160287373	-3.2379647469876
a_HQ_transcript/44427	-3.67450952919445	1.28264148315902	-2.49605248560782	0.0178008182404399	0.439560731809809	-3.22347512374466
a_HQ_transcript/82655	4.25573771843028	4.01588937994127	2.49563783152348	0.0178182165374981	0.439799376085305	-3.37089882632222
a_HQ_transcript/79819	-1.24569020893517	3.21292135605508	-2.49546162386907	0.0178256146789323	0.439799376085305	-3.3239152809361
a_HQ_transcript/8617	2.11166434436972	0.897140938059447	2.49429009320825	0.0178748734458672	0.440827595953063	-3.10881550340094
a_HQ_transcript/146138	1.99314181016685	0.499826115852726	2.49388101756477	0.0178921030130025	0.441039645133985	-3.1645126757768
a_HQ_transcript/47462	3.39944053007827	0.756312932442722	2.49372575277083	0.017898646484845	0.441039645133985	-3.172339468225
a_HQ_transcript/63142	1.68061694247597	3.20270475297448	2.49340813350298	0.017912039048619	0.441182629583951	-3.24171895727151
a_HQ_transcript/61438	0.849582973012402	3.47911321371688	2.49320486809025	0.0179206146478243	0.441206898877057	-3.38201383063961
a_HQ_transcript/40615	-0.530086672107097	5.09115194505083	-2.49229677851295	0.0179589721671564	0.441830790784522	-3.58919438625792
a_HQ_transcript/136932	-1.92867442512635	-0.642712002260118	-2.49224509754449	0.0179611574219623	0.441830790784522	-3.1883786088515
a_HQ_transcript/52631	-1.56061914128755	2.29349693682178	-2.4918186835005	0.0179791970231736	0.442087463859151	-3.21460357844372
a_HQ_transcript/32958	1.69794881241571	-0.557095660792671	2.49146845376551	0.0179940260365445	0.442122531063497	-3.18317643196378
a_HQ_transcript/74685	-1.74991385171117	-0.393290783400103	-2.49140095381433	0.0179968853275465	0.442122531063497	-3.23297942477153
a_HQ_transcript/31430	2.47892663775107	2.26725266171652	2.49124622450259	0.0180034412164069	0.442122531063497	-3.19733600477474
a_HQ_transcript/27	0.878122263271523	0.478376606968738	2.48991653784804	0.0180598703811638	0.442954441068159	-3.25739330101124
a_HQ_transcript/133924	-2.93293058299052	2.24262265842504	-2.48991478204697	0.0180599450007654	0.442954441068159	-3.29473922923891
a_HQ_transcript/59822	0.948686564314211	3.24185451470696	2.4898799209259	0.0180614266182976	0.442954441068159	-3.35284298465384
a_HQ_transcript/117052	-2.58497939462612	-0.544018741753275	-2.48973003079593	0.0180677983032722	0.442954441068159	-3.19921476105052
a_HQ_transcript/19582	-3.92149076035486	0.842814072287399	-2.48911651553733	0.0180938997180427	0.443407336766603	-3.23680536002987
a_HQ_transcript/147734	6.39335027922017	3.32100615160689	2.48851634568563	0.0181194667888098	0.443760454727288	-3.41536855031159
a_HQ_transcript/24960	5.77647959932595	1.33096343711503	2.488419919239	0.0181235776135869	0.443760454727288	-3.279708778963
a_HQ_transcript/107879	-2.49198524124388	-0.365743471752019	-2.48816807342752	0.018134318262734	0.443836485042612	-3.18182360972607
a_HQ_transcript/19488	3.98614166455783	1.7194750408923	2.48748751196214	0.0181633718331301	0.444113735453408	-3.2179271401704
a_HQ_transcript/121752	-2.85133998040958	0.471383027692868	-2.48747933853873	0.0181637210203844	0.444113735453408	-3.16472459685515
a_HQ_transcript/89053	2.65250011619268	-0.271563405299404	2.48736592345451	0.018168567005715	0.444113735453408	-3.19449493322537
a_HQ_transcript/127112	-2.63102391472712	0.299996032804911	-2.48697183674313	0.0181854147016952	0.444162600769139	-3.20263050563464
a_HQ_transcript/144583	-3.04836021087517	-0.461862142284458	-2.48696169886339	0.0181858482973877	0.444162600769139	-3.2050230913757
a_HQ_transcript/151038	-3.36301213329001	1.60803509533166	-2.48587163166123	0.0182325256097696	0.444861743219278	-3.24085009898178
a_HQ_transcript/112017	-2.1574290644765	-0.594397071316739	-2.485782613214	0.0182363422741179	0.444861743219278	-3.19752658570223
a_HQ_transcript/152395	-6.1549755659917	0.93266260430085	-2.48575414556349	0.0182375629785549	0.444861743219278	-3.28150562666622
a_HQ_transcript/149643	-0.592200760019852	4.41739210441918	-2.48557872737448	0.018245086633546	0.444861743219278	-3.50181780352747
a_HQ_transcript/121489	2.13881509828253	-0.824282340811155	2.48535393968884	0.0182547318930867	0.444910295799307	-3.21692808537795
a_HQ_transcript/152826	-4.20833311993526	0.737495331052499	-2.4846425496035	0.0182852872244287	0.445468221199326	-3.25115603926205
a_HQ_transcript/30175	-2.50343479737331	0.0347952492959307	-2.4841317419928	0.0183072561057058	0.445487534484043	-3.26988517763903
a_HQ_transcript/112680	3.0019002006463	0.74196678909842	2.48408988964856	0.0183090571678774	0.445487534484043	-3.24994500478686
a_HQ_transcript/119950	2.47848178635155	0.351434841996755	2.48368150094265	0.0183266401733366	0.445487534484043	-3.27460821115703
a_HQ_transcript/104094	2.82470525742673	1.8573647168098	2.48364268312051	0.0183283122630313	0.445487534484043	-3.22539177374464
a_HQ_transcript/142923	2.96963763373826	-0.307313637864045	2.48356061159837	0.0183318479792088	0.445487534484043	-3.21635876212903
a_HQ_transcript/79235	2.71421475696104	1.36175415846684	2.48355561122295	0.0183320634201389	0.445487534484043	-3.14772689758283
a_HQ_transcript/113380	-3.48665384125857	1.60618154518703	-2.48330610459301	0.0183428163459095	0.445562569391989	-3.23891086378626
a_HQ_transcript/47009	1.34420182116657	-0.73224991593166	2.48239662232829	0.0183820609973839	0.446329340708409	-3.25094812741407

a_HQ_transcript/148776	-3.31370090900724	1.64050884301571	-2.48212468954364	0.0183938099669025	0.446428136015076	-3.26189653032927
a_HQ_transcript/96826	-4.4057383620045	-0.38050673444415	-2.48192866346472	0.0184022836201469	0.446447388260393	-3.2033586763032
a_HQ_transcript/12947	-3.75986284864546	0.310309473601198	-2.48113300195607	0.0184367145298891	0.446812261528478	-3.22819025048898
a_HQ_transcript/63817	2.88432463403455	1.52918181674194	2.48095285861156	0.0184445181140003	0.446812261528478	-3.1771398094114
a_HQ_transcript/133342	1.93318821848755	-0.450656278284544	2.48078555830844	0.0184517680620502	0.446812261528478	-3.18544535197759
a_HQ_transcript/137339	3.26487545269109	-0.0393132924501294	2.48054225010519	0.0184623164643455	0.446812261528478	-3.25261075274778
a_HQ_transcript/57210	3.14476059296887	0.419442577463185	2.48048686605454	0.0184647183599875	0.446812261528478	-3.20618575686841
a_HQ_transcript/149362	0.762912434391598	4.61457723046833	2.48033376397626	0.0184713595799666	0.446812261528478	-3.56201565032014
a_HQ_transcript/139076	-2.29493109575193	0.325231249324219	-2.48031505063265	0.0184721714722303	0.446812261528478	-3.20389515173604
a_HQ_transcript/66638	-1.01120636326523	2.4915426199989	-2.48016190499971	0.0184788170367888	0.446812261528478	-3.29461523903423
a_HQ_transcript/93682	3.50903447790478	0.115895955727972	2.47992741945709	0.0184889964873969	0.446872510527821	-3.2601168447699
a_HQ_transcript/13976	-3.51576105438596	2.50637190342546	-2.4795265144794	0.0185064124063537	0.446872611236631	-3.21758505899834
a_HQ_transcript/587	-2.25917289882913	2.29735875475506	-2.47945531233499	0.0185095071054188	0.446872611236631	-3.26281740129721
a_HQ_transcript/80007	1.77186861154558	-0.778649615226634	2.47939649539596	0.0185120638566234	0.446872611236631	-3.22788620054012
a_HQ_transcript/152216	6.10088968067634	3.96461019879218	2.4789501403283	0.018531477290748	0.447155546681859	-3.55474765111734
a_HQ_transcript/29563	-7.18713912336886	3.56052286646906	-2.47866505022896	0.0185438865385879	0.447269309840265	-3.50455927584759
a_HQ_transcript/149633	-3.52759646552061	0.522233269025889	-2.47825776773387	0.0185616277078188	0.447511528577393	-3.22755137562728
a_HQ_transcript/134279	3.37600257796523	2.23059646261584	2.47765958972975	0.0185877124285104	0.447954621908976	-3.29942161945293
a_HQ_transcript/24609	2.26765261408209	-0.0510616392911008	2.47718819419234	0.0186082921883206	0.448264736146995	-3.16771704682331
a_HQ_transcript/139497	-1.2315928073267	4.36713467543155	-2.47674774455167	0.0186275397857902	0.448312850091317	-3.53540466496328
a_HQ_transcript/50205	1.9927634153858	2.25103345041289	2.47673013590797	0.0186283096601443	0.448312850091317	-3.28947507369683
a_HQ_transcript/117828	1.31524208311978	5.17662573705748	2.47661310806381	0.0186334270200355	0.448312850091317	-3.57403627819948
a_HQ_transcript/125644	0.533933564656737	3.27165095420767	2.47640965395214	0.0186423266659892	0.448341400265048	-3.41230544728041
a_HQ_transcript/2829	-2.19118934997509	-0.648101848149093	-2.47615523928355	0.0186534609420855	0.448423646667305	-3.22364051639312
a_HQ_transcript/124204	1.07211304533105	3.31476040853173	2.47592369082214	0.0186635997852716	0.448481905050959	-3.44366515843825
a_HQ_transcript/132974	-0.992715007272251	3.73719206935877	-2.47532610792223	0.0186897895373881	0.448664413650617	-3.45371703539902
a_HQ_transcript/144799	-3.27166558229634	2.4304343989424	-2.47531568144061	0.0186902467882398	0.448664413650617	-3.41330866891501
a_HQ_transcript/14696	2.38662787287436	-0.220667804739949	2.47514428676405	0.0186977647282969	0.448664413650617	-3.2316225258376
a_HQ_transcript/45660	-1.06210773944271	2.56937562130185	-2.47488863184387	0.0187089837456574	0.448664413650617	-3.32988639096405
a_HQ_transcript/113182	2.13120156719471	0.425437861782542	2.47487031729879	0.0187097876873296	0.448664413650617	-3.1918463535445
a_HQ_transcript/136973	1.8676748329877	1.29367120790224	2.47442837423606	0.0187291969633557	0.448944643746781	-3.19042844334559
a_HQ_transcript/134153	1.7189958886897	-0.622552840180427	2.47385809173281	0.0187542698868681	0.4492338548372	-3.26691176690464
a_HQ_transcript/5122	3.34292549851335	-0.101883920960073	2.47380242274661	0.0187567190629281	0.4492338548372	-3.19687047286481
a_HQ_transcript/52093	-5.78493165677396	1.49662822392908	-2.47353600970139	0.0187684440484251	0.449329536922097	-3.24830371394527
a_HQ_transcript/130521	2.41630262226915	0.620084146355873	2.47204062988035	0.0188343811102283	0.450690985953635	-3.19084556776551
a_HQ_transcript/32043	-5.40240153243925	4.19351835123073	-2.47189489449371	0.018840818467302	0.450690985953635	-3.54535370900079
a_HQ_transcript/121166	0.684323021641328	3.69198684978616	2.47140407104514	0.0188625137070984	0.450701948673356	-3.46389713811109
a_HQ_transcript/2256	-1.50678566987037	1.97622288134099	-2.47135725713518	0.0188645841559182	0.450701948673356	-3.18309201839318
a_HQ_transcript/29228	2.26632724197572	1.45737871931568	2.47128319016104	0.0188678603565881	0.450701948673356	-3.21021459320723
a_HQ_transcript/150140	0.406944746930171	10.3833025852604	2.47110264281087	0.0188758486795002	0.450701948673356	-4.13679445126084
a_HQ_transcript/99033	1.81879958541352	2.55265903066717	2.47098046737122	0.0188812560908114	0.450701948673356	-3.36442700533861
a_HQ_transcript/75052	1.97238285819829	2.25417217995485	2.47079062460269	0.0188896612294438	0.450701948673356	-3.2917642257332
a_HQ_transcript/133204	4.07310512069192	2.80168694317167	2.47047036605344	0.0189038481824861	0.450701948673356	-3.41413361462968
a_HQ_transcript/132562	-2.88153259479347	-0.588388739728969	-2.47032839776213	0.0189101402736949	0.450701948673356	-3.25473306271938
a_HQ_transcript/134045	-2.41743032403326	-0.183673745849482	-2.47029905973271	0.0189114407857045	0.450701948673356	-3.27484614247875
a_HQ_transcript/123834	-2.99026330361378	-0.079479929835608	-2.4701327889149	0.0189188128743977	0.450701948673356	-3.2436547166929
a_HQ_transcript/69385	-1.77741776464456	1.38831389866985	-2.4683632152168	0.0189974349912242	0.45238955394096	-3.17093782666597
a_HQ_transcript/65031	-2.22522924820688	-0.52341363073699	-2.46791705619868	0.0190173049467875	0.452632103393387	-3.23555217591159
a_HQ_transcript/130135	-2.01671196325994	0.324767026973859	-2.46769566759609	0.0190271716741681	0.452632103393387	-3.18851939122035
a_HQ_transcript/131745	-4.57335852999394	1.99429544391821	-2.46761022354437	0.0190309809505477	0.452632103393387	-3.38667669654796

a_HQ_transcript/5972	-3.44676995871389	0.299663578398423	-2.46683654219802	0.0190655050671728	0.452999663904112	-3.24756331367345
a_HQ_transcript/45441	-2.72729133871201	1.28454731365735	-2.46661672983717	0.019075324236281	0.452999663904112	-3.21071713117863
a_HQ_transcript/55050	3.56101481238005	1.6772922790004	2.46653145612315	0.0190791347166813	0.452999663904112	-3.29862017245692
a_HQ_transcript/65977	-1.15832669754986	1.47671608586117	-2.46616256917047	0.0190956265571116	0.452999663904112	-3.17612998234507
a_HQ_transcript/127940	2.41745233256245	2.33899485497814	2.46613661910341	0.0190967871982596	0.452999663904112	-3.30749672316238
a_HQ_transcript/100838	1.85143358140375	0.967848863014784	2.46604190457259	0.0191010239422042	0.452999663904112	-3.15532365990839
a_HQ_transcript/147579	0.903357262046994	6.07786749088071	2.46591221120045	0.0191068267443883	0.452999663904112	-3.70806885907233
a_HQ_transcript/111757	2.67918270542867	0.827612673298925	2.46586855915748	0.0191087802073507	0.452999663904112	-3.21393302356315
a_HQ_transcript/11503	2.87065453255143	2.73230059381296	2.46546904932852	0.0191266670710848	0.453063103770938	-3.38824777635013
a_HQ_transcript/126788	1.99163541828815	-0.446023934423143	2.46546061763242	0.0191270447401232	0.453063103770938	-3.20806354319205
a_HQ_transcript/19518	-3.92490601158575	1.09555371414691	-2.46519778564138	0.0191388208220055	0.453157383601439	-3.28084467592301
a_HQ_transcript/3423	3.25227866306875	0.248240924551183	2.46496836597604	0.0191491052972799	0.453216283680898	-3.24667181128402
a_HQ_transcript/121846	-2.21889346607729	0.324249307998864	-2.4644521645643	0.0191722641690088	0.453579719827491	-3.25800794683957
a_HQ_transcript/8207	0.910246853972336	3.1766933029362	2.46397030136932	0.0191939056005192	0.453765253670364	-3.33139455471348
a_HQ_transcript/34372	-1.97958212931901	-0.687577242152585	-2.46364754385742	0.0192084137698901	0.453765253670364	-3.23076666546298
a_HQ_transcript/124222	-2.76653509350123	0.222534482947569	-2.46355417489154	0.0192126126379242	0.453765253670364	-3.29245444471451
a_HQ_transcript/5652	1.19869479131322	2.81865393198348	2.46338592874792	0.0192201809040655	0.453765253670364	-3.3758159667638
a_HQ_transcript/116276	2.2110437554145	0.070671472824453	2.46323997167632	0.0192267487424051	0.453765253670364	-3.22550767406398
a_HQ_transcript/22648	-2.67457425286177	3.36625113852705	-2.46323562591793	0.0192269443261441	0.453765253670364	-3.47503450354613
a_HQ_transcript/130420	-3.23746514057435	1.70661807318198	-2.4624574120588	0.0192619977026305	0.453815528458977	-3.3342758920755
a_HQ_transcript/104027	-3.58443978906423	3.1527067457826	-2.46234116077237	0.0192672390659457	0.453815528458977	-3.42246619546546
a_HQ_transcript/51256	-0.923839351779098	3.09762380453083	-2.46223981536437	0.0192718094392912	0.453815528458977	-3.32881213254825
a_HQ_transcript/59831	1.58240950146945	-0.804014415854375	2.46222693820376	0.0192723902315063	0.453815528458977	-3.26211206977503
a_HQ_transcript/350	-2.99281063081391	2.82507482000489	-2.46202543795173	0.0192814804825275	0.453815528458977	-3.22889678965378
a_HQ_transcript/31951	3.93986646328762	1.50995958420648	2.46199257642609	0.0192829633313922	0.453815528458977	-3.25907551841504
a_HQ_transcript/76270	2.79994228449383	1.15770972881341	2.46197570231516	0.0192837248020519	0.453815528458977	-3.20225637455975
a_HQ_transcript/117768	-6.65286152955876	3.09263040709023	-2.46118488938652	0.0193194423232391	0.454393183306962	-3.45443481259211
a_HQ_transcript/87872	0.804973542018439	3.33930699240139	2.46108617657027	0.0193239049878683	0.454393183306962	-3.48597975319806
a_HQ_transcript/139674	-3.99601643688232	1.30621775053285	-2.46079560171643	0.0193370469302105	0.454518343695622	-3.31611621360156
a_HQ_transcript/147621	3.26609958014439	1.71966652952519	2.45995909290927	0.0193749256717873	0.455124986552649	-3.27039742170951
a_HQ_transcript/135755	-2.10369940481978	4.18476297229773	-2.45987989597893	0.0193785153749967	0.455124986552649	-3.58632977645911
a_HQ_transcript/96717	2.48568755817305	3.50460020521286	2.45940331839073	0.019400129710097	0.455156660548967	-3.38635065980823
a_HQ_transcript/54833	-1.96207630698819	1.42591232383828	-2.45928917782932	0.0194053096236485	0.455156660548967	-3.30418737118075
a_HQ_transcript/15450	1.92843882123036	0.545838658668681	2.45920557675258	0.0194091043999528	0.455156660548967	-3.22489812026577
a_HQ_transcript/133807	-3.89792477054087	0.905815961881942	-2.45906445227573	0.0194155117873317	0.455156660548967	-3.27207049018347
a_HQ_transcript/147120	2.66500740942853	0.90056554360572	2.45897244020449	0.0194196903957052	0.455156660548967	-3.19039327187892
a_HQ_transcript/110476	-1.73934234648057	-0.272715081845862	-2.45858823405782	0.0194371475014612	0.455156660548967	-3.22454904415951
a_HQ_transcript/55477	3.1030655880178	0.208276125541004	2.45856142555427	0.0194383661298126	0.455156660548967	-3.26292208562283
a_HQ_transcript/148373	-1.06091947928706	2.94933571144574	-2.45847036460365	0.0194425059892493	0.455156660548967	-3.31117527749798
a_HQ_transcript/124652	-2.41035464808979	1.00568530852813	-2.4578835531482	0.019469203246345	0.455593015702878	-3.17509772250899
a_HQ_transcript/144349	-1.93901876032744	-0.667055149070424	-2.45771625082009	0.0194768208784347	0.455593015702878	-3.26278312251605
a_HQ_transcript/143697	2.24622294983146	0.45499510639156	2.45737264664791	0.0194924744529973	0.455747225370123	-3.31364456383589
a_HQ_transcript/118976	-1.09060419062766	3.06217770187204	-2.45722741490455	0.0194990942316181	0.455747225370123	-3.46881553904938
a_HQ_transcript/119350	1.11710027959366	2.25392623655015	2.45705317418722	0.0195070389735789	0.455749662964709	-3.26883480522452
a_HQ_transcript/151625	3.07322017150113	0.359031700789036	2.45650449476257	0.0195320760859377	0.456151273090954	-3.28087656574491
a_HQ_transcript/70184	1.89475696941817	0.493112950404814	2.4557726425498	0.0195655173734437	0.456748752563669	-3.30716097006416
a_HQ_transcript/144803	0.901684434511581	3.97302005633759	2.45512412595657	0.0195951944349098	0.456937935483058	-3.56454999943066
a_HQ_transcript/29094	2.39091974768362	0.632120278965259	2.45508640607616	0.019596921816592	0.456937935483058	-3.25910269201544
a_HQ_transcript/40327	-4.0342111762322	1.69884065017746	-2.45494388537484	0.01960344980738	0.456937935483058	-3.31333214708795
a_HQ_transcript/78316	4.93893356913003	2.17195181727864	2.4549086314978	0.019605064875701	0.456937935483058	-3.27735827996339

a_HQ_transcript/151226	1.47223571355189	-0.8348761696644	2.45443417787196	0.019626812615826	0.457203849549778	-3.27341886253681
a_HQ_transcript/119471	5.15397308854164	2.36049709561188	2.45411133229244	0.0196416236317717	0.457203849549778	-3.34001558570183
a_HQ_transcript/39033	-3.34368193387051	0.483342205388406	-2.45409558171332	0.0196423464739636	0.457203849549778	-3.21126446492273
a_HQ_transcript/93300	1.6860496804214	-0.769629868474312	2.45385252538491	0.0196535041534029	0.457203849549778	-3.27809742532803
a_HQ_transcript/84895	3.35080597049968	2.76281225158193	2.4536714228701	0.0196618215621327	0.457203849549778	-3.31291964729828
a_HQ_transcript/94908	-1.69843521729587	1.75812976371678	-2.45339643908599	0.0196744567602171	0.457203849549778	-3.27792180075997
a_HQ_transcript/82877	-2.27129141109451	-0.129191425092112	-2.45331546062105	0.0196781790440981	0.457203849549778	-3.27935254951658
a_HQ_transcript/129355	-3.04703810280968	-0.263735171930602	-2.45319959441802	0.0196835061086579	0.457203849549778	-3.29879693754879
a_HQ_transcript/152908	6.38554639686779	7.89509680223918	2.45273584397117	0.0197048406918994	0.457203849549778	-3.92311770430665
a_HQ_transcript/36767	-4.35625365739947	3.93434299400628	-2.45267422392498	0.0197076770776237	0.457203849549778	-3.63113425007295
a_HQ_transcript/19952	-2.12657217982769	1.70011758063452	-2.45260950192491	0.0197106566486806	0.457203849549778	-3.25071031260407
a_HQ_transcript/8562	1.63729924878484	0.33556045729174	2.45260509370461	0.0197108596024591	0.457203849549778	-3.20986445002984
a_HQ_transcript/40389	0.610167174687344	3.74382839922835	2.4515564629664	0.0197591926788778	0.45814214281524	-3.52769330882148
a_HQ_transcript/113962	3.17396161204915	7.27000634878741	2.45132393773821	0.0197699247955575	0.458208209137228	-3.84443101924435
a_HQ_transcript/28727	-0.99724978854298	3.47156409646265	-2.45091330069967	0.0197888906037396	0.458322912192117	-3.53503926464642
a_HQ_transcript/103055	-3.75929360268604	0.218008998449115	-2.45087537199391	0.0197906432287747	0.458322912192117	-3.28798967815414
a_HQ_transcript/109226	5.43014841738434	2.1913205295142	2.45024085352327	0.0198199843510792	0.458813821838693	-3.36327061692275
a_HQ_transcript/7854	-1.85698476095352	0.970519806744218	-2.45007091464455	0.0198278493326935	0.458813821838693	-3.23612079825202
a_HQ_transcript/142861	-1.64895257987327	1.85324684561038	-2.44990522332061	0.0198355204768895	0.458813821838693	-3.25858151497963
a_HQ_transcript/145976	-0.873742630947215	4.66314367078111	-2.44960954511237	0.0198492164636457	0.458947993078281	-3.62221746289462
a_HQ_transcript/26007	1.07273414141521	4.28755175981544	2.44899135040716	0.019877879514861	0.459253455548818	-3.58493483813386
a_HQ_transcript/130591	-2.10783422270838	0.401893553467053	-2.44867563814311	0.0198925323241918	0.459253455548818	-3.21488269018459
a_HQ_transcript/1835	-1.99438443967457	1.8772807697444	-2.44826843485098	0.019911445974117	0.459253455548818	-3.27786200976634
a_HQ_transcript/2672	-2.01855785476881	-0.595101779907216	-2.4480864085068	0.0199199059868036	0.459253455548818	-3.27717292220698
a_HQ_transcript/85941	1.08108096968908	1.91986924425247	2.4477866926295	0.0199338429921923	0.459253455548818	-3.25076710205179
a_HQ_transcript/140042	-1.86961164952309	1.54978193923686	-2.44775712055921	0.0199352185974412	0.459253455548818	-3.2801926499389
a_HQ_transcript/145914	1.51365748169322	-0.708265416496787	2.44774421279561	0.0199358190556068	0.459253455548818	-3.25953114309426
a_HQ_transcript/150636	0.401775977741587	11.1537946600316	2.4476303453493	0.0199411167877818	0.459253455548818	-4.28469691298169
a_HQ_transcript/123853	-6.08398367093469	2.01962061087069	-2.44754641476039	0.0199450225177719	0.459253455548818	-3.40743059394588
a_HQ_transcript/11883	2.97539917099798	0.71403075124622	2.44729598898477	0.0199566802933586	0.459253455548818	-3.22769413075654
a_HQ_transcript/58526	3.69839954529127	0.171481954584229	2.44726908411742	0.019957933134005	0.459253455548818	-3.20716532136273
a_HQ_transcript/118131	2.101101357671	-0.153275780747526	2.44695161363769	0.0199727217547142	0.459253455548818	-3.25020039732875
a_HQ_transcript/111898	1.42105010689839	-0.316119298463646	2.44694892886106	0.0199728468614222	0.459253455548818	-3.27334960802302
a_HQ_transcript/110790	-2.6480096858806	1.614006809763	-2.44694483344057	0.0199730377034719	0.459253455548818	-3.236942831315
a_HQ_transcript/72820	-2.05624790937858	0.426572950550689	-2.44663821924832	0.0199873303146738	0.459292050808443	-3.19838510653613
a_HQ_transcript/139176	1.84059825427935	-0.795078653884797	2.44656984081729	0.0199905190019502	0.459292050808443	-3.29293675183611
a_HQ_transcript/123939	-2.79241841362249	1.15650551356615	-2.44637078578559	0.0199998041679609	0.459323831163663	-3.27926890397229
a_HQ_transcript/22372	2.04985705516503	1.10099513851271	2.44580189290122	0.0200263625893052	0.45958782897399	-3.22819568708082
a_HQ_transcript/131754	2.07923944847767	0.623697667602495	2.44578584855498	0.0200271120766432	0.45958782897399	-3.29082663169238
a_HQ_transcript/43741	-2.28521989993643	0.740731115232313	-2.44561558239522	0.0200350673807186	0.459588948976485	-3.31823412973512
a_HQ_transcript/58277	1.22454559512238	2.94438692736297	2.44526766270991	0.0200513321196211	0.459748234832576	-3.3895449117782
a_HQ_transcript/4654	-1.15685751100756	2.52124150616987	-2.44512874520083	0.0200578296782173	0.459748234832576	-3.29657167562718
a_HQ_transcript/81169	-2.75645803083359	-0.262091367115348	-2.44466943571171	0.0200793265501224	0.460059556052627	-3.29582457838094
a_HQ_transcript/45630	-5.07965329370848	1.13658944022223	-2.44382019894594	0.0201191284373023	0.460787271807838	-3.36836239218298
a_HQ_transcript/147759	2.96536741532318	1.27136264530461	2.44342970733084	0.020137454097038	0.460787271807838	-3.31487755652353
a_HQ_transcript/44720	2.34409975864971	1.73285888274972	2.44338939818971	0.0201393466609541	0.460787271807838	-3.26184232737788
a_HQ_transcript/88774	-2.34378270552517	1.78676103127649	-2.44322140716686	0.0201472357954302	0.460787271807838	-3.31269552217913
a_HQ_transcript/13184	2.4256876716609	-0.483281337864571	2.44306018410809	0.020154809748169	0.460787271807838	-3.31020709649616
a_HQ_transcript/62805	2.28484112636546	-0.145738722039779	2.4429784505056	0.0201586504302115	0.460787271807838	-3.28845785095883
a_HQ_transcript/87387	-2.43790422239586	3.148517972013	-2.44278427035138	0.0201677776826911	0.460814693844132	-3.45461154206756

a_HQ_transcript/75618	1.17508098609013	2.58052482175018	2.44257016283232	0.0201778459749431	0.460863587753041	-3.3087977594679
a_HQ_transcript/33320	2.76802715983902	1.23658549659824	2.44176014276919	0.020215978267651	0.461553175468191	-3.21941952305896
a_HQ_transcript/64289	2.32885226463822	-0.181562621512004	2.44141957507356	0.0202320303769889	0.461738304575425	-3.25403442924314
a_HQ_transcript/97438	-2.96240288095147	4.11266925126776	-2.44123736857882	0.0202406231628958	0.461753117430459	-3.42379743743128
a_HQ_transcript/18911	2.91625455805985	0.494804276799242	2.44057354841931	0.0202719568406025	0.462286507348193	-3.23637362348595
a_HQ_transcript/142409	2.67749852357107	0.448022898710313	2.43928779509032	0.0203327729986001	0.463491540730442	-3.31718282337173
a_HQ_transcript/121052	-2.65032070588204	-0.312118313455542	-2.43842558454479	0.0203736487942332	0.463841322098666	-3.35030746084526
a_HQ_transcript/147109	-0.920686833025705	3.23968232272404	-2.43841860648889	0.0203739799165594	0.463841322098666	-3.51943065521772
a_HQ_transcript/61864	-4.20900733223873	1.69246554385398	-2.43825785564266	0.0203816092167201	0.463841322098666	-3.28623802790283
a_HQ_transcript/115090	3.41127990334255	3.19678698718981	2.43819778492534	0.0203844608663388	0.463841322098666	-3.50009312504914
a_HQ_transcript/92738	1.76298722082101	-0.767638093763712	2.43798894790838	0.0203943775150616	0.463841322098666	-3.30876422478311
a_HQ_transcript/152356	5.00457549272081	1.36813398281218	2.43769613186641	0.0204082893308119	0.463841322098666	-3.35942663762031
a_HQ_transcript/82032	1.18354965280336	2.24834355098723	2.43751800061214	0.0204167566589069	0.463841322098666	-3.30997651618234
a_HQ_transcript/136848	-4.42476681008662	0.996618865394951	-2.43748963191352	0.0204181054384081	0.463841322098666	-3.35949427470609
a_HQ_transcript/94874	2.54750798319774	2.38935241923172	2.43730315208464	0.0204269735784005	0.463841322098666	-3.27527101951056
a_HQ_transcript/8825	-1.76857932985255	1.99105162853143	-2.43728338132188	0.0204279139927846	0.463841322098666	-3.25505728888444
a_HQ_transcript/116955	-1.90408336073312	1.12200542741744	-2.43697631658085	0.0204425248800475	0.463913048239105	-3.22139647178451
a_HQ_transcript/129632	-2.06067008120957	3.3261987987396	-2.43678820702746	0.0204514802995519	0.463913048239105	-3.45776027559539
a_HQ_transcript/32923	-2.70586978657494	1.00638132475654	-2.43649683712065	0.0204653587497869	0.463913048239105	-3.3160052454489
a_HQ_transcript/150553	-1.31419879283605	2.22835770099009	-2.43639144693881	0.0204703807814496	0.463913048239105	-3.30684723038656
a_HQ_transcript/144088	2.03230444512881	3.48180664877014	2.43625587042425	0.020476842899803	0.463913048239105	-3.42395974187663
a_HQ_transcript/72250	2.06237419136737	-0.0793209897459025	2.43610895553567	0.0204838475516183	0.463913048239105	-3.33131556381144
a_HQ_transcript/121587	-3.24003543638983	1.13970405549812	-2.43604412870283	0.0204869390797857	0.463913048239105	-3.36483417423932
a_HQ_transcript/139378	-2.57084875360938	0.23896130188987	-2.43579056124587	0.020499035553089	0.464006206631604	-3.22896895235893
a_HQ_transcript/78492	-2.4618322111388	0.0982944184261384	-2.43535132521828	0.0205200047949801	0.464157147352698	-3.27796043636674
a_HQ_transcript/91439	-2.55298056145499	-0.481635946497242	-2.43531637763844	0.0205216740417085	0.464157147352698	-3.33444125041663
a_HQ_transcript/136354	-3.20843569692587	1.14281215188766	-2.43483675004299	0.0205445956392788	0.464225612285326	-3.36401484549499
a_HQ_transcript/41507	3.07100898842575	1.90137218205415	2.43477524521893	0.0205475366688391	0.464225612285326	-3.29552666261583
a_HQ_transcript/83949	-4.02800536062893	1.73956355618852	-2.43475175837105	0.0205486598611709	0.464225612285326	-3.443882274445
a_HQ_transcript/147209	3.3800862209947	-0.280272243987501	2.43344659745828	0.0206111636141757	0.465442917115356	-3.31977402022703
a_HQ_transcript/144592	-2.60093657350708	-0.18561939629838	-2.43326196941835	0.0206200193825416	0.465442917115356	-3.30576084487397
a_HQ_transcript/9936	-1.08725573386598	2.68630754820804	-2.43311136091874	0.0206272459584226	0.465442917115356	-3.37251574146542
a_HQ_transcript/125525	1.85812780763022	-0.564342374991027	2.4329587299487	0.0206345719344597	0.465442917115356	-3.28221369394371
a_HQ_transcript/145818	-3.26054827984292	1.52850596392333	-2.43199805708586	0.0206807367831376	0.46630328461219	-3.39388188960848
a_HQ_transcript/144915	3.66305262770411	2.67838676507957	2.43159349624954	0.0207002060077247	0.466314207708764	-3.43915952748012
a_HQ_transcript/104417	-2.90260525703711	4.95556242995464	-2.43156515224924	0.0207015706705685	0.466314207708764	-3.72301050277382
a_HQ_transcript/139776	-3.25466275981985	3.80992917297494	-2.43134385747897	0.0207122280485199	0.466314207708764	-3.55715947773442
a_HQ_transcript/121484	-2.50489308941449	2.75503578387255	-2.43116166912928	0.0207210058481929	0.466314207708764	-3.53895924474136
a_HQ_transcript/104307	3.500695187598	0.0475370555814256	2.43115489672525	0.0207213322067117	0.466314207708764	-3.33039074157564
a_HQ_transcript/61415	1.0252741002572	4.13385501997232	2.43071144627473	0.0207427120523752	0.466520100961953	-3.55324454368043
a_HQ_transcript/17540	-1.24452310930832	3.28068984548465	-2.43063224122537	0.020746532841086	0.466520100961953	-3.47185688078278
a_HQ_transcript/126277	2.11137576639299	0.145256510362652	2.43023242695204	0.0207658293634597	0.466598026626902	-3.33352879093405
a_HQ_transcript/133245	0.80065375737121	3.90418940656703	2.43022780737983	0.0207660524168008	0.466598026626902	-3.52560954138481
a_HQ_transcript/118197	3.30405398072984	2.55200268269475	2.42963689069658	0.0207946025052051	0.467046023944285	-3.47663563634488
a_HQ_transcript/126682	-3.99301576531121	2.11240410099811	-2.42948265636847	0.0208020602118042	0.467046023944285	-3.33302986228948
a_HQ_transcript/137271	2.77751148485478	0.527468504701366	2.42872357001904	0.0208387999373203	0.467686498365088	-3.2355297657407
a_HQ_transcript/58694	-2.67675415218191	1.31086259051868	-2.42824902653243	0.0208617978553567	0.467686498365088	-3.31312280944059
a_HQ_transcript/143472	1.90347999121665	-0.534124925960965	2.42823743732388	0.0208623597954158	0.467686498365088	-3.32748444496218
a_HQ_transcript/61617	3.12770457056321	1.36039456413709	2.4280594544516	0.0208709916013444	0.467686498365088	-3.341427235986
a_HQ_transcript/20958	2.32537610014072	1.00263722939112	2.42779502372626	0.0208838219648665	0.467686498365088	-3.34118598130445

a_HQ_transcript/110330	3.33192713572379	1.17325051476079	2.42768323650271	0.0208892481214921	0.467686498365088	-3.2509172057719
a_HQ_transcript/43408	2.12673834751534	2.60752639216309	2.42767899923814	0.0208894538237806	0.467686498365088	-3.34076901003719
a_HQ_transcript/108611	3.16571872066202	1.81845873934183	2.42756573279445	0.0208949531422745	0.467686498365088	-3.37704937623381
a_HQ_transcript/136874	0.569138426023349	4.56695382744637	2.42714411657194	0.0209154350795304	0.467852410678792	-3.66746327431727
a_HQ_transcript/133304	-1.84095690621317	1.74548802028991	-2.42708182010549	0.0209184629671446	0.467852410678792	-3.25451628014034
a_HQ_transcript/15182	1.31745596434199	3.21060751524987	2.42661774698241	0.020941031582609	0.468177032243807	-3.4914846849767
a_HQ_transcript/150854	0.527174213256066	10.5355300754324	2.42609738562688	0.020966363984144	0.468213610962402	-4.2471089924645
a_HQ_transcript/131667	-3.2558787855458	1.3415583729351	-2.42602137030307	0.0209700669247847	0.468213610962402	-3.30195422442672
a_HQ_transcript/134750	-4.0789374959877	1.76423801484448	-2.42593041230948	0.020974498552445	0.468213610962402	-3.34183084612201
a_HQ_transcript/17860	-1.87090584826666	0.723354797684967	-2.42558908221132	0.0209911363482174	0.468213610962402	-3.32539635689801
a_HQ_transcript/96470	-1.75865129805515	1.41420746860752	-2.42552924430843	0.0209940543264024	0.468213610962402	-3.25852054010485
a_HQ_transcript/71225	3.28884402175724	1.77907493187251	2.4254338728469	0.0209987058527588	0.468213610962402	-3.31403909901038
a_HQ_transcript/135600	4.56118693446602	3.36873112703673	2.42542678030557	0.0209990518128782	0.468213610962402	-3.53457669864186
a_HQ_transcript/119622	-2.28135243217611	0.151690385739905	-2.4249152492514	0.0210240169902023	0.468265917502887	-3.33093421748696
a_HQ_transcript/136151	1.20123737444082	4.15986960850542	2.42488039803093	0.0210257188815792	0.468265917502887	-3.62661419918199
a_HQ_transcript/18864	-2.96076761336412	0.370144913612965	-2.42487018268629	0.0210262177518104	0.468265917502887	-3.34147716964164
a_HQ_transcript/152269	-3.6388629133686	0.821452821366014	-2.42463379636617	0.0210377647815495	0.468265917502887	-3.33647547622077
a_HQ_transcript/107981	0.737394914783543	4.30611836053208	2.42452686764204	0.0210429899483366	0.468265917502887	-3.61298903968881
a_HQ_transcript/115727	-4.13607471340328	3.31305442469567	-2.42438892557948	0.0210497323567823	0.468265917502887	-3.55782220759124
a_HQ_transcript/122721	4.14689597449617	1.2420854341082	2.4240565900777	0.02106598452339	0.468442982120269	-3.37958537761848
a_HQ_transcript/108686	-2.75633513114138	-0.295033570225604	-2.42362618292622	0.021087049675668	0.468442982120269	-3.37454177674939
a_HQ_transcript/122141	3.87344357484163	0.316601390119509	2.42360187938251	0.0210882397215368	0.468442982120269	-3.44804335332504
a_HQ_transcript/132148	0.608768421004211	3.28275841282999	2.42330036312501	0.0211030088401464	0.468442982120269	-3.50924221443154
a_HQ_transcript/19144	1.24212784881996	3.03294385761118	2.4232324113982	0.0211063386093104	0.468442982120269	-3.47796406519211
a_HQ_transcript/40654	-2.39700010483076	-0.331767585952744	-2.4231884619342	0.0211084924750848	0.468442982120269	-3.34729234974608
a_HQ_transcript/139055	2.66767410762443	-0.0115611040284528	2.42300762220352	0.0211173571375367	0.468442982120269	-3.27336547110706
a_HQ_transcript/144652	3.02899988968534	4.41309956186419	2.42286880184789	0.0211241643356559	0.468442982120269	-3.49871351903342
a_HQ_transcript/144663	3.34559382258027	2.05056120936663	2.42263320000663	0.0211357218905072	0.468442982120269	-3.50545210812596
a_HQ_transcript/76349	2.55259198721706	-0.0954446209562645	2.42258106778285	0.0211382800389049	0.468442982120269	-3.33676595370292
a_HQ_transcript/78683	-2.45119264193127	-0.324344934009399	-2.42163281853518	0.021184860271418	0.469249257237643	-3.30233698445356
a_HQ_transcript/52610	2.03138390663221	2.58484029133474	2.42151187099874	0.0211908082206306	0.469249257237643	-3.38663132833075
a_HQ_transcript/18849	0.751807902737061	3.20469053185957	2.42109743911999	0.0212112006652574	0.469521962022117	-3.51550424858101
a_HQ_transcript/108462	2.68846289506236	3.568161430761	2.42086443148975	0.0212226738402419	0.469597101250697	-3.55435828603309
a_HQ_transcript/112462	-1.93411856529842	1.74110154286603	-2.42037472834824	0.0212468050035975	0.469952161814733	-3.28372989421007
a_HQ_transcript/32485	2.48441550012864	-0.252994495111627	2.41975900741598	0.0212771814427065	0.470445037239119	-3.33813723356586
a_HQ_transcript/89031	-1.59901234109605	3.20665865721753	-2.41920476456536	0.0213045587133528	0.470775889829709	-3.50840531727644
a_HQ_transcript/52066	2.32022978608804	0.022575661122852	2.41912820264655	0.0213083430729075	0.470775889829709	-3.3756404815128
a_HQ_transcript/127667	-2.04735312773954	0.863576254048055	-2.41880652701332	0.0213242497894278	0.470948325136725	-3.27437599221671
a_HQ_transcript/97599	6.15247198152712	5.26016146973112	2.41850948686746	0.0213389478992763	0.471093947394278	-3.72221051908574
a_HQ_transcript/120917	2.74599175837293	0.924370654293236	2.41807040876849	0.0213606912231621	0.471155989743478	-3.29100268772975
a_HQ_transcript/24654	2.78775093264836	1.70572276285648	2.41805980235054	0.0213612167068401	0.471155989743478	-3.33319971887345
a_HQ_transcript/102484	2.92783136955418	0.0739562795824906	2.41794244503623	0.0213670318361176	0.471155989743478	-3.33986848638267
a_HQ_transcript/150718	-2.80950971994644	1.74675949758752	-2.41779822167056	0.0213741801705469	0.471155989743478	-3.33503039765375
a_HQ_transcript/45617	3.57943334115504	1.2573166094183	2.41755292190606	0.0213863432933296	0.471183939236455	-3.35837006556456
a_HQ_transcript/138098	-2.89915195349773	3.78833724886754	-2.41744573605187	0.0213916600544489	0.471183939236455	-3.68213990567359
a_HQ_transcript/104408	-2.94262727716532	0.582439012804069	-2.416960458283	0.021415746454051	0.471286059905151	-3.29194983017956
a_HQ_transcript/149770	2.44286503978624	0.140818727364926	2.41690491121155	0.0214185050667566	0.471286059905151	-3.34470187484231
a_HQ_transcript/131045	1.44364790279028	-0.776601527365985	2.41686233967636	0.0214206194995425	0.471286059905151	-3.34043030209237
a_HQ_transcript/121891	0.751787093445998	2.99718633326458	2.41575864238031	0.0214755040849461	0.471342747384958	-3.45353113820575
a_HQ_transcript/46446	1.74811498193878	1.74099782460607	2.41574278207486	0.0214762937179433	0.471342747384958	-3.30166803952537

a_HQ_transcript/46331	-2.89471645172959	-0.419608688328112	-2.41559660553711	0.0214835726173128	0.471342747384958	-3.36375581991465
a_HQ_transcript/91919	1.42150948798441	-0.836918722303201	2.41558040662076	0.0214843793849345	0.471342747384958	-3.35359736060991
a_HQ_transcript/138739	-2.96498770326991	4.48907482281911	-2.4154691360487	0.021489921828343	0.471342747384958	-3.72565968149593
a_HQ_transcript/137885	0.942959326657666	3.20894059718123	2.41546275503379	0.0214902397094014	0.471342747384958	-3.52888683550535
a_HQ_transcript/33955	4.42588108325309	2.98093358622203	2.41543531292768	0.0214916068331851	0.471342747384958	-3.36212127366099
a_HQ_transcript/140558	1.36087219625499	2.96589835647059	2.41530823801923	0.0214979385437357	0.471342747384958	-3.53858267856243
a_HQ_transcript/64058	2.29227959703088	1.63700327865481	2.41530587267054	0.0214980564171237	0.471342747384958	-3.29921214755935
a_HQ_transcript/65640	5.00093865993218	4.03817164493704	2.41501699311228	0.0215124566973285	0.471342747384958	-3.576272311182
a_HQ_transcript/118518	1.25303882189729	0.314340185239928	2.41498246727616	0.021514178354199	0.471342747384958	-3.31428957093267
a_HQ_transcript/132305	2.41398150117136	8.68382316203961	2.41485570606164	0.0215205004741205	0.471342747384958	-3.99883449781821
a_HQ_transcript/92814	2.37722225359912	0.756460670002641	2.414506285045	0.0215379363457441	0.471427269097612	-3.3098148895322
a_HQ_transcript/87705	-1.89580238137363	1.5347297164701	-2.41445333036683	0.0215405798706864	0.471427269097612	-3.26642345222169
a_HQ_transcript/30127	0.684351891573372	2.94347193320591	2.41382495761982	0.0215719711302524	0.471431159753208	-3.47812369411884
a_HQ_transcript/29740	4.18638131145437	1.57832609147501	2.41371986897559	0.0215772250486052	0.471431159753208	-3.43431848101972
a_HQ_transcript/16911	3.51403064890687	1.04639510797581	2.41367695587121	0.0215793708290825	0.471431159753208	-3.32168463822032
a_HQ_transcript/60388	-2.15877226563878	2.04650051354734	-2.41351869844237	0.0215872858424955	0.471431159753208	-3.31221994856215
a_HQ_transcript/147117	-3.26834353647838	0.641990392980446	-2.41344739096783	0.0215908530453264	0.471431159753208	-3.34375278694908
a_HQ_transcript/30129	2.34395263763812	0.0135991302543586	2.41323610806473	0.0216014257621869	0.471431159753208	-3.28747930363806
a_HQ_transcript/91194	-4.79745315290225	1.77713716381877	-2.41315916210223	0.0216052773531002	0.471431159753208	-3.45195818947346
a_HQ_transcript/120613	-5.70525016308406	2.49144308360528	-2.41315192900431	0.0216056394436854	0.471431159753208	-3.50482609601748
a_HQ_transcript/127789	1.04038038762208	3.83728115387433	2.41250228525816	0.0216381833103716	0.471669942313909	-3.57328077974621
a_HQ_transcript/38057	2.4166351739973	2.18168052516723	2.41242355188656	0.0216421304856354	0.471669942313909	-3.34535562656288
a_HQ_transcript/49950	-2.86112473843706	-0.469724617042042	-2.41239683506866	0.0216434700407868	0.471669942313909	-3.36642987458735
a_HQ_transcript/146691	-4.1869004418468	3.09286752858893	-2.41228575783433	0.021649040154375	0.471669942313909	-3.55065774073829
a_HQ_transcript/1881	0.892581603883748	4.01636186019015	2.41187934908823	0.0216694311814781	0.471847330132208	-3.61497562903977
a_HQ_transcript/91680	2.82603381087993	0.153051232568586	2.4117999534576	0.0216734167948836	0.471847330132208	-3.34559028776559
a_HQ_transcript/29112	2.40357311572797	-0.482284915200681	2.41127472278095	0.0216997998812003	0.472244839945492	-3.36250275449838
a_HQ_transcript/93870	5.41259568315739	2.32363391561787	2.41092179524445	0.0217175443586757	0.472328180479629	-3.54741528465173
a_HQ_transcript/33058	-2.19993692635528	-0.531930325295441	-2.41087534593752	0.0217198807187938	0.472328180479629	-3.35636901892178
a_HQ_transcript/1178	1.61472723508544	2.1946536984117	2.41061394291023	0.021733033334433	0.472437457615528	-3.31594530291803
a_HQ_transcript/38020	3.37430721684632	1.23850206549858	2.41024398886509	0.0217516601399368	0.472665607706261	-3.43409911669941
a_HQ_transcript/58782	1.66047149197602	2.42635696138933	2.40965490815056	0.0217813497490992	0.473133893204648	-3.38433407702822
a_HQ_transcript/37639	2.23060647122124	0.497152489519489	2.40943101020909	0.0217926438562223	0.47320239151083	-3.31187118257339
a_HQ_transcript/28331	2.08771500862105	1.9217127621914	2.4091433713602	0.0218071610770667	0.473292918504905	-3.35495196135816
a_HQ_transcript/101100	-3.0811191925056	-0.227030586959791	-2.40902580044227	0.0218130974517382	0.473292918504905	-3.36862847274697
a_HQ_transcript/49643	-3.51100323576333	0.909402645785378	-2.40839462571369	0.0218449917963735	0.473808090723732	-3.28898277570597
a_HQ_transcript/130272	-1.98200680367767	3.74452544385241	-2.4082267716572	0.0218534808939742	0.473815418651598	-3.64346934585371
a_HQ_transcript/121003	2.59904133833552	0.853694969297724	2.40799524057936	0.021865195339066	0.473835265865119	-3.28257467378435
a_HQ_transcript/138800	0.719360478007577	4.71085416971305	2.4077696789143	0.0218766132552531	0.473835265865119	-3.70661178651101
a_HQ_transcript/56438	-3.01621278988752	1.43820050843166	-2.40772548378207	0.0218788510456575	0.473835265865119	-3.38460280120087
a_HQ_transcript/61472	3.09510079256758	0.013194157215661	2.40679013874332	0.0219262605022599	0.474685165912613	-3.35508824726593
a_HQ_transcript/92887	3.67771994926219	2.48753609596525	2.40636684358321	0.021947746612685	0.474973423344062	-3.39940625707812
a_HQ_transcript/41381	-1.11398052634388	3.59988013394482	-2.4059382466578	0.0219695213603106	0.475165002399211	-3.60477059866774
a_HQ_transcript/56817	1.69157999375981	1.62187484020721	2.40587083287505	0.0219729480878248	0.475165002399211	-3.33957492463152
a_HQ_transcript/41501	-1.33758979397193	5.86112081719108	-2.40539327600778	0.0219972368376392	0.475513344328111	-3.82883371661176
a_HQ_transcript/3446	-1.90791026613737	3.31908983829065	-2.40522909554348	0.0220055927702473	0.475517136263544	-3.46577107842114
a_HQ_transcript/140053	1.76783418283835	-0.724314534873195	2.40472923014871	0.0220310511114758	0.475616942708991	-3.36976191433856
a_HQ_transcript/142692	-2.47116702313165	0.959382926677772	-2.40450051810791	0.0220427084411891	0.475616942708991	-3.32033347752266
a_HQ_transcript/88955	0.831210153147851	5.33291513232428	2.40447679680448	0.0220439178245119	0.475616942708991	-3.77204754078038
a_HQ_transcript/99847	2.27808895964719	1.90587771179946	2.40439776795043	0.0220479473886641	0.475616942708991	-3.43479995496202

a_HQ_transcript/102325	3.48440245689211	0.248207051527542	2.40429349137273	0.0220532653226106	0.475616942708991	-3.39603746447591
a_HQ_transcript/145558	4.65843238686838	0.944023026730841	2.40417509143453	0.0220593049398472	0.475616942708991	-3.41714458478309
a_HQ_transcript/98134	3.52205608724187	0.199166671784206	2.40353794165902	0.022091831998193	0.475994822221452	-3.37042728594501
a_HQ_transcript/133724	-2.24093215760068	1.66271246081607	-2.40351099491633	0.022093208614669	0.475994822221452	-3.44347844396363
a_HQ_transcript/22299	-2.82664768178569	1.27100338906749	-2.40283747847274	0.0221276416231921	0.476369231140271	-3.35827307586858
a_HQ_transcript/128830	0.719571021162302	3.16460983971467	2.40268046271215	0.0221356759486354	0.476369231140271	-3.52511344078382
a_HQ_transcript/52212	-1.96917466980751	1.79305920824162	-2.40253728967421	0.0221430042706505	0.476369231140271	-3.34258840412363
a_HQ_transcript/128398	-3.31087122654992	3.26368237146973	-2.402200240608	0.0221602648672106	0.476369231140271	-3.52546232100151
a_HQ_transcript/146674	2.07200940793875	0.317521968374333	2.40194750412801	0.0221732157684699	0.476369231140271	-3.35456400769831
a_HQ_transcript/138445	1.06991549419046	3.30375952218101	2.40167995182163	0.0221869333764206	0.476369231140271	-3.58293222593246
a_HQ_transcript/104993	0.722850347466191	4.26662048534874	2.40156373377451	0.0221928943681834	0.476369231140271	-3.67313510843924
a_HQ_transcript/136110	-2.45697600801634	0.0140849919280341	-2.40089558454099	0.0222271929873663	0.476369231140271	-3.40245273886978
a_HQ_transcript/133406	2.99802501331175	0.536770036920525	2.40076489136103	0.0222339075976142	0.476369231140271	-3.35314136521296
a_HQ_transcript/106449	2.13435145192051	-0.215434218446499	2.40060867279112	0.0222419360444491	0.476369231140271	-3.30642711522454
a_HQ_transcript/3700	1.95375957069085	3.19049729116483	2.4005897271863	0.0222429098838045	0.476369231140271	-3.45824495494859
a_HQ_transcript/80482	1.51702977542869	-0.653368547941772	2.40053973572266	0.0222454797243218	0.476369231140271	-3.36914788577187
a_HQ_transcript/1712	2.25296328375455	2.27908189271751	2.40052529734231	0.0222462219880059	0.476369231140271	-3.39687049549774
a_HQ_transcript/127666	3.03900895297124	8.37105782433989	2.4004929905595	0.0222478829312299	0.476369231140271	-4.0772330082999
a_HQ_transcript/40743	-2.44685973131998	1.07438140287916	-2.40034335278338	0.0222555775194647	0.476369231140271	-3.3534907423457
a_HQ_transcript/3609	0.956110171047863	4.23268442740676	2.40030345447228	0.022257629556409	0.476369231140271	-3.64260115656936
a_HQ_transcript/72322	2.5770997566746	0.340138019620181	2.40019111291299	0.0222634083955959	0.476369231140271	-3.36192052960427
a_HQ_transcript/144370	-1.4982910343897	6.04793452237314	-2.40017633233196	0.0222641688088841	0.476369231140271	-3.75482846638043
a_HQ_transcript/115035	2.86423175671419	-0.675120919411784	2.40013500504109	0.0222662950902855	0.476369231140271	-3.38645313256837
a_HQ_transcript/59819	8.61200516826213	4.85487900313877	2.39964323244508	0.022291610880389	0.476642484731244	-3.63271040842273
a_HQ_transcript/127984	-2.69848909769432	2.35052042095976	-2.399455155411	0.0223012997523437	0.476642484731244	-3.46222682587298
a_HQ_transcript/4607	-1.00435137143476	2.82360024909609	-2.39916147078609	0.022316436707807	0.476642484731244	-3.43450819883647
a_HQ_transcript/38234	-2.00086307568904	2.07262004935169	-2.39913499866582	0.0223178015805179	0.476642484731244	-3.42417351974922
a_HQ_transcript/118996	1.31008460337086	7.69020826470351	2.39909106713408	0.022320066808396	0.476642484731244	-3.97893179846431
a_HQ_transcript/134476	4.11091659459101	1.49429585787687	2.39871272828331	0.0223395836347095	0.476732403511341	-3.42880874349585
a_HQ_transcript/16831	-2.24012969458281	1.79384928265678	-2.39860963310492	0.0223449045492176	0.476732403511341	-3.41635965423614
a_HQ_transcript/76914	5.7435812844222	0.909604598363383	2.39853258718274	0.0223488817707198	0.476732403511341	-3.48925422855554
a_HQ_transcript/99522	-5.48827424843499	0.456596160261717	-2.39781071038465	0.0223861773833557	0.477184362096028	-3.3949507665093
a_HQ_transcript/52407	4.23900843207083	1.21293296680322	2.39757433920035	0.0223984017481815	0.477184362096028	-3.46310190248111
a_HQ_transcript/105325	2.66013885153633	1.04010629059917	2.397491557598	0.0224026843843125	0.477184362096028	-3.37017623781908
a_HQ_transcript/142371	-2.12375432051712	-0.115914068144346	-2.39747124174942	0.0224037355213546	0.477184362096028	-3.47526078267654
a_HQ_transcript/120483	4.00996862593749	0.790299979733402	2.39721153860354	0.0224171764510859	0.477184362096028	-3.42388512018077
a_HQ_transcript/133661	-4.1211614337461	-0.122402217444768	-2.3971700480765	0.0224193244715843	0.477184362096028	-3.46174412836084
a_HQ_transcript/136228	-2.9847566364474	1.60029034025264	-2.39636523162995	0.0224610279542669	0.477636633538114	-3.3490185418072
a_HQ_transcript/104943	2.35787374509013	1.9049030584254	2.3960451318868	0.0224776342866728	0.477636633538114	-3.35593260462436
a_HQ_transcript/126107	-0.855865472561477	3.95422155852367	-2.39588815754803	0.0224857819781654	0.477636633538114	-3.64935786958103
a_HQ_transcript/77318	2.06328563899136	-0.440158974482778	2.39588473098598	0.0224859598624752	0.477636633538114	-3.38401996473003
a_HQ_transcript/88641	-3.26087350424509	1.36791976480677	-2.39559820052643	0.0225008391466221	0.477636633538114	-3.36555543132386
a_HQ_transcript/137406	-4.97322428920805	1.3346955759343	-2.39552846033311	0.0225044620504114	0.477636633538114	-3.43821350536716
a_HQ_transcript/14444	2.25737864865048	0.811861983813882	2.39548866368575	0.0225065296673198	0.477636633538114	-3.29829366768155
a_HQ_transcript/61248	1.62960018797983	0.795484310588961	2.39529300543872	0.0225166975161984	0.477636633538114	-3.38195928134703
a_HQ_transcript/54403	2.00946201696144	0.771510391994146	2.3952608149411	0.0225183707725233	0.477636633538114	-3.33682999425503
a_HQ_transcript/34959	2.66071518393117	0.536311399038827	2.3951767088134	0.0225227431277176	0.477636633538114	-3.36295022347703
a_HQ_transcript/114238	-1.45721508129399	2.18845753829035	-2.39463998127018	0.0225506637233162	0.478030861841508	-3.39443030038775
a_HQ_transcript/114228	2.24288591348774	1.02192466662778	2.3944055977467	0.0225628662368964	0.478030861841508	-3.32337429582003
a_HQ_transcript/111178	-4.80806403958738	0.446817399406371	-2.39412597438738	0.0225774318883339	0.478030861841508	-3.42002085206919

a_HQ_transcript/58235	-2.2354589118781	-0.16056666116243	-2.39406425456672	0.0225806480410326	0.478030861841508	-3.35488937822767
a_HQ_transcript/75879	0.77853735497165	3.80325082693569	2.39389876593341	0.0225892735296758	0.478030861841508	-3.64316788573631
a_HQ_transcript/134740	-1.70269323321821	0.879023481761993	-2.39371683615436	0.0225987594097989	0.478030861841508	-3.31753579908775
a_HQ_transcript/29535	-2.13969460798183	0.388663838116577	-2.39356011192233	0.0226069339679885	0.478030861841508	-3.38246369367685
a_HQ_transcript/127306	-4.33847871647222	-0.326706226869007	-2.39348708291617	0.0226107439963254	0.478030861841508	-3.41869844610267
a_HQ_transcript/3678	2.04586027396876	-0.134393886820298	2.39322825209297	0.0226242522747958	0.478030861841508	-3.30495777494174
a_HQ_transcript/89498	2.8133442781586	0.393433536919549	2.39321984751284	0.0226246910294876	0.478030861841508	-3.40198011366063
a_HQ_transcript/60381	2.90662488878289	0.957264369780682	2.39293908095933	0.0226393526798785	0.478030861841508	-3.41099823217109
a_HQ_transcript/114099	4.11842469114217	0.0202312631879612	2.39286323303221	0.0226433149460643	0.478030861841508	-3.39350105639115
a_HQ_transcript/113013	-4.20655207566588	1.98022440622955	-2.39268950747805	0.0226523926773365	0.478030861841508	-3.34331727174279
a_HQ_transcript/14063	-1.82512341304692	0.298455219304335	-2.39253280236993	0.0226605838725698	0.478030861841508	-3.37285632985759
a_HQ_transcript/119606	-2.62373523903437	0.768380517574551	-2.39242551759804	0.0226661933505108	0.478030861841508	-3.40463661203856
a_HQ_transcript/68126	-2.8653667195776	0.10104954283512	-2.39229703212666	0.0226729129868056	0.478030861841508	-3.36876130542963
a_HQ_transcript/16075	2.16467382518544	1.74896936423603	2.39187663203745	0.022694912067502	0.478260517755361	-3.3577082571582
a_HQ_transcript/132920	0.558121107701208	4.69810968423242	2.39175442774309	0.0227013105249597	0.478260517755361	-3.75324460663718
a_HQ_transcript/98963	1.82960455995238	0.786051238806178	2.39161736957278	0.0227084886630333	0.478260517755361	-3.2894621411464
a_HQ_transcript/11773	-1.64661662621083	2.8804160040814	-2.3913645419619	0.0227217354000151	0.478349945047772	-3.41460205980985
a_HQ_transcript/19817	-2.86072622406685	-0.335869423967888	-2.39122225918749	0.0227291933013685	0.478349945047772	-3.41402914075776
a_HQ_transcript/15316	-1.99136703683688	0.983052802702731	-2.39076893899076	0.0227529693980803	0.478677019606085	-3.32660161192811
a_HQ_transcript/151757	-2.51902051867519	-0.546682475603371	-2.38973434959825	0.0228073170626896	0.479369159144832	-3.40622945762788
a_HQ_transcript/44284	2.3455071329943	0.816419112162716	2.38968467048068	0.0228099297061414	0.479369159144832	-3.41406759163682
a_HQ_transcript/75807	1.46621312910375	-0.688529150573	2.38965897187399	0.0228112813122736	0.479369159144832	-3.39761940789303
a_HQ_transcript/98786	-1.70021551825252	0.245788158900032	-2.38926623942272	0.0228319459497569	0.479369159144832	-3.33600564199456
a_HQ_transcript/53847	-1.11068365890211	1.57815876304481	-2.38908719155132	0.0228413726639269	0.479369159144832	-3.33408323350457
a_HQ_transcript/139963	3.03823898465613	-0.167281787442789	2.38901823257118	0.0228450042375296	0.479369159144832	-3.42748923073832
a_HQ_transcript/150694	-2.42927380448269	-0.45556957778694	-2.3889399666674	0.0228491265756707	0.479369159144832	-3.38859101770026
a_HQ_transcript/132493	1.20365464369539	2.07743777312935	2.38888839688832	0.0228518431735193	0.479369159144832	-3.38359192383937
a_HQ_transcript/5482	2.0053053101729	-0.188456161106188	2.38816609968751	0.0228899232775719	0.479855838516473	-3.35039295614825
a_HQ_transcript/149857	-2.93768568413772	0.440440883945696	-2.38797920246518	0.0228997860407078	0.479855838516473	-3.37168923215623
a_HQ_transcript/94186	3.19278081459235	-0.230790547278423	2.38797876796821	0.0228998089740692	0.479855838516473	-3.41247466465007
a_HQ_transcript/98176	2.38807938496653	0.259840749673893	2.38700539532464	0.0229512373819482	0.480760189743382	-3.40324377254243
a_HQ_transcript/113620	1.00781224488436	4.07440655946938	2.38673609691022	0.0229654843610873	0.480885329589799	-3.68872834809827
a_HQ_transcript/142664	-4.76557247097847	1.55445846310575	-2.38653868098548	0.0229759335693537	0.480930884594667	-3.46249804852909
a_HQ_transcript/78894	-2.78118985582023	-0.0161457503307691	-2.38616604578121	0.0229956688938522	0.481057809802282	-3.46413280385495
a_HQ_transcript/86389	-2.91277219831291	0.210538989259816	-2.3861099186549	0.022998642805862	0.481057809802282	-3.40697544877994
a_HQ_transcript/143525	3.47482275437219	0.162189321416026	2.38585705924327	0.0230120449690323	0.481057809802282	-3.45219108455181
a_HQ_transcript/122417	-1.95481561598059	0.584906377550206	-2.38579942675546	0.0230151006237983	0.481057809802282	-3.33811534586997
a_HQ_transcript/76589	-1.47342987777977	2.74783026480027	-2.38520754157029	0.0230465035161076	0.48154103392678	-3.46791183712334
a_HQ_transcript/148977	2.00775711862542	-0.298946869681516	2.38503573871503	0.023055625927085	0.48155854254028	-3.43652638965408
a_HQ_transcript/3493	1.74381634722964	0.523884286490679	2.38466349472747	0.0230754026442682	0.48179849314153	-3.31251973648592
a_HQ_transcript/127299	-1.33909707786569	3.30019777251091	-2.38444978484859	0.0230867636655373	0.481862620592586	-3.56487113979694
a_HQ_transcript/138431	-1.03898748388108	3.65763653605831	-2.38416510495709	0.0231019054140778	0.482005584317844	-3.59371477427499
a_HQ_transcript/80156	-2.31695093931976	-0.328843722105017	-2.38299256942087	0.023164366190884	0.483135370629244	-3.40907142633362
a_HQ_transcript/2241	2.37236300797809	1.93149092216366	2.38256308607116	0.0231872830598846	0.48343988152976	-3.3754698548557
a_HQ_transcript/85302	0.606204382327314	3.20167359924056	2.38222706157172	0.0232052274017428	0.48360840208856	-3.56764354195626
a_HQ_transcript/2375	2.14704384578769	1.46085334197296	2.38210020125704	0.0232120052612697	0.48360840208856	-3.35028237054117
a_HQ_transcript/149805	3.36204195462841	2.50483594872753	2.3814768176398	0.0232453373740024	0.483852361222872	-3.5321572366796
a_HQ_transcript/55720	-2.39923132336837	1.27727275733974	-2.38142424768058	0.0232481502588886	0.483852361222872	-3.38291316178747
a_HQ_transcript/4159	2.22765298809262	-0.466486528121899	2.3814142278134	0.0232486864315903	0.483852361222872	-3.34873208292772
a_HQ_transcript/89551	0.852570701002051	3.11154783381167	2.38118927400589	0.0232607268826707	0.483929682260516	-3.56772361434132

a_HQ_transcript/79057	1.8161696974214	1.25575455558913	2.38100909722798	0.0232703747680207	0.483957189450987	-3.40395345228486
a_HQ_transcript/122302	-1.83472052711635	0.440327983222764	-2.38012315052632	0.0233178672515203	0.484547862861923	-3.32848473085132
a_HQ_transcript/149034	-1.90417059063973	2.37191463118093	-2.38005487628369	0.0233215308441925	0.484547862861923	-3.40847606761146
a_HQ_transcript/10194	1.88844455646614	0.747525810574083	2.37974446010408	0.0233381943544159	0.484547862861923	-3.34052093066408
a_HQ_transcript/10416	3.35162107778794	0.444856110100048	2.37972823740828	0.0233390655052936	0.484547862861923	-3.38191087790554
a_HQ_transcript/35087	1.75517104182962	-0.604611057411235	2.37967953763934	0.0233416808366058	0.484547862861923	-3.43723837953936
a_HQ_transcript/124139	-2.59626340820597	0.220757612240221	-2.37954715217875	0.0233487916989445	0.484547862861923	-3.3231985561742
a_HQ_transcript/125696	2.48528764265893	0.893892585267514	2.37895793547711	0.0233804643526755	0.485031988541157	-3.38523772869366
a_HQ_transcript/118207	4.62516424336052	1.47198447833359	2.37868786331379	0.0233949948016964	0.485160277500182	-3.43438665124739
a_HQ_transcript/115278	0.782131838865327	2.80094909074636	2.37839131287837	0.0234109592827852	0.485318202992061	-3.52086656774407
a_HQ_transcript/90475	3.91499266420931	0.212024813621886	2.37785226757366	0.0234400035564594	0.485747068352896	-3.46844398420219
a_HQ_transcript/9188	-3.71922050632329	1.30692807094721	-2.37735541299404	0.0234668035279192	0.48609360120604	-3.54388309752474
a_HQ_transcript/85607	-2.07177711714695	-0.491123540530764	-2.37723225953964	0.0234734506362743	0.48609360120604	-3.42002799043271
a_HQ_transcript/144307	3.19603319985017	0.349357718222562	2.37676785894863	0.0234985316646223	0.486406715960713	-3.48660221407625
a_HQ_transcript/121097	2.11832270173269	0.0113623994004295	2.37661243794322	0.0235069309682919	0.486406715960713	-3.39912729077861
a_HQ_transcript/151922	0.848923241436791	6.99949695676221	2.37648303605682	0.0235139262192016	0.486406715960713	-3.97007604998867
a_HQ_transcript/952	0.867451779229905	4.58923099232078	2.3763329430612	0.0235220423645328	0.486406715960713	-3.81910452615001
a_HQ_transcript/48500	3.07055886332336	0.265292440603483	2.37584585452633	0.0235483987674064	0.486778564563229	-3.4399622682633
a_HQ_transcript/43082	2.27983084369237	-0.216920690320758	2.3755415263306	0.0235648795986506	0.486946079385126	-3.42400659576282
a_HQ_transcript/32884	-2.04742430850979	0.198837214531462	-2.37488223522951	0.0236006192944025	0.487511300051539	-3.42318854197012
a_HQ_transcript/25968	2.31203973534497	1.06363528391486	2.37437435933881	0.0236281844263293	0.487589683682806	-3.41028925156096
a_HQ_transcript/40952	-1.84539225771417	-0.432813277790293	-2.37433204566672	0.0236304823332913	0.487589683682806	-3.39274004027888
a_HQ_transcript/152867	-2.5911319970413	-0.514552976101572	-2.37427486617216	0.0236335878731676	0.487589683682806	-3.43742028274059
a_HQ_transcript/67436	-2.50987435826534	1.40255839808295	-2.37419425429607	0.0236379667048264	0.487589683682806	-3.34028811252341
a_HQ_transcript/23636	1.16198723752803	2.35394155965285	2.37394530658507	0.0236514941771469	0.487695655739338	-3.40188130258668
a_HQ_transcript/104273	-3.15219451942055	2.25034716694742	-2.37365587753091	0.0236672301832387	0.487847076628121	-3.46824757654096
a_HQ_transcript/62204	2.88759157924598	-0.338545804352328	2.37334189695382	0.0236843117806052	0.488026116690187	-3.43793409266629
a_HQ_transcript/136291	2.80377088329195	0.944695117227876	2.37288290572667	0.0237093025807778	0.488367944867275	-3.5099805635557
a_HQ_transcript/140444	-2.20954782974172	-0.438711098521835	-2.37245938495502	0.0237323833417216	0.488670201518808	-3.42403550284902
a_HQ_transcript/92359	-7.67515146038626	2.00021934576969	-2.37205437862827	0.0237544741879605	0.488951868129521	-3.36978561930214
a_HQ_transcript/93410	3.7088740542265	0.482663543511584	2.37169015940553	0.0237743562604675	0.489087903516119	-3.43853985258108
a_HQ_transcript/44871	-1.81222942973247	-0.511377712982897	-2.37162506785768	0.0237779110813472	0.489087903516119	-3.45030370388028
a_HQ_transcript/25007	-2.53200303006628	2.80947254742153	-2.37138962047187	0.023790773511852	0.489179371311261	-3.59200334316911
a_HQ_transcript/111749	-4.02367189390275	-0.12901935670224	-2.3710504975811	0.0238093108061848	0.489387418154848	-3.50204106970203
a_HQ_transcript/150779	3.28359586800588	-0.0205883520072698	2.37088338700427	0.0238184503030948	0.489402219589357	-3.4778682739424
a_HQ_transcript/121684	3.78859816306512	3.7992646596411	2.37056903876769	0.0238356510487553	0.48956803838821	-3.59942623607812
a_HQ_transcript/133066	1.79639544940272	-0.129588673781847	2.37012318017697	0.0238600672108154	0.48956803838821	-3.49955937974955
a_HQ_transcript/121799	1.21231024104513	2.11744173719818	2.3699550970268	0.0238692776898578	0.48956803838821	-3.37114680804084
a_HQ_transcript/88857	0.523377258256682	4.67959995697953	2.36992094846118	0.0238711493284988	0.48956803838821	-3.79368998825069
a_HQ_transcript/31198	1.55191979305868	2.02549519018928	2.36975183793399	0.0238804200202801	0.48956803838821	-3.4027424809167
a_HQ_transcript/80684	2.13394372962791	4.41611891403234	2.3696896053184	0.0238838324539247	0.48956803838821	-3.76208712291614
a_HQ_transcript/55418	2.13303931575336	2.97849578910493	2.36957051615566	0.0238903637659745	0.48956803838821	-3.5206571614683
a_HQ_transcript/32324	-4.18643438942813	2.16418459026176	-2.36950607986744	0.0238938983778446	0.48956803838821	-3.50265537496185
a_HQ_transcript/139988	2.10214638333972	-0.729096602503011	2.36904629075776	0.023919133595359	0.489912402266043	-3.43090755124047
a_HQ_transcript/4453	-2.09942526590803	2.5763783349412	-2.36859934939259	0.023943686849136	0.490042329583698	-3.50425488948898
a_HQ_transcript/64913	-2.41832319489466	1.53596929064485	-2.36849313908702	0.0239495250000911	0.490042329583698	-3.4916477205383
a_HQ_transcript/99956	3.28300357264317	0.665521691371788	2.36843580657951	0.023952676980861	0.490042329583698	-3.40929358997006
a_HQ_transcript/123897	3.80679847508808	0.354251910906369	2.36825946921047	0.023962373874992	0.490042329583698	-3.44257253094945
a_HQ_transcript/81402	-2.32950561355706	-0.497140689006551	-2.36790808707083	0.0239817072032173	0.490042329583698	-3.43665947217153
a_HQ_transcript/95647	-1.48538660958689	0.80433313791122	-2.36784020128737	0.0239854439644556	0.490042329583698	-3.38448448089764

a_HQ_transcript/111050	3.61407270017014	-0.279865535998191	2.36773038069462	0.023991490138567	0.490042329583698	-3.45748778861206
a_HQ_transcript/130422	-2.34212487515016	0.262401148162928	-2.36770440779043	0.0239929202793009	0.490042329583698	-3.37234173607671
a_HQ_transcript/30698	-0.605119796104019	3.91203353672289	-2.36713904754812	0.0240240697665058	0.490506191565665	-3.67032635835048
a_HQ_transcript/16161	1.58262997784375	2.82022986306648	2.36695409463823	0.0240342680242229	0.490542110853944	-3.4337015803704
a_HQ_transcript/98869	4.20894566520809	1.80327594982469	2.36597265622603	0.0240884500385473	0.491295963967783	-3.49356716648068
a_HQ_transcript/72974	-3.65049811928918	0.783687025197991	-2.36593095475429	0.024090754692927	0.491295963967783	-3.40482852637957
a_HQ_transcript/128061	2.82494349903225	3.2406512026885	2.36569198408666	0.0241039653937158	0.491295963967783	-3.57838971682574
a_HQ_transcript/133307	1.95753093641004	-0.253253112573559	2.36567307194902	0.0241050111690772	0.491295963967783	-3.37632159822627
a_HQ_transcript/91604	-1.38457004385145	4.00754014393216	-2.36542171208888	0.0241189144033082	0.491303725639245	-3.6584483737475
a_HQ_transcript/11625	-1.58240796352965	1.11898559740415	-2.36536059182721	0.024122296190724	0.491303725639245	-3.34038344640322
a_HQ_transcript/146662	-6.45171319414097	1.06600648894558	-2.36487002159096	0.0241494550526264	0.491672413699166	-3.54061690114186
a_HQ_transcript/137310	-3.37242599083633	4.07287165812792	-2.36472813857569	0.0241573151239475	0.491672413699166	-3.87562256324495
a_HQ_transcript/147216	2.16738495231502	0.127319851133412	2.36443496959768	0.0241735635246482	0.491768044451687	-3.40018866868501
a_HQ_transcript/141100	-4.47391969351503	0.0425955442364651	-2.36417057115094	0.0241882258670434	0.491768044451687	-3.47192890437674
a_HQ_transcript/34497	-2.61650082234928	0.243725220395074	-2.36407644541951	0.0241934476011099	0.491768044451687	-3.37799597645209
a_HQ_transcript/103877	1.97465364960959	2.93606529390524	2.36393283774671	0.0242014163731352	0.491768044451687	-3.45278815242493
a_HQ_transcript/152285	2.41998052584867	0.63131629950459	2.36388062595631	0.0242043141889671	0.491768044451687	-3.39396248454216
a_HQ_transcript/135561	3.60566436542663	0.863202774569922	2.36342497538801	0.0242296166916058	0.492069318146995	-3.41916027420628
a_HQ_transcript/148286	-2.55226166087114	-0.237252530447676	-2.3631935590856	0.0242424765373279	0.492069318146995	-3.46991762878264
a_HQ_transcript/37764	-2.0117429738043	1.68326609412666	-2.36293439302929	0.0242568857894474	0.492069318146995	-3.3905913532824
a_HQ_transcript/85897	-2.36962463512546	0.739491722454655	-2.36288749818604	0.0242594939035688	0.492069318146995	-3.3684503256457
a_HQ_transcript/22000	-2.78098138107114	0.9545117141845	-2.36262665459962	0.0242740056809238	0.492069318146995	-3.53399640023811
a_HQ_transcript/91726	2.72578816021946	0.433331449143595	2.36251994578073	0.0242799445901408	0.492069318146995	-3.46374592869417
a_HQ_transcript/83305	2.93356849898986	1.3878772127784	2.36248355065988	0.0242819704721213	0.492069318146995	-3.45928007469652
a_HQ_transcript/85542	2.99948817306874	4.26666243333708	2.36239563608457	0.0242868647427011	0.492069318146995	-3.72939671698847
a_HQ_transcript/134943	3.46073928543929	3.20839781786532	2.36146954622203	0.0243384752339447	0.49250789773612	-3.65048996921521
a_HQ_transcript/146118	-2.55442204293335	-0.545694469251741	-2.36127256466319	0.0243494657286423	0.49250789773612	-3.45080152689246
a_HQ_transcript/110390	2.51993817513398	0.180814324505127	2.36117726482647	0.0243547845539164	0.49250789773612	-3.36051722887413
a_HQ_transcript/74143	4.69286503188288	0.770786801771874	2.36109021695711	0.0243596437452269	0.49250789773612	-3.52570440661993
a_HQ_transcript/74281	-2.02433994482343	2.21928107210851	-2.36084089316179	0.0243735663780191	0.49250789773612	-3.41183338708355
a_HQ_transcript/90361	-1.78894727399883	-0.106465390522652	-2.360371459081	0.0243997998982027	0.49250789773612	-3.45991763946869
a_HQ_transcript/120439	1.43470752487286	1.1318086118932	2.36031700159698	0.0244028448180978	0.49250789773612	-3.39157283050503
a_HQ_transcript/119361	3.11324372185319	0.513459422380718	2.36026488081402	0.0244057594070319	0.49250789773612	-3.4175798869575
a_HQ_transcript/2833	2.54538995987354	0.632427496604013	2.36022998044486	0.0244077112085851	0.49250789773612	-3.41734469912762
a_HQ_transcript/67579	-2.57205107157836	0.276852491903591	-2.36017295398829	0.0244109007151178	0.49250789773612	-3.45548806050694
a_HQ_transcript/149740	-4.0766629630107	0.180534298690711	-2.36012057201937	0.0244138307870614	0.49250789773612	-3.58695260691419
a_HQ_transcript/133368	1.27109278180625	-0.73980571874932	2.36004916971821	0.0244178253062748	0.49250789773612	-3.48233195778698
a_HQ_transcript/110496	1.33693970155703	2.1935443261415	2.35988374558438	0.0244270820450673	0.49250789773612	-3.42554727375912
a_HQ_transcript/99606	-2.52269351061626	-0.514657464104425	-2.35961423990278	0.0244421697525089	0.49250789773612	-3.42092361966143
a_HQ_transcript/88622	-1.9272279341829	0.0219502891583466	-2.3595326366975	0.0244467398020691	0.49250789773612	-3.49656319183927
a_HQ_transcript/107433	-2.84754556152553	1.00155186148242	-2.3594748656874	0.0244499756381651	0.49250789773612	-3.51034034586291
a_HQ_transcript/25283	1.97215302191865	0.723422363128544	2.35942891955891	0.0244525494231084	0.49250789773612	-3.3685008309235
a_HQ_transcript/108088	2.29480119891516	0.440143358068668	2.35893513105938	0.024480225704789	0.492876787313185	-3.40225297443754
a_HQ_transcript/134658	0.983441020589385	2.73949873033606	2.35858859212947	0.0244996657711788	0.492876787313185	-3.53254640572903
a_HQ_transcript/125931	-2.50646174803662	1.66425034753675	-2.35832019312678	0.0245147319767227	0.492876787313185	-3.49849418394495
a_HQ_transcript/131450	-3.69958364263463	-0.46607779916297	-2.35824956295594	0.0245186980990242	0.492876787313185	-3.46052315340538
a_HQ_transcript/40482	2.61778745933507	1.33911619806352	2.35810077741164	0.0245270548124549	0.492876787313185	-3.43711349510159
a_HQ_transcript/34750	1.2887699577148	2.26657974380154	2.35808989179411	0.024527666317195	0.492876787313185	-3.47211059118318
a_HQ_transcript/149302	2.45162689401742	1.4274341392218	2.35791556712581	0.0245374609713242	0.492876787313185	-3.42116540554908
a_HQ_transcript/94353	0.736916269806137	3.72604451492987	2.35789355959878	0.0245386977443634	0.492876787313185	-3.70391733148088

a_HQ_transcript/120084	2.33458662084309	-0.386484946175061	2.35764199911579	0.0245528388873794	0.492990472830947	-3.45911972246448
a_HQ_transcript/113675	-3.09621770053867	1.08315328182199	-2.35733007403322	0.0245703836057094	0.493172395798576	-3.5845325424563
a_HQ_transcript/70744	2.6379958010211	0.58842295011432	2.35669880099701	0.0246059253290708	0.493580130722605	-3.46299224031221
a_HQ_transcript/137937	0.748021986070964	4.80667955193312	2.3564557039449	0.0246196245212225	0.493580130722605	-3.82908422861415
a_HQ_transcript/57680	-2.62785300597884	-0.0822909165779144	-2.35641129195668	0.0246221280064609	0.493580130722605	-3.46986345710754
a_HQ_transcript/1845	-5.26657541869514	0.722855442042226	-2.35625113701795	0.024631157789908	0.493580130722605	-3.47842428553485
a_HQ_transcript/126981	4.01948194190749	1.72557334409289	2.35621574385057	0.0246331537163893	0.493580130722605	-3.43508725825449
a_HQ_transcript/150702	6.63594801946096	4.03780736655348	2.35547540516222	0.0246749372441786	0.494246985571885	-3.70558321621234
a_HQ_transcript/56042	3.38620358277674	1.94335109374715	2.35530165494387	0.0246847527296063	0.494273271328472	-3.53345096718817
a_HQ_transcript/103212	2.29005361617984	1.87521884301834	2.35506589724877	0.0246980767953779	0.494369768581862	-3.4640144133745
a_HQ_transcript/39898	2.33233970823961	-0.506119650198758	2.3545645798957	0.0247264308801592	0.494638203509585	-3.47084047379331
a_HQ_transcript/96597	-2.41812363719742	0.223073135847474	-2.35446792430518	0.0247319010293175	0.494638203509585	-3.3684288035702
a_HQ_transcript/126620	-0.88371669863113	5.05693086645082	-2.3543775646842	0.0247370158547062	0.494638203509585	-3.87528571726642
a_HQ_transcript/14185	2.00767577058932	1.17277762532139	2.35380559598988	0.0247694145073584	0.495115724375422	-3.47958868926479
a_HQ_transcript/88453	3.06530445830836	2.17777808501954	2.35352344863969	0.0247854106485665	0.49526515991058	-3.44506496851829
a_HQ_transcript/145179	-1.8627926205143	-0.503956483007915	-2.35334212963223	0.0247956953395105	0.495300405049851	-3.42159406975734
a_HQ_transcript/48715	-3.14526713571645	2.59091771835831	-2.35282532254412	0.0248250306489126	0.495624064026	-3.48458455436547
a_HQ_transcript/148697	-1.96215436154112	-0.284172888585488	-2.35266716535473	0.0248340143430874	0.495624064026	-3.48508183882466
a_HQ_transcript/76935	3.05883932748296	2.21601612921126	2.35251597073	0.0248426053013997	0.495624064026	-3.45422023415429
a_HQ_transcript/82309	-3.40817688635674	1.16279365804103	-2.35234646657575	0.0248522398183973	0.495624064026	-3.46055186882236
a_HQ_transcript/54154	-2.6508386974717	1.33013181371141	-2.35230617508746	0.024854530460979	0.495624064026	-3.41115462453647
a_HQ_transcript/123028	2.04006463234919	3.13030727379031	2.35197823229419	0.0248731817065269	0.49582589377126	-3.59391993721205
a_HQ_transcript/120271	1.76935639129828	6.31396714505936	2.35122816092176	0.0249158885494919	0.496257308735279	-4.02187944842942
a_HQ_transcript/19713	-1.04247156166869	2.7994912598751	-2.35114805231916	0.0249204536203056	0.496257308735279	-3.60309707304397
a_HQ_transcript/116859	1.7649955767988	-0.733247681973748	2.3509995036288	0.0249289208250647	0.496257308735279	-3.47723278059194
a_HQ_transcript/68356	-1.93269724013656	-0.234973821593236	-2.35099858872607	0.0249289729821603	0.496257308735279	-3.4358336482022
a_HQ_transcript/135867	2.28542700936002	2.99653694879985	2.35064118032379	0.0249493558100328	0.496493034757132	-3.63656015708616
a_HQ_transcript/4903	2.64499872597032	2.84135191681048	2.35017130649203	0.0249761754580653	0.496856648537447	-3.48257893489924
a_HQ_transcript/70777	1.23844153876875	1.95510082402677	2.349931569967	0.0249898692956258	0.496958988168367	-3.46025288151999
a_HQ_transcript/120899	-2.92389325431624	1.75665693425437	-2.34968539218984	0.0250039381372937	0.497068712737555	-3.56186947463151
a_HQ_transcript/36297	-1.01267174393453	2.01127563775571	-2.34917069646573	0.0250333757254489	0.497483782621843	-3.50214596457255
a_HQ_transcript/78647	-1.25306674453352	0.00555427382192739	-2.34880118146659	0.0250545291797564	0.497733995953822	-3.39569187977572
a_HQ_transcript/34908	-1.4162282820146	1.93356816344416	-2.34817003813979	0.0250906973967985	0.498229931576478	-3.41752329407119
a_HQ_transcript/117314	-1.49868673142948	-0.821663802471935	-2.34806649230231	0.0250966356946038	0.498229931576478	-3.46718261106739
a_HQ_transcript/6278	1.33206839692515	1.54731871086247	2.34779381939868	0.025112279428321	0.498370289726678	-3.4007600965014
a_HQ_transcript/5229	2.42139353111453	0.0928110090605126	2.34749924518	0.025129189603627	0.498535676887245	-3.39931252680241
a_HQ_transcript/85891	3.35922982657618	0.949741927691241	2.3473243757967	0.0251392329425716	0.498564767139476	-3.5914924444387
a_HQ_transcript/152462	2.16385349716728	-0.722099618302625	2.34679021788826	0.0251699339503249	0.499003383582702	-3.48383412573739
a_HQ_transcript/5259	2.88190946289228	0.858195934211011	2.34625764952648	0.0252005773971713	0.499374566344705	-3.44748477330182
a_HQ_transcript/104715	-2.3758471785953	-0.53174171713333	-2.346166273881	0.0252058384540215	0.499374566344705	-3.48604227042909
a_HQ_transcript/10216	2.15176995505525	0.644194553601594	2.34561946025826	0.0252373426614077	0.499715119558985	-3.39790496384231
a_HQ_transcript/119414	-2.51814961118941	0.870531804839965	-2.34556952482317	0.0252402214255639	0.499715119558985	-3.52192566504483
a_HQ_transcript/138727	4.01208330598508	-0.108818797650087	2.3450754487013	0.025268720808503	0.49987923147075	-3.48116852650198
a_HQ_transcript/42555	2.98982773655379	2.28256741344806	2.34501310834958	0.0252723188047116	0.49987923147075	-3.59694741904611
a_HQ_transcript/51647	-2.15900805796582	-0.41455382036416	-2.3449786205976	0.0252743094772319	0.49987923147075	-3.4820723556895
a_HQ_transcript/89757	2.08075430976351	1.47586671449569	2.34481029079565	0.0252840277039043	0.499901347745765	-3.4034287811491
a_HQ_transcript/30033	2.30630939326765	-0.0659205904613699	2.3444417206187	0.0253053182740865	0.499944665849253	-3.45937422509129
a_HQ_transcript/37595	2.50204371676325	0.448063870033069	2.3443527751791	0.0253104586656206	0.499944665849253	-3.47668479122527
a_HQ_transcript/140661	1.94174392249241	-0.545939859641167	2.34417699736873	0.0253206201093792	0.499944665849253	-3.48052127897187
a_HQ_transcript/71580	2.10901790763931	-0.271509718541725	2.34417697042886	0.0253206216670142	0.499944665849253	-3.50182399972813

a_HQ_transcript/84176	2.72559089845121	0.35005206504028	2.3438820034777	0.0253376815517302	0.500111630980976	-3.53467659631345
a_HQ_transcript/95087	2.60516222638114	0.972304516419072	2.34372530519511	0.0253467486792335	0.500120776383735	-3.42329337705711
a_HQ_transcript/21303	2.23036568384016	1.2183416050912	2.34357479416373	0.0253554605559226	0.500122908447461	-3.39924674331796
a_HQ_transcript/80384	-2.10580574935366	1.64422787647148	-2.34303755108194	0.0253865793627379	0.500303109590702	-3.43783951639469
a_HQ_transcript/3608	1.53334356823444	2.60381353465572	2.34268669800126	0.0254069205343444	0.500303109590702	-3.56224519564767
a_HQ_transcript/19412	-1.51821928610617	1.0303099394083	-2.34211592893717	0.0254400431537277	0.500303109590702	-3.39969260155379
a_HQ_transcript/37426	1.8227444369765	0.534056435357573	2.34200592744581	0.0254464312028873	0.500303109590702	-3.41002066079664
a_HQ_transcript/79350	-4.42975935052284	1.65924085886268	-2.34187192104049	0.0254542152366044	0.500303109590702	-3.60292395726319
a_HQ_transcript/68661	-1.80410914168982	0.426724583399161	-2.34155203908712	0.0254728049426327	0.500303109590702	-3.47399218552048
a_HQ_transcript/36409	-2.53033707654726	-0.207079445792696	-2.34154214829352	0.0254733799346974	0.500303109590702	-3.50435773030025
a_HQ_transcript/147316	3.08261037794284	1.66346339001708	2.34150608260578	0.0254754766793568	0.500303109590702	-3.49964992764221
a_HQ_transcript/120727	0.536830653755278	7.46912280723881	2.34132599479846	0.0254859487509175	0.500303109590702	-4.08921452288743
a_HQ_transcript/29785	2.90061533080204	1.68089097215819	2.34122168302772	0.0254920162424351	0.500303109590702	-3.45623019541252
a_HQ_transcript/103294	-4.02762223019565	2.97182429155368	-2.34121732089363	0.025492270002698	0.500303109590702	-3.79843720147963
a_HQ_transcript/1064	1.05814612553973	3.0988927190838	2.34111324489456	0.0254983251377336	0.500303109590702	-3.66057200681892
a_HQ_transcript/20725	-2.40522938200943	-0.0907881127586346	-2.34109827836418	0.0254991959965831	0.500303109590702	-3.47296070989729
a_HQ_transcript/117232	-1.55129200224355	-0.0856038032264867	-2.34077023999697	0.0255182903576126	0.500303109590702	-3.45317745241836
a_HQ_transcript/142190	-5.86064060577297	4.7221635537572	-2.34076443725012	0.0255186282386597	0.500303109590702	-3.74092207423236
a_HQ_transcript/58311	-2.57743097215169	0.232029053879415	-2.3406357918756	0.025526120012875	0.500303109590702	-3.49613755953098
a_HQ_transcript/134521	2.92421935709412	2.87744772731253	2.34051115460412	0.0255333802704013	0.500303109590702	-3.50884833230603
a_HQ_transcript/38077	-2.7539596432115	0.29573058054926	-2.34046996844557	0.0255357798200614	0.500303109590702	-3.50070069700519
a_HQ_transcript/149426	2.41956770803764	0.162303922932735	2.34039169924766	0.0255403404297692	0.500303109590702	-3.48383627287803
a_HQ_transcript/21962	1.44452084405732	3.98091756900235	2.34021035592731	0.0255509098217941	0.500303109590702	-3.74593926598338
a_HQ_transcript/138787	0.626427185177153	5.83727141750922	2.34018121573893	0.0255526085941266	0.500303109590702	-3.94396864016245
a_HQ_transcript/58774	-2.56963905760703	-0.44577958656491	-2.34015822854473	0.0255539487402765	0.500303109590702	-3.49247037411718
a_HQ_transcript/131819	2.56283664820527	0.314521880964084	2.3397495383601	0.0255777858708002	0.500601190942046	-3.43557245620783
a_HQ_transcript/38946	2.01967209945852	1.04495395205297	2.33932952049721	0.0256023046630317	0.500912408432415	-3.4061084332376
a_HQ_transcript/54631	2.01069547819496	-0.128131965120884	2.33904809271715	0.025618745076001	0.500996512033601	-3.52129542640169
a_HQ_transcript/91264	-1.67637587186802	-0.567672929516957	-2.33883263800924	0.0256313379387791	0.500996512033601	-3.47948402518009
a_HQ_transcript/74915	2.32121040500543	1.07425656804714	2.33847542355573	0.0256522286963153	0.500996512033601	-3.44321972141568
a_HQ_transcript/65775	-2.78889524592871	0.827042573336295	-2.33843397681269	0.0256546535985724	0.500996512033601	-3.52060506454642
a_HQ_transcript/135593	3.06601448899364	1.26661798752454	2.33841657659259	0.0256556716856946	0.500996512033601	-3.54276992645365
a_HQ_transcript/12905	1.86707631131637	2.321320577972	2.33837137827464	0.0256583164107492	0.500996512033601	-3.44343449415341
a_HQ_transcript/95515	-2.28722927111657	0.358175493753355	-2.33819668385724	0.0256685407636285	0.501027850069912	-3.43182277826344
a_HQ_transcript/51605	2.80487904000948	2.96018285788889	2.33796720992672	0.025681976803344	0.50112183538932	-3.63582597962477
a_HQ_transcript/54478	-2.13631514289568	-0.444952883555465	-2.33777148097241	0.0256934420534518	0.501177315329881	-3.49348412935446
a_HQ_transcript/10325	-2.78578406028534	0.538556325401385	-2.33749517245614	0.0257096353114868	0.501291118897909	-3.47645309780163
a_HQ_transcript/77836	-2.37775882707926	3.92041676771514	-2.33737767446856	0.0257165241631153	0.501291118897909	-3.7732975743062
a_HQ_transcript/46501	-1.37720180982986	3.30775822768091	-2.33701736487405	0.0257376593601084	0.501377940589005	-3.58823825668759
a_HQ_transcript/58151	-1.96411592960866	-0.452362998595771	-2.3366904654090	0.025756848368431	0.501377940589005	-3.45709288609552
a_HQ_transcript/34181	2.24336024479241	0.708255013111016	2.33668254984356	0.0257573131728339	0.501377940589005	-3.47748965513716
a_HQ_transcript/45641	3.69109942290154	0.17589692603652	2.33654957347747	0.0257651227201416	0.501377940589005	-3.47634797883182
a_HQ_transcript/73369	-1.32866714173685	1.35756939942018	-2.3365286510057	0.0257663516676164	0.501377940589005	-3.39857349184042
a_HQ_transcript/5257	-1.54013261316069	2.57298501670768	-2.33642006541612	0.0257727306372135	0.501377940589005	-3.57042259274814
a_HQ_transcript/32222	-1.65579364617554	0.834507854817817	-2.33587950807753	0.0258045074882714	0.501600569732774	-3.57268583793061
a_HQ_transcript/78093	-1.82406650099806	0.440642458688717	-2.3356012584736	0.0258208783037017	0.501600569732774	-3.40328635479844
a_HQ_transcript/143156	3.46134512573712	3.99791807165411	2.33558600295774	0.0258217761336282	0.501600569732774	-3.84652841593368
a_HQ_transcript/1992	1.92270234644634	2.27194021685822	2.33513774849968	0.0258481697774129	0.501600569732774	-3.49260132470248
a_HQ_transcript/125279	4.17032517881533	2.0147944969212	2.33501195769344	0.0258555808426432	0.501600569732774	-3.56122332459778
a_HQ_transcript/136088	2.08117236203197	1.19739143782485	2.33484942734616	0.0258651592935782	0.501600569732774	-3.399549911294

a_HQ_transcript/130930	-2.78820900401511	1.28875124074521	-2.33474924172112	0.0258710651629663	0.501600569732774	-3.50306349823392
a_HQ_transcript/27555	1.55238941589277	-0.436792145593458	2.33467216410246	0.0258756096625508	0.501600569732774	-3.51716137023191
a_HQ_transcript/21543	2.08924252802893	1.54368811092045	2.33467097644911	0.0258756796922903	0.501600569732774	-3.5039999913977
a_HQ_transcript/81572	3.97127587644245	1.45883376978244	2.33445129742877	0.0258886359706516	0.501600569732774	-3.53156782836105
a_HQ_transcript/116166	-2.64852785178363	1.34683347324834	-2.33443567253072	0.0258895577228969	0.501600569732774	-3.45313177438359
a_HQ_transcript/28881	2.64533317860451	0.54767411409905	2.33433278659444	0.0258956279658543	0.501600569732774	-3.48452060935494
a_HQ_transcript/150773	-2.55996254088398	2.9368849460426	-2.33432046937549	0.0258963547647959	0.501600569732774	-3.67811366037665
a_HQ_transcript/115738	-0.523686061655312	4.66095141707458	-2.3340205889468	0.0259140554260315	0.501690012142915	-3.86195820524457
a_HQ_transcript/15589	-1.14764115781113	3.94079906224815	-2.33394982341724	0.0259182340088283	0.501690012142915	-3.80827283040938
a_HQ_transcript/131656	2.33543917912145	1.09979813792896	2.33336828683347	0.0259525958353003	0.502162103249991	-3.41596811166434
a_HQ_transcript/127719	2.76907161549173	1.54688437302189	2.33308079819063	0.0259695981772904	0.502162103249991	-3.47017024810073
a_HQ_transcript/51100	3.03367477434059	2.63772427928975	2.33306644051156	0.0259704475676922	0.502162103249991	-3.65571743103419
a_HQ_transcript/149692	-1.78782576022321	-0.530162692939273	-2.33295267547724	0.0259771787171883	0.502162103249991	-3.48066654127942
a_HQ_transcript/54472	5.78747979423051	2.16954416354801	2.33248280405612	0.0260049963774207	0.502496686566544	-3.70716396295977
a_HQ_transcript/38699	-2.15226816410271	0.557003733946034	-2.33236835331869	0.0260117762503222	0.502496686566544	-3.47806320554117
a_HQ_transcript/146929	2.7295427624443	0.124692823475033	2.33189668170596	0.0260397341727384	0.50286965714051	-3.43715378093645
a_HQ_transcript/151224	5.61886393533855	5.39686450172441	2.3316199383005	0.0260561505443834	0.502887512147465	-3.85144248829833
a_HQ_transcript/28553	-0.507182940804348	3.98396948247315	-2.33158941913696	0.0260579615088798	0.502887512147465	-3.75528024408627
a_HQ_transcript/133183	0.878002250843871	4.6742964324646	2.33098356613577	0.0260939355467512	0.50324761558676	-3.8862175688926
a_HQ_transcript/17144	1.71241465239044	-0.792080479788042	2.33098355805245	0.0260939360270179	0.50324761558676	-3.51554522187504
a_HQ_transcript/7734	0.78965673617323	2.86739932899008	2.330685354433	0.026111659102069	0.503311203996281	-3.65913185834362
a_HQ_transcript/22491	1.23650982992245	1.27471269642669	2.33050879276549	0.026122157785857	0.503311203996281	-3.45398935293925
a_HQ_transcript/122307	-3.64965141827682	0.85408426234795	-2.33019482956575	0.0261408360488283	0.503311203996281	-3.59578796903663
a_HQ_transcript/49739	-2.780982007286	0.357458348492381	-2.33010752454065	0.0261460321351713	0.503311203996281	-3.45592691459049
a_HQ_transcript/55467	2.51471591535535	-0.242520436751269	2.33005009799886	0.0261494504690551	0.503311203996281	-3.51526455705194
a_HQ_transcript/50475	2.31864688456371	1.9471305495624	2.32969305290934	0.0261707127650583	0.503311203996281	-3.49470817085457
a_HQ_transcript/31791	2.83097231113645	2.18535519532083	2.32962643635164	0.0261746815598305	0.503311203996281	-3.47575126158649
a_HQ_transcript/108248	-1.94258918068754	-0.480089821228142	-2.32955820263603	0.0261787472637453	0.503311203996281	-3.5055363742105
a_HQ_transcript/11941	4.01521028442235	0.924314060185083	2.32952729840224	0.0261805888794804	0.503311203996281	-3.51363546208531
a_HQ_transcript/126125	-3.98173206620971	2.86827993094107	-2.32947308390874	0.0261838198610782	0.503311203996281	-3.73798239940223
a_HQ_transcript/60864	0.931598302081906	2.12557419938105	2.32921561543003	0.0261991689446731	0.50343976608792	-3.48615008459244
a_HQ_transcript/61854	-2.97666162486242	1.18685074957755	-2.32878386826159	0.0262249259814391	0.503745283326505	-3.56586225114295
a_HQ_transcript/85111	-2.2468432059114	0.37325246817371	-2.32865865096552	0.0262324004374713	0.503745283326505	-3.50008690626399
a_HQ_transcript/141315	2.58452633124433	0.0361644314754536	2.32825813773291	0.026256320761246	0.503818843527314	-3.47469931507845
a_HQ_transcript/103813	4.55796462269991	2.53493494757026	2.32819132475839	0.0262603130300411	0.503818843527314	-3.55034572752236
a_HQ_transcript/107647	2.34591711366325	-0.0819326611295031	2.32815919157394	0.0262622332763515	0.503818843527314	-3.4854573045396
a_HQ_transcript/65952	2.29490882586234	1.50460835791818	2.32787160258231	0.026279424954464	0.503982320604779	-3.47058085352613
a_HQ_transcript/44115	2.08360534938972	1.06736914562732	2.32750587800803	0.0263013021679225	0.504235518607189	-3.4052144121644
a_HQ_transcript/117150	1.99030564650675	2.60012403022111	2.32678487527733	0.0263444799359064	0.504738909380836	-3.54858029827725
a_HQ_transcript/100472	2.27731629601585	0.238085460580431	2.32677743440892	0.0263449258715499	0.504738909380836	-3.49701395826497
a_HQ_transcript/112716	1.79090003437759	-0.191787552292668	2.32659539574717	0.0263558376820004	0.504781592349035	-3.5023891913021
a_HQ_transcript/35002	-4.38898547030536	5.03118912558024	-2.32624291746946	0.0263769776450624	0.50502007791574	-4.02261911518977
a_HQ_transcript/117982	2.42184272726462	-0.0975094353144497	2.32587812274669	0.0263988724294031	0.505272853663597	-3.51588713773771
a_HQ_transcript/22455	-2.22006340632948	1.76480511601105	-2.32513313514924	0.0264436372281186	0.50596304963663	-3.55137608585569
a_HQ_transcript/24271	-0.875507738619272	4.3426626134843	-2.32457278313321	0.0264773528482605	0.506441449938693	-3.83572067848906
a_HQ_transcript/123380	2.00832501818563	1.95675111895764	2.32436797128925	0.0264896857916588	0.506510676216298	-3.46233566505735
a_HQ_transcript/113273	-0.839882150687881	3.56225754229499	-2.32364719687388	0.0265331292187978	0.507138593989369	-3.78565170715747
a_HQ_transcript/146846	3.87277634013998	2.96445488055571	2.32353373331493	0.0265399739009713	0.507138593989369	-3.65963784702135
a_HQ_transcript/74002	3.47262817112143	0.964433586981989	2.32303117761729	0.0265703097331633	0.507369190811479	-3.49095853019805
a_HQ_transcript/124384	1.9289662958291	0.621624934900413	2.32285863693338	0.026580732051865	0.507369190811479	-3.46349966728731

a_HQ_transcript/45525	1.79914673163365	2.74129069867792	2.32279362552408	0.026584660023737	0.507369190811479	-3.50812067320066
a_HQ_transcript/149713	-3.3652075344535	-0.60721086068847	-2.32275563569799	0.0265869556016337	0.507369190811479	-3.53989550547123
a_HQ_transcript/108311	0.989680662621243	2.99549276716635	2.32251196857304	0.0266016837209232	0.507399472442996	-3.64262283809035
a_HQ_transcript/126422	3.5324592505222	-0.252439872404547	2.32236501701407	0.0266105695664542	0.507399472442996	-3.52271668383462
a_HQ_transcript/116788	0.893971476007469	3.54989833770263	2.32229623786024	0.0266147294157497	0.507399472442996	-3.77082558554305
a_HQ_transcript/76846	-0.779882257424905	6.12031461739548	-2.32120045238725	0.0266810831980236	0.508452587595079	-4.03221376136897
a_HQ_transcript/3400	2.33131906370347	0.904292186731356	2.32109522557979	0.0266874629223883	0.508452587595079	-3.45362286625649
a_HQ_transcript/133256	-2.76217840496676	-0.551968606300375	-2.32082405727254	0.0267039097539661	0.508504959711054	-3.52820658674039
a_HQ_transcript/73293	0.710241070298756	2.96979879422402	2.32076145731557	0.0267077078516008	0.508504959711054	-3.68441727410532
a_HQ_transcript/19010	2.03662218640836	-0.12359434143003	2.32042697947047	0.0267280097403476	0.508630285088321	-3.46295805250943
a_HQ_transcript/113229	5.71997316355718	2.52078378185681	2.32036471524131	0.0267317905474955	0.508630285088321	-3.5690207556458
a_HQ_transcript/12453	0.821570518399127	3.81531293432004	2.32000333704815	0.0267537436885108	0.508881417907643	-3.79858800302525
a_HQ_transcript/19935	-2.22719466714094	1.82044616175746	-2.31930471960194	0.0267962297560633	0.508935136596254	-3.48815578000361
a_HQ_transcript/29595	5.66836005259775	3.69939143090711	2.31927107038656	0.026798277653913	0.508935136596254	-3.67497023657785
a_HQ_transcript/98243	2.83032584475451	0.949479778367986	2.31906887349965	0.0268105863738954	0.508935136596254	-3.51332921831348
a_HQ_transcript/90657	-2.40039017148793	-0.221549354888588	-2.31901819086523	0.0268136724758281	0.508935136596254	-3.54672353864388
a_HQ_transcript/131473	1.2435359312983	2.81628598402902	2.31895744177501	0.0268173719543213	0.508935136596254	-3.65446616415002
a_HQ_transcript/113991	-1.70567260399811	1.86970199392141	-2.31885204375818	0.026823791542748	0.508935136596254	-3.49242317596021
a_HQ_transcript/122238	0.738181287809818	3.04267948082525	2.31853346409466	0.0268432040517805	0.508935136596254	-3.7092050160549
a_HQ_transcript/89086	3.82353384088947	1.35738257447583	2.31847258813568	0.0268469149449917	0.508935136596254	-3.56107708018248
a_HQ_transcript/134067	-2.38815395674181	-0.472936091139405	-2.31832619586979	0.026855840661668	0.508935136596254	-3.5061972916991
a_HQ_transcript/152192	-2.41239920568419	-0.689319999677433	-2.31805490633983	0.0268723886054448	0.508935136596254	-3.53054941852464
a_HQ_transcript/87579	-2.00432490169211	-0.173501913389187	-2.31804576443964	0.0268729463975615	0.508935136596254	-3.54015587365284
a_HQ_transcript/42918	3.01226509382216	0.571841461469253	2.31801970931407	0.0268745362057984	0.508935136596254	-3.53240687850982
a_HQ_transcript/25540	2.58877201334038	0.71888784029018	2.31800973387323	0.0268751449007731	0.508935136596254	-3.50551998126783
a_HQ_transcript/81850	2.4976194910505	0.836137959570579	2.31794420442761	0.0268791437749536	0.508935136596254	-3.55572979876667
a_HQ_transcript/12176	-2.44413741283709	1.26297863912162	-2.31775954248821	0.0268904154916008	0.508982026390873	-3.48560026504853
a_HQ_transcript/131131	1.87371475173728	-0.796124597747565	2.31761677855403	0.0268991326911774	0.508982026390873	-3.53427609937449
a_HQ_transcript/102854	2.24798301734331	-0.251198289315361	2.31717133648727	0.0269263478985853	0.509331191229731	-3.49128385865121
a_HQ_transcript/101970	-3.42829082723716	2.74763897912145	-2.31679849146713	0.0269491467571445	0.509335033766177	-3.50381448455607
a_HQ_transcript/94151	-2.99161669374901	2.22576273193097	-2.31667659547819	0.0269566042771152	0.509335033766177	-3.63483232659395
a_HQ_transcript/29074	-1.62682810834617	-0.516734881227048	-2.31667589554647	0.0269566471038798	0.509335033766177	-3.56234608926361
a_HQ_transcript/119297	-3.50677825778192	1.33562900693586	-2.31652463733776	0.0269659035907315	0.509335033766177	-3.55680399585983
a_HQ_transcript/20071	1.86505435180448	-0.381349266146401	2.31640430938668	0.0269732693014051	0.509335033766177	-3.53335830497324
a_HQ_transcript/7239	1.19138556285116	2.48875520659813	2.31630867305278	0.0269791248445854	0.509335033766177	-3.57844905240076
a_HQ_transcript/149924	3.15154898174565	0.37789347551465	2.3156397039969	0.0270201160469899	0.509823595912194	-3.54649221788852
a_HQ_transcript/112916	3.38902408965272	0.0331121650348788	2.31560009178103	0.0270225450558332	0.509823595912194	-3.56703620769184
a_HQ_transcript/146460	2.65359510657168	-0.39439287521266	2.31488902851622	0.027066180790213	0.510065273650852	-3.53229408235872
a_HQ_transcript/58418	-2.25017576679103	1.15899343951445	-2.31485547748588	0.0270682412834629	0.510065273650852	-3.48379946641459
a_HQ_transcript/115950	2.5051301922308	0.301344992807575	2.31473414298651	0.0270756940677308	0.510065273650852	-3.60602277770623
a_HQ_transcript/67792	-3.12327179772007	2.74036060709732	-2.31469640304525	0.0270780125631436	0.510065273650852	-3.79700261296231
a_HQ_transcript/136587	-1.52578517006296	-0.761493868770773	-2.31467660020078	0.0270792291922401	0.510065273650852	-3.53001418833424
a_HQ_transcript/72912	2.6581877647393	0.684809234737842	2.31424491251458	0.0271057630964897	0.510399675177438	-3.43477108297736
a_HQ_transcript/132873	2.36416220427881	-0.408077741107008	2.31397659505659	0.0271222671953606	0.510545060729249	-3.5458389936094
a_HQ_transcript/97164	1.38198761364581	2.62436256606141	2.31266080109589	0.0272033325797629	0.511500441887476	-3.60296442967961
a_HQ_transcript/33509	2.38463111520298	0.190027383529391	2.31263971505044	0.0272046334592144	0.511500441887476	-3.45187004951042
a_HQ_transcript/63368	1.79189759615245	-0.418556735596797	2.31256496971136	0.0272092452398415	0.511500441887476	-3.52076263877587
a_HQ_transcript/152002	-5.30349672326431	0.465277577698674	-2.31252691617888	0.0272115934104831	0.511500441887476	-3.56810640223398
a_HQ_transcript/3085	-2.54311197738673	1.07575171020261	-2.31230253565503	0.027225442987654	0.511500441887476	-3.47974434410694
a_HQ_transcript/143823	-2.19011780754443	0.203253987855706	-2.31229645626385	0.0272258183181917	0.511500441887476	-3.53537148125191

a_HQ_transcript/64288	2.37455517305656	-0.0705688421860635	2.31185073128124	0.027253349297199	0.511803160717501	-3.55591229013948
a_HQ_transcript/10465	-2.70334315133274	2.40285205387993	-2.31166518045355	0.0272648175743431	0.511803160717501	-3.6113133286198
a_HQ_transcript/59698	1.6327090582506	-0.358693766018262	2.31149994215112	0.0272750340694006	0.511803160717501	-3.51270705974813
a_HQ_transcript/2229	1.42574619979298	3.93329564011625	2.31146572210923	0.0272771502873455	0.511803160717501	-3.86516410352327
a_HQ_transcript/136325	-2.6158320479805	-0.477754451909488	-2.31085190881129	0.0273151345923471	0.512222417774771	-3.54916987937198
a_HQ_transcript/122489	2.26939819178347	-0.534990891461593	2.31074076486698	0.0273220175617406	0.512222417774771	-3.5753580923913
a_HQ_transcript/120101	0.469295903447423	6.44655949597563	2.31060585838205	0.0273303742113902	0.512222417774771	-4.06605647550809
a_HQ_transcript/98601	-1.53240975483442	1.13213381798004	-2.31053534551198	0.0273347429799295	0.512222417774771	-3.45196524504045
a_HQ_transcript/86122	-2.12283581399206	-0.639698081335546	-2.31020508382296	0.0273552134078414	0.512440813719306	-3.54049102033647
a_HQ_transcript/142446	0.58704582056539	5.22803019006398	2.30989507933804	0.0273744408343881	0.512635791501712	-3.95791238331162
a_HQ_transcript/140688	-2.47137255423454	0.0861485889251987	-2.30943363205817	0.0274030838005054	0.512953330254991	-3.55174614596948
a_HQ_transcript/76686	1.1817643630146	5.76962762778558	2.30924427416174	0.027414845454194	0.512953330254991	-4.0095804086625
a_HQ_transcript/87955	-1.87604833141959	0.299930606429552	-2.30919557826976	0.0274178708557366	0.512953330254991	-3.50437157918895
a_HQ_transcript/93443	3.64763366019056	0.698489912876028	2.30853580357375	0.0274588913634024	0.513417053620516	-3.61442038204846
a_HQ_transcript/36770	1.34557501273594	2.02478927243067	2.30839824365227	0.0274674509214695	0.513417053620516	-3.58022075470545
a_HQ_transcript/39373	-2.05831140787595	-0.548059726877899	-2.30774509120808	0.0275081256905449	0.513417053620516	-3.54999344409999
a_HQ_transcript/131861	2.25286695257065	3.10909160519338	2.30767657231767	0.0275123958218628	0.513417053620516	-3.65066008332275
a_HQ_transcript/41463	-2.61364668449429	0.503674312999761	-2.30760345013051	0.0275169534927605	0.513417053620516	-3.51437875239883
a_HQ_transcript/52654	-2.64255665005024	0.70676307908016	-2.30756813169284	0.0275191551180876	0.513417053620516	-3.55248944861691
a_HQ_transcript/99335	-1.70312271534385	-0.617021364058213	-2.3073799042911	0.0275308912238205	0.513417053620516	-3.51715945815346
a_HQ_transcript/6558	-1.82310706662887	1.40746933906996	-2.30729168864199	0.0275363930835147	0.513417053620516	-3.50119054624405
a_HQ_transcript/99611	2.42087761973061	-0.0954942375369324	2.30726857573727	0.0275378347600252	0.513417053620516	-3.53258251276279
a_HQ_transcript/23815	-1.42156315224756	2.08213041110498	-2.30723463164096	0.0275399521598408	0.513417053620516	-3.5570289147491
a_HQ_transcript/110631	1.14422145012165	1.82651990904405	2.30711864639911	0.0275471883155325	0.513417053620516	-3.48921071713481
a_HQ_transcript/102073	1.33709306764463	3.39528607123582	2.30688576998347	0.0275617223278672	0.513417053620516	-3.72971969186498
a_HQ_transcript/106377	1.69468472459421	2.28855401232084	2.3067915157901	0.02756760677798	0.513417053620516	-3.48964203458786
a_HQ_transcript/395	1.40275430331524	3.98325958864277	2.30674144749417	0.0275707330881743	0.513417053620516	-3.81694363410415
a_HQ_transcript/90225	-2.4410023022161	0.989775819200305	-2.30655041392644	0.0275826643381928	0.513417053620516	-3.51777256875694
a_HQ_transcript/137483	-2.36763649432198	0.958435774364552	-2.30652938924423	0.0275839777466431	0.513417053620516	-3.47927922255884
a_HQ_transcript/66261	-1.91089906394918	2.9204824362025	-2.3061291774232	0.0276089896774077	0.513602907613148	-3.67727054513139
a_HQ_transcript/9947	1.30718003188354	2.56649018954407	2.30608687825709	0.0276116344324781	0.513602907613148	-3.57709170941288
a_HQ_transcript/150693	-2.69447942260799	0.371141564229502	-2.30565343949654	0.027638748364084	0.513671119750235	-3.54426994518149
a_HQ_transcript/79209	-2.65438759827777	-0.460581596264331	-2.30550584052769	0.0276479869573963	0.513671119750235	-3.55972735874843
a_HQ_transcript/34622	1.53314785941076	2.53564813757591	2.30532777580577	0.0276591361877426	0.513671119750235	-3.60358110113494
a_HQ_transcript/68034	2.20582707476783	0.0310962332086883	2.3046727672586	0.027700183388102	0.513671119750235	-3.52742546968127
a_HQ_transcript/48214	-2.19547859296851	-0.437408489101725	-2.3046196014468	0.0277035175224883	0.513671119750235	-3.55289140773669
a_HQ_transcript/96171	0.920270229169976	6.79875447250096	2.30458591068839	0.0277056305247421	0.513671119750235	-4.09072042084507
a_HQ_transcript/84945	2.46131363311178	1.17962171192319	2.30438705933908	0.0277181049587695	0.513671119750235	-3.57584152136932
a_HQ_transcript/3706	1.760731426407	0.790484452072801	2.30417213638058	0.0277315933023324	0.513671119750235	-3.46456689276601
a_HQ_transcript/144857	-2.74871689057722	1.09727689406321	-2.30408711061079	0.0277369310666368	0.513671119750235	-3.52650278140582
a_HQ_transcript/143495	-3.42226662695535	1.34427982056725	-2.30377731908928	0.0277563870608312	0.513671119750235	-3.61808226604917
a_HQ_transcript/86760	4.39395142647232	2.6020248635089	2.30377421387318	0.0277565821416021	0.513671119750235	-3.64142118913742
a_HQ_transcript/43892	3.63411320806149	1.31016084780004	2.30365948562115	0.0277637906475091	0.513671119750235	-3.68600707494074
a_HQ_transcript/133459	-3.59873330012514	1.3046478550521	-2.30363040169117	0.0277656182920573	0.513671119750235	-3.57566461071956
a_HQ_transcript/117685	0.773344015113818	2.30962434290382	2.30361113304003	0.0277668292007503	0.513671119750235	-3.61897730175348
a_HQ_transcript/122121	0.902696007328407	2.6186244795222	2.30360334656589	0.0277673185432367	0.513671119750235	-3.6663565766354
a_HQ_transcript/40505	-1.26809678650051	1.73380489410338	-2.30359089418481	0.0277681011317246	0.513671119750235	-3.48774192413376
a_HQ_transcript/116517	2.05440085926823	2.62552932942181	2.30353085726332	0.0277718745209629	0.513671119750235	-3.59711187195946
a_HQ_transcript/53883	-4.51462494775034	1.72944673668355	-2.30349122512949	0.0277743656994045	0.513671119750235	-3.52208268290584
a_HQ_transcript/12606	1.23289201842663	1.74315686286527	2.30307346165418	0.0278006375486044	0.513993466738319	-3.56129189966917

a_HQ_transcript/97523	2.96062331771251	4.04775203809965	2.30270351180322	0.02782392126082	0.514260379983766	-3.67740926673157
a_HQ_transcript/146432	-2.16010099590153	3.21680148329397	-2.302385290875	0.027843963356583	0.514305103636646	-3.80075382545252
a_HQ_transcript/1884	-1.28402697104167	2.50955676204298	-2.30234206205008	0.0278466869860097	0.514305103636646	-3.5921110150733
a_HQ_transcript/14996	2.05462310699388	1.38664722038436	2.30224371181611	0.0278528844317396	0.514305103636646	-3.51672640350295
a_HQ_transcript/119515	1.54321865444569	5.21861358076489	2.30197476266569	0.0278698383581783	0.514454736133435	-3.9838514519145
a_HQ_transcript/129263	2.36876837223382	1.03607612086997	2.30163039103076	0.0278915603318097	0.514568813972408	-3.60211942134323
a_HQ_transcript/151827	-3.60005198105115	1.43276383883925	-2.3015961175592	0.0278937230392764	0.514568813972408	-3.64374896373243
a_HQ_transcript/37380	1.56278265741234	-0.641006711705974	2.30138385248588	0.0279071206533704	0.514652636211648	-3.55740733254597
a_HQ_transcript/133639	1.74835282928509	2.38907130778713	2.30083130905301	0.0279420229666515	0.514895115585346	-3.57322120664495
a_HQ_transcript/84084	2.37301569569559	-0.0555616454294507	2.3003940445622	0.0279696713963651	0.514895115585346	-3.5725657699653
a_HQ_transcript/90656	-2.64574964811609	3.94463074504589	-2.30036590782028	0.0279714513397958	0.514895115585346	-3.88585282450491
a_HQ_transcript/70228	2.51478627621506	0.114768500669283	2.30027735668783	0.0279770537924696	0.514895115585346	-3.55556730805518
a_HQ_transcript/86477	0.687361447842532	4.25152733163778	2.29994447845003	0.0279981233851147	0.514895115585346	-3.89436778683489
a_HQ_transcript/51421	1.95909581864501	5.04998381393315	2.29989189007036	0.0280014532854213	0.514895115585346	-3.98919622253251
a_HQ_transcript/149576	2.68118148214145	1.73953444482882	2.29980075415096	0.0280072248647642	0.514895115585346	-3.52911946986749
a_HQ_transcript/60210	3.13414965317372	0.647341057141781	2.2997025915818	0.0280134426374029	0.514895115585346	-3.58774337798415
a_HQ_transcript/117852	3.60384942174145	4.01536123011467	2.29968090359869	0.0280148165565016	0.514895115585346	-3.73415103024018
a_HQ_transcript/114814	1.83167750702785	2.59442807449828	2.29967962480199	0.0280148975693068	0.514895115585346	-3.65509202310597
a_HQ_transcript/144120	4.46718027064423	2.30214540140877	2.2996292024235	0.0280180920361693	0.514895115585346	-3.50609385860435
a_HQ_transcript/122927	-1.41050672480659	5.104733137641	-2.29949549452699	0.028026564576659	0.514895115585346	-3.83801696354328
a_HQ_transcript/149104	6.59636115385341	3.35571278426289	2.2992222488225	0.0280438862618516	0.515040913376721	-3.78367351690345
a_HQ_transcript/67121	2.53765032054161	2.05793740728447	2.29909081643505	0.028052221506859	0.515040913376721	-3.52465155624859
a_HQ_transcript/121488	1.83590733135666	2.00940454036104	2.29862228438269	0.0280819532873136	0.515293322480305	-3.5359885298592
a_HQ_transcript/91379	-1.9812786388148	3.01021023849434	-2.29816090590767	0.0281112588856866	0.515293322480305	-3.75421326697489
a_HQ_transcript/126420	-2.15162818958324	-0.297682946559749	-2.29813750702576	0.0281127458581777	0.515293322480305	-3.60241941098933
a_HQ_transcript/139417	-3.98154448705496	2.45503159699529	-2.29801491721964	0.0281205374609553	0.515293322480305	-3.72414215911531
a_HQ_transcript/62990	2.97516128500958	0.0548269985380808	2.29785413479497	0.0281307594741952	0.515293322480305	-3.58579196871143
a_HQ_transcript/60021	2.77792826638137	1.56868767940592	2.29781647279504	0.028133154383489	0.515293322480305	-3.49438348413502
a_HQ_transcript/102034	-2.94166467772404	0.891433921346606	-2.29772073317669	0.0281392432495745	0.515293322480305	-3.50702793006449
a_HQ_transcript/133433	2.08696414084383	2.97865803777079	2.29769885350349	0.0281406349238982	0.515293322480305	-3.71296188903241
a_HQ_transcript/86498	3.22522606173828	0.572946251254053	2.29753212338678	0.0281512419663798	0.515293322480305	-3.52708995260528
a_HQ_transcript/39188	-0.909489618932016	2.70901972421936	-2.29747907962839	0.0281546172618608	0.515293322480305	-3.65042644034756
a_HQ_transcript/110379	-3.92817011719028	1.79666223830145	-2.2967974045659	0.0281980262896161	0.515875762318732	-3.51048530610173
a_HQ_transcript/134135	3.30931202728482	5.60785031094006	2.29670068485392	0.0282041902809134	0.515875762318732	-3.98981364024093
a_HQ_transcript/48772	1.24294544849414	-0.684737475738369	2.29634575243725	0.028226820695431	0.515996522334564	-3.57039822410898
a_HQ_transcript/122029	2.66798410769932	2.18049454148447	2.29614406260888	0.0282396876884906	0.515996522334564	-3.63224107954102
a_HQ_transcript/134852	2.36579835240536	-0.335373315670098	2.29605317678016	0.0282454875638131	0.515996522334564	-3.56845464462634
a_HQ_transcript/51735	-2.60959783203711	1.76028028294517	-2.29604044493182	0.0282463001319258	0.515996522334564	-3.62047257088911
a_HQ_transcript/20133	1.2870458961117	2.74063272934959	2.2956711852604	0.0282698760799135	0.516178176163278	-3.69340291556789
a_HQ_transcript/145014	-3.83686013536311	1.80718996513434	-2.29560655776996	0.028274004144369	0.516178176163278	-3.71698570450457
a_HQ_transcript/67360	2.37074819988669	-0.410290791281405	2.29542238063884	0.0282857713927097	0.516230869549586	-3.58283314809473
a_HQ_transcript/145832	5.5627854181543	3.0107979137527	2.29519072816343	0.0283005781533596	0.516338985216098	-3.7881498857683
a_HQ_transcript/26697	2.52526616444807	0.214726086843511	2.29477598434488	0.0283271051984171	0.516648980480813	-3.61945866480116
a_HQ_transcript/140807	-2.29419583683551	0.912435301804739	-2.29452830286762	0.0283429576110439	0.516648980480813	-3.49561603844377
a_HQ_transcript/82375	2.17393338135502	-0.400727692312885	2.29450837518477	0.0283442333944624	0.516648980480813	-3.57866353241824
a_HQ_transcript/84929	1.09213261607279	2.48939520808427	2.29425125176388	0.0283606992490787	0.516787061426472	-3.60824409214767
a_HQ_transcript/9745	-2.29966024092996	-0.412330935641259	-2.29390112072229	0.0283831350566973	0.517033805883954	-3.5768195676914
a_HQ_transcript/47786	-1.76451941721312	-0.390906979272733	-2.29334869980266	0.0284185657918465	0.517418784227166	-3.53188542755167
a_HQ_transcript/137269	-1.92554967145329	-0.356251571476699	-2.29329407247202	0.028422071601248	0.517418784227166	-3.57496325777528
a_HQ_transcript/139777	3.41816858654963	2.55994325230846	2.293146406226	0.0284315503051874	0.517429291214756	-3.61315251459179

a_HQ_transcript/115768	2.27691437870909	2.28519747458822	2.29263237065271	0.0284645684999605	0.517587803497673	-3.62737407268431
a_HQ_transcript/36257	-2.472027953291	0.35721176758185	-2.2925954511421	0.028466941291126	0.517587803497673	-3.59185160000431
a_HQ_transcript/127183	2.44461670614308	0.0459364824523656	2.29249273947969	0.0284735434357692	0.517587803497673	-3.53010560890081
a_HQ_transcript/133135	-1.1381095063778	3.42011860138682	-2.2924564359759	0.0284758772981276	0.517587803497673	-3.73925940411339
a_HQ_transcript/113846	-3.88046319695483	4.66999653635109	-2.29223621934837	0.0284900381748629	0.517593681847198	-4.05789260793052
a_HQ_transcript/63498	1.05439005814295	3.36243492764815	2.29217145625941	0.0284942039276215	0.517593681847198	-3.77765020818655
a_HQ_transcript/67175	-2.42070106792508	1.31907721169787	-2.29201076914518	0.0285045421670989	0.517593681847198	-3.586276486511
a_HQ_transcript/136862	1.05076032185773	2.95415259076675	2.29189770909929	0.0285118182162594	0.517593681847198	-3.73170169219493
a_HQ_transcript/84115	-1.94516352023518	0.476535188074803	-2.29154417125556	0.0285345811637679	0.517657399049859	-3.49971587880822
a_HQ_transcript/33366	-2.12271087912024	1.17423736954056	-2.29152108145679	0.0285360683970227	0.517657399049859	-3.53736416710345
a_HQ_transcript/45090	1.86129315028633	0.862606312548179	2.29142771741908	0.0285420827650856	0.517657399049859	-3.54597854403081
a_HQ_transcript/74916	1.82642498875208	-0.654186382361202	2.29129009930268	0.0285509499957653	0.517657399049859	-3.58794154333718
a_HQ_transcript/128950	3.36593340195496	3.63558573885266	2.29083686933212	0.0285801707874353	0.517743522978888	-3.76821369118721
a_HQ_transcript/140555	-3.15253649893269	-0.0108633845833945	-2.29079890036094	0.0285826199583519	0.517743522978888	-3.618726369125
a_HQ_transcript/90474	2.97900646556089	1.56293914973958	2.29077281066924	0.0285843029714622	0.517743522978888	-3.5618086197074
a_HQ_transcript/151593	6.68815719749586	0.534712132310577	2.29066392658972	0.0285913279101677	0.517743522978888	-3.62819154150446
a_HQ_transcript/34401	2.65291151863748	2.13722050037225	2.29036594225466	0.0286105610971536	0.517930456385969	-3.63851252450477
a_HQ_transcript/13013	1.57856455106725	3.75599053788033	2.28966316708252	0.0286559673966508	0.518525093189318	-3.89554045392138
a_HQ_transcript/129783	-2.26855355161001	-0.199027030294642	-2.28958147499642	0.028661249731924	0.518525093189318	-3.58594106260522
a_HQ_transcript/9377	-2.37861846642172	1.57826163962603	-2.28891793568825	0.0287041877116541	0.519140330834795	-3.59133372752919
a_HQ_transcript/73230	-4.17947293555227	-0.430547160219809	-2.28814928435528	0.0287539999903829	0.519626301108935	-3.57574622360708
a_HQ_transcript/125132	2.43471741132236	0.744607309338867	2.28801823228079	0.0287625005570747	0.519626301108935	-3.51708412925213
a_HQ_transcript/120342	2.97288757050544	0.503537056747061	2.28797464108871	0.028765328559502	0.519626301108935	-3.55161724122852
a_HQ_transcript/84047	-1.59814364305737	-0.721683335055216	-2.28795172656803	0.0287668152520051	0.519626301108935	-3.58050087207644
a_HQ_transcript/127153	3.0422784113656	1.14382817775341	2.28777911236053	0.0287780166745531	0.519667149194913	-3.54633934933198
a_HQ_transcript/46235	1.9931762165148	-0.364243662831339	2.28759253030477	0.028790128928923	0.519724414403862	-3.5446051715443
a_HQ_transcript/133757	-0.629372575857507	4.19245953946784	-2.28727473850144	0.0288107694332613	0.519935549710218	-3.90226271872366
a_HQ_transcript/45785	4.37960430855644	0.930482306792881	2.28655648729891	0.0288574687995237	0.520295987387255	-3.59836022629986
a_HQ_transcript/87851	4.11151365544815	0.697324529501294	2.2864705626106	0.0288630600277377	0.520295987387255	-3.61612988916174
a_HQ_transcript/82614	5.54356255806182	1.55278325434616	2.28641358084709	0.0288667684412904	0.520295987387255	-3.63663032948628
a_HQ_transcript/122549	1.85742360842273	-0.615811062495567	2.28616907860173	0.0288826856926966	0.520295987387255	-3.61543600744938
a_HQ_transcript/8886	3.16337197435448	1.44240924913049	2.28612921885285	0.0288852813369473	0.520295987387255	-3.52818426739829
a_HQ_transcript/123376	-2.00006991008214	-0.596999371889174	-2.28598936335522	0.0288943903096032	0.520295987387255	-3.584417883933
a_HQ_transcript/1068	-1.43245287877527	2.79271028118127	-2.28593211513037	0.028898119708063	0.520295987387255	-3.7394714738762
a_HQ_transcript/63650	1.45262151249942	2.67752482827576	2.28579332630008	0.0289071628159885	0.520295987387255	-3.66297448967169
a_HQ_transcript/130719	-1.65776657541906	-0.681876094955002	-2.28565821182865	0.0289159689608951	0.520295987387255	-3.55731148216714
a_HQ_transcript/140958	1.9695682532343	1.67747161961434	2.28559252894992	0.028920250744017	0.520295987387255	-3.52240686960262
a_HQ_transcript/19033	-0.805594594196957	3.23014345217143	-2.28543900625107	0.0289302609202797	0.520315039224635	-3.72360619361202
a_HQ_transcript/9102	1.6535939383908	-0.757528161896897	2.28526922531674	0.0289413348196978	0.520353204577603	-3.59934261551618
a_HQ_transcript/96563	5.75608985432419	2.78929073926372	2.28508564884629	0.0289533128232817	0.520397430413418	-3.74435508662003
a_HQ_transcript/109169	-2.01466561319657	-0.300974060888023	-2.28495714926524	0.0289616998243085	0.520397430413418	-3.57305802423576
a_HQ_transcript/61460	2.43178975745909	1.64066624509235	2.28466315566297	0.0289808966354282	0.520581446113773	-3.67098808473935
a_HQ_transcript/55765	-1.98810126398773	0.557673593346675	-2.28435495024834	0.0290010337336346	0.520632744375723	-3.58140357576274
a_HQ_transcript/108285	2.97471598479325	0.409930222141377	2.28434528024632	0.0290016657426471	0.520632744375723	-3.5675099953575
a_HQ_transcript/132994	-2.84155316570772	1.25355986498581	-2.28388203124292	0.0290319571616651	0.52101562392506	-3.59180394490185
a_HQ_transcript/35834	1.95833441451241	2.03703609877118	2.28295662035871	0.0290925542320418	0.521433368956969	-3.67776766020315
a_HQ_transcript/114247	4.34067065920216	2.84193526560243	2.28290833493978	0.0290957191460674	0.521433368956969	-3.63818900727658
a_HQ_transcript/25222	-2.64661578770827	1.36846996303488	-2.2827940605316	0.0291032106066815	0.521433368956969	-3.71864934800499
a_HQ_transcript/73268	2.20672249093881	0.417629967515413	2.28274618051091	0.0291063499822489	0.521433368956969	-3.51953755577544
a_HQ_transcript/124298	1.6165195515281	-0.736870063344472	2.28273700140478	0.0291069518685635	0.521433368956969	-3.59744881574034

a_HQ_transcript/117871	-0.803157463195976	2.41876777437169	-2.28253640186957	0.0291201082483457	0.521433368956969	-3.57476008127932
a_HQ_transcript/7732	2.31581772489061	1.10932950522229	2.28252684959765	0.0291207348704554	0.521433368956969	-3.52262903043781
a_HQ_transcript/119069	8.44407201812331	3.69576255054094	2.28243138174647	0.0291269981592912	0.521433368956969	-3.83280226286343
a_HQ_transcript/151121	3.00375771610999	-0.413858033533393	2.28203777553778	0.0291528340023345	0.521735201627986	-3.64338199450506
a_HQ_transcript/152307	0.349260144591192	11.3015658412455	2.2810792136614	0.0292158391922976	0.522701846897469	-4.6444240144208
a_HQ_transcript/9222	-2.09366441514721	1.16086720517653	-2.28090728841294	0.0292271526013574	0.522743361972832	-3.52568776074516
a_HQ_transcript/37454	-2.62003195626082	2.81368008920565	-2.2796577520762	0.0293094959466459	0.523967105245924	-3.73443052856561
a_HQ_transcript/138995	-6.23109829759712	2.13532872906988	-2.27946376086328	0.0293222985072092	0.523967105245924	-3.74217413868903
a_HQ_transcript/80758	2.50062312002603	0.273855124648733	2.27945895869628	0.0293226154927916	0.523967105245924	-3.63517923368113
a_HQ_transcript/1862	-1.7497830866471	-0.426170941521809	-2.27919016497245	0.0293403631798137	0.524123119519425	-3.62015906507083
a_HQ_transcript/14075	-3.72876434611675	3.69317882595967	-2.27877678261238	0.0293676765140233	0.524443671547907	-3.79697758230608
a_HQ_transcript/66242	-1.70122060518737	3.57604687805596	-2.27843857431245	0.0293900399058723	0.524443671547907	-3.71079443224033
a_HQ_transcript/145555	5.49185589499875	3.29238120800905	2.278387272427	0.0293934334856895	0.524443671547907	-3.70175379693412
a_HQ_transcript/64230	3.1338360397791	0.747184584081669	2.278372714628	0.0293943965370059	0.524443671547907	-3.52818438190535
a_HQ_transcript/95325	-3.11273977438511	2.18684543385785	-2.27792419527173	0.0294240816423362	0.524784867856683	-3.68888263575337
a_HQ_transcript/17654	1.60514990956012	4.22958595710184	2.27775972098719	0.0294349740778267	0.524784867856683	-3.82143822712388
a_HQ_transcript/122949	-1.08822263109959	3.17489847963337	-2.27767472497308	0.0294406044261052	0.524784867856683	-3.76786580395556
a_HQ_transcript/149515	3.64330405174817	3.02307462791344	2.27720178824407	0.0294719506409354	0.525182571077957	-3.67077590167504
a_HQ_transcript/36380	-3.66499246963915	1.2389472673176	-2.27696480432022	0.0294876692130273	0.52520094536404	-3.77485276258475
a_HQ_transcript/128546	4.55518587348194	2.01200616000454	2.27678090290077	0.029499872149001	0.52520094536404	-3.73294983025415
a_HQ_transcript/120730	-2.66445064215901	1.87315946851261	-2.27677765801275	0.0295000875071152	0.52520094536404	-3.70693481218099
a_HQ_transcript/134028	-2.00889840389404	-0.367875246260422	-2.27658550344175	0.0295128430266312	0.525267158436013	-3.61109900086919
a_HQ_transcript/14169	0.917537510893367	3.33923021482645	2.27641134264339	0.0295244083791033	0.525312154961898	-3.83485235359854
a_HQ_transcript/107030	-2.63871760652261	0.787763358688189	-2.27619125877453	0.0295390291402358	0.52536086695121	-3.63032595597898
a_HQ_transcript/126738	1.80823092603102	-0.642060690236644	2.27609806574836	0.02954522216597	0.52536086695121	-3.63834464454586
a_HQ_transcript/117866	3.20124814682442	0.712592719716804	2.2756599206996	0.0295743542079698	0.525571241734909	-3.60508542071424
a_HQ_transcript/108407	4.42565011499313	1.90528705993624	2.27555910531507	0.0295810610285141	0.525571241734909	-3.77493000875065
a_HQ_transcript/64356	2.41111209529009	-0.00913584232241956	2.27551225763776	0.0295841780717834	0.525571241734909	-3.64585107496764
a_HQ_transcript/67694	-1.41758184562992	3.30476661850616	-2.27533958373751	0.0295956696039492	0.52557778716301	-3.84249751858861
a_HQ_transcript/79000	1.76471061860291	2.22662591996549	2.27523502524501	0.0296026299747401	0.52557778716301	-3.62445396464081
a_HQ_transcript/104523	-2.11286952372088	1.09819599739042	-2.27500291238479	0.0296180867967736	0.5256916486482	-3.6256516288728
a_HQ_transcript/39016	2.16559049250054	0.686639793077406	2.27448934878596	0.0296523117514746	0.526138454667191	-3.5395237502996
a_HQ_transcript/81889	0.766165513394327	3.41450733461406	2.27413254666342	0.0296761107365832	0.526400050319227	-3.80814733597567
a_HQ_transcript/129647	1.70505115122714	1.77886630877505	2.27399600103717	0.0296852229841046	0.526401049914593	-3.56657596845981
a_HQ_transcript/59491	2.58632413072215	0.341099170809833	2.27371924786496	0.0297036995758511	0.526555225195788	-3.54979341193828
a_HQ_transcript/40622	-1.52706045834226	2.1766744527241	-2.2734696041792	0.0297203751592659	0.526555225195788	-3.69809807614263
a_HQ_transcript/105749	2.04531770163263	0.376362972205109	2.27332453982258	0.0297300689694252	0.526555225195788	-3.52289707364158
a_HQ_transcript/35879	2.94204371485895	0.265778821898991	2.27295394263344	0.0297548467482026	0.526555225195788	-3.64202731548659
a_HQ_transcript/81684	-2.84952535191524	0.582547351421933	-2.27294453160845	0.0297554762022554	0.526555225195788	-3.57250399294532
a_HQ_transcript/106002	2.0469003788963	1.27799432470824	2.27222349873866	0.0298037379360995	0.526555225195788	-3.5938076786061
a_HQ_transcript/144600	3.40893319697295	3.46713743427319	2.27216933694456	0.0298073660523288	0.526555225195788	-3.82683561379533
a_HQ_transcript/8976	-2.92823468895071	2.44481259364475	-2.27215561496899	0.0298082853043466	0.526555225195788	-3.62032606048423
a_HQ_transcript/1627	0.999555131784925	3.04747791604517	2.27209031205761	0.0298126603761449	0.526555225195788	-3.70367822563681
a_HQ_transcript/83006	1.88762620330469	-0.492262320507917	2.2720230297733	0.0298171686632977	0.526555225195788	-3.61032280383371
a_HQ_transcript/14272	-2.55786575918581	0.145345056653371	-2.27192878017094	0.0298234849418809	0.526555225195788	-3.59565981106885
a_HQ_transcript/113121	-2.74929802929141	0.574633604941905	-2.27186052186588	0.0298280601260765	0.526555225195788	-3.64486022769438
a_HQ_transcript/44662	2.30255964183347	1.17836720927396	2.27182838960747	0.0298302140898763	0.526555225195788	-3.52234266597095
a_HQ_transcript/75800	-2.22430720805528	0.499270597175571	-2.27179715149943	0.029832308249045	0.526555225195788	-3.61428792728345
a_HQ_transcript/152879	-2.00746254927491	-0.448291515423673	-2.27172344605806	0.0298372498830824	0.526555225195788	-3.60364073613218
a_HQ_transcript/84096	1.85406447581971	0.245928616526695	2.27169952103682	0.0298388541115285	0.526555225195788	-3.54208509798405

a_HQ_transcript/20152	2.43725507441257	-0.381202866957938	2.27135723608019	0.0298618136119531	0.526800455731596	-3.62226527090869
a_HQ_transcript/23064	2.51091035027891	0.103164563671048	2.27103728796793	0.0298832891874195	0.52701936707716	-3.61028864419727
a_HQ_transcript/29649	4.73336029165726	1.78189578198596	2.27025279960099	0.0299360044578175	0.527788919358209	-3.72911187635212
a_HQ_transcript/58653	2.39098411818544	0.124787922079055	2.2700457263742	0.0299499331032448	0.527874381875505	-3.59022089992034
a_HQ_transcript/58648	1.75528061077501	-0.749026213097668	2.2698204552536	0.0299650924370635	0.527981477169332	-3.62615486669868
a_HQ_transcript/42525	-1.92522135190359	-0.446317281745951	-2.26955944891349	0.0299826651560463	0.528023844462879	-3.63973850678021
a_HQ_transcript/101153	2.21358793418605	3.37025133659385	2.26919152385508	0.0300074521088111	0.528023844462879	-3.93724292927153
a_HQ_transcript/142178	-2.43812839093236	0.81295150942173	-2.26912284066487	0.0300120813063163	0.528023844462879	-3.59947668661332
a_HQ_transcript/7066	-0.630366408677083	3.7808922988854	-2.26908188927366	0.030014841706152	0.528023844462879	-3.87869449669341
a_HQ_transcript/27341	-3.53877730391933	1.40813637648282	-2.26906346061858	0.0300160839962268	0.528023844462879	-3.64900247909971
a_HQ_transcript/102530	-2.06328523764817	1.64094525074276	-2.26887426590908	0.0300288404340443	0.528023844462879	-3.72935765395758
a_HQ_transcript/5670	-2.03027284229228	2.69327924990206	-2.26875975639754	0.0300365635965279	0.528023844462879	-3.67370195984648
a_HQ_transcript/56408	-4.40894705710632	1.73236730101862	-2.26870633194348	0.0300401674518088	0.528023844462879	-3.67909022909778
a_HQ_transcript/114454	0.849587810560481	-0.658599811028516	2.26853710974542	0.0300515852460793	0.528064857068954	-3.67184206718166
a_HQ_transcript/61765	-1.69564515457935	0.634187461429602	-2.26792512639967	0.0300929096916649	0.528631204157479	-3.55299186731019
a_HQ_transcript/151555	-2.44188614626877	2.05050074949606	-2.26769126455306	0.0301087148048255	0.528727148458954	-3.6694082196036
a_HQ_transcript/123746	-3.7629981314335	2.46026578160075	-2.26740968327139	0.030127754849632	0.528727148458954	-3.73163139168884
a_HQ_transcript/24632	1.96510217671894	2.50100919445222	2.26724634912332	0.0301388041858775	0.528727148458954	-3.64105207272231
a_HQ_transcript/148569	-2.26184071924034	-0.612300652842578	-2.26719215216325	0.0301424713425782	0.528727148458954	-3.63105510820655
a_HQ_transcript/94369	2.42349690726776	0.792822470973217	2.26717176395939	0.0301438509839143	0.528727148458954	-3.65680465825299
a_HQ_transcript/56731	2.8057413155336	0.973556438956069	2.26678964291265	0.0301697190884406	0.529021246205995	-3.55035978383107
a_HQ_transcript/137074	-2.88103412094307	0.261417045346599	-2.26576338749535	0.0302392913836337	0.529584129229103	-3.66537907688631
a_HQ_transcript/96307	1.72211124292631	-0.518455040328095	2.2657516985763	0.0302400846333071	0.529584129229103	-3.65340030742752
a_HQ_transcript/147023	-3.35984897239097	1.19086755547439	-2.2656817255206	0.0302448336332634	0.529584129229103	-3.71076512835603
a_HQ_transcript/94976	2.07223067024136	1.60054263702266	2.26566481192829	0.0302459816421417	0.529584129229103	-3.55002274542435
a_HQ_transcript/107139	-0.957245176869676	4.73425542885136	-2.2655373058868	0.0302546373665128	0.529584129229103	-3.97599385810653
a_HQ_transcript/117415	2.1958254669609	-0.282960931155902	2.26526191960163	0.0302733395129881	0.529584129229103	-3.61226595929873
a_HQ_transcript/100446	6.03655185950182	2.37317031756626	2.26516680701479	0.0302798012521501	0.529584129229103	-3.66652823228766
a_HQ_transcript/45732	-1.1414237351641	2.59342376195695	-2.26515994905894	0.0302802672144471	0.529584129229103	-3.69182780255193
a_HQ_transcript/144382	3.13537544187331	0.0552001801523609	2.26510772449087	0.0302838158126469	0.529584129229103	-3.62844451513325
a_HQ_transcript/130832	1.96356168119373	0.762188719192445	2.26496674841894	0.0302933968381891	0.529592352303836	-3.62122783604802
a_HQ_transcript/103159	-2.68513753945325	1.97697205245796	-2.26479027243129	0.0303053943517679	0.529642803030536	-3.61816436626703
a_HQ_transcript/10279	1.28665888967397	2.10459518499765	2.2645172008832	0.0303239672193429	0.529729795236327	-3.70621837267166
a_HQ_transcript/152478	3.54803440747487	2.46404801788615	2.26444913571754	0.0303285982408908	0.529729795236327	-3.76457365328179
a_HQ_transcript/116096	2.58234716643321	0.575621281762112	2.26411493422947	0.0303513458853467	0.52996786831584	-3.68260022780081
a_HQ_transcript/59915	2.51323797287658	-0.443228798388009	2.26386138841475	0.0303686138776156	0.530110146389801	-3.63106445450421
a_HQ_transcript/127630	-3.09188951914332	2.38643081587587	-2.2629122907983	0.0304333315435088	0.531080365043854	-3.75881924587794
a_HQ_transcript/141222	1.50520892570918	4.39347191229227	2.26264672745649	0.0304514621238747	0.531160378205848	-3.99026263911356
a_HQ_transcript/68864	-2.63107876749428	2.05480616677532	-2.26252302952451	0.0304599105610404	0.531160378205848	-3.62025156734841
a_HQ_transcript/105803	-2.30919251938451	0.789026448124008	-2.26244369727976	0.0304653299776063	0.531160378205848	-3.5507188082389
a_HQ_transcript/40980	-1.38598919547694	2.87781932790907	-2.26225217787448	0.0304784167969394	0.531222484396603	-3.80617768843071
a_HQ_transcript/2360	3.82341442977905	1.84622554168093	2.26212412260277	0.030487169831184	0.531222484396603	-3.82780392389282
a_HQ_transcript/117883	2.63321938047007	-0.228153724418118	2.2618217689629	0.0305078457446093	0.531357776148311	-3.65077305163374
a_HQ_transcript/151920	1.00463893262606	2.99892243892391	2.26174325797204	0.0305132166388498	0.531357776148311	-3.78204579941642
a_HQ_transcript/130026	-4.09781342821171	0.497421121679785	-2.26122600862406	0.0305486226301989	0.531815015348369	-3.642609518855
a_HQ_transcript/141689	3.49374632885519	-0.00251369786488605	2.26093740876824	0.0305683934842197	0.531985212587612	-3.63460177285357
a_HQ_transcript/17293	-4.0768143598656	2.78809181752306	-2.2608161620283	0.030576703056276	0.531985212587612	-3.86665528038206
a_HQ_transcript/49984	-3.83585516126023	0.959097304711553	-2.26035579123093	0.0306082727780612	0.532012664182645	-3.70270817958643
a_HQ_transcript/35401	2.34579956312455	2.95139046306616	2.26029515626751	0.0306124329755835	0.532012664182645	-3.64464555434134
a_HQ_transcript/23823	-2.28578901357762	-0.225269199069934	-2.26021153193368	0.0306181713197115	0.532012664182645	-3.64898837095623

a_HQ_transcript/42968	-2.07875224069778	1.37739262758108	-2.26012166385865	0.0306243391899268	0.532012664182645	-3.62564176941647
a_HQ_transcript/125115	1.51414274745583	2.81115276675385	2.26005452969551	0.0306289475034983	0.532012664182645	-3.62928532672579
a_HQ_transcript/111375	-1.4939123800199	1.66259881595795	-2.25997801791089	0.0306342002880629	0.532012664182645	-3.56618590801399
a_HQ_transcript/149507	8.13295027025048	1.05922504322584	2.25969911379525	0.0306533548222848	0.532012664182645	-3.71316618269151
a_HQ_transcript/131519	3.06654080240635	0.376398290418633	2.25939124664541	0.0306745109694923	0.532012664182645	-3.67702394374686
a_HQ_transcript/67686	-1.1914056113124	3.30828306765086	-2.25934581140149	0.0306776343187529	0.532012664182645	-3.75225286416735
a_HQ_transcript/116529	-4.05379907374311	0.594042673816807	-2.25925101368021	0.0306841519065859	0.532012664182645	-3.65427903592012
a_HQ_transcript/133872	-3.18115643161908	1.08228794969688	-2.25899222742442	0.0307019504630013	0.532012664182645	-3.76342009589414
a_HQ_transcript/119308	-4.09180919204676	1.78820630886573	-2.25897482834431	0.0307031474531832	0.532012664182645	-3.71463410549936
a_HQ_transcript/51482	3.26298513634709	1.33300154962241	2.25895558495152	0.030704471373853	0.532012664182645	-3.62167776679133
a_HQ_transcript/131398	-2.79189175519991	2.83986168244286	-2.25892733609061	0.0307064149520502	0.532012664182645	-3.88837856915252
a_HQ_transcript/17453	-1.28587615081972	2.46414965611957	-2.25869428465213	0.0307224535753894	0.532131937255731	-3.67431601336479
a_HQ_transcript/90633	3.15730288986438	0.805324829884637	2.25837908364271	0.0307441577131647	0.532195810599742	-3.65170814702837
a_HQ_transcript/38050	-2.6588234797153	1.31847969288902	-2.2583748046716	0.0307444524496617	0.532195810599742	-3.59608137924486
a_HQ_transcript/53422	-0.894277072078048	3.51225668552559	-2.25814088073061	0.0307605690465093	0.532295685985639	-3.83062252893574
a_HQ_transcript/50258	-2.25588655762598	0.0498500761282084	-2.25802527418912	0.0307685367621757	0.532295685985639	-3.60347688430852
a_HQ_transcript/141540	-2.05942016305494	-0.0628571177140611	-2.25732921945777	0.0308165487308857	0.532455091073338	-3.68210072720288
a_HQ_transcript/86821	-1.66648585430793	3.44262604510778	-2.25715525036209	0.030828559154648	0.532455091073338	-3.89715644970648
a_HQ_transcript/1228	2.10578508609464	1.25633073122538	2.25707668140446	0.0308339847517819	0.532455091073338	-3.60942223957228
a_HQ_transcript/23802	-4.2395148504162	2.03840876894043	-2.25685039779737	0.0308496156100301	0.532455091073338	-3.63362568143163
a_HQ_transcript/134273	-3.38189804047652	3.79941881745801	-2.25676505806853	0.030855512419774	0.532455091073338	-3.90309208220828
a_HQ_transcript/27259	1.32227565912168	3.7307270658075	2.2566241875352	0.0308652485153669	0.532455091073338	-3.90358164779461
a_HQ_transcript/100695	-1.68163314383011	-0.159368922312758	-2.25661581629215	0.0308658271704999	0.532455091073338	-3.64626278116164
a_HQ_transcript/15294	1.06582346873969	-0.730322750566291	2.2563514642032	0.0308841052992475	0.532455091073338	-3.6818590133204
a_HQ_transcript/136620	-3.98442194940774	2.46734454107676	-2.25634421074851	0.0308846069627574	0.532455091073338	-3.7393123575195
a_HQ_transcript/124812	-2.7067851255277	0.962480704659328	-2.25631467791374	0.0308866495885552	0.532455091073338	-3.56020087354947
a_HQ_transcript/13275	-3.32979908562057	0.863853716881846	-2.25618601755889	0.0308955497424062	0.532455091073338	-3.71653847850664
a_HQ_transcript/2604	0.796335169484626	3.72215168748462	2.25612451040011	0.0308998053491457	0.532455091073338	-3.88444260778221
a_HQ_transcript/108322	2.66299625514076	0.531609230224667	2.25588466792125	0.0309164047929186	0.532455091073338	-3.56745676589671
a_HQ_transcript/113328	4.07546037718717	0.33273690644299	2.25579131999792	0.0309228675464474	0.532455091073338	-3.70222940715377
a_HQ_transcript/37990	4.13557469578353	1.96569088313547	2.2557334780707	0.0309268727234057	0.532455091073338	-3.7785518167037
a_HQ_transcript/14918	3.31510466822358	0.263125303040007	2.25567388713173	0.0309309994952875	0.532455091073338	-3.6385963680966
a_HQ_transcript/90118	-1.76262780995675	2.81151432002053	-2.25561032498495	0.0309354018250178	0.532455091073338	-3.71712375264208
a_HQ_transcript/584	-0.671303962426266	2.84225656648271	-2.25531144759575	0.0309561096905032	0.532455091073338	-3.72811691339142
a_HQ_transcript/76519	2.86761364509372	0.922063586439385	2.25521447030015	0.0309628314841617	0.532455091073338	-3.6238462701094
a_HQ_transcript/101631	-2.83856449516213	-0.289103395691303	-2.25512562175271	0.0309689909999308	0.532455091073338	-3.69635830891705
a_HQ_transcript/149102	4.41365758040306	4.01539911146996	2.25504768051221	0.0309743952616293	0.532455091073338	-3.95973764659951
a_HQ_transcript/126902	-1.74137704283138	2.64175703764689	-2.25446520664896	0.031014809447121	0.532455091073338	-3.78123556609203
a_HQ_transcript/27400	3.10966665131834	1.73176228435885	2.25442019732388	0.0310179343302017	0.532455091073338	-3.65644751604949
a_HQ_transcript/44292	1.55732173771031	-0.731188538978144	2.25432256141335	0.0310247139152883	0.532455091073338	-3.65699909728338
a_HQ_transcript/126544	1.32603598177258	3.50007456721518	2.25431508223599	0.0310252333048859	0.532455091073338	-3.85462396514608
a_HQ_transcript/85166	2.97399996112425	0.840617589948049	2.25410275907783	0.0310399812909654	0.532455091073338	-3.61631787299053
a_HQ_transcript/12530	1.52349317762609	1.26500519398302	2.25394550769147	0.0310509080441739	0.532455091073338	-3.68987230429437
a_HQ_transcript/35627	-0.524764676469564	3.5306852145372	-2.25382321380266	0.0310594081318548	0.532455091073338	-3.85730841467373
a_HQ_transcript/105386	1.85191406310554	1.74321469009021	2.25372201331031	0.0310664436940934	0.532455091073338	-3.60738242400028
a_HQ_transcript/81512	3.95846753387378	4.39785093243081	2.25363211831343	0.0310726944868097	0.532455091073338	-3.91053270453072
a_HQ_transcript/137365	3.80226201702626	0.961125042355962	2.25335720117955	0.0310918176873971	0.532455091073338	-3.76201108785131
a_HQ_transcript/96010	-1.77206804901421	-0.668419384931801	-2.2530731519853	0.031111587212302	0.532455091073338	-3.65696216369019
a_HQ_transcript/50956	3.13361146798106	1.26663779329148	2.25305415923002	0.0311129094905618	0.532455091073338	-3.6022822011803
a_HQ_transcript/52549	1.74936687733256	-0.0129687526270566	2.25300654405577	0.0311162246874337	0.532455091073338	-3.71902791964985

a_HQ_transcript/127027	3.25931468286707	1.32921874263349	2.25285294165488	0.0311269213855499	0.532455091073338	-3.59971992331074
a_HQ_transcript/4941	1.42215458340752	0.937781490717948	2.25278366522892	0.0311317467980308	0.532455091073338	-3.56379304691526
a_HQ_transcript/108206	-2.04829792512003	-0.140659273494319	-2.25276500485712	0.0311330466910895	0.532455091073338	-3.65165811305639
a_HQ_transcript/4095	1.64832683002379	1.21442586723739	2.25266462574127	0.0311400399986691	0.532455091073338	-3.6590439293593
a_HQ_transcript/108349	3.87352287220807	1.76926108304004	2.25263687443542	0.0311419736518547	0.532455091073338	-3.75225038968822
a_HQ_transcript/45287	-2.39672415478116	1.21239579467628	-2.25220506685439	0.0311720749969367	0.532455091073338	-3.70751074980036
a_HQ_transcript/52333	-2.94970675436311	0.734897520717604	-2.25217449550731	0.031174207118137	0.532455091073338	-3.6360206782062
a_HQ_transcript/129829	4.76776749503284	2.06143828245827	2.25207980624221	0.0311808118120251	0.532455091073338	-3.67886275342553
a_HQ_transcript/110893	1.46422826694379	3.31745138681316	2.25201837345981	0.0311850974968299	0.532455091073338	-3.8019645132885
a_HQ_transcript/42038	1.90500330935093	0.619792781872336	2.25199863887917	0.0311864743364177	0.532455091073338	-3.58506385599666
a_HQ_transcript/46680	-2.00706163850417	1.06637512138029	-2.25175064121048	0.0312037812574606	0.532455091073338	-3.6167087553327
a_HQ_transcript/49025	0.549638783324508	3.97972238144786	2.2515938758825	0.0312147258286328	0.532455091073338	-3.93897072194025
a_HQ_transcript/141868	0.623173418845647	8.34903964183895	2.25144778301708	0.0312249284040653	0.532455091073338	-4.35976217278441
a_HQ_transcript/60696	-2.84047385557116	1.54891333772218	-2.25119616212025	0.0312425076784184	0.532455091073338	-3.59894070323773
a_HQ_transcript/148221	2.70930797623695	0.999387391654286	2.25101889346755	0.0312548977321752	0.532455091073338	-3.63333963768405
a_HQ_transcript/98853	2.52566417161095	1.67076173613794	2.25101888533007	0.0312548983010391	0.532455091073338	-3.58786389413418
a_HQ_transcript/6164	1.73463288774125	1.53182566679865	2.25093303387202	0.0312609004068959	0.532455091073338	-3.62797651692689
a_HQ_transcript/144644	-1.36987726533731	3.94441139600877	-2.25085319136351	0.0312664833395641	0.532455091073338	-3.81499953849981
a_HQ_transcript/116366	-1.51935068027604	1.67669222309636	-2.2508174972091	0.0312689795183434	0.532455091073338	-3.5808756579029
a_HQ_transcript/137239	2.86914071907748	1.88337206762696	2.25076868513378	0.0312723933547409	0.532455091073338	-3.64272329654512
a_HQ_transcript/72519	-2.44411906577104	1.74762928446893	-2.25055788726157	0.0312871400544941	0.532550183627418	-3.73862808724193
a_HQ_transcript/143744	4.06366190634962	1.33937095637148	2.25025475555895	0.0313083570565457	0.532605899085722	-3.72364371700077
a_HQ_transcript/97770	-2.90378904485528	1.55318056060963	-2.25024930609716	0.0313087385971634	0.532605899085722	-3.7709462880077
a_HQ_transcript/78155	1.60390379418854	-0.734644806932157	2.2499245556557	0.0313314833307038	0.532713591424738	-3.6648518862628
a_HQ_transcript/110216	-0.997753795216777	2.70278873149344	-2.24989722508047	0.0313333981743941	0.532713591424738	-3.77226845708047
a_HQ_transcript/112517	-1.32546825282579	2.17493669860927	-2.24964679782991	0.0313509485818131	0.532856122562466	-3.61968833914277
a_HQ_transcript/120746	4.24796673518457	4.0432010167652	2.24882032722766	0.0314089318070474	0.533655850203284	-3.94076114019431
a_HQ_transcript/46090	-2.57633718161148	2.83084394699086	-2.24871451199823	0.0314163624999249	0.533655850203284	-3.93428797315929
a_HQ_transcript/44081	1.7349613980434	2.65836063864998	2.24826644981091	0.0314478443862612	0.533879865310569	-3.71273284162848
a_HQ_transcript/83191	1.61987305247496	-0.15968346714237	2.24821296468722	0.0314516042658235	0.533879865310569	-3.6349405019521
a_HQ_transcript/92211	1.97884800793455	-0.401169092275462	2.24813474072104	0.0314571039548703	0.533879865310569	-3.64083874934092
a_HQ_transcript/2660	-2.86679993414142	0.854618947522825	-2.24784192121942	0.0314776988662703	0.534073461675003	-3.63958020639148
a_HQ_transcript/943	-2.62704453135641	0.697031627719132	-2.24732995776704	0.0315137359320747	0.534423565139589	-3.67209001817817
a_HQ_transcript/112183	2.28020919967878	1.70063894358908	2.24721952043863	0.0315215144596385	0.534423565139589	-3.63402735617307
a_HQ_transcript/7323	-0.881851206934302	2.39412625053257	-2.24697921985665	0.0315384457111803	0.534423565139589	-3.66683604237863
a_HQ_transcript/111529	0.724612698971224	3.02965405595048	2.2466288297674	0.0315631483450532	0.534423565139589	-3.80085911108798
a_HQ_transcript/115708	1.42199838285268	-0.668408447522179	2.24657358302597	0.0315670448454099	0.534423565139589	-3.66457425477301
a_HQ_transcript/104987	-2.54047331937057	0.278168486400325	-2.24656443671398	0.0315676899677768	0.534423565139589	-3.66761304872917
a_HQ_transcript/48386	2.05917047698171	0.854859581264634	2.2465388599043	0.0315694940551938	0.534423565139589	-3.60404541175745
a_HQ_transcript/129509	4.15592146099258	3.95323591859034	2.24645525136338	0.0315753921179132	0.534423565139589	-3.83295192825078
a_HQ_transcript/152188	-0.938485227696934	7.87660009166364	-2.24637465308106	0.0315810787616035	0.534423565139589	-4.30136366364372
a_HQ_transcript/100136	-2.08807988602186	3.45339493915419	-2.24585851020895	0.031617517217244	0.534863654062657	-3.82134740810697
a_HQ_transcript/61868	2.01532592935799	0.0881895119364266	2.24533676319097	0.0316543896482328	0.534863654062657	-3.62843250924893
a_HQ_transcript/32791	3.94344165272847	1.67468141607662	2.24527286155608	0.0316589082975048	0.534863654062657	-3.67370433880253
a_HQ_transcript/75545	1.60598787438458	1.86163404395401	2.24507627825661	0.0316728128381519	0.534863654062657	-3.6381935493845
a_HQ_transcript/132171	-2.47294309066667	0.898427952410692	-2.24503114708328	0.0316760057860738	0.534863654062657	-3.6325670742166
a_HQ_transcript/138678	-2.60291108065045	1.20825488892041	-2.24497558271089	0.031679937260385	0.534863654062657	-3.6940866079138
a_HQ_transcript/149066	-3.95971566920807	-0.0226661309213103	-2.24487012991746	0.0316873998091912	0.534863654062657	-3.74804481268479
a_HQ_transcript/137878	0.75751626234542	3.86059786354117	2.24480620346905	0.0316919244423651	0.534863654062657	-3.958720883485
a_HQ_transcript/89810	2.38259806629785	0.155644056103164	2.24478711557778	0.0316932755715421	0.534863654062657	-3.63385514310763

a_HQ_transcript/126455	-0.805492873316888	4.28835171146259	-2.2447048392861	0.0316991000592805	0.534863654062657	-3.99600718014286
a_HQ_transcript/42635	-3.78532471662117	1.99008861049719	-2.24454005531731	0.0317107683027901	0.534905264046601	-3.82207380997256
a_HQ_transcript/90538	2.63237509888036	1.3547309947947	2.24435566230518	0.0317238296179018	0.534921750602506	-3.64966734628696
a_HQ_transcript/138992	3.09497458538771	0.776051536601994	2.24426644986019	0.0317301506344178	0.534921750602506	-3.73979002647616
a_HQ_transcript/146880	-1.97716438080645	3.82165171159876	-2.24410861746349	0.031741336384493	0.534955175806845	-3.99856623343308
a_HQ_transcript/88242	1.71784806889158	-0.622643401617032	2.24396730883254	0.0317513540789937	0.534966942756361	-3.63938241405749
a_HQ_transcript/135935	-1.5545626003333	1.38901958942482	-2.24383916297897	0.0317604410860893	0.534966942756361	-3.7233796128946
a_HQ_transcript/18283	3.3640301215015	1.19117604938879	2.24310905242433	0.0318122588037802	0.535482214101915	-3.66064718404274
a_HQ_transcript/124224	1.48821223468647	3.12271432081754	2.24307917893661	0.0318143806117452	0.535482214101915	-3.80153391804409
a_HQ_transcript/135125	1.15170175439124	2.13255384738278	2.24293091328046	0.0318249132745248	0.535482214101915	-3.67292142786449
a_HQ_transcript/75645	2.94520575349762	2.55374900412848	2.24282312293655	0.0318325725699622	0.535482214101915	-3.71738739364404
a_HQ_transcript/147183	1.57501976290027	-0.488533786242636	2.24237074738508	0.0318647352092084	0.535482214101915	-3.66076741549844
a_HQ_transcript/100243	-2.47370774463716	1.02728033527871	-2.24197523781309	0.0318928787149407	0.535482214101915	-3.76415863657604
a_HQ_transcript/142088	1.60361420666115	0.868142227335969	2.24193984045436	0.03189539859213	0.535482214101915	-3.61691394536484
a_HQ_transcript/151611	0.355789852063654	10.5795535332608	2.24192039450742	0.0318967829916995	0.535482214101915	-4.63092470692108
a_HQ_transcript/136299	1.54226411722567	-0.727698171997005	2.24191537800546	0.0318971401362109	0.535482214101915	-3.65638423877581
a_HQ_transcript/105613	3.03424248598551	-0.100137590613162	2.24177087531913	0.0319074293908001	0.535482214101915	-3.6651936363335
a_HQ_transcript/112988	0.621886613636062	3.85758663216415	2.24160778177953	0.0319190459754463	0.535482214101915	-3.94367164311705
a_HQ_transcript/25423	4.60456411283933	0.985107635141741	2.24121924396119	0.0319467354543061	0.535482214101915	-3.72033003164843
a_HQ_transcript/88267	1.36641869810387	-0.621211879354121	2.24115888256691	0.0319510390933908	0.535482214101915	-3.72884005907467
a_HQ_transcript/139812	0.496211690960821	4.3154625448982	2.24114518039782	0.0319520161013257	0.535482214101915	-3.99272168063749
a_HQ_transcript/103303	1.11477741817118	3.61533164463041	2.24101408578673	0.0319613649157295	0.535482214101915	-3.92580155383048
a_HQ_transcript/15171	3.09921663529233	0.351746349036252	2.24097642017229	0.031964051435839	0.535482214101915	-3.68759889665952
a_HQ_transcript/140026	-4.7704274313027	0.171907590132829	-2.24093854204573	0.0319667533177262	0.535482214101915	-3.79868491434109
a_HQ_transcript/141531	2.439238039836	-0.0492346259228228	2.24089885946036	0.0319695841330284	0.535482214101915	-3.70113981047221
a_HQ_transcript/85954	-1.87735248884075	-0.27731392280671	-2.24080147541386	0.0319765321193859	0.535482214101915	-3.64773274896736
a_HQ_transcript/112433	3.68534947597992	0.912223680123995	2.2406194981079	0.0319895191472112	0.535482214101915	-3.6086632615843
a_HQ_transcript/41828	-1.812116544502	0.60071060234151	-2.24059631248224	0.0319911741567773	0.535482214101915	-3.62762979509597
a_HQ_transcript/22855	3.18308746450764	0.2529610897374	2.2405609445364	0.0319936988985678	0.535482214101915	-3.62042668394566
a_HQ_transcript/125808	2.60941808100216	0.209650968348278	2.24041424372963	0.0320041730475654	0.53550333071643	-3.7093395299323
a_HQ_transcript/41645	2.42265557135148	0.861803283412776	2.24010878631369	0.032025991974316	0.535714204743321	-3.59059279439856
a_HQ_transcript/11469	-0.999910192775507	-0.732912813749768	-2.23973225657053	0.0320529059772455	0.535772004477549	-3.69532085575043
a_HQ_transcript/723	2.77191524364989	2.36628107428662	2.23953049765051	0.0320673358652651	0.535772004477549	-3.70093718955156
a_HQ_transcript/86581	-2.33329477539492	0.186202694123963	-2.239463873783	0.0320721021132468	0.535772004477549	-3.6686835478076
a_HQ_transcript/98561	1.39178989592731	-0.805276381262406	2.23941856016557	0.0320753441964055	0.535772004477549	-3.68544229689788
a_HQ_transcript/90268	3.86772475231237	0.694536275044636	2.23938490684223	0.0320777522030158	0.535772004477549	-3.75575350269406
a_HQ_transcript/97919	-0.970126494613372	2.42249343273993	-2.23928712142587	0.0320847499928838	0.535772004477549	-3.76012144626575
a_HQ_transcript/103918	-0.648900877494137	-0.824998489467312	-2.23905201091994	0.0321015807415896	0.535782814222937	-3.71168156958652
a_HQ_transcript/49361	2.59559152956858	0.0297383917254891	2.23897240711682	0.0321072810965299	0.535782814222937	-3.64836442198768
a_HQ_transcript/101778	-2.41893215651279	0.7502048282285	-2.23889186991812	0.0321130492147109	0.535782814222937	-3.56656006625227
a_HQ_transcript/72108	-0.531453829154545	5.0127380217715	-2.23860934263706	0.0321332913207597	0.535818736630482	-4.08717914584657
a_HQ_transcript/142283	-4.73315164412407	0.794834577403442	-2.23852347605393	0.0321394456320411	0.535818736630482	-3.70182701634898
a_HQ_transcript/69654	1.48098411170105	-0.782515920424788	2.2384440363966	0.032145140247738	0.535818736630482	-3.68329303655786
a_HQ_transcript/147688	1.65627824563472	-0.662731157121188	2.23827226768507	0.0321574565459961	0.535818736630482	-3.70836405131867
a_HQ_transcript/143458	4.07204500682855	1.56483717964473	2.23821879096326	0.0321612918404857	0.535818736630482	-3.83359384248905
a_HQ_transcript/870	1.40527853026933	-0.633744633682827	2.23800195092855	0.0321768475841453	0.535924296954498	-3.67846150866536
a_HQ_transcript/7634	-1.00891542219875	3.34023902457538	-2.2378699317382	0.0321863217249367	0.535928533150135	-3.85344048933211
a_HQ_transcript/13616	2.45685518858834	0.28881356794008	2.23726782816924	0.0322295624225225	0.53632294156662	-3.65520071686743
a_HQ_transcript/54292	-1.8462731056502	-0.470716183630807	-2.23710902076128	0.0322409760190445	0.53632294156662	-3.66934780079116
a_HQ_transcript/139330	2.05774078516619	0.30617735255778	2.23705506466468	0.0322448547050897	0.53632294156662	-3.64397349918486

a_HQ_transcript/90136	-2.51304253837409	1.79252907976588	-2.23702640507713	0.0322469150972911	0.53632294156662	-3.65164597098376
a_HQ_transcript/129038	3.21878585554913	0.60524864758335	2.23679735531897	0.0322633861611602	0.53644339553087	-3.71027120920833
a_HQ_transcript/140743	-2.72315875118633	0.393713566330395	-2.23646990202383	0.0322869465631123	0.53646732878667	-3.65296065506313
a_HQ_transcript/123590	3.34225694885011	2.96099167325413	2.23646397658344	0.0322873730430716	0.53646732878667	-3.89042057483609
a_HQ_transcript/80694	3.29704583248397	0.184342145173007	2.23636264991206	0.0322946667500511	0.53646732878667	-3.71029622021159
a_HQ_transcript/106641	1.48136306399373	4.36891785813602	2.23626437968536	0.0323017418585423	0.53646732878667	-4.01321065446017
a_HQ_transcript/92069	-2.03109936645769	0.0747371455511826	-2.2361286925254	0.0323115131375738	0.536476331237044	-3.68555176185998
a_HQ_transcript/34193	2.90837238892663	1.51165180513175	2.23592552057088	0.0323261491729447	0.53656607627782	-3.75026197222158
a_HQ_transcript/145808	2.58144711204798	2.50430310604722	2.23568472894948	0.0323435029367791	0.536586398463898	-3.81252706512934
a_HQ_transcript/126982	1.10401826740012	3.89503341761078	2.23565236896322	0.0323458357455529	0.536586398463898	-3.92815218103981
a_HQ_transcript/85653	-2.26941814403695	0.24558238362248	-2.23541309063934	0.0323630898337649	0.536719453882193	-3.64959684060851
a_HQ_transcript/22485	2.09227854420987	0.813150880549206	2.23497478526058	0.0323947169350254	0.537090731887951	-3.593728345275
a_HQ_transcript/119969	2.26233371631006	1.90532743330469	2.23470976770299	0.0324138534250706	0.537254768147277	-3.74324830650915
a_HQ_transcript/137754	4.86712674802522	1.99995093421782	2.23453654750935	0.0324263668506412	0.537307031565729	-3.7592633510235
a_HQ_transcript/128809	-1.53382974686616	0.754739946927692	-2.23441024557651	0.0324354936306796	0.537307031565729	-3.59523248175039
a_HQ_transcript/142653	2.35057577651969	3.40542751823504	2.23407077437644	0.0324600357750523	0.537560387331122	-3.80009174078574
a_HQ_transcript/84632	-1.22925173145992	1.69858903701948	-2.23392121413617	0.0324708535456223	0.537586378496136	-3.63780345462096
a_HQ_transcript/28796	2.14266816772014	0.367010614980498	2.23364938765005	0.032490523170997	0.537676722676646	-3.66736308434382
a_HQ_transcript/86108	2.2490674531439	2.12396297138214	2.23350144955356	0.0325012326002658	0.537676722676646	-3.65909080871872
a_HQ_transcript/150823	-0.946254956514773	2.67899171406818	-2.23346239875617	0.0325040600654716	0.537676722676646	-3.75004621936136
a_HQ_transcript/16355	2.32695755036077	1.02484310396095	2.23273961444826	0.0325564329333444	0.538375724510171	-3.65009760924207
a_HQ_transcript/7987	1.87167973263777	0.774655390458525	2.23262368117153	0.0325648404792486	0.538375724510171	-3.6011264619831
a_HQ_transcript/62368	-1.64348679095725	2.44586532119443	-2.23167212016343	0.0326339217694758	0.538882252021895	-3.75244949117151
a_HQ_transcript/152794	-2.63238561817826	2.44091998834086	-2.23153151479558	0.0326441405452687	0.538882252021895	-3.77522885035927
a_HQ_transcript/118650	-2.18919244749452	-0.182239664657366	-2.23138207044631	0.032655004854746	0.538882252021895	-3.6888998853796
a_HQ_transcript/152833	-4.08984202451432	-0.576654442153546	-2.2312684244667	0.0326632688618359	0.538882252021895	-3.72393723519861
a_HQ_transcript/108145	2.45516744776682	2.70025211387298	2.23122218030285	0.0326666321400949	0.538882252021895	-3.84664832498531
a_HQ_transcript/1863	-2.96875149197276	0.0549365960627755	-2.23114759283479	0.0326720574440783	0.538882252021895	-3.69270711609018
a_HQ_transcript/33548	-1.73935284604665	2.16083952332257	-2.23107848007867	0.0326770852519418	0.538882252021895	-3.67548001199546
a_HQ_transcript/143344	-4.9421487563064	0.828335429445497	-2.231068420088	0.0326778171529308	0.538882252021895	-3.72189684003079
a_HQ_transcript/9673	2.03736446411528	2.14734243135409	2.23105333828327	0.0326789144367117	0.538882252021895	-3.75759240347458
a_HQ_transcript/22715	-3.35323171169221	2.20128029033354	-2.23074677884841	0.0327012254687859	0.539097230303342	-3.67989943732705
a_HQ_transcript/112292	2.68730165967382	0.562993772657303	2.23012781824183	0.0327463142873787	0.539477889674558	-3.72580208526741
a_HQ_transcript/84868	2.11989648499006	-0.321296885705544	2.23012625890409	0.0327464279492328	0.539477889674558	-3.69114394613204
a_HQ_transcript/6601	-2.2973263130004	4.32113110198453	-2.23004764642362	0.032752158558036	0.539477889674558	-3.96033067043777
a_HQ_transcript/144341	-2.2967637127751	-0.193229553860638	-2.2295700289899	0.032786994719957	0.539898705122283	-3.70710331989524
a_HQ_transcript/140731	4.55887645177543	0.756793006588168	2.22929665401178	0.032806948906813	0.540074292284119	-3.66439337819277
a_HQ_transcript/87170	-2.31006427423265	-0.346065657753227	-2.22916267116911	0.0328167325546641	0.540082398057054	-3.73107484504263
a_HQ_transcript/132272	-3.51250539711388	1.37418319962505	-2.22900079400184	0.032828556578546	0.540124069288911	-3.73681051838797
a_HQ_transcript/88497	-0.798684284057634	3.94247716226698	-2.22868134084727	0.0328519016518232	0.540263653758365	-4.00150560261373
a_HQ_transcript/148939	-2.03682407245218	2.20538922878236	-2.22863035159148	0.0328556292326559	0.540263653758365	-3.75117497119754
a_HQ_transcript/135853	-2.05057976960156	1.29312663114987	-2.2283480019329	0.0328762773253919	0.540387036821837	-3.72108055565508
a_HQ_transcript/64423	-1.9202435193042	1.98596324826037	-2.22827021927214	0.0328819675751317	0.540387036821837	-3.65252584316021
a_HQ_transcript/11129	2.2147367866617	-0.0613386141203917	2.22814647277155	0.0328910221627384	0.540387036821837	-3.6611747902265
a_HQ_transcript/136097	2.27948763312145	2.94651326320047	2.22777963303506	0.032917877117638	0.540632435622651	-3.89555626112022
a_HQ_transcript/36571	-1.25746947550351	-0.818711849868178	-2.22768838662462	0.0329245599728906	0.540632435622651	-3.6890767239396
a_HQ_transcript/150438	-2.22658533321767	0.0609900580414412	-2.22747906837665	0.0329398949597644	0.540731492296296	-3.71583209835731
a_HQ_transcript/18031	1.63245477094351	2.48849821092149	2.22704414220639	0.0329717787981862	0.541102077352052	-3.75006444793625
a_HQ_transcript/70017	-3.30798425802331	1.69759053135629	-2.22626909196142	0.033028665186025	0.541882473933748	-3.71230782019883
a_HQ_transcript/29036	1.5583280273125	1.33851459465034	2.22614234599417	0.0330379763215869	0.541882473933748	-3.61700454071072

a_HQ_transcript/110425	3.02528827285075	0.823307505827791	2.22597512994155	0.0330502641063618	0.541931100698053	-3.62748451643835
a_HQ_transcript/144402	-4.08205459979438	1.9835142676474	-2.22563013087045	0.0330756291177038	0.542194069191733	-3.80536808690603
a_HQ_transcript/143456	-0.847973309841967	5.20487243505611	-2.22550099311996	0.0330851280624951	0.542196877365864	-4.09907121024427
a_HQ_transcript/152492	-5.07238066125273	1.31374863946525	-2.22514463421463	0.033111353315236	0.542217944205547	-3.78167913564962
a_HQ_transcript/125101	-4.42182000474971	0.614050417544298	-2.22507009904845	0.0331168408789211	0.542217944205547	-3.69822168552705
a_HQ_transcript/54116	-0.887045622048333	2.09235789910299	-2.22502348884946	0.0331202729136916	0.542217944205547	-3.71560550486396
a_HQ_transcript/23511	-1.27392418837103	-0.641476980565025	-2.22491849309126	0.0331280052016044	0.542217944205547	-3.71245999811832
a_HQ_transcript/17299	-1.77617994666758	-0.524228974524795	-2.22480345233502	0.0331364790972362	0.542217944205547	-3.68323007957369
a_HQ_transcript/152360	2.61278186972396	3.5162859997962	2.22472333655375	0.0331423815676147	0.542217944205547	-3.87001313203321
a_HQ_transcript/116814	1.88435981756556	-0.198934454916627	2.2245812943037	0.0331528487385999	0.542236576105047	-3.72814149327692
a_HQ_transcript/94601	-1.63236749160296	2.3000807481461	-2.22427727803517	0.0331752618116942	0.542450525623111	-3.74891573870653
a_HQ_transcript/28969	2.05509502173308	2.44007947466599	2.22405526579514	0.0331916378501967	0.542565670685105	-3.78970985592714
a_HQ_transcript/65546	2.48161274557467	1.07802927662048	2.22376233473867	0.0332132560682572	0.542633372449015	-3.66713302757468
a_HQ_transcript/7585	2.58444690345937	0.818824583742111	2.22370127470282	0.0332177638667844	0.542633372449015	-3.70872823946701
a_HQ_transcript/37865	-1.98134629373156	1.71436215724612	-2.22350076008078	0.033232570847895	0.542633372449015	-3.64400897154458
a_HQ_transcript/151480	-3.78952647287672	-0.0142192255032215	-2.22349332353003	0.0332331201128284	0.542633372449015	-3.83566852483258
a_HQ_transcript/84974	-1.86598007055129	1.75985900785646	-2.22330771874436	0.0332468315478099	0.542648463861314	-3.77556138572713
a_HQ_transcript/25338	1.41458993550006	2.68031757727679	2.22322809592487	0.0332527151850055	0.542648463861314	-3.70683364534406
a_HQ_transcript/17096	1.65283446248616	1.69479196647305	2.22276163055036	0.033287202841737	0.543058806282483	-3.6289891697045
a_HQ_transcript/39215	2.40191615147218	0.356957404895668	2.22223288009278	0.0333263342024583	0.54320492213917	-3.63386363701791
a_HQ_transcript/128745	1.00058751850897	2.8265383801107	2.22211882569059	0.0333347804513007	0.54320492213917	-3.82092205862212
a_HQ_transcript/142310	-2.20764493765048	1.58378247800509	-2.22210160866364	0.0333360556171515	0.54320492213917	-3.8313380308696
a_HQ_transcript/60492	-2.64796093310861	1.32477373257939	-2.22198124099158	0.0333449717760066	0.54320492213917	-3.6800746486824
a_HQ_transcript/45593	1.47619216925821	1.45840467570655	2.22193295004847	0.0333485494969395	0.54320492213917	-3.64319559588656
a_HQ_transcript/138288	0.732105647593094	2.90059567811625	2.22188329016553	0.0333522289966057	0.54320492213917	-3.84132656823366
a_HQ_transcript/74703	3.19691102466153	0.0272272357631742	2.22174950369362	0.0333621435806965	0.543214196655105	-3.6994073365432
a_HQ_transcript/104586	5.13818175097146	1.49873844996987	2.22157896408927	0.0333747856733989	0.543267863798188	-3.83289797059436
a_HQ_transcript/119732	0.865792248955745	2.66422743448816	2.22128905496442	0.033396286464224	0.543465660580182	-3.76990993672373
a_HQ_transcript/61211	1.54081635193358	2.96785205411986	2.22081917890437	0.0334311606378488	0.54358237544106	-3.80141814057664
a_HQ_transcript/118447	-2.31101143383983	0.161422953863837	-2.22076051368607	0.0334355170550772	0.54358237544106	-3.72609428564924
a_HQ_transcript/101325	2.69212322321324	1.12527360955516	2.2206898509406	0.0334407650706307	0.54358237544106	-3.66374582412834
a_HQ_transcript/24036	1.30362511412539	3.61668518995321	2.22068851157714	0.0334408645502551	0.54358237544106	-3.94682883033729
a_HQ_transcript/150904	-2.83464283282219	1.38427834785896	-2.22044862614047	0.0334586860277482	0.543720017171078	-3.75955614475788
a_HQ_transcript/133449	3.7020944299092	1.20253125783492	2.22029729438187	0.0334699330815188	0.543749871103693	-3.69551782659415
a_HQ_transcript/150882	-2.12024749407573	1.11453174235315	-2.22017221169684	0.033479231844896	0.543749871103693	-3.68138887101742
a_HQ_transcript/75772	3.02513824826013	0.882372954210411	2.21993600473626	0.0334967979966657	0.543883205014017	-3.6837316616301
a_HQ_transcript/108732	1.59285980881888	-0.753033654863558	2.21968813093073	0.0335152406538226	0.544030692188048	-3.70176362182296
a_HQ_transcript/109519	-3.71758117068109	1.4884701000963	-2.21891726211523	0.0335726540159433	0.54462051244262	-3.68920205374943
a_HQ_transcript/97600	-2.67345147494154	0.813218014556642	-2.21872653959994	0.0335868723697561	0.54462051244262	-3.68464612893428
a_HQ_transcript/18364	1.37485308766831	-0.286965282877011	2.21867866757704	0.033590442072567	0.54462051244262	-3.64813587152558
a_HQ_transcript/117601	0.597149343021109	4.61754550400634	2.21867510117301	0.0335907080244166	0.54462051244262	-4.08502208439181
a_HQ_transcript/110519	2.25036751759724	-0.425934749693353	2.21844740350027	0.0336076916666193	0.54462051244262	-3.716519743206
a_HQ_transcript/67617	2.26184960349925	1.75312998125872	2.2183348880374	0.0336160868697055	0.54462051244262	-3.70587193702777
a_HQ_transcript/103547	3.02822444177882	1.48016745018617	2.21832047921758	0.0336171621016398	0.54462051244262	-3.60265701606577
a_HQ_transcript/125627	-2.16085511522413	1.12706752941622	-2.21787124849473	0.033650700567127	0.544807148947436	-3.64237939043275
a_HQ_transcript/1464	-1.29576764076107	1.36962267935379	-2.21784226865643	0.0336528651585602	0.544807148947436	-3.63879768911184
a_HQ_transcript/122301	3.5736496316309	1.33418514621656	2.21763210823152	0.0336685664021859	0.544807148947436	-3.61818721371511
a_HQ_transcript/122065	-5.18831325359757	2.30747461299283	-2.21709580472839	0.033708663751626	0.544807148947436	-3.75116099687115
a_HQ_transcript/69360	1.3022183186464	1.10711635871946	2.21708384079777	0.0337095587357016	0.544807148947436	-3.6329403317513
a_HQ_transcript/23508	2.36875361999062	0.744584782363273	2.21692400184664	0.0337215178253071	0.544807148947436	-3.67632802498282

a_HQ_transcript/89635	-5.28870946792239	7.25178686623244	-2.21685219086095	0.0337268919317187	0.544807148947436	-4.27653852382852
a_HQ_transcript/138153	-3.50070872430944	1.00924883739789	-2.21665036808633	0.0337419998115012	0.544807148947436	-3.70905069804197
a_HQ_transcript/43934	-1.64690619655665	-0.246656585781914	-2.21661010734003	0.0337450143409586	0.544807148947436	-3.75085556568063
a_HQ_transcript/72476	1.65131389555203	2.67180687686222	2.21651946723257	0.0337518019146001	0.544807148947436	-3.75602965860728
a_HQ_transcript/2019	0.924882009397715	1.98308317154558	2.21646101454031	0.0337561797853339	0.544807148947436	-3.68952271294548
a_HQ_transcript/63088	-2.97038817898274	0.605224769116128	-2.21620563090026	0.0337753129486166	0.544807148947436	-3.6834908640126
a_HQ_transcript/128290	2.9741389202345	1.92487116932537	2.2160724277554	0.0337852962844379	0.544807148947436	-3.81082466949283
a_HQ_transcript/65626	-2.19592882492475	-0.471546892991542	-2.21602311180397	0.0337889930957794	0.544807148947436	-3.72430335393637
a_HQ_transcript/10694	1.03664449219327	1.79182993410767	2.21600540473432	0.0337903205374763	0.544807148947436	-3.66334703445475
a_HQ_transcript/74467	-2.37372487109026	2.0868862131922	-2.21596450817797	0.0337933865982502	0.544807148947436	-3.75000285156203
a_HQ_transcript/88413	2.73610520331419	1.63757440707667	2.21567153505382	0.0338153584016245	0.544807148947436	-3.75541252802034
a_HQ_transcript/144777	-1.66139570108457	1.0129122486668	-2.21562222967826	0.0338190573621923	0.544807148947436	-3.59799239164001
a_HQ_transcript/51642	-2.19353781008085	-0.484174329869428	-2.2156044322442	0.0338203926403395	0.544807148947436	-3.70931029021882
a_HQ_transcript/10807	-2.27129813364228	-0.0625858172944164	-2.21553561437654	0.0338255562438619	0.544807148947436	-3.67188710168836
a_HQ_transcript/42373	-2.25475680638863	1.35223693786322	-2.2153822846761	0.0338370635508231	0.544807148947436	-3.72730265903212
a_HQ_transcript/152769	-3.05035894171422	-0.414975838732561	-2.21530487343726	0.0338428745506227	0.544807148947436	-3.73106597881028
a_HQ_transcript/115700	5.0451936856132	1.07005761267086	2.2152865404755	0.0338442508747797	0.544807148947436	-3.7750759186672
a_HQ_transcript/85396	3.10429362179861	1.15392432168611	2.21511690935955	0.033856988093778	0.544861296764986	-3.72575920537738
a_HQ_transcript/79777	3.59516714362915	0.38590908763444	2.21493670188839	0.033870524164981	0.544901042087107	-3.68796254230262
a_HQ_transcript/106880	-0.70174659242151	3.76925136816079	-2.21483445950512	0.0338782061330651	0.544901042087107	-3.94893327725665
a_HQ_transcript/97444	1.98985547286519	-0.520456670939349	2.21463033258254	0.0338935478449083	0.544946314640231	-3.72859949755199
a_HQ_transcript/134227	2.53511103037993	1.04597844074065	2.21451377047616	0.0339023111731261	0.544946314640231	-3.7041811402875
a_HQ_transcript/137852	0.872362197442599	3.87274083687125	2.2141841156718	0.0339271061286288	0.544946314640231	-4.00569632656532
a_HQ_transcript/1350	-1.28048110830009	3.42578342152063	-2.21412646079474	0.033931444301026	0.544946314640231	-3.92841661649822
a_HQ_transcript/63614	1.90158478384329	1.24409322216358	2.21405273409201	0.0339369925016871	0.544946314640231	-3.64696352067852
a_HQ_transcript/124211	1.3317036326013	3.81130150952022	2.2140490403664	0.0339372704892244	0.544946314640231	-3.91297550572207
a_HQ_transcript/54717	-3.91805293951402	1.93204712734942	-2.2138690052624	0.0339508222906323	0.544974309307592	-3.74855867030173
a_HQ_transcript/31205	1.52668259303387	2.12857264091923	2.21366782086624	0.0339659717914615	0.544974309307592	-3.72180760944172
a_HQ_transcript/56450	2.38664061564816	-0.0626560454030767	2.21365230847792	0.0339671401496939	0.544974309307592	-3.69206383597422
a_HQ_transcript/2633	-2.76916536248557	2.22166069519852	-2.21340987873729	0.0339854040842875	0.545116878755922	-3.76403903656867
a_HQ_transcript/137696	9.41140789122155	5.39526423573331	2.21327120120504	0.0339958555853999	0.545134094749827	-3.83540408797915
a_HQ_transcript/11412	-2.85665884237403	1.32650991903201	-2.21282099272866	0.0340298055890633	0.545163784970204	-3.71943847492201
a_HQ_transcript/103824	3.55798896416568	2.62441489821239	2.2126764529738	0.0340407116907062	0.545163784970204	-3.85903548556543
a_HQ_transcript/73802	3.59854905677767	0.897390740779172	2.21256179931452	0.0340493649902867	0.545163784970204	-3.79160547603821
a_HQ_transcript/24831	2.16306009853687	-0.0919233656939604	2.21254548329821	0.0340505965751276	0.545163784970204	-3.71688242683766
a_HQ_transcript/145062	0.872184745347318	4.00204630703483	2.21252018567149	0.0340525061992829	0.545163784970204	-4.02086261276445
a_HQ_transcript/114489	5.0522637619528	2.17208641452846	2.21250067298736	0.0340539792049754	0.545163784970204	-3.91455246907177
a_HQ_transcript/52442	4.04344124783121	0.55315344634513	2.21164914286251	0.034118316446785	0.545835574478252	-3.81603502906159
a_HQ_transcript/99770	2.24143897816325	-0.645917976276991	2.21155650290661	0.0341253223979411	0.545835574478252	-3.73495874516687
a_HQ_transcript/58578	-1.03791660029303	4.21697412542118	-2.21146474631483	0.0341322628122903	0.545835574478252	-4.03299692077014
a_HQ_transcript/101041	-3.04289948389869	2.20333557621359	-2.21140561441032	0.0341367361834026	0.545835574478252	-3.75374710824484
a_HQ_transcript/150715	2.59086097065028	0.122271206762234	2.2113242263039	0.0341428941096016	0.545835574478252	-3.66701636251337
a_HQ_transcript/8443	-0.984631583591125	2.89301330986003	-2.21115270495458	0.0341558748826857	0.545892959906724	-3.70979549951415
a_HQ_transcript/75711	-1.83073415583572	-0.355481268335414	-2.21075211940204	0.0341862084750584	0.546036282958701	-3.75615930350549
a_HQ_transcript/78768	4.31632515110407	1.83755999854612	2.21052677490736	0.0342032828505066	0.546036282958701	-3.77349849054695
a_HQ_transcript/92175	2.8517192958553	2.09598973696247	2.21046228388871	0.0342081707448028	0.546036282958701	-3.66217310016198
a_HQ_transcript/105846	1.64293443350325	2.73957334786262	2.21035187526642	0.0342165402696788	0.546036282958701	-3.7777334220988
a_HQ_transcript/6450	2.47351471505223	2.32797219603194	2.21017230777742	0.034230156292557	0.546036282958701	-3.70738268822752
a_HQ_transcript/2960	-2.59490815348392	1.38313281346119	-2.21014270867696	0.0342324011618185	0.546036282958701	-3.65499747311163
a_HQ_transcript/137692	-4.28362860221136	0.13413157169292	-2.20996149141367	0.0342461479992317	0.546036282958701	-3.80819244225338

a_HQ_transcript/79098	2.15284863379245	-0.403304538398683	2.20989487921028	0.0342512023296575	0.546036282958701	-3.73773069652045
a_HQ_transcript/21357	-1.59178557806377	-0.367766822227598	-2.20980376049855	0.0342581172192115	0.546036282958701	-3.74920627284763
a_HQ_transcript/101744	-4.95273591236905	4.21127350421152	-2.2095417366872	0.0342780088473958	0.546036282958701	-3.91787562644204
a_HQ_transcript/8122	-1.27289648813992	3.99865904954498	-2.2094164937072	0.0342875203585734	0.546036282958701	-4.05734856288151
a_HQ_transcript/22424	1.81424050453463	1.22778620062141	2.20939006643072	0.0342895276654226	0.546036282958701	-3.66933520892436
a_HQ_transcript/85126	2.37907814703125	1.65760694513175	2.20935444163003	0.0342922337445273	0.546036282958701	-3.68515273095491
a_HQ_transcript/95197	4.13977193883797	3.03346499331634	2.20930020355213	0.0342963540648606	0.546036282958701	-3.7871582327076
a_HQ_transcript/112253	-3.22330227871499	0.126337533714104	-2.20916443972585	0.0343066696168251	0.546050956047866	-3.73782981623139
a_HQ_transcript/101847	-0.824799457401836	4.42154049583076	-2.20877918575636	0.0343359569333641	0.546184622136732	-4.05887153212143
a_HQ_transcript/131319	-0.52504201864687	3.58914092685077	-2.2087715471513	0.0343365378521834	0.546184622136732	-3.96543038354235
a_HQ_transcript/92669	-0.815114801814217	3.46951659154972	-2.20867862346528	0.0343436054374727	0.546184622136732	-3.97197601115895
a_HQ_transcript/144742	-2.15473626992218	3.50940085591877	-2.20839980061907	0.0343648199386175	0.546184622136732	-3.84557363507066
a_HQ_transcript/35931	2.89162390160625	1.38811579586564	2.20835849386682	0.0343679637982433	0.546184622136732	-3.63677340314116
a_HQ_transcript/100870	2.62487943392432	2.10126066896065	2.20831276038579	0.0343714448764135	0.546184622136732	-3.71108376993722
a_HQ_transcript/120580	-2.41907037483085	0.826697426439643	-2.20811289499945	0.0343866616538975	0.5462032540585	-3.76672102953822
a_HQ_transcript/127403	-2.96939258521623	0.876565167598145	-2.20786056661263	0.0344058813095959	0.5462032540585	-3.76086630975883
a_HQ_transcript/130406	3.10243955315291	0.161448357937312	2.20785078584587	0.0344066264962342	0.5462032540585	-3.68210956025211
a_HQ_transcript/9	-0.912129918977311	4.34063350640377	-2.20754150688159	0.0344301975873525	0.5462032540585	-4.07832151936357
a_HQ_transcript/27481	1.46796349442333	1.06585982370532	2.20750393966436	0.0344330616822405	0.5462032540585	-3.65410972484365
a_HQ_transcript/108738	2.34243694541502	0.251710472371583	2.2074761399006	0.0344351812513208	0.5462032540585	-3.67402923831373
a_HQ_transcript/111198	-1.94188479618157	1.0949517279376	-2.20741760787603	0.0344396443564916	0.5462032540585	-3.71586787482059
a_HQ_transcript/33516	-1.88212352029778	2.19629026005027	-2.20731079990528	0.0344477898668191	0.5462032540585	-3.85116257899099
a_HQ_transcript/17755	2.1130747847076	0.286278806765132	2.20691288414393	0.0344781513296503	0.546443047423375	-3.64697688237629
a_HQ_transcript/67618	1.83061813372746	0.709042450576318	2.2068662065204	0.03448171445687	0.546443047423375	-3.61214640128843
a_HQ_transcript/20068	1.47191470588705	-0.679623651024172	2.20662063174687	0.0345004657803752	0.546591189665209	-3.7690746955339
a_HQ_transcript/38967	-3.69750066951806	3.59202629177889	-2.20626874578643	0.0345273505812093	0.546868074818674	-3.92145571177742
a_HQ_transcript/40641	2.69370060642101	0.547043086256648	2.20589202987668	0.0345561531925565	0.547175176457893	-3.76523801651445
a_HQ_transcript/139467	2.56300507589307	-0.109901774919006	2.20565681601095	0.0345741478540142	0.547255250669546	-3.73563458283036
a_HQ_transcript/640	2.48870784198408	-0.0110551532653355	2.20557982831101	0.0345800394940346	0.547255250669546	-3.6839081122573
a_HQ_transcript/149168	0.496783210198893	7.97118717664432	2.20515593764741	0.0346124946610609	0.547619784882457	-4.385777317358
a_HQ_transcript/129644	4.8520678363933	-0.37106142076696	2.20466569094568	0.0346500643205888	0.548065017367942	-3.7662727602319
a_HQ_transcript/59995	-2.54242767045633	-0.179098717284957	-2.20439475311397	0.034670843053748	0.548112416984076	-3.73687612998618
a_HQ_transcript/59900	-1.03599483964433	4.10359392134851	-2.20438071647194	0.0346719198536067	0.548112416984076	-3.98978770297986
a_HQ_transcript/100056	1.99546699319883	4.33137951366096	2.2041480190813	0.0346897752383824	0.548193126062553	-4.06394590105686
a_HQ_transcript/148001	8.07383498809639	1.24629024828703	2.20406839509314	0.034695886849438	0.548193126062553	-3.85620446965814
a_HQ_transcript/111427	-4.01274020520161	2.56085719430445	-2.20394366328717	0.0347054626859198	0.548195417121507	-4.043200890512
a_HQ_transcript/47724	2.74373648485204	0.360209680415683	2.20354586829916	0.0347360177452313	0.548528997417769	-3.69572564035037
a_HQ_transcript/19894	4.09653271893329	0.282465682819779	2.20302643047482	0.034775952506621	0.548808870537056	-3.76157331040501
a_HQ_transcript/152337	-3.13179670270297	-0.253831618131511	-2.2029528465292	0.0347816130103632	0.548808870537056	-3.75353283296108
a_HQ_transcript/151154	-0.665969679260912	4.1271780555834	-2.20288323254266	0.0347869688796211	0.548808870537056	-4.04558394221238
a_HQ_transcript/55899	2.50458957416989	-0.0699913847483947	2.20282426182877	0.0347915064672628	0.548808870537056	-3.70418694226785
a_HQ_transcript/96706	-1.62095595337059	-0.618435398856843	-2.20265585263685	0.0348044678700324	0.548864380995454	-3.75907015723512
a_HQ_transcript/44884	1.87493000249659	-0.427619121836105	2.20229774402963	0.0348320435926224	0.549074991835708	-3.71894701404291
a_HQ_transcript/94232	2.45585949852957	0.391432427994449	2.20217077977167	0.0348418250087363	0.549074991835708	-3.75705760574463
a_HQ_transcript/118432	-1.50952085772412	1.73077896535001	-2.20182363240886	0.0348685820158251	0.549074991835708	-3.7442747878616
a_HQ_transcript/30715	2.01555632256744	2.23805512901125	2.20152363166464	0.0348917198979372	0.549074991835708	-3.74906332632159
a_HQ_transcript/121337	-3.91673054349619	2.69705617056406	-2.20151767680784	0.0348921793115141	0.549074991835708	-3.93683051599174
a_HQ_transcript/119798	3.18337109499195	0.674216008054479	2.20151408473181	0.0348924564406061	0.549074991835708	-3.68523291064921
a_HQ_transcript/146822	1.45674907785863	2.01896209979841	2.20146845142763	0.0348959772266058	0.549074991835708	-3.72077565201431
a_HQ_transcript/61821	2.23135462937059	2.45951015948952	2.20146333815137	0.0348963717554279	0.549074991835708	-3.74157457186227

a_HQ_transcript/136804	-1.80905319722065	0.930346664274878	-2.20137955613254	0.0349028367539385	0.549074991835708	-3.72874846446144
a_HQ_transcript/46973	3.46625243382134	1.04475601282869	2.20110267128281	0.0349242100497354	0.549262576236748	-3.82042968859248
a_HQ_transcript/66172	1.44556177092696	-0.711332136089949	2.20091374818982	0.0349388001111487	0.549343406237721	-3.75481467073521
a_HQ_transcript/13875	1.24383697604296	2.49658299016269	2.20070276440944	0.0349551003001323	0.549420052589283	-3.87438159517252
a_HQ_transcript/2966	1.19407386097325	4.76985681920341	2.20060599613416	0.034962578697491	0.549420052589283	-4.06049148811862
a_HQ_transcript/15481	-1.77250248654839	1.16204302311059	-2.20035291104547	0.0349821442498164	0.549445803790829	-3.67044124164642
a_HQ_transcript/138646	2.11132289789807	0.262773102283705	2.20034026599354	0.0349831220724927	0.549445803790829	-3.69026215325434
a_HQ_transcript/81873	-1.8474934173142	3.6785443226758	-2.20005531915324	0.0350051630539974	0.549554016578327	-3.92493320567704
a_HQ_transcript/142732	4.76247603408576	3.91028635733935	2.20000676035808	0.035008920372102	0.549554016578327	-3.91012640636307
a_HQ_transcript/69296	0.69700828009725	3.46970904633261	2.19986238167411	0.0350200940425002	0.549581000675608	-3.97988558213114
a_HQ_transcript/104546	2.14589109849137	2.51723642387982	2.19945072958656	0.0350519698699936	0.549923080203347	-3.79815753452149
a_HQ_transcript/103865	2.43978790581628	0.473961423027968	2.19933658715906	0.0350608129513075	0.549923080203347	-3.71779581558463
a_HQ_transcript/119785	3.0136999370296	2.26875920789184	2.19913721085392	0.0350762642303719	0.550017018393056	-3.87244347387149
a_HQ_transcript/72916	-1.04973228134862	2.6980385914012	-2.19864682296225	0.0351142942245244	0.550199305306936	-3.77223082367789
a_HQ_transcript/135789	3.51063085533774	3.52745908701026	2.19813703113788	0.0351538680449285	0.550199305306936	-3.87781507291985
a_HQ_transcript/92736	-2.29031631954863	2.36902243037547	-2.19811961178034	0.035155220968049	0.550199305306936	-3.82141823712261
a_HQ_transcript/120581	-2.21321663696804	4.84377636971681	-2.19809525762899	0.0351571125792765	0.550199305306936	-4.19615279949792
a_HQ_transcript/114731	-5.2848078194398	1.72504151883642	-2.19806860376675	0.0351591829155203	0.550199305306936	-3.84542602909581
a_HQ_transcript/150046	1.03808902955642	-0.471157675707782	2.19806694461486	0.0351593117935865	0.550199305306936	-3.78339500446179
a_HQ_transcript/102697	2.61831211244389	0.592677603467126	2.19790533280843	0.03517186734821	0.550199305306936	-3.67713943260083
a_HQ_transcript/117918	3.32663598967484	3.00714057818735	2.19784568624659	0.0351765022765116	0.550199305306936	-3.77639303294272
a_HQ_transcript/41219	-6.04312897245169	2.34128251033164	-2.19782298205576	0.0351782666840826	0.550199305306936	-3.87276388527773
a_HQ_transcript/140543	0.493862729313117	4.45818384719245	2.19767225399733	0.0351899821964603	0.550199305306936	-4.11867210411826
a_HQ_transcript/76949	-1.20584886682049	2.84708914887523	-2.19764620048454	0.0351920075889621	0.550199305306936	-3.83363625259841
a_HQ_transcript/3461	-0.894655283290091	2.48992456530428	-2.19718162963033	0.0352281406740497	0.550566050149106	-3.79054795041992
a_HQ_transcript/127337	3.47114738912937	4.79966583224622	2.1970841098242	0.0352357297107547	0.550566050149106	-4.17697435803695
a_HQ_transcript/119675	3.61521404249654	0.719694334194331	2.19688314057553	0.0352513738355994	0.550566050149106	-3.76286105589763
a_HQ_transcript/95328	-0.823114049950902	4.90241789662895	-2.19685773555238	0.0352533518898805	0.550566050149106	-4.18740995894092
a_HQ_transcript/5834	2.06970167539693	2.18515171773294	2.19608347211287	0.0353136841838673	0.551327417091256	-3.75420342749054
a_HQ_transcript/653	1.95264511010447	2.19099432816652	2.19598873693024	0.0353210724822433	0.551327417091256	-3.74750817298595
a_HQ_transcript/55185	-5.1437853735474	1.66426270965144	-2.1957932866083	0.0353363198107464	0.551413165136164	-3.90456486157503
a_HQ_transcript/118784	-2.43757409926629	0.0765927142367714	-2.19567515378894	0.0353455383515233	0.551413165136164	-3.74381433792834
a_HQ_transcript/15206	-2.1610223737037	1.60450644119179	-2.19544348557013	0.0353636229061204	0.551547269194249	-3.81987455840274
a_HQ_transcript/16908	-2.94722019619696	0.57606760648576	-2.19529225638526	0.0353754326605683	0.55158346290062	-3.90389666685618
a_HQ_transcript/7816	1.45685343474567	2.83466725467202	2.194764543355	0.0354166702895627	0.552020434518779	-3.81312155792148
a_HQ_transcript/120378	1.15575954106654	4.27301205417922	2.19469061454838	0.0354224508112277	0.552020434518779	-4.03780154904059
a_HQ_transcript/61922	-1.4812903733133	3.20122758435705	-2.19434714075391	0.0354493182069557	0.552258877790551	-3.84604402594511
a_HQ_transcript/59105	-1.19908761304526	1.65097383822958	-2.1939830173512	0.0354778207317718	0.552258877790551	-3.70795803344103
a_HQ_transcript/111175	1.76637743889701	-0.634712778631875	2.19394638790606	0.0354806891107772	0.552258877790551	-3.76503760094249
a_HQ_transcript/3283	0.85123046150644	3.60566046126883	2.19386755426301	0.0354868631188187	0.552258877790551	-3.97454197723284
a_HQ_transcript/58840	0.665551186748126	2.87002488440717	2.19372365689347	0.0354981351904263	0.552258877790551	-3.86407175474219
a_HQ_transcript/56487	-1.48403795593381	2.27122071383374	-2.19343838604486	0.0355204910760195	0.552258877790551	-3.80185814540356
a_HQ_transcript/146216	2.56996272693689	-0.574595494167599	2.19317002975778	0.0355415328852376	0.552258877790551	-3.75828021881811
a_HQ_transcript/95486	2.36118311384776	5.43387684080703	2.19296808893379	0.0355573743955327	0.552258877790551	-4.09812741326544
a_HQ_transcript/116082	-6.26093230394868	2.45949429016248	-2.19295583913635	0.0355583355495245	0.552258877790551	-3.9916613804155
a_HQ_transcript/11680	2.17588642629433	2.72655009599195	2.19281310053458	0.0355695369362799	0.552258877790551	-3.8159769343174
a_HQ_transcript/137096	-1.30585546734908	-0.816346411195265	-2.19268637837613	0.03557948407458	0.552258877790551	-3.75230179044081
a_HQ_transcript/61942	-2.49543304200626	1.54596713068303	-2.19253152722411	0.0355916425872247	0.552258877790551	-3.94057477241905
a_HQ_transcript/135921	4.24839333741344	4.06832950147649	2.1924640990573	0.0355969380316605	0.552258877790551	-3.88577756100789
a_HQ_transcript/1664	-1.44777909713855	4.11380550057317	-2.19229891869006	0.0356099133779937	0.552258877790551	-4.05113848836592

a_HQ_transcript/35186	-1.36775793583986	1.91366911231742	-2.19206498863371	0.0356282964130471	0.552258877790551	-3.74632267468764
a_HQ_transcript/56395	-1.77411552192447	2.15965943974054	-2.19203306837628	0.0356308054741706	0.552258877790551	-3.78807415175776
a_HQ_transcript/36332	-2.60742300139744	2.48403432875525	-2.19199384157637	0.0356338890750147	0.552258877790551	-3.89061182407231
a_HQ_transcript/45285	-2.00076345763096	2.92703379097624	-2.19193728022909	0.0356383357560245	0.552258877790551	-3.90533872029658
a_HQ_transcript/66358	1.41411248297896	-0.340482959804199	2.19183801567771	0.0356461408302345	0.552258877790551	-3.70324782382597
a_HQ_transcript/68802	-1.18994978928342	3.68883192589564	-2.19168692458123	0.0356580239030946	0.552258877790551	-3.99090748997755
a_HQ_transcript/129210	1.349368625718	2.15741698438093	2.19152772121723	0.0356705488178985	0.552258877790551	-3.74466247804662
a_HQ_transcript/51901	-0.482801755821299	4.85462704294849	-2.19148730066198	0.0356737294259264	0.552258877790551	-4.14489995419106
a_HQ_transcript/125673	2.75745826586261	0.218846956687099	2.19144218212092	0.0356772800074578	0.552258877790551	-3.76565857193884
a_HQ_transcript/43607	2.57219073409444	1.59468179300015	2.19141584116646	0.0356793530417977	0.552258877790551	-3.74548520501664
a_HQ_transcript/145655	-3.58813403389675	3.52618192386269	-2.19139798096913	0.0356807587013102	0.552258877790551	-4.05431968157448
a_HQ_transcript/50550	-1.68670557571367	-0.038722161830108	-2.19134700965826	0.035684770592164	0.552258877790551	-3.73148106609563
a_HQ_transcript/79049	-3.00763430243694	0.514299776876211	-2.19105127706421	0.0357080552951335	0.552427931685837	-3.77011695355675
a_HQ_transcript/41257	-2.81492726776252	-0.174039315351961	-2.1908700490283	0.0357223311049004	0.552427931685837	-3.81458837335
a_HQ_transcript/129333	2.52408410843601	0.649576915468302	2.19080147522838	0.0357277341718155	0.552427931685837	-3.82257255344291
a_HQ_transcript/48471	-2.56592908280152	1.06948726585597	-2.19060993765849	0.0357428296637351	0.552427931685837	-3.78491810937302
a_HQ_transcript/81497	-1.38701059971416	3.76567764262651	-2.19060508213873	0.0357432124117539	0.552427931685837	-3.90628064960042
a_HQ_transcript/113165	0.667860225312298	4.2671985817271	2.19044501440141	0.0357558321838341	0.552476080058987	-4.09352898752232
a_HQ_transcript/147143	2.06666882041903	0.503327303523323	2.19030620021719	0.03576677954058	0.552498368624723	-3.66393095053178
a_HQ_transcript/151286	0.524301733137929	7.24235793022897	2.19006087307658	0.0357861341837888	0.552633480312785	-4.34210146341376
a_HQ_transcript/8954	2.26621389666843	0.0731293210133558	2.18995435614281	0.0357945405549414	0.552633480312785	-3.78958290124722
a_HQ_transcript/117698	-1.67226137481045	1.67056794883204	-2.18925707823284	0.0358496136200254	0.552884488273898	-3.67503966826244
a_HQ_transcript/80802	-2.54670675209603	0.208172685059429	-2.18911696190129	0.0358606895394079	0.552884488273898	-3.80113001966436
a_HQ_transcript/113428	-4.1658688577395	6.90624975540092	-2.18910677034401	0.0358614952809936	0.552884488273898	-4.40551380498554
a_HQ_transcript/117953	5.22943725370634	2.7938354854439	2.18894601869443	0.0358742063970265	0.552884488273898	-4.02335829410246
a_HQ_transcript/106010	4.97593436326583	2.22410936206418	2.18884637632982	0.0358820874362066	0.552884488273898	-3.8898917810756
a_HQ_transcript/147168	-5.93701735862088	1.91105686689145	-2.18871477132879	0.0358924988718538	0.552884488273898	-3.93429034229157
a_HQ_transcript/133098	1.78522024139198	-0.566140932606392	2.18855995794264	0.03590474980186	0.552884488273898	-3.76729379329505
a_HQ_transcript/54856	-2.02978326064062	1.04860696822414	-2.188551262699	0.0359054379978384	0.552884488273898	-3.7316707200825
a_HQ_transcript/79618	-0.742969346962044	3.36311568159143	-2.18833939396908	0.0359222102518718	0.552884488273898	-3.98461101671067
a_HQ_transcript/59234	2.46976266984599	0.362024299158931	2.18777839098234	0.0359666549315599	0.552884488273898	-3.73489178762787
a_HQ_transcript/76275	2.54760471144736	0.517402891494994	2.18769248718318	0.0359734648696275	0.552884488273898	-3.75649345461041
a_HQ_transcript/64532	-2.5574745660614	1.37160873596138	-2.18762958471119	0.0359784521298987	0.552884488273898	-3.78019576825863
a_HQ_transcript/73733	-1.74026664521372	2.50927750088722	-2.18754964246306	0.0359847912891307	0.552884488273898	-3.78309735609956
a_HQ_transcript/64331	-3.13499464649539	2.15627363655041	-2.18739009308669	0.0359974460124889	0.552884488273898	-3.84107734162287
a_HQ_transcript/149349	-1.19855929343195	1.7236195065655	-2.18737079527702	0.0359989768951693	0.552884488273898	-3.70260411875642
a_HQ_transcript/131577	2.24727472930416	1.5057460884089	2.18735324131096	0.0360003694903136	0.552884488273898	-3.72917844802977
a_HQ_transcript/70670	-2.28918307620555	-0.45886762193096	-2.18732649090127	0.0360024917521905	0.552884488273898	-3.77736261003682
a_HQ_transcript/113353	1.93026648275703	1.23657017132593	2.1873006339853	0.0360045432342135	0.552884488273898	-3.68562380836175
a_HQ_transcript/86285	2.91658317325684	0.0849573723650878	2.18727743878401	0.0360063836251098	0.552884488273898	-3.73233509222236
a_HQ_transcript/109784	3.85676588189017	1.92182484656357	2.18714256385168	0.0360170867512971	0.552884488273898	-3.82818996588275
a_HQ_transcript/106537	-0.972152565147269	2.83039783000754	-2.18706552093511	0.0360232018374158	0.552884488273898	-3.88147812455151
a_HQ_transcript/119020	3.36146881603134	0.182842749153785	2.18698884845541	0.0360292884405161	0.552884488273898	-3.81306310512773
a_HQ_transcript/40268	-2.58847576187406	0.434698049694918	-2.18698538936046	0.0360295630605135	0.552884488273898	-3.7899141902597
a_HQ_transcript/113249	2.21324824931192	-0.692762175537421	2.18660385162537	0.036059865075065	0.553203440771544	-3.77065062185905
a_HQ_transcript/106319	-5.77627674579057	2.20869837646688	-2.18647214895555	0.0360703302770914	0.553217983732656	-3.89959899384556
a_HQ_transcript/19809	-1.32708079063998	1.68331613039969	-2.18632161318828	0.0360822952903373	0.553255515863025	-3.74928795032173
a_HQ_transcript/98457	-2.27812939117253	1.63955976960796	-2.18602072179098	0.0361062216095573	0.55340381663325	-3.8111831321651
a_HQ_transcript/83794	2.46590101468703	1.54600488558349	2.18596054994255	0.0361110080596256	0.55340381663325	-3.8203675209104
a_HQ_transcript/114062	3.96459004253471	0.353302761826207	2.18545764175701	0.0361510347003023	0.5538712032312	-3.84503441351286

a_HQ_transcript/109828	3.27607711322157	1.29200700896145	2.18529837280677	0.0361637192203036	0.553919544357789	-3.7958459481891
a_HQ_transcript/144621	4.67110507851954	2.21209359517447	2.18462221610039	0.0362176139955235	0.554044490517958	-3.91638789851593
a_HQ_transcript/8478	-2.27344461633375	0.716497203707844	-2.18461329254205	0.0362183257489015	0.554044490517958	-3.6694448932901
a_HQ_transcript/120018	-2.98354257630698	0.191120633238206	-2.18436014079022	0.0362385226239146	0.554044490517958	-3.87903658721846
a_HQ_transcript/145669	3.13086544829401	1.78603083800308	2.18427302029957	0.0362454755667552	0.554044490517958	-3.83889263016221
a_HQ_transcript/89744	1.69095864650387	-0.702468576916694	2.18419391166477	0.0362517901271533	0.554044490517958	-3.77908877074317
a_HQ_transcript/96691	-2.41799559614014	1.54232908909708	-2.18414029003753	0.0362560708372759	0.554044490517958	-3.74224490881353
a_HQ_transcript/60773	1.4002798815832	2.56601145851024	2.18356643142042	0.0363019112278862	0.554044490517958	-3.85053061734248
a_HQ_transcript/45163	-2.1580864689988	0.16741692693703	-2.18356466449373	0.036302052451504	0.554044490517958	-3.81765647720052
a_HQ_transcript/142552	0.751467047353112	5.17823105420662	2.18356071533772	0.0363023680940429	0.554044490517958	-4.23596320166818
a_HQ_transcript/127365	-0.930271870128102	4.54824810568363	-2.18353164930378	0.0363046913180621	0.554044490517958	-4.15016533741779
a_HQ_transcript/78567	0.803568027480778	2.85424647777733	2.18331724036499	0.0363218329434604	0.554044490517958	-3.86197495628524
a_HQ_transcript/58030	-3.0287877501891	3.2538191578184	-2.18315779662335	0.0363345848746351	0.554044490517958	-4.01826936053091
a_HQ_transcript/37334	-2.04577422255957	1.20396813358925	-2.18308636239497	0.036340299309098	0.554044490517958	-3.72334109904641
a_HQ_transcript/111237	-2.05296815198768	-0.0606050779370009	-2.1830157850924	0.03634594598014	0.554044490517958	-3.75475290735655
a_HQ_transcript/38423	2.520571624018	0.128698087829724	2.18298778322847	0.0363481865391782	0.554044490517958	-3.83671437745575
a_HQ_transcript/138180	-2.41359834895209	0.743535509020958	-2.18287363893792	0.0363573210276793	0.554044490517958	-3.7573444747318
a_HQ_transcript/128941	-2.45086208427375	2.52873860059687	-2.18286028886346	0.0363583895117932	0.554044490517958	-4.00452938457907
a_HQ_transcript/49563	4.04934203691221	1.37956560551261	2.18279916472838	0.0363632819894532	0.554044490517958	-3.90275559591273
a_HQ_transcript/56917	2.29546792354319	0.0977782242825267	2.18268851183602	0.03637214032464	0.554044490517958	-3.75270731192148
a_HQ_transcript/79933	2.05056704108086	2.25170887071939	2.18267141676083	0.0363735090452291	0.554044490517958	-3.82148283776825
a_HQ_transcript/28333	1.07125109644222	3.06391696264687	2.18255431371597	0.0363828861586202	0.554044490517958	-3.9121695977471
a_HQ_transcript/151933	-3.09661996890341	0.710536419892569	-2.18251929483921	0.0363856907398506	0.554044490517958	-3.78275181706017
a_HQ_transcript/43051	2.31045824500342	1.15311191256335	2.18240060235375	0.0363951979817457	0.554044490517958	-3.68179315008132
a_HQ_transcript/134366	1.35663136528827	3.06918286790976	2.18233278008269	0.0364006315250495	0.554044490517958	-3.90273471428199
a_HQ_transcript/121928	-2.52609182212718	1.06372116378454	-2.1820231747293	0.0364254445726151	0.554277026732191	-3.76754864695307
a_HQ_transcript/61498	-2.01818801787325	1.3244483301054	-2.18162178868758	0.0364576357506381	0.554293703911468	-3.73581562341275
a_HQ_transcript/99862	2.13697914098534	0.195461085186091	2.18157409012086	0.0364614628634171	0.554293703911468	-3.7568777529135
a_HQ_transcript/79889	-1.47034594280704	1.8558386546292	-2.18128983137964	0.0364842779009641	0.554293703911468	-3.73705107665117
a_HQ_transcript/88598	0.975452891052868	4.697949645165	2.18126810565988	0.0364860221636058	0.554293703911468	-4.1645850648403
a_HQ_transcript/91469	0.52614556908912	4.16559467864873	2.18126582546985	0.0364862052343616	0.554293703911468	-4.10866643727132
a_HQ_transcript/81112	-3.666195751085	1.49248337875138	-2.18114866333411	0.0364956129914281	0.554293703911468	-3.78456300498902
a_HQ_transcript/3221	1.68532635275156	-0.580073888460852	2.18102075048229	0.0365058864669918	0.554293703911468	-3.78282870634649
a_HQ_transcript/131007	-0.730099082041267	3.30542770646802	-2.18099500701863	0.0365079543960701	0.554293703911468	-3.9705989610857
a_HQ_transcript/111136	-2.80707392203347	0.80627967479442	-2.18088831500048	0.0365165258994996	0.554293703911468	-3.910095221032
a_HQ_transcript/84219	-2.059073164965	-0.624794074320455	-2.18074594106565	0.0365279668379174	0.554293703911468	-3.78370228572106
a_HQ_transcript/132826	6.98497098400434	1.03244794259018	2.18063721565775	0.0365367059871027	0.554293703911468	-3.86461833106717
a_HQ_transcript/83483	2.38906595291857	-0.159494737036455	2.18058418126652	0.0365409694706294	0.554293703911468	-3.80978384612467
a_HQ_transcript/90687	-2.22158898283307	6.01056370669424	-2.18040853629387	0.0365550928985829	0.554363276808981	-4.2170255567188
a_HQ_transcript/151736	-3.73404494783673	-0.311699604312983	-2.1798955360657	0.036596370573901	0.55460647423927	-3.81532258672407
a_HQ_transcript/39097	2.25025379590621	1.44574002529413	2.17949860706146	0.0366283372843486	0.55460647423927	-3.73520012144861
a_HQ_transcript/52409	3.47997380964803	1.32659794439861	2.1794809133451	0.0366297628285578	0.55460647423927	-3.74566563453437
a_HQ_transcript/121239	2.45924192770136	1.22588718599705	2.17940953396235	0.0366355142122122	0.55460647423927	-3.70380319744301
a_HQ_transcript/69036	0.780839287407981	-0.63110017896714	2.17939188299304	0.0366369365606919	0.55460647423927	-3.88961637054623
a_HQ_transcript/94842	-2.45528754172766	-0.0390145160646089	-2.17935662944998	0.036639777505172	0.55460647423927	-3.77757587996163
a_HQ_transcript/150719	1.52647582244694	-0.778320700228933	2.17933294290885	0.0366416864207143	0.55460647423927	-3.79356292612567
a_HQ_transcript/82923	3.13886345429199	0.956075468296916	2.17919080313234	0.0366531434306603	0.55460647423927	-3.77483504983357
a_HQ_transcript/37845	5.19627182656797	4.25005154725776	2.17914297074654	0.0366569996219942	0.55460647423927	-4.2941248763909
a_HQ_transcript/41499	-0.783071955117899	2.58466265954313	-2.17891897110699	0.0366750630247812	0.554735379522374	-3.7858550954272
a_HQ_transcript/18902	-2.21944165658216	0.0786666558257796	-2.17843266257661	0.0367143064116865	0.555184496123443	-3.69884690166147

a_HQ_transcript/21912	1.39469696738115	1.06402653040122	2.1782205764303	0.0367314327340706	0.5552990174164	-3.75058168797791
a_HQ_transcript/52449	1.92962439584059	-0.755772698233894	2.17790606277866	0.0367568433627926	0.555494219023736	-3.78759300106041
a_HQ_transcript/83089	-1.07147718174713	3.09842790803853	-2.17772536760137	0.0367714494262545	0.555494219023736	-3.95422360031732
a_HQ_transcript/59882	-4.11374688602816	1.98892953780127	-2.17770601630654	0.0367730139485848	0.555494219023736	-3.86816681684815
a_HQ_transcript/126320	-2.64318144395769	-0.327676584770074	-2.17712388933343	0.0368201057583033	0.556061082753613	-3.79583534177014
a_HQ_transcript/134488	0.883225728735743	2.85504467941776	2.17699388334722	0.0368306300756548	0.556075549360432	-3.91062127963835
a_HQ_transcript/143331	-3.88867736538333	1.85553995507328	-2.17661500906676	0.0368613162095368	0.556308115765554	-3.88766190620211
a_HQ_transcript/70011	-0.742688153695802	4.32282070113276	-2.1764608505432	0.0368738084776918	0.556308115765554	-4.10481710158975
a_HQ_transcript/130635	-1.31969494710702	1.9929759977101	-2.17644929699099	0.0368747448741515	0.556308115765554	-3.80336756423267
a_HQ_transcript/120645	0.529260870738097	4.30071229809084	2.17565261676583	0.0369393656276209	0.556802615465439	-4.12431214092021
a_HQ_transcript/5756	2.79917278792536	0.55264305819264	2.1754114633887	0.0369589460735341	0.556802615465439	-3.8018754587219
a_HQ_transcript/97581	-2.93978553500362	-0.256114613913939	-2.17532314889386	0.0369661190810836	0.556802615465439	-3.80743669431471
a_HQ_transcript/84211	3.39629848848497	2.74358457403015	2.17532235330591	0.0369661837053135	0.556802615465439	-3.83881206270605
a_HQ_transcript/36981	-5.30052498549875	1.26959253608986	-2.17529898450766	0.0369680819571736	0.556802615465439	-3.8048075864322
a_HQ_transcript/61379	-1.42745884490254	1.14627900239879	-2.17482364548961	0.0370067126891169	0.556802615465439	-3.69801324539334
a_HQ_transcript/122414	-3.24735169626006	2.20465888305685	-2.17441780782988	0.0370397235130513	0.556802615465439	-3.80303635359944
a_HQ_transcript/15094	0.801269719692497	2.27905649781879	2.1743740364988	0.0370432854737132	0.556802615465439	-3.84578589989452
a_HQ_transcript/8802	2.11722361441386	1.27985253902308	2.17434660204511	0.0370455180940052	0.556802615465439	-3.6990051367109
a_HQ_transcript/101314	-1.5756216176114	2.8238360936501	-2.17431422851383	0.0370481528527306	0.556802615465439	-3.96074096715672
a_HQ_transcript/21509	0.602044318182249	3.29438367897563	2.17410005197577	0.0370655880735113	0.556802615465439	-4.00704779468311
a_HQ_transcript/57568	2.26244966669653	0.456201003156744	2.1740625144631	0.0370686445989412	0.556802615465439	-3.75217931827745
a_HQ_transcript/104722	1.96475205756686	-0.391616036157807	2.1740386254704	0.0370705898983824	0.556802615465439	-3.76048955813521
a_HQ_transcript/144170	-3.11767837858159	2.82988963876109	-2.17399492343898	0.0370741488244944	0.556802615465439	-3.85093743251843
a_HQ_transcript/13874	2.83898706721304	0.390183636039797	2.17397260279066	0.0370759666500585	0.556802615465439	-3.82766964404129
a_HQ_transcript/88587	-2.2046897726131	0.144954596197415	-2.17396227083095	0.0370768081265366	0.556802615465439	-3.77882227193585
a_HQ_transcript/106050	3.2380711280471	2.53712926821295	2.17395659004563	0.0370772707998585	0.556802615465439	-3.9685127353562
a_HQ_transcript/110546	4.95617656469446	0.377834168677597	2.17382646784989	0.0370878700507222	0.556802615465439	-3.81834398907549
a_HQ_transcript/30558	-2.20479724145978	2.74171901244724	-2.17380619223491	0.0370895218669519	0.556802615465439	-3.79355855629529
a_HQ_transcript/152419	-3.48232103067009	1.21504130108472	-2.17324675037442	0.037135124401885	0.557235557168043	-3.81013901784785
a_HQ_transcript/120822	3.52035310201232	1.12659863940501	2.1732172128619	0.0371375335203172	0.557235557168043	-3.88002224133722
a_HQ_transcript/12540	0.604934448925329	4.18003955004794	2.17282330983596	0.0371696740802174	0.557573887725129	-4.13239686246238
a_HQ_transcript/52844	-2.6249248746516	4.08108887317493	-2.17196243310818	0.0372400036700312	0.55787086239141	-4.17074795371571
a_HQ_transcript/152385	-1.177548412381	8.28490852979101	-2.17165880564286	0.0372648368679189	0.55787086239141	-4.52701341831441
a_HQ_transcript/24612	3.56500752865566	0.773920176581468	2.17156726441539	0.0372723267694965	0.55787086239141	-3.81075233591504
a_HQ_transcript/103431	-1.23819179208075	1.91520920461171	-2.17156602062917	0.037272428545294	0.55787086239141	-3.7763583831329
a_HQ_transcript/33540	-0.84317191395778	5.05523784511141	-2.17142571215791	0.0372839112122048	0.55787086239141	-4.20763490611006
a_HQ_transcript/150667	2.63468512699876	0.311462057643867	2.17119241360047	0.0373030110441704	0.55787086239141	-3.76460402624985
a_HQ_transcript/125315	-2.32105032775663	0.940397249507408	-2.17118840148623	0.0373033395866471	0.55787086239141	-3.79011078518659
a_HQ_transcript/54133	5.42579632125725	3.6385356847838	2.17106080866864	0.0373137892027066	0.55787086239141	-4.01797729286604
a_HQ_transcript/72011	-1.79775597453632	2.67552046933298	-2.17096341658401	0.0373217671887987	0.55787086239141	-3.90006768937553
a_HQ_transcript/93559	-2.52295715279865	1.03548596127035	-2.17085801967009	0.0373304026112802	0.55787086239141	-3.70352687294434
a_HQ_transcript/142402	-3.56540715160419	0.767844859219825	-2.17084399086996	0.0373315521587672	0.55787086239141	-3.7937929974848
a_HQ_transcript/104085	2.88586825491923	0.802115217097987	2.17080965944479	0.0373343654762708	0.55787086239141	-3.79272366074622
a_HQ_transcript/107695	1.85354389199259	1.68994895406643	2.17079094262363	0.0373358993214444	0.55787086239141	-3.70834635917687
a_HQ_transcript/104899	-3.00009793165904	1.21307650221039	-2.1707906301787	0.0373359249268109	0.55787086239141	-3.79221558985528
a_HQ_transcript/152747	-3.70301452123987	-0.446835237633686	-2.17072185014533	0.0373415619421976	0.55787086239141	-3.83164402728198
a_HQ_transcript/68544	-0.894747392134755	3.46833955717839	-2.17070396482205	0.0373430278964522	0.55787086239141	-4.06208428274824
a_HQ_transcript/110116	-2.82831494066079	0.573931617010864	-2.17034581804149	0.0373723938620621	0.558166112644899	-3.81475240315001
a_HQ_transcript/144817	-4.39834580632966	3.07880319083177	-2.16987588429743	0.0374109569058387	0.558598536609965	-3.98087878089312
a_HQ_transcript/23996	-2.41257506411488	-0.430078699894601	-2.16938483108016	0.0374512908797655	0.559057174180537	-3.78322273191382

a_HQ_transcript/51145	0.509495345278862	5.4004974299947	2.16911911966565	0.0374731319404543	0.559192782685878	-4.25480675649887
a_HQ_transcript/31890	1.51455633976003	0.976839210699928	2.16904027104592	0.0374796153548063	0.559192782685878	-3.72551145794726
a_HQ_transcript/147480	-3.02054362470955	3.71854678260207	-2.16845010449134	0.0375281741705437	0.55977359717356	-3.99005733982011
a_HQ_transcript/1628	1.72283328379307	-0.638780969662816	2.16787496210617	0.0375755506845169	0.560336482860339	-3.81073791235598
a_HQ_transcript/136390	2.99549911806475	0.47415655008958	2.16773548324135	0.0375870480715992	0.560364178072819	-3.91373707935225
a_HQ_transcript/13912	-1.00614692773931	1.67371480200103	-2.16759082607164	0.0375989756201126	0.56038254595513	-3.745498665351
a_HQ_transcript/91074	3.12246043680149	1.70910732008756	2.16748672672452	0.0376075611025833	0.56038254595513	-3.78939546545166
a_HQ_transcript/34136	-2.53943439835282	-0.278234504021221	-2.16704429575671	0.0376440695984805	0.560616621120046	-3.83069345080863
a_HQ_transcript/49357	2.27799121960155	2.42922270173997	2.16700911338553	0.0376469741293357	0.560616621120046	-3.81169667228281
a_HQ_transcript/135495	-2.04877511938529	1.17708881519888	-2.16692714513294	0.0376537419092458	0.560616621120046	-3.82376686824905
a_HQ_transcript/21481	2.03408894651974	1.79278398800686	2.16682898410485	0.037661848084809	0.560616621120046	-3.8030906983422
a_HQ_transcript/6041	1.9829919577143	1.57690352298574	2.16630678457228	0.0377049976430295	0.560991817607678	-3.7312259939199
a_HQ_transcript/118673	-4.22255311925923	1.44287623938378	-2.16629036054073	0.0377063554808904	0.560991817607678	-3.82542562726442
a_HQ_transcript/89654	1.60155503500761	-0.77534067784514	2.16571282426521	0.0377541303100794	0.561512299649007	-3.8175639839175
a_HQ_transcript/150954	4.6131032479347	2.88153440616728	2.16543113626293	0.0377774515852406	0.561512299649007	-4.07582674523455
a_HQ_transcript/58820	2.24013427760393	0.553982219821627	2.16524029755346	0.037793258627504	0.561512299649007	-3.80147065389777
a_HQ_transcript/39443	1.79191869932017	1.65403493655624	2.16518609577492	0.0377977491977301	0.561512299649007	-3.77521222046072
a_HQ_transcript/47563	-1.36322602411104	2.05849446906282	-2.16516308966951	0.0377996553771187	0.561512299649007	-3.79490655109083
a_HQ_transcript/136929	-0.606707137282193	3.66772252481553	-2.16515484612714	0.0378003384196978	0.561512299649007	-4.08729460935394
a_HQ_transcript/22651	-2.09755936189796	2.70147319024985	-2.16496445563501	0.0378161168323289	0.561512299649007	-3.88612582509804
a_HQ_transcript/73741	-1.61214100859055	0.00154347013577636	-2.16493427634336	0.0378186184476648	0.561512299649007	-3.76810471720752
a_HQ_transcript/50736	3.22189721196109	2.3312684834724	2.16464894165061	0.0378422776295974	0.561720100626465	-3.92090656453443
a_HQ_transcript/117035	2.93629907231394	1.1065156496999	2.16435013942921	0.0378670676216842	0.561944576643671	-3.86857916614087
a_HQ_transcript/115748	2.63356589816605	-0.225779107747864	2.16421522058116	0.0378782658387208	0.561967288584268	-3.83290445956049
a_HQ_transcript/115568	3.23550144254086	1.28113685750319	2.16399851932569	0.037896258135101	0.562090761132216	-3.81198352855885
a_HQ_transcript/113237	-0.929624390135758	3.63274344154733	-2.16382662908249	0.0379105352663626	0.562146941642815	-4.06011765640552
a_HQ_transcript/3055	-2.07057734936003	0.521172007648386	-2.16372008077076	0.0379193875272068	0.562146941642815	-3.76184168361752
a_HQ_transcript/4536	1.62776207539038	2.11610990458006	2.16356491486567	0.0379322823312618	0.56219472395502	-3.86549037807263
a_HQ_transcript/134926	1.79262262842748	-0.455079669619594	2.16336464989276	0.0379489307862836	0.562298100623271	-3.81010836993981
a_HQ_transcript/55147	-2.48820036105591	1.33545093679615	-2.16304979142807	0.0379751187908544	0.562441021290019	-3.768636251415
a_HQ_transcript/114287	0.992154573234951	2.67798060138778	2.16301602575081	0.0379779281682378	0.562441021290019	-3.83343977489788
a_HQ_transcript/71475	-2.68578103012627	0.510070266833211	-2.16265676713348	0.038007830715928	0.562735857020944	-3.84373653260087
a_HQ_transcript/91041	1.95453064593626	0.918780917248114	2.16254427216593	0.0380171984331346	0.562735857020944	-3.73949702780361
a_HQ_transcript/99466	-1.37847665787574	-0.715536621565615	-2.16213278474413	0.0380514814493983	0.563099927110648	-3.78505618541883
a_HQ_transcript/45507	-1.06807391803856	1.9634576734501	-2.16196064891937	0.0380658310796541	0.563168905318944	-3.82059337350932
a_HQ_transcript/66117	2.49138565850378	1.35043957633615	2.1609929304864	0.0381465919207963	0.564190181849352	-3.79205916925545
a_HQ_transcript/70828	-1.82981616502426	-0.687586071292362	-2.16090098182278	0.0381542734112614	0.564190181849352	-3.82378342392791
a_HQ_transcript/37500	2.32217903011621	0.356817099082885	2.16054839606094	0.0381837415627047	0.564253731325248	-3.81444179299826
a_HQ_transcript/134682	-2.13722830483195	0.0774193187218544	-2.16034025792968	0.0382011466793916	0.564253731325248	-3.8600703747986
a_HQ_transcript/43580	1.75747261929099	-0.432900947797318	2.16033094240386	0.0382019258357694	0.564253731325248	-3.82661431741458
a_HQ_transcript/150983	0.419189736815408	7.8405698058878	2.16004864436803	0.0382255441255975	0.564253731325248	-4.47615432697439
a_HQ_transcript/139771	-2.38125162489981	2.91851987190936	-2.16000863070509	0.0382288928946887	0.564253731325248	-3.96716153414827
a_HQ_transcript/150105	6.8415880720392	3.24900942266772	2.15999515134348	0.0382300210498609	0.564253731325248	-3.99742425915121
a_HQ_transcript/11823	1.82857056104129	2.03373965531739	2.15998173199486	0.0382311442116628	0.564253731325248	-3.75069145356126
a_HQ_transcript/45229	-2.449981491604	-0.580608843175	-2.15992099890436	0.0382362277678598	0.564253731325248	-3.82841086589554
a_HQ_transcript/2310	-2.08602693961561	2.26740650593996	-2.15943198710378	0.0382771815546007	0.564480825856835	-3.847980185028
a_HQ_transcript/111070	-2.43263159825495	0.723797680367711	-2.15922669880102	0.0382943856751529	0.564480825856835	-3.82629894465745
a_HQ_transcript/89595	4.08844390160633	0.751224267865865	2.1592062109698	0.0382961030287012	0.564480825856835	-3.93323644904278
a_HQ_transcript/88526	1.38365477200521	5.96789716871769	2.15896766274977	0.0383161039231495	0.564480825856835	-4.32162985055549
a_HQ_transcript/150774	2.08043571352739	2.52754096484596	2.15890632610687	0.0383212481487992	0.564480825856835	-3.81405305371873

a_HQ_transcript/129368	4.13005299389059	3.02928980412057	2.15880020334653	0.0383301499796026	0.564480825856835	-3.83385119075419
a_HQ_transcript/146777	-3.67988936405034	2.29508187124985	-2.15878528387878	0.038331401607669	0.564480825856835	-4.05159892672102
a_HQ_transcript/125123	1.59657398431998	0.947435434040202	2.15867139632117	0.0383409570916859	0.564480825856835	-3.70077465528602
a_HQ_transcript/42273	1.49766288058641	1.81693213316696	2.15866916755684	0.0383411441123698	0.564480825856835	-3.76276243082263
a_HQ_transcript/92499	-2.3475522614844	-0.351807459488523	-2.15857881331085	0.0383487266258712	0.564480825856835	-3.7900030028651
a_HQ_transcript/89584	-1.96446965261628	-0.0723458286591956	-2.15832512315654	0.0383700233919001	0.56460383031356	-3.8057262348242
a_HQ_transcript/16655	-2.24401178998749	3.44651112457091	-2.158247886759	0.0383765093168331	0.56460383031356	-4.07152490769504
a_HQ_transcript/142429	3.5939372469821	0.592522775280233	2.15785308470492	0.0384096780444663	0.564891086865857	-3.85975363911631
a_HQ_transcript/105301	-2.82071940319427	1.90776406046752	-2.15778416971731	0.0384154704510861	0.564891086865857	-3.84010146860237
a_HQ_transcript/134389	-2.34528397857273	1.34855665427955	-2.15753380248496	0.0384365207256341	0.565057682534057	-3.92626194653506
a_HQ_transcript/13985	-3.51411836128255	0.541711591782797	-2.15731133340789	0.0384552339969119	0.56518984621302	-3.8500911913741
a_HQ_transcript/44117	2.9636293968159	2.30366338769401	2.15702561858509	0.0384792791494262	0.565400287764876	-3.96989578879907
a_HQ_transcript/90626	1.0395958055414	4.26557154462247	2.15637382541612	0.0385341827245049	0.565753055772013	-4.12682583438767
a_HQ_transcript/14875	1.11527968606774	3.7817355205853	2.156146753927	0.0385533263686305	0.565753055772013	-4.08752741126556
a_HQ_transcript/139862	-2.36130766561295	1.30753902649287	-2.15587081899076	0.0385766009096074	0.565753055772013	-3.84071641780698
a_HQ_transcript/129465	5.51539624653541	2.40843433396996	2.1558651505501	0.038577079161781	0.565753055772013	-4.00021372909984
a_HQ_transcript/80272	3.1918978926793	1.92461175962384	2.15576218894107	0.0385857670563391	0.565753055772013	-3.78273538427739
a_HQ_transcript/142988	1.38873521264126	2.89627752064476	2.15570939314138	0.0385902226375463	0.565753055772013	-3.96279171245734
a_HQ_transcript/113650	2.53470471636091	1.39432754876699	2.15547932635594	0.0386096439402261	0.565753055772013	-3.76254021545139
a_HQ_transcript/43381	-3.992870257244	1.71172330540642	-2.15537179406421	0.0386187243589355	0.565753055772013	-3.83311660291055
a_HQ_transcript/136197	2.9920080492707	3.88521197322154	2.15533069841611	0.0386221951267266	0.565753055772013	-4.12334629800375
a_HQ_transcript/29632	-1.41078689791969	2.26111679645595	-2.15532163169112	0.0386229609020125	0.565753055772013	-3.79935580541487
a_HQ_transcript/148712	3.66314446590663	1.46061387678441	2.15513764876413	0.0386385030103856	0.565753055772013	-3.82761999143956
a_HQ_transcript/8635	3.21560424529585	1.58896307268261	2.15511234309845	0.0386406411621624	0.565753055772013	-3.88708629726479
a_HQ_transcript/121762	1.95108271651972	2.39881600180633	2.1550771803445	0.038643612343604	0.565753055772013	-3.8221360369584
a_HQ_transcript/136613	1.93243804586172	0.755624670129229	2.15502694029469	0.0386478578784247	0.565753055772013	-3.77375055123578
a_HQ_transcript/138175	3.32746475141773	0.953132395936989	2.1550101050875	0.0386492806301724	0.565753055772013	-3.84857654999202
a_HQ_transcript/8117	-2.53868476072818	1.27038507070754	-2.15480561996405	0.0386665654880075	0.565863574694587	-3.89181634550387
a_HQ_transcript/92295	1.36926752295107	-0.779641029846662	2.15465066865068	0.0386796678948786	0.565912845555879	-3.83712069148059
a_HQ_transcript/15131	-2.71198369905925	1.07343648082282	-2.1541175280404	0.0387247794743639	0.566136110477575	-3.84130038573827
a_HQ_transcript/119105	-4.72421090872972	2.27455886884017	-2.15406196627786	0.03872948351129	0.566136110477575	-3.98359205747892
a_HQ_transcript/97658	-2.32850349690068	0.345214794289901	-2.15400996079066	0.0387338869227902	0.566136110477575	-3.79861843944567
a_HQ_transcript/150399	0.666216381918032	5.15184958597463	2.15389855981839	0.0387433209699494	0.566136110477575	-4.25903298620048
a_HQ_transcript/57918	2.2105659885924	-0.102510417200745	2.15365071257428	0.0387643173659789	0.566136110477575	-3.79578822633234
a_HQ_transcript/124068	2.3407211776822	1.42484465151688	2.15362599361904	0.0387664119885617	0.566136110477575	-3.8132338617837
a_HQ_transcript/108746	-1.99484478044791	1.14241108991735	-2.15362067053954	0.0387668630662011	0.566136110477575	-3.74342201074744
a_HQ_transcript/149418	-7.1349731371343	1.45234833565804	-2.15355010001183	0.0387728436521337	0.566136110477575	-3.8621936319193
a_HQ_transcript/108636	-1.75080442664798	2.34547163691003	-2.1533228454014	0.0387921081963046	0.566190189216665	-3.87579114968074
a_HQ_transcript/24376	2.42217318212036	0.184562214422665	2.15287214405421	0.0388303396601097	0.566190189216665	-3.84858816334395
a_HQ_transcript/31015	2.16371627193711	-0.181891570275085	2.15284462895723	0.0388326747566712	0.566190189216665	-3.79710286197465
a_HQ_transcript/147725	2.53091941118936	0.233086401770909	2.15282772998633	0.0388341089668156	0.566190189216665	-3.77718438510169
a_HQ_transcript/125793	-1.83762312406869	2.79565275351674	-2.15271740929488	0.0388434730049275	0.566190189216665	-3.94101233400722
a_HQ_transcript/151111	-1.58879193982837	3.92585616668235	-2.15254287003447	0.0388582920250428	0.566190189216665	-4.19381847761556
a_HQ_transcript/147574	2.79549424235953	0.57958623422573	2.15253346313688	0.0388590908476698	0.566190189216665	-3.8064093902776
a_HQ_transcript/84628	3.59118780129199	0.758927130526044	2.15247952810766	0.0388636712283853	0.566190189216665	-3.85835301425178
a_HQ_transcript/35152	3.74453005087587	1.51548246425549	2.15247317295766	0.0388642109650167	0.566190189216665	-3.9150919071319
a_HQ_transcript/92970	1.88064458103029	-0.306953356944928	2.15208210786086	0.0388974365603447	0.566532245647637	-3.78750307547817
a_HQ_transcript/129592	-0.763319392669615	3.3017104185062	-2.15172298650301	0.0389279703928784	0.56683493562055	-4.05550743672734
a_HQ_transcript/112278	-2.33775363471902	0.11622980679497	-2.1515623348382	0.038941636489458	0.566891922328879	-3.87531348915211
a_HQ_transcript/4012	-0.69658562049818	3.76264733997459	-2.15121933520395	0.0389708285853022	0.56705384930257	-4.09634918779035

a_HQ_transcript/90128	-2.93013021216111	2.29655010526429	-2.1510910613191	0.0389817507457416	0.56705384930257	-4.19624734843748
a_HQ_transcript/51590	1.18393832020775	0.791859666763752	2.15108783419855	0.0389820255610561	0.56705384930257	-3.84731776005621
a_HQ_transcript/83444	-2.13964522077889	-0.425142376172006	-2.15079025524825	0.0390073742068608	0.567280622440931	-3.83028094770844
a_HQ_transcript/87832	1.84568764029557	-0.0683549304214584	2.15049724766746	0.0390323477615449	0.56743893374734	-3.90755544900504
a_HQ_transcript/93491	2.09177348093645	2.31717461978055	2.15043349039628	0.0390377837884602	0.56743893374734	-3.86695960589316
a_HQ_transcript/113103	-3.10190600453103	1.82456558272929	-2.15018419558286	0.0390590454434515	0.567606048384237	-3.85657092537716
a_HQ_transcript/133777	1.95879104747072	-0.473823567629626	2.14982333879488	0.0390898401342547	0.567911578936256	-3.84510365134538
a_HQ_transcript/75429	2.45781298577074	1.18791072838767	2.14958755376629	0.0391099731397286	0.567948561555094	-3.74220493209673
a_HQ_transcript/95243	-3.41829060047935	0.861207918917033	-2.14950746161798	0.0391168140741037	0.567948561555094	-3.88203226872518
a_HQ_transcript/127648	1.65842453323395	1.95979367077046	2.14937955205434	0.0391277414564446	0.567948561555094	-3.82128701448487
a_HQ_transcript/112029	0.617012594361223	4.22499347762541	2.14933593511946	0.0391314682945939	0.567948561555094	-4.14898888955036
a_HQ_transcript/44477	-1.15619344023585	3.03422183475246	-2.14904342153254	0.0391564701968107	0.568054256130761	-3.99375123571251
a_HQ_transcript/48750	2.44559239341246	1.04981649568211	2.14874502201415	0.039181989808602	0.568054256130761	-3.74831823010426
a_HQ_transcript/115199	3.23750143465642	0.558070152877055	2.14874057391726	0.0391823703288328	0.568054256130761	-3.83710761711795
a_HQ_transcript/80558	3.15180547558984	0.133240613836717	2.1485932445173	0.0391949757356518	0.568054256130761	-3.86385394651408
a_HQ_transcript/137553	0.513170406036411	4.96026494727655	2.14857319803721	0.0391966911777872	0.568054256130761	-4.24217084776309
a_HQ_transcript/150623	-2.87543679546258	-0.416357062166328	-2.14856508508474	0.0391973854483293	0.568054256130761	-3.85827491755771
a_HQ_transcript/32585	0.68761060901121	4.06648019079743	2.1483997315375	0.0392115380534144	0.568117717838702	-4.15078191494135
a_HQ_transcript/82393	2.36878980164112	1.47972104910949	2.14803149473787	0.0392430717437981	0.568427707506256	-3.86372416450029
a_HQ_transcript/42745	-2.91206076958959	3.92110826936199	-2.14792154749404	0.0392524913626843	0.568427707506256	-4.13280457666974
a_HQ_transcript/70694	-1.80347753737707	-0.667771364114377	-2.1477208997381	0.0392696868341073	0.56853508251382	-3.83684826323341
a_HQ_transcript/56047	-0.743731431289085	2.98246290258034	-2.14742559039243	0.0392950069570154	0.568748561435605	-4.00032993187496
a_HQ_transcript/80559	2.90168072338726	3.44054023807348	2.14732073305636	0.0393040010199358	0.568748561435605	-4.09741030233571
a_HQ_transcript/85316	-1.48729427636841	3.75257367323179	-2.14718517447927	0.0393156311701242	0.568775263478591	-4.02961809078307
a_HQ_transcript/131426	2.53109406786168	2.65016763780943	2.14657513123635	0.0393680072328652	0.569358338983859	-4.01697593360363
a_HQ_transcript/106256	2.3807500721253	1.01235867662256	2.14648762711167	0.0393755250948787	0.569358338983859	-3.85180400637066
a_HQ_transcript/24452	2.45685284668873	0.3626246054326	2.14592329905542	0.0394240396248619	0.56984053453968	-3.84528794229105
a_HQ_transcript/121111	1.80490727998042	-0.508301644409726	2.14587168988751	0.0394284790448423	0.56984053453968	-3.85226162404752
a_HQ_transcript/25359	2.30642775664621	0.60996301972305	2.14574449016064	0.03943942265965	0.569857012269484	-3.81722464497635
a_HQ_transcript/110632	2.452524624817	0.343869273624207	2.14562632301502	0.0394495915745323	0.569862291015014	-3.79177902958632
a_HQ_transcript/13203	-1.60221186781168	-0.712034673286735	-2.14536239183663	0.0394723126738809	0.570048842511143	-3.83869635155085
a_HQ_transcript/106099	1.14438947619724	3.30711836761193	2.14509183733923	0.0394956160195127	0.570243707894245	-4.03005982053725
a_HQ_transcript/18571	-1.020483977608	3.29650193401714	-2.1449350994051	0.0395091217287268	0.570297051861791	-3.95424985432052
a_HQ_transcript/122178	-2.80516831093548	0.4597044775997	-2.14463016743136	0.0395354086940834	0.570534815434379	-3.84240710383367
a_HQ_transcript/150242	-0.904045983456246	6.22340695140081	-2.14449353733171	0.0395471920664788	0.570563211824344	-4.3615113436093
a_HQ_transcript/133349	2.2082555074902	0.0229023822580873	2.14435826340022	0.0395588615515838	0.570589951431877	-3.83421557044253
a_HQ_transcript/53070	2.01171595130539	2.07096970169187	2.14417014962605	0.0395750943797178	0.570682482288325	-3.89128843428067
a_HQ_transcript/87850	2.18783238793954	-0.442984262306738	2.14338736382279	0.0396427065478113	0.571422221617997	-3.90809581543988
a_HQ_transcript/84614	-1.69197250348721	2.11278911725692	-2.14334864062894	0.0396460538773979	0.571422221617997	-3.97035345278858
a_HQ_transcript/16176	4.41391750949502	1.28506407244764	2.14297494539976	0.0396783699263134	0.571727720378759	-3.90691250349133
a_HQ_transcript/18023	-3.42314345129625	0.202774963057296	-2.14272852165353	0.039699692712041	0.571727720378759	-3.8306708670534
a_HQ_transcript/101924	-1.62034054758486	-0.545581917078771	-2.14266020302311	0.039705606051863	0.571727720378759	-3.80494991457109
a_HQ_transcript/146059	2.07428195956261	0.981399023592242	2.14264802292925	0.03970666038573	0.571727720378759	-3.82513245554352
a_HQ_transcript/2666	2.99393252921757	0.287103043782784	2.14253519551536	0.0397164281394411	0.571727720378759	-3.84314013986104
a_HQ_transcript/5469	-3.36212982564482	1.89444700847964	-2.14239847750386	0.039728267021321	0.57175654999142	-3.89415896620155
a_HQ_transcript/30785	2.91222221165739	1.60394698828866	2.14195289149734	0.0397668735932523	0.572170502036775	-3.75886979961799
a_HQ_transcript/62929	-2.42262763702145	-0.0726548884424199	-2.14175684171714	0.0397838703354401	0.57227340135077	-3.84866429645238
a_HQ_transcript/73692	1.35901025040966	1.33038408547651	2.14157134684307	0.0397999579467521	0.572363175539042	-3.76765543275764
a_HQ_transcript/52418	1.49485795457697	-0.76366179722619	2.14130313980056	0.0398232292369917	0.572556188248294	-3.80988273098235
a_HQ_transcript/121377	-2.36191023219281	2.99275792336528	-2.14094270018984	0.0398545222163201	0.572864408355651	-3.94949074743162

a_HQ_transcript/143028	-0.758287740155981	3.60764057100168	-2.14052907105364	0.0398904599507545	0.573054010407614	-4.08155365809873
a_HQ_transcript/98890	-1.39492582814009	-0.6518998328228	-2.140393825094	0.0399022168935835	0.573054010407614	-3.85018909676667
a_HQ_transcript/146549	-9.52825926153	3.70133545184573	-2.14023936586989	0.039915647809529	0.573054010407614	-4.07074827904331
a_HQ_transcript/85715	1.97400553560901	0.94172295093625	2.14013660204642	0.0399245858036292	0.573054010407614	-3.81857649823737
a_HQ_transcript/51771	3.42943632913232	0.851983973104062	2.14006863524335	0.0399304982654836	0.573054010407614	-3.85046734722261
a_HQ_transcript/60873	2.18363046028778	0.128706169808097	2.13993939619004	0.0399417429711991	0.573054010407614	-3.8630037156711
a_HQ_transcript/110662	-2.21437607800169	-0.551524600213387	-2.13991541953177	0.0399438294180162	0.573054010407614	-3.85342976678188
a_HQ_transcript/149622	-2.30577924600535	1.04167501771981	-2.13983135875682	0.0399511451438766	0.573054010407614	-3.7781902423806
a_HQ_transcript/6391	4.11459804836629	2.01469752620178	2.13977053508275	0.0399564393094904	0.573054010407614	-3.81506217733874
a_HQ_transcript/37063	-2.12859639881848	-0.237459334225681	-2.13951570808141	0.0399786265280236	0.573230785106304	-3.85315823094756
a_HQ_transcript/2284	2.94533040898615	-0.010241474783165	2.13917738446752	0.0400081005073332	0.573511927568499	-3.84503076911138
a_HQ_transcript/146156	1.70282304424341	0.768293027627474	2.13888229466011	0.0400338238018328	0.573625218730051	-3.76515491085775
a_HQ_transcript/133138	0.710874787582732	4.178271924616	2.13880706211509	0.0400403842511547	0.573625218730051	-4.1885892585419
a_HQ_transcript/16516	3.54714794072372	1.14344550678801	2.13862514368125	0.0400562518964144	0.573625218730051	-3.8586225982132
a_HQ_transcript/2947	2.37648459672354	0.549718765029425	2.13842194439933	0.0400739823395969	0.573625218730051	-3.77309865284056
a_HQ_transcript/37938	2.36964620868087	1.65110060073561	2.13833879351698	0.0400812397965196	0.573625218730051	-3.90616721685388
a_HQ_transcript/139118	-1.82220881239236	-0.66977525463138	-2.13829837131689	0.040084768290776	0.573625218730051	-3.85384048438489
a_HQ_transcript/126571	1.19387873598854	5.52216947638642	2.13829477921678	0.0400850818621227	0.573625218730051	-4.29523475522202
a_HQ_transcript/141542	-1.22171498151045	3.32688433711686	-2.13799283359871	0.0401114479019031	0.57375451573228	-4.07975441308509
a_HQ_transcript/63097	-0.775655665071153	2.95068771647399	-2.13786981503576	0.0401221943551309	0.57375451573228	-3.99867035285207
a_HQ_transcript/67955	-4.22216385235635	3.31340811056107	-2.13777742642631	0.0401302667672152	0.57375451573228	-4.1280720737094
a_HQ_transcript/121013	1.09471373711188	4.12072794293159	2.13752479227663	0.0401523479191159	0.57375451573228	-4.17005456628679
a_HQ_transcript/115977	-1.75575739024204	-0.281213686873194	-2.13741080343206	0.040162314491965	0.57375451573228	-3.82612082141601
a_HQ_transcript/99630	2.38569971779253	-0.0837582757603922	2.13725780937761	0.0401756949217223	0.57375451573228	-3.8161405411255
a_HQ_transcript/125387	-3.85925922821177	-0.097489680498439	-2.137136057244	0.0401863458474055	0.57375451573228	-3.87106682383012
a_HQ_transcript/111594	1.61659115780408	1.89525173296024	2.13709391198825	0.0401900333151049	0.57375451573228	-3.80886603345508
a_HQ_transcript/30391	-0.802682679076998	3.34304212540394	-2.13706080827956	0.0401929299099291	0.57375451573228	-4.0589523093497
a_HQ_transcript/141428	-0.855818651743503	3.542552321225	-2.13696886721196	0.0402009757815398	0.57375451573228	-4.13198554184797
a_HQ_transcript/43773	2.46354454143432	0.719526215561103	2.13694924571911	0.0402026930666388	0.57375451573228	-3.89064034107809
a_HQ_transcript/130747	-2.63631712513198	2.09231014507276	-2.13669986300539	0.0402245248667764	0.573817969396753	-4.11071584462102
a_HQ_transcript/40849	2.37025089785784	0.632132555866786	2.13643224943058	0.0402479643587577	0.573817969396753	-3.82142257136287
a_HQ_transcript/108547	2.18913902372623	0.996175342671398	2.13611556253927	0.040275717695499	0.573817969396753	-3.83321519792665
a_HQ_transcript/110082	3.06931427379307	0.763611599939453	2.13611349975008	0.0402758985268119	0.573817969396753	-3.91907511736096
a_HQ_transcript/94815	1.52716358509624	5.17674042175638	2.1356558394502	0.040316036443297	0.573817969396753	-4.26255641911358
a_HQ_transcript/101145	-2.15409680661831	0.544030452275104	-2.13564121663459	0.0403173194845221	0.573817969396753	-3.79222349952722
a_HQ_transcript/87150	-2.23762156965228	-0.396757825002415	-2.13562582982955	0.0403186695991455	0.573817969396753	-3.83502249656171
a_HQ_transcript/82926	-2.46375238354433	0.828704198518089	-2.13548256476741	0.0403312423122153	0.573817969396753	-3.83251719274589
a_HQ_transcript/7220	3.37775121867727	3.50590183430058	2.13544445916668	0.0403345869844707	0.573817969396753	-4.05359570952926
a_HQ_transcript/15813	2.27819702383099	0.558599212488119	2.13540198602428	0.0403383153023413	0.573817969396753	-3.75374160044552
a_HQ_transcript/79331	2.54022869874022	0.416890226203327	2.13535187253742	0.0403427146874288	0.573817969396753	-3.83544177328239
a_HQ_transcript/116333	-3.28238572190895	-0.0947700770813938	-2.13529130563909	0.0403480323293967	0.573817969396753	-3.90872113230765
a_HQ_transcript/137533	3.66520124397083	2.1634529547944	2.13515980340981	0.0403595800800749	0.573817969396753	-3.97706990662787
a_HQ_transcript/133905	1.46827752883423	1.83728134113293	2.1351513919479	0.0403603188248481	0.573817969396753	-3.82455471984552
a_HQ_transcript/137477	2.05935597057188	0.342001009331522	2.13514869225232	0.0403605559307652	0.573817969396753	-3.86529186642218
a_HQ_transcript/88022	-1.90918612166985	0.860555003205861	-2.13509712369533	0.0403650852749677	0.573817969396753	-3.85475181628007
a_HQ_transcript/50171	3.20880156905347	2.76526983222277	2.13448694095474	0.0404187127918084	0.573874545827349	-4.01514534452799
a_HQ_transcript/1197	1.69225776213901	0.870552852608976	2.13445763174283	0.040421290299258	0.573874545827349	-3.7723871626322
a_HQ_transcript/147218	1.26657400293899	3.07026579084875	2.13443700022307	0.0404231047615176	0.573874545827349	-4.04878358894217
a_HQ_transcript/18999	2.1019305751539	0.626641195682204	2.13436678319552	0.0404292806185852	0.573874545827349	-3.8845416102572
a_HQ_transcript/7705	-2.19114931655292	0.251031402826934	-2.13407492220011	0.0404549598845394	0.573874545827349	-3.84444709338967

a_HQ_transcript/71315	1.59006706823641	-0.758258312135396	2.13396733171377	0.0404644298372502	0.573874545827349	-3.87408979346957
a_HQ_transcript/81735	2.35431686306241	0.745617521756406	2.13395641181196	0.0404653911004885	0.573874545827349	-3.80892705456044
a_HQ_transcript/8235	2.52574745190168	1.42092520315099	2.13382593570326	0.0404768782916491	0.573874545827349	-3.91858981410917
a_HQ_transcript/5634	1.80806163846014	0.990568306798736	2.13375556307464	0.0404830751388329	0.573874545827349	-3.76931992243245
a_HQ_transcript/22132	1.73427392143461	1.71763167316759	2.13372963174357	0.0404853588026745	0.573874545827349	-3.80430710267141
a_HQ_transcript/152014	4.1961474119139	0.500556726237393	2.13363674456637	0.0404935399255811	0.573874545827349	-3.87826727810931
a_HQ_transcript/118769	-2.36583257693484	-0.481629922778407	-2.13357069040633	0.0404993585981949	0.573874545827349	-3.8752822333166
a_HQ_transcript/151608	1.83550106167562	3.43808123524323	2.13350422042792	0.040505214648675	0.573874545827349	-3.9695805088097
a_HQ_transcript/25603	-0.823335950588832	3.63572749167741	-2.13348076186984	0.0405072815429675	0.573874545827349	-4.13887087676201
a_HQ_transcript/67024	-2.96308311727349	1.04022840110401	-2.13278784138077	0.0405683758169721	0.574592732135899	-3.86315751306338
a_HQ_transcript/142490	2.25916139666904	0.270602582739764	2.13253419606706	0.0405907599578295	0.574592732135899	-3.82246446217016
a_HQ_transcript/145704	0.828524459979088	3.27340914516076	2.13243136143019	0.0405998382114404	0.574592732135899	-4.0684054373234
a_HQ_transcript/99721	-2.86462421206875	1.64862551639285	-2.13232120973174	0.0406095644136181	0.574592732135899	-3.9373793752914
a_HQ_transcript/99761	2.94704397688041	2.11952319858268	2.13222620085688	0.0406179551908599	0.574592732135899	-3.94544381123607
a_HQ_transcript/10923	2.23927911763523	1.00251794215287	2.13209930703295	0.0406291643086267	0.574592732135899	-3.7706008958316
a_HQ_transcript/139716	4.86705134899676	5.13992192694377	2.13209053896281	0.0406299389340975	0.574592732135899	-4.19781824234468
a_HQ_transcript/59514	-2.90697190449188	0.0920637497022046	-2.13201000055962	0.0406370548068174	0.574592732135899	-3.96367231600788
a_HQ_transcript/104349	0.886780563186966	2.43716217725469	2.13129212779801	0.0407005304310551	0.575350299828884	-3.97671189847425
a_HQ_transcript/106419	3.69430786353074	1.35038960540431	2.13114456824399	0.0407135888062807	0.575394964777896	-3.93096367539642
a_HQ_transcript/49788	1.13744714356177	2.8641936457846	2.13097004185719	0.0407290384241351	0.57547243075415	-3.99824057482596
a_HQ_transcript/133277	-2.40666143838125	0.598262655014605	-2.13085900682569	0.0407388702957839	0.57547243075415	-3.81816019173945
a_HQ_transcript/142701	6.5259900219908	3.97941272837811	2.1304511818513	0.0407750002314196	0.575651718123916	-4.19656215500529
a_HQ_transcript/126955	2.76256630916179	0.333795043623237	2.13026577357931	0.0407914352711083	0.575651718123916	-3.88225630942711
a_HQ_transcript/93626	-1.1569734918075	2.53961898064885	-2.13014448352051	0.0408021898945722	0.575651718123916	-3.94025060099936
a_HQ_transcript/91033	-1.17880740142747	5.41534580517157	-2.13007388187817	0.040808451203173	0.575651718123916	-4.31233866386998
a_HQ_transcript/50421	-2.61616580546614	3.50070604839223	-2.13003704293925	0.0408117186037292	0.575651718123916	-4.1290378863487
a_HQ_transcript/135642	-2.06974384175173	2.36620541737775	-2.13003271985419	0.0408121020515487	0.575651718123916	-3.96217973519298
a_HQ_transcript/79197	2.20015358188029	1.92845707007363	2.12988820518972	0.0408249220122305	0.575651718123916	-3.92556849815241
a_HQ_transcript/36812	1.33114976917272	1.71494243208297	2.12965654958669	0.0408454797342399	0.575651718123916	-3.82473229790004
a_HQ_transcript/129130	1.63569565852184	-0.0725914165421328	2.12948347926634	0.0408608444353895	0.575651718123916	-3.78748695641077
a_HQ_transcript/131890	1.60324356254548	0.592562659448276	2.12925835107781	0.0408808383575024	0.575651718123916	-3.81958172491229
a_HQ_transcript/82829	-2.88391177107697	0.880930800362315	-2.12914098388604	0.0408912653272649	0.575651718123916	-3.92921301193145
a_HQ_transcript/89829	-1.59074211216343	2.70546976230837	-2.12900609177238	0.0409032521335896	0.575651718123916	-4.02301885920991
a_HQ_transcript/30634	1.95539363961365	0.71042500244662	2.12887661355269	0.0409147607797237	0.575651718123916	-3.81620230600924
a_HQ_transcript/107601	-0.774614378593061	2.78638402891261	-2.12886625181357	0.0409156819049253	0.575651718123916	-4.01339621599476
a_HQ_transcript/86518	2.22023782751136	0.851452125430425	2.12880529576766	0.040921101073115	0.575651718123916	-3.88034026772068
a_HQ_transcript/143834	1.81145723370308	2.2915354920565	2.12876760114354	0.0409244525522741	0.575651718123916	-3.91890520643818
a_HQ_transcript/12350	3.46590427510625	0.697785253375342	2.12875650450862	0.0409254392152057	0.575651718123916	-3.85431384264359
a_HQ_transcript/148889	4.78553061550343	1.49347983815568	2.12868305241295	0.0409319707774515	0.575651718123916	-4.00094327675481
a_HQ_transcript/85572	-1.68304461469344	2.27180003419456	-2.12852117464193	0.0409463686566224	0.575651718123916	-3.78669003541571
a_HQ_transcript/148991	2.82715312726632	-0.32722562351729	2.12840289607695	0.040956891534436	0.575651718123916	-3.83702292625062
a_HQ_transcript/126668	0.867028893662911	3.22627338353864	2.12837325397099	0.0409595290765297	0.575651718123916	-4.05067129441999
a_HQ_transcript/109105	1.95764606493096	1.10433446831563	2.1281143614181	0.0409825716283707	0.575836336382386	-3.82273720033292
a_HQ_transcript/88932	-2.35669058494835	1.75269682471472	-2.12797027542484	0.0409954008791874	0.575877395433882	-3.88563499364512
a_HQ_transcript/103919	-2.45543139185236	2.81507594588428	-2.12749877380668	0.0410374077309595	0.576094616630822	-4.12953311736871
a_HQ_transcript/69880	-1.81906869632892	-0.312790799751123	-2.12742850028396	0.0410436717803962	0.576094616630822	-3.87288087637482
a_HQ_transcript/105908	-2.0427401973371	1.23202078649702	-2.12735964054464	0.0410498106300105	0.576094616630822	-3.96914362257669
a_HQ_transcript/82761	2.51649464320342	1.24708907182009	2.12735182389102	0.0410505075365549	0.576094616630822	-3.90877149620137
a_HQ_transcript/145876	0.979397215840411	8.6481686917514	2.12700184752579	0.0410817210010328	0.576351570943327	-4.62037752682421
a_HQ_transcript/148375	1.97199324423329	1.47010935091661	2.12689943758223	0.0410908586503493	0.576351570943327	-3.82543895890762

a_HQ_transcript/108614	2.48818901476945	2.60806149651768	2.12681310862694	0.0410985628537037	0.576351570943327	-3.99320453186149
a_HQ_transcript/84249	0.875255918757858	4.1191312012454	2.12667069016222	0.0411112754180788	0.576368918466942	-4.17630972589312
a_HQ_transcript/94639	2.19853069565877	1.36578809307086	2.12657710522943	0.0411196308987478	0.576368918466942	-3.78703757881329
a_HQ_transcript/13311	-1.3183921202837	2.20628647601907	-2.12626629397887	0.0411473916526124	0.576618992763514	-3.870760253574
a_HQ_transcript/59481	1.80745228640465	2.33631015069537	2.12607224571269	0.041164731899354	0.576722953927609	-3.86223965273598
a_HQ_transcript/118070	-3.20746588086681	1.46114744293259	-2.12596065126694	0.0411747069659926	0.576723702775715	-3.81426970537029
a_HQ_transcript/1424	-2.25532952984898	1.618646400829	-2.12524990153707	0.0412382888746951	0.577167271766184	-3.83709353839218
a_HQ_transcript/117767	-1.58287864492487	-0.740115122849258	-2.12523670361909	0.0412394703501533	0.577167271766184	-3.86751825763069
a_HQ_transcript/151203	-2.6372452731368	0.273318096002014	-2.12517123442825	0.0412453315849126	0.577167271766184	-3.81284719439553
a_HQ_transcript/150687	2.17716252818028	1.59647247867103	2.12516273915931	0.0412460921916585	0.577167271766184	-3.85964364298137
a_HQ_transcript/55886	-2.32983011630303	0.124720556539241	-2.12487625241626	0.0412717494825536	0.577353709316607	-3.8267367643182
a_HQ_transcript/150778	0.319876298671805	11.0631424002664	2.12474269837841	0.0412837151998926	0.577353709316607	-4.91779117205096
a_HQ_transcript/20779	1.99250074082013	0.751542086069443	2.12468134748631	0.0412892129374679	0.577353709316607	-3.74346493764422
a_HQ_transcript/137682	-1.75323959300029	2.48701093438021	-2.12444198459886	0.041310668778266	0.577370276051742	-3.96261181195915
a_HQ_transcript/29612	2.84623984825435	1.29175645624373	2.12431402913688	0.0413221424112946	0.577370276051742	-3.91381911301739
a_HQ_transcript/151004	-1.67561191282079	-0.688340395866289	-2.12425945491674	0.0413270368837768	0.577370276051742	-3.87086366144683
a_HQ_transcript/74905	2.9659922349444	1.76556339940348	2.12422498404394	0.0413301286583282	0.577370276051742	-3.94508938525399
a_HQ_transcript/67957	-1.54462383442176	4.68165251374698	-2.12385389430323	0.0413634255620723	0.577589183839948	-4.26427188517408
a_HQ_transcript/101665	2.81675809210695	1.74751245442481	2.12377913390281	0.041370136491652	0.577589183839948	-3.80655134195111
a_HQ_transcript/41982	2.02620430203861	0.294362406314152	2.12371818495397	0.0413756083386585	0.577589183839948	-3.88568592592722
a_HQ_transcript/112481	-4.51382998017676	2.05303223908276	-2.12338087248771	0.0414059030210522	0.57787330871734	-3.89789710158505
a_HQ_transcript/110335	-1.78256665322227	1.5010423925975	-2.12319171131547	0.0414229005502674	0.57797176264665	-3.83448752163355
a_HQ_transcript/136404	0.508330877370619	4.45463439003407	2.12299192517558	0.0414408595242014	0.578026922314732	-4.25703913389534
a_HQ_transcript/123868	1.98271895307767	2.47774791028718	2.12292650304451	0.0414467418835639	0.578026922314732	-3.96308296108488
a_HQ_transcript/117093	-1.7767346768432	0.880112423026989	-2.12226755030723	0.0415060321631868	0.578609376762717	-3.79069183607022
a_HQ_transcript/138270	-2.40994986811314	-0.352891154705707	-2.12224109366456	0.041508414208308	0.578609376762717	-3.855508888171
a_HQ_transcript/22712	1.09000666157864	1.21516709933396	2.12199907796353	0.0415302099087239	0.578666844225085	-3.83991265021413
a_HQ_transcript/106580	1.66128265989832	-0.692063389178478	2.12191729023476	0.0415375779245126	0.578666844225085	-3.89506408005167
a_HQ_transcript/52597	1.51056958569357	2.00531205122072	2.12152285433972	0.0415731277554749	0.578666844225085	-3.91202311712031
a_HQ_transcript/3512	1.9637062973631	2.00740837640407	2.12133761472841	0.0415898323834449	0.578666844225085	-3.81848334867127
a_HQ_transcript/122615	2.95913152764341	1.38905179241251	2.12113504777231	0.0416081063712351	0.578666844225085	-3.96558223121828
a_HQ_transcript/144952	1.58675346855427	2.83059057072333	2.1210197599793	0.0416185098992995	0.578666844225085	-3.93350313284704
a_HQ_transcript/62556	3.25805941576411	1.80598935711516	2.12100457509409	0.0416198803494848	0.578666844225085	-3.79056233125881
a_HQ_transcript/107029	1.41920909999812	2.54901899009035	2.12083194717771	0.0416354629920976	0.578666844225085	-3.97090555862524
a_HQ_transcript/111933	-2.64876496568952	-0.490389701975732	-2.1207029457443	0.0416471109669794	0.578666844225085	-3.90513888599014
a_HQ_transcript/31232	-2.53649453058937	3.8991542037743	-2.12065078718214	0.0416518213590512	0.578666844225085	-4.02298182225962
a_HQ_transcript/104378	-2.181019638162	-0.187550092926853	-2.12042458179115	0.0416722552228826	0.578666844225085	-3.84634252494057
a_HQ_transcript/12158	-2.30692915078528	2.09746171315685	-2.12020114514474	0.0416924476937241	0.578666844225085	-3.8852261297271
a_HQ_transcript/143310	0.426005091311961	10.8656151971974	2.12015107464834	0.0416969738652587	0.578666844225085	-4.91400142706222
a_HQ_transcript/138709	3.75092677369143	0.595943318110993	2.12011834018651	0.0416999331642521	0.578666844225085	-3.92714315256402
a_HQ_transcript/71411	1.07014529147795	2.85407216699774	2.1200455995648	0.0417065098127629	0.578666844225085	-3.99871437196359
a_HQ_transcript/133706	4.28699332934035	2.47358081522492	2.12002913642855	0.0417079984106264	0.578666844225085	-4.08092547141441
a_HQ_transcript/87375	2.27407869148142	-0.298678481311094	2.11999965224804	0.0417106644897805	0.578666844225085	-3.89375020635124
a_HQ_transcript/26144	-1.57701084744855	5.23952913652593	-2.11981490846006	0.0417273732085488	0.578666844225085	-4.30177153717524
a_HQ_transcript/127675	2.32374798989992	0.421581805978913	2.11971528947928	0.0417363854737261	0.578666844225085	-3.76240768592234
a_HQ_transcript/144760	0.870731336190332	2.84717018194384	2.11951817619711	0.0417542228705463	0.578666844225085	-4.01257268856041
a_HQ_transcript/35234	-0.777715971484367	3.17323134029304	-2.11941003315134	0.0417640119407259	0.578666844225085	-4.016836347566
a_HQ_transcript/150171	-2.38969603947055	-0.487835910899262	-2.11932160164006	0.0417720182403823	0.578666844225085	-3.90103158312788
a_HQ_transcript/51191	-0.676125902644102	3.03860474952123	-2.11918292315101	0.0417845764743577	0.578666844225085	-3.98699912930264
a_HQ_transcript/22650	1.49134240240368	-0.80763724497809	2.11914518541275	0.0417879944493085	0.578666844225085	-3.9044350971578

a_HQ_transcript/138068	-6.74432302703577	3.37321503787847	-2.11897949152179	0.0418030045741296	0.578666844225085	-4.0483732748312
a_HQ_transcript/78071	-2.87476480209404	1.56974900819572	-2.11887209038274	0.0418127365388388	0.578666844225085	-3.96454372547651
a_HQ_transcript/27969	1.25057985935898	3.69405543885115	2.11877152916795	0.0418218505347279	0.578666844225085	-4.14451411688551
a_HQ_transcript/73986	2.46534125066028	0.197766426815307	2.11852128371341	0.0418445382481527	0.578666844225085	-3.87238855744939
a_HQ_transcript/138583	0.526110848418777	5.01634222757109	2.11849013846326	0.0418473626964034	0.578666844225085	-4.31850169565299
a_HQ_transcript/146593	2.61850240488981	2.27008187335007	2.11847803130668	0.0418484606955013	0.578666844225085	-3.99354046684862
a_HQ_transcript/112489	-2.61640396470099	0.71982601038005	-2.11845949702814	0.041850141620279	0.578666844225085	-3.8307515849345
a_HQ_transcript/152378	-2.2257274964219	0.77434551320572	-2.1184424231279	0.0418516901522374	0.578666844225085	-3.87791711763278
a_HQ_transcript/60521	2.65992699157879	3.36290973969747	2.11832903771099	0.0418619750268635	0.578666844225085	-4.03608962136509
a_HQ_transcript/29133	-2.71610751138418	1.58946283455316	-2.11823949650401	0.0418700986414137	0.578666844225085	-3.86523416544864
a_HQ_transcript/114821	2.47230119068126	1.17862523535359	2.11822996730941	0.0418709632585595	0.578666844225085	-3.91285551452558
a_HQ_transcript/150246	2.37594646191108	-0.654161111192919	2.1180657566904	0.0418858651462216	0.578666844225085	-3.89048097512624
a_HQ_transcript/40768	2.3742654555974	0.954581983160491	2.11793245116573	0.0418979658927815	0.578666844225085	-3.8233941535152
a_HQ_transcript/128040	-1.5879594748702	-0.328443363207528	-2.11778531002444	0.0419113261552005	0.578666844225085	-3.86566761115631
a_HQ_transcript/152579	3.49942057750178	1.74666491723619	2.11774924893672	0.041914601039942	0.578666844225085	-3.93612713567005
a_HQ_transcript/80977	2.6403191721092	0.473590433436209	2.11758953148262	0.0419291084920304	0.578666844225085	-3.81401909748803
a_HQ_transcript/136281	0.751549136552215	2.74620952165587	2.11732166349689	0.0419534494527656	0.578666844225085	-4.0046113923705
a_HQ_transcript/117489	2.00586247631069	0.816955407335896	2.11726607661519	0.0419585021579246	0.578666844225085	-3.79938781783352
a_HQ_transcript/115492	-2.08936237907989	0.0855028061920126	-2.11722546448837	0.0419621940368617	0.578666844225085	-3.80652237319762
a_HQ_transcript/82303	4.71016731437963	1.95520894845675	2.11699577024603	0.0419830799960789	0.578666844225085	-4.02275711848664
a_HQ_transcript/95105	2.63504037293852	0.445576471605047	2.11697402070007	0.0419850581461814	0.578666844225085	-3.83772949728815
a_HQ_transcript/66300	-2.56040160342596	0.920926942895672	-2.11696689628744	0.0419857061389205	0.578666844225085	-3.8228077128991
a_HQ_transcript/72461	-2.67062417166088	0.607391008998397	-2.11694490186758	0.0419877066718947	0.578666844225085	-3.90396624372305
a_HQ_transcript/140075	2.66940823113144	0.551665541619014	2.11686133434808	0.0419953084411449	0.578666844225085	-3.9305716681342
a_HQ_transcript/151852	-1.48459341637464	-0.627069792878019	-2.11680609469844	0.042000334017782	0.578666844225085	-3.88766527288089
a_HQ_transcript/77219	1.90577939642578	-0.501265284080557	2.11643618054313	0.0420340016839128	0.578901660887516	-3.91233307952678
a_HQ_transcript/36126	2.25770621435061	1.6512808882487	2.11627170611048	0.042048978965563	0.578901660887516	-3.88186511310826
a_HQ_transcript/100020	-3.24531418749533	-0.550070703401013	-2.11616362389395	0.042058823662218	0.578901660887516	-3.92138729878755
a_HQ_transcript/151315	2.97637945217886	0.343210265549405	2.11608986788633	0.0420655429186215	0.578901660887516	-3.95477302197646
a_HQ_transcript/73120	0.669103481563537	4.06231224613277	2.11604596340427	0.042069543118238	0.578901660887516	-4.23920754260328
a_HQ_transcript/15108	1.8399203771018	0.553756780603605	2.11596268343478	0.0420771317996449	0.578901660887516	-3.79087163589065
a_HQ_transcript/136784	2.38846019622656	0.614457521214286	2.1156131806268	0.0421089925963459	0.579202915674489	-3.85094762532045
a_HQ_transcript/46964	-2.22914679810955	0.809245843384388	-2.1152353356203	0.0421434611020846	0.579539888086579	-3.8893422653306
a_HQ_transcript/71944	2.278461393117	-0.30724182452014	2.11463004380497	0.0421987302558877	0.580147952476074	-3.89153404287654
a_HQ_transcript/141194	-2.91206630375736	0.734545994501245	-2.11452780683196	0.0422080718381516	0.580147952476074	-3.81726049388218
a_HQ_transcript/115995	3.14593745990534	0.423782004498697	2.11442332524457	0.0422176204062379	0.580147952476074	-3.90447305604437
a_HQ_transcript/120771	2.71340982061319	0.76266362555649	2.11373800667227	0.0422802990603952	0.580611827456591	-3.91047230091198
a_HQ_transcript/128260	2.23423965818504	0.512359852587246	2.11372560453876	0.0422814341073636	0.580611827456591	-3.82808770005845
a_HQ_transcript/1689	2.16778088385429	1.40974924682704	2.11356672706828	0.0422959770069727	0.580611827456591	-3.88969648780509
a_HQ_transcript/16706	-0.806874133658469	4.03921057133988	-2.11356273004946	0.0422963429324428	0.580611827456591	-4.24116337310448
a_HQ_transcript/60977	1.6485896393506	2.96524859172315	2.11350837583264	0.0423013193173455	0.580611827456591	-4.0674567429966
a_HQ_transcript/15305	1.97938187885784	0.179724571411283	2.11302100189748	0.0423459638606603	0.581087390767815	-3.89372923144524
a_HQ_transcript/72581	1.2901465911143	0.928406688220449	2.11280257942138	0.0423659853771861	0.58122492282395	-3.85309244649819
a_HQ_transcript/57690	2.969259913067	3.30370772822892	2.1124686197112	0.0423966137168286	0.581400821757846	-4.05105273159944
a_HQ_transcript/71594	0.512489764476843	4.28533178013704	2.11241832331838	0.0424012282312552	0.581400821757846	-4.27676205766593
a_HQ_transcript/139538	0.652679650397181	3.71402117457265	2.11233566243491	0.0424088130376629	0.581400821757846	-4.14905731961866
a_HQ_transcript/143687	-2.24576773333196	-0.244549109955946	-2.11169281392122	0.0424678405927414	0.582072774811335	-3.92559700202121
a_HQ_transcript/63753	-2.35465211625187	2.30594285930095	-2.11155259469007	0.0424807254399071	0.582112118899321	-3.97808468928582
a_HQ_transcript/35446	1.50035076686069	1.30474323761076	2.11138787986256	0.0424958656237999	0.582182189841935	-3.85304296583955
a_HQ_transcript/106399	2.84076945351054	0.532888425836725	2.11127906715964	0.0425058700400697	0.582182189841935	-3.92892419665849

a_HQ_transcript/130768	1.2895689993918	2.39026242676459	2.11094829386754	0.0425362946707529	0.582399582435294	-3.86998885477244
a_HQ_transcript/101544	2.96316862820374	0.220468434973906	2.11071690356162	0.0425575894766123	0.582399582435294	-3.89854627723691
a_HQ_transcript/145699	1.58186527644616	2.01361135440244	2.11067573240002	0.0425613794391465	0.582399582435294	-3.82863273741278
a_HQ_transcript/116265	-1.64715057689946	-0.744491373585718	-2.11052500437455	0.0425752570771886	0.582399582435294	-3.90050028963726
a_HQ_transcript/132421	-3.2043110876189	0.524019308501227	-2.11051881664276	0.0425758268716643	0.582399582435294	-3.91638077944429
a_HQ_transcript/89014	1.4877831947156	1.9672111304046	2.11016544648741	0.042608377999358	0.582399582435294	-3.87167777696417
a_HQ_transcript/1117	-1.87210639947961	0.275251109533763	-2.11011758539662	0.0426127884754703	0.582399582435294	-3.9187108986216
a_HQ_transcript/18798	0.782276881929374	2.99726629580265	2.11011323516142	0.0426131893766307	0.582399582435294	-4.08574645182559
a_HQ_transcript/77833	-1.80953600812223	-0.668975732492567	-2.11005598267946	0.042618465858981	0.582399582435294	-3.90326539363554
a_HQ_transcript/54803	1.33521180357331	3.51563682352722	2.11001834678818	0.0426219347591478	0.582399582435294	-4.16645499712701
a_HQ_transcript/24102	-1.30449614835077	3.79815010110174	-2.10949259216674	0.0426704196896219	0.582925065973759	-4.14488347569757
a_HQ_transcript/58940	2.03437020001847	2.38204345029677	2.10936008903768	0.0426826467835906	0.582955096860093	-3.89647743891267
a_HQ_transcript/79304	2.75640075704352	0.508170147864045	2.10915546373764	0.0427015352406178	0.583076072461036	-3.91582973334467
a_HQ_transcript/16647	1.46467934347497	-0.771209876828777	2.10880573265089	0.0427338351753471	0.583380077753071	-3.91916564748487
a_HQ_transcript/112458	-2.81445417041471	2.68758635560774	-2.10867594555847	0.0427458273529645	0.583406774447786	-4.05975446565826
a_HQ_transcript/9425	-3.81194521519648	1.51882670357042	-2.10838730886233	0.0427725077235732	0.583469297136497	-3.89538445144308
a_HQ_transcript/78018	-2.13775769347332	0.316064098534126	-2.10833823160824	0.0427770456849856	0.583469297136497	-3.9516243974156
a_HQ_transcript/119469	-2.22030726903392	-0.290991802386907	-2.10830064613568	0.0427805213390405	0.583469297136497	-3.93528437466607
a_HQ_transcript/139244	-3.85718686095822	2.4817146512767	-2.10807240650521	0.0428016327840861	0.583599135623411	-4.15208954348504
a_HQ_transcript/123833	-1.34507032651492	1.76241290088732	-2.10780936500052	0.0428259747225861	0.583599135623411	-3.82836080936929
a_HQ_transcript/16704	2.48183786538004	0.686303078107872	2.10777132732818	0.0428294957532378	0.583599135623411	-3.86592789886844
a_HQ_transcript/86327	0.608079564132148	3.3669622496494	2.10776371095795	0.0428302008080352	0.583599135623411	-4.1382086033469
a_HQ_transcript/1211	-1.06246507042568	2.27144680224981	-2.10759988077315	0.0428453692132502	0.583637938535436	-3.923230460148
a_HQ_transcript/18292	2.70982923025666	3.28948834915637	2.10751608180127	0.0428531296736382	0.583637938535436	-4.07087135118494
a_HQ_transcript/148415	5.05178015260307	2.14574829432077	2.10710658868342	0.0428910699224373	0.584017829105512	-4.0185564179992
a_HQ_transcript/144800	1.78433528383277	-0.343675006808606	2.10667712232493	0.0429308926427143	0.584143927660694	-3.91075301706999
a_HQ_transcript/149547	1.96742310315172	-0.302096956774502	2.10659297862031	0.0429386987863416	0.584143927660694	-3.89468845930497
a_HQ_transcript/150433	2.90104338642677	0.551178809990915	2.106535942402	0.0429439908397251	0.584143927660694	-3.94338946708569
a_HQ_transcript/33796	-2.38585102978841	0.475877806115191	-2.10647556560445	0.0429495934742006	0.584143927660694	-3.85354155834087
a_HQ_transcript/19382	0.595904428559361	5.17211243821086	2.10646496606485	0.0429505771198356	0.584143927660694	-4.34195804980725
a_HQ_transcript/152426	0.346413709341236	10.849199700992	2.10632726724103	0.042963357490047	0.584181062966422	-4.92831377783038
a_HQ_transcript/117730	2.8053110692083	3.91683200556376	2.10586773173849	0.0430060331342441	0.58462457764905	-4.1397247107426
a_HQ_transcript/76443	1.83295198560517	1.7755759059521	2.10542132241449	0.0430475256829607	0.585051805681351	-3.8576573458091
a_HQ_transcript/118565	2.46285217090521	1.63084234071285	2.10475002667248	0.0431099874646009	0.585734099372053	-3.89429299372742
a_HQ_transcript/65084	1.49761619185572	-0.116103808696729	2.1046643572366	0.0431179644772841	0.585734099372053	-3.85307936904461
a_HQ_transcript/101976	2.60937678856747	2.6433634073083	2.10455705069078	0.0431279580462494	0.585734099372053	-3.95344551344373
a_HQ_transcript/109858	-3.19994888511027	2.4953766782886	-2.10429654129915	0.0431522280757286	0.585926819337993	-3.93915987497925
a_HQ_transcript/150990	0.923702101888689	4.85848824257657	2.1038547385613	0.0431934156788663	0.586341059612755	-4.3253111160828
a_HQ_transcript/150565	3.18101309019172	1.08310886418762	2.10375294857646	0.0432029101004925	0.586341059612755	-3.87552638429191
a_HQ_transcript/32851	2.14993397496727	0.820398790445305	2.10358245171349	0.0432188172635033	0.586420030320476	-3.83606045701002
a_HQ_transcript/35823	-2.66850769917021	0.486456104652222	-2.10338531393285	0.0432372164487851	0.586532769599763	-3.91016452772593
a_HQ_transcript/9327	1.68015145845166	-0.742030300425397	2.10299727629381	0.0432734528561175	0.586749968288859	-3.92821117156353
a_HQ_transcript/7314	2.48820347593364	0.326776693304067	2.10293618820608	0.0432791599358266	0.586749968288859	-3.82979353778241
a_HQ_transcript/131734	-2.34690415510746	0.785010257613222	-2.10273950083259	0.0432975397330882	0.586749968288859	-3.94657068954921
a_HQ_transcript/119343	3.58021565802484	4.66538686721701	2.10259239488356	0.0433112908171267	0.586749968288859	-4.09770950737095
a_HQ_transcript/30565	-1.45505509027311	-0.720780067805859	-2.10253935007201	0.0433162502560562	0.586749968288859	-3.90790208038982
a_HQ_transcript/46848	-0.943681283948533	4.79522825741516	-2.10249479544795	0.0433204162905431	0.586749968288859	-4.32631580193841
a_HQ_transcript/6103	-3.36665955837256	1.50238206837472	-2.1024576873236	0.043323886318053	0.586749968288859	-3.88934737909854
a_HQ_transcript/74514	2.11927097931487	0.0486325454439552	2.10221081075659	0.0433469783129886	0.586925962119124	-3.89037641295666
a_HQ_transcript/113695	1.95182810445077	-0.16723832121576	2.10204236296329	0.043362740590992	0.587002651391053	-3.88014926777464

a_HQ_transcript/150977	-2.35444442727816	0.809773224578597	-2.10134816785529	0.0434277524513436	0.58723160391093	-3.91830632030475
a_HQ_transcript/15531	-2.60329370085242	0.403860406529977	-2.10129732909688	0.0434325169208565	0.58723160391093	-3.84524251391867
a_HQ_transcript/66354	2.6830615122496	0.944882384984255	2.10126596645539	0.0434354563723376	0.58723160391093	-3.92566851049369
a_HQ_transcript/64797	-1.50783573751396	1.23907658355783	-2.10107550300732	0.0434533112623354	0.58723160391093	-3.90631718325191
a_HQ_transcript/7096	-1.12297423093463	1.91508314323112	-2.10099191833427	0.0434611489089604	0.58723160391093	-3.86394572395198
a_HQ_transcript/114913	2.10833577869374	-0.365975213883444	2.10095510640465	0.0434646011210823	0.58723160391093	-3.87140945946995
a_HQ_transcript/44558	3.13318493401109	2.20469086586676	2.10085230367428	0.0434742432149983	0.58723160391093	-3.97497695460155
a_HQ_transcript/5531	-1.4432779118647	-0.719974248982908	-2.10061554543082	0.0434964564817784	0.58723160391093	-3.91571239268307
a_HQ_transcript/108096	2.69521209553361	0.163523165299755	2.10057923182438	0.0434998644047998	0.58723160391093	-3.95687173407294
a_HQ_transcript/111547	-2.47188669704233	0.6754567831008	-2.10052900449368	0.0435045784780428	0.58723160391093	-4.04540622986494
a_HQ_transcript/52853	1.61593014210636	-0.707980056497221	2.10052230373815	0.0435052074098694	0.58723160391093	-3.93301607257913
a_HQ_transcript/34620	2.74671825810986	1.51428383953253	2.10052165588853	0.0435052682173559	0.58723160391093	-3.86726237308132
a_HQ_transcript/87133	2.10648388527832	1.25452698198417	2.10034485217648	0.0435218659091698	0.58723160391093	-3.89759864666183
a_HQ_transcript/9103	0.739621119979697	2.9389827687656	2.10021361017624	0.0435341900513063	0.58723160391093	-4.06275006216691
a_HQ_transcript/133618	3.05692724667533	0.570262145656468	2.1000854984734	0.0435462232208806	0.58723160391093	-3.98848136331784
a_HQ_transcript/74787	1.28937244086694	-0.714412106973712	2.10008111066744	0.0435466354071308	0.58723160391093	-3.93607654432124
a_HQ_transcript/95118	-0.840899375055761	2.64050995461486	-2.10000386727084	0.0435538921417969	0.58723160391093	-3.96882928395088
a_HQ_transcript/80765	-1.25439408951534	-0.818406931380882	-2.09992293446754	0.0435614966292308	0.58723160391093	-3.91626282248704
a_HQ_transcript/116970	2.83247168338975	2.17436110918456	2.09939922896293	0.0436107326341987	0.587759023083863	-3.87378186724858
a_HQ_transcript/16975	2.26661997167513	0.268908299806469	2.09916198233836	0.0436330534804861	0.587879593623485	-3.97453635969277
a_HQ_transcript/2267	-1.53388158616079	1.42806010485391	-2.09908917066546	0.0436399058368658	0.587879593623485	-3.80482578495733
a_HQ_transcript/41859	1.92200797772803	4.81455400681183	2.09860244978649	0.0436857359016445	0.588314895957275	-4.28914674448973
a_HQ_transcript/13371	1.85436218270338	1.31993558491909	2.09853106039131	0.0436924615649524	0.588314895957275	-3.81179421132513
a_HQ_transcript/38006	-1.62664489756473	-0.594052633827088	-2.09829650841586	0.0437145653730546	0.588415666124703	-3.92070436493239
a_HQ_transcript/8204	2.14576352007223	1.43859244797946	2.09813586332839	0.0437297100131751	0.588415666124703	-3.83747206303568
a_HQ_transcript/125066	4.11824333286714	0.664804746106023	2.0981294602763	0.0437303137499779	0.588415666124703	-3.92555765537332
a_HQ_transcript/555	1.7496675609664	-0.258712683810787	2.09794750464914	0.0437474732263479	0.588510327169902	-3.92972806709793
a_HQ_transcript/136713	1.66124766695587	0.78497377002371	2.09781500580674	0.0437599723828055	0.588542266235011	-3.88708522167339
a_HQ_transcript/115999	1.48900566243688	-0.771839982662627	2.09762599887965	0.0437778076382222	0.588645940873139	-3.93855896202719
a_HQ_transcript/143604	1.48444474232358	8.63892706563751	2.09715499371187	0.0438222810331375	0.58904788281248	-4.67748853202606
a_HQ_transcript/109849	-2.79435913572031	2.26990544942059	-2.09709480070686	0.0438279674711666	0.58904788281248	-3.87123784460835
a_HQ_transcript/19321	1.5602173816798	2.64302462887497	2.09680086344457	0.0438557451133781	0.58919132621339	-4.07049854558086
a_HQ_transcript/42161	-2.96109982436935	0.960752525915691	-2.09672646473908	0.0438627784016667	0.58919132621339	-3.94731771023573
a_HQ_transcript/81586	2.47983899422768	3.32892580941107	2.09666014643274	0.0438690486486986	0.58919132621339	-3.90734325324137
a_HQ_transcript/146160	0.494554303991348	6.43192468000786	2.09629038508128	0.0439040232724733	0.589300043326676	-4.4902404867732
a_HQ_transcript/105580	2.61108477439949	1.98757102169005	2.09628547793806	0.0439044875900916	0.589300043326676	-3.98030357244021
a_HQ_transcript/16380	2.53311283959943	1.88370240707426	2.09618892367482	0.0439136245102692	0.589300043326676	-3.9621467424886
a_HQ_transcript/19740	-2.15159398492653	0.65546368433836	-2.09606390074314	0.0439254579133099	0.589300043326676	-3.87173080850915
a_HQ_transcript/147953	-3.58099598270583	0.173438010161327	-2.09603880891054	0.0439278331911383	0.589300043326676	-3.94165592610122
a_HQ_transcript/80436	-1.54034492848991	1.95341030370166	-2.09586973693319	0.0439438410751965	0.589378771116525	-3.92607248882258
a_HQ_transcript/122264	3.28125610810892	0.634236873310743	2.09572969705793	0.0439571040757397	0.589420656450888	-3.90141235198303
a_HQ_transcript/8841	2.48667638605402	1.52489080683508	2.09539941904335	0.0439883982962836	0.589704247270843	-3.99864125714594
a_HQ_transcript/5796	-1.82739241954177	0.021670670063453	-2.09516374747728	0.044010740477025	0.589753761771049	-3.88900942161874
a_HQ_transcript/85647	2.56935725489824	1.53241843694464	2.0951464223116	0.0440123833361343	0.589753761771049	-3.93274496352127
a_HQ_transcript/129744	-2.87012724644595	0.878460753679567	-2.0949395974455	0.0440319996908553	0.589756037224116	-3.89823396668461
a_HQ_transcript/64958	1.42288221992765	-0.800170256099016	2.09487847947189	0.0440377979179289	0.589756037224116	-3.94382339272175
a_HQ_transcript/129618	1.93903621837133	1.39926709498147	2.09481622858144	0.0440437043174214	0.589756037224116	-3.8538658476097
a_HQ_transcript/137868	-3.48561204645447	3.61205521457621	-2.09471683363161	0.0440531364166514	0.589756037224116	-4.2331271191702
a_HQ_transcript/15400	1.77707161338908	1.29906446923283	2.09460403795806	0.0440638423407066	0.589763533866128	-3.87737134701286
a_HQ_transcript/72739	1.70425567386859	0.88386328251702	2.09430816753335	0.0440919355995416	0.590003690729777	-3.84063926903306

a_HQ_transcript/67375	-2.52096798465021	0.205582097634002	-2.09410302155031	0.0441114237473118	0.590128616705119	-3.95590111490001
a_HQ_transcript/140282	-2.8364750506268	0.768271520321523	-2.09397519174418	0.0441235709765216	0.590142540497536	-3.92568491562425
a_HQ_transcript/125730	4.98431511474098	5.27787925358853	2.09387114145159	0.0441334607008369	0.590142540497536	-4.27250647042612
a_HQ_transcript/108823	3.45540679774662	1.64671790946859	2.09377161862498	0.0441429219323439	0.590142540497536	-4.01497718786592
a_HQ_transcript/108842	-2.43523026951961	0.983092415143156	-2.09338540484518	0.0441796546756981	0.590497807999306	-3.91127871125533
a_HQ_transcript/106075	0.858061557081443	3.60046029653141	2.09325021161836	0.0441925192631847	0.590533967754115	-4.20489988609873
a_HQ_transcript/60080	-3.28882940131624	1.48314762422397	-2.09285231446404	0.0442304011771632	0.590791425647375	-3.94384196610839
a_HQ_transcript/61271	-1.02183085479006	-0.483844077709226	-2.0928343376345	0.0442321133432662	0.590791425647375	-3.95237499320541
a_HQ_transcript/125598	-6.25683990635586	1.59451196669191	-2.09257288621566	0.0442570213614937	0.590921149370015	-3.93182955924091
a_HQ_transcript/130619	-3.03362384966108	-0.219677554897174	-2.09251899121705	0.044262157382966	0.590921149370015	-3.94628913798026
a_HQ_transcript/104309	2.46057578241842	0.314298581592883	2.09215623437135	0.044296740648651	0.591247058650927	-3.89451645140056
a_HQ_transcript/127045	1.73209871366848	-0.652528991928406	2.09164274515255	0.0443457347087067	0.591765121016461	-3.94656088183349
a_HQ_transcript/10289	-0.867178195533863	2.88153049197842	-2.0913859586514	0.0443702536773184	0.591953845792635	-4.02050106597296
a_HQ_transcript/91724	2.72638867244024	0.866697657922337	2.09128135957722	0.044380244631921	0.591953845792635	-3.988235420255
a_HQ_transcript/63841	1.83695428506126	2.97828636478094	2.09113822685515	0.0443939194106226	0.592000400894854	-4.06488611848603
a_HQ_transcript/64861	-0.633611124469867	3.00528050157135	-2.09097267019079	0.0444097411936174	0.592075558739127	-4.10599609506634
a_HQ_transcript/8700	-2.39450443301735	-0.300750343132206	-2.09062554362119	0.0444429312628345	0.592321099314815	-3.96011028041291
a_HQ_transcript/29575	-0.993531209165769	1.81464914299607	-2.09056692346824	0.0444485383156349	0.592321099314815	-3.88085902756883
a_HQ_transcript/97865	-0.873793308649246	3.08507641487974	-2.09030458067252	0.0444736392136157	0.59243918539029	-4.14768729039566
a_HQ_transcript/149254	0.355630848551294	10.4369574592955	2.0902396416179	0.0444798544991869	0.59243918539029	-4.90746626543951
a_HQ_transcript/40606	1.39704402590971	-0.580080981840969	2.09015480239961	0.0444879755750863	0.59243918539029	-3.97654629060165
a_HQ_transcript/132280	3.11170300157306	1.37200289541979	2.08960701384459	0.044540443130251	0.593002033503259	-4.05888925203676
a_HQ_transcript/63838	-2.88989765328811	0.922878523566924	-2.08940952449738	0.0445593721841731	0.593118201584981	-3.97616633112186
a_HQ_transcript/126029	-2.95403362272245	0.65642215546484	-2.08913284747167	0.0445859031996313	0.593155643460721	-3.98672121243023
a_HQ_transcript/72129	1.23187567806607	-0.679881153162186	2.08906260951796	0.0445926406514463	0.593155643460721	-3.9875770222538
a_HQ_transcript/147929	3.96138047168601	0.178850557240678	2.08901826879263	0.0445968944200298	0.593155643460721	-3.95631530229232
a_HQ_transcript/26620	1.50819012464858	1.61509928940417	2.08895460760456	0.0446030022978051	0.593155643460721	-3.8286524311841
a_HQ_transcript/102	-1.12582847252713	3.43509220000387	-2.08883371824036	0.0446146028799993	0.593174207732982	-4.14533674663865
a_HQ_transcript/92978	-4.74529390584481	0.703889244836468	-2.08844896044374	0.0446515420898225	0.593233924948669	-3.89923521768914
a_HQ_transcript/132492	2.71146387638666	0.572238466045329	2.08842009496495	0.0446543144486982	0.593233924948669	-3.97319594295886
a_HQ_transcript/135753	2.56658774090484	0.990411891661471	2.08787076592457	0.0447071032255015	0.593233924948669	-3.91864631509701
a_HQ_transcript/130523	3.03390599196156	3.55500417740416	2.08783976111933	0.0447100843302091	0.593233924948669	-4.12763971458581
a_HQ_transcript/42118	2.33859987000976	2.17463002166529	2.08781953436253	0.0447120292226587	0.593233924948669	-3.96433642807578
a_HQ_transcript/134995	-3.48651801079791	2.10335341227778	-2.08775617947054	0.0447181215599678	0.593233924948669	-4.07883528367646
a_HQ_transcript/73126	-2.08417539932579	-0.269585934254453	-2.08775457879574	0.0447182754936518	0.593233924948669	-3.91740364210881
a_HQ_transcript/81324	1.68176520417832	-0.742355070078244	2.0877256677815	0.0447210558883956	0.593233924948669	-3.90700560537914
a_HQ_transcript/115308	-1.96379067819098	0.230371617994469	-2.08760929498734	0.0447322490936981	0.593233924948669	-3.94181317827824
a_HQ_transcript/11981	-1.33835066131874	1.51095193675773	-2.08751292372812	0.0447415203420769	0.593233924948669	-3.86966434558107
a_HQ_transcript/108629	0.673384700546087	3.6204965252003	2.08749253558192	0.0447434819695628	0.593233924948669	-4.19662990317314
a_HQ_transcript/150319	2.23285211584525	0.678180351845246	2.0873261168048	0.0447594966437769	0.593233924948669	-3.90577628245728
a_HQ_transcript/148493	2.03446282917362	-0.449836208054297	2.08721960715442	0.0447697488470116	0.593233924948669	-3.94621004497741
a_HQ_transcript/134888	-3.24971849223913	0.0865336558370112	-2.08720456134833	0.044771197264619	0.593233924948669	-4.02509121764754
a_HQ_transcript/78315	2.05913665072121	0.964498468478671	2.08696070317737	0.0447946785750742	0.593233924948669	-3.8281998091551
a_HQ_transcript/9487	2.30871306039718	0.784161054589518	2.08696042327278	0.0447947055335688	0.593233924948669	-3.98115723700815
a_HQ_transcript/139864	3.0565057037537	0.878130858141547	2.08695256720015	0.0447954621825448	0.593233924948669	-3.84156874337724
a_HQ_transcript/36068	-1.92109989046143	-0.482948057604366	-2.08687642748407	0.0448027960797663	0.593233924948669	-3.8982260662411
a_HQ_transcript/117242	1.97048378403378	0.348847349015765	2.08672030006355	0.0448178378325365	0.593297945235637	-3.95663923890941
a_HQ_transcript/106684	3.06292574942571	1.26519356941787	2.08621662236967	0.0448663939339627	0.593803814533409	-3.85135589516063
a_HQ_transcript/58027	1.69539960378865	-0.53774658498319	2.08611203606106	0.0448764821986868	0.593803814533409	-3.91128500096896
a_HQ_transcript/116833	-3.98094836912328	0.263182454588477	-2.0857050642834	0.0449157572386555	0.594188242323297	-4.01746291590225

a_HQ_transcript/35414	-2.10682437012151	1.42845602820158	-2.08552246984715	0.0449333884714732	0.594286235510761	-3.92879846096923
a_HQ_transcript/31821	-2.52359188424894	4.58360901242482	-2.0852365806792	0.044961006060517	0.594516233004034	-4.38809222782715
a_HQ_transcript/68324	-1.72941777534799	1.77818626039965	-2.08368826444352	0.0451108375201624	0.596361783800773	-3.93027703205275
a_HQ_transcript/125918	1.39355429630772	-0.711151153402208	2.08343603022644	0.0451352880501235	0.596438952541469	-3.94729923774422
a_HQ_transcript/84308	-1.04861116061148	2.51783000615323	-2.08335629934386	0.0451430192596819	0.596438952541469	-4.0401840015336
a_HQ_transcript/78546	-1.70873661194198	1.31302479575074	-2.08327904878514	0.0451505110755772	0.596438952541469	-3.95507509783323
a_HQ_transcript/10914	-1.59638187425906	-0.764637153812481	-2.08320474681855	0.0451577179695673	0.596438952541469	-3.95862807694797
a_HQ_transcript/24041	1.5493471377361	0.539794968587385	2.08277310962248	0.0451996045454225	0.596505850717813	-3.87614529114445
a_HQ_transcript/49426	2.13011685952953	2.59813716689579	2.08267151866501	0.0452094680308922	0.596505850717813	-4.19282489827695
a_HQ_transcript/83378	-1.59897227242137	-0.434129657416057	-2.0824535094893	0.0452306409921452	0.596505850717813	-3.96972889989605
a_HQ_transcript/55461	2.80941830746791	-0.0375335275704244	2.08234013976347	0.0452416548694444	0.596505850717813	-3.98510612467037
a_HQ_transcript/151135	3.16775157914248	1.046231552482	2.08210230537319	0.0452647681849657	0.596505850717813	-4.03989513687092
a_HQ_transcript/36514	2.52807783883458	-0.623009517857108	2.08208393189134	0.0452665541972149	0.596505850717813	-3.96684966690324
a_HQ_transcript/66196	-1.92784253563321	2.53210186560537	-2.08195311505192	0.0452792721725932	0.596505850717813	-3.94943350021511
a_HQ_transcript/102322	2.66049201789005	1.55628706856569	2.08189508401994	0.0452849149417906	0.596505850717813	-3.96654426929774
a_HQ_transcript/60472	2.23171602413567	-0.122518978760994	2.0818802331156	0.0452863591003096	0.596505850717813	-3.84554555395962
a_HQ_transcript/152137	1.23253143926099	-0.624573458217603	2.08185799014513	0.0452885221674805	0.596505850717813	-4.00118943514687
a_HQ_transcript/140270	-2.36148964104967	1.18122433657944	-2.0818093191331	0.0452932556062187	0.596505850717813	-3.87659277143291
a_HQ_transcript/133232	4.20911972871195	1.84041998132949	2.08180700011919	0.0452934811499214	0.596505850717813	-4.0305809595422
a_HQ_transcript/13455	-1.10747054919587	4.59910348927421	-2.08177916780597	0.0452961881549069	0.596505850717813	-4.35422065213195
a_HQ_transcript/79919	2.13862081129157	0.0333318945591351	2.08132219410892	0.0453406543866962	0.596753466405891	-3.87838763069918
a_HQ_transcript/152373	2.99541665788629	0.150768834618688	2.08116869070782	0.0453555998069279	0.596753466405891	-4.01006755398609
a_HQ_transcript/18352	-1.39857288901982	1.61584162103181	-2.08101083097505	0.0453709739000171	0.596753466405891	-3.88525732272481
a_HQ_transcript/81010	2.98881209613648	0.468461860649507	2.08096868234934	0.045375079567646	0.596753466405891	-3.94433422228396
a_HQ_transcript/133565	-2.97357002535805	3.14607242528312	-2.08075840655701	0.0453955672778835	0.596753466405891	-4.03465540694422
a_HQ_transcript/78251	-2.09138334862094	0.12302294332966	-2.080750338047	0.0453963535760152	0.596753466405891	-3.8896913041387
a_HQ_transcript/68941	2.86016957916255	0.183892773197677	2.08074797660103	0.0453965837075868	0.596753466405891	-3.97865128038625
a_HQ_transcript/14579	2.35512022016771	0.71498619237741	2.08074246704509	0.0453971206380204	0.596753466405891	-3.91412479474492
a_HQ_transcript/122938	-1.58915273481745	1.54902013923196	-2.08021036471363	0.0454490027433083	0.597300391468014	-3.91729831749747
a_HQ_transcript/67896	-1.97708139237693	-0.18402334014433	-2.07973699576244	0.0454952020483303	0.597493610815382	-3.91218034889073
a_HQ_transcript/113775	-2.56462852758352	2.16810567572029	-2.0796957177108	0.0454992326171619	0.597493610815382	-4.06504594352885
a_HQ_transcript/78848	2.18842589670436	1.37573542624903	2.07967284863859	0.0455014657885629	0.597493610815382	-3.82678678636875
a_HQ_transcript/48635	1.50366033937619	3.62876168627387	2.07920971448566	0.0455467117717059	0.597493610815382	-4.20774206640663
a_HQ_transcript/4752	1.49101758044124	-0.758004240396097	2.0790158202924	0.0455656660808125	0.597493610815382	-3.97096213787112
a_HQ_transcript/75378	2.47923425931444	0.997876276646052	2.07894608491084	0.0455724848288061	0.597493610815382	-3.91704957017394
a_HQ_transcript/121437	-2.07318020009748	4.2599014756826	-2.07890912404307	0.0455760992390893	0.597493610815382	-4.27580246035485
a_HQ_transcript/149611	0.357761255353146	8.83248967944437	2.07887468759346	0.045579467013475	0.597493610815382	-4.7294590171106
a_HQ_transcript/12361	-2.1101173874152	2.47730621264116	-2.07883880123676	0.0455829768175825	0.597493610815382	-3.99013276534464
a_HQ_transcript/28963	-5.94767756613228	1.20328049496961	-2.07872825964196	0.0455937896478434	0.597493610815382	-3.98793706960906
a_HQ_transcript/63425	2.40699007802178	0.532484451843951	2.07864418275605	0.0456020152977447	0.597493610815382	-3.99569101468597
a_HQ_transcript/118994	2.54218134602511	3.73412002753209	2.07857672203931	0.0456086162531706	0.597493610815382	-4.1093228839156
a_HQ_transcript/13708	-2.21219377452814	-0.274489237031263	-2.07854660036808	0.0456115638969291	0.597493610815382	-3.90510936390575
a_HQ_transcript/83048	2.73884004644532	-0.107423459241093	2.07854558168693	0.0456116635858716	0.597493610815382	-3.97444503226901
a_HQ_transcript/56543	-3.09673556289644	0.326977535936994	-2.07840709291639	0.0456252179947972	0.597493610815382	-3.98672552053424
a_HQ_transcript/7298	-4.29485505965617	1.25813525405132	-2.07809348838183	0.0456559247583333	0.597493610815382	-4.01812783563859
a_HQ_transcript/117386	1.23526301008908	2.35622325909956	2.07791919534095	0.0456729986344197	0.597493610815382	-3.97322551665979
a_HQ_transcript/96261	4.96992446426731	4.12312986460341	2.07790441703324	0.0456744465872866	0.597493610815382	-4.0293307733494
a_HQ_transcript/145038	-2.37827794872319	-0.271145875916947	-2.07776157429105	0.045688444156574	0.597493610815382	-3.96610524632299
a_HQ_transcript/104024	2.03528152912884	0.694131330635321	2.0777261882647	0.0456919123190003	0.597493610815382	-3.82551026591586
a_HQ_transcript/21034	-0.810617540979076	3.60048168351697	-2.07768205166965	0.0456962384444802	0.597493610815382	-4.28635674196043

a_HQ_transcript/64312	2.78548730088169	1.53805650600405	2.07766194502939	0.0456982093516657	0.597493610815382	-3.87944406666408
a_HQ_transcript/137147	3.52010379306148	0.343887567621781	2.07764245064673	0.0457001203152558	0.597493610815382	-3.96610401180476
a_HQ_transcript/6914	1.8263894497045	-0.679557894854858	2.07719792778771	0.0457437143992791	0.597924271208581	-3.97406540419465
a_HQ_transcript/149301	-0.776162598001028	3.60081656290013	-2.07709291665172	0.0457540181280563	0.597924271208581	-4.19801519955361
a_HQ_transcript/4703	1.64201227498274	1.99995329733808	2.07694588557954	0.045768448304977	0.597924271208581	-3.93567302971105
a_HQ_transcript/44554	0.876382336239533	3.32373808271002	2.07688723795205	0.0457742053206404	0.597924271208581	-4.18815143517538
a_HQ_transcript/19671	0.832260647441905	3.32704872001467	2.07671996815385	0.0457906284999347	0.598004415512065	-4.2482755843794
a_HQ_transcript/111077	1.39797426073835	-0.790830818028537	2.07649482180656	0.0458127424172599	0.598070427181733	-3.97603296577729
a_HQ_transcript/138234	1.69637934739494	1.53452389582318	2.07645900847979	0.0458162608766904	0.598070427181733	-4.01091887397877
a_HQ_transcript/47729	1.43457254465311	-0.393194632044416	2.07624148906287	0.0458376360620062	0.598215112036888	-4.04849849829209
a_HQ_transcript/84768	-2.1899129951633	-0.47160897066105	-2.07596434132619	0.0458648835333509	0.598339399172734	-3.9711907130174
a_HQ_transcript/144360	4.87165921203057	1.33365778833887	2.07593523009918	0.0458677463995614	0.598339399172734	-4.03502398127215
a_HQ_transcript/106894	2.00794527668284	-0.503743769451073	2.07549040174225	0.0459115114557747	0.598569510376185	-3.97521113353126
a_HQ_transcript/55002	-3.27075709888645	3.13090833457177	-2.07548550258375	0.0459119936711225	0.598569510376185	-4.1884085194709
a_HQ_transcript/4950	-3.24314576273474	2.56384093023014	-2.07544197027327	0.0459162786740884	0.598569510376185	-4.22639871431656
a_HQ_transcript/67902	1.66996695488294	0.882934088339669	2.07487376421503	0.0459722410641791	0.599164670084889	-3.87593216805964
a_HQ_transcript/72808	-0.763099769292674	3.01888547359354	-2.07465976040599	0.0459933337919749	0.599305202120582	-4.12929961508952
a_HQ_transcript/148508	6.20088194278311	3.22029615741395	2.07436766011882	0.046022137659919	0.599425766626841	-4.26106947283466
a_HQ_transcript/14638	-1.88026441531685	1.23400855815505	-2.07432165556425	0.0460266755954016	0.599425766626841	-3.97993835740373
a_HQ_transcript/126877	-1.21203830181318	8.82247935388001	-2.07419953375787	0.0460387237257192	0.599425766626841	-4.76664934881365
a_HQ_transcript/22968	2.37605387456665	1.21836381352704	2.07396805576899	0.046061568198757	0.599425766626841	-3.94248703047514
a_HQ_transcript/103667	-0.88477612627401	2.24353983866993	-2.07392131704836	0.046066182036814	0.599425766626841	-3.96164590878842
a_HQ_transcript/10652	-1.4998298060362	0.0975541521949087	-2.07380050599609	0.046078109851871	0.599425766626841	-3.90372236833926
a_HQ_transcript/148076	4.27036686263732	1.8039534790453	2.07371983374715	0.0460860762316611	0.599425766626841	-4.11812158220469
a_HQ_transcript/151023	-1.70410146927417	2.91591540240912	-2.07363215787375	0.0460947355951279	0.599425766626841	-4.07849805028744
a_HQ_transcript/111319	1.1696335394312	5.99429101624175	2.07346512566741	0.0461112366047205	0.599425766626841	-4.48323949594945
a_HQ_transcript/80815	-2.49966231809007	0.181020044465639	-2.07342417521177	0.0461152828695566	0.599425766626841	-3.89579736353821
a_HQ_transcript/49945	2.09584521920534	0.766644810700378	2.07340164030817	0.046117509649485	0.599425766626841	-3.94252206853146
a_HQ_transcript/116303	1.88148682957486	-0.630212900400287	2.0733123631679	0.0461263324752591	0.599425766626841	-3.98286267138046
a_HQ_transcript/3344	-0.713756612108321	2.64912109798941	-2.07310181823691	0.046147145496908	0.599562197908867	-4.01562075320654
a_HQ_transcript/4295	1.67736765234583	1.92360689173843	2.07276573030648	0.0461803859809598	0.599708693973338	-3.87748379719364
a_HQ_transcript/108392	2.00833523359015	-0.143407796371511	2.07271273178027	0.0461856296791716	0.599708693973338	-3.92873741995319
a_HQ_transcript/102793	2.76115911728956	3.79065363913691	2.07239209536976	0.0462173647793896	0.599708693973338	-4.20098805466928
a_HQ_transcript/7264	-1.55619319216674	1.77374005382071	-2.07233547488032	0.0462229708073402	0.599708693973338	-3.94447602522722
a_HQ_transcript/136258	1.06323953460323	4.39211566130973	2.07224418207861	0.0462320110229912	0.599708693973338	-4.36369683256208
a_HQ_transcript/112535	1.00958625647679	3.98981807268411	2.07213595491597	0.0462427301715334	0.599708693973338	-4.27367379701918
a_HQ_transcript/92849	-2.39262532624537	0.536930312033151	-2.07209779321058	0.0462465103454153	0.599708693973338	-3.91207230188551
a_HQ_transcript/97067	-1.63291535833808	-0.423409576565664	-2.07206913474291	0.0462493493385946	0.599708693973338	-3.93476724008623
a_HQ_transcript/43012	-2.16754366848162	0.355709823670061	-2.07189874835392	0.0462662314920748	0.599708693973338	-3.9301375356749
a_HQ_transcript/60733	-2.02114951503215	0.164508054001157	-2.07189183791735	0.0462669163036219	0.599708693973338	-3.91759669565063
a_HQ_transcript/8291	0.955128418830622	4.50485717363069	2.07184146519882	0.0462719084171212	0.599708693973338	-4.31888538688954
a_HQ_transcript/152748	-1.88251395875625	-0.456337398924677	-2.07156788138045	0.046299029823327	0.59989261180396	-3.98222772714659
a_HQ_transcript/37463	1.60083526706183	-0.137408052482935	2.07149013752363	0.0463067394227286	0.59989261180396	-3.89924395498365
a_HQ_transcript/95360	5.84754540932388	3.56326566119684	2.07132068424306	0.0463235474592295	0.599976641423818	-4.06333863085633
a_HQ_transcript/98154	2.45390425322508	1.00761121135489	2.07111035997402	0.0463444169554671	0.60008764985378	-3.84173584499361
a_HQ_transcript/125159	1.410579929771	2.69169043687068	2.07096286849122	0.0463590567872676	0.60008764985378	-3.99265077123659
a_HQ_transcript/93802	4.50153223714311	2.2109971051109	2.07092225281151	0.0463630889673364	0.60008764985378	-3.96945484337221
a_HQ_transcript/1079	2.58476425733309	1.99202170063483	2.07063716258676	0.0463914004051746	0.600320419134459	-4.15217121453446
a_HQ_transcript/135063	-0.721601350653588	5.88552566961524	-2.07039836304408	0.0464151265757043	0.600464094790017	-4.46099293735764
a_HQ_transcript/142631	-1.84318462166654	2.54671142079626	-2.07028611067599	0.0464262832159017	0.600464094790017	-4.07949134662137

a_HQ_transcript/148768	2.05460929006283	0.312092155327815	2.0702135778567	0.0464334934296918	0.600464094790017	-3.89984548722805
a_HQ_transcript/149743	-2.29860447885774	3.17178128138368	-2.06988694619455	0.0464659748728529	0.600675581192738	-4.20100028915978
a_HQ_transcript/121702	2.42155095028084	0.089713985691873	2.0697970523835	0.0464749177520956	0.600675581192738	-3.99286046791492
a_HQ_transcript/116090	2.22481545858139	1.62226852228852	2.06973744446549	0.0464808485446761	0.600675581192738	-4.00540690173667
a_HQ_transcript/58042	-1.26291486646327	2.35761334203024	-2.0693775258455	0.0465166734340149	0.600979945686166	-3.92603870941326
a_HQ_transcript/29397	1.8297589044538	1.69940315056492	2.06929312008868	0.0465250783716582	0.600979945686166	-3.96181312629781
a_HQ_transcript/152110	-1.89507862222516	-0.0723277877716991	-2.06899790971328	0.046554485284788	0.601226198763421	-3.91192064331568
a_HQ_transcript/116157	0.624434996906755	3.25926186498311	2.06878882844088	0.0465753224848159	0.601299913028036	-4.19024698119402
a_HQ_transcript/129507	5.60888273072562	2.30159261121097	2.0687190659935	0.0465822768910353	0.601299913028036	-4.0946869726199
a_HQ_transcript/122947	2.23194850572467	-0.117794276811854	2.06862930334535	0.0465912264017044	0.601299913028036	-3.97877532509108
a_HQ_transcript/139533	2.93316057032353	0.769540411713427	2.06834870948894	0.0466192119202145	0.601433777968478	-4.02495147980737
a_HQ_transcript/151678	-2.36963413557462	1.94466108051203	-2.0681741429153	0.046636630090747	0.601433777968478	-3.99874130460414
a_HQ_transcript/79718	1.80866565082549	2.22741334959063	2.06806662288931	0.0466473612399447	0.601433777968478	-3.97927128282247
a_HQ_transcript/5201	0.819096106974782	2.87688644718371	2.06787515265116	0.0466664765120168	0.601433777968478	-4.08882819540655
a_HQ_transcript/34472	2.66882862729159	0.337381630487029	2.0678266970454	0.0466713151306465	0.601433777968478	-3.92984584980222
a_HQ_transcript/136027	-1.34253138380105	-0.340263447288554	-2.06764597942371	0.0466893648988163	0.601433777968478	-3.94735453354509
a_HQ_transcript/66666	3.41262159639966	2.77014082830756	2.06760488333494	0.0466934703638583	0.601433777968478	-4.1484727375275
a_HQ_transcript/150141	-2.50913476859848	0.0433756164896809	-2.06753605326557	0.0467003471424878	0.601433777968478	-4.02037284361458
a_HQ_transcript/23983	-2.34319314835061	2.85545591346125	-2.06748112987548	0.046705835151263	0.601433777968478	-4.05379013353759
a_HQ_transcript/69694	1.97579596642981	4.33106882115978	2.0672412727942	0.0467298086042615	0.601433777968478	-4.352092285514
a_HQ_transcript/2487	1.15212950540258	2.66249578341464	2.06714345853015	0.0467395881426574	0.601433777968478	-4.04907243761525
a_HQ_transcript/100202	-1.411416129476	1.16937369509272	-2.06699431732577	0.0467545028528597	0.601433777968478	-3.87687451952673
a_HQ_transcript/144666	0.809510224966772	2.75699262642636	2.06691253194725	0.0467626834929965	0.601433777968478	-4.14074429025804
a_HQ_transcript/143562	-2.06227878193815	1.19059010165034	-2.06681755289421	0.0467721854212995	0.601433777968478	-3.9743826292074
a_HQ_transcript/29214	3.18095383200984	0.550719206726326	2.06680990428789	0.0467729506798541	0.601433777968478	-4.0540400280038
a_HQ_transcript/39065	2.49021091616604	1.46927714428075	2.06667983697754	0.046785965865461	0.601433777968478	-3.89306457187814
a_HQ_transcript/73880	1.96844994391438	0.650089451172316	2.0665324605236	0.0468007169437632	0.601433777968478	-3.9421486037611
a_HQ_transcript/52946	-1.63486155678183	1.34643419617003	-2.06650363277222	0.0468036028252176	0.601433777968478	-3.99576085965128
a_HQ_transcript/58564	2.35503744073139	1.29459101759432	2.06648166110652	0.0468058024645401	0.601433777968478	-3.97499568261122
a_HQ_transcript/44255	-1.32834592430053	1.37101563333858	-2.06645438680058	0.0468085330912709	0.601433777968478	-3.90973991547469
a_HQ_transcript/21704	-2.11267545465617	-0.305959847338087	-2.06616195140923	0.0468378197141343	0.601541182055685	-3.97795201450868
a_HQ_transcript/1947	-3.08365644679326	0.0609610543556014	-2.06614394208731	0.0468396238267949	0.601541182055685	-3.95281050925301
a_HQ_transcript/93147	2.37866557954565	1.31530601871315	2.06582483165825	0.0468716013725035	0.601541182055685	-3.91484441023973
a_HQ_transcript/121943	-0.736397147020326	2.56832988810242	-2.06577963795878	0.0468761317141602	0.601541182055685	-4.14901666342752
a_HQ_transcript/22340	-1.50123370250085	1.95477955655735	-2.06576642953077	0.046877455836373	0.601541182055685	-3.92734174938868
a_HQ_transcript/111774	1.66890161477722	-0.78246359792219	2.06572346454336	0.0468817632302906	0.601541182055685	-3.98243424346023
a_HQ_transcript/39539	-2.20307824737906	1.10021254081448	-2.06564797578284	0.0468893320928694	0.601541182055685	-4.03258716082942
a_HQ_transcript/11946	-2.39649409950535	0.0421598287978192	-2.06542777915121	0.0469114162012695	0.601617390723104	-3.97953012593928
a_HQ_transcript/138969	1.25650964257955	6.04632678607175	2.06538236345253	0.0469159722018275	0.601617390723104	-4.44728884684613
a_HQ_transcript/80309	-2.04185526641055	1.62772654658261	-2.06521785833407	0.0469324782454709	0.601674589037177	-3.92925800188732
a_HQ_transcript/33222	0.899575773117765	2.55490853154958	2.06513160821611	0.0469411344151458	0.601674589037177	-4.08185722195846
a_HQ_transcript/152517	1.78061853370331	1.96261616194849	2.06485152130329	0.046969253980683	0.601808232153055	-3.93183893425913
a_HQ_transcript/132746	2.83654939534101	0.633679591816119	2.06482151642346	0.0469722672254062	0.601808232153055	-3.99778882462061
a_HQ_transcript/72792	1.81039529808568	-0.504789881503428	2.06463824224003	0.0469906762579651	0.60191142122587	-3.9496310744346
a_HQ_transcript/1532	-1.3907101346493	1.64837867634083	-2.0643307700973	0.0470215746574578	0.602174507972838	-3.93206402592884
a_HQ_transcript/134996	1.10150678647209	1.71586845708846	2.0640278197639	0.0470520361413571	0.602415028751844	-3.89918356469183
a_HQ_transcript/11637	2.69031454126346	2.92880518758502	2.06393787655952	0.0470610832225799	0.602415028751844	-4.09924070714298
a_HQ_transcript/87537	-2.38421611950482	2.78162248153134	-2.06374698882171	0.0470802890442654	0.602528190569146	-4.07375080150727
a_HQ_transcript/92557	-2.16393388092836	-0.434580553657851	-2.06340086659133	0.0471151310962278	0.602841369218914	-3.991078195184
a_HQ_transcript/133509	3.35134629660379	4.20136491530117	2.06313616290982	0.0471417925423045	0.603049761641523	-4.32976393163843

a_HQ_transcript/20452	3.00532576553152	0.352476579806867	2.06298709923812	0.0471568123550655	0.603109172403795	-3.95292159765987
a_HQ_transcript/128498	1.56331070057067	3.27232469199467	2.06285082936588	0.0471705467356766	0.603152120688827	-4.20709950535837
a_HQ_transcript/82934	1.82207655394969	-0.554704366867666	2.06216430165355	0.0472397941096342	0.603705309582891	-3.99736090371824
a_HQ_transcript/124611	-2.20730684382358	-0.516112990648442	-2.06213420022494	0.0472428323694318	0.603705309582891	-3.99428511728226
a_HQ_transcript/18321	3.55584906331823	2.40935754449594	2.06211305052023	0.0472449671981243	0.603705309582891	-4.06359455976944
a_HQ_transcript/6568	2.73332795520782	-0.0431801035425254	2.06198818976861	0.0472575722395398	0.603725587601643	-3.96488026208511
a_HQ_transcript/89911	3.01394081959973	0.861041382350506	2.06176338901132	0.0472802739638499	0.603725587601643	-3.96999188056645
a_HQ_transcript/144327	2.25827485822548	-0.695845960609535	2.06174107700488	0.0472825276870327	0.603725587601643	-4.003000119202
a_HQ_transcript/54460	1.84286094552083	-0.247483416250609	2.06168592777909	0.0472880986848039	0.603725587601643	-3.99546103247657
a_HQ_transcript/103769	2.74823171931134	0.292450462304277	2.06123818873874	0.0473333492286509	0.604057408587095	-4.02264112671772
a_HQ_transcript/40282	1.96147129355971	1.85945281288045	2.0612212928133	0.0473350575534543	0.604057408587095	-3.97152451788755
a_HQ_transcript/150401	-1.72567489341068	4.54339001997534	-2.06106238220564	0.0473511274507068	0.604057408587095	-4.36715537240666
a_HQ_transcript/62968	1.36561891729959	-0.682531827266601	2.06101760262292	0.0473556566703033	0.604057408587095	-4.00145534829466
a_HQ_transcript/22865	1.00498328787715	2.68238519483269	2.0608213198185	0.0473755141425351	0.604178123316647	-4.0456386075307
a_HQ_transcript/13480	1.74368178287572	0.873076272687057	2.06056559422755	0.0474013962922196	0.604305325523758	-3.89793743401384
a_HQ_transcript/31372	1.6652700087633	-0.686081401638949	2.06051734820444	0.0474062806975698	0.604305325523758	-3.95763214656015
a_HQ_transcript/7885	-1.95958556901229	3.18646380169901	-2.06022917855902	0.0474354640830095	0.604464040440558	-4.1428859003254
a_HQ_transcript/21853	2.28836912047738	0.393845408435853	2.05991864034239	0.0474669304602371	0.604464040440558	-3.92003756917077
a_HQ_transcript/123100	-1.2617435728737	-0.792665525855541	-2.05984911803249	0.0474739775680404	0.604464040440558	-3.94198910480275
a_HQ_transcript/136492	2.215935585858	2.62083544997645	2.05983939486042	0.0474749632277805	0.604464040440558	-4.13460727510457
a_HQ_transcript/70041	-1.2253154112283	2.45887407971169	-2.05978799595348	0.0474801739492803	0.604464040440558	-4.13342912249381
a_HQ_transcript/130640	-1.80309490097376	-0.517373305919415	-2.05977861956204	0.0474811245639208	0.604464040440558	-3.99079556367265
a_HQ_transcript/29145	3.12170398889627	0.890533884431855	2.05963038291411	0.0474961555905471	0.604522998065978	-3.97356228232304
a_HQ_transcript/4565	-0.909198090473736	2.90261009052255	-2.05941264859167	0.047518241191572	0.604671699646169	-4.12492858193746
a_HQ_transcript/27929	0.897356583510458	1.93133506976232	2.05881909794845	0.0475784931247731	0.605130110767123	-3.95208094261531
a_HQ_transcript/14171	2.35830575291242	-0.196413597321292	2.05878106824501	0.0475823558486698	0.605130110767123	-4.01332616684205
a_HQ_transcript/61505	1.96387143459725	1.37706963373051	2.05866606789567	0.0475940382543727	0.605130110767123	-4.08061926579113
a_HQ_transcript/3959	2.13566923169582	4.68025032957485	2.05863472525233	0.0475972226602209	0.605130110767123	-4.3805106077615
a_HQ_transcript/109468	2.13663691194035	1.4830121645469	2.05854522366761	0.047606317033754	0.605130110767123	-3.92750319902701
a_HQ_transcript/145409	0.499405988632195	7.16899570562719	2.05837158158856	0.0476239653926046	0.605200154993568	-4.60686642958585
a_HQ_transcript/65914	1.54270495533345	1.84363213679103	2.05828614951637	0.0476326505143059	0.605200154993568	-3.98064525627462
a_HQ_transcript/9843	1.44749237989543	2.40549830380206	2.05796728297714	0.0476650791544042	0.605234355394172	-3.94485182492132
a_HQ_transcript/51407	-2.39395375499487	-0.179536603281153	-2.05790859560971	0.0476710497590413	0.605234355394172	-3.92146814610537
a_HQ_transcript/124707	-1.37840775315468	2.06306268811048	-2.05777650377702	0.0476844906301005	0.605234355394172	-4.13641517673814
a_HQ_transcript/89259	5.52946417765585	3.44546048400954	2.05771527215063	0.0476907223229661	0.605234355394172	-4.26123103539687
a_HQ_transcript/111002	1.18592393557719	2.63632639204131	2.05766440153373	0.0476959000951793	0.605234355394172	-4.04817765966299
a_HQ_transcript/25906	1.69543663503804	-0.287753128455338	2.05764559058803	0.0476978148579119	0.605234355394172	-3.97819788641759
a_HQ_transcript/97131	-2.12760251807929	-0.456476624880258	-2.05746111173323	0.0477165965097529	0.605340532937345	-3.99595201580484
a_HQ_transcript/114719	2.11765089121354	1.84334216364123	2.0571591119586	0.0477473569163396	0.605574628100118	-4.02219331375086
a_HQ_transcript/133573	-1.36289848473562	2.55807676969135	-2.05698310392307	0.0477652923851783	0.605574628100118	-4.13944521388866
a_HQ_transcript/116888	-2.50577734862818	2.08288744410811	-2.05697318655105	0.0477663031557776	0.605574628100118	-4.02028921817622
a_HQ_transcript/52482	-2.20960289392878	1.5505104527191	-2.05670265460198	0.0477938828110035	0.605781444693404	-3.97752119705862
a_HQ_transcript/136651	-1.94469589709331	1.70502823613452	-2.0566087490726	0.0478034593794496	0.605781444693404	-3.96581486906879
a_HQ_transcript/71802	2.43474128368336	0.289236561575118	2.05601539707833	0.047864008962826	0.606416545987609	-4.0232578195794
a_HQ_transcript/26122	-1.85613824053096	0.121510828189824	-2.05577698325529	0.0478883572814687	0.60659281587649	-4.02448602623385
a_HQ_transcript/1566	-1.04794490869194	2.5424791972665	-2.05520555883165	0.047946759074638	0.607103313471952	-3.9973951524359
a_HQ_transcript/141207	-2.0582119499002	-0.0544095583178016	-2.05490428029514	0.0479775761155885	0.607103313471952	-3.97231618099077
a_HQ_transcript/33661	-1.95096821351583	0.64416344690561	-2.05478290217347	0.0479899965057694	0.607103313471952	-3.89118877700627
a_HQ_transcript/60721	-2.91658531153622	1.27747273194077	-2.05472328335992	0.0479960982197405	0.607103313471952	-3.96310438168632
a_HQ_transcript/84644	2.92797582296595	2.89262145109136	2.05451582472559	0.0480173359825014	0.607103313471952	-4.09272588131536

a_HQ_transcript/150381	1.89352423749579	4.62273275000762	2.05446951057233	0.0480220783399738	0.607103313471952	-4.24271823992064
a_HQ_transcript/133410	3.85067640580639	1.51041769865978	2.05446929293239	0.0480221006262804	0.607103313471952	-4.14864149286433
a_HQ_transcript/146070	0.441145277936823	10.1070032650202	2.05442600664412	0.0480265333183054	0.607103313471952	-4.92375353679676
a_HQ_transcript/117750	2.98756918448566	-0.0291433796352406	2.05438538429917	0.0480306935388862	0.607103313471952	-4.00864586081341
a_HQ_transcript/85684	-0.802145472650872	2.96513583673545	-2.05436187078752	0.0480331017522968	0.607103313471952	-4.14068051883948
a_HQ_transcript/60970	2.64397356200123	1.41468837077935	2.05410847636256	0.0480590607101483	0.607207735890231	-4.04394442886992
a_HQ_transcript/81311	-0.855997722633299	4.14243683846386	-2.05398649700457	0.0480715612661106	0.607207735890231	-4.35829372819943
a_HQ_transcript/136165	-3.00348211474583	1.97202588485445	-2.05396908214747	0.0480733461897257	0.607207735890231	-4.08392646422128
a_HQ_transcript/39364	-2.85772160768048	1.65718091857334	-2.05387346267217	0.0480831476793066	0.607207735890231	-4.11191316373435
a_HQ_transcript/8273	2.01819126972664	2.8098858542665	2.05335831186969	0.0481359835282878	0.607742930176436	-4.18961318371263
a_HQ_transcript/140828	2.22027557073195	0.148954925165702	2.05294049189898	0.0481788742291325	0.60812837296689	-4.04262147369771
a_HQ_transcript/114816	1.89879828847865	1.88771507931643	2.05285712549864	0.0481874361045537	0.60812837296689	-4.02757497787079
a_HQ_transcript/104222	-1.4442357566797	-0.727986254860409	-2.05247033758824	0.0482271774004647	0.608497800723727	-3.9987964614453
a_HQ_transcript/138376	-3.37062176116032	1.3087051565628	-2.05224810095793	0.0482500245757987	0.608595407948576	-4.04240926638361
a_HQ_transcript/64675	1.90257377179008	-0.254507881568091	2.05219142011746	0.0482558532073181	0.608595407948576	-3.9875315039379
a_HQ_transcript/95260	-2.08856696061681	2.73747211536897	-2.05166283958098	0.0483102382579809	0.608664253408361	-4.07237579009034
a_HQ_transcript/150927	2.07049581187054	-0.64719840966433	2.0516040989912	0.0483162853350977	0.608664253408361	-4.0169726140543
a_HQ_transcript/104196	2.03635951038163	0.479225509436429	2.0515839310534	0.0483183616861658	0.608664253408361	-3.96903296270548
a_HQ_transcript/148878	6.33295405168236	1.2096186394046	2.05154783421105	0.0483220781626128	0.608664253408361	-4.06670203539236
a_HQ_transcript/146580	2.55130088044091	0.459743307954424	2.0514592624067	0.0483311984465522	0.608664253408361	-4.0933207313985
a_HQ_transcript/93516	2.61808218387592	1.38135224822874	2.05124046127154	0.048353734996094	0.608664253408361	-3.96989786679289
a_HQ_transcript/54978	1.50961521039959	-0.702507590983224	2.05122012253897	0.0483558303576469	0.608664253408361	-4.01885197146281
a_HQ_transcript/47767	1.56157124778976	2.43715114870296	2.05107111888794	0.0483711836262535	0.608664253408361	-3.981224641149
a_HQ_transcript/102924	0.752106046054694	2.73062053684296	2.05104575048535	0.0483737980016793	0.608664253408361	-4.11594566226396
a_HQ_transcript/40038	-3.23289221135562	1.88758434982596	-2.05092359704912	0.0483863884297642	0.608664253408361	-4.10599160026632
a_HQ_transcript/140940	1.74788181591449	-0.304509633818473	2.05091948158117	0.0483868126636609	0.608664253408361	-4.0896397774387
a_HQ_transcript/83461	3.26349883351592	0.242478897660542	2.05091800153343	0.0483869652319027	0.608664253408361	-4.04975668511851
a_HQ_transcript/126610	1.46135866568026	-0.568112198637941	2.05070767018682	0.0484086511857897	0.608708150786394	-4.00745342494865
a_HQ_transcript/106981	-3.78435006896384	1.29579407356421	-2.05064819460733	0.0484147848908436	0.608708150786394	-4.04609795292019
a_HQ_transcript/101931	3.68680374349598	3.16846982236326	2.05057949697957	0.0484218705139741	0.608708150786394	-3.9955638734797
a_HQ_transcript/40955	3.39534758957583	0.437811611473403	2.05031411101736	0.0484492515614614	0.60886344914665	-3.99863006043087
a_HQ_transcript/2750	4.51504327730542	4.32418961827038	2.05022434982709	0.0484585157004756	0.60886344914665	-4.313077823325
a_HQ_transcript/19643	-1.15825103550129	3.00939640665401	-2.05013747601062	0.048467483319556	0.60886344914665	-4.11102950654866
a_HQ_transcript/33977	-2.43824920324308	-0.153607465377769	-2.05005379830505	0.0484761223962754	0.60886344914665	-3.96523065622902
a_HQ_transcript/126448	0.657339410608549	3.16698432632988	2.0499126105084	0.0484907020132933	0.608914998191557	-4.19682051746954
a_HQ_transcript/37685	1.93357837440759	-0.667035740987816	2.04965104346491	0.0485177226547258	0.609122717596954	-4.02055720286599
a_HQ_transcript/150146	-2.56276701194183	3.93694986065015	-2.04950444986703	0.0485328719869471	0.6091813394207	-4.39400111956255
a_HQ_transcript/82432	2.65357309094711	0.743992127979762	2.04922445073333	0.0485618193168	0.609221796478643	-3.97820496609732
a_HQ_transcript/146724	-2.19790776768169	0.16528589357629	-2.04913976088929	0.0485705778488102	0.609221796478643	-3.91116164332544
a_HQ_transcript/81719	-2.86537817124483	0.29744601193527	-2.049059126879	0.0485789182206703	0.609221796478643	-4.05688076263128
a_HQ_transcript/56063	2.60017796232691	-0.283667675192474	2.04903777982642	0.0485811264618534	0.609221796478643	-4.00217520618009
a_HQ_transcript/152939	-1.95454236393985	-0.566191838733094	-2.04888350452457	0.0485970880587133	0.609221796478643	-4.00065336859553
a_HQ_transcript/132659	4.0120617377102	1.08910322676977	2.04886522760838	0.0485989793261675	0.609221796478643	-4.02248572200325
a_HQ_transcript/70969	2.29578184241345	1.92070495834553	2.04820482443357	0.0486673602376801	0.609947459227224	-4.07978681515093
a_HQ_transcript/49955	1.15242639727721	-0.736868132852571	2.04778907087979	0.0487104524466069	0.610355934429051	-4.01550216357322
a_HQ_transcript/130165	0.506560054117713	5.06530830243729	2.04755919268678	0.0487342933510713	0.610461963032035	-4.45547855650361
a_HQ_transcript/260	-3.73425203211385	2.50366152123911	-2.04750497042864	0.0487399182912138	0.610461963032035	-4.08864226004974
a_HQ_transcript/43048	1.20322251890748	1.87241703198786	2.04730585964277	0.0487605786543361	0.610589167604319	-3.95875078571771
a_HQ_transcript/12706	-2.24854139720397	0.483525376907232	-2.04715449675835	0.0487762896911963	0.610654354333375	-3.98987393532397
a_HQ_transcript/106329	0.922647694050943	4.46423091876836	2.04675906354089	0.0488173555191499	0.61091201992555	-4.38524300788506

a_HQ_transcript/135448	-2.04946208451154	-0.370615254689041	-2.04675391506837	0.0488178903893852	0.61091201992555	-4.03214058382747
a_HQ_transcript/93257	3.2634458168292	2.76313037114032	2.04629180509107	0.0488659195550199	0.611135461134965	-4.25137790060725
a_HQ_transcript/9244	1.89581552329279	2.31983893993003	2.04620690306372	0.0488747483158364	0.611135461134965	-3.98658932945184
a_HQ_transcript/144409	2.87350489672351	1.09280802896855	2.04614923500976	0.0488807458791964	0.611135461134965	-4.10525254102361
a_HQ_transcript/108200	5.42165500348906	3.06517359707166	2.04608862490885	0.0488870501165266	0.611135461134965	-4.1634756757831
a_HQ_transcript/137901	2.33880727382712	0.383627246107016	2.04607647815032	0.0488883136229973	0.611135461134965	-3.94336856007835
a_HQ_transcript/151809	2.34867823786782	1.26088967362927	2.04595255009897	0.0489012062646775	0.611140656181064	-4.05240371278561
a_HQ_transcript/100837	5.06335634042883	3.09388619169286	2.04587037731177	0.0489097566156467	0.611140656181064	-4.05234339092523
a_HQ_transcript/122848	2.66131042826018	0.433058394964001	2.04533266081399	0.0489657402154927	0.611667344830092	-3.94943762947821
a_HQ_transcript/109840	3.04231487875923	0.745567114270578	2.04526342026377	0.0489729531867473	0.611667344830092	-4.01391633115405
a_HQ_transcript/48582	0.625792722520748	2.88147733436674	2.04504025058562	0.0489962077133419	0.611809160144544	-4.14260854821628
a_HQ_transcript/38262	2.22826526679601	0.895957377800508	2.04492926488482	0.049007776155823	0.611809160144544	-3.97167651218738
a_HQ_transcript/116820	0.974503477233501	4.46362292108475	2.04481709756781	0.0490194701994954	0.611809160144544	-4.34653852926869
a_HQ_transcript/129727	4.7230776809006	2.80951136418131	2.04470691100802	0.049030960124446	0.611809160144544	-4.1368523971153
a_HQ_transcript/100659	3.12512016364533	0.153795442254749	2.04464963514817	0.04903693361398	0.611809160144544	-4.02595566541344
a_HQ_transcript/113186	2.73918754114263	1.17055528195339	2.04436449689642	0.0490666811397991	0.611995154874834	-4.01945447872198
a_HQ_transcript/67172	2.19178014534131	-0.117729855002972	2.04430492543451	0.0490728980331614	0.611995154874834	-4.0144857252398
a_HQ_transcript/13913	0.592557623329592	3.82218118123381	2.0441709569412	0.049086881547094	0.612038234785389	-4.32179676763687
a_HQ_transcript/115817	-2.6235027803361	0.020591875187918	-2.04376364177406	0.0491294182404056	0.612437234286574	-4.06045537761577
a_HQ_transcript/12622	1.1985014202686	2.32992665944598	2.04337699719041	0.0491698262057457	0.612805487761905	-4.09490245620918
a_HQ_transcript/23739	2.23684529135676	0.194135112190149	2.04327927171858	0.0491800440477788	0.612805487761905	-3.98104845592588
a_HQ_transcript/85880	0.7027979842642	4.29400287642044	2.04279511453703	0.0492306933735513	0.613305131679766	-4.39560771044903
a_HQ_transcript/144764	4.45111096282609	3.59877435912337	2.04254188182421	0.0492572031434975	0.613503900648215	-4.1006073987671
a_HQ_transcript/63988	1.87543357939211	1.23794687343385	2.04226281409499	0.0492864319774467	0.613736443441522	-4.00554003076452
a_HQ_transcript/152536	3.02104609610229	2.84135456099642	2.04210408463915	0.0493030636835134	0.613811150815715	-4.06995653574722
a_HQ_transcript/53903	1.84119860832476	0.325473429918817	2.04197602072268	0.0493164858388128	0.613811150815715	-3.97318711283148
a_HQ_transcript/133535	-1.47724025689544	3.47716161217823	-2.0418142057632	0.0493334499691331	0.613811150815715	-4.14972179785828
a_HQ_transcript/97959	1.84074754835547	0.176934344060685	2.04173107971557	0.0493421666129531	0.613811150815715	-3.98464380860388
a_HQ_transcript/67679	-2.96731210100132	3.04540991622053	-2.04160485525149	0.049355405165979	0.613811150815715	-4.06099781931723
a_HQ_transcript/55424	2.54702556101987	3.32985399306993	2.04133203203963	0.0493840298013064	0.613811150815715	-4.21565429459391
a_HQ_transcript/148801	-0.835259632209914	3.96002460041867	-2.0410843319362	0.0494100311385016	0.613811150815715	-4.37669216974139
a_HQ_transcript/148671	0.736504803515687	8.9913145421429	2.0410718269588	0.0494113441175578	0.613811150815715	-4.814411540179
a_HQ_transcript/152521	-2.21206124638124	-0.283715997452754	-2.04103436042838	0.049415278156047	0.613811150815715	-4.04604241534968
a_HQ_transcript/64240	3.89083619171734	-0.103023109248922	2.04096813552266	0.0494222325369517	0.613811150815715	-4.00416387607419
a_HQ_transcript/97266	-1.00864662070174	4.32632016360392	-2.040899493648	0.0494294416342129	0.613811150815715	-4.35618689437059
a_HQ_transcript/123171	-3.69414933232808	2.55560919464699	-2.04079248229018	0.0494406823188739	0.613811150815715	-4.01800359565506
a_HQ_transcript/131982	-1.29161469513199	3.6927534707821	-2.04075429940822	0.0494446936673987	0.613811150815715	-4.19930622738952
a_HQ_transcript/77564	-2.00552670707423	1.0408912774248	-2.04072381222411	0.0494478967402462	0.613811150815715	-3.98850090919796
a_HQ_transcript/143879	0.830394831254279	2.7813430027348	2.04053855480199	0.0494673643437282	0.613811150815715	-4.18528174771696
a_HQ_transcript/147883	-0.653366489958333	5.22506222641486	-2.04053284541666	0.049467964416004	0.613811150815715	-4.47616813295966
a_HQ_transcript/46310	2.50045545576959	1.62900608558052	2.04049497000392	0.0494719453889971	0.613811150815715	-4.04855587569326
a_HQ_transcript/33412	-2.56224111288506	0.712620155009475	-2.04009391977704	0.049514115851724	0.614203270642128	-3.99266072271592
a_HQ_transcript/96039	-4.02192121633415	2.99605709504554	-2.03996908266949	0.0495272489205235	0.61423510246473	-4.26823299084146
a_HQ_transcript/24756	-2.44200613081823	0.757627386204967	-2.03981312889183	0.0495436598093498	0.614307563438115	-4.10016461168062
a_HQ_transcript/124471	2.81096988250948	3.92009483427198	2.03970385754256	0.0495551611875301	0.614319131906323	-4.15724722134015
a_HQ_transcript/6151	1.24360755053699	2.06636713530765	2.03957334703591	0.0495689011682502	0.614358440747985	-3.98581373163659
a_HQ_transcript/1912	-0.885010043282064	2.20238299338931	-2.03928474034866	0.0495992971866463	0.614604124251839	-4.08142646421067
a_HQ_transcript/77667	1.67871286212484	2.40926889896506	2.03907123270486	0.0496217943090884	0.614751845609269	-4.0166779727017
a_HQ_transcript/141622	-0.669539061601531	3.35853149320456	-2.03864709950776	0.0496665114349907	0.615173311458646	-4.29803263221726
a_HQ_transcript/107419	-4.5663861335765	-0.200889199320306	-2.038547850643	0.0496769805255107	0.615173311458646	-4.06858407466476

a_HQ_transcript/134893	2.03526990599883	-0.460710685956443	2.03812176587211	0.0497219473268327	0.615508939834151	-4.08565003863928
a_HQ_transcript/59044	-3.21073577011412	2.73779978380385	-2.03799297000785	0.0497355468096802	0.615508939834151	-4.18060952772479
a_HQ_transcript/96518	-2.81898015437318	0.786749770318045	-2.03792734281857	0.0497424776028099	0.615508939834151	-4.00799968689418
a_HQ_transcript/141130	-1.98636443877193	-0.697277904255367	-2.03783675125869	0.0497520462395645	0.615508939834151	-4.04655367113953
a_HQ_transcript/25922	3.32618237811052	2.35553044313932	2.03773155879496	0.0497631591076551	0.615508939834151	-4.11775807197071
a_HQ_transcript/125126	0.98443805668501	3.40674758631285	2.03768937082676	0.0497676165913245	0.615508939834151	-4.23517066411979
a_HQ_transcript/55897	1.09393585996225	1.46793381277573	2.03734605120572	0.0498039039803064	0.615826702949851	-3.99903889700598
a_HQ_transcript/67811	-1.12822184785829	3.21777366107947	-2.03714289455423	0.0498253876847819	0.61596132185049	-4.22545319852779
a_HQ_transcript/148485	-0.882406858847155	8.56456400247503	-2.03696493813088	0.0498442131590442	0.616063028388034	-4.7758665032814
a_HQ_transcript/2940	1.78010086265783	2.51669497568711	2.03678282954903	0.0498634843378679	0.616170199317939	-4.10511553663054
a_HQ_transcript/99055	1.5135582372811	-0.722145281441115	2.03648742442683	0.049894758737833	0.616323142069692	-3.98824922014381
a_HQ_transcript/138955	0.511847998803168	4.03464161500822	2.03646562861843	0.0498970669312547	0.616323142069692	-4.34774164578646
a_HQ_transcript/131657	0.665832400086275	3.39871715348345	2.03605667089779	0.0499403932505598	0.616704912092505	-4.26543249350895
a_HQ_transcript/71931	2.95251583923314	0.507069999865243	2.03597364222231	0.0499491936094742	0.616704912092505	-3.99426639535421
a_HQ_transcript/123017	2.08576496806484	1.83920305238257	2.03579616903612	0.0499680088740523	0.616806205103188	-3.96459267642321
a_HQ_transcript/144297	4.88323484193007	0.702538584993647	2.0356042014296	0.0499883678003562	0.616911777730071	-4.05470260276971
a_HQ_transcript/61318	1.59000104328777	0.158559243749244	2.03551540588633	0.0499977873810619	0.616911777730071	-4.01624939255867
a_HQ_transcript/27113	2.79721860256012	0.948079711483728	2.03527683384171	0.0500231032169432	0.617093154455534	-4.01290900099993
a_HQ_transcript/69514	2.68063218839605	0.080578961363366	2.03503267188812	0.0500490238619458	0.617204154512303	-4.05956140017511
a_HQ_transcript/62797	-2.45135491093912	4.89156687451267	-2.03493360879028	0.0500595439267895	0.617204154512303	-4.50429136550257
a_HQ_transcript/114941	-2.76140322718017	1.71096832839802	-2.03480717050499	0.0500729739335897	0.617204154512303	-4.12601637727474
a_HQ_transcript/137344	2.3720285020818	2.5748336105592	2.034713376749	0.0500829385492445	0.617204154512303	-4.02747630334354
a_HQ_transcript/127046	-0.91019286507115	3.7509478295438	-2.03469217508571	0.0500851912474975	0.617204154512303	-4.32963458594691
a_HQ_transcript/54644	-2.18125167989382	0.675662412962508	-2.03446988007008	0.050108815669972	0.617364399589685	-3.99459182231424
a_HQ_transcript/34398	-1.6483588036182	-0.0305854626627458	-2.03427769998089	0.0501292475025961	0.617415592928435	-3.91869395345437
a_HQ_transcript/11533	0.710054673731399	3.49988145974809	2.03423099473629	0.0501342141243843	0.617415592928435	-4.2977964170484
a_HQ_transcript/142019	-1.53053476553959	4.24438354475689	-2.03412235992927	0.0501457679867743	0.617427070861092	-4.42180473441591
a_HQ_transcript/139915	1.97565564982115	0.217167929183003	2.03370958949841	0.0501896895110946	0.617815121823279	-3.98635030045637
a_HQ_transcript/143671	-4.52354886078659	2.63323901199767	-2.03356117977815	0.0502054895382182	0.617815121823279	-4.20697595493084
a_HQ_transcript/11675	2.03853673250491	0.396568184685186	2.03352156002872	0.0502097082820156	0.617815121823279	-3.97540492956811
a_HQ_transcript/112022	-2.46217441748542	1.02655399611611	-2.03342681043076	0.0502197985586119	0.617815121823279	-3.96509657460462
a_HQ_transcript/146163	-2.35182752550967	1.71701670266166	-2.03320920101558	0.0502429794176937	0.617968791013677	-4.17737589357
a_HQ_transcript/69994	-0.87480457754541	2.16280825226098	-2.03302962004096	0.0502621163607626	0.617968791013677	-4.0082522530759
a_HQ_transcript/74830	1.08740318528048	1.49449118433344	2.03299299943633	0.0502660195989379	0.617968791013677	-3.95316258254474
a_HQ_transcript/66882	4.88696067948047	1.49198528959342	2.03288427219937	0.0502776099498306	0.617968791013677	-4.06482395880593
a_HQ_transcript/19457	-1.89069898423659	2.38368649668913	-2.03278460575762	0.0502882364763532	0.617968791013677	-4.09892142870621
a_HQ_transcript/88264	1.41842126089021	-0.749999294953304	2.03270467090598	0.0502967606260123	0.617968791013677	-4.05118791353835
a_HQ_transcript/119838	-1.35037839683862	2.09493105465689	-2.0325520996607	0.0503130341443589	0.617968791013677	-4.02256594013124
a_HQ_transcript/123750	2.19193170530506	0.908036338977268	2.03241644205461	0.0503275075015715	0.617968791013677	-3.91607011736981
a_HQ_transcript/138632	-2.89318935027722	-0.101850840820791	-2.03241210499833	0.0503279702838348	0.617968791013677	-4.04769710811457
a_HQ_transcript/37847	2.58471149844531	0.358798436650792	2.03221398907459	0.0503491140702579	0.618053132622836	-3.99770478826898
a_HQ_transcript/13770	3.4788157990291	1.24863799580987	2.03208052292923	0.0503633625440435	0.618053132622836	-4.02396758329566
a_HQ_transcript/80689	-3.97338640869497	0.0591564474795985	-2.03204891894091	0.0503667370154551	0.618053132622836	-4.0443719856614
a_HQ_transcript/116419	0.769936165449379	3.41659390968166	2.03188527251029	0.0503842133041517	0.61813709390961	-4.25457193990169
a_HQ_transcript/15107	-1.5186985891643	2.46842781139002	-2.03161152234964	0.0504134597838801	0.618365391499765	-4.03593872152385
a_HQ_transcript/3513	-2.07435636776894	-0.412298013154571	-2.03123196443255	0.0504540350086379	0.618564235103452	-4.0683456467785
a_HQ_transcript/92388	-1.82589326195849	2.02093191757464	-2.03122662383169	0.05045460612988	0.618564235103452	-4.07787075237738
a_HQ_transcript/26981	1.99958642541702	0.340877780804192	2.03108098030627	0.050470183362342	0.618564235103452	-3.94129158621382
a_HQ_transcript/6952	1.2716932086952	1.66609775084567	2.03103073175642	0.0504755586497817	0.618564235103452	-3.99294120923529
a_HQ_transcript/149390	-1.15498490968376	6.83752663077475	-2.03093251341792	0.0504860669058752	0.618564235103452	-4.63288972462417

a_HQ_transcript/116011	-2.10402163433893	1.15902542838406	-2.03086286748976	0.0504935193979817	0.618564235103452	-3.96368237560399
a_HQ_transcript/136689	1.97033168100733	-0.259542197763816	2.03059869524203	0.0505217960293509	0.618644125430565	-4.02025977639335
a_HQ_transcript/52365	-2.49815492774554	0.318309393791696	-2.0305862714189	0.050523126199886	0.618644125430565	-4.05630646282496
a_HQ_transcript/115098	1.95817927753018	-0.695399636158922	2.03050368458219	0.0505319692324581	0.618644125430565	-4.05292508967276
a_HQ_transcript/51935	-1.50572573166764	1.41008548849914	-2.03036082070518	0.0505472696672989	0.618701135232838	-4.01012767570627
a_HQ_transcript/85340	2.0385869909627	0.654515941809549	2.03000455070174	0.0505854431641877	0.619038029525875	-3.91491272553252
a_HQ_transcript/101613	2.44879306513887	0.919567734507747	2.02973474132168	0.0506143694217563	0.619158423874647	-4.10765086868694
a_HQ_transcript/142794	1.04468974450324	3.5124491333945	2.02971408474098	0.0506165846107267	0.619158423874647	-4.23729185122948
a_HQ_transcript/28593	-2.3222522954799	-0.455819752503447	-2.02947652720705	0.0506420661283348	0.619162159594851	-4.04400015531944
a_HQ_transcript/113007	1.68921590223202	-0.580644865383865	2.02934515555006	0.0506561624866303	0.619162159594851	-4.0487851198337
a_HQ_transcript/97408	1.87289480444216	0.492891706656911	2.02933943151916	0.0506567767611527	0.619162159594851	-3.91511073328566
a_HQ_transcript/22204	-3.08564492082682	1.4816119435072	-2.02922532843872	0.0506690230985502	0.619162159594851	-4.05586686726113
a_HQ_transcript/17230	2.57144261133991	1.46557226274652	2.02921484373291	0.050670148520381	0.619162159594851	-3.9940931991515
a_HQ_transcript/85999	1.30975738701752	-0.444251398893082	2.02910790396616	0.0506816286202435	0.619172280041512	-4.00118174229739
a_HQ_transcript/85978	1.74016812419439	-0.56898198584928	2.02888834080542	0.0507052061093002	0.619245280052618	-4.08734099178161
a_HQ_transcript/101422	2.02449836649653	-0.651528010392673	2.02881387993325	0.0507132041707234	0.619245280052618	-4.05267639145524
a_HQ_transcript/72438	-1.95394719559997	0.464027140492563	-2.02875468419012	0.0507195633486532	0.619245280052618	-4.03521550569084
a_HQ_transcript/146203	1.09087674013733	1.85237999480824	2.02855942426658	0.0507405443492562	0.619371348579077	-3.96263531281477
a_HQ_transcript/67966	3.02983358416281	0.014809909912079	2.02821907923476	0.050777133169384	0.619687843138768	-4.05333435832004
a_HQ_transcript/49489	-2.40578662950012	0.218492928462999	-2.02767410085632	0.0508357692725206	0.620251645287023	-4.09361553840057
a_HQ_transcript/149352	0.519225832100412	8.78461794121947	2.02759140556625	0.0508446719273442	0.620251645287023	-4.81120554458467
a_HQ_transcript/149549	-3.79875684175844	0.287886779954342	-2.02732435093588	0.0508734313265171	0.620472265242927	-4.15543063689959
a_HQ_transcript/126381	4.20000962591457	1.0972335453248	2.0270071626404	0.0509076081938386	0.620758852337204	-4.10861660422173
a_HQ_transcript/149255	3.1220189545191	1.03241956281322	2.02672886575205	0.0509376111020179	0.620840819820754	-4.11270406462786
a_HQ_transcript/113268	1.44877187401697	-0.823623806044075	2.02666000857678	0.0509450369156843	0.620840819820754	-4.05810659016105
a_HQ_transcript/148186	1.49410204772669	2.45296776657731	2.02663376286511	0.0509478676004474	0.620840819820754	-4.09643747685696
a_HQ_transcript/93251	2.00480945356536	-0.513441583927593	2.02654860998348	0.0509570525627033	0.620840819820754	-4.00577401057344
a_HQ_transcript/23042	-2.67350089472346	0.00432510486762761	-2.02634194261	0.0509793506438788	0.62098233324128	-4.10405188369021
a_HQ_transcript/8835	-1.87514659995224	-0.299541727958256	-2.02591667453933	0.051025261233849	0.621296453080055	-4.02147520066906
a_HQ_transcript/48972	-1.95483495124178	-0.414968100908574	-2.02573389864401	0.0510450042670444	0.621296453080055	-4.0017147921
a_HQ_transcript/9719	1.07690044341124	2.5693735442913	2.02563508229008	0.0510556809702641	0.621296453080055	-4.04080828382379
a_HQ_transcript/44230	-2.63914963617964	-0.400562169272397	-2.02557752586424	0.0510619006085359	0.621296453080055	-4.06094953487268
a_HQ_transcript/119847	1.80759977468449	1.38077402899344	2.02552463263176	0.0510676169200987	0.621296453080055	-3.95446233756024
a_HQ_transcript/34703	5.93181040301159	2.82509425989803	2.02550934932996	0.0510692687313601	0.621296453080055	-4.25016743766136
a_HQ_transcript/72014	2.93497383335579	0.615828501857216	2.0252268555877	0.0510998089374058	0.621426143268443	-3.99485769221597
a_HQ_transcript/112375	-3.27798895970753	0.0534203431796645	-2.02508040188784	0.051115648244185	0.621426143268443	-4.11765496175222
a_HQ_transcript/85723	0.58287884771091	5.20281628954841	2.0250050356785	0.0511238009504157	0.621426143268443	-4.49596949909488
a_HQ_transcript/69985	-1.74912296804516	1.90051554929719	-2.02481958377429	0.0511438669784105	0.621426143268443	-4.1083047782929
a_HQ_transcript/67345	0.621647924710533	4.52131555282536	2.0247154772635	0.0511551344005158	0.621426143268443	-4.4221267484332
a_HQ_transcript/79688	-2.32959652844004	0.996854963748445	-2.02470842524686	0.051155897717181	0.621426143268443	-4.02897472612562
a_HQ_transcript/54471	-0.730172747852532	4.30572877843063	-2.0247022241636	0.0511565689362926	0.621426143268443	-4.38026457763026
a_HQ_transcript/33028	1.50844604722475	1.96573232261213	2.0245840383985	0.0511693631025412	0.621426143268443	-4.0260804178373
a_HQ_transcript/46346	1.83228424746479	1.92812107250625	2.02452140268938	0.051176144849746	0.621426143268443	-4.00664053231819
a_HQ_transcript/114747	-1.90013553727356	-0.488139944046153	-2.0242318208157	0.0512075089429736	0.621645916224241	-4.06034632975298
a_HQ_transcript/135958	2.20341845506728	-0.175495171191437	2.02406055876693	0.0512260659511773	0.621645916224241	-4.01017653821931
a_HQ_transcript/83932	1.42280365784679	-0.776934463661478	2.02397638680416	0.051235188518341	0.621645916224241	-4.06606921597894
a_HQ_transcript/150992	1.02393582516114	5.62734299745421	2.02395947603908	0.0512370214807036	0.621645916224241	-4.51746466311559
a_HQ_transcript/139222	1.63339207974713	2.35045567927724	2.02363120977055	0.0512726137312409	0.621872429545159	-4.09470190205274
a_HQ_transcript/119131	-2.27094329825775	1.29390858977268	-2.02358996045692	0.0512770877169341	0.621872429545159	-4.07776295864721
a_HQ_transcript/73786	2.24419998173046	2.38512710490362	2.02348170956885	0.051288830457956	0.621875440877301	-4.16097039477257

a_HQ_transcript/32808	-2.25839350770961	-0.375633269254182	-2.02339044152598	0.0512987327794979	0.621875440877301	-4.04704367059918
a_HQ_transcript/146306	1.66410918221477	-0.727891933178983	2.02300639059246	0.0513404195584854	0.622181513759405	-4.05235123003349
a_HQ_transcript/110376	1.85012738330293	1.44362166308155	2.02289149779356	0.0513528963530461	0.622181513759405	-3.99935165659242
a_HQ_transcript/143389	1.80657326639814	3.23823917920543	2.02285697436109	0.0513566459473764	0.622181513759405	-4.17543120079613
a_HQ_transcript/3393	-3.33992786039583	3.01843984244799	-2.02253006270706	0.0513921637507872	0.622181513759405	-4.19584244249329
a_HQ_transcript/21553	1.56583767232121	-0.653892250828849	2.02249813099118	0.051395634172651	0.622181513759405	-4.06758971022692
a_HQ_transcript/98964	-2.17207750198598	1.481205947125	-2.02248466487105	0.0513970977671101	0.622181513759405	-4.00253292640289
a_HQ_transcript/3000	-2.30102042554582	2.66784315376454	-2.02241566982861	0.0514045972155216	0.622181513759405	-4.19778039874726
a_HQ_transcript/118921	8.85890602666515	5.4218378045665	2.02236955696849	0.0514096100087122	0.622181513759405	-4.50348566365972
a_HQ_transcript/10309	-1.7468009136553	-0.200356676938866	-2.02210196499521	0.0514387075952476	0.622314191157224	-4.04066112780682
a_HQ_transcript/125278	2.19011617310716	-0.666825193159555	2.02207183556065	0.0514419847321508	0.622314191157224	-4.0570817973565
a_HQ_transcript/6180	3.55095739482467	0.705512639504892	2.02184395943352	0.0514667764248336	0.622396716896409	-3.98880102218878
a_HQ_transcript/123789	-2.59722930625921	0.603847352931344	-2.02177138796743	0.0514746740050937	0.622396716896409	-3.98723907600341
a_HQ_transcript/46436	2.07057793006682	0.884090563725615	2.0217139213837	0.051480928551437	0.622396716896409	-3.9825838857797
a_HQ_transcript/140068	-4.07625527531738	1.68592863473488	-2.02151001529606	0.0515031266467953	0.622401755623568	-4.16680072655383
a_HQ_transcript/145450	-1.92159642471388	7.49691275235708	-2.02127025735913	0.0515292384507503	0.622401755623568	-4.75393115296971
a_HQ_transcript/126175	-2.24694531848401	-0.0435908192375133	-2.02118797716277	0.051538202178485	0.622401755623568	-4.08801157592948
a_HQ_transcript/84742	-1.31277020336791	4.79585906022073	-2.02115313760218	0.0515419980638208	0.622401755623568	-4.46399975465311
a_HQ_transcript/70249	2.61967682585461	2.84008363783197	2.02111356573738	0.0515463098463017	0.622401755623568	-4.05412016328069
a_HQ_transcript/68531	3.16897941147751	2.71390263980006	2.02106661157989	0.0515514264191201	0.622401755623568	-4.07439110932277
a_HQ_transcript/140744	2.53332312147252	1.25145410122607	2.02098695526367	0.051560107548259	0.622401755623568	-4.09814171910492
a_HQ_transcript/16948	-2.53558674874915	-0.465030687500786	-2.02084986352142	0.0515750511200493	0.622401755623568	-4.06191787278095
a_HQ_transcript/21492	2.23719822490756	0.78176797585637	2.02072530327771	0.0515886319943897	0.622401755623568	-3.96451406342136
a_HQ_transcript/14721	2.10615195784576	1.44460511925711	2.02057460134237	0.0516050672983026	0.622401755623568	-4.11820278238878
a_HQ_transcript/94072	1.48361672725156	0.678574457015984	2.02051277179645	0.0516118116526182	0.622401755623568	-4.01841760588985
a_HQ_transcript/83409	1.8430967678833	1.4958070173702	2.02042790032977	0.0516210706717751	0.622401755623568	-3.96633300062603
a_HQ_transcript/94373	1.60914004597512	-0.744086883908628	2.02021946841895	0.0516438156408605	0.622401755623568	-4.07123121932338
a_HQ_transcript/68426	-0.951864431368686	1.58513740376548	-2.02006120254017	0.0516610921415386	0.622401755623568	-3.97154369093501
a_HQ_transcript/150630	-2.53432410557726	0.924828395441957	-2.01993372815677	0.0516750110832963	0.622401755623568	-4.1034447879591
a_HQ_transcript/96495	2.74363251472181	-0.19229552969673	2.01981597225455	0.051687871780405	0.622401755623568	-4.02858000182212
a_HQ_transcript/47602	-2.21850033873252	1.10948461446244	-2.01973363058022	0.0516968663815636	0.622401755623568	-4.01250235080198
a_HQ_transcript/15225	1.89147840477971	2.048956220524	2.01959648600267	0.0517118504254343	0.622401755623568	-4.21219834663556
a_HQ_transcript/3741	-2.37881109762099	2.16689215750116	-2.01929426926125	0.0517448832276439	0.622401755623568	-4.19146757660697
a_HQ_transcript/134147	2.75353881478811	1.89817846104657	2.01919043767259	0.0517562364592499	0.622401755623568	-3.992280909608
a_HQ_transcript/22535	1.12642242219127	3.67443770455114	2.01908375208433	0.0517679040264737	0.622401755623568	-4.22760764128551
a_HQ_transcript/24712	-2.37600143447047	1.64180186385433	-2.01904965638184	0.0517716333558538	0.622401755623568	-4.07622704308107
a_HQ_transcript/124319	1.82424797144754	-0.536491732436401	2.01901413478134	0.0517755188976643	0.622401755623568	-4.06105280528938
a_HQ_transcript/9300	1.60544284967838	0.815994606439592	2.01901064849306	0.0517759002601981	0.622401755623568	-3.96824126770997
a_HQ_transcript/29562	1.78614474926547	-0.674101306324079	2.0189292275988	0.051784807531852	0.622401755623568	-4.06217847516642
a_HQ_transcript/151243	2.72904333985639	1.72900707944924	2.01886488051796	0.0517918479138361	0.622401755623568	-4.13402337217605
a_HQ_transcript/151232	-1.77692141398701	1.99795527131784	-2.01873480873562	0.0518060819657624	0.622401755623568	-4.13974922394464
a_HQ_transcript/13271	3.35436011690051	0.792798729522991	2.01871290612036	0.0518084791557803	0.622401755623568	-4.00042629206661
a_HQ_transcript/134805	-3.43719422870367	-0.217379334153529	-2.01864260326679	0.0518161742947642	0.622401755623568	-4.03142593234317
a_HQ_transcript/113049	-2.07835325766664	0.287075197972233	-2.01857825016448	0.0518232190689677	0.622401755623568	-3.95568413060785
a_HQ_transcript/86462	1.36890592197647	-0.668637672549297	2.0184316786963	0.0518392674685343	0.622401755623568	-4.02563952175131
a_HQ_transcript/113949	1.56488283649515	-0.670951918510168	2.01835019103919	0.0518481915946257	0.622401755623568	-4.02660583820411
a_HQ_transcript/122046	-2.62441353196221	3.00844249237092	-2.01834532971344	0.0518487240255371	0.622401755623568	-4.07576379459602
a_HQ_transcript/99990	-2.37252351221064	1.32406903032711	-2.01829610652383	0.0518541154063321	0.622401755623568	-4.01832279340369
a_HQ_transcript/51024	-2.09756076585729	-0.270633143345289	-2.01818374996827	0.0518664235781527	0.622401755623568	-4.01481790030828
a_HQ_transcript/145192	-3.32026294016783	0.226187112020883	-2.0181801957536	0.0518668129686307	0.622401755623568	-4.13111117193146

a_HQ_transcript/23761	3.56668692505643	0.504080899224988	2.0179785472182	0.0518889092471296	0.622538393543271	-4.01713105771273
a_HQ_transcript/138721	4.77745168926683	0.882986494818969	2.0177227455011	0.0519169513884566	0.622746295977756	-4.12693076170912
a_HQ_transcript/72417	0.881075382253381	2.80619539708131	2.01735550986251	0.0519572326116039	0.623100890705036	-4.1819372261793
a_HQ_transcript/99202	2.61001367981774	-0.123119637903561	2.01711923526552	0.0519831634820373	0.623198778659108	-4.05916867471381
a_HQ_transcript/120041	2.56189082290867	1.00716995183827	2.01708576897556	0.0519868372852672	0.623198778659108	-4.07145069479743
a_HQ_transcript/74450	-0.949380173373306	2.61482183643444	-2.01680899186076	0.0520172295464545	0.623434540015733	-4.07841010952811
a_HQ_transcript/66243	2.38297506476765	2.5139292151939	2.01635306608282	0.0520673276185906	0.623906332676445	-4.23812063736002
a_HQ_transcript/131171	-3.28975894400466	1.26234523283762	-2.01616701776079	0.0520877831231789	0.623933774791126	-4.11207406750327
a_HQ_transcript/106986	-3.26658606557661	1.34866638181154	-2.01613698913883	0.0520910853471878	0.623933774791126	-4.1832454522825
a_HQ_transcript/144131	-1.05487130200408	4.88729466351809	-2.01586397567942	0.0521211168291943	0.624164870013887	-4.50385759962107
a_HQ_transcript/140728	1.68266521467857	0.197302945389792	2.01556559134655	0.0521539564351859	0.624249861211367	-4.00162414416133
a_HQ_transcript/45275	1.48952570450567	2.16342622455485	2.01551692489794	0.0521593142879978	0.624249861211367	-4.04103733541349
a_HQ_transcript/90060	2.05970808566171	0.290868516772096	2.01546567691258	0.0521649568715162	0.624249861211367	-4.05332959380786
a_HQ_transcript/71078	-1.03037382886506	3.50460512666715	-2.01533582405886	0.052179256519097	0.624249861211367	-4.29434592480677
a_HQ_transcript/32748	-1.28783348930965	1.07878712850114	-2.01531172989996	0.0521819101917498	0.624249861211367	-3.95614157069901
a_HQ_transcript/48642	2.24738611746836	1.14787048643959	2.01498564844399	0.0522178356340392	0.624439048040942	-4.06441171522846
a_HQ_transcript/118512	2.35477906433627	0.161382076313598	2.01496778187146	0.0522198046770151	0.624439048040942	-4.15454929241796
a_HQ_transcript/78418	2.17719042217713	0.868706680167282	2.01487571683749	0.0522299520295737	0.624439048040942	-3.97359948308073
a_HQ_transcript/24572	2.24405969081231	0.103560768805581	2.01411182742193	0.0523142140296969	0.62531783531117	-4.04344406104203
a_HQ_transcript/128526	2.06427537104275	2.01637333561888	2.01385697557161	0.0523423523032032	0.625414359821847	-4.23664347240435
a_HQ_transcript/135488	-1.29885833397007	1.86377315977538	-2.01380144507276	0.0523484851998051	0.625414359821847	-4.14635149555134
a_HQ_transcript/34894	2.50040306347994	2.3705040313781	2.01374638217603	0.052354567074269	0.625414359821847	-4.13660015035931
a_HQ_transcript/7172	2.05711077731202	3.23303512497091	2.01332387508038	0.0524012549189141	0.625745962829451	-4.28596661968386
a_HQ_transcript/28622	-2.11772138036846	0.157744552283701	-2.01330034512626	0.0524038560943739	0.625745962829451	-4.14487606389923
a_HQ_transcript/99981	-2.28809087529487	-0.312677388448333	-2.01314417696103	0.0524211229433338	0.625823584812098	-4.08170505692323
a_HQ_transcript/66394	2.18802220959469	0.851004981997051	2.0130141856603	0.0524354993233214	0.625866674469409	-4.04791148632145
a_HQ_transcript/33916	-1.64215334580025	-0.620673861178604	-2.01261887940025	0.0524792393896429	0.626255226874262	-4.09184333532127
a_HQ_transcript/79388	2.3534265211445	0.940721896338506	2.01252528686302	0.0524895999403283	0.626255226874262	-4.03599691470137
a_HQ_transcript/70382	2.16105318359969	0.983854247871846	2.01238997106124	0.0525045823559966	0.626305430574465	-3.99762078896935
a_HQ_transcript/82390	-0.967095427614517	3.41432690499784	-2.01199035723345	0.0525488501758292	0.626704875465859	-4.31272621233425
a_HQ_transcript/80682	-1.61747382340651	2.53612955001999	-2.01147479720006	0.0526060103365307	0.627168691959958	-4.02913341518498
a_HQ_transcript/11739	2.36422175580643	1.07438117801021	2.01144496217009	0.0526093198113947	0.627168691959958	-4.01909290509147
a_HQ_transcript/122357	-2.64736901397314	1.0702897342106	-2.01061767963212	0.0527011593549738	0.628046211423961	-4.08379637472395
a_HQ_transcript/27365	1.55089487251179	0.103401666477583	2.01058726381087	0.0527045385928654	0.628046211423961	-3.95650716990999
a_HQ_transcript/100951	2.21984080954251	0.226227909168117	2.01031716308893	0.0527345554536218	0.628106906274908	-4.09102060762723
a_HQ_transcript/128679	-1.80511779394725	-0.382267706571917	-2.01028485636656	0.0527381467684156	0.628106906274908	-4.06835646403359
a_HQ_transcript/131619	-1.46406433359078	-0.472666100034499	-2.01024975716376	0.0527420487463498	0.628106906274908	-4.07040535513085
a_HQ_transcript/7048	-1.07957865990795	2.80373813595464	-2.00990312345998	0.0527805975713076	0.628437233843705	-4.21752198702288
a_HQ_transcript/114226	0.834400544842431	3.01852329827301	2.00918148581674	0.0528609292954076	0.629259205628902	-4.22077582304244
a_HQ_transcript/146058	1.6344183745111	0.663307810083907	2.00904094777548	0.0528765862310604	0.629259205628902	-4.01107398354965
a_HQ_transcript/71445	-2.17112005614126	3.25801572162164	-2.00899138551605	0.0528821087857347	0.629259205628902	-4.31502690380459
a_HQ_transcript/21460	-1.96716436824857	0.780817394207327	-2.0086056368224	0.0529251086963858	0.629511112119712	-4.0430100615396
a_HQ_transcript/43943	-1.87522943718722	2.70041654345597	-2.00851802025739	0.0529348796886028	0.629511112119712	-4.29352041777589
a_HQ_transcript/143932	1.85490959636338	1.10983003014443	2.00851005642927	0.0529357678922577	0.629511112119712	-4.13616255593696
a_HQ_transcript/5197	-1.42981850023732	1.35906182898719	-2.00825025941302	0.0529647501319234	0.629726937138155	-3.97327982479281
a_HQ_transcript/109759	-1.9247749605021	-0.597666413714811	-2.0081214053887	0.052979129878048	0.629769092341754	-4.08112075131931
a_HQ_transcript/35521	1.82490786198266	-0.54610641195706	2.00767202245102	0.0530293064603698	0.63003571940283	-4.08695124473348
a_HQ_transcript/6010	-0.672980660142982	3.70861275239415	-2.00767156889873	0.0530293571234665	0.63003571940283	-4.34938676338701
a_HQ_transcript/152090	-3.34951582380894	3.88449813798645	-2.00756361042046	0.0530414176003742	0.63003571940283	-4.52556522153309
a_HQ_transcript/18601	0.944219530113761	3.38177889351494	2.00737373143966	0.0530626355645969	0.63003571940283	-4.27387315916273

a_HQ_transcript/84070	1.86452646757674	-0.113574799908669	2.0070938231761	0.0530939273524278	0.63003571940283	-4.07758802195811
a_HQ_transcript/143650	1.72731366169564	-0.645129531171799	2.00694565202709	0.0531104983802271	0.63003571940283	-4.09323443939583
a_HQ_transcript/9674	-1.73223409645362	0.357044812286746	-2.00684134396502	0.0531221666001508	0.63003571940283	-4.08240269127442
a_HQ_transcript/22030	1.66606003357869	-0.340167057099634	2.00681008033416	0.0531256642822805	0.63003571940283	-4.09792164959504
a_HQ_transcript/5743	1.16027695178658	-0.710576824978093	2.00678872911949	0.0531280531084239	0.63003571940283	-4.09589820901834
a_HQ_transcript/140676	3.34492118476415	4.83504563644867	2.00677885395925	0.0531291579972717	0.63003571940283	-4.25309919898914
a_HQ_transcript/85991	-2.57483560862025	1.4307929493323	-2.00677878936801	0.0531291652241719	0.63003571940283	-4.10568004120012
a_HQ_transcript/34638	1.92280971554003	0.74952855845494	2.00675680551629	0.0531316249744129	0.63003571940283	-4.02326571254614
a_HQ_transcript/69439	-2.21025432484493	1.30574864487824	-2.00655595863029	0.0531541021326013	0.630172708320181	-4.09452742933724
a_HQ_transcript/37706	1.91050953223277	0.476544970302987	2.00645986004919	0.0531648596477114	0.630172708320181	-3.98155149334114
a_HQ_transcript/126797	1.65213080573877	0.355477566365049	2.00634745593694	0.0531774448594205	0.630193377123016	-4.11598499704478
a_HQ_transcript/44695	-1.98328599848474	2.12327935004583	-2.00617387280586	0.0531968850332874	0.630295257483679	-4.21402483319758
a_HQ_transcript/83539	-1.6316272861753	2.42917502479028	-2.00598096850259	0.0532184963390387	0.630318697534768	-4.04391653844724
a_HQ_transcript/133734	2.80067771733493	1.67341816755314	2.0059018070117	0.0532273671184104	0.630318697534768	-4.1853672784093
a_HQ_transcript/45236	1.25677299171177	-0.434431147189672	2.00586587311582	0.0532313942712321	0.630318697534768	-4.02274816220168
a_HQ_transcript/73342	-1.10581469079815	2.78158433583561	-2.00574307303813	0.0532451586265979	0.630353275080832	-4.1985700477201
a_HQ_transcript/81080	2.52134274093169	-0.0422383688476553	2.0054491534676	0.0532781159741641	0.630615012287968	-4.02826128700684
a_HQ_transcript/15381	2.14049458427791	2.28768854631526	2.00498223196921	0.053330508773089	0.630911385171721	-4.20678975161395
a_HQ_transcript/71605	3.39503129380663	1.40262806319133	2.00487736304078	0.0533422822019749	0.630911385171721	-4.0550936777199
a_HQ_transcript/143836	1.90874965254102	-0.252312890569294	2.00476426116617	0.0533549824734617	0.630911385171721	-4.05159625901727
a_HQ_transcript/121471	-0.878408102702131	3.52745235908589	-2.00474335219481	0.0533573306427123	0.630911385171721	-4.34281548128732
a_HQ_transcript/91332	-1.46776018077884	2.51148289251483	-2.00462084353634	0.0533710907175935	0.630911385171721	-4.08047754405665
a_HQ_transcript/104674	2.74815410818725	1.7487225945133	2.00457397345009	0.0533763559484308	0.630911385171721	-4.07006547945346
a_HQ_transcript/124195	-2.25822019007386	0.536592424115956	-2.00454926206239	0.0533791321269358	0.630911385171721	-4.06033917450589
a_HQ_transcript/84216	1.57176298229681	3.30772810716161	2.00421686417526	0.0534164873328789	0.631117371214708	-4.28100275963391
a_HQ_transcript/99338	-1.12610852899975	3.85171874265076	-2.00420096556053	0.0534182746073556	0.631117371214708	-4.38460276170468
a_HQ_transcript/140433	-1.38730096582247	1.74830557242684	-2.00406901676767	0.0534331099107037	0.631164359457303	-4.12337298152905
a_HQ_transcript/1389	2.0235530876301	1.41447651720351	2.00350794085574	0.0534962331639342	0.631698182131431	-3.97381427383126
a_HQ_transcript/76778	3.0889805005614	0.631110242360386	2.00347414866454	0.0535000369982287	0.631698182131431	-4.05088897773956
a_HQ_transcript/46930	1.51643947312932	-0.332437313161912	2.00329875916299	0.0535197835987525	0.631803001833526	-4.0613313673498
a_HQ_transcript/103419	-2.61548236523173	0.508893488655147	-2.00306455772676	0.053546161595394	0.631928620409194	-4.15990232607092
a_HQ_transcript/144247	3.51622239232689	1.11544181174403	2.00301124865973	0.0535521673571375	0.631928620409194	-4.20599924363337
a_HQ_transcript/144701	7.51036780388364	3.85888164617133	2.00268666907576	0.0535887469656131	0.632231922796257	-4.42995345843177
a_HQ_transcript/28124	-2.61791750810106	0.296688827762339	-2.00221667084697	0.0536417537176474	0.632512829679064	-4.09589232081482
a_HQ_transcript/33693	-2.03457990032086	-0.0486282541272242	-2.00216674809557	0.0536473867331557	0.632512829679064	-4.09987652250021
a_HQ_transcript/87844	1.14735523162911	2.38841069235478	2.00213001906994	0.0536515313693705	0.632512829679064	-4.08709392915044
a_HQ_transcript/143637	3.19685306601582	2.54612955707617	2.0020896906816	0.053656082492903	0.632512829679064	-4.14110569616684
a_HQ_transcript/120871	-2.59478511065524	1.39031512980528	-2.00170621689004	0.0536993749811093	0.632807239227941	-4.07890555973676
a_HQ_transcript/109396	-1.89707932835477	-0.107906220141482	-2.00167562350311	0.0537028301526189	0.632807239227941	-4.03682681467119
a_HQ_transcript/146068	-1.44208599303603	-0.0638341855221259	-2.00153210596548	0.0537190414019686	0.63282717785705	-4.06108624245532
a_HQ_transcript/1083	-0.786349586807118	2.82363305005953	-2.00138094903355	0.0537361201932915	0.63282717785705	-4.28140866743346
a_HQ_transcript/89077	3.52654815158854	0.287655294762211	2.00129806412951	0.0537454871359527	0.63282717785705	-4.1066739325748
a_HQ_transcript/151602	-2.96170257616025	1.08092568560422	-2.0012752169208	0.0537480693827459	0.63282717785705	-4.12616444488234
a_HQ_transcript/117539	3.26728103736213	0.97581887375375	2.00096819096599	0.0537827807139907	0.632992227244715	-4.11713043659353
a_HQ_transcript/118158	2.4156768750235	0.895373120720599	2.000958587042	0.0537838668173969	0.632992227244715	-4.16749098917507
a_HQ_transcript/2296	1.36984004788457	-0.61851432582497	2.00058934262923	0.0538256390312537	0.633355616519983	-4.0280096316602
a_HQ_transcript/141484	-3.35618700499277	-0.0447159844464023	-2.00030570155432	0.0538577462759083	0.633404247210885	-4.10770893064528
a_HQ_transcript/147407	-2.34776811194463	2.87208107000027	-2.00015031242812	0.0538753429096278	0.633404247210885	-4.32279655189684
a_HQ_transcript/148211	1.78593314150928	0.369271619304699	1.99996095367042	0.0538967931329986	0.633404247210885	-4.12268370299983
a_HQ_transcript/73012	-3.07693856923299	0.0114242517270853	-1.9999081188196	0.053902779501758	0.633404247210885	-4.12856200877654

a_HQ_transcript/3116	2.04013881693275	2.20970424829538	1.99980584735326	0.0539143688576453	0.633404247210885	-3.99263760243961
a_HQ_transcript/124237	3.98095885955066	4.38242872518107	1.99968097099994	0.0539285227398826	0.633404247210885	-4.42744855212056
a_HQ_transcript/105097	-3.85123869794924	1.78464060952045	-1.99963194178203	0.0539340807541891	0.633404247210885	-4.30080624593584
a_HQ_transcript/67695	2.13597116050418	1.41739583734724	1.99962898767225	0.0539344156517978	0.633404247210885	-3.9746770219192
a_HQ_transcript/150717	1.79496076738226	0.69241044290364	1.99950035171139	0.0539490004447079	0.633404247210885	-4.10264861661765
a_HQ_transcript/68535	2.50456588774707	2.70130927689376	1.99945627143851	0.0539539990747167	0.633404247210885	-4.1470143840033
a_HQ_transcript/134768	-2.22481790304798	-0.37988733462734	-1.99945134758729	0.0539545574564105	0.633404247210885	-4.05116175927193
a_HQ_transcript/1477	0.593622367992424	2.49845439652646	1.99936579594657	0.0539642601129262	0.633404247210885	-4.21147758282657
a_HQ_transcript/35385	1.66518661888105	2.20542756278676	1.99930259229896	0.0539714291982438	0.633404247210885	-4.17552005226097
a_HQ_transcript/150263	6.72310419169044	2.70459973040606	1.99897973777756	0.0540080630305418	0.633706234929215	-4.3106018351983
a_HQ_transcript/124335	1.99775780625989	0.839040517907246	1.99855090940252	0.054056755136663	0.634045909349574	-3.97176840515713
a_HQ_transcript/143876	-1.90036828558924	0.540132130312899	-1.99847129004174	0.0540657998816321	0.634045909349574	-4.04507967219735
a_HQ_transcript/119628	2.27320862832698	3.09718108689849	1.99843665098264	0.054069735284989	0.634045909349574	-4.37347855029417
a_HQ_transcript/102257	-1.7155634776081	0.0617649786002532	-1.99828934489149	0.0540864737783713	0.63411426942097	-4.15681576878566
a_HQ_transcript/99852	0.534490152866641	3.24249716776606	1.99800672912177	0.0541186002728786	0.634266410900744	-4.29573178141333
a_HQ_transcript/114414	2.66711049486509	0.0400073941874542	1.99783039540124	0.0541386535406231	0.634266410900744	-4.09857704547054
a_HQ_transcript/152817	-1.86479173634421	0.150576255214654	-1.99770485697213	0.0541529341493733	0.634266410900744	-4.13186940071943
a_HQ_transcript/2642	-1.58980680692972	1.82109183507737	-1.99768266889677	0.0541554584931945	0.634266410900744	-4.09362954042326
a_HQ_transcript/95962	-2.75096698868346	0.722169291579933	-1.99763908087018	0.0541604178149016	0.634266410900744	-4.1060781597272
a_HQ_transcript/53380	1.1427553966079	4.04721392466098	1.9975995146	0.0541649198959416	0.634266410900744	-4.29640676792995
a_HQ_transcript/104841	0.715254602744987	4.94627463556936	1.99711679383522	0.0542198729977759	0.634782029731061	-4.52950898037656
a_HQ_transcript/34179	2.19674364820289	0.504990820797476	1.99681887238653	0.0542538127665496	0.634912433125031	-4.0770643392426
a_HQ_transcript/20075	-1.20387689479222	2.75974108030686	-1.99672856009797	0.0542641049743293	0.634912433125031	-4.22160488696899
a_HQ_transcript/72521	-2.1937291419042	0.44522854740456	-1.9966597862788	0.0542719437490601	0.634912433125031	-4.05913076182647
a_HQ_transcript/46703	1.28183302604818	1.69392873060365	1.9966355883575	0.0542747020402952	0.634912433125031	-3.97474065086244
a_HQ_transcript/150126	2.35853919487856	0.214911994877841	1.99628490305757	0.0543146899668212	0.635161393585058	-4.10024090224197
a_HQ_transcript/98767	1.02155269582365	3.12229657684319	1.99625730503481	0.0543178380042548	0.635161393585058	-4.26237830465986
a_HQ_transcript/9295	1.96920416467951	0.963037701507225	1.99577145016596	0.0543732843446503	0.635681872965775	-3.97296325062043
a_HQ_transcript/49952	-2.45563389857597	0.563451263576945	-1.99557003461341	0.0543962846117197	0.635822889987944	-4.09704890966567
a_HQ_transcript/114481	-0.917755745326689	3.66878782307674	-1.99536118247882	0.0544201430536694	0.635856917027338	-4.36357558369235
a_HQ_transcript/67734	1.40728223198953	2.69453201724753	1.99531271091277	0.0544256815594916	0.635856917027338	-4.08486143142404
a_HQ_transcript/119832	-2.19813245171428	-0.316129074981095	-1.99525730996003	0.0544320124402703	0.635856917027338	-4.09804124272402
a_HQ_transcript/127383	1.18049621422169	5.52297552837276	1.99494537294332	0.0544676706879405	0.636042083115442	-4.61735027745276
a_HQ_transcript/9496	-1.86842130030283	1.40594548666699	-1.99492720949936	0.0544697476216052	0.636042083115442	-4.02707468513552
a_HQ_transcript/110950	-0.731321927867901	3.44064383884543	-1.99435204687226	0.0545355514513093	0.636343775773642	-4.37184645111593
a_HQ_transcript/130707	2.2272075562403	2.54499023525138	1.99430812690166	0.0545405791451341	0.636343775773642	-4.12895630119592
a_HQ_transcript/36564	1.50175520753185	1.93116794883573	1.99424542591123	0.0545477574780479	0.636343775773642	-4.02864917437201
a_HQ_transcript/54534	-1.67559002066005	1.24186781500934	-1.99418613602138	0.0545545460490537	0.636343775773642	-4.00032916504899
a_HQ_transcript/27014	2.19325854771587	1.10252107883701	1.99418591266636	0.0545545716241403	0.636343775773642	-3.98902802393811
a_HQ_transcript/18305	2.40447739202989	0.416213352208759	1.99412743549977	0.0545612678649847	0.636343775773642	-4.00666685108231
a_HQ_transcript/136122	-0.828441478000803	3.00011382098621	-1.99393888860147	0.0545828633218018	0.636398794166197	-4.24376817676776
a_HQ_transcript/33308	-3.11975439518682	3.33698239694673	-1.99380524617736	0.0545981747426849	0.636398794166197	-4.37116021495949
a_HQ_transcript/124455	3.90871978956104	0.5982948291954	1.99379952819603	0.0545988299357766	0.636398794166197	-4.07354249276639
a_HQ_transcript/145067	1.72077723280556	-0.759274483868411	1.99337209306172	0.0546478268881477	0.636732642398052	-4.11836631579544
a_HQ_transcript/11263	2.42527353666466	-0.0836398551051731	1.99335855079354	0.0546493798672564	0.636732642398052	-4.09434198761901
a_HQ_transcript/145767	1.62200570897203	-0.623730726536934	1.99290681689922	0.0547012051761497	0.637188040853017	-4.15822845059425
a_HQ_transcript/3541	2.95845127851684	1.34760474197475	1.99282680229345	0.0547103893458816	0.637188040853017	-4.20635917697192
a_HQ_transcript/151254	-4.75578403944892	-0.282026957381066	-1.99242618986311	0.0547563923437491	0.637479843358417	-4.13369799796619
a_HQ_transcript/96590	1.54216607403134	-0.765412772038833	1.99232899585386	0.0547675583814261	0.637479843358417	-4.11872076842647
a_HQ_transcript/14841	2.09766610963261	2.70001282980164	1.99230480080206	0.0547703383148728	0.637479843358417	-4.16522272002655

a_HQ_transcript/119997	-1.74152920611802	2.12075722026377	-1.99222671050967	0.0547793114776922	0.637479843358417	-4.05938532685723
a_HQ_transcript/57940	1.45896853947509	-0.704052791909039	1.99176196700197	0.0548327405450973	0.637973887591157	-4.12023527726168
a_HQ_transcript/71643	-2.32193331749909	0.355737482922676	-1.99136857706898	0.0548780020015818	0.638372723703811	-4.12683111257575
a_HQ_transcript/143941	3.33073482233272	0.611282521690809	1.99110688789014	0.0549081286857488	0.638595379000641	-4.18216442862853
a_HQ_transcript/141593	-4.08140792261509	3.50663755196513	-1.99089051265443	0.0549330495475854	0.638661558215419	-4.22579463702976
a_HQ_transcript/152784	-1.8761489041765	-0.604155252293912	-1.99086590720618	0.0549358840871088	0.638661558215419	-4.0928053372864
a_HQ_transcript/137197	2.92311786495536	0.386083760063098	1.99077133096652	0.054946780426564	0.638661558215419	-4.13662798727184
a_HQ_transcript/61033	1.245293552203	-0.456581982394408	1.99039370137581	0.054990306769757	0.638761225001566	-4.07821766152401
a_HQ_transcript/151473	1.96956439615149	-0.561780344740474	1.99030793579906	0.0550001964722989	0.638761225001566	-4.14291711944297
a_HQ_transcript/131847	1.40368684815117	4.991895179529	1.99022224311581	0.0550100793189609	0.638761225001566	-4.44884454474647
a_HQ_transcript/1057	-1.86684695610106	1.09966646894468	-1.9901278553917	0.0550209667483196	0.638761225001566	-4.04034738924969
a_HQ_transcript/37310	0.908685738441619	2.20156358063017	1.99000792413019	0.0550348032835478	0.638761225001566	-4.13295903728053
a_HQ_transcript/107716	4.36421709563441	1.74884787158509	1.98993059987192	0.0550437258347535	0.638761225001566	-4.13951682953678
a_HQ_transcript/6931	-2.18663901196975	0.375666969234335	-1.9897113372625	0.0550690337075817	0.638761225001566	-4.09895362148312
a_HQ_transcript/141141	1.69856504190004	0.168323301025498	1.98970721909868	0.0550695091340795	0.638761225001566	-4.03483020739562
a_HQ_transcript/129272	1.54160787540755	-0.604229071791191	1.98958500207031	0.0550836202594218	0.638761225001566	-4.1419279201595
a_HQ_transcript/56618	-4.1564648104707	2.97724693756884	-1.98923077676568	0.0551245367793613	0.638761225001566	-4.17137794081386
a_HQ_transcript/78684	-1.85224593411212	0.741441131144257	-1.98919682107014	0.0551284603894757	0.638761225001566	-4.09274938125048
a_HQ_transcript/57811	-2.08800148416978	0.993662776632895	-1.98908634405699	0.055141227789867	0.638761225001566	-4.04165240882956
a_HQ_transcript/17746	1.28412295009693	1.68841326016317	1.98907366312927	0.0551426934405415	0.638761225001566	-4.05414151798536
a_HQ_transcript/79757	-0.97529804457143	1.28686959779687	-1.98893557880231	0.0551586553073904	0.638761225001566	-4.03377871789321
a_HQ_transcript/139960	1.84435710749374	-0.650058947670878	1.98889147072446	0.0551637548326932	0.638761225001566	-4.12336202911422
a_HQ_transcript/47709	1.95930430065166	1.28818968635243	1.98881635373221	0.0551724403789039	0.638761225001566	-4.10433929141395
a_HQ_transcript/116644	2.47665917295523	3.62012593155829	1.9887881374335	0.0551757032506736	0.638761225001566	-4.25846780866393
a_HQ_transcript/99623	1.75684286713277	0.616991129880467	1.98876848302765	0.0551779761431101	0.638761225001566	-3.99627896645131
a_HQ_transcript/85252	2.08510923147346	0.04541362525661	1.98866788255763	0.0551896111516518	0.638761225001566	-4.1066458693894
a_HQ_transcript/83375	2.73834523893951	3.33485463627418	1.98860083133221	0.0551973671902115	0.638761225001566	-4.35674925922327
a_HQ_transcript/142416	2.0121920535685	1.03427962563392	1.98857270619324	0.0552006208017015	0.638761225001566	-4.1239329488985
a_HQ_transcript/94452	-2.46559913555039	1.67190969369385	-1.98836937570718	0.0552241477381964	0.638761225001566	-4.19430011996445
a_HQ_transcript/133886	1.85939701304184	-0.214834496528099	1.98833892238446	0.0552276721800294	0.638761225001566	-4.0749033337679
a_HQ_transcript/66203	-1.47381498232932	-0.413887960029502	-1.98833628844345	0.0552279770220548	0.638761225001566	-4.03131028371785
a_HQ_transcript/13108	2.5239256890978	0.073151032587852	1.98816915254777	0.0552473236777388	0.638761225001566	-4.0640158809795
a_HQ_transcript/7083	1.10216405379782	-0.504172062085649	1.98808516165046	0.055257048194895	0.638761225001566	-4.10874732199449
a_HQ_transcript/31456	3.0548368052217	-0.215141383765861	1.9878922585535	0.0552793882841785	0.638761225001566	-4.12464771330168
a_HQ_transcript/103836	1.76520564287299	0.194636897115722	1.98770549897697	0.0553010243967739	0.638761225001566	-4.03792614832645
a_HQ_transcript/130991	4.33313162266955	0.255282265365244	1.98761318567247	0.0553117216278175	0.638761225001566	-4.14451952220867
a_HQ_transcript/11065	2.42341030044569	1.91102143462258	1.98741936283336	0.0553341876174557	0.638761225001566	-4.1724607889498
a_HQ_transcript/126157	1.77892053835907	4.13919910166533	1.98740744335141	0.0553355694633281	0.638761225001566	-4.41510586861779
a_HQ_transcript/46955	-2.47248736992129	0.762121835862291	-1.98739981485513	0.0553364538637055	0.638761225001566	-4.05627690183799
a_HQ_transcript/134487	0.824959863348006	3.37188382341851	1.98728740453363	0.0553494874458017	0.638761225001566	-4.33392393242452
a_HQ_transcript/81201	3.26800522116286	1.43520138656057	1.98725264299429	0.0553535184660977	0.638761225001566	-4.08928032410858
a_HQ_transcript/9687	-1.25553385145729	4.35034479092834	-1.98721119276338	0.05535832545558	0.638761225001566	-4.45451081083328
a_HQ_transcript/112813	-1.90950176248131	1.95692121085108	-1.98719443813244	0.0553602685959413	0.638761225001566	-4.20328072994574
a_HQ_transcript/83838	-2.32179774198527	-0.494463704054312	-1.98707385925864	0.0553742546437443	0.638761225001566	-4.11369909231356
a_HQ_transcript/102993	0.703055392488285	3.19018044941743	1.98701782419298	0.0553807552479854	0.638761225001566	-4.31733676814373
a_HQ_transcript/40880	1.63604024935075	1.64358833167504	1.98699053546316	0.0553839212429103	0.638761225001566	-4.08455819426275
a_HQ_transcript/65734	-2.00437973961128	2.38845202717231	-1.98687750588801	0.0553970364346946	0.638785743677034	-4.11854236141561
a_HQ_transcript/106732	1.83395132146015	1.49709588188937	1.98656804262518	0.055432958328703	0.639022268384171	-4.01147071365578
a_HQ_transcript/33279	-2.38703004247859	2.0331089829088	-1.98644281743701	0.0554474999962319	0.639022268384171	-4.09185343542645
a_HQ_transcript/122622	1.36266861237787	0.129786312409673	1.98637862472527	0.0554549556092372	0.639022268384171	-4.06115098990308

a_HQ_transcript/5479	1.26850272697673	2.46420205038048	1.98627458818688	0.055467040709032	0.639022268384171	-4.15443519104279
a_HQ_transcript/79744	3.14192567467885	0.304934849003804	1.98620278119221	0.0554753832976252	0.639022268384171	-4.13284565677388
a_HQ_transcript/150476	-2.35998957412381	0.978001255814865	-1.98613285339689	0.0554835086109089	0.639022268384171	-4.13366532477962
a_HQ_transcript/126386	-1.42879816513368	-0.560054155019834	-1.98582290542771	0.0555195357438932	0.639071638749545	-4.12958616638389
a_HQ_transcript/52179	0.89131719833194	4.0912413154792	1.98581331267662	0.0555206510928646	0.639071638749545	-4.43271672374301
a_HQ_transcript/139074	-2.14121090320078	2.20535134786335	-1.98581222236927	0.0555207778641137	0.639071638749545	-4.3442566406878
a_HQ_transcript/44820	1.68952300704807	0.225042726359463	1.98536846860731	0.0555723945654766	0.639461493077489	-4.00902246991573
a_HQ_transcript/87365	-0.747825486656461	2.77737125706461	-1.98533190738278	0.0555766491712681	0.639461493077489	-4.24472074995873
a_HQ_transcript/135828	3.00346204124558	0.608096727556274	1.98488799588505	0.0556283295212081	0.639824526292282	-4.11672969665965
a_HQ_transcript/143708	1.35530861234507	3.68391393704605	1.9848718045372	0.0556302153158752	0.639824526292282	-4.33289715748805
a_HQ_transcript/32653	-1.67845421508362	-0.511220175408555	-1.98457279447383	0.0556650508221776	0.639872969498527	-4.10836846505571
a_HQ_transcript/78256	-3.05896968420785	2.81204794614674	-1.98455818842852	0.055666752954203	0.639872969498527	-4.42218998882539
a_HQ_transcript/78856	2.615778456668	0.0748393361774717	1.98446977800007	0.0556770569320724	0.639872969498527	-3.98015999189453
a_HQ_transcript/17269	0.565514666466508	4.06459049692135	1.9844152221935	0.0556834160818559	0.639872969498527	-4.46693575535786
a_HQ_transcript/83766	2.15278325774995	1.74254661741701	1.98435673800402	0.0556902338360813	0.639872969498527	-4.03656436368268
a_HQ_transcript/144186	-0.933250039309553	2.19533006935734	-1.9841797728463	0.0557108678599267	0.639872969498527	-4.06794137564899
a_HQ_transcript/80225	-4.12403085979456	0.102042404097478	-1.98417449586602	0.0557114832547489	0.639872969498527	-4.13194771928444
a_HQ_transcript/33991	2.51711022461168	-0.153510277906797	1.98397379128667	0.0557348935699653	0.639959394907917	-4.1355814469653
a_HQ_transcript/3701	-3.64336967275047	1.99324119841202	-1.98382964530302	0.0557517121431366	0.639959394907917	-4.2414319898252
a_HQ_transcript/34086	-1.71299922860003	1.44857863459608	-1.98374553194727	0.0557615283112897	0.639959394907917	-4.11322363061727
a_HQ_transcript/146914	2.52744894333642	4.06884772776466	1.98371187403579	0.0557654566673796	0.639959394907917	-4.24869901421499
a_HQ_transcript/117895	-1.91639239823433	0.219751196219486	-1.98362362501285	0.0557757577265557	0.639959394907917	-4.08467467979209
a_HQ_transcript/136242	2.83960060968997	0.825894286341278	1.98354390258976	0.055785064925654	0.639959394907917	-4.19880667402561
a_HQ_transcript/62169	1.22492655760716	0.813251529627638	1.98329383323769	0.0558142680702262	0.640095023089634	-4.01378886374081
a_HQ_transcript/144942	3.40694356823463	2.37061821126714	1.98325408390957	0.0558189112310996	0.640095023089634	-4.21102278925546
a_HQ_transcript/61880	-1.59110625539964	1.54200204989139	-1.98310563041685	0.0558362552165311	0.64016762193817	-4.04927704143941
a_HQ_transcript/14710	1.66220491221566	-0.383558157309739	1.98296929713295	0.0558521873216904	0.640224008013256	-4.10482575228246
a_HQ_transcript/31864	-1.60538403158802	2.15318874104276	-1.98280555826849	0.0558713273192649	0.640253290433615	-4.10949939618523
a_HQ_transcript/141793	-2.2703823439268	0.638436612038479	-1.98271810707702	0.0558815521306548	0.640253290433615	-4.05705224293891
a_HQ_transcript/121575	2.39926148488701	0.291308088941995	1.98266480088153	0.0558877855019984	0.640253290433615	-4.1263126696289
a_HQ_transcript/2380	-3.74433815299412	1.34254756755396	-1.98247395167611	0.0559101074631751	0.640315442641752	-4.04799871302408
a_HQ_transcript/136036	2.65520724058496	1.85209353878781	1.98243006161831	0.0559152419978243	0.640315442641752	-4.06865589944222
a_HQ_transcript/116500	1.39843565668326	-0.652326604546874	1.98218088882798	0.0559443995852975	0.640523155228318	-4.13587458410095
a_HQ_transcript/125469	-2.19969121960449	0.742966494733377	-1.98203215689325	0.0559618101448534	0.640596317467515	-4.04581654192501
a_HQ_transcript/8317	-1.51943027694646	1.92887092072062	-1.98184994208169	0.0559831466454472	0.640691061915992	-4.0904784592733
a_HQ_transcript/141851	2.6712054753388	0.550824821201807	1.9817732327846	0.055992131064775	0.640691061915992	-4.17895900032859
a_HQ_transcript/107017	2.75004620851143	2.3433469010318	1.98152084526062	0.0560217003047697	0.640785376729997	-4.24134565358945
a_HQ_transcript/122473	1.95224501652144	2.26599599397658	1.98151469572459	0.0560224209424347	0.640785376729997	-4.13405132945146
a_HQ_transcript/114423	2.85847557434971	1.72295389475819	1.98136720281805	0.0560397074217917	0.640831936438871	-4.12603015544569
a_HQ_transcript/120449	1.84584386558623	2.38143796439394	1.98129185152284	0.0560485405459541	0.640831936438871	-4.15701113946731
a_HQ_transcript/12228	-2.18841660629848	1.56678908406831	-1.98115357791254	0.0560647529515444	0.640891240819543	-4.0261592178097
a_HQ_transcript/81730	1.75544834143146	-0.374786040602268	1.98072255971602	0.0561153155116622	0.641343110511581	-4.11232589297425
a_HQ_transcript/100941	2.29534903208925	0.799840296824119	1.98035757626017	0.0561581626265024	0.641706639896468	-4.11557423465073
a_HQ_transcript/71514	-3.10220139134196	2.86004811070452	-1.98019749941546	0.0561769637862812	0.641777306077947	-4.36401715076411
a_HQ_transcript/42754	1.82252005333571	0.937480812403343	1.9800952594542	0.0561889748251037	0.641777306077947	-4.00184782351768
a_HQ_transcript/44509	2.10018776320999	1.55754151490414	1.98002296566712	0.0561974691703955	0.641777306077947	-4.11527331771535
a_HQ_transcript/1364	-2.30636381927096	1.14823761496224	-1.97940720907432	0.0562698644375121	0.642477839328954	-4.06061903825819
a_HQ_transcript/145684	2.2026683888088	0.0106232121791131	1.97904044552171	0.0563130238799586	0.642844354299731	-4.16660329688787
a_HQ_transcript/72530	-3.41729174716365	1.76116261177023	-1.9789186964663	0.0563273572533901	0.642881724410968	-4.13424676021632
a_HQ_transcript/49665	2.68555306431359	0.83723233819326	1.97868190328767	0.05635524373776	0.643055555128523	-4.13469040994665

a_HQ_transcript/12670	1.22885920758222	4.20614031187183	1.97859341851621	0.0563656674331435	0.643055555128523	-4.43594595919468
a_HQ_transcript/152096	-2.85574623440966	0.355206858330728	-1.97850762218879	0.0563757760276451	0.643055555128523	-4.16062708402191
a_HQ_transcript/94149	0.913842161243796	4.43365014573056	1.97834159252664	0.0563953422648703	0.643113115718738	-4.48842548846578
a_HQ_transcript/77688	3.05833852882361	2.19064790664998	1.97820262588328	0.0564117237307802	0.643113115718738	-4.19442021460659
a_HQ_transcript/149955	-2.4682329409383	2.53013252463184	-1.97818320415108	0.0564140135055368	0.643113115718738	-4.30678838191838
a_HQ_transcript/52312	-2.10620474105987	2.62822318739655	-1.97789422133765	0.056448093437672	0.643375446146078	-4.46279152330802
a_HQ_transcript/19646	0.665291105542529	3.86142093199902	1.97703572095181	0.0565494427305525	0.644404235844257	-4.43750454835071
a_HQ_transcript/118138	-3.1732114922366	2.33699453407933	-1.97685865087677	0.0565703662405386	0.644481382511962	-4.28466751824232
a_HQ_transcript/129375	-1.71459183500388	-0.358544737493789	-1.97665763793692	0.0565941271331718	0.644481382511962	-4.15872357662324
a_HQ_transcript/130780	2.8426315842902	1.32401449055461	1.97654696610379	0.0566072128936239	0.644481382511962	-4.08613316709685
a_HQ_transcript/129832	-0.927106593455459	2.86761872744001	-1.97645282393019	0.0566183462724929	0.644481382511962	-4.16584579153117
a_HQ_transcript/94626	1.99997726704854	0.94642413602658	1.97644702542064	0.0566190320742924	0.644481382511962	-4.12172703428194
a_HQ_transcript/27132	3.13834678060547	1.87102933914738	1.97641570624642	0.0566227363833022	0.644481382511962	-4.03538288466109
a_HQ_transcript/88346	-2.21765400937479	1.93296391285966	-1.9762717243161	0.056639768716332	0.644549036010758	-4.11256965346181
a_HQ_transcript/125986	-1.89895101315632	-0.099068243213256	-1.97604276521859	0.0566668626058053	0.644622747176603	-4.10852071829623
a_HQ_transcript/134051	1.7887291283411	1.92763641237906	1.97597831557234	0.0566744912940233	0.644622747176603	-4.13163349794723
a_HQ_transcript/79565	-1.60398995115137	3.55348937485079	-1.9759131233391	0.0566822087895314	0.644622747176603	-4.45736155585686
a_HQ_transcript/59119	-0.649166076734479	2.49973069550921	-1.97584220711697	0.0566906049333676	0.644622747176603	-4.23991949298756
a_HQ_transcript/137636	2.10614703689071	-0.463352469695242	1.97567328197663	0.0567106092297994	0.644724094134516	-4.17350545568024
a_HQ_transcript/142307	-1.61762483471044	-0.179736297671087	-1.9754535128975	0.0567366437194516	0.644893943317224	-4.15282634899596
a_HQ_transcript/100326	1.55342059981414	1.59462495520607	1.97508907914376	0.0567798385469936	0.645091276367279	-4.08386758476234
a_HQ_transcript/104560	-1.17371024674805	2.33813172543082	-1.97504376928551	0.056785210938007	0.645091276367279	-4.20632309704941
a_HQ_transcript/50391	-2.2852356561111	3.01721602532419	-1.97502616791682	0.056787298052081	0.645091276367279	-4.16187777777412
a_HQ_transcript/18203	-0.756386334093524	2.52348223522752	-1.97458655991227	0.056839447034124	0.645557518014764	-4.16708038873024
a_HQ_transcript/148974	2.69962331958018	1.19581986647116	1.97439839708894	0.0568617807943667	0.645685015435622	-4.17954827835345
a_HQ_transcript/152095	1.86941178330742	-0.147703809511619	1.97416704561044	0.0568892512511705	0.645692423895559	-4.13738620329579
a_HQ_transcript/95154	3.41231096590371	1.25496265742319	1.97413141272463	0.0568934832930917	0.645692423895559	-4.24608387046806
a_HQ_transcript/4265	4.70356403958498	3.47005260556592	1.97411226487482	0.0568957575556196	0.645692423895559	-4.20903018535733
a_HQ_transcript/44190	1.82369682281853	1.21101629167013	1.97399949573055	0.0569091531789499	0.645718379072028	-4.09192341120933
a_HQ_transcript/136815	-0.865254107217052	2.4156700456645	-1.97356007527277	0.0569613772614111	0.646184804342566	-4.16959728916913
a_HQ_transcript/1242	-0.711591025170605	3.57140981932085	-1.97314294128676	0.0570109912419802	0.646621443690503	-4.38254265587183
a_HQ_transcript/52634	-1.0281632954445	2.23222831395297	-1.97304709485917	0.0570223965439169	0.64662463252142	-4.12699572387669
a_HQ_transcript/148921	-0.87155676950976	5.99165896557073	-1.97277366586143	0.05705494430311	0.646867525346436	-4.68385284391562
a_HQ_transcript/80978	-3.15835020083242	0.139130010600133	-1.97238136196116	0.0571016706394148	0.64727104347268	-4.2031830924482
a_HQ_transcript/8651	-2.06811517920387	0.264051727158337	-1.97211141519713	0.0571338426642785	0.647444403064458	-4.08521439514301
a_HQ_transcript/97106	2.12680845911881	1.20276575480444	1.97206613407806	0.0571392407741651	0.647444403064458	-4.00872530833354
a_HQ_transcript/104236	-1.55855264830616	-0.472580401474345	-1.97167675124952	0.0571856786942012	0.647756538880117	-4.13660579208727
a_HQ_transcript/88098	-1.97418121789978	0.536745504768313	-1.97153917350221	0.0572020941108888	0.647756538880117	-4.16648522496025
a_HQ_transcript/45288	2.16124640729132	0.495200566884286	1.97133683663062	0.0572262438671809	0.647756538880117	-4.11105948802414
a_HQ_transcript/80371	1.87385457700115	-0.120683194095061	1.97133122231112	0.0572269140863094	0.647756538880117	-4.17636916247765
a_HQ_transcript/138849	2.03748455704319	-0.683533846565047	1.9712412883178	0.057237651043893	0.647756538880117	-4.1419209993403
a_HQ_transcript/38807	-4.48971033484824	-0.326341266983363	-1.97118154126764	0.0572447850393655	0.647756538880117	-4.18338361271531
a_HQ_transcript/55399	-1.9638601993892	-0.0493211218590425	-1.97087977610271	0.0572808286230184	0.647756538880117	-4.1369966602109
a_HQ_transcript/50231	-1.65957959072673	1.16997377607412	-1.97083939524194	0.0572856533115294	0.647756538880117	-4.02834442191181
a_HQ_transcript/16837	-1.80187072453875	4.62526071824924	-1.97073900338826	0.0572976496210396	0.647756538880117	-4.53950837731063
a_HQ_transcript/46859	-2.19707942107865	1.19212193190395	-1.97071726403263	0.0573002476500016	0.647756538880117	-4.10035303320322
a_HQ_transcript/15608	-1.98099121416102	-0.157865066472164	-1.97067522311897	0.057305272170272	0.647756538880117	-4.22396839608109
a_HQ_transcript/11309	-0.427635097136118	3.48786398871885	-1.97052680210266	0.0573230137757581	0.647756538880117	-4.38508372159161
a_HQ_transcript/133812	-2.29755486367037	1.58554413479526	-1.97051251410536	0.0573247219532488	0.647756538880117	-4.18083959369948
a_HQ_transcript/26678	1.93328184391673	0.0401149927928168	1.97048070614237	0.057328524859833	0.647756538880117	-4.17826005552946

a_HQ_transcript/146108	-1.84881214302471	2.24413132821234	-1.97043539798687	0.0573339422057907	0.647756538880117	-4.26468180514904
a_HQ_transcript/152824	1.58010990388531	2.61247920459536	1.97004066612121	0.0573811578344929	0.647822146011744	-4.21378530209834
a_HQ_transcript/95262	2.5366281411683	0.771813800184563	1.9700060286893	0.057385302585465	0.647822146011744	-4.10176878560902
a_HQ_transcript/45014	-2.1293116582857	0.459709934126124	-1.96988554227572	0.0573997221435644	0.647822146011744	-4.02959422438418
a_HQ_transcript/137076	1.93070118972077	-0.208197022365545	1.96986879128555	0.0574017271160862	0.647822146011744	-4.14925614900078
a_HQ_transcript/88424	1.75849644843609	1.31936759346883	1.96985428851258	0.0574034630424636	0.647822146011744	-4.03473964954512
a_HQ_transcript/61701	-2.07326655267427	1.35082557774951	-1.96957525734373	0.057436870899196	0.647822146011744	-4.17829830408476
a_HQ_transcript/120159	2.93493718965686	0.665168267487417	1.96955618330478	0.0574391552141896	0.647822146011744	-4.04200482969277
a_HQ_transcript/26771	-0.811152406680553	2.60997673580366	-1.9694536210701	0.0574514394661234	0.647822146011744	-4.22661098689294
a_HQ_transcript/110941	3.2452777712549	2.72092476510638	1.969437350502	0.057453388461303	0.647822146011744	-4.20050249198739
a_HQ_transcript/152379	-2.48915544209217	-0.00609621231022329	-1.969391623788	0.0574588662145129	0.647822146011744	-4.0687179554063
a_HQ_transcript/119547	3.14090186524458	1.20619492907988	1.96935710487502	0.057463001649404	0.647822146011744	-4.11504203559162
a_HQ_transcript/9614	-2.81132878710177	1.82231147968779	-1.96924458799937	0.0574764831941078	0.647822146011744	-4.12503647293191
a_HQ_transcript/103674	2.41898343617758	-0.317513675834476	1.9689653415134	0.0575099538337409	0.647822146011744	-4.14977029517392
a_HQ_transcript/11089	1.45322062329304	3.01061736738244	1.9688629106935	0.0575222354990265	0.647822146011744	-4.26890917957575
a_HQ_transcript/52103	-1.75088691930389	-0.502413047043217	-1.96884150749236	0.0575248020749011	0.647822146011744	-4.13145385489413
a_HQ_transcript/50574	-2.60424150372465	-0.00722484586325681	-1.9686543357571	0.0575472511152006	0.647822146011744	-4.20565485770106
a_HQ_transcript/127921	-3.66275359721981	5.88204378450867	-1.96864122618503	0.0575488237391808	0.647822146011744	-4.78148664292686
a_HQ_transcript/18490	-2.05593073582315	0.950280283434654	-1.96847555030256	0.057568701436436	0.647822146011744	-4.13267753080172
a_HQ_transcript/5608	2.01843019355211	0.70388442036252	1.96831736924574	0.0575876854820872	0.647822146011744	-4.1248203332437
a_HQ_transcript/138059	-2.9916122028384	0.264540465367205	-1.9682880757056	0.0575912017338999	0.647822146011744	-4.20431851258183
a_HQ_transcript/141743	-0.732497787259821	3.35008028098062	-1.96825955490737	0.0575946254090296	0.647822146011744	-4.38564813742997
a_HQ_transcript/72167	-0.794579477998105	2.06753636714097	-1.96819301346491	0.0576026138220379	0.647822146011744	-4.23038810074727
a_HQ_transcript/110378	-2.36532836615212	1.42493408195161	-1.96813980081635	0.0576090027842898	0.647822146011744	-4.15107315204755
a_HQ_transcript/148371	-3.16984471089506	0.477231578228574	-1.96804029613148	0.0576209514414182	0.647822146011744	-4.1646612521173
a_HQ_transcript/147714	-1.30403618496778	5.13174724477326	-1.96799025698625	0.0576269610242397	0.647822146011744	-4.55599183612029
a_HQ_transcript/127527	-1.93374526587225	2.33278213702916	-1.96796901127941	0.057629512748189	0.647822146011744	-4.28031241619817
a_HQ_transcript/61247	1.69868392072377	1.23297432090028	1.96787561765027	0.0576407309923138	0.647822971987861	-4.04980179181733
a_HQ_transcript/93011	2.35474142267876	1.36091897613247	1.96765373749634	0.0576673903819316	0.647853740782454	-4.05940280431974
a_HQ_transcript/95986	2.26072075000107	0.977086206967642	1.96760684856634	0.0576730255627681	0.647853740782454	-4.04395769693966
a_HQ_transcript/68912	-2.10707791635779	0.946128686444296	-1.96757457467552	0.0576769045649119	0.647853740782454	-4.1249499330262
a_HQ_transcript/131937	-5.89481344742661	4.83940204495105	-1.96731528333112	0.0577080770535706	0.64795617869597	-4.33603260022713
a_HQ_transcript/20966	1.53409070498667	2.86987933311032	1.9672648887158	0.057714137288653	0.64795617869597	-4.3917953778108
a_HQ_transcript/125834	-1.4816700721144	1.36336226727299	-1.96722058474743	0.0577194655465135	0.64795617869597	-4.13629569033721
a_HQ_transcript/138073	1.40209259304603	2.78886733556631	1.96661149175881	0.057792762065968	0.648528889639402	-4.2635450040974
a_HQ_transcript/76367	1.38985296236557	-0.784240380213963	1.96661120957392	0.0577927960420469	0.648528889639402	-4.14959031530065
a_HQ_transcript/19901	2.08272626284074	2.32103077200039	1.96637737645256	0.0578209563662477	0.648674469325625	-4.28659090187808
a_HQ_transcript/52465	1.76510722249737	1.8040194901553	1.96631817355782	0.0578280880134426	0.648674469325625	-4.19774216776348
a_HQ_transcript/4726	1.6699024837066	3.83308522750713	1.9659651931346	0.0578706243108659	0.648916375023292	-4.45847294027399
a_HQ_transcript/22114	-2.42987584549023	-0.17583294572924	-1.96595394193399	0.0578719805966272	0.648916375023292	-4.14169073137548
a_HQ_transcript/123943	3.23024720188067	0.605230793492662	1.96564435029523	0.0579093114365489	0.649023625890316	-4.22145957777355
a_HQ_transcript/14470	-1.84736575961334	-0.335447478215175	-1.96561038067997	0.0579134087981069	0.649023625890316	-4.09839181463534
a_HQ_transcript/21532	-2.54344399436414	0.808946645967652	-1.96558285918437	0.0579167285824757	0.649023625890316	-4.14173881017796
a_HQ_transcript/93371	-2.40324625644493	1.39698220069321	-1.96534223044956	0.0579457615075649	0.649023625890316	-4.14479116934072
a_HQ_transcript/22094	2.83888866917048	-0.112859581995744	1.96525969180957	0.0579557230754899	0.649023625890316	-4.14587232522951
a_HQ_transcript/148257	-2.19779199462753	0.0373641157286538	-1.96525428073552	0.0579563761886797	0.649023625890316	-4.16674885315176
a_HQ_transcript/70094	-1.34852595706421	2.39254885347564	-1.96509262222275	0.0579758912200937	0.649023625890316	-4.17721036275365
a_HQ_transcript/125920	-3.81681103047534	0.941818325760653	-1.96500294611226	0.0579867191707261	0.649023625890316	-4.12825678649449
a_HQ_transcript/131185	-2.70218539816047	-0.132467846013268	-1.96475401298722	0.0580167828410771	0.649023625890316	-4.15581686047006
a_HQ_transcript/124789	-3.96304054732055	0.515561715369153	-1.96474990383579	0.0580172822667995	0.649023625890316	-4.10463666555034

a_HQ_transcript/104606	2.57061089893243	0.0777438866852245	1.96471141794741	0.0580219319181935	0.649023625890316	-4.19556546792649
a_HQ_transcript/115447	2.02235390988543	2.06587243849543	1.96469585174786	0.0580238126320375	0.649023625890316	-4.12403247897125
a_HQ_transcript/58907	-2.24869652910184	1.15948218574463	-1.96467198787224	0.0580266959770158	0.649023625890316	-4.13267736968388
a_HQ_transcript/2865	-2.47795094239827	2.29560273735755	-1.96433964667839	0.0580668639436066	0.649045115578064	-4.10757415969343
a_HQ_transcript/40209	-2.91549507442409	1.40968563403494	-1.96430657944249	0.0580708618921899	0.649045115578064	-4.17287736822345
a_HQ_transcript/23116	3.22670237118196	2.99447234190436	1.96418868837606	0.0580851172967657	0.649045115578064	-4.38444059802818
a_HQ_transcript/124494	2.68863794010495	0.759761025524173	1.96418557035084	0.0580854943701156	0.649045115578064	-4.18222666021207
a_HQ_transcript/79693	2.0207389136144	1.56673595978843	1.96409733404198	0.0580961659681151	0.649045115578064	-4.07411865914186
a_HQ_transcript/53487	2.07048337736995	0.541795183550948	1.9637104802849	0.0581429734935399	0.649045115578064	-4.15794597584384
a_HQ_transcript/134760	2.59265979671567	1.53093018348092	1.96363586240933	0.0581520056842356	0.649045115578064	-4.15089999114501
a_HQ_transcript/57496	2.16129398862975	0.571501204438162	1.96362714700315	0.0581530607283001	0.649045115578064	-4.04990303350412
a_HQ_transcript/49918	5.6128666869236	3.33804155793985	1.96358308456821	0.058158394963985	0.649045115578064	-4.42663916788721
a_HQ_transcript/115154	2.01755537833565	0.898703189582779	1.96356694006079	0.0581603495382047	0.649045115578064	-4.12851807610066
a_HQ_transcript/89181	1.90332847729296	-0.15921076446934	1.96356268831262	0.0581608642959802	0.649045115578064	-4.11906344281527
a_HQ_transcript/152023	2.31747915764513	0.499896622220388	1.96354829612911	0.0581626067823791	0.649045115578064	-4.14336374370054
a_HQ_transcript/75417	-2.21545547506689	-0.332381160770458	-1.96331481931622	0.0581908805621256	0.64905032440491	-4.15150452494084
a_HQ_transcript/118161	2.79201834136727	1.17063089540699	1.96324644140948	0.0581991633115711	0.64905032440491	-4.17308460117751
a_HQ_transcript/114531	3.29979858322751	0.705727301873691	1.96323684559482	0.058200325753353	0.64905032440491	-4.19813166134391
a_HQ_transcript/5488	2.13226576630752	0.477905539845376	1.96308298375705	0.0582189674098688	0.64905032440491	-4.08397850991663
a_HQ_transcript/16823	-2.11733853093561	0.575775503590471	-1.9630128295384	0.0582274689066177	0.64905032440491	-4.0960200577894
a_HQ_transcript/146047	-3.26163505969363	0.0613866671928728	-1.96279350150367	0.0582540546912209	0.64905032440491	-4.24088236428559
a_HQ_transcript/139544	2.44767410974338	2.88857330565885	1.96274248029707	0.0582602407247011	0.64905032440491	-4.14777412986084
a_HQ_transcript/113373	0.928478529473935	3.11243814799902	1.96272244505294	0.0582626700411672	0.64905032440491	-4.3383622186973
a_HQ_transcript/86632	-2.25622621190162	0.680005086607345	-1.96269393939619	0.0582661265651552	0.64905032440491	-4.19677471330741
a_HQ_transcript/72344	2.44271837495602	1.60158912506018	1.9626229744664	0.0582747323676924	0.64905032440491	-4.27788484708606
a_HQ_transcript/120804	3.18980656954471	0.718130145378451	1.96248827556682	0.0582910701248677	0.649107916516917	-4.14636394661212
a_HQ_transcript/127329	1.69756230544872	0.931316937896556	1.96211996886919	0.0583357627215422	0.649481175153765	-4.15730652074911
a_HQ_transcript/105189	-2.34475033478774	-0.0740190948791222	-1.96193328556774	0.0583584273922087	0.649495073403617	-4.12332152483156
a_HQ_transcript/132629	-2.30510938379196	3.25827837347761	-1.96192562079806	0.0583593581129076	0.649495073403617	-4.38344054413486
a_HQ_transcript/92016	-0.753047197068026	3.09567141031294	-1.96173607855671	0.0583823780282036	0.649594637215675	-4.34081108793759
a_HQ_transcript/74872	2.10770936672155	-0.00601607021280902	1.96158415957178	0.0584008342944016	0.649594637215675	-4.13539269866116
a_HQ_transcript/147654	-0.986287145376175	3.75808088449831	-1.9615759648323	0.0584018299974043	0.649594637215675	-4.42601608905566
a_HQ_transcript/86062	-0.825437060446939	3.52817179672922	-1.96101129880403	0.0584704753699743	0.650229078279169	-4.47463141033059
a_HQ_transcript/110694	1.40256748751068	1.69598982814414	1.96092278841717	0.0584812417637541	0.650229078279169	-4.10184915663995
a_HQ_transcript/28492	2.07720745942184	1.43123204074163	1.96076571809339	0.0585003520223641	0.650317166256642	-4.1529802943731
a_HQ_transcript/90555	3.03622439468402	0.197362350507062	1.96064141638939	0.0585154792713233	0.65036095202361	-4.19809622654592
a_HQ_transcript/120661	0.620353657394026	3.88943393201886	1.96025307356292	0.0585627616567412	0.650762035859883	-4.45433370147668
a_HQ_transcript/109422	5.59902643978491	0.617867307951121	1.9597011119679	0.0586300224485222	0.651200648465138	-4.06761303453655
a_HQ_transcript/18235	0.512767296889402	4.87054139892282	1.95967169120692	0.0586336094794946	0.651200648465138	-4.58373966975613
a_HQ_transcript/118492	-1.23398986632408	2.74807074566109	-1.95959470388198	0.0586429968120754	0.651200648465138	-4.20675233807371
a_HQ_transcript/94645	1.90821902772799	-0.0502785962860882	1.95956151244262	0.0586470443626995	0.651200648465138	-4.17036820188452
a_HQ_transcript/78810	3.05844160566588	0.686263717379623	1.95944347204933	0.0586614408382945	0.651236102568446	-4.07778289944906
a_HQ_transcript/625	2.18533294347846	2.30291851842664	1.95899014060828	0.0587167587199714	0.651725749641874	-4.17434065553538
a_HQ_transcript/38079	1.16083050245604	2.3528179076074	1.95845868050379	0.0587816679793202	0.651903965039801	-4.18648311307016
a_HQ_transcript/121769	1.6493568481652	-0.0853818103969239	1.95841420984166	0.0587871021770842	0.651903965039801	-4.15568561592734
a_HQ_transcript/10081	1.30875051854326	0.731534978215678	1.95841055390017	0.0587875489429174	0.651903965039801	-4.08704980728335
a_HQ_transcript/120651	2.19659722985074	0.907118322478121	1.95837760185413	0.058791575904244	0.651903965039801	-4.13003018947809
a_HQ_transcript/64904	-2.74115316671638	2.01776321605063	-1.95825144035073	0.0588069959025348	0.651903965039801	-4.2266904380291
a_HQ_transcript/70217	1.93797899276474	-0.0828118176811575	1.95823405642265	0.0588091209159477	0.651903965039801	-4.11418629749037
a_HQ_transcript/145076	0.621941350092075	4.01756203175483	1.9581263511626	0.0588222883067003	0.651903965039801	-4.4393994251191

a_HQ_transcript/53579	0.533804592622636	4.54945127893185	1.95808203331923	0.0588277070807033	0.651903965039801	-4.55552137740103
a_HQ_transcript/114	-2.6433754169759	2.84298765900047	-1.9579647017613	0.0588420553870788	0.651903965039801	-4.28902867941104
a_HQ_transcript/28612	-1.55090105145135	3.11918270904335	-1.95788225186263	0.0588521398845697	0.651903965039801	-4.39603509287063
a_HQ_transcript/112590	-0.848411783971252	2.64427635678121	-1.95783663124539	0.0588577204153272	0.651903965039801	-4.28654351667708
a_HQ_transcript/116476	3.65914978892224	2.40931024831935	1.95775755418709	0.0588673945859812	0.651903965039801	-4.27369831802744
a_HQ_transcript/125949	1.14720078280441	3.49793696736888	1.95722726133046	0.0589323055298639	0.652491992695911	-4.45784265855813
a_HQ_transcript/105294	1.37047673558971	-0.495191255383507	1.95714039759404	0.0589429440828212	0.652491992695911	-4.17875192559489
a_HQ_transcript/113764	-1.42826326927831	0.171352259719106	-1.9569251780481	0.0589693100779866	0.652659568966718	-4.06768924386996
a_HQ_transcript/117115	1.43219231020006	-0.597453763126918	1.95669612894212	0.0589973815494062	0.652845953684349	-4.17324391634758
a_HQ_transcript/16552	-2.37525361425067	1.07185530584502	-1.95655951409903	0.0590141301205764	0.652890718871546	-4.18209115618217
a_HQ_transcript/10659	-2.0374336947869	1.5017424529981	-1.95643768801235	0.0590290691176924	0.652890718871546	-4.24607372485665
a_HQ_transcript/84371	1.36687849738979	-0.594566742871663	1.95633123854893	0.0590421252359481	0.652890718871546	-4.20002245149719
a_HQ_transcript/86856	1.39548576994772	3.43487954895347	1.95629675872763	0.0590463547534358	0.652890718871546	-4.44672419407331
a_HQ_transcript/145255	1.39190250168273	1.9712685608135	1.95566162976695	0.0591243108537974	0.653467913513294	-4.09327634625203
a_HQ_transcript/142366	-3.58203224473411	2.3111321389917	-1.95542376654722	0.0591535293225706	0.653467913513294	-4.23075365959519
a_HQ_transcript/43024	-0.4948138052559	4.22637602645428	-1.95540287291843	0.0591560964379276	0.653467913513294	-4.51579792539063
a_HQ_transcript/74354	1.69711607644591	0.295938275754711	1.95537659551865	0.0591593251727296	0.653467913513294	-4.13011122839653
a_HQ_transcript/151250	-3.75186485461613	6.85945998786983	-1.95526164875956	0.0591734506142614	0.653467913513294	-4.94093729739859
a_HQ_transcript/76831	1.87574387870344	-0.42519943765084	1.95523487355521	0.0591767413544995	0.653467913513294	-4.1544815783252
a_HQ_transcript/2997	0.571100680878643	2.9601727041343	1.95520060961481	0.0591809527111281	0.653467913513294	-4.30433082908862
a_HQ_transcript/70173	1.7003742214442	1.10903772758364	1.95513928839568	0.059188490308414	0.653467913513294	-4.09756123958635
a_HQ_transcript/50043	-3.36682923157959	0.312050570099192	-1.95473721281126	0.0592379340511471	0.653681110501005	-4.19049156040081
a_HQ_transcript/77552	4.46488315502732	1.58680596242587	1.95465568111548	0.0592479644795011	0.653681110501005	-4.3216386849345
a_HQ_transcript/90922	3.44838963534134	1.39589634938861	1.95461916261264	0.0592524576427016	0.653681110501005	-4.22018879160537
a_HQ_transcript/26939	2.11533108658532	-0.244858106248522	1.95453075423906	0.0592633364565726	0.653681110501005	-4.12543370152781
a_HQ_transcript/686	-2.56051352057318	3.30473999324852	-1.95452513020107	0.0592640285635201	0.653681110501005	-4.4620626509025
a_HQ_transcript/41095	5.55020607035363	0.961684163056334	1.95409808147786	0.0593166026581143	0.654136877122153	-4.19614846863459
a_HQ_transcript/65281	-1.36447465103054	-0.789959956411567	-1.95383047017788	0.0593495690004559	0.65437627975313	-4.16395861305602
a_HQ_transcript/140087	-2.7267179606477	1.14676624905437	-1.95371738466385	0.0593635044925817	0.654405801089473	-4.08778278275245
a_HQ_transcript/130500	-2.60245677492619	-0.0407932291844511	-1.9533155261116	0.0594130483830887	0.654827773305305	-4.16284273463943
a_HQ_transcript/143644	2.29113792195394	-0.524617897851781	1.95314168790839	0.0594344914754256	0.654939928053752	-4.17521514802919
a_HQ_transcript/9554	1.87102589029692	0.962255260832805	1.95286659311657	0.0594684383845943	0.654986011384255	-4.14257605296649
a_HQ_transcript/132069	2.28136005989997	0.277062900131637	1.9528064526913	0.0594758620012187	0.654986011384255	-4.15520103462045
a_HQ_transcript/4304	1.69037319691426	2.2668216510835	1.9527259786271	0.0594857968210252	0.654986011384255	-4.2257812130385
a_HQ_transcript/4918	-2.15971443572878	0.9188283883405	-1.95267838525438	0.0594916730762336	0.654986011384255	-4.16775090327235
a_HQ_transcript/112796	0.706873269006519	2.78996393630585	1.95265133272599	0.0594950134205352	0.654986011384255	-4.35126052493345
a_HQ_transcript/145137	2.46120095496692	-0.119087886377793	1.95230494338708	0.0595377986630238	0.654987929973255	-4.16706914197883
a_HQ_transcript/126469	2.25114560690163	0.645547684759291	1.95222673011461	0.0595474630884359	0.654987929973255	-4.14806029913286
a_HQ_transcript/78750	-2.27535113010113	0.689477855901712	-1.95217856364676	0.0595534154570522	0.654987929973255	-4.20603031447808
a_HQ_transcript/4579	-2.19350756764318	0.365936580201981	-1.95215864391869	0.0595558772700322	0.654987929973255	-4.241321881899
a_HQ_transcript/40757	3.16110852128033	1.04591412521508	1.95214556152521	0.0595574941276183	0.654987929973255	-4.13410944664567
a_HQ_transcript/96236	2.03018776521492	0.241882861857762	1.95210266605146	0.0595627958615233	0.654987929973255	-4.15927754644917
a_HQ_transcript/47094	-1.99706632367725	0.424330574444049	-1.95159101770046	0.0596260654418377	0.655338079101013	-4.25557977170508
a_HQ_transcript/138670	-2.38069651030872	1.83273064539402	-1.95152559391805	0.0596341598426001	0.655338079101013	-4.12765135162948
a_HQ_transcript/28397	2.48940537289436	1.89160326279153	1.95150031060817	0.0596372882150633	0.655338079101013	-4.13446274941719
a_HQ_transcript/84892	-2.54431767816236	0.331559645944859	-1.95148054744177	0.059639733664402	0.655338079101013	-4.15211364016812
a_HQ_transcript/106458	-2.34659065985266	0.251947311234388	-1.95127083471363	0.0596656884037825	0.655383877778769	-4.20483289920556
a_HQ_transcript/65842	1.42620069270429	2.14459489939225	1.95114472676808	0.0596813006592149	0.655383877778769	-4.21006978330385
a_HQ_transcript/112385	3.73315877479184	2.03829972061552	1.95108439425361	0.059688771122281	0.655383877778769	-4.23276767355912
a_HQ_transcript/76824	1.64165318841811	-0.0275505859216729	1.95107685307142	0.0596897049399115	0.655383877778769	-4.17961087196845

a_HQ_transcript/92656	1.82379624693707	-0.613178116009922	1.95086593696706	0.0597158276237103	0.655383877778769	-4.17928431273996
a_HQ_transcript/43394	1.05403010444553	3.51687477484787	1.95086072985256	0.0597164726682097	0.655383877778769	-4.34611385463637
a_HQ_transcript/88126	-0.639468388994701	5.08702207851551	-1.9506446093078	0.0597432504829903	0.655383877778769	-4.61591012653678
a_HQ_transcript/102820	-2.08873956391561	-0.0318140441458757	-1.95062646523743	0.0597454990476556	0.655383877778769	-4.23171790232099
a_HQ_transcript/15139	2.33035776542293	3.04026801015889	1.95055291424241	0.0597546148533322	0.655383877778769	-4.32552986159937
a_HQ_transcript/104724	2.51028938973632	1.18249400962964	1.95053649482256	0.0597566500176761	0.655383877778769	-4.13399726874998
a_HQ_transcript/123103	0.814882392640949	2.77890074029602	1.9503055335353	0.0597852837244199	0.655531581620947	-4.29982410618233
a_HQ_transcript/35679	1.90261063236343	1.75269259390764	1.95024595374574	0.0597926721331245	0.655531581620947	-4.10287067075074
a_HQ_transcript/108353	0.898354282155345	3.216194856897	1.95008070488479	0.0598131685666785	0.655632634818761	-4.46251958835339
a_HQ_transcript/135094	1.87700741557404	-0.0377921642223226	1.94949096436408	0.0598863660037322	0.656311214755834	-4.17593624788015
a_HQ_transcript/145797	4.7727413094732	2.51559160749814	1.94931526584324	0.0599081883831177	0.656365946123036	-4.45135778053194
a_HQ_transcript/28475	1.02625245707133	2.00175790164876	1.94926893859898	0.0599139435406143	0.656365946123036	-4.09430191194464
a_HQ_transcript/31400	2.23298235924231	-0.0358878982591261	1.94893669714322	0.0599552314105908	0.656394077339538	-4.09638174380372
a_HQ_transcript/47135	-0.74017578832661	2.46449754143107	-1.94887196633096	0.0599632784252777	0.656394077339538	-4.20811543217616
a_HQ_transcript/77943	-3.48711170012362	1.90981355031382	-1.94882418074377	0.0599692194945811	0.656394077339538	-4.2366167067468
a_HQ_transcript/78326	1.92033076149217	0.14025271930773	1.94865147290478	0.0599906961117356	0.656394077339538	-4.20038174718017
a_HQ_transcript/148607	1.53419727820948	-0.687521577211529	1.94864425233265	0.0599915941518128	0.656394077339538	-4.19156807810319
a_HQ_transcript/121535	1.07149212779673	5.36444971068332	1.94856048518472	0.0600020133278909	0.656394077339538	-4.65715734929914
a_HQ_transcript/119596	2.59494439556791	0.244939411465218	1.94854987976904	0.0600033325690904	0.656394077339538	-4.1896346373494
a_HQ_transcript/82890	2.00545787074039	2.29381429270874	1.94841918181813	0.0600195925668595	0.656394077339538	-4.20939063826127
a_HQ_transcript/77550	2.86262923917583	0.88299637646722	1.94835438010545	0.0600276558989105	0.656394077339538	-4.20313820351446
a_HQ_transcript/972	0.945993016751911	1.52596464079122	1.94827039827374	0.0600381072277585	0.656394077339538	-4.12533345676052
a_HQ_transcript/110993	-2.5120090408155	0.346911598795595	-1.94816281744858	0.0600514976988582	0.656394077339538	-4.18609079750304
a_HQ_transcript/68254	1.75221967783105	-0.485001592911891	1.94815863756362	0.0600520180169912	0.656394077339538	-4.18437832919434
a_HQ_transcript/47827	1.51712327469948	0.16912721956176	1.94800042025376	0.0600717160132133	0.656485938788506	-4.14992075075974
a_HQ_transcript/119250	0.549215663177008	4.94816547204359	1.94788612370081	0.060085949378846	0.656518057423601	-4.60526287426287
a_HQ_transcript/35497	-2.14329244605073	0.528810502590246	-1.94767721652282	0.0601119721800502	0.656586794113024	-4.15042370132231
a_HQ_transcript/31538	1.75660028936236	0.221275506634731	1.94765426810083	0.0601148313767808	0.656586794113024	-4.11116836382729
a_HQ_transcript/85204	-1.28525513825327	4.26763706249703	-1.94718091059239	0.060173834400426	0.656707587377423	-4.46136870783335
a_HQ_transcript/70952	1.19661163417761	3.68544418747974	1.94702897496245	0.0601927835065919	0.656707587377423	-4.41151226593401
a_HQ_transcript/117941	1.20556833703457	0.987725407569382	1.94698628948986	0.0601981080842247	0.656707587377423	-4.18002326268786
a_HQ_transcript/138668	0.438881749903317	6.07911171496528	1.94689619674103	0.060209347577642	0.656707587377423	-4.71207799656617
a_HQ_transcript/63868	1.06683387198776	4.00751154392073	1.9468592342308	0.060213959350946	0.656707587377423	-4.47602743377387
a_HQ_transcript/102461	-4.05303934119916	4.18364818703685	-1.94679364984567	0.060222142999524	0.656707587377423	-4.4423915838408
a_HQ_transcript/114194	1.92380864204763	0.585923255406958	1.94679290630495	0.060222235784395	0.656707587377423	-4.11366901997304
a_HQ_transcript/59574	2.25403777817845	5.02477331203964	1.94670181807738	0.060233603429444	0.656707587377423	-4.56791544823261
a_HQ_transcript/151701	1.91346327168865	-0.229193674719758	1.94668887478988	0.0602352188791997	0.656707587377423	-4.1762201466568
a_HQ_transcript/37969	-2.09445984005407	0.0155032430059021	-1.94665964716724	0.0602388669126139	0.656707587377423	-4.19645412675254
a_HQ_transcript/122454	-1.30887683628322	3.94063954769705	-1.94633404968199	0.0602795191867798	0.657013657705161	-4.51659226869012
a_HQ_transcript/122288	1.46908681499602	-0.753584511725249	1.94597944283019	0.060323820476871	0.657013657705161	-4.19640386173183
a_HQ_transcript/105320	1.3145515612995	2.01403519029571	1.94596519420401	0.0603256011580393	0.657013657705161	-4.18418041374709
a_HQ_transcript/129043	1.93048919123903	-0.407388573691172	1.94588879386719	0.0603351498485309	0.657013657705161	-4.21773937233458
a_HQ_transcript/147775	2.74160084853522	0.0186560333399737	1.94573047769112	0.060354940742646	0.657013657705161	-4.17660819862996
a_HQ_transcript/101115	1.97477199868286	1.84682639958814	1.94570646953926	0.0603579424613184	0.657013657705161	-4.17127279034438
a_HQ_transcript/150933	-1.5971563897996	1.8000785321644	-1.94564152104135	0.0603660635653679	0.657013657705161	-4.13796035962297
a_HQ_transcript/8573	2.55877439286325	1.99289147363309	1.94552701036146	0.0603803841902895	0.657013657705161	-4.1195541054263
a_HQ_transcript/135292	2.3961602783579	0.142754091367529	1.94545153121612	0.0603898251701035	0.657013657705161	-4.237890311452
a_HQ_transcript/122780	1.27208918164541	1.98631084421424	1.94537666476038	0.0603991907790303	0.657013657705161	-4.1613616564068
a_HQ_transcript/56692	0.969709641856565	2.05734461562227	1.94527774585416	0.0604115672219659	0.657013657705161	-4.18009871255954
a_HQ_transcript/144809	-1.89095866560974	0.375240607009989	-1.94518544347814	0.0604231178077519	0.657013657705161	-4.1796001113104

a_HQ_transcript/9006	1.42777174758386	-0.773408896612649	1.94477062796251	0.0604750508698401	0.657013657705161	-4.18561349386932
a_HQ_transcript/114729	-1.85809505898317	-0.44266224393028	-1.94476555954634	0.0604756856523593	0.657013657705161	-4.17879028663865
a_HQ_transcript/133417	2.565983604141	0.418529881803557	1.94475183592018	0.0604774044664223	0.657013657705161	-4.19286474211964
a_HQ_transcript/97188	-0.970186286618525	2.92041959767331	-1.94467716031873	0.0604867579464625	0.657013657705161	-4.33923836414505
a_HQ_transcript/78650	2.60678334531225	0.519833239093352	1.94454171661963	0.0605037261257276	0.657013657705161	-4.18499900624181
a_HQ_transcript/143361	-3.24844172143529	-0.111431530677297	-1.94440990978561	0.0605202426470811	0.657013657705161	-4.21843464338022
a_HQ_transcript/82593	1.29704701192821	-0.80542190692518	1.94437443740482	0.0605246883068363	0.657013657705161	-4.20066951066535
a_HQ_transcript/13390	-0.702281799000104	2.71920645450688	-1.94434609848226	0.0605282401526115	0.657013657705161	-4.31285898895192
a_HQ_transcript/108199	-2.75786722310084	0.465508029347787	-1.94419717587066	0.0605469082702478	0.657013657705161	-4.21864024237997
a_HQ_transcript/141872	0.616552183892456	3.55822533655885	1.94412790520289	0.0605555933604681	0.657013657705161	-4.48484008483827
a_HQ_transcript/122730	2.15738171845463	0.221137540229004	1.94412640359407	0.0605557816427118	0.657013657705161	-4.19853389627495
a_HQ_transcript/72517	1.82452280534127	0.311077956466138	1.9441004383015	0.0605590374334489	0.657013657705161	-4.12490942776591
a_HQ_transcript/54467	-0.506088871038937	2.96561286691803	-1.94398531401997	0.0605734747076644	0.657013657705161	-4.36332567190543
a_HQ_transcript/126654	-0.402863104547063	3.43908761773002	-1.94392802895898	0.0605806597092746	0.657013657705161	-4.42495894290527
a_HQ_transcript/93871	2.31147350357306	3.55500335489835	1.9437895333614	0.0605980336280297	0.657013657705161	-4.33822583351912
a_HQ_transcript/45632	2.21549316317541	0.223152540204655	1.94373105468122	0.0606053709262111	0.657013657705161	-4.19455564111904
a_HQ_transcript/151866	-7.6128918505818	3.1605069808111	-1.94353761466304	0.0606296472620469	0.657013657705161	-4.37021198685474
a_HQ_transcript/4678	0.91007164626982	2.33155296784129	1.94351084792851	0.0606330070977512	0.657013657705161	-4.19844583758264
a_HQ_transcript/116799	1.4369551744143	2.00807713089212	1.94351050894873	0.0606330496484803	0.657013657705161	-4.13394250318431
a_HQ_transcript/85202	2.31520608910606	1.11382047038515	1.94330549176708	0.060658789350879	0.657013657705161	-4.17825288398595
a_HQ_transcript/152558	-2.0772532089249	-0.149017378297626	-1.94322134996781	0.0606693560115366	0.657013657705161	-4.20154229261075
a_HQ_transcript/110134	-4.26303132060436	1.56414551925656	-1.94319167192084	0.0606730834084831	0.657013657705161	-4.24695162630084
a_HQ_transcript/130197	-2.2937342488542	0.720983828798259	-1.94306611303217	0.0606888551007067	0.657013657705161	-4.13836114049821
a_HQ_transcript/137369	-1.70114133149389	2.37263012108941	-1.94304774281592	0.0606911629167701	0.657013657705161	-4.23180420134545
a_HQ_transcript/86109	-2.33755839918997	1.01474094255629	-1.94288161698248	0.0607120364514863	0.657013657705161	-4.13276875398997
a_HQ_transcript/75764	1.53326918050597	-0.140703119525503	1.94281088806991	0.060720925353283	0.657013657705161	-4.10387359817552
a_HQ_transcript/151668	1.97581037418515	-0.323517551011899	1.94280362771995	0.0607218378663106	0.657013657705161	-4.19992819357077
a_HQ_transcript/126428	2.64721850826694	3.21217679657026	1.94277578941258	0.0607253368186076	0.657013657705161	-4.45473229055477
a_HQ_transcript/152584	-3.26800467373855	1.52492308256314	-1.94273582419461	0.0607303602884983	0.657013657705161	-4.17948509401865
a_HQ_transcript/8404	-2.31179804307343	0.297531006732399	-1.94249339698498	0.0607608401522613	0.657141800393176	-4.21622123525751
a_HQ_transcript/117561	2.10539879060324	0.710572965973324	1.94246178859667	0.0607648151856819	0.657141800393176	-4.17641380034839
a_HQ_transcript/4960	1.79622251605369	0.0644724368843171	1.94204080758548	0.060817778759557	0.657235979328623	-4.09757327971763
a_HQ_transcript/137718	4.48043369147528	1.27012849199517	1.94197236723719	0.0608263930130258	0.657235979328623	-4.20895530715229
a_HQ_transcript/116029	0.573117071759219	3.34359976323365	1.94194682444257	0.0608296082312748	0.657235979328623	-4.43127161893354
a_HQ_transcript/53376	2.69437951455866	1.24460157325091	1.94185483122368	0.0608411891669156	0.657235979328623	-4.40650140348601
a_HQ_transcript/2847	0.539324361500631	2.99696315499031	1.9417438311122	0.0608551654059741	0.657235979328623	-4.38971227824628
a_HQ_transcript/94931	-1.33661481250027	3.83927698659648	-1.94172708498926	0.0608572741843513	0.657235979328623	-4.46487110175476
a_HQ_transcript/139272	2.09070011829152	-0.350237177476424	1.94160576915743	0.0608725529377761	0.657235979328623	-4.19127197393435
a_HQ_transcript/63314	-2.31847649386587	1.31450556622016	-1.9415517792319	0.0608793536038496	0.657235979328623	-4.13093392866489
a_HQ_transcript/57714	1.7490843645827	-0.319035920094121	1.94153286878689	0.0608817357525341	0.657235979328623	-4.14935886034619
a_HQ_transcript/53455	-1.6432596909136	-0.160717422521445	-1.94149432965479	0.0608865907769858	0.657235979328623	-4.24528508705997
a_HQ_transcript/107307	0.775161262529906	3.23832143691102	1.94127226130793	0.0609145727060762	0.657415945462087	-4.35794263729539
a_HQ_transcript/44784	3.11283716622356	1.13437745914316	1.94113252355483	0.0609321862055159	0.657483965055546	-4.17367002916511
a_HQ_transcript/141373	0.749374870500158	4.07693006217205	1.94097388935109	0.0609521868927644	0.657577713382073	-4.53911434264971
a_HQ_transcript/16815	2.10712359900203	2.66170963316283	1.94085567273054	0.0609670954092442	0.657616500639923	-4.25949759265418
a_HQ_transcript/123083	1.61934097308515	-0.450826984848117	1.94070723866973	0.0609858191787272	0.657696418779416	-4.19694455985744
a_HQ_transcript/123732	1.55914099529848	3.06161684941662	1.94052045587629	0.0610093874159114	0.657828542332053	-4.33938564589947
a_HQ_transcript/38053	0.612057165398378	3.62223122127897	1.94040140425863	0.0610244134580572	0.657868528466237	-4.42137268223971
a_HQ_transcript/2334	-2.15038250022764	3.62661352286757	-1.93997794656427	0.0610778859632536	0.658221789334675	-4.36389460622131
a_HQ_transcript/37574	1.45785958133902	-0.622329323809638	1.93996256108487	0.0610798295429266	0.658221789334675	-4.2063876838155

a_HQ_transcript/129461	-2.06101439104118	-0.493881721542097	-1.93983942634037	0.061095386542698	0.658267401103605	-4.19868575058153
a_HQ_transcript/141393	3.85600004696454	0.531887084794711	1.93962549692031	0.0611224228226989	0.658436655640815	-4.34274332442552
a_HQ_transcript/122676	-2.92870309292984	0.916759081443619	-1.9390488293004	0.0611953534036874	0.659043462672922	-4.16489715553964
a_HQ_transcript/133591	-0.568661921499693	5.01179470589601	-1.93898794026026	0.0612030583798319	0.659043462672922	-4.61073269941343
a_HQ_transcript/12836	-0.726407370587323	2.40896413143138	-1.93891123562248	0.0612127658782533	0.659043462672922	-4.26104348157471
a_HQ_transcript/102498	1.91075153657828	-0.159584005631003	1.93842607872814	0.0612741967268381	0.659343819123374	-4.09439911841852
a_HQ_transcript/134980	2.03560749310346	-0.212024881602281	1.93838403632582	0.0612795226746206	0.659343819123374	-4.1688120927822
a_HQ_transcript/100325	-1.14054710653805	1.83139163237686	-1.93837329849808	0.0612808830110975	0.659343819123374	-4.29602681771838
a_HQ_transcript/102713	3.36212815303356	0.24378886845738	1.93830089650077	0.061290056040754	0.659343819123374	-4.20376469850945
a_HQ_transcript/105719	-1.25858136181004	3.05048453810323	-1.93812163097593	0.0613127733514844	0.659343819123374	-4.29622677768089
a_HQ_transcript/22945	2.07587728497325	-0.283889479749753	1.93782226814972	0.0613507261818703	0.659343819123374	-4.11909598656581
a_HQ_transcript/152242	1.59252045571253	-0.751875135967566	1.93770764319773	0.0613652635725629	0.659343819123374	-4.20924295264055
a_HQ_transcript/104762	-0.752769839635463	3.83616950891888	-1.93769699498538	0.0613666141911258	0.659343819123374	-4.51404704207988
a_HQ_transcript/149372	-2.58801927298645	-0.482527847372243	-1.93768791722801	0.0613677656336182	0.659343819123374	-4.20711215292742
a_HQ_transcript/13118	0.848387847472273	2.29906865825242	1.93762540921433	0.061375694794285	0.659343819123374	-4.27124707198869
a_HQ_transcript/141304	2.34212328795374	1.44384061909634	1.93760647026855	0.061378097380559	0.659343819123374	-4.14114841688858
a_HQ_transcript/108176	0.569750594968515	3.73853517966631	1.93753755866039	0.0613868401643277	0.659343819123374	-4.52338190738436
a_HQ_transcript/2100	-2.03976656037958	3.04049744070339	-1.93752745668582	0.0613881218878286	0.659343819123374	-4.38594113153139
a_HQ_transcript/31601	2.4672540372592	1.65850027848808	1.93694301512624	0.0614623144474709	0.659931089728607	-4.31668888363353
a_HQ_transcript/147482	1.79018597196751	1.31477752099151	1.93676411595396	0.0614850405176511	0.659931089728607	-4.06081134814664
a_HQ_transcript/5349	-0.733273471393264	2.65802887770178	-1.93665405715188	0.061499025216495	0.659931089728607	-4.25343639473312
a_HQ_transcript/135576	-1.45723269566663	2.73633523524523	-1.93660702007358	0.0615050028562541	0.659931089728607	-4.29640932540072
a_HQ_transcript/78671	-1.58612560543772	1.09383516995214	-1.93658960405617	0.0615072162735738	0.659931089728607	-4.14681272920223
a_HQ_transcript/65201	-3.03559616328028	1.93430862963759	-1.93654666208847	0.0615126741017074	0.659931089728607	-4.33604399061188
a_HQ_transcript/72238	0.850088076542569	2.76791407159769	1.93647116120382	0.0615222711127051	0.659931089728607	-4.35495601397893
a_HQ_transcript/131279	4.40370490687314	4.95100364078432	1.93608885220176	0.0615708867985895	0.660233472085679	-4.62544156251417
a_HQ_transcript/95258	4.11419408123767	2.47080255356199	1.93607084609197	0.0615731773358187	0.660233472085679	-4.32688435474323
a_HQ_transcript/58676	-2.10956751926732	-0.0922694644145703	-1.935874006134	0.0615982219425788	0.660380200125087	-4.21296871976001
a_HQ_transcript/33538	1.12791408784137	3.49117332640019	1.93572951674753	0.0616166114262104	0.660455539181774	-4.42024061046692
a_HQ_transcript/16586	-4.26213024495497	2.52162762937502	-1.93547793999711	0.061648641503446	0.660677034165249	-4.18518071910212
a_HQ_transcript/45097	1.98924512688308	-0.700584563655418	1.93528316998802	0.0616734490023737	0.660753054604922	-4.20128429858525
a_HQ_transcript/58020	-1.02003608393879	3.29639422065564	-1.93520834236909	0.0616829819577005	0.660753054604922	-4.41092880226322
a_HQ_transcript/117331	3.15217373060261	0.794799413070244	1.93515454325263	0.0616898366938637	0.660753054604922	-4.12863820296594
a_HQ_transcript/140321	-2.36585206052654	0.864500159854934	-1.93474301492481	0.0617422928062359	0.660883825206248	-4.1865969271207
a_HQ_transcript/105584	-0.80859435976671	2.37549778468382	-1.93467392305878	0.0617511034970387	0.660883825206248	-4.24283217678722
a_HQ_transcript/93760	2.65805472560508	0.73099494743621	1.93466890947166	0.0617517428790095	0.660883825206248	-4.17454285404217
a_HQ_transcript/41370	2.53445847002797	1.73259939169696	1.93451872112439	0.061770899033783	0.660883825206248	-4.11177407171727
a_HQ_transcript/50369	-3.33303424327825	1.37238392267478	-1.93446084438234	0.0617782824437011	0.660883825206248	-4.17510127202609
a_HQ_transcript/75160	3.38819080886439	0.470747740039329	1.93437063695111	0.0617897918449185	0.660883825206248	-4.20636832945187
a_HQ_transcript/13370	1.94752631327965	0.701986656661164	1.93430155258001	0.0617986074490946	0.660883825206248	-4.11831205692563
a_HQ_transcript/145297	1.23900157313133	2.67027977386218	1.93405229836975	0.0618304229230699	0.660883825206248	-4.16008113095454
a_HQ_transcript/20589	-0.733460346811535	3.20559905689042	-1.93405009539255	0.0618307041801966	0.660883825206248	-4.40185624036145
a_HQ_transcript/84422	5.15058413632036	1.16910696858968	1.9339809389934	0.061839534036044	0.660883825206248	-4.24007712891873
a_HQ_transcript/36967	-1.95318872183252	2.5653117579895	-1.9339805175551	0.0618395878484314	0.660883825206248	-4.28946555626309
a_HQ_transcript/41354	-1.25359034481778	0.70468158987012	-1.93393924973164	0.0618448574285035	0.660883825206248	-4.04829033237236
a_HQ_transcript/35993	1.87635055641545	1.06441892823563	1.93389728366985	0.0618502165669583	0.660883825206248	-4.07781111296444
a_HQ_transcript/149393	-1.95014264179258	-0.564106338837548	-1.93381114233125	0.0618612182243875	0.660883825206248	-4.19209490925785
a_HQ_transcript/149952	-0.7341568498134	3.80327171643728	-1.93357292984765	0.0618916506766969	0.661023793827229	-4.4919926146297
a_HQ_transcript/59288	-1.44389573351844	0.11485888357732	-1.93353057080017	0.0618970635459951	0.661023793827229	-4.11023615560082
a_HQ_transcript/50578	1.36064717004393	4.01373918386726	1.93338680967611	0.0619154371802971	0.661098554815634	-4.31311293660712

a_HQ_transcript/2690	-2.10090724880707	1.05982479537922	-1.93321386532764	0.0619375468840427	0.6611289084852	-4.11923294478776
a_HQ_transcript/116984	0.787950041240157	3.83930564430845	1.93318664593412	0.0619410273123176	0.6611289084852	-4.48094151583153
a_HQ_transcript/77300	0.575841852453569	4.14168729466371	1.93288912089861	0.0619790816187089	0.661413632519242	-4.53511804888963
a_HQ_transcript/86468	0.668471290241223	5.61084209471287	1.93274845551093	0.0619970801723354	0.661484265098662	-4.68261051917623
a_HQ_transcript/2233	-2.8983617807606	2.04659227839293	-1.93252514254507	0.0620256630238088	0.661649744058298	-4.31455098371501
a_HQ_transcript/51794	0.787555841961125	2.658357433338	1.93244944239559	0.0620353548224217	0.661649744058298	-4.30730208958767
a_HQ_transcript/46925	1.8956639492574	0.0454086592311459	1.93228190648104	0.0620568089050727	0.661709229893578	-4.1792487853288
a_HQ_transcript/110646	2.00297239412711	0.0218898903832494	1.93216177169485	0.0620721969139168	0.661709229893578	-4.17969771513015
a_HQ_transcript/106382	3.90217490250156	-0.136168155552435	1.9321392419758	0.0620750831029741	0.661709229893578	-4.22582059758463
a_HQ_transcript/66356	-1.78552804975853	1.89993340805765	-1.93198498530938	0.0620948474072964	0.661798549705046	-4.19997214017596
a_HQ_transcript/21276	1.75134531059071	1.75700536305682	1.93181272798988	0.0621169245035149	0.661912481675584	-4.17980743439493
a_HQ_transcript/64655	2.51112798467839	0.410040828273971	1.93160958633951	0.062142968574609	0.661997449623901	-4.17149532364948
a_HQ_transcript/45803	2.05288620385004	3.19600161616934	1.93157288041383	0.0621476755195023	0.661997449623901	-4.23431933594355
a_HQ_transcript/29342	1.24529957152238	3.01064550807641	1.93138031032411	0.0621723745951951	0.66213920675513	-4.3575608875767
a_HQ_transcript/113550	3.36907195942462	1.56806397347312	1.93109808787099	0.0622085878521753	0.662366092547822	-4.15891081393013
a_HQ_transcript/22970	-3.67989018165399	1.87870427662998	-1.93103669219434	0.062216468230648	0.662366092547822	-4.18966946599037
a_HQ_transcript/3606	-3.01789092636547	-0.312620096263822	-1.9306060174244	0.0622717713396653	0.662832053974088	-4.21253980540717
a_HQ_transcript/69000	1.96560481783557	1.99755697725459	1.93050970216481	0.0622841450387827	0.662832053974088	-4.21325461051452
a_HQ_transcript/13368	-1.01787176315568	2.44487529207951	-1.93034173525162	0.0623057289756568	0.662832053974088	-4.25623064450465
a_HQ_transcript/121069	3.00079461450234	3.04622911635918	1.93034080775172	0.0623058481784066	0.662832053974088	-4.37272523268276
a_HQ_transcript/128227	1.5030230086016	2.0453676204138	1.92975923032688	0.0623806316771458	0.663384923032731	-4.2675848139173
a_HQ_transcript/117659	-2.08002659979314	0.124688933938592	-1.92973700740927	0.062383490803517	0.663384923032731	-4.144075752753
a_HQ_transcript/86543	3.04934531160028	0.275160115577942	1.92965939479164	0.0623934770734879	0.663384923032731	-4.2198376074941
a_HQ_transcript/89283	-1.82634594526418	0.533445176556833	-1.92951417344121	0.0624121661486687	0.663384923032731	-4.14396898089664
a_HQ_transcript/77790	1.59535350999023	-0.340162408323341	1.92934907592291	0.0624334190465107	0.663384923032731	-4.21918233479058
a_HQ_transcript/28292	2.17793076468793	0.816405139883744	1.92933391070195	0.0624353715697486	0.663384923032731	-4.13958979059444
a_HQ_transcript/84407	1.15349875895389	1.85551394448822	1.92931578783087	0.0624377049599517	0.663384923032731	-4.14813887765714
a_HQ_transcript/87093	-0.828880647320042	2.19569775137674	-1.9288318442287	0.0625000425045001	0.663925890113593	-4.19165186528628
a_HQ_transcript/43856	-3.19014959696126	3.7187457108039	-1.92860650112548	0.0625290876815306	0.663957845702561	-4.51395080128716
a_HQ_transcript/101548	-1.14655333586546	2.50076135837187	-1.92857565209592	0.0625330648182277	0.663957845702561	-4.23592660921336
a_HQ_transcript/27677	-2.11541275414479	-0.0115369657041828	-1.92847937776264	0.0625454781619504	0.663957845702561	-4.15874061349197
a_HQ_transcript/114873	1.58839254225601	3.24609390358565	1.92845408204563	0.0625487400747871	0.663957845702561	-4.40467014519781
a_HQ_transcript/12688	2.57392199698447	1.49302288135061	1.92825235925588	0.0625747577369551	0.664066400437062	-4.3006190374925
a_HQ_transcript/69225	-3.5122685214725	1.44125755718889	-1.92811598392622	0.0625923523648131	0.664066400437062	-4.30548314820692
a_HQ_transcript/74798	3.73026781708099	1.74626352133136	1.92802779591661	0.0626037323264001	0.664066400437062	-4.25795256926075
a_HQ_transcript/39452	2.79686709136059	0.0055748037993195	1.92802058122037	0.0626046634048152	0.664066400437062	-4.22970647779242
a_HQ_transcript/6264	-1.63457998854304	-0.60531631817894	-1.9278144748464	0.0626312671179396	0.66422651267739	-4.20841913429374
a_HQ_transcript/134286	2.65527754160593	1.92942969290397	1.92772660696908	0.0626426118651502	0.66422651267739	-4.22369249236945
a_HQ_transcript/134705	-1.37160186400209	2.99777937910483	-1.92745583232029	0.062677583149738	0.664476117696146	-4.44262980560012
a_HQ_transcript/92809	-0.549403550568482	5.14503872875393	-1.92726561557241	0.0627021602618172	0.664615458004908	-4.67884006088965
a_HQ_transcript/144336	4.73578461826243	0.877706053135617	1.92689378085276	0.0627502275552026	0.664859922714274	-4.23623062676242
a_HQ_transcript/29642	2.1433525442324	0.0273321848930142	1.92688357156821	0.06275154776498	0.664859922714274	-4.19254755401647
a_HQ_transcript/36169	1.49475627052425	-0.444511929073763	1.9268217902752	0.0627595375022918	0.664859922714274	-4.17696201366312
a_HQ_transcript/144105	5.61546775978768	5.06318587356643	1.9265199266617	0.0627985880432795	0.665130726574489	-4.66090106435028
a_HQ_transcript/43951	1.36501767098537	-0.670531763208945	1.92644731091261	0.0628079851047236	0.665130726574489	-4.20639512309468
a_HQ_transcript/103114	2.74469082614758	4.36788413068123	1.92635053021759	0.0628205111953913	0.665142199410875	-4.63431423748942
a_HQ_transcript/37261	0.980396181750746	2.67433547508239	1.92613639998862	0.0628482332323163	0.665314533113837	-4.35278818683776
a_HQ_transcript/25469	-2.71102038056464	2.56100228201043	-1.92585967902461	0.0628840741558114	0.665489624858575	-4.1974682952256
a_HQ_transcript/109415	2.31674118130163	2.21676878941582	1.92578972768208	0.0628931370589758	0.665489624858575	-4.11910586828516
a_HQ_transcript/81775	-2.13155250421634	0.956168310861206	-1.92574355999978	0.0628991191675786	0.665489624858575	-4.20002198347125

a_HQ_transcript/11276	-1.99563601327476	1.14237534292423	-1.92531298582452	0.0629549338787867	0.665958943859165	-4.18809168098116
a_HQ_transcript/123630	-2.30117932133895	0.376729756509856	-1.92516990451958	0.062973490783613	0.666034037894807	-4.26868280664304
a_HQ_transcript/130931	1.23357491227497	2.81547068626628	1.92498890799889	0.0629969718735277	0.666115950629475	-4.30955169972414
a_HQ_transcript/30500	3.30779254755258	0.680660031179973	1.92493355415775	0.0630041545651124	0.666115950629475	-4.21495819459399
a_HQ_transcript/129862	-1.39598572275009	3.61225661014971	-1.92471331186102	0.0630327401309703	0.666296984603208	-4.4203769433473
a_HQ_transcript/148596	-1.92794939744386	-0.478212191817621	-1.92428634628249	0.06308818856318	0.666492909370195	-4.21936587324901
a_HQ_transcript/4181	2.03439781566232	-0.233548044424121	1.92420409741009	0.0630988747629417	0.666492909370195	-4.20979975572854
a_HQ_transcript/136544	1.04850981207667	2.6196518224143	1.92408547348661	0.0631142897548561	0.666492909370195	-4.26144940408443
a_HQ_transcript/16724	-1.40552359406782	1.6107769546753	-1.92400428496737	0.0631248419522088	0.666492909370195	-4.20471624516258
a_HQ_transcript/87312	-1.67459073408149	-0.665436918868233	-1.92393841524348	0.0631334042631045	0.666492909370195	-4.20968484966919
a_HQ_transcript/48364	1.53139494637513	-0.0673497322504485	1.92375306470434	0.0631575031034549	0.666492909370195	-4.19940133294702
a_HQ_transcript/3645	-0.507216237852048	3.06065980534414	-1.9237080121869	0.0631633619272774	0.666492909370195	-4.37013446084803
a_HQ_transcript/25245	0.776659079366724	3.24299059882502	1.92352242216889	0.0631875018105038	0.666492909370195	-4.35884396797314
a_HQ_transcript/20406	2.25990040551753	1.07739528341365	1.92350207147231	0.0631901493317186	0.666492909370195	-4.07581115442139
a_HQ_transcript/10054	1.38864625714073	1.63449654206999	1.92326359417988	0.0632211811530853	0.666492909370195	-4.16137467656668
a_HQ_transcript/108863	3.0670057801998	1.82650749249356	1.92323304341444	0.0632251575188651	0.666492909370195	-4.2954733593296
a_HQ_transcript/131618	-1.7137384750239	-0.511702349951913	-1.92312970609631	0.0632386090950105	0.666492909370195	-4.22944230382623
a_HQ_transcript/133467	-0.426023397093341	3.76623892788093	-1.92312519578088	0.0632391962660026	0.666492909370195	-4.50605960329695
a_HQ_transcript/62687	5.16015495506644	8.16304646476127	1.9231147283346	0.0632405589783982	0.666492909370195	-4.69600977104071
a_HQ_transcript/90355	3.03530925081274	1.54176557548142	1.9229793831283	0.0632581812831471	0.666492909370195	-4.23578518498667
a_HQ_transcript/101342	3.56480474827075	4.75856194393878	1.92293255197088	0.0632642798127406	0.666492909370195	-4.73515397923829
a_HQ_transcript/129970	2.63265086668358	1.09744217885565	1.92286548009722	0.0632730150500538	0.666492909370195	-4.1912220876214
a_HQ_transcript/100928	-3.04302411693064	1.21700254677957	-1.92275169986871	0.0632878358270056	0.666492909370195	-4.20602461351663
a_HQ_transcript/30747	2.3302868918381	1.17661981235539	1.92265558257209	0.063300358205882	0.666492909370195	-4.20098863297447
a_HQ_transcript/117974	3.36271445910379	0.30385461722848	1.92242196162627	0.0633308038035885	0.666492909370195	-4.24698130921092
a_HQ_transcript/74634	1.72239142204754	-0.314928370070414	1.92225650650556	0.0633523736551178	0.666492909370195	-4.21084599140943
a_HQ_transcript/85077	-1.74066403345902	1.7822077583257	-1.92222252677072	0.0633568042698928	0.666492909370195	-4.15436497861889
a_HQ_transcript/115215	1.83455142831681	-0.569515754844278	1.92221363194256	0.0633579641100326	0.666492909370195	-4.23347084821
a_HQ_transcript/562	0.873755285121862	2.73238964992775	1.92204949319618	0.0633793702590882	0.666492909370195	-4.39975669421739
a_HQ_transcript/70589	-1.58649357526181	-0.571857093761789	-1.9220122708039	0.0633842254858175	0.666492909370195	-4.21796488314196
a_HQ_transcript/142743	1.23205498961508	3.94767409634201	1.92191156398121	0.0633973631276976	0.666492909370195	-4.47901503360935
a_HQ_transcript/37167	2.38838396972582	-0.0764416365610908	1.92185205665237	0.0634051272234041	0.666492909370195	-4.2205231281181
a_HQ_transcript/85626	-1.87375133359169	4.07959665916029	-1.92178897517104	0.0634133585468728	0.666492909370195	-4.67130222820251
a_HQ_transcript/102342	-0.740879381636788	2.68827004118651	-1.92176630794279	0.0634163165547805	0.666492909370195	-4.26301359770351
a_HQ_transcript/129392	-1.17471782467332	1.59706961316506	-1.92171161225401	0.0634234546756254	0.666492909370195	-4.12513157091973
a_HQ_transcript/102485	2.92962880495234	0.0393464926828456	1.92168570332615	0.0634268361911166	0.666492909370195	-4.23168123196383
a_HQ_transcript/151874	2.88023074483315	1.02320846852756	1.92162891687382	0.0634342482459503	0.666492909370195	-4.27192930368735
a_HQ_transcript/8907	-2.62458503359148	3.04623529557113	-1.92161540592423	0.0634360118736691	0.666492909370195	-4.40261010495728
a_HQ_transcript/109338	2.24642732041247	1.169819651296	1.92157629552055	0.0634411173194552	0.666492909370195	-4.17936752021514
a_HQ_transcript/118542	-2.09593560441321	-0.17814370963138	-1.92135649105371	0.063469817055512	0.66661540896328	-4.20128667276463
a_HQ_transcript/3805	1.26355156807548	2.45019581730057	1.92131134034395	0.0634757137457294	0.66661540896328	-4.2414487829382
a_HQ_transcript/117388	1.31037226182442	-0.250618606081713	1.92121877415527	0.0634878043862085	0.66662194605519	-4.17260903789228
a_HQ_transcript/100285	1.1514047705007	-0.648304786656668	1.92102517536717	0.0635130979537037	0.666697354605229	-4.27405577366407
a_HQ_transcript/76107	3.72756548752874	3.51093612883987	1.92098823566936	0.0635179250929631	0.666697354605229	-4.35219000438151
a_HQ_transcript/89391	4.69602005928403	1.47092006889672	1.92080415809721	0.0635419843784447	0.66682947606973	-4.33366995630024
a_HQ_transcript/72127	-1.49826975630181	0.109377183498282	-1.9207123160062	0.0635539912582672	0.666835090949559	-4.14546368073882
a_HQ_transcript/57624	0.872630072100988	2.65307599041892	1.92029113141417	0.0636090795096281	0.666940930602552	-4.19700534254773
a_HQ_transcript/75258	2.32471288134529	3.47923044766655	1.92025850600121	0.0636133484269257	0.666940930602552	-4.45222990384802
a_HQ_transcript/149408	2.14138443381918	0.646102530975565	1.92018738691421	0.0636226549605511	0.666940930602552	-4.18676072205586
a_HQ_transcript/87324	1.92395425111875	1.53346883823185	1.92011827803587	0.0636316995688017	0.666940930602552	-4.17270127334558

a_HQ_transcript/151586	4.43349851407408	2.15845515115922	1.91998066771873	0.0636497125966117	0.666940930602552	-4.29301945796964
a_HQ_transcript/91354	1.2716219044074	1.82909772423146	1.91992652059767	0.0636568015989721	0.666940930602552	-4.21976015879957
a_HQ_transcript/141626	1.57872351374093	-0.547998439116187	1.9199236367832	0.0636571791703645	0.666940930602552	-4.23844932418627
a_HQ_transcript/23474	1.51388408713583	-0.328728842855428	1.91991505699005	0.0636583025149828	0.666940930602552	-4.23832700415658
a_HQ_transcript/116821	-3.11957954364775	2.5927299166766	-1.91978109083655	0.0636758448058106	0.666940930602552	-4.42680048427687
a_HQ_transcript/69034	1.58117770963512	2.54738155474127	1.91975841049612	0.0636788151122379	0.666940930602552	-4.3003229960382
a_HQ_transcript/146330	-4.31234304733429	-0.071228663190527	-1.91917567746315	0.0637551731955436	0.667474770320939	-4.26934150279848
a_HQ_transcript/95692	2.10216684440339	1.63250786671629	1.91913787638297	0.0637601291700377	0.667474770320939	-4.20762265309774
a_HQ_transcript/21330	3.0625877819151	0.800817235194479	1.9191065684846	0.0637642340970303	0.667474770320939	-4.27531247284829
a_HQ_transcript/114774	3.03556892697235	-0.00615147301490745	1.91892118057551	0.0637885458655488	0.667609037463561	-4.24367814831323
a_HQ_transcript/31502	1.01462333412481	1.91568617324937	1.91874254368211	0.0638119798841631	0.667734071414335	-4.23818998541596
a_HQ_transcript/146116	2.10988338836828	0.123067337965789	1.91861892302129	0.0638282010960838	0.667750361203792	-4.23177966700042
a_HQ_transcript/3686	5.17184121512622	3.02457781500588	1.91855559836889	0.0638365117879417	0.667750361203792	-4.33659129571936
a_HQ_transcript/20230	1.76417736009106	-0.660731833183598	1.91844241766423	0.0638513678894168	0.66778559062181	-4.18671797968682
a_HQ_transcript/142068	-2.32388146784033	1.36975797534051	-1.91827734080514	0.0638730412377719	0.667892092295234	-4.3204704693024
a_HQ_transcript/112753	-0.885575381772752	3.4848068298214	-1.91799220462705	0.0639104924446301	0.668060586172143	-4.35448340516503
a_HQ_transcript/96741	1.4051278658345	-0.592773477193327	1.91797965822294	0.0639121407876288	0.668060586172143	-4.26356202575507
a_HQ_transcript/85680	-0.944484178854624	3.69250424412203	-1.91785727810775	0.0639282209791869	0.668108527342354	-4.5821777439079
a_HQ_transcript/50772	3.97813896732766	1.52702400326985	1.91747108302464	0.0639789881848755	0.668518897215611	-4.37799005024093
a_HQ_transcript/146528	-1.19353094744086	2.12271901685603	-1.9173559146859	0.0639941343597735	0.668556980960624	-4.18996656853711
a_HQ_transcript/25173	-1.61992816465615	-0.651726174201465	-1.91702824443948	0.0640372443219159	0.668678417837602	-4.22650734485166
a_HQ_transcript/17601	0.553513962519672	4.31567284581838	1.91695002646537	0.0640475387848617	0.668678417837602	-4.60185227545065
a_HQ_transcript/74706	-2.40463943303121	0.552416755061036	-1.91694008771452	0.0640488469510632	0.668678417837602	-4.18934792482826
a_HQ_transcript/122281	-2.59186101969399	0.491190114549381	-1.91688331022131	0.0640563206062648	0.668678417837602	-4.26916145142425
a_HQ_transcript/66516	2.09404739770976	-0.180821528459195	1.91683047490104	0.0640632760276907	0.668678417837602	-4.23745015993984
a_HQ_transcript/21329	1.40699569595132	-0.386441285690747	1.9160706385697	0.0641633756589142	0.669495911288171	-4.24492500772997
a_HQ_transcript/122810	1.17819971049183	3.92995339918096	1.91606110937205	0.0641646318776906	0.669495911288171	-4.60179023401582
a_HQ_transcript/133733	-0.73856063623132	3.88504841612386	-1.91562550311223	0.0642220798388522	0.669799178382383	-4.51936366333669
a_HQ_transcript/40544	2.64195811766273	0.807997583560352	1.91547822793865	0.0642415126066275	0.669799178382383	-4.27288072228807
a_HQ_transcript/70686	0.42797872243225	3.34199885540899	1.9154659234625	0.064243136396153	0.669799178382383	-4.45848884046483
a_HQ_transcript/17900	-1.83838974113584	2.27846528383391	-1.91540664791439	0.0642509593319577	0.669799178382383	-4.20424304196409
a_HQ_transcript/3778	-1.84326911196734	0.22991486887352	-1.91529169113869	0.0642661331857669	0.669799178382383	-4.20633718714695
a_HQ_transcript/10957	3.03797482836019	0.0490424489252743	1.91525563198195	0.0642708934939806	0.669799178382383	-4.23817166313379
a_HQ_transcript/143347	-2.39197711081367	1.03312764898453	-1.91522939837352	0.0642743568850973	0.669799178382383	-4.20212620734336
a_HQ_transcript/93367	-0.629287502348865	3.2396899515485	-1.91484157658362	0.0643255763741047	0.670037330139663	-4.42310053056782
a_HQ_transcript/14267	-1.29862829497587	-0.734428032363965	-1.9145650297336	0.06436212134985	0.670037330139663	-4.22792802629094
a_HQ_transcript/139275	-1.38859956006864	4.13966741340731	-1.91447977916122	0.064373390615596	0.670037330139663	-4.5886550169602
a_HQ_transcript/146738	2.61381440359616	3.4028531101974	1.9144571408652	0.0643763834563271	0.670037330139663	-4.41486874281722
a_HQ_transcript/133636	1.48323279750087	1.34551394033567	1.91434795688018	0.0643908195464551	0.670037330139663	-4.08905781807231
a_HQ_transcript/11793	-2.95773849008731	4.1329741049062	-1.91428728390586	0.0643988428153528	0.670037330139663	-4.43706048614974
a_HQ_transcript/100091	1.88245816664822	1.87550509683611	1.91409638502556	0.0644240925216705	0.670037330139663	-4.2453239914141
a_HQ_transcript/117107	1.91631485535829	0.079610926586194	1.91406360915707	0.0644284285618232	0.670037330139663	-4.15616499901515
a_HQ_transcript/131353	1.05847598392839	2.64351264591608	1.91388034251172	0.0644526782251785	0.670037330139663	-4.38229382809274
a_HQ_transcript/69006	-3.39299196883624	0.820720126032477	-1.91371138241226	0.0644750418434233	0.670037330139663	-4.18789314987326
a_HQ_transcript/147017	0.599468242492059	4.83996920046611	1.91347883006401	0.0645058335131738	0.670037330139663	-4.65462624642618
a_HQ_transcript/138365	-4.57391763130956	2.02179363541605	-1.91342696450562	0.0645127026326384	0.670037330139663	-4.39861670355916
a_HQ_transcript/112830	0.62521796878099	4.29138228816193	1.91339496567519	0.0645169409005257	0.670037330139663	-4.55383657004438
a_HQ_transcript/63947	1.52251100826985	-0.255769000503118	1.91338782423835	0.0645178868217436	0.670037330139663	-4.17189396219293
a_HQ_transcript/128757	-1.92262736465994	-0.448364850204068	-1.91329821775577	0.0645297566939564	0.670037330139663	-4.22959502309819
a_HQ_transcript/15774	-0.616880334936788	3.72730943326719	-1.91328247330827	0.0645318425032388	0.670037330139663	-4.5324464731816

a_HQ_transcript/69125	-2.50418208146773	-0.239537130646954	-1.91311048919113	0.0645546305856696	0.670037330139663	-4.21021194845188
a_HQ_transcript/15848	1.83255740143075	1.33825192454817	1.91295567272813	0.0645751498780778	0.670037330139663	-4.1876863166207
a_HQ_transcript/73737	-2.13715263023918	0.937770079963958	-1.91289237415454	0.0645835410596488	0.670037330139663	-4.24765003974099
a_HQ_transcript/117269	0.614068956137465	4.82527161529801	1.91287958610455	0.0645852364230608	0.670037330139663	-4.68383471549967
a_HQ_transcript/103251	2.53787874617632	1.0348512770704	1.91281995208881	0.0645931428527372	0.670037330139663	-4.1624840104918
a_HQ_transcript/9679	1.42503176756195	-0.757012586327128	1.91279165777387	0.0645968944774081	0.670037330139663	-4.25542132374476
a_HQ_transcript/2845	-3.18647425481329	2.5350171269611	-1.91276230077721	0.0646007872049947	0.670037330139663	-4.27854737395457
a_HQ_transcript/15784	3.52836240997307	-0.099735848614086	1.91266402735068	0.0646138197013246	0.670037330139663	-4.20886935551588
a_HQ_transcript/102411	2.04579876189296	0.590857284360018	1.91260574158148	0.0646215503208507	0.670037330139663	-4.19652588318622
a_HQ_transcript/147250	-1.88371001836284	-0.49056332182314	-1.91258078329584	0.0646248608586828	0.670037330139663	-4.21461210221647
a_HQ_transcript/71342	3.19829981273285	2.24940512274212	1.91254014757376	0.0646302512094867	0.670037330139663	-4.25162500404679
a_HQ_transcript/39430	0.541777945507474	3.21711732856201	1.91238504684928	0.0646508289745294	0.670037330139663	-4.42312038855196
a_HQ_transcript/5947	1.73285793147561	-0.469015438021356	1.91224131932846	0.0646699028660554	0.670037330139663	-4.23123302156466
a_HQ_transcript/94868	-0.944920994762897	3.95057847246996	-1.91213823201008	0.0646835864415019	0.670037330139663	-4.53664337967065
a_HQ_transcript/126945	1.88721633750823	1.49299409624862	1.91213332108833	0.0646842383684283	0.670037330139663	-4.18971075211144
a_HQ_transcript/33701	-1.06849913446576	2.36343408486273	-1.91212935622604	0.0646847647097028	0.670037330139663	-4.23730561793979
a_HQ_transcript/116235	1.07373787391735	2.79951245472743	1.91206866957165	0.0646928214134142	0.670037330139663	-4.35657365052586
a_HQ_transcript/13885	-0.562472228572281	3.01947898590663	-1.91203680748626	0.0646970517410477	0.670037330139663	-4.39729602857331
a_HQ_transcript/127542	-1.81612080164084	-0.573376227912825	-1.91200968789924	0.0647006525955465	0.670037330139663	-4.24820741307721
a_HQ_transcript/111177	1.41008772810019	-0.780902987772408	1.91187075506461	0.0647191023796749	0.670043557360974	-4.2528217889426
a_HQ_transcript/133451	1.50570901449251	3.86279553349111	1.91177043165757	0.064732427793861	0.670043557360974	-4.57242772833166
a_HQ_transcript/48419	2.5959297848762	0.0735428316415761	1.91171638913021	0.0647396069514034	0.670043557360974	-4.23736303929045
a_HQ_transcript/32742	0.784142087501856	2.16338003562406	1.91136730801714	0.0647859964067565	0.670043557360974	-4.30647595445136
a_HQ_transcript/121843	4.26110350569829	2.63006152514151	1.91132791254625	0.064791233480012	0.670043557360974	-4.62688581001335
a_HQ_transcript/150314	-0.959517764698055	4.02334977200088	-1.91128634002883	0.0647967603572916	0.670043557360974	-4.51348561034641
a_HQ_transcript/16617	1.81546841220351	0.0507290479357669	1.91126716649298	0.0647993095289018	0.670043557360974	-4.2444523944863
a_HQ_transcript/60925	2.43308620700265	-0.0349360306656246	1.91122951124382	0.0648043161444979	0.670043557360974	-4.2503105403747
a_HQ_transcript/54614	-1.49695364175804	-0.688523838561086	-1.9112243888623	0.0648049972385666	0.670043557360974	-4.22186779708737
a_HQ_transcript/131142	1.67210367181735	-0.289098596924117	1.9108028187505	0.0648610722165563	0.670504073591319	-4.27985245905458
a_HQ_transcript/136990	-2.16359897138713	3.36176923842931	-1.91060324551776	0.0648876329744819	0.670566120460536	-4.50889726097835
a_HQ_transcript/142183	-0.537955118441224	4.77170822508089	-1.91056454655385	0.0648927844202715	0.670566120460536	-4.64877468500172
a_HQ_transcript/78306	2.308969904143	1.00344191410603	1.91049770903315	0.0649016823856141	0.670566120460536	-4.2271609858028
a_HQ_transcript/117157	-1.33695117119589	-0.77065720540329	-1.9102880103519	0.0649296060501319	0.670735407940655	-4.22120526227471
a_HQ_transcript/28135	2.16342688677466	2.27263763697253	1.9098455595575	0.064988557220986	0.671074100751351	-4.32479397791097
a_HQ_transcript/100789	0.89569761244025	4.06911435811943	1.90972086067423	0.0650051801687904	0.671074100751351	-4.53557218664202
a_HQ_transcript/118274	-0.960722540097189	4.33359480858886	-1.90963832772862	0.0650161842169255	0.671074100751351	-4.61643787543308
a_HQ_transcript/80586	0.834620360707297	2.27908982348313	1.90949897404292	0.065034767775685	0.671074100751351	-4.28144257458329
a_HQ_transcript/84888	-3.71948126183711	0.0951511113386983	-1.90945552838016	0.0650405624243983	0.671074100751351	-4.26153631036256
a_HQ_transcript/72185	-0.991443253824291	1.86071990375598	-1.90933899416531	0.0650561075999053	0.671074100751351	-4.21746989723356
a_HQ_transcript/98685	2.24451902141956	0.0604878671219628	1.90927048187815	0.0650652483484264	0.671074100751351	-4.23730020473268
a_HQ_transcript/88981	1.91764329016751	-0.471692338535376	1.90904703895884	0.0650950672812938	0.671074100751351	-4.25523551125598
a_HQ_transcript/28002	-0.680600006756352	3.22099990645944	-1.90904006841067	0.065095997705452	0.671074100751351	-4.43959937840124
a_HQ_transcript/149537	3.07212701038718	-0.448538019264006	1.90895491985915	0.065107364204242	0.671074100751351	-4.26357153725468
a_HQ_transcript/15948	1.47308817882159	0.274244084758761	1.90884673265798	0.0651218086081767	0.671074100751351	-4.14906353344539
a_HQ_transcript/94821	1.23896241880216	1.69904497916729	1.90859007231187	0.0651560871792454	0.671074100751351	-4.2127080546452
a_HQ_transcript/118487	1.32043049779806	2.34381890182987	1.9085114769258	0.0651665871912501	0.671074100751351	-4.19864550113675
a_HQ_transcript/98383	2.22883333645584	1.11857209316573	1.90846498136482	0.0651727994894752	0.671074100751351	-4.19825449644595
a_HQ_transcript/53900	1.47347007375749	-0.324126237164389	1.90836353701184	0.0651863553008874	0.671074100751351	-4.27092954888716
a_HQ_transcript/134803	-0.740407252438662	2.55842179339744	-1.90828791650393	0.0651964619050464	0.671074100751351	-4.26055315891912
a_HQ_transcript/42060	-1.16188634974228	-0.447416941742959	-1.90828530386708	0.0651968111054755	0.671074100751351	-4.24311993534364

a_HQ_transcript/70890	2.30590429901183	0.407009556371013	1.90825781740838	0.0652004849948381	0.671074100751351	-4.1462923688043
a_HQ_transcript/13068	1.32439371165296	1.82394306967957	1.90812626819618	0.0652180705721681	0.671074100751351	-4.20496776657628
a_HQ_transcript/14707	-1.71309825585618	0.59125291254313	-1.90810793157036	0.0652205221482676	0.671074100751351	-4.26376410579879
a_HQ_transcript/143668	4.04612334509	3.19810757072404	1.90793418780324	0.0652437553452726	0.671074100751351	-4.34330125859166
a_HQ_transcript/115874	-0.545516106962147	7.7249795492416	-1.90780756539983	0.065260691923295	0.671074100751351	-4.89092580924783
a_HQ_transcript/142973	-1.91086379887797	0.219388412262241	-1.90759409271956	0.0652892538857692	0.671074100751351	-4.26083852509591
a_HQ_transcript/74957	-3.015263605379	-0.275483885016736	-1.90731503527787	0.0653266071455318	0.671074100751351	-4.27318327841903
a_HQ_transcript/43062	1.40205781128107	1.53278679606695	1.9072595087202	0.0653340418572484	0.671074100751351	-4.25476303412642
a_HQ_transcript/136438	2.64273583151391	1.36849879377636	1.90725419752348	0.0653347530365884	0.671074100751351	-4.3178960692242
a_HQ_transcript/152762	-3.53051609514569	-0.294301970460072	-1.90717816366951	0.0653449348465117	0.671074100751351	-4.28320288594014
a_HQ_transcript/75795	1.93819256952568	0.0633637665993006	1.90713090747794	0.0653512636854154	0.671074100751351	-4.15616555400899
a_HQ_transcript/97669	-2.61177043655255	2.98588380459102	-1.9070978122182	0.0653556963203997	0.671074100751351	-4.34528183879198
a_HQ_transcript/8560	-0.756473699341149	2.37524501381583	-1.9067416111264	0.0654034207671434	0.671074100751351	-4.30948327680576
a_HQ_transcript/4508	-1.17567231073381	1.82249674600361	-1.90671410582137	0.0654071072263522	0.671074100751351	-4.24586141159296
a_HQ_transcript/145383	-2.22549439364166	1.92540376541875	-1.90657985454402	0.0654251031231591	0.671074100751351	-4.15773023203819
a_HQ_transcript/112978	-1.57236156960223	0.623744481060647	-1.90653429179551	0.0654312116151804	0.671074100751351	-4.16068402734722
a_HQ_transcript/40350	-0.705247212588718	3.0861502353588	-1.90648548165456	0.0654377560234765	0.671074100751351	-4.44173876257691
a_HQ_transcript/94061	1.79967323362233	-0.163929150208899	1.90643640994163	0.065444336072317	0.671074100751351	-4.25813181237086
a_HQ_transcript/16452	-1.86037129083434	0.120529587499614	-1.9063840514436	0.0654513574771642	0.671074100751351	-4.25747265888779
a_HQ_transcript/93745	1.81319266085138	-0.151646885264961	1.90637995300978	0.0654519071147629	0.671074100751351	-4.18385322165392
a_HQ_transcript/116372	-0.917619728670945	4.60191655969715	-1.90634690645535	0.0654563391065789	0.671074100751351	-4.66004353886712
a_HQ_transcript/125090	0.484949807904967	3.92603092876437	1.90633532611974	0.0654578922481389	0.671074100751351	-4.56488097733858
a_HQ_transcript/78597	2.58704340429558	0.54187029456893	1.90631630085168	0.065460443964673	0.671074100751351	-4.29594245613345
a_HQ_transcript/24339	2.58316238786663	0.288238097520053	1.90629960485626	0.0654626833440352	0.671074100751351	-4.29421756786772
a_HQ_transcript/152569	-4.42369690889477	2.06279649041577	-1.90619823950936	0.065476280565871	0.671074100751351	-4.36858916505556
a_HQ_transcript/39058	-2.98566466728548	1.66086359067245	-1.90608282882238	0.0654917648061975	0.671074100751351	-4.24587630878048
a_HQ_transcript/92209	1.79480122575111	-0.468321143718077	1.90588919220161	0.0655177514376907	0.671074100751351	-4.28551353529313
a_HQ_transcript/109380	2.16430338564889	1.77328945972304	1.90587758696656	0.065519309178514	0.671074100751351	-4.24596863888769
a_HQ_transcript/65189	1.79336299556363	-0.0344933877162737	1.90586420754104	0.0655211051040073	0.671074100751351	-4.17157819859104
a_HQ_transcript/19908	-3.02847805392414	2.48218141863203	-1.90584080587867	0.0655242464203512	0.671074100751351	-4.45233545013467
a_HQ_transcript/151030	3.24770751079724	-0.382542019198185	1.90575420040508	0.0655358730147832	0.671074100751351	-4.26298227161343
a_HQ_transcript/76034	2.74517591619657	1.59669317890435	1.90567996662856	0.0655458401525418	0.671074100751351	-4.20317933856977
a_HQ_transcript/372	-0.838547280258652	4.1326347118506	-1.90554262503412	0.0655642840280405	0.671074100751351	-4.63047889672894
a_HQ_transcript/143277	-1.78864669825683	0.137368579616224	-1.90550517678671	0.0655693138042847	0.671074100751351	-4.21559809238324
a_HQ_transcript/104367	-1.87354175318278	2.00642429511413	-1.90538857539492	0.0655849769869939	0.671074100751351	-4.31895469676178
a_HQ_transcript/87235	2.44386880836927	-0.0515267328568894	1.9049351062527	0.065645922642876	0.671074100751351	-4.24132500997512
a_HQ_transcript/78537	2.4929347881211	1.24517151256804	1.90493487459621	0.0656459537896828	0.671074100751351	-4.20916086204616
a_HQ_transcript/2587	-3.74480345857752	0.422916879632008	-1.90477329204634	0.0656676820822424	0.671074100751351	-4.23124229728485
a_HQ_transcript/109339	5.85912938234167	2.34776720587179	1.90475293319178	0.0656704202135778	0.671074100751351	-4.27547227107119
a_HQ_transcript/48314	2.47098502381839	0.169683875500521	1.90443979515902	0.0657125476161636	0.671074100751351	-4.26255605042755
a_HQ_transcript/129085	-2.23498073365892	0.363434933897824	-1.90438612324888	0.0657197705993569	0.671074100751351	-4.34432738074022
a_HQ_transcript/113316	-3.73456378896865	1.3232872088336	-1.90435901445184	0.0657234190693693	0.671074100751351	-4.30019117360542
a_HQ_transcript/4920	2.18184351174138	0.721442672769088	1.90420505337249	0.065744143419858	0.671074100751351	-4.15780481476014
a_HQ_transcript/76430	2.7069184578627	0.638167430774765	1.90408921230181	0.0657597402382636	0.671074100751351	-4.2096269358341
a_HQ_transcript/42468	-2.64579856545618	1.03752444512714	-1.90400271966442	0.0657713876703224	0.671074100751351	-4.13921433291945
a_HQ_transcript/147397	-2.50062295535232	3.13063134398888	-1.90381799946575	0.0657962687622815	0.671074100751351	-4.56419212933492
a_HQ_transcript/68775	2.31753808136988	0.485045517436016	1.90380048816169	0.0657986278879897	0.671074100751351	-4.18204984583781
a_HQ_transcript/134180	2.05494621277361	0.283452749081214	1.90373611957593	0.0658073002611726	0.671074100751351	-4.25925910828687
a_HQ_transcript/140269	1.54725589679636	0.0958161137509144	1.90353616698035	0.0658342461420885	0.671074100751351	-4.13440264559184
a_HQ_transcript/121448	-2.38428766399417	0.179106503731198	-1.90348106291677	0.0658416737121226	0.671074100751351	-4.24641972778333

a_HQ_transcript/22949	1.97444522617461	1.80093784608874	1.90340548050734	0.0658518627685023	0.671074100751351	-4.23640327433286
a_HQ_transcript/131048	1.89780440766582	0.0606357654866739	1.90328885515541	0.0658675873787198	0.671074100751351	-4.26465638680123
a_HQ_transcript/87279	-2.07565164385366	0.890446207697108	-1.90322854107886	0.0658757208031545	0.671074100751351	-4.22115556258431
a_HQ_transcript/107464	-2.19828995000615	-0.345176316592838	-1.90314107467265	0.0658875172900037	0.671074100751351	-4.24350032106771
a_HQ_transcript/21254	0.674392503672947	3.40745162471221	1.90313474258982	0.065888371360951	0.671074100751351	-4.50709415903363
a_HQ_transcript/84946	-2.74379699346274	-0.0827424040570974	-1.9030929124796	0.0658940136435288	0.671074100751351	-4.22877539089493
a_HQ_transcript/81605	1.68048706271986	3.2695868796738	1.90306756213449	0.06589743324466	0.671074100751351	-4.36950444212429
a_HQ_transcript/88615	1.27294500785359	2.90585516053538	1.90298087408818	0.0659091280699607	0.671074100751351	-4.37244313705929
a_HQ_transcript/134748	2.19423589051437	-0.327274953817512	1.90297264730843	0.0659102380132144	0.671074100751351	-4.24423375356745
a_HQ_transcript/62625	-2.38381560044824	0.0906253604836256	-1.90291711442977	0.0659177308372132	0.671074100751351	-4.22975923217757
a_HQ_transcript/14456	1.05829616502135	1.65663525914288	1.90287475231993	0.0659234470771542	0.671074100751351	-4.1740471281413
a_HQ_transcript/24790	1.70056995218822	-0.343926344720828	1.90276436674369	0.0659383442461533	0.671074100751351	-4.28758799315703
a_HQ_transcript/33293	-0.69285827596336	2.0340763897392	-1.90274856994286	0.065940476352784	0.671074100751351	-4.2133904957506
a_HQ_transcript/112702	-2.20865069406817	0.710566104435168	-1.9027380553492	0.0659418955487125	0.671074100751351	-4.17885792849556
a_HQ_transcript/107518	-0.800133543147677	3.77729979878912	-1.9027072885609	0.0659460484140427	0.671074100751351	-4.5259608183546
a_HQ_transcript/116901	3.1491564139137	4.09625116349415	1.90260278711145	0.0659601555821877	0.671074100751351	-4.5213197595528
a_HQ_transcript/11102	-1.72143357578338	1.16232242487032	-1.9025763767079	0.0659637212652486	0.671074100751351	-4.1997100887786
a_HQ_transcript/99913	-1.87356795916848	1.74017077388772	-1.90257606838405	0.0659637628932022	0.671074100751351	-4.35347775942718
a_HQ_transcript/90403	-2.41658277616462	0.0754214099114051	-1.90255452089687	0.0659666721559613	0.671074100751351	-4.3579081035318
a_HQ_transcript/64609	1.28112938264353	-0.319968351333995	1.90255366815942	0.0659667872917221	0.671074100751351	-4.32729447178232
a_HQ_transcript/97752	-2.65947536949516	1.14581086876328	-1.90237806064517	0.0659905013358619	0.671197876054414	-4.24548091214647
a_HQ_transcript/121308	1.3608342188956	-0.704509909952916	1.90219932586875	0.0660146452333704	0.671209174982388	-4.21766995712555
a_HQ_transcript/106700	2.66540417293148	0.802650419462554	1.90219887316128	0.0660147063957871	0.671209174982388	-4.19539408387104
a_HQ_transcript/92994	-0.978332257159929	2.30049846610873	-1.90187498450943	0.0660584774487657	0.671536757107704	-4.19559767521717
a_HQ_transcript/55681	1.36734346837267	-0.508634700811385	1.90168909895192	0.0660836097480041	0.671654773615506	-4.21746629674321
a_HQ_transcript/144555	-2.2479891997471	-0.472032364655782	-1.90148883736502	0.0661106949551366	0.671654773615506	-4.24762323396315
a_HQ_transcript/149970	-0.598651782444261	3.88834838754876	-1.90146763722029	0.0661135628166061	0.671654773615506	-4.55835404243915
a_HQ_transcript/140729	1.73661221493101	1.36544286892751	1.90131660568215	0.0661339967966608	0.671654773615506	-4.16213097960096
a_HQ_transcript/48844	-1.09388779352778	3.89900712499527	-1.90130587437345	0.0661354489081779	0.671654773615506	-4.58917114667751
a_HQ_transcript/57936	1.29871609654471	0.277340640640707	1.90123115964333	0.0661455597250218	0.671654773615506	-4.14051152602612
a_HQ_transcript/44596	-1.79859193332134	0.214143531861773	-1.90110181953267	0.0661630659053775	0.671654773615506	-4.18605220463402
a_HQ_transcript/32302	2.36027072877021	0.546771730778201	1.90095172893916	0.0661833856698428	0.671654773615506	-4.15536132003575
a_HQ_transcript/142529	2.35868583812105	-0.239363159845521	1.90084581861018	0.0661977274002975	0.671654773615506	-4.27854041594396
a_HQ_transcript/103528	-2.51719952511955	4.10257564966309	-1.90084237289171	0.0661981940434632	0.671654773615506	-4.68835751271412
a_HQ_transcript/51120	1.18936711608325	0.769518997383505	1.90078710771183	0.0662056788260701	0.671654773615506	-4.28451958783297
a_HQ_transcript/108683	-2.14128056520461	-0.105560211668779	-1.90076447936633	0.0662087436831965	0.671654773615506	-4.25475331338482
a_HQ_transcript/11630	2.57750832464759	2.68720688951155	1.90065802999252	0.066223163175673	0.6716838298858	-4.40406282416933
a_HQ_transcript/38373	2.9996764302832	0.50729201259339	1.90055556148609	0.0662370459822254	0.671707433505722	-4.2290476005165
a_HQ_transcript/104777	-1.40868136728957	1.85237677000342	-1.9001350640768	0.0662940427785131	0.671803004219677	-4.20122126747783
a_HQ_transcript/47909	2.16278289858533	0.24643259309574	1.9000738104185	0.066302349001608	0.671803004219677	-4.19351747746262
a_HQ_transcript/123116	0.711292771911723	4.40038287009318	1.90003038937792	0.0663082375989826	0.671803004219677	-4.62689199343331
a_HQ_transcript/151965	-2.84269444351012	0.617571156998799	-1.89992174366413	0.0663229736951853	0.671803004219677	-4.26511665193009
a_HQ_transcript/120697	-2.27640265773265	0.787414718257725	-1.8998837650966	0.0663281255612151	0.671803004219677	-4.32884260166497
a_HQ_transcript/113730	3.13313037473743	2.17339719122656	1.89987884949903	0.0663287923967304	0.671803004219677	-4.52494543045715
a_HQ_transcript/101780	-1.05539105515832	4.51921523483234	-1.89985802130857	0.0663316179519355	0.671803004219677	-4.64985356549091
a_HQ_transcript/98390	-3.1494847721006	2.60102040738343	-1.89980413509895	0.0663389286440433	0.671803004219677	-4.37833351946985
a_HQ_transcript/116684	0.644843403281992	2.9632713682327	1.8997140985437	0.0663511453697174	0.671809680900703	-4.40186051322624
a_HQ_transcript/95861	1.78823729241609	1.52066695982036	1.89959211894972	0.0663676994208057	0.671811162211492	-4.23810673216412
a_HQ_transcript/139407	4.15396947031042	1.99702278813666	1.89938446051061	0.0663958892774263	0.671811162211492	-4.38558929925644
a_HQ_transcript/109717	-3.63118201690768	1.66020339741961	-1.8993660075282	0.0663983947888621	0.671811162211492	-4.30140182429139

a_HQ_transcript/57452	-1.8580840609073	0.682377431637706	-1.8993131042798	0.0664055783446895	0.671811162211492	-4.18644255950925
a_HQ_transcript/60045	1.59380755831803	1.08998156899078	1.89921751033384	0.0664185604268513	0.671811162211492	-4.17364246083883
a_HQ_transcript/90858	-2.63278454550046	0.994808787387487	-1.89920222577212	0.0664206363409965	0.671811162211492	-4.27084424867355
a_HQ_transcript/37859	1.92160259867117	0.781101129973857	1.89910258002632	0.0664341713685515	0.671831160979673	-4.13222174358607
a_HQ_transcript/111462	3.40364539512431	0.515844798852794	1.89898592239114	0.0664500201664341	0.671874547266391	-4.15673712527489
a_HQ_transcript/126931	0.602216295338213	3.70218622937176	1.898742461281	0.0664831066461526	0.672092177935227	-4.58330949542742
a_HQ_transcript/39344	1.32676842936433	3.33918697704434	1.89863193550016	0.0664981318371424	0.672127179173954	-4.46953901978837
a_HQ_transcript/99397	1.76972773599321	0.913899565159702	1.89846869276649	0.0665203288745998	0.672234644788376	-4.18745021037993
a_HQ_transcript/59788	-2.11356425817567	0.362932180746212	-1.8981665172165	0.0665614342424349	0.672533121787633	-4.18472914131664
a_HQ_transcript/134525	1.86879683109085	-0.181353311551246	1.89791219930853	0.0665960464291746	0.672765899693267	-4.23247499586402
a_HQ_transcript/139706	-0.684072965779245	2.23125877058614	-1.89769410476174	0.066625741037227	0.672948927022056	-4.26588789972037
a_HQ_transcript/150446	1.9105153272006	-0.707335285795767	1.89752312013176	0.066649029384582	0.673017795783381	-4.274504289617
a_HQ_transcript/69262	-2.24911101246725	1.16028300218761	-1.89747403723677	0.0666557158396113	0.673017795783381	-4.14119906195893
a_HQ_transcript/151027	3.07560703136995	4.3605088463798	1.89721392000543	0.0666911606882491	0.6731797172409	-4.51563058420564
a_HQ_transcript/141006	-2.95264536848187	1.32373535624253	-1.89716589209967	0.0666977069818855	0.6731797172409	-4.30587694663057
a_HQ_transcript/1943	2.06646398264092	1.82393399972389	1.89710142003857	0.0667064955152007	0.6731797172409	-4.25806787047004
a_HQ_transcript/116783	-1.75484002979482	3.34765276491251	-1.89696563821804	0.0667250079288627	0.67324965472816	-4.45959197393392
a_HQ_transcript/131521	-1.00601329797951	6.00659872343087	-1.89683286546342	0.0667431143686107	0.673315472408643	-4.83052240574484
a_HQ_transcript/50678	-2.3274044372065	1.28333368102803	-1.89647990776051	0.0667912683795494	0.673596875965781	-4.36281596023279
a_HQ_transcript/42104	0.97471768795425	2.0544001846341	1.89643265980996	0.0667977166873729	0.673596875965781	-4.17669590372422
a_HQ_transcript/131262	2.12520711722442	0.998520508176046	1.89637363308808	0.0668057732924362	0.673596875965781	-4.2174300180703
a_HQ_transcript/83026	2.66460717637495	2.26811317508045	1.89626891432338	0.0668200685015148	0.67362416612141	-4.26101822327724
a_HQ_transcript/9025	1.8856179327919	0.490140431580227	1.89592299642353	0.0668673086531727	0.673983512639435	-4.23594566526014
a_HQ_transcript/28696	2.64080531362424	2.48718264225684	1.89573989275721	0.0668923257820219	0.67408720938037	-4.35943580272233
a_HQ_transcript/107285	-1.11951120529638	2.05004338847223	-1.89542109147266	0.0669359022761918	0.67408720938037	-4.30184266061995
a_HQ_transcript/85180	-2.38046842824001	1.57832817833722	-1.89540580722023	0.0669379920730776	0.67408720938037	-4.26013865511267
a_HQ_transcript/23851	1.7359642975047	-0.0463659101597047	1.89524490970759	0.0669599948007332	0.67408720938037	-4.18848174474002
a_HQ_transcript/38124	3.22857606809476	1.58730912056559	1.8952025091831	0.0669657941076624	0.67408720938037	-4.15785078593206
a_HQ_transcript/139204	1.39354105003307	2.55871937016849	1.89517211744603	0.0669699511859143	0.67408720938037	-4.33005183528276
a_HQ_transcript/53215	0.579736132036012	3.610995912602	1.89517149034421	0.066970036965232	0.67408720938037	-4.44200001819758
a_HQ_transcript/87533	-2.48637603970518	1.70266503043342	-1.89515250827919	0.0669726335079707	0.67408720938037	-4.25719464211315
a_HQ_transcript/126656	2.13692760085439	-0.264974916551328	1.89508251888851	0.0669822080546943	0.67408720938037	-4.26547592771226
a_HQ_transcript/148842	1.43298818990236	-0.666534176114775	1.89499952875502	0.0669935626305807	0.67408720938037	-4.27673802587095
a_HQ_transcript/143891	-1.89700878837257	1.5534977496834	-1.89485451459557	0.067013407213093	0.674170185961002	-4.31964629991137
a_HQ_transcript/4658	-2.47093663957496	1.0382412237039	-1.89472224097514	0.0670315127213869	0.674235641368537	-4.19402458238759
a_HQ_transcript/137167	-1.82642130499382	-0.571634755349373	-1.89457776864272	0.0670512927937628	0.674317914795128	-4.25708574032551
a_HQ_transcript/42395	-1.22943474180773	3.62774093567343	-1.89441767361452	0.0670732176809907	0.674421725888363	-4.46143811152145
a_HQ_transcript/121838	-1.09965654602041	1.82049013160003	-1.89421952585621	0.0671003624099871	0.674574305734772	-4.2629843884991
a_HQ_transcript/75469	-2.0158279759832	1.12665545834694	-1.89410785057821	0.0671156652384337	0.674574305734772	-4.1953731497809
a_HQ_transcript/48923	-0.56427677477515	3.28601596884893	-1.89405282022889	0.0671232071354583	0.674574305734772	-4.50824509970301
a_HQ_transcript/90890	1.70186037489059	-0.452757452910146	1.89379765180164	0.0671581874508988	0.67477287913708	-4.21872966711962
a_HQ_transcript/149199	1.9906460869578	0.204377041004466	1.89373934415401	0.067166182884103	0.67477287913708	-4.30569836818385
a_HQ_transcript/131442	-2.54428821391606	1.13274770873863	-1.89335736667175	0.0672185818058881	0.674855301794423	-4.30206007655233
a_HQ_transcript/125235	2.2117026232399	1.03956544918347	1.89326806636027	0.0672308369232966	0.674855301794423	-4.26608847993271
a_HQ_transcript/19142	0.873913950427789	2.29778800686085	1.89325326834941	0.0672328679123525	0.674855301794423	-4.36732832086762
a_HQ_transcript/91503	-0.812250247948951	2.89774333798709	-1.89314054406438	0.0672483407657751	0.674855301794423	-4.40208762773124
a_HQ_transcript/145877	4.85689087929954	1.53923071286613	1.8931015562087	0.0672536930625199	0.674855301794423	-4.31791760159791
a_HQ_transcript/113318	3.09925887790354	0.426505596705612	1.89307147614418	0.0672578227383495	0.674855301794423	-4.32289254229267
a_HQ_transcript/23696	-2.03853859511804	2.44697001140233	-1.89303869839082	0.067262323027047	0.674855301794423	-4.26582145668428
a_HQ_transcript/148744	1.33812586767593	-0.727629427237804	1.8930027009697	0.0672672656653744	0.674855301794423	-4.28404985366331

a_HQ_transcript/150442	0.39876098586805	8.02072652064514	1.89271598592042	0.0673066443416142	0.675133843363132	-4.98852657859432
a_HQ_transcript/134316	1.59743987211257	-0.714440618837706	1.89242125930639	0.067347144049257	0.675423531624432	-4.27922560990938
a_HQ_transcript/139344	-0.748714867493206	2.60031005587197	-1.89203563148435	0.0674001665883496	0.675439210458564	-4.35660749698231
a_HQ_transcript/78286	-2.27464062461521	0.299114116275726	-1.89202683884978	0.0674013759652629	0.675439210458564	-4.16412041176902
a_HQ_transcript/119125	-2.62402320628812	0.756387883848967	-1.891774887429	0.067436038390584	0.675439210458564	-4.35980397347273
a_HQ_transcript/28663	-1.27998491426871	-0.09792258254897	-1.89174015323661	0.0674408181794329	0.675439210458564	-4.22369958640097
a_HQ_transcript/69553	2.01884732575864	-0.242310908210799	1.89154677400708	0.0674674345207067	0.675439210458564	-4.30944146248893
a_HQ_transcript/46679	0.825736041571436	3.63992968054932	1.89154038993034	0.0674683133668116	0.675439210458564	-4.55344178542713
a_HQ_transcript/147156	1.44654137058474	1.14754818473534	1.89144620881939	0.0674812796918595	0.675439210458564	-4.15562066663375
a_HQ_transcript/100080	3.43522713636994	2.87886677020747	1.89132809467812	0.0674975440141734	0.675439210458564	-4.35007219834189
a_HQ_transcript/37688	2.59358908708651	0.0447425000942337	1.89121875322027	0.0675126033456848	0.675439210458564	-4.25326836127674
a_HQ_transcript/103935	-3.09235155479809	0.355601378237819	-1.89107472485422	0.0675324444347183	0.675439210458564	-4.27431918329774
a_HQ_transcript/113515	1.85402518351703	-0.164612980675344	1.89090103513749	0.0675563782950966	0.675439210458564	-4.19983875838121
a_HQ_transcript/45562	-0.942837431099393	1.85404644394441	-1.89084344445086	0.0675643157103974	0.675439210458564	-4.23274808852145
a_HQ_transcript/60314	-1.41992948364927	0.137906351513572	-1.89083467142119	0.0675655249204632	0.675439210458564	-4.17256325945875
a_HQ_transcript/134556	2.16382369824026	0.327159966700417	1.89078581828368	0.0675722588193664	0.675439210458564	-4.27410034339298
a_HQ_transcript/129290	-2.78026661632186	3.44015865805062	-1.89076724159696	0.067574819574796	0.675439210458564	-4.51185439439578
a_HQ_transcript/45122	1.10014340134946	-0.279403725474585	1.89068176233122	0.0675866037803281	0.675439210458564	-4.20802933132261
a_HQ_transcript/94473	-1.88286164393411	2.05974886544277	-1.8905747275048	0.0676013621384724	0.675439210458564	-4.43135197816143
a_HQ_transcript/9419	-1.17027461111801	2.21536166125474	-1.890560968188	0.0676032595252348	0.675439210458564	-4.24929157221694
a_HQ_transcript/64141	3.07025636318271	1.43289927893944	1.89056035036061	0.0676033447236736	0.675439210458564	-4.24735040275036
a_HQ_transcript/15820	1.56916486825821	-0.689604430270695	1.89052119776948	0.0676087440571537	0.675439210458564	-4.28624817599041
a_HQ_transcript/50294	-1.47703094885911	2.117902280804	-1.89049574916975	0.0676122537423682	0.675439210458564	-4.30885453836057
a_HQ_transcript/141390	-5.19312156625889	1.35968066411587	-1.8904745454719	0.0676151781214601	0.675439210458564	-4.26023635601039
a_HQ_transcript/76522	2.28051316882677	2.07336063198121	1.89042581418994	0.0676218994723034	0.675439210458564	-4.39090036005407
a_HQ_transcript/118618	3.13106885604833	1.34426954733133	1.89038460309567	0.0676275840364169	0.675439210458564	-4.40120346590254
a_HQ_transcript/131287	-1.51531645273632	-0.664563356446184	-1.89016932434988	0.0676572857818145	0.675547399265677	-4.2743295372925
a_HQ_transcript/6223	2.195686201186	0.636430937694183	1.89013762855618	0.0676616597599224	0.675547399265677	-4.15764021403678
a_HQ_transcript/52859	2.55064386909791	1.50001682727023	1.88967237951357	0.0677258915337892	0.675981320753874	-4.2745416344874
a_HQ_transcript/76865	2.53497483648873	1.16318975140537	1.88948515380752	0.0677517545161199	0.675981320753874	-4.29302158309829
a_HQ_transcript/99687	1.32963173081144	-0.699702585696396	1.88947725504964	0.0677528458214982	0.675981320753874	-4.28986438447946
a_HQ_transcript/39879	-0.870866911425897	3.57308107776404	-1.88942401864975	0.0677602014446877	0.675981320753874	-4.53153033050513
a_HQ_transcript/130134	1.50269985627552	-0.223203700454703	1.88937393881297	0.0677671215559222	0.675981320753874	-4.30040557834207
a_HQ_transcript/51369	-1.47625501679539	1.37108467994942	-1.88931768370162	0.0677748957017888	0.675981320753874	-4.23179120756486
a_HQ_transcript/29208	1.48545420411125	5.2306703070263	1.88918463605795	0.0677932852063106	0.676048736056343	-4.70000954403828
a_HQ_transcript/31102	-2.02924340725828	-0.48260181364275	-1.8889363132594	0.0678276192352173	0.67618767030754	-4.27135087888269
a_HQ_transcript/6768	1.24879005697557	1.04764923962603	1.88891560708403	0.0678304828234803	0.67618767030754	-4.20214124938533
a_HQ_transcript/149772	2.29881792131664	0.293051140878907	1.88872047450803	0.0678574740554011	0.676245924818538	-4.28288003965742
a_HQ_transcript/128680	1.75524639620834	-0.478277113602071	1.88859249770425	0.0678751811495194	0.676245924818538	-4.30222643064565
a_HQ_transcript/78842	2.6098312861926	0.74915512498503	1.88854578207908	0.0678816457956498	0.676245924818538	-4.33569165555047
a_HQ_transcript/74473	-0.740532241301722	3.41093473872452	-1.88853699767683	0.067882861466353	0.676245924818538	-4.55081549557672
a_HQ_transcript/141305	1.65419283275666	-0.492221911609919	1.88815568613896	0.0679356491130033	0.676608031605103	-4.26766917493077
a_HQ_transcript/65003	2.1654728551711	0.526319929542294	1.88810628695655	0.0679424903743288	0.676608031605103	-4.32598662911074
a_HQ_transcript/40181	0.473900412345997	3.29929453857557	1.88800697343836	0.0679562460343036	0.676629097204865	-4.52010384946292
a_HQ_transcript/40769	2.11938680587907	-0.173006412540631	1.88785321810634	0.0679775470171372	0.676631583890089	-4.28231888564214
a_HQ_transcript/93647	-3.45511193845367	1.07800217657418	-1.88772168404591	0.0679957740653046	0.676631583890089	-4.29947288967644
a_HQ_transcript/44940	-2.17833083954255	1.60007520931022	-1.88769214934924	0.0679998673501872	0.676631583890089	-4.20260416587592
a_HQ_transcript/98937	1.59573208279765	4.15147025600199	1.8876591908856	0.0680044353935271	0.676631583890089	-4.69505597750792
a_HQ_transcript/17447	-1.75121710242428	1.0895487801625	-1.88758515562308	0.068014697644333	0.676631583890089	-4.22747517785515
a_HQ_transcript/45	-0.619998772036036	3.44154005668649	-1.88740671497502	0.0680394373167398	0.676761877540631	-4.47881451649966

a_HQ_transcript/151645	5.5321134838714	6.17718256842215	1.88731924256284	0.0680515676438982	0.676766727802312	-4.53218671495262
a_HQ_transcript/22874	-1.69147267956391	-0.485833105759715	-1.88713768862941	0.0680767507626681	0.676894071212775	-4.26665445534548
a_HQ_transcript/117339	-0.596391387171383	3.6201476172473	-1.88702574243277	0.0680922826755114	0.676894071212775	-4.54603026495494
a_HQ_transcript/151478	-4.07159161154872	2.99649789988099	-1.88697512047529	0.0680993071919266	0.676894071212775	-4.39861838844413
a_HQ_transcript/126692	5.18871548021816	1.25856655374676	1.88667717185959	0.0681406644348311	0.676945197194985	-4.31435598060306
a_HQ_transcript/67641	-2.02041432094629	0.799243221020422	-1.88664368870947	0.0681453134684306	0.676945197194985	-4.21402254941721
a_HQ_transcript/36453	2.54577203812336	0.858071063656352	1.88650884176824	0.0681640393189874	0.676945197194985	-4.18559328268069
a_HQ_transcript/141782	-1.99505727900415	-0.113746555864611	-1.88645557394712	0.0681714377050828	0.676945197194985	-4.27815377082021
a_HQ_transcript/278	2.42901805481959	1.69245626571382	1.88644893727169	0.0681723595236271	0.676945197194985	-4.23284668985608
a_HQ_transcript/125384	1.82193166768894	0.569954422503625	1.88638045170294	0.0681818726337171	0.676945197194985	-4.26356742361297
a_HQ_transcript/17330	-1.96186339988929	3.74468654584358	-1.88634766438458	0.0681864274189174	0.676945197194985	-4.54462214644411
a_HQ_transcript/99489	2.12821203554743	2.56255594137132	1.88619412714046	0.0682077601600777	0.676945197194985	-4.32171485001151
a_HQ_transcript/136559	1.46677963817294	-0.550890010926551	1.88611069550684	0.0682193547090718	0.676945197194985	-4.18870102470527
a_HQ_transcript/81946	2.59843802485694	2.72386047440306	1.88607778717015	0.0682239284685453	0.676945197194985	-4.43093121602677
a_HQ_transcript/99253	-1.89953009630477	2.04143699324438	-1.88593676776412	0.0682435310137757	0.676945197194985	-4.22659315733357
a_HQ_transcript/145000	-4.7003934087659	2.22121204479135	-1.8859319559888	0.0682441999649499	0.676945197194985	-4.42218806782935
a_HQ_transcript/55104	-1.30085346592538	1.3059780088601	-1.88576136041767	0.0682679204510696	0.677028392856848	-4.13753918468758
a_HQ_transcript/28307	3.13720956733534	1.55331973072912	1.8857041168212	0.0682758814844282	0.677028392856848	-4.28956937429556
a_HQ_transcript/138130	-1.60524839818347	-0.489409059511013	-1.88553397287433	0.0682995486124794	0.677104803466982	-4.23066351993713
a_HQ_transcript/3128	2.67927164906975	2.22489919556306	1.88542516736125	0.06831468722582	0.677104803466982	-4.32600046178862
a_HQ_transcript/91248	2.34260712109236	0.321550259369805	1.88537992732504	0.0683209825304375	0.677104803466982	-4.27735747204387
a_HQ_transcript/12980	-1.57903083263183	1.01827576930241	-1.88531382870695	0.0683301812747268	0.677104803466982	-4.24651435253461
a_HQ_transcript/150	-3.10990370832665	0.852153560535154	-1.88507656071618	0.0683632099181747	0.677253255184616	-4.36725469128721
a_HQ_transcript/150457	-2.29641744079983	0.0957420844224082	-1.88494732552783	0.0683812057443005	0.677253255184616	-4.32864494314228
a_HQ_transcript/70586	1.39614413738049	-0.556578733101356	1.88479294634638	0.0684027081785376	0.677253255184616	-4.32536865531589
a_HQ_transcript/68880	-2.37673160082913	1.7937824690087	-1.88472429748037	0.0684122716836718	0.677253255184616	-4.28677432764063
a_HQ_transcript/148807	3.06193959513209	-0.0226419490809533	1.88454918055294	0.0684366725123221	0.677253255184616	-4.33474895781428
a_HQ_transcript/139325	3.50485760863824	0.212655449012124	1.88452953060154	0.0684394110076419	0.677253255184616	-4.29241992586467
a_HQ_transcript/139410	-2.99547145794816	0.459435776122367	-1.88451358474172	0.0684416333553145	0.677253255184616	-4.34958793717495
a_HQ_transcript/139000	-2.5169629595581	-0.287176290811828	-1.88445715591134	0.0684494982449975	0.677253255184616	-4.28789798040912
a_HQ_transcript/118690	-3.44950058588639	0.240005456371613	-1.88445339885254	0.0684500219207545	0.677253255184616	-4.28877033096126
a_HQ_transcript/34292	-5.82339630590242	3.13604226451909	-1.8840435825598	0.0685071646508535	0.677703278901432	-4.39770755606096
a_HQ_transcript/132548	1.2248281220929	2.24024488348314	1.88386246266791	0.0685324321709064	0.677766529832164	-4.34181312936889
a_HQ_transcript/141503	2.56790700922398	-0.177064141080595	1.88383059828509	0.068536878309136	0.677766529832164	-4.24531705109855
a_HQ_transcript/75394	1.31037493256526	1.16600462144682	1.88368518022175	0.0685571721014615	0.677851896566381	-4.17443067408218
a_HQ_transcript/98756	-1.04178533790419	-0.638095651479996	-1.88352895335242	0.0685789800728214	0.677952203005606	-4.293739980369
a_HQ_transcript/101498	1.46562362786831	-0.575864248891861	1.88335469627881	0.0686033119487685	0.678077421689851	-4.33395783464627
a_HQ_transcript/100412	-2.21425831133856	1.74211569918782	-1.88282854695771	0.0686768243219411	0.678637806536201	-4.32286274207129
a_HQ_transcript/150260	5.61018889262702	0.279236202577854	1.88274127215603	0.0686890247040469	0.678637806536201	-4.32178353788294
a_HQ_transcript/7766	0.601982166272764	2.8636661970857	1.88268920187221	0.0686963046384075	0.678637806536201	-4.43045539675228
a_HQ_transcript/117040	2.45950976168698	0.228788009484187	1.88246715842999	0.0687273559279935	0.678637806536201	-4.32838229362865
a_HQ_transcript/83660	-1.78715669367654	1.73510992507107	-1.88242111465635	0.0687337963521738	0.678637806536201	-4.22933136387469
a_HQ_transcript/145601	-2.98023002767028	1.05591221398892	-1.88233048586642	0.0687464746712299	0.678637806536201	-4.20928890397302
a_HQ_transcript/19499	-1.75918092218871	-0.372550490919853	-1.88227474548888	0.0687542733497419	0.678637806536201	-4.19284433632894
a_HQ_transcript/23532	-2.52313991226301	0.994086684959779	-1.88215434542847	0.0687711212096786	0.678637806536201	-4.34687973914809
a_HQ_transcript/129249	2.12437832950411	0.0476401104025612	1.88200473906787	0.0687920609207019	0.678637806536201	-4.2378058100177
a_HQ_transcript/138655	1.29185117682583	4.13063595122543	1.88192728946434	0.0688029033372346	0.678637806536201	-4.48843591419267
a_HQ_transcript/12813	1.76631852092402	0.180537590769815	1.88190881545027	0.0688054897902933	0.678637806536201	-4.18502468887184
a_HQ_transcript/18493	1.40861489947338	-0.144628292090834	1.88188805087763	0.0688083970326323	0.678637806536201	-4.25683553075904
a_HQ_transcript/139029	1.96625069668756	1.34353117773185	1.88180361219669	0.0688202203580946	0.678637806536201	-4.31905257025538

a_HQ_transcript/91409	3.45224256484757	1.51039693254723	1.88178050402222	0.0688234563296674	0.678637806536201	-4.36364436657231
a_HQ_transcript/63538	1.40719880850408	0.587424808885737	1.88157632497804	0.0688520543893977	0.678719867269101	-4.26695266772156
a_HQ_transcript/85889	1.82235891469955	-0.608120488617515	1.88144918133776	0.0688698677515321	0.678719867269101	-4.29340957638563
a_HQ_transcript/8644	-2.07915145846857	0.388276796078713	-1.88143424758953	0.0688719602928672	0.678719867269101	-4.24843191035575
a_HQ_transcript/91447	-0.858185846830487	3.51046140390337	-1.8813850304994	0.0688788570593242	0.678719867269101	-4.43837680783532
a_HQ_transcript/48062	-2.12041848569704	-0.217650659493161	-1.88128826401487	0.0688924186301847	0.678719867269101	-4.25895991970699
a_HQ_transcript/34404	1.9928399205533	0.238542933500199	1.88122107578072	0.0689018362364947	0.678719867269101	-4.19815320946497
a_HQ_transcript/35551	1.19017593987692	0.3831575830765	1.88112340861268	0.0689155279722923	0.678739717040564	-4.19617783254441
a_HQ_transcript/137177	2.83972596968221	1.2579135921594	1.88083098580864	0.0689565360393804	0.679028549364239	-4.35306629945437
a_HQ_transcript/128264	2.42539489091522	0.328481823776095	1.88052571139939	0.0689993687367128	0.67912858161781	-4.32567068846723
a_HQ_transcript/41974	-2.34870533846461	1.22245016617759	-1.88043370264126	0.0690122828631419	0.67912858161781	-4.32086753819801
a_HQ_transcript/61369	-1.65497423906534	0.0612372711838409	-1.88042823979186	0.0690130496807906	0.67912858161781	-4.23580442776834
a_HQ_transcript/1215	-2.44997802187877	1.79853917173315	-1.88034838384956	0.069024259858635	0.67912858161781	-4.28196377104761
a_HQ_transcript/146060	3.72358918997702	-0.0246898457911692	1.88030240939375	0.0690307144630795	0.67912858161781	-4.28191779329215
a_HQ_transcript/137143	2.10055057961135	-0.65334737854649	1.88024567326862	0.0690386806721558	0.67912858161781	-4.30199018154833
a_HQ_transcript/120766	2.04346133561143	2.05740429299484	1.88005992957976	0.0690647661110713	0.67912858161781	-4.18590178296823
a_HQ_transcript/124563	4.14633300406203	1.17017150150023	1.87996211654633	0.0690785061653715	0.67912858161781	-4.35966171821512
a_HQ_transcript/64929	1.37928861343122	-0.647998315118224	1.87995220230362	0.0690798989762814	0.67912858161781	-4.29745857655335
a_HQ_transcript/60319	-1.47405975457038	0.182532622269093	-1.87990565092765	0.0690864391091169	0.67912858161781	-4.30451497023491
a_HQ_transcript/868	1.37549172510575	-0.773108731430832	1.87984321946702	0.0690952111149142	0.67912858161781	-4.30490372224509
a_HQ_transcript/138658	-3.63600530460878	1.72126725553959	-1.87948419162264	0.0691456753295361	0.679407597506408	-4.32850861619919
a_HQ_transcript/125587	2.04236485763655	0.24044113090578	1.87938670764187	0.0691593829443795	0.679407597506408	-4.19208006792437
a_HQ_transcript/33266	2.08590351904857	0.752081329298419	1.87936390320442	0.0691625899051684	0.679407597506408	-4.21191505783709
a_HQ_transcript/94491	1.65898079019917	1.21076791366271	1.87930871855913	0.0691703509847737	0.679407597506408	-4.28350828222798
a_HQ_transcript/46086	-0.577608876462047	2.98688011533116	-1.87909624018857	0.0692002405902782	0.679457915111081	-4.48866343278146
a_HQ_transcript/132836	2.45844362900115	-0.208148448433514	1.87903972717764	0.0692081922167386	0.679457915111081	-4.34044399750609
a_HQ_transcript/89113	0.549813203291174	4.14879524664587	1.87897488384842	0.0692173169226378	0.679457915111081	-4.64152513002145
a_HQ_transcript/65827	1.94959083739815	0.330361587834896	1.87893997434248	0.0692222297909411	0.679457915111081	-4.30073407287332
a_HQ_transcript/106463	1.55192085850311	4.06261563378054	1.8786579323975	0.0692619329788468	0.679490320566168	-4.49343482183207
a_HQ_transcript/37424	2.06982984875495	1.45258466701453	1.87862780686498	0.0692661749219538	0.679490320566168	-4.19272983101352
a_HQ_transcript/23552	-1.00053857100431	3.93651193451488	-1.87848272887593	0.0692866063207681	0.679490320566168	-4.60116762465752
a_HQ_transcript/125473	-2.80211631473156	-0.341494232552242	-1.87837872941631	0.0693012557985563	0.679490320566168	-4.31794411455755
a_HQ_transcript/116130	1.3148886084048	-0.279166916431934	1.87831588435961	0.0693101095122209	0.679490320566168	-4.26493272528362
a_HQ_transcript/102566	-2.28928733857404	2.83229246234498	-1.87824942926511	0.0693194728712475	0.679490320566168	-4.4620097867726
a_HQ_transcript/51413	3.72872657815985	1.92077004206955	1.87823910016067	0.0693209283142081	0.679490320566168	-4.51475915918
a_HQ_transcript/73085	-2.75337616496998	1.05013410492702	-1.87805586101033	0.0693467523558	0.679490320566168	-4.21959576010948
a_HQ_transcript/144662	-2.71028836316839	2.07387589975011	-1.87796059752818	0.0693601811811393	0.679490320566168	-4.40202605546685
a_HQ_transcript/8061	-0.730252441782305	2.91110876583368	-1.87795532517737	0.0693609244637997	0.679490320566168	-4.47626249324894
a_HQ_transcript/88157	7.24318238112136	2.6535867977166	1.87794001763193	0.0693630825215635	0.679490320566168	-4.29946201259984
a_HQ_transcript/23010	2.01824192509119	0.201512092592278	1.87791971512299	0.0693659448582938	0.679490320566168	-4.27316486829927
a_HQ_transcript/75377	2.68593958409222	2.94161141430506	1.87780135998473	0.0693826331052135	0.679490320566168	-4.43008360425587
a_HQ_transcript/140732	4.09787529110779	0.753003721233565	1.87770559264311	0.0693961389649482	0.679490320566168	-4.31024240992605
a_HQ_transcript/102023	3.44962830201837	1.48863996242339	1.8776476696572	0.0694043088138979	0.679490320566168	-4.29861792304553
a_HQ_transcript/38862	2.14397845885868	2.21711646601366	1.87758273097727	0.0694134691863529	0.679490320566168	-4.24696279938292
a_HQ_transcript/108480	-2.73821058926213	0.875183620270951	-1.8774901582502	0.0694265294655829	0.679490320566168	-4.36320593168939
a_HQ_transcript/16256	2.00856439106428	2.63374735096148	1.87705022958079	0.0694886240302974	0.679490320566168	-4.34407067052448
a_HQ_transcript/123747	2.30500507141095	0.820629770436398	1.87700834631786	0.0694945382096633	0.679490320566168	-4.30495343058329
a_HQ_transcript/27071	0.654477372908453	4.206273741439	1.87696920394424	0.0695000657492185	0.679490320566168	-4.6395421504133
a_HQ_transcript/90614	-1.4107018561977	-0.532345950573252	-1.87692441784501	0.0695063907379544	0.679490320566168	-4.31027554263317
a_HQ_transcript/26948	1.64086370316401	2.18504859095734	1.87691995556081	0.0695070209582923	0.679490320566168	-4.30739979074177

a_HQ_transcript/38399	-2.60346723707176	0.60216739020475	-1.87691603535912	0.0695075746229189	0.679490320566168	-4.23498215744162
a_HQ_transcript/41140	-2.07739443556561	1.57372613630737	-1.87688524299807	0.0695119236740695	0.679490320566168	-4.34583961133423
a_HQ_transcript/66894	1.78568697641301	0.0101005579170068	1.87681300712387	0.0695221270412367	0.679490320566168	-4.31251126193059
a_HQ_transcript/36396	1.48022334143445	0.906401997878708	1.87676110119195	0.0695294595844957	0.679490320566168	-4.17570833489533
a_HQ_transcript/142953	2.24396158551166	0.227940324699926	1.87667527206858	0.0695415857789656	0.679494587016257	-4.24321095877239
a_HQ_transcript/28255	1.90661102301969	1.83506004151446	1.87641993035775	0.0695776719611994	0.679730535788664	-4.34547097879073
a_HQ_transcript/6632	-0.567505620933454	3.00079736800962	-1.87633894289261	0.0695891208794099	0.679730535788664	-4.50364833648566
a_HQ_transcript/109776	2.0440497912639	-0.341867733456807	1.87613913973697	0.069617373283498	0.679892250373517	-4.2498526446412
a_HQ_transcript/42705	-1.30139855874699	1.8578706458359	-1.87573283614741	0.0696748554885473	0.679895083353792	-4.33335403281194
a_HQ_transcript/25709	1.8982089786308	0.271419088178601	1.87564562074968	0.0696871996877177	0.679895083353792	-4.33401004904659
a_HQ_transcript/151433	-5.90497408731939	3.16638924310661	-1.8755680817004	0.0696981759038369	0.679895083353792	-4.33818163455572
a_HQ_transcript/4766	1.3353700000225	1.4284493886192	1.87554595605917	0.0697013082215341	0.679895083353792	-4.18439788631187
a_HQ_transcript/101352	-1.25860891190831	2.6996636787749	-1.87546757941053	0.0697124049435016	0.679895083353792	-4.44972870190038
a_HQ_transcript/76415	-2.30392971161633	1.1834630593256	-1.8753703353474	0.0697261750610376	0.679895083353792	-4.28944706650561
a_HQ_transcript/145058	-2.40059746084962	0.675384766812415	-1.87534786268807	0.0697293576047137	0.679895083353792	-4.26061209363438
a_HQ_transcript/52566	2.04756429227461	-0.587041290477572	1.87507489728922	0.069768024511075	0.679895083353792	-4.2993687381112
a_HQ_transcript/30265	-1.43513611774296	1.98130476877423	-1.87502356989144	0.0697752973490776	0.679895083353792	-4.41669660605887
a_HQ_transcript/77172	-1.70203515235276	1.30307051777917	-1.87495533217928	0.0697849673032153	0.679895083353792	-4.30851924540508
a_HQ_transcript/13280	0.885357324214449	1.87854514745969	1.8749504765812	0.069785655433027	0.679895083353792	-4.2369273420372
a_HQ_transcript/7587	3.72203519511136	3.46632845631937	1.87494680454181	0.069786175834125	0.679895083353792	-4.5609641285581
a_HQ_transcript/66772	2.54301044973527	1.2374442080403	1.87485924594616	0.069798585617931	0.679895083353792	-4.33177490389475
a_HQ_transcript/148756	0.878192931159111	3.18312151696325	1.8748586244251	0.0697986737136238	0.679895083353792	-4.4545579224486
a_HQ_transcript/152430	-0.612894153781642	4.49682559224954	-1.87478957746029	0.0698084611692749	0.679895083353792	-4.69265377228352
a_HQ_transcript/89900	-1.45305098345453	-0.340163729705521	-1.87473651738043	0.0698159832737604	0.679895083353792	-4.27317948323021
a_HQ_transcript/1728	-2.01352024168091	1.73414851500615	-1.87473284344376	0.0698165041380881	0.679895083353792	-4.3485864329251
a_HQ_transcript/12937	-1.24577617341748	4.25615607971139	-1.87441443914957	0.0698616578906974	0.680164653802125	-4.62421387832667
a_HQ_transcript/68890	2.57508346988317	0.743757286174508	1.87425110289985	0.0698848307668724	0.680164653802125	-4.34918902430084
a_HQ_transcript/58301	-1.523231294196	2.42634459922059	-1.87422232298647	0.069888914521036	0.680164653802125	-4.33918716199199
a_HQ_transcript/123188	-1.41982981730547	1.76833839022859	-1.87420054938652	0.0698920042436341	0.680164653802125	-4.21367630960304
a_HQ_transcript/93818	2.23275777045667	2.77195015379996	1.87412524271756	0.0699026913331595	0.680164653802125	-4.44661400342776
a_HQ_transcript/45868	2.27001707434505	0.671982112848051	1.87403451362254	0.0699155689487323	0.680176099054713	-4.38281230009506
a_HQ_transcript/103862	2.31457617382503	2.13949163059977	1.87382780531435	0.0699449156591532	0.680347733841241	-4.29463992443715
a_HQ_transcript/68478	-1.80140114547491	2.28933926602771	-1.87373720509983	0.0699577816511907	0.680359031591168	-4.35999322652129
a_HQ_transcript/152729	-2.44437572240328	-0.15291978244625	-1.87359134253966	0.0699784996308977	0.680446675567886	-4.3247165945418
a_HQ_transcript/76427	0.79831837933937	2.7254504412185	1.87311744145612	0.0700458478142989	0.680944837337466	-4.33612956377141
a_HQ_transcript/20671	-2.18150696605205	0.487043797415194	-1.87306600702635	0.0700531607362724	0.680944837337466	-4.36118776541682
a_HQ_transcript/10214	1.47263270309797	-0.648794121745595	1.87256427362587	0.0701245313413856	0.681266400255484	-4.31107900301789
a_HQ_transcript/12815	2.6109948198647	1.67676224961725	1.87247280842861	0.0701375488147695	0.681266400255484	-4.27582226857508
a_HQ_transcript/7365	2.49041856728945	-0.181809422609483	1.87247115533085	0.0701377841053771	0.681266400255484	-4.30371766004032
a_HQ_transcript/22369	1.87436601527118	0.0173815895260714	1.87246539429595	0.0701386040969103	0.681266400255484	-4.24745523681705
a_HQ_transcript/139702	0.83331695662253	4.25902872308017	1.87239926063672	0.0701480177592691	0.681266400255484	-4.65788856106772
a_HQ_transcript/73675	-1.78965717319239	0.471893376690002	-1.87224728664289	0.0701696543018441	0.681266400255484	-4.20862885221546
a_HQ_transcript/39032	1.41355836815877	0.105343693410562	1.87221427609446	0.0701743547721372	0.681266400255484	-4.21602456254259
a_HQ_transcript/98789	0.752209397032842	1.91649418176754	1.87201857265114	0.0702022271209526	0.681266400255484	-4.38097760492065
a_HQ_transcript/92832	2.34129930000889	4.589862566824	1.87199350012355	0.0702057986709829	0.681266400255484	-4.77646830501136
a_HQ_transcript/7939	2.16020974086277	0.275102134114602	1.87199198021713	0.070206015184756	0.681266400255484	-4.33948270000312
a_HQ_transcript/60768	2.55164354778875	0.889461292247946	1.87192776599247	0.0702151631559766	0.681266400255484	-4.27027372377684
a_HQ_transcript/95474	-1.03236452713117	1.70362264475859	-1.87181194088221	0.0702316662120106	0.681268654976221	-4.24631222684618
a_HQ_transcript/143346	1.28683624609332	-0.488882631961047	1.87176162887159	0.070238835832516	0.681268654976221	-4.29670267520344
a_HQ_transcript/31808	0.607102166983917	3.00454885294068	1.87148642816135	0.070278063923353	0.681520381411939	-4.48512394647958

a_HQ_transcript/19539	-1.91801834027147	0.950639235435415	-1.87128875301781	0.0703062528494284	0.681520381411939	-4.18906075172278
a_HQ_transcript/90598	-1.13246926888473	2.00222110122298	-1.87116432527328	0.0703240015069199	0.681520381411939	-4.27098051545268
a_HQ_transcript/109217	-1.57563349721479	-0.498525058060323	-1.87111632756094	0.0703308490381588	0.681520381411939	-4.28673155270317
a_HQ_transcript/54806	-1.81485596782299	0.105908668113845	-1.87099254566606	0.0703485108628033	0.681520381411939	-4.31466226037989
a_HQ_transcript/121240	0.882869442039457	5.12737269666531	1.87083499999067	0.0703709957807095	0.681520381411939	-4.77489390111022
a_HQ_transcript/66670	2.86526772223953	1.06568092840193	1.87065899085559	0.0703961230993347	0.681520381411939	-4.2464253224214
a_HQ_transcript/139698	1.17895366650449	3.66051342363571	1.87063346646208	0.0703997676380176	0.681520381411939	-4.45755435712875
a_HQ_transcript/149879	2.37652875845941	0.50190116322108	1.87050168679753	0.070418586571291	0.681520381411939	-4.29441693042513
a_HQ_transcript/81551	0.895975605765658	3.43552862383613	1.87041352944598	0.0704311783848922	0.681520381411939	-4.56378809576816
a_HQ_transcript/32853	2.33785648087099	3.36552234703404	1.87039942082881	0.0704331937459417	0.681520381411939	-4.5053920981513
a_HQ_transcript/81625	-0.544970775909733	3.57336221223891	-1.87038745549223	0.0704349029864949	0.681520381411939	-4.51111208392923
a_HQ_transcript/32142	-2.23909299902594	1.19344869831416	-1.87024183788153	0.0704557072209964	0.681520381411939	-4.29591501411745
a_HQ_transcript/86264	-1.98297692394138	0.104670068846586	-1.87023572201556	0.0704565811037223	0.681520381411939	-4.51639672040147
a_HQ_transcript/94607	-1.4140628043455	2.7814466484269	-1.87018463721154	0.0704638808631827	0.681520381411939	-4.43934685508488
a_HQ_transcript/59086	-1.26559588508197	2.2861314969921	-1.87017049109653	0.0704659023860553	0.681520381411939	-4.41565052118443
a_HQ_transcript/140777	-2.50568905043873	0.0431214347004114	-1.87003610880045	0.0704851085077038	0.681520381411939	-4.3132318992157
a_HQ_transcript/23700	2.08477544978705	0.975375597693328	1.86998548855328	0.0704923443951937	0.681520381411939	-4.24066270749458
a_HQ_transcript/49141	1.87857428590114	1.17944529277718	1.86993081776815	0.0705001600010777	0.681520381411939	-4.23126179672454
a_HQ_transcript/151171	-4.77645123217236	1.17575529876669	-1.86988956105306	0.070506058455698	0.681520381411939	-4.48215262997407
a_HQ_transcript/83057	2.25055812369241	1.03126069238126	1.86985497977346	0.070511002852522	0.681520381411939	-4.39002739157038
a_HQ_transcript/50553	0.832961150844317	3.85691764587503	1.8696309405594	0.0705430429658798	0.681712004438412	-4.6287895353891
a_HQ_transcript/112117	1.12790519348582	3.27788511509787	1.86955235897101	0.0705542839716055	0.681712004438412	-4.55380715216105
a_HQ_transcript/108856	5.12810875496698	1.31464813205769	1.86904739199381	0.0706265556038931	0.682296896151421	-4.44606777620093
a_HQ_transcript/30890	1.44073108041104	-0.775328036735793	1.86879931819707	0.070662083565165	0.682449809598774	-4.31284852741771
a_HQ_transcript/119681	-1.70317502954218	1.69949650006394	-1.86871239994859	0.0706745352153076	0.682449809598774	-4.35916502313966
a_HQ_transcript/48585	1.02918810713311	1.50451078916573	1.86850547992088	0.0707041855411928	0.682449809598774	-4.25521501447765
a_HQ_transcript/101584	0.534898257022951	4.93555794633127	1.86849625125169	0.0707055081995074	0.682449809598774	-4.74805913375528
a_HQ_transcript/18973	-4.60543459169376	4.53434490381731	-1.86841507282803	0.0707171436539519	0.682449809598774	-4.73680655566265
a_HQ_transcript/143294	1.78720396314718	3.15953497298546	1.8683778617458	0.0707224777367669	0.682449809598774	-4.55426691678653
a_HQ_transcript/21112	3.36523836917018	1.28290204625647	1.86836328480424	0.0707245673861652	0.682449809598774	-4.41258470180535
a_HQ_transcript/38763	1.53402864593423	-0.55829847679931	1.86809847057557	0.0707625385453974	0.682495817611879	-4.33781414159149
a_HQ_transcript/65906	1.69792382633899	1.38102802679673	1.86809457799973	0.0707630968241517	0.682495817611879	-4.18545273931034
a_HQ_transcript/121618	3.17424877616193	1.37872521884886	1.86796740241721	0.0707813386058013	0.682495817611879	-4.34954766687745
a_HQ_transcript/128127	0.564686284864505	4.11961611707664	1.8677664742796	0.0708101675129864	0.682495817611879	-4.67042021338701
a_HQ_transcript/60971	2.66098519473212	0.419618678396553	1.86767437733382	0.070823384829683	0.682495817611879	-4.27063510857797
a_HQ_transcript/138370	-1.81375799296208	-0.648057619495043	-1.86758245627825	0.0708365790142263	0.682495817611879	-4.30013615895757
a_HQ_transcript/143918	-0.634817219314684	4.50301810497814	-1.86750760382765	0.070847324759556	0.682495817611879	-4.69413984633902
a_HQ_transcript/9779	0.477109589337767	3.48419674523054	1.86729262822617	0.0708781942258414	0.682495817611879	-4.53973588095617
a_HQ_transcript/11404	1.63411368229175	0.669309353222703	1.86719017724035	0.0708929097564529	0.682495817611879	-4.19616068485393
a_HQ_transcript/117225	1.65263395658658	2.21537129280855	1.86716206317506	0.0708969483739578	0.682495817611879	-4.27796158703042
a_HQ_transcript/105709	1.09906331467449	4.21691244441227	1.86706478044367	0.0709109246724668	0.682495817611879	-4.60945303875225
a_HQ_transcript/137128	1.91362843251782	9.18428658088583	1.86703064308235	0.0709158296381191	0.682495817611879	-5.24516256163144
a_HQ_transcript/106047	0.751680709924952	3.22506334340056	1.86702092349082	0.0709172262334663	0.682495817611879	-4.52904143722039
a_HQ_transcript/36905	-2.27204526692966	1.28958040036505	-1.86691559140066	0.0709323627768173	0.682495817611879	-4.32139796092837
a_HQ_transcript/29011	-2.45447364716274	0.677116955852764	-1.86681432803732	0.0709469172448665	0.682495817611879	-4.36311656902712
a_HQ_transcript/144433	1.62043458222069	2.14813052247689	1.86679597169938	0.070949555854744	0.682495817611879	-4.32328081389644
a_HQ_transcript/22011	-2.02670499230267	-0.524175599546264	-1.86669188500752	0.0709645192642189	0.682495817611879	-4.25963621353242
a_HQ_transcript/26358	2.23712364834819	0.468106398454669	1.86667481406362	0.0709669736262189	0.682495817611879	-4.26974913159183
a_HQ_transcript/108783	1.50864123734928	0.905756565655764	1.86664580562744	0.070971144459128	0.682495817611879	-4.15720162554925
a_HQ_transcript/139175	-1.66833016146898	4.86372366650707	-1.8665466361328	0.0709854046372962	0.682495817611879	-4.73420119328641

a_HQ_transcript/132401	2.80604304517098	2.69395968803047	1.86650731661332	0.0709910593080057	0.6824958176118 79	-4.48464554594199
a_HQ_transcript/139329	1.13309055709778	-0.626063921415617	1.86649797285074	0.0709924031223671	0.682495817611879	-4.35230171160011
a_HQ_transcript/26511	-2.05494624536598	0.215825044890951	-1.8664388378882	0.0710009083835159	0.682495817611879	-4.26739368393812
a_HQ_transcript/100602	-1.75675385542236	1.34609084638914	-1.86636780863469	0.0710111255318718	0.682495817611879	-4.32034663016485
a_HQ_transcript/24615	-2.24234621902058	2.1165161723625	-1.86585821449435	0.0710844647013179	0.683087744116087	-4.41279364535499
a_HQ_transcript/53405	1.28533518232082	-0.661056132277455	1.86573201248342	0.0711026373285201	0.683149438451606	-4.31698705667188
a_HQ_transcript/128135	-0.967049659933883	3.12305523264837	-1.865602167902	0.0711213386317451	0.683190437053389	-4.53789469656488
a_HQ_transcript/9070	2.40314148687997	1.35244051928223	1.86553918896781	0.0711304109043338	0.683190437053389	-4.29518212240044
a_HQ_transcript/127636	-5.32083790263566	0.0630042975180938	-1.86540382356884	0.0711499139875992	0.683233913114617	-4.34766791750535
a_HQ_transcript/121790	-4.28728567559027	2.08814096193601	-1.86521975046577	0.0711764421054845	0.683233913114617	-4.45473810710695
a_HQ_transcript/130198	2.89488585096954	1.77213316476269	1.86520325172478	0.0711788202736593	0.683233913114617	-4.27623710653003
a_HQ_transcript/133563	-2.11756397016009	1.0138448999136	-1.86515479609567	0.0711858051785117	0.683233913114617	-4.27881159258309
a_HQ_transcript/21129	3.13082501119819	0.574592856697563	1.86506268424561	0.0711990847720418	0.683233913114617	-4.22928460822811
a_HQ_transcript/20818	-1.96192634337269	-0.073645087256531	-1.86501846073025	0.0712054611486435	0.683233913114617	-4.28157225207397
a_HQ_transcript/15353	-2.86115172999366	1.79469968185304	-1.86473441634396	0.0712464278235688	0.683387911549151	-4.55192244794514
a_HQ_transcript/89710	-0.629572879547183	3.49702062558873	-1.86464071087274	0.071259947052016	0.683387911549151	-4.54631647741701
a_HQ_transcript/148065	1.97370606897572	0.10006527305413	1.86461952408224	0.07126300405213	0.683387911549151	-4.30494958176684
a_HQ_transcript/131807	2.29090105040043	0.333684576531973	1.86439928538926	0.0712947885198537	0.683387911549151	-4.265385914974
a_HQ_transcript/131751	-2.21339302362279	3.82380084873431	-1.86433520551127	0.0713040387002279	0.683387911549151	-4.62521658510932
a_HQ_transcript/1573	-1.811182395612	1.90886788749912	-1.86430367770009	0.071308590240549	0.683387911549151	-4.34764839002936
a_HQ_transcript/23388	1.86625046106861	-0.47620461190919	1.86420537244046	0.0713227837659703	0.683387911549151	-4.28165807588234
a_HQ_transcript/69284	1.49602835776472	0.51331425348136	1.86417586772417	0.0713270441932378	0.683387911549151	-4.39474728369351
a_HQ_transcript/44773	-1.46069814753771	1.71069050356046	-1.86417395744606	0.0713273200414378	0.683387911549151	-4.23591665610221
a_HQ_transcript/48958	2.0978965678276	1.0159073525791	1.86399322741976	0.0713534219778208	0.683525331695249	-4.18330125565316
a_HQ_transcript/143014	-3.18997339100423	2.15013116033621	-1.86386524175492	0.0713719112631985	0.683589793690427	-4.29587539243508
a_HQ_transcript/152166	3.18196758166408	1.63741806597558	1.86356283186798	0.0714156148445621	0.683895693523016	-4.30045805309006
a_HQ_transcript/68224	1.46174117107176	-0.332109336338212	1.86302905709779	0.0714928107483319	0.684460579324114	-4.31565369077186
a_HQ_transcript/128110	2.6571222714645	0.195591018649407	1.86282389515541	0.0715225008352726	0.684460579324114	-4.31094029613998
a_HQ_transcript/149801	-2.68383955662412	0.22299672010304	-1.86281454842904	0.0715238537020189	0.684460579324114	-4.27397184664377
a_HQ_transcript/123657	0.98164584252011	4.57325860893111	1.86272690400058	0.0715365406264306	0.684460579324114	-4.63026801248731
a_HQ_transcript/119099	0.509385946985667	3.01536069341039	1.86257142680024	0.071559051396618	0.684460579324114	-4.52930940010477
a_HQ_transcript/21613	1.70956711245525	3.7068026473845	1.86244177844399	0.0715778271805979	0.684460579324114	-4.650316837374
a_HQ_transcript/76851	1.91293392964725	1.20729133673388	1.86233110308864	0.0715938586203853	0.684460579324114	-4.32287330413464
a_HQ_transcript/29524	1.03815353171681	0.850124569414912	1.86227444033005	0.0716020674710695	0.684460579324114	-4.30677158738232
a_HQ_transcript/152694	-2.28753066802168	2.75282651401678	-1.86227192303558	0.0716024321754315	0.684460579324114	-4.52685431531733
a_HQ_transcript/32281	2.87125079737714	0.95850161203468	1.86220331014125	0.0716123733905243	0.684460579324114	-4.41303095657911
a_HQ_transcript/5087	4.47419000241144	2.43575840654682	1.86219999737781	0.0716128534015897	0.684460579324114	-4.41075660896751
a_HQ_transcript/39631	1.0354355300649	2.8507498138006	1.8621283270494	0.0716232389268166	0.684460579324114	-4.39265934762643
a_HQ_transcript/22707	1.41947082034632	2.22283136366242	1.86204990956009	0.0716346036415609	0.684460579324114	-4.34897519604494
a_HQ_transcript/116485	-3.54840342217837	1.43097169497082	-1.86192176390958	0.0716531785756825	0.684460579324114	-4.39158178463498
a_HQ_transcript/75521	2.6629113732804	1.21929634081557	1.86188040254463	0.071659174858646	0.684460579324114	-4.37680834799202
a_HQ_transcript/98883	2.82890360147483	-0.0116395030572285	1.86180076037305	0.0716707220383629	0.684460579324114	-4.31803917084464
a_HQ_transcript/65271	2.80069451011101	0.514667629056737	1.86170418662497	0.0716847262377914	0.684460579324114	-4.41434373410763
a_HQ_transcript/119966	1.15843243111327	1.94057954433654	1.86169158394003	0.0716865539314136	0.684460579324114	-4.29827228069559
a_HQ_transcript/124594	-0.875663188684616	2.22616667338742	-1.86145344235568	0.0717210977217375	0.68467793863839	-4.28241226270097
a_HQ_transcript/112855	-2.08533742755861	-0.0521030453164393	-1.86119545164936	0.0717585368425429	0.684904106678077	-4.32623908393052
a_HQ_transcript/114622	3.15829815987983	-0.531994289429636	1.86112781746216	0.0717683545584945	0.684904106678077	-4.33347622438583
a_HQ_transcript/101224	1.90392915353897	0.0703028095085962	1.86099716775516	0.0717873228093882	0.684972669117551	-4.26860765067957
a_HQ_transcript/9410	1.29398696906736	0.459596416464467	1.86081721053966	0.0718134567592037	0.685021857583737	-4.24438839026518
a_HQ_transcript/95022	-1.38333305803271	1.25916277185322	-1.8607993749341	0.0718160473457764	0.685021857583737	-4.39893275495412

a_HQ_transcript/121188	-1.27280554795544	0.629595405218607	-1.86062025374237	0.0718420687882969	0.685144689402815	-4.2224403461492
a_HQ_transcript/130942	-1.09827735467046	-0.815991822480582	-1.86054847612952	0.0718524983931936	0.685144689402815	-4.31488560479113
a_HQ_transcript/21949	1.18821116271647	1.95031944339297	1.86044913752502	0.0718669348731728	0.685169950845955	-4.30505151501865
a_HQ_transcript/119752	-2.41876967075542	2.25524768347263	-1.86027772653608	0.0718918511892229	0.685252369960946	-4.42042677167909
a_HQ_transcript/2524	-2.42191016175633	2.61145186985671	-1.86009764752288	0.071918035465009	0.685252369960946	-4.48350789588117
a_HQ_transcript/85255	2.12668344377322	0.403110768335067	1.85995711820854	0.0719384747195819	0.685252369960946	-4.31008182392112
a_HQ_transcript/105441	4.42754610552433	1.42934471297954	1.85990264260857	0.0719463992499393	0.685252369960946	-4.21717277753029
a_HQ_transcript/50339	1.8074183518145	-0.699560474188331	1.85982235140365	0.0719580805236972	0.685252369960946	-4.33451894685254
a_HQ_transcript/130974	-0.742122690590997	2.67892764406095	-1.8597739204259	0.0719651273554489	0.685252369960946	-4.42289903397289
a_HQ_transcript/87468	1.81386679602282	0.814340553793589	1.8596955474169	0.0719765320826826	0.685252369960946	-4.19803441269432
a_HQ_transcript/96280	1.74640535868709	1.95573510311247	1.85960582525829	0.0719895902234806	0.685252369960946	-4.26857385987583
a_HQ_transcript/131006	2.78554580158891	2.76322804376612	1.85959443509595	0.0719912480901209	0.685252369960946	-4.49008266631954
a_HQ_transcript/17281	1.88181579083496	-0.382955367829592	1.85957919383213	0.0719934665454083	0.685252369960946	-4.26980066395564
a_HQ_transcript/127819	0.716600206432209	6.00914807371057	1.85924932753866	0.0720414948761068	0.68559725182684	-4.85039319934154
a_HQ_transcript/133023	5.78731156632135	2.77964056807486	1.8590587190915	0.0720692598649899	0.685749212216751	-4.49134473211242
a_HQ_transcript/108773	1.02585018124962	3.02821871224719	1.85882141159114	0.0721038400887572	0.685784245401017	-4.40082358363884
a_HQ_transcript/127772	1.83714950920573	0.758940195294112	1.85873683323522	0.0721161682029384	0.685784245401017	-4.20301983617045
a_HQ_transcript/22798	-1.20831573546273	3.32627738874051	-1.85869554166103	0.0721221875057622	0.685784245401017	-4.42176543495385
a_HQ_transcript/41335	1.29849380930334	-0.0728526932801248	1.85856214341577	0.07214163665344З	0.685784245401017	-4.24873678941539
a_HQ_transcript/147389	1.70229329020099	1.02998612496236	1.85853419673877	0.0721457117820052	0.685784245401017	-4.30435191756195
a_HQ_transcript/35683	0.653582266135594	3.60657598283023	1.85851611882918	0.0721483479717036	0.685784245401017	-4.57985180733406
a_HQ_transcript/125561	-0.890339093428791	3.50668203247506	-1.85846507015839	0.0721557925291386	0.685784245401017	-4.57187368407046
a_HQ_transcript/53280	1.70878684460163	3.25067373411902	1.85838600337816	0.0721673243379786	0.685784245401017	-4.59901702668978
a_HQ_transcript/3662	-2.90959224252991	0.960021057035715	-1.85820619538723	0.0721935550285666	0.685812592636891	-4.37420658831036
a_HQ_transcript/76214	0.663473746581178	3.08167012781092	1.85820380330008	0.0721939040452989	0.685812592636891	-4.50354262224919
a_HQ_transcript/144569	-1.16519404063086	1.94873995633191	-1.85789469961692	0.0722390158878764	0.685849356761256	-4.35838987457751
a_HQ_transcript/115908	-1.81696320718464	-0.225490262667858	-1.85785640761967	0.0722446060579725	0.685849356761256	-4.30749028092339
a_HQ_transcript/107991	1.01601762428277	3.45037274994405	1.85775437818024	0.0722595029360315	0.685849356761256	-4.58711042107642
a_HQ_transcript/25746	-2.15412986343784	1.15550147441848	-1.85772174608059	0.0722642679635068	0.685849356761256	-4.33299560674605
a_HQ_transcript/144901	-2.74242708662829	-0.15368551131684	-1.8577125387869	0.0722656124861388	0.685849356761256	-4.39854461117643
a_HQ_transcript/29509	-1.08655855002916	3.00458827701195	-1.85769230117975	0.0722685678186535	0.685849356761256	-4.39901244096606
a_HQ_transcript/1324	1.01257178789225	2.20797276959926	1.85758919658693	0.072283625967082	0.685862126925538	-4.40200436712607
a_HQ_transcript/151113	0.631753847421917	5.44187323108788	1.85752151760637	0.0722935117615051	0.685862126925538	-4.79261973549544
a_HQ_transcript/51622	1.71977205654394	1.25083351049246	1.85743364234137	0.072306349333488	0.685871976836976	-4.19531970570737
a_HQ_transcript/102955	0.509434283824831	3.69626611153135	1.85727510127483	0.0723295153121065	0.685952461054421	-4.60202043696809
a_HQ_transcript/148281	-2.02403395362692	1.26320865279524	-1.85708329886298	0.0723575499292808	0.685952461054421	-4.22361925211664
a_HQ_transcript/149008	-1.18363566654329	4.01187124587499	-1.85706006600598	0.0723609463697576	0.685952461054421	-4.65095127804704
a_HQ_transcript/21784	3.49203882518982	1.65476498116878	1.85705260538301	0.0723620370765502	0.685952461054421	-4.4342374255235
a_HQ_transcript/139145	-2.68031425026192	0.133838357825497	-1.85693568928556	0.0723791314813087	0.686002634069054	-4.3041482570817
a_HQ_transcript/141226	2.2638059193586	-0.0769950887947291	1.85668688521876	0.0724155208563491	0.686235636833691	-4.29172112200923
a_HQ_transcript/108798	1.0110277805374	1.25909483565584	1.85652987419868	0.0724384929067139	0.686341436948894	-4.22735896596868
a_HQ_transcript/113461	3.04080741930629	0.012254503174523	1.85635248530879	0.0724644539234422	0.686356492537657	-4.34348836239144
a_HQ_transcript/7738	-1.33462125489599	-0.675016167030631	-1.85633211367043	0.0724674358404043	0.686356492537657	-4.29589904181457
a_HQ_transcript/30351	1.78810925802153	0.432439863029899	1.85627699139161	0.0724755049407538	0.686356492537657	-4.21847589887389
a_HQ_transcript/27545	-2.06965311899497	0.917895032436008	-1.85607428923157	0.0725051842098133	0.686435425486345	-4.31368915494958
a_HQ_transcript/90348	2.00268678278823	1.64583196980688	1.85605876345227	0.0725074578944081	0.686435425486345	-4.26890401997874
a_HQ_transcript/32806	-1.65072699393914	1.70637110946347	-1.85590444263803	0.0725300608445315	0.686535102010735	-4.35798670100365
a_HQ_transcript/94446	-1.77760150061946	0.595541027439873	-1.85582561996464	0.072541608115709	0.686535102010735	-4.19249317183346
a_HQ_transcript/20798	0.688383694763309	4.4682689141934	1.85573838079955	0.0725543902120442	0.686544293382013	-4.69046771596401
a_HQ_transcript/142327	-0.716337711300312	2.9666210775651	-1.85515738377289	0.0726395658725769	0.687083568844176	-4.48259454938644

a_HQ_transcript/74343	1.63055437601753	-0.213513414213193	1.85514567767726	0.0726412828961318	0.687083568844176	-4.3351633238355
a_HQ_transcript/111101	2.51636040590039	1.15171749894153	1.85502991779716	0.0726582641643873	0.687083568844176	-4.39245529608241
a_HQ_transcript/138899	4.25662993303388	5.487491558113	1.85495347140573	0.0726694802475766	0.687083568844176	-4.49047152546644
a_HQ_transcript/82198	2.20226652532123	0.91430441181182	1.85494664375282	0.0726704820612096	0.687083568844176	-4.18736881861667
a_HQ_transcript/135769	1.84047965158622	1.0613729951472	1.85471665859351	0.0727042344285929	0.687152253273109	-4.22612851899244
a_HQ_transcript/143945	0.888446826710659	5.02029432062945	1.85467696645711	0.0727100609608901	0.687152253273109	-4.76501047944472
a_HQ_transcript/28224	2.74939752573717	-0.212598202732268	1.85465481244896	0.0727133131906175	0.687152253273109	-4.39113951449124
a_HQ_transcript/140390	-2.85947532955871	1.89831955154939	-1.85453394064036	0.0727310594826911	0.687152253273109	-4.46412580939679
a_HQ_transcript/44625	1.92278327762524	0.25215272730394	1.85449448315218	0.0727368533992128	0.687152253273109	-4.42058244633008
a_HQ_transcript/9338	-1.11407775494246	1.93138858730496	-1.85429293599509	0.0727664546427864	0.687275452981241	-4.40695869764186
a_HQ_transcript/67874	3.14554970679909	0.324446257243787	1.85424469469226	0.0727735413759211	0.687275452981241	-4.39032189099368
a_HQ_transcript/144620	-2.48095791642407	2.81447701639548	-1.85400488807814	0.0728087781659939	0.687361345265761	-4.54342486015973
a_HQ_transcript/11357	-0.748419112052076	2.79275312211261	-1.85396085461895	0.0728152499576309	0.687361345265761	-4.47854256943043
a_HQ_transcript/8638	1.8203786895785	-0.515271961331201	1.85391643554878	0.0728217789233134	0.687361345265761	-4.33230490927864
a_HQ_transcript/110462	2.1365467399809	0.899644057478257	1.85386094393842	0.0728299360977811	0.687361345265761	-4.30730676506401
a_HQ_transcript/139623	-1.94960430948508	1.85255334574929	-1.85348480275413	0.0728852488559888	0.687732056066803	-4.36721486592415
a_HQ_transcript/108771	2.0458469316374	-0.477200948979991	1.85343294351715	0.0728928777426984	0.687732056066803	-4.33942218420866
a_HQ_transcript/122962	0.97287529487	-0.568058149532234	1.85314275031613	0.0729355799804826	0.688023270546169	-4.32955529410317
a_HQ_transcript/137742	3.6089737516286	3.80060749183699	1.85304691570835	0.0729496868488453	0.688028670526524	-4.68370592039863
a_HQ_transcript/23231	2.3369606417437	0.278057383821876	1.85297805021891	0.0729598253014984	0.688028670526524	-4.23053046065361
a_HQ_transcript/85762	2.46308556435293	1.15143260813397	1.85282306992043	0.0729826460831441	0.688132238689537	-4.26719689561791
a_HQ_transcript/94395	1.74669610960044	0.308383791612569	1.85248947013303	0.0730317892436485	0.688365647875791	-4.32171515670611
a_HQ_transcript/92317	1.77440174961524	-0.658384548294019	1.85244850842791	0.0730378253355749	0.688365647875791	-4.33390696986422
a_HQ_transcript/32729	1.44914413471683	0.858702658868283	1.85241388365735	0.0730429279537895	0.688365647875791	-4.30017181309919
a_HQ_transcript/102936	1.37161575703632	-0.757488963363874	1.85217254434834	0.0730785023672153	0.688502904350105	-4.34916616050795
a_HQ_transcript/126083	0.386677005060305	4.64697671356669	1.85195764421693	0.07311019201981	0.688502904350105	-4.75859664839361
a_HQ_transcript/85531	2.00397787373211	3.1059236135219	1.85184623519199	0.0731266252728383	0.688502904350105	-4.39528149992221
a_HQ_transcript/147014	-2.09466284829914	-0.294305872260297	-1.85180667592257	0.0731324611755666	0.688502904350105	-4.28083826098268
a_HQ_transcript/53181	-1.10717901382155	2.42954868979849	-1.85176004244418	0.0731393411989545	0.688502904350105	-4.47856438256415
a_HQ_transcript/122297	1.57109254875316	0.322591038508375	1.85165037599866	0.073155522911481	0.688502904350105	-4.21877469074436
a_HQ_transcript/137730	-3.4824369341415	1.23624613553381	-1.8516419052151	0.0731567729361883	0.688502904350105	-4.31034944898515
a_HQ_transcript/107407	1.84577478664515	0.395154585876855	1.85155982876383	0.0731688858195288	0.688502904350105	-4.32661120934136
a_HQ_transcript/97483	2.13349517834649	-0.65227035226776	1.85146069426201	0.0731835184275208	0.688502904350105	-4.34884909926768
a_HQ_transcript/151633	-1.24688552265912	2.97083563480215	-1.85121977038529	0.073219090097356	0.688502904350105	-4.39539388373537
a_HQ_transcript/131742	3.00665628128812	-0.42436529910639	1.85118833895499	0.0732237319426228	0.688502904350105	-4.34354008980245
a_HQ_transcript/31089	2.34425282756349	0.236605829593977	1.85109631733274	0.0732373232952975	0.688502904350105	-4.28024395031936
a_HQ_transcript/116903	-1.94645231967305	1.01174107588586	-1.85109091415711	0.0732381213973057	0.688502904350105	-4.389990130028
a_HQ_transcript/131634	1.93863507380299	1.25078432335752	1.85102698621858	0.0732475647456394	0.688502904350105	-4.32909016357618
a_HQ_transcript/40217	0.640331179771332	4.36686146235522	1.85099369444122	0.073252482974519	0.688502904350105	-4.72087144982756
a_HQ_transcript/86544	2.03360911822932	0.437053860016886	1.85092105475343	0.0732632150923509	0.688502904350105	-4.20227786186215
a_HQ_transcript/118892	1.71113741158252	1.25778032292038	1.85080731980289	0.0732800215145247	0.688502904350105	-4.23504565771973
a_HQ_transcript/9548	-1.98243821722547	1.13381307459686	-1.8508022760465	0.0732807668983243	0.688502904350105	-4.40239611273951
a_HQ_transcript/116778	0.528252009888588	4.59140614566697	1.85075926086595	0.07328712409447	0.688502904350105	-4.7046115728787
a_HQ_transcript/118603	2.39150539418866	1.07361055082113	1.85071013931262	0.0732943843262877	0.688502904350105	-4.34163078459967
a_HQ_transcript/90108	-2.31770935051528	0.262051993527204	-1.85045746070222	0.0733317402942929	0.688742510878439	-4.34178881807114
a_HQ_transcript/70216	-2.10648745521998	0.702507154405005	-1.85025437145707	0.0733617767890578	0.688913305524128	-4.2810995151277
a_HQ_transcript/50624	0.809213900636987	2.69504799123924	1.85008761447532	0.0733864476863803	0.68903366679275	-4.34674453868791
a_HQ_transcript/17948	-0.656523936795359	3.54393019131592	-1.84998473002091	0.0734016724905961	0.689035011034801	-4.5534526644183
a_HQ_transcript/16667	-1.44328460586433	2.401170833406	-1.84990794672154	0.0734130366167958	0.689035011034801	-4.30736626811361
a_HQ_transcript/79686	-0.586028624401377	2.48561621174391	-1.84984636391752	0.0734221521185928	0.689035011034801	-4.41718715752017

a_HQ_transcript/147789	-3.57185937205187	1.02997169926628	-1.84972685845816	0.0734398441098368	0.689055427718016	-4.39305212892013
a_HQ_transcript/147252	-3.11181976581855	1.7641243933217	-1.84963995984438	0.0734527111645766	0.689055427718016	-4.51139320294061
a_HQ_transcript/143696	2.55003178477656	1.11378742490661	1.84954304982354	0.0734670628811368	0.689055427718016	-4.35084648538664
a_HQ_transcript/139121	2.07835284269906	2.63654396901188	1.84944494121369	0.0734815945449507	0.689055427718016	-4.44243640598034
a_HQ_transcript/136543	4.85345619541008	5.71637422547813	1.84942637702087	0.07348434451521	0.689055427718016	-4.71855490390035
a_HQ_transcript/26724	3.75612725657297	1.45069487344794	1.84931992834755	0.0735001147808826	0.689055427718016	-4.29874180618784
a_HQ_transcript/82680	1.57348728281473	-0.631340906926246	1.84923133875478	0.0735132414494635	0.689055427718016	-4.34732245216408
a_HQ_transcript/146782	1.51304819602958	-0.467949843853655	1.84919139640995	0.0735191605200099	0.689055427718016	-4.36164533409152
a_HQ_transcript/135381	-3.32164369933903	1.79422725941235	-1.84908977039474	0.0735342223549184	0.689071314028108	-4.41540904661446
a_HQ_transcript/80336	-2.15539705208286	0.970775633542672	-1.84889316526616	0.0735633683931502	0.689071314028108	-4.26118721291195
a_HQ_transcript/145338	2.21646720359308	0.187182878942876	1.84879548117161	0.0735778534018028	0.689071314028108	-4.2760257094104
a_HQ_transcript/67768	1.24591962532623	-0.180093375900253	1.84879016731886	0.0735786414322392	0.689071314028108	-4.25441297250516
a_HQ_transcript/46862	1.44621727630177	-0.23288950779908	1.84870232938723	0.0735916686136873	0.689071314028108	-4.27051182079292
a_HQ_transcript/9214	1.56675885280856	0.402623668930942	1.84870021790808	0.0735919817899548	0.689071314028108	-4.31110817764574
a_HQ_transcript/50393	-1.33502801316544	2.29877373420521	-1.84861256569659	0.0736049834420033	0.689082054681393	-4.37293003286585
a_HQ_transcript/135011	-2.43187845018636	3.56669052176782	-1.8482843190446	0.0736536904786508	0.689427008074559	-4.48794596471967
a_HQ_transcript/98138	-0.666127540400872	2.62081573957249	-1.84814142621104	0.0736749023142786	0.689499894333123	-4.4458074998436
a_HQ_transcript/12417	2.07115349778259	0.746269165601295	1.84802250777027	0.0736925592376015	0.689499894333123	-4.32044451733784
a_HQ_transcript/142942	-2.11732433661419	3.05979756016816	-1.84799218307698	0.0736970624052383	0.689499894333123	-4.55785314040304
a_HQ_transcript/72144	-2.26487343847719	1.1976101314283	-1.84753842338381	0.0737644731054475	0.690019519258682	-4.40157453351483
a_HQ_transcript/105936	1.51370907540281	0.199938039768482	1.84741046140232	0.0737834927157334	0.69008638207243	-4.35023539952696
a_HQ_transcript/103897	-1.86607083561513	1.64380604196151	-1.84721279244885	0.073812881457647	0.690250188766105	-4.38697663153165
a_HQ_transcript/808	1.41352527987998	3.0615712840829	1.84690845671767	0.0738581486279047	0.69051368080677	-4.55044466731074
a_HQ_transcript/100180	3.14024385422041	1.17652373037157	1.84686363983867	0.0738648167364523	0.69051368080677	-4.34774800707617
a_HQ_transcript/142897	-5.47077116276936	2.73783894609257	-1.84612292590335	0.0739750989559779	0.691143914174043	-4.43359626451973
a_HQ_transcript/53498	1.7848858038415	1.57890105905277	1.84602691965567	0.0739894032840967	0.691143914174043	-4.2676446539192
a_HQ_transcript/109656	0.631304224695015	3.05496428237407	1.84600020264645	0.0739933843714942	0.691143914174043	-4.50177702686924
a_HQ_transcript/52293	-1.88008736777777	0.310512559592963	-1.84594352347752	0.0740018307110906	0.691143914174043	-4.27832221958498
a_HQ_transcript/42218	2.92233029717661	0.725855515662497	1.84594023845664	0.0740023202707556	0.691143914174043	-4.30535977603962
a_HQ_transcript/127348	1.97309623400504	-0.551555158932669	1.84593182901683	0.0740035735242782	0.691143914174043	-4.35642316559728
a_HQ_transcript/13780	1.42601047904017	2.16274871105422	1.84541656365266	0.0740803978376999	0.691297156213951	-4.4043921089744
a_HQ_transcript/34580	-0.654845513173889	3.51904471548074	-1.84525944278291	0.0741038375915834	0.691297156213951	-4.62578885119134
a_HQ_transcript/118808	1.8577153874418	-0.263673404127113	1.84522045363599	0.0741096550894811	0.691297156213951	-4.30072175004334
a_HQ_transcript/152309	4.50433575734985	0.049399048559747	1.84521985266245	0.0741097447626787	0.691297156213951	-4.45124975802083
a_HQ_transcript/42181	1.80703989768568	-0.0859438030715466	1.84521537332345	0.0741104131422026	0.691297156213951	-4.34751657554142
a_HQ_transcript/147645	0.620838761316709	9.46719154977675	1.84511160482568	0.0741258982848239	0.691297156213951	-5.26298751102706
a_HQ_transcript/109646	2.22767865862973	-0.0489373445168054	1.84510833050546	0.0741263869493939	0.691297156213951	-4.34483856183578
a_HQ_transcript/100530	2.95324118987177	0.243621040250356	1.84494427494546	0.0741508743800984	0.691297156213951	-4.2229765025682
a_HQ_transcript/38734	-1.35672118646301	2.09846307332787	-1.84487717442788	0.0741608920002082	0.691297156213951	-4.40182744470224
a_HQ_transcript/144272	-2.48462048845066	0.0586566559579777	-1.84477036438661	0.0741768403513245	0.691297156213951	-4.3617848931151
a_HQ_transcript/74777	1.41222988139352	-0.696288739738329	1.84466912347756	0.0741919598544517	0.691297156213951	-4.36508254756606
a_HQ_transcript/98054	1.53087500795321	0.608591207236059	1.84462679896075	0.0741982814565976	0.691297156213951	-4.22224399196026
a_HQ_transcript/126631	-3.14057631419603	2.5688185957909	-1.84459158979145	0.0742035406593381	0.691297156213951	-4.52541374660963
a_HQ_transcript/151992	-2.08268658813011	1.02424541471892	-1.84452775565645	0.0742130763935017	0.691297156213951	-4.38171700071388
a_HQ_transcript/50747	-2.30780399476189	0.473127191833019	-1.84446235431753	0.0742228473280701	0.691297156213951	-4.39500810164555
a_HQ_transcript/2417	0.898144303938141	3.11396345231709	1.84436970727113	0.0742366906436917	0.691297156213951	-4.48497349926944
a_HQ_transcript/51319	-0.851811061374366	3.8086639605869	-1.84434981536612	0.0742396631787313	0.691297156213951	-4.65415099683645
a_HQ_transcript/6462	-1.39508601220006	0.885583013912473	-1.84433551103656	0.0742418008006563	0.691297156213951	-4.19677800329166
a_HQ_transcript/71221	2.39934254737742	1.77769708946851	1.84430218166095	0.0742467817069262	0.691297156213951	-4.3727791250578
a_HQ_transcript/139248	-2.54454577684032	0.546193250855446	-1.84422822496424	0.0742578351809784	0.691297156213951	-4.29479227261193

a_HQ_transcript/61094	-1.41551063349727	-0.682125314122075	-1.84367734056857	0.074340213949507	0.691898239879247	-4.34166661268099
a_HQ_transcript/103340	-3.44555422145442	2.03290582942919	-1.84358850031616	0.0743535063570347	0.691898239879247	-4.41479313654255
a_HQ_transcript/50344	-2.48990306658092	1.17372573804493	-1.84355772494149	0.0743581114871603	0.691898239879247	-4.31790383517488
a_HQ_transcript/33069	-2.53438982406352	-0.476679738648667	-1.84341867470575	0.0743789215667211	0.691981106406908	-4.37428303139947
a_HQ_transcript/122040	4.41376029423649	0.144066223044812	1.84320645570459	0.0744106915888375	0.691986502078004	-4.36961455887768
a_HQ_transcript/108017	1.08313563331742	-0.696576843382374	1.8431352893913	0.0744213480642379	0.691986502078004	-4.33233484386386
a_HQ_transcript/70299	1.39651944602722	-0.655384227244369	1.84310347204911	0.0744261128292705	0.691986502078004	-4.38728059291564
a_HQ_transcript/66221	3.18820001971012	4.14345738168167	1.84309674909748	0.0744271196495954	0.691986502078004	-4.58686915471384
a_HQ_transcript/81104	-2.21496218582769	0.726015135583625	-1.84293436444438	0.0744514416976228	0.691991392006224	-4.33110986705746
a_HQ_transcript/7830	-1.60143744821225	0.765128724247838	-1.8429027743253	0.0744561740696619	0.691991392006224	-4.27758799185369
a_HQ_transcript/61170	2.20221465760533	0.997264016943635	1.84285481298901	0.0744633594308927	0.691991392006224	-4.28354663224235
a_HQ_transcript/135061	2.93345823100594	2.18967698527701	1.84270265283253	0.0744861593353924	0.692092626254426	-4.55668759643287
a_HQ_transcript/108402	-1.46936846468151	2.57216513940914	-1.84252853784605	0.0745122563061236	0.692224458136863	-4.52380614695611
a_HQ_transcript/22398	-0.691089099793042	2.65013379990409	-1.84231172740765	0.0745447635486611	0.692267648260344	-4.45365525061162
a_HQ_transcript/24678	-1.55525277364845	0.695750706728861	-1.84228503737302	0.074548766128584	0.692267648260344	-4.34130636756979
a_HQ_transcript/4302	2.55495409004373	0.492368838198739	1.84225925032736	0.0745526334659674	0.692267648260344	-4.27441482823655
a_HQ_transcript/98376	-2.80706381598743	2.46565541265992	-1.84208085073457	0.0745793931252342	0.692405520457374	-4.47234422136578
a_HQ_transcript/1158	-2.24348373920925	0.394862898188013	-1.84166251113394	0.0746421756126177	0.692548583130147	-4.30828050462961
a_HQ_transcript/30392	0.823986434615009	2.57820811528048	1.8416558117057	0.0746431813994825	0.692548583130147	-4.43299741249877
a_HQ_transcript/43705	1.16278196727865	2.14212011076914	1.84151994180795	0.074663582081803	0.692548583130147	-4.41134809175479
a_HQ_transcript/94939	1.27611670310344	1.44371394235738	1.84147535725875	0.0746702774303228	0.692548583130147	-4.34039316402358
a_HQ_transcript/40419	2.17468483135072	2.2289646255881	1.84141150801996	0.0746798666884853	0.692548583130147	-4.43443296298914
a_HQ_transcript/134141	2.58959783201471	1.70075572810165	1.84138990788425	0.0746831109635143	0.692548583130147	-4.43841070450364
a_HQ_transcript/109511	-3.45355821161104	0.943362187322438	-1.84136982265263	0.0746861278126213	0.692548583130147	-4.28819250135268
a_HQ_transcript/124798	1.40124819364881	-0.722420581704293	1.84134327107174	0.0746901160824885	0.692548583130147	-4.36600944688285
a_HQ_transcript/141236	-2.20023929215809	2.46135421279924	-1.84109388774921	0.0747275844317013	0.69271891347973	-4.44555860870701
a_HQ_transcript/93217	1.17370027106957	-0.817634362555094	1.84098531346973	0.0747439020853424	0.69271891347973	-4.35701574074172
a_HQ_transcript/138256	2.80663653658689	2.95557357031637	1.84098308328254	0.0747442372926106	0.69271891347973	-4.51631525458715
a_HQ_transcript/122181	-1.73335929709233	0.372252255336103	-1.8408797799308	0.074759765663952	0.692752376616324	-4.35815661459459
a_HQ_transcript/116769	1.12693023845527	3.01483816605424	1.84040734074721	0.0748308170037789	0.693056489022321	-4.48985482172901
a_HQ_transcript/83330	2.34851355240855	0.148526215655769	1.84037545287537	0.0748356147809409	0.693056489022321	-4.30755862770143
a_HQ_transcript/28142	3.14514424257231	4.14395513353017	1.84034740517509	0.0748398349918687	0.693056489022321	-4.57760562556981
a_HQ_transcript/32215	3.03345271351139	0.126077094257717	1.84034447220614	0.0748402763142222	0.693056489022321	-4.37058206479131
a_HQ_transcript/101608	2.06891903907859	1.3801695612504	1.84010061372791	0.074876977356572	0.693285909490732	-4.22281465630167
a_HQ_transcript/56955	3.80915736452761	-0.0435066971477102	1.83991040935094	0.0749056140573987	0.693440601039731	-4.37131198647337
a_HQ_transcript/94966	-0.463984203466275	4.48207539399352	-1.83979571595101	0.0749228865351362	0.693490055495923	-4.73868486526857
a_HQ_transcript/114819	-1.35546311213935	1.11744198218552	-1.83964892474954	0.074944997809773	0.693584275224723	-4.25548332516648
a_HQ_transcript/128426	-1.52870997234626	-0.112287326509124	-1.83930065606099	0.0749974801073717	0.693921974278229	-4.21951893008508
a_HQ_transcript/67138	-1.86121801571313	-0.033977148640444	-1.83905247246046	0.0750348992584263	0.693921974278229	-4.31328063706876
a_HQ_transcript/64632	-2.0822294665485	0.850983721561516	-1.83900617492479	0.0750418813981577	0.693921974278229	-4.32455003925473
a_HQ_transcript/122526	-2.00764420485397	1.63239861575301	-1.83885217150066	0.0750651106770592	0.693921974278229	-4.29519558436472
a_HQ_transcript/62920	2.18028327337192	1.07681508840685	1.83883561962177	0.0750676076638524	0.693921974278229	-4.22273911592696
a_HQ_transcript/111709	-1.50120242148104	1.05245664503141	-1.83873930113013	0.0750821395070618	0.693921974278229	-4.31435532040122
a_HQ_transcript/127685	5.06523162075187	1.13873986712951	1.83871683962269	0.0750855286837968	0.693921974278229	-4.43820837685777
a_HQ_transcript/65590	1.33563981387919	-0.769701324703123	1.83870792285317	0.0750868741554663	0.693921974278229	-4.3708232589514
a_HQ_transcript/105682	-1.74505502680242	-0.240588105053796	-1.83864484209568	0.0750963931430403	0.693921974278229	-4.34151601903904
a_HQ_transcript/110367	-1.92621108859313	-0.320526142097575	-1.83857185979152	0.0751074075763873	0.693921974278229	-4.33445323415092
a_HQ_transcript/79211	-0.777390884522011	3.43106236106061	-1.83853610709622	0.0751128038494119	0.693921974278229	-4.58917850167918
a_HQ_transcript/5655	-0.500523285038089	3.50766943384138	-1.83845351126331	0.0751252715793348	0.693926868959729	-4.62184069260449
a_HQ_transcript/78666	-2.32180381030612	0.204377255029231	-1.83803366220481	0.0751886745699773	0.694240974796812	-4.29874452608575

a_HQ_transcript/122776	-3.03136710941685	3.75927593393071	-1.83773794742504	0.0752333590083114	0.694240974796812	-4.54180442089494
a_HQ_transcript/78271	-1.67463812722146	-0.482632843320837	-1.83763682077066	0.0752486451122248	0.694240974796812	-4.37544989975174
a_HQ_transcript/62912	1.04870429717381	3.16536904269479	1.83758209518491	0.0752569184292687	0.694240974796812	-4.54183333774784
a_HQ_transcript/46511	1.22128054508649	-0.107160068918358	1.83757183987151	0.0752584688958191	0.694240974796812	-4.31544835443961
a_HQ_transcript/145931	-1.60872403110036	2.32511131769998	-1.83735957430332	0.0752905667455872	0.694240974796812	-4.3910758176688
a_HQ_transcript/121472	-4.21517455581088	0.866916197002612	-1.83732246137049	0.0752961799981898	0.694240974796812	-4.3960221236939
a_HQ_transcript/151241	2.04785369729237	0.328927325569736	1.83730307170527	0.0752991127862198	0.694240974796812	-4.31850249334278
a_HQ_transcript/80874	-2.50754511288777	0.792823472846054	-1.83727596531755	0.0753032129323509	0.694240974796812	-4.39223070099557
a_HQ_transcript/55544	1.7883042117038	0.494110786095931	1.83720276612124	0.075314286087283	0.694240974796812	-4.2793340158112
a_HQ_transcript/14704	-0.67028171609787	1.88549405174619	-1.83716344288095	0.0753202352560062	0.694240974796812	-4.30115934988118
a_HQ_transcript/78501	-1.65010550815778	-0.352057173549227	-1.83713086796455	0.0753251637824041	0.694240974796812	-4.29278088321023
a_HQ_transcript/94745	1.66555950462056	0.906991937566479	1.83706834031113	0.0753346248760496	0.694240974796812	-4.3248803410946
a_HQ_transcript/64715	-2.31674453339746	2.33635658247784	-1.83692234366284	0.0753567196613821	0.694240974796812	-4.4772872591728
a_HQ_transcript/20448	1.69033290279389	-0.582458313664225	1.83687848700578	0.0753633579045173	0.694240974796812	-4.32143680287764
a_HQ_transcript/83505	-0.765499019473219	2.7175199890187	-1.83679112805233	0.0753765822423662	0.694240974796812	-4.37792429491868
a_HQ_transcript/131555	2.04194305360148	1.46107724556302	1.83664339689395	0.0753989501928945	0.694240974796812	-4.35753917319209
a_HQ_transcript/140722	2.87510510598795	4.06856163155479	1.83663511481411	0.0754002043490615	0.694240974796812	-4.71818506156041
a_HQ_transcript/122279	2.26746585815358	-0.161915920949282	1.83659131739566	0.0754068368927255	0.694240974796812	-4.29393408447801
a_HQ_transcript/9908	1.95295794946678	0.0200691408599067	1.83658748910093	0.0754074166613532	0.694240974796812	-4.3237171323418
a_HQ_transcript/126801	-0.60217216898467	3.19229899545897	-1.83656985932908	0.0754100866168984	0.694240974796812	-4.60284613394417
a_HQ_transcript/23350	-1.58586227220293	-0.39787777962456	-1.8359742930472	0.0755003300998758	0.694502578091975	-4.32277762023809
a_HQ_transcript/97302	2.27458233791041	0.637689865362036	1.83592876809926	0.075507232083774	0.694502578091975	-4.25634926399706
a_HQ_transcript/6658	1.71229543614685	1.28036008261189	1.83578510843621	0.0755290156899342	0.694502578091975	-4.25403419859215
a_HQ_transcript/6744	0.915390345470745	2.82268837747788	1.83574239857167	0.0755354929677351	0.694502578091975	-4.52376530148488
a_HQ_transcript/19512	2.39275603346746	0.192410359512197	1.83558449263897	0.0755594447302373	0.694502578091975	-4.27822017346424
a_HQ_transcript/73415	1.61619920935469	0.567319061607399	1.83536771676554	0.0755923366693963	0.694502578091975	-4.4098868000614
a_HQ_transcript/111674	1.63484879340047	2.61924841529286	1.8353600966644	0.07559349310861	0.694502578091975	-4.4471761860275
a_HQ_transcript/152860	3.11807632933171	1.03516272235684	1.83525182598739	0.0756099260789577	0.694502578091975	-4.39696731586425
a_HQ_transcript/145150	2.33287735489426	1.04762296283038	1.83518861272006	0.0756195217929791	0.694502578091975	-4.28166105355828
a_HQ_transcript/117764	-2.00914678641283	0.413812663220845	-1.8351206951644	0.0756298327724006	0.694502578091975	-4.42161266198652
a_HQ_transcript/140829	1.40110223010887	3.22693381476949	1.83508383546723	0.0756354291709973	0.694502578091975	-4.56322238553819
a_HQ_transcript/133723	0.860595509112838	6.44017608282652	1.83503186883157	0.075643319853021	0.694502578091975	-4.96254003352407
a_HQ_transcript/121135	1.95629635942826	1.24265900432851	1.83478470498529	0.0756808591584788	0.694502578091975	-4.25114457341171
a_HQ_transcript/112602	1.90803304136798	2.44578258419741	1.83476247746196	0.0756842358596558	0.694502578091975	-4.28885122349695
a_HQ_transcript/13006	-1.88783768845707	0.82905733284955	-1.83465882085926	0.075699984583888	0.694502578091975	-4.45511211491644
a_HQ_transcript/133683	-3.17829204857932	2.07207859437248	-1.83462200556236	0.0757055786680326	0.694502578091975	-4.51637952045232
a_HQ_transcript/11072	-0.585385560445611	2.95675562369909	-1.8346191448649	0.0757060133657751	0.694502578091975	-4.50325348112803
a_HQ_transcript/242	-2.1630949911906	0.0482999139127171	-1.83459730267505	0.0757093324695009	0.694502578091975	-4.31221425261991
a_HQ_transcript/50782	-1.52361660696991	0.988970204889564	-1.83456482440381	0.0757142680446374	0.694502578091975	-4.29423646022005
a_HQ_transcript/127416	0.567357690012083	5.48294704476433	1.83453993489419	0.0757180505761364	0.694502578091975	-4.84492770116938
a_HQ_transcript/7874	1.38578212245046	0.944856474131387	1.8345390757319	0.0757181811484212	0.694502578091975	-4.32367604861776
a_HQ_transcript/10145	-1.24987527911683	2.93116317378367	-1.83452552037936	0.0757202412658547	0.694502578091975	-4.4601125689618
a_HQ_transcript/131958	-2.17191213775607	-0.179573100664744	-1.83449858359684	0.0757243352103403	0.694502578091975	-4.39126219046905
a_HQ_transcript/6115	-3.03911772146439	0.862435039264105	-1.83449256385796	0.0757252501367144	0.694502578091975	-4.44805655828607
a_HQ_transcript/152396	1.43077503593524	1.32256637519788	1.83437851441347	0.0757425860350616	0.694518169854712	-4.22673165166296
a_HQ_transcript/26965	2.49050454032561	3.28986148196777	1.83432417924846	0.0757508463542333	0.694518169854712	-4.72322791384519
a_HQ_transcript/149904	4.72666108573452	0.58227621132899	1.83413970144013	0.0757788973856949	0.694580316589254	-4.42781901446173
a_HQ_transcript/133596	2.52039386037253	4.4255009911387	1.83408064599453	0.0757878790179994	0.694580316589254	-4.60352043160376
a_HQ_transcript/106098	8.09702948513447	2.58985834721346	1.83404387352948	0.0757934721326321	0.694580316589254	-4.41204444518286
a_HQ_transcript/50984	1.33562641939483	3.0412734621769	1.83388601110485	0.0758174871090035	0.694621586511586	-4.53248424392996

a_HQ_transcript/71185	1.90440016511903	-0.101749658524449	1.83374618171547	0.0758387642176493	0.694621586511586	-4.42330466413148
a_HQ_transcript/111937	-1.44173613656448	0.437384037465298	-1.83365578679525	0.0758525218539701	0.694621586511586	-4.35263580169838
a_HQ_transcript/51682	-2.77313995973696	0.872874696414875	-1.833645681847	0.0758540599068934	0.694621586511586	-4.53617217596253
a_HQ_transcript/7667	-0.669678569072946	2.98775476777926	-1.83362160389375	0.0758577248688334	0.694621586511586	-4.55592347135118
a_HQ_transcript/82276	2.10401809004276	1.23677697972812	1.83327760550402	0.0759101021845024	0.69497579347753	-4.26047057650782
a_HQ_transcript/71014	-2.19616120139453	0.126622935572411	-1.8332105297745	0.0759203187548568	0.69497579347753	-4.31601660541765
a_HQ_transcript/65942	-0.962359197600759	1.67804373585667	-1.8331209335276	0.0759339673457084	0.694991285446597	-4.38688179469067
a_HQ_transcript/66517	1.74064715734517	-0.321905981843119	1.83297614839104	0.0759560275306198	0.695032867303535	-4.33645427423963
a_HQ_transcript/81688	-3.48009925449612	-0.11103778786561	-1.83293417119835	0.0759624244078475	0.695032867303535	-4.32032369000104
a_HQ_transcript/92539	0.676523701071157	3.9090386923528	1.8326481652717	0.0760060209084544	0.695307617451627	-4.66254218608568
a_HQ_transcript/27161	2.98302985613588	3.78914325016031	1.83254693083849	0.0760214574087784	0.695307617451627	-4.698869150276
a_HQ_transcript/112353	1.55881960862626	-0.633491762483669	1.83246561621082	0.0760338584212697	0.695307617451627	-4.37423561015057
a_HQ_transcript/132398	-1.74915456883639	1.41210373851352	-1.83242338649315	0.076040299410611	0.695307617451627	-4.38214658096278
a_HQ_transcript/126700	-0.543991905167539	2.73458792509964	-1.83183107670069	0.0761306891719879	0.696024646144906	-4.42411030156506
a_HQ_transcript/87414	-2.40924972948021	2.51803065162413	-1.83153393457999	0.0761760692420913	0.696299336265443	-4.48328555127983
a_HQ_transcript/137391	2.07692182123632	0.379216459199589	1.83138224652966	0.0761992442091219	0.696299336265443	-4.31820575204453
a_HQ_transcript/131761	2.80601643757895	1.45756546207304	1.83126044953725	0.0762178567624947	0.696299336265443	-4.30574402475164
a_HQ_transcript/16602	-2.17027103478218	0.955256715405652	-1.83122265423849	0.0762236332851536	0.696299336265443	-4.28744532727546
a_HQ_transcript/56716	1.30370153173986	1.48697300573661	1.83120357101129	0.0762265500516049	0.696299336265443	-4.2961775706694
a_HQ_transcript/42287	-2.03399665077334	1.15854607170766	-1.83108236690161	0.076245077657843	0.696299336265443	-4.37557576105431
a_HQ_transcript/90596	0.609834607431885	4.83779040222296	1.83089982637401	0.0762729885767258	0.696299336265443	-4.82424851223551
a_HQ_transcript/141165	-2.4792081007613	0.903698255264532	-1.8308361466599	0.076282727421019	0.696299336265443	-4.48249867043154
a_HQ_transcript/56250	2.20161449838652	1.17353699116375	1.83078647556519	0.0762903245966011	0.696299336265443	-4.29234886902237
a_HQ_transcript/141619	-2.73759405196151	-0.146525223013046	-1.83071974272291	0.0763005323766349	0.696299336265443	-4.37034319315284
a_HQ_transcript/86665	3.20739803582397	2.83333080818181	1.83063536968229	0.0763134401556104	0.696299336265443	-4.44979849082069
a_HQ_transcript/124735	1.25006492454643	1.78671798316128	1.83037907783252	0.0763526602988799	0.696299336265443	-4.44364725566055
a_HQ_transcript/132017	2.95848254285842	0.264997292863454	1.83027286530285	0.0763689189604702	0.696299336265443	-4.37648754960163
a_HQ_transcript/147931	2.36651231441095	3.6125921034305	1.83022173584954	0.0763767467408639	0.696299336265443	-4.69166311078237
a_HQ_transcript/44833	1.74795591200736	-0.0864862449972668	1.83019074077752	0.0763814923354975	0.696299336265443	-4.37961714934101
a_HQ_transcript/130250	-2.07960245495259	2.62494164045139	-1.83018296213812	0.076382683347202	0.696299336265443	-4.46852682453181
a_HQ_transcript/148063	1.50244263820374	-0.52011437740051	1.83011304963112	0.076393388580186	0.696299336265443	-4.38572844768486
a_HQ_transcript/130614	2.15458631611438	0.374460718623598	1.83007718281982	0.0763988811211495	0.696299336265443	-4.32836921748562
a_HQ_transcript/148586	-0.566226269663446	8.75711854410776	-1.82997964553798	0.0764138194059118	0.696299336265443	-5.17721979777017
a_HQ_transcript/112906	-1.92561123664285	1.21609320939706	-1.82991328696763	0.0764239839517226	0.696299336265443	-4.24635300194314
a_HQ_transcript/149266	2.38885749654011	0.535570854214642	1.829817344403	0.0764386820977309	0.696299336265443	-4.35232230314669
a_HQ_transcript/106787	2.14653096789255	0.791727065518258	1.82976760361884	0.0764463032030749	0.696299336265443	-4.27123137871288
a_HQ_transcript/95004	0.834535875293207	3.93591714583833	1.8297636796626	0.0764469044452285	0.696299336265443	-4.69824019792557
a_HQ_transcript/118973	1.23610511427219	3.07700944056492	1.82974817603043	0.0764492800048623	0.696299336265443	-4.53452826433145
a_HQ_transcript/45612	1.76162419883745	-0.22744228626999	1.82957011426485	0.0764765682143021	0.696299336265443	-4.38416878752736
a_HQ_transcript/61393	1.60365982265283	-0.725287489235654	1.82948691540706	0.0764893214017649	0.696299336265443	-4.38326181432725
a_HQ_transcript/71	1.04698541388601	3.47856158952523	1.82944381871216	0.0764959282182059	0.696299336265443	-4.52916631179541
a_HQ_transcript/130493	3.00487699089969	0.433912274596567	1.82940837371978	0.0765013623770193	0.696299336265443	-4.42269201733995
a_HQ_transcript/111786	2.02932809846556	0.928828748382251	1.82934507232043	0.0765110680893552	0.696299336265443	-4.32452748439705
a_HQ_transcript/151417	-4.31033973938585	5.94916469651856	-1.82923643739388	0.0765277270314696	0.696299336265443	-5.04182196115913
a_HQ_transcript/126301	-2.64189915181851	1.32717819610642	-1.82910413847306	0.0765480189775381	0.696299336265443	-4.40568531881701
a_HQ_transcript/14118	1.42656887802558	1.54274581142368	1.82908775185686	0.0765505326684832	0.696299336265443	-4.3210611750721
a_HQ_transcript/109993	1.71795638473064	-0.564639615845047	1.82905190024939	0.0765560325156971	0.696299336265443	-4.36964196511692
a_HQ_transcript/126232	1.39999034213751	-0.7726916240976	1.82869872505052	0.0766102296864947	0.6966832652091	-4.38597300911507
a_HQ_transcript/78041	-1.6674631742451	-0.439063470300438	-1.82849851043502	0.0766409685359806	0.696756086529767	-4.38048166989914
a_HQ_transcript/113719	-1.28601337242171	1.22048731552273	-1.82849042186189	0.0766422105916483	0.696756086529767	-4.23548183069401

a_HQ_transcript/44063	-0.609428030138961	2.910542920821	-1.82825639828236	0.0766781539475779	0.696973844044536	-4.5242501644514
a_HQ_transcript/113934	2.67080124730235	1.16542862343765	1.82810218871174	0.076701846639852	0.697080197229725	-4.4582253641117
a_HQ_transcript/95422	0.478244419563664	5.67611144978673	1.82798898409796	0.0767192433227974	0.69712930684189	-4.90769168269183
a_HQ_transcript/42794	2.93679748746335	0.0890347243303459	1.82746936739384	0.0767991384417308	0.697667485871908	-4.36464110922353
a_HQ_transcript/130953	2.27195132697918	1.15774266769093	1.82744768009684	0.0768024745749783	0.697667485871908	-4.40746927988607
a_HQ_transcript/35721	1.68812353123844	1.96779691916153	1.82726034300572	0.0768312975813897	0.697784265538648	-4.34509826479746
a_HQ_transcript/17465	-2.05760183635963	0.191253276981692	-1.82718672179328	0.076842627199587	0.697784265538648	-4.41197112051933
a_HQ_transcript/22261	-1.71515513191405	1.99902379963436	-1.82713009154966	0.0768513430356872	0.697784265538648	-4.48164303062923
a_HQ_transcript/136665	1.31225311878715	-0.506023134392091	1.82682845108429	0.0768977820636336	0.698087849121135	-4.4294144328043
a_HQ_transcript/32190	-1.25469144779202	2.54608559142074	-1.82675692359707	0.0769087975807141	0.698087849121135	-4.36480744602707
a_HQ_transcript/104786	4.32561203474077	1.60860918746114	1.82666537022288	0.0769228991276186	0.69810683536147	-4.46875878502194
a_HQ_transcript/34796	1.67910437407212	-0.285606822394245	1.82643711421341	0.0769580659578225	0.698316961910132	-4.38293961781953
a_HQ_transcript/109434	1.69172058520745	-0.603047753050747	1.82631523396342	0.0769768493565233	0.698353458735321	-4.3814356539115
a_HQ_transcript/100434	-1.04947657321816	3.4391609639446	-1.82616117343039	0.0770005977674317	0.698353458735321	-4.51693711552946
a_HQ_transcript/21098	-1.70914383842863	-0.344141968730723	-1.82613523926805	0.0770045961295162	0.698353458735321	-4.34401041201109
a_HQ_transcript/72215	1.87441381859751	-0.0362673011158108	1.82609925435342	0.0770101443451247	0.698353458735321	-4.28928634035804
a_HQ_transcript/130677	5.00318341949712	1.59827330111731	1.82596118425051	0.0770314353928956	0.698437572378449	-4.23419657751912
a_HQ_transcript/120343	-1.26967731204763	2.24738512051227	-1.82564739393759	0.0770798419773146	0.698598103855676	-4.48985825555114
a_HQ_transcript/143176	2.14201834839476	-0.428790886495598	1.82563248012951	0.077082143288264	0.698598103855676	-4.38124530287364
a_HQ_transcript/106628	-5.41823616313939	1.37401270991443	-1.82559707102806	0.0770876074093395	0.698598103855676	-4.38908370042072
a_HQ_transcript/108281	1.11796485646929	-0.217380792298459	1.82547572462502	0.0771063353670915	0.698598103855676	-4.3062488862457
a_HQ_transcript/12295	-2.28592931587651	0.92138946641629	-1.82545695872701	0.0771092319422313	0.698598103855676	-4.33611150223959
a_HQ_transcript/77912	-1.43645618755213	1.19930923673607	-1.82532114410776	0.0771301981199962	0.698679157911663	-4.3050019925403
a_HQ_transcript/57962	4.1479600784564	0.821243195839658	1.82513186945068	0.0771594252182689	0.698835006090298	-4.38915237285783
a_HQ_transcript/145155	3.31656219086848	1.82141969747434	1.82470240060036	0.0772257772473004	0.699116534158413	-4.39677698580151
a_HQ_transcript/12306	-1.35396040698642	3.89701222569097	-1.82462630136572	0.0772375394905315	0.699116534158413	-4.59170179765126
a_HQ_transcript/35675	-2.04397991270271	0.192824291677393	-1.82462241153976	0.0772381407607246	0.699116534158413	-4.31219586473177
a_HQ_transcript/147642	-1.86794633066622	1.81322375767255	-1.82461932465869	0.0772386179184787	0.699116534158413	-4.38374980304113
a_HQ_transcript/67287	1.97269020234133	0.0967532290258041	1.8244214597161	0.0772692083355721	0.699135413998696	-4.36723682090576
a_HQ_transcript/132158	1.86718172324524	0.292474631439666	1.82442004266191	0.0772694274529562	0.699135413998696	-4.37293344500301
a_HQ_transcript/49089	6.20171359108911	1.74915523057457	1.82437245414686	0.0772767863154009	0.699135413998696	-4.45749256424041
a_HQ_transcript/78547	-3.70092883465577	3.34333568459461	-1.82426204091559	0.0772938623956552	0.699181082062659	-4.47415601439773
a_HQ_transcript/97877	2.21439869512994	-0.0682045369537596	1.82389960619216	0.0773499377512177	0.699515441285265	-4.40610166907407
a_HQ_transcript/114007	0.787471383592927	4.44949540007421	1.82386758541887	0.0773548936241	0.699515441285265	-4.73231810054701
a_HQ_transcript/128282	0.553934676870713	3.86997475997817	1.82347325220258	0.0774159469913229	0.699696702775443	-4.70971163836432
a_HQ_transcript/121002	-2.34856963810905	1.84743199251584	-1.82346772276971	0.0774168033879145	0.699696702775443	-4.5345739434984
a_HQ_transcript/95898	1.51589806838042	-0.403373774379444	1.82343375323363	0.0774220647547585	0.699696702775443	-4.28884787913122
a_HQ_transcript/52441	0.702910636923437	3.07617427176031	1.82341566667365	0.0774248662134201	0.699696702775443	-4.52945643978391
a_HQ_transcript/146454	2.53185545122078	-0.160719423307683	1.82334944578847	0.0774351240186214	0.699696702775443	-4.38553223682196
a_HQ_transcript/719	1.35449773387204	-0.340666803723455	1.82297984155409	0.0774923980551573	0.699986715006456	-4.35109637059238
a_HQ_transcript/52124	-2.72076814916287	0.973929647416815	-1.82292577669771	0.07750077899589	0.699986715006456	-4.46166023595443
a_HQ_transcript/135440	-2.17900084179819	-0.563339325655181	-1.8228523284926	0.0775121659117847	0.699986715006456	-4.36386010201427
a_HQ_transcript/146198	3.87027981789652	3.6177087533595	1.82283154575607	0.077515388186357	0.699986715006456	-4.71568931180902
a_HQ_transcript/53862	-1.90857824557493	0.506198597341985	-1.8225843122426	0.0775537294460973	0.700214533946175	-4.25668871504982
a_HQ_transcript/51528	-1.92138296761993	1.49425087288644	-1.82248882324525	0.0775685423170521	0.700214533946175	-4.35193135711835
a_HQ_transcript/133260	-1.62188038146245	0.9352824187275	-1.8224118588314	0.0775804832898337	0.700214533946175	-4.29538300450609
a_HQ_transcript/86499	-1.15489203129156	3.28713449848632	-1.8223582550425	0.0775888008042134	0.700214533946175	-4.60887018254213
a_HQ_transcript/83396	1.75056809182533	0.0923177408538135	1.82215324149897	0.0776206190585812	0.700269216270914	-4.36218535339957
a_HQ_transcript/1046	-0.902271240593124	3.73655815389481	-1.82205708050375	0.0776355471475193	0.700269216270914	-4.70017798488381
a_HQ_transcript/8447	0.512324583496217	4.53893200692112	1.82203144823943	0.0776395267281839	0.700269216270914	-4.79326494463775

a_HQ_transcript/128716	4.90680169130028	1.46449382291398	1.82200876832818	0.0776430480812353	0.700269216270914	-4.32506031987408
a_HQ_transcript/152101	-2.10256929899953	1.81861292461302	-1.82189700860809	0.0776604022284931	0.70031707426898	-4.4813477695702
a_HQ_transcript/135236	1.97069102064615	3.40165175154536	1.82152606443824	0.0777180264610385	0.700660577504019	-4.48608384809372
a_HQ_transcript/129782	0.986369446535936	1.30948238251446	1.82149662097875	0.0777226019086485	0.700660577504019	-4.29369191765666
a_HQ_transcript/7215	1.56092334698553	1.67761302044347	1.82123026806716	0.0777640029860468	0.700831710935678	-4.41826893787665
a_HQ_transcript/61398	2.5564334397273	1.48033432934863	1.82121936113929	0.0777656987258313	0.700831710935678	-4.37088353309861
a_HQ_transcript/108249	-1.87945844681605	0.72014800916308	-1.82106750926301	0.0777893109633975	0.700916978276726	-4.25797749455903
a_HQ_transcript/53876	3.06509356947142	3.11429253341735	1.82097167370278	0.0778042160771289	0.700916978276726	-4.57058929502624
a_HQ_transcript/81958	-2.91935449528916	2.3074871234484	-1.82075303340161	0.0778382298771199	0.700916978276726	-4.59394908418489
a_HQ_transcript/118450	4.25885574008224	1.45393880774432	1.82069063186082	0.0778479399882596	0.700916978276726	-4.43519156071599
a_HQ_transcript/143979	-0.747244069816413	2.6276219802134	-1.82064365783144	0.0778552501528759	0.700916978276726	-4.52748576716483
a_HQ_transcript/55354	1.61211303981748	0.306122522596024	1.82061167331254	0.0778602279634729	0.700916978276726	-4.2624436117157
a_HQ_transcript/5444	-1.28726053931155	-0.428895698306195	-1.82055848694374	0.0778685060581031	0.700916978276726	-4.33432120327791
a_HQ_transcript/148843	2.691613246098	0.203056280854515	1.82053844607554	0.0778716254767254	0.700916978276726	-4.43608408592416
a_HQ_transcript/24471	1.61994139849222	-0.527348962136672	1.82030395517138	0.0779081325708832	0.700969858176241	-4.42573299122897
a_HQ_transcript/99790	-2.02851795769543	1.16837889998805	-1.8201359029342	0.0779343050236295	0.700969858176241	-4.35533468301754
a_HQ_transcript/122620	2.2956096787659	1.19189773712269	1.82007322058791	0.0779440690892648	0.700969858176241	-4.48977947753552
a_HQ_transcript/150756	3.41818173093755	0.954055372666516	1.82005660851497	0.0779466569362601	0.700969858176241	-4.43955500232569
a_HQ_transcript/63932	0.847946690759949	2.27963002240967	1.81999725961226	0.0779559029715579	0.700969858176241	-4.40875963521213
a_HQ_transcript/81632	2.35694056286416	0.846907710242548	1.81999211647388	0.0779567042711264	0.700969858176241	-4.35751249305423
a_HQ_transcript/114108	1.32611954903719	1.99172098930074	1.81995867937499	0.077961913933206	0.700969858176241	-4.35317733540747
a_HQ_transcript/60566	-1.59667453787479	1.52423446743669	-1.81982145831213	0.0779832967464656	0.701053676659225	-4.37334241271174
a_HQ_transcript/150077	0.513747128754775	8.36702070078066	1.81948561725565	0.0780356510941631	0.701372550212546	-5.15635724426189
a_HQ_transcript/8618	-1.55424801623215	1.68661164055048	-1.81938295648999	0.0780516608815383	0.701372550212546	-4.33282402441349
a_HQ_transcript/147090	1.80614827807095	0.864983696155596	1.81912144353504	0.078092456064905	0.701372550212546	-4.25486426530905
a_HQ_transcript/64415	-2.60200169958869	0.591959090127803	-1.81906503249638	0.0781012583875728	0.701372550212546	-4.48701755080654
a_HQ_transcript/84719	1.69419079471475	-0.339626677947393	1.81904820893647	0.0781038836828344	0.701372550212546	-4.34558318893589
a_HQ_transcript/77356	-2.21424835935107	2.19964647407581	-1.81904795062033	0.0781039239933265	0.701372550212546	-4.53251199100984
a_HQ_transcript/99601	1.52109386680341	-0.532285734282634	1.8189874697142	0.0781133625873615	0.701372550212546	-4.39562021986899
a_HQ_transcript/121440	-2.97144008318532	1.54563071747572	-1.81882781009279	0.0781382835878205	0.701372550212546	-4.48350357022284
a_HQ_transcript/100021	-2.13315506721283	-8.81395862485356e-05	-1.81882005256365	0.0781394946199471	0.701372550212546	-4.3892541555934
a_HQ_transcript/134783	-2.62936112318283	1.98287264276559	-1.81880095526226	0.0781424759782619	0.701372550212546	-4.52918400238531
a_HQ_transcript/140195	-2.0413671009868	0.0120846404183386	-1.81869537391218	0.078158960466125	0.701372550212546	-4.38922282104959
a_HQ_transcript/57852	1.63683510178622	-0.104532639227977	1.8186659216836	0.0781635593909454	0.701372550212546	-4.38813289389655
a_HQ_transcript/74932	2.38762199099167	0.700424292719347	1.81848683585312	0.0781915283584317	0.701461372128076	-4.34688623866279
a_HQ_transcript/57189	-1.55790812473133	-0.533236895104699	-1.81843348828872	0.078199861632966	0.701461372128076	-4.3894012765312
a_HQ_transcript/133795	2.435447806291	0.462785373230853	1.81834605246171	0.0782135213761336	0.701461372128076	-4.41246003187005
a_HQ_transcript/132252	-2.66470621039997	0.926200225824389	-1.81825565777304	0.0782276455068833	0.701461372128076	-4.38956079271853
a_HQ_transcript/144604	3.26425517754514	0.595680605415644	1.81820029815178	0.0782362964962249	0.701461372128076	-4.48442805501019
a_HQ_transcript/70905	-3.28200215115298	3.95488914365575	-1.8179113675264	0.0782814606048456	0.701461372128076	-4.77421700180358
a_HQ_transcript/95893	2.60021212434721	0.402313636400597	1.81789876344701	0.0782834313133228	0.701461372128076	-4.42241248637326
a_HQ_transcript/50138	-2.04880809191837	0.288094591202809	-1.81783759062041	0.0782929965794184	0.701461372128076	-4.40251015825396
a_HQ_transcript/89714	1.63106155801262	3.04191747344767	1.81781098876369	0.0782971564793274	0.701461372128076	-4.41690910899962
a_HQ_transcript/135490	1.52938140809269	2.89749303505933	1.81778017108947	0.0783019758685391	0.701461372128076	-4.51171921249098
a_HQ_transcript/117585	2.23954089007711	-0.631319652344633	1.81766176682252	0.0783204947441612	0.701461372128076	-4.40437271321892
a_HQ_transcript/37750	1.57346604947674	1.83314881312409	1.81760338607494	0.0783296270870915	0.701461372128076	-4.30374379956185
a_HQ_transcript/102108	1.84962569263399	1.00660808330823	1.81743701630784	0.0783556568361117	0.701461372128076	-4.2841888205226
a_HQ_transcript/100988	-1.69977975377114	0.839887937641553	-1.8173873133105	0.0783634346572769	0.701461372128076	-4.31117618501076
a_HQ_transcript/10966	-1.77955382426406	1.03298812777499	-1.81736145739896	0.0783674810043574	0.701461372128076	-4.26719625548949
a_HQ_transcript/38135	-1.42298125662397	1.50691051294607	-1.81736039320384	0.0783676475504634	0.701461372128076	-4.35642029679294

a_HQ_transcript/2827	-2.22119360159232	1.35624543561546	-1.81729037494958	0.0783786060413545	0.701461372128076	-4.47529089067547
a_HQ_transcript/68877	1.5815420277576	1.01840502513898	1.81660731438256	0.0784855796876399	0.702310618239399	-4.2930794476285
a_HQ_transcript/96077	2.2949533408933	1.81167732185996	1.81638164388492	0.078520949101804	0.702518967121697	-4.42224097277341
a_HQ_transcript/4519	-1.82987320761333	-0.315107617536113	-1.81619599903152	0.0785500554574496	0.702559408140334	-4.41208675234618
a_HQ_transcript/93081	1.38530517070338	-0.391937224414307	1.81600766557528	0.0785795927332029	0.702559408140334	-4.43741438573931
a_HQ_transcript/150776	-2.28120214688297	-0.157801461778229	-1.81595285222211	0.078588191161511	0.702559408140334	-4.38941442193966
a_HQ_transcript/63989	1.20643715156311	1.62702212796037	1.81572764060717	0.078623527930757	0.702559408140334	-4.34778102498832
a_HQ_transcript/17998	-1.30877954281757	2.19658937270005	-1.81572678768427	0.0786236617841109	0.702559408140334	-4.38271317667208
a_HQ_transcript/107223	-2.73537703249727	2.46236246256863	-1.81572528356104	0.0786238978339844	0.702559408140334	-4.54072480293356
a_HQ_transcript/80801	1.53346059352671	-0.570657808664246	1.81570642242329	0.0786268578615524	0.702559408140334	-4.45209431023415
a_HQ_transcript/8081	-2.58186396445129	-0.250456764150659	-1.81545522328898	0.0786662895769051	0.702559408140334	-4.42624281855126
a_HQ_transcript/34470	2.54667158428022	1.46037053238935	1.81537358801176	0.0786791078115986	0.702559408140334	-4.5214654347275
a_HQ_transcript/113478	1.35361186719998	-0.583175137130011	1.81520986469248	0.0787048207329488	0.702559408140334	-4.38623058893112
a_HQ_transcript/78246	1.53781057450388	-0.447775352752182	1.81516102289092	0.0787124927751478	0.702559408140334	-4.40191639758752
a_HQ_transcript/62140	-1.88316172403454	1.81761058300677	-1.81515322952589	0.0787137170113604	0.702559408140334	-4.33377056101108
a_HQ_transcript/64421	2.45133931339737	0.988813263864863	1.81509276574501	0.0787232156351649	0.702559408140334	-4.3769136652255
a_HQ_transcript/75462	-0.934232402553716	3.05629200717185	-1.81504865283798	0.0787301462162318	0.702559408140334	-4.55106683980331
a_HQ_transcript/105730	3.26045664786918	0.23948154802249	1.81500781656402	0.0787365624683171	0.702559408140334	-4.32235302244171
a_HQ_transcript/123543	-2.48257740577158	0.928672662843972	-1.81500402706839	0.0787371579017059	0.702559408140334	-4.43438151950583
a_HQ_transcript/29894	-0.475867704151828	3.22960857378118	-1.81496377084038	0.0787434834926169	0.702559408140334	-4.60529160029662
a_HQ_transcript/37910	2.26755494597441	3.2796540832058	1.81493587459987	0.0787478671726164	0.702559408140334	-4.52597940454524
a_HQ_transcript/48423	-1.89400569402266	0.00734165314015853	-1.81488977902683	0.0787551111932703	0.702559408140334	-4.42549178532748
a_HQ_transcript/61670	2.12256994775734	0.758839162116624	1.8147706295474	0.0787738384243961	0.702618640468513	-4.37357103613189
a_HQ_transcript/140815	-2.34111719972513	4.21259969182857	-1.81423050579937	0.078858779525912	0.702815428637714	-4.79194486271532
a_HQ_transcript/136441	-1.58271371412176	-0.625886814176595	-1.81422819552531	0.0788591430123112	0.702815428637714	-4.39843920424839
a_HQ_transcript/79621	1.55085822937033	0.466849877785373	1.81420839952423	0.0788622576697286	0.702815428637714	-4.35948143584682
a_HQ_transcript/25687	-2.51932575936171	1.99953314613926	-1.81413738366211	0.0788734320043047	0.702815428637714	-4.48537155130623
a_HQ_transcript/116223	1.82026253993041	0.151283993613452	1.81396564931368	0.078900459947337	0.702815428637714	-4.34814057109714
a_HQ_transcript/43883	-3.11331298595717	1.88355853527445	-1.81394053340416	0.078904413407134	0.702815428637714	-4.47993327336645
a_HQ_transcript/64196	-1.03112871364169	1.08924063107357	-1.81390056500789	0.078910705123543	0.702815428637714	-4.30388701061236
a_HQ_transcript/148139	1.42915037058869	-0.709043793460923	1.81384308598366	0.0789197540641194	0.702815428637714	-4.41330825907997
a_HQ_transcript/120249	5.54041616560984	0.0150786026946238	1.81383014910364	0.0789217908429122	0.702815428637714	-4.41973316859022
a_HQ_transcript/41455	2.4246301649483	0.663616197018449	1.81371639531562	0.0789397021341631	0.702815428637714	-4.39980670869486
a_HQ_transcript/114067	0.487789105096935	3.57271523797109	1.81370446965361	0.0789415801094119	0.702815428637714	-4.70877883940945
a_HQ_transcript/5048	-0.675851332891866	2.71464610546207	-1.81365047020831	0.0789500840647994	0.702815428637714	-4.48428725751001
a_HQ_transcript/75008	1.95936260514032	2.5536824805848	1.81363143489973	0.0789530819743371	0.702815428637714	-4.49197619015437
a_HQ_transcript/8218	-1.78390532459273	-0.114600406027651	-1.81348664072073	0.0789758890765148	0.702870324381953	-4.46176396489925
a_HQ_transcript/23780	1.71911897692551	-0.0189414434385591	1.8133763036857	0.078993272462542	0.702870324381953	-4.42131538934259
a_HQ_transcript/1523	2.20827605894133	2.25835973902075	1.81330461827908	0.0790045681045858	0.702870324381953	-4.39441373092614
a_HQ_transcript/140712	2.31452357020102	2.09989671235382	1.81328527731325	0.0790076159426039	0.702870324381953	-4.41180018089244
a_HQ_transcript/115339	0.727756266917162	2.55913257124746	1.81308326317627	0.0790394562378769	0.702900926422425	-4.41591299205295
a_HQ_transcript/40288	-3.03489042886355	3.32607543144558	-1.81308217716008	0.0790396274389517	0.702900926422425	-4.76848756152467
a_HQ_transcript/105628	2.53836042519897	0.911449772680461	1.81303330072016	0.0790473327144128	0.702900926422425	-4.4816113857553
a_HQ_transcript/39710	1.15941746364158	3.0477009501213	1.81281993844509	0.0790809763482538	0.703007694728605	-4.59278734199844
a_HQ_transcript/38314	-1.40495710477561	1.83116510080633	-1.81280375942338	0.079083528004238	0.703007694728605	-4.37290886816831
a_HQ_transcript/93318	2.90850023782696	0.264712066758571	1.81272483844435	0.0790959759411358	0.703010839374058	-4.39558781042092
a_HQ_transcript/37607	-0.596843729857593	2.1792426632203	-1.81242517466514	0.0791432560743504	0.703323526844494	-4.41063912902877
a_HQ_transcript/16944	-1.37573899645176	1.72378924150577	-1.81222228752494	0.0791752807063195	0.703500568159116	-4.36255882273255
a_HQ_transcript/128169	2.28435180014181	1.60538166923048	1.81203825254176	0.0792043391652594	0.703521388946868	-4.31324945862655
a_HQ_transcript/48903	1.37952044165289	4.33313595575822	1.81200536962083	0.0792095322152135	0.703521388946868	-4.76008322117708

a_HQ_transcript/126854	0.660579309109967	3.27979524578069	1.81197750557668	0.0792139328835889	0.703521388946868	-4.65750100745202
a_HQ_transcript/44904	1.06714873502977	3.12681762358205	1.81189674852677	0.0792266883092687	0.703527182713286	-4.54699679445843
a_HQ_transcript/77276	-1.63710139543637	3.03936234171416	-1.81166163700772	0.0792638336876522	0.703547917094105	-4.56500614028477
a_HQ_transcript/77561	1.79674857953545	-0.335154917655406	1.81155573855724	0.0792805694731962	0.703547917094105	-4.39353067196665
a_HQ_transcript/128598	1.4815366110391	-0.703093168843842	1.81129318220829	0.0793220758420795	0.703547917094105	-4.41251846502703
a_HQ_transcript/132163	2.45478106635895	3.34396597241376	1.81116019395603	0.0793431064185598	0.703547917094105	-4.56724687795809
a_HQ_transcript/34963	3.62968391027306	2.07137242211148	1.81112252189498	0.0793490646879073	0.703547917094105	-4.55193325391375
a_HQ_transcript/93665	2.35991573866699	1.44531712220985	1.81104451935458	0.0793614028962144	0.703547917094105	-4.37712000262209
a_HQ_transcript/134143	3.4453159753757	2.21105423005972	1.81087264665142	0.0793885949668058	0.703547917094105	-4.52360877427756
a_HQ_transcript/19160	-1.67514342882461	3.7831364436416	-1.81086412226752	0.079389943820361	0.703547917094105	-4.74829596973128
a_HQ_transcript/100332	-2.31752898465675	0.464155404467543	-1.81082717144882	0.0793957909484992	0.703547917094105	-4.38656752029405
a_HQ_transcript/96141	2.39822802816615	1.25415639833188	1.81078939294828	0.0794017694284908	0.703547917094105	-4.43785339299251
a_HQ_transcript/128527	6.84461235721429	4.53550735420552	1.81077767948737	0.0794036231714736	0.703547917094105	-4.72535131126595
a_HQ_transcript/148129	-3.36517515685192	3.53419273654559	-1.81074622474921	0.0794086013017476	0.703547917094105	-4.5803613874416
a_HQ_transcript/22197	-1.65368153653373	0.487503976185695	-1.8106869781065	0.0794179785913021	0.703547917094105	-4.30385523787128
a_HQ_transcript/80209	-0.720017768050732	2.20221211875912	-1.81066540236391	0.0794213937358668	0.703547917094105	-4.37747937658833
a_HQ_transcript/65573	2.00121843544908	0.423063720173664	1.81050010515611	0.0794475621657058	0.703547917094105	-4.26847899412625
a_HQ_transcript/121549	3.82148027877905	0.836104129531169	1.81036013766265	0.0794697263485141	0.703547917094105	-4.35274053308798
a_HQ_transcript/118954	-2.87368442550528	0.875719589952646	-1.81034864549143	0.0794715463946057	0.703547917094105	-4.37677789629368
a_HQ_transcript/126761	-0.410928029101101	4.72984854794593	-1.81034451265454	0.0794722009317789	0.703547917094105	-4.80129084447217
a_HQ_transcript/42811	1.73564922249256	1.59294254660272	1.81033926793855	0.0794730315691735	0.703547917094105	-4.31441328284107
a_HQ_transcript/136832	-2.24502659487996	0.0612151111380979	-1.81030014795927	0.0794792274699183	0.703547917094105	-4.34442983365082
a_HQ_transcript/93163	-1.70846058034424	0.836901174198557	-1.81021915164057	0.0794920571355056	0.703547917094105	-4.2854668648505
a_HQ_transcript/67013	-2.3209139409811	0.251960282892938	-1.81016879338983	0.0795000346765452	0.703547917094105	-4.47468597627099
a_HQ_transcript/110153	-0.596502938515462	4.30162911233675	-1.80998524165367	0.079529117931786	0.703547917094105	-4.76082041288293
a_HQ_transcript/137991	1.35128840411093	-0.785144228928464	1.80994261235963	0.079535873718245	0.703547917094105	-4.41584015907916
a_HQ_transcript/62040	-1.84891403165274	-0.0732513383284976	-1.80991982409771	0.0795394853466037	0.703547917094105	-4.46735042962603
a_HQ_transcript/41868	1.74834913686766	0.924300021302226	1.80989315416364	0.0795437123441793	0.703547917094105	-4.39908355133047
a_HQ_transcript/17695	0.521935856655689	3.89492582840077	1.80980501397123	0.0795576833061194	0.703564432560187	-4.72707741857565
a_HQ_transcript/72683	1.65589113820241	-0.258369932061173	1.80954311888411	0.0795992081970633	0.703626881541649	-4.34594072364598
a_HQ_transcript/83599	-1.95557071541953	1.50213304624853	-1.80950626988674	0.07960505228534	0.703626881541649	-4.3024731388438
a_HQ_transcript/91314	-1.56496938766615	-0.304400276068844	-1.8094340599773	0.0796165055165207	0.703626881541649	-4.32920758955034
a_HQ_transcript/65771	2.79608280905758	0.149093645737371	1.80943038293494	0.0796170887706027	0.703626881541649	-4.41502600808337
a_HQ_transcript/127013	3.52246578235299	1.34470841017314	1.80931570633498	0.0796352806477617	0.703626881541649	-4.39913636588408
a_HQ_transcript/42162	-2.25633845155981	0.0576981787066309	-1.80930251425496	0.079637373617921	0.703626881541649	-4.40119096681769
a_HQ_transcript/10382	0.676837247834292	3.84849438543985	1.8091450379725	0.0796623614003193	0.70374069049814	-4.69666305613542
a_HQ_transcript/75634	0.870488032800461	3.96339032995458	1.80888217667899	0.0797040861620353	0.704002297588024	-4.75403916439381
a_HQ_transcript/21824	2.34814204057825	0.874553235606097	1.80869474287502	0.0797338494434262	0.704088996152356	-4.33082909182743
a_HQ_transcript/13241	1.60340021287098	2.81142170273438	1.80866780825	0.0797381272651899	0.704088996152356	-4.51552806318423
a_HQ_transcript/63087	2.5127369882807	0.138503016051352	1.80858237677969	0.0797516969877679	0.704101859432712	-4.44417534418095
a_HQ_transcript/79420	2.62251148763335	0.493078114952787	1.80842611026657	0.0797765230545764	0.70417548726999	-4.44117945053345
a_HQ_transcript/44481	1.40839411466381	3.20839555060969	1.80837738706334	0.0797842650557418	0.70417548726999	-4.6075075784029
a_HQ_transcript/77686	1.26787742015029	-0.398145178210539	1.80809397241175	0.0798293116399912	0.704466103994141	-4.4482662362973
a_HQ_transcript/148141	3.18265588041796	4.06101319069337	1.80788838783141	0.0798620012656162	0.704564070749017	-4.63667900131298
a_HQ_transcript/2800	-1.34050509547559	1.94157541325575	-1.80780563018295	0.0798751636202056	0.704564070749017	-4.37945597491164
a_HQ_transcript/106445	-2.17143137412062	1.11390675588977	-1.8077934416267	0.0798771023291977	0.704564070749017	-4.38113490381175
a_HQ_transcript/63433	3.81070570930254	2.17536906215839	1.80769954736187	0.0798920384712054	0.704564070749017	-4.40080135133919
a_HQ_transcript/215	-0.620164207666138	2.14907503486642	-1.80764310797071	0.0799010176571965	0.704564070749017	-4.39829990917226
a_HQ_transcript/116499	-2.00676733801261	-0.120653154256573	-1.80738534618458	0.0799420370012723	0.704818857395716	-4.36704544751531
a_HQ_transcript/127590	1.9947717137545	-0.492672733763485	1.80707994088157	0.079990661350867	0.705053569823253	-4.44647633928063

a_HQ_transcript/127805	0.636708160073739	3.39418967717114	1.80706577570767	0.0799929172341101	0.705053569823253	-4.66897391807845
a_HQ_transcript/15198	-1.01560032684092	1.76978249530437	-1.80690288050314	0.080018863095598	0.705170673715717	-4.37514416463614
a_HQ_transcript/46586	-2.04110703071845	0.672956771175686	-1.80683004809267	0.0800304661179222	0.705170673715717	-4.33675022172837
a_HQ_transcript/136071	2.72016975691609	0.705852117413942	1.80674571485253	0.0800439031390251	0.705177070018712	-4.41386358444754
a_HQ_transcript/39030	-2.849100915458	1.80155810233997	-1.80667322319354	0.080055454945181	0.705177070018712	-4.33185519995148
a_HQ_transcript/87109	2.01312384483944	2.03011180211146	1.80659612690372	0.0800677420711799	0.70517844107781	-4.36888356090068
a_HQ_transcript/136507	2.51004379843134	0.920292171144417	1.80648280933647	0.0800858048289388	0.70518118213746	-4.51757969579199
a_HQ_transcript/90010	1.8516448423357	1.02124607514548	1.80639758680183	0.0800993915364686	0.70518118213746	-4.34066746728321
a_HQ_transcript/116715	-0.746044217923341	2.53681829105058	-1.80636587413514	0.0801044478676997	0.70518118213746	-4.50553201399263
a_HQ_transcript/24171	2.66126137572123	0.606368918506853	1.80615113847522	0.0801386928929598	0.705247036099355	-4.41478049386331
a_HQ_transcript/127371	1.95090406434984	-0.182555651237492	1.80612188038144	0.0801433597984219	0.705247036099355	-4.36457977957271
a_HQ_transcript/119685	3.56545058276594	2.25480881806429	1.80609074462584	0.0801483264600939	0.705247036099355	-4.48901144429513
a_HQ_transcript/48716	2.48074640713081	2.61149222409729	1.80579590541532	0.0801953711037828	0.705533419624438	-4.52956841960934
a_HQ_transcript/145407	1.73153419731831	0.712040556119144	1.80573465054862	0.0802051478956491	0.705533419624438	-4.39341188571684
a_HQ_transcript/124535	1.36326714633776	-0.656702065498605	1.80556570161257	0.0802321188183691	0.705663882988978	-4.38379715435751
a_HQ_transcript/66477	1.30178380761735	0.392387073544419	1.80532475534009	0.0802705966980464	0.705877041063202	-4.29576265784888
a_HQ_transcript/112479	1.74165650539741	1.69170044704253	1.80526187267484	0.0802806413169011	0.705877041063202	-4.3860778249296
a_HQ_transcript/141897	-2.80869343743911	0.402279447932202	-1.80483102217956	0.0803494923509593	0.706193046521859	-4.4805998029132
a_HQ_transcript/738	0.912058240944208	3.0396087836831	1.80474479288593	0.0803632780434977	0.706193046521859	-4.52672003334185
a_HQ_transcript/61097	-0.734067056142707	4.49139029323351	-1.80473494043537	0.0803648533073475	0.706193046521859	-4.81730187847525
a_HQ_transcript/42157	-1.06648744146811	4.01265613424517	-1.80468772857531	0.0803724021627892	0.706193046521859	-4.72737299424174
a_HQ_transcript/83774	1.70956090505234	1.04819523178772	1.80464910461902	0.0803785783190335	0.706193046521859	-4.38275047521725
a_HQ_transcript/82391	-2.67862472691452	0.844634500733216	-1.80455639980976	0.080393403909733	0.706193046521859	-4.49040697725149
a_HQ_transcript/135891	-1.94147774615675	-0.378929691437713	-1.80436449949291	0.0804241004857904	0.706193046521859	-4.39681082756451
a_HQ_transcript/109655	-2.19863659774172	0.339463422095052	-1.80435332666503	0.0804258880099165	0.706193046521859	-4.28516089912452
a_HQ_transcript/92378	2.33236698799686	3.98974077740556	1.80435311701397	0.0804259215520008	0.706193046521859	-4.67633607942534
a_HQ_transcript/37738	-1.7561108671499	0.339791389690696	-1.80414835759794	0.0804586866989491	0.706200845446603	-4.3480292698317
a_HQ_transcript/60734	-1.3236735431643	-0.737837141933198	-1.80404368388757	0.0804754407353775	0.706200845446603	-4.39655096747167
a_HQ_transcript/124704	1.4694759710761	3.21634367287887	1.80389572817315	0.0804991275314133	0.706200845446603	-4.45998344200967
a_HQ_transcript/144118	-2.61983184790649	1.98324598732863	-1.8038672485138	0.0805036876289591	0.706200845446603	-4.41776983099913
a_HQ_transcript/61000	-0.664587210841146	2.63813913552565	-1.80383948443351	0.0805081333609671	0.706200845446603	-4.48661363874439
a_HQ_transcript/117951	0.464636587067962	3.26697376770157	1.80383140404396	0.0805094272750609	0.706200845446603	-4.6372072824777
a_HQ_transcript/24745	-1.64642671097787	2.07725586572385	-1.80379486693229	0.0805152781893393	0.706200845446603	-4.46329595961087
a_HQ_transcript/24883	-2.6325833099425	0.6800623648627	-1.80374039203004	0.0805240022643147	0.706200845446603	-4.44903259319416
a_HQ_transcript/27133	0.382436068172121	3.51949200437149	1.80364616756739	0.0805390940691102	0.706226649577498	-4.68482766037144
a_HQ_transcript/130948	-1.44874931137233	4.45922148205741	-1.80353425099962	0.080557022717647	0.706257548253216	-4.80594126303326
a_HQ_transcript/1752	-2.26445080799811	-0.0149438384318723	-1.80347249118768	0.0805669178789409	0.706257548253216	-4.32966566025571
a_HQ_transcript/110989	2.00659008256521	2.0229994874179	1.80300384367024	0.0806420382795832	0.706783319528437	-4.43928768098323
a_HQ_transcript/102897	1.91070969876693	-0.0918326337540164	1.80294662624032	0.0806512138458595	0.706783319528437	-4.51196742544837
a_HQ_transcript/86049	0.852540122385754	2.73233453240803	1.80278582895001	0.0806770045529745	0.706902761630283	-4.63401868223557
a_HQ_transcript/55313	-1.24307600837173	-0.633784602852899	-1.80262728358921	0.0807024409284097	0.707002208771447	-4.36104478961346
a_HQ_transcript/8929	-0.848486201520973	3.68259592126841	-1.80256347587195	0.0807126799030987	0.707002208771447	-4.58768459751895
a_HQ_transcript/36410	3.03667419212734	3.19330959456691	1.80242188454181	0.0807354044521942	0.707094709958889	-4.6190637080158
a_HQ_transcript/54087	-1.52942411286544	0.377597630028734	-1.80221192980336	0.0807691109205884	0.707141970308897	-4.42604424450608
a_HQ_transcript/122098	3.70756551322919	3.18481638204652	1.8021450135157	0.0807798562804789	0.707141970308897	-4.53920547794906
a_HQ_transcript/132042	2.33825414514862	1.55112030283093	1.80207642094666	0.0807908720768136	0.707141970308897	-4.50557665989049
a_HQ_transcript/21179	1.9508579932929	-0.381533507429556	1.80203800616816	0.0807970419524437	0.707141970308897	-4.41213870761088
a_HQ_transcript/120376	-1.94189575850722	0.0740993541569013	-1.80200946123395	0.0808016268715885	0.707141970308897	-4.41507543592115
a_HQ_transcript/114299	0.628985251836648	4.32126025796238	1.80176646352749	0.0808406663854373	0.707373485085523	-4.7863745779692
a_HQ_transcript/152212	-0.934054073070375	1.74054448487808	-1.80151287075574	0.0808814251683686	0.707373485085523	-4.36318234220607

a_HQ_transcript/142454	4.47242033287143	1.45583513949109	1.8014754642624	0.0808874388168428	0.707373485085523	-4.52648902788949
a_HQ_transcript/55608	-4.48133729506428	1.1366025997457	-1.80138563833147	0.0809018812185361	0.707373485085523	-4.43102271482736
a_HQ_transcript/151553	1.71562671709446	-0.257002566250868	1.80137653193317	0.0809033454866638	0.707373485085523	-4.36531806131005
a_HQ_transcript/107486	3.09952593952556	1.43082038307046	1.80134270281316	0.0809087852550787	0.707373485085523	-4.50793597862644
a_HQ_transcript/34088	2.83309593031244	1.64204672328731	1.80131484170744	0.0809132655920322	0.707373485085523	-4.36135425679717
a_HQ_transcript/150720	4.18347310425885	2.32615441307857	1.8012120037132	0.0809298047672029	0.707411682933529	-4.55514349558741
a_HQ_transcript/79961	1.4708681823478	3.09225096979694	1.80108333433729	0.0809505023830107	0.707486212978446	-4.67273936559576
a_HQ_transcript/41590	-1.8582729402685	1.87188818991494	-1.80076044464852	0.0810024618692144	0.707599635920176	-4.47420627239338
a_HQ_transcript/59380	1.78301099435857	1.00084669137852	1.80074039974307	0.0810056884339883	0.707599635920176	-4.31469802471339
a_HQ_transcript/145360	-4.55227713796411	4.24036564406851	-1.80070037767946	0.0810121309850828	0.707599635920176	-4.61123565362248
a_HQ_transcript/61903	-0.586927206052879	2.55884724350737	-1.80070011829874	0.0810121727403106	0.707599635920176	-4.48608535175798
a_HQ_transcript/137348	3.4152471027262	0.792257237765684	1.80051886557621	0.0810413553569014	0.707664162812928	-4.41385650619933
a_HQ_transcript/152777	-1.86537873749059	1.19469578275098	-1.80050300870404	0.0810439088192551	0.707664162812928	-4.38301066373885
a_HQ_transcript/28427	-1.11036987893789	1.63766820117783	-1.80027039596324	0.0810813747476964	0.707782981040757	-4.34623493289331
a_HQ_transcript/138851	-4.49178716602117	2.25671765146966	-1.80026732905447	0.0810818688196808	0.707782981040757	-4.55398404773724
a_HQ_transcript/78897	-2.74664627216979	0.531585524198126	-1.80002678075868	0.0811206285666674	0.708014999598084	-4.46105894475413
a_HQ_transcript/92877	2.41226172384704	0.533905392011458	1.79994452366722	0.0811338863306877	0.708024402436603	-4.32019112774367
a_HQ_transcript/136657	-5.27548457542934	2.43523495549708	-1.79968064275366	0.0811764296866266	0.708241465017605	-4.5431858861306
a_HQ_transcript/7377	-1.59635176903828	-0.60985684555936	-1.79963910444679	0.0811831282929449	0.708241465017605	-4.41397661423228
a_HQ_transcript/74500	-1.90166912741408	1.87123256781911	-1.79952609503429	0.0812013549449836	0.708260741313318	-4.39155000906923
a_HQ_transcript/134152	-1.98137248848315	1.78662808047016	-1.79940515266674	0.0812208649085922	0.708260741313318	-4.44208681090082
a_HQ_transcript/79426	0.501382512645853	3.01227895728004	1.79939879030611	0.0812218913706747	0.708260741313318	-4.57309592512934
a_HQ_transcript/113626	2.71988296943263	3.03005384898116	1.79914323273598	0.081263130484748	0.708445186283213	-4.51171000848801
a_HQ_transcript/136272	2.52718521161479	0.229667740449217	1.7990967994631	0.0812706252923358	0.708445186283213	-4.32509886973003
a_HQ_transcript/141421	-1.812308521464	-0.629764724341763	-1.79904116420617	0.0812796061678149	0.708445186283213	-4.43251168777869
a_HQ_transcript/142158	-0.757413298394896	4.57373615962131	-1.79874873773531	0.0813268247283274	0.708591927939011	-4.83466053317024
a_HQ_transcript/89275	0.733436713387009	2.39792566185663	1.79850071783376	0.0813668911458682	0.708591927939011	-4.48507172961969
a_HQ_transcript/50045	-0.466320521580676	3.8495152449089	-1.798395514951	0.0813838912258498	0.708591927939011	-4.69908710470256
a_HQ_transcript/28247	1.11726337380278	4.08613010382624	1.79837561245431	0.081387107675024	0.708591927939011	-4.74733603202634
a_HQ_transcript/69686	-2.52536976167396	0.0285023525149859	-1.79832991988285	0.0813944924752377	0.708591927939011	-4.40249347328745
a_HQ_transcript/141506	-2.45298222967839	0.656279206068236	-1.79822475219635	0.0814114917623245	0.708591927939011	-4.39966575660821
a_HQ_transcript/143290	-3.60958687878766	5.02102764764615	-1.79817086137773	0.081420203833304	0.708591927939011	-4.92035543446781
a_HQ_transcript/53464	-0.716377415606263	2.94264701115003	-1.79813815272101	0.081425491950108	0.708591927939011	-4.57661554503569
a_HQ_transcript/128418	-2.55680579337058	-0.333260917749699	-1.79808059927274	0.0814347975146571	0.708591927939011	-4.41379187392778
a_HQ_transcript/138718	-3.15673307574524	1.85306254424795	-1.79793341187134	0.0814585997076244	0.708591927939011	-4.51373457720559
a_HQ_transcript/150609	5.21328815004871	2.37593363408106	1.79788646982431	0.0814661921136563	0.708591927939011	-4.56617649783975
a_HQ_transcript/39714	-0.746050182209078	2.54260850529545	-1.79781977800725	0.0814769798804644	0.708591927939011	-4.42690774759221
a_HQ_transcript/144137	-2.23683822106513	0.23154039490066	-1.79773575038988	0.0814905735347868	0.708591927939011	-4.42292150643553
a_HQ_transcript/72949	0.784469464640027	2.25706158856222	1.79773160306404	0.0814912445225594	0.708591927939011	-4.38035597451637
a_HQ_transcript/128436	-1.90816508620712	-0.418096355770586	-1.79758951756407	0.0815142350924797	0.708591927939011	-4.42440425861664
a_HQ_transcript/122612	0.661013145276566	4.84206464038887	1.79758158817778	0.081515518292809	0.708591927939011	-4.84346521016904
a_HQ_transcript/130545	2.04213930831128	0.133443665733681	1.79747983223779	0.0815319868216479	0.708591927939011	-4.43021658928797
a_HQ_transcript/117291	0.725073714139948	3.94099351627545	1.79744783553323	0.0815371658613449	0.708591927939011	-4.80962953201859
a_HQ_transcript/72983	1.45278678193019	2.13240937364527	1.79743450304736	0.0815393239620731	0.708591927939011	-4.37746683280246
a_HQ_transcript/127493	-0.680864757850567	2.85508250717096	-1.79742880833484	0.0815402457676859	0.708591927939011	-4.5621719892136
a_HQ_transcript/70149	-1.85350575781951	-0.170809174444635	-1.7971163431046	0.0815908382148372	0.708795216078194	-4.38808550771477
a_HQ_transcript/15070	-0.674050789847592	4.51346743175171	-1.79706709488067	0.0815988146149257	0.708795216078194	-4.82280799842466
a_HQ_transcript/85785	-1.18868464154924	0.915982187619943	-1.79705841834663	0.0816002199627593	0.708795216078194	-4.3700877352563
a_HQ_transcript/18041	1.57322411703087	2.84353075455856	1.79682043533474	0.0816387743470422	0.708876308910043	-4.58225299984352
a_HQ_transcript/107592	1.97531612504546	3.11557481743981	1.79679482739303	0.0816429238772937	0.708876308910043	-4.64784003739408

a_HQ_transcript/77489	-2.09490372504911	1.66253336851708	-1.79674458401524	0.0816510658725434	0.708876308910043	-4.43217972280506
a_HQ_transcript/2113	2.30411053699646	2.16312086025947	1.79669972343082	0.081658336162635	0.708876308910043	-4.42271536918461
a_HQ_transcript/24415	1.41989320723078	3.82616323991099	1.796577871623	0.081678086739552	0.708937469255828	-4.73551955658418
a_HQ_transcript/62833	1.04722314041594	-0.819317632208447	1.79647014996224	0.0816955503823772	0.708937469255828	-4.44135532618142
a_HQ_transcript/40112	-1.83103577929797	0.32607811429388	-1.79643055709698	0.0817019699033992	0.708937469255828	-4.32509670844407
a_HQ_transcript/148800	-4.74446397298178	0.931502807478584	-1.796271455941	0.0817277706169438	0.708986155484032	-4.49690358300762
a_HQ_transcript/88056	-2.04284586803404	1.02490135466368	-1.79619053586758	0.0817408957118086	0.708986155484032	-4.48216062899762
a_HQ_transcript/146806	-2.054947062524	0.832850506431901	-1.79617033999954	0.0817441717253988	0.708986155484032	-4.45068583060415
a_HQ_transcript/73014	2.25259007697477	0.163032938761181	1.79582216919399	0.0818006667583602	0.709285882691847	-4.41647597038793
a_HQ_transcript/129424	-4.80957284029914	5.54151543894978	-1.79580697024085	0.0818031337318701	0.709285882691847	-4.82193818245789
a_HQ_transcript/143394	-2.37454377280383	-0.153346421040824	-1.79543498668858	0.0818635308407429	0.709585515222219	-4.48011572470173
a_HQ_transcript/84046	-1.18839703795088	1.08949777369249	-1.79530728973496	0.0818842730812587	0.709585515222219	-4.38284389846553
a_HQ_transcript/4952	-0.459330120122572	2.99877214593504	-1.79528413478179	0.0818880346949656	0.709585515222219	-4.59553226100552
a_HQ_transcript/139900	3.68029800709852	1.39736583995137	1.7952451803588	0.0818943633259435	0.709585515222219	-4.57760502086456
a_HQ_transcript/27478	0.818144752402702	4.68146805233027	1.79521832029523	0.0818987273194651	0.709585515222219	-4.87164752114539
a_HQ_transcript/4511	-1.94908627861233	0.0337146413695399	-1.7950842430215	0.0819205140004853	0.709668500420299	-4.36986106225017
a_HQ_transcript/119075	-3.7197193507134	-0.130887359269806	-1.79493630883666	0.0819445580466207	0.709678015936723	-4.46322819102711
a_HQ_transcript/148879	5.19584515749749	5.60834464214816	1.79483304372232	0.0819613454866395	0.709678015936723	-4.61673663427598
a_HQ_transcript/96100	1.76377288798614	-0.565261511842341	1.79481519350673	0.0819642476281493	0.709678015936723	-4.43874117100673
a_HQ_transcript/10548	1.74298863983148	0.795731273357881	1.7947625131327	0.0819728130696317	0.709678015936723	-4.37425100936638
a_HQ_transcript/35775	0.8194084144376	4.57205707487325	1.79470197634788	0.0819826568437774	0.709678015936723	-4.85435876354299
a_HQ_transcript/63265	1.69640593853376	0.0443818432494827	1.79451904153769	0.0820124096300682	0.709829861074539	-4.31566548948201
a_HQ_transcript/140138	5.91598682075516	0.796571034337417	1.79416361283411	0.0820702432750087	0.710185674020779	-4.49828029790811
a_HQ_transcript/142424	2.76039041927233	1.78882660253693	1.79410924838729	0.0820790922466013	0.710185674020779	-4.39369394317394
a_HQ_transcript/118509	1.84686867043127	0.072221685379986	1.79400485955061	0.0820960860230493	0.710185674020779	-4.45453051127352
a_HQ_transcript/84142	1.96204238641387	-0.258009329175999	1.793901499586	0.0821129152478622	0.710185674020779	-4.42440386828582
a_HQ_transcript/71233	2.8806146420427	2.00902460303441	1.79376260659163	0.0821355346239917	0.710185674020779	-4.50754065378548
a_HQ_transcript/123511	1.67033976509472	0.932837083527661	1.79368565428733	0.0821480689439779	0.710185674020779	-4.34222637025971
a_HQ_transcript/41361	3.46098199727518	0.851360449956784	1.79368561273247	0.0821480757130491	0.710185674020779	-4.34682476649285
a_HQ_transcript/149548	2.35386034728808	2.86450177748427	1.79363397854313	0.0821564870208148	0.710185674020779	-4.49589153033103
a_HQ_transcript/76796	-1.70427530638245	-0.196729001594342	-1.79359106577786	0.082163478148048	0.710185674020779	-4.44988758860609
a_HQ_transcript/54165	2.94784932074217	0.9554509063927	1.79344332223998	0.0821875516270389	0.710288135738406	-4.44691986839336
a_HQ_transcript/47778	2.66969422959526	1.66322335388699	1.79324816352594	0.0822193601515166	0.710294101125121	-4.40765955954219
a_HQ_transcript/33050	-2.01923177652456	-0.358982536700932	-1.79318894105639	0.0822290147734734	0.710294101125121	-4.45391242067478
a_HQ_transcript/146351	-0.726841532116131	3.11427755279154	-1.79308540772007	0.0822458953868812	0.710294101125121	-4.66649096084461
a_HQ_transcript/69644	-0.73527587673421	3.06253434602959	-1.79300483614022	0.0822590342304722	0.710294101125121	-4.57421626835182
a_HQ_transcript/108825	1.13116746962694	2.87172306086731	1.792991770735	0.0822611649797446	0.710294101125121	-4.49275292154092
a_HQ_transcript/103570	4.83029114617812	2.14028211082473	1.79292911803072	0.0822713832396041	0.710294101125121	-4.56970444759865
a_HQ_transcript/38551	-2.15777395110971	-0.0828760673050801	-1.79285782190918	0.0822830124961485	0.710294101125121	-4.41749158990651
a_HQ_transcript/146868	-0.490006697318401	4.47441527224591	-1.79283952145331	0.0822859977459497	0.710294101125121	-4.82387544787978
a_HQ_transcript/67997	1.67731708960429	1.31747543271641	1.79276441600425	0.082298250235249	0.710294385994737	-4.35435250383376
a_HQ_transcript/94909	-1.27835324822803	2.27105728378546	-1.79264621721268	0.0823175359846669	0.710355363972197	-4.45154246527136
a_HQ_transcript/120204	-2.43228930443974	2.51862706575873	-1.79255736822601	0.0823320354362448	0.710375026842517	-4.54313405574608
a_HQ_transcript/64116	1.81228607803496	-0.562460172572017	1.79242774929453	0.0823531921063931	0.710425635005747	-4.42955573278469
a_HQ_transcript/149007	-2.51646234664695	0.374989139880222	-1.79237168550346	0.0823623443831497	0.710425635005747	-4.40916921751496
a_HQ_transcript/4728	-2.51795451798238	0.892313175684055	-1.79209796272161	0.0824070413497287	0.710647611784842	-4.31832969088401
a_HQ_transcript/112967	2.01135179919547	0.493102496048899	1.79199605007755	0.0824236882095356	0.710647611784842	-4.30899355448675
a_HQ_transcript/32717	-2.6523979150662	1.60502997636275	-1.79188156613937	0.0824423919220139	0.710647611784842	-4.3932838366036
a_HQ_transcript/93054	2.57770985304447	1.24636641239229	1.79180670360082	0.0824546244701132	0.710647611784842	-4.44871314211331
a_HQ_transcript/140854	-1.8123431335543	-0.557342861885293	-1.79168407571716	0.0824746652081493	0.710647611784842	-4.42976516966707

a_HQ_transcript/36798	2.05419159253641	0.208509838998226	1.79164168012535	0.0824815947643299	0.710647611784842	-4.41305555396959
a_HQ_transcript/45675	1.9527219220752	1.53129096553642	1.79162691811456	0.0824840077297648	0.710647611784842	-4.36690517253864
a_HQ_transcript/66995	0.740597974538488	5.14444337143786	1.7915266870751	0.0825003928560311	0.710647611784842	-4.91777058213286
a_HQ_transcript/144020	3.51543773555504	0.361556527933695	1.79151221095215	0.0825027595477469	0.710647611784842	-4.42086573985856
a_HQ_transcript/150083	5.42329184619385	8.10789564608642	1.79146587946021	0.08251033463969	0.710647611784842	-4.95601037633935
a_HQ_transcript/48011	1.61495068247314	-0.441631004926946	1.79130361705986	0.0825368688188053	0.710770831214743	-4.47621572556311
a_HQ_transcript/676	-0.509677312814226	3.45111514352141	-1.79120932502438	0.0825522913766313	0.710798339970497	-4.68635976720641
a_HQ_transcript/147262	-1.91155794847778	3.21193129387615	-1.79080627493095	0.0826182424596082	0.711108631358834	-4.71450409033006
a_HQ_transcript/109593	4.55884250760476	4.41745740301548	1.79069966601017	0.0826356943478584	0.711108631358834	-4.79240476646144
a_HQ_transcript/81099	-1.62939041772771	1.5102476246311	-1.79069117266347	0.0826370848438038	0.711108631358834	-4.37950794233223
a_HQ_transcript/35635	-0.93635331050769	2.36297916710256	-1.79058139439116	0.0826550590789883	0.711108631358834	-4.44221045241179
a_HQ_transcript/833	1.26781838101198	4.40953180769166	1.79058138418309	0.082655060750533	0.711108631358834	-4.70839748312406
a_HQ_transcript/44362	1.49519109015542	1.53532585443303	1.79054065869115	0.0826617296671422	0.711108631358834	-4.40175075686284
a_HQ_transcript/4198	-1.86862473868533	0.903401542778868	-1.79039446520821	0.082685673030572	0.711127602747907	-4.51255816146645
a_HQ_transcript/67257	-2.1089117727788	0.32453177171993	-1.79037780120506	0.0826884026110154	0.711127602747907	-4.43044654779164
a_HQ_transcript/141688	0.767118837437339	3.56821927281399	1.79021061518225	0.0827157920790604	0.711257923368583	-4.69382919761727
a_HQ_transcript/7509	1.47928072565614	-0.0305919126334047	1.7900558487627	0.0827411537470193	0.711284567741866	-4.36675840369124
a_HQ_transcript/147821	-2.75705090921824	3.96155235279757	-1.79004236450434	0.0827433637329772	0.711284567741866	-4.67045144903831
a_HQ_transcript/127612	0.509320617670252	4.43193585019165	1.78989521732877	0.0827674834924197	0.711386704191538	-4.83073244450141
a_HQ_transcript/144090	-2.04514843690845	-0.406177744111164	-1.78943005119892	0.0828437708303904	0.711829236475223	-4.3676216191697
a_HQ_transcript/25942	2.18002232052011	0.708022092289421	1.7894236651364	0.0828448185606804	0.711829236475223	-4.39285009506133
a_HQ_transcript/22454	1.50863341508338	1.94742864417747	1.78929406594738	0.0828660836987099	0.711829236475223	-4.42202745270924
a_HQ_transcript/20859	1.88999798422292	0.384710931478421	1.78927336397927	0.0828694809866938	0.711829236475223	-4.42150450337621
a_HQ_transcript/96430	-1.71131434146911	2.53298408889704	-1.78915290727703	0.0828892508273295	0.711829236475223	-4.57097156636374
a_HQ_transcript/102673	2.36538234315275	5.89103407910502	1.78913344146396	0.082892446010542	0.711829236475223	-4.94368637016761
a_HQ_transcript/130120	-0.576554715867466	2.53687637088429	-1.78905114943799	0.0829059548510363	0.71184008029262	-4.53467620612744
a_HQ_transcript/133073	0.79365912984129	2.8301557618621	1.78896143110821	0.0829206849008	0.711861404801905	-4.53363226129209
a_HQ_transcript/5504	-2.04525369078229	1.57346785800489	-1.78869440480245	0.082964538691982	0.712132708961537	-4.5719194924477
a_HQ_transcript/45167	1.60171403359569	3.28993422429869	1.78843087058713	0.0830078382551994	0.712397633077896	-4.683138519984
a_HQ_transcript/4989	2.50654064085979	3.82820356606992	1.78835739417018	0.0830199140942346	0.712397633077896	-4.61256065622583
a_HQ_transcript/35442	-1.69304578001568	3.27459086691719	-1.78824978549631	0.0830376022529754	0.712444242621543	-4.58673052392175
a_HQ_transcript/74950	-2.55250427783075	-0.16131238850675	-1.78811341516782	0.0830600226942493	0.712531434352321	-4.39174963250644
a_HQ_transcript/125961	2.18422112316978	2.2450688635704	1.78799913405092	0.0830788154337332	0.712587484658705	-4.51652594865549
a_HQ_transcript/132843	-1.88508904710165	1.08485817792135	-1.78777428376211	0.0831158010324831	0.712799540043697	-4.4372205877754
a_HQ_transcript/823	4.72816990071885	4.52152822094842	1.78764682616636	0.0831367727163213	0.712874218093277	-5.03465121476432
a_HQ_transcript/129590	-2.35672587302294	1.02843812416118	-1.7874499540532	0.0831691745774539	0.713046870182632	-4.43089036250038
a_HQ_transcript/56746	2.45706122669438	1.42873359729542	1.78706720908305	0.0832321986432959	0.713294644015462	-4.45510061168545
a_HQ_transcript/148115	1.92832341919268	0.376226898973942	1.78704329695334	0.0832361374377255	0.713294644015462	-4.34914744538108
a_HQ_transcript/116480	0.832070345844743	3.1019974193039	1.78699549531994	0.0832440117732607	0.713294644015462	-4.60878888604616
a_HQ_transcript/145322	-3.04688572042749	0.990436617309539	-1.78695561016261	0.0832505825154828	0.713294644015462	-4.56199409463838
a_HQ_transcript/126823	-2.01535944606674	-0.428859556960383	-1.78683582092181	0.0832703194224284	0.713294644015462	-4.43908760412373
a_HQ_transcript/114651	0.734712419274922	2.56152860492642	1.78682743467439	0.0832717013193112	0.713294644015462	-4.50486783766719
a_HQ_transcript/149064	2.8538810410659	0.608024842035506	1.78659265187409	0.0833103970286896	0.713520960436668	-4.48296604842478
a_HQ_transcript/124643	2.33188668983419	1.44723767807197	1.78643333994815	0.0833366626904053	0.713640767364154	-4.47249402155682
a_HQ_transcript/10395	-1.13294619690896	3.268143057006	-1.78628602513945	0.0833609566399972	0.713743657028982	-4.52125535307752
a_HQ_transcript/47081	-0.645514441612396	4.24176606254053	-1.78606385443881	0.0833976065613603	0.713952293696429	-4.74264414021696
a_HQ_transcript/127992	2.05337949224246	1.48216852026126	1.78591607903151	0.0834219915818261	0.71405588671306	-4.42201723347548
a_HQ_transcript/14035	2.41338150501502	0.810578940124417	1.7856874081286	0.0834597374066376	0.714273795049033	-4.33726114129065
a_HQ_transcript/21706	2.08716757478557	-0.588163643584057	1.78538626145152	0.0835094686091607	0.714594112719794	-4.44252204373125
a_HQ_transcript/151943	-1.82970629390063	0.143703059385668	-1.78529488864607	0.0835245628305741	0.714594112719794	-4.41597326224417

a_HQ_transcript/117966	-3.29503925075276	2.86356490870421	-1.78523033310976	0.0835352283989317	0.714594112719794	-4.52796568002472
a_HQ_transcript/49901	1.07658290832991	1.59559150087232	1.78516309169783	0.0835463389423981	0.714594112719794	-4.37059730752888
a_HQ_transcript/127225	1.86691230213522	1.00185290630989	1.7849606711883	0.0835797931798351	0.714613511549693	-4.31127086275793
a_HQ_transcript/141408	0.575335488956873	10.4747707649473	1.78490803285933	0.083588494627944	0.714613511549693	-5.45117183944015
a_HQ_transcript/56089	5.38809408891604	1.49216404419438	1.78487623477212	0.083593751424427	0.714613511549693	-4.49328214278095
a_HQ_transcript/45454	1.15821854095701	0.298683844506348	1.78485185450842	0.0835977821108229	0.714613511549693	-4.40753776999112
a_HQ_transcript/50533	2.0757296460602	0.941127958904447	1.78477614807506	0.0836102993865318	0.714615421664508	-4.40150724475193
a_HQ_transcript/45318	1.96903973938892	2.02011408772357	1.78462665184153	0.0836350217069022	0.714721632134491	-4.48482848383657
a_HQ_transcript/60176	-0.767101881723537	3.10631918092151	-1.78445460704053	0.083663480568747	0.714771146120688	-4.58195470544068
a_HQ_transcript/67809	1.89346202183721	2.93949238491987	1.78430415410764	0.0836883745275336	0.714771146120688	-4.434357925819
a_HQ_transcript/152765	-1.57774457137868	0.862841571460592	-1.78424882831571	0.083697530317265	0.714771146120688	-4.50758496406309
a_HQ_transcript/121646	1.15581889437551	-0.00097170602467236	1.78423885901555	0.0836991802133683	0.714771146120688	-4.31157738095203
a_HQ_transcript/92119	-3.80247072543548	1.60171335871845	-1.78413228355141	0.0837168199280338	0.714771146120688	-4.48020251663924
a_HQ_transcript/139648	2.40497398880445	1.08566159611132	1.78411321136629	0.0837199769709392	0.714771146120688	-4.49124260794305
a_HQ_transcript/115556	-2.10951287410658	0.0133084603674778	-1.7840714433312	0.0837268912389169	0.714771146120688	-4.3890029846296
a_HQ_transcript/144596	0.607921247846124	4.45400231580315	1.78394483695661	0.0837478525760925	0.714805389697247	-4.83413885210833
a_HQ_transcript/144321	-2.11413669951984	-0.431376556510013	-1.78389867347238	0.083755496649256	0.714805389697247	-4.43388129844476
a_HQ_transcript/53990	1.41468171160551	4.20848889969375	1.78379975481333	0.0837718782843199	0.714840243821337	-4.8363475513042
a_HQ_transcript/106790	0.808190805555892	2.97421351159935	1.78363941921906	0.0837984367675052	0.714961915811176	-4.56396899743794
a_HQ_transcript/84930	-2.54173870379421	3.12269310291254	-1.783135727753	0.0838819161859553	0.715430564662417	-4.64298276663626
a_HQ_transcript/72140	0.530648686959525	4.08350856128071	1.78296199597536	0.0839107259965394	0.715430564662417	-4.76396484021584
a_HQ_transcript/121235	-2.02247145002004	-0.384527622755277	-1.78278527050301	0.0839400408487668	0.715430564662417	-4.46562677454857
a_HQ_transcript/80086	-1.81272488632829	1.95690865549933	-1.78255481077659	0.0839782820593212	0.715430564662417	-4.34464763937667
a_HQ_transcript/114903	0.781805421119344	4.5349212119364	1.78244121624812	0.0839971367351653	0.715430564662417	-4.85338371834636
a_HQ_transcript/123279	1.30508245337653	2.82083097293984	1.78231499740093	0.0840180910293303	0.715430564662417	-4.43229147651372
a_HQ_transcript/61370	1.61228147924323	1.08803612478532	1.7820714402427	0.0840585378256037	0.715430564662417	-4.4510269118484
a_HQ_transcript/25435	1.50347002819242	1.83174815707598	1.78206769096402	0.0840591605857761	0.715430564662417	-4.35334565114635
a_HQ_transcript/141147	1.86612559774435	-0.107387419545688	1.78204435779877	0.0840630363433751	0.715430564662417	-4.40675261586036
a_HQ_transcript/1355	2.18903535425548	0.390171359589281	1.78198443671135	0.0840729902346644	0.715430564662417	-4.575469048569
a_HQ_transcript/108359	2.00296475566722	-0.210404983910394	1.78183161437625	0.0840983810912484	0.715430564662417	-4.44148232631432
a_HQ_transcript/107211	-1.79605819599529	0.987287337013865	-1.78180101465531	0.0841034659002065	0.715430564662417	-4.49584916934505
a_HQ_transcript/147848	-1.86438036739094	0.95468308982312	-1.78176644081571	0.0841092114086944	0.715430564662417	-4.38821866831965
a_HQ_transcript/72003	1.89438909995976	2.09816431235766	1.78158492840294	0.084139380741667	0.715430564662417	-4.42624273243693
a_HQ_transcript/66649	-4.36969153552994	0.376177123213011	-1.78155150321696	0.0841449373694757	0.715430564662417	-4.49915872138939
a_HQ_transcript/125750	0.873803483895482	6.65829859820027	1.78154111944889	0.0841466636380819	0.715430564662417	-5.09976798001344
a_HQ_transcript/142169	1.0421842716781	2.83361092288008	1.78148150142828	0.0841565755259588	0.715430564662417	-4.61253096332187
a_HQ_transcript/121818	-2.35111397802113	4.49916091563539	-1.78135897565309	0.0841769493459597	0.715430564662417	-4.86512663726427
a_HQ_transcript/103631	0.818212413955238	2.87197656209622	1.78130316342583	0.0841862312944066	0.715430564662417	-4.50553211363195
a_HQ_transcript/146140	-2.20779191424714	0.778242290550657	-1.78118962893985	0.0842051155173822	0.715430564662417	-4.32585503508373
a_HQ_transcript/71563	1.48260324974548	4.19348746143934	1.78114845810698	0.0842119643598339	0.715430564662417	-4.83765179054452
a_HQ_transcript/147467	3.10017573129352	0.528800375320494	1.78112590640196	0.0842157160766884	0.715430564662417	-4.43263834779814
a_HQ_transcript/113506	1.35595215217674	-0.547087164824586	1.78110204359122	0.0842196860641006	0.715430564662417	-4.49014649221297
a_HQ_transcript/23704	-2.32537630388062	1.25149522340914	-1.7810252361115	0.0842324653803577	0.715430564662417	-4.34296432045487
a_HQ_transcript/89540	-2.53926451544378	3.20745323080748	-1.78098316045989	0.0842394666715088	0.715430564662417	-4.62187671797209
a_HQ_transcript/114701	-2.83862707429977	3.05250339254069	-1.78093912951202	0.0842467938471235	0.715430564662417	-4.62907521245245
a_HQ_transcript/95050	2.27820949784611	0.95779582786212	1.78093465647361	0.0842475382341779	0.715430564662417	-4.4066917997735
a_HQ_transcript/93782	1.92362370526393	-0.175832703684338	1.78088177268811	0.0842563393868269	0.715430564662417	-4.40572196458541
a_HQ_transcript/143009	4.28042009679544	2.65409877423006	1.78085485917224	0.0842608187516909	0.715430564662417	-4.49081227843926
a_HQ_transcript/137903	1.26756779406829	-0.719108222277628	1.78084697272678	0.0842621313745041	0.715430564662417	-4.46196490452676
a_HQ_transcript/57092	1.02115200069155	4.54650147131104	1.78078408084742	0.0842725997411374	0.715430564662417	-4.79716094753948

a_HQ_transcript/59405	4.75616819535654	1.84328798645991	1.78067233298029	0.0842912029141878	0.715430564662417	-4.48481971752923
a_HQ_transcript/67331	-2.43391996734152	0.427275233881322	-1.78048709467437	0.0843220480329934	0.715430564662417	-4.46236436813711
a_HQ_transcript/48406	2.40384374831027	2.18093767348949	1.78047568077538	0.0843239489409516	0.715430564662417	-4.50832287385693
a_HQ_transcript/149274	1.76716237014578	1.67278828128397	1.78045592240706	0.0843272396498184	0.715430564662417	-4.524007968788
a_HQ_transcript/112897	2.30915783123902	0.0143905462797453	1.78038685525682	0.0843387434725051	0.715430564662417	-4.44000734443801
a_HQ_transcript/87393	-0.924681572856532	2.41416723748969	-1.78020062463332	0.0843697686624958	0.715430564662417	-4.47154867088761
a_HQ_transcript/152077	-2.36386920318249	1.02000465795687	-1.7801116939673	0.0843845875230951	0.715430564662417	-4.5190767144865
a_HQ_transcript/12193	-3.30866234876845	3.82910626531025	-1.7800555526547	0.0843939437017459	0.715430564662417	-4.72416417492574
a_HQ_transcript/145998	3.36179079290276	6.40322873272218	1.78005320755677	0.0843943345409884	0.715430564662417	-5.09045178062814
a_HQ_transcript/109069	1.81612842434958	-0.0102249239186283	1.77996443830947	0.0844091301484114	0.715430564662417	-4.4436479357017
a_HQ_transcript/147147	-6.34163455925842	2.86849957392484	-1.77993220803152	0.0844145026709345	0.715430564662417	-4.65873522259843
a_HQ_transcript/22386	1.34505083370064	-0.359426581349044	1.77992350234891	0.0844159538863619	0.715430564662417	-4.46283397129036
a_HQ_transcript/97973	-1.06736342426845	2.30759607708561	-1.77989946430283	0.0844199610779621	0.715430564662417	-4.40694274515648
a_HQ_transcript/141320	-2.14849212243505	0.830795303582174	-1.77989851409746	0.0844201194824553	0.715430564662417	-4.3849721360309
a_HQ_transcript/81944	-0.515410455247045	4.40086148948422	-1.77984509597287	0.0844290249842028	0.715430564662417	-4.82039110924476
a_HQ_transcript/103591	1.47890713839677	-0.104715613268163	1.77977932068707	0.0844399916794254	0.715430564662417	-4.44607443582114
a_HQ_transcript/27510	-1.95163009504506	2.24950691553604	-1.77975442821378	0.0844441423091942	0.715430564662417	-4.61023918153119
a_HQ_transcript/32609	-1.66450423334381	1.81004001504435	-1.77909847744435	0.0845535793557105	0.716178530943559	-4.38734556019971
a_HQ_transcript/1933	0.901103233429019	4.57977932552672	1.77907757786521	0.0845570681576116	0.716178530943559	-4.85039549308852
a_HQ_transcript/145824	-2.54889388022282	1.08554329002772	-1.77866879115322	0.0846253321184731	0.716558899826211	-4.55229238472284
a_HQ_transcript/291	2.82215597135866	4.83144686312086	1.7786610115617	0.0846266316974081	0.716558899826211	-4.75474663730428
a_HQ_transcript/97081	1.43673035093112	0.163159544997663	1.77828740761978	0.0846890620381729	0.716612710934363	-4.35374517724419
a_HQ_transcript/87464	1.97494134884652	0.38130247273692	1.77826694492276	0.0846924825421151	0.716612710934363	-4.31276681022909
a_HQ_transcript/18445	2.55918930515709	0.857953495104792	1.77814350246744	0.0847131194193275	0.716612710934363	-4.48170171383348
a_HQ_transcript/36831	0.831011955921007	2.89070417005125	1.77795285935918	0.0847449991379498	0.716612710934363	-4.58431664463716
a_HQ_transcript/108056	0.694026368326601	4.24843974471027	1.77785320136169	0.0847616681871712	0.716612710934363	-4.7830568010869
a_HQ_transcript/60373	-3.0113660595669	1.98622546745002	-1.77781581718556	0.0847679218749509	0.716612710934363	-4.56621257899852
a_HQ_transcript/20633	2.33820294206676	0.420111944849707	1.7777152155682	0.084784752622008	0.716612710934363	-4.33509431639242
a_HQ_transcript/150862	4.18025001585612	1.98471488338381	1.77766195221913	0.0847936647765171	0.716612710934363	-4.60632850147338
a_HQ_transcript/146811	3.3048109193459	1.1546941461468	1.77763043191426	0.0847989392048584	0.716612710934363	-4.54201207274304
a_HQ_transcript/142961	0.419689986073424	5.0380040430615	1.77756481352611	0.084809920303069	0.716612710934363	-4.90265179101619
a_HQ_transcript/139066	0.696933136913088	-0.770364206695736	1.77754888841562	0.0848125855180484	0.716612710934363	-4.51245428653248
a_HQ_transcript/151392	4.1827674133358	3.29885916457944	1.77748636902712	0.0848230494031482	0.716612710934363	-4.62396764098141
a_HQ_transcript/139615	2.18817420653576	-0.168814393538207	1.77733856656217	0.0848477914823464	0.716612710934363	-4.37717297765475
a_HQ_transcript/124469	0.479399807633071	3.23213646530204	1.77732023677682	0.0848508603074654	0.716612710934363	-4.69472110670651
a_HQ_transcript/19232	-3.04309435797643	2.65900009801457	-1.7772875494227	0.0848563331509692	0.716612710934363	-4.51050545127079
a_HQ_transcript/107749	1.78012226789362	0.833984833180391	1.77717865555183	0.0848745674065823	0.716612710934363	-4.37217337623036
a_HQ_transcript/9749	-1.4718615125927	4.34655412216043	-1.77711780773221	0.0848847578052061	0.716612710934363	-4.7027061169907
a_HQ_transcript/133738	2.51133805922154	0.491687541336556	1.77703023931764	0.084899425013329	0.716612710934363	-4.3506528816187
a_HQ_transcript/96539	2.67149057424033	0.187414428729205	1.77701281933171	0.0849023430170034	0.716612710934363	-4.41136826043873
a_HQ_transcript/94347	2.66977600992869	0.228887465128678	1.77698034364154	0.0849077832126084	0.716612710934363	-4.4482148250547
a_HQ_transcript/3291	-2.11331047946562	1.69874007519448	-1.7769270393191	0.0849167131755119	0.716612710934363	-4.45886820041168
a_HQ_transcript/144562	3.09402307605104	1.80596615016213	1.77691726404489	0.0849183508932281	0.716612710934363	-4.52344838260184
a_HQ_transcript/144269	1.34936680705225	-0.682809953415057	1.7768839420411	0.0849239337544568	0.716612710934363	-4.44053678060089
a_HQ_transcript/128050	-1.83731483273996	0.373111858409186	-1.77685452100288	0.0849288632952592	0.716612710934363	-4.44725360065723
a_HQ_transcript/78687	1.45784277486262	-0.592793779914574	1.77670949606786	0.0849531659880432	0.716713734768211	-4.47058664156242
a_HQ_transcript/125445	-2.56514121516946	1.05637684602236	-1.77654548565929	0.0849806572807612	0.716841626239456	-4.39673276588146
a_HQ_transcript/5603	1.88027145466727	0.618045803887989	1.77645939147211	0.0849950913320832	0.716859354171697	-4.4696726224138
a_HQ_transcript/106476	2.02820610865614	0.319569456857084	1.7763644099978	0.0850110177812198	0.716889662206114	-4.3932559679034
a_HQ_transcript/82538	-1.31862567837886	-0.432916801652044	-1.7762573477911	0.0850289729494257	0.716937066957386	-4.41054671122581

a_HQ_transcript/43217	-1.7403200114785	0.217856069441881	-1.7761350130395	0.085049493370945	0.716940245240235	-4.39716511569429
a_HQ_transcript/23641	2.57085649235375	1.21397352142934	1.77607644260571	0.0850593194542023	0.716940245240235	-4.53238177453751
a_HQ_transcript/78606	-2.7576881710737	0.524755906156004	-1.77600453105571	0.0850713850268362	0.716940245240235	-4.44122127188963
a_HQ_transcript/14849	3.04935729146108	0.335930207568416	1.77595977576134	0.085078894958547	0.716940245240235	-4.51088619542177
a_HQ_transcript/109605	-1.12534130512994	2.38809006664197	-1.77588752987545	0.0850910189910607	0.716940245240235	-4.50827013253616
a_HQ_transcript/121368	0.4622842011864	4.5408282914516	1.77581300874942	0.0851035263774631	0.716941707430315	-4.87801532639448
a_HQ_transcript/58541	4.04558760317388	1.29337132444932	1.77550792389966	0.0851547470599099	0.717115749752098	-4.50132025118073
a_HQ_transcript/4404	-1.97169570930873	0.346891142999825	-1.77543824243982	0.0851664495356141	0.717115749752098	-4.41536358519211
a_HQ_transcript/140520	1.17340254444729	3.56342582486991	1.77531080188657	0.085187855735477	0.717115749752098	-4.70758745134158
a_HQ_transcript/81885	2.36641452053589	0.467707476823903	1.7752999944197	0.0851896712759174	0.717115749752098	-4.41493438498563
a_HQ_transcript/125268	-2.84056896984871	1.08112696396047	-1.77527926546341	0.0851931536135443	0.717115749752098	-4.54889606750345
a_HQ_transcript/25507	1.55669404329608	1.62197477519771	1.77524918685149	0.0851982068501924	0.717115749752098	-4.34039286564181
a_HQ_transcript/142712	0.655616388502511	5.02327108957353	1.7750756291082	0.085227369676764	0.717257354071368	-4.92098909911501
a_HQ_transcript/24127	0.679078381210348	3.69834104606649	1.77488212355728	0.0852598942808467	0.717427205378844	-4.78184361008621
a_HQ_transcript/146347	-2.79632075534686	5.81393129979924	-1.77446545272533	0.0853299643763046	0.717759195085476	-5.04876974701213
a_HQ_transcript/148017	-0.870494507803563	2.54413840720135	-1.77437644542956	0.085344938739036	0.717759195085476	-4.58204623914104
a_HQ_transcript/112092	1.7159754879136	-0.356960614027328	1.77434658941985	0.0853499621366002	0.717759195085476	-4.41654607604087
a_HQ_transcript/57958	2.93605086483124	2.39281543806567	1.77419833468787	0.0853749103155697	0.717759195085476	-4.53994837197262
a_HQ_transcript/21889	-2.13938930655344	0.873061469458614	-1.77414784098119	0.0853834087613511	0.717759195085476	-4.41895697237818
a_HQ_transcript/108339	1.88797080903035	1.47071421009871	1.77411575149496	0.0853888100193471	0.717759195085476	-4.42866960741194
a_HQ_transcript/83399	2.36287670611874	-0.365719849861675	1.77393701744549	0.0854188995761531	0.717759195085476	-4.46050854563054
a_HQ_transcript/1123	1.43719692112375	0.758279039550111	1.77387550433287	0.0854292572727202	0.717759195085476	-4.35979028969325
a_HQ_transcript/135960	2.52330677341647	1.18260102469144	1.77386935406659	0.0854302929248377	0.717759195085476	-4.44251947318476
a_HQ_transcript/130437	-1.57554420943862	1.01339516269947	-1.77381785417201	0.0854389654823955	0.717759195085476	-4.37083483564344
a_HQ_transcript/27815	1.88497862224185	0.603395863951055	1.77377236489742	0.0854466264738216	0.717759195085476	-4.35583634468183
a_HQ_transcript/133673	1.57269449244623	-0.107537300930402	1.77374726288326	0.0854508542315468	0.717759195085476	-4.35421366474115
a_HQ_transcript/82066	-1.98039060962005	1.46946954609766	-1.77369372917766	0.0854598711324418	0.717759195085476	-4.38693671102996
a_HQ_transcript/85658	0.720437811841392	2.92020634396188	1.77347138557348	0.0854973299880118	0.717897721253352	-4.61425868991036
a_HQ_transcript/78853	2.68946006728947	1.75469422869247	1.77342957575792	0.0855043753568627	0.717897721253352	-4.67048114469437
a_HQ_transcript/1415	-2.89443277452036	0.062679553220344	-1.77332843652399	0.0855214203522247	0.717897721253352	-4.47660648980809
a_HQ_transcript/121463	3.43233838469636	1.15340189879572	1.77330265429833	0.0855257658904394	0.717897721253352	-4.53662335600967
a_HQ_transcript/39177	1.18482404127018	2.37113896590039	1.77317969946577	0.0855464922299179	0.717900749092193	-4.42468674503838
a_HQ_transcript/34786	1.04116445924624	2.28776020933279	1.77310592226481	0.0855589307978772	0.717900749092193	-4.51260845022916
a_HQ_transcript/150346	1.95320330822043	1.45492930741933	1.77305989983946	0.0855666907862246	0.717900749092193	-4.47233888077427
a_HQ_transcript/81306	-2.31579244452909	0.807059429965735	-1.77286491186486	0.0855995749362182	0.717900749092193	-4.3629097360892
a_HQ_transcript/5139	2.10067595916153	0.831134047343854	1.77265997102072	0.08563414912829	0.717900749092193	-4.35134592783226
a_HQ_transcript/53292	-1.56273237383982	0.498783477718219	-1.77264293293929	0.0856370240401345	0.717900749092193	-4.33046378677644
a_HQ_transcript/18173	-2.05605012496531	0.496589997673425	-1.77258194646615	0.0856473152300791	0.717900749092193	-4.45216442162513
a_HQ_transcript/143956	-0.369094198690356	7.3060084648285	-1.77253639965575	0.0856550017293803	0.717900749092193	-5.11517113853223
a_HQ_transcript/22446	-0.588944787092368	5.19957451761121	-1.77234213206513	0.0856877929624445	0.717900749092193	-4.94429372165817
a_HQ_transcript/41082	-2.35728661643475	1.95150603427528	-1.77223190747404	0.085706402948778	0.717900749092193	-4.65638953457048
a_HQ_transcript/55779	-1.15650211027475	2.45703091844208	-1.77221859868449	0.0857086501957662	0.717900749092193	-4.49919726530066
a_HQ_transcript/49390	-3.22619974605613	0.410279198770906	-1.77221758183136	0.0857088218978888	0.717900749092193	-4.47148724955262
a_HQ_transcript/118667	-1.21998232471458	-0.599267106049554	-1.77217962185695	0.0857152318892429	0.717900749092193	-4.44666507458004
a_HQ_transcript/62671	-2.09865197277755	5.1039873215165	-1.77217623279106	0.0857158041928771	0.717900749092193	-5.03024819073457
a_HQ_transcript/2879	2.80098270145832	0.569542428426289	1.77212584514428	0.0857243134181729	0.717900749092193	-4.39866503096874
a_HQ_transcript/150261	1.83382021949124	-0.556771428103618	1.77193607085346	0.0857563680065015	0.717900749092193	-4.45721986346969
a_HQ_transcript/117931	2.61144599239006	0.265517092121972	1.77190859478704	0.0857610098016415	0.717900749092193	-4.46258299464224
a_HQ_transcript/125878	1.01000018469346	-0.829318340103552	1.77184567046037	0.085771641011441	0.717900749092193	-4.48012814361643
a_HQ_transcript/113970	1.73508793498172	-0.43230965186131	1.77183619154536	0.0857732425925235	0.717900749092193	-4.51435446969083

a_HQ_transcript/13073	-1.10417834827778	1.96237086974859	-1.77182433165603	0.0857752465045682	0.717900749092193	-4.36947281073928
a_HQ_transcript/60117	1.76607546306519	0.148876970573965	1.77174705632642	0.085788304338057	0.717900749092193	-4.36851783638058
a_HQ_transcript/146884	-3.91969333555379	2.80882205613116	-1.77169066580706	0.0857978341581246	0.717900749092193	-4.73419076157588
a_HQ_transcript/99134	-5.53741261971144	1.31617156997329	-1.77159584519846	0.0858138605602498	0.717931503547237	-4.47019430363851
a_HQ_transcript/50068	-1.23901345820964	2.59820549187951	-1.77132469009395	0.0858597046594206	0.718101870664145	-4.4312863309072
a_HQ_transcript/122438	-1.71518961958315	2.81796339103382	-1.77121720644542	0.0858778826127473	0.718101870664145	-4.51489790944039
a_HQ_transcript/92138	1.94892966563796	-0.401131566584028	1.77119806988591	0.0858811193856588	0.718101870664145	-4.45089795414544
a_HQ_transcript/130748	0.729257970448877	6.02733211278253	1.77112260845848	0.0858938839969282	0.718101870664145	-5.03095562620822
a_HQ_transcript/117033	2.63790074409769	0.697113191867257	1.77101818526245	0.0859115502563264	0.718101870664145	-4.48790339507619
a_HQ_transcript/117014	4.25971702136743	4.56408557207134	1.77101805723415	0.0859115719179703	0.718101870664145	-4.97696867296971
a_HQ_transcript/82141	1.91141636773442	0.665854215486217	1.77096410314394	0.0859207010471568	0.718101870664145	-4.34518099030129
a_HQ_transcript/90223	1.69267617472785	2.21609681795277	1.77083421319642	0.0859426820241416	0.718182320399555	-4.43131506282916
a_HQ_transcript/39691	1.47826113448721	1.32605525997888	1.770565026828	0.08598825095044	0.718323447620158	-4.33840419325927
a_HQ_transcript/50179	2.6304670330094	1.09711887143508	1.77052255574468	0.0859954424876894	0.718323447620158	-4.42804038057241
a_HQ_transcript/111576	1.68782393378918	-0.648992193930594	1.77051546538725	0.0859966431322027	0.718323447620158	-4.47707430393756
a_HQ_transcript/147176	2.3507506171021	1.25706820256094	1.77032288801552	0.086029258609906	0.718397234311754	-4.50849658523499
a_HQ_transcript/136134	1.52432661009063	4.46777441882475	1.77031736276588	0.0860301945369549	0.718397234311754	-4.89766811434097
a_HQ_transcript/14656	1.3823380472996	-0.516670524434862	1.76999047730783	0.0860855812822483	0.718704136112017	-4.41303936270167
a_HQ_transcript/108518	1.93577249446558	0.558414531358933	1.76995452226778	0.0860916752640376	0.718704136112017	-4.36132743269062
a_HQ_transcript/136330	-2.06612838367373	0.570159938211958	-1.76977671004324	0.0861218178316943	0.718852531148869	-4.41486914580038
a_HQ_transcript/149634	2.10028874720327	3.35163994119954	1.76930489870765	0.086201842106786	0.719417182768594	-4.67652720838353
a_HQ_transcript/103488	4.06755302266855	1.29032504327298	1.76914093397032	0.0862296670016492	0.719457517638769	-4.48626377851889
a_HQ_transcript/13282	1.4761432677821	2.02206902177738	1.76913055041074	0.0862314293522795	0.719457517638769	-4.41818697148262
a_HQ_transcript/120070	1.58722941164685	-0.506799808273531	1.76898848572523	0.086255544352245	0.719551686938339	-4.46943262583606
a_HQ_transcript/27642	0.981676046420105	5.19708705659374	1.7688903758553	0.086272201498142	0.719551686938339	-4.92856431145059
a_HQ_transcript/150386	-1.41360441688777	3.45384297213637	-1.76884531797725	0.086279852359624	0.719551686938339	-4.57920707719029
a_HQ_transcript/50010	-1.54968962069376	-0.613163619729629	-1.76831238003611	0.0863703891340328	0.72011099337742	-4.45950547146018
a_HQ_transcript/124213	2.58248180747573	2.2746019602541	1.76808182336097	0.0864095815278287	0.72011099337742	-4.4724884048005
a_HQ_transcript/103624	3.0913701302947	0.51937971510708	1.76802269465764	0.0864196352538063	0.72011099337742	-4.50565677702273
a_HQ_transcript/135980	-1.49392379067869	0.478209090477746	-1.7679723986729	0.0864281879198417	0.72011099337742	-4.401953205923
a_HQ_transcript/52772	-1.93000279414395	2.03993978886197	-1.76781351055461	0.0864552110185683	0.72011099337742	-4.57898267856825
a_HQ_transcript/87407	-0.668338144616175	5.61435625214797	-1.76775048044136	0.0864659329230382	0.72011099337742	-4.9998868922236
a_HQ_transcript/30343	-0.567581988478284	3.65693494152745	-1.76773585417004	0.0864684211243047	0.72011099337742	-4.7131173539268
a_HQ_transcript/16558	1.4546737251562	0.209023548109252	1.76767071381786	0.0864795034467285	0.72011099337742	-4.43524277833976
a_HQ_transcript/144894	-4.15269471356908	2.85889768908941	-1.76761762193695	0.0864885368515522	0.72011099337742	-4.75148644688072
a_HQ_transcript/144276	-2.81799325446403	1.66947842084245	-1.76761124060613	0.0864896226671892	0.72011099337742	-4.51540402946244
a_HQ_transcript/7572	1.97711688066305	-0.238591195329866	1.76759810505204	0.0864918577842528	0.72011099337742	-4.44567730760853
a_HQ_transcript/121545	-2.2750146400757	-0.53652781125651	-1.76744794647197	0.086517411907102	0.72011099337742	-4.47486452051107
a_HQ_transcript/140992	-2.88366346726325	0.237366992797498	-1.76730776380631	0.0865412740775876	0.72011099337742	-4.52455408105161
a_HQ_transcript/93245	-1.9247579494823	0.75701281110823	-1.76725027950051	0.0865510607774265	0.72011099337742	-4.42109537229578
a_HQ_transcript/97041	-1.81338422792737	-0.142106036831846	-1.76724549484428	0.0865518754070381	0.72011099337742	-4.46640613494923
a_HQ_transcript/90067	-1.65527363979782	1.64300489037737	-1.76722097052018	0.0865560509896307	0.72011099337742	-4.42579762220747
a_HQ_transcript/49355	-1.03458705298981	2.59135662878125	-1.76712571885123	0.086572270429661	0.72011099337742	-4.489953867406
a_HQ_transcript/90377	1.86844161438877	0.413195278760316	1.76694397701159	0.0866032245276865	0.72011099337742	-4.40632590448042
a_HQ_transcript/116273	2.14821329673176	3.87460290847144	1.76690693811066	0.0866095341051994	0.72011099337742	-4.78145672658135
a_HQ_transcript/136850	2.71619415411562	0.713055533321236	1.76687699090009	0.0866146358970882	0.72011099337742	-4.51630839104383
a_HQ_transcript/11992	-2.07818916342969	0.970922236935925	-1.76686843406709	0.0866160936815411	0.72011099337742	-4.41673546289038
a_HQ_transcript/143416	-1.3081599688345	4.73047903206131	-1.76684866550396	0.0866194616311402	0.72011099337742	-4.89367794035163
a_HQ_transcript/19847	2.29580996861003	1.15145686160801	1.76672875777188	0.0866398925582076	0.720177845649005	-4.377650091546
a_HQ_transcript/2914	-1.56080558188501	2.95345411427055	-1.76651112546643	0.0866769850533302	0.720271400486223	-4.47577929504201

a_HQ_transcript/5996	1.47098945179136	1.87963284532523	1.76649761678608	0.0866792878681188	0.720271400486223	-4.37044413104856
a_HQ_transcript/152669	1.8582184695309	0.807779107096263	1.76644170503877	0.0866888196522184	0.720271400486223	-4.3993088474936
a_HQ_transcript/81140	-3.56789248514788	2.37898919237928	-1.7663457316031	0.0867051831813673	0.720271400486223	-4.55399457872997
a_HQ_transcript/74522	1.62676467488403	3.1985404774842	1.76624242954226	0.0867227991615723	0.720271400486223	-4.64099298592823
a_HQ_transcript/63080	-2.24501649782935	0.189181413884378	-1.76617689172337	0.086733976817161	0.720271400486223	-4.56631315327192
a_HQ_transcript/84991	2.83360102579937	1.05258324833154	1.76602388461159	0.0867600773394931	0.720271400486223	-4.39886870849005
a_HQ_transcript/86459	1.66859167596886	-0.297278144854341	1.76593320206272	0.0867755494374511	0.720271400486223	-4.44425633139728
a_HQ_transcript/124420	0.456376023516552	3.63454830138647	1.76592007228716	0.0867777898099699	0.720271400486223	-4.78475991259675
a_HQ_transcript/150303	3.13205159258986	5.30617940553946	1.76590496978689	0.086780366854782	0.720271400486223	-4.88495903372039
a_HQ_transcript/18916	-1.71967361883987	1.15597029211524	-1.76583023887338	0.0867931196625465	0.720271400486223	-4.367431321292
a_HQ_transcript/11358	1.88511644565744	1.90730887090636	1.76579085557525	0.0867998410474469	0.720271400486223	-4.34813762105561
a_HQ_transcript/41223	2.11235314671834	0.486858476900331	1.76549251350818	0.086850772139864	0.720483228625054	-4.41852728320582
a_HQ_transcript/149234	4.087168167359	4.45603150227309	1.76547400799207	0.0868539321171986	0.720483228625054	-4.71507421420884
a_HQ_transcript/12058	-2.46145304690901	0.862611770282056	-1.76542352658399	0.0868625527491808	0.720483228625054	-4.3983425109352
a_HQ_transcript/44012	-1.74995867067904	-0.160410039185207	-1.76529200723282	0.0868850154999001	0.720496954145907	-4.46657704110931
a_HQ_transcript/111271	1.10643545769624	1.21816750974463	1.76516321611096	0.0869070170396746	0.720496954145907	-4.35223093414534
a_HQ_transcript/141870	-2.29357567329227	1.01874512524987	-1.76491960196411	0.0869486468026225	0.720496954145907	-4.50345454378194
a_HQ_transcript/118011	-0.799762859924738	3.43506076441939	-1.76489870812696	0.0869522180094728	0.720496954145907	-4.71362862994736
a_HQ_transcript/36424	0.883767544151164	2.6730904029607	1.76486359153378	0.0869582204708079	0.720496954145907	-4.61350360584576
a_HQ_transcript/32833	1.39178384167001	2.06126965378156	1.76482708358166	0.0869644611274713	0.720496954145907	-4.60132464307226
a_HQ_transcript/12994	-5.21566597369084	3.10150440739404	-1.76462886556789	0.0869983510489922	0.720496954145907	-4.52362990634692
a_HQ_transcript/5397	-2.37848626227138	2.27233654967881	-1.76460623318689	0.0870022212837107	0.720496954145907	-4.59476563383087
a_HQ_transcript/118700	0.480856430324317	4.17486154959557	1.76458554248326	0.0870057596104675	0.720496954145907	-4.84125266228883
a_HQ_transcript/127956	-1.34802485586093	3.14277091632795	-1.76457639175539	0.0870073245194173	0.720496954145907	-4.58398574759784
a_HQ_transcript/132906	-2.27428495892365	0.736847074336697	-1.76452943950869	0.087015354417568	0.720496954145907	-4.37110350505245
a_HQ_transcript/52425	-2.55976396888512	2.01632499515003	-1.76433511660142	0.0870485946944026	0.720496954145907	-4.44091358629362
a_HQ_transcript/142290	4.14512979823092	3.37236053380826	1.76432871441922	0.0870496900143908	0.720496954145907	-4.91738617672299
a_HQ_transcript/127401	0.76294043931616	4.6694864749129	1.76431395820866	0.0870522146311071	0.720496954145907	-4.86340599385311
a_HQ_transcript/50485	2.0197129328648	0.146828370056596	1.76425998392238	0.0870614495331158	0.720496954145907	-4.4239639126656
a_HQ_transcript/92873	-2.101108639526	-0.417635227473943	-1.76425368321076	0.0870625276273199	0.720496954145907	-4.46626538807011
a_HQ_transcript/57711	-2.20877462595644	-0.270085446707623	-1.76379553565421	0.0871409499830375	0.720955773219979	-4.47093330868791
a_HQ_transcript/6623	-0.692598646331978	4.11435979912236	-1.7637468313832	0.087149290331661	0.720955773219979	-4.79497902936572
a_HQ_transcript/49690	-1.33640717729095	-0.600818543261577	-1.76354408550462	0.08718401673464	0.720955773219979	-4.46503863103201
a_HQ_transcript/151409	1.94235144607123	-0.538964805405236	1.76348641165746	0.0871938972710065	0.720955773219979	-4.48491500355587
a_HQ_transcript/86193	1.7503632497635	-0.394024624085217	1.76344505286336	0.0872009833362703	0.720955773219979	-4.47233083442272
a_HQ_transcript/123962	2.32542463363843	-0.370014908466153	1.76337915292776	0.087212275077464	0.720955773219979	-4.45990989912255
a_HQ_transcript/117555	1.77440998394988	-0.364933071116031	1.76331824245655	0.0872227129880552	0.720955773219979	-4.43662129414317
a_HQ_transcript/133075	0.45132014537472	4.35943898494488	1.76324380635267	0.0872354701482271	0.720955773219979	-4.8360644879055
a_HQ_transcript/123843	-2.16581050606454	-0.136274706665812	-1.76321472255254	0.087240455075434	0.720955773219979	-4.47585735826611
a_HQ_transcript/76964	3.3418312497144	2.25825589853887	1.76309866547359	0.0872603495096619	0.720955773219979	-4.61206029492994
a_HQ_transcript/26424	-0.692897847103162	3.69392623701668	-1.76308951575612	0.0872619181115942	0.720955773219979	-4.76540344369772
a_HQ_transcript/109841	2.23703076452098	-0.0335508654455934	1.76306101330544	0.0872668046445047	0.720955773219979	-4.34100999249211
a_HQ_transcript/7689	2.0647720126711	1.74100870809097	1.76289339995494	0.0872955453801084	0.721090729267435	-4.44200124788963
a_HQ_transcript/68773	-0.962391720421777	2.39241464144729	-1.76265140337287	0.0873370547409819	0.721235600849383	-4.42523653045635
a_HQ_transcript/12112	2.07675538460642	1.088974436506	1.76264648228038	0.0873378990224816	0.721235600849383	-4.41774573408548
a_HQ_transcript/91529	1.67766054288452	2.32192638564436	1.76240246057558	0.0873797729658264	0.721298769244143	-4.48902028438244
a_HQ_transcript/114000	3.88294999562405	1.98848509687032	1.76231021998101	0.0873956057974412	0.721298769244143	-4.4909388143615
a_HQ_transcript/19626	1.99164403008023	-0.665199600590178	1.76228790094224	0.087399437159899	0.721298769244143	-4.48641117799413
a_HQ_transcript/143242	-1.99622167412691	2.85171143155874	-1.76226895160891	0.0874026901785161	0.721298769244143	-4.54856702751542
a_HQ_transcript/37120	0.561539381610998	3.82825584292501	1.76224039658746	0.0874075923918893	0.721298769244143	-4.80333873535708

a_HQ_transcript/2078	-1.27039433700956	1.77104337945318	-1.76211806631302	0.0874285961980845	0.721302136572845	-4.42377779939005
a_HQ_transcript/23764	-2.23774957494992	-0.109295233485123	-1.76209347974744	0.0874328181649509	0.721302136572845	-4.46925250350456
a_HQ_transcript/47129	2.30066163067226	0.937147644001904	1.76195816297528	0.0874560576316969	0.721391474104623	-4.56397276428233
a_HQ_transcript/43635	-2.41047046610259	0.646923738873395	-1.76180162030515	0.0874829489658048	0.721453691361533	-4.42041302510196
a_HQ_transcript/31480	-1.10099289070794	2.68845669270485	-1.76156846006957	0.0875230148191317	0.721453691361533	-4.62443573796109
a_HQ_transcript/150310	-3.11282053578163	0.607206036726857	-1.76152459446306	0.0875305543404528	0.721453691361533	-4.55211460133255
a_HQ_transcript/94073	0.728175801484015	3.64127100564953	1.76151747380186	0.0875317782753328	0.721453691361533	-4.70911031243461
a_HQ_transcript/112526	1.93475133283677	1.25768617833983	1.76146908979817	0.0875400951426203	0.721453691361533	-4.3586880381694
a_HQ_transcript/68972	1.21261783203632	1.66867915639907	1.76135856207819	0.0875590965768292	0.721453691361533	-4.40524945266914
a_HQ_transcript/97255	4.25450280216004	3.55356714836876	1.76107090086261	0.0876085663372914	0.721453691361533	-4.65471443775973
a_HQ_transcript/127059	1.18497024810171	1.96954050256708	1.76098935964054	0.0876225934596939	0.721453691361533	-4.42391882271767
a_HQ_transcript/103568	2.32313782574795	0.515249951728227	1.76091275796517	0.0876357725839219	0.721453691361533	-4.57390530595216
a_HQ_transcript/36748	1.66426753389135	-0.450455953238778	1.76089849420388	0.087638226812705	0.721453691361533	-4.4673773704356
a_HQ_transcript/103050	1.32012482632666	1.61829745670356	1.76087410472899	0.0876424234104521	0.721453691361533	-4.54052190118718
a_HQ_transcript/147774	1.46942906761553	4.41801428885192	1.76081498090233	0.0876525973108431	0.721453691361533	-4.83829010810962
a_HQ_transcript/10133	2.13787464712399	1.42342189561519	1.7607931662078	0.0876563513881193	0.721453691361533	-4.36500655815826
a_HQ_transcript/61795	-1.79416512007064	1.41409351973286	-1.76078146807342	0.0876583645687236	0.721453691361533	-4.48176944437927
a_HQ_transcript/150910	-1.48619733892391	-0.687663622906817	-1.76060578933704	0.0876886025474638	0.721453691361533	-4.42982141983055
a_HQ_transcript/86347	1.72124330728014	0.245317735087369	1.76059952519668	0.0876896808993562	0.721453691361533	-4.34198139539003
a_HQ_transcript/91484	-2.10979272446148	0.586475996192355	-1.76047619223453	0.0877109145573439	0.721453691361533	-4.58344637683542
a_HQ_transcript/77849	0.73249760745591	2.64052513726939	1.76043182773648	0.0877185536462497	0.721453691361533	-4.58608188314314
a_HQ_transcript/100989	-0.985761800940145	0.628712719359201	-1.76025079389699	0.0877497315455387	0.721453691361533	-4.44862846108906
a_HQ_transcript/120187	2.14373424201641	0.684454931457831	1.76022638375222	0.0877539362100827	0.721453691361533	-4.50711419786976
a_HQ_transcript/76917	1.82878441100924	-0.528437599088847	1.76003418194683	0.0877870490467368	0.721453691361533	-4.48665449003358
a_HQ_transcript/62349	2.95542983881191	0.879463649515755	1.75998188976617	0.0877960598524662	0.721453691361533	-4.43379225172566
a_HQ_transcript/66628	-2.75987042608462	-0.0859733873838788	-1.7599705303597	0.0877980173688682	0.721453691361533	-4.47807088592573
a_HQ_transcript/143655	1.59927283594754	-0.53100548568016	1.75980962677986	0.0878257491293627	0.721453691361533	-4.49005873823873
a_HQ_transcript/120668	1.5706922070158	-0.678208624462007	1.75973540676377	0.0878385434534754	0.721453691361533	-4.49086129521626
a_HQ_transcript/5590	-3.13336989628892	1.67778499308232	-1.75970738592356	0.0878433741997819	0.721453691361533	-4.60057676630076
a_HQ_transcript/7860	0.635613873344839	3.51915716292801	1.75956136062972	0.0878685523467353	0.721453691361533	-4.75738291896744
a_HQ_transcript/20030	2.46957014469506	1.11599380424299	1.75953165291389	0.0878736753913369	0.721453691361533	-4.36938553946877
a_HQ_transcript/139678	-3.13987211523963	1.76507275516078	-1.75952028591361	0.0878756356776995	0.721453691361533	-4.63711428013574
a_HQ_transcript/5269	1.69072396747617	2.94210208929195	1.75949831201023	0.0878794252731383	0.721453691361533	-4.67365454708003
a_HQ_transcript/10183	2.35888714136968	0.720156980428279	1.75944732028412	0.0878882197820301	0.721453691361533	-4.367608038173
a_HQ_transcript/73333	2.32314950287588	0.194375700429466	1.75941487256723	0.0878938164047294	0.721453691361533	-4.39752358999188
a_HQ_transcript/55171	2.10158869033326	0.594450607126709	1.75939634784091	0.0878970117075042	0.721453691361533	-4.46256799967282
a_HQ_transcript/80169	3.09961899046406	1.80181033778322	1.75935935699809	0.0879033924968424	0.721453691361533	-4.43006064262497
a_HQ_transcript/141340	-2.1546031767309	2.1167950610227	-1.75916820338858	0.0879363720468112	0.721453691361533	-4.51460967603997
a_HQ_transcript/119801	0.769079207154771	3.18307696721542	1.75916683186588	0.0879366087120367	0.721453691361533	-4.63843142050315
a_HQ_transcript/27888	-3.20455589533006	0.613738918108806	-1.75913795429365	0.0879415918510725	0.721453691361533	-4.48170594771404
a_HQ_transcript/66047	-1.92084311692017	0.642734111602202	-1.75909266030467	0.0879494083012808	0.721453691361533	-4.34122141569053
a_HQ_transcript/6338	1.30805312128022	-0.44925187512949	1.75906005876988	0.0879550347588725	0.721453691361533	-4.45292054180182
a_HQ_transcript/10590	1.64131537482636	1.91768389637997	1.75895337085579	0.0879734493597462	0.721453691361533	-4.49399643038253
a_HQ_transcript/107260	1.79419665330501	3.44427948694932	1.7589004655482	0.0879825821547501	0.721453691361533	-4.69867473116583
a_HQ_transcript/113170	0.957262879845465	5.5655216628159	1.75888714380275	0.0879848819512439	0.721453691361533	-4.95896945808964
a_HQ_transcript/51957	-1.74358775018644	0.211924874938696	-1.75876782950991	0.0880054820088119	0.7215208262635	-4.45929968786531
a_HQ_transcript/7907	2.7239272129624	1.97722641681923	1.75831964648889	0.0880828988379858	0.721755169838855	-4.48851423111614
a_HQ_transcript/97539	0.648887771657136	3.56602126049783	1.75830135835993	0.0880860590551003	0.721755169838855	-4.7632183784408
a_HQ_transcript/56574	1.49917489435704	1.62427030207096	1.75828501313397	0.088088883616515	0.721755169838855	-4.41766141649646
a_HQ_transcript/111991	1.86848002569901	0.109138971535117	1.75827710377854	0.088090250432041	0.721755169838855	-4.41731303827492

a_HQ_transcript/19838	1.55003169504772	1.18197618767556	1.75816673795731	0.0881093246139857	0.721755169838855	-4.42050992600096
a_HQ_transcript/50457	3.05202961026191	1.82724100907884	1.75810886756719	0.0881193275662605	0.721755169838855	-4.48473293738281
a_HQ_transcript/5499	-1.27043535311619	1.35667091357065	-1.75809929678883	0.0881209819767814	0.721755169838855	-4.40339857372216
a_HQ_transcript/54659	1.16847245291716	-0.682696615120734	1.75793954674132	0.0881486003359541	0.7217967014348	-4.49172192663612
a_HQ_transcript/136117	1.11767750096735	-0.665237346386542	1.75792631973477	0.0881508874120156	0.7217967014348	-4.48892435323357
a_HQ_transcript/92538	-3.29160394381847	0.808655143281689	-1.75775970263085	0.0881797013920053	0.721800483334076	-4.45307638603055
a_HQ_transcript/104332	2.87133795752985	0.707685667052023	1.7577050267849	0.088189158507625	0.721800483334076	-4.557446188126
a_HQ_transcript/81277	2.27285723713123	0.603851148964179	1.75759529431216	0.0882081411833732	0.721800483334076	-4.35420931756079
a_HQ_transcript/10448	2.15260295432879	-0.0177021158558255	1.75758706565348	0.0882095648021145	0.721800483334076	-4.38831313659609
a_HQ_transcript/45156	-2.40030866369935	0.477609631631276	-1.75756468774403	0.0882134364438012	0.721800483334076	-4.49685014323027
a_HQ_transcript/113201	-1.35187182424638	-0.415547323160588	-1.75746836418598	0.0882301031895871	0.721820261754972	-4.49048641478455
a_HQ_transcript/150646	0.262617457452232	10.7041683169053	1.75740719169373	0.0882406891692615	0.721820261754972	-5.52549559317186
a_HQ_transcript/8858	1.34368683539239	0.0323306118632134	1.7573103884397	0.0882574432882034	0.721855728641718	-4.36551371206302
a_HQ_transcript/111787	1.64623790953672	2.78131830063966	1.75716380120331	0.0882828188248474	0.721961690018392	-4.59912500721197
a_HQ_transcript/119519	1.61413315734685	4.05163482584352	1.75707626053951	0.0882979758168262	0.721984067840832	-4.75839127595335
a_HQ_transcript/60828	-2.33061371597284	1.2604367206487	-1.75687939687005	0.0883320692647889	0.722019321837646	-4.48505166104346
a_HQ_transcript/148571	-4.1664563052664	2.08441787229556	-1.75679254566369	0.0883471139501996	0.722019321837646	-4.63120823400305
a_HQ_transcript/101693	-1.06354880224225	3.62380646134551	-1.7567289064788	0.0883581391359782	0.722019321837646	-4.75545742105286
a_HQ_transcript/3351	1.51147796745975	1.25179135123831	1.75665289527323	0.088371309236369	0.722019321837646	-4.34854564397818
a_HQ_transcript/87819	3.45445832006263	1.49473130620506	1.75664981289685	0.0883718433400121	0.722019321837646	-4.42618191396572
a_HQ_transcript/146796	-1.37339041135263	-0.619113600450239	-1.75656335179136	0.0883868261342261	0.722019321837646	-4.43323349437588
a_HQ_transcript/140569	-1.87948795114102	4.79387549944428	-1.75649073273933	0.0883994119026596	0.722019321837646	-4.89976548475139
a_HQ_transcript/14587	1.99268345873306	-0.233383372364301	1.75647778004699	0.0884016569212519	0.722019321837646	-4.4777887743256
a_HQ_transcript/130818	3.8115831658633	1.30753931694096	1.75638739892694	0.0884173235199948	0.722045824890455	-4.40219573013616
a_HQ_transcript/23108	1.91292632023532	0.307721492462205	1.75617941345842	0.0884533844678927	0.722238844268811	-4.38676829211542
a_HQ_transcript/123349	-2.01825328584336	2.22827078467801	-1.75593686730781	0.0884954532852861	0.722323286408481	-4.38024788854601
a_HQ_transcript/26459	2.18073271650354	0.296390091459316	1.75586407277847	0.0885080825444627	0.722323286408481	-4.36184873510215
a_HQ_transcript/109510	0.476752984491109	4.08590546918535	1.75585856775709	0.0885090376827503	0.722323286408481	-4.81477114775807
a_HQ_transcript/131605	-2.0897918384694	3.507114444163	-1.75579563195417	0.0885199578576134	0.722323286408481	-4.75435971218177
a_HQ_transcript/4997	1.49381926455083	-0.248565889443687	1.75564574531211	0.0885459697021367	0.722323286408481	-4.37794575728995
a_HQ_transcript/28452	1.58551564011642	0.0305057373337017	1.75563533211919	0.0885477770829944	0.722323286408481	-4.43739652137931
a_HQ_transcript/131717	1.91746937744491	2.08178951568077	1.75556272471851	0.0885603801561041	0.722323286408481	-4.47862704969718
a_HQ_transcript/144987	-2.00096187857526	3.42147123973934	-1.75539971955864	0.0885886798341329	0.722323286408481	-4.62167362112633
a_HQ_transcript/118107	-2.33806338895199	0.188455869193393	-1.75536534524702	0.0885946486062635	0.722323286408481	-4.44654959459305
a_HQ_transcript/41258	1.34143929879926	-0.696177445858052	1.75507004653551	0.0886459383903187	0.722323286408481	-4.50039297682767
a_HQ_transcript/32370	1.37718527846897	-0.665738584476301	1.75506151459063	0.0886474206571808	0.722323286408481	-4.50008346150511
a_HQ_transcript/143919	7.10981957966544	6.65213656825509	1.75505848783221	0.0886479465052275	0.722323286408481	-4.77321932957909
a_HQ_transcript/149003	1.61719761067974	-0.269739882452618	1.75500283457949	0.0886576157848057	0.722323286408481	-4.4354634614572
a_HQ_transcript/14348	-1.54868462625487	1.35834513835377	-1.75497343310987	0.0886627243992619	0.722323286408481	-4.44940507026172
a_HQ_transcript/131120	-1.12682622650662	3.34066774070598	-1.75492510793401	0.0886711216172114	0.722323286408481	-4.68691473658508
a_HQ_transcript/133510	0.53839751570373	5.28839667487577	1.75487439039969	0.0886799352642881	0.722323286408481	-4.98303496833728
a_HQ_transcript/43526	2.14004723077636	-0.26412287210978	1.75475126994399	0.0887013340987896	0.722323286408481	-4.48979451527764
a_HQ_transcript/89966	-0.943158951052554	2.87757321605664	-1.75471728944392	0.0887072408141582	0.722323286408481	-4.63968431081739
a_HQ_transcript/81465	2.1425537671867	0.10738088457597	1.75462298595565	0.088723635008018	0.722323286408481	-4.48003705886579
a_HQ_transcript/58033	2.14670169628918	1.30652753425515	1.75455908051325	0.0887347461045503	0.722323286408481	-4.43694129804666
a_HQ_transcript/4861	2.44933329300645	0.657179642807184	1.75438715085154	0.0887646449556143	0.722323286408481	-4.47024886715025
a_HQ_transcript/127804	-2.21275501751015	0.320880979513164	-1.75425982064471	0.0887867933628359	0.722323286408481	-4.51181170110608
a_HQ_transcript/74901	-2.27502510555598	2.17670645440091	-1.75419830855909	0.088797494729726	0.722323286408481	-4.42539750938765
a_HQ_transcript/45602	-0.548403425820264	3.57262873077833	-1.75416847866346	0.0888026846812649	0.722323286408481	-4.7983697009808
a_HQ_transcript/140212	1.92794513146545	0.743701246781249	1.75413561918083	0.0888084020312558	0.722323286408481	-4.56026372643418

a_HQ_transcript/90764	-1.09403468885604	2.69322612024194	-1.75398808928075	0.0888340751609773	0.722323286408481	-4.69890721715372
a_HQ_transcript/22475	2.70619255935685	-0.139976437695757	1.75393213645614	0.0888438136991604	0.722323286408481	-4.5040449932545
a_HQ_transcript/73316	-1.62526870238393	0.347121524065665	-1.75389792340527	0.0888497688929764	0.722323286408481	-4.43048770739821
a_HQ_transcript/73672	2.94653766561656	4.37549198849128	1.75387595407773	0.0888535930966376	0.722323286408481	-4.75182630852903
a_HQ_transcript/30691	2.13425526648305	0.618635918677801	1.75381602093823	0.0888640263716166	0.722323286408481	-4.39097038415857
a_HQ_transcript/130196	2.09830906859467	-0.0315824265860388	1.75378729997081	0.0888690265383629	0.722323286408481	-4.56455320616536
a_HQ_transcript/108271	1.45472355394119	-0.363105901153892	1.75377349106728	0.0888714306790184	0.722323286408481	-4.46768137223697
a_HQ_transcript/142605	1.88630609990488	1.84822459191581	1.75375989254836	0.0888737982451392	0.722323286408481	-4.54021318805708
a_HQ_transcript/12180	-1.4946331924938	2.26447506449327	-1.75358897987428	0.0889035594803808	0.722446763111571	-4.61331822041278
a_HQ_transcript/77934	-3.08126859242759	-0.0353747363865818	-1.75352990754318	0.088913847772161	0.722446763111571	-4.54314576031353
a_HQ_transcript/93645	2.26287992054524	1.15292180836238	1.75345406175092	0.0889270588739836	0.722453120646669	-4.42854916631029
a_HQ_transcript/134402	-2.0444556342293	1.0064524672045	-1.75322947806951	0.0889661873992751	0.722538642820895	-4.49752012212219
a_HQ_transcript/100302	2.60927701915736	3.15310863652475	1.75303089326574	0.0890007983103092	0.722538642820895	-4.73009051938876
a_HQ_transcript/37483	2.24792031113359	0.610765622798402	1.75301754501995	0.089003125153947	0.722538642820895	-4.3599612922649
a_HQ_transcript/59560	-2.75175455739968	0.600991433519152	-1.75298177984081	0.0890093599322911	0.722538642820895	-4.590130996179
a_HQ_transcript/22282	-1.87063115559638	2.91833765678303	-1.75296910800435	0.0890115690432957	0.722538642820895	-4.67820213215979
a_HQ_transcript/52047	3.14717383315379	1.63847244740994	1.75296568025292	0.0890121666191927	0.722538642820895	-4.63845367892429
a_HQ_transcript/15958	1.40452508702072	2.37039158648607	1.75287013686511	0.0890288245046658	0.722571776230692	-4.46666320976432
a_HQ_transcript/23652	1.38685908875425	0.138938716682318	1.75279044729436	0.0890427203043628	0.722571776230692	-4.47385020739283
a_HQ_transcript/115	2.85440605123211	1.67368995039856	1.75259718437121	0.0890764279526548	0.722571776230692	-4.3979416776125
a_HQ_transcript/33130	-1.28150566934987	2.67684333243394	-1.75254942297396	0.0890847598388962	0.722571776230692	-4.62126790229681
a_HQ_transcript/81534	3.0989778173519	2.24793026405743	1.75252238678287	0.0890894765429431	0.722571776230692	-4.66783665720451
a_HQ_transcript/149448	0.327916069790083	8.34110194103482	1.7523551108513	0.089118663997907	0.722571776230692	-5.26265915445456
a_HQ_transcript/139461	3.46519727700876	1.78677526082602	1.75232667010782	0.0891236273360649	0.722571776230692	-4.54024070273117
a_HQ_transcript/149587	-1.51918422284761	2.86555716449966	-1.75229208473317	0.0891296633188707	0.722571776230692	-4.53120386620337
a_HQ_transcript/23194	-1.91087944856441	2.13706906078221	-1.75225878899557	0.0891354745544176	0.722571776230692	-4.43151012236868
a_HQ_transcript/109239	2.44207271585131	4.07868922166362	1.75217042914448	0.0891508978938333	0.722571776230692	-4.80287802471874
a_HQ_transcript/152310	-2.07801766205539	-0.35340165201717	-1.752158466558	0.0891529861534291	0.722571776230692	-4.51695537452181
a_HQ_transcript/32056	-1.57723292735346	-0.416158529867605	-1.7520216097953	0.0891768796106089	0.722618473633233	-4.47906618565173
a_HQ_transcript/85735	2.12103085226759	0.551044759581966	1.75198306039542	0.0891836108217179	0.722618473633233	-4.50525970525313
a_HQ_transcript/108736	1.52515738243943	0.753590166149443	1.75142768222744	0.0892806343871682	0.7233003543141	-4.35801107906229
a_HQ_transcript/54233	1.6377487284448	-0.175603719944248	1.75125879584054	0.0893101561679814	0.7233003543141	-4.49300903032577
a_HQ_transcript/25849	-1.24211648559605	2.62331915476024	-1.75116286185105	0.0893269293430695	0.7233003543141	-4.51503856918988
a_HQ_transcript/2017	1.42079213949973	1.19961552863036	1.75114646620543	0.0893297962364158	0.7233003543141	-4.35214629018799
a_HQ_transcript/134007	-3.42038877381991	3.33443884674762	-1.75114539893458	0.0893299828588791	0.7233003543141	-4.78813533392166
a_HQ_transcript/57137	2.20465318340101	1.30497893806424	1.75088946495344	0.0893747448383061	0.723411421634769	-4.47460746238612
a_HQ_transcript/101618	2.09414553164404	0.558044696742432	1.75085236529816	0.0893812350096499	0.723411421634769	-4.40964885597096
a_HQ_transcript/40010	2.33652611726976	-0.0333392306149526	1.75080321251733	0.089389834352695	0.723411421634769	-4.4693716758743
a_HQ_transcript/138062	-2.2350800733093	0.629116074676367	-1.75074586001747	0.0893998691286226	0.723411421634769	-4.43891568674627
a_HQ_transcript/17838	2.32716771782059	0.172745582799388	1.75071124668378	0.0894059257676882	0.723411421634769	-4.52903454937603
a_HQ_transcript/75047	1.20489978531439	-0.799231604599579	1.75061599438339	0.0894225947945933	0.723430435741862	-4.50912493469282
a_HQ_transcript/76702	1.49677435188879	-0.157486886910069	1.75055559105867	0.0894331666536096	0.723430435741862	-4.52430298225312
a_HQ_transcript/42029	2.67910753755304	1.34094691226827	1.75037464470818	0.0894648423942678	0.723575816582654	-4.53218418703205
a_HQ_transcript/151304	4.0199023338683	4.52642151479184	1.75021183444914	0.0894933513829821	0.723575816582654	-4.74192433575115
a_HQ_transcript/115176	1.85717839288228	0.30900765937712	1.75011156727515	0.0895109125355758	0.723575816582654	-4.46519319104571
a_HQ_transcript/102314	1.33326463905188	-0.682712878212241	1.75009393302795	0.0895140013611198	0.723575816582654	-4.50585251328431
a_HQ_transcript/71779	-0.813238567990564	2.15093297355699	-1.74994968212957	0.0895392718092319	0.723575816582654	-4.42501264541788
a_HQ_transcript/143926	1.13642409183496	-0.601174250183814	1.74992384480909	0.0895437987288483	0.723575816582654	-4.46658613438371
a_HQ_transcript/128071	1.85949135656253	0.525282244113217	1.74991830552015	0.0895447692847697	0.723575816582654	-4.41519314464538
a_HQ_transcript/4913	1.03151840412859	3.27896859156905	1.74982421201416	0.0895612570526288	0.723575816582654	-4.67207280591395

a_HQ_transcript/21473	1.65778304470193	1.75307944291581	1.74981329066669	0.089563170938484	0.723575816582654	-4.5193180529591
a_HQ_transcript/117643	-2.62369168035564	2.22966345076421	-1.74945837506445	0.0896253860472556	0.723871323715963	-4.65723878083248
a_HQ_transcript/126691	-2.04945872633016	1.0750345841347	-1.74940103710179	0.0896354405498343	0.723871323715963	-4.37604163918629
a_HQ_transcript/118082	-4.34200441861621	0.473466930750321	-1.74930524136042	0.0896522409391161	0.723871323715963	-4.52117654390813
a_HQ_transcript/150914	4.39834385076522	5.80897851324879	1.74918812912508	0.0896727833595408	0.723871323715963	-4.97996414655129
a_HQ_transcript/5731	-2.04782940984635	1.33950772237088	-1.74916119839159	0.0896775077858116	0.723871323715963	-4.56588376877945
a_HQ_transcript/77140	2.17680207367214	0.815770057561414	1.74910453540757	0.0896874487898935	0.723871323715963	-4.4222006062401
a_HQ_transcript/63563	0.640033416040316	7.02945632166177	1.74886370374755	0.0897297108685255	0.723871323715963	-5.11004469887033
a_HQ_transcript/56655	-1.29837676122986	1.47535112049001	-1.74882309634232	0.0897368384669257	0.723871323715963	-4.42338164385911
a_HQ_transcript/85347	-1.80984323124099	-0.0970546393445749	-1.74873412392749	0.0897524569814908	0.723871323715963	-4.52875508845774
a_HQ_transcript/79026	-2.05025030335059	0.537083896249105	-1.74871859559149	0.0897551831121042	0.723871323715963	-4.51351126567443
a_HQ_transcript/91741	1.57448934822543	0.274953780955009	1.74859542092192	0.0897768099375312	0.723871323715963	-4.3723028534605
a_HQ_transcript/150411	1.59865100965961	-0.296732485452591	1.74853080747298	0.0897881564260995	0.723871323715963	-4.58616191344089
a_HQ_transcript/139888	0.510816323243678	5.85025734984081	1.74845742492797	0.0898010442827671	0.723871323715963	-5.02346758791757
a_HQ_transcript/33075	0.915186566276068	2.78261618224555	1.74834643680569	0.0898205396038504	0.723871323715963	-4.62397380884196
a_HQ_transcript/12209	1.40018659051198	1.08127117410509	1.74819031560391	0.0898479686894808	0.723871323715963	-4.44253452952246
a_HQ_transcript/120649	-1.48675714294627	-0.286891505514449	-1.74817544063722	0.0898505824549407	0.723871323715963	-4.53210383180985
a_HQ_transcript/129574	2.26665284905143	0.272900550240172	1.7480224751135	0.0898774646178948	0.723871323715963	-4.47778767156543
a_HQ_transcript/26553	4.75571254244918	0.455379849100586	1.74801372284989	0.0898790029456855	0.723871323715963	-4.46354371826971
a_HQ_transcript/116128	-1.40186712087601	-0.392123356191579	-1.74798109262765	0.0898847383406357	0.723871323715963	-4.42135120576761
a_HQ_transcript/14999	2.05224063204201	3.77996505293625	1.74792656639682	0.0898943230721023	0.723871323715963	-4.84544231378629
a_HQ_transcript/34105	1.48908415244972	-0.374079397055288	1.74790364405079	0.0898983526656035	0.723871323715963	-4.50005710260633
a_HQ_transcript/102788	1.22299906506716	1.84074446784248	1.74788915469788	0.0899008998743926	0.723871323715963	-4.43555063663227
a_HQ_transcript/72471	-1.472621927949	1.17197265627676	-1.74775865607042	0.0899238440973932	0.723871323715963	-4.41750123723925
a_HQ_transcript/113469	-1.82704259110471	1.21716632918845	-1.74774857521057	0.0899256167158438	0.723871323715963	-4.41053926835334
a_HQ_transcript/100153	-3.62143829711211	-0.211251175202921	-1.74767425075478	0.0899386868369926	0.723871323715963	-4.46747083367477
a_HQ_transcript/106851	1.35565732125107	0.842315502670471	1.74761308148673	0.089949444789318	0.723871323715963	-4.37282450858257
a_HQ_transcript/127457	1.10017835860019	3.13786045195822	1.74761063811234	0.0899498745326029	0.723871323715963	-4.68293280017491
a_HQ_transcript/85188	2.28192755693101	1.26055966260304	1.74760652648355	0.0899505976941462	0.723871323715963	-4.36044074175524
a_HQ_transcript/37116	3.62813065385079	3.81338003132181	1.74739039665621	0.0899886179375674	0.723871323715963	-4.65337020456371
a_HQ_transcript/45182	0.569225790641457	2.72876968293408	1.7473625626361	0.0899935153125698	0.723871323715963	-4.61882728563919
a_HQ_transcript/61574	1.58207877821504	-0.234973586794401	1.7473415939058	0.0899972048932203	0.723871323715963	-4.49093374678644
a_HQ_transcript/105931	1.38887205554024	-0.307154282367532	1.7472661705841	0.0900104771567536	0.723871323715963	-4.50800083526465
a_HQ_transcript/141767	-1.40125933337432	-0.294724377818591	-1.74726490837983	0.0900106992812238	0.723871323715963	-4.43638210610494
a_HQ_transcript/119254	1.75838583934069	1.85084768693225	1.74713686543718	0.0900332348561082	0.723952396696066	-4.5185483856154
a_HQ_transcript/135954	-4.68693276559729	3.28526077626594	-1.7467251285459	0.0901057328127902	0.724435136506482	-4.50345870644696
a_HQ_transcript/118425	2.25881601902666	-0.30233991303476	1.7466491811075	0.0901191108822953	0.724442494449739	-4.54851671782542
a_HQ_transcript/60783	2.00718118655666	1.13267807771694	1.74630145874618	0.090180383210056	0.724551434409903	-4.42466732334824
a_HQ_transcript/68875	-0.579750253085198	3.71259850892839	-1.74605780386606	0.0902233386557715	0.724551434409903	-4.73628588672337
a_HQ_transcript/16929	-1.50900777206919	1.67367077007408	-1.74596267377387	0.0902401144117313	0.724551434409903	-4.38633919825884
a_HQ_transcript/50152	0.73190179734611	3.05870995026859	1.74593570071737	0.0902448714645823	0.724551434409903	-4.73336471561233
a_HQ_transcript/87216	0.584588838804677	3.77456782988712	1.74591396942034	0.0902487042178798	0.724551434409903	-4.81262329142199
a_HQ_transcript/137847	-1.97127584010812	1.36424294592736	-1.74584860222551	0.0902602338696058	0.724551434409903	-4.47955399013493
a_HQ_transcript/226	2.6749260921344	2.17901788644749	1.74579480187411	0.0902697242590414	0.724551434409903	-4.66517897901569
a_HQ_transcript/147699	0.546276131961451	7.55573090461681	1.74578845373784	0.0902708441265754	0.724551434409903	-5.20415433623537
a_HQ_transcript/63968	-1.91490002128008	2.27141991540948	-1.74578373380389	0.0902716767723622	0.724551434409903	-4.56403730391025
a_HQ_transcript/94139	1.31323594530389	-0.66868614979892	1.74574151338192	0.0902791251846806	0.724551434409903	-4.51505867653432
a_HQ_transcript/150541	1.22032283870455	9.77217495660005	1.74567351416548	0.0902911225098528	0.724551434409903	-5.37059754851278
a_HQ_transcript/129431	-2.15037214982355	2.37324943577606	-1.74563292045204	0.0902982852319635	0.724551434409903	-4.63904147785355
a_HQ_transcript/108481	1.67837330502927	-0.458030540142556	1.74561931959298	0.0903006851976029	0.724551434409903	-4.50085578331529

a_HQ_transcript/125779	-2.47536523805454	1.02749596243532	-1.74539525475467	0.0903402307189918	0.724551434409903	-4.54154878048062
a_HQ_transcript/16593	-0.840713881231215	2.19480772016133	-1.7453184898663	0.0903537824161636	0.724551434409903	-4.51878804581598
a_HQ_transcript/95975	-1.67442369399134	1.28130986674784	-1.74527566699635	0.0903613428994152	0.724551434409903	-4.45333804589774
a_HQ_transcript/40737	-2.4140419601493	1.68428669432192	-1.74522500113331	0.0903702887703213	0.724551434409903	-4.49938211527253
a_HQ_transcript/34533	-2.05326527366118	0.69862326611033	-1.74514238453893	0.0903848776546413	0.724551434409903	-4.39365230688245
a_HQ_transcript/1246	-1.52363242726895	2.61969820112222	-1.74497125456342	0.0904151030171404	0.724551434409903	-4.52241842540474
a_HQ_transcript/98047	3.16679555077833	1.14434192542304	1.74495584703183	0.0904178247476563	0.724551434409903	-4.39258784911318
a_HQ_transcript/14914	-1.43166012953145	-0.246803188305135	-1.74493501588154	0.0904215046665882	0.724551434409903	-4.47310849954381
a_HQ_transcript/144523	2.33997390701846	0.28384180118847	1.7449341732977	0.0904216535155675	0.724551434409903	-4.53902652461851
a_HQ_transcript/17955	-1.14504738275138	3.79719758341298	-1.74484192937857	0.0904379503680165	0.724551434409903	-4.85765618663892
a_HQ_transcript/90404	-1.74645128408777	-0.317669877181151	-1.74481139170829	0.0904433460424855	0.724551434409903	-4.4954591743003
a_HQ_transcript/46299	2.0563841863174	1.34868737388527	1.74480609622481	0.0904442817244402	0.724551434409903	-4.33564622201197
a_HQ_transcript/94248	-1.71749011883498	-0.606205998905064	-1.74465656694202	0.0904707060663636	0.724653461097526	-4.47921581754615
a_HQ_transcript/101011	1.78985403629176	0.0497654641768518	1.74459294823277	0.0904819505340945	0.724653461097526	-4.46516293414253
a_HQ_transcript/105513	-1.40663230673089	2.64079667987167	-1.7442483180492	0.0905428836163826	0.725041567551052	-4.68390332417228
a_HQ_transcript/99604	2.23847107995054	1.82503400934695	1.74406660472556	0.090575025737855	0.725199049048214	-4.4902976298242
a_HQ_transcript/56355	0.949737553749035	3.05588259649955	1.74393803891407	0.0905977727330737	0.725281274401337	-4.64696668638135
a_HQ_transcript/104037	-3.90720233531466	3.85472369035077	-1.74370556391133	0.0906389164659532	0.725499948478929	-4.99224504843695
a_HQ_transcript/76211	3.65687269117111	1.98650449083958	1.74361867135749	0.0906542988621143	0.725499948478929	-4.6842277111864
a_HQ_transcript/52958	2.28631977663574	1.26357142909112	1.74360746347044	0.0906562831294891	0.725499948478929	-4.397294754601
a_HQ_transcript/103422	2.09191772741451	1.17427808190657	1.7433696376688	0.090698396917338	0.725590856808593	-4.42698319069249
a_HQ_transcript/31967	-1.97375699844581	0.448924458495863	-1.74328659211288	0.09071310636113	0.725590856808593	-4.43126971300369
a_HQ_transcript/129209	0.506148462618362	5.53439569445463	1.74324546061672	0.0907203925207054	0.725590856808593	-4.99641893611966
a_HQ_transcript/90476	3.37159446682964	3.2783192833791	1.74322609949562	0.0907238223796596	0.725590856808593	-4.80814398686897
a_HQ_transcript/100546	1.96368066068625	1.99911922851414	1.74303307961411	0.0907580221820939	0.725623592067676	-4.52310846225023
a_HQ_transcript/68270	1.41494736170631	1.83893298175656	1.74297663903247	0.0907680245321844	0.725623592067676	-4.39308295264204
a_HQ_transcript/107395	1.52163518162408	-0.61423895585785	1.74275612644367	0.0908071124722348	0.725623592067676	-4.51258632626657
a_HQ_transcript/12789	1.22352279295973	2.09637870255235	1.74274369760878	0.0908093160223227	0.725623592067676	-4.43471317856211
a_HQ_transcript/136560	0.779008947382768	2.90279976579396	1.74270313456661	0.0908165078940719	0.725623592067676	-4.64813995139301
a_HQ_transcript/119971	-3.6835747998506	3.63467956879814	-1.74266059809247	0.090824050172351	0.725623592067676	-4.82553106584217
a_HQ_transcript/31019	-0.616352118596378	4.29280403479888	-1.74264803092541	0.0908262785978122	0.725623592067676	-4.86772031134114
a_HQ_transcript/121353	1.71880605261559	-0.481502798730645	1.74263677588665	0.0908282743941331	0.725623592067676	-4.49222593358872
a_HQ_transcript/93927	3.15570935367686	0.639639095020463	1.74256916508672	0.0908402642354197	0.725623592067676	-4.62504096343231
a_HQ_transcript/9598	0.934642980761729	2.23961382131813	1.74229138712732	0.0908895382972499	0.7259174336552	-4.54354413471436
a_HQ_transcript/39877	1.01625771989219	3.46047146277237	1.74213810720019	0.0909167377165084	0.726034912760709	-4.77506660192079
a_HQ_transcript/124997	-2.95239106862401	0.360126574341577	-1.74194670054522	0.0909507123144089	0.726206456787357	-4.54878884664575
a_HQ_transcript/53282	-0.874128970496169	2.31358833620971	-1.74172471712428	0.0909901276537466	0.726394037475073	-4.45422119756937
a_HQ_transcript/110885	1.5568726923559	1.02914076722672	1.74167364529799	0.0909991979922495	0.726394037475073	-4.37820222273387
a_HQ_transcript/4821	-3.16840323443465	0.430890666369609	-1.74149202519377	0.0910314598229218	0.726551791375367	-4.46838951229977
a_HQ_transcript/110675	-1.95370845396401	-0.0922181638370286	-1.74127305723531	0.0910703686821025	0.726627712123657	-4.45669933037389
a_HQ_transcript/150658	0.60546458898838	3.4709595726438	1.74111424250889	0.0910985975456484	0.726627712123657	-4.8116646752681
a_HQ_transcript/106835	1.7140509864662	-0.250111308782474	1.74105066578777	0.0911099001893063	0.726627712123657	-4.49728029780111
a_HQ_transcript/90266	4.17735888377537	4.957015353466	1.7410383058462	0.0911120976716746	0.726627712123657	-4.95130402835356
a_HQ_transcript/133096	1.92605680303466	1.83744795860751	1.74096301478373	0.0911254846837413	0.726627712123657	-4.38893689944402
a_HQ_transcript/100113	0.672355730866649	5.34745746760825	1.74092363221916	0.0911324876994871	0.726627712123657	-5.01798510487554
a_HQ_transcript/113931	1.63394881046767	2.94124115850824	1.74086815181551	0.0911423540045171	0.726627712123657	-4.82242271533629
a_HQ_transcript/12603	-2.33804383844354	0.262510633091289	-1.74080342982505	0.0911538649140328	0.726627712123657	-4.54656350066585
a_HQ_transcript/52101	1.21408932934673	1.47897907615401	1.74074180874291	0.0911648254585989	0.726627712123657	-4.41027424714113
a_HQ_transcript/15384	-1.44975434787923	-0.53443530678791	-1.7406296516294	0.0911847776948508	0.726627712123657	-4.48610324734426
a_HQ_transcript/151562	-2.99924472512087	1.32104156250595	-1.74062021250494	0.0911864570393975	0.726627712123657	-4.6036109615319

a_HQ_transcript/31336	-0.825787159767921	2.17557450384973	-1.7405948030744	0.0911909778410074	0.726627712123657	-4.50678440838929
a_HQ_transcript/92164	1.3959553247969	-0.470608178854129	1.74049720652101	0.091208343802768	0.726666475954947	-4.51448595120751
a_HQ_transcript/24940	2.31440915890039	0.115172321924083	1.74026792569569	0.0912491521116611	0.726891971213736	-4.53951036239726
a_HQ_transcript/758	0.615470461362763	2.60755634112175	1.7397545593673	0.0913405787807913	0.72742332245952	-4.63486819197623
a_HQ_transcript/50771	-0.77897377945121	2.71522344467255	-1.7397528534623	0.0913408827180023	0.72742332245952	-4.56987877836847
a_HQ_transcript/34249	-1.26586590464974	2.58658730375079	-1.7396609065518	0.0913572659484398	0.727454130828892	-4.6236306857422
a_HQ_transcript/3835	-1.15469913033332	3.81691646139848	-1.73956507879668	0.0913743433036213	0.727490457136406	-4.7507984479161
a_HQ_transcript/105899	-3.88198490226193	0.69242992603667	-1.73947142104028	0.0913910365403109	0.727523715696411	-4.53995516858101
a_HQ_transcript/29726	-1.41334324093882	1.63905493725809	-1.73923490261911	0.0914332041897533	0.727674428743803	-4.48062859235227
a_HQ_transcript/32559	1.56360059975245	0.875096569838251	1.73922479842426	0.0914350059789557	0.727674428743803	-4.58109081240535
a_HQ_transcript/12918	1.2799992061875	-0.531656224446265	1.73887968723772	0.0914965644506598	0.728064654125661	-4.50265603718752
a_HQ_transcript/71879	1.03604664421921	2.52859874408016	1.738671409164	0.0915337324707009	0.728073839835865	-4.61841586638475
a_HQ_transcript/29190	-0.964425249030352	1.81025142117983	-1.73859111210587	0.0915480651792646	0.728073839835865	-4.44846911126355
a_HQ_transcript/91985	-1.24561611763975	1.25798533703541	-1.7385267970539	0.0915595465239517	0.728073839835865	-4.35962796325462
a_HQ_transcript/5250	-2.04591267363506	3.83230921973846	-1.73841652698572	0.0915792344486156	0.728073839835865	-4.7428740238279
a_HQ_transcript/8821	-0.69826770938786	2.79218421757	-1.7381237226311	0.0916315298310898	0.728073839835865	-4.59551057559461
a_HQ_transcript/117070	-1.50900319510282	0.366879186061257	-1.73810193902404	0.091635421425785	0.728073839835865	-4.5228827657429
a_HQ_transcript/41670	-2.01774577982126	-0.23542238775605	-1.73808662950564	0.0916381565215859	0.728073839835865	-4.49563528452722
a_HQ_transcript/54085	-2.12980848049077	0.145818857356825	-1.73803973778155	0.0916465343094293	0.728073839835865	-4.55075370279111
a_HQ_transcript/11477	-1.31581141757882	-0.688949572188333	-1.73788898907998	0.0916734717975363	0.728073839835865	-4.48821055030487
a_HQ_transcript/138659	-1.5689533642181	-0.442761837330377	-1.73786673792599	0.0916774484499951	0.728073839835865	-4.51036357346374
a_HQ_transcript/76767	1.36087069028226	0.816254396994093	1.73785950274091	0.0916787415295312	0.728073839835865	-4.39725807002224
a_HQ_transcript/82412	-2.48857623142511	4.41072769952256	-1.73783593011649	0.0916829545590021	0.728073839835865	-4.90644495246622
a_HQ_transcript/19772	1.96724114259805	0.914720720003182	1.7378221168018	0.0916854234264532	0.728073839835865	-4.36212176093409
a_HQ_transcript/47414	-1.80541559855249	1.86553521564454	-1.73776075672569	0.0916963910495162	0.728073839835865	-4.54532466203749
a_HQ_transcript/112444	2.06401573540886	1.28416884420163	1.73774892669488	0.0916985056997954	0.728073839835865	-4.61168337379441
a_HQ_transcript/143935	-2.78896792122683	1.78928508235506	-1.73771840350494	0.0917039619926568	0.728073839835865	-4.59512102564156
a_HQ_transcript/40330	-1.73750119769239	2.25566057277778	-1.73768099279432	0.0917106498637181	0.728073839835865	-4.64115406462616
a_HQ_transcript/149661	-3.44113704544655	0.602421277453697	-1.73759539867927	0.0917259529709366	0.7280958888836	-4.53343211202045
a_HQ_transcript/123931	5.33930247453758	1.68671020698231	1.73746936678371	0.0917484897322105	0.728131196158618	-4.54209178634308
a_HQ_transcript/107050	1.1385529013966	2.58433423009994	1.73729303998049	0.0917800279376534	0.728131196158618	-4.53352763586804
a_HQ_transcript/78744	1.64744479664257	0.212232035616957	1.73727025429542	0.091784104101118	0.728131196158618	-4.41351676785485
a_HQ_transcript/21231	-1.52878396900741	0.631354461222883	-1.73721516756448	0.0917939592732376	0.728131196158618	-4.37256926634478
a_HQ_transcript/71319	1.42728974088067	1.07001707899922	1.7370833470668	0.0918175459454356	0.728131196158618	-4.52555430987861
a_HQ_transcript/50840	-0.864703726771101	2.89907615337804	-1.73702128176106	0.0918286530692724	0.728131196158618	-4.55990981519348
a_HQ_transcript/4395	-1.91991103388022	-0.20203476254547	-1.7369901378419	0.0918342269689003	0.728131196158618	-4.52115550663495
a_HQ_transcript/141282	0.786316148132738	2.0139903236724	1.73697115160625	0.091837625119204	0.728131196158618	-4.48081166581412
a_HQ_transcript/748	2.15615137115551	-0.206639956159784	1.73694035070925	0.0918431380786366	0.728131196158618	-4.52194125267762
a_HQ_transcript/42681	1.66361066550977	0.33750227313136	1.73676560458518	0.091874420626549	0.728182618957757	-4.47014282198256
a_HQ_transcript/149242	-1.89393361525972	1.60343844764988	-1.73676416279926	0.0918746787681701	0.728182618957757	-4.4262983593838
a_HQ_transcript/39304	-1.0806380240506	1.91335984012522	-1.73648152592063	0.091925294689449	0.728360000267553	-4.42847392933532
a_HQ_transcript/19607	-1.0309106027693	2.47794382481468	-1.73645570659186	0.0919299197028177	0.728360000267553	-4.50552505213826
a_HQ_transcript/80355	2.65285161416658	1.16623106034036	1.73631001837695	0.0919560204821931	0.728360000267553	-4.54362839851791
a_HQ_transcript/14099	2.47460328710672	0.29391539740229	1.73630359522455	0.0919571713657534	0.728360000267553	-4.51440384893508
a_HQ_transcript/91749	2.6060472976992	-0.0109376323824606	1.73626154248088	0.0919647065649615	0.728360000267553	-4.53793531251863
a_HQ_transcript/92500	4.07854918122277	2.86862251475838	1.73621949909486	0.0919722406063143	0.728360000267553	-4.59977615283293
a_HQ_transcript/151270	-1.40119753355702	-0.70181981425715	-1.73611573023168	0.0919908378780194	0.728408040345118	-4.49339669057159
a_HQ_transcript/6979	-1.72023925663154	0.527980015803487	-1.7360187347442	0.0920082240956719	0.728446479192757	-4.46437897008005
a_HQ_transcript/3728	-1.9432484142433	0.995352523554839	-1.73591156422262	0.0920274373768131	0.728468409692774	-4.39034698956036
a_HQ_transcript/88854	1.54708882860907	-0.310201787477441	1.73582303771478	0.092043310746166	0.728468409692774	-4.51992536721163

a_HQ_transcript/13256	2.36076156572331	2.31278971419166	1.73562343646794	0.0920791089718221	0.728468409692774	-4.557594073277
a_HQ_transcript/150648	1.69894981363402	-0.52695099040316	1.73559781532913	0.0920837049373875	0.728468409692774	-4.46803264594334
a_HQ_transcript/91333	2.48943942077281	0.201621629217519	1.73558857572742	0.0920853624009226	0.728468409692774	-4.52867928574253
a_HQ_transcript/53289	-1.32883634001715	0.539173811381803	-1.73558397935345	0.0920861869395558	0.728468409692774	-4.38738820914601
a_HQ_transcript/101229	1.18642318664131	-0.808490501098659	1.73528073552072	0.0921405992401351	0.728799666979259	-4.53263838264368
a_HQ_transcript/147863	-2.855221455578	1.91484686720581	-1.7349496467016	0.0922000387399018	0.728803349819431	-4.65217666751098
a_HQ_transcript/30423	-0.701115102463897	3.83616747594266	-1.73492015438882	0.092205334979665	0.728803349819431	-4.79367849497918
a_HQ_transcript/144000	1.82337118038047	3.0361815172421	1.73489432069251	0.092209974413896	0.728803349819431	-4.65779116997307
a_HQ_transcript/81466	-1.87556542188844	2.53947404261274	-1.73485224068836	0.0922175319179181	0.728803349819431	-4.53362110644705
a_HQ_transcript/150047	2.17455404808462	0.52709198656147	1.73483400811851	0.0922208066210661	0.728803349819431	-4.51633461567569
a_HQ_transcript/52180	-1.72165400124932	-0.423919137681579	-1.73474751991919	0.0922363418668597	0.728803349819431	-4.44813269991029
a_HQ_transcript/68445	1.81873769454285	-0.0682187538896809	1.73464504030716	0.092254752380768	0.728803349819431	-4.52907792257031
a_HQ_transcript/130928	3.49904195788894	0.948336008192557	1.73446826514095	0.0922865173869517	0.728803349819431	-4.5837683507826
a_HQ_transcript/57850	-1.28711812916209	2.50252409013383	-1.7344474634487	0.0922902558799601	0.728803349819431	-4.53084697879264
a_HQ_transcript/4799	-1.27147614119153	4.36336652293663	-1.73444336674583	0.0922909921569387	0.728803349819431	-4.9563726805279
a_HQ_transcript/73245	-1.83066293698833	-0.148211783907938	-1.73444079873184	0.0922914536939226	0.728803349819431	-4.52514767880133
a_HQ_transcript/116547	1.27520933533918	-0.624214362160442	1.73443028273058	0.0922933437052295	0.728803349819431	-4.53254546689808
a_HQ_transcript/129155	1.49236195923254	3.83669147781883	1.7343706740487	0.0923040576206071	0.728803349819431	-4.80212992332436
a_HQ_transcript/148623	-0.611091370373646	6.72338247788822	-1.73397649314563	0.0923749330225033	0.728857347202834	-5.13598594108171
a_HQ_transcript/113308	0.635619545681428	4.51456335040717	1.73393440669617	0.0923825030464044	0.728857347202834	-4.92312384606369
a_HQ_transcript/145688	2.62141518525945	2.93662984756497	1.73392420497256	0.0923843380929846	0.728857347202834	-4.50500154094249
a_HQ_transcript/121059	1.40909840199877	-0.704061233176376	1.73388561139465	0.0923912804336148	0.728857347202834	-4.52177515577087
a_HQ_transcript/112537	1.62084691160928	-0.141842100226518	1.73379270063594	0.0924079953267415	0.728857347202834	-4.43874383265806
a_HQ_transcript/62814	-1.67751035879304	1.57039112506541	-1.733772893717	0.0924115589728286	0.728857347202834	-4.54250850108977
a_HQ_transcript/68722	0.449961218159946	4.57090539578653	1.73370534410515	0.0924237133171523	0.728857347202834	-4.93026513353401
a_HQ_transcript/148134	-1.87465289455883	2.0816513470346	-1.73359382044907	0.0924437829437575	0.728857347202834	-4.51264328461741
a_HQ_transcript/141729	-1.01318978816942	3.70810115858051	-1.73358797330978	0.0924448352870287	0.728857347202834	-4.85793331055346
a_HQ_transcript/125581	1.41464193841777	-0.44218301490114	1.73354854409139	0.0924519318529307	0.728857347202834	-4.41539345862371
a_HQ_transcript/28203	-1.39802207955557	0.683625259649417	-1.7335385015964	0.0924537393985626	0.728857347202834	-4.43305873190108
a_HQ_transcript/65434	-3.4191773374931	2.20832012943118	-1.73349615053126	0.0924613624806238	0.728857347202834	-4.64204388867561
a_HQ_transcript/119034	2.33915599313262	0.580286262547463	1.73333964597095	0.0924895374784573	0.728883054788896	-4.4431322661746
a_HQ_transcript/106943	1.67526336599335	0.389562436839066	1.73333873085236	0.0924897022454394	0.728883054788896	-4.55031617816286
a_HQ_transcript/59308	1.03136638724677	2.43370528044019	1.73298591681657	0.0925532447463594	0.729284941116495	-4.59163707168935
a_HQ_transcript/100542	-1.33226804258096	-0.678598992833297	-1.7327671928658	0.0925926557744148	0.729496597296717	-4.51162836671029
a_HQ_transcript/101174	2.17980505659699	-0.617917836074971	1.73233474773501	0.0926706178977689	0.729897766354403	-4.521238663671
a_HQ_transcript/115168	2.05039737487812	2.73665239933215	1.73229054813695	0.0926785894002927	0.729897766354403	-4.64617090862844
a_HQ_transcript/106744	-1.8573735512998	1.22120514764403	-1.73222680612137	0.0926900864386647	0.729897766354403	-4.51483137359545
a_HQ_transcript/62380	2.88153533495976	1.10029003272525	1.73202552906424	0.0927263983028101	0.729897766354403	-4.50391388866585
a_HQ_transcript/122278	1.71549506553082	-0.611558238548495	1.73199937476285	0.0927311176086833	0.729897766354403	-4.52086296476092
a_HQ_transcript/64345	2.4106668446953	3.27917313049761	1.7319206035881	0.0927453323699178	0.729897766354403	-4.81052300910438
a_HQ_transcript/121611	3.18926430590759	3.15868308495442	1.73188897851688	0.0927510398308099	0.729897766354403	-4.73916163101376
a_HQ_transcript/28168	2.58666533211836	2.49571507996762	1.7318572053375	0.0927567743183011	0.729897766354403	-4.55255158743343
a_HQ_transcript/52413	-1.0139316429369	1.51488452899665	-1.7317595245131	0.0927744058157897	0.729897766354403	-4.45743539869679
a_HQ_transcript/15233	2.27935455850779	0.160136073601032	1.73174994881137	0.0927761343921181	0.729897766354403	-4.46254800442474
a_HQ_transcript/76289	-1.85060643198481	0.421494604191572	-1.73171372927667	0.0927826728762149	0.729897766354403	-4.4840830383048
a_HQ_transcript/74761	1.71295942186043	0.698165050691507	1.73161681743654	0.092800169662822	0.729897766354403	-4.43928864972058
a_HQ_transcript/105480	0.93957852864733	2.217135464688	1.7315126329144	0.0928189825750027	0.729897766354403	-4.468646944286
a_HQ_transcript/49720	-0.486117985218224	3.20957392167276	-1.73151049237745	0.0928193691317738	0.729897766354403	-4.71803701175414
a_HQ_transcript/149783	-1.86547759732674	-0.300638359355644	-1.731392257848	0.0928407230513585	0.729966934874796	-4.52281550026184
a_HQ_transcript/41130	1.28755108701369	-0.206546291232354	1.73119200105014	0.092876900135739	0.730046565531606	-4.40458812769464

a_HQ_transcript/146842	0.936639665591492	2.4893258507396	1.7311400464347	0.0928862878512811	0.730046565531606	-4.50587868532937
a_HQ_transcript/128682	2.36267424662565	1.36758040643536	1.73108288465398	0.0928966173739472	0.730046565531606	-4.48983304224252
a_HQ_transcript/22692	1.91665532861104	2.40138210832381	1.73105814645489	0.0929010880339473	0.730046565531606	-4.54334147444162
a_HQ_transcript/114390	2.50918224524	0.395926899773843	1.730977626484	0.092915640765469	0.73006222849624	-4.60188902805925
a_HQ_transcript/5816	1.99768483175179	0.637723393669247	1.73069275662599	0.0929671419292699	0.73036816138189	-4.49086413720461
a_HQ_transcript/152705	0.656787739101008	5.74744815060885	1.73059773825307	0.0929843254794725	0.730404442090645	-5.05962431181544
a_HQ_transcript/15761	-2.16523386663193	2.36739206528393	-1.73033204349373	0.0930323890741388	0.730683247142486	-4.55955937672099
a_HQ_transcript/92134	0.752109036930744	2.78951315762434	1.73014514433316	0.0930662112183697	0.730737977777874	-4.63822825623723
a_HQ_transcript/136377	-0.486544397298127	3.11111448889023	-1.73014411370922	0.0930663977535116	0.730737977777874	-4.68090691595348
a_HQ_transcript/144197	-2.19875758485034	2.82252807587397	-1.73007356612544	0.093079167079217	0.730737977777874	-4.50589630501654
a_HQ_transcript/116362	2.37973322210008	2.79180605683045	1.72995541364729	0.0931005563301396	0.730737977777874	-4.55002950747204
a_HQ_transcript/17604	0.746028479392315	3.96283722942871	1.72994626080365	0.0931022134500229	0.730737977777874	-4.875544845774
a_HQ_transcript/72980	-0.679772706599072	3.76242022854571	-1.7297757244776	0.0931330935364719	0.730810210445013	-4.77537463434861
a_HQ_transcript/137153	4.91419358884759	3.8808917723251	1.72969059980311	0.0931485108151585	0.730810210445013	-4.81513537783001
a_HQ_transcript/11345	-2.54584117077395	1.93338990042009	-1.72968716034717	0.0931491337941629	0.730810210445013	-4.41358441449018
a_HQ_transcript/98085	-1.69736235412414	0.226773196662488	-1.72960091541455	0.0931647562407151	0.730823462199611	-4.36066101748869
a_HQ_transcript/121275	6.69740318144994	3.35210734341875	1.72953902651628	0.0931759681799015	0.730823462199611	-4.63679140875198
a_HQ_transcript/49138	2.10526606619542	-0.159729452482646	1.72943015055657	0.0931956951561557	0.730879569351999	-4.58760320379459
a_HQ_transcript/135683	2.34570675513724	2.09374530419776	1.72925173075673	0.093228030197984	0.730951537426156	-4.51943330998558
a_HQ_transcript/136010	1.30299826994066	2.75872769589248	1.72924074392819	0.0932300216501088	0.730951537426156	-4.64795138352475
a_HQ_transcript/99070	-1.92301320134921	1.13833653001786	-1.72900545135511	0.0932726789189895	0.731187360782606	-4.47818544829728
a_HQ_transcript/141098	-1.02297502946429	2.57714628922278	-1.72879195397253	0.093311399017873	0.731270237138564	-4.54160999638228
a_HQ_transcript/57645	-1.64588760352639	0.502297488804511	-1.72867735544789	0.0933321882865601	0.731270237138564	-4.42534025989188
a_HQ_transcript/112430	0.852535678389323	0.278043380943017	1.72864719680878	0.0933376599985281	0.731270237138564	-4.57162748638161
a_HQ_transcript/11840	-0.951571373406679	2.29013848247893	-1.7286298386281	0.0933408094326719	0.731270237138564	-4.61632379553708
a_HQ_transcript/116203	-2.69037455021013	1.83848329280858	-1.72856224681723	0.0933530740079827	0.731270237138564	-4.7452262911316
a_HQ_transcript/52217	1.24724604732732	-0.544975407420409	1.72846833180095	0.0933701171908956	0.731270237138564	-4.51750268503628
a_HQ_transcript/40175	1.66112508031199	1.55402535370714	1.72846174123232	0.0933713133092903	0.731270237138564	-4.49494952569398
a_HQ_transcript/146652	1.37926161301799	-0.406322598983237	1.72819000400199	0.0934206419393679	0.731558005476435	-4.52686416919814
a_HQ_transcript/49228	-0.690726853343919	3.30557878038422	-1.72809201551837	0.0934384352166597	0.731598782633889	-4.75906876211295
a_HQ_transcript/101691	-2.30417271862733	-0.199353465153517	-1.72794438356434	0.0934652483952351	0.731710162790334	-4.53742675011352
a_HQ_transcript/89797	-1.44707948737631	0.536374050519993	-1.72777636272534	0.093495772498113	0.731850560701631	-4.5021004029565
a_HQ_transcript/110065	1.95442334765866	-0.575079074137024	1.72752068276206	0.0935422376530046	0.732115683999237	-4.5244907470898
a_HQ_transcript/113295	1.59039265793539	-0.0731181295566157	1.72744260970881	0.093556429746988	0.73212818367433	-4.43063062397224
a_HQ_transcript/138963	2.06255961781543	-0.139563593598852	1.72731520858391	0.0935795927096724	0.732210871588079	-4.56548521360082
a_HQ_transcript/266	0.794074338849187	-0.693038165427876	1.72717139417013	0.09360574557728	0.732316928521687	-4.59865022919739
a_HQ_transcript/59209	-0.851541939504456	2.83564818625177	-1.7269731390646	0.0936418086245193	0.732492287243339	-4.54557459090874
a_HQ_transcript/74296	1.44066559211607	-0.770887045255265	1.72690963345676	0.0936533629024308	0.732492287243339	-4.51860841381074
a_HQ_transcript/104240	-2.95215507724151	0.763853707772122	-1.72670070626153	0.093691383783196	0.732691074471898	-4.46520950975641
a_HQ_transcript/128832	-0.892487881663848	1.89098485642603	-1.72653450464968	0.0937216386502455	0.732829084135253	-4.55486899445266
a_HQ_transcript/71869	-2.23046844111121	4.93556572527605	-1.72628675449918	0.0937667536019086	0.733034033033489	-5.02465254873167
a_HQ_transcript/136522	2.22600149204105	2.40691235565888	1.72625207043508	0.0937730709750382	0.733034033033489	-4.65927326388407
a_HQ_transcript/2657	2.63974014199923	3.38551868056531	1.72597107462198	0.0938242648663991	0.733213135539871	-4.71676807049745
a_HQ_transcript/113307	1.258453516808	2.20837062548719	1.72595019582534	0.0938280696556212	0.733213135539871	-4.56923088807655
a_HQ_transcript/129881	4.38747465804214	2.95605302495069	1.72591862077094	0.0938338238934954	0.733213135539871	-4.63984275719153
a_HQ_transcript/137296	1.91878215003502	0.326203497192782	1.72558310567134	0.0938949864592652	0.733358574021905	-4.57559716241173
a_HQ_transcript/37672	-0.929760651235727	1.66373862900446	-1.72555848853149	0.0938994753461251	0.733358574021905	-4.42354816697133
a_HQ_transcript/36678	1.24580457548405	1.88281751704126	1.72548100474363	0.0939136055373741	0.733358574021905	-4.48819051738129
a_HQ_transcript/43374	0.74705143965543	4.09935002146868	1.72538921772081	0.0939303464209751	0.733358574021905	-4.88600500611213
a_HQ_transcript/145336	-2.00058128212841	1.2109048920377	-1.72525662012344	0.0939545350995501	0.733358574021905	-4.55140360918669

a_HQ_transcript/43038	1.288950365213	-0.414698190083344	1.72522274612949	0.0939607152890885	0.733358574021905	-4.511815482792
a_HQ_transcript/20155	-1.96222578713099	0.315963163822081	-1.72514767530845	0.0939744129086537	0.733358574021905	-4.45638417283334
a_HQ_transcript/142884	1.42079260230733	-0.48303349558266	1.72514591932147	0.0939747333307128	0.733358574021905	-4.51809918434793
a_HQ_transcript/144259	2.37115373156426	0.206256305055641	1.72505265767987	0.0939917524776446	0.733358574021905	-4.45052237916669
a_HQ_transcript/22590	-1.45671727513385	1.9372016601997	-1.72499320790671	0.0940026027080365	0.733358574021905	-4.51659948057411
a_HQ_transcript/89761	-0.874545134350027	2.29832053005019	-1.72469951665208	0.094056219978629	0.733358574021905	-4.56142611176854
a_HQ_transcript/135278	1.90589570136918	0.705022084593902	1.72455413669163	0.0940827705298768	0.733358574021905	-4.64216680604106
a_HQ_transcript/65155	1.46089819826281	-0.650927599529833	1.72434553697098	0.0941208778070448	0.733358574021905	-4.54999362063103
a_HQ_transcript/142704	-3.15940089291789	1.90169553567252	-1.72416980565977	0.0941529906878811	0.733358574021905	-4.52219031169429
a_HQ_transcript/126720	-2.67964431174287	1.31322437875274	-1.72410301082048	0.0941651990890523	0.733358574021905	-4.5764824647871
a_HQ_transcript/122789	0.52367059790818	3.43097094308035	1.72409303471542	0.0941670225818149	0.733358574021905	-4.79975974765698
a_HQ_transcript/111505	1.18507077748111	-0.770554502023197	1.7240734787401	0.0941705972271067	0.733358574021905	-4.4927022603956
a_HQ_transcript/140994	-2.90302320806057	0.56866389913957	-1.72406595286563	0.0941719729153306	0.733358574021905	-4.52284467781968
a_HQ_transcript/7018	1.21370098138171	-0.490162694291084	1.72405289635503	0.094174359613243	0.733358574021905	-4.53132275957356
a_HQ_transcript/46337	-0.946305054108953	2.42509528636057	-1.72396070524002	0.0941912133657316	0.733358574021905	-4.55238659598218
a_HQ_transcript/53034	1.50842453518738	-0.229292914103937	1.72392935853425	0.0941969445336581	0.733358574021905	-4.54285481309509
a_HQ_transcript/105887	0.921157665827415	2.54925503488762	1.72382506208675	0.0942160153245284	0.733358574021905	-4.47923185666409
a_HQ_transcript/56458	-1.97467982411064	0.167473673822414	-1.72376039215407	0.0942278419634621	0.733358574021905	-4.49310534373967
a_HQ_transcript/151902	-1.27881970290322	1.88690781849674	-1.72374956211096	0.0942298226502653	0.733358574021905	-4.50443230143162
a_HQ_transcript/124547	-1.49953673940888	3.34850842045206	-1.72358736199414	0.0942594913122866	0.733358574021905	-4.65495543592758
a_HQ_transcript/128522	-1.72561861900323	0.752962498452215	-1.72355656314785	0.09426512573567	0.733358574021905	-4.45006936604016
a_HQ_transcript/150827	4.26276445248208	0.341503969137557	1.72340300624621	0.0942932220623586	0.733358574021905	-4.56568652176405
a_HQ_transcript/88028	1.71959683468068	-0.126345242881786	1.72333797149581	0.0943051235966666	0.733358574021905	-4.48215823410753
a_HQ_transcript/43353	1.59425923133991	0.23497565279675	1.72329312424902	0.0943133314975496	0.733358574021905	-4.49470985690063
a_HQ_transcript/135117	4.33699844497558	1.49276809002197	1.7232853187096	0.0943147601212473	0.733358574021905	-4.51138806726132
a_HQ_transcript/47615	0.819443508618686	3.23307544921148	1.7231595462987	0.0943377823588081	0.733358574021905	-4.76730928342122
a_HQ_transcript/87169	1.78282008436432	1.04142462945415	1.72315370096224	0.0943388524435718	0.733358574021905	-4.48215641024363
a_HQ_transcript/15066	2.7900220307877	1.60203575787482	1.72309073338893	0.0943503803367897	0.733358574021905	-4.52448224508817
a_HQ_transcript/75648	2.03946060148685	0.879078468084921	1.72293028033931	0.09437976088742	0.733358574021905	-4.45206588724553
a_HQ_transcript/142888	0.821401087610123	5.40036745282934	1.72283224986564	0.0943977150192017	0.733358574021905	-5.01715305957702
a_HQ_transcript/107013	2.01780616812413	0.168231946089836	1.72268902424632	0.0944239517232236	0.733358574021905	-4.574761474412
a_HQ_transcript/107709	0.875339797364877	2.42531834066998	1.72261284500658	0.0944379090656561	0.733358574021905	-4.58341125994255
a_HQ_transcript/152246	4.15481408092793	1.10677544259865	1.72260089892268	0.0944400979499269	0.733358574021905	-4.6172656239423
a_HQ_transcript/121089	1.67401036672628	0.204974940387719	1.72257967880308	0.0944439862232577	0.733358574021905	-4.49325536474607
a_HQ_transcript/86139	0.57933657281869	2.22200763818176	1.72250622286442	0.0944574469753688	0.733358574021905	-4.51166682200365
a_HQ_transcript/44742	0.980101114024169	-0.547189376778049	1.72247103001663	0.0944638966115246	0.733358574021905	-4.588590890875
a_HQ_transcript/35075	2.28727722166351	1.56594241501066	1.72231672399038	0.0944921799485358	0.733358574021905	-4.55462752457207
a_HQ_transcript/145961	2.53830024531198	-0.475499696555987	1.72217066587016	0.0945189580381262	0.733358574021905	-4.55018737508218
a_HQ_transcript/49729	2.04540589999086	0.757677857297572	1.7220556927428	0.0945400415254389	0.733358574021905	-4.5103783568738
a_HQ_transcript/78992	2.32633983141889	0.873523699138703	1.72201245844838	0.0945479707436097	0.733358574021905	-4.49560036380789
a_HQ_transcript/33283	2.33880530467637	0.00945360231717933	1.72200061986142	0.0945501420510243	0.733358574021905	-4.47992642847444
a_HQ_transcript/124914	2.16552448679288	1.13037216442918	1.72196961595876	0.0945558286544978	0.733358574021905	-4.55741461453487
a_HQ_transcript/28858	1.6863861360384	0.396652326074448	1.72180027906659	0.0945868927660267	0.733358574021905	-4.50068399680139
a_HQ_transcript/141543	3.82346260643181	4.39334235201381	1.72179522514637	0.0945878200170763	0.733358574021905	-4.6069618752483
a_HQ_transcript/137209	1.82116965703705	1.09157766879781	1.72175849300577	0.0945945595523199	0.733358574021905	-4.42034836400499
a_HQ_transcript/104147	3.3970892408378	0.0658710476987819	1.72173677427553	0.0945985446483183	0.733358574021905	-4.50308371955162
a_HQ_transcript/121731	1.58860968280454	0.527612814463908	1.72168026752372	0.0946089135398213	0.733358574021905	-4.37997569207991
a_HQ_transcript/33343	-2.32920814759305	0.722150216582776	-1.72163698862328	0.0946168557879272	0.733358574021905	-4.41572852720111
a_HQ_transcript/127851	-1.64395264238386	1.69490552556966	-1.72161729401802	0.0946204701924264	0.733358574021905	-4.53221838130647
a_HQ_transcript/65741	1.89484966251904	0.241208700116424	1.72153823388461	0.0946349806763212	0.733358574021905	-4.51548664025201

a_HQ_transcript/76493	1.61878426796436	0.380777754104753	1.72148827664622	0.0946441506553352	0.733358574021905	-4.47364006998443
a_HQ_transcript/65837	1.6674617371045	0.83522829419091	1.72147575120638	0.0946464498988547	0.733358574021905	-4.44126350186331
a_HQ_transcript/76486	-0.864037554846469	1.82744978270698	-1.72133212496051	0.094672818126684	0.733358574021905	-4.47401417373362
a_HQ_transcript/93135	-3.05907860085457	1.56377192747741	-1.72121087568156	0.0946950829799431	0.733358574021905	-4.53133191495252
a_HQ_transcript/117529	0.680928780542978	4.07290029913846	1.72120578612353	0.0946960176651234	0.733358574021905	-4.88533494090933
a_HQ_transcript/105129	2.01287755044566	0.0321053315526079	1.72119527086115	0.0946979487924524	0.733358574021905	-4.52368781464425
a_HQ_transcript/133609	3.15202851300676	2.35704930072597	1.72111168268719	0.0947133009303896	0.733358574021905	-4.83385081643222
a_HQ_transcript/52014	1.46368148238429	-0.692346530909253	1.7210917966193	0.0947169535923448	0.733358574021905	-4.55491479097679
a_HQ_transcript/107847	1.22558674396743	2.4033223246606	1.72095206246327	0.0947426232160087	0.733358574021905	-4.54564568319675
a_HQ_transcript/99549	-2.23887250645078	1.42126569599282	-1.72093228969416	0.0947462560097364	0.733358574021905	-4.58655747838945
a_HQ_transcript/16017	1.1193316579151	0.620820672374702	1.72092238703238	0.0947480754410632	0.733358574021905	-4.41470495392967
a_HQ_transcript/30466	1.43398302157982	-0.67994042674167	1.72086461082222	0.0947586913373771	0.733358574021905	-4.53795434041791
a_HQ_transcript/76947	-1.74460929973759	1.98072878814225	-1.72082185928409	0.0947665472176242	0.733358574021905	-4.44531874555004
a_HQ_transcript/60606	-1.51793495690319	1.78127772412791	-1.72077102171813	0.094775889670181	0.733358574021905	-4.50135090017224
a_HQ_transcript/48031	0.533555448243624	3.30835689899763	1.72074437530597	0.0947807868071254	0.733358574021905	-4.77300946548256
a_HQ_transcript/122066	2.28371955783064	2.25797797073343	1.72073014026798	0.094783403041039	0.733358574021905	-4.5504105140111
a_HQ_transcript/147616	2.15534594582578	0.561257101852707	1.72070817332523	0.0947874404276607	0.733358574021905	-4.4921602865122
a_HQ_transcript/69189	1.02325055699915	2.04950853876882	1.72069312663852	0.0947902059977331	0.733358574021905	-4.47420416815791
a_HQ_transcript/117437	4.92616451319884	3.60668988843055	1.72057748797762	0.0948114625575237	0.733358574021905	-4.53523043337982
a_HQ_transcript/12354	-2.46546146048091	1.98705544050582	-1.72053853891643	0.0948186230268846	0.733358574021905	-4.64584122557765
a_HQ_transcript/47245	1.67331632376912	-0.667262427136083	1.72032289728928	0.094858275197739	0.733358574021905	-4.55140203810541
a_HQ_transcript/76945	3.19567891726671	3.13507991506643	1.72024122109612	0.0948732974413686	0.733358574021905	-4.76579520633243
a_HQ_transcript/121579	1.93807021874675	0.0163070807919398	1.72022418287097	0.0948764314378769	0.733358574021905	-4.48690028090167
a_HQ_transcript/89110	-0.98994037000052	4.75158430949225	-1.72010516401861	0.094898326084266	0.733358574021905	-4.97585402179756
a_HQ_transcript/4152	-1.11682181552485	2.13959973238749	-1.72008917006096	0.0949012686472648	0.733358574021905	-4.55769648229099
a_HQ_transcript/148962	1.30177924651062	-0.512750013421027	1.72007668722855	0.0949035652877597	0.733358574021905	-4.58013102164862
a_HQ_transcript/3665	3.04101809369118	3.48459307884912	1.72006412656717	0.0949058762945664	0.733358574021905	-4.64498952793104
a_HQ_transcript/141355	2.26970661017922	2.4908912328615	1.72005349201652	0.0949078329576303	0.733358574021905	-4.61916201829501
a_HQ_transcript/26334	-1.62629141110552	-0.0396402434001167	-1.71997859310147	0.0949216146520664	0.733358574021905	-4.42597201438388
a_HQ_transcript/74393	1.67827685645716	0.204572252607067	1.71996117684318	0.0949248195523811	0.733358574021905	-4.57370423244655
a_HQ_transcript/11213	1.45932149691056	0.481116554951611	1.71977028070078	0.0949599537739921	0.733532517338819	-4.54194362166809
a_HQ_transcript/20582	-1.33354038528725	2.91477723734012	-1.71966472583972	0.0949793857082802	0.733549485686525	-4.71729104930098
a_HQ_transcript/27950	-1.61793068391035	0.532737925769536	-1.71958972802774	0.0949931943225296	0.733549485686525	-4.46762028133119
a_HQ_transcript/109650	1.52430186075168	0.261290225885012	1.71953102713446	0.0950040035184694	0.733549485686525	-4.41136849584165
a_HQ_transcript/85398	-2.90462609508379	3.09308466694632	-1.71948419117355	0.0950126286425448	0.733549485686525	-4.77848294820998
a_HQ_transcript/29416	-0.866635597228889	3.0302115249018	-1.71936838649534	0.0950339575890254	0.733571414750359	-4.78541347471768
a_HQ_transcript/95671	-1.81657313698152	-0.330427402753755	-1.71933173528604	0.0950407088577785	0.733571414750359	-4.52655795190923
a_HQ_transcript/19363	-1.92572937893432	0.868770138712806	-1.71892804563071	0.0951150963743443	0.733958812629302	-4.49689043802403
a_HQ_transcript/49780	2.18410988549174	0.892638913405245	1.71892231395605	0.0951161528959629	0.733958812629302	-4.46747382774974
a_HQ_transcript/32264	-0.6463172744595	3.0158533508855	-1.7186942921647	0.0951581922139542	0.734185744227865	-4.64692681438755
a_HQ_transcript/91344	2.14074874083904	0.82639994043169	1.71851793223748	0.0951907175537223	0.734339220963871	-4.54587342091635
a_HQ_transcript/20374	-0.950075049606504	1.34467708620398	-1.71821003702357	0.0952475237417432	0.734654614768932	-4.41186750971891
a_HQ_transcript/141784	2.57386314047837	1.25489818008857	1.71815934823941	0.095256878466719	0.734654614768932	-4.54571407812363
a_HQ_transcript/100672	1.13006304258717	2.32637340158505	1.71802063555167	0.095282482130737	0.734741495314176	-4.55926843034242
a_HQ_transcript/87307	2.02605671251732	1.50011834249444	1.71796136702799	0.0952934237058487	0.734741495314176	-4.54974226436013
a_HQ_transcript/36889	-1.82393599950905	-0.09058725571334	-1.71759488195614	0.0953611039833787	0.735155101300478	-4.50696919779646
a_HQ_transcript/96473	-2.3852035315841	0.104462503063399	-1.71753394521752	0.095372361321685	0.735155101300478	-4.55033100473713
a_HQ_transcript/1092	0.814737659336904	2.57372509162918	1.71734087329443	0.0954080364192475	0.735332582998942	-4.56858584164618
a_HQ_transcript/1610	-1.07677046347404	2.09639356616174	-1.71712262435663	0.0954483770941842	0.735545971593629	-4.47467985255453
a_HQ_transcript/57730	0.818287067558962	2.73690017232612	1.71687960370693	0.0954933133373468	0.735765436125534	-4.73767710320081

a_HQ_transcript/47434	-1.82393081322938	0.509512769725062	-1.71671493653139	0.0955237715406845	0.735765436125534	-4.57706546681371
a_HQ_transcript/1789	0.496665074797042	3.839519694401	1.71670732332239	0.0955251799396631	0.735765436125534	-4.86299294510241
a_HQ_transcript/118657	0.384213576847182	4.58972702190848	1.71669485419775	0.0955274866921176	0.735765436125534	-4.95344267260733
a_HQ_transcript/140038	-0.826849946863147	3.64197134371953	-1.71662460169078	0.095540484077577	0.735768052260386	-4.68786589082209
a_HQ_transcript/95912	-1.96841915422056	0.351116030289387	-1.71653304849786	0.0955574245172318	0.735801029585064	-4.44921717623417
a_HQ_transcript/51559	2.50106168483186	0.653846959352898	1.71630544253215	0.0955995502307483	0.735986042699123	-4.61736738511313
a_HQ_transcript/147653	0.617413166841034	4.68794224391891	1.71622996702233	0.0956135228059327	0.735986042699123	-4.96930056871467
a_HQ_transcript/95783	2.30756560962473	2.98665681465638	1.71619802683461	0.095619436320943	0.735986042699123	-4.54222641519284
a_HQ_transcript/118843	1.48330471665683	2.26036606106013	1.71592955804834	0.0956691536582863	0.736108668104885	-4.62357604844217
a_HQ_transcript/137857	-3.47769675146336	2.05576587112775	-1.71587505233074	0.0956792501358	0.736108668104885	-4.80833489739868
a_HQ_transcript/16437	1.77630701422582	0.0310460918161647	1.71584182438456	0.0956854056199806	0.736108668104885	-4.52593475876196
a_HQ_transcript/32614	-0.526558201604981	2.77755723775808	-1.71578848484585	0.0956952874747323	0.736108668104885	-4.62264594625541
a_HQ_transcript/111854	-2.12738550507022	-0.386056817216666	-1.7157701429931	0.0956986857429917	0.736108668104885	-4.54220615641814
a_HQ_transcript/45672	1.63092879076169	2.90010831005314	1.71568794606318	0.09571391593793	0.736128407732204	-4.66115495847995
a_HQ_transcript/135729	2.17460106425856	0.626893258066488	1.71547187486317	0.0957539612601187	0.736338968134433	-4.60363685824332
a_HQ_transcript/23441	0.737388822629244	4.67482139513239	1.71529164198411	0.0957873752612815	0.736498485342298	-4.96896516984052
a_HQ_transcript/26256	0.831170200737345	2.24819114628877	1.7151994813482	0.0958044650163533	0.736532461641394	-4.55342615785128
a_HQ_transcript/24418	-1.36047504636688	1.22062897296816	-1.71509665094427	0.0958235363284211	0.736581660896385	-4.43558884521625
a_HQ_transcript/48384	0.834356979315393	3.64785328164059	1.71483263897612	0.0958725155413041	0.736860714449944	-4.84707233552798
a_HQ_transcript/36649	0.807107122834582	2.12709150062281	1.71468819574342	0.0958993213661676	0.736871182872373	-4.50000975051865
a_HQ_transcript/145364	-1.61692609906378	-0.575233880536675	-1.71463896567821	0.0959084589328993	0.736871182872373	-4.54468835670995
a_HQ_transcript/118551	-1.1195814112259	2.25823228438637	-1.71462038603774	0.0959119076798165	0.736871182872373	-4.5903988467736
a_HQ_transcript/27739	2.28840707139426	0.0804643526006884	1.71449942237689	0.0959343634554187	0.736937406438299	-4.59938101355305
a_HQ_transcript/45735	1.26042482804856	2.75530061927567	1.71443737821297	0.0959458830843147	0.736937406438299	-4.72316037668696
a_HQ_transcript/151713	0.254825935814306	11.0267166558663	1.71423651992115	0.0959831840305228	0.737126505658109	-5.63294482263476
a_HQ_transcript/127592	1.86976294624035	-0.583186555159612	1.71378818376723	0.0960664873512988	0.737649583445725	-4.54469746481539
a_HQ_transcript/65238	-1.44901074819537	1.51072999102254	-1.71369500615079	0.0960838078616228	0.737649583445725	-4.40391839867034
a_HQ_transcript/99077	2.38923792936716	0.673038257281093	1.71361227779017	0.0960991881824726	0.737649583445725	-4.5825855319091
a_HQ_transcript/112809	3.50763478549676	1.39470613476111	1.7135968549267	0.0961020557293297	0.737649583445725	-4.65581268988151
a_HQ_transcript/141334	-1.65649146930077	1.50994278957563	-1.71328679441354	0.0961597199571621	0.737994745401362	-4.51976188186545
a_HQ_transcript/127287	1.88699642071152	2.94456247858864	1.71312073531476	0.096190615098012	0.738134399263002	-4.61189371145064
a_HQ_transcript/109110	-2.72511558022806	2.28788990502327	-1.71278839455026	0.0962524717518082	0.738511573124222	-4.67955270290861
a_HQ_transcript/68600	1.96594415984078	0.660564064316254	1.71249066737955	0.0963079142589957	0.738706995228103	-4.6263228799904
a_HQ_transcript/126121	-2.27350814939467	1.59610720362731	-1.71245067194218	0.0963153642130724	0.738706995228103	-4.54808201386259
a_HQ_transcript/17193	-2.88959455669118	2.64561871221809	-1.71244690150779	0.0963160665571462	0.738706995228103	-4.68915289998395
a_HQ_transcript/2246	-2.29238523130969	0.02822437618888	-1.7123551461729	0.0963331597600476	0.738740621442222	-4.55632302822298
a_HQ_transcript/18634	1.80128332822277	-0.129147735949315	1.71222093282673	0.0963581670874154	0.73876895784758	-4.54050443138858
a_HQ_transcript/106005	-2.01054147137513	2.42941690598602	-1.71207872868816	0.096384669230529	0.73876895784758	-4.73861476713036
a_HQ_transcript/104215	2.40123054838702	4.47244766432689	1.71198893975801	0.0964014060570745	0.73876895784758	-5.01384842001478
a_HQ_transcript/37128	-2.89350617926852	0.25347089928719	-1.71193739684458	0.0964110148529153	0.73876895784758	-4.5124558754712
a_HQ_transcript/43831	-1.3422621993536	1.28953958335892	-1.71191725705412	0.0964147695953375	0.73876895784758	-4.57152063562851
a_HQ_transcript/89725	3.06791204232643	2.28943914220879	1.7118185224164	0.0964331788636175	0.73876895784758	-4.4833291606303
a_HQ_transcript/139609	-0.739336187350093	3.39993956783929	-1.71178392845067	0.0964396296729793	0.73876895784758	-4.72681546490713
a_HQ_transcript/36607	1.57602270667145	1.0200371108461	1.71177285206471	0.0964416951862568	0.73876895784758	-4.43697546083684
a_HQ_transcript/103956	0.528283179486956	3.45958367889247	1.71172167556666	0.0964512390088302	0.73876895784758	-4.82500401287422
a_HQ_transcript/118352	-1.79686630042363	2.67912558548757	-1.71132445412961	0.0965253430820722	0.739239149232502	-4.71762164325932
a_HQ_transcript/107969	0.958512715441492	4.64379204617604	1.71116165980229	0.0965557271191281	0.739374431034209	-4.92559748387866
a_HQ_transcript/9688	-1.00049880525999	2.26985346994553	-1.71104697997588	0.0965771358410677	0.739440957872706	-4.51246243120046
a_HQ_transcript/31330	-1.30475472349578	2.90944198490067	-1.7106810787056	0.0966454697867722	0.739866701931449	-4.56324798111508
a_HQ_transcript/111568	2.56151017670909	0.809609663724335	1.71051432539144	0.0966766252482281	0.740007752492627	-4.58227236220225

a_HQ_transcript/117272	0.861751579737404	2.34381848833691	1.71015924335917	0.0967429952342022	0.740268665495261	-4.55501175598483
a_HQ_transcript/152012	2.15128469173008	-0.127344772634056	1.71014065866045	0.0967464700340855	0.740268665495261	-4.53779207901598
a_HQ_transcript/42326	-1.55516847966678	1.96164326276999	-1.71012757118347	0.0967489170755488	0.740268665495261	-4.50673490405634
a_HQ_transcript/40028	-1.73720384057996	2.42952118993044	-1.70995258287748	0.0967816406307429	0.74038158917458	-4.80208361007768
a_HQ_transcript/17677	1.32779036178288	3.10917852789423	1.70991243510398	0.0967891497408759	0.74038158917458	-4.67615020145733
a_HQ_transcript/127434	3.7156293923524	1.48259513699777	1.70953670789006	0.0968594481844344	0.740755590359344	-4.62630159314723
a_HQ_transcript/81685	1.89411395333244	0.0271755699747169	1.70951490135313	0.0968635294921789	0.740755590359344	-4.541291960919
a_HQ_transcript/68413	-2.31203530240454	0.556677424698185	-1.70941922521487	0.0968814379228615	0.740795083343869	-4.53382662788715
a_HQ_transcript/120072	-2.53164195229717	1.14979645319369	-1.70934569225394	0.096895203529538	0.74080289237998	-4.49716800355797
a_HQ_transcript/142718	-2.33701091648918	-0.140744534466919	-1.70922088398678	0.0969185717849855	0.740884105828201	-4.55293173459617
a_HQ_transcript/150771	-1.70029994391164	2.02808691613994	-1.70913399937145	0.096934842256524	0.740905388549629	-4.6680577036676
a_HQ_transcript/105119	2.03557840219593	2.38712748630044	1.70905801390617	0.0969490735691848	0.740905388549629	-4.45640755787875
a_HQ_transcript/127694	-1.43858887692095	-0.231333323896835	-1.70898249227597	0.0969632197424864	0.740905388549629	-4.46403105213299
a_HQ_transcript/139958	2.5201846281921	-0.0428158650979177	1.70885886132473	0.0969863811387605	0.740905388549629	-4.52477627644502
a_HQ_transcript/42182	1.23743599099962	2.40591541964657	1.70883737253723	0.0969904073854037	0.740905388549629	-4.6299127893305
a_HQ_transcript/117374	-1.85690230789645	0.878303086685953	-1.70875649160549	0.0970055628949391	0.740905388549629	-4.56231433410126
a_HQ_transcript/59037	2.50485642900303	2.05971854386804	1.70871062767121	0.0970141577822911	0.740905388549629	-4.77892112688087
a_HQ_transcript/43668	-1.88954414550293	2.23579610689711	-1.70866171506995	0.0970233246910228	0.740905388549629	-4.76186355164152
a_HQ_transcript/125362	-2.42757593499799	-0.206290228463653	-1.70850436313502	0.0970528195706552	0.741033271509661	-4.5462177499828
a_HQ_transcript/137199	-0.972699509604177	1.78890892269934	-1.70814858615462	0.0971195359492887	0.741235052203472	-4.51108140073588
a_HQ_transcript/75553	-2.03312497075918	0.193121845321187	-1.70814618917908	0.0971199855675087	0.741235052203472	-4.50989857551086
a_HQ_transcript/147335	1.15930245423574	-0.773834770951271	1.70808359294209	0.0971317278177163	0.741235052203472	-4.56051591036851
a_HQ_transcript/112394	4.09092041034605	1.54862102143413	1.7080200273965	0.0971436531132331	0.741235052203472	-4.72134411154302
a_HQ_transcript/64253	2.53405921037817	1.35976570068398	1.70796302836152	0.0971543475317414	0.741235052203472	-4.53346353218793
a_HQ_transcript/136737	-2.29384008148168	0.640468392829598	-1.70791066839006	0.0971641724159972	0.741235052203472	-4.55526398429091
a_HQ_transcript/61623	4.80820597508994	1.67195341493532	1.7077919159281	0.0971864583426761	0.741235052203472	-4.69831869032344
a_HQ_transcript/59762	1.5501050684282	0.128022035228718	1.70772092631425	0.0971997827976975	0.741235052203472	-4.46341110330463
a_HQ_transcript/91367	1.36861823747532	-0.65297093216425	1.70759273557053	0.097223847538213	0.741235052203472	-4.57631189839788
a_HQ_transcript/89905	-0.548020026202302	4.03276373056344	-1.7075116580486	0.0972390704731608	0.741235052203472	-4.95697694865137
a_HQ_transcript/84180	-0.754427441205259	2.23706275230795	-1.70750312331181	0.0972406730523227	0.741235052203472	-4.55090217805419
a_HQ_transcript/109303	2.58613093899114	-0.168153875696984	1.70742250973349	0.0972558110637646	0.741235052203472	-4.54701826852628
a_HQ_transcript/141558	0.596652884481788	2.51596542988431	1.70738594624639	0.0972626777837707	0.741235052203472	-4.70161481830426
a_HQ_transcript/112948	1.7668363814043	-0.557573416344152	1.70723133936826	0.0972917178495233	0.741235052203472	-4.56040141755602
a_HQ_transcript/143556	0.386552103844704	10.3214500766553	1.70715627484555	0.0973058199584296	0.741235052203472	-5.5628056095083
a_HQ_transcript/81941	-0.596362310468962	3.99920399079243	-1.70715040417133	0.0973069229335158	0.741235052203472	-4.93022129683373
a_HQ_transcript/147436	-2.30054904714422	-0.0497936181486589	-1.70708733848995	0.0973187722961639	0.741235052203472	-4.58999246161479
a_HQ_transcript/21041	1.26202033949795	1.62209077925309	1.70704554202754	0.0973266260665415	0.741235052203472	-4.45307178107451
a_HQ_transcript/35644	0.358902740383121	3.82042802945869	1.70696352904123	0.0973420382695079	0.741235052203472	-4.8912771856874
a_HQ_transcript/150012	-3.57727195826824	0.374167435353493	-1.7069472302088	0.0973451014533645	0.741235052203472	-4.56802999147557
a_HQ_transcript/104178	-6.49031868765724	2.53604294244152	-1.70688329163233	0.097357118774595	0.741235052203472	-4.71282427643041
a_HQ_transcript/144110	2.7234848219555	3.52634106313963	1.70685409790434	0.0973626061784385	0.741235052203472	-4.88279093618785
a_HQ_transcript/45695	2.11872909519157	0.409287391900859	1.70680126554187	0.0973725374797983	0.741235052203472	-4.54394807994886
a_HQ_transcript/85899	1.54288650457807	-0.164324521845324	1.70668920977065	0.0973936042606171	0.741298340770087	-4.51359919398904
a_HQ_transcript/145534	-2.41725390140228	0.783367555344136	-1.70628870461118	0.0974689314534279	0.741421392940804	-4.52102464790333
a_HQ_transcript/39091	-0.45673373985995	3.71270169416928	-1.70627453461888	0.0974715974442788	0.741421392940804	-4.85594148749864
a_HQ_transcript/136860	-4.06318200512043	2.35251171281485	-1.70623876049047	0.0974783283825901	0.741421392940804	-4.60236923980892
a_HQ_transcript/11216	-1.05918184212756	2.5116334737275	-1.70620668121672	0.0974843644608642	0.741421392940804	-4.66092448674948
a_HQ_transcript/62548	2.29838410086819	0.782132978150693	1.70620197276057	0.0974852504364642	0.741421392940804	-4.64142224370871
a_HQ_transcript/7130	1.78275378091376	0.355211510367422	1.70619638950246	0.0974863010295652	0.741421392940804	-4.53013087712602
a_HQ_transcript/93750	2.09205581770427	0.502043068502341	1.70587734808432	0.0975463502954194	0.741781037411321	-4.53807235011494

a_HQ_transcript/944	-1.56632016302148	3.37685786652923	-1.70576013004024	0.0975684205840656	0.741851818405568	-4.69077541633667
a_HQ_transcript/53335	-1.32924690636733	0.51943127518309	-1.70551591040859	0.0976144166607043	0.741884478470115	-4.47431779027718
a_HQ_transcript/133568	-3.27094838049809	1.68265503547315	-1.70541074499872	0.0976342289915787	0.741884478470115	-4.59658957471786
a_HQ_transcript/143827	-0.810875383344956	2.7505228317886	-1.70534252473207	0.097647082947997	0.741884478470115	-4.66727378637137
a_HQ_transcript/123126	-1.96960422616662	1.26495141793489	-1.70531530526631	0.0976522119922937	0.741884478470115	-4.63343660187619
a_HQ_transcript/83190	2.37568552435595	1.12641808969861	1.70511485341012	0.0976899906662797	0.741884478470115	-4.55796127424702
a_HQ_transcript/28297	-1.47403954449234	2.70025466776502	-1.7048714666608	0.0977358776026646	0.741884478470115	-4.68186661240001
a_HQ_transcript/23966	4.03283971933798	3.2860236488833	1.7048282735874	0.0977440228942571	0.741884478470115	-4.7105599943866
a_HQ_transcript/74843	-2.85646680111609	0.47631574945159	-1.70482631187081	0.0977443928456033	0.741884478470115	-4.56528386698635
a_HQ_transcript/145536	3.38606367622817	2.02824648067985	1.70475470063333	0.0977578984896534	0.741884478470115	-4.70482966340419
a_HQ_transcript/114784	-2.35561971104967	0.726589355011649	-1.70475003317895	0.0977587788103716	0.741884478470115	-4.51537494724188
a_HQ_transcript/76520	-1.49301457844989	2.37346156349538	-1.70471830402368	0.0977647633686585	0.741884478470115	-4.7009286873743
a_HQ_transcript/136153	1.448056412676	-0.544956853886995	1.70463772807302	0.0977799624860479	0.741884478470115	-4.53470597433579
a_HQ_transcript/34132	2.56535552785816	1.53237202203297	1.70463354557123	0.0977807514892328	0.741884478470115	-4.46405221455043
a_HQ_transcript/142614	2.00206619389666	0.475324397143792	1.70460140359534	0.0977868150525269	0.741884478470115	-4.5526802994623
a_HQ_transcript/130293	0.896155908200697	1.51182348665826	1.70459960404943	0.0977871545450111	0.741884478470115	-4.45088279746088
a_HQ_transcript/148585	5.76835966904751	2.50469814270261	1.70459240712975	0.0977885122863487	0.741884478470115	-4.65089651982263
a_HQ_transcript/140781	5.42672486100134	1.36635708724827	1.70450044073365	0.0978058636769642	0.741884478470115	-4.65065357017063
a_HQ_transcript/149651	5.62154177869298	2.21459556834163	1.704428360542	0.0978194649171554	0.741884478470115	-4.71150759161218
a_HQ_transcript/26598	-2.31730011252706	-0.491710314814768	-1.70435339540686	0.097833612212095	0.741884478470115	-4.57145155448421
a_HQ_transcript/45505	-3.21185210930068	1.45882363968724	-1.70429768642661	0.0978441266256471	0.741884478470115	-4.64053863187904
a_HQ_transcript/136726	2.84909701628705	3.3530264290691	1.70427728621602	0.0978479771613255	0.741884478470115	-4.85275645036643
a_HQ_transcript/103432	-2.09877850364108	0.118300896043117	-1.70424802287049	0.0978535008331676	0.741884478470115	-4.56828766802779
a_HQ_transcript/65683	-2.11226526423642	2.37439304771058	-1.70403003458354	0.0978946559314873	0.742099707875	-4.55129203239789
a_HQ_transcript/90839	0.490078800484724	2.6874234693297	1.70379941527788	0.0979382114549408	0.742279785824849	-4.73109806017804
a_HQ_transcript/90739	1.13103874029689	-0.730764964771927	1.70371667049134	0.0979538428571591	0.742279785824849	-4.563409655637
a_HQ_transcript/147619	2.16627687893366	0.406613042021411	1.70368063154083	0.0979606516641411	0.742279785824849	-4.59553811558061
a_HQ_transcript/147544	2.14663087772514	0.647317272729491	1.70363385324277	0.097969490036613	0.742279785824849	-4.57360910022326
a_HQ_transcript/114245	-0.621745639097349	2.89031665810504	-1.70354428097661	0.0979864158357461	0.742311270650365	-4.70911844217453
a_HQ_transcript/85017	1.53609305854121	1.7941800975675	1.70329839622253	0.0980328913991307	0.742566576915386	-4.5545413921521
a_HQ_transcript/142996	-1.92789101142426	-0.0817906412230682	-1.70318989420387	0.0980534056156045	0.74262519369692	-4.55464224930614
a_HQ_transcript/76922	1.67879279362765	-0.0516269031121331	1.7029856143212	0.0980920380609867	0.742651664607154	-4.50473313606992
a_HQ_transcript/48189	1.61185337823436	-0.0219186767827547	1.7029009604713	0.098108051125004	0.742651664607154	-4.52658803673475
a_HQ_transcript/54920	2.23483224869691	-0.474495467961454	1.70272706482121	0.0981409519609265	0.742651664607154	-4.51204348079525
a_HQ_transcript/84985	0.414799437351921	3.82600337786398	1.70269038616918	0.098147892692872	0.742651664607154	-4.9170871895323
a_HQ_transcript/87894	-1.90035962128121	1.04493035101751	-1.70263391028746	0.0981585804733372	0.742651664607154	-4.46538219893223
a_HQ_transcript/22072	2.08330111958712	0.491348421570578	1.70260179709868	0.0981646581688363	0.742651664607154	-4.47648628160736
a_HQ_transcript/150591	4.06563144020714	3.00186001932819	1.70258047192087	0.0981686943148213	0.742651664607154	-4.77941419873898
a_HQ_transcript/150614	1.99622321419636	6.50853962991745	1.70248531368433	0.0981867062904592	0.742651664607154	-5.21117380772602
a_HQ_transcript/351	-0.318629869142982	4.93274543953617	-1.70244871811542	0.0981936339984119	0.742651664607154	-5.00794188034609
a_HQ_transcript/133184	1.6634756220327	2.68855982781093	1.70231712436822	0.0982185486703	0.742651664607154	-4.65889484924325
a_HQ_transcript/77516	1.69285427105643	1.96678172268073	1.7022574769381	0.0982298434708797	0.742651664607154	-4.63846058511238
a_HQ_transcript/3912	-2.01602346429013	0.786953217554432	-1.7021665578921	0.0982470619337964	0.742651664607154	-4.46435992517602
a_HQ_transcript/23819	-1.87206121836792	1.80957797433648	-1.7021629051587	0.0982477537497476	0.742651664607154	-4.56134403825639
a_HQ_transcript/98217	-2.05652313787811	0.175793598700298	-1.70211629617409	0.0982565816988863	0.742651664607154	-4.55297812385634
a_HQ_transcript/92439	-0.989800328691678	3.15107054954157	-1.7020967871673	0.0982602769884184	0.742651664607154	-4.73929225569124
a_HQ_transcript/73962	-1.17030713172262	-0.513002270801835	-1.70209128590442	0.0982613190285855	0.742651664607154	-4.52146339358486
a_HQ_transcript/151855	1.38701796233154	-0.232719196215268	1.70197347405576	0.0982836369720274	0.742658670638803	-4.5048713562416
a_HQ_transcript/96028	-2.07705894195474	3.24811429593089	-1.70188434641806	0.0983005238743008	0.742658670638803	-4.79620788469084
a_HQ_transcript/35908	-1.28260682194935	1.04161151333636	-1.7017488677641	0.09832619749002	0.742658670638803	-4.4888334408723

a_HQ_transcript/147732	1.60807090660021	-0.642882653028724	1.70154158654795	0.098365488754308	0.742658670638803	-4.57020015778826
a_HQ_transcript/134665	1.94378424745517	0.499896930725568	1.70149969759105	0.098373430623853	0.742658670638803	-4.50436584694183
a_HQ_transcript/140930	-2.31455662228357	0.176712885739091	-1.70149413490343	0.0983744853129846	0.742658670638803	-4.5570338659268
a_HQ_transcript/27690	-1.40207989754634	3.44431850670494	-1.70149219770183	0.0983748526098664	0.742658670638803	-4.87008365566913
a_HQ_transcript/101784	1.18080201497535	0.440522618140674	1.70137325860231	0.098397405870737	0.742658670638803	-4.47401899805236
a_HQ_transcript/120760	2.24921824564603	1.57548119672565	1.70136534262287	0.0983989070540167	0.742658670638803	-4.72191109957634
a_HQ_transcript/113081	0.537601013801471	4.48727599251389	1.70131974897782	0.0984075537878061	0.742658670638803	-4.96673857047336
a_HQ_transcript/118181	1.75979224349884	0.174237699817219	1.70127433804818	0.0984161665011447	0.742658670638803	-4.56272267918231
a_HQ_transcript/146119	-2.66739682879756	0.932156397129126	-1.70121563313415	0.0984273015074155	0.742658670638803	-4.6378695282186
a_HQ_transcript/9777	1.60722763591621	1.43026494270847	1.70121017188179	0.0984283374380908	0.742658670638803	-4.55085567185159
a_HQ_transcript/107812	2.16286964554468	0.360886972414502	1.70112797962946	0.0984439293718624	0.742679912592812	-4.52676383081446
a_HQ_transcript/84959	2.14621659377633	2.12581007814557	1.70098723898957	0.0984706327727512	0.74278496519783	-4.49542618211825
a_HQ_transcript/47549	-1.96789282948253	1.13658332257549	-1.70054910397831	0.0985538009949118	0.743315861454811	-4.54026871408565
a_HQ_transcript/133546	4.73360216453118	4.66609560935125	1.70046914405072	0.0985689855813506	0.743333937969998	-4.65389030929582
a_HQ_transcript/20317	2.75407283853128	1.67507786475159	1.70033220921574	0.098594994382689	0.743433627380587	-4.55002094074416
a_HQ_transcript/128310	1.42615717270855	1.34758796619019	1.70012854610581	0.0986336878651691	0.743598439956199	-4.44135085116549
a_HQ_transcript/22147	-1.33813603704259	-0.520479504663325	-1.70008250496882	0.0986424368721141	0.743598439956199	-4.534152752329
a_HQ_transcript/110092	-1.03366154247238	3.07841326415651	-1.69990659002868	0.0986758712324847	0.743754025285512	-4.89420521051332
a_HQ_transcript/64678	-1.96020260636359	0.510867165805875	-1.69941722145555	0.0987689303357877	0.744348514491989	-4.48734821681561
a_HQ_transcript/23429	1.58742905339313	-0.202998504151862	1.69935717241997	0.0987803544039225	0.744348514491989	-4.54647811385613
a_HQ_transcript/12698	-1.32268670475036	1.31350524742562	-1.69926027893434	0.0987987902960712	0.744390937437463	-4.55458309021758
a_HQ_transcript/147025	-3.57216894470913	6.94858529910674	-1.69905419615493	0.0988380111575757	0.744432172279836	-5.29109627513195
a_HQ_transcript/23659	1.98724651500345	0.119366220630226	1.69904396064897	0.0988399594776569	0.744432172279836	-4.51271424700886
a_HQ_transcript/65798	-1.54552410331253	-0.368559649278373	-1.6989649397875	0.0988550021130799	0.744432172279836	-4.57033495588605
a_HQ_transcript/13161	-1.56886601847313	1.30331074463262	-1.69884209593706	0.0988783908141425	0.744432172279836	-4.48116629442271
a_HQ_transcript/133753	1.87807743375067	-0.0274781386137399	1.69874578009124	0.0988967319815392	0.744432172279836	-4.51636755854539
a_HQ_transcript/97044	3.75053867682572	1.48696339022163	1.69872985728669	0.0988997643920792	0.744432172279836	-4.70821298231798
a_HQ_transcript/79878	2.13554941352882	2.14196980223325	1.69868662510771	0.0989079981144556	0.744432172279836	-4.5872579393817
a_HQ_transcript/6980	3.22962953006404	0.851365870423389	1.6986615301491	0.0989127778012808	0.744432172279836	-4.65205836710375
a_HQ_transcript/123488	1.29451245126656	2.51311498112183	1.69858960871232	0.0989264773163998	0.744432172279836	-4.62742354516111
a_HQ_transcript/84054	-1.88813505521766	2.37592787035915	-1.69855888034216	0.0989323309052735	0.744432172279836	-4.69645074807741
a_HQ_transcript/102550	-1.67201950566363	0.879496909032203	-1.69845113310393	0.0989528584590554	0.744464287980332	-4.56751135188428
a_HQ_transcript/61721	-1.88479660294857	-0.306842956215753	-1.698123681608	0.0990152649852183	0.744464287980332	-4.53444682302393
a_HQ_transcript/119073	-2.6257271162705	0.174126936088528	-1.69798808639067	0.0990411166835894	0.744464287980332	-4.62823661507199
a_HQ_transcript/110492	3.23713914690137	1.85820322095682	1.69798199685765	0.0990422778064251	0.744464287980332	-4.63171166916814
a_HQ_transcript/32511	2.1300947550175	0.562432235788096	1.6979560107662	0.0990472328369867	0.744464287980332	-4.47416433302896
a_HQ_transcript/132931	1.62148641513592	0.299481193605551	1.69793417179859	0.0990513972536602	0.744464287980332	-4.47407139143621
a_HQ_transcript/53846	-2.89264637056004	1.80945855697605	-1.6979129270405	0.0990554485023579	0.744464287980332	-4.60442180605513
a_HQ_transcript/100631	-2.27414373620637	0.887908795847023	-1.69765756290821	0.0991041557492102	0.744464287980332	-4.46058605103209
a_HQ_transcript/113771	1.29183452031308	-0.729785010578226	1.6975861827445	0.0991177741240938	0.744464287980332	-4.57303476204146
a_HQ_transcript/97861	1.0144986701034	1.29394000486781	1.69756563659474	0.0991216943426796	0.744464287980332	-4.48591615820333
a_HQ_transcript/1496	1.92634073786064	1.08121831184046	1.69739596456262	0.0991540728254144	0.744464287980332	-4.45706941525221
a_HQ_transcript/90322	1.51955798706474	-0.302560188062067	1.69734905320487	0.0991630264695852	0.744464287980332	-4.58003600153779
a_HQ_transcript/99578	-2.3067731813872	1.03582942441181	-1.69731548490966	0.0991694338307364	0.744464287980332	-4.71611476213427
a_HQ_transcript/131267	2.45128671039799	2.89250294524364	1.69730089990076	0.0991722178576077	0.744464287980332	-4.66880645078354
a_HQ_transcript/145801	1.65508263730846	-0.281955340369551	1.69727457223186	0.0991772435211623	0.744464287980332	-4.5557288880891
a_HQ_transcript/72038	-1.73881632299145	-0.556256967932395	-1.6972647099242	0.0991791261824006	0.744464287980332	-4.57848780046994
a_HQ_transcript/61723	1.51743190410301	0.354225758948767	1.69721635148922	0.0991883579782451	0.744464287980332	-4.47742112355204
a_HQ_transcript/151912	-1.28942861991779	1.36819710768687	-1.6971950800431	0.099192418999584	0.744464287980332	-4.53495166830641
a_HQ_transcript/93532	1.17533764762847	3.11656835401296	1.69717158851036	0.0991969040283398	0.744464287980332	-4.7260335806731

a_HQ_transcript/8609	-1.76385474731693	-0.225516696227719	-1.69713835489476	0.099203249315293	0.744464287980332	-4.56765442827084
a_HQ_transcript/132687	1.84877613673967	1.08550757630137	1.69712411497016	0.0992059682447035	0.744464287980332	-4.58178644944738
a_HQ_transcript/94618	2.20303461768307	0.422171550485854	1.69705135316424	0.0992198621417004	0.744464287980332	-4.58472009065612
a_HQ_transcript/147905	-2.48266136958746	1.23331651800957	-1.69697422748186	0.0992345910965909	0.744464287980332	-4.68409526278025
a_HQ_transcript/99738	-1.84897091792947	0.977405095579938	-1.69690538333917	0.0992477400397138	0.744464287980332	-4.56508696575334
a_HQ_transcript/35122	1.11630523873952	-0.292326461398267	1.6968580459166	0.0992567821333536	0.744464287980332	-4.47035746834272
a_HQ_transcript/97641	0.830055180938579	2.91192748439092	1.69659974368451	0.0993061335020562	0.744530933845345	-4.71333856882248
a_HQ_transcript/143910	2.16427288519785	3.38684074934367	1.69648415530676	0.0993282245166196	0.744530933845345	-4.82932293971666
a_HQ_transcript/16584	2.06102354098227	1.48103762535564	1.69628015500188	0.0993672226488324	0.744530933845345	-4.58361837801102
a_HQ_transcript/12026	1.17717672940478	-0.518580047400236	1.69627549508406	0.0993681136207982	0.744530933845345	-4.50164998437935
a_HQ_transcript/71228	0.674797067324648	3.53880533413117	1.69623144287349	0.099376536692166	0.744530933845345	-4.84191304409003
a_HQ_transcript/26247	-0.711085920450451	1.75913246149822	-1.69621824047473	0.0993790611934309	0.744530933845345	-4.48399173405195
a_HQ_transcript/135270	1.42380440485091	-0.721082205483202	1.69619915481785	0.0993827107585854	0.744530933845345	-4.58335731042549
a_HQ_transcript/108354	-2.99686767247101	0.89277463224337	-1.69618247773003	0.0993858998480314	0.744530933845345	-4.55339453807144
a_HQ_transcript/148545	-0.866148775717245	3.01401646837876	-1.6961801333928	0.0993863481526424	0.744530933845345	-4.70369654805743
a_HQ_transcript/44974	-0.620512788324648	2.78634810473161	-1.69611345596431	0.0993990994988933	0.744530933845345	-4.69339819897309
a_HQ_transcript/147830	3.73808188437475	1.83420082459543	1.69607039846276	0.0994073345120932	0.744530933845345	-4.63938973207756
a_HQ_transcript/136483	-1.35791936123357	-0.588784093036893	-1.695986887538	0.0994233081161273	0.744530933845345	-4.56632292830735
a_HQ_transcript/138862	2.10210428233818	3.28258574322936	1.69594052041003	0.099432177942496	0.744530933845345	-4.6182172038804
a_HQ_transcript/79280	1.37742489332541	2.15984396497799	1.69584193901425	0.099451038322603	0.744576243639396	-4.69470257452077
a_HQ_transcript/102643	-2.29207347085625	3.07428058584044	-1.69575674851961	0.099467339189902	0.744602381510705	-4.69356653414056
a_HQ_transcript/88187	-1.73145997284841	0.530086256473123	-1.69560180192772	0.0994969933309404	0.744728461027672	-4.49044570975134
a_HQ_transcript/22863	0.975974554548748	3.55329379192063	1.69535159673551	0.0995448939278532	0.744869094290429	-4.77456587836832
a_HQ_transcript/33824	1.50150509712209	-0.505250522603711	1.69534329664175	0.0995464832712535	0.744869094290429	-4.57642449989319
a_HQ_transcript/71977	-1.76560457765585	0.230841671761445	-1.69529341083852	0.0995560361002272	0.744869094290429	-4.56295261486618
a_HQ_transcript/82575	1.25797890287251	-0.762793383411477	1.69523595581114	0.0995670393379547	0.744869094290429	-4.59141417150152
a_HQ_transcript/24233	-0.636127878375225	3.69160650967027	-1.69502734415854	0.0996069991826011	0.744972910923453	-4.85700709040682
a_HQ_transcript/145149	-2.45577621536713	0.374183112909356	-1.69501528268843	0.0996093099828509	0.744972910923453	-4.63447603712652
a_HQ_transcript/55643	1.76731959544206	0.0571160620531449	1.694962803253	0.0996193647916322	0.744972910923453	-4.64992729159134
a_HQ_transcript/88536	1.46908894676735	2.18919627355813	1.69478712601424	0.0996530298752243	0.745039495105062	-4.52393111565325
a_HQ_transcript/98470	0.491006427373651	3.62474424053262	1.69476113068845	0.0996580121739807	0.745039495105062	-4.81562955918347
a_HQ_transcript/24189	1.55182968257167	2.71621326730081	1.69455186338949	0.0996981281975564	0.745039495105062	-4.66242347836769
a_HQ_transcript/68104	2.07236063530683	1.14836816182948	1.69447308284304	0.0997132337250704	0.745039495105062	-4.44395144577232
a_HQ_transcript/107962	0.57060293328322	6.46816980714019	1.69438651297809	0.0997298349937106	0.745039495105062	-5.15971980674803
a_HQ_transcript/18461	0.961601045657853	2.47421008437014	1.69438272406635	0.0997305616357461	0.745039495105062	-4.57048107325034
a_HQ_transcript/146249	3.08584234431667	3.5896344853477	1.694358715404	0.0997351661485071	0.745039495105062	-4.8061651314975
a_HQ_transcript/31185	-1.2809864642311	2.55549904489175	-1.69423045252081	0.0997597681127774	0.745039495105062	-4.60092480167635
a_HQ_transcript/26739	-0.420889792712893	3.11083318456395	-1.69419890900278	0.0997658192166059	0.745039495105062	-4.73339933885697
a_HQ_transcript/41684	-1.88458359445935	-0.199647355091472	-1.69418869388301	0.0997677788845649	0.745039495105062	-4.55846005938341
a_HQ_transcript/89439	-2.8197184279831	2.21932275700546	-1.69418098376177	0.0997692580150324	0.745039495105062	-4.61887085534423
a_HQ_transcript/103628	1.39164615501894	-0.681984390941804	1.69398293767654	0.099807257983792	0.745227526278981	-4.59147972629387
a_HQ_transcript/101014	-0.606046436546382	4.91277171782759	-1.69373392152626	0.0998550549431929	0.745412499878509	-5.04664397459147
a_HQ_transcript/129484	1.44564985296201	-0.481384617841928	1.6937202559419	0.0998576785121448	0.745412499878509	-4.57934986715041
a_HQ_transcript/131966	0.592178306682311	3.38753348772657	1.6935491044477	0.0998905416751443	0.745462978115844	-4.84009850214195
a_HQ_transcript/127487	-1.76287547912961	0.398168987348768	-1.69344944883757	0.0999096809183204	0.745462978115844	-4.49849779890369
a_HQ_transcript/45543	0.674582908482631	2.93980735161231	1.69342942113683	0.0999135276845424	0.745462978115844	-4.75114793836276
a_HQ_transcript/51088	2.35292596842578	2.5789299335198	1.69335047753457	0.0999286917662937	0.745462978115844	-4.7761633873104
a_HQ_transcript/9386	-2.20313384572851	0.930510428910327	-1.69328964483096	0.0999403782803382	0.745462978115844	-4.57468048953417
a_HQ_transcript/108723	-2.1542846982474	0.891320581177213	-1.69322650362196	0.0999525094853972	0.745462978115844	-4.47563182960291
a_HQ_transcript/117774	1.04603847933774	4.72516800680333	1.69312778371433	0.0999714788231763	0.745462978115844	-4.96723116145454

a_HQ_transcript/110431	-1.29525739290158	3.72836764530199	-1.69309731647335	0.0999773338050272	0.745462978115844	-4.87054173673099
a_HQ_transcript/10911	-0.763445664190382	2.02697939210488	-1.69304404971286	0.0999875709269335	0.745462978115844	-4.49580753173365
a_HQ_transcript/129855	-1.48841910417862	-0.159556156726112	-1.69295606225881	0.100004482793288	0.745462978115844	-4.52685485913803
a_HQ_transcript/117970	1.70410353889157	1.99651165833697	1.69292778976445	0.100009917491123	0.745462978115844	-4.59800387273012
a_HQ_transcript/142755	0.606121473863813	3.57635164726097	1.69284816834024	0.10002522409327	0.745462978115844	-4.8281519795542
a_HQ_transcript/101729	2.9590350652565	2.39974576710624	1.69281729735972	0.100031159326032	0.745462978115844	-4.65200040850633
a_HQ_transcript/61174	-1.81672057020646	1.72629113532099	-1.69269926596114	0.100053854668444	0.745480671458719	-4.61716969568765
a_HQ_transcript/111407	2.41593178388887	1.83165753571663	1.69267155737667	0.100059183159939	0.745480671458719	-4.47932443985155
a_HQ_transcript/141468	-1.86552516903322	0.202646180253816	-1.6924233354025	0.100106927978438	0.745567637563049	-4.58711399019725
a_HQ_transcript/58981	0.471541464702283	3.61212687364949	1.69237968640097	0.100115325709439	0.745567637563049	-4.85879818805606
a_HQ_transcript/17946	2.02271049406139	-0.264543184001624	1.6923449184527	0.10012201521487	0.745567637563049	-4.63473948610188
a_HQ_transcript/152827	-1.83663931628526	-0.499717163297113	-1.69234416039621	0.100122161072412	0.745567637563049	-4.59893208751857
a_HQ_transcript/55246	-0.403011709992575	3.84614843071048	-1.69196353824794	0.100195418920073	0.745711719104592	-4.85719435880168
a_HQ_transcript/112624	0.527530706930171	3.92534710352383	1.69196198132527	0.100195718670753	0.745711719104592	-4.92904249970201
a_HQ_transcript/68858	-2.53119368975188	0.523385718573935	-1.69188811217774	0.100209941384837	0.745711719104592	-4.53456775903194
a_HQ_transcript/70148	-1.4030827208676	1.1211720284485	-1.69187845023065	0.100211801813935	0.745711719104592	-4.652622601724
a_HQ_transcript/125157	-0.634605361825794	4.19226204574851	-1.69152381250898	0.100280108026081	0.745711719104592	-4.93197277850931
a_HQ_transcript/114592	-0.927783813522545	3.98099841836153	-1.69150999810518	0.100282769582352	0.745711719104592	-4.86396592179502
a_HQ_transcript/122300	-1.66775976938821	2.37330933667838	-1.6914878339589	0.100287039967391	0.745711719104592	-4.74977524929782
a_HQ_transcript/19974	1.33009279457767	-0.653362317204007	1.69142409478076	0.100299321494818	0.745711719104592	-4.57912681992092
a_HQ_transcript/47449	-2.19764178309664	0.0504145630576124	-1.69138940520577	0.10030600615225	0.745711719104592	-4.65020714394256
a_HQ_transcript/140372	-2.53183577081029	-0.644096381279233	-1.69126791688992	0.100329419797745	0.745711719104592	-4.6103129956491
a_HQ_transcript/19720	-3.08451374097979	0.946346894268839	-1.69125115503464	0.100332650557929	0.745711719104592	-4.44273045771925
a_HQ_transcript/132448	0.847472260962676	2.47764592883189	1.69115170609898	0.100351820606502	0.745711719104592	-4.58041650941912
a_HQ_transcript/148971	-2.39249257975351	1.31587553792312	-1.69113664748033	0.100354723613302	0.745711719104592	-4.60748070796869
a_HQ_transcript/108073	-1.76925001446229	1.11696114131825	-1.69104129391065	0.100373107540545	0.745711719104592	-4.43959567500399
a_HQ_transcript/76183	-2.70676896171015	0.306894332995929	-1.69098861470675	0.100383265162526	0.745711719104592	-4.62712870100879
a_HQ_transcript/128864	1.53585005210914	1.68290467865895	1.69098182236245	0.100384574927062	0.745711719104592	-4.59446378734893
a_HQ_transcript/87512	-1.71213024907696	1.99974087831479	-1.69090335217865	0.100399707329078	0.745711719104592	-4.63947568225076
a_HQ_transcript/41864	1.23257688623249	1.35685412808929	1.69087718374737	0.100404754143028	0.745711719104592	-4.48285296532612
a_HQ_transcript/126748	-1.7942003407317	2.73067610642157	-1.69081424784388	0.100416892756372	0.745711719104592	-4.62970187417006
a_HQ_transcript/119050	2.61835996460674	-0.159747587939787	1.69068188832535	0.100442425279191	0.745711719104592	-4.55308374340096
a_HQ_transcript/32771	1.3439581816616	0.875329242487047	1.69060965622967	0.100456361336981	0.745711719104592	-4.42509389997346
a_HQ_transcript/100872	1.53347564174535	0.181353117453211	1.69057532502585	0.100462985573417	0.745711719104592	-4.50816403291111
a_HQ_transcript/61788	0.755389501784241	-0.62622845546623	1.69054612663474	0.100468619715749	0.745711719104592	-4.57425817994066
a_HQ_transcript/12115	-1.32269766762895	1.48174332832027	-1.69049605378696	0.100478282421597	0.745711719104592	-4.5507559673233
a_HQ_transcript/4656	1.3565266226231	-0.222467439232058	1.69038413825024	0.100499881896641	0.745711719104592	-4.55448919865659
a_HQ_transcript/58712	0.489935836252478	4.92314550890946	1.69028585289564	0.100518853967449	0.745711719104592	-5.03100322879657
a_HQ_transcript/66945	1.74801243473225	1.8684106800165	1.69024383254607	0.100526966087866	0.745711719104592	-4.53729454029164
a_HQ_transcript/21578	1.70127499743021	1.49395290275683	1.69024368188917	0.100526995173493	0.745711719104592	-4.47070960804489
a_HQ_transcript/35614	-0.847629738327765	2.39894863126385	-1.6902116875715	0.100533172114133	0.745711719104592	-4.57914955737487
a_HQ_transcript/134581	3.0175013527531	4.49973415494999	1.69016757216599	0.10054168971571	0.745711719104592	-4.89814541310784
a_HQ_transcript/76495	-0.757589090995433	4.09721251747423	-1.69012790124928	0.10054934970958	0.745711719104592	-5.003241692801
a_HQ_transcript/18466	-1.69689591969748	-0.429951944172726	-1.68998635879648	0.100576683881771	0.745711719104592	-4.52028151577257
a_HQ_transcript/99985	-2.33042981649872	1.92897681117961	-1.68990582524563	0.100592238998905	0.745711719104592	-4.78395703489612
a_HQ_transcript/143171	-0.553307642631375	4.02753174329463	-1.68980687937584	0.100611353216301	0.745711719104592	-4.91469740178683
a_HQ_transcript/10557	1.53040109496132	0.705020547133685	1.68980583051375	0.100611555850158	0.745711719104592	-4.45115368187078
a_HQ_transcript/11608	-2.24087809770833	0.99482778855473	-1.68955660289363	0.100659714779066	0.745711719104592	-4.55515131974331
a_HQ_transcript/145078	1.59829373840629	0.926894582709514	1.68954762731053	0.100661449513883	0.745711719104592	-4.50488437248098
a_HQ_transcript/31849	-1.97162236786396	0.569152847172796	-1.68946157964505	0.100678081440222	0.745711719104592	-4.70108777160286

a_HQ_transcript/92835	1.80435821808912	-0.212845356277189	1.68940472872463	0.100689071261818	0.745711719104592	-4.51333400454991
a_HQ_transcript/138071	1.94916346845777	0.264222877340352	1.68939669906857	0.100690623551038	0.745711719104592	-4.64619396652872
a_HQ_transcript/85973	-2.38301711884426	1.10601903778301	-1.6892144654967	0.100725858222945	0.745711719104592	-4.55068435868582
a_HQ_transcript/5123	1.80555347171736	0.2038367591383	1.68918579093355	0.100731403356005	0.745711719104592	-4.61943767236563
a_HQ_transcript/93194	1.82483495932829	-0.10361808908672	1.6891811481521	0.100732301208479	0.745711719104592	-4.58248036950777
a_HQ_transcript/80218	2.59213603372606	0.57655556605791	1.68916110241946	0.100736177864428	0.745711719104592	-4.62577395340061
a_HQ_transcript/111593	-1.78086094528637	2.18118066709486	-1.68906303716419	0.100755144554425	0.745711719104592	-4.53999233744135
a_HQ_transcript/141281	2.00764131427549	1.21664158995777	1.68898528390726	0.100770184840157	0.745711719104592	-4.70988411831468
a_HQ_transcript/44797	2.34080622758735	1.14320381064376	1.68898419495117	0.100770395496854	0.745711719104592	-4.57635937208408
a_HQ_transcript/102529	8.16707727190562	4.48432944212741	1.68892726239371	0.100781409514353	0.745711719104592	-4.84153110991539
a_HQ_transcript/21389	-0.453162861408646	2.89031487727696	-1.68873673220522	0.100818276266383	0.745711719104592	-4.6766419753735
a_HQ_transcript/76401	2.29260343805457	1.08872973438412	1.68873563810905	0.100818488001637	0.745711719104592	-4.54876302040265
a_HQ_transcript/141471	1.17837925378541	2.57109250832653	1.68870115950395	0.100825160672896	0.745711719104592	-4.71652295739039
a_HQ_transcript/63307	1.36802479677503	2.33837392050267	1.68866475952875	0.100832205587976	0.745711719104592	-4.69432415682067
a_HQ_transcript/116568	0.723321069388527	2.80323560124244	1.68864095668021	0.100836812654997	0.745711719104592	-4.70435633122954
a_HQ_transcript/141698	-2.24847754416229	-0.150945950592873	-1.68852206701348	0.100859826505938	0.745711719104592	-4.58726852622563
a_HQ_transcript/22935	-1.68575523388245	-0.109600217189273	-1.68832494749366	0.100897993207895	0.745711719104592	-4.52236640578865
a_HQ_transcript/40775	2.83218247613692	0.257062796310181	1.68829744333065	0.100903319579777	0.745711719104592	-4.59099758474081
a_HQ_transcript/81464	-1.10821034162524	1.72571832065335	-1.68828020127518	0.100906658742915	0.745711719104592	-4.49049315634753
a_HQ_transcript/93088	3.43011853331649	2.39401593179033	1.68822733522102	0.100916897561691	0.745711719104592	-4.7434126670255
a_HQ_transcript/137980	-1.23942205748564	3.86560064392272	-1.68817238939591	0.100927540097469	0.745711719104592	-4.93189753469389
a_HQ_transcript/132579	1.6410763803934	0.98163580149066	1.68814317321296	0.10093319940294	0.745711719104592	-4.57460873980421
a_HQ_transcript/152883	-2.56917210257991	-0.00116062530307504	-1.68808736608471	0.100944010228919	0.745711719104592	-4.62009970682878
a_HQ_transcript/66971	-2.38184912429132	2.30362635454698	-1.6880444235961	0.100952329604275	0.745711719104592	-4.62740249673984
a_HQ_transcript/6848	0.848784739993523	2.9675010382606	1.6880028514964	0.100960384034669	0.745711719104592	-4.71711783843545
a_HQ_transcript/69318	-2.69860931380867	1.40808964134782	-1.68795432620908	0.10096978629538	0.745711719104592	-4.6183692598821
a_HQ_transcript/23332	1.78646887885874	3.57756422520264	1.68792545410108	0.100975380902014	0.745711719104592	-4.86937414013659
a_HQ_transcript/150219	5.52020323843896	1.8054577312085	1.68760963442366	0.101036594794113	0.746069001802868	-4.64499596299927
a_HQ_transcript/140084	-1.83573759858912	0.15919697067187	-1.68736788605427	0.101083472681582	0.746320348029341	-4.59388870760087
a_HQ_transcript/151876	-3.80835154403732	0.429587781584227	-1.68726068810002	0.101104265442887	0.746379062949471	-4.66032755636283
a_HQ_transcript/46011	-1.08489983658894	2.00737583786518	-1.68708746808863	0.101137871775305	0.746532344197452	-4.50875639532884
a_HQ_transcript/23913	0.67654327325905	4.2478159345722	1.68701800322218	0.101151351236592	0.746537042239794	-4.97420153188849
a_HQ_transcript/140578	1.35541773702976	0.976774089243441	1.68674345983236	0.101204640261925	0.746804045521363	-4.47415614266015
a_HQ_transcript/88029	0.456297010698888	3.55023769070993	1.68669924919596	0.101213223758211	0.746804045521363	-4.87606569933979
a_HQ_transcript/28744	1.59362767730117	-0.577021507888874	1.6864905766386	0.101253745732237	0.747008215753706	-4.58709963804824
a_HQ_transcript/143633	-0.982531972410201	3.07551740520585	-1.68626575219263	0.10129741936095	0.747108218231161	-4.7432258810842
a_HQ_transcript/25847	2.0023434783881	1.64317170881656	1.68626117102108	0.101298309446873	0.747108218231161	-4.54096333878352
a_HQ_transcript/16200	1.63907744405131	2.470371713209	1.68622231530703	0.101305859071326	0.747108218231161	-4.53836254350468
a_HQ_transcript/89986	-1.7723100526962	0.110202440340646	-1.68615419151609	0.101319096584193	0.747111054959533	-4.56539903640152
a_HQ_transcript/126148	0.884854704795841	2.0176785777899	1.68591357174759	0.101365864288876	0.747361105959384	-4.59465398342214
a_HQ_transcript/150022	4.11109439437152	1.14251550283546	1.68568812827139	0.101409698598717	0.747509537237255	-4.63036005087716
a_HQ_transcript/24323	1.09072802975073	-0.68890582963637	1.68567775606823	0.101411715707628	0.747509537237255	-4.60573026183834
a_HQ_transcript/12942	-1.65577781057234	-0.00310009261138849	-1.68546367373226	0.101453356315095	0.747721655367545	-4.49917178598844
a_HQ_transcript/86342	1.72401809454507	-0.0194345352368882	1.6853479601539	0.101475869398366	0.747792765769296	-4.56782316075138
a_HQ_transcript/17569	2.20930486667489	0.512719231175627	1.6852781068664	0.101489461978048	0.747798129783237	-4.51953667311365
a_HQ_transcript/72661	-0.985692109103233	3.0482499981909	-1.6851134280291	0.101521512433035	0.747921519406886	-4.85039145276438
a_HQ_transcript/150912	-6.39672142412417	1.40775866311219	-1.68505985040476	0.101531941743045	0.747921519406886	-4.62817748005034
a_HQ_transcript/36218	1.31326111183322	4.92330725647186	1.68484022638668	0.101574702632693	0.748141702310337	-5.04142598824777
a_HQ_transcript/6809	2.33180488620147	1.06594448147422	1.68439873040898	0.101660707488355	0.748680299617773	-4.59179590563674
a_HQ_transcript/75123	2.03846588932836	1.55799699664691	1.68428134461119	0.101683584831929	0.748736955937244	-4.68143309547596

a_HQ_transcript/68081	-1.44418448550596	0.427901128701975	-1.68414627930421	0.10170991304547	0.748736955937244	-4.51631880275971
a_HQ_transcript/79217	1.89250173953815	-0.218634494290349	1.68410978984699	0.101717026891674	0.748736955937244	-4.59357807809944
a_HQ_transcript/149781	-2.02266572305897	0.363007112190076	-1.68397595503841	0.101743122366835	0.748736955937244	-4.59742327287633
a_HQ_transcript/54848	-2.27142288829428	1.52848180615779	-1.68397115236947	0.101744058907948	0.748736955937244	-4.60004163957802
a_HQ_transcript/19274	-1.58240171784809	2.80478366882322	-1.68388833373646	0.101760210027559	0.748736955937244	-4.50228949198975
a_HQ_transcript/25462	2.81033814991942	1.63067430793308	1.68388520844595	0.10176081955711	0.748736955937244	-4.7579185353714
a_HQ_transcript/10129	1.81427193228674	2.56321902003708	1.68383071844248	0.10177144730354	0.748736955937244	-4.58925272370376
a_HQ_transcript/116026	6.76900417299262	3.28838074818136	1.68360818065035	0.10181486074024	0.748961557214459	-4.80426329473853
a_HQ_transcript/35954	1.96874361192613	1.64802628861551	1.6834457608536	0.101846555889619	0.749099911521162	-4.63000614563925
a_HQ_transcript/27425	-1.49414245788941	3.37897956461185	-1.68337177546583	0.101860996373284	0.749111335676397	-4.75422301396661
a_HQ_transcript/98107	1.34377019016645	-0.658417942474009	1.68320717608923	0.101893129032509	0.749160793732741	-4.60659841048331
a_HQ_transcript/28979	-2.1231879530242	2.43022619216596	-1.68320528775956	0.101893497716265	0.749160793732741	-4.80055966183015
a_HQ_transcript/73922	2.53154501744571	1.68591217195707	1.68309808016566	0.101914431105325	0.749219938192786	-4.72644891872591
a_HQ_transcript/64294	2.51907252553642	0.939138796024041	1.68303161209173	0.101927411481569	0.749220608826585	-4.55230064985013
a_HQ_transcript/63084	1.78377693909737	0.277890646250886	1.68290947573007	0.101951266762055	0.749271780850362	-4.51956723605801
a_HQ_transcript/63478	0.761321294049153	3.86450140377275	1.68286398413323	0.101960153222653	0.749271780850362	-4.9409262257279
a_HQ_transcript/147454	1.82270821816825	2.13165159269804	1.6826370665936	0.102004489589379	0.749359563193083	-4.5403368828645
a_HQ_transcript/39783	1.65177269023704	-0.482864549635882	1.68258822475272	0.102014034670137	0.749359563193083	-4.60640359866095
a_HQ_transcript/51670	-1.09681164782818	1.50524049721415	-1.68225744478917	0.102078698036159	0.749359563193083	-4.54692222779648
a_HQ_transcript/141074	0.602489269092846	3.79134947661627	1.68219119647964	0.10209165285829	0.749359563193083	-4.84735043692872
a_HQ_transcript/2300	1.70051717817312	0.333711016666749	1.68205819251472	0.102117665847726	0.749359563193083	-4.46138974575619
a_HQ_transcript/54335	2.4119093103949	3.60616071200021	1.68204335140447	0.102120568822621	0.749359563193083	-4.89821624713932
a_HQ_transcript/89421	1.71087361182458	-0.355589741950151	1.68204258570245	0.102120718598573	0.749359563193083	-4.58869109137812
a_HQ_transcript/83146	2.33057796614259	2.05972612560551	1.68203945781655	0.102121330433895	0.749359563193083	-4.68285003609906
a_HQ_transcript/46118	-2.37986274568611	1.17971122146005	-1.68195243925928	0.102138353067341	0.749359563193083	-4.60514555711587
a_HQ_transcript/83544	-1.95455746273849	0.176678663216373	-1.68179960557365	0.102168256223768	0.749359563193083	-4.57977619572783
a_HQ_transcript/142080	1.56882555427806	-0.611234854172483	1.681657299388	0.10219610614327	0.749359563193083	-4.55306898331994
a_HQ_transcript/95218	2.87260570804461	2.96859435753798	1.68160229298261	0.102206872822259	0.749359563193083	-4.87024598624538
a_HQ_transcript/39083	-3.14621667881321	4.18171255945978	-1.68142153978303	0.102242259202391	0.749359563193083	-5.06866797131935
a_HQ_transcript/46496	1.52611039488872	-0.279550486390243	1.6814166751676	0.102243211697892	0.749359563193083	-4.59886600540408
a_HQ_transcript/2016	-1.62484854279065	1.57474027545625	-1.68137813375585	0.102250758397609	0.749359563193083	-4.5415507643565
a_HQ_transcript/151482	1.88055260730324	0.527800225691152	1.68130703568598	0.102264681154089	0.749359563193083	-4.74534966832416
a_HQ_transcript/115122	1.76772899221365	0.0116306292811529	1.68126761823245	0.102272400744311	0.749359563193083	-4.58819120818386
a_HQ_transcript/46926	1.81920362151716	0.106601347017606	1.68117453872591	0.102290631540356	0.749359563193083	-4.5068518553746
a_HQ_transcript/30664	-1.76682277534403	0.069436493822335	-1.68117280443035	0.102290971249705	0.749359563193083	-4.53337981188173
a_HQ_transcript/143837	2.88495465193387	0.763234897113683	1.68116869867365	0.102291775478505	0.749359563193083	-4.65932461974023
a_HQ_transcript/25843	1.63626183219528	0.823591557871212	1.68112781757873	0.102299783486633	0.749359563193083	-4.48317004666113
a_HQ_transcript/149423	2.62624154169832	3.15393228437386	1.68111359956193	0.10230256871036	0.749359563193083	-4.77889281149401
a_HQ_transcript/95042	0.818642978534166	4.51623396746067	1.68104713747862	0.102315589069004	0.749359563193083	-4.94191743845986
a_HQ_transcript/8107	-1.88881407283848	2.45339910869113	-1.68104345181311	0.102316311155463	0.749359563193083	-4.66962080992412
a_HQ_transcript/104004	-2.05208371687767	5.8167521540284	-1.68097001948354	0.102330698720104	0.749359563193083	-5.13581604608886
a_HQ_transcript/7512	0.775034968937617	2.25968320839593	1.68094260983741	0.102336069511965	0.749359563193083	-4.5724921824713
a_HQ_transcript/131930	-2.26490219271046	-0.469626405971471	-1.68093831392773	0.102336911296633	0.749359563193083	-4.52508329114396
a_HQ_transcript/29060	1.78251751447373	0.192289539558664	1.68093237869426	0.102338074316729	0.749359563193083	-4.5309269638149
a_HQ_transcript/139122	-3.26746960465044	1.29663692929587	-1.68087012316721	0.102350274066782	0.749359563193083	-4.61137634760994
a_HQ_transcript/42387	4.40322762231018	2.51742790889226	1.68082639016127	0.102358844820965	0.749359563193083	-4.63436008833634
a_HQ_transcript/116	2.63422235796938	3.1779134056478	1.68060635100409	0.102401976959745	0.749547301747017	-4.86731540275951
a_HQ_transcript/35437	1.86981591937181	0.516485403564326	1.68056400928556	0.102410278531427	0.749547301747017	-4.55932563973893
a_HQ_transcript/87015	-1.55129170149302	1.9686833969692	-1.6803578661321	0.102450703227583	0.74963260895119	-4.6041705796591
a_HQ_transcript/139149	2.3188992224928	6.3183448556457	1.68014371681405	0.102492712001418	0.74963260895119	-5.10106936564566

a_HQ_transcript/43551	-1.64468784663904	-0.118073435592229	-1.68009706173608	0.102501866034612	0.74963260895119	-4.54264141002933
a_HQ_transcript/108418	-0.636930808098497	4.7210072918481	-1.68000603782356	0.102519727484359	0.74963260895119	-5.01537516969867
a_HQ_transcript/38972	1.51520817194969	-0.483263498244288	1.6800043518542	0.102520058343369	0.74963260895119	-4.57722795817065
a_HQ_transcript/17475	-1.53173130785901	2.3291396467404	-1.67999928646598	0.102521052393666	0.74963260895119	-4.70970509450742
a_HQ_transcript/72366	2.93420880268499	1.54078659813346	1.67996299367577	0.102528174858169	0.74963260895119	-4.70688257810145
a_HQ_transcript/85570	1.65916024954678	2.20847353727035	1.6799587060521	0.102529016332044	0.74963260895119	-4.62428624527461
a_HQ_transcript/106360	3.82086839258654	0.546867321884537	1.67991293187791	0.102538000168093	0.74963260895119	-4.65067595403258
a_HQ_transcript/3829	-1.62517623390271	2.0958277210871	-1.67977314696349	0.102565439012119	0.749738913342113	-4.63824839800477
a_HQ_transcript/114616	3.60955052240225	2.03149708934392	1.67968732728141	0.102582287869928	0.749767789425775	-4.6196729241562
a_HQ_transcript/134842	3.6750874022786	5.85488561644723	1.67945941201061	0.102627045340364	0.749867722238026	-5.18347062181303
a_HQ_transcript/58909	2.7719546943456	2.9180268821778	1.67944064747823	0.102630730999704	0.749867722238026	-4.77063501142511
a_HQ_transcript/109328	1.57355927952493	0.756255696378154	1.67939738811253	0.102639228262478	0.749867722238026	-4.537317383243
a_HQ_transcript/41824	-1.03705633151341	2.04163862869895	-1.67935496572958	0.102647561688824	0.749867722238026	-4.65435735760504
a_HQ_transcript/79123	2.084941458843	1.04867105918288	1.67906912640184	0.102703726470442	0.750078317036905	-4.55269801250286
a_HQ_transcript/19325	-0.400062420179287	3.64291885873815	-1.67895156528089	0.10272683356473	0.750078317036905	-4.8595262804204
a_HQ_transcript/149682	-1.57820808786201	0.483811530070129	-1.67894199475357	0.102728714879453	0.750078317036905	-4.52390543959606
a_HQ_transcript/38848	1.60991201262457	0.561407479365972	1.67889058825326	0.102738820540193	0.750078317036905	-4.51629212588533
a_HQ_transcript/74437	2.46576752671024	3.67046782180757	1.67887996482289	0.102740909032615	0.750078317036905	-4.85433100080549
a_HQ_transcript/16045	1.07679585028705	0.438462723336944	1.67872686134667	0.102771012034257	0.750121116036101	-4.50667370510469
a_HQ_transcript/101108	0.769445500325899	-0.186222203261479	1.67869433084074	0.102777409084522	0.750121116036101	-4.63668409465246
a_HQ_transcript/141470	2.15450051507842	0.0560940440072982	1.67865326381668	0.102785485294996	0.750121116036101	-4.59277232394906
a_HQ_transcript/56512	-1.69049081232789	1.68568979942755	-1.67844342313309	0.102826760653249	0.750168444850158	-4.60594209705809
a_HQ_transcript/121352	-1.76688218354793	2.13309184245676	-1.67833644675598	0.102847808059745	0.750168444850158	-4.55311509997337
a_HQ_transcript/111380	-1.26041775627847	-0.665586909837699	-1.67816889414193	0.102880780935589	0.750168444850158	-4.59370188537643
a_HQ_transcript/97884	-1.38509921671786	1.48031578415603	-1.6781190964294	0.102890582381665	0.750168444850158	-4.55409800635589
a_HQ_transcript/18387	3.64178302625805	1.19550200498537	1.67809169277985	0.102895976442684	0.750168444850158	-4.65407233971495
a_HQ_transcript/123640	1.19398306470299	-0.757420513738144	1.67807850375942	0.102898572618046	0.750168444850158	-4.61780073768003
a_HQ_transcript/31838	-1.45500234662316	0.491006521952623	-1.67805332342561	0.102903529359089	0.750168444850158	-4.49195200242448
a_HQ_transcript/14798	-0.665031294661092	2.58248657651943	-1.67795390700623	0.102923101391987	0.750168444850158	-4.63597001194715
a_HQ_transcript/115546	-2.65878967013256	2.63101754458815	-1.67791072392874	0.102931603775739	0.750168444850158	-4.676469503207
a_HQ_transcript/150675	2.13975960460224	0.0333193914041454	1.67789120693697	0.10293544669859	0.750168444850158	-4.53037638739258
a_HQ_transcript/48049	-1.83114595819323	-0.35579583714181	-1.67787302507059	0.102939026840817	0.750168444850158	-4.59135724042168
a_HQ_transcript/118342	-2.62054533257925	1.96095061811055	-1.67783336769606	0.102946836026867	0.750168444850158	-4.63358228349503
a_HQ_transcript/49900	0.574797361496697	2.66184112976564	1.67774664067752	0.102963915714088	0.750198858439273	-4.71981624774839
a_HQ_transcript/130885	0.557497743489265	5.25933639671513	1.67741021031706	0.103030193339036	0.750587677454754	-5.10604060023909
a_HQ_transcript/139964	-1.95971504338095	1.27799934302461	-1.67727074577373	0.103057678610322	0.750641261975825	-4.49672357754014
a_HQ_transcript/31731	2.80451440204572	1.61064899101644	1.677241840929	0.10306337585723	0.750641261975825	-4.65861452339367
a_HQ_transcript/21244	1.18924742630745	4.08316712570612	1.67716416414249	0.103078687524421	0.750658725685233	-4.73006998052049
a_HQ_transcript/103269	1.76307307694352	0.116564871880682	1.6770771027043	0.103095851345997	0.750660894250074	-4.53596405968247
a_HQ_transcript/15193	-2.53489257415008	-0.168231070372998	-1.67703164973513	0.103104813165645	0.750660894250074	-4.61526702449518
a_HQ_transcript/3100	-1.38090068080729	2.83633551807177	-1.67677605533233	0.103155219985279	0.750809023435428	-4.60205086373713
a_HQ_transcript/35809	2.51064011050384	1.84603847375214	1.67674591860792	0.103161164723265	0.750809023435428	-4.48179139479353
a_HQ_transcript/77393	-2.28995514398521	2.24566119669327	-1.67668574020366	0.103173036303708	0.750809023435428	-4.70237823951428
a_HQ_transcript/94810	-1.0669280708771	2.33518430089603	-1.67657766913171	0.103194358672691	0.750809023435428	-4.78262690743155
a_HQ_transcript/125433	2.03175247410656	-0.0397305706919331	1.67638663455794	0.103232058667599	0.750809023435428	-4.58514685577312
a_HQ_transcript/85148	-2.05015856820314	0.480547416266248	-1.67625280568924	0.103258476144395	0.750809023435428	-4.4493729088644
a_HQ_transcript/110906	1.77566506531439	-0.596761558487795	1.67624674964213	0.103259671725621	0.750809023435428	-4.61521805044634
a_HQ_transcript/7116	2.17336980153289	-0.16910553813874	1.67622126394892	0.103264703222259	0.750809023435428	-4.63525805768458
a_HQ_transcript/143594	3.31633094384335	2.53177575897612	1.67615281842038	0.103278217046276	0.750809023435428	-4.86745578248139
a_HQ_transcript/58211	1.87420243434143	1.47602209877969	1.67594469142507	0.103319318493136	0.750809023435428	-4.64417672603321

a_HQ_transcript/30776	-1.14893090505541	1.59816444169887	-1.67585070979603	0.10333788268259	0.750809023435428	-4.50612046792113
a_HQ_transcript/123899	0.691225942481828	2.86276668310024	1.67581482427406	0.103344971881073	0.750809023435428	-4.7945756311322
a_HQ_transcript/148040	-1.27678253214806	-0.442869068924025	-1.6757920379795	0.103349473532205	0.750809023435428	-4.57191262579558
a_HQ_transcript/144138	1.76894283805644	-0.464279977947102	1.67577094856004	0.103353640094729	0.750809023435428	-4.61522239076013
a_HQ_transcript/122528	1.54706883399905	0.00155813031709255	1.67573875902483	0.103359999937535	0.750809023435428	-4.54537164304263
a_HQ_transcript/141520	-3.07824356320367	5.85419546050186	-1.67564424184328	0.103378676037477	0.750809023435428	-4.99553768508436
a_HQ_transcript/96672	-2.14093758078289	-0.367349740689345	-1.67561310160506	0.103384829800161	0.750809023435428	-4.58897409507726
a_HQ_transcript/14869	1.64197100433635	-0.410003595362202	1.67556024766671	0.103395275202381	0.750809023435428	-4.55605221516024
a_HQ_transcript/79126	-1.60777752583608	0.205048026070191	-1.67537642531983	0.103431610425876	0.750809023435428	-4.5049166662527
a_HQ_transcript/83450	2.43053539672283	1.65901061319747	1.67537209713278	0.10343246608445	0.750809023435428	-4.75695042836916
a_HQ_transcript/82607	1.89798680237552	1.26283326806427	1.67531438683641	0.103443875652616	0.750809023435428	-4.60714832027075
a_HQ_transcript/72865	-1.48662944228722	2.20575422715454	-1.67523999383779	0.103458585005667	0.750809023435428	-4.58868201962243
a_HQ_transcript/24980	-7.30349613925726	3.81964989215474	-1.67518463626773	0.1034695317082	0.750809023435428	-5.01128654975144
a_HQ_transcript/70555	-1.90783509154168	0.734847611912057	-1.67515966700376	0.103474469579514	0.750809023435428	-4.51254814348701
a_HQ_transcript/37202	2.5912259053158	0.729726234572411	1.67504581909304	0.103496986397162	0.750809023435428	-4.57020188095739
a_HQ_transcript/47056	2.63639594411185	1.29828416794345	1.67503716535097	0.103498698099236	0.750809023435428	-4.56558285181317
a_HQ_transcript/31639	1.80522825521443	1.40987421024036	1.67499994259729	0.103506060993499	0.750809023435428	-4.57413139182617
a_HQ_transcript/137123	0.870551730840322	4.1477319464006	1.67492121436312	0.103521635367452	0.750809023435428	-4.99843605439319
a_HQ_transcript/102823	2.14472245893889	1.86160164032893	1.67491058369392	0.103523738523681	0.750809023435428	-4.6555216386645
a_HQ_transcript/81790	2.7668271517697	6.25258275932097	1.67489324602419	0.103527168659441	0.750809023435428	-5.16293789160718
a_HQ_transcript/146285	-1.92699198049559	0.434763791090599	-1.67477154788678	0.103551248438761	0.750809023435428	-4.58743510984998
a_HQ_transcript/43638	3.96152709211663	4.45091902498793	1.67464860167642	0.103575579897733	0.750809023435428	-4.62345158416208
a_HQ_transcript/127661	4.26215410091718	2.97925361083683	1.67464327419268	0.103576634331742	0.750809023435428	-4.69995008484332
a_HQ_transcript/143662	0.551173619631611	3.00369620990477	1.67458982387236	0.10358721389815	0.750809023435428	-4.7219035970128
a_HQ_transcript/66543	1.15321294196976	3.07512210516571	1.67458202713477	0.103588757202608	0.750809023435428	-4.80943092459299
a_HQ_transcript/120600	-3.60845414279315	0.914448981306943	-1.67457497964843	0.103590152214976	0.750809023435428	-4.68145586611712
a_HQ_transcript/24621	1.49441070895217	-0.452718413370188	1.67442803326246	0.103619243028516	0.750840284107836	-4.56924579695237
a_HQ_transcript/23362	2.08533981876222	0.0900354313753139	1.67442269824344	0.10362029932413	0.750840284107836	-4.63511920956378
a_HQ_transcript/142084	-2.33917833641314	1.43079677656051	-1.67413646022077	0.103676985536402	0.750992304217788	-4.60918557832179
a_HQ_transcript/90768	1.81880201164614	4.34178092225845	1.67393831438838	0.10371624118692	0.750992304217788	-5.006892990026
a_HQ_transcript/104217	-0.444078089751665	4.05862838721985	-1.67393665777816	0.103716569438255	0.750992304217788	-4.98118170964447
a_HQ_transcript/69565	1.54978417221009	1.61951533662984	1.67380742304848	0.103742179496054	0.750992304217788	-4.58705827617377
a_HQ_transcript/75552	2.15582621709728	0.375030503723646	1.67379084880243	0.103745464344976	0.750992304217788	-4.62484106539535
a_HQ_transcript/67211	1.31492097275644	3.38054595271494	1.67374763762464	0.103754028772635	0.750992304217788	-4.66956961690474
a_HQ_transcript/46122	2.80271793209097	1.94713004847616	1.6737276049518	0.103757999434966	0.750992304217788	-4.7839160443638
a_HQ_transcript/29656	2.50757495357524	1.17916871323564	1.67372756961805	0.103758006438556	0.750992304217788	-4.64098552194796
a_HQ_transcript/94792	1.7071642355948	-0.427906339869076	1.67368831668838	0.103765787101609	0.750992304217788	-4.6367409403769
a_HQ_transcript/20835	2.56303530309122	3.13987280030506	1.67360099510554	0.103783097608762	0.750992304217788	-4.7403026851928
a_HQ_transcript/41926	-4.21681889127579	3.01976936225722	-1.67359949545774	0.103783394917793	0.750992304217788	-4.87059087053843
a_HQ_transcript/17693	1.9734638275003	0.0903991408704285	1.67350154023015	0.103802816327547	0.751039346214546	-4.54496991332508
a_HQ_transcript/37150	0.524402176115688	2.68135257390769	1.67332662581905	0.103837503814689	0.75119681664471	-4.66979520897239
a_HQ_transcript/151836	5.52674103217019	6.30484222973705	1.67317328342992	0.103867921256121	0.751254788040753	-5.36570076596322
a_HQ_transcript/56707	-1.90942941202922	1.21410796321423	-1.67315592263596	0.103871365460424	0.751254788040753	-4.6555820087878
a_HQ_transcript/58890	-2.69046631965057	2.54014224701768	-1.67299590330423	0.103903116133997	0.751319522323847	-4.77564804804165
a_HQ_transcript/100474	2.47449726994732	0.483446961157619	1.6729152989959	0.103919112514202	0.751319522323847	-4.58223578666628
a_HQ_transcript/132125	2.99965424680942	0.963553017051055	1.67289165575835	0.103923805036587	0.751319522323847	-4.68119685528141
a_HQ_transcript/143822	3.1155448563886	1.31689117324417	1.67285028242838	0.103932016911059	0.751319522323847	-4.6406163438191
a_HQ_transcript/117818	1.9385811568571	0.119777372035845	1.67259099443002	0.103983493280046	0.751598171771016	-4.5809323100206
a_HQ_transcript/131063	-0.410368413966473	3.6273578791668	-1.67242360306792	0.104016736695017	0.751706524617431	-4.89928888378667
a_HQ_transcript/99934	2.24075722913978	1.00527303872824	1.67238528537021	0.104024347715776	0.751706524617431	-4.52315048803554

a_HQ_transcript/132038	1.81262005297838	0.272875378867208	1.6722407756173	0.104053055764901	0.751820512803251	-4.65615240245191
a_HQ_transcript/21265	1.42127622534586	1.51181311883106	1.6719679043897	0.104107281835126	0.751929320060324	-4.54759882025259
a_HQ_transcript/135592	-1.74229177654624	1.46036773240821	-1.67194146920945	0.104112536388615	0.751929320060324	-4.56064819965209
a_HQ_transcript/24005	2.26313320542931	0.0766355865296054	1.67193283659173	0.104114252352473	0.751929320060324	-4.60618610788865
a_HQ_transcript/117810	1.89997910916775	-0.490883594808499	1.67186788447287	0.104127164078723	0.751929320060324	-4.6098196364657
a_HQ_transcript/107895	1.01258318952741	2.14955326631116	1.67183956739242	0.104132793601803	0.751929320060324	-4.56703049178769
a_HQ_transcript/103686	0.398361106593157	5.18392456679989	1.67167504033345	0.104165507094977	0.752072114820128	-5.10343845199462
a_HQ_transcript/26712	-0.715550767483269	2.89724196920436	-1.67148582624188	0.104203139758608	0.752152254279365	-4.70328470049518
a_HQ_transcript/82162	-1.26680122034907	2.60495913189482	-1.67146032752423	0.104208212045419	0.752152254279365	-4.69910957268206
a_HQ_transcript/139452	2.26044601052608	0.580996144500628	1.67142406657648	0.104215425543043	0.752152254279365	-4.68512234586906
a_HQ_transcript/123662	1.21126980875406	-0.47867372794026	1.67126689160864	0.104246697623116	0.75223390188371	-4.59542304702514
a_HQ_transcript/11593	-0.819267669611515	3.98021880366499	-1.6712371282987	0.104252620313363	0.75223390188371	-4.96677557817905
a_HQ_transcript/62333	4.85394385988158	0.963919504003644	1.67116809823302	0.104266357889593	0.75223964892718	-4.6462847811649
a_HQ_transcript/48609	-3.75376743913945	0.918817818737535	-1.67095650090507	0.104308476950912	0.75238644796244	-4.75632452645245
a_HQ_transcript/103767	-1.12312722782701	3.02749309557518	-1.67093181644612	0.10431339138493	0.75238644796244	-4.71966109978128
a_HQ_transcript/73100	-3.71011832648504	5.35856207687871	-1.67087081881937	0.104325536240319	0.75238644796244	-4.91148129991709
a_HQ_transcript/55392	1.70676786017422	0.0686469776233025	1.67063888033688	0.10437172678912	0.752507829822891	-4.52145086567874
a_HQ_transcript/71778	-1.89562965242095	2.03993615157801	-1.67057924546561	0.104383605820187	0.752507829822891	-4.69199133554375
a_HQ_transcript/61507	1.18944269309128	0.173428005903043	1.67053070920497	0.104393274881187	0.752507829822891	-4.48866296723937
a_HQ_transcript/22880	1.56277300178137	0.328338746917588	1.67050035328321	0.104399322558083	0.752507829822891	-4.54999345249325
a_HQ_transcript/85483	1.19053447408754	1.58361589281731	1.67040843870865	0.104417636070411	0.752507829822891	-4.57980888290861
a_HQ_transcript/67606	-1.81839502988133	1.34191806379266	-1.67032351479679	0.104434559101506	0.752507829822891	-4.62004242007762
a_HQ_transcript/20035	2.15409750725945	1.60545087910481	1.67019680647489	0.104459812868967	0.752507829822891	-4.58354337447894
a_HQ_transcript/112685	1.37067968764525	-0.466592066371191	1.67016114909702	0.104466920524127	0.752507829822891	-4.64503074408893
a_HQ_transcript/67535	1.51444225256082	-0.479285442883052	1.67013759193031	0.104471616440892	0.752507829822891	-4.62546261356552
a_HQ_transcript/52390	0.946965898298001	2.60956944536261	1.67012301574538	0.104474522164719	0.752507829822891	-4.66207256505258
a_HQ_transcript/144973	2.4440929882611	1.02133391147326	1.67007161288996	0.104484769723723	0.752507829822891	-4.66809559188892
a_HQ_transcript/56927	-1.29448855093292	5.24573328217184	-1.6699279756271	0.104513409357336	0.75252317363945	-5.09299831049224
a_HQ_transcript/16764	-0.642640415616214	4.22708028325983	-1.66969441945827	0.104559991723151	0.75252317363945	-4.99966086219912
a_HQ_transcript/27494	-1.43123809453514	-0.336372097508859	-1.66968393336778	0.10456208356001	0.75252317363945	-4.57981471422458
a_HQ_transcript/110303	-2.1002680912865	1.86423154314952	-1.66967752904979	0.104563361154151	0.75252317363945	-4.83008098770529
a_HQ_transcript/5905	-0.979771969008571	2.23067098161516	-1.6696044861241	0.104577933367925	0.75252317363945	-4.68123447326958
a_HQ_transcript/124637	1.91161596702704	1.06789496942868	1.66958642799032	0.104581536262873	0.75252317363945	-4.45920170781228
a_HQ_transcript/65902	-1.74236608184019	1.56564724603675	-1.66955345919241	0.104588114346805	0.75252317363945	-4.58863188502857
a_HQ_transcript/12969	-1.26571929239081	4.30951981640329	-1.66949112405418	0.104600552675532	0.75252317363945	-5.01379898068722
a_HQ_transcript/147107	-3.33827244292028	0.845522782782414	-1.66942471600431	0.10461380506361	0.75252317363945	-4.61964621126995
a_HQ_transcript/116112	2.75409744087994	2.52852966497842	1.66939840135538	0.10461905679994	0.75252317363945	-4.81140365594299
a_HQ_transcript/673	-2.21578913619784	2.06986430624282	-1.66934705013715	0.104629305831166	0.75252317363945	-4.71389380480625
a_HQ_transcript/99772	2.50512885866102	0.595464944214629	1.66926184436513	0.10464631362888	0.752552383845047	-4.52684154051918
a_HQ_transcript/14243	2.03631244409548	1.52199871516486	1.66894147491854	0.104710282655553	0.752790953264651	-4.56054761139079
a_HQ_transcript/132897	3.60156280259026	1.19151060096132	1.66890601868353	0.104717364293426	0.752790953264651	-4.68646722749726
a_HQ_transcript/13123	1.22094268358343	1.83383132913088	1.66883786060339	0.104730978555328	0.752790953264651	-4.65019653800933
a_HQ_transcript/151785	-0.715903553273715	3.48021019314196	-1.66883630028137	0.104731290239665	0.752790953264651	-4.78514352310341
a_HQ_transcript/109757	-2.18423266375884	0.134444918523548	-1.66870955281493	0.10475661143357	0.752803690268387	-4.59659144184386
a_HQ_transcript/130112	-2.33193980931806	-0.0403708638633359	-1.6686977788874	0.10475896384842	0.752803690268387	-4.61541896754514
a_HQ_transcript/152894	2.95582107384913	5.94684830194602	1.66854216910041	0.104790058597301	0.752934057619764	-4.98799646647436
a_HQ_transcript/68580	1.83745955298431	0.556137716417199	1.66838595810606	0.104821281194252	0.753065311242057	-4.60475213689701
a_HQ_transcript/105960	-1.36121589984278	2.8507685155621	-1.66795288229126	0.104907882428214	0.753517196306269	-4.71009691287973
a_HQ_transcript/1636	-1.52577969574109	1.48285343547296	-1.66790352867525	0.104917755335465	0.753517196306269	-4.5097903954836
a_HQ_transcript/141702	1.58209473033331	2.06494197168586	1.66787696506899	0.10492306955173	0.753517196306269	-4.65335581068101

a_HQ_transcript/9579	1.44821972755416	0.609746861230298	1.6676809790464	0.104962284697173	0.75356361440693	-4.56958972690358
a_HQ_transcript/142096	1.27274669295253	1.18129268343786	1.6675646273832	0.104985571439536	0.75356361440693	-4.60542324371183
a_HQ_transcript/93174	1.4322365726616	0.075938508473057	1.66755287449484	0.104987923913392	0.75356361440693	-4.46371664522523
a_HQ_transcript/31881	-1.0088016731474	2.37366856128167	-1.66738588076513	0.105021354332793	0.75356361440693	-4.57118284423184
a_HQ_transcript/54813	-1.73032580832963	0.600291772101221	-1.66736948108512	0.105024637856028	0.75356361440693	-4.59523165853643
a_HQ_transcript/7147	-1.60932434745762	-0.308120214894032	-1.6672994507675	0.105038660197337	0.75356361440693	-4.63217802923166
a_HQ_transcript/150600	5.31390265425585	7.02264806391144	1.66724348109845	0.105049868258676	0.75356361440693	-5.07333850173892
a_HQ_transcript/150804	0.654894064406528	7.99337570408627	1.66722167316143	0.10505423561962	0.75356361440693	-5.33832524051547
a_HQ_transcript/58398	-2.1490082278132	1.3820246946111	-1.66717893859666	0.105062794283633	0.75356361440693	-4.61025608588394
a_HQ_transcript/104007	2.12024636928868	1.37066288238958	1.66713392868883	0.105071809268109	0.75356361440693	-4.76437376857932
a_HQ_transcript/141678	1.523692505293	1.14418278765257	1.66705732998399	0.105087152614687	0.75356361440693	-4.53184960055755
a_HQ_transcript/97471	-1.97944375247227	4.13532304847068	-1.66703989941408	0.105090644360185	0.75356361440693	-5.01337872613831
a_HQ_transcript/107822	-0.950086721566196	3.06412169363838	-1.66690883764874	0.105116902143324	0.75356361440693	-4.81666326379979
a_HQ_transcript/53206	-1.47474582826268	1.94436011090057	-1.66685272306225	0.105128146177446	0.75356361440693	-4.68982090404342
a_HQ_transcript/98552	1.63053846391904	-0.397697439066688	1.66676197221441	0.105146332612003	0.75356361440693	-4.52046454066704
a_HQ_transcript/92228	2.05393414190219	1.55116445087841	1.66675050058572	0.105148631707931	0.75356361440693	-4.75365452179696
a_HQ_transcript/127281	-0.667148687827098	2.55207508669262	-1.6666748705285	0.105163790213209	0.75356361440693	-4.70920920200276
a_HQ_transcript/136618	-2.61291439764564	1.05698772861996	-1.66660395701388	0.105178005032102	0.75356361440693	-4.67488955583449
a_HQ_transcript/107144	2.27553446623858	-0.485552637536291	1.6665460631999	0.105189611197461	0.75356361440693	-4.61795388608437
a_HQ_transcript/123575	2.55719526726597	0.0625149948611586	1.66652398548125	0.105194037471243	0.75356361440693	-4.72571359291799
a_HQ_transcript/28583	1.9260770913413	1.52056580611611	1.66643924166991	0.105211028856415	0.75356361440693	-4.48628698713346
a_HQ_transcript/22682	2.68440136290067	1.22849814440622	1.66642074363673	0.105214738069891	0.75356361440693	-4.55492475578585
a_HQ_transcript/24349	1.49267615393764	-0.670677108255806	1.66634475813442	0.105229975772213	0.753579897953332	-4.63545052696873
a_HQ_transcript/39423	2.70148792221476	0.063469833699537	1.66613406932641	0.105272235698124	0.753690886892214	-4.69204280113678
a_HQ_transcript/143055	-1.68024158068793	-0.608134242706672	-1.66611788604055	0.105275482321642	0.753690886892214	-4.63208859384214
a_HQ_transcript/7186	-2.78258560216798	0.319119141775651	-1.66607357399044	0.105284372446408	0.753690886892214	-4.57786117143884
a_HQ_transcript/43365	-1.80570168894909	0.234906153218409	-1.66595317858896	0.105308529975312	0.753713219208857	-4.62400706676778
a_HQ_transcript/42983	1.17712346631943	-0.474862948171139	1.66592878591008	0.10531342496584	0.753713219208857	-4.58836050039814
a_HQ_transcript/122453	-0.669071166307068	2.67059060466562	-1.66580651003847	0.105337965472125	0.753714965392307	-4.70678481528369
a_HQ_transcript/125892	-0.486590450918385	3.12707925942875	-1.66578113717252	0.105343058346971	0.753714965392307	-4.78459884895351
a_HQ_transcript/144447	1.64821837811783	-0.574957666978131	1.66573376069337	0.105352568363138	0.753714965392307	-4.5708211783162
a_HQ_transcript/147337	-3.12345843009268	-0.125961047086258	-1.6656640610104	0.105366560675699	0.753722303588113	-4.64208407752066
a_HQ_transcript/120796	0.650005222603443	3.1896943394675	1.66553569876168	0.105392333621164	0.753813900422172	-4.83045711761225
a_HQ_transcript/37469	3.05468431272871	2.39237526448175	1.66545478468397	0.105408582474458	0.753837362460053	-4.66132321232963
a_HQ_transcript/148496	2.3221473495379	-0.00542500917528891	1.66537803593169	0.105423996785266	0.753854851166676	-4.62736423328987
a_HQ_transcript/126356	2.69722222463143	0.100036253467466	1.66513314991445	0.105473192497797	0.753936423006647	-4.63346760004857
a_HQ_transcript/63958	2.28441151441923	1.12163275990477	1.6650833871455	0.105483191784101	0.753936423006647	-4.67061179830017
a_HQ_transcript/7210	1.4595608959637	-0.256208313851038	1.66506839941975	0.105486203558529	0.753936423006647	-4.51930743163568
a_HQ_transcript/100685	-0.65702975607302	4.22372206673595	-1.66506301563416	0.105487285444417	0.753936423006647	-4.95463625202308
a_HQ_transcript/40661	2.34630511809865	0.352251327468157	1.66484024134368	0.105532060602455	0.754097475140732	-4.69293709814719
a_HQ_transcript/7823	-0.56407456418984	2.33885332703305	-1.66482181294466	0.10553576521246	0.754097475140732	-4.57700622050086
a_HQ_transcript/146350	0.318601583240939	6.25813749036438	1.66469976229052	0.105560303442847	0.75412067405227	-5.18899923153259
a_HQ_transcript/136317	2.44444208206821	0.637025262382807	1.66465165036321	0.105569977626173	0.75412067405227	-4.66890778684643
a_HQ_transcript/87659	3.33478727384931	0.562074076974268	1.66461209297417	0.10557793224328	0.75412067405227	-4.63386727737633
a_HQ_transcript/117528	-1.91821169178934	0.41399161402044	-1.66454104265028	0.105592221041445	0.754130068152984	-4.72989170792197
a_HQ_transcript/84647	-1.88215660061596	1.00810035166042	-1.6641514914572	0.105670591488448	0.754589970643826	-4.55842914085009
a_HQ_transcript/74098	-0.977120026600028	4.92396992772878	-1.66400855050188	0.105699360653763	0.754589970643826	-5.10070849155161
a_HQ_transcript/25080	0.87441304034407	1.97185012957101	1.6639566520316	0.105709807663963	0.754589970643826	-4.57287945490777
a_HQ_transcript/5408	2.1128786959277	0.730482760735382	1.6638478394844	0.105731714093659	0.754589970643826	-4.49553395030498
a_HQ_transcript/120595	-1.15207697745358	0.847550974409378	-1.6638392473344	0.105733444048523	0.754589970643826	-4.50777459525048

a_HQ_transcript/89076	-0.817087538544888	3.19581585539997	-1.66381464242447	0.105738398163661	0.754589970643826	-4.81915195370572
a_HQ_transcript/78840	1.42673208741524	-0.239778519810904	1.66374698419753	0.10575202191146	0.754589970643826	-4.65000023561164
a_HQ_transcript/12410	-2.29533505512701	0.828509198270581	-1.66370504229158	0.105760468119241	0.754589970643826	-4.53359898894621
a_HQ_transcript/133286	-3.02221117168116	4.20499152622361	-1.66324809737317	0.105852523391816	0.755154084403473	-4.81459959078077
a_HQ_transcript/127840	-1.75067879131065	-0.529600349772625	-1.66301262414235	0.105899987349314	0.75539998338949	-4.60482885439219
a_HQ_transcript/109723	1.46141707421663	0.458926919220048	1.66281659110814	0.105939514882207	0.755554242440493	-4.56892012358294
a_HQ_transcript/15392	-1.46084540813503	1.66363492369513	-1.66277645538014	0.105947609243952	0.755554242440493	-4.56590619313248
a_HQ_transcript/134530	2.38045919222356	0.192658999526258	1.66251707036806	0.105999933021431	0.755834654890792	-4.64982540370543
a_HQ_transcript/17680	1.27802711358894	-0.773743023859257	1.66241472461608	0.106020584355248	0.755889185257187	-4.62814973007123
a_HQ_transcript/52055	1.12713447061958	2.71497407658291	1.66229371744317	0.106045005499314	0.75597057636303	-4.80070561325976
a_HQ_transcript/152325	3.57195625476365	1.18854776411318	1.66210809260336	0.106082476573618	0.756144966066372	-4.72467820580789
a_HQ_transcript/122161	2.02561639843654	-0.11276650958508	1.66193759444657	0.106116903786494	0.756179947829223	-4.56995304349199
a_HQ_transcript/44364	2.15039385573048	0.739655262409535	1.66193318128811	0.106117795020348	0.756179947829223	-4.5003102122596
a_HQ_transcript/2178	-2.23308937891603	1.91985399812722	-1.66189051914067	0.10612641092745	0.756179947829223	-4.67293754296577
a_HQ_transcript/20643	-1.74763761497292	1.6861266468583	-1.6615017361595	0.106204955010947	0.756646847024921	-4.66232069829881
a_HQ_transcript/63155	1.75139083427894	0.300196641173675	1.66139158495242	0.106227217131908	0.75665812970304	-4.54437804649373
a_HQ_transcript/116881	-1.19389359222885	0.0229468754169462	-1.66136508798247	0.106232572882372	0.75665812970304	-4.59085025274194
a_HQ_transcript/42687	-1.62417950989944	0.201570987963576	-1.66117505636033	0.106270989950804	0.75676533287598	-4.58308968959558
a_HQ_transcript/32815	0.790407869673153	5.12967314655456	1.66105652926949	0.106294957395489	0.75676533287598	-5.08067058181935
a_HQ_transcript/16163	-1.79307466714886	0.803593339870809	-1.6609361639282	0.106319301143222	0.75676533287598	-4.57032178494523
a_HQ_transcript/87074	2.85146401160034	4.91500390574573	1.6609140291971	0.106323778369826	0.75676533287598	-5.03733167560237
a_HQ_transcript/32584	1.76048437918857	-0.194230506223153	1.66082451063284	0.106341887025006	0.75676533287598	-4.64711954512709
a_HQ_transcript/115795	2.11776786527041	0.0488582475029622	1.66078358893811	0.106350165901514	0.75676533287598	-4.69082420198155
a_HQ_transcript/91282	4.78288475799183	3.67168885482554	1.66075823507288	0.106355295515478	0.75676533287598	-4.89277132919316
a_HQ_transcript/151148	2.24906608208309	0.661722453466075	1.66073827297455	0.106359334407341	0.75676533287598	-4.56821284786799
a_HQ_transcript/37137	2.27944876347063	1.90608421717203	1.66071128806839	0.106364794412276	0.75676533287598	-4.76042907417575
a_HQ_transcript/123482	-2.4679024949151	0.596049839317729	-1.66034602622866	0.106438722732735	0.756950721089122	-4.61532961040668
a_HQ_transcript/53969	-2.28170578546229	1.58433047577263	-1.66024439938757	0.10645929939506	0.756950721089122	-4.62731921716876
a_HQ_transcript/138987	2.57280464706178	0.82498673229833	1.65996653677826	0.10651557583638	0.756950721089122	-4.62905598097959
a_HQ_transcript/144192	0.873201690546576	2.25017927741021	1.65995499214646	0.106517914542981	0.756950721089122	-4.68888793162061
a_HQ_transcript/135567	2.21887020733434	2.20859098116884	1.65994086748412	0.106520775969008	0.756950721089122	-4.68106039274447
a_HQ_transcript/151144	1.93011671337005	0.907937112621082	1.65989578284534	0.106529909807434	0.756950721089122	-4.62942404852094
a_HQ_transcript/10457	-1.06322276236	2.2805741013989	-1.65988611076808	0.106531869388543	0.756950721089122	-4.54950329822412
a_HQ_transcript/53902	2.23601615325922	0.852593437720716	1.6598691774322	0.106535300186345	0.756950721089122	-4.54870498482002
a_HQ_transcript/92706	0.526694137989791	2.92131310194769	1.65983977646252	0.106541257220986	0.756950721089122	-4.79770970739147
a_HQ_transcript/117543	1.66306901421716	0.828873917933488	1.65983293045482	0.10654264435452	0.756950721089122	-4.59829324413034
a_HQ_transcript/120976	-2.56372424995671	1.68486739501279	-1.65980492829292	0.106548318289906	0.756950721089122	-4.67144695279759
a_HQ_transcript/119232	2.53865867741852	3.56890838242954	1.65978199833048	0.106552964658002	0.756950721089122	-4.76923176182113
a_HQ_transcript/58643	-2.14071541447926	0.738522759771301	-1.65961724652647	0.106586353759519	0.756950721089122	-4.54910296203257
a_HQ_transcript/110477	1.45331006138671	-0.421105562821171	1.65954410608449	0.106601179404998	0.756950721089122	-4.67447712326658
a_HQ_transcript/150234	0.646509571397347	3.57162260359757	1.65945819109082	0.106618596650203	0.756950721089122	-4.86505213440974
a_HQ_transcript/64996	2.1321516110173	0.887515475403709	1.65942978439181	0.10662435595979	0.756950721089122	-4.52508978625443
a_HQ_transcript/23426	-3.03823566261153	3.30092479997224	-1.65942690628828	0.106624939494617	0.756950721089122	-4.61412883329842
a_HQ_transcript/11077	0.678504539618899	2.47961439809584	1.65942537717489	0.106625249523082	0.756950721089122	-4.69117655196593
a_HQ_transcript/118705	-2.31608049162295	0.0206954222550013	-1.65906873862122	0.106697578583549	0.757371699585361	-4.62797976814948
a_HQ_transcript/121165	1.61055773891408	-0.356676834262674	1.65892280552172	0.106727186675496	0.757478346298243	-4.6568223279128
a_HQ_transcript/72315	1.62672879947426	-0.416206349070415	1.65885408470259	0.106741131672756	0.757478346298243	-4.61247182902802
a_HQ_transcript/92048	-0.917625613774599	2.40180353928455	-1.6587900225765	0.106754132677391	0.757478346298243	-4.57397568804026
a_HQ_transcript/84404	0.832979948150729	2.89815167153523	1.6587082056281	0.106770738820787	0.757478346298243	-4.7888768913698
a_HQ_transcript/151253	-4.45130757687796	4.99589383155874	-1.65864275488479	0.106784024706262	0.757478346298243	-5.16661175936448

a_HQ_transcript/15078	1.60370323173391	2.13414429392219	1.65854085398195	0.106804712360134	0.757478346298243	-4.72059813993232
a_HQ_transcript/144912	2.0021027925809	0.37099781545286	1.65847605087681	0.106817870245228	0.757478346298243	-4.66072103020573
a_HQ_transcript/29655	-2.7987984664337	1.80978287897644	-1.65841134195219	0.106831010348749	0.757478346298243	-4.85047112112109
a_HQ_transcript/48487	2.31488011256286	2.26438124664259	1.65833875794763	0.106845751198595	0.757478346298243	-4.7517607222971
a_HQ_transcript/4054	3.3113852173828	2.05617540720728	1.65815901731929	0.106882261398884	0.757478346298243	-4.5606119173356
a_HQ_transcript/129396	2.25533857345805	0.658220894125399	1.65805817089475	0.10690275057488	0.757478346298243	-4.69771807099763
a_HQ_transcript/74083	0.847398917173169	2.01104119662445	1.65801731716773	0.106911051837686	0.757478346298243	-4.54778223458383
a_HQ_transcript/60199	-2.08190213755583	0.853032223653933	-1.65801273067918	0.106911983821445	0.757478346298243	-4.66538369319298
a_HQ_transcript/85416	-1.2315522043041	2.8900510113893	-1.65794026795928	0.106926709286837	0.757478346298243	-4.67680948573252
a_HQ_transcript/62441	1.79135907278376	0.205340599671876	1.65791561156417	0.106931720203882	0.757478346298243	-4.55043910135302
a_HQ_transcript/58011	-2.50818135301147	0.339200345438532	-1.65790647985724	0.1069335760893	0.757478346298243	-4.62662148568211
a_HQ_transcript/101720	-2.46772906184219	1.97450399077551	-1.65776948048324	0.106961422407535	0.757478346298243	-4.63846905007126
a_HQ_transcript/91303	1.67239229475333	-0.421664363280881	1.65774636639622	0.106966121140127	0.757478346298243	-4.62689457480402
a_HQ_transcript/141776	2.49069471889857	0.517633190531589	1.65760726576377	0.106994401738514	0.757478346298243	-4.63066824582951
a_HQ_transcript/111715	2.35332813364195	0.625606930787955	1.65740422756931	0.10703569263312	0.757478346298243	-4.71732827012626
a_HQ_transcript/7723	1.27159124858752	2.02002024709495	1.65737108611696	0.107042433703871	0.757478346298243	-4.60820334796842
a_HQ_transcript/101993	1.92136449345078	-0.0071505177230471	1.65736592835781	0.107043482839194	0.757478346298243	-4.64228111127431
a_HQ_transcript/71882	3.02264681300429	0.933363190751042	1.65736573142657	0.107043522896975	0.757478346298243	-4.60224133113168
a_HQ_transcript/146943	-1.23841962724757	3.0522024850586	-1.65731448938275	0.107053946463046	0.757478346298243	-4.84184050182939
a_HQ_transcript/128233	-1.07160312740346	2.3000203452608	-1.65727201203289	0.107062587768445	0.757478346298243	-4.71196203827765
a_HQ_transcript/117857	0.856941708898688	2.35111514072351	1.65721110450644	0.107074979393652	0.757478346298243	-4.66177115894612
a_HQ_transcript/38556	0.919037421815291	1.72528745064609	1.65720098039217	0.107077039258105	0.757478346298243	-4.55042099484681
a_HQ_transcript/117042	1.00073182431853	3.92072020911019	1.65714092298541	0.107089259285729	0.757478346298243	-4.93852136934398
a_HQ_transcript/32205	-0.977023944224451	1.07289520019376	-1.65713478621415	0.107090508014708	0.757478346298243	-4.6074817865495
a_HQ_transcript/105434	2.3411003956419	-0.193752266051229	1.65698239983518	0.107121519932775	0.757534752329458	-4.69975356435815
a_HQ_transcript/150681	-2.12351198775875	3.14744001155893	-1.65694063712911	0.107130020296629	0.757534752329458	-4.67785636329348
a_HQ_transcript/9155	0.674430580359073	2.59528271683349	1.656903502581	0.107137579116785	0.757534752329458	-4.61401503629428
a_HQ_transcript/110587	0.557788457629077	3.642146070073	1.65652201703023	0.107215256956063	0.75799178500876	-4.91788474385596
a_HQ_transcript/62734	1.74500658971161	2.32099041786265	1.65644426992488	0.10723109349634	0.758011553296276	-4.60080335507021
a_HQ_transcript/14908	-0.914314590420441	1.98203971138844	-1.65624273784008	0.107272153196612	0.758178301608201	-4.65642817935187
a_HQ_transcript/90237	-1.91749893509202	0.394404763241377	-1.65612186134617	0.107296786557241	0.758178301608201	-4.51959613475714
a_HQ_transcript/152260	-2.60381241055858	0.730938616088351	-1.65611549957187	0.107298083149931	0.758178301608201	-4.58850373688153
a_HQ_transcript/20702	1.11317506856486	2.91578415262376	1.65599320642735	0.107323010229955	0.758178301608201	-4.76954897067379
a_HQ_transcript/591	-0.848123342176753	2.70527389582549	-1.65598445234659	0.107324794763206	0.758178301608201	-4.82469237109376
a_HQ_transcript/108292	-1.55568449227954	-0.042342693037054	-1.65588288412995	0.107345501403418	0.758178301608201	-4.63813250029317
a_HQ_transcript/140960	2.49550229254099	3.4364238526653	1.65588051171326	0.107345985105895	0.758178301608201	-4.92017942972412
a_HQ_transcript/133981	-0.454206195537614	4.72386474250735	-1.65561933076157	0.107399247285434	0.758251921054099	-5.05744622516701
a_HQ_transcript/43162	-1.78213611799445	1.05065316562452	-1.65557374073047	0.107408546626502	0.758251921054099	-4.5572474515785
a_HQ_transcript/34591	2.89015613902926	1.33550189214887	1.65555838432601	0.107411679137906	0.758251921054099	-4.63853278654875
a_HQ_transcript/14573	-0.986680115883103	1.22685682515109	-1.65554052636983	0.107415322029301	0.758251921054099	-4.54037135586917
a_HQ_transcript/18245	-1.84887134675324	2.30031306053013	-1.65550959984997	0.107421631053546	0.758251921054099	-4.69288518153731
a_HQ_transcript/17599	1.23561394042531	2.70201705086665	1.65525554878387	0.107473469211894	0.758525718595067	-4.7611231669947
a_HQ_transcript/151948	3.93399326200057	2.91242755408387	1.65511395383139	0.107502370122044	0.758637582912973	-4.74964049433969
a_HQ_transcript/50524	-1.65596966541515	2.52183546373254	-1.65503260857992	0.107518976393553	0.758651877534765	-4.80965862534249
a_HQ_transcript/3618	-1.99296311937351	0.821879281938677	-1.65494440473096	0.107536985219423	0.758651877534765	-4.52484876720431
a_HQ_transcript/68467	3.61646534443314	3.13926438836943	1.65489361912804	0.107547355391533	0.758651877534765	-4.73635938862031
a_HQ_transcript/107984	0.909591681052019	1.77817759449469	1.65484834257521	0.107556601341247	0.758651877534765	-4.63336900348948
a_HQ_transcript/141669	1.51976743281187	-0.659402370620651	1.65477258170581	0.107572073983228	0.758668953712335	-4.64700591185251
a_HQ_transcript/116849	-1.83783010178126	-0.322512158270011	-1.65451853265263	0.107623971883297	0.758942889437377	-4.66614359108284
a_HQ_transcript/16182	-1.93373706185452	1.69806506366501	-1.65406982941805	0.107715685002825	0.759198674348429	-4.65846376849791

a_HQ_transcript/105916	3.77982766365552	3.63297583878933	1.65402745559135	0.107724349386379	0.759198674348429	-5.05916850532542
a_HQ_transcript/77288	2.51832273010101	0.716379999311359	1.65399938776798	0.107730088869399	0.759198674348429	-4.68038863823037
a_HQ_transcript/135719	1.80511171327611	1.22516446665694	1.65389014456857	0.107752430010146	0.759198674348429	-4.76213842334837
a_HQ_transcript/117662	-0.702408211863159	2.22314266674089	-1.65375492345185	0.107780089166856	0.759198674348429	-4.59089702107923
a_HQ_transcript/145858	0.722077210394964	8.13791302301395	1.6537237795994	0.107786460400898	0.759198674348429	-5.41340017559759
a_HQ_transcript/33658	1.77998639914694	0.0379806959299176	1.65370973204444	0.107789334272738	0.759198674348429	-4.66090241342464
a_HQ_transcript/135001	-2.40204915548574	1.30068005940459	-1.65370420698653	0.107790464615544	0.759198674348429	-4.61019362205643
a_HQ_transcript/119566	0.455403442251583	6.5499082247568	1.65368500505419	0.107794393115191	0.759198674348429	-5.25958556216489
a_HQ_transcript/129581	1.54147175385452	-0.56663259151781	1.65367978227347	0.107795461657976	0.759198674348429	-4.64562669273164
a_HQ_transcript/50767	1.36184918680945	1.81904819475219	1.65356336126268	0.107819282824498	0.759198674348429	-4.61717682113301
a_HQ_transcript/150408	1.91114938749075	-0.0274488289200584	1.65352449070207	0.107827237188961	0.759198674348429	-4.64201387418694
a_HQ_transcript/50142	-0.97894853149369	3.21564081078629	-1.65334648600646	0.107863669790835	0.759198674348429	-4.84388097836984
a_HQ_transcript/15573	1.9342346171402	0.26276487502683	1.65329496731897	0.107874216133518	0.759198674348429	-4.60301262331097
a_HQ_transcript/55843	-2.09362153965162	0.282230462100388	-1.65324283802173	0.107884888343046	0.759198674348429	-4.603348668342
a_HQ_transcript/21720	1.54425802812171	0.0505199446118809	1.65324258968503	0.107884939186057	0.759198674348429	-4.52838135475312
a_HQ_transcript/92395	1.46547900134451	1.69867803115664	1.65318887129044	0.107895937644089	0.759198674348429	-4.57165058002944
a_HQ_transcript/3726	-0.902960122671975	4.49197055807217	-1.65297690930529	0.107939344412101	0.759198674348429	-5.07302697279506
a_HQ_transcript/147369	-1.31883287291484	4.41454114967912	-1.65286459305515	0.107962351028702	0.759198674348429	-4.99712241564768
a_HQ_transcript/148733	3.75768149530166	0.444545903682761	1.65272370440784	0.107991216096482	0.759198674348429	-4.68820227470242
a_HQ_transcript/24040	-1.38467480215786	1.28456193635542	-1.65272045797858	0.107991881295683	0.759198674348429	-4.53798034412689
a_HQ_transcript/16691	3.90570720345417	4.36651971013427	1.65268745383298	0.107998644097188	0.759198674348429	-4.98619620713927
a_HQ_transcript/145629	-1.16785148739901	2.23713505349996	-1.65250857048256	0.108035304765701	0.759198674348429	-4.6600778799056
a_HQ_transcript/18226	1.80300141282463	0.0489062594595178	1.65249666203388	0.108037745670031	0.759198674348429	-4.55551824952855
a_HQ_transcript/45195	1.29163304680938	2.70009656460137	1.65246828460435	0.108043562446432	0.759198674348429	-4.71379077596449
a_HQ_transcript/27164	1.85605656600069	0.479078858466831	1.65246316042641	0.10804461282282	0.759198674348429	-4.47011518853549
a_HQ_transcript/118807	0.533143194550905	5.45898488078851	1.65232134635593	0.108073685847202	0.759198674348429	-5.16073404683992
a_HQ_transcript/29481	0.386478507909982	3.98372436798012	1.65231743447832	0.108074487905383	0.759198674348429	-4.99381066998463
a_HQ_transcript/152036	2.37390484805136	1.65419010243262	1.65228571091714	0.108080992417213	0.759198674348429	-4.59536687013601
a_HQ_transcript/69308	-2.55454876178742	1.94545337945872	-1.65215596930631	0.108107597655546	0.759198674348429	-4.72027420461756
a_HQ_transcript/117091	1.61351777330043	-0.01045945127067	1.65193901277554	0.108152099589579	0.759198674348429	-4.59240840190461
a_HQ_transcript/129448	-1.9305968382253	-0.524383855633056	-1.65190054230859	0.108159992201627	0.759198674348429	-4.6605059115695
a_HQ_transcript/21378	1.84049129522889	0.227830781924068	1.65189834515308	0.108160442985082	0.759198674348429	-4.59525639363169
a_HQ_transcript/142296	1.67354085243616	0.455406479975952	1.65189520431492	0.108161087383628	0.759198674348429	-4.54384460367305
a_HQ_transcript/99637	-4.17522502982858	1.70600606662858	-1.65185277614151	0.108169792586042	0.759198674348429	-4.83216332168534
a_HQ_transcript/94167	1.16234364880214	1.44523685146977	1.65184133105719	0.108172140931114	0.759198674348429	-4.50490885381836
a_HQ_transcript/137506	2.49865086078523	0.448313712000682	1.65173638722454	0.108193675666625	0.759198674348429	-4.58786996588109
a_HQ_transcript/134247	-1.84515942701873	0.726347304590119	-1.65172074209425	0.108196886390376	0.759198674348429	-4.62174808968426
a_HQ_transcript/132322	-1.67594371327029	1.51552284913549	-1.65168929404753	0.108203340457776	0.759198674348429	-4.54193855611146
a_HQ_transcript/33065	-1.61753314928221	0.78620616096015	-1.65161219840234	0.108219164108679	0.759198674348429	-4.5151507594987
a_HQ_transcript/71560	2.29751451950271	0.475107275704276	1.65159578912028	0.108222532312158	0.759198674348429	-4.62925244564443
a_HQ_transcript/110696	-1.87562092535482	0.938202034803248	-1.65157762816998	0.108226260167932	0.759198674348429	-4.58134549079694
a_HQ_transcript/76084	1.59946659195648	0.363146619319133	1.65154718796782	0.108232508795512	0.759198674348429	-4.52756475428806
a_HQ_transcript/106690	1.6254413314157	2.08672066647173	1.65153544346281	0.108234919734473	0.759198674348429	-4.67378486408853
a_HQ_transcript/30205	-2.38426181062188	0.664333570669903	-1.65139700178752	0.108263342711846	0.759204156000482	-4.71182132280849
a_HQ_transcript/149257	-2.36438551713485	4.96427310312785	-1.65134914928408	0.108273168579807	0.759204156000482	-5.17339033953057
a_HQ_transcript/54538	-1.85320287916937	-0.128451601008319	-1.65126136877234	0.108291195047172	0.759204156000482	-4.65006442619279
a_HQ_transcript/104100	3.62107164281015	0.456435701686885	1.65124175908403	0.108295222401429	0.759204156000482	-4.63096896785566
a_HQ_transcript/104280	1.50481391202891	-0.22155691368931	1.65121360035443	0.108301005738302	0.759204156000482	-4.69914454322662
a_HQ_transcript/69791	2.02704265446136	0.559752970538294	1.65079694796072	0.108386609136262	0.759580652449006	-4.56732964591333
a_HQ_transcript/129745	-1.91287421669671	0.708647610748624	-1.65078880960614	0.108388281761198	0.759580652449006	-4.53386023636264

a_HQ_transcript/7506	-1.14569970397484	1.84256679201163	-1.65070109231701	0.108406311101775	0.759580652449006	-4.56816883038017
a_HQ_transcript/103359	0.527769450456642	2.81630436886298	1.65058688288615	0.108429789337179	0.759580652449006	-4.78030240946663
a_HQ_transcript/90508	3.06385579273179	0.917549343055956	1.65049007608584	0.108449693379686	0.759580652449006	-4.78399033289039
a_HQ_transcript/72174	2.51064852269801	2.28112351268151	1.65047882783924	0.108452006280928	0.759580652449006	-4.56912900395829
a_HQ_transcript/90802	1.28336940323824	0.425543059653197	1.65046876023476	0.108454076449317	0.759580652449006	-4.47814714502109
a_HQ_transcript/77674	1.48824837008246	1.44951925919289	1.65040173615054	0.108467859225258	0.759580652449006	-4.56805414050834
a_HQ_transcript/87633	2.07674911451816	0.893661389132089	1.65038004717182	0.108472319639059	0.759580652449006	-4.58454426498194
a_HQ_transcript/133425	1.63460131774208	1.64575463650624	1.65012580797027	0.108524616138804	0.759785791534994	-4.63613841488645
a_HQ_transcript/121204	-3.19255381102114	0.107979767719582	-1.65011054457416	0.108527756453259	0.759785791534994	-4.71081735518302
a_HQ_transcript/142305	2.2024052818198	0.203052648117122	1.64984923925582	0.108581529479129	0.7600707063539	-4.65044796219358
a_HQ_transcript/48484	1.86529462037867	1.56851118534453	1.6496228878284	0.108628127304756	0.760259071871325	-4.5667784170385
a_HQ_transcript/79992	1.09503999309367	4.46334985641267	1.64959146758203	0.108634596940601	0.760259071871325	-4.99325125974714
a_HQ_transcript/76190	-1.56429223225214	-0.525562754596542	-1.64938276732497	0.108677577795865	0.760386119536287	-4.62511474362489
a_HQ_transcript/110938	-2.62513838698406	-0.304564597669084	-1.64937628293525	0.108678913451477	0.760386119536287	-4.65038105815893
a_HQ_transcript/32295	-0.657288523403874	2.37745263439506	-1.6492877610726	0.108697148557693	0.760422174914139	-4.70040256378119
a_HQ_transcript/2738	-3.68296882349588	0.251320361278387	-1.64913548655259	0.108728522350895	0.760495306878449	-4.6703238067064
a_HQ_transcript/100867	-1.91944658371434	2.42859206407651	-1.64906671607394	0.108742693893921	0.760495306878449	-4.91845026442362
a_HQ_transcript/123195	1.51452551695261	0.393794069345531	1.64904654123234	0.108746851616668	0.760495306878449	-4.56193711117093
a_HQ_transcript/107932	1.26512744380672	0.418222182586119	1.6488565210803	0.108786018287245	0.760604067616822	-4.60488001986919
a_HQ_transcript/130589	-2.38919819076734	-0.0659157061817447	-1.64876165426396	0.108805576463579	0.760604067616822	-4.57984601237159
a_HQ_transcript/5309	-0.77442383136827	2.78295663669974	-1.64863620787669	0.108831443532953	0.760604067616822	-4.78542250582357
a_HQ_transcript/44711	1.15243349755149	2.47057328569779	1.6486054237643	0.108837792000644	0.760604067616822	-4.63520124416001
a_HQ_transcript/63787	-1.37070800296201	1.84798470332013	-1.64859686335814	0.108839557428799	0.760604067616822	-4.61289214510988
a_HQ_transcript/137232	2.21965049445164	1.49824678633197	1.64856474533315	0.108846181398891	0.760604067616822	-4.5501981056989
a_HQ_transcript/136837	0.691321702171024	2.63438448011752	1.64852684304387	0.108853998735624	0.760604067616822	-4.70768302949248
a_HQ_transcript/137954	0.442139003162243	3.3183498720777	1.64844752004007	0.108870360587292	0.760610729620277	-4.87182976421529
a_HQ_transcript/25794	-1.04316985267341	3.27642348244511	-1.64839535180147	0.108881122370808	0.760610729620277	-4.85794793735415
a_HQ_transcript/76411	-2.4942877056949	2.94612280310109	-1.64832500925898	0.108895634724643	0.760620698783229	-4.67480155406397
a_HQ_transcript/51506	2.23008772855911	2.58388963603593	1.64818134597608	0.108925278837428	0.760657186687199	-4.76352348122582
a_HQ_transcript/127008	-1.78186232435709	1.04644348391115	-1.64813220137757	0.108935421082786	0.760657186687199	-4.70609254126772
a_HQ_transcript/46615	-1.82014241045698	0.281182070311122	-1.64810945191646	0.10894011628081	0.760657186687199	-4.58293245973117
a_HQ_transcript/123850	2.68242196140684	2.19095959110349	1.64795212749292	0.108972590615429	0.760680158468675	-4.74927150225998
a_HQ_transcript/77199	-2.15627853886805	5.40257874885834	-1.64783715625562	0.108996327625961	0.760680158468675	-5.21527936107757
a_HQ_transcript/13314	-1.62131936848386	1.38943826463836	-1.6478154732994	0.109000804777895	0.760680158468675	-4.5686291054937
a_HQ_transcript/149563	-1.22125533534121	-0.736876584238887	-1.64773740549344	0.109016925677446	0.760680158468675	-4.6367985078291
a_HQ_transcript/57724	1.75512133889034	1.47255145709243	1.64773736812153	0.109016933395168	0.760680158468675	-4.58930224787158
a_HQ_transcript/70059	2.01383148438701	0.900827796883273	1.64769636076004	0.109025402152441	0.760680158468675	-4.60568921459441
a_HQ_transcript/64325	-2.85262921056899	0.575617936618292	-1.64764983930531	0.109035010328258	0.760680158468675	-4.65361938775604
a_HQ_transcript/150688	-3.38514376017133	5.08690389849805	-1.64748691609086	0.109068664733525	0.760791511614551	-5.2270119979599
a_HQ_transcript/5145	-1.45628525160456	1.56114573819308	-1.64744585469433	0.109077147980245	0.760791511614551	-4.5679955832499
a_HQ_transcript/112332	-2.77814580934926	2.50027555036109	-1.64713473318645	0.109141443141123	0.761007703651518	-4.72105036661624
a_HQ_transcript/135842	0.440480944887261	3.59230349495471	1.64713324522561	0.10914175071266	0.761007703651518	-4.93299813417212
a_HQ_transcript/57741	2.35517775081347	1.11130072342566	1.64703950070357	0.10916112978335	0.761007703651518	-4.74281561433917
a_HQ_transcript/84924	2.01684193099431	1.9494249348005	1.64698844372394	0.109171685590398	0.761007703651518	-4.62941188675127
a_HQ_transcript/60334	1.55161486957132	1.4790313041733	1.64695018624263	0.109179595710741	0.761007703651518	-4.5886685825161
a_HQ_transcript/105174	1.90887139388195	-0.109478333060954	1.64691584886545	0.109186695713833	0.761007703651518	-4.68643300331241
a_HQ_transcript/125965	0.839016303835805	5.37227999798184	1.64678386615508	0.109213989579884	0.761042392791189	-5.1936524689018
a_HQ_transcript/86491	-1.15081802901491	3.59615407779106	-1.64676516292326	0.109217857842418	0.761042392791189	-4.87685069961139
a_HQ_transcript/23319	-1.66770625540747	0.992604459537876	-1.64667782824863	0.109235922181242	0.761077032788112	-4.5011024517629
a_HQ_transcript/62014	0.495993206991739	4.69499385967626	1.64653341113351	0.109265798898611	0.761193954743347	-5.0738414418128

a_HQ_transcript/35870	-0.468938463345224	3.41703278661084	-1.64644707965531	0.109283662206901	0.761227167976361	-4.90458897282594
a_HQ_transcript/11558	1.44222949729428	-0.197490989039548	1.64617757721096	0.109339441945712	0.761362475605971	-4.56632112509031
a_HQ_transcript/140887	-2.15642922223499	0.884079399073147	-1.64589435048091	0.109398087624158	0.761362475605971	-4.58839469268923
a_HQ_transcript/148249	1.41337859021522	-0.621056317250128	1.64586327272834	0.109404524250174	0.761362475605971	-4.65491484684591
a_HQ_transcript/110103	-1.77181952212653	-0.379917316601088	-1.64580610073855	0.109416366166607	0.761362475605971	-4.59757626416266
a_HQ_transcript/44818	2.46014284256209	0.586884417479297	1.64562516292871	0.109453850430779	0.761362475605971	-4.67235136072312
a_HQ_transcript/85630	-1.41009218627039	-0.621895080148256	-1.64561002243473	0.109456987516475	0.761362475605971	-4.61578852511007
a_HQ_transcript/119509	-1.31336713347171	-0.407983484253209	-1.64549518335823	0.109480784407842	0.761362475605971	-4.61357257812805
a_HQ_transcript/127294	2.68036803009817	1.34172464869771	1.64539717982762	0.109501096031	0.761362475605971	-4.64411577023176
a_HQ_transcript/111233	1.08767823431439	2.54138772690801	1.64534782889226	0.109511325389505	0.761362475605971	-4.68707923844521
a_HQ_transcript/128711	-1.89909582681413	1.55683239172644	-1.64533030429032	0.109514958042471	0.761362475605971	-4.58265098859883
a_HQ_transcript/52592	-2.38534218210695	0.70150852556652	-1.64523381699322	0.1095349605597	0.761362475605971	-4.67668763853584
a_HQ_transcript/123401	2.90081890817605	0.888477258759986	1.64521945959762	0.109537937210332	0.761362475605971	-4.60151353584787
a_HQ_transcript/53024	-3.08986788150214	2.04352596227901	-1.6451980182557	0.109542382666485	0.761362475605971	-4.7690410411493
a_HQ_transcript/108401	-0.793582674122848	4.92203019411813	-1.6451699954458	0.109548192890349	0.761362475605971	-5.12590955310329
a_HQ_transcript/105046	-3.12240449353995	3.62803092021751	-1.64508862196686	0.109565066238218	0.761362475605971	-4.9842902535488
a_HQ_transcript/63382	-1.23611000859714	-0.528935620528106	-1.64507753477868	0.10956736540898	0.761362475605971	-4.62456422523792
a_HQ_transcript/133575	0.999115171621079	-0.831690143033333	1.64507230789544	0.109568449331286	0.761362475605971	-4.65489184394806
a_HQ_transcript/122548	-1.67293731832858	-0.559617816848079	-1.64506830494395	0.109569279447353	0.761362475605971	-4.64376942248755
a_HQ_transcript/39210	-2.23036144794357	1.57286623656718	-1.64499489731778	0.10958450334968	0.761362475605971	-4.61943232229106
a_HQ_transcript/131399	-2.09625247791453	0.733885144710999	-1.64498245937222	0.109587083010709	0.761362475605971	-4.60893103670005
a_HQ_transcript/11947	2.07260455780918	0.642359619106926	1.64498143026408	0.109587296452561	0.761362475605971	-4.56529510024002
a_HQ_transcript/39463	2.82308864899924	2.51957073207367	1.64493414833074	0.109597103318041	0.761362475605971	-4.71239623200542
a_HQ_transcript/77774	1.57972210879456	1.18406488231071	1.64489925031011	0.109604342070444	0.761362475605971	-4.51142111421515
a_HQ_transcript/69148	-6.21322759166621	3.07459484186622	-1.64469116041105	0.109647513509272	0.761367619198776	-4.82733869296499
a_HQ_transcript/142935	-3.80683509865509	2.63495873363528	-1.64460927184872	0.109664506402569	0.761367619198776	-4.85478959015356
a_HQ_transcript/983	0.492980507378337	3.88283353933193	1.64460476162286	0.109665442393713	0.761367619198776	-4.94767195438775
a_HQ_transcript/21730	2.19333605911744	1.1574201879396	1.64458055123045	0.109670466803332	0.761367619198776	-4.65968366662236
a_HQ_transcript/34707	2.98903867253597	1.00223455602859	1.64455563975927	0.109675636907351	0.761367619198776	-4.74105779814957
a_HQ_transcript/132717	2.40495457607468	0.518356648580056	1.64445442107004	0.109696645815407	0.761367619198776	-4.71251182854801
a_HQ_transcript/65149	-2.07987187690491	-0.0918669813252346	-1.64434746181095	0.109718849850525	0.761367619198776	-4.58873770035165
a_HQ_transcript/15050	1.40473081219635	2.90370986586982	1.6442759179232	0.109733703963279	0.761367619198776	-4.94727749947617
a_HQ_transcript/109294	2.20540291777854	0.9311351235508	1.64426415190772	0.109736147010745	0.761367619198776	-4.72348613938981
a_HQ_transcript/110339	2.25927530432459	0.572365351610103	1.64413369206181	0.109763238175702	0.761367619198776	-4.56423130649513
a_HQ_transcript/69771	-1.62655190233435	-0.44137897848395	-1.64405022942217	0.10978057284752	0.761367619198776	-4.58612504577058
a_HQ_transcript/44041	1.96461934251795	0.607989747359976	1.6440013714362	0.109790721398268	0.761367619198776	-4.51722881144318
a_HQ_transcript/141218	-0.524306695510798	3.60940193035952	-1.64399140419028	0.109792791842931	0.761367619198776	-4.83190911672324
a_HQ_transcript/113520	-2.50680809187665	0.0668429041895906	-1.6439856919286	0.109793978436188	0.761367619198776	-4.64407418139073
a_HQ_transcript/134506	-1.0497049282562	-0.813357642183609	-1.64394922263158	0.10980155435837	0.761367619198776	-4.64277638497178
a_HQ_transcript/62892	-1.04959329135297	-0.29042517736247	-1.64352193341815	0.109890349173036	0.761729721359721	-4.6573861034773
a_HQ_transcript/58891	1.54356856593104	0.220121989189751	1.64351981052492	0.109890790478897	0.761729721359721	-4.48651004843746
a_HQ_transcript/130790	0.964856085908114	4.57630383525926	1.64347546046121	0.109900010280494	0.761729721359721	-5.0451160158872
a_HQ_transcript/126997	-1.63888703023116	0.829641457552925	-1.64332995466533	0.109930263535569	0.761729721359721	-4.64475873380712
a_HQ_transcript/30402	-1.3579004958722	-0.391159967658652	-1.64326021571709	0.109944765950187	0.761729721359721	-4.64172883847227
a_HQ_transcript/84814	-2.11728172446395	1.2760568217997	-1.64318740427832	0.109959908986092	0.761729721359721	-4.55407464892312
a_HQ_transcript/38433	-1.5310149452638	-0.361628419598989	-1.64316581134883	0.109964400129965	0.761729721359721	-4.64105506739934
a_HQ_transcript/108975	1.92247589709312	0.1659133313992	1.64313237085511	0.109971355764776	0.761729721359721	-4.56461650700213
a_HQ_transcript/85524	2.30060198584372	1.57618785171169	1.64313064611815	0.109971714520554	0.761729721359721	-4.76245802996396
a_HQ_transcript/120357	-4.71181845754425	2.57180253920386	-1.64301656953738	0.109995445292035	0.761803317518812	-4.77881859892889
a_HQ_transcript/132963	2.30391650794474	0.0283994481375807	1.64264039369967	0.110073729244224	0.762033614502796	-4.63233070436817

a_HQ_transcript/34879	-0.8457466338735	1.75600197479659	-1.64263770609364	0.110074288713305	0.762033614502796	-4.53512323874617
a_HQ_transcript/126844	0.991436367405959	2.96736884105764	1.64256319286941	0.110089800793826	0.762033614502796	-4.77248024786523
a_HQ_transcript/9870	1.49804428293209	-0.414292049975016	1.64247985127146	0.110107152896608	0.762033614502796	-4.65558939034637
a_HQ_transcript/37441	1.70973736634466	-0.575201912404794	1.642405450117	0.110122645468364	0.762033614502796	-4.66146692658416
a_HQ_transcript/47481	-1.93012598654633	-0.391551257287422	-1.64236962012681	0.110130106999459	0.762033614502796	-4.6414264775152
a_HQ_transcript/44803	3.39775818855859	2.37422458857298	1.64228002278481	0.110148767314427	0.762033614502796	-4.76629500725118
a_HQ_transcript/5602	1.20945057207457	2.00646363741168	1.64223989919803	0.110157124645919	0.762033614502796	-4.67381488486824
a_HQ_transcript/95226	-1.48154584949404	4.03082695606475	-1.64223057647226	0.110159066549104	0.762033614502796	-5.04052648629941
a_HQ_transcript/136659	-0.766382332134738	3.15011944359441	-1.64219270183622	0.110166956043174	0.762033614502796	-4.81945390267476
a_HQ_transcript/146745	-4.35918242532789	4.30839768230067	-1.64208560650613	0.110189267114749	0.762033614502796	-5.05507675251876
a_HQ_transcript/68612	1.83923905451819	0.706540993944365	1.64201601701624	0.110203766631265	0.762033614502796	-4.56232489525695
a_HQ_transcript/115389	2.35603775560694	2.38873772955959	1.64198628673854	0.110209961648867	0.762033614502796	-4.65955548297349
a_HQ_transcript/9555	-1.20195432132623	2.42082724554466	-1.64179949923382	0.110248889899509	0.762033614502796	-4.67336943199506
a_HQ_transcript/1287	2.19580422515276	1.99025346970629	1.64174570949074	0.110260102289237	0.762033614502796	-4.62178458496974
a_HQ_transcript/17622	-1.51841395298216	1.43135315160908	-1.64167629366791	0.110274573304557	0.762033614502796	-4.54395235483894
a_HQ_transcript/108638	-2.30160595531525	0.847472220337037	-1.6416697005656	0.110275947840613	0.762033614502796	-4.72839968975472
a_HQ_transcript/132713	1.34625995755279	1.57676470723937	1.64163455767124	0.11028327470262	0.762033614502796	-4.67765876760221
a_HQ_transcript/125414	1.3413815419962	1.43533764766607	1.64159424665765	0.110291679549555	0.762033614502796	-4.59858053102163
a_HQ_transcript/54454	-1.0781585736184	2.60659893877085	-1.64154167809402	0.110302640891326	0.762033614502796	-4.74412365265326
a_HQ_transcript/53054	-1.51301445951123	-0.101379060047665	-1.64152532462389	0.110306051021194	0.762033614502796	-4.63803646684512
a_HQ_transcript/104717	-2.95341630309199	4.31561951407472	-1.64126038733679	0.110361309577104	0.762033614502796	-5.0395799496826
a_HQ_transcript/71307	0.599319069543629	2.95178750714037	1.64125440031845	0.110362558567089	0.762033614502796	-4.78949127149513
a_HQ_transcript/12990	2.07379760876503	-0.474332109955739	1.64123857184766	0.110365860701362	0.762033614502796	-4.64451586989293
a_HQ_transcript/34983	2.00170976009168	-0.451124762818234	1.64123288754618	0.110367046579799	0.762033614502796	-4.65034357923338
a_HQ_transcript/148784	-2.4350172152716	2.15305075904021	-1.64116089134776	0.110382067584746	0.762033614502796	-4.72867626626327
a_HQ_transcript/37555	0.594772463583349	4.51554789972369	1.64114552897172	0.110385272949475	0.762033614502796	-5.01453021048687
a_HQ_transcript/121369	0.729551038322639	4.34622205638872	1.6410466987648	0.110405895743689	0.762033614502796	-5.02300492851717
a_HQ_transcript/117985	0.874212518115268	1.28029945729915	1.64085216115398	0.110446499003821	0.762033614502796	-4.55620714664312
a_HQ_transcript/55348	-1.34060689978418	-0.554658615926436	-1.64074279349343	0.110469331286853	0.762033614502796	-4.63037929389928
a_HQ_transcript/3632	0.850804950645213	3.34765402495452	1.64056564696719	0.110506321790231	0.762033614502796	-4.92309417450009
a_HQ_transcript/136051	4.89474581937524	3.16678876730338	1.64052604845568	0.11051459187526	0.762033614502796	-4.83906316326604
a_HQ_transcript/44496	-1.41822307931992	0.610396028415389	-1.64040679692175	0.110539500454841	0.762033614502796	-4.52107960225571
a_HQ_transcript/102616	-2.2210868684523	-0.210931557999157	-1.64038493312527	0.110544067743094	0.762033614502796	-4.66219929943516
a_HQ_transcript/105546	-1.78568655019656	3.73603209012519	-1.64032399206602	0.110556798989578	0.762033614502796	-4.96201589746665
a_HQ_transcript/125773	3.10407142020857	4.37991959821108	1.6402887573082	0.110564160464446	0.762033614502796	-4.86842787999493
a_HQ_transcript/126021	-1.37950231039053	1.68528345255087	-1.64012793796607	0.110597765031835	0.762033614502796	-4.60033511384464
a_HQ_transcript/21399	1.4467347563848	1.57516870234136	1.64002652128973	0.110618961247145	0.762033614502796	-4.5466041981573
a_HQ_transcript/106936	2.50370987377292	-0.22248214195091	1.64001272656993	0.110621844620606	0.762033614502796	-4.63485016781416
a_HQ_transcript/123298	-6.83858573354768	1.70737183466765	-1.64000646375656	0.110623153694869	0.762033614502796	-4.58063451400471
a_HQ_transcript/137734	-2.80647507857739	0.39976804579679	-1.63993776777849	0.110637513599037	0.762033614502796	-4.69355797535798
a_HQ_transcript/117352	-1.29612994933536	2.9519127873224	-1.63988861866124	0.110647788458925	0.762033614502796	-4.85810229229555
a_HQ_transcript/144027	-1.67738944519742	1.39821763396041	-1.63982440401023	0.110661214029388	0.762033614502796	-4.52791304454341
a_HQ_transcript/22140	1.631515539899	0.916919509042779	1.63979882150887	0.110666563022911	0.762033614502796	-4.66823876248259
a_HQ_transcript/98152	1.9606487905142	0.165048615549628	1.63976878416651	0.110672843742835	0.762033614502796	-4.48280223043979
a_HQ_transcript/109088	-4.42647230445041	4.30672559587539	-1.63970527952336	0.110686123346568	0.762033614502796	-5.01683549056643
a_HQ_transcript/3597	-1.3978830600346	-0.513169065155766	-1.63970298537464	0.110686603105986	0.762033614502796	-4.64010804817647
a_HQ_transcript/105832	3.41471933636048	2.18271199925392	1.63958085715692	0.110712145414272	0.762033614502796	-4.83676498887028
a_HQ_transcript/96906	-0.573642275993805	5.08197180088316	-1.63950412190048	0.110728196576532	0.762033614502796	-5.11970243525154
a_HQ_transcript/97066	1.7961224735223	1.6493733380465	1.63939565329775	0.110750888868457	0.762033614502796	-4.56018846328499
a_HQ_transcript/25316	1.15368449988401	1.77784312823036	1.63930318153302	0.110770237558725	0.762033614502796	-4.5878218268079

a_HQ_transcript/938	-1.72584316782608	1.16752131293791	-1.63924752014501	0.110781885432818	0.762033614502796	-4.51277951964255
a_HQ_transcript/120523	0.766593099905506	5.06478067641254	1.63923657726861	0.11078417549199	0.762033614502796	-5.11998090457978
a_HQ_transcript/124102	0.498050034092125	3.97328338122488	1.63920919737884	0.11078990556155	0.762033614502796	-5.00741234351228
a_HQ_transcript/148081	0.41349175110697	4.33127434924824	1.63919986971984	0.11079185771207	0.762033614502796	-5.05251418893403
a_HQ_transcript/54392	1.64225475347698	-0.459881880589733	1.63919550307185	0.1107927716011	0.762033614502796	-4.64661001642073
a_HQ_transcript/82343	-1.97869275815941	0.0201470063115376	-1.63915951876053	0.110800302939338	0.762033614502796	-4.65075857380326
a_HQ_transcript/136423	2.89347149156081	0.221809154395759	1.63910375493712	0.110811974867217	0.762033614502796	-4.66780075243783
a_HQ_transcript/151573	0.76816568634208	4.91714187238186	1.63910167041368	0.11081241119859	0.762033614502796	-5.1029058681461
a_HQ_transcript/69490	1.61602209846446	-0.467544233884666	1.63906859268828	0.110819335201021	0.762033614502796	-4.59654817760134
a_HQ_transcript/103648	-0.4432337703027	5.13925821941938	-1.63891406761638	0.110851685932663	0.762033614502796	-5.10582513768765
a_HQ_transcript/100645	-1.19792653156075	3.48176078534753	-1.63873637779774	0.110888895984578	0.762033614502796	-4.89764508268943
a_HQ_transcript/123814	1.47572313396203	-0.497273506883342	1.63867739013233	0.110901250881804	0.762033614502796	-4.64118954487509
a_HQ_transcript/87429	-1.39696716328443	-0.353403415047638	-1.63867242979778	0.110902289869883	0.762033614502796	-4.68677719503929
a_HQ_transcript/122432	-1.61847577474773	0.928063124391518	-1.63856640348466	0.110924499987691	0.762033614502796	-4.56953069869477
a_HQ_transcript/148305	-2.67466434970366	2.2819058062231	-1.63855953282269	0.110925939362957	0.762033614502796	-4.89979777576744
a_HQ_transcript/96181	-2.27101646545173	0.9682447073559	-1.63853851568786	0.110930342461574	0.762033614502796	-4.67476981832557
a_HQ_transcript/119848	1.49007222068981	3.04273952491753	1.63847839612498	0.110942938961204	0.762033614502796	-4.69023011761084
a_HQ_transcript/25016	1.02345749495695	1.64183212127804	1.63845762965306	0.110947289467205	0.762033614502796	-4.53422652416542
a_HQ_transcript/145627	2.22550841007569	0.261568126923952	1.63843117517188	0.110952832596937	0.762033614502796	-4.70618150352651
a_HQ_transcript/93646	-2.8908607996843	1.13218882991425	-1.63839946352427	0.110959477586562	0.762033614502796	-4.64699671476772
a_HQ_transcript/120739	1.20171248405297	-0.57135518867087	1.63822408094895	0.110996233913282	0.762195993492175	-4.66742442118855
a_HQ_transcript/482	2.63829046058644	0.20333755886742	1.6380890851575	0.111024532909534	0.762300265646752	-4.62874802994722
a_HQ_transcript/22679	1.76658098137607	0.804673874527323	1.63779268624632	0.111086687546118	0.762546998928592	-4.50901711286329
a_HQ_transcript/18857	-3.3683573364586	1.83291283206496	-1.63779260314031	0.111086704977417	0.762546998928592	-4.88184462268435
a_HQ_transcript/89190	3.5778347220523	3.17360929763562	1.63772271035477	0.11110136563294	0.762547313500611	-4.84156747679231
a_HQ_transcript/85959	0.875074470969395	1.57620209130337	1.63763022946117	0.111120766808601	0.762547313500611	-4.57860753264636
a_HQ_transcript/80568	-1.43247042220203	1.90484129788517	-1.63760478086359	0.111126106053256	0.762547313500611	-4.55089032284111
a_HQ_transcript/97206	0.686414643923222	3.64686669737376	1.63733119828029	0.111183518450039	0.762761565570196	-4.91407003128276
a_HQ_transcript/95383	3.20128856500392	3.21726854770812	1.63733093726782	0.111183573236242	0.762761565570196	-4.99047385548524
a_HQ_transcript/133919	-2.1038964588285	1.64509547573746	-1.63689664226924	0.111274762124285	0.76322836407355	-4.85827046878521
a_HQ_transcript/10676	-2.51811244078612	1.60370122646551	-1.63687867062384	0.111278536959843	0.76322836407355	-4.62323334358192
a_HQ_transcript/96615	1.46500636287497	1.23345486043854	1.63681930884505	0.111291006294512	0.76322836407355	-4.57598726512352
a_HQ_transcript/78079	1.15880288442412	0.373892626201289	1.63671384270638	0.1113131630016	0.763290260582402	-4.57899220481752
a_HQ_transcript/28544	3.18317743155147	1.83998870707641	1.63646960724894	0.111364486854752	0.763496553857876	-4.69543196492767
a_HQ_transcript/120731	-2.2197407499499	-0.0511679480863851	-1.63644567556485	0.111369516930927	0.763496553857876	-4.61830505706392
a_HQ_transcript/124555	1.52680360245623	3.51189031471341	1.63627271609881	0.111405875959587	0.763509789897607	-4.72851524951697
a_HQ_transcript/120007	1.6966900643989	2.13207669053902	1.63620036913383	0.111421087432492	0.763509789897607	-4.65865443243124
a_HQ_transcript/108491	-0.563690586849011	2.80069271015983	-1.63614693317627	0.111432323830457	0.763509789897607	-4.77216175210893
a_HQ_transcript/113673	1.30939600268651	0.494014481425055	1.6360421365703	0.111454362951889	0.763509789897607	-4.55762807347189
a_HQ_transcript/116532	-0.598481128714999	5.00182526181631	-1.63600502778728	0.11146216793179	0.763509789897607	-5.1582819089693
a_HQ_transcript/38281	1.59638400108966	1.75840872077777	1.63596544233108	0.111470494320508	0.763509789897607	-4.67780394897771
a_HQ_transcript/134209	1.11618748726407	-0.796632002398661	1.63592279294217	0.111479465750353	0.763509789897607	-4.66918784794527
a_HQ_transcript/89526	0.392469065403331	3.38005101848409	1.63590807344523	0.1114825621801	0.763509789897607	-4.89661083548647
a_HQ_transcript/116494	-1.95786290763068	1.68811570325346	-1.63569313814822	0.111527784590569	0.763509789897607	-4.58598809523271
a_HQ_transcript/42819	0.812238607847009	2.82165848916272	1.63568933146983	0.111528585652615	0.763509789897607	-4.73501339960683
a_HQ_transcript/2289	0.422503876487239	4.77631462523066	1.63566557439924	0.111533585102073	0.763509789897607	-5.1080338418124
a_HQ_transcript/33052	1.21206068372985	4.7735117873631	1.63562822853252	0.111541444558461	0.763509789897607	-5.01679182447984
a_HQ_transcript/15177	3.44927711953196	1.58620766459852	1.63562462803624	0.111542202308878	0.763509789897607	-4.70026227687985
a_HQ_transcript/42798	-0.785804902805818	2.71963249005147	-1.63550249078422	0.111567909492442	0.763555612389625	-4.85279976346005
a_HQ_transcript/147116	2.06572796796764	0.70837897992849	1.63546800863621	0.111575168105517	0.763555612389625	-4.68494610326651

a_HQ_transcript/123166	3.03763027786024	2.7266340018366	1.63508195844614	0.111656459646652	0.763897896372661	-4.8671933502858
a_HQ_transcript/115065	1.59663134782651	5.42156014611335	1.63506626823179	0.11165976460762	0.763897896372661	-5.19161086978132
a_HQ_transcript/33534	0.599108051157483	4.39029894169361	1.63504326756255	0.11166460957677	0.763897896372661	-5.07297674368986
a_HQ_transcript/112330	1.72516339430474	-0.266364024036727	1.63450770517566	0.111777472036582	0.764265794106985	-4.60565493540453
a_HQ_transcript/45565	-0.496566432101666	2.85057089276291	-1.63450730599487	0.111777556193591	0.764265794106985	-4.83368783384191
a_HQ_transcript/114425	1.21716035119175	-0.642599955783326	1.63438611247121	0.111803109151511	0.764265794106985	-4.67273844120403
a_HQ_transcript/75351	1.97572048555698	1.96191892366013	1.63435738779404	0.111809166291462	0.764265794106985	-4.80113697003615
a_HQ_transcript/14703	-2.1140380629322	1.25413597678649	-1.63432722017279	0.11181552799503	0.764265794106985	-4.68551038234396
a_HQ_transcript/99677	3.32154003825662	0.994505980795317	1.63428905665158	0.111823576290303	0.764265794106985	-4.65440988130282
a_HQ_transcript/122507	1.90512319155005	1.09637678250901	1.63427535848049	0.11182646521074	0.764265794106985	-4.59049004120667
a_HQ_transcript/9564	0.894025735437291	3.26040242262755	1.634223803637	0.111837338589773	0.764265794106985	-4.83210158434814
a_HQ_transcript/71948	2.59619537447872	1.21917460031699	1.63418352728379	0.111845833841244	0.764265794106985	-4.69960748130993
a_HQ_transcript/118580	1.70841595513629	1.51918556916448	1.63416440340441	0.111849867713806	0.764265794106985	-4.58269789720848
a_HQ_transcript/124565	-2.80507329393266	2.24261327626163	-1.6340950544861	0.111864496752112	0.764275912929801	-4.87732230920713
a_HQ_transcript/81954	1.09466182155092	3.62984566729178	1.63394716170395	0.111895699762695	0.764382640214579	-4.91980514735742
a_HQ_transcript/40023	0.67407629263648	4.12768618547745	1.63389636815135	0.111906418046829	0.764382640214579	-5.04110382476263
a_HQ_transcript/128470	1.35265448833331	0.0671701858262116	1.63378985872233	0.111928896058909	0.764446348268392	-4.54386868641178
a_HQ_transcript/136371	-1.87105348967289	0.223192038062046	-1.63345102700677	0.11200042872493	0.764460321796158	-4.59293038202349
a_HQ_transcript/142632	0.816919672736202	5.88060472575014	1.63339877400148	0.112011463507645	0.764460321796158	-5.19689884855289
a_HQ_transcript/13828	0.716740712314797	2.90103193568752	1.63336292154648	0.112019035343372	0.764460321796158	-4.81957277346272
a_HQ_transcript/132073	-2.6539843436778	2.54635227706611	-1.63335927455209	0.112019805591631	0.764460321796158	-4.79963261223327
a_HQ_transcript/10398	1.7238159645186	-0.561872004504613	1.63327539919435	0.112037521343547	0.764460321796158	-4.68012240760639
a_HQ_transcript/35792	-1.64467928810992	0.959677974532264	-1.63326643475417	0.112039414906496	0.764460321796158	-4.66665751321174
a_HQ_transcript/75373	3.35389590143645	2.11828291063244	1.63325550273997	0.112041724116784	0.764460321796158	-4.63067484753991
a_HQ_transcript/147139	2.10383875788941	1.90965001127253	1.63322100687167	0.112049011065731	0.764460321796158	-4.69976332001613
a_HQ_transcript/27641	1.23442791331257	-0.579209971109092	1.63321962028002	0.112049303979206	0.764460321796158	-4.60473911836594
a_HQ_transcript/69417	-1.2677137239517	1.46450386601607	-1.6330862013644	0.112077491295144	0.764469699118746	-4.70852975626936
a_HQ_transcript/140413	-1.66995353927249	4.99767059384956	-1.63306756568982	0.112081428906122	0.764469699118746	-5.12681364409304
a_HQ_transcript/146921	1.84581207812539	0.763895913736801	1.63297395628279	0.112101209763196	0.764469699118746	-4.5620954024448
a_HQ_transcript/140897	2.34842799957369	3.69661287267889	1.63296207609942	0.1121037204029	0.764469699118746	-4.91257359885555
a_HQ_transcript/80789	1.8244366471821	-0.124956542361847	1.63288008983588	0.11212104782894	0.764469699118746	-4.60525332316677
a_HQ_transcript/117236	2.06962911442597	1.2458230582353	1.63280173538247	0.112137609753825	0.764469699118746	-4.66169597657753
a_HQ_transcript/50611	1.95338763534491	3.4650987953194	1.63273073392425	0.112152619206174	0.764469699118746	-4.94887642945177
a_HQ_transcript/53090	3.63022278345432	2.81107383220689	1.63269263789275	0.112160673253314	0.764469699118746	-4.8582754340204
a_HQ_transcript/19218	-0.999772551131548	2.35839203805088	-1.63265305648917	0.112169041834981	0.764469699118746	-4.6452342254153
a_HQ_transcript/136995	4.42047245772051	1.57253449322919	1.63258365399531	0.112183716647338	0.764480079868286	-4.58394953820431
a_HQ_transcript/128995	1.47596039626014	-0.24165271016744	1.63225606311979	0.112253005499301	0.764862583948347	-4.73121619430885
a_HQ_transcript/10787	1.84251454039712	1.81470224607429	1.63215539686354	0.11227430454342	0.764918046706504	-4.71083519437152
a_HQ_transcript/120644	-1.50131551669798	4.00752278685989	-1.63207206167528	0.112291939189648	0.764948534069593	-5.09654271234865
a_HQ_transcript/72701	-1.04376938394367	2.3846571830205	-1.6320056864716	0.112305986550891	0.76495458005978	-4.79758003344948
a_HQ_transcript/37450	-1.77130361222266	1.2369560440637	-1.63189960851148	0.112328439442234	0.764980172802686	-4.55110453023087
a_HQ_transcript/56805	2.93607258571231	2.50509107211108	1.63186358833165	0.112336064461941	0.764980172802686	-4.76016352415092
a_HQ_transcript/119270	1.81282031305256	3.33478775824552	1.63179645546434	0.112350276786567	0.76498733618948	-4.88745537659541
a_HQ_transcript/140377	1.89950940789061	0.00636234145151747	1.63166755727784	0.112377569261129	0.765007702651968	-4.5974144980952
a_HQ_transcript/115698	2.41452111169738	1.58598422411199	1.63162878189454	0.112385780502413	0.765007702651968	-4.64453043557082
a_HQ_transcript/140916	2.08068683849655	1.14991905290724	1.63158618941919	0.112394800637976	0.765007702651968	-4.68905389458921
a_HQ_transcript/49709	-0.59179736399512	2.50473754727364	-1.63153373141897	0.112405910892349	0.765007702651968	-4.78680866376627
a_HQ_transcript/132099	-3.46403761192201	0.0592994438179858	-1.63142216220133	0.11242954352104	0.765016055257893	-4.7064343037924
a_HQ_transcript/110567	-0.96587788462148	1.28622606130589	-1.6314036746715	0.112433459951627	0.765016055257893	-4.55679417901199
a_HQ_transcript/7670	-0.641528767983156	3.19435496932474	-1.63128287599456	0.112459052924741	0.765093356773836	-4.91484159403981

a_HQ_transcript/6562	-1.40140966305771	3.39285305109511	-1.6311713057758	0.112482694982836	0.765093356773836	-4.99358388882219
a_HQ_transcript/35416	0.827775911770589	1.95572456389945	1.6311571649915	0.112485691749458	0.765093356773836	-4.60358093432614
a_HQ_transcript/71527	-0.78308293692472	3.81986474097417	-1.63107440717021	0.112503231409781	0.765093356773836	-4.94942162512851
a_HQ_transcript/18458	1.62688594313083	1.32278676269424	1.63101940952016	0.112514888838146	0.765093356773836	-4.5977298537205
a_HQ_transcript/77413	1.46727959749111	0.160834034396524	1.63097739897033	0.112523794162186	0.765093356773836	-4.73785005255996
a_HQ_transcript/32984	-1.29848994589565	-0.65872826560397	-1.63078670837719	0.11256422373596	0.76517069810278	-4.66042226557155
a_HQ_transcript/23163	0.6154103173698	3.21203015253365	1.63066165698048	0.11259074321491	0.76517069810278	-4.88323500021186
a_HQ_transcript/73308	1.45325132235518	0.00384495877608024	1.63056702321138	0.112610815494858	0.76517069810278	-4.7092707586557
a_HQ_transcript/48970	1.88879008952754	0.535608757216423	1.63036919996842	0.112652784295877	0.76517069810278	-4.68364381208996
a_HQ_transcript/19429	-0.557046033694398	3.50386143914028	-1.63029454133489	0.112668626704507	0.76517069810278	-4.95624703236823
a_HQ_transcript/131628	2.03741037995567	2.5610517364115	1.63021771475216	0.112684931065997	0.76517069810278	-4.74554252720418
a_HQ_transcript/134823	-1.98093444216049	0.89087530402209	-1.63013620234067	0.112702231995507	0.76517069810278	-4.67349786439408
a_HQ_transcript/86438	0.835893860811496	3.34715903957864	1.63003301186279	0.112724137215807	0.76517069810278	-4.87821026895222
a_HQ_transcript/149640	2.34734251492001	0.579536640978728	1.62995714077806	0.112740245329829	0.76517069810278	-4.71116458968304
a_HQ_transcript/105334	0.970522278540738	0.709553808988061	1.62989007611963	0.11275448534105	0.76517069810278	-4.64577921065321
a_HQ_transcript/107321	0.618717984674132	2.71849959622839	1.62979848925197	0.112773934612254	0.76517069810278	-4.79842228897892
a_HQ_transcript/97390	-1.53707681068046	-0.312785603082406	-1.62965119633317	0.112805219353148	0.76517069810278	-4.64466031514923
a_HQ_transcript/46145	-2.58890891277774	0.501954420166889	-1.62962214126669	0.112811391440174	0.76517069810278	-4.72000681538406
a_HQ_transcript/149441	5.33684119189927	6.56428082655718	1.62956540880977	0.112823443761193	0.76517069810278	-5.13577263790283
a_HQ_transcript/49849	1.09489171515083	2.34607161294268	1.62946552376641	0.112844666057696	0.76517069810278	-4.74015331384836
a_HQ_transcript/92954	1.59544067868732	-0.182802675439664	1.62938075375508	0.112862679488073	0.76517069810278	-4.64816119134068
a_HQ_transcript/102103	-2.99035665918718	3.06081126777834	-1.62934586429997	0.112870094105035	0.76517069810278	-4.90322655821882
a_HQ_transcript/124528	1.5721371169321	1.80944660808959	1.62933823570458	0.11287171536796	0.76517069810278	-4.59245192022547
a_HQ_transcript/144869	-3.07833268641965	-0.210840703713859	-1.62933655356347	0.112872072866634	0.76517069810278	-4.74783825332079
a_HQ_transcript/144081	-2.06254937415489	0.374412716799744	-1.6293202551454	0.112875536752125	0.76517069810278	-4.63799720023275
a_HQ_transcript/72775	-2.27197185175011	0.842259594587394	-1.62931435529968	0.112876790661683	0.76517069810278	-4.59018349008537
a_HQ_transcript/126922	-0.827622330009275	2.25836891567585	-1.62930785416092	0.112878172378918	0.76517069810278	-4.67176521082518
a_HQ_transcript/3433	-1.65643276242489	0.983034842845861	-1.62929468281677	0.112880971788663	0.76517069810278	-4.61039288459621
a_HQ_transcript/27358	-2.10202666900787	1.05925158717743	-1.62927161610128	0.112885874477729	0.76517069810278	-4.78344074848584
a_HQ_transcript/151788	0.231608789399139	8.90797770622627	1.62923350660805	0.112893974801778	0.76517069810278	-5.50205613812313
a_HQ_transcript/19425	1.3662875765891	0.325008989154227	1.62920377369363	0.112900294983634	0.76517069810278	-4.56143633854703
a_HQ_transcript/10937	-4.47552783885426	0.4754738430208	-1.62918663677314	0.112903937828519	0.76517069810278	-4.67352696987282
a_HQ_transcript/144499	-1.58809953397629	2.75252324850099	-1.62918571708781	0.112904133331436	0.76517069810278	-4.97718724645852
a_HQ_transcript/147971	6.07863176511331	6.02938745199064	1.62912560749475	0.1129169117865	0.76517069810278	-4.93216523735946
a_HQ_transcript/105947	-1.76260956400961	-0.523607688534183	-1.62891305941621	0.112962106087438	0.765387726151134	-4.61143235804154
a_HQ_transcript/124799	0.441668109633673	3.14077271894622	1.62882782295942	0.112980234186571	0.765406908727315	-4.88947938060587
a_HQ_transcript/14314	-1.92880686927802	1.57538818480263	-1.62877592780058	0.112991272429622	0.765406908727315	-4.6385279698365
a_HQ_transcript/41483	-0.491410461891395	3.4885715282085	-1.6285638327112	0.11303639488349	0.765623346747557	-4.96265632863884
a_HQ_transcript/15550	2.28417528120268	0.875110779076013	1.62844201905985	0.113062317008722	0.765666538367366	-4.5524761174159
a_HQ_transcript/102074	1.76157434512861	2.14704304215058	1.62841007371535	0.113069115836524	0.765666538367366	-4.65122209184848
a_HQ_transcript/39903	1.80519813770249	-0.490802815861883	1.62828678702682	0.113095357718086	0.76575503243295	-4.67208356960621
a_HQ_transcript/148678	0.492949755176228	2.98210206854804	1.62790388671814	0.113176891030666	0.766031916761534	-4.84065085011954
a_HQ_transcript/20872	-1.99393590970852	1.87687514503774	-1.62786716079711	0.113184713852853	0.766031916761534	-4.63539847292202
a_HQ_transcript/99661	1.93750143923078	0.500818915217343	1.62782160318053	0.113194418494026	0.766031916761534	-4.59686857650668
a_HQ_transcript/9148	-2.46642858023546	3.86188942626153	-1.62775679976235	0.11320822403994	0.766031916761534	-4.85160636475348
a_HQ_transcript/131268	-0.770210354337095	2.68891708606731	-1.62774005612029	0.113211791286078	0.766031916761534	-4.78179987623977
a_HQ_transcript/24461	-0.879041898932441	1.98185072785718	-1.62772348763383	0.113215321306399	0.766031916761534	-4.64333959189907
a_HQ_transcript/99823	1.58557631267626	-0.368211990975299	1.62763453592348	0.113234274579571	0.766070986122123	-4.63418468505484
a_HQ_transcript/138199	3.69480797944754	1.76282699141122	1.62755986038843	0.113250187994513	0.766089483037946	-4.7863607409194
a_HQ_transcript/34308	0.768006441100145	2.42537605640859	1.62733893447011	0.113297278276919	0.766167671437681	-4.75285166687452

a_HQ_transcript/10618	1.08460650855724	-0.547035797896051	1.62715165008906	0.113337210518404	0.766167671437681	-4.72223291443155
a_HQ_transcript/122155	-2.2956972231971	2.13052130141377	-1.62711363853167	0.113345316650464	0.766167671437681	-4.70238046208421
a_HQ_transcript/100189	0.625683916241143	3.1920288857081	1.62710287953866	0.113347611139898	0.766167671437681	-4.89375920789913
a_HQ_transcript/119393	1.73117573623909	1.61762635836231	1.6270609177922	0.113356560371527	0.766167671437681	-4.62318418708383
a_HQ_transcript/7400	-0.651531523071239	2.48134014418571	-1.62691287715077	0.113388137827831	0.766167671437681	-4.72472625560411
a_HQ_transcript/104017	-1.18351503964837	-0.322970522405934	-1.62685399931892	0.113400698638727	0.766167671437681	-4.65856097965119
a_HQ_transcript/110354	1.00325563743255	1.73211050261469	1.62681765176716	0.113408453483335	0.766167671437681	-4.61098165169611
a_HQ_transcript/123462	2.7841709299439	1.30402728181938	1.62678019685354	0.113416445043789	0.766167671437681	-4.71178060715801
a_HQ_transcript/118629	1.72512569758169	2.56643204583654	1.6267368937507	0.113425684980583	0.766167671437681	-4.7811396174853
a_HQ_transcript/107967	1.88707147469943	0.358903426387338	1.6267050396898	0.11343248233787	0.766167671437681	-4.68013214180091
a_HQ_transcript/123688	-2.11182964194608	1.14184516586012	-1.62670235613592	0.113433054998388	0.766167671437681	-4.56894441669295
a_HQ_transcript/124635	-1.49762168566433	2.01786548394225	-1.62658971890525	0.113457093517974	0.766167671437681	-4.77608884713146
a_HQ_transcript/3876	-2.1563423571522	2.19342249346923	-1.62657409446102	0.113460428346588	0.766167671437681	-4.59584269943561
a_HQ_transcript/9029	2.33713043832684	1.24819917776426	1.62656123123183	0.113463173891428	0.766167671437681	-4.64392929868012
a_HQ_transcript/145154	-1.32995774243794	1.56842438906937	-1.6265168941089	0.113472637685917	0.766167671437681	-4.68384719395084
a_HQ_transcript/80181	1.77610535543308	-0.336194024882632	1.6264195961017	0.113493408304288	0.766218912649439	-4.66132229353254
a_HQ_transcript/5695	-0.421994999464931	3.14457087709527	-1.62629940553105	0.113519070221652	0.766229512142204	-4.87221939040389
a_HQ_transcript/19456	1.51568038380434	1.13257725395931	1.62622795045824	0.113534328877947	0.766229512142204	-4.63788291112266
a_HQ_transcript/4074	-2.16440370193552	2.02276535649875	-1.62621622982509	0.113536831886298	0.766229512142204	-4.79136524436228
a_HQ_transcript/134271	-0.43467027297623	3.47096181295322	-1.62616531593381	0.113547705367344	0.766229512142204	-4.91114257566158
a_HQ_transcript/150923	1.63095981128116	0.0119192922641623	1.6260801144474	0.113565903436185	0.766263358669596	-4.74366040864615
a_HQ_transcript/2630	-0.745284191724472	5.06783333991372	-1.6258356938178	0.113618122243409	0.766327647396254	-5.15911100389974
a_HQ_transcript/84676	0.650884848967003	2.69926198863822	1.62574012361226	0.113638545549354	0.766327647396254	-4.77085671794928
a_HQ_transcript/101291	3.13403338581056	1.22184712810844	1.6256743254329	0.113652608346443	0.766327647396254	-4.67314250895324
a_HQ_transcript/28934	-0.702149904522302	3.05968492031727	-1.62562033657018	0.113664148253924	0.766327647396254	-4.72609314741014
a_HQ_transcript/79437	1.20680692991826	-0.549530808033231	1.62557210687581	0.113674457978381	0.766327647396254	-4.68905618934653
a_HQ_transcript/73542	1.53025125914589	-0.464014240443558	1.62544852047895	0.113700879695617	0.766327647396254	-4.67475865819028
a_HQ_transcript/22722	-2.23522607226089	0.0136014758779772	-1.62543918806119	0.11370287509241	0.766327647396254	-4.67720079825933
a_HQ_transcript/75757	-1.67906599971888	1.03447036025162	-1.62540743515644	0.113709664507867	0.766327647396254	-4.57257906049576
a_HQ_transcript/61797	2.19517449926711	1.92948113808185	1.62532359331624	0.113727593200579	0.766327647396254	-4.71076104555418
a_HQ_transcript/22534	-1.84609734923981	1.12903569450181	-1.62530585287067	0.113731387106231	0.766327647396254	-4.64739266704902
a_HQ_transcript/22951	-1.71885794666093	0.386380536944902	-1.62528890058566	0.113735012556249	0.766327647396254	-4.58791820543264
a_HQ_transcript/124386	-1.50647693670437	1.71962046508702	-1.62518972582051	0.113756224180823	0.766327647396254	-4.70854929284523
a_HQ_transcript/115858	-1.25627713436407	0.783595866918316	-1.6251164205538	0.113771904899481	0.766327647396254	-4.65087478401931
a_HQ_transcript/69332	-2.65345374055235	2.91130740275517	-1.62511095215678	0.113773074714858	0.766327647396254	-4.79225512670243
a_HQ_transcript/102122	-1.54835107401415	1.80077347027081	-1.62511044471645	0.113773183268471	0.766327647396254	-4.68468489983562
a_HQ_transcript/82773	-2.63987589070544	2.6898043965593	-1.62495508314739	0.113806422834056	0.766462721179237	-4.75926445930669
a_HQ_transcript/9260	1.44218339203785	-0.433851605536491	1.62480642030722	0.113838236700021	0.766588162986427	-4.68286166216383
a_HQ_transcript/55503	-0.6348967531838	3.15478381079899	-1.62459456864264	0.113883585643931	0.766748357021859	-4.88538805971418
a_HQ_transcript/145920	3.89280356926939	5.84868424948177	1.62457204994103	0.113888406869783	0.766748357021859	-5.04956822139692
a_HQ_transcript/7691	1.79050111030718	0.19143773562137	1.6244207348472	0.113920807603978	0.766808099962596	-4.53086564915591
a_HQ_transcript/132242	-1.38680740651194	0.613189899073672	-1.62440739602933	0.113923664176937	0.766808099962596	-4.60241918834919
a_HQ_transcript/151284	-1.02052235114055	3.67940767599012	-1.62426542267591	0.113954072120733	0.7669239671453	-4.95146097149438
a_HQ_transcript/138144	4.67641900026013	1.67491606390046	1.62406742027606	0.113996491567628	0.767109456384852	-4.68206733818995
a_HQ_transcript/149160	-1.64563709409812	0.972603891465497	-1.62401358650429	0.114008027004348	0.767109456384852	-4.54043620465339
a_HQ_transcript/94179	1.77509314250232	0.109422939838718	1.62390155953866	0.114032035092474	0.767139714981997	-4.64745909489849
a_HQ_transcript/16456	2.64422992486117	3.35261478916819	1.62386944183165	0.11403891888865	0.767139714981997	-4.99235748721163
a_HQ_transcript/127531	-1.52362342653738	0.821421628884571	-1.62380000279203	0.1140538029456	0.767151059664641	-4.64740944365974
a_HQ_transcript/4287	1.82571557473958	-0.270982975359735	1.62365511009373	0.11408486542942	0.767198959570269	-4.66560426138687
a_HQ_transcript/5105	2.49770464773826	0.65465984348705	1.6236205644812	0.114092272438952	0.767198959570269	-4.64749908259008

a_HQ_transcript/67764	-3.26164199546084	1.28723303096227	-1.62356862054326	0.114103410611814	0.767198959570269	-4.89272340450276
a_HQ_transcript/37109	-2.12109619961067	1.06449676498224	-1.6235205544063	0.114113718078113	0.767198959570269	-4.75850736409506
a_HQ_transcript/111763	-0.749752926717004	4.21343587694435	-1.62338110976345	0.11414362540335	0.767311282230361	-4.96262247221018
a_HQ_transcript/18678	1.56564737416807	-0.391088393585822	1.62304276029707	0.114216219589742	0.767469952602035	-4.59539721377084
a_HQ_transcript/133750	-2.70042099490381	1.71654897753199	-1.62299073310546	0.114227385597669	0.767469952602035	-4.82781475578042
a_HQ_transcript/152084	2.9565329072101	0.477132362227127	1.62291631740629	0.11424335816065	0.767469952602035	-4.73092630154161
a_HQ_transcript/107251	-0.731685819264883	3.36340422865929	-1.62283574788554	0.114260653652142	0.767469952602035	-4.89752917866437
a_HQ_transcript/4885	-1.83579300974034	0.831111356712349	-1.62262491515929	0.114305922357823	0.767469952602035	-4.53826108823976
a_HQ_transcript/18816	-0.443973135283728	3.80457169950954	-1.62261808986622	0.114307388089565	0.767469952602035	-4.95817042712561
a_HQ_transcript/121163	2.01140188998795	-0.0101670525917727	1.62258889209911	0.114313658485053	0.767469952602035	-4.64752821311612
a_HQ_transcript/19424	-1.61832743933362	-0.0904443852109445	-1.62257748695562	0.114316107884921	0.767469952602035	-4.68425607812863
a_HQ_transcript/47214	1.92364965225116	-0.224193015535388	1.62256357949854	0.114319094747004	0.767469952602035	-4.66223863180962
a_HQ_transcript/19722	1.88301986095106	0.891115471518531	1.62253237717395	0.114325796208841	0.767469952602035	-4.60094920205045
a_HQ_transcript/102197	-1.55877350642922	0.669244876997083	-1.62250230185962	0.114332255923914	0.767469952602035	-4.57471194459946
a_HQ_transcript/70015	-1.77569629226736	-0.225281556240259	-1.622344654383	0.114366121095769	0.767469952602035	-4.67877863489296
a_HQ_transcript/104646	2.02968033769305	-0.0257648554733919	1.62234446059975	0.11436616272856	0.767469952602035	-4.63650313939098
a_HQ_transcript/50113	2.76312302924636	1.77209044761855	1.62232180708927	0.114371029741231	0.767469952602035	-4.62477287587507
a_HQ_transcript/107920	2.39592710492165	2.47727830381614	1.62229554715727	0.114376671792073	0.767469952602035	-4.65696508191494
a_HQ_transcript/140811	0.51222510603643	4.03414680555194	1.62228713828737	0.114378478519671	0.767469952602035	-5.03878788006988
a_HQ_transcript/19742	1.0542889906862	5.12495142553808	1.62219726909355	0.114397789259289	0.767510929601102	-5.13342303449547
a_HQ_transcript/1745	1.06163214089552	1.02886299850768	1.62208891168485	0.114421076244339	0.767578571255737	-4.53443936553774
a_HQ_transcript/101197	1.76522647547416	1.99569814325239	1.62190121570479	0.114461423042381	0.767635837422329	-4.64125594790054
a_HQ_transcript/4889	-2.16340111268937	1.29845798374218	-1.62183812779965	0.114474986938183	0.767635837422329	-4.51966272238628
a_HQ_transcript/6390	0.544239344155751	2.57750824587469	1.62179872057911	0.114483460159851	0.767635837422329	-4.7143357943734
a_HQ_transcript/122082	1.88967152916019	2.81833318503709	1.62175553696012	0.114492745964408	0.767635837422329	-4.83763061537852
a_HQ_transcript/42919	-1.59687177907556	-0.0257197989467906	-1.62174206695929	0.11449564255525	0.767635837422329	-4.64780762200795
a_HQ_transcript/103289	1.0538897695621	3.03619113319857	1.62167610484282	0.114509827929257	0.767642403168246	-4.96357794852099
a_HQ_transcript/149974	0.641356207965615	3.58364277632487	1.62139302085361	0.114570722523373	0.76776307410221	-5.00233589268231
a_HQ_transcript/121931	-1.65388257422284	-0.436864807667906	-1.62135635376305	0.114578611982973	0.76776307410221	-4.67570758141139
a_HQ_transcript/56918	-2.23303105049089	-0.113592155376505	-1.6212748027572	0.114596160475842	0.76776307410221	-4.67108139501628
a_HQ_transcript/76645	0.774918189875236	3.20221669592716	1.6212685333188	0.11459750965191	0.76776307410221	-4.94279400915886
a_HQ_transcript/111292	1.29156841468444	-0.717677159737652	1.6211299275484	0.114627340798461	0.76776307410221	-4.67474267788672
a_HQ_transcript/92311	1.72498696069521	-0.186353149985611	1.62103737162252	0.114647264520616	0.76776307410221	-4.64131278911551
a_HQ_transcript/54986	0.948686499876005	-0.587096720344276	1.62095315728595	0.11466539509765	0.76776307410221	-4.65497494749214
a_HQ_transcript/103939	3.31692922206143	0.856796248718957	1.62087973247179	0.114681204715526	0.76776307410221	-4.70504779182249
a_HQ_transcript/5952	1.74665338022495	-0.133529621225273	1.62087245346633	0.114682772107633	0.76776307410221	-4.65084167402197
a_HQ_transcript/82950	-2.52622818372981	0.461196133784939	-1.62086053901766	0.114685337690246	0.76776307410221	-4.74367485016992
a_HQ_transcript/117426	-2.49640760066275	1.01152019375696	-1.62068041108705	0.114724131066737	0.76776307410221	-4.82958901880276
a_HQ_transcript/69663	1.41545707744568	1.83427706505019	1.62067600128508	0.114725080922262	0.76776307410221	-4.61463363420081
a_HQ_transcript/27364	1.23751981543104	1.01554278124403	1.62059845981655	0.114741784134369	0.76776307410221	-4.60533167348861
a_HQ_transcript/110483	4.09897345902591	2.09531501421882	1.62057762006738	0.114746273567259	0.76776307410221	-4.78072145474247
a_HQ_transcript/29394	-2.20106252422992	0.950627852183608	-1.62057243874118	0.114747389784344	0.76776307410221	-4.559868036584
a_HQ_transcript/63536	1.04407142414689	3.245282097087	1.6204516340611	0.114773417363585	0.76776307410221	-4.76050441402278
a_HQ_transcript/74312	1.08542429921543	-0.736694074524967	1.62034964009393	0.114795395925885	0.76776307410221	-4.70312125521349
a_HQ_transcript/117333	-1.86974626816124	2.43625528247288	-1.62033024319399	0.114799576133591	0.76776307410221	-4.75367261062703
a_HQ_transcript/148490	-3.75499250887931	1.44766957557793	-1.62032636229559	0.114800412517409	0.76776307410221	-4.73905365083751
a_HQ_transcript/50645	-2.19074842631172	1.82297421243944	-1.62023107626919	0.114820949462681	0.76776307410221	-4.73549427245405
a_HQ_transcript/49943	1.30283117792988	1.68001075230881	1.62022042895956	0.114823244459371	0.76776307410221	-4.60379914796005
a_HQ_transcript/118802	-7.34239664505946	3.09339280761859	-1.62018762357908	0.114830315801355	0.76776307410221	-4.73150337061573
a_HQ_transcript/152394	-2.41351868026152	-0.312589501037051	-1.62009011604534	0.114851336089481	0.76776307410221	-4.65029387838814

a_HQ_transcript/47285	-1.52517131130834	2.13958355004688	-1.62008456395229	0.114852533083035	0.76776307410221	-4.64094528433954
a_HQ_transcript/30966	0.553006735223268	3.45845651306662	1.61998080211463	0.114874905320097	0.76776307410221	-4.95446432290364
a_HQ_transcript/108290	2.76543340822409	1.19795177852902	1.61986117357632	0.114900703051222	0.76776307410221	-4.8662112552789
a_HQ_transcript/17534	1.56747216816179	1.69784692444324	1.61979905065174	0.114914101656442	0.76776307410221	-4.8100550825841
a_HQ_transcript/125029	1.70670722894094	0.772688828461351	1.61964404646365	0.11494753840042	0.76776307410221	-4.65815502604257
a_HQ_transcript/56974	1.8851871652223	1.01370147304507	1.61959601577537	0.114957900970326	0.76776307410221	-4.7205426572469
a_HQ_transcript/151723	0.520943646261916	4.22146201173491	1.61954120873423	0.11496972647052	0.76776307410221	-5.06225758142075
a_HQ_transcript/139656	-2.31792147840683	0.383084528807095	-1.61946326542362	0.114986545717387	0.76776307410221	-4.68927049802228
a_HQ_transcript/9624	0.820744424199242	4.53847644256171	1.61946065855104	0.114987108284735	0.76776307410221	-5.04314266772753
a_HQ_transcript/10170	1.23125658549255	1.24800328827605	1.61943261461026	0.114993160356109	0.76776307410221	-4.57265713982108
a_HQ_transcript/151363	-1.43045844656356	0.677820573103301	-1.61934933769667	0.115011133620512	0.76776307410221	-4.53424051403109
a_HQ_transcript/1773	1.27583869638087	-0.553130846185065	1.61929319461322	0.11502325202292	0.76776307410221	-4.64741329265468
a_HQ_transcript/151294	-1.84325330912922	1.04313835022644	-1.61922587475927	0.115037784300557	0.76776307410221	-4.65679211538881
a_HQ_transcript/55861	-1.31184810533924	1.92526487222268	-1.61921438365908	0.115040265025234	0.76776307410221	-4.67854689345779
a_HQ_transcript/126359	1.82288574434135	-0.530494536738696	1.61915420008128	0.115053258311245	0.76776307410221	-4.68344984929867
a_HQ_transcript/149509	5.00301288057721	0.317048570763529	1.6191154019082	0.115061635252519	0.76776307410221	-4.72712900744197
a_HQ_transcript/63011	-0.856800482904222	2.51129980101702	-1.61905864359624	0.115073890884592	0.76776307410221	-4.82795972499778
a_HQ_transcript/5264	1.54249087811274	0.324029657816734	1.61899457045272	0.115087727274771	0.76776307410221	-4.56611619899783
a_HQ_transcript/118041	-0.972782578743728	2.64078291988987	-1.61896733420324	0.115093609270369	0.76776307410221	-4.78201189539853
a_HQ_transcript/125715	0.357594824926299	5.38502311219705	1.61892977903545	0.115101720166263	0.76776307410221	-5.18294405237541
a_HQ_transcript/100710	-1.42031622477647	-0.404056074607594	-1.61888909886165	0.115110506509349	0.76776307410221	-4.68899858622815
a_HQ_transcript/129498	-2.09758024652762	1.62535880240487	-1.61878330696438	0.115133358649733	0.76776307410221	-4.79200019078229
a_HQ_transcript/93955	2.40822028025887	1.7938847686065	1.61877384276865	0.115135403195516	0.76776307410221	-4.70650655243718
a_HQ_transcript/128085	-1.06948686191677	10.3668466371729	-1.61840946481813	0.11521414231898	0.768200007423454	-5.74242991745233
a_HQ_transcript/16966	-1.01158524225606	1.84559321400349	-1.61821674998522	0.1152558043471	0.768389654213584	-4.64045152156989
a_HQ_transcript/25346	2.18670487033456	-0.443464889824988	1.61807892621018	0.115285607363017	0.768454933503593	-4.68571808207798
a_HQ_transcript/14134	-1.61556071536768	0.971935730552381	-1.61804920036639	0.115292036111424	0.768454933503593	-4.53656421987351
a_HQ_transcript/129490	-1.79614339905848	3.59523176787444	-1.61793235387515	0.115317309125877	0.768468300384369	-5.09702846673145
a_HQ_transcript/73433	2.12012569068594	0.661531060325355	1.61790498260384	0.11532322998434	0.768468300384369	-4.56251305361937
a_HQ_transcript/131811	-2.70174361521723	2.29283333213472	-1.6178003163668	0.115345873328998	0.768468300384369	-4.72955935957029
a_HQ_transcript/9234	0.731486483745823	3.26287009453915	1.61779546632814	0.11534692266814	0.768468300384369	-4.90866745997407
a_HQ_transcript/147449	-0.749347789409401	2.46522835321228	-1.61769266945112	0.115369165325517	0.768486745085037	-4.72423896062174
a_HQ_transcript/133873	1.91542850330897	2.14638971754954	1.61763838635332	0.115380912245743	0.768486745085037	-4.77439121892573
a_HQ_transcript/19930	0.462457589790745	4.49985141303898	1.61759938402576	0.115389352998593	0.768486745085037	-5.10992341786637
a_HQ_transcript/21611	1.93414146682736	2.91902688943847	1.61720366978412	0.115475020879089	0.768774257269391	-4.78469465606393
a_HQ_transcript/29896	1.06506506810874	1.88303328807968	1.61715767185455	0.115484982330629	0.768774257269391	-4.62984319555512
a_HQ_transcript/46317	1.63917624714919	-0.192175722965612	1.61703811029377	0.115510878253259	0.768774257269391	-4.6499993601165
a_HQ_transcript/129367	1.57939441272751	-0.450646754177573	1.61702609181105	0.115513481609134	0.768774257269391	-4.71227272713974
a_HQ_transcript/4192	-2.01182284642445	0.951508952665395	-1.61701437911262	0.1155160187747	0.768774257269391	-4.72412317105003
a_HQ_transcript/113281	-1.35938248052913	1.12383681413056	-1.61691396177188	0.115537772724852	0.768774257269391	-4.65103176191003
a_HQ_transcript/27550	-1.06074990681065	5.20321347563069	-1.61682110609203	0.115557891550852	0.768774257269391	-5.21314225648558
a_HQ_transcript/124496	2.09752995430128	0.0600618368236152	1.61676125248693	0.115570861421075	0.768774257269391	-4.67701719474029
a_HQ_transcript/40277	1.89380169179551	0.22845230562374	1.61672984799424	0.115577667040643	0.768774257269391	-4.65564037389369
a_HQ_transcript/42515	-0.50144881067514	3.1299146290553	-1.61670292823549	0.115583501042818	0.768774257269391	-4.95389613491023
a_HQ_transcript/95031	2.08939135699504	2.9518113202391	1.61663707299219	0.115597774095164	0.768774257269391	-4.80621962090418
a_HQ_transcript/142830	1.46004916541836	-0.39115186496209	1.61661399366902	0.115602776506016	0.768774257269391	-4.69514482121668
a_HQ_transcript/135040	-2.71037592814291	0.00689348809550776	-1.61652707463123	0.115621617683619	0.768774257269391	-4.68519630932365
a_HQ_transcript/43697	1.50571362433768	4.70403769535433	1.61642288494025	0.115644205897583	0.768774257269391	-4.95659494742489
a_HQ_transcript/119402	-1.36559620368489	-0.377242378014149	-1.61634899034539	0.115660228367296	0.768774257269391	-4.66779301188302
a_HQ_transcript/148121	-0.45721757151437	4.28384683902413	-1.61623058297605	0.115685906292079	0.768774257269391	-5.0601853437173

a_HQ_transcript/49296	-2.77360329597156	0.30128816864833	-1.61622830052033	0.115686401313472	0.768774257269391	-4.7460576339613
a_HQ_transcript/129688	5.26530029095074	5.71715244124781	1.61622083776021	0.115688019856888	0.768774257269391	-5.11455487429563
a_HQ_transcript/78819	1.78113150300246	1.18556142638475	1.6161795784761	0.115696968617189	0.768774257269391	-4.69798944195566
a_HQ_transcript/138368	-1.52810067226096	1.11777313807853	-1.61614450256218	0.115704576709013	0.768774257269391	-4.72415829142439
a_HQ_transcript/37428	-0.643451369886162	3.77602635177662	-1.6161183031816	0.115710259717346	0.768774257269391	-5.03172429601087
a_HQ_transcript/75990	1.86168561825054	-0.314684763212903	1.61604154223446	0.115726911551309	0.768797018817654	-4.7486559510613
a_HQ_transcript/151779	-2.64162549123966	0.254014583709571	-1.61591331502983	0.115754732412041	0.7688939647637	-4.69032570008316
a_HQ_transcript/104374	0.618926177252803	4.76617174994371	1.61581608270788	0.115775832124419	0.76894624882635	-5.10560968518136
a_HQ_transcript/111556	-2.11288920612906	2.04965540313721	-1.61566523476196	0.115808572852963	0.76904659693244	-4.835870797572
a_HQ_transcript/14633	-1.90585735495308	-0.0313143156448718	-1.61559714695047	0.115823353437518	0.76904659693244	-4.65437173885278
a_HQ_transcript/4791	0.858788417374355	1.921883055648	1.6155636220066	0.115830631642986	0.76904659693244	-4.6634040674232
a_HQ_transcript/41618	2.51636240445631	1.2884985515124	1.61543775841536	0.11585795976132	0.769062828354175	-4.68518210742711
a_HQ_transcript/118208	2.36355075403392	5.24383872291164	1.61536831883663	0.115873039091989	0.769062828354175	-4.97764328240333
a_HQ_transcript/135840	1.57404795973368	1.29678193665177	1.61530361654342	0.115887091137981	0.769062828354175	-4.67458146146457
a_HQ_transcript/68888	1.34935486084318	0.785778048137749	1.61525047775891	0.115898632869013	0.769062828354175	-4.55311960856456
a_HQ_transcript/149718	1.61596064079946	1.23036692297079	1.61524773380506	0.11589922888079	0.769062828354175	-4.66522245510285
a_HQ_transcript/119316	3.57093277542108	1.76960851919301	1.61491353909584	0.115971837868757	0.769329418932832	-4.80888373677898
a_HQ_transcript/146705	-1.27716904409616	-0.669845651669025	-1.61478194786922	0.116000438368835	0.769329418932832	-4.68254661716034
a_HQ_transcript/120136	-1.02908917471484	1.89930763166891	-1.61470858304287	0.116016386256582	0.769329418932832	-4.7217985041118
a_HQ_transcript/129345	2.53486969771626	0.394907997903152	1.61463182659023	0.116033073338754	0.769329418932832	-4.63544492049455
a_HQ_transcript/144928	3.02419521401845	0.596963832581774	1.61445068296894	0.116072462329091	0.769329418932832	-4.61844674579478
a_HQ_transcript/36565	2.07053385957683	-0.504994337001232	1.61427565808759	0.116110531266843	0.769329418932832	-4.68939497218599
a_HQ_transcript/64096	1.58234797458833	2.25150347908747	1.6141797234079	0.11613140197782	0.769329418932832	-4.603316868858
a_HQ_transcript/50383	2.21637436323203	0.778761923036341	1.61416508117304	0.116134587685876	0.769329418932832	-4.68749500125188
a_HQ_transcript/132767	2.02232206947667	4.1259352731953	1.61416094613617	0.116135487358015	0.769329418932832	-4.97622990748023
a_HQ_transcript/131644	0.56712410518875	6.82770928191083	1.61398935948543	0.116172825023919	0.769329418932832	-5.31352250785878
a_HQ_transcript/122994	-1.05872661295855	3.18030330439502	-1.61374044416592	0.116227007138128	0.769329418932832	-4.89325739996681
a_HQ_transcript/36605	2.10038961576176	0.934501368098388	1.61373943728992	0.116227226349911	0.769329418932832	-4.73794952504091
a_HQ_transcript/43060	1.44622238211645	-0.657919832991962	1.61368247129248	0.116239629243066	0.769329418932832	-4.70720531631085
a_HQ_transcript/128661	1.07761999610422	2.75037682812523	1.61366072861915	0.116244363442622	0.769329418932832	-4.86267030071736
a_HQ_transcript/53678	1.45974858533007	-0.650583765366132	1.61354915542959	0.116268659625777	0.769329418932832	-4.70729181486536
a_HQ_transcript/10790	-0.668394482452807	4.10126710257915	-1.61344709539466	0.116290887880173	0.769329418932832	-5.05408454083936
a_HQ_transcript/4484	-1.82787438669941	-0.0259282709954253	-1.613406004234	0.116299838351866	0.769329418932832	-4.68000078777069
a_HQ_transcript/45435	-1.6951956222153	1.30428974792594	-1.61334305908776	0.116313550154397	0.769329418932832	-4.73575874033129
a_HQ_transcript/3123	2.35255028856752	1.07620325810686	1.61333539840235	0.116315219028364	0.769329418932832	-4.72019757296397
a_HQ_transcript/84268	-2.66363918727932	1.25082141468617	-1.61312070675163	0.116361997434009	0.769329418932832	-4.76505595279998
a_HQ_transcript/135957	1.37943397049268	-0.560834729814387	1.6130789084361	0.116371106519857	0.769329418932832	-4.6998819513796
a_HQ_transcript/109085	1.39466058460147	0.813208703438172	1.61302589921007	0.116382659636955	0.769329418932832	-4.64217261806382
a_HQ_transcript/97992	-1.95853038871451	1.10936113593844	-1.61299514990667	0.11638936173838	0.769329418932832	-4.60775525172769
a_HQ_transcript/5117	-0.661233967617169	4.07491767768441	-1.61299299347146	0.116389831765728	0.769329418932832	-5.06308147091752
a_HQ_transcript/86843	-3.07700658631483	0.692815088174836	-1.61298759967679	0.116391007430889	0.769329418932832	-4.70564715875118
a_HQ_transcript/80489	-1.217327193149	1.3929270028071	-1.6129675808905	0.116395370935892	0.769329418932832	-4.59066208713352
a_HQ_transcript/109053	-2.20924575460074	2.44388931262933	-1.61285087423811	0.116420812221601	0.769329418932832	-4.89114068714016
a_HQ_transcript/90464	-4.01116488663927	3.30782526699415	-1.61284585688074	0.116421906075124	0.769329418932832	-4.69575523563966
a_HQ_transcript/129414	2.12300522131475	0.836318825781504	1.61283207673603	0.116424910381342	0.769329418932832	-4.56004129568717
a_HQ_transcript/106642	-2.24849854711915	0.247632535259857	-1.61269131974216	0.116455601453514	0.769329418932832	-4.72132306159242
a_HQ_transcript/61538	2.14185858634902	1.29630829514273	1.61267506716507	0.116459145641873	0.769329418932832	-4.58001185250638
a_HQ_transcript/140727	-3.55007645148666	0.671872302258707	-1.61264384568155	0.116465954338181	0.769329418932832	-4.86210638026515
a_HQ_transcript/20802	1.18638971934263	-0.641920014300467	1.61258754671664	0.116478232690539	0.769329418932832	-4.67285343247669
a_HQ_transcript/120473	1.37725319844314	-0.183615447343653	1.61257349870085	0.116481296615904	0.769329418932832	-4.62490101790279

a_HQ_transcript/68604	1.56335482594006	-0.290679557547355	1.6124612785304	0.116505774638023	0.769329418932832	-4.66426778592074
a_HQ_transcript/65336	-3.55488943237969	1.82462496756736	-1.61246083826386	0.116505870679479	0.769329418932832	-4.74008992583748
a_HQ_transcript/78427	-2.04194610940356	0.935874610220855	-1.61244300422457	0.116509761120888	0.769329418932832	-4.72379236802982
a_HQ_transcript/130704	-0.647545683148612	3.75551825930941	-1.61239476201609	0.116520285547037	0.769329418932832	-5.03796530464119
a_HQ_transcript/35415	-1.98285260748784	1.69980611322852	-1.61232425228106	0.116535669219133	0.769329418932832	-4.61481841332638
a_HQ_transcript/139600	2.53660867374241	1.80303607048016	1.61226325764335	0.116548978253177	0.769329418932832	-4.75049262441138
a_HQ_transcript/561	-1.30528433688399	2.09904093661363	-1.61213926142173	0.116576038088702	0.769329418932832	-4.69158806107163
a_HQ_transcript/33067	-2.40862999679688	1.35009711885458	-1.61211517594654	0.11658129488809	0.769329418932832	-4.79953439463902
a_HQ_transcript/18762	-0.556488551349881	3.96692844143605	-1.61204314789126	0.116597016605685	0.769329418932832	-5.00663955401226
a_HQ_transcript/51773	-1.70918487621947	1.10494114815935	-1.61202573753584	0.116600817062694	0.769329418932832	-4.6068748052189
a_HQ_transcript/133561	-2.53876651582147	0.536191667180035	-1.61201887432918	0.116602315240553	0.769329418932832	-4.77004347283093
a_HQ_transcript/148703	-0.519283976129176	3.58515316730593	-1.61200257130945	0.116605874110297	0.769329418932832	-4.9961466182102
a_HQ_transcript/1682	1.17165696495948	3.3598190363197	1.61194222747797	0.116619047651417	0.769329418932832	-5.04039499702021
a_HQ_transcript/140355	1.82694288205237	6.34695284327255	1.61192656867349	0.116622466293589	0.769329418932832	-5.30022505256272
a_HQ_transcript/26698	-0.779363694329408	2.10895273756903	-1.6118820546061	0.116632185088741	0.769329418932832	-4.65670039981627
a_HQ_transcript/133450	0.701119391995951	4.25072381959048	1.61182918710618	0.116643728560896	0.769329418932832	-5.06736265851361
a_HQ_transcript/10146	-1.20298845228789	-0.599875559218946	-1.61178853727653	0.116652604977175	0.769329418932832	-4.68160332032304
a_HQ_transcript/108510	1.41799508072365	0.111253135449206	1.61164137861913	0.116684743617174	0.769329418932832	-4.63869355065244
a_HQ_transcript/7970	0.432883531328315	4.10379068860631	1.61164080870108	0.116684868098281	0.769329418932832	-5.04731348929173
a_HQ_transcript/135968	1.23828982652433	2.75988086995465	1.61146171550231	0.116723990917665	0.769329418932832	-4.69346269204811
a_HQ_transcript/15712	0.559735135084883	5.09456813269985	1.61141385650048	0.116734447517785	0.769329418932832	-5.12855064169795
a_HQ_transcript/97173	-0.71038749131932	2.77967776108481	-1.61140377670802	0.116736649925945	0.769329418932832	-4.82072350009469
a_HQ_transcript/29987	-0.834648354839139	1.66669053834685	-1.61131591788296	0.116755848294424	0.769329418932832	-4.69587100854774
a_HQ_transcript/23327	1.94332522639892	0.422134875998049	1.61127161608875	0.116765529833244	0.769329418932832	-4.64741830407849
a_HQ_transcript/119837	1.02602294544823	4.00938172355641	1.61123325400763	0.116773913863672	0.769329418932832	-4.98504924367724
a_HQ_transcript/47393	-2.06442538647195	-0.163511739970701	-1.61121414572473	0.116778090162288	0.769329418932832	-4.74538942110394
a_HQ_transcript/115460	-0.978561175477717	3.18243287252906	-1.61120928796504	0.116779151891857	0.769329418932832	-4.92437496223082
a_HQ_transcript/25486	-0.403463264109611	4.12741469041592	-1.61110063251085	0.116802902095129	0.769329418932832	-4.98299207471555
a_HQ_transcript/57752	2.18361769238802	1.43317629215167	1.61107239612506	0.116809074730536	0.769329418932832	-4.58954180878602
a_HQ_transcript/127581	2.09382954502363	1.38479670468927	1.61096075071206	0.11683348368179	0.769329418932832	-4.55822120039872
a_HQ_transcript/86251	1.71085960707786	0.574165592418535	1.61090974387349	0.116844636662749	0.769329418932832	-4.73377909117473
a_HQ_transcript/26361	1.7695419893942	0.792296317556951	1.61089308409246	0.116848279623064	0.769329418932832	-4.61654101003434
a_HQ_transcript/59848	1.71416360752397	-0.453943963229035	1.61087529775677	0.116852169027801	0.769329418932832	-4.70403677041357
a_HQ_transcript/22999	1.75717509923778	3.17196803756041	1.61077355666524	0.116874419178751	0.769329418932832	-4.88519742620278
a_HQ_transcript/143609	2.38151562677967	0.650298126290177	1.61065713931203	0.116899883208795	0.769329418932832	-4.77364501968625
a_HQ_transcript/146508	5.14006562741818	2.19114799882463	1.61059568587931	0.116913326789395	0.769329418932832	-4.7450345210954
a_HQ_transcript/121816	2.5490453240433	1.69156947716099	1.61052479330833	0.116928836858184	0.769329418932832	-4.68094563601065
a_HQ_transcript/145461	1.64387156334975	0.429031721047584	1.61050123333144	0.116933991748076	0.769329418932832	-4.79833730696953
a_HQ_transcript/85495	1.48054450104532	3.01635101014512	1.61050027303459	0.116934201863641	0.769329418932832	-4.75755246104214
a_HQ_transcript/10697	2.31328783520746	1.90953500545859	1.61039191454219	0.116957912989299	0.769329418932832	-4.63583264452286
a_HQ_transcript/147897	-0.558557953655166	2.71578610393941	-1.61029823587632	0.116978415042973	0.769329418932832	-4.86188450110779
a_HQ_transcript/75208	-3.42546024528414	0.699850745792425	-1.6102483855636	0.116989326241851	0.769329418932832	-4.71311363321071
a_HQ_transcript/142828	2.12411534912317	-0.660209991775647	1.61023778349628	0.116991646922191	0.769329418932832	-4.7103987190341
a_HQ_transcript/80125	1.5439271925827	6.28087139602431	1.61023032538119	0.11699327944732	0.769329418932832	-5.29382290377028
a_HQ_transcript/148167	0.820052887057836	3.65427103206655	1.61019542327971	0.117000919502799	0.769329418932832	-5.05655784662298
a_HQ_transcript/80054	-2.8290942264854	0.504122682919878	-1.61012480338538	0.117016379422146	0.769329418932832	-4.76494963966251
a_HQ_transcript/77753	0.461305065428082	4.66444434614632	1.61009014562555	0.117023967220671	0.769329418932832	-5.13948164207591
a_HQ_transcript/126916	3.4033622042331	3.11648719563555	1.61002889055235	0.117037379092115	0.769329418932832	-4.86398460668064
a_HQ_transcript/28884	1.66767507715456	2.90961136824003	1.61002643296254	0.117037917210691	0.769329418932832	-4.8064211981092
a_HQ_transcript/121975	2.75980193886078	1.51336429398194	1.6099235812631	0.117060439638756	0.769390460800726	-4.72402729406883

a_HQ_transcript/134894	1.6235733569777	2.42934634159761	1.6097860044067	0.117090571736409	0.769501498461729	-4.72417444666487
a_HQ_transcript/112601	2.64248925325085	0.318059116966239	1.60965593159185	0.117119066158328	0.769543349039269	-4.75351296659909
a_HQ_transcript/136175	2.32577463105476	2.1489509644539	1.60959161228483	0.11713315838247	0.769543349039269	-4.81035472192059
a_HQ_transcript/146716	1.71129979961604	0.142092605491779	1.6095756485803	0.117136656212143	0.769543349039269	-4.6497685934029
a_HQ_transcript/38643	1.51774173832403	1.02809322457161	1.6093805848107	0.117179403839127	0.769737189101681	-4.5739472569516
a_HQ_transcript/81138	2.83680837938168	1.84963496183622	1.60877315914498	0.117312601409317	0.770525072849802	-4.96922668881826
a_HQ_transcript/89682	-3.41285988287687	1.32842950566585	-1.60849766606616	0.117373053089353	0.770674361248823	-4.72626518571472
a_HQ_transcript/103259	-0.434958090306167	3.20758463136918	-1.60849070812933	0.11737458020655	0.770674361248823	-4.92107492300149
a_HQ_transcript/62658	1.54028837363668	1.68603480579062	1.60841489159538	0.117391221359777	0.770674361248823	-4.66939613886471
a_HQ_transcript/54759	-1.74429978042072	0.745729490545817	-1.60837677970613	0.117399587361827	0.770674361248823	-4.56787531781883
a_HQ_transcript/50871	-1.10086964300108	3.30166056567641	-1.60836751226783	0.117401621746118	0.770674361248823	-4.75880782195566
a_HQ_transcript/52140	-0.544899078668457	3.00852023139116	-1.60826693068503	0.117423703236856	0.770680187234642	-4.91347360102946
a_HQ_transcript/124888	1.11363768879255	4.20864739739161	1.60824268811911	0.117429025914142	0.770680187234642	-5.10502137715043
a_HQ_transcript/111112	1.82892604689556	-0.34232335788988	1.60807728333683	0.117465347337094	0.770831531949719	-4.70133628699371
a_HQ_transcript/97622	-0.909768375463283	4.78304699042636	-1.60784231865667	0.117516959359523	0.771083171198823	-5.11523396019444
a_HQ_transcript/147539	-2.06398255157869	-0.132409303581779	-1.60766209217596	0.11755656028377	0.771255952164219	-4.64572500295595
a_HQ_transcript/74531	1.03005487356578	-0.664539404372701	1.60753087183488	0.117585400048278	0.771257545611291	-4.71628559063278
a_HQ_transcript/67288	0.926428585460038	2.86550425807361	1.60746194674788	0.117600550814215	0.771257545611291	-4.8524342338944
a_HQ_transcript/19042	-1.55805674204198	1.73449995871601	-1.6073782273895	0.117618955738667	0.771257545611291	-4.57889273342512
a_HQ_transcript/152276	2.55992203628939	1.20279203933125	1.60722206099717	0.117653293793052	0.771257545611291	-4.80132875883631
a_HQ_transcript/141952	-2.08938207336659	2.05671243288502	-1.60715914231795	0.117667130754279	0.771257545611291	-4.82559202303251
a_HQ_transcript/68119	-1.87730852741145	-0.211880060070026	-1.60705343723275	0.117690380232277	0.771257545611291	-4.64859402122745
a_HQ_transcript/127627	-3.36516672205613	1.89936946938091	-1.60687140056501	0.117730427426718	0.771257545611291	-4.66935374332998
a_HQ_transcript/135119	2.18566389120596	0.163831014532247	1.60680778167876	0.117744425915124	0.771257545611291	-4.752000779893
a_HQ_transcript/2576	-2.04164080151569	0.482724920022071	-1.6067958515409	0.1177470511352	0.771257545611291	-4.66929144249219
a_HQ_transcript/96323	-0.561336788144508	2.72398882307808	-1.60676957834798	0.117752832705885	0.771257545611291	-4.75842249896721
a_HQ_transcript/66483	-2.69150976538194	1.85990208224683	-1.60674682677508	0.117757839511688	0.771257545611291	-4.75711264144022
a_HQ_transcript/16681	-1.2414014518949	-0.383596527697311	-1.60672454474828	0.117762743156648	0.771257545611291	-4.63935486471057
a_HQ_transcript/151007	2.26451008424465	0.606614960093259	1.60662672178591	0.11778427321248	0.771257545611291	-4.73743136951967
a_HQ_transcript/101003	-3.62133310026857	1.68478561123053	-1.60662523361687	0.117784600771604	0.771257545611291	-4.76771844291765
a_HQ_transcript/74431	1.29229931045987	-0.570711855622705	1.60660494967798	0.117789065519926	0.771257545611291	-4.75513316172604
a_HQ_transcript/47209	-1.24202803601725	0.152833923179862	-1.60657378741366	0.117795924994291	0.771257545611291	-4.62037415776199
a_HQ_transcript/92220	-1.78564176371698	1.03372006047018	-1.60653990165861	0.117803384338879	0.771257545611291	-4.67346054070358
a_HQ_transcript/68310	1.00386551324187	-0.322396723306934	1.60653078320035	0.117805391671052	0.771257545611291	-4.65641090244871
a_HQ_transcript/68309	-2.41592175928339	0.0425618602234954	-1.60651484600975	0.11780890014249	0.771257545611291	-4.7072484496557
a_HQ_transcript/31699	-1.29112721939385	1.19420272685254	-1.60643310832879	0.117826895522938	0.771288489071773	-4.63956620737959
a_HQ_transcript/6472	0.380391658917456	5.03485394582842	1.60635353566473	0.117844416420897	0.771316319976791	-5.18851227948903
a_HQ_transcript/129044	4.06960421564861	0.964006981075149	1.60606642665665	0.117907651990652	0.771604817857447	-4.8839624726735
a_HQ_transcript/130615	1.09708020078208	-0.757092072938776	1.60598530623701	0.117925523756274	0.771604817857447	-4.72726931121424
a_HQ_transcript/70385	2.34181879855745	0.714533770464177	1.60597262922313	0.117928316849807	0.771604817857447	-4.66100689884215
a_HQ_transcript/86827	-0.920342188236822	1.6353130253845	-1.60549775505359	0.118032983770343	0.771942338112147	-4.58584819921215
a_HQ_transcript/47744	-0.736083247438141	2.40900043197469	-1.60534655703885	0.11806632529586	0.771942338112147	-4.77295515169222
a_HQ_transcript/29407	1.0431031105297	3.82438475006308	1.60529007967221	0.118078781421065	0.771942338112147	-4.78701631791302
a_HQ_transcript/32334	1.51062693401682	0.915076913074819	1.60522045821239	0.118094137970296	0.771942338112147	-4.60287275562744
a_HQ_transcript/89363	1.13114437339288	2.87431384294747	1.6051866204687	0.118101602223328	0.771942338112147	-4.84757009552085
a_HQ_transcript/87520	1.26655398450613	1.23790051380884	1.60517983799548	0.118103098412711	0.771942338112147	-4.64771413993906
a_HQ_transcript/137144	-2.57757577558228	1.22791177520327	-1.60517977508151	0.11810311229138	0.771942338112147	-4.68431657879738
a_HQ_transcript/89389	1.18900621577062	2.78601821127637	1.6051313886392	0.118113786620301	0.771942338112147	-4.76478549445186
a_HQ_transcript/15060	2.03858836743058	1.00397018112401	1.60511904949533	0.118116508833499	0.771942338112147	-4.62019594947078
a_HQ_transcript/68767	-1.34509693440264	-0.0573856004481497	-1.60507972067624	0.118125185745932	0.771942338112147	-4.65927627282002

a_HQ_transcript/45108	1.80681055557262	0.377429526900573	1.60507611047512	0.118125982272013	0.771942338112147	-4.70430212063247
a_HQ_transcript/76453	-1.3292264614688	1.6209351094222	-1.60479490098179	0.118188039646921	0.772225219659795	-4.58744052847952
a_HQ_transcript/107132	-0.578049899698917	2.84245022960266	-1.60475648750763	0.118196518818083	0.772225219659795	-4.81078517389308
a_HQ_transcript/119842	-3.44799262091928	4.3331279073457	-1.60464298641306	0.118221575319447	0.772225219659795	-4.90820943438414
a_HQ_transcript/124975	1.34155655445154	-0.629175296561506	1.60463920727103	0.118222409677823	0.772225219659795	-4.64958727808664
a_HQ_transcript/95447	-1.59761947919405	1.63663545994361	-1.60451877544239	0.118249001128989	0.772237059592084	-4.81733652983953
a_HQ_transcript/66149	1.47744312214818	-0.354566983655244	1.6045106631027	0.118250792517017	0.772237059592084	-4.62507550641824
a_HQ_transcript/121734	2.23365857954021	1.95709545363956	1.6044313854057	0.118268299997449	0.772264630673076	-4.76072424677335
a_HQ_transcript/49848	1.09837713359557	0.836458083231448	1.60423131988974	0.118312491404265	0.772321392247221	-4.57743417280326
a_HQ_transcript/51965	2.12085009117551	0.581342793853305	1.60422125666875	0.118314714573495	0.772321392247221	-4.79747408719936
a_HQ_transcript/40139	1.99844671346957	3.48708231851768	1.60415468306053	0.118329422894873	0.772321392247221	-4.88403211235496
a_HQ_transcript/40166	-1.58734576741344	0.920477650573593	-1.60413057904056	0.118334748643527	0.772321392247221	-4.63311374721051
a_HQ_transcript/145928	1.31866183609532	0.798582869550106	1.60409130975821	0.118343425556462	0.772321392247221	-4.61056129218479
a_HQ_transcript/101712	-1.6010382290007	0.780506811057556	-1.60402213446321	0.118358711750875	0.772334440576432	-4.79907604997668
a_HQ_transcript/69017	-2.27684044175089	0.230181106206148	-1.60390050251074	0.118385593623798	0.7723544149006	-4.6193587842853
a_HQ_transcript/122755	1.22896787047342	2.04050148935834	1.60379948473207	0.118407923367424	0.7723544149006	-4.71597576065882
a_HQ_transcript/25647	-2.04089777529419	2.64630771603396	-1.60374483771265	0.11842000440374	0.7723544149006	-4.86204225023642
a_HQ_transcript/68198	4.56450978136108	1.46114547966273	1.60363029093239	0.118445331003295	0.7723544149006	-4.7106807759453
a_HQ_transcript/35525	-1.87260265395622	0.480690644161237	-1.60362929771118	0.118445550626596	0.7723544149006	-4.64201420571483
a_HQ_transcript/150592	-1.37585957149101	-0.328097966938443	-1.60354508660374	0.118464172790953	0.7723544149006	-4.73298484576731
a_HQ_transcript/148787	-1.63496545487469	-0.311333877637211	-1.60354479218716	0.11846423790147	0.7723544149006	-4.69769413515442
a_HQ_transcript/90546	-1.66805924126902	0.252736931186036	-1.60352746520238	0.118468069833019	0.7723544149006	-4.68572196137991
a_HQ_transcript/23314	1.97088678934033	-0.539509625235265	1.60299356712273	0.118586193375279	0.772867969665348	-4.71672812216342
a_HQ_transcript/48986	-1.97533766650828	1.19542898814317	-1.60291037014513	0.118604609187131	0.772867969665348	-4.79034985711819
a_HQ_transcript/38138	-2.4288897539097	1.5597094793057	-1.6028371218007	0.118620824792101	0.772867969665348	-4.75051042518437
a_HQ_transcript/25490	1.29084679330349	1.58196308219368	1.6028336240617	0.118621599161643	0.772867969665348	-4.57940810948633
a_HQ_transcript/135689	2.82523595157972	0.443379879491521	1.60280092254528	0.118628839198734	0.772867969665348	-4.66631774405838
a_HQ_transcript/132032	2.80665814364411	0.606614663259169	1.60276357729807	0.118637107790089	0.772867969665348	-4.74531216364886
a_HQ_transcript/77545	-0.794754880549139	1.53402107398937	-1.60275090563368	0.118639913524014	0.772867969665348	-4.63901152950101
a_HQ_transcript/85306	1.38144485786282	-0.648546560680786	1.60263179041964	0.118666290431492	0.772884440368445	-4.71609060979874
a_HQ_transcript/111635	1.82900728325751	-0.464938893488656	1.60261940030916	0.118669034377381	0.772884440368445	-4.71048946135742
a_HQ_transcript/134720	-4.26105614086953	0.681573370703485	-1.60217569085044	0.118767333737518	0.773399583830415	-4.77745495740517
a_HQ_transcript/7809	-0.609366515461025	3.39562697542915	-1.60207056471192	0.118790633159794	0.773399583830415	-4.9549944706774
a_HQ_transcript/135702	2.960880538549	-0.00659317950541791	1.60204008542186	0.118797389077646	0.773399583830415	-4.79854391390299
a_HQ_transcript/139170	1.24723694749597	-0.616922399931043	1.60202221553049	0.118801350193053	0.773399583830415	-4.71798718732733
a_HQ_transcript/137066	1.66156154409359	1.52319024180695	1.60188866936866	0.118830956028467	0.773476446145466	-4.61417146247733
a_HQ_transcript/6406	1.40286501099363	-0.457718395005402	1.60184891730936	0.118839769827366	0.773476446145466	-4.66176998409318
a_HQ_transcript/117894	1.76458680836619	-0.460713329626135	1.60170817128176	0.118870980249208	0.773521082095772	-4.71658335250255
a_HQ_transcript/121656	1.98766305790591	0.528193100084911	1.60169797171048	0.118873242264684	0.773521082095772	-4.7887588104083
a_HQ_transcript/49447	1.42994721589912	-0.24414368068758	1.60155366528083	0.118905249685578	0.773586287025966	-4.61759611531563
a_HQ_transcript/114963	1.47559048809692	0.219390882495026	1.60148360528161	0.118920791666065	0.773586287025966	-4.63057656704291
a_HQ_transcript/12022	0.854935952285493	1.90919001930605	1.6013979479094	0.11893979600291	0.773586287025966	-4.67822644970028
a_HQ_transcript/113296	-1.71874246212164	-0.399231003426075	-1.6012886622955	0.118964046226153	0.773586287025966	-4.65993467130317
a_HQ_transcript/11821	0.701625815654119	2.78102356895674	1.60116072334271	0.118992440734451	0.773586287025966	-4.90491659372862
a_HQ_transcript/56492	0.971824346361438	2.82516721755732	1.601099047009	0.119006131038858	0.773586287025966	-4.76500173747769
a_HQ_transcript/38625	3.2782296813529	0.797217122429084	1.60104563788496	0.119017987313281	0.773586287025966	-4.63983446083283
a_HQ_transcript/1449	1.28394664235534	0.0866458561703417	1.60100354635159	0.11902733188196	0.773586287025966	-4.66186833762056
a_HQ_transcript/21163	1.87916002268193	0.987085576549454	1.60090642307659	0.119048896118027	0.773586287025966	-4.63514459375438
a_HQ_transcript/141219	1.46130542621147	2.26634066938022	1.60088219742206	0.119054275428218	0.773586287025966	-4.75569821866923
a_HQ_transcript/101	-1.82678058005422	4.72032665966885	-1.60080897671206	0.119070535309599	0.773586287025966	-5.14113197215512

a_HQ_transcript/147418	2.56660380000261	0.272339070266929	1.60077753819119	0.119077517317014	0.773586287025966	-4.71744698983906
a_HQ_transcript/125287	2.24345513914683	1.86161058408329	1.60062900388373	0.119110509021566	0.773586287025966	-4.6324788723976
a_HQ_transcript/96708	-1.46103828953792	2.33693525752511	-1.60048416144573	0.119142687925201	0.773586287025966	-4.71422575591106
a_HQ_transcript/89558	0.640011653212905	3.78844866792888	1.60046307853986	0.119147372400905	0.773586287025966	-5.02251745420808
a_HQ_transcript/128761	1.50329331886965	2.74634117065086	1.60042525047239	0.119155777914216	0.773586287025966	-4.71378886216182
a_HQ_transcript/18233	-0.987527234194632	2.9654469370239	-1.6004147259082	0.11915811659132	0.773586287025966	-4.96879597968238
a_HQ_transcript/142558	-1.36264048102586	1.79739323901202	-1.60036181963069	0.119169873534329	0.773586287025966	-4.71396804235807
a_HQ_transcript/67094	1.57788257572872	0.0463383939420974	1.60033900974015	0.119174942689112	0.773586287025966	-4.71272942006005
a_HQ_transcript/29459	1.39994248248627	-0.10497610809266	1.60033873742757	0.119175003207551	0.773586287025966	-4.74836005880892
a_HQ_transcript/147118	1.40820880961313	-0.666107749030273	1.60026362076892	0.119191698011068	0.773586287025966	-4.71700010257609
a_HQ_transcript/40412	1.94341958319608	0.975349272384559	1.60019874409612	0.119206118504009	0.773586287025966	-4.64568993270047
a_HQ_transcript/138760	-3.8461342015569	1.94980301434115	-1.60018248826972	0.119209731999606	0.773586287025966	-4.69706269060272
a_HQ_transcript/112110	-1.83565619990037	0.981520704525948	-1.60013356648754	0.119220607328229	0.773586287025966	-4.61309640080262
a_HQ_transcript/76609	-1.72823344922204	2.03198925020308	-1.60012904869357	0.119221611676381	0.773586287025966	-4.6763071368021
a_HQ_transcript/16903	1.46699981414456	-0.0905811422345653	1.60009455907992	0.119229279271016	0.773586287025966	-4.75643762279418
a_HQ_transcript/140417	-2.00921285178778	0.0447957174671151	-1.59998505523683	0.119253626412906	0.773616449616357	-4.70477850461149
a_HQ_transcript/103511	4.01626268514419	1.5667035074861	1.59995393800441	0.119260545778492	0.773616449616357	-4.71761580380101
a_HQ_transcript/106903	1.62903683382367	3.12514004264708	1.59983812534566	0.119286301286894	0.773680631963594	-4.97757760868416
a_HQ_transcript/89379	1.49745343449054	0.612204527011376	1.59978975355687	0.119297060010489	0.773680631963594	-4.67762709044625
a_HQ_transcript/106471	1.9804651295329	0.610087550992049	1.59963406323936	0.119331693635487	0.773719374709815	-4.68793172806047
a_HQ_transcript/151755	1.63694551705075	0.501037067798893	1.59954567794934	0.119351358789785	0.773719374709815	-4.7163796158359
a_HQ_transcript/131355	-4.32741547182922	5.63570440498509	-1.59953626205259	0.119353453922405	0.773719374709815	-5.39997455794921
a_HQ_transcript/12607	0.473092238497682	5.01533962583512	1.59952077461839	0.119356900099338	0.773719374709815	-5.18121513185468
a_HQ_transcript/142425	-0.78362031303348	4.23604888536963	-1.59940406138661	0.119382873096788	0.773719374709815	-5.08421651859689
a_HQ_transcript/121780	-2.81714056796533	2.44489679030793	-1.59903605680311	0.119464798028141	0.773719374709815	-4.8444541875633
a_HQ_transcript/7241	-1.15812548571234	0.725039676113147	-1.59901011803606	0.119470574237383	0.773719374709815	-4.67338408040932
a_HQ_transcript/146275	0.457742421282959	3.31507161644288	1.5989852700521	0.119476107758853	0.773719374709815	-4.93497190649102
a_HQ_transcript/152386	-4.88365903976982	3.02326252735346	-1.59893194151509	0.119487984466128	0.773719374709815	-4.91301963316663
a_HQ_transcript/84752	1.47480085707017	2.51259947408061	1.59889564524469	0.119496068499999	0.773719374709815	-4.81246987076344
a_HQ_transcript/25303	2.27834469985759	2.78260341748131	1.59888963925593	0.119497406218317	0.773719374709815	-4.77885107565293
a_HQ_transcript/83385	1.95146401120682	-0.164373784926324	1.59887807996398	0.119499980862519	0.773719374709815	-4.75706000569467
a_HQ_transcript/70072	5.15420164445807	1.48757601767423	1.59880838046059	0.119515506256668	0.773719374709815	-4.76930045404652
a_HQ_transcript/148013	-1.92333149846075	-0.153688676800702	-1.59878948905672	0.119519714555566	0.773719374709815	-4.76616113036947
a_HQ_transcript/152349	2.41446868400251	1.21714145633479	1.59873366923597	0.119532149835674	0.773719374709815	-4.82437669792904
a_HQ_transcript/136461	-1.68225433719551	-0.0121902484987342	-1.59872490803947	0.119534101710591	0.773719374709815	-4.64917618563896
a_HQ_transcript/27359	-2.54217928542594	-0.105974231113559	-1.59870438133807	0.119538674881629	0.773719374709815	-4.71152816967146
a_HQ_transcript/152935	1.18324347226012	-0.638543936019047	1.59868665186357	0.119542624970218	0.773719374709815	-4.74369711269626
a_HQ_transcript/48700	0.779584931353756	4.35963315133266	1.59858844273356	0.119564507690173	0.773774850034998	-5.10785673777504
a_HQ_transcript/90826	-3.53605887496921	0.599015585338006	-1.59838665323057	0.119609480242694	0.773780488330553	-4.72293756885496
a_HQ_transcript/114581	2.26909042799364	0.476536846447766	1.59834512452901	0.119618737408361	0.773780488330553	-4.74338594097091
a_HQ_transcript/18828	0.677366432733525	0.590911277653729	1.59831482635006	0.119625491548014	0.773780488330553	-4.60490997028287
a_HQ_transcript/127028	-1.23696621461137	2.08541836994559	-1.59827628961601	0.119634082696974	0.773780488330553	-4.63264917527811
a_HQ_transcript/114752	-2.05224880655138	2.542425690988	-1.59825655194235	0.119638483091724	0.773780488330553	-4.73403412692352
a_HQ_transcript/89523	1.84856773890501	-0.33894202591334	1.59810930326595	0.119671315479101	0.773780488330553	-4.72032410144848
a_HQ_transcript/56214	-0.409473507462242	3.47261383391939	-1.59810749341253	0.119671719072363	0.773780488330553	-4.97658087488481
a_HQ_transcript/3334	-1.04728129942629	3.23750507664139	-1.5979502004324	0.119706799317143	0.773780488330553	-4.99741825858683
a_HQ_transcript/73574	1.76306670111711	-0.122533118243095	1.59792937842126	0.119711443774944	0.773780488330553	-4.71597758238583
a_HQ_transcript/15476	-1.83926765183864	0.322663561407832	-1.59792913683722	0.119711497662386	0.773780488330553	-4.61933674931433
a_HQ_transcript/106901	-2.29575222290824	-0.0136549975302517	-1.59790782888005	0.119716250667642	0.773780488330553	-4.75074615024969
a_HQ_transcript/8718	2.77960605827737	1.3310417503019	1.59775546182968	0.119750242543922	0.773780488330553	-4.67048578856509

a_HQ_transcript/61185	1.20780293927882	1.99541127986587	1.5976278571661	0.119778716215235	0.773780488330553	-4.71846656560437
a_HQ_transcript/121913	0.396912624588763	3.81585082540507	1.59758835311515	0.119787532263637	0.773780488330553	-5.05155382883268
a_HQ_transcript/39301	1.70861402702469	-0.320458194970877	1.59755984376686	0.119793894974307	0.773780488330553	-4.70570673214358
a_HQ_transcript/29073	2.7816271440225	1.75998723304995	1.59754142899522	0.119798004926614	0.773780488330553	-4.66505078643256
a_HQ_transcript/127069	-1.2435515844355	1.69978853488748	-1.59751588408071	0.119803706431152	0.773780488330553	-4.61292651916507
a_HQ_transcript/25100	0.808876185036344	1.13162108023428	1.59717187136588	0.119880510114623	0.773780488330553	-4.59485401039615
a_HQ_transcript/46000	-1.80962821403877	0.604500260636121	-1.59714351072796	0.119886843661633	0.773780488330553	-4.69070370392295
a_HQ_transcript/59638	2.08058514704424	0.284447067263958	1.59697411403254	0.119924679337375	0.773780488330553	-4.76198628097865
a_HQ_transcript/19578	1.43793149032802	3.78031579469906	1.59694398608121	0.119931409604483	0.773780488330553	-4.84826254944874
a_HQ_transcript/54951	-1.19615770531915	1.91032895318548	-1.59694125768058	0.119932019115741	0.773780488330553	-4.72856671838947
a_HQ_transcript/45004	1.21723289875493	1.99516193624944	1.5969364109135	0.119933101865887	0.773780488330553	-4.65776291016289
a_HQ_transcript/1142	-1.3143688847966	1.41360363286384	-1.59693118079786	0.119934270263693	0.773780488330553	-4.63760577372579
a_HQ_transcript/63797	1.42633976609899	0.782052824911131	1.59691439239528	0.119938020823671	0.773780488330553	-4.64302166630287
a_HQ_transcript/40633	1.61814677484933	0.0595158834831839	1.5968717063783	0.119947557391163	0.773780488330553	-4.67452934251034
a_HQ_transcript/147199	-2.59039131115432	1.09597842105624	-1.59676922121999	0.119970456340093	0.773780488330553	-4.84607525855124
a_HQ_transcript/134883	-2.41243457549214	-0.0528103771621639	-1.59674904637172	0.119974964564275	0.773780488330553	-4.70892117010727
a_HQ_transcript/75955	1.53231512123441	0.568124920089115	1.59665590142698	0.119995780314824	0.773780488330553	-4.7289534069937
a_HQ_transcript/114320	1.51223880171515	0.999189818850957	1.59657974984784	0.120012800638218	0.773780488330553	-4.56996681972904
a_HQ_transcript/82978	-1.45818603761412	1.30863303137167	-1.59655969817124	0.120017282634256	0.773780488330553	-4.64475883127309
a_HQ_transcript/108254	-2.35023907606977	2.16497088217305	-1.59653283287209	0.120023287841583	0.773780488330553	-4.90144573924752
a_HQ_transcript/72616	1.82353472066821	-0.0329123581109032	1.59652795775983	0.120024377603094	0.773780488330553	-4.67544803696206
a_HQ_transcript/67169	2.26620152458363	2.11464323452827	1.59651846499872	0.120026499597121	0.773780488330553	-4.64073730115761
a_HQ_transcript/7182	1.48443645491026	1.27735079374155	1.5964925948623	0.120032282714983	0.773780488330553	-4.71716860366132
a_HQ_transcript/150243	-2.33960554954147	1.0710497069473	-1.59638198225087	0.120057012093342	0.773780488330553	-4.68024735997787
a_HQ_transcript/137920	1.45050558970259	0.127278084944076	1.59627671962658	0.120080549265404	0.773780488330553	-4.69180444878354
a_HQ_transcript/51059	-2.88413703104314	1.22915627514281	-1.59627559653594	0.120080800413651	0.773780488330553	-4.72288173263445
a_HQ_transcript/55974	-1.83809828077647	0.684071882621644	-1.59615405785733	0.120107981730624	0.773780488330553	-4.70823988349919
a_HQ_transcript/38816	2.51112546691035	-0.530575297235269	1.59607489606935	0.120125688448935	0.773780488330553	-4.72601927226549
a_HQ_transcript/121019	1.398041213064	1.78425967058925	1.59600917402162	0.120140390623423	0.773780488330553	-4.80092249249244
a_HQ_transcript/99069	2.48391355656827	1.781617336369	1.59599592888941	0.120143353768713	0.773780488330553	-4.64265309081993
a_HQ_transcript/110495	0.680065298757549	2.11834480145615	1.59598417003081	0.120145984461298	0.773780488330553	-4.72030889101558
a_HQ_transcript/19939	2.95306246288493	1.80126001450058	1.59596133967528	0.120151092204644	0.773780488330553	-4.69462849498016
a_HQ_transcript/140280	1.64271629730331	1.05596859930005	1.59586111097584	0.120173518072371	0.773839177856517	-4.65772333896362
a_HQ_transcript/102404	-1.87830643611958	1.59267971641354	-1.59571274179335	0.120206721523065	0.773893374650752	-4.68421637340536
a_HQ_transcript/10497	1.24534253913472	0.991335242717946	1.59563559490083	0.12022398915001	0.773893374650752	-4.58101978610053
a_HQ_transcript/89234	1.65158261051075	0.899001439940127	1.59562586988313	0.120226166024531	0.773893374650752	-4.59515847149672
a_HQ_transcript/147221	0.819190490969666	3.04662624375532	1.59558515837593	0.120235279350051	0.773893374650752	-4.79755196474403
a_HQ_transcript/139996	-1.16694310144468	2.18665432894062	-1.59544201444748	0.120267326804024	0.773893374650752	-4.64718857998426
a_HQ_transcript/114294	1.59996050250657	-0.201120141906001	1.59542527350398	0.120271075269252	0.773893374650752	-4.72028241608496
a_HQ_transcript/19029	0.515966772645416	3.90744533318953	1.59533746033205	0.120290739087399	0.773893374650752	-5.02647805699326
a_HQ_transcript/91184	-2.51277434484777	1.66005289837095	-1.5953190562429	0.120294860610047	0.773893374650752	-4.82558982751211
a_HQ_transcript/97876	-1.71536129768196	1.31457678202672	-1.59523746604417	0.120313133804246	0.773893374650752	-4.72127743561947
a_HQ_transcript/138568	1.16173999314137	0.739243009668677	1.59522881858383	0.120315070649581	0.773893374650752	-4.6633334955011
a_HQ_transcript/132489	0.518123998785482	3.94844080102444	1.5951564674808	0.120331276743206	0.773911977708461	-5.03516165624252
a_HQ_transcript/72761	2.13204772140521	2.30767590484104	1.59487999308238	0.120393221367472	0.773948996612043	-4.64165177879058
a_HQ_transcript/63909	1.45440316761717	4.27093100753764	1.59487286172087	0.120394819508344	0.773948996612043	-5.19321604448938
a_HQ_transcript/133851	0.957482297507364	3.04051130835996	1.59486556553232	0.120396454605008	0.773948996612043	-4.84486924021279
a_HQ_transcript/121406	2.27012699662167	0.431073304294355	1.59482203282306	0.120406210787362	0.773948996612043	-4.67337990448203
a_HQ_transcript/113728	-1.75618514290348	-0.115586297836178	-1.59474627649172	0.120423190199261	0.773948996612043	-4.70271047371798
a_HQ_transcript/137453	2.15412279074893	0.781837813358143	1.59473761090303	0.120425132559284	0.773948996612043	-4.72620877156549

a_HQ_transcript/142624	4.02331548139956	2.4786658620406	1.59466111578124	0.120442279774357	0.773948996612043	-4.89147348488034
a_HQ_transcript/43692	-1.1180011665562	1.0179248095189	-1.59461571148592	0.120452458587727	0.773948996612043	-4.58701877407849
a_HQ_transcript/52802	-1.54000549030955	0.431673465957053	-1.59458194751979	0.1204600283088	0.773948996612043	-4.64024507359195
a_HQ_transcript/45432	1.9696268619616	1.16613206929634	1.5943202098788	0.12051872186592	0.773948996612043	-4.65952055843902
a_HQ_transcript/60848	0.891796182783308	-0.531145402215251	1.59430796190734	0.120521468995243	0.773948996612043	-4.73831095905996
a_HQ_transcript/38492	-1.7494260394339	0.323749650404317	-1.59430281736107	0.120522622894182	0.773948996612043	-4.6708892548603
a_HQ_transcript/150863	-1.6466767427737	1.02807588356213	-1.59417245188078	0.120551866314018	0.773948996612043	-4.69720642689074
a_HQ_transcript/140599	-3.23672311192995	1.76974611434952	-1.59416296384413	0.120553994885272	0.773948996612043	-4.79335766071152
a_HQ_transcript/124795	0.619137267544342	3.78971898082177	1.59413801111401	0.120559592993728	0.773948996612043	-5.04096757526192
a_HQ_transcript/83459	-0.667380107035551	3.92480965691264	-1.59408464501318	0.120571566314908	0.773948996612043	-5.04005555205929
a_HQ_transcript/35441	-0.549690738533122	3.67524957840009	-1.5940677163593	0.120575364663775	0.773948996612043	-4.89802276382628
a_HQ_transcript/25512	1.28894771419911	2.09121752868429	1.5939156968173	0.120609478280055	0.773948996612043	-4.79604455945509
a_HQ_transcript/15509	-1.3123202304604	0.591905456880195	-1.59389761205873	0.12061353707723	0.773948996612043	-4.6987190678397
a_HQ_transcript/126217	2.8680717876087	2.12359443211788	1.59369843389413	0.120658246417231	0.773948996612043	-4.76374420701032
a_HQ_transcript/123656	1.28248233603372	-0.2541191599728	1.59367298565853	0.120663959737087	0.773948996612043	-4.73200476409626
a_HQ_transcript/117769	-1.69904427892932	2.77910320472814	-1.59363847256024	0.120671708540635	0.773948996612043	-4.99363870900451
a_HQ_transcript/75884	1.73131702360999	4.26635693102661	1.59363404801317	0.120672701959508	0.773948996612043	-5.20907154083918
a_HQ_transcript/146847	1.22224736576785	-0.697578411482547	1.59361145753993	0.120677774176843	0.773948996612043	-4.73596491144338
a_HQ_transcript/148205	1.81163573456745	-0.475693016683817	1.59355123514925	0.12069129670574	0.773948996612043	-4.7243123676797
a_HQ_transcript/106139	1.38981539639287	0.25344191885569	1.59341634822778	0.120721589150513	0.773948996612043	-4.70262419832331
a_HQ_transcript/10828	-0.645537217537071	4.22965240538154	-1.59338829573006	0.120727889866822	0.773948996612043	-5.12954934549553
a_HQ_transcript/30018	1.38886407511188	-0.452245556401205	1.59337334249466	0.120731248540326	0.773948996612043	-4.70887520126172
a_HQ_transcript/47666	-1.47565771238274	-0.316933412510985	-1.59323836900316	0.120761568645395	0.773948996612043	-4.70358456381582
a_HQ_transcript/69878	1.76337127615654	0.359561977574579	1.59323440013968	0.120762460295252	0.773948996612043	-4.63576878998327
a_HQ_transcript/4441	-0.99323878688942	3.15493520812471	-1.59315372322363	0.120780586441284	0.773948996612043	-4.93292156015915
a_HQ_transcript/145619	1.49552158462275	-0.574150028522033	1.59314050761613	0.120783555880073	0.773948996612043	-4.70667304130072
a_HQ_transcript/77383	-3.82303984769696	3.1379657219393	-1.59312097055898	0.120787945806353	0.773948996612043	-4.90657678505828
a_HQ_transcript/121610	3.28381395265201	5.99978482013418	1.59311304011041	0.120789727794943	0.773948996612043	-5.4698899356829
a_HQ_transcript/81075	-2.07799765125922	1.34393904092919	-1.59305312177169	0.12080319226838	0.773949957034763	-4.74506673446422
a_HQ_transcript/66705	-2.63326194547685	0.985820288284867	-1.5929116716593	0.12083498292629	0.774068314694666	-4.7625065183852
a_HQ_transcript/42988	0.566090610158149	3.50260667341898	1.59273828324221	0.120873961005654	0.774112835168537	-5.02868607130865
a_HQ_transcript/124461	1.62938290040506	-0.183090845675014	1.5926719628495	0.120888872690667	0.774112835168537	-4.76914123285014
a_HQ_transcript/115282	-1.42661269964196	-0.463550391266327	-1.59265772336026	0.120892074538486	0.774112835168537	-4.73005202444302
a_HQ_transcript/10854	1.78657025431802	2.14321056736702	1.59264381377619	0.120895202271882	0.774112835168537	-4.77240157609863
a_HQ_transcript/131086	-1.38866375911125	-0.268599860730385	-1.59250996786613	0.120925302483751	0.774121273863441	-4.71933249768495
a_HQ_transcript/113202	2.9512055136275	4.52499951500709	1.59239873614479	0.120950321725836	0.774121273863441	-4.94763735741702
a_HQ_transcript/147019	-1.71879035104745	-0.542573314951019	-1.59229522086183	0.120973609124303	0.774121273863441	-4.72264489446039
a_HQ_transcript/72546	1.47117715672835	-0.574395543380385	1.59227628312228	0.120977869865244	0.774121273863441	-4.73721548841643
a_HQ_transcript/102453	3.92184797519185	4.05309452493722	1.59226822563269	0.120979682731097	0.774121273863441	-5.02417935789754
a_HQ_transcript/18002	-1.1097724155954	1.1448451064391	-1.592231211652	0.12098801084415	0.774121273863441	-4.61772330227306
a_HQ_transcript/34063	2.39917646331556	2.38914215152473	1.59217963371499	0.120999616617293	0.774121273863441	-4.69976749789852
a_HQ_transcript/52360	1.83026216010055	0.623424367680446	1.59216432981217	0.121003060389541	0.774121273863441	-4.70932672949194
a_HQ_transcript/17405	-0.724270958733253	2.7741208509187	-1.59204858475227	0.121029108607333	0.774194067960026	-4.85204743111828
a_HQ_transcript/105713	-1.43808045930478	-0.487040500334307	-1.59199541274419	0.121041076411036	0.774194067960026	-4.72313674656491
a_HQ_transcript/117593	1.91494868075493	0.615697545649642	1.59180942628722	0.121082945333131	0.774376658193887	-4.76883612160927
a_HQ_transcript/137154	-1.24738964462531	7.53283000657261	-1.59147564944758	0.121158114280632	0.774771697210716	-5.5009200878219
a_HQ_transcript/113782	-0.537229701349823	3.74549340960429	-1.59139340749858	0.121176641624484	0.774771697210716	-4.95587305846227
a_HQ_transcript/86044	1.26461904747465	0.127755811786541	1.5913048459137	0.121196595239846	0.774771697210716	-4.66992622874535
a_HQ_transcript/93821	-1.57316521410801	1.8984500051061	-1.5912191931113	0.121215896042312	0.774771697210716	-4.61682282845046
a_HQ_transcript/58306	-2.55998731288898	1.48881798095483	-1.59117231825351	0.121226459778501	0.774771697210716	-4.67243935668427

a_HQ_transcript/38268	1.81765818848092	1.56305048024702	1.59113926466042	0.121233909202133	0.774771697210716	-4.67971692488306
a_HQ_transcript/77596	-0.474628302919454	3.66978952949244	-1.59106974010273	0.121249579459404	0.774771697210716	-5.00946446374903
a_HQ_transcript/3262	-0.520648639405922	3.1857138531386	-1.59106191137005	0.121251344094514	0.774771697210716	-4.935681509353
a_HQ_transcript/95629	-0.589182955929753	2.68884657665128	-1.59093720596323	0.121279456145264	0.77485422857091	-4.85713385497339
a_HQ_transcript/57974	1.95131118898412	0.199180183405051	1.59073020747915	0.121326131110825	0.77485422857091	-4.75691891553135
a_HQ_transcript/101830	-1.69716797871687	1.15773105382879	-1.59066411211814	0.121341037691769	0.77485422857091	-4.64773584025898
a_HQ_transcript/118773	-1.27089866126451	2.86073865067367	-1.59057536506574	0.121361055298579	0.77485422857091	-4.85586364589359
a_HQ_transcript/1865	1.25328766543758	2.59544714058334	1.59055489067404	0.121365673844915	0.77485422857091	-4.70993325490222
a_HQ_transcript/38638	1.44075475927423	1.86355558923005	1.59053561545001	0.121370022018393	0.77485422857091	-4.58235980018791
a_HQ_transcript/143710	0.892805444662138	1.91428735164175	1.59051253397088	0.121375228988164	0.77485422857091	-4.70109382549742
a_HQ_transcript/81147	1.65135588695222	-0.597882963333274	1.59043074396071	0.121393681535348	0.77485422857091	-4.73862965934583
a_HQ_transcript/142805	4.91794424346583	0.326871933851226	1.59037125465239	0.121407104289897	0.77485422857091	-4.7386521588946
a_HQ_transcript/136367	-3.2857209645289	0.00611152129903543	-1.59027662007057	0.121428459483076	0.77485422857091	-4.73025148084002
a_HQ_transcript/44847	-2.41257036828383	1.19033307655266	-1.590240553983	0.121436598947681	0.77485422857091	-4.77355252360414
a_HQ_transcript/134635	2.84984075184708	0.974076681540256	1.59022764548802	0.121439512270376	0.77485422857091	-4.83485239419134
a_HQ_transcript/73179	1.9625941172561	1.0689500479596	1.59014747335165	0.121457607628419	0.77485422857091	-4.62323501159321
a_HQ_transcript/18040	1.64597129874945	0.560132010094338	1.59008045852796	0.121472734990926	0.77485422857091	-4.74358620897906
a_HQ_transcript/84583	1.60908233599507	1.84869125052314	1.59005858763395	0.121477672276251	0.77485422857091	-4.67000556067612
a_HQ_transcript/127839	-1.29223446377915	1.63520327515801	-1.59004356206913	0.121481064345278	0.77485422857091	-4.65826983402683
a_HQ_transcript/141807	-1.45149462385613	-0.389294329144395	-1.58989708603645	0.121514135836536	0.77485422857091	-4.69985609104393
a_HQ_transcript/62132	-2.32879480701702	1.15678873690366	-1.58984196384468	0.121526583282799	0.77485422857091	-4.68725811020043
a_HQ_transcript/15433	1.6145253616811	-0.541068700051762	1.58980361528135	0.121535243598936	0.77485422857091	-4.73054373616636
a_HQ_transcript/68305	-1.17731351646854	-0.525737913902719	-1.58963011779481	0.121574431119586	0.77485422857091	-4.7263441576533
a_HQ_transcript/95239	1.55070672610525	-0.112436959238169	1.58953214812109	0.121596563895796	0.77485422857091	-4.70599316009519
a_HQ_transcript/31092	2.48524340213049	3.43394254097029	1.5893173947147	0.121645091353447	0.77485422857091	-4.91555807943099
a_HQ_transcript/119167	1.76997739924467	-0.0438546114175041	1.5893171354829	0.121645149941186	0.77485422857091	-4.74115224453853
a_HQ_transcript/92329	-2.20449917979616	1.16037997735377	-1.5892644789103	0.121657051079823	0.77485422857091	-4.75348736489766
a_HQ_transcript/101892	1.11661024938389	-0.73967549421512	1.58915081572199	0.121682743834151	0.77485422857091	-4.74884767683275
a_HQ_transcript/138921	0.955909392442828	3.53976672499316	1.58914682224334	0.121683646612292	0.77485422857091	-5.03376801916945
a_HQ_transcript/81200	0.762947721924168	1.86389404930954	1.58912552866679	0.12168846039661	0.77485422857091	-4.67076490319639
a_HQ_transcript/42786	0.554641432315391	3.67917602138638	1.58912112517512	0.121689455902136	0.77485422857091	-5.02924061011769
a_HQ_transcript/136158	0.419463465143282	5.48972887110069	1.58906956281967	0.121701113195297	0.77485422857091	-5.24018615234322
a_HQ_transcript/4140	-1.78147234470923	1.58115260873017	-1.58904842171816	0.121705893070733	0.77485422857091	-4.67215464230876
a_HQ_transcript/92208	0.819570280950486	3.70448069219683	1.58897453448025	0.121722599735773	0.77485422857091	-4.98838193603085
a_HQ_transcript/74493	2.19904155528134	2.15225215402308	1.58889300150061	0.121741037359384	0.77485422857091	-4.66549450204092
a_HQ_transcript/147805	-2.81064843444365	1.32668129922673	-1.58885983928586	0.121748537216901	0.77485422857091	-4.802344817509
a_HQ_transcript/143959	-0.938198654291258	2.24691456101542	-1.58881542359261	0.121758582716347	0.77485422857091	-4.74332448030758
a_HQ_transcript/121144	-2.04326481710015	0.469046766927642	-1.58881295239471	0.121759141647208	0.77485422857091	-4.74145291421296
a_HQ_transcript/114345	0.692065195665473	3.39299866043634	1.58872967638771	0.1217779780841	0.77485422857091	-5.01331667119906
a_HQ_transcript/85956	2.03791529939689	0.0589188090212698	1.58863495995652	0.121799405160972	0.77485422857091	-4.74049331293522
a_HQ_transcript/145947	1.31208538379376	1.15130162774891	1.58852719681952	0.121823787459514	0.77485422857091	-4.58929913298197
a_HQ_transcript/27688	2.62527759714488	1.64552623753354	1.58852229827636	0.121824895890182	0.77485422857091	-4.81675331249503
a_HQ_transcript/108444	1.44194266188417	-0.612135962010024	1.58849065764532	0.121832055655526	0.77485422857091	-4.7369659357854
a_HQ_transcript/102518	-1.70725226337196	0.349790333603883	-1.58840188159846	0.121852146083508	0.77485422857091	-4.61358042793624
a_HQ_transcript/134574	2.03375152979381	1.22865432834158	1.58828376103395	0.121878881513085	0.77485422857091	-4.60328746844948
a_HQ_transcript/9213	-3.04344996319576	1.37221153782415	-1.5881772825587	0.121902985982664	0.77485422857091	-4.81905742364725
a_HQ_transcript/4000	1.41123431596417	-0.610306062683739	1.588148625509	0.121909473997227	0.77485422857091	-4.74354756555492
a_HQ_transcript/132084	2.31822670359912	2.00847837236729	1.58811503259539	0.121917079861115	0.77485422857091	-4.82126521209761
a_HQ_transcript/98424	2.86576937672135	2.0292203781758	1.58807754446414	0.121925568110247	0.77485422857091	-4.7232121609664
a_HQ_transcript/43796	1.81198190879419	0.111130653208773	1.5880657797555	0.121928232033756	0.77485422857091	-4.69018273259442

a_HQ_transcript/17973	-0.918095084969047	3.11292317110978	-1.58806489337437	0.121928432742019	0.77485422857091	-4.94864941145705
a_HQ_transcript/126118	-2.06661141713356	0.731002739512962	-1.58800197163794	0.121942681155088	0.77485422857091	-4.6251984143536
a_HQ_transcript/45646	1.2034766642841	4.47712360852968	1.58795407659886	0.121953527736655	0.77485422857091	-5.07198821138807
a_HQ_transcript/62173	1.09025694343138	2.62744041805866	1.5879306405441	0.121958835485801	0.77485422857091	-4.81447970449891
a_HQ_transcript/140669	1.91041730274192	0.628526524605325	1.58792871261457	0.121959272127701	0.77485422857091	-4.71956276413451
a_HQ_transcript/61851	-1.84057830132493	1.53997759261504	-1.58779849079634	0.121988768024079	0.77485422857091	-4.68901383554899
a_HQ_transcript/43224	-2.41166988093636	1.23845568512298	-1.58776912009969	0.121995421440921	0.77485422857091	-4.73397287926754
a_HQ_transcript/115218	1.24162106679252	-0.649038432098269	1.58772795049641	0.122004748193046	0.77485422857091	-4.7320593599948
a_HQ_transcript/132914	-1.06385108914664	4.6776494055095	-1.58764439590774	0.122023678828683	0.77485422857091	-5.17878506341697
a_HQ_transcript/138373	1.11953209012917	-0.0922460772556527	1.58764263401139	0.122024078040499	0.77485422857091	-4.71379550256419
a_HQ_transcript/135267	5.06618638086914	2.26269188961221	1.58733412834483	0.122093995961571	0.774975428767637	-4.81684575869292
a_HQ_transcript/111255	1.97180081232909	1.26588679223136	1.58727284516426	0.122107888723806	0.774975428767637	-4.6670558672224
a_HQ_transcript/55048	3.49971323077574	3.29718216237249	1.58726476628593	0.12210972028421	0.774975428767637	-4.94896620059478
a_HQ_transcript/37694	-1.359243212353	1.92907540894599	-1.5872255081167	0.122118620813446	0.774975428767637	-4.70138626069536
a_HQ_transcript/144501	-4.05105410810265	1.61339486802715	-1.58715803741009	0.122133918869225	0.774975428767637	-4.6944014950911
a_HQ_transcript/123977	0.568327565476304	2.83728107252823	1.58715244513903	0.122135186910199	0.774975428767637	-4.88463868193688
a_HQ_transcript/146478	0.345772259510206	11.8160847297605	1.58702968103115	0.122163026242072	0.774975428767637	-5.94041348646511
a_HQ_transcript/135934	1.04737410200539	-0.707154310175073	1.58693558590773	0.122184367794777	0.774975428767637	-4.76060820244435
a_HQ_transcript/23076	-2.12716195414028	0.0363342759248267	-1.58688381408082	0.122196111373926	0.774975428767637	-4.67126925216736
a_HQ_transcript/70822	-1.07756876628193	2.97329349610482	-1.58687532260114	0.122198037613193	0.774975428767637	-4.96858678186716
a_HQ_transcript/83066	1.18484300112581	1.58582217219875	1.58683478732919	0.122207233129332	0.774975428767637	-4.6583278855312
a_HQ_transcript/126561	0.675248111066049	4.07697019656204	1.58681027043668	0.122212795115302	0.774975428767637	-5.07587003381529
a_HQ_transcript/101284	-1.82058099235908	2.13017002083763	-1.58679401256767	0.122216483545158	0.774975428767637	-4.77882158445887
a_HQ_transcript/44856	2.04181483139023	0.472994095239852	1.58658518186867	0.122263869152854	0.77519133792726	-4.62413151390496
a_HQ_transcript/105410	1.71706290247849	0.285201987539275	1.58645354062305	0.12229374748332	0.775228340422693	-4.71225464404333
a_HQ_transcript/17414	1.86575212923298	-0.308835196008169	1.58639032044577	0.122308098553258	0.775228340422693	-4.72808990975531
a_HQ_transcript/14952	1.35283474004	1.8693064767352	1.58636769366405	0.122313235199463	0.775228340422693	-4.74173601392887
a_HQ_transcript/127881	-0.513901836923871	4.584964767493	-1.58629322930496	0.122330141061596	0.775228340422693	-5.17310950277343
a_HQ_transcript/135227	1.25982002959851	-0.760969036538939	1.58626571549289	0.122336388086591	0.775228340422693	-4.74825692049932
a_HQ_transcript/63463	-2.29378391906842	1.95874035662975	-1.58610185826099	0.122373597357613	0.775300987870137	-4.76935243956743
a_HQ_transcript/125183	2.17872330038894	2.06301428533397	1.58607313756387	0.122380120306699	0.775300987870137	-4.80871620252005
a_HQ_transcript/84713	-1.12428116189289	1.35625192866844	-1.58597181031691	0.122403135684622	0.775300987870137	-4.6425587057549
a_HQ_transcript/124288	-1.14988163870315	2.13371813584906	-1.58593098302484	0.12241241015859	0.775300987870137	-4.71489528959514
a_HQ_transcript/40905	4.40911255142594	2.31335576021609	1.58590051669882	0.122419331372761	0.775300987870137	-4.69267015360092
a_HQ_transcript/126650	-3.73306456294621	3.61122145202855	-1.58586289174956	0.122427879295004	0.775300987870137	-4.73324295908419
a_HQ_transcript/49446	-2.52870103487402	2.75902473158051	-1.58580299775422	0.122441487475381	0.775302699778751	-4.86753106109834
a_HQ_transcript/41738	2.09861976451359	0.26684250993104	1.5856072649851	0.122485967450906	0.775482744029797	-4.58268784047818
a_HQ_transcript/136879	0.436582749745754	4.55250322167164	1.58551432118959	0.122507093413682	0.775482744029797	-5.16134917009581
a_HQ_transcript/5663	1.48742352975026	-0.347550847063679	1.58548592417904	0.122513548598845	0.775482744029797	-4.7197532008125
a_HQ_transcript/11470	1.48549088863861	-0.661190761974227	1.58542471134423	0.12252746439616	0.775482744029797	-4.73411683962544
a_HQ_transcript/43103	-1.3733366367582	3.12321922417523	-1.58538441377822	0.12253662613394	0.775482744029797	-4.85441836014106
a_HQ_transcript/120110	-2.18846289376406	2.94912536622713	-1.58521122736395	0.122576006811838	0.775498229823512	-4.91963079751809
a_HQ_transcript/79045	0.554119185963966	2.79460184143974	1.58513932807655	0.122592358955045	0.775498229823512	-4.90537277951626
a_HQ_transcript/33503	-1.48492660636844	1.49649815630425	-1.5849708357148	0.122630686366785	0.775498229823512	-4.75040909715573
a_HQ_transcript/52779	3.3040586789192	1.87601915977983	1.58493748705866	0.12263827343673	0.775498229823512	-4.76675621829371
a_HQ_transcript/74424	1.78653479560699	0.186477041711172	1.58481223528117	0.122666772585008	0.775498229823512	-4.6275727548338
a_HQ_transcript/60189	-1.26613969458091	0.236766281007195	-1.584761869943	0.122678233982412	0.775498229823512	-4.61245210501912
a_HQ_transcript/10907	1.36348674193724	3.85427924257645	1.5847323601589	0.122684949788575	0.775498229823512	-5.09543839184763
a_HQ_transcript/46961	-2.34093652552723	1.43009983745759	-1.58469969295202	0.122692384508253	0.775498229823512	-4.60136124696153
a_HQ_transcript/84984	1.19601589250486	1.24343642147653	1.58463146893382	0.122707912779657	0.775498229823512	-4.64692517235883

a_HQ_transcript/15530	0.668475045260101	2.61916061392968	1.58439067813268	0.122762731319442	0.775498229823512	-4.80694885861285
a_HQ_transcript/133619	0.5297076724124	5.68292445619626	1.58436584196016	0.122768386672149	0.775498229823512	-5.25505749233232
a_HQ_transcript/32024	1.68299806876106	-0.439231192674776	1.58433935441983	0.122774418286366	0.775498229823512	-4.74699877816455
a_HQ_transcript/16126	-1.67276155084402	0.0647118843602403	-1.58433424695196	0.122775581362036	0.775498229823512	-4.62908857506451
a_HQ_transcript/151096	4.80365936586139	1.79545757608225	1.58431847822037	0.12277917228395	0.775498229823512	-4.80178744516542
a_HQ_transcript/24210	1.30980282090047	-0.600911742793392	1.58431745255872	0.122779405854911	0.775498229823512	-4.73422579749943
a_HQ_transcript/126702	3.21474401096862	0.800706033671388	1.58428488774319	0.122786821934995	0.775498229823512	-4.74967488436864
a_HQ_transcript/122004	-1.74583150497139	0.91193999372529	-1.58426182318905	0.122792074714451	0.775498229823512	-4.70775384566343
a_HQ_transcript/97602	2.38870089759513	1.4560805372787	1.58401413226967	0.122848496045158	0.775498229823512	-4.71128506772068
a_HQ_transcript/78233	1.79913450286448	0.567965933913477	1.58400979296973	0.122849484679912	0.775498229823512	-4.61301353358494
a_HQ_transcript/13519	1.24984560917951	1.37010808717863	1.583996463941	0.122852521510203	0.775498229823512	-4.61286108674698
a_HQ_transcript/36272	-2.62969141249422	1.35205719042051	-1.58395659382732	0.122861605716832	0.775498229823512	-4.87155097445439
a_HQ_transcript/151983	1.68878172172041	0.054084118853561	1.58395136581132	0.122862796934929	0.775498229823512	-4.74335790620131
a_HQ_transcript/135852	1.77503233664391	-0.205541623174927	1.58389095932172	0.122876561407594	0.775498229823512	-4.74727611764272
a_HQ_transcript/7625	-4.09853669210735	3.27494510350915	-1.58382226143303	0.122892216724533	0.775498229823512	-4.93404664790087
a_HQ_transcript/144004	1.69935556939797	0.289088449118309	1.58368799777221	0.122922818298261	0.775498229823512	-4.66604112030453
a_HQ_transcript/64703	1.64829532704055	0.569064309224765	1.5835849873919	0.122946300799736	0.775498229823512	-4.70545515621248
a_HQ_transcript/89223	1.74329318814738	-0.554267324575475	1.58357628992923	0.122948283662714	0.775498229823512	-4.7246964513353
a_HQ_transcript/254	1.26583346751509	-0.59551104059736	1.58350687473562	0.122964109998521	0.775498229823512	-4.75022899297733
a_HQ_transcript/71485	4.08352323920047	1.19154464210332	1.58350185050148	0.122965255564757	0.775498229823512	-4.77559201167583
a_HQ_transcript/100922	1.22804005541093	0.0561281292904957	1.58347220541172	0.122972015063829	0.775498229823512	-4.72111238199492
a_HQ_transcript/17718	-0.662947593867175	2.84145362461193	-1.58345333742897	0.122976317388474	0.775498229823512	-4.76917336518539
a_HQ_transcript/147500	1.92728749628833	0.269495575095761	1.58343284002248	0.122980991397812	0.775498229823512	-4.71756641996658
a_HQ_transcript/47660	-0.604695225326353	2.70485054770752	-1.58331878771064	0.123007001318678	0.775498229823512	-4.85743106197081
a_HQ_transcript/6580	1.489371024745	-0.103583069226824	1.58329318565774	0.123012840550027	0.775498229823512	-4.79213303426871
a_HQ_transcript/126367	-1.07756901138949	2.10086922030142	-1.58327483518096	0.123017026005312	0.775498229823512	-4.60838586281376
a_HQ_transcript/22943	-1.99900955015681	0.152106927457198	-1.58317491649873	0.123039817928213	0.775498229823512	-4.78695686943585
a_HQ_transcript/107121	2.0458088677514	0.766089983568454	1.58300139090436	0.123079408135865	0.775498229823512	-4.66531776573087
a_HQ_transcript/62370	-7.29718541339084	3.22988396745818	-1.58291061267379	0.123100123515992	0.775498229823512	-4.70312473398746
a_HQ_transcript/128475	-2.37442477586137	-0.0939815349542585	-1.58286616517593	0.123110267370178	0.775498229823512	-4.74313473361171
a_HQ_transcript/89082	-2.9922694803993	4.78872543905162	-1.58285378032231	0.123113093976034	0.775498229823512	-5.14160063345677
a_HQ_transcript/29645	1.40182806409933	-0.283116267794781	1.58282462520107	0.123119748283951	0.775498229823512	-4.65745224048255
a_HQ_transcript/145310	-2.52535269624	1.1304834666567	-1.5827775837076	0.123130485562221	0.775498229823512	-4.82175781680958
a_HQ_transcript/56751	1.32954988401647	1.07504290424748	1.58267226117587	0.123154528333116	0.775498229823512	-4.60172128858388
a_HQ_transcript/24047	1.70651908674038	1.7811684186146	1.58256682482223	0.123178600929377	0.775498229823512	-4.71825626854965
a_HQ_transcript/502	2.37910449634233	2.05452009165807	1.5825521562709	0.123181950269742	0.775498229823512	-4.80381021565098
a_HQ_transcript/67338	1.80573464688677	0.013431331044652	1.58250480689298	0.123192762287113	0.775498229823512	-4.68893390751224
a_HQ_transcript/149775	-1.98678404508454	0.0939926073995028	-1.58248847389635	0.12319649203327	0.775498229823512	-4.72934021177413
a_HQ_transcript/93978	0.662937017901801	2.60190307383714	1.58247734469493	0.123199033511754	0.775498229823512	-4.78901765220444
a_HQ_transcript/52737	2.02771729588175	1.77027836191535	1.58242340342496	0.123211352215298	0.775498229823512	-4.6354081787578
a_HQ_transcript/43331	-1.06433965295548	1.6600072876158	-1.58233502749458	0.123231537024209	0.775498229823512	-4.61780253025556
a_HQ_transcript/19912	-0.704651381112733	3.90244953051415	-1.58230910770979	0.123237457541092	0.775498229823512	-5.09302692069958
a_HQ_transcript/59330	-1.69132930881669	0.78515892343325	-1.5822815184858	0.123243759641457	0.775498229823512	-4.69371491659415
a_HQ_transcript/142892	1.46399627886378	-0.605474538091333	1.5822710211882	0.123246157567947	0.775498229823512	-4.73873939213783
a_HQ_transcript/7834	0.772123173184091	4.85816504042717	1.58204575846264	0.12329762413753	0.77573809891399	-5.14528890496311
a_HQ_transcript/148347	1.70693187738311	2.68982488063509	1.58193013083926	0.123324048808006	0.775745515441982	-4.8309453235209
a_HQ_transcript/42906	1.33522735465242	2.09052625331184	1.58192380816061	0.123325493879015	0.775745515441982	-4.76401659497507
a_HQ_transcript/65791	-2.04732883695501	2.24133568358328	-1.58185496919542	0.123341228168103	0.77576054002981	-4.86501282424303
a_HQ_transcript/123616	1.95123478930179	-0.534110072188795	1.58150059572187	0.123422252068034	0.775919026859345	-4.73464481402177
a_HQ_transcript/73976	0.9464468810825	2.94508285765694	1.58147310841775	0.1234285385781	0.775919026859345	-4.92455053053323

a_HQ_transcript/59695	-1.68283254059026	1.29471301871918	-1.5814100463751	0.123442962231147	0.775919026859345	-4.6461140567584
a_HQ_transcript/139592	1.72769639358411	1.19485290062288	1.58138662250026	0.123448320128644	0.775919026859345	-4.68966521028494
a_HQ_transcript/143858	-1.10474705460752	2.43899834945004	-1.58123826229839	0.123482259949351	0.775919026859345	-4.83498379029628
a_HQ_transcript/90969	0.956010623026854	3.55546290450187	1.58122271088097	0.123485818031588	0.775919026859345	-5.02985318758728
a_HQ_transcript/3737	-1.54651476166934	1.0351367762343	-1.58117738044459	0.123496189874089	0.775919026859345	-4.67235189096121
a_HQ_transcript/2837	-0.730968574232648	4.01717738046702	-1.58115010011218	0.123502432100779	0.775919026859345	-5.10798311741195
a_HQ_transcript/121735	1.58661770272178	0.564781175384692	1.58110487934465	0.123512779986791	0.775919026859345	-4.79289950198333
a_HQ_transcript/47121	1.5674659429513	1.3711380355584	1.58109095354367	0.123515966775943	0.775919026859345	-4.66431497170541
a_HQ_transcript/92735	-0.544574477969801	2.80243754802381	-1.58102296993757	0.123531525151941	0.775919026859345	-4.8834820059934
a_HQ_transcript/26814	-0.847685403844197	3.54507498989255	-1.58088563204322	0.123562960474923	0.775919026859345	-5.04689233785102
a_HQ_transcript/65634	1.39076679041393	0.684656239997938	1.58081342579387	0.12357949041157	0.775919026859345	-4.67115830972098
a_HQ_transcript/122687	0.485686820484236	4.55627015389618	1.58078628877879	0.123585703265212	0.775919026859345	-5.15813903581765
a_HQ_transcript/149317	2.39224268588799	-0.400073331779888	1.58076552256983	0.123590457734078	0.775919026859345	-4.75707185484998
a_HQ_transcript/6402	-1.84373676378504	1.33509966924139	-1.58065441262632	0.123615899136551	0.775919026859345	-4.81784890433258
a_HQ_transcript/64071	1.94200353294511	0.828659542275769	1.58062455246058	0.123622737098113	0.775919026859345	-4.76552911020925
a_HQ_transcript/32278	0.837570311621913	1.80328343649874	1.5805336224131	0.123643561932054	0.775919026859345	-4.68663951559202
a_HQ_transcript/64146	-1.96953338156619	2.67041293464053	-1.58049275253691	0.123652922899926	0.775919026859345	-4.74839191792198
a_HQ_transcript/40773	-1.47252639489188	0.48651842106112	-1.58047755507728	0.123656403922598	0.775919026859345	-4.67967937468343
a_HQ_transcript/120299	-1.68805904378992	2.11393669571928	-1.58043068626883	0.123667139864617	0.775919026859345	-4.92084096065514
a_HQ_transcript/61732	0.742025880996011	2.53543370975688	1.58039158136526	0.123676097960562	0.775919026859345	-4.77757569982172
a_HQ_transcript/125677	-3.34346797271987	0.260894777229435	-1.58036189056144	0.123682899841838	0.775919026859345	-4.77329822836248
a_HQ_transcript/29629	1.77676308249207	0.0877940974394883	1.58034491225242	0.12368678954856	0.775919026859345	-4.76107429563532
a_HQ_transcript/138176	0.412914452675542	4.12264245880191	1.58022077937263	0.123715231252545	0.776013700469185	-5.13455940485974
a_HQ_transcript/95416	-1.81800188593109	-0.067061471566361	-1.58005177686763	0.123753962208592	0.776172886843015	-4.72488764332148
a_HQ_transcript/19711	3.06839971329907	1.55870915835074	1.57989118383918	0.1237907751081	0.776302547696783	-4.71765167121702
a_HQ_transcript/105017	1.72338422358905	-0.297869974529449	1.57976402008031	0.123819931327186	0.776302547696783	-4.7972659300364
a_HQ_transcript/39353	-2.77974251539466	3.83321557002046	-1.57975129028182	0.123822850335423	0.776302547696783	-5.21726466140588
a_HQ_transcript/94794	-0.480850929229459	2.7484479653512	-1.57962836424706	0.123851040798868	0.776302547696783	-4.81208703804789
a_HQ_transcript/64977	1.29225020317152	-0.499263346967197	1.57961787487196	0.123853446555944	0.776302547696783	-4.72298304689894
a_HQ_transcript/112106	-3.52075968089517	3.57662927310928	-1.57959233094254	0.123859305261802	0.776302547696783	-4.99423565674829
a_HQ_transcript/125936	-1.43999532130224	-0.609368528507659	-1.57955389679572	0.123868120869248	0.776302547696783	-4.71895787329299
a_HQ_transcript/70520	1.31744744344081	-0.391152302624007	1.57947379469916	0.123886495465556	0.776334002632797	-4.75439542661232
a_HQ_transcript/134986	-1.67366763222474	-0.426166870999769	-1.57930810845508	0.123924509247931	0.776407327562378	-4.76917608076023
a_HQ_transcript/11166	-1.42718653701955	1.44092474781313	-1.57930636071699	0.123924910286329	0.776407327562378	-4.65588267123086
a_HQ_transcript/124167	-2.3818921186739	0.394671609066532	-1.57904456297558	0.123984994728229	0.776700051035943	-4.73165600063718
a_HQ_transcript/143976	-1.73380075225019	2.59649842418467	-1.57886938148021	0.124025213409024	0.776794456219986	-4.7944096613267
a_HQ_transcript/125642	-2.94384931836981	3.57194839856905	-1.57880171998214	0.124040750185462	0.776794456219986	-5.13252605922386
a_HQ_transcript/95375	-4.88267129495393	4.47140925528167	-1.57872711509079	0.124057883181244	0.776794456219986	-5.13916619877394
a_HQ_transcript/46078	1.47601534830021	0.824198382207703	1.57870897043346	0.124062050389645	0.776794456219986	-4.6578242317975
a_HQ_transcript/121679	1.60938800554996	-0.467205727515974	1.57868793239488	0.124066882252036	0.776794456219986	-4.78728034503694
a_HQ_transcript/59786	1.17532093500011	-0.41088890054246	1.57842824780579	0.124126537358677	0.777084261021558	-4.80281436816986
a_HQ_transcript/4850	-0.717813146030615	3.89950875299013	-1.57832159088039	0.124151045520298	0.77709594720599	-4.94818966954706
a_HQ_transcript/85052	-0.678714979322589	2.46709824352904	-1.57824705562018	0.124168174950141	0.77709594720599	-4.80281982700729
a_HQ_transcript/149832	-1.00134864216089	3.05653913684277	-1.5782042971608	0.124178002418947	0.77709594720599	-4.89468151077267
a_HQ_transcript/74203	-0.65054093557871	2.2469335580489	-1.57807004786333	0.12420886197896	0.77709594720599	-4.71923046627293
a_HQ_transcript/150113	0.713287814330358	3.93264441341233	1.57802391878465	0.124219467003401	0.77709594720599	-5.04297594927425
a_HQ_transcript/140867	0.505929997828136	4.732413605642	1.57802226277975	0.124219847730851	0.77709594720599	-5.19199845011557
a_HQ_transcript/28925	1.19290864545511	0.748773155954793	1.57796399779323	0.124233243877167	0.77709594720599	-4.63929919358598
a_HQ_transcript/146945	0.725546966936603	3.90187858829789	1.57795482201694	0.124235353657192	0.77709594720599	-5.00861283532786
a_HQ_transcript/93557	2.59550303174155	2.07674552637532	1.57788112794426	0.124252299146218	0.777118317707269	-4.85177703690901

a_HQ_transcript/26780	1.97093267653576	-0.298554181611	1.57777532186126	0.124276631889578	0.777180399114997	-4.77838637940594
a_HQ_transcript/151576	-2.26265345837286	3.53321365148537	-1.57763630915156	0.124308607236361	0.777180399114997	-5.12780579808078
a_HQ_transcript/123478	0.418490975773418	4.70339493334057	1.5775419434407	0.124330316821642	0.777180399114997	-5.17570165148109
a_HQ_transcript/120996	0.92270419800707	2.26283925642681	1.57741969979505	0.12435844454985	0.777180399114997	-4.74699647501039
a_HQ_transcript/78973	-1.84669933269417	1.54241471709272	-1.5773312874277	0.124378791085706	0.777180399114997	-4.85269823588118
a_HQ_transcript/41659	1.21219484379679	2.8920874053653	1.57728130252086	0.124390295426484	0.777180399114997	-4.84415278557541
a_HQ_transcript/13242	-1.31346486933983	-0.621379545492329	-1.57722227982438	0.124403880989737	0.777180399114997	-4.71784161429413
a_HQ_transcript/97565	-0.69181003039812	2.10761621678989	-1.57719108303153	0.124411062208225	0.777180399114997	-4.7633085125008
a_HQ_transcript/144001	-1.47878444833559	0.0473642292111367	-1.57717519293179	0.124414720094986	0.777180399114997	-4.73867153841879
a_HQ_transcript/135436	-0.488789315865909	3.86608375591895	-1.57716289577671	0.124417550949352	0.777180399114997	-5.08046081086282
a_HQ_transcript/73020	1.26420184490729	1.20579882818897	1.57714637919934	0.124421353214367	0.777180399114997	-4.59658154357987
a_HQ_transcript/28860	-2.5863778628783	0.153182507244147	-1.57712759786042	0.124425676963153	0.777180399114997	-4.78365540280262
a_HQ_transcript/16005	1.25181949406971	3.35430442862934	1.57698872068438	0.124457652399767	0.777180399114997	-5.0300411052498
a_HQ_transcript/73652	1.8555089676536	2.25428930086716	1.5769548866819	0.124465443443093	0.777180399114997	-4.7050276714012
a_HQ_transcript/80759	2.13761693702107	1.75365560049898	1.57692724971957	0.124471807773327	0.777180399114997	-4.7069041138905
a_HQ_transcript/94580	1.70034181362738	0.510018995174432	1.57690840417759	0.124476147738794	0.777180399114997	-4.6808116619777
a_HQ_transcript/68328	1.42632322081584	0.318927575192671	1.57656693544381	0.124554806433225	0.777587991445655	-4.75154519616867
a_HQ_transcript/130457	1.80158448101986	-0.0207809343751706	1.57647487185096	0.124576020593381	0.777636912054562	-4.72552120184644
a_HQ_transcript/69939	1.76468649722347	2.01366232710562	1.57624860604869	0.124628171420483	0.777833208502323	-4.68537398676872
a_HQ_transcript/122508	2.86246852164747	1.66343712017542	1.57622232611714	0.124634229699811	0.777833208502323	-4.70153689865929
a_HQ_transcript/143521	0.460650995586595	4.33894694039748	1.57613268867338	0.12465489551411	0.777871652090468	-5.11155103128642
a_HQ_transcript/131143	3.10932740548516	3.68685034011058	1.57607952478912	0.124667153710343	0.777871652090468	-5.02486516189748
a_HQ_transcript/152322	1.32994835643276	-0.47196191140737	1.57593245318285	0.12470106968717	0.777999761594486	-4.77816556390688
a_HQ_transcript/134612	1.69735225377521	0.370069444597768	1.57568397124075	0.124758388866315	0.778071305537032	-4.73072558763873
a_HQ_transcript/95910	1.95238021897114	1.43892354232972	1.57567977518297	0.124759356986756	0.778071305537032	-4.74393065785315
a_HQ_transcript/131471	1.73508230977822	3.05842533833174	1.57565323354794	0.124765480852709	0.778071305537032	-4.79858154929084
a_HQ_transcript/1103	1.93438365022734	0.455856712295926	1.57562445727864	0.124772120585758	0.778071305537032	-4.64638135285718
a_HQ_transcript/147951	4.16383510888283	0.941181529854237	1.57551676147928	0.124796972486616	0.778071305537032	-4.75428643738653
a_HQ_transcript/113195	-2.38572256340213	1.50468168708981	-1.57545716714624	0.124810726220792	0.778071305537032	-4.88502997734025
a_HQ_transcript/136075	-2.28774184395514	1.32834327497863	-1.57543052793293	0.124816874666072	0.778071305537032	-4.85164974948886
a_HQ_transcript/53988	-1.13935823460835	3.03249634877714	-1.57539675151211	0.124824670764951	0.778071305537032	-4.9866362016382
a_HQ_transcript/121167	1.85335793214178	1.11700186514682	1.57536063951045	0.124833006390008	0.778071305537032	-4.61224597431738
a_HQ_transcript/94983	1.8896634366056	0.271849656687357	1.57528903600459	0.124849535755681	0.77809089893923	-4.75966369966909
a_HQ_transcript/83241	1.64552193202271	-0.394979587680414	1.57512632813028	0.124887102818089	0.778227928519261	-4.78011545617035
a_HQ_transcript/113126	2.40973502666162	3.13648009931417	1.57498260109733	0.124920295130556	0.778227928519261	-5.04400441629979
a_HQ_transcript/83297	-1.85116754282059	2.22786814293662	-1.57495491123162	0.124926690653331	0.778227928519261	-4.71923715444897
a_HQ_transcript/149645	1.7977121695704	0.33311023537489	1.57493483290222	0.124931328308162	0.778227928519261	-4.76508099701071
a_HQ_transcript/130776	1.5205024649539	3.42377104916312	1.57490394120355	0.124938463889034	0.778227928519261	-5.07631044896647
a_HQ_transcript/78435	1.63796098038666	0.781236751144724	1.57483420486046	0.124954573300444	0.778241441180315	-4.69456757063332
a_HQ_transcript/74134	-3.11607221043477	1.54358401099273	-1.5747786408137	0.124967410060161	0.778241441180315	-4.93077100349528
a_HQ_transcript/123118	-1.52238171246418	2.23142460325141	-1.57471849322312	0.124981306952	0.778244607445728	-4.7323310961815
a_HQ_transcript/126783	1.90159632356509	2.26232114939321	1.57462948858477	0.125001873477682	0.778270696923611	-4.72514797068154
a_HQ_transcript/49082	0.622454818992908	3.29961715467624	1.57458448081773	0.125012274586701	0.778270696923611	-4.97747527653267
a_HQ_transcript/9842	1.78095538714354	0.28899852853211	1.57439690563757	0.125055630039112	0.778398219289053	-4.71180726298351
a_HQ_transcript/90261	-0.881794282550531	4.89993244250419	-1.57437998985213	0.12505954049581	0.778398219289053	-5.20031346288518
a_HQ_transcript/148470	-5.54971857703045	2.99796378432367	-1.57421471144854	0.125097753491271	0.778551805598688	-4.95542167897294
a_HQ_transcript/123307	-1.92225124462301	-0.183886344764625	-1.57415318636327	0.125111980757538	0.778551805598688	-4.72644386653947
a_HQ_transcript/56764	0.628868412800831	5.83799959428579	1.57409949620162	0.125124397328361	0.778551805598688	-5.26202982113172
a_HQ_transcript/96383	-1.57495219145998	0.993201959007549	-1.57385654772515	0.12518059499176	0.778669437851542	-4.66916534921141
a_HQ_transcript/64223	-0.816514824024506	2.9721085449705	-1.57382185955625	0.125188620570181	0.778669437851542	-4.8737406739548

a_HQ_transcript/38496	-1.58238913358381	0.81822932127722	-1.57375559616291	0.125203952671733	0.778669437851542	-4.73162254204411
a_HQ_transcript/25799	-0.657986459287525	3.04941605352295	-1.57361041458685	0.125237550308965	0.778669437851542	-4.86230474382847
a_HQ_transcript/97558	-1.36121411129042	-0.617438718124486	-1.57348088312606	0.125267532434066	0.778669437851542	-4.79123856573783
a_HQ_transcript/114673	1.709032338476	2.59228450290797	1.5734727044029	0.12526942572663	0.778669437851542	-4.86253380779642
a_HQ_transcript/881	-0.783388425897235	2.07082018258756	-1.57336054365414	0.12529539217231	0.778669437851542	-4.67901782605657
a_HQ_transcript/58466	2.94834061560306	2.31539853708027	1.57328404891648	0.125313104062406	0.778669437851542	-4.8695721296722
a_HQ_transcript/44885	-1.84977908129395	1.14858247748584	-1.57324022653786	0.125323251786978	0.778669437851542	-4.6545310754976
a_HQ_transcript/87009	2.51920862264286	0.994590469481474	1.57318902956039	0.125335108058642	0.778669437851542	-4.89215663778317
a_HQ_transcript/57218	-1.41100663346348	3.96171491981199	-1.57308991213253	0.125358064418933	0.778669437851542	-5.1709736954479
a_HQ_transcript/48699	-1.18169531075506	1.96634835811171	-1.57304222941187	0.125369109326178	0.778669437851542	-4.73541301617293
a_HQ_transcript/45768	1.76744847965219	0.99130362481366	1.5730322603891	0.1253714185844	0.778669437851542	-4.70163344642755
a_HQ_transcript/96435	-2.37507385630702	0.785851319556454	-1.57302381312299	0.125373375364902	0.778669437851542	-4.74014418635747
a_HQ_transcript/122099	-2.16469406083286	1.59906754679833	-1.57295064019676	0.125390326667119	0.778669437851542	-4.72082088795527
a_HQ_transcript/80035	-1.21961260239211	1.9245548251648	-1.57293425640099	0.125394122407259	0.778669437851542	-4.75237084222563
a_HQ_transcript/53208	-2.16775995767852	-0.106452231627264	-1.572896220247	0.125402934850844	0.778669437851542	-4.73344692665215
a_HQ_transcript/139950	-2.22220761360029	3.90107968968949	-1.57285115993201	0.125413375347645	0.778669437851542	-5.11573683908645
a_HQ_transcript/93873	2.09234225913627	2.97849139874961	1.57281770767433	0.125421126710417	0.778669437851542	-5.02984414270926
a_HQ_transcript/75916	2.15053030791645	-0.257446411665835	1.57279186521041	0.125427115043488	0.778669437851542	-4.74714685864727
a_HQ_transcript/56310	-1.59669625934261	-0.436876549924856	-1.57279162525493	0.125427170648153	0.778669437851542	-4.77077022129185
a_HQ_transcript/6021	1.42851481984619	0.466215197587388	1.57271346538517	0.125445283633669	0.778669437851542	-4.67515554734391
a_HQ_transcript/8992	-1.41147820681253	2.52865753614054	-1.57266664108038	0.12545613584995	0.778669437851542	-4.86262564769156
a_HQ_transcript/18429	2.1945465156587	1.14214168143197	1.57262925555432	0.125464801041108	0.778669437851542	-4.70837803557177
a_HQ_transcript/135135	2.05506784351946	0.40180995825967	1.57245352019642	0.12550553939931	0.778839115426828	-4.71970461574102
a_HQ_transcript/99447	2.09000400228401	2.64999151710504	1.57224333956989	0.125554276904233	0.77905839110688	-4.85160406593413
a_HQ_transcript/121430	0.922200077907373	2.42218840396118	1.57214698553027	0.125576625014069	0.779081097357481	-4.79635675948116
a_HQ_transcript/7936	1.47774199009641	-0.407436073239809	1.57211199260195	0.125584741987331	0.779081097357481	-4.75822412377134
a_HQ_transcript/63587	-0.536766338136454	4.38888536472398	-1.57203047985879	0.125603651373893	0.77909179620837	-5.14373725909423
a_HQ_transcript/148357	2.98163506183012	1.60001816536017	1.57198900913028	0.125613272675214	0.77909179620837	-4.85115093303039
a_HQ_transcript/126899	-1.47830271938921	3.07519091596447	-1.57184326606117	0.125647090158677	0.779165468538058	-4.98971374444678
a_HQ_transcript/42976	1.38817715935827	-0.582320802103123	1.57182228329597	0.125651959504469	0.779165468538058	-4.75395145835073
a_HQ_transcript/150603	3.89417064234412	0.915265545774254	1.57151729897303	0.125722752778986	0.779521298510604	-4.67363463896134
a_HQ_transcript/150295	-6.13037127499997	2.49454877058811	-1.57139910896131	0.125750195907335	0.779608296469877	-4.81041334789635
a_HQ_transcript/104529	0.731525458648447	2.04108457227286	1.57124701614232	0.125785518283576	0.779627137093357	-4.73228375719827
a_HQ_transcript/39479	-1.76153796313858	0.198675604979196	-1.57120047243836	0.125796329313718	0.779627137093357	-4.67746704609428
a_HQ_transcript/74801	-3.22234123245492	2.87829083759658	-1.5711956184798	0.125797456820084	0.779627137093357	-4.95065454744225
a_HQ_transcript/10007	1.22146002679743	3.90627681929635	1.57110946775769	0.12581746979302	0.779627137093357	-5.05473916482762
a_HQ_transcript/38995	0.826864717369685	3.77071375925179	1.57098919536857	0.125845413633784	0.779627137093357	-5.04074688070002
a_HQ_transcript/145856	2.33728154419581	0.581706019451079	1.57095146257742	0.125854181435826	0.779627137093357	-4.80522630589256
a_HQ_transcript/101476	1.01050533994886	1.58802828560312	1.5709072839769	0.125864447654972	0.779627137093357	-4.61888555708205
a_HQ_transcript/5993	1.60379445750614	0.960294210208742	1.5708815982137	0.125870416824109	0.779627137093357	-4.70037832972072
a_HQ_transcript/150296	1.54449642940405	0.240912270666615	1.57086641619346	0.125873945114595	0.779627137093357	-4.80079342575479
a_HQ_transcript/68947	1.46616938975118	-0.689169625375597	1.57079415134213	0.125890740518826	0.779648089162402	-4.75199922631676
a_HQ_transcript/93098	1.91574807503746	3.24029758891919	1.5706563084432	0.125922782282405	0.779701536743013	-4.88538189189826
a_HQ_transcript/37002	1.29020550224985	1.27075714912654	1.57061509266131	0.125932364236186	0.779701536743013	-4.72215615246461
a_HQ_transcript/105205	1.17797391247684	0.863569588662337	1.57058392165496	0.12593961134875	0.779701536743013	-4.59827109953041
a_HQ_transcript/149395	-0.665954699579156	2.16086947238478	-1.5705144173203	0.125955772004242	0.779718542605172	-4.77536562177191
a_HQ_transcript/50914	1.68566446038627	0.49925485528825	1.57036110893346	0.125991424160627	0.779774086744134	-4.75377097392391
a_HQ_transcript/57455	1.30932563317473	-0.638993275695766	1.57028710571896	0.126008636692018	0.779774086744134	-4.76963767193682
a_HQ_transcript/134184	2.07698501806018	8.72872537452092	1.57025802343569	0.12601540151352	0.779774086744134	-5.56791419762643
a_HQ_transcript/82889	-1.26079547922691	2.47968297309157	-1.57024415096713	0.126018628488696	0.779774086744134	-4.97116595279871

a_HQ_transcript/50437	0.96891533575343	2.42280938512262	1.57018745139545	0.126031818486119	0.779774086744134	-4.9526925835638
a_HQ_transcript/1722	1.68426939475173	0.673558839841167	1.56997249763245	0.126081833308986	0.780000511556486	-4.80474663283448
a_HQ_transcript/130728	0.652853841357946	3.01793485566859	1.5698799566842	0.126103370451289	0.780050730828194	-4.87711588671521
a_HQ_transcript/86351	1.82274924801689	0.735291280836185	1.56973391765944	0.126137364350992	0.780177986273087	-4.62070567465055
a_HQ_transcript/77651	1.0626780482664	-0.755797209099599	1.56957767208859	0.126173742325445	0.780261654163661	-4.74933876421604
a_HQ_transcript/149154	1.37510399991226	-0.554445434120757	1.56956051305923	0.126177737908382	0.780261654163661	-4.74899198265799
a_HQ_transcript/23359	-0.979747791079861	2.83046466107876	-1.56931958466578	0.126233850419724	0.780525609743401	-4.85430457139532
a_HQ_transcript/145573	2.00082251902	0.825964733620034	1.56926114231058	0.126247464778524	0.780526763736016	-4.615131793664
a_HQ_transcript/139281	1.4409487628904	-0.164746920609376	1.56901886534248	0.126303916840412	0.780792734031636	-4.80648571734176
a_HQ_transcript/77373	-1.77815329001742	-0.53413227001472	-1.56893762546241	0.126322850849487	0.780826741192999	-4.74167808871031
a_HQ_transcript/68178	1.7717092436628	0.918652751629889	1.56884009225968	0.126345585284161	0.780884229813685	-4.77477395113573
a_HQ_transcript/63193	1.29477585124202	-0.39066842452055	1.56876769351669	0.126362463175118	0.780901364329513	-4.76536681248209
a_HQ_transcript/141444	1.8788827602953	0.624904330777025	1.56871295203036	0.12637522595389	0.780901364329513	-4.70885662084513
a_HQ_transcript/78039	0.826945615824361	2.0952335184097	1.56854645237991	0.126414051198848	0.780965497706493	-4.76773208401175
a_HQ_transcript/136897	-1.15830826523401	-0.713207397746442	-1.56852015527164	0.126420184182467	0.780965497706493	-4.74771365077029
a_HQ_transcript/150844	1.45982799240641	-0.436165957264181	1.56840283174476	0.126447549204305	0.780965497706493	-4.7835896081455
a_HQ_transcript/45367	-1.07418612643621	0.61972695333025	-1.56838633785919	0.126451396692916	0.780965497706493	-4.70132918635201
a_HQ_transcript/152230	-2.97530691816984	3.01988413384161	-1.56838040277167	0.126452781179699	0.780965497706493	-5.17211731824046
a_HQ_transcript/136101	2.06292983883202	0.901722205423454	1.56820821815106	0.126492952321781	0.781113472499924	-4.64827894913623
a_HQ_transcript/125219	1.66611448684249	-0.0935230003288653	1.56806774554708	0.126525732664587	0.781113472499924	-4.7592854788193
a_HQ_transcript/25681	1.57591316632127	-0.237181882924256	1.56800398389976	0.12654061421001	0.781113472499924	-4.77452690007656
a_HQ_transcript/113711	-2.83773167394039	0.725941153776448	-1.56799143778931	0.126543542556838	0.781113472499924	-4.82100851990706
a_HQ_transcript/96892	2.1707848567837	3.33139006305307	1.56795724220058	0.126551524318282	0.781113472499924	-5.00700452876509
a_HQ_transcript/39283	-1.19410412169434	1.9056631141026	-1.56793220794943	0.126557367946674	0.781113472499924	-4.81075212042733
a_HQ_transcript/67759	1.76749185368028	-0.177610725295385	1.56780689104509	0.126586623391347	0.781168861750743	-4.72590117678477
a_HQ_transcript/131189	-0.648945546993293	2.72201271437976	-1.56777863852576	0.126593219751101	0.781168861750743	-4.73023227116618
a_HQ_transcript/94510	2.53044430527831	0.606259308564345	1.5675466030471	0.12664740567363	0.781420273537496	-4.61362467023228
a_HQ_transcript/23002	-2.17946277059536	1.01991392406634	-1.56711897680813	0.126747316286918	0.781890652545909	-4.80146992133587
a_HQ_transcript/112176	1.70091467183944	-0.310918881564672	1.56710516668882	0.12675054395369	0.781890652545909	-4.76827362410543
a_HQ_transcript/107276	-1.97564198211565	1.28163695401554	-1.56681512317756	0.126818347672921	0.782144282424489	-4.77674344412874
a_HQ_transcript/29521	2.6140941299054	3.95178395714693	1.56681417050697	0.126818570428207	0.782144282424489	-4.90554740347655
a_HQ_transcript/100440	-1.16328547608739	2.21973261629878	-1.56662288986914	0.126863302494293	0.782256848850838	-4.83216692180865
a_HQ_transcript/6195	0.62808107695218	2.85926241210284	1.56662103141131	0.126863737168264	0.782256848850838	-4.87831096967134
a_HQ_transcript/38136	1.43523957241954	1.58116074410024	1.56619257744667	0.126963980478128	0.782789096597924	-4.74399975412664
a_HQ_transcript/74196	-1.41975613535766	-0.669476602596543	-1.56613699921835	0.126976988573869	0.782789096597924	-4.6915016076135
a_HQ_transcript/122555	-1.33784093721484	-0.448692106573679	-1.56591152911773	0.127029771030641	0.782893844284083	-4.74325174918218
a_HQ_transcript/2723	1.55445541639313	0.521855350022311	1.56590140748658	0.127032140919279	0.782893844284083	-4.80641433080728
a_HQ_transcript/74100	1.71012545545157	0.402365511677515	1.56582067933992	0.127051043975493	0.782893844284083	-4.74033732820869
a_HQ_transcript/146186	-0.644372296580814	4.20209552547058	-1.56577774019343	0.127061099408056	0.782893844284083	-5.13237295267934
a_HQ_transcript/13309	1.59516348823171	0.615704257742334	1.56577678957362	0.127061322030279	0.782893844284083	-4.74293278524052
a_HQ_transcript/110269	4.12231220844745	1.25297792832432	1.56564865494238	0.127091332324197	0.782995756792892	-4.76215312107012
a_HQ_transcript/33119	1.54654632746625	0.693257360690018	1.56554458985264	0.127115709560172	0.783062946726754	-4.60027004520866
a_HQ_transcript/89653	3.20027149595159	1.0965461355523	1.56545262493237	0.12713725550178	0.783112682823725	-4.90114044123067
a_HQ_transcript/137299	1.6771206474047	0.385417555016511	1.56532524553647	0.127167103412715	0.783213539645509	-4.69671267026772
a_HQ_transcript/128928	2.0224818475217	-0.0673526500751648	1.56524038617503	0.127186991071954	0.783218815633813	-4.88838060096282
a_HQ_transcript/4538	1.94313022182862	-0.3460067879448	1.56520660761924	0.127194908126603	0.783218815633813	-4.75146679933071
a_HQ_transcript/42961	1.74441285148097	0.0430455332535702	1.56484497704913	0.127279692639549	0.783631711549185	-4.77440148219756
a_HQ_transcript/32130	-0.693984063980958	1.99049170436144	-1.56467813166725	0.127318825152479	0.783631711549185	-4.67185762456761
a_HQ_transcript/129188	3.31423032323415	0.37096561963069	1.5646623452878	0.127322528253606	0.783631711549185	-4.74537411316824
a_HQ_transcript/66785	0.854594558301571	3.2826821077964	1.56448828174456	0.127363365146537	0.783631711549185	-4.92078741109458

a_HQ_transcript/132750	-0.627767567840133	3.91419371202011	-1.56446274337981	0.127369357576481	0.783631711549185	-5.04445615349589
a_HQ_transcript/130637	1.15721851840499	0.510411361655486	1.5644173453812	0.127380010521606	0.783631711549185	-4.62973637746733
a_HQ_transcript/45744	-1.76994771342388	0.509929700432438	-1.56439768636266	0.127384623867335	0.783631711549185	-4.67474786905258
a_HQ_transcript/123987	0.876294672030935	-0.790021896625955	1.56436244199162	0.1273928949396	0.783631711549185	-4.78595117237134
a_HQ_transcript/64023	-2.95668955793315	2.9143341645941	-1.56435966574351	0.127393546482039	0.783631711549185	-4.94904841023251
a_HQ_transcript/123107	-1.20540846981899	3.10906251487138	-1.56434591369375	0.127396773915149	0.783631711549185	-4.98035846822876
a_HQ_transcript/1630	1.25713144677158	0.831888825671776	1.56424429068596	0.127420625620355	0.783695495297853	-4.69263211834624
a_HQ_transcript/50069	1.78243174304332	0.166001918445635	1.56413288492363	0.127446777590659	0.783750937534478	-4.74333742885962
a_HQ_transcript/23016	1.73518941096006	0.00695170291009151	1.56404442513911	0.127467546214368	0.783750937534478	-4.71856162894731
a_HQ_transcript/121227	1.64020865838531	2.02759548054084	1.56403359325787	0.127470089517117	0.783750937534478	-4.7801670120217
a_HQ_transcript/148069	2.00055996007091	0.551660820482438	1.5638234836801	0.127519430973013	0.78388840440229	-4.75744775314365
a_HQ_transcript/114314	2.45551846326756	3.34571469812371	1.56365662194751	0.127558627319554	0.78388840440229	-4.87397458019554
a_HQ_transcript/117843	-1.39405263291261	2.51095310014753	-1.56357647959361	0.127577456496807	0.78388840440229	-4.92223102470329
a_HQ_transcript/116839	1.1263299839435	2.70063242986828	1.56347637032492	0.127600980011691	0.78388840440229	-4.99275361019652
a_HQ_transcript/128261	-1.28165706412466	0.743346926241197	-1.56341294751155	0.12761588482908	0.78388840440229	-4.70170529916263
a_HQ_transcript/2278	-1.83911252530174	2.03210360231564	-1.56339648697287	0.127619753405689	0.78388840440229	-4.88942779722229
a_HQ_transcript/115468	1.71923013866954	0.45493160116291	1.56338229821682	0.127623088141676	0.78388840440229	-4.73679803021401
a_HQ_transcript/124127	4.69246689246281	4.4557510224376	1.56332376701696	0.127636845284014	0.78388840440229	-5.12359833005248
a_HQ_transcript/93313	1.89932269733136	1.23543933159503	1.56325096790092	0.127653957634036	0.78388840440229	-4.70117814602409
a_HQ_transcript/110039	-2.75238183052356	0.585295436193716	-1.56316742965224	0.127673596653367	0.78388840440229	-4.81943236939123
a_HQ_transcript/76015	1.88410879895674	3.035736903292	1.56307154972302	0.127696140111023	0.78388840440229	-4.92363451433925
a_HQ_transcript/81336	1.14280070053304	1.39658398729016	1.56306780904913	0.127697019690507	0.78388840440229	-4.75295276192407
a_HQ_transcript/4331	-0.973314425083736	3.01424450714767	-1.56300697874403	0.127711323978692	0.78388840440229	-4.83425493854212
a_HQ_transcript/11702	1.13096920672737	-0.518189501704993	1.56298815426895	0.127715750831201	0.78388840440229	-4.78273478865977
a_HQ_transcript/129801	2.70461988204117	2.43590553841378	1.56293818949078	0.127727501390183	0.78388840440229	-4.84344841478085
a_HQ_transcript/52354	1.62932482938998	0.808106362115938	1.56290444830632	0.127735437033256	0.78388840440229	-4.67118709651187
a_HQ_transcript/146917	0.630847108344707	5.18618508756701	1.5628584801018	0.127746249014421	0.78388840440229	-5.22829762045898
a_HQ_transcript/8374	-1.0688518652045	2.39345963334788	-1.56282520724485	0.127754075442834	0.78388840440229	-4.7494498764709
a_HQ_transcript/26554	-0.990946470953883	1.78054024571395	-1.56277549668547	0.127765769068208	0.78388840440229	-4.7007120165387
a_HQ_transcript/16289	-2.76007438222767	0.561012981068761	-1.56276391442777	0.127768493736847	0.78388840440229	-4.78351630905696
a_HQ_transcript/71585	-0.303282551439233	5.06989246620394	-1.56272066097572	0.127778669314811	0.78388840440229	-5.26760142963028
a_HQ_transcript/97345	1.79474749710331	-0.199843509757448	1.56267620062259	0.127789129509292	0.78388840440229	-4.81665137850357
a_HQ_transcript/21067	-3.27805423262577	0.581100432605234	-1.5624598397859	0.127840042705784	0.784117970075109	-4.8441935283017
a_HQ_transcript/78845	-2.51008600147246	1.26378064963388	-1.56232713630104	0.127871278143717	0.784226804804598	-4.92045661416027
a_HQ_transcript/5598	1.72711450346421	-0.160840628708614	1.5622404945106	0.127891675037909	0.784269151450954	-4.75999367348788
a_HQ_transcript/128437	-2.22988291761455	1.38698822274071	-1.56193997269829	0.127962443304913	0.784592928880985	-4.67948127294575
a_HQ_transcript/102614	4.61084197189628	1.36915028336447	1.56190165361724	0.127971469149474	0.784592928880985	-4.84046683757279
a_HQ_transcript/146552	1.85526498620964	0.794887983088154	1.561764746461	0.128003721093808	0.78464559934239	-4.8268551020453
a_HQ_transcript/136519	1.25471151828409	3.84721545558762	1.56175058670704	0.128007057159439	0.78464559934239	-5.11911458938097
a_HQ_transcript/125056	4.08281435787353	1.47076501138665	1.56159792062676	0.128043030078873	0.78474006928467	-4.87719743207978
a_HQ_transcript/93697	1.26954290784625	-0.540226876150648	1.56151875774942	0.128061686575114	0.78474006928467	-4.77059020716014
a_HQ_transcript/148165	1.46134353566975	-0.643072866302428	1.56150683408922	0.128064496843011	0.78474006928467	-4.780113402986
a_HQ_transcript/133879	1.05468861827646	-0.610008788931831	1.56144496259937	0.128079080038571	0.78474006928467	-4.80978537945339
a_HQ_transcript/7785	1.72583790233614	-0.414604018324681	1.5613296389112	0.128106265597243	0.78474006928467	-4.71084055126951
a_HQ_transcript/23647	1.27823802331639	0.42396245335559	1.56131271697068	0.128110255046079	0.78474006928467	-4.67349622758789
a_HQ_transcript/21651	-1.95799051561709	0.92669285061479	-1.56117084384238	0.128143706462148	0.78474006928467	-4.83026449222357
a_HQ_transcript/4777	-2.12769474261223	1.39856245227951	-1.56114644957732	0.128149458955867	0.78474006928467	-4.78114649408202
a_HQ_transcript/102332	1.29198354544246	-0.571345187620159	1.56114584650462	0.128149601171132	0.78474006928467	-4.76088827347896
a_HQ_transcript/34549	1.50251395639737	-0.250056683629826	1.56111247460257	0.128157471059031	0.78474006928467	-4.74167462408357
a_HQ_transcript/21447	1.40261126352994	1.03913769910086	1.56103589561838	0.128175531690471	0.784767990952572	-4.65700712186166

a_HQ_transcript/110002	-2.74136084612077	3.63874301210618	-1.56077959854252	0.128235992671075	0.784953245858569	-4.9896060223271
a_HQ_transcript/7719	1.44512706086815	1.19828892735158	1.56075576800153	0.128241615520919	0.784953245858569	-4.65585892742747
a_HQ_transcript/148982	-7.24832877485919	1.94824587346387	-1.56073591146534	0.128246300851893	0.784953245858569	-4.8219010592079
a_HQ_transcript/115882	5.30812751266474	3.4876399625146	1.56067534100297	0.12826059386567	0.784958075407839	-4.88149056646599
a_HQ_transcript/80544	-3.66049105894551	0.44385613461061	-1.56044839465416	0.128314158675404	0.785092981397657	-4.86630857786257
a_HQ_transcript/135169	2.61464217805682	2.22149066335468	1.56044544922151	0.128314853988119	0.785092981397657	-4.79174449643799
a_HQ_transcript/36712	-2.42013756572232	0.739017621467563	-1.56041028153875	0.128323156073822	0.785092981397657	-4.7382898322801
a_HQ_transcript/136416	-1.73556603804158	-0.0170101266857262	-1.56014999944115	0.128384614829668	0.785376232460096	-4.73758486958032
a_HQ_transcript/14958	4.24754439040448	2.48174384462596	1.56009977967567	0.128396475658345	0.785376232460096	-4.68187505221293
a_HQ_transcript/1232	0.768031799870891	3.33613671098008	1.55983210720272	0.128459709176551	0.785679804187838	-5.00360955029382
a_HQ_transcript/109438	1.08701316831585	5.07239145395459	1.55977527768515	0.12847313753794	0.785679804187838	-5.26427291072605
a_HQ_transcript/130661	2.11921958676203	1.38536483379135	1.5596615027826	0.1285000250701	0.785696811221408	-4.71436367109308
a_HQ_transcript/136100	0.660720287538788	3.49707253496823	1.55964911931046	0.128502951835293	0.785696811221408	-4.9756385976117
a_HQ_transcript/36265	-2.57476846682473	0.527082935915488	-1.55954738928092	0.128526997217612	0.785761179455759	-4.73658814542348
a_HQ_transcript/132624	0.500818189272466	3.36741135900709	1.5593744963733	0.128567871382425	0.785908377438163	-4.98386528606838
a_HQ_transcript/122447	2.42161702215536	0.372114437628978	1.55933117402946	0.128578115012334	0.785908377438163	-4.80641264893248
a_HQ_transcript/62189	1.93605647304171	0.177503689615704	1.55922648508563	0.128602871602169	0.785977049783504	-4.80983715518129
a_HQ_transcript/82398	-1.79916766486036	2.28303852065985	-1.55915086758153	0.128620755858878	0.785981238164349	-4.82916836793982
a_HQ_transcript/59143	1.9180157321852	0.255188842528858	1.55910924848831	0.128630600031954	0.785981238164349	-4.75186281497705
a_HQ_transcript/148543	-2.09996005877802	3.69549414499738	-1.55904779568343	0.128645136597364	0.785987439576578	-5.22129881690689
a_HQ_transcript/146087	-4.06544005635166	2.26256859945329	-1.55895309876094	0.12866753962247	0.786041696602728	-4.94688560629392
a_HQ_transcript/80465	-2.54685814408044	1.77461692416527	-1.55883674435769	0.12869507063288	0.786094038121887	-4.82620513045854
a_HQ_transcript/128198	0.955430657970227	3.69718012413033	1.55880258361526	0.128703154431702	0.786094038121887	-4.99957883027133
a_HQ_transcript/90636	-3.33835923737816	2.22386081122692	-1.55863506159044	0.128742802824675	0.786133730500928	-4.96815534770982
a_HQ_transcript/105501	5.21532959008808	5.92466837426912	1.55851841797282	0.1287704153996	0.786133730500928	-5.14841100845832
a_HQ_transcript/57638	-1.62657569903874	0.368343022332242	-1.55845479771019	0.128785477998149	0.786133730500928	-4.69411451764896
a_HQ_transcript/121522	1.60535781844324	-0.474155599087931	1.55845083888396	0.128786415328801	0.786133730500928	-4.7869281467363
a_HQ_transcript/102204	2.21353020762364	0.188275000280837	1.55841398020062	0.128795142619602	0.786133730500928	-4.82253404644039
a_HQ_transcript/82351	-0.423741784847197	3.1531658376406	-1.55834960602995	0.12881038610001	0.786133730500928	-4.95251120951347
a_HQ_transcript/48454	1.57723922941375	0.391701558857337	1.55834801190009	0.128810763600569	0.786133730500928	-4.84455476120073
a_HQ_transcript/45369	2.63273536935256	0.807062498635462	1.55831810243072	0.128817846528718	0.786133730500928	-4.84173794959912
a_HQ_transcript/19132	1.1130532734639	1.11551175058545	1.55821356760293	0.128842604141949	0.786202277300356	-4.60021861508097
a_HQ_transcript/75095	1.60640986949736	1.03865021865049	1.55802387339152	0.128887540444973	0.786393927887625	-4.82274495542096
a_HQ_transcript/34096	-1.04332777527932	2.76585143993145	-1.55789747901735	0.128917488842213	0.786431240333908	-4.79533223285501
a_HQ_transcript/107589	-1.24827886253862	2.4154372425833	-1.55788386709886	0.128920714442985	0.786431240333908	-4.85123487206274
a_HQ_transcript/120309	-1.77161457037285	1.8219116315657	-1.55772444536984	0.128958497312532	0.786529645081559	-4.76624169289308
a_HQ_transcript/131127	1.49205678131892	0.181879313084941	1.55764721616433	0.128976803831859	0.786529645081559	-4.64784495418861
a_HQ_transcript/100293	-1.47862842960037	0.612410370226977	-1.55763688647394	0.128979252556354	0.786529645081559	-4.73357667964997
a_HQ_transcript/115066	-3.10538623898092	1.31608450610359	-1.55758745968512	0.128990970041331	0.786529645081559	-4.77585391262302
a_HQ_transcript/43768	1.94523798422089	0.854496549855421	1.5574768539954	0.129017194190107	0.786607034181091	-4.71471313318182
a_HQ_transcript/42840	-0.874476622501521	3.6816316705554	-1.55740258122287	0.129034806387652	0.786631906208853	-5.07238905357312
a_HQ_transcript/43542	3.5107947238183	2.85439206776079	1.55718761184626	0.129085792787014	0.786824972475952	-4.7674673652474
a_HQ_transcript/41002	1.2414711349272	-0.717186123672446	1.55709159842436	0.129108570523862	0.786824972475952	-4.76858359771342
a_HQ_transcript/101027	-1.71481872017845	-0.333237279975103	-1.55707615946316	0.129112233489544	0.786824972475952	-4.75700948425352
a_HQ_transcript/152547	-0.788447520334008	4.1156350718436	-1.55704081171881	0.12912062022516	0.786824972475952	-5.16549993582799
a_HQ_transcript/115886	2.45566181549522	1.46501873853389	1.55693962350586	0.129144630960389	0.786888795436633	-4.75537368473061
a_HQ_transcript/48287	1.74725205353257	-0.365671208088102	1.55663308231505	0.129217391617341	0.787182742291429	-4.77965050138787
a_HQ_transcript/15306	-0.97197938705617	2.55012571658945	-1.55647871352893	0.129254045224864	0.787182742291429	-4.83464726454892
a_HQ_transcript/129009	-1.9960009072569	2.3875515227153	-1.5564737362656	0.129255227176002	0.787182742291429	-4.89732501892883
a_HQ_transcript/135413	-4.46428240487913	3.03940137625536	-1.55647212914083	0.129255608821931	0.787182742291429	-4.85025892854016

a_HQ_transcript/96333	2.01049312916175	-0.18170314056184	1.55635981856291	0.129282281623822	0.787182742291429	-4.83281892302099
a_HQ_transcript/48158	0.499780578181386	3.25040443288471	1.55630366450016	0.129295619410946	0.787182742291429	-5.01802337435289
a_HQ_transcript/100567	2.3762537133568	2.87601931717205	1.55613485527762	0.129335721934766	0.787182742291429	-4.94596785211914
a_HQ_transcript/91776	-1.28103628374642	2.80209619261732	-1.55594398834374	0.129381076690858	0.787182742291429	-4.89028366958798
a_HQ_transcript/53274	1.45675076052523	-0.248947232410716	1.55587021241275	0.129398611160049	0.787182742291429	-4.78197758803384
a_HQ_transcript/62211	2.69953213038331	1.29744776349531	1.55573681922681	0.129430319880844	0.787182742291429	-4.76403390017425
a_HQ_transcript/132725	1.55562536244921	-0.560890766536367	1.555700776103	0.129438888728426	0.787182742291429	-4.78738368008107
a_HQ_transcript/110953	-0.851701887981229	2.39791727320739	-1.55569171418331	0.129441043170504	0.787182742291429	-4.83917912890178
a_HQ_transcript/124467	-0.47846448079487	5.04603649476227	-1.55566877842874	0.129446496202135	0.787182742291429	-5.18792585961786
a_HQ_transcript/30496	-3.60571406799234	2.13725430330258	-1.55566418274168	0.129447588860398	0.787182742291429	-4.90570023541716
a_HQ_transcript/150156	0.31581888433184	6.28518171537226	1.55562826804788	0.129456128100107	0.787182742291429	-5.36325194037817
a_HQ_transcript/137589	2.48963716262327	-0.0376410459824209	1.55549761956311	0.129487195534981	0.787182742291429	-4.76076951129599
a_HQ_transcript/144527	2.37565735219731	2.3787064111768	1.55547807908336	0.129491842665972	0.787182742291429	-4.84361995860377
a_HQ_transcript/113349	-1.56857376131859	2.99990120449837	-1.55545083484141	0.129498322137447	0.787182742291429	-4.94989223671271
a_HQ_transcript/89644	-1.49101547520453	-0.494828965791419	-1.55526130423168	0.129543405309175	0.787182742291429	-4.745945311153
a_HQ_transcript/68374	-1.41039689407012	3.40416477289215	-1.55521479168067	0.12955447108195	0.787182742291429	-5.06932374821548
a_HQ_transcript/92826	-1.45560613752813	-0.0887040837463918	-1.55518206459065	0.129562257624397	0.787182742291429	-4.67529239112985
a_HQ_transcript/99188	-1.3428791100366	1.58426391792625	-1.55518151277329	0.129562388917952	0.787182742291429	-4.70912683796002
a_HQ_transcript/131225	0.391853137922954	4.15483966700982	1.55516981519746	0.12956517214019	0.787182742291429	-5.17840550476494
a_HQ_transcript/1195	1.74145831400988	-0.0382947299208034	1.55516353795908	0.129566665713218	0.787182742291429	-4.73613319045739
a_HQ_transcript/121597	-1.01943412332954	2.97294481202925	-1.55515429049036	0.129568866032688	0.787182742291429	-4.91181890859849
a_HQ_transcript/104545	-1.97071166587929	1.89916650886968	-1.55513280159551	0.129573979163583	0.787182742291429	-4.83059072511159
a_HQ_transcript/97377	1.46946333439936	-0.347664594533844	1.5551213624403	0.12957670109671	0.787182742291429	-4.80719710008361
a_HQ_transcript/120484	-2.14176868287469	0.552598809002208	-1.55497573828215	0.12961135624933	0.787182742291429	-4.85909161499129
a_HQ_transcript/26871	2.67205962983296	0.823701877442633	1.55492245152897	0.129624039135389	0.787182742291429	-4.63423143157886
a_HQ_transcript/26160	2.54839165851907	1.2711104262072	1.55487403260353	0.129635564294467	0.787182742291429	-4.85257531031322
a_HQ_transcript/85465	0.89231172916312	0.807507176090597	1.55476965422126	0.129660412317657	0.787182742291429	-4.69136532714607
a_HQ_transcript/12745	-2.31783285193533	2.26730968509429	-1.55476212817413	0.129662204096756	0.787182742291429	-4.7821575594437
a_HQ_transcript/67697	-1.59778075334503	4.24263551815038	-1.55474364216771	0.12966660527603	0.787182742291429	-5.13945339628432
a_HQ_transcript/55797	-2.97530849318292	4.03454818363055	-1.55473785287468	0.129667983625538	0.787182742291429	-5.21413150101751
a_HQ_transcript/54922	1.9007831877166	0.936507777736159	1.55469661714906	0.129677801618076	0.787182742291429	-4.73664296947754
a_HQ_transcript/36660	1.34741896624467	2.26624990139313	1.55468572968934	0.129680393961305	0.787182742291429	-4.82811286659614
a_HQ_transcript/122997	1.84087926456777	0.973520021582817	1.5545425141049	0.129714498025605	0.787241995914715	-4.70846253879986
a_HQ_transcript/40913	0.501191304526748	3.90419849668376	1.55448252937494	0.129728784411754	0.787241995914715	-5.09683587176179
a_HQ_transcript/28555	1.76769261088167	-0.37452155945541	1.55434533018821	0.129761465542893	0.787241995914715	-4.82870773685875
a_HQ_transcript/90002	-2.38411325640296	1.12266914520313	-1.55432654259479	0.129765941307268	0.787241995914715	-4.89033060622473
a_HQ_transcript/139355	0.362272494724361	6.10985346054436	1.55424048933422	0.12978644336002	0.787241995914715	-5.34335342737137
a_HQ_transcript/142710	1.86073674079805	2.02722648705685	1.55422098194381	0.129791091330679	0.787241995914715	-4.8475847203347
a_HQ_transcript/7576	1.73857164587458	-0.205985008880765	1.55421361835639	0.129792845866953	0.787241995914715	-4.83008995285465
a_HQ_transcript/39064	1.68286972915991	2.14914273205873	1.55417295513169	0.129802535119453	0.787241995914715	-4.87440811056119
a_HQ_transcript/124906	1.76272470825867	1.22325927375149	1.55412387280835	0.129814231261453	0.787241995914715	-4.60668950079677
a_HQ_transcript/100903	-1.80851778095733	1.0710834839346	-1.55407621867737	0.129825587889459	0.787241995914715	-4.82978171485421
a_HQ_transcript/51723	-0.546312021667629	4.20361891984323	-1.55397999206653	0.129848522460805	0.787298591753234	-5.18303265454297
a_HQ_transcript/11184	-0.924182706101807	2.61395229123617	-1.55392341023873	0.129862009663673	0.787298591753234	-4.94599536609485
a_HQ_transcript/139608	0.729075131868924	2.54299428383934	1.55371927764499	0.129910677460656	0.787328813662813	-4.86371282819209
a_HQ_transcript/21875	-0.567080685978903	3.63056150935146	-1.55370677880697	0.129913657823294	0.787328813662813	-5.07745480162745
a_HQ_transcript/8591	-0.654356452102149	3.47313364923976	-1.55364923010058	0.129927381096647	0.787328813662813	-5.00687099167889
a_HQ_transcript/143760	0.53212690233878	2.98957992719615	1.55363036463833	0.129931880079501	0.787328813662813	-4.946863171401
a_HQ_transcript/14112	1.50429721302605	1.275060293376	1.55361846318102	0.129934718370963	0.787328813662813	-4.61362488249535
a_HQ_transcript/9903	-0.889778576250514	1.86640592858071	-1.55352799017951	0.129956296261639	0.787345582045948	-4.69187140384607

a_HQ_transcript/50862	2.13733196229404	1.61488206828528	1.55343976978719	0.129977339705778	0.787345582045948	-4.82696433249876
a_HQ_transcript/89188	1.46773122780638	1.65539109797757	1.55343649547025	0.129978120790547	0.787345582045948	-4.7476582127953
a_HQ_transcript/61522	-2.07926431796717	1.61283724272309	-1.55329291221861	0.130012376169517	0.787468713432214	-4.78748014644013
a_HQ_transcript/74664	-3.84662990152597	3.40139037737633	-1.55320292953636	0.13003384752823	0.787468713432214	-4.97053234998272
a_HQ_transcript/26028	2.00855571784331	0.796886467527509	1.55312019812226	0.13005359115541	0.787468713432214	-4.74448140457128
a_HQ_transcript/106713	-1.7993853877949	3.58517591051061	-1.55304526707317	0.130071475347844	0.787468713432214	-4.9321701300205
a_HQ_transcript/69046	2.30505924810638	1.24396782267956	1.55302424113053	0.130076494080381	0.787468713432214	-4.68144333684841
a_HQ_transcript/51380	-1.42665777439575	2.42051046729347	-1.55301068166842	0.130079730704241	0.787468713432214	-4.93351725600609
a_HQ_transcript/6880	-2.55685225401475	4.9921167746867	-1.55283778098035	0.130121007581498	0.787549377900654	-5.22426934880115
a_HQ_transcript/6659	-1.66822666100735	-0.260360711427326	-1.55283120837465	0.130122576880753	0.787549377900654	-4.75116760018021
a_HQ_transcript/127556	2.65380196756824	8.12041150220442	1.55278461949541	0.130133701051744	0.787549377900654	-5.56402604460314
a_HQ_transcript/113924	2.00183684893588	1.39669790522292	1.55270633987273	0.130152393858756	0.787572061097547	-4.63330050461388
a_HQ_transcript/39449	-3.20986622352941	1.69121037099304	-1.55265545100787	0.130164547050718	0.787572061097547	-4.80816662030457
a_HQ_transcript/5020	-3.00637662478307	2.97565341813768	-1.55247893553982	0.130206709316557	0.787745170602919	-5.01984829189763
a_HQ_transcript/95495	-0.6591386104543	2.85669580457393	-1.55234810726738	0.130237965951197	0.787791569270799	-4.84967804911119
a_HQ_transcript/44330	1.48490211129472	-0.482602175766857	1.55225675623922	0.130259794553404	0.787791569270799	-4.73723109694146
a_HQ_transcript/36559	1.5837929389107	0.478227640871611	1.55219936632643	0.130273509564661	0.787791569270799	-4.68192245284645
a_HQ_transcript/46797	1.38776377770768	-0.484077954872542	1.55217836911853	0.130278527759378	0.787791569270799	-4.75930444985537
a_HQ_transcript/150187	-1.69709596505068	1.48305588939987	-1.55213010914521	0.130290062170879	0.787791569270799	-4.70629351212198
a_HQ_transcript/29029	3.44927360841363	0.291797113672515	1.5521065435141	0.130295694793722	0.787791569270799	-4.81925824224664
a_HQ_transcript/51033	1.37760799908777	-0.629400656320335	1.55201108532082	0.130318513094868	0.787847584937958	-4.77961325529712
a_HQ_transcript/64953	-1.97736948601761	4.08979660394926	-1.55188173130281	0.130349439022518	0.787896846393905	-5.12948453150956
a_HQ_transcript/128180	1.67727235524065	1.54849231213691	1.55180904964714	0.130366818350038	0.787896846393905	-4.69488282813629
a_HQ_transcript/151056	-2.56920550941435	2.69702110689628	-1.55177294527177	0.130375452173703	0.787896846393905	-5.06244577032006
a_HQ_transcript/75241	1.26124551762482	-0.477310401640976	1.55174698261475	0.13038166104343	0.787896846393905	-4.78554971036961
a_HQ_transcript/144922	2.62864079878948	4.02309387864949	1.55169357490333	0.130394434047436	0.787896846393905	-5.0502461285914
a_HQ_transcript/62464	2.35866627285177	2.03157031558759	1.55159145529447	0.130418859831801	0.787932325266179	-5.05477462981181
a_HQ_transcript/58943	1.9308278560233	0.0813945410454975	1.5515556874919	0.130427415939154	0.787932325266179	-4.79485984220823
a_HQ_transcript/150814	1.73972429689362	-0.401977097200189	1.55147563174504	0.130446567930298	0.787966133290282	-4.73748665095695
a_HQ_transcript/110812	-0.917130477254049	4.300969996148	-1.55138032034244	0.130469372557907	0.787973989632059	-5.13421318047518
a_HQ_transcript/130698	1.99619676452069	2.85544114130561	1.55123893169762	0.130503207792287	0.787973989632059	-4.93587494651174
a_HQ_transcript/122077	1.03336767883382	1.56062781064074	1.55117659494233	0.130518127651223	0.787973989632059	-4.79036361011809
a_HQ_transcript/149809	-1.67123625133857	1.24980594916696	-1.55112055023667	0.130531542735002	0.787973989632059	-4.67263955738315
a_HQ_transcript/97757	1.92769156233039	-0.436559586698995	1.55107999367484	0.130541251213152	0.787973989632059	-4.78053292097791
a_HQ_transcript/18969	2.01372616528958	2.65279974346068	1.55107802615284	0.130541722215824	0.787973989632059	-4.80626062149105
a_HQ_transcript/135406	1.84960576208058	-0.123243904736678	1.5510736934121	0.130542759430167	0.787973989632059	-4.8128961957425
a_HQ_transcript/9602	0.851304594269505	2.08066413020042	1.55095793380134	0.130570473588376	0.788059442295202	-4.81925906237612
a_HQ_transcript/146802	1.77239713510056	0.934372044186599	1.55075753318288	0.130618463051652	0.788267236323344	-4.72073023614777
a_HQ_transcript/109753	1.56660537043	0.887668692517104	1.5505372136925	0.130671238962811	0.788330885930501	-4.70861975730646
a_HQ_transcript/62822	1.07483069542561	-0.571730805508289	1.55047878728662	0.130685237481453	0.788330885930501	-4.84265528532895
a_HQ_transcript/91491	-1.47579845399874	-0.683839906177028	-1.55046186418528	0.130689292354063	0.788330885930501	-4.79227332247948
a_HQ_transcript/117370	2.76001911654006	1.08460877196009	1.55044742268064	0.130692752702566	0.788330885930501	-4.77840718062846
a_HQ_transcript/124588	-1.51501045687254	-0.49052229600994	-1.55035654826163	0.130714528951754	0.788330885930501	-4.77071329711003
a_HQ_transcript/86930	-1.60239767861347	1.11899581608043	-1.55033371757538	0.130720000335051	0.788330885930501	-4.6704676713549
a_HQ_transcript/14545	-1.27848988252821	-0.626743674837841	-1.55031726267641	0.130723943873591	0.788330885930501	-4.77271086272122
a_HQ_transcript/31280	1.49199544555133	1.313260785198	1.55023591523475	0.130743440809889	0.788366672966514	-4.64488007213302
a_HQ_transcript/145569	-2.13694628577978	1.92613967746184	-1.55017119113369	0.130758955235484	0.788378441025641	-4.80161513728489
a_HQ_transcript/36662	-2.05920844038297	1.04904344507066	-1.55009384666422	0.130777496734539	0.788408455733799	-4.79093427950155
a_HQ_transcript/1549	-0.582066594191812	3.671401795823	-1.54996950099288	0.13080731012834	0.788475105719461	-5.03631028626077
a_HQ_transcript/140606	3.89438892782214	1.99850769249982	1.54993459087387	0.130815681247565	0.788475105719461	-4.94253627615138

a_HQ_transcript/128278	4.19183140709621	2.46275376493136	1.54971451679304	0.130868462959599	0.788672890678965	-4.73803729537817
a_HQ_transcript/85170	-2.44249521886401	1.10294627367149	-1.54960172183008	0.130895521963893	0.788672890678965	-4.78967426303165
a_HQ_transcript/141182	2.04648403173342	0.750772571130318	1.54939714257529	0.130944611193238	0.788672890678965	-4.69688214966533
a_HQ_transcript/22435	2.02932117714159	0.678488744350343	1.54924306807887	0.130981591560221	0.788672890678965	-4.69066857351771
a_HQ_transcript/110189	3.07078723930608	1.43229831545873	1.54921768950608	0.130987683640389	0.788672890678965	-4.84415800099376
a_HQ_transcript/47080	3.81459299755877	1.17687486591005	1.54918128485859	0.130996422911204	0.788672890678965	-4.7042427726278
a_HQ_transcript/113184	-1.64696492805175	0.56662986209195	-1.54904148029908	0.13102998868067	0.788672890678965	-4.67598623363672
a_HQ_transcript/149335	-0.396034494145703	5.00547715349161	-1.54899582173277	0.131040952387944	0.788672890678965	-5.25077836364329
a_HQ_transcript/98209	0.385822107205718	3.0708365067323	1.54896643986625	0.131048008066286	0.788672890678965	-4.99783318606001
a_HQ_transcript/106477	0.832927111237473	5.74273506146533	1.54889870952778	0.13106427381264	0.788672890678965	-5.33980046420413
a_HQ_transcript/125639	1.00471141597156	3.07333687606324	1.54878088343287	0.131092574184879	0.788672890678965	-4.88840524972042
a_HQ_transcript/32196	-1.12511756418256	2.39107394583998	-1.54852083253628	0.131155052746468	0.788672890678965	-4.80703212345474
a_HQ_transcript/68915	-0.68317727356315	2.74059497410087	-1.54847971682382	0.131164933218916	0.788672890678965	-4.98354393738934
a_HQ_transcript/64799	1.92230242923304	0.617933496982932	1.54837491306414	0.131190121228997	0.788672890678965	-4.81793236184251
a_HQ_transcript/114389	1.31129467568406	1.20592325805403	1.54832911520714	0.131201129288444	0.788672890678965	-4.69087161444993
a_HQ_transcript/144831	-1.34819896871393	-0.216212386806322	-1.54828013813636	0.131212902338997	0.788672890678965	-4.83376983989515
a_HQ_transcript/81871	2.02365005031233	-0.350249903305897	1.54826547976524	0.131216426067622	0.788672890678965	-4.78061425470582
a_HQ_transcript/124982	-1.65524184053878	0.494072370331446	-1.5482598299079	0.131217784258405	0.788672890678965	-4.74353112123411
a_HQ_transcript/115664	1.80027932016294	2.08917267921206	1.54825174150774	0.131219728679662	0.788672890678965	-4.75561912878776
a_HQ_transcript/141918	-0.918286713170657	2.63636853566423	-1.54824506291739	0.131221334205512	0.788672890678965	-4.93501057250322
a_HQ_transcript/53303	-0.480234570246864	2.89172114532771	-1.54820768275452	0.131230320650244	0.788672890678965	-4.90711667084187
a_HQ_transcript/107354	2.03771434661107	0.532801698042918	1.54814423550602	0.131245574942217	0.788672890678965	-4.79991344802256
a_HQ_transcript/130987	1.95037059563054	0.399547653646328	1.54813160687094	0.131248611350933	0.788672890678965	-4.8198627090344
a_HQ_transcript/31519	-1.29810632254586	-0.315365073088334	-1.5480793787862	0.131261169592948	0.788672890678965	-4.73668212543186
a_HQ_transcript/144126	-0.733143336633422	2.99002327919351	-1.54806208022733	0.131265329246025	0.788672890678965	-4.985918265072
a_HQ_transcript/96657	1.05948838976647	-0.756803555554833	1.54805465982537	0.131267113606508	0.788672890678965	-4.7988422981397
a_HQ_transcript/141028	2.54267770978061	0.557914560359262	1.5479282535484	0.131297513145383	0.788672890678965	-4.78699820645436
a_HQ_transcript/142848	2.93951750751254	4.09366985092154	1.54788120185966	0.131308830099331	0.788672890678965	-5.01463401374428
a_HQ_transcript/35293	-1.52107653856821	1.01709618656608	-1.54777283349561	0.131334898056894	0.788672890678965	-4.64018259499145
a_HQ_transcript/39986	-1.45687831650322	-0.312622444316044	-1.54774894400227	0.13134064522771	0.788672890678965	-4.8006317422766
a_HQ_transcript/149382	0.531790896768722	7.86098975002167	1.54765792936302	0.131362542776141	0.788672890678965	-5.50705355547669
a_HQ_transcript/9591	1.89436487999094	0.359410812112315	1.54760216359272	0.131375961132148	0.788672890678965	-4.79919813001384
a_HQ_transcript/7988	0.691204079966915	1.79078080047154	1.54754987573442	0.131388543643253	0.788672890678965	-4.73985888144421
a_HQ_transcript/75868	2.92467778484028	0.933604683066648	1.54753209881456	0.131392821690628	0.788672890678965	-4.93004976929622
a_HQ_transcript/94342	1.53404240219266	-0.270299771420781	1.54749470720653	0.131401820416396	0.788672890678965	-4.73976600289847
a_HQ_transcript/56419	-1.25603936043642	0.635363875878597	-1.54749360864597	0.131402084805344	0.788672890678965	-4.71077943386527
a_HQ_transcript/138125	-2.97128685137096	3.06977988935239	-1.54746835562164	0.131408162532395	0.788672890678965	-4.92713550079061
a_HQ_transcript/37507	1.47846903470049	-0.147039948684697	1.54745833711076	0.131410573782996	0.788672890678965	-4.74060650836952
a_HQ_transcript/125710	-2.26365482039969	0.576702544934556	-1.5474389333798	0.131415243966137	0.788672890678965	-4.8157272587805
a_HQ_transcript/104166	-1.3952260271079	1.98734986294634	-1.54738246056006	0.131428836881726	0.788672890678965	-4.7633733844662
a_HQ_transcript/7079	0.548963263444889	2.38718609994687	1.54736662260034	0.131432649257574	0.788672890678965	-4.85783798319385
a_HQ_transcript/115281	1.90403603041957	0.928932463731424	1.5473409197149	0.131438836423426	0.788672890678965	-4.62228188608836
a_HQ_transcript/136375	-2.19137172679429	1.06684449379245	-1.54731561478258	0.13144492802532	0.788672890678965	-4.79731646884262
a_HQ_transcript/133951	0.498268702856463	2.86568491474843	1.54728532337838	0.131452220311009	0.788672890678965	-4.92756159641428
a_HQ_transcript/4485	1.35677050242543	1.10027697169414	1.54724465541167	0.131462011143475	0.788672890678965	-4.66254710741705
a_HQ_transcript/113974	-1.76736906548173	1.06141676380056	-1.54720060274715	0.131472617510996	0.788672890678965	-4.72336021531825
a_HQ_transcript/70018	2.56201575491412	1.65007140644446	1.54691338930326	0.13154178565365	0.788930268709704	-4.86936752576244
a_HQ_transcript/133756	1.68340533683659	2.06960165529143	1.54690838345669	0.131542991447695	0.788930268709704	-4.84097180472462
a_HQ_transcript/76053	1.37136455110136	-0.429580556335188	1.54677650785422	0.13157476049607	0.788930268709704	-4.76941777520973
a_HQ_transcript/125527	1.76238936881229	0.0570201899465	1.54675798490707	0.131579223202737	0.788930268709704	-4.78754780791111

a_HQ_transcript/122798	2.87073998288748	1.04914891811002	1.54672369309584	0.131587485402886	0.788930268709704	-4.79052587800942
a_HQ_transcript/115474	1.5456697335319	-0.46887463698126	1.54664670174294	0.131606037077858	0.788930268709704	-4.78108165231921
a_HQ_transcript/123070	1.06110693924026	2.81100996155371	1.5466280621312	0.131610528758567	0.788930268709704	-4.99016695762123
a_HQ_transcript/144280	1.0611453216277	1.95430906246477	1.54653946991097	0.131631878970037	0.788976888097578	-4.7334538430593
a_HQ_transcript/106396	1.01806271206253	-0.820868339446342	1.54633956967413	0.131680064088583	0.789184324707818	-4.80753061014693
a_HQ_transcript/13753	1.6486919744598	0.154918504219763	1.54607103266318	0.131744816330646	0.789490998316268	-4.73503982103197
a_HQ_transcript/143194	1.17089711128704	-0.722914782288096	1.54601288883527	0.131758839933273	0.789493644741913	-4.80549705381869
a_HQ_transcript/2561	0.512975891429958	2.63640730990556	1.54594492701162	0.131775233054612	0.789510487218974	-4.86353647934463
a_HQ_transcript/69049	3.36435756420076	2.62106772728102	1.54578957268076	0.131812712398667	0.789578596258464	-4.8328485506267
a_HQ_transcript/45811	-1.59454113442913	0.421877115558141	-1.54575136988438	0.131821930174876	0.789578596258464	-4.65734939965454
a_HQ_transcript/148236	-2.15739585101001	1.15459782679057	-1.54572012652975	0.1318294691281	0.789578596258464	-4.8076680843219
a_HQ_transcript/117012	-2.5831849528408	0.42371737493707	-1.54560217506765	0.131857933709858	0.789578596258464	-4.93358643988205
a_HQ_transcript/85779	-2.52726687152423	1.69896941096923	-1.54556621455055	0.131866612856213	0.789578596258464	-4.74301138451442
a_HQ_transcript/129780	2.06090357583914	1.70772847574288	1.54556004620475	0.131868101645974	0.789578596258464	-4.81274325376797
a_HQ_transcript/494	1.67919401814071	0.657355233742151	1.54544151830443	0.131896712145493	0.789668563558886	-4.73101443387424
a_HQ_transcript/31959	1.44130316650586	1.82835020804911	1.54534899306253	0.131919049569806	0.789720959154862	-4.78912070760769
a_HQ_transcript/123646	0.438549803909601	4.4435820723664	1.54515936527883	0.131964839054631	0.789913723052991	-5.19107567551305
a_HQ_transcript/127047	1.89020403410927	-0.462185366221456	1.54499490729475	0.132004561213389	0.789999138538567	-4.78793772045415
a_HQ_transcript/17619	-1.70234561360147	2.88552296087619	-1.54498775013599	0.132006290129113	0.789999138538567	-4.93818188464814
a_HQ_transcript/75032	1.56978264541468	1.29549922965707	1.5449089418743	0.132025328624271	0.790031737932017	-4.78249014398268
a_HQ_transcript/24274	0.499511716582136	3.79887554386539	1.54482784394936	0.132044922580291	0.790067654117052	-5.04172420894841
a_HQ_transcript/149641	-1.39738412775805	-0.404546309783384	-1.54476914532831	0.13205910614264	0.790071194098333	-4.79094340539736
a_HQ_transcript/2155	1.98313063127954	0.118347119128845	1.54467534986885	0.132081772846136	0.79012548029229	-4.71876637117491
a_HQ_transcript/27081	1.04533597103482	3.72675919171301	1.54461738660413	0.132095781882842	0.79012796967337	-5.12715199675278
a_HQ_transcript/150430	1.3083983963963	4.26776754738654	1.54454715304833	0.132112758118117	0.790148204948033	-5.13946918784727
a_HQ_transcript/9438	0.487096444257309	2.97167167659483	1.54429235919591	0.13217435948747	0.790435305379387	-4.94637050721436
a_HQ_transcript/86418	-0.970072109498499	2.20871193374914	-1.54421638581081	0.132192732036986	0.790463854320124	-4.78585365658786
a_HQ_transcript/50081	2.10668418761055	0.305495171405906	1.54404571208365	0.132234013402364	0.790571171545264	-4.74026198692456
a_HQ_transcript/77402	1.08749338088111	2.64949276788721	1.54376349533402	0.132302296938407	0.790571171545264	-4.93599837846385
a_HQ_transcript/106457	1.69537123299661	0.809001909390456	1.54375548358209	0.132304235832805	0.790571171545264	-4.88287544213331
a_HQ_transcript/148666	-2.06532480854788	0.589602422831583	-1.54373698399432	0.132308712937485	0.790571171545264	-4.88550363451026
a_HQ_transcript/73105	1.71203037076556	-0.199803518225837	1.54373321291054	0.132309625596501	0.790571171545264	-4.71627213197746
a_HQ_transcript/21880	1.12873067602143	-0.499180330278675	1.54366798793183	0.132325411830206	0.790571171545264	-4.83127982784002
a_HQ_transcript/112376	-3.41717852586832	2.41584115100043	-1.54365319494134	0.132328992351999	0.790571171545264	-4.86284095138136
a_HQ_transcript/119521	3.55116242556136	4.06461687486793	1.54362562068279	0.132335666684321	0.790571171545264	-5.14708369911756
a_HQ_transcript/119369	1.79086252177529	-0.0732507401847961	1.54361466429005	0.132338318748196	0.790571171545264	-4.76396633325785
a_HQ_transcript/41635	-2.49285473187396	0.818237051496689	-1.54358010472246	0.132346684391463	0.790571171545264	-4.74477724002561
a_HQ_transcript/127644	0.620947453600667	3.18417121126658	1.54342623234583	0.13238393662911	0.790685871676939	-5.053412842881
a_HQ_transcript/69256	1.29046811080738	-0.701931893528451	1.54337323777185	0.132396768491691	0.790685871676939	-4.79803365925878
a_HQ_transcript/27275	2.79837176561113	0.989132993911539	1.54331278723799	0.13241140693694	0.790685871676939	-4.72675887724927
a_HQ_transcript/147431	2.03101974613805	1.87318128321181	1.54327608220619	0.132420295912039	0.790685871676939	-4.77713535441741
a_HQ_transcript/138573	1.72612677794587	0.983489593122215	1.54314691804162	0.132451579857019	0.790713365306586	-4.75257556801434
a_HQ_transcript/2119	-0.923711491914945	2.60217580017302	-1.54313386475114	0.132454741736048	0.790713365306586	-4.8878337046224
a_HQ_transcript/50440	-1.89456054933364	2.5346116966066	-1.5430648496583	0.132471460175549	0.790713365306586	-4.96008076512034
a_HQ_transcript/124546	0.86764988760711	2.09182665976761	1.5430113153237	0.132484429683852	0.790713365306586	-4.78237365796724
a_HQ_transcript/59489	1.84480262174082	0.372194343330704	1.54295052472072	0.132499158381493	0.790713365306586	-4.75573814886971
a_HQ_transcript/103347	1.68498984462216	0.508075970634319	1.54291804388948	0.132507028568413	0.790713365306586	-4.80440427351701
a_HQ_transcript/20500	0.95213702214165	4.21974806371062	1.54282223908661	0.132530244519363	0.790713365306586	-5.18558302537973
a_HQ_transcript/44545	-0.787384677349969	3.26821342376957	-1.54280788088001	0.132533724163606	0.790713365306586	-4.94021499696835
a_HQ_transcript/37693	-1.5199414786028	-0.332680236762443	-1.54273614442021	0.132551110268316	0.790732123328108	-4.72837888545507

a_HQ_transcript/140518	-1.27851011698812	3.09841118688858	-1.54267873627682	0.132565025082412	0.790732123328108	-4.86722731568615
a_HQ_transcript/138750	-1.79647834545166	1.34101566900657	-1.54262653805128	0.132577678123378	0.790732123328108	-4.76550090114448
a_HQ_transcript/66176	-3.8241623573282	2.39582766570239	-1.54244830507098	0.132620889826804	0.790908698456185	-4.81843405504171
a_HQ_transcript/122294	-1.50542656378249	0.436725803379502	-1.54228248161763	0.132661103150101	0.790937527191174	-4.73387522584236
a_HQ_transcript/100414	1.40135815295029	-0.624236268506902	1.54219727574402	0.132681770000691	0.790937527191174	-4.80337022275159
a_HQ_transcript/128638	-1.44003145589993	1.5599244643209	-1.54216642932668	0.132689252502463	0.790937527191174	-4.68585870852188
a_HQ_transcript/134332	-1.22292666507555	1.52940651234779	-1.54213480535824	0.132696923971942	0.790937527191174	-4.66258845124227
a_HQ_transcript/152130	0.70923672316559	2.95222633067192	1.54203573070455	0.132720960226696	0.790937527191174	-5.01726300584849
a_HQ_transcript/52393	1.3773076752672	0.272427466950051	1.54196380736998	0.132738411577627	0.790937527191174	-4.77903612407735
a_HQ_transcript/114175	1.02903125847003	2.05827268909829	1.54192711909584	0.132747314271786	0.790937527191174	-4.78429745083358
a_HQ_transcript/112042	0.549615062081876	4.00756035049991	1.54191380072359	0.13275054619776	0.790937527191174	-5.18588083070159
a_HQ_transcript/119106	1.49924876032025	0.428000974767483	1.54189581859418	0.132754909963632	0.790937527191174	-4.75856532957695
a_HQ_transcript/144465	2.27909416619025	2.19332649789105	1.5418674596668	0.132761792127792	0.790937527191174	-4.81647631676852
a_HQ_transcript/111706	-1.73413121596505	-0.3821367208011	-1.54180506556485	0.132776934989409	0.790941987998682	-4.77636414855327
a_HQ_transcript/38382	1.64751973495232	0.155764579569061	1.54170148167409	0.132802077579527	0.790941987998682	-4.6560794821672
a_HQ_transcript/50481	1.47895428909565	1.48967466553881	1.54169619213449	0.132803361596255	0.790941987998682	-4.74765928375312
a_HQ_transcript/125922	0.354227525170228	4.76941717042504	1.54136582705281	0.13288357646707	0.791338646949888	-5.24831067812452
a_HQ_transcript/123729	-1.46090297903031	3.59442214847527	-1.54114155137977	0.13293805447964	0.791581974061927	-5.04402068814059
a_HQ_transcript/139542	-1.7775093671518	1.97621246068924	-1.54091963975798	0.132991976056005	0.791757562245072	-4.79919215174693
a_HQ_transcript/105177	0.96078613195986	1.57139356800462	1.54090808343306	0.132994784574747	0.791757562245072	-4.68528007078204
a_HQ_transcript/34188	-1.70144739471831	0.116166166681536	-1.54074306181303	0.133034894808157	0.791915244793504	-4.71091884802336
a_HQ_transcript/2133	0.802214394607024	1.97263257614999	1.54065668464995	0.133055893589158	0.79195914218212	-4.72640303803514
a_HQ_transcript/121480	0.852648530894655	4.91982792153487	1.5403866951693	0.133121546884285	0.792247638833987	-5.22113944691323
a_HQ_transcript/135550	-1.20336349611186	1.68399148691157	-1.54021787227437	0.133162612850336	0.792247638833987	-4.79404585640496
a_HQ_transcript/71346	2.47285117914061	0.726970786993971	1.54021780036464	0.133162630344477	0.792247638833987	-4.82465858234659
a_HQ_transcript/11074	1.90304815017753	2.17770375845062	1.54017914768035	0.133172034007849	0.792247638833987	-4.68793915499638
a_HQ_transcript/45059	2.56622761271577	1.26829890009589	1.54017718997146	0.133172510305651	0.792247638833987	-4.79164847947652
a_HQ_transcript/137038	-5.58160594510981	1.24560021937625	-1.53991518148575	0.133236267711232	0.792326702822636	-4.71978485905021
a_HQ_transcript/79218	2.11212186159122	1.4897112844108	1.53985539974639	0.133250818522554	0.792326702822636	-4.72739434771266
a_HQ_transcript/115517	1.58969805880749	-0.0105371432104519	1.53981273708284	0.133261203356107	0.792326702822636	-4.84045110782155
a_HQ_transcript/93197	1.65802403412804	0.0113129305603249	1.53980382216913	0.133263373483792	0.792326702822636	-4.77045998735142
a_HQ_transcript/150703	8.68161223846895	4.12088080128727	1.53978459053437	0.133268055073911	0.792326702822636	-4.93197039477974
a_HQ_transcript/119377	-2.86598125796894	1.36945542229341	-1.53977087097968	0.133271394930249	0.792326702822636	-4.87838667115959
a_HQ_transcript/82277	0.738483721576855	1.83055779541106	1.53969265359574	0.133290437282369	0.792326702822636	-4.71586036638868
a_HQ_transcript/33301	0.888673871297099	1.52443248706165	1.53967454454939	0.133294846320234	0.792326702822636	-4.71282761652858
a_HQ_transcript/89147	0.407689513502618	3.84029266842845	1.53941667249002	0.133357643651589	0.792509841601023	-5.14962728328888
a_HQ_transcript/145300	-2.16301524937677	1.82816571074435	-1.53936470615184	0.13337030146037	0.792509841601023	-4.86036149767097
a_HQ_transcript/40254	2.07923169544622	0.166066293426783	1.53932870052644	0.133379072175961	0.792509841601023	-4.89067459297675
a_HQ_transcript/126452	0.336025950961685	4.94354633820793	1.5393241049782	0.133380191652608	0.792509841601023	-5.25927698271083
a_HQ_transcript/125298	1.5378713403115	1.30563432150054	1.53923021557099	0.13340306480039	0.792564733311229	-4.76641066842091
a_HQ_transcript/26381	-2.69018540218961	0.810635813269044	-1.53906484945832	0.133443358682844	0.792605930409482	-4.82582612490216
a_HQ_transcript/104422	-2.8506422426336	1.61473497997097	-1.53898061704849	0.133463886935039	0.792605930409482	-4.88528612894234
a_HQ_transcript/13385	1.54017257097876	-0.002479268558855	1.53894981093982	0.133471395319997	0.792605930409482	-4.80641656039698
a_HQ_transcript/70181	4.76069332430435	3.23628907185677	1.53886486911331	0.133492099996516	0.792605930409482	-4.96597463438227
a_HQ_transcript/84516	-1.00456273547363	2.12203318874168	-1.538821405391	0.133502695339022	0.792605930409482	-4.84107771164962
a_HQ_transcript/48637	2.71410811487207	0.762149102559885	1.53881072340413	0.133505299438122	0.792605930409482	-4.84751660281138
a_HQ_transcript/140192	-0.551675452219371	2.53348823013142	-1.53875672238358	0.133518464660617	0.792605930409482	-4.84712837476452
a_HQ_transcript/115466	1.58143154657259	0.766256553678681	1.53860917973745	0.133554440299187	0.792605930409482	-4.73186825232661
a_HQ_transcript/88985	1.45766396172773	-0.620506190489566	1.53860310802461	0.133555920946023	0.792605930409482	-4.80345319626154
a_HQ_transcript/83132	1.83521132369322	0.885028118782299	1.53854914385495	0.133569081223066	0.792605930409482	-4.76641245519631

a_HQ_transcript/102258	-1.8135201590988	1.67117701016581	-1.53848232201347	0.133585378563892	0.792605930409482	-4.8903944613819
a_HQ_transcript/16525	0.543691543072739	3.48272232238253	1.53842920764258	0.133598333904968	0.792605930409482	-5.07560858504614
a_HQ_transcript/149369	-1.1950264974847	2.26688700025423	-1.5383551675639	0.133616395017471	0.792605930409482	-4.90486968983626
a_HQ_transcript/119980	-1.70168998798152	0.395943722640404	-1.53834972595642	0.133617722504697	0.792605930409482	-4.74919493801984
a_HQ_transcript/113258	1.89163067490668	-0.182465943052866	1.53834708157652	0.133618367608468	0.792605930409482	-4.82273731980144
a_HQ_transcript/106407	3.0709427825035	0.48979854199346	1.53824428721586	0.133643446530705	0.792605930409482	-4.79533312775009
a_HQ_transcript/93142	1.37516684941104	4.42718874342068	1.53822009723791	0.133649348756363	0.792605930409482	-5.25220341074283
a_HQ_transcript/122368	1.74310969964473	1.02694763908876	1.53819513958083	0.133655438512829	0.792605930409482	-4.66969160745236
a_HQ_transcript/132834	2.52253705688269	0.141200226418389	1.53789878629788	0.133727766933336	0.792923262894217	-4.81316640566125
a_HQ_transcript/2392	-2.05101989617908	2.3282133260145	-1.53782023190778	0.133746944345927	0.792923262894217	-4.79022398905276
a_HQ_transcript/59450	1.42236678069372	-0.525861512545159	1.53778937668097	0.133754477613666	0.792923262894217	-4.81300250171337
a_HQ_transcript/56833	-1.21631019064533	1.11213198845094	-1.53774262072216	0.133765893681261	0.792923262894217	-4.68361148632018
a_HQ_transcript/56499	1.25080195857522	-0.679830878193701	1.53757800839093	0.133806092172471	0.792923262894217	-4.75496754406584
a_HQ_transcript/79548	1.31661022866129	-0.361664510081347	1.53744151180015	0.133839432188367	0.792923262894217	-4.79060229433334
a_HQ_transcript/127137	-1.99390535045789	-0.0975799954455979	-1.53723109471877	0.133890840848931	0.792923262894217	-4.7763292425082
a_HQ_transcript/130396	-4.07951013699656	0.940364432538758	-1.5371642146149	0.133907184202992	0.792923262894217	-4.78310715489759
a_HQ_transcript/9089	1.47319883559474	2.50062026474014	1.53713025298519	0.133915483955084	0.792923262894217	-4.67413007713656
a_HQ_transcript/85940	1.79183782298388	1.959936887028	1.53711598343658	0.133918971359427	0.792923262894217	-4.78934590962818
a_HQ_transcript/48884	1.47403030705179	-0.620221418513904	1.53705847843668	0.133933026030913	0.792923262894217	-4.81347245594669
a_HQ_transcript/57146	-1.13509538151365	1.74923115535218	-1.53702180111855	0.133941990877741	0.792923262894217	-4.7460427692471
a_HQ_transcript/27493	1.00371474359348	1.16386241821569	1.53699040022502	0.133949666421354	0.792923262894217	-4.6952960845122
a_HQ_transcript/80294	2.36910882307242	1.46551956368661	1.53695374288491	0.133958627285963	0.792923262894217	-4.79670488380741
a_HQ_transcript/87541	2.85240544977806	3.86962874950905	1.53692311995376	0.133966113414917	0.792923262894217	-4.91817857087516
a_HQ_transcript/72866	1.48496615674133	1.38149340487221	1.53687893361794	0.133976915871476	0.792923262894217	-4.84536657965855
a_HQ_transcript/152144	-1.79339882249485	-0.0348314503961712	-1.53684632983544	0.133984887134786	0.792923262894217	-4.81378115347584
a_HQ_transcript/28707	2.15673904843247	0.271385466938732	1.53682448514935	0.133990228133077	0.792923262894217	-4.80515822716907
a_HQ_transcript/67434	-1.13157920962489	1.96287262141887	-1.53682431951372	0.133990268631438	0.792923262894217	-4.76845346492727
a_HQ_transcript/44913	3.68063051291958	1.6500279881217	1.53678542924905	0.133999777681066	0.792923262894217	-4.94797736809096
a_HQ_transcript/32970	-1.43463773391837	-0.347807473959719	-1.53677522182179	0.13400227358724	0.792923262894217	-4.71228797136515
a_HQ_transcript/2873	-1.24232720254493	1.98405466691969	-1.53672112718418	0.134015501362233	0.792923262894217	-4.75679942199938
a_HQ_transcript/132331	1.35369628424669	0.878613574664931	1.53665885140268	0.13403073098299	0.792923262894217	-4.70058909912948
a_HQ_transcript/56852	-1.34757774960657	-0.42203151898157	-1.53660396855281	0.134044153814172	0.792923262894217	-4.79660666122241
a_HQ_transcript/146335	4.92589261332491	4.09480378422325	1.53648171723742	0.134074057024546	0.792923262894217	-5.15768235917821
a_HQ_transcript/106464	-0.855932666793269	2.70807945408998	-1.53647788528852	0.13407499442339	0.792923262894217	-4.91423468148072
a_HQ_transcript/60011	-1.60425835401496	1.68617099645045	-1.53641324280079	0.134090808532197	0.792923262894217	-4.69745856308352
a_HQ_transcript/141623	1.00593024225312	2.8924582226361	1.53632959540198	0.134111274234319	0.792923262894217	-4.90539344670061
a_HQ_transcript/143284	-1.78790293796642	0.17061235097977	-1.5363195087755	0.134113742262991	0.792923262894217	-4.78750208573827
a_HQ_transcript/95144	2.18073208458749	1.8787181102439	1.53630137574656	0.1341181792041	0.792923262894217	-4.8537671951032
a_HQ_transcript/100934	1.2890699969593	-0.690072258157985	1.53613071210349	0.134159944431765	0.792984971420234	-4.82187158509531
a_HQ_transcript/92588	-1.49089678585939	-0.346299486836189	-1.53612114474138	0.134162286091743	0.792984971420234	-4.78229229455969
a_HQ_transcript/139068	-2.26673333366796	1.43275374750605	-1.53608801290906	0.134170395529878	0.792984971420234	-4.88460711646231
a_HQ_transcript/30292	1.87645083274813	0.88957404938953	1.53603574674907	0.134183189144982	0.792984971420234	-4.85010298338151
a_HQ_transcript/62880	-1.27998427280212	2.53767242778872	-1.53598003574811	0.134196827068897	0.792984971420234	-4.96731093970332
a_HQ_transcript/122642	2.01682517791738	0.909497028639377	1.53585242992278	0.134228068906202	0.79299350369341	-4.9349040589364
a_HQ_transcript/48004	3.7215357298322	3.2096931140786	1.53584534367898	0.134229804009477	0.79299350369341	-4.96169971197745
a_HQ_transcript/2328	1.08802204283029	2.93215731351527	1.53580698122103	0.1342391975699	0.79299350369341	-4.97497836248929
a_HQ_transcript/112478	0.471788868684826	3.79431700414863	1.53562855394332	0.13428289486396	0.792996760447398	-5.06523905260345
a_HQ_transcript/56294	-1.30627232449056	-0.0659281037922126	-1.53559242299403	0.134291744826513	0.792996760447398	-4.75845307511349
a_HQ_transcript/105806	-1.60234240366595	0.610529890976357	-1.5355698332842	0.13429727821972	0.792996760447398	-4.80622667311406
a_HQ_transcript/48359	2.04968899870094	1.09323168555089	1.53545947452954	0.134324313462843	0.792996760447398	-4.7084005103858

a_HQ_transcript/94444	1.2756266440877	0.0519298285567846	1.53541632330932	0.134334885672439	0.792996760447398	-4.67652711957852
a_HQ_transcript/98133	-1.33847982711586	2.06504905331653	-1.53538972468722	0.134341402769285	0.792996760447398	-4.74056045941907
a_HQ_transcript/102555	-2.10001337105981	4.17613236357826	-1.53537567538644	0.134344845180688	0.792996760447398	-5.15388640050254
a_HQ_transcript/98187	-2.08349805867738	1.90490269533344	-1.53535918059789	0.134348886885597	0.792996760447398	-4.91840412428915
a_HQ_transcript/54060	1.17750791235711	-0.622182019374416	1.53518812837828	0.134390805475204	0.793108562651218	-4.82461727511183
a_HQ_transcript/16052	1.87253344181497	-0.0778781908275274	1.53517053862251	0.134395116675518	0.793108562651218	-4.64659611625458
a_HQ_transcript/116840	1.4456816596816	0.0683799519302768	1.53505197736776	0.134424178629061	0.793199538661053	-4.6731054926446
a_HQ_transcript/70901	0.798451546056892	4.21271991933799	1.53485547883178	0.134472355909291	0.79338620126842	-5.1125308766758
a_HQ_transcript/19286	1.27684448491689	0.955257715188597	1.53481162276028	0.134483110395984	0.79338620126842	-4.64850009735439
a_HQ_transcript/95468	1.28883375546157	1.81459189809375	1.53452764495681	0.134552764927556	0.793716573950576	-4.79044150332952
a_HQ_transcript/72945	-2.04478036833132	0.915474100292532	-1.53441688050866	0.134579941319993	0.793791940645898	-4.80358004585705
a_HQ_transcript/149957	-4.75817013678961	0.725392669700699	-1.53430496127096	0.134607405553415	0.793791940645898	-4.90480784976946
a_HQ_transcript/147370	-1.53565533307459	-0.423717123333157	-1.53424293581554	0.134622628138399	0.793791940645898	-4.7312604051776
a_HQ_transcript/137207	1.30993863008508	-0.211254495322054	1.53419846209217	0.134633543950267	0.793791940645898	-4.78969741320889
a_HQ_transcript/49430	-0.414673258653314	3.39368203855085	-1.53417937235647	0.134638229632392	0.793791940645898	-5.0417899289055
a_HQ_transcript/89491	-1.72502634895537	3.92472626402929	-1.53414170021207	0.134647476857428	0.793791940645898	-5.2258475032541
a_HQ_transcript/146908	-3.28449843593021	0.754101410387413	-1.53405188235288	0.134669526147442	0.79381416951334	-4.82782404678049
a_HQ_transcript/112086	1.48563482077468	-0.193394179799423	1.53398334991081	0.134686352067602	0.79381416951334	-4.86317101849
a_HQ_transcript/107312	-0.495547920398188	3.60991287867284	-1.53386041013386	0.134716540206972	0.79381416951334	-5.11402736292061
a_HQ_transcript/128819	2.59249379925021	0.666187542763188	1.53375385384406	0.134742709771483	0.79381416951334	-4.79556692199927
a_HQ_transcript/96303	0.488645711396503	4.01860061116516	1.53373582380935	0.134747138243	0.79381416951334	-5.14802178656716
a_HQ_transcript/11531	1.05106650227425	1.10214730316927	1.53368937497597	0.13475854737873	0.79381416951334	-4.70235490685769
a_HQ_transcript/58792	-0.680251214595427	1.93235584960298	-1.53362939855663	0.134773280424645	0.79381416951334	-4.73365545319672
a_HQ_transcript/129020	-1.91390740932558	0.103675580208609	-1.53350112960396	0.134804793720054	0.79381416951334	-4.87686204100325
a_HQ_transcript/55212	1.3303156017504	1.09150534346001	1.53349316732027	0.134806750101469	0.79381416951334	-4.63575616182176
a_HQ_transcript/58828	3.78243498554203	3.79041557944748	1.53346792420169	0.134812952640683	0.79381416951334	-5.01092228426059
a_HQ_transcript/1638	1.59964061631981	0.642009835032154	1.5334191450629	0.134824938918364	0.79381416951334	-4.69659712074938
a_HQ_transcript/124479	3.96652312134208	1.44882901891091	1.53340934166312	0.134827347967577	0.79381416951334	-4.79495653897546
a_HQ_transcript/131145	1.67907590909489	1.46251033089085	1.53335181221347	0.134841485731675	0.79381416951334	-4.95315699597109
a_HQ_transcript/58772	2.88245422729619	1.53620929896511	1.53334794625488	0.134842435827393	0.79381416951334	-4.83586079027398
a_HQ_transcript/6657	2.82874386980895	2.36084416633992	1.53326921503149	0.134861785944661	0.793847685406709	-4.92029074107185
a_HQ_transcript/25299	-1.09982490144254	3.3564656569818	-1.53319512858651	0.134879996545275	0.793874487564171	-4.9602426476543
a_HQ_transcript/91181	1.81847072772579	1.13469250229113	1.53308045522515	0.134908187414757	0.793893557257934	-4.742352308008
a_HQ_transcript/122882	-1.82758466729481	1.65772492373416	-1.53306817424913	0.134911206806351	0.793893557257934	-4.76619393818816
a_HQ_transcript/142713	-2.21993849860917	0.795669932376399	-1.53301528988843	0.134924209540172	0.793893557257934	-4.85390147772642
a_HQ_transcript/11132	1.50071607457293	3.46683117263584	1.53289647839602	0.134953425543916	0.793985093119103	-5.1397638816427
a_HQ_transcript/5560	-0.896527503118304	3.02120090533727	-1.53283547504129	0.134968428388858	0.793992997205497	-4.95762632467063
a_HQ_transcript/11315	1.00574397557175	-0.783423295016214	1.53269086054085	0.135003999508861	0.794048504782428	-4.8165514882696
a_HQ_transcript/106354	1.3095003759751	-0.525807295579745	1.53268604291614	0.135005184640186	0.794048504782428	-4.82133846480474
a_HQ_transcript/130679	-1.4360248002273	2.36022190616575	-1.53258333180965	0.135030453481321	0.794116774581369	-4.83313938865376
a_HQ_transcript/13225	-2.29683757319737	1.59818366582411	-1.53218832324757	0.135127668568004	0.794608105060285	-4.84605869322984
a_HQ_transcript/129084	1.78487786662543	-0.416402618863134	1.53208863731324	0.135152211095501	0.794672033841721	-4.80862116399829
a_HQ_transcript/29911	0.487038755357547	3.42454872379729	1.53182039620095	0.135218269552773	0.794980031613593	-5.02834204610219
a_HQ_transcript/86185	1.4680796469172	-0.353284830708323	1.53155713212298	0.135283127719766	0.795166160055409	-4.73737051770597
a_HQ_transcript/79269	1.98779373543223	2.45212608461309	1.5315238484115	0.135291329339722	0.795166160055409	-4.89112269029268
a_HQ_transcript/84571	1.82176073803862	1.41727205825195	1.53136871593252	0.135329561679021	0.795166160055409	-4.76953923236007
a_HQ_transcript/141928	-1.81776798532945	0.333436338131405	-1.53134399248547	0.135335655569809	0.795166160055409	-4.95648117133637
a_HQ_transcript/51731	-1.40441289507174	-0.325780305542713	-1.53126311142053	0.13535559286641	0.795166160055409	-4.75481198716525
a_HQ_transcript/149998	0.824989261950031	4.16436371622062	1.53119474067322	0.135372448203913	0.795166160055409	-5.08498829122975
a_HQ_transcript/21475	1.22112339465146	2.30181212648682	1.53112124510842	0.135390568847234	0.795166160055409	-4.82286431448729

a_HQ_transcript/144391	2.14126802653484	1.7981613750526	1.53102305020904	0.13541478227621	0.795166160055409	-4.89857792492362
a_HQ_transcript/129804	-1.84420766775958	-0.225821321268955	-1.53101535828967	0.135416679139093	0.795166160055409	-4.77507673036242
a_HQ_transcript/56667	2.56502504642167	0.743592234647509	1.53095679106462	0.135431122792	0.795166160055409	-4.77226628586672
a_HQ_transcript/47041	-0.90476074111776	3.37408527197916	-1.530865951861	0.135453527715859	0.795166160055409	-4.9837069289328
a_HQ_transcript/142242	1.89392602804894	-0.434472502905495	1.53081906249596	0.135465093858108	0.795166160055409	-4.78597770555632
a_HQ_transcript/131035	0.493610997728103	4.12091352904977	1.53081678383992	0.135465655951742	0.795166160055409	-5.1953856776801
a_HQ_transcript/109424	1.70063906931284	0.20904307955148	1.53077097001371	0.135476957601098	0.795166160055409	-4.7672139564839
a_HQ_transcript/151045	-3.30219437895133	6.84208320181444	-1.53071426401947	0.135490947256005	0.795166160055409	-5.5082607801495
a_HQ_transcript/82046	1.2865030596259	-0.272306765702947	1.53071152543196	0.13549162290892	0.795166160055409	-4.73749796494818
a_HQ_transcript/133991	-1.84774488607271	0.521320482011611	-1.53066698882129	0.135502611178461	0.795166160055409	-4.72967566657272
a_HQ_transcript/72831	-1.87782901919726	0.531039543554323	-1.53065828558669	0.135504758563123	0.795166160055409	-4.85459271550339
a_HQ_transcript/58862	1.93592836892378	0.0631027072241708	1.53063769110154	0.135509840034216	0.795166160055409	-4.82057210436738
a_HQ_transcript/148887	1.60306077409296	-0.386593818841552	1.53042307952659	0.135562802333554	0.795322655010832	-4.86734113005452
a_HQ_transcript/135790	1.95014843028509	0.788092583502269	1.53041873780612	0.135563873965169	0.795322655010832	-4.77594872415097
a_HQ_transcript/132638	2.51739745468023	-0.197781127505069	1.53030262874043	0.135592534763278	0.795347785512196	-4.80195051230343
a_HQ_transcript/45263	1.35604232232805	2.53164608549461	1.53029052455459	0.135595522887866	0.795347785512196	-4.85075522217181
a_HQ_transcript/101025	2.43008231675032	0.639260166798716	1.53013092984434	0.135634926539792	0.795498638876504	-4.90009837174665
a_HQ_transcript/136872	1.37907913264628	-0.598999122167818	1.53004360910211	0.13565648979341	0.795500677641025	-4.81466307630765
a_HQ_transcript/123455	-1.40819581079806	-0.0978609898894483	-1.53000302259704	0.135666513293371	0.795500677641025	-4.73331014554965
a_HQ_transcript/77857	-1.15007298136732	2.29454982291096	-1.52996327522526	0.135676330135789	0.795500677641025	-4.83600286355262
a_HQ_transcript/135539	-2.80033145218039	0.613288804841466	-1.52986696131036	0.135700120216014	0.795559918095455	-4.81534960088294
a_HQ_transcript/92206	2.94606848054705	2.8523653336039	1.52980159711162	0.135716267462008	0.795574343992698	-4.78793910045751
a_HQ_transcript/32874	1.71230085494156	1.3071312218343	1.52966072878278	0.135751072151354	0.795656144651413	-4.71271685581259
a_HQ_transcript/149599	2.86466005730336	1.63748588492766	1.52963432031151	0.135757597761023	0.795656144651413	-4.82642265051074
a_HQ_transcript/46858	1.58112583784254	3.08917512539274	1.52957196450129	0.135773007068115	0.795666231964453	-4.87475804571863
a_HQ_transcript/117545	1.09869292405542	1.09390367428075	1.52942547253178	0.135809213581732	0.795798182165211	-4.68479378595927
a_HQ_transcript/109951	-1.50567939331268	2.58770832054485	-1.5291419854596	0.135879301550324	0.796047923981402	-4.86835851058879
a_HQ_transcript/109395	-1.20084328774375	1.66110852134014	-1.52910950744743	0.135887333122226	0.796047923981402	-4.71249166578036
a_HQ_transcript/106795	1.24322323881003	4.25046350411034	1.52897089267591	0.135921615841136	0.796047923981402	-5.19410014960604
a_HQ_transcript/34314	-2.34203262199544	0.389615399800877	-1.52895250073856	0.135926165128336	0.796047923981402	-4.86470635955268
a_HQ_transcript/91677	-2.22289838789641	1.18053683012962	-1.52888687247832	0.135942399429359	0.796047923981402	-4.82788371575588
a_HQ_transcript/10501	-1.438211192984	1.08161986303445	-1.52878516064023	0.135967562731594	0.796047923981402	-4.72138892424709
a_HQ_transcript/80477	1.55769933268614	0.699466853159546	1.52878265446132	0.135968182802672	0.796047923981402	-4.71338573171758
a_HQ_transcript/129995	-0.563615540533415	3.20435667231065	-1.52877686524209	0.135969615162223	0.796047923981402	-5.07174230866591
a_HQ_transcript/31873	1.72058620679586	0.49493203548289	1.52874329886841	0.135977920342085	0.796047923981402	-4.83530209019276
a_HQ_transcript/41000	1.60831231566386	0.00519783705399295	1.52869940511441	0.135988781398557	0.796047923981402	-4.68947731502435
a_HQ_transcript/12811	1.18838188774909	2.14179092785069	1.52861101618247	0.136010654454472	0.796095793185936	-4.78652521023023
a_HQ_transcript/325	0.67774190311716	3.35822233688836	1.52849956771465	0.136038237960907	0.796177073720462	-5.15430677272544
a_HQ_transcript/28965	1.87739604594907	0.875938829531825	1.52824222141303	0.136101948462431	0.796429810728065	-4.84399079784906
a_HQ_transcript/102036	3.74874840525204	1.47464398735957	1.5282144535503	0.136108824314833	0.796429810728065	-4.83909674227781
a_HQ_transcript/47111	-1.66387189991778	0.572786949568349	-1.52810805097701	0.136135174219261	0.796503815502486	-4.79116600756976
a_HQ_transcript/123418	-0.797112411228016	4.18402114080311	-1.52803224711897	0.136153949064764	0.796533489456182	-5.22911221704705
a_HQ_transcript/47055	0.960321931920931	5.56146712880707	1.52788485395473	0.136190460897403	0.796666912654146	-5.34433026122634
a_HQ_transcript/46433	1.98129799786942	0.519689367584483	1.52772595036415	0.136229832921198	0.796750603379636	-4.74236535859022
a_HQ_transcript/17109	-0.797585684563769	2.67512006945726	-1.52768717635466	0.136239441470302	0.796750603379636	-4.93079412598954
a_HQ_transcript/1157	-0.864146386159865	2.56959877616174	-1.52764805059034	0.136249137742331	0.796750603379636	-4.74493244898119
a_HQ_transcript/136257	-0.835014815397741	2.25700330063404	-1.52754783914757	0.13627397500484	0.796750603379636	-4.7257006822442
a_HQ_transcript/91807	0.571523765279477	2.99275952875952	1.52754108647905	0.136275648775575	0.796750603379636	-4.95854564353097
a_HQ_transcript/108284	0.412586855814906	4.3330304280547	1.52744795142034	0.136298735674342	0.796750603379636	-5.22156971858339
a_HQ_transcript/151291	-3.37715219462497	0.34369981598192	-1.52739916310705	0.13631083088638	0.796750603379636	-4.91374104971526

a_HQ_transcript/101595	-2.61305457834184	0.184926110697261	-1.52738467636373	0.136314422491923	0.796750603379636	-4.85038012375651
a_HQ_transcript/85114	1.59511231828448	2.98629075354882	1.52724950396127	0.136347938604658	0.796853235979116	-4.91608616516648
a_HQ_transcript/94199	0.99479446335485	3.10062299513081	1.52720329015866	0.136359398883228	0.796853235979116	-4.9112737480317
a_HQ_transcript/96871	1.79493368830116	1.53784978511835	1.52693331466015	0.136426364007022	0.797164423703272	-4.73679848201297
a_HQ_transcript/42360	0.954492866944649	2.42810699835606	1.52679814809189	0.136459900899875	0.797280241180815	-4.91815453533339
a_HQ_transcript/50543	-1.34505257125687	1.74712982479323	-1.52669912939425	0.136484473178313	0.797343664010953	-4.76886421679581
a_HQ_transcript/144472	-2.33439436702303	0.806515473878113	-1.52645121440863	0.136546010930346	0.797557013111614	-4.81339364346103
a_HQ_transcript/8479	-1.61498668559055	-0.15412897304376	-1.5264414529301	0.136548434394557	0.797557013111614	-4.79037667803694
a_HQ_transcript/114807	1.62348430371385	-0.0987984341477976	1.52626826958444	0.136591436078326	0.797619228979275	-4.7689863265634
a_HQ_transcript/25697	1.64926232629859	-0.349641592162513	1.52624526879757	0.13659714803066	0.797619228979275	-4.86028349885273
a_HQ_transcript/21052	-1.01251820936035	3.83054936423795	-1.52621687896496	0.136604198549805	0.797619228979275	-5.18824054069072
a_HQ_transcript/123546	-2.02716105990554	0.390079102504123	-1.52617752136018	0.136613973364259	0.797619228979275	-4.72723452620747
a_HQ_transcript/7628	-1.05679243923558	1.8366457545208	-1.52597597980757	0.136664036869874	0.7978313884877	-4.78739495389036
a_HQ_transcript/87650	1.48417479614787	1.24828770208018	1.52588321218674	0.13668708559392	0.797885811548841	-4.63041794662724
a_HQ_transcript/150881	-2.93237874164356	0.169535241957707	-1.52580036612269	0.136707671895199	0.797891030821094	-4.82875788543515
a_HQ_transcript/120608	3.16058028449294	0.96681052202602	1.52576913704651	0.136715432613854	0.797891030821094	-4.67391247273368
a_HQ_transcript/22570	0.598084936275128	5.04154353331312	1.52565878645007	0.136742858622627	0.797970975405691	-5.28847496953387
a_HQ_transcript/9918	0.978964126543649	3.87026522413622	1.52551535477006	0.136778513084827	0.798094981778113	-5.19484378246756
a_HQ_transcript/56769	0.398039533115523	3.77097763413591	1.52546284199318	0.136791568666655	0.798094981778113	-5.12284370483308
a_HQ_transcript/20061	0.898050324957349	1.1322307133885	1.52536133351471	0.136816808276946	0.798162126808744	-4.71814566348476
a_HQ_transcript/9056	-0.435762478593409	3.00567645115085	-1.52517992272365	0.136861924589147	0.798345203463914	-4.97030066861455
a_HQ_transcript/94170	1.66982474225104	0.462632879854646	1.5250913854097	0.136883947911047	0.798371384070939	-4.82018333459331
a_HQ_transcript/1660	-0.824965060300228	3.5604644359541	-1.52505145169052	0.136893882208833	0.798371384070939	-5.10814397723265
a_HQ_transcript/13833	-1.40388232747117	2.1789611871247	-1.52494510058717	0.136920341976007	0.798445589725255	-4.79489099536402
a_HQ_transcript/22264	-1.20349759802809	1.26634504624331	-1.52472162231719	0.136975956009079	0.798484008183048	-4.68144619694462
a_HQ_transcript/71590	-1.42017626347115	0.922160643261113	-1.52463203723991	0.136998254962602	0.798484008183048	-4.72757527055271
a_HQ_transcript/97964	0.741537872355411	3.00493801369743	1.5245449009251	0.13701994719686	0.798484008183048	-5.00013208749203
a_HQ_transcript/68487	1.38788046869779	3.01008464713537	1.52447489625947	0.137037376584585	0.798484008183048	-5.05273394202489
a_HQ_transcript/40863	1.11775949071336	1.74619757648721	1.52439628561616	0.137056950775823	0.798484008183048	-4.74648703647811
a_HQ_transcript/146482	2.98702099296752	5.36452725470014	1.52439378878766	0.137057572527578	0.798484008183048	-5.39074803189121
a_HQ_transcript/22859	-1.78271393186346	0.0970795835209534	-1.52420202740727	0.137105331097958	0.798484008183048	-4.83432481905159
a_HQ_transcript/111875	-1.73974456190695	0.234846379994352	-1.52415258002702	0.137117648249005	0.798484008183048	-4.85513917643772
a_HQ_transcript/40826	2.14168892472917	-0.389488759925219	1.52408097322427	0.137135486809647	0.798484008183048	-4.88554383362788
a_HQ_transcript/22972	0.482969542317291	2.80571934075756	1.52407063610275	0.137138062129881	0.798484008183048	-5.02004990372883
a_HQ_transcript/80672	-1.56919331852731	2.95345423394688	-1.52401228010294	0.137152601279461	0.798484008183048	-4.93501365466057
a_HQ_transcript/30810	-1.58203548478013	2.58337607058456	-1.52401191381339	0.137152692542874	0.798484008183048	-4.92361749250274
a_HQ_transcript/5174	1.51301787002544	2.96180004042072	1.52399082714105	0.137157946506571	0.798484008183048	-4.88176425417089
a_HQ_transcript/111672	-2.76870582032445	0.755956762760062	-1.52391988758562	0.13717562302706	0.798484008183048	-4.87562899067337
a_HQ_transcript/109308	1.8926375507233	1.07550357478655	1.52379217579795	0.137207450528684	0.798484008183048	-4.74968410511956
a_HQ_transcript/64744	3.15840039844213	2.77499867156176	1.52379004136889	0.137207982507855	0.798484008183048	-4.88106202625429
a_HQ_transcript/127888	0.345830193807297	5.25191537710898	1.52378356594017	0.137209596435909	0.798484008183048	-5.30782395675549
a_HQ_transcript/133169	1.43184052719712	-0.595992794234879	1.52375421687163	0.137216911554498	0.798484008183048	-4.81587106846508
a_HQ_transcript/142078	-1.50883606945127	-0.383277090327833	-1.52374053354844	0.137220322166633	0.798484008183048	-4.80205646633615
a_HQ_transcript/94205	4.25508762678203	1.95689702069704	1.5236337965684	0.137246929049293	0.798484008183048	-4.76594449192868
a_HQ_transcript/49870	2.41151280349397	2.37554471761493	1.52362440719747	0.137249269785894	0.798484008183048	-4.85670689126883
a_HQ_transcript/103798	2.182578313432	2.48505995707553	1.5235113296301	0.137277462149704	0.798484008183048	-4.95084954572035
a_HQ_transcript/23674	0.950874022287737	1.28339753879765	1.52337522987588	0.137311400575158	0.798484008183048	-4.650441936811
a_HQ_transcript/96428	4.45771988069709	2.0117732004864	1.52326401843727	0.137339137769044	0.798484008183048	-4.77242191287242
a_HQ_transcript/4284	2.01667479860181	0.514674623192567	1.52322705547436	0.13734835768577	0.798484008183048	-4.64604815607106
a_HQ_transcript/116211	2.12867044129794	-0.0215030462630966	1.52316552124358	0.137363707684544	0.798484008183048	-4.82098476161982

a_HQ_transcript/152416	-2.66525073095713	1.33566502687697	-1.52316461386631	0.137363934044364	0.798484008183048	-4.87376612251447
a_HQ_transcript/4830	1.24087355929475	3.37130454429256	1.52308246216604	0.137384429351461	0.798484008183048	-5.0508651355969
a_HQ_transcript/483	0.898818440483002	1.87487732796326	1.52303363894067	0.13739661099874	0.798484008183048	-4.69733161650585
a_HQ_transcript/141810	2.23382857244978	1.04521858326477	1.52297203482619	0.137411982787695	0.798484008183048	-4.8611372841693
a_HQ_transcript/12554	-1.92236372303755	0.668802480008546	-1.52283569637653	0.137446007623016	0.798484008183048	-4.78015669791142
a_HQ_transcript/150458	6.53765185135529	3.23815651224765	1.52282534631	0.137448590879311	0.798484008183048	-4.8200640108239
a_HQ_transcript/4745	-1.60031093074077	0.766275675216375	-1.52279199554937	0.137456915108002	0.798484008183048	-4.82825888960137
a_HQ_transcript/104	2.64195017336931	2.82616516223845	1.52277813693577	0.137460374287252	0.798484008183048	-4.90094077491688
a_HQ_transcript/45590	1.02532866264602	-0.141941917742849	1.52273588842357	0.13747092016062	0.798484008183048	-4.87124292622373
a_HQ_transcript/141109	-2.12059339270993	2.47377108226347	-1.5227261005911	0.137473363446111	0.798484008183048	-4.89945291343218
a_HQ_transcript/63676	-1.78359715844752	0.886756273732504	-1.52263521923738	0.137496051356875	0.798484008183048	-4.82866245956521
a_HQ_transcript/58481	1.24188447189841	0.342933942793215	1.52243255066147	0.137546657067204	0.798484008183048	-4.77274456579801
a_HQ_transcript/138469	0.501256718536065	4.16055089314891	1.52240167163502	0.137554368782785	0.798484008183048	-5.24756764500506
a_HQ_transcript/152818	1.61474668039853	0.357030487180482	1.52220790457634	0.137602768052807	0.798484008183048	-4.79390059537635
a_HQ_transcript/135652	1.09248032100577	3.86391865032643	1.52215842023946	0.137615130486955	0.798484008183048	-5.09351551636167
a_HQ_transcript/143790	2.32627247698166	1.16991857504858	1.52209042534277	0.137632118786898	0.798484008183048	-4.73456631956808
a_HQ_transcript/6865	1.63095398323203	1.54178120193367	1.52207196405233	0.137636731571275	0.798484008183048	-4.76328705991345
a_HQ_transcript/150489	1.44588464880295	-0.522769801051443	1.52205819100021	0.137640173021895	0.798484008183048	-4.82183243392183
a_HQ_transcript/648	0.863095646556268	2.02018475138328	1.52198350312586	0.137658836371223	0.798484008183048	-4.77045313222235
a_HQ_transcript/149053	1.67321290591855	-0.498263049193199	1.52195972206944	0.137664779319426	0.798484008183048	-4.82713671167129
a_HQ_transcript/65572	1.36904813414949	3.34978558400154	1.52195118213868	0.137666913520971	0.798484008183048	-5.05453282031683
a_HQ_transcript/121005	-1.25508032941219	2.03874912401898	-1.52189434704499	0.137681117775687	0.798484008183048	-4.80197567902647
a_HQ_transcript/58724	1.82402426672669	3.56162402246791	1.52188807585518	0.137682685146972	0.798484008183048	-4.95771146274855
a_HQ_transcript/89717	-2.09760895412292	1.75565273716847	-1.52179808580813	0.13770517812873	0.798484008183048	-4.91563864740445
a_HQ_transcript/128624	1.64941974298866	2.34943459502039	1.52176766654592	0.137712782083387	0.798484008183048	-4.74135638128729
a_HQ_transcript/50987	-1.848101596627	1.38983329715092	-1.52172523486729	0.137723389367976	0.798484008183048	-4.87303311376249
a_HQ_transcript/31442	-1.56009516381445	1.11273451211694	-1.52168159815915	0.137734298579363	0.798484008183048	-4.78633154315344
a_HQ_transcript/68209	4.25074761461872	2.2044130622775	1.52167534179063	0.137735862733555	0.798484008183048	-4.94744110956937
a_HQ_transcript/108722	-1.84728159863055	0.0514297333452406	-1.52161633026134	0.137750616906297	0.798484008183048	-4.85989930014445
a_HQ_transcript/114992	1.42366766928455	2.28086782836832	1.52160432929564	0.137753617566154	0.798484008183048	-4.8209721741897
a_HQ_transcript/67804	1.58572648939616	0.0962611259137335	1.52148669283307	0.137783033575137	0.798484008183048	-4.75801676330484
a_HQ_transcript/88354	1.2090994367878	2.53114801099533	1.52148541010295	0.137783354360758	0.798484008183048	-4.79650400596123
a_HQ_transcript/6242	-2.55498560396818	1.35618128676304	-1.52141097190995	0.13780197092353	0.798484008183048	-4.8374054600798
a_HQ_transcript/66519	1.53395827439746	0.0233493751483173	1.52137130200376	0.13781189296707	0.798484008183048	-4.77865594383483
a_HQ_transcript/11333	1.22405283842421	1.93310484311944	1.5213672185389	0.137812914336106	0.798484008183048	-4.81202237031496
a_HQ_transcript/126762	1.35081514513005	-0.479560583342819	1.5212953755622	0.137830884925517	0.798484008183048	-4.83611393424262
a_HQ_transcript/14178	-1.47639168276855	1.80736105943482	-1.5212741056315	0.137836205685636	0.798484008183048	-4.90785986597113
a_HQ_transcript/129256	0.454217733810954	3.3507313232318	1.52127221406132	0.13783667887767	0.798484008183048	-5.07258629240211
a_HQ_transcript/148424	1.97105038953372	-0.108889115949597	1.52126555988742	0.137838343485108	0.798484008183048	-4.81684897440064
a_HQ_transcript/87190	1.33939352587935	0.871547877577398	1.52124677099714	0.137843043799445	0.798484008183048	-4.80351374552116
a_HQ_transcript/36010	-1.1515929290416	0.0452661966642185	-1.52121714263133	0.137850456029026	0.798484008183048	-4.7626668203865
a_HQ_transcript/16906	2.29361361233048	3.00844633784792	1.52111251267456	0.137876634235534	0.798484008183048	-4.98631252584094
a_HQ_transcript/60960	1.02520687505288	1.99798198262191	1.52101809930131	0.137900259714853	0.798484008183048	-4.80047712713973
a_HQ_transcript/67418	-1.87543694256225	0.403134290647012	-1.52094985817187	0.137917338029882	0.798484008183048	-4.81179178464362
a_HQ_transcript/132712	1.77371578309788	0.778013959029287	1.52087617049473	0.137935781336725	0.798484008183048	-4.75373071369398
a_HQ_transcript/62762	1.20902495324512	1.34129604944571	1.52081755392198	0.137950453913731	0.798484008183048	-4.68888810714761
a_HQ_transcript/99412	1.97970892315451	0.702822439477692	1.52079093151149	0.137957118304306	0.798484008183048	-4.70728572029701
a_HQ_transcript/30014	0.757073153126647	2.07276948102904	1.52077288793815	0.137961635301394	0.798484008183048	-4.80061398851551
a_HQ_transcript/87417	-1.29178938339624	2.93533349409463	-1.5207722539687	0.137961794010384	0.798484008183048	-5.0202311250361
a_HQ_transcript/144111	-3.4978570938816	2.90993864748001	-1.52073581866169	0.137970915534519	0.798484008183048	-4.95687119438325

a_HQ_transcript/87789	1.33277108295495	0.01832749071061	1.52056604977737	0.138013423332183	0.798650499895781	-4.77879879778258
a_HQ_transcript/102847	0.668390429471138	2.27703708364847	1.52050086849321	0.13802974663674	0.798665450179218	-4.850723593029
a_HQ_transcript/39040	-2.15812045288322	0.452646919551224	-1.52041674572896	0.138050815744741	0.798676766095454	-4.76688336411862
a_HQ_transcript/55168	1.28291841702754	0.221208685260947	1.52036421315737	0.138063974193601	0.798676766095454	-4.81804463087109
a_HQ_transcript/71519	1.30934087338908	-0.571964651628616	1.52032849221401	0.138072922214651	0.798676766095454	-4.81938567912281
a_HQ_transcript/96806	-2.93323826013842	0.419447092123279	-1.52021184516382	0.138102145312321	0.798766318678072	-4.81263527776655
a_HQ_transcript/4060	1.82484716210386	2.07990604609685	1.52012850778011	0.138123026533227	0.798780200465562	-4.75564146685122
a_HQ_transcript/30636	1.0432548458198	1.78510129933886	1.52009258233911	0.138132028885904	0.798780200465562	-4.76095099141605
a_HQ_transcript/85051	0.652021276971699	3.43546438968585	1.51979337137415	0.138207024848901	0.798995892481567	-5.05736079982556
a_HQ_transcript/40173	-1.5352303074974	0.367527150529893	-1.51976783608034	0.138213426682663	0.798995892481567	-4.82897040540135
a_HQ_transcript/53099	1.496064155447	2.04776978185861	1.51975147588195	0.138217528396956	0.798995892481567	-4.78004874068948
a_HQ_transcript/137643	-0.441378401071311	4.1595149766035	-1.51972442725849	0.138224310053582	0.798995892481567	-5.22771789463984
a_HQ_transcript/38381	-0.610376131474522	5.03786456061992	-1.51957597105423	0.138261535912222	0.799131605797518	-5.23537666917397
a_HQ_transcript/16153	-1.53140506362884	2.37052348312156	-1.51951844572872	0.138275962740922	0.799135530145587	-4.93585093779074
a_HQ_transcript/134583	4.11875764651037	0.850920326959126	1.5194323696189	0.138297552113763	0.799180843947588	-4.85135628672233
a_HQ_transcript/7393	-0.574812192472785	2.70546808880517	-1.51933203941472	0.138322720089583	0.799246826378456	-4.97124425313543
a_HQ_transcript/8170	0.475654575304329	3.1319183142336	1.51880779513665	0.138454287588274	0.799759670950333	-5.06346759142908
a_HQ_transcript/84442	3.63455356371311	0.836140302710584	1.51874459370756	0.138470155811833	0.799759670950333	-4.85823677676466
a_HQ_transcript/35377	2.35395309926873	0.0611377268373934	1.51870392782867	0.138480366724148	0.799759670950333	-4.82687082164059
a_HQ_transcript/76028	0.405582455171101	3.84144346080462	1.51869343129819	0.138483002426692	0.799759670950333	-5.18738383571967
a_HQ_transcript/77065	3.21141481856272	2.41367336754716	1.51859782458079	0.138507011351545	0.799759670950333	-4.97346456419028
a_HQ_transcript/127129	1.96789280296939	1.02761070575567	1.51858910688611	0.138509200721097	0.799759670950333	-4.80388382559848
a_HQ_transcript/90734	1.38287105730327	-0.402295195088876	1.5185867677574	0.138509788176698	0.799759670950333	-4.87888365408282
a_HQ_transcript/3712	1.47064264487457	1.78389296256534	1.51853995620333	0.138521544989127	0.799759670950333	-4.81374262306371
a_HQ_transcript/72662	-1.48617597187486	0.877516714844927	-1.5183192386846	0.138576989460798	0.800000322115134	-4.7498058753884
a_HQ_transcript/5081	-1.64538326092433	1.1408332238965	-1.51812256869609	0.138626408238674	0.800206142810094	-4.79796688259672
a_HQ_transcript/111525	2.10283286684555	1.41974764100528	1.51761572690683	0.138753831748796	0.800862151910833	-4.72605733454212
a_HQ_transcript/11324	-2.07312363746008	0.408183234041103	-1.51746544423044	0.138791631987248	0.800903314093508	-4.89871521198469
a_HQ_transcript/111512	1.40316148379838	-0.514914645362345	1.51742779752819	0.138801102471177	0.800903314093508	-4.83387451898631
a_HQ_transcript/20499	-1.90583778296012	0.781886210549576	-1.51737116319756	0.138815350504112	0.800903314093508	-4.74752658734038
a_HQ_transcript/116883	-2.82671062052174	2.9635387020222	-1.51736827793329	0.138816076408824	0.800903314093508	-4.98359884358717
a_HQ_transcript/45618	1.81445836191136	0.0253119958586185	1.51707195716001	0.13889064415559	0.801254005902754	-4.83647239228272
a_HQ_transcript/21937	1.43812225863301	1.37395274840063	1.51697358709382	0.138915405661811	0.80131732661603	-4.86306170343651
a_HQ_transcript/70349	-1.59941143216154	4.57536447663973	-1.51679087474541	0.138961407070541	0.80150314250808	-5.26719584837684
a_HQ_transcript/43185	1.0594197159776	3.67470772312011	1.51669131141369	0.138986479254159	0.801556956386702	-5.13415398631928
a_HQ_transcript/9166	-1.70413009576177	2.96261179838786	-1.51661469520405	0.139005775341786	0.801556956386702	-4.92057036541955
a_HQ_transcript/9857	-2.54163144968211	2.79605598769456	-1.51655007834365	0.13902205102512	0.801556956386702	-5.07767746603141
a_HQ_transcript/90479	0.415709951467641	2.69861160798167	1.51653481742244	0.139025895167402	0.801556956386702	-4.95367926869911
a_HQ_transcript/142778	1.58335467115165	-0.282407128948127	1.51633960467877	0.139075075741836	0.801594962257195	-4.86146448666762
a_HQ_transcript/137243	3.77597380710308	1.30442749593114	1.51633203747138	0.139076982454941	0.801594962257195	-4.94562455615273
a_HQ_transcript/9037	0.899339799342252	2.08310811322163	1.5162863554856	0.139088493415592	0.801594962257195	-4.8379630625965
a_HQ_transcript/65072	1.40000451131741	0.0467987373862276	1.51624286115073	0.139099453844704	0.801594962257195	-4.73802060992656
a_HQ_transcript/91797	1.38042571926289	-0.361519679950175	1.51619737639505	0.139110916597729	0.801594962257195	-4.80716140066018
a_HQ_transcript/151087	0.758813631445048	5.54001234378765	1.51618026891078	0.139115228104366	0.801594962257195	-5.36344132464509
a_HQ_transcript/77660	1.33740150749691	1.37174578668136	1.51583937912624	0.139201163161651	0.801918390615331	-4.72537492575918
a_HQ_transcript/82395	-2.19619738629007	1.72980546602078	-1.51580669125737	0.139209405707773	0.801918390615331	-4.80520513329343
a_HQ_transcript/89624	-0.709905618310993	3.15111429603456	-1.5157934462909	0.139212745659567	0.801918390615331	-5.02520969664425
a_HQ_transcript/124076	3.09317564714834	1.469747928135	1.51558787500138	0.139264592361944	0.802006031567395	-4.86665148883932
a_HQ_transcript/87744	-0.673893415790518	2.74454417430346	-1.51556917837507	0.139269308570386	0.802006031567395	-4.85138767242413
a_HQ_transcript/88906	2.02922638431978	1.18293974598473	1.51556900691821	0.139269351820831	0.802006031567395	-4.72882138400237

a_HQ_transcript/90450	-1.74209502478794	0.257793203950276	-1.51542791650889	0.139304945917546	0.802131539999517	-4.79092512939911
a_HQ_transcript/116635	-2.43592478386245	1.04122594999825	-1.51536614062015	0.139320532961552	0.802138142810227	-4.75139185723729
a_HQ_transcript/79690	1.37751834884339	-0.445127469840912	1.51531399334814	0.139333691645246	0.802138142810227	-4.78000034401769
a_HQ_transcript/76911	-1.77411578070594	1.63044693249395	-1.51524604645458	0.139350838659463	0.802157412319003	-4.82007911940383
a_HQ_transcript/49925	1.48589561092736	-0.068858958283116	1.51514950897075	0.139375203691492	0.802218223604221	-4.84269648322368
a_HQ_transcript/83617	-0.661888573900251	2.73216122154818	-1.51501168872632	0.139409993999149	0.80233902288936	-4.8699957545436
a_HQ_transcript/4099	1.74591242472298	0.135178480529974	1.51476863112788	0.139471366681338	0.802612098152465	-4.85556919996466
a_HQ_transcript/18945	1.4722335063784	-0.0679167147370954	1.51471442284615	0.139485057382607	0.802612098152465	-4.7887492262392
a_HQ_transcript/130857	-5.66357472537784	0.468939468507364	-1.51450869685329	0.139537024848888	0.802772891488458	-4.87657454828318
a_HQ_transcript/27506	0.78568286667479	3.10914640269938	1.51445061397984	0.139551699710406	0.802772891488458	-5.05929558643678
a_HQ_transcript/11278	-1.26644476644174	1.33105859254703	-1.51443979708623	0.139554432777506	0.802772891488458	-4.80948382042668
a_HQ_transcript/6578	-0.733991807423179	1.76264432861327	-1.51432731816197	0.139582854993339	0.802856935983851	-4.74679128275267
a_HQ_transcript/100470	-2.10397441811394	1.43925386963485	-1.51416695074502	0.139623386178863	0.803010605699511	-4.84297633280389
a_HQ_transcript/995	1.41563603673011	0.127433704377783	1.51405386535701	0.139651973024987	0.80309555678635	-4.73868034824269
a_HQ_transcript/98380	-2.38933610398074	1.94421335298359	-1.51372523627006	0.139735073899161	0.80349395346824	-4.92101361310312
a_HQ_transcript/91926	1.0910651092529	-0.64108242901932	1.51332458280645	0.139836441538628	0.803997297107554	-4.85238145867314
a_HQ_transcript/56439	0.488652236319238	2.5074104844069	1.51325261799331	0.139854655323442	0.804020965060385	-4.90921388462653
a_HQ_transcript/74727	0.488454181441426	3.65071212689684	1.51319902001827	0.13986822183269	0.804020965060385	-5.13052406203892
a_HQ_transcript/73579	1.67100030898853	-0.50740957698497	1.51312737934036	0.139886356894901	0.804045698960425	-4.82445830009986
a_HQ_transcript/132810	1.40995836407695	-0.184009113116865	1.5129940106636	0.139920122775047	0.804111508655744	-4.86560969957708
a_HQ_transcript/136108	0.397091808116522	3.95260447220692	1.51297287963765	0.139925473266805	0.804111508655744	-5.19461203756823
a_HQ_transcript/73294	0.407697981438328	3.31706660241263	1.51286028338117	0.139953986034659	0.804130401311675	-5.09807808842169
a_HQ_transcript/39421	1.86776257076854	1.63032902028241	1.51280070220456	0.13996907567799	0.804130401311675	-4.74992108610984
a_HQ_transcript/92665	0.321286214377567	6.34821341968385	1.51278716824988	0.139972503495935	0.804130401311675	-5.43877250526241
a_HQ_transcript/12802	-1.35185401347444	1.40250264542816	-1.51272779085903	0.139987543127817	0.804130401311675	-4.94506162791332
a_HQ_transcript/31163	-3.3152876956049	1.86086698194617	-1.51265848430332	0.14000509935217	0.804130401311675	-4.77799424644253
a_HQ_transcript/54941	1.50324509670308	-0.0817457473602735	1.51253666089712	0.140035963053326	0.804130401311675	-4.78296944567799
a_HQ_transcript/21511	1.67681637425678	0.271433875531771	1.5124153501657	0.140066702312398	0.804130401311675	-4.82480500772194
a_HQ_transcript/17349	2.0451810484612	0.180192432722961	1.51236376706892	0.140079774740766	0.804130401311675	-4.75706284409982
a_HQ_transcript/33992	-2.53486198845645	1.44713640328768	-1.51233348589936	0.140087449193167	0.804130401311675	-4.85220681934097
a_HQ_transcript/145401	1.62054370115341	-0.358532432653137	1.51224216579559	0.140110595388179	0.804130401311675	-4.84211529896196
a_HQ_transcript/12760	1.35916305487812	1.16144885455498	1.51223626251207	0.140112091753385	0.804130401311675	-4.77167540119115
a_HQ_transcript/31252	-1.5198140261357	0.0898567103577346	-1.51221153251428	0.140118360456836	0.804130401311675	-4.6987297329846
a_HQ_transcript/19259	-2.39454727190562	0.966673004163833	-1.51221059847182	0.140118597227766	0.804130401311675	-4.86315708500378
a_HQ_transcript/120375	0.490370845829952	2.85845303986524	1.51219249066083	0.140123187450285	0.804130401311675	-4.97679368544662
a_HQ_transcript/104087	1.2706827396141	-0.419148552501408	1.51214089369535	0.140136267638441	0.804130401311675	-4.82574909304161
a_HQ_transcript/14305	-0.750706896760774	2.74899456340089	-1.51204985027503	0.140159350173533	0.804183467267511	-4.90916864036466
a_HQ_transcript/8601	-1.15972124797078	0.474212707862684	-1.51188787720816	0.140200423299319	0.804240004946888	-4.72988482986537
a_HQ_transcript/143169	2.05544613628224	1.5203674371967	1.51184106726864	0.140212295166987	0.804240004946888	-4.74338630451264
a_HQ_transcript/12032	1.62580573966954	1.01386871347265	1.51181683427716	0.140218441420359	0.804240004946888	-4.76153874511854
a_HQ_transcript/121595	-0.581828285735196	2.57901392369155	-1.51179276371028	0.140224546692415	0.804240004946888	-4.9278352282974
a_HQ_transcript/42839	-2.18036549889817	4.50393689816518	-1.51163447668698	0.140264700032039	0.804245726035805	-5.3136449251679
a_HQ_transcript/152520	1.56030717646593	-0.396020336808747	1.51158090863932	0.140278290961464	0.804245726035805	-4.87898618722205
a_HQ_transcript/152781	-2.85631457884433	2.54885787665039	-1.51155109645173	0.140285855170887	0.804245726035805	-4.99983075821441
a_HQ_transcript/98329	1.396076530387	2.73901770936781	1.51145985455061	0.140309007905518	0.804245726035805	-5.02543621292891
a_HQ_transcript/130427	2.04711111595217	1.09832204921883	1.51135898464248	0.140334607337201	0.804245726035805	-4.80438272668091
a_HQ_transcript/138079	-1.31355306101364	0.187106836848302	-1.51134650049853	0.140337775907144	0.804245726035805	-4.81133552884284
a_HQ_transcript/137660	1.68637214053274	0.0542021332653489	1.51127348014274	0.140356310177885	0.804245726035805	-4.78157782205578
a_HQ_transcript/35491	2.07216550140033	2.43605225315882	1.51125615912276	0.14036070694615	0.804245726035805	-4.86883035765496
a_HQ_transcript/43974	2.11715376701905	0.274039706081414	1.51124711205136	0.140363003499116	0.804245726035805	-4.67716408698057

a_HQ_transcript/80560	-0.86160114158338	3.41564535615706	-1.51117075491878	0.140382387572882	0.804245726035805	-5.10675528828584
a_HQ_transcript/1900	1.89582556594909	2.00980535294337	1.51114965904853	0.140387743364735	0.804245726035805	-4.91446277979111
a_HQ_transcript/81292	1.84725019247695	0.249161750684156	1.51102141040114	0.140420306502005	0.804245726035805	-4.85295645555006
a_HQ_transcript/39801	1.91185613991189	1.84856539672849	1.51094311440928	0.140440189334478	0.804245726035805	-4.79949735978823
a_HQ_transcript/15885	-1.4013689648759	-0.646564174532705	-1.5109211997316	0.140445754851045	0.804245726035805	-4.82632590453346
a_HQ_transcript/12459	-1.20851834753596	0.749510683416583	-1.51086883729493	0.140459053689598	0.804245726035805	-4.75259670220999
a_HQ_transcript/145736	2.15153858707854	1.77908172251193	1.51086269165304	0.140460614605857	0.804245726035805	-4.82209855099091
a_HQ_transcript/62172	-0.967936073584254	2.59044004162021	-1.51086214945693	0.140460752317566	0.804245726035805	-4.92678246987782
a_HQ_transcript/70264	2.19023281194641	2.40612009988252	1.51073011336843	0.140494291232982	0.804354672608312	-4.99695769758276
a_HQ_transcript/148060	0.43362088702536	5.55745355385969	1.51067829705197	0.140507455024512	0.804354672608312	-5.37982787025702
a_HQ_transcript/16112	0.798181874148853	3.16854800955927	1.51052630954065	0.140546072756152	0.804496515723251	-5.05102340420921
a_HQ_transcript/4855	1.04829492165525	2.29303248148948	1.51045614972066	0.140563902188254	0.804519348798622	-4.84680641141704
a_HQ_transcript/26833	-1.24637288587544	0.809616206491642	-1.51035718687025	0.140589054312636	0.804564019804771	-4.7242945806188
a_HQ_transcript/3599	1.41689619555335	2.26903309507395	1.510294085632	0.140605093839648	0.804564019804771	-4.82651332030884
a_HQ_transcript/7234	-2.0861190451269	0.217402420146285	-1.51026207634821	0.140613230752764	0.804564019804771	-4.84028095446184
a_HQ_transcript/93939	-0.698661671851096	2.19295572924008	-1.51019944758043	0.140629152381223	0.804575922206272	-4.77546863392346
a_HQ_transcript/77800	1.08911045845944	-0.388396697366952	1.51001788291489	0.140675318364494	0.804760841048255	-4.87470355521067
a_HQ_transcript/76207	1.62401761044869	1.82501868348436	1.50961720330732	0.140777241294285	0.805211104427859	-4.81856899392827
a_HQ_transcript/137485	0.611367976091762	4.09920419830235	1.50959955909211	0.140781730909378	0.805211104427859	-5.15315703210457
a_HQ_transcript/89189	1.58177927690711	0.137479462570103	1.5095217662103	0.140801526878163	0.805245095865199	-4.70266666571799
a_HQ_transcript/74880	-0.700108431692496	2.37432871643842	-1.5094592772739	0.140817430071163	0.805256819987856	-4.80558148409678
a_HQ_transcript/152157	-3.16295406928489	1.48523618987949	-1.50926182901243	0.140867689388668	0.805443439755319	-4.92224717372922
a_HQ_transcript/32408	1.88398897158842	-0.00716096089064427	1.50914537976511	0.140897337638581	0.805443439755319	-4.83677250653113
a_HQ_transcript/72942	-1.38713457060157	-0.243271427770931	-1.50904772852919	0.140922203740688	0.805443439755319	-4.84607022562699
a_HQ_transcript/121869	-3.34446529965873	1.45571066290665	-1.50899783282759	0.140934910642674	0.805443439755319	-4.81907973567536
a_HQ_transcript/100257	1.33579418120606	4.73901090497721	1.50898662562709	0.140937764899029	0.805443439755319	-5.32816250081878
a_HQ_transcript/105841	3.15171456875049	2.52757855909328	1.50893687383211	0.140950436276601	0.805443439755319	-4.9275672508472
a_HQ_transcript/102243	2.42632481467814	4.00947687130521	1.50889638846177	0.140960748247492	0.805443439755319	-5.09053750204148
a_HQ_transcript/18090	1.05939003198468	2.06414585203924	1.50889573072796	0.140960915782942	0.805443439755319	-4.7787012788564
a_HQ_transcript/89794	1.5245599798776	1.61920231438643	1.50878181603296	0.140989934116313	0.805527606894493	-4.74739660589015
a_HQ_transcript/139303	-1.95863711825798	3.3184641940391	-1.50869518170491	0.141012006336769	0.805527606894493	-5.25520333759563
a_HQ_transcript/138050	0.558166383442274	2.74769388994388	1.50867472192336	0.141017219373767	0.805527606894493	-4.92148321936097
a_HQ_transcript/118189	1.73520017803641	0.454230836974163	1.50857425332162	0.141042820456868	0.805548131508933	-4.76143206427489
a_HQ_transcript/75689	1.62579572449946	0.827045730005178	1.50855185353653	0.14104852880708	0.805548131508933	-4.7041486827552
a_HQ_transcript/75184	1.56264227645257	0.0686159394371549	1.50836793563311	0.141095405382521	0.805736685732898	-4.91167424751547
a_HQ_transcript/91840	1.38890942932927	-0.674820721971635	1.50812741333186	0.14115672806645	0.805938936934824	-4.84707483460743
a_HQ_transcript/88400	1.67606454254932	-0.365432277782208	1.5081101052906	0.141161141687845	0.805938936934824	-4.85341228723688
a_HQ_transcript/89704	1.58978621558334	-0.194381629376633	1.50794450513514	0.141203376017305	0.805938936934824	-4.82652822399653
a_HQ_transcript/97745	-0.647641388747454	3.38883392861013	-1.50790353428603	0.14121382670808	0.805938936934824	-5.11177709573386
a_HQ_transcript/93208	2.04315294451151	2.25913179852763	1.50789886989541	0.141215016522817	0.805938936934824	-4.9677619216177
a_HQ_transcript/16004	-0.46614653565438	2.20195664002764	-1.50774008815248	0.141255524126908	0.805938936934824	-4.80816772596002
a_HQ_transcript/134589	4.6806836029695	2.16775035418583	1.5076849138022	0.141269602118808	0.805938936934824	-4.77875242372368
a_HQ_transcript/88133	-0.518473984572088	3.5726150309844	-1.50766212165562	0.141275417970269	0.805938936934824	-5.11588639901183
a_HQ_transcript/117330	-1.90756579383216	-0.276449340028984	-1.50764576527676	0.141279591731366	0.805938936934824	-4.82935988355845
a_HQ_transcript/7888	-1.3957317231512	1.46538089320661	-1.50758490776766	0.141295121999469	0.805938936934824	-4.68096018696188
a_HQ_transcript/83202	1.78007619231687	-0.515648328250483	1.50754274800281	0.141305881582803	0.805938936934824	-4.85372120702779
a_HQ_transcript/120404	1.44036148956796	-0.269460976943481	1.50752652998771	0.141310020753977	0.805938936934824	-4.89071870996347
a_HQ_transcript/111074	1.505937288315	-0.610200972316775	1.50751652940851	0.141312573156173	0.805938936934824	-4.8491242399394
a_HQ_transcript/92253	2.10987083132873	3.35627390543366	1.50746811247232	0.141324930914132	0.805938936934824	-4.99723083351106
a_HQ_transcript/15219	1.261682867607	0.11718502219162	1.50738951866297	0.141344992754864	0.805974272989476	-4.68649984581481

a_HQ_transcript/139343	-2.33791549767344	1.39375736956158	-1.50732664473876	0.141361043589577	0.805986732886214	-4.97291215766654
a_HQ_transcript/131551	1.48706359494967	1.1571980101975	1.50727174346687	0.141375060316218	0.805987593768252	-4.75519990673711
a_HQ_transcript/65955	-0.573007286930223	2.8750012887729	-1.50717387140395	0.141400050596001	0.806051009222749	-5.02975283910891
a_HQ_transcript/79877	1.109787585355	-0.507702436351178	1.50706910693941	0.141426804691509	0.806124465886255	-4.87756996609331
a_HQ_transcript/123554	-1.77120959434434	1.99741454334814	-1.50700394682505	0.141443446928195	0.806140276795972	-4.73953143955621
a_HQ_transcript/88780	1.36582344742323	1.52203901342055	1.50686060613854	0.141480062437424	0.806176006765478	-4.83745681357354
a_HQ_transcript/2063	1.21457177224487	1.17555868274886	1.50680580007262	0.141494064328657	0.806176006765478	-4.69839255591196
a_HQ_transcript/102641	1.56767303193443	0.205367575085222	1.50679506355606	0.141496807431773	0.806176006765478	-4.83761192723027
a_HQ_transcript/45398	0.485087196844885	2.84251081264878	1.50675811764672	0.141506247173847	0.806176006765478	-4.99757615711354
a_HQ_transcript/143117	-1.35186347294671	2.9480068884302	-1.50670796950794	0.141519060917887	0.806176006765478	-5.08938221219516
a_HQ_transcript/59097	1.77483348403435	2.16961115359919	1.50653876438204	0.141562302730715	0.806224663413962	-4.79717643948097
a_HQ_transcript/56976	-0.623795113362483	3.82543355198481	-1.50650462629336	0.141571028298142	0.806224663413962	-5.19245535716791
a_HQ_transcript/40453	-2.18099432353862	2.97577737632969	-1.50647452587084	0.141578722211964	0.806224663413962	-4.99545098263837
a_HQ_transcript/510	-1.12989808927179	2.960221339523	-1.50645747170038	0.14158308154641	0.806224663413962	-5.17408808826046
a_HQ_transcript/103559	-1.77893539123816	1.58329830659027	-1.50639592726263	0.141598814243207	0.80623527028398	-4.77235869126314
a_HQ_transcript/65021	1.59107719886357	1.08101884507871	1.50624093888114	0.14163844038215	0.806307516079924	-4.79064453436213
a_HQ_transcript/142642	1.99640273735993	0.687557876903721	1.5062153026916	0.141644995688868	0.806307516079924	-4.84342896168768
a_HQ_transcript/30536	1.83523634416548	0.159223135273645	1.50611047857428	0.141671802297478	0.806307516079924	-4.84363367062502
a_HQ_transcript/135481	0.949759115530987	-0.813031171279037	1.50608232552881	0.141679002553659	0.806307516079924	-4.86326683183062
a_HQ_transcript/105913	0.475819873883111	4.71520988881664	1.50607431939305	0.141681050209254	0.806307516079924	-5.26897780134221
a_HQ_transcript/79347	1.90203505935316	1.9865552359598	1.50602083435683	0.141694730194681	0.806307516079924	-4.81209115843559
a_HQ_transcript/129786	-3.21905102098983	2.73316809139033	-1.50592848145825	0.141718353998589	0.806322161175788	-4.90799541530689
a_HQ_transcript/49673	1.78071518881125	0.406236862997838	1.5058375590337	0.14174161498133	0.806322161175788	-4.7074080530855
a_HQ_transcript/96775	1.28914457837523	0.600033006444069	1.5057957326096	0.141752316603567	0.806322161175788	-4.72888016925961
a_HQ_transcript/27147	-1.68485011010675	2.29293633394732	-1.50576024384138	0.141761397195648	0.806322161175788	-5.01219698105943
a_HQ_transcript/150957	-0.757376572137608	2.79955699316853	-1.50569284939706	0.14177864285306	0.806322161175788	-4.99742610669528
a_HQ_transcript/23891	-1.64954547990704	1.22055022373851	-1.50564889592908	0.141789891075272	0.806322161175788	-4.87657082111449
a_HQ_transcript/117713	-1.70964946111802	-0.135143186672367	-1.50558212144627	0.141806980842969	0.806322161175788	-4.93392314038819
a_HQ_transcript/79544	1.34182498212027	-0.366464972963018	1.50557706208668	0.141808275765553	0.806322161175788	-4.83048376989398
a_HQ_transcript/76855	-1.25379711186974	-0.070287980803659	-1.50547736908892	0.14183379372337	0.806388376521132	-4.75791157343698
a_HQ_transcript/86356	1.90289914259215	0.484446747786389	1.50535749499873	0.141864482229933	0.806403845834925	-4.83669731675217
a_HQ_transcript/140459	2.82538335956883	2.21996591702187	1.50533612106715	0.141869954648905	0.806403845834925	-4.79359705530672
a_HQ_transcript/35531	0.522492518199164	5.04346327951393	1.50530417856383	0.141878133280928	0.806403845834925	-5.30570577245532
a_HQ_transcript/139685	1.68463779818165	0.78456772369193	1.5050720397515	0.141937582022064	0.806662863490274	-4.74247774025875
a_HQ_transcript/47942	-1.76735979224214	0.278050982803833	-1.50493584281884	0.141972470196267	0.806782260980407	-4.83657863362058
a_HQ_transcript/150044	3.96438128767252	2.31938267701325	1.50476924767178	0.142015154541181	0.806866141850188	-4.92726273573854
a_HQ_transcript/131195	-1.05436762673718	3.82100787078406	-1.50475615661062	0.14201850911776	0.806866141850188	-5.22719820638546
a_HQ_transcript/20759	-0.861193153486799	2.41785860988972	-1.50466240801647	0.142042533999915	0.806866141850188	-4.8344759816263
a_HQ_transcript/46664	1.06287695675153	1.95609670017497	1.50454587407967	0.142072402618347	0.806866141850188	-4.8369344091275
a_HQ_transcript/124359	-3.6047374709075	2.128673592661	-1.50453416074022	0.142075405123948	0.806866141850188	-4.81870369694338
a_HQ_transcript/82215	2.02923690503541	1.2367361101702	1.50451395039144	0.142080585806208	0.806866141850188	-4.7149792848613
a_HQ_transcript/56566	1.63459999785931	1.17895611466643	1.50446921222481	0.142092054442516	0.806866141850188	-4.72881761757253
a_HQ_transcript/107704	1.90680233939567	1.31048271620672	1.50444493651054	0.142098277837193	0.806866141850188	-4.81392422588763
a_HQ_transcript/143692	-1.47014533933427	-0.101784592285428	-1.50430042357923	0.142135330137799	0.806969757169645	-4.8515576973175
a_HQ_transcript/127837	1.33988586735359	-0.104661342586664	1.50426547883693	0.142144290938274	0.806969757169645	-4.81509688345932
a_HQ_transcript/76267	-1.74674225022952	-0.0860603273814094	-1.50403012051408	0.142204655161854	0.807140053206534	-4.80875164670691
a_HQ_transcript/129328	-1.82447143416864	3.04650878727569	-1.50402862521596	0.142205038738716	0.807140053206534	-5.09080339975702
a_HQ_transcript/76759	2.31617072878984	0.272772321458112	1.50398611242594	0.14221594455239	0.807140053206534	-4.8246362808257
a_HQ_transcript/47015	-3.92690829187438	-0.0892447037575164	-1.50387057204998	0.142245587541248	0.80720462708623	-4.8731567838182
a_HQ_transcript/7206	1.29808040544427	-0.360738066243869	1.50374517911531	0.142277763916046	0.80720462708623	-4.89584928326058

a_HQ_transcript/31350	2.73144761735891	0.919725854808197	1.50374075511615	0.142278899240356	0.80720462708623	-4.78738163328976
a_HQ_transcript/81947	1.27809699030335	0.131622079057636	1.50372528645058	0.142282868998168	0.80720462708623	-4.80892233676475
a_HQ_transcript/193	-0.453414593035426	4.47317307101538	-1.50365280742346	0.142301470636453	0.807231373587952	-5.26215855134596
a_HQ_transcript/104492	-2.38043394912909	1.90044923537478	-1.50356249730081	0.142324651334058	0.807284087894818	-4.86749467807566
a_HQ_transcript/115720	-1.33997694869882	2.31940237086977	-1.50349955487328	0.142340809117533	0.807296383133908	-4.81637953489704
a_HQ_transcript/42734	1.57440475819725	1.27416722023583	1.50344585475181	0.142354595498255	0.807296383133908	-4.86107994122741
a_HQ_transcript/139680	1.53505894484295	0.150968016362795	1.50337862559765	0.142371856686887	0.807315509266937	-4.86456914625371
a_HQ_transcript/92506	1.49074475770104	-0.217265401065001	1.50330262331907	0.142391372413778	0.807347414715967	-4.8599159750948
a_HQ_transcript/132438	0.65057926125568	4.31014415266335	1.50319952147377	0.142417850153559	0.807418784134015	-5.1993093907843
a_HQ_transcript/57499	-1.88675631975629	0.307862147114966	-1.50313975762187	0.142433200009103	0.807427057746161	-4.78262307877524
a_HQ_transcript/137308	1.62249270787428	0.117501098364903	1.50303496545648	0.142460118226517	0.807443759767463	-4.82633501308174
a_HQ_transcript/67587	-1.36335982913882	-0.093390247792412	-1.50297120827141	0.142476497688781	0.807443759767463	-4.77480321219732
a_HQ_transcript/86107	-0.574154675887377	2.83287846297879	-1.50291768558589	0.142490249037488	0.807443759767463	-5.02449844575919
a_HQ_transcript/4211	1.80477570090185	0.309707748172079	1.50281313213048	0.142517114573781	0.807443759767463	-4.75333043602797
a_HQ_transcript/108211	-1.53451795948851	-0.467340568076011	-1.50277352011124	0.142527294144228	0.807443759767463	-4.83158959159431
a_HQ_transcript/30358	1.34817206996021	-0.376131425931982	1.50275117307123	0.142533037185881	0.807443759767463	-4.83801081636613
a_HQ_transcript/16632	4.32002410265969	2.71074888211692	1.50259702509478	0.142572657261561	0.807443759767463	-5.00597616869624
a_HQ_transcript/51544	-2.52109822784799	0.302887301080408	-1.50254884026563	0.142585043842126	0.807443759767463	-4.82660713694665
a_HQ_transcript/92076	-0.871671984731229	1.82340290009634	-1.50249772688697	0.142598184191938	0.807443759767463	-4.84366115068775
a_HQ_transcript/118398	0.674315146994786	-0.587324106125346	1.50246929025338	0.14260549517095	0.807443759767463	-4.91295345365734
a_HQ_transcript/134210	1.20769114730836	3.84086965696724	1.50240341435111	0.142622432837992	0.807443759767463	-5.16475935740289
a_HQ_transcript/71845	-2.90083412650731	0.868460398945144	-1.50238174900351	0.142628003671836	0.807443759767463	-4.88397233688409
a_HQ_transcript/34296	-1.39587937506124	1.48865117449248	-1.50237901998403	0.14262870539991	0.807443759767463	-4.71168623432422
a_HQ_transcript/88253	1.01007930346674	2.8086891658835	1.5022967072812	0.142649872229846	0.807443759767463	-5.11793523250783
a_HQ_transcript/119706	1.55093584907665	0.66931774226582	1.50224929492125	0.142662065531648	0.807443759767463	-4.97256583479691
a_HQ_transcript/82695	2.78335399376781	2.14393360774284	1.50220211957922	0.142674198709131	0.807443759767463	-4.79800840931503
a_HQ_transcript/128361	-0.910958376561334	3.83046119930019	-1.50219550881237	0.142675899019666	0.807443759767463	-5.21725728057906
a_HQ_transcript/125391	-1.39946820950337	2.23736584363923	-1.50210035954817	0.142700373520109	0.807443759767463	-4.8388574581033
a_HQ_transcript/136038	-1.41157064595363	1.41799879131922	-1.5020602087911	0.14271070219675	0.807443759767463	-4.9130942786744
a_HQ_transcript/111891	-1.78613620210556	1.38707230438286	-1.50204753709805	0.142713962081113	0.807443759767463	-4.86834210584723
a_HQ_transcript/108149	0.671749586245059	3.37298253049866	1.50196081792183	0.142736272819469	0.807491393328478	-4.99755870146435
a_HQ_transcript/116139	-1.34860263718985	2.45197353553488	-1.50176315818513	0.142787136340088	0.80763281866685	-4.9268306687315
a_HQ_transcript/85224	1.27374496933809	0.00898091292023688	1.5017556839475	0.142789059961451	0.80763281866685	-4.81744869016917
a_HQ_transcript/100105	-2.85888705669323	2.32403456200615	-1.50167688222508	0.142809342182983	0.807668947500722	-4.99182709793294
a_HQ_transcript/57490	1.15716733860131	1.62819127185803	1.50130878142099	0.142904115712324	0.808078184236712	-4.75673503305307
a_HQ_transcript/64965	-0.479193605061556	3.53524298468616	-1.5012878534715	0.142909505469884	0.808078184236712	-5.07152999323077
a_HQ_transcript/146	-0.774023249645494	4.19268524619269	-1.50121818181504	0.142927449796289	0.808101040925852	-5.22731979757248
a_HQ_transcript/12599	-1.83655309401208	2.09928778164374	-1.50100274410126	0.142982948437757	0.808259149214572	-4.92881890968977
a_HQ_transcript/139130	-1.71633022950392	0.907143636819031	-1.50092984814114	0.143001730993272	0.808259149214572	-4.7650285423964
a_HQ_transcript/140265	1.38662037803078	2.24662357520462	1.50091375032557	0.143005879063786	0.808259149214572	-4.82341045718996
a_HQ_transcript/48075	-1.39101865736146	1.92538034270603	-1.50089374790736	0.143011033403384	0.808259149214572	-4.82880147529383
a_HQ_transcript/10868	-3.89557772213249	2.12208518526806	-1.50083064076976	0.143027296195864	0.808272474555043	-4.84186038906254
a_HQ_transcript/31341	-1.59716155663009	3.32297193605578	-1.50075556048404	0.14304664641873	0.808303243222315	-5.23816173713717
a_HQ_transcript/144065	-0.616101389294489	1.69335098204159	-1.50058192772829	0.143091404334093	0.808477561346437	-4.78079693843492
a_HQ_transcript/48869	-1.17014809744177	1.02537732647558	-1.50052471027755	0.143106155937124	0.808482324065811	-4.7359721850784
a_HQ_transcript/149463	0.741922619562235	4.3552118891031	1.50034614413087	0.14315220108853	0.808663862475614	-5.18166743704796
a_HQ_transcript/8667	1.01957091134774	1.53497806946006	1.50023637147248	0.143180513021855	0.80874520075157	-4.67913926505955
a_HQ_transcript/68241	3.96227855860879	2.10358628315256	1.50011385529488	0.143212116999089	0.808786952313818	-4.93875392739093
a_HQ_transcript/33306	0.966807494923147	1.96820377459585	1.5000998407112	0.14321573252419	0.808786952313818	-4.73520056366383
a_HQ_transcript/79853	-0.543560390270449	2.89265893180818	-1.49998109840748	0.143246368967324	0.808881380933805	-5.01267563378812

a_HQ_transcript/138084	-2.3629944455902	0.737303179133464	-1.49979856147509	0.143293475184534	0.809055766948048	-4.89139828068822
a_HQ_transcript/56611	-2.03494943213216	-0.126424273785484	-1.49975356771812	0.143305088365282	0.809055766948048	-4.86835751118278
a_HQ_transcript/11303	-1.16826802627456	2.16735469858825	-1.49955490794564	0.143356372775401	0.809241469109006	-4.90869936781149
a_HQ_transcript/40105	1.93836453944936	0.245218181011493	1.49929611013475	0.143423204035308	0.809241469109006	-4.79947367135844
a_HQ_transcript/78932	0.919686296975403	2.47838772630078	1.49926335239174	0.143431665090809	0.809241469109006	-4.9179155670471
a_HQ_transcript/117482	-0.77278314916551	4.0934009531966	-1.49926188115682	0.143432045108087	0.809241469109006	-5.19357932407653
a_HQ_transcript/140337	2.90461882398165	2.52342822711371	1.49921194840866	0.143444943124393	0.809241469109006	-5.04116765387327
a_HQ_transcript/137094	-0.959872605965892	2.84239586733738	-1.49918299931379	0.14345242132657	0.809241469109006	-5.02411652548988
a_HQ_transcript/123975	-2.00220938490819	1.47868806813061	-1.49909854390092	0.143474239847067	0.809241469109006	-4.89382089258984
a_HQ_transcript/72754	4.10346214141441	2.45778678321369	1.49893404587579	0.143516744515764	0.809241469109006	-4.96748788051883
a_HQ_transcript/111705	1.66782637538756	-0.478192844932715	1.49890096711081	0.143525292963119	0.809241469109006	-4.81097953472051
a_HQ_transcript/45224	-1.86166334609122	0.32093120937292	-1.49883479143118	0.143542395774827	0.809241469109006	-4.83926825196618
a_HQ_transcript/48038	-1.08164189081739	-0.724371776757971	-1.49883043546591	0.143543521612164	0.809241469109006	-4.84274571579759
a_HQ_transcript/107232	-1.38402551325594	-0.354151849330891	-1.49881418992393	0.143547720477134	0.809241469109006	-4.85340222765828
a_HQ_transcript/72459	-1.37434840051234	1.22059756910998	-1.49875779472443	0.143562297291965	0.809241469109006	-4.8911895185829
a_HQ_transcript/96552	-1.73390526529651	0.597560253576111	-1.49874378626107	0.143565918330042	0.809241469109006	-4.75960005123981
a_HQ_transcript/10765	2.0373159325687	1.3925033467642	1.49865815938305	0.143588053556455	0.809241469109006	-4.72028393512366
a_HQ_transcript/73187	-1.43288114633414	2.45754526727385	-1.49856716170806	0.143611580178614	0.809241469109006	-4.96942194184662
a_HQ_transcript/137839	0.457782751276139	4.43620053810612	1.49850504948236	0.143627640506784	0.809241469109006	-5.2413430739917
a_HQ_transcript/129622	1.17065368908237	2.92720522787309	1.49847730527958	0.143634814777606	0.809241469109006	-4.91523978478715
a_HQ_transcript/132748	-1.65822729302052	-0.00802964635363598	-1.49841431045649	0.143651105443895	0.809241469109006	-4.85706380797873
a_HQ_transcript/77678	0.729264842096621	5.47334096466054	1.49832663896428	0.143673780045011	0.809241469109006	-5.35383895102866
a_HQ_transcript/123599	0.501999168567945	2.52870866744904	1.49831346278719	0.143677188066461	0.809241469109006	-4.92958769166819
a_HQ_transcript/103993	2.11268376177836	1.71652596712414	1.4982877790274	0.143683831363742	0.809241469109006	-4.94891081470932
a_HQ_transcript/28405	2.67672495304627	2.03442460443425	1.49828444173748	0.14368469459693	0.809241469109006	-4.75250645408208
a_HQ_transcript/91215	-1.16303982197055	2.3909528076141	-1.4981924942594	0.143708479638064	0.809241469109006	-4.89484516388642
a_HQ_transcript/145453	1.24134701171058	-0.397987637197336	1.49816622056222	0.143715276717753	0.809241469109006	-4.74615316232649
a_HQ_transcript/5424	2.05240401942605	1.52515331547311	1.4981314902533	0.143724261942709	0.809241469109006	-4.82267128934477
a_HQ_transcript/47944	1.95593952751175	1.75961603383632	1.49803505905944	0.143749212427942	0.809241469109006	-4.90003159096642
a_HQ_transcript/1792	-0.39071857327236	4.2077087601503	-1.49801806088967	0.143753610873243	0.809241469109006	-5.23781081877758
a_HQ_transcript/86861	2.01824083799137	0.328807845039788	1.49800330338119	0.143757429612757	0.809241469109006	-4.85122220735026
a_HQ_transcript/119717	-1.16022540885976	3.33721161173915	-1.49792026977476	0.143778917393745	0.809241469109006	-5.07156095307506
a_HQ_transcript/27385	-1.28647469465675	1.74124203100788	-1.49792003219615	0.143778978879021	0.809241469109006	-4.83296855767233
a_HQ_transcript/140618	1.7684772556149	3.96571561519192	1.49785703770674	0.143795282579167	0.809241469109006	-5.11113191181054
a_HQ_transcript/131066	0.785734179114482	3.05977379061684	1.49782427631301	0.143803762191959	0.809241469109006	-5.02783099362124
a_HQ_transcript/6210	1.12442123721425	-0.502949709856226	1.49777218445261	0.143817245924625	0.809241469109006	-4.848585698906
a_HQ_transcript/148684	4.25698090120523	5.67327625764842	1.4977412982702	0.143825241146524	0.809241469109006	-5.04003720487355
a_HQ_transcript/11251	-1.62641000716776	0.0100798246612541	-1.49767267500237	0.143843006297107	0.809263092338197	-4.82364845585926
a_HQ_transcript/36753	1.715654895519	0.649888678170599	1.49750172865089	0.143887268442923	0.809406583673084	-4.69720009093346
a_HQ_transcript/14434	1.15950230454991	-0.422717256388713	1.49741263771693	0.143910340572532	0.809406583673084	-4.94198162071174
a_HQ_transcript/139152	1.46667122545248	-0.365084156246639	1.49727173022469	0.143946837842091	0.809406583673084	-4.85471171161858
a_HQ_transcript/105799	-1.23052233479818	0.913676006662984	-1.49726901443929	0.143947541346415	0.809406583673084	-4.73647848650041
a_HQ_transcript/126919	1.81555226512359	0.128601622510217	1.4971888999313	0.143968295664138	0.809406583673084	-4.88661820150284
a_HQ_transcript/43100	1.91047982597866	0.86401232897881	1.49714816431189	0.143978849479806	0.809406583673084	-4.72452079261422
a_HQ_transcript/122282	1.68467606788957	0.229128325259678	1.49701087155284	0.14401442396523	0.809406583673084	-4.84983975014225
a_HQ_transcript/18504	1.57947251576209	4.15443255578662	1.49688458299891	0.144047153332856	0.809406583673084	-5.12660220012382
a_HQ_transcript/12618	-1.05035973131307	1.34108808445718	-1.49681080653648	0.144066276250939	0.809406583673084	-4.77899390761099
a_HQ_transcript/140950	-2.34782563828445	5.7966382431603	-1.49680772828404	0.144067074180695	0.809406583673084	-5.32415865717262
a_HQ_transcript/49985	2.64872930296437	3.51156126240225	1.4967775305395	0.144074902082427	0.809406583673084	-5.08466853775491
a_HQ_transcript/149961	0.847861856212206	3.59271885892557	1.49677229707796	0.144076258742369	0.809406583673084	-5.13357034334002

a_HQ_transcript/6910	-1.0841579197433	3.47024869851292	-1.49677140575799	0.144076489798512	0.809406583673084	-5.13694102061759
a_HQ_transcript/24153	1.45295336591528	-0.269925262508013	1.49671426784355	0.144091302233498	0.809406583673084	-4.8354684973643
a_HQ_transcript/108113	2.16086675095986	-0.23188604397184	1.4966635719986	0.144104445649003	0.809406583673084	-4.84736657511322
a_HQ_transcript/115195	-2.55727675005815	3.36513803755933	-1.49659143267043	0.144123150164564	0.809406583673084	-5.02194662177571
a_HQ_transcript/36978	1.34655082372905	-0.535224407380983	1.4965484831273	0.144134287184317	0.809406583673084	-4.80447301380688
a_HQ_transcript/74147	1.44669617760025	-0.220195066040981	1.49650830893365	0.144144705167725	0.809406583673084	-4.75876497747748
a_HQ_transcript/127876	1.63627678490759	1.31660711636213	1.49648511841209	0.14415071921541	0.809406583673084	-4.72937742820563
a_HQ_transcript/125963	0.788595734940258	1.99319847309257	1.49647654128828	0.144152943590312	0.809406583673084	-4.81533098654961
a_HQ_transcript/66782	-0.835621932408346	2.79292480627858	-1.49644575862898	0.144160926933104	0.809406583673084	-4.94322196352058
a_HQ_transcript/99501	1.47928950433976	0.868911055182601	1.49626510851926	0.144207784866502	0.809464496172878	-4.74458886413077
a_HQ_transcript/150226	-2.71430221192846	-0.111803720747003	-1.49625724889863	0.144209823811713	0.809464496172878	-4.8842254940019
a_HQ_transcript/87385	1.83999835655291	0.763228989853746	1.49621398839974	0.144221046877632	0.809464496172878	-4.73117353594746
a_HQ_transcript/138057	-1.41140760327017	2.33409663213129	-1.49619125932416	0.144226943759677	0.809464496172878	-5.07222584948431
a_HQ_transcript/74336	-2.20891987161892	0.00183460163047852	-1.49605125921168	0.144263269965661	0.809590206271862	-4.88649468546763
a_HQ_transcript/40319	-3.44017977190752	1.70200663427424	-1.49595252164285	0.144288894081336	0.809655838899495	-4.94216758496253
a_HQ_transcript/95715	2.81760791642576	1.26829389024845	1.49579761081802	0.1443291034903	0.809705603540382	-4.9727775555225
a_HQ_transcript/144228	1.46152455100614	-0.541623188347429	1.49567133828162	0.144361886053935	0.809705603540382	-4.87399649398267
a_HQ_transcript/149914	1.72617715490157	0.0943318467145509	1.49566580778614	0.144363322004	0.809705603540382	-4.8430639653826
a_HQ_transcript/22159	-1.704323345725	1.06832056666687	-1.49565778818379	0.144365404251622	0.809705603540382	-4.79040230272954
a_HQ_transcript/41932	3.25272620015272	2.519556144834	1.4955804170223	0.144385494504552	0.809705603540382	-4.96829052332138
a_HQ_transcript/98930	0.299984089062507	-0.500487324602008	1.49555495236142	0.144392107167639	0.809705603540382	-5.07527354885615
a_HQ_transcript/58536	0.979070003152888	-0.5981280918441	1.49544974669993	0.144419429548388	0.809705603540382	-4.85274696882648
a_HQ_transcript/30731	1.31411828966816	1.96747501183545	1.49542261507451	0.144426476425352	0.809705603540382	-4.76080591365635
a_HQ_transcript/70277	1.88022959250228	-0.21675942998129	1.49540394486059	0.144431325786355	0.809705603540382	-4.9105505607554
a_HQ_transcript/74649	2.41827512078863	2.5702506927036	1.4953818699433	0.14443705964613	0.809705603540382	-4.95481711957013
a_HQ_transcript/118520	1.19942584214148	2.47081418065377	1.49497559612436	0.145542620032327	0.810162698912878	-4.92262889149334
a_HQ_transcript/88775	-1.11068622135923	-0.749630087172027	-1.49496077370525	0.144546472447451	0.810162698912878	-4.84585891481935
a_HQ_transcript/26079	1.90836397392854	0.747013896800184	1.49483563780739	0.144578999126685	0.810266878252598	-4.7264554996483
a_HQ_transcript/129947	4.22829482447733	1.33609086450432	1.49473264335163	0.14460577496554	0.810338811066897	-4.87792945884221
a_HQ_transcript/24631	-1.65447700227168	2.36786227570551	-1.49466684114055	0.144622883884584	0.810356563952489	-5.04441797283742
a_HQ_transcript/36693	2.20531072138982	0.970764771382781	1.49447473408472	0.144672842010816	0.810372402300893	-4.95569795212689
a_HQ_transcript/33298	-1.02370733414555	3.47186698361157	-1.49447048849254	0.144673946248499	0.810372402300893	-5.1813617035283
a_HQ_transcript/50230	1.77901197161099	-0.446136023464938	1.49447005644906	0.144674058619231	0.810372402300893	-4.81415866100501
a_HQ_transcript/107035	3.33875904745131	1.61501814152328	1.49442902878241	0.144684729875495	0.810372402300893	-4.73737736974198
a_HQ_transcript/32256	1.77843287475996	0.251873046099203	1.49436023802339	0.144702623701396	0.810372402300893	-4.74664365280246
a_HQ_transcript/136736	3.72149274048788	1.94551992436958	1.49431005961631	0.144715677208642	0.810372402300893	-4.99962865851432
a_HQ_transcript/142422	-1.13988376260043	3.76420264570756	-1.49428076376211	0.144723298724979	0.810372402300893	-5.15495070777895
a_HQ_transcript/139644	1.49069131816721	-0.320925191063483	1.49420545374966	0.14474289261559	0.810404051431226	-4.88056506323898
a_HQ_transcript/24722	1.40316465705772	0.509277364802756	1.49405476894265	0.144782103637106	0.810432502252555	-4.71236328654559
a_HQ_transcript/48455	-2.52808089360721	0.187552588207781	-1.49404342396152	0.144785056159335	0.810432502252555	-4.81597466788319
a_HQ_transcript/23104	-1.74099218985473	-0.421161585652339	-1.49402519353688	0.144789800713817	0.810432502252555	-4.83863394966123
a_HQ_transcript/129625	-1.60234808379853	0.356615705603092	-1.49396030713163	0.144806688719984	0.810448989208093	-4.86550511635613
a_HQ_transcript/17597	-0.871032110273475	2.47177712974778	-1.49372046043603	0.14486912740717	0.810656982255156	-4.87009566718859
a_HQ_transcript/128378	1.17444165718067	1.40726636538831	1.49362563519194	0.144893818977377	0.810656982255156	-4.77348864101607
a_HQ_transcript/68550	-1.77369309226666	2.24732644785628	-1.49350364479085	0.144925589051922	0.810656982255156	-4.84527328324293
a_HQ_transcript/95181	1.98058163553264	0.750739985348277	1.49349211243787	0.144928592722317	0.810656982255156	-4.74010591861531
a_HQ_transcript/15917	1.6368733255596	0.684588885526996	1.49342168440337	0.144946937208692	0.810656982255156	-4.71610960347384
a_HQ_transcript/68781	0.784027821696668	1.71194727611718	1.4934052164534	0.144951226906622	0.810656982255156	-4.76003324832266
a_HQ_transcript/125746	1.36972295699774	-0.648280860724081	1.49332116072956	0.144973123971815	0.810656982255156	-4.86667334183826
a_HQ_transcript/133814	2.7174045647661	3.9602979956814	1.49331092221872	0.144975791351934	0.810656982255156	-5.21398093222597

a_HQ_transcript/128461	-2.10132949721511	0.146272905332206	-1.49322592858454	0.144997935771675	0.810656982255156	-4.95363275633335
a_HQ_transcript/7562	1.45473904677125	0.382880207801086	1.49316699154259	0.145013292944141	0.810656982255156	-4.7969231738939
a_HQ_transcript/134687	-1.57210132645567	0.311427288614216	-1.49310690442542	0.145028951132893	0.810656982255156	-4.73766114716025
a_HQ_transcript/16901	-1.00565504510704	3.09187607252127	-1.49309353108707	0.145032436294855	0.810656982255156	-5.15777600954535
a_HQ_transcript/37914	1.59889006962392	-0.235021224053214	1.49307436486296	0.145037431230212	0.810656982255156	-4.8639575099545
a_HQ_transcript/124069	1.78938885212985	0.562430172721992	1.49297208788429	0.145064088102043	0.810656982255156	-4.91450343971168
a_HQ_transcript/9189	-2.40994304343064	1.94512167487471	-1.49296400533005	0.145066194859001	0.810656982255156	-4.83532047078285
a_HQ_transcript/110585	0.491648078117139	3.12749862065825	1.49292616980966	0.145076057196976	0.810656982255156	-5.05277314726178
a_HQ_transcript/70880	1.97452636299252	0.791007089311304	1.49290745775917	0.14508093494358	0.810656982255156	-4.88204957352577
a_HQ_transcript/36107	-2.11884082017583	1.31450072362838	-1.49281026287816	0.145106273244848	0.810686856848632	-4.84703233898669
a_HQ_transcript/2253	-1.033367768982	3.27959481007656	-1.49277995666098	0.145114174675028	0.810686856848632	-5.09736708957361
a_HQ_transcript/63683	0.485270089797538	2.21431957153847	1.49270924791025	0.145132611185688	0.810704086416374	-4.87129310115575
a_HQ_transcript/130643	-1.33321197356076	1.06021593871242	-1.4926611526793	0.145145152548431	0.810704086416374	-4.79220281131358
a_HQ_transcript/55258	-1.02465243528483	2.32225830208704	-1.49255029506141	0.145174063205396	0.810787658147891	-4.86053523845305
a_HQ_transcript/139487	2.00751016894331	3.07524929806867	1.49247932716006	0.145192573410597	0.8107909846245	-5.06317024344212
a_HQ_transcript/7935	-2.39279168392064	3.55017216268004	-1.49238680127218	0.145216709319059	0.8107909846245	-4.85059666653297
a_HQ_transcript/128622	3.23677397058425	3.89361754810327	1.49233912547788	0.14522914707869	0.8107909846245	-4.97841153265614
a_HQ_transcript/86675	1.20188222922687	1.31182647674351	1.49233412251835	0.145230452310596	0.8107909846245	-4.78335690244198
a_HQ_transcript/138756	-1.69958597421709	-0.161449076427192	-1.49227908309704	0.145244812273625	0.810793282228109	-4.84244949007446
a_HQ_transcript/137231	2.11348165561714	0.633355282971678	1.49203776439806	0.145307786535459	0.811066930644626	-4.71607900173078
a_HQ_transcript/44470	1.63275166971361	2.77071439795486	1.49187240369073	0.145350951516287	0.811214908870709	-4.97119964245875
a_HQ_transcript/65353	1.02845429833667	1.26418156031229	1.49182928347281	0.145362209104	0.811214908870709	-4.72865877210793
a_HQ_transcript/91392	-1.72203336495821	0.971202977373054	-1.49176789656783	0.145378236862605	0.811226471378468	-4.80501388651744
a_HQ_transcript/152622	1.83662188326746	0.182262584156203	1.49158529898972	0.145425920384	0.811292616452363	-4.95979903549062
a_HQ_transcript/115233	-1.28864052408004	0.497784274100161	-1.49156076267092	0.145432328751996	0.811292616452363	-4.78217335148938
a_HQ_transcript/138988	2.28067500332911	1.79319581447561	1.49148589549244	0.145451883875626	0.811292616452363	-4.74316360339244
a_HQ_transcript/144628	1.55878862632055	1.92332712767031	1.49146565773621	0.145457170289715	0.811292616452363	-4.78616242972137
a_HQ_transcript/174	-1.43206875051591	4.28593451715157	-1.49143955667745	0.145463988516185	0.811292616452363	-5.11602916307201
a_HQ_transcript/35821	-1.42090607058103	4.57462853630021	-1.49134640347495	0.145488324468817	0.811292616452363	-5.34442528871725
a_HQ_transcript/144725	-0.757331577455957	3.1749493135497	-1.49130596230368	0.145498890600743	0.811292616452363	-5.07659070422419
a_HQ_transcript/121121	1.35765727089094	-0.483113452358042	1.49123370208073	0.145517771679943	0.811292616452363	-4.85856236799363
a_HQ_transcript/110215	-2.75593237903495	1.82970599719801	-1.49122062413663	0.145521189062952	0.811292616452363	-4.91757246216556
a_HQ_transcript/139512	0.855967752796359	0.0663000324649499	1.49118820554332	0.145529660606743	0.811292616452363	-4.80349139786409
a_HQ_transcript/138387	-4.46385191872729	0.285553384334919	-1.49112766984922	0.145545480697166	0.811303001722755	-4.90918307261227
a_HQ_transcript/126727	1.76411363836444	0.0581587369999169	1.49092503459319	0.145598446390538	0.811501214269615	-4.77842501040623
a_HQ_transcript/143137	1.90634436583453	1.37877366348946	1.4908848163316	0.145608960652905	0.811501214269615	-4.80779659665901
a_HQ_transcript/8221	1.87430963624183	1.20333805325884	1.49065661229583	0.145668631565757	0.811717842434615	-4.84467920705461
a_HQ_transcript/96579	-1.15030541334525	-0.673047151631017	-1.49052865167292	0.145702099349018	0.811717842434615	-4.84927974459884
a_HQ_transcript/101415	0.380341003390117	4.67736703520583	1.49048232383374	0.145714217799092	0.811717842434615	-5.27353330129332
a_HQ_transcript/64596	2.7695859991384	0.682952831823599	1.49046599118426	0.145718490290952	0.811717842434615	-4.82883004931047
a_HQ_transcript/93587	0.892748234749579	2.21407015007239	1.49042886820388	0.145728201742147	0.811717842434615	-4.85422854720652
a_HQ_transcript/39683	1.616906587257	2.12975408789136	1.4903862880119	0.145739341450006	0.811717842434615	-4.88804991659892
a_HQ_transcript/144355	2.01495239499802	0.299625718619977	1.49036243993779	0.145745580812863	0.811717842434615	-4.85417264809359
a_HQ_transcript/77444	1.2855684572183	3.15698202713169	1.49011673287992	0.14580987752062	0.811813596416752	-5.11351160698603
a_HQ_transcript/13734	0.712397409549351	2.73626741124912	1.49010962745718	0.14581173720808	0.811813596416752	-4.93365193726172
a_HQ_transcript/110090	-1.38491545924947	1.58445635273005	-1.48998696132528	0.14584384535413	0.811813596416752	-4.79274405931668
a_HQ_transcript/150013	2.04229106251054	-0.430764260131299	1.48998506026067	0.145844343006786	0.811813596416752	-4.84715693326868
a_HQ_transcript/7427	-1.84823899634604	1.12297199437941	-1.48992064668446	0.145861205724326	0.811813596416752	-4.79384928438394
a_HQ_transcript/8572	-2.53007886047192	1.60431948490472	-1.48989237939142	0.145868606262607	0.811813596416752	-4.93836453191881
a_HQ_transcript/26136	-2.62044449628063	1.02017557912872	-1.48989005698734	0.145869214294587	0.811813596416752	-4.76677371649711

a_HQ_transcript/141115	1.17560591679518	2.61794198098973	1.48982637986691	0.145885886484853	0.811813596416752	-4.9395267033263
a_HQ_transcript/148888	3.49189853684618	4.38312918222923	1.48981652325935	0.145888467316429	0.811813596416752	-5.35369105787337
a_HQ_transcript/110787	0.962643182781949	-0.42972054038157	1.48973170828588	0.145910676586339	0.811855517813695	-4.79770217482477
a_HQ_transcript/41692	-1.7868503843181	-0.0301080957854994	-1.48959559750429	0.145946323607435	0.811855517813695	-4.71311144068073
a_HQ_transcript/63019	-1.22305990570671	0.12092552823248	-1.48958098284797	0.145950151558429	0.811855517813695	-4.80122216078634
a_HQ_transcript/79359	-0.620113537666505	3.16751272141306	-1.48957443122199	0.14595186762237	0.811855517813695	-5.0372721603829
a_HQ_transcript/106829	-1.69295673633842	6.30538178863086	-1.48947330388963	0.145978357901206	0.811883938205943	-5.14239461426043
a_HQ_transcript/60906	1.01531898767865	4.39101111762445	1.48928131628282	0.146028659600606	0.811883938205943	-5.27684024896563
a_HQ_transcript/78414	1.13348342308275	4.89727247043401	1.48924000976365	0.146039483926724	0.811883938205943	-5.35533173116181
a_HQ_transcript/10481	1.41644822747083	-0.489346499933105	1.48922720593736	0.146042839285223	0.811883938205943	-4.88318379948057
a_HQ_transcript/137561	-1.42215490364608	1.96741850698379	-1.4891518143644	0.146062597581001	0.811883938205943	-4.98008058636265
a_HQ_transcript/137459	-0.873699480424818	3.63652715019871	-1.48913306750335	0.146067511009207	0.811883938205943	-5.17666542216224
a_HQ_transcript/110226	0.63335921291849	2.99861207701334	1.48912820522674	0.146068785401346	0.811883938205943	-5.13479322806792
a_HQ_transcript/149071	1.32769839854859	-0.228021243060459	1.4890382627785	0.146092360727511	0.811883938205943	-4.83799506373719
a_HQ_transcript/143620	-3.43050025095338	1.2246473287567	-1.48891199404504	0.146125462884494	0.811883938205943	-4.94929162463156
a_HQ_transcript/45850	-1.68959712260729	0.946908321032409	-1.48890987205009	0.146126019230375	0.811883938205943	-4.93524663267324
a_HQ_transcript/139736	-0.832667508138241	3.08854460833823	-1.48880701469149	0.14615298846681	0.811883938205943	-4.9948762892389
a_HQ_transcript/104639	1.63638273486919	-0.316663305682283	1.48878009276425	0.146160048063966	0.811883938205943	-4.86838554211282
a_HQ_transcript/136812	2.10587582440528	0.118367596391553	1.48877319627006	0.146161856539755	0.811883938205943	-4.80834795747082
a_HQ_transcript/116936	-4.18148202428302	2.84112861031696	-1.48873116890557	0.146172877812351	0.811883938205943	-5.07994068706553
a_HQ_transcript/61226	2.67214474943456	2.85453246595129	1.48872647846324	0.146174107877075	0.811883938205943	-5.18382497273659
a_HQ_transcript/124402	-3.02918774609438	0.623408763381739	-1.48870228885272	0.146180451714551	0.811883938205943	-4.79223407069079
a_HQ_transcript/107259	1.38606502929716	1.32116826937802	1.4885781970785	0.146212998824083	0.811987121013308	-4.70676974395163
a_HQ_transcript/141677	1.81129864642994	-0.0609114849617621	1.48846890791142	0.146241668273359	0.812003369945557	-4.77929001347601
a_HQ_transcript/61286	-1.42202413519559	3.4923832907646	-1.48841684198711	0.146255328130216	0.812003369945557	-5.16552827757773
a_HQ_transcript/140778	2.74197943098707	3.3416648885653	1.48814671302639	0.146326214740659	0.812003369945557	-5.07626270976126
a_HQ_transcript/82496	-0.799806264460014	1.72865095865796	-1.48806231953801	0.146348366721519	0.812003369945557	-4.87601829982876
a_HQ_transcript/65349	-1.2175981390123	1.95208266407537	-1.48805423711753	0.146350488372335	0.812003369945557	-4.91029993957557
a_HQ_transcript/121638	1.63487517431084	0.979565558137131	1.48804373883916	0.146353244227227	0.812003369945557	-4.76106415768336
a_HQ_transcript/139051	0.995728218720097	2.75069587792049	1.48794349123784	0.146379561857908	0.812003369945557	-4.9475916215719
a_HQ_transcript/15461	1.92657777926682	0.0814126112958593	1.48775324967203	0.146429515688112	0.812003369945557	-4.86797161849644
a_HQ_transcript/67847	-1.45539287015135	2.76560209008467	-1.48768068662276	0.146448572965195	0.812003369945557	-4.94335204988016
a_HQ_transcript/41832	-1.63168342225903	1.3224943167321	-1.48765744466791	0.146454677433564	0.812003369945557	-4.71605228030793
a_HQ_transcript/59111	1.46079828267584	-0.00700294481198637	1.48765491628165	0.146455341523123	0.812003369945557	-4.80896807418073
a_HQ_transcript/138707	1.22515358599667	-0.179373157465933	1.48765019884728	0.14645658058032	0.812003369945557	-4.85426342712661
a_HQ_transcript/72686	1.11151619577987	4.32776053133272	1.48762449317459	0.146463332448378	0.812003369945557	-5.26911232442714
a_HQ_transcript/66277	2.59272650442289	0.0760347040363902	1.48762249763682	0.146463856608	0.812003369945557	-4.8692375097653
a_HQ_transcript/2634	1.33169354484618	0.255660539754181	1.48752410592923	0.146489702611913	0.812003369945557	-4.82538499332688
a_HQ_transcript/125444	-2.23607070934364	0.577762331641321	-1.48751428781872	0.146492281880443	0.812003369945557	-4.95643969928081
a_HQ_transcript/94544	-1.37277811966511	1.05080533435123	-1.48749210005302	0.146498110855438	0.812003369945557	-4.79441549077254
a_HQ_transcript/35288	2.8118903723516	2.5185870649091	1.48747979169123	0.146501344480249	0.812003369945557	-4.8958425881015
a_HQ_transcript/84360	0.492680245205181	5.49985063541004	1.4873874595933	0.146525603581097	0.812003369945557	-5.37275518800314
a_HQ_transcript/58619	-1.6259790784451	4.34382109905472	-1.48736995169994	0.146530203923208	0.812003369945557	-5.30226054769866
a_HQ_transcript/129819	0.726202773590278	6.05129773628018	1.48734996695573	0.146535455219445	0.812003369945557	-5.41626500387514
a_HQ_transcript/151835	-3.0831032942614	4.16733328861356	-1.48733026398732	0.146540632622279	0.812003369945557	-5.16835403276931
a_HQ_transcript/48061	1.67789901511276	1.49481999694248	1.48731228216032	0.146545357883893	0.812003369945557	-4.8833306736042
a_HQ_transcript/18477	2.07128220427514	-0.164634129823185	1.48725180935899	0.146561249811791	0.812003369945557	-4.81489464515284
a_HQ_transcript/83018	-1.01366208547903	5.09809451444317	-1.48714681696751	0.146588844526157	0.812003369945557	-5.3475561241015
a_HQ_transcript/116406	0.706793326249617	2.30435569246985	1.48714449152493	0.146589455759652	0.812003369945557	-4.87801645477436
a_HQ_transcript/148149	1.23113734090423	0.323913814925993	1.48709693148614	0.146601957177561	0.812003369945557	-4.88516805869448

a_HQ_transcript/126696	2.03954491380407	-0.00321510903032165	1.48698271199819	0.146631983884469	0.812003369945557	-4.79683543110356
a_HQ_transcript/102562	-3.75202827331629	0.317665349835494	-1.48697811939092	0.146633191319674	0.812003369945557	-4.89381799973477
a_HQ_transcript/138034	-6.50652661775365	5.5132517828742	-1.48697123376933	0.1466350016226	0.812003369945557	-5.18974767186613
a_HQ_transcript/1692	1.87618109510996	0.742356310297357	1.48684861683887	0.146667241909788	0.812099422475603	-4.90192401571294
a_HQ_transcript/98454	-1.84174109256797	1.45501064297756	-1.48679900216463	0.146680288958357	0.812099422475603	-4.88949407644913
a_HQ_transcript/121947	-0.721389733112158	3.70073556745129	-1.48662434055839	0.146726226682289	0.812244467980058	-5.13481533608531
a_HQ_transcript/123217	2.26602046078016	1.51679706558978	1.48659314119789	0.146734433633681	0.812244467980058	-4.85914352985999
a_HQ_transcript/98952	-2.28010108299476	0.846205726491448	-1.48649571957585	0.146760062630143	0.812250260433764	-4.82603435442392
a_HQ_transcript/152741	2.23392446046628	0.0952180496158362	1.48648293181045	0.146763427011695	0.812250260433764	-4.96658069789115
a_HQ_transcript/80437	-2.2140847567934	-0.311781381197973	-1.48638396618985	0.146789466338574	0.812282219862173	-4.83682023865046
a_HQ_transcript/60952	1.47446679973353	1.27365581684307	1.48616386441123	0.146847391647221	0.812282219862173	-4.71251239336782
a_HQ_transcript/66545	0.740085627251584	4.46345729901046	1.48610651578119	0.146862487382834	0.812282219862173	-5.2803904576268
a_HQ_transcript/79214	1.73861722670943	0.800466449973909	1.48600845302279	0.146888303073489	0.812282219862173	-4.73937186382351
a_HQ_transcript/135680	-1.71706106380033	1.86632913774321	-1.48599829385064	0.146890977752512	0.812282219862173	-4.87617221982791
a_HQ_transcript/53553	-0.372643547233971	2.92278999178772	-1.4859731650549	0.146897593760478	0.812282219862173	-5.04726144894387
a_HQ_transcript/114707	4.38647690757657	3.36478882543158	1.48595944590647	0.146901205892358	0.812282219862173	-4.85679402695666
a_HQ_transcript/116150	2.33368608959889	-0.197228665080766	1.48595247447226	0.146903041437294	0.812282219862173	-4.93709012404572
a_HQ_transcript/145973	0.606835775807721	4.59471017239734	1.48594099468218	0.146906064050542	0.812282219862173	-5.24604289823489
a_HQ_transcript/5925	0.979262714206824	3.39615067662252	1.48593006470308	0.146908941945552	0.812282219862173	-5.15945285114051
a_HQ_transcript/137187	0.864524745235367	2.03723307053119	1.48577669738863	0.146949328760053	0.812418040047224	-4.8177860800304
a_HQ_transcript/138769	1.19333025399743	-0.607995016684632	1.48559924039664	0.146996070290975	0.812418040047224	-4.8643718765869
a_HQ_transcript/76104	1.06864518003965	1.0083407268604	1.48557847334134	0.147001541035883	0.812418040047224	-4.77042883082174
a_HQ_transcript/11219	0.688392698405491	2.20328350896932	1.48546624105372	0.14703110963683	0.812418040047224	-4.80820563596542
a_HQ_transcript/115923	1.54444542559205	0.260322277958718	1.4854550853046	0.147034048978832	0.812418040047224	-4.77403385775025
a_HQ_transcript/40147	0.94412709112848	0.916146115431721	1.48544146211444	0.147037638511546	0.812418040047224	-4.88376082637235
a_HQ_transcript/8576	-2.03302534504247	1.38697286341177	-1.48541834728776	0.14704372912697	0.812418040047224	-4.88417351044906
a_HQ_transcript/81706	-1.73507446870521	0.54794314928907	-1.48541232035702	0.14704531721953	0.812418040047224	-4.77420372391787
a_HQ_transcript/27697	-1.7419663327659	0.397857570072561	-1.48535841307665	0.147059522365281	0.812419303939276	-4.80452970904384
a_HQ_transcript/139405	4.83675210486245	3.2969255727711	1.48512175894804	0.147121896254571	0.812574387389573	-5.31329707245469
a_HQ_transcript/118291	2.86366699756768	0.805038500909521	1.48506865790547	0.147135894770445	0.812574387389573	-4.89515066586515
a_HQ_transcript/49956	-2.13154903351783	0.95378347630383	-1.48505100919573	0.14714054756559	0.812574387389573	-5.00532745020487
a_HQ_transcript/145134	2.5677172528292	1.1203612206176	1.48492647058182	0.147173383491106	0.812574387389573	-4.89939963612031
a_HQ_transcript/128559	4.68268924137132	3.42097402086587	1.48488505974362	0.147184303196268	0.812574387389573	-4.98431891474483
a_HQ_transcript/27553	2.68277595055628	1.53619183392965	1.48487467986483	0.14718704038847	0.812574387389573	-4.76295059779647
a_HQ_transcript/114696	-1.35538235393676	1.95242028336638	-1.48479388889623	0.147208346500472	0.812574387389573	-4.82520562804993
a_HQ_transcript/27775	1.71246671718173	1.26279796418284	1.48474809269128	0.147220424924784	0.812574387389573	-4.71190908855867
a_HQ_transcript/80735	1.83641519499848	3.1783126015497	1.48471985192843	0.147227873620948	0.812574387389573	-5.01541759284896
a_HQ_transcript/38275	-1.02419395953139	1.8417391304497	-1.4845627329433	0.147269320343334	0.812574387389573	-4.78816353635414
a_HQ_transcript/38178	3.44479715973602	2.98401007821552	1.48454059385008	0.147275161208004	0.812574387389573	-5.12560392135151
a_HQ_transcript/14877	-1.06702118506082	-0.76268454941428	-1.48446607629475	0.147294822225698	0.812574387389573	-4.8542611974847
a_HQ_transcript/135422	2.62493575947071	8.09327300492858	1.48444018710638	0.147301653426901	0.812574387389573	-5.63251023656269
a_HQ_transcript/127724	1.64080635149548	1.39666995009099	1.48437182549629	0.147319692750712	0.812574387389573	-4.7882525466645
a_HQ_transcript/103962	-1.25988555739015	1.39050106097083	-1.48434060547302	0.147327931706678	0.812574387389573	-4.78901583627121
a_HQ_transcript/126355	-2.324709602604	2.40822729919314	-1.48433470489847	0.147329488908173	0.812574387389573	-5.04119523990965
a_HQ_transcript/15118	0.935419646684308	2.44014501086791	1.48426577787917	0.147347680189589	0.812574387389573	-4.86001029993577
a_HQ_transcript/107730	2.41903899756906	0.920868220753145	1.48421873827069	0.147360095957245	0.812574387389573	-4.90192204565813
a_HQ_transcript/17643	-0.527033341566429	3.98632671418096	-1.48420625060573	0.147363392127522	0.812574387389573	-5.26025981387458
a_HQ_transcript/152277	-1.33465122795992	2.68241816158479	-1.48414820064562	0.147378715426437	0.812574387389573	-5.04925631134038
a_HQ_transcript/51338	2.47461868004389	3.71411847359876	1.48413895989752	0.147381154800583	0.812574387389573	-5.27583728575971
a_HQ_transcript/32668	2.67731194567948	0.809888677254481	1.48401005167883	0.147415187380619	0.812595922853609	-4.86735723516459

a_HQ_transcript/33518	1.51058537068698	-0.131972451951387	1.48401004124648	0.147415190135081	0.812595922853609	-4.82938820594088
a_HQ_transcript/135738	1.41605928991271	1.74680772743691	1.48387998009357	0.147449533468153	0.812595922853609	-4.90440888201314
a_HQ_transcript/35260	1.78861948167601	0.202920437966559	1.48387597834996	0.147450590251129	0.812595922853609	-4.88595679995654
a_HQ_transcript/54801	2.26498458758135	1.17503606234125	1.48385943942576	0.147454957924922	0.812595922853609	-4.81505141229077
a_HQ_transcript/51480	1.58471141752539	0.403807290552986	1.48374371343927	0.147485522261598	0.812658740439354	-4.82006878214135
a_HQ_transcript/80411	-1.67627303524048	1.02703280352203	-1.48371041395107	0.147494317912799	0.812658740439354	-4.73939666708056
a_HQ_transcript/118159	4.27614142757943	0.824382131710573	1.48363428895883	0.147514426971348	0.812692504121932	-4.9796006579538
a_HQ_transcript/107995	-1.09849868328843	1.31298174765393	-1.48346628417616	0.147558814611525	0.812722456450829	-4.93383368339652
a_HQ_transcript/48025	2.13717503290882	1.34828191950305	1.48341367840555	0.147572715495283	0.812722456450829	-4.79382127633854
a_HQ_transcript/1015	2.37486784447226	0.247253294869886	1.48338011762305	0.147581584357364	0.812722456450829	-4.87124356155335
a_HQ_transcript/144129	1.911317800501	-0.675996446488209	1.48334847833067	0.147589945831715	0.812722456450829	-4.88355406987643
a_HQ_transcript/109685	-3.64666582348386	1.59253411115504	-1.48333311830914	0.147594005238196	0.812722456450829	-4.9533554225187
a_HQ_transcript/14557	1.6594418758067	0.63394390432472	1.48329623481793	0.147603753315982	0.812722456450829	-4.82443383540234
a_HQ_transcript/75449	1.85060767738226	0.606916007379865	1.48320188447131	0.147628691870339	0.812782780928118	-4.8816595936219
a_HQ_transcript/129695	-1.3296758633568	-0.241166044745305	-1.48312117449154	0.147650027695832	0.812786938141536	-4.8592938232447
a_HQ_transcript/137624	3.20996652825795	0.76179966138228	1.48300637185314	0.147680380224339	0.812786938141536	-4.86687406092098
a_HQ_transcript/45289	1.3057880636037	-0.183407847858114	1.48298878376871	0.147685030757148	0.812786938141536	-4.83965246115862
a_HQ_transcript/3786	-1.58060066842538	0.667557480197124	-1.48298747129461	0.147685377798151	0.812786938141536	-4.91567095260546
a_HQ_transcript/151405	1.73434022610733	0.347837737093437	1.48271741518128	0.147756799210492	0.813103022295512	-4.84620213123471
a_HQ_transcript/137935	1.68831443606458	0.639288229448498	1.48252757949844	0.147807021353429	0.813302398450599	-4.79224832540813
a_HQ_transcript/4069	-1.81569069988912	1.38299764992943	-1.48240941858576	0.147838288407611	0.8133974470949	-4.9499959193664
a_HQ_transcript/131933	-0.519688130302888	3.79974146344773	-1.48230152033013	0.147866844436755	0.813477563261374	-5.22836918955402
a_HQ_transcript/68028	1.5493037828443	0.39296882374819	1.48216845075458	0.147902068305733	0.813594343378033	-4.84202111439682
a_HQ_transcript/36236	-1.71345799227678	3.57966635068491	-1.48204153601973	0.147935669222653	0.813702174543375	-5.07120157248191
a_HQ_transcript/124274	1.46107287346233	0.585931350155499	1.48194461591569	0.147961333114176	0.813766332790312	-4.82765354015606
a_HQ_transcript/55930	2.02566170446454	1.68495322426984	1.48173992281722	0.148015546378731	0.813967237462623	-4.96295427409789
a_HQ_transcript/141466	1.8466696212653	0.764966404184139	1.4817009574635	0.148025868208392	0.813967237462623	-4.94725947070231
a_HQ_transcript/60817	1.13998073376983	1.42010241576299	1.48164333254798	0.148041133966477	0.81397418040138	-4.9088179970129
a_HQ_transcript/42893	0.992161675918923	2.01738771485878	1.48148201730652	0.148083875637396	0.81409004024884	-4.83367178736666
a_HQ_transcript/11867	-0.762330074047129	3.22548229807459	-1.48145809009037	0.148090216171058	0.81409004024884	-5.09598759107951
a_HQ_transcript/9935	-0.594024263440818	2.2426161995881	-1.48130706833782	0.148130240816363	0.814177468818822	-4.94395808120966
a_HQ_transcript/83641	1.17030651756476	-0.130050877515027	1.48129238225135	0.148134133467999	0.814177468818822	-4.90878030607923
a_HQ_transcript/32770	-0.870209309199159	2.80203820656666	-1.48109805000271	0.148185650312936	0.814325313274954	-5.01700283128437
a_HQ_transcript/11541	-1.12479636298919	2.06934868706851	-1.4810120627306	0.148208449828274	0.814325313274954	-4.87140029679661
a_HQ_transcript/114388	2.48826887201179	1.61991190053123	1.48096479876161	0.14822098305844	0.814325313274954	-4.75771262762146
a_HQ_transcript/131149	1.4945589936192	0.480579476380144	1.48093886650665	0.14822786000803	0.814325313274954	-4.75271586444971
a_HQ_transcript/19265	-1.8754507651352	0.131108448575498	-1.48092672967639	0.148231078649916	0.814325313274954	-4.87942402497459
a_HQ_transcript/35347	2.72616011221683	1.12422041677008	1.4808323424197	0.148256111703255	0.814349603033049	-4.93903089539843
a_HQ_transcript/110384	1.66234193481841	3.78919293891182	1.48079888895034	0.148264984924766	0.814349603033049	-5.15307415792089
a_HQ_transcript/121349	0.970577920003504	2.53835913857047	1.4807259931039	0.148284321340135	0.814349603033049	-4.84961661885319
a_HQ_transcript/80722	3.16962599521583	1.20680973877019	1.48068725589023	0.148294597627159	0.814349603033049	-4.94427969447458
a_HQ_transcript/63393	-0.740042340045975	2.64452425794822	-1.48064597979111	0.14830554806131	0.814349603033049	-5.01718130522395
a_HQ_transcript/86851	0.587310222585709	4.43826039585982	1.48046038497826	0.148354793835229	0.814420073689047	-5.26174884369895
a_HQ_transcript/24345	0.849781664907111	-0.468407723101323	1.48044787411785	0.148358113940211	0.814420073689047	-4.85342320585315
a_HQ_transcript/120750	1.4886808894619	-0.603494476787687	1.48043447950847	0.148361668638406	0.814420073689047	-4.88921922214152
a_HQ_transcript/104319	-1.34703803291745	0.799888440301432	-1.48034064382864	0.148386572917551	0.814420073689047	-4.79079906686352
a_HQ_transcript/71141	0.942888422016944	1.81918269497602	1.48025624146667	0.148408976419198	0.814420073689047	-4.94502370448026
a_HQ_transcript/133280	1.1814866534642	0.175566005689669	1.48018376373154	0.14842821684221	0.814420073689047	-4.83187175481014
a_HQ_transcript/128005	-0.646743311282723	2.31737735559425	-1.48014893370326	0.14843746376304	0.814420073689047	-4.93082239527867
a_HQ_transcript/125838	1.90984292596787	2.10929180030394	1.4801044177908	0.148449282830765	0.814420073689047	-4.8599158715384

a_HQ_transcript/14357	1.15142225324519	-0.571867900220396	1.47991752059158	0.148498912635191	0.814420073689047	-4.82630930766915
a_HQ_transcript/93089	1.82627543970642	1.42156938552227	1.47988057126676	0.148508725950467	0.814420073689047	-4.8069070371442
a_HQ_transcript/17626	-0.419235172204826	2.92517386774096	-1.4798631246259	0.148513359757542	0.814420073689047	-5.05188513120459
a_HQ_transcript/34389	1.15055017673456	1.88067424693258	1.47976234384496	0.148540129274727	0.814420073689047	-4.80979546666439
a_HQ_transcript/108196	1.69082526971164	-0.482163592438916	1.47974617597084	0.148544424164592	0.814420073689047	-4.88887844849468
a_HQ_transcript/43560	-2.46090798472028	2.88487195720727	-1.47964352582378	0.148571694819074	0.814420073689047	-5.01759226088278
a_HQ_transcript/149687	2.94120146204328	5.31861221895442	1.4796419420404	0.148572115607816	0.814420073689047	-5.07895443197876
a_HQ_transcript/138428	-1.1934336692425	1.364771083306	-1.47963365640296	0.148574316999486	0.814420073689047	-4.86336945171255
a_HQ_transcript/135501	1.34549410893675	1.53481235138169	1.47958513771129	0.148587208340629	0.814420073689047	-4.72560493474313
a_HQ_transcript/26880	1.41769504815945	0.0327345888327179	1.47957789873925	0.148589131800902	0.814420073689047	-4.91414332179944
a_HQ_transcript/85981	1.6284645527301	0.0806075901902628	1.47953396633277	0.148600805465017	0.814420073689047	-4.91270307191686
a_HQ_transcript/102270	1.6012221713605	0.496372516826892	1.47943814746287	0.14862626887766	0.814420073689047	-4.86754759656552
a_HQ_transcript/77582	1.67511422473309	1.65596926208655	1.47936872237344	0.148644720445851	0.814420073689047	-4.7587329087179
a_HQ_transcript/146663	-2.54152374217644	2.2126586150694	-1.47930037358831	0.148662887745533	0.814420073689047	-4.96260136402248
a_HQ_transcript/82238	1.14146158120375	-0.460672573052789	1.47928865849932	0.148666001827305	0.814420073689047	-4.88517595690809
a_HQ_transcript/56837	1.69607899657933	2.07770304061165	1.47922140738703	0.148683879393352	0.814420073689047	-4.97658617041485
a_HQ_transcript/94678	1.53704244273247	-0.475226245824188	1.47921070037565	0.148686725828995	0.814420073689047	-4.9067259890831
a_HQ_transcript/22743	2.16851997764384	1.16543506383047	1.47919373605503	0.148691235845812	0.814420073689047	-4.81361139479435
a_HQ_transcript/26099	-1.41414914039189	1.49872616193185	-1.47916173934008	0.148699742568411	0.814420073689047	-4.78153030331868
a_HQ_transcript/62179	1.07436942115313	-0.0170388247145403	1.47907264470674	0.14872343152809	0.814420073689047	-4.84249789211415
a_HQ_transcript/144752	1.0619037989323	0.931637739824341	1.47906789235529	0.148724695193525	0.814420073689047	-4.75331231187431
a_HQ_transcript/59277	2.83260270548305	1.43199618631731	1.47900347062445	0.148741825983996	0.814437157196472	-4.8510736026218
a_HQ_transcript/140954	-1.15753993643326	2.13041567985067	-1.47886479894167	0.148778706398897	0.814497255574198	-4.85324119498368
a_HQ_transcript/98745	2.17665827217754	0.864401807082216	1.47885682961473	0.148780826102512	0.814497255574198	-4.84235993115549
a_HQ_transcript/117500	1.6761474156337	0.706364340397022	1.47879503274096	0.148797263824873	0.814510533158696	-4.80522304003457
a_HQ_transcript/89046	3.01105955399968	0.292692153753587	1.47873091867417	0.148814319444935	0.814527190272616	-4.86595976823661
a_HQ_transcript/26990	0.434983392121545	4.12104067088365	1.47867388993266	0.148829491539727	0.814533535846084	-5.27095362439635
a_HQ_transcript/136127	1.42122681958138	-0.219367866439138	1.4785130218578	0.148872296017295	0.81463425259122	-4.885447620966
a_HQ_transcript/109048	1.85196117994435	-0.445408473269345	1.47848312926406	0.148880251052255	0.81463425259122	-4.89002209609606
a_HQ_transcript/142654	0.446062345831597	4.36771954834991	1.4784375206583	0.148892389096096	0.81463425259122	-5.27744424861776
a_HQ_transcript/90512	-4.72374360394322	1.18537726406833	-1.47833490164122	0.148919702493266	0.81463425259122	-4.91923148613461
a_HQ_transcript/34874	-1.42232186805185	-0.20368867183952	-1.47832480125452	0.148922391060007	0.81463425259122	-4.82482234669291
a_HQ_transcript/83600	1.98787582416203	0.0679655556752843	1.47828877433603	0.148931981184402	0.81463425259122	-4.92365946328932
a_HQ_transcript/35663	1.53533884035038	2.47734171279989	1.47818917390712	0.148958496718843	0.814676948967093	-4.96710520226375
a_HQ_transcript/102840	-1.7799956199727	0.442107188400197	-1.47808525775255	0.148986165201501	0.814676948967093	-4.76275653367975
a_HQ_transcript/39080	-5.1343205161377	0.456103442529954	-1.4780785484701	0.148987951741105	0.814676948967093	-4.8889441571521
a_HQ_transcript/111217	-0.43697191231403	3.37398874666604	-1.47804889565752	0.148995847861085	0.814676948967093	-5.1041792182825
a_HQ_transcript/148713	-2.70288103774617	0.156741315850588	-1.47780477567918	0.149060866225806	0.814909776039437	-4.8930817554514
a_HQ_transcript/4194	1.26763736168302	-0.490030933319471	1.4777823690667	0.149066835087843	0.814909776039437	-4.81977795773816
a_HQ_transcript/147096	2.11106137025462	3.01900527533441	1.47768837304604	0.149091876619388	0.814909776039437	-4.93748493634866
a_HQ_transcript/45808	0.931477571637736	2.32551556414746	1.47767369538626	0.149095787206094	0.814909776039437	-4.93949447061133
a_HQ_transcript/85681	1.31360399474685	1.32297008442479	1.47756092002479	0.149125836815727	0.814909776039437	-4.89588622317286
a_HQ_transcript/24286	-0.378208694854372	2.82410455621711	-1.47753285311457	0.149133316148626	0.814909776039437	-5.01442906036172
a_HQ_transcript/150780	-0.509186727572145	4.74374916607959	-1.47748458076142	0.149146180573875	0.814909776039437	-5.32871402061395
a_HQ_transcript/97092	1.5759730595635	2.55096065840562	1.47743058212482	0.149160572085331	0.814909776039437	-4.85592247824119
a_HQ_transcript/147270	1.79859447658416	0.315437570696267	1.47741546009306	0.149164602550571	0.814909776039437	-4.92034591786913
a_HQ_transcript/36644	-1.90316159893677	2.33112904412425	-1.47736187254423	0.149178885905222	0.814911218860894	-4.90069061492133
a_HQ_transcript/44850	-1.57472077668905	0.048236947643987	-1.47723061365111	0.149213876582401	0.81502576752319	-4.79581948419586
a_HQ_transcript/137227	1.65411042413462	-0.321308505288771	1.47714889823505	0.149235663390129	0.815048446169267	-4.89247596900428
a_HQ_transcript/2761	1.46904395339659	0.523007119938513	1.47710986298544	0.149246071790285	0.815048446169267	-4.79821891144693

a_HQ_transcript/85313	-0.528734311447691	2.30809391209631	-1.47688832295556	0.149305154440407	0.815051392986201	-4.90433769809477
a_HQ_transcript/50413	1.4348971350004	0.286695500081627	1.47688188015523	0.149306872954355	0.815051392986201	-4.74140979385805
a_HQ_transcript/105750	-1.08288953299552	1.36948009865757	-1.47687039876723	0.149309935470141	0.815051392986201	-4.7751291025831
a_HQ_transcript/75306	0.959770643716565	0.38170591366696	1.47681122182581	0.149325720970299	0.815051392986201	-4.78893780108837
a_HQ_transcript/136721	0.599679724109804	2.48490721100857	1.47679354272159	0.149330437145146	0.815051392986201	-4.96559305477354
a_HQ_transcript/6609	1.36787284589289	2.5634964911701	1.47672242819378	0.149349409248402	0.815051392986201	-4.969846385241
a_HQ_transcript/90880	0.582675340963569	2.60349447338783	1.47665084839124	0.149368507422513	0.815051392986201	-4.97456190147134
a_HQ_transcript/146104	2.1716922482175	-0.360623827793128	1.47663810680824	0.149371907202862	0.815051392986201	-4.87991966305203
a_HQ_transcript/91780	1.3872377199896	2.14088339199568	1.47659038340792	0.149384641576304	0.815051392986201	-4.8308368132624
a_HQ_transcript/17092	1.43830873941763	0.254907963158024	1.47658218969616	0.149386828049735	0.815051392986201	-4.81394639137138
a_HQ_transcript/105423	1.33995929865604	-0.386645146590375	1.47650259320686	0.149408069522656	0.815090780404782	-4.91901511855226
a_HQ_transcript/121507	-2.84239306179088	2.37876622754389	-1.47633556214516	0.149452652015214	0.81515118311095	-4.94451307008481
a_HQ_transcript/146158	-2.24571928231188	0.249585168494023	-1.47631414943981	0.149458368078227	0.81515118311095	-4.87596566531044
a_HQ_transcript/59488	0.923584158385408	3.88947115341386	1.47627581315322	0.149468602280922	0.81515118311095	-5.24499157880602
a_HQ_transcript/12625	-2.37641996040398	0.27791738526289	-1.47625096873372	0.149475235012036	0.81515118311095	-4.83261740562143
a_HQ_transcript/110416	3.03237954915312	1.48746239218885	1.47608927409943	0.149518408477978	0.81531013583564	-4.75819922973919
a_HQ_transcript/148698	-2.34237271930914	2.42616528254522	-1.47596263346402	0.149552229262209	0.815418064211019	-4.97921705290091
a_HQ_transcript/146746	-1.8440043803458	-0.124761115864323	-1.47587049483727	0.149576839741422	0.815474587123469	-4.91457016143521
a_HQ_transcript/111771	0.601663630397414	3.92151285956984	1.47581878209609	0.149590653772666	0.815474587123469	-5.21667468088714
a_HQ_transcript/8586	-2.6281725066197	2.099048323154	-1.47571205926483	0.149619165876629	0.815511153541581	-5.18158156930939
a_HQ_transcript/61012	-1.0556687707039	2.51750829442361	-1.47568864931243	0.149625420666821	0.815511153541581	-4.88986830457428
a_HQ_transcript/99090	1.72009440843196	0.341932084737567	1.47553267867644	0.149667099024008	0.815638226240797	-4.8854925893023
a_HQ_transcript/51922	1.86635981076437	0.836326064941326	1.47549638479561	0.149676798777019	0.815638226240797	-4.73586640054891
a_HQ_transcript/147784	3.91781756755076	8.17867936642705	1.47528078025289	0.149734430704989	0.815700935416997	-5.64756463663691
a_HQ_transcript/144664	1.44632065697121	0.31099853193086	1.47525627472608	0.149740982248092	0.815700935416997	-4.84829830925651
a_HQ_transcript/95751	1.44372714959653	-0.376045316753164	1.47519403491282	0.149757623066774	0.815700935416997	-4.84230354447741
a_HQ_transcript/143781	1.37590698110614	-0.648846468799546	1.47518818271304	0.149759187822774	0.815700935416997	-4.8866738365589
a_HQ_transcript/90504	1.25216761738089	1.68212224797066	1.47505432463886	0.149794982239804	0.815700935416997	-4.86197446887242
a_HQ_transcript/12911	1.83424305287363	2.47423991491149	1.47504347888404	0.149797882755937	0.815700935416997	-4.89381095379582
a_HQ_transcript/150391	1.85511239528738	-0.0174116029314621	1.47502832170196	0.149801936366451	0.815700935416997	-4.87276232565405
a_HQ_transcript/92377	-1.01409320643698	2.1934453087431	-1.47494678066854	0.149823745061815	0.815700935416997	-4.89402019260581
a_HQ_transcript/22315	1.63094841947472	1.08580542225944	1.47492157550061	0.149830486865511	0.815700935416997	-4.7776128679669
a_HQ_transcript/34123	1.43173698964527	0.187324944664651	1.47485892033817	0.149847246732045	0.815700935416997	-4.77943802767909
a_HQ_transcript/80419	-3.69299811093083	2.28190808104558	-1.47482191781393	0.149857145380138	0.815700935416997	-5.0551821475899
a_HQ_transcript/72043	2.50956455790052	3.53740196043236	1.47479612154768	0.149864046519785	0.815700935416997	-4.92710181547297
a_HQ_transcript/18572	1.43641031718942	-0.567751791208594	1.4747711271345	0.149870733385864	0.815700935416997	-4.88895624163961
a_HQ_transcript/113313	1.56927095367481	0.955592381275637	1.47459014936682	0.149919158275372	0.815792980922529	-4.72157376066498
a_HQ_transcript/128429	2.61737043924254	4.76402907584414	1.47445654944948	0.149954914110575	0.815792980922529	-5.14370848817453
a_HQ_transcript/142378	3.27902145497534	4.76666638906666	1.47442362733525	0.149963726221812	0.815792980922529	-5.23002388054702
a_HQ_transcript/93740	-0.516586706154194	3.73259049676377	-1.47437885621365	0.149975710565697	0.815792980922529	-5.23609141206963
a_HQ_transcript/142095	-1.82662364872214	2.92101068871486	-1.47437605999026	0.149976459084766	0.815792980922529	-5.19970126001731
a_HQ_transcript/124230	1.80916593705014	-0.20121733470394	1.4743338342954	0.149987762813716	0.815792980922529	-4.82278442667371
a_HQ_transcript/38213	-1.79087123483092	0.35863045631877	-1.47425115243043	0.150009898542893	0.815792980922529	-4.86345752238966
a_HQ_transcript/53006	-1.10082896682211	1.11299955604119	-1.47424880766214	0.150010526326255	0.815792980922529	-4.71587982240235
a_HQ_transcript/92690	1.57031660680577	1.28170668024151	1.4742360019209	0.150013954945653	0.815792980922529	-4.73595729054638
a_HQ_transcript/101790	1.65045677535725	1.0714074842865	1.47411149099659	0.150047294860596	0.815897956562839	-4.79438399068264
a_HQ_transcript/8257	-1.48334998133932	0.581954547675837	-1.47397885738272	0.150082816268226	0.816014773551532	-4.82484895617887
a_HQ_transcript/130936	-1.86721987146602	0.784097713401682	-1.47378341935115	0.150135169935368	0.816060316715518	-4.72576560497912
a_HQ_transcript/134024	0.806898144993368	2.93551809655586	1.47378319598512	0.150135229778685	0.816060316715518	-5.0334240606481
a_HQ_transcript/122304	-1.12456100952225	-0.534599711709012	-1.47374732093951	0.150144841521886	0.816060316715518	-4.91875914707389

a_HQ_transcript/1167	0.955653336587095	-0.353025762489197	1.47373796327267	0.150147348735075	0.816060316715518	-4.84185989383484
a_HQ_transcript/63796	1.22603343537783	1.26708812719221	1.47365992780452	0.150168258191805	0.816097654466459	-4.71488186175715
a_HQ_transcript/99477	1.83854039443301	2.60570011206745	1.47355535675021	0.150196281452059	0.816143119612297	-4.9400182275558
a_HQ_transcript/115750	1.76819989772882	1.54359125822496	1.47352269087108	0.150205036206264	0.816143119612297	-4.89925449116337
a_HQ_transcript/7856	0.639238888096412	3.77425663517049	1.47347154169549	0.150218745470891	0.816143119612297	-5.19788688951228
a_HQ_transcript/125200	2.16235559757505	-0.235761606167579	1.47331713514474	0.150260136363861	0.816291701547525	-4.88178701817738
a_HQ_transcript/148581	-1.1598804867077	1.857587302229	-1.47318111913238	0.150296604947579	0.816407031989923	-4.91533038985522
a_HQ_transcript/1802	-1.58933034502943	-0.351358574875878	-1.47313319527079	0.150309455965389	0.816407031989923	-4.80448061848512
a_HQ_transcript/150988	-5.31591694791348	2.35774160278195	-1.47297320291663	0.150352365046969	0.816563792903881	-4.98006602743557
a_HQ_transcript/152331	4.01463489120267	5.68643684058132	1.47276302515051	0.150408748436069	0.816690356516582	-5.52390823367491
a_HQ_transcript/19767	1.99989081504573	0.114883307550749	1.47274203900263	0.150414379216287	0.816690356516582	-4.79501636447505
a_HQ_transcript/10642	-3.30107935412078	2.96777090012704	-1.47272522535742	0.150418890596268	0.816690356516582	-5.02616907881615
a_HQ_transcript/1918	1.37370721745125	3.06772428562491	1.47264999346033	0.150439077884714	0.816690356516582	-4.9269773411702
a_HQ_transcript/137401	-2.5379175684007	2.72851977102274	-1.47256224055227	0.150462627719225	0.816690356516582	-5.0131910050009
a_HQ_transcript/112267	0.613510064907469	3.52727475093699	1.47250392929181	0.150478278063918	0.816690356516582	-5.19823565317126
a_HQ_transcript/104721	-2.27712134915177	2.57408077948333	-1.47238827243182	0.150509323419	0.816690356516582	-5.13336123965475
a_HQ_transcript/55408	-1.49203657137285	0.152551052695155	-1.47237479462834	0.150512941549727	0.816690356516582	-4.89262467876204
a_HQ_transcript/67488	-4.15757216604781	2.85657947214823	-1.47235481331242	0.150518305682995	0.816690356516582	-4.97005260268532
a_HQ_transcript/72437	-2.24526246091027	2.27249279940005	-1.4723225739083	0.150526960913009	0.816690356516582	-5.05759133375754
a_HQ_transcript/137921	1.06389118303676	-0.740626481426761	1.47231044420421	0.150530217446302	0.816690356516582	-4.90078347219325
a_HQ_transcript/12121	1.47032299100115	0.220419582989076	1.47222129901842	0.150554152505233	0.81674398290473	-4.78384564049933
a_HQ_transcript/135510	1.83071371431196	0.977607435966113	1.47211216259124	0.150583459251067	0.816826737527622	-4.9270158929691
a_HQ_transcript/1374	-0.850992672657484	2.71326378750447	-1.47189810463873	0.150640954118433	0.817062366426822	-4.97832544944185
a_HQ_transcript/94142	0.816617538076461	-0.812027653014932	1.47171507922428	0.150690127691249	0.817159214211117	-4.91081212074892
a_HQ_transcript/102884	2.6741791521871	1.19619311201806	1.4716297593574	0.150713055017735	0.817159214211117	-4.86416292494595
a_HQ_transcript/126256	1.95062814596892	0.708751966019498	1.47161522356928	0.150716961381382	0.817159214211117	-4.81700039355759
a_HQ_transcript/81945	0.811203270551789	1.67525692114029	1.4715915427637	0.150723325560012	0.817159214211117	-4.85768306853064
a_HQ_transcript/133088	1.03365761833289	-0.692626813985528	1.47139771754722	0.150775423834412	0.817159214211117	-4.89956208455792
a_HQ_transcript/140980	-1.83431409206333	0.593394899554883	-1.4713102321353	0.15079894373985	0.817159214211117	-4.79062020214066
a_HQ_transcript/106962	3.70028784403654	1.72310563070212	1.47128457165209	0.150805842955156	0.817159214211117	-5.00312643798846
a_HQ_transcript/39921	1.70959303069953	1.59255267991155	1.47123647414433	0.150818775387531	0.817159214211117	-4.99426299418307
a_HQ_transcript/145181	1.35444625607753	-0.363911454725055	1.47120851428211	0.150826293626989	0.817159214211117	-4.95030535238961
a_HQ_transcript/109885	-1.83824809137049	1.54395052632942	-1.47120571846446	0.150827045422045	0.817159214211117	-4.84717379906004
a_HQ_transcript/117630	-1.1069017593917	0.742022819801113	-1.47106792092497	0.150864102869019	0.817159214211117	-4.72414046133063
a_HQ_transcript/87403	1.30169811221953	-0.507955899036149	1.47106627406687	0.150864545797151	0.817159214211117	-4.89714503574755
a_HQ_transcript/28332	-1.33342938326394	-0.451828194221506	-1.47097176192136	0.150889966900433	0.817159214211117	-4.84265836773089
a_HQ_transcript/41757	-1.74015212084084	0.578887041887552	-1.47092955787945	0.150901319701449	0.817159214211117	-4.85234846448692
a_HQ_transcript/114877	1.16522525629891	0.198643901324474	1.47092537006088	0.150902446253235	0.817159214211117	-4.80695177203144
a_HQ_transcript/123932	-3.11543022714124	1.8393908477401	-1.47089205927081	0.150911407322039	0.817159214211117	-5.08950446273032
a_HQ_transcript/20862	-0.967133318818997	2.50165757160459	-1.47086742936985	0.15091803338305	0.817159214211117	-4.91418582403607
a_HQ_transcript/83615	-2.01014214109794	1.26857057004334	-1.47084049054003	0.150925280869482	0.817159214211117	-4.81367472902033
a_HQ_transcript/25331	1.36350764220879	1.69674853983089	1.47079704912353	0.150936968712996	0.817159214211117	-4.88252080615239
a_HQ_transcript/18801	0.971533922800455	-0.323085689310676	1.47075382866168	0.150948597825322	0.817159214211117	-4.83984339096814
a_HQ_transcript/128954	0.797912261682949	1.45668256366089	1.47072487943371	0.150956387448077	0.817159214211117	-4.80234413571437
a_HQ_transcript/77551	-1.12546017436524	0.581697803152543	-1.47068141185738	0.150968084252223	0.817159214211117	-4.73314367059393
a_HQ_transcript/64052	1.54530829444729	1.93315614172111	1.47058486355473	0.150994067273796	0.817223756284097	-5.01178908039484
a_HQ_transcript/48201	1.32032670791664	0.392705067265923	1.47047460536899	0.151023744252393	0.817308277246356	-4.80492102760818
a_HQ_transcript/147148	3.45959402494716	0.826099930235484	1.47032450937337	0.151064151409856	0.81745084650457	-4.9467774134913
a_HQ_transcript/109115	1.81269302061467	0.130556813652214	1.47023325130024	0.151088723096679	0.817507706987224	-4.84433052415041
a_HQ_transcript/128567	-1.63558824811811	-0.345936466655986	-1.47017062015338	0.15110558868631	0.817511084162403	-4.84422432513031

a_HQ_transcript/110983	-1.61242533581527	0.67311820838485	-1.47012648232626	0.151117475215473	0.817511084162403	-4.82957061266705
a_HQ_transcript/151545	2.34494135656336	0.0920365433374466	1.47003970979525	0.151140845649091	0.817512700145866	-4.9410326879988
a_HQ_transcript/35041	1.22605751854607	0.851577666015709	1.47002093757161	0.15114590194859	0.817512700145866	-4.75695058041506
a_HQ_transcript/80986	-1.32305269253291	1.88543702522157	-1.46993902034661	0.151167967936205	0.817555976944151	-4.78577304493826
a_HQ_transcript/91868	-1.68860741873847	1.09914873536218	-1.46978148905671	0.151210409252436	0.817590335995446	-4.75145989431666
a_HQ_transcript/53947	-1.29199689456018	-0.306410553052956	-1.46976119053984	0.151215878671293	0.817590335995446	-4.89608945569544
a_HQ_transcript/696	1.24055562462377	1.37652048155235	1.4697588214299	0.151216517036317	0.817590335995446	-4.78506478803904
a_HQ_transcript/31434	1.2145043303236	0.688704871776833	1.46963019211374	0.151251179884299	0.817701691249492	-4.78364495103862
a_HQ_transcript/137943	2.63194183312679	2.9921081101432	1.46948278932458	0.151290909570215	0.817840415837204	-4.95036270586081
a_HQ_transcript/136268	-2.7868590575399	1.256767431641	-1.46940616514308	0.15131156548245	0.817876016213862	-4.96218012318446
a_HQ_transcript/102576	1.86633049897993	-0.052865565828791	1.46928795500742	0.151343436309979	0.81795606084438	-4.85620488322703
a_HQ_transcript/5026	1.68834971450982	-0.10583740705297	1.46919561763962	0.151368335260946	0.81795606084438	-4.81798161675149
a_HQ_transcript/134245	1.81500535460594	-0.207102991418372	1.46914315320666	0.151382483852169	0.81795606084438	-4.89193203782865
a_HQ_transcript/49590	-1.75531241750905	0.503112650195087	-1.46913232685795	0.151385403629587	0.81795606084438	-4.84204660575139
a_HQ_transcript/112555	1.41541185292793	0.421049123760312	1.4690903222227	0.151396732360045	0.81795606084438	-4.81582269042113
a_HQ_transcript/70260	1.31068793483108	1.97294170000004	1.46895861822773	0.151432257560859	0.818071957945877	-4.86184152534352
a_HQ_transcript/143514	-2.47407501941362	1.37246839193102	-1.46878673081085	0.151478631639097	0.818246436197141	-4.93723048056048
a_HQ_transcript/11107	2.3143911303856	1.4927553733822	1.46855426024249	0.151541368654396	0.818252008046328	-4.87909051710969
a_HQ_transcript/145565	2.00452391963569	0.649270779707078	1.4685481328792	0.151543022530841	0.818252008046328	-4.77348326757715
a_HQ_transcript/139203	3.76074515461625	1.55634245722803	1.46848127405458	0.151561069765636	0.818252008046328	-5.05238865668937
a_HQ_transcript/93210	3.77223425919059	1.73392730521228	1.46846032705525	0.151566724351684	0.818252008046328	-4.84441088034871
a_HQ_transcript/93020	-2.22603384022803	2.68804416474813	-1.46844922416544	0.151569721615066	0.818252008046328	-5.00212374403213
a_HQ_transcript/81369	3.38952319527001	1.09390866909892	1.46843578763741	0.151573348915311	0.818252008046328	-4.96087986493531
a_HQ_transcript/21222	1.85812490694719	1.30338645163531	1.46834571170719	0.15159766743339	0.818252008046328	-4.73231546735913
a_HQ_transcript/2623	-1.56115816423742	1.68941067034286	-1.46833857114623	0.151599595360669	0.818252008046328	-4.81418261156048
a_HQ_transcript/48606	1.1594054625673	2.42978219811497	1.46831354059477	0.151606353679104	0.818252008046328	-4.90211121641631
a_HQ_transcript/106426	-2.46163949163412	2.37957606253978	-1.46825909880338	0.151621053943159	0.818255373095158	-4.88893201579159
a_HQ_transcript/558	1.76888388107234	0.553037908555455	1.46819213713838	0.151639136356474	0.818274872677857	-4.93448848639741
a_HQ_transcript/70679	1.16235815148887	0.268014959006881	1.46807457384335	0.151670887456055	0.818274872677857	-4.83112741346368
a_HQ_transcript/74921	-3.34079681026993	3.0509083424848	-1.46804618279701	0.151678556008646	0.818274872677857	-5.30312160839292
a_HQ_transcript/93648	-1.10173235582242	2.52276174577923	-1.4679759668837	0.151697522977888	0.818274872677857	-5.04694354236457
a_HQ_transcript/13340	1.72562928539125	0.277361108796127	1.46794381398186	0.151706208863637	0.818274872677857	-4.88790199655074
a_HQ_transcript/73531	1.74286778867258	-0.431481856875313	1.46793300138922	0.15170912990038	0.818274872677857	-4.90414852619414
a_HQ_transcript/29610	-1.39305447901873	1.52399752122451	-1.46783175753471	0.151736483241447	0.818323842584353	-4.88939900208573
a_HQ_transcript/72286	-1.3858595317342	0.151063824390701	-1.46774105839109	0.151760991025338	0.818323842584353	-4.79469232864635
a_HQ_transcript/71776	0.446150262312583	3.72987917481157	1.46770751635153	0.151770055207079	0.818323842584353	-5.20252813187612
a_HQ_transcript/442	-1.76621361283209	2.80608330953152	-1.46769099187754	0.151774520831646	0.818323842584353	-4.9842630020265
a_HQ_transcript/60600	1.51741389242914	-0.24895356993036	1.4674957423169	0.151827293600371	0.818426802800508	-4.89630949254747
a_HQ_transcript/107060	1.26548633023026	1.45978753881449	1.46723257528245	0.151898446488064	0.818426802800508	-4.82542950262319
a_HQ_transcript/10870	-0.562856902649834	2.96652792106643	-1.46719851632031	0.151907657005113	0.818426802800508	-5.09498197040105
a_HQ_transcript/96582	1.61148195747052	0.507273951296932	1.4671947045722	0.15190868783846	0.818426802800508	-4.94023525321435
a_HQ_transcript/53720	1.1291460323965	1.0195595202325	1.4671897085212	0.15191003895821	0.818426802800508	-4.78256387074786
a_HQ_transcript/67206	2.00697902642298	0.426922166563166	1.46713368939793	0.151925189287804	0.818426802800508	-4.89489178546007
a_HQ_transcript/114015	-1.19550644301055	4.85799709386525	-1.46713045470045	0.151926064146059	0.818426802800508	-5.43274601381473
a_HQ_transcript/64354	1.37186047420956	0.370533778235491	1.46711986924323	0.151928927123346	0.818426802800508	-4.86072962518412
a_HQ_transcript/116180	1.63291909155151	3.71493367980062	1.46707391870447	0.151941355554271	0.818426802800508	-5.15444979747801
a_HQ_transcript/76546	-1.66273759204965	1.26786160911847	-1.46694821605589	0.151975359005065	0.818426802800508	-4.96584118158221
a_HQ_transcript/70376	1.87464362092274	0.400830362415023	1.46692250540253	0.151982314664512	0.818426802800508	-4.87723185870083
a_HQ_transcript/123792	1.67083070275307	1.95192170053307	1.46691342075083	0.151984772451241	0.818426802800508	-4.87490818508589
a_HQ_transcript/1823	0.505345091270222	4.20473721963554	1.46677192356285	0.152023057578721	0.818426802800508	-5.32501245948538

a_HQ_transcript/56662	1.52852195158355	0.41176483944674	1.46676132536045	0.152025925461099	0.818426802800508	-4.82757590306108
a_HQ_transcript/142577	-2.33612179802974	0.439514950652884	-1.46675471218588	0.152027715013476	0.818426802800508	-4.92233627072894
a_HQ_transcript/50698	4.99966517458718	1.43290548008719	1.46674922230195	0.152029200611511	0.818426802800508	-5.01628430095843
a_HQ_transcript/109829	1.2437871346837	0.957249406720603	1.46663066006336	0.152061287140953	0.818426802800508	-4.74076566520837
a_HQ_transcript/70074	-0.61268478020785	2.5190826378703	-1.46658250438326	0.15207432106578	0.818426802800508	-4.89785488175667
a_HQ_transcript/142115	2.13096038397245	2.24488142732598	1.46657987690814	0.152075032249719	0.818426802800508	-4.82202439288951
a_HQ_transcript/94714	1.77672200179475	-0.4481920239467	1.46657683196765	0.152075856433156	0.818426802800508	-4.88471898161813
a_HQ_transcript/2004	2.08958054162848	1.08518371601392	1.46652719884414	0.15208929128563	0.818426802800508	-4.8482089187498
a_HQ_transcript/5354	1.90552617970609	-0.113672444435947	1.46644282292512	0.15211213259903	0.81847394647009	-4.92858262879558
a_HQ_transcript/97835	0.89876170023352	2.15213673584269	1.46629810727462	0.152151314778708	0.818542323036461	-4.86705418107595
a_HQ_transcript/136598	0.705927520159024	4.31526212580212	1.46629186998724	0.152153003722973	0.818542323036461	-5.27251765246874
a_HQ_transcript/125383	-1.24789857502655	1.33280088030089	-1.46622693365319	0.152170588191323	0.818550573276813	-4.83503168031454
a_HQ_transcript/122272	0.589876163448564	5.31910377034489	1.46618220656195	0.152182701028807	0.818550573276813	-5.38393915656085
a_HQ_transcript/147702	1.27632197018119	-0.216569611951782	1.46600460946673	0.15223080484515	0.81873355144697	-4.86732317342169
a_HQ_transcript/113897	2.7847379536395	2.84858307314586	1.46582139333432	0.152280443321993	0.818924748766842	-5.2713793666741
a_HQ_transcript/109135	-1.55839500508619	0.610579200239717	-1.46558865960195	0.152343516118604	0.819040894719317	-4.84192157331665
a_HQ_transcript/78344	1.22575609696774	0.20308047505256	1.46557050366702	0.152348437404312	0.819040894719317	-4.84085398992575
a_HQ_transcript/112336	0.631057441192697	2.79600094975759	1.46556112538149	0.152350979499949	0.819040894719317	-5.0214169693888
a_HQ_transcript/90287	-0.482031841758827	2.84944070476825	-1.46549476844824	0.15236896729979	0.819040894719317	-4.99431188718223
a_HQ_transcript/123250	2.25956782642776	0.721976058503031	1.46546369765671	0.152377390440136	0.819040894719317	-4.86321980424196
a_HQ_transcript/57545	2.14777105107469	1.32019839755184	1.4653705954659	0.152402632211533	0.819040894719317	-5.00843378557209
a_HQ_transcript/112563	2.41148020847171	1.73968780032559	1.46532919443153	0.152413857887602	0.819040894719317	-5.05137713702229
a_HQ_transcript/122952	1.41545898005802	-0.277380738295974	1.46532585550216	0.152414763249705	0.819040894719317	-4.88435090145336
a_HQ_transcript/142610	-1.46570764998826	-0.417867086433815	-1.46525054901128	0.152435183999705	0.819074909921876	-4.80004081963749
a_HQ_transcript/94727	-3.05320400055631	3.20102432602062	-1.46518407800886	0.152453210653844	0.819096055909663	-5.30817603046575
a_HQ_transcript/42321	-2.89752261361813	3.04245016474318	-1.46508996326629	0.152478737076859	0.819102638082939	-5.16334294404341
a_HQ_transcript/83816	2.68050651060787	1.93146707797796	1.46507565383092	0.152482618474668	0.819102638082939	-4.85912490802229
a_HQ_transcript/41023	-2.09730899991479	1.7192296926777	-1.46499178836117	0.15250536835125	0.819149145400248	-4.99485239453637
a_HQ_transcript/122306	0.369246671198904	4.43033733635885	1.46484808127705	0.152544357526116	0.819171483683268	-5.30587729362276
a_HQ_transcript/113001	1.77620758132417	1.00855828816017	1.46482730180164	0.152549995863272	0.819171483683268	-4.94456512556741
a_HQ_transcript/38357	-1.02740155599525	4.30164718240174	-1.46482063520024	0.152551804825065	0.819171483683268	-5.24056614964182
a_HQ_transcript/141838	-1.99213075929897	1.67144839072512	-1.46475241014816	0.152570318465138	0.819195221849392	-4.88652411454417
a_HQ_transcript/45030	1.42728452343371	-0.0840110041715034	1.46467560902077	0.152591161461001	0.819202094806831	-4.91759862309296
a_HQ_transcript/63355	-0.773631884791237	1.95499371581105	-1.46464068625178	0.152600639872961	0.819202094806831	-4.84735314670382
a_HQ_transcript/54907	-0.655201336416902	2.3239919330717	-1.4645919148743	0.152613877729548	0.819202094806831	-4.85892070554966
a_HQ_transcript/94321	1.91820394514435	-0.0450545309639017	1.46449204059688	0.152640989133984	0.81927196827149	-4.82875157497247
a_HQ_transcript/144312	-1.41760933158654	-0.604455172716741	-1.46420644157645	0.152718537673193	0.819566722297955	-4.87895639863498
a_HQ_transcript/42063	1.21070919579293	0.212232064693245	1.46418594493686	0.152724104320318	0.819566722297955	-4.76735461801006
a_HQ_transcript/129544	0.326449093044466	3.71724225668883	1.46377015650097	0.152837062471331	0.820097181513295	-5.24654054061888
a_HQ_transcript/120977	2.0445217888838	2.44810736242381	1.46359253350194	0.152885337971602	0.820273135698707	-4.89581751231537
a_HQ_transcript/101273	-1.38158178925696	2.70005658247475	-1.46339216636643	0.152939809586915	0.820273135698707	-5.18116978208145
a_HQ_transcript/22628	1.0070679177087	2.21437436380996	1.46338307525186	0.152942281454877	0.820273135698707	-4.90109941549999
a_HQ_transcript/72635	1.75695789792198	0.236921244140381	1.46336511617419	0.152947164609538	0.820273135698707	-4.8368833301685
a_HQ_transcript/95178	1.95795732189444	-0.36220254880167	1.46329153440826	0.152967173121435	0.820273135698707	-4.84312242390118
a_HQ_transcript/48486	2.68945106771302	4.00823074495096	1.46327640828157	0.152971286508496	0.820273135698707	-5.39171098667547
a_HQ_transcript/59960	1.32378107656647	-0.385190587210363	1.46326683492762	0.15297388992438	0.820273135698707	-4.83798683206239
a_HQ_transcript/147833	0.70980752486705	-0.801193714903051	1.46316840818321	0.153000658529636	0.820273135698707	-4.92332710189996
a_HQ_transcript/95045	1.19686925998053	3.3678467213227	1.46313064349199	0.153010930184325	0.820273135698707	-5.19265896990273
a_HQ_transcript/120403	2.25087967053635	0.432242290016894	1.46313050051132	0.153010969074819	0.820273135698707	-4.92166544268126
a_HQ_transcript/129246	1.37626048246333	-0.596388984993434	1.46288143912513	0.153078725283275	0.820399489873208	-4.91328684089128

a_HQ_transcript/98932	1.9732588529046	2.56815566438678	1.46287763376945	0.153079760701109	0.820399489873208	-4.96726539288013
a_HQ_transcript/146171	-2.17571317904911	5.31696791083508	-1.4628297235345	0.153092797308131	0.820399489873208	-5.49653910822755
a_HQ_transcript/45166	0.92433233706515	2.51350571627733	1.46265716193506	0.153139759479018	0.820399489873208	-5.07828340600934
a_HQ_transcript/95170	1.45834753379889	2.61385601802962	1.4626483171302	0.153142166877541	0.820399489873208	-5.02966589480282
a_HQ_transcript/84559	-3.99287084083974	4.284264158463	-1.46263368270646	0.153146150173473	0.820399489873208	-5.13481148611009
a_HQ_transcript/142716	2.18229377372675	1.16621363679276	1.46258667557684	0.153158945448199	0.820399489873208	-5.01139793702528
a_HQ_transcript/123446	-0.848187363574517	4.09603070445834	-1.4625854041735	0.153159291534223	0.820399489873208	-5.30075075046549
a_HQ_transcript/58221	1.17865003262049	-0.645355624894351	1.46257706405236	0.15316156179645	0.820399489873208	-4.85299058991896
a_HQ_transcript/14121	-1.74952749525296	0.901483590515258	-1.46240376322949	0.153208742029824	0.820515673982308	-4.828874527114
a_HQ_transcript/124095	-1.43666436797525	-0.508327345969579	-1.46237822465833	0.153215695748694	0.820515673982308	-4.89053614228375
a_HQ_transcript/98126	-0.372789116411897	4.33481528705301	-1.46230722496891	0.153235029077301	0.820515673982308	-5.32571595882215
a_HQ_transcript/81219	-2.65093825581458	1.40829975234695	-1.46227454180095	0.153243929408614	0.820515673982308	-5.01069592455349
a_HQ_transcript/148223	-3.36655092137789	0.940974610773069	-1.4622381846587	0.153253830725742	0.820515673982308	-4.96393527548426
a_HQ_transcript/58373	1.19190851891199	-0.487710211584345	1.4620338407253	0.153309490174631	0.820670162794033	-4.96639222191098
a_HQ_transcript/120923	1.59032192764776	-0.282469886358458	1.46202858330713	0.153310922408829	0.820670162794033	-4.85251442888027
a_HQ_transcript/137213	1.31651198273818	3.15018220477848	1.46196723755398	0.153327635103252	0.820684049105142	-5.04751504699437
a_HQ_transcript/72770	1.12966059469887	-0.252783670778946	1.46182596431591	0.153366128309443	0.820724999726075	-4.99219932056842
a_HQ_transcript/95493	1.41739331104446	2.56392797978562	1.46175279076478	0.153386069182194	0.820724999726075	-4.95243156981192
a_HQ_transcript/90754	-0.733668735847163	2.90435763936031	-1.46174715608186	0.153387604802013	0.820724999726075	-5.12246699800563
a_HQ_transcript/111743	-2.43525422401215	0.302542348149135	-1.46173189870829	0.153391762954584	0.820724999726075	-4.80143953760058
a_HQ_transcript/148534	3.59657115616675	4.2689273733595	1.46161089950762	0.153424742523558	0.82080975647848	-5.19946985006575
a_HQ_transcript/15286	1.69709426743924	0.440481495934482	1.46154101766816	0.153443792103457	0.82080975647848	-4.9055928109852
a_HQ_transcript/33560	1.23110741671137	0.421805105771076	1.46151837071027	0.153449966000063	0.82080975647848	-4.90562731319265
a_HQ_transcript/106199	0.685342455747032	-0.735241072565648	1.46137725121354	0.153488441721459	0.820940020278336	-4.96126566931961
a_HQ_transcript/62960	-4.37856434331809	3.46232838980306	-1.46124038247734	0.15352576582105	0.82106410117269	-5.08179847801372
a_HQ_transcript/123581	1.88742511563656	0.932407898340952	1.4610946213777	0.153565522808255	0.821132784346434	-4.82789723728601
a_HQ_transcript/29282	0.847146841807123	2.1602464456326	1.46108971553297	0.153566861041668	0.821132784346434	-4.91767710834082
a_HQ_transcript/151547	0.81688124345599	1.66480864549526	1.46093199201902	0.153609890356004	0.821246408218813	-4.86651005662874
a_HQ_transcript/105398	-1.66869109233666	0.579906098373225	-1.46081850073876	0.153640858395032	0.821246408218813	-4.86368079118079
a_HQ_transcript/11114	2.0848259005388	1.30316185978487	1.46071202479045	0.153669916696477	0.821246408218813	-4.74075179919906
a_HQ_transcript/81588	-2.65669364708907	2.58465482405438	-1.46066088339132	0.1536838752262	0.821246408218813	-4.92024842124399
a_HQ_transcript/28569	1.31299165294122	-0.0939192442589915	1.46061509267596	0.153696374196607	0.821246408218813	-4.9534763056761
a_HQ_transcript/123728	2.02744645839409	2.79705235003657	1.46056225378056	0.153710798033498	0.821246408218813	-5.00379989297472
a_HQ_transcript/1227	1.05910410511563	3.1913233756136	1.46049520541829	0.15372910228729	0.821246408218813	-5.1002013713881
a_HQ_transcript/35583	2.31120954642743	0.504299854165748	1.4604913937468	0.153730142928591	0.821246408218813	-4.81945411858304
a_HQ_transcript/1297	-2.87202152233544	1.83481837109396	-1.46048966976266	0.153730613602969	0.821246408218813	-5.00005022605577
a_HQ_transcript/37671	-1.8445428231	1.54184102201246	-1.46046801856966	0.153736524811699	0.821246408218813	-4.86984998842256
a_HQ_transcript/3648	1.04295481122072	3.01772978982656	1.46044239262962	0.153743521439532	0.821246408218813	-4.99349442592108
a_HQ_transcript/144086	-1.87170258128205	0.141078343875732	-1.46036434945583	0.15376483105898	0.821247182501959	-4.91460509961288
a_HQ_transcript/57450	-1.39819393934654	-0.283343355404473	-1.46028359163695	0.153786884383469	0.821247182501959	-4.8449295282536
a_HQ_transcript/116540	0.979098712996149	2.12896993742476	1.46019456727599	0.15381119804871	0.821247182501959	-4.85761470694899
a_HQ_transcript/53259	1.54389469538829	0.943037297133805	1.46016907452838	0.153818160999747	0.821247182501959	-4.79858612314698
a_HQ_transcript/139554	2.31387241698995	2.08881539847382	1.46015744772837	0.153821336764197	0.821247182501959	-4.8564886740401
a_HQ_transcript/145588	1.56155344782917	0.951490838808311	1.45991903857836	0.153886467756122	0.821247182501959	-4.93788119923736
a_HQ_transcript/144854	1.85171742848784	0.285905730309913	1.45990649427789	0.153889895340749	0.821247182501959	-4.98852839107242
a_HQ_transcript/63990	-1.14862892345262	3.20687018052841	-1.45987689710349	0.153897982666088	0.821247182501959	-5.10685973303202
a_HQ_transcript/114769	1.63581680615865	0.494941584568518	1.45985917958342	0.153902824078803	0.821247182501959	-4.80623596532477
a_HQ_transcript/110430	-0.639780993328868	4.06480795310766	-1.45982899209249	0.153911073260739	0.821247182501959	-5.26964876540036
a_HQ_transcript/13528	1.35622382143102	0.971501752430996	1.45982341592763	0.153912597069398	0.821247182501959	-4.71535520902311
a_HQ_transcript/26267	-1.76761858200542	0.428264590361453	-1.45982118967003	0.153913205446194	0.821247182501959	-4.89130957871994

a_HQ_transcript/104544	-3.25646599561066	1.89563755844786	-1.4597385709326	0.153935784295124	0.821292268885451	-4.91109666350042
a_HQ_transcript/44145	-0.459162791695403	3.18531420170209	-1.45957537607379	0.153980391508751	0.821335986849992	-5.06381750041846
a_HQ_transcript/130757	2.45285628509487	0.214453705134202	1.45957121274319	0.153981529636045	0.821335986849992	-4.90659189892417
a_HQ_transcript/123979	1.98078197831352	2.88267459687034	1.45954710950559	0.153988118855755	0.821335986849992	-5.06401766907715
a_HQ_transcript/23099	-0.533924917427136	2.24311658084314	-1.45950183075787	0.154000497534373	0.821335986849992	-4.93705646425903
a_HQ_transcript/152268	1.5886251589524	-0.428527099163299	1.45940420200575	0.154027190783247	0.821390481866368	-4.89216022023365
a_HQ_transcript/142361	-0.985409420213456	1.40729636769863	-1.45930731054322	0.154053686083602	0.821390481866368	-4.75102493457405
a_HQ_transcript/35913	0.996271979638097	1.62797553952287	1.45927993149438	0.15406117363455	0.821390481866368	-4.86771746268252
a_HQ_transcript/15539	-4.18997610560619	3.94840483065878	-1.45925775641643	0.154067238229236	0.821390481866368	-5.0451683871965
a_HQ_transcript/654	1.04976112114979	3.48911119356004	1.4590491152367	0.154124308169061	0.821604578529457	-5.2007009329239
a_HQ_transcript/24747	1.54689086812456	0.367046462284817	1.45900760368376	0.154135664892371	0.821604578529457	-4.78405112987289
a_HQ_transcript/19185	1.69369312586882	-0.383134557634939	1.45870706254895	0.154217906734544	0.821885042125934	-4.90600944364675
a_HQ_transcript/121732	-1.23781116318375	0.506726453327629	-1.45868802510465	0.154223117427643	0.821885042125934	-4.88251760612355
a_HQ_transcript/67775	1.95430321258727	1.9605754642665	1.45866032849476	0.154230698450139	0.821885042125934	-4.95777571532399
a_HQ_transcript/98651	2.22645425002926	0.161898114127717	1.45853652627418	0.154264588798503	0.821990284872983	-4.91177931733423
a_HQ_transcript/18343	-0.841513522067554	4.07544376997163	-1.45835357271687	0.154314682423016	0.822077934446969	-5.31127253531959
a_HQ_transcript/140700	-3.47781812423063	2.59033036629348	-1.45831983458804	0.154323921508489	0.822077934446969	-5.11567798905868
a_HQ_transcript/93285	2.11327227152273	1.09729281171228	1.45831728189498	0.154324620573732	0.822077934446969	-4.78482297800039
a_HQ_transcript/29742	-1.60216047860199	0.957001208610526	-1.45826985762725	0.154337608357758	0.822077934446969	-4.89612846319914
a_HQ_transcript/24829	-0.748239314096451	2.02657249865904	-1.45817103089673	0.154364676201512	0.822146774623554	-4.84892803294762
a_HQ_transcript/69923	2.82077449428393	5.26843793509369	1.45793070139058	0.154430516263531	0.822326760592338	-5.24537998865071
a_HQ_transcript/104554	-0.599382351106891	3.51000711697555	-1.45789536762878	0.154440198093625	0.822326760592338	-5.24490878467273
a_HQ_transcript/125251	1.76363968656305	1.29369529085165	1.45789024778419	0.15444160102604	0.822326760592338	-4.85355788687305
a_HQ_transcript/149164	-1.98472714681255	1.43724733141278	-1.45784114241014	0.154455057324304	0.822326760592338	-4.83736399988928
a_HQ_transcript/123926	2.13114072103534	0.253807575977376	1.45765127518083	0.154507095227826	0.822528476179416	-4.84646263693121
a_HQ_transcript/120077	1.86031906811538	1.0162997538876	1.45754003732999	0.154537589241306	0.822548244141465	-4.890702663332
a_HQ_transcript/31238	-1.81911276757113	0.419870338212533	-1.45753449101522	0.154539109796809	0.822548244141465	-4.9021870191614
a_HQ_transcript/139796	3.5383777937971	0.324324034631508	1.45746342206759	0.15455859482827	0.822576634332329	-4.93726102682614
a_HQ_transcript/46731	1.35528105674561	0.170698936109303	1.45730178098979	0.154602919367293	0.822737205619517	-4.85866619786017
a_HQ_transcript/123542	-2.42734825468749	2.84104240593226	-1.45706882152139	0.154666818311778	0.822970875248222	-5.0916217207314
a_HQ_transcript/49429	-1.83935770577987	0.814612440437343	-1.45698783532769	0.154689037102831	0.822970875248222	-4.94573888607132
a_HQ_transcript/66819	0.824641470429838	2.79061478924892	1.45694442693772	0.15470094736162	0.822970875248222	-4.94962405528082
a_HQ_transcript/148858	3.76546489437844	3.58266802683121	1.45686507576514	0.154722721373231	0.822970875248222	-4.98794163115864
a_HQ_transcript/65848	-1.4427824757293	0.992470223244927	-1.45661460410715	0.154791466933724	0.822970875248222	-4.76889920299691
a_HQ_transcript/84564	1.4762153779126	-0.523604834917027	1.45656421224037	0.154805300641677	0.822970875248222	-4.92524729705569
a_HQ_transcript/36960	1.12676568283757	1.65141102781091	1.45655878266118	0.154806791242692	0.822970875248222	-4.88125723613599
a_HQ_transcript/102317	4.51888543661808	1.0866517951355	1.45652303169747	0.154816606362343	0.822970875248222	-5.08602913808404
a_HQ_transcript/143936	-1.93304793591458	0.289451125376854	-1.45650707266443	0.154820987937295	0.822970875248222	-4.89385309895052
a_HQ_transcript/125694	-2.81233469338392	0.468860772144985	-1.45647706044515	0.154829228101233	0.822970875248222	-4.91536865382095
a_HQ_transcript/635	-1.90406957541895	0.264258329896411	-1.45635855890806	0.154861767324179	0.822970875248222	-4.9291262258638
a_HQ_transcript/128126	3.15853418140219	1.73116736957589	1.45632630012793	0.154870626172622	0.822970875248222	-5.02099023230932
a_HQ_transcript/144393	-2.04058781047976	-0.0865007362554283	-1.45630375799307	0.154876816892392	0.822970875248222	-4.95939376207282
a_HQ_transcript/138476	0.745814695714123	2.48679902552323	1.45628168182653	0.154882879834449	0.822970875248222	-4.91779825508266
a_HQ_transcript/26869	1.69596669064327	1.12791114120155	1.45624810443701	0.154892101804674	0.822970875248222	-4.84944616876303
a_HQ_transcript/152542	1.19975329928549	-0.135957256306587	1.45620455852668	0.154904062261322	0.822970875248222	-4.92673469313959
a_HQ_transcript/30240	-1.81484539675489	1.68772634725753	-1.45615392996632	0.154917968983647	0.822970875248222	-4.96552202544538
a_HQ_transcript/37819	0.572042919331771	2.27064615731028	1.45606945797493	0.154941174074717	0.822970875248222	-4.87762903506865
a_HQ_transcript/22574	1.22879109315023	-0.310525855908753	1.45603298421902	0.154951194543487	0.822970875248222	-4.8781961611083
a_HQ_transcript/119287	0.771090648556488	4.50499594123692	1.45594415794672	0.154975600021939	0.822970875248222	-5.35715099791332
a_HQ_transcript/146640	2.05938881112559	-0.220128583521501	1.4559386108346	0.154977124221061	0.822970875248222	-4.89501161787553

a_HQ_transcript/131155	1.92395329773	-0.0610866038466513	1.45583030073614	0.15500688734746	0.822970875248222	-4.91065770857508
a_HQ_transcript/105649	1.53965450488234	-0.112656316039432	1.45578189323416	0.155020190979222	0.822970875248222	-4.7960808786145
a_HQ_transcript/91948	1.60192057872723	1.88677211645213	1.4557793208436	0.155020897963972	0.822970875248222	-5.00609929780868
a_HQ_transcript/67631	1.58300387769235	0.121014644732756	1.45574598642933	0.15503005968266	0.822970875248222	-4.80041216983227
a_HQ_transcript/23921	0.319641008625048	3.89975174561777	1.45572326621981	0.155036304411774	0.822970875248222	-5.24527416890759
a_HQ_transcript/39595	-0.705136819478692	3.34541684026499	-1.45570037780805	0.155042595573974	0.822970875248222	-5.1691754190376
a_HQ_transcript/12363	-0.707261335311759	4.84266154015646	-1.45569799574813	0.155043250324169	0.822970875248222	-5.39086310743632
a_HQ_transcript/132323	1.44388736346633	-0.619416767431302	1.45544373274632	0.155113151524466	0.823218815242431	-4.92233939476981
a_HQ_transcript/114031	2.88637742704133	2.23365646925919	1.45539402966311	0.155126818668343	0.823218815242431	-4.96341691552676
a_HQ_transcript/134981	1.72072140654323	-0.433798987763792	1.45532829137661	0.15514489657436	0.823218815242431	-4.9100969280751
a_HQ_transcript/10800	1.45886453992127	0.444780204171007	1.45532206258146	0.155146609568209	0.823218815242431	-4.82829817123624
a_HQ_transcript/149778	-1.99588552983096	0.547604391428203	-1.45526141603309	0.155163288886504	0.823232170171111	-5.01159894133906
a_HQ_transcript/97194	-0.638692751981054	3.32516353109743	-1.45489961718715	0.155262822226471	0.823624704056022	-5.12796127931392
a_HQ_transcript/140740	2.02798968626965	-0.124049172106443	1.45488947835674	0.155265612218653	0.823624704056022	-4.99494923078948
a_HQ_transcript/58888	-1.53008205692331	-0.0392041398306313	-1.45483611003679	0.155280298710789	0.823627448075623	-4.88042128043017
a_HQ_transcript/95393	-1.5701191549298	0.403970690060973	-1.45475322983484	0.155303108801068	0.823666978050749	-4.92571620356645
a_HQ_transcript/29187	0.696469075864853	2.33472863807127	1.45468145200382	0.155322865474379	0.823666978050749	-4.90819673196065
a_HQ_transcript/137407	-1.63188276944136	-0.071152452247391	-1.45465458511334	0.155330261034137	0.823666978050749	-4.97597459300812
a_HQ_transcript/135573	1.70355456807766	2.82305267822412	1.45449502560948	0.155374188213346	0.823818208344879	-4.95074566307709
a_HQ_transcript/6282	0.937187656794985	0.245420584738939	1.45437096983664	0.155408347934345	0.823818208344879	-4.75731054119171
a_HQ_transcript/7124	1.49838986144332	2.44923649926928	1.45429999704839	0.15542789352581	0.823818208344879	-4.96129717018612
a_HQ_transcript/124154	2.01707234102219	0.523332021545387	1.45427056867148	0.15543599854313	0.823818208344879	-4.8336188627089
a_HQ_transcript/19695	-2.03229845357095	3.27747606886463	-1.45418070316815	0.15546075093147	0.823818208344879	-4.99085639762322
a_HQ_transcript/152523	2.61323815586999	0.876088664752254	1.45408779392064	0.155486344974912	0.823818208344879	-4.99644336235568
a_HQ_transcript/131995	1.39092918142989	-0.305845968822618	1.45407997025789	0.155488500340111	0.823818208344879	-4.95581653093839
a_HQ_transcript/147749	1.02709693122193	1.59747096087907	1.45407179277578	0.155490753205477	0.823818208344879	-4.83977896648485
a_HQ_transcript/73935	-1.73937160828303	0.031032173998822	-1.45406239162837	0.155493343218038	0.823818208344879	-4.79499196103893
a_HQ_transcript/149307	-1.30266610618517	0.896846135144246	-1.45402151452953	0.155504605241769	0.823818208344879	-4.73496523089899
a_HQ_transcript/48522	1.3404871673436	1.29094566881928	1.45397597546382	0.155517152444006	0.823818208344879	-4.89165568931297
a_HQ_transcript/87075	1.38385495507768	2.00609999354898	1.45388924046458	0.155541052426477	0.823818208344879	-4.95299637975763
a_HQ_transcript/1776	-1.56331918749751	2.47390350138058	-1.45385360533249	0.155550872595657	0.823818208344879	-5.0057675787023
a_HQ_transcript/122736	1.62730686453554	1.88500744412856	1.45383066224113	0.155557195410016	0.823818208344879	-4.81840188182486
a_HQ_transcript/132077	0.409262467151937	3.6639034405898	1.45364694482008	0.155607832878271	0.824011306326693	-5.26225999944044
a_HQ_transcript/118255	-1.74464939236841	0.536141251648712	-1.45345176959092	0.15566164276831	0.824110851174635	-4.87076922464851
a_HQ_transcript/79687	-0.442096762080594	2.86860902734232	-1.45337558378051	0.155682651237022	0.824110851174635	-5.04594334340161
a_HQ_transcript/91580	1.20883262120179	-0.429913700630486	1.45337459770012	0.155682923166446	0.824110851174635	-4.92128239945931
a_HQ_transcript/141976	1.79601921452757	-0.0833450809273513	1.45337308184702	0.155683341190969	0.824110851174635	-4.90142868289691
a_HQ_transcript/142097	1.41967340674687	-0.36537759618409	1.45325541145329	0.155715793698603	0.824207581143	-4.81944527544456
a_HQ_transcript/108400	0.993452746136971	2.71735042360091	1.45317622075809	0.155737636852352	0.824248143035008	-5.06304649029642
a_HQ_transcript/90769	1.77408619252257	-0.401715903540142	1.4531148943539	0.155754554174061	0.824262629736872	-4.92334965747972
a_HQ_transcript/112515	0.525944597683569	3.20144382101721	1.4530292674093	0.15577817741279	0.824312598693733	-5.12198975042993
a_HQ_transcript/53064	-1.56715686529035	-0.455379137623036	-1.45284097327438	0.15583013505597	0.824512478657696	-4.85766572264735
a_HQ_transcript/76832	0.895586507179467	2.74585401687221	1.45271261268711	0.155865562600028	0.824528049964354	-4.97510297502314
a_HQ_transcript/68159	1.19253164943087	2.6564613843405	1.45251706250154	0.1559195467779	0.824528049964354	-4.93241640106992
a_HQ_transcript/19178	2.13194191469739	2.18428042461668	1.45249239209487	0.155926358419781	0.824528049964354	-4.78729957099786
a_HQ_transcript/79770	0.467366029649201	2.44468415437208	1.45243830047892	0.15594129425459	0.824528049964354	-5.02380317693476
a_HQ_transcript/135027	-0.607698028277006	2.59537792091668	-1.45243325379517	0.155942687808237	0.824528049964354	-4.93032431097429
a_HQ_transcript/44957	1.04562208136065	0.58444919989896	1.45237470846195	0.155958854803027	0.824528049964354	-4.82263855832294
a_HQ_transcript/120797	1.37862922739656	0.019403049660842	1.45228110510572	0.155984705656579	0.824528049964354	-4.93894753455036
a_HQ_transcript/23686	1.74431593005343	0.569957480481035	1.4522589078992	0.155990836456463	0.824528049964354	-4.83267274451048

a_HQ_transcript/32487	-1.65180502542357	0.926860204543274	-1.45223247171801	0.155998138297673	0.824528049964354	-4.85162326837013
a_HQ_transcript/30104	1.28059084842371	-0.502385174890553	1.45220521027941	0.156005668364432	0.824528049964354	-4.90776792616475
a_HQ_transcript/73167	0.978097862342954	-0.746994022109938	1.45220439821102	0.156005892675774	0.824528049964354	-4.92795664638448
a_HQ_transcript/140697	1.34298631270151	-0.543574729940826	1.4521245421462	0.15602795194679	0.824528049964354	-4.92105574060293
a_HQ_transcript/128901	0.846602213344799	4.11596501583606	1.45211677917508	0.156030096505635	0.824528049964354	-5.29302567007407
a_HQ_transcript/58723	1.35948203073161	-0.363477441512452	1.45211110609967	0.156031663735341	0.824528049964354	-4.91310932449976
a_HQ_transcript/5994	0.818273342633558	4.67075020995465	1.45203312814835	0.156053206996858	0.824566931761966	-5.35762122336007
a_HQ_transcript/141057	3.22343826993777	2.82581013537457	1.45182481780422	0.156110769258912	0.824721359022631	-5.09013113377641
a_HQ_transcript/144098	-1.65968384997103	2.33849298637998	-1.45181103469541	0.156114578530901	0.824721359022631	-4.90569075390558
a_HQ_transcript/141569	-1.46135395000642	2.34729840528891	-1.45171575693504	0.156140912700165	0.824721359022631	-5.04977631987917
a_HQ_transcript/75779	-0.556595105892571	4.34457576166662	-1.45170940924442	0.156142667286924	0.824721359022631	-5.36406064495873
a_HQ_transcript/48914	-0.935059563409049	2.72741069445872	-1.45167067894242	0.156153373200576	0.824721359022631	-4.94675886434799
a_HQ_transcript/95813	1.66135571558081	-0.0549124652926457	1.45160496489842	0.156171539351576	0.824736695122885	-4.8979249077762
a_HQ_transcript/50266	1.54779040815688	0.00803532954113783	1.45154905061153	0.156186997754797	0.824736695122885	-4.84421972666861
a_HQ_transcript/53710	-0.691451299000468	2.16555175186338	-1.4514575829597	0.156212288075885	0.824736695122885	-5.04708492777757
a_HQ_transcript/70550	-1.69920404155085	0.29226345495674	-1.45141989463416	0.156222709643842	0.824736695122885	-4.90426242423033
a_HQ_transcript/126113	-2.34406289426548	0.552782407399945	-1.45140358995961	0.156227218380094	0.824736695122885	-4.9517250349111
a_HQ_transcript/52574	1.4372953811757	-0.582986914460397	1.45129770857227	0.156256500303503	0.824783373089871	-4.86071294375818
a_HQ_transcript/141950	-1.71926142417156	3.68316979081302	-1.45108718766941	0.156314733647993	0.824783373089871	-5.3134525341989
a_HQ_transcript/62572	1.63056401014653	0.851348069155644	1.45106791447869	0.156320065771866	0.824783373089871	-4.94846655224215
a_HQ_transcript/186	0.405786722636841	3.28073599196684	1.45102080068245	0.156333100889715	0.824783373089871	-5.16224457997531
a_HQ_transcript/146032	0.899087462034269	2.54022490080922	1.45101294232798	0.15633527516859	0.824783373089871	-5.14910114039748
a_HQ_transcript/107761	2.17895788747068	1.32190334800668	1.45098985122731	0.156341664239487	0.824783373089871	-4.91080617095425
a_HQ_transcript/10786	1.43661830685874	1.50330351603423	1.45090057305704	0.156366368543916	0.824783373089871	-4.81432007480315
a_HQ_transcript/142622	3.05889449258637	3.9392125821872	1.45084738307343	0.156381088301648	0.824783373089871	-5.14994263338242
a_HQ_transcript/25698	1.65866136491355	2.79772782378135	1.45084525242987	0.156381677957322	0.824783373089871	-5.09722999384905
a_HQ_transcript/101065	1.53471807599473	0.565611679892464	1.45076717011318	0.156403288458324	0.824783373089871	-4.9362801993255
a_HQ_transcript/83651	-0.60309480617673	3.73174254880047	-1.45076015092783	0.156405231243637	0.824783373089871	-5.20554088018699
a_HQ_transcript/83880	-1.24167580140437	-0.692896874168484	-1.45075618298914	0.156406329506772	0.824783373089871	-4.9047871848843
a_HQ_transcript/117416	0.535055076261574	4.17949633942946	1.45067239037561	0.156429523416098	0.824786485947241	-5.20181069719365
a_HQ_transcript/129941	-1.48208358543234	1.3796944017972	-1.45065152973803	0.156435298093317	0.824786485947241	-4.91264488563403
a_HQ_transcript/18276	1.69412486490265	1.76252850245348	1.45056163292527	0.156460185417708	0.824842885721072	-4.83416695087802
a_HQ_transcript/49495	1.03172321845057	-0.594621066258239	1.45041052695349	0.156502025163613	0.824944930968831	-4.91785225052214
a_HQ_transcript/127733	2.67559900565158	1.46382042119887	1.45033213807616	0.156523733764573	0.824944930968831	-5.00237651667366
a_HQ_transcript/94635	-0.783430985756969	2.92012290620424	-1.45029810671764	0.156533158972862	0.824944930968831	-5.08767144236614
a_HQ_transcript/142900	-1.07100086782766	-0.22829479542303	-1.45028673217549	0.156536309327453	0.824944930968831	-4.87424723908334
a_HQ_transcript/5386	1.0790107989404	2.68434645114907	1.45021393339918	0.156556473261084	0.824976400844917	-4.98133696176669
a_HQ_transcript/76280	-1.86566609312091	1.94162846897917	-1.45015060610466	0.156574015443339	0.824991094585514	-4.94359622847122
a_HQ_transcript/5932	1.56260304762992	1.52196371716076	1.45010139995129	0.156587647030037	0.824991094585514	-4.8504410410479
a_HQ_transcript/5719	1.34045051043878	-0.215912151586278	1.4499843528894	0.156620076376523	0.825037788651259	-4.8797544977337
a_HQ_transcript/108862	1.87641575323834	0.543429323422759	1.44990466280474	0.156642158556477	0.825037788651259	-4.92877471851233
a_HQ_transcript/125600	1.78193436327945	0.955296561972675	1.44990376487129	0.156642407388561	0.825037788651259	-4.86818809309006
a_HQ_transcript/147788	1.31388816707182	-0.368513165211755	1.44986357853004	0.156653544003788	0.825037788651259	-4.99035514479054
a_HQ_transcript/1172	-1.35554741550432	2.97057758919895	-1.44981330417272	0.156667477138945	0.825037788651259	-5.21255920946075
a_HQ_transcript/136041	0.854403696690333	2.92013948493613	1.44973774319742	0.156688420107209	0.825073329467512	-4.95600198348969
a_HQ_transcript/43300	1.89022856698077	3.48965791419604	1.44967056097933	0.156707042630556	0.825096646198275	-5.11204084453397
a_HQ_transcript/89508	-2.05736999383933	1.34562719565818	-1.44935059239375	0.156795760178509	0.825488990821153	-4.87588417298237
a_HQ_transcript/143551	-2.0341946777459	2.73320930154473	-1.44920118975287	0.156837198628226	0.825594237965316	-5.18671686472611
a_HQ_transcript/98464	-1.4158584596701	-0.181058730966246	-1.44913721261673	0.156854946040567	0.825594237965316	-4.90729385196743
a_HQ_transcript/102379	1.38442722420316	1.79434335926733	1.44908096142832	0.156870551572609	0.825594237965316	-4.90122912156717

a_HQ_transcript/80137	1.38832240371005	0.257949665520348	1.44907371041776	0.156872563279778	0.825594237965316	-4.8379194360057
a_HQ_transcript/3146	-1.219062112632	1.65718426934542	-1.44892964630358	0.156912536408498	0.825665211237002	-4.91099189931024
a_HQ_transcript/135412	-1.26005575850661	-0.583332538471518	-1.44892272319262	0.156914457551183	0.825665211237002	-4.92611911071646
a_HQ_transcript/141090	2.51749574367684	0.67707545920871	1.44884702011975	0.156935466146232	0.825700886342091	-4.96413585660607
a_HQ_transcript/34710	0.96443984165911	2.16541473169219	1.44879592413544	0.15694964721294	0.825700886342091	-4.99189682125446
a_HQ_transcript/46585	-2.3217942065323	-0.0357411962115577	-1.44865753174113	0.156988061438084	0.825763053579107	-4.87466722611416
a_HQ_transcript/97941	1.77741334732172	0.629866328079074	1.44861787175163	0.156999071422038	0.825763053579107	-4.90853886678161
a_HQ_transcript/52082	1.96414280779578	0.802899761788318	1.4485946964544	0.157005505384808	0.825763053579107	-4.82807136498912
a_HQ_transcript/99709	-3.14926379791453	2.52875253396748	-1.44854865623117	0.157018287765102	0.825763053579107	-5.08049301488684
a_HQ_transcript/20599	1.4830079020734	-0.0893441882764554	1.44830784628461	0.157085158501335	0.82600721062941	-4.90423145273472
a_HQ_transcript/16547	1.41643019505323	-0.536170579736221	1.44827912967607	0.157093134350195	0.82600721062941	-4.92073161367531
a_HQ_transcript/125904	-1.55059270349585	4.46542109260883	-1.44808264969247	0.157147713989446	0.82604387594106	-5.21231002510089
a_HQ_transcript/129283	1.59078824491176	0.680832736587744	1.44803511722013	0.157160920164662	0.82604387594106	-4.91928532372642
a_HQ_transcript/17090	1.43985201729038	-0.521216918737897	1.44781004681825	0.157223464508676	0.82604387594106	-4.9322150716365
a_HQ_transcript/70286	1.18596856748313	3.79724731903754	1.44768061136293	0.157259441997097	0.82604387594106	-5.18329168389131
a_HQ_transcript/25671	0.731198923159315	3.73312761215103	1.4476569438585	0.157266021251242	0.82604387594106	-5.30843630366496
a_HQ_transcript/11573	0.447074451250366	5.02065807346905	1.44750989515301	0.157306903744207	0.82604387594106	-5.38077103413911
a_HQ_transcript/28841	1.23912255084879	3.17438931088754	1.44749649377433	0.157310630015622	0.82604387594106	-5.034956461027
a_HQ_transcript/133800	1.16714741019582	0.0295703935345217	1.44741374732403	0.157333639328252	0.82604387594106	-4.88666527276516
a_HQ_transcript/97817	-1.75881591764995	3.42981766912386	-1.44740996864067	0.157334690130784	0.82604387594106	-5.32074755556012
a_HQ_transcript/39453	0.887714819450502	-0.669426232828627	1.44740310452333	0.157336598966481	0.82604387594106	-4.91252925580019
a_HQ_transcript/15299	1.07679718468009	0.691753779624604	1.44737192913758	0.157345268730225	0.82604387594106	-4.82554823684393
a_HQ_transcript/13171	1.69690011495002	-0.0206994478578764	1.44731049231981	0.157362355199419	0.82604387594106	-4.84781579947906
a_HQ_transcript/115563	1.07005221704388	3.12108812734881	1.4472619430475	0.157375858496284	0.82604387594106	-5.04277997042583
a_HQ_transcript/45640	0.719039392150931	2.94956140807106	1.44725285886344	0.15737838523629	0.82604387594106	-5.09635862314459
a_HQ_transcript/39336	1.82533701569259	0.469484265220687	1.4472360786057	0.157383052701942	0.82604387594106	-4.90885639190948
a_HQ_transcript/147307	3.36366647692299	3.09989445668703	1.44706412460123	0.157430888413359	0.82604387594106	-5.10481984897729
a_HQ_transcript/91699	2.0792925237047	1.09101993827664	1.446995418615	0.157450004885928	0.82604387594106	-4.78639385686579
a_HQ_transcript/108117	-2.38131834981305	0.86016692886693	-1.44691152521307	0.157473349538864	0.82604387594106	-4.9072192659474
a_HQ_transcript/39775	-2.02133558973294	1.99591118064564	-1.44683792678232	0.15749383171484	0.82604387594106	-4.87080595587522
a_HQ_transcript/138004	1.32480452337929	-0.5951470883174	1.44682529305498	0.157497347847696	0.82604387594106	-4.89598770018924
a_HQ_transcript/116341	-1.80719320935367	0.038573486853004	-1.4468018437804	0.157503874254908	0.82604387594106	-4.82562686167321
a_HQ_transcript/112662	-2.89270371557508	2.88430744369129	-1.44676853264495	0.15751314578607	0.82604387594106	-5.16717135576087
a_HQ_transcript/50036	0.896068702915587	-0.788335833935537	1.44674494684496	0.157519710713788	0.82604387594106	-4.94235627111278
a_HQ_transcript/142658	1.85404575835156	-0.338604521636257	1.44674245196044	0.157520405158576	0.82604387594106	-4.8519477898385
a_HQ_transcript/130318	-1.83478550579571	3.43350716389191	-1.44659351656825	0.157561865345781	0.82604387594106	-5.4003494459459
a_HQ_transcript/17458	-0.82177770229314	3.83924449457823	-1.44656280581419	0.157570415589203	0.82604387594106	-5.22150614807384
a_HQ_transcript/103560	-0.662948288440821	3.97314020210079	-1.44649969666622	0.157587987090058	0.82604387594106	-5.30571150738547
a_HQ_transcript/12748	0.666476424734957	2.83411000576485	1.44647271322346	0.157595500573051	0.82604387594106	-5.06380105256742
a_HQ_transcript/47140	1.94689698806235	2.06939796316537	1.44647024810479	0.157596186994327	0.82604387594106	-4.83665488298515
a_HQ_transcript/59174	1.95075383912977	1.43545983385261	1.44644614271058	0.157602899354125	0.82604387594106	-5.02059964362231
a_HQ_transcript/151100	2.52512531486819	2.27837208080057	1.44644004784272	0.157604596559782	0.82604387594106	-5.07604098558058
a_HQ_transcript/31950	-1.42203881775322	0.64169194860371	-1.44631928199098	0.157638228576909	0.82604387594106	-4.8171256251619
a_HQ_transcript/32180	2.17852621347327	3.31419870550931	1.44627218999393	0.157651344743789	0.82604387594106	-5.10535667294014
a_HQ_transcript/125442	0.713013050115739	4.16997857711133	1.44623631136083	0.157661338320525	0.82604387594106	-5.27834901675241
a_HQ_transcript/7301	1.62482594219889	-0.0923153534556899	1.4461080282288	0.157697074206056	0.82604387594106	-4.82632767010068
a_HQ_transcript/63217	1.40721328124194	0.179747645467854	1.446103412907	0.157698360017837	0.82604387594106	-4.84484657170903
a_HQ_transcript/135460	0.388748508960338	3.22750712611615	1.44609845021593	0.157699742614701	0.82604387594106	-5.14103507547491
a_HQ_transcript/121781	1.7960162647368	1.00068129967013	1.44609283064847	0.157701308227756	0.82604387594106	-4.95510043435826
a_HQ_transcript/135970	-1.71271128748224	0.118650106503223	-1.44606775025272	0.157708295783202	0.82604387594106	-4.8235563328948

a_HQ_transcript/142363	-0.719008553611096	2.39245337621156	-1.4460499405085	0.157713257838596	0.82604387594106	-4.99215912733686
a_HQ_transcript/14312	1.4325570986529	-0.494302717341666	1.44604555592347	0.157714479467142	0.82604387594106	-4.8974771695472
a_HQ_transcript/80594	-1.41034035350617	0.460725466955734	-1.44588366166686	0.157759591530009	0.82604387594106	-4.8209216114338
a_HQ_transcript/10546	-1.23792764522865	3.61846068657803	-1.44585824922078	0.157766673672735	0.82604387594106	-5.32606228563235
a_HQ_transcript/118442	1.99887291450159	0.911479655552691	1.44577387348525	0.157790189981939	0.82604387594106	-4.96058979159938
a_HQ_transcript/65756	0.618712851291296	2.37501534365	1.44567890463185	0.157816662016363	0.82604387594106	-5.05858344604956
a_HQ_transcript/89745	1.79037693114053	0.465486224915493	1.44566752326265	0.157819834745326	0.82604387594106	-4.89934743937173
a_HQ_transcript/127624	3.92513288374738	1.35638277322324	1.44566544843775	0.157820413139624	0.82604387594106	-4.92938026545375
a_HQ_transcript/17108	-1.22787039491514	1.18957740885745	-1.44563871523538	0.157827865644997	0.82604387594106	-4.86773518033066
a_HQ_transcript/78130	1.45697898432905	1.23809137858985	1.44557085594042	0.157846784263329	0.82604387594106	-4.84454033008143
a_HQ_transcript/96913	1.45513874636687	0.380236467594797	1.44548191058877	0.157871584224652	0.82604387594106	-4.856974298976
a_HQ_transcript/14408	1.68832315387374	1.73206710730156	1.44546323789684	0.157876790982709	0.82604387594106	-4.9017237027647
a_HQ_transcript/11015	0.508030648678958	3.80928411994871	1.44532517117802	0.157915294208881	0.82604387594106	-5.17907772786798
a_HQ_transcript/69274	2.03674626236216	0.946622164967931	1.44531100433478	0.157919245394964	0.82604387594106	-4.906294077343
a_HQ_transcript/61060	-1.03127818536204	2.35019810849708	-1.44525669383112	0.15793439352559	0.82604387594106	-4.89072089514181
a_HQ_transcript/22918	0.620597351277326	3.27924498162021	1.44508104472245	0.157983392959635	0.82604387594106	-5.1803596058382
a_HQ_transcript/151997	2.24314578389986	10.7451779180568	1.44502969135236	0.157997720876756	0.82604387594106	-5.9677362846492
a_HQ_transcript/107707	-2.93595633510696	0.721389386276086	-1.4449162979372	0.158029362009232	0.82604387594106	-5.00651369689276
a_HQ_transcript/129061	4.94911824252152	0.84859822022289	1.44482051352582	0.15805609346379	0.82604387594106	-5.02239349138631
a_HQ_transcript/103206	0.643958875888831	3.97269898792725	1.44475293640432	0.158074955001251	0.82604387594106	-5.2429620236172
a_HQ_transcript/26691	-0.535610265555915	3.01038705153516	-1.44473777951906	0.158079185703942	0.82604387594106	-5.09796901406881
a_HQ_transcript/23738	1.14636484637714	2.36527991612174	1.44473642835276	0.158079562855898	0.82604387594106	-4.8812639145677
a_HQ_transcript/130590	-2.66897974294183	2.0231234175874	-1.44471717926333	0.158084935944603	0.82604387594106	-4.92375120209485
a_HQ_transcript/91836	-1.89586631458324	0.608031473714086	-1.44468068456735	0.158095123277775	0.82604387594106	-4.87883395638505
a_HQ_transcript/81249	1.24088524251276	0.853648794267912	1.44455212366595	0.158131014630258	0.82604387594106	-4.8945836060947
a_HQ_transcript/116254	1.87256904027806	1.22653163628714	1.44452088571748	0.158139736547761	0.82604387594106	-4.85080731447568
a_HQ_transcript/145546	1.84697057417437	-0.29302539698243	1.44449098569567	0.158148085261149	0.82604387594106	-4.91937909771019
a_HQ_transcript/75499	1.54830855126562	0.211715273586239	1.44447092341088	0.158153687267776	0.82604387594106	-4.80311599969819
a_HQ_transcript/100835	-1.11708440494176	1.69165539896024	-1.44445211359351	0.158158939689838	0.82604387594106	-4.92041169400601
a_HQ_transcript/111306	1.38361978637341	-0.144242288358004	1.44441151111294	0.158170277930118	0.82604387594106	-4.98444581539861
a_HQ_transcript/81252	-1.65642621411454	1.53197590987732	-1.44440458742522	0.158172211433786	0.82604387594106	-4.93906293875741
a_HQ_transcript/138948	2.38787001489826	-0.364254879546316	1.44440361268929	0.158172483639292	0.82604387594106	-4.90781358338595
a_HQ_transcript/134919	-2.73938387730709	3.97300876670335	-1.44438620357132	0.158177345385477	0.82604387594106	-5.25999496228703
a_HQ_transcript/151981	1.62497591496483	2.64459259784663	1.44429948652118	0.158201564131072	0.82604387594106	-5.15764462346149
a_HQ_transcript/86660	-1.20698637538172	5.45706332630578	-1.44423865796415	0.158218554370082	0.82604387594106	-5.2144739890512
a_HQ_transcript/145315	-1.2573811862869	0.947357073228893	-1.44423199981686	0.158220414168499	0.82604387594106	-4.86359838426163
a_HQ_transcript/38072	-0.575831227106323	3.16617062994591	-1.44422266070905	0.15822302286001	0.82604387594106	-5.18314746743372
a_HQ_transcript/128634	-1.43816882958314	1.43798129012371	-1.44418685321233	0.158233025278283	0.82604387594106	-4.82609779010746
a_HQ_transcript/151792	5.99611603706753	4.44056318881526	1.44416949042146	0.158237875557465	0.82604387594106	-5.28273182917675
a_HQ_transcript/109820	0.917792338803215	-0.635755198785461	1.44409799625782	0.158257848625699	0.82604387594106	-4.99124967075478
a_HQ_transcript/18725	-2.21854010476405	1.99467783947593	-1.44405961623415	0.158268571539382	0.82604387594106	-4.90735398742247
a_HQ_transcript/10802	0.757064500653044	3.49865029379607	1.4440117252989	0.158281952493933	0.82604387594106	-5.18399635488777
a_HQ_transcript/6386	1.1631918749427	0.769178845854581	1.44394583746188	0.158300363333267	0.82604387594106	-4.78332756561904
a_HQ_transcript/114720	-2.81634742270002	2.537537973308	-1.44393725989511	0.158302760261676	0.82604387594106	-5.11987456512743
a_HQ_transcript/112834	1.91866512977806	3.66018365035946	1.44388830174373	0.158316441753077	0.82604387594106	-5.1882641764259
a_HQ_transcript/99848	-2.61825900951453	5.41023701507061	-1.44384820583906	0.158327647362889	0.82604387594106	-5.47941360769408
a_HQ_transcript/69450	-1.78220327800339	-0.454531227205592	-1.44376095297079	0.158352034109718	0.82604387594106	-4.962585729814
a_HQ_transcript/78855	-1.03602106903404	1.86611918793828	-1.44371422629499	0.158365095209075	0.82604387594106	-4.85234524087542
a_HQ_transcript/7071	2.93389428951646	0.455873766851788	1.44367694426803	0.158375516940489	0.82604387594106	-4.89343149046816
a_HQ_transcript/124943	-2.1111949982462	1.36113047324256	-1.44361893090223	0.158391734942994	0.82604387594106	-4.97215701835434

a_HQ_transcript/112245	-2.4779736374659	1.79526063764794	-1.44359019729126	0.158399768092522	0.82604387594106	-5.23303850733396
a_HQ_transcript/144520	-2.09392549035456	0.600595868468274	-1.44357217971687	0.158404805489348	0.82604387594106	-4.92606064019492
a_HQ_transcript/119433	0.468186266988607	3.9225613711441	1.44351923552875	0.158419608487402	0.82604387594106	-5.26648982861951
a_HQ_transcript/109763	2.74481280377176	0.701218562254566	1.44348258391597	0.158429856783987	0.82604387594106	-4.85281156156562
a_HQ_transcript/104117	-2.28297096790846	2.95498665389861	-1.44346089961899	0.15843592025989	0.82604387594106	-5.20693981632606
a_HQ_transcript/70644	-1.65963082350007	0.0752814461658984	-1.4433560410404	0.158465243955313	0.826055258425283	-4.9044516914074
a_HQ_transcript/73701	2.88863641910348	2.04730485362692	1.44335145918168	0.158466525369879	0.826055258425283	-4.97471122135974
a_HQ_transcript/111858	-1.07736144507999	3.14720703869459	-1.44322184456488	0.158502778264495	0.826074086707553	-5.00478354611314
a_HQ_transcript/22153	-0.602879417260408	2.27970749555781	-1.44319969101186	0.158508975217104	0.826074086707553	-5.02146798610565
a_HQ_transcript/58157	1.26728620185122	0.957152802324929	1.44318612112279	0.158512771179742	0.826074086707553	-4.84477834512814
a_HQ_transcript/25152	-0.700908939315855	2.09303155561905	-1.44299542389134	0.158566123343019	0.826206264484479	-4.87127451388104
a_HQ_transcript/119626	1.13992303007408	-0.153678855276981	1.44299385800982	0.158566561495129	0.826206264484479	-4.95064855378724
a_HQ_transcript/132174	0.847734181883397	3.28320565356155	1.44287317242375	0.158600333630724	0.826308164675692	-5.0940225598688
a_HQ_transcript/107626	2.68780703417249	3.24025864155069	1.4427237717937	0.158642149153519	0.826396655244507	-5.24490904574073
a_HQ_transcript/110677	-1.58511992960541	3.41557404728799	-1.44267174535084	0.158656712808142	0.826396655244507	-5.17830545161638
a_HQ_transcript/129983	1.54271476809149	0.637306712363743	1.44265514403314	0.158661360203579	0.826396655244507	-4.82556271076045
a_HQ_transcript/89818	1.2460402761376	0.257352284508456	1.44260933091896	0.158674185748416	0.826396655244507	-4.79248018302758
a_HQ_transcript/133382	1.15566393175142	-0.576279840008772	1.44251430860444	0.158700790202734	0.826424826450279	-4.93085813697896
a_HQ_transcript/36789	-0.545713553846896	3.07219722444391	-1.44248845448033	0.158708029480878	0.826424826450279	-5.1678723700556
a_HQ_transcript/122917	1.48278587142771	0.381360657973509	1.44241192185719	0.15872946051381	0.826462386308378	-4.79908207754699
a_HQ_transcript/147649	0.811519605293179	1.71111089363632	1.44234682277563	0.158747691675585	0.826483280046432	-4.8707948010968
a_HQ_transcript/88529	1.83085902873325	0.336631529850778	1.44209616721348	0.158817903885247	0.826597299399598	-4.92290264084228
a_HQ_transcript/118165	-0.722156876629134	1.91199207168559	-1.44205455757622	0.158829561720079	0.826597299399598	-4.8861479798593
a_HQ_transcript/117693	2.49998632427444	0.968303330156231	1.44194132220557	0.158861290476584	0.826597299399598	-5.02086534677895
a_HQ_transcript/127423	-1.02440018610191	-0.71389838402033	-1.44193326014216	0.158863549672809	0.826597299399598	-4.91661026110023
a_HQ_transcript/117016	1.43879916154566	-0.456236417315108	1.44188543196247	0.158876952874439	0.826597299399598	-4.93105124787346
a_HQ_transcript/136709	0.401026896354018	6.13611398352922	1.44179914088565	0.15890113704923	0.826597299399598	-5.49197584913503
a_HQ_transcript/149523	-2.0271689403299	1.6184562426102	-1.44176300984773	0.15891126409931	0.826597299399598	-4.92245887334821
a_HQ_transcript/16408	-1.88629160613059	0.450743597224919	-1.44172870915949	0.158920878599638	0.826597299399598	-4.77304563554118
a_HQ_transcript/29213	-0.406734354090315	4.00647759089644	-1.44167234051931	0.158936679758703	0.826597299399598	-5.27357516302141
a_HQ_transcript/73814	0.883244162202144	2.50297684418385	1.44158882897665	0.158960091856028	0.826597299399598	-5.05116450602937
a_HQ_transcript/28401	0.856144934380557	1.67778252252514	1.44144568085088	0.159000229162683	0.826597299399598	-4.85014900033319
a_HQ_transcript/101346	1.17254375440822	1.87499556058096	1.44141885064029	0.159001303225762	0.826597299399598	-4.83920483511871
a_HQ_transcript/60236	0.659448185641792	-0.419440850233973	1.44141152752218	0.159009806601135	0.826597299399598	-4.91330464580759
a_HQ_transcript/150084	0.312587412133544	9.44685449199647	1.44138605864159	0.159016949002812	0.826597299399598	-5.86725541849564
a_HQ_transcript/84761	-0.981714316506127	1.9028780091035	-1.44126811015384	0.159050029367089	0.826597299399598	-4.76500573417466
a_HQ_transcript/66267	-1.34423572428756	1.10100463056259	-1.44119099077783	0.159071661560938	0.826597299399598	-4.74963016841494
a_HQ_transcript/25391	1.24906148734678	-0.423635399169761	1.44117728839185	0.159075505361055	0.826597299399598	-4.9919044310053
a_HQ_transcript/89364	-1.95958797196525	2.25238818092009	-1.44117219621826	0.159076933838863	0.826597299399598	-5.12836229451198
a_HQ_transcript/113247	0.441022302735913	2.97961865119254	1.44113087973457	0.15908852448703	0.826597299399598	-5.09909123705631
a_HQ_transcript/152757	1.17171112547798	-0.662801057363536	1.44108700962392	0.159100832244503	0.826597299399598	-4.93106798023008
a_HQ_transcript/71804	0.869394675183828	-0.834697089030488	1.44107993748651	0.159102816402886	0.826597299399598	-4.9338435067568
a_HQ_transcript/77168	1.84561876754962	1.62018467411517	1.44107396029949	0.159104493377343	0.826597299399598	-4.86887958836096
a_HQ_transcript/150363	-0.618283037054651	5.45373339118736	-1.44105386027789	0.159110132792063	0.826597299399598	-5.44674712017368
a_HQ_transcript/122057	-1.90408369314182	0.50772477754728	-1.44105120016387	0.159110879145715	0.826597299399598	-4.85613642786106
a_HQ_transcript/22071	-1.87387469234742	3.98085173496663	-1.44079147306291	0.159183764661717	0.826828875823638	-5.13340948237562
a_HQ_transcript/41026	1.17395286457596	-0.472512041407851	1.44079097799307	0.159183903615165	0.826828875823638	-4.87639775186834
a_HQ_transcript/119276	-0.549636684777538	2.96460577914253	-1.44066443092861	0.159219425290596	0.826939488321277	-5.09470235835055
a_HQ_transcript/147382	2.37612594196306	6.9680694466415	1.44060416615673	0.159236343777206	0.826953470122525	-5.62088110160224
a_HQ_transcript/906	-0.451974766759236	2.98179553476025	-1.4403190106929	0.159316416462615	0.827295395402794	-5.09628903148304

a_HQ_transcript/115658	-2.48728385781262	3.44931071469098	-1.44021581169345	0.159345402969453	0.827372003913209	-5.28986566108135
a_HQ_transcript/125151	2.58638753283297	-0.400424007084422	1.4401074074942	0.159375856007592	0.827456212755388	-4.94369529386461
a_HQ_transcript/84808	2.59446112307558	2.10894402473564	1.43996895050352	0.159414758212231	0.827525884949369	-5.0270790684905
a_HQ_transcript/126260	3.10362500642965	1.93224594078078	1.43995831040495	0.159417748067421	0.827525884949369	-5.15026289948339
a_HQ_transcript/105811	0.345765916210136	3.83713121680688	1.43985352523874	0.15944719494718	0.827604835064709	-5.26158561246169
a_HQ_transcript/25835	2.16218988117205	0.690520121155255	1.43974436443502	0.159477876053966	0.827642836836894	-4.80109875472083
a_HQ_transcript/135500	-0.750242114509838	3.23523183968106	-1.43972616049151	0.159482992970858	0.827642836836894	-5.11109608999825
a_HQ_transcript/136792	0.643867108647256	4.18971650578005	1.43960484700427	0.159517096100696	0.827723622327372	-5.36437845053176
a_HQ_transcript/93749	-0.525568091752517	2.84887766172113	-1.43956948035568	0.159527039308656	0.827723622327372	-5.07319823049979
a_HQ_transcript/119590	-1.52211869361936	0.33348416428126	-1.43949821281169	0.159547077416529	0.827753705468404	-4.81421255140584
a_HQ_transcript/41726	1.1281295951075	2.24248775102755	1.43939253913318	0.159576793073819	0.827833987308788	-4.87421068189263
a_HQ_transcript/137778	0.877203436495585	2.74083447638107	1.43929534994715	0.159604126741269	0.827901898912771	-5.04479020507003
a_HQ_transcript/141344	-3.25989745586916	4.02859574145625	-1.43906874013966	0.159667873298272	0.828126692166933	-5.17044101748249
a_HQ_transcript/108424	-2.9376180156033	3.30185378945856	-1.43900671751841	0.15968532410695	0.828126692166933	-4.93634581578759
a_HQ_transcript/25780	1.30051391378015	0.291372134409216	1.43898937988826	0.159690202527167	0.828126692166933	-4.90966348502602
a_HQ_transcript/70748	1.91184293788697	0.0727496097735416	1.43888489219642	0.159719605517347	0.828143521116513	-4.83034474513566
a_HQ_transcript/68694	-2.90919610077905	1.04958762958984	-1.43885787534554	0.159727208796027	0.828143521116513	-4.96382122661896
a_HQ_transcript/134744	1.47282779236157	-0.475737859917385	1.43870182582652	0.159771130992317	0.828143521116513	-4.92628573937112
a_HQ_transcript/98643	1.65043006987794	1.57441640260113	1.43857882773216	0.159805757164425	0.828143521116513	-4.98944616005225
a_HQ_transcript/91088	-0.495036851177409	4.26588102261763	-1.43850077324562	0.159827733983821	0.828143521116513	-5.3215558307427
a_HQ_transcript/32547	1.73505929480281	0.116384854146536	1.43847470740105	0.159835073548292	0.828143521116513	-4.95838479274386
a_HQ_transcript/142656	1.94448067706479	-0.0705699452444117	1.4384603177456	0.159839125470793	0.828143521116513	-4.9335512042121
a_HQ_transcript/136434	-1.06082062810467	2.25106665638602	-1.43838497380333	0.159860342581367	0.828143521116513	-4.93543109513482
a_HQ_transcript/19308	1.04452898515306	2.41952814353644	1.43835311076752	0.159869315991205	0.828143521116513	-4.93405388121174
a_HQ_transcript/32571	-1.93511352207735	0.0879201926078694	-1.43831441932253	0.15988021298386	0.828143521116513	-4.86467191725975
a_HQ_transcript/90757	0.823659423301996	-0.514622460568189	1.43829284422095	0.159886289613956	0.828143521116513	-4.97412122082809
a_HQ_transcript/10320	1.61480135924473	0.677137808861049	1.43826521076614	0.159894072846945	0.828143521116513	-4.78434031235139
a_HQ_transcript/26393	-2.14370118981819	0.110525405590337	-1.43824403494761	0.159900037426377	0.828143521116513	-4.92209966399104
a_HQ_transcript/15532	0.709376953816089	3.19547505408218	1.43824392851927	0.159900067404422	0.828143521116513	-5.11721597421576
a_HQ_transcript/144410	1.81673250104093	0.292456241929359	1.43821878024683	0.159907151132187	0.828143521116513	-4.92017970918795
a_HQ_transcript/8623	1.99273914661512	1.7749669719136	1.43794173635251	0.159985204878685	0.828246909962272	-5.01498237196103
a_HQ_transcript/41788	1.08324439793945	4.05516992707545	1.43791132035668	0.159993776051612	0.828246909962272	-5.27194258283065
a_HQ_transcript/543	-1.73839019498172	1.16938506032476	-1.43788858111592	0.160000184166115	0.828246909962272	-4.83731751821584
a_HQ_transcript/120802	3.07597570661513	0.757879877328756	1.43782747909295	0.160017404250047	0.828246909962272	-5.0222417864153
a_HQ_transcript/3105	1.78077882612171	0.698495002998386	1.43777772940983	0.160031426042608	0.828246909962272	-4.90782968495489
a_HQ_transcript/142053	-1.25250324791807	3.90597866165678	-1.43773914964733	0.160042300297204	0.828246909962272	-5.34114152059456
a_HQ_transcript/102908	1.8081565721076	0.317933205128802	1.43773077944798	0.160044659634076	0.828246909962272	-4.82550872888827
a_HQ_transcript/36855	-1.50031255157201	-0.249672736078228	-1.4376932467156	0.160055239452769	0.828246909962272	-4.8874656997984
a_HQ_transcript/6664	-0.363454818234396	3.42544236040213	-1.4376928451034	0.160055352663195	0.828246909962272	-5.18423035289308
a_HQ_transcript/129675	1.51523418058466	1.36709784514957	1.43756210802853	0.160092209491917	0.82836389116487	-4.84134428039317
a_HQ_transcript/91035	-1.51165879959028	0.570738366264804	-1.43745761297209	0.160121673112706	0.828376849509268	-4.8762926874208
a_HQ_transcript/99714	2.68711002612046	0.457221400804516	1.43745214255213	0.160123215680673	0.828376849509268	-4.9639549194333
a_HQ_transcript/40900	-2.00498863469303	1.57795724268149	-1.4372127043276	0.160190744820133	0.828484548442988	-4.90576209350123
a_HQ_transcript/69802	-1.12690604140982	-0.432334532866285	-1.4371596982413	0.160205697255516	0.828484548442988	-4.85793446164575
a_HQ_transcript/73312	-0.96169778490169	2.39827983341164	-1.43711547249439	0.160218173698374	0.828484548442988	-4.98486180088718
a_HQ_transcript/18517	1.06983639845707	2.33508707783357	1.43709350778309	0.160224370407714	0.828484548442988	-4.92777356893558
a_HQ_transcript/115496	1.19011604009506	-0.278884428488751	1.43706021065859	0.160233764591911	0.828484548442988	-4.92397499011977
a_HQ_transcript/121158	1.54414448786537	-0.355929186244957	1.43701752331733	0.160245808694047	0.828484548442988	-4.9308983498375
a_HQ_transcript/139941	3.63207724285148	2.89643286718707	1.43697196538817	0.160258663513723	0.828484548442988	-5.14590768244742
a_HQ_transcript/89265	1.89648803840511	0.563946526335137	1.4369447472233	0.1602663438965	0.828484548442988	-4.88910621408367

a_HQ_transcript/110687	1.63898940927045	0.4215115470501	1.43675876894278	0.1603188307733	0.828484548442988	-4.90597443045984
a_HQ_transcript/20773	-1.32228181997002	1.90910046116704	-1.43672513183988	0.160328325303945	0.828484548442988	-4.89175263785191
a_HQ_transcript/150878	-5.47701101839876	1.71363905014526	-1.4366851015651	0.16033962497407	0.828484548442988	-5.11069934449741
a_HQ_transcript/123727	1.91965109248363	0.804883811449191	1.4366634970637	0.160345723713517	0.828484548442988	-4.88832111746112
a_HQ_transcript/22877	-1.13576703551917	2.26948041012852	-1.43660427379555	0.160362442803791	0.828484548442988	-5.03331120144414
a_HQ_transcript/115171	0.623255381449066	2.99703438052207	1.43659535095065	0.160364961896918	0.828484548442988	-5.12334121151258
a_HQ_transcript/23217	-1.94923123399487	2.69211236589355	-1.43658585333595	0.160367643293155	0.828484548442988	-5.06996597254876
a_HQ_transcript/69757	-1.57122800303805	0.0505137229317867	-1.43657014636372	0.160372077812423	0.828484548442988	-4.95552763887794
a_HQ_transcript/112642	2.19701967284286	1.22042447214335	1.43634516177003	0.160435607925268	0.828721879335572	-4.79692120708464
a_HQ_transcript/86580	0.515738497742441	3.41599211681195	1.43623962792638	0.160465414943224	0.828721879335572	-5.18223855164268
a_HQ_transcript/48489	2.28925875505616	1.35665414630971	1.43618370634471	0.160481211230723	0.828721879335572	-5.04417431572981
a_HQ_transcript/42175	0.721149165335518	1.25409698004235	1.43616331919624	0.160486970337582	0.828721879335572	-4.8383137730162
a_HQ_transcript/151777	0.502001087014921	3.23426098515779	1.43609115853764	0.160507356107401	0.828721879335572	-5.19117753979371
a_HQ_transcript/110412	1.52415653017987	-0.291113305133076	1.43606423412437	0.160514962921938	0.828721879335572	-4.93476949776939
a_HQ_transcript/677	0.729839481907453	2.28220274418253	1.43605413399278	0.160517816533154	0.828721879335572	-4.99078008568373
a_HQ_transcript/86807	-1.61292069875002	1.27357365742178	-1.43599443223067	0.16053468501586	0.828735361509939	-4.9338260916644
a_HQ_transcript/34440	1.61454534122177	0.335471975496622	1.43593646055659	0.160551066010983	0.82874632493441	-4.81015462697206
a_HQ_transcript/263	-0.951226314021888	3.76579057499351	-1.43584542197392	0.160576793355703	0.828805526920643	-5.20321634182849
a_HQ_transcript/145866	2.59488763755084	0.529525872250721	1.43571565681963	0.160613470397485	0.828921229447306	-5.01715871201597
a_HQ_transcript/150449	0.707809297608661	2.65406401465271	1.43557477132813	0.160653298010432	0.829011563199361	-5.09054422162489
a_HQ_transcript/144335	1.29235140580852	0.670265986119918	1.43554003013832	0.160663120368611	0.829011563199361	-4.89616552565813
a_HQ_transcript/55559	-1.55239716778321	-0.41375592083413	-1.43550240324789	0.160673759135081	0.829011563199361	-4.90749064641746
a_HQ_transcript/77347	1.63584905715322	0.939324801521483	1.43539698626248	0.160703568090913	0.829091772964283	-4.8342951928876
a_HQ_transcript/130569	-1.19514103449404	-0.198712794824846	-1.43528299635153	0.160735806149466	0.829184499454755	-4.85168794132882
a_HQ_transcript/56933	1.71958358035239	0.765845413943574	1.43514474261382	0.160774913244568	0.829240432149478	-4.83575421451639
a_HQ_transcript/48829	1.32297663698367	0.307740795521341	1.43505171687597	0.160801231171138	0.829240432149478	-4.90636238536769
a_HQ_transcript/10635	1.23432058967899	0.978812412913031	1.43504415076477	0.160803371850665	0.829240432149478	-4.81336803579644
a_HQ_transcript/33550	-0.940934419887815	-0.107901570582276	-1.43503553217946	0.1608058103341	0.829240432149478	-4.90529634453058
a_HQ_transcript/110752	1.0179850542582	0.576096838157754	1.43496508973671	0.160825741931924	0.829240432149478	-4.77728273886359
a_HQ_transcript/1012	1.84332472383237	0.41060029460558	1.43489005287957	0.16084697566183	0.829240432149478	-4.94136248141107
a_HQ_transcript/25943	-2.04864357779448	1.14288511441129	-1.43488601900544	0.160848117219563	0.829240432149478	-4.88473321600911
a_HQ_transcript/89131	-1.00753426524548	1.03817935193488	-1.43473534467557	0.160890761573212	0.829240432149478	-4.76399667975435
a_HQ_transcript/141009	-1.52509810995709	-0.167387828476352	-1.43472951149469	0.160892412679313	0.829240432149478	-4.94111518899048
a_HQ_transcript/107964	0.528466648101352	4.84364535072909	1.43472747164219	0.160892990071206	0.829240432149478	-5.38686653636514
a_HQ_transcript/1327	-2.31199405280399	0.160396672876686	-1.43465408021445	0.160913765023494	0.829240432149478	-4.9999432168886
a_HQ_transcript/43142	1.78942271314572	0.334145563824504	1.4346396933037	0.160917837782929	0.829240432149478	-4.94278185817544
a_HQ_transcript/102407	2.8102776002204	0.134497179139335	1.43457963844645	0.160934839462641	0.829254529588192	-4.93181074015116
a_HQ_transcript/151152	1.85429190657788	0.0662707365746233	1.43452383357554	0.160950639233525	0.829262431959434	-4.93407389466422
a_HQ_transcript/93255	1.03543896993188	-0.106118020633297	1.43436161925038	0.160996573176272	0.829425578799108	-5.01938262112816
a_HQ_transcript/138155	1.35260793181732	-0.122785156796286	1.43424435028261	0.161029786475371	0.829470410177386	-4.7782049065333
a_HQ_transcript/54755	-1.35708864263397	-0.0704517152088152	-1.43420187854223	0.161041816796051	0.829470410177386	-4.89552711754455
a_HQ_transcript/76284	1.49460541209132	0.204006461899933	1.43417975248602	0.161048084387248	0.829470410177386	-4.88071735756376
a_HQ_transcript/53861	0.567525381952548	2.99401180465888	1.43404749055925	0.161085553905494	0.829589889024411	-5.04125993749053
a_HQ_transcript/62913	1.58237995529256	-0.0932249368916077	1.43394439966912	0.161114764110674	0.829666815044761	-4.90744889165301
a_HQ_transcript/28293	2.17822706017394	0.768407142889785	1.43388164752423	0.161132546616445	0.829684885261823	-4.89390149109422
a_HQ_transcript/72406	0.537527265437592	3.91053694502617	1.43370988550655	0.161181227932372	0.82981119322706	-5.29107693776141
a_HQ_transcript/141863	1.59773994280544	-0.425627423521706	1.43363630518612	0.16120208584798	0.82981119322706	-4.9932145388274
a_HQ_transcript/57938	1.44027262091038	0.0606725440382681	1.43362135111882	0.16120632515904	0.82981119322706	-4.84211167858927
a_HQ_transcript/110965	1.4330038307504	-0.2160764299594	1.4335936472083	0.16121417914108	0.82981119322706	-4.94729935458545
a_HQ_transcript/23793	-0.867269015997516	2.38562868490411	-1.43343030406046	0.161260492620558	0.829976085979084	-4.92382640708591

a_HQ_transcript/118149	1.08575498198604	1.76340671985157	1.43329633433198	0.161298485559741	0.830001677982346	-4.88131508995776
a_HQ_transcript/85878	0.786309065858767	2.30683369449033	1.43322871706867	0.161317664059734	0.830001677982346	-4.96040366862206
a_HQ_transcript/13492	-0.551497689236055	2.88531881200142	-1.43320870569955	0.161323340293755	0.830001677982346	-5.17729675155216
a_HQ_transcript/95225	1.93275556388905	1.43084374924268	1.43311980730212	0.161348558273088	0.830001677982346	-4.98658812490101
a_HQ_transcript/7276	1.66468965334272	0.258811373566788	1.43302902350765	0.161374314301708	0.830001677982346	-5.00690160230164
a_HQ_transcript/49931	1.22800087228066	1.24089323006795	1.4330262176643	0.161375110391814	0.830001677982346	-4.91319261343336
a_HQ_transcript/1537	2.15467055749082	1.69829012921712	1.43298117347587	0.161387891014368	0.830001677982346	-4.90642845459818
a_HQ_transcript/150655	2.12907867872343	3.67357299924049	1.43297547602399	0.161389507639094	0.830001677982346	-5.05291827882172
a_HQ_transcript/137780	2.08378446075722	1.14341632619465	1.43295973275183	0.16139397478383	0.830001677982346	-4.98741758316448
a_HQ_transcript/148209	-2.42789375551718	1.62041905022058	-1.43266009721227	0.161479014830845	0.830299911930567	-5.00549186033059
a_HQ_transcript/75959	-1.30719551183941	0.292781352292378	-1.43257749548631	0.161502464385146	0.830299911930567	-4.97437477099302
a_HQ_transcript/7391	1.91513832407804	2.37100537182733	1.43252445314739	0.161517523833078	0.830299911930567	-4.94172740091123
a_HQ_transcript/70432	0.921221279276383	2.31061633902276	1.43250725260259	0.161522407542036	0.830299911930567	-4.945359301646
a_HQ_transcript/24637	2.17273896080011	0.201094769108983	1.43238743776844	0.161556429514489	0.830299911930567	-4.92750123486128
a_HQ_transcript/95816	2.85345834249504	0.365834325384027	1.43228326970862	0.161586013114721	0.830299911930567	-4.93275103735958
a_HQ_transcript/10262	-0.765396726164385	2.25988426787526	-1.43203909647847	0.161655374785891	0.830299911930567	-4.99158643593425
a_HQ_transcript/111989	0.462528361190214	3.38178710009278	1.43202166948541	0.161660326126924	0.830299911930567	-5.22849675405079
a_HQ_transcript/72345	1.8618017394989	2.03376598262964	1.43199815747608	0.161667006526986	0.830299911930567	-5.08037357679435
a_HQ_transcript/65224	2.19480632981442	1.54724084717482	1.43191075401772	0.161691842134414	0.830299911930567	-4.90012534452451
a_HQ_transcript/15520	1.24061987022506	2.02343185257895	1.43186096806227	0.161705990112523	0.830299911930567	-4.97062534868318
a_HQ_transcript/94030	-1.3219424503776	1.91723058309016	-1.43182779638513	0.161715417252906	0.830299911930567	-4.85876173379934
a_HQ_transcript/9649	0.716470638514262	2.20208682569083	1.43181678167931	0.161718547645221	0.830299911930567	-5.00587686360634
a_HQ_transcript/121174	1.01456293872982	3.0330497076112	1.43178528111419	0.161727500407015	0.830299911930567	-5.18163995937346
a_HQ_transcript/78236	1.58702205994953	-0.514556450611839	1.43176596756463	0.161732989696093	0.830299911930567	-4.94053631898463
a_HQ_transcript/122358	2.36667451007793	1.8621048268763	1.43160328382586	0.16177923344308	0.830299911930567	-4.98684932314489
a_HQ_transcript/2965	0.937798902917107	1.62231249105985	1.43155367582163	0.161793336869038	0.830299911930567	-4.898643323661
a_HQ_transcript/43730	1.45458234873165	0.737909857134574	1.43151429038054	0.161804534738679	0.830299911930567	-4.91748794759441
a_HQ_transcript/36518	0.959483128115681	1.78915094063195	1.43151266744479	0.161804996176707	0.830299911930567	-4.88149507705945
a_HQ_transcript/106150	1.00674401619076	1.06687365333197	1.43146016323341	0.161819924894571	0.830299911930567	-4.88539004189586
a_HQ_transcript/126196	1.47628175276467	-0.386319981292783	1.4314353830525	0.161826971113596	0.830299911930567	-4.8784305156085
a_HQ_transcript/151351	1.98089052997579	4.71390784008016	1.43138570369424	0.161841098118196	0.830299911930567	-5.32855602615486
a_HQ_transcript/48946	0.950275848331236	1.41985171956917	1.43136581769039	0.161846753248238	0.830299911930567	-4.78054712313394
a_HQ_transcript/37843	0.546259149012611	3.13014541277754	1.43133651300784	0.161855087122134	0.830299911930567	-5.16902323135006
a_HQ_transcript/19314	1.8446485022842	0.784765567143944	1.43133441458468	0.161855683899631	0.830299911930567	-4.96345062134829
a_HQ_transcript/115982	1.34656653534318	0.704004171891115	1.43130561659525	0.161863874030634	0.830299911930567	-4.86030262462599
a_HQ_transcript/17823	1.22178932337154	1.58567906170581	1.4312957270693	0.161866686681426	0.830299911930567	-4.85881117435149
a_HQ_transcript/112423	1.34765607039857	0.408701190857199	1.43123325392083	0.161884455376881	0.830299911930567	-4.8509983487486
a_HQ_transcript/123737	2.84043796811789	1.4578173552139	1.43122106781308	0.161887921545334	0.830299911930567	-4.9537463163581
a_HQ_transcript/150467	-1.46183281474692	2.97809616067142	-1.43116398106184	0.161904159856405	0.830299911930567	-5.20242939223667
a_HQ_transcript/148566	1.4210623771515	-0.44762560479342	1.4310907937024	0.161924979863208	0.830299911930567	-4.95890995754259
a_HQ_transcript/11748	-1.99505376303474	1.93545732306058	-1.43107577038094	0.16192925389909	0.830299911930567	-4.95035623623545
a_HQ_transcript/121190	-3.03155299386238	1.38521296942136	-1.43101701584653	0.161945969992176	0.830299911930567	-5.18997918487812
a_HQ_transcript/18047	0.767370903751913	2.57245279329482	1.43097471121416	0.161958006818196	0.830299911930567	-4.99535482489026
a_HQ_transcript/48605	-1.7424883408369	-0.337762179835681	-1.4309621813799	0.161961572034695	0.830299911930567	-4.93600155759125
a_HQ_transcript/46338	1.44022436447244	-0.210086648747651	1.43094595147118	0.161966190155875	0.830299911930567	-4.9020763988223
a_HQ_transcript/97057	2.56515373226602	3.53849517689094	1.43050402812552	0.162091976716142	0.830770521506378	-5.09831882188474
a_HQ_transcript/87215	2.4998585694815	1.25929029022207	1.4304282945507	0.162113540834175	0.830770521506378	-4.96854799309841
a_HQ_transcript/30250	1.34846817643834	0.854671131151609	1.43026498689841	0.162160048204603	0.830770521506378	-4.93121882851815
a_HQ_transcript/143109	1.37328276149301	-0.273774965985246	1.43024668725317	0.162165260303178	0.830770521506378	-4.92504413326911
a_HQ_transcript/58376	4.47168511411281	2.78113165359224	1.43023984548345	0.162167209007622	0.830770521506378	-5.12391902978133

a_HQ_transcript/32221	0.557562372878776	2.74302173163555	1.43021603545152	0.162173990835027	0.830770521506378	-5.06978406630549
a_HQ_transcript/92801	1.8226492506823	-0.141568951365109	1.43016087062895	0.162189704329141	0.830770521506378	-4.87957265283535
a_HQ_transcript/40852	1.44372558622885	1.12745693695928	1.43015379708925	0.162191719287531	0.830770521506378	-4.85188782929362
a_HQ_transcript/115619	1.32576866188334	-0.627379251706235	1.43012960417781	0.162198610995072	0.830770521506378	-4.94641647824883
a_HQ_transcript/27115	1.1470479214674	1.88068625415573	1.42999365050464	0.162237343704794	0.830770521506378	-4.89156958249784
a_HQ_transcript/118150	1.99128983502314	0.734822297642397	1.42996096479858	0.162246656833235	0.830770521506378	-4.80591393550181
a_HQ_transcript/106164	2.85482640318185	0.761351003515011	1.4299357886948	0.162253830540764	0.830770521506378	-4.91614103924275
a_HQ_transcript/31305	1.8943712631065	1.42254059814369	1.42989289957399	0.162266051992222	0.830770521506378	-4.92587355288942
a_HQ_transcript/62629	-1.51836437537741	0.80712547709377	-1.42988936889391	0.162267058108069	0.830770521506378	-4.92553071337143
a_HQ_transcript/144070	-1.6700844965705	0.0784296008123026	-1.42987071785743	0.16227237305917	0.830770521506378	-4.93455856898008
a_HQ_transcript/25920	-1.21496712945481	1.73941608486664	-1.42963955551437	0.162338258372219	0.831034012689037	-4.81404759260549
a_HQ_transcript/5137	-0.643544499616803	3.60399687944668	-1.42955925641314	0.162361149963746	0.831034012689037	-5.23387839352714
a_HQ_transcript/41345	-1.92175291417912	0.047679767387389	-1.42953968408245	0.162366730011516	0.831034012689037	-4.98867238963628
a_HQ_transcript/95076	1.44673120081723	-0.152010315215484	1.42939286331468	0.162408593263093	0.831036670366335	-4.84055984464404
a_HQ_transcript/59233	1.39521940668718	0.0380623719150854	1.42935497826434	0.162419396874124	0.831036670366335	-4.91838492477216
a_HQ_transcript/130856	-1.93188871515447	1.31081480326624	-1.42932513526726	0.162427907546955	0.831036670366335	-4.99849695542022
a_HQ_transcript/26071	-0.45874906725974	2.97564875427281	-1.42932228827128	0.162428719476143	0.831036670366335	-5.11817613834022
a_HQ_transcript/44008	1.38838154919294	0.802755720542514	1.42928717858418	0.162438732602228	0.831036670366335	-4.91544935087121
a_HQ_transcript/67668	-1.19729977183584	0.33332696039924	-1.429178329665	0.162469778925896	0.831097276111355	-4.95656821317841
a_HQ_transcript/4877	-1.74981371964929	1.55584111369322	-1.42897709919793	0.162527187019859	0.831097276111355	-4.90319287936089
a_HQ_transcript/110343	1.332202889217	0.827855326430523	1.42896812016588	0.162529748978889	0.831097276111355	-4.86671229973959
a_HQ_transcript/3087	1.65991200669156	0.456071439587188	1.42894423755535	0.162536563484369	0.831097276111355	-4.87849889949731
a_HQ_transcript/51557	2.05221220897934	0.532576850281401	1.42892430794359	0.162542250240084	0.831097276111355	-4.93877248667873
a_HQ_transcript/39943	1.43528474934106	1.18990217611991	1.42882159223859	0.162571561838076	0.831097276111355	-4.81768535285486
a_HQ_transcript/36121	1.98246993063286	2.2315089770229	1.42876295093454	0.162588297959638	0.831097276111355	-5.13814632713647
a_HQ_transcript/83704	1.25388760404551	0.197891010021575	1.42874454528074	0.162593551180212	0.831097276111355	-4.92861240602067
a_HQ_transcript/116636	1.46275312991701	-0.25815916468746	1.42871433778618	0.162602173094913	0.831097276111355	-4.96679632081668
a_HQ_transcript/13143	-0.477354185659785	2.71052174354711	-1.42870874700041	0.162603768873521	0.831097276111355	-5.08450705161402
a_HQ_transcript/135225	1.42427996013157	-0.149933395742835	1.42867569045525	0.162613204459573	0.831097276111355	-5.00276010307147
a_HQ_transcript/86164	1.06653457856625	2.19787872028053	1.42864434711005	0.162622151432882	0.831097276111355	-4.97747051698502
a_HQ_transcript/131019	1.06405073653273	-0.747516903644405	1.42854593452441	0.162650245879463	0.831107893983388	-4.88596189721858
a_HQ_transcript/52892	-1.4642621563338	0.467639143503414	-1.4284996546697	0.162663458998928	0.831107893983388	-4.82408282027605
a_HQ_transcript/149350	1.35594433627915	0.965139873735623	1.42843337271586	0.16268238428894	0.831107893983388	-4.88993929361018
a_HQ_transcript/150266	-4.51457987693276	3.28445105947606	-1.42837505696427	0.162699036450099	0.831107893983388	-5.13786313228895
a_HQ_transcript/52041	0.686509442205934	3.3766510785498	1.42836061409478	0.162703160843701	0.831107893983388	-5.19424194261749
a_HQ_transcript/21302	1.13599710302402	0.639597818371374	1.42833660782018	0.162710016403987	0.831107893983388	-4.80475601506926
a_HQ_transcript/143518	-3.93057417643164	0.484615023255563	-1.42814212451572	0.162765564124261	0.831183372579556	-4.95683294539816
a_HQ_transcript/129161	-0.743146234777162	1.114919597002	-1.42811336863834	0.162773778559133	0.831183372579556	-4.8351657525685
a_HQ_transcript/127540	0.717422050420292	3.00563677358695	1.42808566457605	0.162781692840859	0.831183372579556	-5.11942103220274
a_HQ_transcript/114763	1.12968945098574	1.22457745577073	1.42799035742542	0.162808921768493	0.831183372579556	-4.84803103328403
a_HQ_transcript/70212	1.44762648209925	2.6609781721721	1.42797799850512	0.162812452933136	0.831183372579556	-5.00441632048138
a_HQ_transcript/93288	-1.71018189218678	1.96258847834336	-1.42796835440045	0.162815208468375	0.831183372579556	-5.06082505308159
a_HQ_transcript/149279	1.99566833217221	-0.154469983472568	1.4278738300856	0.162842218116284	0.831183372579556	-4.94687765229282
a_HQ_transcript/108658	-1.86132936711185	3.18538659930576	-1.42786304223792	0.162845300891536	0.831183372579556	-5.16435607442488
a_HQ_transcript/147495	1.66232211320926	0.688412732246228	1.42782375404996	0.162856528418784	0.831183372579556	-4.82477893228399
a_HQ_transcript/32591	2.82867843292071	1.40833647352223	1.42775800706681	0.162875318538089	0.831183372579556	-4.99792110057643
a_HQ_transcript/119226	1.42050277027128	0.798159299445659	1.42767551729333	0.162898896073124	0.831183372579556	-4.82408341041656
a_HQ_transcript/83405	-1.2998975650675	1.85057502715787	-1.42762500757732	0.162913334278335	0.831183372579556	-4.9696341692072
a_HQ_transcript/79675	2.78353971045624	2.55044066369321	1.4275580648772	0.162932471409267	0.831183372579556	-4.92803925930041
a_HQ_transcript/47848	1.12617907421666	0.692834475264699	1.42750305321929	0.162948199101707	0.831183372579556	-4.79223175030809

a_HQ_transcript/136673	0.987013818224764	3.01218075876371	1.42749722965917	0.162949864112793	0.831183372579556	-5.02771943970196
a_HQ_transcript/75473	-1.1048157012556	-0.607397475585192	-1.42745507898287	0.162961915791335	0.831183372579556	-4.93206458449816
a_HQ_transcript/140548	0.422858539599013	5.50001035054922	1.4274342219936	0.162967879460659	0.831183372579556	-5.47956088926054
a_HQ_transcript/86197	-3.84572804449263	4.57337706749489	-1.42737893554124	0.162983688429888	0.831191072210259	-5.33672201633392
a_HQ_transcript/113818	1.76129628215086	-0.0387041452101164	1.42725415782529	0.163019372632904	0.831300122151545	-4.93679960496491
a_HQ_transcript/59598	1.95178564734618	1.30022483804235	1.42718627656132	0.163038788053893	0.831326199297958	-4.89609788425682
a_HQ_transcript/95623	2.28248196715134	1.01579325663305	1.42707342870589	0.163071068872081	0.831417866099144	-4.99174829909243
a_HQ_transcript/124983	4.05925429949463	0.252507154044263	1.42691186147012	0.163117294946003	0.831468706601706	-4.98106245411267
a_HQ_transcript/7307	1.39021930817072	0.757329884846843	1.42690648911731	0.163118832209868	0.831468706601706	-4.87797988970189
a_HQ_transcript/135977	0.277234464230682	5.18699936940389	1.42685613353112	0.163133241689591	0.831468706601706	-5.45639292823504
a_HQ_transcript/130918	-0.724936605297545	2.13245367419692	-1.42683860802142	0.163138256929405	0.831468706601706	-4.91987543089521
a_HQ_transcript/137931	-1.61767738939956	0.160158846572208	-1.42670911943176	0.163175316188683	0.831470257086542	-4.99292483186292
a_HQ_transcript/104179	-1.4835188772045	1.29474273211924	-1.4266492854283	0.163192442750857	0.831470257086542	-4.89114002207972
a_HQ_transcript/145678	2.2361755221501	3.51934886407175	1.42664555827358	0.163193509638431	0.831470257086542	-5.01960299728472
a_HQ_transcript/89324	2.94509461232255	0.682628779322501	1.42663763508356	0.163195777647611	0.831470257086542	-4.99560681880809
a_HQ_transcript/120624	-1.74681564737671	0.817980968448091	-1.42648927985391	0.163238248850564	0.831613753653427	-5.04985516082821
a_HQ_transcript/135512	-1.60198167970619	-0.267374933097688	-1.4264010106944	0.163263522723579	0.831669621319448	-4.84494304944889
a_HQ_transcript/60114	1.30976630448434	-0.611293657501486	1.42625646598891	0.163304916490758	0.831717804458704	-4.92878908215002
a_HQ_transcript/101522	1.49906193933433	2.70019058419783	1.42607424726709	0.163357110894147	0.831717804458704	-4.90919200600525
a_HQ_transcript/88337	0.981831098217912	1.93429040067382	1.42598827750459	0.163381740487373	0.831717804458704	-4.84307864356433
a_HQ_transcript/145480	2.47876865502504	0.997317107544713	1.42594659186761	0.16339368411633	0.831717804458704	-5.06096473708378
a_HQ_transcript/124561	0.909760104769869	2.84651712412888	1.42594283316227	0.163394761081835	0.831717804458704	-5.10654861249333
a_HQ_transcript/53561	0.470905366181266	3.37573407226413	1.42594026376208	0.163395497284126	0.831717804458704	-5.20022947502052
a_HQ_transcript/124153	1.7329920416565	0.78502195599118	1.42579775596842	0.163436333699237	0.831717804458704	-4.88322175633354
a_HQ_transcript/14925	-3.08363697774303	0.821673059687566	-1.42575889355006	0.163447471342287	0.831717804458704	-4.82964449173835
a_HQ_transcript/24311	-1.32423990618556	4.33189734978172	-1.42575147775916	0.163449596713767	0.831717804458704	-5.35989412748897
a_HQ_transcript/5165	0.495628602870616	3.5512894062802	1.42572935365303	0.163455937629454	0.831717804458704	-5.1222899458811
a_HQ_transcript/15422	-0.503052901012705	2.7451568691197	-1.42565945811275	0.163475971430217	0.831717804458704	-5.10772874089831
a_HQ_transcript/26803	-2.78266170475055	1.99326722430483	-1.42557534821878	0.163500081986152	0.831717804458704	-5.12768040628645
a_HQ_transcript/8836	-0.97674256701968	1.83645239309158	-1.4255606790294	0.163504287275865	0.831717804458704	-4.85634483750986
a_HQ_transcript/99158	1.46100505423099	-0.0235974236577168	1.42545529088317	0.163534501929967	0.831717804458704	-4.90493680179727
a_HQ_transcript/134681	0.422890876039838	4.55769870649627	1.42541899332587	0.16354490941537	0.831717804458704	-5.39441396008816
a_HQ_transcript/152434	1.55373567016781	2.03182466973664	1.42538733354517	0.163553987552126	0.831717804458704	-5.09943348286515
a_HQ_transcript/107397	-1.36849682448585	2.56467717720506	-1.42532801470815	0.163570997726541	0.831717804458704	-4.9915740491782
a_HQ_transcript/139880	1.55191859398886	0.833573585054389	1.42513100728847	0.163627501270921	0.831717804458704	-4.83444617909637
a_HQ_transcript/135756	2.71821214818674	4.72284993366432	1.42505634762436	0.163648918373082	0.831717804458704	-5.36377368115489
a_HQ_transcript/137900	1.83978711887813	0.0944296417674057	1.42502950345688	0.16365661951437	0.831717804458704	-5.04291859451106
a_HQ_transcript/15625	-1.62076315196293	0.888623318095756	-1.42502288598578	0.163658517999725	0.831717804458704	-4.934589781656
a_HQ_transcript/131283	0.351720073633259	4.14691468947773	1.4250112952532	0.163661843305222	0.831717804458704	-5.34582766350995
a_HQ_transcript/115289	1.2317908785462	2.5028960043993	1.42493558994717	0.163683563973942	0.831717804458704	-5.06059530245628
a_HQ_transcript/126012	-2.94483392722253	3.47561206825484	-1.42493341911559	0.163684186842461	0.831717804458704	-5.42818756898352
a_HQ_transcript/112991	-2.0108490726248	1.67937245488188	-1.42489782159846	0.163694400970509	0.831717804458704	-4.91440524611065
a_HQ_transcript/52368	-0.924694339126728	1.66239131233879	-1.4248749043814	0.163700976958853	0.831717804458704	-4.83331917255018
a_HQ_transcript/88283	2.53288755565549	0.142343615071034	1.42486993653761	0.163702402485893	0.831717804458704	-4.95926637511004
a_HQ_transcript/69485	-1.73650401324685	0.273119860668823	-1.42475357916117	0.163735794135109	0.831717804458704	-4.97099614237305
a_HQ_transcript/152228	4.66399425248308	2.13679523443554	1.42474598211121	0.163737974484737	0.831717804458704	-5.11405383331627
a_HQ_transcript/149908	1.0521510607634	3.70699003851118	1.42474180117723	0.163739174420515	0.831717804458704	-5.30698264039278
a_HQ_transcript/131329	-4.14675950858399	1.3912784876929	-1.42473844142018	0.163740138682021	0.831717804458704	-5.09206542747153
a_HQ_transcript/131178	0.406404861025901	2.78314784617654	1.42471050952632	0.16374815540234	0.831717804458704	-5.08872660859845
a_HQ_transcript/53269	-1.50936636502992	-0.131020485402563	-1.42463278858289	0.163770463684389	0.831717804458704	-5.01029218004369

a_HQ_transcript/122146	1.72512264765945	0.492373305042377	1.42460788921257	0.163777611070262	0.831717804458704	-4.97384878448269
a_HQ_transcript/13562	1.11540968943613	3.36020919692328	1.4245697615463	0.163788556126724	0.831717804458704	-5.19944533430238
a_HQ_transcript/133501	1.74108302987019	-0.384362260562289	1.42455685929713	0.163792260020505	0.831717804458704	-4.88280881175862
a_HQ_transcript/33980	1.7430995809772	0.267640529258305	1.42452156744918	0.16380239171214	0.831717804458704	-4.88826767683732
a_HQ_transcript/120431	-2.11381203068458	0.870014679047415	-1.42442800323952	0.163829254803623	0.831781546268232	-4.97308845504274
a_HQ_transcript/138401	1.70425285203888	2.34368827051553	1.42429401342837	0.163867730483705	0.831904230354306	-4.99766391758284
a_HQ_transcript/143631	1.3253347947648	-0.620958284550402	1.42417817442014	0.16390099983495	0.832000464449043	-4.96909586442595
a_HQ_transcript/140250	0.942999413060776	1.65828767059379	1.42407605653012	0.163930332852133	0.832076701714006	-4.81707732456798
a_HQ_transcript/37264	-0.622681357177132	3.1415559945072	-1.42392466386881	0.163973827497384	0.832224800905258	-5.13430232402
a_HQ_transcript/78164	-3.47711120622157	1.55195634750721	-1.42362133832231	0.164060999371316	0.83227142278174	-4.89428519683275
a_HQ_transcript/75201	0.954349269414705	2.17994237564555	1.42356450283155	0.164077337226652	0.83227142278174	-4.96455984596687
a_HQ_transcript/15591	1.79151924830065	2.32933698817448	1.42353710536827	0.164085213323569	0.83227142278174	-4.8890541321278
a_HQ_transcript/59401	-2.00635605658463	4.14243286423149	-1.42351498417391	0.164091572840425	0.83227142278174	-5.29055208564947
a_HQ_transcript/10327	2.55325032726572	2.86666122854075	1.42347114312684	0.164104177068533	0.83227142278174	-5.08203465087413
a_HQ_transcript/135658	1.44545213225058	2.59547196303217	1.42332752204149	0.164145473237794	0.83227142278174	-4.94531989303178
a_HQ_transcript/150154	-1.79435596785629	0.783453805074359	-1.42331614973938	0.164148743529073	0.83227142278174	-4.95770776567396
a_HQ_transcript/144016	-1.00912279637885	4.9304783396591	-1.42323683565474	0.164171553018377	0.83227142278174	-5.42082111531789
a_HQ_transcript/14653	-2.04485964180013	0.327434968301847	-1.42316483072659	0.164192262672531	0.83227142278174	-5.05068147457599
a_HQ_transcript/24192	0.620528934882118	-0.710871652994937	1.42316399867207	0.16419250199543	0.83227142278174	-5.01029235488966
a_HQ_transcript/93634	1.5533464408273	3.89570305633306	1.42313361314968	0.164201241937141	0.83227142278174	-5.38783095687818
a_HQ_transcript/150942	1.77352773799739	0.626072038071416	1.42311546213803	0.164206462979534	0.83227142278174	-4.91019803806105
a_HQ_transcript/26005	-5.10925714739414	0.861822364485525	-1.42304832472755	0.164225775837471	0.83227142278174	-5.04541939532077
a_HQ_transcript/139474	1.91032799372483	1.02068970266301	1.42301557634471	0.164235196940866	0.83227142278174	-4.90488559477571
a_HQ_transcript/29251	-1.35650481637334	1.44889222818252	-1.42301360578575	0.164235763847782	0.83227142278174	-4.87325873378128
a_HQ_transcript/68452	1.69211776488905	3.18652588006052	1.42276734628545	0.164306621987557	0.83227142278174	-5.2271994254835
a_HQ_transcript/146129	-1.57675269714163	0.642480332119093	-1.422715347925	0.16432158695666	0.83227142278174	-5.01546232908737
a_HQ_transcript/147567	-1.4949534035376	2.2312073162184	-1.4227102492369	0.164323054401339	0.83227142278174	-4.95058397579322
a_HQ_transcript/9804	-0.887558481064596	1.79229800123736	-1.42270441420246	0.16432473378527	0.83227142278174	-4.8725030486934
a_HQ_transcript/128657	-1.47038363400522	-0.168978085967056	-1.42270046888023	0.164325869297932	0.83227142278174	-4.95652111765873
a_HQ_transcript/116033	-1.52147534775665	0.753790333289235	-1.42258420095387	0.164359335425926	0.83227142278174	-4.83935868357116
a_HQ_transcript/61100	0.85050361826656	-0.640757830552727	1.42241491489541	0.164408071701251	0.83227142278174	-4.91750087058509
a_HQ_transcript/106179	-1.95672816103933	0.799099573267866	-1.42235887881901	0.164424206605766	0.83227142278174	-4.97908550974541
a_HQ_transcript/13523	1.87018893109192	1.11643768843192	1.42235364339476	0.164425714146051	0.83227142278174	-4.93781240851118
a_HQ_transcript/130137	1.28462197900266	2.86225699680628	1.42233594453351	0.164430810613382	0.83227142278174	-5.06816930162219
a_HQ_transcript/150392	1.52908928272371	3.34006798821856	1.42230770577346	0.164438942350156	0.83227142278174	-5.1261652824783
a_HQ_transcript/49698	-1.21934252994647	2.13679117522343	-1.42225803548888	0.164453246356302	0.83227142278174	-4.89178357845384
a_HQ_transcript/45255	-0.542771109071429	2.6081573067345	-1.42225754736868	0.164453386929606	0.83227142278174	-5.17731703716423
a_HQ_transcript/23980	2.47158440975954	0.330062674799938	1.42221066769126	0.164466888207746	0.83227142278174	-4.9083265563782
a_HQ_transcript/92694	1.41894882934586	1.16941281199553	1.42216288149262	0.164480651461762	0.83227142278174	-4.85657434696589
a_HQ_transcript/120087	1.41430406227252	2.23414730796159	1.42212284356799	0.164492183777375	0.83227142278174	-5.20486719591781
a_HQ_transcript/85257	0.787932148839012	2.19861723758798	1.42211011132899	0.164495851238834	0.83227142278174	-4.83798252833686
a_HQ_transcript/35865	-1.75830393577348	0.249253675235485	-1.42208606088213	0.164502779032582	0.83227142278174	-4.80673372618043
a_HQ_transcript/71996	-0.751659713876588	3.13598381700201	-1.42202427997642	0.164520576237143	0.83227142278174	-5.23037663181772
a_HQ_transcript/14330	1.55699115692933	0.457296964342908	1.42191261656467	0.164552746928206	0.83227142278174	-5.00405076751124
a_HQ_transcript/8585	-0.748467100141407	2.14500712395493	-1.42191132279274	0.164553119698305	0.83227142278174	-5.0638026375682
a_HQ_transcript/52098	1.74863152681392	-0.442771545451265	1.42190804715754	0.164554063498892	0.83227142278174	-4.94296413563152
a_HQ_transcript/17005	1.60450543668862	0.838301869992225	1.42189477116987	0.164557888719741	0.83227142278174	-4.84810921083802
a_HQ_transcript/16105	2.14301981601223	1.25629535117842	1.42184415015517	0.16457247483585	0.83227142278174	-4.96993377050932
a_HQ_transcript/3333	1.97050688201025	0.562129101316944	1.42174215399422	0.164601867460925	0.83227142278174	-4.86755331969945
a_HQ_transcript/36771	1.27554994833598	0.0021699720978112	1.42165294089412	0.164627579733883	0.83227142278174	-4.92949581779371

a_HQ_transcript/83758	-0.994726676825293	3.02192151271898	-1.42159137832127	0.164645324642741	0.83227142278174	-5.05082815094073
a_HQ_transcript/148146	4.00122930971315	1.93906838972618	1.42153585568169	0.164661329882831	0.83227142278174	-5.20330159649379
a_HQ_transcript/916	2.72501861119786	3.3516746803761	1.42150775289415	0.164669431403441	0.83227142278174	-5.24650543651979
a_HQ_transcript/16634	1.34096449037148	0.320764257252031	1.42150328947886	0.164670718153491	0.83227142278174	-4.92584875613535
a_HQ_transcript/141738	0.522868022750903	3.092996887413	1.4214699350577	0.164680334091046	0.83227142278174	-5.15218675260572
a_HQ_transcript/143903	1.26400416869513	-0.457698075374977	1.42142187664902	0.164694189903646	0.83227142278174	-4.98027107952541
a_HQ_transcript/13858	2.98832886884018	3.97501803158377	1.42135887816824	0.164712354508669	0.83227142278174	-5.23405294871378
a_HQ_transcript/136695	1.04911352499497	3.24808274991464	1.42135413653752	0.164713721745982	0.83227142278174	-5.11448473866036
a_HQ_transcript/11791	-1.54813132543517	2.50851229579542	-1.42134385863312	0.16471668538461	0.83227142278174	-5.02968494998299
a_HQ_transcript/127534	1.30081824753358	-0.53322625885068	1.42124757839476	0.16474444987765	0.83227142278174	-4.95031046813173
a_HQ_transcript/32001	-1.05644230749526	2.08518737689038	-1.42124733872704	0.16474451899563	0.83227142278174	-4.90018655220482
a_HQ_transcript/127968	1.23098590962902	1.2286933945227	1.42121652334307	0.164753406061248	0.83227142278174	-4.84116735096053
a_HQ_transcript/54168	-1.5446794438064	-0.324519977512031	-1.42120690329599	0.164756180531749	0.83227142278174	-4.94091736506146
a_HQ_transcript/146062	2.30379699316444	1.5510899711393	1.42112051573186	0.164781096794297	0.83232214222432	-4.91534860360873
a_HQ_transcript/63802	-1.92252994034697	1.41769409095938	-1.42104102716018	0.164804025843348	0.83232214222432	-4.8826321520587
a_HQ_transcript/139677	-2.17711696759073	0.848593050013697	-1.4209200889257	0.164838916167087	0.83232214222432	-4.90807968530661
a_HQ_transcript/15898	1.34719336326034	-0.296645812858618	1.4208763913261	0.164851524226846	0.83232214222432	-4.91208361396684
a_HQ_transcript/96085	1.46684323201135	-0.435102692498912	1.42087302407994	0.164852495809242	0.83232214222432	-4.95634163438864
a_HQ_transcript/11655	1.24106247997455	0.47614782573518	1.42085000622681	0.164859137482209	0.83232214222432	-4.76209151980279
a_HQ_transcript/48896	-1.24593717418865	2.32355786392114	-1.42075871941068	0.164885479859228	0.83232214222432	-5.11251570710315
a_HQ_transcript/149002	-3.4246902283504	-0.00834697745337384	-1.42073095255678	0.164893493119059	0.83232214222432	-4.97272954337342
a_HQ_transcript/105498	3.29152250140278	3.45432450928123	1.42065055060783	0.164916698115955	0.83232214222432	-5.14811168524922
a_HQ_transcript/152115	3.69474404872547	5.21279852026122	1.42062587296671	0.164923820904392	0.83232214222432	-5.46837030020357
a_HQ_transcript/100416	-2.22330254546236	2.45486828172719	-1.4205524080957	0.164945026745641	0.83232214222432	-5.15745729608131
a_HQ_transcript/10809	-1.52591805302969	1.292579397241	-1.42052969633788	0.164951582992974	0.83232214222432	-4.8684491550645
a_HQ_transcript/124700	-2.32442268160622	1.23009773945877	-1.4205269870637	0.164952365098131	0.83232214222432	-4.84151428009269
a_HQ_transcript/138608	0.448504924110753	3.620927963373	1.42039349281419	0.164990905436819	0.832339935804205	-5.27958406542807
a_HQ_transcript/45906	-1.62655768540727	-0.401701884183757	-1.42039192462537	0.164991358221531	0.832339935804205	-4.93128379060458
a_HQ_transcript/144554	-2.81791268568021	1.43383157661797	-1.42036598217751	0.164998848752268	0.832339935804205	-5.00431805585366
a_HQ_transcript/126361	0.650183397344304	5.3977491170944	1.4201292219854	0.165067222433487	0.832453388455395	-5.46510367818711
a_HQ_transcript/122012	-1.35610033093648	-0.262518824873725	-1.42009240475919	0.165077856839386	0.832453388455395	-4.8974125040136
a_HQ_transcript/146720	2.2247914874479	2.15414422420352	1.4199906542535	0.165107249589158	0.832453388455395	-5.12862389485218
a_HQ_transcript/5247	-1.99209117291096	0.63031456824229	-1.41999838689355	0.165109209815997	0.832453388455395	-4.99831363826817
a_HQ_transcript/122158	2.31514845468612	0.129092946609237	1.41998174449208	0.165109823555335	0.832453388455395	-5.04371638547773
a_HQ_transcript/68206	1.65044704884872	0.222616359444145	1.41995761495653	0.165116794563829	0.832453388455395	-4.96288796045617
a_HQ_transcript/81994	4.34437878571644	3.36667667747116	1.4199063191446	0.165131614663728	0.832453388455395	-5.22091995988494
a_HQ_transcript/19682	-1.85194707197357	0.633284140739309	-1.41989146184616	0.165135907347219	0.832453388455395	-4.98469764809858
a_HQ_transcript/87331	-0.913919620217502	2.81399298702555	-1.41967444713279	0.165198618905412	0.832574328710826	-5.04148847902533
a_HQ_transcript/137829	1.60029864497164	-0.195110103340646	1.41964164124324	0.165208100578689	0.832574328710826	-4.93844347834017
a_HQ_transcript/61963	-1.37746755209839	0.703635819636561	-1.41963343945713	0.165210471154658	0.832574328710826	-4.98049988847082
a_HQ_transcript/147037	1.67450191898909	-0.275865794922219	1.41960224977041	0.165219486206692	0.832574328710826	-4.96990547919592
a_HQ_transcript/36069	1.61310178140101	0.0966975458456109	1.41952989531448	0.165240400995786	0.832574328710826	-4.84707343761475
a_HQ_transcript/125096	-0.823055484876521	3.18088383165949	-1.41944254165717	0.16526565423457	0.832574328710826	-5.1068014171161
a_HQ_transcript/11168	1.30511700516076	1.25214293956032	1.4193537322217	0.165291331443091	0.832574328710826	-4.86009420198389
a_HQ_transcript/70499	2.84208599033465	0.938750328769205	1.41930640779735	0.165305015496674	0.832574328710826	-4.91161967825997
a_HQ_transcript/137950	-2.09855582352769	3.03166763804565	-1.41928059228135	0.165312480536293	0.832574328710826	-5.2798538397977
a_HQ_transcript/112893	-1.24885918117576	2.71385085914857	-1.41923540276578	0.16532554856965	0.832574328710826	-5.10308271501609
a_HQ_transcript/121143	1.30783749050233	-0.33555980600626	1.41922727556197	0.165327898904008	0.832574328710826	-4.92369850255148
a_HQ_transcript/143203	-1.54507129567196	1.64254735162421	-1.41921386912088	0.165331776016878	0.832574328710826	-4.88717672697756
a_HQ_transcript/79063	-1.42608943386079	0.741524046040948	-1.41913049939391	0.165355887963263	0.832623618808778	-4.83667823928236

a_HQ_transcript/55103	3.02077865694373	1.1415628464027	1.41875395981261	0.165464824131308	0.832953820177176	-4.98718435350137
a_HQ_transcript/121494	1.10721015860447	0.252285152805169	1.41874930593981	0.165466170890572	0.832953820177176	-4.92409864149158
a_HQ_transcript/149906	1.80935691765347	5.61845073686553	1.41870682097102	0.165478465786687	0.832953820177176	-5.54406972566779
a_HQ_transcript/87981	1.80054295302799	2.01073211983695	1.41868848295613	0.165483772920895	0.832953820177176	-5.08847723319073
a_HQ_transcript/131371	1.42052190359906	0.327018627894508	1.4186562110156	0.165493112944299	0.832953820177176	-4.94364487918995
a_HQ_transcript/101866	1.24105945349992	-0.239437353652117	1.41855786516304	0.165521578390346	0.832953894467892	-4.95315658777319
a_HQ_transcript/101227	0.830049286522606	2.62788753633801	1.41855356958309	0.165522821800598	0.832953894467892	-5.04135230515056
a_HQ_transcript/16759	-1.77353985827879	1.21866771924073	-1.41840127990628	0.165566908737255	0.832953894467892	-4.92929494945261
a_HQ_transcript/124973	1.41402038476992	-0.43865300968237	1.41839870696788	0.165567653666714	0.832953894467892	-4.96350499061743
a_HQ_transcript/128322	-1.99333947954466	5.20442460186982	-1.41837849584392	0.165573505380013	0.832953894467892	-5.53609076407113
a_HQ_transcript/15970	3.37118414876424	2.45165484526929	1.41835915385931	0.165579105604468	0.832953894467892	-5.09919240676576
a_HQ_transcript/4697	-1.00738331741654	3.63862972089333	-1.41824396054376	0.165612461446056	0.833016022114606	-5.22397700247317
a_HQ_transcript/104448	0.964116477167899	-0.439695576248366	1.41821752452217	0.165620117113585	0.833016022114606	-4.98940827432008
a_HQ_transcript/15453	-1.45614112641596	2.27830306744085	-1.41814548822791	0.16564097968178	0.833023310810626	-5.02869729470543
a_HQ_transcript/138460	-2.04234360717163	2.47718727255289	-1.41807707692586	0.165660794324619	0.833023310810626	-5.0521023825249
a_HQ_transcript/79998	-1.24035214765268	-0.344439008009498	-1.41806408062259	0.165664558783561	0.833023310810626	-4.95123955959558
a_HQ_transcript/105415	1.14867546216525	-0.542306136142279	1.41789569206622	0.165713339651397	0.833196523419807	-4.97479998297035
a_HQ_transcript/145115	3.01424995293902	0.80676698431806	1.41782083088743	0.165735029989318	0.833233059061688	-5.06791285578099
a_HQ_transcript/146479	-1.41624069186496	-0.123316846460582	-1.41774232480213	0.165757778806362	0.833233059061688	-4.84461520006136
a_HQ_transcript/94808	-2.4412482209128	0.720836697060493	-1.41767462483371	0.165777398293882	0.833233059061688	-5.03101678208546
a_HQ_transcript/150915	0.93937817983614	2.59023795682203	1.41765276265953	0.165783734354295	0.833233059061688	-5.0144766993952
a_HQ_transcript/1544	-1.04348335146183	-0.530746439857906	-1.4174311586962	0.165847970021963	0.833233059061688	-4.95473531453608
a_HQ_transcript/22367	1.17757811029578	2.63872301191545	1.41739077954311	0.165859676714452	0.833233059061688	-5.0616380858926
a_HQ_transcript/80444	1.23788908604315	-0.542444308635056	1.41738339443719	0.165861817868951	0.833233059061688	-4.96191977516973
a_HQ_transcript/8007	-0.370523171041102	4.1651765792189	-1.41733306507563	0.165876410377065	0.833233059061688	-5.3694099179856
a_HQ_transcript/144476	-1.35538598563783	1.59184464878043	-1.41733203254222	0.165876709760648	0.833233059061688	-4.83939158967089
a_HQ_transcript/62973	2.24117635902742	1.96233357858156	1.4173152874925	0.16588156505565	0.833233059061688	-4.96746156822047
a_HQ_transcript/106393	-2.2984412679782	1.35011362245721	-1.41718556377414	0.165919182763798	0.833233059061688	-5.03772849668606
a_HQ_transcript/12850	1.75204922963504	1.07764724677708	1.41716837122928	0.165924168818106	0.833233059061688	-4.93933834219166
a_HQ_transcript/61576	0.64939861664471	3.34092956799801	1.41713086460812	0.165935046619425	0.833233059061688	-5.1789829286859
a_HQ_transcript/111803	1.28624117436561	3.19393595873523	1.41707457525332	0.165951372908184	0.833233059061688	-5.13603091710217
a_HQ_transcript/100305	-0.573685021620763	2.40254208560715	-1.41707189553976	0.165952150169749	0.833233059061688	-5.0471439437866
a_HQ_transcript/116145	-3.24828842216553	4.25397913461628	-1.4170577567456	0.165956251230625	0.833233059061688	-5.36737055943802
a_HQ_transcript/113	1.79493252973006	3.14084173284727	1.41703003668137	0.165964291869946	0.833233059061688	-5.15741784104413
a_HQ_transcript/111495	-0.449470422933745	3.0704284218593	-1.41678460914166	0.166035495353433	0.833518548571063	-5.13919717568105
a_HQ_transcript/134611	2.97541897322725	5.98370131833396	1.41667020271794	0.166068695181801	0.833584795136772	-5.44181660592909
a_HQ_transcript/146825	2.57472734500314	0.0164264117632212	1.41664030346994	0.166077372565355	0.833584795136772	-4.9526901794779
a_HQ_transcript/141478	0.409782399533312	3.15031776802638	1.41656613357251	0.166098899752641	0.83361654317578	-5.18866380887674
a_HQ_transcript/136349	0.380528097250624	4.57929114847909	1.41651969279956	0.166112379913382	0.83361654317578	-5.38135538042142
a_HQ_transcript/63187	1.09302597994029	-0.611403862822447	1.41641532494003	0.166142677458854	0.833617275830504	-4.94985864417413
a_HQ_transcript/150598	0.626307809377882	4.08805976203567	1.41640333870441	0.166146157290446	0.833617275830504	-5.26395226525229
a_HQ_transcript/86205	-1.8374375145722	2.29409148484106	-1.41635710702688	0.166159579760788	0.833617275830504	-5.02682671447558
a_HQ_transcript/82848	3.00433725144106	0.747640295359243	1.41617408153507	0.16621272603718	0.833617275830504	-4.97257824148229
a_HQ_transcript/117043	3.6343641268393	1.1495855437743	1.41613182111023	0.166224999366417	0.833617275830504	-4.96625788134962
a_HQ_transcript/43342	1.60682942562922	0.902621693538839	1.41611382518803	0.16623022598278	0.833617275830504	-4.90095896994347
a_HQ_transcript/59451	-1.6579532818808	2.60416556176954	-1.41599000328903	0.166266191500712	0.833617275830504	-5.13060812015519
a_HQ_transcript/101205	-1.05974905432058	1.42409787779329	-1.41595598890392	0.166276072448505	0.833617275830504	-4.90796431619588
a_HQ_transcript/67180	0.766747681661668	2.65131260859113	1.41590115917763	0.166292001084269	0.833617275830504	-4.9994053452472
a_HQ_transcript/107765	-1.32977096379706	-0.209127168833638	-1.41586294248407	0.166303104162262	0.833617275830504	-4.96666400914129
a_HQ_transcript/33705	-0.937296665957764	-0.155974097178756	-1.41586173748098	0.166303454260731	0.833617275830504	-4.89051692621083

a_HQ_transcript/149711	-1.41229716472454	2.53975571024758	-1.41581836109805	0.166316057108533	0.833617275830504	-5.09493555771314
a_HQ_transcript/30683	-1.49841481616173	2.49726117204769	-1.41575645594865	0.166334044722609	0.833617275830504	-4.94506479105103
a_HQ_transcript/26592	2.1549115790363	1.67322090879075	1.41559879028981	0.166379864126707	0.833617275830504	-4.9415780300146
a_HQ_transcript/89811	1.2454238197002	0.240476254017974	1.41552121910743	0.166402410824833	0.833617275830504	-4.89783002143158
a_HQ_transcript/27917	-0.611982755331619	2.14599500206448	-1.41541873524611	0.166432202282801	0.833617275830504	-5.02849904744109
a_HQ_transcript/126939	-1.72571147040585	0.453819277948035	-1.41539382547575	0.166439444041018	0.833617275830504	-4.95860809328024
a_HQ_transcript/55373	3.10030586819431	1.19849670702052	1.4153411205098	0.166454767224326	0.833617275830504	-5.19270847963562
a_HQ_transcript/112415	-2.47960029227382	6.28841633987902	-1.41526215722015	0.166477726701047	0.833617275830504	-5.5710789766826
a_HQ_transcript/133176	2.04039354554527	1.61356388474399	1.41525361701611	0.166480210012043	0.833617275830504	-4.9821881197051
a_HQ_transcript/137376	-1.39247806820523	-0.20390415194191	-1.41516732681358	0.166505303015763	0.833617275830504	-4.97566475235182
a_HQ_transcript/139113	0.777317958676684	2.63590415196707	1.41502904529848	0.166545521185435	0.833617275830504	-5.13478780647237
a_HQ_transcript/55756	1.27188204956228	-0.54566455926579	1.41501593915122	0.166549333409209	0.833617275830504	-4.91767541795093
a_HQ_transcript/81274	1.70246655317331	2.06151164892272	1.41500633752388	0.166552126306493	0.833617275830504	-5.00617556526404
a_HQ_transcript/129763	-0.704408867222383	3.25506666446528	-1.41495780479197	0.166566243949665	0.833617275830504	-5.25970998404822
a_HQ_transcript/123836	1.3933577577573	-0.452729123996902	1.41489812405253	0.166583605717456	0.833617275830504	-4.9748717205619
a_HQ_transcript/92869	-0.905600468417628	3.24401941119455	-1.4147430803724	0.166628716241984	0.833617275830504	-5.14016659135842
a_HQ_transcript/81560	1.83529710824815	0.512106243954418	1.41474023458374	0.166629544324382	0.833617275830504	-5.04828868801431
a_HQ_transcript/7010	2.19222549648577	3.0790904995636	1.41471927090827	0.166635644543576	0.833617275830504	-5.07386255552278
a_HQ_transcript/115422	-2.87388186375537	3.88803437988808	-1.41468783772157	0.166644791614674	0.833617275830504	-5.12545296338054
a_HQ_transcript/61490	2.16321848411841	1.38768973177328	1.41467223289766	0.166649332771986	0.833617275830504	-4.84011903059232
a_HQ_transcript/34677	1.12391309846189	1.85254725200316	1.41464623798984	0.166656897762107	0.833617275830504	-4.85097738688342
a_HQ_transcript/77476	1.69580423685009	2.13402203329412	1.4145235250187	0.166692613112336	0.833617275830504	-4.96774505988525
a_HQ_transcript/78151	1.62534343294909	0.172735269434768	1.41449056369187	0.166702207462416	0.833617275830504	-4.87150354351116
a_HQ_transcript/126701	1.37721036847778	1.23807356792577	1.41447699230574	0.166706157934176	0.833617275830504	-4.94555867866343
a_HQ_transcript/15341	1.43262349928047	-0.0710068119565169	1.41444044669041	0.16671679630149	0.833617275830504	-4.87102984795964
a_HQ_transcript/104605	-2.01031742398534	0.218929505265873	-1.4144190419778	0.166723027425402	0.833617275830504	-4.93491728909321
a_HQ_transcript/28652	-0.812997307005373	4.09862598936809	-1.41437943566893	0.166734557697426	0.833617275830504	-5.30784230515128
a_HQ_transcript/127657	-1.78220067126872	0.887613960418976	-1.41434290651325	0.166745192698166	0.833617275830504	-4.93409249909776
a_HQ_transcript/68994	-1.80046622539019	1.65226797608018	-1.41428236926154	0.16676281852666	0.833617275830504	-4.88742287413481
a_HQ_transcript/4002	1.51947520762434	1.00460390334444	1.41412169197792	0.166809607906779	0.833617275830504	-4.96341424869816
a_HQ_transcript/120362	3.52963457842091	2.42407892242667	1.41404550907925	0.166831796048699	0.833617275830504	-4.9902072362487
a_HQ_transcript/18339	1.83752970463328	0.343810007779322	1.41403024902084	0.16683624079426	0.833617275830504	-4.87340476523909
a_HQ_transcript/98741	-0.878577521750572	1.70998163902595	-1.41402379063335	0.166838121934974	0.833617275830504	-4.87932212426788
a_HQ_transcript/137363	-1.30874851595584	1.59839934360776	-1.41401617463003	0.166840340276997	0.833617275830504	-4.8332921837887
a_HQ_transcript/9587	-1.11623818656101	1.54292171288467	-1.41398112012177	0.166850551035016	0.833617275830504	-4.88577502242046
a_HQ_transcript/117466	-2.18986899497743	-0.0505498735009722	-1.41395053516062	0.166859460294537	0.833617275830504	-5.00481737287049
a_HQ_transcript/17050	0.692980420721716	5.07132632845341	1.41391954695213	0.166868487399762	0.833617275830504	-5.44289896780217
a_HQ_transcript/29231	2.2273070941889	0.715623426996526	1.41391378582997	0.166870165701627	0.833617275830504	-4.92148391882634
a_HQ_transcript/132957	2.25520697163993	1.15041274048746	1.41380399709468	0.16690215135502	0.833617275830504	-4.96668871123449
a_HQ_transcript/22178	2.0501539579365	1.05201117507661	1.41369854387555	0.16693287844683	0.833617275830504	-5.04233053440412
a_HQ_transcript/364	-1.2543598175232	3.09627074170538	-1.4136257957759	0.166954078472873	0.833617275830504	-5.22047638685744
a_HQ_transcript/122157	-2.37783245856369	1.60783005487296	-1.41361212256893	0.166958063312906	0.833617275830504	-5.0730370487189
a_HQ_transcript/129164	-0.471638894485213	3.15868190095484	-1.41356575707766	0.166971576359285	0.833617275830504	-5.1778751665571
a_HQ_transcript/10236	-0.692429322121964	2.08164088640388	-1.4135523273779	0.166975490554934	0.833617275830504	-5.01909686613026
a_HQ_transcript/90359	-1.6141702992564	0.54650339735732	-1.41337630698146	0.167026799808117	0.833617275830504	-4.8682839684734
a_HQ_transcript/12319	-1.09930029255374	0.483700961153731	-1.41336064598892	0.167031365526436	0.833617275830504	-4.83387286442381
a_HQ_transcript/141394	-3.05615345981556	1.74171596484871	-1.41333894615338	0.167037691937536	0.833617275830504	-4.94677098728722
a_HQ_transcript/81379	0.533785684420727	2.58018503211063	1.41332802260874	0.167040876679703	0.833617275830504	-5.01789602133597
a_HQ_transcript/106958	-0.883468535095328	2.34809011002422	-1.41332288240932	0.167042375313241	0.833617275830504	-5.00135679363192
a_HQ_transcript/149828	-2.29168151219428	0.550742206967055	-1.41331970692607	0.167043301135849	0.833617275830504	-4.98560956225843

a_HQ_transcript/142954	1.82362979904774	1.1158506756194	1.41330601439568	0.167047293284442	0.833617275830504	-4.9578668007516
a_HQ_transcript/122860	-2.10907835781463	1.64552268673855	-1.41326141046314	0.167060298378858	0.833617275830504	-4.95833479403125
a_HQ_transcript/136245	3.2093774948338	3.01414396587725	1.41321917104912	0.167072614788538	0.833617275830504	-5.22453331258858
a_HQ_transcript/134551	1.67839871294743	0.312140989830657	1.41319058993023	0.167080949039518	0.833617275830504	-4.8200119482508
a_HQ_transcript/35665	1.44887463330814	0.610551619432594	1.41315031938038	0.167092692482054	0.833617275830504	-4.91238229163279
a_HQ_transcript/95809	-0.695854232889003	3.45388703953939	-1.41294221691509	0.167153388347818	0.833617275830504	-5.12769807629657
a_HQ_transcript/145472	2.21959603836069	-0.157007311514454	1.41291461139787	0.16716144116817	0.833617275830504	-5.03601183092077
a_HQ_transcript/119733	-2.53316164586571	1.04145431703618	-1.41287541733597	0.167172875013174	0.833617275830504	-4.94469413361677
a_HQ_transcript/81677	1.17036910531034	0.729070151949158	1.41278442089534	0.167199423221896	0.833617275830504	-4.97854724359778
a_HQ_transcript/148331	1.48454010595548	-0.0872417723179675	1.4127686519546	0.167204024146857	0.833617275830504	-4.95032447685945
a_HQ_transcript/97277	-1.60457754772304	0.534029403441528	-1.41271382297404	0.167220022446778	0.833617275830504	-5.03220358538616
a_HQ_transcript/133647	1.57813125580319	-0.262446152324454	1.41269411780296	0.167225772423405	0.833617275830504	-4.925544543045
a_HQ_transcript/124587	-1.98998455872353	2.8888055704914	-1.41259573783532	0.167254482062022	0.833617275830504	-5.08898276974742
a_HQ_transcript/133328	1.72958398713704	0.974821116287461	1.41256444773378	0.167263614077807	0.833617275830504	-4.86464415983454
a_HQ_transcript/112712	-1.67516809495453	0.0848707567467185	-1.41255660623247	0.167265902681308	0.833617275830504	-4.99864959419604
a_HQ_transcript/76965	-0.323496911587889	4.28123974345047	-1.412554309576	0.167266572983124	0.833617275830504	-5.3662891312683
a_HQ_transcript/118744	0.32756952905811	3.69693891099248	1.41246016696665	0.167294051248892	0.833617275830504	-5.30708893372574
a_HQ_transcript/83010	-3.01160372440673	0.456143976620644	-1.4124566200644	0.167295086585115	0.833617275830504	-4.93343441714039
a_HQ_transcript/10306	-0.653788947278076	3.76311048000667	-1.41238484000182	0.167316040176003	0.833617275830504	-5.22360679321803
a_HQ_transcript/83393	-1.12212304624194	0.988436856878114	-1.41236273973201	0.167322491965184	0.833617275830504	-4.84988626462149
a_HQ_transcript/136353	-1.48625731678422	-0.245686538218871	-1.41235563579764	0.167324565876739	0.833617275830504	-4.97094085368666
a_HQ_transcript/78122	1.24099601531395	2.64760205786475	1.41232298421163	0.16733409838992	0.833617275830504	-5.18745356345374
a_HQ_transcript/128870	-1.74340570744707	2.2259910103017	-1.41228805454291	0.167344296453835	0.833617275830504	-5.0333572031836
a_HQ_transcript/110408	2.08394004580072	1.53356964108776	1.41228619709545	0.167344838767762	0.833617275830504	-4.87780738474491
a_HQ_transcript/140116	0.568498309561935	3.38394080885172	1.41227066990353	0.167349372254181	0.833617275830504	-5.1871392644209
a_HQ_transcript/102848	2.19725234485286	2.12925626654833	1.41223359190486	0.167360198337152	0.833617275830504	-5.07203728331105
a_HQ_transcript/31404	-1.17426178002926	0.892918111290278	-1.41212627268479	0.167391536647856	0.833632147368553	-4.86858997050694
a_HQ_transcript/129482	-2.22266830606876	1.2031335004124	-1.41212514269625	0.167391866640616	0.833632147368553	-5.02544326812198
a_HQ_transcript/69119	-2.10632983995697	0.0544245581550281	-1.41196474437575	0.167438713264327	0.833645623003893	-5.01908371279695
a_HQ_transcript/6788	1.27902921048361	-0.162015263369426	1.41195632229024	0.167441173339629	0.833645623003893	-4.8928079910649
a_HQ_transcript/118814	-2.7344392593388	3.91317162268653	-1.41195068171328	0.167442820957414	0.833645623003893	-5.25240617575906
a_HQ_transcript/55970	1.98176104622186	0.552820152334095	1.41191903429775	0.16745206543333	0.833645623003893	-4.93187908227394
a_HQ_transcript/152577	2.91590588947123	0.623437980306829	1.41185018523926	0.16747217820753	0.833645623003893	-5.01888357386499
a_HQ_transcript/106694	2.07096255103564	0.71907452181941	1.41182128340101	0.167480621824929	0.833645623003893	-4.91380170876218
a_HQ_transcript/21038	1.41583161552543	0.970034783113	1.41140094874305	0.167603459628844	0.834017631978177	-4.86539869133161
a_HQ_transcript/239	-2.56185240600236	2.82841571156142	-1.4113367233217	0.167622234979566	0.834017631978177	-5.02615692868793
a_HQ_transcript/28805	3.41753835997813	2.95274752027685	1.41130846962882	0.167630495051799	0.834017631978177	-4.95958959518109
a_HQ_transcript/73297	1.45294212910806	0.0621628159368986	1.41130373612734	0.167631878939859	0.834017631978177	-4.97815339610698
a_HQ_transcript/21485	1.26131966390436	0.845149175260828	1.41128622839637	0.167636997583906	0.834017631978177	-4.84401625760754
a_HQ_transcript/113881	1.39384439689283	-0.635563244098821	1.41121585490175	0.167657573555053	0.834017631978177	-4.98460271828499
a_HQ_transcript/135049	1.91681203839988	1.24806997312569	1.41113892470972	0.167680068860087	0.834017631978177	-4.91219893250281
a_HQ_transcript/82914	1.55364368115635	0.297477077575616	1.41112250512714	0.167684870448909	0.834017631978177	-4.94981664034898
a_HQ_transcript/99863	1.41018585453444	-0.487969183821111	1.41096933730596	0.167729666616859	0.834017631978177	-4.96516980483105
a_HQ_transcript/21120	-1.7215626949361	0.264721490915476	-1.410925601456	0.167742459531483	0.834017631978177	-4.87392999455173
a_HQ_transcript/65272	1.05915302835232	-0.487522656360103	1.41088826505272	0.167753381188566	0.834017631978177	-4.92028603778586
a_HQ_transcript/136996	-2.21188468112288	0.478741502428626	-1.41088097316805	0.167755514278321	0.834017631978177	-4.98132221263189
a_HQ_transcript/53395	-1.28778066780562	0.0546073679779756	-1.41084446362176	0.167766194710081	0.834017631978177	-4.98618537096663
a_HQ_transcript/112116	4.15616309824648	2.6722614564543	1.41082750450933	0.167771156077665	0.834017631978177	-5.16203724795545
a_HQ_transcript/51212	2.02439497356868	0.773256687868663	1.41079289051082	0.16778128271841	0.834017631978177	-4.82232021930197
a_HQ_transcript/144190	4.45358276615636	1.8946330976654	1.41078043718794	0.16778492616902	0.834017631978177	-5.00793880686686

a_HQ_transcript/85699	-1.81315620116931	0.302194719295593	-1.41063563668142	0.167827294808027	0.834074165408375	-5.04512329246311
a_HQ_transcript/108559	0.878069411184894	2.86070898948046	1.41062462323258	0.167830517688015	0.834074165408375	-5.02175718512459
a_HQ_transcript/133684	-0.870856365373671	2.15377512419677	-1.41054309299893	0.167854377500025	0.834074165408375	-4.98764770775848
a_HQ_transcript/63333	0.554453680609508	3.23984290527961	1.4105327302699	0.167857410342219	0.834074165408375	-5.16656905702319
a_HQ_transcript/43088	1.15547965691723	-0.058579554649932	1.41048788032937	0.167870536994174	0.834074165408375	-4.94655256238399
a_HQ_transcript/134700	-2.08316805821476	0.399478396271799	-1.41030859435221	0.167923018357122	0.834074165408375	-5.03240247554935
a_HQ_transcript/123391	1.9686839834327	-0.340159286235657	1.41020864666337	0.167952281071421	0.834074165408375	-4.96957504386705
a_HQ_transcript/68348	1.55761299368002	-0.369272070889632	1.41017656107572	0.167961675948986	0.834074165408375	-5.03604589189007
a_HQ_transcript/92811	-1.24178293203827	1.61782422736723	-1.41016206242902	0.167965921386479	0.834074165408375	-4.96708295327354
a_HQ_transcript/121211	1.93190481525931	2.77580910279824	1.41013797583546	0.167972974515782	0.834074165408375	-5.02241679231031
a_HQ_transcript/44260	1.55285034444048	1.60094976554653	1.41009740048339	0.167984856473212	0.834074165408375	-4.85135689078165
a_HQ_transcript/57837	-1.06889242248497	0.484054561952343	-1.41000384310583	0.168012256035955	0.834074165408375	-4.80853565729192
a_HQ_transcript/146856	2.0308156472892	0.088315191515009	1.40998601421109	0.16801747787093	0.834074165408375	-4.92117653278376
a_HQ_transcript/72047	1.75368171028959	1.99487871540217	1.40988294854221	0.168047666869358	0.834074165408375	-5.00720954287919
a_HQ_transcript/6212	1.58178574775642	0.802757571358186	1.40984786436678	0.168057944358804	0.834074165408375	-5.03800989871478
a_HQ_transcript/24421	-1.2859714797995	1.92671903996275	-1.40984441018486	0.168058956246775	0.834074165408375	-4.96213955734311
a_HQ_transcript/81346	1.92597104488625	1.1529450701397	1.40983397443361	0.168062013385007	0.834074165408375	-4.88946266308935
a_HQ_transcript/36294	1.70007732234529	1.16043726236867	1.40974855205656	0.168087039389474	0.834074165408375	-4.8037959927078
a_HQ_transcript/128913	1.9000526099176	4.48796461233535	1.40971015460549	0.168098289559414	0.834074165408375	-5.43690574541467
a_HQ_transcript/39205	-1.71522274952478	1.58549399319179	-1.4097035673996	0.168100219621601	0.834074165408375	-5.07777004681881
a_HQ_transcript/18927	0.73042787692773	2.78476239217221	1.40967580167298	0.16810835521665	0.834074165408375	-5.06585725067595
a_HQ_transcript/93774	1.19673806495506	-0.638119344520371	1.40965807103984	0.168113550605443	0.834074165408375	-4.98458495722189
a_HQ_transcript/81170	-1.62070451243462	0.046850958221677	-1.40958941414952	0.168133669477395	0.834074165408375	-4.98811250751276
a_HQ_transcript/10554	2.56100930763321	1.76816328903359	1.40933907728734	0.168207042963442	0.834074165408375	-5.01696103792733
a_HQ_transcript/27949	1.06094300615219	1.62629237582648	1.40927625381257	0.168225460409569	0.834074165408375	-4.88880368408051
a_HQ_transcript/125858	1.66050346563239	-0.419704356014774	1.40923850635302	0.168236527286542	0.834074165408375	-4.96229423215239
a_HQ_transcript/132653	-1.62080070442407	-0.227796703729233	-1.40922289185616	0.168241105343714	0.834074165408375	-4.9794123892168
a_HQ_transcript/144254	1.63716257836316	0.343448139685959	1.40913710496045	0.16826625918491	0.834074165408375	-4.97157617136941
a_HQ_transcript/103029	1.6165582790752	0.400072124415375	1.40913488464065	0.168266910250971	0.834074165408375	-4.92815878070959
a_HQ_transcript/119151	1.19852600538279	1.84477445037187	1.40911861584447	0.168271680822789	0.834074165408375	-4.88229377417383
a_HQ_transcript/120930	1.86007759872283	0.321046045513953	1.40907053717787	0.168285779766044	0.834074165408375	-4.87859294953347
a_HQ_transcript/75529	-1.58018694784508	0.883685450863718	-1.40905146118426	0.168291374008418	0.834074165408375	-4.93218099530893
a_HQ_transcript/99766	-1.15468288407144	1.72915748694157	-1.40904032664632	0.168294639400156	0.834074165408375	-5.00988896258404
a_HQ_transcript/139935	-2.56674691496552	2.73017311710595	-1.40903883078442	0.168295078090774	0.834074165408375	-5.21525400149675
a_HQ_transcript/61016	-1.30001483144753	-0.410075619299822	-1.4090271229736	0.168298511665201	0.834074165408375	-4.9639542230085
a_HQ_transcript/62119	1.45975996130462	0.224036718238597	1.40895038323672	0.168321018652493	0.83411459268816	-4.91292951971074
a_HQ_transcript/133078	1.123268620612	1.50167189531116	1.40889647036485	0.168336832165524	0.834121846399931	-4.86484933228118
a_HQ_transcript/26860	1.52837970910183	-0.205452639660919	1.40882681394532	0.168357265242388	0.834151987215268	-5.0094045433949
a_HQ_transcript/91865	2.08360159501052	0.489262993971222	1.40875783735116	0.168377500818385	0.834181144427773	-4.87832049023865
a_HQ_transcript/137488	3.67317904551636	2.15191746714208	1.40851989379043	0.168447320681761	0.834455927781611	-5.12243527278752
a_HQ_transcript/35512	-1.85414014358214	1.46109552115715	-1.40845638469079	0.168465960023576	0.834466019763587	-5.04938249683762
a_HQ_transcript/120010	-1.5822917785358	0.0934687100237585	-1.4083966010733	0.168483507450502	0.834466019763587	-4.98054481995721
a_HQ_transcript/564	-0.677238826534043	5.12975902353961	-1.40832564340276	0.168504336499832	0.834466019763587	-5.44666886124839
a_HQ_transcript/116837	1.5158479412916	-0.111044765308543	1.40826768888553	0.168521350079265	0.834466019763587	-4.99609154360052
a_HQ_transcript/113958	-1.65986502566107	0.243835514946103	-1.4082014474098	0.168540798095076	0.834466019763587	-4.91561234873859
a_HQ_transcript/11874	-2.61790044856582	1.67944023858653	-1.40814900882214	0.168556194933241	0.834466019763587	-4.97873132054924
a_HQ_transcript/124523	0.641052495703104	4.02308938875462	1.40813123751526	0.168561413133996	0.834466019763587	-5.34029395831908
a_HQ_transcript/142636	1.70094833106369	0.329598103777389	1.40811648456549	0.168565745148907	0.834466019763587	-4.81871170339248
a_HQ_transcript/125796	1.34144027155993	-0.384587510516605	1.40807284836854	0.168578558871522	0.834466019763587	-5.00520988642041
a_HQ_transcript/34006	1.75067979359261	2.12145685167905	1.40784115576308	0.168646607971928	0.834693209369208	-5.14690143493926

a_HQ_transcript/18666	0.381862283594541	3.68287013210791	1.40781168249716	0.168655265941426	0.834693209369208	-5.29562461410065
a_HQ_transcript/26791	1.26814513524706	0.739833194191098	1.40773160494926	0.168678791020589	0.834693209369208	-4.82509146930531
a_HQ_transcript/116158	1.72068870244998	-0.0364323377885591	1.40772104353489	0.16868189393167	0.834693209369208	-4.93284322554477
a_HQ_transcript/25076	2.87072876951072	0.529833910153995	1.40757693204736	0.16872423792246	0.834779023656735	-4.94958874681375
a_HQ_transcript/129162	2.37084544286069	2.86311646175727	1.40754436282836	0.168733808827346	0.834779023656735	-5.10509974872465
a_HQ_transcript/82178	-1.58957215869941	0.94289112876527	-1.40751540390158	0.168742319157147	0.834779023656735	-4.83556820660603
a_HQ_transcript/116217	0.427769450133065	3.0719501764251	1.40741604005896	0.16877152235515	0.834852442469591	-5.18573842354122
a_HQ_transcript/21386	-1.69292536358138	1.14869950072896	-1.40720722995636	0.168832904922821	0.835085015091366	-4.9351224871348
a_HQ_transcript/109223	-0.531672754453841	2.75120361490194	-1.40715742078471	0.168847549594873	0.835086391802157	-5.09018324854159
a_HQ_transcript/41210	1.89642792074899	1.29830258160887	1.40703458630732	0.168883669106596	0.835128830729659	-4.8547568050719
a_HQ_transcript/118812	4.5916094426437	3.77051373316807	1.40703052129924	0.168884864527029	0.835128830729659	-5.20656428899511
a_HQ_transcript/114761	-1.53240562236911	1.48118565253351	-1.40684981142383	0.168938013637716	0.835320590058963	-4.92391122611511
a_HQ_transcript/27893	1.70840699120601	0.519219766543825	1.40679002831119	0.168955599514557	0.835336487929078	-4.83794720698911
a_HQ_transcript/110945	1.09417239714063	-0.0410003526258824	1.40670164403862	0.168981601378186	0.835376305017686	-4.90067369769058
a_HQ_transcript/13576	-0.471690870047158	4.74915262571022	-1.40662957238885	0.169002806539135	0.835376305017686	-5.43463121230825
a_HQ_transcript/131054	-2.18420635743511	0.817954574108741	-1.40660960535744	0.169008681675183	0.835376305017686	-4.96614424302853
a_HQ_transcript/3964	2.42097788407091	3.07266481953011	1.40656727273293	0.169021138234809	0.835376305017686	-5.05204989360674
a_HQ_transcript/132224	2.01549275393598	0.988541775576674	1.40648472261652	0.169045431040153	0.835425337145214	-4.8201735170309
a_HQ_transcript/67967	0.481469951860033	4.674790110764	1.40642344860153	0.169063464497693	0.835443429764677	-5.44657704578762
a_HQ_transcript/63460	-2.18137463677157	3.09468057789471	-1.40629885174253	0.169100139058018	0.835465657487213	-5.11583307302444
a_HQ_transcript/131456	-0.642403153804612	2.90130044993846	-1.40626081430438	0.169111336461839	0.835465657487213	-5.17169921425893
a_HQ_transcript/48783	-0.7291629677102	4.40666321968405	-1.40620138636649	0.169128831933828	0.835465657487213	-5.37697951561579
a_HQ_transcript/123152	-1.08876989883236	1.96731429612485	-1.40609866862828	0.169159075190946	0.835465657487213	-4.93726636160694
a_HQ_transcript/17052	2.12395161305193	1.97690530014677	1.40608966305756	0.169161726909963	0.835465657487213	-5.07040655146604
a_HQ_transcript/41759	1.06843398961793	1.60941573786818	1.4060358309925	0.169177578613672	0.835465657487213	-4.88321461378434
a_HQ_transcript/125131	-1.97152960789958	1.83876738866184	-1.40598182785688	0.169193481863324	0.835465657487213	-4.94886412231515
a_HQ_transcript/148089	0.481674370486972	4.30672235018279	1.40592648976727	0.169209779457548	0.835465657487213	-5.38346225858456
a_HQ_transcript/143072	0.495359015173815	3.7403573639091	1.40586266682943	0.169228577450431	0.835465657487213	-5.29018552647644
a_HQ_transcript/120814	3.43914213549156	2.47406569997059	1.40581005633384	0.169244074235969	0.835465657487213	-5.15558541602578
a_HQ_transcript/113654	1.15313547772159	-0.225363665419827	1.40572929485726	0.169267865255018	0.835465657487213	-4.96381253232849
a_HQ_transcript/63047	2.34321990501082	1.1049158689262	1.40571481760067	0.169272130297059	0.835465657487213	-4.97348150823203
a_HQ_transcript/88944	2.32512818987685	0.310724340570109	1.40570815876453	0.169274092037939	0.835465657487213	-5.0196270300029
a_HQ_transcript/109767	0.64741910605619	2.39169064558911	1.40570089702845	0.169276233273858	0.835465657487213	-4.99211975654441
a_HQ_transcript/121920	-1.77494289984702	2.79352984536926	-1.40557510102577	0.169313295542857	0.835465657487213	-5.09766442374488
a_HQ_transcript/124393	1.60240123283372	2.75927993186028	1.40552461034933	0.169328173742698	0.835465657487213	-5.02496838472581
a_HQ_transcript/97789	-1.43174814708753	1.10227493780963	-1.40544898418659	0.169350460591595	0.835465657487213	-4.94067121746459
a_HQ_transcript/53270	1.68170050146062	1.08339662555706	1.40538196083909	0.169370214135653	0.835465657487213	-4.93901528137478
a_HQ_transcript/126502	0.901038575610136	3.61686637155605	1.40534124273361	0.169382215715692	0.835465657487213	-5.22461593819888
a_HQ_transcript/109599	1.31871179769352	0.228963717332875	1.40531169474107	0.169390925345203	0.835465657487213	-4.94562919458014
a_HQ_transcript/132035	1.18145697094107	-0.559591713332741	1.40526159821298	0.169405692708113	0.835465657487213	-4.99041605326644
a_HQ_transcript/150168	0.675923441390304	3.06798413617272	1.40517004374616	0.169432683576818	0.835465657487213	-5.11077930302147
a_HQ_transcript/88199	-3.32695897653042	2.52450219008825	-1.40511771567692	0.16944811175694	0.835465657487213	-5.04200563343849
a_HQ_transcript/12178	1.43681037937271	1.43786995593925	1.40511741511577	0.169448200376267	0.835465657487213	-4.8987720113856
a_HQ_transcript/32283	-0.927211250964644	1.66885891720477	-1.40501135187513	0.16947947499512	0.835465657487213	-4.91472663966393
a_HQ_transcript/40671	-1.84225028240517	0.302218111735309	-1.40495404304786	0.169496375399419	0.835465657487213	-4.84069146548094
a_HQ_transcript/80362	-0.801475910790203	1.90322934155586	-1.40494144722299	0.169500090092287	0.835465657487213	-4.94374246495948
a_HQ_transcript/147073	2.01517679088315	-0.333392395698999	1.40489339816648	0.169514261048064	0.835465657487213	-4.92021780732839
a_HQ_transcript/29706	1.3257350919167	1.31887285028411	1.40489123178531	0.169514899993893	0.835465657487213	-4.83121662563311
a_HQ_transcript/92874	1.94199876682759	-0.10176903407879	1.40488448193324	0.169516890786706	0.835465657487213	-5.04039993168563
a_HQ_transcript/884	-0.598088795844262	3.29950858255971	-1.40485761194959	0.169524815967512	0.835465657487213	-5.21524324414999

a_HQ_transcript/137733	0.634421540370786	2.44377608087039	1.40484717583398	0.169527894131256	0.835465657487213	-5.06222429502805
a_HQ_transcript/5760	0.350087921118361	3.61815959134496	1.40479781347317	0.169542454302645	0.835466580510696	-5.31987418139739
a_HQ_transcript/54070	-1.45846524118934	0.680717736186986	-1.40470294569407	0.169570439737289	0.83549549604022	-4.79359602345119
a_HQ_transcript/144853	-1.14695316196496	4.26270716293757	-1.40468047523236	0.169577068921733	0.83549549604022	-5.37384938633666
a_HQ_transcript/93016	1.7856781687435	6.06827176949803	1.40453140686738	0.169621051880749	0.835566502034286	-5.57631447629164
a_HQ_transcript/102303	1.69777277395009	1.06862566950847	1.40431854909181	0.169683871565336	0.835566502034286	-4.96695775701383
a_HQ_transcript/151498	0.506336774769261	4.17649813901172	1.40430458205824	0.169687994226419	0.835566502034286	-5.34633063992972
a_HQ_transcript/93176	1.24767707814725	-0.56671936073502	1.40427231064815	0.169697520106331	0.835566502034286	-4.98571661488967
a_HQ_transcript/6762	-1.6331358356075	2.1581402583167	-1.4042110062785	0.169715617095361	0.835566502034286	-5.08245266878675
a_HQ_transcript/132023	1.53516430345595	0.210676269154587	1.4041192450296	0.169742707754253	0.835566502034286	-4.95804352416886
a_HQ_transcript/15389	1.46648056782049	1.86778657142681	1.40403564845199	0.169767390911722	0.835566502034286	-4.90444879814058
a_HQ_transcript/56741	0.841575737159002	1.81203423598552	1.4039738193369	0.169785648707219	0.835566502034286	-4.91338213550033
a_HQ_transcript/129380	1.18281642789728	-0.375613682666249	1.40394313212753	0.169794711042308	0.835566502034286	-5.00671778549099
a_HQ_transcript/83755	1.25787525269937	-0.191029082480745	1.40386996014943	0.169816321217562	0.835566502034286	-5.03748339432926
a_HQ_transcript/111410	1.79766877740291	1.09210145199018	1.4038313355167	0.169827729256098	0.835566502034286	-4.91098246912446
a_HQ_transcript/54442	-1.84674490158668	0.25195545131165	-1.403713685511	0.16986248166145	0.835566502034286	-5.0001735551435
a_HQ_transcript/95221	-0.515468505680582	2.6189016130591	-1.40365898169603	0.16987864241442	0.835566502034286	-5.06717819949584
a_HQ_transcript/64217	0.936974330831752	1.91689575862634	1.40359703166471	0.1698969453211	0.835566502034286	-5.00836501288741
a_HQ_transcript/82568	-0.614898085663225	3.84093135081959	-1.40357393520896	0.169903769479034	0.835566502034286	-5.14018817528992
a_HQ_transcript/58452	1.21749558170678	2.24359329374864	1.40354523049425	0.169912250972043	0.835566502034286	-5.03063906320011
a_HQ_transcript/86702	0.918433418856359	1.80841984642207	1.40352811200145	0.169917309197374	0.835566502034286	-4.86595895102207
a_HQ_transcript/19805	1.39501493934077	1.83740588960265	1.40348990419598	0.169928599384237	0.835566502034286	-4.91506508444506
a_HQ_transcript/67749	-3.08983265815066	3.50842170060844	-1.40343499062865	0.169944827058337	0.835566502034286	-5.18436626810976
a_HQ_transcript/110702	-2.03338836340618	-0.144681201846631	-1.40342184352187	0.169948712379911	0.835566502034286	-4.96337899276461
a_HQ_transcript/74234	1.20705791101505	0.259200665953445	1.40332844301231	0.169976316733639	0.835566502034286	-4.83695462953254
a_HQ_transcript/7035	-1.1858581171006	0.12749951259895	-1.40331107794515	0.169981449335921	0.835566502034286	-4.90124337405119
a_HQ_transcript/129278	0.73956666304994	3.59542103828763	1.40330985055017	0.169981812122318	0.835566502034286	-5.31744361216259
a_HQ_transcript/1602	-0.780229652738783	2.2479890400682	-1.40326581531281	0.169994828208013	0.835566502034286	-5.00680751014536
a_HQ_transcript/39340	1.98624774102042	4.35197162954161	1.40326506492528	0.169995050016896	0.835566502034286	-5.18741562598091
a_HQ_transcript/28941	1.20584398917188	-0.183964361716635	1.40319507701437	0.170015738912232	0.835566502034286	-4.83841846933405
a_HQ_transcript/110916	2.60038913336736	1.70135245873342	1.40316896374358	0.170023458675909	0.835566502034286	-5.04812338503051
a_HQ_transcript/94067	-1.61977657912431	0.501407321648826	-1.40313227507036	0.170034305268769	0.835566502034286	-5.08098281206622
a_HQ_transcript/35787	1.6183633124583	-0.296411399445578	1.40302322730481	0.170066547215934	0.835566502034286	-4.96721226644253
a_HQ_transcript/145576	1.87327646275882	1.5349872179703	1.40301649655054	0.170068537442465	0.835566502034286	-5.00687624127945
a_HQ_transcript/57063	1.28775378639012	0.114558727556972	1.403001897615	0.170072854285754	0.835566502034286	-4.89906459202242
a_HQ_transcript/25559	1.81836440372891	1.51691806411468	1.40294715292923	0.170089042822429	0.835566502034286	-4.8665186415032
a_HQ_transcript/133918	-2.11728552530583	0.032378031907916	-1.40292505404813	0.170095578019159	0.835566502034286	-4.96430722525082
a_HQ_transcript/5447	-1.527147481488	2.00462460899683	-1.4029010451821	0.170102678269827	0.835566502034286	-5.02107598936628
a_HQ_transcript/129951	0.608963303170379	3.2287524980284	1.40284964885945	0.170117878718509	0.835566502034286	-5.17840067618638
a_HQ_transcript/116570	-1.26345357281184	0.733964244077734	-1.40281316457634	0.170128669581998	0.835566502034286	-4.93439820323158
a_HQ_transcript/94823	-1.24073176138905	-0.0476725727585803	-1.40277498747881	0.170139961699663	0.835566502034286	-4.85468465000584
a_HQ_transcript/143849	-1.31023862896359	-0.544543668870126	-1.40272461926509	0.170154860635603	0.835566502034286	-4.95833334901978
a_HQ_transcript/140919	-1.41139147561199	-0.419354704436647	-1.40264572821343	0.170178198693811	0.835566502034286	-4.96277694797805
a_HQ_transcript/69934	-1.77757191998046	0.51808086092648	-1.40262048977091	0.170185665422174	0.835566502034286	-4.9525540776622
a_HQ_transcript/141936	-1.55197118669966	0.221691475408892	-1.40250473378779	0.170219914823925	0.835566502034286	-4.91373456395412
a_HQ_transcript/71943	2.29660765523007	1.44223110275052	1.40249244209619	0.170223551956265	0.835566502034286	-5.02562688524242
a_HQ_transcript/14250	2.43681474199418	1.89689257378459	1.40243875177498	0.170239439727667	0.835566502034286	-5.07846515459997
a_HQ_transcript/124880	1.33288439443971	-0.496469149592789	1.40239370504107	0.170252770629065	0.835566502034286	-5.03438215785863
a_HQ_transcript/13243	2.35269810964529	2.40932381513502	1.40236735265409	0.170260569600003	0.835566502034286	-4.97278958615726
a_HQ_transcript/86178	1.4000599952216	-0.273712686425022	1.40230889026843	0.170277872503326	0.835566502034286	-4.90353882814276

a_HQ_transcript/55724	-1.58161892981498	1.28423912061261	-1.40224363916009	0.17029718626903	0.835566502034286	-4.88002566067834
a_HQ_transcript/147640	3.34526939899084	0.452865247924395	1.40222861459131	0.170301633655206	0.835566502034286	-4.97011245804502
a_HQ_transcript/138413	-0.92960673629473	1.82002875627869	-1.40214007096002	0.170327845090823	0.835566502034286	-4.87575590828583
a_HQ_transcript/1581	-1.5393781912092	0.863201095093303	-1.40213789857953	0.170328488217051	0.835566502034286	-4.91758540424552
a_HQ_transcript/36799	0.999773858417313	-0.539039512693238	1.40211504516525	0.170335254011924	0.835566502034286	-5.02812259307216
a_HQ_transcript/104260	2.06581869405303	3.35184224475695	1.40210252846125	0.170338959694232	0.835566502034286	-5.1783893794275
a_HQ_transcript/115577	-1.94300853982732	0.0309742506467234	-1.40187470121318	0.170406421039163	0.835793157121233	-5.00098225341686
a_HQ_transcript/17829	-0.820460605576475	1.63691381901258	-1.40184937135965	0.170413922691317	0.835793157121233	-4.8304847036764
a_HQ_transcript/118899	-2.00379016086684	0.800918827700399	-1.40179423059015	0.170430253995874	0.835802733845621	-4.94432882038984
a_HQ_transcript/114102	1.79087856616109	0.0177127029238521	1.40133501692435	0.170566309165363	0.836335048185984	-4.96079462476863
a_HQ_transcript/65215	-1.79991923655972	0.468086717099558	-1.40133074602026	0.17056757494228	0.836335048185984	-4.89154520423876
a_HQ_transcript/47443	1.79419734678618	1.4929689985349	1.40126365614558	0.170587459477397	0.836361997680678	-4.88309118582195
a_HQ_transcript/92310	2.45740293426061	1.69402226739775	1.40096939341826	0.170674696453084	0.836435976414212	-5.0706523588528
a_HQ_transcript/71254	0.979114161262167	2.40848015919969	1.4009169409296	0.170690250162602	0.836435976414212	-4.90780069808791
a_HQ_transcript/126665	-0.536862060102506	2.67310301734655	-1.40087035563143	0.170704065008766	0.836435976414212	-5.03397473372744
a_HQ_transcript/140182	-1.14125459413406	2.30764030830604	-1.40086777272142	0.170704830995044	0.836435976414212	-5.00800412162918
a_HQ_transcript/10412	1.03289675106806	2.27745309564987	1.40085003193497	0.170710092265074	0.836435976414212	-5.00636261630204
a_HQ_transcript/2772	1.55918274387795	1.35402347323302	1.4007691855927	0.170734069950249	0.836435976414212	-4.91681637580402
a_HQ_transcript/26159	-0.636360937932503	1.76246459653521	-1.40075255859213	0.170739001570332	0.836435976414212	-4.92238338592953
a_HQ_transcript/112874	-2.08683217715198	0.448182497149222	-1.40071553931192	0.170749982005052	0.836435976414212	-4.91901268281776
a_HQ_transcript/48275	-1.47086539313412	1.70498513586386	-1.40062490573107	0.170776867525554	0.836435976414212	-5.04376647540623
a_HQ_transcript/31920	1.3038096569921	2.03141178199217	1.40062002941459	0.170778314128797	0.836435976414212	-4.9037473078583
a_HQ_transcript/119165	-5.30019210604403	3.63170174906549	-1.40061436983314	0.170779993106699	0.836435976414212	-5.12887465255661
a_HQ_transcript/2251	-0.859905757365981	2.85258520336438	-1.40061433557178	0.170780003270751	0.836435976414212	-5.14552394836874
a_HQ_transcript/151570	-6.24719635510231	4.98590071918882	-1.40058194464667	0.170789612648986	0.836435976414212	-5.11269297995554
a_HQ_transcript/130720	1.6209809712131	-0.119243127119517	1.40047164945266	0.170822336975125	0.836496218246929	-4.97451017668681
a_HQ_transcript/69093	1.69590319042119	2.04172374932394	1.4004434837974	0.170830694446898	0.836496218246929	-5.13828145737713
a_HQ_transcript/46499	1.38492485038469	0.513956781659877	1.40037934890018	0.170849726110385	0.836518941993298	-5.04471622162814
a_HQ_transcript/84855	-1.33555854945701	2.43932511660263	-1.40006455836172	0.170943162497243	0.836869172683611	-5.08975795988735
a_HQ_transcript/76188	-1.46741422692803	2.06989062259293	-1.39998323095601	0.170967308673581	0.836869172683611	-4.95014100102224
a_HQ_transcript/121247	2.08213490517597	2.33109017504531	1.39997619758628	0.170969397012647	0.836869172683611	-4.97148873789646
a_HQ_transcript/128507	1.56841541830088	1.60531596647948	1.39990379865952	0.170990894770632	0.836869172683611	-4.84922430418508
a_HQ_transcript/91689	0.4769297426058	2.69798972900796	1.39987497527189	0.170999454025903	0.836869172683611	-5.14456483794645
a_HQ_transcript/42119	2.4434802495315	2.78622045618985	1.39984741372344	0.171007638885493	0.836869172683611	-5.15487607312982
a_HQ_transcript/102714	-0.741206849493614	5.16129736608011	-1.39962744001667	0.171072974735591	0.837056609543466	-5.498362581055
a_HQ_transcript/145483	2.60316312341466	1.33605627219795	1.39962149560484	0.171074740596208	0.837056609543466	-4.98449494131372
a_HQ_transcript/86173	0.407358865388347	5.71758932258394	1.39953257296659	0.171101157864189	0.837106375216689	-5.55683100589714
a_HQ_transcript/120597	1.03123035567976	0.64244250622831	1.39947629399141	0.171117878956557	0.837106375216689	-4.84567143440668
a_HQ_transcript/19446	0.631206234149008	2.83840858805504	1.39941877177839	0.171134970751557	0.837106375216689	-5.15225566404072
a_HQ_transcript/100684	-1.48737152973694	2.2621453479865	-1.39925723259777	0.17118297665926	0.837106375216689	-4.87402179010024
a_HQ_transcript/132623	-0.857275991096995	0.516593215409346	-1.39919873699676	0.171200362873258	0.837106375216689	-4.89377019949522
a_HQ_transcript/15692	2.03527994177835	1.66442494403012	1.39912500754936	0.171222278901557	0.837106375216689	-4.91580826359791
a_HQ_transcript/120350	1.26353994489173	-0.418250972794932	1.3991148599418	0.171225295443124	0.837106375216689	-4.97958802482652
a_HQ_transcript/74633	0.828803872837462	3.1733755844721	1.39910392552273	0.171228545923832	0.837106375216689	-5.24017705842081
a_HQ_transcript/96048	-2.87468596487009	3.39090232867543	-1.39897699772372	0.171266281357908	0.837106375216689	-5.09325313996569
a_HQ_transcript/53357	2.48976554290806	2.0008578622973	1.39894790719101	0.171274930845065	0.837106375216689	-5.15750317358351
a_HQ_transcript/113884	-2.79551895203573	1.73479133198893	-1.39892461191908	0.171281857474295	0.837106375216689	-4.92114805425158
a_HQ_transcript/39791	3.19733587028038	0.937567169096084	1.39883985970778	0.171307059596173	0.837106375216689	-4.97428805872418
a_HQ_transcript/70571	2.08613152273111	3.36809070729812	1.39881509159792	0.171314425249605	0.837106375216689	-5.23137947163169
a_HQ_transcript/11842	-1.46702963574213	0.162144514042308	-1.39875650442056	0.171331849159152	0.837106375216689	-4.87062682675945

a_HQ_transcript/3255	-0.589744188366595	2.8534311363413	-1.3987540460279	0.171332580318962	0.837106375216689	-5.02994596644808
a_HQ_transcript/147461	-0.524756304962127	3.73264165767524	-1.39870671059379	0.171346659005227	0.837106375216689	-5.2878583091675
a_HQ_transcript/73900	3.43038701556345	1.33985265689787	1.39869050642223	0.171351478720288	0.837106375216689	-4.87783252241737
a_HQ_transcript/40636	1.02955026335634	2.13902202859538	1.39863252283265	0.171368726036955	0.837106375216689	-4.99802139874971
a_HQ_transcript/152703	6.82376176004822	2.94341697749331	1.39862318259085	0.171371504434841	0.837106375216689	-5.11694070135357
a_HQ_transcript/55445	0.966267224306714	2.34033385813842	1.39861837927194	0.171372933269312	0.837106375216689	-4.93851191414278
a_HQ_transcript/9980	3.45632350717227	1.87432984762585	1.39834779364244	0.171453438953519	0.837429249600048	-5.12715797668812
a_HQ_transcript/84728	-1.92309202033689	1.7676899574181	-1.39820519899999	0.171495876182549	0.837461879799144	-4.9626797508986
a_HQ_transcript/73500	-1.07553249223413	3.26357763840166	-1.39814318449704	0.171514334728324	0.837461879799144	-5.26289369527633
a_HQ_transcript/151804	-0.362796383172492	6.11766164083328	-1.39810015430633	0.171527143529873	0.837461879799144	-5.55022253160606
a_HQ_transcript/71136	-0.563008033094622	2.65411787484118	-1.39806839018737	0.171536599239333	0.837461879799144	-5.06734911621218
a_HQ_transcript/115298	-0.646386253042881	2.30548891625149	-1.39803268900049	0.171547227443181	0.837461879799144	-4.91176289761546
a_HQ_transcript/119792	1.62501496190934	1.0847656932193	1.398030429071	0.171547900239048	0.837461879799144	-4.87787044682859
a_HQ_transcript/80972	1.33892942996362	-0.532115513169355	1.39798652893451	0.171560970008399	0.837461879799144	-4.98460903416722
a_HQ_transcript/97100	1.6901541245364	1.09087444339198	1.39790487936305	0.171585280459763	0.837510217697966	-4.98412574266087
a_HQ_transcript/8300	0.871284119340543	3.24978311612702	1.39774364697634	0.171633293928806	0.837629204837881	-5.22017838886985
a_HQ_transcript/112291	1.89106576302866	1.90195294709501	1.39772624008077	0.171638478165841	0.837629204837881	-4.86395547746713
a_HQ_transcript/118608	1.29867703239618	2.25945962140557	1.39748218575587	0.171711176958524	0.837843841195234	-5.08588926784535
a_HQ_transcript/63834	1.49935658771524	-0.209142852046313	1.39743263404592	0.171725940344603	0.837843841195234	-4.98056199169012
a_HQ_transcript/65095	1.12495557387389	0.508986626712512	1.39732025914183	0.171759424893	0.837843841195234	-4.79744563964674
a_HQ_transcript/136886	1.65515820542943	0.913965077358397	1.39730684493966	0.171763422288093	0.837843841195234	-4.85737985178542
a_HQ_transcript/94704	-0.744006115109595	2.64549191598335	-1.39726984037013	0.171774449925044	0.837843841195234	-4.99493435782516
a_HQ_transcript/29205	-1.35424455785292	0.909551153210646	-1.39725411908245	0.171779135153528	0.837843841195234	-4.9093990584345
a_HQ_transcript/35320	-1.95665007243311	0.605812997760991	-1.39721512749815	0.171790755783823	0.837843841195234	-4.86917559488349
a_HQ_transcript/55020	-1.58873790269892	1.15574679104412	-1.39706299161923	0.171836102604103	0.837843841195234	-5.01503490039292
a_HQ_transcript/12478	-0.990943558100638	2.75963950926464	-1.39704418422304	0.171841709135977	0.837843841195234	-5.18869155121533
a_HQ_transcript/14400	-0.541645204612082	2.65481407477658	-1.39694844429599	0.17187025166861	0.837843841195234	-4.98942831271021
a_HQ_transcript/57046	0.984089830693452	-0.652907687935045	1.39686261481883	0.171895842797357	0.837843841195234	-5.00127780762634
a_HQ_transcript/42452	-1.03979795719823	1.75209319346439	-1.3967470927176	0.171930291862998	0.837843841195234	-4.89160257737143
a_HQ_transcript/96232	0.752605307225659	2.89981976313427	1.39667598736876	0.171951498396588	0.837843841195234	-5.0707103030001
a_HQ_transcript/131108	-1.46110515959343	0.744113340831173	-1.39666295825993	0.171955384433658	0.837843841195234	-4.88160289569182
a_HQ_transcript/146891	0.273359146399335	6.4218262452562	1.39661187394566	0.1719706214069	0.837843841195234	-5.58259109429356
a_HQ_transcript/144337	1.27529766801477	-0.335119303550327	1.39657772570105	0.171980807430023	0.837843841195234	-4.9152445300152
a_HQ_transcript/117993	3.13530960867159	1.51661439906385	1.39655896670431	0.171986403221021	0.837843841195234	-5.1165066674945
a_HQ_transcript/133155	-0.621355996419907	2.20421970025517	-1.39653194357218	0.171994464446409	0.837843841195234	-5.04760270525477
a_HQ_transcript/126449	1.15336577960368	1.78199182946978	1.3965266698336	0.171996037681054	0.837843841195234	-4.95755453490439
a_HQ_transcript/67196	1.49999698085649	1.46165034612238	1.39643764925644	0.172022595543512	0.837843841195234	-4.94413734569031
a_HQ_transcript/28535	1.52617970944202	4.20947914638491	1.39641224528884	0.172030174998966	0.837843841195234	-5.38567206223913
a_HQ_transcript/142509	2.94153545509404	1.76447012210221	1.39632425685465	0.172056428999011	0.837843841195234	-4.92974841391411
a_HQ_transcript/64078	1.21022418481822	2.43418195260805	1.39631832638994	0.17205819864466	0.837843841195234	-5.11287749826057
a_HQ_transcript/42472	-1.28388656501888	0.399355102192216	-1.39630316245197	0.172062723615718	0.837843841195234	-5.05039491601124
a_HQ_transcript/45756	-2.38314435765626	1.7842659554648	-1.3962910925712	0.172066325376102	0.837843841195234	-5.08037406942756
a_HQ_transcript/40423	-1.50134738686783	1.70846103267932	-1.39627410749749	0.172071393974013	0.837843841195234	-4.94327495484673
a_HQ_transcript/149290	-2.29620879306327	1.07875803623958	-1.39626933687063	0.17207281762085	0.837843841195234	-5.11005727840043
a_HQ_transcript/114032	-1.5140401904034	1.30267175788786	-1.39620638356203	0.172091604963147	0.837843841195234	-4.92850344576985
a_HQ_transcript/1181	1.63035418900805	-0.064032413772081	1.39617671797247	0.172100458709591	0.837843841195234	-4.93545332194373
a_HQ_transcript/9382	1.72133637331711	0.0186098035596797	1.39594808167153	0.172168707575435	0.837990594313281	-5.00003266679799
a_HQ_transcript/50376	2.65524385341445	0.967773442125067	1.39592017986035	0.172177037830088	0.837990594313281	-5.01982976238044
a_HQ_transcript/142130	3.77649929413219	3.76504206613205	1.3958966773648	0.172184054887264	0.837990594313281	-5.10523052898279
a_HQ_transcript/52235	-1.45879756280791	0.586744437173036	-1.39586015468845	0.172194959778181	0.837990594313281	-4.93901834463185

a_HQ_transcript/125945	-2.50128907502857	2.84631749879296	-1.39574478219263	0.172229411092461	0.837990594313281	-5.29551428970498
a_HQ_transcript/47207	-2.32436548045609	2.12436364160173	-1.39571697053821	0.172237716721625	0.837990594313281	-5.12107775912411
a_HQ_transcript/131770	-1.04765469682903	-0.606022464131843	-1.39571577059678	0.172238075077321	0.837990594313281	-4.97574881508671
a_HQ_transcript/70348	0.793705454745586	2.90307918899638	1.39568368352063	0.172247657916948	0.837990594313281	-5.13581969594795
a_HQ_transcript/118836	-1.04623802149093	2.11988559103617	-1.39560906639304	0.172269944015816	0.837990594313281	-4.95774288013839
a_HQ_transcript/96018	1.06476598443923	-0.476998466260182	1.39557228760787	0.172280929665319	0.837990594313281	-4.98838691201018
a_HQ_transcript/34532	1.44550366990656	0.217442491885505	1.39550659173388	0.172300554077433	0.837990594313281	-4.95912572652785
a_HQ_transcript/41017	1.26824290813703	-0.642724721742563	1.39549640001057	0.172303598665571	0.837990594313281	-4.99051968223774
a_HQ_transcript/108810	1.63635974739239	0.993068380849542	1.39537449627743	0.17234001840732	0.838097598090915	-4.96895356815562
a_HQ_transcript/119609	0.43402822858105	3.15612378946079	1.395215410022	0.172387555795977	0.838239092851783	-5.19186172648507
a_HQ_transcript/70850	-2.31703360059765	0.469365617334156	-1.39518061159906	0.172397955461104	0.838239092851783	-5.02006306122442
a_HQ_transcript/4339	-1.33492664362739	1.7818253941431	-1.39473994160882	0.172529694157844	0.838797253361664	-4.85694632753941
a_HQ_transcript/29075	0.865706851802095	0.802644062005104	1.39467105009741	0.172550296457919	0.838797253361664	-4.99974526385391
a_HQ_transcript/151867	-1.2472694075302	1.92839593086181	-1.39465184195984	0.172556041076569	0.838797253361664	-4.95250852497456
a_HQ_transcript/113912	-1.33278563601679	-0.137829220770729	-1.39439849913208	0.1726318228632	0.839036739878962	-4.95754772233438
a_HQ_transcript/978	-1.6242016160066	2.78560239662367	-1.39439063233569	0.17263417645459	0.839036739878962	-5.18369928493891
a_HQ_transcript/6018	1.1716801641502	-0.611802343297611	1.39433271479124	0.172651505022446	0.839050805446429	-4.96897123171092
a_HQ_transcript/65548	1.94789805206316	-0.185793261084914	1.39414425940904	0.172707899117372	0.839200870774823	-4.97895994220192
a_HQ_transcript/92455	0.447106212438923	2.73870896036857	1.3941330356649	0.172711258207391	0.839200870774823	-5.04707281259326
a_HQ_transcript/15630	1.85173530065059	1.60303129927746	1.39404697690448	0.172737015939174	0.839203197128357	-5.06952343421211
a_HQ_transcript/126389	0.600504191533727	2.83459698825887	1.39396429167803	0.172761766790187	0.839203197128357	-5.13776627305036
a_HQ_transcript/43186	-2.71519016191206	1.61367847173691	-1.39391570980899	0.172776310496081	0.839203197128357	-5.18953209290456
a_HQ_transcript/141929	0.767606172112991	4.38689523046534	1.39389446281743	0.172782671400489	0.839203197128357	-5.40159664240369
a_HQ_transcript/92116	-0.42074203879477	4.98224695119248	-1.39389028297999	0.172783922777873	0.839203197128357	-5.45958226285027
a_HQ_transcript/7520	1.54805317557212	0.275964724400947	1.39384180357845	0.172798437263954	0.83920357266932	-4.94768060069383
a_HQ_transcript/89689	-1.27436924148679	0.27532155808726	-1.39374628141081	0.172827038906649	0.839272357357199	-4.91292331859022
a_HQ_transcript/136735	-2.26667103301698	0.133366058652762	-1.39349608416682	0.172901971558602	0.839355148619712	-4.92902727549879
a_HQ_transcript/16989	0.766114743946219	2.10065507491305	1.39349550948546	0.172902143701657	0.839355148619712	-4.98009273194706
a_HQ_transcript/127176	2.01330673639596	1.9505743371521	1.3934817085395	0.172906277749347	0.839355148619712	-4.9813251381035
a_HQ_transcript/57198	-2.14063263648772	-0.255180275663654	-1.39345535682885	0.172914171570063	0.839355148619712	-4.97260569293175
a_HQ_transcript/1003	-2.22796497208488	3.27456745556906	-1.39341152729322	0.172927301607713	0.839355148619712	-5.09846898162528
a_HQ_transcript/143082	-0.933096423951136	2.01759216655174	-1.39340009562876	0.172930726325861	0.839355148619712	-4.90001376811327
a_HQ_transcript/51043	1.39357520604469	0.189195764135967	1.39329196348205	0.172963123369558	0.839376849057233	-4.96252233204657
a_HQ_transcript/143106	-2.1223836774072	2.53211755208895	-1.39328877904292	0.172964077518709	0.839376849057233	-5.04981763222298
a_HQ_transcript/52229	1.29367933220681	0.00691791111194688	1.39316234837231	0.173001963095594	0.8394281095096	-4.89738639026641
a_HQ_transcript/60760	0.711372747759648	2.33466283254278	1.39315714506344	0.173003522431961	0.8394281095096	-4.99245469298815
a_HQ_transcript/18087	-1.62201021857079	4.7088246034957	-1.39303730186536	0.173039440286578	0.839512605694854	-5.27357030560115
a_HQ_transcript/122694	1.11648161985965	-0.683380372384107	1.3929714841186	0.173059168812667	0.839512605694854	-5.00681593464996
a_HQ_transcript/96493	0.850749805612536	3.07105239859256	1.39295448572095	0.173064264279546	0.839512605694854	-5.17422214295894
a_HQ_transcript/8550	2.10892949189435	-0.399590725822935	1.39289272477042	0.173082778828806	0.839532357122899	-4.999487791785
a_HQ_transcript/109851	1.85246100889193	-0.0988523954306431	1.39276692687426	0.173120495011848	0.839645234380366	-4.88092477770038
a_HQ_transcript/88555	-1.88162723533688	1.1749715205589	-1.39267252601556	0.173148802092675	0.839712461875774	-5.0531654148814
a_HQ_transcript/112018	1.5877973690845	0.944990154176906	1.39229494891664	0.173262058721592	0.840191620035764	-4.82532354672808
a_HQ_transcript/140635	2.6664383119657	0.44009527885154	1.39216951439583	0.173299696415623	0.840304033470747	-5.05149301506697
a_HQ_transcript/151431	0.635588336841187	3.91003945742459	1.39211625802507	0.173315678376075	0.84031143153261	-5.3139094193913
a_HQ_transcript/54929	2.88545338469493	2.59345284477062	1.39203615162587	0.173339720061714	0.840357902230803	-5.00203371857544
a_HQ_transcript/107068	1.45437296031462	-0.0303798714903996	1.39172868988733	0.173432020284733	0.840735257702524	-5.02365364088555
a_HQ_transcript/119400	1.45001555204833	1.46834125649274	1.39158830388628	0.173474177037257	0.840823108608668	-4.9372627816208
a_HQ_transcript/11534	0.848196740073475	3.44642122969538	1.39156697375077	0.173480583001378	0.840823108608668	-5.21052202961051
a_HQ_transcript/79973	1.64685278257248	0.960075138188981	1.39149154900647	0.173503236390118	0.840823108608668	-4.9376681198936

a_HQ_transcript/26821	-1.53059547496854	0.124576763576425	-1.3914135043768	0.173526679081479	0.840823108608668	-4.86656295125678
a_HQ_transcript/68643	-1.59659278868319	-0.428466088906847	-1.39140096900719	0.173530444629716	0.840823108608668	-4.97251181968487
a_HQ_transcript/66368	1.71728766592968	0.224129828302898	1.39137937039	0.173536932871907	0.840823108608668	-5.02777583395847
a_HQ_transcript/47937	1.32866539052224	-0.351482045747507	1.39124084106595	0.173578551703955	0.840868677000934	-4.83250626264449
a_HQ_transcript/126435	-1.19967207472907	-0.526576535795619	-1.39123828305934	0.173579320287891	0.840868677000934	-4.96027999035281
a_HQ_transcript/121965	1.53412980922283	-0.519393690004474	1.39119456469989	0.173592456407668	0.840868677000934	-4.99588356959702
a_HQ_transcript/47977	-2.02765519317264	0.554628513268114	-1.39111162137531	0.17361738064678	0.840868677000934	-4.96086926309998
a_HQ_transcript/75729	-0.639859214851947	3.34164789813717	-1.39110734168101	0.173618666758967	0.840868677000934	-5.26387820479442
a_HQ_transcript/46392	-0.535424835624948	2.960318157801	-1.39083862139563	0.173699436145994	0.841189771248385	-5.20493115375223
a_HQ_transcript/133614	1.63703835648985	4.73241196134216	1.39044889528371	0.173816628510407	0.841687186109041	-5.20147223266794
a_HQ_transcript/144757	4.39126828278245	3.84732569928421	1.39032482067759	0.173853951262441	0.841779739873767	-5.15774988843231
a_HQ_transcript/148813	-3.00028190913455	3.08152084048171	-1.39028907602406	0.17386470473652	0.841779739873767	-5.29143532977204
a_HQ_transcript/62263	2.29799618879675	2.12309233066906	1.39007762419901	0.173928328850671	0.842017647991321	-4.9230876157546
a_HQ_transcript/104044	1.40193159139603	-0.0623282567448448	1.38996526461388	0.17396214432952	0.842023093757554	-4.99413843616249
a_HQ_transcript/17037	-0.53472958977332	4.32433481700671	-1.38993538759157	0.173971136913416	0.842023093757554	-5.40751686827799
a_HQ_transcript/149243	-2.05393260063824	1.1136798826195	-1.38990041964148	0.173981662259958	0.842023093757554	-5.01763893512203
a_HQ_transcript/37841	-0.599992501931206	4.90039155146921	-1.38988136995891	0.173987396420348	0.842023093757554	-5.44763115188982
a_HQ_transcript/123095	-0.752546600022172	2.49823736303081	-1.38980370327383	0.174010776461121	0.84206613504263	-5.00893423678242
a_HQ_transcript/36056	-2.10171413958542	2.00055755028635	-1.38974863416314	0.174027355430743	0.842076260424393	-4.96032355816371
a_HQ_transcript/31804	-1.0714377801458	-0.138679500611644	-1.38954604260223	0.174088357768819	0.84218097229264	-4.89230424170053
a_HQ_transcript/28285	-0.814450575832895	1.32993048464253	-1.3895022958572	0.174101532543638	0.84218097229264	-4.87277373187893
a_HQ_transcript/99031	2.32954187460916	4.04544702587208	1.38946049922873	0.17411412074907	0.84218097229264	-5.46840447354566
a_HQ_transcript/26269	-0.650810551972121	2.95741242181888	-1.38941809516674	0.17412689262678	0.84218097229264	-5.09862480169848
a_HQ_transcript/108697	3.76885369284671	1.93299424316881	1.38936887175758	0.174141719373871	0.84218097229264	-5.12518938081742
a_HQ_transcript/93280	2.79770458037475	0.629177298322626	1.38933847762702	0.174150874983939	0.84218097229264	-5.07404023635533
a_HQ_transcript/120183	1.49060104779305	-0.45121439525081	1.38931573588805	0.174157725713639	0.84218097229264	-4.95509233190994
a_HQ_transcript/140876	-1.47742480255014	2.11377923483584	-1.38929191163855	0.174164902765102	0.84218097229264	-4.98621440566732
a_HQ_transcript/82605	-2.22184933148599	0.0426378672992066	-1.38914801575102	0.174208256293384	0.842313707002363	-4.99575432972017
a_HQ_transcript/66355	1.47284675555769	0.79443187436139	1.38910461486761	0.174221333940432	0.842313707002363	-4.86920197058374
a_HQ_transcript/46464	-1.53156114772412	2.27562563018433	-1.38879510458846	0.174314618486349	0.842375419711648	-5.01863925251512
a_HQ_transcript/144168	1.76637666720249	1.12089848680951	1.38877458415715	0.174320804600971	0.842375419711648	-4.9842034931686
a_HQ_transcript/105360	-1.10141121088017	4.78607759426205	-1.38872633068865	0.174335351827313	0.842375419711648	-5.40016012275602
a_HQ_transcript/104959	2.193815120972	1.12817488618214	1.38865373136189	0.174357240514609	0.842375419711648	-4.92913951234257
a_HQ_transcript/80065	2.49156829752329	0.992082254363872	1.388630479202	0.174364251491735	0.842375419711648	-5.04773022882753
a_HQ_transcript/51776	1.06530167869624	1.75348173063743	1.38861148800837	0.17436997786834	0.842375419711648	-4.92189891356757
a_HQ_transcript/77527	-1.29938495261142	0.761549069226076	-1.38859319545668	0.174375493723781	0.842375419711648	-4.94985279550194
a_HQ_transcript/96535	-3.42644939586738	2.41277216279462	-1.38858014790888	0.174379428107166	0.842375419711648	-5.18479586428545
a_HQ_transcript/119408	-0.797330851876743	2.36528217368895	-1.38857713866375	0.174380335530741	0.842375419711648	-5.02931160060364
a_HQ_transcript/39400	0.782589713397818	2.01499280150499	1.38855753962075	0.17438624561934	0.842375419711648	-4.96397298523133
a_HQ_transcript/137579	1.0223578369999	2.6619316511101	1.38853345865363	0.174393507445809	0.842375419711648	-4.99633178956721
a_HQ_transcript/5617	0.902723186138598	4.18214586586012	1.3883629055372	0.174444945994662	0.842504701288159	-5.24355393528741
a_HQ_transcript/132740	-2.27678246101232	0.221821819040951	-1.38834860354953	0.174449259990096	0.842504701288159	-4.99687530751234
a_HQ_transcript/46945	-1.74297924375391	0.614773888036405	-1.38825076465848	0.174478773964909	0.842505053390321	-4.85844244406966
a_HQ_transcript/111982	-2.07620843890799	1.86375937390384	-1.38823581073798	0.174483285292212	0.842505053390321	-4.93139763925354
a_HQ_transcript/74044	-0.843630010878409	2.43735101496307	-1.38815991809615	0.174506182134377	0.842505053390321	-5.04497361071263
a_HQ_transcript/5703	-0.955323938471482	0.53077994033045	-1.38812240152194	0.17451750176776	0.842505053390321	-4.8986658636547
a_HQ_transcript/35818	1.09037861998426	-0.425787519646789	1.38810814744353	0.174521802709071	0.842505053390321	-4.91339887998615
a_HQ_transcript/122449	0.581911359448985	3.04525467181492	1.38801623499924	0.174549537815086	0.842548944624538	-5.24287572255638
a_HQ_transcript/133115	1.43491942288309	-0.490665979866338	1.38797655413925	0.17456151280589	0.842548944624538	-4.98426282949282
a_HQ_transcript/79068	-1.69644144721455	0.349008178171929	-1.38793392328537	0.174574378768544	0.842548944624538	-4.86676365488871

a_HQ_transcript/54694	-0.957850695337459	2.11516347970373	-1.38787346937535	0.17459262498761	0.842567049006209	-4.99511724023455
a_HQ_transcript/139619	-1.7327857810689	1.9336197825375	-1.38776609188909	0.174625037369293	0.842653509231469	-5.03295000295697
a_HQ_transcript/19478	-1.77178291320913	2.02705387028828	-1.38760275107992	0.174674351548171	0.842791498574607	-5.00831916989169
a_HQ_transcript/72765	1.85614984078641	1.56587604134397	1.38757533100767	0.174682631001013	0.842791498574607	-4.97390948420141
a_HQ_transcript/120847	1.82756068433508	1.33086684931196	1.38743940918796	0.17472367693272	0.842879171515699	-4.86712126765518
a_HQ_transcript/144951	-0.773370737408974	1.90819520867463	-1.38737878771225	0.174741985951489	0.842879171515699	-4.96485409329773
a_HQ_transcript/3704	-1.04886675984688	1.20400417232849	-1.38737111336503	0.174744303879984	0.842879171515699	-4.82446852381392
a_HQ_transcript/61136	1.29601380108137	0.955223412592211	1.38724221423982	0.174783239635055	0.842997025680922	-4.92485249449734
a_HQ_transcript/140737	-1.87665497297207	-0.267995792055314	-1.38719306970307	0.174798086203125	0.842998685374203	-4.93507186061019
a_HQ_transcript/74581	1.35578625847919	-0.356694833163454	1.38711451224594	0.174821820462382	0.84304320390222	-5.021691936845
a_HQ_transcript/123185	0.434292670067926	3.38562030458256	1.38705899186545	0.174838596137336	0.843054161449281	-5.26643391886832
a_HQ_transcript/151521	-0.506585961889475	4.37946798909671	-1.38697441626574	0.174864153363813	0.843107457426318	-5.40393707008788
a_HQ_transcript/49530	1.41555663728085	-0.153762363497079	1.38681607416397	0.174912009347038	0.843212143027653	-4.97558912299607
a_HQ_transcript/70865	1.19110234404332	0.864669194587741	1.38680262583337	0.174916074328462	0.843212143027653	-4.9362439895447
a_HQ_transcript/136187	-1.73528198924126	0.769660058060805	-1.38675859467723	0.174929383993436	0.843212143027653	-4.87964988847956
a_HQ_transcript/84922	0.450969300651406	5.3443511505529	1.38657725044157	0.174984208766322	0.843382786602905	-5.5024398176183
a_HQ_transcript/23887	1.93569364775013	-0.266950574107345	1.38651426992029	0.175003252448946	0.843382786602905	-4.99312949001199
a_HQ_transcript/99899	2.06312578138451	3.1532615667292	1.38643660951585	0.175026737177461	0.843382786602905	-5.06301862557016
a_HQ_transcript/33589	-0.889391454953649	3.30223292552812	-1.38639865308691	0.17503821620558	0.843382786602905	-5.26916071057132
a_HQ_transcript/151930	-2.29119109791406	1.99143100382673	-1.38637063362319	0.175046690409768	0.843382786602905	-5.09066009005203
a_HQ_transcript/61192	-1.33574831256341	1.94186373675743	-1.38635360931943	0.175051839395198	0.843382786602905	-5.08553594635717
a_HQ_transcript/151128	0.662624714854496	2.64451425764022	1.38613438960362	0.175118152770203	0.843632354071469	-5.04450007847375
a_HQ_transcript/79074	0.767928520976927	4.11427796026024	1.38597303380275	0.175166974998617	0.843797623495451	-5.34634367568782
a_HQ_transcript/63792	-2.18718562639542	0.822074646433987	-1.38578823071413	0.175222904846722	0.84399710083943	-4.91490565353073
a_HQ_transcript/40746	0.464013835469646	3.4140163271712	1.38567837892998	0.175256157617045	0.84402106225771	-5.27308866942838
a_HQ_transcript/63622	0.605019601915831	2.24707706504082	1.38567586194136	0.175256919581795	0.84402106225771	-5.06024830707306
a_HQ_transcript/145458	2.04492173110483	-0.143278905336313	1.38538966413975	0.175343576746534	0.844112687172633	-5.04965215581414
a_HQ_transcript/149886	-1.77284865385553	2.23094684760744	-1.38528234784772	0.175376079418989	0.844112687172633	-5.09426599263809
a_HQ_transcript/59474	1.3417258265486	0.974935425788301	1.38526877774269	0.175380189704756	0.844112687172633	-4.89505740316319
a_HQ_transcript/38731	1.34925799300094	2.08483701605039	1.38513692575002	0.175420130626628	0.844112687172633	-4.90048525514075
a_HQ_transcript/2529	-0.654839265354313	2.2094654603523	-1.38511244559852	0.1754275469944	0.844112687172633	-4.98109574243195
a_HQ_transcript/144061	-1.33427410784211	0.654510389971243	-1.38506780346268	0.175441072152745	0.844112687172633	-4.92394408615494
a_HQ_transcript/142000	0.583603582110239	3.82098859414591	1.38506438497501	0.175442107880184	0.844112687172633	-5.3631812819134
a_HQ_transcript/16682	-1.69061585812657	0.00388290284360911	-1.38506399892838	0.175442224844218	0.844112687172633	-4.89823840810946
a_HQ_transcript/142796	1.13966493786487	1.652976928518	1.38498871280248	0.175465036125554	0.844112687172633	-4.89772612178418
a_HQ_transcript/18244	1.45281201141114	-0.0139033149275461	1.38495573641485	0.175475028518496	0.844112687172633	-4.95757544993135
a_HQ_transcript/88411	2.43389727192657	1.23790706217645	1.38494623462046	0.175477907802389	0.844112687172633	-5.03555798779569
a_HQ_transcript/13276	-0.645528259867857	2.11954418380454	-1.38494494339001	0.175478299080703	0.844112687172633	-5.05373211782388
a_HQ_transcript/94937	1.17239825020275	-0.482808893233794	1.38492265167981	0.17548505418892	0.844112687172633	-5.00823895281447
a_HQ_transcript/32886	1.17757704795418	-0.579522628433189	1.38491077594117	0.175488653004198	0.844112687172633	-4.99210877887969
a_HQ_transcript/111334	-1.02914162305823	-0.805599406559592	-1.38483282462753	0.175512276744375	0.844112687172633	-4.98951417650377
a_HQ_transcript/118273	-0.903009606345617	2.14827615644206	-1.38481785559625	0.175516813507605	0.844112687172633	-4.97437652521613
a_HQ_transcript/101907	0.842253083175258	3.55150710823325	1.38476936635134	0.175531510092054	0.844112687172633	-5.2880053278502
a_HQ_transcript/125978	0.517293734968178	4.63241227288522	1.38471714403998	0.175547339204406	0.844112687172633	-5.45436311794573
a_HQ_transcript/68021	-1.30537538434108	1.46904969929934	-1.38460888704432	0.175580156547168	0.844112687172633	-4.88176950210057
a_HQ_transcript/102871	-1.67724513096735	0.624364834038615	-1.38457999411119	0.175588916045119	0.844112687172633	-5.03086815084486
a_HQ_transcript/25674	0.843811459186944	2.8958612110733	1.38453456684229	0.175602688963112	0.844112687172633	-5.28447829910457
a_HQ_transcript/81982	-0.692293105756745	1.81312735146949	-1.38451842781069	0.175607582296706	0.844112687172633	-4.92181697721654
a_HQ_transcript/148547	0.402631606981598	5.02672741268386	1.38443629326157	0.175632487035403	0.844112687172633	-5.47943990115684
a_HQ_transcript/113153	-2.68260387590029	4.1879043671999	-1.38443232092232	0.175633691593197	0.844112687172633	-5.49212898274985

a_HQ_transcript/116611	-1.02903880087205	2.8379578130586	-1.38439529290108	0.175644920146706	0.844112687172633	-5.32483788408439
a_HQ_transcript/103139	1.05380756439794	0.562476242299517	1.38436655273227	0.175653635838603	0.844112687172633	-4.84560070440755
a_HQ_transcript/53112	-1.09948652539084	3.18674697061955	-1.38422940112578	0.175695232837384	0.844112687172633	-5.18734759335584
a_HQ_transcript/98290	-0.659128997975183	2.75572406969802	-1.38422858666928	0.175695479878568	0.844112687172633	-5.0275614936443
a_HQ_transcript/34275	0.966924245329646	1.76461211492882	1.38421854330466	0.175698526257161	0.844112687172633	-4.92056810299313
a_HQ_transcript/99778	1.73896971951121	0.422501926812978	1.38405657297011	0.175747661199931	0.844112687172633	-5.01176862935826
a_HQ_transcript/128939	3.33519469735264	2.82820953194771	1.3840521674739	0.175748997790783	0.844112687172633	-5.0257918718175
a_HQ_transcript/151205	-1.69681598238887	0.231693524663655	-1.38403198807363	0.175755120154126	0.844112687172633	-4.97532350134346
a_HQ_transcript/133816	-2.09546753264915	1.72855749545657	-1.38398020925795	0.175770830437191	0.844112687172633	-4.93118960315824
a_HQ_transcript/27684	1.344359910405	0.0175646099758769	1.38395570975121	0.175778264248847	0.844112687172633	-4.9459275841962
a_HQ_transcript/120646	1.10844257515859	1.25418941245086	1.38393614225678	0.175784201731089	0.844112687172633	-4.82919429536827
a_HQ_transcript/113497	-1.66591550734224	2.26286248955573	-1.38380596713478	0.175823705533859	0.844232641274752	-5.17651532695403
a_HQ_transcript/59748	1.90254493545325	1.10802677857848	1.38359906516569	0.175886507629545	0.844239871963807	-5.0093013330994
a_HQ_transcript/106695	1.30314041237645	-0.630383613320588	1.38353619573752	0.175905594201204	0.844239871963807	-4.99923367952437
a_HQ_transcript/86771	1.52169897532485	-0.128795071411749	1.38353048859328	0.175907326916952	0.844239871963807	-5.05056946894113
a_HQ_transcript/144036	0.501030165606541	2.88860198885359	1.38352665867546	0.175908489705427	0.844239871963807	-5.14982455991099
a_HQ_transcript/33558	1.68391705462731	2.30089398382027	1.38350931670096	0.175913754919076	0.844239871963807	-5.04602966815909
a_HQ_transcript/141878	0.941507725086447	3.06683927178169	1.38349504490044	0.175918088086482	0.844239871963807	-5.1569587389687
a_HQ_transcript/51348	1.9732120289348	0.152588624066192	1.38346609485386	0.17592687808109	0.844239871963807	-5.02408116456553
a_HQ_transcript/7638	1.86864822182226	2.14769929897145	1.38327345084233	0.175985378592247	0.844430078027242	-4.93227472623255
a_HQ_transcript/147165	1.12586258901971	-0.117843247084957	1.38323990086087	0.175995568319915	0.844430078027242	-4.93479789854318
a_HQ_transcript/3515	0.932869617594992	2.40161827280019	1.38318754030909	0.176011472073151	0.844434312053015	-4.96587390720056
a_HQ_transcript/83517	1.50823700174561	-0.323143124705472	1.38314134196307	0.176025505077525	0.844434312053015	-4.93866082355459
a_HQ_transcript/3231	-1.0946755259829	1.45528518426933	-1.38287075417434	0.176107715153927	0.844680981342719	-4.97867435949509
a_HQ_transcript/110657	-2.16030309736365	3.74554787458954	-1.38268422315219	0.176164404515508	0.844680981342719	-5.54619475669542
a_HQ_transcript/37523	1.97624658190104	0.954404319740248	1.38262725374296	0.176181721146029	0.844680981342719	-5.13370046055657
a_HQ_transcript/144149	1.96062991537437	-0.0527170471088915	1.3826061846466	0.176188125722195	0.844680981342719	-5.01516289529754
a_HQ_transcript/44944	-1.51451048831767	0.870453102439969	-1.38260413195828	0.176188749707347	0.844680981342719	-4.99744538078715
a_HQ_transcript/112122	0.769670076921158	-0.672820666855959	1.38260110608084	0.176189669529917	0.844680981342719	-5.01970847385054
a_HQ_transcript/132352	3.05065799134978	2.60695068295832	1.38257682412028	0.176197051027119	0.844680981342719	-5.25633063701927
a_HQ_transcript/94623	1.41420575779893	0.888958022770414	1.38249952460761	0.176220550988974	0.844680981342719	-4.9045432706765
a_HQ_transcript/13415	1.45385084446604	-0.0344727378339913	1.38249680646448	0.176221377380986	0.844680981342719	-5.00282116362234
a_HQ_transcript/126539	0.353656918252297	3.60817688360838	1.38249397517188	0.176222238176837	0.844680981342719	-5.30221432222468
a_HQ_transcript/134062	1.15330600114472	-0.640269993572188	1.38215302594034	0.176325920710541	0.845048639188736	-5.01794228442434
a_HQ_transcript/134120	1.66042149867365	0.012970661174221	1.38211470221011	0.176337577917072	0.845048639188736	-5.04580429097841
a_HQ_transcript/63358	-3.44966902280273	2.30047715736614	-1.38209834283896	0.176342554248544	0.845048639188736	-4.98583910974226
a_HQ_transcript/132637	2.27751438957407	1.95476191799109	1.38195782983333	0.176385301190993	0.84514175729983	-4.98824581129787
a_HQ_transcript/105932	1.22507161360918	-0.62460121236291	1.38193888722534	0.176391064539663	0.84514175729983	-4.99705664422321
a_HQ_transcript/139887	-1.28931035404999	1.42459629643331	-1.38165911657185	0.176476202756975	0.84547998960335	-4.9950650869136
a_HQ_transcript/149251	3.48828131621822	2.13788476870647	1.3815845244815	0.176498907609785	0.845485507789122	-5.13600908409127
a_HQ_transcript/133460	-1.39681717833158	2.80432460428195	-1.38155976348562	0.176506445035456	0.845485507789122	-4.98247646719428
a_HQ_transcript/146902	1.6858461992731	0.096357352841547	1.38132581903048	0.176577671806493	0.845756996272892	-4.95345196219956
a_HQ_transcript/148243	-1.27356119767398	1.44079625542767	-1.38119847995603	0.176616450904681	0.845873037668889	-4.96942820905179
a_HQ_transcript/28685	-0.680318669749825	2.48148482664035	-1.38101167823198	0.17667335042889	0.84594974355824	-5.15831283555435
a_HQ_transcript/138259	-0.60048905664817	2.73555177992399	-1.38100577796659	0.176675147873725	0.84594974355824	-5.20093384766844
a_HQ_transcript/148634	1.64054520403272	-0.026688603607148	1.38100256524585	0.176676126596426	0.84594974355824	-5.00072546363425
a_HQ_transcript/132011	1.41713571033	3.1676450198037	1.38083621879832	0.176726808127346	0.846122716648249	-5.10261989584975
a_HQ_transcript/49238	0.955360081221976	-0.447983815073373	1.38076863004431	0.176747403940153	0.846151630393116	-4.97223851950443
a_HQ_transcript/104277	-1.75846003353759	1.74933949001013	-1.38066237074983	0.176779787320327	0.846171904038333	-4.9537896505984
a_HQ_transcript/112008	-0.700391803838946	2.26553226900977	-1.38065920274389	0.176780752866803	0.846171904038333	-4.9841607475401

a_HQ_transcript/5222	1.46162814684842	3.73341035479188	1.38057848611366	0.176805355109966	0.846219982036401	-5.09325179600004
a_HQ_transcript/82717	-1.08738801892746	-0.141662695378957	-1.38046471247514	0.176840037586861	0.846316293829167	-4.94316452982417
a_HQ_transcript/130084	2.43474368617251	2.63393486285077	1.38030546601058	0.176888590797125	0.846343663414297	-5.2292145357699
a_HQ_transcript/113348	2.44770746937689	1.59328148650865	1.3802880639386	0.176893897205496	0.846343663414297	-4.88471611963079
a_HQ_transcript/41884	1.50732023113028	-0.5327416124138	1.38023202959372	0.176910984584037	0.846343663414297	-4.95006499943718
a_HQ_transcript/136509	-1.26979663278271	1.68820608756144	-1.38022895536629	0.176911922090752	0.846343663414297	-4.89632564273152
a_HQ_transcript/101475	-2.39895168462386	3.08281158676164	-1.38020720038193	0.176918556532947	0.846343663414297	-5.20020754701764
a_HQ_transcript/37767	1.1419122184184	-0.230464311865806	1.38007551268592	0.17695872040911	0.846364018758485	-5.02599070734389
a_HQ_transcript/140515	1.08198431941551	5.04239640262934	1.38006642263555	0.17696149307661	0.846364018758485	-5.44137307187262
a_HQ_transcript/151261	-3.20704131398787	0.395754423552341	-1.38005003211068	0.176966492636778	0.846364018758485	-5.03167287945644
a_HQ_transcript/13599	1.58017060434419	-0.0860143341882213	1.37990199115624	0.177011654182437	0.846471928640494	-4.93187088478926
a_HQ_transcript/3974	-1.49332991546707	1.23633983974593	-1.37982003959001	0.177036658285961	0.846471928640494	-4.99230274387602
a_HQ_transcript/143877	1.070099392045	2.85253046512527	1.37970473032528	0.177071844763147	0.846471928640494	-5.11456501879189
a_HQ_transcript/142789	-2.66207770959665	3.76282534679369	-1.37966890732997	0.177082777215032	0.846471928640494	-5.18608738408441
a_HQ_transcript/29644	1.25472255298718	-0.184416912280525	1.37962790123002	0.17709529209126	0.846471928640494	-4.9516130180939
a_HQ_transcript/126020	1.55266179562268	-0.332420829456212	1.37958185013018	0.177109347500577	0.846471928640494	-4.93416999762996
a_HQ_transcript/102663	-0.814203858075975	3.41571907201507	-1.37955968811073	0.177116111954176	0.846471928640494	-5.37267811515855
a_HQ_transcript/69057	-0.761998743339488	1.03594338794517	-1.37952180981777	0.177127673909954	0.846471928640494	-4.86343490504948
a_HQ_transcript/50776	-0.427639140055521	3.82876721425226	-1.37949950678941	0.177134481953074	0.846471928640494	-5.34120234298706
a_HQ_transcript/86091	1.50297523678165	3.81849879144345	1.37949886583806	0.177134677607744	0.846471928640494	-5.17254975069309
a_HQ_transcript/94812	0.67460057495873	4.48857781896474	1.37941900108318	0.177159058174752	0.846518843697656	-5.34348339657508
a_HQ_transcript/1328	0.655621847301399	3.62595772116652	1.37930423829103	0.177194096756297	0.846616674030086	-5.21593615477223
a_HQ_transcript/131368	-1.35637410307124	1.41108042512836	-1.37908022729789	0.177262505805231	0.846740355237029	-4.96194845583807
a_HQ_transcript/43372	-1.52410858664797	1.91438573349093	-1.37908022079922	0.177262507790108	0.846740355237029	-5.00080804520791
a_HQ_transcript/134340	2.12602712982499	-0.347891518494111	1.37907637208453	0.177263683300293	0.846740355237029	-5.00482434885715
a_HQ_transcript/49684	4.76579787486777	2.63591682689308	1.3789687871622	0.177296545345272	0.84682773934511	-5.30138564435298
a_HQ_transcript/104889	-2.57268195825057	0.573200295731263	-1.37890448806135	0.177316187910252	0.846851973613272	-4.99507541597076
a_HQ_transcript/122594	1.58776182591061	0.603612338970971	1.37876104075749	0.177360015366713	0.846942703118736	-4.88621230956298
a_HQ_transcript/67992	1.60334678732794	0.458847387682189	1.37874693448141	0.177364325713329	0.846942703118736	-5.03195461160992
a_HQ_transcript/139431	0.420173926003925	5.87244798990051	1.37867532862571	0.177386207024681	0.846977611461366	-5.58365897178879
a_HQ_transcript/90195	1.73212343425451	0.633530900858422	1.37860432652132	0.177407905919062	0.847011643142772	-4.96715006093276
a_HQ_transcript/111116	-0.696699999833055	2.22686865635306	-1.37846499788487	0.177450492040887	0.847032916973588	-4.9619802825786
a_HQ_transcript/6460	-0.839904147621384	3.06425234457054	-1.37844902008225	0.17745537620257	0.847032916973588	-5.11889924438907
a_HQ_transcript/93622	0.788390019448276	3.44063426760708	1.37844672648465	0.177456077327697	0.847032916973588	-5.34230739533555
a_HQ_transcript/78347	1.65659074706487	1.81310440597561	1.3783981118666	0.177470938738014	0.847034298954204	-4.94884369747395
a_HQ_transcript/16834	2.6340740178971	1.3904789456377	1.37830785585978	0.177498532425631	0.847074809107163	-5.03413489780395
a_HQ_transcript/132652	-0.425215115198751	3.31872812677722	-1.37820167170473	0.177531000056785	0.847074809107163	-5.22873192537255
a_HQ_transcript/146208	-1.65073728643806	3.88224307109272	-1.37818291779147	0.177536734869066	0.847074809107163	-5.32356252674972
a_HQ_transcript/92221	1.42313204718562	2.84598655586423	1.37817970689763	0.177537716751868	0.847074809107163	-4.95603274763604
a_HQ_transcript/37862	0.679953994703164	1.7914751355859	1.37810174410279	0.17756155886608	0.847119032646708	-4.87974429835781
a_HQ_transcript/58684	0.83757918084902	0.379724164761068	1.37804612046362	0.177578570878748	0.847130666232239	-4.94749096319016
a_HQ_transcript/8528	-1.46219491268745	1.62390123508743	-1.37791753690307	0.177617901918753	0.847198176535072	-4.96400549011773
a_HQ_transcript/134890	1.72855420970687	1.07076512793641	1.37790455878929	0.177621872031257	0.847198176535072	-4.85339612037496
a_HQ_transcript/81001	1.64761676115057	0.799117286454363	1.37772397098288	0.177677122512542	0.847345903396972	-4.96456495166812
a_HQ_transcript/17361	1.78157117746977	0.498828633815917	1.37759940111051	0.17771524222174	0.847345903396972	-4.98089910471476
a_HQ_transcript/58995	-0.805449070307822	3.1471530316632	-1.37758457570305	0.177719779378781	0.847345903396972	-5.23437265924474
a_HQ_transcript/113365	-0.74635707126405	3.83776146476767	-1.3775239965591	0.177738319914314	0.847345903396972	-5.33885439764347
a_HQ_transcript/115670	-2.04838130645212	3.44292521177721	-1.37751810628539	0.177740122741025	0.847345903396972	-5.44838602454186
a_HQ_transcript/107899	-2.05689299345661	0.669000749733951	-1.37751750231176	0.177740307599079	0.847345903396972	-4.90604163905137
a_HQ_transcript/10819	1.07122119347411	2.31768251284532	1.37745459227116	0.177759563279485	0.84736820520829	-4.95827072342898

a_HQ_transcript/69558	1.50399190944508	0.760762610618789	1.3772977787314	0.177807568279337	0.847479792968061	-4.87189425775565
a_HQ_transcript/40929	3.05713001660327	1.84586339678974	1.37728287558601	0.177812131073467	0.847479792968061	-4.95104370932802
a_HQ_transcript/136935	-0.343988372574332	6.10121848959297	-1.3771714270415	0.177846255404163	0.847502777379511	-5.59100738333467
a_HQ_transcript/44202	-1.9733309944361	3.9347879587753	-1.37712604495268	0.177860152367232	0.847502777379511	-5.49926058309536
a_HQ_transcript/47568	-0.8769768444979	2.35460745448868	-1.37712427846964	0.1778606933191	0.847502777379511	-5.04286831469452
a_HQ_transcript/13826	1.4270354589398	0.358584942774486	1.37703309262897	0.177888618991216	0.847566364321428	-4.95563405052767
a_HQ_transcript/39968	1.25994207197545	0.602155603891029	1.37697095886512	0.177907649418729	0.847587562118831	-4.9667756648653
a_HQ_transcript/145712	1.43755612146936	-0.331931162191807	1.37683333397558	0.177949807034571	0.847718929954561	-5.07351173637936
a_HQ_transcript/46719	-2.33663412183093	0.466415386796401	-1.37676003616704	0.177972262986612	0.847756428983513	-4.98550624003123
a_HQ_transcript/62798	1.6375266359256	1.49042368797065	1.37650891738009	0.178049213966368	0.847991077797696	-4.8633770180452
a_HQ_transcript/113613	1.04622257448646	-0.348089802844148	1.37625541660704	0.178126921147742	0.847991077797696	-5.05882551932408
a_HQ_transcript/67681	1.35696988139893	2.58621083406899	1.37622829784443	0.178135235595872	0.847991077797696	-5.02581289400401
a_HQ_transcript/59227	-1.21245468016357	1.63988271131272	-1.37618015530633	0.178149996550652	0.847991077797696	-4.94712665530006
a_HQ_transcript/144153	-1.64289746466904	0.885599069709813	-1.3761751889702	0.178151519330204	0.847991077797696	-5.03584436806569
a_HQ_transcript/105197	-0.651395744960561	3.38260866032989	-1.37617511559092	0.178151541829859	0.847991077797696	-5.25037202488615
a_HQ_transcript/44311	-1.42077002095399	1.21369095048196	-1.3761740955633	0.178151854592335	0.847991077797696	-4.88946858963918
a_HQ_transcript/152507	-1.51419866734625	2.2545007175749	-1.37617172344835	0.178152581935614	0.847991077797696	-5.07387085735782
a_HQ_transcript/17227	1.00860741433786	2.14244154395107	1.37615657863808	0.178157225726287	0.847991077797696	-5.04263065197769
a_HQ_transcript/16020	-1.12414809918081	2.39435213203577	-1.37612337554501	0.17816740698361	0.847991077797696	-5.12867642454067
a_HQ_transcript/10112	-0.945284932385557	3.20913142982237	-1.37592690021409	0.178227662630449	0.848208414391905	-5.13148157844259
a_HQ_transcript/112846	-1.06199632624821	-0.581735955909585	-1.3758015229906	0.178266121989703	0.848262839802072	-5.02180939812813
a_HQ_transcript/139143	3.14609609784176	1.32690655435697	1.37579447331008	0.178268284665258	0.848262839802072	-5.11620067000305
a_HQ_transcript/3842	-1.31481312469161	1.33371214365546	-1.37571196287812	0.17829359843696	0.848286718672515	-4.82252339345858
a_HQ_transcript/128790	-0.667775671094951	2.33179493965385	-1.37565439094735	0.178311262864057	0.848286718672515	-5.0797340905565
a_HQ_transcript/150128	1.6677817036395	-0.328936739185138	1.3755927184086	0.178330186966368	0.848286718672515	-5.02167418110457
a_HQ_transcript/19032	-2.36071056346916	0.588887175579555	-1.3755878637288	0.178331676682118	0.848286718672515	-5.01513322467255
a_HQ_transcript/111600	-0.995483059228489	3.53896280190298	-1.3754861618225	0.178362887339577	0.848288833656288	-5.2743811409503
a_HQ_transcript/148034	0.711801631458078	4.91688939354009	1.37541061651328	0.178386073716861	0.848288833656288	-5.50193695810582
a_HQ_transcript/104944	2.07940353829812	3.35189975354139	1.37530952747188	0.178417103651706	0.848288833656288	-5.03743342024766
a_HQ_transcript/10265	0.845604729423858	-0.365616268627999	1.3752565753527	0.178433359324363	0.848288833656288	-5.00373980260964
a_HQ_transcript/134048	3.52856990395232	2.45122419171724	1.37523015389017	0.17844147083131	0.848288833656288	-5.15449756664921
a_HQ_transcript/83780	1.74966872051957	0.579285150063156	1.37520036546127	0.1784506163563	0.848288833656288	-4.981497425516
a_HQ_transcript/73466	-1.36733279638696	0.931066860682216	-1.37519843410081	0.178451293327507	0.848288833656288	-4.90424903952582
a_HQ_transcript/15458	1.74539974889142	0.652468461907832	1.37518530800096	0.178455239376758	0.848288833656288	-4.86950831957654
a_HQ_transcript/117912	1.1519024194883	0.140971048672163	1.37515852561576	0.178463462475618	0.848288833656288	-4.95009224985087
a_HQ_transcript/145524	0.610374562755011	3.57882413037723	1.37493659218601	0.178531614903653	0.848543394204377	-5.31492774899259
a_HQ_transcript/95219	1.43659415631096	0.105735586294723	1.37483906052577	0.178561571822552	0.848561618886136	-5.01436386314102
a_HQ_transcript/80721	-1.56308260677619	1.13469729379605	-1.37482905369925	0.178564645647798	0.848561618886136	-5.11292333644532
a_HQ_transcript/6959	0.811517654442014	0.0540719342739949	1.37473900646163	0.178592307566207	0.848600202321978	-4.92445398392314
a_HQ_transcript/80379	1.56146295500496	0.142365989508395	1.374679622289	0.178610551818471	0.848600202321978	-4.88189667627192
a_HQ_transcript/125705	-1.34559614032021	-0.371091161333573	-1.37457210456046	0.17864358755356	0.848600202321978	-4.91689903328681
a_HQ_transcript/122425	-0.635975993793021	3.62132926110396	-1.37453508089969	0.178654964488717	0.848600202321978	-5.23814288739722
a_HQ_transcript/109693	2.08037893376157	1.45347460451569	1.37447860295502	0.178672320582833	0.848600202321978	-5.03607001417927
a_HQ_transcript/145296	1.45962126738755	1.43807637147268	1.37427977281702	0.178733433013945	0.848600202321978	-4.95056249638887
a_HQ_transcript/141266	0.971520727334093	2.23220056291028	1.3742562359111	0.178740668394329	0.848600202321978	-4.96669363205717
a_HQ_transcript/114022	1.27912348843138	0.136289580047688	1.37425228007191	0.178741884464448	0.848600202321978	-4.93960829297289
a_HQ_transcript/143017	1.70765776579051	1.55325730453464	1.37418618915993	0.178762202517885	0.848600202321978	-5.00578335310858
a_HQ_transcript/70811	-1.34156155637045	0.960596013008339	-1.37416591115258	0.178768436861539	0.848600202321978	-4.93514569262403
a_HQ_transcript/31803	-0.649598251064682	2.8670693181996	-1.3741514969216	0.178772868527567	0.848600202321978	-5.14567066770199
a_HQ_transcript/5976	-0.639777278966185	3.26990676822608	-1.37411162740202	0.17878512688641	0.848600202321978	-5.23717215619354

a_HQ_transcript/71079	2.6119650730187	2.76500958316905	1.37410869104568	0.178786029730021	0.848600202321978	-5.23409491531046
a_HQ_transcript/151550	1.39298517146981	-0.265014854864318	1.37409896327508	0.17878902076005	0.848600202321978	-4.94087611891271
a_HQ_transcript/66665	1.31818277669557	5.67314346374696	1.37407905862448	0.178795141030665	0.848600202321978	-5.6038056517017
a_HQ_transcript/121766	-0.86261377521557	2.13990379545875	-1.37404261876216	0.17880634596132	0.848600202321978	-4.90950074332517
a_HQ_transcript/99524	-2.29789132254582	1.57225002381313	-1.37396830639158	0.178829198042337	0.848639368422315	-5.0398445189766
a_HQ_transcript/114232	-2.26803924799654	0.429589400798106	-1.37387622295319	0.178857518126079	0.848655882599911	-4.96590794804333
a_HQ_transcript/53414	1.11625896084347	1.65668296355981	1.37386163484789	0.178862004990015	0.848655882599911	-5.05606994559384
a_HQ_transcript/36309	-2.42681570539354	1.37323091670793	-1.37379188251957	0.178883459926476	0.848655882599911	-5.12491656694867
a_HQ_transcript/81003	1.35137927688215	2.32486451745205	1.37373408091519	0.178901240488684	0.848655882599911	-5.00849350388702
a_HQ_transcript/118696	-1.1274571522846	2.30490487879133	-1.37361873097923	0.178936727814638	0.848655882599911	-5.05361039769603
a_HQ_transcript/93155	1.71715241525238	1.64878595209398	1.37355160061334	0.178957382946032	0.848655882599911	-4.91083872968389
a_HQ_transcript/31281	-0.931176451789789	2.12137830724418	-1.37355020992212	0.178957810863063	0.848655882599911	-4.96395224733818
a_HQ_transcript/141173	2.09043449806923	0.794197096500966	1.37352571041458	0.178965349515791	0.848655882599911	-5.08089710459839
a_HQ_transcript/113604	1.2362582142147	1.43691429194604	1.37348822381551	0.178976884856772	0.848655882599911	-4.94657783764895
a_HQ_transcript/134811	-1.99762054706703	0.843298473991471	-1.37348240404262	0.178978675763699	0.848655882599911	-5.02160101057393
a_HQ_transcript/96327	2.30838335756938	1.96566737852121	1.37338875948469	0.179007494731718	0.848723299654592	-4.98754282182477
a_HQ_transcript/109243	-1.76407365798865	2.0051643406224	-1.37323906113265	0.179053571669244	0.848868818309262	-5.14484971426034
a_HQ_transcript/14590	2.1211468675485	-0.018058522062467	1.37315600864165	0.179079139087254	0.848868818309262	-5.05897311823688
a_HQ_transcript/75381	2.21844841784319	0.410116898688662	1.3731467258009	0.179081996953731	0.848868818309262	-4.99065126168829
a_HQ_transcript/77829	1.38364064972984	1.17665345767813	1.37301592510684	0.179122269749408	0.848986128071011	-4.92720364965111
a_HQ_transcript/143295	1.87002235426986	0.86927291464498	1.37295822673335	0.179140036993076	0.848986128071011	-5.15696804783527
a_HQ_transcript/33160	1.80488204760931	2.46732717213901	1.372880840953	0.179163868795705	0.848986128071011	-5.12193635585413
a_HQ_transcript/141068	-1.344022645415	2.53775432662064	-1.37286761891569	0.179167940914357	0.848986128071011	-5.07680080955072
a_HQ_transcript/67027	2.15006325022941	-0.104631732276996	1.3728292032832	0.179179772556688	0.848986128071011	-5.07019850853092
a_HQ_transcript/71629	-0.94636695510064	1.84960523694686	-1.37273072042789	0.179210107098449	0.849010484149769	-4.97325468738226
a_HQ_transcript/83645	-1.09113744342706	-0.32542408467109	-1.37268144605479	0.179225286016952	0.849010484149769	-5.01259190331176
a_HQ_transcript/77959	-1.30271508617211	-0.49541932448132	-1.37267026464594	0.179228730577378	0.849010484149769	-4.99374195085703
a_HQ_transcript/103253	-1.41402117432234	2.78155492480105	-1.37244102238375	0.179299362623735	0.849259968905449	-5.06334498813754
a_HQ_transcript/34663	1.35344357533049	0.532055989067777	1.3723167426619	0.179337663616736	0.849259968905449	-4.94041756351665
a_HQ_transcript/116669	0.857689494175284	1.79437575816812	1.37225978166985	0.179355220197183	0.849259968905449	-4.91152385478034
a_HQ_transcript/22356	1.45133144132736	2.14363508769367	1.37225047482257	0.179358088891116	0.849259968905449	-4.95458962237987
a_HQ_transcript/78195	-1.66819434657164	1.54736551240942	-1.37211290023106	0.179400498327929	0.849259968905449	-5.00698404326252
a_HQ_transcript/77426	1.41470684976012	1.91026826998857	1.37211169403954	0.179400870189105	0.849259968905449	-5.01623574345991
a_HQ_transcript/43336	1.62096482610874	1.20136577744164	1.37206254115393	0.179416024221239	0.849259968905449	-4.89030063269883
a_HQ_transcript/133773	1.5864400042801	-0.23002993252905	1.37192377176176	0.179458812757136	0.849259968905449	-5.01647712819071
a_HQ_transcript/105327	-1.57381456901896	1.17605217790286	-1.37186176314963	0.179477935229187	0.849259968905449	-4.93298105587145
a_HQ_transcript/3925	1.50944104445284	1.4984280585787	1.37177665949425	0.179504182426781	0.849259968905449	-4.94197203282968
a_HQ_transcript/131026	-2.5498866531584	2.79949113136656	-1.37168871543563	0.179531308782915	0.849259968905449	-5.14021131503302
a_HQ_transcript/14990	1.55767584484286	3.37933195693441	1.37166726751468	0.179537924880544	0.849259968905449	-5.3604437994425
a_HQ_transcript/48697	1.55295251579276	1.38197626264564	1.37158208378239	0.179564203607218	0.849259968905449	-4.97498987570561
a_HQ_transcript/126638	-1.02213486263827	0.74215525025657	-1.37158118658823	0.179564480402748	0.849259968905449	-4.94972016762733
a_HQ_transcript/78725	-1.72261811927551	2.2082194170751	-1.37156427148016	0.179569698985591	0.849259968905449	-5.23285371189809
a_HQ_transcript/62247	1.76048202661722	4.65239818185557	1.37155132737783	0.179573692528992	0.849259968905449	-5.48474194346788
a_HQ_transcript/144860	-1.13183518786461	-0.0623978402442207	-1.37154862565233	0.179574526080101	0.849259968905449	-5.03538893134071
a_HQ_transcript/110847	1.34607299002644	0.384901478436248	1.37150063719899	0.179589332241374	0.849259968905449	-4.90242407514505
a_HQ_transcript/78324	-0.373785083874065	2.83558202146635	-1.37146129334424	0.17960147194195	0.849259968905449	-5.16019945174697
a_HQ_transcript/84938	1.41741391998047	-0.118371935030405	1.37145698963803	0.179602799906099	0.849259968905449	-5.00085208619481
a_HQ_transcript/80975	-1.15530476956791	2.43241218895783	-1.37144603585907	0.179606179870559	0.849259968905449	-5.00378529027765
a_HQ_transcript/53120	1.1347665094571	1.46268076268018	1.37133920139764	0.179639147954504	0.849259968905449	-4.87334199769195
a_HQ_transcript/89501	-1.54691840744684	2.13504727003536	-1.37133797040324	0.179639527854926	0.849259968905449	-5.00425044887518

a_HQ_transcript/20829	-1.71732929039546	0.998683669677542	-1.37133394470502	0.179640770240621	0.849259968905449	-4.92323462815641
a_HQ_transcript/8436	-3.05956961631047	2.35247077256384	-1.37131488689963	0.1796466518315	0.849259968905449	-4.93499167042604
a_HQ_transcript/147994	1.50760623548529	4.47510759770209	1.37114794387833	0.179698179935682	0.849434480222925	-5.52297535294688
a_HQ_transcript/40244	-2.53767744372741	1.68899421143775	-1.37100551386428	0.179742151019832	0.849573243981198	-5.32147823103414
a_HQ_transcript/148661	2.96053630968155	3.17736677452516	1.37087892961787	0.179781237211867	0.849646817597925	-5.18632910709515
a_HQ_transcript/481	3.26702652729424	3.48176911618515	1.37081415483505	0.179801240674436	0.849646817597925	-5.27522293221041
a_HQ_transcript/14446	1.41443522139057	0.88017358364455	1.37081309725102	0.17980156728723	0.849646817597925	-5.01551747228582
a_HQ_transcript/143021	1.72242981700498	2.72577788519894	1.37071719439861	0.179831186803315	0.849690789667298	-5.12154032518415
a_HQ_transcript/150604	0.576336295874695	2.76100750605464	1.37064650769139	0.179853020763127	0.849690789667298	-5.18516760678436
a_HQ_transcript/34494	-1.28670841835079	-0.334735936120642	-1.37062213922702	0.179860548258332	0.849690789667298	-4.99425018023469
a_HQ_transcript/17811	1.69431979164919	0.90136592012086	1.37057047710849	0.179876507659396	0.849690789667298	-4.94964224019096
a_HQ_transcript/87391	0.835247863085678	4.90738718901139	1.37053716645102	0.179886798532589	0.849690789667298	-5.44636864201057
a_HQ_transcript/3490	0.909072122020697	-0.360376817662618	1.37049903880384	0.179898578110987	0.849690789667298	-5.03085992257469
a_HQ_transcript/34078	0.898468797009782	-0.707148572918945	1.37026337751325	0.179971399237697	0.849848848248135	-5.03900214671301
a_HQ_transcript/115352	3.60815596718094	0.970707050249307	1.37025581389421	0.179973736832965	0.849848848248135	-4.86792457212595
a_HQ_transcript/101929	0.501042678208461	-0.720514175582602	1.37020108005065	0.179990653456454	0.849848848248135	-5.09428759972332
a_HQ_transcript/32992	-1.22492475022867	0.905344707614819	-1.37016892321035	0.18000059276793	0.849848848248135	-4.82909642236443
a_HQ_transcript/122905	1.29557222963867	-0.147426039949143	1.37011775355645	0.180016409602497	0.849848848248135	-5.05480495385278
a_HQ_transcript/128318	-1.44460979932886	1.31195535162603	-1.37010690057932	0.180019764459112	0.849848848248135	-4.94464875075798
a_HQ_transcript/42052	1.27974783455463	0.588307930330303	1.37004845378872	0.180037832282517	0.849865122211965	-4.96936069426622
a_HQ_transcript/144436	1.1736759193923	-0.0278445074324197	1.3699830031121	0.180058066910818	0.849891621063094	-4.92981794947637
a_HQ_transcript/152661	-0.979208740000546	2.37355624430839	-1.36993093854289	0.180074164373975	0.849898589374018	-5.01955956751894
a_HQ_transcript/34575	0.976817176689836	2.07077884904912	1.36987083849462	0.180092747659459	0.84991728797183	-4.94994382054094
a_HQ_transcript/37490	-1.62207251298647	-0.494570553935074	-1.36981789894827	0.18010911811118	0.849925541286465	-4.98201048909343
a_HQ_transcript/65682	0.761738361115512	2.63878524258936	1.36976019044792	0.1801269645806	0.849927595679326	-5.09901656321249
a_HQ_transcript/13702	-0.815750168268684	2.65699985234929	-1.36972193222016	0.180138796772112	0.849927595679326	-5.11660086003259
a_HQ_transcript/56578	-0.507060707041711	2.50014911268077	-1.36954028381074	0.180194983751472	0.850123692517292	-5.05558977598448
a_HQ_transcript/141788	-2.05522801941925	1.30829960041355	-1.36933908004134	0.18025723544377	0.850165879599658	-4.87763284462011
a_HQ_transcript/89625	-0.562678770163613	5.28132495450083	-1.36931231881022	0.180265516528023	0.850165879599658	-5.53956823132022
a_HQ_transcript/124183	1.59863556250195	-0.106567432446189	1.36923695908632	0.180288837680414	0.850165879599658	-4.91563378739716
a_HQ_transcript/23292	0.837941015165988	-0.493739043040551	1.3692106564982	0.180296977947455	0.850165879599658	-5.03669922018289
a_HQ_transcript/5416	0.600799058169329	3.1033552030311	1.3692016522307	0.180299764702445	0.850165879599658	-5.19138161007403
a_HQ_transcript/136489	-1.96936410194694	0.253070283463984	-1.36916395012057	0.180312473549998	0.850165879599658	-5.03079032130761
a_HQ_transcript/121644	1.77644912669411	0.815744003011103	1.36913753977467	0.180319608007101	0.850165879599658	-5.06393789386042
a_HQ_transcript/139009	1.27093670863164	-0.614967565804295	1.3691022660761	0.180330526215601	0.850165879599658	-5.03368795205337
a_HQ_transcript/24773	1.34001592411957	-0.272163356401458	1.36908601144674	0.180335557656616	0.850165879599658	-4.96478902478092
a_HQ_transcript/72815	5.06733958870808	2.63157152879447	1.3689576699259	0.180375288189702	0.850213053731014	-4.94784390816354
a_HQ_transcript/83215	1.3956789189616	0.570583422063366	1.36888160996682	0.18039883719238	0.850213053731014	-4.90888320867444
a_HQ_transcript/88937	-1.555087060642	0.569335991117051	-1.36886991110522	0.180402459501085	0.850213053731014	-4.96959670037528
a_HQ_transcript/139783	2.54386697879833	6.21498139942297	1.36886470846388	0.180404070408724	0.850213053731014	-5.4678244474177
a_HQ_transcript/31513	1.49481572168838	-0.621953822364669	1.36880920749244	0.180421256017619	0.850225113075977	-5.02338818801801
a_HQ_transcript/130506	2.04712374753868	0.510161736106231	1.36873635968158	0.180443814923223	0.850262489774406	-5.12838190103677
a_HQ_transcript/120098	1.3057575922124	2.31893137151174	1.36866803644998	0.180464974687068	0.850293268104877	-4.95020262784486
a_HQ_transcript/8818	-1.1809955715873	-0.633631224745273	-1.36857412311503	0.180494062875492	0.850334381382124	-5.00337062296634
a_HQ_transcript/69218	1.39937677257021	-0.240545718114992	1.36854540750346	0.180502957815064	0.850334381382124	-5.01188824237046
a_HQ_transcript/67204	1.37792270580269	0.409511501917259	1.36813918222066	0.180628826546835	0.850858381646224	-4.98102763970989
a_HQ_transcript/147674	1.40049221678442	0.471543757881627	1.36789483739894	0.180704569535249	0.851146197062228	-5.08391260505362
a_HQ_transcript/150602	0.762275799070593	0.415609134534996	1.36772046387082	0.180758637631923	0.851290629156159	-4.97445720031592
a_HQ_transcript/683	-1.52757568322711	2.66460594848513	-1.36769195711945	0.180767477931613	0.851290629156159	-5.02119341962766
a_HQ_transcript/129761	-3.56083713481131	2.41416844530383	-1.36764935000289	0.180780691557021	0.851290629156159	-5.1413930217088

a_HQ_transcript/25592	-0.843146319088542	1.94437147582787	-1.36758212222092	0.180801542246684	0.851290629156159	-5.05733768235697
a_HQ_transcript/122482	1.37371576403324	1.54576817156782	1.36750444034982	0.180825637598153	0.851290629156159	-4.95041778277138
a_HQ_transcript/85587	-1.51510767602424	2.48164554334261	-1.36746006086039	0.180839404341639	0.851290629156159	-5.28026323077194
a_HQ_transcript/25286	0.415355222807334	4.47830278313964	1.36744270973131	0.18084478697163	0.851290629156159	-5.45591422644171
a_HQ_transcript/119107	-2.37289773881692	1.643623538794	-1.36741818768141	0.180852394361571	0.851290629156159	-5.07504068845674
a_HQ_transcript/53470	0.325029850380084	5.387070923974	1.36717368154612	0.180928260247166	0.851521380421062	-5.56532078362819
a_HQ_transcript/30268	-1.43234597346795	1.82556914167811	-1.36712072277975	0.180944695663712	0.851521380421062	-4.97218870014081
a_HQ_transcript/49338	1.31317871072194	-0.114035866406357	1.36708870932757	0.18095463139766	0.851521380421062	-5.02016520770845
a_HQ_transcript/130811	1.20209177181224	-0.228989812568345	1.36706297525921	0.180962618561728	0.851521380421062	-4.97317925917273
a_HQ_transcript/123622	-1.36943236924599	1.36910078291251	-1.36701175392607	0.180978517103836	0.851521380421062	-5.00072300465097
a_HQ_transcript/146955	1.89639189697079	0.682500406641291	1.36693831239676	0.181001314445486	0.851521380421062	-5.08317346186284
a_HQ_transcript/119444	2.1425554172416	0.661052769985011	1.36692979079687	0.18100395982113	0.851521380421062	-5.22451182422946
a_HQ_transcript/152633	-1.2580488984622	-0.573631729295061	-1.3666791544313	0.181081778755549	0.851733576794579	-5.00326574735468
a_HQ_transcript/150543	0.478201183328675	4.10009760861123	1.36665233313504	0.181090107915994	0.851733576794579	-5.42967259301226
a_HQ_transcript/133947	-0.702037849804542	2.1348040202865	-1.36664294438105	0.181093023596274	0.851733576794579	-5.07371562896447
a_HQ_transcript/124499	-2.28354498747156	2.93216379319822	-1.36650596652152	0.181135566257861	0.851864740731143	-5.01625057192984
a_HQ_transcript/41863	-0.414169357782676	2.68595737270905	-1.36636962071591	0.18117792032556	0.851994996576666	-5.12674545036687
a_HQ_transcript/72890	1.27403124619438	1.96506350673824	1.36621853837341	0.181224861093343	0.852038869584026	-4.99400498735312
a_HQ_transcript/1772	0.544427873388918	3.10269561919171	1.36619020946875	0.181233663838921	0.852038869584026	-5.09454400886793
a_HQ_transcript/121307	1.52124147399912	0.707469160385805	1.36618577664815	0.181235041295752	0.852038869584026	-5.02109557614732
a_HQ_transcript/98267	1.68634058604075	0.835291004983656	1.3661508912398	0.181245881888358	0.852038869584026	-4.87287675124717
a_HQ_transcript/141459	2.18096917365128	0.483972443940351	1.36592131333815	0.181317235469563	0.852168754564413	-5.09992215254313
a_HQ_transcript/114940	2.34200224481061	1.45910368669841	1.3658807058597	0.181329858680571	0.852168754564413	-5.15784120364657
a_HQ_transcript/151466	5.30851821013073	6.70290119117215	1.36583240233506	0.181344875167741	0.852168754564413	-5.38123454133142
a_HQ_transcript/136078	2.31105518973834	2.28738127009322	1.3657985124625	0.181355411348877	0.852168754564413	-5.02208562742979
a_HQ_transcript/152727	-1.55640433240204	0.743124425387848	-1.36577162262344	0.181363771595111	0.852168754564413	-4.92372380219641
a_HQ_transcript/74792	2.42700787382037	0.951327559430223	1.36574543686921	0.18137191322393	0.852168754564413	-5.0400906683626
a_HQ_transcript/114056	2.36684734641439	2.42771592625593	1.36573186677345	0.181376132525993	0.852168754564413	-5.24443633798429
a_HQ_transcript/45028	0.944840924340065	-0.717905932709703	1.36561087067971	0.181413756785416	0.852211689193892	-5.03535468006798
a_HQ_transcript/72695	-1.79622999607088	2.22015328668806	-1.3655485924257	0.181433124841006	0.852211689193892	-4.99341979686194
a_HQ_transcript/144689	0.68497828457432	5.20936213282375	1.36548480051009	0.181452965298533	0.852211689193892	-5.51480890854714
a_HQ_transcript/108822	-1.2966665241057	1.22076475609994	-1.36545834410934	0.181461194219436	0.852211689193892	-4.8358641914321
a_HQ_transcript/127953	1.26740203155257	2.63581498681772	1.36544287789576	0.181466004919149	0.852211689193892	-5.04987529875433
a_HQ_transcript/79440	0.965512496589853	1.83588985402366	1.36541517480115	0.181474622096069	0.852211689193892	-5.01648405861404
a_HQ_transcript/118204	-1.45094379748554	1.47547216115683	-1.36536807440493	0.181489273623663	0.852211689193892	-4.94976028712406
a_HQ_transcript/44363	1.49263435649224	1.10655505165635	1.36532537040441	0.181502558357769	0.852211689193892	-4.8748159941551
a_HQ_transcript/95725	2.98310958653452	2.87241295281544	1.3652339564057	0.181530998762771	0.852276382851332	-5.11627041063829
a_HQ_transcript/135286	2.34603968156548	0.995597058539601	1.36506123002599	0.181584746239758	0.852459871541322	-5.06835024490406
a_HQ_transcript/131254	0.981457390811845	4.44773458019709	1.36493220799974	0.181624902242283	0.852567733350469	-5.45884389935692
a_HQ_transcript/139187	0.869158259869774	-0.758051014781651	1.36489117343334	0.181637675027361	0.852567733350469	-5.03216269359669
a_HQ_transcript/134219	-1.4812525554886	-0.291802476831869	-1.36484444148587	0.18165222207954	0.852567733350469	-5.0159081156332
a_HQ_transcript/76215	2.21968390382563	0.626088294717829	1.36479892876846	0.181666390470667	0.852567733350469	-5.02306523341565
a_HQ_transcript/85356	-1.55315580471634	0.187407703182447	-1.3645868448061	0.181732424839088	0.852589529972568	-4.96039582826399
a_HQ_transcript/37650	0.560732679032681	3.92436522598121	1.36454345425592	0.181745937197763	0.852589529972568	-5.31122611720589
a_HQ_transcript/10537	-1.48300560627568	0.178448804128174	-1.36448909247838	0.181762867234357	0.852589529972568	-4.94524260227725
a_HQ_transcript/132247	1.70696808456628	0.0665596049665325	1.36448615889891	0.181763780881829	0.852589529972568	-4.88204750652588
a_HQ_transcript/14217	0.454391744319854	2.47865049135795	1.36446263375676	0.181771107789169	0.852589529972568	-5.14491139710058
a_HQ_transcript/127015	0.601760559965307	3.45751045933538	1.36439965359962	0.181790724092666	0.852589529972568	-5.22329387779275
a_HQ_transcript/23466	1.48178551879274	2.97064492043483	1.3643800807198	0.181796820753123	0.852589529972568	-5.21737117243839
a_HQ_transcript/132338	-5.1792001596307	3.82732979860029	-1.36431899584183	0.181815848803326	0.852589529972568	-5.09576520507045

a_HQ_transcript/94915	0.400548679242784	4.2095665237347	1.36430828835206	0.181819184365064	0.852589529972568	-5.4312432616896
a_HQ_transcript/122574	-1.20189352701335	2.33655663112062	-1.36426044743739	0.181834088188648	0.852589529972568	-4.91883582253892
a_HQ_transcript/2205	1.28273847588379	-0.358982594656298	1.36421809836123	0.181847281937654	0.852589529972568	-4.94185742586425
a_HQ_transcript/93879	2.58076224452719	1.36808309163983	1.36415294280692	0.18186758243939	0.852589529972568	-5.14146461999667
a_HQ_transcript/105871	-1.72632385436293	2.7988168843777	-1.36408496730375	0.18188876342717	0.852589529972568	-5.16519532888912
a_HQ_transcript/146815	1.90409934644807	0.144730718395742	1.36403859987359	0.181903212494946	0.852589529972568	-5.07312970209765
a_HQ_transcript/27765	0.70569840015984	3.11698326532718	1.36401978698127	0.181909075242519	0.852589529972568	-5.23761645667465
a_HQ_transcript/115980	1.63133562215764	0.3318886673703	1.36399146408385	0.181917901913076	0.852589529972568	-5.03023882454572
a_HQ_transcript/133617	4.40558996961562	2.41001043500881	1.36398350756798	0.181920381576	0.852589529972568	-5.09045151224299
a_HQ_transcript/46349	-0.924931082038006	2.08366454543129	-1.36390557753892	0.181944670001148	0.852634616077614	-4.96971690839213
a_HQ_transcript/117508	-1.04199444744101	2.12101350935405	-1.3637547330689	0.181991690796914	0.852710366376854	-5.10577787533913
a_HQ_transcript/23606	-1.47607338710687	0.545851132371336	-1.3637378217626	0.181996962927709	0.852710366376854	-4.95949979993827
a_HQ_transcript/48151	1.79643191907778	2.50057398745429	1.36371254562891	0.182004843029824	0.852710366376854	-5.0211424226578
a_HQ_transcript/27193	2.07925355667983	4.38427231176813	1.36366043920568	0.182021088594344	0.852717749662478	-5.42292845777298
a_HQ_transcript/103188	0.682837558409481	3.08997917703673	1.36360645034582	0.182037922245892	0.852727886558887	-5.18972862326737
a_HQ_transcript/84941	-1.13509774859314	1.16381544728216	-1.36352010056641	0.18206484849922	0.852785295210531	-4.87047006390908
a_HQ_transcript/88874	-1.72095653046404	0.330915122340673	-1.36340369476393	0.182101151945904	0.852886613513136	-4.9452774054129
a_HQ_transcript/61591	1.52749469433769	0.489011172444346	1.36323875607017	0.18215260095307	0.853058845327107	-4.96138505661572
a_HQ_transcript/95348	-0.723390929068473	2.89142575358312	-1.36307441623326	0.182203874379904	0.853120871534772	-5.12556755350127
a_HQ_transcript/129293	0.981903016985225	2.20199515166109	1.36298889655246	0.182230560637741	0.853120871534772	-5.01556906016506
a_HQ_transcript/149820	-1.56326518466969	1.47277653130059	-1.36289486166728	0.182259907550728	0.853120871534772	-5.05655703279805
a_HQ_transcript/10960	0.306936932996789	3.51259354915534	1.36285847278353	0.182271264973633	0.853120871534772	-5.32702891159396
a_HQ_transcript/49521	0.933902698610156	2.04279140872111	1.36280412976606	0.182288227127413	0.853120871534772	-5.02163751075396
a_HQ_transcript/78586	1.45349961672844	0.473867235372275	1.36277380529643	0.18229769287442	0.853120871534772	-4.86304520161393
a_HQ_transcript/140264	-2.4139106284118	2.6420469707415	-1.36275602295572	0.182303243788275	0.853120871534772	-5.3525400412091
a_HQ_transcript/34339	-1.39250582419418	0.454629555043872	-1.3627419988099	0.182307621641098	0.853120871534772	-4.89743917469232
a_HQ_transcript/133602	2.65928715694889	3.38039915478759	1.36258776660149	0.182355772979775	0.853120871534772	-5.18113185368971
a_HQ_transcript/52064	1.77020489041792	1.1461253230129	1.36257154528224	0.182360837853716	0.853120871534772	-4.85661693507048
a_HQ_transcript/54881	2.09119783773885	0.664304099601305	1.36255711042335	0.182365345022862	0.853120871534772	-4.9140036291777
a_HQ_transcript/88243	0.378116611364083	3.32784442839631	1.36254045945404	0.182370544262118	0.853120871534772	-5.28347918079435
a_HQ_transcript/14930	1.32134862425909	2.59662503530736	1.36251777358198	0.182377628075483	0.853120871534772	-5.19722573674346
a_HQ_transcript/41365	-1.55069555144802	2.05229344612201	-1.36245700428221	0.1823966047427	0.853120871534772	-5.1060905433681
a_HQ_transcript/95341	1.99793802030556	0.0132592841800972	1.36241809651373	0.182408755428458	0.853120871534772	-4.91108883285962
a_HQ_transcript/50050	1.71484356974814	0.514370353043316	1.36234894302653	0.182430353238947	0.853120871534772	-5.07458247653851
a_HQ_transcript/6257	0.507344438967684	3.97031562893073	1.36231925056081	0.18243962731002	0.853120871534772	-5.36701034329442
a_HQ_transcript/113399	1.56098001389506	3.27346409011848	1.36227963580314	0.182452001054765	0.853120871534772	-5.33890389811106
a_HQ_transcript/50239	-0.524945639802343	2.31135673448596	-1.36223268477849	0.182466667139674	0.853120871534772	-5.01817653520295
a_HQ_transcript/2058	0.488232389606743	3.23653393012748	1.36219442369093	0.182478619426622	0.853120871534772	-5.27771596895183
a_HQ_transcript/82116	2.10916941155617	1.14743827661085	1.3621746645009	0.182484792189217	0.853120871534772	-5.08367353595003
a_HQ_transcript/7893	1.19757244057398	1.15831513381038	1.36216205866806	0.182488730330707	0.853120871534772	-4.94023702671698
a_HQ_transcript/84209	-0.730048489617277	3.14196909750351	-1.36200618948851	0.182537430291993	0.853279915401124	-5.30305415639474
a_HQ_transcript/152597	0.423362389020229	10.2430094008101	1.36183238082424	0.18259174717901	0.853389772951704	-6.04913088328365
a_HQ_transcript/69316	1.34965082797192	3.14304305344612	1.36180682378914	0.18259973505683	0.853389772951704	-5.1078025814058
a_HQ_transcript/2188	1.59299936561204	2.64746079677252	1.36177301596739	0.182610302143107	0.853389772951704	-5.04170319957393
a_HQ_transcript/63961	1.40267470177303	-0.140487451622202	1.36174308976052	0.18261965637466	0.853389772951704	-5.05799080564043
a_HQ_transcript/88333	-1.1330393026783	2.23319880897307	-1.36167732444715	0.182640214377896	0.853417233228163	-5.00041475811798
a_HQ_transcript/139180	1.37633622933136	-0.63679802790732	1.36162505036203	0.182656556351844	0.853424990565068	-5.04143958571221
a_HQ_transcript/834	1.5705230478802	2.01135146476126	1.36155560002523	0.182678269736569	0.853457841444081	-5.18492232657952
a_HQ_transcript/54753	0.299984089062508	-0.735657115579295	1.36149101402113	0.182698464103056	0.853483590885031	-5.17130411765664
a_HQ_transcript/7901	-2.76453152871367	1.63960294831458	-1.36126987984196	0.182767620135236	0.853672513218505	-5.11655395742477

a_HQ_transcript/149968	3.29119971654071	2.10854796203331	1.36126777856914	0.182768277370704	0.853672513218505	-5.12282281878056
a_HQ_transcript/137159	-1.88144759520825	3.28959115203503	-1.36115530995834	0.182803457943534	0.853768231025369	-5.44813288618614
a_HQ_transcript/2506	-0.819808067191928	5.287738279938	-1.36110505159026	0.182819180634026	0.853773064344391	-5.51860898156728
a_HQ_transcript/83260	-0.524122663122162	4.00371039834446	-1.36101939731169	0.182845978901648	0.853829616130704	-5.39560948696955
a_HQ_transcript/105521	0.420483510128182	-0.807518985687392	1.36084462124652	0.18290066975229	0.853961547043017	-5.09682853146283
a_HQ_transcript/44365	1.71239769751467	0.876312496558853	1.36081559119001	0.182909755053672	0.853961547043017	-4.92165674359805
a_HQ_transcript/98895	0.826734599785499	2.01112455553029	1.36068980762664	0.182949124559747	0.853961547043017	-5.00303754175242
a_HQ_transcript/47173	-1.12908067474515	-0.558508110036621	-1.36067933615801	0.182952402363382	0.853961547043017	-5.01299574179063
a_HQ_transcript/42675	-0.705492368405002	3.20910438395566	-1.36067021089782	0.182955258810842	0.853961547043017	-5.18027715812336
a_HQ_transcript/120514	-1.42499465807761	0.216827215192928	-1.36064746836759	0.182962377974018	0.853961547043017	-5.00084112872259
a_HQ_transcript/57908	1.70819279887631	0.21404782806651	1.36036237359059	0.183051640285877	0.854099835605271	-5.0491478409318
a_HQ_transcript/80219	1.96916343271056	1.93960247462586	1.36030178689451	0.183070614147726	0.854099835605271	-5.05152122209189
a_HQ_transcript/108670	0.995810214302966	-0.117710458861698	1.36026883869908	0.183080933134216	0.854099835605271	-5.04160925616853
a_HQ_transcript/132799	3.36108203156905	3.71076885893977	1.36025816103855	0.183084277348426	0.854099835605271	-5.08384301598099
a_HQ_transcript/115322	-0.315687633064975	5.29905438743102	-1.36024124554165	0.18308957533298	0.854099835605271	-5.53778454980811
a_HQ_transcript/120232	-1.07649740145273	2.17303592522601	-1.36022458585791	0.183094793312289	0.854099835605271	-4.91137860508514
a_HQ_transcript/17029	1.78447776762003	0.763159957400467	1.3602243713287	0.183094860505734	0.854099835605271	-4.85210361381282
a_HQ_transcript/23089	0.692608000712946	1.85800806287289	1.36014715723065	0.183119046245132	0.854144111710564	-5.00409844835614
a_HQ_transcript/109900	2.45510296122228	3.94938684804425	1.35992757216995	0.183187840322664	0.854275470990608	-5.37171023389428
a_HQ_transcript/4173	-0.366096393474426	3.78439614038731	-1.35992342108339	0.183189141014737	0.854275470990608	-5.34991525099002
a_HQ_transcript/97699	1.68746779996739	1.24108548679662	1.35978133514672	0.183233666218631	0.854275470990608	-4.97119927935374
a_HQ_transcript/24278	1.03258268357559	1.086039796324	1.35977795169977	0.18323472658535	0.854275470990608	-4.90793728950776
a_HQ_transcript/76847	1.10219795746415	0.282274097332392	1.3597122937641	0.183255304616695	0.854275470990608	-4.97013123018554
a_HQ_transcript/38725	-0.509277606942818	4.14838465822853	-1.35971204371887	0.183255382987474	0.854275470990608	-5.36852143232499
a_HQ_transcript/111478	1.59807734459061	1.52942628068135	1.35969035354332	0.183262181360114	0.854275470990608	-5.0447587015013
a_HQ_transcript/131875	-1.20506563773313	0.380801680773458	-1.35968206323127	0.183264779852272	0.854275470990608	-5.08313125801324
a_HQ_transcript/151317	-0.722690369489434	2.70007539979674	-1.35945503850335	0.183335948949942	0.854395960447372	-5.20313918892521
a_HQ_transcript/114925	-1.43307877724605	0.665493865203177	-1.35942498473618	0.183345371994304	0.854395960447372	-4.89364136442316
a_HQ_transcript/41806	-0.898396000378122	0.0458510919377994	-1.35942127559396	0.183346534983086	0.854395960447372	-4.92812201666159
a_HQ_transcript/94624	-0.789283203586569	2.30187198931323	-1.35933176112298	0.183374603672177	0.854395960447372	-5.04363742478277
a_HQ_transcript/6561	0.651929792387802	4.19943441026198	1.35929759992518	0.18338531633851	0.854395960447372	-5.42756662511039
a_HQ_transcript/81672	-1.21373025691556	0.962876970009321	-1.35925539102716	0.183398553366551	0.854395960447372	-4.98788101920395
a_HQ_transcript/41853	-1.83734675603692	1.03214799844374	-1.35924772611419	0.183400957220412	0.854395960447372	-4.92052528530647
a_HQ_transcript/47907	1.90118176829518	0.169310976847064	1.35922458047635	0.183408216254857	0.854395960447372	-5.01768597005221
a_HQ_transcript/85515	-1.9346801508263	2.26465623158227	-1.35914161749379	0.183434237292292	0.85444477552291	-5.01099201868861
a_HQ_transcript/113872	-1.46711438357828	-0.46254907559993	-1.35907616737364	0.183454767503771	0.85444477552291	-5.01625080848096
a_HQ_transcript/3223	0.51917300693381	2.98288970228047	1.3590305824697	0.183469067501927	0.85444477552291	-5.20553311130644
a_HQ_transcript/66420	-0.487270059883931	2.7578281068426	-1.35895852707719	0.183491673062879	0.85444477552291	-5.24542398100334
a_HQ_transcript/107254	4.15301134388665	1.90059698086679	1.35895687324627	0.183492191935943	0.85444477552291	-5.05841545404043
a_HQ_transcript/88808	1.30441237371362	1.02967036901022	1.35885048258722	0.183525573341159	0.854452339829444	-4.88494754398502
a_HQ_transcript/776	-1.25782511529665	2.32912041949327	-1.35884841438701	0.183526222311591	0.854452339829444	-5.02074094618868
a_HQ_transcript/18684	2.90112731382409	0.854652781995605	1.35881115243461	0.183537914862208	0.854452339829444	-4.9507236140143
a_HQ_transcript/137933	-2.69500381233703	2.47944853283501	-1.35874268419676	0.183559401241245	0.854483933318739	-5.28342642579265
a_HQ_transcript/45357	-0.846974740192673	2.11098471620757	-1.35855033495631	0.183619773805047	0.854605228866408	-5.14037241361924
a_HQ_transcript/128314	1.29836460518531	-0.058043332566033	1.35847510071254	0.183643391728148	0.854605228866408	-5.07004440234851
a_HQ_transcript/20121	1.17504985868442	2.22476523287356	1.35841532498492	0.183662158512528	0.854605228866408	-5.01199483090662
a_HQ_transcript/127043	1.69898500865528	-0.0517409015647482	1.35838635691565	0.183671253666968	0.854605228866408	-5.02151120568295
a_HQ_transcript/74232	-1.9615604654685	-0.0768611338992859	-1.35835841253641	0.18368002774252	0.854605228866408	-5.00528965095202
a_HQ_transcript/152680	1.42602059418925	-0.2267666216518	1.35833921974664	0.183686054151087	0.854605228866408	-5.05421139481757
a_HQ_transcript/81305	-1.38972545390858	2.59224169208704	-1.35833183574371	0.183688372719806	0.854605228866408	-5.31245145797898

a_HQ_transcript/116013	3.25381134306798	4.84139198214657	1.35812274762318	0.183754035573458	0.85484230330644	-5.34417750774921
a_HQ_transcript/109779	1.33988200772243	0.526666943999625	1.35795089370658	0.183808018872026	0.854945605816646	-4.93857623564209
a_HQ_transcript/116535	-1.53846050219483	0.150082651242571	-1.357942960438	0.183810511192338	0.854945605816646	-5.0101010918304
a_HQ_transcript/119446	1.50203607265913	-0.235676445748675	1.35791159548084	0.183820365082171	0.854945605816646	-5.02223190833294
a_HQ_transcript/63857	1.18938214748113	1.30599950092188	1.35775762108314	0.183868744966742	0.855019542054592	-4.9134379260357
a_HQ_transcript/125523	1.21373419736951	1.8194861338702	1.35768026621497	0.18389305415727	0.855019542054592	-4.95736884574667
a_HQ_transcript/123026	-2.10038810888413	1.13258828075615	-1.35755447397078	0.183932590376506	0.855019542054592	-4.90247585241557
a_HQ_transcript/73823	1.23316854863309	0.756431850031206	1.35752717392881	0.183941171588109	0.855019542054592	-4.98537104974436
a_HQ_transcript/141008	-1.01698110787393	3.26872412963789	-1.35747431118336	0.183957788796388	0.855019542054592	-5.26004086844429
a_HQ_transcript/117922	1.39287966795857	3.28444321982277	1.35741952715133	0.183975011181481	0.855019542054592	-5.2840356142003
a_HQ_transcript/10476	1.23831120574014	-0.266176560242797	1.35741906965187	0.183975155010254	0.855019542054592	-4.98509398733756
a_HQ_transcript/56161	2.04340197981956	1.39083732817625	1.35738608265935	0.183985525697246	0.855019542054592	-5.10076530064058
a_HQ_transcript/100447	2.51684815540472	2.78146316935567	1.35738361490658	0.183986301545251	0.855019542054592	-5.23513041802115
a_HQ_transcript/87669	-0.912780864704137	2.4278422688066	-1.35731543294708	0.18400773858396	0.855019542054592	-5.16962144118024
a_HQ_transcript/95943	1.21793923429603	-0.376177010105878	1.35730404866761	0.184011318095277	0.855019542054592	-5.03584522577825
a_HQ_transcript/64972	0.951124872318155	-0.580736478873717	1.35729942141888	0.184012773037141	0.855019542054592	-5.00343219342421
a_HQ_transcript/42614	-0.610651598334642	2.03646749624894	-1.35721355743843	0.184039772792598	0.855076645582936	-4.99141477675732
a_HQ_transcript/9455	-0.866227203379781	2.15253000616365	-1.35713044811911	0.184065909273936	0.855129729401801	-5.08053591809191
a_HQ_transcript/32744	0.693127803862848	2.48215530270087	1.35695035417055	0.184122555656664	0.855273981173656	-5.11142244533927
a_HQ_transcript/133597	4.7036566944786	0.751387559449133	1.35690136990267	0.184137965404604	0.855273981173656	-5.04238667363633
a_HQ_transcript/128910	0.609316635681679	2.22773849236941	1.35687485032096	0.184146308501482	0.855273981173656	-5.01977136186304
a_HQ_transcript/148609	-1.07184378967625	-0.737513066967342	-1.35671759779083	0.184195786381384	0.855273981173656	-5.00927362608092
a_HQ_transcript/28716	2.51165040969739	2.73322354329665	1.3566751741175	0.18420913631483	0.855273981173656	-5.18951238497199
a_HQ_transcript/10842	-1.12797350854485	2.34890942334526	-1.35650484554666	0.18426274306486	0.855273981173656	-5.15926480621721
a_HQ_transcript/4279	0.841555350396215	1.45663176636653	1.356470580548	0.184273528594851	0.855273981173656	-4.93618142740749
a_HQ_transcript/147979	-2.26056139228073	1.98505764818877	-1.35646943273567	0.184273889898071	0.855273981173656	-5.16319848816855
a_HQ_transcript/36031	0.571765303616597	2.57840250550408	1.3564588308285	0.184277227144844	0.855273981173656	-5.15152798772273
a_HQ_transcript/97937	-1.49322693802978	1.04631360581599	-1.35644728487784	0.184280861608969	0.855273981173656	-4.86930000864442
a_HQ_transcript/61141	1.39141127045019	1.4840859030465	1.35635981469822	0.184308397501671	0.855273981173656	-4.93624357877721
a_HQ_transcript/133065	0.90512720020528	2.58796347056188	1.35629478820885	0.18432887011434	0.855273981173656	-5.10218171594598
a_HQ_transcript/59454	0.825428779032429	0.418419397462412	1.35627088270046	0.184336396847145	0.855273981173656	-4.98659926543891
a_HQ_transcript/99310	1.22108543694699	-0.498096561690576	1.35624268611763	0.184345274945754	0.855273981173656	-5.0429229197465
a_HQ_transcript/48694	1.37541080370525	1.14043573100552	1.35619214345729	0.184361189856471	0.855273981173656	-4.96010216129353
a_HQ_transcript/119851	0.575778244158985	2.74325648280907	1.35613517777647	0.184379128528503	0.855273981173656	-5.11582525654446
a_HQ_transcript/117391	-1.73093373024361	0.656403173556134	-1.35612167482075	0.184383380849953	0.855273981173656	-4.94206019468909
a_HQ_transcript/141353	4.35891869137012	4.70210017356223	1.3561188218876	0.184384279299179	0.855273981173656	-5.15536510686456
a_HQ_transcript/93477	1.43142893545447	3.1005998396548	1.35604591837669	0.184407239313316	0.855273981173656	-5.14843346263884
a_HQ_transcript/123300	1.5728288440786	0.81744978122478	1.35595901402335	0.184434611604434	0.855273981173656	-4.89594177691164
a_HQ_transcript/146485	-2.04373510208342	0.71515750848661	-1.35592587270189	0.184445050965651	0.855273981173656	-4.96412645995678
a_HQ_transcript/118731	-1.83744260062593	-0.246118029180168	-1.355924035446	0.184445629706052	0.855273981173656	-5.01460474840675
a_HQ_transcript/50054	1.23804698221795	-0.209424592366275	1.35591622642491	0.184448089583769	0.855273981173656	-5.05529490535472
a_HQ_transcript/21063	1.30786219398868	2.74558075923918	1.35588103072229	0.184459176708057	0.855273981173656	-5.25421248978471
a_HQ_transcript/71365	0.944674553680541	2.05620408015932	1.35580965691436	0.184481662011704	0.855273981173656	-4.98306997186425
a_HQ_transcript/142663	1.93224054414927	-0.0272321538237744	1.35572469911056	0.184508429527712	0.855273981173656	-5.04175714170289
a_HQ_transcript/20276	-1.30754901717224	3.83615432784143	-1.35571446458015	0.18451165430745	0.855273981173656	-5.42562620717229
a_HQ_transcript/87598	1.81984444107302	-0.602427853241716	1.35570669123748	0.184514103625152	0.855273981173656	-5.03900655787046
a_HQ_transcript/152289	-0.778964870482802	3.02895447743652	-1.35567638011329	0.184523654656944	0.855273981173656	-5.25739323621654
a_HQ_transcript/3049	1.40211798198061	0.0491057997777523	1.35538552066481	0.184615323904369	0.85541331618539	-4.92057371133118
a_HQ_transcript/137176	2.01594814065131	2.4857542853663	1.35528511768129	0.184646975781106	0.85541331618539	-5.01156448252248
a_HQ_transcript/124226	-1.35290563010643	1.82223896234768	-1.35527409740529	0.184650450161082	0.85541331618539	-4.98793220731747

a_HQ_transcript/140905	1.40384521406046	0.232230151712042	1.35526564966217	0.184653113528801	0.85541331618539	-5.06848208381777
a_HQ_transcript/17316	-2.87454331369759	1.49983792725031	-1.35523090067682	0.184664069350702	0.85541331618539	-4.93238620261779
a_HQ_transcript/138928	0.846426210039147	4.22658989676894	1.35522043589853	0.184667368833357	0.85541331618539	-5.44288150437876
a_HQ_transcript/148078	-1.82188932496769	0.529147938047113	-1.35517745408467	0.184680921223737	0.85541331618539	-5.03134120373585
a_HQ_transcript/121911	1.7762417445579	1.64890098287746	1.35517446207977	0.184681864647181	0.85541331618539	-5.02126706857185
a_HQ_transcript/75143	-0.843793981715313	3.56944598606434	-1.35516083927644	0.184686160165955	0.85541331618539	-5.26913706772073
a_HQ_transcript/137491	-1.15450969998031	3.07603635272021	-1.35509129861778	0.184708088811733	0.85544672029706	-5.15978867218085
a_HQ_transcript/75964	-3.54708150432113	3.95612410507667	-1.35503644317931	0.184725388107266	0.855458680680304	-5.47899924168303
a_HQ_transcript/14175	-1.39712443623756	-0.21878963979904	-1.35491568107024	0.184763476253438	0.855566904139238	-5.03122038663093
a_HQ_transcript/15544	-1.78316716925766	0.275009145500034	-1.35482473455605	0.184792164630472	0.855631587194526	-5.04165030357302
a_HQ_transcript/142052	1.19588494065447	-0.375660999706842	1.35473255238944	0.184821246304442	0.855688794978662	-4.99638227255248
a_HQ_transcript/36567	-0.558868069958775	4.79873707884519	-1.35469225193087	0.184833961425683	0.855688794978662	-5.49338160834255
a_HQ_transcript/140866	0.571526065291163	3.89902684635146	1.35461101862337	0.184859593251184	0.85570918013187	-5.3630677834468
a_HQ_transcript/122390	0.504872424362226	4.62403445783103	1.3545849892245	0.184867806979356	0.85570918013187	-5.47344128466661
a_HQ_transcript/80676	1.24815168580556	1.49448741602672	1.35441710519176	0.184920790551459	0.855790518685204	-4.96497876926237
a_HQ_transcript/81919	2.92811553503306	0.309071256039746	1.35441418135931	0.184921713406189	0.855790518685204	-4.96402725869155
a_HQ_transcript/59102	1.14725642256518	-0.494452150865865	1.35434305956749	0.184944162809368	0.855790518685204	-4.97229642750444
a_HQ_transcript/78584	0.793141809314759	-0.704964334564497	1.35434272167423	0.184944269469508	0.855790518685204	-5.04930040144007
a_HQ_transcript/106112	-1.49721984539383	-0.511622219074972	-1.35406258521195	0.185032714358284	0.855945717697457	-5.02719203850153
a_HQ_transcript/150045	0.517943412169939	2.90317941077782	1.35405133952676	0.185036265537788	0.855945717697457	-5.11998095566968
a_HQ_transcript/51555	2.19419961288801	2.99729850567463	1.35401829754776	0.185046699889244	0.855945717697457	-5.2369209921186
a_HQ_transcript/22145	0.400476235552843	3.22857915376907	1.35401318170361	0.185048315466252	0.855945717697457	-5.27212292145915
a_HQ_transcript/81749	-1.75459086246864	0.15411095481935	-1.3538797213547	0.185090465931316	0.855945717697457	-4.91063143947055
a_HQ_transcript/131762	-3.35133226835281	0.142005713880941	-1.35385246676852	0.185099074596417	0.855945717697457	-5.05618710343797
a_HQ_transcript/62664	-1.39350983798941	1.71628526333997	-1.3538059537855	0.185113766956905	0.855945717697457	-5.05119357667481
a_HQ_transcript/16752	1.82500616516447	1.96945652027041	1.35378905313489	0.185119105699685	0.855945717697457	-4.9570685224319
a_HQ_transcript/78068	-0.799239618219977	2.97840194720487	-1.35376436580635	0.185126904388658	0.855945717697457	-5.12844319454583
a_HQ_transcript/14259	-0.845522109433451	1.81379994320291	-1.35373694893393	0.1851355656344	0.855945717697457	-4.98456409569289
a_HQ_transcript/144230	-1.47471716297634	0.568280695923022	-1.35371178282438	0.185143516119278	0.855945717697457	-4.95877640226675
a_HQ_transcript/28651	0.718217457544324	1.97586043678249	1.35367697937946	0.185154511669554	0.855945717697457	-5.00189353725221
a_HQ_transcript/146967	-0.887210530014769	2.21789235151664	-1.35362810942363	0.185169952145467	0.855949024120217	-5.03041086773535
a_HQ_transcript/104303	1.41254909258652	-0.119272346768072	1.35351314354218	0.185206279577735	0.855957401698128	-5.05269724430978
a_HQ_transcript/147136	4.0882403163608	3.86051698849019	1.35349002002219	0.185213586917134	0.855957401698128	-5.1722749945682
a_HQ_transcript/95403	1.4560070232844	0.779677425176043	1.35347162182742	0.185219401150436	0.855957401698128	-4.94602289516159
a_HQ_transcript/134035	1.34986512506185	1.4149104505645	1.35339103223482	0.185244870895149	0.855957401698128	-5.06456934311638
a_HQ_transcript/135874	-0.386347019483425	5.56364399901751	-1.35338240582115	0.185247597370009	0.855957401698128	-5.58116718630667
a_HQ_transcript/105989	1.27418635659972	2.78146700688413	1.35332257933281	0.185266507057877	0.855957401698128	-5.22687049527217
a_HQ_transcript/120776	1.53377722200398	-0.282006737202131	1.35326801797025	0.185283753871058	0.855957401698128	-5.10590258494703
a_HQ_transcript/127232	2.00127008051024	1.38721198315068	1.35321928135932	0.185299160534632	0.855957401698128	-5.03231153184108
a_HQ_transcript/134602	-1.37205279097889	-0.483501619234203	-1.35318611127858	0.185309646859041	0.855957401698128	-5.02320720766172
a_HQ_transcript/11368	-1.64221897183039	2.1920401289963	-1.35315646874695	0.185319018379627	0.855957401698128	-4.9584921545011
a_HQ_transcript/14168	0.687046143150001	2.42303892152705	1.35310862839285	0.185334143936558	0.855959249860498	-5.12651343441019
a_HQ_transcript/61541	-1.62421160993795	0.251233172168464	-1.35305275311177	0.185351811084601	0.855972835046136	-5.0233350139099
a_HQ_transcript/130960	-0.747390570167011	2.29197055994387	-1.35296489098347	0.185379594757585	0.856033133465914	-5.06480763501319
a_HQ_transcript/129532	1.54268445701049	3.00661567816457	1.3528792821148	0.185406669007422	0.856090146640991	-5.23651985767886
a_HQ_transcript/37355	-0.850542840786017	2.55419830096779	-1.35281591894001	0.18542670991163	0.856114677819159	-5.16696984975066
a_HQ_transcript/138541	1.76560086725408	0.00178615249058889	1.35274834849644	0.18544808336451	0.856145356986121	-5.08673076658705
a_HQ_transcript/28493	1.9330164623194	0.271894122878999	1.35265625709726	0.185477216200138	0.856168537592671	-5.13601796886453
a_HQ_transcript/51182	1.52320336790916	2.53076688573573	1.35263935815007	0.185482562515561	0.856168537592671	-5.13603830715495
a_HQ_transcript/90691	1.23191012068014	0.00255018644275031	1.35243153113129	0.185548322446912	0.856404072351445	-5.03900279634332

a_HQ_transcript/19038	-0.916554152574797	2.76551346792026	-1.35227709683511	0.185597199714809	0.856529472319667	-5.12096239412545
a_HQ_transcript/119142	3.27089680018599	2.43139588185706	1.35224039813769	0.185608816035495	0.856529472319667	-5.03217201528546
a_HQ_transcript/106349	1.02188567447343	0.600145398073569	1.35220552104429	0.185619856281194	0.856529472319667	-5.00239738911189
a_HQ_transcript/23484	-0.663552555297176	6.11413944424319	-1.35215947596807	0.185634432498678	0.856529472319667	-5.64210565684958
a_HQ_transcript/18971	-1.43843612972835	1.04480303780884	-1.35190246642626	0.185715808759263	0.856796064240478	-4.97643472518002
a_HQ_transcript/129167	1.28465397782833	2.46231671534875	1.35188389444775	0.185721690225537	0.856796064240478	-5.13210829802246
a_HQ_transcript/52717	1.31870489309329	0.907271349748163	1.35177918039698	0.185754854287638	0.856872156186485	-4.94047926761529
a_HQ_transcript/140557	1.1744627957917	-0.698537369900491	1.35172877867033	0.185770818688041	0.856872156186485	-5.0459474277026
a_HQ_transcript/124654	-1.64009021541288	0.759725278902988	-1.35169219328691	0.185782407521647	0.856872156186485	-4.9666224577094
a_HQ_transcript/18418	1.0333843255439	3.21115683123127	1.35154656404712	0.185828542773492	0.856967600241861	-5.28935915651017
a_HQ_transcript/24494	-0.596473647516064	3.16244103614804	-1.35153380036242	0.185832586723243	0.856967600241861	-5.27712419918534
a_HQ_transcript/10114	1.11215099029375	-0.529231445396543	1.35142526960407	0.185866975542385	0.857058190887493	-5.02845721382566
a_HQ_transcript/140626	1.67763543487509	4.35592297682059	1.35127403938316	0.18591490222954	0.857162612027386	-5.46866878684957
a_HQ_transcript/2980	1.48625696843023	0.490414966539441	1.35125115001435	0.185922156980458	0.857162612027386	-4.92476957410768
a_HQ_transcript/126362	-0.412476835857327	6.5321567814806	-1.35115255297005	0.185953409668443	0.857162612027386	-5.66575350755894
a_HQ_transcript/89127	-1.59308863689026	0.050580029441478	-1.35111347420573	0.185965797740646	0.857162612027386	-5.0398928077933
a_HQ_transcript/101258	1.30170233675262	-0.178527820108782	1.35091717584851	0.186028034499852	0.857162612027386	-5.15187336672309
a_HQ_transcript/98434	1.05535295595948	3.37752363135844	1.35090847306609	0.186030794106146	0.857162612027386	-5.34799141093993
a_HQ_transcript/13584	-1.09996661014061	3.3878250533313	-1.35089038105705	0.186036531087903	0.857162612027386	-5.21451786411321
a_HQ_transcript/151504	1.71539986836437	0.190676286135363	1.35072917612634	0.186087655264672	0.857162612027386	-5.08476580962861
a_HQ_transcript/150185	1.71521821627881	1.14244837085918	1.35071352786973	0.186092618496901	0.857162612027386	-4.96777475147598
a_HQ_transcript/113235	1.56239533706993	0.65591611286575	1.35069840036115	0.186097416658328	0.857162612027386	-5.05189582151486
a_HQ_transcript/131589	-2.76391809758522	0.578944080791894	-1.35068847300959	0.186100565479681	0.857162612027386	-5.10755405150586
a_HQ_transcript/48400	0.681182515949293	3.1595557083797	1.35060888321302	0.186125811773979	0.857162612027386	-5.20184868615065
a_HQ_transcript/56592	-1.0938525879488	1.28767985586707	-1.35056826672122	0.186138696555679	0.857162612027386	-4.92471891991676
a_HQ_transcript/96673	-1.1704120616536	2.36616224088155	-1.3504622691827	0.186172325435715	0.857162612027386	-5.11692683814357
a_HQ_transcript/90092	-1.68065932993754	1.54844942146448	-1.35040710057662	0.186189830139476	0.857162612027386	-5.09731505356973
a_HQ_transcript/106523	1.45352906180827	-0.0558133652614038	1.35037830229498	0.186198968185943	0.857162612027386	-5.04243523268411
a_HQ_transcript/91488	1.01655115269961	0.339205168382602	1.3503683664475	0.186202121032174	0.857162612027386	-4.92001535697933
a_HQ_transcript/77502	-1.49433355374794	0.984060734223466	-1.35036832881228	0.186202132974673	0.857162612027386	-4.94431709890559
a_HQ_transcript/145515	1.43075307223244	-0.179137860467435	1.35036798161575	0.186202243147935	0.857162612027386	-5.07758389507391
a_HQ_transcript/124443	1.18626739657462	-0.512289354911117	1.3503188830655	0.186217823729312	0.857162612027386	-5.06726134408528
a_HQ_transcript/137498	1.42567240931941	2.65405280165985	1.35030471200622	0.18622232085901	0.857162612027386	-5.17130271466447
a_HQ_transcript/145075	1.00764559977107	4.62686445388876	1.35021540844214	0.186250662924951	0.857162612027386	-5.5134975648241
a_HQ_transcript/577	0.860344663464939	1.89952117267517	1.35020509654098	0.186253935804047	0.857162612027386	-4.94739669459274
a_HQ_transcript/96231	-1.63791645120079	2.7923851401732	-1.3501650517581	0.186266645980364	0.857162612027386	-5.16171757493138
a_HQ_transcript/93156	-1.60248917295105	0.41830914029789	-1.35014305682059	0.18627362743818	0.857162612027386	-5.0660406552763
a_HQ_transcript/125111	-2.02343592593216	0.105555201169463	-1.35000372441614	0.186317857921386	0.857162612027386	-5.0764934185012
a_HQ_transcript/48022	1.53020778809483	-0.00338527106208021	1.35000363010535	0.1863178878627	0.857162612027386	-4.97160899045515
a_HQ_transcript/33292	0.619817417824679	1.92223715908588	1.34997148383513	0.186328093714676	0.857162612027386	-4.97217321177239
a_HQ_transcript/151245	-3.42898182942481	2.48218346573544	-1.34994930159663	0.186335136421174	0.857162612027386	-5.15567903733556
a_HQ_transcript/42465	0.941307420358076	1.45575793458519	1.3499172092204	0.186345325891857	0.857162612027386	-4.95411187394809
a_HQ_transcript/46420	-1.04210452342895	-0.619847923453038	-1.34987639284589	0.186358285892893	0.857162612027386	-5.01796461675663
a_HQ_transcript/43906	-0.424753879987131	3.2828716622961	-1.34986628045717	0.186361496882778	0.857162612027386	-5.22890707577505
a_HQ_transcript/98225	1.48928267598564	-0.566706252164287	1.34970874997965	0.186411523108219	0.857219453654457	-5.04241996188527
a_HQ_transcript/147448	2.73868207638405	1.31675077313561	1.34966842336476	0.186424331114128	0.857219453654457	-5.15011459779183
a_HQ_transcript/36258	-1.3440174156907	0.393369121994831	-1.34959531810796	0.186447551573584	0.857219453654457	-4.96712060869754
a_HQ_transcript/120355	1.38649186025254	-0.0980161955223339	1.34959461515347	0.186447774864227	0.857219453654457	-5.06827047739514
a_HQ_transcript/79663	0.984707130211253	-0.673599117287409	1.34957079693241	0.186455340747769	0.857219453654457	-5.0489596084935
a_HQ_transcript/6026	1.42213219414455	0.475607202361756	1.34953205660109	0.186467647162774	0.857219453654457	-5.07553716566436

a_HQ_transcript/129907	1.51180232471863	0.122308574962214	1.34950234812421	0.186477084906768	0.857219453654457	-4.95919018650048
a_HQ_transcript/112890	-0.308910467102117	3.39924951791396	-1.34935641979018	0.186523448562266	0.857271202572148	-5.31002740831643
a_HQ_transcript/121708	1.09937683228773	0.116699575043748	1.34935497971486	0.186523906140549	0.857271202572148	-4.99056007179973
a_HQ_transcript/128914	-1.89488535651916	0.214382077840323	-1.34932766266177	0.18653258619138	0.857271202572148	-5.01448224850432
a_HQ_transcript/94665	1.37411654148024	1.97346471143318	1.34925600955838	0.186555355599079	0.857308064689958	-5.04825523172685
a_HQ_transcript/10627	-1.27373263451703	1.90058159514268	-1.34917524230916	0.186581023810789	0.857358241270637	-5.03269863312014
a_HQ_transcript/77375	0.690223893419445	2.8063116056128	1.34910708193324	0.186602687623324	0.857390010763465	-5.24938313158847
a_HQ_transcript/23423	1.65789452348389	0.708060564610035	1.3490533485066	0.186619767404693	0.857392623722333	-4.83275562552592
a_HQ_transcript/20173	0.678860642558266	1.74300254651332	1.34901248712893	0.186632756467772	0.857392623722333	-4.96321287719013
a_HQ_transcript/97607	1.76804709073829	0.161562806585935	1.34887123348531	0.186677663727615	0.857470707926367	-5.08512266383526
a_HQ_transcript/26682	-1.42958978047029	0.324224766412636	-1.34882465700874	0.186692473119825	0.857470707926367	-4.97044529609796
a_HQ_transcript/28989	1.45832849141593	0.133870627517671	1.34881829634435	0.186694495618383	0.857470707926367	-4.9588840245197
a_HQ_transcript/54375	1.30686444928177	-0.174937639043488	1.34869070746843	0.186735068592908	0.857470707926367	-4.94253355804665
a_HQ_transcript/132730	1.2200789681415	5.49165180436297	1.34864288920402	0.186750276449004	0.857470707926367	-5.53761595079923
a_HQ_transcript/70196	1.1507903483335	-0.592423660780937	1.34863646426425	0.186752319874243	0.857470707926367	-5.02114407099818
a_HQ_transcript/5299	0.819738752202986	2.29163726183364	1.34863428372289	0.186753013390239	0.857470707926367	-4.96497018848879
a_HQ_transcript/12084	-1.56765042553092	-0.715708731338152	-1.34857093394631	0.186773162505173	0.857471771806316	-5.03473212016216
a_HQ_transcript/15835	0.860453688949113	1.34249990476913	1.34854079860996	0.186782747980518	0.857471771806316	-4.93976353524603
a_HQ_transcript/103989	-4.08154581121634	3.42569084243555	-1.34832748252866	0.186850610628417	0.857683076990791	-5.34748619671628
a_HQ_transcript/109756	1.19531377072911	-0.157427283666383	1.34830335783992	0.186858286660669	0.857683076990791	-4.91165044735676
a_HQ_transcript/89517	0.563133751584113	4.93664702609518	1.34824771669704	0.186875991578377	0.857696615749538	-5.51146080676948
a_HQ_transcript/70010	-1.23784871350829	0.764237565358496	-1.34810273859476	0.186922129456732	0.857763688441538	-5.05099986080339
a_HQ_transcript/121675	-1.47871317561669	0.743228920892295	-1.34806736858205	0.186933386956992	0.857763688441538	-5.07711848500227
a_HQ_transcript/111367	-1.33508088334903	1.26199904015543	-1.34796818324936	0.186964958270915	0.857763688441538	-5.07546437917622
a_HQ_transcript/93507	-1.864985768735	0.129088937033285	-1.34791503881837	0.186981876172455	0.857763688441538	-5.03051900826324
a_HQ_transcript/68201	-0.740901474130934	1.83008281505011	-1.34776177384425	0.187030672892136	0.857763688441538	-4.90366075626943
a_HQ_transcript/138606	-0.49566888420275	5.00845780247951	-1.3477594651952	0.187031407998328	0.857763688441538	-5.50319124001889
a_HQ_transcript/139249	3.02899976247797	1.83822122941247	1.34772159021474	0.187043468241403	0.857763688441538	-5.07679842615802
a_HQ_transcript/149581	0.348673586030568	3.81991930535484	1.34769479615473	0.187052000433916	0.857763688441538	-5.38505135640855
a_HQ_transcript/115624	-2.46120537682513	1.91060122893203	-1.3476640994932	0.187061775724834	0.857763688441538	-5.05141577247217
a_HQ_transcript/54395	1.45152014428438	2.20347925422968	1.3476639839588	0.187061812517282	0.857763688441538	-5.01538704706691
a_HQ_transcript/151758	-0.588230335349592	8.33430812595402	-1.34765220509448	0.187065563578619	0.857763688441538	-5.84270239728233
a_HQ_transcript/132194	-3.05175078679304	0.55151538608013	-1.34764554984034	0.187067683016333	0.857763688441538	-5.05362973290763
a_HQ_transcript/136654	-2.60638884460709	0.597571407407183	-1.34749074266603	0.187116988273149	0.85779620781584	-5.03316150870556
a_HQ_transcript/114457	-1.44533254867654	-0.46976549559084	-1.34747098133104	0.187123282875374	0.85779620781584	-5.04181019681606
a_HQ_transcript/119970	1.91323029502524	0.868247979142789	1.34730582816439	0.187175895714518	0.85779620781584	-5.14729940777217
a_HQ_transcript/62228	-0.8412013494756	-0.832501794147861	-1.34728674969954	0.187181974276959	0.85779620781584	-5.02454506420135
a_HQ_transcript/31735	1.54345605081691	2.21261940476414	1.34726713156353	0.187188224942334	0.85779620781584	-5.03182341382105
a_HQ_transcript/116184	4.7951356422663	1.71928408759221	1.34726444372641	0.187189081344641	0.85779620781584	-5.11153493256507
a_HQ_transcript/9187	1.60512480346693	2.21614902102372	1.3472134241819	0.187205337835232	0.85779620781584	-4.96320707227124
a_HQ_transcript/14668	0.999486055771832	-0.377321485103048	1.34713756485075	0.187229511108823	0.85779620781584	-5.03633021353029
a_HQ_transcript/148849	-3.75409141097786	1.87488897706735	-1.34709066939147	0.187244455984633	0.85779620781584	-5.18428464954384
a_HQ_transcript/70627	-0.817481178915348	2.4080036039543	-1.34706531587397	0.18725253615228	0.85779620781584	-5.1295046300804
a_HQ_transcript/15875	1.04292478715295	1.08572486288823	1.34706394628421	0.187252972648295	0.85779620781584	-4.88734765038341
a_HQ_transcript/67356	-0.533238133232454	1.43922635099323	-1.34705443305884	0.187256004588804	0.85779620781584	-4.97146399207659
a_HQ_transcript/111087	-1.37929406886754	1.97010479035566	-1.34699299200995	0.187275587252911	0.85779620781584	-4.90619190823664
a_HQ_transcript/82839	-1.0893391219937	-0.555143584646234	-1.34686768889162	0.187315529119897	0.85779620781584	-5.02929200520406
a_HQ_transcript/144892	2.47947180300513	2.29647393147118	1.34682255291853	0.18732991836336	0.85779620781584	-5.17125548291362
a_HQ_transcript/126617	-0.665712929294142	2.90015083037701	-1.34680997515979	0.187333928275808	0.85779620781584	-5.14312357929605
a_HQ_transcript/55207	-1.10978884761762	2.46720768402303	-1.34676475220763	0.187348346342978	0.85779620781584	-5.18618155363894

a_HQ_transcript/2061	1.52693291614378	0.770560085224139	1.34667059814777	0.187378367469291	0.85779620781584	-4.97619925459216
a_HQ_transcript/74593	2.30525858143511	1.05315125331915	1.34658187491072	0.187406660373067	0.85779620781584	-4.96739308738358
a_HQ_transcript/10231	1.05143534371499	-0.66361924514438	1.34656435538065	0.187412247556481	0.85779620781584	-5.06034239497422
a_HQ_transcript/3215	1.26831154122011	2.19611801775745	1.34647997314431	0.187439159843053	0.85779620781584	-5.03581228235507
a_HQ_transcript/120728	0.746283759053864	5.58098965218001	1.34647335386261	0.187441271077243	0.85779620781584	-5.62382970356824
a_HQ_transcript/124032	1.21527328443621	-0.14966092728593	1.34645839780683	0.187446041411177	0.85779620781584	-5.14167943324793
a_HQ_transcript/16184	1.60882058311662	0.745746991486358	1.34645552461872	0.187446957844465	0.85779620781584	-4.89998423325787
a_HQ_transcript/38711	-2.45084266961069	1.05867390529364	-1.34645431749025	0.187447342871701	0.85779620781584	-4.95425683275279
a_HQ_transcript/133093	2.60533724946464	0.886250995582271	1.34641947143415	0.187458457677619	0.85779620781584	-5.01462024911558
a_HQ_transcript/14768	1.34787270361332	1.3412160222434	1.34633315603161	0.187485991793715	0.857854670260158	-4.89901299136217
a_HQ_transcript/17023	-0.63476049307313	2.26550530911076	-1.34627182762212	0.187505557101545	0.8578766645715	-5.08882322050245
a_HQ_transcript/15823	1.02510371913384	-0.737521994122038	1.34617048635063	0.187537890977199	0.857957069630303	-5.06420789316056
a_HQ_transcript/138049	1.69902404681299	0.51935481506138	1.34601204463574	0.187588451912004	0.858110154287729	-4.9372877766912
a_HQ_transcript/81146	0.855463065250765	3.67572156478525	1.34597013487518	0.187601827655087	0.858110154287729	-5.36360783476158
a_HQ_transcript/140378	-0.388158445629886	2.95720072275887	-1.3459268579885	0.18761564049757	0.858110154287729	-5.20781555623166
a_HQ_transcript/124318	0.455721193006635	3.03234023226432	1.34582625720283	0.187647752654651	0.858189501676597	-5.3089848769282
a_HQ_transcript/87317	-2.64188750865746	2.26512279859352	-1.34572650294922	0.187679598787457	0.858191309041184	-5.19608710195812
a_HQ_transcript/12430	-0.978137676411425	2.52347378145876	-1.34569003672178	0.187691241521665	0.858191309041184	-5.10018451393867
a_HQ_transcript/63636	-1.65800662176383	2.859250955458	-1.3456098539152	0.187716843804015	0.858191309041184	-5.02142118362662
a_HQ_transcript/132452	1.21335539393346	-0.313650202064226	1.3455775002696	0.187727175051404	0.858191309041184	-5.07357498571013
a_HQ_transcript/148094	1.18107043134934	-0.47630822362883	1.34552119233674	0.187745156489271	0.858191309041184	-5.09603784258794
a_HQ_transcript/107192	2.00671700073996	1.0576833647053	1.34531972042028	0.187809505650677	0.858191309041184	-5.19335384411866
a_HQ_transcript/149670	0.469654899597328	4.26900727557599	1.34531471592964	0.187811104277717	0.858191309041184	-5.48785186372801
a_HQ_transcript/90350	1.28069587226528	-0.410052383245813	1.34529226832098	0.187818275037713	0.858191309041184	-5.08588877716327
a_HQ_transcript/131821	1.06596711985899	3.16553263516468	1.34527326286719	0.187824346386351	0.858191309041184	-5.32985571266095
a_HQ_transcript/148051	1.51879623881686	-0.442560780882582	1.34527269921599	0.187824526448714	0.858191309041184	-5.03694105050024
a_HQ_transcript/127265	-1.7428635787114	1.49938959160661	-1.34526673513958	0.187826431723287	0.858191309041184	-4.980834056716
a_HQ_transcript/152435	-3.35805768442672	0.802804657488567	-1.34521927344487	0.187841594294502	0.858191309041184	-5.06238665400051
a_HQ_transcript/150700	2.74383739854259	-0.286103324785431	1.34519595710348	0.187849043504304	0.858191309041184	-5.13330314764246
a_HQ_transcript/126405	1.0647304243677	3.41308830799656	1.34514843642427	0.187864226331346	0.858191309041184	-5.3443098838552
a_HQ_transcript/107092	1.07077669937078	0.74892776949869	1.34513160202226	0.187869605139504	0.858191309041184	-4.88779486756125
a_HQ_transcript/74439	1.21781330098597	-0.550592666425895	1.34481610168499	0.18797043356225	0.858485896999795	-5.05960624131799
a_HQ_transcript/124533	-1.36452913344629	1.55401774699593	-1.34478496390955	0.187980386919175	0.858485896999795	-5.06239450387056
a_HQ_transcript/30791	-0.847476503622767	1.45565859990007	-1.34470772422771	0.188005078754726	0.858485896999795	-4.95961652281448
a_HQ_transcript/67575	1.18818378795112	3.13835985441696	1.34469560025287	0.188008954751505	0.858485896999795	-5.30565284405192
a_HQ_transcript/91086	0.907301890381151	2.88557474941569	1.34462706702718	0.188030865769344	0.858485896999795	-5.1021403201282
a_HQ_transcript/106994	-0.308521708170849	4.50162387013243	-1.34462308407345	0.188032139235234	0.858485896999795	-5.45567726229495
a_HQ_transcript/70684	0.689623599532081	-0.628197451164677	1.3445435469065	0.18805757097003	0.858485896999795	-5.09210757878225
a_HQ_transcript/115801	-2.28549552629577	1.27923102879646	-1.34448209898888	0.188077220548317	0.858485896999795	-5.15702868469292
a_HQ_transcript/112922	-1.06673441467835	-0.408939910599333	-1.34427266973769	0.188144202936645	0.858485896999795	-5.01093952285925
a_HQ_transcript/4497	2.12394161354273	0.76217041008235	1.34426205519043	0.188147598309639	0.858485896999795	-5.00557024644703
a_HQ_transcript/110860	1.51097690849663	-0.185019002947307	1.34423694832267	0.188155629662968	0.858485896999795	-5.04857425680167
a_HQ_transcript/31922	1.20927429491467	-0.235616682358937	1.34421949476904	0.188161212978781	0.858485896999795	-5.13817905897954
a_HQ_transcript/107072	1.47985246551741	-0.455910809515848	1.34405433215338	0.188214054113402	0.858485896999795	-5.06976882972486
a_HQ_transcript/37705	0.66240071157507	4.27054180684559	1.34404226034755	0.188217916743666	0.858485896999795	-5.41796542065523
a_HQ_transcript/128251	-1.99179880095858	2.03686698452517	-1.34398783611149	0.18823533169203	0.858485896999795	-5.05974549343942
a_HQ_transcript/61057	-1.09031550341512	1.97976901947184	-1.34398544256121	0.18823609762118	0.858485896999795	-5.00491439646246
a_HQ_transcript/61484	-1.12877117333681	3.07031958853518	-1.34397098984741	0.188240722498815	0.858485896999795	-5.13025305987736
a_HQ_transcript/121559	3.33022531876983	5.23623354098026	1.34396883263907	0.188241412814384	0.858485896999795	-5.39652973414002
a_HQ_transcript/133364	0.387562589867214	3.80196946846507	1.34392643781597	0.188254979729329	0.858485896999795	-5.41375892320282

a_HQ_transcript/61414	1.01052949892798	0.845576379814497	1.34388347603729	0.188268728847801	0.858485896999795	-4.85260881956521
a_HQ_transcript/101700	2.8166748515682	1.19138648241581	1.34385256672496	0.188278621278023	0.858485896999795	-5.18884087462812
a_HQ_transcript/11882	-0.894443672522176	4.60066391887971	-1.34383108654012	0.188285496181363	0.858485896999795	-5.4006326226804
a_HQ_transcript/113726	1.41093633324367	1.61429463177119	1.34382954660974	0.188285989055726	0.858485896999795	-5.02935314815441
a_HQ_transcript/116434	1.16204987293744	-0.0170221085049335	1.34382155245641	0.188288547702491	0.858485896999795	-4.96406502244615
a_HQ_transcript/98880	1.47291558938819	1.69370267577196	1.34348615038943	0.188395922275066	0.858665695761496	-5.02128794798094
a_HQ_transcript/129191	1.10808196097269	3.2158734776353	1.34346583533903	0.188402427389895	0.858665695761496	-5.3035698916852
a_HQ_transcript/115169	0.833657089612448	5.69611494513983	1.34346425052275	0.188402934873743	0.858665695761496	-5.54685672865935
a_HQ_transcript/79679	-1.15565548497716	-0.0170441478597083	-1.34344003624034	0.188410688810524	0.858665695761496	-5.04768827333652
a_HQ_transcript/48283	1.14441919358508	0.819819849495386	1.34343619317379	0.188411919466178	0.858665695761496	-4.93470799769633
a_HQ_transcript/5317	-1.28409834476202	0.871882849023152	-1.34342153277394	0.188416614186586	0.858665695761496	-5.00206260524046
a_HQ_transcript/25538	0.535397032915241	4.27187513845032	1.34332339396508	0.188448043640526	0.858741602440931	-5.41983942065893
a_HQ_transcript/77214	1.80122359886162	0.577788729758982	1.34321379604154	0.188483147717492	0.85880815058203	-4.97166079528567
a_HQ_transcript/1565	-0.586643783242347	4.34798107390386	-1.34318554822423	0.1884921962759	0.85880815058203	-5.43351934566506
a_HQ_transcript/43355	-3.05804759708636	1.30767364288065	-1.34300058902363	0.188551452117253	0.859010800899107	-4.97224268917935
a_HQ_transcript/151515	-1.96069569716603	0.264419601351787	-1.34285293826608	0.188598765678817	0.859123106304822	-5.04216023653902
a_HQ_transcript/63531	1.22317591005653	-0.512978271126925	1.34283141731827	0.188605662667834	0.859123106304822	-5.06127239837872
a_HQ_transcript/77709	1.67075210603449	0.278471754918514	1.3426272511985	0.188671103088928	0.859353853655637	-5.06836805125681
a_HQ_transcript/54346	-1.54264393147212	1.56757915480925	-1.34256404741209	0.188691365057622	0.859378803421567	-5.08969434434431
a_HQ_transcript/100709	-1.88803338082892	0.790179919916592	-1.34243594459243	0.188732437611018	0.859434012195407	-5.11035976916074
a_HQ_transcript/58718	2.29581378337503	1.01625712914867	1.34243401139764	0.188733057488205	0.859434012195407	-5.12043143621769
a_HQ_transcript/138296	-0.523496478458262	2.33572510999481	-1.34230967884424	0.188772927910671	0.859483064091422	-5.07027204286956
a_HQ_transcript/23400	0.362741320650779	3.87812686751268	1.34227870429606	0.18878286170533	0.859483064091422	-5.40809733454197
a_HQ_transcript/9552	1.80295373254577	-0.226610772890618	1.34220098793795	0.18880778776216	0.859483064091422	-4.96240793624139
a_HQ_transcript/151888	-1.77639724538019	0.859691124024649	-1.34218345909359	0.188813410159546	0.859483064091422	-5.14291200329852
a_HQ_transcript/141286	1.5993637052895	-0.567424137490193	1.34216989928464	0.188817759572795	0.859483064091422	-5.06318262869521
a_HQ_transcript/87097	-0.575776475201852	3.33924667366786	-1.34198810914998	0.18887607762637	0.859599381926504	-5.17840993188966
a_HQ_transcript/24145	-1.75267200798395	2.98269355603177	-1.34181361170293	0.188932069267113	0.859599381926504	-5.12053897446128
a_HQ_transcript/121338	2.56446089332922	0.411956013262446	1.34181281049791	0.188932326382264	0.859599381926504	-4.97030700438398
a_HQ_transcript/90805	1.59205006976618	1.25413097986113	1.34179557092373	0.188937858809033	0.859599381926504	-4.90442055376769
a_HQ_transcript/151627	-3.16742405338709	4.09440741526017	-1.34170446236913	0.188967098927679	0.859599381926504	-5.2464960169447
a_HQ_transcript/21325	-0.628288095563179	3.35610667880465	-1.34163466098589	0.188989503145824	0.859599381926504	-5.37389083903871
a_HQ_transcript/116179	1.2583588554762	-0.591930173973574	1.34162592688098	0.188992306684265	0.859599381926504	-5.05999997458048
a_HQ_transcript/49846	1.96032773610482	2.21361007521865	1.34154653640828	0.189017791504539	0.859599381926504	-5.12499151034197
a_HQ_transcript/78861	1.33822173090343	0.0599554369734481	1.34153709967808	0.189020820928113	0.859599381926504	-4.93493085869642
a_HQ_transcript/57110	1.09705866846272	2.72314751337131	1.3415342868741	0.189021723914943	0.859599381926504	-5.21250609256308
a_HQ_transcript/143004	1.41553582727138	0.182297504870102	1.3415228423082	0.189025397967195	0.859599381926504	-5.11523532588246
a_HQ_transcript/118488	-3.18290516552793	1.66327313081095	-1.34146946054937	0.189042535856309	0.859599381926504	-5.27223797261006
a_HQ_transcript/95390	1.28015773361139	1.73245335544658	1.3414523185077	0.189048039458842	0.859599381926504	-4.95929452186877
a_HQ_transcript/21155	1.11604335698284	1.84641703819199	1.34144513518685	0.189050345763632	0.859599381926504	-5.04295865576534
a_HQ_transcript/154	-1.44602538606601	0.783619915696951	-1.34135792051185	0.18907834897645	0.859619165100627	-4.99667427728755
a_HQ_transcript/143405	-4.46393440186632	2.23024230326818	-1.34133947041166	0.189084273413443	0.859619165100627	-5.16300743020064
a_HQ_transcript/66757	1.46718553696308	1.14703838514775	1.34122361281462	0.18912147925077	0.859654159297875	-5.00049961130736
a_HQ_transcript/10024	2.16226354252624	1.23153725421106	1.3411953163455	0.189130567072977	0.859654159297875	-4.92589652969841
a_HQ_transcript/87346	-2.28642492584372	0.386793268125725	-1.34113897730996	0.189148662175977	0.859654159297875	-5.04666909355012
a_HQ_transcript/149485	3.23943174811839	3.03324162987507	1.34113130409884	0.189151126779174	0.859654159297875	-5.28464065857666
a_HQ_transcript/114387	1.94476183726657	-0.00362319319872035	1.34094756334023	0.189210150939008	0.859781380944539	-5.05376308841424
a_HQ_transcript/151138	-2.77260174194545	1.19306817077633	-1.3409316730247	0.189215256149001	0.859781380944539	-5.19467918747027
a_HQ_transcript/94851	-1.30281214043787	-0.168195789217506	-1.34090603567462	0.189223493091513	0.859781380944539	-5.00062787500678
a_HQ_transcript/132086	0.834777422596404	3.89693648159207	1.34080812599768	0.189254952724896	0.859857112083223	-5.26136853286783

a_HQ_transcript/152393	-1.48037642600948	-0.202160552820884	-1.34057567878548	0.189329657150854	0.860018697481801	-5.01810892621183
a_HQ_transcript/50693	0.503699914449448	3.77253515325019	1.34054974101484	0.189337994502343	0.860018697481801	-5.35941470298572
a_HQ_transcript/112173	-1.66486771033859	0.860674697468529	-1.34051830990975	0.189348097992643	0.860018697481801	-4.92124167437578
a_HQ_transcript/120002	-0.904503634532032	1.28523056696165	-1.34050417968645	0.18935264027114	0.860018697481801	-4.91697471515777
a_HQ_transcript/131039	1.23501366128232	-0.201489895010707	1.34037284757705	0.18939486209729	0.860018697481801	-5.05423369526102
a_HQ_transcript/15253	1.32033171181304	-0.48573392359088	1.34030238582543	0.189417517766865	0.860018697481801	-5.05039733373002
a_HQ_transcript/59580	-1.69395908089973	1.17133287250927	-1.3402394262691	0.18943776301284	0.860018697481801	-4.97128872011146
a_HQ_transcript/34447	-0.552484515595333	4.68340420960692	-1.34019390056669	0.189452403276404	0.860018697481801	-5.47974439317971
a_HQ_transcript/123517	-1.20248575706126	-0.564886723383649	-1.34013877905634	0.189470130550557	0.860018697481801	-5.04358581336469
a_HQ_transcript/22392	2.01618535460078	-0.107204436070163	1.34011619234018	0.189477394888448	0.860018697481801	-5.01102494354753
a_HQ_transcript/143893	-1.1877805798607	1.32211440357014	-1.34009397544978	0.189484540492558	0.860018697481801	-4.97150743106352
a_HQ_transcript/90393	-4.33194753135583	3.69778239701167	-1.34007859202445	0.18948948837548	0.860018697481801	-5.42683028918137
a_HQ_transcript/92611	0.921530012822528	3.0152557655052	1.34005410781239	0.189497363616096	0.860018697481801	-5.27349640430734
a_HQ_transcript/144670	-0.507450348406027	4.99729979506153	-1.34005321327673	0.189497651344393	0.860018697481801	-5.51616158224863
a_HQ_transcript/92181	-0.981536949656667	0.603615742891752	-1.33992218722143	0.189539799645927	0.860142827216679	-4.95576371800233
a_HQ_transcript/80532	2.01295518271192	1.57069292630465	1.33985554884697	0.18956123857015	0.86015322594377	-5.28736745236416
a_HQ_transcript/4406	1.95587053221508	0.554993719383551	1.33982307658525	0.189571686236678	0.86015322594377	-5.05834561135084
a_HQ_transcript/84654	-1.66825862403792	-0.56705604800118	-1.33971939095612	0.189605049154678	0.860157424687016	-5.06508240760394
a_HQ_transcript/85521	0.721611393845455	4.15444073616301	1.33968539209617	0.189615989951283	0.860157424687016	-5.45201653084495
a_HQ_transcript/93167	-1.92573137121564	0.899184089017597	-1.33968223930531	0.189617004540659	0.860157424687016	-5.14982737168189
a_HQ_transcript/144750	-1.92250151278507	1.34399000345612	-1.33951777970948	0.18966993455337	0.86030897105287	-5.03684281207131
a_HQ_transcript/86483	3.94571075067098	1.91708848801957	1.33948647004599	0.189680012610336	0.86030897105287	-5.21396882546122
a_HQ_transcript/25247	0.690747449027651	3.19287141051855	1.33943646122697	0.189696110469137	0.860314855999846	-5.29565122039346
a_HQ_transcript/6738	-1.6325616563644	0.453936028517697	-1.33936043534689	0.18972058524834	0.860358728297357	-5.04246445392552
a_HQ_transcript/70590	2.44815867174745	1.16232160719066	1.3391403103732	0.18979146313912	0.860482523567289	-5.06491045692102
a_HQ_transcript/47813	-1.4744167272666	-0.118877414706966	-1.33911773642767	0.189798732862544	0.860482523567289	-5.02608687951725
a_HQ_transcript/112936	-1.55778639997472	1.95817271352377	-1.33908643085221	0.189808814879257	0.860482523567289	-5.23307472804174
a_HQ_transcript/61058	1.42955482546874	-0.54258111718112	1.33901425147817	0.189832061949614	0.860482523567289	-5.05145839592276
a_HQ_transcript/96151	1.03543252354894	-0.259530641304851	1.33899720467443	0.189837552595373	0.860482523567289	-5.04616759895663
a_HQ_transcript/150322	-1.14714460777584	1.42748837499933	-1.33893558154283	0.189857401958423	0.860482523567289	-4.89576008903725
a_HQ_transcript/128122	0.49342811234141	4.31037670664428	1.33891440236772	0.189864224330025	0.860482523567289	-5.38525463768519
a_HQ_transcript/16508	1.41301772195242	-0.10683978626367	1.33887436578067	0.189877121688353	0.860482523567289	-4.93346831343197
a_HQ_transcript/10178	2.62297843598816	2.34623438029471	1.33886197654391	0.189881112885315	0.860482523567289	-5.20756357965418
a_HQ_transcript/24866	2.62549370466627	0.964609235470442	1.33869537997224	0.189934788311823	0.860534770464092	-5.149123894844
a_HQ_transcript/19445	1.50864913516219	2.80615622071583	1.33866615956142	0.189944203984965	0.860534770464092	-5.1444678502624
a_HQ_transcript/60823	-0.544210408801084	3.13721295266384	-1.33861517054066	0.189960635004288	0.860534770464092	-5.19410235133959
a_HQ_transcript/91030	1.4807807457142	1.30900850170719	1.33860954962637	0.189962446389734	0.860534770464092	-5.00499559817394
a_HQ_transcript/68859	-0.363444425991784	3.04764347325274	-1.33858062590444	0.189971767505433	0.860534770464092	-5.25963833477991
a_HQ_transcript/6915	1.11219957198126	1.38371453385205	1.33855052945765	0.189981466924128	0.860534770464092	-4.90380599539124
a_HQ_transcript/11408	-1.77253044641192	3.39466154393657	-1.33847663849272	0.190005281965944	0.860575582835936	-5.37119434318402
a_HQ_transcript/39217	-1.5347224855264	-0.239682179403647	-1.33837433450709	0.190038258316802	0.860576213159015	-5.02962149640847
a_HQ_transcript/146414	-2.09349697173634	1.05887788995866	-1.33833116733573	0.190052174012965	0.860576213159015	-5.01731296997723
a_HQ_transcript/23360	1.88344855810325	2.25729803621591	1.3383211769789	0.19005539469195	0.860576213159015	-4.91789008484416
a_HQ_transcript/129852	1.18443445642123	2.81082098077189	1.33825007835853	0.19007831659313	0.860576213159015	-5.28314483551364
a_HQ_transcript/63550	1.32673869197395	-0.538200096786007	1.33824657730041	0.190079445374838	0.860576213159015	-5.04553896091445
a_HQ_transcript/70304	1.44179757411761	-0.426050389955963	1.33803898671617	0.190146384221182	0.860706927921477	-5.06940318666167
a_HQ_transcript/139332	-1.19786738453881	1.6031912041878	-1.33797924484504	0.190165651717387	0.860706927921477	-5.05414786650927
a_HQ_transcript/99629	1.57203727106284	0.62609907010091	1.3379742754936	0.190167254462799	0.860706927921477	-5.0123573695458
a_HQ_transcript/18974	-0.980760999992943	-0.711110311617193	-1.33797337363733	0.190167545336078	0.860706927921477	-5.04739443270537
a_HQ_transcript/109174	1.61424105224735	-0.264876917393089	1.33787521231387	0.190199207093843	0.860775706907721	-4.98347585542757

a_HQ_transcript/118142	-0.904064181616324	-0.580895999169772	-1.33776470408026	0.19023485618667	0.860775706907721	-4.98861300322392
a_HQ_transcript/134657	1.74217487468074	0.728923616434907	1.33776469913735	0.19023485778133	0.860775706907721	-4.99195122833478
a_HQ_transcript/44087	0.413763462204812	3.33386075013023	1.33774263932192	0.190241974722053	0.860775706907721	-5.30296324334519
a_HQ_transcript/93409	1.83096912229646	2.78921099072459	1.33768269229116	0.190261315877962	0.860777804870847	-5.21148872949204
a_HQ_transcript/143630	3.02111894279331	2.38066732906042	1.33764940880689	0.190272055030029	0.860777804870847	-5.14163138162673
a_HQ_transcript/93284	-0.994304359182387	2.66429125669024	-1.33746810453652	0.190330562341253	0.860778269694578	-5.13070598121175
a_HQ_transcript/92988	1.49365359357516	-0.327707129926295	1.33742693947811	0.190343848332812	0.860778269694578	-5.04712314155661
a_HQ_transcript/57574	1.3260612081913	1.94381760633672	1.33742427357767	0.190344708774808	0.860778269694578	-5.01466295843782
a_HQ_transcript/59370	-1.39881243197028	-0.129155840567156	-1.33742379309169	0.190344863856057	0.860778269694578	-4.92630021560624
a_HQ_transcript/27184	1.17141111021347	-0.341428928902571	1.33741965521143	0.190346199398811	0.860778269694578	-5.0181418973452
a_HQ_transcript/149240	1.54971946368466	0.199924572261389	1.33733425942975	0.190373763364483	0.860835948413121	-5.0471827956409
a_HQ_transcript/82849	-3.76484865734887	1.82393978441349	-1.337073713759	0.190457881046788	0.861079350680709	-5.10549871559753
a_HQ_transcript/78213	1.72965813509075	-0.180010565842822	1.33702489695617	0.190473644832915	0.861079350680709	-5.04504354032978
a_HQ_transcript/121259	1.35572577312529	-0.460518852993442	1.33699194296614	0.190484286812073	0.861079350680709	-5.07156575747629
a_HQ_transcript/62264	3.55869725048803	3.02886422549525	1.33698401912707	0.190486845761135	0.861079350680709	-5.23301816198052
a_HQ_transcript/108936	-1.05126999878823	0.492943494534378	-1.33690327102439	0.190512924313154	0.861130269399303	-4.98502481823959
a_HQ_transcript/89454	-1.75802569633914	0.291749757087303	-1.33673053277507	0.190568721404912	0.861270957439488	-5.05560924743363
a_HQ_transcript/125348	2.23280961491414	2.10839753197524	1.33671517392443	0.19057368315763	0.861270957439488	-4.99380016713153
a_HQ_transcript/6349	-1.02223460735436	-0.68702567463596	-1.33643382483322	0.190664591987043	0.861572045030911	-5.048422661144
a_HQ_transcript/30106	1.50555497611502	0.379671073611413	1.33641724934859	0.190669948859029	0.861572045030911	-4.92342231406615
a_HQ_transcript/113164	2.01311583389609	2.02571149476556	1.33620579138148	0.190738298107885	0.861669455591702	-4.98508101615515
a_HQ_transcript/89650	1.14920816066714	-0.639451685724669	1.33619567005315	0.190741570083296	0.861669455591702	-5.06427832378111
a_HQ_transcript/56010	-1.51663627486971	1.96914490667663	-1.33617374511826	0.190748658021391	0.861669455591702	-4.96205548998749
a_HQ_transcript/138215	1.33854508257812	0.382390852134131	1.33611741551251	0.190766869302641	0.861669455591702	-4.96846793145639
a_HQ_transcript/16958	-1.10907946605829	-0.199108715109774	-1.33611023465246	0.190769190960714	0.861669455591702	-5.02279900645609
a_HQ_transcript/105608	1.49004064783723	0.109844799419779	1.33605475093982	0.190787130243464	0.861669455591702	-5.14394648260599
a_HQ_transcript/87176	2.2235129887895	0.893199498350448	1.33602957136239	0.190795271864176	0.861669455591702	-5.03862027449715
a_HQ_transcript/126575	-1.96665766825196	1.62553925141622	-1.33581638478466	0.19086421481374	0.861712980672366	-5.0861134586029
a_HQ_transcript/138086	1.16395354081763	3.11511042045501	1.33579843397112	0.190870020848812	0.861712980672366	-5.2136376303818
a_HQ_transcript/16858	2.80344287429713	1.53299852086195	1.33578889491304	0.190873106229835	0.861712980672366	-5.03118471623627
a_HQ_transcript/116046	-1.33090556220997	1.89631683461724	-1.33575273939675	0.190884800976815	0.861712980672366	-5.08224305954143
a_HQ_transcript/112220	1.53637614698573	0.128594740124412	1.33565045389009	0.190917888907128	0.861712980672366	-5.058705995118
a_HQ_transcript/136787	3.43516286370828	0.688733411851872	1.33564621018509	0.190919261781761	0.861712980672366	-5.07515399316796
a_HQ_transcript/144898	2.37071130755312	2.88817893723937	1.33562355738824	0.190926590282694	0.861712980672366	-5.00305563968777
a_HQ_transcript/20263	-1.20930140964756	0.734681525170598	-1.33562124320431	0.190927338966204	0.861712980672366	-4.896477486632
a_HQ_transcript/152477	-1.52050418360901	-0.292890175321516	-1.33544560018086	0.190984169499518	0.861712980672366	-5.02476227560101
a_HQ_transcript/21191	-1.64690357486833	1.56149204767008	-1.33542554243424	0.19099066015304	0.861712980672366	-5.03723687186238
a_HQ_transcript/116267	0.585126072644612	2.5381246930627	1.33541602697976	0.190993739397736	0.861712980672366	-5.12092920212631
a_HQ_transcript/8161	1.43577492000786	0.0902481230511172	1.33535163006246	0.191014579540453	0.861712980672366	-5.05224860183936
a_HQ_transcript/93783	1.39417129936019	1.1022535270708	1.33533326427396	0.191020523401406	0.861712980672366	-4.95076901574026
a_HQ_transcript/114046	-0.433859833699356	3.42632024005564	-1.33530373812422	0.191030079474233	0.861712980672366	-5.28440790164753
a_HQ_transcript/136034	1.73003815385066	0.346581324879427	1.33527125240509	0.191040593830519	0.861712980672366	-5.00408829182619
a_HQ_transcript/133210	2.22033361348268	0.828479568115177	1.33525606970384	0.191045508029726	0.861712980672366	-5.02746076250041
a_HQ_transcript/59958	-3.18083353161951	5.77705682361566	-1.3352207996836	0.191056924286152	0.861712980672366	-5.84398423686091
a_HQ_transcript/140397	-0.445029378722105	5.71163182062176	-1.3350529230579	0.191111270062279	0.86181571964491	-5.61714551550698
a_HQ_transcript/116320	1.80347937349236	3.41911128329939	1.3350416182787	0.191114930124024	0.86181571964491	-5.21186307006416
a_HQ_transcript/46196	1.26942956953069	0.950784109022629	1.33500550385865	0.191126622974788	0.86181571964491	-5.02758634583786
a_HQ_transcript/109131	0.681441711250998	3.82414629028604	1.33491275454463	0.191156655162798	0.86181571964491	-5.23943218728107
a_HQ_transcript/56848	-3.65934299583374	0.624230761295913	-1.33488215712535	0.191166563391152	0.86181571964491	-5.0705969867459
a_HQ_transcript/29303	0.930407425805077	1.86788183816926	1.33484767019178	0.191177731617993	0.86181571964491	-4.95590800716799

a_HQ_transcript/144480	1.54705224387086	-0.380480481011244	1.33478566361157	0.19119781305369	0.86181571964491	-5.09406951525638
a_HQ_transcript/143441	-1.4817611926361	1.96023877503393	-1.33474827045158	0.191209923978343	0.86181571964491	-5.10849208101689
a_HQ_transcript/132903	-2.97254793778644	3.59406361708493	-1.33473834459805	0.191213138870431	0.86181571964491	-5.31703370193306
a_HQ_transcript/62974	0.965284090086084	3.34097135453781	1.33468303152332	0.191231055025653	0.861829645412558	-5.14908522349949
a_HQ_transcript/113061	0.399889827889091	3.57189901318733	1.33446994828181	0.191300085733736	0.86198840947708	-5.37037992647426
a_HQ_transcript/62164	1.4694359169795	0.235255158956885	1.33435785970276	0.191336405787645	0.86198840947708	-4.9239126515526
a_HQ_transcript/56094	1.32940264464305	2.10201268498715	1.33427534534292	0.191363146302312	0.86198840947708	-4.98963489393694
a_HQ_transcript/60731	1.25860456466058	2.91345281776493	1.33426289797599	0.191367180383258	0.86198840947708	-5.29745708821595
a_HQ_transcript/50842	1.04918328008769	1.01860913659302	1.33419900909283	0.191387887232772	0.86198840947708	-4.95043533804469
a_HQ_transcript/56793	1.46570469257292	2.2220219219809	1.33418149178956	0.191393565018902	0.86198840947708	-5.15282933545728
a_HQ_transcript/132412	0.745288104277425	2.91398034422252	1.33416999143811	0.191397292634404	0.86198840947708	-5.23498325616631
a_HQ_transcript/115672	1.67306630063313	0.0265459935906671	1.33416643656696	0.191398444888134	0.86198840947708	-5.06471128137915
a_HQ_transcript/138723	-1.59230819054784	0.260398089440757	-1.33416242429055	0.191399745408765	0.86198840947708	-5.0459267128504
a_HQ_transcript/143764	-0.367912096740956	4.61246411032917	-1.33406545218588	0.19143117956557	0.862038989703704	-5.50046594071215
a_HQ_transcript/132606	-0.441429786262375	3.2393579282181	-1.33399017834753	0.191455582824131	0.862038989703704	-5.28506583218683
a_HQ_transcript/64728	-1.50224031297439	-0.0773349956466598	-1.33395993570675	0.191465387953826	0.862038989703704	-4.97915287300683
a_HQ_transcript/102271	0.877641162198426	2.63844547481101	1.33394479632657	0.191470296519139	0.862038989703704	-5.15944255310458
a_HQ_transcript/87049	2.02778540557297	0.284629363810795	1.33385762283829	0.1914985622271	0.862099475304902	-5.07218684003395
a_HQ_transcript/122559	2.15436130749217	2.53198031724793	1.33374154101389	0.191536206351892	0.862135398203298	-5.10520880234457
a_HQ_transcript/68500	1.00570702682742	0.85005682656727	1.33369075948319	0.191552676063662	0.862135398203298	-4.88504047592262
a_HQ_transcript/84780	0.462132579916564	3.13322618848877	1.33368848359146	0.191553414217404	0.862135398203298	-5.25989111488324
a_HQ_transcript/13436	-1.55328022148758	1.31858983882784	-1.33365008534515	0.19156586848324	0.862135398203298	-4.96024254569376
a_HQ_transcript/52038	-2.9793520634887	2.32674411546245	-1.33357558671367	0.191590033493858	0.862153807663742	-5.20846046023164
a_HQ_transcript/23529	-1.67620345217806	2.12915110671097	-1.33354050835945	0.191601412620147	0.862153807663742	-4.95585449806021
a_HQ_transcript/63324	0.635163935863669	3.29475023235021	1.33350030462601	0.191614455016651	0.862153807663742	-5.20532087737108
a_HQ_transcript/132059	1.30299702328838	-0.229595892036739	1.33341239507028	0.191642975925227	0.862215395091453	-4.98215557913695
a_HQ_transcript/109036	1.75777009342002	0.79717771078106	1.33328937725264	0.191682892657389	0.862282641593607	-5.10162071171965
a_HQ_transcript/39257	1.13939215419565	2.89694970682656	1.33327489864485	0.19168759108644	0.862282641593607	-5.0937849173033
a_HQ_transcript/105490	1.57556024570759	0.186629455387006	1.33322023958283	0.191705329207211	0.862288809552968	-5.0594683585621
a_HQ_transcript/65642	1.19588925323576	-0.176879192641875	1.33317925380992	0.191718630860559	0.862288809552968	-5.10485731859066
a_HQ_transcript/58560	1.39561661819507	0.0427733368542036	1.33306067240985	0.191757119647048	0.862358543086367	-4.94471183687332
a_HQ_transcript/92651	0.299984089062507	-0.777422126402508	1.33294491185118	0.191794698590026	0.862358543086367	-5.18872014561322
a_HQ_transcript/8338	-0.563601374074378	3.12155020646238	-1.33294396428084	0.191795006219838	0.862358543086367	-5.24262868494535
a_HQ_transcript/78244	0.482106568485547	2.89548567115987	1.33294160277055	0.191795772888554	0.862358543086367	-5.26487351682388
a_HQ_transcript/131753	1.65333945214794	0.683240385488976	1.33290297823571	0.191808312750544	0.862358543086367	-5.01556246075913
a_HQ_transcript/128681	-1.54177975372243	-0.0281752695185338	-1.33281545287721	0.191836731115017	0.862419606052106	-5.03237222323208
a_HQ_transcript/117990	1.43291369531574	1.02218316064831	1.33256485512216	0.191918114922395	0.862427612117475	-4.97592675214993
a_HQ_transcript/32846	0.572052887261032	2.18704309832388	1.33255069614898	0.191922713965393	0.862427612117475	-5.08153664108331
a_HQ_transcript/137196	1.59209571811753	-0.174332479664338	1.33253307880899	0.19192843645528	0.862427612117475	-5.06426473143071
a_HQ_transcript/126426	1.58650897639235	1.22591890689266	1.33250543596918	0.191937415707188	0.862427612117475	-4.99128982447666
a_HQ_transcript/136975	-1.22967292312018	3.57891992347847	-1.33247402740348	0.191947618576036	0.862427612117475	-5.38227498189692
a_HQ_transcript/144290	0.496502868058472	3.97554666376368	1.33245072343365	0.1919551889892	0.862427612117475	-5.44176160931024
a_HQ_transcript/52615	1.25365009934942	-0.565985677228682	1.33241520719427	0.1919667270622	0.862427612117475	-5.05480844510246
a_HQ_transcript/141879	2.58445078901706	1.42291846050767	1.33241503649893	0.191966782516864	0.862427612117475	-5.13430498131979
a_HQ_transcript/150752	-1.53751113293125	0.0975356589527258	-1.33236182437078	0.191984070412226	0.862427612117475	-5.02815682791076
a_HQ_transcript/143806	1.23796494390556	0.274537534845442	1.33235317997869	0.19198687897055	0.862427612117475	-5.13059354615797
a_HQ_transcript/20261	0.893148767108412	2.08644117375911	1.33229080777648	0.19200714460247	0.862451997639469	-5.02374214013694
a_HQ_transcript/49959	1.77006118580623	0.814408766908973	1.33215337987466	0.192051802727662	0.862585936405004	-4.97798293062498
a_HQ_transcript/111038	1.16182584428963	0.81872255762102	1.33205366514653	0.192084210705509	0.862598193435672	-4.99358584263244
a_HQ_transcript/92162	1.00525434401307	-0.441620227324116	1.33205366430276	0.192084210979757	0.862598193435672	-5.11137930103319

a_HQ_transcript/119094	2.7285586035908	1.25378593636177	1.33199130986443	0.192104478742811	0.862622567814763	-5.06755127677567
a_HQ_transcript/82646	-1.16245995830502	1.14839266285099	-1.33192672934864	0.192125471807468	0.862650195058281	-4.91601181044977
a_HQ_transcript/108494	-1.14189034513446	1.5651570228687	-1.33183792002214	0.192154343767983	0.862713191824001	-5.03394466913027
a_HQ_transcript/152592	-3.64143543216311	0.190747079640902	-1.33159393579016	0.192233680358922	0.862956312206671	-5.10575636342246
a_HQ_transcript/89192	2.38470634900302	1.67241015820953	1.33150729865306	0.192261858303994	0.862956312206671	-4.9298888819201
a_HQ_transcript/15121	0.877739660794027	2.53135176542927	1.33150253358725	0.192263408190733	0.862956312206671	-5.05457948427823
a_HQ_transcript/39957	1.40247137479386	1.42191121868985	1.3314474522747	0.192281324652763	0.862956312206671	-4.93568736685251
a_HQ_transcript/60516	-0.932883832625715	2.3422494781688	-1.33141190694758	0.192292887268715	0.862956312206671	-5.15730408332033
a_HQ_transcript/72104	-1.69505206822663	0.339109528468881	-1.33132419287638	0.192321422256365	0.862956312206671	-5.00999480573
a_HQ_transcript/51671	-1.41217135928499	1.9059851756603	-1.33127006544977	0.19233903252105	0.862956312206671	-5.09564004042231
a_HQ_transcript/135269	-1.43650963839113	3.37584698121101	-1.33126309718549	0.192341299723915	0.862956312206671	-5.31082065853794
a_HQ_transcript/17741	1.84052096570022	-0.0841679095170523	1.33120330534287	0.192360754514975	0.862956312206671	-5.09290086492127
a_HQ_transcript/72495	2.37642747474985	1.99508780676235	1.33114927226833	0.192378336847015	0.862956312206671	-5.11099235530977
a_HQ_transcript/94833	-1.62095725600599	0.950155304644247	-1.33113441999409	0.192383169985299	0.862956312206671	-5.04623305360921
a_HQ_transcript/19557	-2.49191390225533	1.40829735715469	-1.3311237441012	0.192386644128239	0.862956312206671	-5.14433258568808
a_HQ_transcript/125480	1.30530481556329	0.113961956812914	1.33105516243617	0.19240896308517	0.862976203926218	-5.04905296977754
a_HQ_transcript/82379	1.33081622467945	-0.576214259122645	1.33099969158097	0.192427016766867	0.862976203926218	-5.06253141488016
a_HQ_transcript/11422	-0.295183437511939	3.29231566000559	-1.33092682797009	0.192450733117797	0.862976203926218	-5.33175327851395
a_HQ_transcript/88229	-1.13461132241196	1.6300678729009	-1.33090134779801	0.192459027180593	0.862976203926218	-4.94891297056836
a_HQ_transcript/8677	1.24158054343439	1.92466306639639	1.33088204861469	0.192465309449826	0.862976203926218	-5.0183600874095
a_HQ_transcript/48584	-2.06551139829951	2.07240929060602	-1.33078695818266	0.192496265586091	0.862981057648531	-4.99042663317547
a_HQ_transcript/6072	-2.07873706887589	0.303323960132298	-1.3307579214125	0.192505719100736	0.862981057648531	-5.02695354883634
a_HQ_transcript/133330	-0.669281043308639	2.36174211922499	-1.33074191456383	0.192510930610523	0.862981057648531	-5.01933863021498
a_HQ_transcript/48234	-0.622022136501455	2.77869795372324	-1.33061763868242	0.192551396044062	0.863095893680541	-5.12975985920971
a_HQ_transcript/151681	0.38122159083638	10.6988771521576	1.33045496686422	0.192604373453457	0.863200571816387	-6.1408242125085
a_HQ_transcript/150276	-1.40996805439481	2.15616731479556	-1.33045473465307	0.192604449085785	0.863200571816387	-5.15237910431975
a_HQ_transcript/54245	0.904134061269612	1.09459580074367	1.3303125349154	0.192650768539389	0.86326382487628	-4.95670269737556
a_HQ_transcript/108711	2.7554476999232	1.12747138094659	1.33029902738259	0.192655168863196	0.86326382487628	-5.04984897590851
a_HQ_transcript/113923	1.4327854549652	2.08293119949341	1.33023302289722	0.192676672133211	0.86326382487628	-5.07696103057325
a_HQ_transcript/5782	1.47238599736975	0.101527662649179	1.33013424364529	0.192708856378132	0.86326382487628	-5.07067888277571
a_HQ_transcript/89705	1.93128010406122	1.37452643721022	1.33009557549463	0.192721456356652	0.86326382487628	-5.09786445760591
a_HQ_transcript/118780	-1.79125613645316	-0.260687793701415	-1.33004159216146	0.192739047831837	0.86326382487628	-5.03415811694249
a_HQ_transcript/111966	1.55963536989932	1.22661426339283	1.3300252356518	0.192744378149097	0.86326382487628	-5.06997025235495
a_HQ_transcript/54497	-3.59449951536442	2.92848870510424	-1.32997194627347	0.192761745066642	0.86326382487628	-5.1956268628815
a_HQ_transcript/55745	-1.00772912099176	3.29490350096	-1.32988692704826	0.192789455178625	0.86326382487628	-5.3062306229881
a_HQ_transcript/59020	-1.70266474924571	1.71545459986284	-1.32988596972661	0.192789767213615	0.86326382487628	-4.99112049078609
a_HQ_transcript/1986	1.19325513701124	1.62700977856019	1.32966405029187	0.19286211140551	0.86326382487628	-5.01734633541042
a_HQ_transcript/66966	-0.811450688054739	1.98104657865624	-1.32965278954861	0.192865782885897	0.86326382487628	-4.98967611187577
a_HQ_transcript/119603	1.75157206617079	0.466900691978727	1.32962931634467	0.192873436319735	0.86326382487628	-5.04218336586882
a_HQ_transcript/88694	-1.33252388206863	0.907626168101765	-1.32956319371128	0.192894996847716	0.86326382487628	-5.02679391962516
a_HQ_transcript/148560	0.331244963534096	8.94841698435387	1.3295246615028	0.192907561853467	0.86326382487628	-5.94387261116228
a_HQ_transcript/104286	0.320906580329124	5.57644027094245	1.32952033320713	0.192908973310974	0.86326382487628	-5.6170379696026
a_HQ_transcript/104173	1.40774816439155	-0.299914795057687	1.3293277553347	0.192971781004891	0.86326382487628	-5.01763550637083
a_HQ_transcript/6967	0.588371979365269	3.88011908818361	1.32925329151954	0.192996070982312	0.86326382487628	-5.39254142298958
a_HQ_transcript/88405	-0.721567685266032	3.60411523930512	-1.3292243771334	0.19300550344243	0.86326382487628	-5.27885176351011
a_HQ_transcript/86283	-0.930153418181664	3.53448480025058	-1.32906137733222	0.193058683919869	0.86326382487628	-5.37861613985714
a_HQ_transcript/2414	2.02296369570036	4.23085003402423	1.32904157116306	0.19306514666789	0.86326382487628	-5.54518122215874
a_HQ_transcript/12512	1.05270057072249	0.235075453033631	1.32903903023173	0.193065975785183	0.86326382487628	-4.87209353432632
a_HQ_transcript/54071	-2.63260564093993	1.98272649794721	-1.32903062998388	0.193068716843227	0.86326382487628	-5.11482648431333
a_HQ_transcript/150280	1.19146237749512	-0.560439200803707	1.32900822886579	0.193076026626794	0.86326382487628	-5.06712962858342

a_HQ_transcript/54230	0.60030007287391	3.68379385006285	1.32898772292954	0.193082718173785	0.86326382487628	-5.38240192277499
a_HQ_transcript/60119	-0.954815579434106	0.261324522370028	-1.328972211006	0.193087780180849	0.86326382487628	-4.95330889551226
a_HQ_transcript/69351	0.572135192645707	2.52338384207421	1.32883997453219	0.193130937060607	0.86326382487628	-5.14882278302409
a_HQ_transcript/98211	1.65429242603304	1.00517571211054	1.32882673801377	0.193135257357451	0.86326382487628	-4.92709435844821
a_HQ_transcript/111193	1.16006720978923	1.21072900283494	1.32882564988152	0.193135612518672	0.86326382487628	-4.93499068102636
a_HQ_transcript/146413	0.313280988863689	8.84724689965164	1.32878011952935	0.193150473860447	0.86326382487628	-5.94434728152553
a_HQ_transcript/42913	0.804223436760843	1.61280959513926	1.32875932757095	0.19315726075529	0.86326382487628	-5.13693358198795
a_HQ_transcript/97765	1.16435331197757	0.424808591523723	1.32875072533742	0.193160068743265	0.86326382487628	-5.00281781055532
a_HQ_transcript/51625	0.638642322789515	2.42885455718526	1.32873904021489	0.19316388311494	0.86326382487628	-5.0909344827855
a_HQ_transcript/25911	-1.7799519097414	2.7872073480748	-1.3286930197526	0.193178906126243	0.86326382487628	-5.36338960498928
a_HQ_transcript/18130	-1.07705954759903	0.988185160424477	-1.32865171146134	0.193192391651983	0.86326382487628	-4.97139072665024
a_HQ_transcript/16626	1.03323024948855	2.60106679462102	1.32845987802272	0.193255027177883	0.86326382487628	-5.06361562320277
a_HQ_transcript/56401	1.71554379186479	0.640172328462387	1.32845751122385	0.19325580005873	0.86326382487628	-5.05837532683142
a_HQ_transcript/57825	0.608415020444381	3.59946086159547	1.32845633927823	0.193256182759778	0.86326382487628	-5.37176039775084
a_HQ_transcript/144267	0.472985104890586	4.68700784959307	1.3284477543125	0.19325898621425	0.86326382487628	-5.50036371602864
a_HQ_transcript/131718	-0.94212167910238	3.44703392155803	-1.32836041271643	0.193287509734366	0.86326382487628	-5.28717568546887
a_HQ_transcript/141329	1.32681035676124	0.0439583348031519	1.32832624914737	0.19329866755821	0.86326382487628	-5.00596796367488
a_HQ_transcript/55622	-1.15237129189669	0.61057031741995	-1.32832416255887	0.193299349054189	0.86326382487628	-4.93161020499524
a_HQ_transcript/87562	1.34642718684699	0.597118721761867	1.32829315109712	0.193309477855709	0.86326382487628	-4.99246057850911
a_HQ_transcript/112218	3.03363621118436	2.27101724580525	1.32826423447199	0.193318922820194	0.86326382487628	-5.20154541544702
a_HQ_transcript/38623	-1.34691576858521	2.051893070401	-1.32818192139469	0.193345810475399	0.86326382487628	-5.10934730680578
a_HQ_transcript/101315	1.51617365396489	-0.262516701510288	1.32816810724076	0.193350323165294	0.86326382487628	-5.1100687526785
a_HQ_transcript/143419	-0.778485804695717	3.93374388340814	-1.32811184783015	0.193368702345586	0.86326382487628	-5.44497433957438
a_HQ_transcript/95812	1.29646816589011	-0.356230955498091	1.32808881895158	0.19337622595269	0.86326382487628	-5.06434351228822
a_HQ_transcript/148637	-2.70805670320156	2.67123051319356	-1.3280808908714	0.193378816133496	0.86326382487628	-5.26583018228995
a_HQ_transcript/37780	2.35384924859497	1.23495122403757	1.32804541285116	0.193390407474	0.86326382487628	-5.01335573091625
a_HQ_transcript/98985	0.99437722181005	2.9794944559459	1.32802817857402	0.19339603843115	0.86326382487628	-5.11933571788015
a_HQ_transcript/58503	-1.29125285102352	0.443302524728213	-1.32800385955598	0.193403984400793	0.86326382487628	-4.96434905214695
a_HQ_transcript/129082	-0.645202522973389	2.79754996854269	-1.32799870050312	0.193405670096404	0.86326382487628	-5.31898754917683
a_HQ_transcript/62765	1.17514004809548	-0.0607066503059222	1.32792442002232	0.193429942136626	0.863265493224091	-5.00161294377091
a_HQ_transcript/121296	-1.13870971657461	2.82043059135432	-1.32790665903674	0.193435746099019	0.863265493224091	-5.25668621900295
a_HQ_transcript/5572	0.921231245722874	3.87138119927422	1.32780651014059	0.193468475415712	0.86334112892707	-5.29523636650068
a_HQ_transcript/111930	-1.46684797643146	0.16359868935858	-1.32773364105323	0.193492292186664	0.86334112892707	-5.05926645337952
a_HQ_transcript/121730	-0.4599774699822	2.90552000637959	-1.32771846894842	0.193497251370456	0.86334112892707	-5.23039909268044
a_HQ_transcript/48318	-0.473994513663169	3.36908672502752	-1.3272047379492	0.193665228144023	0.864024282873266	-5.26098818369084
a_HQ_transcript/44377	-1.36921250750353	0.730641544010526	-1.32713231549528	0.193688917425663	0.864063652503372	-4.96321709373764
a_HQ_transcript/53628	1.59365784411344	2.03629671106663	1.32702706345632	0.193723349187107	0.864124440725143	-4.97793861038278
a_HQ_transcript/138666	2.71907405550978	1.1653373039904	1.32685727052432	0.193778904529844	0.864124440725143	-5.08258222297114
a_HQ_transcript/37399	0.629860670069093	2.28077484784582	1.32684255308636	0.193783720573588	0.864124440725143	-5.08598164565103
a_HQ_transcript/110900	-0.840749942646539	1.921813210939	-1.32681403152657	0.19379305405493	0.864124440725143	-5.11404898426132
a_HQ_transcript/81287	1.49588411925405	-0.126246383492367	1.32680979390434	0.193794440816693	0.864124440725143	-5.03075533432999
a_HQ_transcript/132293	1.03128867090564	2.25921278584153	1.32678609178807	0.193802197474214	0.864124440725143	-5.1561407655743
a_HQ_transcript/97082	-1.19417103901956	0.839706147531968	-1.32677262407034	0.193806604970646	0.864124440725143	-4.90527822647064
a_HQ_transcript/121306	1.52329062932043	1.83392875121542	1.32667766059435	0.193837685264385	0.864196730253733	-5.03087608477014
a_HQ_transcript/142458	-0.57268529153219	2.30224678234992	-1.32656271050756	0.193875312035344	0.864256719652382	-5.10937551476691
a_HQ_transcript/97847	0.909812181991026	0.834885194690524	1.32650539154706	0.193894076426533	0.864256719652382	-4.90298199196819
a_HQ_transcript/1013	1.99859508059959	2.54791495398075	1.3264751839585	0.193903965984586	0.864256719652382	-5.15955245997869
a_HQ_transcript/123225	1.8688905971392	2.39466539103078	1.32645488003338	0.193910613434255	0.864256719652382	-5.16432379427914
a_HQ_transcript/39528	-1.47320233833702	0.703237883058478	-1.32630225212093	0.193960589000913	0.864296694711151	-5.00648396034194
a_HQ_transcript/151178	1.00488064732991	-0.663240788343752	1.32621821395289	0.193988110177324	0.864296694711151	-5.04805824530877

a_HQ_transcript/97634	1.14993932738354	0.811690148392656	1.32620555607778	0.193992255691992	0.864296694711151	-5.02980656415034
a_HQ_transcript/149299	-2.18053267295961	3.64626480975183	-1.32612569980376	0.194018410572838	0.864296694711151	-5.50418544926503
a_HQ_transcript/12626	1.13582661870052	0.372512460058695	1.32609749887222	0.194027647714134	0.864296694711151	-4.90184757541392
a_HQ_transcript/92797	1.95195662451976	1.77554395402741	1.32605567312695	0.194041348249566	0.864296694711151	-4.9877598037603
a_HQ_transcript/21344	1.58735056155554	0.952907762006225	1.32603030253644	0.194049659059487	0.864296694711151	-4.99378469340654
a_HQ_transcript/146641	-2.67318039768012	1.91892542242244	-1.32594867255958	0.194076400978979	0.864296694711151	-5.17626846037637
a_HQ_transcript/78341	0.870226697145424	-0.121503916810932	1.32590853816679	0.194089550014113	0.864296694711151	-4.95507388279389
a_HQ_transcript/138617	0.931184184562779	6.28561257932249	1.32588880453923	0.194096015497095	0.864296694711151	-5.66456927084301
a_HQ_transcript/126729	1.00013204253078	-0.539169785210563	1.32587466688817	0.194100647628177	0.864296694711151	-5.08912066993569
a_HQ_transcript/36749	0.819625601420257	1.52993705359285	1.32586553140405	0.194103640869295	0.864296694711151	-4.94233764793016
a_HQ_transcript/56968	0.725169392366489	4.22957426569628	1.32583734122089	0.194112877605527	0.864296694711151	-5.43659406711891
a_HQ_transcript/110904	-1.95665689604309	-0.196021474805	-1.32578529448854	0.194129932015675	0.864306425260966	-5.07422578629953
a_HQ_transcript/151198	1.6536138328069	0.701398113727775	1.32560524721698	0.194188937872609	0.864430774206979	-5.22641041332891
a_HQ_transcript/105968	-1.66202485312564	3.07157252797668	-1.32556172820513	0.194203202175074	0.864430774206979	-5.14927947005742
a_HQ_transcript/143317	-2.64259694330051	4.68209371654317	-1.32555166127531	0.194206501944837	0.864430774206979	-5.45269946289713
a_HQ_transcript/130346	-1.51028158854194	1.00027666616124	-1.32544576604237	0.194241215224746	0.864430774206979	-4.95841605912724
a_HQ_transcript/120497	1.3415342289444	-0.27272477195315	1.32543914716254	0.194243385103198	0.864430774206979	-5.10135630820644
a_HQ_transcript/1409	0.428286771518815	3.53802166888157	1.32542785000368	0.194247088712695	0.864430774206979	-5.35860823329185
a_HQ_transcript/96678	1.04243746315294	-0.653603549016796	1.32520849443259	0.194319012002286	0.864684645921218	-5.0850675306083
a_HQ_transcript/34907	1.27954482837846	-0.464878589157472	1.32506951771812	0.194364590893956	0.864821259911505	-5.07829867511462
a_HQ_transcript/87390	-1.31475956248945	0.184654436324626	-1.32495401706915	0.194402476806753	0.864839376629013	-5.0546588970497
a_HQ_transcript/98940	-1.51715490777167	0.514622425062495	-1.32493698117955	0.19440806530915	0.864839376629013	-5.07967214346014
a_HQ_transcript/106598	1.0013133299753	1.06511400828676	1.32492103299324	0.194413297110021	0.864839376629013	-4.95876825477527
a_HQ_transcript/69211	-1.52425107560466	0.010027970678729	-1.32485125833369	0.194436187951178	0.864875017847113	-5.07289289305761
a_HQ_transcript/118831	2.11647702870811	-0.213733121400707	1.32474793264375	0.194470089617343	0.864901344019375	-5.08599992033935
a_HQ_transcript/64641	0.386771201267427	4.76796147456431	1.32469305554265	0.194488096909049	0.864901344019375	-5.51973276762891
a_HQ_transcript/118666	1.58047238187887	0.0461252263029179	1.32468660292491	0.194490214345721	0.864901344019375	-4.97496470135633
a_HQ_transcript/49308	-0.715275996820306	4.62152455392508	-1.3246198245064	0.194512128828448	0.864901344019375	-5.44055619208239
a_HQ_transcript/84776	-2.17377030155942	1.04105544936726	-1.32460649694106	0.194516502724423	0.864901344019375	-4.96387603356375
a_HQ_transcript/132066	4.60607910071935	1.01853430373391	1.32449994148556	0.194551475253835	0.864979848737937	-5.02007218055387
a_HQ_transcript/116735	1.67029042159721	1.70348913897422	1.32445777839341	0.194565314920871	0.864979848737937	-4.98858829773939
a_HQ_transcript/39106	-1.9025742466551	3.34125899062887	-1.32436949270054	0.194594296377597	0.864979848737937	-5.36567680214231
a_HQ_transcript/126961	-2.27837189368254	1.34343211379627	-1.32431665935498	0.194611641512678	0.864979848737937	-4.91612167272088
a_HQ_transcript/126055	-1.85013327913026	2.01380491240022	-1.32431137650978	0.194613375930809	0.864979848737937	-5.03348724654824
a_HQ_transcript/131806	0.366411438143268	4.32847191532142	1.32409865499293	0.194683224683466	0.864979848737937	-5.49445719094768
a_HQ_transcript/132616	1.59384958779477	1.01848843849149	1.32402744527431	0.194706611243817	0.864979848737937	-4.96288469656572
a_HQ_transcript/74352	-0.87347237535718	1.75699632848955	-1.32401902921989	0.194709375370912	0.864979848737937	-4.97783312436092
a_HQ_transcript/87919	1.38306701315676	3.49320055677023	1.32401481015149	0.194710761072109	0.864979848737937	-5.36042459279747
a_HQ_transcript/92415	2.2095863331874	2.34340235129068	1.32400082816016	0.194715353339555	0.864979848737937	-5.11169736595023
a_HQ_transcript/25389	-1.68839202333489	0.429077106745102	-1.32398591775773	0.194720250627429	0.864979848737937	-5.01139075446634
a_HQ_transcript/43209	1.66504627747014	1.43079041158321	1.32395971519824	0.194728857027792	0.864979848737937	-5.01827570922937
a_HQ_transcript/114683	1.56161556628031	0.669608426710097	1.3239496838862	0.194732151954495	0.864979848737937	-5.00248027106728
a_HQ_transcript/124697	-4.78306475091819	1.25515892914912	-1.32391822017944	0.194742486932862	0.864979848737937	-5.07489458160363
a_HQ_transcript/117412	-1.25061184077401	3.69810001257382	-1.32378114557851	0.194787517152551	0.864995354680757	-5.27593012473733
a_HQ_transcript/52699	-2.96451123630425	1.18295813235915	-1.32377378803381	0.194789934397124	0.864995354680757	-5.02588017909322
a_HQ_transcript/1386	-2.01068732487844	1.29982222665906	-1.3237556285203	0.194795900614439	0.864995354680757	-5.17557217773816
a_HQ_transcript/128276	-4.5444723668176	2.81239451757289	-1.32370958762934	0.19481102774753	0.864995354680757	-5.27186940836825
a_HQ_transcript/130957	-2.26240358673343	2.29401209263809	-1.32368111745847	0.194820382319716	0.864995354680757	-5.06881134243098
a_HQ_transcript/93825	-1.54462237233246	-0.341061396739756	-1.32360419094652	0.194845660134578	0.865041513198101	-4.97203823148086
a_HQ_transcript/112926	0.314052255026089	3.58734827661392	1.32345891544801	0.194893404082517	0.865071332445457	-5.42017281542641

a_HQ_transcript/146677	-1.12034205976914	2.38150129166709	-1.32345445044204	0.194894871622996	0.865071332445457	-5.12668140417197
a_HQ_transcript/101521	0.849036175745082	2.4514264978075	1.32344790402622	0.194897023288359	0.865071332445457	-5.02419498119945
a_HQ_transcript/127954	-1.10538757393142	1.94493570895199	-1.32336958515426	0.194922766422482	0.865116629100366	-5.06046223273322
a_HQ_transcript/9569	0.849920701669916	4.09692808790998	1.32332630262134	0.194936994356534	0.865116629100366	-5.43370316217838
a_HQ_transcript/125158	1.01451638777659	5.41849253347077	1.32323310507376	0.194967633175471	0.865158132312709	-5.57385262131255
a_HQ_transcript/96221	-2.26464299971801	2.89099642850462	-1.32316737297411	0.194989244914196	0.865158132312709	-5.22538101578634
a_HQ_transcript/40342	-1.43994395758548	3.29849458171731	-1.32316204331849	0.194990997306441	0.865158132312709	-5.50145110706438
a_HQ_transcript/113509	-1.78254633809786	0.225016903528911	-1.32309755870221	0.195012200824043	0.86518617077545	-5.06115017699222
a_HQ_transcript/6777	0.778667710810793	2.02021094733035	1.32296925044082	0.195054395785549	0.865259215747499	-4.95789804685749
a_HQ_transcript/31148	-1.57194575240912	-0.108191014326451	-1.32291457518883	0.19507237820764	0.865259215747499	-5.06205212165618
a_HQ_transcript/118136	-4.20738159852029	0.445037682915032	-1.32284515983403	0.195095210410279	0.865259215747499	-5.1030532157228
a_HQ_transcript/118876	1.94836584836849	0.760653675574548	1.32282330687707	0.195102398742118	0.865259215747499	-5.18830430166141
a_HQ_transcript/100932	2.29980256875561	0.689454403293059	1.32281998226053	0.195103492362363	0.865259215747499	-5.16589972148896
a_HQ_transcript/133448	1.6337385096559	0.919764402923981	1.32277594844192	0.195117977567063	0.865259215747499	-4.92460862841418
a_HQ_transcript/1318	-1.82940862188269	0.523060464180427	-1.32264021617119	0.195162632753805	0.865335195272352	-5.0878249154515
a_HQ_transcript/7498	1.12018187586752	-0.188301341210823	1.3226165085347	0.195170433243158	0.865335195272352	-5.05433732514474
a_HQ_transcript/144728	0.85895697374583	-0.815317523044467	1.32258812885022	0.195179771248335	0.865335195272352	-5.09770181742154
a_HQ_transcript/96131	-0.432027523475432	4.78724926148045	-1.32253985869606	0.195195654866024	0.86533961455554	-5.52912548221251
a_HQ_transcript/129812	-1.76828947235698	2.92789390760695	-1.32244752775723	0.195226039674564	0.865408314970112	-5.35045829878035
a_HQ_transcript/74774	1.92351053875375	0.905409034069335	1.322328265567	0.195265292549476	0.865476224226354	-5.10553981191858
a_HQ_transcript/141691	1.06437646249213	3.58150436645553	1.32225508793212	0.195289380569925	0.865476224226354	-5.36477115700956
a_HQ_transcript/149928	-1.5023513511786	1.2678241063111	-1.32225400094814	0.195289738391683	0.865476224226354	-4.97194610475505
a_HQ_transcript/56168	1.28525431398949	0.271553830687574	1.32221155010862	0.195303713079424	0.865476224226354	-4.99501122085038
a_HQ_transcript/10137	2.35625857712484	2.54566315196187	1.32203905843992	0.195360504707637	0.865476224226354	-5.1037122547088
a_HQ_transcript/129646	-1.75294871653375	-0.0345923069607002	-1.32197201366856	0.195382582130659	0.865476224226354	-5.04561219629672
a_HQ_transcript/96737	1.62160960921013	0.187562611509848	1.32186082774166	0.195419199179141	0.865476224226354	-4.99353267932134
a_HQ_transcript/57117	1.12554263201985	-0.180661778948997	1.32185624638656	0.195420708077465	0.865476224226354	-5.1688423383849
a_HQ_transcript/124138	-2.29449452258899	0.177466698830553	-1.32185558156164	0.195420927042508	0.865476224226354	-5.05661162935032
a_HQ_transcript/109992	-1.02560283424443	3.8315376440852	-1.32179869898594	0.195439662439764	0.865476224226354	-5.37438595942965
a_HQ_transcript/124626	3.67068818438032	2.3662676497874	1.3217983600104	0.195439774092168	0.865476224226354	-5.17499604053195
a_HQ_transcript/144418	1.59200414132429	-0.377424418412933	1.32179403021725	0.195441200251933	0.865476224226354	-5.07284118254463
a_HQ_transcript/95419	-1.98597000439653	1.11086365632311	-1.32173591473798	0.195460343269027	0.865476224226354	-5.08705114053835
a_HQ_transcript/45223	1.48410272442205	1.36916388414535	1.32167797161119	0.195479430945979	0.865476224226354	-4.95368189091387
a_HQ_transcript/70151	-1.14651787692429	1.12686426554992	-1.32163834574363	0.195492485358298	0.865476224226354	-4.88811878477796
a_HQ_transcript/20687	-1.14132931680926	2.05293585578294	-1.32155111818748	0.195521224107392	0.865476224226354	-5.12541302727249
a_HQ_transcript/21547	-1.80472308680492	0.516034660891429	-1.32148892899134	0.195541715479741	0.865476224226354	-5.03567820462411
a_HQ_transcript/148810	-1.61293965530694	0.581890670336892	-1.32130109967643	0.195603615338835	0.865476224226354	-5.02233163275704
a_HQ_transcript/103486	1.39398598881945	1.99792708674113	1.32128587017191	0.195608634938208	0.865476224226354	-5.17464862230556
a_HQ_transcript/26830	1.43376003398891	1.43287682416457	1.32128455322214	0.195609069005577	0.865476224226354	-5.00990895046548
a_HQ_transcript/31668	2.1488861613629	0.687275894724436	1.32128226629749	0.195609822778952	0.865476224226354	-5.09382308578543
a_HQ_transcript/42257	-1.67102884675405	-0.0562154915400094	-1.32127748680286	0.195611398113873	0.865476224226354	-5.08234914395448
a_HQ_transcript/147320	0.737304720704	6.12027731673526	1.32125856011293	0.195617636499866	0.865476224226354	-5.68622348647792
a_HQ_transcript/96559	-1.50543481253523	1.65372130081577	-1.32121327250079	0.195632564272832	0.865476224226354	-5.01192920850198
a_HQ_transcript/25054	1.16812467543183	2.38111728923344	1.32113625713144	0.195657952204912	0.865476224226354	-5.17721014616931
a_HQ_transcript/95514	0.593503029233738	3.80485372447956	1.32105270492497	0.195685497847761	0.865476224226354	-5.30726292200385
a_HQ_transcript/116446	-2.67716200562325	0.485082136825846	-1.32097902093782	0.195709792586513	0.865476224226354	-5.08390195126357
a_HQ_transcript/91076	3.55684575912032	4.05596054745991	1.32092399289786	0.195727937682867	0.865476224226354	-5.19632297285404
a_HQ_transcript/101844	3.58628232476758	4.6925009585851	1.32081246828465	0.195764716071432	0.865476224226354	-5.35777386547112
a_HQ_transcript/144578	-0.474001556692591	3.72012057210396	-1.32080305403392	0.19576782092889	0.865476224226354	-5.39287955636802
a_HQ_transcript/47716	-2.43118918723371	1.1734778112219	-1.32080108609524	0.195768469967693	0.865476224226354	-5.15070606962694

a_HQ_transcript/32471	0.501896839875541	3.45720461389045	1.32076378605703	0.195780772071699	0.865476224226354	-5.35143691033822
a_HQ_transcript/15787	-0.879123937870475	2.16751424607499	-1.32072293494964	0.195794246051107	0.865476224226354	-5.12309118707597
a_HQ_transcript/148517	0.563091207043585	3.33711053801769	1.32070897398782	0.195798850978256	0.865476224226354	-5.27203330103456
a_HQ_transcript/57085	-1.18074065766771	-0.244725734057333	-1.32070713063158	0.195799459002829	0.865476224226354	-4.98317256640025
a_HQ_transcript/147504	1.55171189684414	-0.103875003287011	1.32067610123694	0.195809694156899	0.865476224226354	-5.07282875583394
a_HQ_transcript/148906	3.32011203900542	2.57546907027092	1.32066282019424	0.195814075080573	0.865476224226354	-5.06370815653882
a_HQ_transcript/6341	-1.42411094109276	3.39854118677763	-1.32059619666148	0.195836052848819	0.865476224226354	-5.34958111404713
a_HQ_transcript/35868	1.30377182923174	1.87310652691746	1.32059118060583	0.195837707621629	0.865476224226354	-4.97898178742991
a_HQ_transcript/135138	-1.24211876098301	0.953406344450005	-1.32057893945187	0.195841745964895	0.865476224226354	-5.02624222388971
a_HQ_transcript/51019	1.49993430750029	-0.274769027171621	1.3204686220031	0.195878142451643	0.865476224226354	-5.09464224527256
a_HQ_transcript/5917	1.08552152200555	2.50755164019162	1.32036629878271	0.195911906079589	0.865476224226354	-5.06614630243634
a_HQ_transcript/129311	-1.6785755201765	1.6156242869687	-1.32032840352194	0.195924411524233	0.865476224226354	-5.15598660470338
a_HQ_transcript/128267	1.78205858972736	0.652505966687032	1.32028237083961	0.195939603143303	0.865476224226354	-5.06658203732438
a_HQ_transcript/72908	-0.806071001835022	2.89584523153457	-1.32009562181577	0.196001242970423	0.865476224226354	-5.21969987874271
a_HQ_transcript/38535	-0.941580776978955	1.36962434868645	-1.3200887916802	0.196003497659636	0.865476224226354	-4.90245635998256
a_HQ_transcript/10233	1.86315903793039	0.260006831085393	1.32005947170196	0.196013176674235	0.865476224226354	-4.9838072771973
a_HQ_transcript/119993	1.59280112999607	1.33861123092126	1.3200154160586	0.196027720867165	0.865476224226354	-5.07381614339757
a_HQ_transcript/36793	0.685240744667377	3.82098400266729	1.31995270534676	0.19604842512899	0.865476224226354	-5.43991598274134
a_HQ_transcript/126423	-2.69953988520834	2.39996326931631	-1.31982682292861	0.196089990913672	0.865476224226354	-5.4116715199103
a_HQ_transcript/109087	-1.33875451647157	1.77114636053384	-1.31979983466374	0.196098903191906	0.865476224226354	-5.07538301147437
a_HQ_transcript/59803	-1.35038353342646	-0.437667810577913	-1.31977798952044	0.196106117295313	0.865476224226354	-5.05482747167975
a_HQ_transcript/38581	0.902194212537604	3.34709386635787	1.31972168970718	0.196124710589071	0.865476224226354	-5.31049053584733
a_HQ_transcript/80247	-0.435180222476223	2.80332175186626	-1.31969469377231	0.196133626610487	0.865476224226354	-5.22686732567297
a_HQ_transcript/148440	1.90486156825781	1.93912691131679	1.31969245252236	0.196134366848223	0.865476224226354	-5.15008206497117
a_HQ_transcript/7052	0.936332076460348	1.57597143342497	1.31958033484784	0.196171399692863	0.865476224226354	-5.004986458538
a_HQ_transcript/147780	1.98104104410609	0.402277807184098	1.31950532832684	0.196196177591715	0.865476224226354	-5.08495911979756
a_HQ_transcript/136640	0.427235997780457	2.75833293160747	1.31950137902569	0.196197482283235	0.865476224226354	-5.20093761688725
a_HQ_transcript/145690	-1.13815956016377	-0.623193699958573	-1.31949034765479	0.196201126643264	0.865476224226354	-5.01571824137114
a_HQ_transcript/21352	-1.26280946718691	0.858868915148655	-1.31948583279406	0.196202618202495	0.865476224226354	-4.93319278540231
a_HQ_transcript/11416	0.994553782623881	0.337825808548543	1.31945854240785	0.196211634219955	0.865476224226354	-4.95081705347728
a_HQ_transcript/43373	0.602998865003874	3.56749943674602	1.31941932321832	0.196224591752904	0.865476224226354	-5.41991787026442
a_HQ_transcript/121954	2.63975444479062	1.77344646542786	1.31934716199632	0.196248434638157	0.865476224226354	-5.07764423898072
a_HQ_transcript/117975	1.32608269585815	-0.151570975886364	1.31930519372572	0.196262302451738	0.865476224226354	-5.03393611338524
a_HQ_transcript/122224	-0.706066782563734	2.75269378219343	-1.31928528361898	0.19626888172394	0.865476224226354	-5.19577605819158
a_HQ_transcript/115757	1.87287743418145	0.173132811280947	1.31925141393038	0.196280074312629	0.865476224226354	-5.04730238367608
a_HQ_transcript/137653	-1.7493183236187	-0.161081971325645	-1.31922976132633	0.196287229895858	0.865476224226354	-5.0571491165983
a_HQ_transcript/104458	1.32833146783601	1.75375508893993	1.31918394080954	0.196302372961267	0.865476224226354	-5.0252977049849
a_HQ_transcript/145615	-2.66337077834757	2.20989772085089	-1.3191453087361	0.19631514103857	0.865476224226354	-4.98696820541528
a_HQ_transcript/33813	1.03712759720814	3.21364673925813	1.31913266295346	0.196319320665995	0.865476224226354	-5.3495721398052
a_HQ_transcript/49169	-1.43748626502518	1.29231693524329	-1.31911731660435	0.196324392964177	0.865476224226354	-5.09414295014068
a_HQ_transcript/6693	-0.691705526559125	2.3352462932767	-1.31910734630698	0.196327688415568	0.865476224226354	-5.06229495415423
a_HQ_transcript/53851	1.4507955951224	2.38474682116167	1.31908257177895	0.196335877246892	0.865476224226354	-5.12384542355544
a_HQ_transcript/80686	1.51240139931374	1.45020072340337	1.31900692022449	0.196360884301205	0.865476224226354	-4.99227018400153
a_HQ_transcript/73681	2.28003969282953	2.93988543595104	1.31897267131602	0.196372206276824	0.865476224226354	-5.23044412202545
a_HQ_transcript/139324	1.93517607140781	0.396910807079478	1.318970469293	0.19637293423688	0.865476224226354	-5.06330212298907
a_HQ_transcript/17274	3.12820255213359	1.78941911161573	1.31881276817016	0.196425073548303	0.865535141528979	-5.09708422274823
a_HQ_transcript/2315	-0.333798953490602	3.53914510261284	-1.31875935529912	0.196442735373628	0.865535141528979	-5.40966948476101
a_HQ_transcript/79573	-5.73715427075341	2.5747039551606	-1.31870580050546	0.196460445350142	0.865535141528979	-5.00714864059287
a_HQ_transcript/38182	1.45982789842509	0.718614336961342	1.3186673783389	0.196473151888623	0.865535141528979	-4.986387747813
a_HQ_transcript/107952	1.51739214389153	1.2595376969919	1.31858113621844	0.196501675188003	0.865535141528979	-5.04563675756842

a_HQ_transcript/107422	1.69543786402962	1.36514405247102	1.31857345071252	0.19650421720887	0.865535141528979	-4.98036679156839
a_HQ_transcript/96818	-1.89744636239539	0.740489290051435	-1.3185225989408	0.196521037330743	0.865535141528979	-5.08297082585049
a_HQ_transcript/118823	1.25992441613349	0.610227097132458	1.31851563398464	0.196523341198965	0.865535141528979	-4.9997292560461
a_HQ_transcript/51503	1.35684524541165	1.97228061570402	1.31845931051134	0.196541972638272	0.865535141528979	-5.18652422745242
a_HQ_transcript/62603	2.19455152754295	1.35732311035176	1.31845725907864	0.196542651264447	0.865535141528979	-5.07676703777221
a_HQ_transcript/118524	2.41795550177297	1.51837948555558	1.31843476042414	0.196550094071403	0.865535141528979	-4.97037163590375
a_HQ_transcript/143306	1.56848551437341	-0.496750990896718	1.31836839784961	0.196572048812883	0.865552417177345	-5.06462397306447
a_HQ_transcript/15083	1.72701071293007	1.60382378006398	1.31833288585293	0.196583798024623	0.865552417177345	-5.2254484179232
a_HQ_transcript/36515	1.6167538189371	1.35580705607823	1.31820812554901	0.196625079475991	0.865571821317775	-4.91679633738014
a_HQ_transcript/400	2.1561547647367	1.22927069147343	1.31809552527919	0.196662343045005	0.865571821317775	-5.11313752931057
a_HQ_transcript/100157	-1.17855385492353	2.7358732816287	-1.31808249673781	0.19666665501767	0.865571821317775	-5.25478592934721
a_HQ_transcript/98143	1.5124337459817	2.99480861831176	1.31808149145795	0.1966669877317	0.865571821317775	-5.13664329072824
a_HQ_transcript/127523	-1.43480425017357	1.50615090686512	-1.31803278558258	0.196683108265136	0.865571821317775	-4.96907211077574
a_HQ_transcript/7810	0.880657152635453	-0.663050904611035	1.31799043708711	0.196697125473908	0.865571821317775	-5.06906246077026
a_HQ_transcript/114630	-1.71439256614517	2.41083553244561	-1.31798610907581	0.196698558074129	0.865571821317775	-5.13739061022872
a_HQ_transcript/37757	1.09455978547138	0.644841750283314	1.31795960461411	0.196707331399804	0.865571821317775	-5.02242213766856
a_HQ_transcript/3337	1.22836527506824	1.62187728562624	1.3178151501892	0.196755152987208	0.865716715830778	-4.98368562953923
a_HQ_transcript/5525	-1.04768455523722	2.20565270152424	-1.31769418419475	0.196795205591633	0.865827407707422	-5.15192832033158
a_HQ_transcript/53506	-1.3111005066572	-0.445988865000586	-1.31756215653108	0.196838927915459	0.865954227039266	-5.01159691513885
a_HQ_transcript/65645	-1.37927913151995	0.114586032799107	-1.31734279501305	0.196911588204086	0.866167427994558	-5.06373129215703
a_HQ_transcript/103825	5.20822577409281	3.54795283736299	1.31732587845571	0.196917192419283	0.866167427994558	-5.10918413605038
a_HQ_transcript/60257	-1.80924635751505	0.424044624911647	-1.31717720341623	0.196966451605557	0.86631854562105	-5.09086111769353
a_HQ_transcript/58615	-1.35554737395973	0.737695052673002	-1.31698847111923	0.197028996207835	0.866355162244658	-5.02211135927517
a_HQ_transcript/63782	-1.41423831448754	0.243531624605538	-1.31696761839946	0.197035907591026	0.866355162244658	-4.99970913187242
a_HQ_transcript/113037	0.793958570653929	4.23849664206024	1.31686324796958	0.197070502706279	0.866355162244658	-5.34135832254819
a_HQ_transcript/136028	-2.00623661933148	1.05950073754059	-1.31684594560337	0.197076238279894	0.866355162244658	-5.1440100998771
a_HQ_transcript/50377	-2.51641146058158	0.334271239344256	-1.31683017249463	0.197081467030301	0.866355162244658	-4.98112853719705
a_HQ_transcript/62306	-1.14517386227793	0.310133848174582	-1.31674670927786	0.197109136669878	0.866355162244658	-5.05458519808004
a_HQ_transcript/30491	0.650170297416692	3.87864727182512	1.31672559347916	0.197116137429392	0.866355162244658	-5.42538605136504
a_HQ_transcript/122588	-2.26864660907539	0.903674830945406	-1.31667057519915	0.197134379156664	0.866355162244658	-5.13846026834986
a_HQ_transcript/124077	0.687766077115161	5.05842750485475	1.31662030071857	0.197151049172118	0.866355162244658	-5.62191518549507
a_HQ_transcript/91341	-1.31289094259302	1.60783515056225	-1.31656338095682	0.197169923933741	0.866355162244658	-5.08004294553225
a_HQ_transcript/144724	1.28493046714367	1.0745057789303	1.31653233303979	0.197180220098482	0.866355162244658	-5.04053941270081
a_HQ_transcript/97166	-3.49547077362615	1.8734770717251	-1.31638891798619	0.197227784995699	0.866355162244658	-5.25064350204186
a_HQ_transcript/82511	-1.30647969651453	-0.225964250554154	-1.31636639438717	0.197235255949168	0.866355162244658	-5.0851055459479
a_HQ_transcript/77170	-1.9338792089534	-0.38083476546602	-1.31633374194927	0.197246086967516	0.866355162244658	-5.06901593806991
a_HQ_transcript/88604	2.56331951970362	2.0617954594189	1.31632630388803	0.197248554282772	0.866355162244658	-5.19039962219205
a_HQ_transcript/128647	0.675988640637373	4.08758364840788	1.31626086492702	0.197270262375878	0.866355162244658	-5.45441373711157
a_HQ_transcript/21642	-0.575693812618419	3.13514213064704	-1.31623078637133	0.197280240961247	0.866355162244658	-5.3662139281147
a_HQ_transcript/116782	3.92730348336396	2.39220284203198	1.31617018970681	0.197300345128166	0.866355162244658	-5.26632830695793
a_HQ_transcript/94710	-1.31036445721057	2.05340782619108	-1.31610377451659	0.197322381510989	0.866355162244658	-5.04942720364333
a_HQ_transcript/20103	-1.27355620390749	0.0225761129222815	-1.31606751371229	0.197334413544653	0.866355162244658	-5.0849603594694
a_HQ_transcript/150026	-3.82114918566932	3.24675568212054	-1.31600219924728	0.19735608756058	0.866355162244658	-5.35404285271988
a_HQ_transcript/55138	-1.64287670185397	1.26862178928022	-1.31596422375499	0.197368690223321	0.866355162244658	-5.02121255030656
a_HQ_transcript/148467	-3.65780401035013	2.00214788316395	-1.31594223029911	0.19737598932056	0.866355162244658	-5.17410441920386
a_HQ_transcript/38966	1.10469495145056	2.6906402429259	1.31593479898028	0.19737845564267	0.866355162244658	-5.34570295581834
a_HQ_transcript/98581	-1.36647888605805	-0.438574783759589	-1.31585068360635	0.197406373679991	0.866355162244658	-5.06569093032319
a_HQ_transcript/120494	1.11342366396497	1.66362468972816	1.31580992113572	0.197419903902037	0.866355162244658	-4.92036319394143
a_HQ_transcript/23670	0.80467781452374	1.40873890140949	1.31579787378643	0.197423902895505	0.866355162244658	-5.03080754143157
a_HQ_transcript/57004	1.10849735278293	0.742478898862105	1.31579581938044	0.1974245848406	0.866355162244658	-5.06722339909002

a_HQ_transcript/116477	1.30850475612351	3.80621151567837	1.31578736970836	0.197427389666622	0.866355162244658	-5.38184593139088
a_HQ_transcript/68289	1.1168334485066	-0.635050477344798	1.31566463408759	0.197468134578205	0.866355162244658	-5.08556999674906
a_HQ_transcript/82911	-0.901445967291606	1.30299383700808	-1.31566083113304	0.197469397159272	0.866355162244658	-5.13607263521372
a_HQ_transcript/5801	-0.458007238510108	2.95174684668323	-1.31566079134357	0.197469410369409	0.866355162244658	-5.19161418102551
a_HQ_transcript/53745	-1.42412082853611	-0.555936035666805	-1.31561760029878	0.197483750232657	0.866355162244658	-5.05995495792284
a_HQ_transcript/100820	1.8734024424387	2.77845152683919	1.3155866869592	0.197494014263394	0.866355162244658	-4.99814646158152
a_HQ_transcript/151786	0.538466909124851	3.27021571131966	1.3155548273033	0.197504592923203	0.866355162244658	-5.35307953903616
a_HQ_transcript/82206	1.48210153290289	-0.173134684342686	1.31553453696287	0.197511330341079	0.866355162244658	-5.07190447150191
a_HQ_transcript/110825	1.25697335655025	2.48268868018467	1.3154383146201	0.197543283415587	0.866383468202564	-5.07521863137078
a_HQ_transcript/147469	0.931351452633658	1.98302092689902	1.31542533771875	0.197547593028434	0.866383468202564	-4.97648731696721
a_HQ_transcript/130192	-1.95599412510702	1.16756280859889	-1.31523210810924	0.197611772860511	0.8664822834276	-5.16488457941521
a_HQ_transcript/140136	1.42703157850012	1.8920704908044	1.31521973066082	0.197615884485517	0.8664822834276	-5.04074016007178
a_HQ_transcript/45823	-1.32765486146159	0.410530438550853	-1.31521812215611	0.197616418814362	0.8664822834276	-4.99173856726119
a_HQ_transcript/74059	1.19323722798651	-0.378596598852712	1.31513002714021	0.197645684769019	0.8664822834276	-5.08576661312677
a_HQ_transcript/44333	-1.87879278501608	0.739470248632821	-1.31506115540526	0.197668566888751	0.8664822834276	-4.97166099308493
a_HQ_transcript/1452	1.11154309890893	3.93534997101132	1.31496482484283	0.197700575401748	0.8664822834276	-5.47741847279145
a_HQ_transcript/49068	1.02341147668381	-0.591929621864264	1.31484697346835	0.197739740201741	0.8664822834276	-5.08654394549859
a_HQ_transcript/141611	-2.03297177308701	1.69804633169409	-1.31479739954844	0.197756216563144	0.8664822834276	-5.21875470435512
a_HQ_transcript/67558	1.42368361646895	-0.326018579957937	1.31479666937796	0.197756459250066	0.8664822834276	-5.14077398839921
a_HQ_transcript/122079	2.12972440930619	1.18861631295236	1.3147604902806	0.19776848439023	0.8664822834276	-5.05532407881333
a_HQ_transcript/101078	-3.76327073526335	3.56908276126262	-1.31468592969742	0.197793268462131	0.8664822834276	-5.28932184881384
a_HQ_transcript/79805	-1.55848619619718	0.431260591843903	-1.31467929413267	0.197795474251015	0.8664822834276	-5.16612177641109
a_HQ_transcript/36075	-0.94470073497686	2.63866011203063	-1.3146662136779	0.197799822500005	0.8664822834276	-5.14218845999434
a_HQ_transcript/125502	1.6271118348072	0.00867652400238915	1.31461604271665	0.19781650117925	0.8664822834276	-5.19199822855576
a_HQ_transcript/146997	0.472778817717972	2.80691156998977	1.31451622442448	0.197849687665916	0.8664822834276	-5.24872592672787
a_HQ_transcript/57927	-2.75993192531429	2.19261944731036	-1.31446177526011	0.197867792121191	0.8664822834276	-5.19497478652029
a_HQ_transcript/12546	0.624660781975159	2.2777691206137	1.31445549071974	0.197869881824986	0.8664822834276	-5.10125095621983
a_HQ_transcript/75010	-1.65498601568327	0.71525916837742	-1.31444859595647	0.197872174456469	0.8664822834276	-5.00244895015391
a_HQ_transcript/47076	0.945552532027728	3.46054248309978	1.31444016234963	0.197874978808481	0.8664822834276	-5.32616810557979
a_HQ_transcript/30539	1.50899423176804	-0.383473990601276	1.31443300658144	0.197877358276071	0.8664822834276	-5.08689577482139
a_HQ_transcript/138044	-2.93657755720325	3.44559784517235	-1.31441555996435	0.197883159793927	0.8664822834276	-5.38789429516108
a_HQ_transcript/83545	-1.33239783452778	0.103721226204232	-1.3143053665074	0.197919805396729	0.866520213151229	-5.04870392868018
a_HQ_transcript/50952	2.21862428328743	1.72552080240274	1.31427056419119	0.197931380229238	0.866520213151229	-4.95975381779263
a_HQ_transcript/102067	-0.885825199944769	1.38001370762465	-1.31425504070342	0.197936543322014	0.866520213151229	-4.9236010710674
a_HQ_transcript/24789	-0.414885984184128	2.7301808056248	-1.3141414063818	0.197974341096266	0.866620415637	-5.10167950364531
a_HQ_transcript/60934	-1.17678744226526	2.2782485869415	-1.31389082616905	0.198057710214152	0.866920073744597	-5.13673895556041
a_HQ_transcript/152433	1.65167241478095	3.13639070762751	1.31384068248389	0.198074396461057	0.866927830546519	-5.26395741697568
a_HQ_transcript/145110	1.4096639698867	0.753363834766854	1.31362409001082	0.198146484010896	0.867178047175529	-5.11838815907
a_HQ_transcript/12943	1.36163826105688	1.37011314854549	1.31341383159121	0.198216482631959	0.867393609889264	-5.03422715740592
a_HQ_transcript/151165	1.87354272074142	0.233759487985062	1.31330686642516	0.198252100426261	0.867393609889264	-5.11345005986601
a_HQ_transcript/69122	-1.12050282095851	2.94520969431091	-1.31330084268091	0.198254106388336	0.867393609889264	-5.29777816122458
a_HQ_transcript/24269	2.56678819294336	0.744448156486119	1.31329501292026	0.198256047766888	0.867393609889264	-5.09648714669213
a_HQ_transcript/149631	1.61753259223261	-0.0368690553677105	1.31322249907433	0.198280196942633	0.867393609889264	-5.04772126623059
a_HQ_transcript/41165	-1.86932884723868	0.647047311438617	-1.31320726095925	0.198285271955143	0.867393609889264	-5.08628461321869
a_HQ_transcript/93942	-2.98788706366362	1.7509062753336	-1.31305540683374	0.198335851987649	0.867479081219694	-5.01452507642062
a_HQ_transcript/100095	0.635379137294762	1.72799766690026	1.31293574261032	0.198375717065744	0.867479081219694	-5.03146121086905
a_HQ_transcript/9052	0.874653588125984	-0.697652755298317	1.31282031832959	0.198414175453985	0.867479081219694	-5.09779156825642
a_HQ_transcript/74235	0.312301783756462	4.04352929420928	1.31280835651374	0.198418161355502	0.867479081219694	-5.4585300127422
a_HQ_transcript/9852	-1.0140124222644	2.24912841409349	-1.31271502779485	0.198449262340118	0.867479081219694	-5.20437879050888
a_HQ_transcript/23131	1.8379622210586	1.34576731646455	1.3126566394041	0.198468721660826	0.867479081219694	-5.05181216802595

a_HQ_transcript/81083	1.60035236804439	0.465921715128016	1.31261041467	0.198484128188988	0.867479081219694	-5.05961199855268
a_HQ_transcript/141269	1.12942384029964	0.966288908217538	1.31256223845649	0.198500186111709	0.867479081219694	-5.09831349603225
a_HQ_transcript/134659	0.649029950788472	3.48320394349622	1.3125376195289	0.198508392388147	0.867479081219694	-5.36505737427217
a_HQ_transcript/1292	1.5772915617514	1.84636489210023	1.31245565145524	0.198535716841293	0.867479081219694	-5.1815609961562
a_HQ_transcript/117564	1.74134257136248	1.84123298519322	1.3124295187716	0.198544428902557	0.867479081219694	-5.08653856779596
a_HQ_transcript/109622	1.02569011699198	2.63287125221456	1.31242523494115	0.198545857065205	0.867479081219694	-5.06416464075674
a_HQ_transcript/40873	1.49342133777237	0.555999604943671	1.31242105497942	0.198547250607203	0.867479081219694	-5.05636040306837
a_HQ_transcript/25461	2.25600546328503	-0.106170903166736	1.31241215396534	0.198550218108345	0.867479081219694	-5.14279561412724
a_HQ_transcript/99782	-1.44759238856172	1.92972341591556	-1.31230271374274	0.198586707060281	0.867479081219694	-5.25920163319376
a_HQ_transcript/147819	1.28848342414844	1.81058142569805	1.31227246770421	0.198596792431561	0.867479081219694	-5.08024762802939
a_HQ_transcript/145205	-1.31040571284789	0.0526743551856996	-1.31217753591803	0.198628449446507	0.867479081219694	-5.07487400201452
a_HQ_transcript/104640	0.887010456100723	-0.367386107214806	1.31217272087102	0.198630055228798	0.867479081219694	-5.06535459551977
a_HQ_transcript/46820	2.57424743421659	0.0246219891461049	1.31213754351394	0.19864178691655	0.867479081219694	-5.03907633757503
a_HQ_transcript/131144	0.345458329125664	4.33224353207731	1.3120797239729	0.198661070949642	0.867479081219694	-5.47598188367008
a_HQ_transcript/27107	1.11691527025396	1.00650872843468	1.31204209022636	0.198673623365179	0.867479081219694	-4.96592584746342
a_HQ_transcript/135569	-1.06661893523654	3.09380731167604	-1.31203245606396	0.19867683685552	0.867479081219694	-5.24571147332433
a_HQ_transcript/24601	3.67317374899474	1.84874718545954	1.31202643151564	0.198678846373494	0.867479081219694	-5.16970103482642
a_HQ_transcript/66659	0.381344737085795	2.9120167402684	1.31201783758461	0.198681712948729	0.867479081219694	-5.29587359956571
a_HQ_transcript/31529	2.01990224620749	-0.0597793620006147	1.3118764485872	0.198728878935701	0.867479081219694	-5.08474988762485
a_HQ_transcript/89925	-1.22440067418885	0.706233151347492	-1.31180842928563	0.198751572561064	0.867479081219694	-4.98922670652595
a_HQ_transcript/140283	2.93735783690052	1.22365256371888	1.31179215038127	0.198757004068683	0.867479081219694	-5.17017671364525
a_HQ_transcript/53663	1.28746981278558	3.00724747644938	1.31177055556683	0.198764209421635	0.867479081219694	-5.27933709912724
a_HQ_transcript/55124	1.05176197854975	-0.613861335635316	1.31175570687293	0.198769163971463	0.867479081219694	-5.09218904757754
a_HQ_transcript/112533	0.967972416528302	2.07707029054853	1.31173412279429	0.198776366079305	0.867479081219694	-5.06468275142309
a_HQ_transcript/152966	0.299984089062508	-0.784339347933669	1.31171423824557	0.198783001269991	0.867479081219694	-5.20095757230199
a_HQ_transcript/17678	1.56291343403413	0.9443900909192	1.31171370404942	0.198783179525974	0.867479081219694	-4.97754306211841
a_HQ_transcript/61938	-1.45467850587338	1.45494278839841	-1.31162913595879	0.198811400605272	0.867479081219694	-5.00780769643417
a_HQ_transcript/105878	-2.14226371168927	1.27191510575851	-1.31158327927537	0.198826704645629	0.867479081219694	-4.97135710351283
a_HQ_transcript/99659	-2.01500234673698	3.82057166440933	-1.31157597785732	0.198829141477119	0.867479081219694	-5.53046056910611
a_HQ_transcript/110534	1.33071100011978	1.7102482827245	1.31151136003919	0.19885070852118	0.867479081219694	-5.04674968870874
a_HQ_transcript/135461	1.52109657102303	-0.213524423080118	1.31149255930037	0.198856983850338	0.867479081219694	-5.0838628466943
a_HQ_transcript/11174	0.466897838362564	5.03341848124222	1.31140121253995	0.198887475818253	0.867495739248789	-5.59362296526893
a_HQ_transcript/85696	-1.0713011957701	1.35741683425138	-1.31139170334449	0.198890650236867	0.867495739248789	-4.94763385435742
a_HQ_transcript/96247	-3.34373244993702	0.707208551170726	-1.31130729794388	0.198918828669988	0.867500302898879	-5.16000323293179
a_HQ_transcript/143107	2.63455283530583	0.236414220748932	1.31127692551029	0.198928969142526	0.867500302898879	-4.98815082841746
a_HQ_transcript/12684	-1.76925679871016	1.67139552781211	-1.31123195660679	0.198943983678088	0.867500302898879	-4.97799740098181
a_HQ_transcript/141528	1.83652422806843	-0.0531910747525072	1.31120976823215	0.198951392408907	0.867500302898879	-5.11949244688674
a_HQ_transcript/77822	1.31436188632267	-0.017571516146904	1.31110382187002	0.198986770964075	0.867549037446713	-5.06031716039725
a_HQ_transcript/114849	-0.672140777723069	3.29906317151843	-1.31108691010837	0.198992418735842	0.867549037446713	-5.28084463060961
a_HQ_transcript/25366	-1.5524379751502	3.23619555366269	-1.31103534915909	0.199009638542821	0.86755904223917	-5.25758095040057
a_HQ_transcript/101192	1.69147274449486	0.205231186034477	1.3108717579166	0.199064280644162	0.867663148868348	-5.13979953895687
a_HQ_transcript/54227	1.1695010387269	1.08075734472101	1.31084885664568	0.199071930951818	0.867663148868348	-4.89455090197145
a_HQ_transcript/94561	1.35202802415094	1.95553615655274	1.31074151029427	0.199107793645108	0.867663148868348	-5.12464805517547
a_HQ_transcript/110576	1.2498994400213	-0.208708427378784	1.3107232259055	0.199113902658406	0.867663148868348	-5.08354060030801
a_HQ_transcript/121844	-0.695662888654349	1.54513537063905	-1.31069509568349	0.199123301551661	0.867663148868348	-5.03244785328453
a_HQ_transcript/76572	1.65375855473171	-0.182641217055418	1.31064988925014	0.199138406673663	0.867663148868348	-5.07748978828522
a_HQ_transcript/38388	-1.26589882831597	1.14408400657839	-1.31061669722066	0.19914949790201	0.867663148868348	-5.05934335715223
a_HQ_transcript/134058	1.74563715321737	0.475888271749724	1.31060641530093	0.199152933735919	0.867663148868348	-5.13794885944475
a_HQ_transcript/129464	-2.30741945592534	0.203537640909244	-1.31055917811257	0.199168719224184	0.867666889834623	-5.02565492794632
a_HQ_transcript/51845	1.06040634996753	2.6684175873788	1.31034315097287	0.199240922291502	0.867844720086091	-5.25218901667222

a_HQ_transcript/41791	-1.3894825274249	1.37523125649819	-1.31033081374984	0.199245046383735	0.867844720086091	-4.98160087712833
a_HQ_transcript/102751	-1.22330293403303	-0.286336998041854	-1.31030150892615	0.199254842674514	0.867844720086091	-5.04855484172381
a_HQ_transcript/128213	-0.98459440072024	-0.407283285529922	-1.31023938070301	0.199275612696645	0.867844720086091	-5.01658269446383
a_HQ_transcript/79572	-1.39927445857162	-0.234195849195849	-1.31021372906883	0.199284188750846	0.867844720086091	-5.07170735088599
a_HQ_transcript/144960	1.46175372599929	-0.297823029384298	1.31003235641883	0.199344834720799	0.867895452871927	-5.0909283846748
a_HQ_transcript/4355	-0.963696029271416	1.51433328635401	-1.31003152294504	0.199345113443826	0.867895452871927	-5.08022326990496
a_HQ_transcript/30868	2.38644366220375	-0.113956027035064	1.31001609586208	0.19935027248796	0.867895452871927	-5.09317805914198
a_HQ_transcript/17856	1.06093193448947	2.74835255163253	1.30997609479408	0.199363649910757	0.867895452871927	-5.20901492173862
a_HQ_transcript/131089	1.44853666819646	-0.193536218883334	1.30988354759764	0.199394602789798	0.867895452871927	-5.12620999102659
a_HQ_transcript/127854	-1.53283825778595	0.475592114703215	-1.30984118331944	0.199408772963313	0.867895452871927	-5.02555008017374
a_HQ_transcript/64582	2.57813388144879	0.893203330879223	1.30982885318532	0.19941289734063	0.867895452871927	-5.0431123066361
a_HQ_transcript/29756	-0.656220571534241	-0.609270745421011	-1.30982171611012	0.199415284691671	0.867895452871927	-5.11163811525902
a_HQ_transcript/18639	-1.15599410897463	-0.327667958408785	-1.30974620080135	0.199440545894775	0.867940409655722	-5.03701502736976
a_HQ_transcript/26474	1.27590274100847	1.9769622997703	1.30965291814713	0.199471753966046	0.867948187555951	-5.14252057093215
a_HQ_transcript/39460	1.97410842141155	2.1887310601244	1.30961015468996	0.199486061893376	0.867948187555951	-5.27365477058752
a_HQ_transcript/122106	1.20448752588117	2.82348901114948	1.30960696792859	0.199487128161084	0.867948187555951	-5.21745094963741
a_HQ_transcript/107593	1.79843482065359	0.259361959830404	1.3095338695147	0.199511587567176	0.867989638657646	-5.03616752391003
a_HQ_transcript/62202	0.974436560548881	1.50865460156856	1.30945277397868	0.199538725567022	0.868041427986571	-4.96712286618857
a_HQ_transcript/147387	3.13982076190548	1.57864202705632	1.30940905145985	0.19955335814383	0.868041427986571	-5.15014489668883
a_HQ_transcript/147314	1.3269183787624	4.19595600026878	1.30916871881858	0.199633804704356	0.86832638430521	-5.41999796150419
a_HQ_transcript/9434	-0.452535738413967	3.53032970435026	-1.30908271870951	0.199662597548395	0.868327202360348	-5.39081688791252
a_HQ_transcript/139649	1.52867556613374	-0.311917848584634	1.30902107380158	0.199683238222083	0.868327202360348	-5.08239019940811
a_HQ_transcript/79147	2.11391571852602	1.50333438825932	1.30901448676758	0.19968544386679	0.868327202360348	-5.03175622739789
a_HQ_transcript/45814	1.26134516003037	-0.0832598590940802	1.30897279620904	0.199699404232722	0.868327202360348	-5.0739797583853
a_HQ_transcript/126344	1.21841597582791	-0.498575737680939	1.30886224514008	0.199736426618996	0.868327202360348	-5.11291568489572
a_HQ_transcript/754	-1.32520259560463	1.07342297739953	-1.30884124604263	0.199743459587983	0.868327202360348	-4.97760166918128
a_HQ_transcript/79916	2.41028442365403	2.38033352449936	1.30879114219039	0.199760241018504	0.868327202360348	-5.19447579366478
a_HQ_transcript/134462	3.05456120669792	1.29447888779982	1.30877491697201	0.199765675609577	0.868327202360348	-5.15462985081319
a_HQ_transcript/134854	1.2310070084872	-0.569897793762357	1.30862096251392	0.199817247844004	0.868327202360348	-5.09759327058563
a_HQ_transcript/26754	-1.84381992009711	2.55863770666311	-1.30852086974931	0.199850782747082	0.868327202360348	-5.28114626978747
a_HQ_transcript/138006	1.62726308144151	0.722961629420835	1.30851768840801	0.199851848688576	0.868327202360348	-5.08869048726811
a_HQ_transcript/96020	0.774685352468525	-0.534178165346203	1.30850772507364	0.199855187035518	0.868327202360348	-5.02813780066147
a_HQ_transcript/73662	1.82767886370613	1.04886862873141	1.30848146257621	0.199863986836814	0.868327202360348	-4.94147892823355
a_HQ_transcript/51561	1.73875049235115	1.07587750804526	1.30845452591377	0.199873012838881	0.868327202360348	-5.11204132217343
a_HQ_transcript/138185	1.74996292604985	0.195315722903003	1.30845395012161	0.199873205780114	0.868327202360348	-5.11711127860785
a_HQ_transcript/67989	1.96043307284359	2.60744893681659	1.30824082014199	0.199944632926603	0.868327202360348	-5.21787038676668
a_HQ_transcript/137860	-1.61598417175395	-0.260291193304328	-1.30823392034424	0.199946945610782	0.868327202360348	-5.05193377809013
a_HQ_transcript/15484	-0.524152391708895	2.46808300302538	-1.30820855267384	0.199955448558933	0.868327202360348	-5.15338596281433
a_HQ_transcript/141364	1.24706441890125	-0.378656128134101	1.30818475494	0.19996342553381	0.868327202360348	-5.08706136217658
a_HQ_transcript/148761	0.384100458815138	5.34834300861372	1.3081767561552	0.199966106772512	0.868327202360348	-5.57626506420018
a_HQ_transcript/23128	-0.460458817241605	2.79247291721504	-1.30816352969127	0.199970540419617	0.868327202360348	-5.23545872832497
a_HQ_transcript/136386	-2.29890590139722	2.53052058280602	-1.30807103936707	0.200001546229512	0.868327202360348	-5.32964972465447
a_HQ_transcript/81090	-1.67577640536598	1.77056857766901	-1.30805429723363	0.200007159137995	0.868327202360348	-5.13171418871408
a_HQ_transcript/148118	-2.44091031256656	1.96234765666265	-1.30798188403215	0.200031437518254	0.868327202360348	-5.27025569613164
a_HQ_transcript/151367	-1.9831298458126	7.082348586896	-1.3079539712622	0.200040796590538	0.868327202360348	-5.76270490860758
a_HQ_transcript/150825	2.63902781877721	2.63105870414508	1.30794687673142	0.200043175419842	0.868327202360348	-5.12585088995809
a_HQ_transcript/120092	-1.1235389151195	1.04113672637803	-1.30791791210555	0.200052887618438	0.868327202360348	-4.93748571548219
a_HQ_transcript/116469	2.27789313735462	1.54980798598113	1.3078831880574	0.200064531497724	0.868327202360348	-5.14010139745226
a_HQ_transcript/26899	1.32466086843957	0.942575384499703	1.30787160507553	0.200068415688926	0.868327202360348	-4.99123047480508
a_HQ_transcript/150125	1.2805064925668	2.20750238288501	1.30779860440523	0.200092896770186	0.868327202360348	-5.07659478062215

a_HQ_transcript/857	1.08196337530402	-0.134770089920051	1.30768569697879	0.20013076525751	0.868327202360348	-5.14643390988061
a_HQ_transcript/80530	1.51558980602836	0.504043357468395	1.30768413708412	0.200131288475361	0.868327202360348	-4.92875577602168
a_HQ_transcript/16772	0.656734483156995	2.16592571027291	1.30758240879499	0.200165412303297	0.868327202360348	-5.03885069897929
a_HQ_transcript/88258	2.08230879727461	3.10956305487943	1.30758159119052	0.200165686579274	0.868327202360348	-5.32925079399045
a_HQ_transcript/150469	-3.5402664546481	1.9615333318556	-1.30757696350094	0.200167239002927	0.868327202360348	-5.22669779444421
a_HQ_transcript/15053	0.487792968823726	1.89694263299602	1.30752073893713	0.20018610105966	0.868327202360348	-5.0758123621341
a_HQ_transcript/137253	-2.84951306400001	2.14135010355977	-1.30751893616696	0.200186705870338	0.868327202360348	-5.18230881614751
a_HQ_transcript/131621	-0.532199265681077	3.02337109368074	-1.30730540713477	0.200258352520465	0.868413385922881	-5.32765657369186
a_HQ_transcript/145044	1.91211077026103	0.271338419190558	1.30730493073672	0.200258512391	0.868413385922881	-4.98950387087863
a_HQ_transcript/10895	-0.549507449147555	3.14427327581207	-1.30729423588464	0.200262101414835	0.868413385922881	-5.37231422228762
a_HQ_transcript/151939	1.02246955868956	-0.496974705507076	1.30728162474037	0.200266333579277	0.868413385922881	-5.02433240855332
a_HQ_transcript/104075	-1.40899868603258	-0.322394563014124	-1.30723677114383	0.200281386517782	0.868413877033092	-5.05953841128627
a_HQ_transcript/142844	0.377043153153384	7.40346684706942	1.30712658092922	0.200318370199699	0.868509452000308	-5.81438100438
a_HQ_transcript/143967	-3.06812280084529	5.03525508880552	-1.30703920045696	0.200347701844944	0.868543903996631	-5.77075274984092
a_HQ_transcript/775	-0.522522561076209	1.59819624016436	-1.30698715960024	0.200365172340621	0.868543903996631	-4.99203554703039
a_HQ_transcript/75606	1.76398017716587	1.29685715930108	1.30696937752702	0.200371142179239	0.868543903996631	-5.0190366841988
a_HQ_transcript/319	1.44604893950148	2.72471079142369	1.30685728715058	0.200408776546728	0.868618378050773	-5.05207299834374
a_HQ_transcript/11740	1.20301315856472	1.31479924899198	1.30682919428294	0.200418209579152	0.868618378050773	-4.95187934887307
a_HQ_transcript/53055	-2.24789336730509	2.06386513537291	-1.30671106277667	0.200457879527383	0.868725536506949	-5.12887044315532
a_HQ_transcript/133202	1.10758952904279	-0.113814546951903	1.30661011761822	0.200491782850337	0.86880768998989	-5.10138328836496
a_HQ_transcript/125593	1.92130605430938	4.53066237810233	1.30651499054637	0.200523736125925	0.868877637620351	-5.54034964905835
a_HQ_transcript/41624	2.2226036892997	2.32909214897755	1.30635004612162	0.200579150337578	0.868877637620351	-5.2308146827977
a_HQ_transcript/67826	-1.03596017232055	2.12207299553338	-1.30634124963563	0.200582105905909	0.868877637620351	-5.05184546431571
a_HQ_transcript/27073	-2.93091421159356	2.55335408188323	-1.30628833271859	0.200599886385405	0.868877637620351	-5.07264312042412
a_HQ_transcript/75720	-2.84169091046772	3.0750670978564	-1.30626014622572	0.200609357748978	0.868877637620351	-5.43294541340561
a_HQ_transcript/113589	-0.506606090686965	3.28904997596771	-1.30619975125607	0.200629653112856	0.868877637620351	-5.31992321476497
a_HQ_transcript/52927	2.10675774227126	0.0159042721188725	1.30616397542273	0.200641676104622	0.868877637620351	-5.11153873758997
a_HQ_transcript/116351	0.752341428254552	3.02303910953435	1.30616358109143	0.200641808628484	0.868877637620351	-5.2140066938656
a_HQ_transcript/120235	1.99075831061118	1.68477152626506	1.30616166261939	0.200642453374931	0.868877637620351	-5.12628537484262
a_HQ_transcript/90493	1.31867715109453	0.180827455863445	1.306069381823	0.200673468317571	0.868943328004378	-4.97566133377095
a_HQ_transcript/28536	1.00605849427659	1.24164461633627	1.30597919838161	0.200703781891068	0.868943328004378	-4.8994025100716
a_HQ_transcript/114348	-0.667031524747353	3.05087335299668	-1.30592917301431	0.200720598544028	0.868943328004378	-5.29845869565301
a_HQ_transcript/2146	1.19513422321969	1.31105986585104	1.30582264304919	0.200756413510001	0.868943328004378	-4.96577517579515
a_HQ_transcript/36973	-2.33975315541985	0.852208087883122	-1.30579007337059	0.200767364285176	0.868943328004378	-5.15000339188084
a_HQ_transcript/114203	-7.58799953473171	3.99362619219236	-1.30577564894229	0.200772214299824	0.868943328004378	-5.22954192283777
a_HQ_transcript/42898	1.52699201210252	-0.327918128736029	1.30576248322655	0.200776641167876	0.868943328004378	-5.09030180854237
a_HQ_transcript/119143	1.40641300679247	0.0745587347787094	1.30576078252734	0.200777213020004	0.868943328004378	-5.14868780409875
a_HQ_transcript/89228	1.26596605259377	1.30307029456199	1.30563167546246	0.200820628296004	0.869031154792454	-5.1061661751688
a_HQ_transcript/136112	-1.18242238536358	1.68669134312798	-1.30561151967867	0.200827406797533	0.869031154792454	-5.00759148592609
a_HQ_transcript/54768	-1.0448903350557	3.01318517313236	-1.30551873424237	0.200858613306213	0.869101494287892	-5.33413965385172
a_HQ_transcript/93584	-0.63591902883713	2.6389516773219	-1.30536619472773	0.200909924940518	0.869237403898227	-5.26670586910131
a_HQ_transcript/115648	0.38308808355341	3.87766361398804	1.30527621642325	0.200940196766972	0.869237403898227	-5.42056348666664
a_HQ_transcript/104439	-1.74212198713125	0.816654968157724	-1.30520641738629	0.200963681985621	0.869237403898227	-5.16819258827401
a_HQ_transcript/148956	1.03588836777257	-0.697768816311487	1.30516544970375	0.200977467320622	0.869237403898227	-5.11419275005987
a_HQ_transcript/108502	1.69887588404314	1.33856898143222	1.30512349982205	0.200991583906848	0.869237403898227	-4.91496147336839
a_HQ_transcript/61372	2.70577312638677	3.28447417887481	1.30506050410791	0.201012784064002	0.869237403898227	-5.1400976981918
a_HQ_transcript/103163	1.55130380237542	-0.474184684650079	1.30505042087678	0.201016177565806	0.869237403898227	-5.09497238136677
a_HQ_transcript/115193	2.35805443480132	-0.141401596990441	1.30503379310807	0.201021773721019	0.869237403898227	-5.08010894981618
a_HQ_transcript/130571	3.31603822291716	2.92235152762685	1.30495864085587	0.201047068055209	0.869237403898227	-5.0696149743648
a_HQ_transcript/76948	1.80423287925724	1.16689199157399	1.3049372838463	0.201054256724112	0.869237403898227	-5.10037824360646

a_HQ_transcript/92408	1.33215529554565	-0.130575092368744	1.30493450602829	0.201055191739005	0.869237403898227	-5.0999896607126
a_HQ_transcript/105929	2.72406162392346	1.98027897167732	1.30487146235974	0.201076413161114	0.869237403898227	-5.20768271217605
a_HQ_transcript/24885	-0.48789863063328	2.96032065784684	-1.30484766686578	0.2010844235174	0.869237403898227	-5.3265301215371
a_HQ_transcript/115948	-1.32917466465284	-0.340605695356762	-1.30474806591164	0.201117955161983	0.869253574940758	-5.088841574991
a_HQ_transcript/126201	-1.55040766879533	-0.157670533853444	-1.30471474009683	0.201129175579618	0.869253574940758	-5.08213641694965
a_HQ_transcript/52119	1.75642223170475	1.32259800528745	1.30466035905342	0.201147486076147	0.869253574940758	-4.95818673675071
a_HQ_transcript/84794	1.31267651946821	1.6165450919791	1.30465888936792	0.201147980947704	0.869253574940758	-5.08083277560337
a_HQ_transcript/78891	1.08378004307866	-0.330623504766931	1.30455204195529	0.201183961029943	0.869309647865215	-5.15947777631814
a_HQ_transcript/9329	0.727698966862295	3.05532355390432	1.30451215804649	0.20119739290279	0.869309647865215	-5.43921254228194
a_HQ_transcript/120218	-0.995216663776417	1.22008191729605	-1.30448713112123	0.201205821675933	0.869309647865215	-4.94710979148866
a_HQ_transcript/20325	1.27221117373668	1.91451115668252	1.30443971580438	0.201221791334312	0.869314030968479	-5.09935938070764
a_HQ_transcript/110382	0.43130714866788	2.65820693207411	1.30430329363376	0.20126774423506	0.869410951605399	-5.17539274071187
a_HQ_transcript/113593	-0.730139209774138	3.30861797548598	-1.30422209329102	0.201295099833149	0.869410951605399	-5.23161541503162
a_HQ_transcript/108569	1.94813814196916	2.49558186831902	1.3042026182147	0.201301661217066	0.869410951605399	-5.22390517943935
a_HQ_transcript/61512	1.06809487476367	-0.539944024974533	1.30419551895063	0.201304053083833	0.869410951605399	-5.11125353662632
a_HQ_transcript/152466	-4.96294397525429	0.469930006178102	-1.30405457273697	0.201351544829821	0.869505853434771	-5.14737260895545
a_HQ_transcript/26011	-0.972899254201484	3.63203649777696	-1.3040415191525	0.201355943660292	0.869505853434771	-5.47813895078244
a_HQ_transcript/136546	-1.30430782963563	0.370627620501107	-1.30384090864112	0.201423555132304	0.86970423988404	-5.00466978037971
a_HQ_transcript/64360	-1.4605272197154	1.77275424988272	-1.30380232884703	0.201436559611114	0.86970423988404	-5.10173735985597
a_HQ_transcript/129007	0.478285415745925	3.59494527023597	1.30377203748172	0.20144677067504	0.86970423988404	-5.4084676648695
a_HQ_transcript/111535	2.43266668295538	1.2475107348603	1.30355164469815	0.201521075846007	0.869795757495329	-5.1788915600832
a_HQ_transcript/29033	-0.507235643388477	2.67968370132216	-1.30354841427693	0.20152216513429	0.869795757495329	-5.12372832789027
a_HQ_transcript/29487	-1.59598321487119	1.17442256756307	-1.303540664359	0.20152477840154	0.869795757495329	-5.12525798095851
a_HQ_transcript/147082	1.83816225467589	1.32336178470194	1.30353163708372	0.201527822425459	0.869795757495329	-5.13151598521872
a_HQ_transcript/116734	2.61476696790174	1.03857047275183	1.30337733377787	0.201579859382657	0.86995575515592	-5.01513287853576
a_HQ_transcript/126096	-1.72682134724858	0.6498719630606	-1.3032575372887	0.201620266395888	0.870065541578334	-5.11189472023346
a_HQ_transcript/7207	-0.459688215869722	3.12486440549141	-1.3031833316071	0.201645298859993	0.870083156418835	-5.33766156088653
a_HQ_transcript/95736	-0.487377425321972	2.50640732685282	-1.30307268758395	0.201682627798984	0.870083156418835	-5.18178738975733
a_HQ_transcript/96104	1.13917320917912	1.74174035654555	1.30305355328652	0.201689083838537	0.870083156418835	-5.10758787299569
a_HQ_transcript/105222	-1.70025281717523	2.06417337078409	-1.30296815767803	0.201717898808699	0.870083156418835	-5.23853331100595
a_HQ_transcript/73216	1.89718342384067	2.513276840128	1.30296516119739	0.201718909965748	0.870083156418835	-5.12310916493749
a_HQ_transcript/124367	2.60941083030593	1.23667255084501	1.30292685031225	0.201731838246443	0.870083156418835	-5.07014419143166
a_HQ_transcript/133675	5.92503962110835	3.97824030428825	1.30292234026395	0.201733360235916	0.870083156418835	-5.17911770135587
a_HQ_transcript/108319	0.621331854947247	2.56714788309633	1.30284629823427	0.201759023180265	0.870083156418835	-5.2439642667614
a_HQ_transcript/11495	-1.63322745726094	2.01095904615647	-1.30281375588931	0.201770006451815	0.870083156418835	-5.03235475873141
a_HQ_transcript/1685	0.469650193974732	4.52030704529804	1.30280182809008	0.20177403228265	0.870083156418835	-5.55263812469472
a_HQ_transcript/34979	1.22490937083719	1.59016391814984	1.30262958465308	0.201832174143287	0.870216184496693	-5.04058437709831
a_HQ_transcript/48645	1.4972353015114	-0.138192559601994	1.30261012684072	0.201838743054285	0.870216184496693	-5.13859646508128
a_HQ_transcript/142328	-2.40099694287277	0.781940745722034	-1.30256348417655	0.201854490170501	0.870216184496693	-4.89815972504235
a_HQ_transcript/89655	1.41654034938181	1.05135557240918	1.30253305325523	0.201864764515439	0.870216184496693	-4.97409145597534
a_HQ_transcript/93267	-2.2369516225909	0.895913169009124	-1.30240628141176	0.201907570590252	0.870297987225877	-5.25321262829578
a_HQ_transcript/1033	0.949813980647226	2.64354259809031	1.30238817634692	0.20191368455354	0.870297987225877	-5.15321217966259
a_HQ_transcript/115188	-1.73602965087624	1.27131357058421	-1.3022908367932	0.201946557912228	0.870375140381257	-4.96451217878913
a_HQ_transcript/66865	2.37419625012634	1.10374530882764	1.30218099386292	0.201983658794477	0.870435254147345	-5.15650695446715
a_HQ_transcript/54552	1.47507229714548	1.32738956039472	1.30215926260551	0.201990999426149	0.870435254147345	-5.06618693443653
a_HQ_transcript/38774	-1.21378715241338	0.149357367532575	-1.30211654687184	0.202005429026419	0.870435254147345	-5.0270108324049
a_HQ_transcript/25905	-1.75619632480502	0.360230080088813	-1.30200313859501	0.202043742756058	0.870449094594696	-5.03662582040975
a_HQ_transcript/137804	2.84921311345815	2.57270189728555	1.30195590225573	0.202059702665017	0.870449094594696	-5.14842945447091
a_HQ_transcript/30308	-2.33593293940103	0.799635216010513	-1.30194763253422	0.202062496883978	0.870449094594696	-4.99971762734302
a_HQ_transcript/5957	-0.965557169854075	1.95378222874113	-1.30192974827489	0.202068539816626	0.870449094594696	-5.10117186504367

a_HQ_transcript/130814	1.26791396491975	0.252233834640863	1.3018295894218	0.202102385152861	0.8705303774854	-4.97717302426144
a_HQ_transcript/9376	-1.34481354065178	-0.47438439050982	-1.30170017391912	0.202146123198787	0.870590686650687	-5.08551119389377
a_HQ_transcript/145166	-0.503113299197989	3.67979861319474	-1.30153717052825	0.202201223083768	0.870590686650687	-5.36855135510925
a_HQ_transcript/79027	1.46137550793329	-0.362447234482062	1.30153088323286	0.202203348601812	0.870590686650687	-5.05056471207227
a_HQ_transcript/132215	2.65616356821387	2.76961477717823	1.3015125195658	0.2022095568228	0.870590686650687	-5.04840606032258
a_HQ_transcript/106026	1.2519291956684	-0.67121262282833	1.30147499378834	0.202222243647292	0.870590686650687	-5.12073078562362
a_HQ_transcript/54224	-1.81465722058608	0.438598717257511	-1.30147386894181	0.202222623948063	0.870590686650687	-5.19383570788306
a_HQ_transcript/122152	0.357069650330711	4.03398316654784	1.30144905709778	0.202231012754225	0.870590686650687	-5.4891553573098
a_HQ_transcript/75426	-2.45772002335329	2.53038540553948	-1.30132503390849	0.202272948582183	0.870590686650687	-5.15917093282633
a_HQ_transcript/90489	1.50507994744074	-0.234574914247891	1.30130752794895	0.2022788683876	0.870590686650687	-5.10300983750427
a_HQ_transcript/32280	0.650507783858814	2.05802524277652	1.30130434851117	0.202279943558668	0.870590686650687	-5.11361655136059
a_HQ_transcript/149583	1.80504578539793	1.48332049015387	1.30130082533029	0.202281134976331	0.870590686650687	-5.21291165157992
a_HQ_transcript/74621	-0.69247181799135	2.25666528506373	-1.30112752020332	0.202339747370331	0.870626800902329	-5.07946868972941
a_HQ_transcript/123855	0.961071157083068	-0.561374702396732	1.30109300000592	0.20235142376732	0.870626800902329	-5.0985175444423
a_HQ_transcript/47936	-0.700983780103866	2.65233241114425	-1.30100868916849	0.202379943922168	0.870626800902329	-5.17144219566886
a_HQ_transcript/129122	-1.99961621259672	2.81317769032617	-1.30089417542748	0.20241868583983	0.870626800902329	-5.3428714048951
a_HQ_transcript/101117	1.35633761542934	-0.101615217068314	1.30087391850456	0.202425539684995	0.870626800902329	-5.06470313235246
a_HQ_transcript/142991	2.15516703953661	1.77264439270215	1.30087380201504	0.202425579099247	0.870626800902329	-5.06941615311541
a_HQ_transcript/1028	0.359143951655377	3.66357709649112	1.30086616414212	0.202428163387937	0.870626800902329	-5.4228207480521
a_HQ_transcript/38854	0.971059482037995	1.2951624452073	1.30084338944696	0.202435869398297	0.870626800902329	-4.93662761188532
a_HQ_transcript/76265	1.74625553054152	1.14831723638472	1.30083456813472	0.20243885422386	0.870626800902329	-5.20616224281012
a_HQ_transcript/5679	1.75944037723792	2.02836508517625	1.30083323999539	0.202439303622968	0.870626800902329	-5.11908966937845
a_HQ_transcript/17115	-0.826062372353201	2.96715014240293	-1.3007477071195	0.202468246765589	0.870686857142129	-5.26454165674414
a_HQ_transcript/145752	2.23088175942806	0.339341437550707	1.30064289360504	0.202503718512087	0.870688462225637	-5.17340386068454
a_HQ_transcript/31152	-0.725456271253413	2.47600917048513	-1.30064137300686	0.202504233158774	0.870688462225637	-5.12244179328248
a_HQ_transcript/4787	1.52095891242728	-0.00986216738530979	1.30058698952404	0.202522639912539	0.870688462225637	-5.14782575820823
a_HQ_transcript/131882	0.65735164270804	2.07905250244445	1.30056957227529	0.202528535262745	0.870688462225637	-4.99143558141842
a_HQ_transcript/4959	-0.941935707781806	3.13797098508225	-1.30047791238239	0.202559562248274	0.870729053462471	-5.27593292456554
a_HQ_transcript/9482	-0.76669323310196	2.42261077609393	-1.3004357119313	0.202573848370797	0.870729053462471	-5.22549683559723
a_HQ_transcript/118449	-2.6320388124384	0.732546329211251	-1.30033773147012	0.202607020667652	0.870729053462471	-5.11835823020196
a_HQ_transcript/146938	2.16109632321349	2.00897221716557	1.30033542752509	0.202607800741867	0.870729053462471	-5.0818408943599
a_HQ_transcript/69009	2.04119026507135	-0.0510547697740134	1.30014282762176	0.202673019691257	0.870729053462471	-5.17234478322416
a_HQ_transcript/114393	1.31034405222708	-0.612609668608968	1.30014277070858	0.202673038965793	0.870729053462471	-5.11278820692733
a_HQ_transcript/55464	-1.22070706804278	2.26654453407428	-1.30007259351741	0.202696806626639	0.870729053462471	-5.05905906354758
a_HQ_transcript/63859	1.51915087309552	-0.00268067906090002	1.30005038097049	0.202704330030494	0.870729053462471	-5.12295533540986
a_HQ_transcript/30097	-0.449968941463917	3.54586435478396	-1.30000708074632	0.20271899645611	0.870729053462471	-5.43531953558295
a_HQ_transcript/56327	-1.4500320664763	0.597204977728217	-1.29999728093297	0.202722315909932	0.870729053462471	-5.04004875589267
a_HQ_transcript/138685	1.13353326142693	3.99595713272072	1.29998742548567	0.202725654250224	0.870729053462471	-5.48430983986939
a_HQ_transcript/125691	3.22588974871061	2.71913990439814	1.29995659801859	0.202736096723196	0.870729053462471	-5.22669310594398
a_HQ_transcript/85362	0.475180925793486	2.91891427403534	1.29988211675214	0.202761328143502	0.870729053462471	-5.25173678554521
a_HQ_transcript/39719	1.59393049057696	0.574419042745582	1.29981451290913	0.202784231826756	0.870729053462471	-5.05258526041894
a_HQ_transcript/46037	1.38132828943282	0.438660636294501	1.29976239127294	0.202801891598982	0.870729053462471	-4.9225128811372
a_HQ_transcript/77954	1.14787653065131	2.71783191208592	1.29969126511191	0.202825992346429	0.870729053462471	-5.33249376158777
a_HQ_transcript/49335	-0.596426152556365	2.99544015685282	-1.29964365958612	0.202842124462737	0.870729053462471	-5.34546437023845
a_HQ_transcript/50986	-1.03806565147794	0.702963931057844	-1.29961883480346	0.20285053724141	0.870729053462471	-5.00851552302198
a_HQ_transcript/77338	1.05346317778489	2.97913100004447	1.2995943542986	0.202858833609511	0.870729053462471	-5.24552909571817
a_HQ_transcript/45256	1.28701959356657	0.890957267568749	1.29956784881849	0.202867816527142	0.870729053462471	-4.9972213982619
a_HQ_transcript/109583	1.51870822715438	0.62652913020278	1.29952423821602	0.202882597166397	0.870729053462471	-4.987413673809
a_HQ_transcript/89673	-0.522113176499863	3.45269935736009	-1.29937245698532	0.202934045727088	0.870729053462471	-5.27511150915461
a_HQ_transcript/6009	-0.477391787302084	2.19152199007066	-1.29935588093137	0.2029396650354	0.870729053462471	-5.05010097707881

a_HQ_transcript/83070	1.39530376322091	0.655550338011446	1.29934684080846	0.202942729701236	0.870729053462471	-4.96072517323293
a_HQ_transcript/118037	6.64578517717176	4.15176623714754	1.29926851200481	0.202969285198678	0.870729053462471	-5.20034534955167
a_HQ_transcript/6265	1.54018708682707	3.12579366813726	1.29925019384957	0.20297549591085	0.870729053462471	-5.17564394944514
a_HQ_transcript/91835	-3.50792829919853	3.21821332286065	-1.29921888890777	0.202986110086919	0.870729053462471	-5.41879056277802
a_HQ_transcript/28798	-1.30868561314526	-0.201194448715242	-1.29920728034876	0.202990046163723	0.870729053462471	-5.02541967751902
a_HQ_transcript/33648	1.79337923097469	0.576561716352316	1.29918269024843	0.202998384042154	0.870729053462471	-4.94750126409663
a_HQ_transcript/111005	1.24067104478436	0.253423592684549	1.29918253308611	0.202998437332744	0.870729053462471	-5.08089576512676
a_HQ_transcript/108852	-2.72887297562309	0.79460621790683	-1.29917101785972	0.203002341944044	0.870729053462471	-5.1113871650745
a_HQ_transcript/74202	-0.308196177264231	4.22688290717193	-1.29910597673826	0.20302439732233	0.870759401427905	-5.46950408823119
a_HQ_transcript/33618	-4.02167891806674	0.660801444437332	-1.29897469916566	0.203068918977054	0.870886094311507	-5.1482484717527
a_HQ_transcript/102371	-1.32868189375007	1.15053099190387	-1.29890688436246	0.203091920696249	0.870920484413985	-5.13972384774351
a_HQ_transcript/94118	0.938694721816309	-0.718142334407181	1.29885216842555	0.20311048093246	0.87093582440558	-5.1268641191478
a_HQ_transcript/95869	1.72754037736017	2.26067894269097	1.29868289725653	0.203167907724845	0.871117809266785	-5.21964922051736
a_HQ_transcript/138503	-2.18541886680142	1.23012704531922	-1.29853869752369	0.203216838562579	0.871247347207502	-5.22093561004371
a_HQ_transcript/103502	-1.93302771443957	1.0388007735983	-1.29848013774794	0.203236712029182	0.871247347207502	-5.13036047354098
a_HQ_transcript/27836	1.45758336931839	1.05559948634594	1.29839772762034	0.203264682112707	0.871247347207502	-5.02367733816926
a_HQ_transcript/137522	-1.83025403656736	-0.115895480940449	-1.29837360690807	0.203272869262559	0.871247347207502	-5.09805446338421
a_HQ_transcript/16727	-1.67748664408299	2.09059239381505	-1.29834316646351	0.203283201840201	0.871247347207502	-5.12992717329256
a_HQ_transcript/128090	-1.36920026009074	1.75164984497749	-1.29832888388774	0.203288049996135	0.871247347207502	-5.02546739284358
a_HQ_transcript/16941	0.569176918123082	2.47371621199746	1.29816037499408	0.203345256232028	0.871351496920767	-5.12724860748096
a_HQ_transcript/63037	1.62706072733116	0.0221916868482792	1.29812512518926	0.203357224561947	0.871351496920767	-5.07859310342175
a_HQ_transcript/95941	-0.9248112057776	1.72586684901011	-1.29812483851911	0.203357321896971	0.871351496920767	-4.92491136028562
a_HQ_transcript/5146	0.529167001379155	2.37390053144122	1.29794384281092	0.203418783659133	0.871528226874354	-5.13072947948022
a_HQ_transcript/47149	2.3512927120434	1.95711485002738	1.29789910497183	0.203433977727839	0.871528226874354	-5.19123715234919
a_HQ_transcript/5427	0.401219512483719	3.2795255140359	1.29787092971798	0.20344354717964	0.871528226874354	-5.37447327144424
a_HQ_transcript/24972	0.549225772501664	2.29410057580365	1.29771297900312	0.203497199959039	0.87153501842632	-5.14354656361593
a_HQ_transcript/148370	-6.13422307017073	4.38412964564395	-1.2977107262411	0.203497965256353	0.87153501842632	-5.26508746012147
a_HQ_transcript/10496	0.881198329166783	1.57193094557838	1.29769388549317	0.203503686382474	0.87153501842632	-4.92464519560832
a_HQ_transcript/10692	0.872264708817329	4.29862372331571	1.2976520043806	0.203517914733582	0.87153501842632	-5.54070336990314
a_HQ_transcript/140836	0.48251485318938	4.69566052365021	1.29764557390733	0.203520099437773	0.87153501842632	-5.5726709723046
a_HQ_transcript/115812	1.82627297599024	1.14480345156744	1.29747620948418	0.203577646139652	0.871628697785381	-5.1011502702095
a_HQ_transcript/41421	1.01586858904986	-0.249571316300763	1.29734978806712	0.203620609731219	0.871628697785381	-4.98231504522958
a_HQ_transcript/40597	0.830522596582312	2.32888913542613	1.29734777540703	0.20362129377803	0.871628697785381	-5.06126713937154
a_HQ_transcript/13031	-0.51604078131521	2.74592431520492	-1.29730923004476	0.203634394605169	0.871628697785381	-5.23944131974763
a_HQ_transcript/10118	-2.32303576163896	2.68148472015578	-1.29730243252385	0.20363670501836	0.871628697785381	-5.08490399421639
a_HQ_transcript/30122	1.12380462136294	-0.391325253046938	1.29726595635638	0.203649103264439	0.871628697785381	-5.11684601698713
a_HQ_transcript/80629	1.35403609864832	-0.52259104461087	1.29723013084448	0.203661280912451	0.871628697785381	-5.11110711757876
a_HQ_transcript/139753	-1.32873044543118	0.252150316270012	-1.29722820583412	0.203661935269079	0.871628697785381	-5.09247494725725
a_HQ_transcript/44867	-1.03915268562412	0.854710411004688	-1.29706703886015	0.203716725421832	0.871798999876334	-5.00108628560007
a_HQ_transcript/131570	-1.5033499361175	3.66497033409771	-1.29696429240746	0.203751660856337	0.871855787130857	-5.47323003056451
a_HQ_transcript/123323	1.36291627403469	3.05565096897487	1.296926082925	0.203764653854455	0.871855787130857	-5.31888784422677
a_HQ_transcript/48285	-2.10547805997669	0.376617899892254	-1.29687340076104	0.203782569271137	0.871855787130857	-5.10477234655793
a_HQ_transcript/115249	1.5200256867301	-0.0106150559799826	1.29685157828644	0.203789990705804	0.871855787130857	-5.16493749058487
a_HQ_transcript/47928	1.27066351402261	0.268842467192991	1.29679184247967	0.203810306842012	0.871878533714488	-5.01114161618951
a_HQ_transcript/47633	-1.43348964664355	-0.537385416692197	-1.2967070426547	0.203839149895078	0.871937751497613	-5.09480801180305
a_HQ_transcript/29054	-1.35304857618939	-0.0425333701136653	-1.29663290534382	0.203864368843031	0.871981459316242	-4.99911595115671
a_HQ_transcript/107038	0.319019902729171	3.62369923734636	1.29657000634303	0.203885766769928	0.87198475742246	-5.41970014508158
a_HQ_transcript/16318	1.72313764207105	1.67127559443058	1.29654244908766	0.203895142149843	0.87198475742246	-5.1230429895496
a_HQ_transcript/14349	1.03001666844404	1.60901196326268	1.29649747978154	0.203910442068241	0.871986035205083	-5.06212912802799
a_HQ_transcript/141526	0.659851533246894	6.1660275093703	1.29637445760843	0.203952302401059	0.87209463748926	-5.71213512120945

a_HQ_transcript/7379	0.85074399263836	2.37596931978139	1.29631539904838	0.203972400379475	0.87209463748926	-5.02359209842262
a_HQ_transcript/25230	3.07069243122214	3.16657475433351	1.29628342515974	0.203983281912781	0.87209463748926	-5.08222728872
a_HQ_transcript/63953	-0.794460677657022	1.48703193086826	-1.29623086533469	0.20400117032817	0.87209463748926	-4.92675681448104
a_HQ_transcript/53783	2.74660975838267	0.268544708864046	1.2962024162482	0.204010853299253	0.87209463748926	-5.09225270651723
a_HQ_transcript/12915	1.57648714193735	1.48369419656129	1.29608854120904	0.204049615472002	0.872196194437569	-4.93695117840479
a_HQ_transcript/145529	-1.9149096969152	2.95131837315824	-1.29598060513044	0.204086361245829	0.872261180922409	-5.2377353956392
a_HQ_transcript/112699	1.38235186601598	-0.266513228748389	1.29588576786554	0.204118651816097	0.872261180922409	-5.10629259334387
a_HQ_transcript/104942	-1.11168430290029	0.898676830746977	-1.29577230638425	0.204157288744786	0.872261180922409	-5.00003741021411
a_HQ_transcript/106920	0.623048577819061	2.47856887011472	1.29571539877459	0.204176669534923	0.872261180922409	-5.23806305432319
a_HQ_transcript/105323	-2.10194821334751	1.38211742235879	-1.29561814333141	0.204209794663143	0.872261180922409	-5.20395096134347
a_HQ_transcript/117380	2.9720812425858	0.755136617995872	1.2955659271225	0.204227581151312	0.872261180922409	-5.09736586391808
a_HQ_transcript/37509	1.9463701641789	1.2519338878155	1.29555172480573	0.204232419112514	0.872261180922409	-4.99516308406512
a_HQ_transcript/33361	1.24743247166093	0.0576025977152872	1.29552968648923	0.204239926547768	0.872261180922409	-5.04900650451039
a_HQ_transcript/83581	-1.42255579579382	1.02514560497433	-1.29550561257947	0.204248127655621	0.872261180922409	-5.07579929017172
a_HQ_transcript/146713	-2.10887295368305	2.62720586471534	-1.29548873308805	0.204253878035622	0.872261180922409	-5.11391372653242
a_HQ_transcript/134239	-2.65552495413187	0.74222841163798	-1.29542253219248	0.204276432055623	0.872261180922409	-5.17785079968296
a_HQ_transcript/117658	1.3712074485458	-0.180301026715489	1.29536205331499	0.204297038295353	0.872261180922409	-5.05144496293512
a_HQ_transcript/10013	0.542043796107881	3.28872846000577	1.29531650335772	0.204312559028576	0.872261180922409	-5.38868311753406
a_HQ_transcript/125983	3.56320215594544	3.63656703943421	1.295293008936	0.204320564889783	0.872261180922409	-5.20473408485236
a_HQ_transcript/49242	-1.93429607741589	0.720504985998713	-1.29520993450412	0.204348874902074	0.872261180922409	-5.03642896398135
a_HQ_transcript/67947	1.36849611192433	1.63804932022967	1.2951981434419	0.204352893289098	0.872261180922409	-5.14997704078832
a_HQ_transcript/67411	1.83394507314976	2.96051634605372	1.29515700193719	0.204366914760678	0.872261180922409	-5.24928903110613
a_HQ_transcript/134213	3.68571507355559	2.42473617933333	1.29512062879363	0.204379311732963	0.872261180922409	-5.14043529181518
a_HQ_transcript/78821	-1.21552861051139	1.77489022556468	-1.29510853095849	0.204383435136299	0.872261180922409	-5.12733454305545
a_HQ_transcript/98861	0.770051703142594	2.22987861778765	1.29508778906763	0.204390504910669	0.872261180922409	-5.17706481360201
a_HQ_transcript/4866	1.48031948255592	0.253022365301265	1.29506541560687	0.204398131006542	0.872261180922409	-5.07676011974684
a_HQ_transcript/150620	1.93321751470878	0.700217928729255	1.29504241174836	0.204405972202197	0.872261180922409	-5.05800167277645
a_HQ_transcript/84267	0.985401510124161	1.04079419103755	1.29498713690686	0.204424814362182	0.872261180922409	-4.94076665614593
a_HQ_transcript/93084	-1.25741467722741	-0.00563518738633298	-1.29498671004491	0.204424959876582	0.872261180922409	-4.98614876329476
a_HQ_transcript/23805	0.714160375819183	2.54769445769172	1.29488933851662	0.20445815524394	0.872338788022589	-5.16556525828334
a_HQ_transcript/151036	1.94756297407746	2.33440656949699	1.29479203963253	0.204491329944281	0.872416295559717	-5.08435410138596
a_HQ_transcript/85741	0.859522638583284	2.40022701536317	1.29462888470399	0.204546967895522	0.87248159029993	-5.25125317573113
a_HQ_transcript/143184	4.60812304003763	1.44144424446643	1.29459686927186	0.20455788692619	0.87248159029993	-5.17729635057837
a_HQ_transcript/29720	-1.19055237702326	0.0183018713504833	-1.29459510765137	0.204558487748955	0.87248159029993	-5.03935946185931
a_HQ_transcript/141342	-1.34284513255665	1.05014850028218	-1.29444460990284	0.204609821852969	0.87248159029993	-4.99117571790817
a_HQ_transcript/112835	-3.01898363688516	1.37211768381699	-1.2944432858409	0.204610273528038	0.87248159029993	-5.04164639140411
a_HQ_transcript/11714	0.668791172405173	3.3858108562596	1.29441388117953	0.204620304487306	0.87248159029993	-5.4578443693532
a_HQ_transcript/76386	0.638813217929116	3.37778299316896	1.29438335480933	0.204630718496686	0.87248159029993	-5.44398723776494
a_HQ_transcript/77237	2.66960856820503	0.321927507728791	1.29438183732799	0.204631236192895	0.87248159029993	-5.12304622885944
a_HQ_transcript/111000	2.53933100707779	1.27871084856124	1.29420398760954	0.204691917402449	0.87248159029993	-5.09069263800839
a_HQ_transcript/45362	-1.36289248088951	3.18989266816181	-1.29420099870697	0.204692937314274	0.87248159029993	-5.27213624302615
a_HQ_transcript/129750	-2.22639836521655	1.15427132397708	-1.2942008144064	0.204693000203815	0.87248159029993	-5.10112725531606
a_HQ_transcript/6330	-1.28054458452887	4.29805529132177	-1.29419403698347	0.204695312898213	0.87248159029993	-5.43992583778412
a_HQ_transcript/95471	-0.880309844152456	3.06864402857633	-1.2941751426875	0.204701760399643	0.87248159029993	-5.33594040549548
a_HQ_transcript/47058	-2.03635063574737	0.184416305997191	-1.29406530531751	0.204739244433575	0.872566894719012	-5.07694821812045
a_HQ_transcript/115420	-2.94485063180324	2.80397684453284	-1.29399314110242	0.204763874649858	0.872566894719012	-5.32744227174553
a_HQ_transcript/132760	-2.38990489118446	0.525719919567654	-1.29398454733278	0.204766807921519	0.872566894719012	-5.13087278328174
a_HQ_transcript/69529	1.4564278855731	1.30900689302643	1.29381077232098	0.20482612860395	0.872732463357551	-5.05212440181518
a_HQ_transcript/47334	1.1417080499274	1.83799982791057	1.29376374763413	0.20484218342754	0.872732463357551	-5.07195230878618
a_HQ_transcript/43244	-1.69003004031654	1.26026693163979	-1.29366909897641	0.20487450058121	0.872732463357551	-5.03478130562827

a_HQ_transcript/16260	-1.25204818439359	1.24390420851327	-1.29362272246424	0.204890336948486	0.872732463357551	-5.05565352191786
a_HQ_transcript/72236	1.30284851772837	1.50663420270583	1.2936123590199	0.204893875921297	0.872732463357551	-5.02341652862463
a_HQ_transcript/74742	-1.43451625259109	2.29202736852551	-1.29360688258151	0.204895746067996	0.872732463357551	-5.14421485579878
a_HQ_transcript/98518	-1.30282011580499	2.01230614756203	-1.29340954682364	0.204963142841583	0.872926593178147	-5.16626402430369
a_HQ_transcript/94383	-1.62012180190206	1.1205382713681	-1.29338549784577	0.204971357526296	0.872926593178147	-5.25049562113456
a_HQ_transcript/15482	-0.517013686362647	3.89096456593363	-1.29330605780071	0.204998494552441	0.872978204494089	-5.49776638349493
a_HQ_transcript/17374	1.56302829717822	0.7151026318195	1.29312190187907	0.205061413439645	0.87312107495649	-5.01988207895725
a_HQ_transcript/123693	-1.75926145632409	2.84802366379553	-1.29311993460096	0.205062085661058	0.87312107495649	-5.29658245465911
a_HQ_transcript/44510	1.58474649167325	0.859065043955452	1.29296242278262	0.205115913089258	0.873223151678424	-5.04929078606101
a_HQ_transcript/89213	-1.32519895773583	0.97593420985884	-1.29292518753754	0.205128639276489	0.873223151678424	-4.95692912719978
a_HQ_transcript/136091	-1.74670297616498	-0.00926367908694848	-1.29291790968157	0.205131126757653	0.873223151678424	-5.04504377430545
a_HQ_transcript/146774	-1.33098953992732	0.649933436605141	-1.29284366763023	0.205156503083677	0.873254297621495	-5.10185993426391
a_HQ_transcript/55929	1.48749358040012	0.150452007392986	1.29280860285041	0.205168489241274	0.873254297621495	-5.11529483861473
a_HQ_transcript/24534	0.676214079634897	5.08505696453325	1.29267477072147	0.205214241837031	0.873377339903253	-5.55271235876624
a_HQ_transcript/34201	1.49577286495775	0.135845934900114	1.29262103068907	0.205232615887161	0.873377339903253	-5.02865075743517
a_HQ_transcript/91474	-0.630212025916768	3.17146155548174	-1.29255098984181	0.205256565165279	0.873377339903253	-5.39072514406414
a_HQ_transcript/29194	1.13962951309229	1.5906815470373	1.2925434474905	0.20525914427099	0.873377339903253	-5.01160733687265
a_HQ_transcript/41234	-1.8415392128324	0.558806367223461	-1.29250432377569	0.205272523014008	0.873377339903253	-5.06920345206955
a_HQ_transcript/126339	1.43784661079677	1.44319320674629	1.29235240294431	0.20532448014055	0.873473964465831	-5.21436912816568
a_HQ_transcript/30719	1.95630164485019	0.981163301877334	1.29235004556926	0.205325286444762	0.873473964465831	-5.12905327504279
a_HQ_transcript/21091	1.33913756554051	3.22705315328121	1.29229458944737	0.205344255060886	0.873490732395111	-5.1393608308697
a_HQ_transcript/104072	0.977600712635281	0.133386952546859	1.29214141128885	0.205396656160072	0.873547772668754	-5.0149228381874
a_HQ_transcript/149884	-1.04762445323784	-0.613410516879167	-1.292020751901	0.205437939992212	0.873547772668754	-5.10072449279563
a_HQ_transcript/2474	0.854178633471621	-0.564514532688824	1.29199474313144	0.205446839767439	0.873547772668754	-5.10419824948362
a_HQ_transcript/82319	1.74505624935612	0.293387231650037	1.291874913704	0.20548784722378	0.873547772668754	-5.03137860903302
a_HQ_transcript/45968	-2.21795083125375	0.260372111532548	-1.29185023605338	0.205496293064857	0.873547772668754	-5.13065391238489
a_HQ_transcript/82124	1.27162652850883	-0.356295642855153	1.29181596761306	0.205508021758578	0.873547772668754	-5.10038130429701
a_HQ_transcript/44572	-1.09200539299817	-0.233644890237043	-1.29179514179244	0.205515149838871	0.873547772668754	-5.01712620438217
a_HQ_transcript/89272	1.77931057539553	-0.298249982294081	1.29178568647621	0.205518386184126	0.873547772668754	-5.12219650190101
a_HQ_transcript/117937	-0.716328184457303	4.44204974248415	-1.29170458169418	0.205546148146181	0.873547772668754	-5.49392491155283
a_HQ_transcript/37915	2.92642325298614	3.62484548442739	1.29169079020347	0.205550869222318	0.873547772668754	-5.41264012666211
a_HQ_transcript/73869	0.550839029844491	3.63893460471712	1.29167551122505	0.205556099588623	0.873547772668754	-5.44241967327261
a_HQ_transcript/104833	-1.62423592614521	-0.0110332380014351	-1.29160265633864	0.205581040981926	0.873547772668754	-5.10971663186593
a_HQ_transcript/134944	-1.65691133964347	0.0521263478639714	-1.29157779726876	0.205589551848298	0.873547772668754	-5.05026448494713
a_HQ_transcript/89914	2.31907261724361	1.29287175010837	1.29157386514775	0.205590898092011	0.873547772668754	-5.08577697235494
a_HQ_transcript/113500	1.54808434925535	0.257359127155941	1.29150126329093	0.20561575605792	0.873547772668754	-5.00618848102337
a_HQ_transcript/115237	-0.676146711764131	4.41605468824467	-1.29135660470375	0.205665292158935	0.873547772668754	-5.53187435911672
a_HQ_transcript/71561	1.99552193281043	0.0204431109340693	1.29123646135794	0.205706440291537	0.873547772668754	-5.10257007226102
a_HQ_transcript/139223	2.53517808467755	1.90228960972728	1.2912253520246	0.205710245465285	0.873547772668754	-5.16163196295562
a_HQ_transcript/44277	1.83437662278101	2.33563565230177	1.29116897917362	0.205729555143894	0.873547772668754	-5.18213763291407
a_HQ_transcript/36640	-1.85370914677996	1.15785246551343	-1.2910925693036	0.205755730404286	0.873547772668754	-5.12539541016483
a_HQ_transcript/57678	-1.13347327679218	-0.451617049552226	-1.2910882144468	0.205757222297132	0.873547772668754	-5.07433120245876
a_HQ_transcript/114965	1.52747506862292	0.643752472193408	1.29101950407487	0.205780762279698	0.873547772668754	-5.13200548222488
a_HQ_transcript/150186	-1.5642246733384	0.459723098532325	-1.290933887858	0.20581009702306	0.873547772668754	-5.04251929804362
a_HQ_transcript/122782	0.308442833230983	4.05996235215232	1.29092572950841	0.20581289248991	0.873547772668754	-5.51947288653169
a_HQ_transcript/127235	-1.37648100939191	2.34277942372291	-1.29089033435404	0.205825021010155	0.873547772668754	-5.1158606610542
[truncated: 770,794 more chars]
